# Supplementary material for: An organism-wide atlas of hormonal signaling based on the mouse lemur single-cell transcriptome
Source: Nat Commun. 2024 Mar 11;15:2188. doi: 10.1038/s41467-024-46070-9 (PMC10928088; doi:10.1038/s41467-024-46070-9)
Supplement: Supplementary file 2 — Description of additional supplementary files [file 41467_2024_46070_MOESM2_ESM.pdf]

DESCRIPTION OF ADDITIONAL SUPPLEMENTARY FILES DOCUMENT

Supplementary Dataset 1. Genes involved in the biosynthesis and sensing of 84 classes of hormones. Rows show hormones and columns show the hormone class name, hormone name(s), type of hormones, symbol of the human genes for the respective hormone ligands, synthases and other enzymes, receptors, and plasma binding proteins, whether the hormone synthesis or maturation requires coordination of multiple tissues, year discovered, classical sites of secretion and targets, approximate plasma concentrations in humans, and references.

Supplementary Dataset 2. Lemur and mouse orthologs of the human hormonal genes in Supplementary Dataset 1. Rows show genes and columns show gene type, NCBI and Ensembl gene IDs, NCBI and Ensembl gene symbols of human, lemur, and mouse genes respectively, as well as the ortholog type for the human-lemur and human-mouse ortholog mapping. Each row shows a unique human gene. Entries for mouse sometimes include multiple genes (separated by commas) for one-to-many mappings. The “all orthology” tab includes the orthology mapping for all hormonal genes. The “non one-to-one orthology” tab shows only the genes with non one-to-one mapping in either lemur or mouse. The “cross-species missing” tabs show the genes with one-to-one orthology mapping but were not included in the cross-species analysis because they were missing from one or more of the analyzed datasets.

Supplementary Dataset 3. Cell types with positive expression of each of the hormones and receptors. Rows are hormone ligands or receptors and columns show the respective hormone class, hormone name(s), hormone type, entry type (ligand or receptor), related genes, and list of positive cell types.

Supplementary Dataset 4. Clusters of mouse lemur cell types according to the expression of hormonal genes. Rows show clusters and columns show cluster ID, name, cell types included in the cluster, and the hormones, receptors, and hormone-related genes that were positively expressed in the

majority (≥50%) of the cluster cell types.

Supplementary Dataset 5. Hormones that regulate or are produced by the anterior pituitary neuroendocrine cells. Rows show the hormones and hormone receptors expressed in individual pituitary neuroendocrine cell types. Columns show the corresponding genes, hormone names, and upstream/downstream cell types.

Supplementary Dataset 6. Generality scores of the hormone ligands, modulators and receptors. Rows show hormone ligands, modulators, or receptors, ordered by descending generality. Columns show entry types, symbols of human and mouse lemur genes, whether the entry includes a single or multiple genes, and generality scores calculated as the percentage of positive cell types and the number of positive clusters.

Supplementary Dataset 7. Two node feedback circuits detected in the mouse lemur hormone cell-cell communication network. Rows show the detected circuits and columns show the two cell types and hormone signaling that connects the cell types.

Supplementary Dataset 8. Measurements of concentrations of mouse lemur seasonallychanging hormones. Rows show individual measurements and columns show the season of measurements, concentration, hormone names and concentration unit, as well as the samples used in the measurements.
