# Supplementary material for: An organism-wide atlas of hormonal signaling based on the mouse lemur single-cell transcriptome
Source: Nat Commun. 2024 Mar 11;15:2188. doi: 10.1038/s41467-024-46070-9 (PMC10928088; doi:10.1038/s41467-024-46070-9)
Supplement: Supplementary file 11 — Reporting Summary [file 41467_2024_46070_MOESM11_ESM.pdf]

nature portfolio

Corresponding author(s): James Ferrell, Mark Krasnow

Last updated by author(s): 2024/1/17

Reporting Summary

Nature Portfolio wishes to improve the reproducibility of the work that we publish. This form provides structure for consistency and transparency in reporting. For further information on Nature Portfolio policies, see our Editorial Policies and the Editorial Policy Checklist.

Statistics

For all statistical analyses, confirm that the following items are present in the figure legend, table legend, main text, or Methods section.

n/a	Confirmed	
☐	☒	The exact sample size (*n*) for each experimental group/condition, given as a discrete number and unit of measurement
☐	☒	A statement on whether measurements were taken from distinct samples or whether the same sample was measured repeatedly
☐	☒	The statistical test(s) used AND whether they are one- or two-sided<br>*Only common tests should be described solely by name; describe more complex techniques in the Methods section.*
☒	☐	A description of all covariates tested
☐	☒	A description of any assumptions or corrections, such as tests of normality and adjustment for multiple comparisons
☐	☒	A full description of the statistical parameters including central tendency (e.g. means) or other basic estimates (e.g. regression coefficient) AND variation (e.g. standard deviation) or associated estimates of uncertainty (e.g. confidence intervals)
☐	☒	For null hypothesis testing, the test statistic (e.g. *F*, *t*, *r*) with confidence intervals, effect sizes, degrees of freedom and *P* value noted<br>*Give P values as exact values whenever suitable.*
☒	☐	For Bayesian analysis, information on the choice of priors and Markov chain Monte Carlo settings
☐	☒	For hierarchical and complex designs, identification of the appropriate level for tests and full reporting of outcomes
☐	☒	Estimates of effect sizes (e.g. Cohen's *d*, Pearson's *r*), indicating how they were calculated

Our web collection on statistics for biologists contains articles on many of the points above.

Software and code

Policy information about availability of computer code

Data collection	Hormone ligands, synthases, processing enzymes, and receptors were manually integrated without software from multiple sources as described in the manuscript. Published scRNAseq data used in this study were downloaded without software from respective data repository (see Data Availability Statement). Protein sequences were extracted from NCBI using Matlab (v2020b) built-in function 'getgenpept'. RNAscope images were taken using a Zeiss LSM 880 Confocal Laser Scanning Microscope with its build-in ZEN software.

Data analysis	Analysis of this study were performed using Matlab (v2020b), with all code and associated data to reproduce the results provided at Stanford Digital Repository at https://purl.stanford.edu/yp860tc1411. https://doi.org/10.25740/yp860tc1411.<br>For data visualization, heatmap figures were generated by the 'imagesc' Matlab built-in function, dotplots and scatterplots by 'plot' and 'scatter', .histograms by 'histogram', bar plots by 'bar', UMAPs by a Matlab umap package (https://www.mathworks.com/matlabcentral/fileexchange/71902).<br>Alignment of protein sequences was performed using 'nwalign' Matlab built-in function. Hierarchical clustering were performed using 'clustergram', 'pdist', 'linkage', 'dendrogram' Matlab built-in functions. Differentially expressed genes were detected with Wilcoxon rank sum test using the 'ranksum' Matlab built-in function. The network graph was generated, analyzed, and visualized using 'digraph', 'centrality', and 'plot' Matlab built-in functions, as well as degree-preserving random rewiring algorithm published previously by Maslov and Sneppen (2002). Distribution fitting and testing were performed using 'ecdf', 'cdffit', 'fitdist', 'kstest' Matlab built-in functions for normal, exponential, lognormal, and gamma distributions, and using 'plfit' and 'plpva' functions from a Matlab package (https://github.com/samuellab/Larval-ORN/blob/master/Figure3/functions/3rdPartyCodes/powerlaws_full_v0.0.10-2012-01-17) for power law distribution. Analysis of the three hormone/receptor genes (LHB, MC1R, SCT) unannotated in the current mouse lemur genome (NCBI MicMur3.0) was performed using previously published TAR (Transcriptionally Active Region) methods (http://github.com/fw262/TAR-scRNA-seq). Trajectory analysis was performed using a custom program developed in Matlab (Trajectory analysis: https://github.com/Shixuan1/scRNAseq_trajectory_analysis).

For manuscripts utilizing custom algorithms or software that are central to the research but not yet described in published literature, software must be made available to editors and reviewers. We strongly encourage code deposition in a community repository (e.g. GitHub). See the Nature Portfolio guidelines for submitting code & software for further information.

Data

Policy information about availability of data

All manuscripts must include a data availability statement. This statement should provide the following information, where applicable:

- Accession codes, unique identifiers, or web links for publicly available datasets
- A description of any restrictions on data availability
- For clinical datasets or third party data, please ensure that the statement adheres to our policy

Tabula Microcebus mouse lemur scRNA-seq gene expression counts/UMI tables, and cellular metadata used in this study are available on figshare (https://figshare.com/projects/Tabula_Microcebus/112227)., scRNA-seq data of hormonal genes and can be explored interactively using cellxgene and in dot plot format on the Tabula Microcebus web portal (https://tabula-microcebus.ds.czbiohub.org/).
For the cross-species comparison, human data were from the 10x data of the Tabula Sapiens for the liver, spleen, and bone marrow (https://figshare.com/projects/Tabula_Sapiens/100973), 10x data of the Human Lung Cell Atlas for the lung (https://www.synapse.org/#!Synapse:syn21041850/wiki/600865), and the drop-seq data from Shami et al. for the testis (https://www.ncbi.nlm.nih.gov/geo/query/acc.cgi?acc=GSE142585). Mouse data were from 10x data of the Tabula Muris Senis (https://figshare.com/articles/dataset/Processed_files_to_use_with_scanpy_/8273102/2) except for the testis which we used 10x data from Ernst et al.(https://www.ebi.ac.uk/biostudies/arrayexpress/studies/E-MTAB-6946).
Metadata generated in this study, including the integrated cross-species meta data, have been deposited in the Stanford Digital Repository at https://purl.stanford.edu/yp860tc1411. https://doi.org/10.25740/yp860tc1411. Selected data discussed in the manuscript are also provided as Supplementary Datasets.

Research involving human participants, their data, or biological material

Policy information about studies with human participants or human data. See also policy information about sex, gender (identity/presentation), and sexual orientation and race, ethnicity and racism.

Reporting on sex and gender	NA
Reporting on race, ethnicity, or other socially relevant groupings	NA
Population characteristics	NA
Recruitment	NA
Ethics oversight	NA

Note that full information on the approval of the study protocol must also be provided in the manuscript.

Field-specific reporting

Please select the one below that is the best fit for your research. If you are not sure, read the appropriate sections before making your selection.

☒ Life sciences ☐ Behavioural & social sciences ☐ Ecological, evolutionary & environmental sciences

For a reference copy of the document with all sections, see nature.com/documents/nr-reporting-summary-flat.pdf

Life sciences study design

All studies must disclose on these points even when the disclosure is negative.

Sample size	This study is not hypothesis driven and focuses on data mining and analysis. A total of 4 mouse lemurs were used in this study. The sample size is determined by the availability of the animals in accordance with the approved animal protocol.
Data exclusions	This study used 10x single-cell RNAseq data with annotated cell information from an accompanying manuscript titled "Tabula Microcebus: A transcriptomic cell atlas of mouse lemur, an emerging primate model organism". In the downstream analysis, we excluded cell types that are 1) low in cell number, 2) technical doublets, or 3) low in transcript and/or gene count. In addition, we excluded several notable cross-contamination genes when clustering the cell types by hormonal genes. In the cross-species analysis, cells that were deemed low quality (i.e., low in transcript and/or gene count) or technical doublets were removed from the integration analysis. In addition, if a dataset includes cells sampled from animals during early or postnatal development, these cells were removed and we only analyzed cells sampled from adult animals for consistency. Cell types (after unification across species) with fewer than 15 cells in any of the species were excluded from the downstream analysis of evolutionary comparisons. Details on data exclusions are described in more detail in the Methods section.
Replication	This study is not hypothesis driven and focuses on data mining and analysis. When clustering cell types by hormonal genes (Fig. 2), we used cell types from different animals and/or tissues to examine the consistency of the clustering pattern. The number of entries ('replicates') with identical cell type designation but were sampled from different animals and/or tissues was based on data availability and were different for each cell type. Details were described in Supplementary Dataset 4. We confirmed that cell types from different animals clustered similarly. We also found consistent clustering pattern for cell types with the same designation but were sampled from different tissues, with a rare exception of the endothelial cell types, which is likely a real biological tissue-specific difference (see Fig. 3h and related manuscript section). When comparing gene expression among cell types (e.g., Fig. 3), comparison were performed by combining scRNAseq data from all sampled animals (n=4) as well as independently for each individual, where available. We identified and report the differentially expressed genes that were consistent among all sampled animals. For RNAscope experiments (Fig. 1h-j), biobanked kidney sample from one of the lemurs profiled by scRNAseq (L4) was used, given the sample availability. Two sections were stained with similar results.
Randomization	This is not applicable as the study does not involve allocation of participants/samples.
Blinding	This is not applicable as the study does not involve allocation of participants/samples.

Reporting for specific materials, systems and methods

We require information from authors about some types of materials, experimental systems and methods used in many studies. Here, indicate whether each material, system or method listed is relevant to your study. If you are not sure if a list item applies to your research, read the appropriate section before selecting a response.

Materials & experimental systems

n/a	Involved in the study
☒	☐ Antibodies
☒	☐ Eukaryotic cell lines
☒	☐ Palaeontology and archaeology
☐	☒ Animals and other organisms
☒	☐ Clinical data
☒	☐ Dual use research of concern
☒	☐ Plants

Methods

n/a	Involved in the study
☒	☐ ChIP-seq
☒	☐ Flow cytometry
☒	☐ MRI-based neuroimaging

Animals and other research organisms

Policy information about studies involving animals; ARRIVE guidelines recommended for reporting animal research, and Sex and Gender in Research

Laboratory animals	Experimental species is gray mouse lemur (Microcebus murinus). Lemur 1: male, age 9.8 yr; Lemur 2: female, age 10.1 yr; Lemur 3: female, age 11.8 yr; Lemur 4: male, age 11.8 yr.
Wild animals	The study did not involve wild animals.
Reporting on sex	Two female and two male animals were sampled in this study. No sex-based analyses were performed given the small sample size.
Field-collected samples	The study did not involve field-collected samples.
Ethics oversight	The use of the animals has been approved by the Stanford University Administrative Panel on Laboratory Animal Care (APLAC #27439) and in accordance with the Guide for the Care and Use of Laboratory Animals.

Note that full information on the approval of the study protocol must also be provided in the manuscript.

Plants

Seed stocks	NA
Novel plant genotypes	NA
Authentication	NA
