# Supplementary material for: An organism-wide atlas of hormonal signaling based on the mouse lemur single-cell transcriptome
Source: Nat Commun. 2024 Mar 11;15:2188. doi: 10.1038/s41467-024-46070-9 (PMC10928088; doi:10.1038/s41467-024-46070-9)
Supplement: Supplementary file 1 — Supplementary information [file 41467_2024_46070_MOESM1_ESM.pdf]

An organism-wide atlas of hormonal signaling based on the mouse lemur single-cell transcriptome

Shixuan Liu[1, 2, 3], Camille Ezran[2, 3], Michael F. Z. Wang[4], Zhengda Li[1], Kyle Awayan[5], The Tabula Microcebus Consortium, Jonathan Z. Long[6, 7], Iwijn De Vlaminck[4], Sheng Wang[8], Jacques Epelbaum[9], Christin S. Kuo[10], Jeremy Terrien[9], Mark A. Krasnow[2, 3]*, James E. Ferrell, Jr.[1, 2]*

1. Department of Chemical and Systems Biology, Stanford University School of Medicine, Stanford, CA, USA;
2. Department of Biochemistry, Stanford University School of Medicine, Stanford, CA, USA;
3. Howard Hughes Medical Institute, Stanford University School of Medicine, Stanford, CA, USA;
4. Meinig School of Biomedical Engineering, Cornell University, Ithaca, NY, USA;
5. Chan Zuckerberg Biohub, San Francisco, CA, USA;
6. Department of Pathology, Stanford University School of Medicine, Stanford, CA, USA;
7. Sarafan ChEM-H, Stanford University, Stanford, Stanford, CA, USA;
8. Paul G. Allen School of Computer Science & Engineering, University of Washington, Seattle, WA, USA;
9. Unité Mécanismes Adaptatifs et Evolution (MECADEV), UMR 7179, CNRS, Museum National d'Histoire Naturelle, Brunoy, France;
10. Department of Pediatrics, Stanford University School of Medicine, Stanford, CA, USA

*Correspondence to James E. Ferrell, Jr. (james.ferrell@stanford.edu) and Mark A. Krasnow (krasnow@stanford.edu)

Supplementary Figures

This large figure is provided at the end of this Supplementary Information File,
and can be accessed interactively at the Tabula Microcebus web portal.

Fig. S1. Dot plot of hormone ligand, receptor, and modulator gene expression for 84 classes of hormones across the mouse lemur cell atlas. Each dot plot shows one of the 84 hormone groups and one of the five cell compartment groups (1. epithelial/neural/germ cells (combined for space saving); 2. endothelial cells; 3. stromal cells; 4. lymphoid cells; and 5. myeloid cells). Cell types are arranged by either their tissue of origin (a) or by their cell type name (b). The hormone group name and cell compartment type is indicated on the top right of the figure. The dot plots are arranged in a polar coordinate style. Radial lines represent individual cell types, with cell type names labeled inside the inner circle and the tissue source labeled outside the outer circle. Circular arcs represent the related genes for this hormone group (red for ligands, light red for synthases and processing enzymes, green for modulators, and blue for receptor genes). The dark colored dots on the intersections of the radial lines and circular arcs depict mean gene expression levels (by dot shade) and percent of positive cells (by dot size) for the respective gene and cell type. The outermost red or blue dots indicate positive expression of one of the hormone(s) (red) or hormone receptor(s) (blue), or both (purple) for the hormone group by the respective cell type (see Methods). The outermost grey dots indicate potentially low-quality cell types (e.g., low cell number (<10), pancreatic cells that show notable signal contamination) with outlines indicating whether or not the cell type expresses one the hormone ligand(s) (red outline), receptor(s) (blue outline), both (purple outline), or neither (no outline).

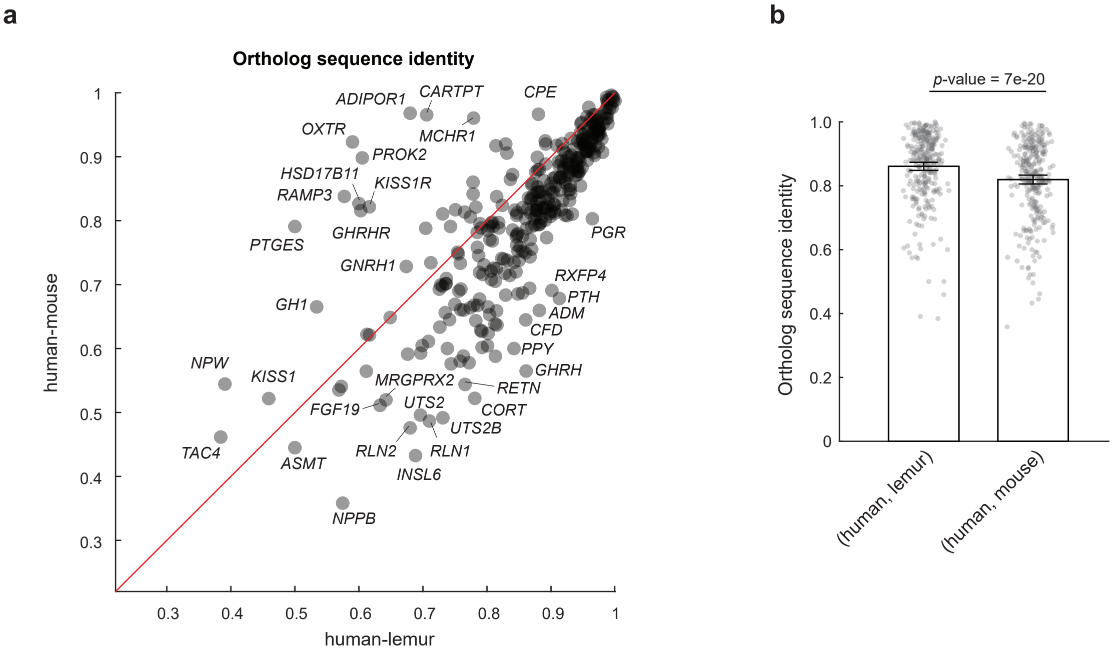

Fig. S2. Protein sequences of hormonal genes are generally more similar between human and mouse lemur than human and mouse. a. Scatter plot comparing pairwise gene sequence identity between human and lemur vs. human and mouse. Each circle is a hormone-related protein. Protein sequences were aligned by the Needleman-Wunsch algorithm and sequence identity was calculated as the percentage of identical amino acids. Points below the red line are proteins more similar between human and mouse lemur than between human and mouse. **b.** Bar plot showing mean and 95% confidence interval of the average sequence identity between human and lemur vs. human and mouse. Grey dots show individual genes (n = 324 orthologs). Comparison used two-tailed paired-sample t-test.

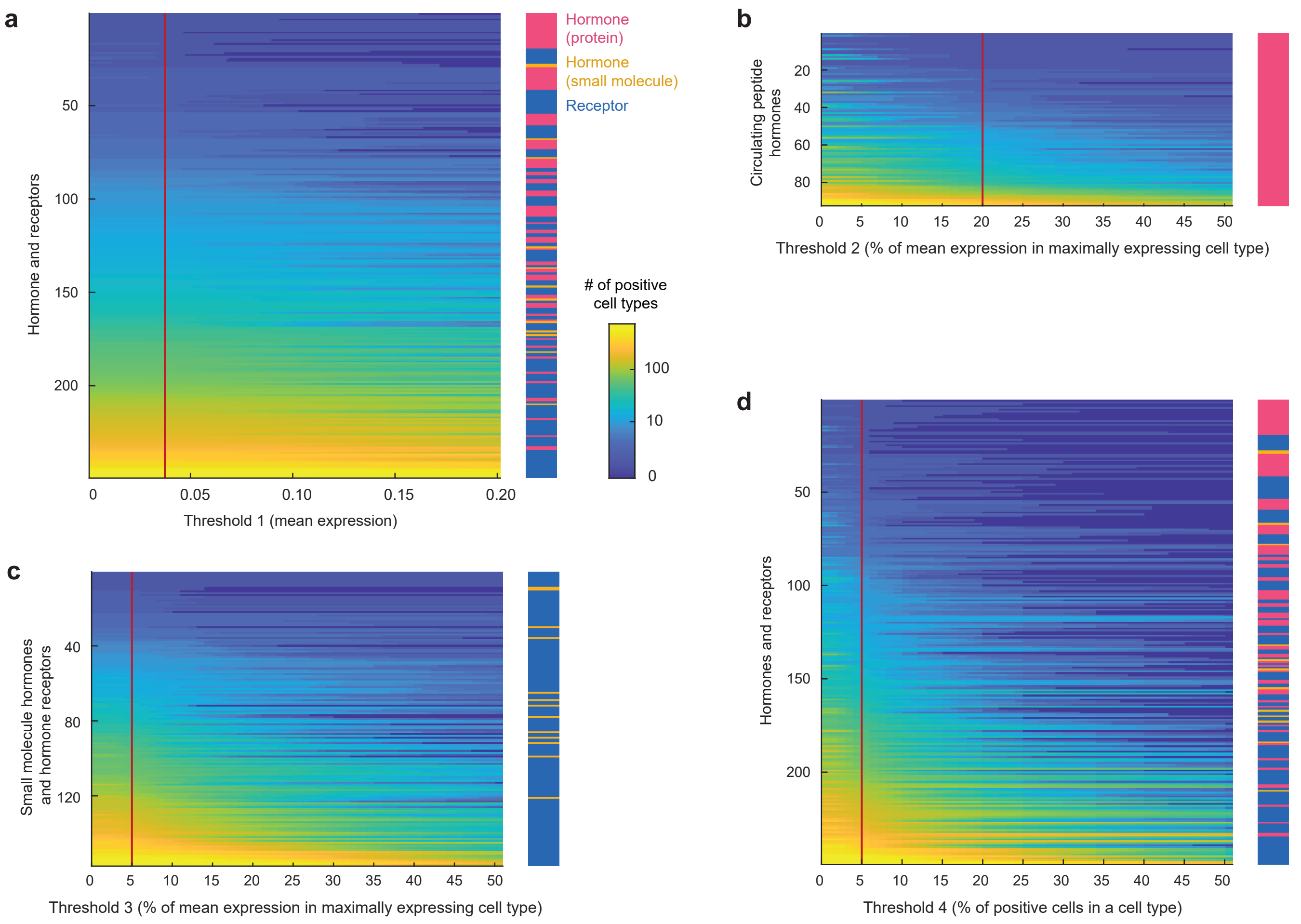

Fig. S3. Number of positive cell types for each hormone ligand and receptor when changing the parameters for adaptive thresholding. Parameters include cell type mean expression (a), percent of mean expression relative to the maximally expressing cell types separately for circulating peptide hormones (ligand genes) (b) or small molecule hormones (synthase genes) and hormone receptors (c), and the percent of positive cells in a cell type (d). Red vertical lines represent the parameters used in the follow-up analysis. Each row represents one hormone or receptor, ordered by the number of positive cell types at the selected parameter.

Fig. S4

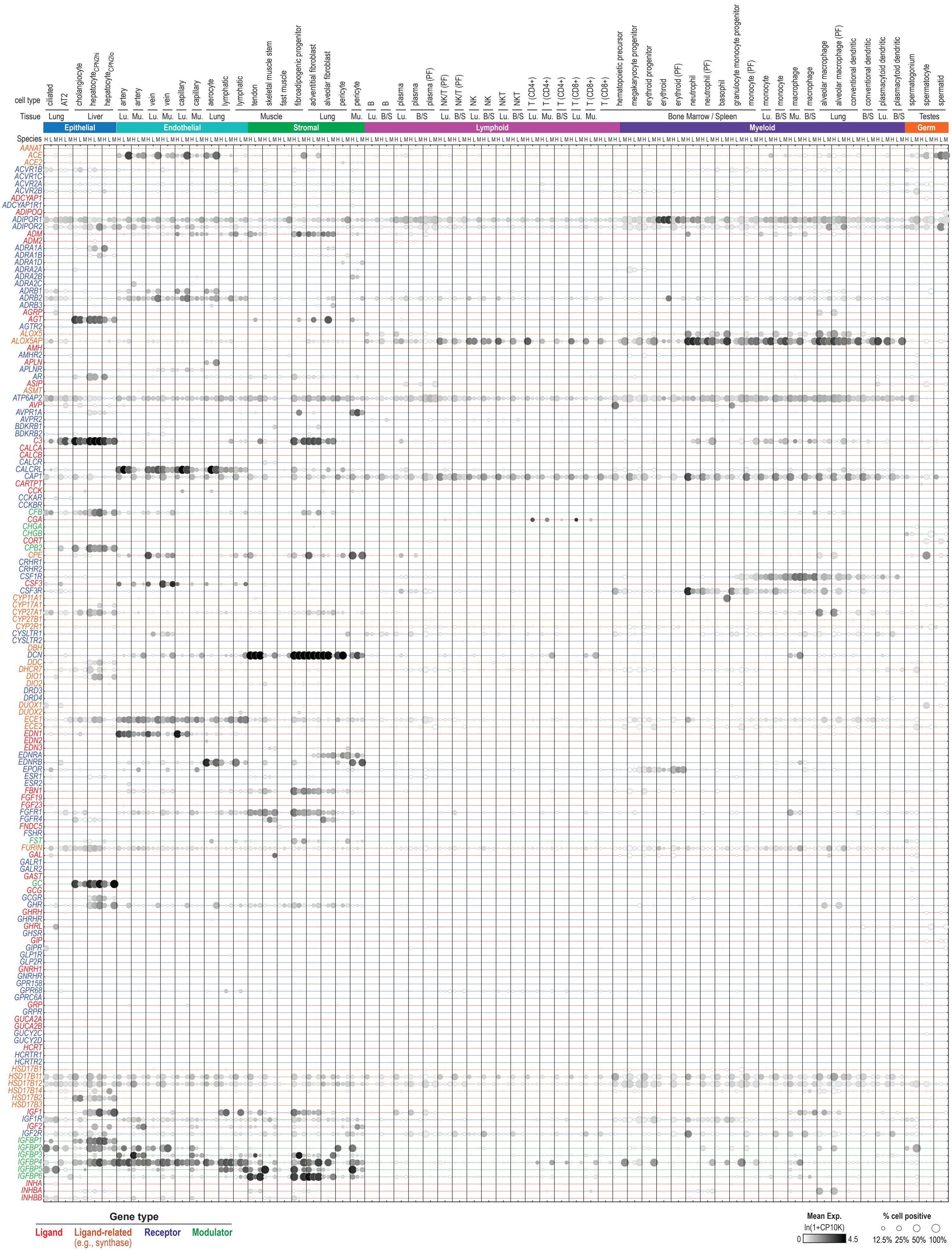
cell type
ciliated
AT2
cholangiocyte
hepatocyte CPN2hi
hepatocyte CPN2lo
artery
artery
vein
vein
capillary
capillary
aerocyte
lymphatic
lymphatic
tendon
skeletal muscle stem
fast muscle
fibroadipogenic progenitor
adventitial fibroblast
alveolar fibroblast
pericyte
pericyte
B
B
plasma
plasma
plasma (PF)
NK/T (PF)
NK/T (PF)
NK
NK
NKT
NKT
T (CD4+)
T (CD4+)
T (CD4+)
T (CD8+)
T (CD8+)
T (CD8+)
hematopoietic precursor
megakaryocyte progenitor
erythroid progenitor
erythroid
erythroid (PF)
neutrophil
neutrophil (PF)
basophil
granulocyte monocyte progenitor
monocyte (PF)
monocyte
monocyte
macrophage
macrophage
alveolar macrophage
alveolar macrophage (PF)
conventional dendritic
conventional dendritic
plasmacytoid dendritic
plasmacytoid dendritic
spermatogonium
spermatocyte
spermatid
Tissue
Lung
Liver
Lu.
Mu.
Lu.
Mu.
Lu.
Mu.
Lung
Muscle
Lung
Mu.
Lu.
B/S
Lu.
B/S
Lu.
B/S
Lu.
B/S
Lu.
B/S
Lu.
Mu.
B/S
Lu.
Mu.
Bone Marrow / Spleen
Lu.
B/S
Mu.
B/S
Lung
B/S
Lu.
B/S
Testes
Epithelial
Endothelial
Stromal
Lymphoid
Myeloid
Germ
Species
AANAT
ACE
ACE2
ACVR1B
ACVR1C
ACVR2A
ACVR2B
ADCYAP1
ADCYAP1R1
ADIPOQ
ADIPOR1
ADIPOR2
ADM
ADM2
ADRA1A
ADRA1B
ADRA1D
ADRA2A
ADRA2B
ADRA2C
ADRB1
ADRB2
ADRB3
AGRP
AGT
AGTR2
ALOX5
ALOX5AP
AMH
AMHR2
APLN
APLNR
AR
ASIP
ASMT
ATP6AP2
AVP
AVPR1A
AVPR2
BDKRB1
BDKRB2
C3
CALCA
CALCB
CALCR
CALCRL
CAP1
CARTPT
CCK
CCKAR
CCKBR
CFB
CGA
CHGA
CHGB
CORT
CPB2
CPE
CRHR1
CRHR2
CSF1R
CSF3
CSF3R
CYP11A1
CYP17A1
CYP27A1
CYP27B1
CYP2R1
CYSLTR1
CYSLTR2
DBH
DCN
DDC
DHCR7
DIO1
DIO2
DRD3
DRD4
DUOX1
DUOX2
ECE1
ECE2
EDN1
EDN2
EDN3
EDNRA
EDNRB
EPOR
ESR1
ESR2
FBN1
FGF19
FGF23
FGFR1
FGFR4
FNDC5
FSHR
FST
FURIN
GAL
GALR1
GALR2
GAST
GC
GCG
GCGR
GHR
GHRH
GHRHR
GHRL
GHSR
GIP
GIPR
GLP1R
GLP2R
GNRH1
GNRHR
GPR158
GPR68
GPRC6A
GRP
GRPR
GUCA2A
GUCA2B
GUCY2C
GUCY2D
HCRT
HCRTR1
HCRTR2
HSD17B1
HSD17B11
HSD17B12
HSD17B14
HSD17B2
HSD17B3
IGF1
IGF1R
IGF2
IGF2R
IGFBP1
IGFBP2
IGFBP3
IGFBP4
IGFBP5
IGFBP6
INHA
INHBA
INHBB
Gene type
Ligand
Ligand-related
(e.g., synthase)
Receptor
Modulator
Mean Exp.
ln(1+CP10K)
0
4.5
% cell positive
12.5%
25%
50%
100%

Fig. S4 - continued

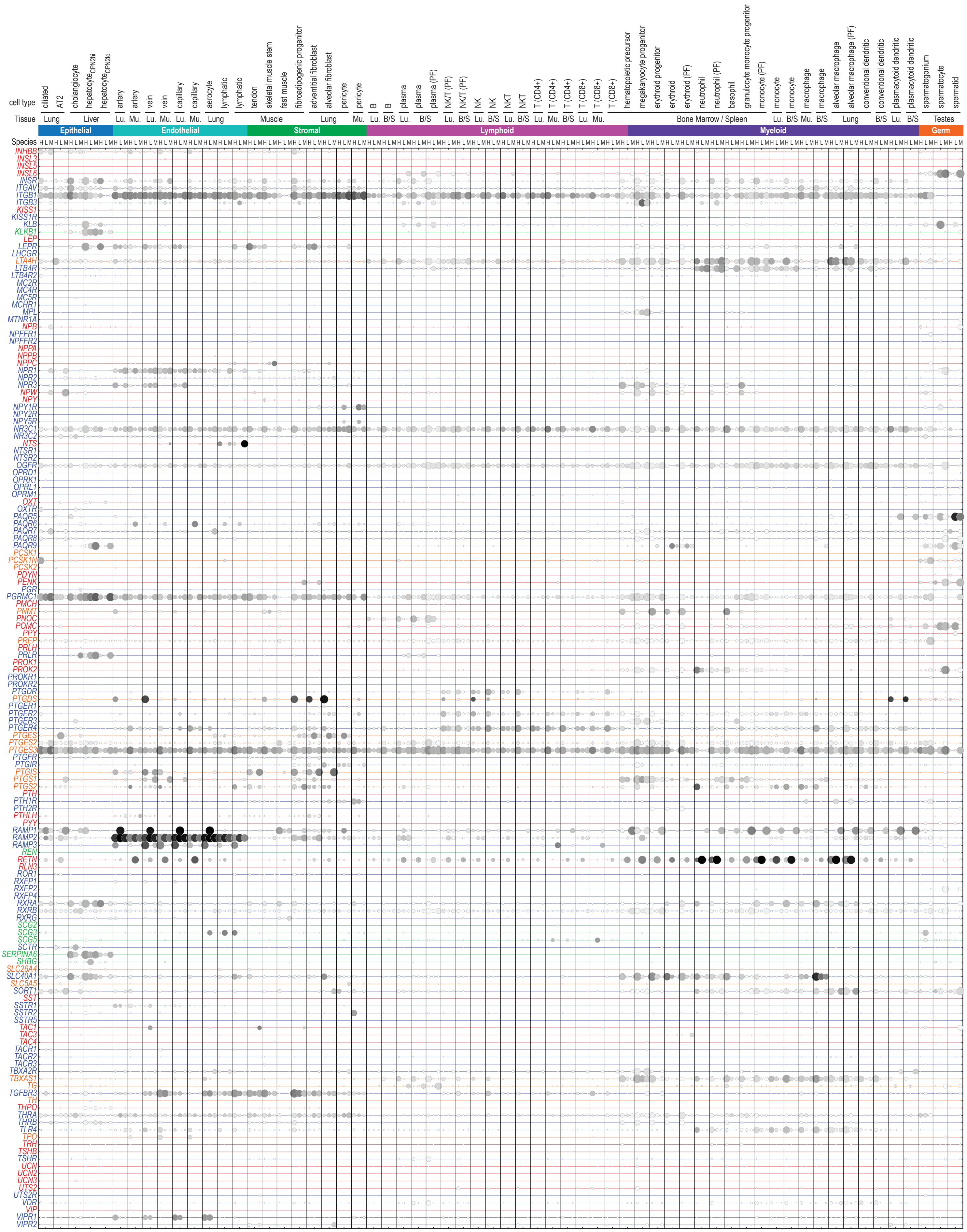

Fig. S4. Expression of the hormonal genes across human, lemur, and mouse cell types. Expression is visualized by a dot plot with dot color indicating the cell type average expression and dot size indicating the percent of positive cells in the cell type. Rows are 295 one-to-one-to-one orthologs of the hormonal genes, labeled with the respective human symbols, and ordered alphabetically. Columns are arranged as triplets showing respective expressions in humans, lemurs, and mice. Triplets of different cell types are segregated by vertical gray lines. The orthologous cell type triplets are ordered first by compartment (e.g., epithelial, stromal), then by tissue, and lastly by the cell type annotation.

Fig. S5

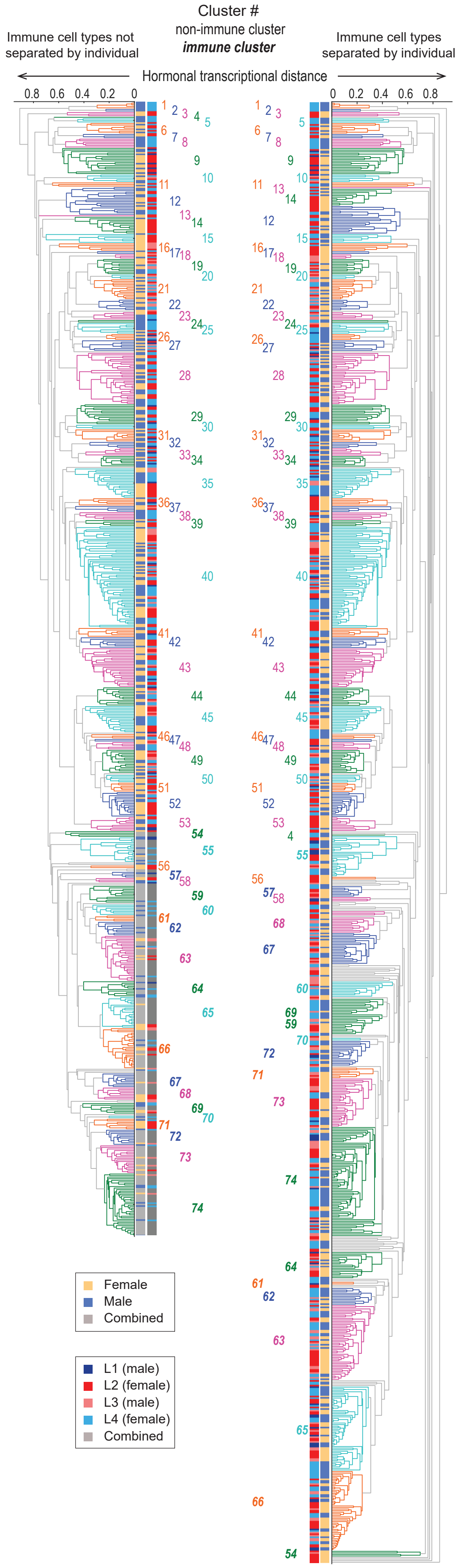

Fig. S5. Cell types clustered similarly in both sexes and across sampled mouse lemurs. Shown here are cell type clustering dendrograms when grouping (left) or separating (right) correspond-ing immune cell types from different lemur individuals. Dendro-grams are colored by clusters with cluster IDs in corresponding color and labeled nearby. The two color bars next to each of the dendrograms indicate the lemur individual and sex the cell type sampled from. Supplementary Dataset 4 lists cell types included in each cluster and the respective lemur individual, sex, and tissue the cell type sampled from.

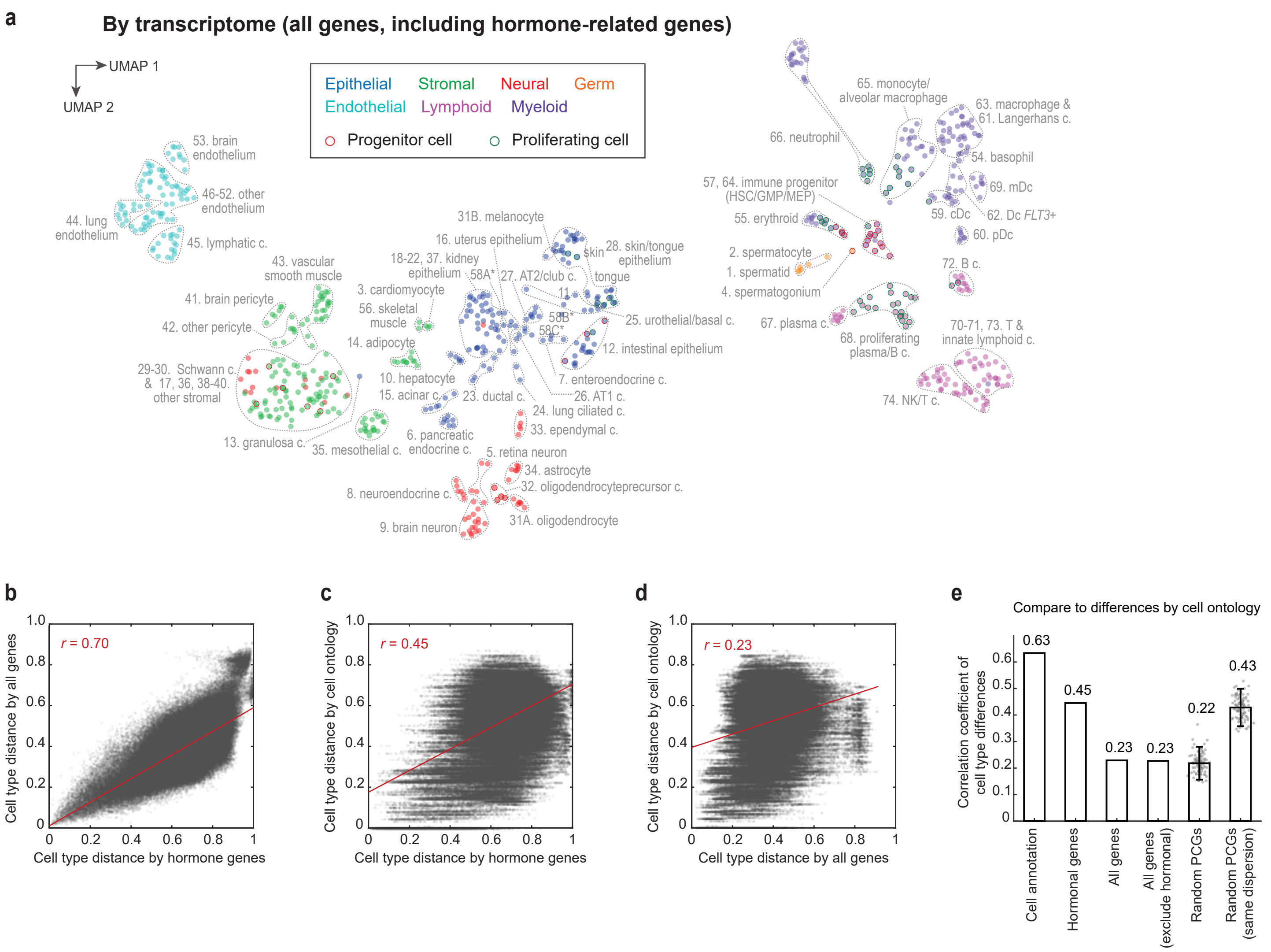

Fig. S6. Comparison of cell type clustering by the hormonal genes with that by the full transcriptome. a. UMAP visualization of mouse lemur cell types based on the full transcriptome. Circles (cell types) are color-coded by cell compartment types (epithelial, neural, germ, stromal, endothelial, lymphoid, and myeloid). Dashed lines circumscribe cell type clusters as in Fig. 2a-b and cluster IDs and names are labeled next to the cell type clusters. Cell types that were clustered by the hormon-al genes but not the full transcriptome are labeled separately (i.e., 31B and 31A; 58A, 58B and 58C). Note some cell types that formed discrete clusters based on hormonal gene expression intermingled with other clusters when mapped based on the non-hormonal transcriptome (e.g., cluster 46-52), and therefore were annotated together. **b-d.** Scatter plots and correlation of cell type pairwise distances based on hormonal genes vs. that of all genes (b), hormonal genes vs. Cell Ontology (c), and all genes vs. Cell Ontology (d). **e.** Bar plot of correlation coefficients comparing cell type pairwise distances of different metrics compared to a benchmark Cell Ontology-based distance. Error bar represents mean and 95% confidence interval of the data (n = 100). Also see Fig. 2e for comparisons with the benchmark of cell annotation-based distances.

Fig. S7

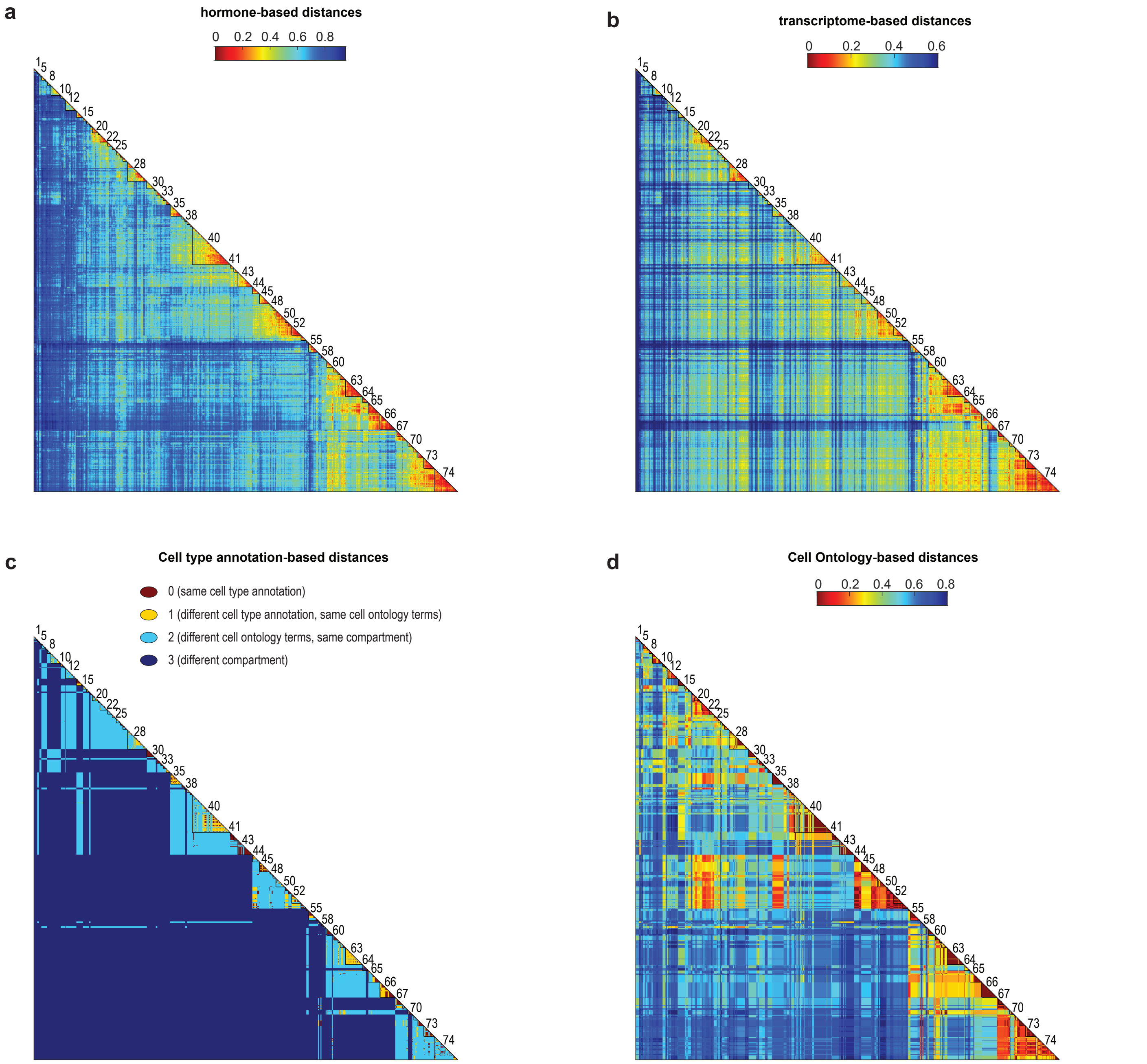

Fig. S7. Heatmap representation of cell type pairwise distances based on the hormonal genes (a), the full transcriptome (b), the cell type annotations (c), and Cell Ontology (d). Cell types were ordered by cluster as in Fig. 2a, and cluster IDs were labeled along the diagonal for large clusters.

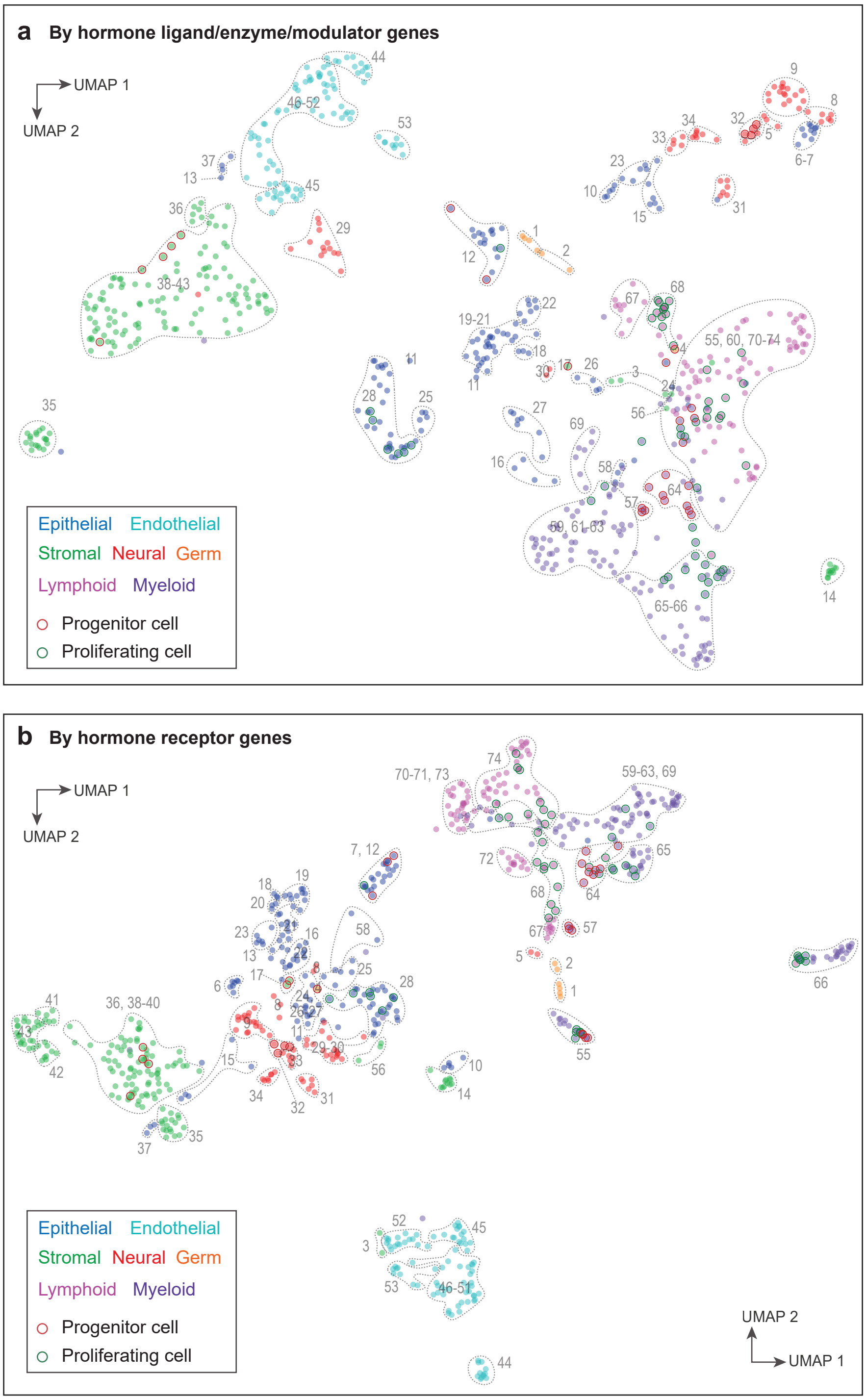

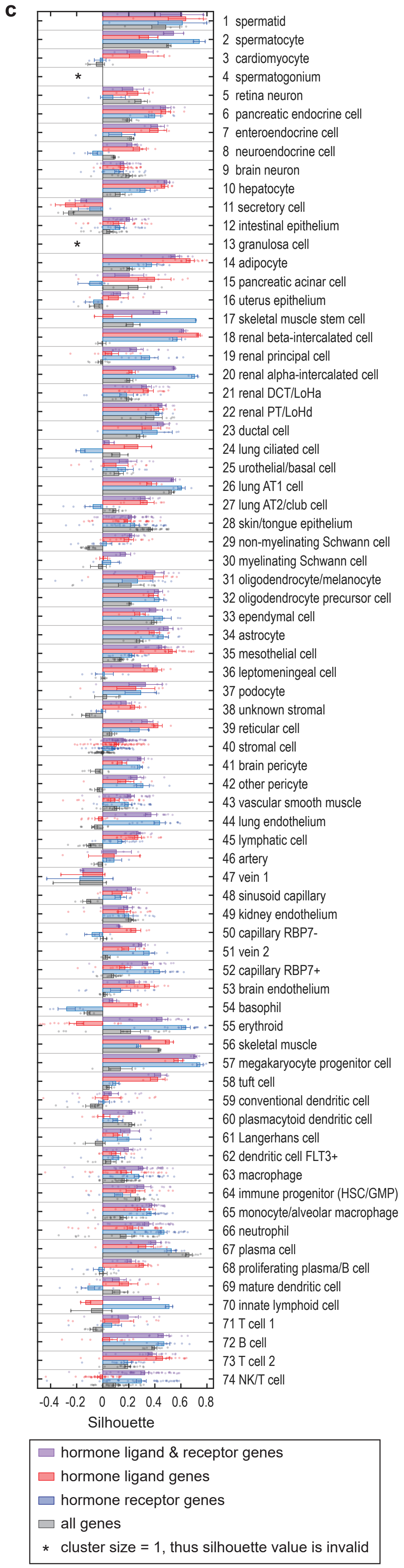

Fig. S8. Classification of mouse lemur cell types using only the hormone ligand or the receptor genes. a-b. UMAP visualization of cell types based on the transcriptional distances of only the hormone ligands/ modulators (a) or the hormone receptors (b). Circles (cell types) are color-coded by the cell compart-ment types (epithelial, neural, germ, stromal, endothelial, lymphoid, and myeloid). Dashed lines circumscribe cell type clusters as in Fig. 2a-b and cluster IDs are annotated nearby. As UMAPs qualitatively display relationships among data points, the extremely distant clusters in panel a (i.e., cluster 35. mesothelial cells by hormone ligand genes) were shifted towards the center of the figure for display purposes. **c.** Cluster mean silhouette values and SEM of the 74 cell type clusters by both hormone ligand and receptor genes (black), only the hormone ligand genes (red), only the hormone receptor genes (blue), or the non-hormonal transcriptome (blank). The silhouette values are a measure of how well each cell type cluster was classified (see Methods). Aster-isks (*) indicate clusters with only a single cell type, thus silhouette values cannot be calculated. See Supplementary Dataset 4 for the cluster cell types.

Fig. S9

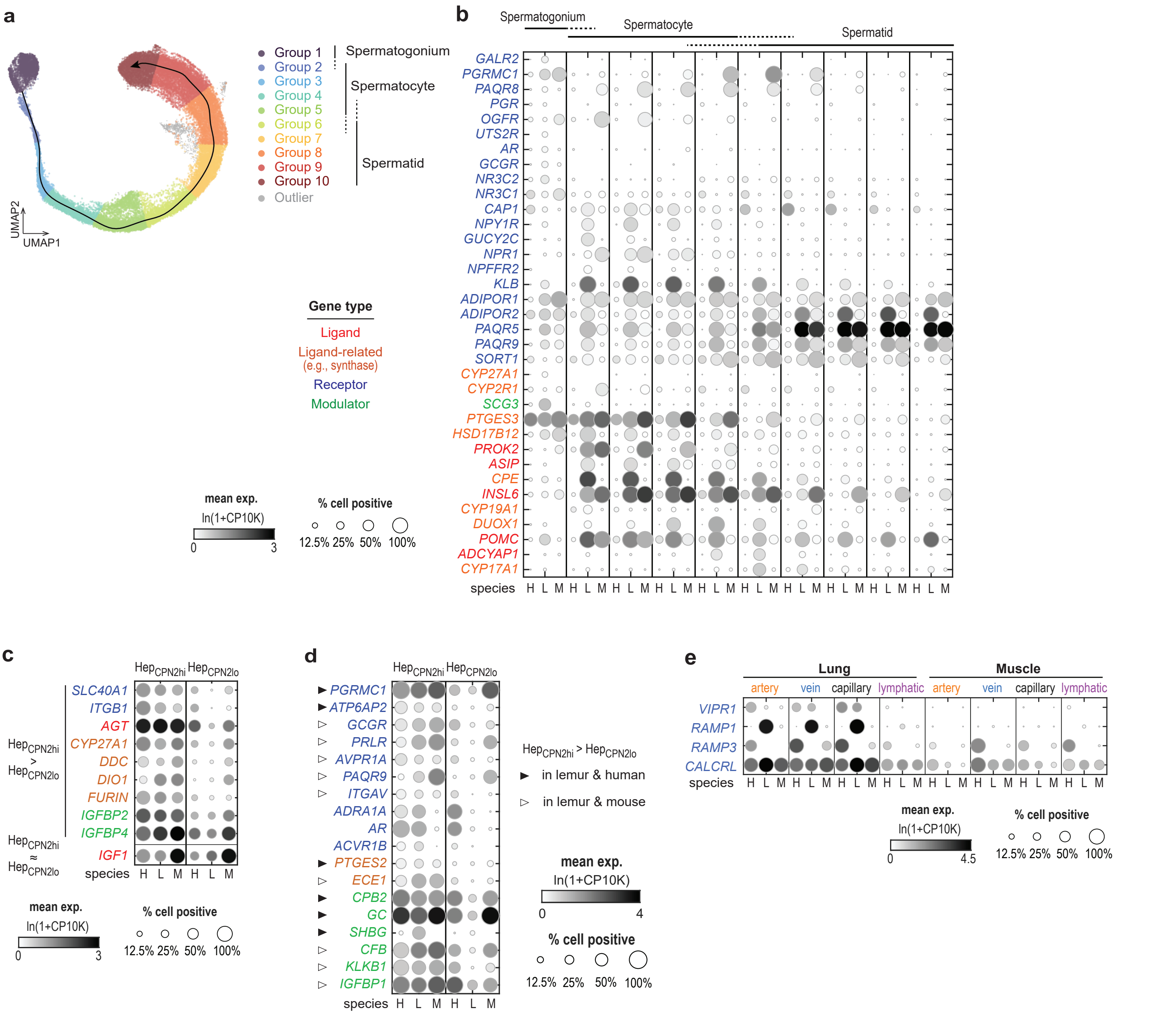

Fig. S9. Cross-species comparison of hormonal gene expression related to examples in Fig. 3. a. Grouping of male germ cells by the species-integrated trajectory, related to Fig. 7g. **b.** Expression of the spermatogenesis stage-specific hormonal genes identified in Fig. 3a across human, lemur, and mouse. Shown here are examples with variable expression patterns across the three species. Also see Fig. 7g-h for additional examples with either conserved or variable expression patterns across the three species. **c-d.** Expression of the hepatocyte subtype related hormonal genes in the two hepatocyte subtypes across human, lemur, and mouse. The two panels separately show the genes with conserved (c) or variable (d) expression patterns across the three species. **e.** Expression of the lung-specific hormone signatures in the lung and muscle endothelial cells across human, lemur, and mouse.

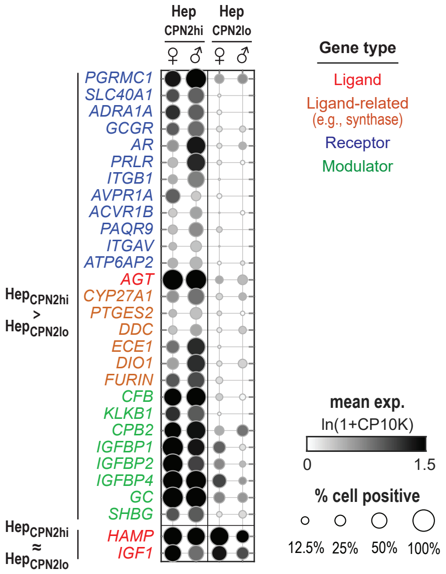

Fig. S10. Hormonal gene expression in two hepatocyte subtypes plotted separately for male and female animals (in comparison to Fig. 3f)**.**

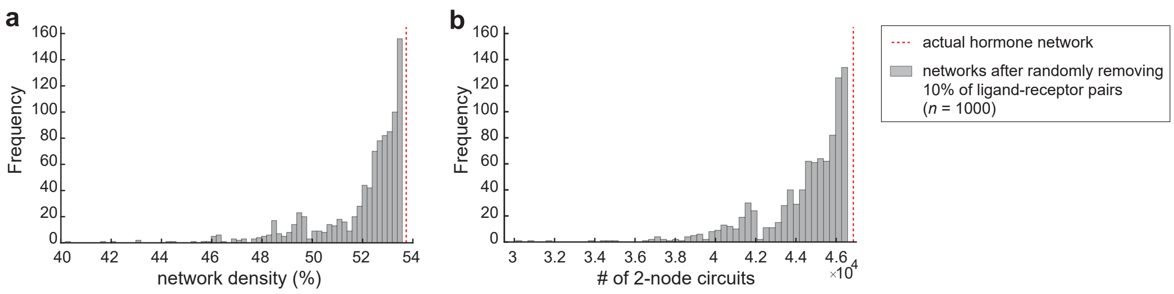

Fig. S11. The hormonal cell-cell communication network is robust to edge removal. Distribution of network density (a) and number of 2-node feedback circuits (b) in networks where 10% of ligand-receptor connections were randomly removed.

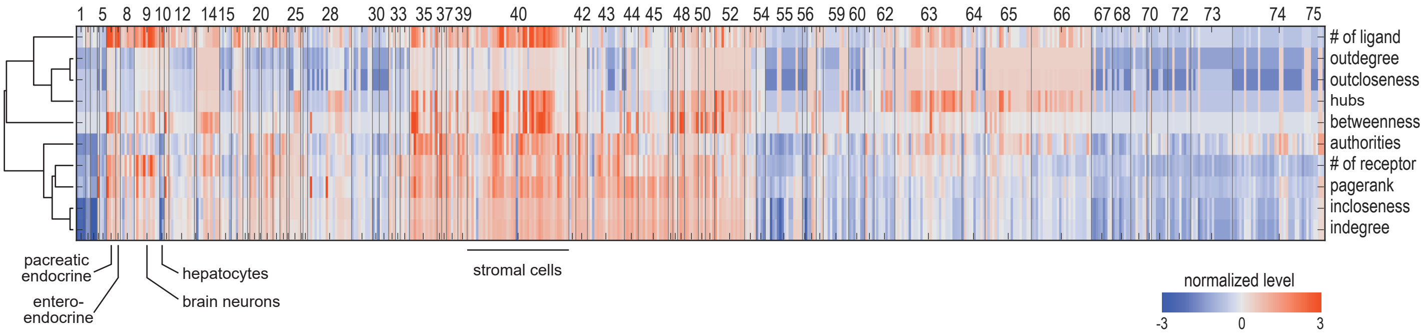

Fig. S12. Node properties of the hormonal cell-cell communication network. Heatmap of indegree, outdegree, number of hormone ligands and receptors expressed, and six node centrality measurements (hubs, authorities, incloseness, outclose-ness, pagerank, and betweenness) for all nodes (cell types) of the hormonal cell-cell communication network. Because the scales of the node properties are different, shown here are normalized values linearly scaled by median and median-abso-lute-deviation. Cell types (columns) are ordered by the clustering of the hormonal genes (as in Fig. 2a) and cluster numbers are labeled as space allowed. Node properties (rows) are ordered by hierarchical clustering of the normalized values.

Fig. S13

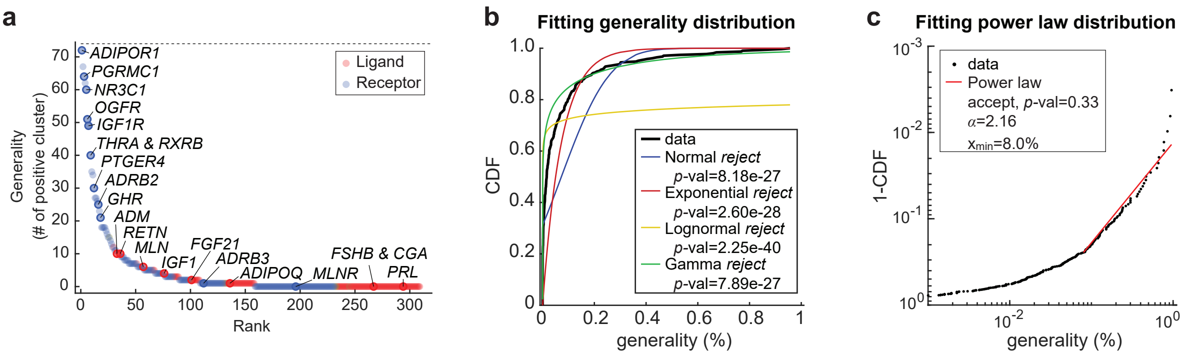

Fig. S13. Generality scores for the hormone ligands and receptors. a. Generality score, defined as the number of clusters with positive expression of the ligand or receptor (see Fig. 4f for comparison), ranked from most generally expressed to most selectively expressed. **b-c.** Fitting the distribution of the ligand and receptor generality scores. Normal, exponential, log-nor-mal, and gamma distributions failed to fit the distribution (b). The tail of the distribution can be approximated by a power law distribution prob(x) ~ $x^{-\alpha}$ for $x \geq x_{\min}$ (c).

Fig. S1a

Acylation stimulating protein (ASP)
- epithelial/neural/germ

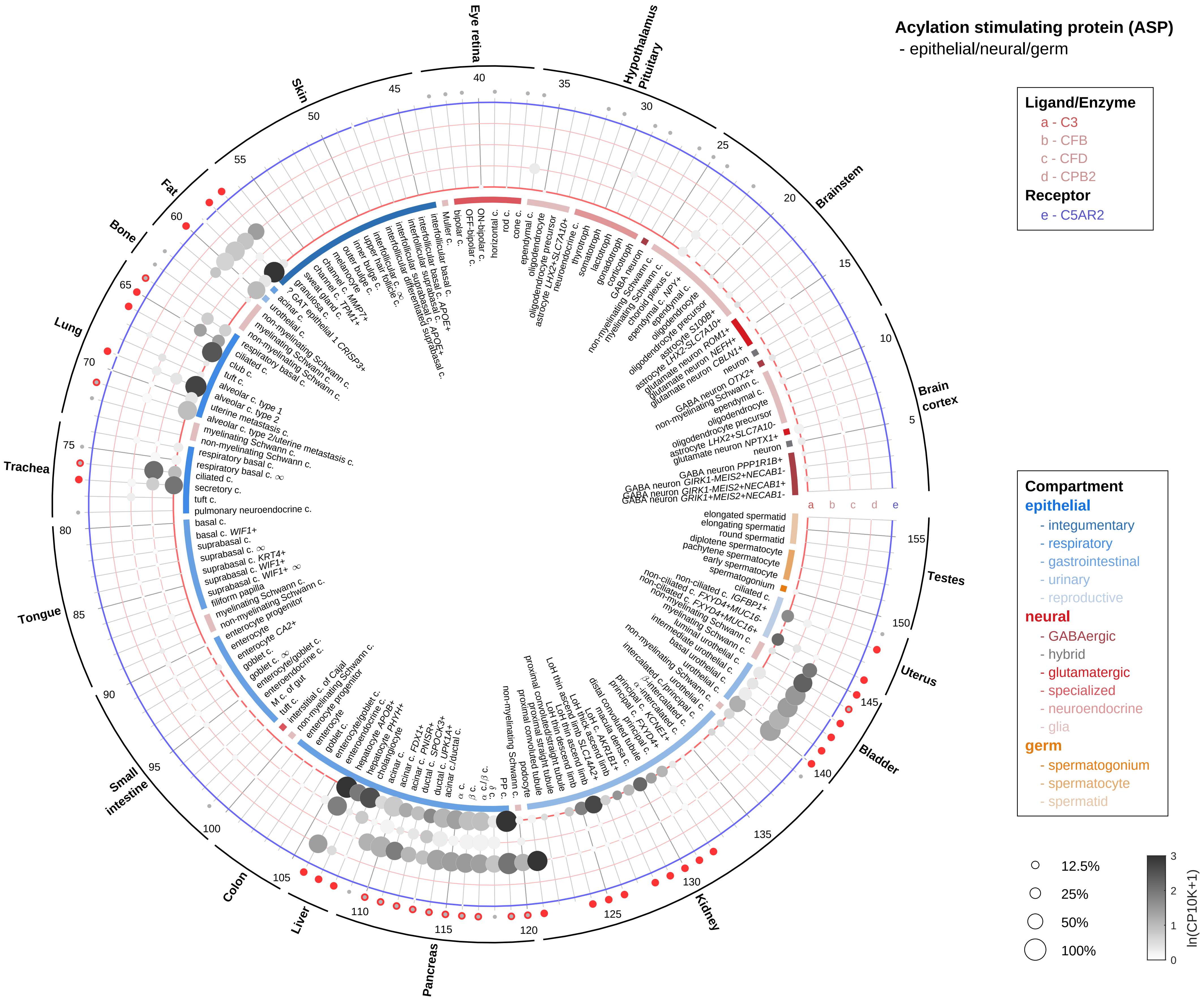

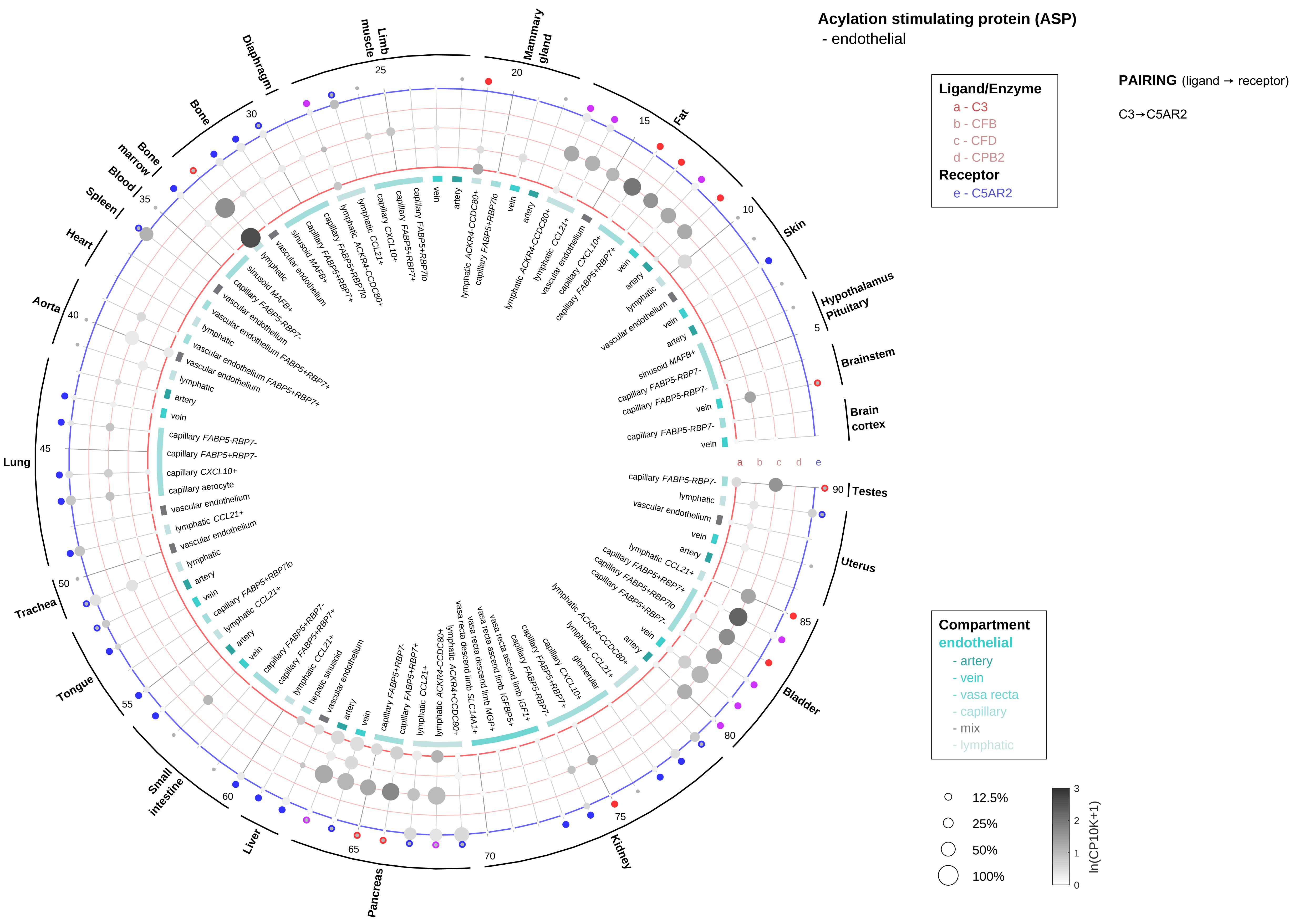
Acylation stimulating protein (ASP)
- endothelial
Ligand/Enzyme
a - C3
b - CFB
c - CFD
d - CPB2
Receptor
e - C5AR2
PAIRING (ligand → receptor)
C3→C5AR2
Compartment
endothelial
- artery
- vein
- vasa recta
- capillary
- mix
- lymphatic
12.5%
25%
50%
100%
ln(CP10K+1)
Brain cortex
Brainstem
Hypothalamus Pituitary
Skin
Fat
Mammary gland
Limb muscle
Diaphragm
Bone
Bone marrow
Blood
Spleen
Heart
Aorta
Lung
Trachea
Tongue
Small intestine
Liver
Pancreas
Kidney
Bladder
Uterus
Testes

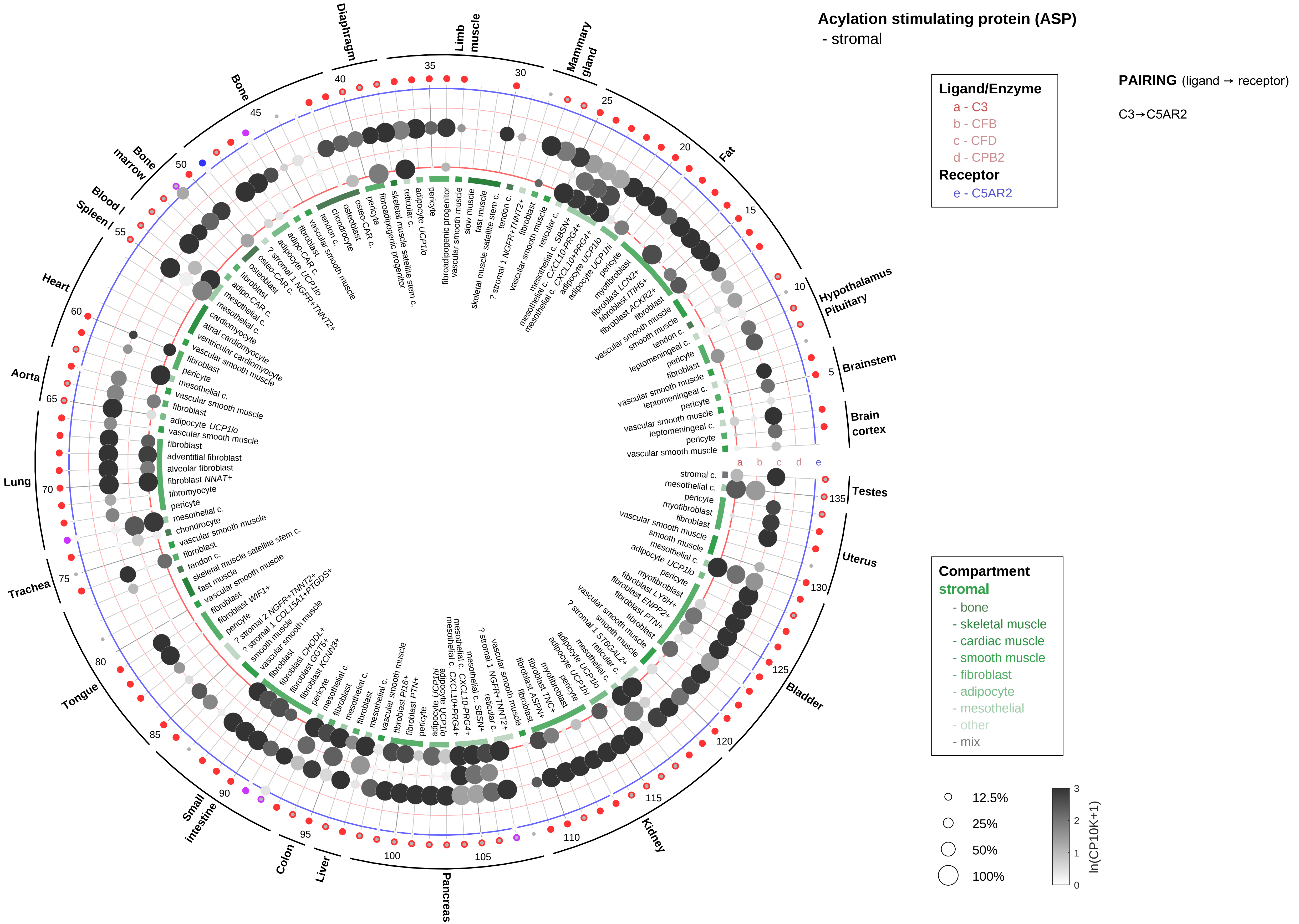

Acylation stimulating protein (ASP)
- stromal
PAIRING (ligand → receptor)
C3→C5AR2
Ligand/Enzyme
a - C3
b - CFB
c - CFD
d - CPB2
Receptor
e - C5AR2
Compartment
stromal
- bone
- skeletal muscle
- cardiac muscle
- smooth muscle
- fibroblast
- adipocyte
- mesothelial
- other
- mix
12.5%
25%
50%
100%
ln(CP10K+1)
Brain cortex
Brainstem
Hypothalamus Pituitary
Fat
Mammary gland
Limb muscle
Diaphragm
Bone
Bone marrow
Blood / Spleen /
Heart
Aorta
Lung
Trachea
Tongue
Small intestine
Colon
Liver
Pancreas
Kidney
Bladder
Uterus
Testes

Acylation stimulating protein (ASP)
- lymphoid

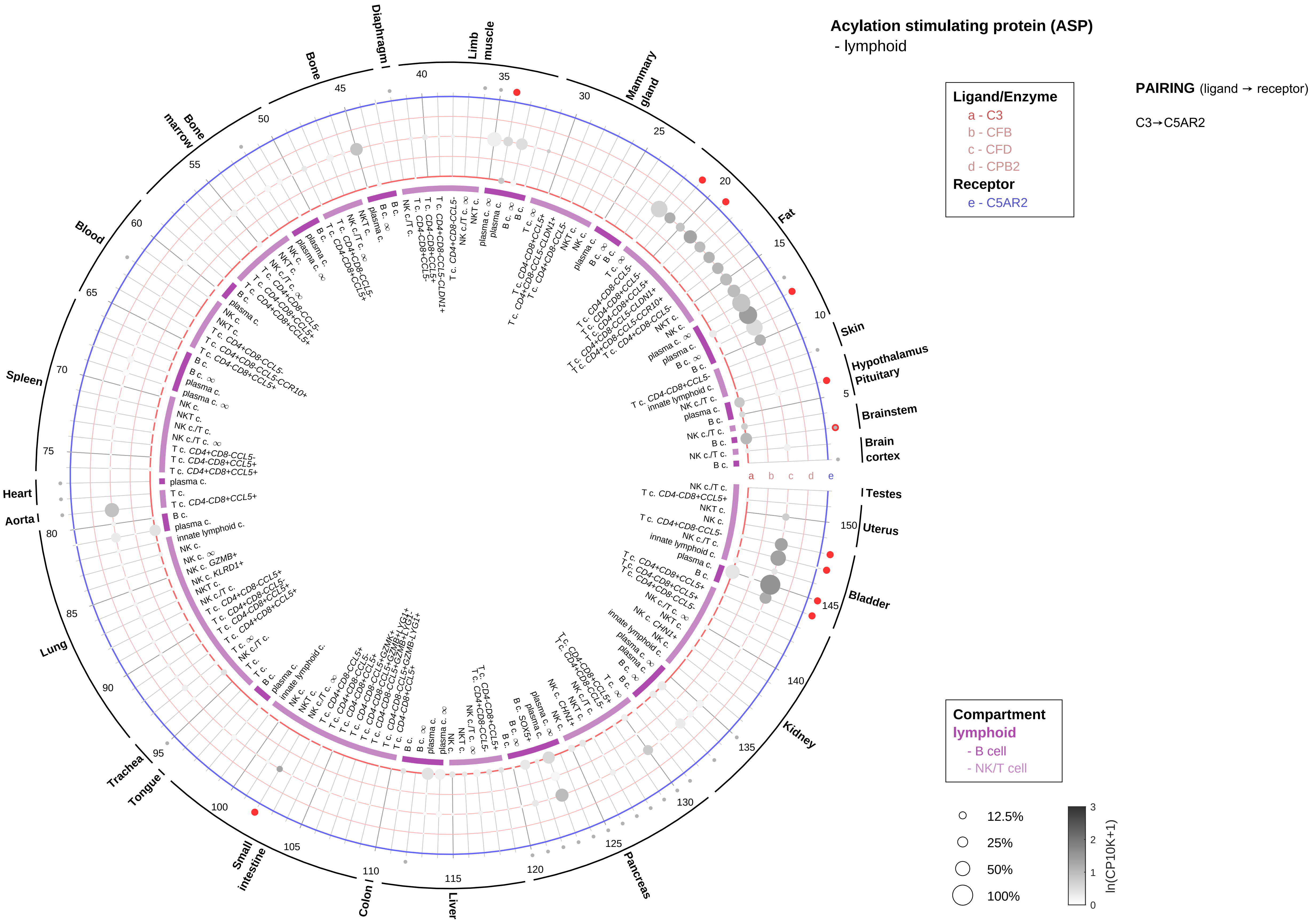

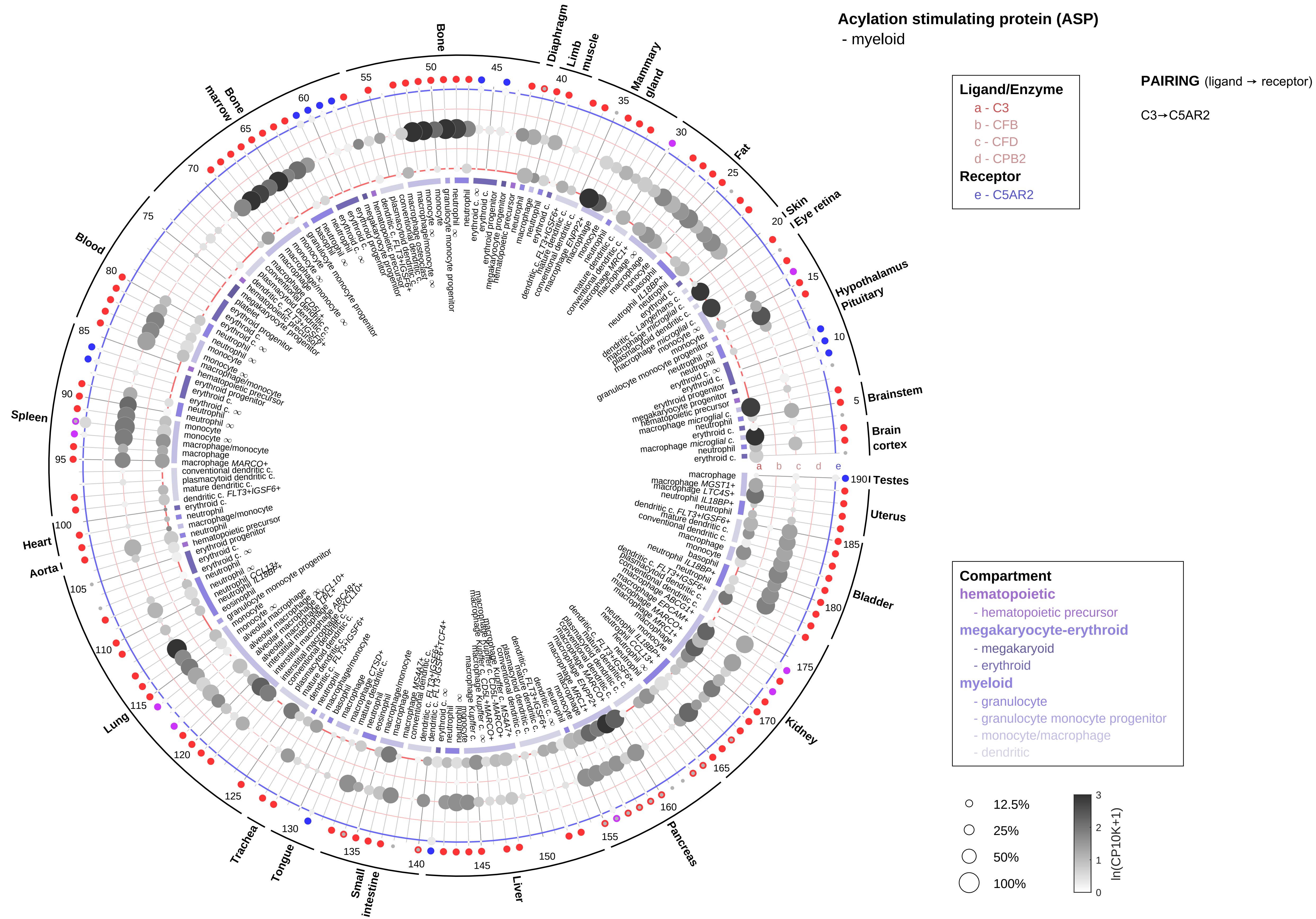

Acylation stimulating protein (ASP)
- myeloid
PAIRING (ligand → receptor)
C3→C5AR2
Ligand/Enzyme
a - C3
b - CFB
c - CFD
d - CPB2
Receptor
e - C5AR2
Compartment
hematopoietic
- hematopoietic precursor
megakaryocyte-erythroid
- megakaryoid
- erythroid
myeloid
- granulocyte
- granulocyte monocyte progenitor
- monocyte/macrophage
- dendritic
12.5%
25%
50%
100%
ln(CP10K+1)
Brain cortex
Brainstem
Pituitary
Hypothalamus
Eye retina
Skin
Fat
Mammary gland
Limb muscle
Diaphragm
Bone
Bone marrow
Blood
Spleen
Heart
Aorta
Lung
Trachea
Tongue
Small intestine
Liver
Pancreas
Kidney
Bladder
Uterus
Testes

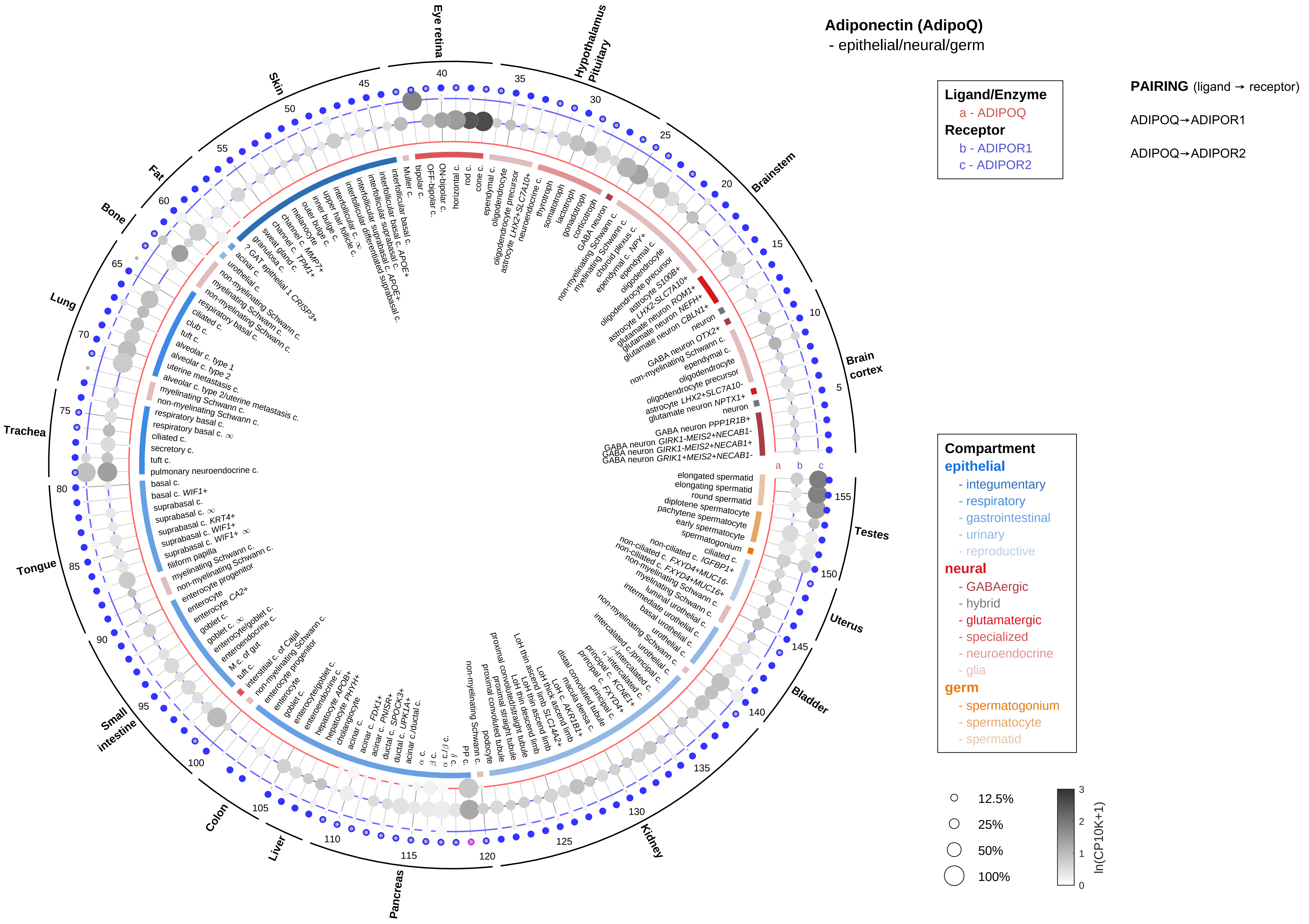
Adiponectin (AdipoQ)
- epithelial/neural/germ
Ligand/Enzyme
a - ADIPOQ
Receptor
b - ADIPOR1
c - ADIPOR2
PAIRING (ligand → receptor)
ADIPOQ→ADIPOR1
ADIPOQ→ADIPOR2
Compartment
epithelial
- integumentary
- respiratory
- gastrointestinal
- urinary
- reproductive
neural
- GABAergic
- hybrid
- glutamatergic
- specialized
- neuroendocrine
- glia
germ
- spermatogonium
- spermatocyte
- spermatid
12.5%
25%
50%
100%
ln(CP10K+1)
Eye retina
Hypothalamus Pituitary
Brainstem
Brain cortex
Testes
Uterus
Bladder
Kidney
Pancreas
Liver
Colon
Small intestine
Tongue
Trachea
Lung
Bone
Fat
Skin

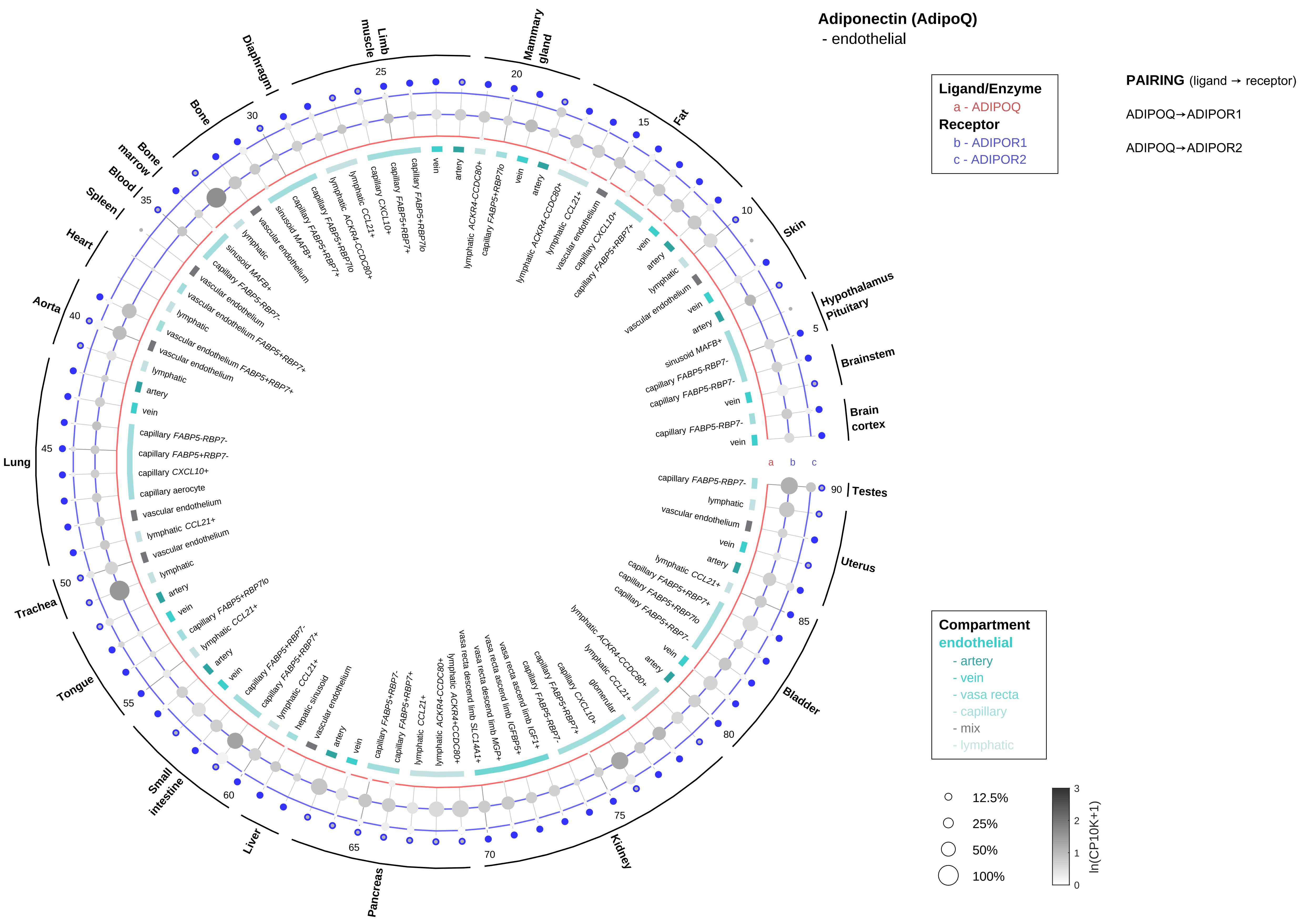

Adiponectin (AdipoQ)
- endothelial
Ligand/Enzyme
a - ADIPOQ
Receptor
b - ADIPOR1
c - ADIPOR2
PAIRING (ligand → receptor)
ADIPOQ→ADIPOR1
ADIPOQ→ADIPOR2
Compartment
endothelial
- artery
- vein
- vasa recta
- capillary
- mix
- lymphatic
12.5%
25%
50%
100%
ln(CP10K+1)
Testes
Uterus
Bladder
Kidney
Pancreas
Liver
Small intestine
Tongue
Trachea
Lung
Aorta
Heart
Spleen
Blood
Bone marrow
Bone
Diaphragm
Limb muscle
Mammary gland
Fat
Skin
Hypothalamus Pituitary
Brainstem
Brain cortex

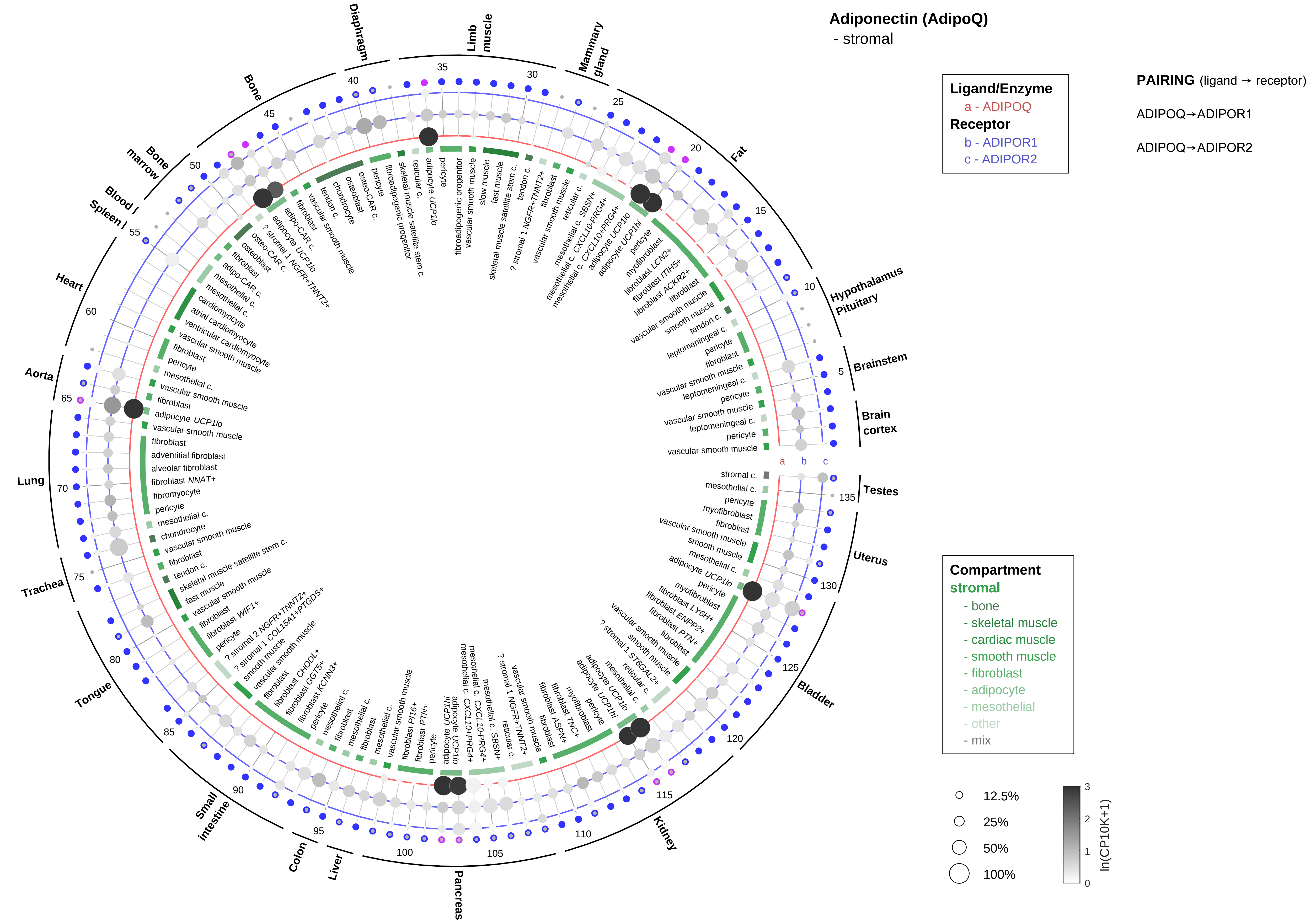
Adiponectin (AdipoQ)
- stromal
Ligand/Enzyme
a - ADIPOQ
Receptor
b - ADIPOR1
c - ADIPOR2
PAIRING (ligand → receptor)
ADIPOQ→ADIPOR1
ADIPOQ→ADIPOR2
Compartment
stromal
- bone
- skeletal muscle
- cardiac muscle
- smooth muscle
- fibroblast
- adipocyte
- mesothelial
- other
- mix
12.5%
25%
50%
100%
ln(CP10K+1)
Limb muscle
Mammary gland
Fat
Hypothalamus Pituitary
Brainstem
Brain cortex
Testes
Uterus
Bladder
Kidney
Pancreas
Liver
Colon
Small intestine
Tongue
Trachea
Lung
Aorta
Heart
Spleen
Blood
Bone marrow
Bone
Diaphragm

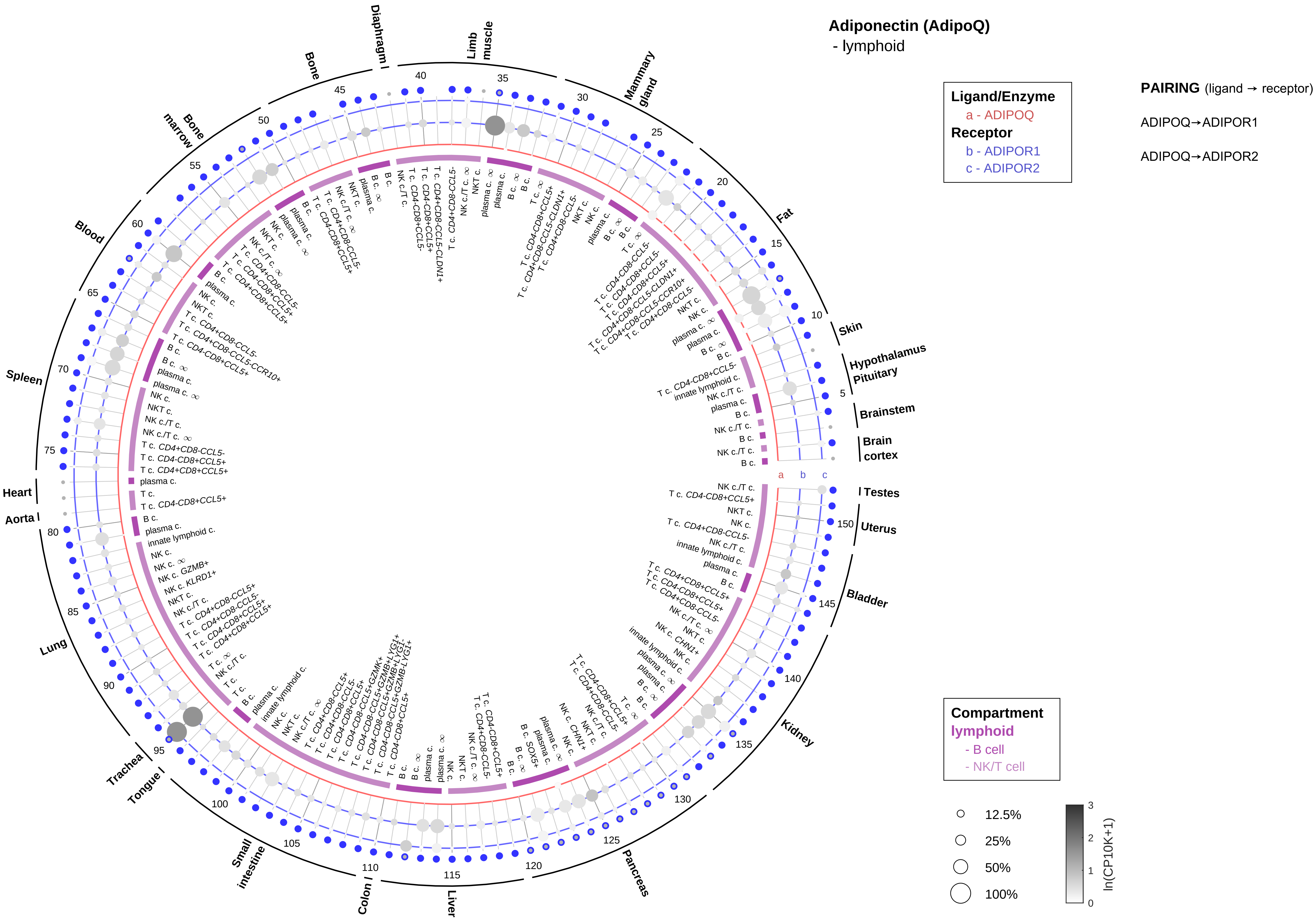
Adiponectin (AdipoQ)
- lymphoid
Ligand/Enzyme
a - ADIPOQ
Receptor
b - ADIPOR1
c - ADIPOR2
PAIRING (ligand → receptor)
ADIPOQ→ADIPOR1
ADIPOQ→ADIPOR2
Compartment
lymphoid
- B cell
- NK/T cell
12.5%
25%
50%
100%
ln(CP10K+1)
Skin
Hypothalamus
Pituitary
Brainstem
Brain cortex
Testes
Uterus
Bladder
Kidney
Pancreas
Liver
Colon
Small intestine
Tongue
Trachea
Lung
Aorta
Heart
Spleen
Blood
Bone marrow
Bone
Diaphragm
Limb muscle
Mammary gland
Fat

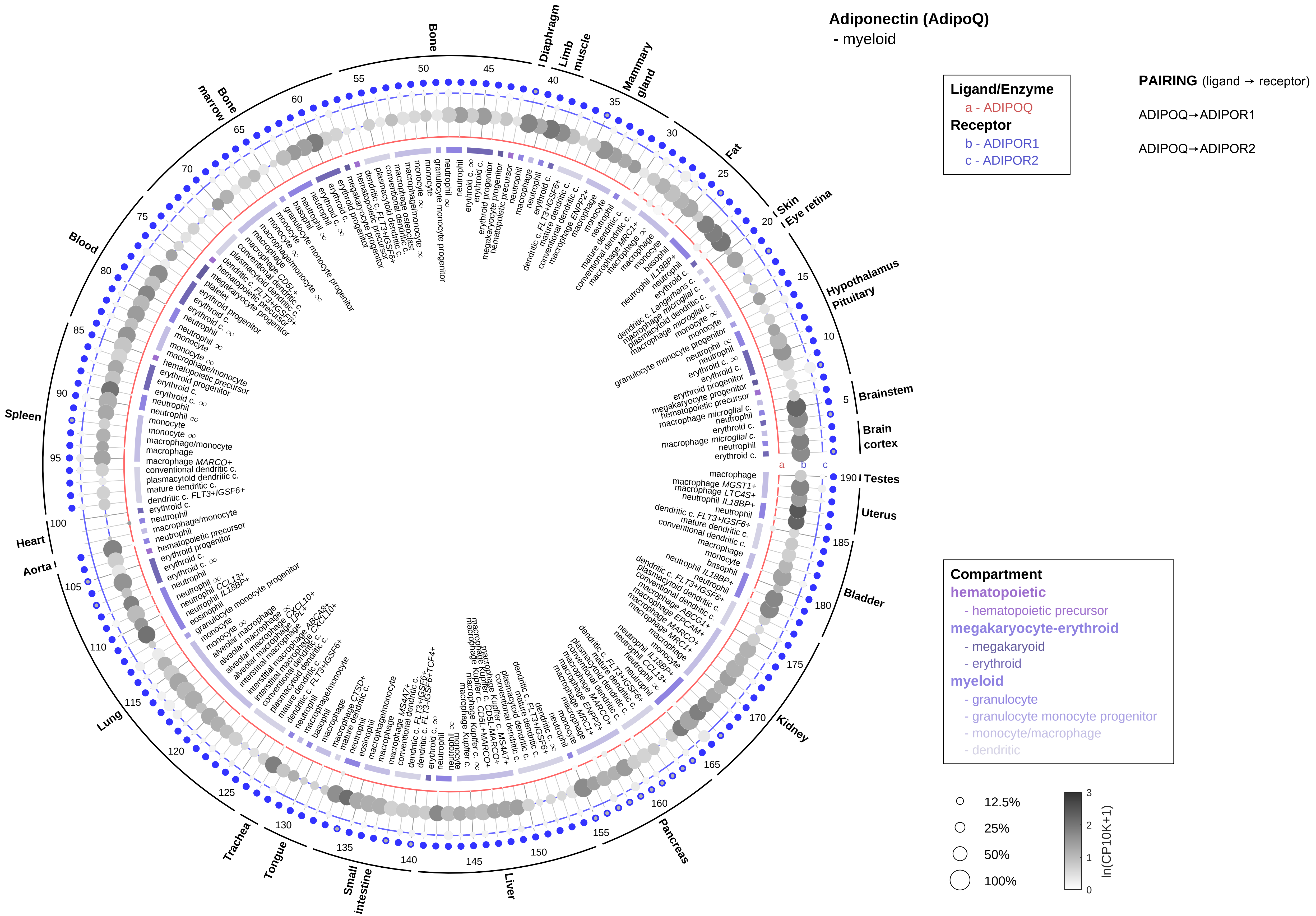
Adiponectin (AdipoQ)
- myeloid
Ligand/Enzyme
a - ADIPOQ
Receptor
b - ADIPOR1
c - ADIPOR2
PAIRING (ligand → receptor)
ADIPOQ→ADIPOR1
ADIPOQ→ADIPOR2
Compartment
hematopoietic
- hematopoietic precursor
megakaryocyte-erythroid
- megakaryoid
- erythroid
myeloid
- granulocyte
- granulocyte monocyte progenitor
- monocyte/macrophage
- dendritic
12.5%
25%
50%
100%
ln(CP10K+1)
Bone
Diaphragm
Limb muscle
Mammary gland
Fat
Skin
Eye retina
Hypothalamus
Pituitary
Brainstem
Brain cortex
Testes
Uterus
Bladder
Kidney
Pancreas
Liver
Small intestine
Tongue
Trachea
Lung
Aorta
Heart
Spleen
Blood
Bone marrow

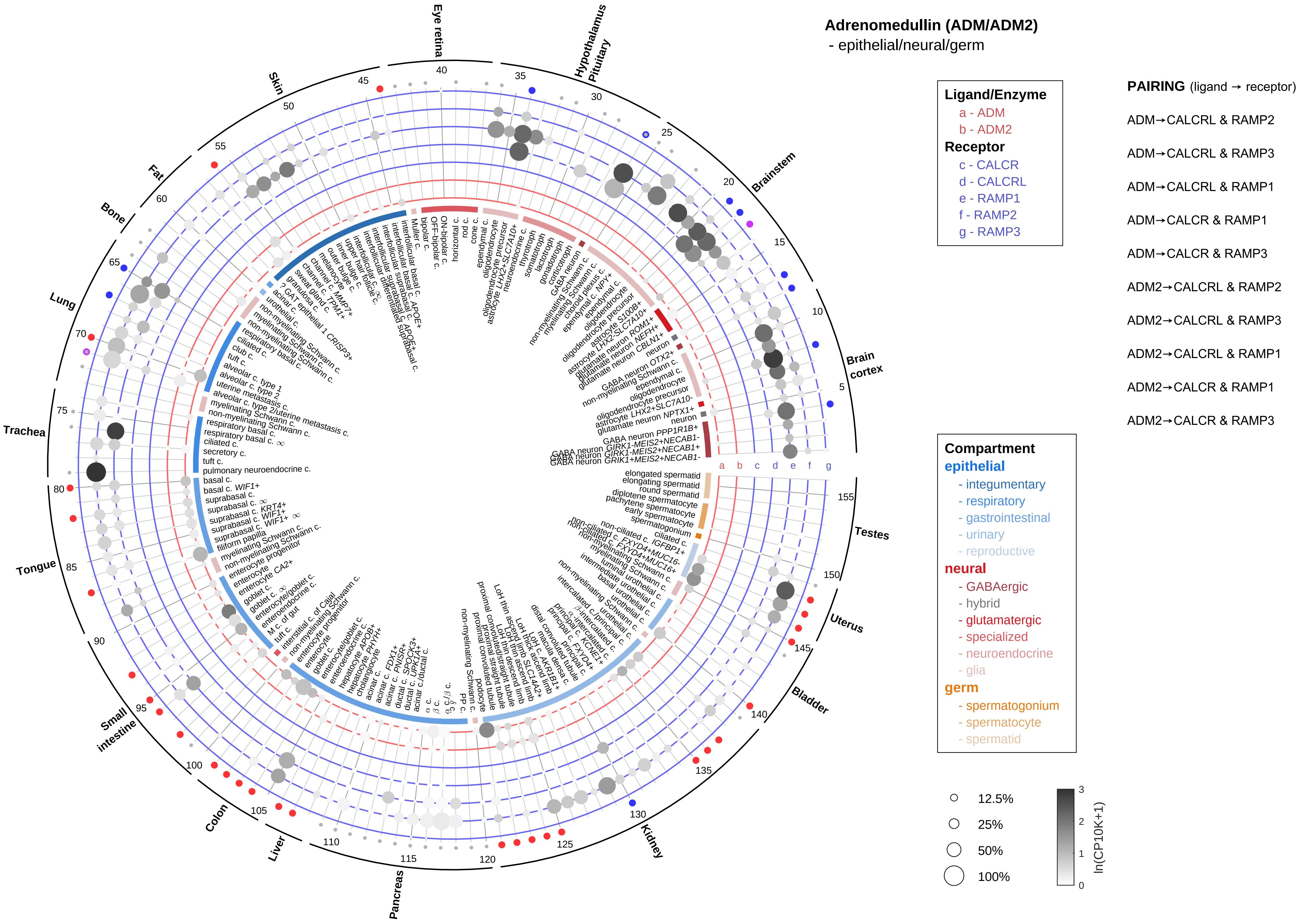

Adrenomedullin (ADM/ADM2)
- epithelial/neural/germ
Ligand/Enzyme
a - ADM
b - ADM2
Receptor
c - CALCR
d - CALCRL
e - RAMP1
f - RAMP2
g - RAMP3
PAIRING (ligand → receptor)
ADM→CALCRL & RAMP2
ADM→CALCRL & RAMP3
ADM→CALCRL & RAMP1
ADM→CALCR & RAMP1
ADM→CALCR & RAMP3
ADM2→CALCRL & RAMP2
ADM2→CALCRL & RAMP3
ADM2→CALCRL & RAMP1
ADM2→CALCR & RAMP1
ADM2→CALCR & RAMP3
Compartment
epithelial
- integumentary
- respiratory
- gastrointestinal
- urinary
- reproductive
neural
- GABAergic
- hybrid
- glutamatergic
- specialized
- neuroendocrine
- glia
germ
- spermatogonium
- spermatocyte
- spermatid
12.5%
25%
50%
100%
ln(CP10K+1)
Eye retina
Hypothalamus Pituitary
Brainstem
Brain cortex
Testes
Uterus
Bladder
Kidney
Pancreas
Liver
Colon
Small intestine
Tongue
Trachea
Lung
Bone
Fat
Skin

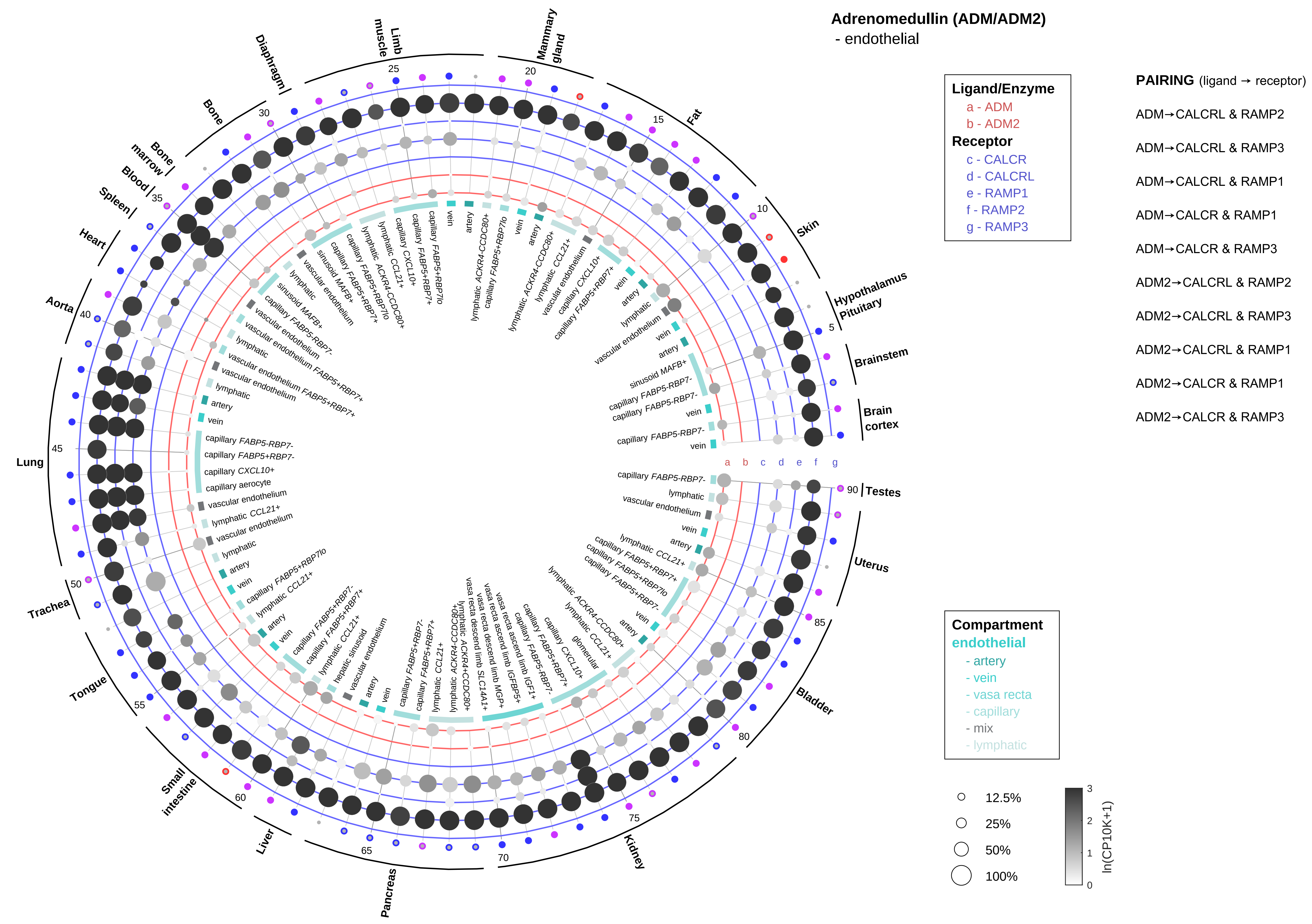
Adrenomedullin (ADM/ADM2)
- endothelial
Ligand/Enzyme
a - ADM
b - ADM2
Receptor
c - CALCR
d - CALCRL
e - RAMP1
f - RAMP2
g - RAMP3
PAIRING (ligand → receptor)
ADM→CALCRL & RAMP2
ADM→CALCRL & RAMP3
ADM→CALCRL & RAMP1
ADM→CALCR & RAMP1
ADM→CALCR & RAMP3
ADM2→CALCRL & RAMP2
ADM2→CALCRL & RAMP3
ADM2→CALCRL & RAMP1
ADM2→CALCR & RAMP1
ADM2→CALCR & RAMP3
Compartment
endothelial
- artery
- vein
- vasa recta
- capillary
- mix
- lymphatic
12.5%
25%
50%
100%
ln(CP10K+1)
0
1
2
3
Brain cortex
Brainstem
Hypothalamus Pituitary
Skin
Fat
Mammary gland
Limb muscle
Diaphragm
Bone
Bone marrow
Blood
Spleen
Heart
Aorta
Lung
Trachea
Tongue
Small intestine
Liver
Pancreas
Kidney
Bladder
Uterus
Testes
a b c d e f g

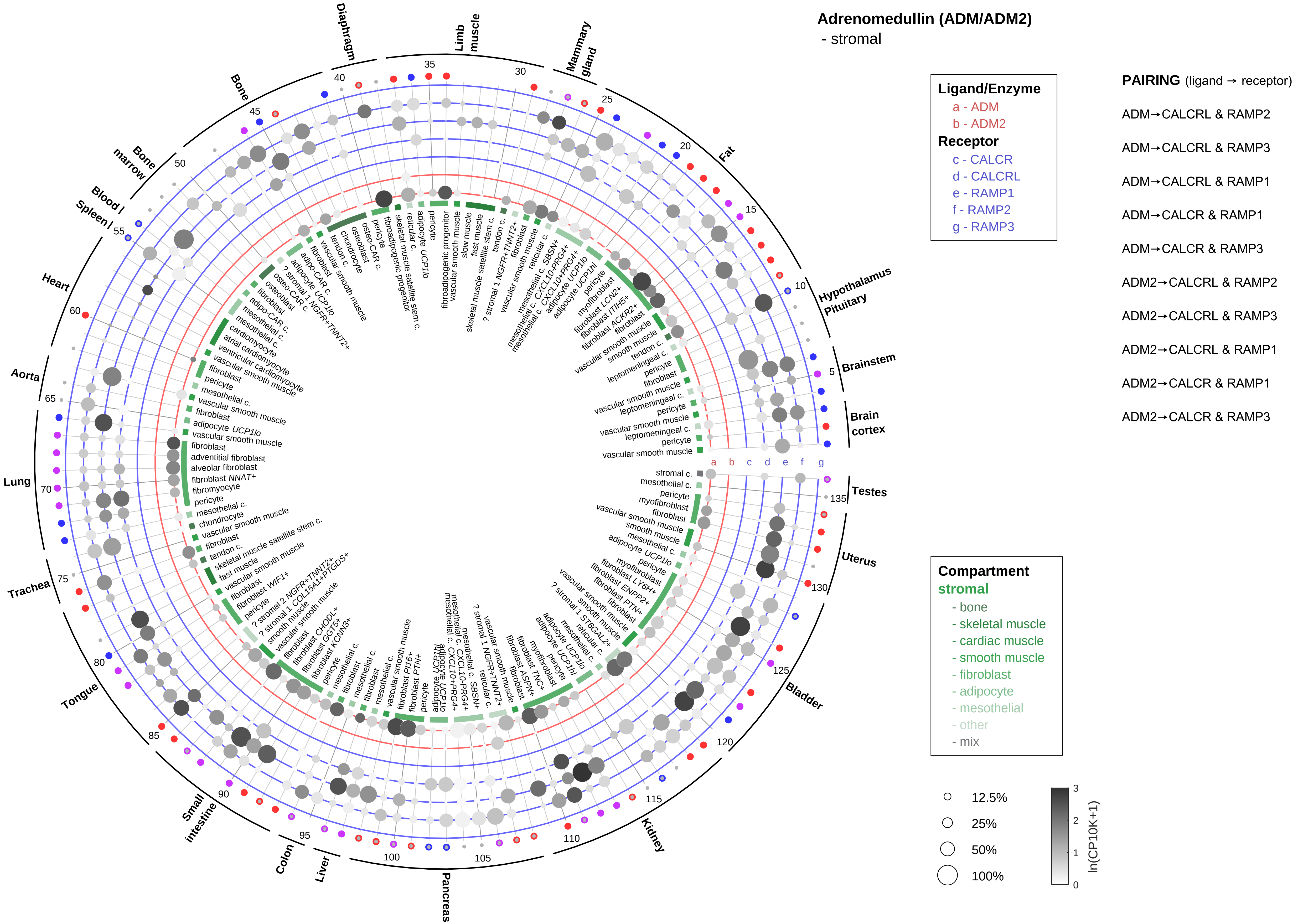
Adrenomedullin (ADM/ADM2)
- stromal
Ligand/Enzyme
a - ADM
b - ADM2
Receptor
c - CALCR
d - CALCRL
e - RAMP1
f - RAMP2
g - RAMP3
PAIRING (ligand → receptor)
ADM→CALCRL & RAMP2
ADM→CALCRL & RAMP3
ADM→CALCRL & RAMP1
ADM→CALCR & RAMP1
ADM→CALCR & RAMP3
ADM2→CALCRL & RAMP2
ADM2→CALCRL & RAMP3
ADM2→CALCRL & RAMP1
ADM2→CALCR & RAMP1
ADM2→CALCR & RAMP3
Compartment
stromal
- bone
- skeletal muscle
- cardiac muscle
- smooth muscle
- fibroblast
- adipocyte
- mesothelial
- other
- mix
12.5%
25%
50%
100%
ln(CP10K+1)
Diaphragm
Limb muscle
Mammary gland
Fat
Hypothalamus Pituitary
Brainstem
Brain cortex
Testes
Uterus
Bladder
Kidney
Pancreas
Liver
Colon
Small intestine
Tongue
Trachea
Lung
Aorta
Heart
Spleen
Blood
Bone marrow
Bone

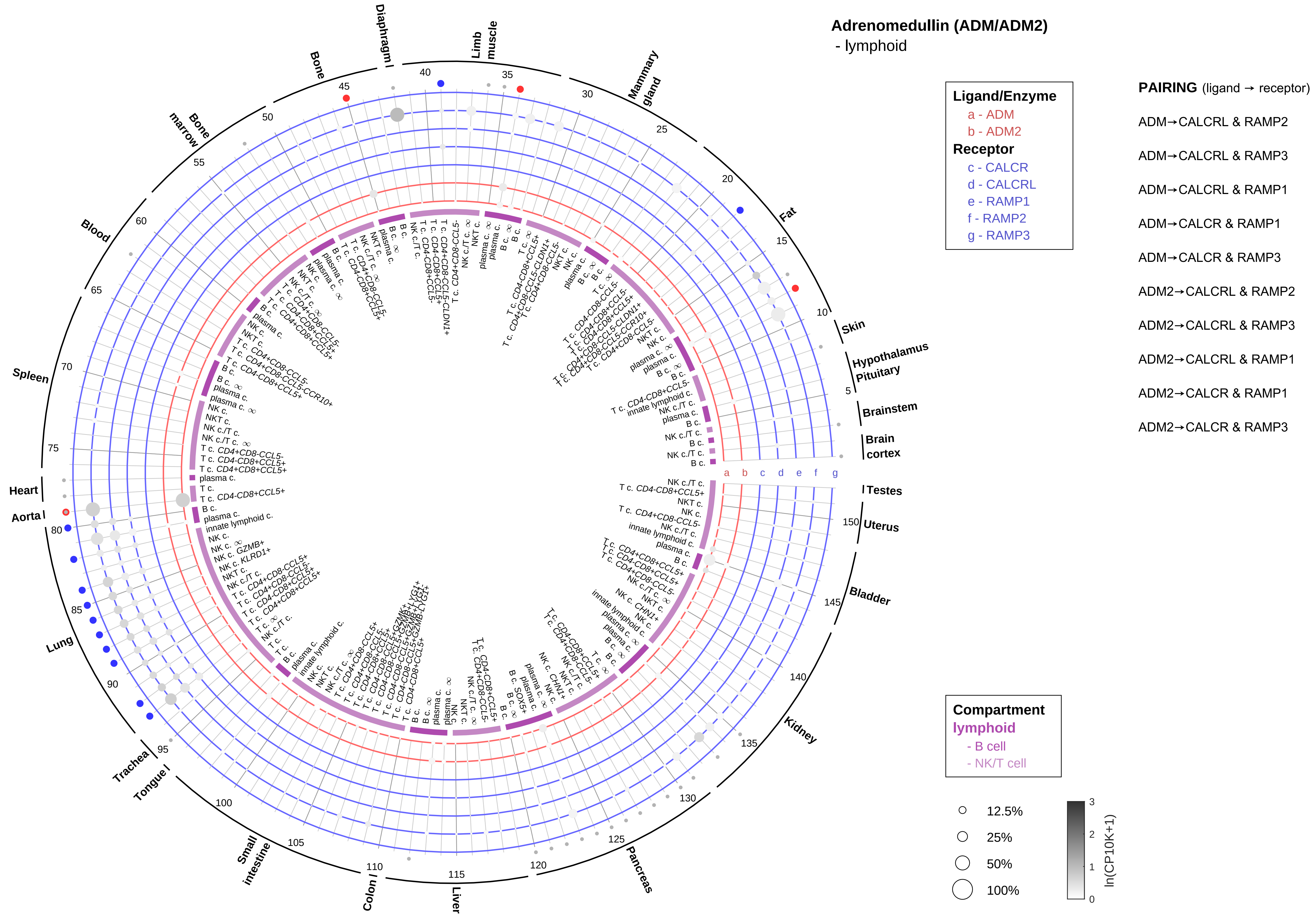

Adrenomedullin (ADM/ADM2)
- lymphoid
Ligand/Enzyme
a - ADM
b - ADM2
Receptor
c - CALCR
d - CALCRL
e - RAMP1
f - RAMP2
g - RAMP3
PAIRING (ligand → receptor)
ADM→CALCRL & RAMP2
ADM→CALCRL & RAMP3
ADM→CALCRL & RAMP1
ADM→CALCR & RAMP1
ADM→CALCR & RAMP3
ADM2→CALCRL & RAMP2
ADM2→CALCRL & RAMP3
ADM2→CALCRL & RAMP1
ADM2→CALCR & RAMP1
ADM2→CALCR & RAMP3
Compartment
lymphoid
- B cell
- NK/T cell
12.5%
25%
50%
100%
ln(CP10K+1)
Diaphragm
Limb muscle
Mammary gland
Fat
Skin
Hypothalamus
Pituitary
Brainstem
Brain cortex
Testes
Uterus
Bladder
Kidney
Pancreas
Liver
Colon
Small intestine
Tongue
Trachea
Lung
Aorta
Heart
Spleen
Blood
Bone marrow
Bone

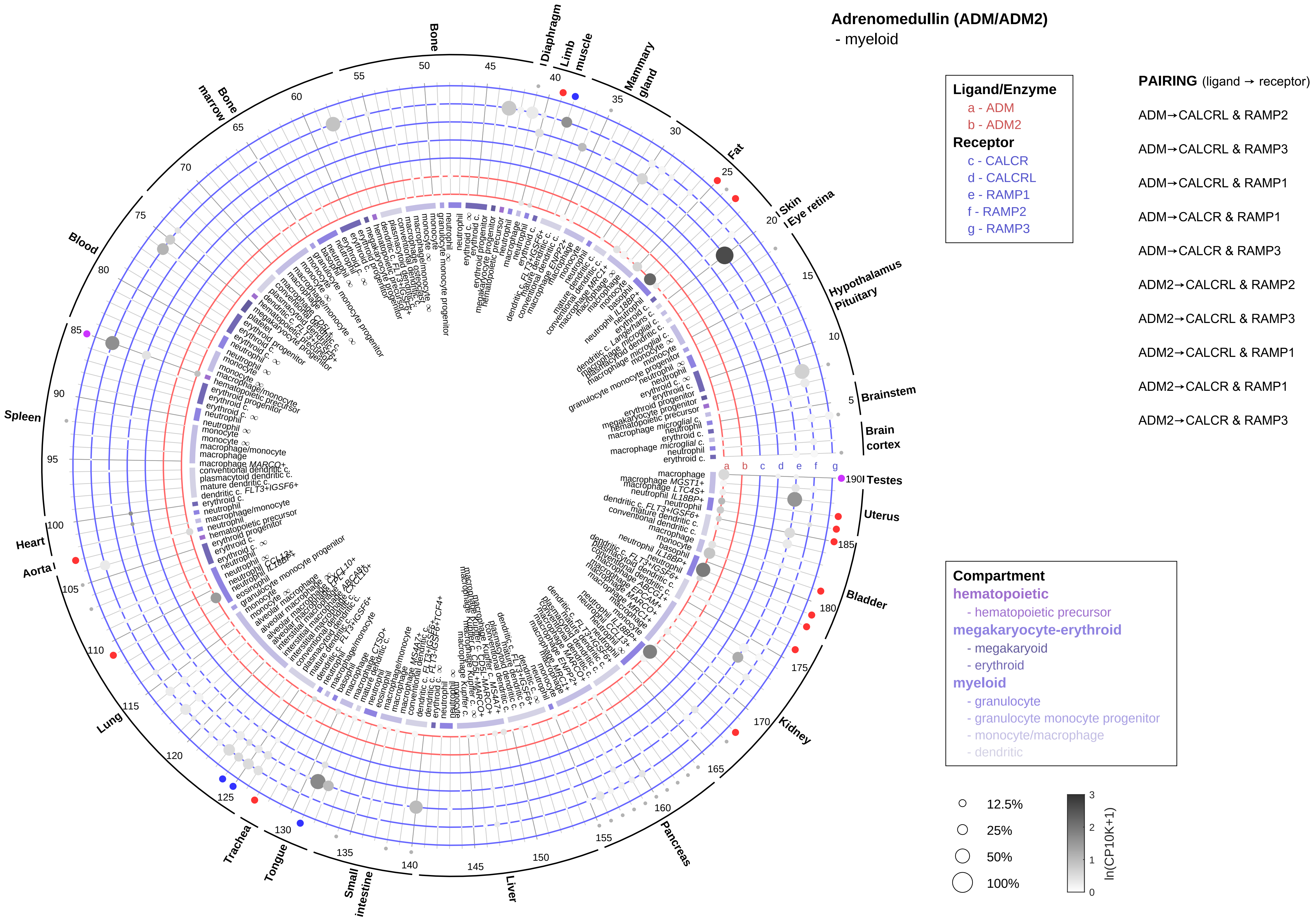

Adrenomedullin (ADM/ADM2)
- myeloid
Ligand/Enzyme
a - ADM
b - ADM2
Receptor
c - CALCR
d - CALCRL
e - RAMP1
f - RAMP2
g - RAMP3
PAIRING (ligand → receptor)
ADM→CALCRL & RAMP2
ADM→CALCRL & RAMP3
ADM→CALCRL & RAMP1
ADM→CALCR & RAMP1
ADM→CALCR & RAMP3
ADM2→CALCRL & RAMP2
ADM2→CALCRL & RAMP3
ADM2→CALCRL & RAMP1
ADM2→CALCR & RAMP1
ADM2→CALCR & RAMP3
Compartment
hematopoietic
- hematopoietic precursor
megakaryocyte-erythroid
- megakaryoid
- erythroid
myeloid
- granulocyte
- granulocyte monocyte progenitor
- monocyte/macrophage
- dendritic
12.5%
25%
50%
100%
ln(CP10K+1)
Diaphragm
Limb muscle
Mammary gland
Fat
Skin
Eye retina
Hypothalamus
Pituitary
Brainstem
Brain cortex
Testes
Uterus
Bladder
Kidney
Pancreas
Liver
Small intestine
Tongue
Trachea
Lung
Aorta
Heart
Spleen
Blood
Bone marrow
Bone

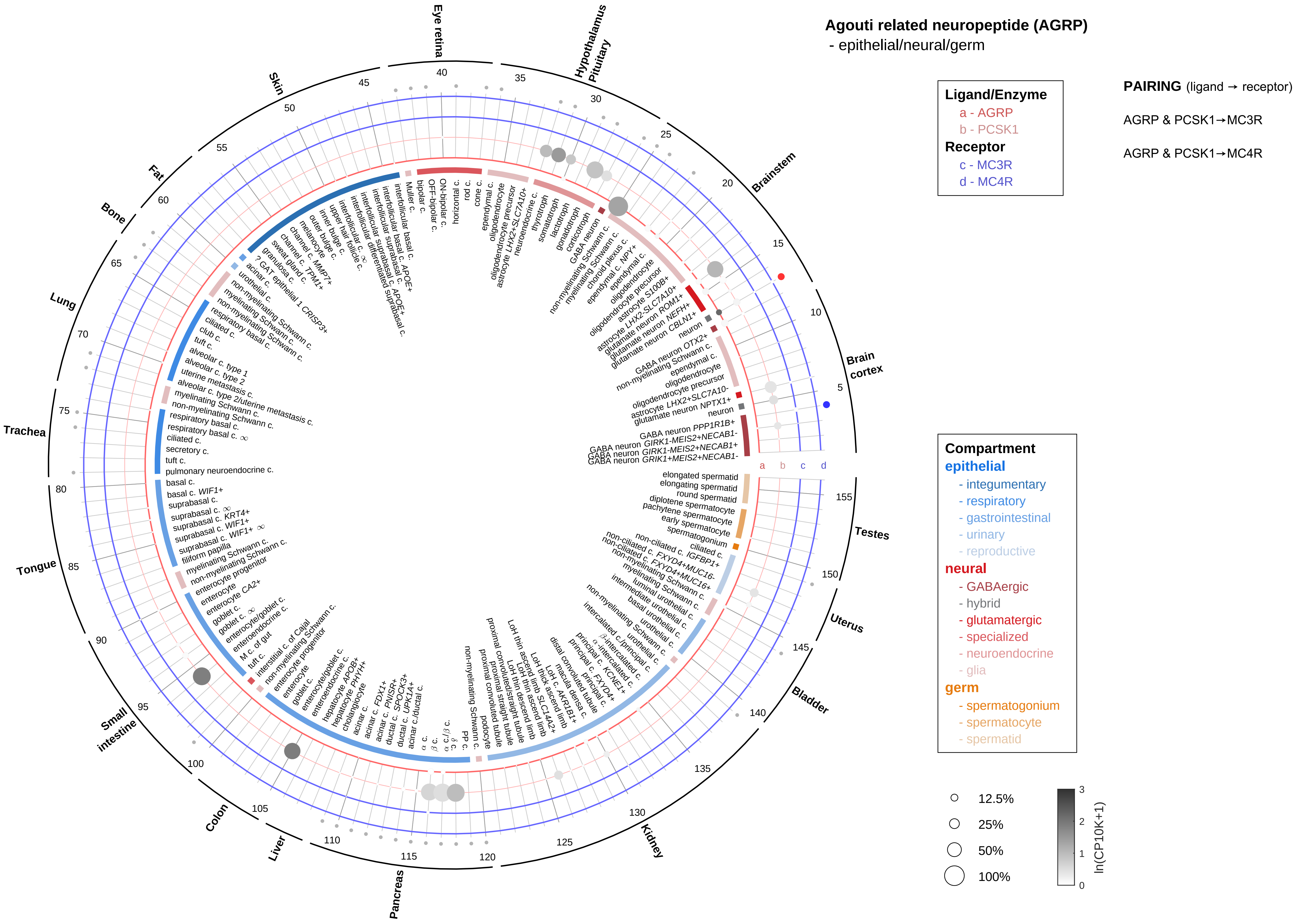

Agouti related neuropeptide (AGRP)
- epithelial/neural/germ
Ligand/Enzyme
a - AGRP
b - PCSK1
Receptor
c - MC3R
d - MC4R
PAIRING (ligand → receptor)
AGRP & PCSK1→MC3R
AGRP & PCSK1→MC4R
Compartment
epithelial
- integumentary
- respiratory
- gastrointestinal
- urinary
- reproductive
neural
- GABAergic
- hybrid
- glutamatergic
- specialized
- neuroendocrine
- glia
germ
- spermatogonium
- spermatocyte
- spermatid
12.5%
25%
50%
100%
ln(CP10K+1)
Eye retina
Hypothalamus Pituitary
Brainstem
Brain cortex
Testes
Uterus
Bladder
Kidney
Pancreas
Liver
Colon
Small intestine
Tongue
Trachea
Lung
Bone
Fat
Skin

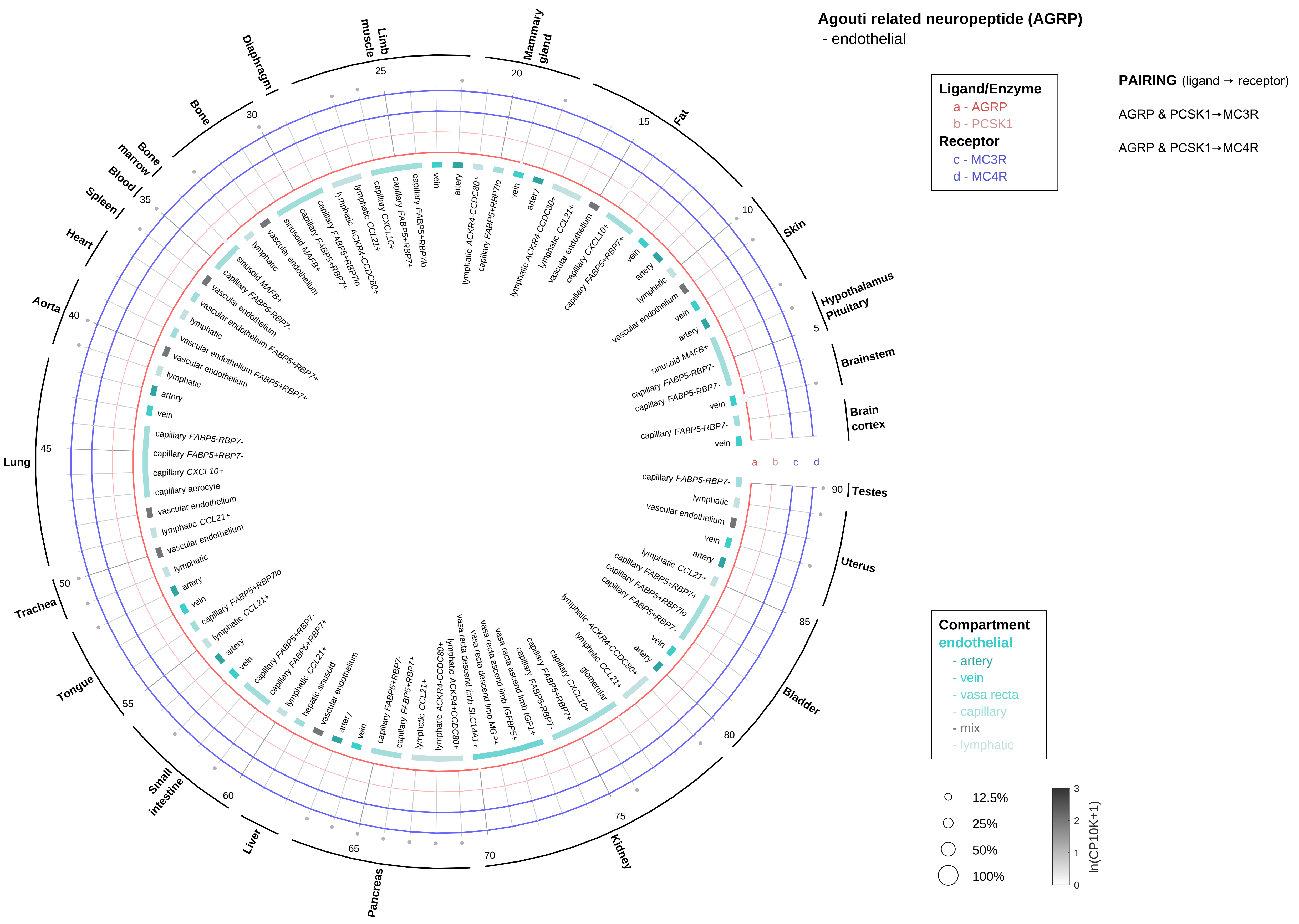

Agouti related neuropeptide (AGRP)
- endothelial
Ligand/Enzyme
a - AGRP
b - PCSK1
Receptor
c - MC3R
d - MC4R
PAIRING (ligand → receptor)
AGRP & PCSK1→MC3R
AGRP & PCSK1→MC4R
Compartment
endothelial
- artery
- vein
- vasa recta
- capillary
- mix
- lymphatic
12.5%
25%
50%
100%
ln(CP10K+1)
Brain cortex
Brainstem
Hypothalamus Pituitary
Skin
Fat
Mammary gland
Limb muscle
Diaphragm
Bone
Bone marrow
Blood
Spleen
Heart
Aorta
Lung
Trachea
Tongue
Small intestine
Liver
Pancreas
Kidney
Bladder
Uterus
Testes

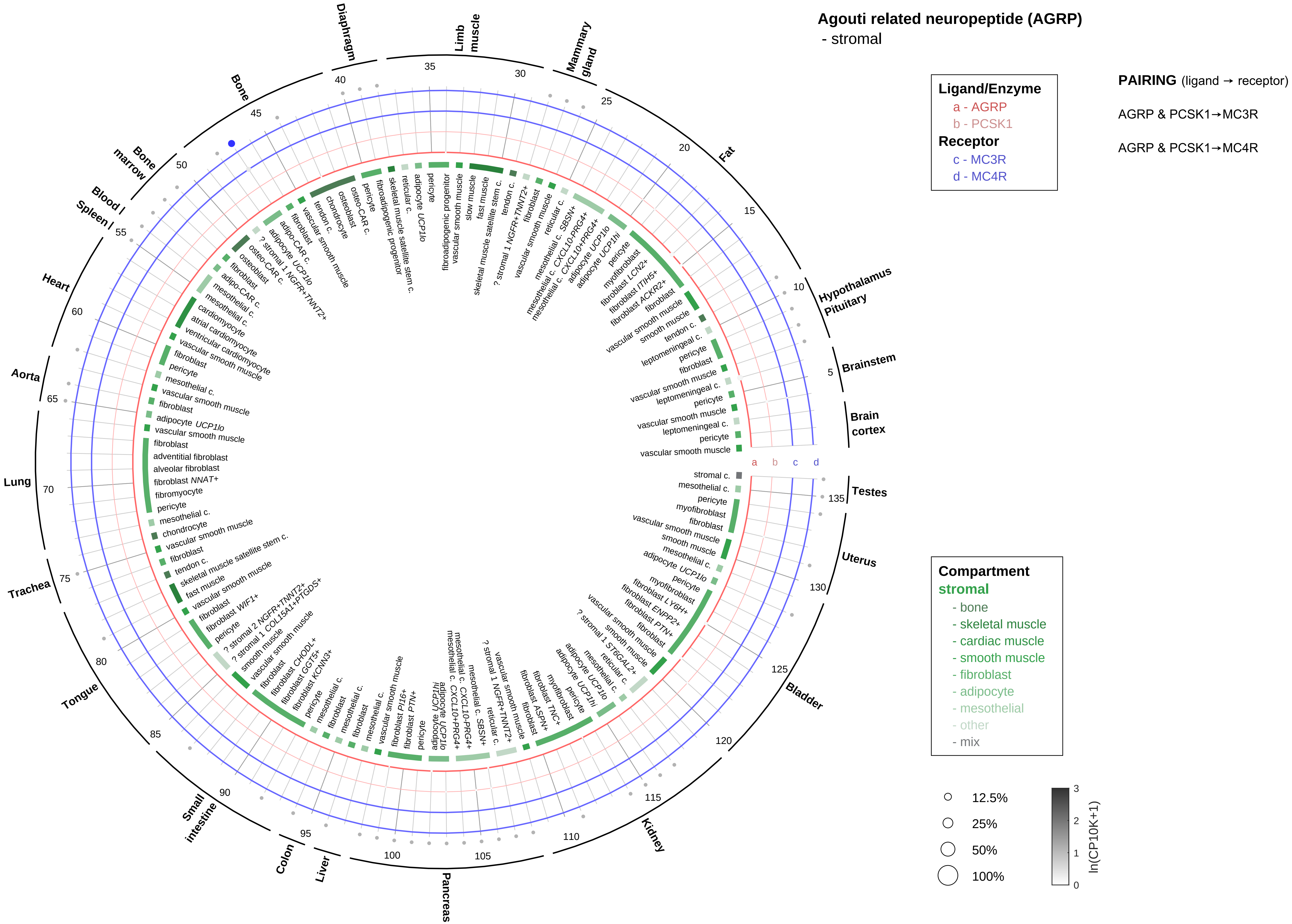
Agouti related neuropeptide (AGRP)
- stromal
Ligand/Enzyme
a - AGRP
b - PCSK1
Receptor
c - MC3R
d - MC4R
PAIRING (ligand → receptor)
AGRP & PCSK1→MC3R
AGRP & PCSK1→MC4R
Compartment
stromal
- bone
- skeletal muscle
- cardiac muscle
- smooth muscle
- fibroblast
- adipocyte
- mesothelial
- other
- mix
12.5%
25%
50%
100%
ln(CP10K+1)
Brain cortex
Brainstem
Hypothalamus Pituitary
Fat
Mammary gland
Limb muscle
Diaphragm
Bone
Bone marrow
Blood / Spleen /
Heart
Aorta
Lung
Trachea
Tongue
Small intestine
Colon
Liver
Pancreas
Kidney
Bladder
Uterus
Testes

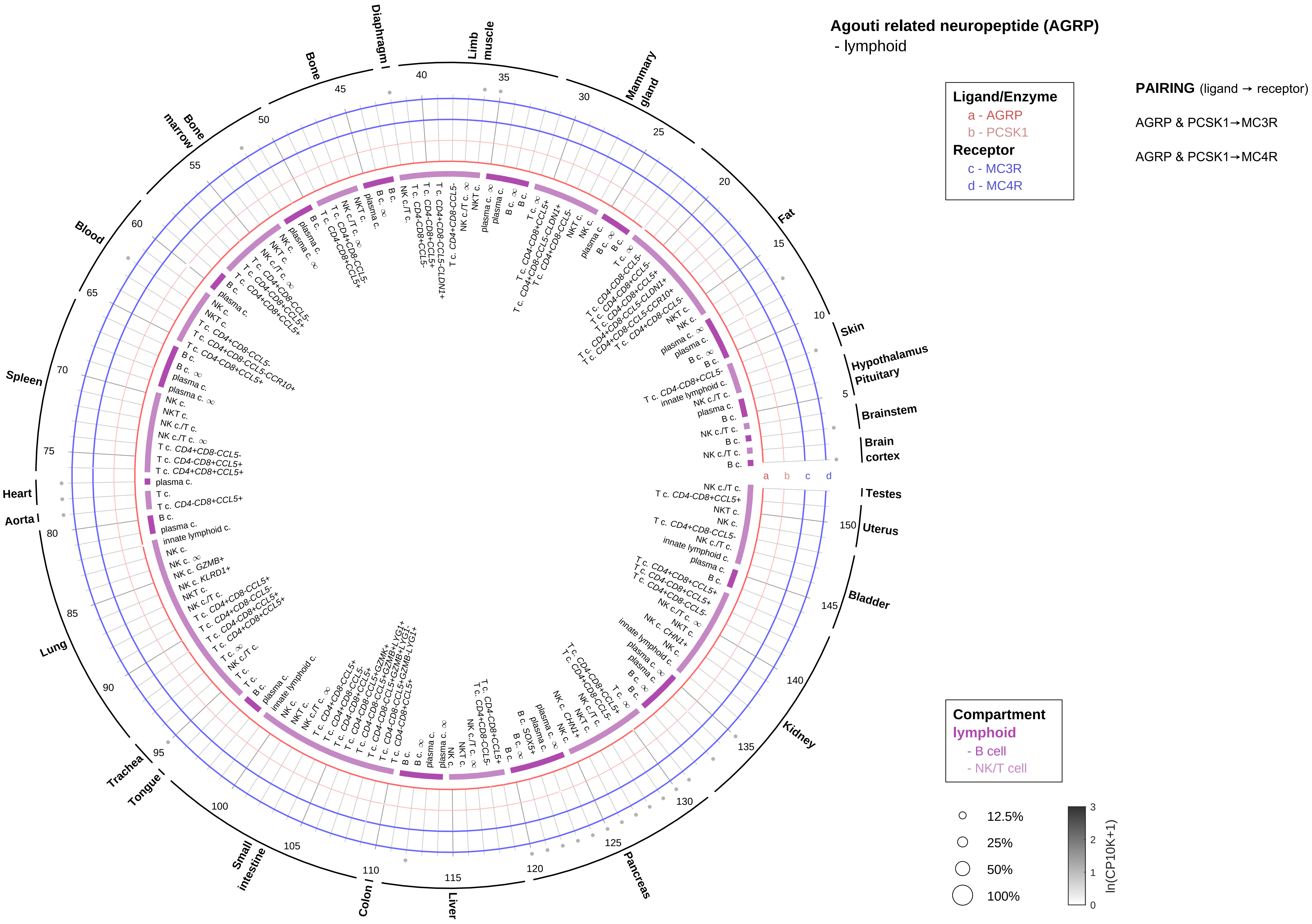

Agouti related neuropeptide (AGRP)
- lymphoid
Ligand/Enzyme
a - AGRP
b - PCSK1
Receptor
c - MC3R
d - MC4R
PAIRING (ligand → receptor)
AGRP & PCSK1→MC3R
AGRP & PCSK1→MC4R
Compartment
lymphoid
- B cell
- NK/T cell
12.5%
25%
50%
100%
ln(CP10K+1)
Diaphragm
Limb muscle
Mammary gland
Fat
Skin
Hypothalamus
Pituitary
Brainstem
Brain cortex
Testes
Uterus
Bladder
Kidney
Pancreas
Liver
Colon
Small intestine
Tongue
Trachea
Lung
Aorta
Heart
Spleen
Blood
Bone marrow
Bone

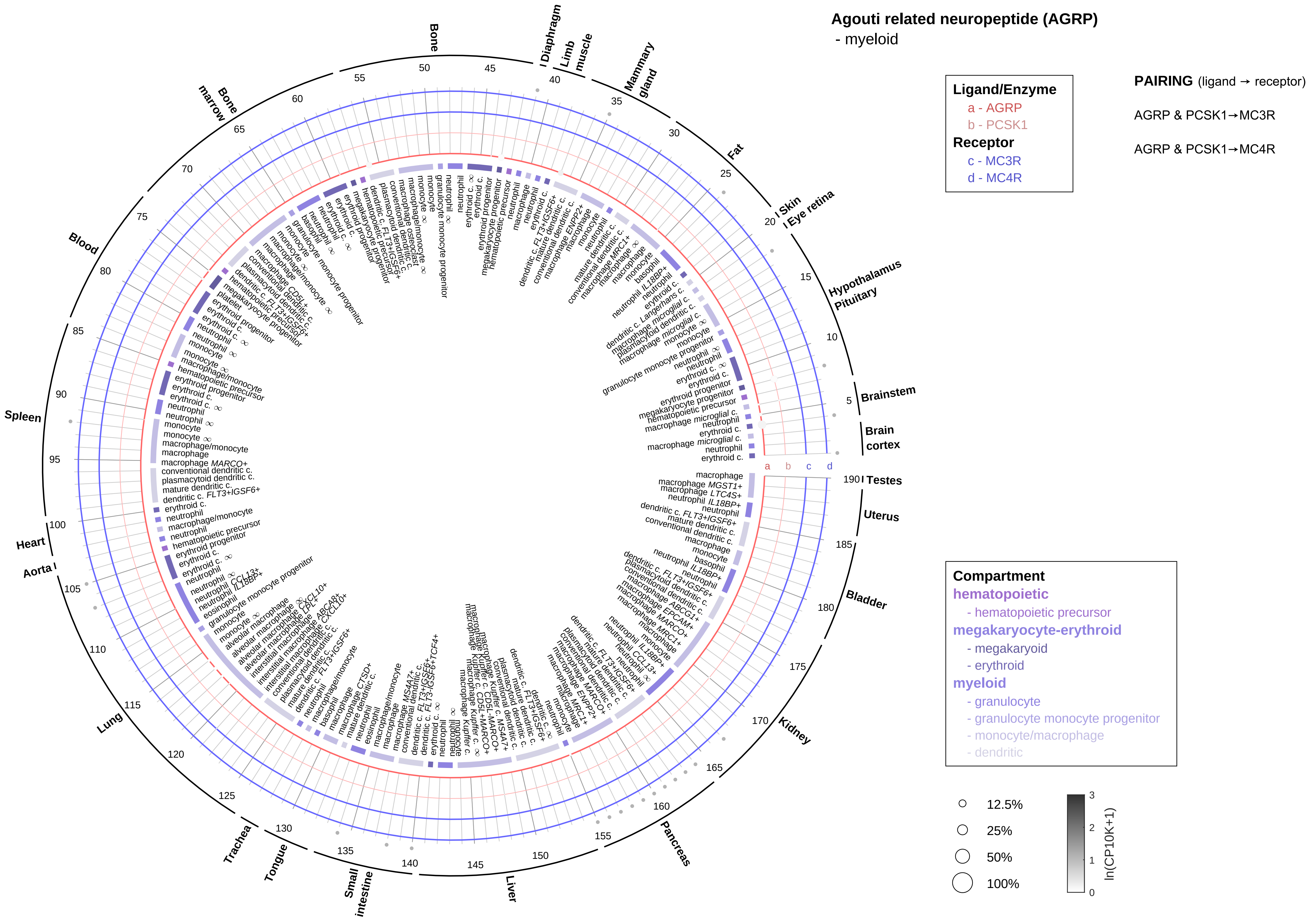

Agouti related neuropeptide (AGRP)
- myeloid
Ligand/Enzyme
a - AGRP
b - PCSK1
Receptor
c - MC3R
d - MC4R
PAIRING (ligand → receptor)
AGRP & PCSK1→MC3R
AGRP & PCSK1→MC4R
Compartment
hematopoietic
- hematopoietic precursor
megakaryocyte-erythroid
- megakaryoid
- erythroid
myeloid
- granulocyte
- granulocyte monocyte progenitor
- monocyte/macrophage
- dendritic
12.5%
25%
50%
100%
ln(CP10K+1)
Bone
Diaphragm
Limb muscle
Mammary gland
Fat
Skin
Eye retina
Hypothalamus
Pituitary
Brainstem
Brain cortex
Testes
Uterus
Bladder
Kidney
Pancreas
Liver
Small intestine
Tongue
Trachea
Lung
Aorta
Heart
Spleen
Blood
Bone marrow

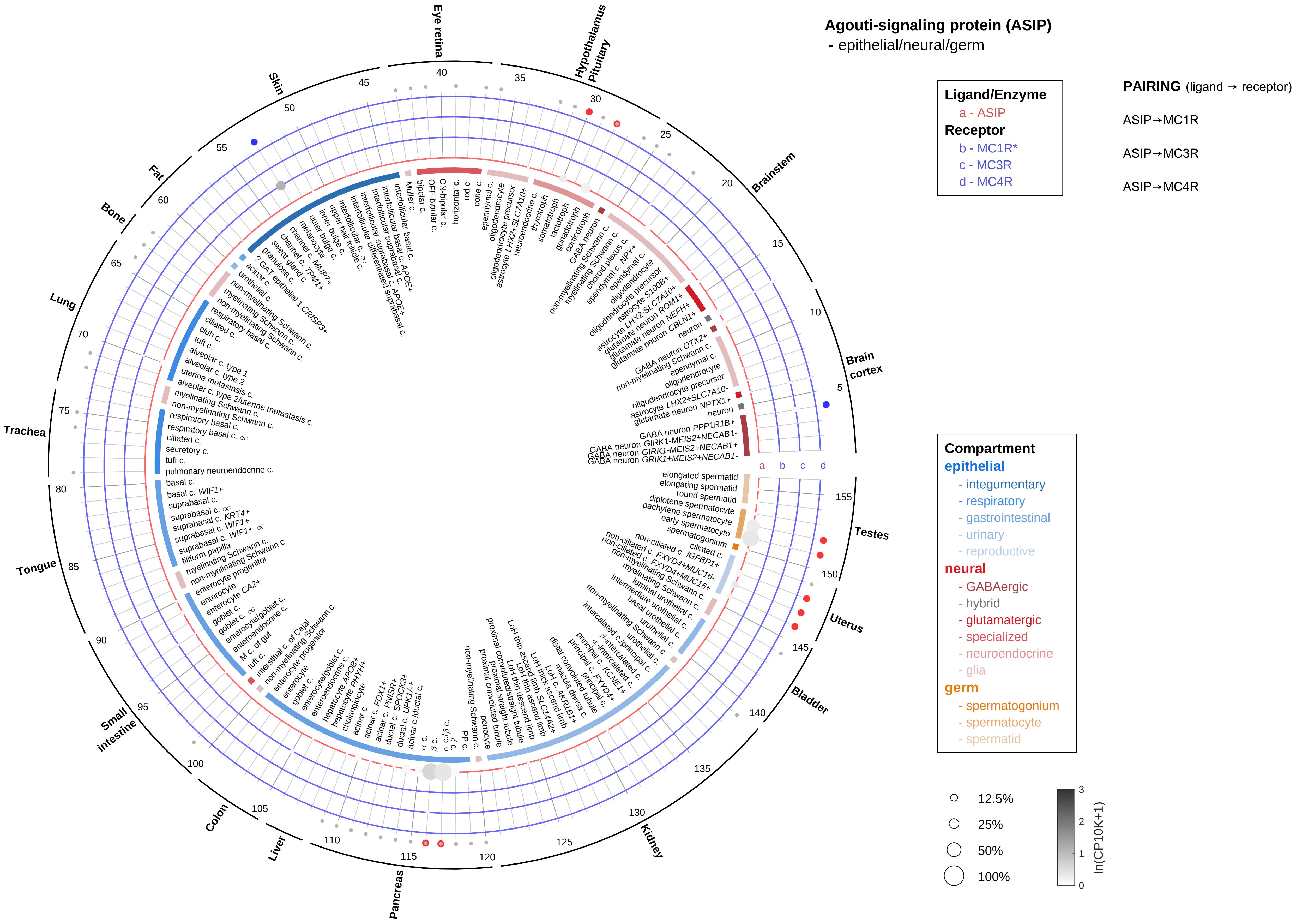

Agouti-signaling protein (ASIP)
- epithelial/neural/germ
Ligand/Enzyme
a - ASIP
Receptor
b - MC1R*
c - MC3R
d - MC4R
PAIRING (ligand → receptor)
ASIP→MC1R
ASIP→MC3R
ASIP→MC4R
Compartment
epithelial
- integumentary
- respiratory
- gastrointestinal
- urinary
- reproductive
neural
- GABAergic
- hybrid
- glutamatergic
- specialized
- neuroendocrine
- glia
germ
- spermatogonium
- spermatocyte
- spermatid
12.5%
25%
50%
100%
ln(CP10K+1)
Eye retina
Hypothalamus Pituitary
Brainstem
Brain cortex
Testes
Uterus
Bladder
Kidney
Pancreas
Liver
Colon
Small intestine
Tongue
Trachea
Lung
Bone
Fat
Skin

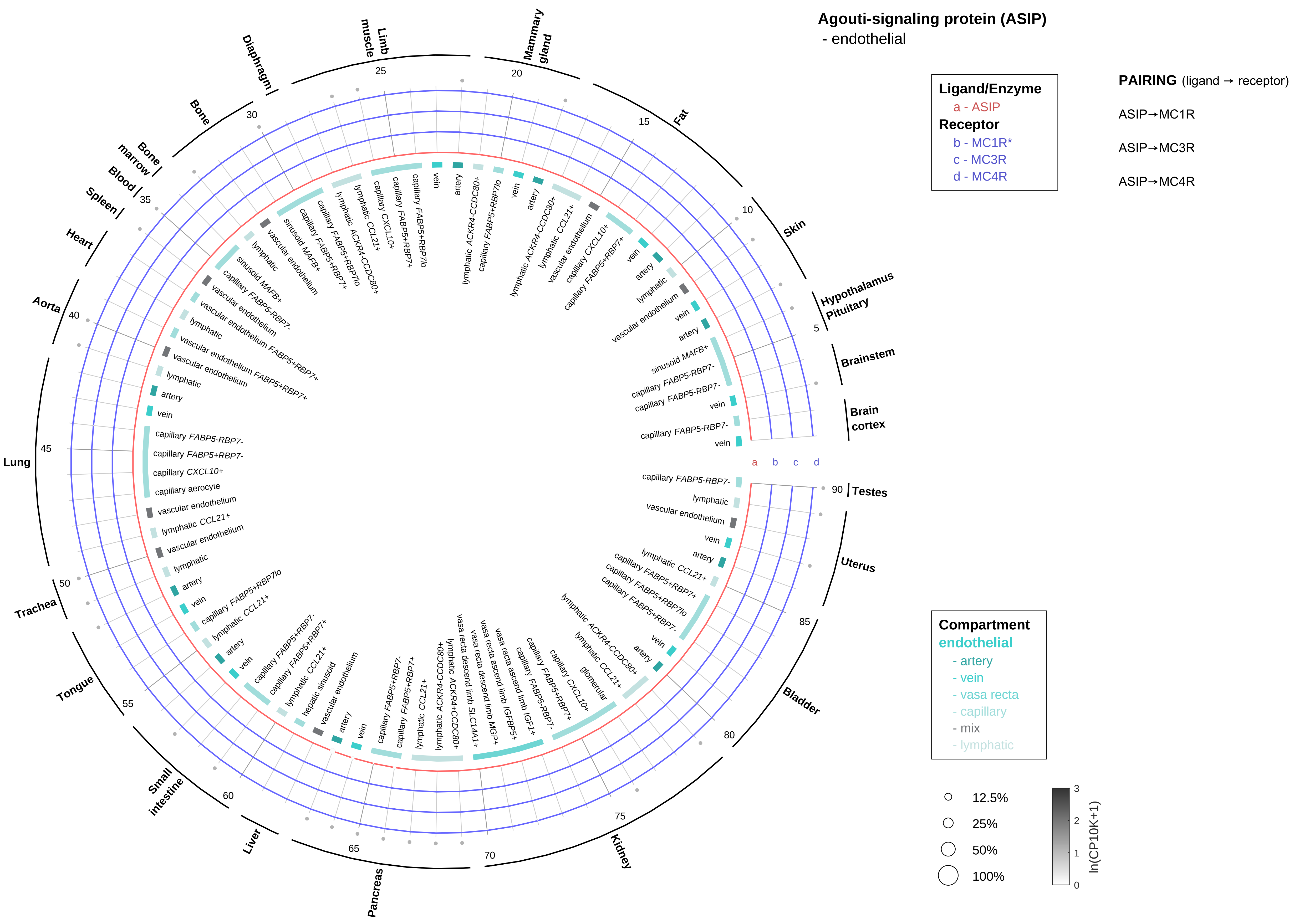

Agouti-signaling protein (ASIP)
- endothelial
Ligand/Enzyme
a - ASIP
Receptor
b - MC1R*
c - MC3R
d - MC4R
PAIRING (ligand → receptor)
ASIP→MC1R
ASIP→MC3R
ASIP→MC4R
Compartment
endothelial
- artery
- vein
- vasa recta
- capillary
- mix
- lymphatic
12.5%
25%
50%
100%
ln(CP10K+1)
Brain cortex
Brainstem
Hypothalamus Pituitary
Skin
Fat
Mammary gland
Limb muscle
Diaphragm
Bone
Bone marrow
Blood
Spleen
Heart
Aorta
Lung
Trachea
Tongue
Small intestine
Liver
Pancreas
Kidney
Bladder
Uterus
Testes

Agouti-signaling protein (ASIP)
- stromal

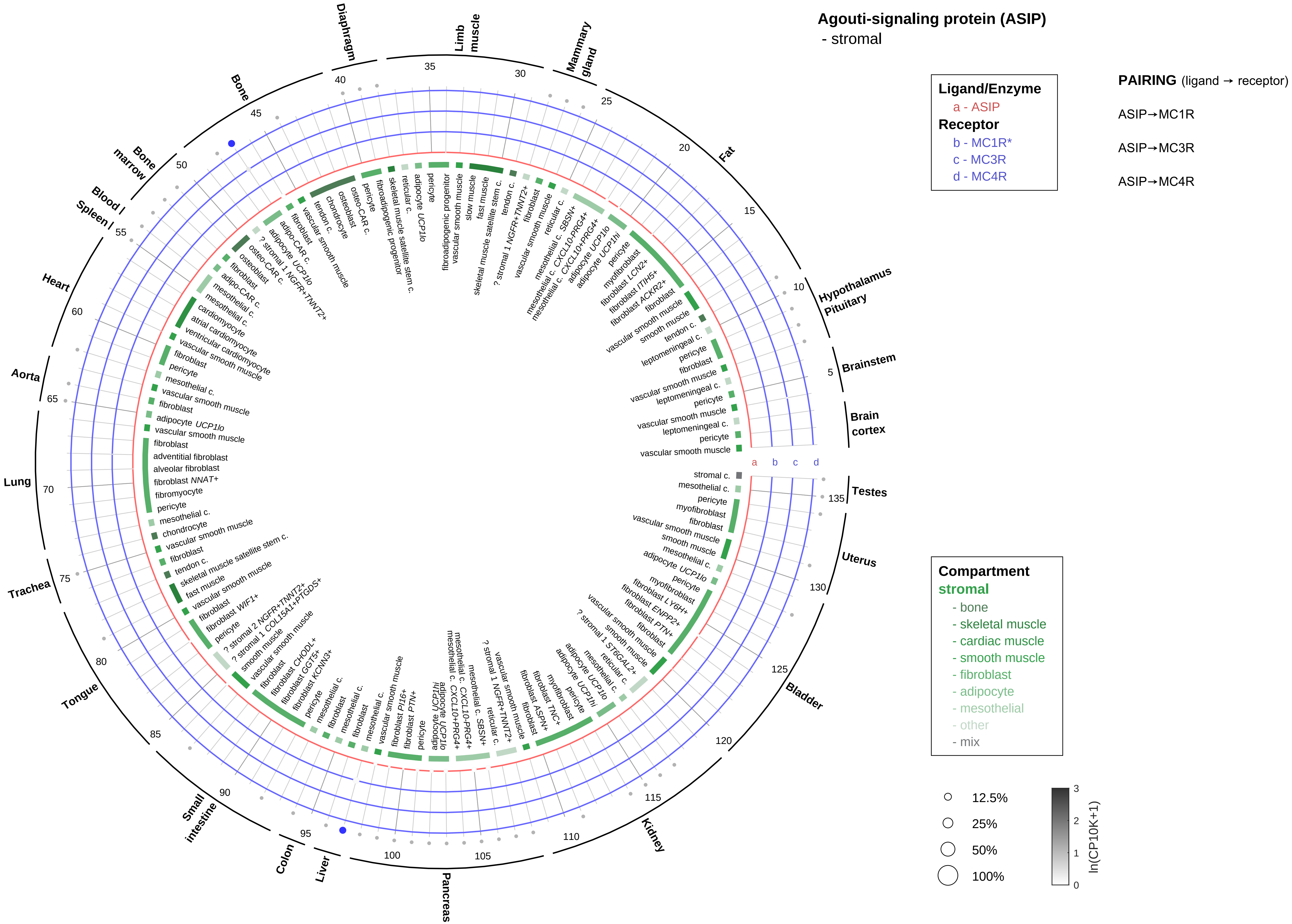

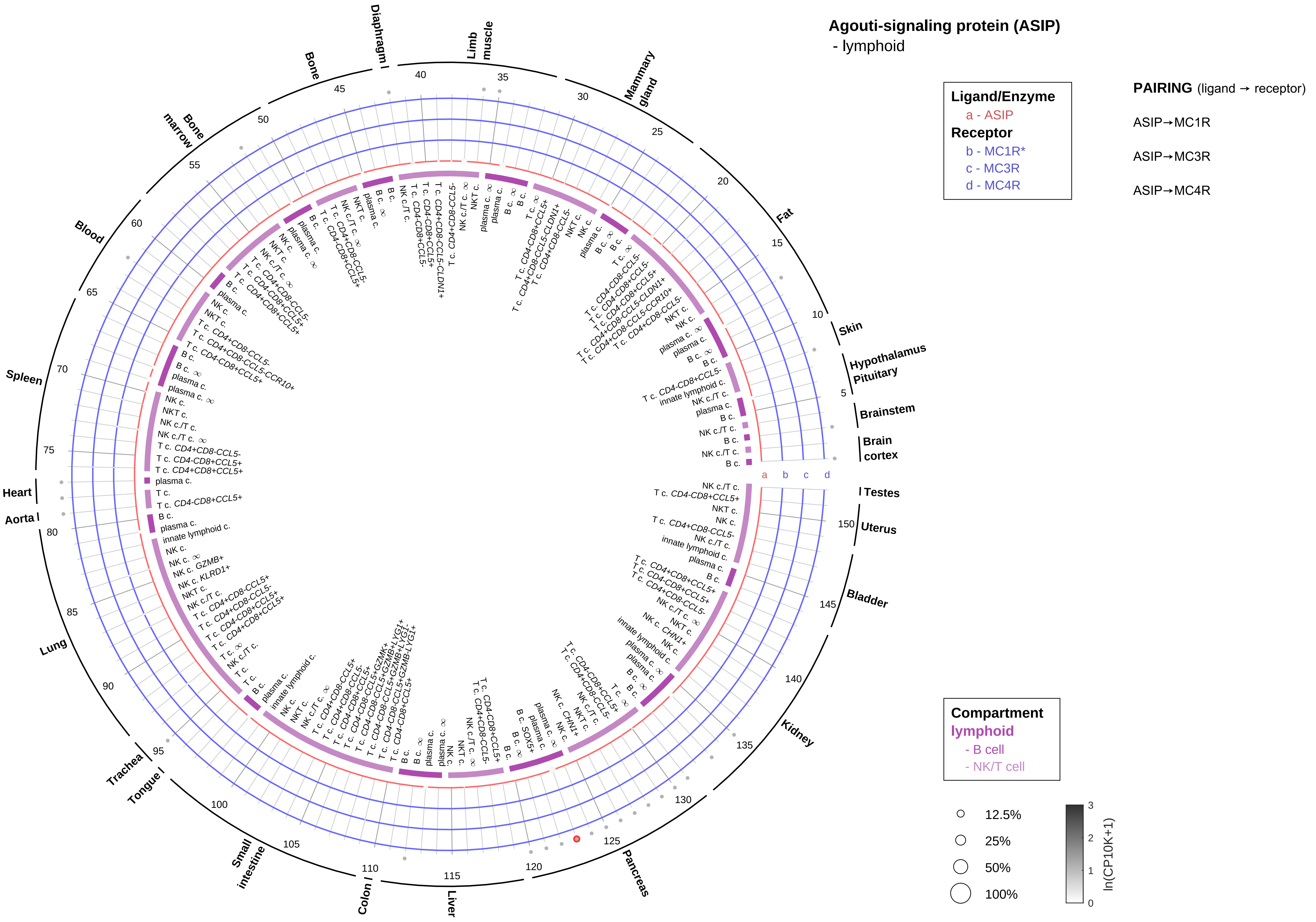

Agouti-signaling protein (ASIP)
- lymphoid
Ligand/Enzyme
a - ASIP
Receptor
b - MC1R*
c - MC3R
d - MC4R
PAIRING (ligand → receptor)
ASIP→MC1R
ASIP→MC3R
ASIP→MC4R
Compartment
lymphoid
- B cell
- NK/T cell
12.5%
25%
50%
100%
ln(CP10K+1)
Skin
Hypothalamus
Pituitary
Brainstem
Brain cortex
Testes
Uterus
Bladder
Kidney
Pancreas
Liver
Colon
Small intestine
Tongue
Trachea
Lung
Aorta
Heart
Spleen
Blood
Bone marrow
Bone
Diaphragm
Limb muscle
Mammary gland
Fat

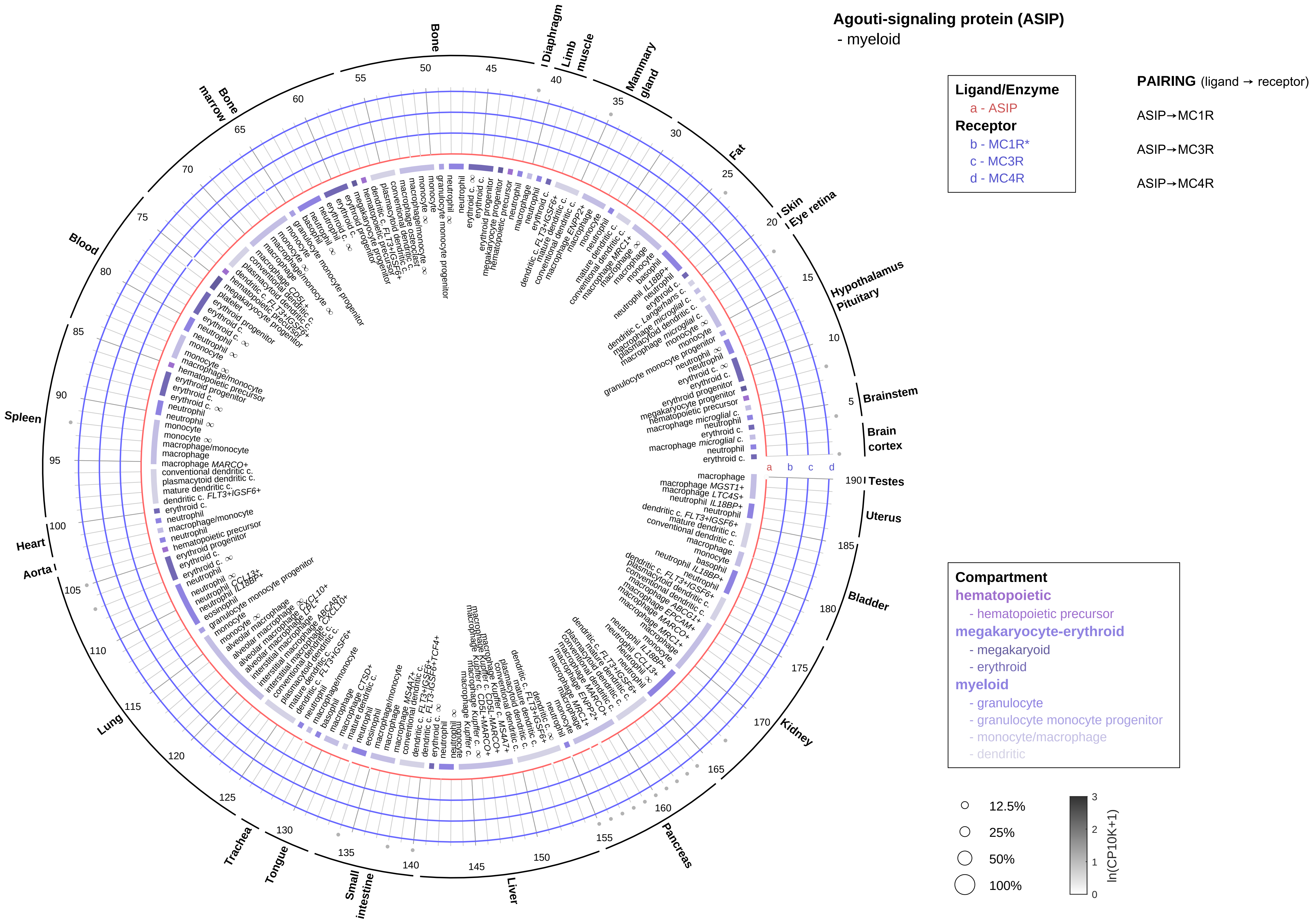
Agouti-signaling protein (ASIP)
- myeloid
Ligand/Enzyme
a - ASIP
Receptor
b - MC1R*
c - MC3R
d - MC4R
PAIRING (ligand → receptor)
ASIP→MC1R
ASIP→MC3R
ASIP→MC4R
Compartment
hematopoietic
- hematopoietic precursor
megakaryocyte-erythroid
- megakaryoid
- erythroid
myeloid
- granulocyte
- granulocyte monocyte progenitor
- monocyte/macrophage
- dendritic
12.5%
25%
50%
100%
ln(CP10K+1)
Bone
Diaphragm
Limb muscle
Mammary gland
Fat
Skin
Eye retina
Hypothalamus
Pituitary
Brainstem
Brain cortex
Testes
Uterus
Bladder
Kidney
Pancreas
Liver
Small intestine
Tongue
Trachea
Lung
Aorta
Heart
Spleen
Blood
Bone marrow

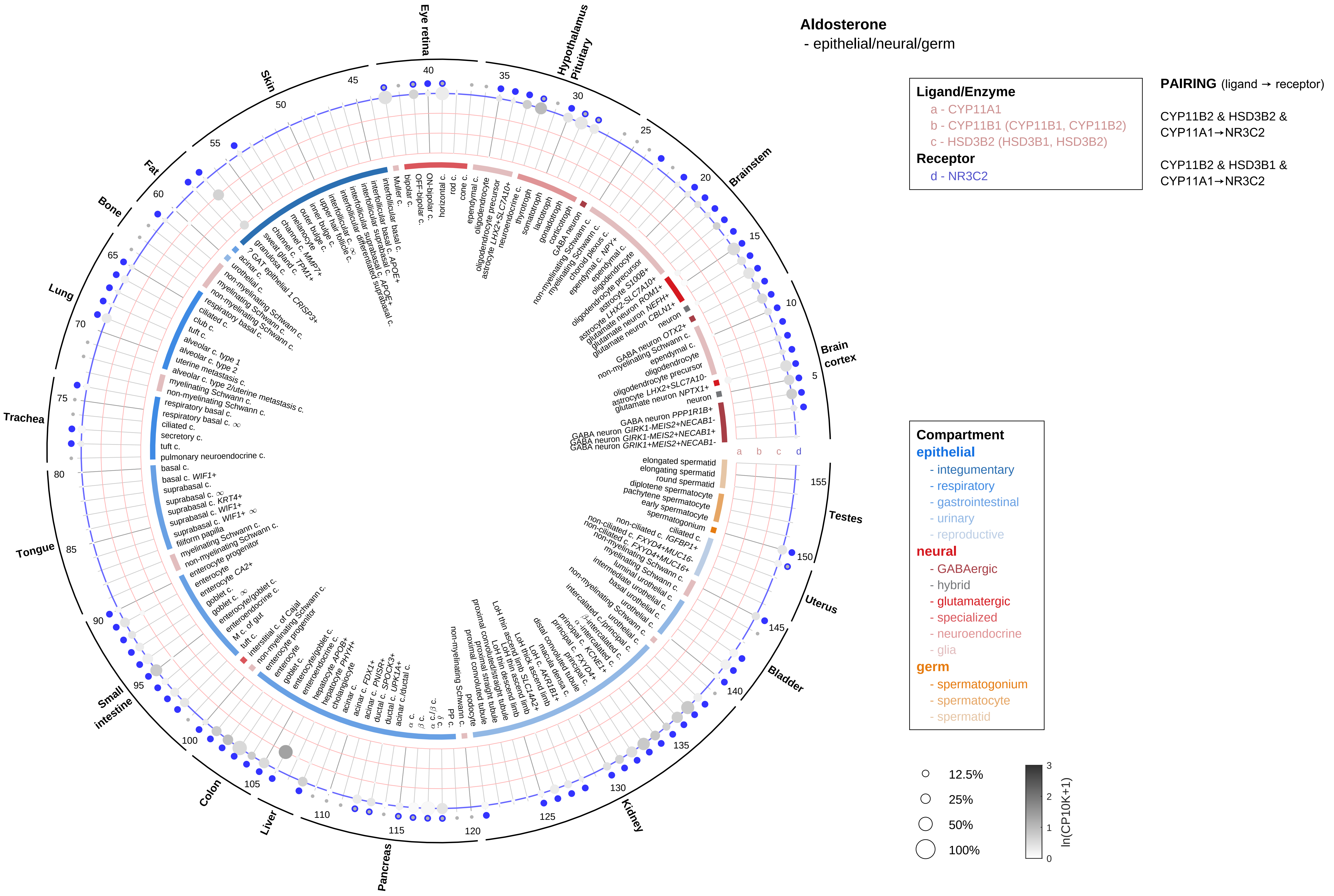

Aldosterone
- epithelial/neural/germ
Ligand/Enzyme
a - CYP11A1
b - CYP11B1 (CYP11B1, CYP11B2)
c - HSD3B2 (HSD3B1, HSD3B2)
Receptor
d - NR3C2
PAIRING (ligand → receptor)
CYP11B2 & HSD3B2 & CYP11A1→NR3C2
CYP11B2 & HSD3B1 & CYP11A1→NR3C2
Compartment
epithelial
- integumentary
- respiratory
- gastrointestinal
- urinary
- reproductive
neural
- GABAergic
- hybrid
- glutamatergic
- specialized
- neuroendocrine
- glia
germ
- spermatogonium
- spermatocyte
- spermatid
12.5%
25%
50%
100%
ln(CP10K+1)
Eye retina
Hypothalamus Pituitary
Brainstem
Brain cortex
Testes
Uterus
Bladder
Kidney
Pancreas
Liver
Colon
Small intestine
Tongue
Trachea
Lung
Bone
Fat
Skin
a
b
c
d

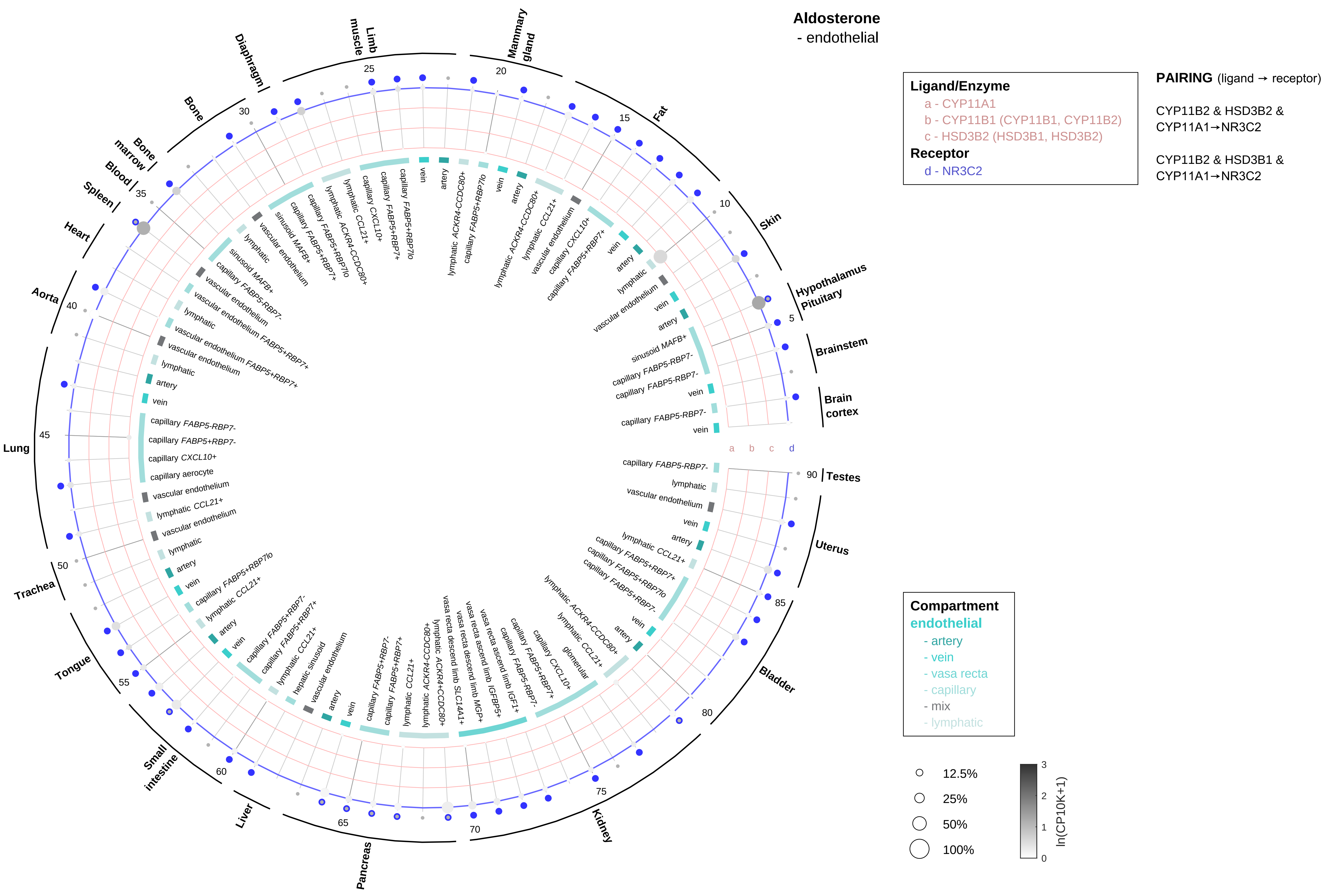

Aldosterone
- endothelial
Ligand/Enzyme
a - CYP11A1
b - CYP11B1 (CYP11B1, CYP11B2)
c - HSD3B2 (HSD3B1, HSD3B2)
Receptor
d - NR3C2
PAIRING (ligand → receptor)
CYP11B2 & HSD3B2 & CYP11A1→NR3C2
CYP11B2 & HSD3B1 & CYP11A1→NR3C2
Compartment
endothelial
- artery
- vein
- vasa recta
- capillary
- mix
- lymphatic
12.5%
25%
50%
100%
ln(CP10K+1)
0
1
2
3
a
b
c
d
Hypothalamus
Pituitary
Brainstem
Brain cortex
Skin
Fat
Mammary gland
Limb muscle
Diaphragm
Bone
Bone marrow
Blood
Spleen
Heart
Aorta
Lung
Trachea
Tongue
Small intestine
Liver
Pancreas
Kidney
Bladder
Uterus
Testes
5
10
15
20
25
30
35
40
45
50
55
60
65
70
75
80
85
90

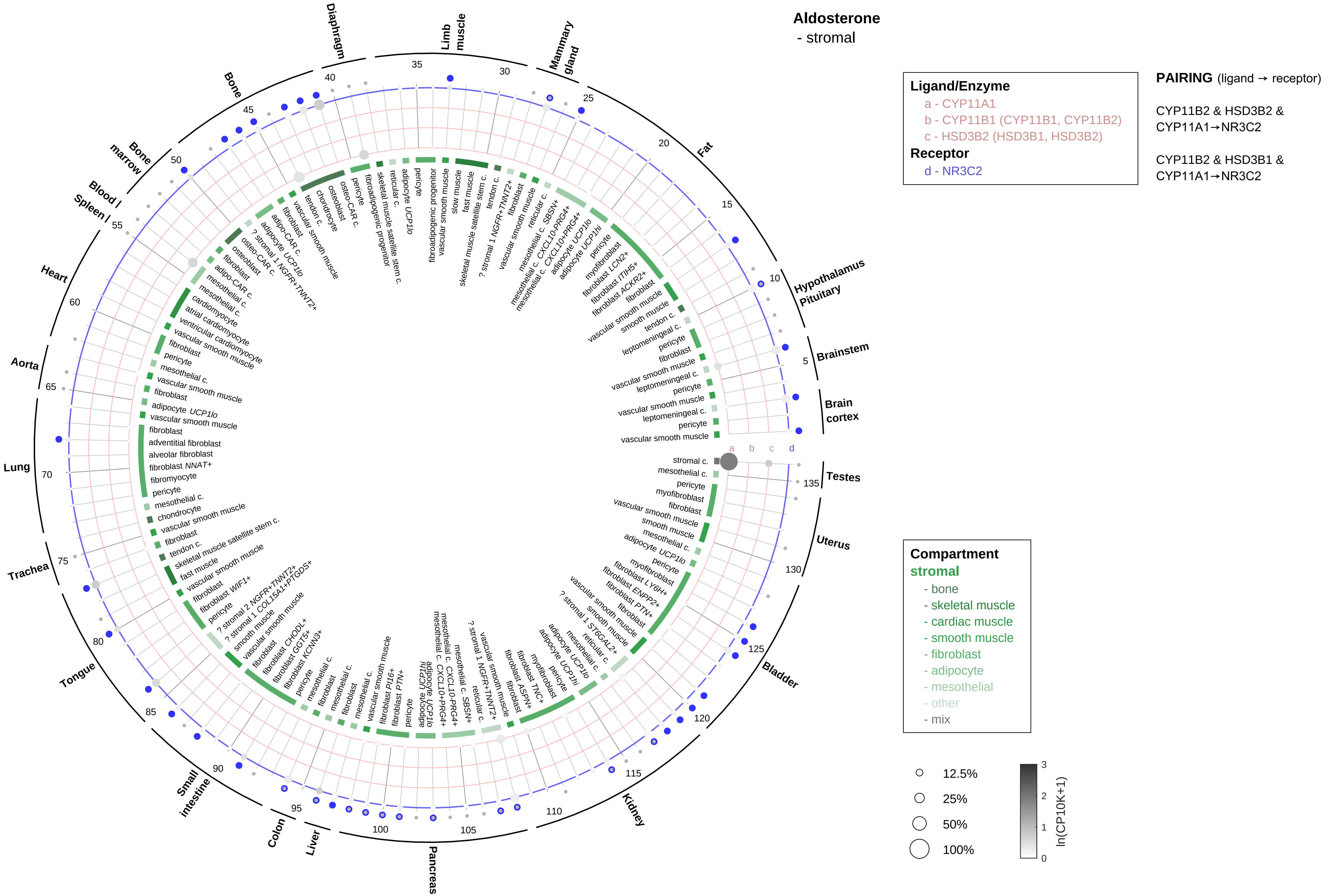

Aldosterone
- stromal
Ligand/Enzyme
a - CYP11A1
b - CYP11B1 (CYP11B1, CYP11B2)
c - HSD3B2 (HSD3B1, HSD3B2)
Receptor
d - NR3C2
PAIRING (ligand → receptor)
CYP11B2 & HSD3B2 & CYP11A1→NR3C2
CYP11B2 & HSD3B1 & CYP11A1→NR3C2
Compartment
stromal
- bone
- skeletal muscle
- cardiac muscle
- smooth muscle
- fibroblast
- adipocyte
- mesothelial
- other
- mix
12.5%
25%
50%
100%
ln(CP10K+1)
Diaphragm
Limb muscle
Mammary gland
Fat
Hypothalamus Pituitary
Brainstem
Brain cortex
Testes
Uterus
Bladder
Kidney
Pancreas
Liver
Colon
Small intestine
Tongue
Trachea
Lung
Aorta
Heart
Spleen
Blood
Bone marrow
Bone

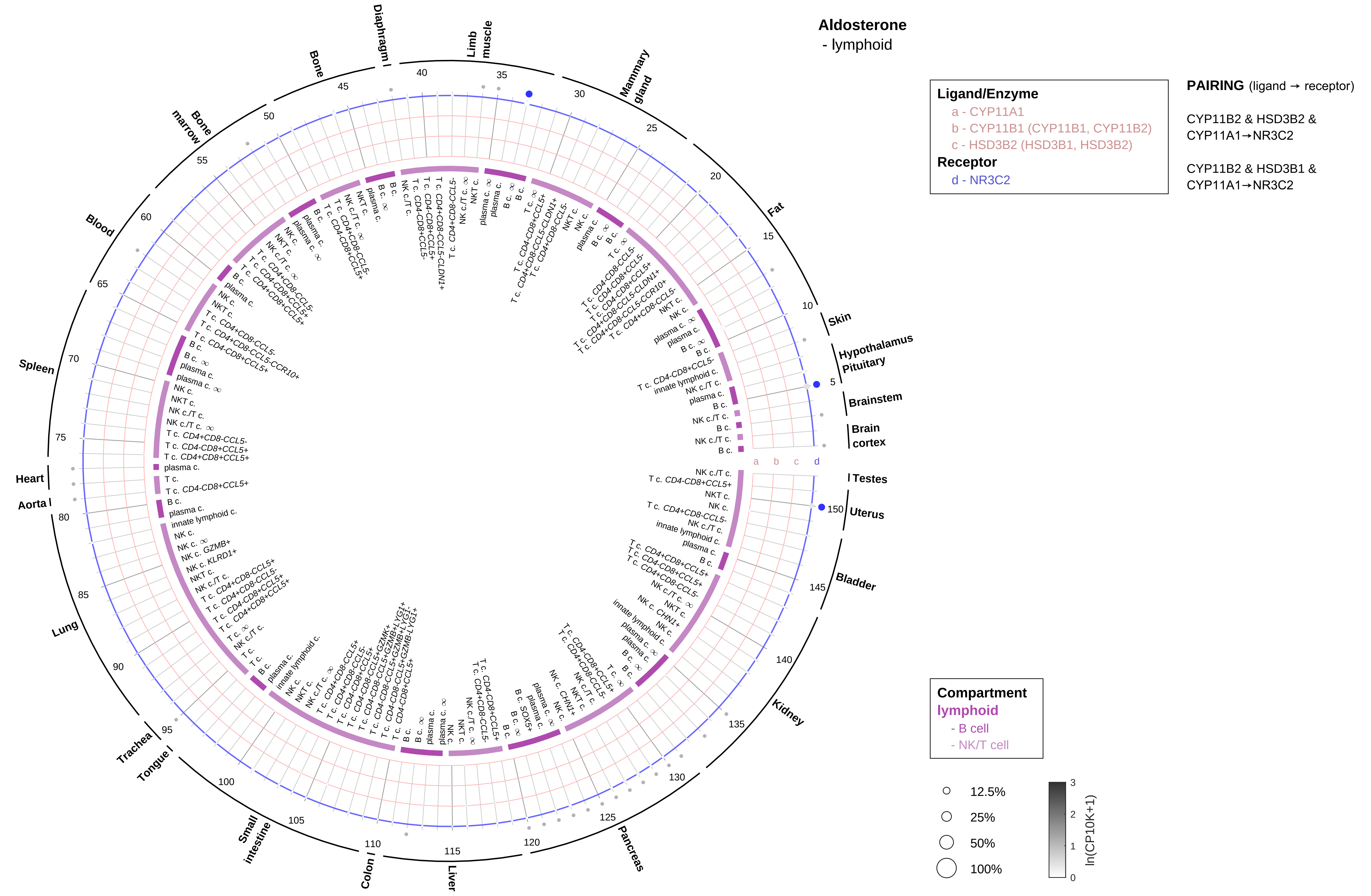
Aldosterone
- lymphoid
Ligand/Enzyme
a - CYP11A1
b - CYP11B1 (CYP11B1, CYP11B2)
c - HSD3B2 (HSD3B1, HSD3B2)
Receptor
d - NR3C2
PAIRING (ligand → receptor)
CYP11B2 & HSD3B2 & CYP11A1→NR3C2
CYP11B2 & HSD3B1 & CYP11A1→NR3C2
Compartment
lymphoid
- B cell
- NK/T cell
12.5%
25%
50%
100%
ln(CP10K+1)
Skin
Hypothalamus
Pituitary
Brainstem
Brain cortex
Testes
Uterus
Bladder
Kidney
Pancreas
Liver
Colon
Small intestine
Tongue
Trachea
Lung
Aorta
Heart
Spleen
Blood
Bone marrow
Bone
Diaphragm
Limb muscle
Mammary gland
Fat

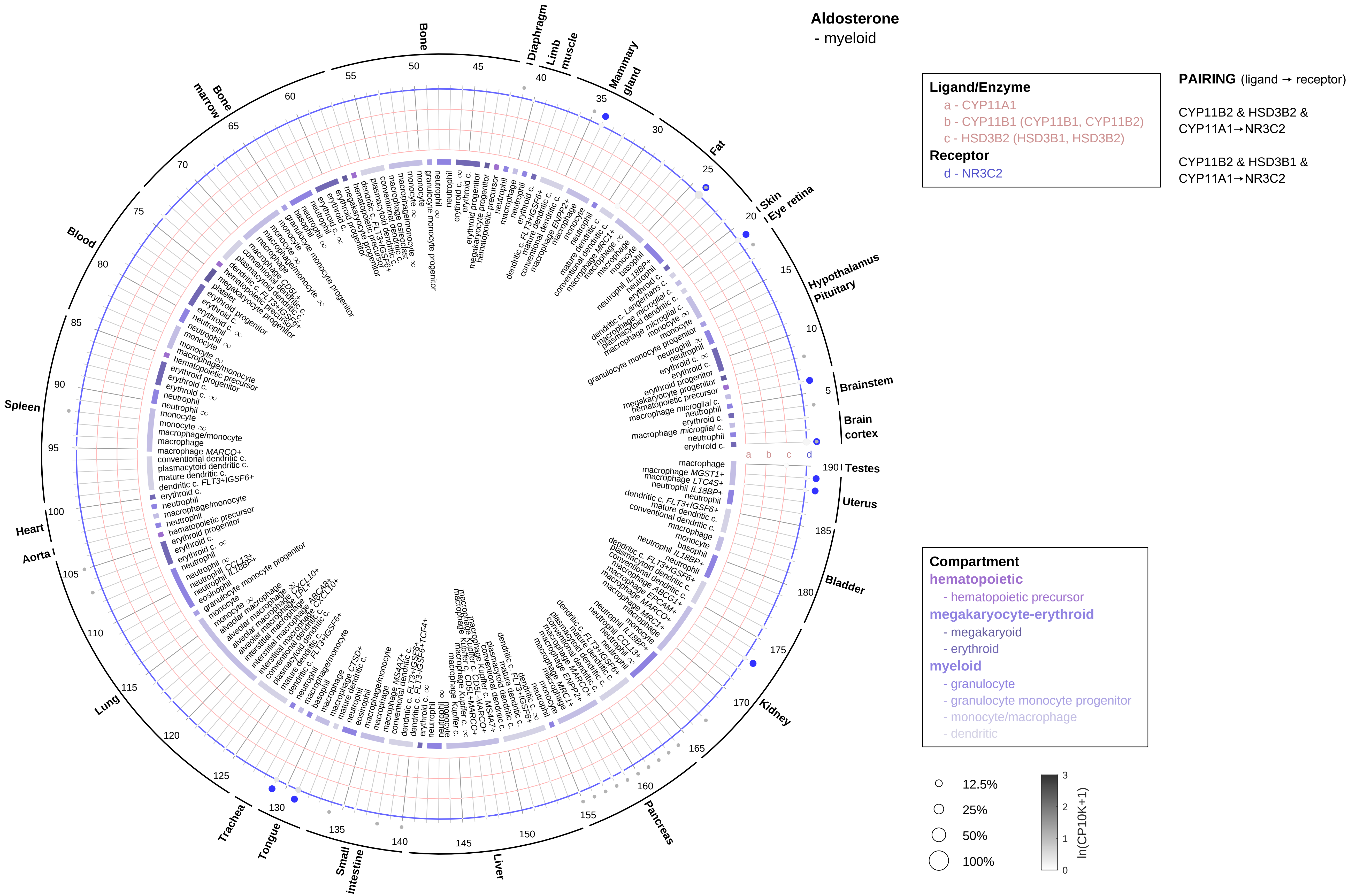
Aldosterone
- myeloid
Ligand/Enzyme
a - CYP11A1
b - CYP11B1 (CYP11B1, CYP11B2)
c - HSD3B2 (HSD3B1, HSD3B2)
Receptor
d - NR3C2
PAIRING (ligand → receptor)
CYP11B2 & HSD3B2 & CYP11A1→NR3C2
CYP11B2 & HSD3B1 & CYP11A1→NR3C2
Compartment
hematopoietic
- hematopoietic precursor
megakaryocyte-erythroid
- megakaryoid
- erythroid
myeloid
- granulocyte
- granulocyte monocyte progenitor
- monocyte/macrophage
- dendritic
12.5%
25%
50%
100%
ln(CP10K+1)
Bone
Diaphragm
Limb muscle
Mammary gland
Fat
Skin
Eye retina
Hypothalamus
Pituitary
Brainstem
Brain cortex
Testes
Uterus
Bladder
Kidney
Pancreas
Liver
Small intestine
Tongue
Trachea
Lung
Aorta
Heart
Spleen
Blood
Bone marrow

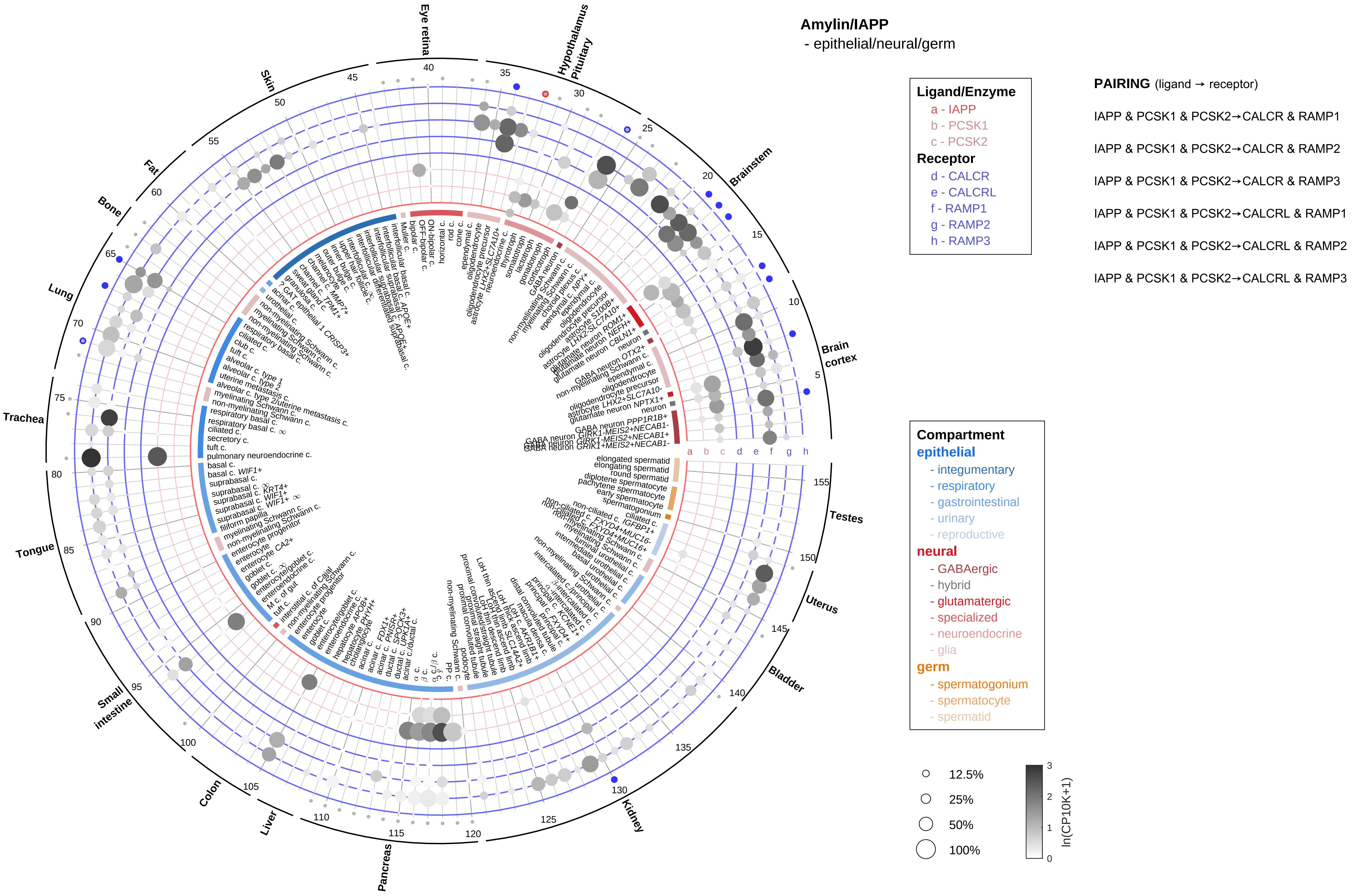
Amylin/IAPP
- epithelial/neural/germ
Ligand/Enzyme
a - IAPP
b - PCSK1
c - PCSK2
Receptor
d - CALCR
e - CALCRL
f - RAMP1
g - RAMP2
h - RAMP3
PAIRING (ligand → receptor)
IAPP & PCSK1 & PCSK2→CALCR & RAMP1
IAPP & PCSK1 & PCSK2→CALCR & RAMP2
IAPP & PCSK1 & PCSK2→CALCR & RAMP3
IAPP & PCSK1 & PCSK2→CALCRL & RAMP1
IAPP & PCSK1 & PCSK2→CALCRL & RAMP2
IAPP & PCSK1 & PCSK2→CALCRL & RAMP3
Compartment
epithelial
- integumentary
- respiratory
- gastrointestinal
- urinary
- reproductive
neural
- GABAergic
- hybrid
- glutamatergic
- specialized
- neuroendocrine
- glia
germ
- spermatogonium
- spermatocyte
- spermatid
12.5%
25%
50%
100%
ln(CP10K+1)
Eye retina
Hypothalamus Pituitary
Brainstem
Brain cortex
Testes
Uterus
Bladder
Kidney
Pancreas
Liver
Colon
Small intestine
Tongue
Trachea
Lung
Bone
Fat
Skin

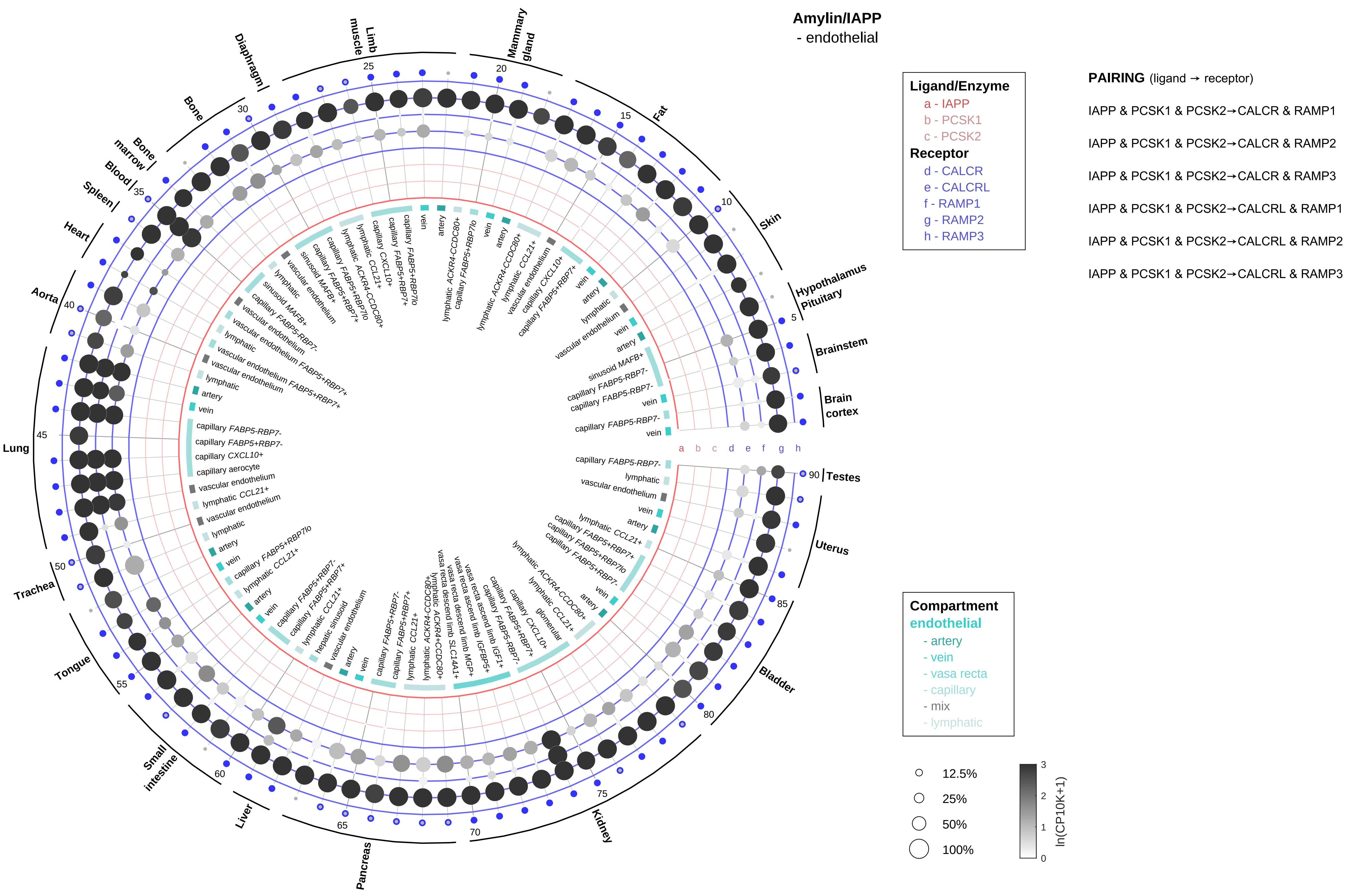
Amylin/IAPP
- endothelial
Ligand/Enzyme
a - IAPP
b - PCSK1
c - PCSK2
Receptor
d - CALCR
e - CALCRL
f - RAMP1
g - RAMP2
h - RAMP3
PAIRING (ligand → receptor)
IAPP & PCSK1 & PCSK2→CALCR & RAMP1
IAPP & PCSK1 & PCSK2→CALCR & RAMP2
IAPP & PCSK1 & PCSK2→CALCR & RAMP3
IAPP & PCSK1 & PCSK2→CALCRL & RAMP1
IAPP & PCSK1 & PCSK2→CALCRL & RAMP2
IAPP & PCSK1 & PCSK2→CALCRL & RAMP3
Compartment
endothelial
- artery
- vein
- vasa recta
- capillary
- mix
- lymphatic
12.5%
25%
50%
100%
ln(CP10K+1)
Brain cortex
Brainstem
Hypothalamus Pituitary
Skin
Fat
Mammary gland
Limb muscle
Diaphragm
Bone
Bone marrow
Blood
Spleen
Heart
Aorta
Lung
Trachea
Tongue
Small intestine
Liver
Pancreas
Kidney
Bladder
Uterus
Testes

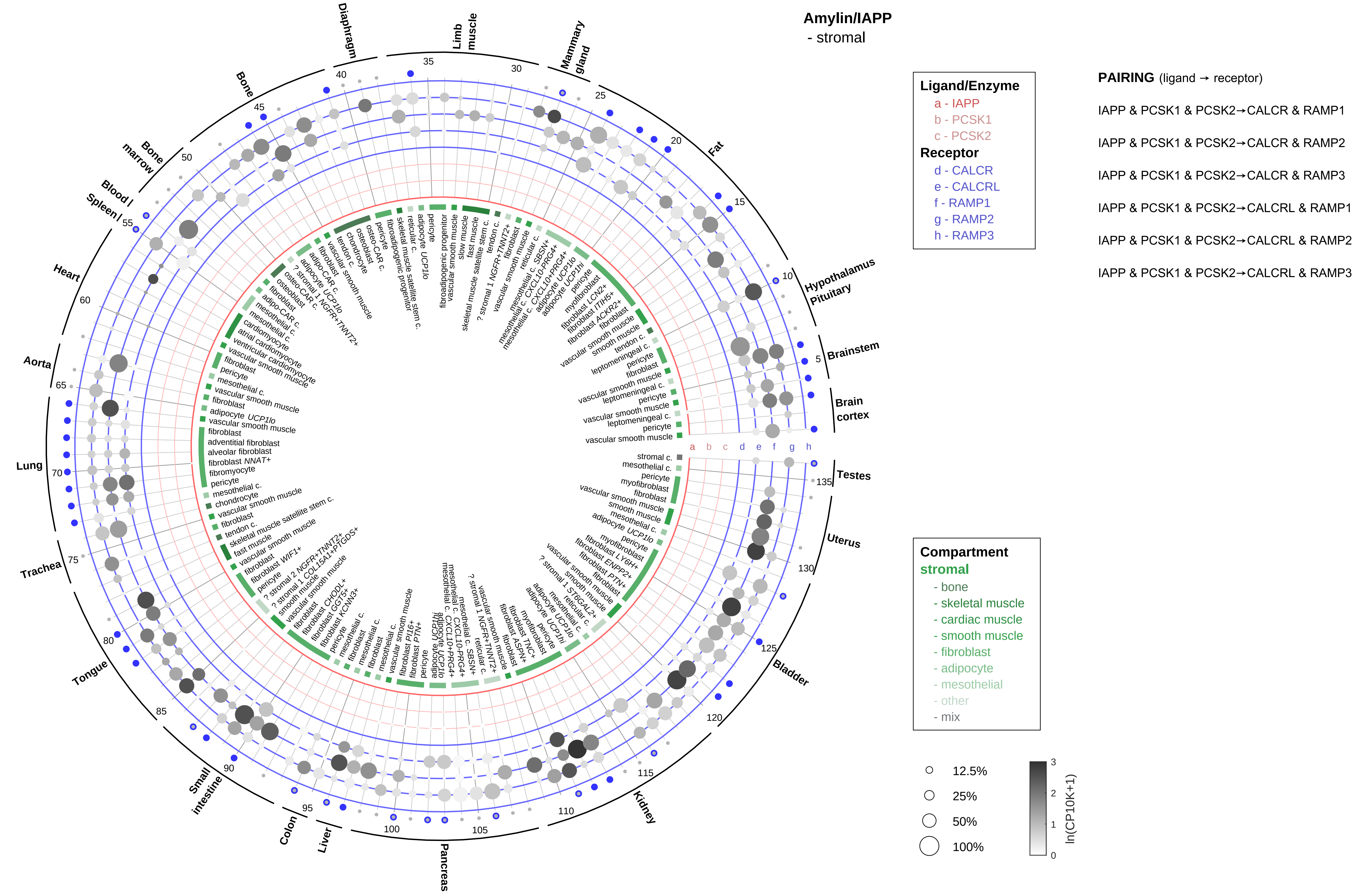

Amylin/IAPP
- stromal
Ligand/Enzyme
a - IAPP
b - PCSK1
c - PCSK2
Receptor
d - CALCR
e - CALCRL
f - RAMP1
g - RAMP2
h - RAMP3
PAIRING (ligand → receptor)
IAPP & PCSK1 & PCSK2→CALCR & RAMP1
IAPP & PCSK1 & PCSK2→CALCR & RAMP2
IAPP & PCSK1 & PCSK2→CALCR & RAMP3
IAPP & PCSK1 & PCSK2→CALCRL & RAMP1
IAPP & PCSK1 & PCSK2→CALCRL & RAMP2
IAPP & PCSK1 & PCSK2→CALCRL & RAMP3
Compartment
stromal
- bone
- skeletal muscle
- cardiac muscle
- smooth muscle
- fibroblast
- adipocyte
- mesothelial
- other
- mix
12.5%
25%
50%
100%
ln(CP10K+1)
Diaphragm
Limb muscle
Mammary gland
Fat
Hypothalamus Pituitary
Brainstem
Brain cortex
Testes
Uterus
Bladder
Kidney
Pancreas
Liver
Colon
Small intestine
Tongue
Trachea
Lung
Aorta
Heart
Spleen
Blood
Bone marrow
Bone

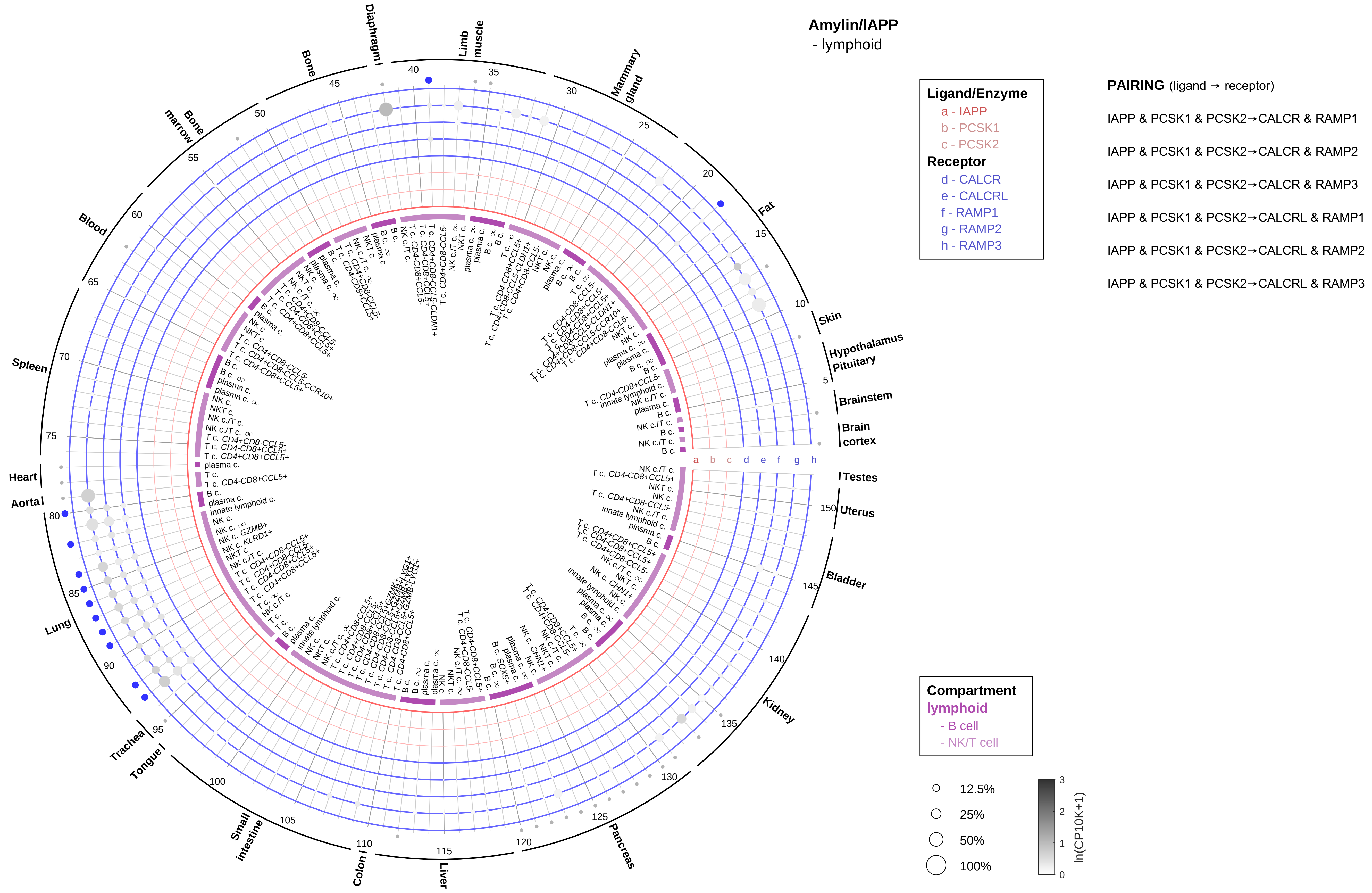

Amylin/IAPP
- lymphoid
Ligand/Enzyme
a - IAPP
b - PCSK1
c - PCSK2
Receptor
d - CALCR
e - CALCRL
f - RAMP1
g - RAMP2
h - RAMP3
PAIRING (ligand → receptor)
IAPP & PCSK1 & PCSK2→CALCR & RAMP1
IAPP & PCSK1 & PCSK2→CALCR & RAMP2
IAPP & PCSK1 & PCSK2→CALCR & RAMP3
IAPP & PCSK1 & PCSK2→CALCRL & RAMP1
IAPP & PCSK1 & PCSK2→CALCRL & RAMP2
IAPP & PCSK1 & PCSK2→CALCRL & RAMP3
Compartment
lymphoid
- B cell
- NK/T cell
12.5%
25%
50%
100%
ln(CP10K+1)
Diaphragm
Limb muscle
Mammary gland
Fat
Skin
Hypothalamus
Pituitary
Brainstem
Brain cortex
Testes
Uterus
Bladder
Kidney
Pancreas
Liver
Colon
Small intestine
Tongue
Trachea
Lung
Aorta
Heart
Spleen
Blood
Bone marrow
Bone

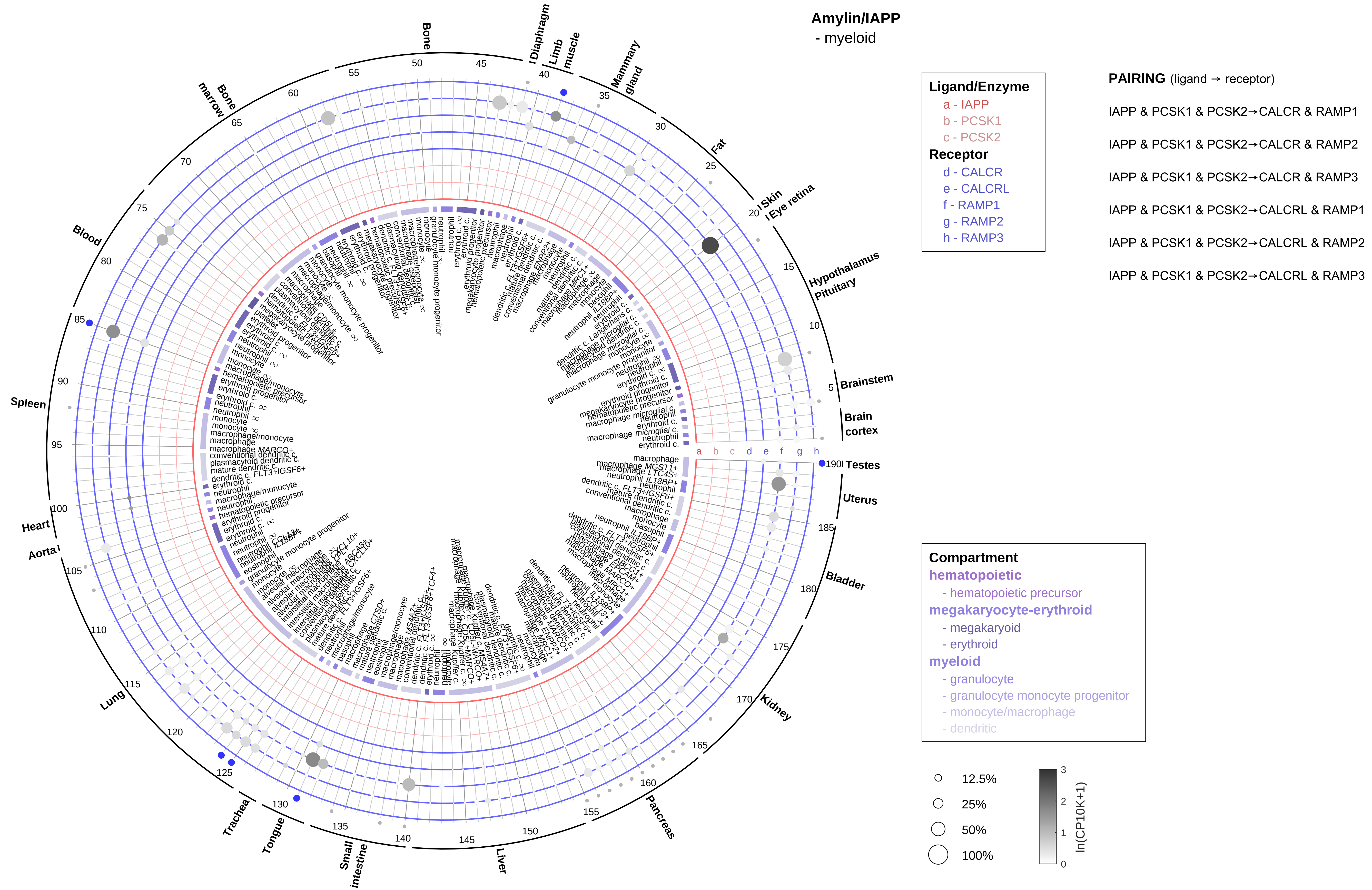

Amylin/IAPP
- myeloid
Ligand/Enzyme
a - IAPP
b - PCSK1
c - PCSK2
Receptor
d - CALCR
e - CALCRL
f - RAMP1
g - RAMP2
h - RAMP3
PAIRING (ligand → receptor)
IAPP & PCSK1 & PCSK2→CALCR & RAMP1
IAPP & PCSK1 & PCSK2→CALCR & RAMP2
IAPP & PCSK1 & PCSK2→CALCR & RAMP3
IAPP & PCSK1 & PCSK2→CALCRL & RAMP1
IAPP & PCSK1 & PCSK2→CALCRL & RAMP2
IAPP & PCSK1 & PCSK2→CALCRL & RAMP3
Compartment
hematopoietic
- hematopoietic precursor
megakaryocyte-erythroid
- megakaryoid
- erythroid
myeloid
- granulocyte
- granulocyte monocyte progenitor
- monocyte/macrophage
- dendritic
12.5%
25%
50%
100%
ln(CP10K+1)
Bone
Diaphragm
Limb muscle
Mammary gland
Fat
Skin
Eye retina
Hypothalamus
Pituitary
Brainstem
Brain cortex
Testes
Uterus
Bladder
Kidney
Pancreas
Liver
Small intestine
Tongue
Trachea
Lung
Aorta
Heart
Spleen
Blood
Bone marrow

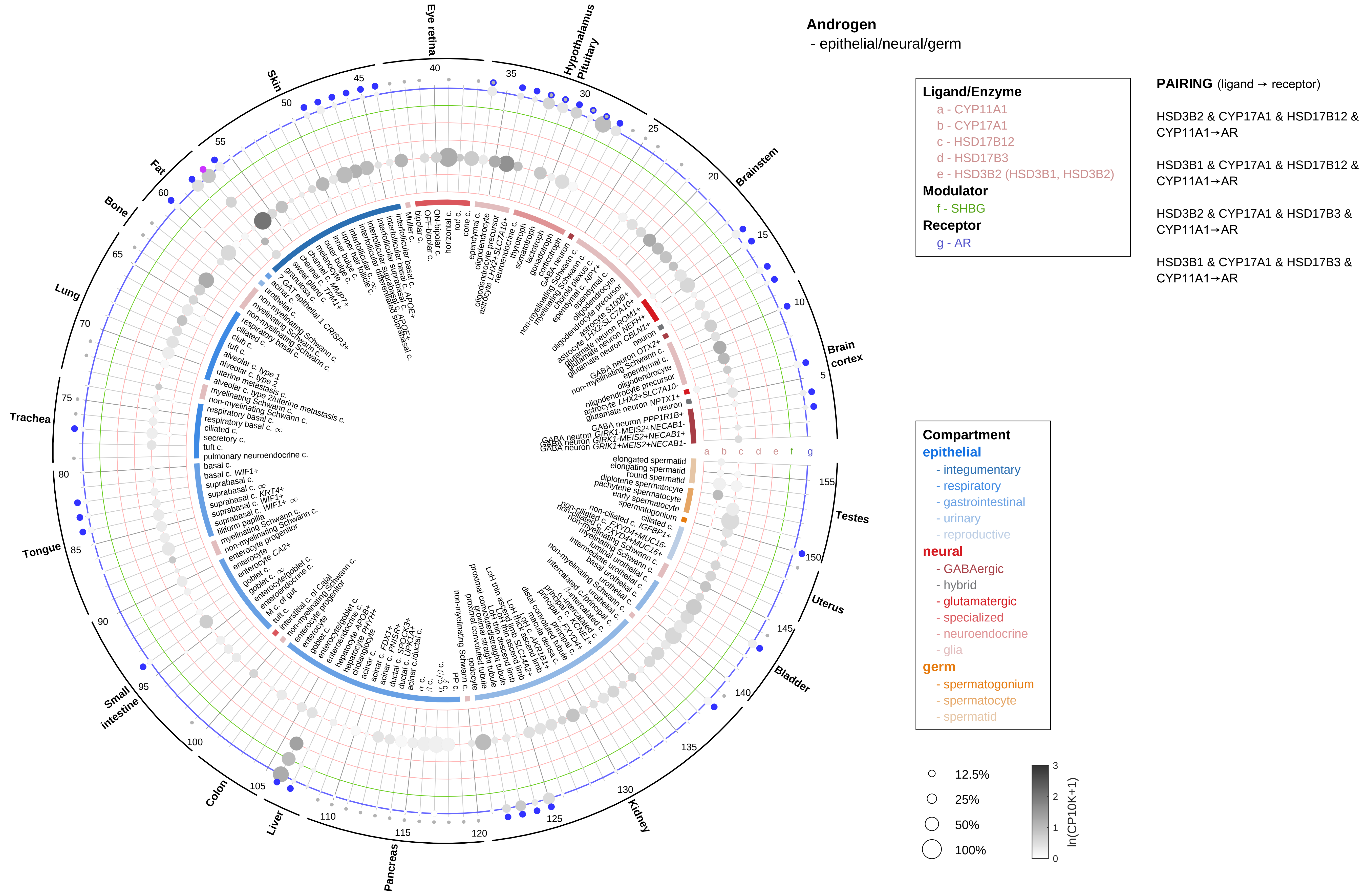
Androgen
- epithelial/neural/germ
Ligand/Enzyme
a - CYP11A1
b - CYP17A1
c - HSD17B12
d - HSD17B3
e - HSD3B2 (HSD3B1, HSD3B2)
Modulator
f - SHBG
Receptor
g - AR
PAIRING (ligand → receptor)
HSD3B2 & CYP17A1 & HSD17B12 & CYP11A1→AR
HSD3B1 & CYP17A1 & HSD17B12 & CYP11A1→AR
HSD3B2 & CYP17A1 & HSD17B3 & CYP11A1→AR
HSD3B1 & CYP17A1 & HSD17B3 & CYP11A1→AR
Compartment
epithelial
- integumentary
- respiratory
- gastrointestinal
- urinary
- reproductive
neural
- GABAergic
- hybrid
- glutamatergic
- specialized
- neuroendocrine
- glia
germ
- spermatogonium
- spermatocyte
- spermatid
12.5%
25%
50%
100%
ln(CP10K+1)
Eye retina
Hypothalamus Pituitary
Brainstem
Brain cortex
Testes
Uterus
Bladder
Kidney
Pancreas
Liver
Colon
Small intestine
Tongue
Trachea
Lung
Bone
Fat
Skin

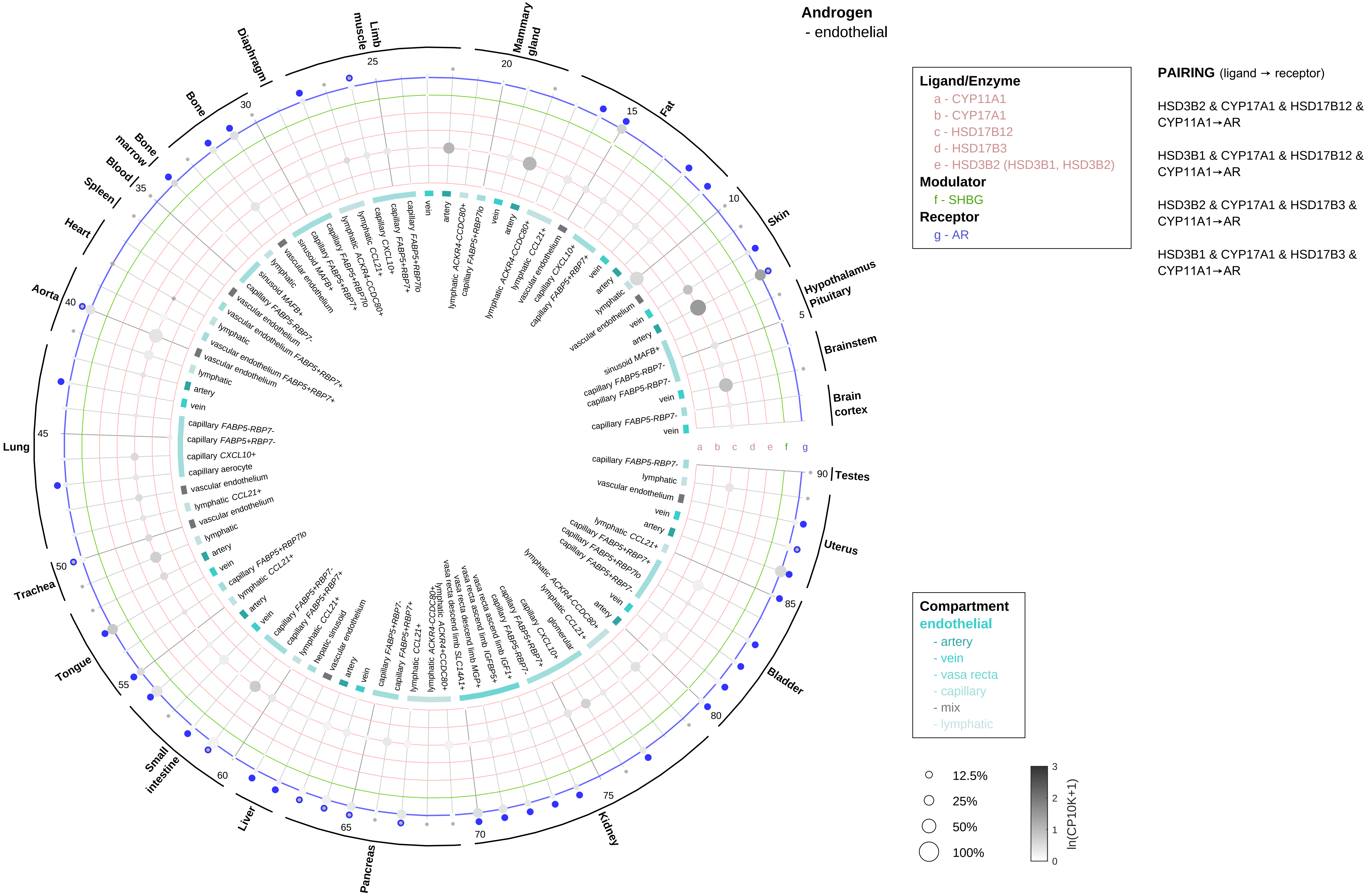

Androgen
- endothelial
Ligand/Enzyme
a - CYP11A1
b - CYP17A1
c - HSD17B12
d - HSD17B3
e - HSD3B2 (HSD3B1, HSD3B2)
Modulator
f - SHBG
Receptor
g - AR
PAIRING (ligand → receptor)
HSD3B2 & CYP17A1 & HSD17B12 & CYP11A1→AR
HSD3B1 & CYP17A1 & HSD17B12 & CYP11A1→AR
HSD3B2 & CYP17A1 & HSD17B3 & CYP11A1→AR
HSD3B1 & CYP17A1 & HSD17B3 & CYP11A1→AR
Compartment
endothelial
- artery
- vein
- vasa recta
- capillary
- mix
- lymphatic
12.5%
25%
50%
100%
ln(CP10K+1)
Brain cortex
Brainstem
Hypothalamus Pituitary
Skin
Fat
Mammary gland
Limb muscle
Diaphragm
Bone
Bone marrow
Blood
Spleen
Heart
Aorta
Lung
Trachea
Tongue
Small intestine
Liver
Pancreas
Kidney
Bladder
Uterus
Testes

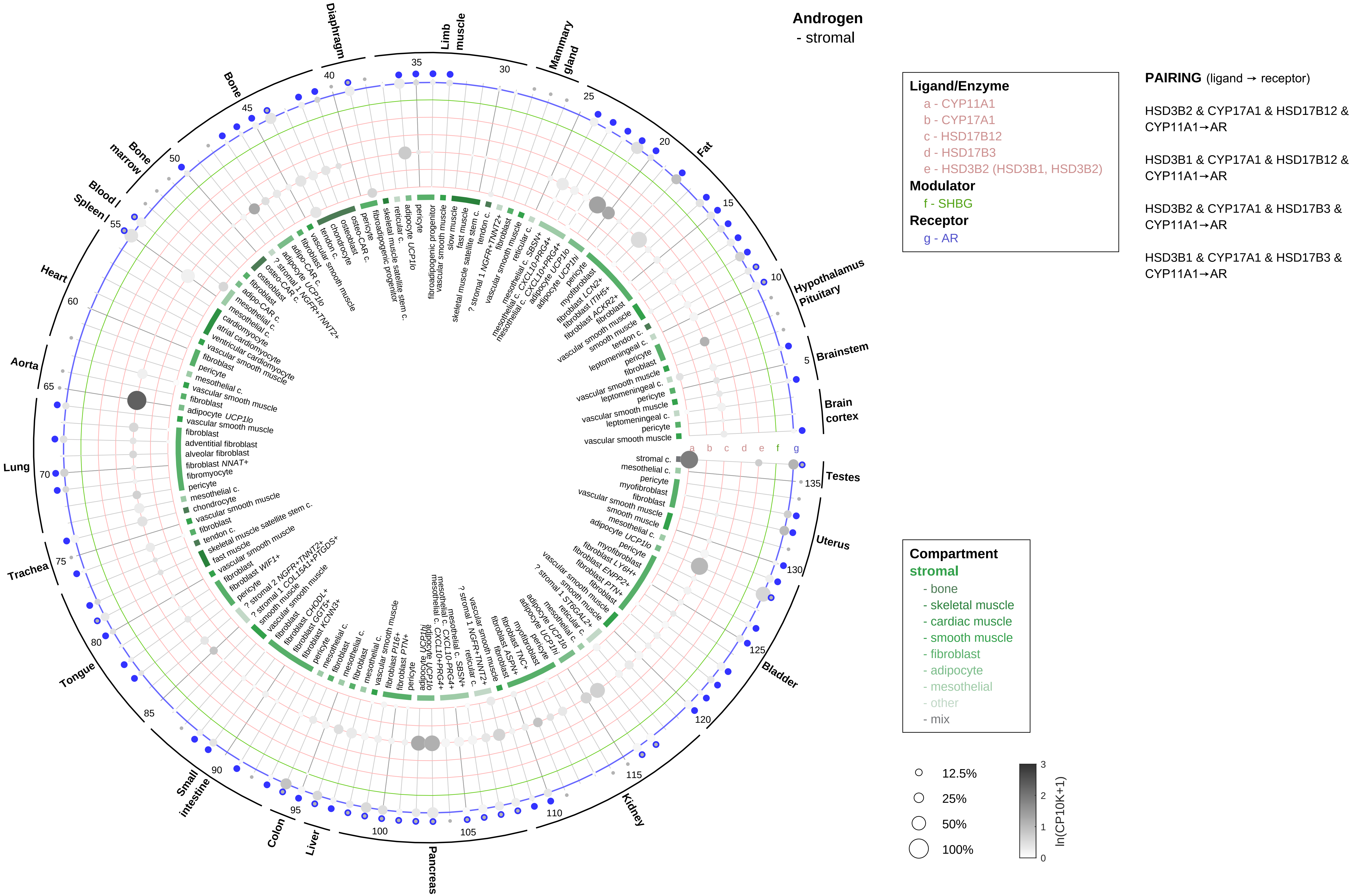

Androgen
- stromal
Ligand/Enzyme
a - CYP11A1
b - CYP17A1
c - HSD17B12
d - HSD17B3
e - HSD3B2 (HSD3B1, HSD3B2)
Modulator
f - SHBG
Receptor
g - AR
PAIRING (ligand → receptor)
HSD3B2 & CYP17A1 & HSD17B12 & CYP11A1→AR
HSD3B1 & CYP17A1 & HSD17B12 & CYP11A1→AR
HSD3B2 & CYP17A1 & HSD17B3 & CYP11A1→AR
HSD3B1 & CYP17A1 & HSD17B3 & CYP11A1→AR
Compartment
stromal
- bone
- skeletal muscle
- cardiac muscle
- smooth muscle
- fibroblast
- adipocyte
- mesothelial
- other
- mix
12.5%
25%
50%
100%
ln(CP10K+1)
Brain cortex
Brainstem
Hypothalamus Pituitary
Fat
Mammary gland
Limb muscle
Diaphragm
Bone
Bone marrow
Blood
Spleen
Heart
Aorta
Lung
Trachea
Tongue
Small intestine
Colon
Liver
Pancreas
Kidney
Bladder
Uterus
Testes

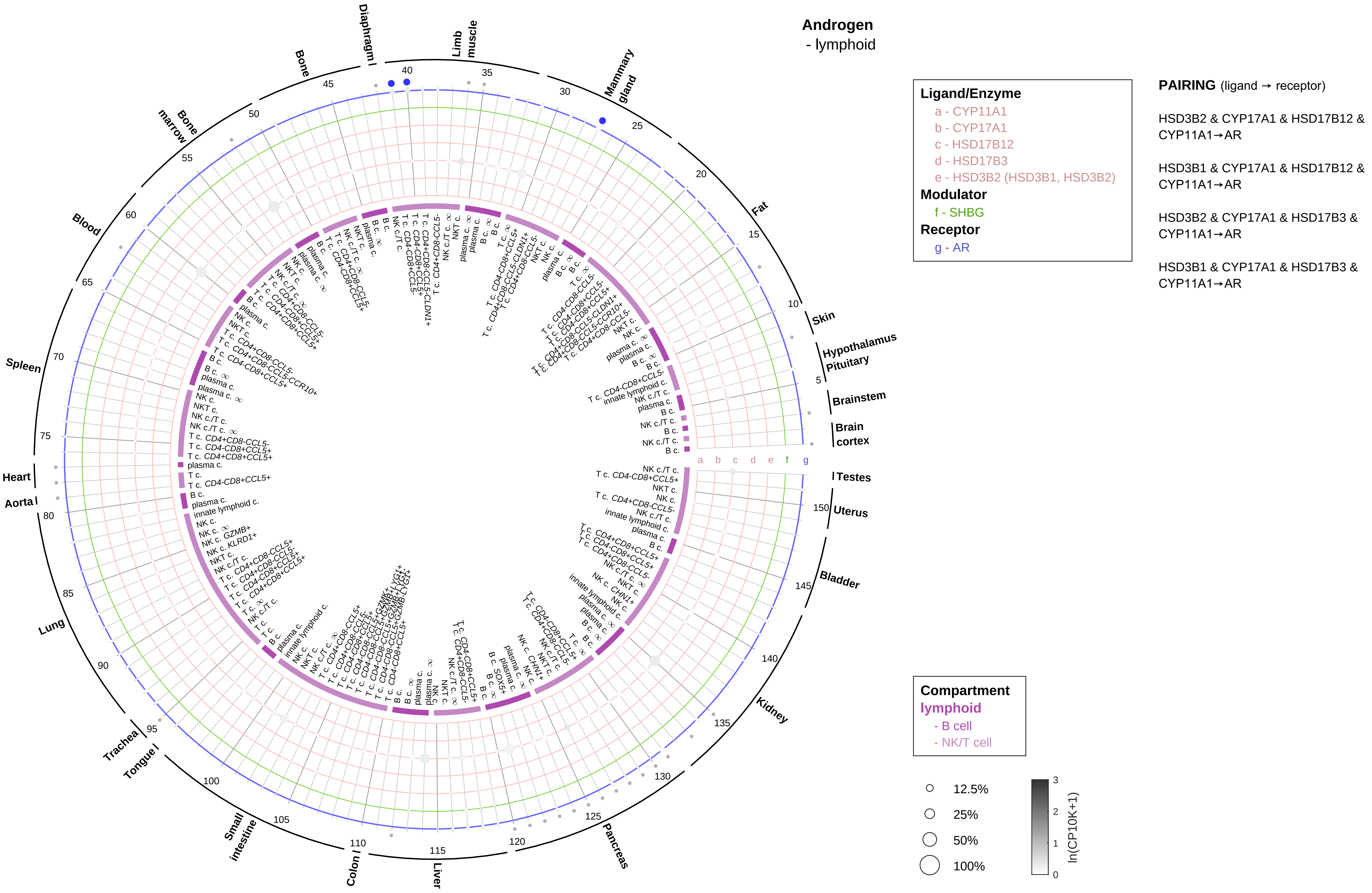

Androgen
- lymphoid
Ligand/Enzyme
a - CYP11A1
b - CYP17A1
c - HSD17B12
d - HSD17B3
e - HSD3B2 (HSD3B1, HSD3B2)
Modulator
f - SHBG
Receptor
g - AR
PAIRING (ligand → receptor)
HSD3B2 & CYP17A1 & HSD17B12 & CYP11A1→AR
HSD3B1 & CYP17A1 & HSD17B12 & CYP11A1→AR
HSD3B2 & CYP17A1 & HSD17B3 & CYP11A1→AR
HSD3B1 & CYP17A1 & HSD17B3 & CYP11A1→AR
Compartment
lymphoid
- B cell
- NK/T cell
12.5%
25%
50%
100%
ln(CP10K+1)
Diaphragm
Limb muscle
Mammary gland
Fat
Skin
Hypothalamus
Pituitary
Brainstem
Brain cortex
Testes
Uterus
Bladder
Kidney
Pancreas
Liver
Colon
Small intestine
Tongue
Trachea
Lung
Aorta
Heart
Spleen
Blood
Bone marrow
Bone

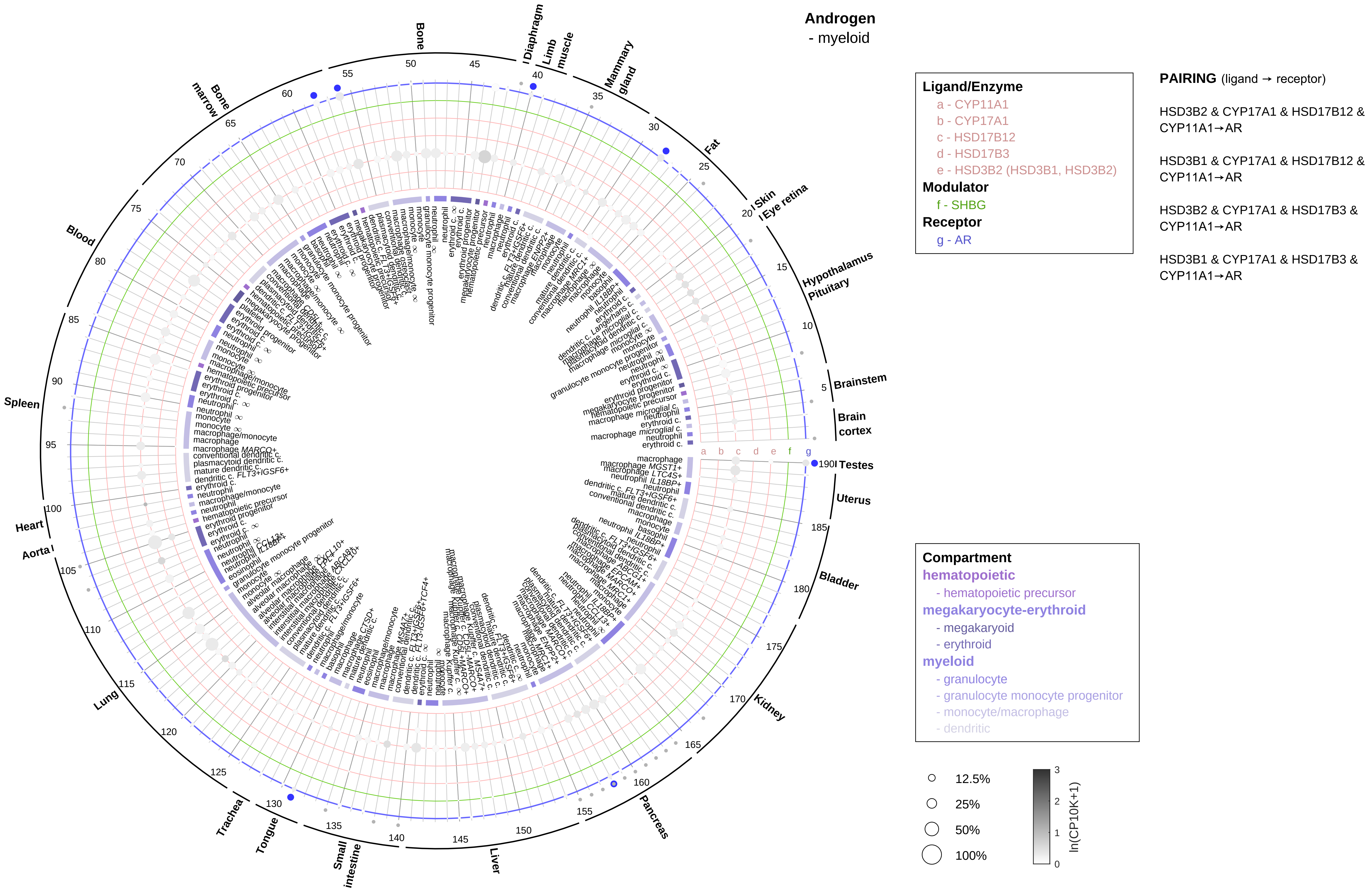
Androgen
- myeloid
Ligand/Enzyme
a - CYP11A1
b - CYP17A1
c - HSD17B12
d - HSD17B3
e - HSD3B2 (HSD3B1, HSD3B2)
Modulator
f - SHBG
Receptor
g - AR
PAIRING (ligand → receptor)
HSD3B2 & CYP17A1 & HSD17B12 & CYP11A1→AR
HSD3B1 & CYP17A1 & HSD17B12 & CYP11A1→AR
HSD3B2 & CYP17A1 & HSD17B3 & CYP11A1→AR
HSD3B1 & CYP17A1 & HSD17B3 & CYP11A1→AR
Compartment
hematopoietic
- hematopoietic precursor
megakaryocyte-erythroid
- megakaryoid
- erythroid
myeloid
- granulocyte
- granulocyte monocyte progenitor
- monocyte/macrophage
- dendritic
12.5%
25%
50%
100%
ln(CP10K+1)
Bone
Diaphragm
Limb muscle
Mammary gland
Fat
Skin
Eye retina
Hypothalamus
Pituitary
Brainstem
Brain cortex
Testes
Uterus
Bladder
Kidney
Pancreas
Liver
Small intestine
Tongue
Trachea
Lung
Aorta
Heart
Spleen
Blood
Bone marrow

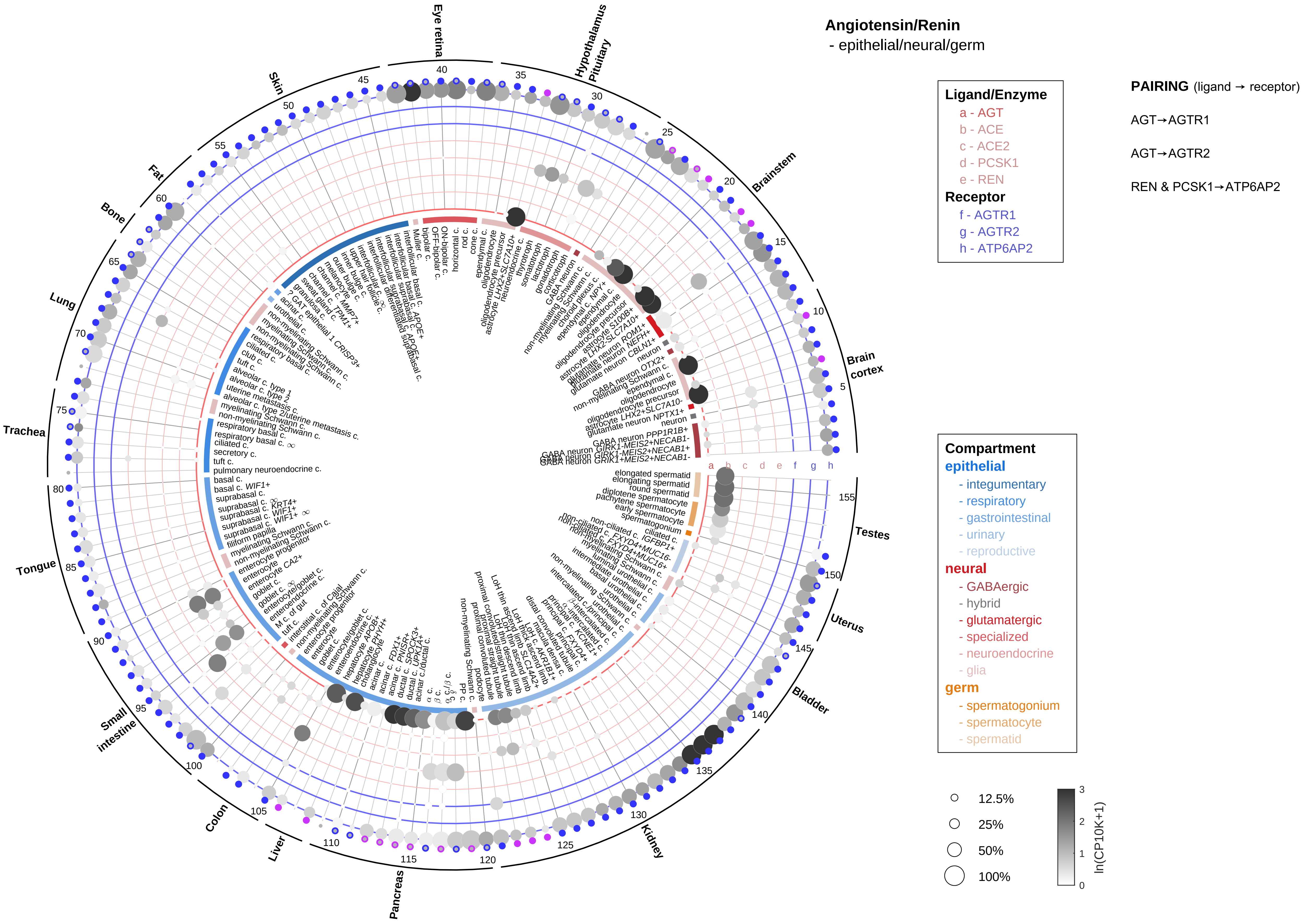
Angiotensin/Renin
- epithelial/neural/germ
Ligand/Enzyme
a - AGT
b - ACE
c - ACE2
d - PCSK1
e - REN
Receptor
f - AGTR1
g - AGTR2
h - ATP6AP2
PAIRING (ligand → receptor)
AGT→AGTR1
AGT→AGTR2
REN & PCSK1→ATP6AP2
Compartment
epithelial
- integumentary
- respiratory
- gastrointestinal
- urinary
- reproductive
neural
- GABAergic
- hybrid
- glutamatergic
- specialized
- neuroendocrine
- glia
germ
- spermatogonium
- spermatocyte
- spermatid
12.5%
25%
50%
100%
ln(CP10K+1)
Eye retina
Hypothalamus Pituitary
Brainstem
Brain cortex
Testes
Uterus
Bladder
Kidney
Pancreas
Liver
Colon
Small intestine
Tongue
Trachea
Lung
Bone
Fat
Skin

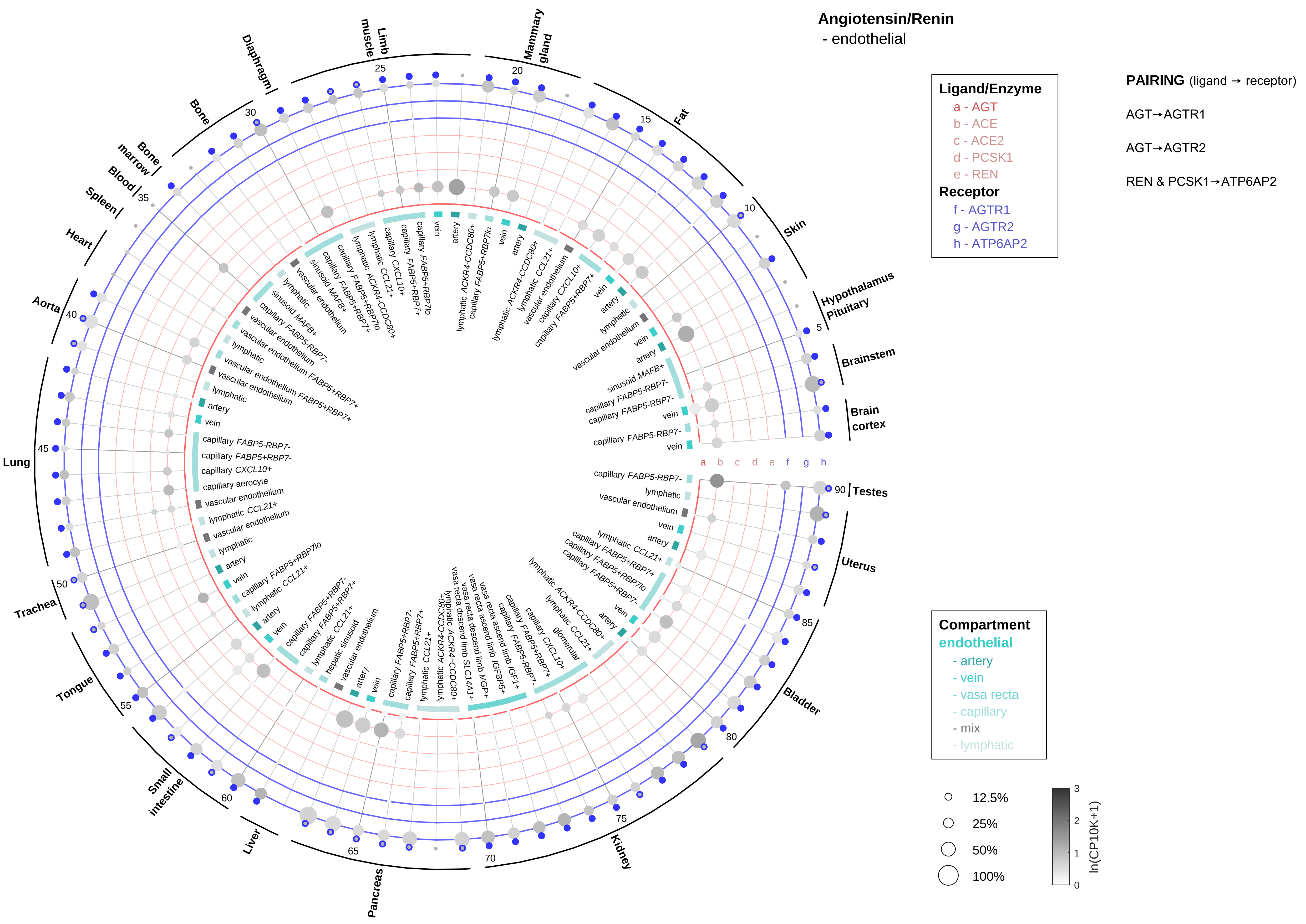
Angiotensin/Renin
- endothelial
Ligand/Enzyme
a - AGT
b - ACE
c - ACE2
d - PCSK1
e - REN
Receptor
f - AGTR1
g - AGTR2
h - ATP6AP2
PAIRING (ligand → receptor)
AGT→AGTR1
AGT→AGTR2
REN & PCSK1→ATP6AP2
Compartment
endothelial
- artery
- vein
- vasa recta
- capillary
- mix
- lymphatic
12.5%
25%
50%
100%
ln(CP10K+1)
Brain cortex
Brainstem
Hypothalamus Pituitary
Skin
Fat
Mammary gland
Limb muscle
Diaphragm
Bone
Bone marrow
Blood
Spleen
Heart
Aorta
Lung
Trachea
Tongue
Small intestine
Liver
Pancreas
Kidney
Bladder
Uterus
Testes

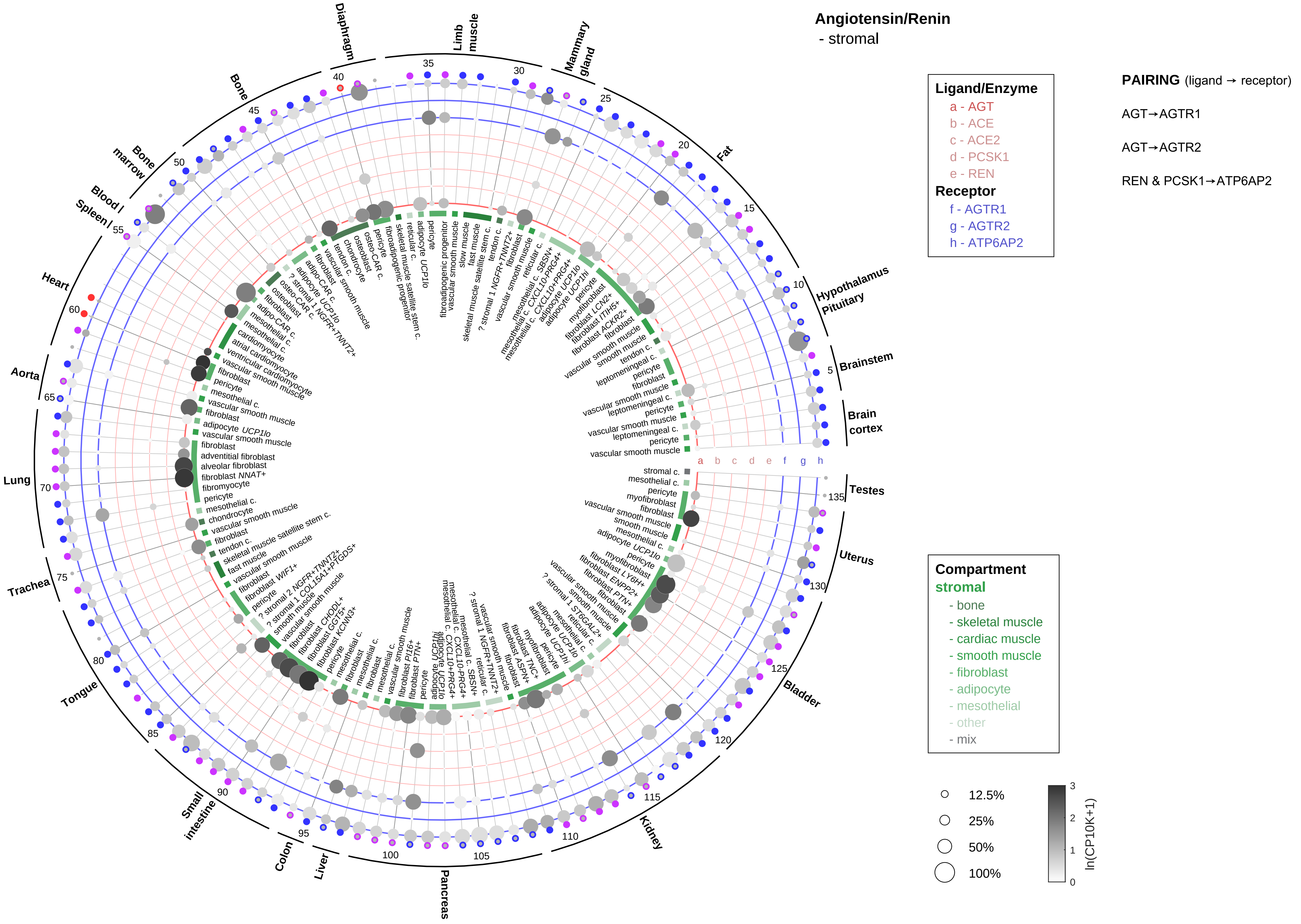

Angiotensin/Renin
- stromal
Ligand/Enzyme
a - AGT
b - ACE
c - ACE2
d - PCSK1
e - REN
Receptor
f - AGTR1
g - AGTR2
h - ATP6AP2
PAIRING (ligand → receptor)
AGT→AGTR1
AGT→AGTR2
REN & PCSK1→ATP6AP2
Compartment
stromal
- bone
- skeletal muscle
- cardiac muscle
- smooth muscle
- fibroblast
- adipocyte
- mesothelial
- other
- mix
12.5%
25%
50%
100%
ln(CP10K+1)
Diaphragm
Limb muscle
Mammary gland
Fat
Hypothalamus Pituitary
Brainstem
Brain cortex
Testes
Uterus
Bladder
Kidney
Pancreas
Liver
Colon
Small intestine
Tongue
Trachea
Lung
Aorta
Heart
Spleen
Blood
Bone marrow
Bone

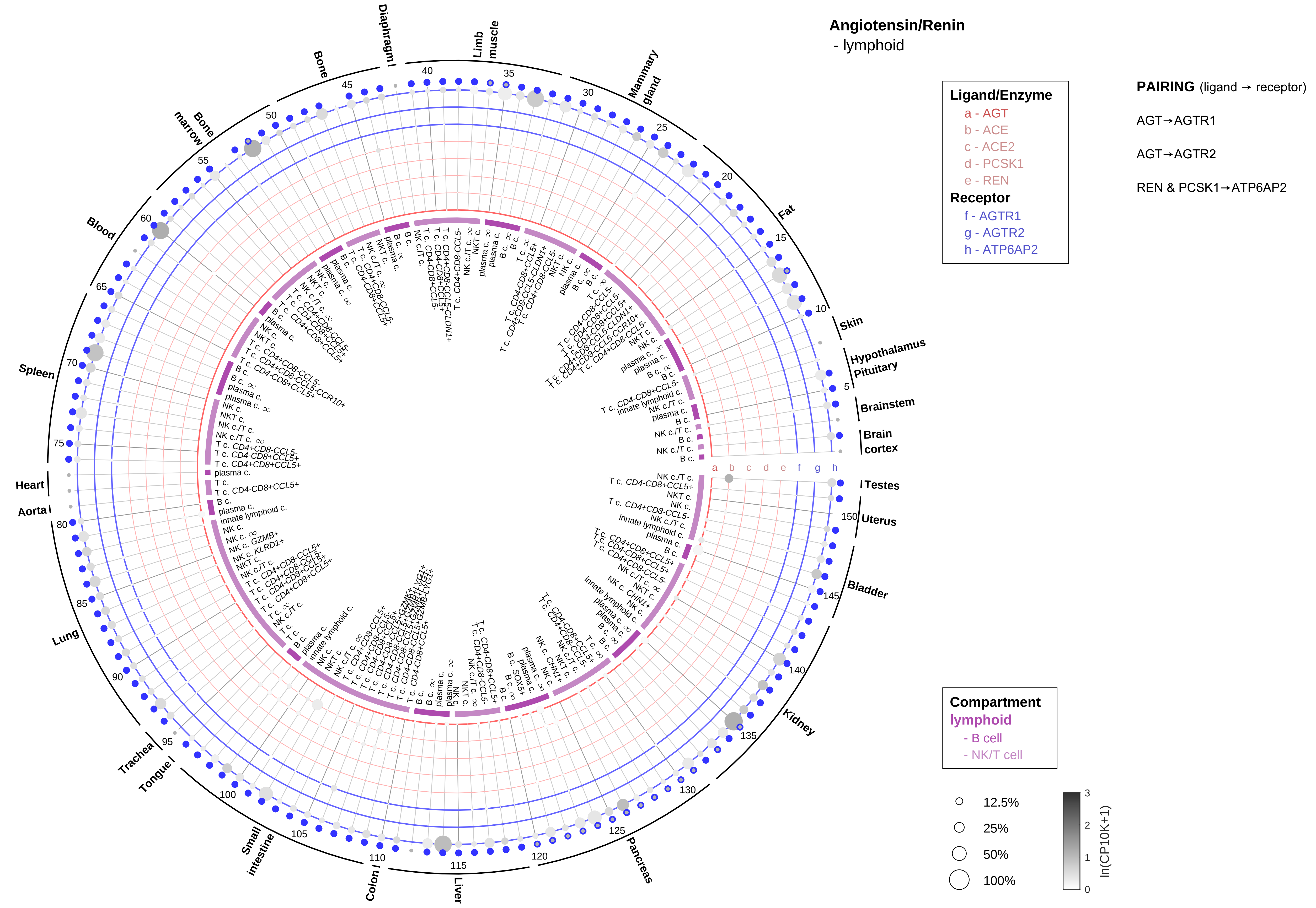
Angiotensin/Renin
- lymphoid
Ligand/Enzyme
a - AGT
b - ACE
c - ACE2
d - PCSK1
e - REN
Receptor
f - AGTR1
g - AGTR2
h - ATP6AP2
PAIRING (ligand → receptor)
AGT→AGTR1
AGT→AGTR2
REN & PCSK1→ATP6AP2
Compartment
lymphoid
- B cell
- NK/T cell
12.5%
25%
50%
100%
ln(CP10K+1)
Skin
Fat
Mammary gland
Limb muscle
Diaphragm
Bone
Bone marrow
Blood
Spleen
Heart
Aorta
Lung
Trachea
Tongue
Small intestine
Colon
Liver
Pancreas
Kidney
Bladder
Uterus
Testes
Brain cortex
Brainstem
Pituitary
Hypothalamus

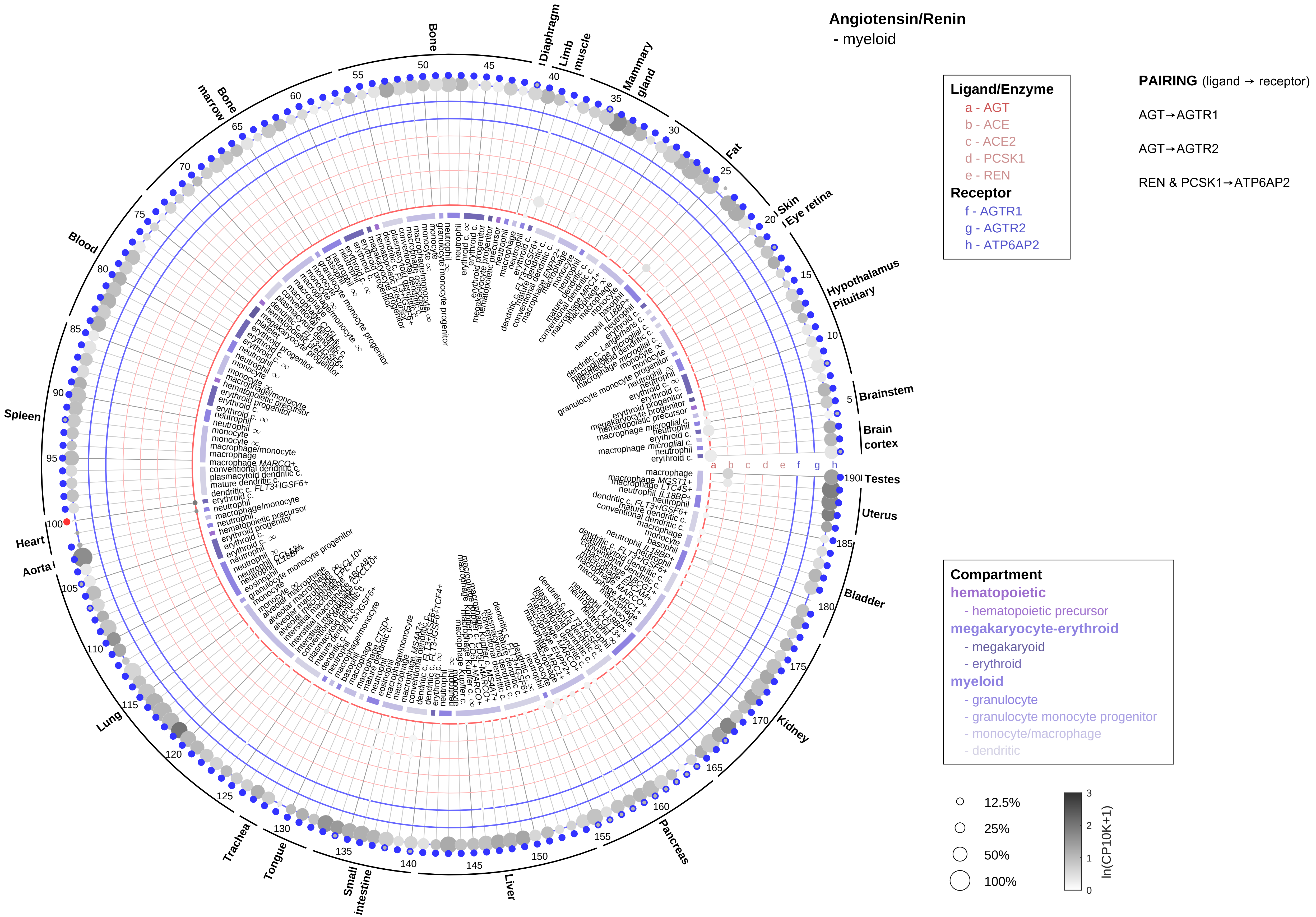
Angiotensin/Renin
- myeloid
Ligand/Enzyme
a - AGT
b - ACE
c - ACE2
d - PCSK1
e - REN
Receptor
f - AGTR1
g - AGTR2
h - ATP6AP2
PAIRING (ligand → receptor)
AGT→AGTR1
AGT→AGTR2
REN & PCSK1→ATP6AP2
Compartment
hematopoietic
- hematopoietic precursor
megakaryocyte-erythroid
- megakaryoid
- erythroid
myeloid
- granulocyte
- granulocyte monocyte progenitor
- monocyte/macrophage
- dendritic
12.5%
25%
50%
100%
ln(CP10K+1)
Testes
Uterus
Bladder
Kidney
Pancreas
Liver
Small intestine
Tongue
Trachea
Lung
Aorta
Heart
Spleen
Blood
Bone marrow
Bone
Diaphragm
Limb muscle
Mammary gland
Fat
Skin
Eye retina
Hypothalamus
Pituitary
Brainstem
Brain cortex

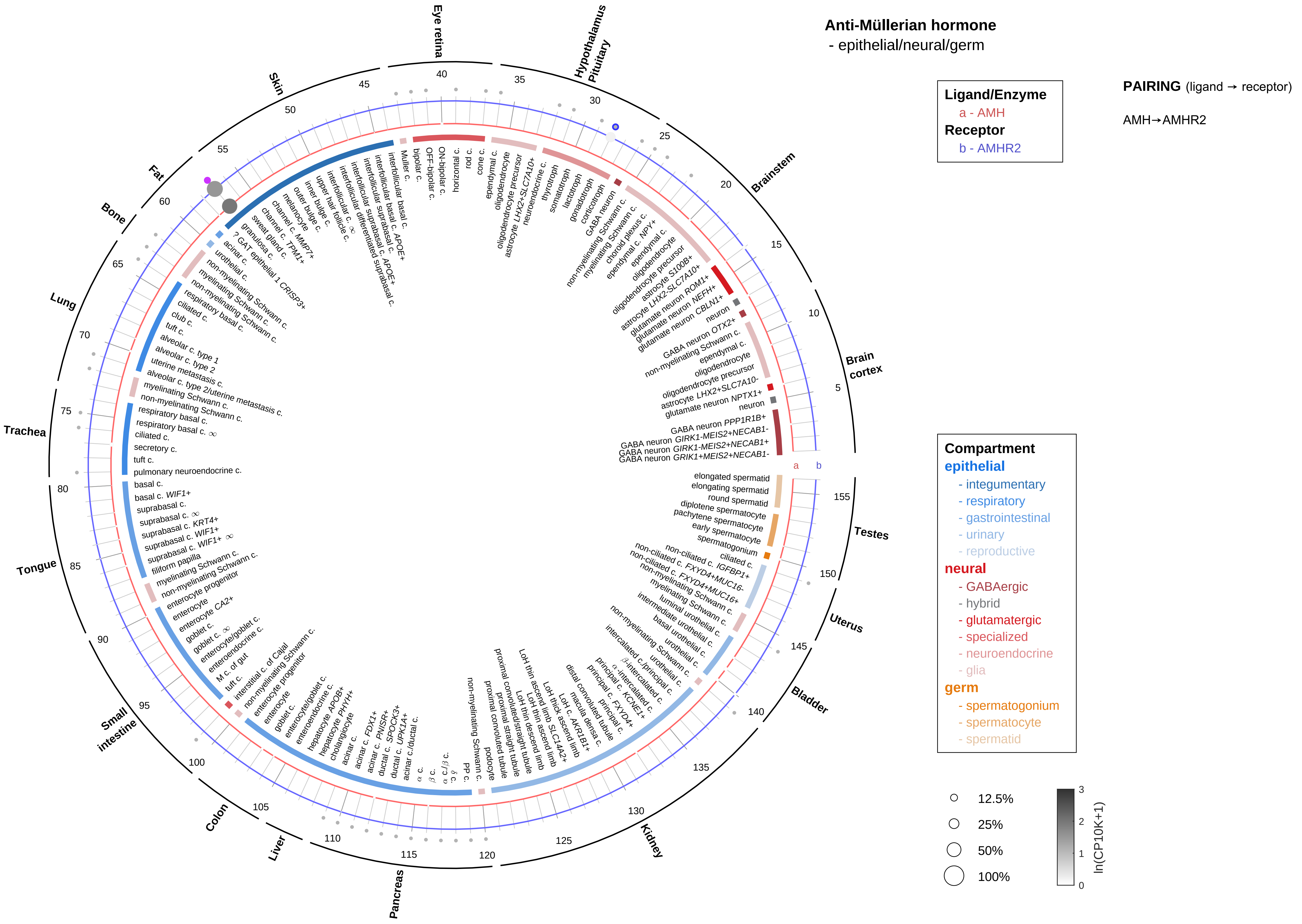
Anti-Müllerian hormone
- epithelial/neural/germ
Ligand/Enzyme
a - AMH
Receptor
b - AMHR2
PAIRING (ligand → receptor)
AMH→AMHR2
Compartment
epithelial
- integumentary
- respiratory
- gastrointestinal
- urinary
- reproductive
neural
- GABAergic
- hybrid
- glutamatergic
- specialized
- neuroendocrine
- glia
germ
- spermatogonium
- spermatocyte
- spermatid
12.5%
25%
50%
100%
ln(CP10K+1)
Eye retina
Hypothalamus Pituitary
Brainstem
Brain cortex
Testes
Uterus
Bladder
Kidney
Pancreas
Liver
Colon
Small intestine
Tongue
Trachea
Lung
Bone
Fat
Skin

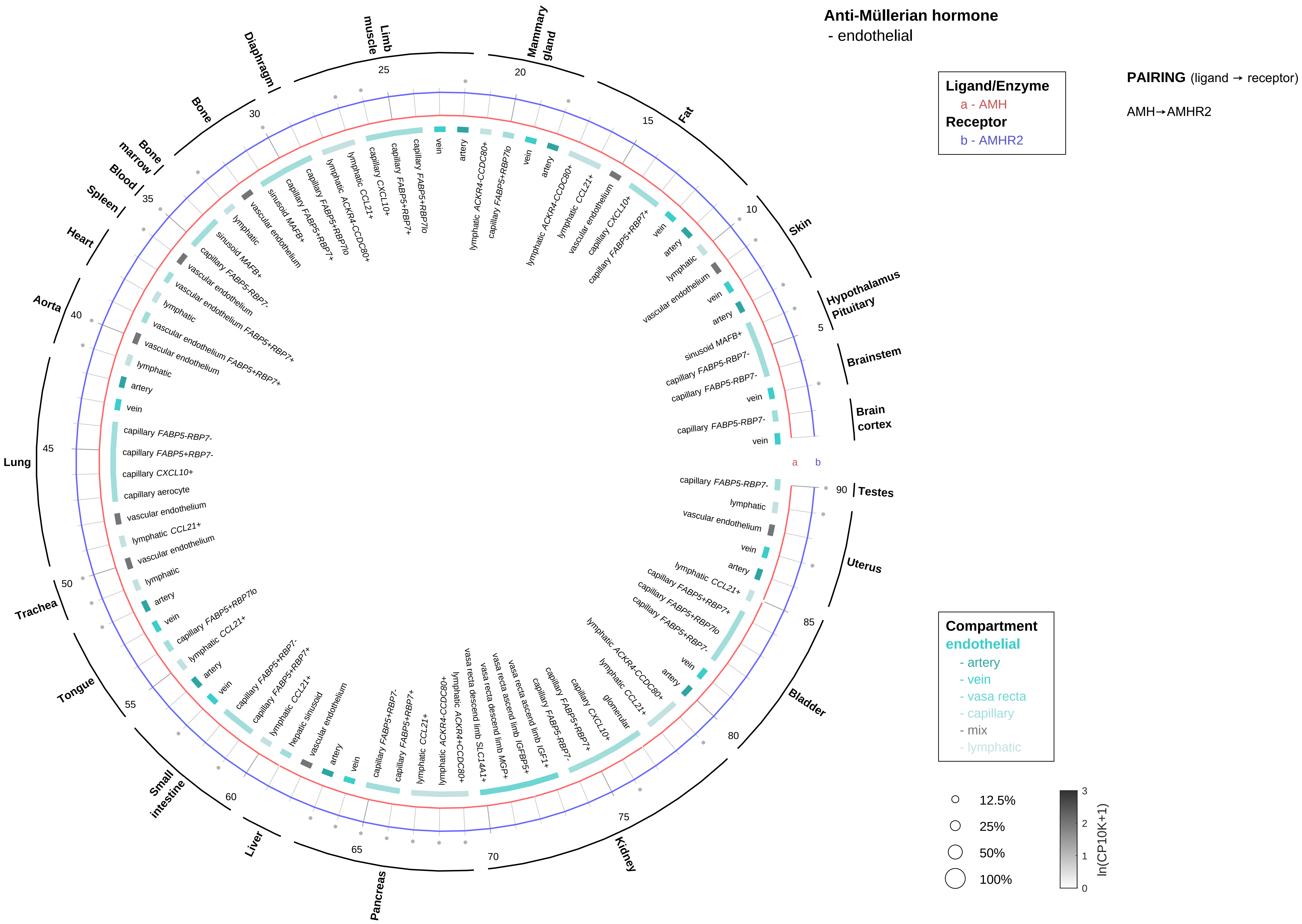
Anti-Müllerian hormone
- endothelial
Ligand/Enzyme
a - AMH
Receptor
b - AMHR2
PAIRING (ligand → receptor)
AMH→AMHR2
Compartment
endothelial
- artery
- vein
- vasa recta
- capillary
- mix
- lymphatic
12.5%
25%
50%
100%
ln(CP10K+1)
Brain cortex
Brainstem
Hypothalamus Pituitary
Skin
Fat
Mammary gland
Limb muscle
Diaphragm
Bone
Bone marrow
Blood
Spleen
Heart
Aorta
Lung
Trachea
Tongue
Small intestine
Liver
Pancreas
Kidney
Bladder
Uterus
Testes

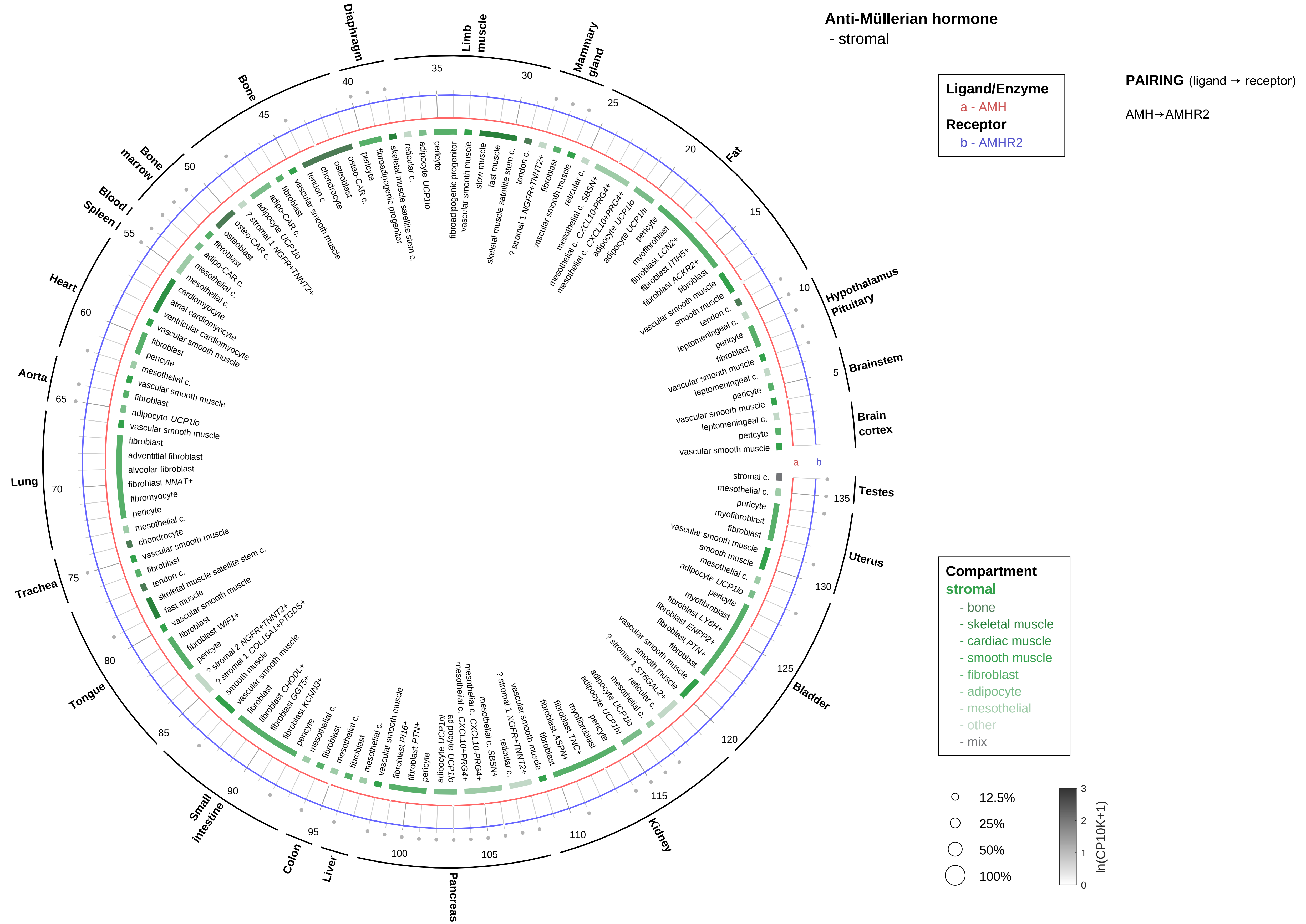

Anti-Müllerian hormone
- stromal
Ligand/Enzyme
a - AMH
Receptor
b - AMHR2
PAIRING (ligand → receptor)
AMH→AMHR2
Compartment
stromal
- bone
- skeletal muscle
- cardiac muscle
- smooth muscle
- fibroblast
- adipocyte
- mesothelial
- other
- mix
12.5%
25%
50%
100%
ln(CP10K+1)
Brain cortex
Brainstem
Hypothalamus Pituitary
Fat
Mammary gland
Limb muscle
Diaphragm
Bone
Bone marrow
Blood
Spleen
Heart
Aorta
Lung
Trachea
Tongue
Small intestine
Colon
Liver
Pancreas
Kidney
Bladder
Uterus
Testes

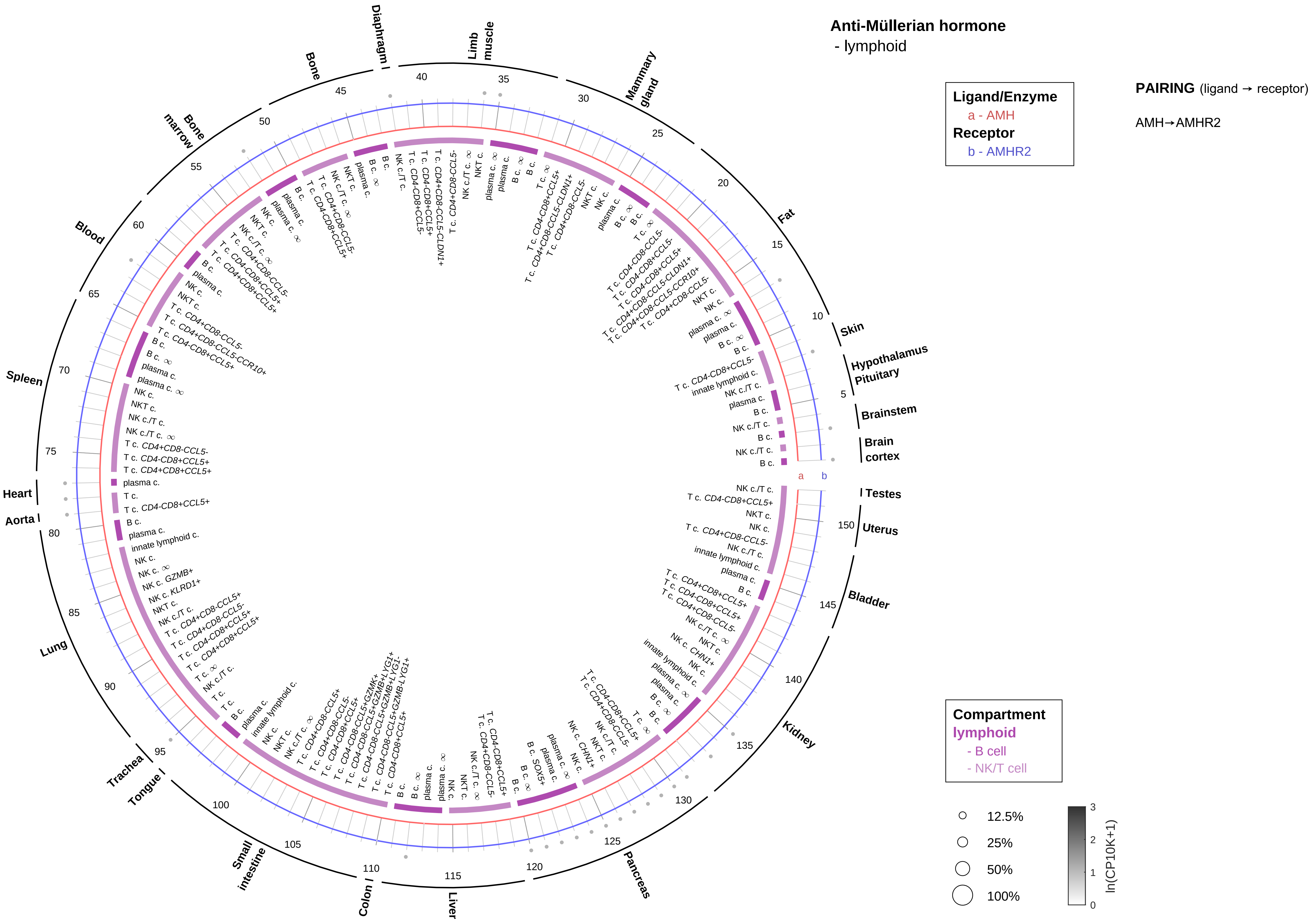

Anti-Müllerian hormone
- lymphoid
Ligand/Enzyme
a - AMH
Receptor
b - AMHR2
PAIRING (ligand → receptor)
AMH→AMHR2
Compartment
lymphoid
- B cell
- NK/T cell
12.5%
25%
50%
100%
ln(CP10K+1)
Skin
Hypothalamus
Pituitary
Brainstem
Brain cortex
Testes
Uterus
Bladder
Kidney
Pancreas
Liver
Colon
Small intestine
Tongue
Trachea
Lung
Aorta
Heart
Spleen
Blood
Bone marrow
Bone
Diaphragm
Limb muscle
Mammary gland
Fat

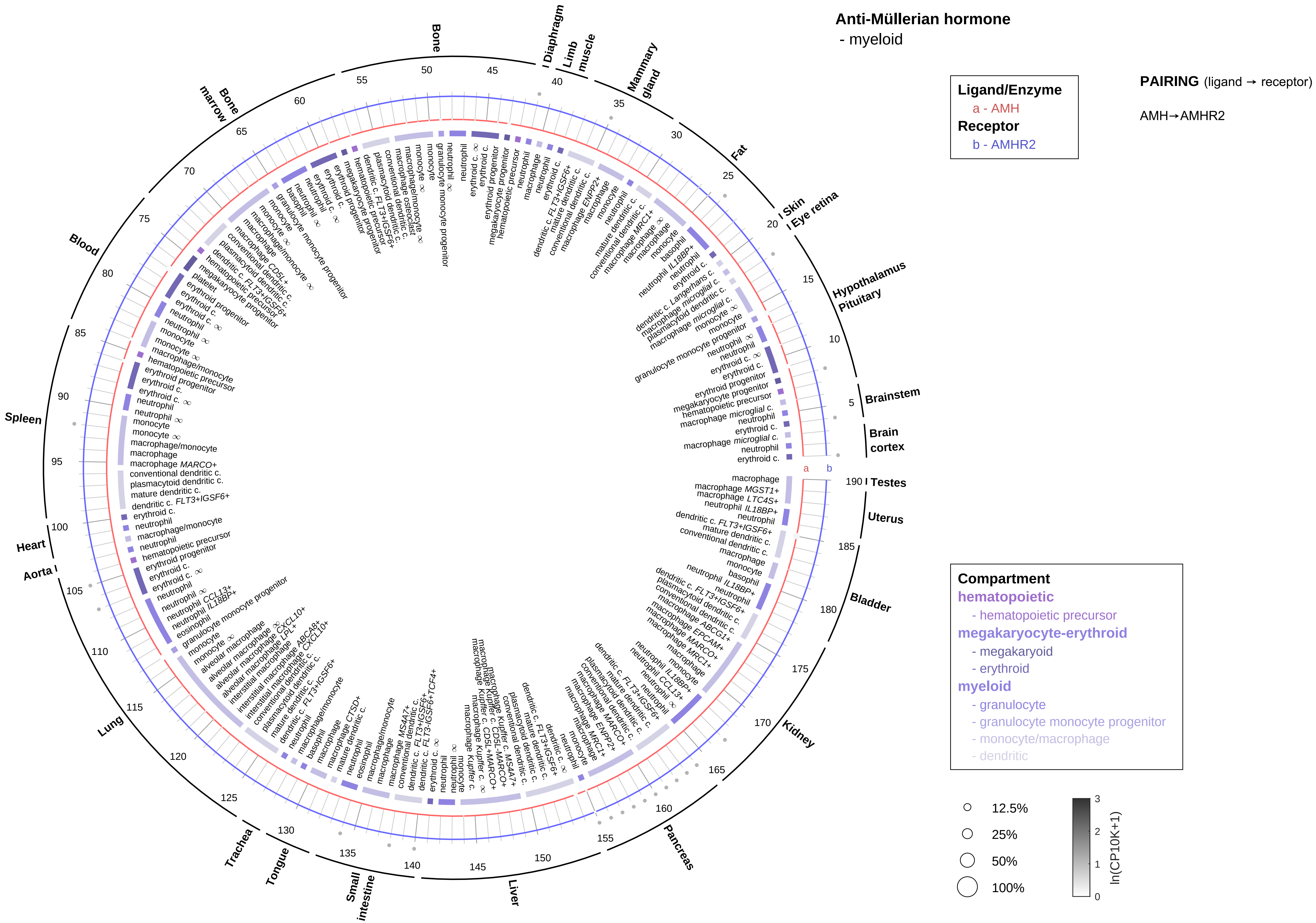

Anti-Müllerian hormone
- myeloid
Ligand/Enzyme
a - AMH
Receptor
b - AMHR2
PAIRING (ligand → receptor)
AMH→AMHR2
Compartment
hematopoietic
- hematopoietic precursor
megakaryocyte-erythroid
- megakaryoid
- erythroid
myeloid
- granulocyte
- granulocyte monocyte progenitor
- monocyte/macrophage
- dendritic
12.5%
25%
50%
100%
ln(CP10K+1)
Brain cortex
Brainstem
Hypothalamus
Pituitary
Eye retina
Skin
Fat
Mammary gland
Limb muscle
Diaphragm
Bone
Bone marrow
Blood
Spleen
Heart
Aorta
Lung
Trachea
Tongue
Small intestine
Liver
Pancreas
Kidney
Bladder
Uterus
Testes

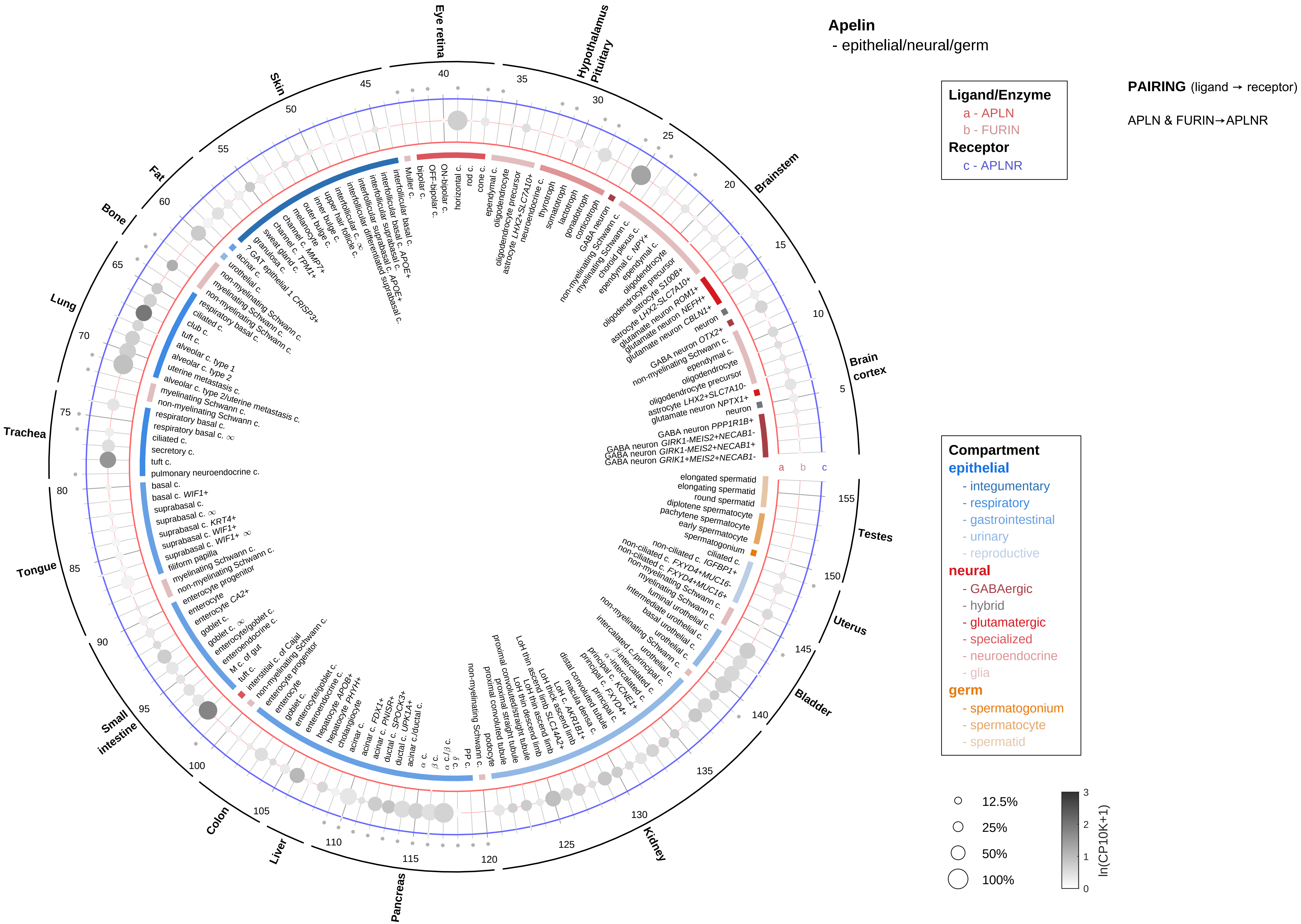

Apelin
- epithelial/neural/germ
Ligand/Enzyme
a - APLN
b - FURIN
Receptor
c - APLNR
PAIRING (ligand → receptor)
APLN & FURIN→APLNR
Compartment
epithelial
- integumentary
- respiratory
- gastrointestinal
- urinary
- reproductive
neural
- GABAergic
- hybrid
- glutamatergic
- specialized
- neuroendocrine
- glia
germ
- spermatogonium
- spermatocyte
- spermatid
12.5%
25%
50%
100%
ln(CP10K+1)
Eye retina
Hypothalamus Pituitary
Brainstem
Brain cortex
Testes
Uterus
Bladder
Kidney
Pancreas
Liver
Colon
Small intestine
Tongue
Trachea
Lung
Bone
Fat
Skin

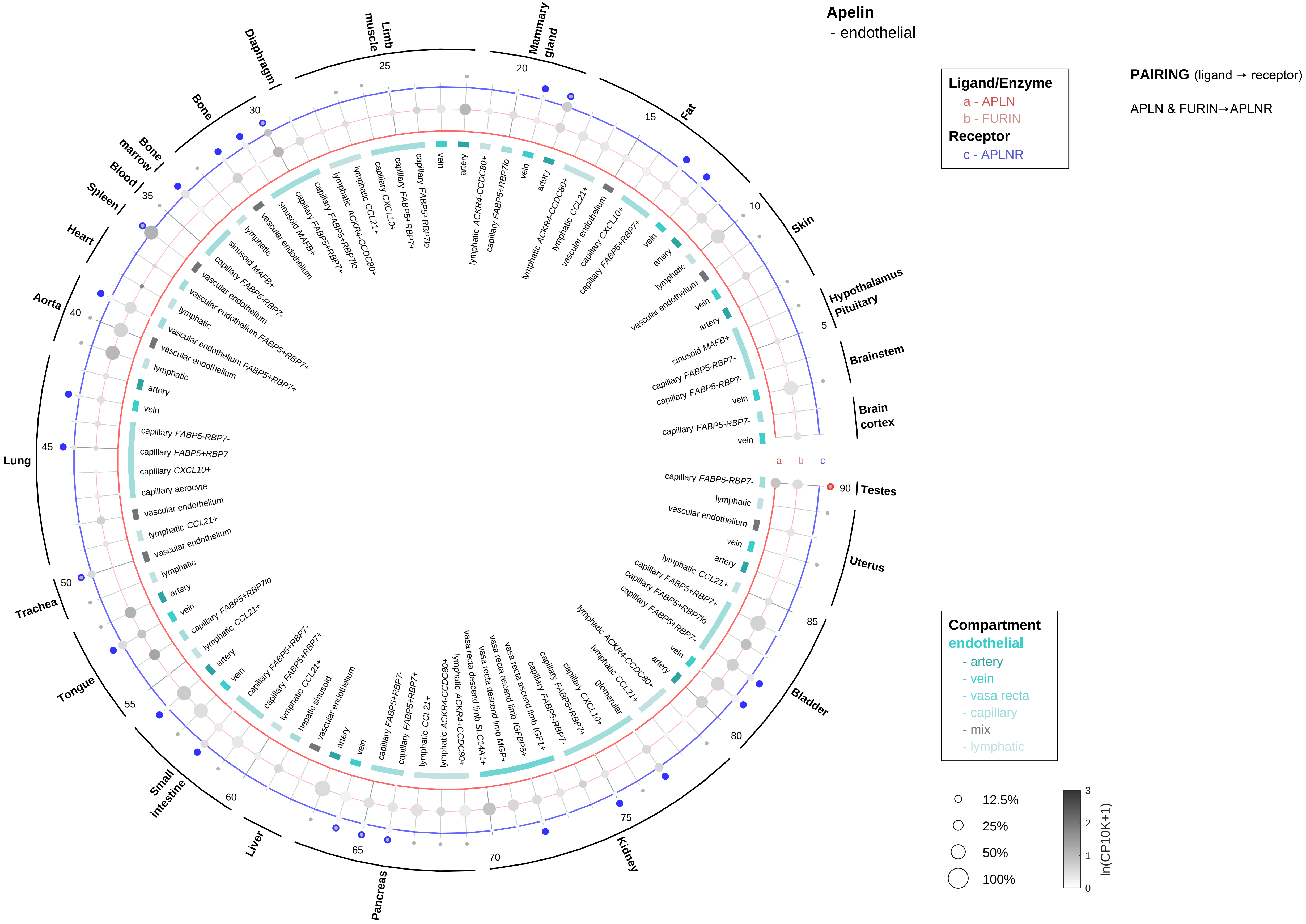

Apelin
- endothelial
Ligand/Enzyme
a - APLN
b - FURIN
Receptor
c - APLNR
PAIRING (ligand → receptor)
APLN & FURIN→APLNR
Compartment
endothelial
- artery
- vein
- vasa recta
- capillary
- mix
- lymphatic
12.5%
25%
50%
100%
ln(CP10K+1)
Brain cortex
Brainstem
Hypothalamus Pituitary
Skin
Fat
Mammary gland
Limb muscle
Diaphragm
Bone
Bone marrow
Blood
Spleen
Heart
Aorta
Lung
Trachea
Tongue
Small intestine
Liver
Pancreas
Kidney
Bladder
Uterus
Testes

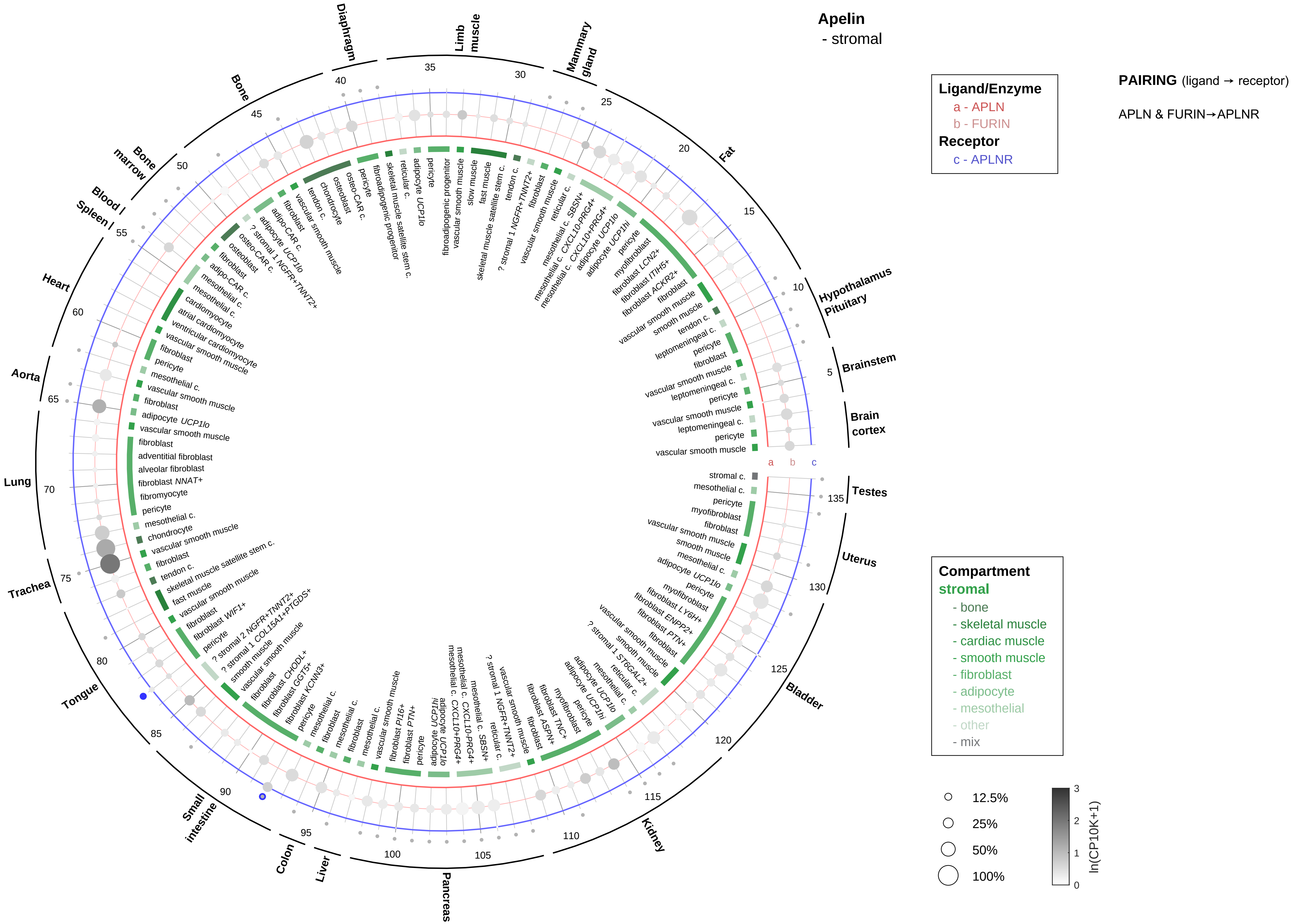
Apelin
- stromal
Ligand/Enzyme
a - APLN
b - FURIN
Receptor
c - APLNR
PAIRING (ligand → receptor)
APLN & FURIN→APLNR
Compartment
stromal
- bone
- skeletal muscle
- cardiac muscle
- smooth muscle
- fibroblast
- adipocyte
- mesothelial
- other
- mix
12.5%
25%
50%
100%
ln(CP10K+1)
Brain cortex
Brainstem
Hypothalamus Pituitary
Fat
Mammary gland
Limb muscle
Diaphragm
Bone
Bone marrow
Blood / Spleen /
Heart
Aorta
Lung
Trachea
Tongue
Small intestine
Colon
Liver
Pancreas
Kidney
Bladder
Uterus
Testes

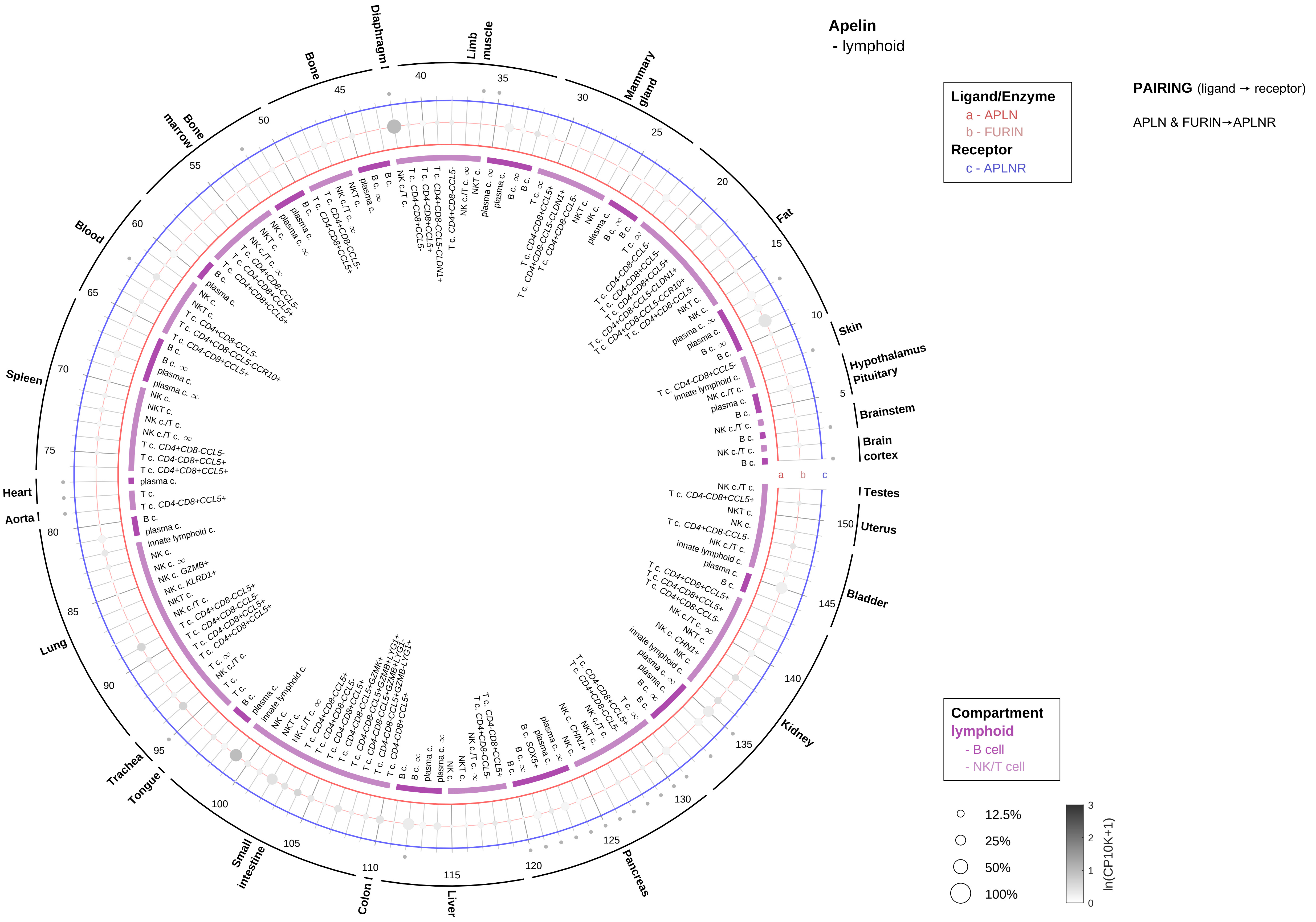

Apelin
- lymphoid
Ligand/Enzyme
a - APLN
b - FURIN
Receptor
c - APLNR
PAIRING (ligand → receptor)
APLN & FURIN→APLNR
Compartment
lymphoid
- B cell
- NK/T cell
12.5%
25%
50%
100%
ln(CP10K+1)
Skin
Hypothalamus
Pituitary
Brainstem
Brain cortex
Testes
Uterus
Bladder
Kidney
Pancreas
Liver
Colon
Small intestine
Tongue
Trachea
Lung
Aorta
Heart
Spleen
Blood
Bone marrow
Bone
Diaphragm
Limb muscle
Mammary gland
Fat

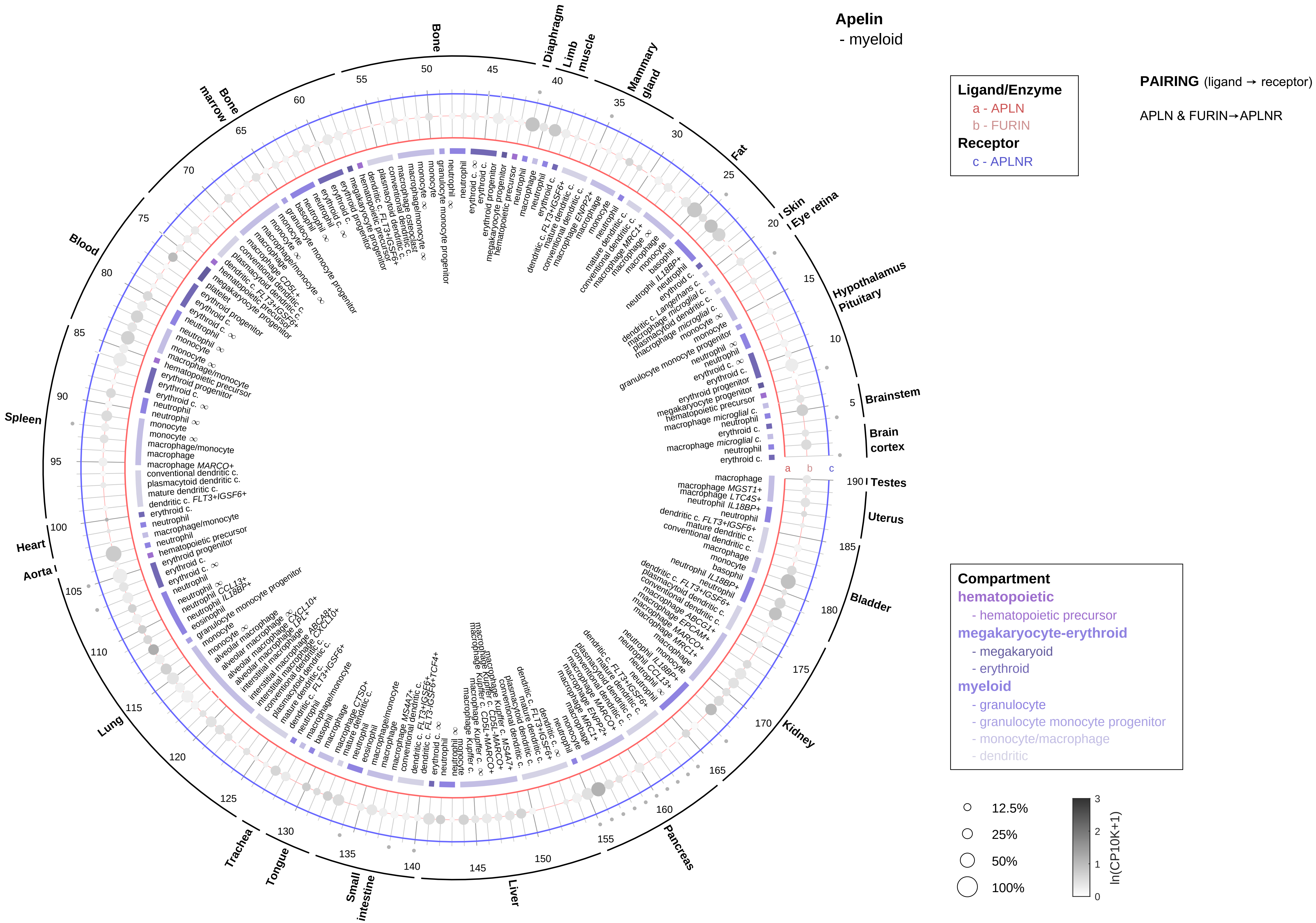
Apelin
- myeloid
Ligand/Enzyme
a - APLN
b - FURIN
Receptor
c - APLNR
PAIRING (ligand → receptor)
APLN & FURIN→APLNR
Compartment
hematopoietic
- hematopoietic precursor
megakaryocyte-erythroid
- megakaryoid
- erythroid
myeloid
- granulocyte
- granulocyte monocyte progenitor
- monocyte/macrophage
- dendritic
12.5%
25%
50%
100%
ln(CP10K+1)
Bone
Diaphragm
Limb muscle
Mammary gland
Fat
Skin
Eye retina
Hypothalamus
Pituitary
Brainstem
Brain cortex
Testes
Uterus
Bladder
Kidney
Pancreas
Liver
Small intestine
Tongue
Trachea
Lung
Aorta
Heart
Spleen
Blood
Bone marrow

Asprosin

- epithelial/neural/germ

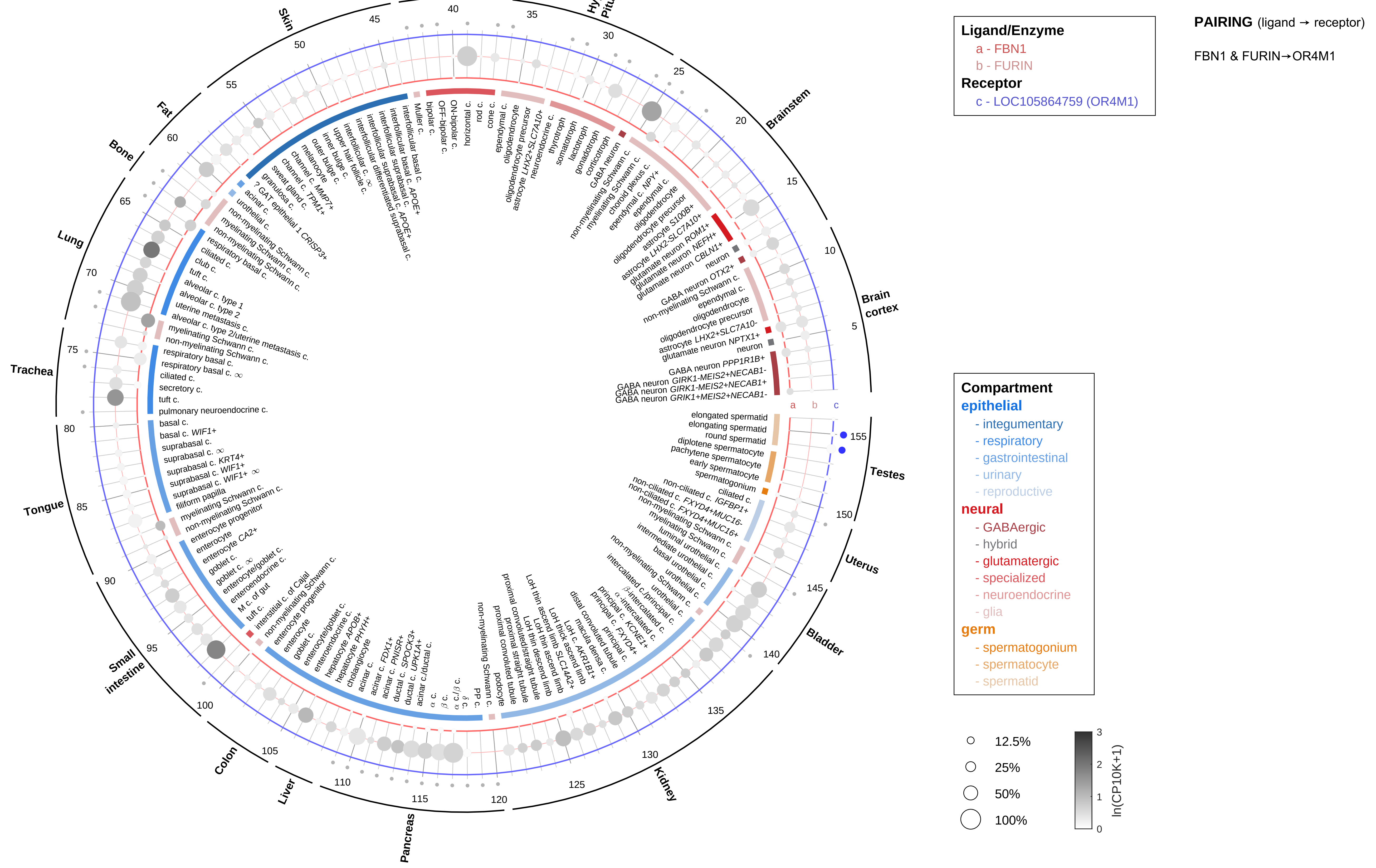

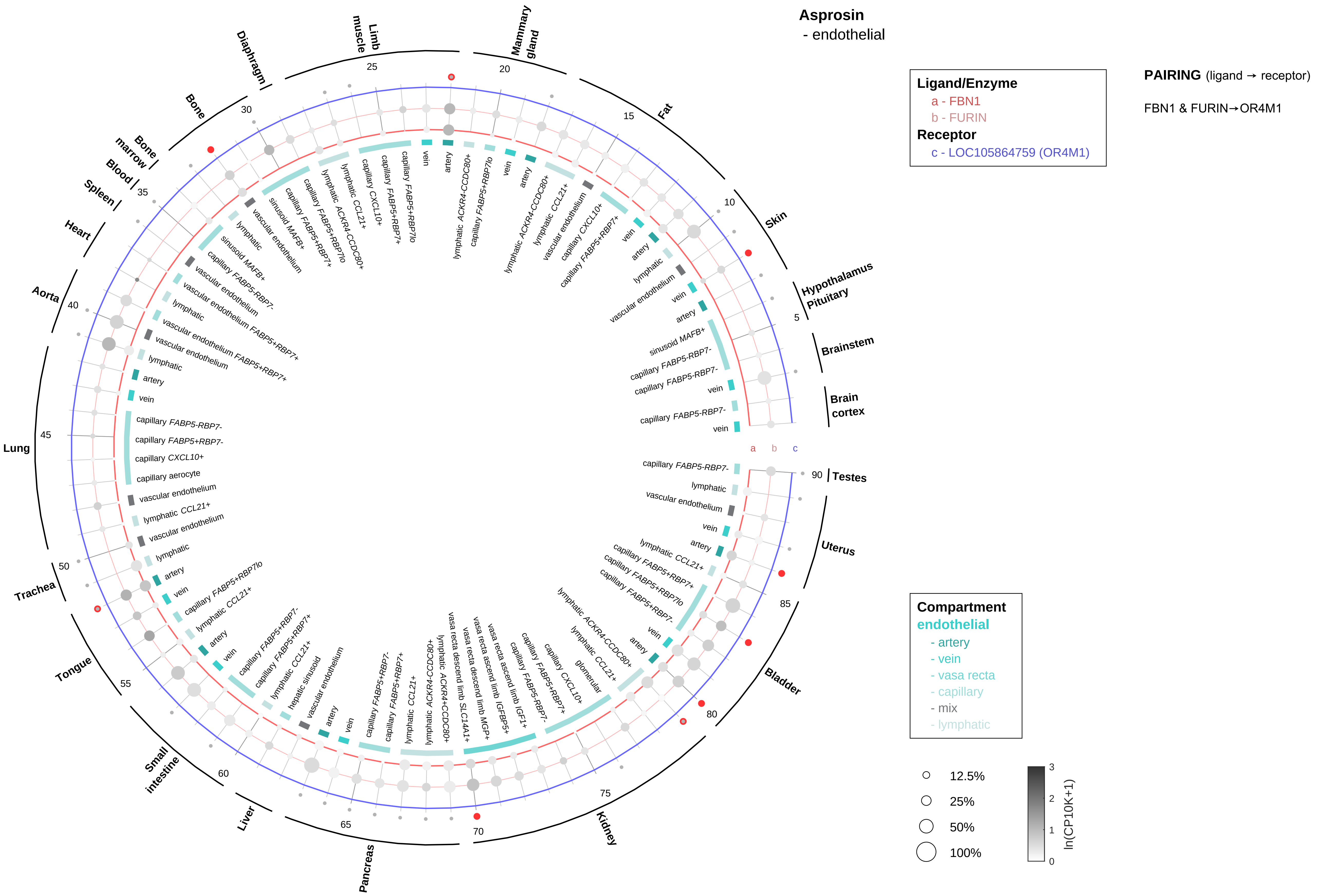
Asprosin
- endothelial
Ligand/Enzyme
a - FBN1
b - FURIN
Receptor
c - LOC105864759 (OR4M1)
PAIRING (ligand → receptor)
FBN1 & FURIN→OR4M1
Brain cortex
Brainstem
Hypothalamus Pituitary
Skin
Fat
Mammary gland
Limb muscle
Diaphragm
Bone
Bone marrow
Blood
Spleen
Heart
Aorta
Lung
Trachea
Tongue
Small intestine
Liver
Pancreas
Kidney
Bladder
Uterus
Testes
a
b
c
5
10
15
20
25
30
35
40
45
50
55
60
65
70
75
80
85
90
Compartment
endothelial
- artery
- vein
- vasa recta
- capillary
- mix
- lymphatic
12.5%
25%
50%
100%
ln(CP10K+1)
0
1
2
3

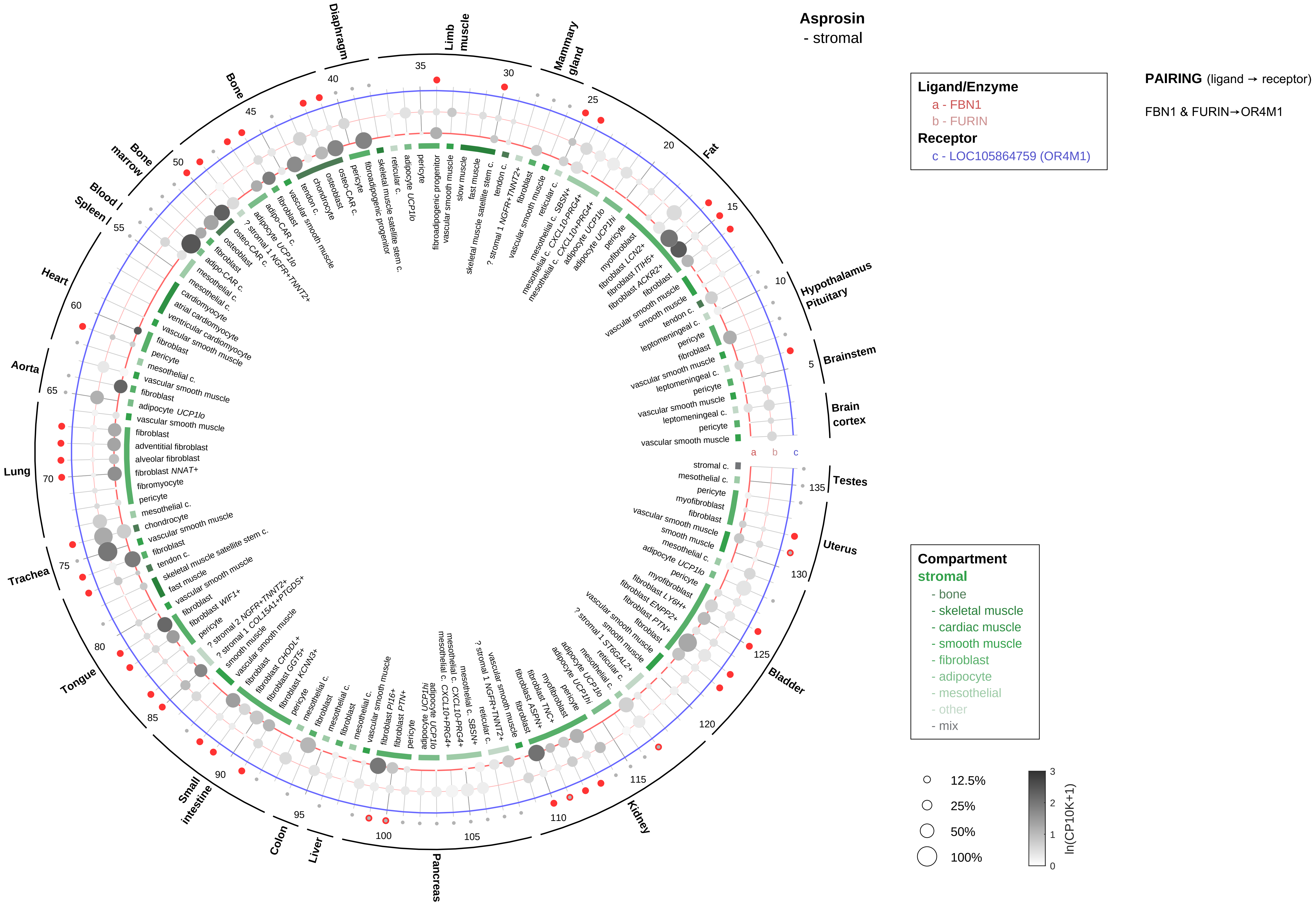
Asprosin
- stromal
Ligand/Enzyme
a - FBN1
b - FURIN
Receptor
c - LOC105864759 (OR4M1)
PAIRING (ligand → receptor)
FBN1 & FURIN→OR4M1
Compartment
stromal
- bone
- skeletal muscle
- cardiac muscle
- smooth muscle
- fibroblast
- adipocyte
- mesothelial
- other
- mix
12.5%
25%
50%
100%
ln(CP10K+1)
Brain cortex
Brainstem
Hypothalamus Pituitary
Fat
Mammary gland
Limb muscle
Diaphragm
Bone
Bone marrow
Blood / Spleen /
Heart
Aorta
Lung
Trachea
Tongue
Small intestine
Colon
Liver
Pancreas
Kidney
Bladder
Uterus
Testes

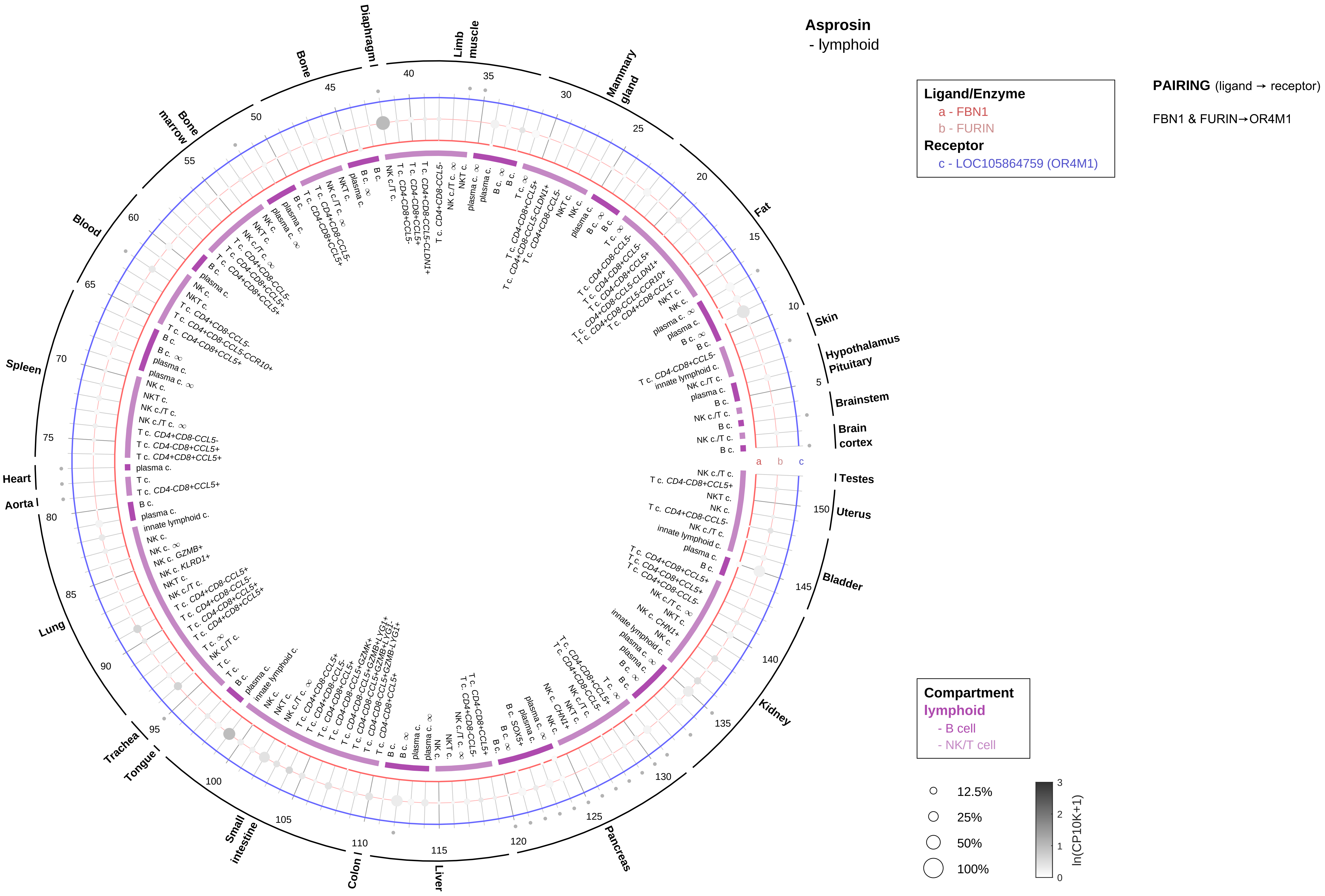

Asprosin
- lymphoid
Ligand/Enzyme
a - FBN1
b - FURIN
Receptor
c - LOC105864759 (OR4M1)
PAIRING (ligand → receptor)
FBN1 & FURIN→OR4M1
Compartment
lymphoid
- B cell
- NK/T cell
12.5%
25%
50%
100%
ln(CP10K+1)
Skin
Hypothalamus
Pituitary
Brainstem
Brain cortex
Testes
Uterus
Bladder
Kidney
Pancreas
Liver
Colon
Small intestine
Tongue
Trachea
Lung
Aorta
Heart
Spleen
Blood
Bone marrow
Bone
Diaphragm
Limb muscle
Mammary gland
Fat

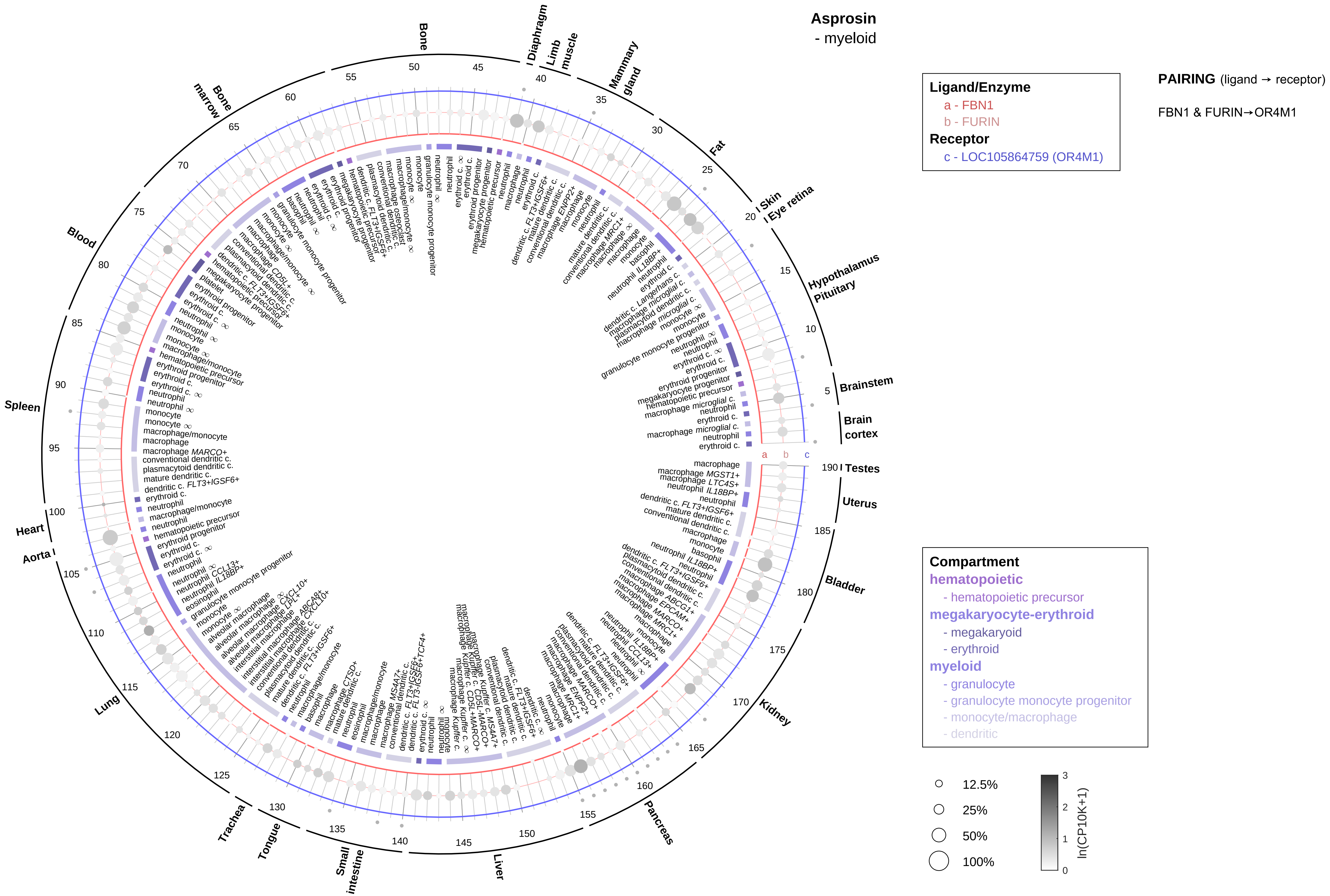

Asprosin
- myeloid
Ligand/Enzyme
a - FBN1
b - FURIN
Receptor
c - LOC105864759 (OR4M1)
PAIRING (ligand → receptor)
FBN1 & FURIN→OR4M1
Compartment
hematopoietic
- hematopoietic precursor
megakaryocyte-erythroid
- megakaryoid
- erythroid
myeloid
- granulocyte
- granulocyte monocyte progenitor
- monocyte/macrophage
- dendritic
12.5%
25%
50%
100%
ln(CP10K+1)
Bone
Diaphragm
Limb muscle
Mammary gland
Fat
Skin
Eye retina
Hypothalamus
Pituitary
Brainstem
Brain cortex
Testes
Uterus
Bladder
Kidney
Pancreas
Liver
Small intestine
Tongue
Trachea
Lung
Aorta
Heart
Spleen
Blood
Bone marrow

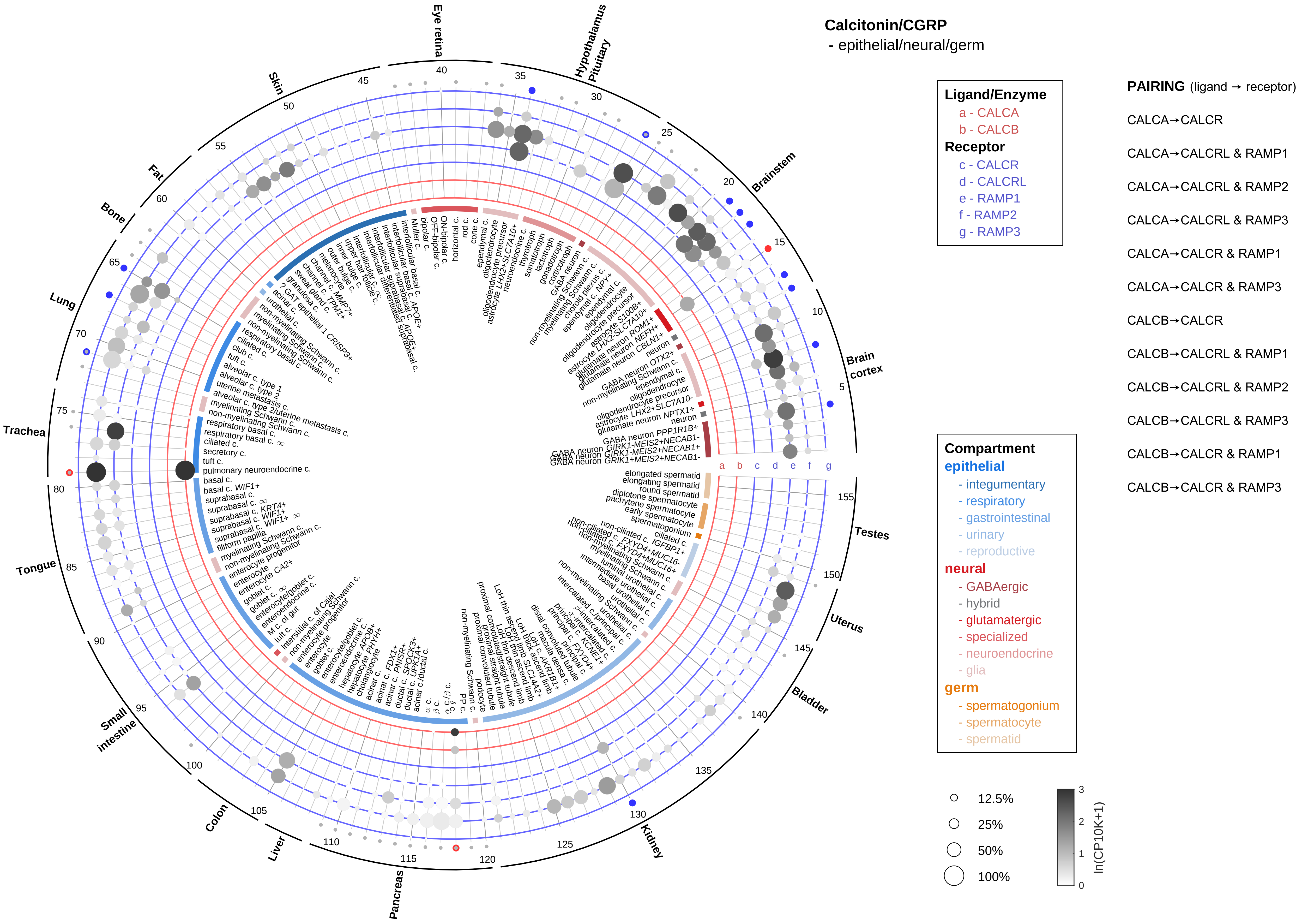
Calcitonin/CGRP
- epithelial/neural/germ
Ligand/Enzyme
a - CALCA
b - CALCB
Receptor
c - CALCR
d - CALCRL
e - RAMP1
f - RAMP2
g - RAMP3
PAIRING (ligand → receptor)
CALCA→CALCR
CALCA→CALCRL & RAMP1
CALCA→CALCRL & RAMP2
CALCA→CALCRL & RAMP3
CALCA→CALCR & RAMP1
CALCA→CALCR & RAMP3
CALCB→CALCR
CALCB→CALCRL & RAMP1
CALCB→CALCRL & RAMP2
CALCB→CALCRL & RAMP3
CALCB→CALCR & RAMP1
CALCB→CALCR & RAMP3
Compartment
epithelial
- integumentary
- respiratory
- gastrointestinal
- urinary
- reproductive
neural
- GABAergic
- hybrid
- glutamatergic
- specialized
- neuroendocrine
- glia
germ
- spermatogonium
- spermatocyte
- spermatid
12.5%
25%
50%
100%
ln(CP10K+1)
0
1
2
3
Eye retina
Hypothalamus Pituitary
Brainstem
Brain cortex
Testes
Uterus
Bladder
Kidney
Pancreas
Liver
Colon
Small intestine
Tongue
Trachea
Lung
Bone
Fat
Skin
a
b
c
d
e
f
g

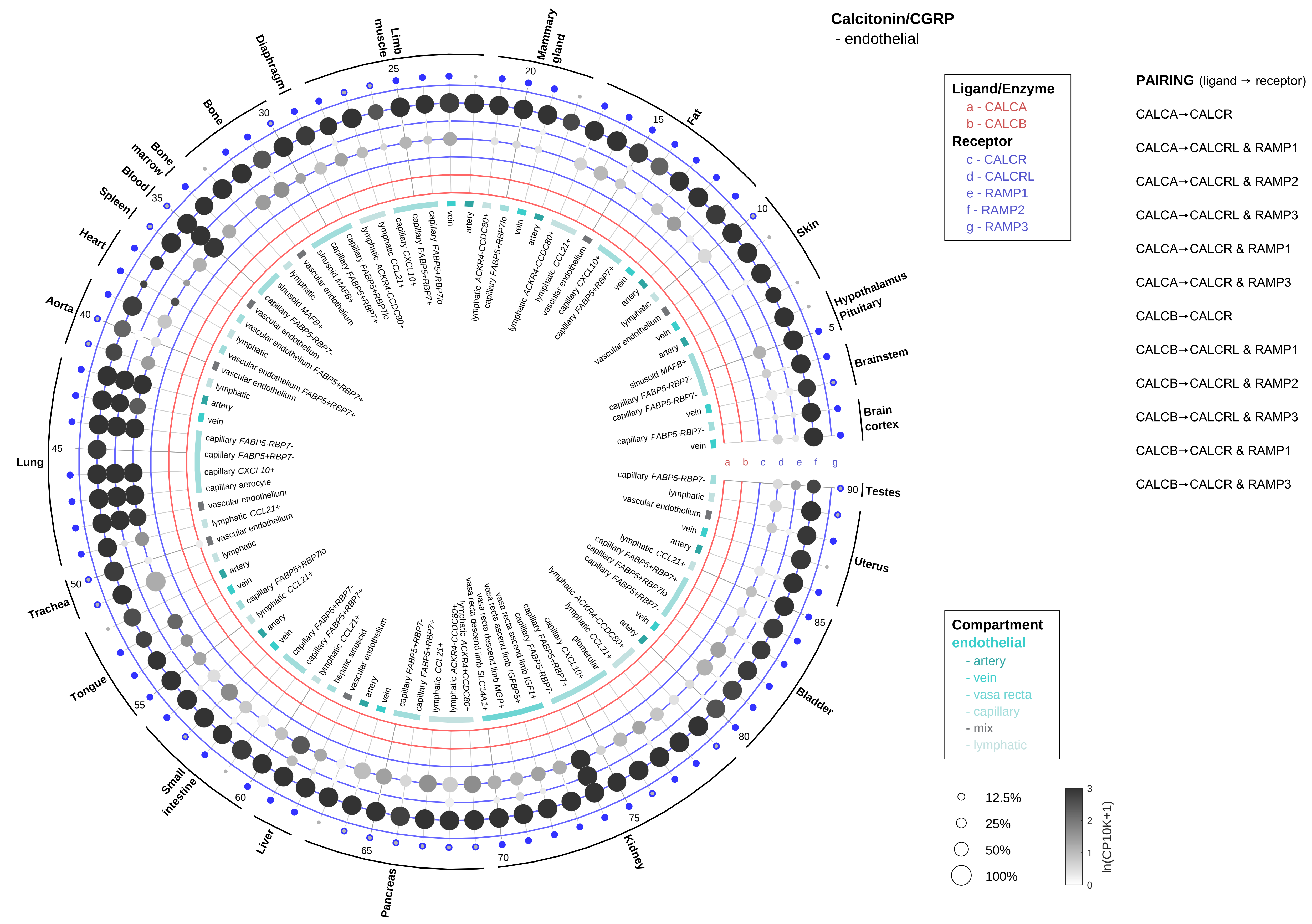
Calcitonin/CGRP
- endothelial
Ligand/Enzyme
a - CALCA
b - CALCB
Receptor
c - CALCR
d - CALCRL
e - RAMP1
f - RAMP2
g - RAMP3
PAIRING (ligand → receptor)
CALCA→CALCR
CALCA→CALCRL & RAMP1
CALCA→CALCRL & RAMP2
CALCA→CALCRL & RAMP3
CALCA→CALCR & RAMP1
CALCA→CALCR & RAMP3
CALCB→CALCR
CALCB→CALCRL & RAMP1
CALCB→CALCRL & RAMP2
CALCB→CALCRL & RAMP3
CALCB→CALCR & RAMP1
CALCB→CALCR & RAMP3
Compartment
endothelial
- artery
- vein
- vasa recta
- capillary
- mix
- lymphatic
12.5%
25%
50%
100%
ln(CP10K+1)
Brain cortex
Brainstem
Hypothalamus Pituitary
Skin
Fat
Mammary gland
Limb muscle
Diaphragm
Bone
Bone marrow
Blood
Spleen
Heart
Aorta
Lung
Trachea
Tongue
Small intestine
Liver
Pancreas
Kidney
Bladder
Uterus
Testes

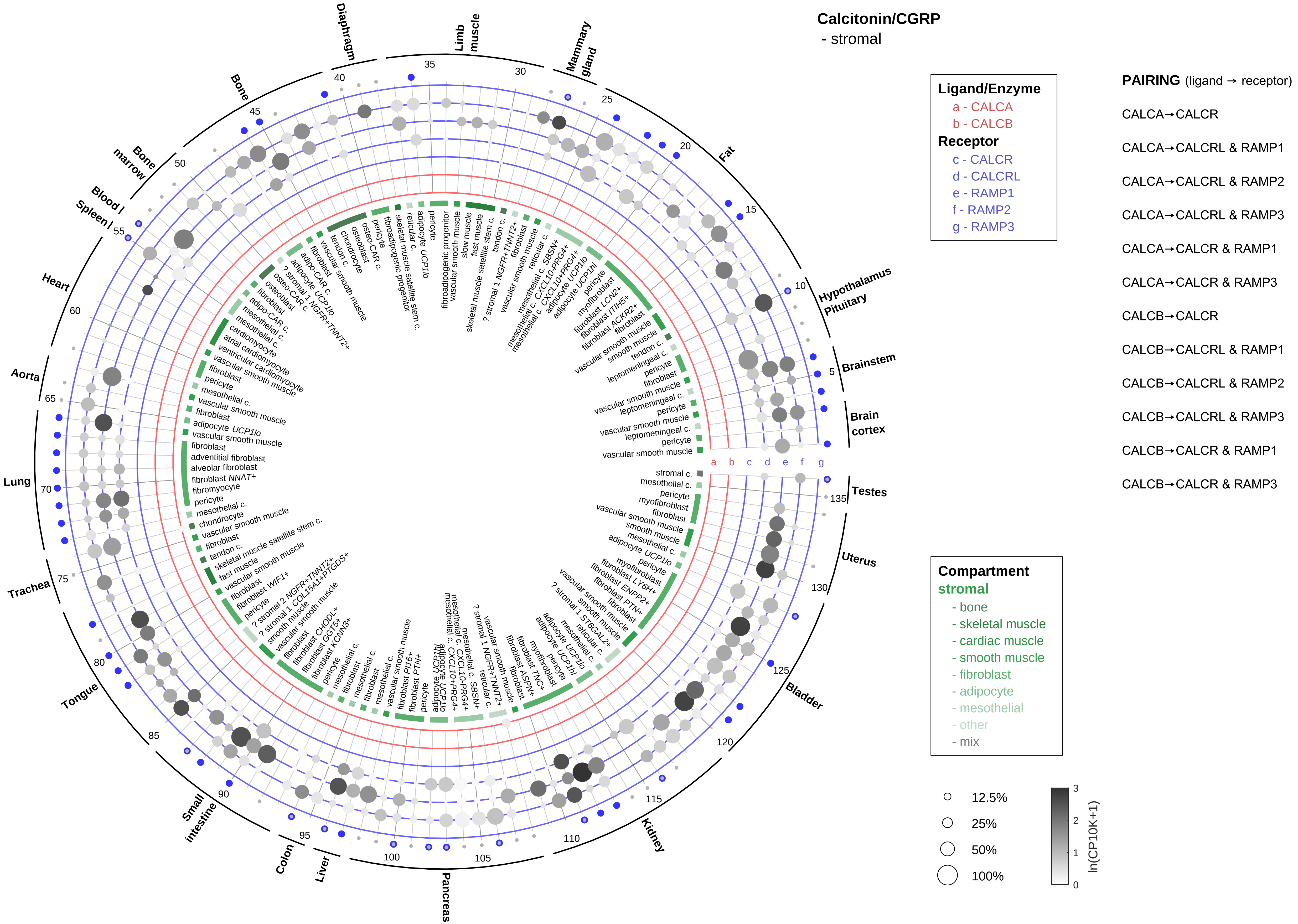

Calcitonin/CGRP
- stromal
Ligand/Enzyme
a - CALCA
b - CALCB
Receptor
c - CALCR
d - CALCRL
e - RAMP1
f - RAMP2
g - RAMP3
PAIRING (ligand → receptor)
CALCA→CALCR
CALCA→CALCRL & RAMP1
CALCA→CALCRL & RAMP2
CALCA→CALCRL & RAMP3
CALCA→CALCR & RAMP1
CALCA→CALCR & RAMP3
CALCB→CALCR
CALCB→CALCRL & RAMP1
CALCB→CALCRL & RAMP2
CALCB→CALCRL & RAMP3
CALCB→CALCR & RAMP1
CALCB→CALCR & RAMP3
Compartment
stromal
- bone
- skeletal muscle
- cardiac muscle
- smooth muscle
- fibroblast
- adipocyte
- mesothelial
- other
- mix
12.5%
25%
50%
100%
ln(CP10K+1)
Diaphragm
Limb muscle
Mammary gland
Fat
Hypothalamus Pituitary
Brainstem
Brain cortex
Testes
Uterus
Bladder
Kidney
Pancreas
Liver
Colon
Small intestine
Tongue
Trachea
Lung
Aorta
Heart
Spleen
Blood
Bone marrow
Bone

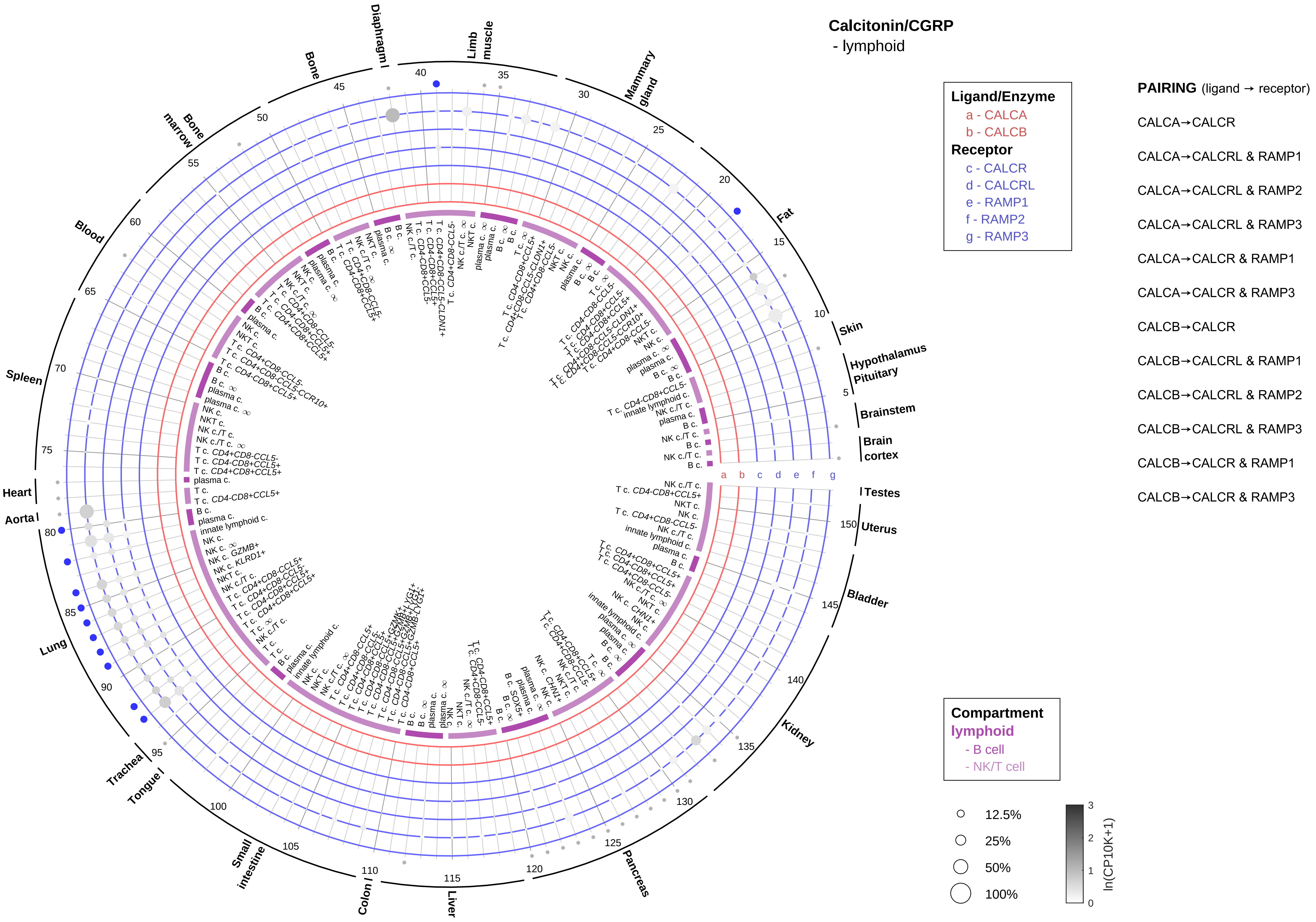

Calcitonin/CGRP
- lymphoid
Ligand/Enzyme
a - CALCA
b - CALCB
Receptor
c - CALCR
d - CALCRL
e - RAMP1
f - RAMP2
g - RAMP3
PAIRING (ligand → receptor)
CALCA→CALCR
CALCA→CALCRL & RAMP1
CALCA→CALCRL & RAMP2
CALCA→CALCRL & RAMP3
CALCA→CALCR & RAMP1
CALCA→CALCR & RAMP3
CALCB→CALCR
CALCB→CALCRL & RAMP1
CALCB→CALCRL & RAMP2
CALCB→CALCRL & RAMP3
CALCB→CALCR & RAMP1
CALCB→CALCR & RAMP3
Compartment
lymphoid
- B cell
- NK/T cell
12.5%
25%
50%
100%
ln(CP10K+1)
0
1
2
3
Diaphragm
Limb muscle
Mammary gland
Fat
Skin
Hypothalamus
Pituitary
Brainstem
Brain cortex
Testes
Uterus
Bladder
Kidney
Pancreas
Liver
Colon
Small intestine
Tongue
Trachea
Lung
Aorta
Heart
Spleen
Blood
Bone marrow
Bone

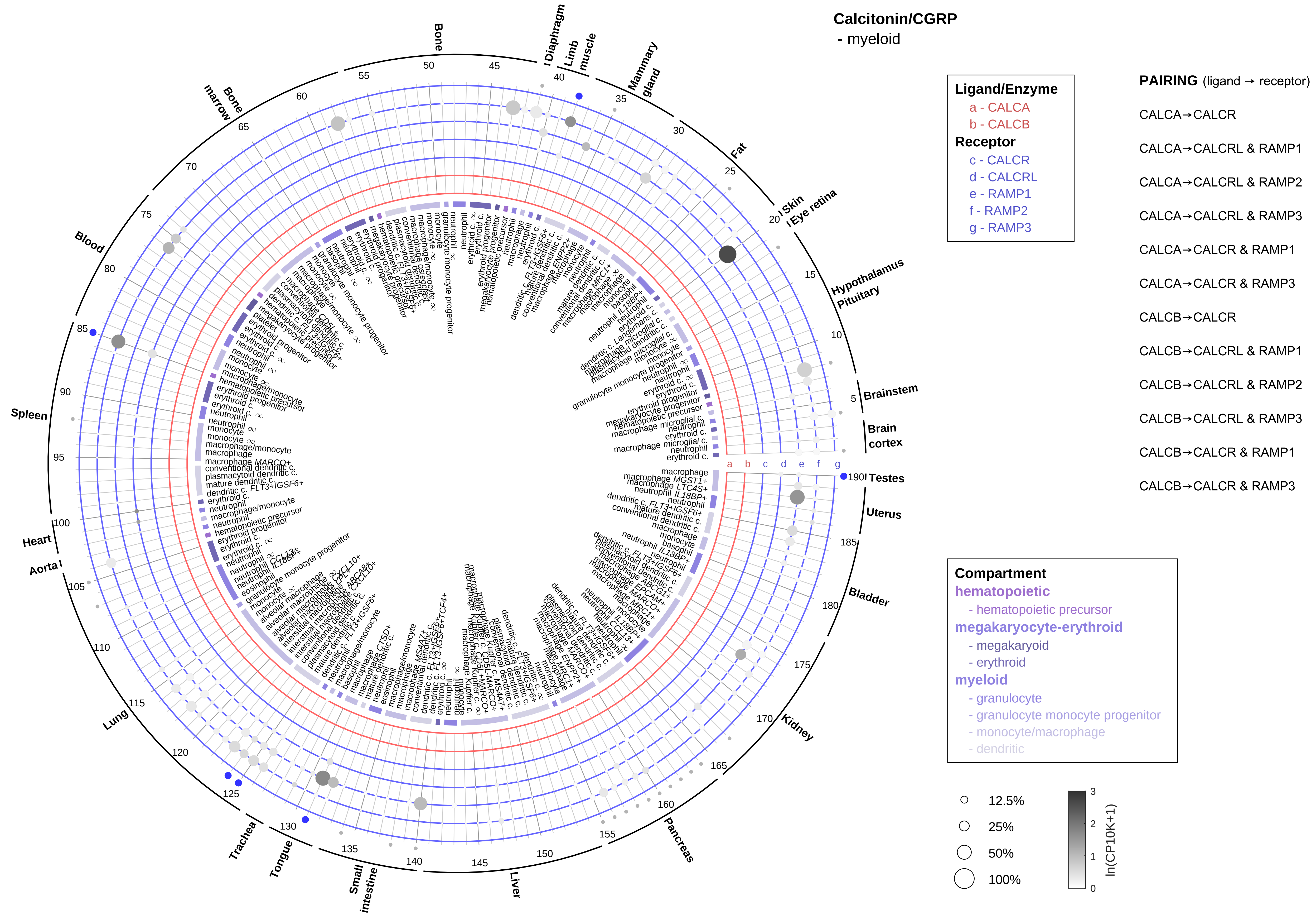
Calcitonin/CGRP
- myeloid
Ligand/Enzyme
a - CALCA
b - CALCB
Receptor
c - CALCR
d - CALCRL
e - RAMP1
f - RAMP2
g - RAMP3
PAIRING (ligand → receptor)
CALCA→CALCR
CALCA→CALCRL & RAMP1
CALCA→CALCRL & RAMP2
CALCA→CALCRL & RAMP3
CALCA→CALCR & RAMP1
CALCA→CALCR & RAMP3
CALCB→CALCR
CALCB→CALCRL & RAMP1
CALCB→CALCRL & RAMP2
CALCB→CALCRL & RAMP3
CALCB→CALCR & RAMP1
CALCB→CALCR & RAMP3
Compartment
hematopoietic
- hematopoietic precursor
megakaryocyte-erythroid
- megakaryoid
- erythroid
myeloid
- granulocyte
- granulocyte monocyte progenitor
- monocyte/macrophage
- dendritic
12.5%
25%
50%
100%
ln(CP10K+1)
Bone
Diaphragm
Limb muscle
Mammary gland
Fat
Skin
Eye retina
Hypothalamus
Pituitary
Brainstem
Brain cortex
Testes
Uterus
Bladder
Kidney
Pancreas
Liver
Small intestine
Tongue
Trachea
Lung
Aorta
Heart
Spleen
Blood
Bone marrow

Cholecalciferol/Calcidiol/Calcitriol
- epithelial/neural/germ

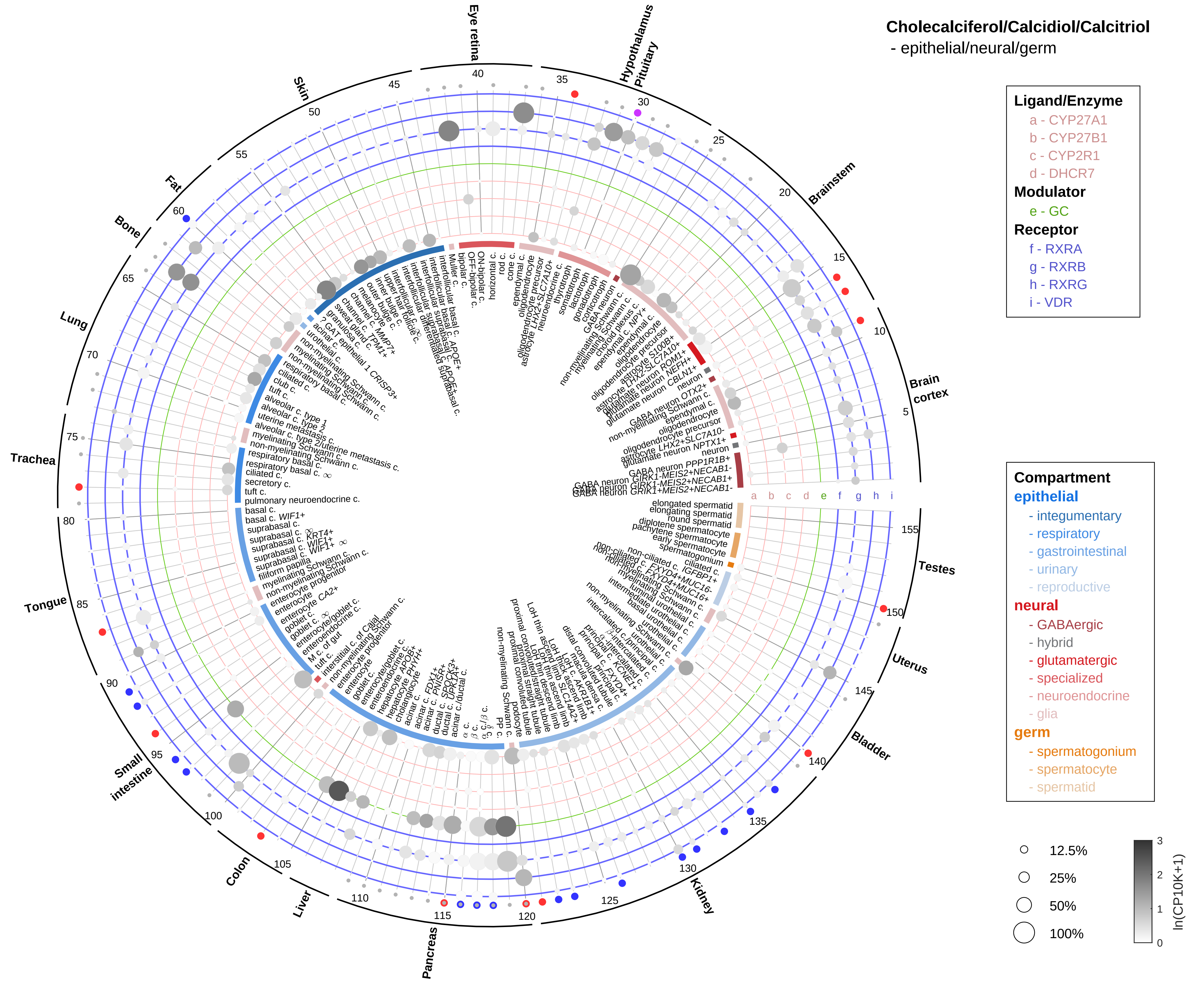

PAIRING (ligand → receptor)

CYP27B1→VDR & RXRA

CYP27B1→VDR & RXRB

CYP27B1→VDR & RXRG

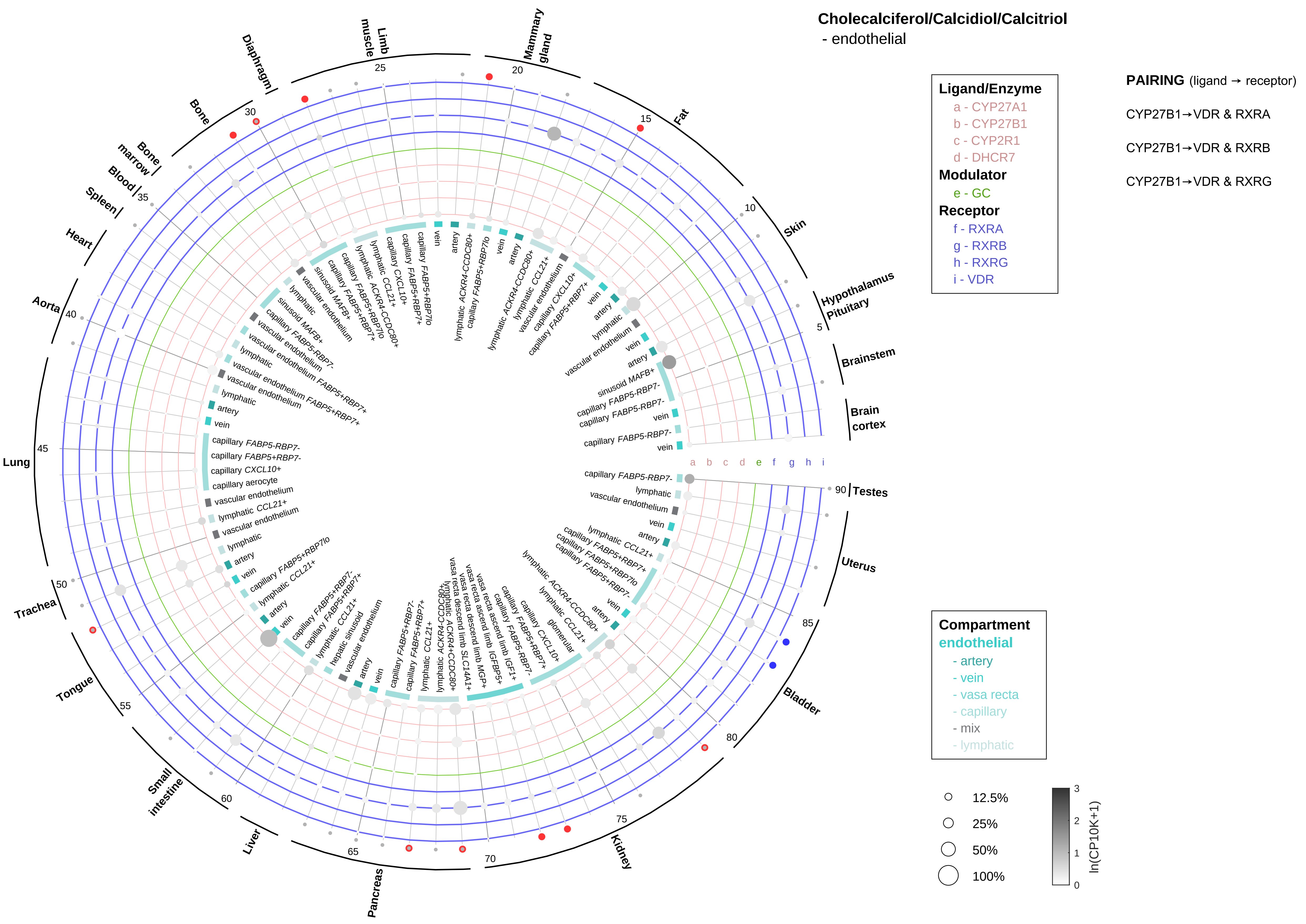

Cholecalciferol/Calcidiol/Calcitriol
- endothelial
Ligand/Enzyme
a - CYP27A1
b - CYP27B1
c - CYP2R1
d - DHCR7
Modulator
e - GC
Receptor
f - RXRA
g - RXRB
h - RXRG
i - VDR
PAIRING (ligand → receptor)
CYP27B1→VDR & RXRA
CYP27B1→VDR & RXRB
CYP27B1→VDR & RXRG
Compartment
endothelial
- artery
- vein
- vasa recta
- capillary
- mix
- lymphatic
12.5%
25%
50%
100%
ln(CP10K+1)
Brain cortex
Brainstem
Hypothalamus Pituitary
Skin
Fat
Mammary gland
Limb muscle
Diaphragm
Bone
Bone marrow
Blood
Spleen
Heart
Aorta
Lung
Trachea
Tongue
Small intestine
Liver
Pancreas
Kidney
Bladder
Uterus
Testes

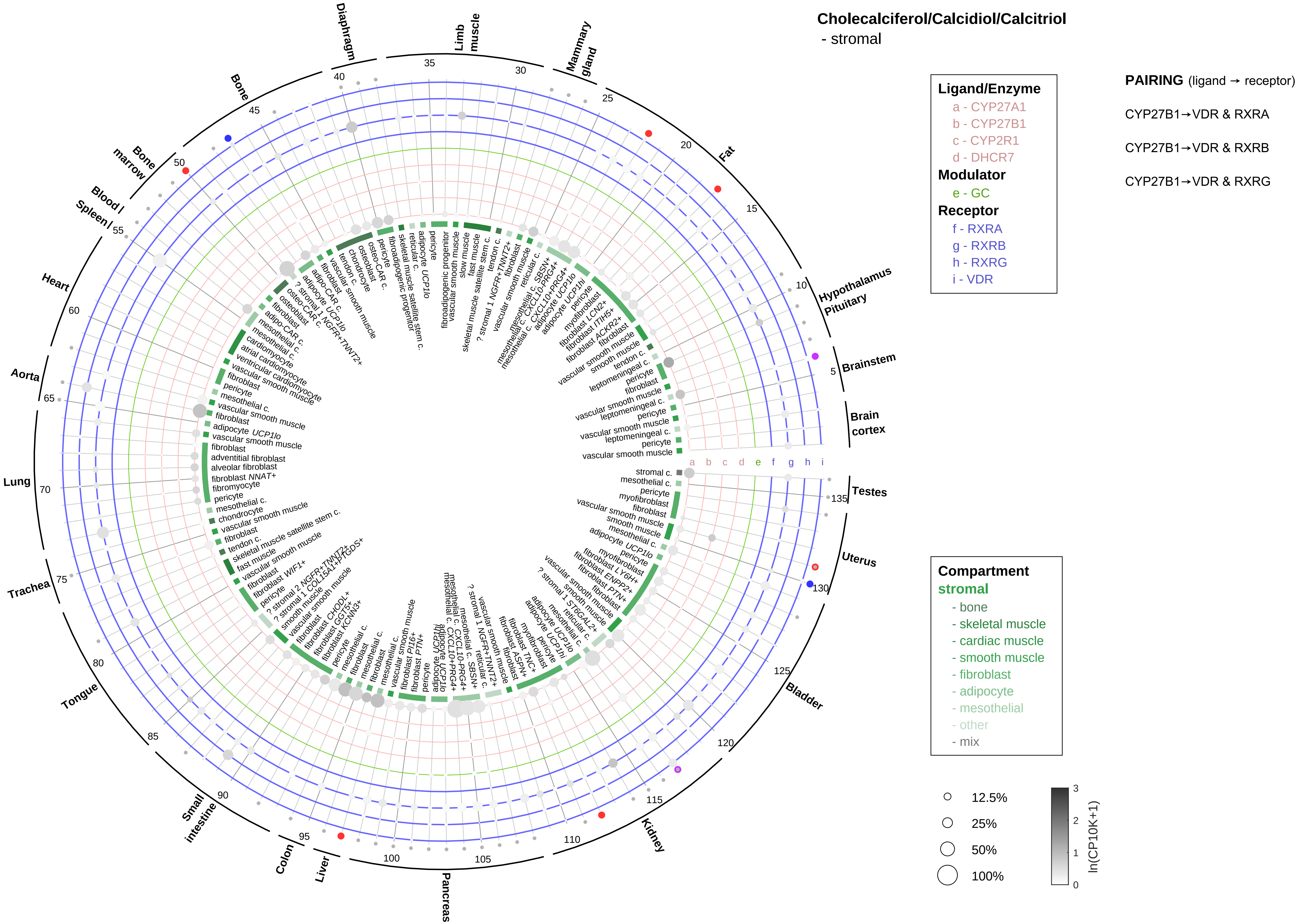
Cholecalciferol/Calcidiol/Calcitriol
- stromal
Ligand/Enzyme
a - CYP27A1
b - CYP27B1
c - CYP2R1
d - DHCR7
Modulator
e - GC
Receptor
f - RXRA
g - RXRB
h - RXRG
i - VDR
PAIRING (ligand → receptor)
CYP27B1→VDR & RXRA
CYP27B1→VDR & RXRB
CYP27B1→VDR & RXRG
Compartment
stromal
- bone
- skeletal muscle
- cardiac muscle
- smooth muscle
- fibroblast
- adipocyte
- mesothelial
- other
- mix
12.5%
25%
50%
100%
ln(CP10K+1)
Brain cortex
Brainstem
Hypothalamus Pituitary
Fat
Mammary gland
Limb muscle
Diaphragm
Bone
Bone marrow
Blood
Spleen
Heart
Aorta
Lung
Trachea
Tongue
Small intestine
Colon
Liver
Pancreas
Kidney
Bladder
Uterus
Testes

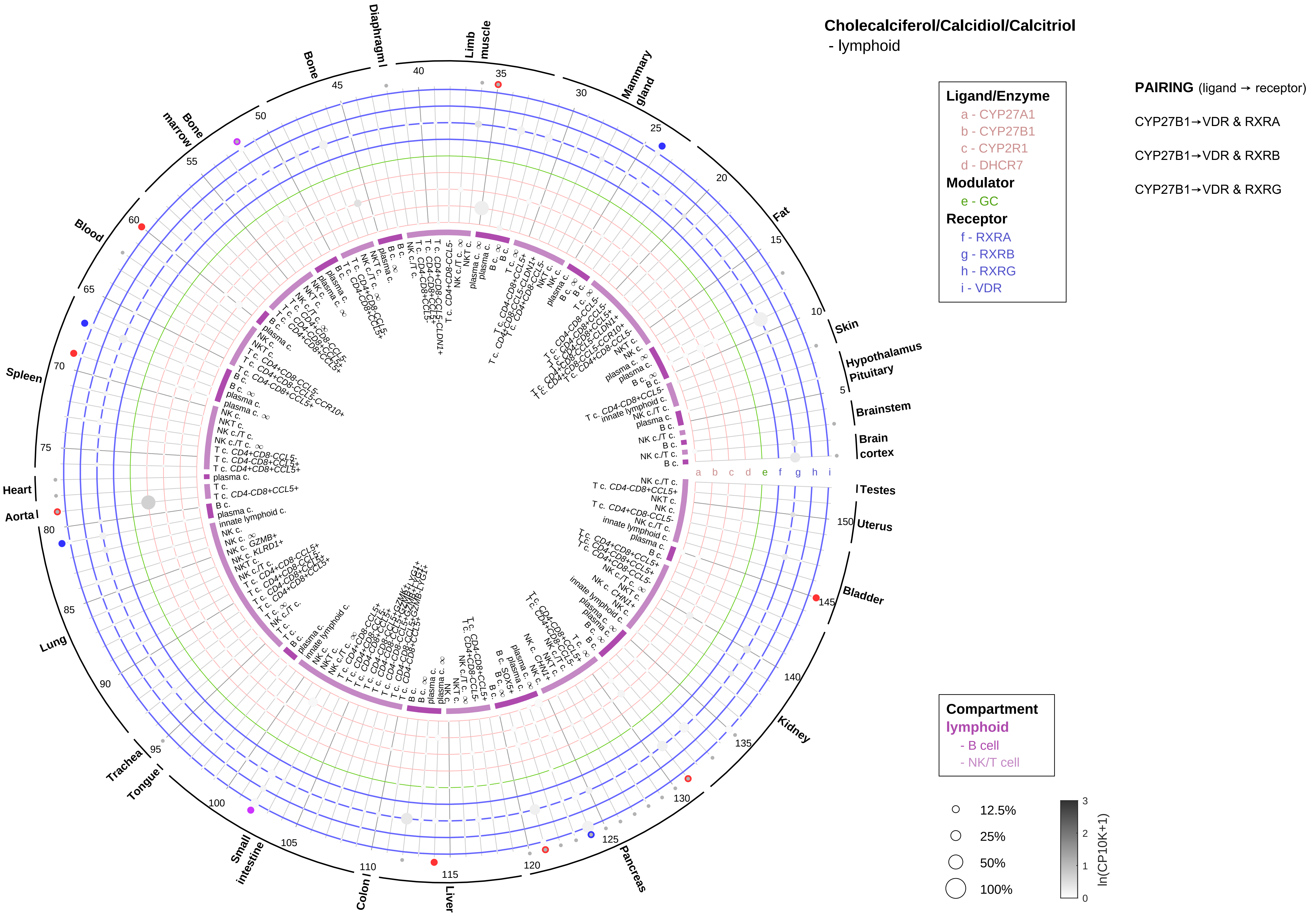

Cholecalciferol/Calcidiol/Calcitriol
- lymphoid
Ligand/Enzyme
a - CYP27A1
b - CYP27B1
c - CYP2R1
d - DHCR7
Modulator
e - GC
Receptor
f - RXRA
g - RXRB
h - RXRG
i - VDR
PAIRING (ligand → receptor)
CYP27B1→VDR & RXRA
CYP27B1→VDR & RXRB
CYP27B1→VDR & RXRG
Compartment
lymphoid
- B cell
- NK/T cell
12.5%
25%
50%
100%
ln(CP10K+1)
Skin
Hypothalamus
Pituitary
Brainstem
Brain cortex
Testes
Uterus
Bladder
Kidney
Pancreas
Liver
Colon
Small intestine
Tongue
Trachea
Lung
Aorta
Heart
Spleen
Blood
Bone marrow
Bone
Diaphragm
Limb muscle
Mammary gland
Fat

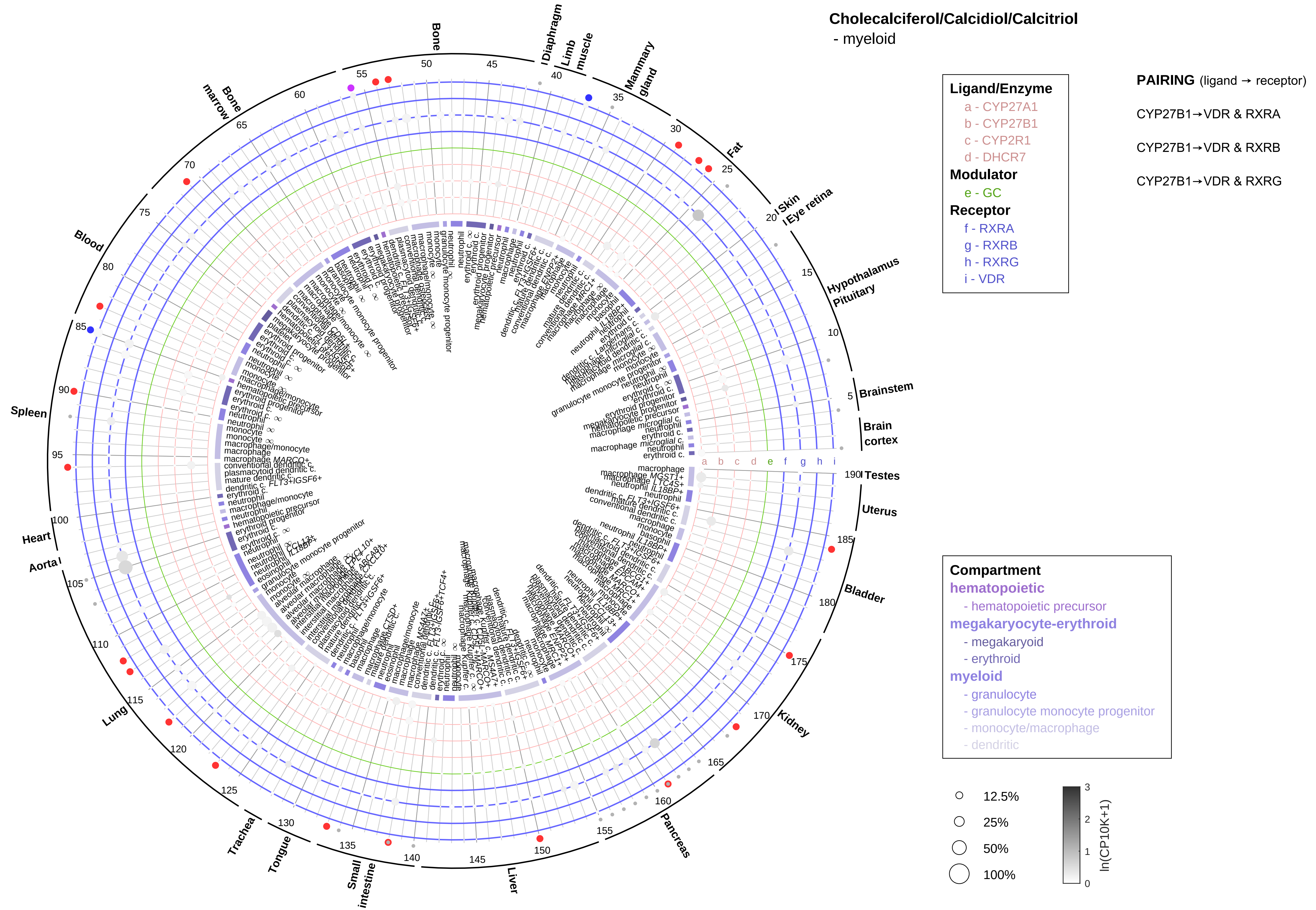

Cholecalciferol/Calcidiol/Calcitriol
- myeloid
Ligand/Enzyme
a - CYP27A1
b - CYP27B1
c - CYP2R1
d - DHCR7
Modulator
e - GC
Receptor
f - RXRA
g - RXRB
h - RXRG
i - VDR
PAIRING (ligand → receptor)
CYP27B1→VDR & RXRA
CYP27B1→VDR & RXRB
CYP27B1→VDR & RXRG
Compartment
hematopoietic
- hematopoietic precursor
megakaryocyte-erythroid
- megakaryoid
- erythroid
myeloid
- granulocyte
- granulocyte monocyte progenitor
- monocyte/macrophage
- dendritic
12.5%
25%
50%
100%
ln(CP10K+1)
Bone
Diaphragm
Limb muscle
Mammary gland
Fat
Skin
Eye retina
Hypothalamus
Pituitary
Brainstem
Brain cortex
Testes
Uterus
Bladder
Kidney
Pancreas
Liver
Small intestine
Tongue
Trachea
Lung
Aorta
Heart
Spleen
Blood
Bone marrow

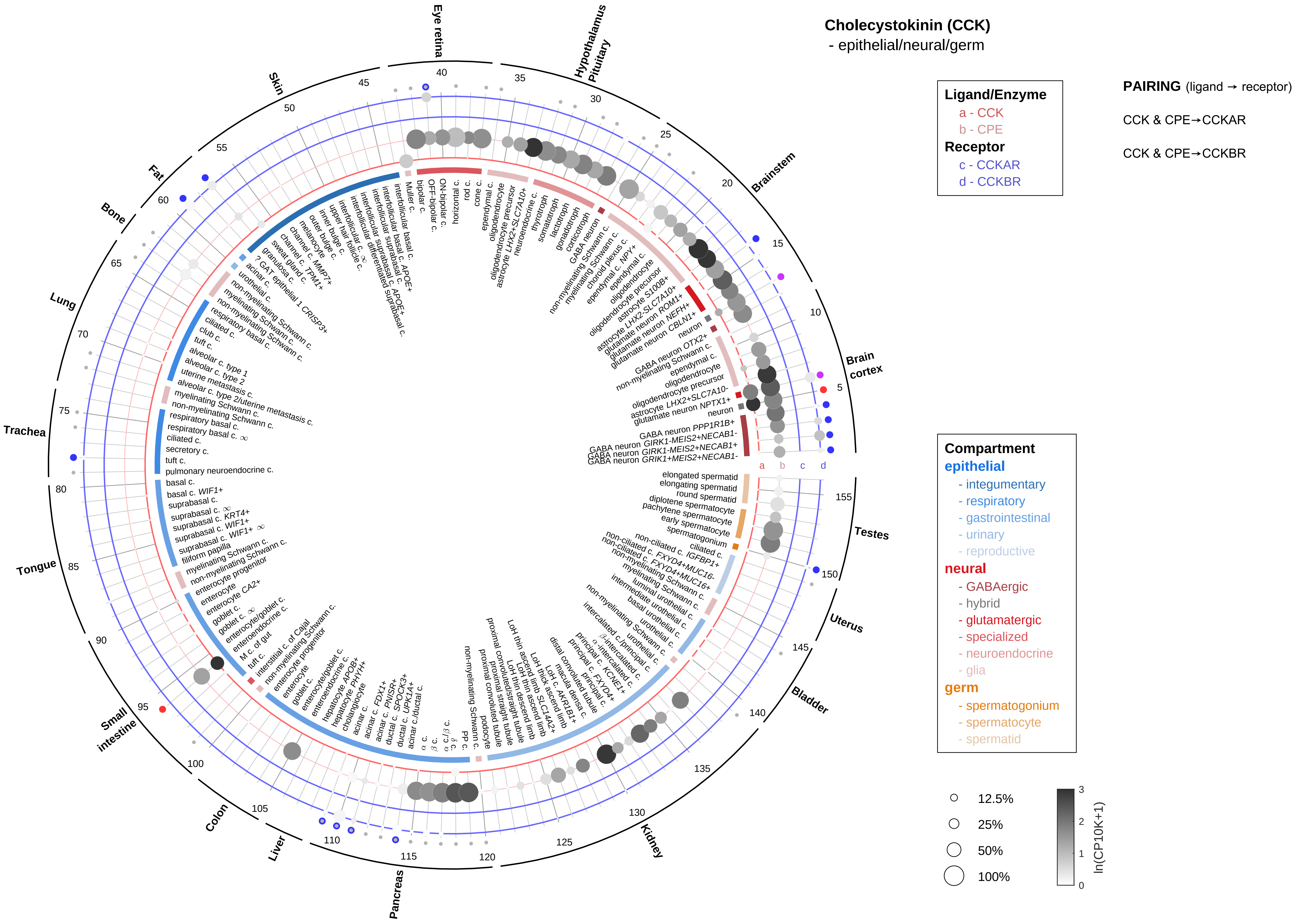
Cholecystokinin (CCK)
- epithelial/neural/germ
Ligand/Enzyme
a - CCK
b - CPE
Receptor
c - CCKAR
d - CCKBR
PAIRING (ligand → receptor)
CCK & CPE→CCKAR
CCK & CPE→CCKBR
Compartment
epithelial
- integumentary
- respiratory
- gastrointestinal
- urinary
- reproductive
neural
- GABAergic
- hybrid
- glutamatergic
- specialized
- neuroendocrine
- glia
germ
- spermatogonium
- spermatocyte
- spermatid
12.5%
25%
50%
100%
ln(CP10K+1)
Eye retina
Hypothalamus Pituitary
Brainstem
Brain cortex
Testes
Uterus
Bladder
Kidney
Pancreas
Liver
Colon
Small intestine
Tongue
Trachea
Lung
Bone
Fat
Skin
a
b
c
d

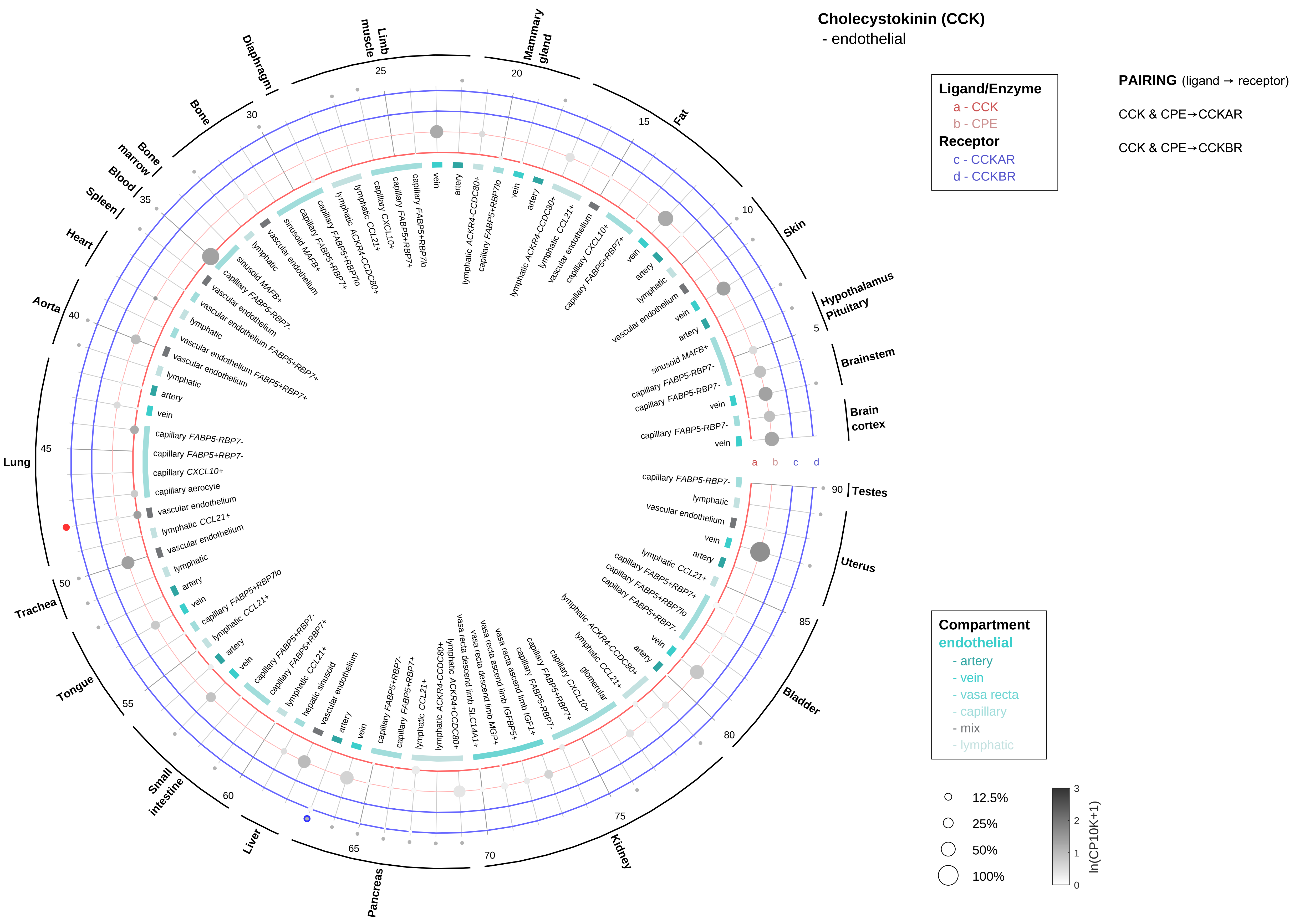

Cholecystokinin (CCK)
- endothelial
Ligand/Enzyme
a - CCK
b - CPE
Receptor
c - CCKAR
d - CCKBR
PAIRING (ligand → receptor)
CCK & CPE→CCKAR
CCK & CPE→CCKBR
Compartment
endothelial
- artery
- vein
- vasa recta
- capillary
- mix
- lymphatic
12.5%
25%
50%
100%
ln(CP10K+1)
Brain cortex
Brainstem
Hypothalamus Pituitary
Skin
Fat
Mammary gland
Limb muscle
Diaphragm
Bone
Bone marrow
Blood
Spleen
Heart
Aorta
Lung
Trachea
Tongue
Small intestine
Liver
Pancreas
Kidney
Bladder
Uterus
Testes
a b c d

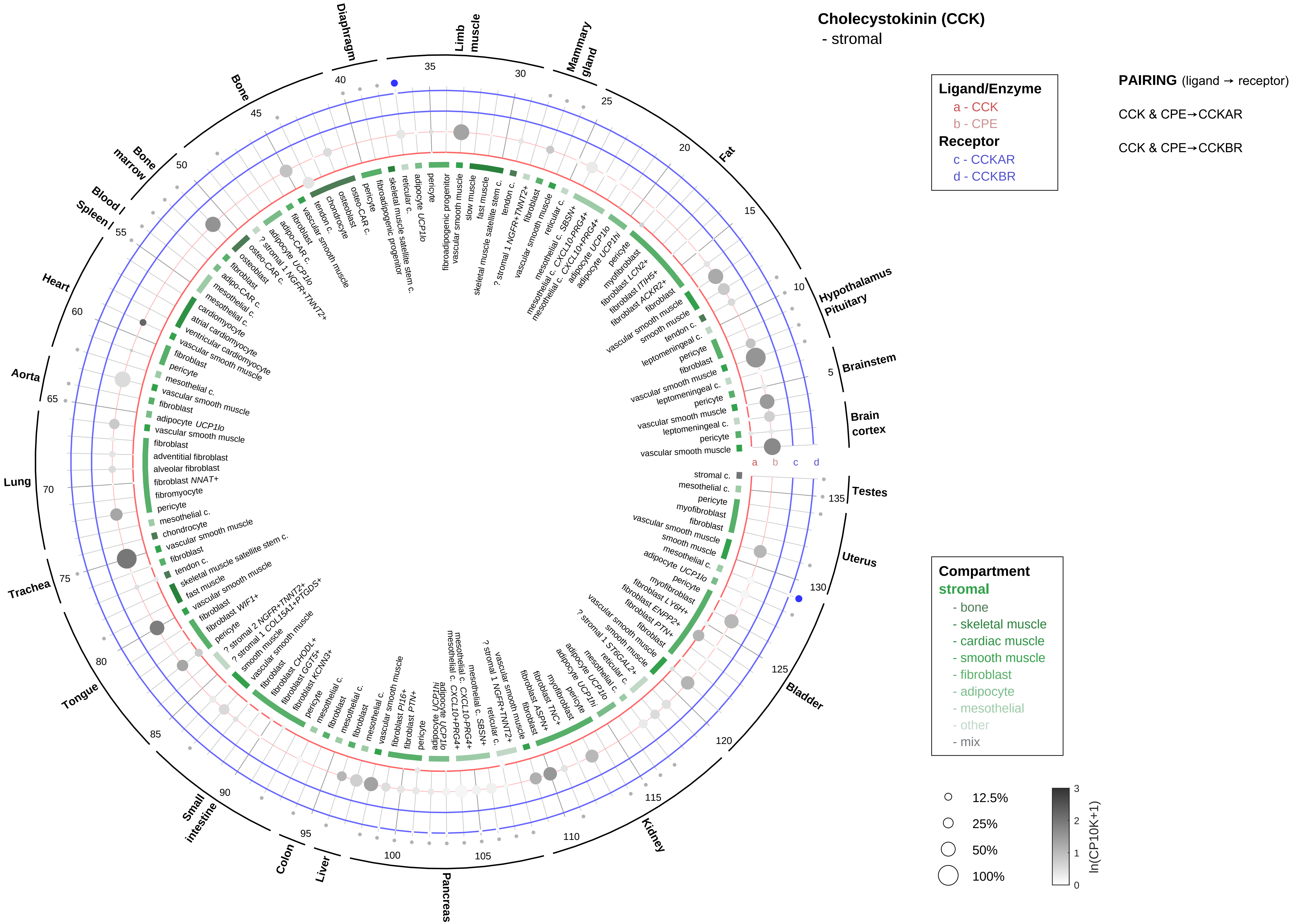

Cholecystokinin (CCK)
- stromal
Ligand/Enzyme
a - CCK
b - CPE
Receptor
c - CCKAR
d - CCKBR
PAIRING (ligand → receptor)
CCK & CPE→CCKAR
CCK & CPE→CCKBR
Compartment
stromal
- bone
- skeletal muscle
- cardiac muscle
- smooth muscle
- fibroblast
- adipocyte
- mesothelial
- other
- mix
12.5%
25%
50%
100%
ln(CP10K+1)
0
1
2
3
a
b
c
d
Brain cortex
Brainstem
Hypothalamus Pituitary
Fat
Mammary gland
Limb muscle
Diaphragm
Bone
Bone marrow
Blood /
Spleen /
Heart
Aorta
Lung
Trachea
Tongue
Small intestine
Colon
Liver
Pancreas
Kidney
Bladder
Uterus
Testes
5
10
15
20
25
30
35
40
45
50
55
60
65
70
75
80
85
90
95
100
105
110
115
120
125
130
135
vascular smooth muscle
pericyte
leptomeningeal c.
vascular smooth muscle
pericyte
leptomeningeal c.
vascular smooth muscle
fibroblast
pericyte
leptomeningeal c.
tendon c.
smooth muscle
vascular smooth muscle
fibroblast
fibroblast ACKR2+
fibroblast ITIH5+
fibroblast LCN2+
myofibroblast
pericyte
adipocyte UCP1hi
adipocyte UCP1lo
mesothelial c. CXCL10+PRG4+
mesothelial c. SBSN+
reticular c.
vascular smooth muscle
fibroblast
? stromal 1 NGFR+TNNT2+
tendon c.
skeletal muscle satellite stem c.
fast muscle
slow muscle
vascular smooth muscle
fibroadipogenic progenitor
pericyte
adipocyte UCP1lo
reticular c.
skeletal muscle satellite stem c.
fibroadipogenic progenitor
pericyte
osteo-CAR c.
osteoblast
chondrocyte
tendon c.
vascular smooth muscle
fibroblast
adipo-CAR c.
adipocyte UCP1lo
? stromal 1 NGFR+TNNT2+
osteo-CAR c.
osteoblast
fibroblast
adipo-CAR c.
mesothelial c.
mesothelial c.
cardiomyocyte
atrial cardiomyocyte
ventricular cardiomyocyte
vascular smooth muscle
fibroblast
pericyte
mesothelial c.
vascular smooth muscle
fibroblast
adipocyte UCP1lo
vascular smooth muscle
fibroblast
adventitial fibroblast
alveolar fibroblast
fibroblast NNAT+
fibromyocyte
pericyte
mesothelial c.
chondrocyte
vascular smooth muscle
fibroblast
tendon c.
skeletal muscle satellite stem c.
fast muscle
vascular smooth muscle
fibroblast
fibroblast WIF1+
pericyte
? stromal 2 NGFR+TNNT2+
? stromal 1 COL15A1+PTGDS+
smooth muscle
vascular smooth muscle
fibroblast
fibroblast CHODL+
fibroblast GGT5+
fibroblast KCNN3+
pericyte
mesothelial c.
fibroblast
mesothelial c.
fibroblast
mesothelial c.
vascular smooth muscle
fibroblast PI16+
fibroblast PTN+
pericyte
adipocyte UCP1hi
adipocyte UCP1lo
mesothelial c. CXCL10+PRG4+
mesothelial c. SBSN+
? stromal 1 NGFR+TNNT2+
reticular c.
vascular smooth muscle
fibroblast
fibroblast ASPN+
fibroblast TNC+
myofibroblast
pericyte
adipocyte UCP1hi
adipocyte UCP1lo
mesothelial c.
reticular c.
? stromal 1 ST6GAL2+
smooth muscle
vascular smooth muscle
fibroblast
fibroblast PTN+
fibroblast ENPP2+
fibroblast LY6H+
myofibroblast
pericyte
adipocyte UCP1lo
mesothelial c.
smooth muscle
vascular smooth muscle
fibroblast
myofibroblast
pericyte
mesothelial c.
stromal c.

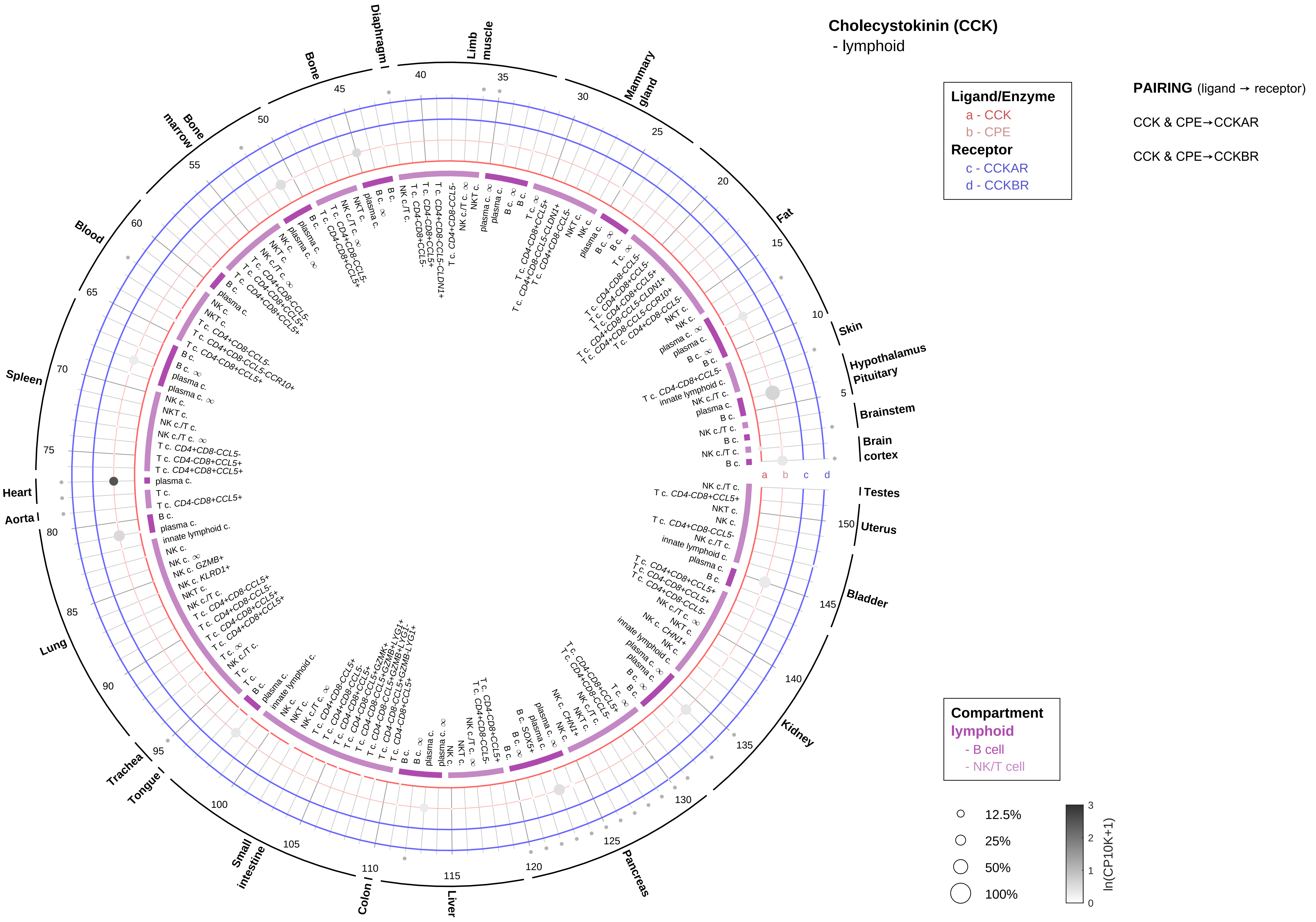

Cholecystokinin (CCK)
- lymphoid
Ligand/Enzyme
a - CCK
b - CPE
Receptor
c - CCKAR
d - CCKBR
PAIRING (ligand → receptor)
CCK & CPE→CCKAR
CCK & CPE→CCKBR
Compartment
lymphoid
- B cell
- NK/T cell
12.5%
25%
50%
100%
ln(CP10K+1)
Hypothalamus
Pituitary
Brainstem
Brain cortex
Testes
Uterus
Bladder
Kidney
Pancreas
Liver
Colon
Small intestine
Tongue
Trachea
Lung
Aorta
Heart
Spleen
Blood
Bone marrow
Bone
Diaphragm
Limb muscle
Mammary gland
Fat
Skin

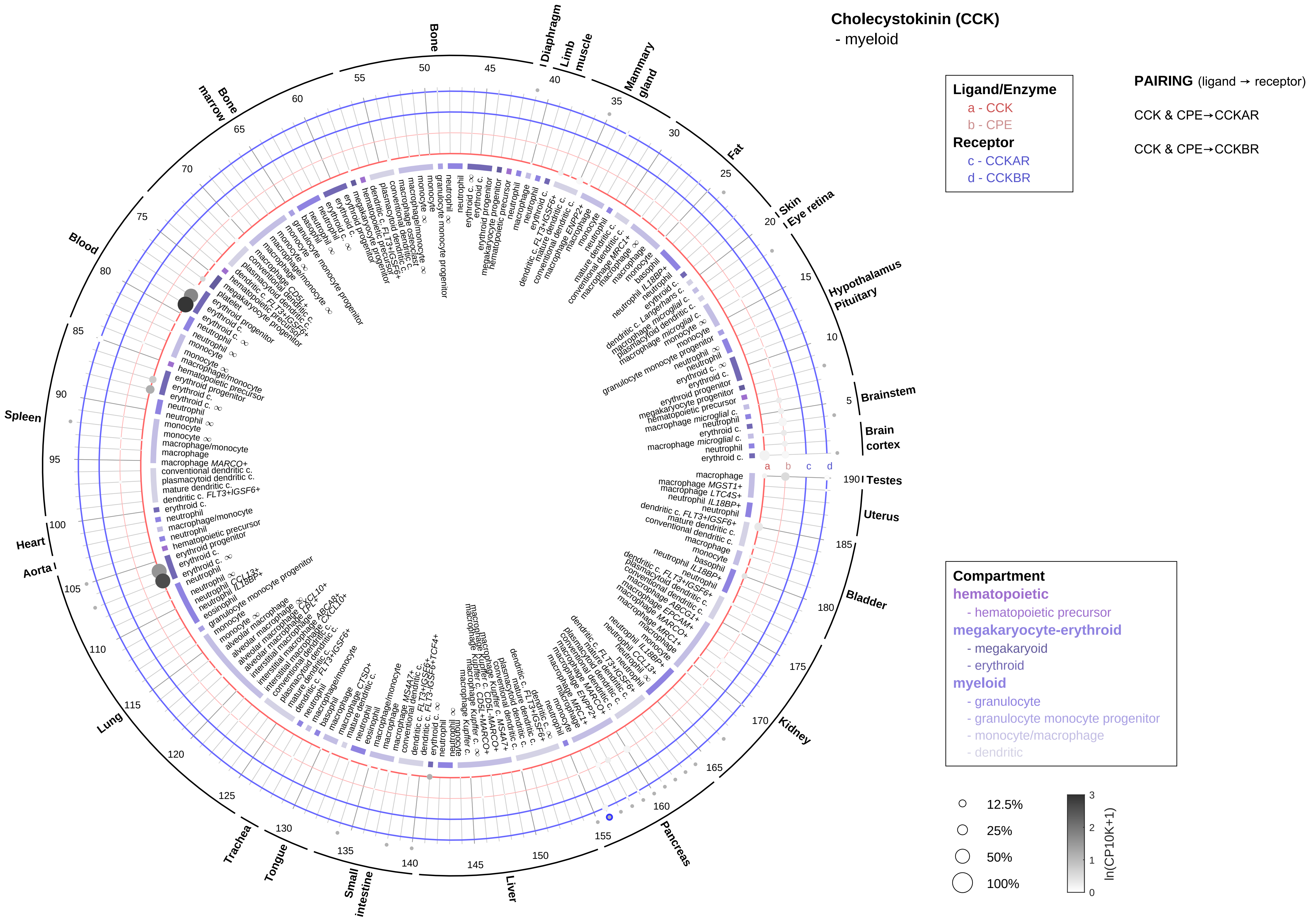
Cholecystokinin (CCK)
- myeloid
Ligand/Enzyme
a - CCK
b - CPE
Receptor
c - CCKAR
d - CCKBR
PAIRING (ligand → receptor)
CCK & CPE→CCKAR
CCK & CPE→CCKBR
Compartment
hematopoietic
- hematopoietic precursor
megakaryocyte-erythroid
- megakaryoid
- erythroid
myeloid
- granulocyte
- granulocyte monocyte progenitor
- monocyte/macrophage
- dendritic
12.5%
25%
50%
100%
ln(CP10K+1)
Bone
Diaphragm
Limb muscle
Mammary gland
Fat
Skin
Eye retina
Hypothalamus
Pituitary
Brainstem
Brain cortex
Testes
Uterus
Bladder
Kidney
Pancreas
Liver
Small intestine
Tongue
Trachea
Lung
Aorta
Heart
Spleen
Blood
Bone marrow

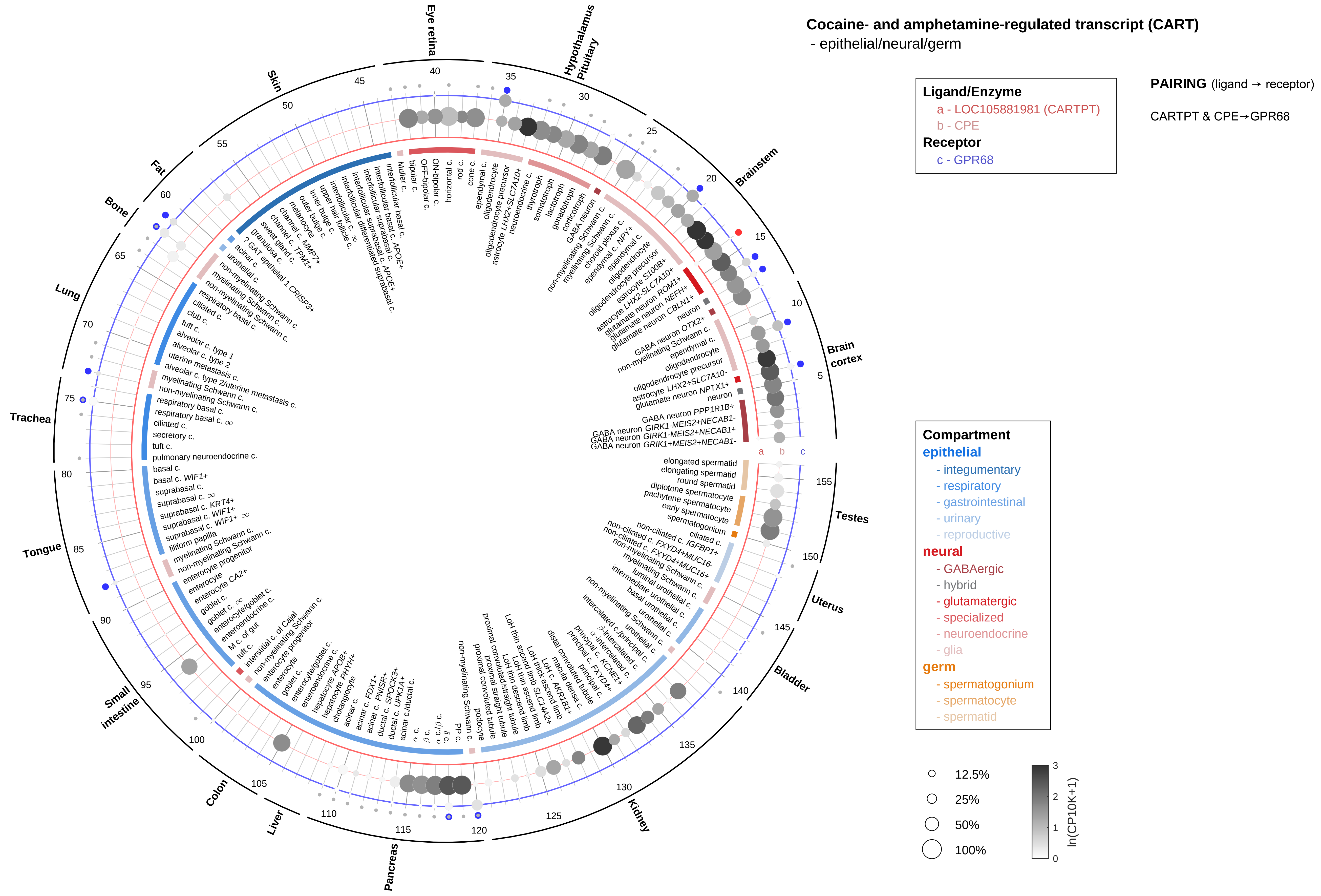
Cocaine- and amphetamine-regulated transcript (CART)
- epithelial/neural/germ
Ligand/Enzyme
a - LOC105881981 (CARTPT)
b - CPE
Receptor
c - GPR68
PAIRING (ligand → receptor)
CARTPT & CPE→GPR68
Compartment
epithelial
- integumentary
- respiratory
- gastrointestinal
- urinary
- reproductive
neural
- GABAergic
- hybrid
- glutamatergic
- specialized
- neuroendocrine
- glia
germ
- spermatogonium
- spermatocyte
- spermatid
12.5%
25%
50%
100%
ln(CP10K+1)
Eye retina
Hypothalamus Pituitary
Brainstem
Brain cortex
Testes
Uterus
Bladder
Kidney
Pancreas
Liver
Colon
Small intestine
Tongue
Trachea
Lung
Bone
Fat
Skin

Cocaine- and amphetamine-regulated transcript (CART)
- endothelial

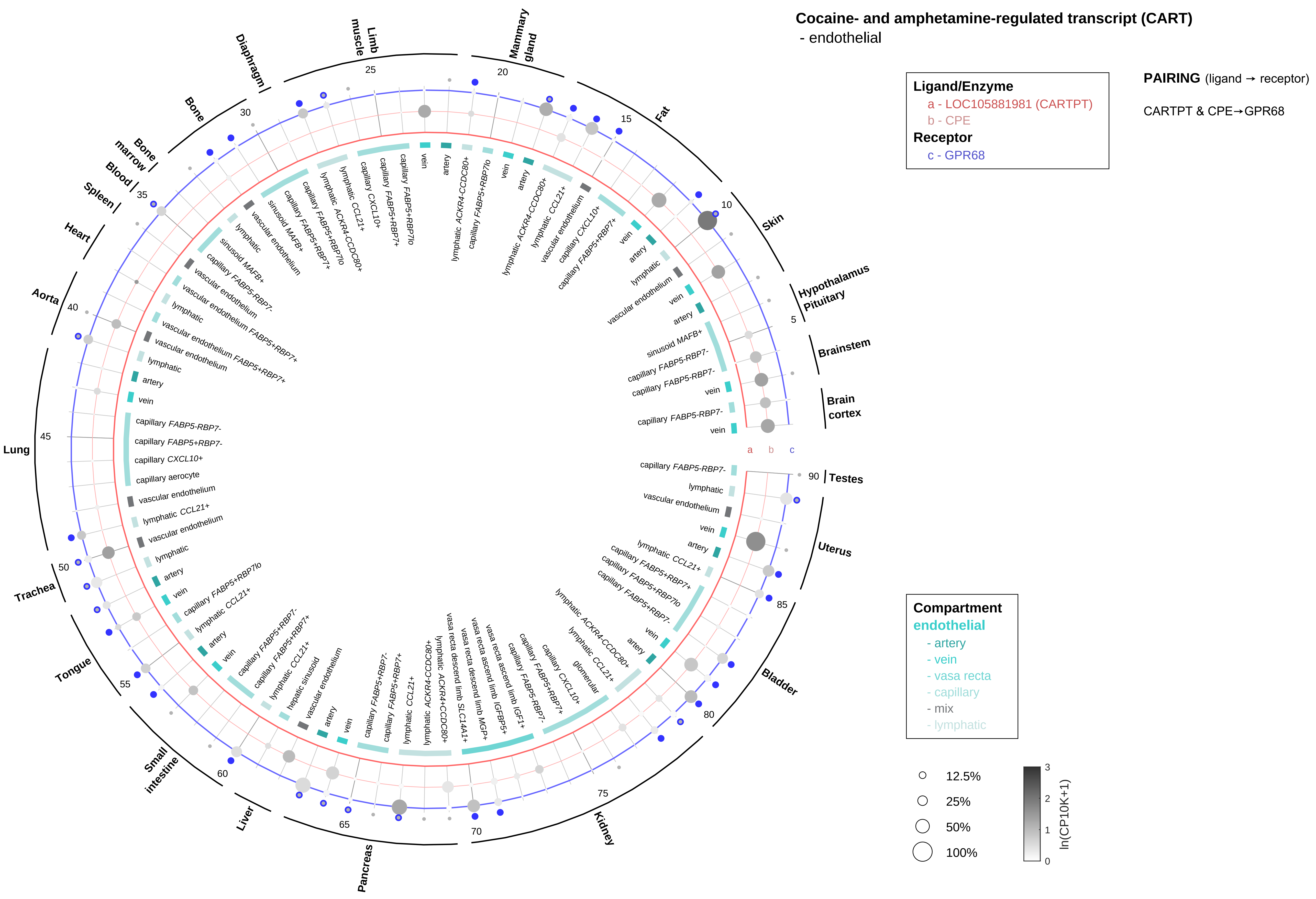

Cocaine- and amphetamine-regulated transcript (CART)
- stromal

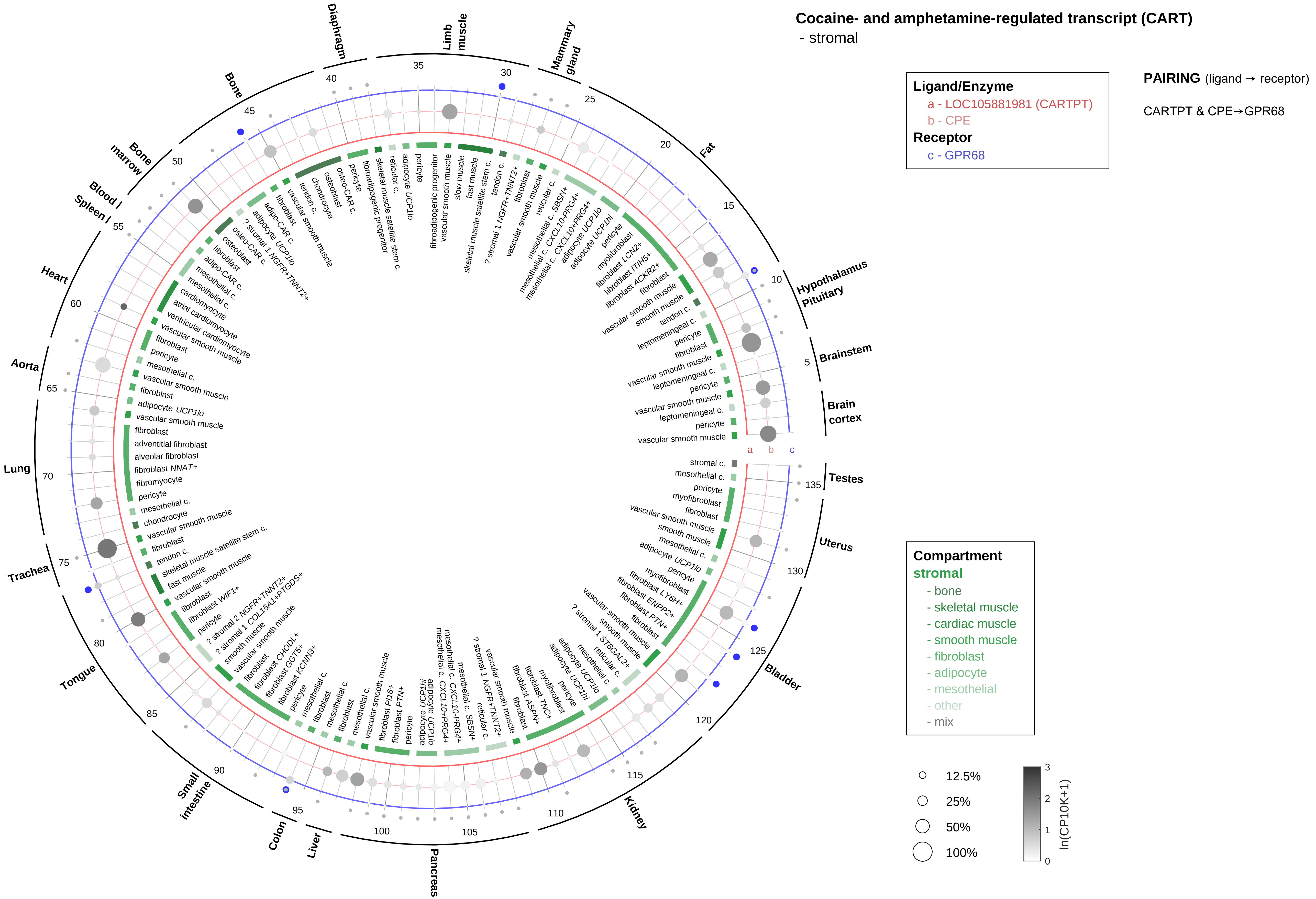

Cocaine- and amphetamine-regulated transcript (CART)
- lymphoid

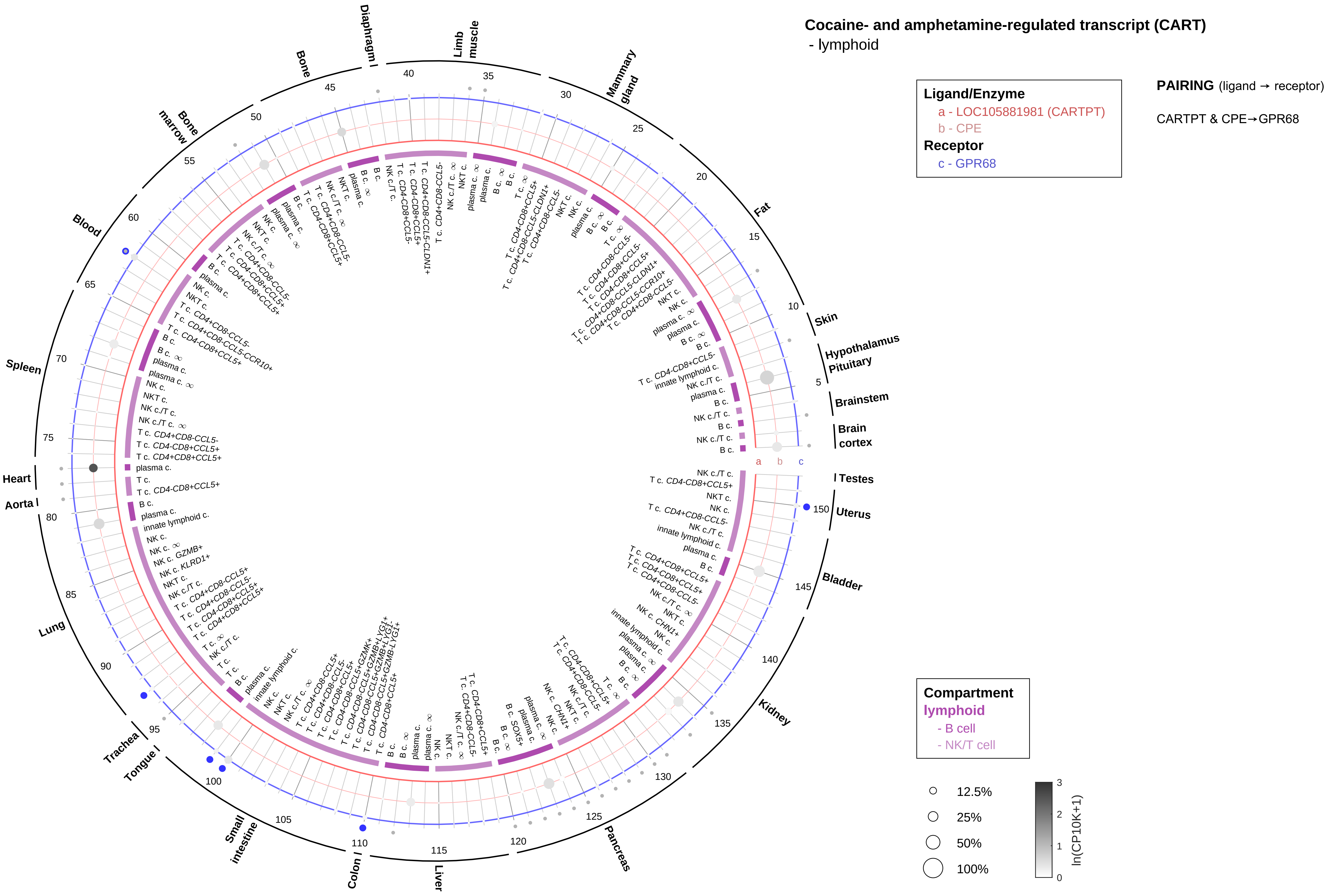

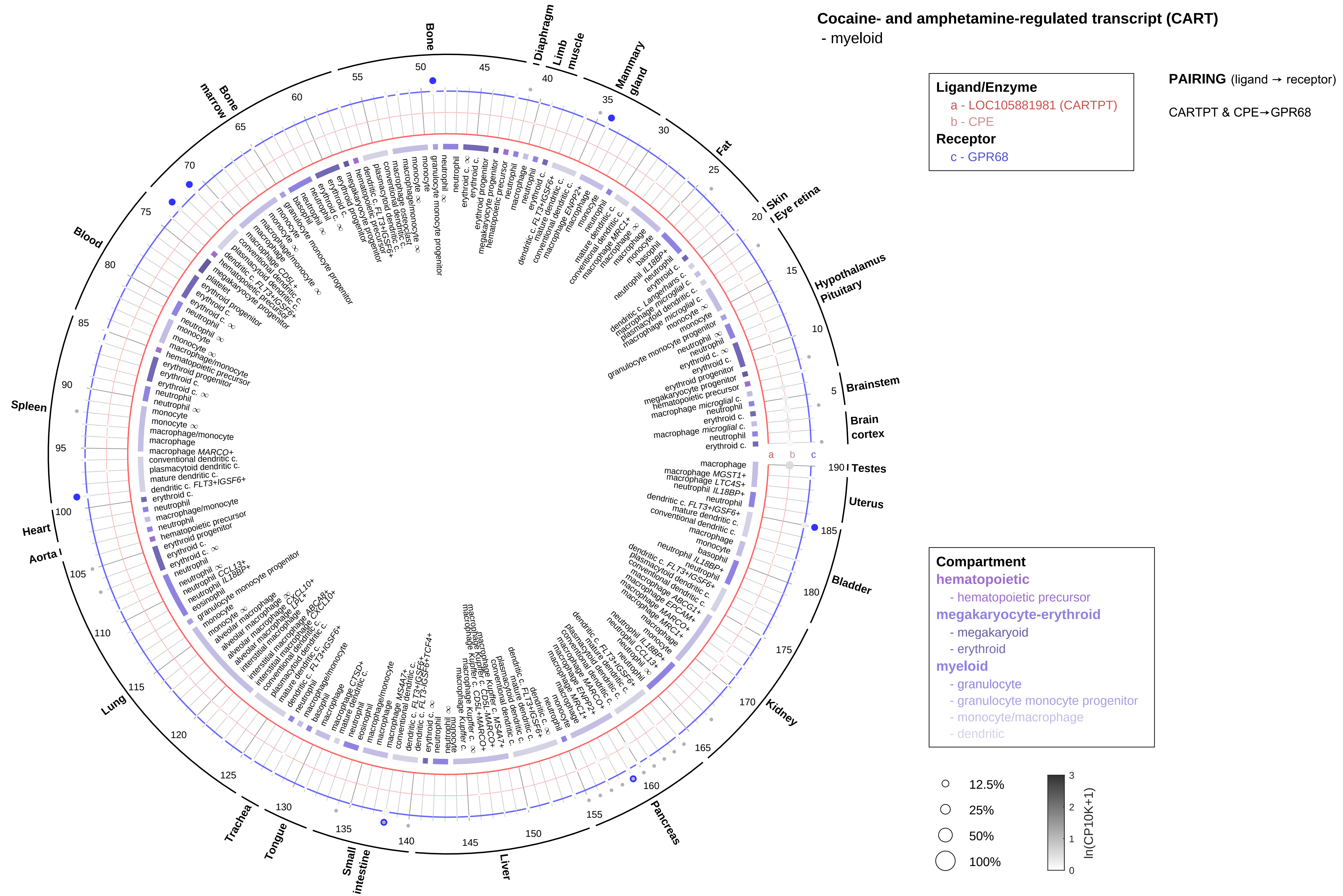
Cocaine- and amphetamine-regulated transcript (CART)
- myeloid
Ligand/Enzyme
a - LOC105881981 (CARTPT)
b - CPE
Receptor
c - GPR68
PAIRING (ligand → receptor)
CARTPT & CPE→GPR68
Compartment
hematopoietic
- hematopoietic precursor
megakaryocyte-erythroid
- megakaryoid
- erythroid
myeloid
- granulocyte
- granulocyte monocyte progenitor
- monocyte/macrophage
- dendritic
12.5%
25%
50%
100%
ln(CP10K+1)
0
1
2
3
Bone
Diaphragm
Limb muscle
Mammary gland
Fat
Skin
Eye retina
Hypothalamus
Pituitary
Brainstem
Brain cortex
Testes
Uterus
Bladder
Kidney
Pancreas
Liver
Small intestine
Tongue
Trachea
Lung
Aorta
Heart
Spleen
Blood
Bone marrow
a
b
c

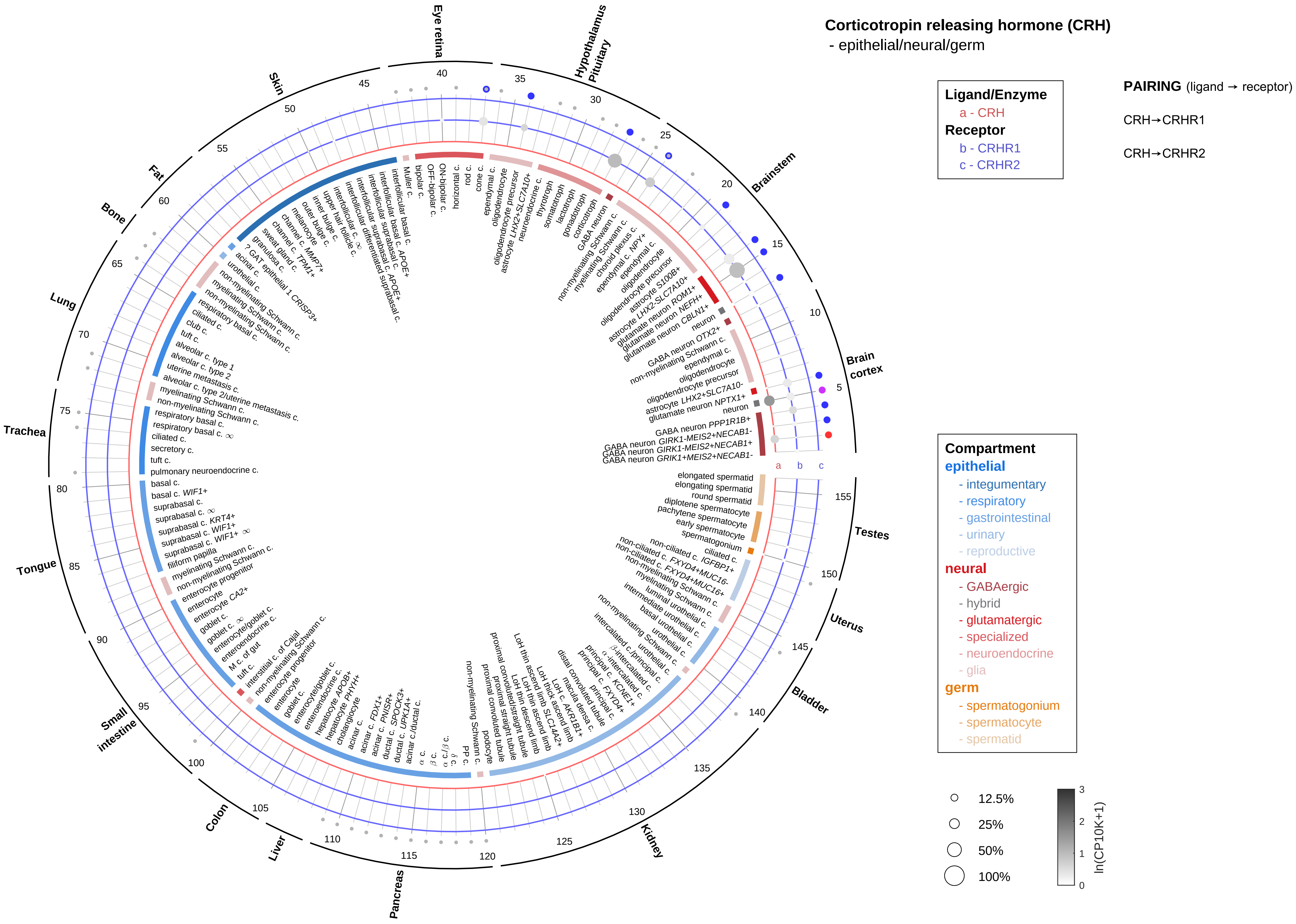
Corticotropin releasing hormone (CRH)
- epithelial/neural/germ
Ligand/Enzyme
a - CRH
Receptor
b - CRHR1
c - CRHR2
PAIRING (ligand → receptor)
CRH→CRHR1
CRH→CRHR2
Compartment
epithelial
- integumentary
- respiratory
- gastrointestinal
- urinary
- reproductive
neural
- GABAergic
- hybrid
- glutamatergic
- specialized
- neuroendocrine
- glia
germ
- spermatogonium
- spermatocyte
- spermatid
12.5%
25%
50%
100%
ln(CP10K+1)
Eye retina
Hypothalamus Pituitary
Brainstem
Brain cortex
Testes
Uterus
Bladder
Kidney
Pancreas
Liver
Colon
Small intestine
Tongue
Trachea
Lung
Bone
Fat
Skin

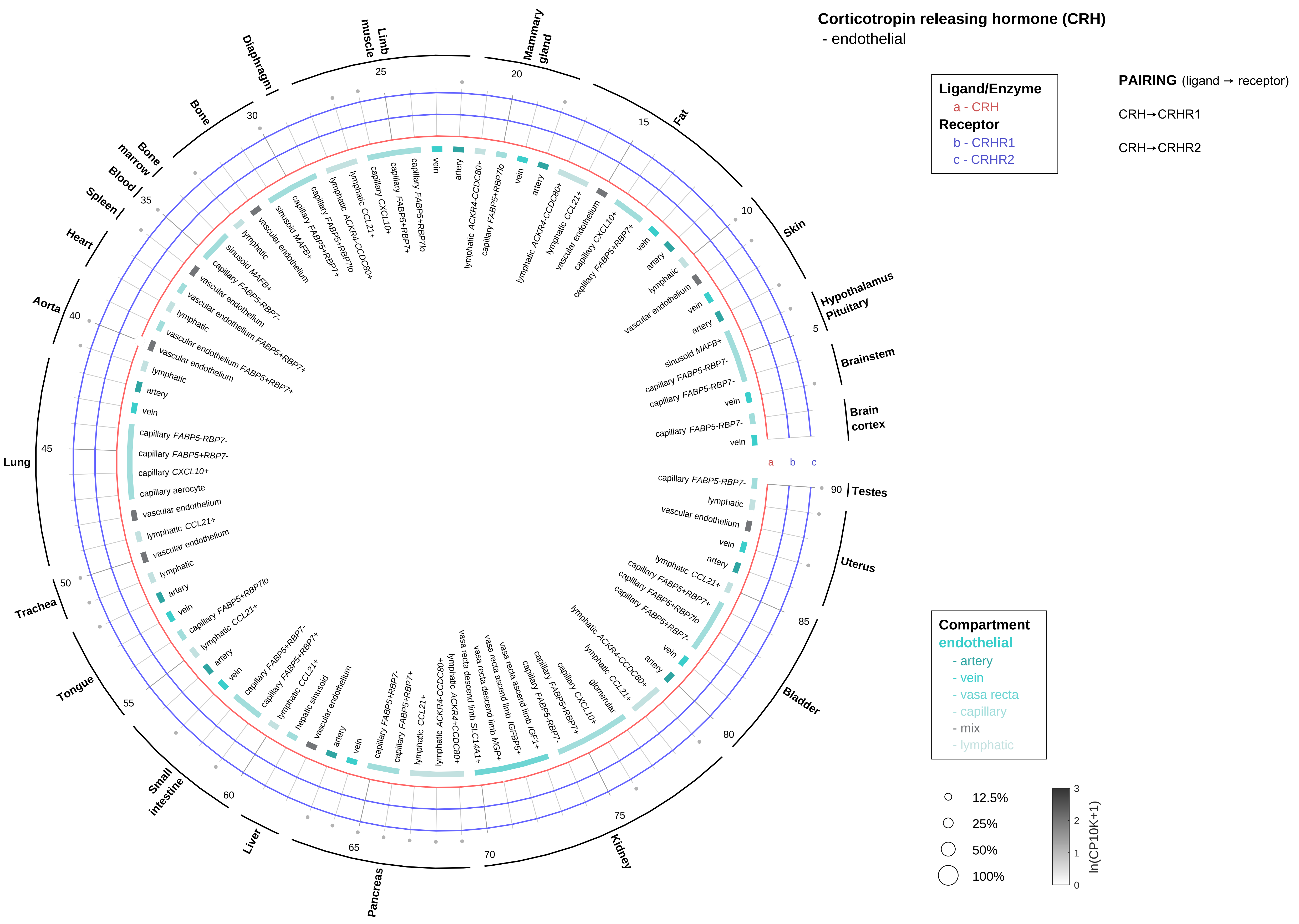

Corticotropin releasing hormone (CRH)
- endothelial
Ligand/Enzyme
a - CRH
Receptor
b - CRHR1
c - CRHR2
PAIRING (ligand → receptor)
CRH→CRHR1
CRH→CRHR2
Compartment
endothelial
- artery
- vein
- vasa recta
- capillary
- mix
- lymphatic
12.5%
25%
50%
100%
ln(CP10K+1)
Testes
Uterus
Bladder
Kidney
Pancreas
Liver
Small intestine
Tongue
Trachea
Lung
Aorta
Heart
Spleen
Blood
Bone marrow
Bone
Diaphragm
Limb muscle
Mammary gland
Fat
Skin
Hypothalamus Pituitary
Brainstem
Brain cortex

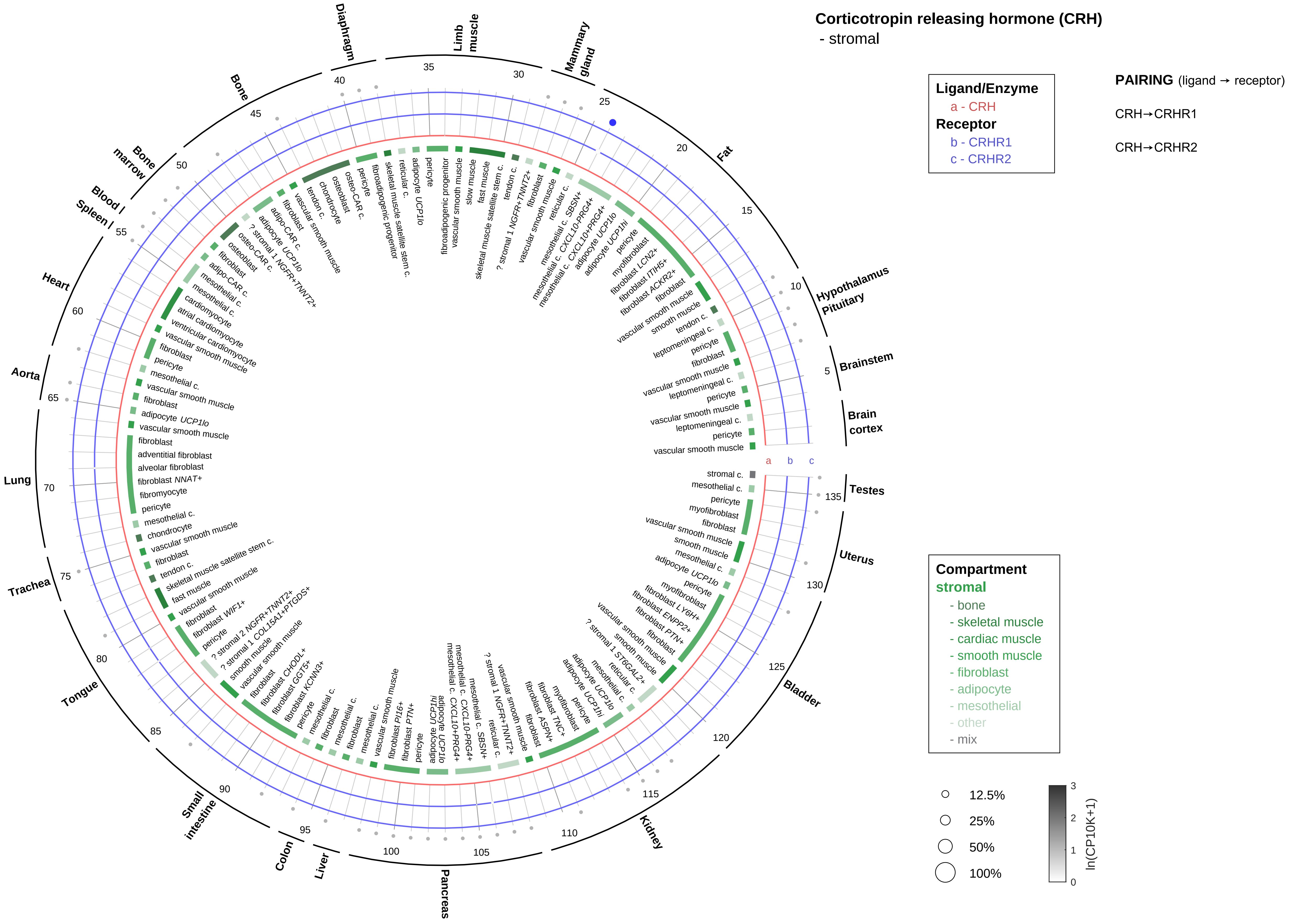
Corticotropin releasing hormone (CRH)
- stromal
Ligand/Enzyme
a - CRH
Receptor
b - CRHR1
c - CRHR2
PAIRING (ligand → receptor)
CRH→CRHR1
CRH→CRHR2
Compartment
stromal
- bone
- skeletal muscle
- cardiac muscle
- smooth muscle
- fibroblast
- adipocyte
- mesothelial
- other
- mix
12.5%
25%
50%
100%
ln(CP10K+1)
Diaphragm
Limb muscle
Mammary gland
Fat
Hypothalamus Pituitary
Brainstem
Brain cortex
Testes
Uterus
Bladder
Kidney
Pancreas
Liver
Colon
Small intestine
Tongue
Trachea
Lung
Aorta
Heart
Spleen
Blood
Bone marrow
Bone

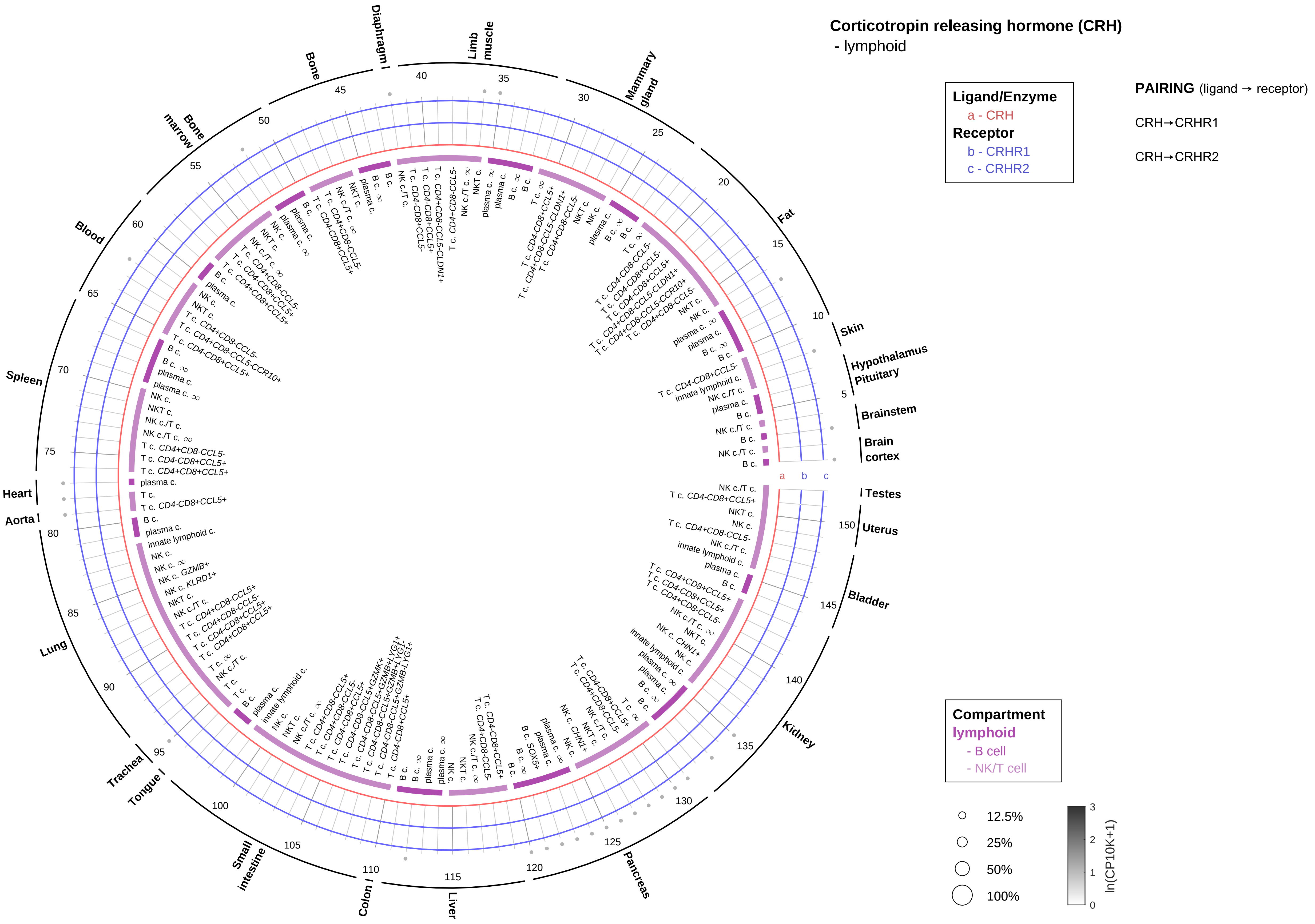

Corticotropin releasing hormone (CRH)
- lymphoid
Ligand/Enzyme
a - CRH
Receptor
b - CRHR1
c - CRHR2
PAIRING (ligand → receptor)
CRH→CRHR1
CRH→CRHR2
Compartment
lymphoid
- B cell
- NK/T cell
12.5%
25%
50%
100%
ln(CP10K+1)
Brain cortex
Brainstem
Hypothalamus Pituitary
Skin
Fat
Mammary gland
Limb muscle
Diaphragm
Bone
Bone marrow
Blood
Spleen
Heart
Aorta
Lung
Trachea
Tongue
Small intestine
Colon
Liver
Pancreas
Kidney
Bladder
Uterus
Testes

Corticotropin releasing hormone (CRH)
- myeloid

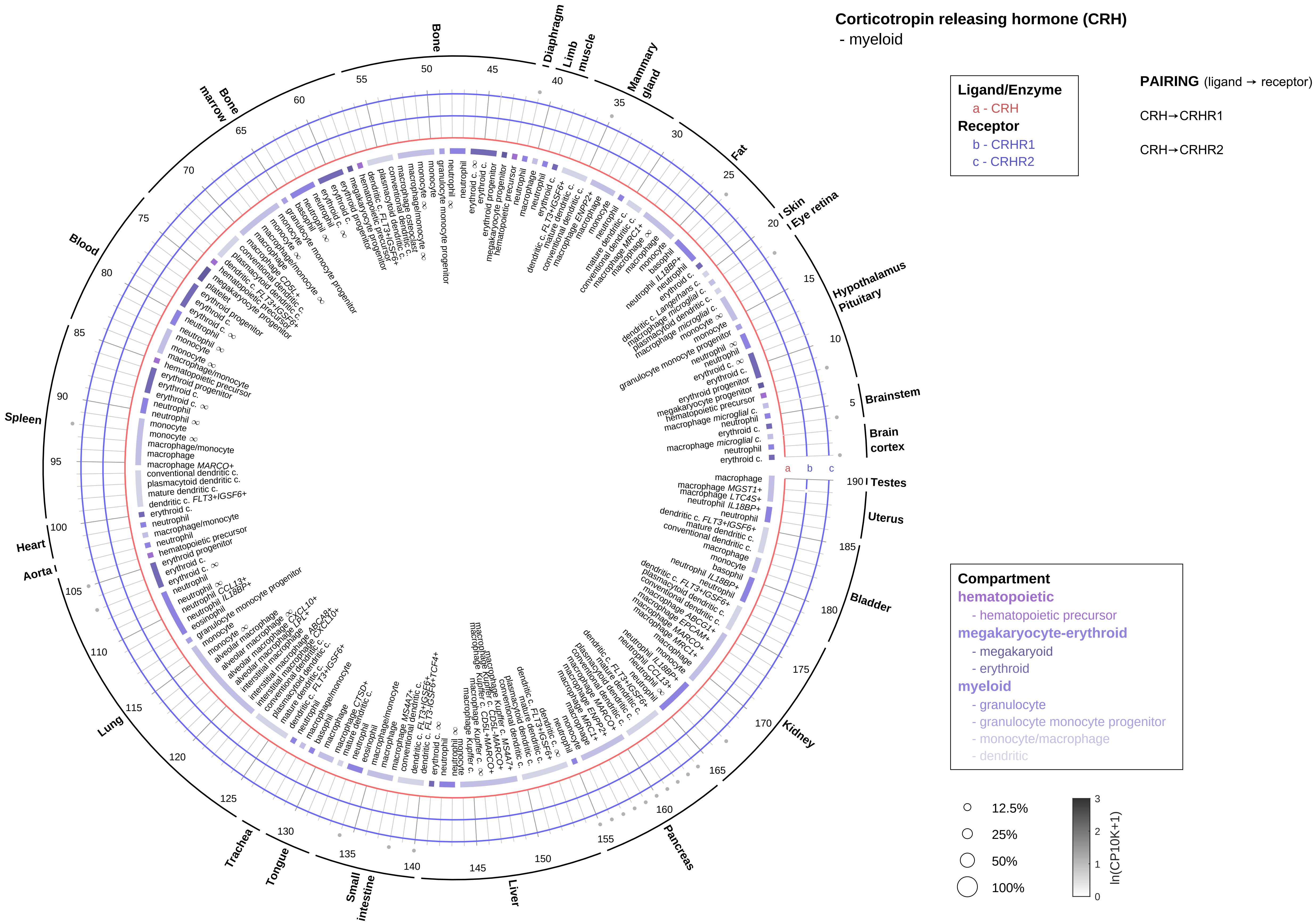

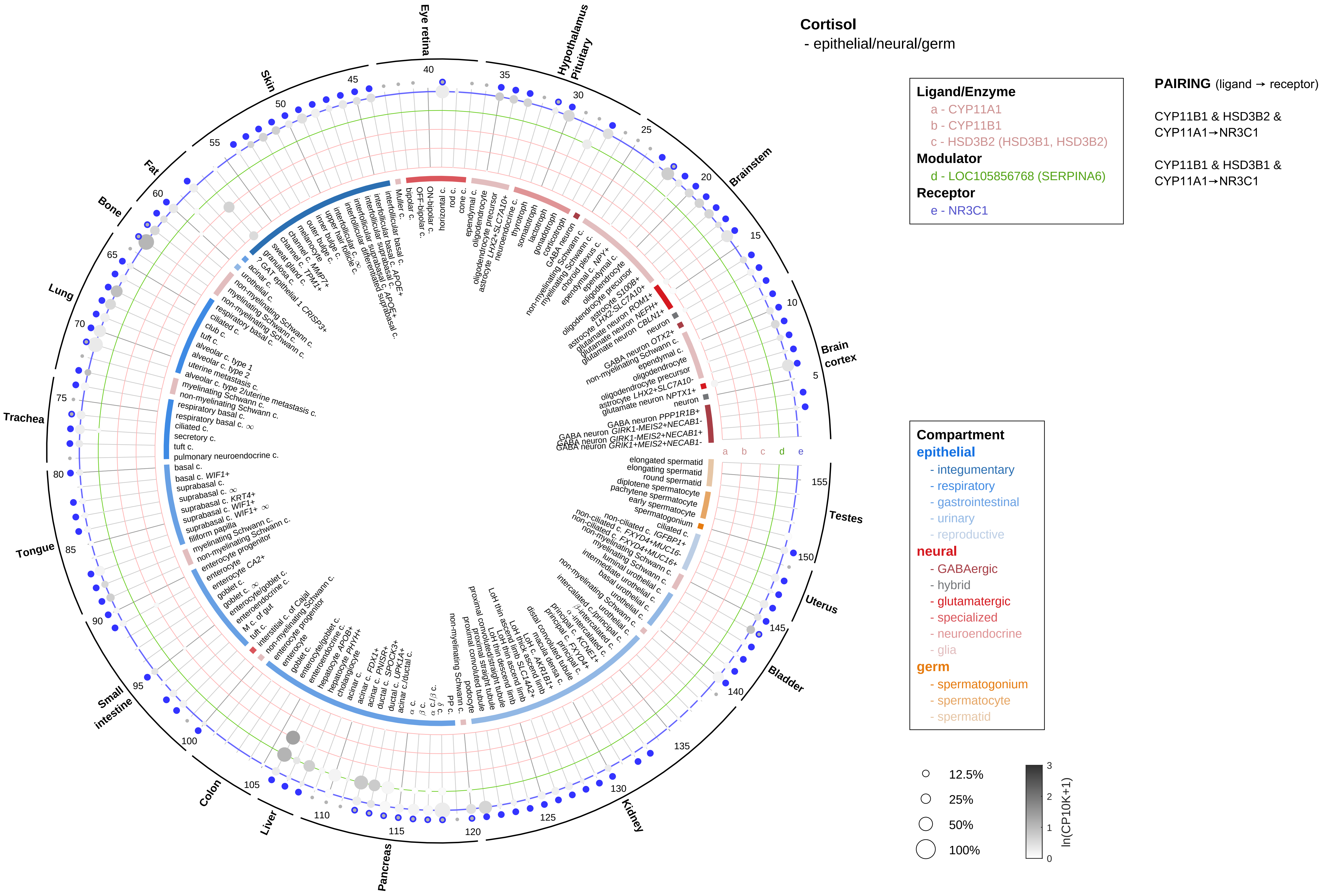
Cortisol
- epithelial/neural/germ
Ligand/Enzyme
a - CYP11A1
b - CYP11B1
c - HSD3B2 (HSD3B1, HSD3B2)
Modulator
d - LOC105856768 (SERPINA6)
Receptor
e - NR3C1
PAIRING (ligand → receptor)
CYP11B1 & HSD3B2 & CYP11A1→NR3C1
CYP11B1 & HSD3B1 & CYP11A1→NR3C1
Compartment
epithelial
- integumentary
- respiratory
- gastrointestinal
- urinary
- reproductive
neural
- GABAergic
- hybrid
- glutamatergic
- specialized
- neuroendocrine
- glia
germ
- spermatogonium
- spermatocyte
- spermatid
12.5%
25%
50%
100%
ln(CP10K+1)
Eye retina
Hypothalamus Pituitary
Brainstem
Brain cortex
Testes
Uterus
Bladder
Kidney
Pancreas
Liver
Colon
Small intestine
Tongue
Trachea
Lung
Bone
Fat
Skin
a
b
c
d
e

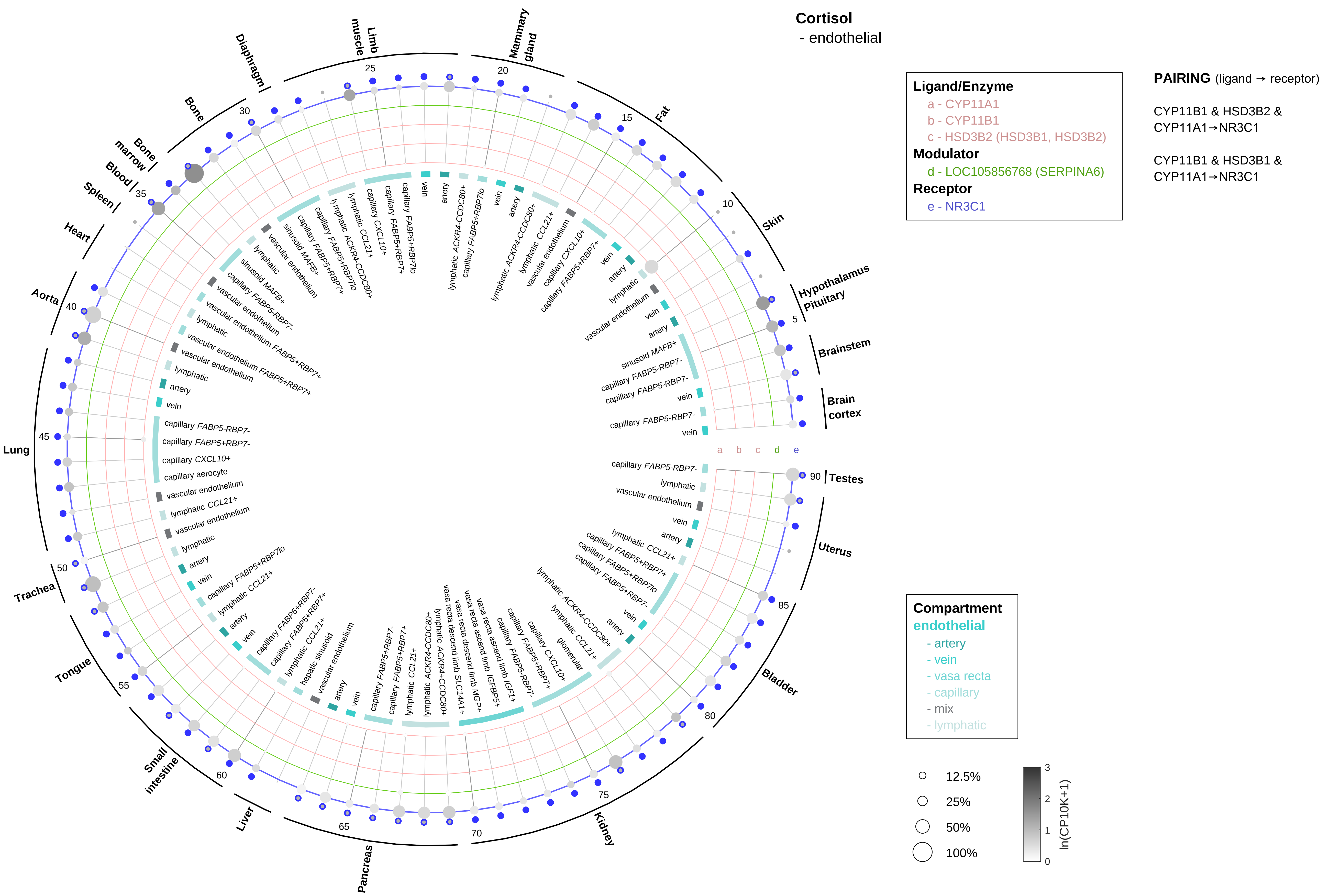
Cortisol
- endothelial
Ligand/Enzyme
a - CYP11A1
b - CYP11B1
c - HSD3B2 (HSD3B1, HSD3B2)
Modulator
d - LOC105856768 (SERPINA6)
Receptor
e - NR3C1
PAIRING (ligand → receptor)
CYP11B1 & HSD3B2 & CYP11A1→NR3C1
CYP11B1 & HSD3B1 & CYP11A1→NR3C1
Compartment
endothelial
- artery
- vein
- vasa recta
- capillary
- mix
- lymphatic
12.5%
25%
50%
100%
ln(CP10K+1)
Hypothalamus Pituitary
Brainstem
Brain cortex
Testes
Uterus
Bladder
Kidney
Pancreas
Liver
Small intestine
Tongue
Trachea
Lung
Aorta
Heart
Spleen
Blood
Bone marrow
Bone
Diaphragm
Limb muscle
Mammary gland
Fat
Skin

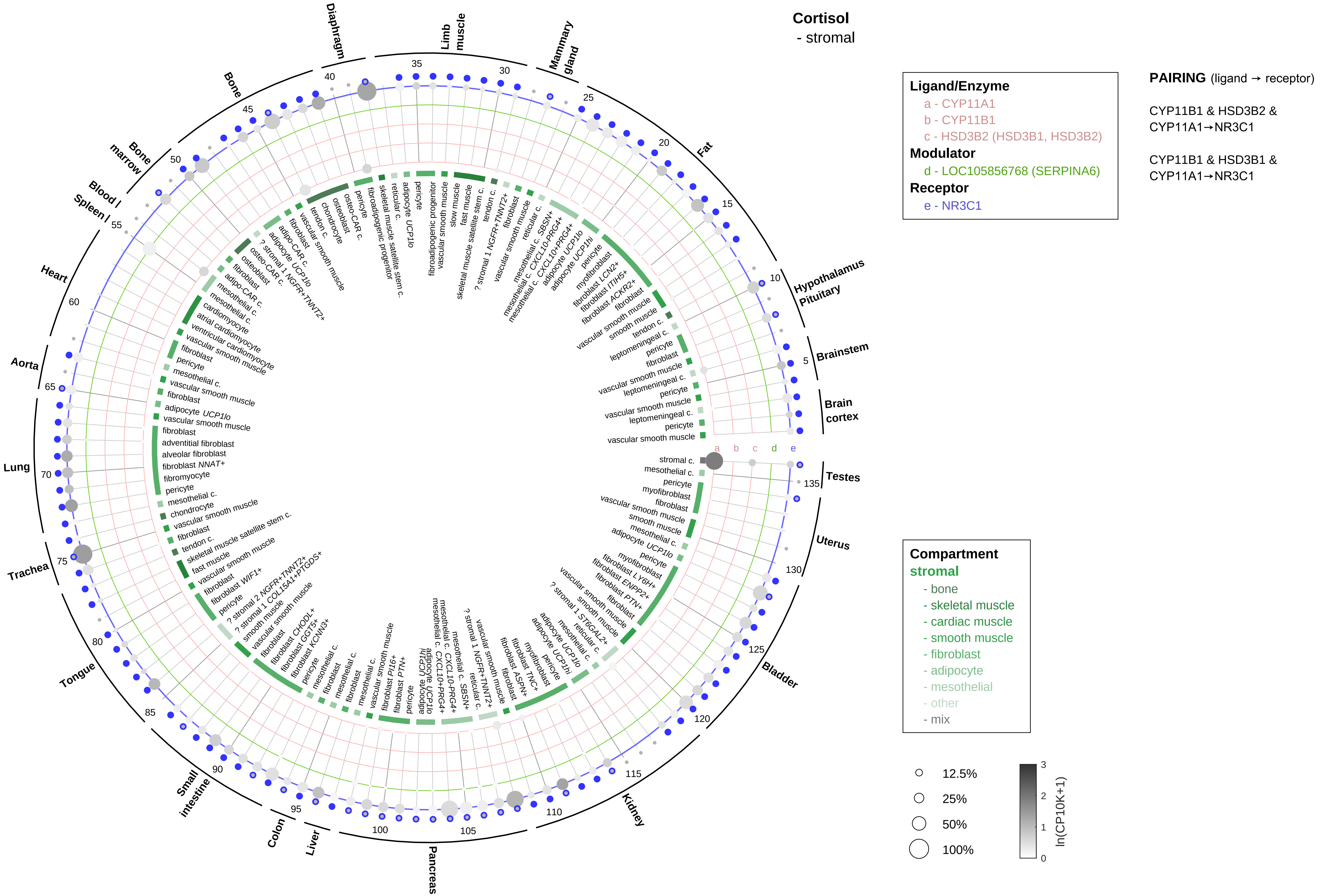

Cortisol
- stromal
Ligand/Enzyme
a - CYP11A1
b - CYP11B1
c - HSD3B2 (HSD3B1, HSD3B2)
Modulator
d - LOC105856768 (SERPINA6)
Receptor
e - NR3C1
PAIRING (ligand → receptor)
CYP11B1 & HSD3B2 & CYP11A1→NR3C1
CYP11B1 & HSD3B1 & CYP11A1→NR3C1
Compartment
stromal
- bone
- skeletal muscle
- cardiac muscle
- smooth muscle
- fibroblast
- adipocyte
- mesothelial
- other
- mix
12.5%
25%
50%
100%
ln(CP10K+1)
Brain cortex
Brainstem
Hypothalamus Pituitary
Fat
Mammary gland
Limb muscle
Diaphragm
Bone
Bone marrow
Blood
Spleen
Heart
Aorta
Lung
Trachea
Tongue
Small intestine
Colon
Liver
Pancreas
Kidney
Bladder
Uterus
Testes

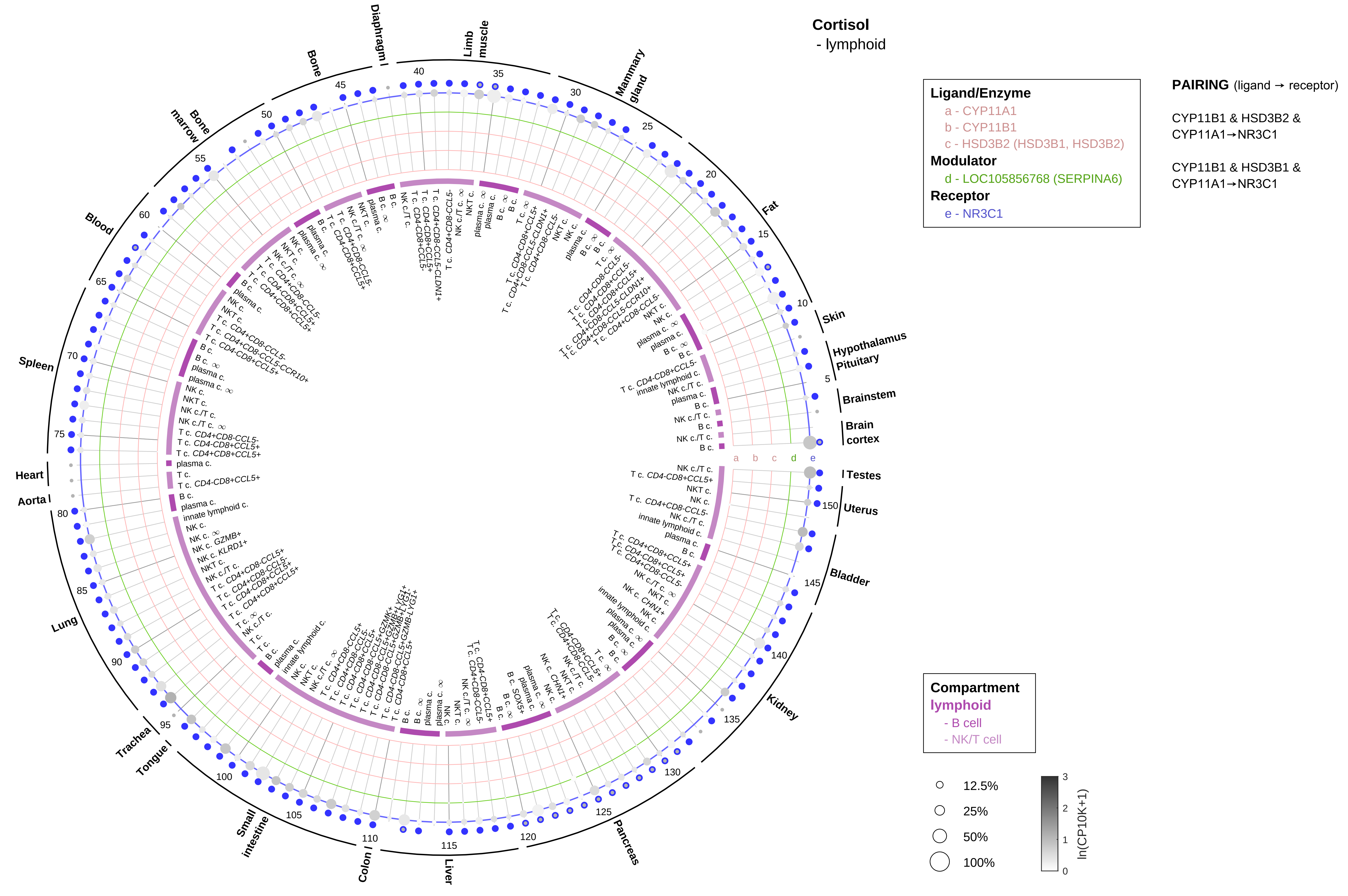
Cortisol
- lymphoid
Ligand/Enzyme
a - CYP11A1
b - CYP11B1
c - HSD3B2 (HSD3B1, HSD3B2)
Modulator
d - LOC105856768 (SERPINA6)
Receptor
e - NR3C1
PAIRING (ligand → receptor)
CYP11B1 & HSD3B2 & CYP11A1→NR3C1
CYP11B1 & HSD3B1 & CYP11A1→NR3C1
Compartment
lymphoid
- B cell
- NK/T cell
12.5%
25%
50%
100%
ln(CP10K+1)
Diaphragm
Limb muscle
Mammary gland
Fat
Skin
Hypothalamus
Pituitary
Brainstem
Brain cortex
Testes
Uterus
Bladder
Kidney
Pancreas
Liver
Colon
Small intestine
Tongue
Trachea
Lung
Aorta
Heart
Spleen
Blood
Bone marrow
Bone
a
b
c
d
e

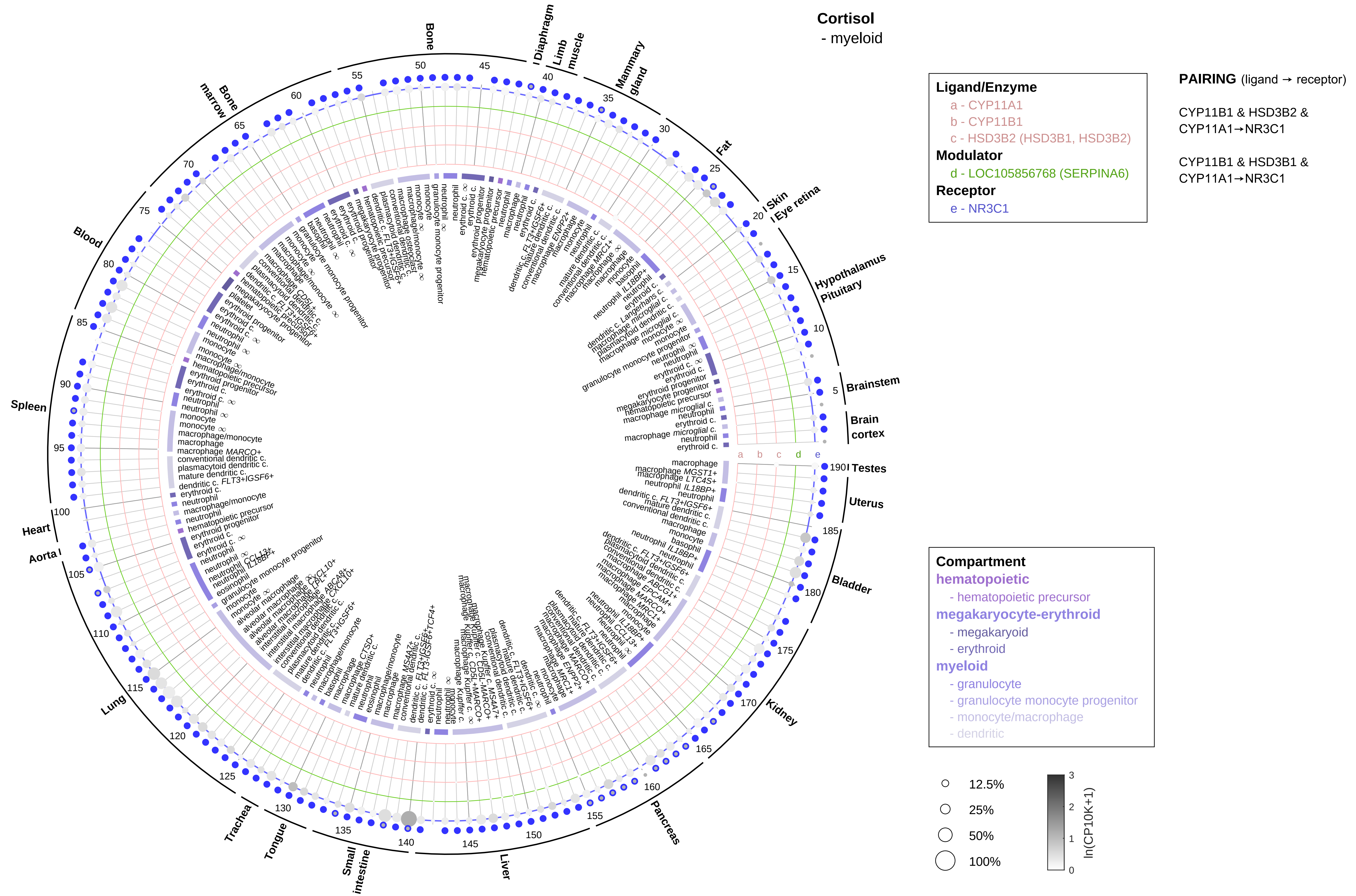

Cortisol
- myeloid
Ligand/Enzyme
a - CYP11A1
b - CYP11B1
c - HSD3B2 (HSD3B1, HSD3B2)
Modulator
d - LOC105856768 (SERPINA6)
Receptor
e - NR3C1
PAIRING (ligand → receptor)
CYP11B1 & HSD3B2 & CYP11A1→NR3C1
CYP11B1 & HSD3B1 & CYP11A1→NR3C1
Compartment
hematopoietic
- hematopoietic precursor
megakaryocyte-erythroid
- megakaryoid
- erythroid
myeloid
- granulocyte
- granulocyte monocyte progenitor
- monocyte/macrophage
- dendritic
12.5%
25%
50%
100%
ln(CP10K+1)
Bone
Diaphragm
Limb muscle
Mammary gland
Fat
Skin
Eye retina
Hypothalamus
Pituitary
Brainstem
Brain cortex
Testes
Uterus
Bladder
Kidney
Pancreas
Liver
Small intestine
Tongue
Trachea
Lung
Aorta
Heart
Spleen
Blood
Bone marrow

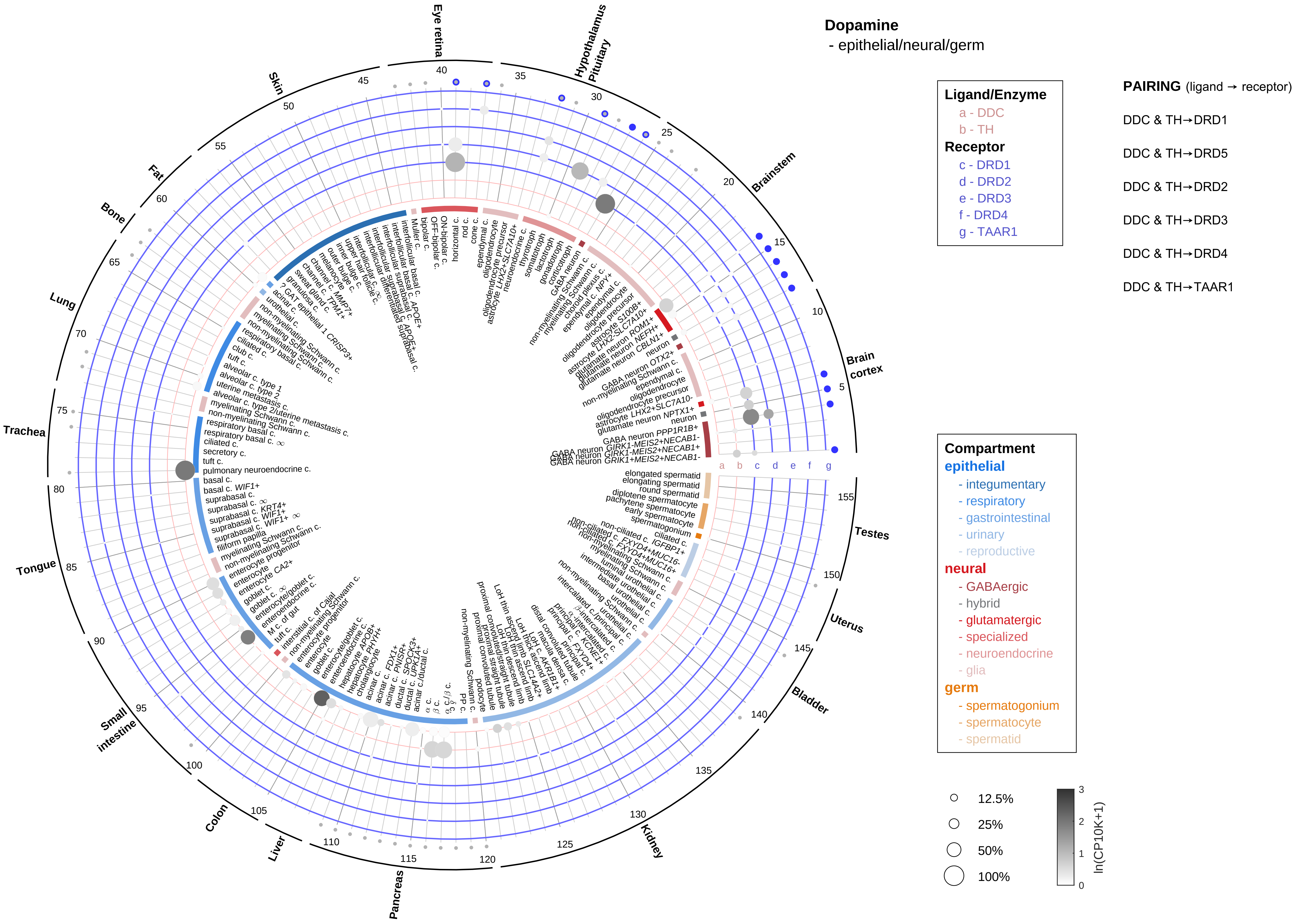

Dopamine
- epithelial/neural/germ
Ligand/Enzyme
a - DDC
b - TH
Receptor
c - DRD1
d - DRD2
e - DRD3
f - DRD4
g - TAAR1
PAIRING (ligand → receptor)
DDC & TH→DRD1
DDC & TH→DRD5
DDC & TH→DRD2
DDC & TH→DRD3
DDC & TH→DRD4
DDC & TH→TAAR1
Compartment
epithelial
- integumentary
- respiratory
- gastrointestinal
- urinary
- reproductive
neural
- GABAergic
- hybrid
- glutamatergic
- specialized
- neuroendocrine
- glia
germ
- spermatogonium
- spermatocyte
- spermatid
12.5%
25%
50%
100%
ln(CP10K+1)
Eye retina
Hypothalamus Pituitary
Brainstem
Brain cortex
Testes
Uterus
Bladder
Kidney
Pancreas
Liver
Colon
Small intestine
Tongue
Trachea
Lung
Bone
Fat
Skin

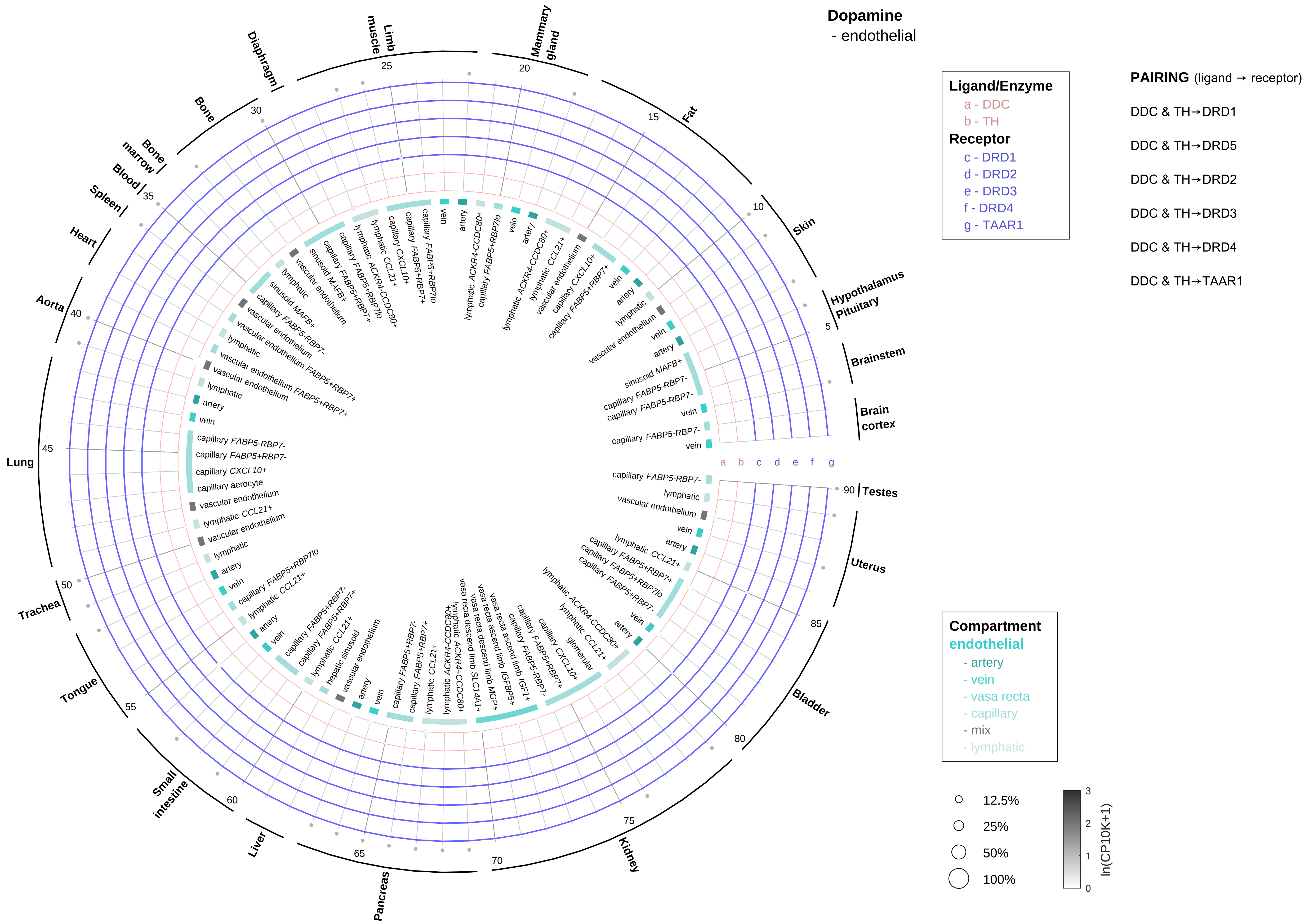

Dopamine
- endothelial
Ligand/Enzyme
a - DDC
b - TH
Receptor
c - DRD1
d - DRD2
e - DRD3
f - DRD4
g - TAAR1
PAIRING (ligand → receptor)
DDC & TH→DRD1
DDC & TH→DRD5
DDC & TH→DRD2
DDC & TH→DRD3
DDC & TH→DRD4
DDC & TH→TAAR1
Compartment
endothelial
- artery
- vein
- vasa recta
- capillary
- mix
- lymphatic
12.5%
25%
50%
100%
ln(CP10K+1)
Brain cortex
Brainstem
Hypothalamus Pituitary
Skin
Fat
Mammary gland
Limb muscle
Diaphragm
Bone
Bone marrow
Blood
Spleen
Heart
Aorta
Lung
Trachea
Tongue
Small intestine
Liver
Pancreas
Kidney
Bladder
Uterus
Testes

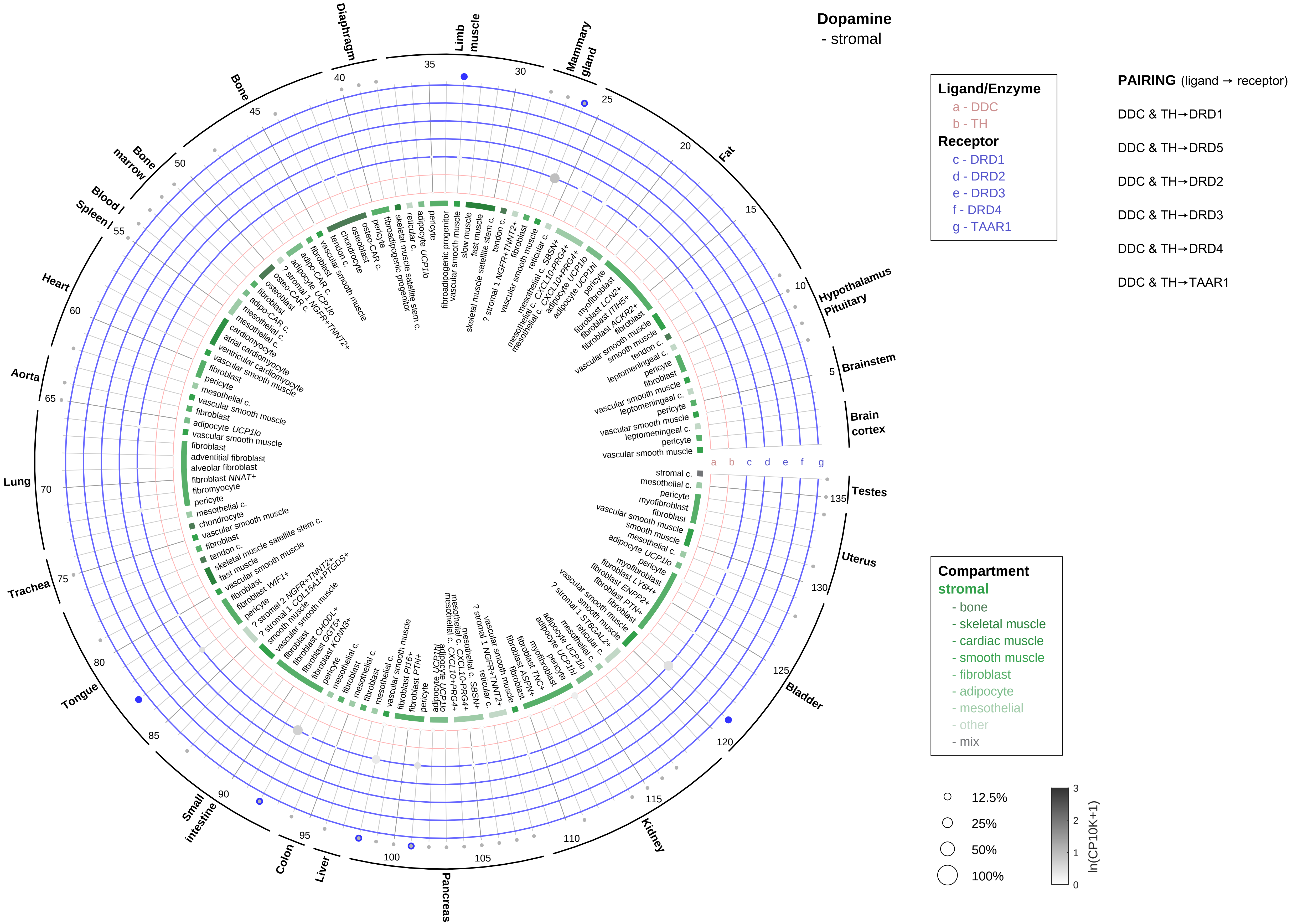

Dopamine
- stromal
Ligand/Enzyme
a - DDC
b - TH
Receptor
c - DRD1
d - DRD2
e - DRD3
f - DRD4
g - TAAR1
PAIRING (ligand → receptor)
DDC & TH→DRD1
DDC & TH→DRD5
DDC & TH→DRD2
DDC & TH→DRD3
DDC & TH→DRD4
DDC & TH→TAAR1
Compartment
stromal
- bone
- skeletal muscle
- cardiac muscle
- smooth muscle
- fibroblast
- adipocyte
- mesothelial
- other
- mix
12.5%
25%
50%
100%
ln(CP10K+1)
Limb muscle
Mammary gland
Fat
Hypothalamus Pituitary
Brainstem
Brain cortex
Testes
Uterus
Bladder
Kidney
Pancreas
Liver
Colon
Small intestine
Tongue
Trachea
Lung
Aorta
Heart
Spleen
Blood
Bone marrow
Bone
Diaphragm

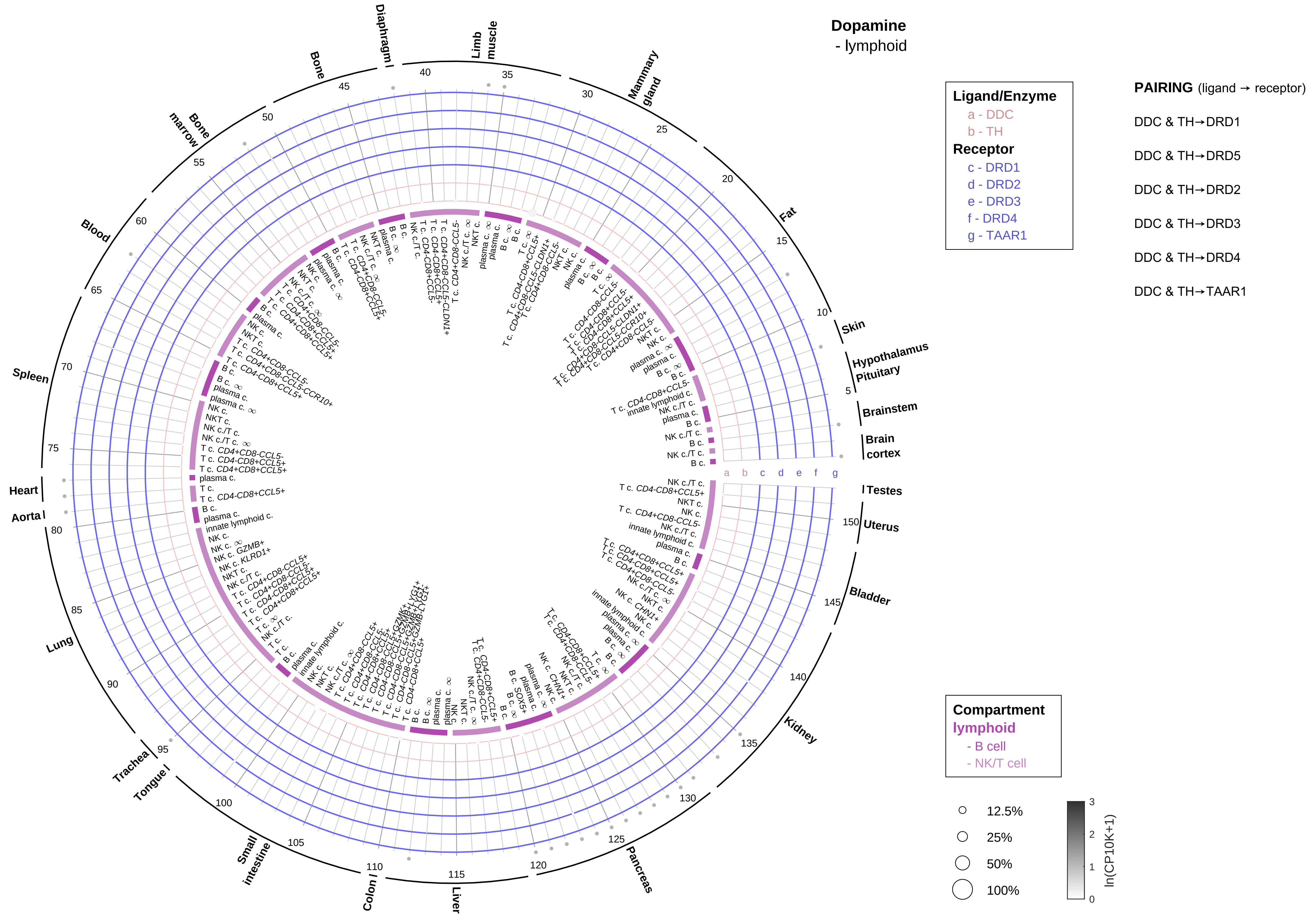
Dopamine
- lymphoid
Ligand/Enzyme
a - DDC
b - TH
Receptor
c - DRD1
d - DRD2
e - DRD3
f - DRD4
g - TAAR1
PAIRING (ligand → receptor)
DDC & TH→DRD1
DDC & TH→DRD5
DDC & TH→DRD2
DDC & TH→DRD3
DDC & TH→DRD4
DDC & TH→TAAR1
Compartment
lymphoid
- B cell
- NK/T cell
12.5%
25%
50%
100%
ln(CP10K+1)
Diaphragm
Limb muscle
Mammary gland
Fat
Skin
Hypothalamus
Pituitary
Brainstem
Brain cortex
Testes
Uterus
Bladder
Kidney
Pancreas
Liver
Colon
Small intestine
Tongue
Trachea
Lung
Aorta
Heart
Spleen
Blood
Bone marrow
Bone

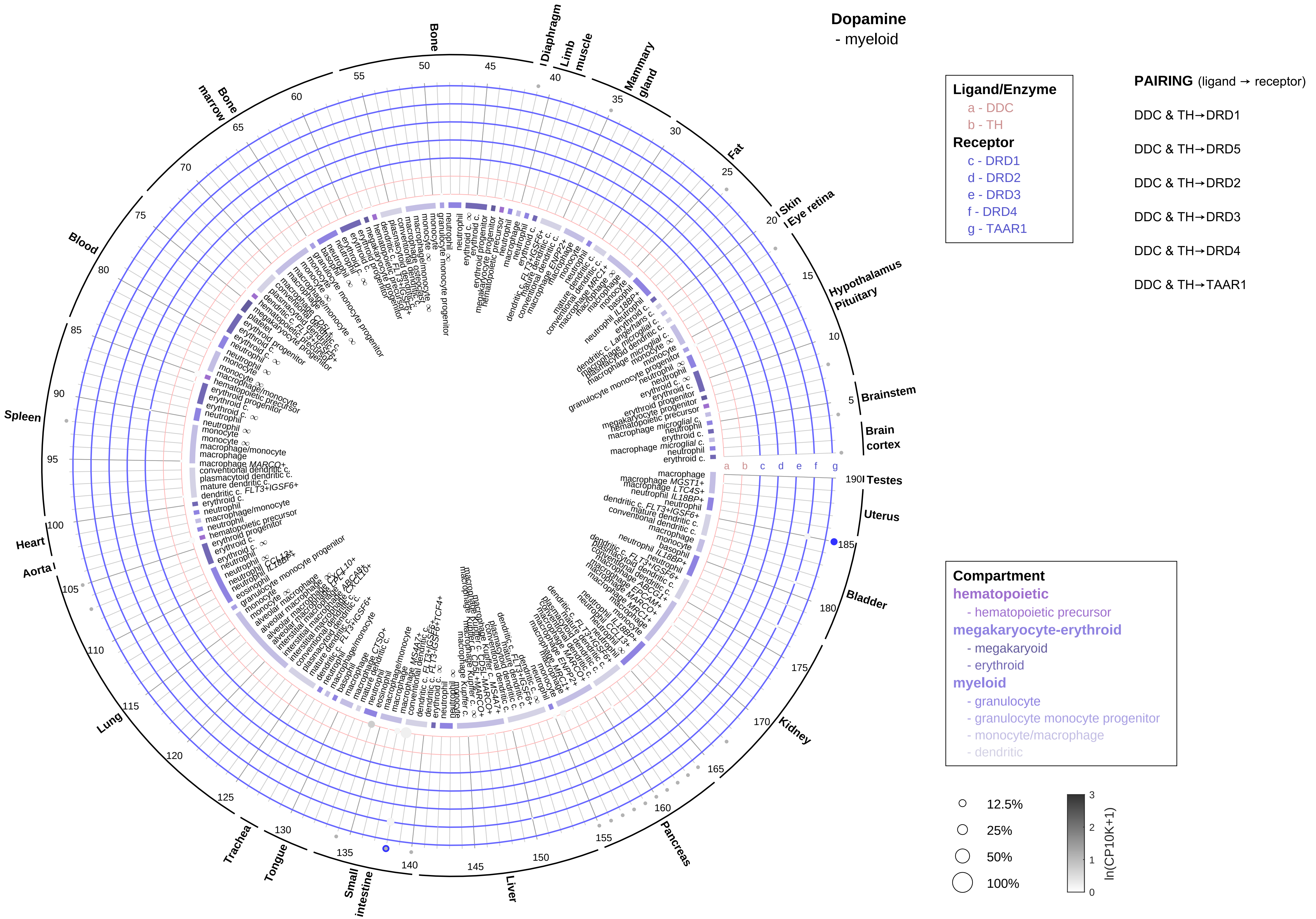

Dopamine
- myeloid
Ligand/Enzyme
a - DDC
b - TH
Receptor
c - DRD1
d - DRD2
e - DRD3
f - DRD4
g - TAAR1
PAIRING (ligand → receptor)
DDC & TH→DRD1
DDC & TH→DRD5
DDC & TH→DRD2
DDC & TH→DRD3
DDC & TH→DRD4
DDC & TH→TAAR1
Compartment
hematopoietic
- hematopoietic precursor
megakaryocyte-erythroid
- megakaryoid
- erythroid
myeloid
- granulocyte
- granulocyte monocyte progenitor
- monocyte/macrophage
- dendritic
12.5%
25%
50%
100%
ln(CP10K+1)
Testes
Brain cortex
Brainstem
Hypothalamus
Pituitary
Eye retina
Skin
Fat
Mammary gland
Limb muscle
Diaphragm
Bone
Bone marrow
Blood
Spleen
Heart
Aorta
Lung
Trachea
Tongue
Small intestine
Liver
Pancreas
Kidney
Bladder
Uterus

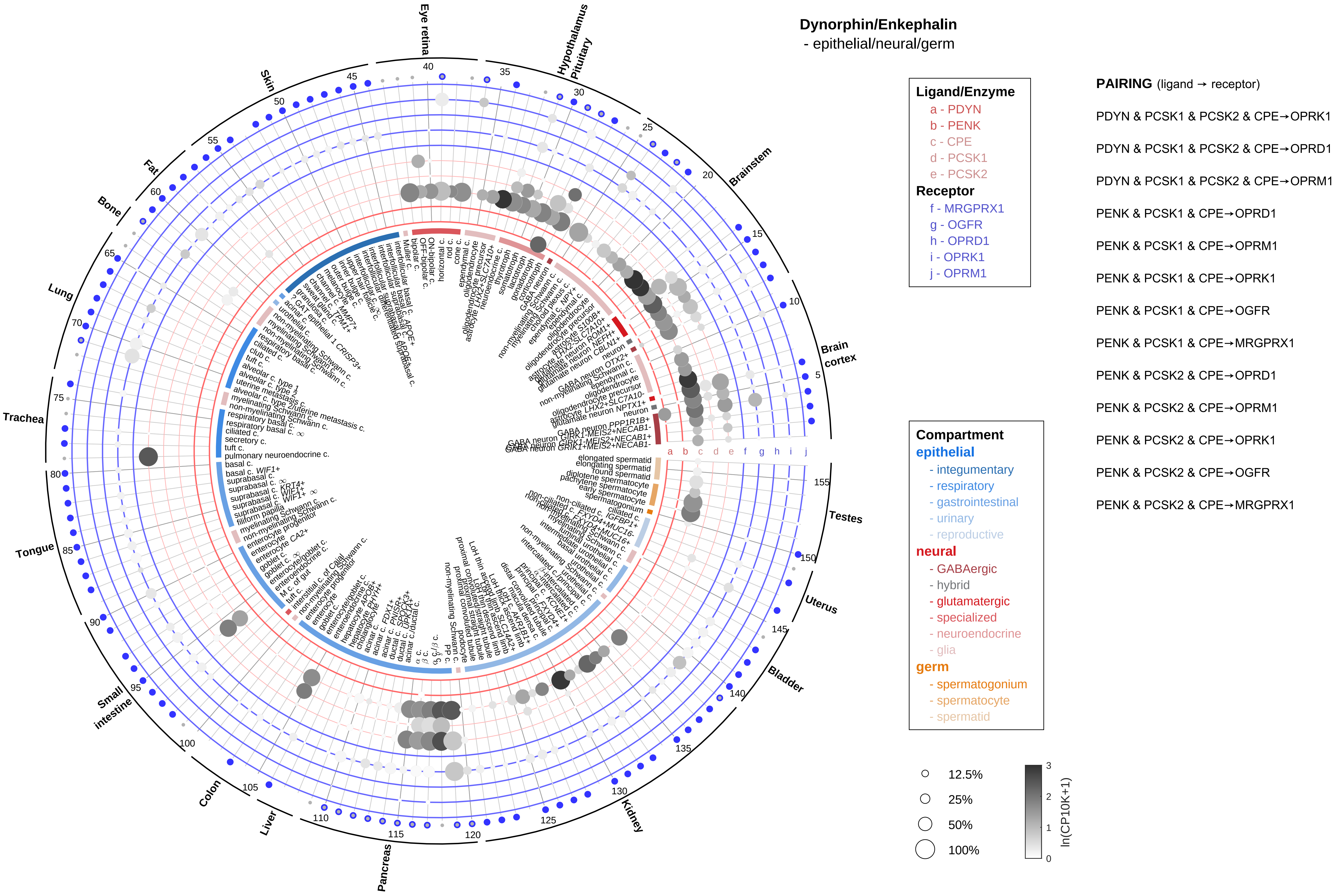

Dynorphin/Enkephalin
- epithelial/neural/germ
Eye retina
Hypothalamus Pituitary
Brainstem
Brain cortex
Testes
Uterus
Bladder
Kidney
Pancreas
Liver
Colon
Small intestine
Tongue
Trachea
Lung
Bone
Fat
Skin
a b c d e f g h i j
Ligand/Enzyme
a - PDYN
b - PENK
c - CPE
d - PCSK1
e - PCSK2
Receptor
f - MRGPRX1
g - OGFR
h - OPRD1
i - OPRK1
j - OPRM1
PAIRING (ligand → receptor)
PDYN & PCSK1 & PCSK2 & CPE→OPRK1
PDYN & PCSK1 & PCSK2 & CPE→OPRD1
PDYN & PCSK1 & PCSK2 & CPE→OPRM1
PENK & PCSK1 & CPE→OPRD1
PENK & PCSK1 & CPE→OPRM1
PENK & PCSK1 & CPE→OPRK1
PENK & PCSK1 & CPE→OGFR
PENK & PCSK1 & CPE→MRGPRX1
PENK & PCSK2 & CPE→OPRD1
PENK & PCSK2 & CPE→OPRM1
PENK & PCSK2 & CPE→OPRK1
PENK & PCSK2 & CPE→OGFR
PENK & PCSK2 & CPE→MRGPRX1
Compartment
epithelial
- integumentary
- respiratory
- gastrointestinal
- urinary
- reproductive
neural
- GABAergic
- hybrid
- glutamatergic
- specialized
- neuroendocrine
- glia
germ
- spermatogonium
- spermatocyte
- spermatid
12.5%
25%
50%
100%
ln(CP10K+1)
0
1
2
3

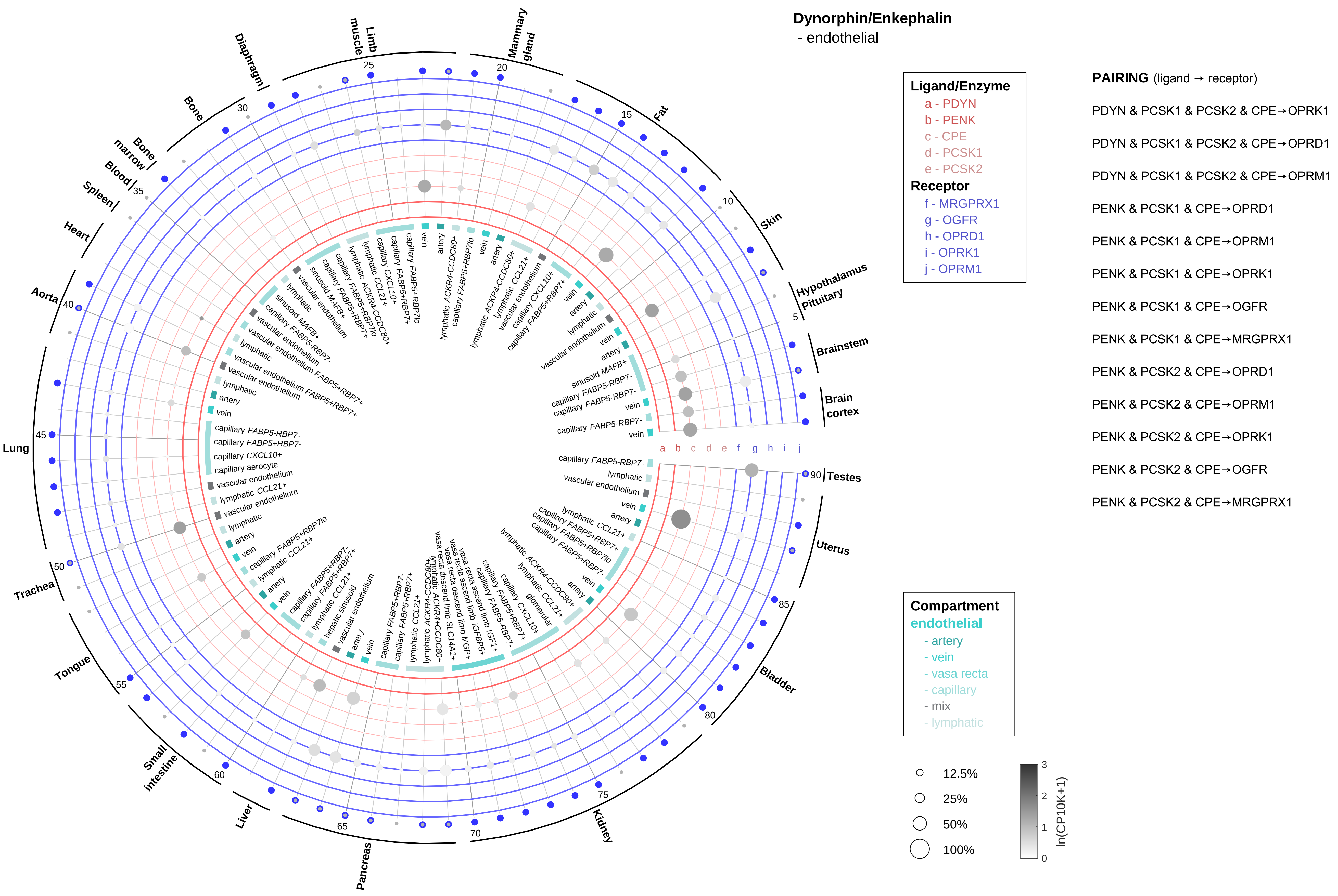
Dynorphin/Enkephalin
- endothelial
Ligand/Enzyme
a - PDYN
b - PENK
c - CPE
d - PCSK1
e - PCSK2
Receptor
f - MRGPRX1
g - OGFR
h - OPRD1
i - OPRK1
j - OPRM1
PAIRING (ligand → receptor)
PDYN & PCSK1 & PCSK2 & CPE→OPRK1
PDYN & PCSK1 & PCSK2 & CPE→OPRD1
PDYN & PCSK1 & PCSK2 & CPE→OPRM1
PENK & PCSK1 & CPE→OPRD1
PENK & PCSK1 & CPE→OPRM1
PENK & PCSK1 & CPE→OPRK1
PENK & PCSK1 & CPE→OGFR
PENK & PCSK1 & CPE→MRGPRX1
PENK & PCSK2 & CPE→OPRD1
PENK & PCSK2 & CPE→OPRM1
PENK & PCSK2 & CPE→OPRK1
PENK & PCSK2 & CPE→OGFR
PENK & PCSK2 & CPE→MRGPRX1
Compartment
endothelial
- artery
- vein
- vasa recta
- capillary
- mix
- lymphatic
12.5%
25%
50%
100%
ln(CP10K+1)
Hypothalamus Pituitary
Brainstem
Brain cortex
Testes
Uterus
Bladder
Kidney
Pancreas
Liver
Small intestine
Tongue
Trachea
Lung
Aorta
Heart
Spleen
Blood
Bone marrow
Bone
Diaphragm
Limb muscle
Mammary gland
Fat
Skin

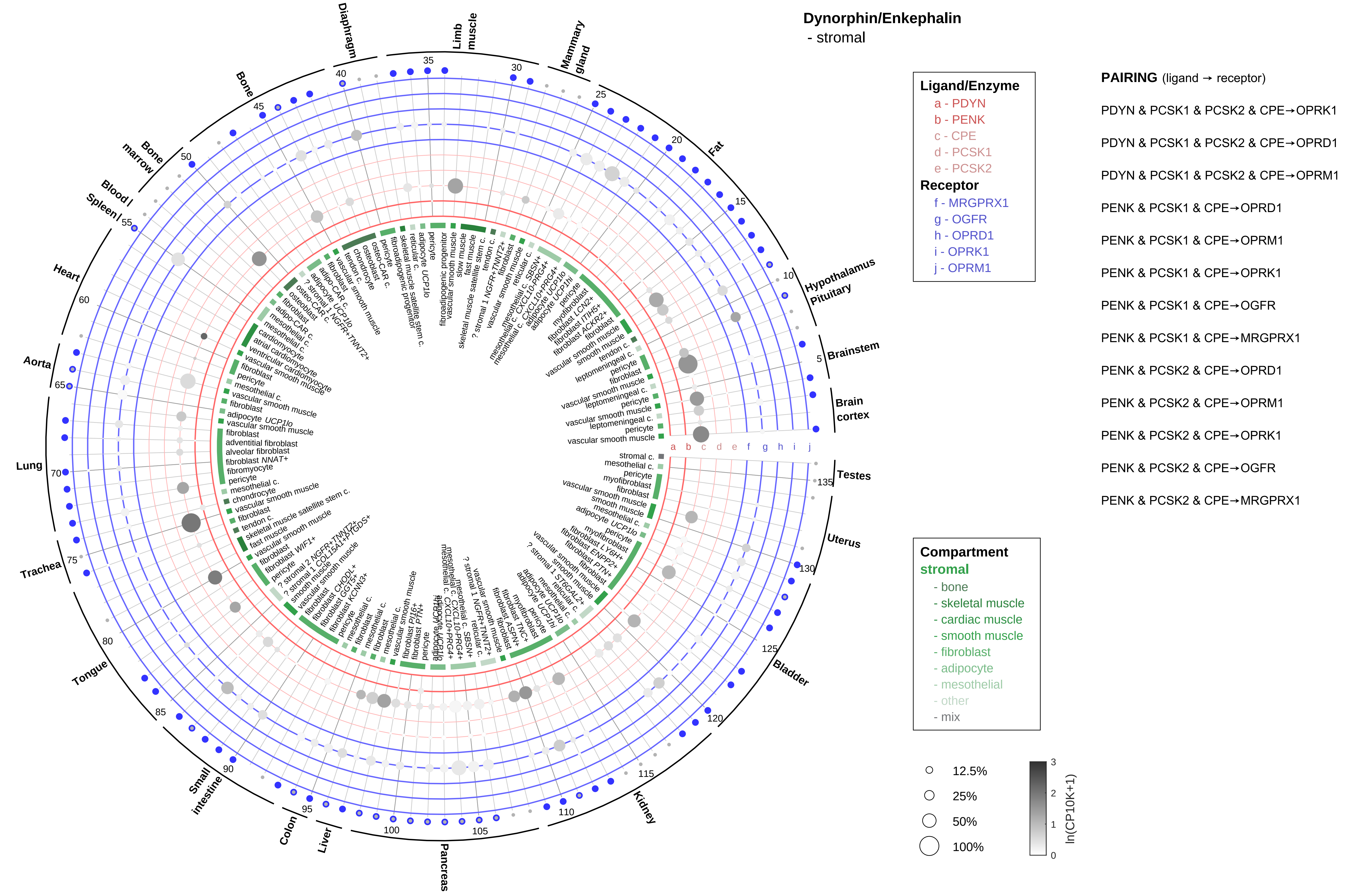
Dynorphin/Enkephalin
- stromal
Ligand/Enzyme
a - PDYN
b - PENK
c - CPE
d - PCSK1
e - PCSK2
Receptor
f - MRGPRX1
g - OGFR
h - OPRD1
i - OPRK1
j - OPRM1
PAIRING (ligand → receptor)
PDYN & PCSK1 & PCSK2 & CPE→OPRK1
PDYN & PCSK1 & PCSK2 & CPE→OPRD1
PDYN & PCSK1 & PCSK2 & CPE→OPRM1
PENK & PCSK1 & CPE→OPRD1
PENK & PCSK1 & CPE→OPRM1
PENK & PCSK1 & CPE→OPRK1
PENK & PCSK1 & CPE→OGFR
PENK & PCSK1 & CPE→MRGPRX1
PENK & PCSK2 & CPE→OPRD1
PENK & PCSK2 & CPE→OPRM1
PENK & PCSK2 & CPE→OPRK1
PENK & PCSK2 & CPE→OGFR
PENK & PCSK2 & CPE→MRGPRX1
Compartment
stromal
- bone
- skeletal muscle
- cardiac muscle
- smooth muscle
- fibroblast
- adipocyte
- mesothelial
- other
- mix
12.5%
25%
50%
100%
ln(CP10K+1)
Limb muscle
Mammary gland
Fat
Hypothalamus Pituitary
Brainstem
Brain cortex
Testes
Uterus
Bladder
Kidney
Pancreas
Liver
Colon
Small intestine
Tongue
Trachea
Lung
Aorta
Heart
Spleen
Blood
Bone marrow
Bone
Diaphragm
a b c d e f g h i j

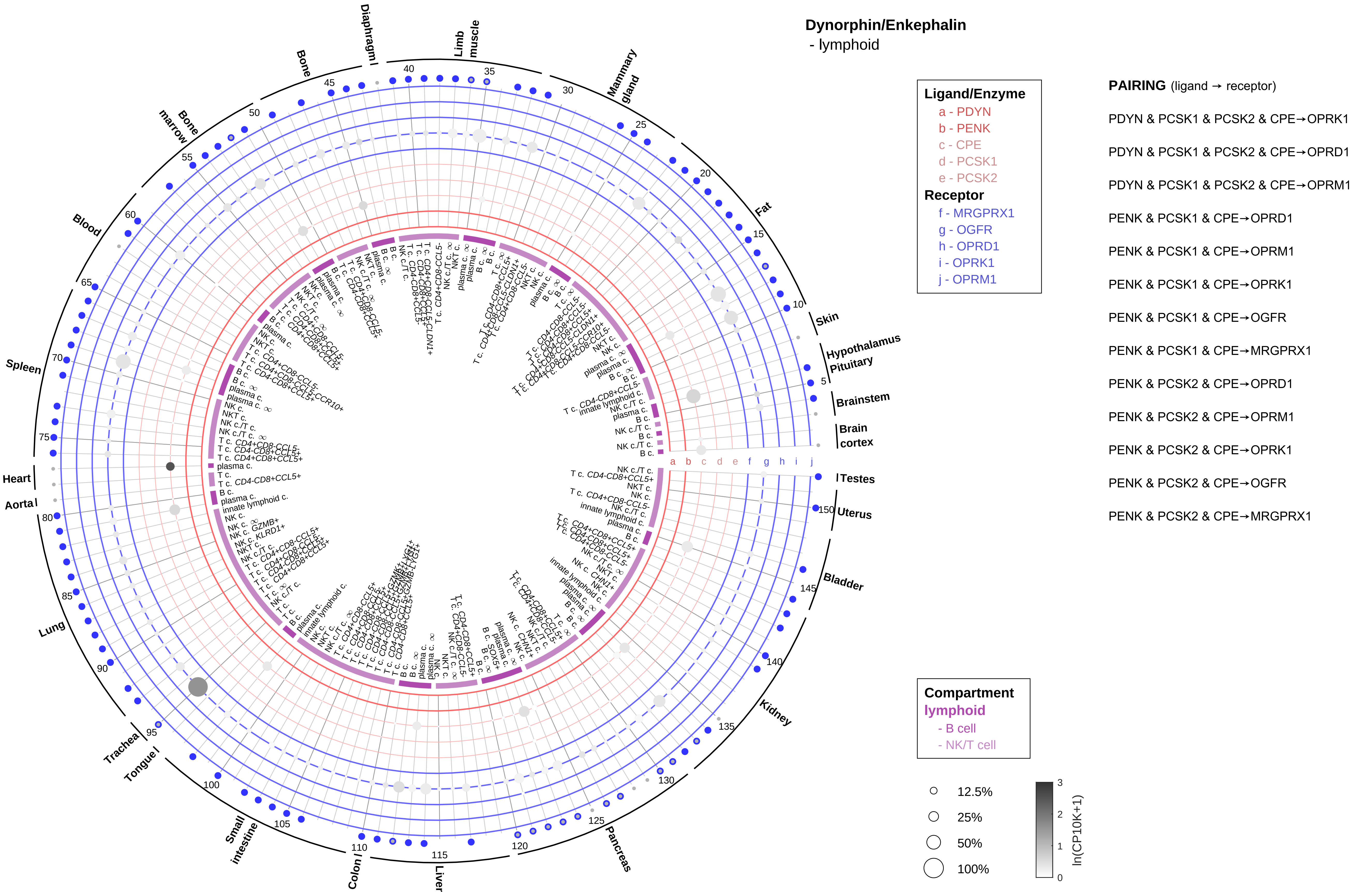
Dynorphin/Enkephalin
- lymphoid
Ligand/Enzyme
a - PDYN
b - PENK
c - CPE
d - PCSK1
e - PCSK2
Receptor
f - MRGPRX1
g - OGFR
h - OPRD1
i - OPRK1
j - OPRM1
PAIRING (ligand → receptor)
PDYN & PCSK1 & PCSK2 & CPE→OPRK1
PDYN & PCSK1 & PCSK2 & CPE→OPRD1
PDYN & PCSK1 & PCSK2 & CPE→OPRM1
PENK & PCSK1 & CPE→OPRD1
PENK & PCSK1 & CPE→OPRM1
PENK & PCSK1 & CPE→OPRK1
PENK & PCSK1 & CPE→OGFR
PENK & PCSK1 & CPE→MRGPRX1
PENK & PCSK2 & CPE→OPRD1
PENK & PCSK2 & CPE→OPRM1
PENK & PCSK2 & CPE→OPRK1
PENK & PCSK2 & CPE→OGFR
PENK & PCSK2 & CPE→MRGPRX1
Compartment
lymphoid
- B cell
- NK/T cell
12.5%
25%
50%
100%
ln(CP10K+1)
Skin
Hypothalamus
Pituitary
Brainstem
Brain cortex
Testes
Uterus
Bladder
Kidney
Pancreas
Liver
Colon
Small intestine
Tongue
Trachea
Lung
Aorta
Heart
Spleen
Blood
Bone marrow
Bone
Diaphragm
Limb muscle
Mammary gland
Fat

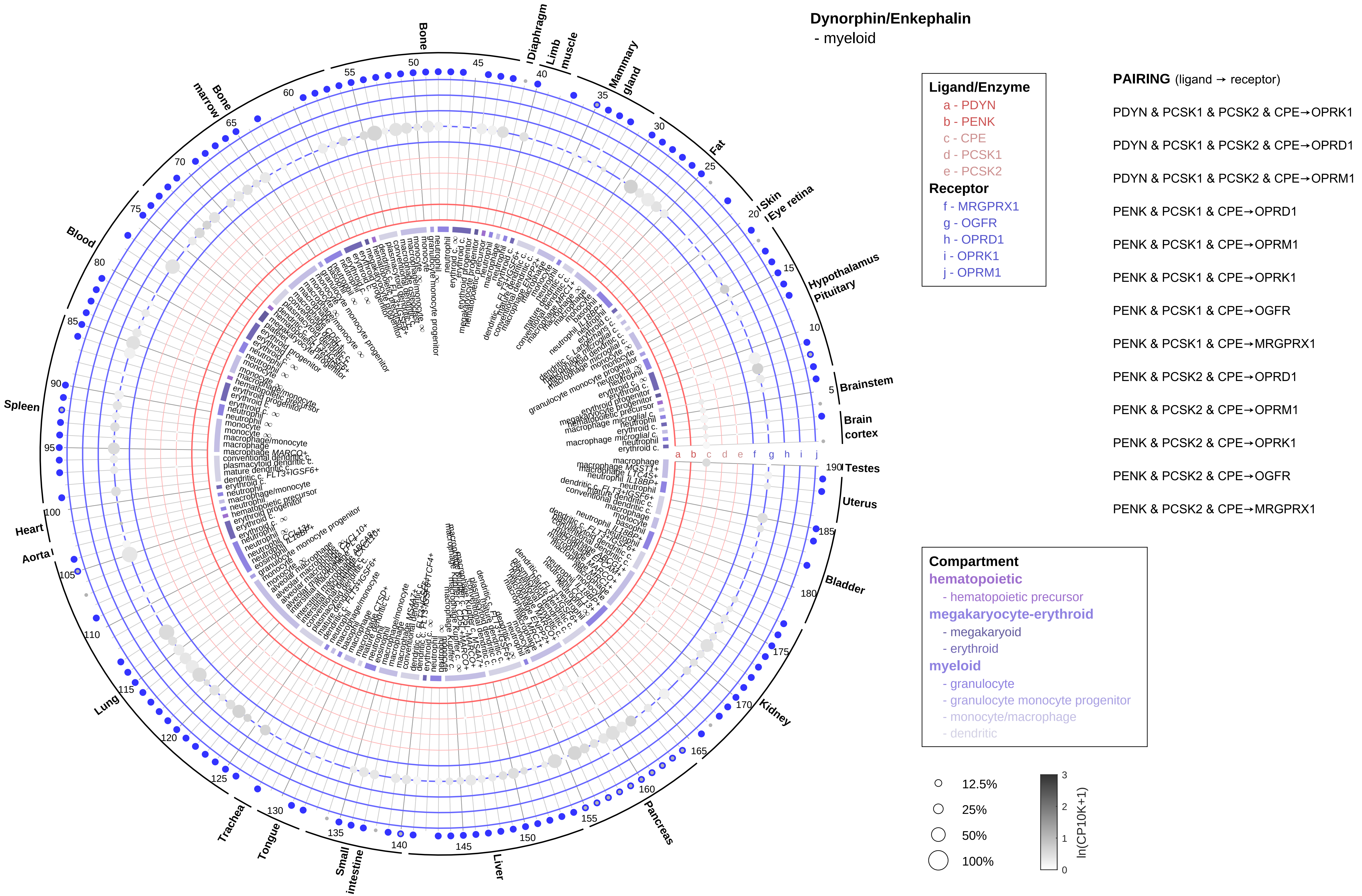

Dynorphin/Enkephalin
- myeloid
Ligand/Enzyme
a - PDYN
b - PENK
c - CPE
d - PCSK1
e - PCSK2
Receptor
f - MRGPRX1
g - OGFR
h - OPRD1
i - OPRK1
j - OPRM1
PAIRING (ligand → receptor)
PDYN & PCSK1 & PCSK2 & CPE→OPRK1
PDYN & PCSK1 & PCSK2 & CPE→OPRD1
PDYN & PCSK1 & PCSK2 & CPE→OPRM1
PENK & PCSK1 & CPE→OPRD1
PENK & PCSK1 & CPE→OPRM1
PENK & PCSK1 & CPE→OPRK1
PENK & PCSK1 & CPE→OGFR
PENK & PCSK1 & CPE→MRGPRX1
PENK & PCSK2 & CPE→OPRD1
PENK & PCSK2 & CPE→OPRM1
PENK & PCSK2 & CPE→OPRK1
PENK & PCSK2 & CPE→OGFR
PENK & PCSK2 & CPE→MRGPRX1
Compartment
hematopoietic
- hematopoietic precursor
megakaryocyte-erythroid
- megakaryoid
- erythroid
myeloid
- granulocyte
- granulocyte monocyte progenitor
- monocyte/macrophage
- dendritic
12.5%
25%
50%
100%
ln(CP10K+1)
0
1
2
3
Bone
Diaphragm
Limb muscle
Mammary gland
Fat
Skin
Eye retina
Hypothalamus
Pituitary
Brainstem
Brain cortex
Testes
Uterus
Bladder
Kidney
Pancreas
Liver
Small intestine
Tongue
Trachea
Lung
Aorta
Heart
Spleen
Blood
Bone marrow

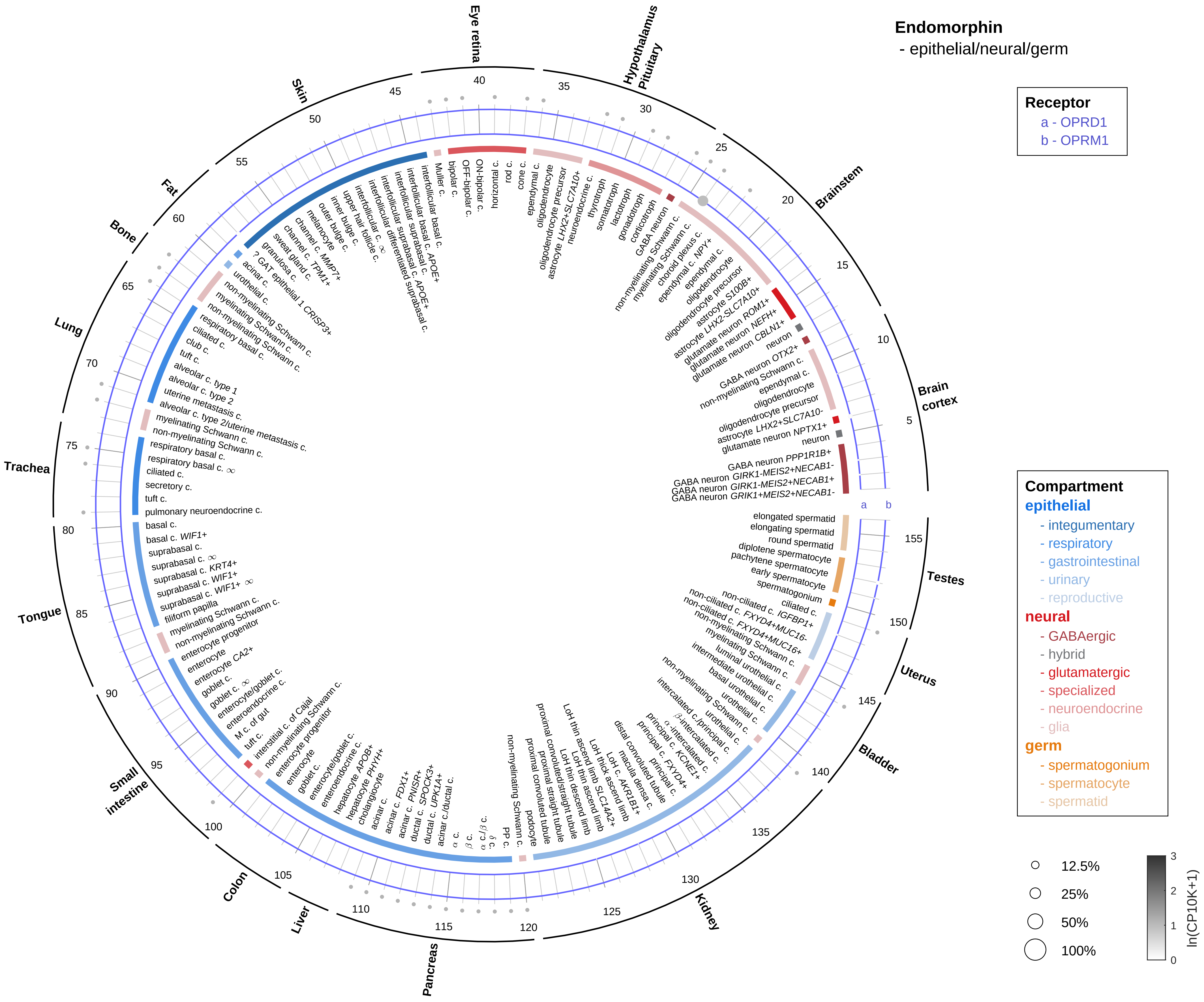

Endomorphin
- epithelial/neural/germ
Receptor
a - OPRD1
b - OPRM1
Compartment
epithelial
- integumentary
- respiratory
- gastrointestinal
- urinary
- reproductive
neural
- GABAergic
- hybrid
- glutamatergic
- specialized
- neuroendocrine
- glia
germ
- spermatogonium
- spermatocyte
- spermatid
12.5%
25%
50%
100%
ln(CP10K+1)
Eye retina
Hypothalamus Pituitary
Brainstem
Brain cortex
Testes
Uterus
Bladder
Kidney
Pancreas
Liver
Colon
Small intestine
Tongue
Trachea
Lung
Bone
Fat
Skin

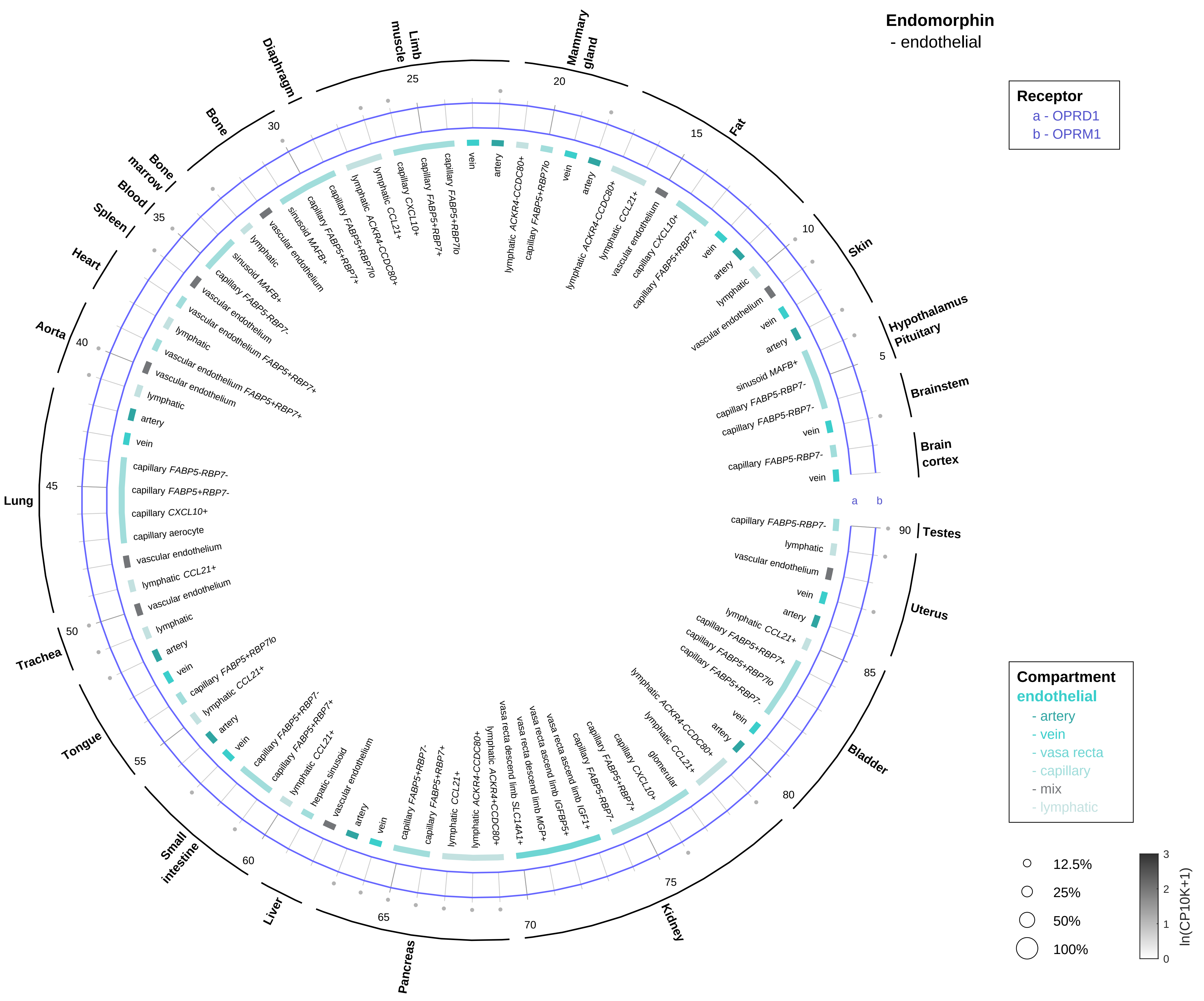

Endomorphin
- endothelial
Receptor
a - OPRD1
b - OPRM1
Compartment
endothelial
- artery
- vein
- vasa recta
- capillary
- mix
- lymphatic
12.5%
25%
50%
100%
ln(CP10K+1)
Brain cortex
Brainstem
Hypothalamus Pituitary
Skin
Fat
Mammary gland
Limb muscle
Diaphragm
Bone
Bone marrow
Blood
Spleen
Heart
Aorta
Lung
Trachea
Tongue
Small intestine
Liver
Pancreas
Kidney
Bladder
Uterus
Testes

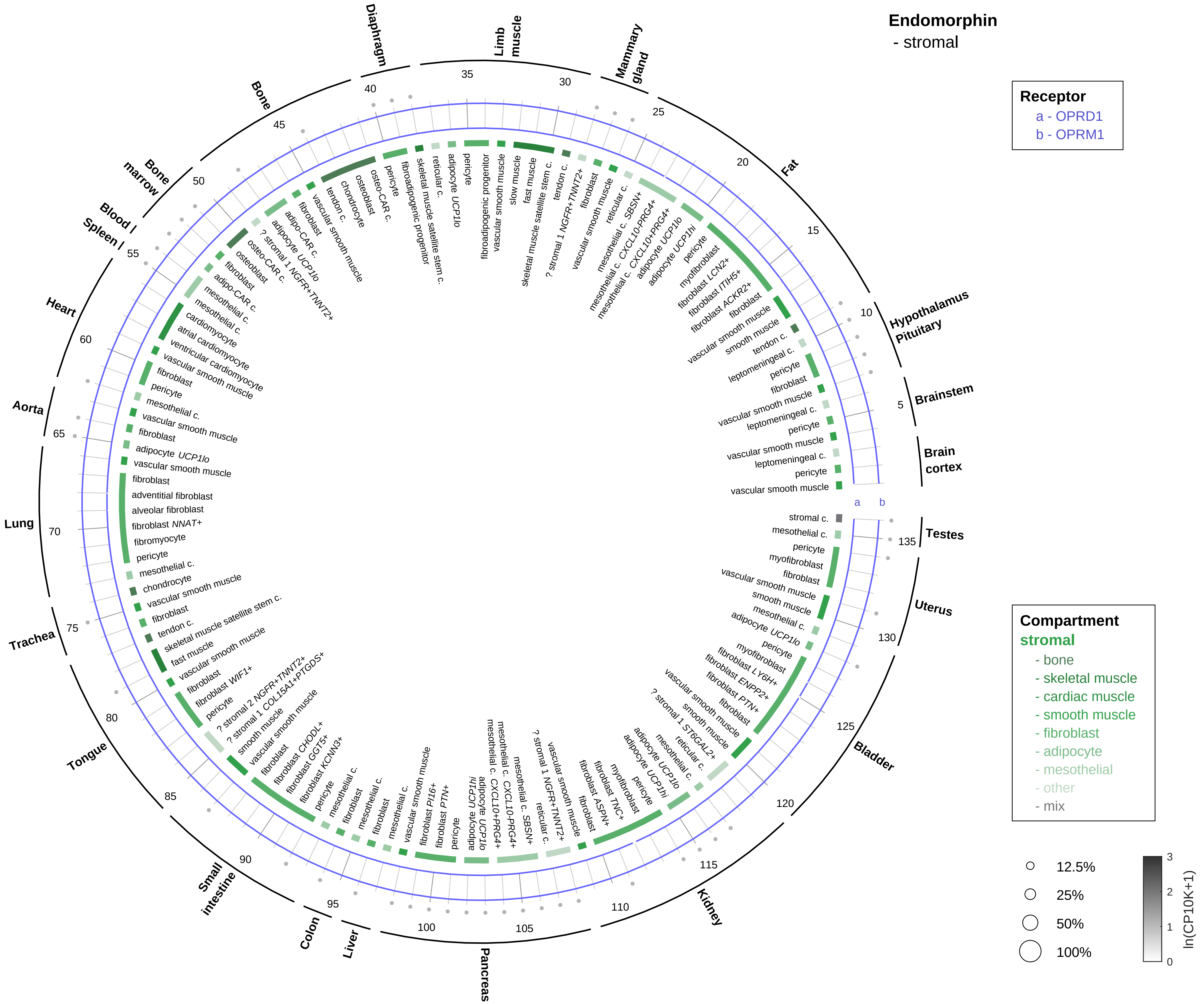

Endomorphin
- stromal
Receptor
a - OPRD1
b - OPRM1
Compartment
stromal
- bone
- skeletal muscle
- cardiac muscle
- smooth muscle
- fibroblast
- adipocyte
- mesothelial
- other
- mix
12.5%
25%
50%
100%
ln(CP10K+1)
Brain cortex
Brainstem
Hypothalamus
Pituitary
Fat
Mammary gland
Limb muscle
Diaphragm
Bone
Bone marrow
Blood
Spleen
Heart
Aorta
Lung
Trachea
Tongue
Small intestine
Colon
Liver
Pancreas
Kidney
Bladder
Uterus
Testes

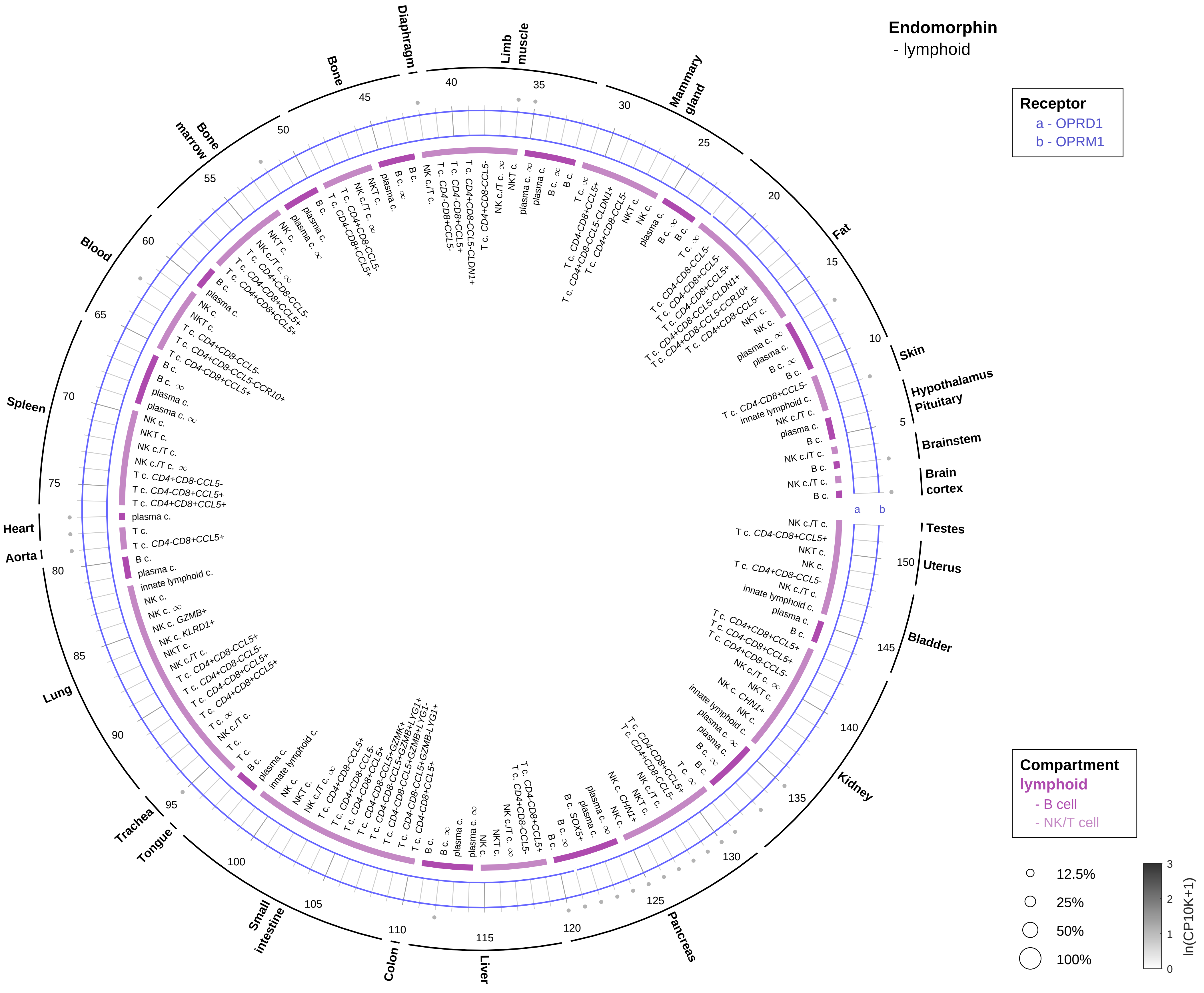
Endomorphin
- lymphoid
Receptor
a - OPRD1
b - OPRM1
Compartment
lymphoid
- B cell
- NK/T cell
12.5%
25%
50%
100%
ln(CP10K+1)
0
1
2
3
Limb muscle
Mammary gland
Fat
Skin
Hypothalamus
Pituitary
Brainstem
Brain cortex
Testes
Uterus
Bladder
Kidney
Pancreas
Liver
Colon
Small intestine
Tongue
Trachea
Lung
Aorta
Heart
Spleen
Blood
Bone marrow
Bone
Diaphragm

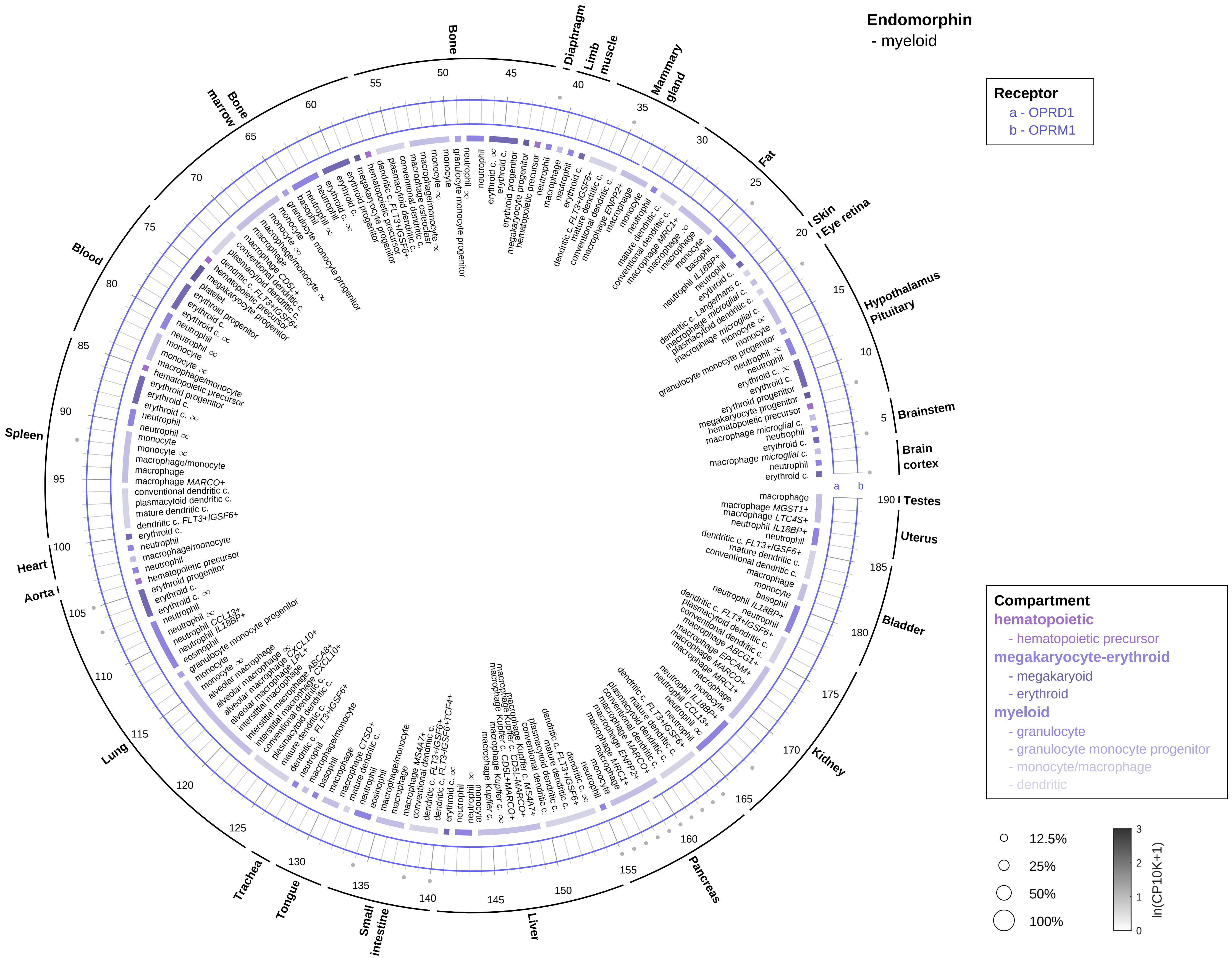

Endomorphin
- myeloid
Receptor
a - OPRD1
b - OPRM1
Compartment
hematopoietic
- hematopoietic precursor
megakaryocyte-erythroid
- megakaryoid
- erythroid
myeloid
- granulocyte
- granulocyte monocyte progenitor
- monocyte/macrophage
- dendritic
12.5%
25%
50%
100%
ln(CP10K+1)
Brain cortex
Brainstem
Pituitary
Hypothalamus
Eye retina
Skin
Fat
Mammary gland
Limb muscle
Diaphragm
Bone
Bone marrow
Blood
Spleen
Heart
Aorta
Lung
Trachea
Tongue
Small intestine
Liver
Pancreas
Kidney
Bladder
Uterus
Testes

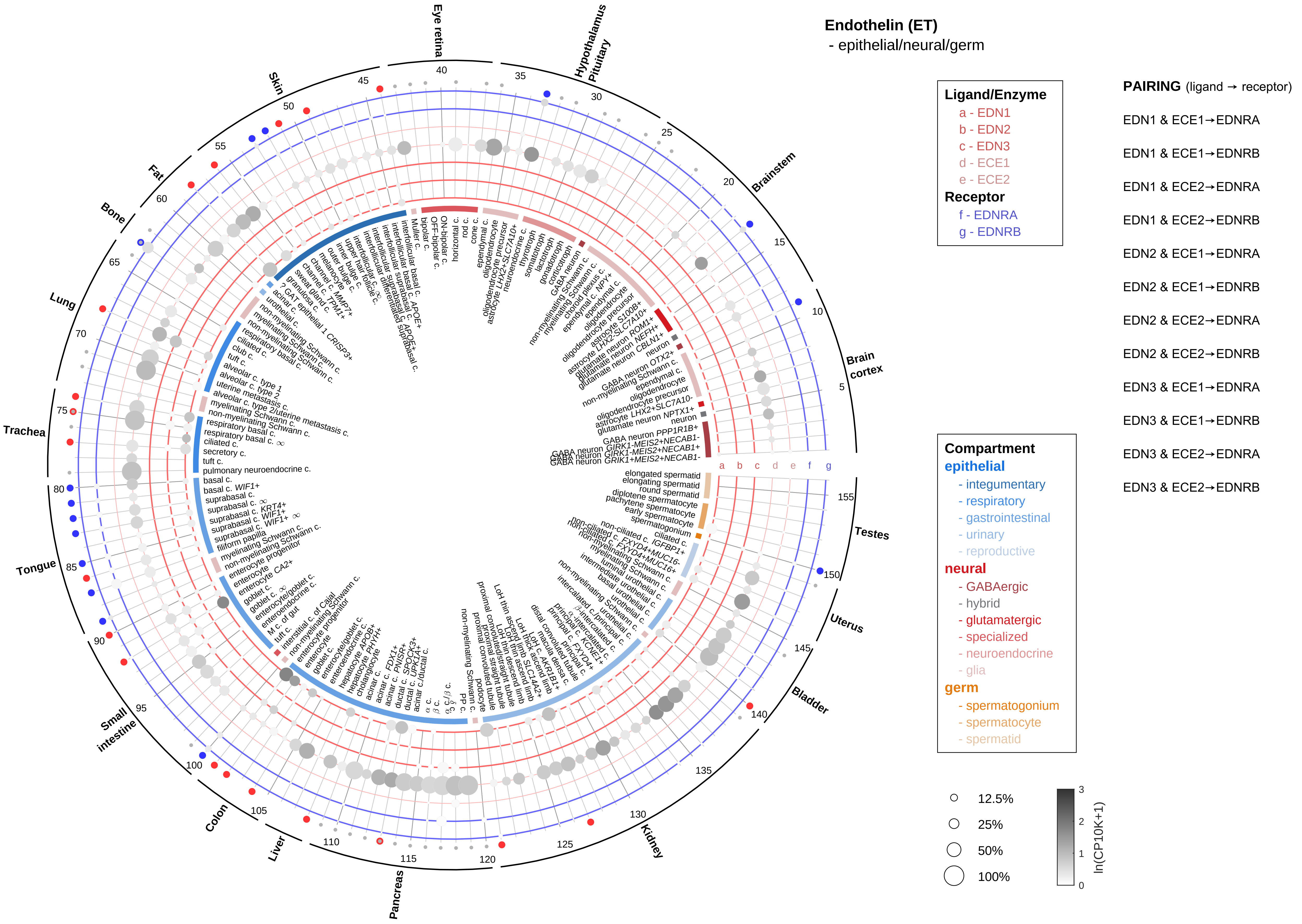
Endothelin (ET)
- epithelial/neural/germ
Ligand/Enzyme
a - EDN1
b - EDN2
c - EDN3
d - ECE1
e - ECE2
Receptor
f - EDNRA
g - EDNRB
PAIRING (ligand → receptor)
EDN1 & ECE1→EDNRA
EDN1 & ECE1→EDNRB
EDN1 & ECE2→EDNRA
EDN1 & ECE2→EDNRB
EDN2 & ECE1→EDNRA
EDN2 & ECE1→EDNRB
EDN2 & ECE2→EDNRA
EDN2 & ECE2→EDNRB
EDN3 & ECE1→EDNRA
EDN3 & ECE1→EDNRB
EDN3 & ECE2→EDNRA
EDN3 & ECE2→EDNRB
Compartment
epithelial
- integumentary
- respiratory
- gastrointestinal
- urinary
- reproductive
neural
- GABAergic
- hybrid
- glutamatergic
- specialized
- neuroendocrine
- glia
germ
- spermatogonium
- spermatocyte
- spermatid
12.5%
25%
50%
100%
ln(CP10K+1)
Eye retina
Hypothalamus Pituitary
Brainstem
Brain cortex
Testes
Uterus
Bladder
Kidney
Pancreas
Liver
Colon
Small intestine
Tongue
Trachea
Lung
Bone
Fat
Skin

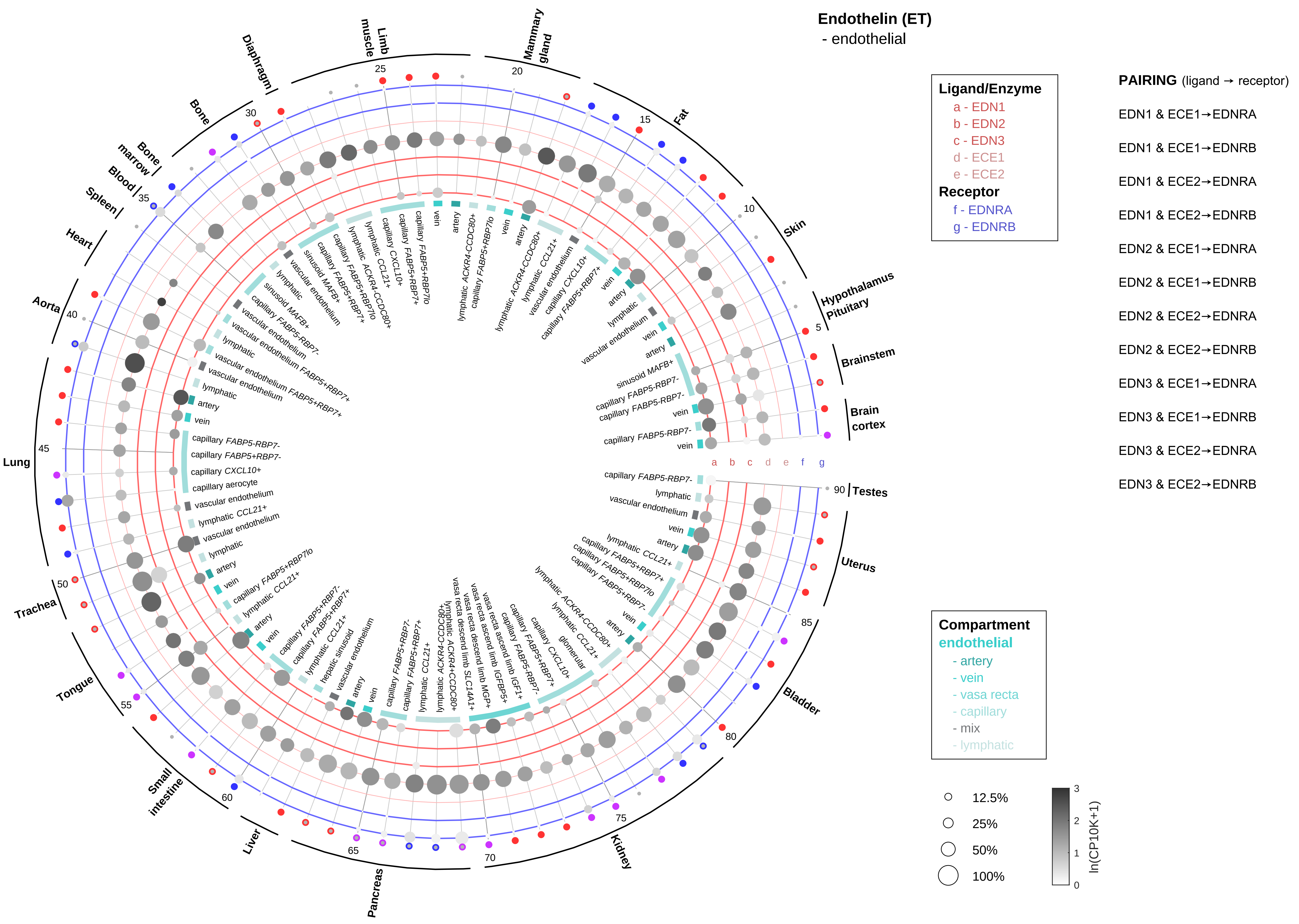
Endothelin (ET)
- endothelial
Ligand/Enzyme
a - EDN1
b - EDN2
c - EDN3
d - ECE1
e - ECE2
Receptor
f - EDNRA
g - EDNRB
PAIRING (ligand → receptor)
EDN1 & ECE1→EDNRA
EDN1 & ECE1→EDNRB
EDN1 & ECE2→EDNRA
EDN1 & ECE2→EDNRB
EDN2 & ECE1→EDNRA
EDN2 & ECE1→EDNRB
EDN2 & ECE2→EDNRA
EDN2 & ECE2→EDNRB
EDN3 & ECE1→EDNRA
EDN3 & ECE1→EDNRB
EDN3 & ECE2→EDNRA
EDN3 & ECE2→EDNRB
Compartment
endothelial
- artery
- vein
- vasa recta
- capillary
- mix
- lymphatic
12.5%
25%
50%
100%
ln(CP10K+1)
Hypothalamus Pituitary
Brainstem
Brain cortex
Testes
Uterus
Bladder
Kidney
Pancreas
Liver
Small intestine
Tongue
Trachea
Lung
Aorta
Heart
Spleen
Blood
Bone marrow
Bone
Diaphragm
Limb muscle
Mammary gland
Fat
Skin
a
b
c
d
e
f
g

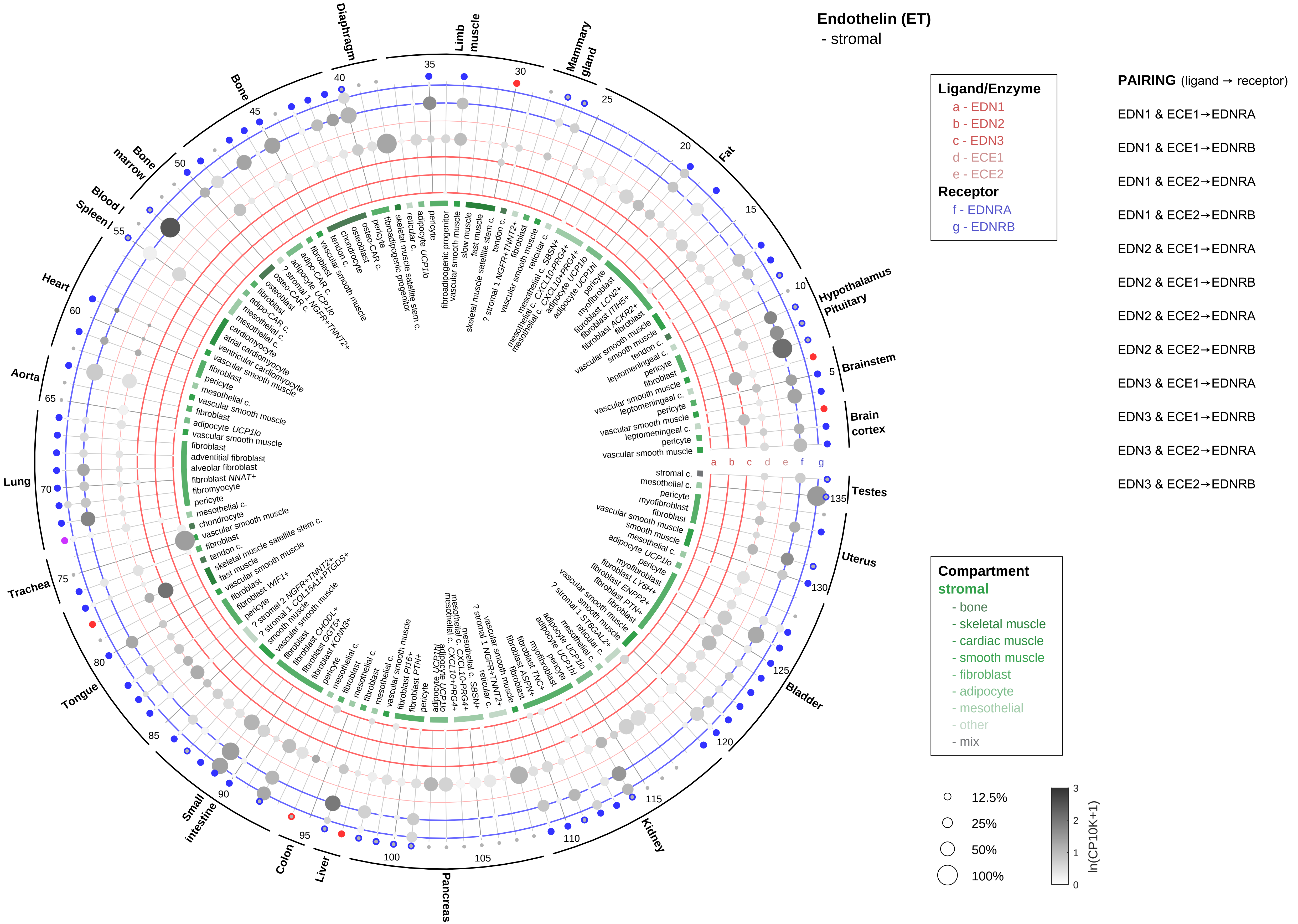

Endothelin (ET)
- stromal
Ligand/Enzyme
a - EDN1
b - EDN2
c - EDN3
d - ECE1
e - ECE2
Receptor
f - EDNRA
g - EDNRB
PAIRING (ligand → receptor)
EDN1 & ECE1→EDNRA
EDN1 & ECE1→EDNRB
EDN1 & ECE2→EDNRA
EDN1 & ECE2→EDNRB
EDN2 & ECE1→EDNRA
EDN2 & ECE1→EDNRB
EDN2 & ECE2→EDNRA
EDN2 & ECE2→EDNRB
EDN3 & ECE1→EDNRA
EDN3 & ECE1→EDNRB
EDN3 & ECE2→EDNRA
EDN3 & ECE2→EDNRB
Compartment
stromal
- bone
- skeletal muscle
- cardiac muscle
- smooth muscle
- fibroblast
- adipocyte
- mesothelial
- other
- mix
12.5%
25%
50%
100%
ln(CP10K+1)
Diaphragm
Limb muscle
Mammary gland
Fat
Hypothalamus Pituitary
Brainstem
Brain cortex
Testes
Uterus
Bladder
Kidney
Pancreas
Liver
Colon
Small intestine
Tongue
Trachea
Lung
Aorta
Heart
Spleen
Blood
Bone marrow
Bone

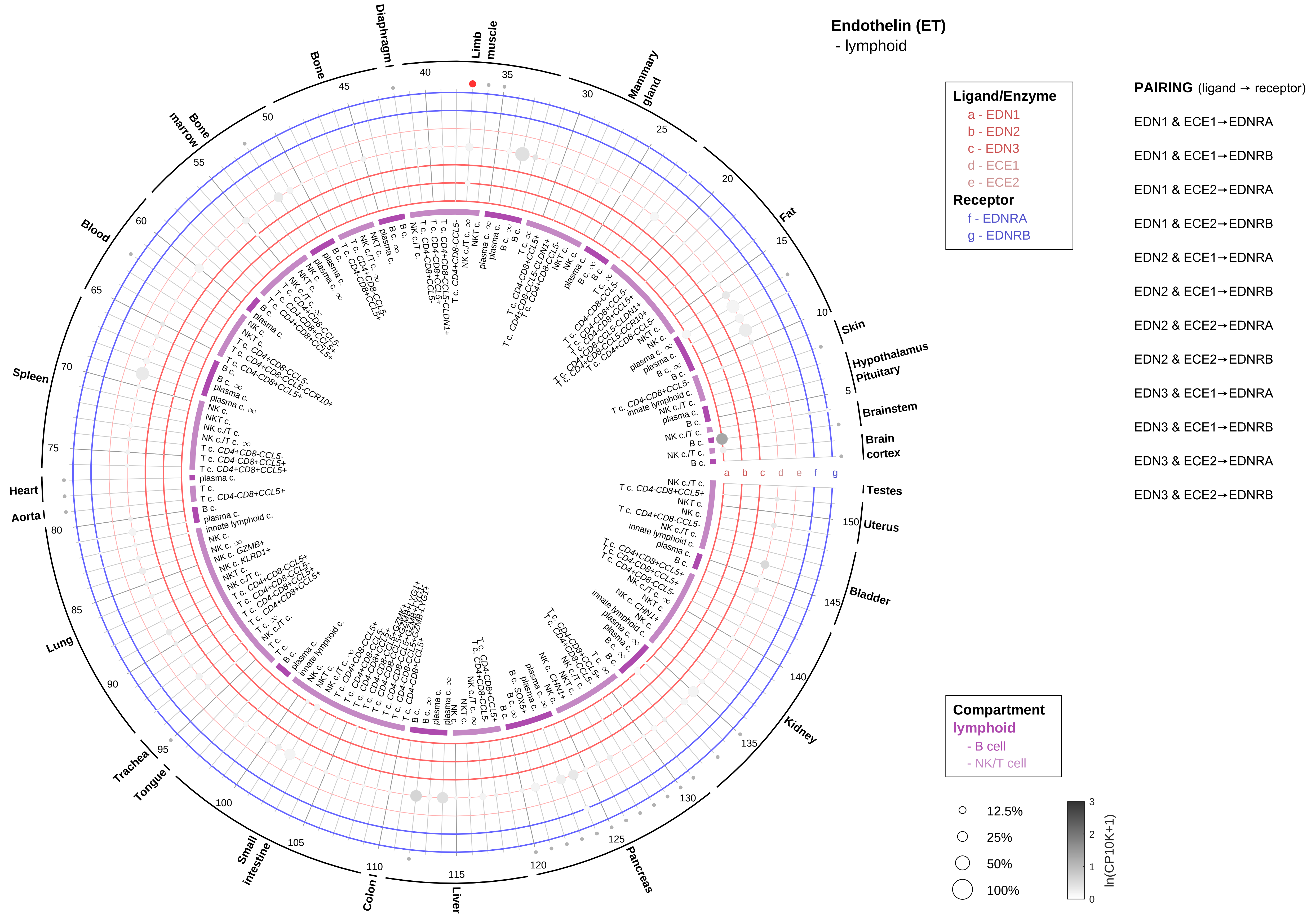

Endothelin (ET)
- lymphoid
Ligand/Enzyme
a - EDN1
b - EDN2
c - EDN3
d - ECE1
e - ECE2
Receptor
f - EDNRA
g - EDNRB
PAIRING (ligand → receptor)
EDN1 & ECE1→EDNRA
EDN1 & ECE1→EDNRB
EDN1 & ECE2→EDNRA
EDN1 & ECE2→EDNRB
EDN2 & ECE1→EDNRA
EDN2 & ECE1→EDNRB
EDN2 & ECE2→EDNRA
EDN2 & ECE2→EDNRB
EDN3 & ECE1→EDNRA
EDN3 & ECE1→EDNRB
EDN3 & ECE2→EDNRA
EDN3 & ECE2→EDNRB
Compartment
lymphoid
- B cell
- NK/T cell
12.5%
25%
50%
100%
ln(CP10K+1)
Skin
Hypothalamus
Pituitary
Brainstem
Brain cortex
Testes
Uterus
Bladder
Kidney
Pancreas
Liver
Colon
Small intestine
Tongue
Trachea
Lung
Aorta
Heart
Spleen
Blood
Bone marrow
Bone
Diaphragm
Limb muscle
Mammary gland
Fat

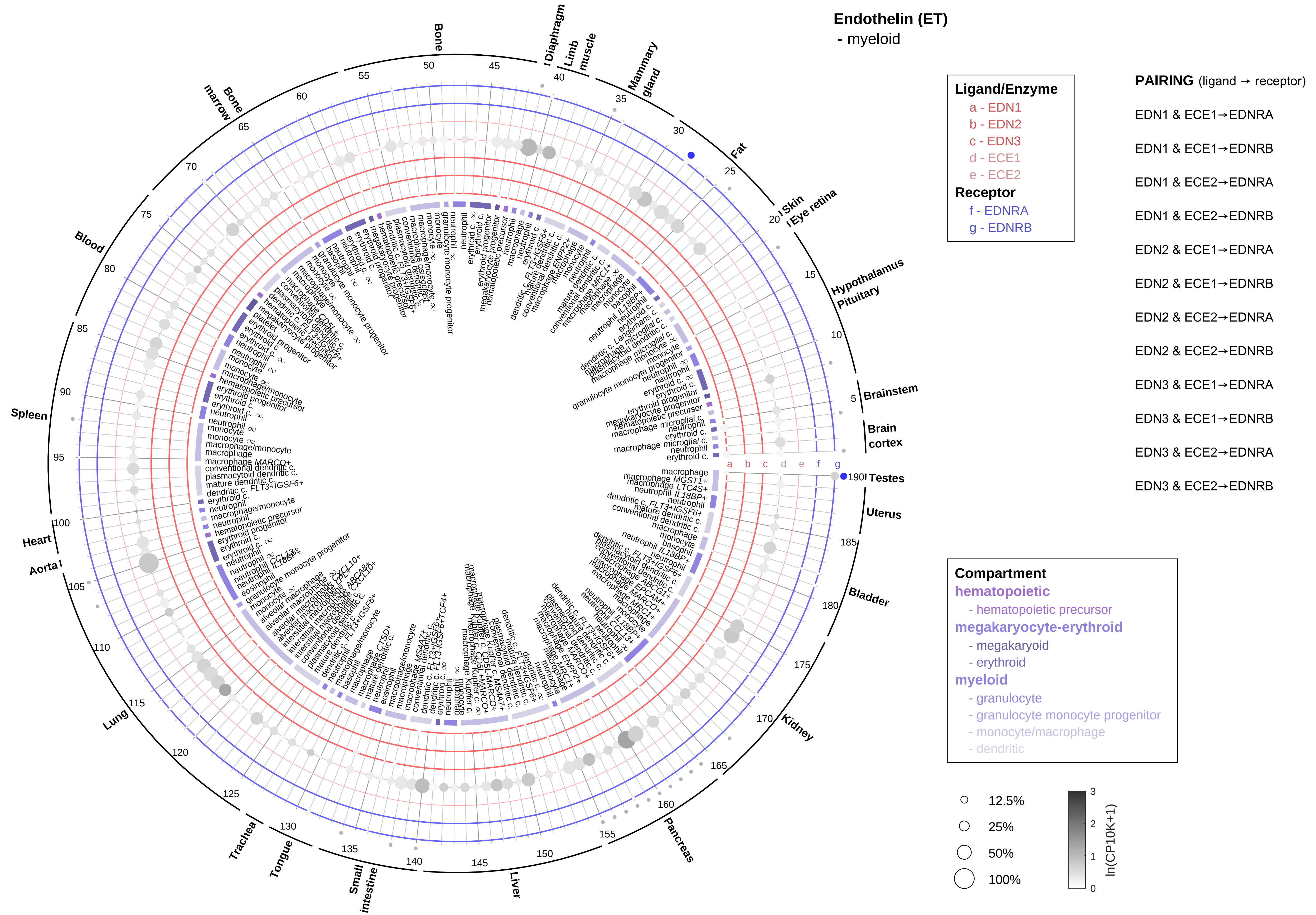

Endothelin (ET)
- myeloid
Ligand/Enzyme
a - EDN1
b - EDN2
c - EDN3
d - ECE1
e - ECE2
Receptor
f - EDNRA
g - EDNRB
PAIRING (ligand → receptor)
EDN1 & ECE1→EDNRA
EDN1 & ECE1→EDNRB
EDN1 & ECE2→EDNRA
EDN1 & ECE2→EDNRB
EDN2 & ECE1→EDNRA
EDN2 & ECE1→EDNRB
EDN2 & ECE2→EDNRA
EDN2 & ECE2→EDNRB
EDN3 & ECE1→EDNRA
EDN3 & ECE1→EDNRB
EDN3 & ECE2→EDNRA
EDN3 & ECE2→EDNRB
Compartment
hematopoietic
- hematopoietic precursor
megakaryocyte-erythroid
- megakaryoid
- erythroid
myeloid
- granulocyte
- granulocyte monocyte progenitor
- monocyte/macrophage
- dendritic
12.5%
25%
50%
100%
ln(CP10K+1)
Bone
Diaphragm
Limb muscle
Mammary gland
Fat
Skin
Eye retina
Hypothalamus
Pituitary
Brainstem
Brain cortex
Testes
Uterus
Bladder
Kidney
Pancreas
Liver
Small intestine
Tongue
Trachea
Lung
Aorta
Heart
Spleen
Blood
Bone marrow

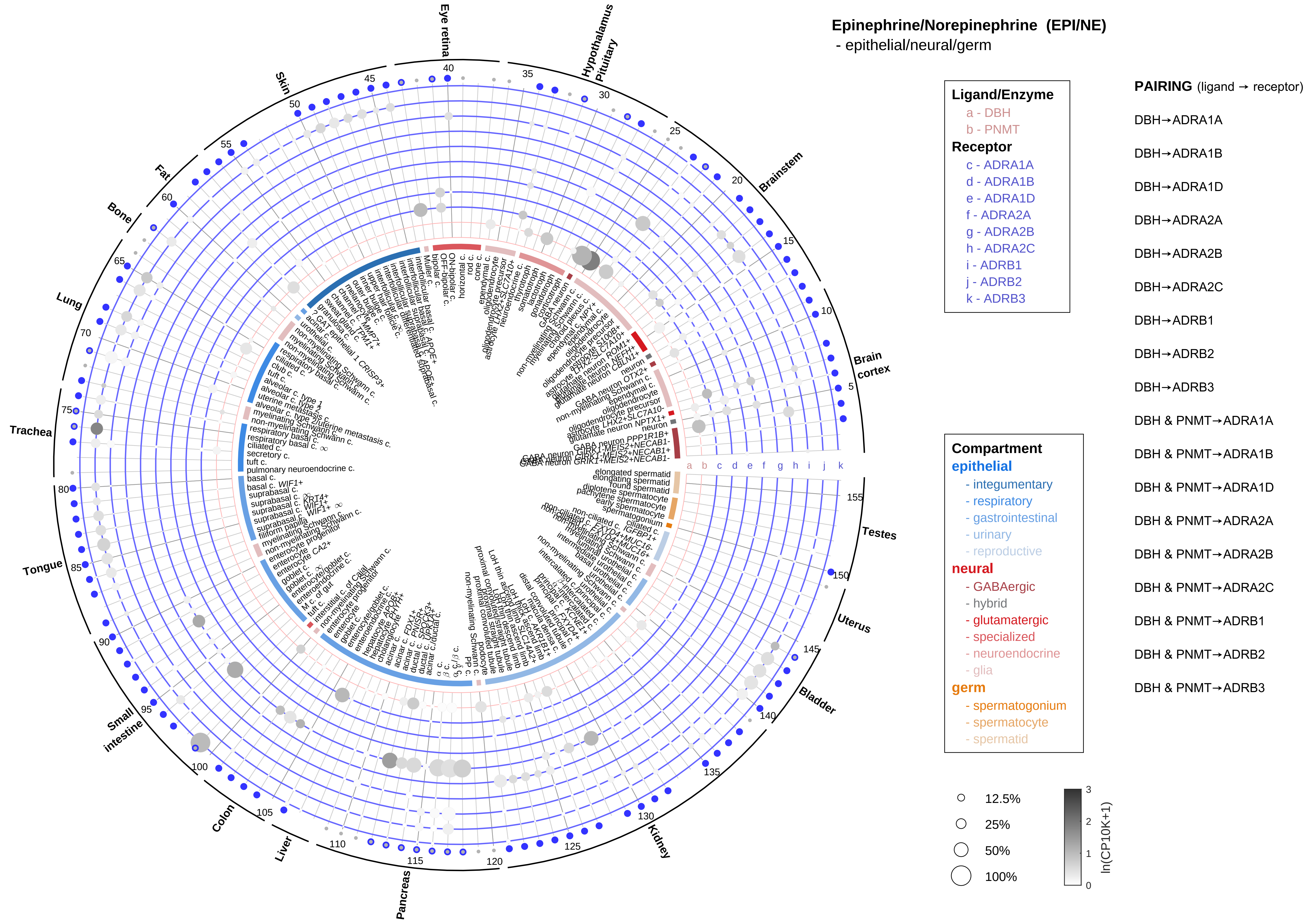

Epinephrine/Norepinephrine (EPI/NE)
- epithelial/neural/germ
Eye retina
Hypothalamus
Pituitary
Brainstem
Brain cortex
Testes
Uterus
Bladder
Kidney
Pancreas
Liver
Colon
Small intestine
Tongue
Trachea
Lung
Bone
Fat
Skin
a b c d e f g h i j k
Ligand/Enzyme
a - DBH
b - PNMT
Receptor
c - ADRA1A
d - ADRA1B
e - ADRA1D
f - ADRA2A
g - ADRA2B
h - ADRA2C
i - ADRB1
j - ADRB2
k - ADRB3
PAIRING (ligand → receptor)
DBH→ADRA1A
DBH→ADRA1B
DBH→ADRA1D
DBH→ADRA2A
DBH→ADRA2B
DBH→ADRA2C
DBH→ADRB1
DBH→ADRB2
DBH→ADRB3
DBH & PNMT→ADRA1A
DBH & PNMT→ADRA1B
DBH & PNMT→ADRA1D
DBH & PNMT→ADRA2A
DBH & PNMT→ADRA2B
DBH & PNMT→ADRA2C
DBH & PNMT→ADRB1
DBH & PNMT→ADRB2
DBH & PNMT→ADRB3
Compartment
epithelial
- integumentary
- respiratory
- gastrointestinal
- urinary
- reproductive
neural
- GABAergic
- hybrid
- glutamatergic
- specialized
- neuroendocrine
- glia
germ
- spermatogonium
- spermatocyte
- spermatid
12.5%
25%
50%
100%
ln(CP10K+1)
0
1
2
3

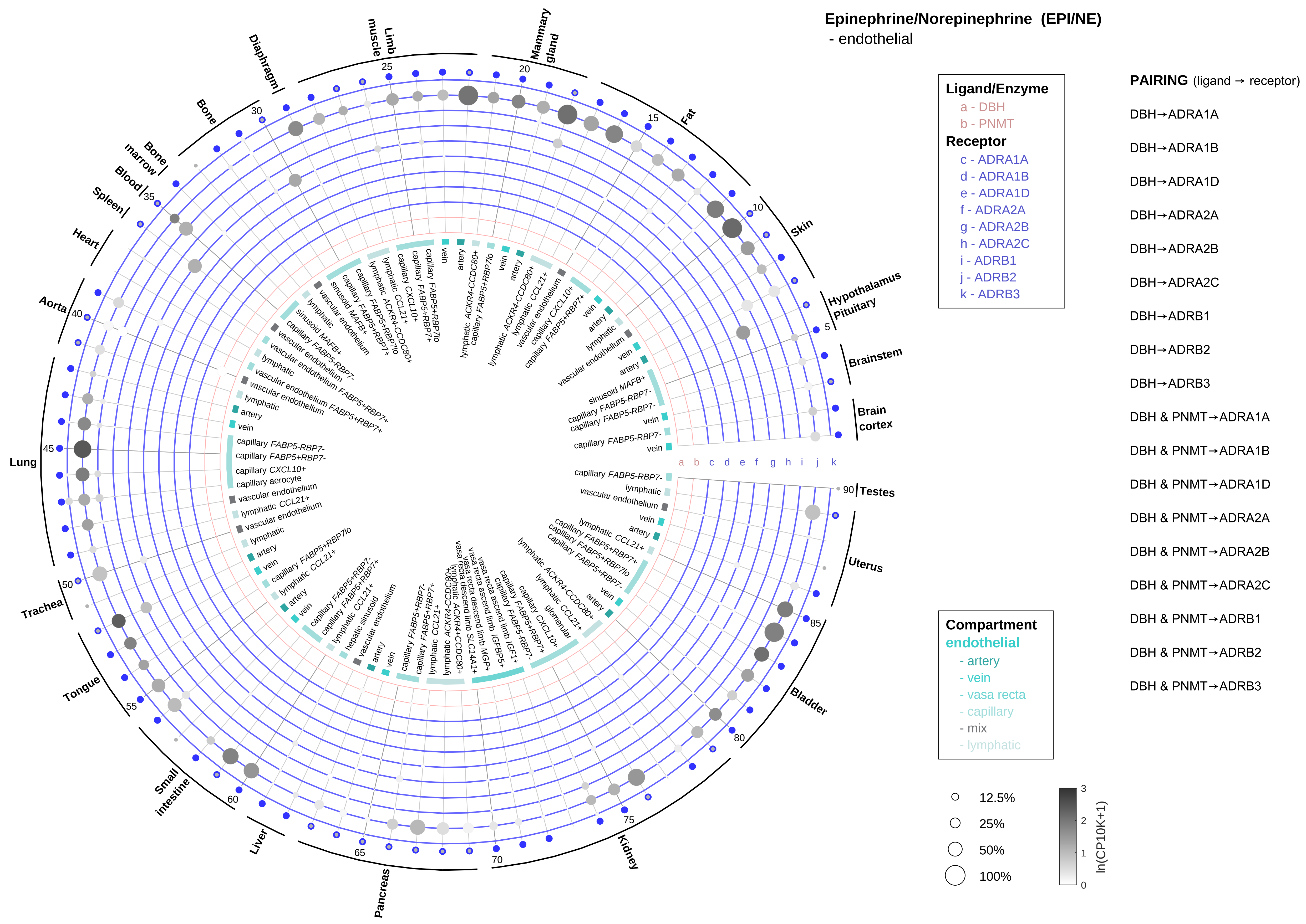

Epinephrine/Norepinephrine (EPI/NE)
- endothelial
Ligand/Enzyme
a - DBH
b - PNMT
Receptor
c - ADRA1A
d - ADRA1B
e - ADRA1D
f - ADRA2A
g - ADRA2B
h - ADRA2C
i - ADRB1
j - ADRB2
k - ADRB3
PAIRING (ligand → receptor)
DBH→ADRA1A
DBH→ADRA1B
DBH→ADRA1D
DBH→ADRA2A
DBH→ADRA2B
DBH→ADRA2C
DBH→ADRB1
DBH→ADRB2
DBH→ADRB3
DBH & PNMT→ADRA1A
DBH & PNMT→ADRA1B
DBH & PNMT→ADRA1D
DBH & PNMT→ADRA2A
DBH & PNMT→ADRA2B
DBH & PNMT→ADRA2C
DBH & PNMT→ADRB1
DBH & PNMT→ADRB2
DBH & PNMT→ADRB3
Compartment
endothelial
- artery
- vein
- vasa recta
- capillary
- mix
- lymphatic
12.5%
25%
50%
100%
ln(CP10K+1)
Mammary gland
Fat
Skin
Hypothalamus Pituitary
Brainstem
Brain cortex
Testes
Uterus
Bladder
Kidney
Pancreas
Liver
Small intestine
Tongue
Trachea
Lung
Aorta
Heart
Spleen
Blood
Bone marrow
Bone
Diaphragm
Limb muscle

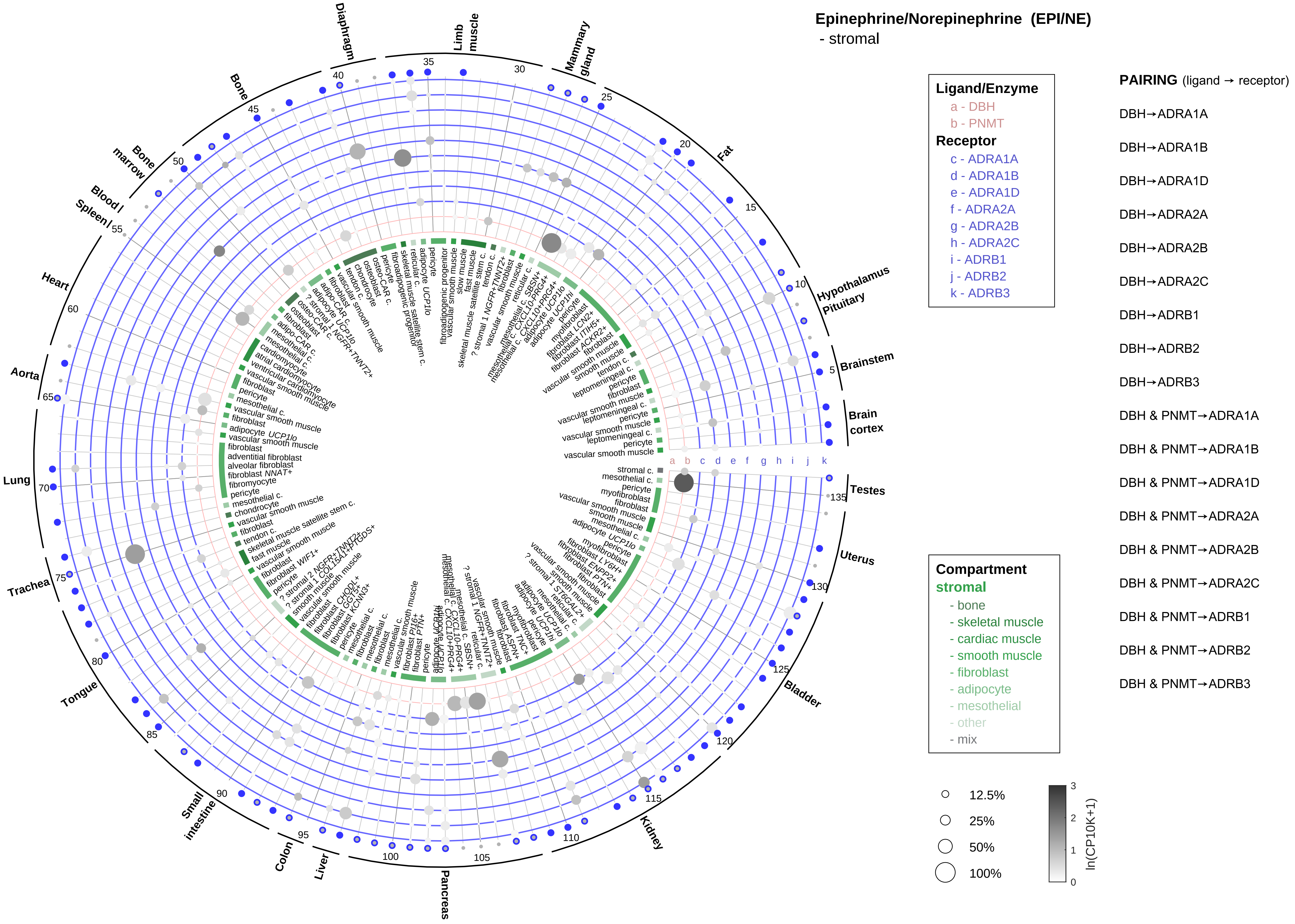

Epinephrine/Norepinephrine (EPI/NE)
- stromal
Ligand/Enzyme
a - DBH
b - PNMT
Receptor
c - ADRA1A
d - ADRA1B
e - ADRA1D
f - ADRA2A
g - ADRA2B
h - ADRA2C
i - ADRB1
j - ADRB2
k - ADRB3
PAIRING (ligand → receptor)
DBH→ADRA1A
DBH→ADRA1B
DBH→ADRA1D
DBH→ADRA2A
DBH→ADRA2B
DBH→ADRA2C
DBH→ADRB1
DBH→ADRB2
DBH→ADRB3
DBH & PNMT→ADRA1A
DBH & PNMT→ADRA1B
DBH & PNMT→ADRA1D
DBH & PNMT→ADRA2A
DBH & PNMT→ADRA2B
DBH & PNMT→ADRA2C
DBH & PNMT→ADRB1
DBH & PNMT→ADRB2
DBH & PNMT→ADRB3
Compartment
stromal
- bone
- skeletal muscle
- cardiac muscle
- smooth muscle
- fibroblast
- adipocyte
- mesothelial
- other
- mix
12.5%
25%
50%
100%
ln(CP10K+1)
Diaphragm
Limb muscle
Mammary gland
Fat
Hypothalamus Pituitary
Brainstem
Brain cortex
Testes
Uterus
Bladder
Kidney
Pancreas
Liver
Colon
Small intestine
Tongue
Trachea
Lung
Aorta
Heart
Spleen
Blood
Bone marrow
Bone

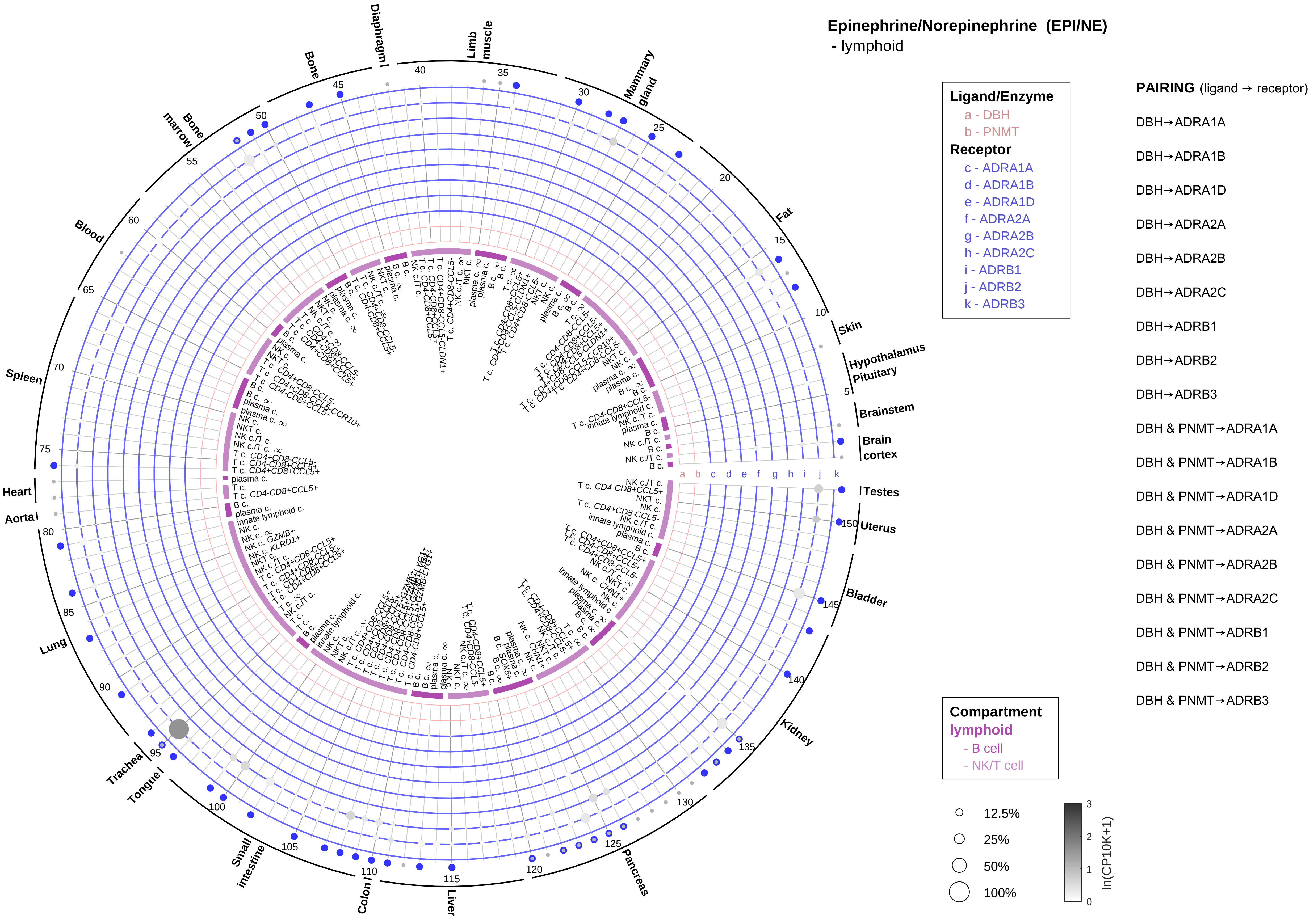

Epinephrine/Norepinephrine (EPI/NE)
- lymphoid
Ligand/Enzyme
a - DBH
b - PNMT
Receptor
c - ADRA1A
d - ADRA1B
e - ADRA1D
f - ADRA2A
g - ADRA2B
h - ADRA2C
i - ADRB1
j - ADRB2
k - ADRB3
PAIRING (ligand → receptor)
DBH→ADRA1A
DBH→ADRA1B
DBH→ADRA1D
DBH→ADRA2A
DBH→ADRA2B
DBH→ADRA2C
DBH→ADRB1
DBH→ADRB2
DBH→ADRB3
DBH & PNMT→ADRA1A
DBH & PNMT→ADRA1B
DBH & PNMT→ADRA1D
DBH & PNMT→ADRA2A
DBH & PNMT→ADRA2B
DBH & PNMT→ADRA2C
DBH & PNMT→ADRB1
DBH & PNMT→ADRB2
DBH & PNMT→ADRB3
Compartment
lymphoid
- B cell
- NK/T cell
12.5%
25%
50%
100%
ln(CP10K+1)
Skin
Hypothalamus
Pituitary
Brainstem
Brain cortex
Testes
Uterus
Bladder
Kidney
Pancreas
Liver
Colon
Small intestine
Tongue
Trachea
Lung
Aorta
Heart
Spleen
Blood
Bone marrow
Bone
Diaphragm
Limb muscle
Mammary gland
Fat

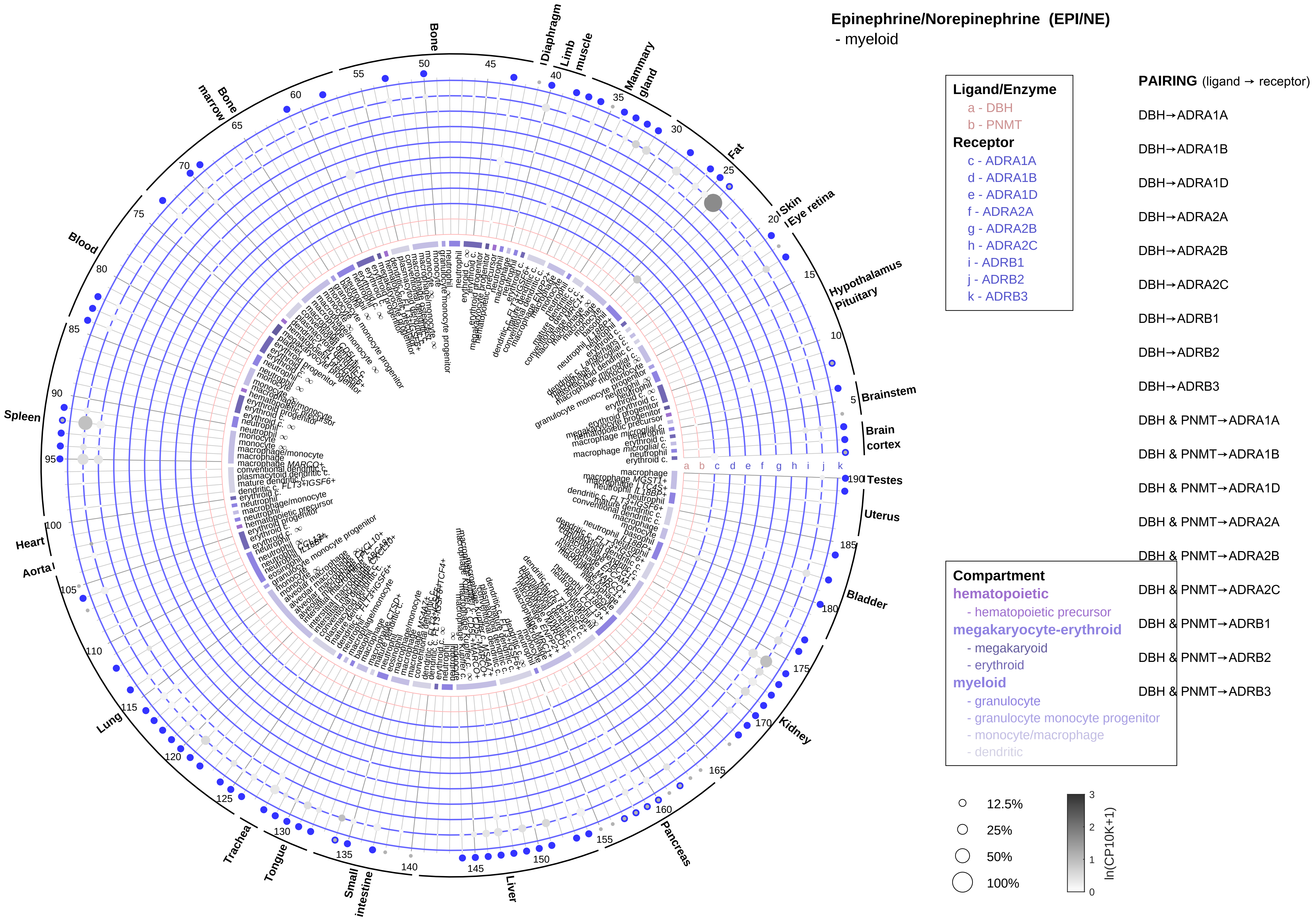

Epinephrine/Norepinephrine (EPI/NE)
- myeloid
Ligand/Enzyme
a - DBH
b - PNMT
Receptor
c - ADRA1A
d - ADRA1B
e - ADRA1D
f - ADRA2A
g - ADRA2B
h - ADRA2C
i - ADRB1
j - ADRB2
k - ADRB3
PAIRING (ligand → receptor)
DBH→ADRA1A
DBH→ADRA1B
DBH→ADRA1D
DBH→ADRA2A
DBH→ADRA2B
DBH→ADRA2C
DBH→ADRB1
DBH→ADRB2
DBH→ADRB3
DBH & PNMT→ADRA1A
DBH & PNMT→ADRA1B
DBH & PNMT→ADRA1D
DBH & PNMT→ADRA2A
DBH & PNMT→ADRA2B
DBH & PNMT→ADRA2C
DBH & PNMT→ADRB1
DBH & PNMT→ADRB2
DBH & PNMT→ADRB3
Compartment
hematopoietic
- hematopoietic precursor
megakaryocyte-erythroid
- megakaryoid
- erythroid
myeloid
- granulocyte
- granulocyte monocyte progenitor
- monocyte/macrophage
- dendritic
12.5%
25%
50%
100%
ln(CP10K+1)
0
1
2
3
Bone
Diaphragm
Limb muscle
Mammary gland
Fat
Skin
Eye retina
Hypothalamus
Pituitary
Brainstem
Brain cortex
Testes
Uterus
Bladder
Kidney
Pancreas
Liver
Small intestine
Tongue
Trachea
Lung
Aorta
Heart
Spleen
Blood
Bone marrow
a b c d e f g h i j k

Erythropoietin (EPO)
- epithelial/neural/germ

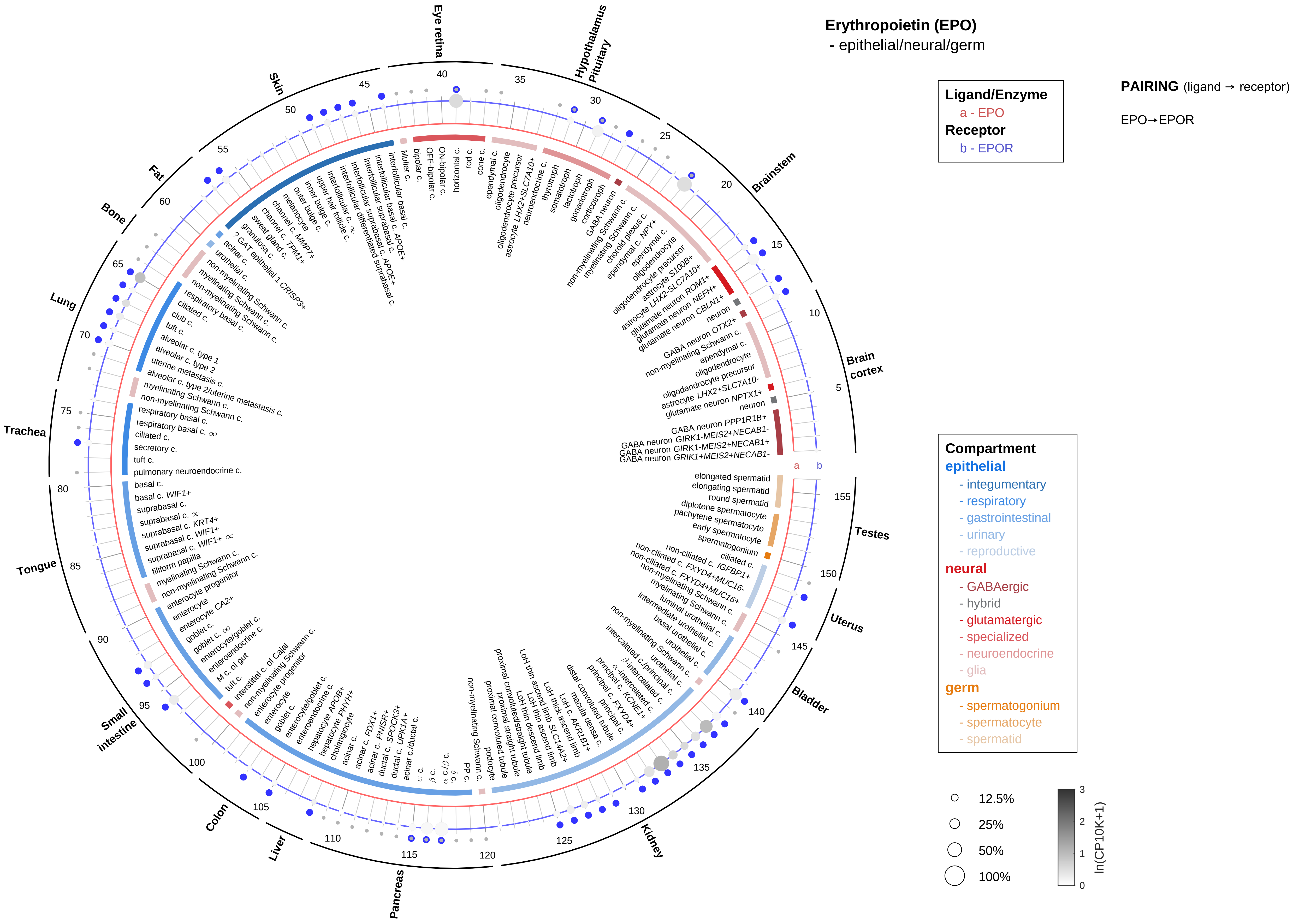

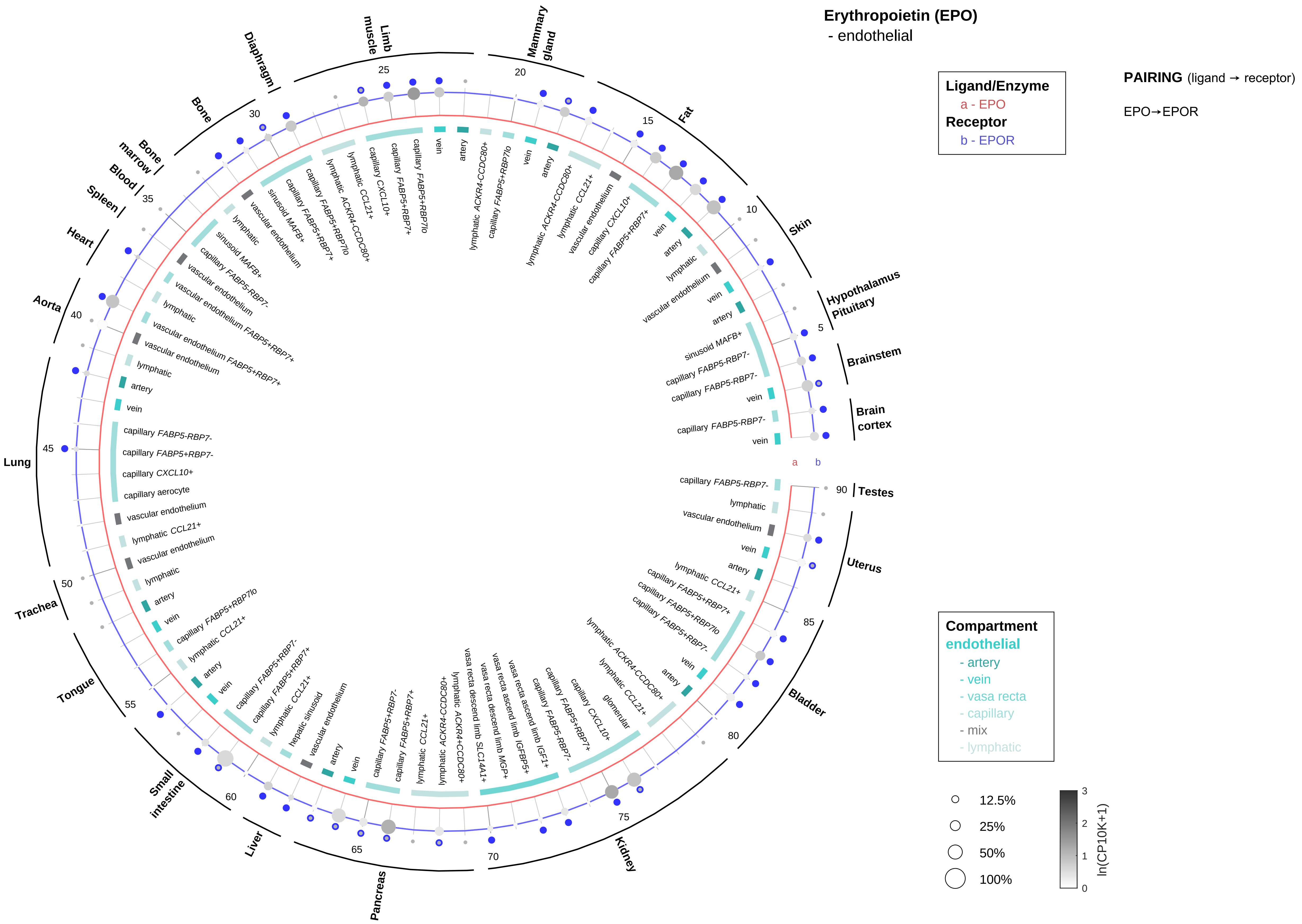

Erythropoietin (EPO)
- endothelial
Ligand/Enzyme
a - EPO
Receptor
b - EPOR
PAIRING (ligand → receptor)
EPO→EPOR
Compartment
endothelial
- artery
- vein
- vasa recta
- capillary
- mix
- lymphatic
12.5%
25%
50%
100%
ln(CP10K+1)
Brain cortex
Brainstem
Hypothalamus Pituitary
Skin
Fat
Mammary gland
Limb muscle
Diaphragm
Bone
Bone marrow
Blood
Spleen
Heart
Aorta
Lung
Trachea
Tongue
Small intestine
Liver
Pancreas
Kidney
Bladder
Uterus
Testes

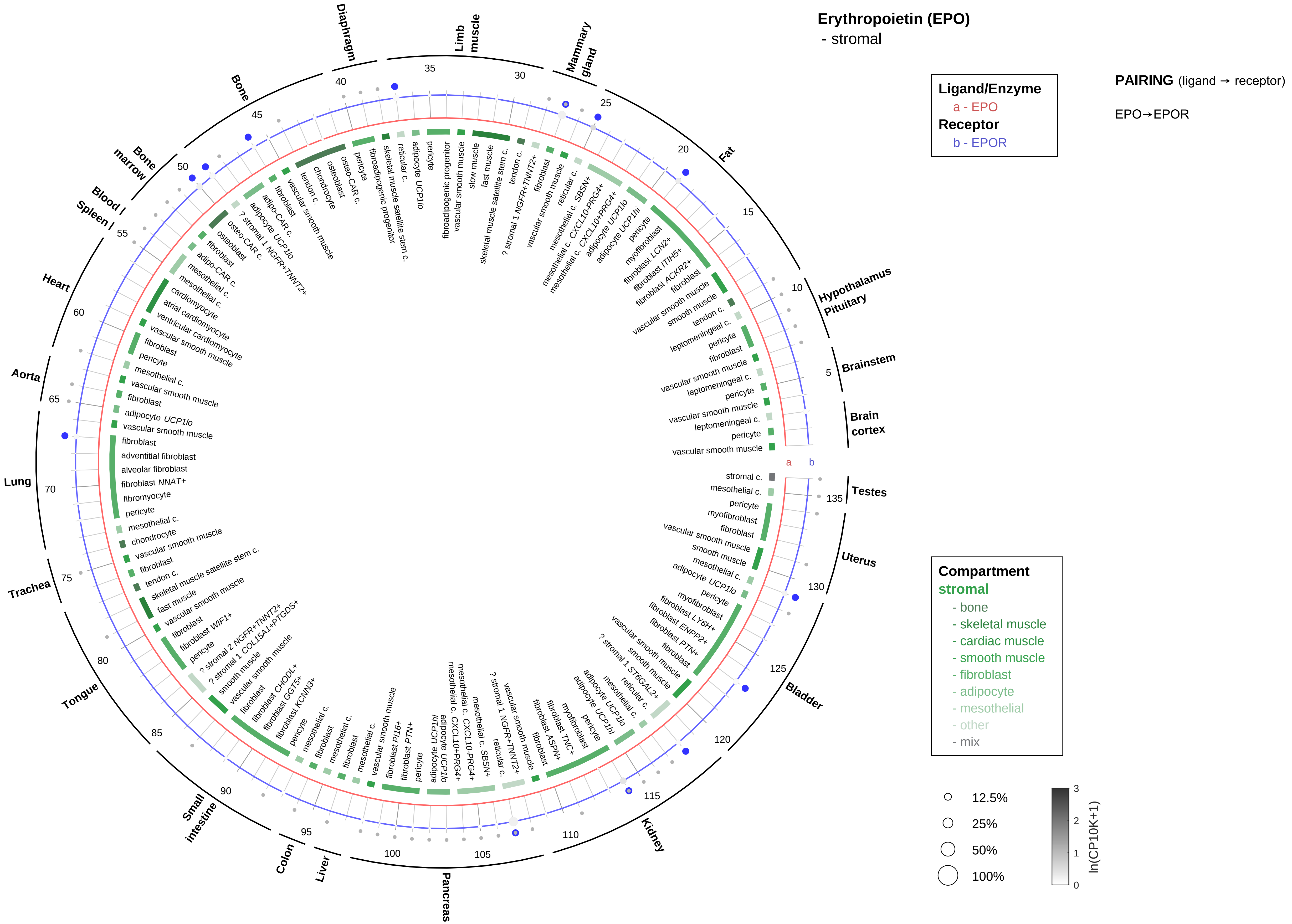

Erythropoietin (EPO)
- stromal
Ligand/Enzyme
a - EPO
Receptor
b - EPOR
PAIRING (ligand → receptor)
EPO→EPOR
Compartment
stromal
- bone
- skeletal muscle
- cardiac muscle
- smooth muscle
- fibroblast
- adipocyte
- mesothelial
- other
- mix
12.5%
25%
50%
100%
ln(CP10K+1)
Brain cortex
Brainstem
Hypothalamus Pituitary
Fat
Mammary gland
Limb muscle
Diaphragm
Bone
Bone marrow
Blood
Spleen
Heart
Aorta
Lung
Trachea
Tongue
Small intestine
Colon
Liver
Pancreas
Kidney
Bladder
Uterus
Testes
vascular smooth muscle
pericyte
leptomeningeal c.
fibroblast
tendon c.
smooth muscle
fibroblast ACKR2+
fibroblast ITIH5+
fibroblast LCN2+
myofibroblast
adipocyte UCP1hi
adipocyte UCP1lo
mesothelial c. CXCL10+PRG4+
mesothelial c. SBSN+
reticular c.
? stromal 1 NGFR+TNNT2+
skeletal muscle satellite stem c.
fast muscle
slow muscle
fibroadipogenic progenitor
osteo-CAR c.
osteoblast
chondrocyte
adipo-CAR c.
mesothelial c.
cardiomyocyte
atrial cardiomyocyte
ventricular cardiomyocyte
adventitial fibroblast
alveolar fibroblast
fibroblast NNAT+
fibromyocyte
fibroblast WIF1+
? stromal 2 NGFR+TNNT2+
? stromal 1 COL15A1+PTGDS+
fibroblast CHODL+
fibroblast GGT5+
fibroblast KCNN3+
fibroblast PI16+
fibroblast PTN+
fibroblast ASPN+
fibroblast TNC+
? stromal 1 ST6GAL2+
fibroblast ENPP2+
fibroblast LY6H+
stromal c.

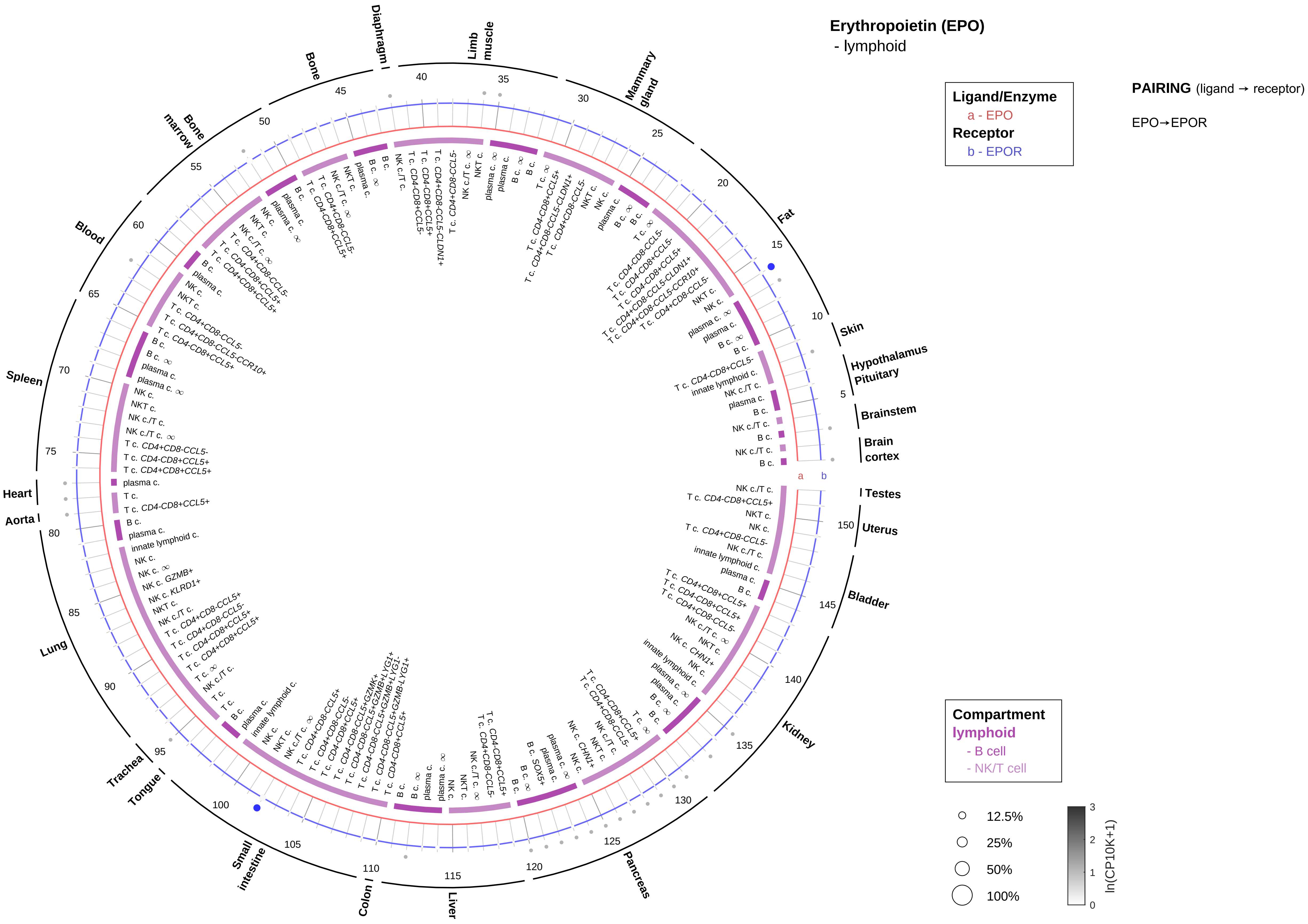

Erythropoietin (EPO)
- lymphoid
Ligand/Enzyme
a - EPO
Receptor
b - EPOR
PAIRING (ligand → receptor)
EPO→EPOR
Compartment
lymphoid
- B cell
- NK/T cell
12.5%
25%
50%
100%
ln(CP10K+1)
Skin
Hypothalamus
Pituitary
Brainstem
Brain cortex
Testes
Uterus
Bladder
Kidney
Pancreas
Liver
Colon
Small intestine
Tongue
Trachea
Lung
Aorta
Heart
Spleen
Blood
Bone marrow
Bone
Diaphragm
Limb muscle
Mammary gland
Fat

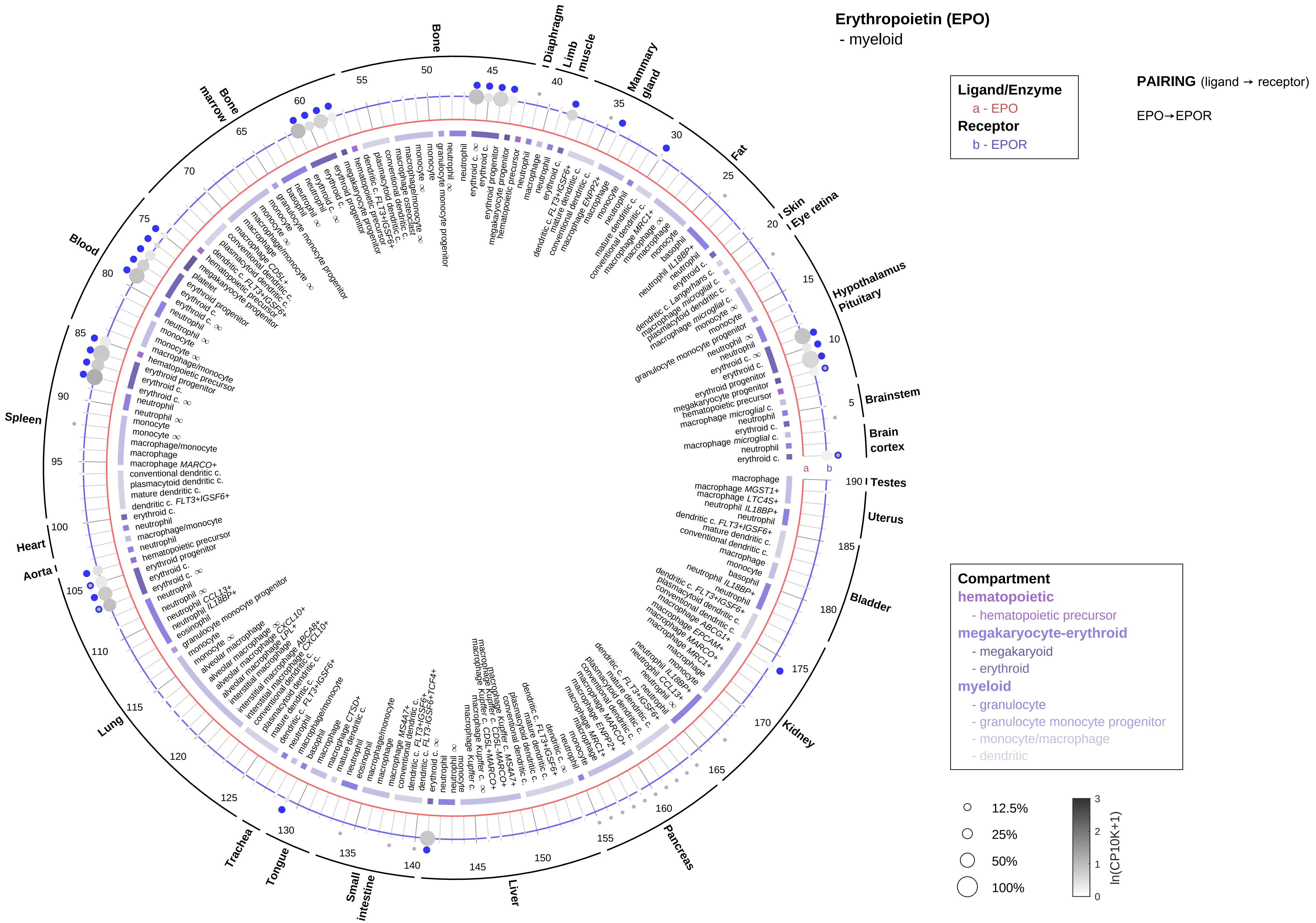

Erythropoietin (EPO)
- myeloid
Ligand/Enzyme
a - EPO
Receptor
b - EPOR
PAIRING (ligand → receptor)
EPO→EPOR
Compartment
hematopoietic
- hematopoietic precursor
megakaryocyte-erythroid
- megakaryoid
- erythroid
myeloid
- granulocyte
- granulocyte monocyte progenitor
- monocyte/macrophage
- dendritic
12.5%
25%
50%
100%
ln(CP10K+1)
Bone
Diaphragm
Limb muscle
Mammary gland
Fat
Skin
Eye retina
Hypothalamus
Pituitary
Brainstem
Brain cortex
Testes
Uterus
Bladder
Kidney
Pancreas
Liver
Small intestine
Tongue
Trachea
Lung
Aorta
Heart
Spleen
Blood
Bone marrow

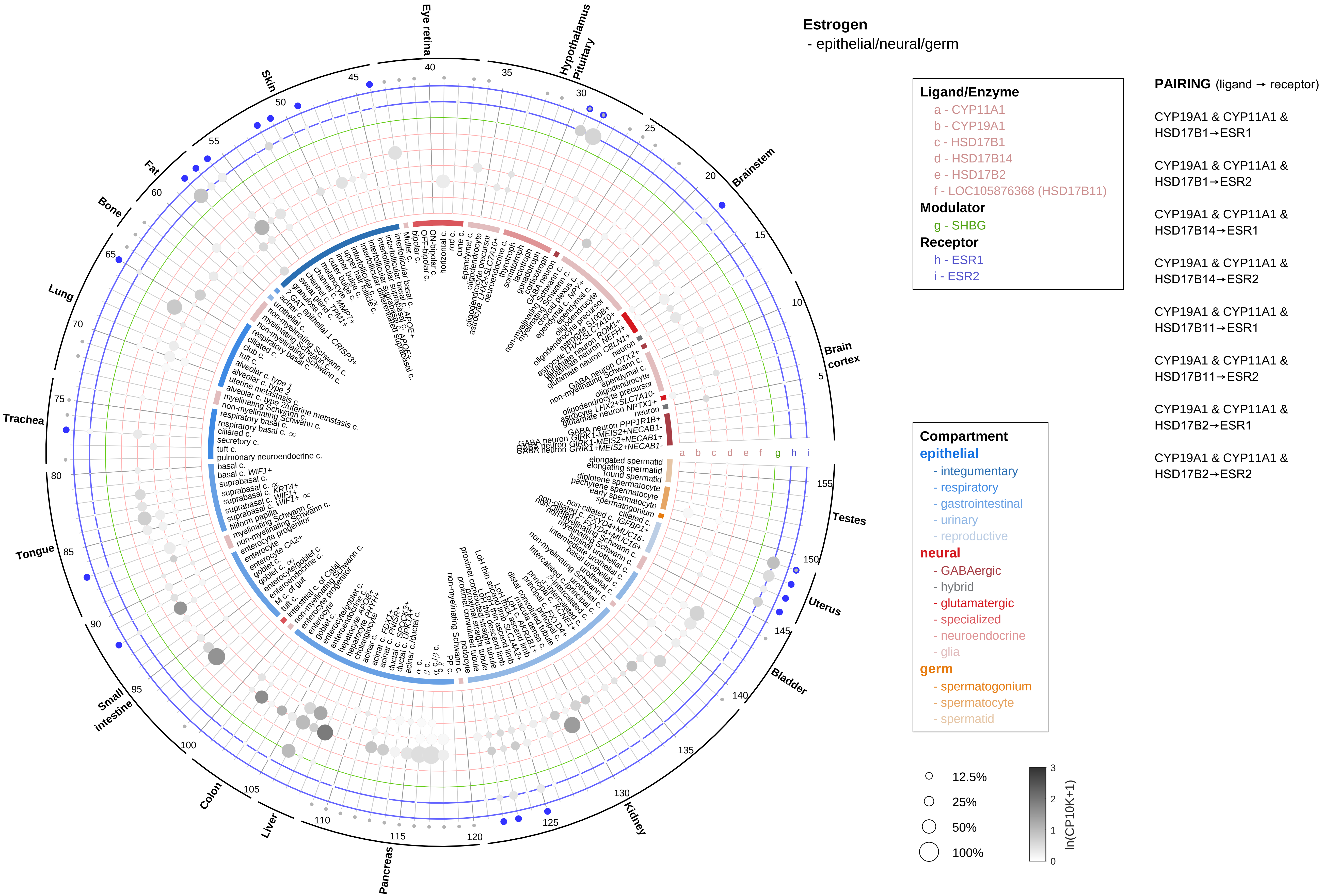
Estrogen
- epithelial/neural/germ
Ligand/Enzyme
a - CYP11A1
b - CYP19A1
c - HSD17B1
d - HSD17B14
e - HSD17B2
f - LOC105876368 (HSD17B11)
Modulator
g - SHBG
Receptor
h - ESR1
i - ESR2
PAIRING (ligand → receptor)
CYP19A1 & CYP11A1 & HSD17B1→ESR1
CYP19A1 & CYP11A1 & HSD17B1→ESR2
CYP19A1 & CYP11A1 & HSD17B14→ESR1
CYP19A1 & CYP11A1 & HSD17B14→ESR2
CYP19A1 & CYP11A1 & HSD17B11→ESR1
CYP19A1 & CYP11A1 & HSD17B11→ESR2
CYP19A1 & CYP11A1 & HSD17B2→ESR1
CYP19A1 & CYP11A1 & HSD17B2→ESR2
Compartment
epithelial
- integumentary
- respiratory
- gastrointestinal
- urinary
- reproductive
neural
- GABAergic
- hybrid
- glutamatergic
- specialized
- neuroendocrine
- glia
germ
- spermatogonium
- spermatocyte
- spermatid
12.5%
25%
50%
100%
ln(CP10K+1)
Eye retina
Hypothalamus Pituitary
Brainstem
Brain cortex
Testes
Uterus
Bladder
Kidney
Pancreas
Liver
Colon
Small intestine
Tongue
Trachea
Lung
Bone
Fat
Skin

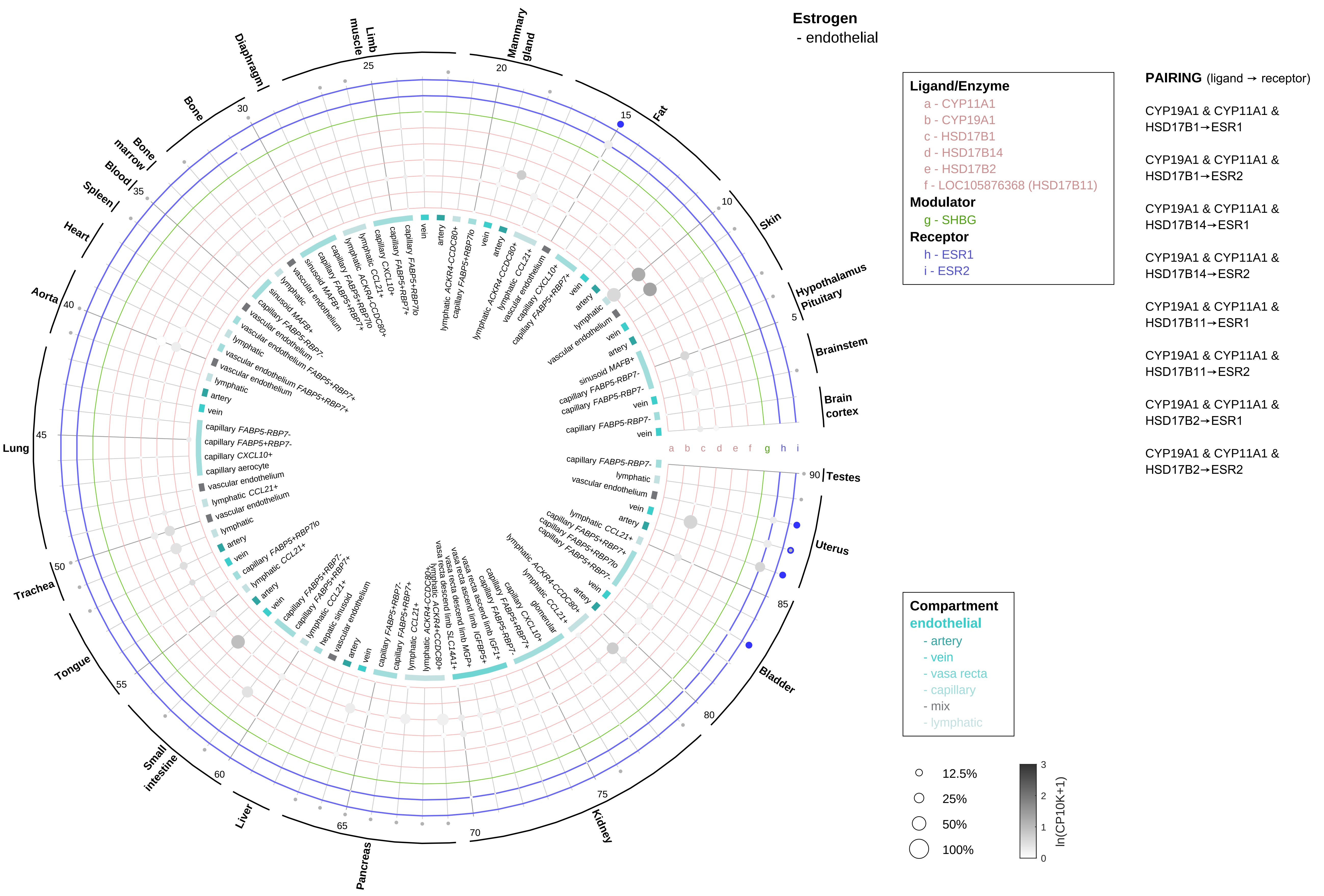

Estrogen
- endothelial
Ligand/Enzyme
a - CYP11A1
b - CYP19A1
c - HSD17B1
d - HSD17B14
e - HSD17B2
f - LOC105876368 (HSD17B11)
Modulator
g - SHBG
Receptor
h - ESR1
i - ESR2
PAIRING (ligand → receptor)
CYP19A1 & CYP11A1 & HSD17B1→ESR1
CYP19A1 & CYP11A1 & HSD17B1→ESR2
CYP19A1 & CYP11A1 & HSD17B14→ESR1
CYP19A1 & CYP11A1 & HSD17B14→ESR2
CYP19A1 & CYP11A1 & HSD17B11→ESR1
CYP19A1 & CYP11A1 & HSD17B11→ESR2
CYP19A1 & CYP11A1 & HSD17B2→ESR1
CYP19A1 & CYP11A1 & HSD17B2→ESR2
Compartment
endothelial
- artery
- vein
- vasa recta
- capillary
- mix
- lymphatic
12.5%
25%
50%
100%
ln(CP10K+1)
0
1
2
3
Brain cortex
Brainstem
Hypothalamus Pituitary
Skin
Fat
Mammary gland
Limb muscle
Diaphragm
Bone
Bone marrow
Blood
Spleen
Heart
Aorta
Lung
Trachea
Tongue
Small intestine
Liver
Pancreas
Kidney
Bladder
Uterus
Testes
a b c d e f g h i
5
10
15
20
25
30
35
40
45
50
55
60
65
70
75
80
85
90

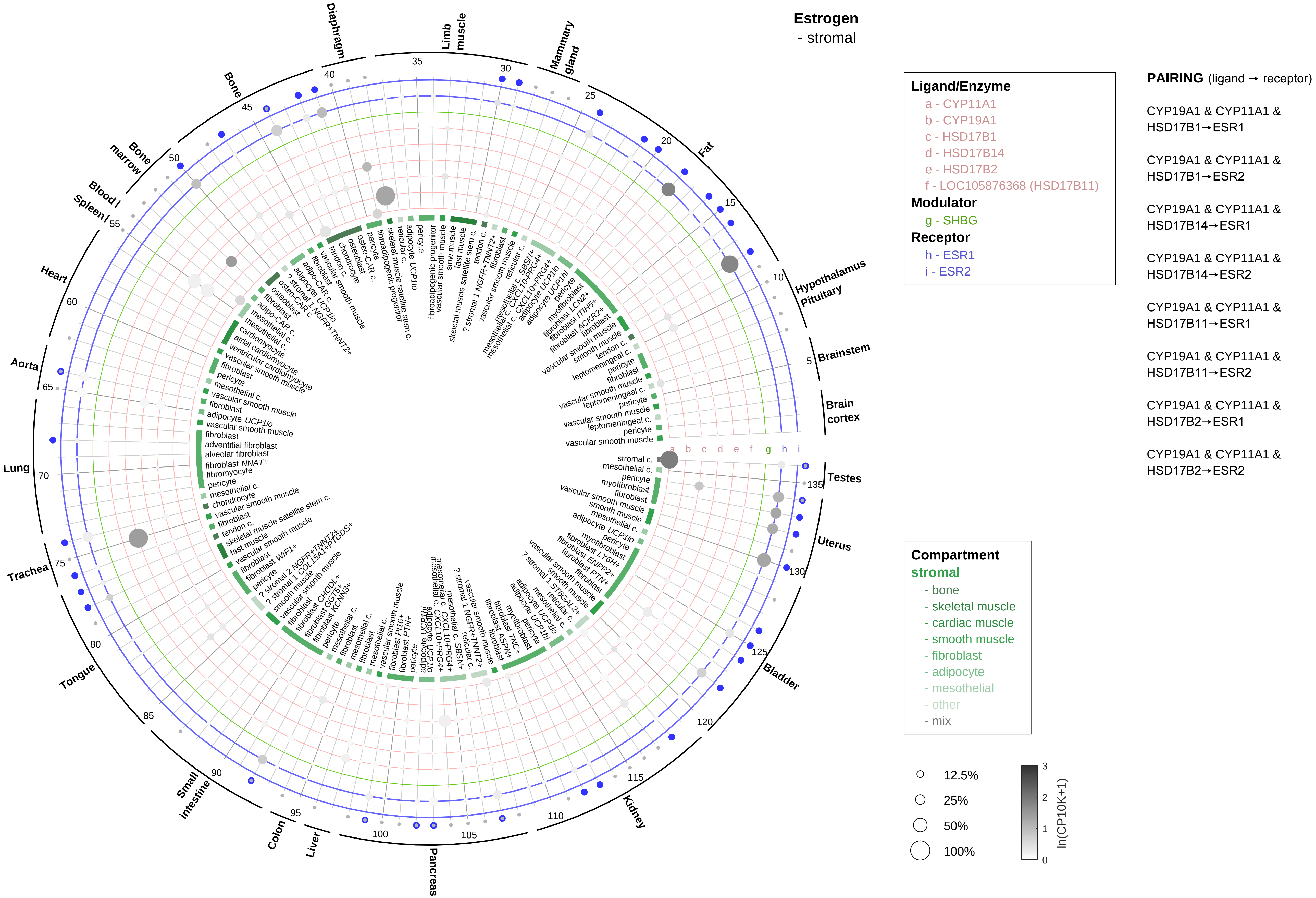

Estrogen
- stromal
Ligand/Enzyme
a - CYP11A1
b - CYP19A1
c - HSD17B1
d - HSD17B14
e - HSD17B2
f - LOC105876368 (HSD17B11)
Modulator
g - SHBG
Receptor
h - ESR1
i - ESR2
PAIRING (ligand → receptor)
CYP19A1 & CYP11A1 & HSD17B1→ESR1
CYP19A1 & CYP11A1 & HSD17B1→ESR2
CYP19A1 & CYP11A1 & HSD17B14→ESR1
CYP19A1 & CYP11A1 & HSD17B14→ESR2
CYP19A1 & CYP11A1 & HSD17B11→ESR1
CYP19A1 & CYP11A1 & HSD17B11→ESR2
CYP19A1 & CYP11A1 & HSD17B2→ESR1
CYP19A1 & CYP11A1 & HSD17B2→ESR2
Compartment
stromal
- bone
- skeletal muscle
- cardiac muscle
- smooth muscle
- fibroblast
- adipocyte
- mesothelial
- other
- mix
12.5%
25%
50%
100%
ln(CP10K+1)
Diaphragm
Limb muscle
Mammary gland
Fat
Hypothalamus
Pituitary
Brainstem
Brain cortex
Testes
Uterus
Bladder
Kidney
Pancreas
Liver
Colon
Small intestine
Tongue
Trachea
Lung
Aorta
Heart
Spleen
Blood
Bone marrow
Bone

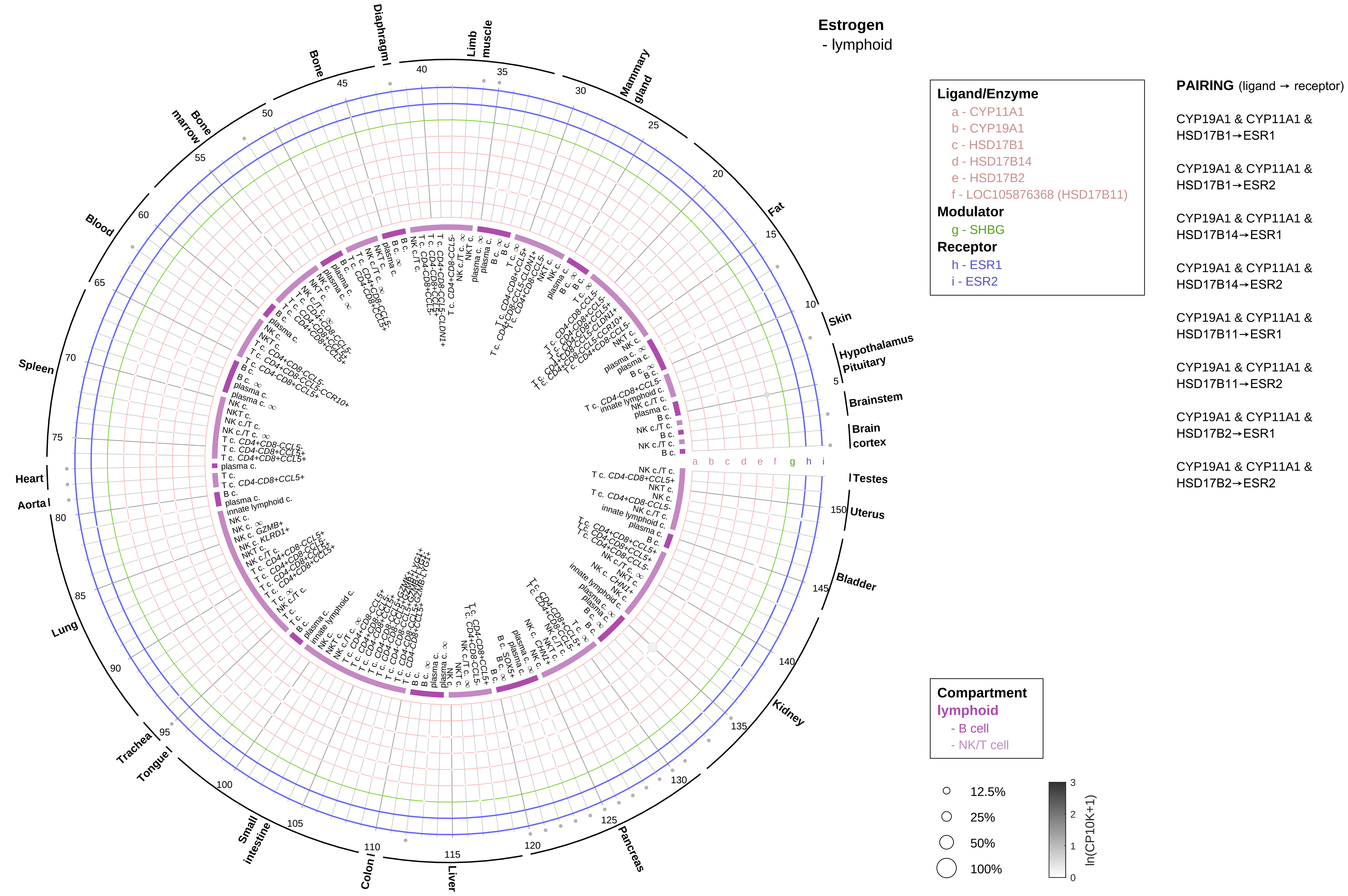
Estrogen
- lymphoid
Ligand/Enzyme
a - CYP11A1
b - CYP19A1
c - HSD17B1
d - HSD17B14
e - HSD17B2
f - LOC105876368 (HSD17B11)
Modulator
g - SHBG
Receptor
h - ESR1
i - ESR2
PAIRING (ligand → receptor)
CYP19A1 & CYP11A1 & HSD17B1→ESR1
CYP19A1 & CYP11A1 & HSD17B1→ESR2
CYP19A1 & CYP11A1 & HSD17B14→ESR1
CYP19A1 & CYP11A1 & HSD17B14→ESR2
CYP19A1 & CYP11A1 & HSD17B11→ESR1
CYP19A1 & CYP11A1 & HSD17B11→ESR2
CYP19A1 & CYP11A1 & HSD17B2→ESR1
CYP19A1 & CYP11A1 & HSD17B2→ESR2
Compartment
lymphoid
- B cell
- NK/T cell
12.5%
25%
50%
100%
ln(CP10K+1)
Diaphragm
Limb muscle
Mammary gland
Fat
Skin
Hypothalamus
Pituitary
Brainstem
Brain cortex
Testes
Uterus
Bladder
Kidney
Pancreas
Liver
Colon
Small intestine
Tongue
Trachea
Lung
Aorta
Heart
Spleen
Blood
Bone marrow
Bone

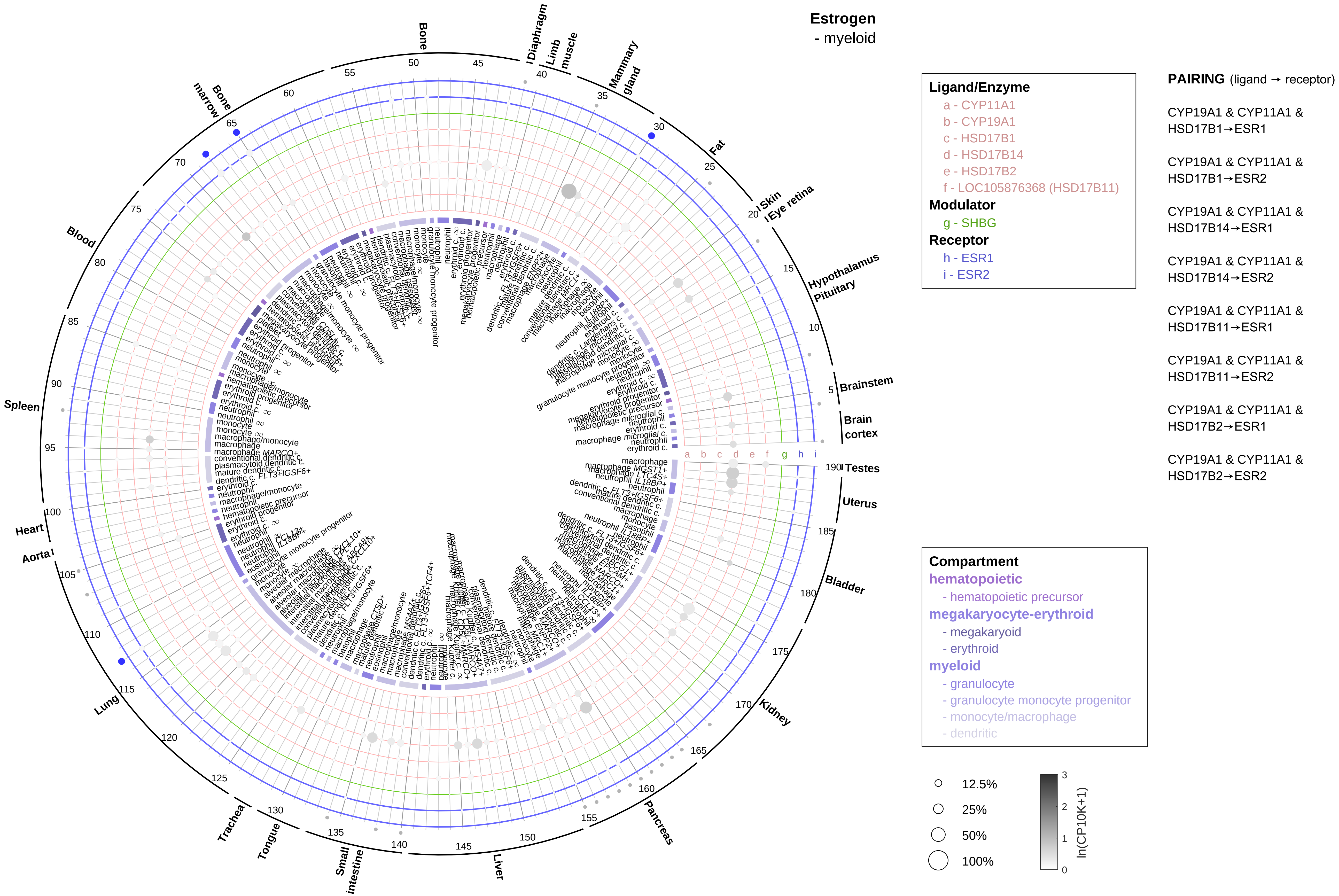

Estrogen
- myeloid
Ligand/Enzyme
a - CYP11A1
b - CYP19A1
c - HSD17B1
d - HSD17B14
e - HSD17B2
f - LOC105876368 (HSD17B11)
Modulator
g - SHBG
Receptor
h - ESR1
i - ESR2
PAIRING (ligand → receptor)
CYP19A1 & CYP11A1 & HSD17B1→ESR1
CYP19A1 & CYP11A1 & HSD17B1→ESR2
CYP19A1 & CYP11A1 & HSD17B14→ESR1
CYP19A1 & CYP11A1 & HSD17B14→ESR2
CYP19A1 & CYP11A1 & HSD17B11→ESR1
CYP19A1 & CYP11A1 & HSD17B11→ESR2
CYP19A1 & CYP11A1 & HSD17B2→ESR1
CYP19A1 & CYP11A1 & HSD17B2→ESR2
Compartment
hematopoietic
- hematopoietic precursor
megakaryocyte-erythroid
- megakaryoid
- erythroid
myeloid
- granulocyte
- granulocyte monocyte progenitor
- monocyte/macrophage
- dendritic
12.5%
25%
50%
100%
ln(CP10K+1)
Bone
Diaphragm
Limb muscle
Mammary gland
Fat
Skin
Eye retina
Hypothalamus
Pituitary
Brainstem
Brain cortex
Testes
Uterus
Bladder
Kidney
Pancreas
Liver
Small intestine
Tongue
Trachea
Lung
Aorta
Heart
Spleen
Blood
Bone marrow

Fibroblast growth factor (endocrine)
- epithelial/neural/germ

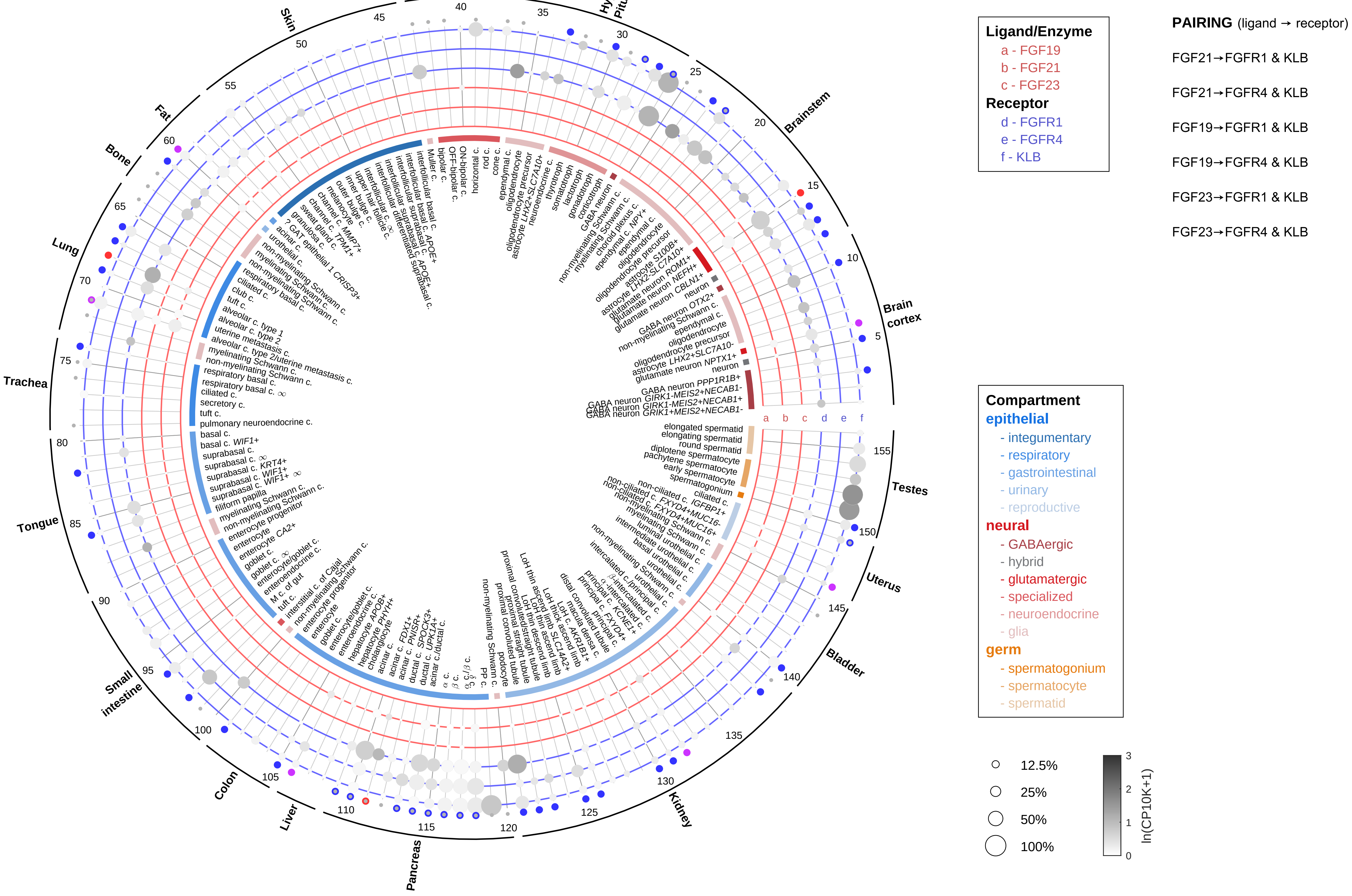

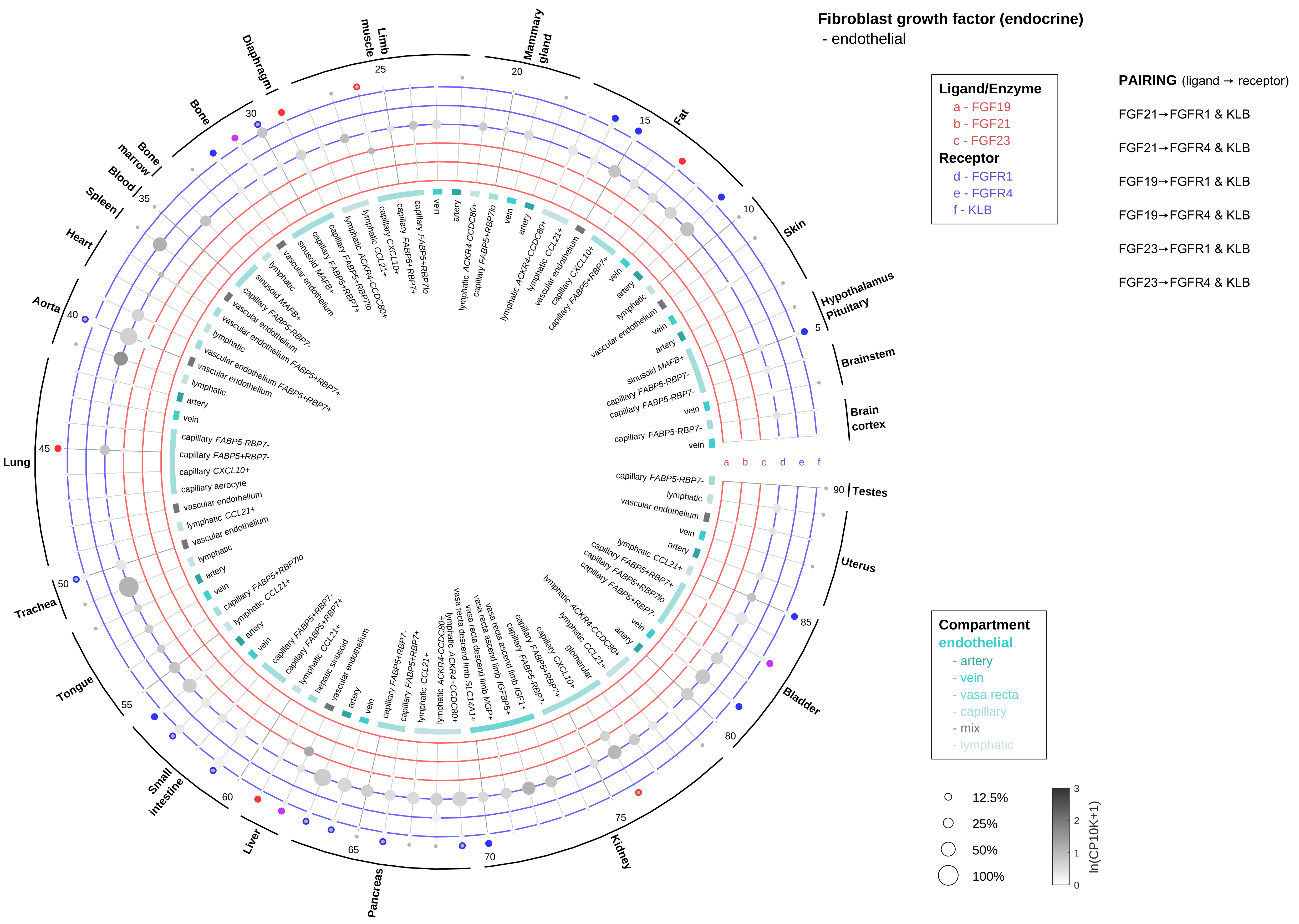

Fibroblast growth factor (endocrine)
- endothelial
Ligand/Enzyme
a - FGF19
b - FGF21
c - FGF23
Receptor
d - FGFR1
e - FGFR4
f - KLB
PAIRING (ligand → receptor)
FGF21→FGFR1 & KLB
FGF21→FGFR4 & KLB
FGF19→FGFR1 & KLB
FGF19→FGFR4 & KLB
FGF23→FGFR1 & KLB
FGF23→FGFR4 & KLB
Compartment
endothelial
- artery
- vein
- vasa recta
- capillary
- mix
- lymphatic
12.5%
25%
50%
100%
ln(CP10K+1)

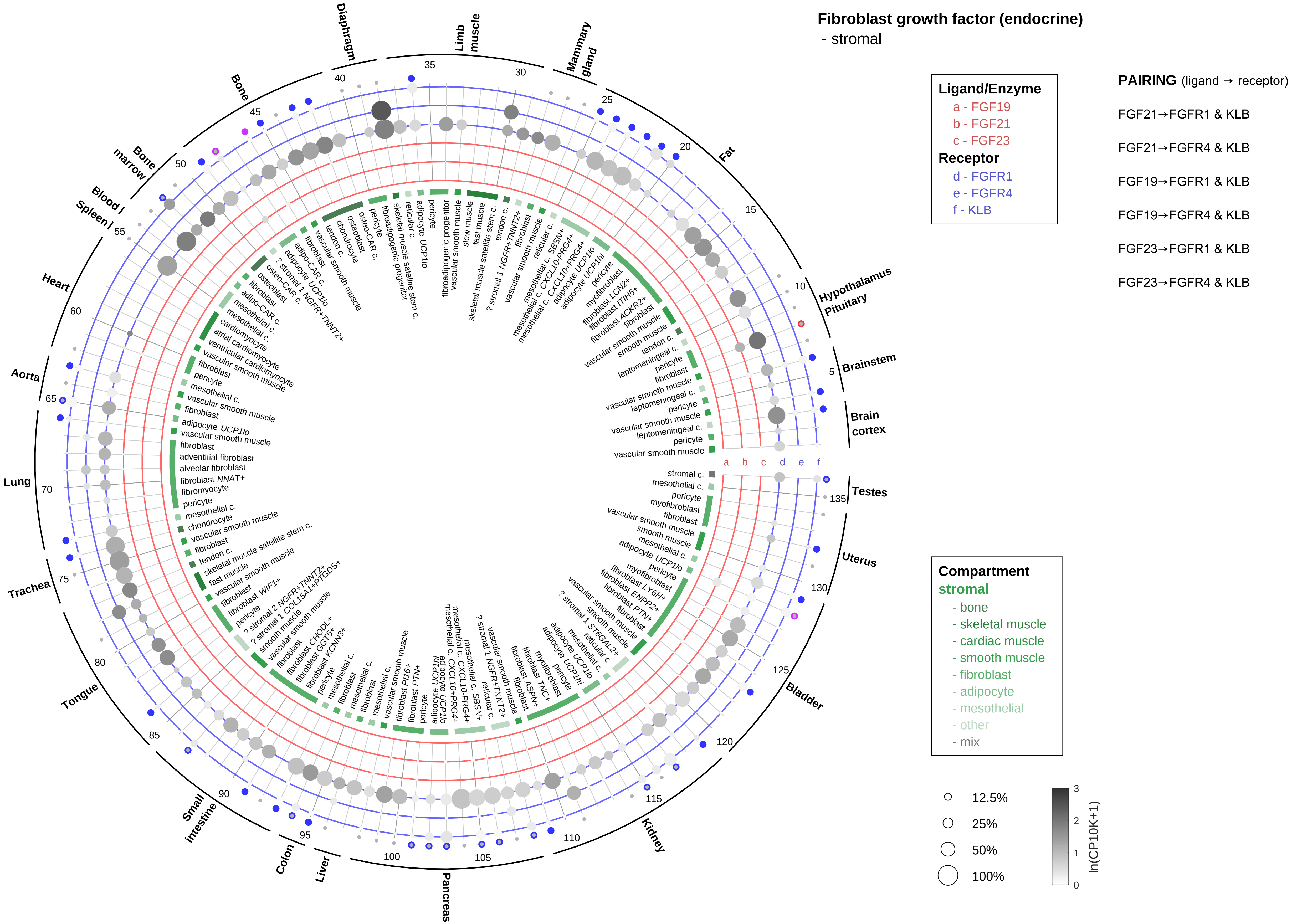
Fibroblast growth factor (endocrine)
- stromal
Ligand/Enzyme
a - FGF19
b - FGF21
c - FGF23
Receptor
d - FGFR1
e - FGFR4
f - KLB
PAIRING (ligand → receptor)
FGF21→FGFR1 & KLB
FGF21→FGFR4 & KLB
FGF19→FGFR1 & KLB
FGF19→FGFR4 & KLB
FGF23→FGFR1 & KLB
FGF23→FGFR4 & KLB
Compartment
stromal
- bone
- skeletal muscle
- cardiac muscle
- smooth muscle
- fibroblast
- adipocyte
- mesothelial
- other
- mix
12.5%
25%
50%
100%
ln(CP10K+1)
Brain cortex
Brainstem
Hypothalamus Pituitary
Fat
Mammary gland
Limb muscle
Diaphragm
Bone
Bone marrow
Blood
Spleen
Heart
Aorta
Lung
Trachea
Tongue
Small intestine
Colon
Liver
Pancreas
Kidney
Bladder
Uterus
Testes

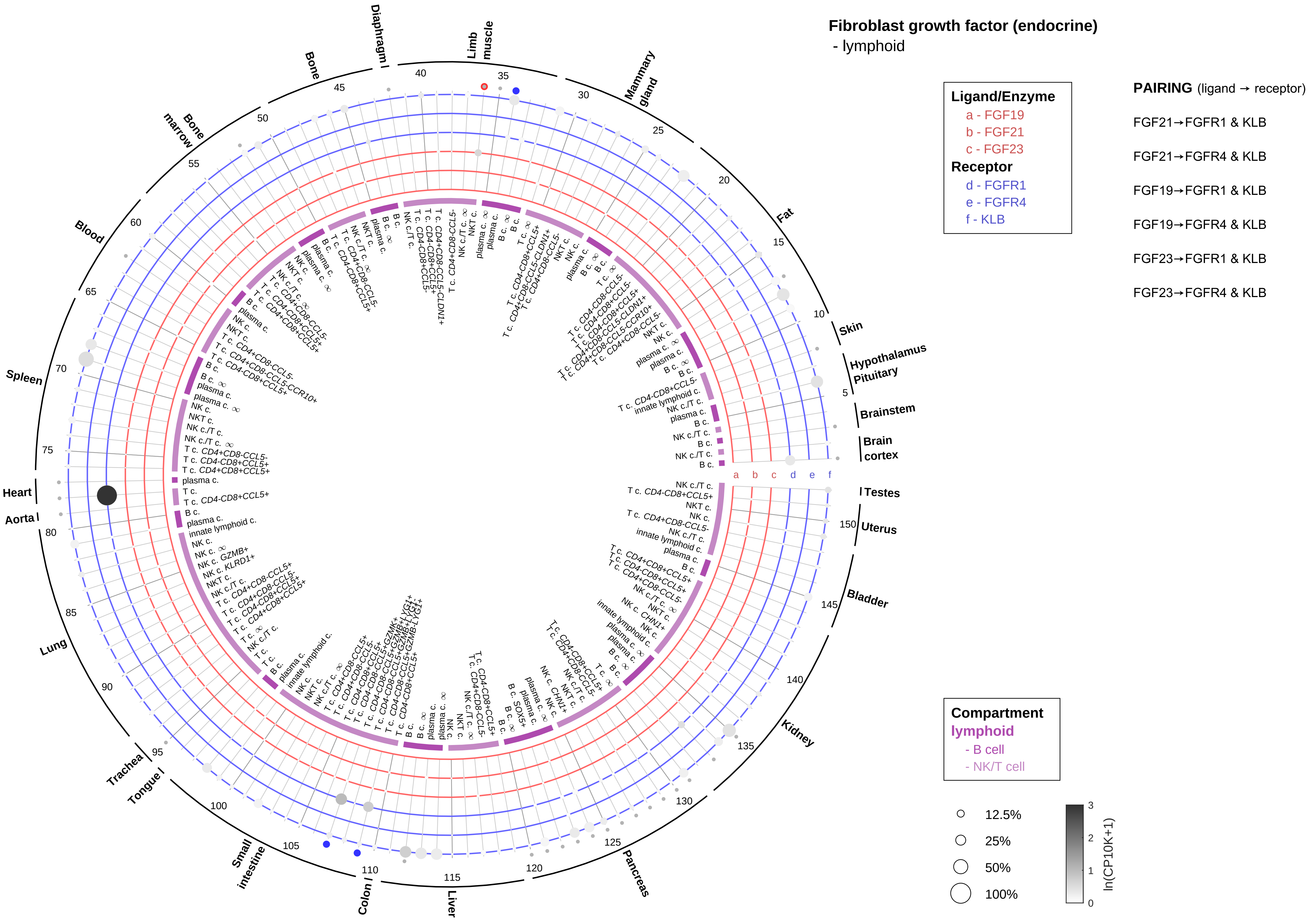

Fibroblast growth factor (endocrine)
- lymphoid
Ligand/Enzyme
a - FGF19
b - FGF21
c - FGF23
Receptor
d - FGFR1
e - FGFR4
f - KLB
PAIRING (ligand → receptor)
FGF21→FGFR1 & KLB
FGF21→FGFR4 & KLB
FGF19→FGFR1 & KLB
FGF19→FGFR4 & KLB
FGF23→FGFR1 & KLB
FGF23→FGFR4 & KLB
Compartment
lymphoid
- B cell
- NK/T cell
12.5%
25%
50%
100%
ln(CP10K+1)
Limb muscle
Mammary gland
Fat
Skin
Hypothalamus
Pituitary
Brainstem
Brain cortex
Testes
Uterus
Bladder
Kidney
Pancreas
Liver
Colon
Small intestine
Tongue
Trachea
Lung
Aorta
Heart
Spleen
Blood
Bone marrow
Bone
Diaphragm

Fibroblast growth factor (endocrine)
- myeloid

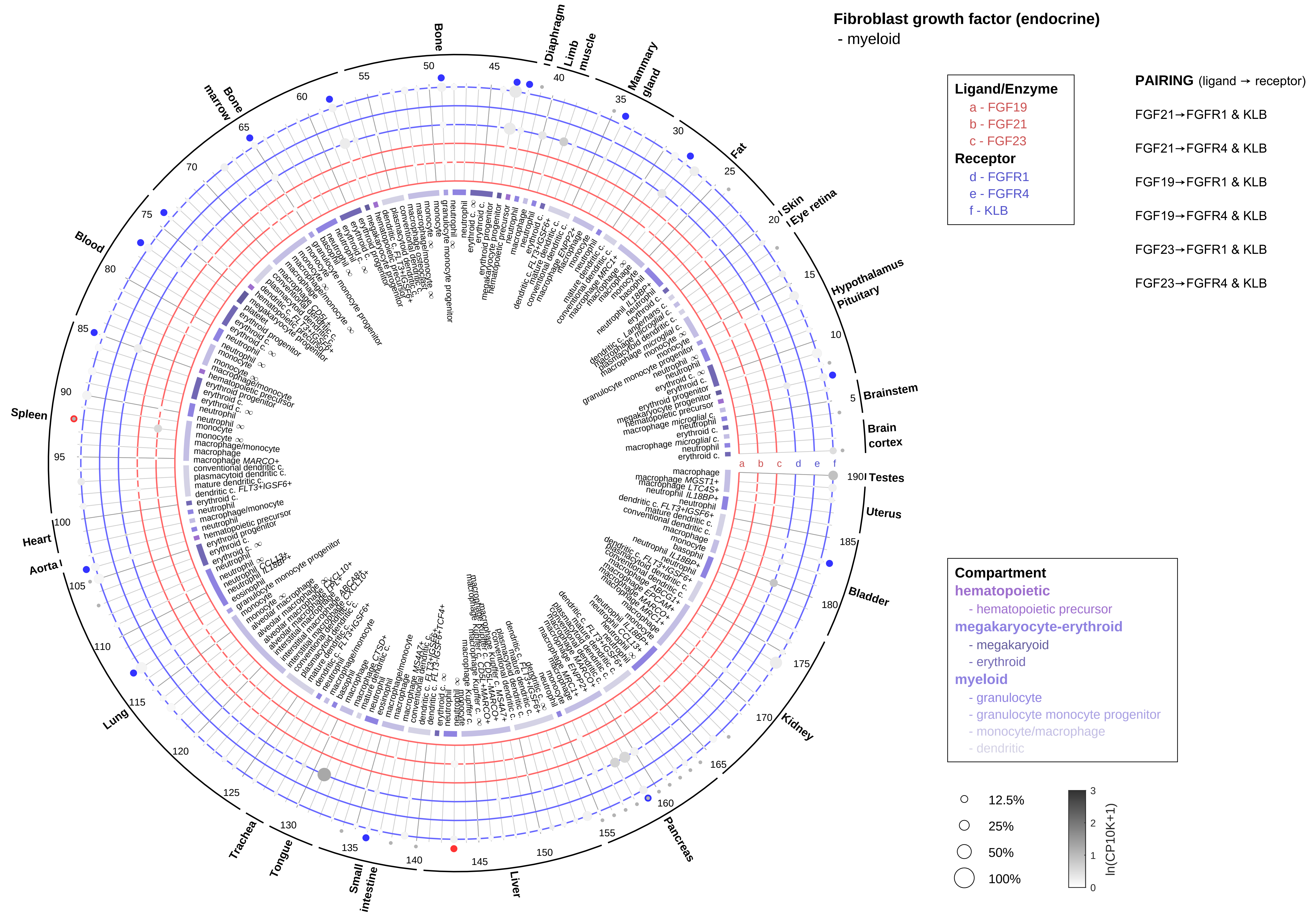

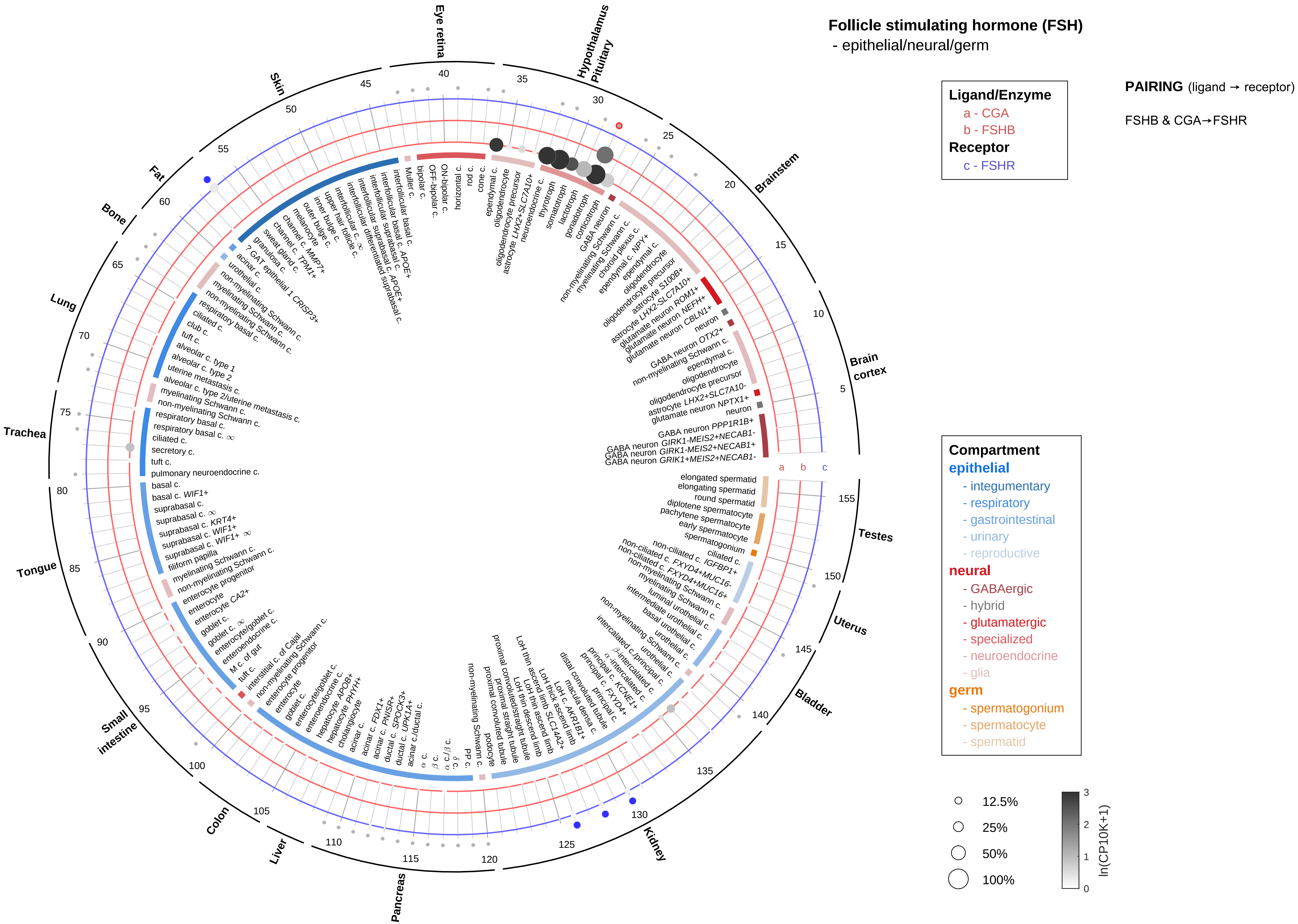
Follicle stimulating hormone (FSH)
- epithelial/neural/germ
Ligand/Enzyme
a - CGA
b - FSHB
Receptor
c - FSHR
PAIRING (ligand → receptor)
FSHB & CGA→FSHR
Compartment
epithelial
- integumentary
- respiratory
- gastrointestinal
- urinary
- reproductive
neural
- GABAergic
- hybrid
- glutamatergic
- specialized
- neuroendocrine
- glia
germ
- spermatogonium
- spermatocyte
- spermatid
12.5%
25%
50%
100%
ln(CP10K+1)
Eye retina
Hypothalamus
Pituitary
Brainstem
Brain cortex
Testes
Uterus
Bladder
Kidney
Pancreas
Liver
Colon
Small intestine
Tongue
Trachea
Lung
Bone
Fat
Skin

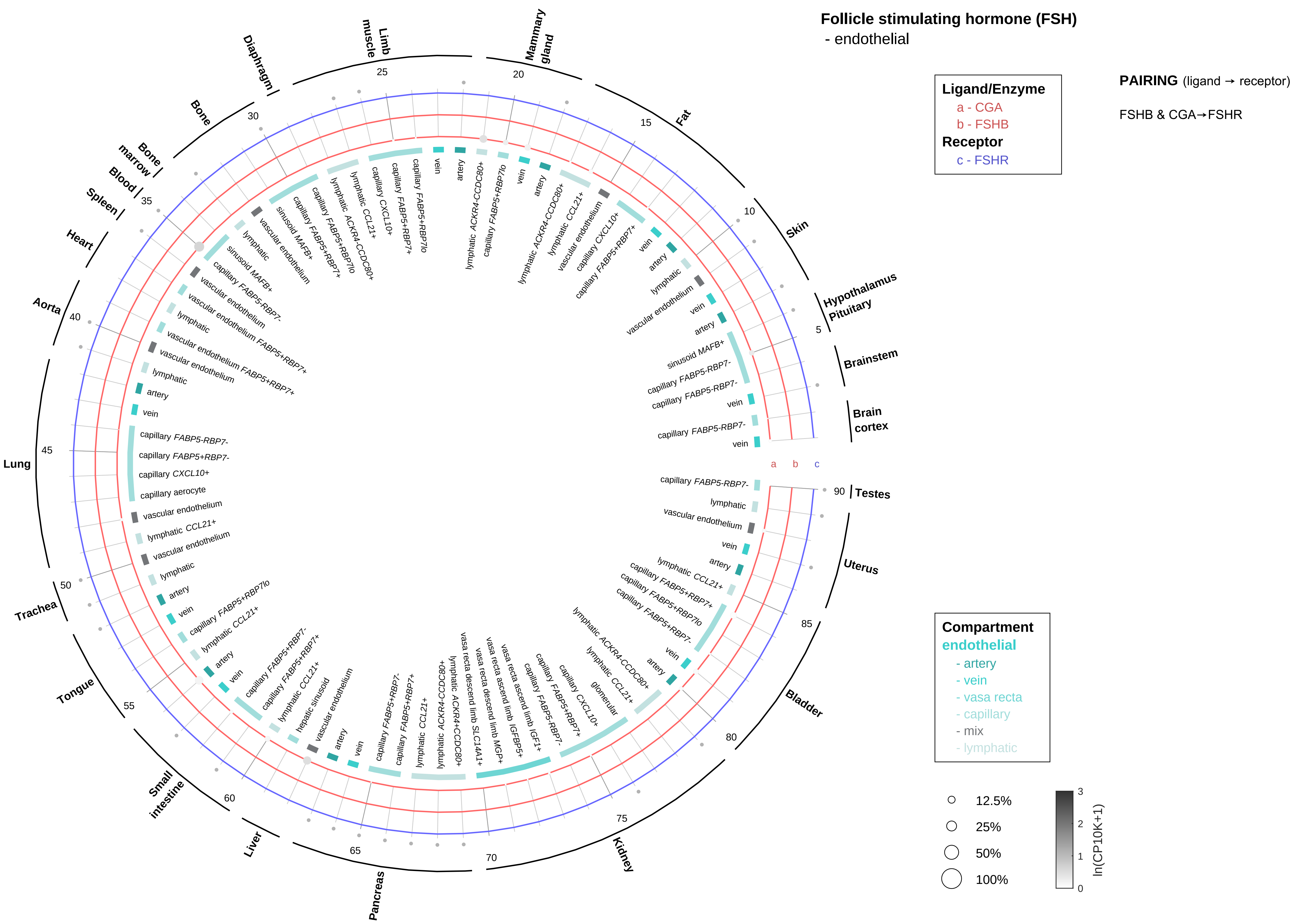

Follicle stimulating hormone (FSH)
- endothelial
Ligand/Enzyme
a - CGA
b - FSHB
Receptor
c - FSHR
PAIRING (ligand → receptor)
FSHB & CGA→FSHR
Compartment
endothelial
- artery
- vein
- vasa recta
- capillary
- mix
- lymphatic
12.5%
25%
50%
100%
ln(CP10K+1)
Brain cortex
Brainstem
Hypothalamus Pituitary
Skin
Fat
Mammary gland
Limb muscle
Diaphragm
Bone
Bone marrow
Blood
Spleen
Heart
Aorta
Lung
Trachea
Tongue
Small intestine
Liver
Pancreas
Kidney
Bladder
Uterus
Testes

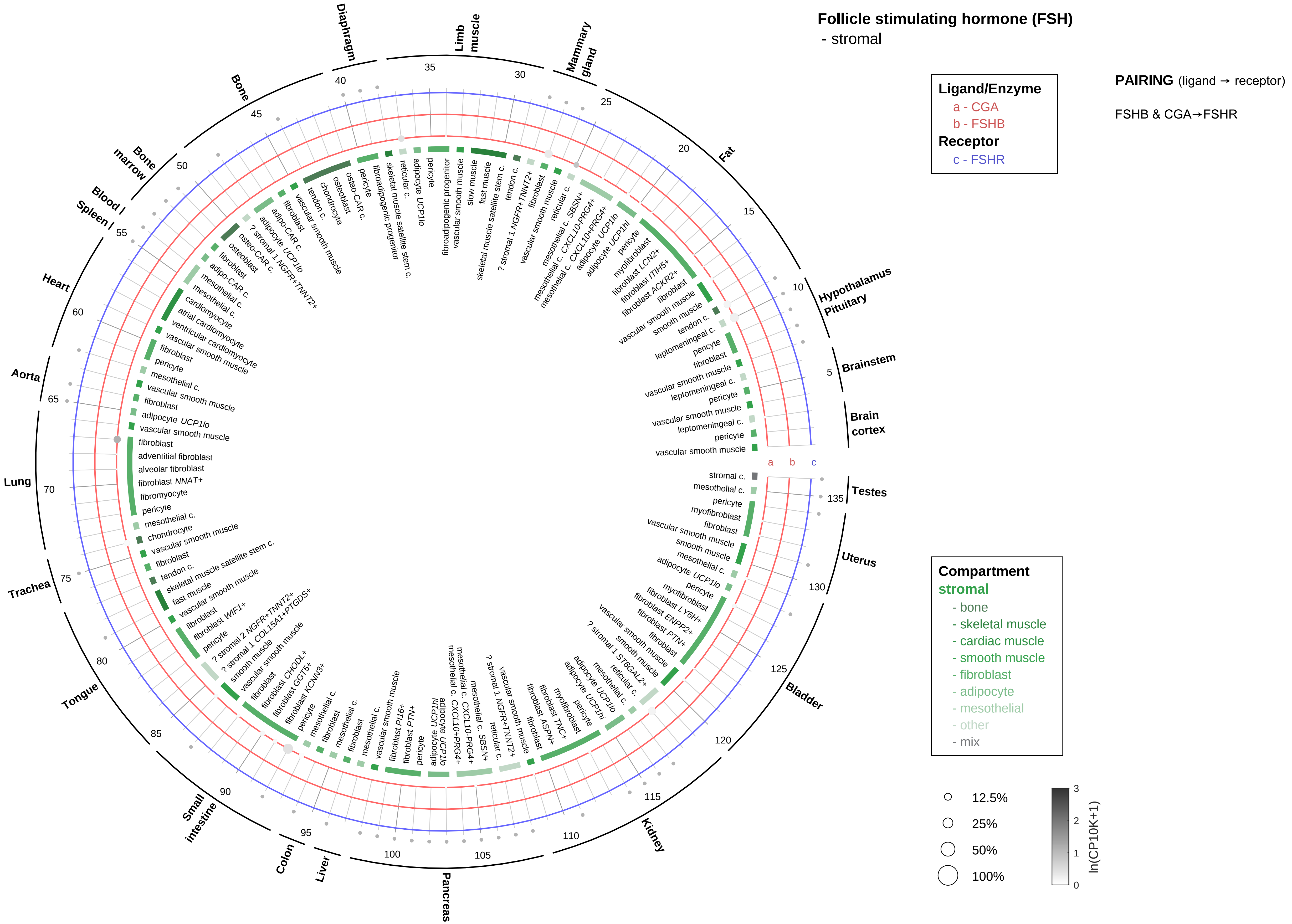

Follicle stimulating hormone (FSH)
- stromal
Ligand/Enzyme
a - CGA
b - FSHB
Receptor
c - FSHR
PAIRING (ligand → receptor)
FSHB & CGA→FSHR
Compartment
stromal
- bone
- skeletal muscle
- cardiac muscle
- smooth muscle
- fibroblast
- adipocyte
- mesothelial
- other
- mix
12.5%
25%
50%
100%
ln(CP10K+1)
Diaphragm
Limb muscle
Mammary gland
Fat
Hypothalamus
Pituitary
Brainstem
Brain cortex
Testes
Uterus
Bladder
Kidney
Pancreas
Liver
Colon
Small intestine
Tongue
Trachea
Lung
Aorta
Heart
Spleen
Blood
Bone marrow
Bone

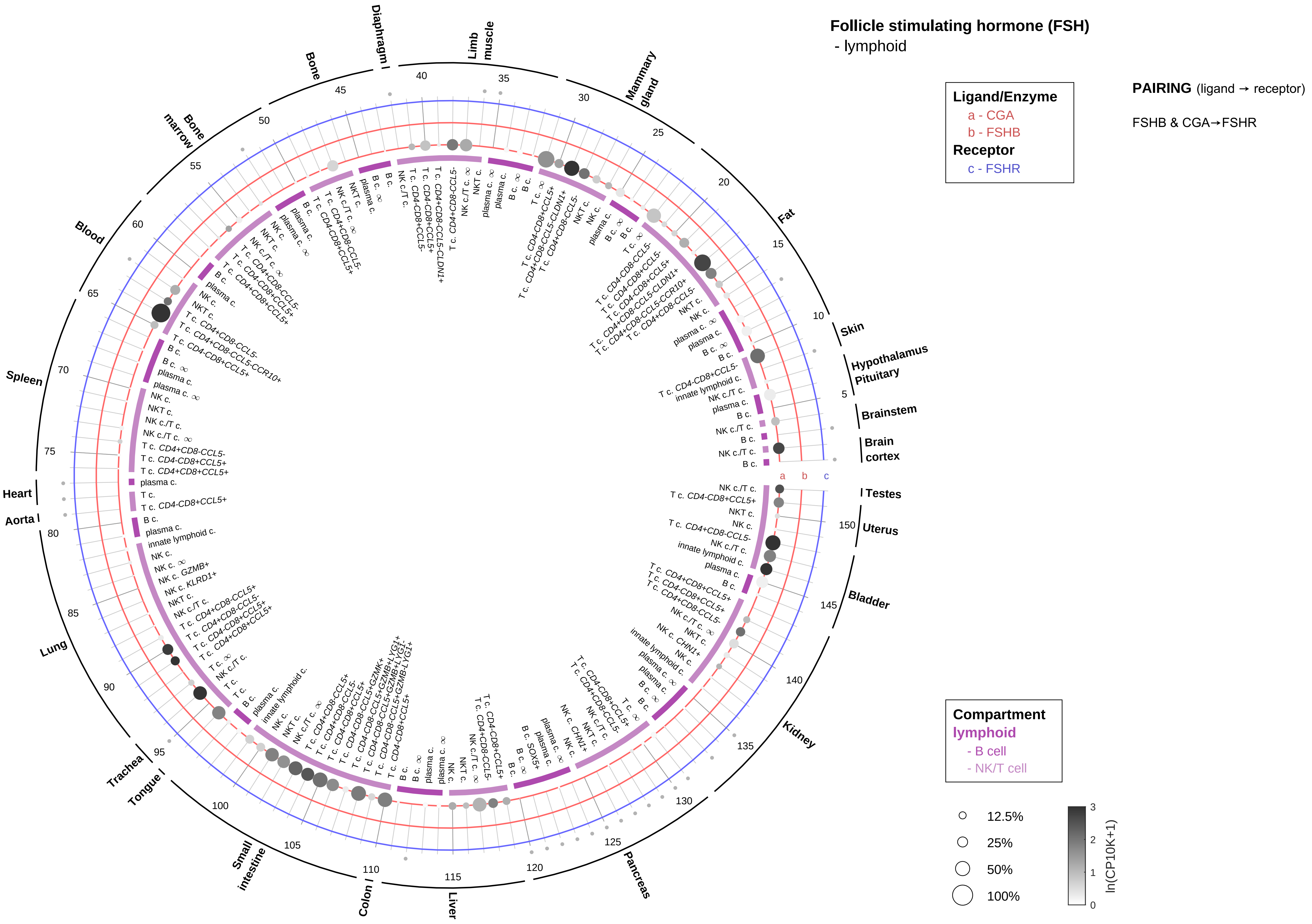

Follicle stimulating hormone (FSH)
- lymphoid
Ligand/Enzyme
a - CGA
b - FSHB
Receptor
c - FSHR
PAIRING (ligand → receptor)
FSHB & CGA→FSHR
Compartment
lymphoid
- B cell
- NK/T cell
12.5%
25%
50%
100%
ln(CP10K+1)
Skin
Hypothalamus
Pituitary
Brainstem
Brain cortex
Testes
Uterus
Bladder
Kidney
Pancreas
Liver
Colon
Small intestine
Tongue
Trachea
Lung
Aorta
Heart
Spleen
Blood
Bone marrow
Bone
Diaphragm
Limb muscle
Mammary gland
Fat

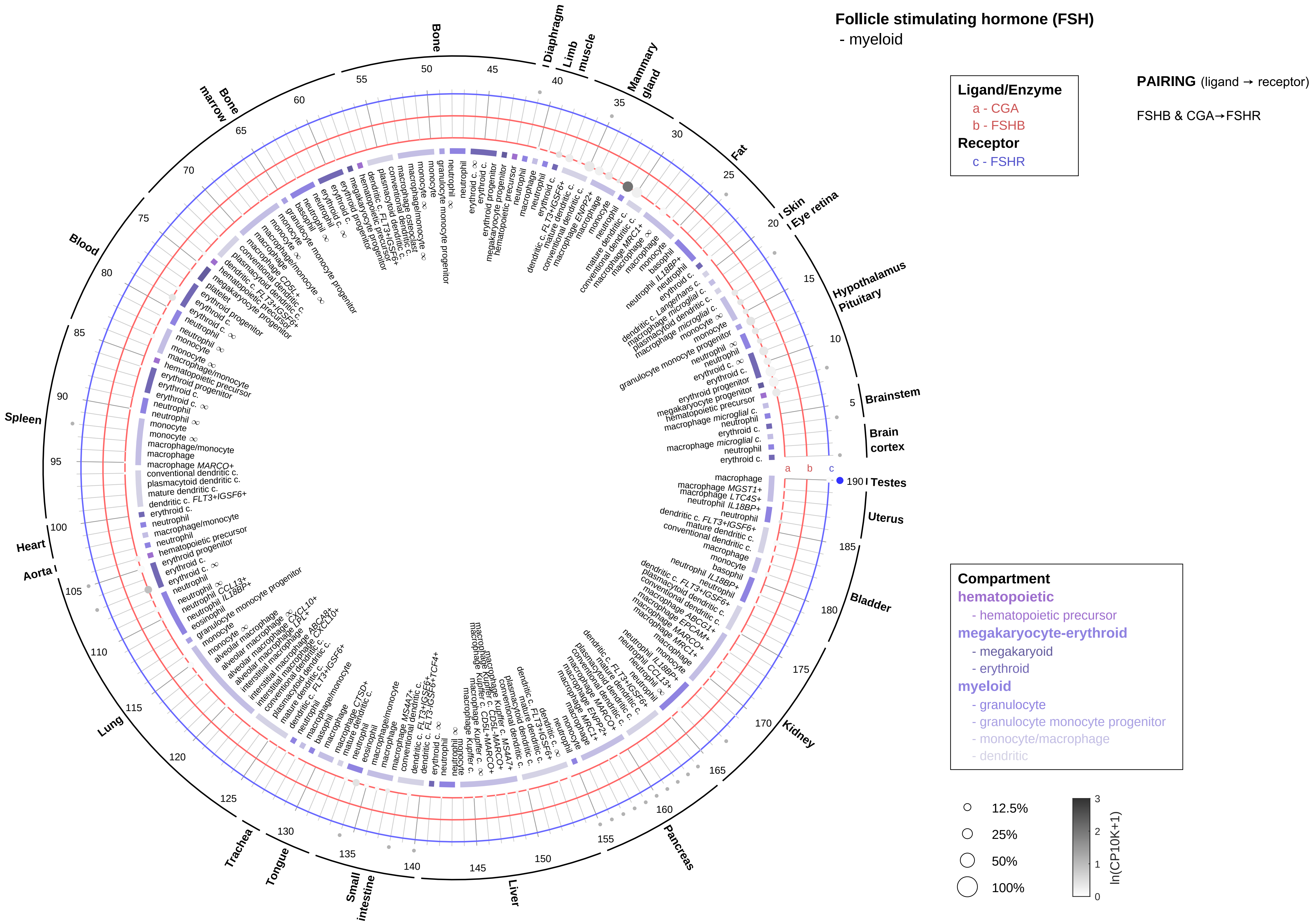
Follicle stimulating hormone (FSH)
- myeloid
Ligand/Enzyme
a - CGA
b - FSHB
Receptor
c - FSHR
PAIRING (ligand → receptor)
FSHB & CGA→FSHR
Compartment
hematopoietic
- hematopoietic precursor
megakaryocyte-erythroid
- megakaryoid
- erythroid
myeloid
- granulocyte
- granulocyte monocyte progenitor
- monocyte/macrophage
- dendritic
12.5%
25%
50%
100%
ln(CP10K+1)
Testes
Uterus
Bladder
Kidney
Pancreas
Liver
Small intestine
Tongue
Trachea
Lung
Aorta
Heart
Spleen
Blood
Bone marrow
Bone
Diaphragm
Limb muscle
Mammary gland
Fat
Skin
Eye retina
Hypothalamus
Pituitary
Brainstem
Brain cortex

Galanin (GAL)/Galanin-like peptide (GALP)
- epithelial/neural/germ

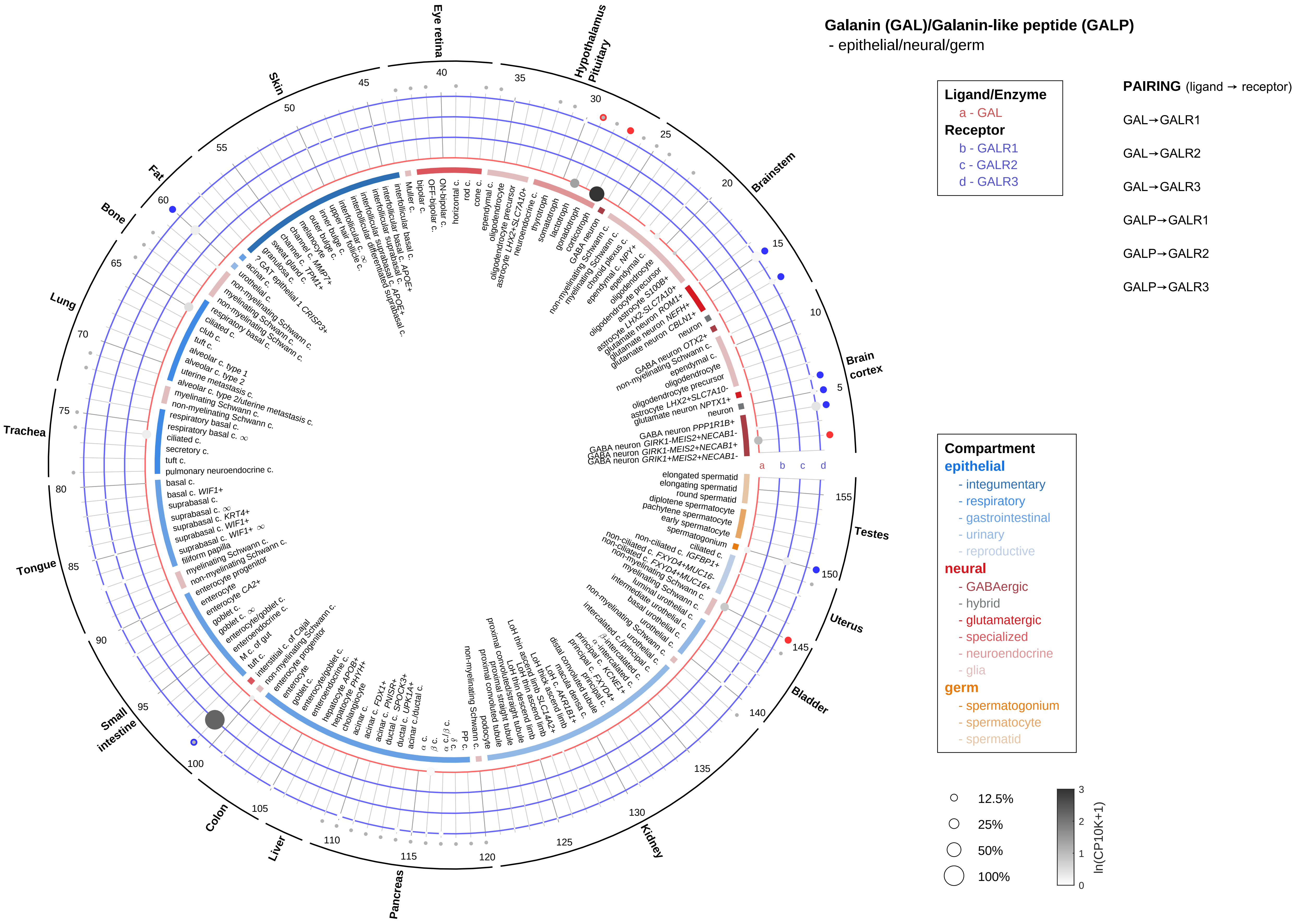

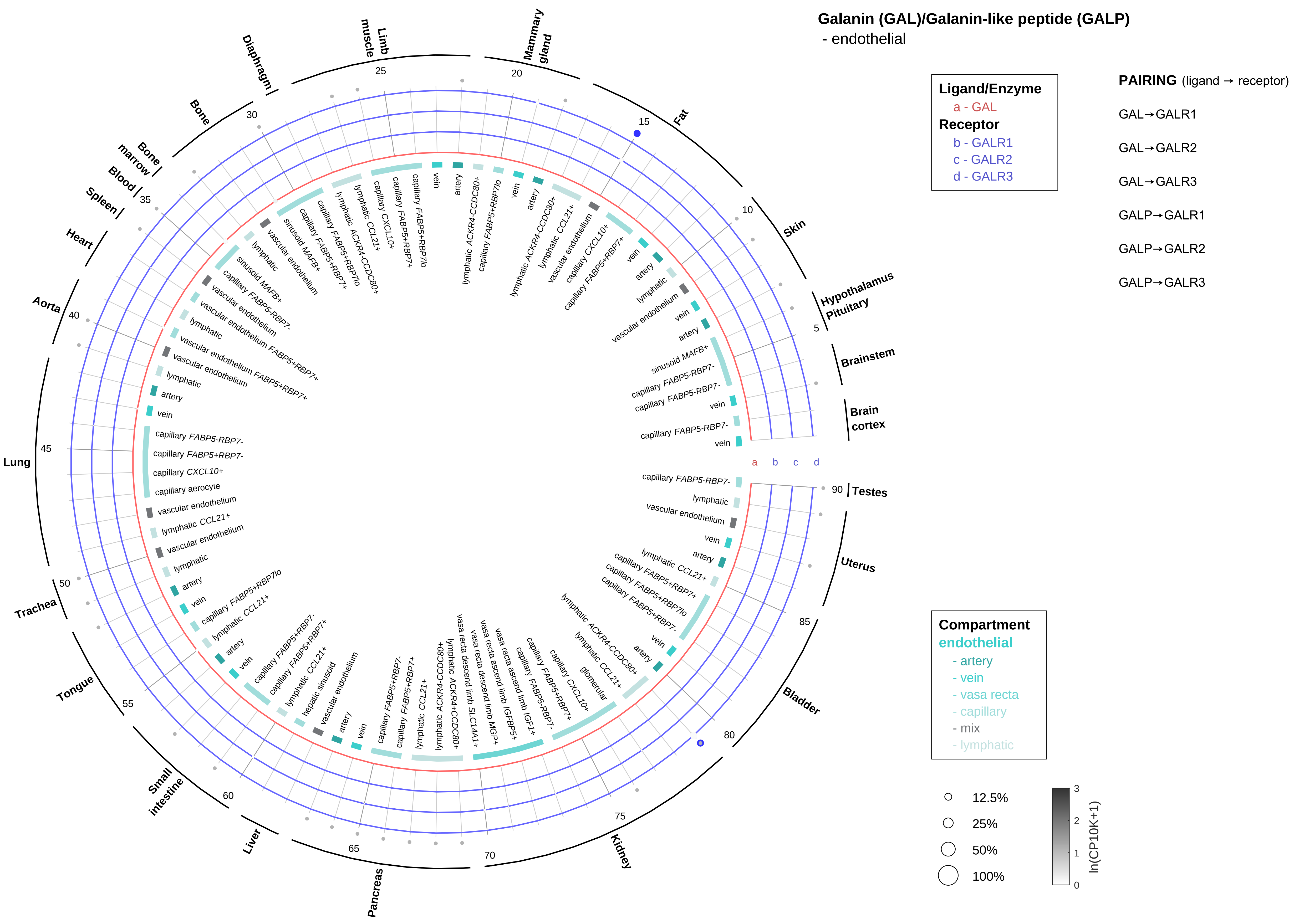

Galanin (GAL)/Galanin-like peptide (GALP)
- endothelial
Ligand/Enzyme
a - GAL
Receptor
b - GALR1
c - GALR2
d - GALR3
PAIRING (ligand → receptor)
GAL→GALR1
GAL→GALR2
GAL→GALR3
GALP→GALR1
GALP→GALR2
GALP→GALR3
Compartment
endothelial
- artery
- vein
- vasa recta
- capillary
- mix
- lymphatic
12.5%
25%
50%
100%
ln(CP10K+1)
Brain cortex
Brainstem
Hypothalamus Pituitary
Skin
Fat
Mammary gland
Limb muscle
Diaphragm
Bone
Bone marrow
Blood
Spleen
Heart
Aorta
Lung
Trachea
Tongue
Small intestine
Liver
Pancreas
Kidney
Bladder
Uterus
Testes

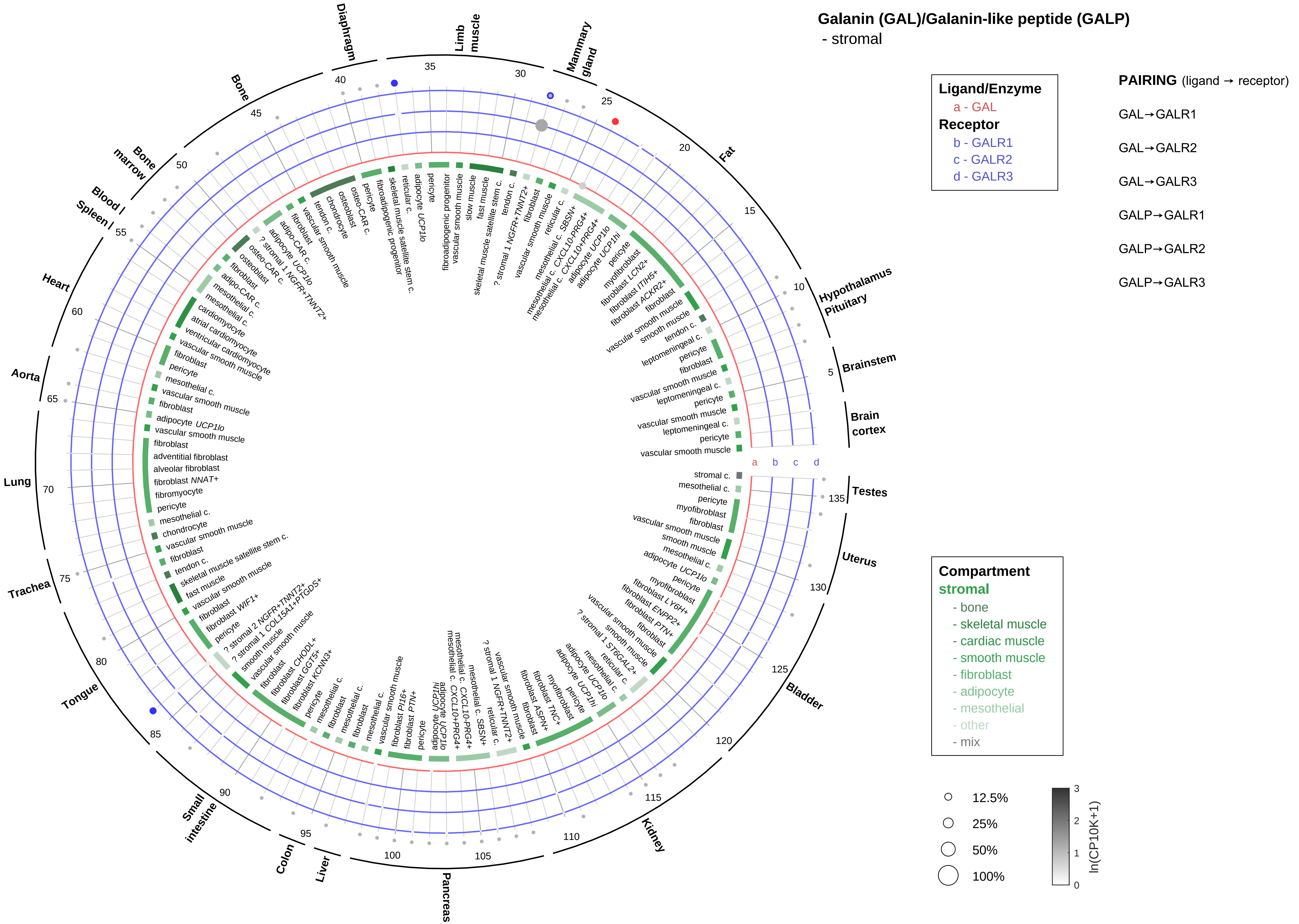

Galanin (GAL)/Galanin-like peptide (GALP)
- stromal
Ligand/Enzyme
a - GAL
Receptor
b - GALR1
c - GALR2
d - GALR3
PAIRING (ligand → receptor)
GAL→GALR1
GAL→GALR2
GAL→GALR3
GALP→GALR1
GALP→GALR2
GALP→GALR3
Compartment
stromal
- bone
- skeletal muscle
- cardiac muscle
- smooth muscle
- fibroblast
- adipocyte
- mesothelial
- other
- mix
12.5%
25%
50%
100%
ln(CP10K+1)
Brain cortex
Brainstem
Hypothalamus Pituitary
Fat
Mammary gland
Limb muscle
Diaphragm
Bone
Bone marrow
Blood / Spleen
Heart
Aorta
Lung
Trachea
Tongue
Small intestine
Colon
Liver
Pancreas
Kidney
Bladder
Uterus
Testes

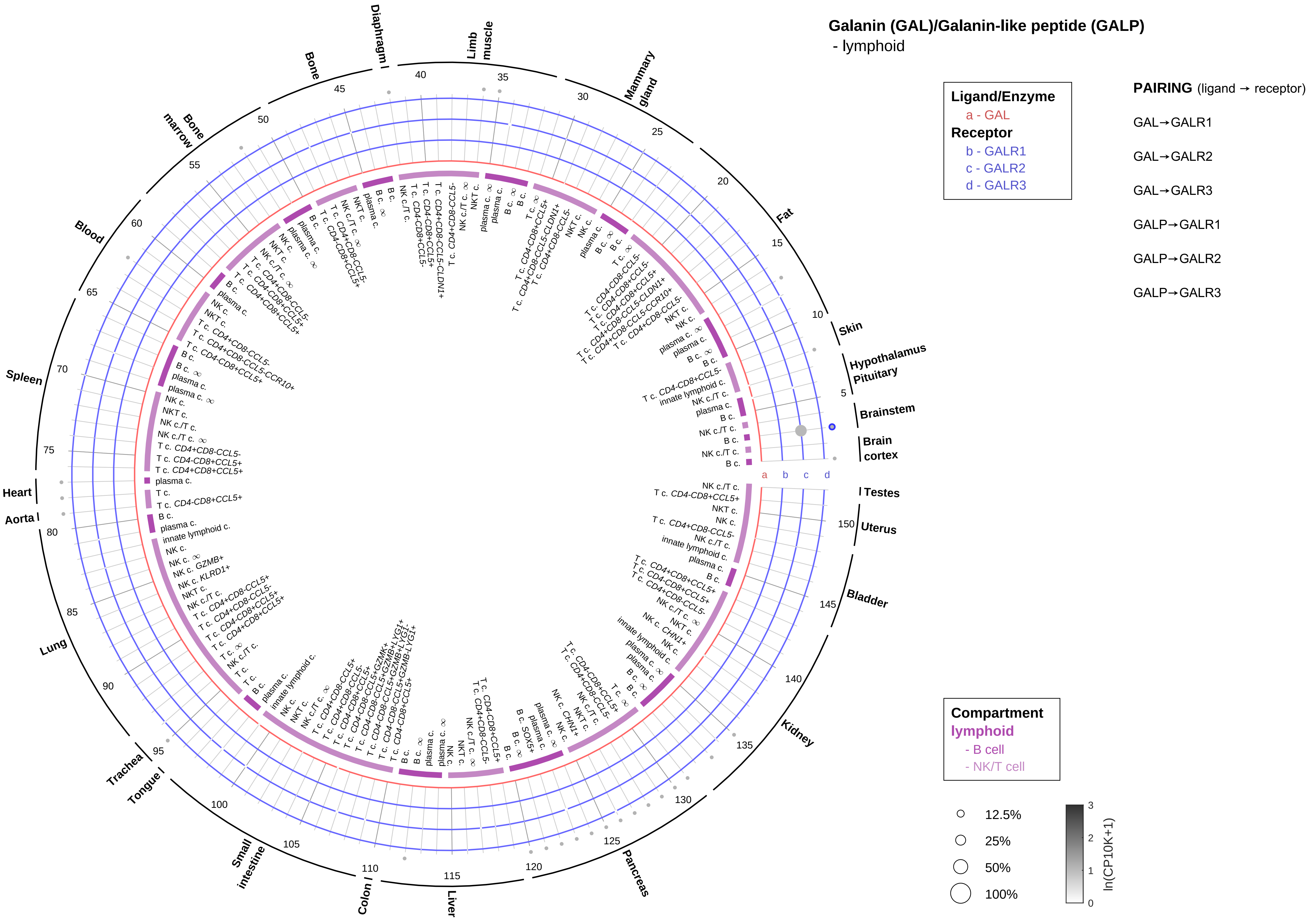

Galanin (GAL)/Galanin-like peptide (GALP)
- lymphoid
Ligand/Enzyme
a - GAL
Receptor
b - GALR1
c - GALR2
d - GALR3
PAIRING (ligand → receptor)
GAL→GALR1
GAL→GALR2
GAL→GALR3
GALP→GALR1
GALP→GALR2
GALP→GALR3
Compartment
lymphoid
- B cell
- NK/T cell
12.5%
25%
50%
100%
ln(CP10K+1)
Skin
Hypothalamus
Pituitary
Brainstem
Brain cortex
Testes
Uterus
Bladder
Kidney
Pancreas
Liver
Colon
Small intestine
Tongue
Trachea
Lung
Aorta
Heart
Spleen
Blood
Bone marrow
Bone
Diaphragm
Limb muscle
Mammary gland
Fat

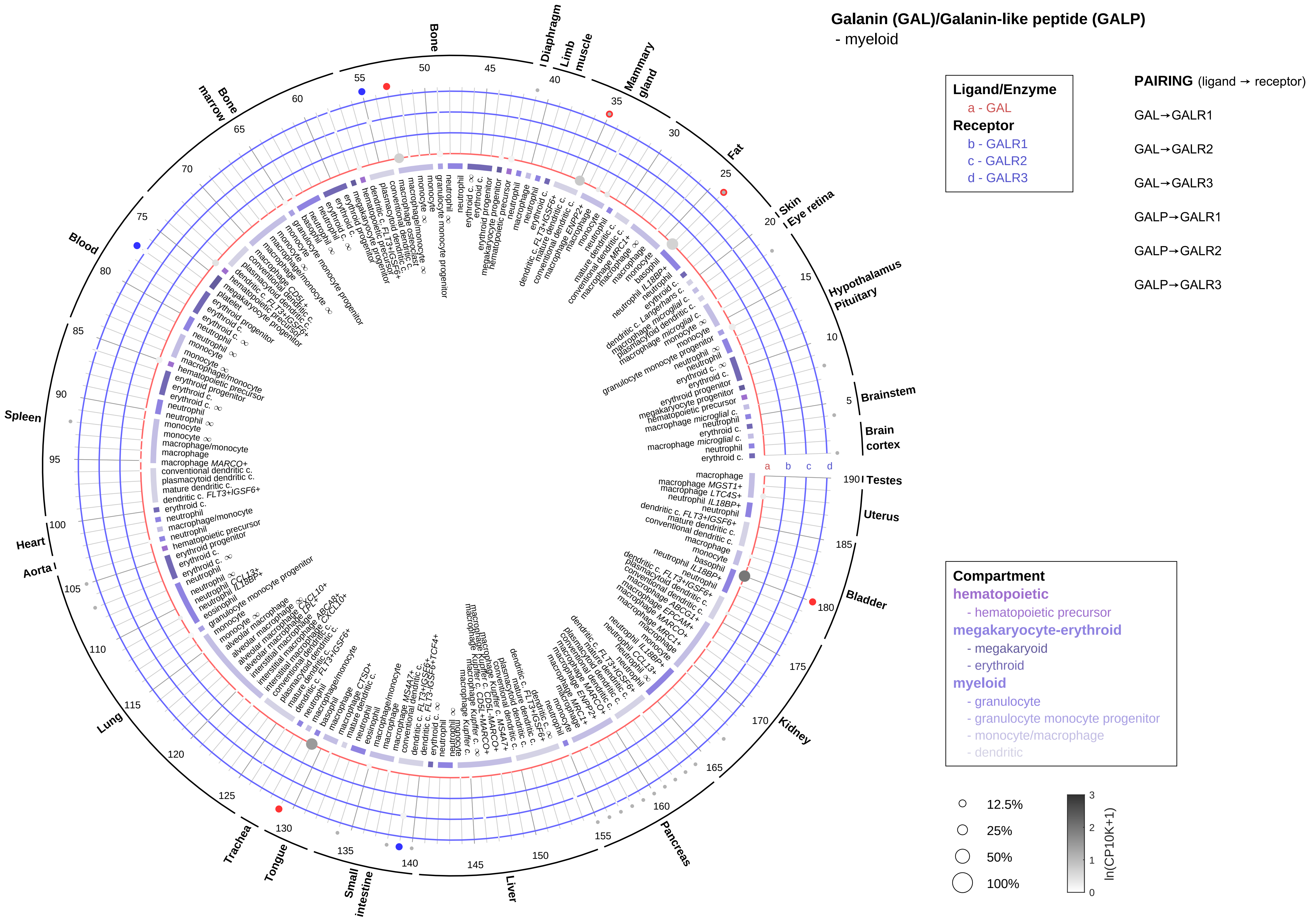

Galanin (GAL)/Galanin-like peptide (GALP)
- myeloid
Ligand/Enzyme
a - GAL
Receptor
b - GALR1
c - GALR2
d - GALR3
PAIRING (ligand → receptor)
GAL→GALR1
GAL→GALR2
GAL→GALR3
GALP→GALR1
GALP→GALR2
GALP→GALR3
Compartment
hematopoietic
- hematopoietic precursor
megakaryocyte-erythroid
- megakaryoid
- erythroid
myeloid
- granulocyte
- granulocyte monocyte progenitor
- monocyte/macrophage
- dendritic
12.5%
25%
50%
100%
ln(CP10K+1)
Bone
Diaphragm
Limb muscle
Mammary gland
Fat
Skin
Eye retina
Hypothalamus
Pituitary
Brainstem
Brain cortex
Testes
Uterus
Bladder
Kidney
Pancreas
Liver
Small intestine
Tongue
Trachea
Lung
Aorta
Heart
Spleen
Blood
Bone marrow

Gastric inhibitory polypeptide (GIP)
- epithelial/neural/germ

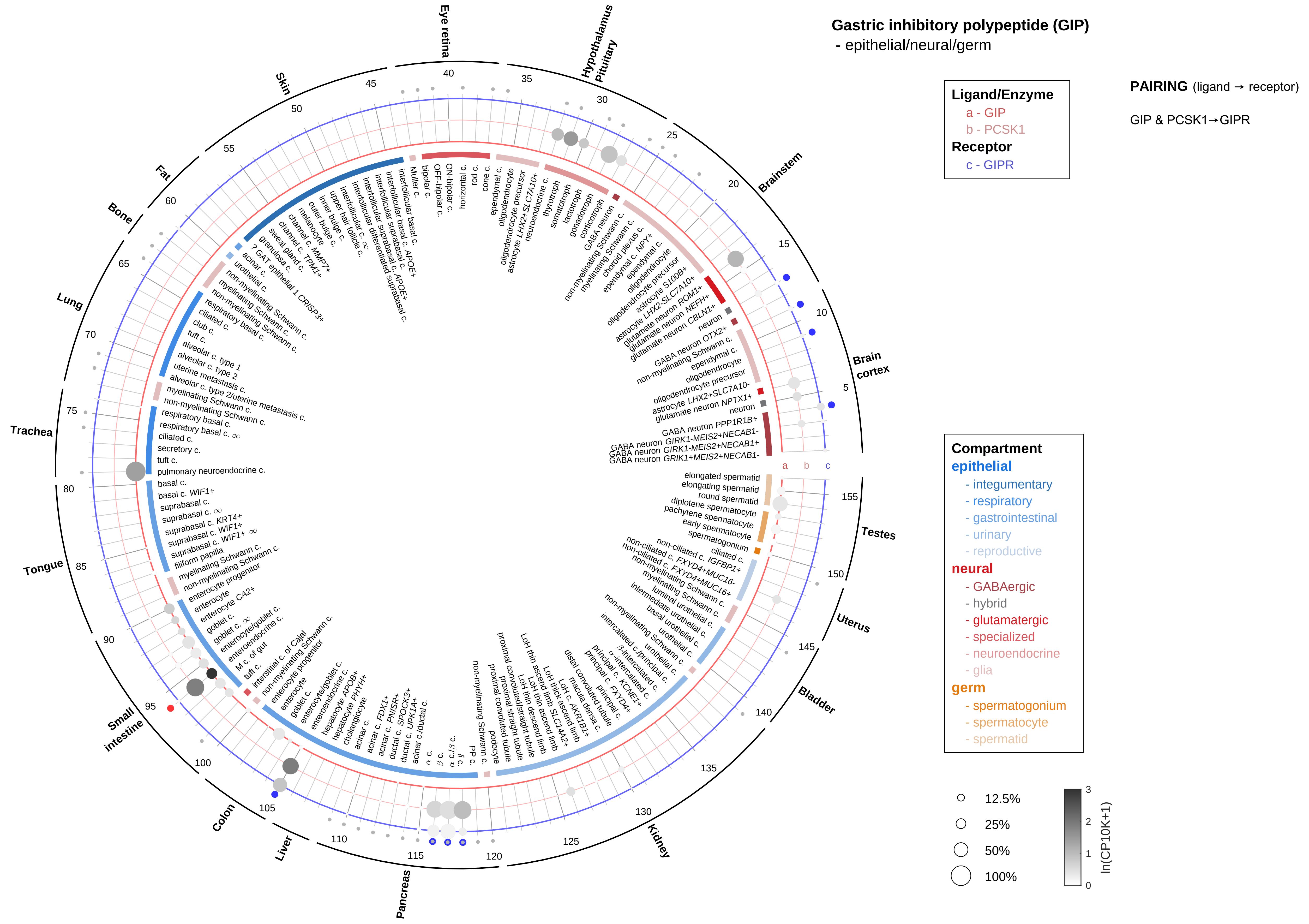

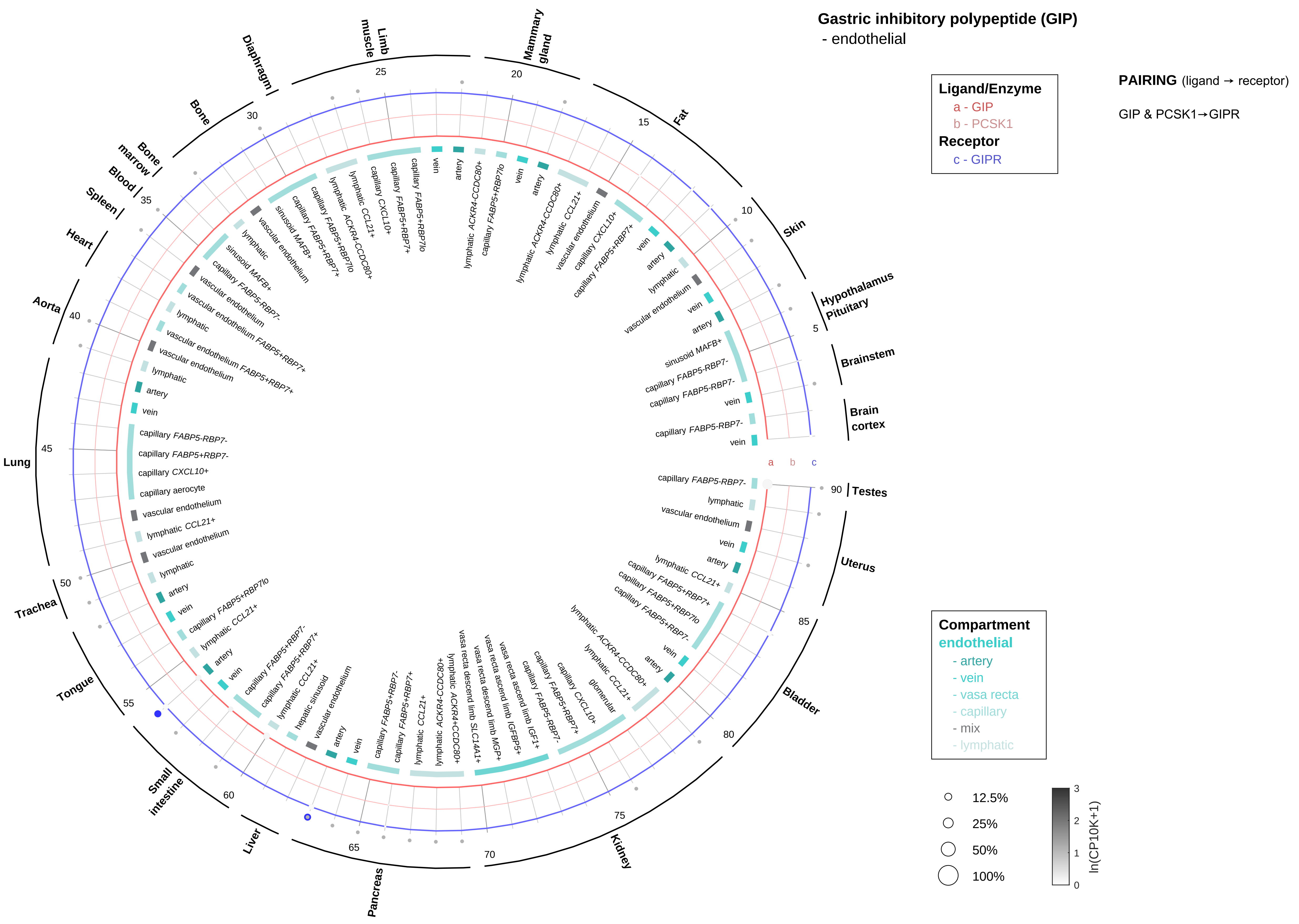

Gastric inhibitory polypeptide (GIP)
- endothelial
Ligand/Enzyme
a - GIP
b - PCSK1
Receptor
c - GIPR
PAIRING (ligand → receptor)
GIP & PCSK1→GIPR
Compartment
endothelial
- artery
- vein
- vasa recta
- capillary
- mix
- lymphatic
12.5%
25%
50%
100%
ln(CP10K+1)
Brain cortex
Brainstem
Hypothalamus Pituitary
Skin
Fat
Mammary gland
Limb muscle
Diaphragm
Bone
Bone marrow
Blood
Spleen
Heart
Aorta
Lung
Trachea
Tongue
Small intestine
Liver
Pancreas
Kidney
Bladder
Uterus
Testes

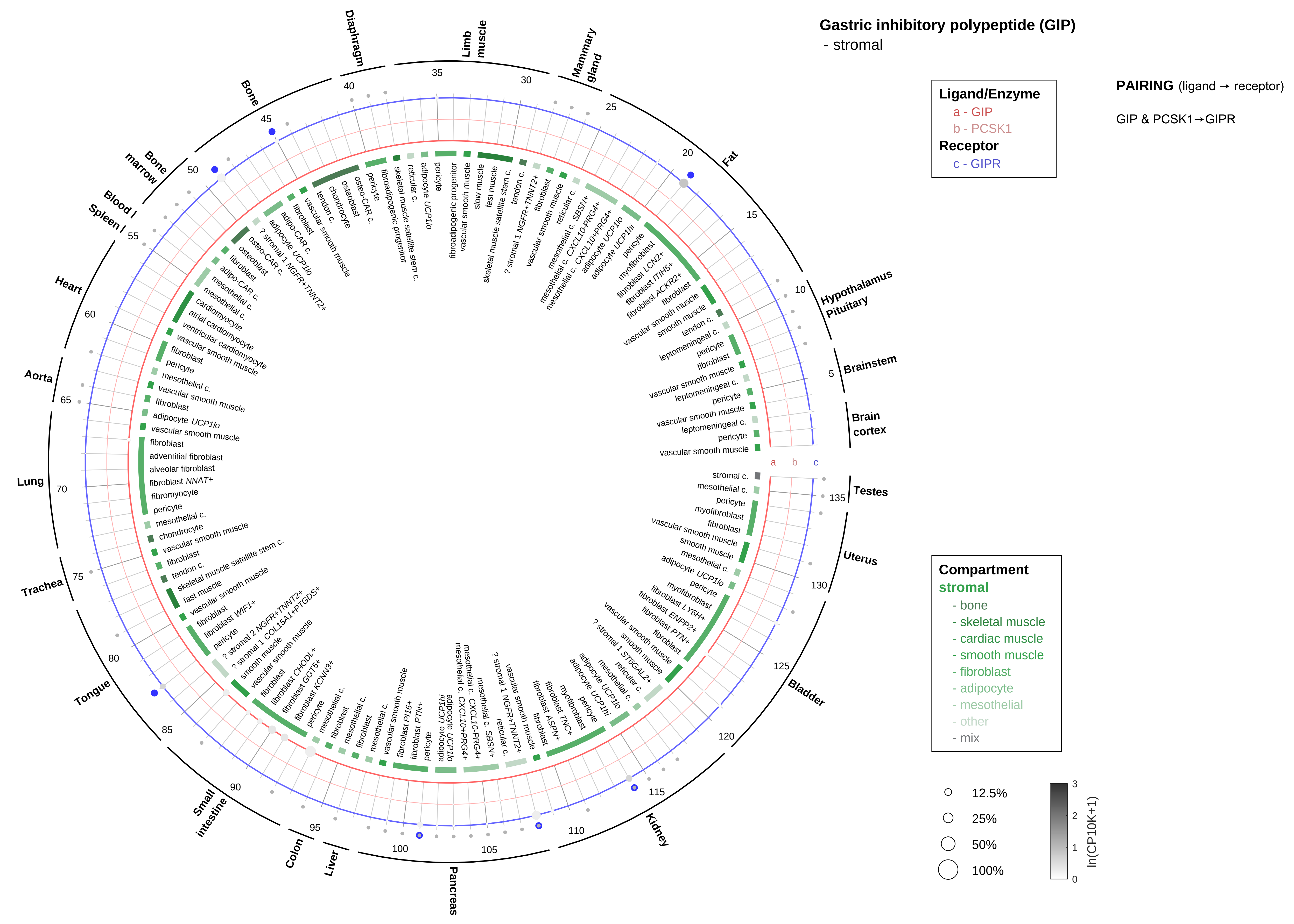
Gastric inhibitory polypeptide (GIP)
- stromal
Ligand/Enzyme
a - GIP
b - PCSK1
Receptor
c - GIPR
PAIRING (ligand → receptor)
GIP & PCSK1→GIPR
Compartment
stromal
- bone
- skeletal muscle
- cardiac muscle
- smooth muscle
- fibroblast
- adipocyte
- mesothelial
- other
- mix
12.5%
25%
50%
100%
ln(CP10K+1)
Brain cortex
Brainstem
Hypothalamus Pituitary
Fat
Mammary gland
Limb muscle
Diaphragm
Bone
Bone marrow
Blood / Spleen /
Heart
Aorta
Lung
Trachea
Tongue
Small intestine
Colon
Liver
Pancreas
Kidney
Bladder
Uterus
Testes

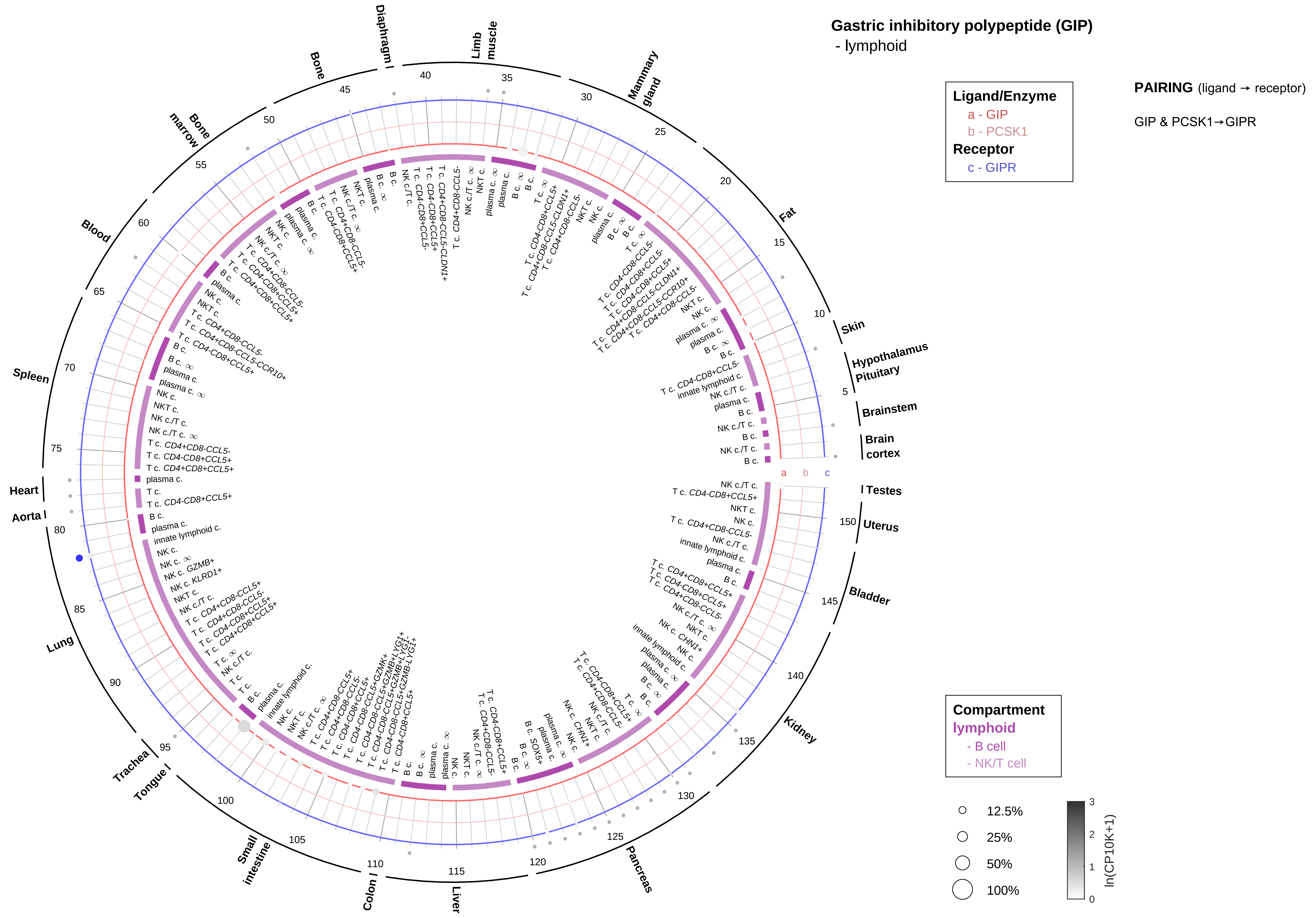
Gastric inhibitory polypeptide (GIP)
- lymphoid
Ligand/Enzyme
a - GIP
b - PCSK1
Receptor
c - GIPR
PAIRING (ligand → receptor)
GIP & PCSK1→GIPR
Compartment
lymphoid
- B cell
- NK/T cell
12.5%
25%
50%
100%
ln(CP10K+1)

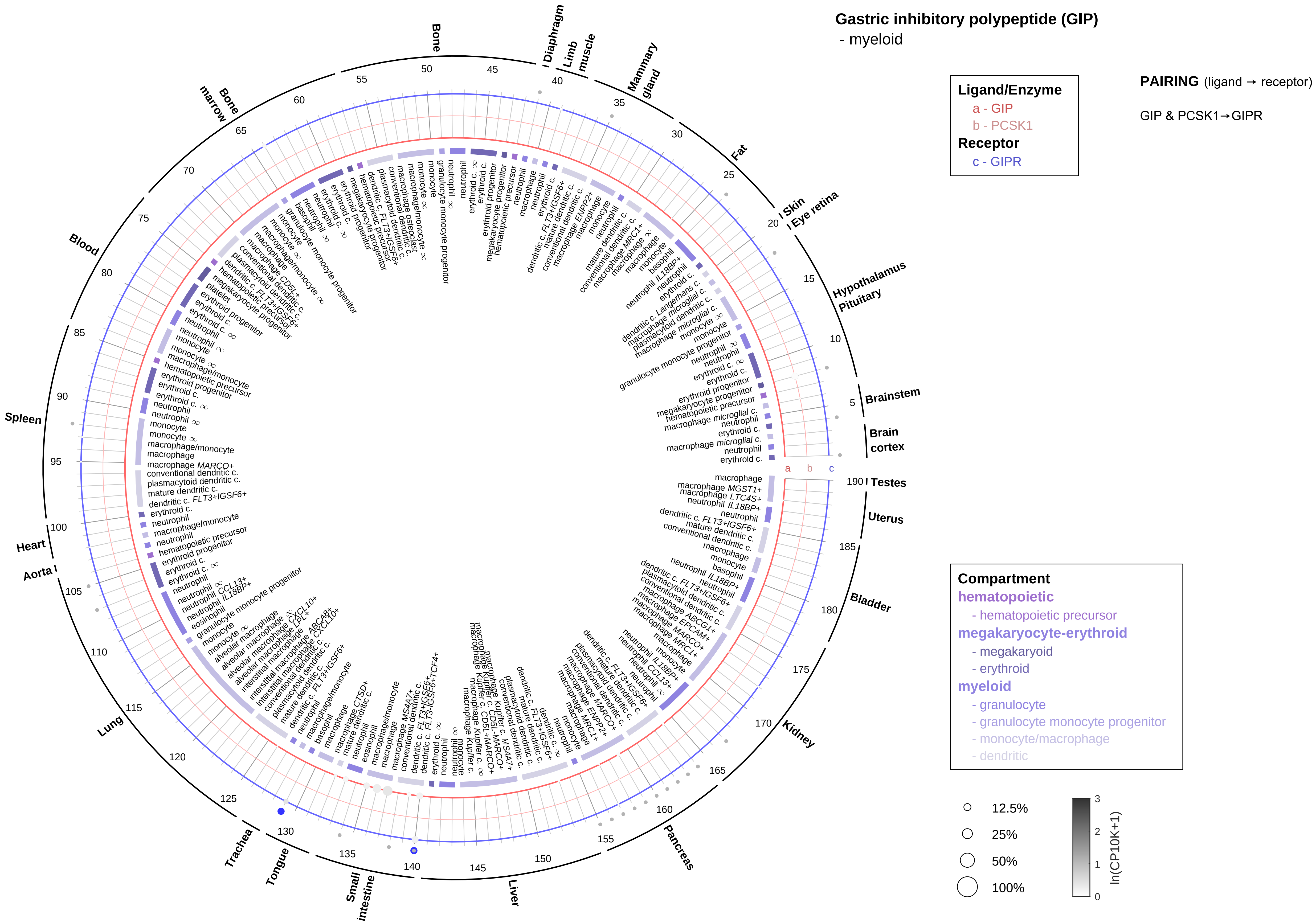
Gastric inhibitory polypeptide (GIP)
- myeloid
PAIRING (ligand → receptor)
GIP & PCSK1→GIPR
Ligand/Enzyme
a - GIP
b - PCSK1
Receptor
c - GIPR
Compartment
hematopoietic
- hematopoietic precursor
megakaryocyte-erythroid
- megakaryoid
- erythroid
myeloid
- granulocyte
- granulocyte monocyte progenitor
- monocyte/macrophage
- dendritic
12.5%
25%
50%
100%
ln(CP10K+1)
0
1
2
3
Bone
Diaphragm
Limb muscle
Mammary gland
Fat
Skin
Eye retina
Hypothalamus
Pituitary
Brainstem
Brain cortex
Testes
Uterus
Bladder
Kidney
Pancreas
Liver
Small intestine
Tongue
Trachea
Lung
Aorta
Heart
Spleen
Blood
Bone marrow
a
b
c

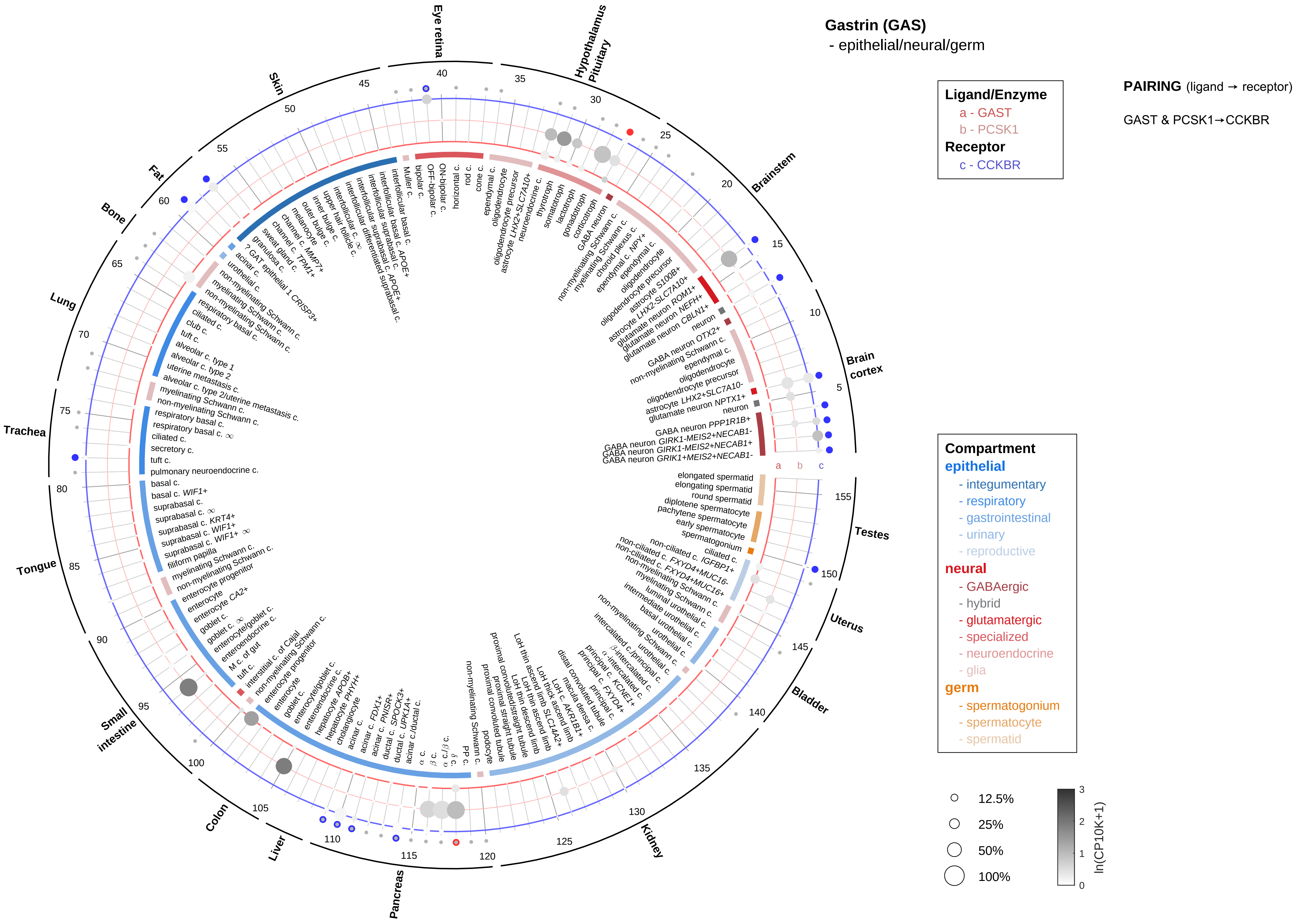

Gastrin (GAS)
- epithelial/neural/germ
Ligand/Enzyme
a - GAST
b - PCSK1
Receptor
c - CCKBR
PAIRING (ligand → receptor)
GAST & PCSK1→CCKBR
Compartment
epithelial
- integumentary
- respiratory
- gastrointestinal
- urinary
- reproductive
neural
- GABAergic
- hybrid
- glutamatergic
- specialized
- neuroendocrine
- glia
germ
- spermatogonium
- spermatocyte
- spermatid
12.5%
25%
50%
100%
ln(CP10K+1)
0
1
2
3
Eye retina
Hypothalamus Pituitary
Brainstem
Brain cortex
Testes
Uterus
Bladder
Kidney
Pancreas
Liver
Colon
Small intestine
Tongue
Trachea
Lung
Bone
Fat
Skin
a
b
c

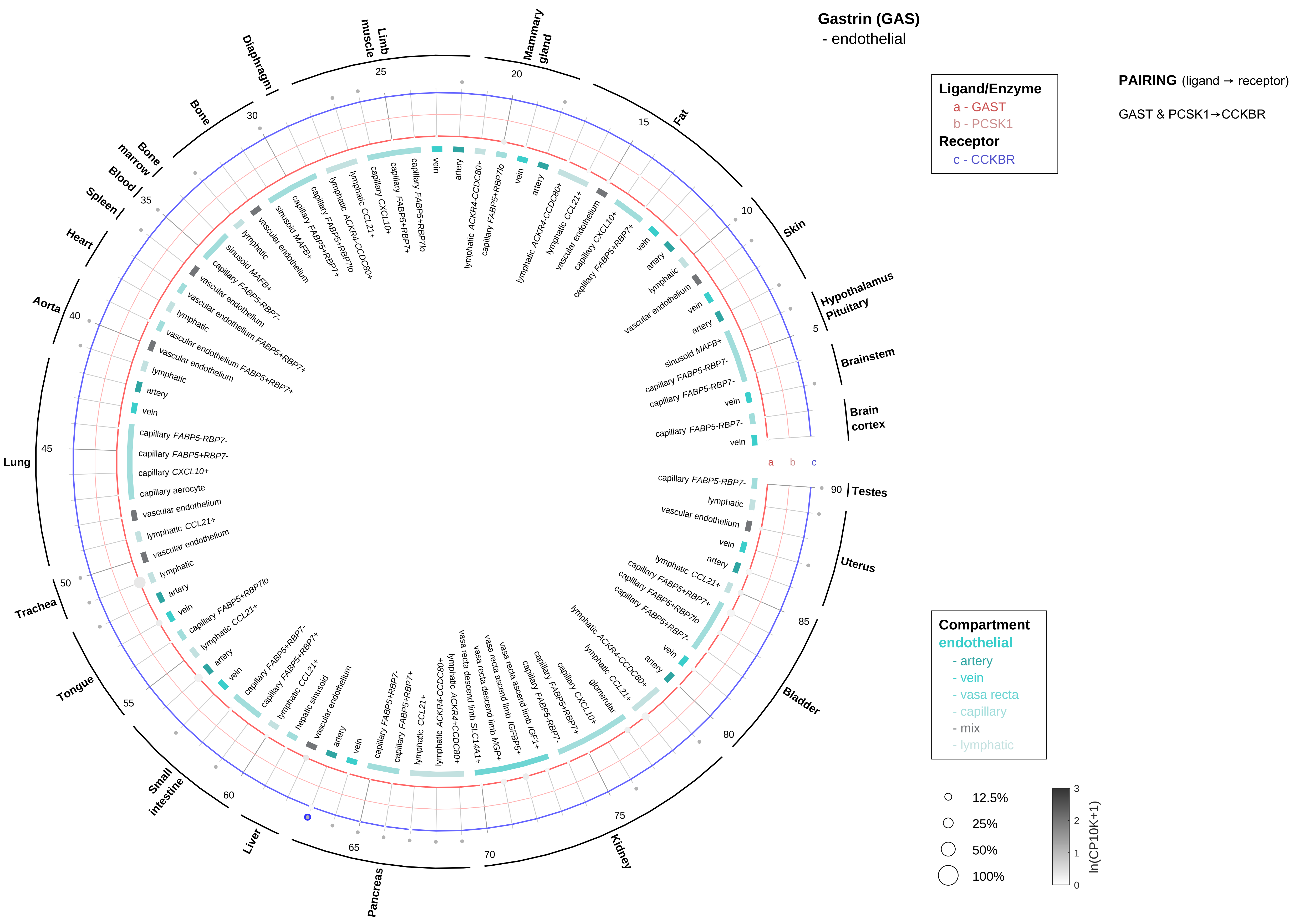
Gastrin (GAS)
- endothelial
Ligand/Enzyme
a - GAST
b - PCSK1
Receptor
c - CCKBR
PAIRING (ligand → receptor)
GAST & PCSK1→CCKBR
Compartment
endothelial
- artery
- vein
- vasa recta
- capillary
- mix
- lymphatic
12.5%
25%
50%
100%
ln(CP10K+1)
Brain cortex
Brainstem
Hypothalamus Pituitary
Skin
Fat
Mammary gland
Limb muscle
Diaphragm
Bone
Bone marrow
Blood
Spleen
Heart
Aorta
Lung
Trachea
Tongue
Small intestine
Liver
Pancreas
Kidney
Bladder
Uterus
Testes

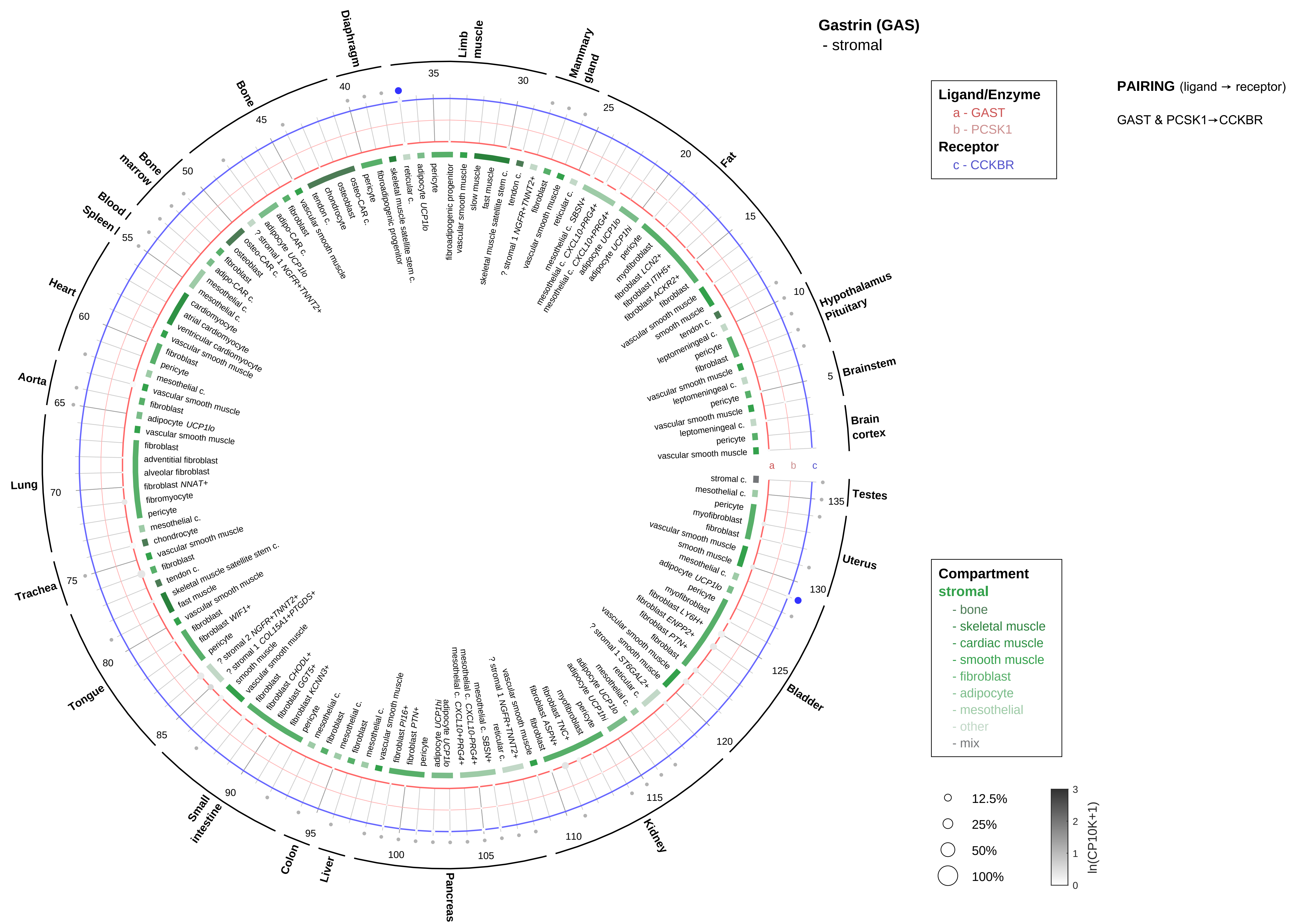

Gastrin (GAS)
- stromal
Ligand/Enzyme
a - GAST
b - PCSK1
Receptor
c - CCKBR
PAIRING (ligand → receptor)
GAST & PCSK1→CCKBR
Compartment
stromal
- bone
- skeletal muscle
- cardiac muscle
- smooth muscle
- fibroblast
- adipocyte
- mesothelial
- other
- mix
12.5%
25%
50%
100%
ln(CP10K+1)
Brain cortex
Brainstem
Hypothalamus Pituitary
Fat
Mammary gland
Limb muscle
Diaphragm
Bone
Bone marrow
Blood
Spleen
Heart
Aorta
Lung
Trachea
Tongue
Small intestine
Colon
Liver
Pancreas
Kidney
Bladder
Uterus
Testes

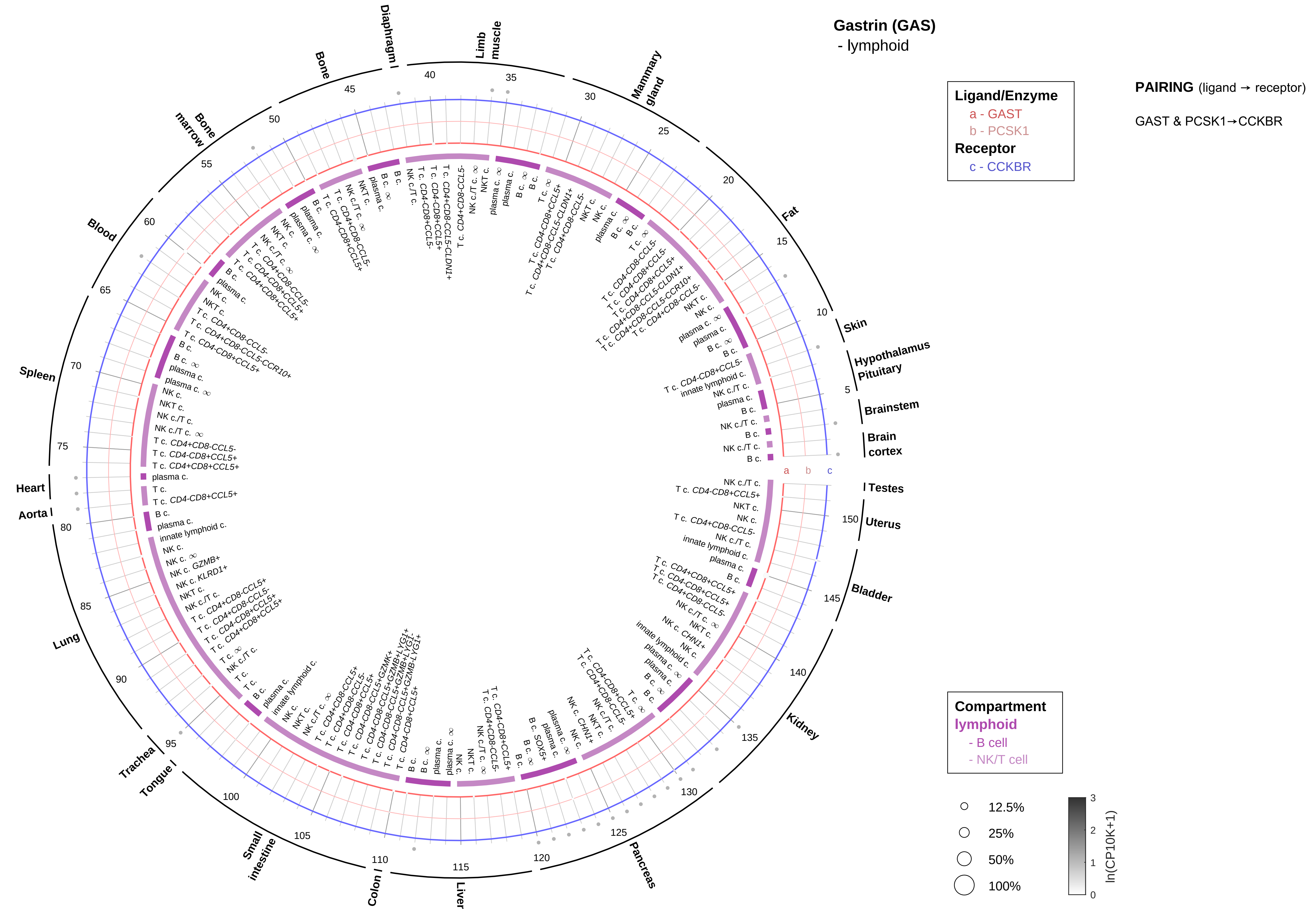

Gastrin (GAS)
- lymphoid
Ligand/Enzyme
a - GAST
b - PCSK1
Receptor
c - CCKBR
PAIRING (ligand → receptor)
GAST & PCSK1→CCKBR
Compartment
lymphoid
- B cell
- NK/T cell
12.5%
25%
50%
100%
ln(CP10K+1)
Brain cortex
Brainstem
Hypothalamus Pituitary
Skin
Fat
Mammary gland
Limb muscle
Diaphragm
Bone
Bone marrow
Blood
Spleen
Heart
Aorta
Lung
Trachea
Tongue
Small intestine
Colon
Liver
Pancreas
Kidney
Bladder
Uterus
Testes

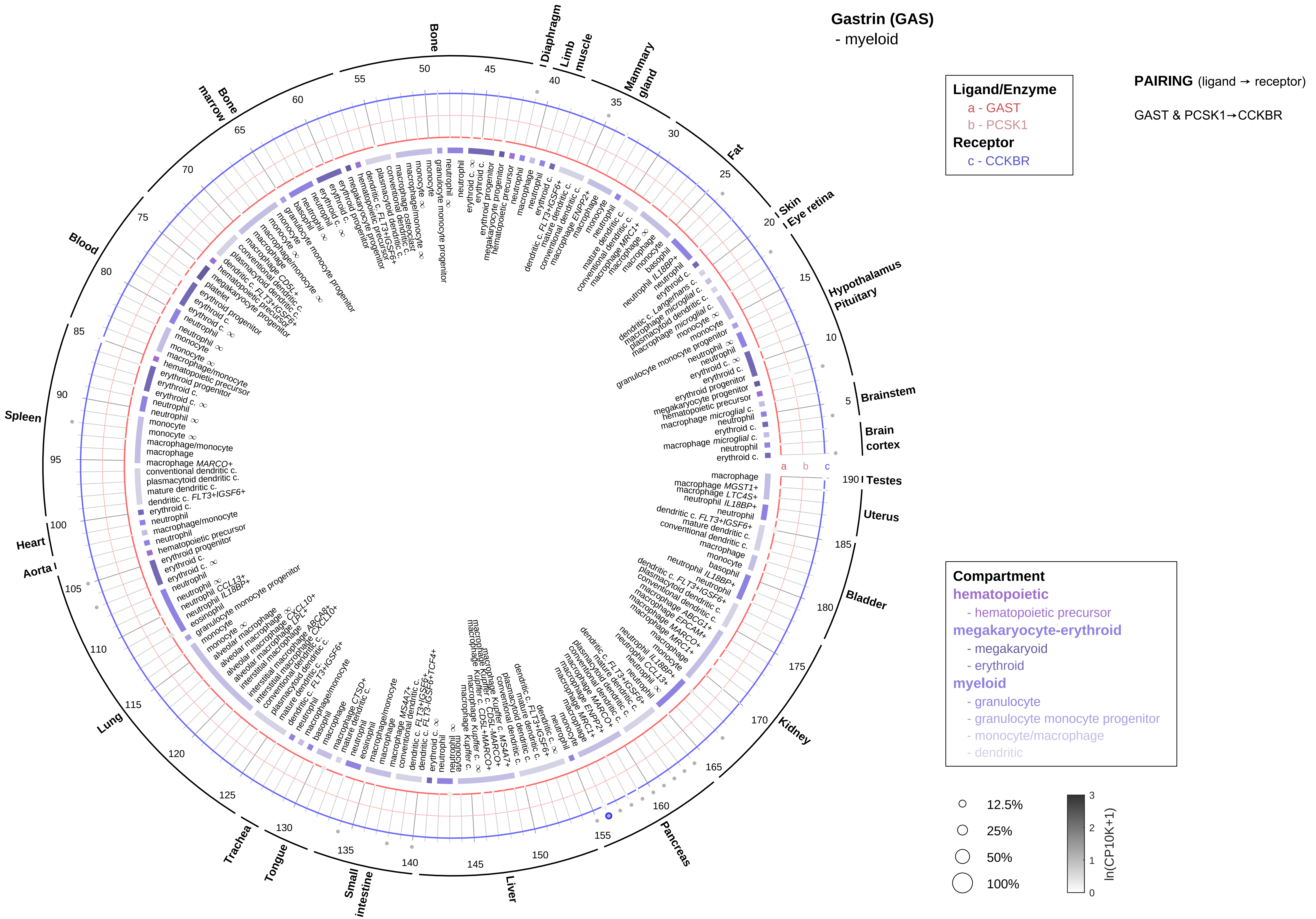

Gastrin (GAS)
- myeloid
Ligand/Enzyme
a - GAST
b - PCSK1
Receptor
c - CCKBR
PAIRING (ligand → receptor)
GAST & PCSK1→CCKBR
Compartment
hematopoietic
- hematopoietic precursor
megakaryocyte-erythroid
- megakaryoid
- erythroid
myeloid
- granulocyte
- granulocyte monocyte progenitor
- monocyte/macrophage
- dendritic
12.5%
25%
50%
100%
ln(CP10K+1)
Bone
Diaphragm
Limb muscle
Mammary gland
Fat
Skin
Eye retina
Hypothalamus
Pituitary
Brainstem
Brain cortex
Testes
Uterus
Bladder
Kidney
Pancreas
Liver
Small intestine
Tongue
Trachea
Lung
Aorta
Heart
Spleen
Blood
Bone marrow

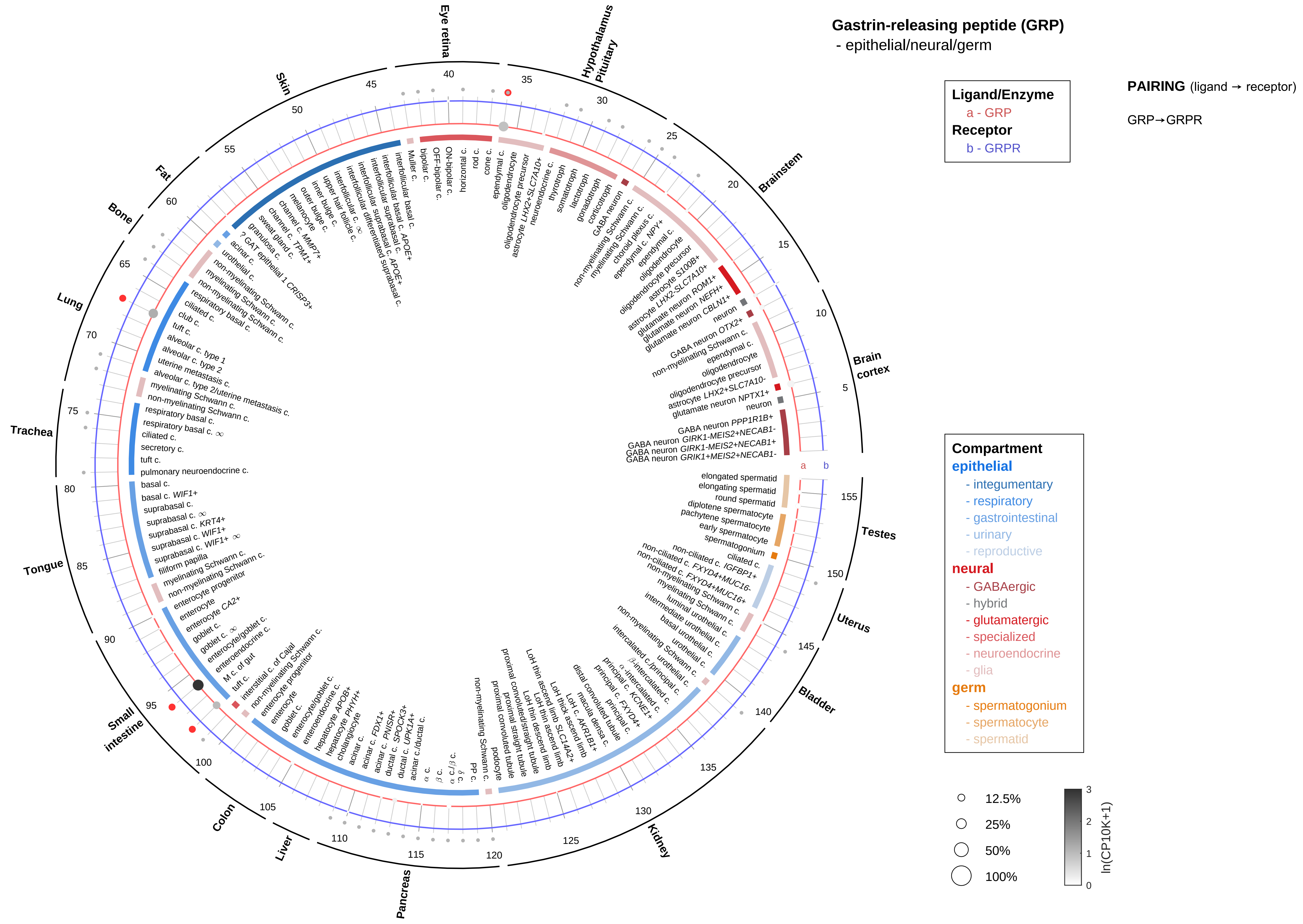

Gastrin-releasing peptide (GRP)
- epithelial/neural/germ
Ligand/Enzyme
a - GRP
Receptor
b - GRPR
PAIRING (ligand → receptor)
GRP→GRPR
Compartment
epithelial
- integumentary
- respiratory
- gastrointestinal
- urinary
- reproductive
neural
- GABAergic
- hybrid
- glutamatergic
- specialized
- neuroendocrine
- glia
germ
- spermatogonium
- spermatocyte
- spermatid
12.5%
25%
50%
100%
ln(CP10K+1)
Eye retina
Hypothalamus Pituitary
Brainstem
Brain cortex
Testes
Uterus
Bladder
Kidney
Pancreas
Liver
Colon
Small intestine
Tongue
Trachea
Lung
Bone
Fat
Skin

Gastrin-releasing peptide (GRP)
- endothelial

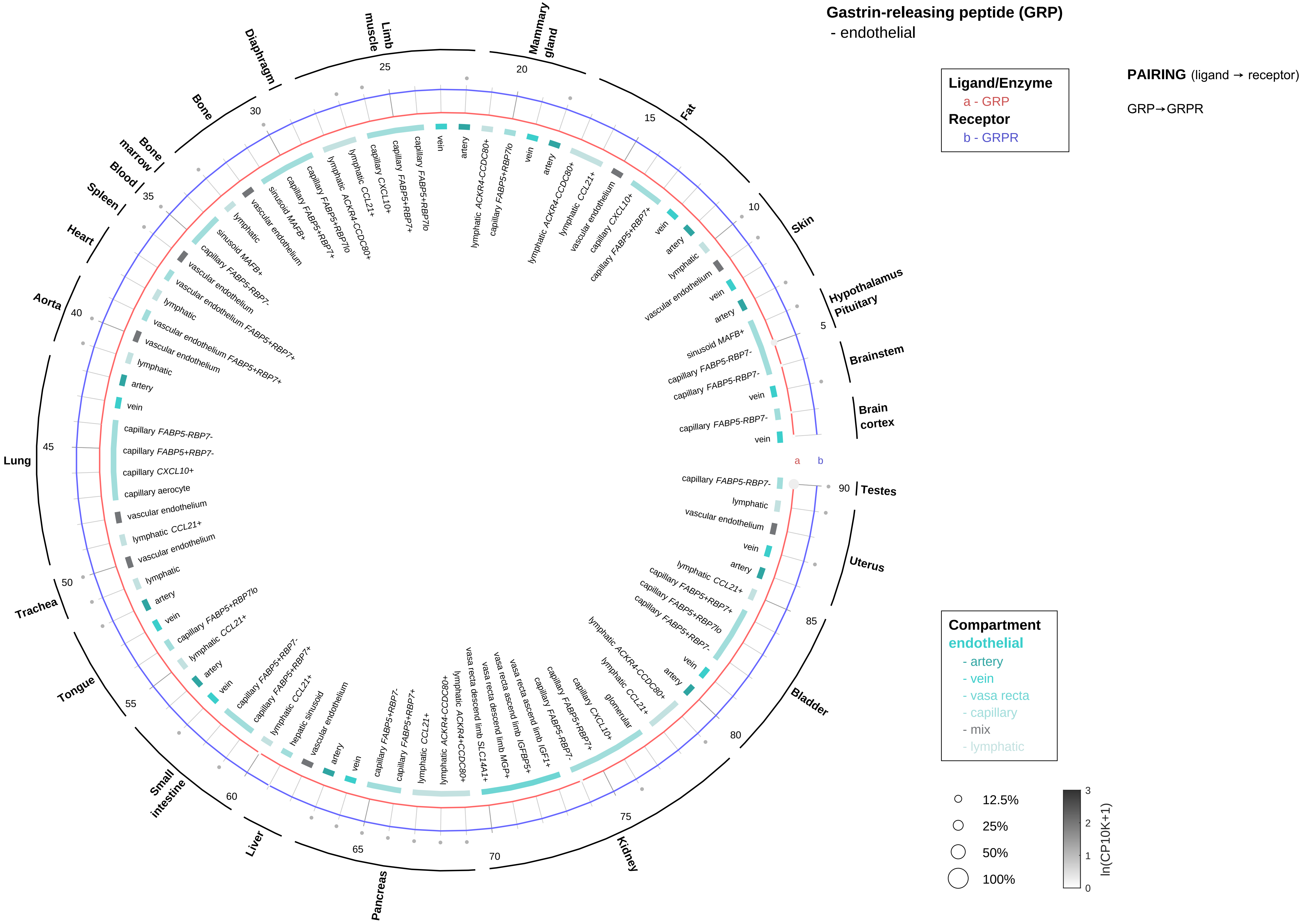

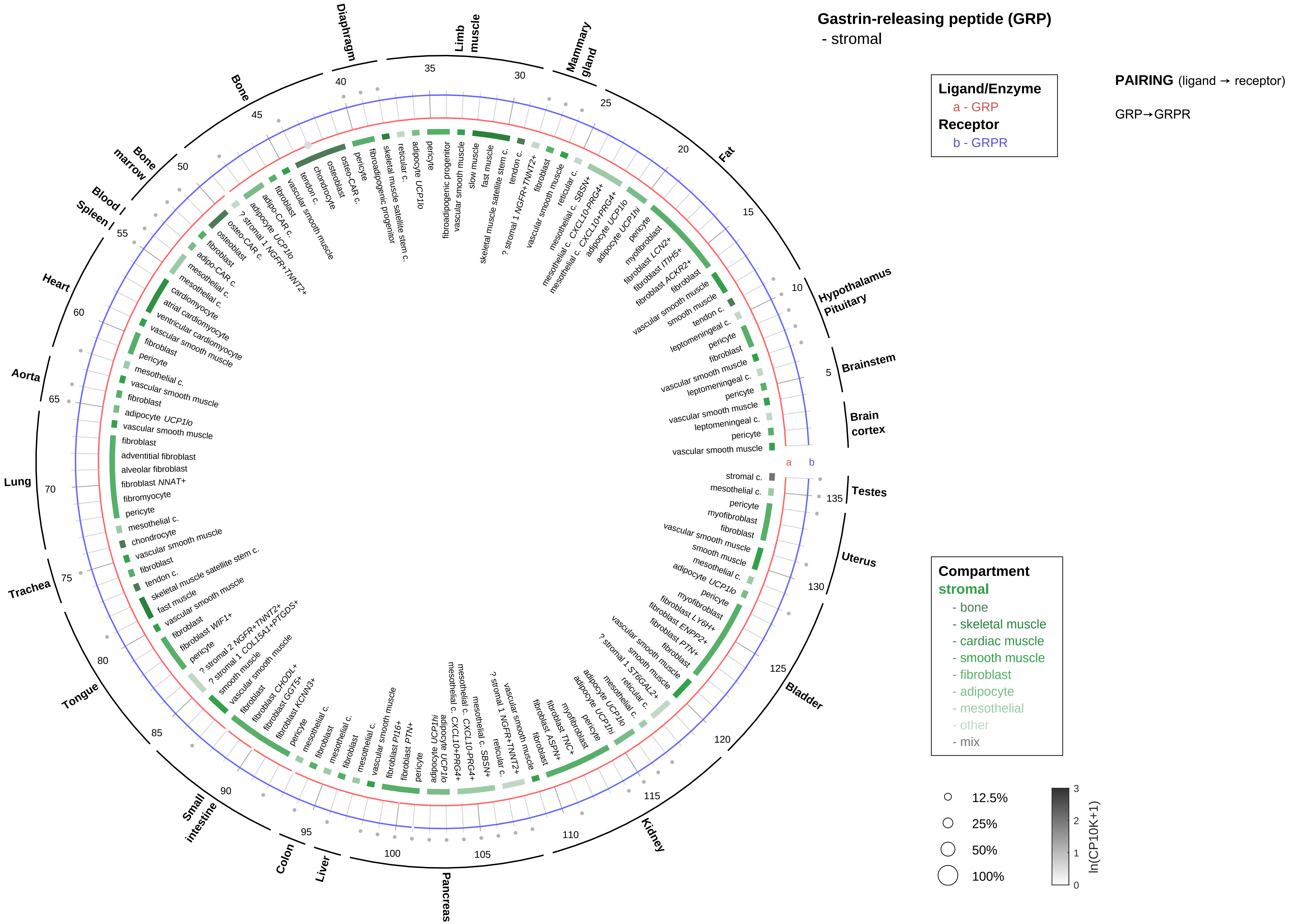
Gastrin-releasing peptide (GRP)
- stromal
Ligand/Enzyme
a - GRP
Receptor
b - GRPR
PAIRING (ligand → receptor)
GRP→GRPR
Compartment
stromal
- bone
- skeletal muscle
- cardiac muscle
- smooth muscle
- fibroblast
- adipocyte
- mesothelial
- other
- mix
12.5%
25%
50%
100%
ln(CP10K+1)
0
1
2
3
a
b
Brain cortex
Brainstem
Hypothalamus Pituitary
Fat
Mammary gland
Limb muscle
Diaphragm
Bone
Bone marrow
Blood
Spleen
Heart
Aorta
Lung
Trachea
Tongue
Small intestine
Colon
Liver
Pancreas
Kidney
Bladder
Uterus
Testes
5
10
15
20
25
30
35
40
45
50
55
60
65
70
75
80
85
90
95
100
105
110
115
120
125
130
135
vascular smooth muscle
pericyte
leptomeningeal c.
vascular smooth muscle
pericyte
leptomeningeal c.
vascular smooth muscle
fibroblast
pericyte
leptomeningeal c.
tendon c.
smooth muscle
vascular smooth muscle
fibroblast
fibroblast ACKR2+
fibroblast ITIH5+
fibroblast LCN2+
myofibroblast
pericyte
adipocyte UCP1hi
adipocyte UCP1lo
mesothelial c. CXCL10-PRG4+
mesothelial c. CXCL10+PRG4+
mesothelial c. SBSN+
reticular c.
vascular smooth muscle
fibroblast
? stromal 1 NGFR+TNNT2+
tendon c.
skeletal muscle satellite stem c.
fast muscle
slow muscle
vascular smooth muscle
fibroadipogenic progenitor
pericyte
adipocyte UCP1lo
reticular c.
skeletal muscle satellite stem c.
fibroadipogenic progenitor
pericyte
osteo-CAR c.
osteoblast
chondrocyte
tendon c.
vascular smooth muscle
fibroblast
adipo-CAR c.
adipocyte UCP1lo
? stromal 1 NGFR+TNNT2+
osteo-CAR c.
osteoblast
fibroblast
adipo-CAR c.
mesothelial c.
mesothelial c.
cardiomyocyte
atrial cardiomyocyte
ventricular cardiomyocyte
vascular smooth muscle
fibroblast
pericyte
mesothelial c.
vascular smooth muscle
fibroblast
adipocyte UCP1lo
vascular smooth muscle
fibroblast
adventitial fibroblast
alveolar fibroblast
fibroblast NNAT+
fibromyocyte
pericyte
mesothelial c.
chondrocyte
vascular smooth muscle
fibroblast
tendon c.
skeletal muscle satellite stem c.
fast muscle
vascular smooth muscle
fibroblast
fibroblast WIF1+
pericyte
? stromal 2 NGFR+TNNT2+
? stromal 1 COL15A1+PTGDS+
smooth muscle
vascular smooth muscle
fibroblast
fibroblast CHODL+
fibroblast GGT5+
fibroblast KCNN3+
pericyte
mesothelial c.
fibroblast
mesothelial c.
fibroblast
mesothelial c.
vascular smooth muscle
fibroblast PI16+
fibroblast PTN+
pericyte
adipocyte UCP1hi
adipocyte UCP1lo
mesothelial c. CXCL10+PRG4+
mesothelial c. CXCL10-PRG4+
mesothelial c. SBSN+
reticular c.
? stromal 1 NGFR+TNNT2+
vascular smooth muscle
fibroblast
fibroblast ASPN+
fibroblast TNC+
myofibroblast
pericyte
adipocyte UCP1lo
adipocyte UCP1hi
mesothelial c.
reticular c.
? stromal 1 ST6GAL2+
smooth muscle
vascular smooth muscle
fibroblast
fibroblast PTN+
fibroblast ENPP2+
fibroblast LY6H+
myofibroblast
pericyte
adipocyte UCP1lo
mesothelial c.
smooth muscle
vascular smooth muscle
fibroblast
myofibroblast
pericyte
mesothelial c.
stromal c.

Gastrin-releasing peptide (GRP)
- lymphoid

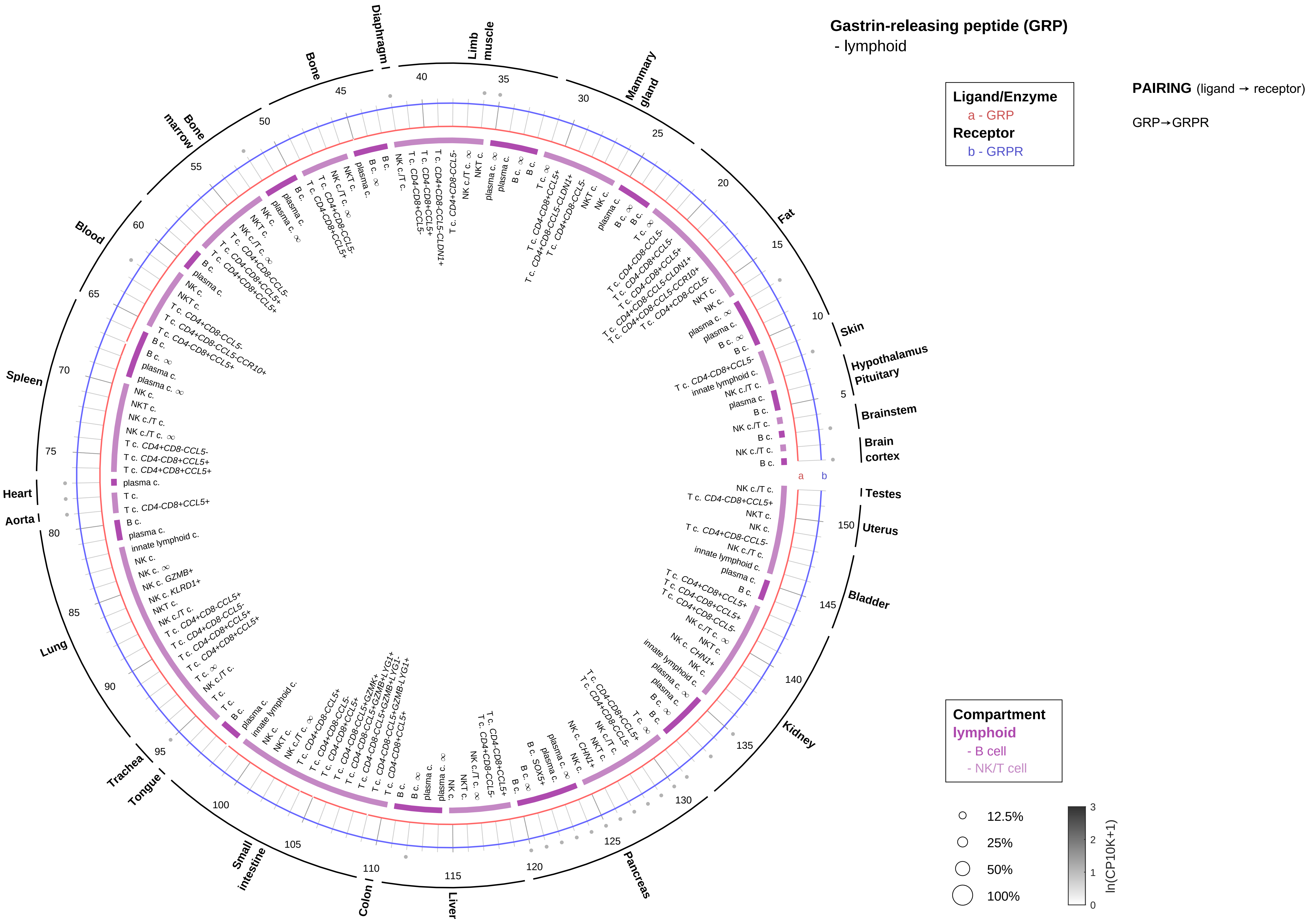

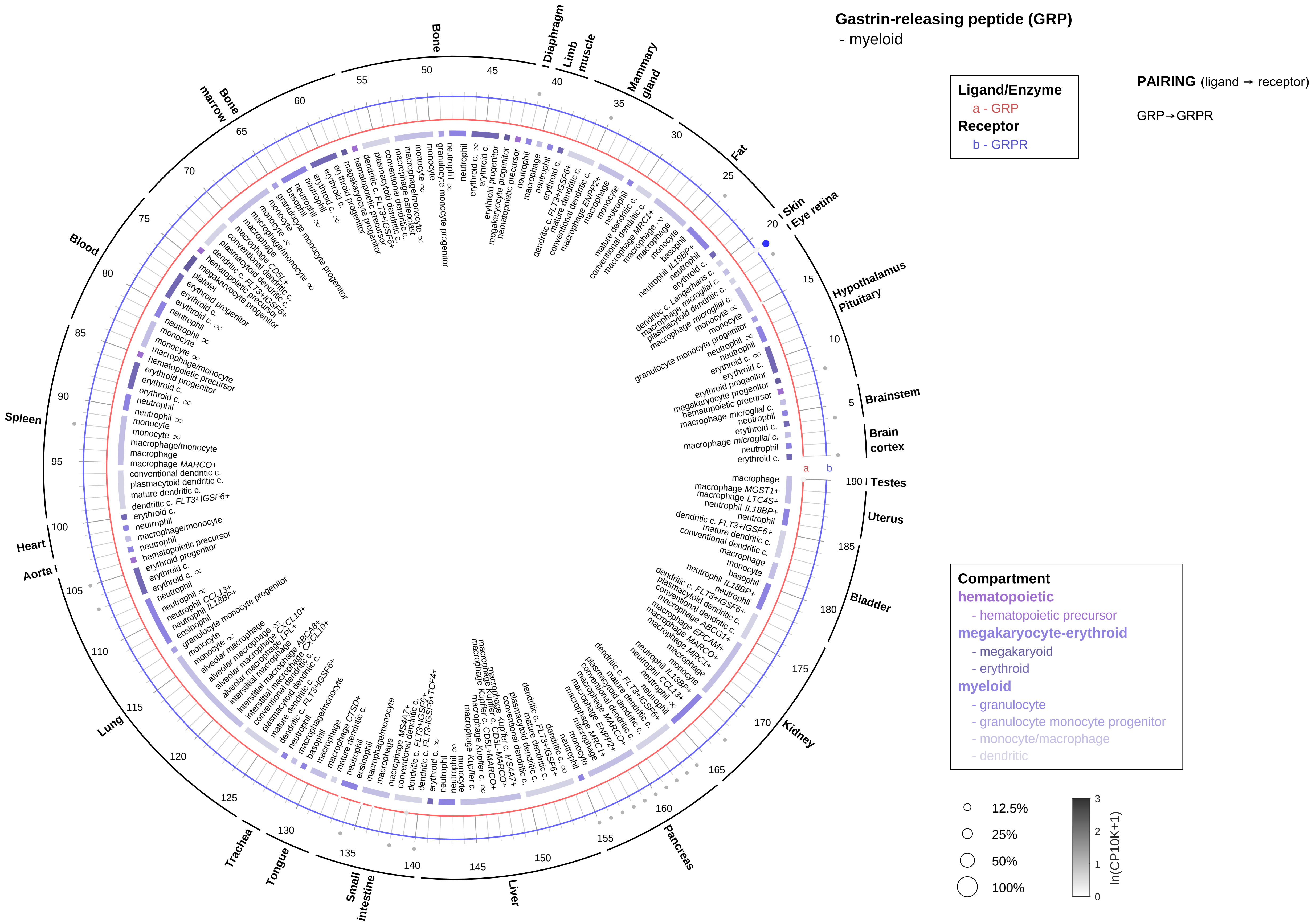
Gastrin-releasing peptide (GRP)
- myeloid
Ligand/Enzyme
a - GRP
Receptor
b - GRPR
PAIRING (ligand → receptor)
GRP→GRPR
Compartment
hematopoietic
- hematopoietic precursor
megakaryocyte-erythroid
- megakaryoid
- erythroid
myeloid
- granulocyte
- granulocyte monocyte progenitor
- monocyte/macrophage
- dendritic
12.5%
25%
50%
100%
ln(CP10K+1)
Bone
Diaphragm
Limb muscle
Mammary gland
Fat
Skin
Eye retina
Hypothalamus
Pituitary
Brainstem
Brain cortex
Testes
Uterus
Bladder
Kidney
Pancreas
Liver
Small intestine
Tongue
Trachea
Lung
Aorta
Heart
Spleen
Blood
Bone marrow

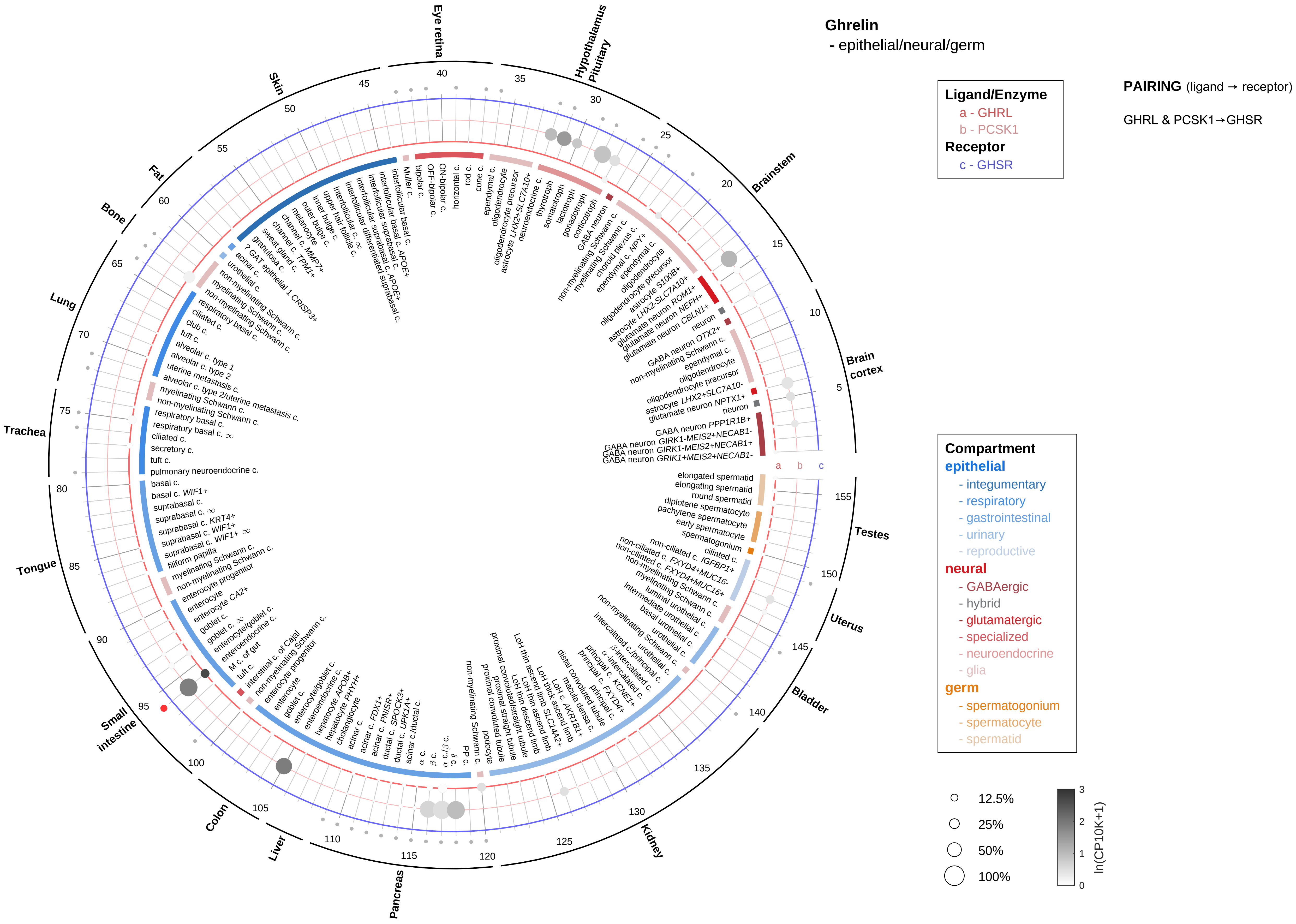
Ghrelin
- epithelial/neural/germ
Ligand/Enzyme
a - GHRL
b - PCSK1
Receptor
c - GHSR
PAIRING (ligand → receptor)
GHRL & PCSK1→GHSR
Compartment
epithelial
- integumentary
- respiratory
- gastrointestinal
- urinary
- reproductive
neural
- GABAergic
- hybrid
- glutamatergic
- specialized
- neuroendocrine
- glia
germ
- spermatogonium
- spermatocyte
- spermatid
12.5%
25%
50%
100%
ln(CP10K+1)
Eye retina
Hypothalamus Pituitary
Brainstem
Brain cortex
Testes
Uterus
Bladder
Kidney
Pancreas
Liver
Colon
Small intestine
Tongue
Trachea
Lung
Bone
Fat
Skin

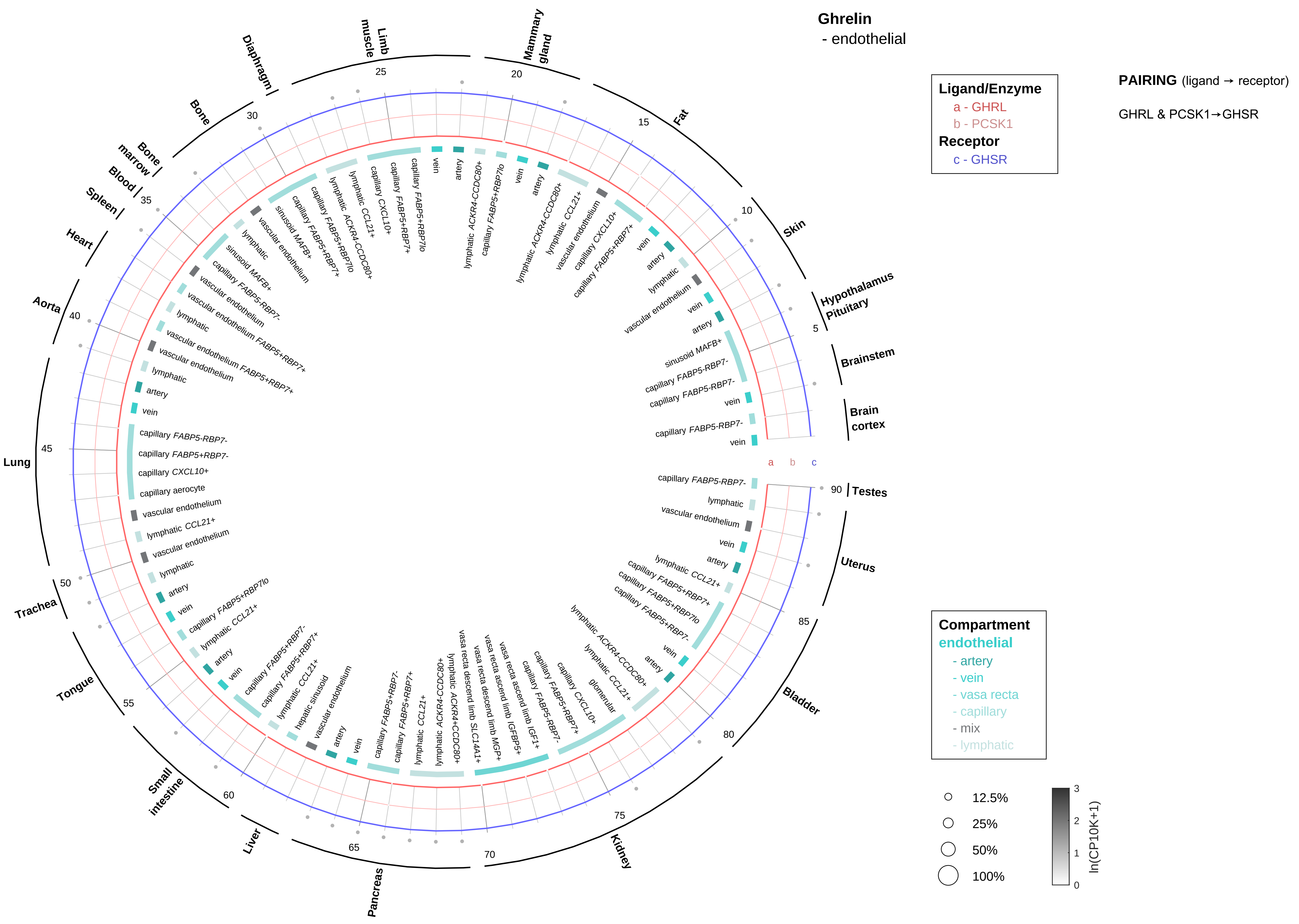

Ghrelin
- endothelial
Ligand/Enzyme
a - GHRL
b - PCSK1
Receptor
c - GHSR
PAIRING (ligand → receptor)
GHRL & PCSK1→GHSR
Compartment
endothelial
- artery
- vein
- vasa recta
- capillary
- mix
- lymphatic
12.5%
25%
50%
100%
ln(CP10K+1)
Brain cortex
Brainstem
Hypothalamus Pituitary
Skin
Fat
Mammary gland
Limb muscle
Diaphragm
Bone
Bone marrow
Blood
Spleen
Heart
Aorta
Lung
Trachea
Tongue
Small intestine
Liver
Pancreas
Kidney
Bladder
Uterus
Testes

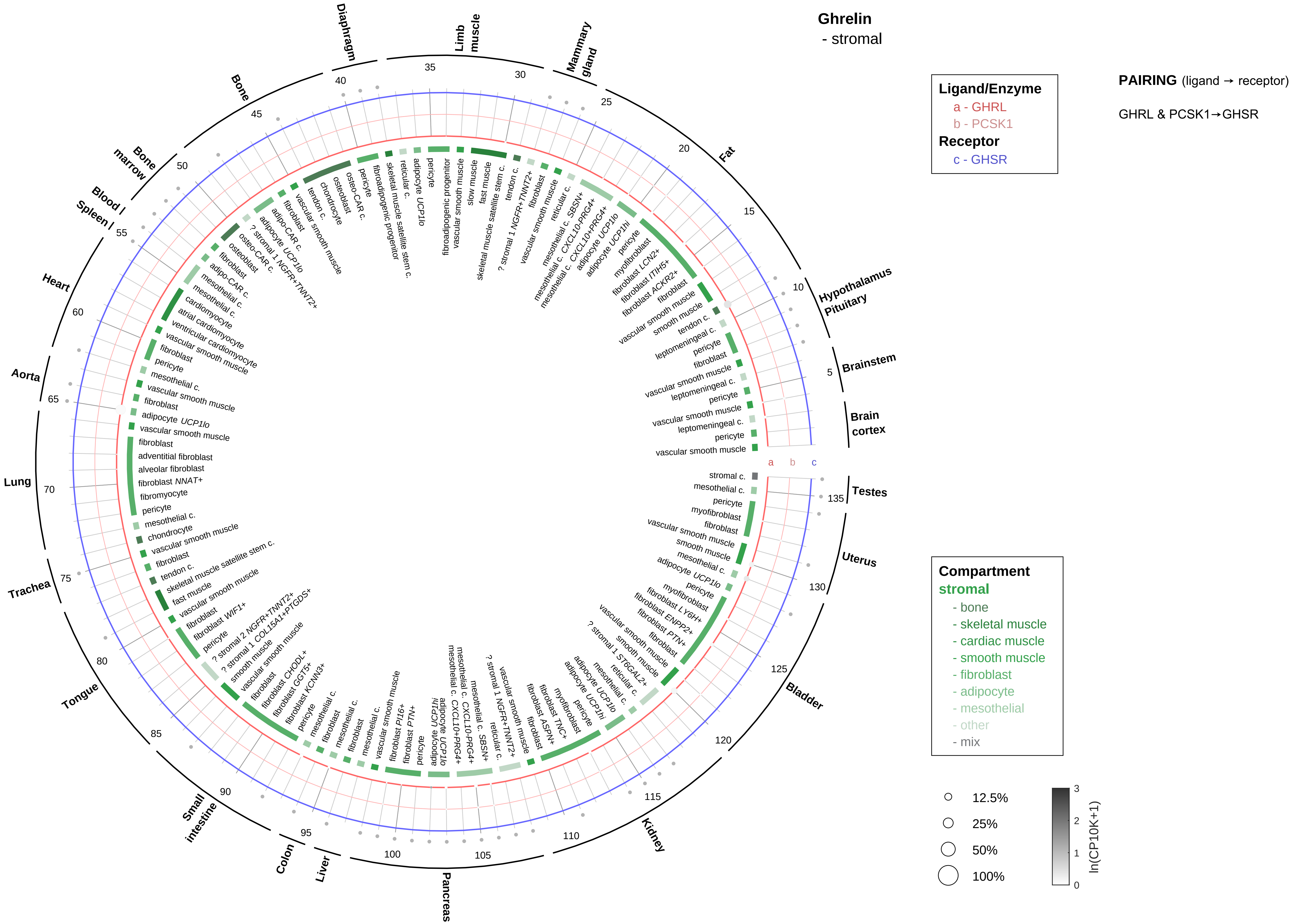
Ghrelin
- stromal
Ligand/Enzyme
a - GHRL
b - PCSK1
Receptor
c - GHSR
PAIRING (ligand → receptor)
GHRL & PCSK1→GHSR
Compartment
stromal
- bone
- skeletal muscle
- cardiac muscle
- smooth muscle
- fibroblast
- adipocyte
- mesothelial
- other
- mix
12.5%
25%
50%
100%
ln(CP10K+1)
Brain cortex
Brainstem
Hypothalamus Pituitary
Fat
Mammary gland
Limb muscle
Diaphragm
Bone
Bone marrow
Blood / Spleen /
Heart
Aorta
Lung
Trachea
Tongue
Small intestine
Colon
Liver
Pancreas
Kidney
Bladder
Uterus
Testes

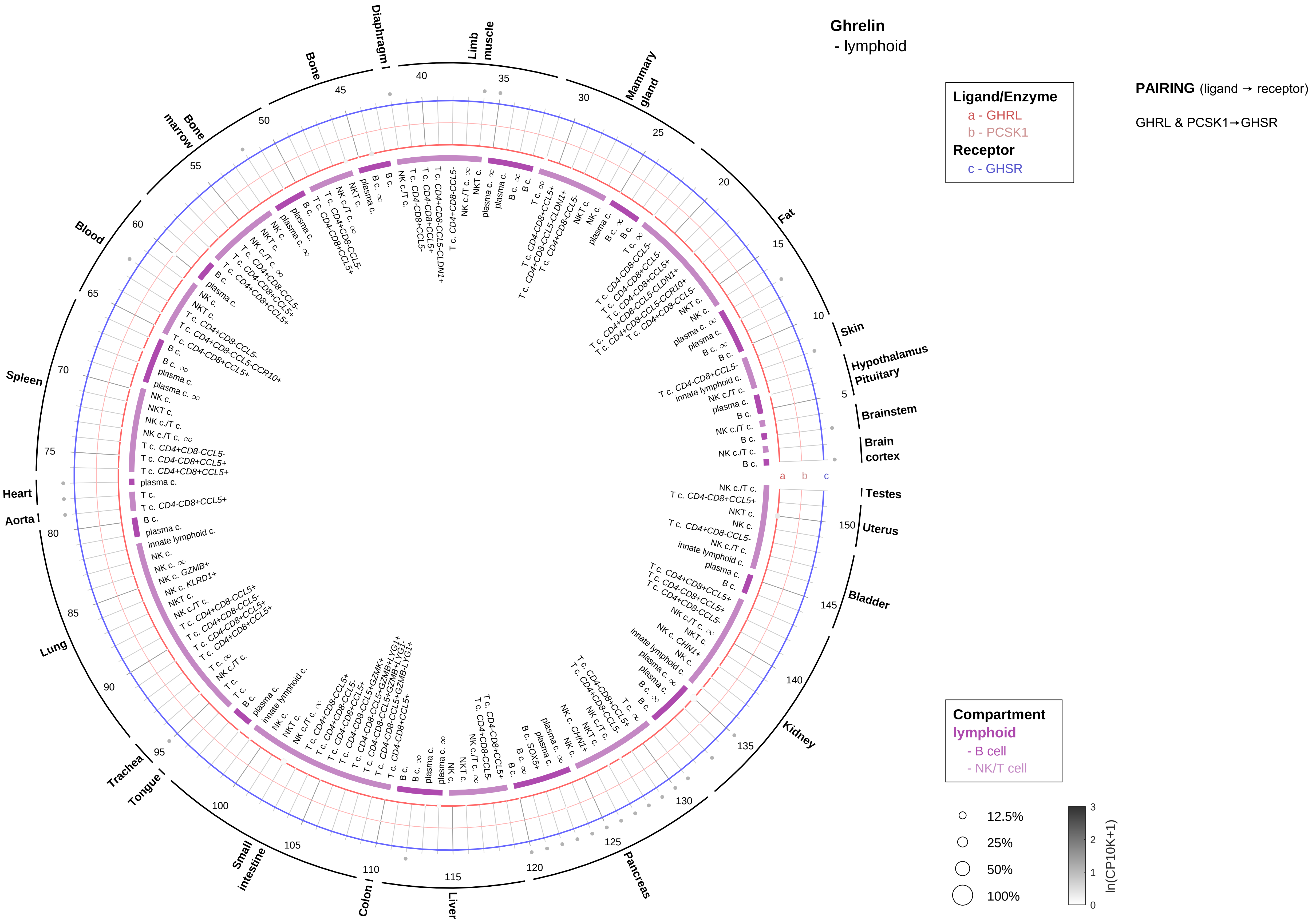

Ghrelin
- lymphoid
Ligand/Enzyme
a - GHRL
b - PCSK1
Receptor
c - GHSR
PAIRING (ligand → receptor)
GHRL & PCSK1→GHSR
Compartment
lymphoid
- B cell
- NK/T cell
12.5%
25%
50%
100%
ln(CP10K+1)
Skin
Hypothalamus
Pituitary
Brainstem
Brain cortex
Testes
Uterus
Bladder
Kidney
Pancreas
Liver
Colon
Small intestine
Tongue
Trachea
Lung
Aorta
Heart
Spleen
Blood
Bone marrow
Bone
Diaphragm
Limb muscle
Mammary gland
Fat

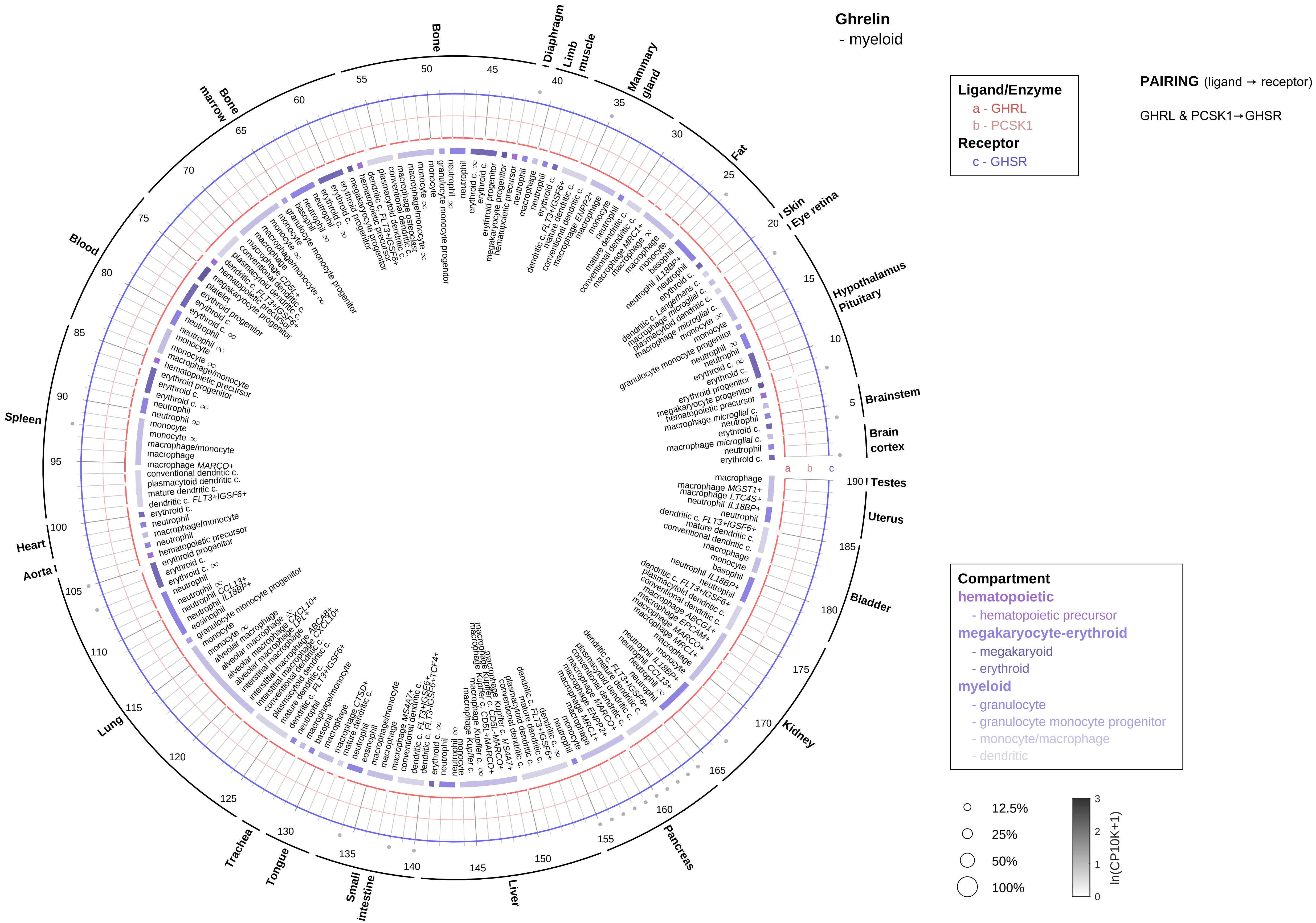

Ghrelin
- myeloid
Ligand/Enzyme
a - GHRL
b - PCSK1
Receptor
c - GHSR
PAIRING (ligand → receptor)
GHRL & PCSK1→GHSR
Compartment
hematopoietic
- hematopoietic precursor
megakaryocyte-erythroid
- megakaryoid
- erythroid
myeloid
- granulocyte
- granulocyte monocyte progenitor
- monocyte/macrophage
- dendritic
12.5%
25%
50%
100%
ln(CP10K+1)
Bone
Diaphragm
Limb muscle
Mammary gland
Fat
Skin
Eye retina
Hypothalamus
Pituitary
Brainstem
Brain cortex
Testes
Uterus
Bladder
Kidney
Pancreas
Liver
Small intestine
Tongue
Trachea
Lung
Aorta
Heart
Spleen
Blood
Bone marrow

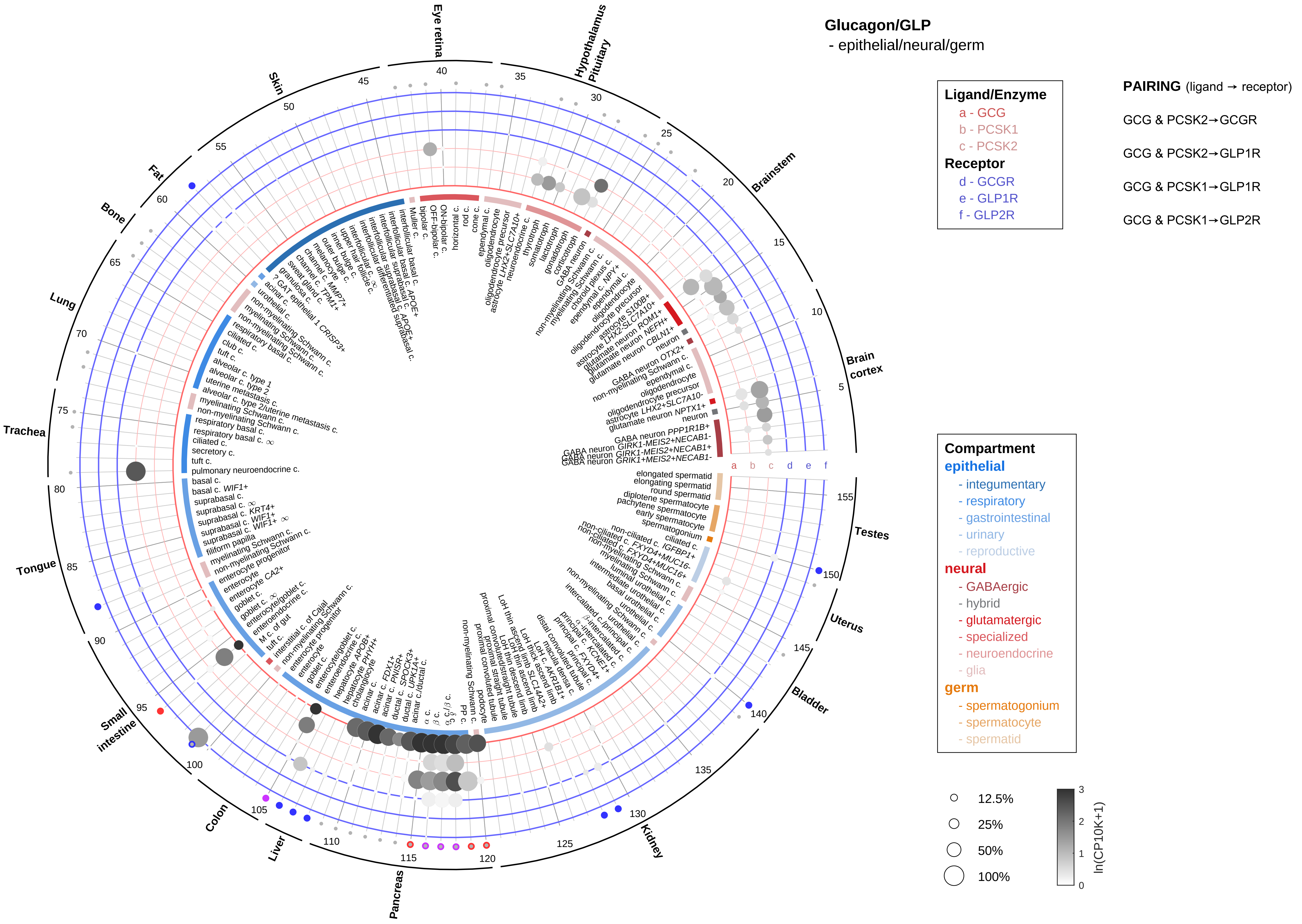
Glucagon/GLP
- epithelial/neural/germ
Ligand/Enzyme
a - GCG
b - PCSK1
c - PCSK2
Receptor
d - GCGR
e - GLP1R
f - GLP2R
PAIRING (ligand → receptor)
GCG & PCSK2→GCGR
GCG & PCSK2→GLP1R
GCG & PCSK1→GLP1R
GCG & PCSK1→GLP2R
Compartment
epithelial
- integumentary
- respiratory
- gastrointestinal
- urinary
- reproductive
neural
- GABAergic
- hybrid
- glutamatergic
- specialized
- neuroendocrine
- glia
germ
- spermatogonium
- spermatocyte
- spermatid
12.5%
25%
50%
100%
ln(CP10K+1)
Eye retina
Hypothalamus Pituitary
Brainstem
Brain cortex
Testes
Uterus
Bladder
Kidney
Pancreas
Liver
Colon
Small intestine
Tongue
Trachea
Lung
Bone
Fat
Skin

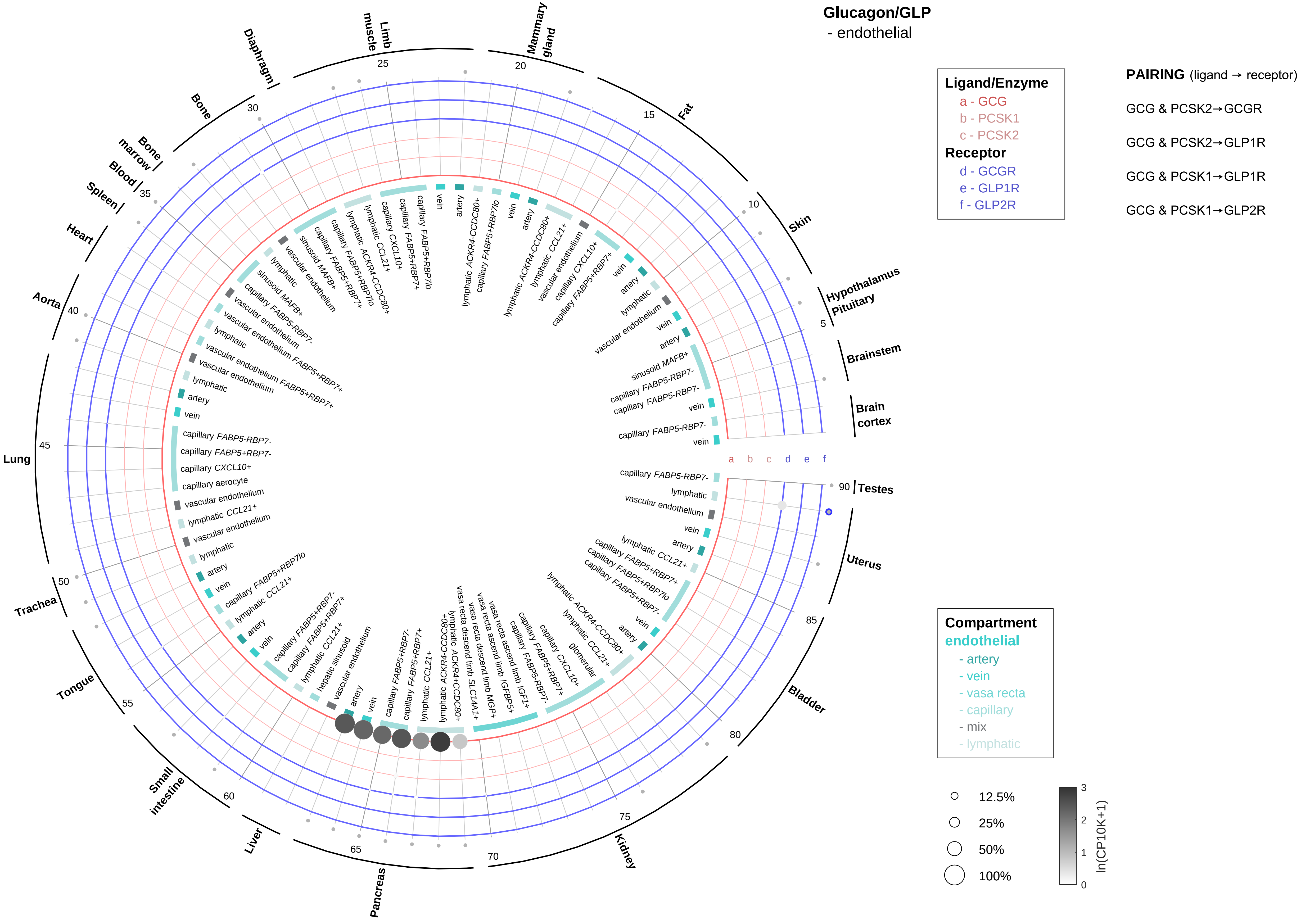
Glucagon/GLP
- endothelial
Ligand/Enzyme
a - GCG
b - PCSK1
c - PCSK2
Receptor
d - GCGR
e - GLP1R
f - GLP2R
PAIRING (ligand → receptor)
GCG & PCSK2→GCGR
GCG & PCSK2→GLP1R
GCG & PCSK1→GLP1R
GCG & PCSK1→GLP2R
Compartment
endothelial
- artery
- vein
- vasa recta
- capillary
- mix
- lymphatic
12.5%
25%
50%
100%
ln(CP10K+1)
Brain cortex
Brainstem
Hypothalamus Pituitary
Skin
Fat
Mammary gland
Limb muscle
Diaphragm
Bone
Bone marrow
Blood
Spleen
Heart
Aorta
Lung
Trachea
Tongue
Small intestine
Liver
Pancreas
Kidney
Bladder
Uterus
Testes

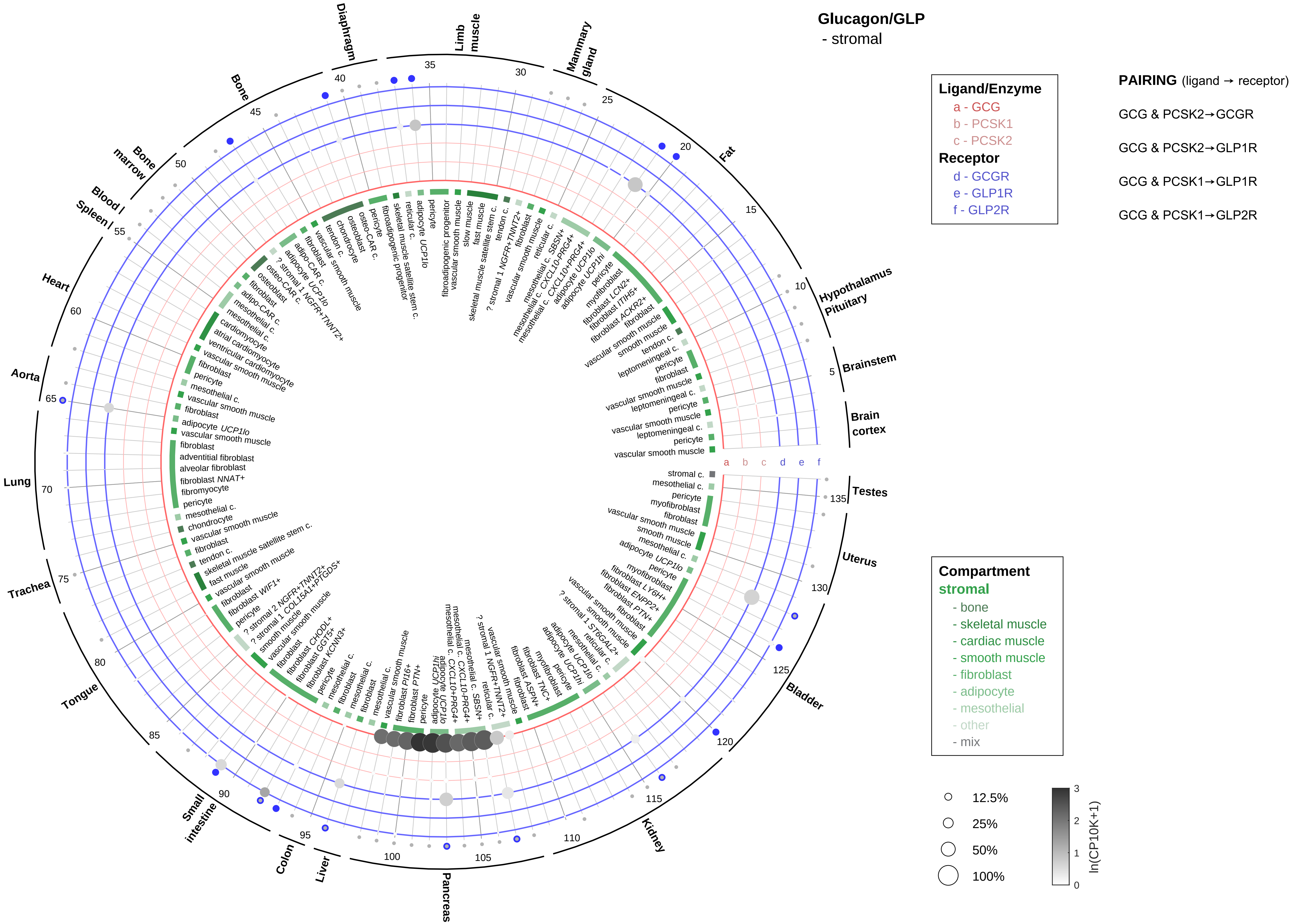

Glucagon/GLP
- stromal
Ligand/Enzyme
a - GCG
b - PCSK1
c - PCSK2
Receptor
d - GCGR
e - GLP1R
f - GLP2R
PAIRING (ligand → receptor)
GCG & PCSK2→GCGR
GCG & PCSK2→GLP1R
GCG & PCSK1→GLP1R
GCG & PCSK1→GLP2R
Compartment
stromal
- bone
- skeletal muscle
- cardiac muscle
- smooth muscle
- fibroblast
- adipocyte
- mesothelial
- other
- mix
12.5%
25%
50%
100%
ln(CP10K+1)
Diaphragm
Limb muscle
Mammary gland
Fat
Hypothalamus Pituitary
Brainstem
Brain cortex
Testes
Uterus
Bladder
Kidney
Pancreas
Liver
Colon
Small intestine
Tongue
Trachea
Lung
Aorta
Heart
Spleen
Blood
Bone marrow
Bone

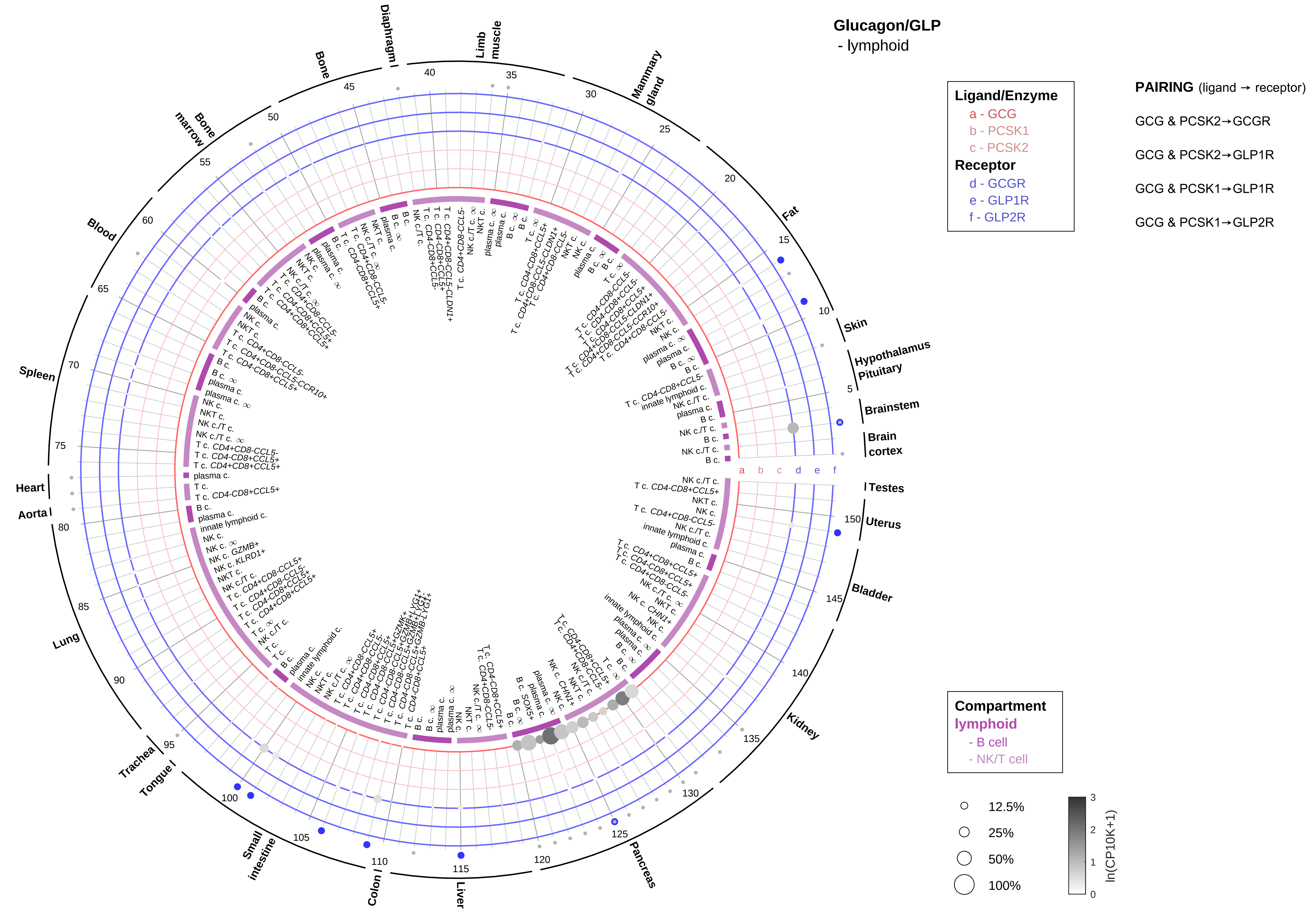
Glucagon/GLP
- lymphoid
Ligand/Enzyme
a - GCG
b - PCSK1
c - PCSK2
Receptor
d - GCGR
e - GLP1R
f - GLP2R
PAIRING (ligand → receptor)
GCG & PCSK2→GCGR
GCG & PCSK2→GLP1R
GCG & PCSK1→GLP1R
GCG & PCSK1→GLP2R
Compartment
lymphoid
- B cell
- NK/T cell
12.5%
25%
50%
100%
ln(CP10K+1)
Skin
Hypothalamus
Pituitary
Brainstem
Brain cortex
Testes
Uterus
Bladder
Kidney
Pancreas
Liver
Colon
Small intestine
Tongue
Trachea
Lung
Aorta
Heart
Spleen
Blood
Bone marrow
Bone
Diaphragm
Limb muscle
Mammary gland
Fat

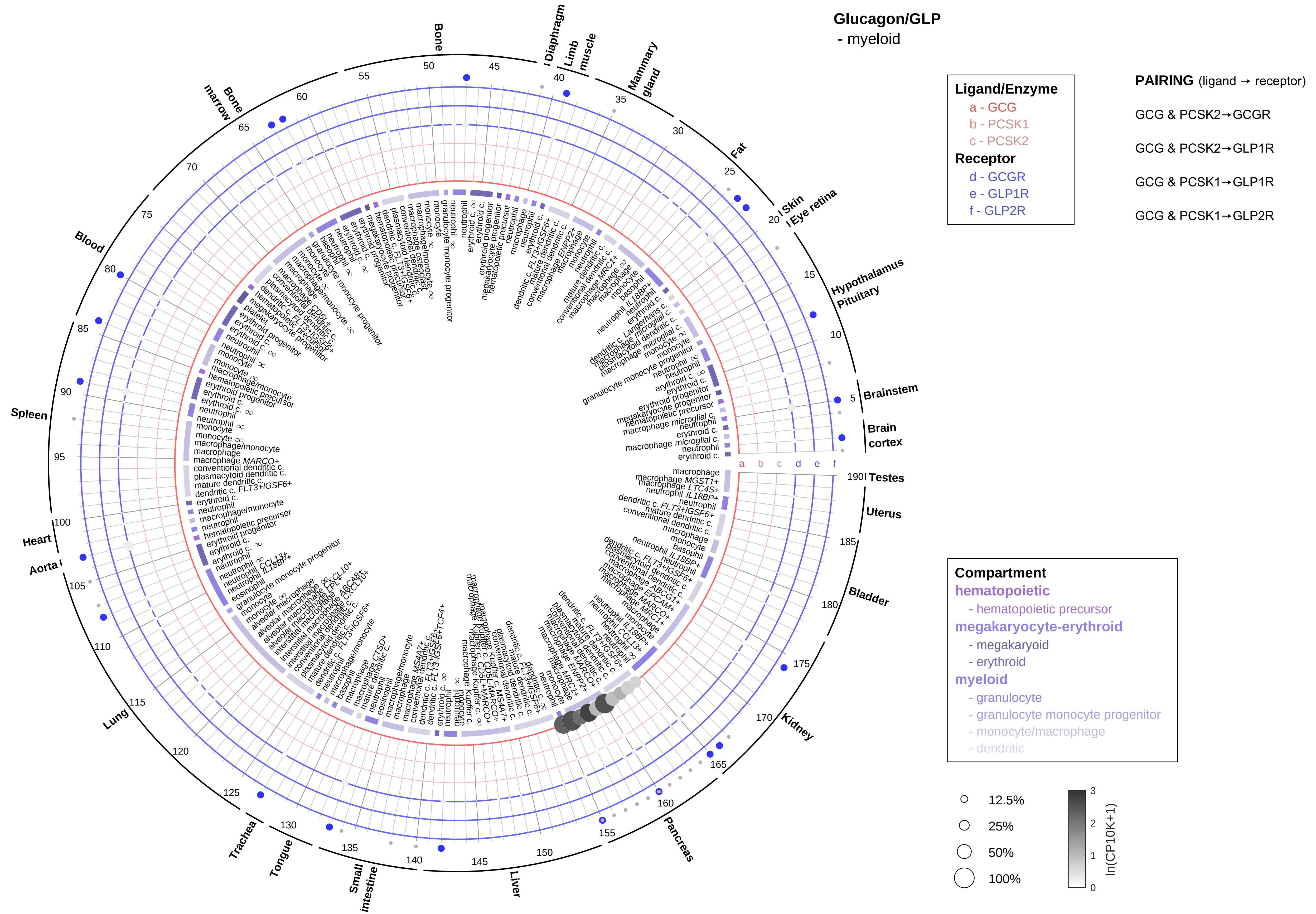
Glucagon/GLP
- myeloid
Ligand/Enzyme
a - GCG
b - PCSK1
c - PCSK2
Receptor
d - GCGR
e - GLP1R
f - GLP2R
PAIRING (ligand → receptor)
GCG & PCSK2→GCGR
GCG & PCSK2→GLP1R
GCG & PCSK1→GLP1R
GCG & PCSK1→GLP2R
Compartment
hematopoietic
- hematopoietic precursor
megakaryocyte-erythroid
- megakaryoid
- erythroid
myeloid
- granulocyte
- granulocyte monocyte progenitor
- monocyte/macrophage
- dendritic
12.5%
25%
50%
100%
ln(CP10K+1)
Bone
Diaphragm
Limb muscle
Mammary gland
Fat
Skin
Eye retina
Hypothalamus
Pituitary
Brainstem
Brain cortex
Testes
Uterus
Bladder
Kidney
Pancreas
Liver
Small intestine
Tongue
Trachea
Lung
Aorta
Heart
Spleen
Blood
Bone marrow

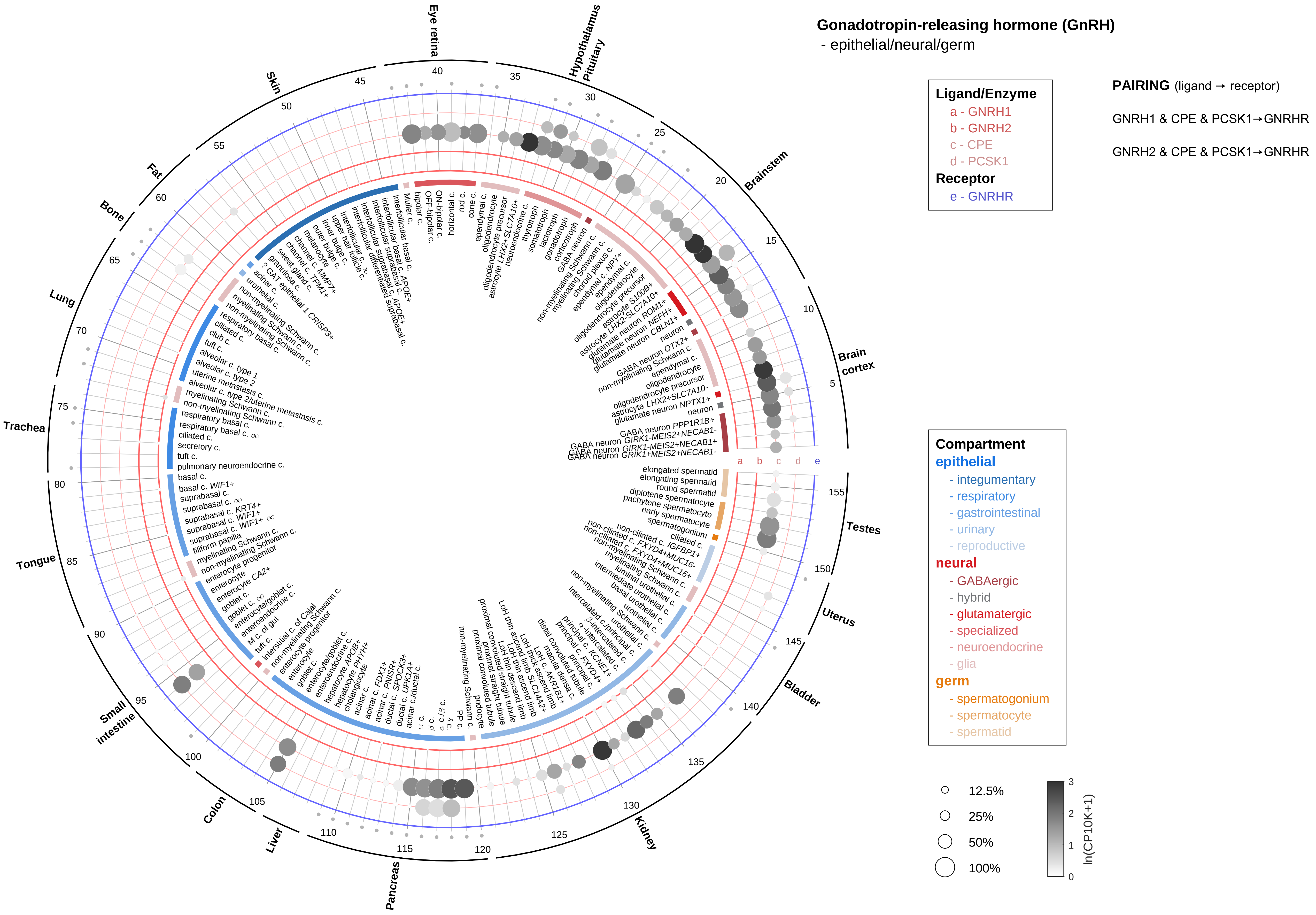
Gonadotropin-releasing hormone (GnRH)
- epithelial/neural/germ
Ligand/Enzyme
a - GNRH1
b - GNRH2
c - CPE
d - PCSK1
Receptor
e - GNRHR
PAIRING (ligand → receptor)
GNRH1 & CPE & PCSK1→GNRHR
GNRH2 & CPE & PCSK1→GNRHR
Compartment
epithelial
- integumentary
- respiratory
- gastrointestinal
- urinary
- reproductive
neural
- GABAergic
- hybrid
- glutamatergic
- specialized
- neuroendocrine
- glia
germ
- spermatogonium
- spermatocyte
- spermatid
12.5%
25%
50%
100%
ln(CP10K+1)
Eye retina
Hypothalamus Pituitary
Brainstem
Brain cortex
Testes
Uterus
Bladder
Kidney
Pancreas
Liver
Colon
Small intestine
Tongue
Trachea
Lung
Bone
Fat
Skin

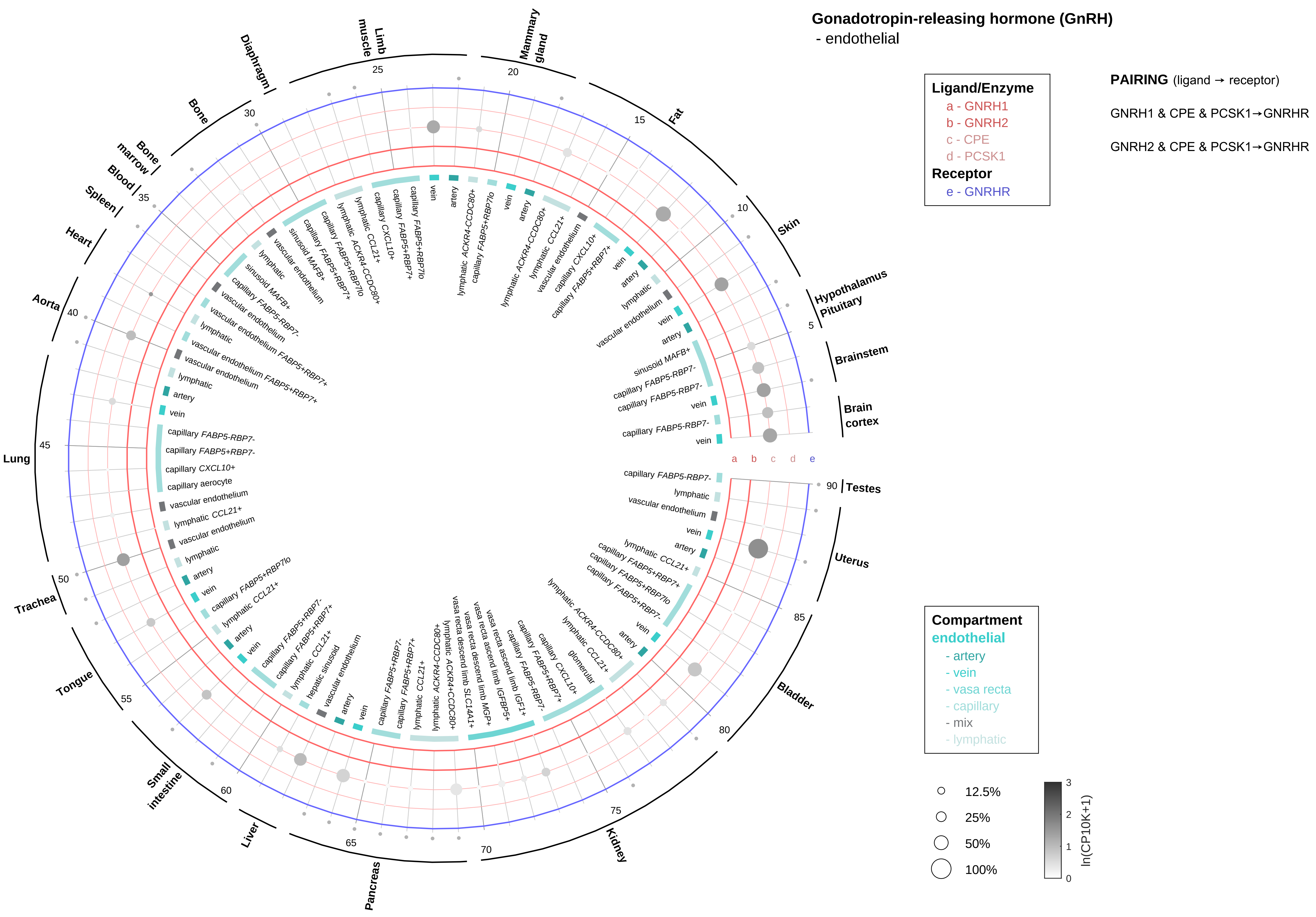
Gonadotropin-releasing hormone (GnRH)
- endothelial
Ligand/Enzyme
a - GNRH1
b - GNRH2
c - CPE
d - PCSK1
Receptor
e - GNRHR
PAIRING (ligand → receptor)
GNRH1 & CPE & PCSK1→GNRHR
GNRH2 & CPE & PCSK1→GNRHR
Compartment
endothelial
- artery
- vein
- vasa recta
- capillary
- mix
- lymphatic
12.5%
25%
50%
100%
ln(CP10K+1)
Brain cortex
Brainstem
Hypothalamus Pituitary
Skin
Fat
Mammary gland
Limb muscle
Diaphragm
Bone
Bone marrow
Blood
Spleen
Heart
Aorta
Lung
Trachea
Tongue
Small intestine
Liver
Pancreas
Kidney
Bladder
Uterus
Testes

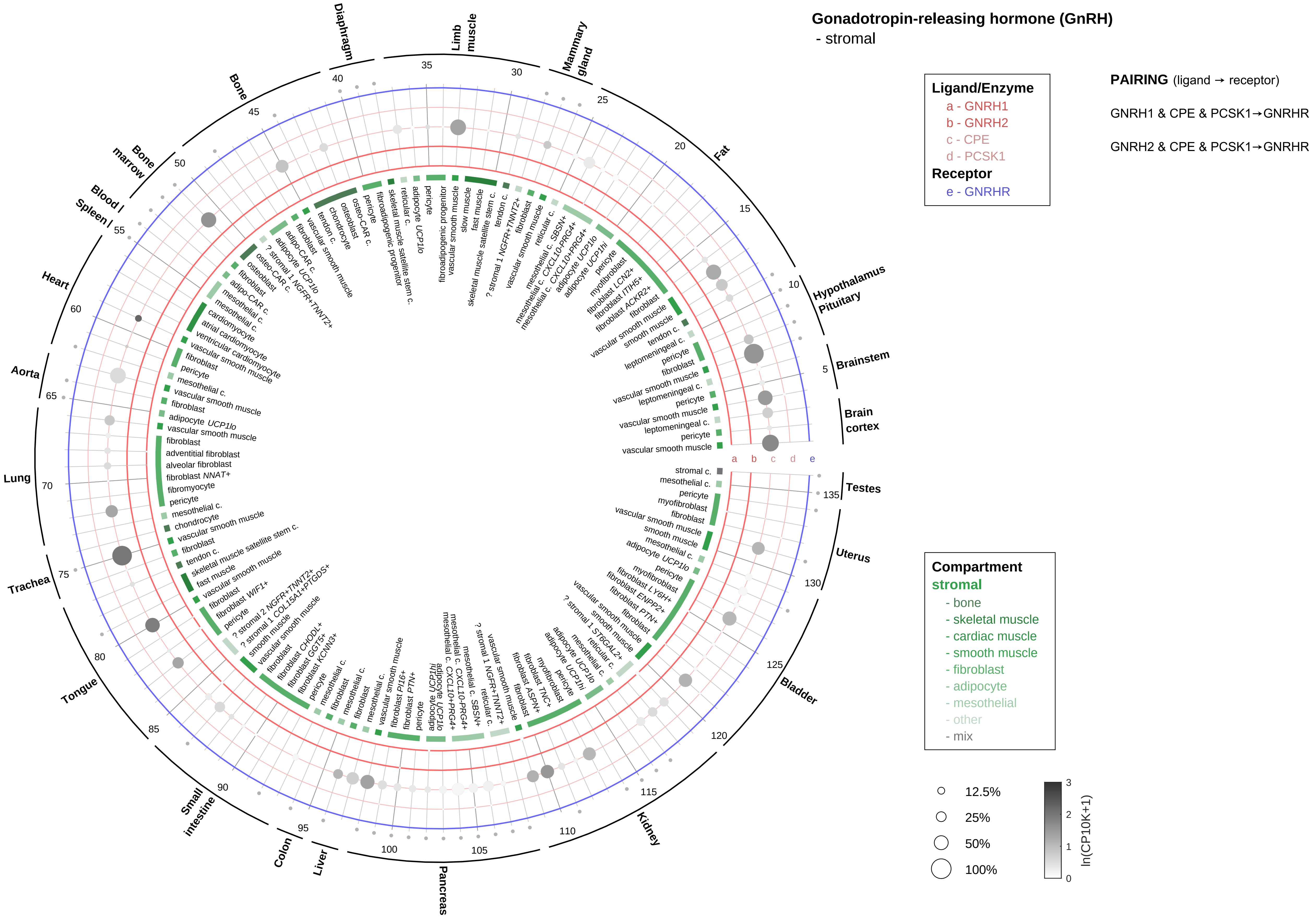

Gonadotropin-releasing hormone (GnRH)
- stromal
Ligand/Enzyme
a - GNRH1
b - GNRH2
c - CPE
d - PCSK1
Receptor
e - GNRHR
PAIRING (ligand → receptor)
GNRH1 & CPE & PCSK1→GNRHR
GNRH2 & CPE & PCSK1→GNRHR
Compartment
stromal
- bone
- skeletal muscle
- cardiac muscle
- smooth muscle
- fibroblast
- adipocyte
- mesothelial
- other
- mix
12.5%
25%
50%
100%
ln(CP10K+1)
Brain cortex
Brainstem
Hypothalamus Pituitary
Fat
Mammary gland
Limb muscle
Diaphragm
Bone
Bone marrow
Blood / Spleen
Heart
Aorta
Lung
Trachea
Tongue
Small intestine
Colon
Liver
Pancreas
Kidney
Bladder
Uterus
Testes

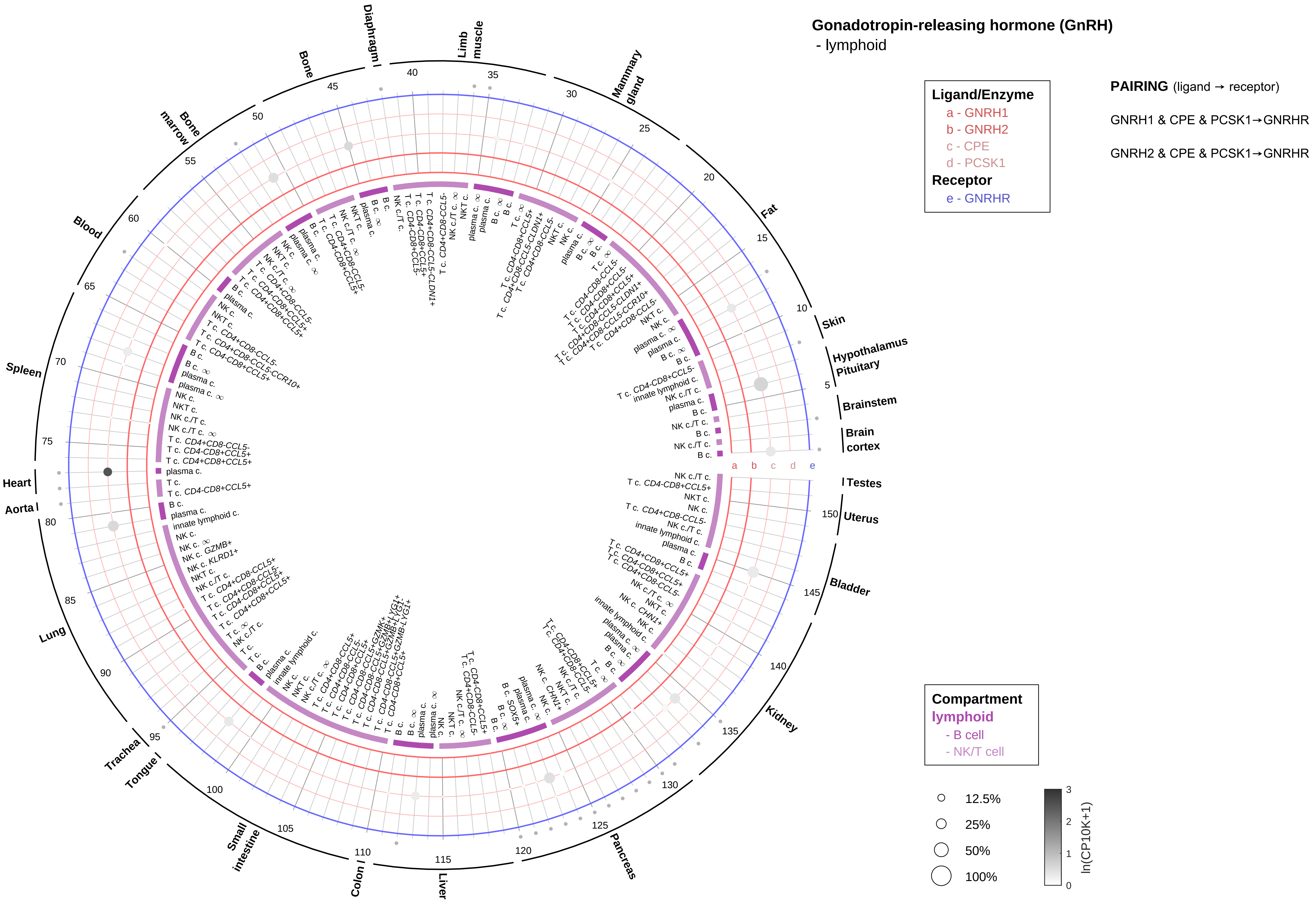

Gonadotropin-releasing hormone (GnRH)
- lymphoid
Ligand/Enzyme
a - GNRH1
b - GNRH2
c - CPE
d - PCSK1
Receptor
e - GNRHR
PAIRING (ligand → receptor)
GNRH1 & CPE & PCSK1→GNRHR
GNRH2 & CPE & PCSK1→GNRHR
Compartment
lymphoid
- B cell
- NK/T cell
12.5%
25%
50%
100%
ln(CP10K+1)
Diaphragm
Limb muscle
Mammary gland
Fat
Skin
Hypothalamus
Pituitary
Brainstem
Brain cortex
Testes
Uterus
Bladder
Kidney
Pancreas
Liver
Colon
Small intestine
Tongue
Trachea
Lung
Aorta
Heart
Spleen
Blood
Bone marrow
Bone

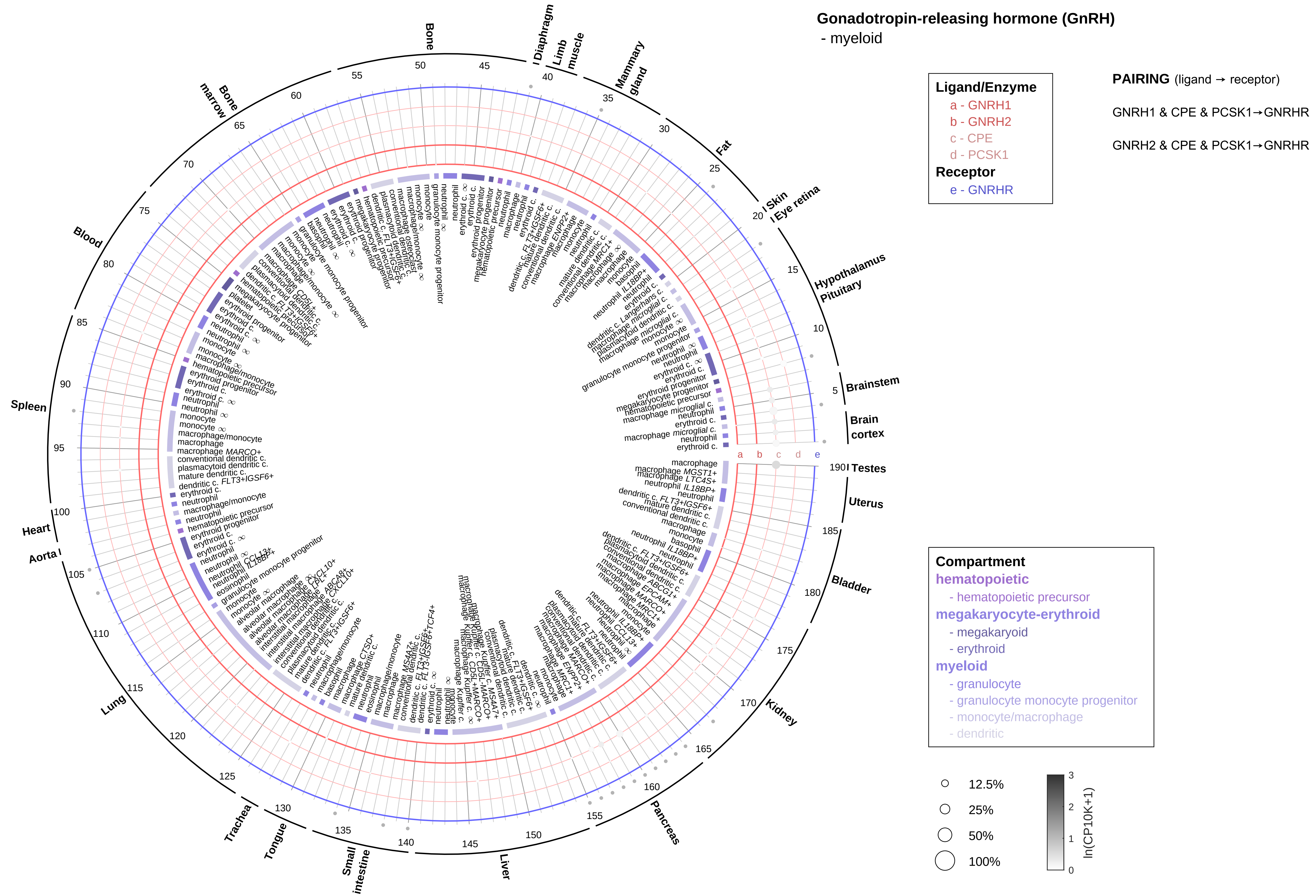
Gonadotropin-releasing hormone (GnRH)
- myeloid
Ligand/Enzyme
a - GNRH1
b - GNRH2
c - CPE
d - PCSK1
Receptor
e - GNRHR
PAIRING (ligand → receptor)
GNRH1 & CPE & PCSK1→GNRHR
GNRH2 & CPE & PCSK1→GNRHR
Compartment
hematopoietic
- hematopoietic precursor
megakaryocyte-erythroid
- megakaryoid
- erythroid
myeloid
- granulocyte
- granulocyte monocyte progenitor
- monocyte/macrophage
- dendritic
12.5%
25%
50%
100%
ln(CP10K+1)
Bone
Diaphragm
Limb muscle
Mammary gland
Fat
Skin
Eye retina
Hypothalamus
Pituitary
Brainstem
Brain cortex
Testes
Uterus
Bladder
Kidney
Pancreas
Liver
Small intestine
Tongue
Trachea
Lung
Aorta
Heart
Spleen
Blood
Bone marrow

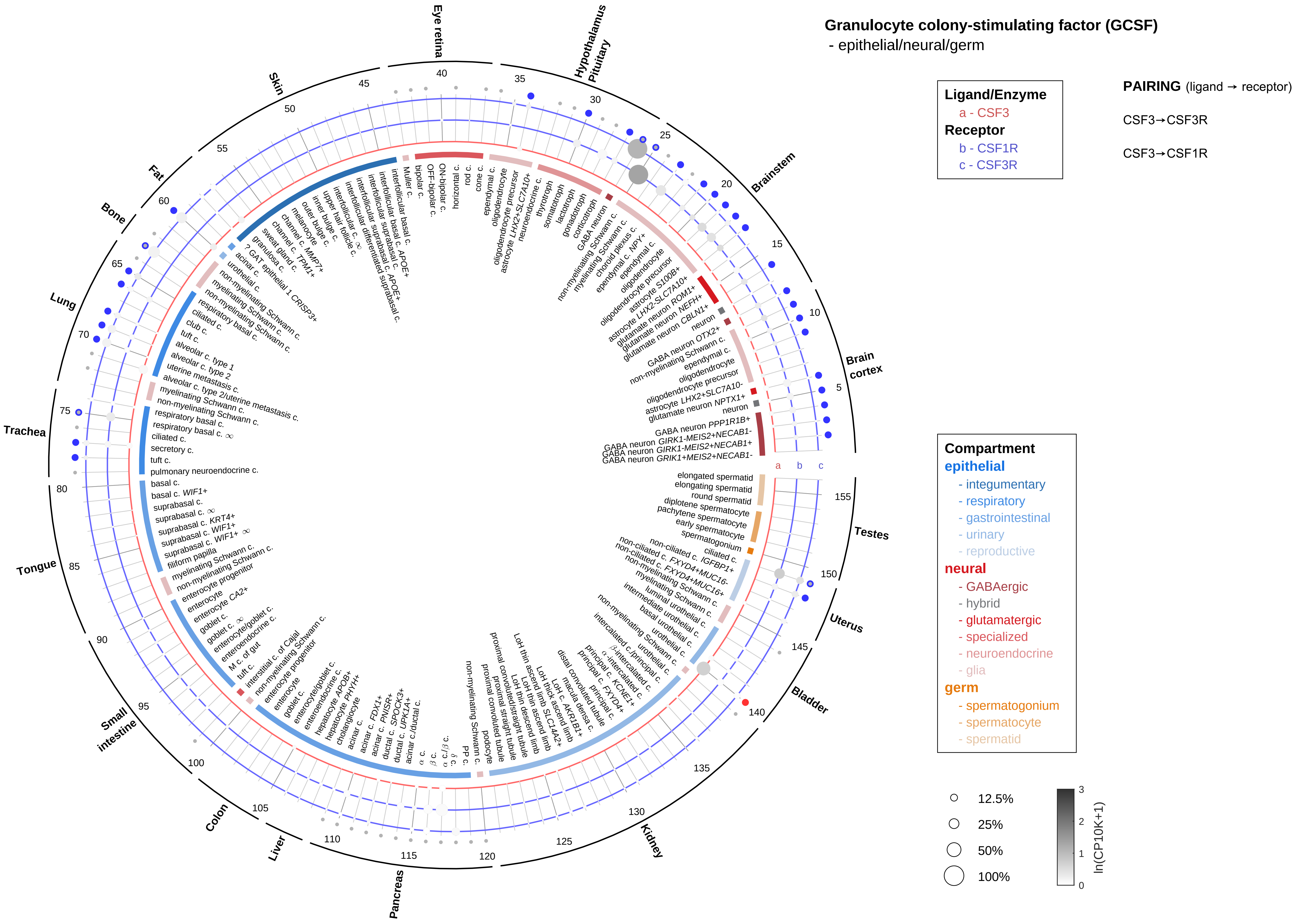
Granulocyte colony-stimulating factor (GCSF)
- epithelial/neural/germ
Ligand/Enzyme
a - CSF3
Receptor
b - CSF1R
c - CSF3R
PAIRING (ligand → receptor)
CSF3→CSF3R
CSF3→CSF1R
Compartment
epithelial
- integumentary
- respiratory
- gastrointestinal
- urinary
- reproductive
neural
- GABAergic
- hybrid
- glutamatergic
- specialized
- neuroendocrine
- glia
germ
- spermatogonium
- spermatocyte
- spermatid
12.5%
25%
50%
100%
ln(CP10K+1)
Eye retina
Hypothalamus Pituitary
Brainstem
Brain cortex
Testes
Uterus
Bladder
Kidney
Pancreas
Liver
Colon
Small intestine
Tongue
Trachea
Lung
Bone
Fat
Skin

Granulocyte colony-stimulating factor (GCSF)
- endothelial

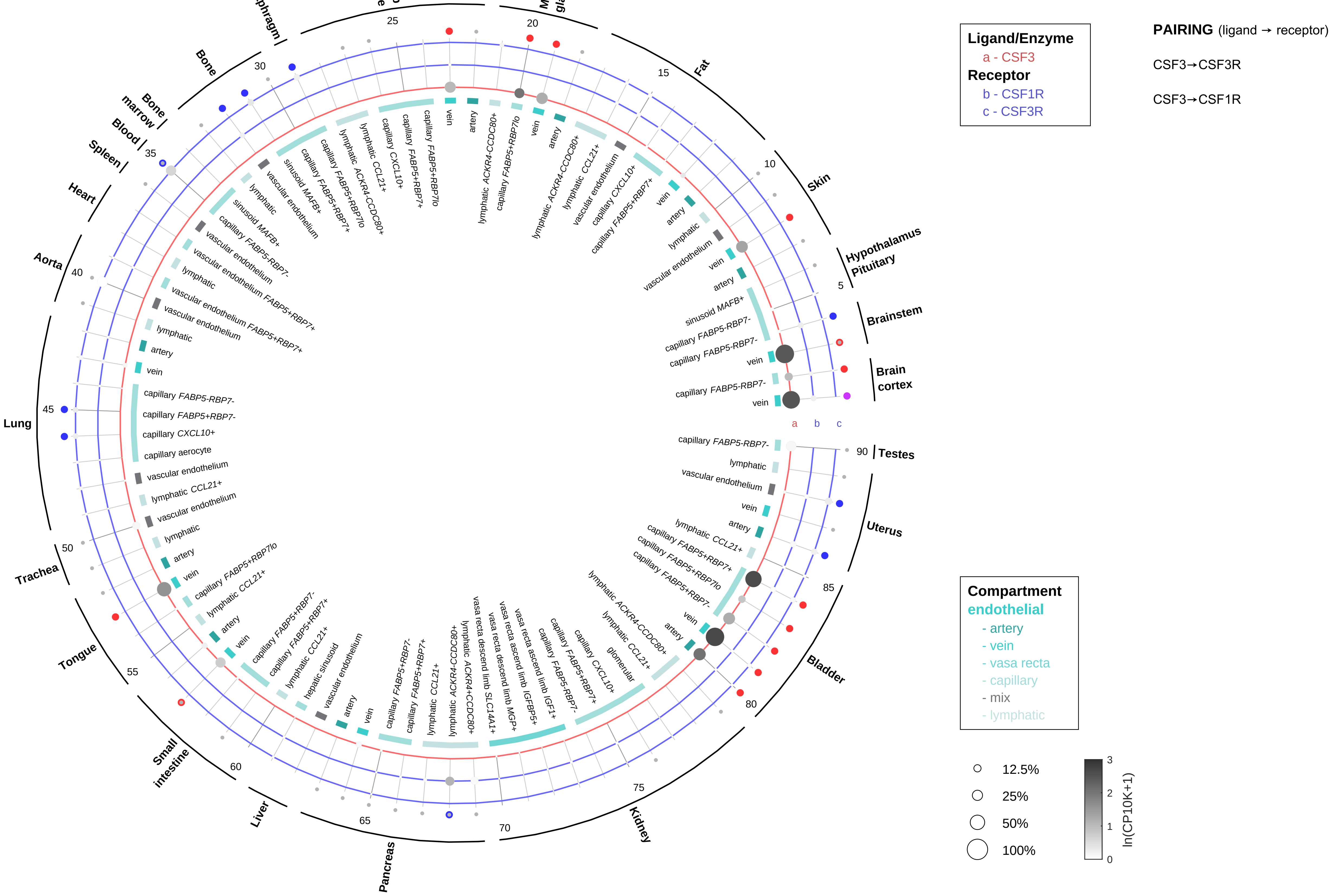

Granulocyte colony-stimulating factor (GCSF)
- stromal

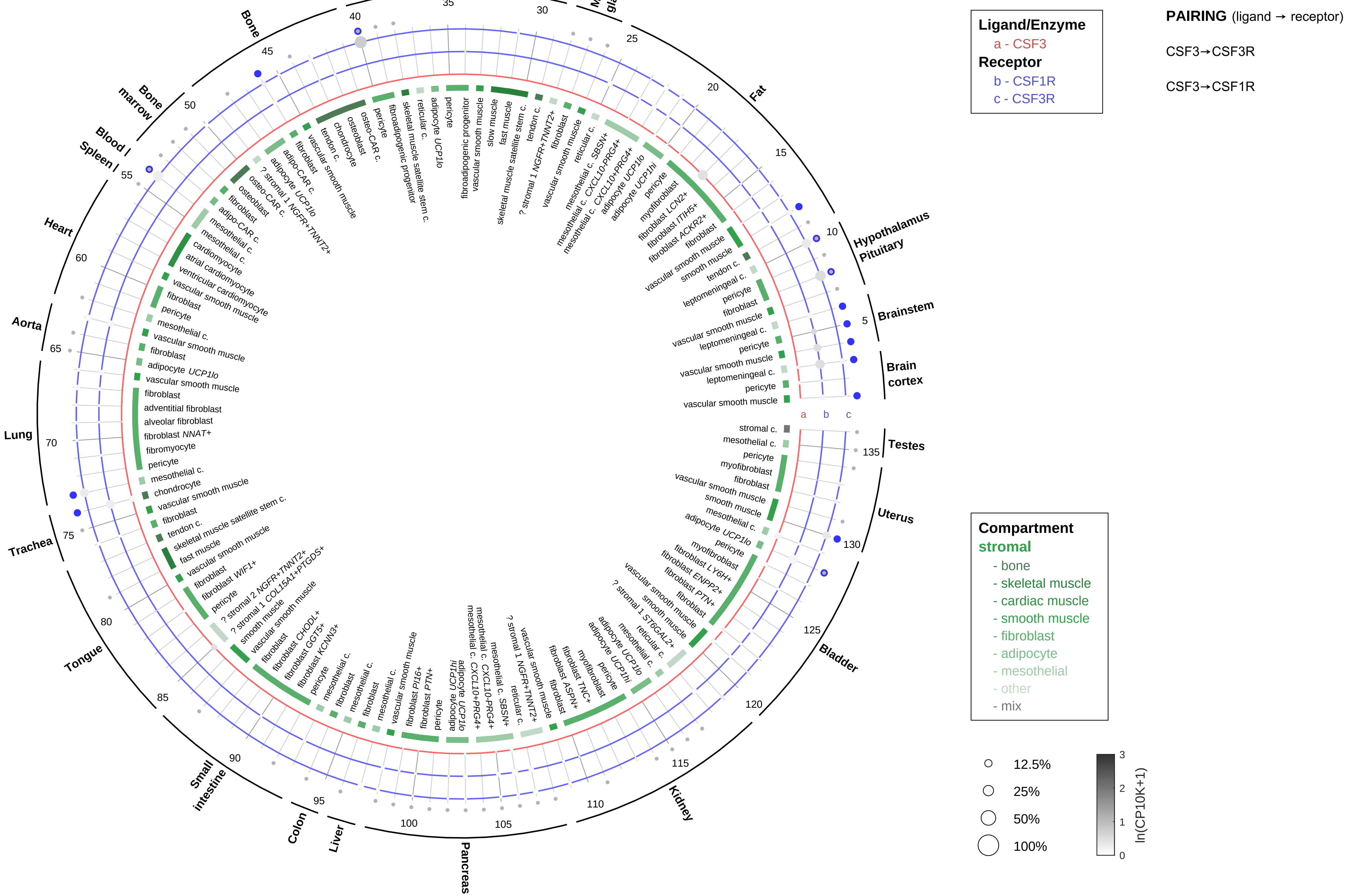

Granulocyte colony-stimulating factor (GCSF)
- lymphoid

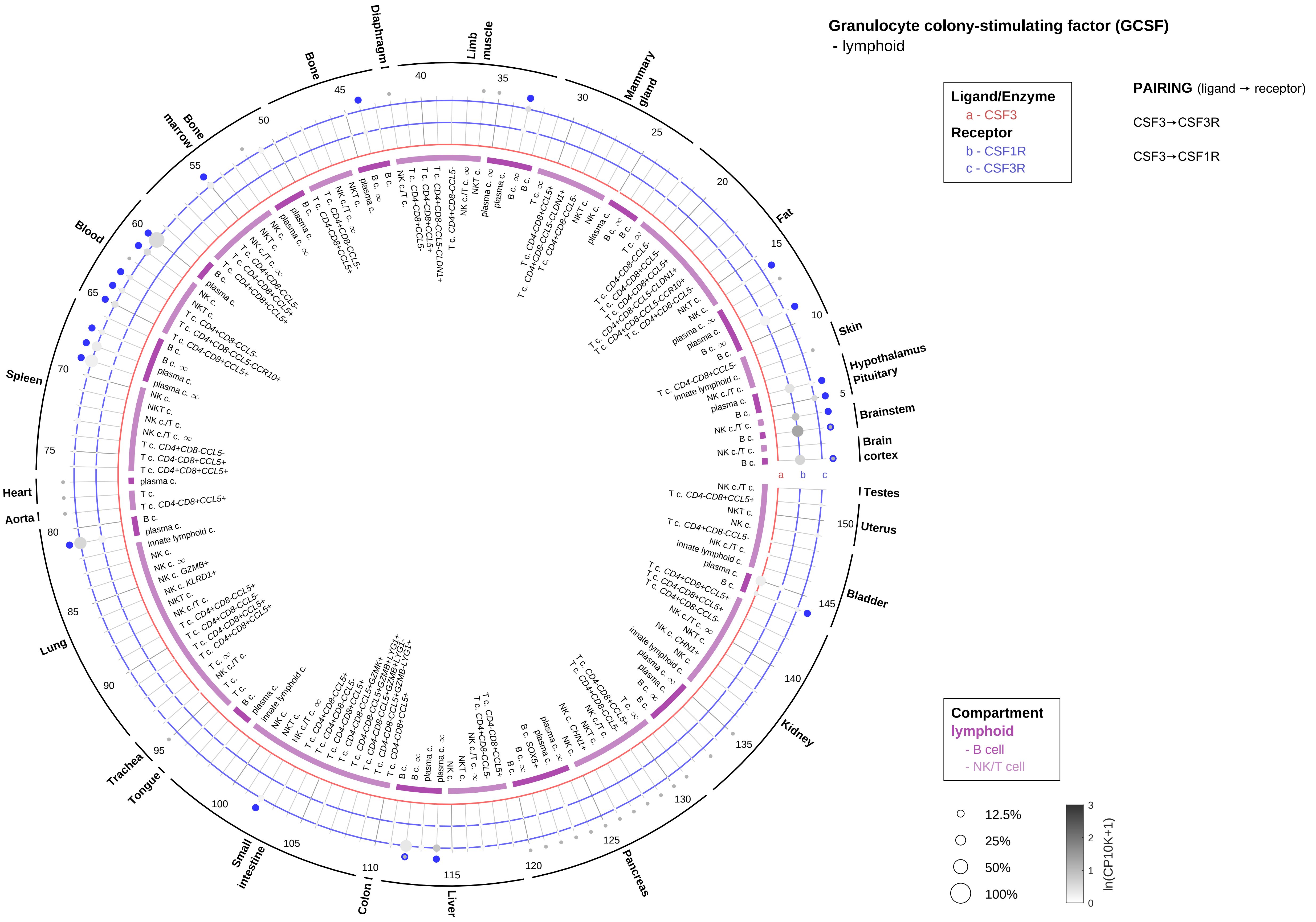

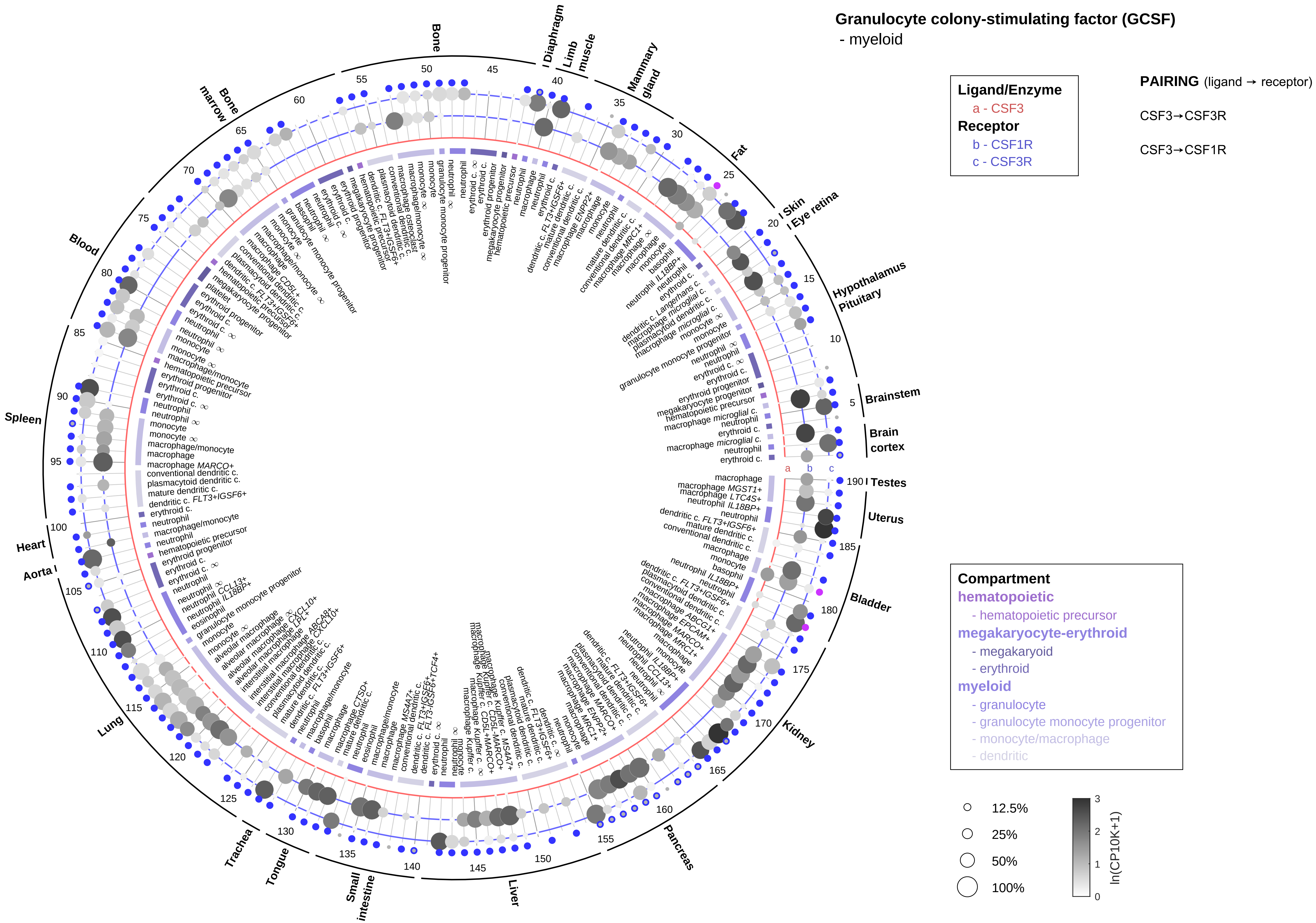

Granulocyte colony-stimulating factor (GCSF)
- myeloid
Ligand/Enzyme
a - CSF3
Receptor
b - CSF1R
c - CSF3R
PAIRING (ligand → receptor)
CSF3→CSF3R
CSF3→CSF1R
Compartment
hematopoietic
- hematopoietic precursor
megakaryocyte-erythroid
- megakaryoid
- erythroid
myeloid
- granulocyte
- granulocyte monocyte progenitor
- monocyte/macrophage
- dendritic
12.5%
25%
50%
100%
ln(CP10K+1)
Brainstem
Brain cortex
Testes
Uterus
Bladder
Kidney
Pancreas
Liver
Small intestine
Tongue
Trachea
Lung
Aorta
Heart
Spleen
Blood
Bone marrow
Bone
Diaphragm
Limb muscle
Mammary gland
Fat
Skin
Eye retina
Hypothalamus
Pituitary

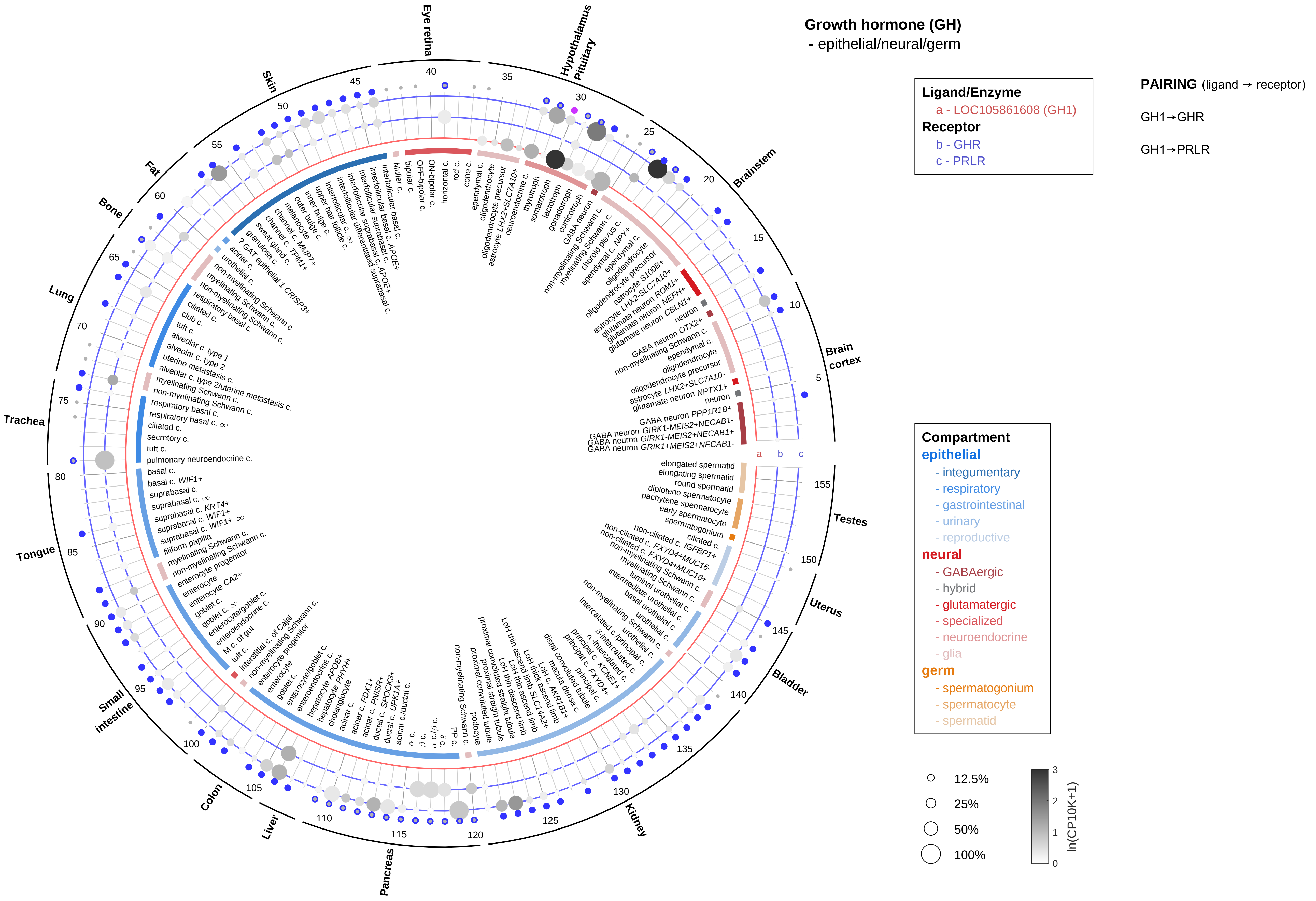

Growth hormone (GH)
- epithelial/neural/germ
Ligand/Enzyme
a - LOC105861608 (GH1)
Receptor
b - GHR
c - PRLR
PAIRING (ligand → receptor)
GH1→GHR
GH1→PRLR
Compartment
epithelial
- integumentary
- respiratory
- gastrointestinal
- urinary
- reproductive
neural
- GABAergic
- hybrid
- glutamatergic
- specialized
- neuroendocrine
- glia
germ
- spermatogonium
- spermatocyte
- spermatid
12.5%
25%
50%
100%
ln(CP10K+1)
Eye retina
Hypothalamus Pituitary
Brainstem
Brain cortex
Testes
Uterus
Bladder
Kidney
Pancreas
Liver
Colon
Small intestine
Tongue
Trachea
Lung
Bone
Fat
Skin

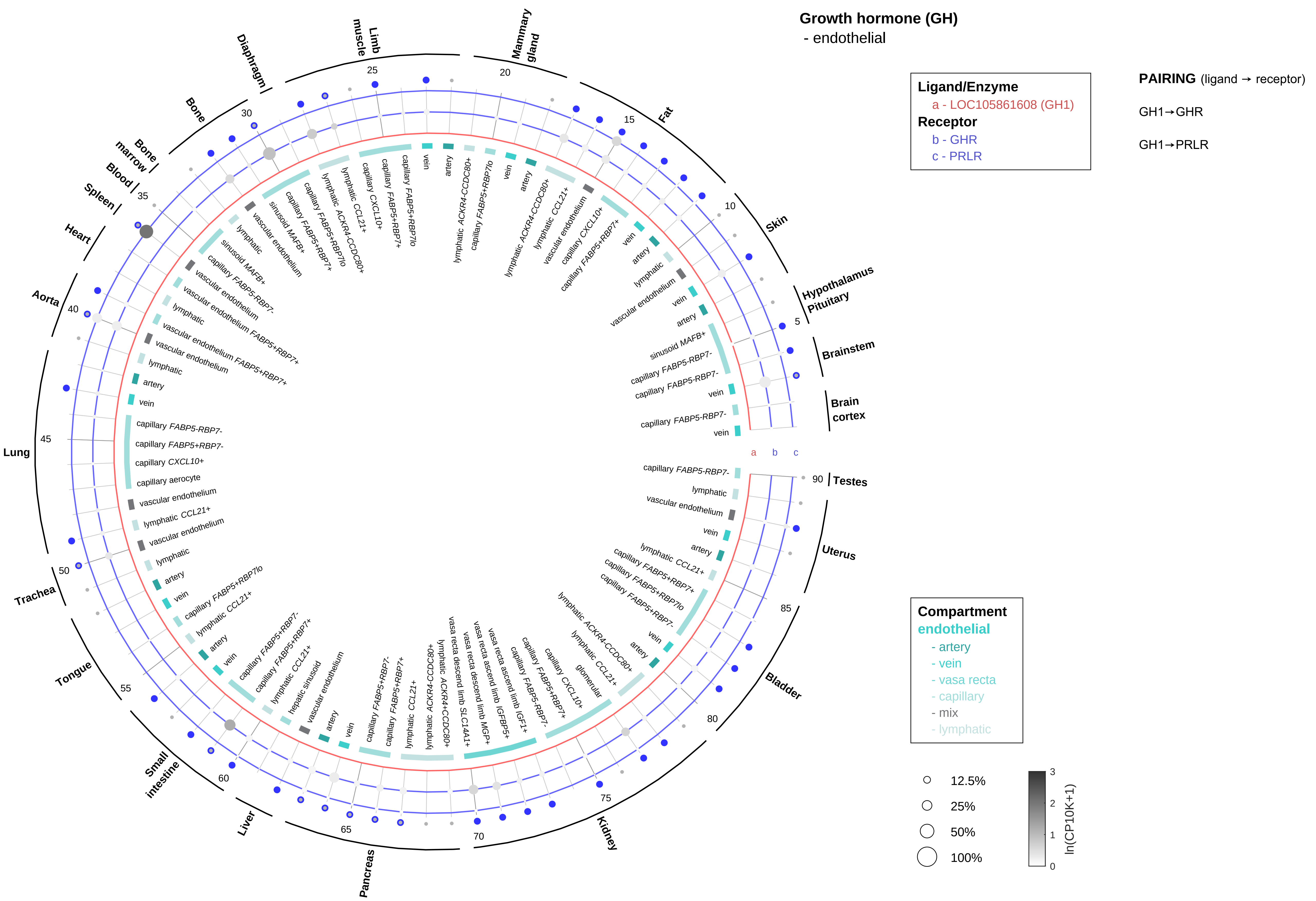

Growth hormone (GH)
- endothelial
Ligand/Enzyme
a - LOC105861608 (GH1)
Receptor
b - GHR
c - PRLR
PAIRING (ligand → receptor)
GH1→GHR
GH1→PRLR
Compartment
endothelial
- artery
- vein
- vasa recta
- capillary
- mix
- lymphatic
12.5%
25%
50%
100%
ln(CP10K+1)
Brain cortex
Brainstem
Hypothalamus Pituitary
Skin
Fat
Mammary gland
Limb muscle
Diaphragm
Bone
Bone marrow
Blood
Spleen
Heart
Aorta
Lung
Trachea
Tongue
Small intestine
Liver
Pancreas
Kidney
Bladder
Uterus
Testes

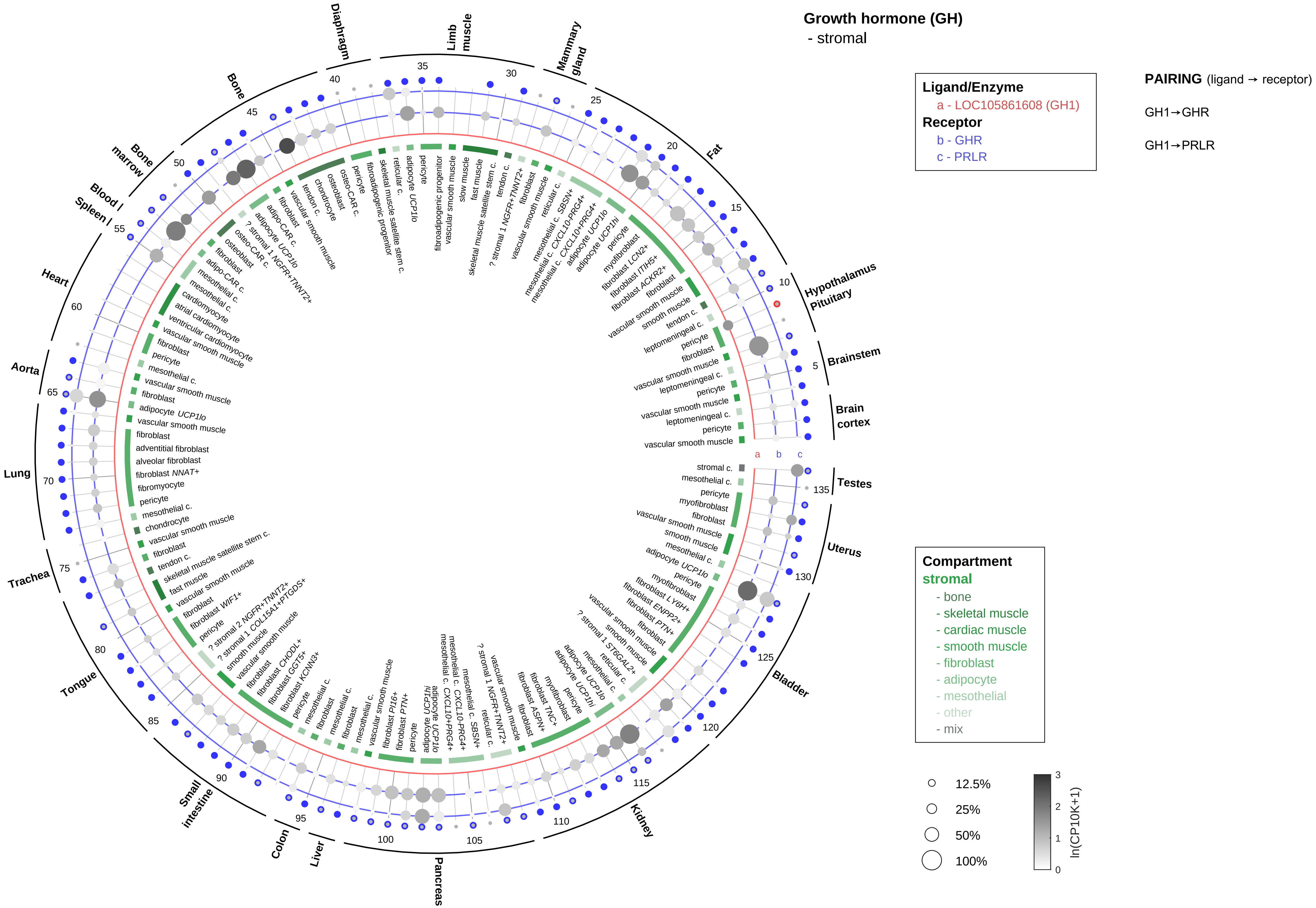
Growth hormone (GH)
- stromal
Ligand/Enzyme
a - LOC105861608 (GH1)
Receptor
b - GHR
c - PRLR
PAIRING (ligand → receptor)
GH1→GHR
GH1→PRLR
Compartment
stromal
- bone
- skeletal muscle
- cardiac muscle
- smooth muscle
- fibroblast
- adipocyte
- mesothelial
- other
- mix
12.5%
25%
50%
100%
ln(CP10K+1)
Hypothalamus Pituitary
Brainstem
Brain cortex
Testes
Uterus
Bladder
Kidney
Pancreas
Liver
Colon
Small intestine
Tongue
Trachea
Lung
Aorta
Heart
Spleen
Blood
Bone marrow
Bone
Diaphragm
Limb muscle
Mammary gland
Fat

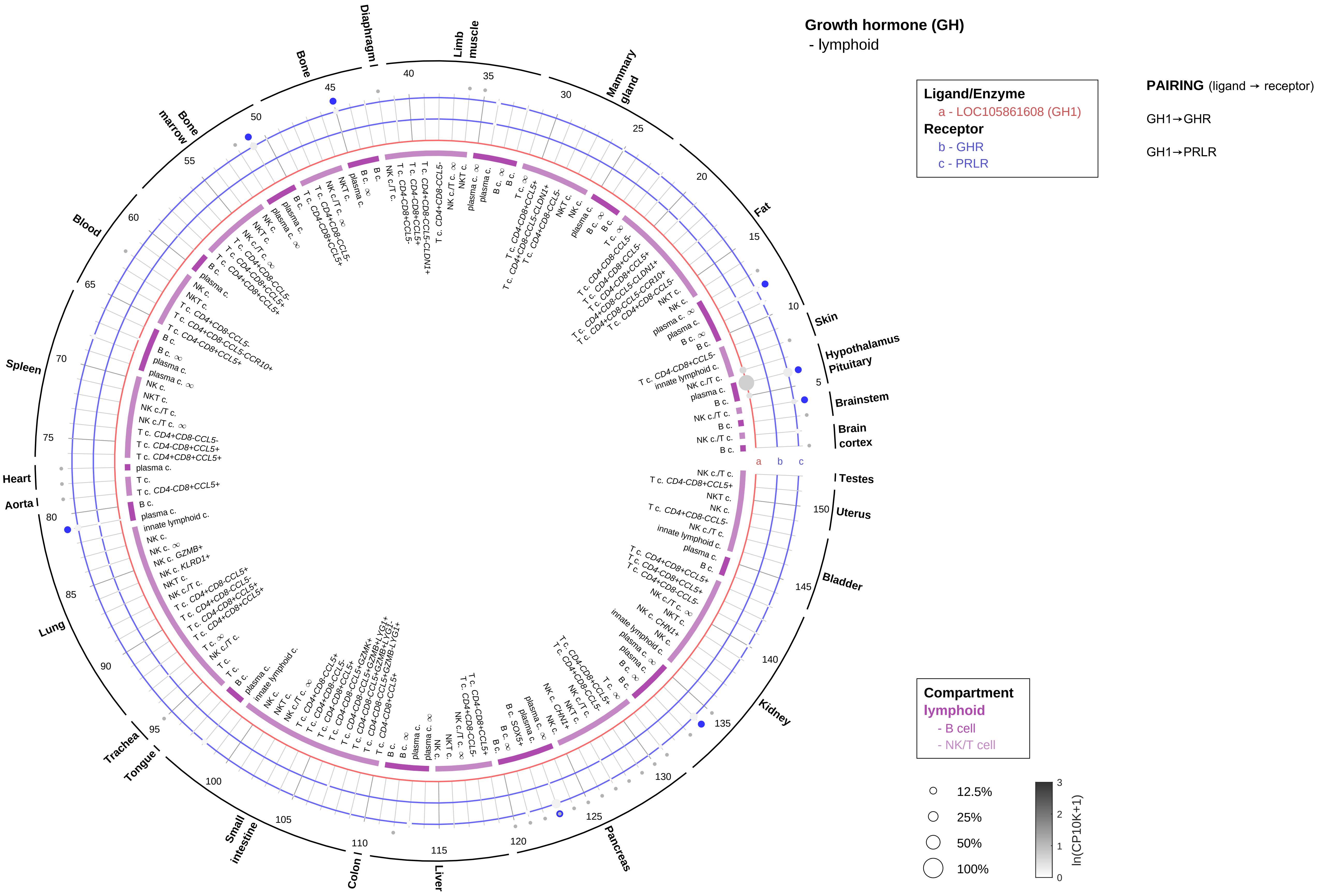

Growth hormone (GH)
- lymphoid
Ligand/Enzyme
a - LOC105861608 (GH1)
Receptor
b - GHR
c - PRLR
PAIRING (ligand → receptor)
GH1→GHR
GH1→PRLR
Compartment
lymphoid
- B cell
- NK/T cell
12.5%
25%
50%
100%
ln(CP10K+1)
Skin
Hypothalamus
Pituitary
Brainstem
Brain cortex
Testes
Uterus
Bladder
Kidney
Pancreas
Liver
Colon
Small intestine
Tongue
Trachea
Lung
Aorta
Heart
Spleen
Blood
Bone marrow
Bone
Diaphragm
Limb muscle
Mammary gland
Fat

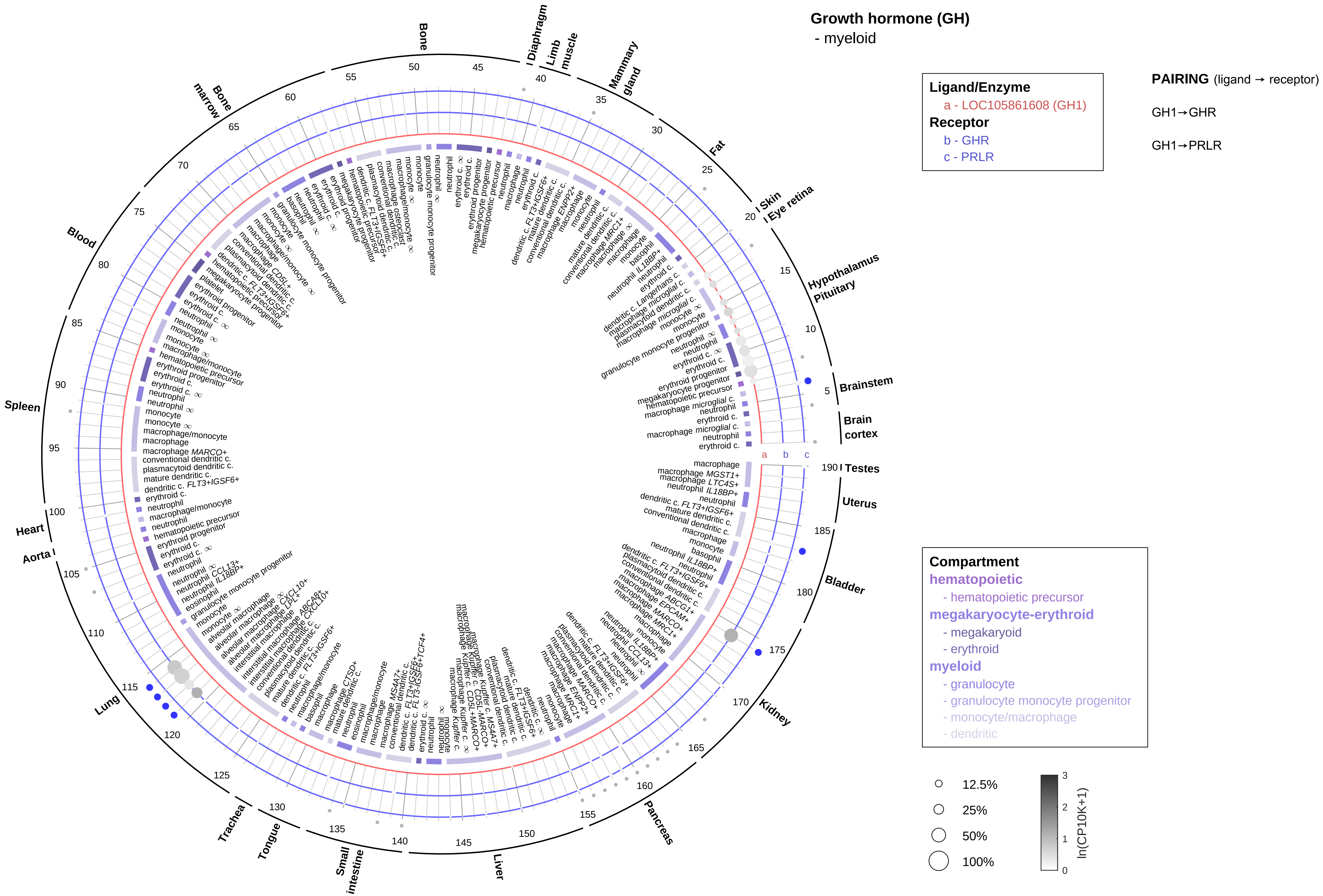

Growth hormone (GH)
- myeloid
Ligand/Enzyme
a - LOC105861608 (GH1)
Receptor
b - GHR
c - PRLR
PAIRING (ligand → receptor)
GH1→GHR
GH1→PRLR
Compartment
hematopoietic
- hematopoietic precursor
megakaryocyte-erythroid
- megakaryoid
- erythroid
myeloid
- granulocyte
- granulocyte monocyte progenitor
- monocyte/macrophage
- dendritic
12.5%
25%
50%
100%
ln(CP10K+1)
0
1
2
3
Bone
Diaphragm
Limb muscle
Mammary gland
Fat
Skin
Eye retina
Hypothalamus
Pituitary
Brainstem
Brain cortex
Testes
Uterus
Bladder
Kidney
Pancreas
Liver
Small intestine
Tongue
Trachea
Lung
Aorta
Heart
Spleen
Blood
Bone marrow
a
b
c

Growth hormone releasing hormone (GHRH)
- epithelial/neural/germ

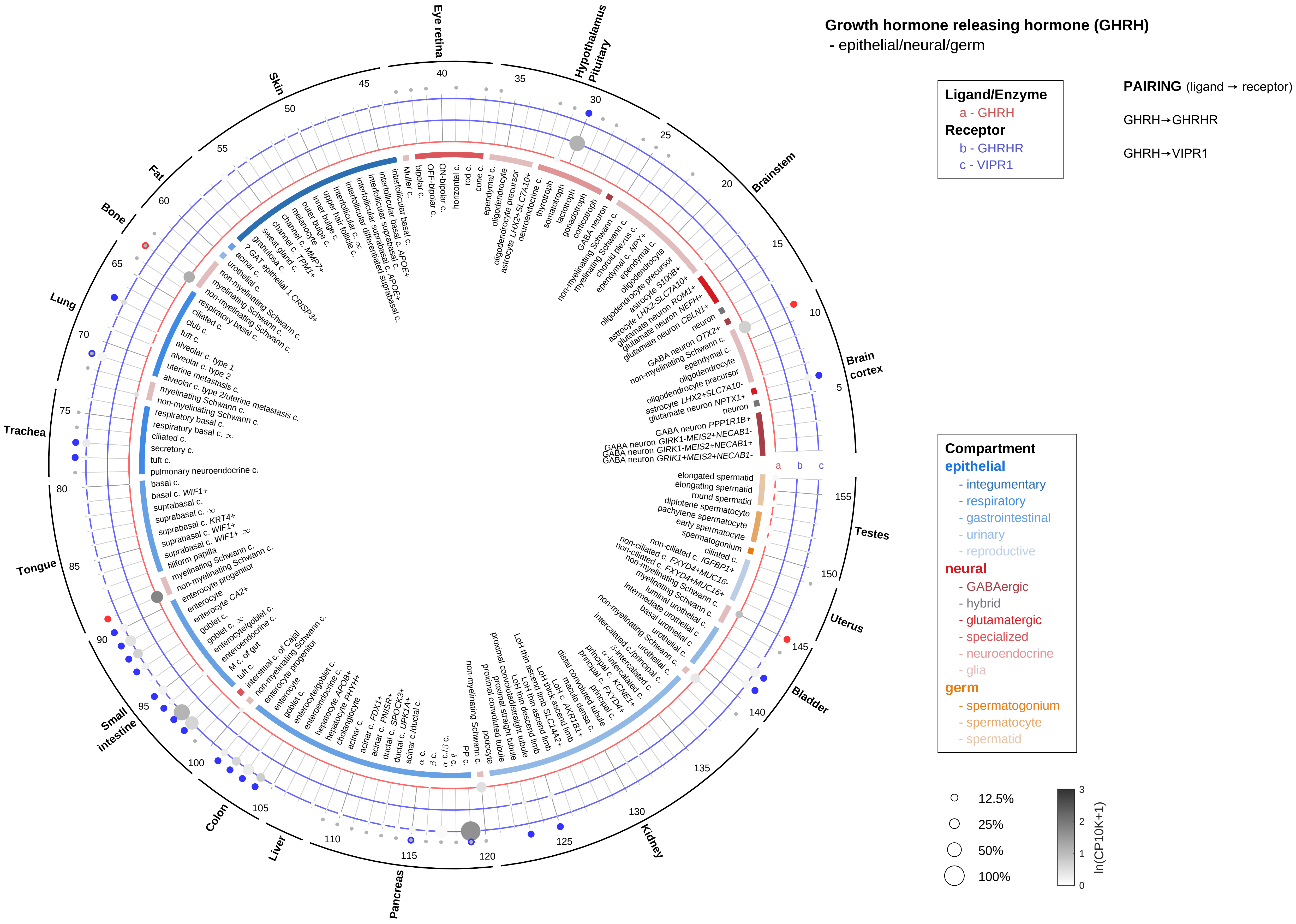

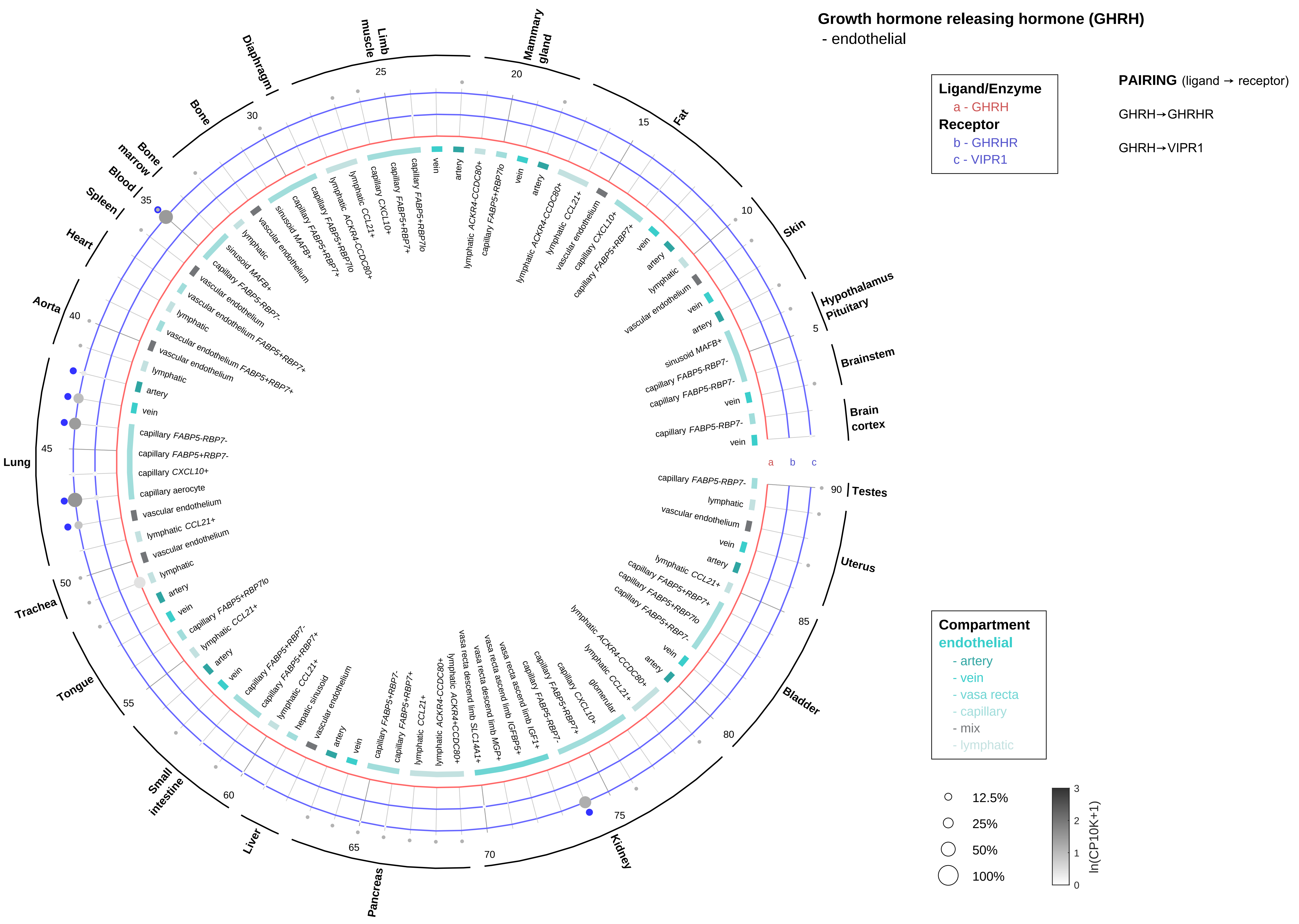
Growth hormone releasing hormone (GHRH)
- endothelial
Ligand/Enzyme
a - GHRH
Receptor
b - GHRHR
c - VIPR1
PAIRING (ligand → receptor)
GHRH→GHRHR
GHRH→VIPR1
Compartment
endothelial
- artery
- vein
- vasa recta
- capillary
- mix
- lymphatic
12.5%
25%
50%
100%
ln(CP10K+1)
Brain cortex
Brainstem
Hypothalamus Pituitary
Skin
Fat
Mammary gland
Limb muscle
Diaphragm
Bone
Bone marrow
Blood
Spleen
Heart
Aorta
Lung
Trachea
Tongue
Small intestine
Liver
Pancreas
Kidney
Bladder
Uterus
Testes

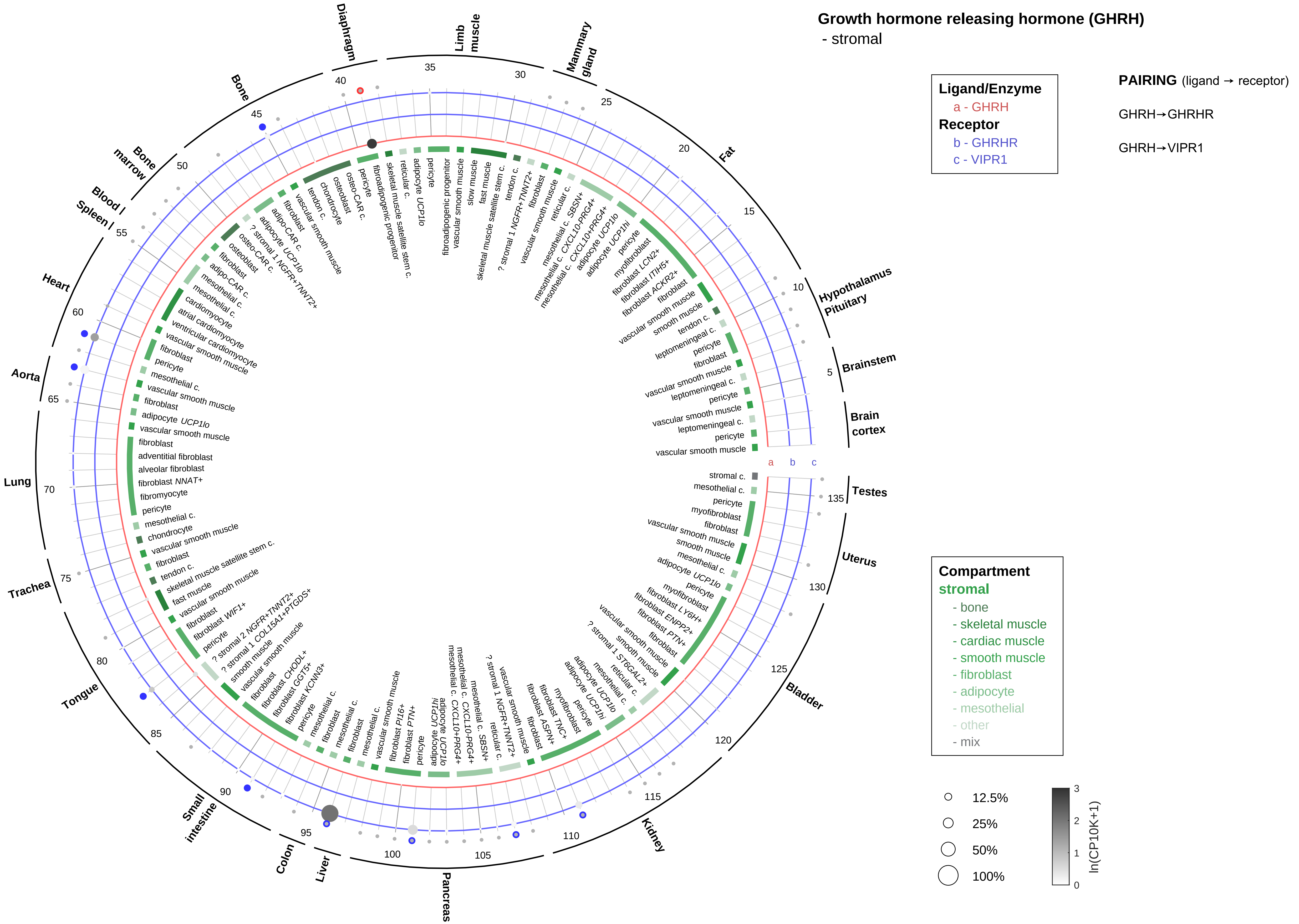
Growth hormone releasing hormone (GHRH)
- stromal
Ligand/Enzyme
a - GHRH
Receptor
b - GHRHR
c - VIPR1
PAIRING (ligand → receptor)
GHRH→GHRHR
GHRH→VIPR1
Compartment
stromal
- bone
- skeletal muscle
- cardiac muscle
- smooth muscle
- fibroblast
- adipocyte
- mesothelial
- other
- mix
12.5%
25%
50%
100%
ln(CP10K+1)
Brain cortex
Brainstem
Hypothalamus Pituitary
Fat
Mammary gland
Limb muscle
Diaphragm
Bone
Bone marrow
Blood / Spleen /
Heart
Aorta
Lung
Trachea
Tongue
Small intestine
Colon
Liver
Pancreas
Kidney
Bladder
Uterus
Testes

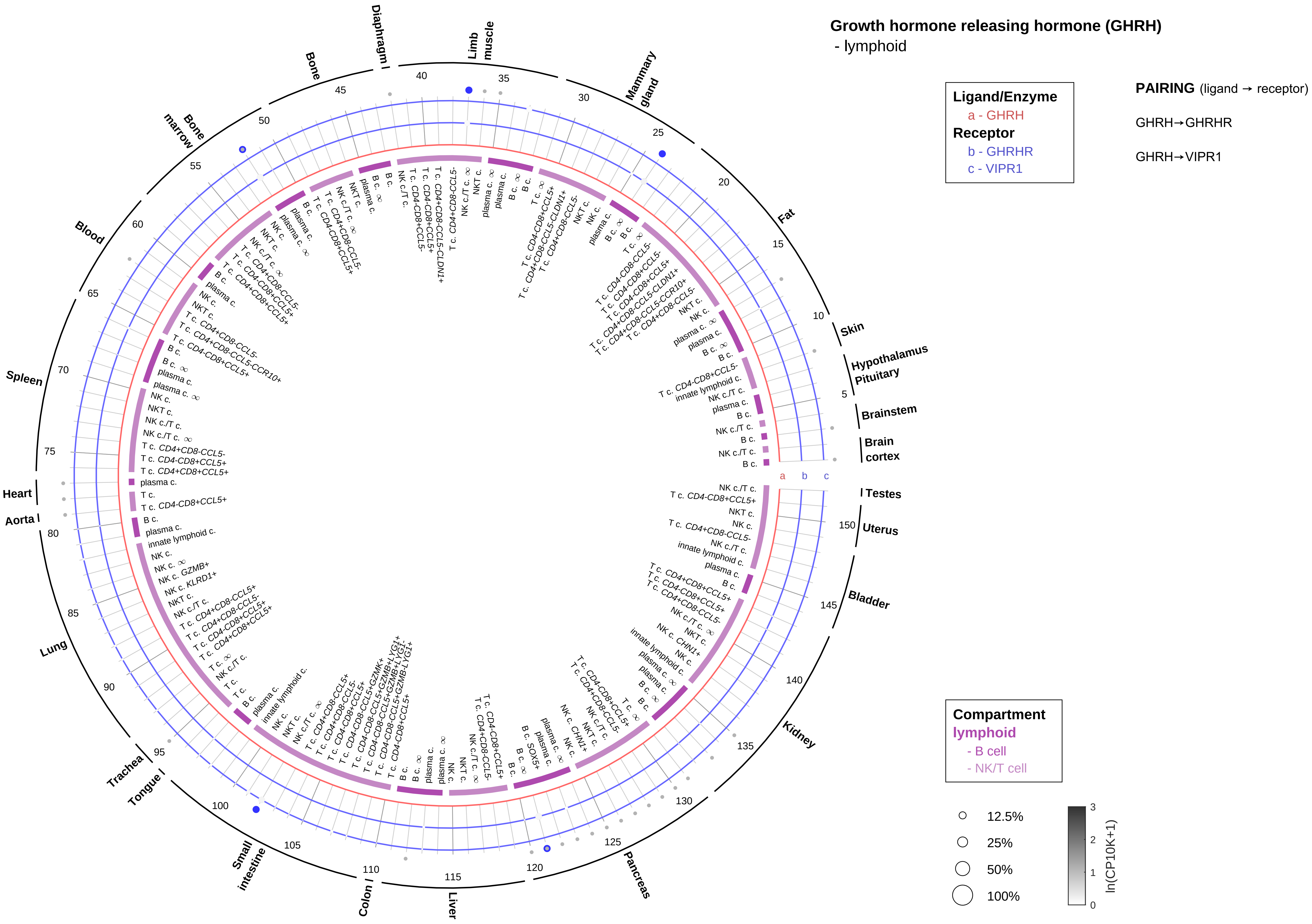

Growth hormone releasing hormone (GHRH)
- lymphoid
Ligand/Enzyme
a - GHRH
Receptor
b - GHRHR
c - VIPR1
PAIRING (ligand → receptor)
GHRH→GHRHR
GHRH→VIPR1
Compartment
lymphoid
- B cell
- NK/T cell
12.5%
25%
50%
100%
ln(CP10K+1)
Brain cortex
Brainstem
Hypothalamus Pituitary
Skin
Fat
Mammary gland
Limb muscle
Diaphragm
Bone
Bone marrow
Blood
Spleen
Heart
Aorta
Lung
Trachea
Tongue
Small intestine
Colon
Liver
Pancreas
Kidney
Bladder
Uterus
Testes

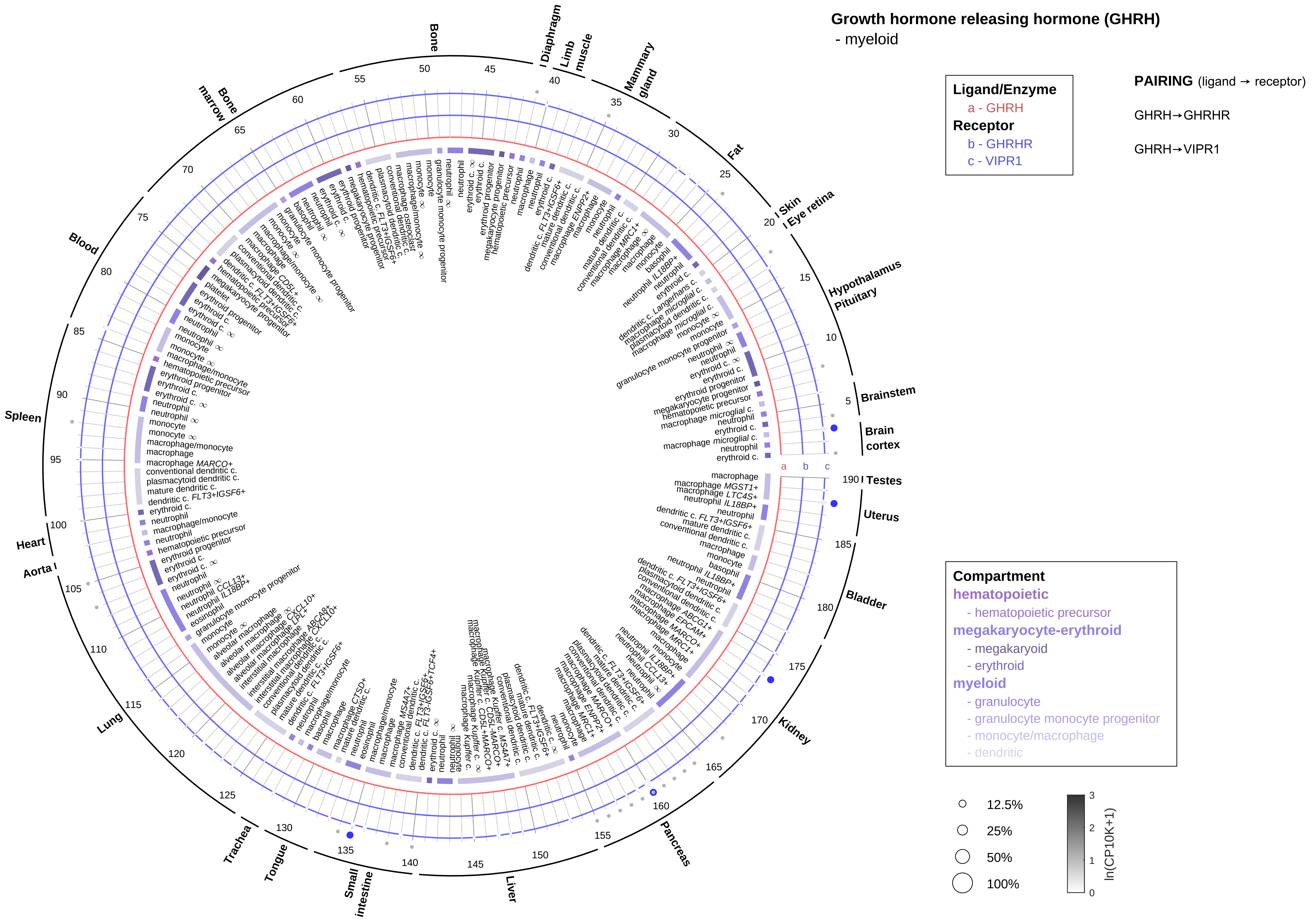
Growth hormone releasing hormone (GHRH)
- myeloid
Ligand/Enzyme
a - GHRH
Receptor
b - GHRHR
c - VIPR1
PAIRING (ligand → receptor)
GHRH→GHRHR
GHRH→VIPR1
Compartment
hematopoietic
- hematopoietic precursor
megakaryocyte-erythroid
- megakaryoid
- erythroid
myeloid
- granulocyte
- granulocyte monocyte progenitor
- monocyte/macrophage
- dendritic
12.5%
25%
50%
100%
ln(CP10K+1)
Bone
Diaphragm
Limb muscle
Mammary gland
Fat
Skin
Eye retina
Hypothalamus
Pituitary
Brainstem
Brain cortex
Testes
Uterus
Bladder
Kidney
Pancreas
Liver
Small intestine
Tongue
Trachea
Lung
Aorta
Heart
Spleen
Blood
Bone marrow

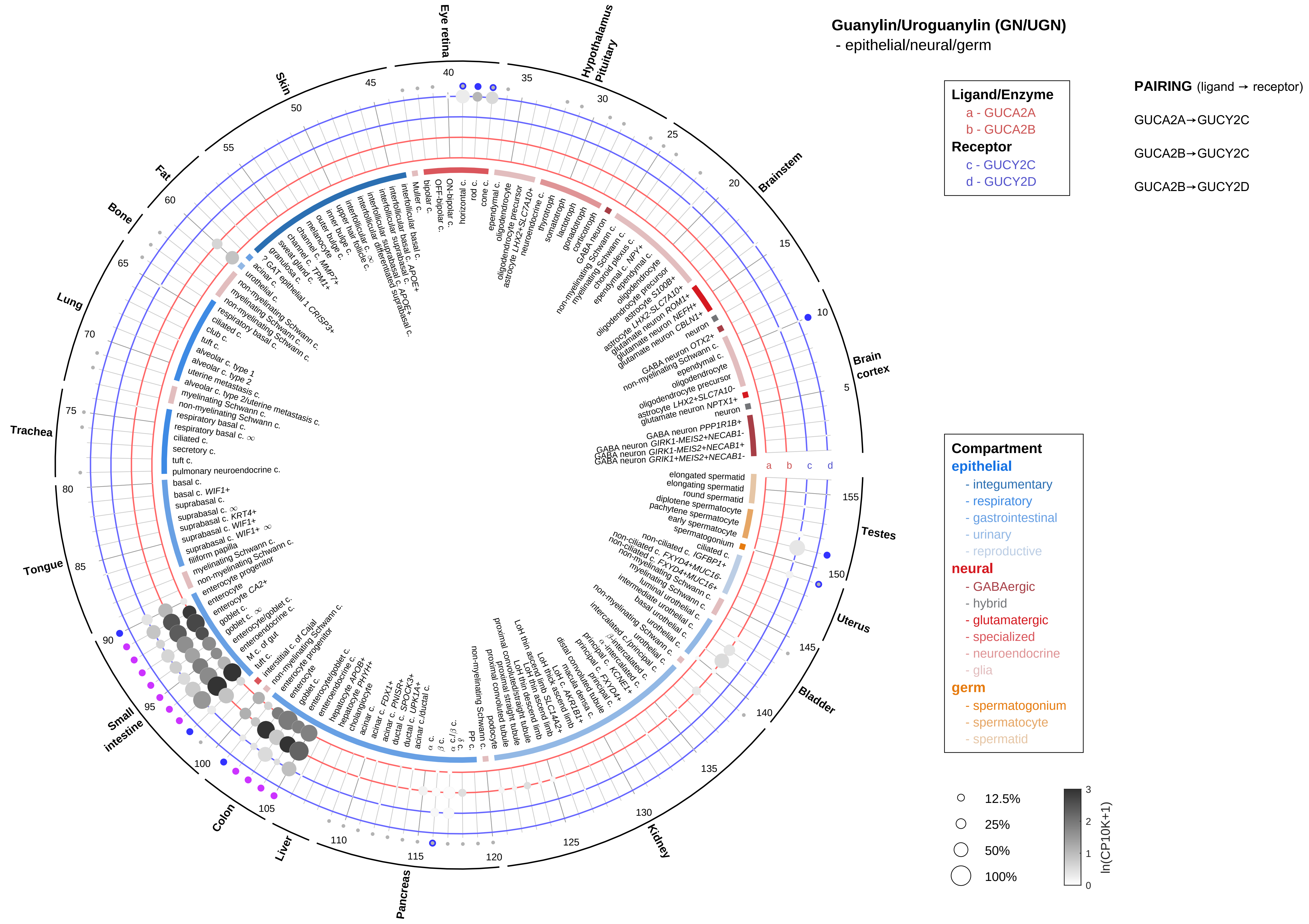
Guanylin/Uroguanylin (GN/UGN)
- epithelial/neural/germ
Ligand/Enzyme
a - GUCA2A
b - GUCA2B
Receptor
c - GUCY2C
d - GUCY2D
PAIRING (ligand → receptor)
GUCA2A→GUCY2C
GUCA2B→GUCY2C
GUCA2B→GUCY2D
Compartment
epithelial
- integumentary
- respiratory
- gastrointestinal
- urinary
- reproductive
neural
- GABAergic
- hybrid
- glutamatergic
- specialized
- neuroendocrine
- glia
germ
- spermatogonium
- spermatocyte
- spermatid
12.5%
25%
50%
100%
ln(CP10K+1)
Eye retina
Hypothalamus Pituitary
Brainstem
Brain cortex
Testes
Uterus
Bladder
Kidney
Pancreas
Liver
Colon
Small intestine
Tongue
Trachea
Lung
Bone
Fat
Skin

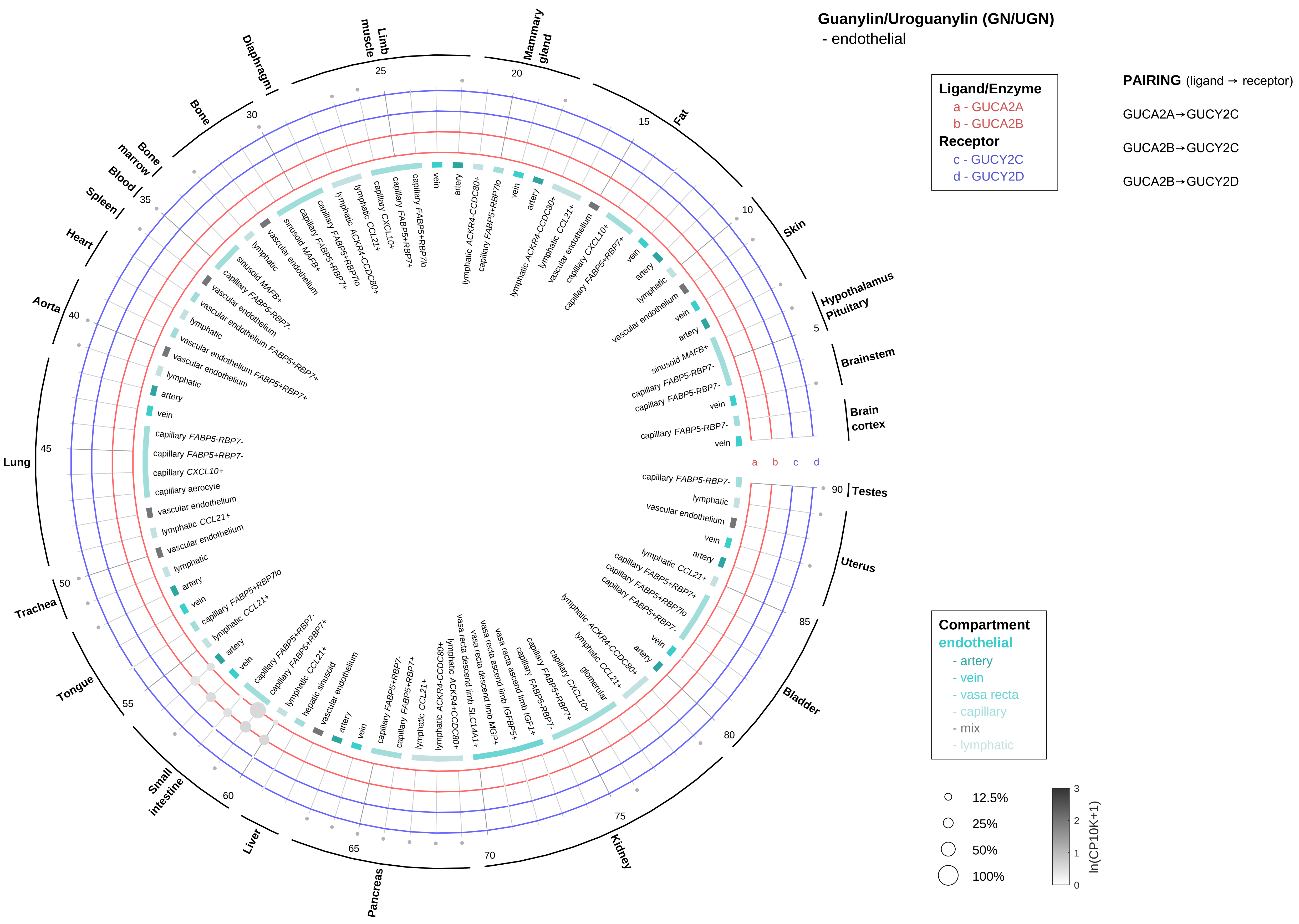

Guanylin/Uroguanylin (GN/UGN)
- endothelial
Ligand/Enzyme
a - GUCA2A
b - GUCA2B
Receptor
c - GUCY2C
d - GUCY2D
PAIRING (ligand → receptor)
GUCA2A→GUCY2C
GUCA2B→GUCY2C
GUCA2B→GUCY2D
Compartment
endothelial
- artery
- vein
- vasa recta
- capillary
- mix
- lymphatic
12.5%
25%
50%
100%
ln(CP10K+1)
Testes
Uterus
Bladder
Kidney
Pancreas
Liver
Small intestine
Tongue
Trachea
Lung
Aorta
Heart
Spleen
Blood
Bone marrow
Bone
Diaphragm
Limb muscle
Mammary gland
Fat
Skin
Hypothalamus Pituitary
Brainstem
Brain cortex

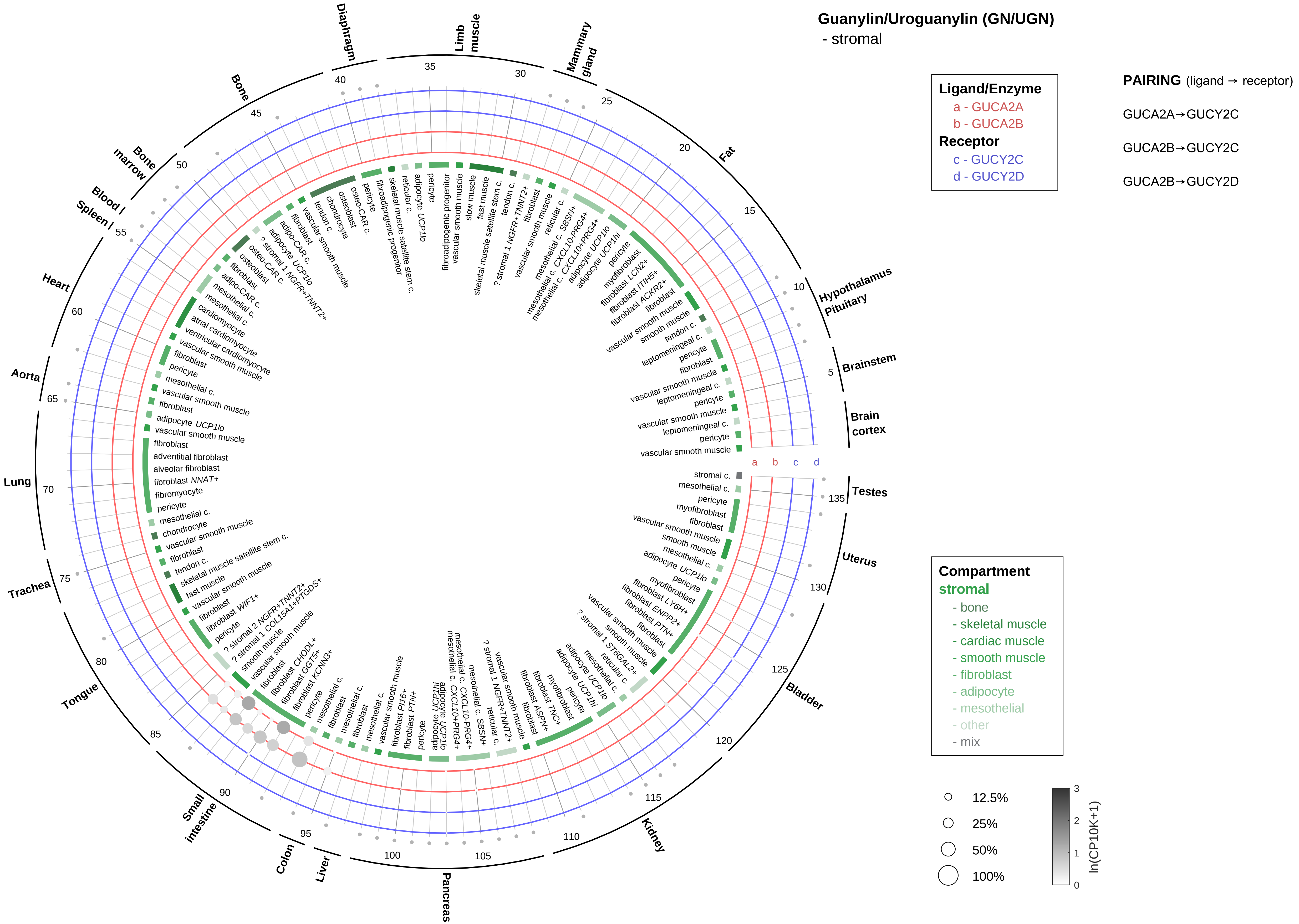

Guanylin/Uroguanylin (GN/UGN)
- stromal
Ligand/Enzyme
a - GUCA2A
b - GUCA2B
Receptor
c - GUCY2C
d - GUCY2D
PAIRING (ligand → receptor)
GUCA2A→GUCY2C
GUCA2B→GUCY2C
GUCA2B→GUCY2D
Compartment
stromal
- bone
- skeletal muscle
- cardiac muscle
- smooth muscle
- fibroblast
- adipocyte
- mesothelial
- other
- mix
12.5%
25%
50%
100%
ln(CP10K+1)
0
1
2
3
a
b
c
d
Brain cortex
Brainstem
Hypothalamus Pituitary
Fat
Mammary gland
Limb muscle
Diaphragm
Bone
Bone marrow
Blood
Spleen
Heart
Aorta
Lung
Trachea
Tongue
Small intestine
Colon
Liver
Pancreas
Kidney
Bladder
Uterus
Testes

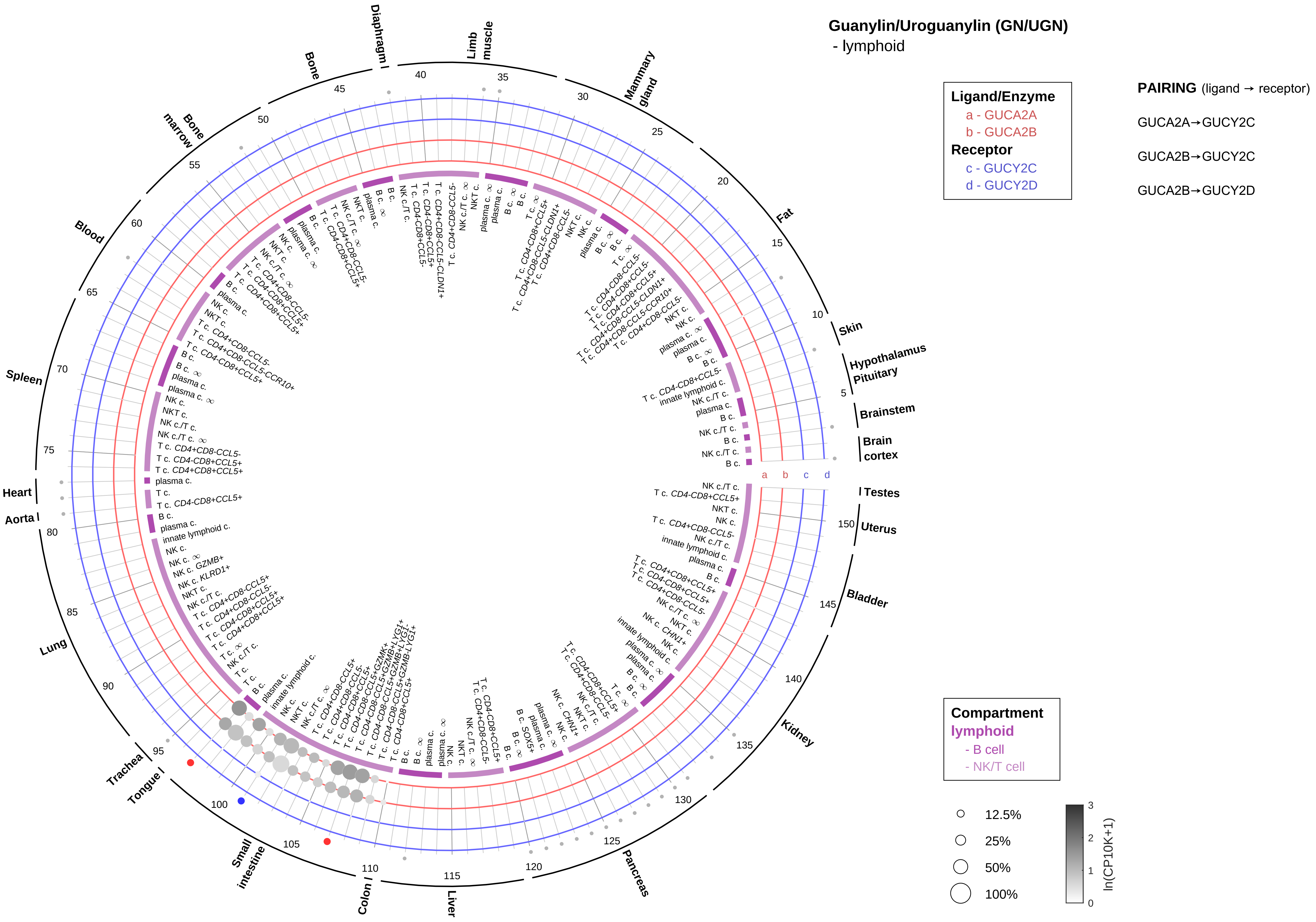
Guanylin/Uroguanylin (GN/UGN)
- lymphoid
Ligand/Enzyme
a - GUCA2A
b - GUCA2B
Receptor
c - GUCY2C
d - GUCY2D
PAIRING (ligand → receptor)
GUCA2A→GUCY2C
GUCA2B→GUCY2C
GUCA2B→GUCY2D
Compartment
lymphoid
- B cell
- NK/T cell
12.5%
25%
50%
100%
ln(CP10K+1)
Limb muscle
Mammary gland
Fat
Skin
Hypothalamus
Pituitary
Brainstem
Brain cortex
Testes
Uterus
Bladder
Kidney
Pancreas
Liver
Colon
Small intestine
Tongue
Trachea
Lung
Aorta
Heart
Spleen
Blood
Bone marrow
Bone
Diaphragm

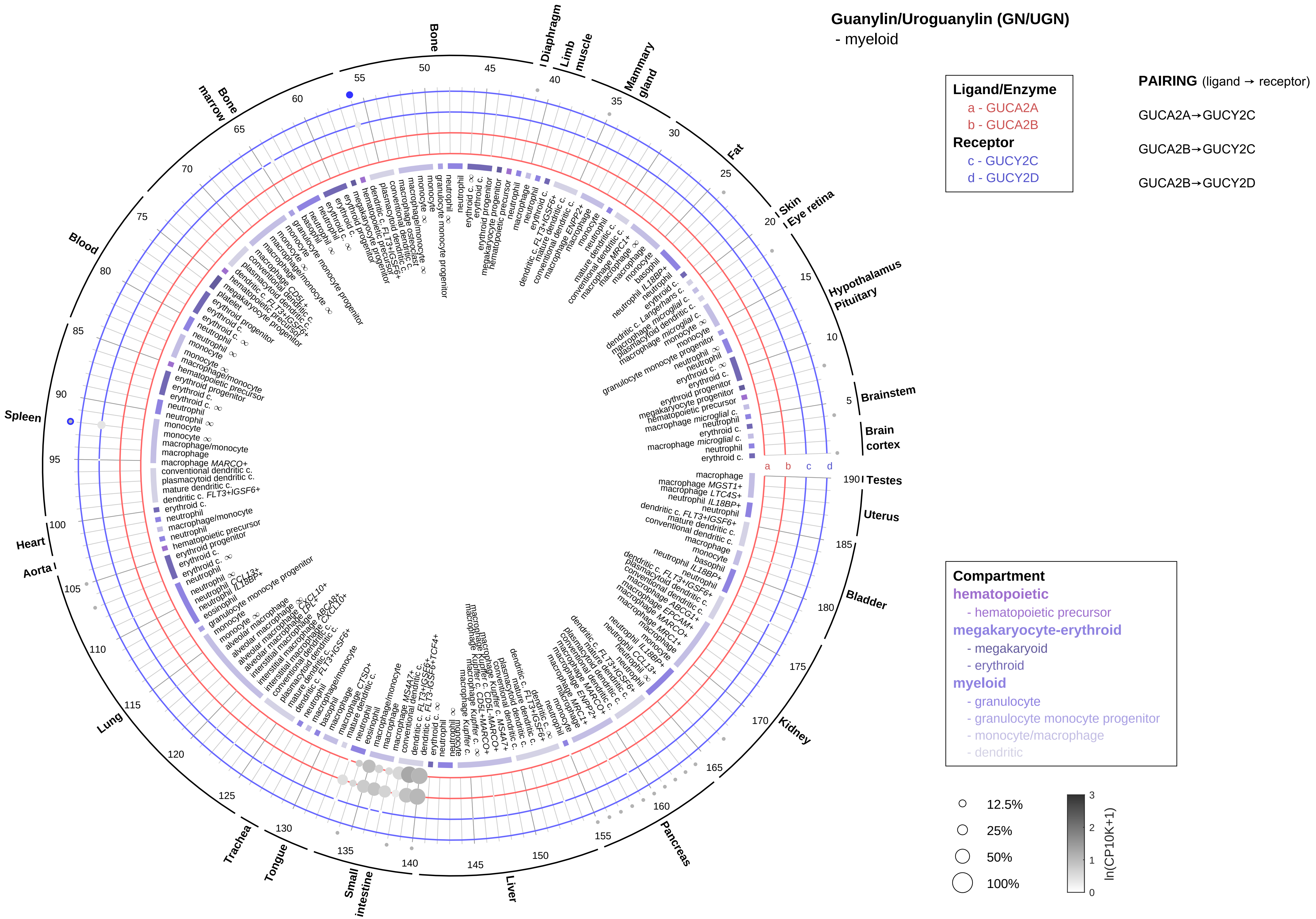
Guanylin/Uroguanylin (GN/UGN)
- myeloid
Ligand/Enzyme
a - GUCA2A
b - GUCA2B
Receptor
c - GUCY2C
d - GUCY2D
PAIRING (ligand → receptor)
GUCA2A→GUCY2C
GUCA2B→GUCY2C
GUCA2B→GUCY2D
Compartment
hematopoietic
- hematopoietic precursor
megakaryocyte-erythroid
- megakaryoid
- erythroid
myeloid
- granulocyte
- granulocyte monocyte progenitor
- monocyte/macrophage
- dendritic
12.5%
25%
50%
100%
ln(CP10K+1)
Bone
Diaphragm
Limb muscle
Mammary gland
Fat
Skin
Eye retina
Hypothalamus
Pituitary
Brainstem
Brain cortex
Testes
Uterus
Bladder
Kidney
Pancreas
Liver
Small intestine
Tongue
Trachea
Lung
Aorta
Heart
Spleen
Blood
Bone marrow

Hepcidin (HAMP)
- epithelial/neural/germ

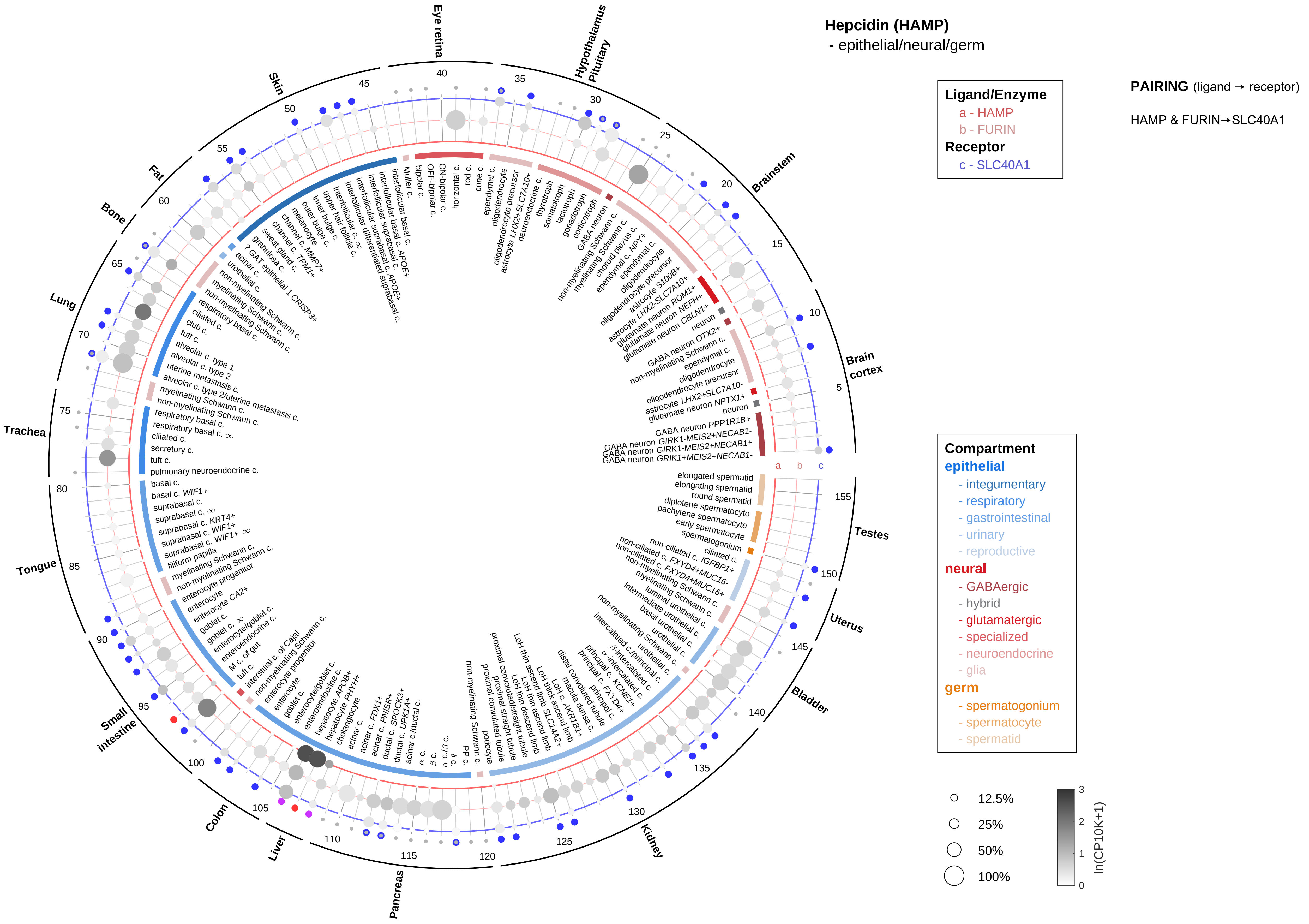

Hepcidin (HAMP)
- endothelial

PAIRING (ligand → receptor)

HAMP & FURIN→SLC40A1

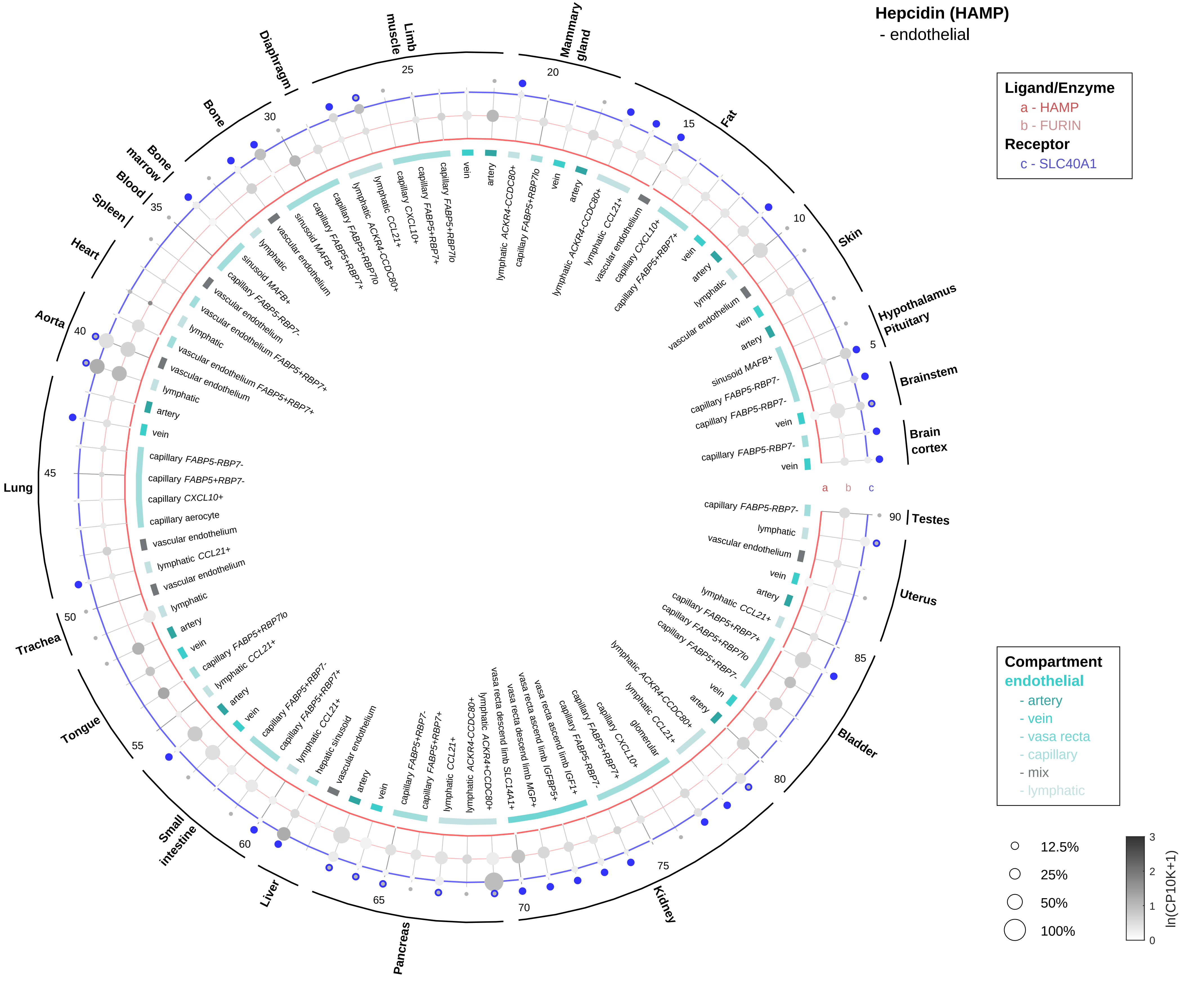

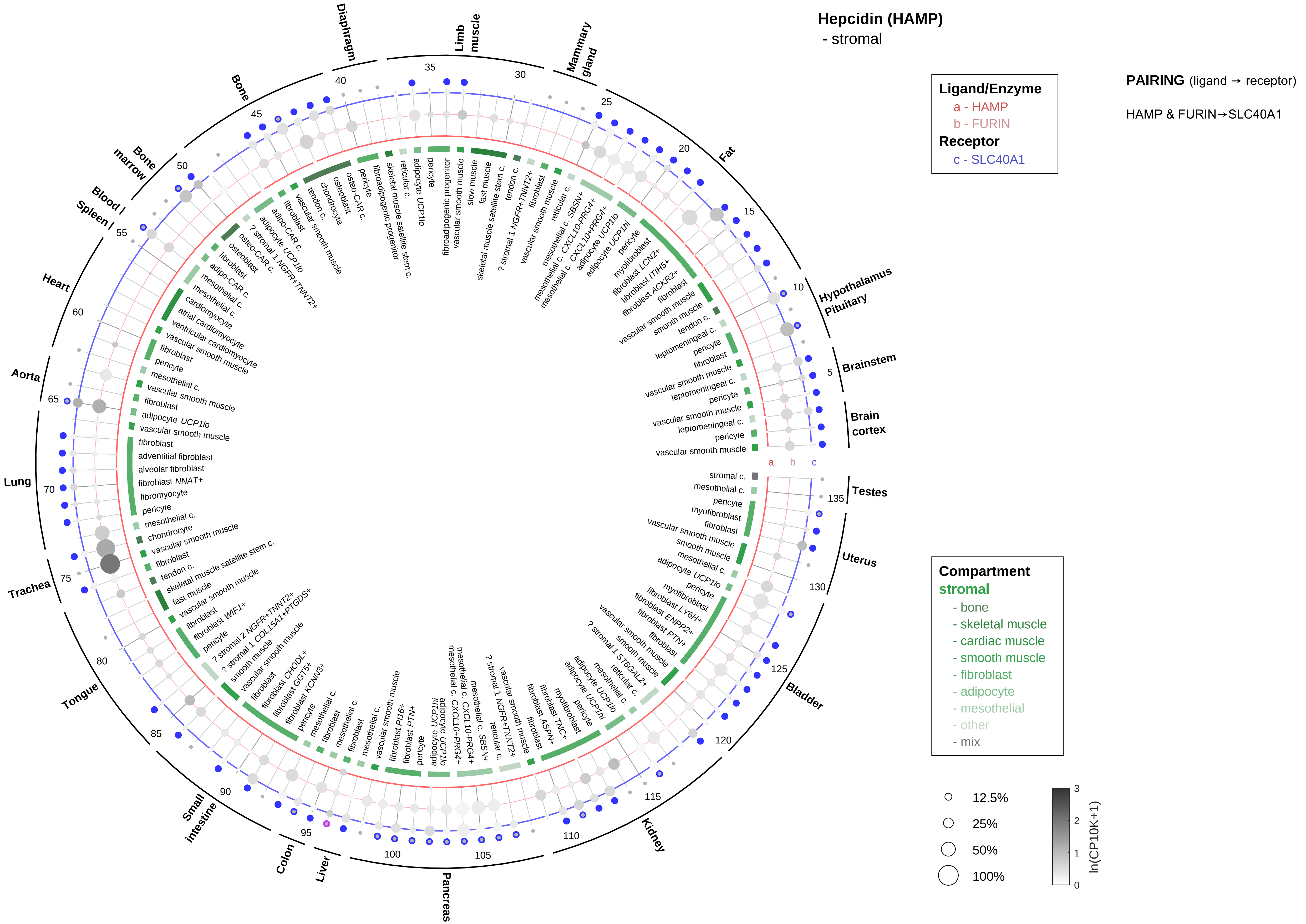

Hepcidin (HAMP)
- stromal
Ligand/Enzyme
a - HAMP
b - FURIN
Receptor
c - SLC40A1
PAIRING (ligand → receptor)
HAMP & FURIN→SLC40A1
Compartment
stromal
- bone
- skeletal muscle
- cardiac muscle
- smooth muscle
- fibroblast
- adipocyte
- mesothelial
- other
- mix
12.5%
25%
50%
100%
ln(CP10K+1)
Brain cortex
Brainstem
Hypothalamus Pituitary
Fat
Mammary gland
Limb muscle
Diaphragm
Bone
Bone marrow
Blood / Spleen /
Heart
Aorta
Lung
Trachea
Tongue
Small intestine
Colon
Liver
Pancreas
Kidney
Bladder
Uterus
Testes

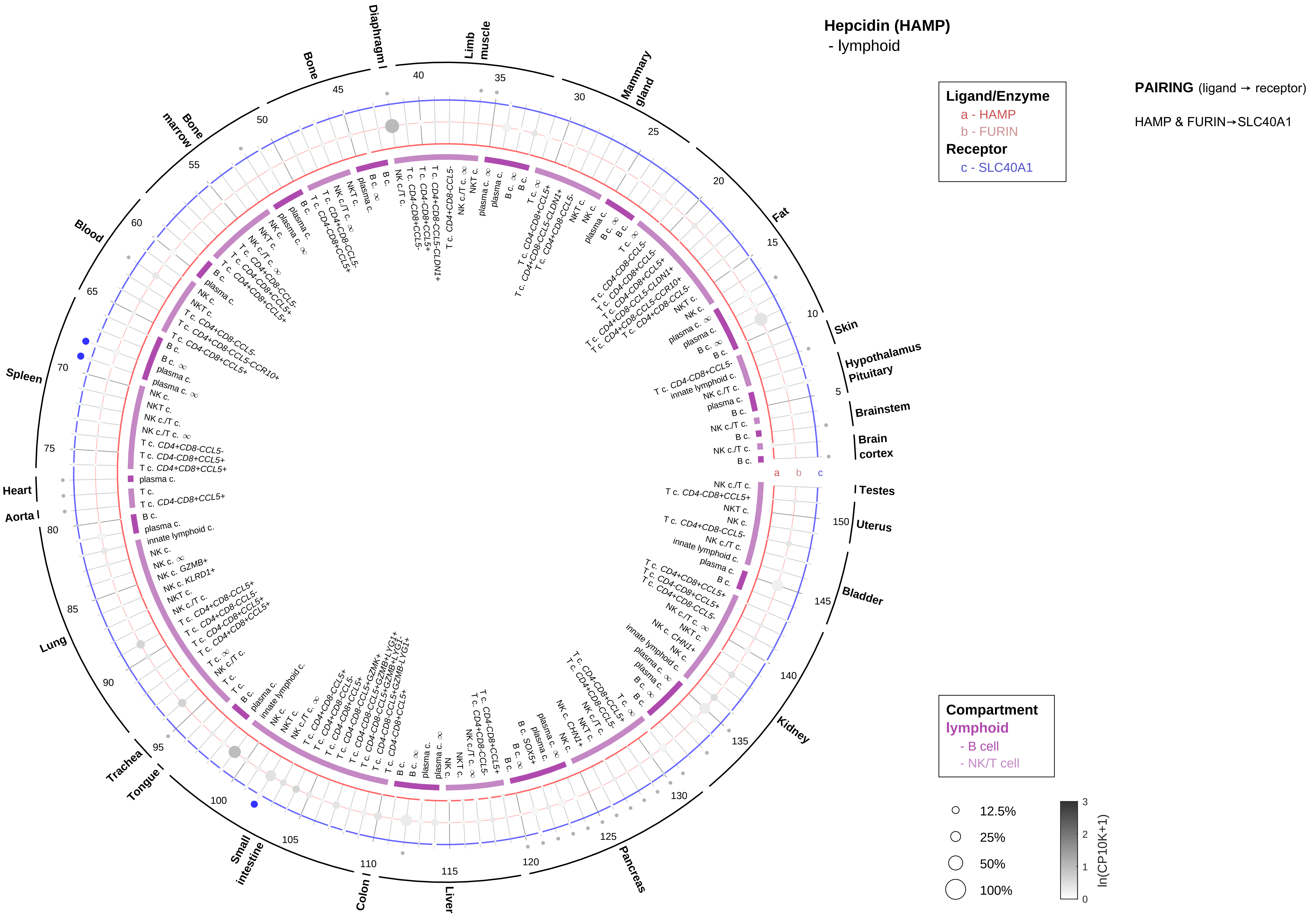
Hepcidin (HAMP)
- lymphoid
Ligand/Enzyme
a - HAMP
b - FURIN
Receptor
c - SLC40A1
PAIRING (ligand → receptor)
HAMP & FURIN→SLC40A1
Compartment
lymphoid
- B cell
- NK/T cell
12.5%
25%
50%
100%
ln(CP10K+1)
Skin
Hypothalamus
Pituitary
Brainstem
Brain cortex
Testes
Uterus
Bladder
Kidney
Pancreas
Liver
Colon
Small intestine
Tongue
Trachea
Lung
Aorta
Heart
Spleen
Blood
Bone marrow
Bone
Diaphragm
Limb muscle
Mammary gland
Fat

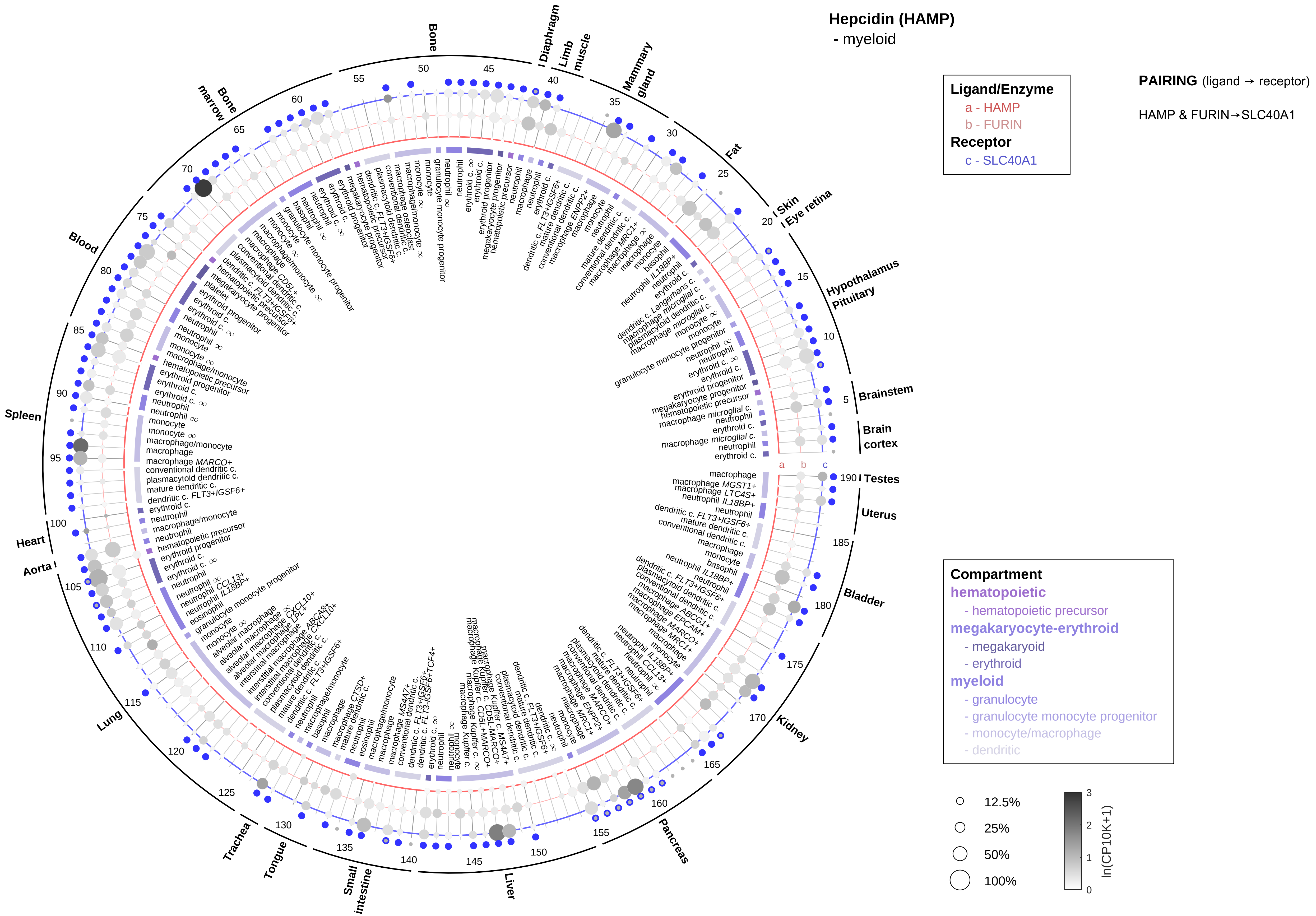
Hepcidin (HAMP)
- myeloid
Ligand/Enzyme
a - HAMP
b - FURIN
Receptor
c - SLC40A1
PAIRING (ligand → receptor)
HAMP & FURIN→SLC40A1
Compartment
hematopoietic
- hematopoietic precursor
megakaryocyte-erythroid
- megakaryoid
- erythroid
myeloid
- granulocyte
- granulocyte monocyte progenitor
- monocyte/macrophage
- dendritic
12.5%
25%
50%
100%
ln(CP10K+1)
Bone
Diaphragm
Limb muscle
Mammary gland
Fat
Skin
Eye retina
Hypothalamus
Pituitary
Brainstem
Brain cortex
Testes
Uterus
Bladder
Kidney
Pancreas
Liver
Small intestine
Tongue
Trachea
Lung
Aorta
Heart
Spleen
Blood
Bone marrow

Hypocretin (Orexin)
- epithelial/neural/germ

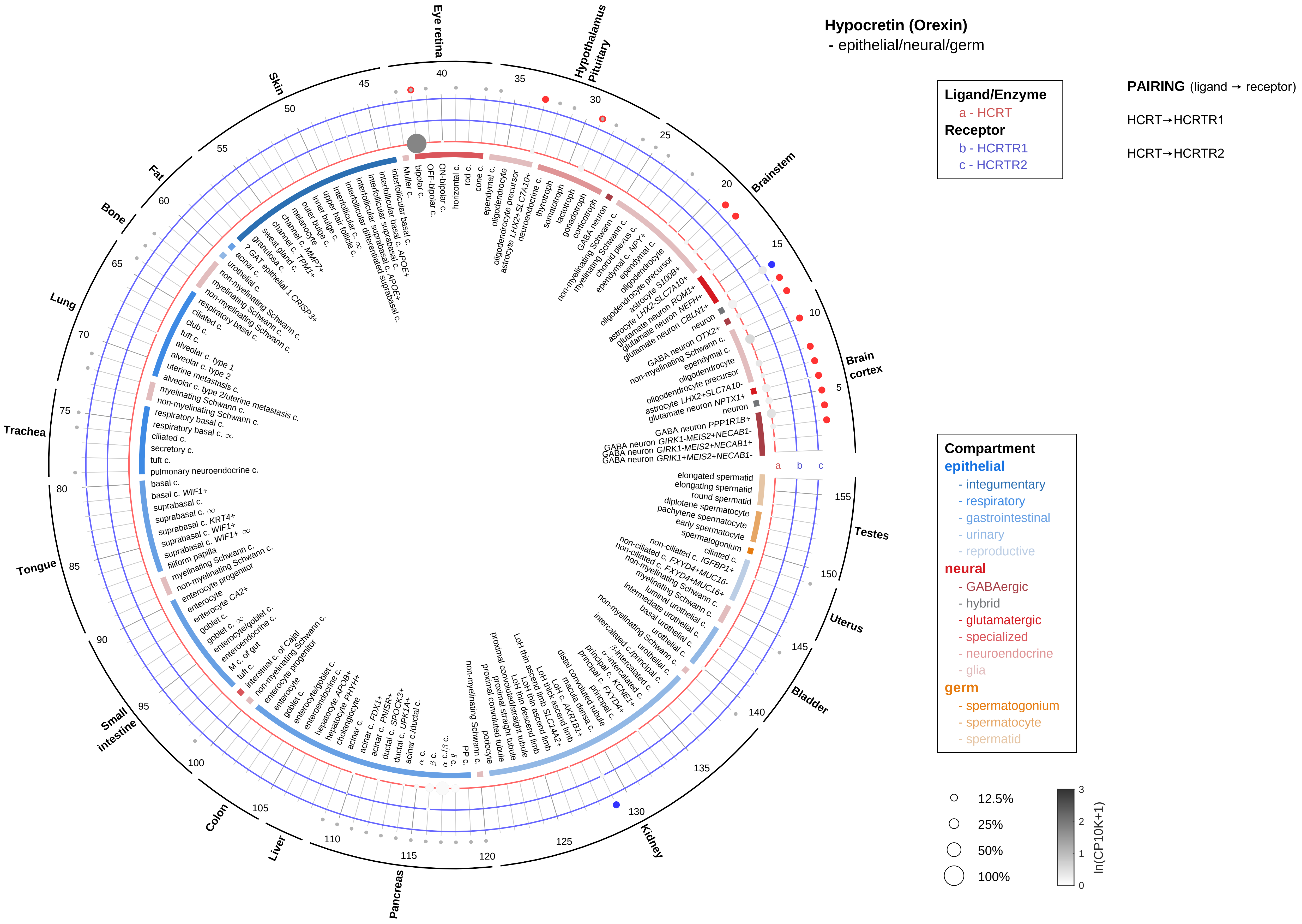

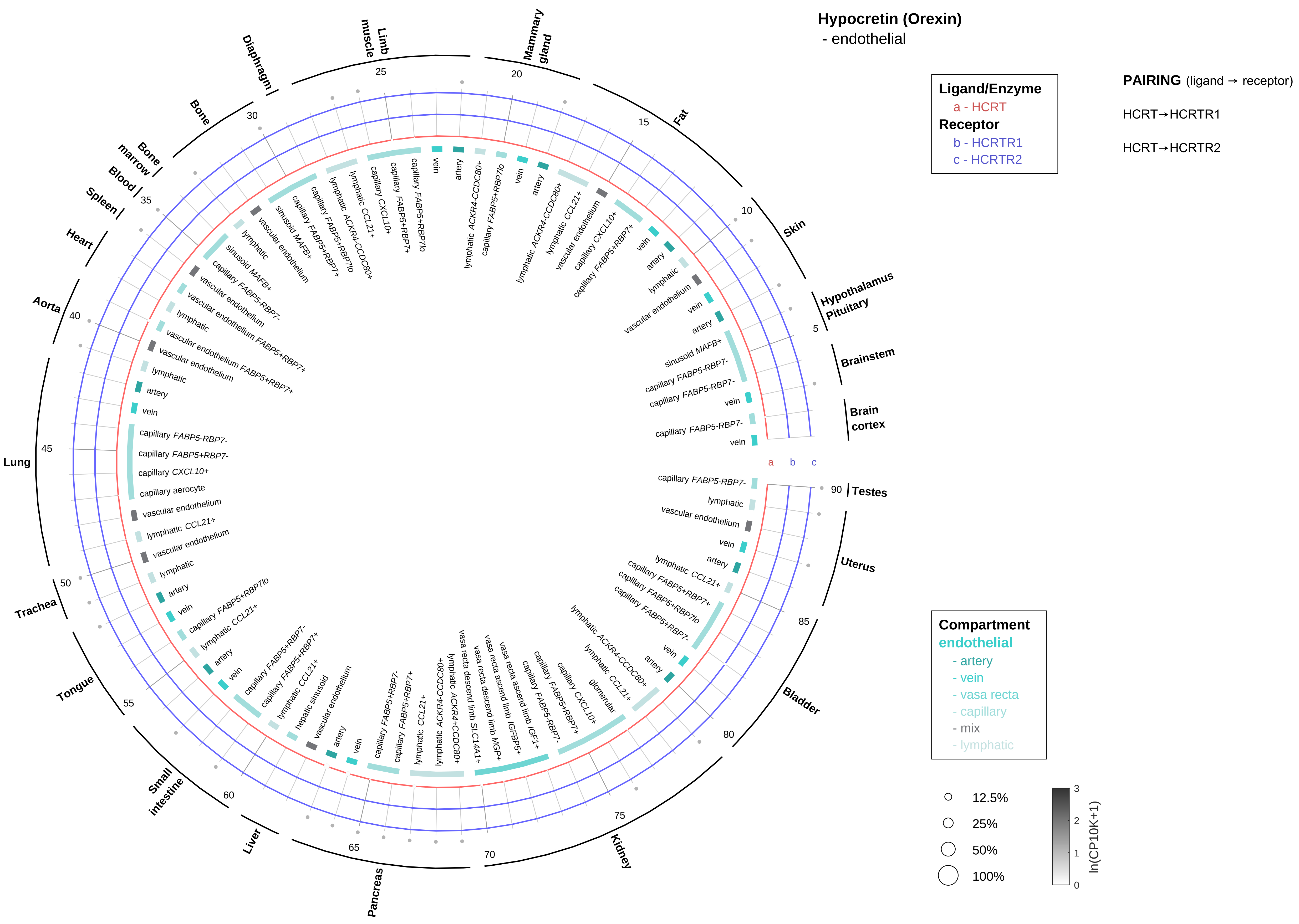

Hypocretin (Orexin)
- endothelial
Ligand/Enzyme
a - HCRT
Receptor
b - HCRTR1
c - HCRTR2
PAIRING (ligand → receptor)
HCRT→HCRTR1
HCRT→HCRTR2
Compartment
endothelial
- artery
- vein
- vasa recta
- capillary
- mix
- lymphatic
12.5%
25%
50%
100%
ln(CP10K+1)
Brain cortex
Brainstem
Hypothalamus Pituitary
Skin
Fat
Mammary gland
Limb muscle
Diaphragm
Bone
Bone marrow
Blood
Spleen
Heart
Aorta
Lung
Trachea
Tongue
Small intestine
Liver
Pancreas
Kidney
Bladder
Uterus
Testes

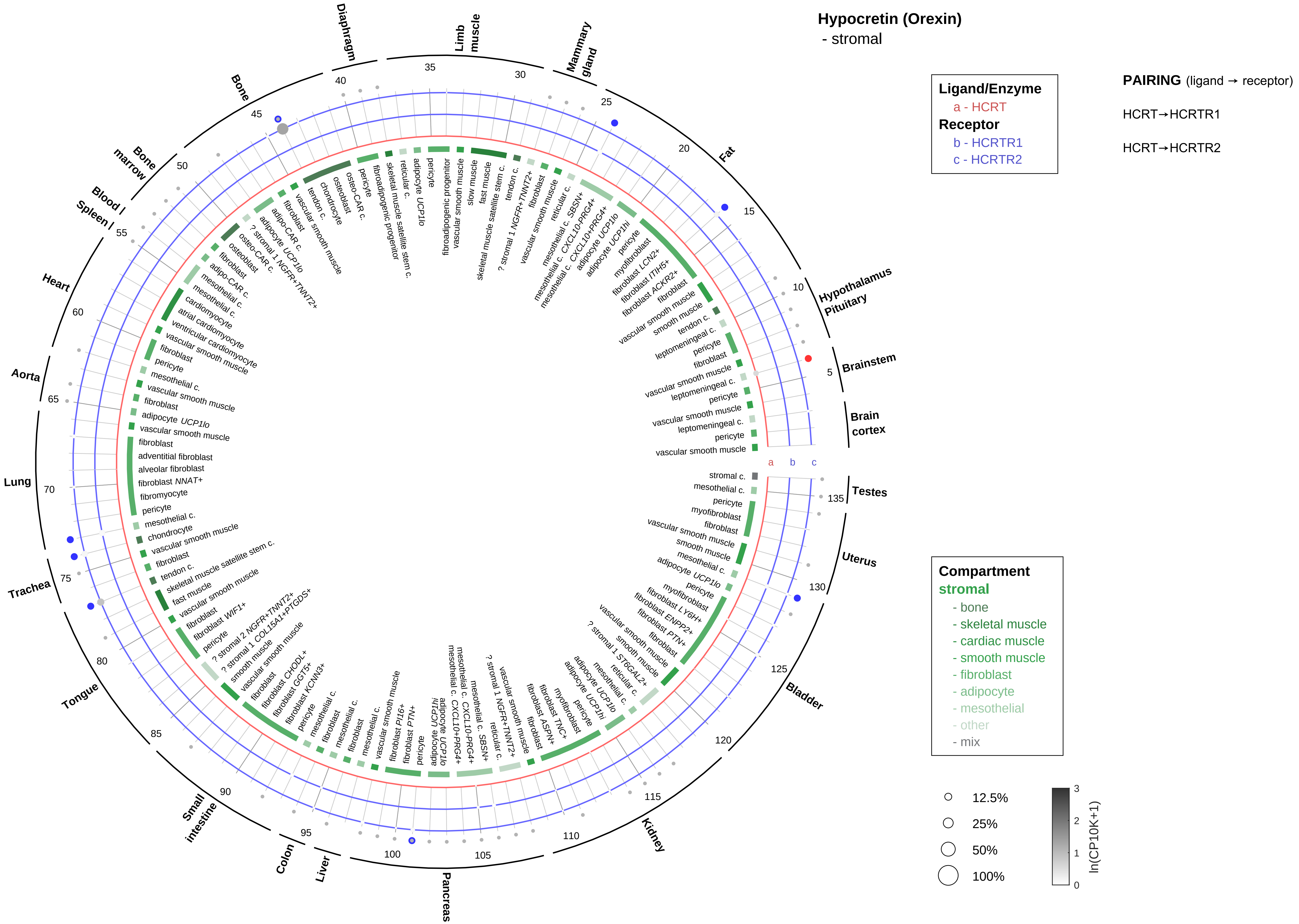

Hypocretin (Orexin)
- stromal
Ligand/Enzyme
a - HCRT
Receptor
b - HCRTR1
c - HCRTR2
PAIRING (ligand → receptor)
HCRT→HCRTR1
HCRT→HCRTR2
Compartment
stromal
- bone
- skeletal muscle
- cardiac muscle
- smooth muscle
- fibroblast
- adipocyte
- mesothelial
- other
- mix
12.5%
25%
50%
100%
ln(CP10K+1)
Brain cortex
Brainstem
Hypothalamus Pituitary
Fat
Mammary gland
Limb muscle
Diaphragm
Bone
Bone marrow
Blood / Spleen
Heart
Aorta
Lung
Trachea
Tongue
Small intestine
Colon
Liver
Pancreas
Kidney
Bladder
Uterus
Testes

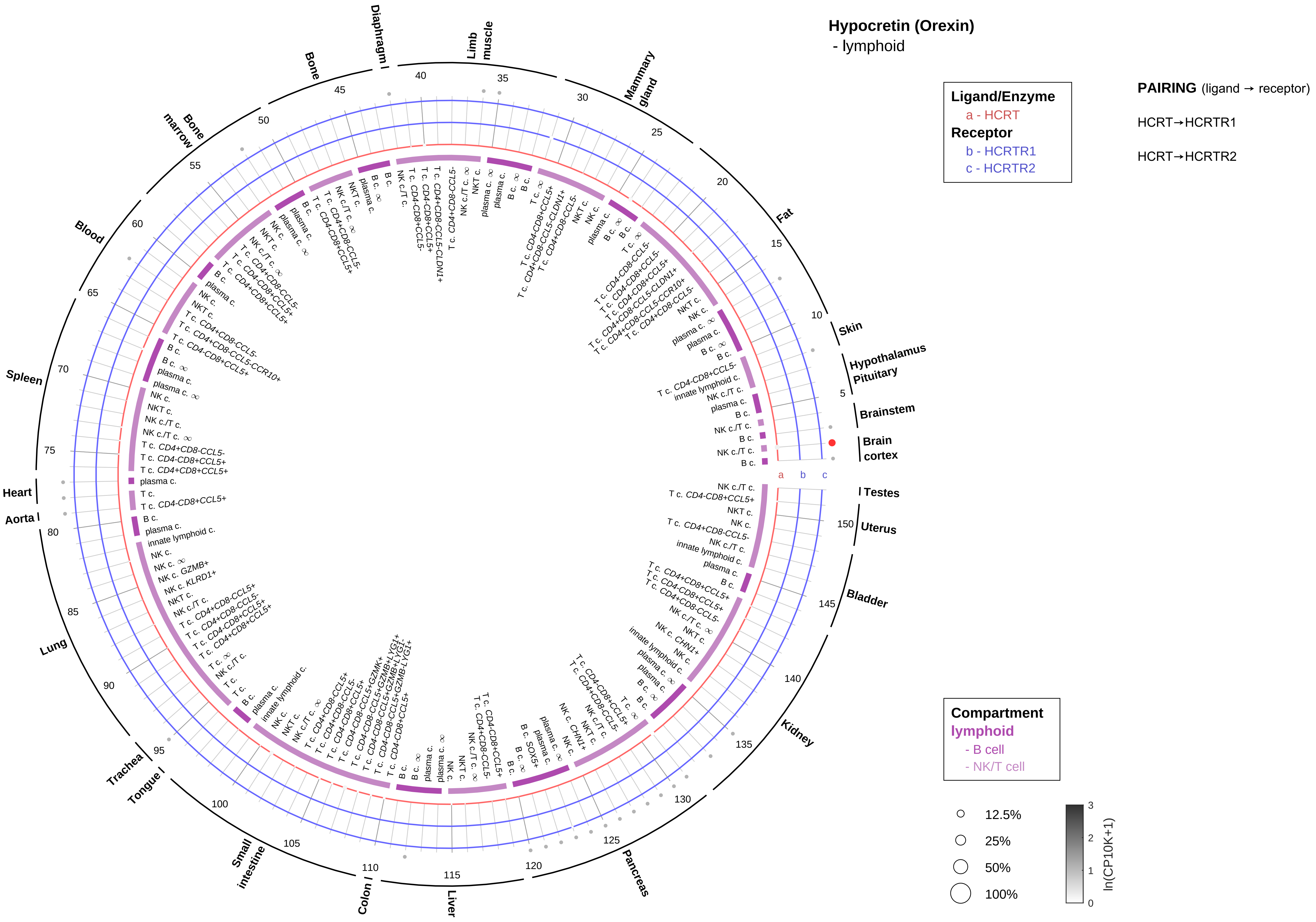

Hypocretin (Orexin)
- lymphoid
Ligand/Enzyme
a - HCRT
Receptor
b - HCRTR1
c - HCRTR2
PAIRING (ligand → receptor)
HCRT→HCRTR1
HCRT→HCRTR2
Compartment
lymphoid
- B cell
- NK/T cell
12.5%
25%
50%
100%
ln(CP10K+1)

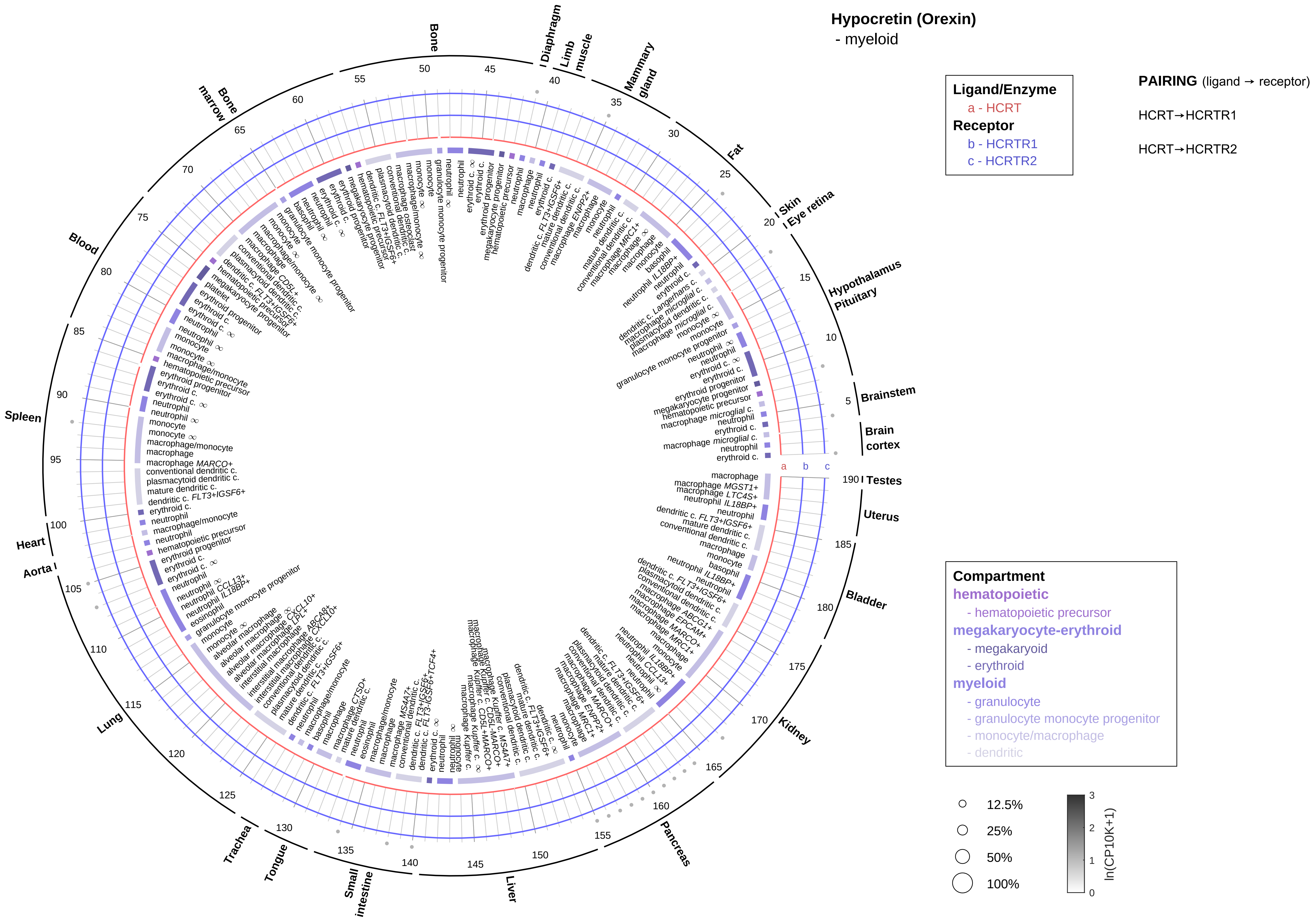

Hypocretin (Orexin)
- myeloid
Ligand/Enzyme
a - HCRT
Receptor
b - HCRTR1
c - HCRTR2
PAIRING (ligand → receptor)
HCRT→HCRTR1
HCRT→HCRTR2
Compartment
hematopoietic
- hematopoietic precursor
megakaryocyte-erythroid
- megakaryoid
- erythroid
myeloid
- granulocyte
- granulocyte monocyte progenitor
- monocyte/macrophage
- dendritic
12.5%
25%
50%
100%
ln(CP10K+1)
Brain cortex
Brainstem
Hypothalamus Pituitary
Eye retina
Skin
Fat
Mammary gland
Limb muscle
Diaphragm
Bone
Bone marrow
Blood
Spleen
Heart
Aorta
Lung
Trachea
Tongue
Small intestine
Liver
Pancreas
Kidney
Bladder
Uterus
Testes

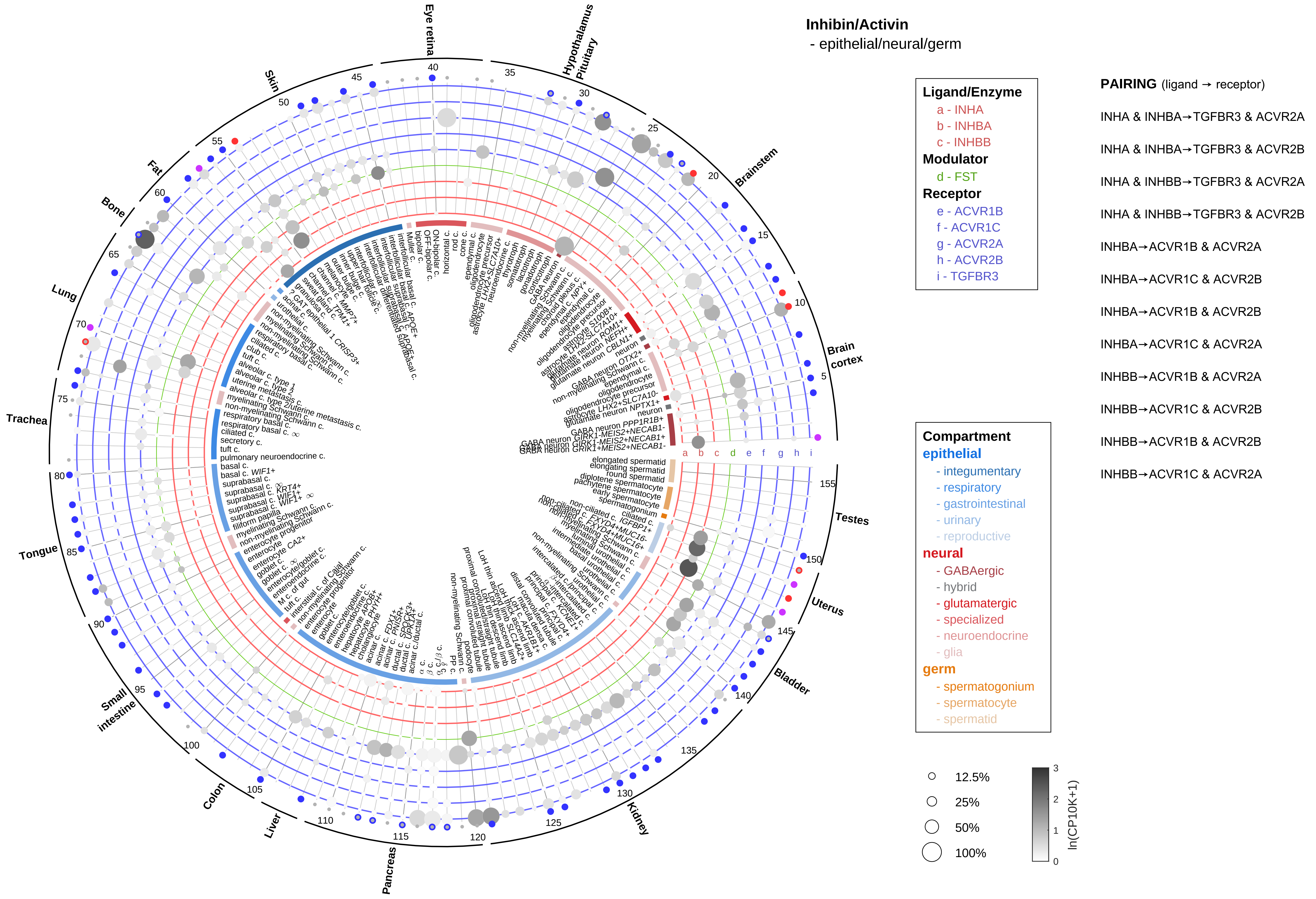
Inhibin/Activin
- epithelial/neural/germ
Eye retina
Hypothalamus Pituitary
Brainstem
Brain cortex
Testes
Uterus
Bladder
Kidney
Pancreas
Liver
Colon
Small intestine
Tongue
Trachea
Lung
Bone
Fat
Skin
a b c d e f g h i
Ligand/Enzyme
a - INHA
b - INHBA
c - INHBB
Modulator
d - FST
Receptor
e - ACVR1B
f - ACVR1C
g - ACVR2A
h - ACVR2B
i - TGFBR3
PAIRING (ligand → receptor)
INHA & INHBA→TGFBR3 & ACVR2A
INHA & INHBA→TGFBR3 & ACVR2B
INHA & INHBB→TGFBR3 & ACVR2A
INHA & INHBB→TGFBR3 & ACVR2B
INHBA→ACVR1B & ACVR2A
INHBA→ACVR1C & ACVR2B
INHBA→ACVR1B & ACVR2B
INHBA→ACVR1C & ACVR2A
INHBB→ACVR1B & ACVR2A
INHBB→ACVR1C & ACVR2B
INHBB→ACVR1B & ACVR2B
INHBB→ACVR1C & ACVR2A
Compartment
epithelial
- integumentary
- respiratory
- gastrointestinal
- urinary
- reproductive
neural
- GABAergic
- hybrid
- glutamatergic
- specialized
- neuroendocrine
- glia
germ
- spermatogonium
- spermatocyte
- spermatid
12.5%
25%
50%
100%
ln(CP10K+1)
0
1
2
3

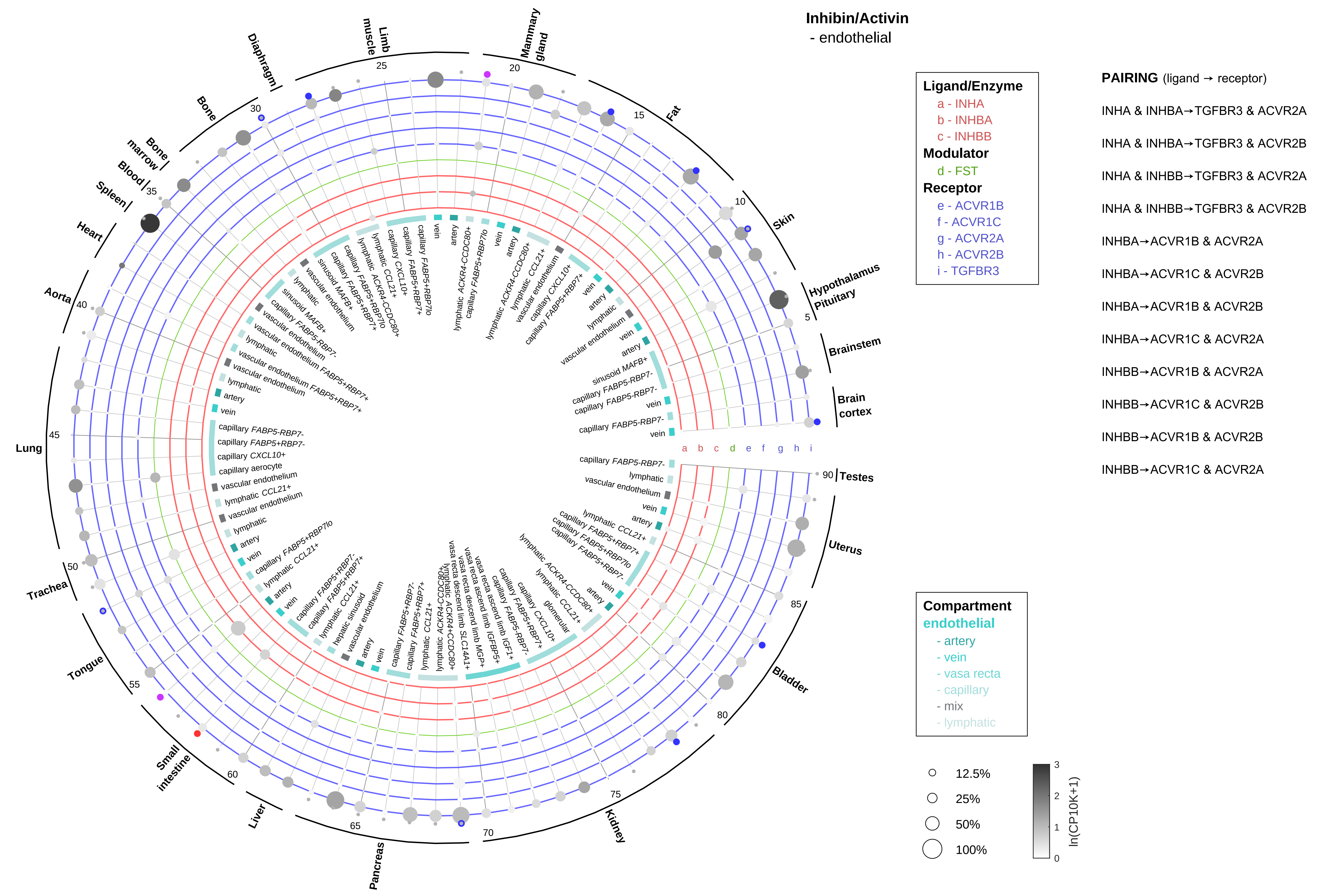
Inhibin/Activin
- endothelial
Ligand/Enzyme
a - INHA
b - INHBA
c - INHBB
Modulator
d - FST
Receptor
e - ACVR1B
f - ACVR1C
g - ACVR2A
h - ACVR2B
i - TGFBR3
PAIRING (ligand → receptor)
INHA & INHBA→TGFBR3 & ACVR2A
INHA & INHBA→TGFBR3 & ACVR2B
INHA & INHBB→TGFBR3 & ACVR2A
INHA & INHBB→TGFBR3 & ACVR2B
INHBA→ACVR1B & ACVR2A
INHBA→ACVR1C & ACVR2B
INHBA→ACVR1B & ACVR2B
INHBA→ACVR1C & ACVR2A
INHBB→ACVR1B & ACVR2A
INHBB→ACVR1C & ACVR2B
INHBB→ACVR1B & ACVR2B
INHBB→ACVR1C & ACVR2A
Compartment
endothelial
- artery
- vein
- vasa recta
- capillary
- mix
- lymphatic
12.5%
25%
50%
100%
ln(CP10K+1)
Limb muscle
Mammary gland
Diaphragm
Bone
Bone marrow
Blood
Spleen
Heart
Aorta
Lung
Trachea
Tongue
Small intestine
Liver
Pancreas
Kidney
Bladder
Uterus
Testes
Brain cortex
Brainstem
Hypothalamus Pituitary
Skin
Fat

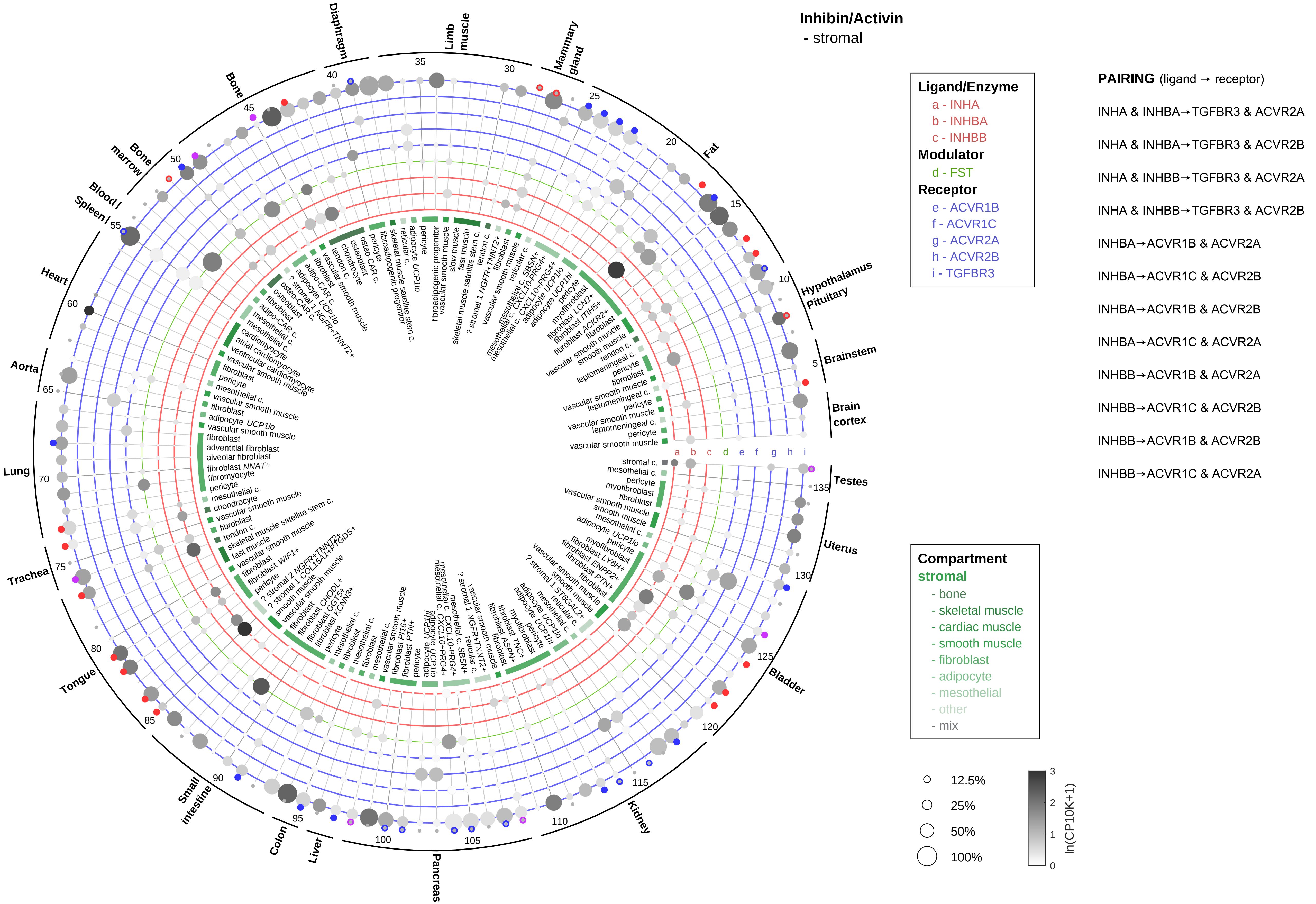

Inhibin/Activin
- stromal
Ligand/Enzyme
a - INHA
b - INHBA
c - INHBB
Modulator
d - FST
Receptor
e - ACVR1B
f - ACVR1C
g - ACVR2A
h - ACVR2B
i - TGFBR3
PAIRING (ligand → receptor)
INHA & INHBA→TGFBR3 & ACVR2A
INHA & INHBA→TGFBR3 & ACVR2B
INHA & INHBB→TGFBR3 & ACVR2A
INHA & INHBB→TGFBR3 & ACVR2B
INHBA→ACVR1B & ACVR2A
INHBA→ACVR1C & ACVR2B
INHBA→ACVR1B & ACVR2B
INHBA→ACVR1C & ACVR2A
INHBB→ACVR1B & ACVR2A
INHBB→ACVR1C & ACVR2B
INHBB→ACVR1B & ACVR2B
INHBB→ACVR1C & ACVR2A
Compartment
stromal
- bone
- skeletal muscle
- cardiac muscle
- smooth muscle
- fibroblast
- adipocyte
- mesothelial
- other
- mix
12.5%
25%
50%
100%
ln(CP10K+1)
Diaphragm
Limb muscle
Mammary gland
Fat
Hypothalamus Pituitary
Brainstem
Brain cortex
Testes
Uterus
Bladder
Kidney
Pancreas
Liver
Colon
Small intestine
Tongue
Trachea
Lung
Aorta
Heart
Spleen
Blood
Bone marrow
Bone

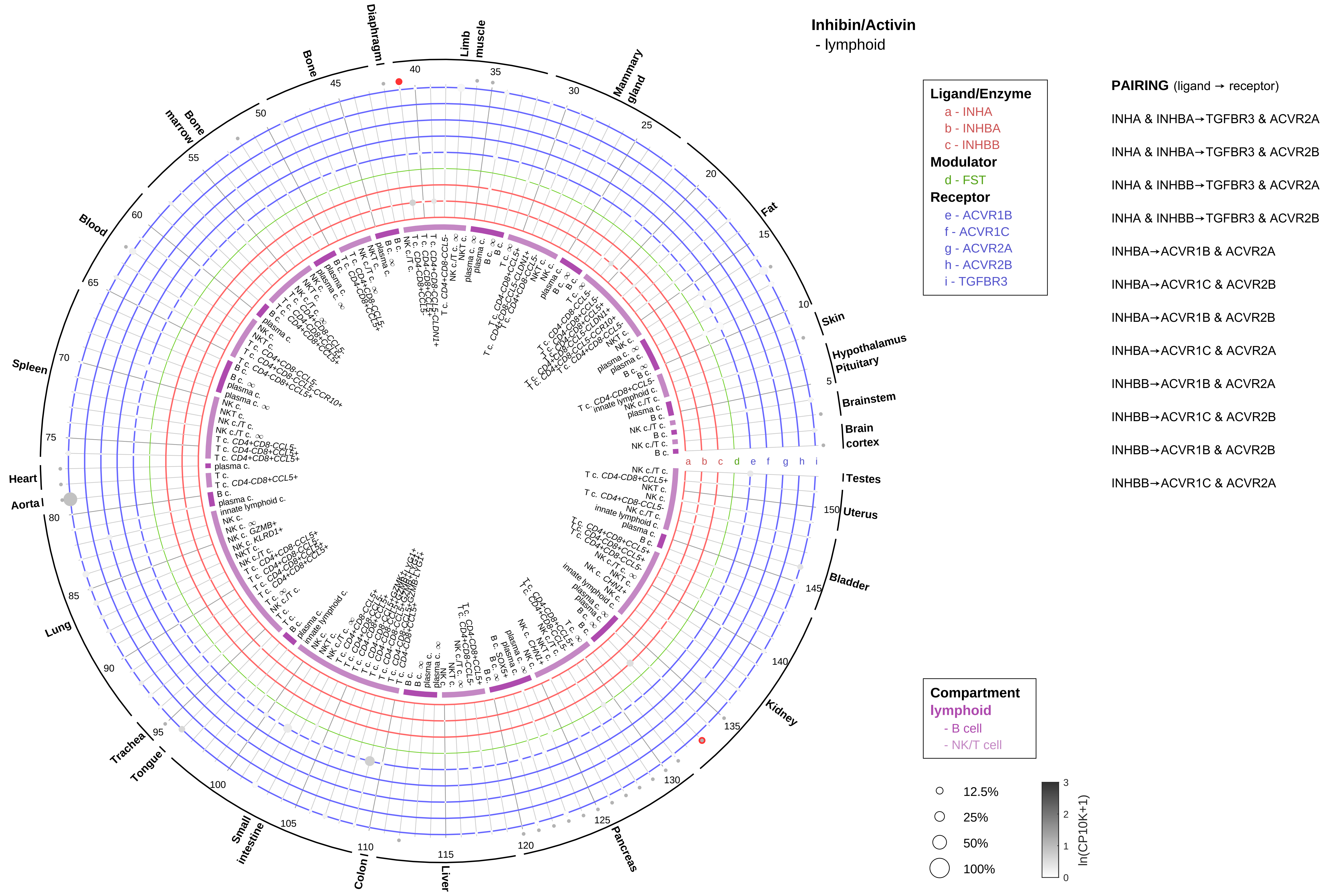

Inhibin/Activin
- lymphoid
Ligand/Enzyme
a - INHA
b - INHBA
c - INHBB
Modulator
d - FST
Receptor
e - ACVR1B
f - ACVR1C
g - ACVR2A
h - ACVR2B
i - TGFBR3
PAIRING (ligand → receptor)
INHA & INHBA→TGFBR3 & ACVR2A
INHA & INHBA→TGFBR3 & ACVR2B
INHA & INHBB→TGFBR3 & ACVR2A
INHA & INHBB→TGFBR3 & ACVR2B
INHBA→ACVR1B & ACVR2A
INHBA→ACVR1C & ACVR2B
INHBA→ACVR1B & ACVR2B
INHBA→ACVR1C & ACVR2A
INHBB→ACVR1B & ACVR2A
INHBB→ACVR1C & ACVR2B
INHBB→ACVR1B & ACVR2B
INHBB→ACVR1C & ACVR2A
Compartment
lymphoid
- B cell
- NK/T cell
12.5%
25%
50%
100%
ln(CP10K+1)
Diaphragm
Limb muscle
Mammary gland
Fat
Skin
Hypothalamus
Pituitary
Brainstem
Brain cortex
Testes
Uterus
Bladder
Kidney
Pancreas
Liver
Colon
Small intestine
Tongue
Trachea
Lung
Aorta
Heart
Spleen
Blood
Bone marrow
Bone

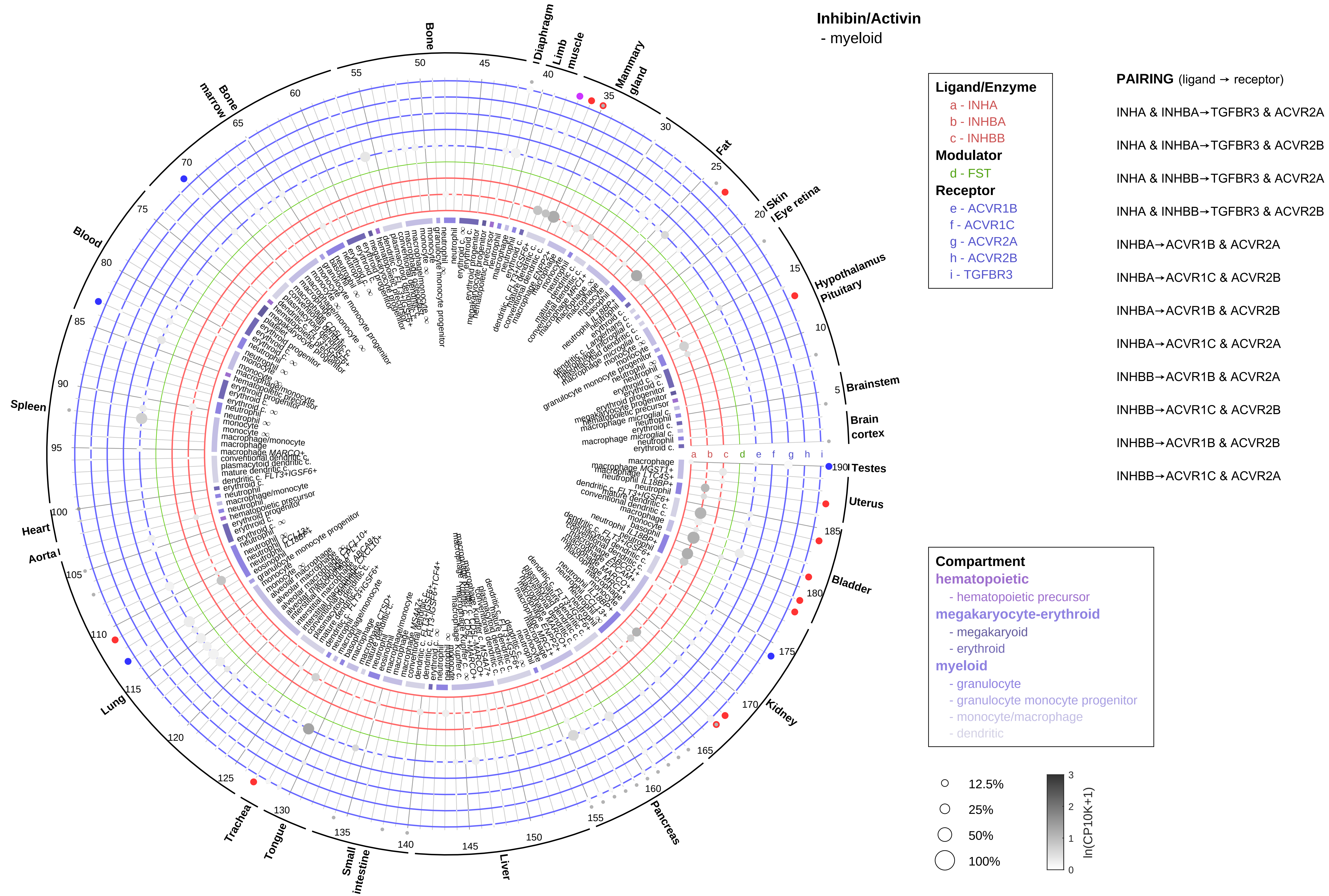

Inhibin/Activin
- myeloid
Ligand/Enzyme
a - INHA
b - INHBA
c - INHBB
Modulator
d - FST
Receptor
e - ACVR1B
f - ACVR1C
g - ACVR2A
h - ACVR2B
i - TGFBR3
PAIRING (ligand → receptor)
INHA & INHBA→TGFBR3 & ACVR2A
INHA & INHBA→TGFBR3 & ACVR2B
INHA & INHBB→TGFBR3 & ACVR2A
INHA & INHBB→TGFBR3 & ACVR2B
INHBA→ACVR1B & ACVR2A
INHBA→ACVR1C & ACVR2B
INHBA→ACVR1B & ACVR2B
INHBA→ACVR1C & ACVR2A
INHBB→ACVR1B & ACVR2A
INHBB→ACVR1C & ACVR2B
INHBB→ACVR1B & ACVR2B
INHBB→ACVR1C & ACVR2A
Compartment
hematopoietic
- hematopoietic precursor
megakaryocyte-erythroid
- megakaryoid
- erythroid
myeloid
- granulocyte
- granulocyte monocyte progenitor
- monocyte/macrophage
- dendritic
12.5%
25%
50%
100%
ln(CP10K+1)
Diaphragm
Limb muscle
Mammary gland
Fat
Skin
Eye retina
Hypothalamus
Pituitary
Brainstem
Brain cortex
Testes
Uterus
Bladder
Kidney
Pancreas
Liver
Small intestine
Tongue
Trachea
Lung
Aorta
Heart
Spleen
Blood
Bone marrow
Bone

Insulin
- epithelial/neural/germ

PAIRING (ligand → receptor)

INS & CPE & PCSK1 & PCSK2→INSR

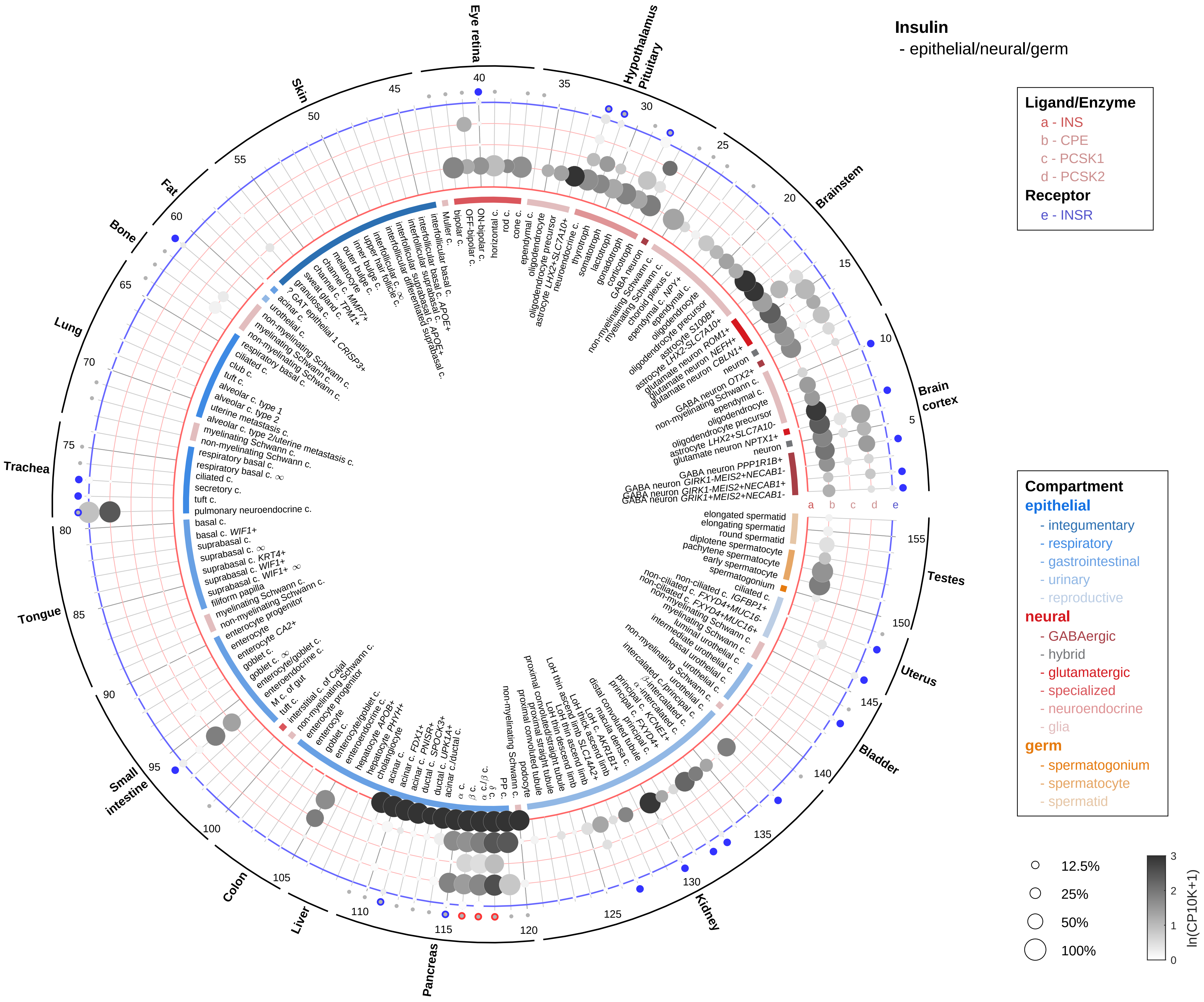

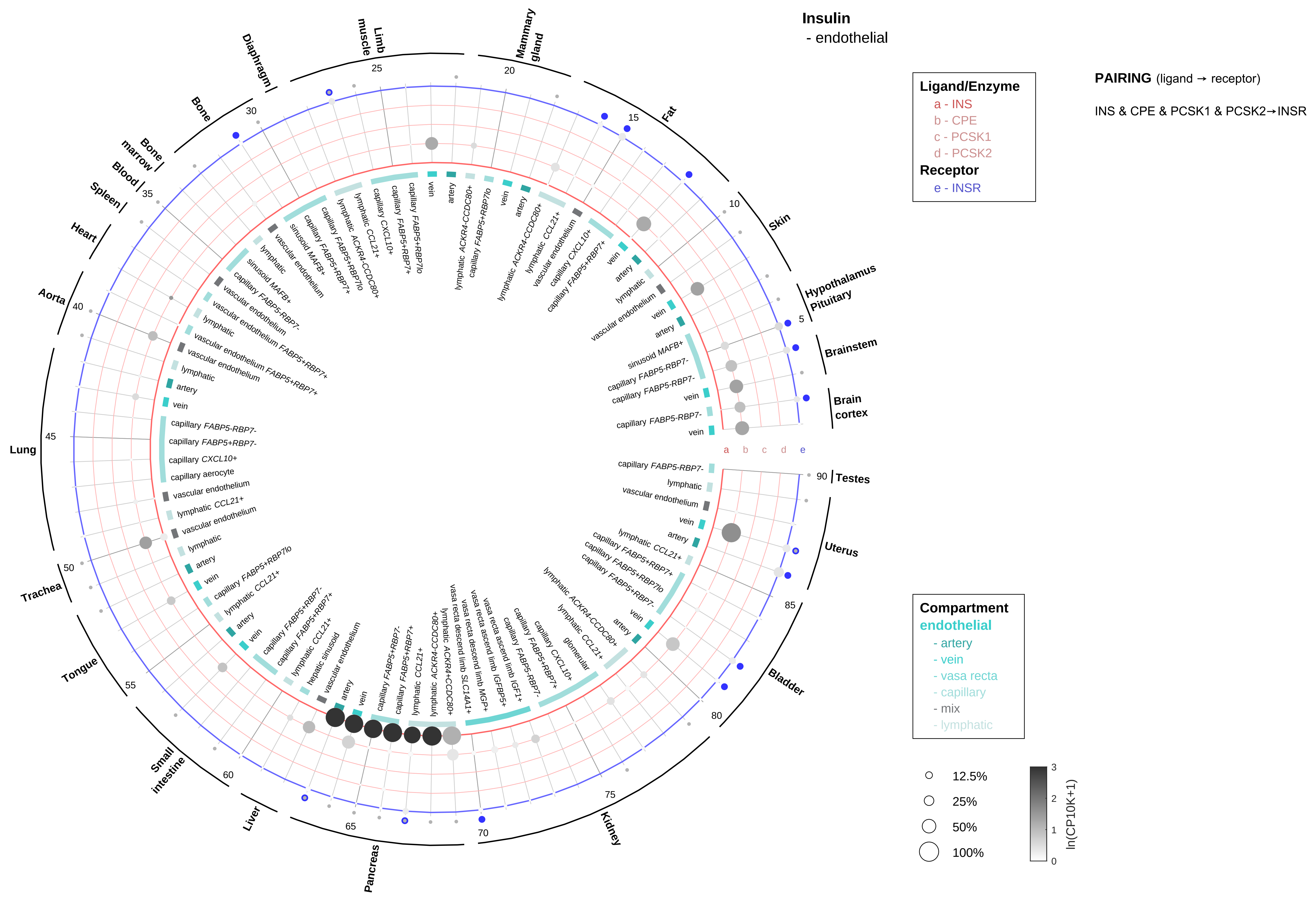
Insulin
- endothelial
Ligand/Enzyme
a - INS
b - CPE
c - PCSK1
d - PCSK2
Receptor
e - INSR
PAIRING (ligand → receptor)
INS & CPE & PCSK1 & PCSK2→INSR
Compartment
endothelial
- artery
- vein
- vasa recta
- capillary
- mix
- lymphatic
12.5%
25%
50%
100%
ln(CP10K+1)
Brain cortex
Brainstem
Hypothalamus Pituitary
Skin
Fat
Mammary gland
Limb muscle
Diaphragm
Bone
Bone marrow
Blood
Spleen
Heart
Aorta
Lung
Trachea
Tongue
Small intestine
Liver
Pancreas
Kidney
Bladder
Uterus
Testes
a
b
c
d
e

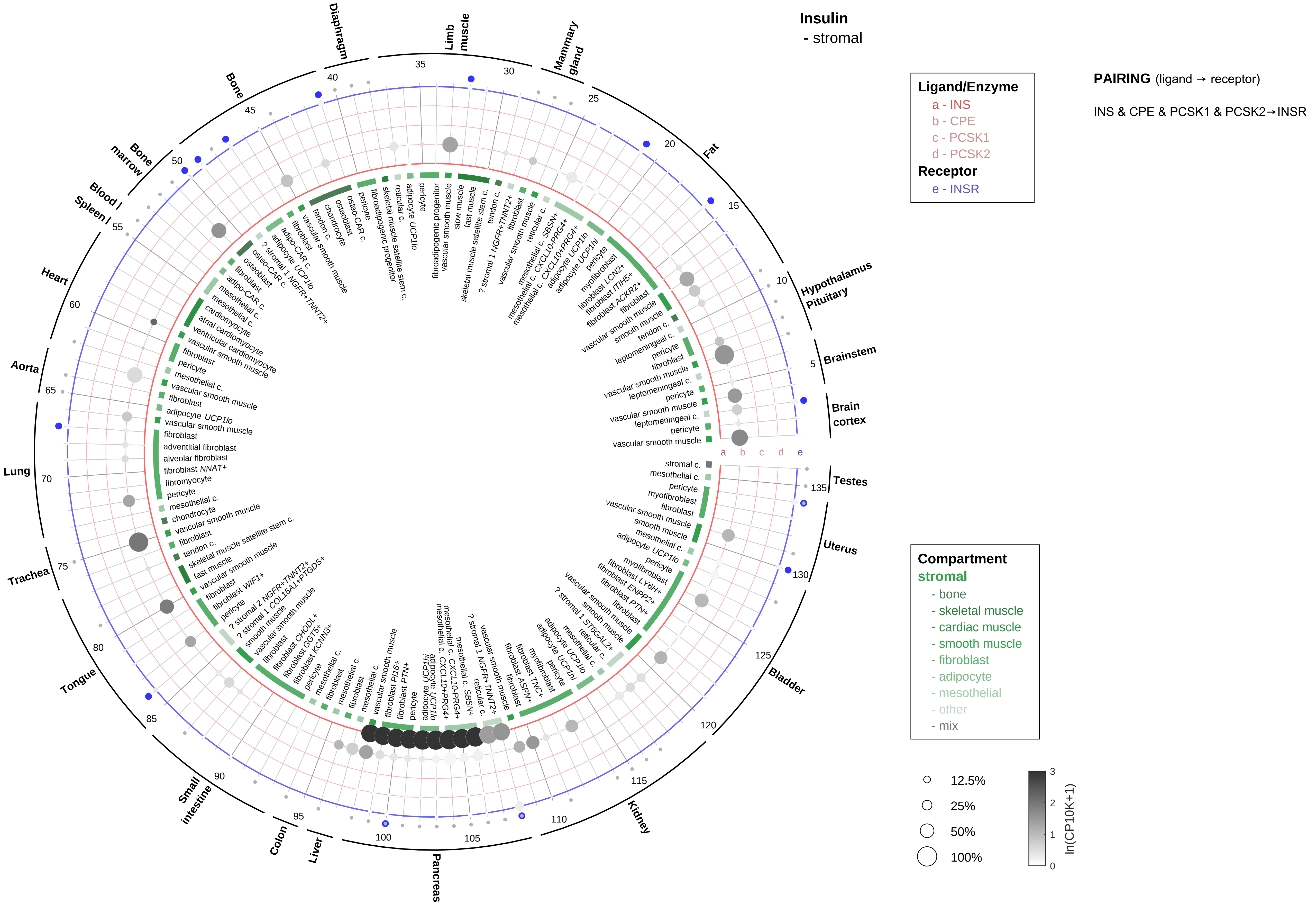

Insulin
- stromal
Ligand/Enzyme
a - INS
b - CPE
c - PCSK1
d - PCSK2
Receptor
e - INSR
PAIRING (ligand → receptor)
INS & CPE & PCSK1 & PCSK2→INSR
Compartment
stromal
- bone
- skeletal muscle
- cardiac muscle
- smooth muscle
- fibroblast
- adipocyte
- mesothelial
- other
- mix
12.5%
25%
50%
100%
ln(CP10K+1)
Brain cortex
Brainstem
Hypothalamus Pituitary
Fat
Mammary gland
Limb muscle
Diaphragm
Bone
Bone marrow
Blood / Spleen /
Heart
Aorta
Lung
Trachea
Tongue
Small intestine
Colon
Liver
Pancreas
Kidney
Bladder
Uterus
Testes

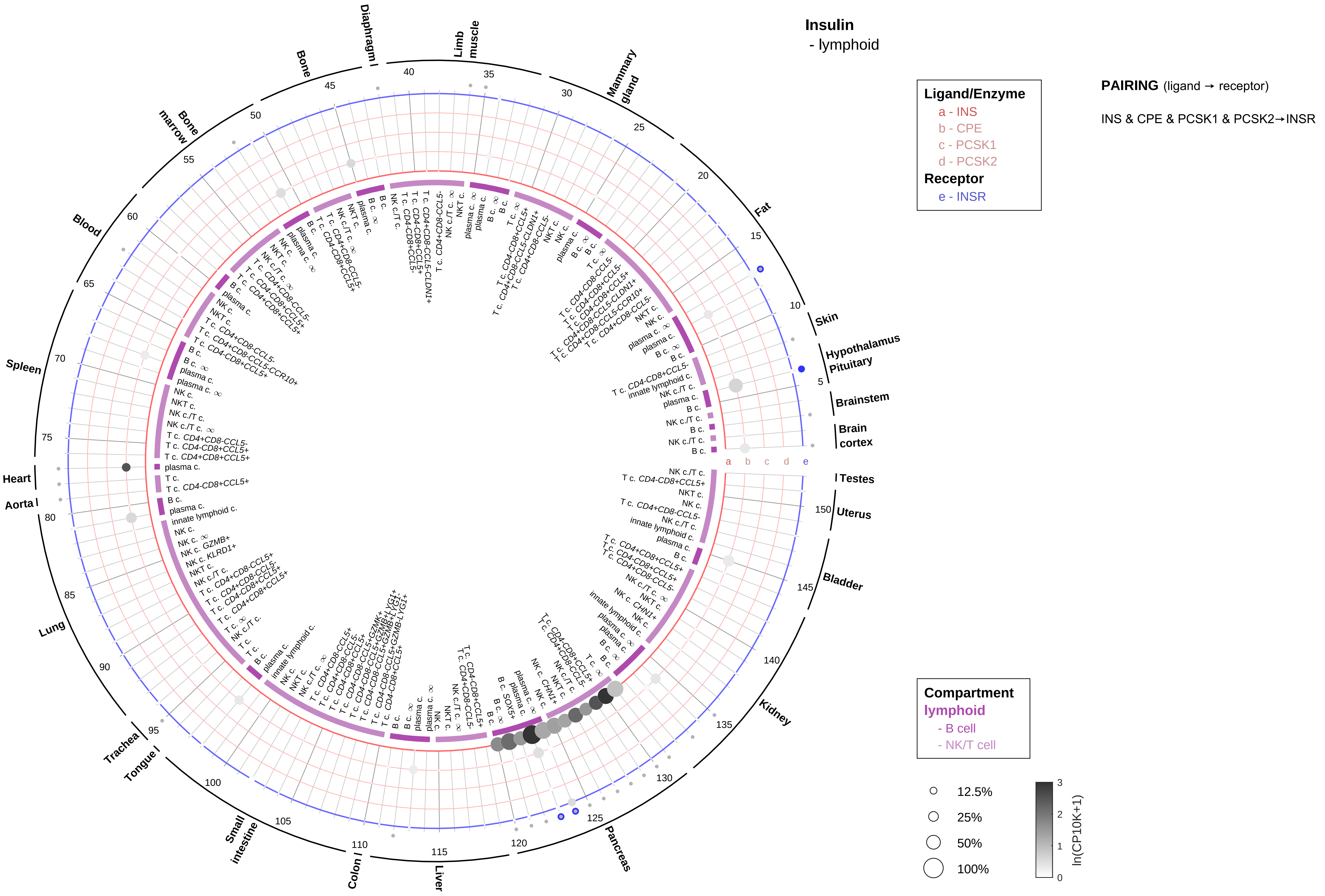
Insulin
- lymphoid
Ligand/Enzyme
a - INS
b - CPE
c - PCSK1
d - PCSK2
Receptor
e - INSR
PAIRING (ligand → receptor)
INS & CPE & PCSK1 & PCSK2→INSR
Compartment
lymphoid
- B cell
- NK/T cell
12.5%
25%
50%
100%
ln(CP10K+1)
Skin
Hypothalamus
Pituitary
Brainstem
Brain cortex
Testes
Uterus
Bladder
Kidney
Pancreas
Liver
Colon
Small intestine
Tongue
Trachea
Lung
Aorta
Heart
Spleen
Blood
Bone marrow
Bone
Diaphragm
Limb muscle
Mammary gland
Fat

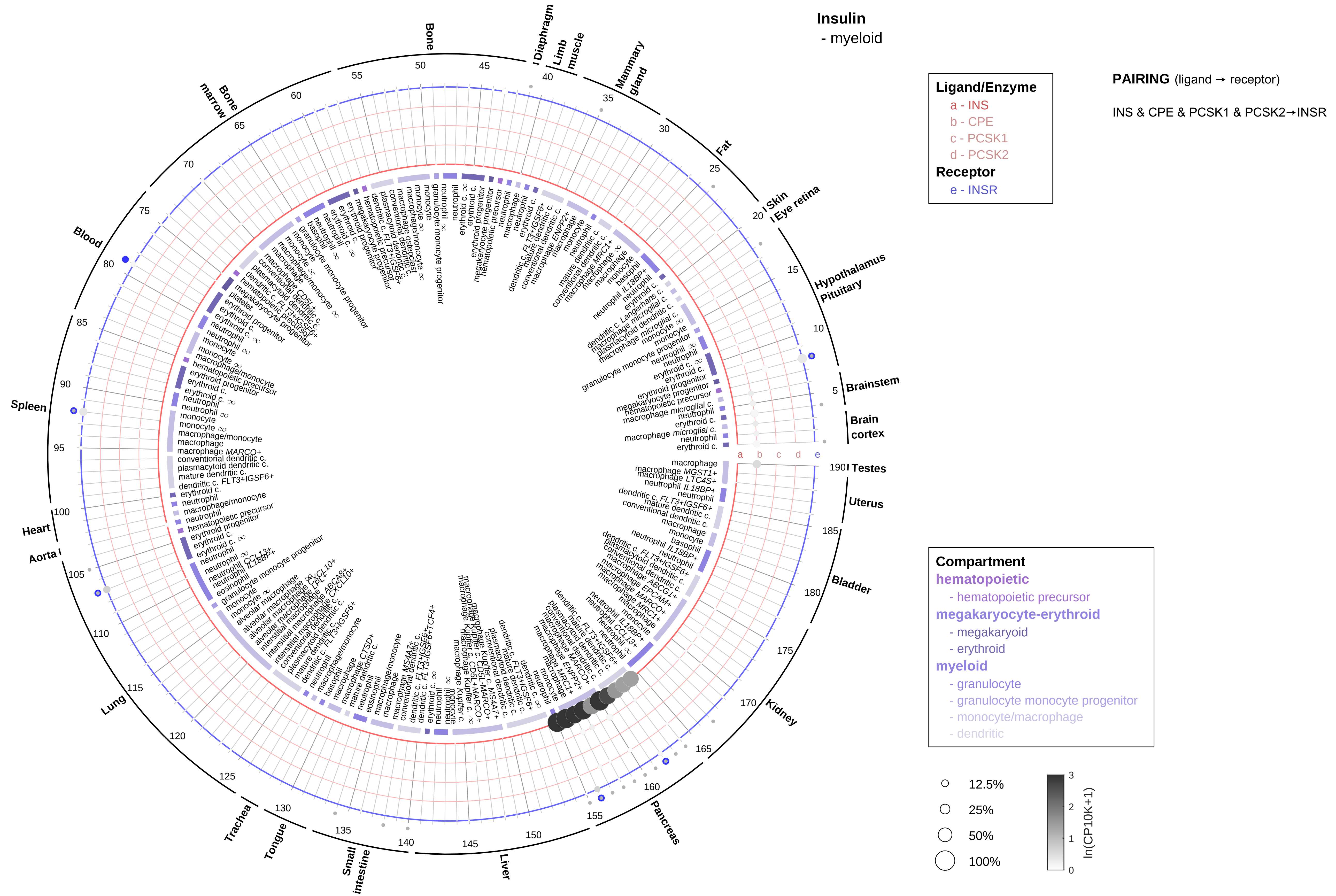
Insulin
- myeloid
Ligand/Enzyme
a - INS
b - CPE
c - PCSK1
d - PCSK2
Receptor
e - INSR
PAIRING (ligand → receptor)
INS & CPE & PCSK1 & PCSK2→INSR
Compartment
hematopoietic
- hematopoietic precursor
megakaryocyte-erythroid
- megakaryoid
- erythroid
myeloid
- granulocyte
- granulocyte monocyte progenitor
- monocyte/macrophage
- dendritic
12.5%
25%
50%
100%
ln(CP10K+1)
Diaphragm
Limb muscle
Mammary gland
Fat
Skin
Eye retina
Hypothalamus
Pituitary
Brainstem
Brain cortex
Testes
Uterus
Bladder
Kidney
Pancreas
Liver
Small intestine
Tongue
Trachea
Lung
Aorta
Heart
Spleen
Blood
Bone marrow
Bone

Insulin-like growth factor (IGF)
- epithelial/neural/germ

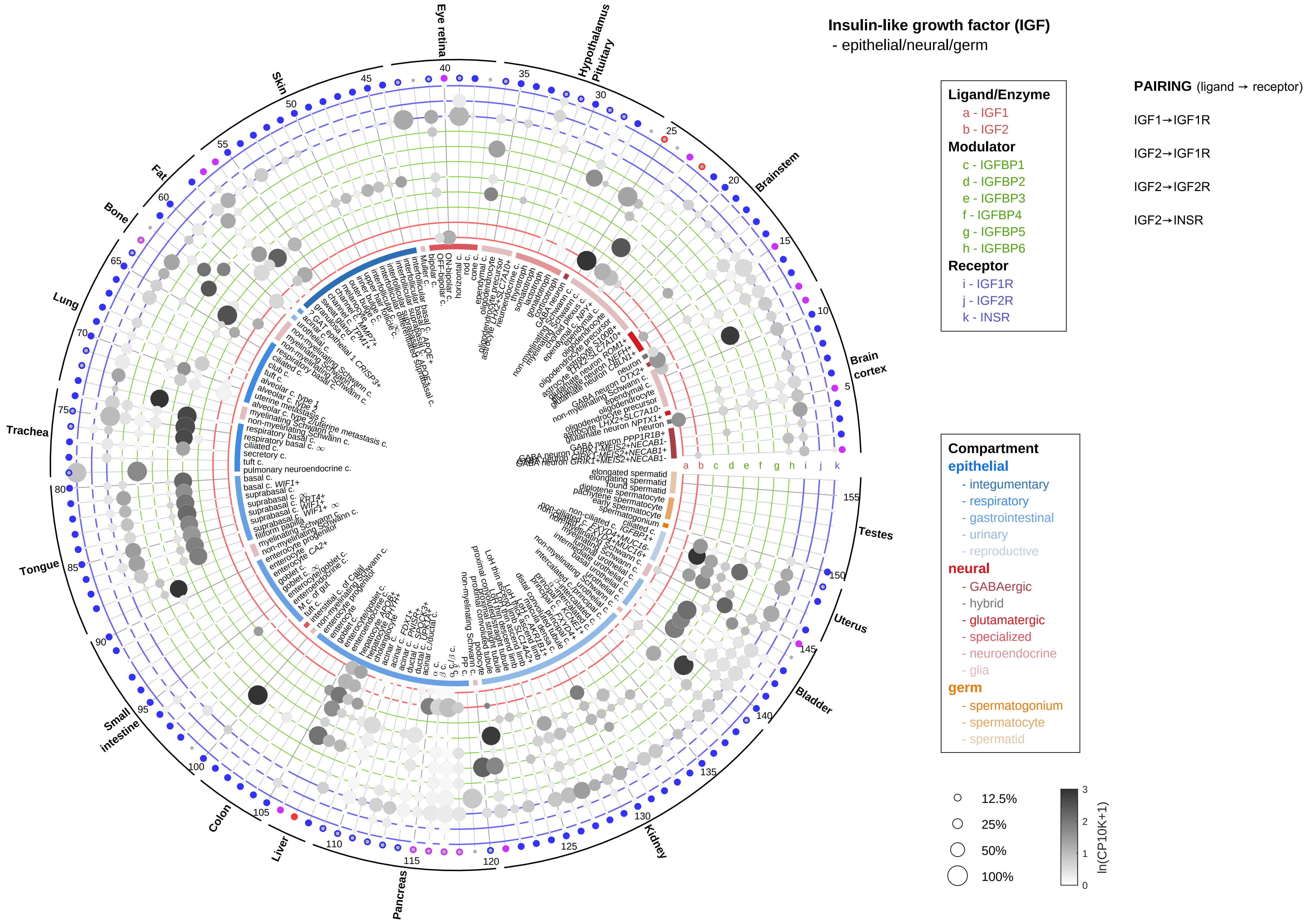

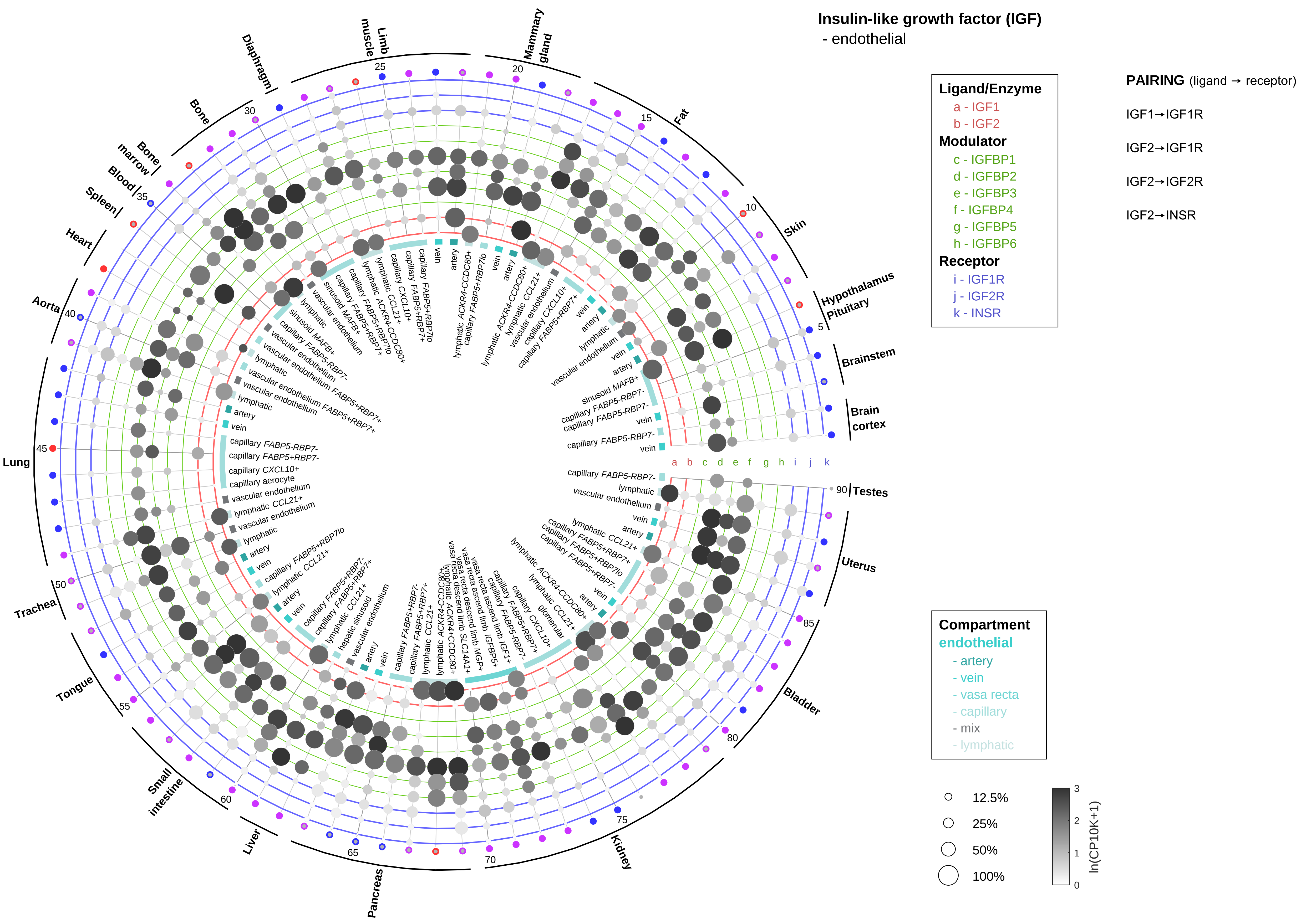

Insulin-like growth factor (IGF)
- endothelial
Ligand/Enzyme
a - IGF1
b - IGF2
Modulator
c - IGFBP1
d - IGFBP2
e - IGFBP3
f - IGFBP4
g - IGFBP5
h - IGFBP6
Receptor
i - IGF1R
j - IGF2R
k - INSR
PAIRING (ligand → receptor)
IGF1→IGF1R
IGF2→IGF1R
IGF2→IGF2R
IGF2→INSR
Compartment
endothelial
- artery
- vein
- vasa recta
- capillary
- mix
- lymphatic
12.5%
25%
50%
100%
ln(CP10K+1)
Brain cortex
Brainstem
Hypothalamus Pituitary
Skin
Fat
Mammary gland
Limb muscle
Diaphragm
Bone
Bone marrow
Blood
Spleen
Heart
Aorta
Lung
Trachea
Tongue
Small intestine
Liver
Pancreas
Kidney
Bladder
Uterus
Testes

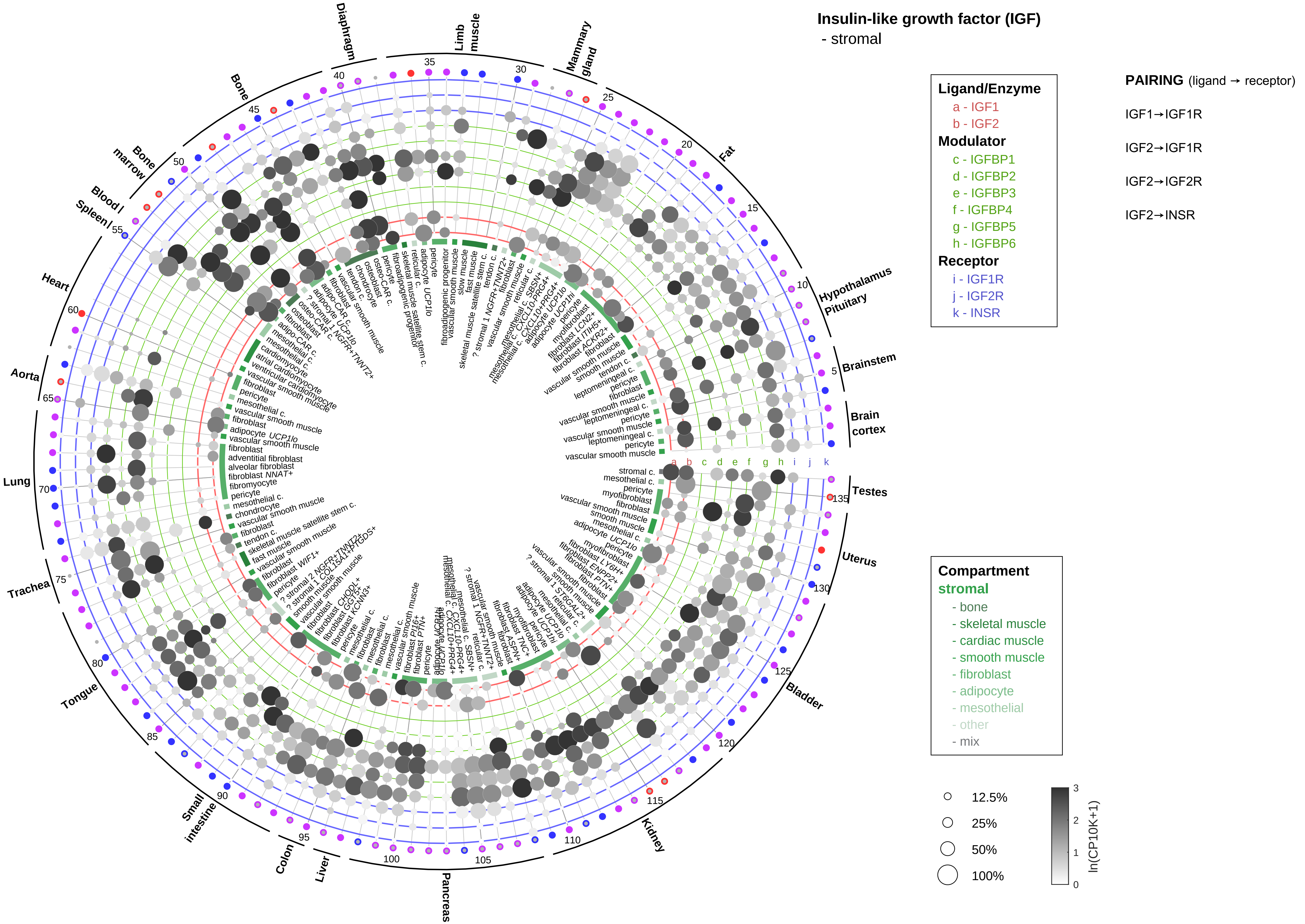

Insulin-like growth factor (IGF)
- stromal
Ligand/Enzyme
a - IGF1
b - IGF2
Modulator
c - IGFBP1
d - IGFBP2
e - IGFBP3
f - IGFBP4
g - IGFBP5
h - IGFBP6
Receptor
i - IGF1R
j - IGF2R
k - INSR
PAIRING (ligand → receptor)
IGF1→IGF1R
IGF2→IGF1R
IGF2→IGF2R
IGF2→INSR
Compartment
stromal
- bone
- skeletal muscle
- cardiac muscle
- smooth muscle
- fibroblast
- adipocyte
- mesothelial
- other
- mix
12.5%
25%
50%
100%
ln(CP10K+1)
Limb muscle
Mammary gland
Fat
Hypothalamus Pituitary
Brainstem
Brain cortex
Testes
Uterus
Bladder
Kidney
Pancreas
Liver
Colon
Small intestine
Tongue
Trachea
Lung
Aorta
Heart
Spleen
Blood
Bone marrow
Bone
Diaphragm

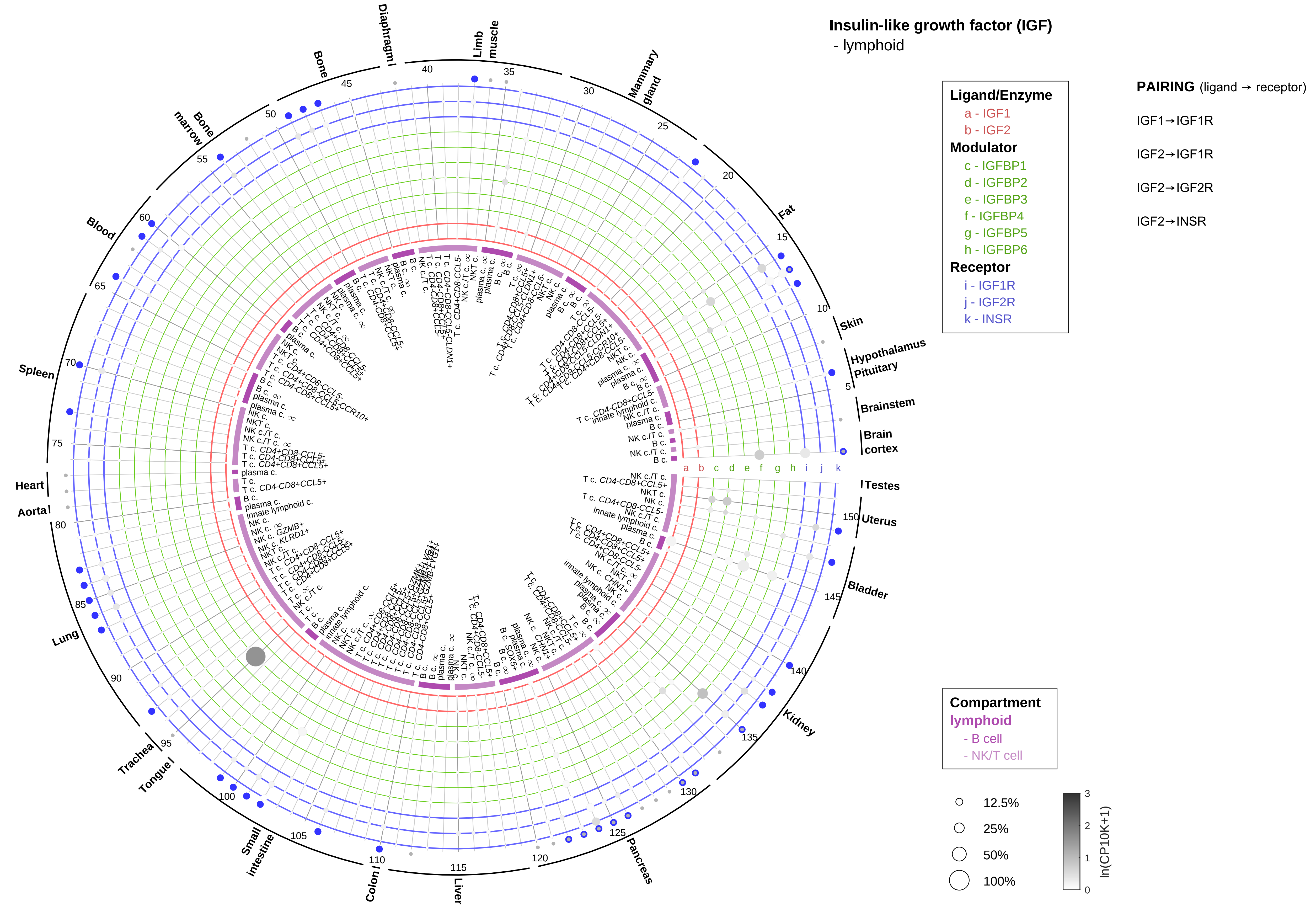
Insulin-like growth factor (IGF)
- lymphoid
Ligand/Enzyme
a - IGF1
b - IGF2
Modulator
c - IGFBP1
d - IGFBP2
e - IGFBP3
f - IGFBP4
g - IGFBP5
h - IGFBP6
Receptor
i - IGF1R
j - IGF2R
k - INSR
PAIRING (ligand → receptor)
IGF1→IGF1R
IGF2→IGF1R
IGF2→IGF2R
IGF2→INSR
Compartment
lymphoid
- B cell
- NK/T cell
12.5%
25%
50%
100%
ln(CP10K+1)
Skin
Hypothalamus
Pituitary
Brainstem
Brain cortex
Testes
Uterus
Bladder
Kidney
Pancreas
Liver
Colon
Small intestine
Tongue
Trachea
Lung
Aorta
Heart
Spleen
Blood
Bone marrow
Bone
Diaphragm
Limb muscle
Mammary gland
Fat

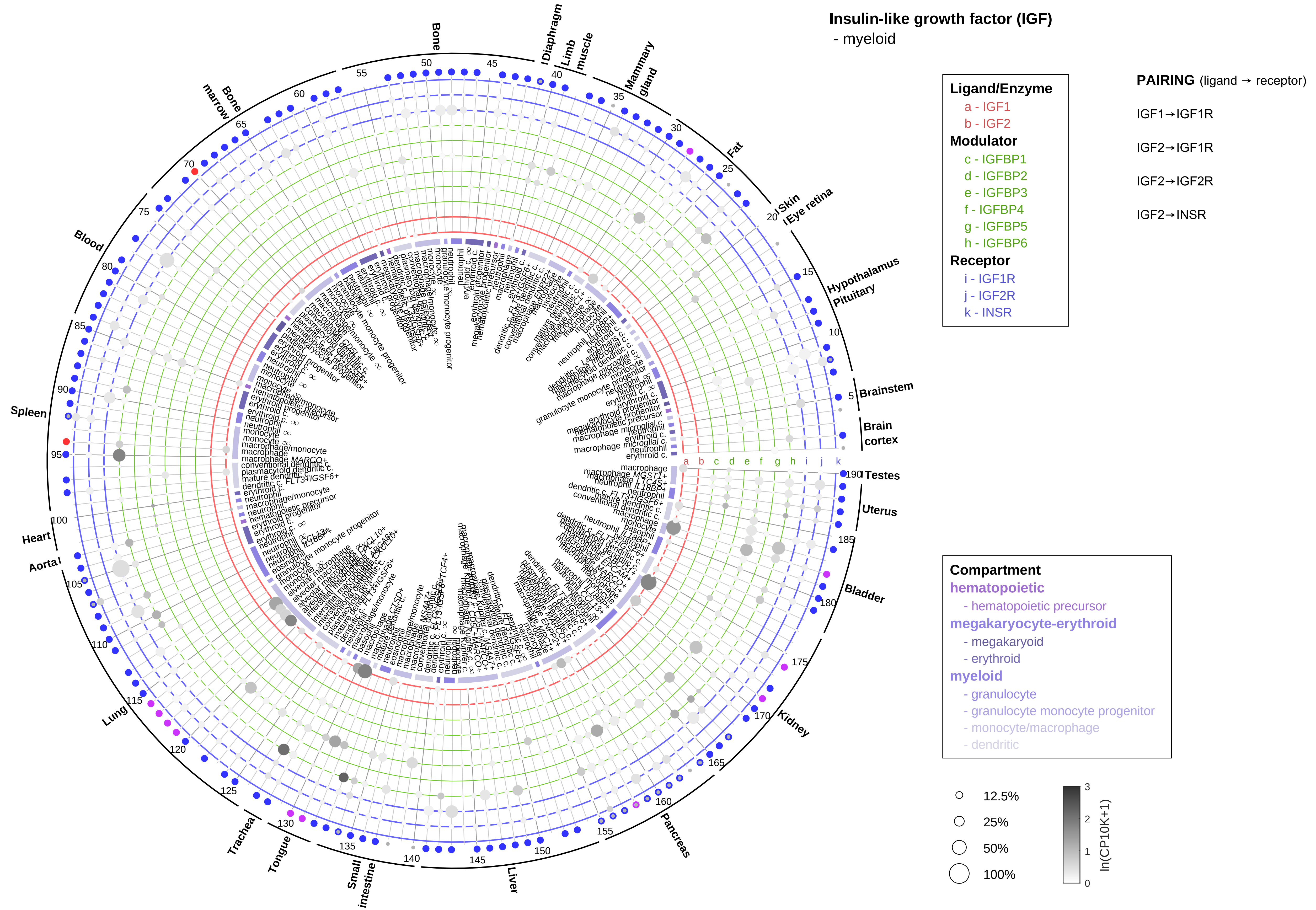

Insulin-like growth factor (IGF)
- myeloid
Ligand/Enzyme
a - IGF1
b - IGF2
Modulator
c - IGFBP1
d - IGFBP2
e - IGFBP3
f - IGFBP4
g - IGFBP5
h - IGFBP6
Receptor
i - IGF1R
j - IGF2R
k - INSR
PAIRING (ligand → receptor)
IGF1→IGF1R
IGF2→IGF1R
IGF2→IGF2R
IGF2→INSR
Compartment
hematopoietic
- hematopoietic precursor
megakaryocyte-erythroid
- megakaryoid
- erythroid
myeloid
- granulocyte
- granulocyte monocyte progenitor
- monocyte/macrophage
- dendritic
12.5%
25%
50%
100%
ln(CP10K+1)
Bone
Diaphragm
Limb muscle
Mammary gland
Fat
Skin
Eye retina
Hypothalamus
Pituitary
Brainstem
Brain cortex
Testes
Uterus
Bladder
Kidney
Pancreas
Liver
Small intestine
Tongue
Trachea
Lung
Aorta
Heart
Spleen
Blood
Bone marrow

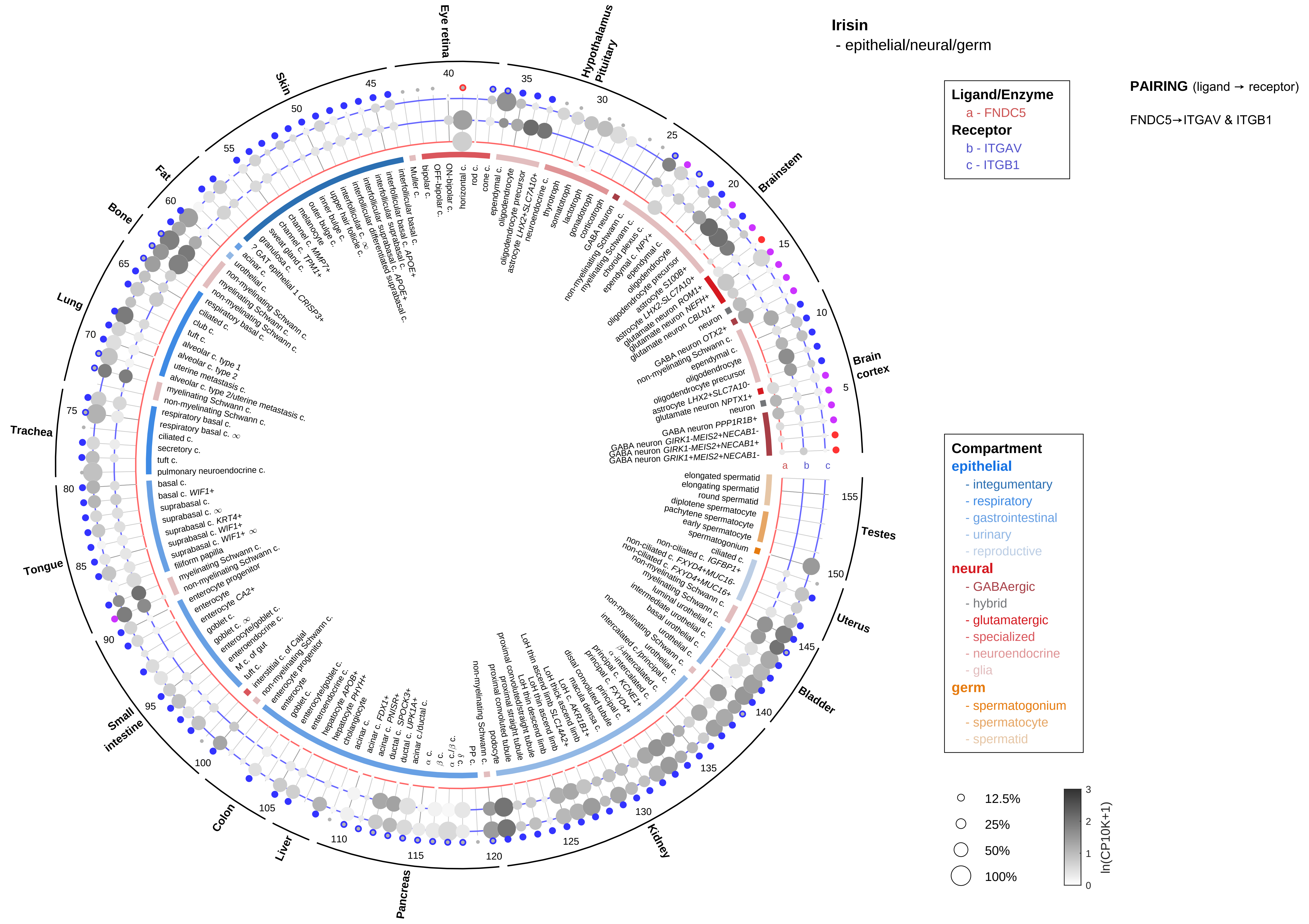

Irisin
- epithelial/neural/germ
Ligand/Enzyme
a - FNDC5
Receptor
b - ITGAV
c - ITGB1
PAIRING (ligand → receptor)
FNDC5→ITGAV & ITGB1
Compartment
epithelial
- integumentary
- respiratory
- gastrointestinal
- urinary
- reproductive
neural
- GABAergic
- hybrid
- glutamatergic
- specialized
- neuroendocrine
- glia
germ
- spermatogonium
- spermatocyte
- spermatid
12.5%
25%
50%
100%
ln(CP10K+1)
Eye retina
Hypothalamus Pituitary
Brainstem
Brain cortex
Testes
Uterus
Bladder
Kidney
Pancreas
Liver
Colon
Small intestine
Tongue
Trachea
Lung
Bone
Fat
Skin

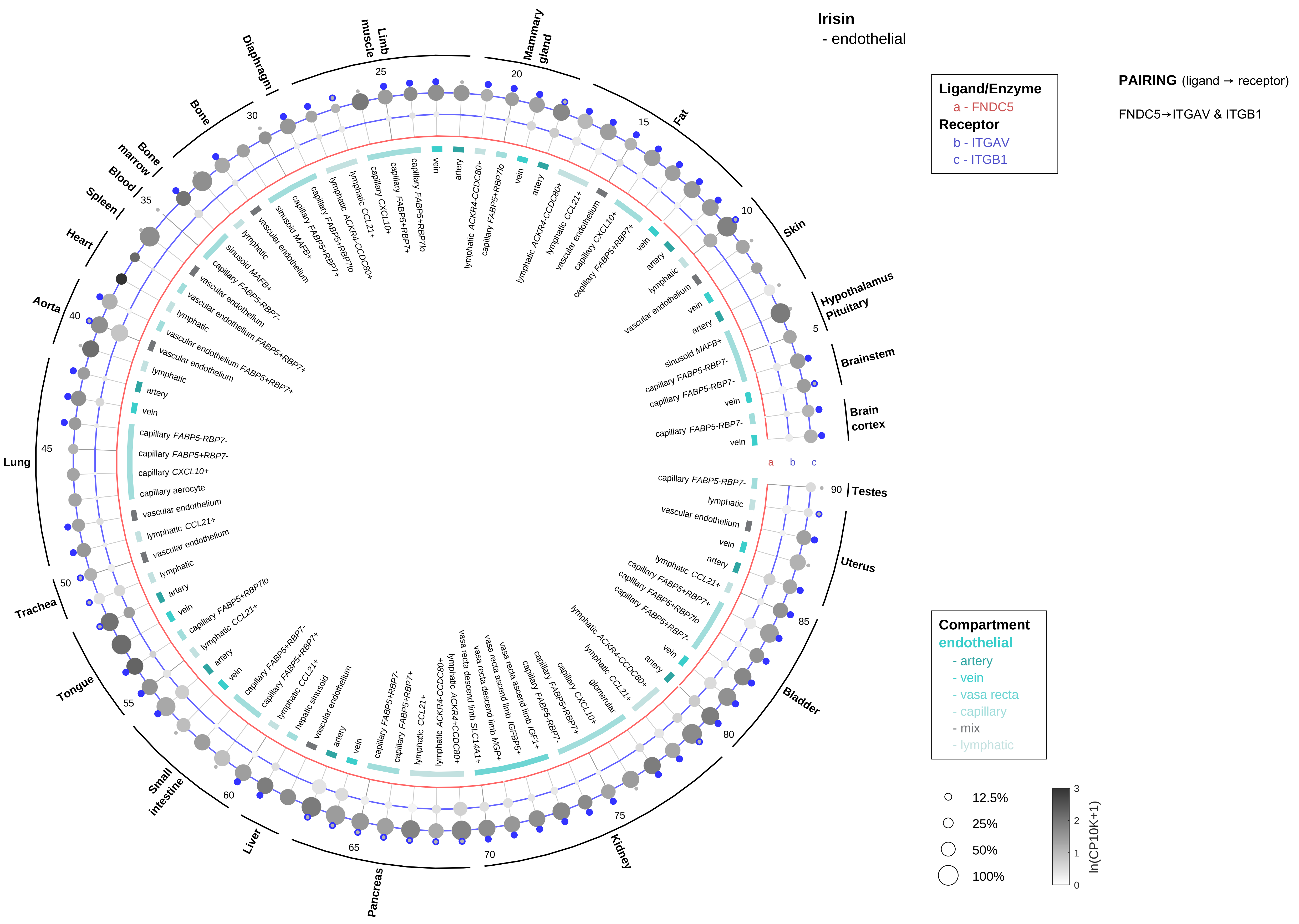

Irisin
- endothelial
Ligand/Enzyme
a - FNDC5
Receptor
b - ITGAV
c - ITGB1
PAIRING (ligand → receptor)
FNDC5→ITGAV & ITGB1
Compartment
endothelial
- artery
- vein
- vasa recta
- capillary
- mix
- lymphatic
12.5%
25%
50%
100%
ln(CP10K+1)
Brain cortex
Brainstem
Hypothalamus Pituitary
Skin
Fat
Mammary gland
Limb muscle
Diaphragm
Bone
Bone marrow
Blood
Spleen
Heart
Aorta
Lung
Trachea
Tongue
Small intestine
Liver
Pancreas
Kidney
Bladder
Uterus
Testes

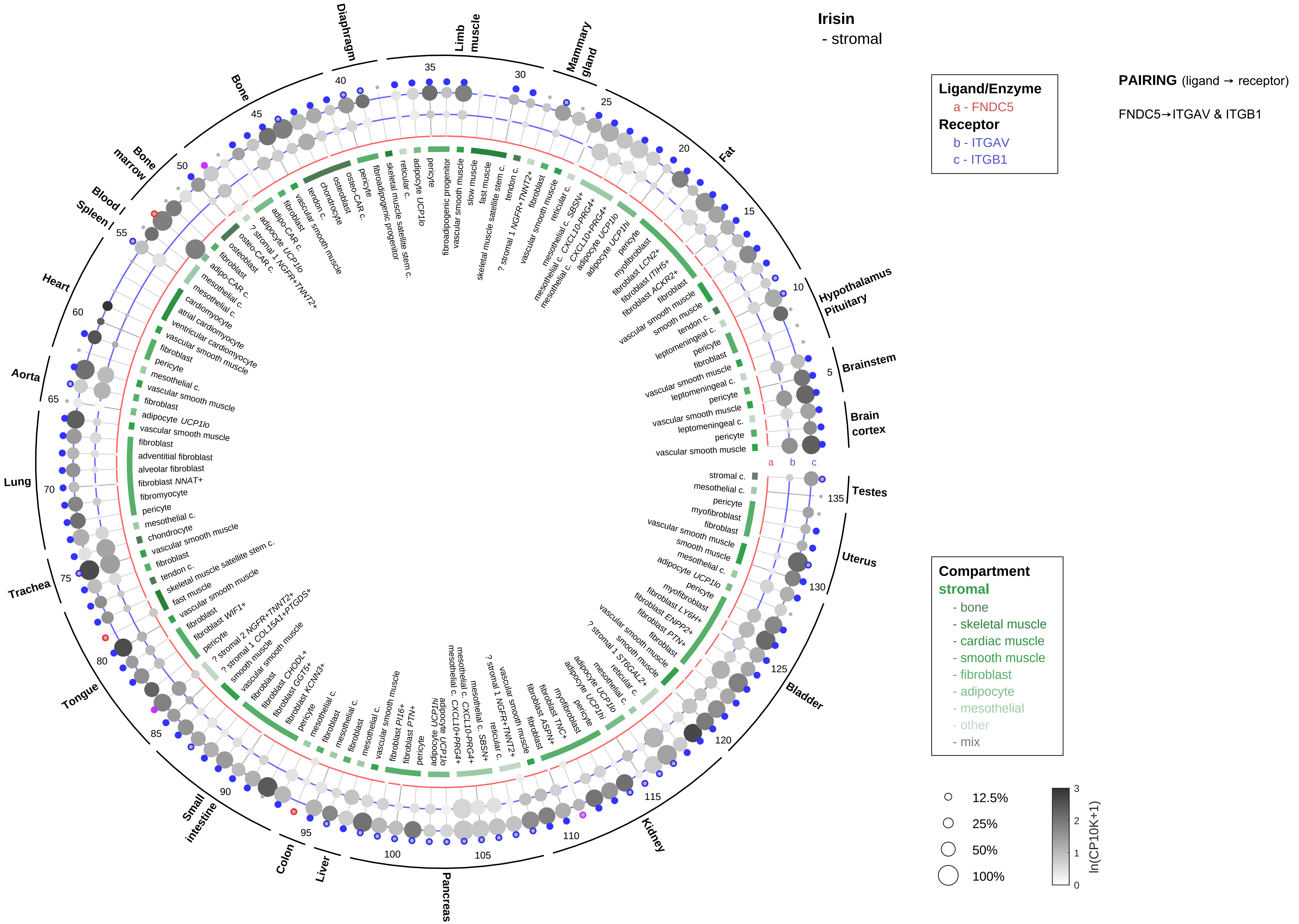

Irisin
- stromal
Ligand/Enzyme
a - FNDC5
Receptor
b - ITGAV
c - ITGB1
PAIRING (ligand → receptor)
FNDC5→ITGAV & ITGB1
Compartment
stromal
- bone
- skeletal muscle
- cardiac muscle
- smooth muscle
- fibroblast
- adipocyte
- mesothelial
- other
- mix
12.5%
25%
50%
100%
ln(CP10K+1)
Brain cortex
Brainstem
Hypothalamus Pituitary
Fat
Mammary gland
Limb muscle
Diaphragm
Bone
Bone marrow
Blood
Spleen
Heart
Aorta
Lung
Trachea
Tongue
Small intestine
Colon
Liver
Pancreas
Kidney
Bladder
Uterus
Testes

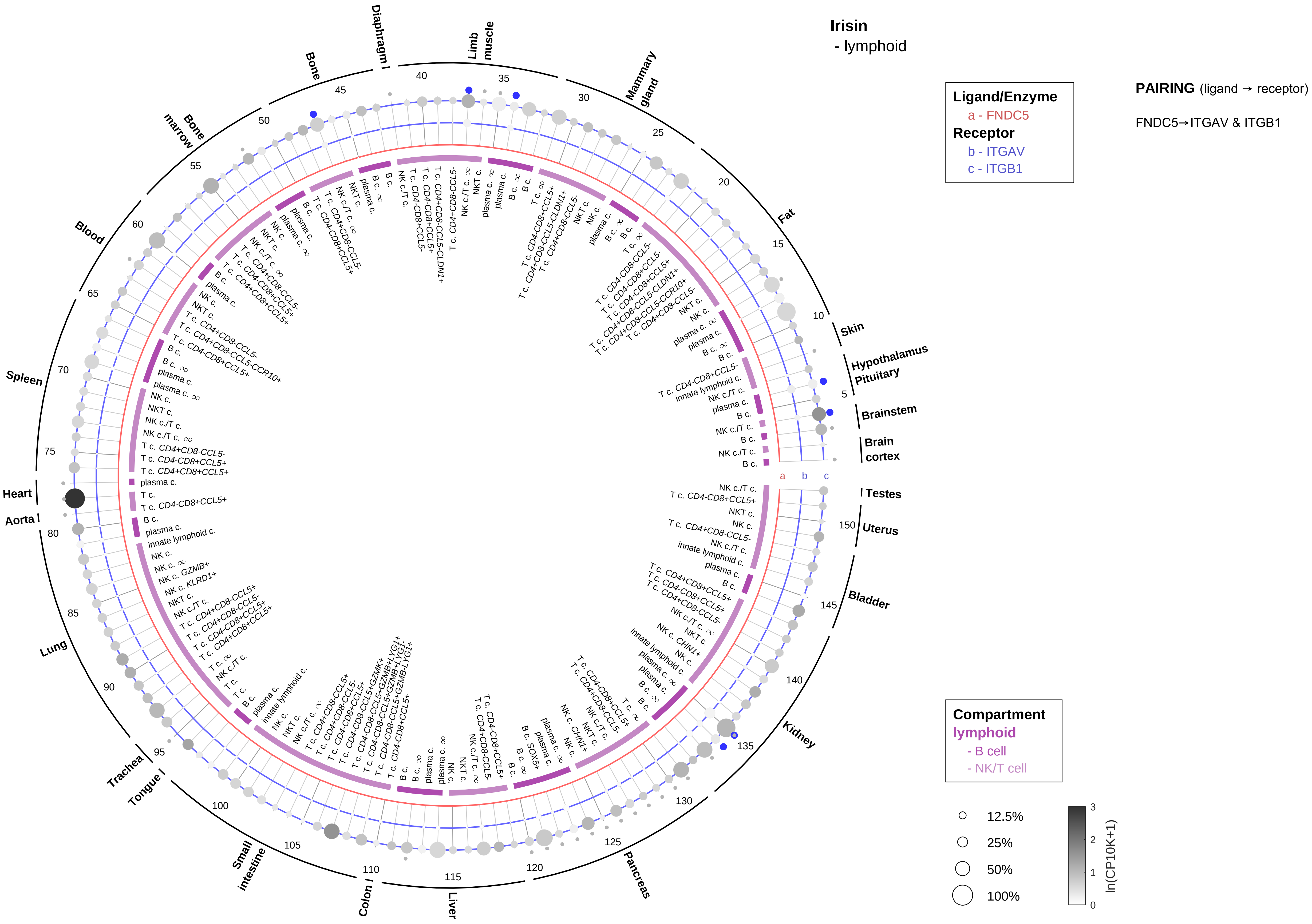
Irisin
- lymphoid
Ligand/Enzyme
a - FNDC5
Receptor
b - ITGAV
c - ITGB1
PAIRING (ligand → receptor)
FNDC5→ITGAV & ITGB1
Compartment
lymphoid
- B cell
- NK/T cell
12.5%
25%
50%
100%
ln(CP10K+1)
Diaphragm
Limb muscle
Mammary gland
Fat
Skin
Hypothalamus
Pituitary
Brainstem
Brain cortex
Testes
Uterus
Bladder
Kidney
Pancreas
Liver
Colon
Small intestine
Tongue
Trachea
Lung
Aorta
Heart
Spleen
Blood
Bone marrow
Bone

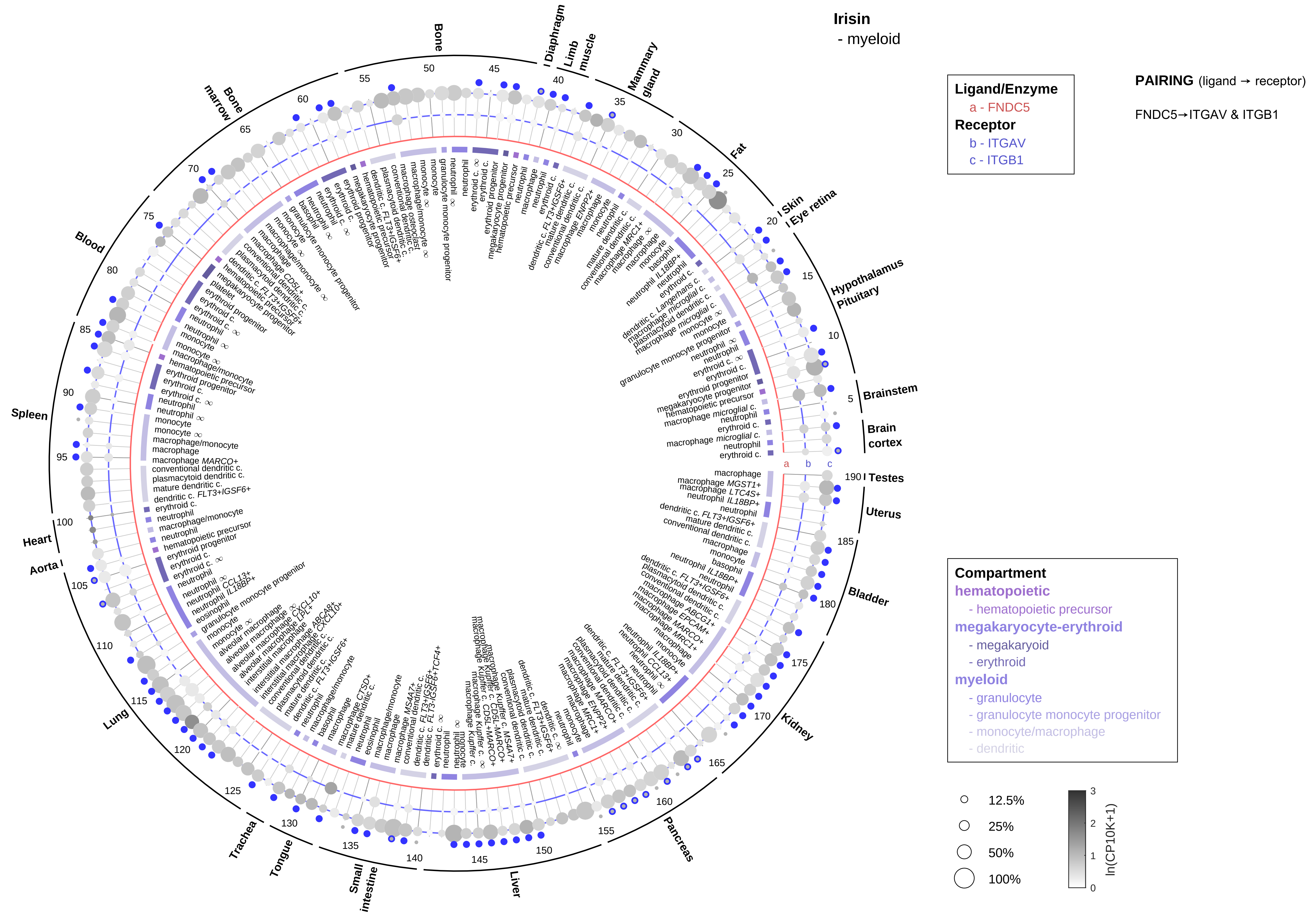
Irisin
- myeloid
Ligand/Enzyme
a - FNDC5
Receptor
b - ITGAV
c - ITGB1
PAIRING (ligand → receptor)
FNDC5→ITGAV & ITGB1
Compartment
hematopoietic
- hematopoietic precursor
megakaryocyte-erythroid
- megakaryoid
- erythroid
myeloid
- granulocyte
- granulocyte monocyte progenitor
- monocyte/macrophage
- dendritic
12.5%
25%
50%
100%
ln(CP10K+1)
Bone
Diaphragm
Limb muscle
Mammary gland
Fat
Skin
Eye retina
Hypothalamus
Pituitary
Brainstem
Brain cortex
Testes
Uterus
Bladder
Kidney
Pancreas
Liver
Small intestine
Tongue
Trachea
Lung
Aorta
Heart
Spleen
Blood
Bone marrow

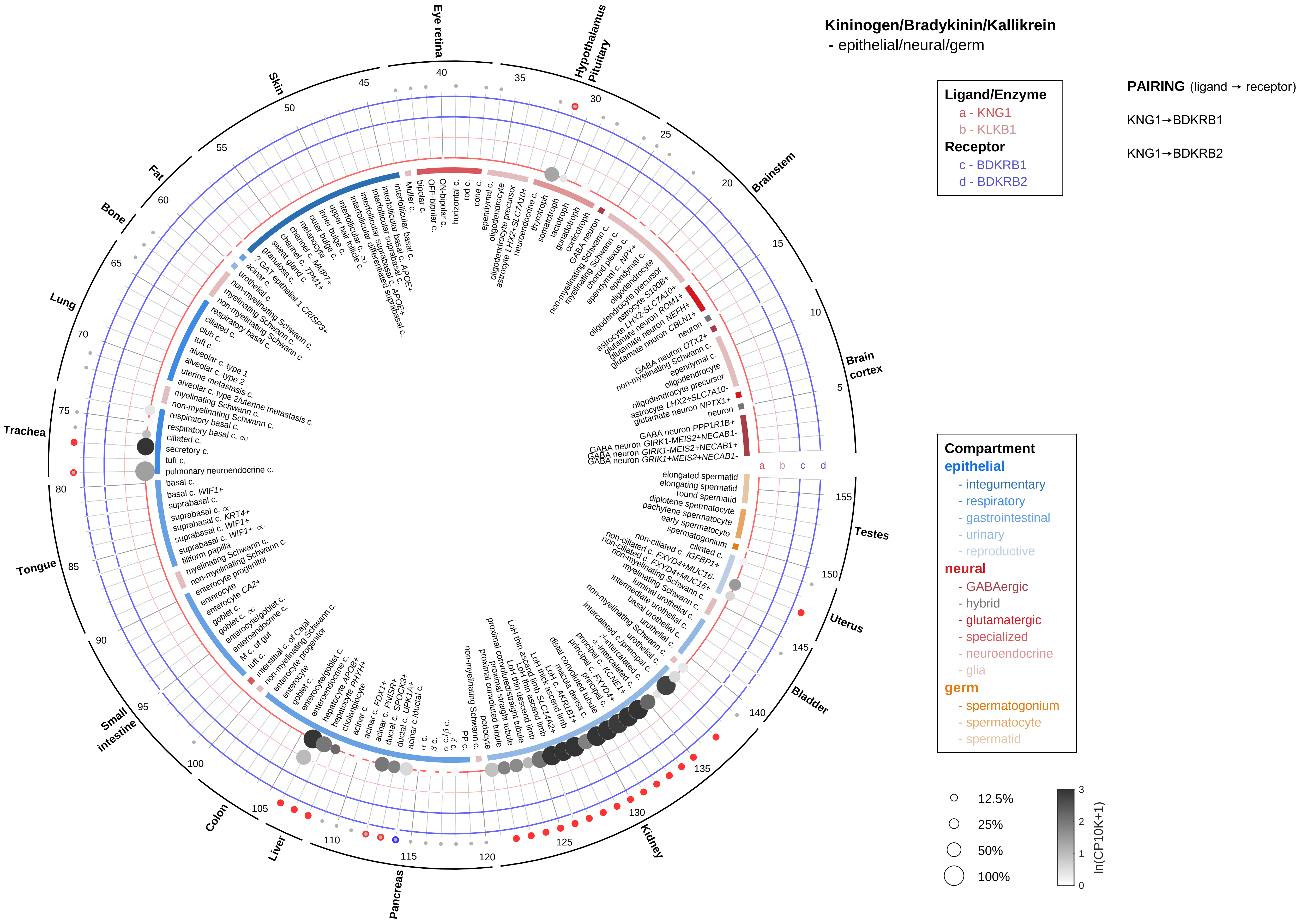

Kininogen/Bradykinin/Kallikrein
- epithelial/neural/germ
Ligand/Enzyme
a - KNG1
b - KLKB1
Receptor
c - BDKRB1
d - BDKRB2
PAIRING (ligand → receptor)
KNG1→BDKRB1
KNG1→BDKRB2
Compartment
epithelial
- integumentary
- respiratory
- gastrointestinal
- urinary
- reproductive
neural
- GABAergic
- hybrid
- glutamatergic
- specialized
- neuroendocrine
- glia
germ
- spermatogonium
- spermatocyte
- spermatid
12.5%
25%
50%
100%
ln(CP10K+1)
Eye retina
Hypothalamus Pituitary
Brainstem
Brain cortex
Testes
Uterus
Bladder
Kidney
Pancreas
Liver
Colon
Small intestine
Tongue
Trachea
Lung
Bone
Fat
Skin

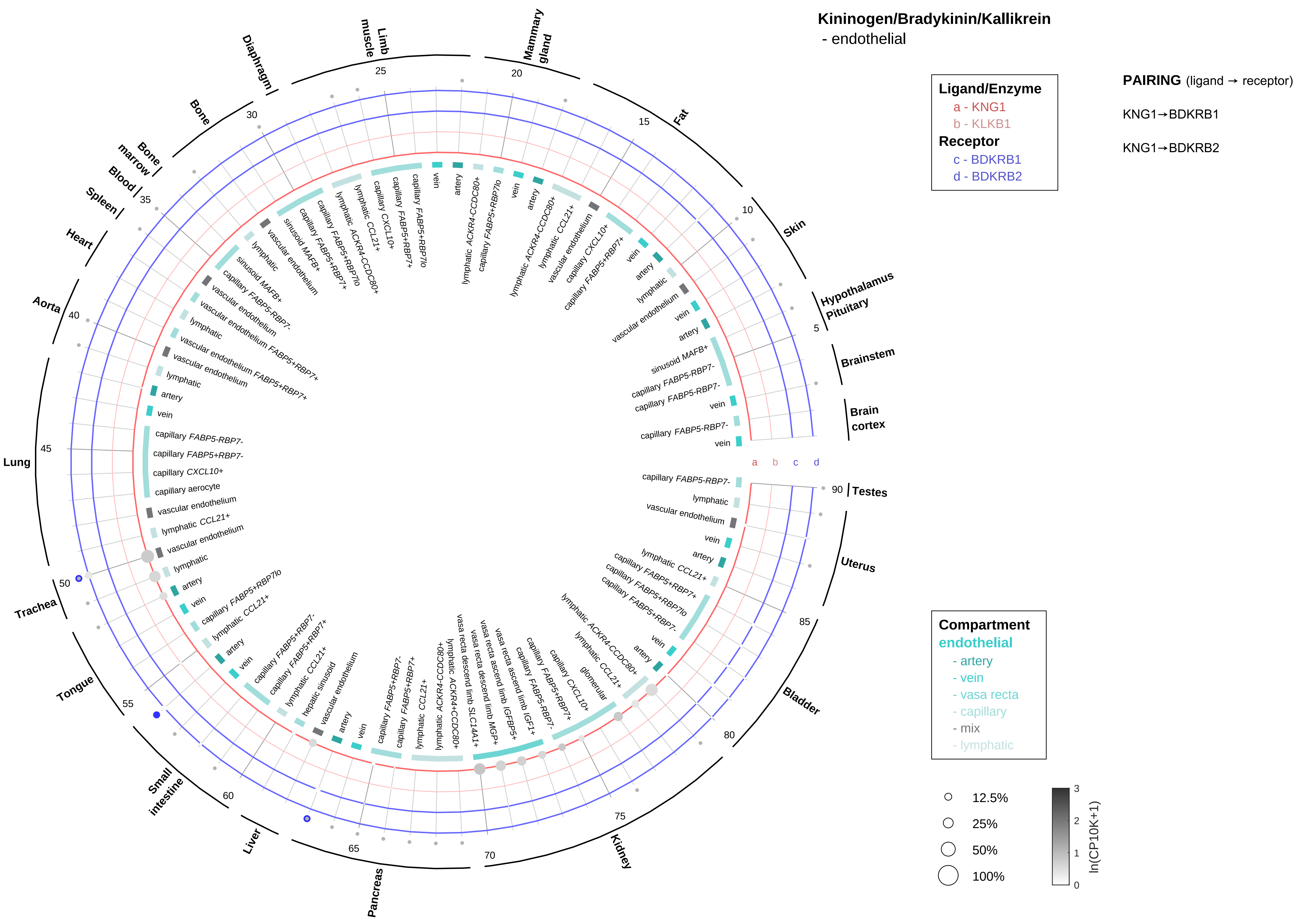

Kininogen/Bradykinin/Kallikrein
- endothelial
Ligand/Enzyme
a - KNG1
b - KLKB1
Receptor
c - BDKRB1
d - BDKRB2
PAIRING (ligand → receptor)
KNG1→BDKRB1
KNG1→BDKRB2
Compartment
endothelial
- artery
- vein
- vasa recta
- capillary
- mix
- lymphatic
12.5%
25%
50%
100%
ln(CP10K+1)

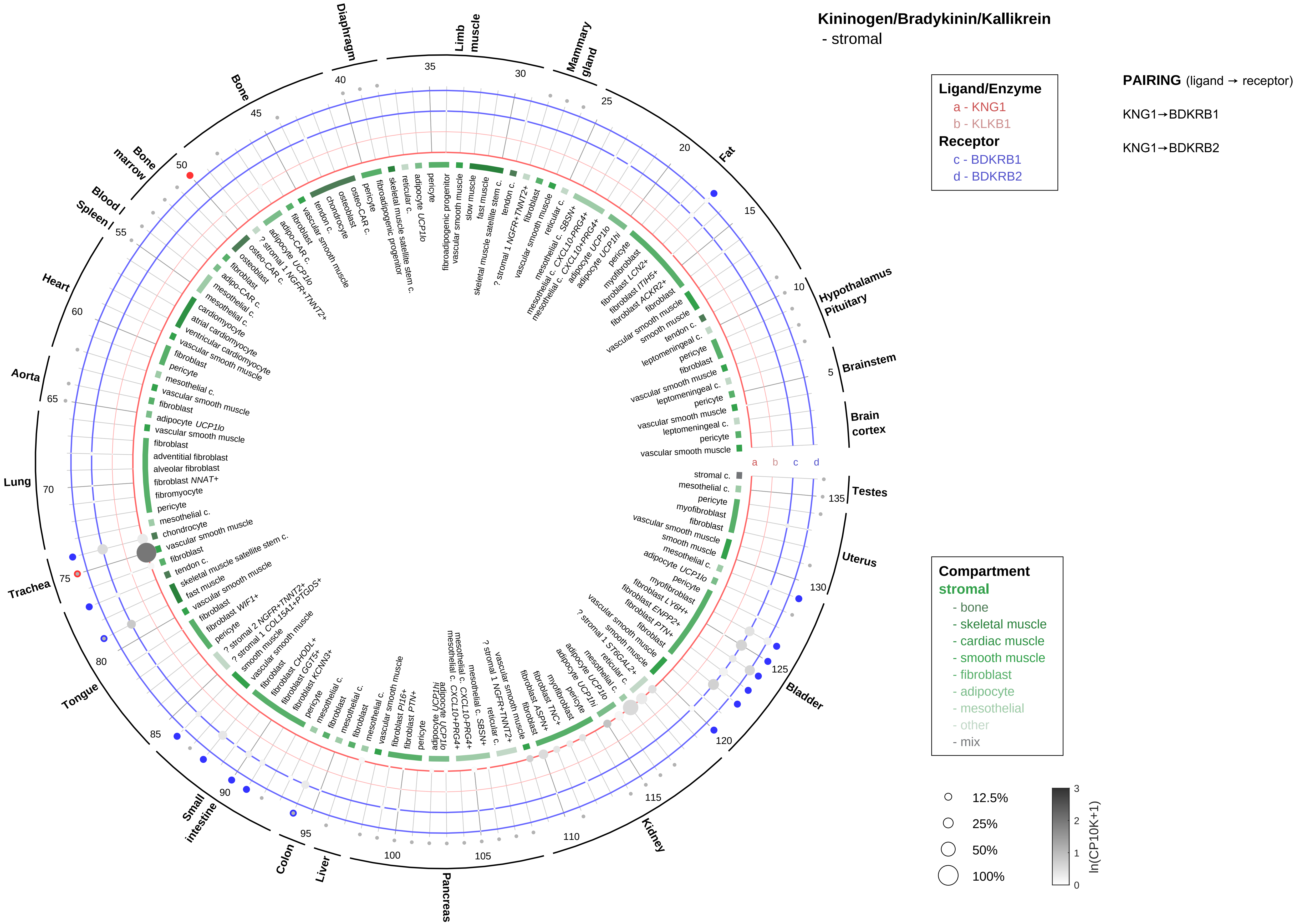
Kininogen/Bradykinin/Kallikrein
- stromal
Ligand/Enzyme
a - KNG1
b - KLKB1
Receptor
c - BDKRB1
d - BDKRB2
PAIRING (ligand → receptor)
KNG1→BDKRB1
KNG1→BDKRB2
Compartment
stromal
- bone
- skeletal muscle
- cardiac muscle
- smooth muscle
- fibroblast
- adipocyte
- mesothelial
- other
- mix
12.5%
25%
50%
100%
ln(CP10K+1)

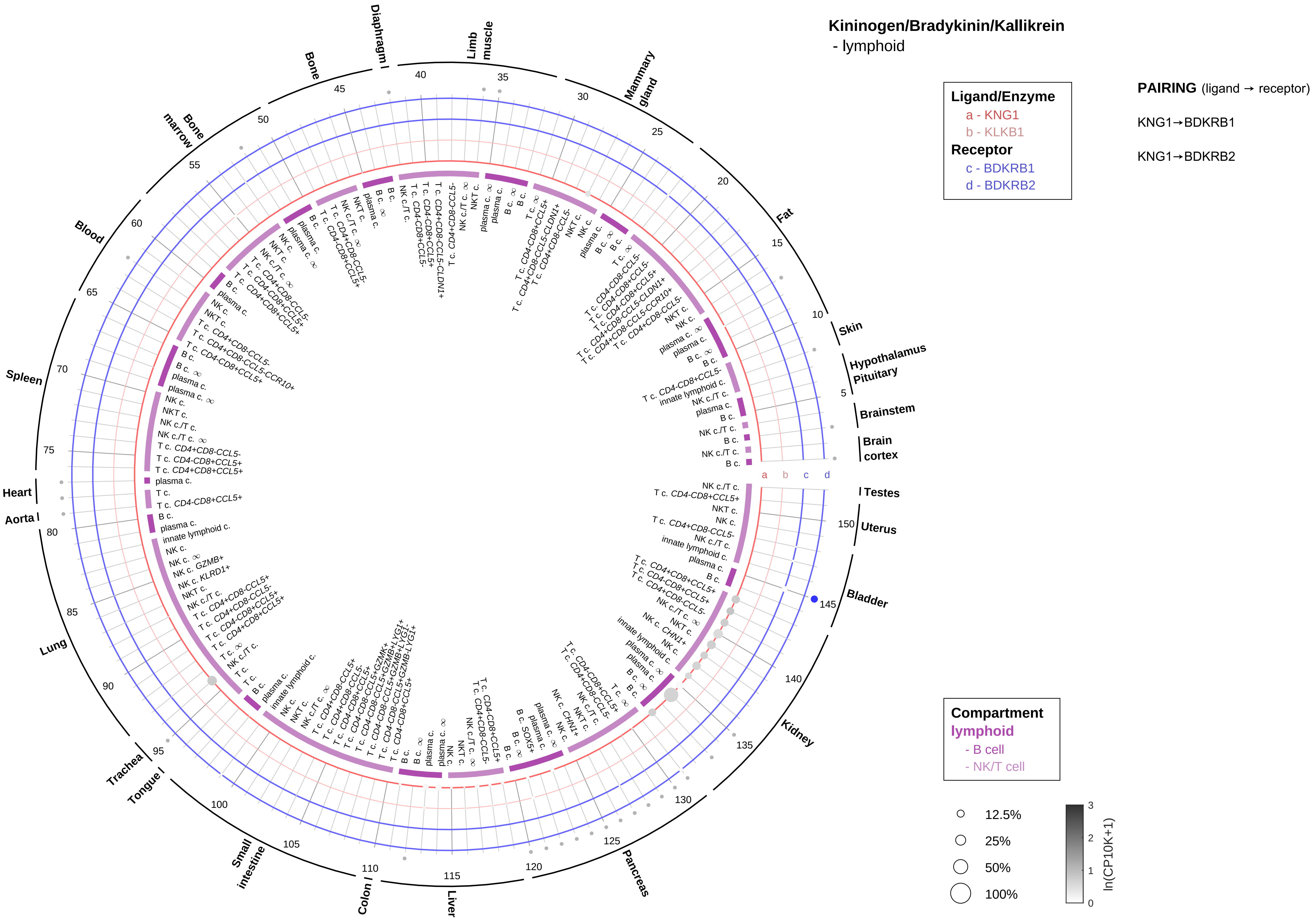

Kininogen/Bradykinin/Kallikrein
- lymphoid
Ligand/Enzyme
a - KNG1
b - KLKB1
Receptor
c - BDKRB1
d - BDKRB2
PAIRING (ligand → receptor)
KNG1→BDKRB1
KNG1→BDKRB2
Compartment
lymphoid
- B cell
- NK/T cell
12.5%
25%
50%
100%
ln(CP10K+1)
Skin
Hypothalamus
Pituitary
Brainstem
Brain cortex
Testes
Uterus
Bladder
Kidney
Pancreas
Liver
Colon
Small intestine
Tongue
Trachea
Lung
Aorta
Heart
Spleen
Blood
Bone marrow
Bone
Diaphragm
Limb muscle
Mammary gland
Fat

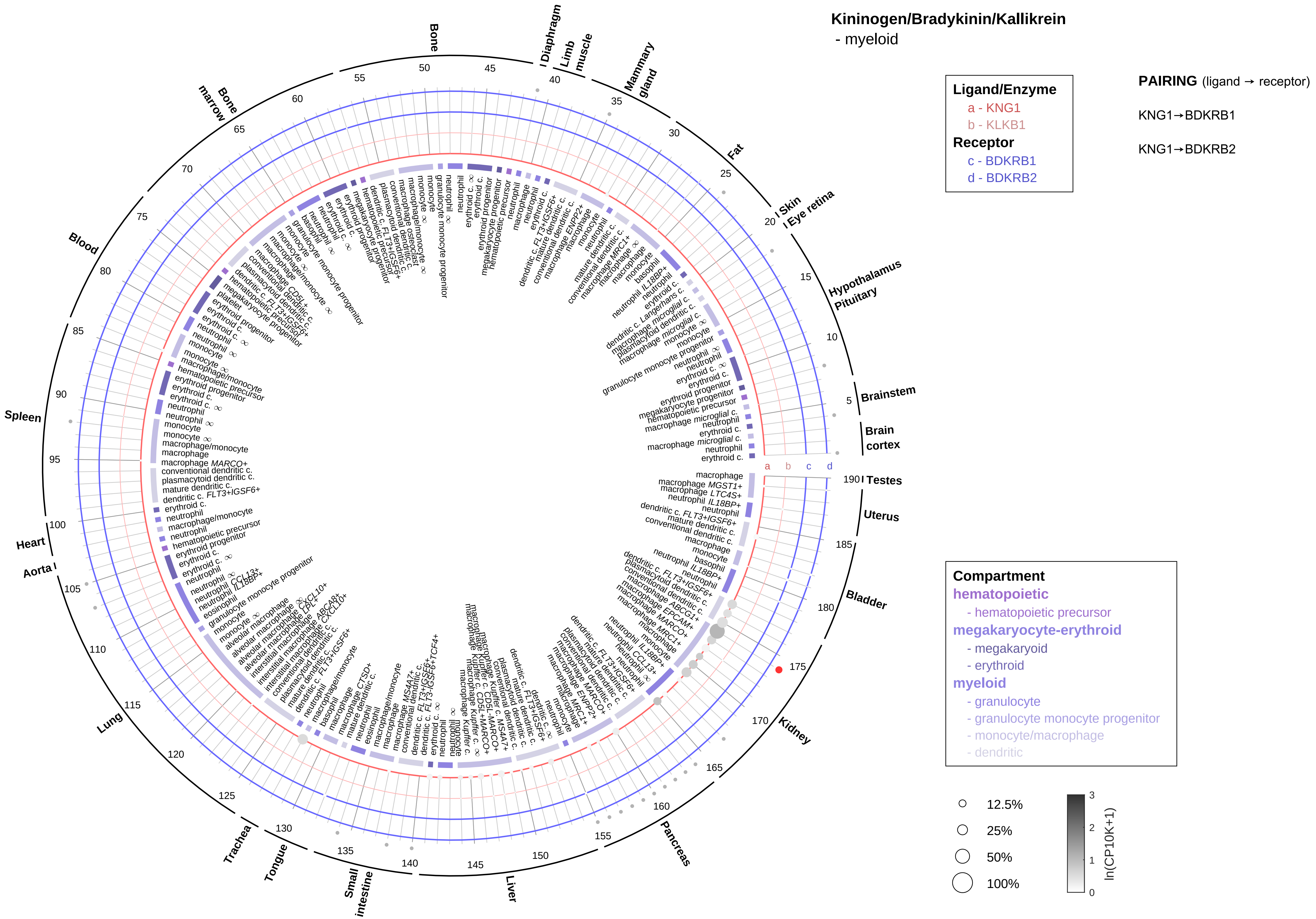
Kininogen/Bradykinin/Kallikrein
- myeloid
Ligand/Enzyme
a - KNG1
b - KLKB1
Receptor
c - BDKRB1
d - BDKRB2
PAIRING (ligand → receptor)
KNG1→BDKRB1
KNG1→BDKRB2
Compartment
hematopoietic
- hematopoietic precursor
megakaryocyte-erythroid
- megakaryoid
- erythroid
myeloid
- granulocyte
- granulocyte monocyte progenitor
- monocyte/macrophage
- dendritic
12.5%
25%
50%
100%
ln(CP10K+1)
Bone
Diaphragm
Limb muscle
Mammary gland
Fat
Skin
Eye retina
Hypothalamus
Pituitary
Brainstem
Brain cortex
Testes
Uterus
Bladder
Kidney
Pancreas
Liver
Small intestine
Tongue
Trachea
Lung
Aorta
Heart
Spleen
Blood
Bone marrow

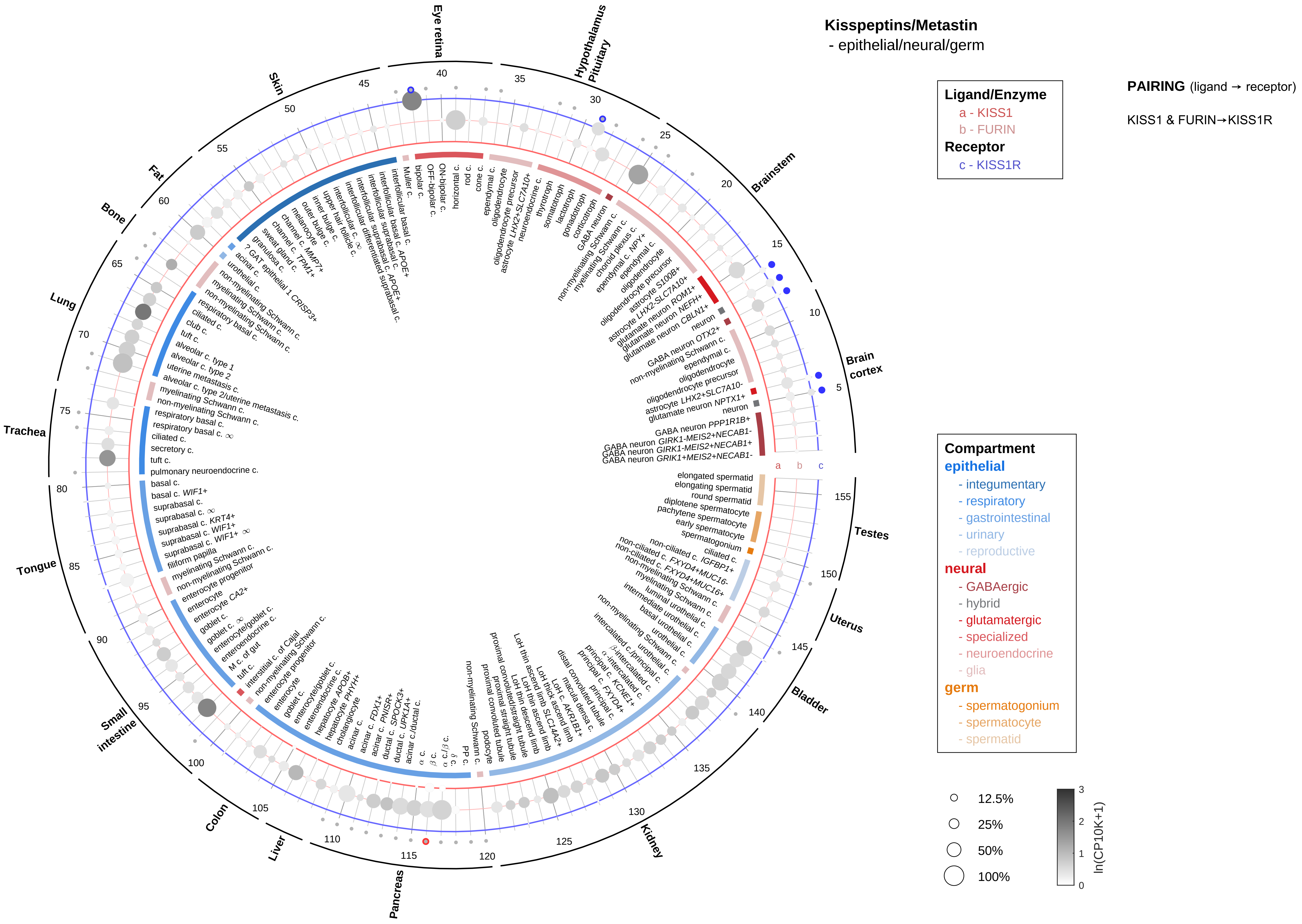
Kisspeptins/Metastin
- epithelial/neural/germ
Ligand/Enzyme
a - KISS1
b - FURIN
Receptor
c - KISS1R
PAIRING (ligand → receptor)
KISS1 & FURIN→KISS1R
Eye retina
Hypothalamus Pituitary
Brainstem
Brain cortex
Testes
Uterus
Bladder
Kidney
Pancreas
Liver
Colon
Small intestine
Tongue
Trachea
Lung
Bone
Fat
Skin
Compartment
epithelial
- integumentary
- respiratory
- gastrointestinal
- urinary
- reproductive
neural
- GABAergic
- hybrid
- glutamatergic
- specialized
- neuroendocrine
- glia
germ
- spermatogonium
- spermatocyte
- spermatid
12.5%
25%
50%
100%
ln(CP10K+1)

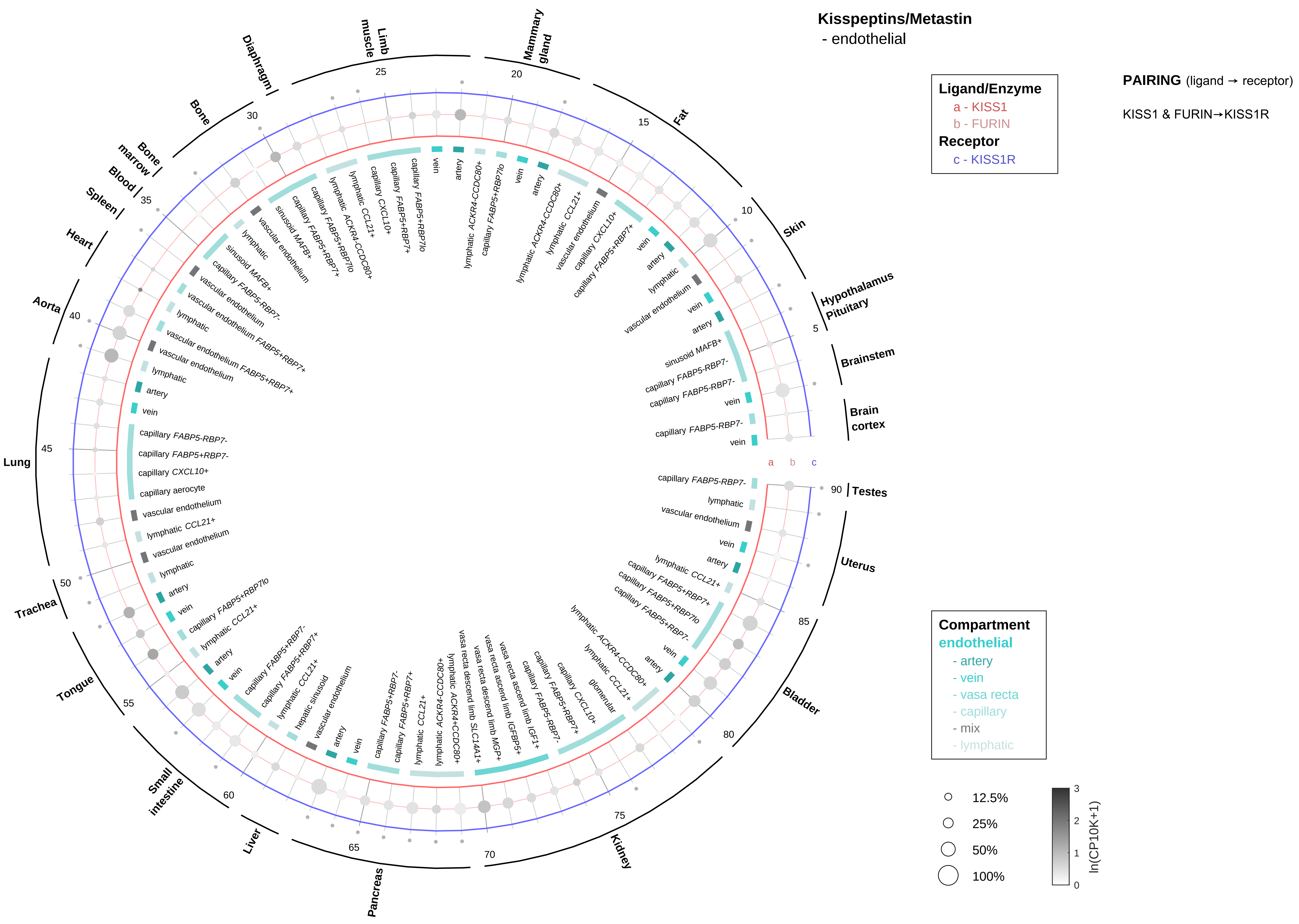
Kisspeptins/Metastin
- endothelial
Ligand/Enzyme
a - KISS1
b - FURIN
Receptor
c - KISS1R
PAIRING (ligand → receptor)
KISS1 & FURIN→KISS1R
Compartment
endothelial
- artery
- vein
- vasa recta
- capillary
- mix
- lymphatic
12.5%
25%
50%
100%
ln(CP10K+1)
Testes
Uterus
Bladder
Kidney
Pancreas
Liver
Small intestine
Tongue
Trachea
Lung
Aorta
Heart
Spleen
Blood
Bone marrow
Bone
Diaphragm
Limb muscle
Mammary gland
Fat
Skin
Hypothalamus
Pituitary
Brainstem
Brain cortex

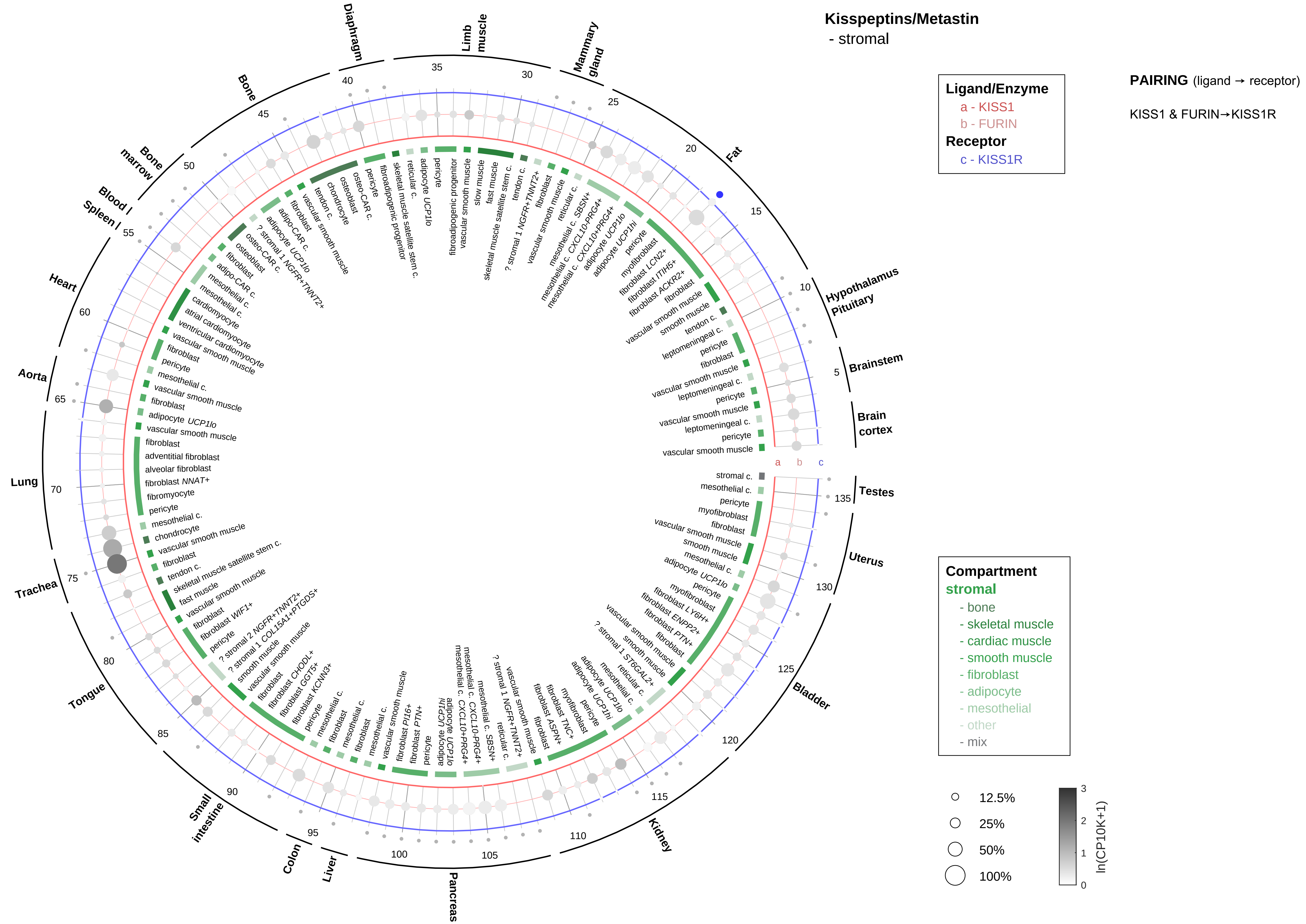

Kisspeptins/Metastin
- stromal
Ligand/Enzyme
a - KISS1
b - FURIN
Receptor
c - KISS1R
PAIRING (ligand → receptor)
KISS1 & FURIN→KISS1R
Compartment
stromal
- bone
- skeletal muscle
- cardiac muscle
- smooth muscle
- fibroblast
- adipocyte
- mesothelial
- other
- mix
12.5%
25%
50%
100%
ln(CP10K+1)
Brain cortex
Brainstem
Hypothalamus Pituitary
Fat
Mammary gland
Limb muscle
Diaphragm
Bone
Bone marrow
Blood / Spleen
Heart
Aorta
Lung
Trachea
Tongue
Small intestine
Colon
Liver
Pancreas
Kidney
Bladder
Uterus
Testes

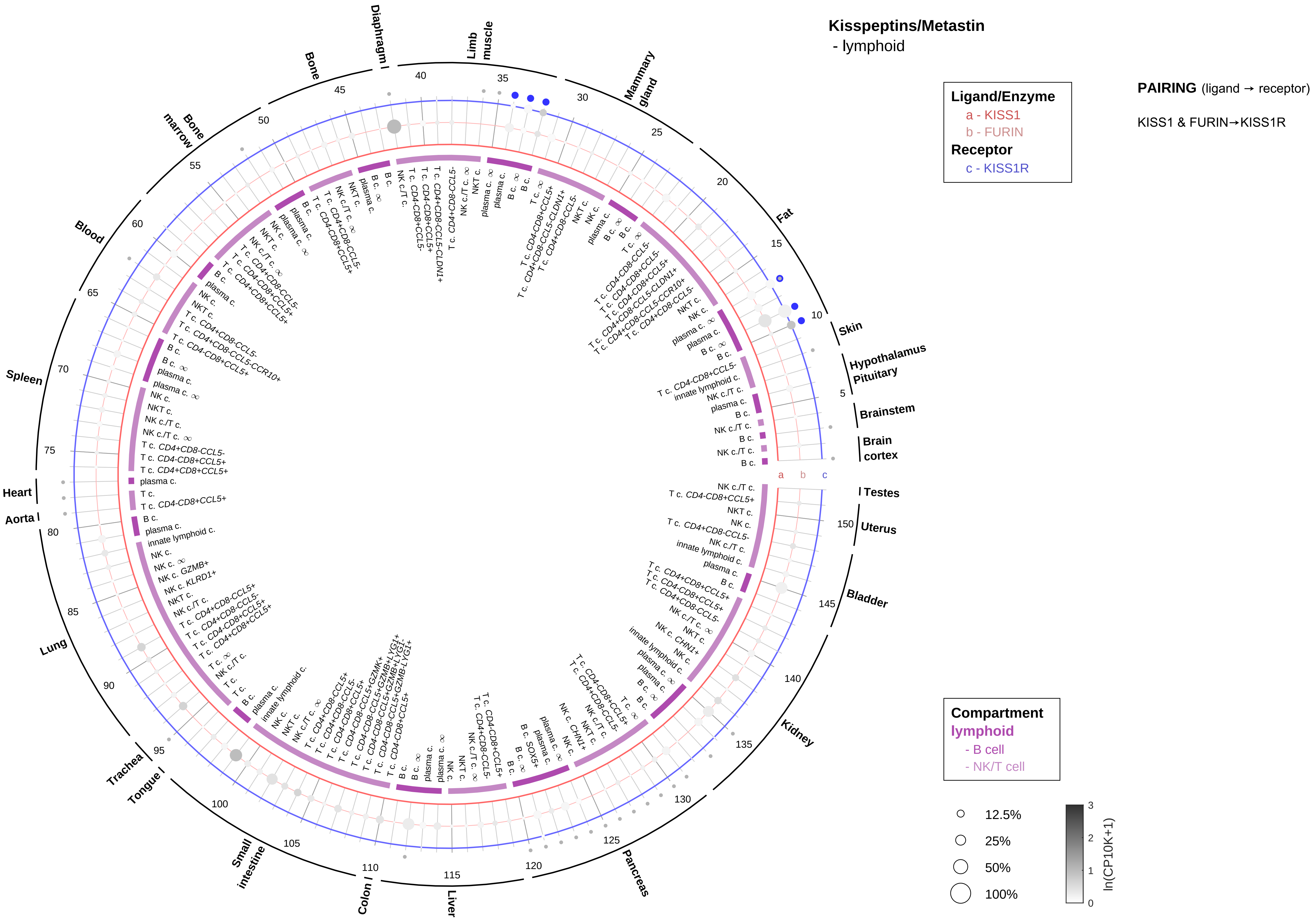
Kisspeptins/Metastin
- lymphoid
Ligand/Enzyme
a - KISS1
b - FURIN
Receptor
c - KISS1R
PAIRING (ligand → receptor)
KISS1 & FURIN→KISS1R
Compartment
lymphoid
- B cell
- NK/T cell
12.5%
25%
50%
100%
ln(CP10K+1)
Diaphragm
Limb muscle
Mammary gland
Fat
Skin
Hypothalamus Pituitary
Brainstem
Brain cortex
Testes
Uterus
Bladder
Kidney
Pancreas
Liver
Colon
Small intestine
Tongue
Trachea
Lung
Aorta
Heart
Spleen
Blood
Bone marrow
Bone

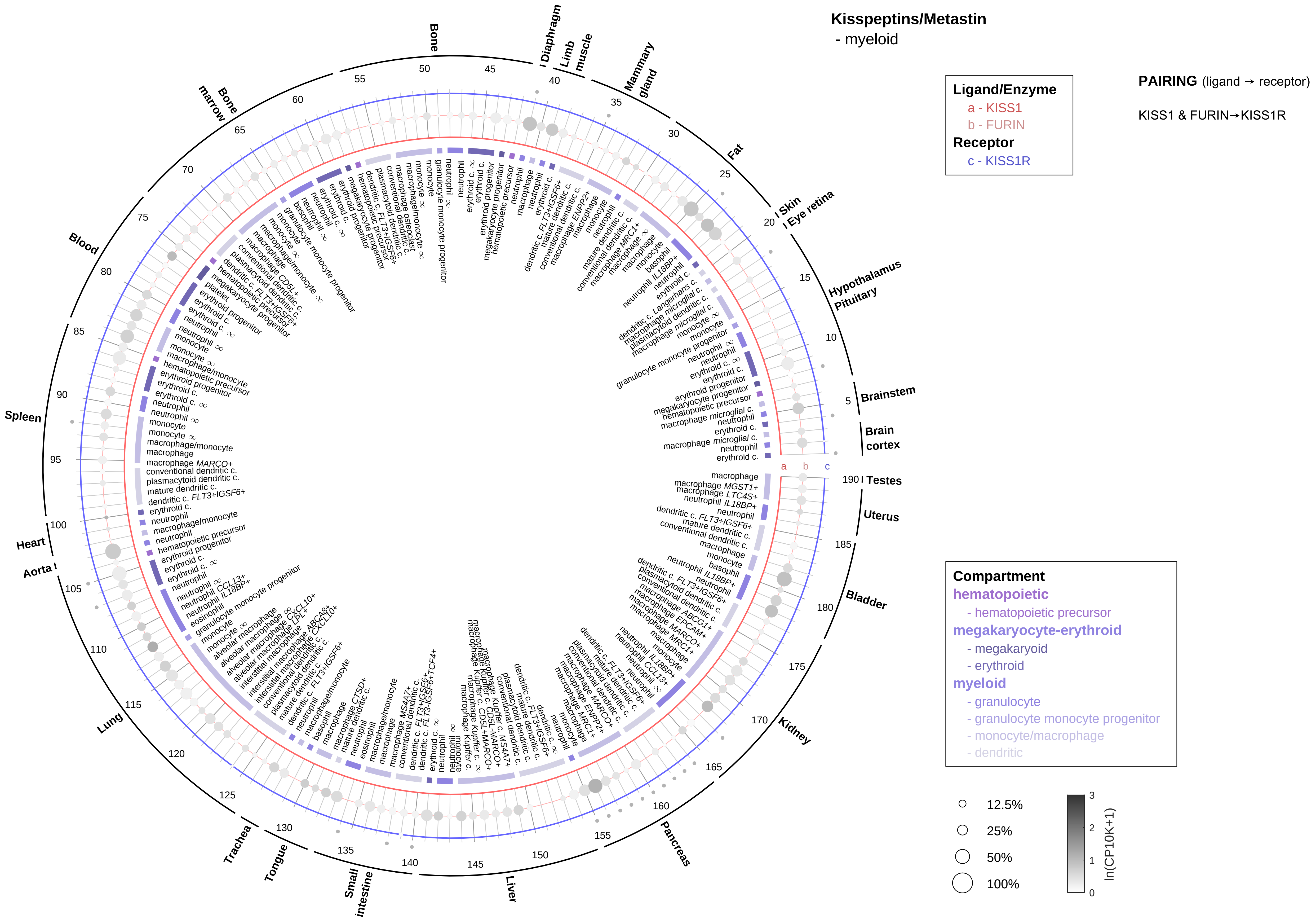
Kisspeptins/Metastin
- myeloid
Ligand/Enzyme
a - KISS1
b - FURIN
Receptor
c - KISS1R
PAIRING (ligand → receptor)
KISS1 & FURIN→KISS1R
Compartment
hematopoietic
- hematopoietic precursor
megakaryocyte-erythroid
- megakaryoid
- erythroid
myeloid
- granulocyte
- granulocyte monocyte progenitor
- monocyte/macrophage
- dendritic
12.5%
25%
50%
100%
ln(CP10K+1)
Bone
Diaphragm
Limb muscle
Mammary gland
Fat
Skin
Eye retina
Hypothalamus
Pituitary
Brainstem
Brain cortex
Testes
Uterus
Bladder
Kidney
Pancreas
Liver
Small intestine
Tongue
Trachea
Lung
Aorta
Heart
Spleen
Blood
Bone marrow

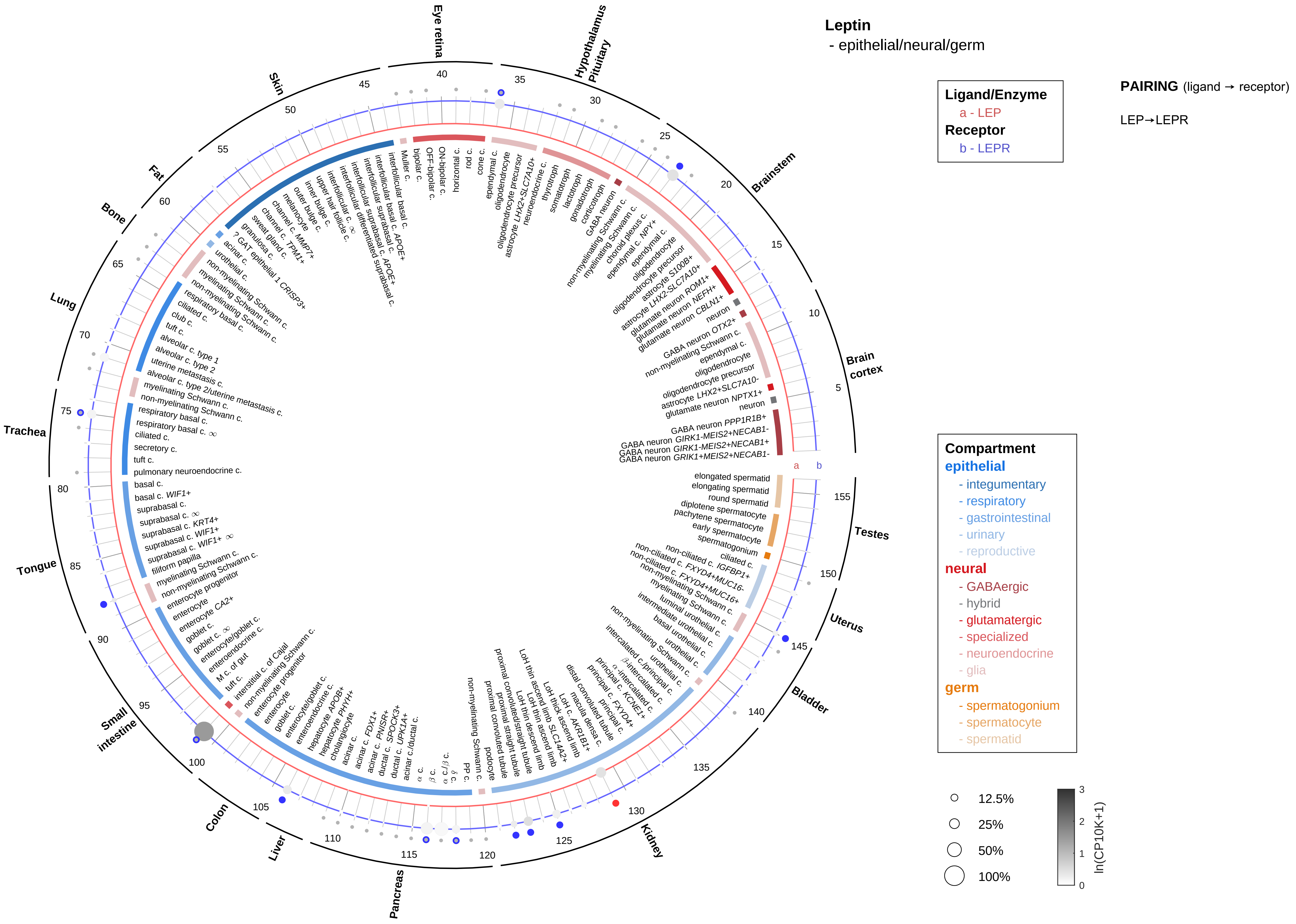

Leptin
- epithelial/neural/germ
Ligand/Enzyme
a - LEP
Receptor
b - LEPR
PAIRING (ligand → receptor)
LEP→LEPR
Compartment
epithelial
- integumentary
- respiratory
- gastrointestinal
- urinary
- reproductive
neural
- GABAergic
- hybrid
- glutamatergic
- specialized
- neuroendocrine
- glia
germ
- spermatogonium
- spermatocyte
- spermatid
12.5%
25%
50%
100%
ln(CP10K+1)
Eye retina
Hypothalamus Pituitary
Brainstem
Brain cortex
Testes
Uterus
Bladder
Kidney
Pancreas
Liver
Colon
Small intestine
Tongue
Trachea
Lung
Bone
Fat
Skin

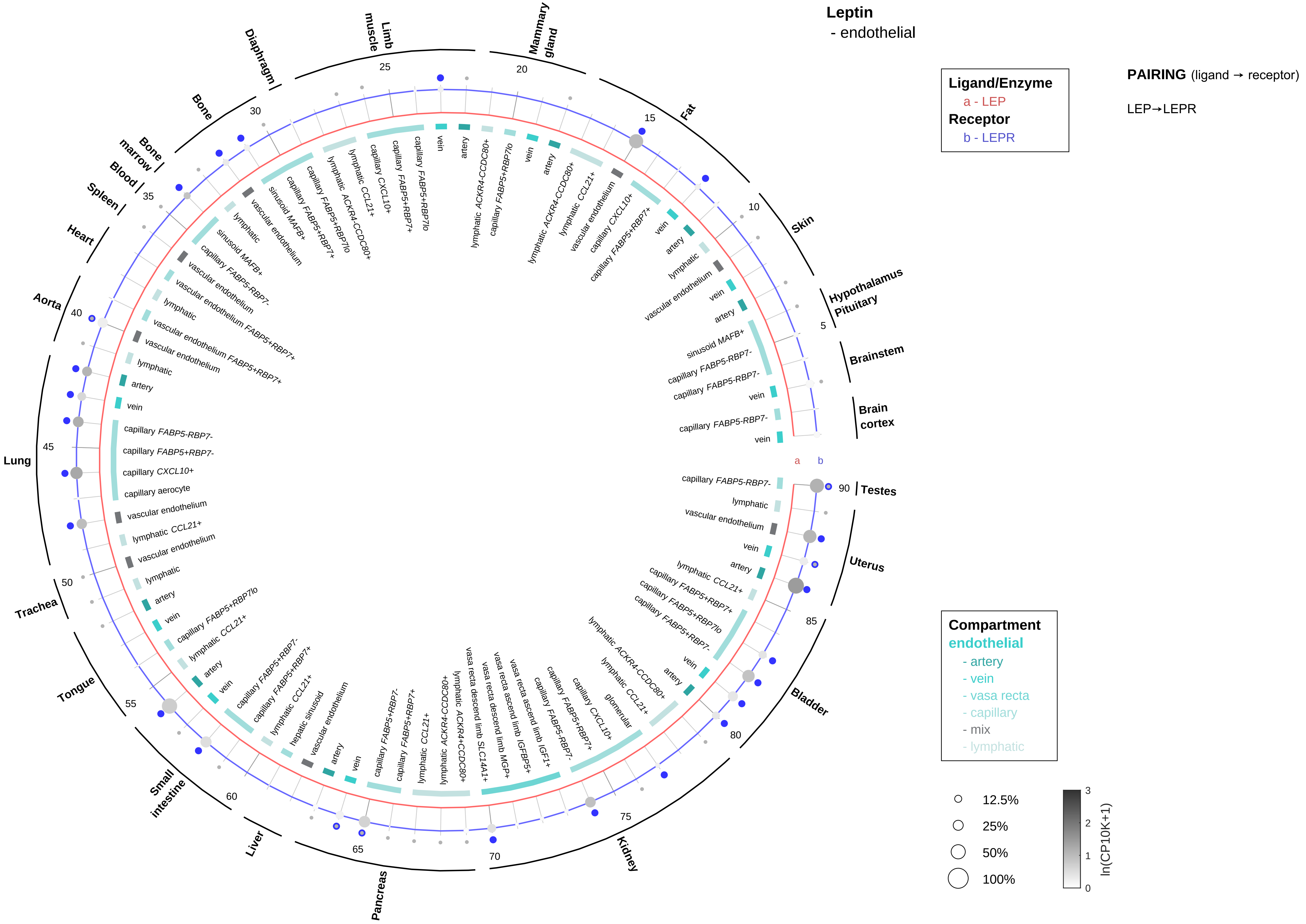

Leptin
- endothelial
Ligand/Enzyme
a - LEP
Receptor
b - LEPR
PAIRING (ligand → receptor)
LEP→LEPR
Compartment
endothelial
- artery
- vein
- vasa recta
- capillary
- mix
- lymphatic
12.5%
25%
50%
100%
ln(CP10K+1)
0
1
2
3
Testes
Uterus
Bladder
Kidney
Pancreas
Liver
Small intestine
Tongue
Trachea
Lung
Aorta
Heart
Spleen
Blood
Bone marrow
Bone
Diaphragm
Limb muscle
Mammary gland
Fat
Skin
Hypothalamus Pituitary
Brainstem
Brain cortex
a
b
5
10
15
20
25
30
35
40
45
50
55
60
65
70
75
80
85
90

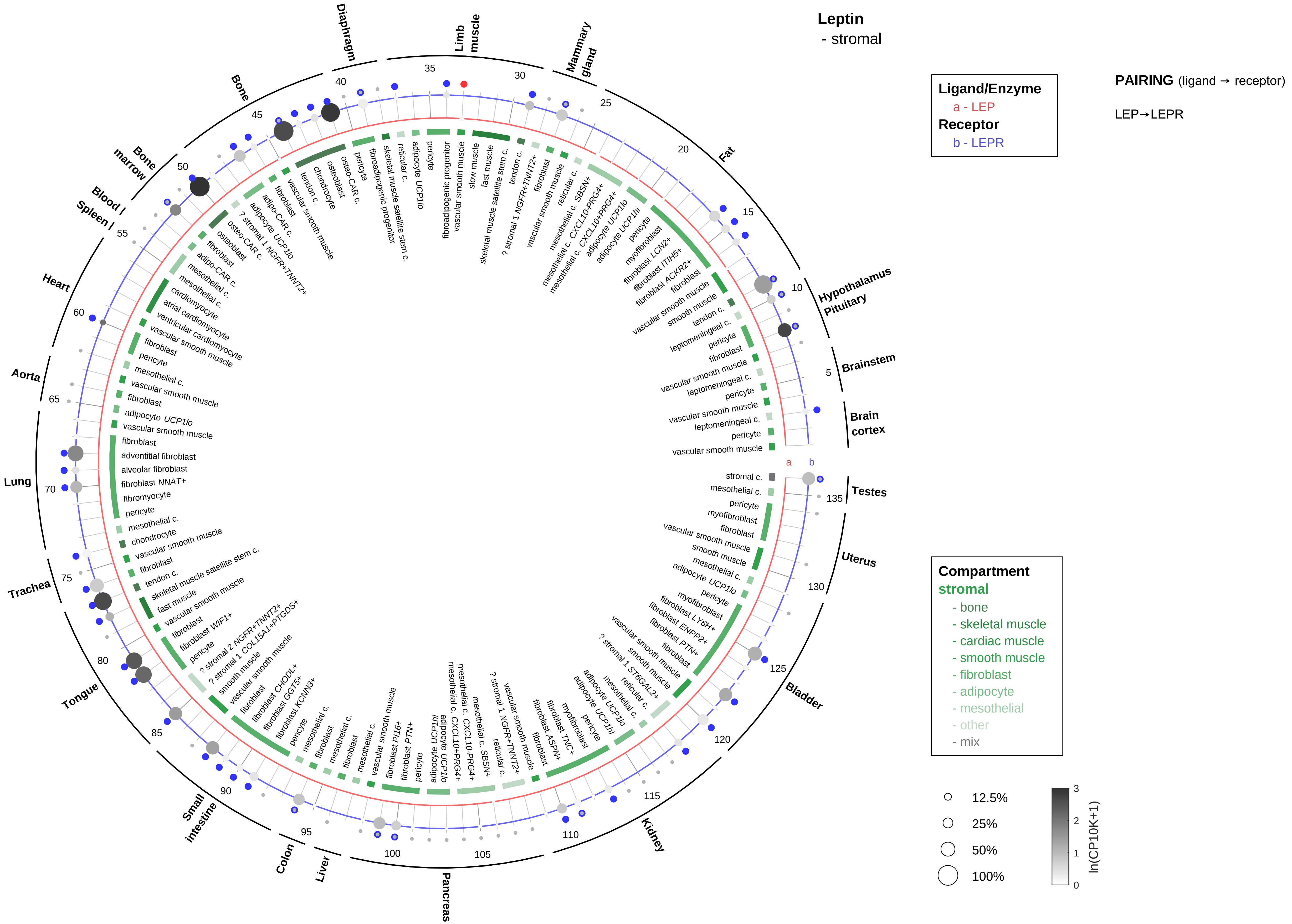

Leptin
- stromal
Ligand/Enzyme
a - LEP
Receptor
b - LEPR
PAIRING (ligand → receptor)
LEP→LEPR
Compartment
stromal
- bone
- skeletal muscle
- cardiac muscle
- smooth muscle
- fibroblast
- adipocyte
- mesothelial
- other
- mix
12.5%
25%
50%
100%
ln(CP10K+1)
0
1
2
3
Limb muscle
Mammary gland
Fat
Hypothalamus
Pituitary
Brainstem
Brain cortex
Testes
Uterus
Bladder
Kidney
Pancreas
Liver
Colon
Small intestine
Tongue
Trachea
Lung
Aorta
Heart
Spleen
Blood
Bone marrow
Bone
Diaphragm
a
b

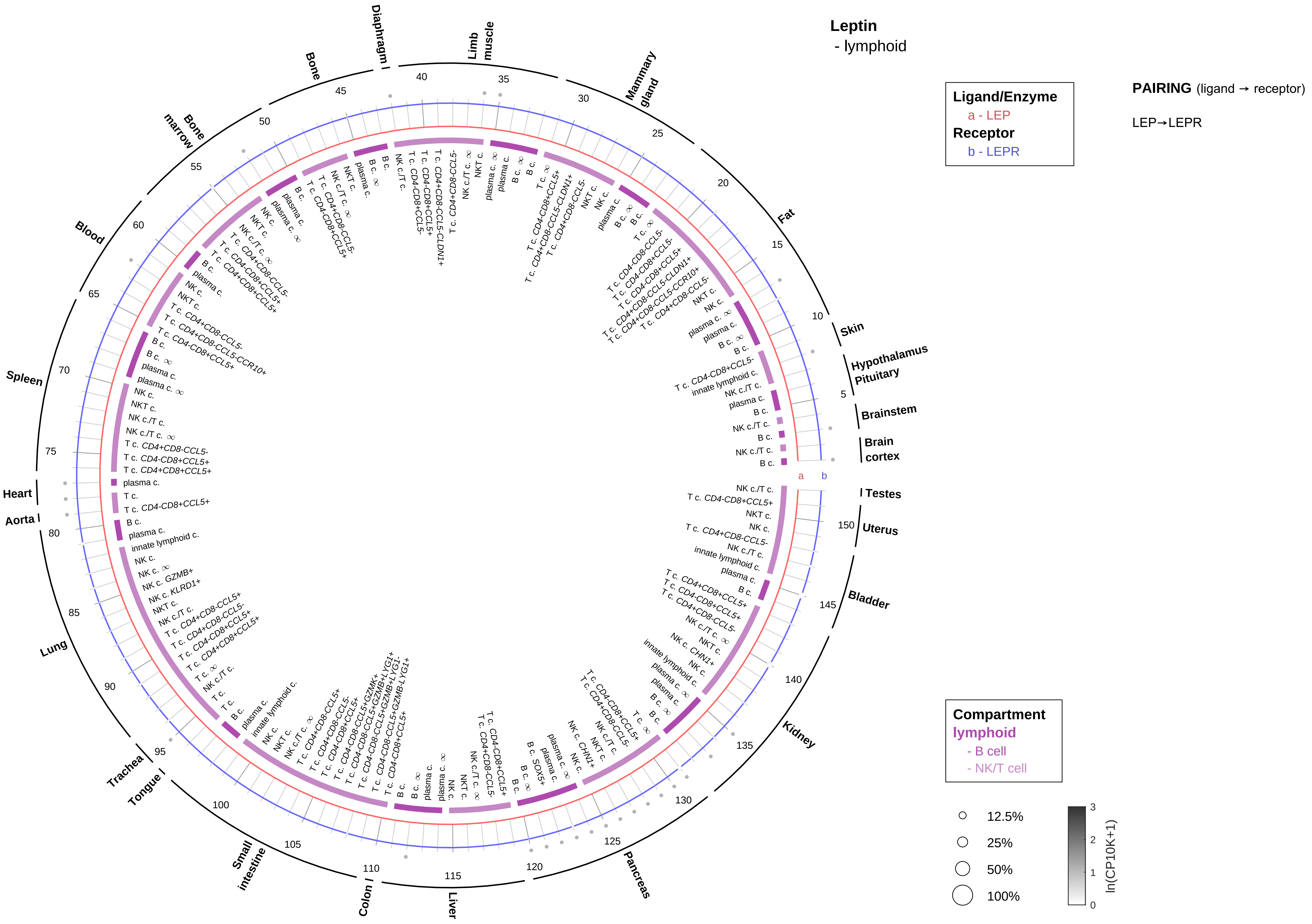

Leptin
- lymphoid
Ligand/Enzyme
a - LEP
Receptor
b - LEPR
PAIRING (ligand → receptor)
LEP→LEPR
Compartment
lymphoid
- B cell
- NK/T cell
12.5%
25%
50%
100%
ln(CP10K+1)
Skin
Hypothalamus
Pituitary
Brainstem
Brain cortex
Testes
Uterus
Bladder
Kidney
Pancreas
Liver
Colon
Small intestine
Tongue
Trachea
Lung
Aorta
Heart
Spleen
Blood
Bone marrow
Bone
Diaphragm
Limb muscle
Mammary gland
Fat

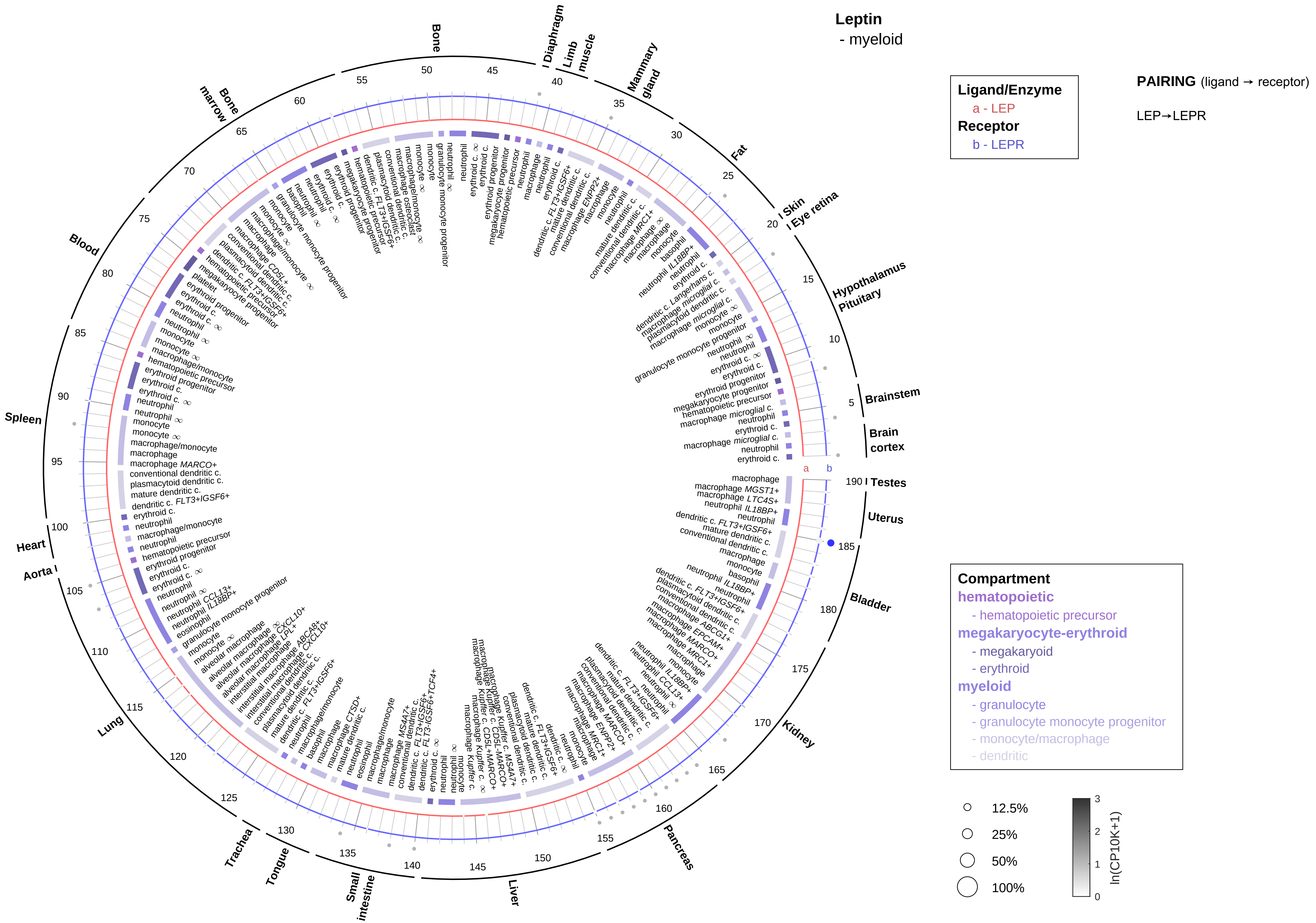
Leptin
- myeloid
Ligand/Enzyme
a - LEP
Receptor
b - LEPR
PAIRING (ligand → receptor)
LEP→LEPR
Compartment
hematopoietic
- hematopoietic precursor
megakaryocyte-erythroid
- megakaryoid
- erythroid
myeloid
- granulocyte
- granulocyte monocyte progenitor
- monocyte/macrophage
- dendritic
12.5%
25%
50%
100%
ln(CP10K+1)
Brain cortex
Brainstem
Hypothalamus
Pituitary
Eye retina
Skin
Fat
Mammary gland
Limb muscle
Diaphragm
Bone
Bone marrow
Blood
Spleen
Heart
Aorta
Lung
Trachea
Tongue
Small intestine
Liver
Pancreas
Kidney
Bladder
Uterus
Testes

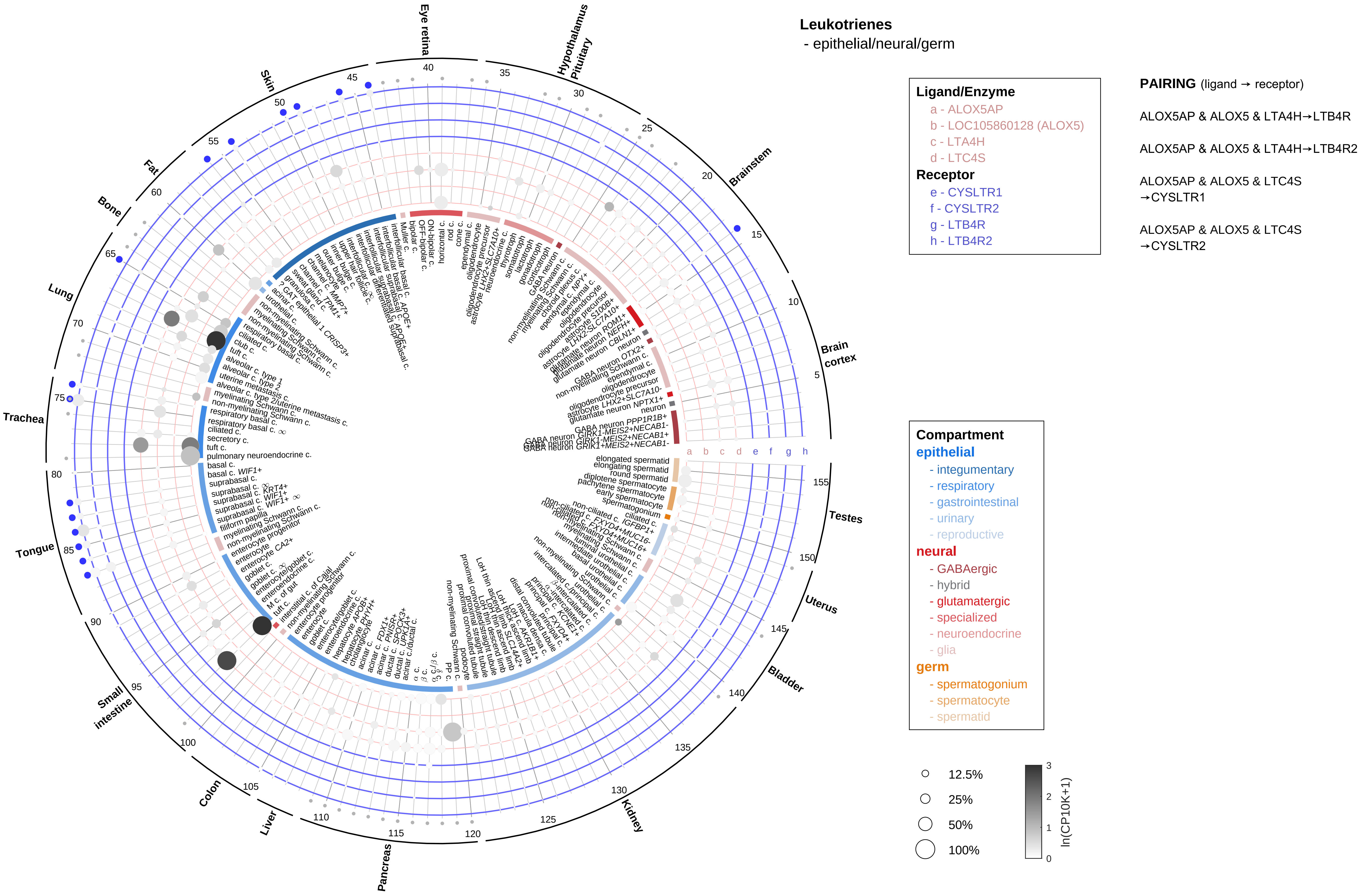
Leukotrienes
- epithelial/neural/germ
Ligand/Enzyme
a - ALOX5AP
b - LOC105860128 (ALOX5)
c - LTA4H
d - LTC4S
Receptor
e - CYSLTR1
f - CYSLTR2
g - LTB4R
h - LTB4R2
PAIRING (ligand → receptor)
ALOX5AP & ALOX5 & LTA4H→LTB4R
ALOX5AP & ALOX5 & LTA4H→LTB4R2
ALOX5AP & ALOX5 & LTC4S →CYSLTR1
ALOX5AP & ALOX5 & LTC4S →CYSLTR2
Compartment
epithelial
- integumentary
- respiratory
- gastrointestinal
- urinary
- reproductive
neural
- GABAergic
- hybrid
- glutamatergic
- specialized
- neuroendocrine
- glia
germ
- spermatogonium
- spermatocyte
- spermatid
12.5%
25%
50%
100%
ln(CP10K+1)
Eye retina
Hypothalamus Pituitary
Brainstem
Brain cortex
Testes
Uterus
Bladder
Kidney
Pancreas
Liver
Colon
Small intestine
Tongue
Trachea
Lung
Bone
Fat
Skin

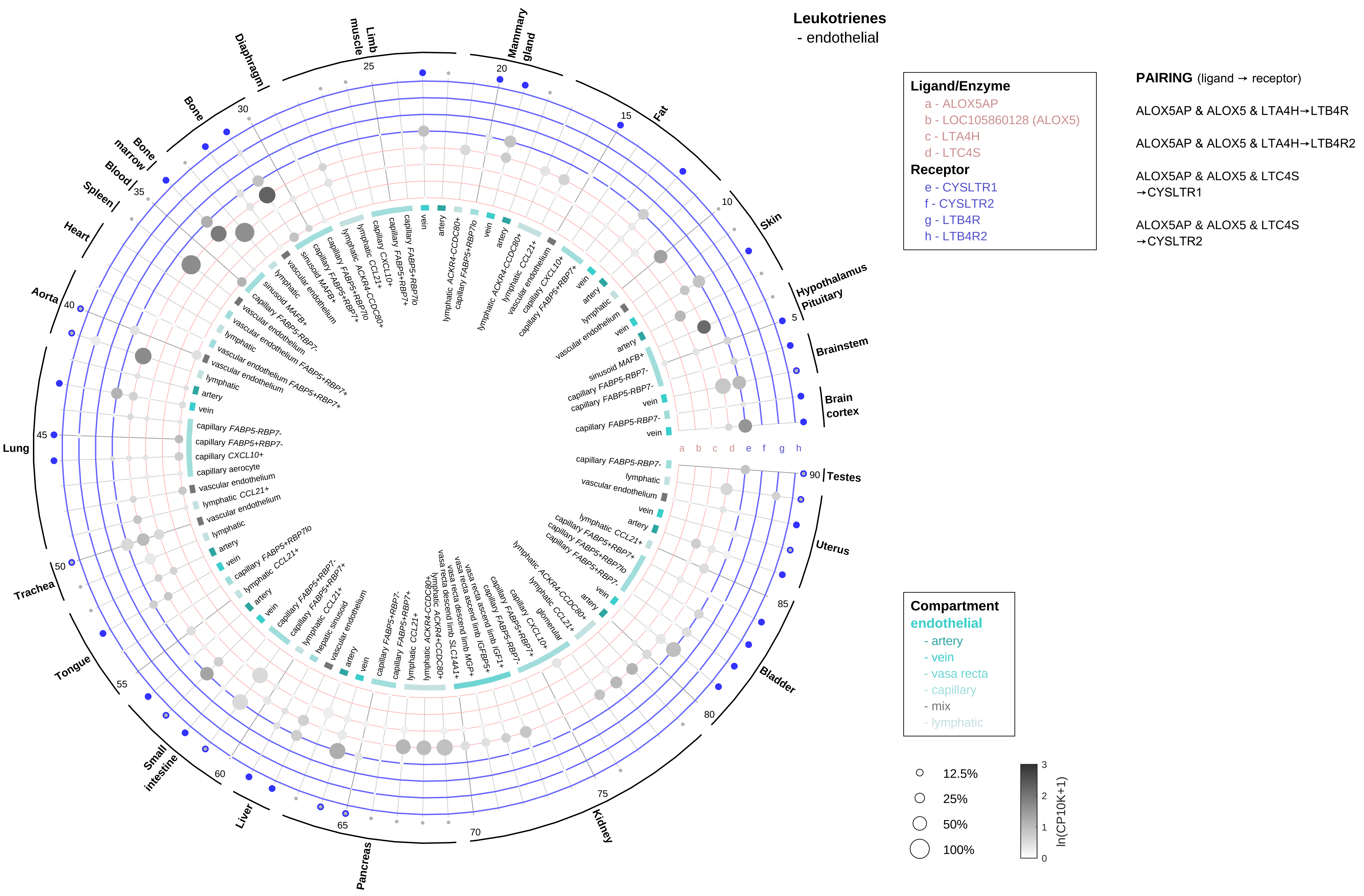

Leukotrienes
- endothelial
Ligand/Enzyme
a - ALOX5AP
b - LOC105860128 (ALOX5)
c - LTA4H
d - LTC4S
Receptor
e - CYSLTR1
f - CYSLTR2
g - LTB4R
h - LTB4R2
PAIRING (ligand → receptor)
ALOX5AP & ALOX5 & LTA4H→LTB4R
ALOX5AP & ALOX5 & LTA4H→LTB4R2
ALOX5AP & ALOX5 & LTC4S →CYSLTR1
ALOX5AP & ALOX5 & LTC4S →CYSLTR2
Compartment
endothelial
- artery
- vein
- vasa recta
- capillary
- mix
- lymphatic
12.5%
25%
50%
100%
ln(CP10K+1)
Brain cortex
Brainstem
Hypothalamus Pituitary
Skin
Fat
Mammary gland
Limb muscle
Diaphragm
Bone
Bone marrow
Blood
Spleen
Heart
Aorta
Lung
Trachea
Tongue
Small intestine
Liver
Pancreas
Kidney
Bladder
Uterus
Testes

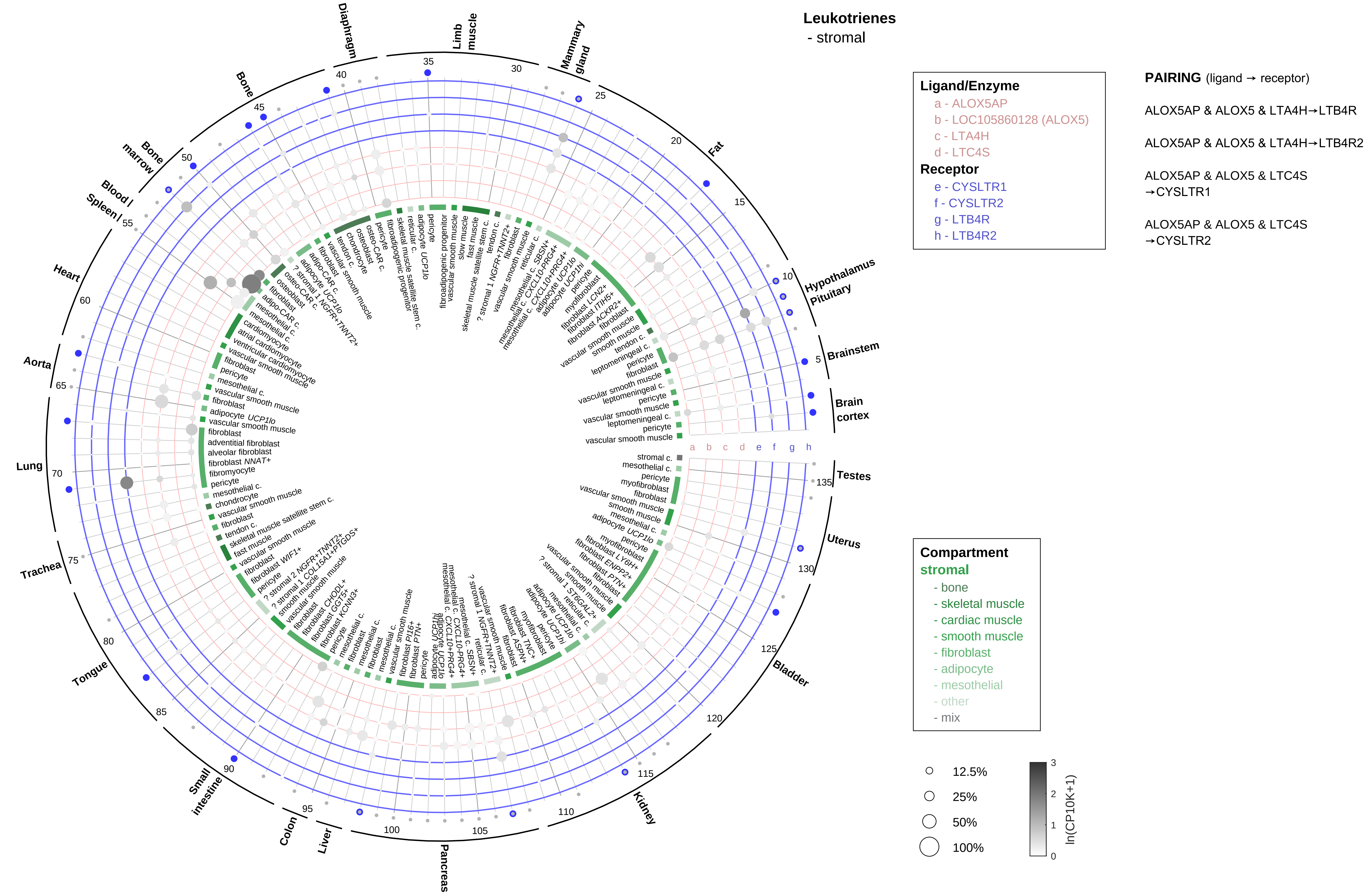

Leukotrienes
- stromal
Ligand/Enzyme
a - ALOX5AP
b - LOC105860128 (ALOX5)
c - LTA4H
d - LTC4S
Receptor
e - CYSLTR1
f - CYSLTR2
g - LTB4R
h - LTB4R2
PAIRING (ligand → receptor)
ALOX5AP & ALOX5 & LTA4H→LTB4R
ALOX5AP & ALOX5 & LTA4H→LTB4R2
ALOX5AP & ALOX5 & LTC4S →CYSLTR1
ALOX5AP & ALOX5 & LTC4S →CYSLTR2
Compartment
stromal
- bone
- skeletal muscle
- cardiac muscle
- smooth muscle
- fibroblast
- adipocyte
- mesothelial
- other
- mix
12.5%
25%
50%
100%
ln(CP10K+1)
Diaphragm
Limb muscle
Mammary gland
Fat
Hypothalamus Pituitary
Brainstem
Brain cortex
Testes
Uterus
Bladder
Kidney
Pancreas
Liver
Colon
Small intestine
Tongue
Trachea
Lung
Aorta
Heart
Spleen
Blood
Bone marrow
Bone

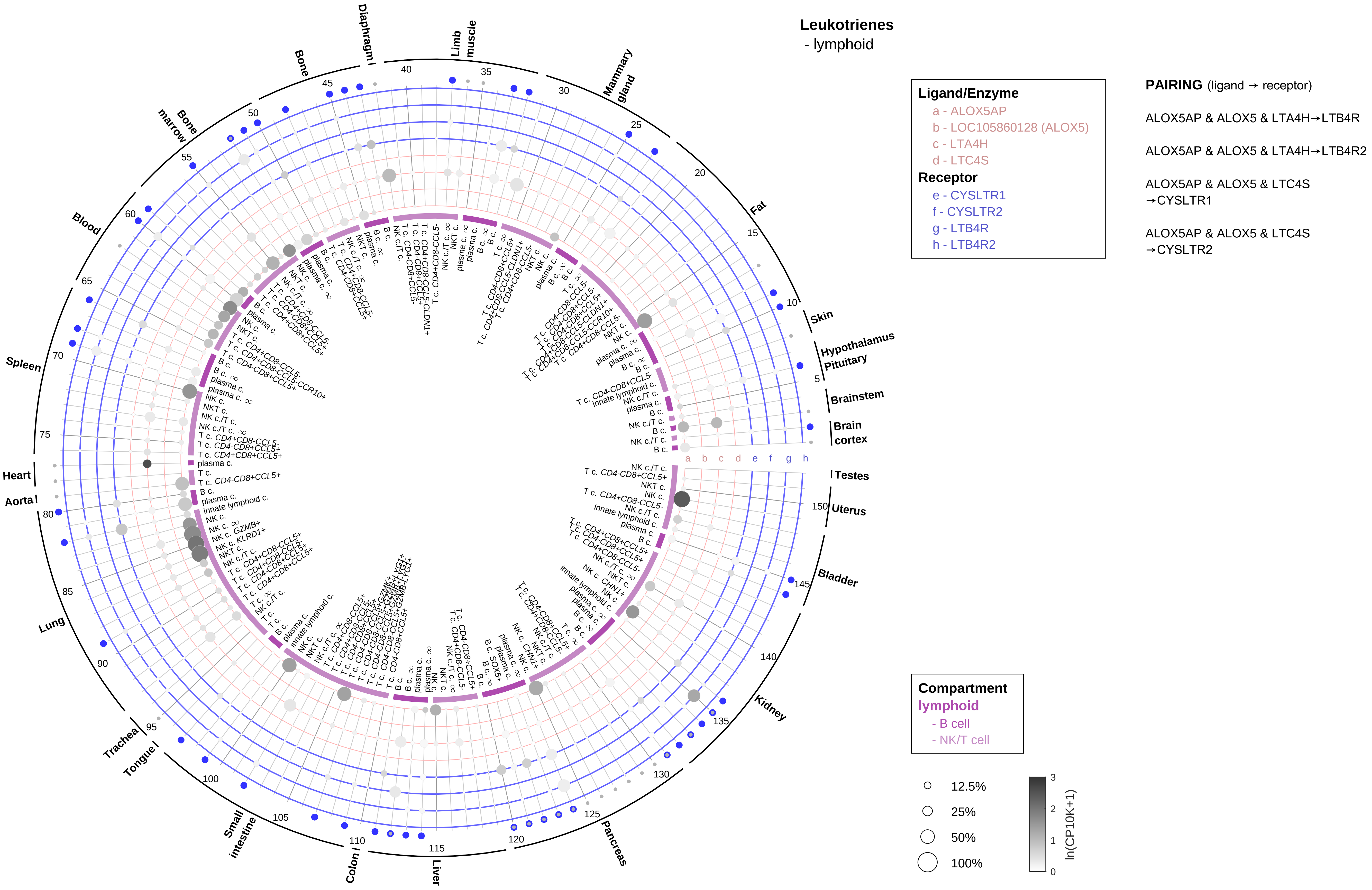
Leukotrienes
- lymphoid
Ligand/Enzyme
a - ALOX5AP
b - LOC105860128 (ALOX5)
c - LTA4H
d - LTC4S
Receptor
e - CYSLTR1
f - CYSLTR2
g - LTB4R
h - LTB4R2
PAIRING (ligand → receptor)
ALOX5AP & ALOX5 & LTA4H→LTB4R
ALOX5AP & ALOX5 & LTA4H→LTB4R2
ALOX5AP & ALOX5 & LTC4S →CYSLTR1
ALOX5AP & ALOX5 & LTC4S →CYSLTR2
Compartment
lymphoid
- B cell
- NK/T cell
12.5%
25%
50%
100%
ln(CP10K+1)
Diaphragm
Limb muscle
Mammary gland
Fat
Skin
Hypothalamus
Pituitary
Brainstem
Brain cortex
Testes
Uterus
Bladder
Kidney
Pancreas
Liver
Colon
Small intestine
Tongue
Trachea
Lung
Aorta
Heart
Spleen
Blood
Bone marrow
Bone

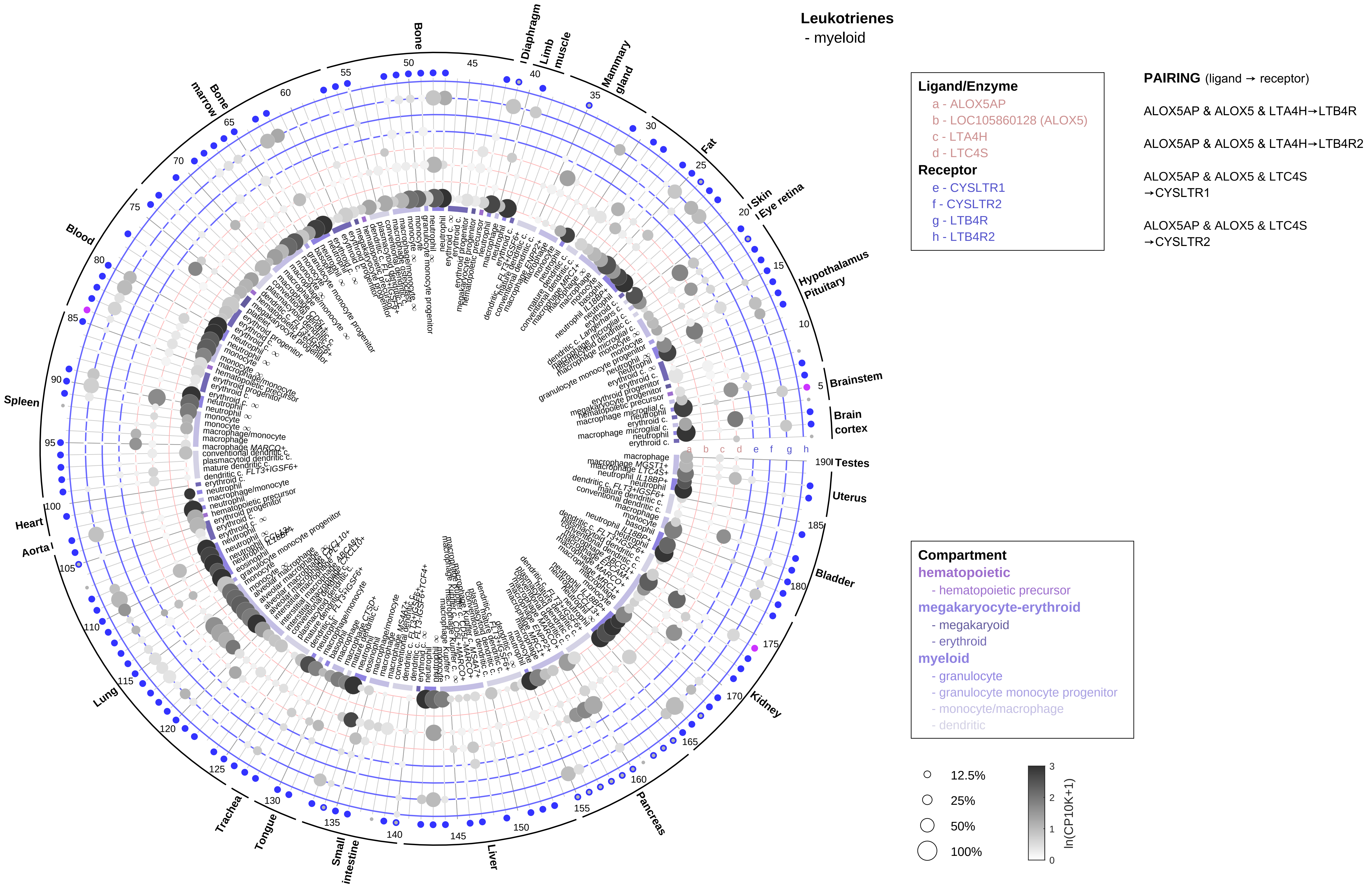
Leukotrienes
- myeloid
Ligand/Enzyme
a - ALOX5AP
b - LOC105860128 (ALOX5)
c - LTA4H
d - LTC4S
Receptor
e - CYSLTR1
f - CYSLTR2
g - LTB4R
h - LTB4R2
PAIRING (ligand → receptor)
ALOX5AP & ALOX5 & LTA4H→LTB4R
ALOX5AP & ALOX5 & LTA4H→LTB4R2
ALOX5AP & ALOX5 & LTC4S →CYSLTR1
ALOX5AP & ALOX5 & LTC4S →CYSLTR2
Compartment
hematopoietic
- hematopoietic precursor
megakaryocyte-erythroid
- megakaryoid
- erythroid
myeloid
- granulocyte
- granulocyte monocyte progenitor
- monocyte/macrophage
- dendritic
12.5%
25%
50%
100%
ln(CP10K+1)
Brainstem
Brain cortex
Testes
Uterus
Bladder
Kidney
Pancreas
Liver
Small intestine
Tongue
Trachea
Lung
Aorta
Heart
Spleen
Blood
Bone marrow
Bone
Diaphragm
Limb muscle
Mammary gland
Fat
Skin
Eye retina
Hypothalamus
Pituitary

Luteinizing hormone (LH)
- epithelial/neural/germ

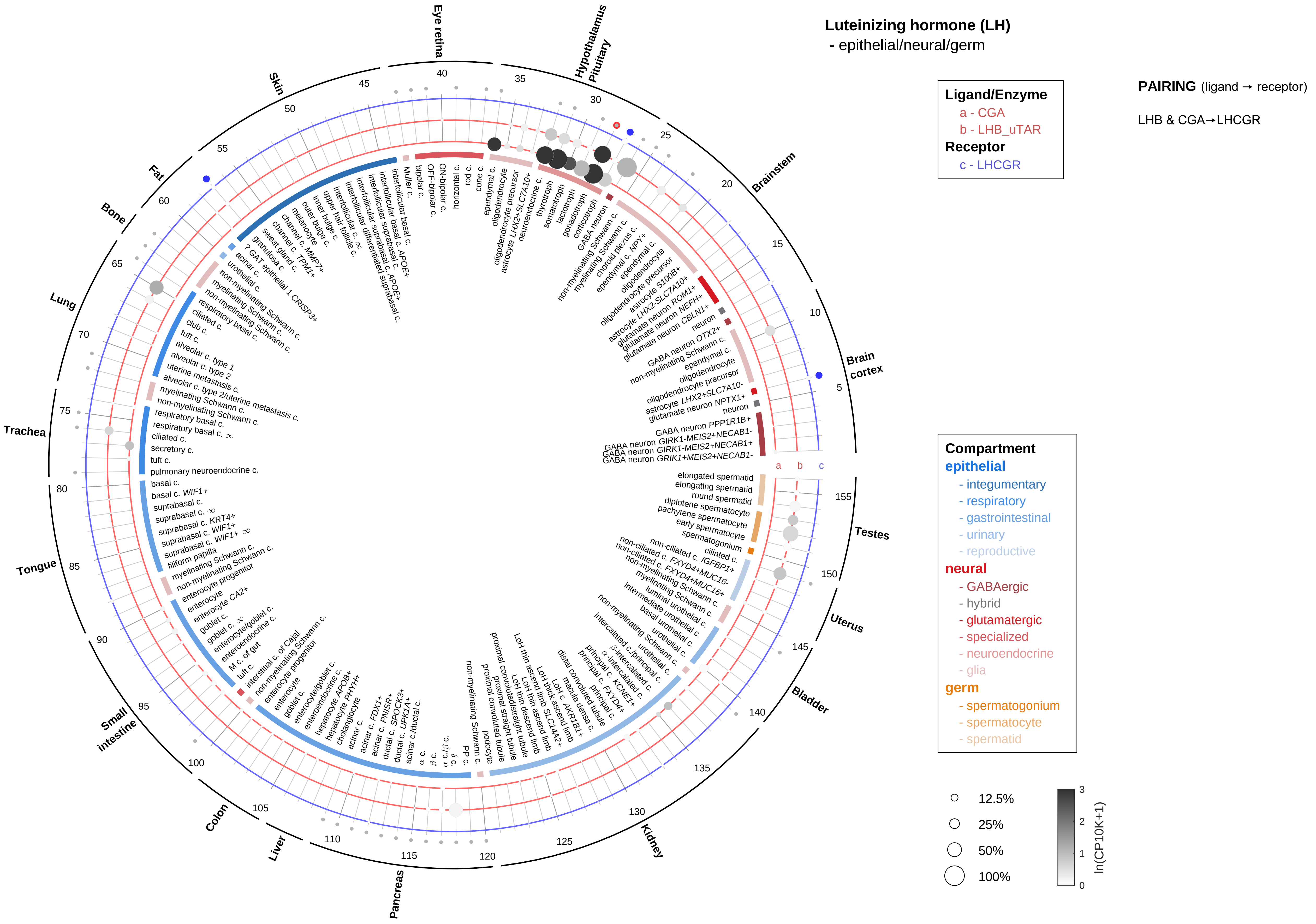

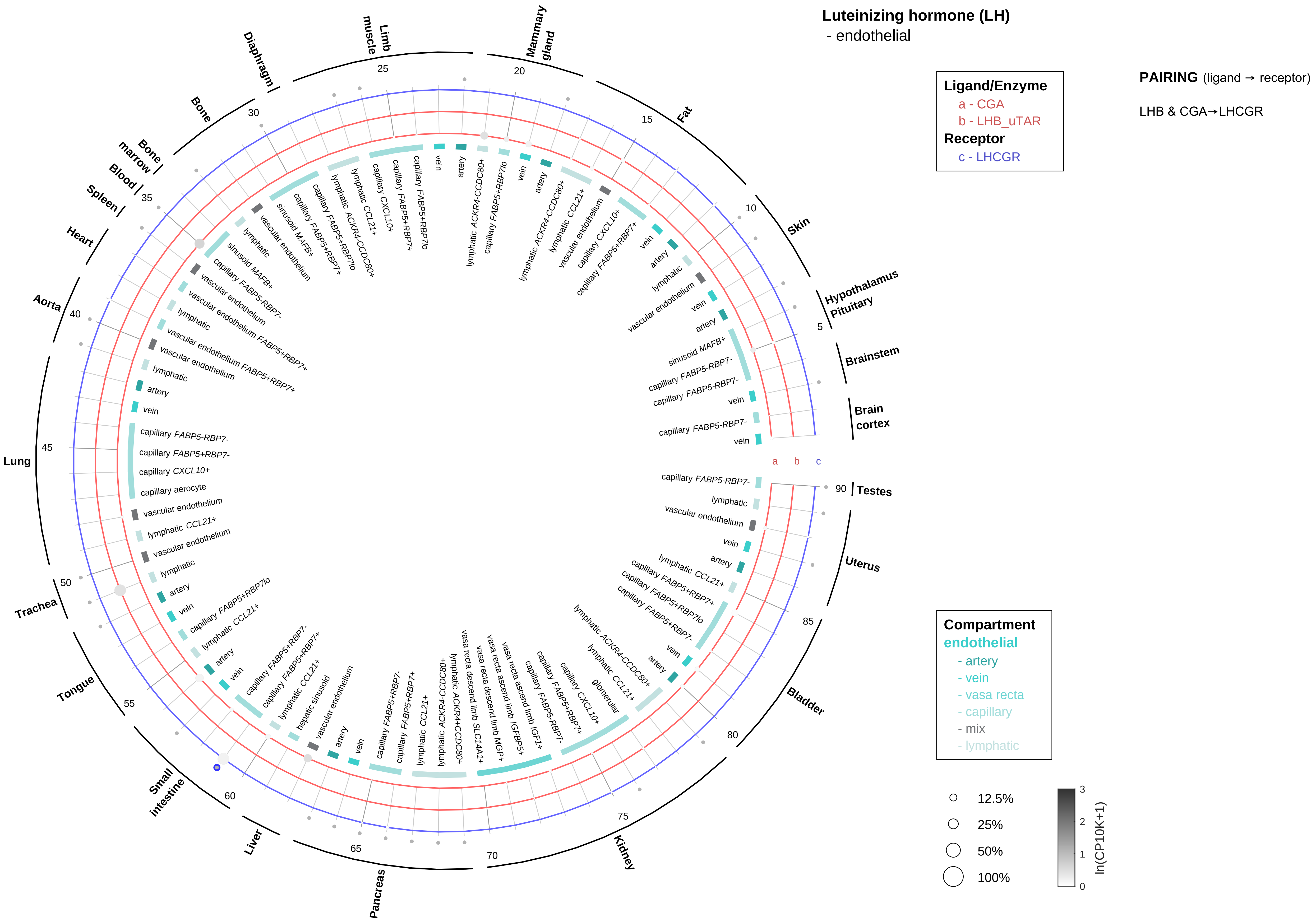

Luteinizing hormone (LH)
- endothelial
Ligand/Enzyme
a - CGA
b - LHB_uTAR
Receptor
c - LHCGR
PAIRING (ligand → receptor)
LHB & CGA→LHCGR
Compartment
endothelial
- artery
- vein
- vasa recta
- capillary
- mix
- lymphatic
12.5%
25%
50%
100%
ln(CP10K+1)
Brain cortex
Brainstem
Hypothalamus Pituitary
Skin
Fat
Mammary gland
Limb muscle
Diaphragm
Bone
Bone marrow
Blood
Spleen
Heart
Aorta
Lung
Trachea
Tongue
Small intestine
Liver
Pancreas
Kidney
Bladder
Uterus
Testes

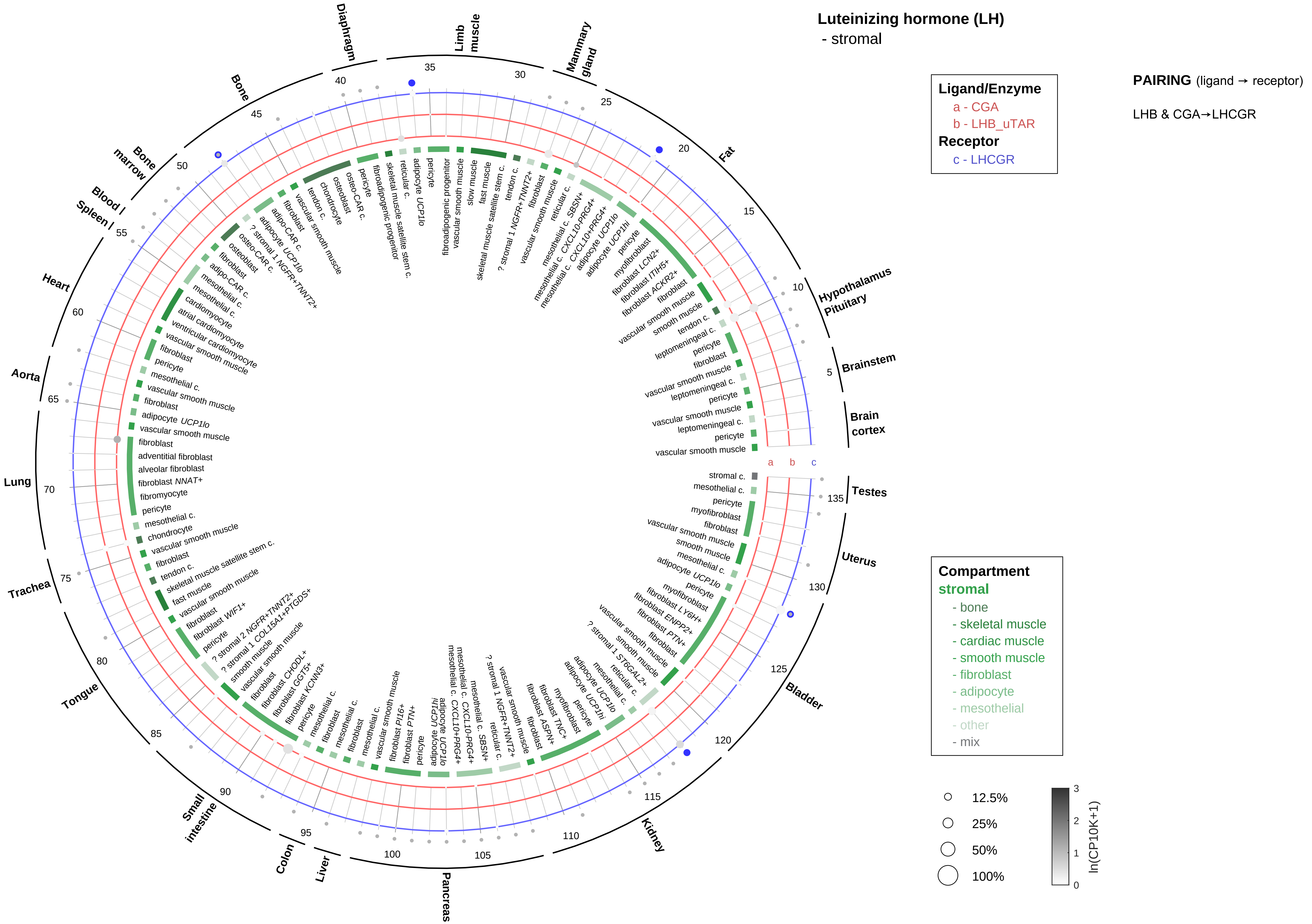
Luteinizing hormone (LH)
- stromal
Ligand/Enzyme
a - CGA
b - LHB_uTAR
Receptor
c - LHCGR
PAIRING (ligand → receptor)
LHB & CGA→LHCGR
Compartment
stromal
- bone
- skeletal muscle
- cardiac muscle
- smooth muscle
- fibroblast
- adipocyte
- mesothelial
- other
- mix
12.5%
25%
50%
100%
ln(CP10K+1)
Brain cortex
Brainstem
Hypothalamus Pituitary
Fat
Mammary gland
Limb muscle
Diaphragm
Bone
Bone marrow
Blood / Spleen /
Heart
Aorta
Lung
Trachea
Tongue
Small intestine
Colon
Liver
Pancreas
Kidney
Bladder
Uterus
Testes

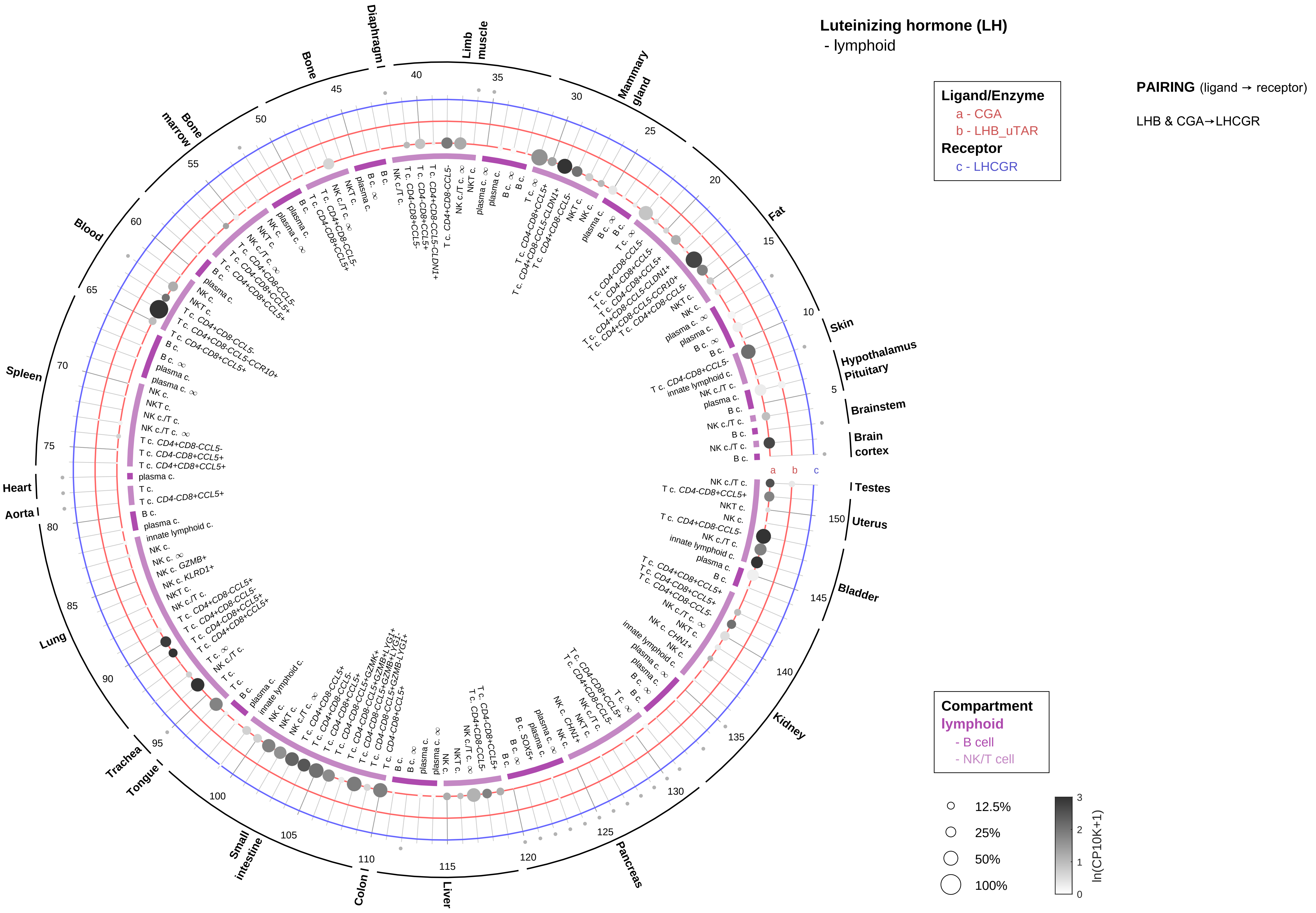

Luteinizing hormone (LH)
- lymphoid
Ligand/Enzyme
a - CGA
b - LHB_uTAR
Receptor
c - LHCGR
PAIRING (ligand → receptor)
LHB & CGA→LHCGR
Compartment
lymphoid
- B cell
- NK/T cell
12.5%
25%
50%
100%
ln(CP10K+1)
Limb muscle
Mammary gland
Fat
Skin
Hypothalamus
Pituitary
Brainstem
Brain cortex
Testes
Uterus
Bladder
Kidney
Pancreas
Liver
Colon
Small intestine
Tongue
Trachea
Lung
Aorta
Heart
Spleen
Blood
Bone marrow
Bone
Diaphragm

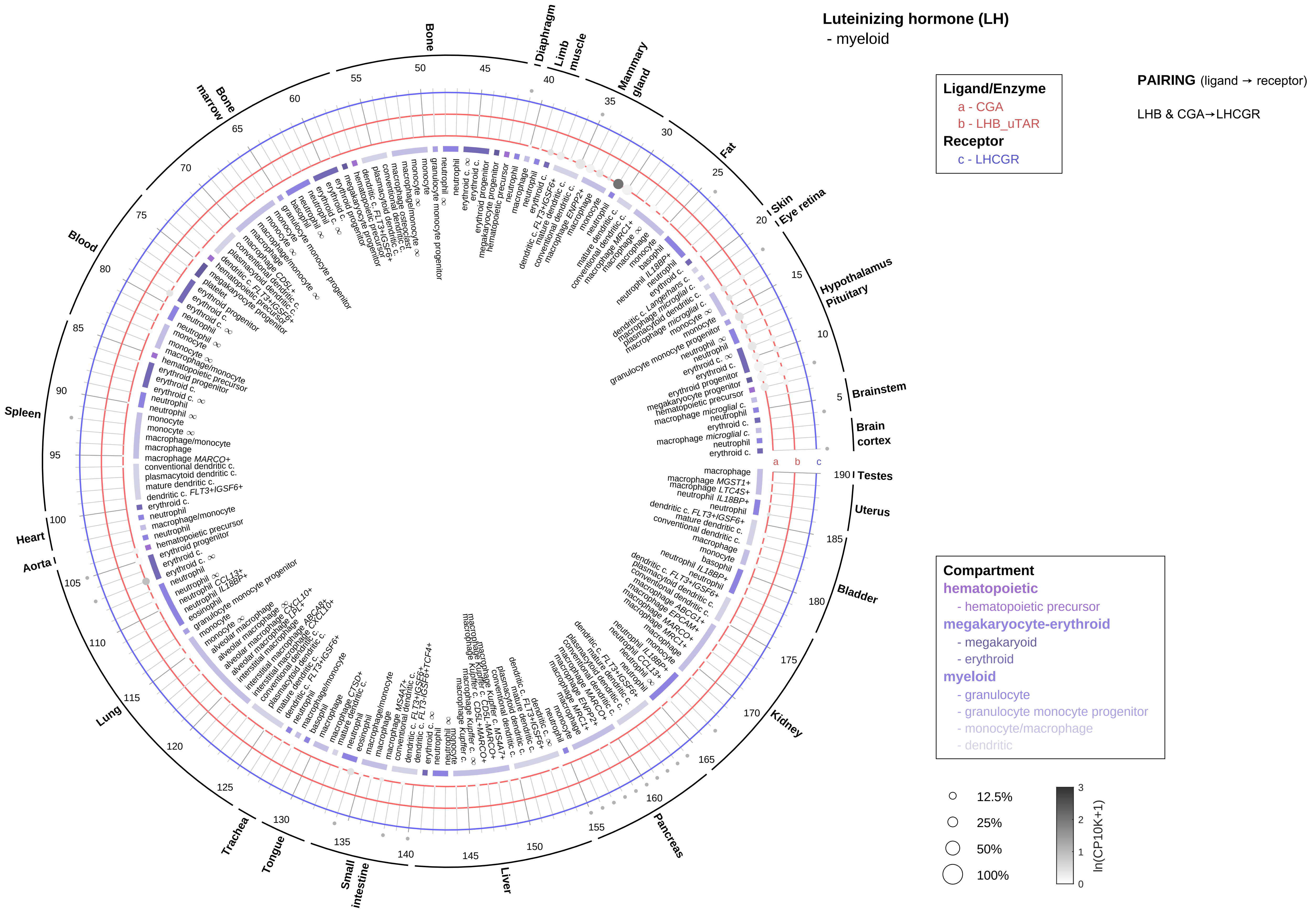
Luteinizing hormone (LH)
- myeloid
Ligand/Enzyme
a - CGA
b - LHB_uTAR
Receptor
c - LHCGR
PAIRING (ligand → receptor)
LHB & CGA→LHCGR
Compartment
hematopoietic
- hematopoietic precursor
megakaryocyte-erythroid
- megakaryoid
- erythroid
myeloid
- granulocyte
- granulocyte monocyte progenitor
- monocyte/macrophage
- dendritic
12.5%
25%
50%
100%
ln(CP10K+1)
Bone
Diaphragm
Limb muscle
Mammary gland
Fat
Skin
Eye retina
Hypothalamus
Pituitary
Brainstem
Brain cortex
Testes
Uterus
Bladder
Kidney
Pancreas
Liver
Small intestine
Tongue
Trachea
Lung
Aorta
Heart
Spleen
Blood
Bone marrow

Melanin-concentrating hormone (MCH)
- epithelial/neural/germ

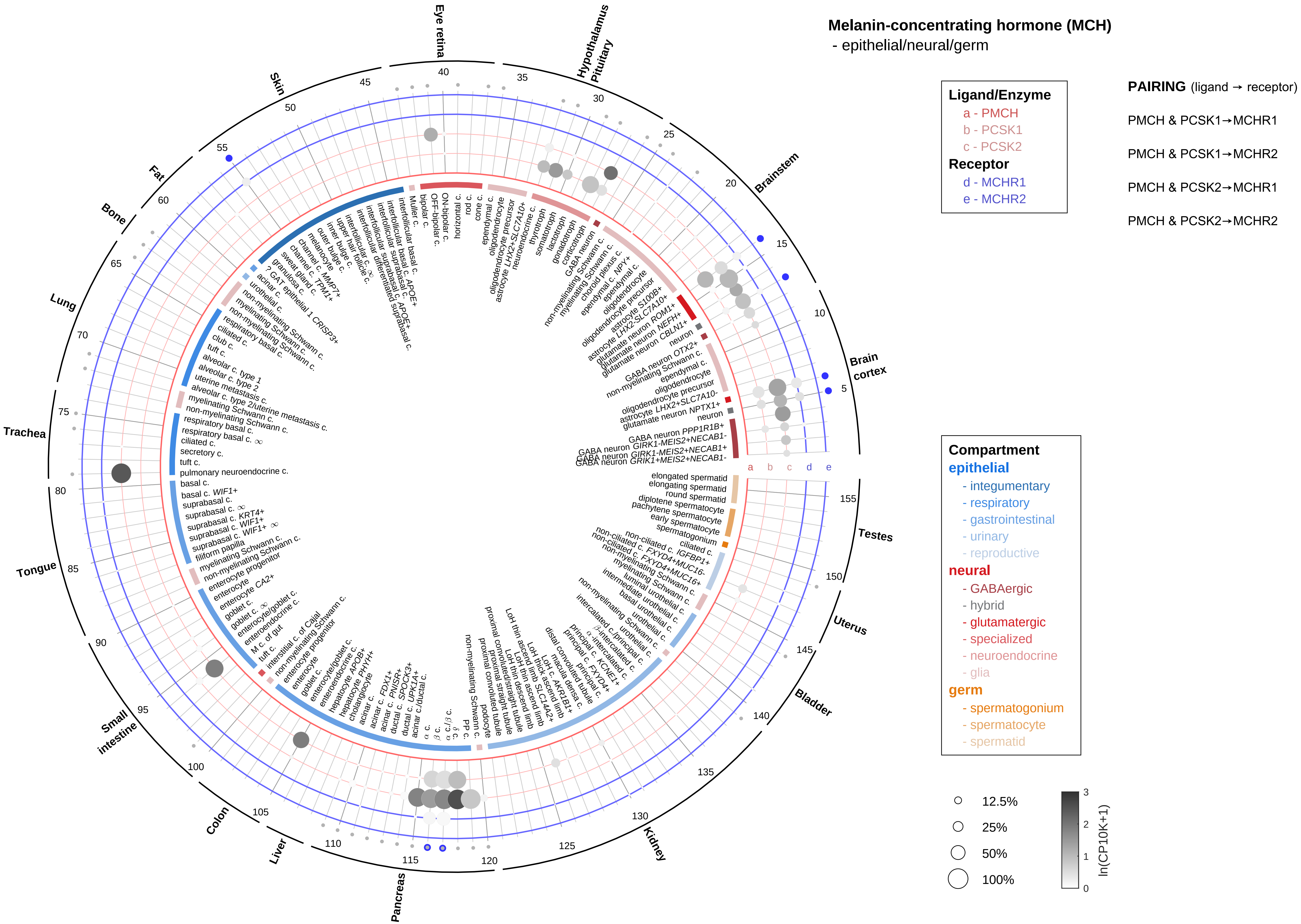

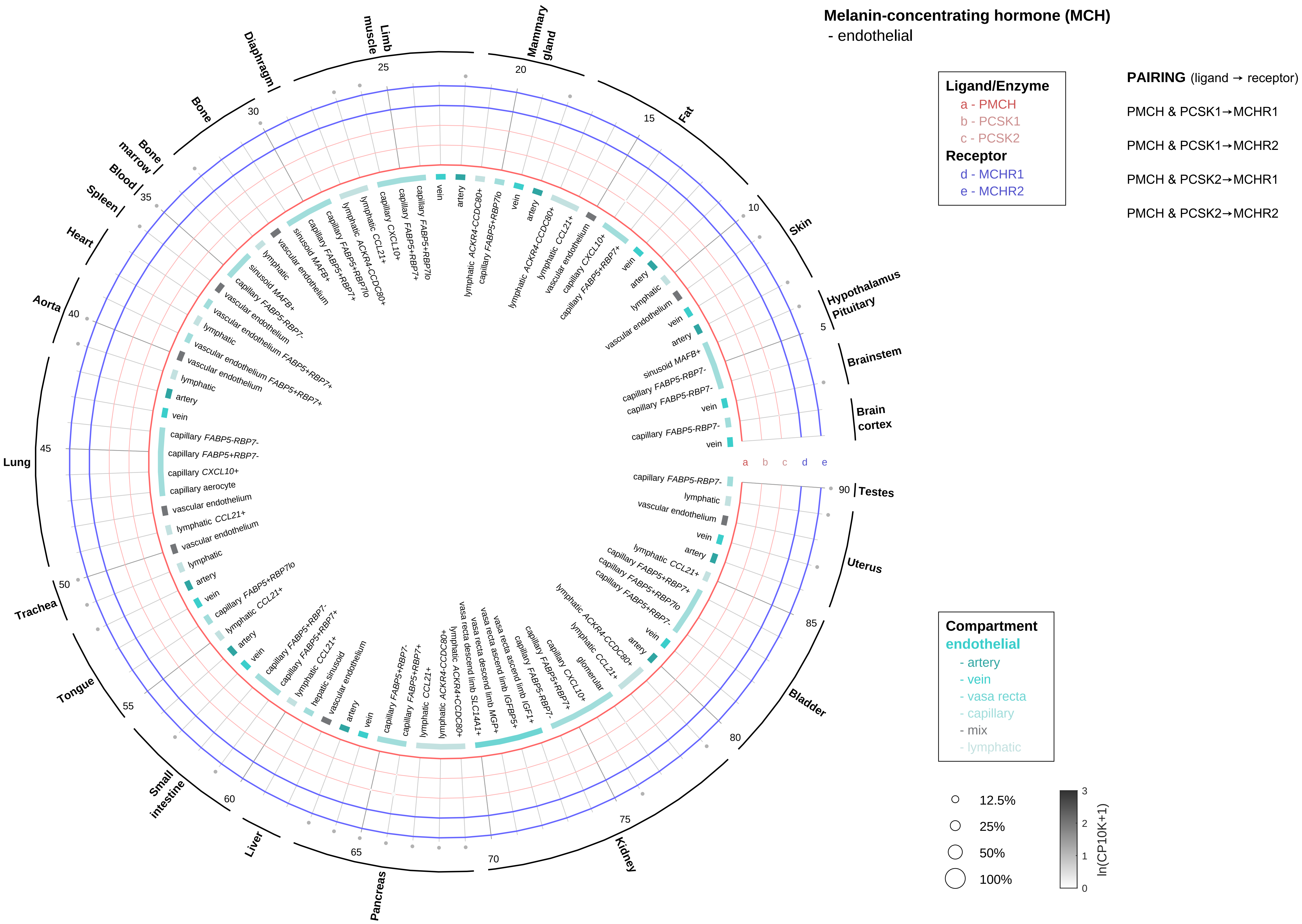

Melanin-concentrating hormone (MCH)
- endothelial
Ligand/Enzyme
a - PMCH
b - PCSK1
c - PCSK2
Receptor
d - MCHR1
e - MCHR2
PAIRING (ligand → receptor)
PMCH & PCSK1→MCHR1
PMCH & PCSK1→MCHR2
PMCH & PCSK2→MCHR1
PMCH & PCSK2→MCHR2
Compartment
endothelial
- artery
- vein
- vasa recta
- capillary
- mix
- lymphatic
12.5%
25%
50%
100%
ln(CP10K+1)
Brain cortex
Brainstem
Hypothalamus Pituitary
Skin
Fat
Mammary gland
Limb muscle
Diaphragm
Bone
Bone marrow
Blood
Spleen
Heart
Aorta
Lung
Trachea
Tongue
Small intestine
Liver
Pancreas
Kidney
Bladder
Uterus
Testes

Melanin-concentrating hormone (MCH)
- stromal

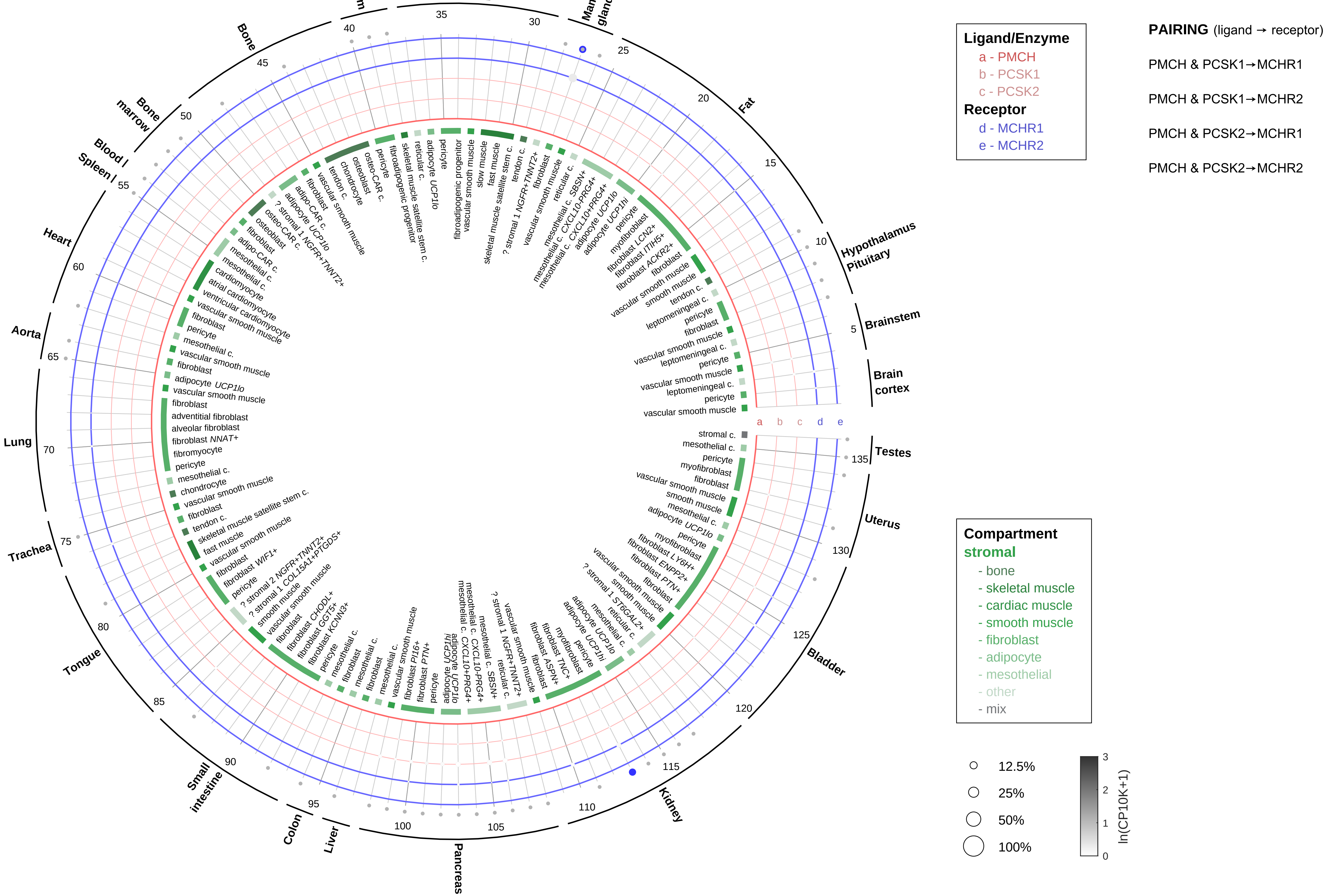

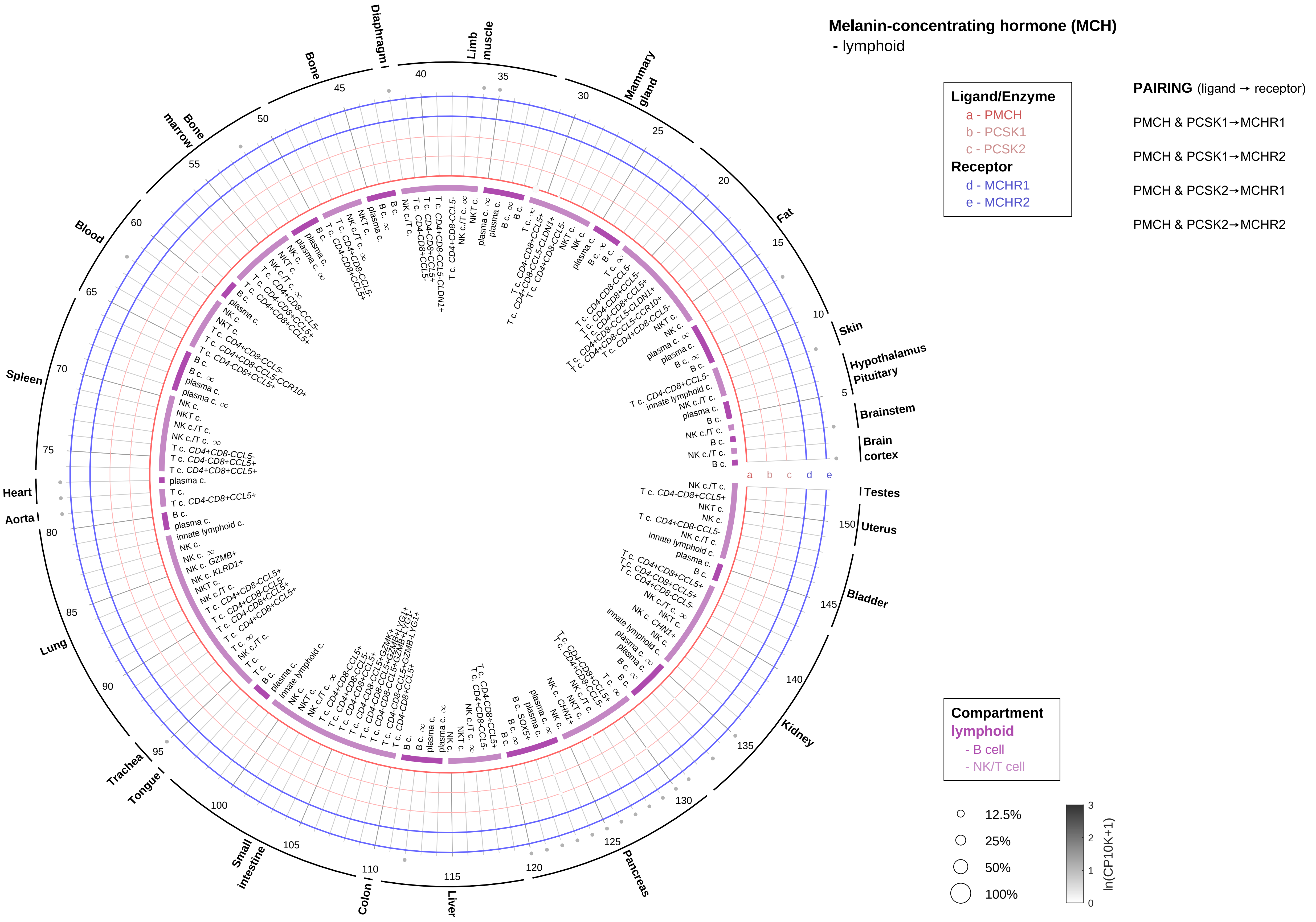

Melanin-concentrating hormone (MCH)
- lymphoid
Ligand/Enzyme
a - PMCH
b - PCSK1
c - PCSK2
Receptor
d - MCHR1
e - MCHR2
PAIRING (ligand → receptor)
PMCH & PCSK1→MCHR1
PMCH & PCSK1→MCHR2
PMCH & PCSK2→MCHR1
PMCH & PCSK2→MCHR2
Compartment
lymphoid
- B cell
- NK/T cell
12.5%
25%
50%
100%
ln(CP10K+1)
Testes
Uterus
Bladder
Kidney
Pancreas
Liver
Colon
Small intestine
Tongue
Trachea
Lung
Aorta
Heart
Spleen
Blood
Bone marrow
Bone
Diaphragm
Limb muscle
Mammary gland
Fat
Skin
Hypothalamus
Pituitary
Brainstem
Brain cortex

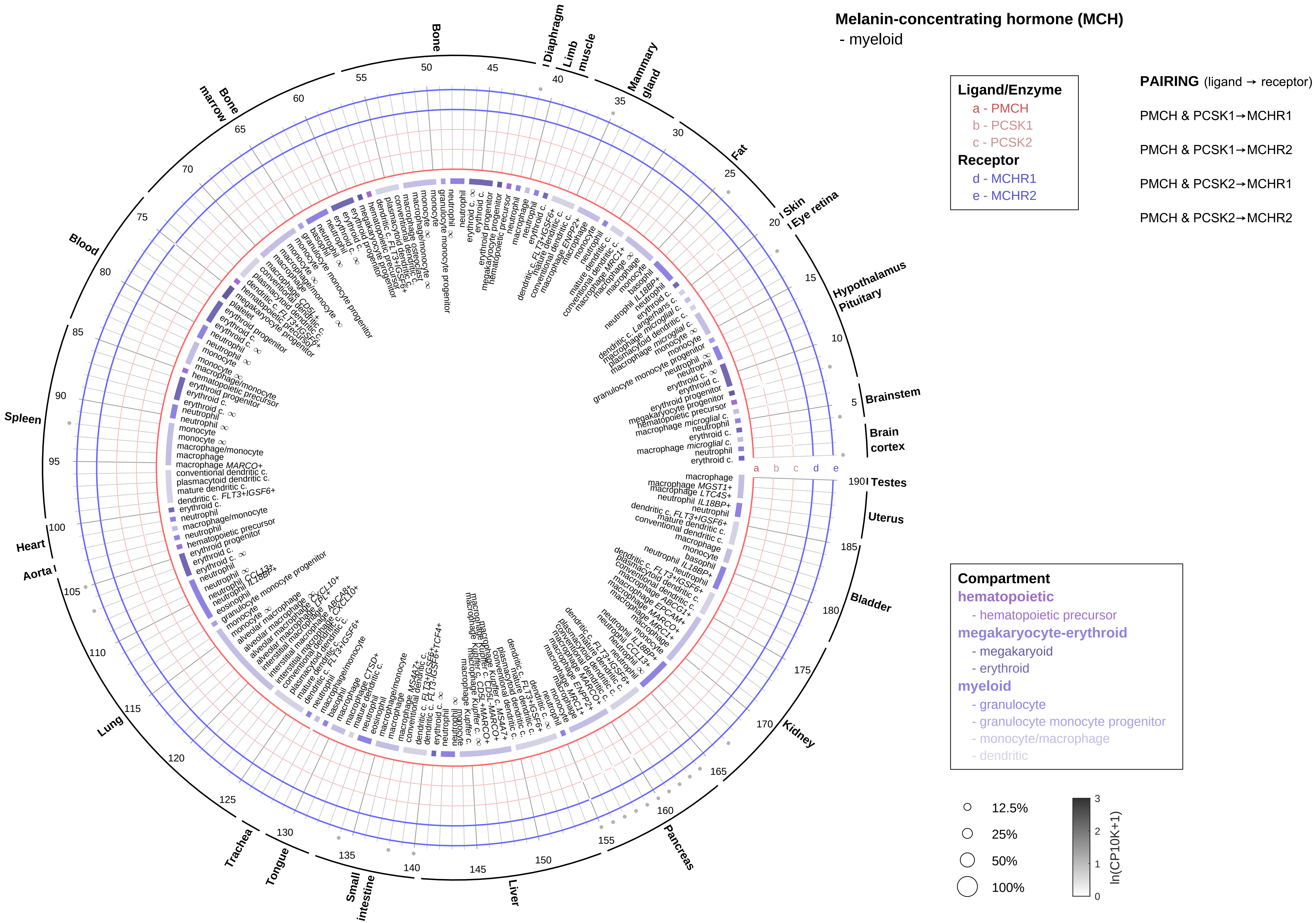

Melanin-concentrating hormone (MCH)
- myeloid
Ligand/Enzyme
a - PMCH
b - PCSK1
c - PCSK2
Receptor
d - MCHR1
e - MCHR2
PAIRING (ligand → receptor)
PMCH & PCSK1→MCHR1
PMCH & PCSK1→MCHR2
PMCH & PCSK2→MCHR1
PMCH & PCSK2→MCHR2
Compartment
hematopoietic
- hematopoietic precursor
megakaryocyte-erythroid
- megakaryoid
- erythroid
myeloid
- granulocyte
- granulocyte monocyte progenitor
- monocyte/macrophage
- dendritic
12.5%
25%
50%
100%
ln(CP10K+1)
Bone
Diaphragm
Limb muscle
Mammary gland
Fat
Skin
Eye retina
Hypothalamus
Pituitary
Brainstem
Brain cortex
Testes
Uterus
Bladder
Kidney
Pancreas
Liver
Small intestine
Tongue
Trachea
Lung
Aorta
Heart
Spleen
Blood
Bone marrow

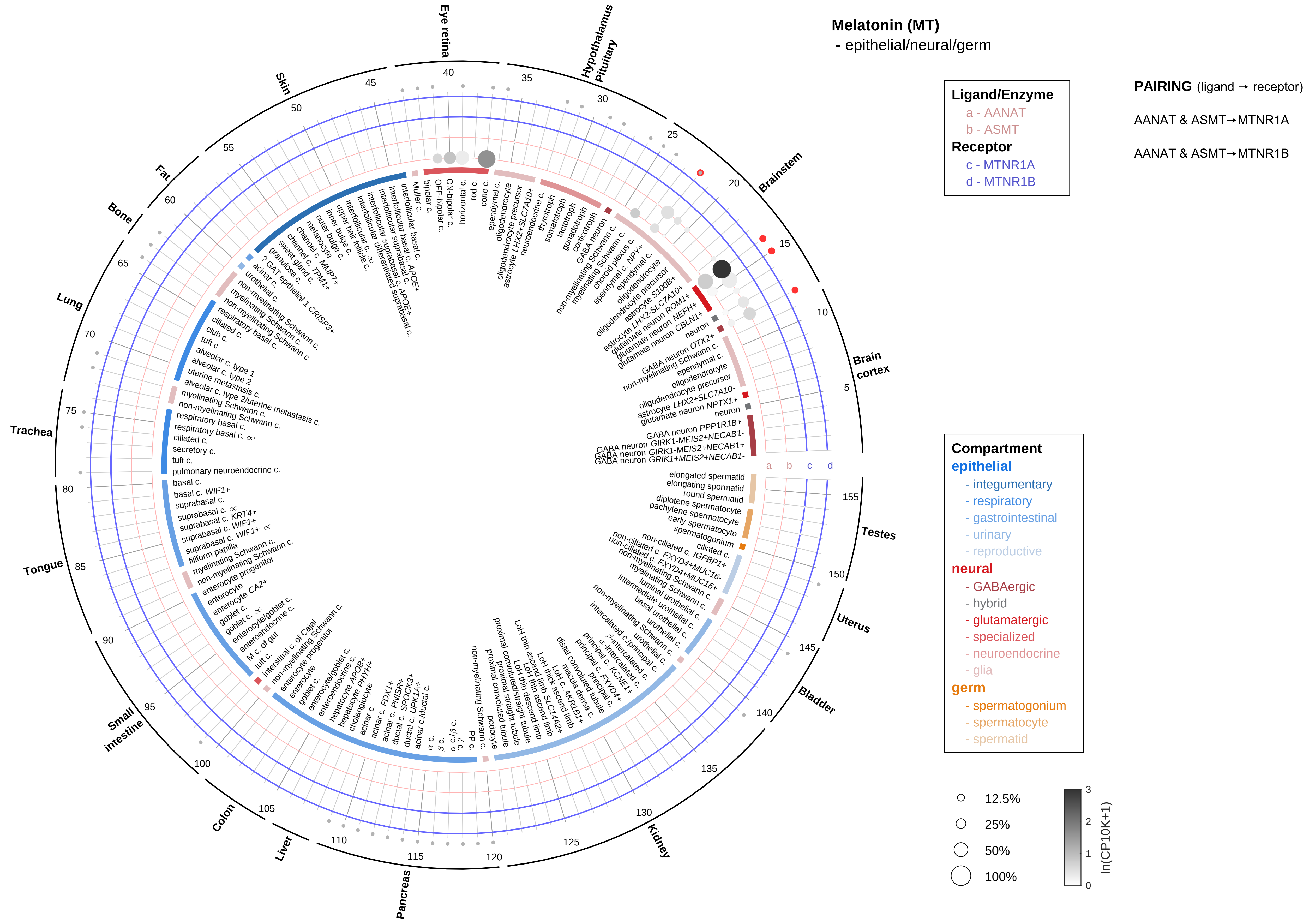

Melatonin (MT)
- epithelial/neural/germ
Ligand/Enzyme
a - AANAT
b - ASMT
Receptor
c - MTNR1A
d - MTNR1B
PAIRING (ligand → receptor)
AANAT & ASMT→MTNR1A
AANAT & ASMT→MTNR1B
Compartment
epithelial
- integumentary
- respiratory
- gastrointestinal
- urinary
- reproductive
neural
- GABAergic
- hybrid
- glutamatergic
- specialized
- neuroendocrine
- glia
germ
- spermatogonium
- spermatocyte
- spermatid
12.5%
25%
50%
100%
ln(CP10K+1)
Eye retina
Hypothalamus Pituitary
Brainstem
Brain cortex
Testes
Uterus
Bladder
Kidney
Pancreas
Liver
Colon
Small intestine
Tongue
Trachea
Lung
Bone
Fat
Skin

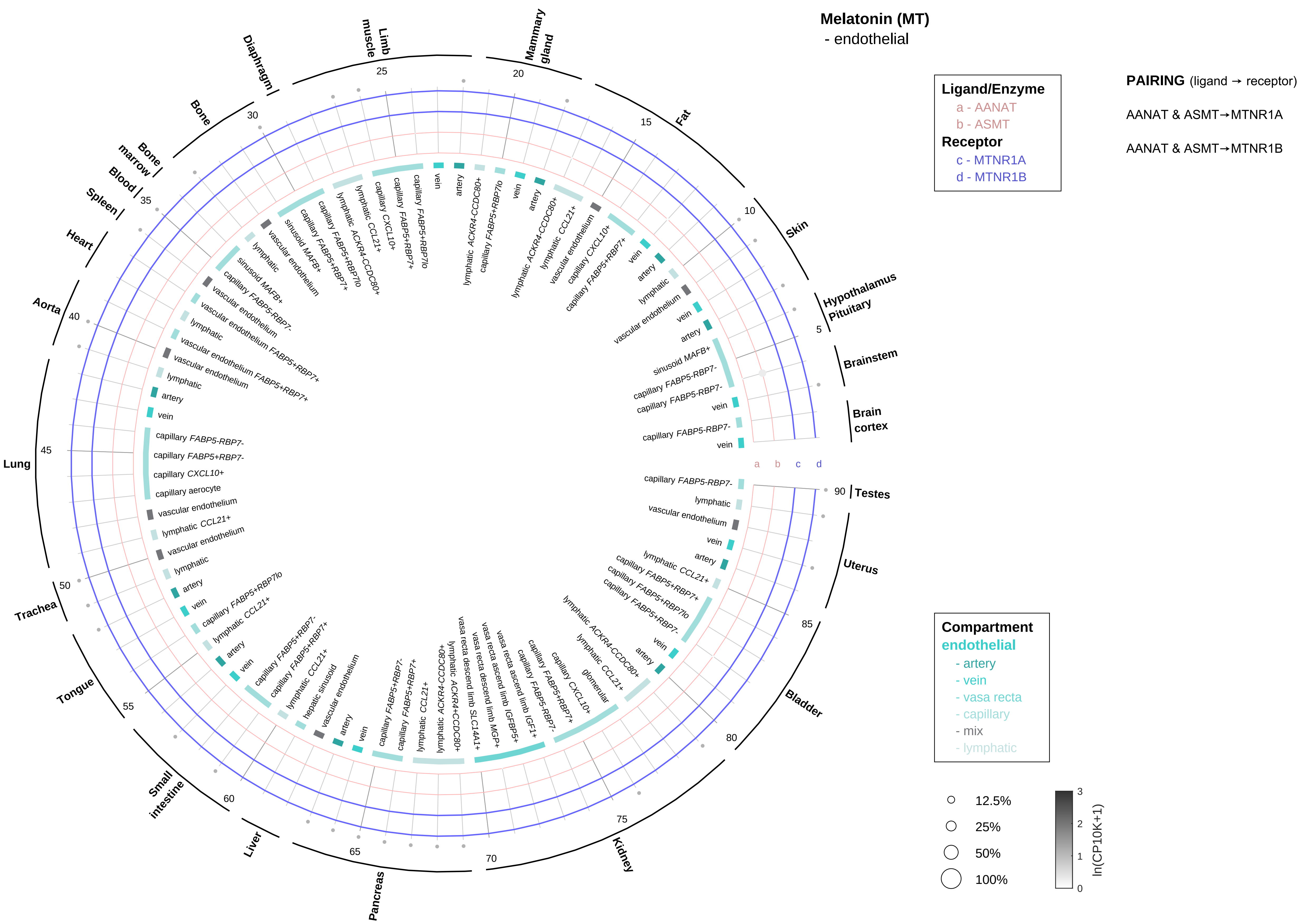

Melatonin (MT)
- endothelial
Ligand/Enzyme
a - AANAT
b - ASMT
Receptor
c - MTNR1A
d - MTNR1B
PAIRING (ligand → receptor)
AANAT & ASMT→MTNR1A
AANAT & ASMT→MTNR1B
Compartment
endothelial
- artery
- vein
- vasa recta
- capillary
- mix
- lymphatic
12.5%
25%
50%
100%
ln(CP10K+1)
Brain cortex
Brainstem
Hypothalamus Pituitary
Skin
Fat
Mammary gland
Limb muscle
Diaphragm
Bone
Bone marrow
Blood
Spleen
Heart
Aorta
Lung
Trachea
Tongue
Small intestine
Liver
Pancreas
Kidney
Bladder
Uterus
Testes

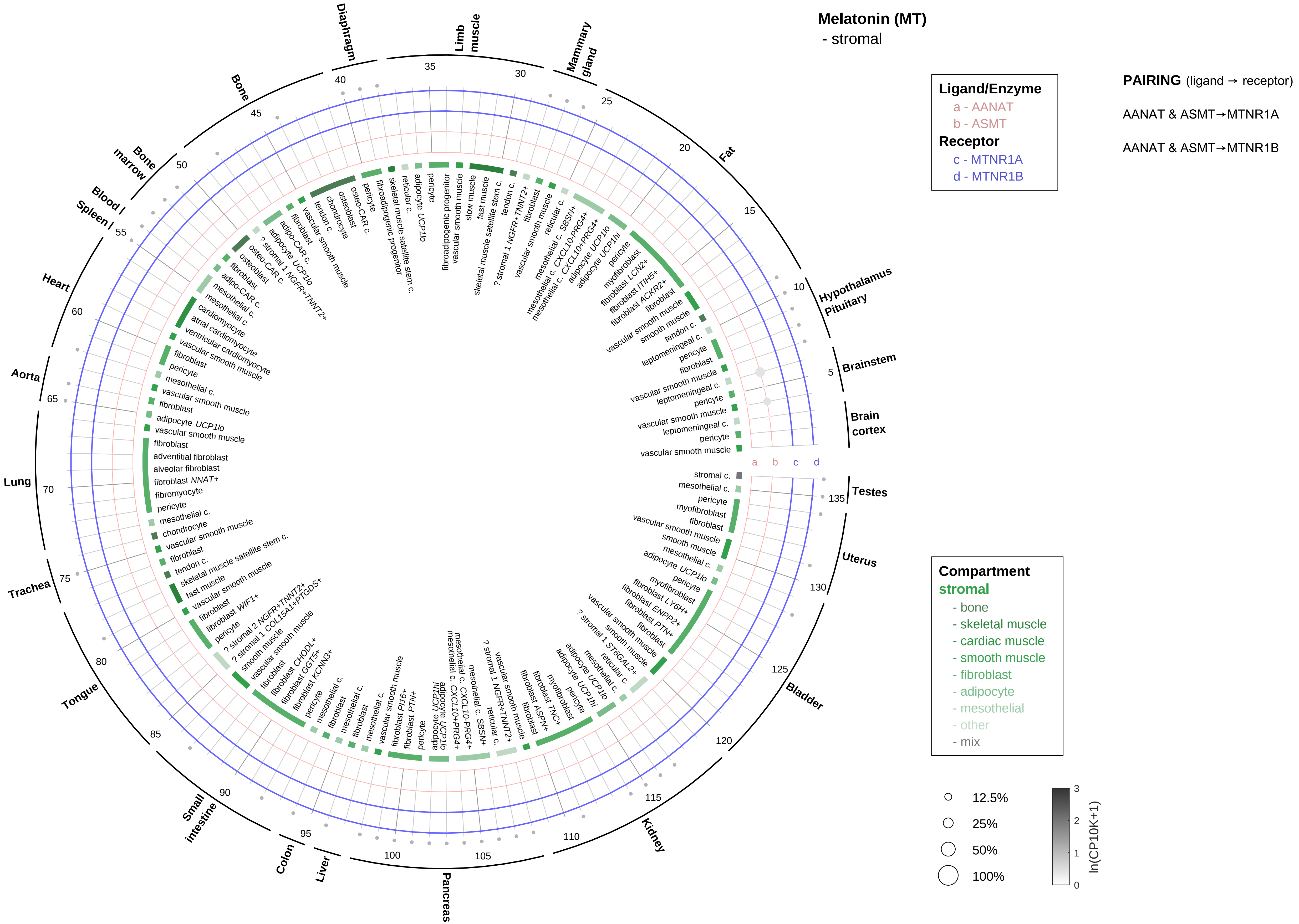

Melatonin (MT)
- stromal
Ligand/Enzyme
a - AANAT
b - ASMT
Receptor
c - MTNR1A
d - MTNR1B
PAIRING (ligand → receptor)
AANAT & ASMT→MTNR1A
AANAT & ASMT→MTNR1B
Compartment
stromal
- bone
- skeletal muscle
- cardiac muscle
- smooth muscle
- fibroblast
- adipocyte
- mesothelial
- other
- mix
12.5%
25%
50%
100%
ln(CP10K+1)
Diaphragm
Limb muscle
Mammary gland
Fat
Hypothalamus Pituitary
Brainstem
Brain cortex
Testes
Uterus
Bladder
Kidney
Pancreas
Liver
Colon
Small intestine
Tongue
Trachea
Lung
Aorta
Heart
Spleen
Blood
Bone marrow
Bone

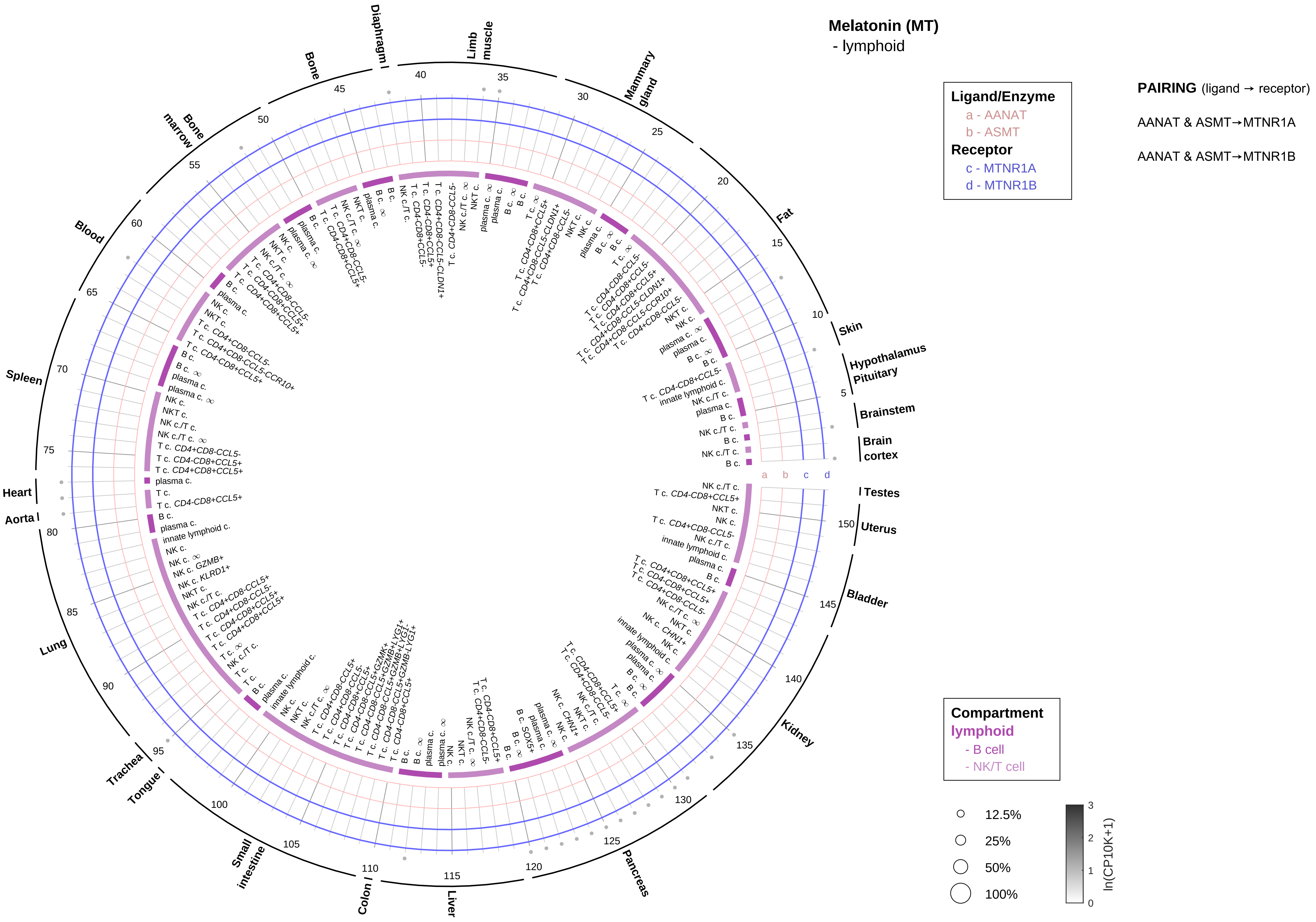
Melatonin (MT)
- lymphoid
Ligand/Enzyme
a - AANAT
b - ASMT
Receptor
c - MTNR1A
d - MTNR1B
PAIRING (ligand → receptor)
AANAT & ASMT→MTNR1A
AANAT & ASMT→MTNR1B
Compartment
lymphoid
- B cell
- NK/T cell
12.5%
25%
50%
100%
ln(CP10K+1)
Limb muscle
Mammary gland
Fat
Skin
Hypothalamus
Pituitary
Brainstem
Brain cortex
Testes
Uterus
Bladder
Kidney
Pancreas
Liver
Colon
Small intestine
Tongue
Trachea
Lung
Aorta
Heart
Spleen
Blood
Bone marrow
Bone
Diaphragm

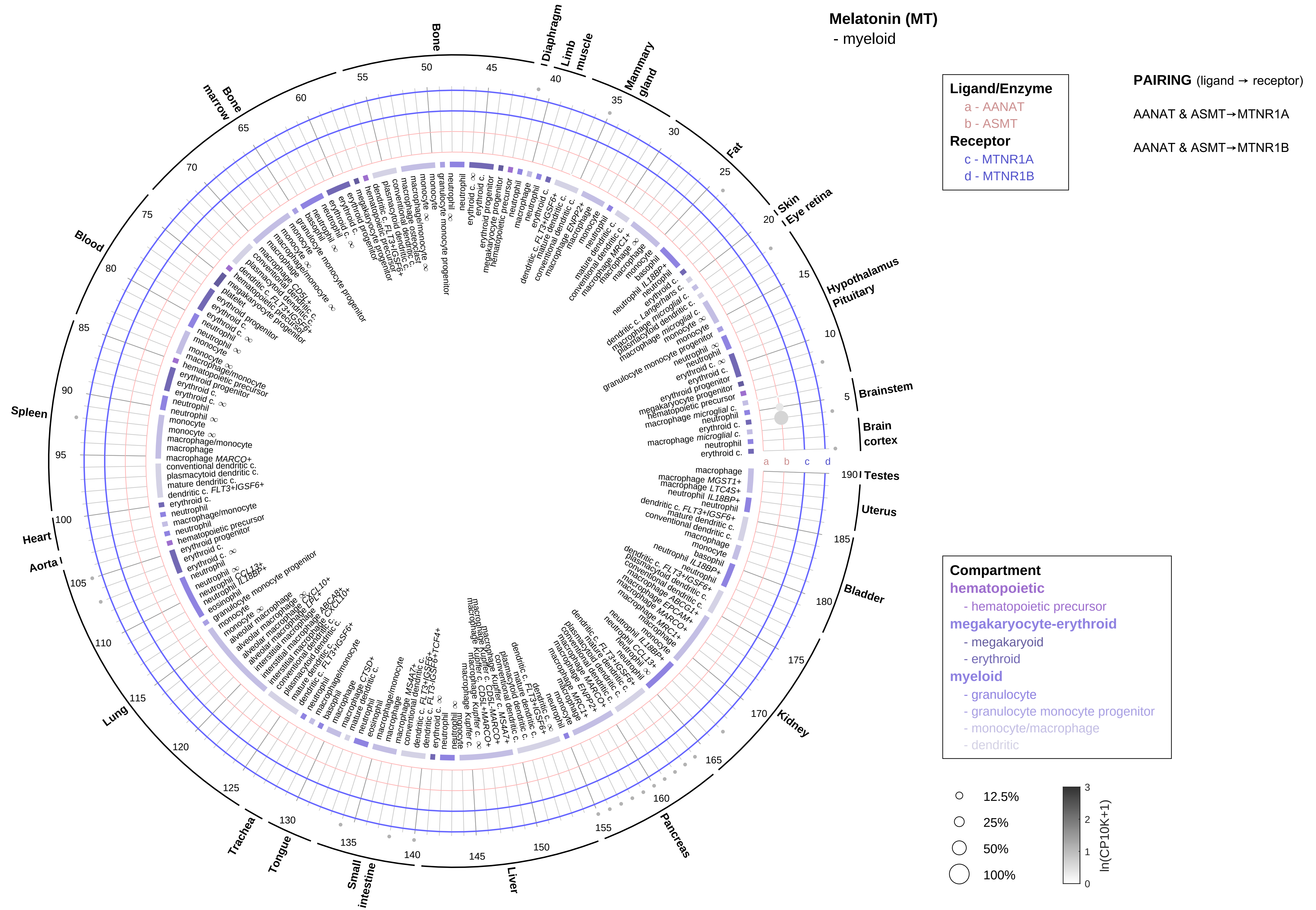
Melatonin (MT)
- myeloid
Ligand/Enzyme
a - AANAT
b - ASMT
Receptor
c - MTNR1A
d - MTNR1B
PAIRING (ligand → receptor)
AANAT & ASMT→MTNR1A
AANAT & ASMT→MTNR1B
Compartment
hematopoietic
- hematopoietic precursor
megakaryocyte-erythroid
- megakaryoid
- erythroid
myeloid
- granulocyte
- granulocyte monocyte progenitor
- monocyte/macrophage
- dendritic
12.5%
25%
50%
100%
ln(CP10K+1)
Bone
Diaphragm
Limb muscle
Mammary gland
Fat
Skin
Eye retina
Hypothalamus
Pituitary
Brainstem
Brain cortex
Testes
Uterus
Bladder
Kidney
Pancreas
Liver
Small intestine
Tongue
Trachea
Lung
Aorta
Heart
Spleen
Blood
Bone marrow

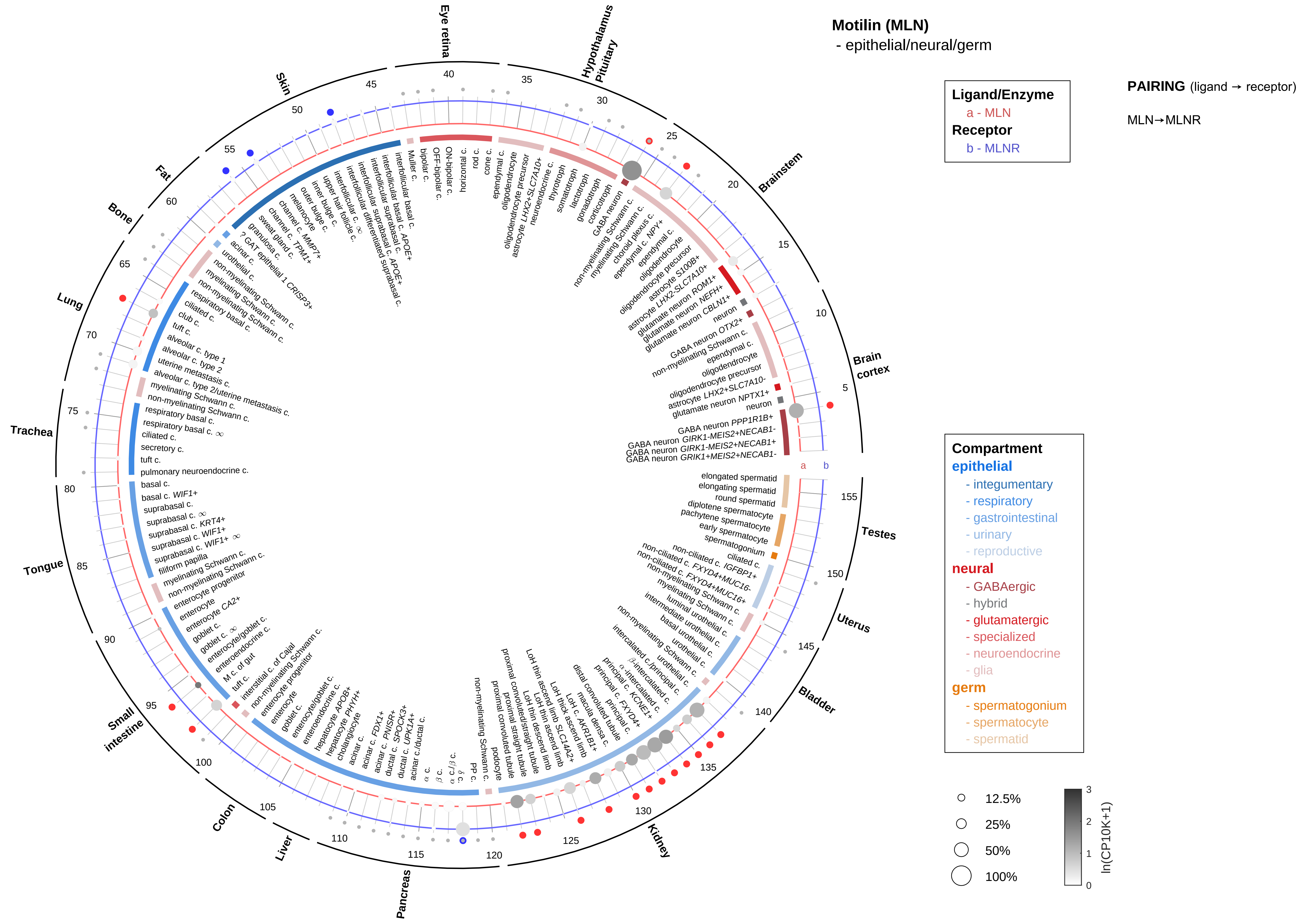
Motilin (MLN)
- epithelial/neural/germ
Ligand/Enzyme
a - MLN
Receptor
b - MLNR
PAIRING (ligand → receptor)
MLN→MLNR
Compartment
epithelial
- integumentary
- respiratory
- gastrointestinal
- urinary
- reproductive
neural
- GABAergic
- hybrid
- glutamatergic
- specialized
- neuroendocrine
- glia
germ
- spermatogonium
- spermatocyte
- spermatid
12.5%
25%
50%
100%
ln(CP10K+1)
Eye retina
Hypothalamus Pituitary
Brainstem
Brain cortex
Testes
Uterus
Bladder
Kidney
Pancreas
Liver
Colon
Small intestine
Tongue
Trachea
Lung
Bone
Fat
Skin

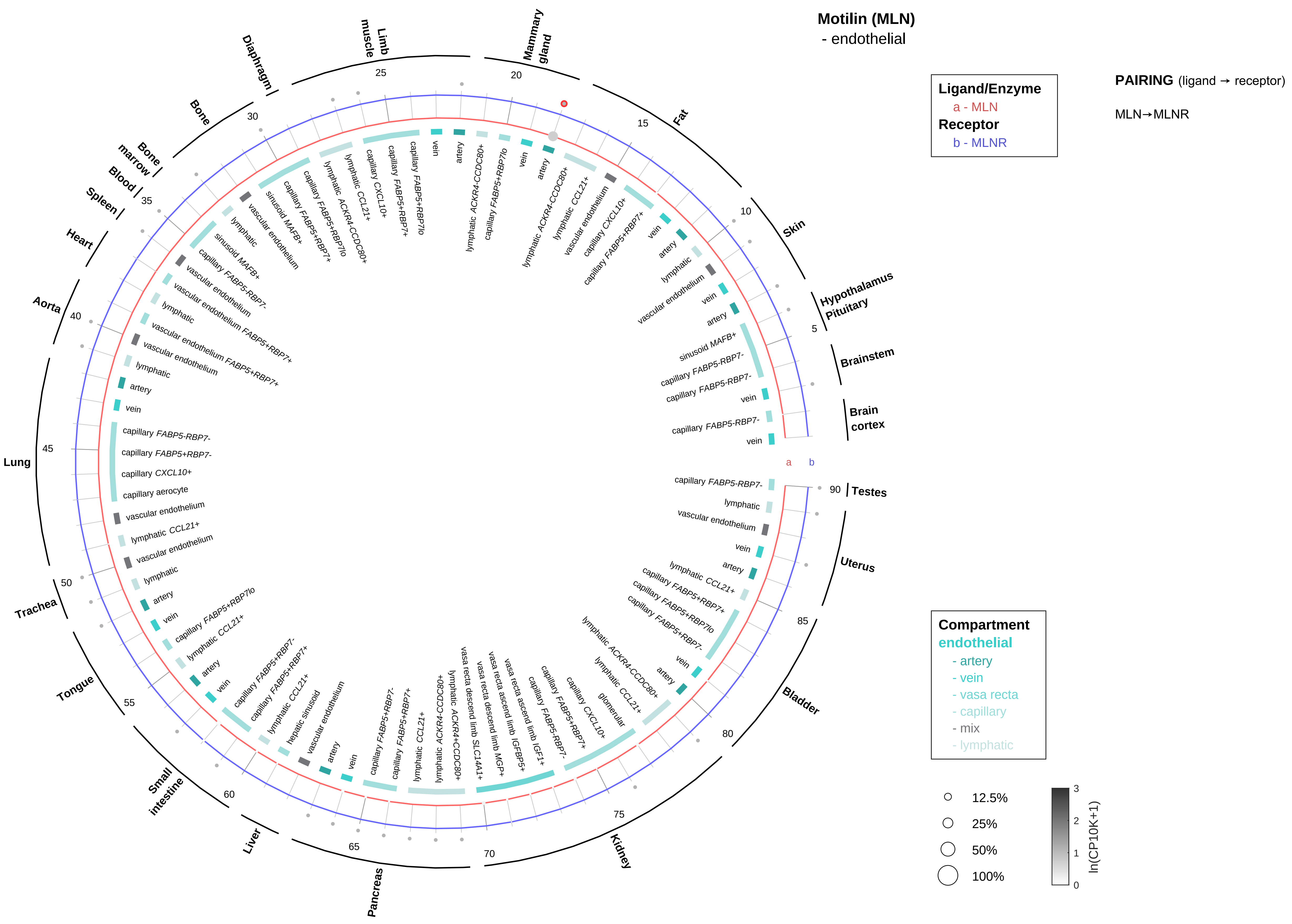
Motilin (MLN)
- endothelial
Ligand/Enzyme
a - MLN
Receptor
b - MLNR
PAIRING (ligand → receptor)
MLN→MLNR
Brain cortex
Brainstem
Hypothalamus Pituitary
Skin
Fat
Mammary gland
Limb muscle
Diaphragm
Bone
Bone marrow
Blood
Spleen
Heart
Aorta
Lung
Trachea
Tongue
Small intestine
Liver
Pancreas
Kidney
Bladder
Uterus
Testes
Compartment
endothelial
- artery
- vein
- vasa recta
- capillary
- mix
- lymphatic
12.5%
25%
50%
100%
ln(CP10K+1)

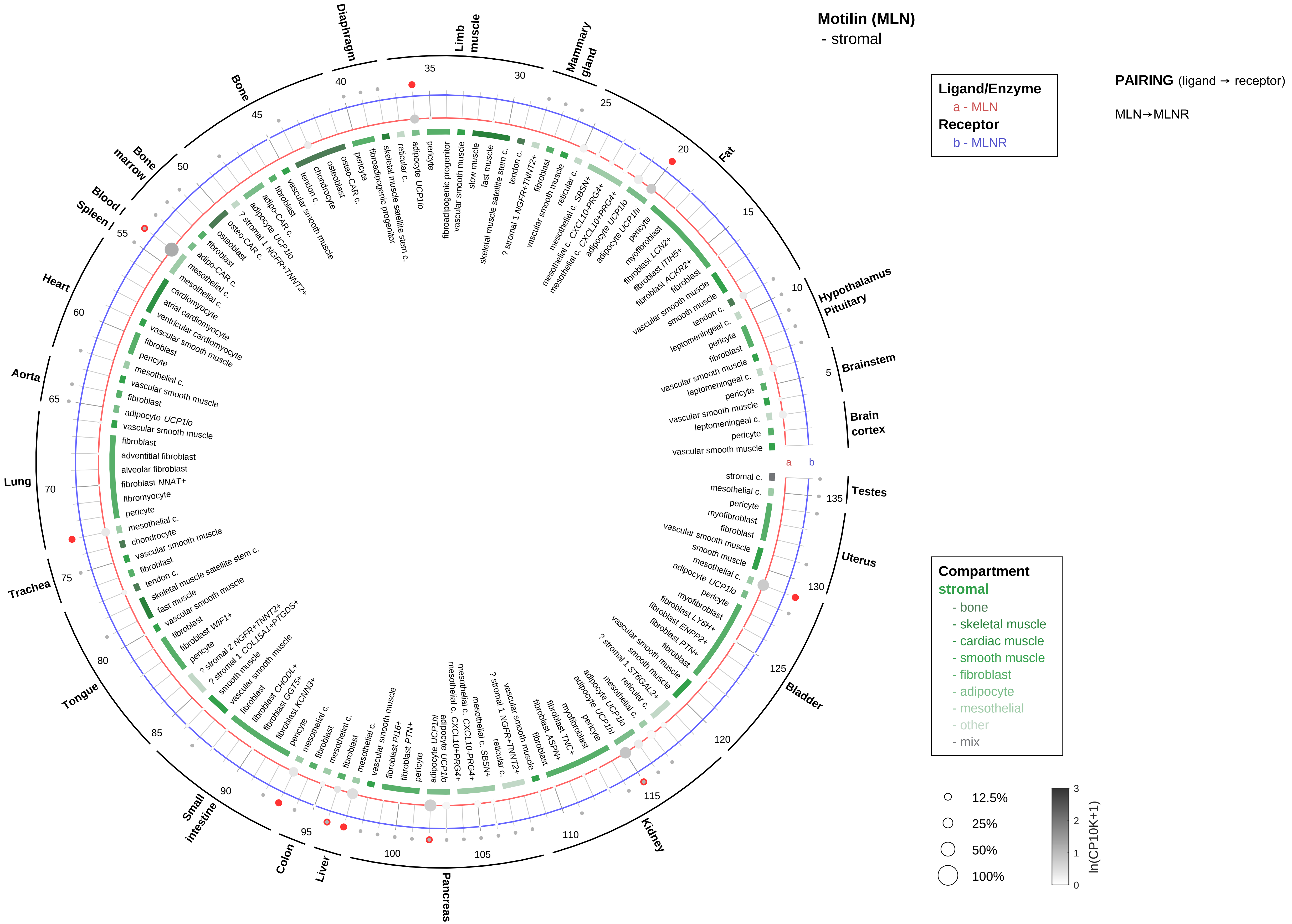
Motilin (MLN)
- stromal
Ligand/Enzyme
a - MLN
Receptor
b - MLNR
PAIRING (ligand → receptor)
MLN→MLNR
Compartment
stromal
- bone
- skeletal muscle
- cardiac muscle
- smooth muscle
- fibroblast
- adipocyte
- mesothelial
- other
- mix
12.5%
25%
50%
100%
ln(CP10K+1)
Brain cortex
Brainstem
Hypothalamus Pituitary
Fat
Mammary gland
Limb muscle
Diaphragm
Bone
Bone marrow
Blood
Spleen
Heart
Aorta
Lung
Trachea
Tongue
Small intestine
Colon
Liver
Pancreas
Kidney
Bladder
Uterus
Testes

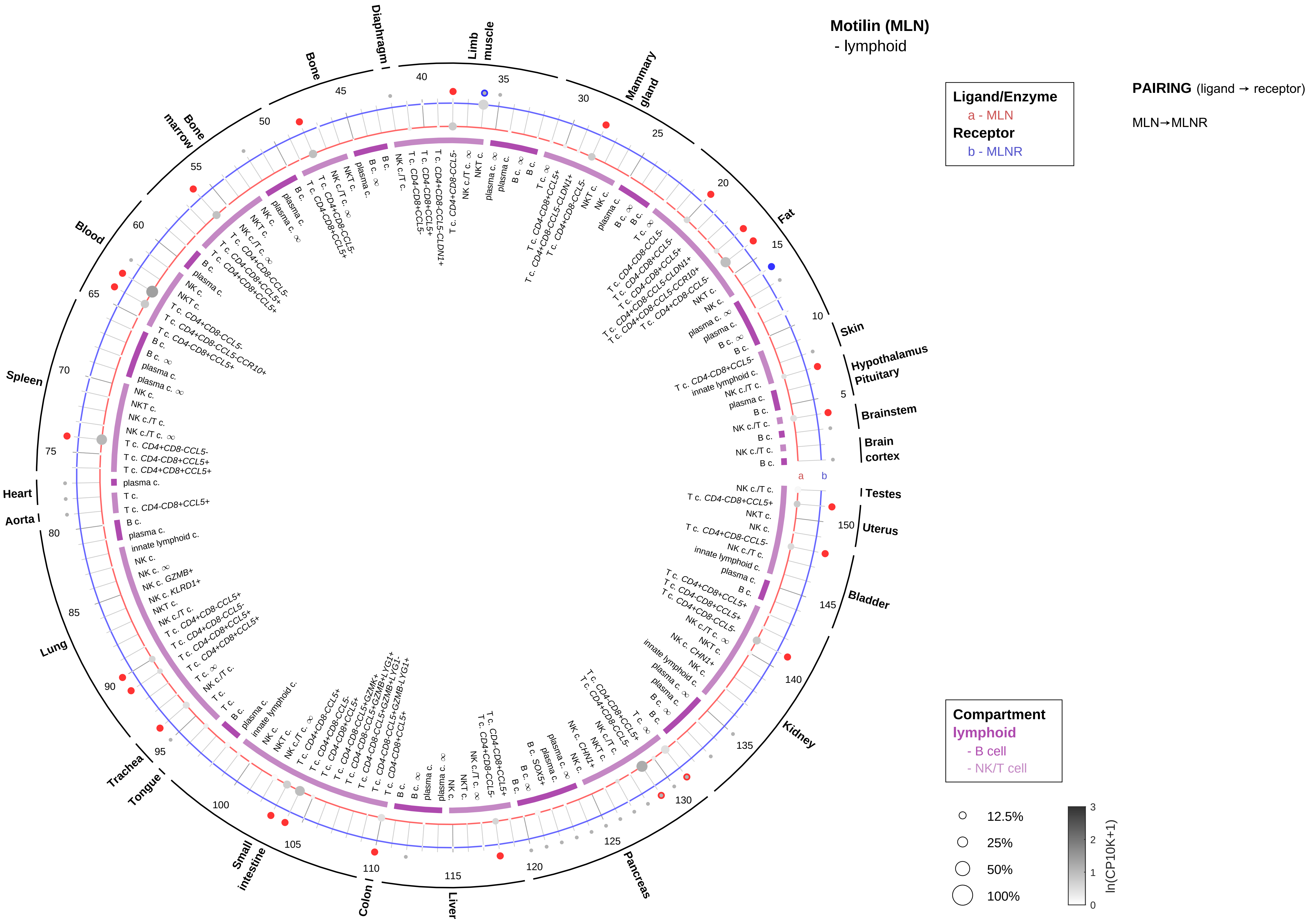

Motilin (MLN)
- lymphoid
Ligand/Enzyme
a - MLN
Receptor
b - MLNR
PAIRING (ligand → receptor)
MLN→MLNR
Compartment
lymphoid
- B cell
- NK/T cell
12.5%
25%
50%
100%
ln(CP10K+1)
Skin
Hypothalamus
Pituitary
Brainstem
Brain cortex
Testes
Uterus
Bladder
Kidney
Pancreas
Liver
Colon
Small intestine
Tongue
Trachea
Lung
Aorta
Heart
Spleen
Blood
Bone marrow
Bone
Diaphragm
Limb muscle
Mammary gland
Fat

Motilin (MLN)
- myeloid

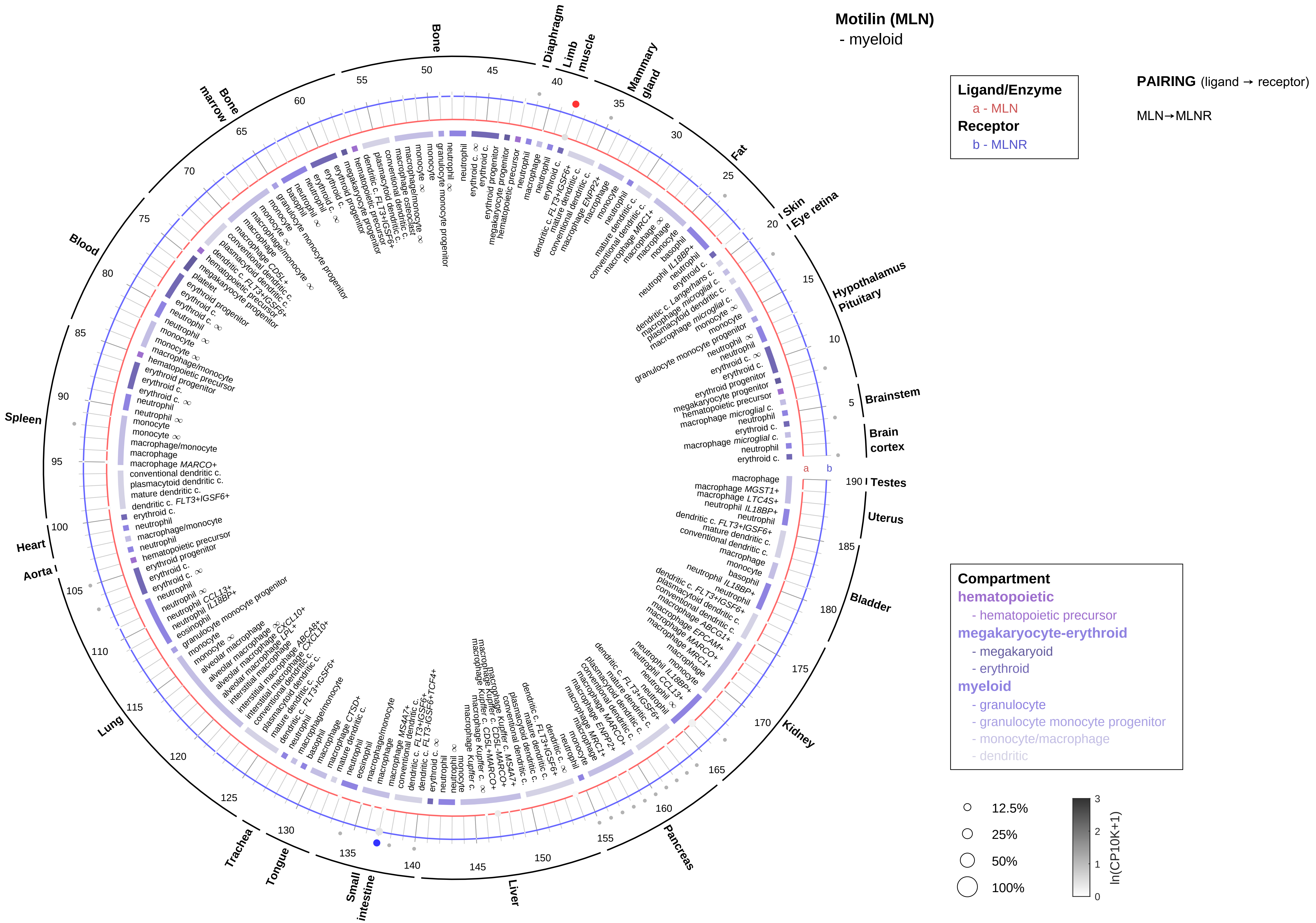

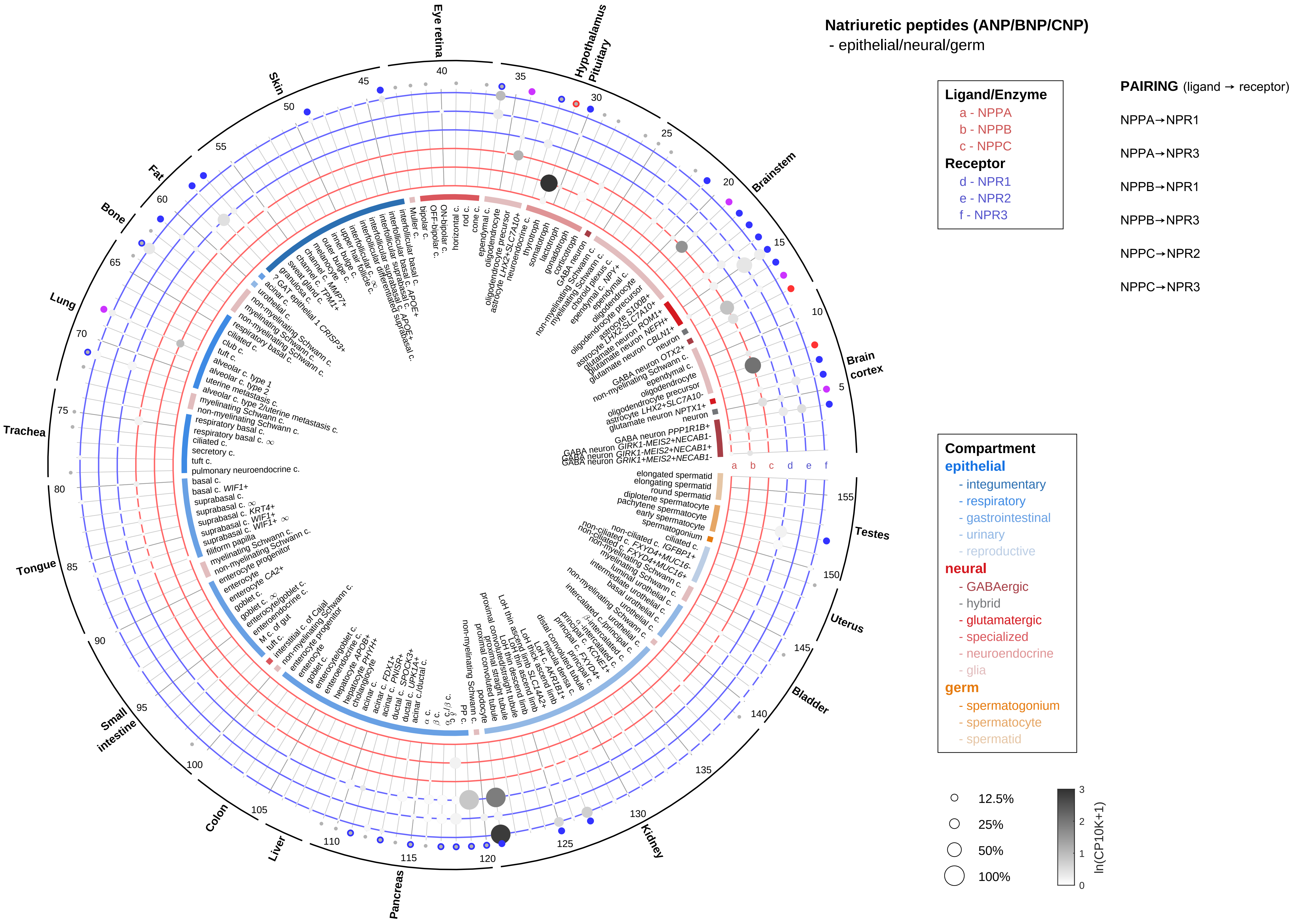

Natriuretic peptides (ANP/BNP/CNP)
- epithelial/neural/germ
Ligand/Enzyme
a - NPPA
b - NPPB
c - NPPC
Receptor
d - NPR1
e - NPR2
f - NPR3
PAIRING (ligand → receptor)
NPPA→NPR1
NPPA→NPR3
NPPB→NPR1
NPPB→NPR3
NPPC→NPR2
NPPC→NPR3
Compartment
epithelial
- integumentary
- respiratory
- gastrointestinal
- urinary
- reproductive
neural
- GABAergic
- hybrid
- glutamatergic
- specialized
- neuroendocrine
- glia
germ
- spermatogonium
- spermatocyte
- spermatid
12.5%
25%
50%
100%
ln(CP10K+1)
Eye retina
Hypothalamus
Pituitary
Brainstem
Brain cortex
Testes
Uterus
Bladder
Kidney
Pancreas
Liver
Colon
Small intestine
Tongue
Trachea
Lung
Bone
Fat
Skin

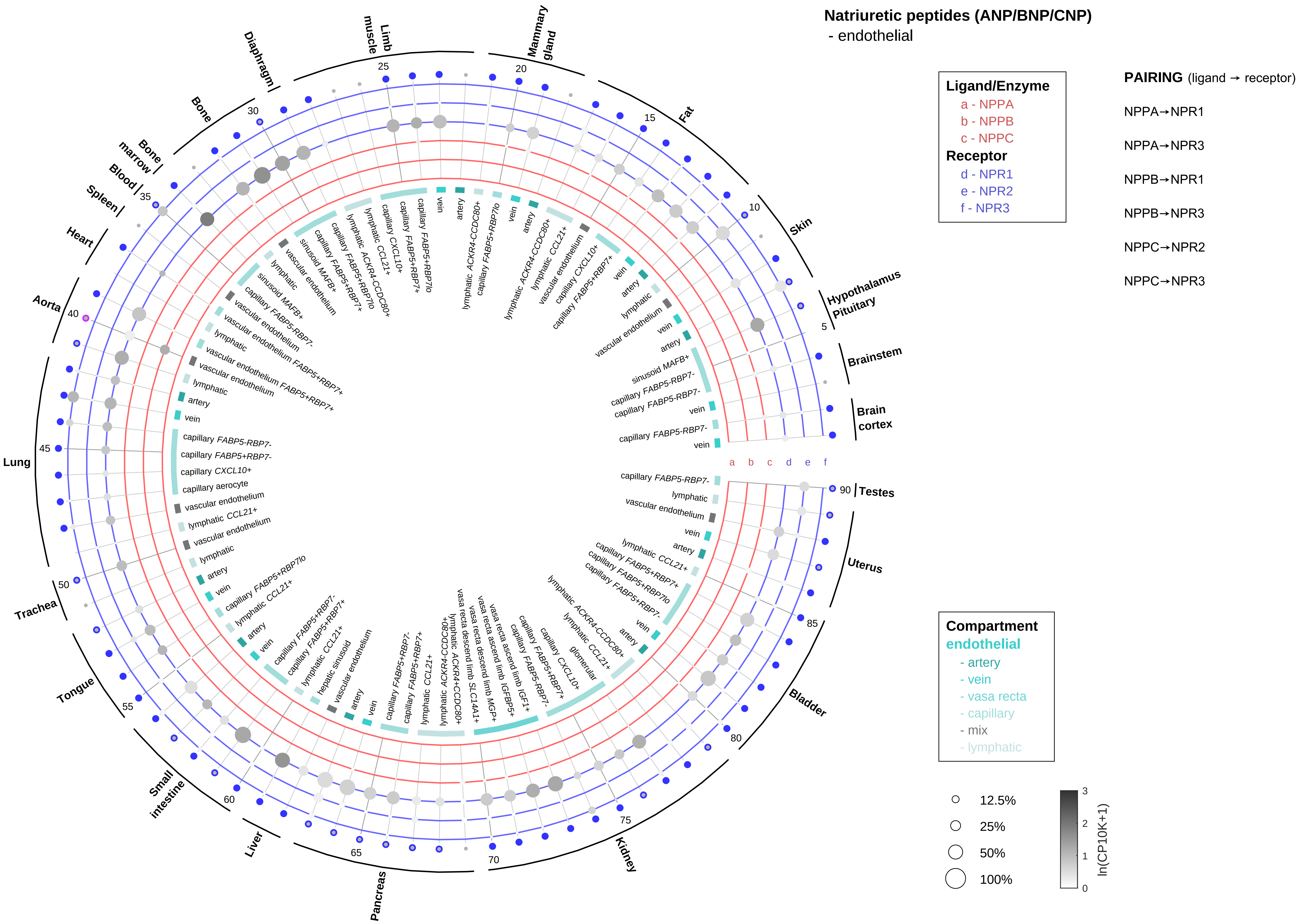
Natriuretic peptides (ANP/BNP/CNP)
- endothelial
Ligand/Enzyme
a - NPPA
b - NPPB
c - NPPC
Receptor
d - NPR1
e - NPR2
f - NPR3
PAIRING (ligand → receptor)
NPPA→NPR1
NPPA→NPR3
NPPB→NPR1
NPPB→NPR3
NPPC→NPR2
NPPC→NPR3
Compartment
endothelial
- artery
- vein
- vasa recta
- capillary
- mix
- lymphatic
12.5%
25%
50%
100%
ln(CP10K+1)
Brain cortex
Brainstem
Hypothalamus Pituitary
Skin
Fat
Mammary gland
Limb muscle
Diaphragm
Bone
Bone marrow
Blood
Spleen
Heart
Aorta
Lung
Trachea
Tongue
Small intestine
Liver
Pancreas
Kidney
Bladder
Uterus
Testes

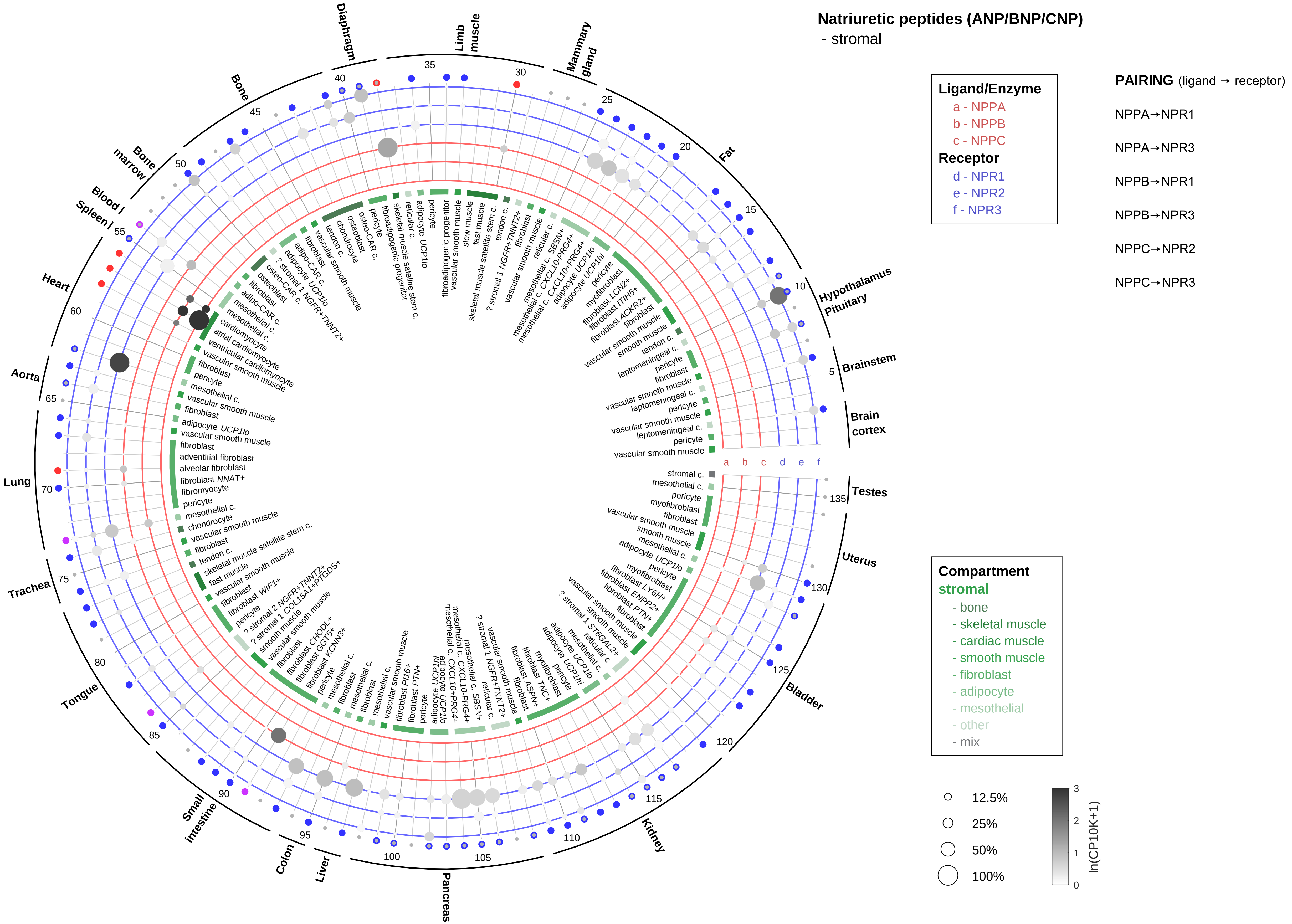

Natriuretic peptides (ANP/BNP/CNP)
- stromal
Ligand/Enzyme
a - NPPA
b - NPPB
c - NPPC
Receptor
d - NPR1
e - NPR2
f - NPR3
PAIRING (ligand → receptor)
NPPA→NPR1
NPPA→NPR3
NPPB→NPR1
NPPB→NPR3
NPPC→NPR2
NPPC→NPR3
Compartment
stromal
- bone
- skeletal muscle
- cardiac muscle
- smooth muscle
- fibroblast
- adipocyte
- mesothelial
- other
- mix
12.5%
25%
50%
100%
ln(CP10K+1)
Brain cortex
Brainstem
Hypothalamus Pituitary
Fat
Mammary gland
Limb muscle
Diaphragm
Bone
Bone marrow
Blood
Spleen
Heart
Aorta
Lung
Trachea
Tongue
Small intestine
Colon
Liver
Pancreas
Kidney
Bladder
Uterus
Testes

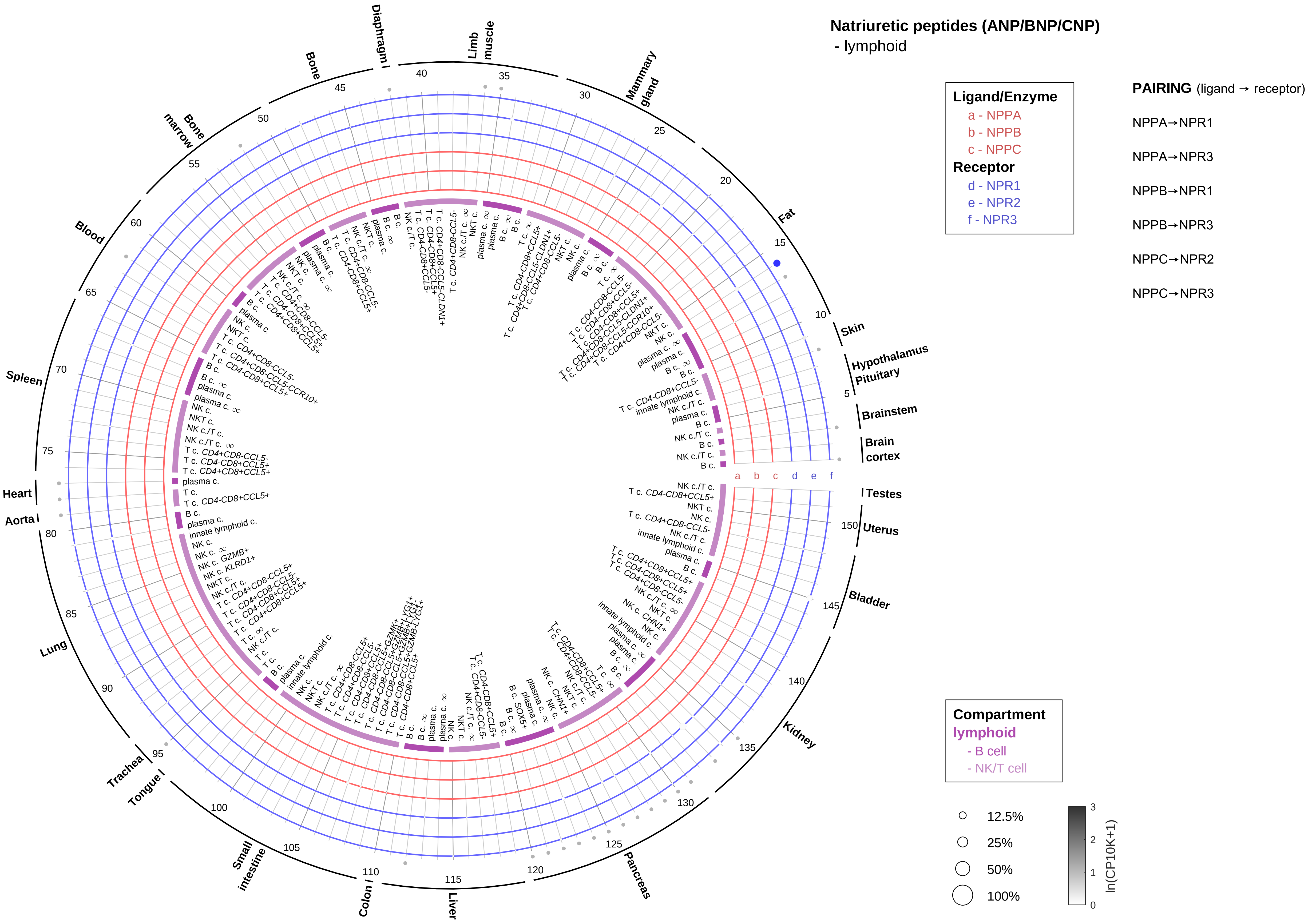
Natriuretic peptides (ANP/BNP/CNP)
- lymphoid
Ligand/Enzyme
a - NPPA
b - NPPB
c - NPPC
Receptor
d - NPR1
e - NPR2
f - NPR3
PAIRING (ligand → receptor)
NPPA→NPR1
NPPA→NPR3
NPPB→NPR1
NPPB→NPR3
NPPC→NPR2
NPPC→NPR3
Compartment
lymphoid
- B cell
- NK/T cell
12.5%
25%
50%
100%
ln(CP10K+1)
Skin
Hypothalamus
Pituitary
Brainstem
Brain cortex
Testes
Uterus
Bladder
Kidney
Pancreas
Liver
Colon
Small intestine
Tongue
Trachea
Lung
Aorta
Heart
Spleen
Blood
Bone marrow
Bone
Diaphragm
Limb muscle
Mammary gland
Fat

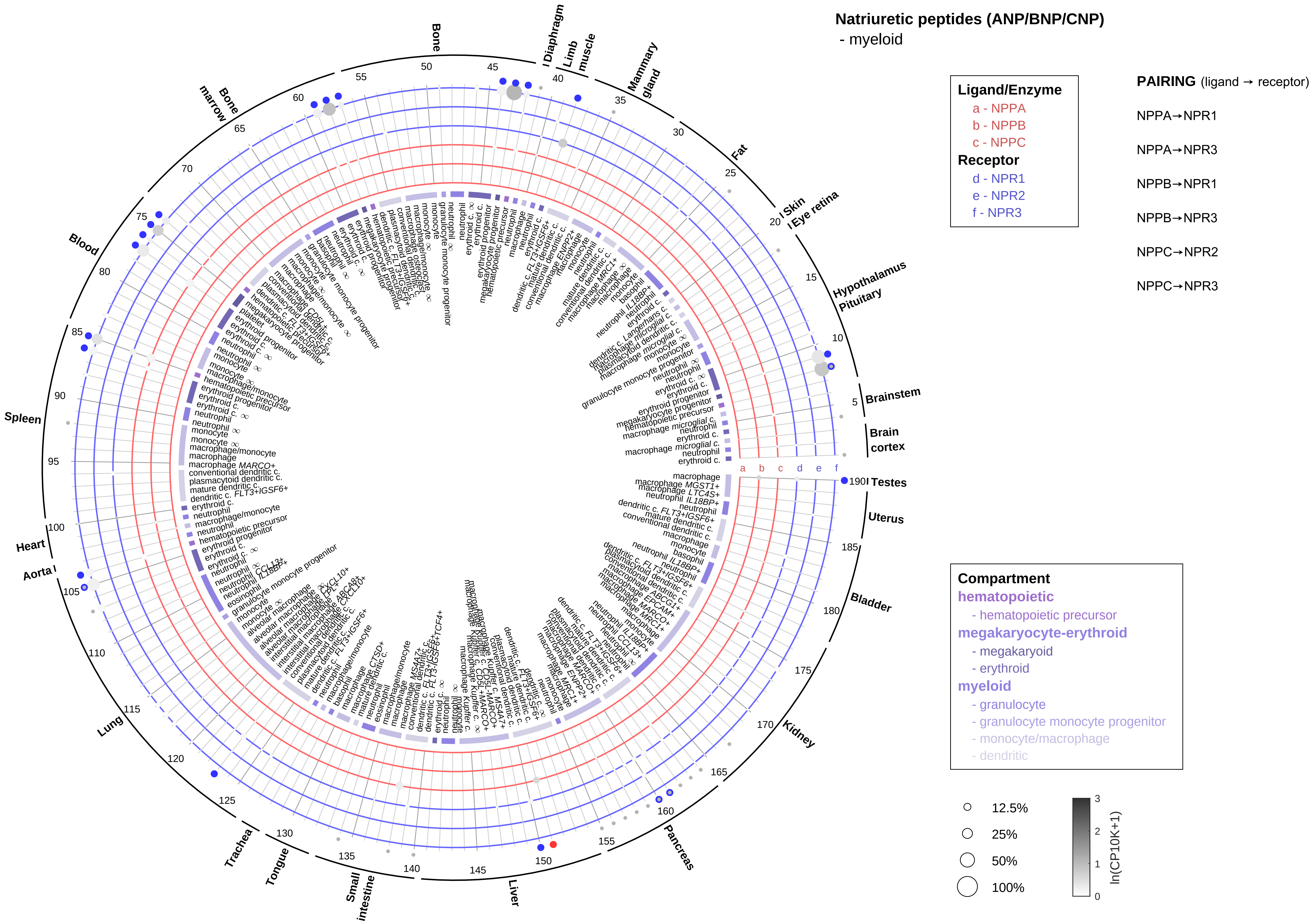

Natriuretic peptides (ANP/BNP/CNP)
- myeloid
Ligand/Enzyme
a - NPPA
b - NPPB
c - NPPC
Receptor
d - NPR1
e - NPR2
f - NPR3
PAIRING (ligand → receptor)
NPPA→NPR1
NPPA→NPR3
NPPB→NPR1
NPPB→NPR3
NPPC→NPR2
NPPC→NPR3
Compartment
hematopoietic
- hematopoietic precursor
megakaryocyte-erythroid
- megakaryoid
- erythroid
myeloid
- granulocyte
- granulocyte monocyte progenitor
- monocyte/macrophage
- dendritic
12.5%
25%
50%
100%
ln(CP10K+1)
Bone
Diaphragm
Limb muscle
Mammary gland
Fat
Skin
Eye retina
Hypothalamus
Pituitary
Brainstem
Brain cortex
Testes
Uterus
Bladder
Kidney
Pancreas
Liver
Small intestine
Tongue
Trachea
Lung
Aorta
Heart
Spleen
Blood
Bone marrow

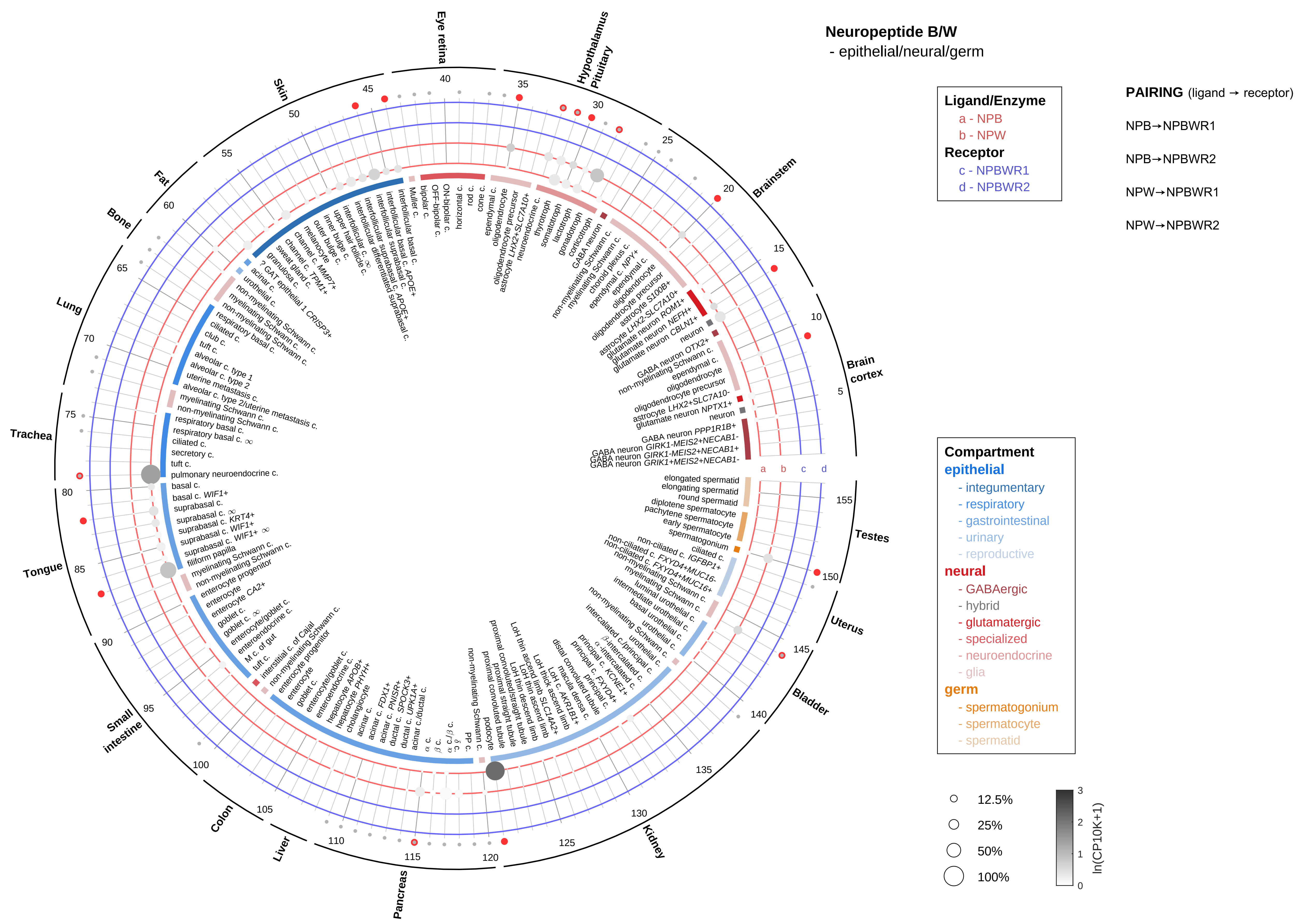

Neuropeptide B/W
- epithelial/neural/germ
Ligand/Enzyme
a - NPB
b - NPW
Receptor
c - NPBWR1
d - NPBWR2
PAIRING (ligand → receptor)
NPB→NPBWR1
NPB→NPBWR2
NPW→NPBWR1
NPW→NPBWR2
Compartment
epithelial
- integumentary
- respiratory
- gastrointestinal
- urinary
- reproductive
neural
- GABAergic
- hybrid
- glutamatergic
- specialized
- neuroendocrine
- glia
germ
- spermatogonium
- spermatocyte
- spermatid
12.5%
25%
50%
100%
ln(CP10K+1)
Eye retina
Hypothalamus Pituitary
Brainstem
Brain cortex
Testes
Uterus
Bladder
Kidney
Pancreas
Liver
Colon
Small intestine
Tongue
Trachea
Lung
Bone
Fat
Skin

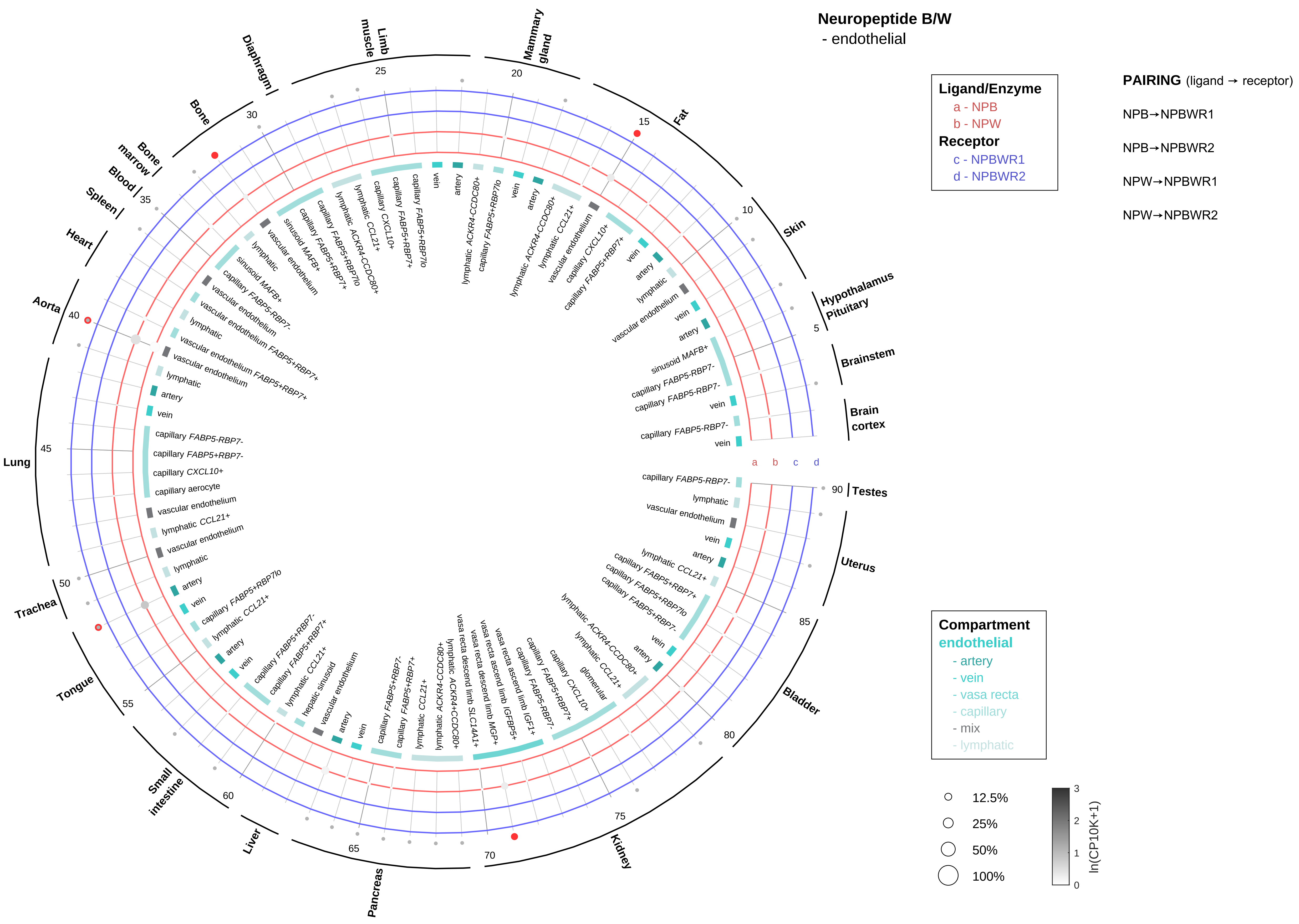

Neuropeptide B/W
- endothelial
Ligand/Enzyme
a - NPB
b - NPW
Receptor
c - NPBWR1
d - NPBWR2
PAIRING (ligand → receptor)
NPB→NPBWR1
NPB→NPBWR2
NPW→NPBWR1
NPW→NPBWR2
Compartment
endothelial
- artery
- vein
- vasa recta
- capillary
- mix
- lymphatic
12.5%
25%
50%
100%
ln(CP10K+1)
Brain cortex
Brainstem
Hypothalamus Pituitary
Skin
Fat
Mammary gland
Limb muscle
Diaphragm
Bone
Bone marrow
Blood
Spleen
Heart
Aorta
Lung
Trachea
Tongue
Small intestine
Liver
Pancreas
Kidney
Bladder
Uterus
Testes

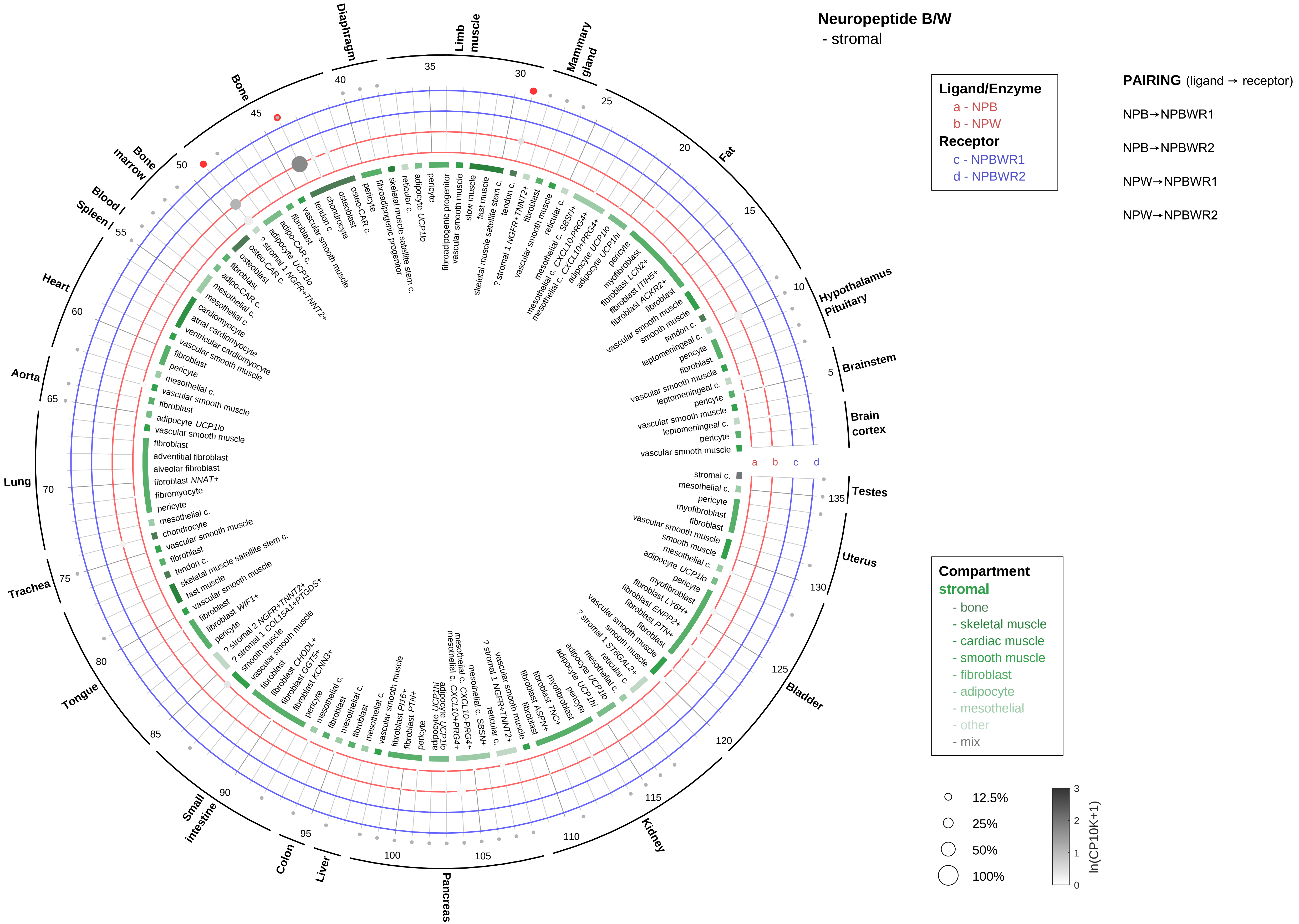
Neuropeptide B/W
- stromal
Ligand/Enzyme
a - NPB
b - NPW
Receptor
c - NPBWR1
d - NPBWR2
PAIRING (ligand → receptor)
NPB→NPBWR1
NPB→NPBWR2
NPW→NPBWR1
NPW→NPBWR2
Compartment
stromal
- bone
- skeletal muscle
- cardiac muscle
- smooth muscle
- fibroblast
- adipocyte
- mesothelial
- other
- mix
12.5%
25%
50%
100%
ln(CP10K+1)
0
1
2
3
Hypothalamus Pituitary
Brainstem
Brain cortex
Testes
Uterus
Bladder
Kidney
Pancreas
Liver
Colon
Small intestine
Tongue
Trachea
Lung
Aorta
Heart
Spleen /
Blood /
Bone marrow
Bone
Diaphragm
Limb muscle
Mammary gland
Fat
a
b
c
d

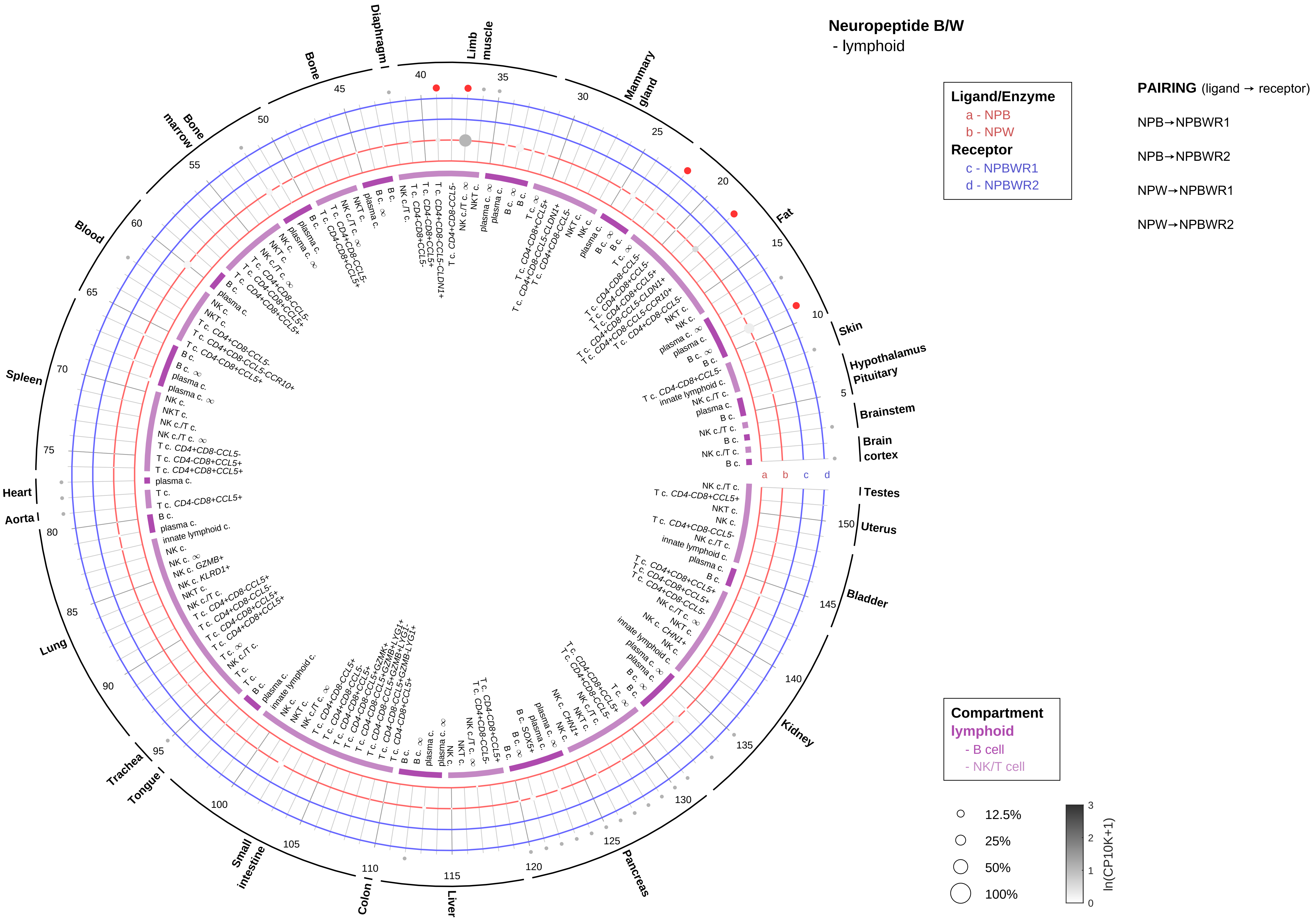
Neuropeptide B/W
- lymphoid
Ligand/Enzyme
a - NPB
b - NPW
Receptor
c - NPBWR1
d - NPBWR2
PAIRING (ligand → receptor)
NPB→NPBWR1
NPB→NPBWR2
NPW→NPBWR1
NPW→NPBWR2
Compartment
lymphoid
- B cell
- NK/T cell
12.5%
25%
50%
100%
ln(CP10K+1)
Skin
Hypothalamus
Pituitary
Brainstem
Brain cortex
Testes
Uterus
Bladder
Kidney
Pancreas
Liver
Colon
Small intestine
Tongue
Trachea
Lung
Aorta
Heart
Spleen
Blood
Bone marrow
Bone
Diaphragm
Limb muscle
Mammary gland
Fat

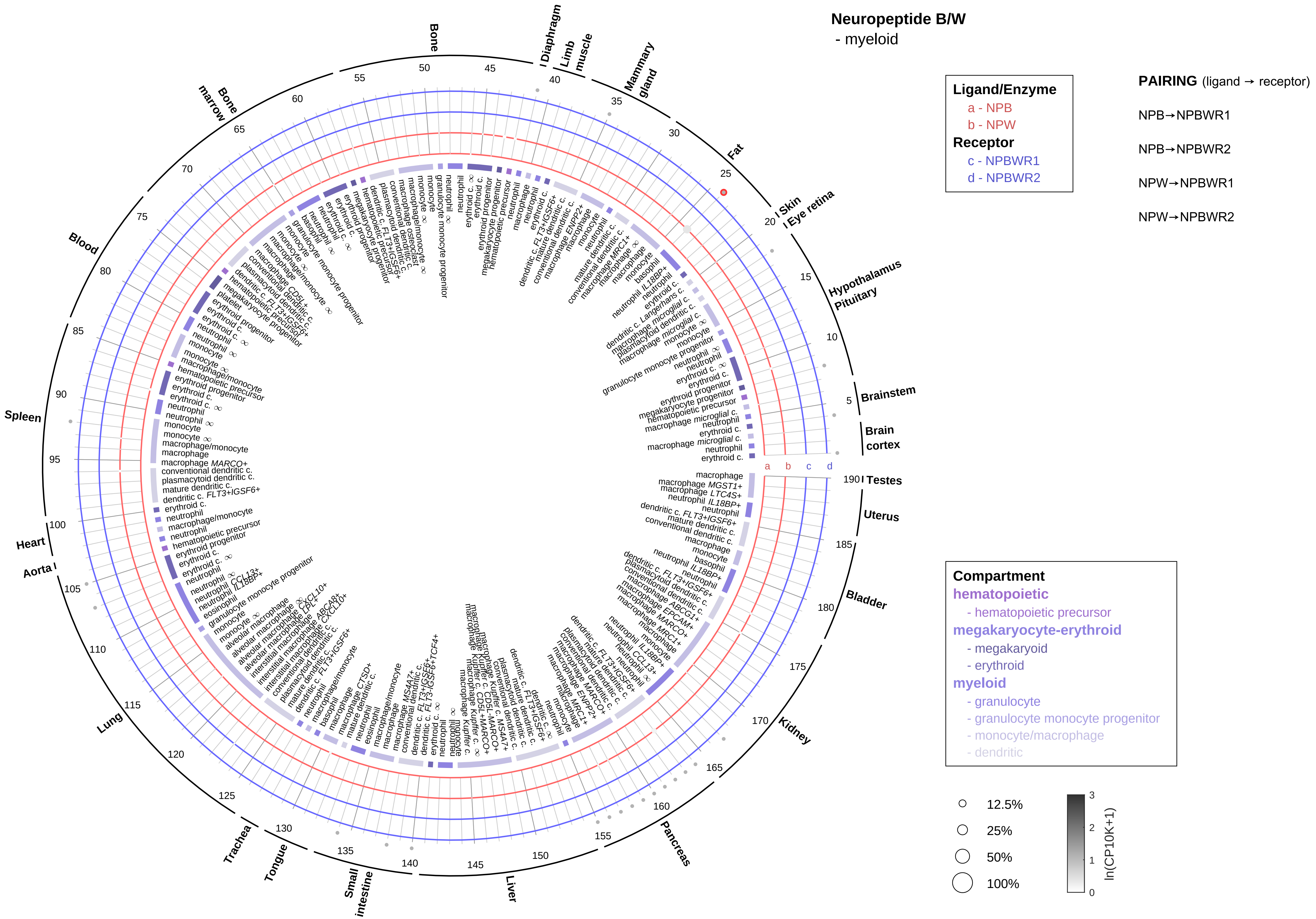
Neuropeptide B/W
- myeloid
Ligand/Enzyme
a - NPB
b - NPW
Receptor
c - NPBWR1
d - NPBWR2
PAIRING (ligand → receptor)
NPB→NPBWR1
NPB→NPBWR2
NPW→NPBWR1
NPW→NPBWR2
Compartment
hematopoietic
- hematopoietic precursor
megakaryocyte-erythroid
- megakaryoid
- erythroid
myeloid
- granulocyte
- granulocyte monocyte progenitor
- monocyte/macrophage
- dendritic
12.5%
25%
50%
100%
ln(CP10K+1)

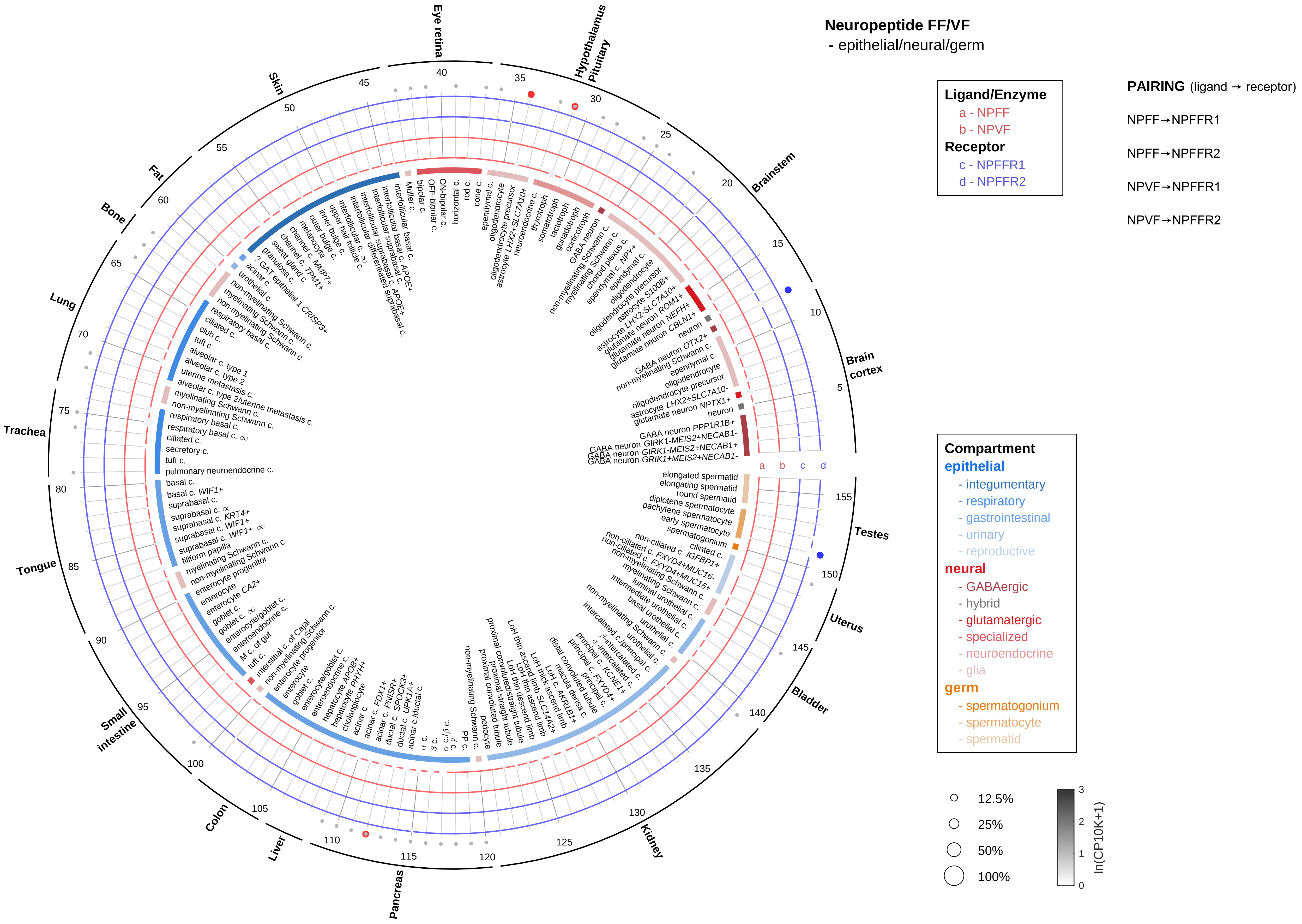
Neuropeptide FF/VF
- epithelial/neural/germ
Ligand/Enzyme
a - NPFF
b - NPVF
Receptor
c - NPFFR1
d - NPFFR2
PAIRING (ligand → receptor)
NPFF→NPFFR1
NPFF→NPFFR2
NPVF→NPFFR1
NPVF→NPFFR2
Eye retina
Hypothalamus
Pituitary
Brainstem
Brain cortex
Testes
Uterus
Bladder
Kidney
Pancreas
Liver
Colon
Small intestine
Tongue
Trachea
Lung
Bone
Fat
Skin
Compartment
epithelial
- integumentary
- respiratory
- gastrointestinal
- urinary
- reproductive
neural
- GABAergic
- hybrid
- glutamatergic
- specialized
- neuroendocrine
- glia
germ
- spermatogonium
- spermatocyte
- spermatid
12.5%
25%
50%
100%
ln(CP10K+1)

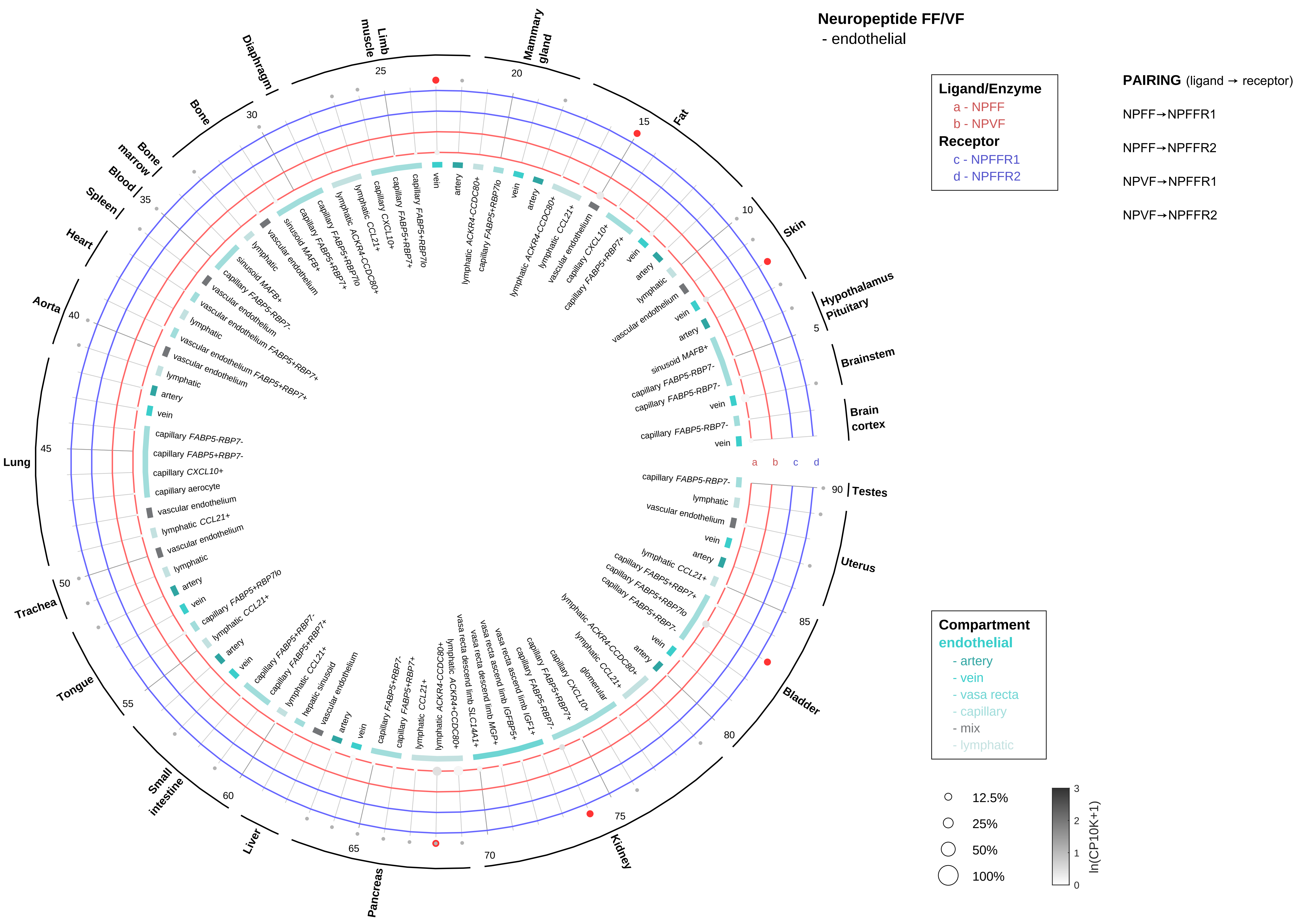

Neuropeptide FF/VF
- endothelial
Ligand/Enzyme
a - NPFF
b - NPVF
Receptor
c - NPFFR1
d - NPFFR2
PAIRING (ligand → receptor)
NPFF→NPFFR1
NPFF→NPFFR2
NPVF→NPFFR1
NPVF→NPFFR2
Compartment
endothelial
- artery
- vein
- vasa recta
- capillary
- mix
- lymphatic
12.5%
25%
50%
100%
ln(CP10K+1)
Brain cortex
Brainstem
Hypothalamus Pituitary
Skin
Fat
Mammary gland
Limb muscle
Diaphragm
Bone
Bone marrow
Blood
Spleen
Heart
Aorta
Lung
Trachea
Tongue
Small intestine
Liver
Pancreas
Kidney
Bladder
Uterus
Testes

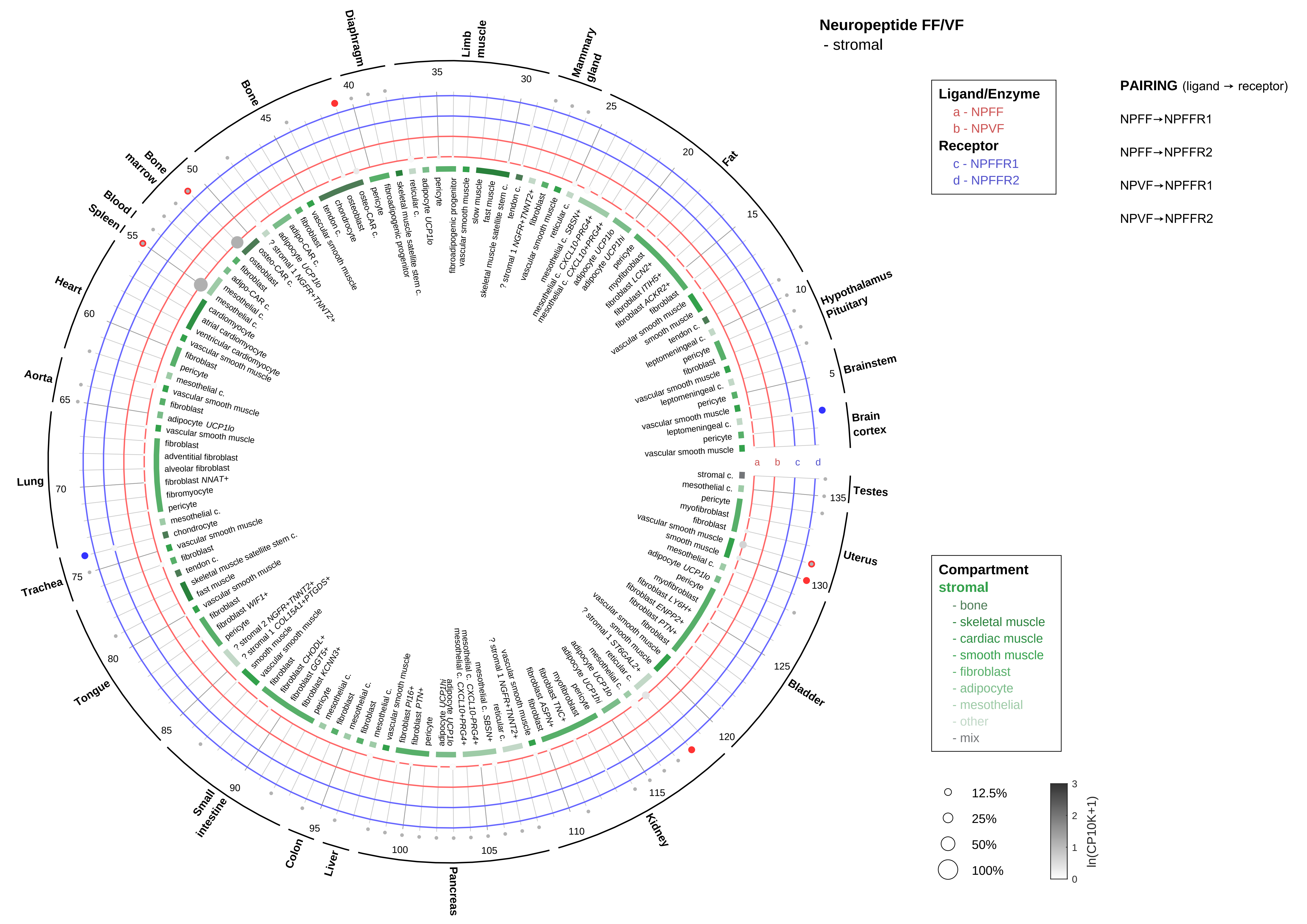

Neuropeptide FF/VF
- stromal
Ligand/Enzyme
a - NPFF
b - NPVF
Receptor
c - NPFFR1
d - NPFFR2
PAIRING (ligand → receptor)
NPFF→NPFFR1
NPFF→NPFFR2
NPVF→NPFFR1
NPVF→NPFFR2
Compartment
stromal
- bone
- skeletal muscle
- cardiac muscle
- smooth muscle
- fibroblast
- adipocyte
- mesothelial
- other
- mix
12.5%
25%
50%
100%
ln(CP10K+1)

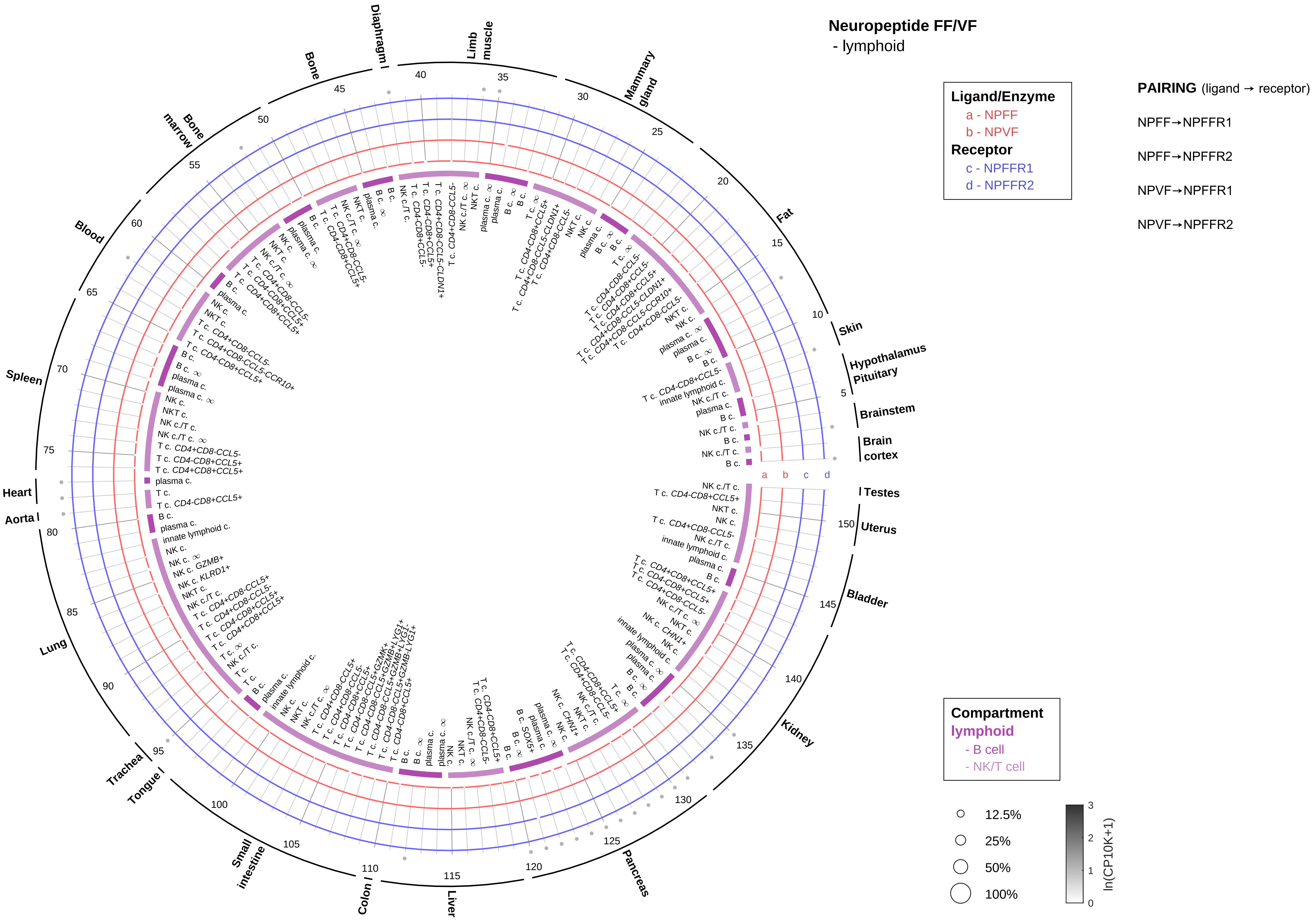

Neuropeptide FF/VF
- lymphoid
Ligand/Enzyme
a - NPFF
b - NPVF
Receptor
c - NPFFR1
d - NPFFR2
PAIRING (ligand → receptor)
NPFF→NPFFR1
NPFF→NPFFR2
NPVF→NPFFR1
NPVF→NPFFR2
Compartment
lymphoid
- B cell
- NK/T cell
12.5%
25%
50%
100%
ln(CP10K+1)
Brain cortex
Brainstem
Hypothalamus Pituitary
Skin
Fat
Mammary gland
Limb muscle
Diaphragm
Bone
Bone marrow
Blood
Spleen
Heart
Aorta
Lung
Trachea
Tongue
Small intestine
Colon
Liver
Pancreas
Kidney
Bladder
Uterus
Testes

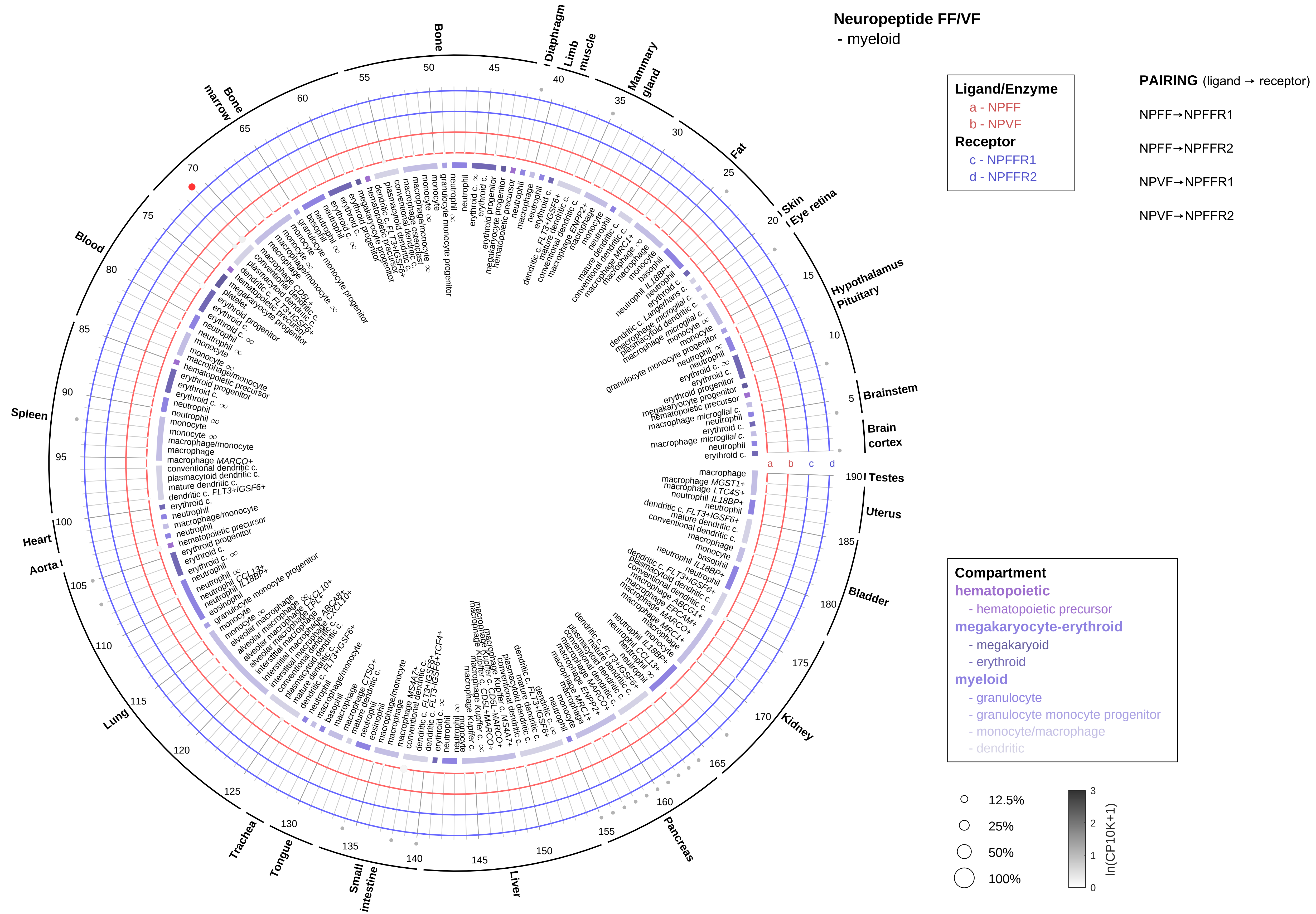
Neuropeptide FF/VF
- myeloid
Ligand/Enzyme
a - NPFF
b - NPVF
Receptor
c - NPFFR1
d - NPFFR2
PAIRING (ligand → receptor)
NPFF→NPFFR1
NPFF→NPFFR2
NPVF→NPFFR1
NPVF→NPFFR2
Compartment
hematopoietic
- hematopoietic precursor
megakaryocyte-erythroid
- megakaryoid
- erythroid
myeloid
- granulocyte
- granulocyte monocyte progenitor
- monocyte/macrophage
- dendritic
12.5%
25%
50%
100%
ln(CP10K+1)
Brain cortex
Brainstem
Hypothalamus
Pituitary
Eye retina
Skin
Fat
Mammary gland
Limb muscle
Diaphragm
Bone
Bone marrow
Blood
Spleen
Heart
Aorta
Lung
Trachea
Tongue
Small intestine
Liver
Pancreas
Kidney
Bladder
Uterus
Testes

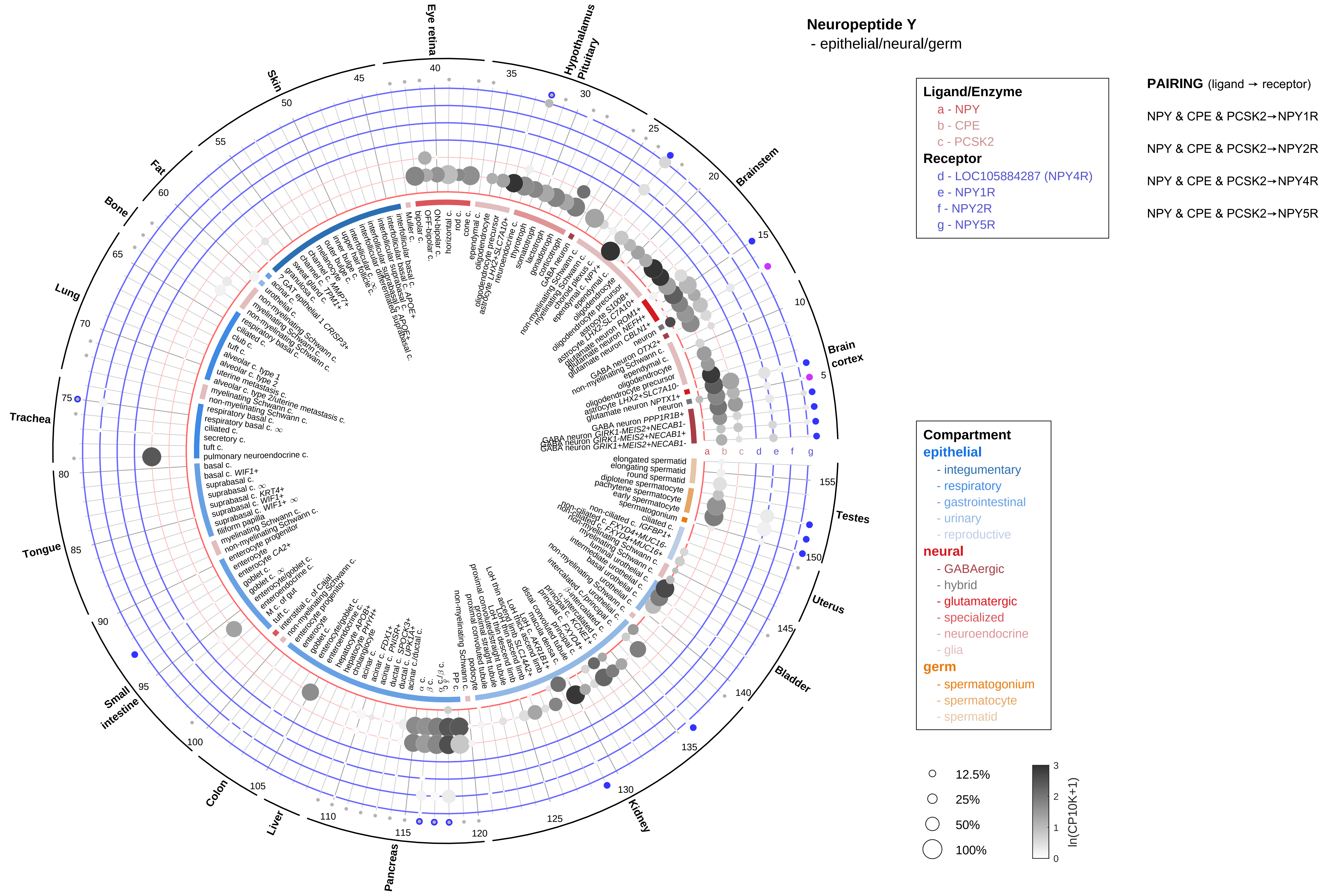
Neuropeptide Y
- epithelial/neural/germ
Ligand/Enzyme
a - NPY
b - CPE
c - PCSK2
Receptor
d - LOC105884287 (NPY4R)
e - NPY1R
f - NPY2R
g - NPY5R
PAIRING (ligand → receptor)
NPY & CPE & PCSK2→NPY1R
NPY & CPE & PCSK2→NPY2R
NPY & CPE & PCSK2→NPY4R
NPY & CPE & PCSK2→NPY5R
Compartment
epithelial
- integumentary
- respiratory
- gastrointestinal
- urinary
- reproductive
neural
- GABAergic
- hybrid
- glutamatergic
- specialized
- neuroendocrine
- glia
germ
- spermatogonium
- spermatocyte
- spermatid
12.5%
25%
50%
100%
ln(CP10K+1)
Eye retina
Hypothalamus Pituitary
Brainstem
Brain cortex
Testes
Uterus
Bladder
Kidney
Pancreas
Liver
Colon
Small intestine
Tongue
Trachea
Lung
Bone
Fat
Skin

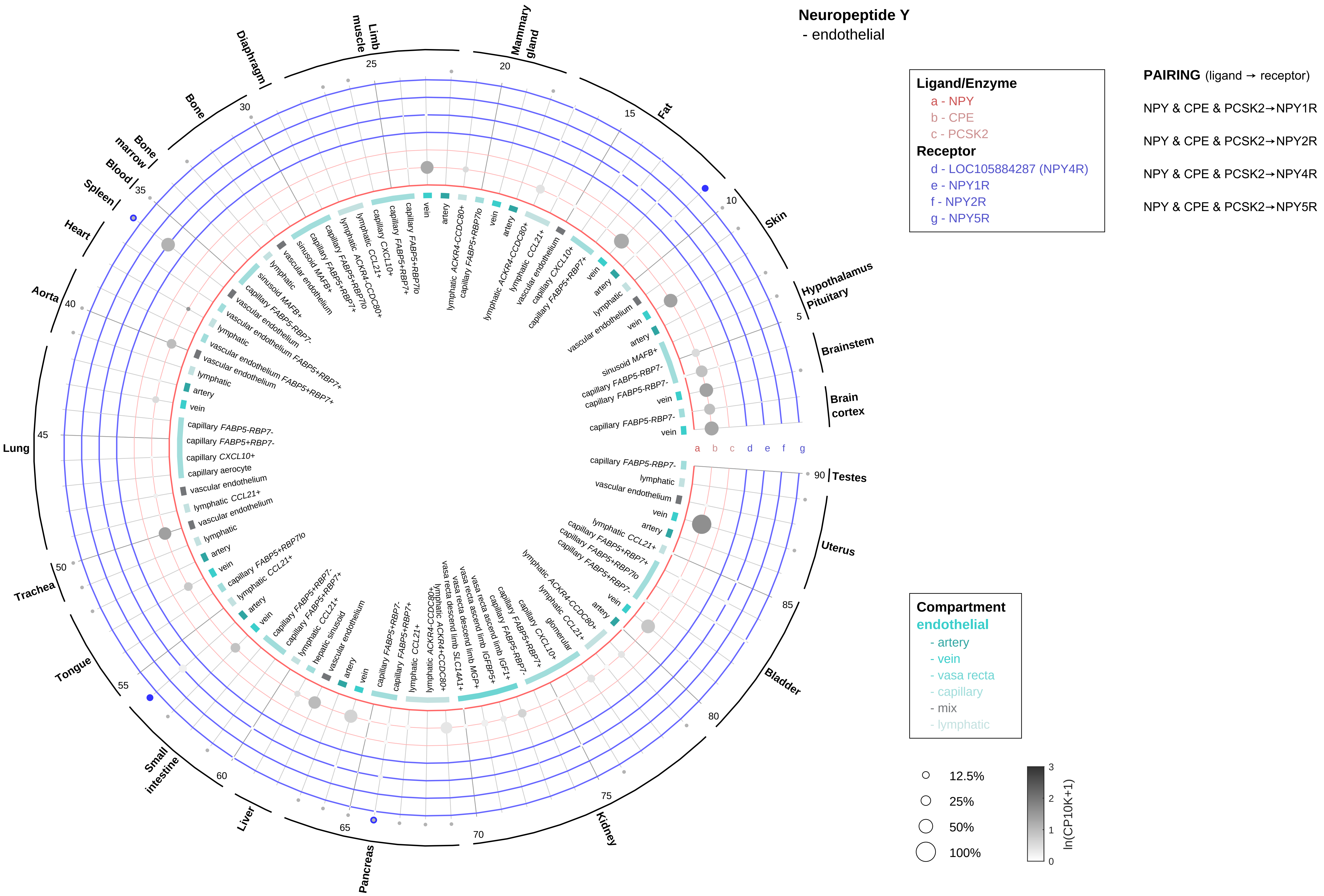

Neuropeptide Y
- endothelial
Ligand/Enzyme
a - NPY
b - CPE
c - PCSK2
Receptor
d - LOC105884287 (NPY4R)
e - NPY1R
f - NPY2R
g - NPY5R
PAIRING (ligand → receptor)
NPY & CPE & PCSK2→NPY1R
NPY & CPE & PCSK2→NPY2R
NPY & CPE & PCSK2→NPY4R
NPY & CPE & PCSK2→NPY5R
Compartment
endothelial
- artery
- vein
- vasa recta
- capillary
- mix
- lymphatic
12.5%
25%
50%
100%
ln(CP10K+1)
Brain cortex
Brainstem
Hypothalamus Pituitary
Skin
Fat
Mammary gland
Limb muscle
Diaphragm
Bone
Bone marrow
Blood
Spleen
Heart
Aorta
Lung
Trachea
Tongue
Small intestine
Liver
Pancreas
Kidney
Bladder
Uterus
Testes
a b c d e f g

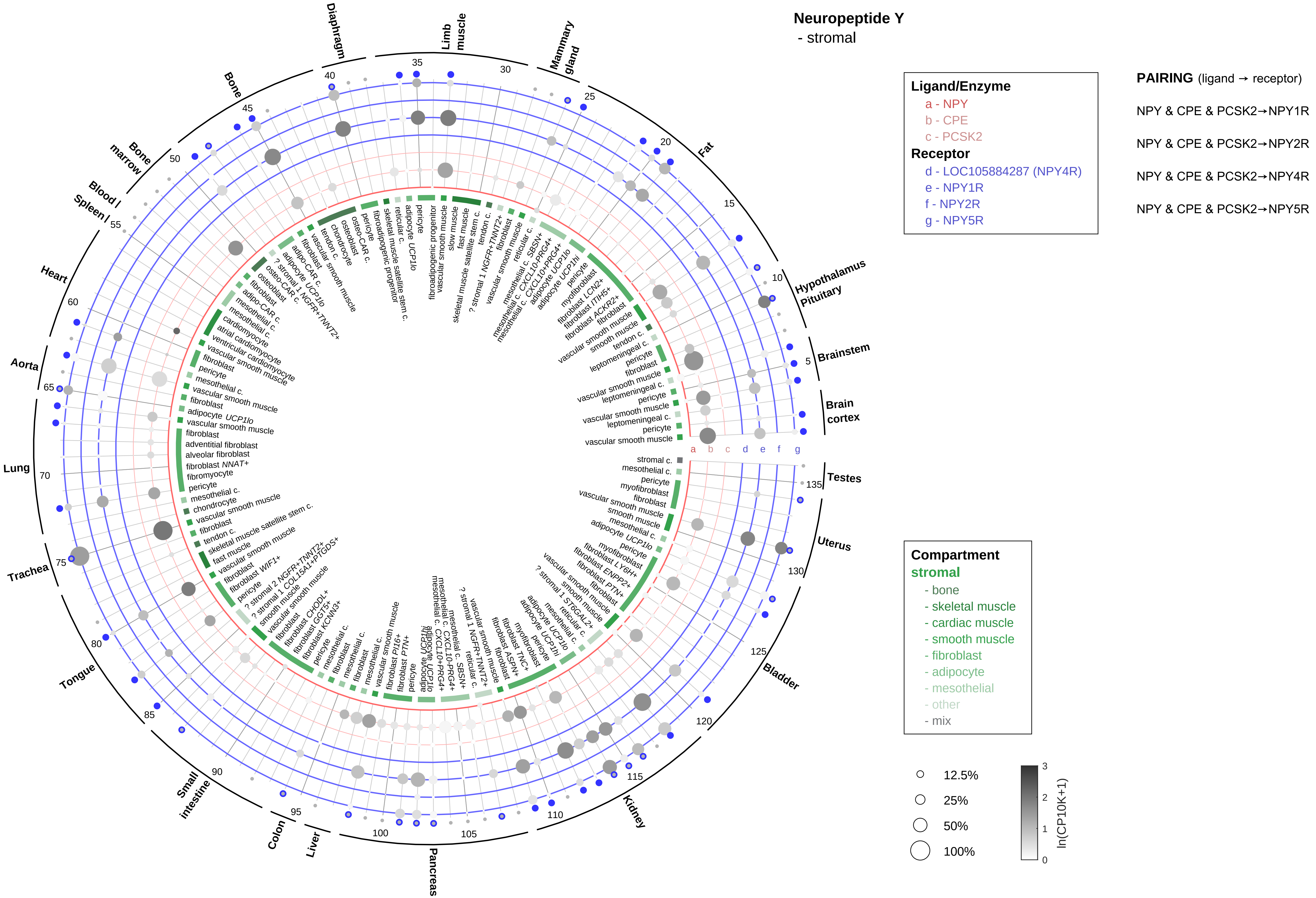

Neuropeptide Y
- stromal
Ligand/Enzyme
a - NPY
b - CPE
c - PCSK2
Receptor
d - LOC105884287 (NPY4R)
e - NPY1R
f - NPY2R
g - NPY5R
PAIRING (ligand → receptor)
NPY & CPE & PCSK2→NPY1R
NPY & CPE & PCSK2→NPY2R
NPY & CPE & PCSK2→NPY4R
NPY & CPE & PCSK2→NPY5R
Compartment
stromal
- bone
- skeletal muscle
- cardiac muscle
- smooth muscle
- fibroblast
- adipocyte
- mesothelial
- other
- mix
12.5%
25%
50%
100%
ln(CP10K+1)
Diaphragm
Limb muscle
Mammary gland
Fat
Hypothalamus Pituitary
Brainstem
Brain cortex
Testes
Uterus
Bladder
Kidney
Pancreas
Liver
Colon
Small intestine
Tongue
Trachea
Lung
Aorta
Heart
Spleen
Blood
Bone marrow
Bone

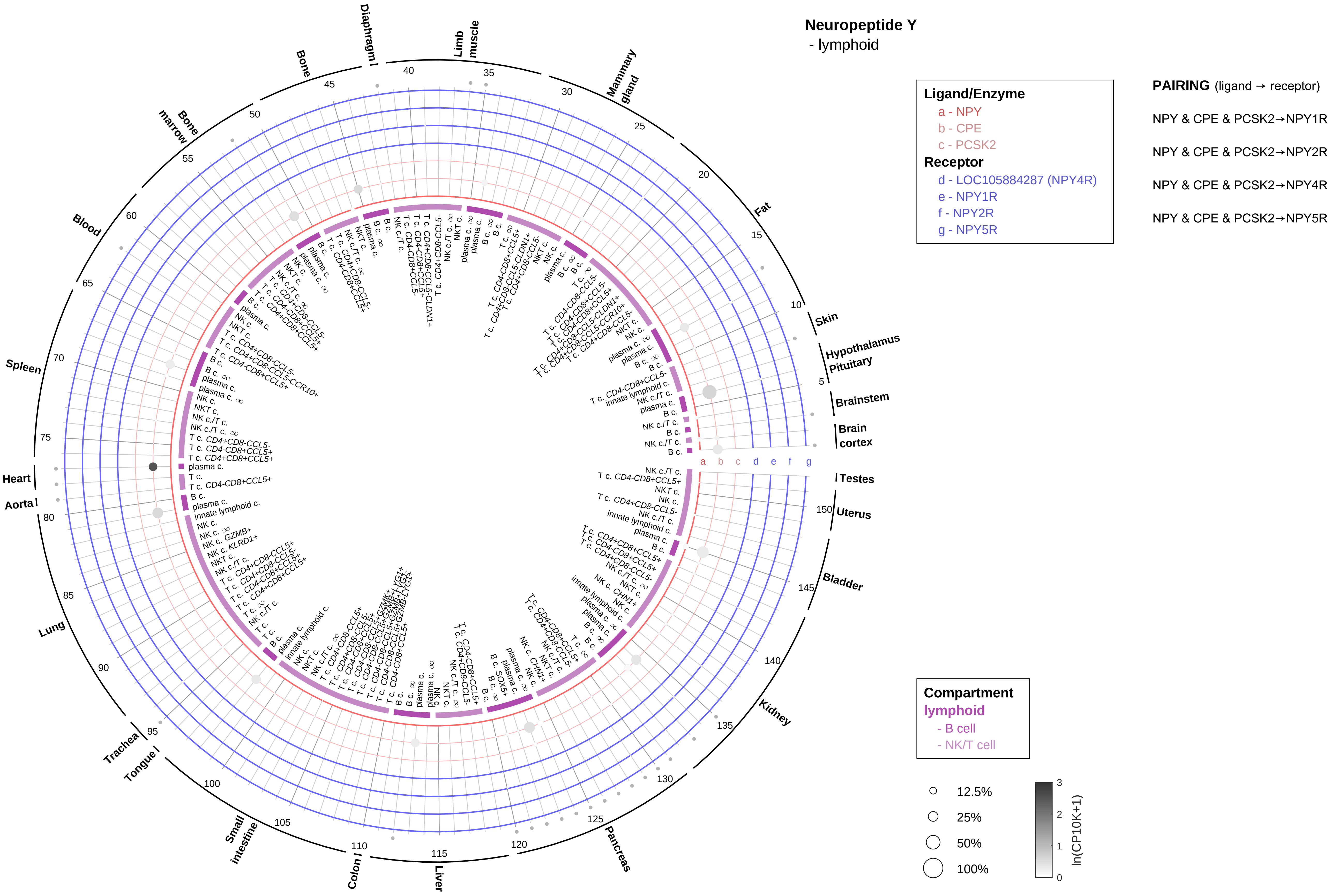

Neuropeptide Y
- lymphoid
Ligand/Enzyme
a - NPY
b - CPE
c - PCSK2
Receptor
d - LOC105884287 (NPY4R)
e - NPY1R
f - NPY2R
g - NPY5R
PAIRING (ligand → receptor)
NPY & CPE & PCSK2→NPY1R
NPY & CPE & PCSK2→NPY2R
NPY & CPE & PCSK2→NPY4R
NPY & CPE & PCSK2→NPY5R
Compartment
lymphoid
- B cell
- NK/T cell
12.5%
25%
50%
100%
ln(CP10K+1)
Skin
Hypothalamus
Pituitary
Brainstem
Brain cortex
Testes
Uterus
Bladder
Kidney
Pancreas
Liver
Colon
Small intestine
Tongue
Trachea
Lung
Aorta
Heart
Spleen
Blood
Bone marrow
Bone
Diaphragm
Limb muscle
Mammary gland
Fat

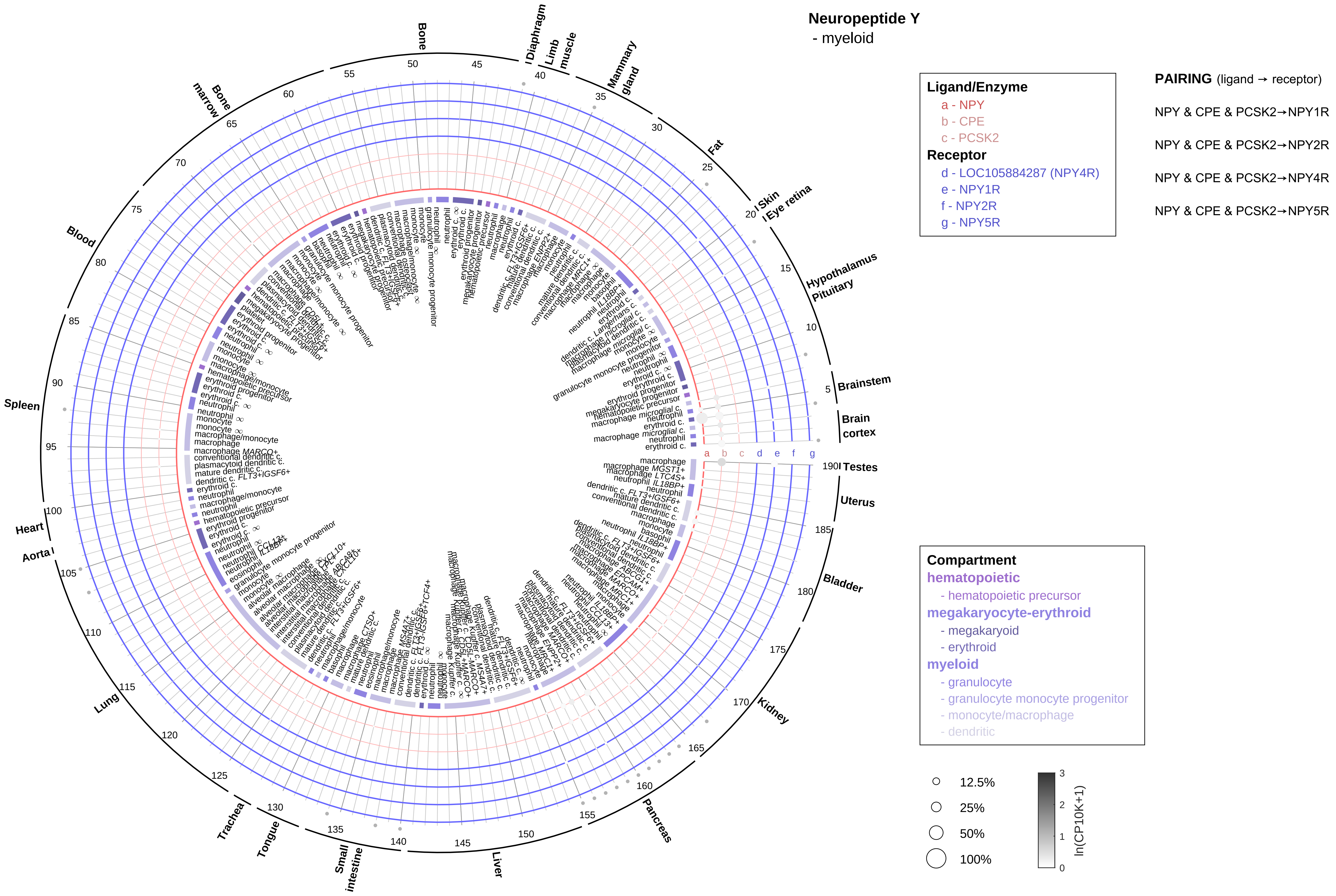

Neuropeptide Y
- myeloid
Ligand/Enzyme
a - NPY
b - CPE
c - PCSK2
Receptor
d - LOC105884287 (NPY4R)
e - NPY1R
f - NPY2R
g - NPY5R
PAIRING (ligand → receptor)
NPY & CPE & PCSK2→NPY1R
NPY & CPE & PCSK2→NPY2R
NPY & CPE & PCSK2→NPY4R
NPY & CPE & PCSK2→NPY5R
Compartment
hematopoietic
- hematopoietic precursor
megakaryocyte-erythroid
- megakaryoid
- erythroid
myeloid
- granulocyte
- granulocyte monocyte progenitor
- monocyte/macrophage
- dendritic
12.5%
25%
50%
100%
ln(CP10K+1)
Bone
Diaphragm
Limb muscle
Mammary gland
Fat
Skin
Eye retina
Hypothalamus
Pituitary
Brainstem
Brain cortex
Testes
Uterus
Bladder
Kidney
Pancreas
Liver
Small intestine
Tongue
Trachea
Lung
Aorta
Heart
Spleen
Blood
Bone marrow

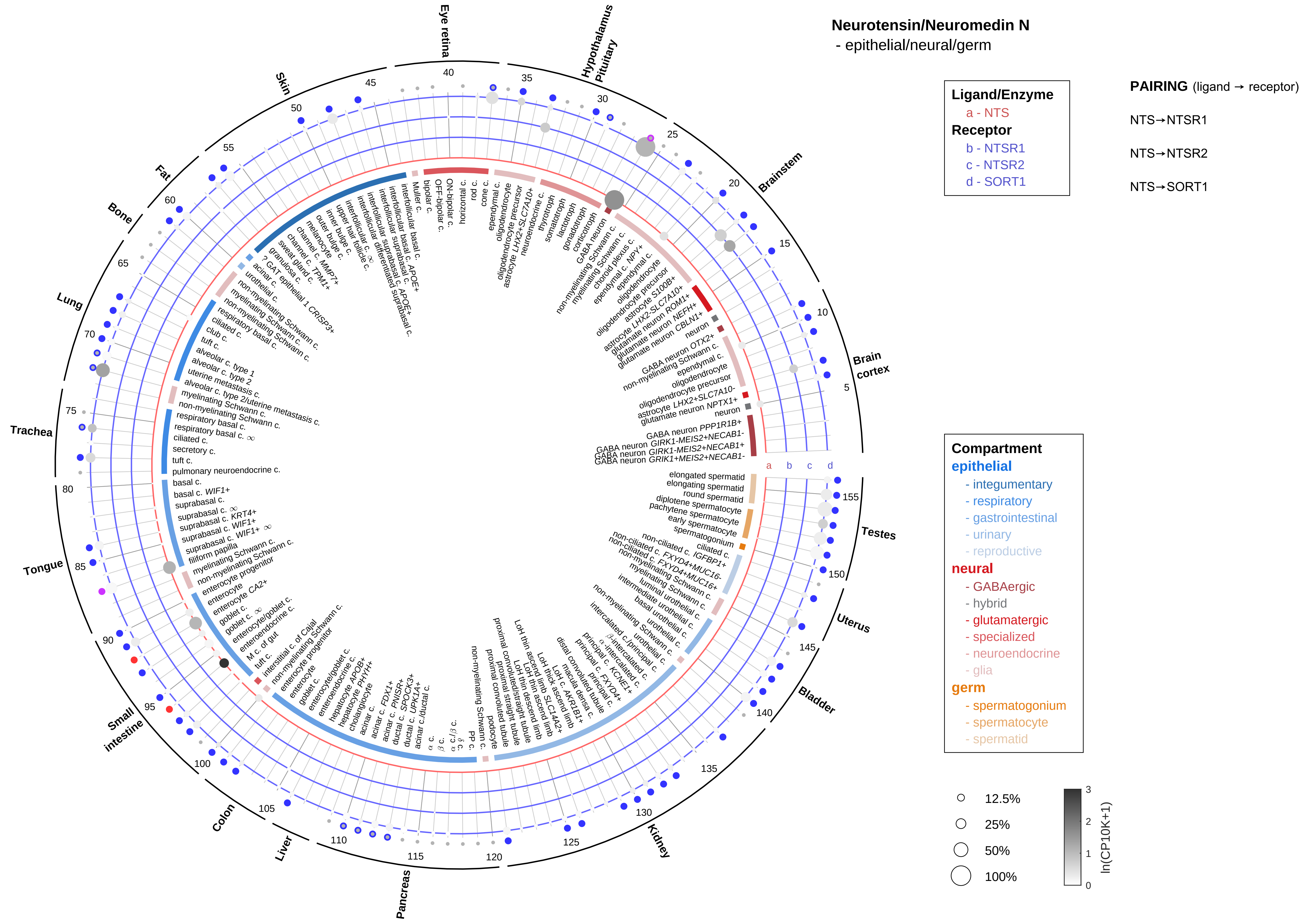

Neurotensin/Neuromedin N
- epithelial/neural/germ
Ligand/Enzyme
a - NTS
Receptor
b - NTSR1
c - NTSR2
d - SORT1
PAIRING (ligand → receptor)
NTS→NTSR1
NTS→NTSR2
NTS→SORT1
Compartment
epithelial
- integumentary
- respiratory
- gastrointestinal
- urinary
- reproductive
neural
- GABAergic
- hybrid
- glutamatergic
- specialized
- neuroendocrine
- glia
germ
- spermatogonium
- spermatocyte
- spermatid
12.5%
25%
50%
100%
ln(CP10K+1)
Eye retina
Hypothalamus Pituitary
Brainstem
Brain cortex
Testes
Uterus
Bladder
Kidney
Pancreas
Liver
Colon
Small intestine
Tongue
Trachea
Lung
Bone
Fat
Skin

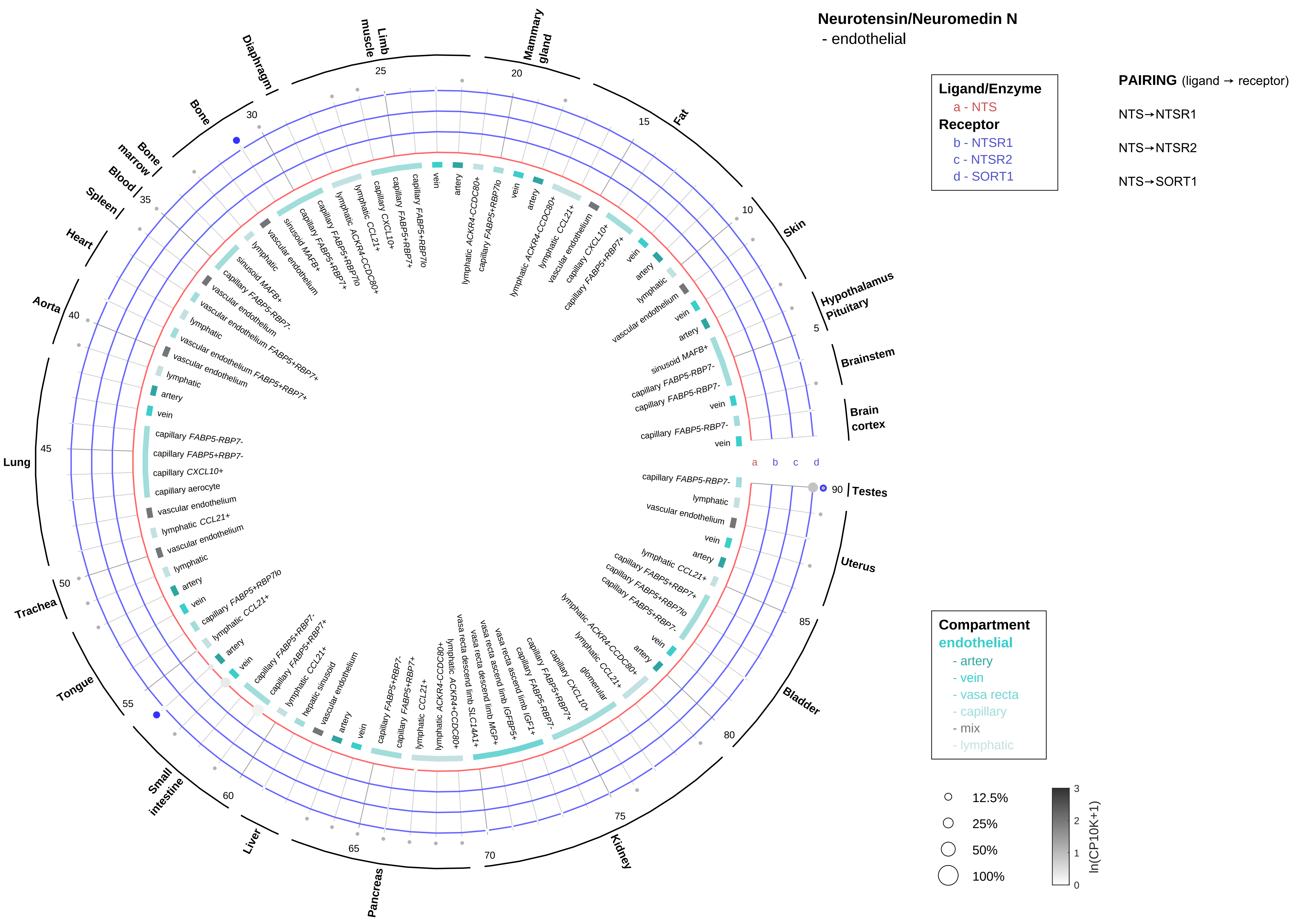

Neurotensin/Neuromedin N
- endothelial
Ligand/Enzyme
a - NTS
Receptor
b - NTSR1
c - NTSR2
d - SORT1
PAIRING (ligand → receptor)
NTS→NTSR1
NTS→NTSR2
NTS→SORT1
Compartment
endothelial
- artery
- vein
- vasa recta
- capillary
- mix
- lymphatic
12.5%
25%
50%
100%
ln(CP10K+1)

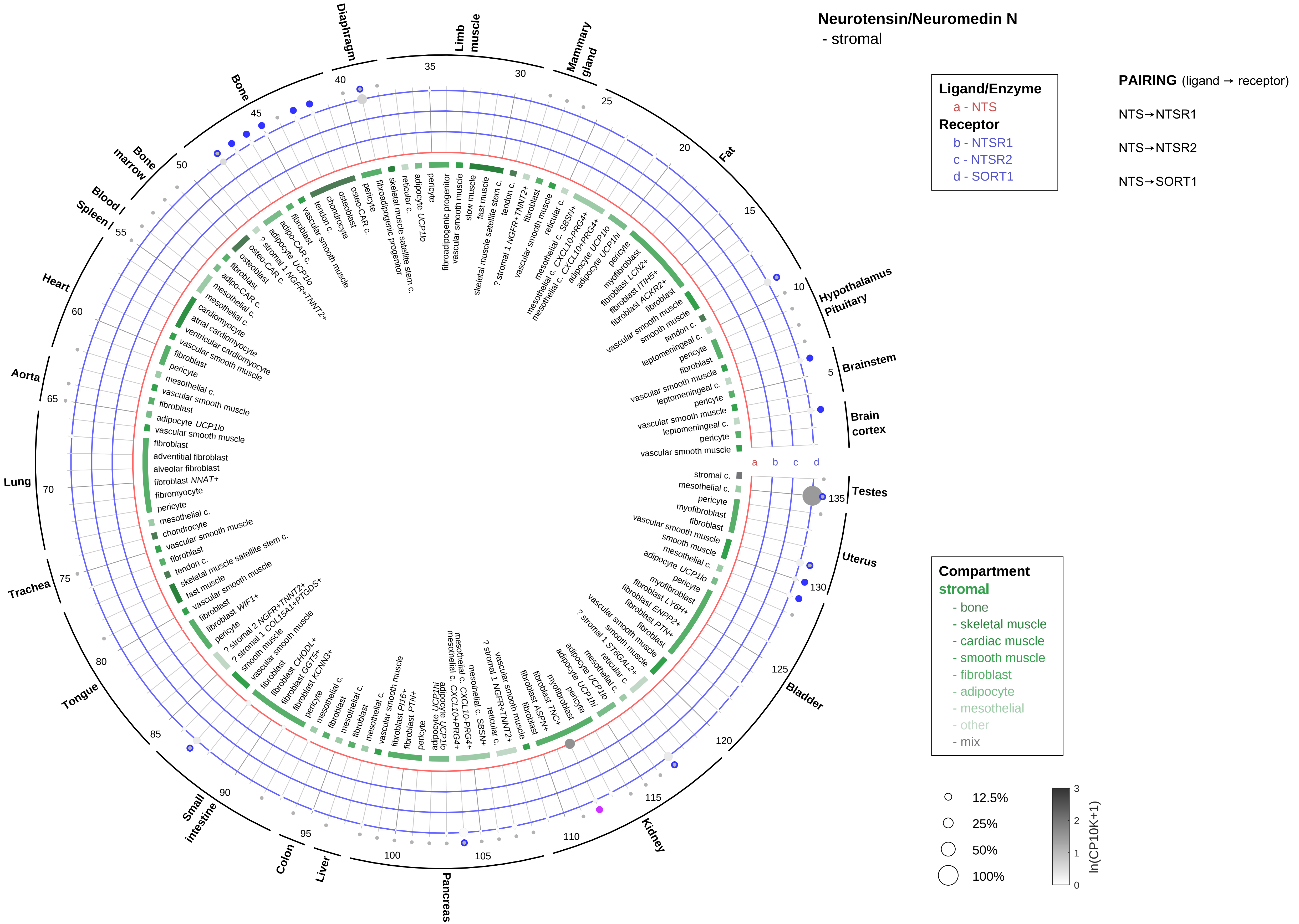
Neurotensin/Neuromedin N
- stromal
Ligand/Enzyme
a - NTS
Receptor
b - NTSR1
c - NTSR2
d - SORT1
PAIRING (ligand → receptor)
NTS→NTSR1
NTS→NTSR2
NTS→SORT1
Compartment
stromal
- bone
- skeletal muscle
- cardiac muscle
- smooth muscle
- fibroblast
- adipocyte
- mesothelial
- other
- mix
12.5%
25%
50%
100%
ln(CP10K+1)
Diaphragm
Limb muscle
Mammary gland
Fat
Hypothalamus Pituitary
Brainstem
Brain cortex
Testes
Uterus
Bladder
Kidney
Pancreas
Liver
Colon
Small intestine
Tongue
Trachea
Lung
Aorta
Heart
Spleen /
Blood /
Bone marrow
Bone

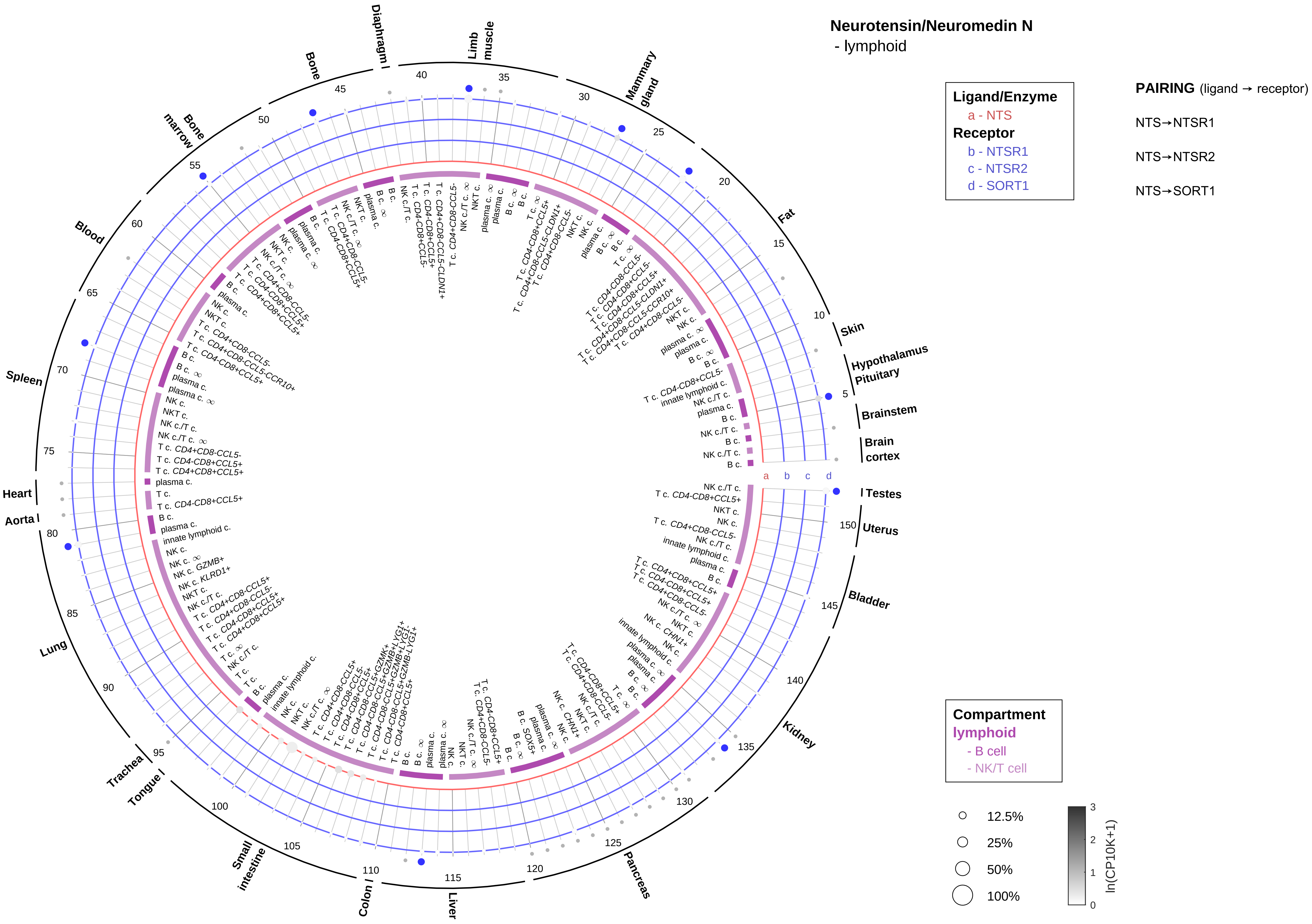

Neurotensin/Neuromedin N
- lymphoid
Ligand/Enzyme
a - NTS
Receptor
b - NTSR1
c - NTSR2
d - SORT1
PAIRING (ligand → receptor)
NTS→NTSR1
NTS→NTSR2
NTS→SORT1
Compartment
lymphoid
- B cell
- NK/T cell
12.5%
25%
50%
100%
ln(CP10K+1)
Skin
Hypothalamus
Pituitary
Brainstem
Brain cortex
Testes
Uterus
Bladder
Kidney
Pancreas
Liver
Colon
Small intestine
Tongue
Trachea
Lung
Aorta
Heart
Spleen
Blood
Bone marrow
Bone
Diaphragm
Limb muscle
Mammary gland
Fat

Neurotensin/Neuromedin N
- myeloid

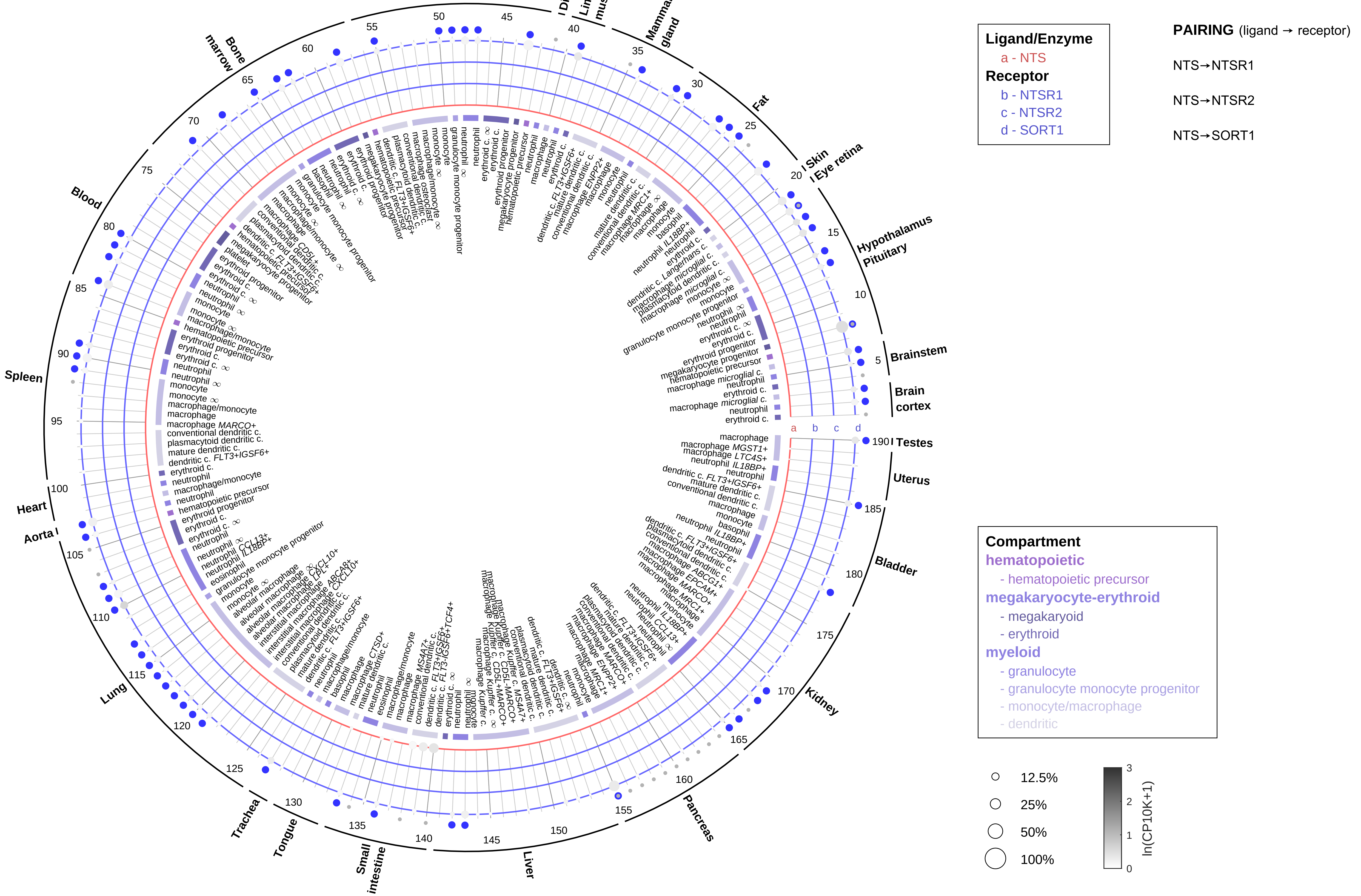

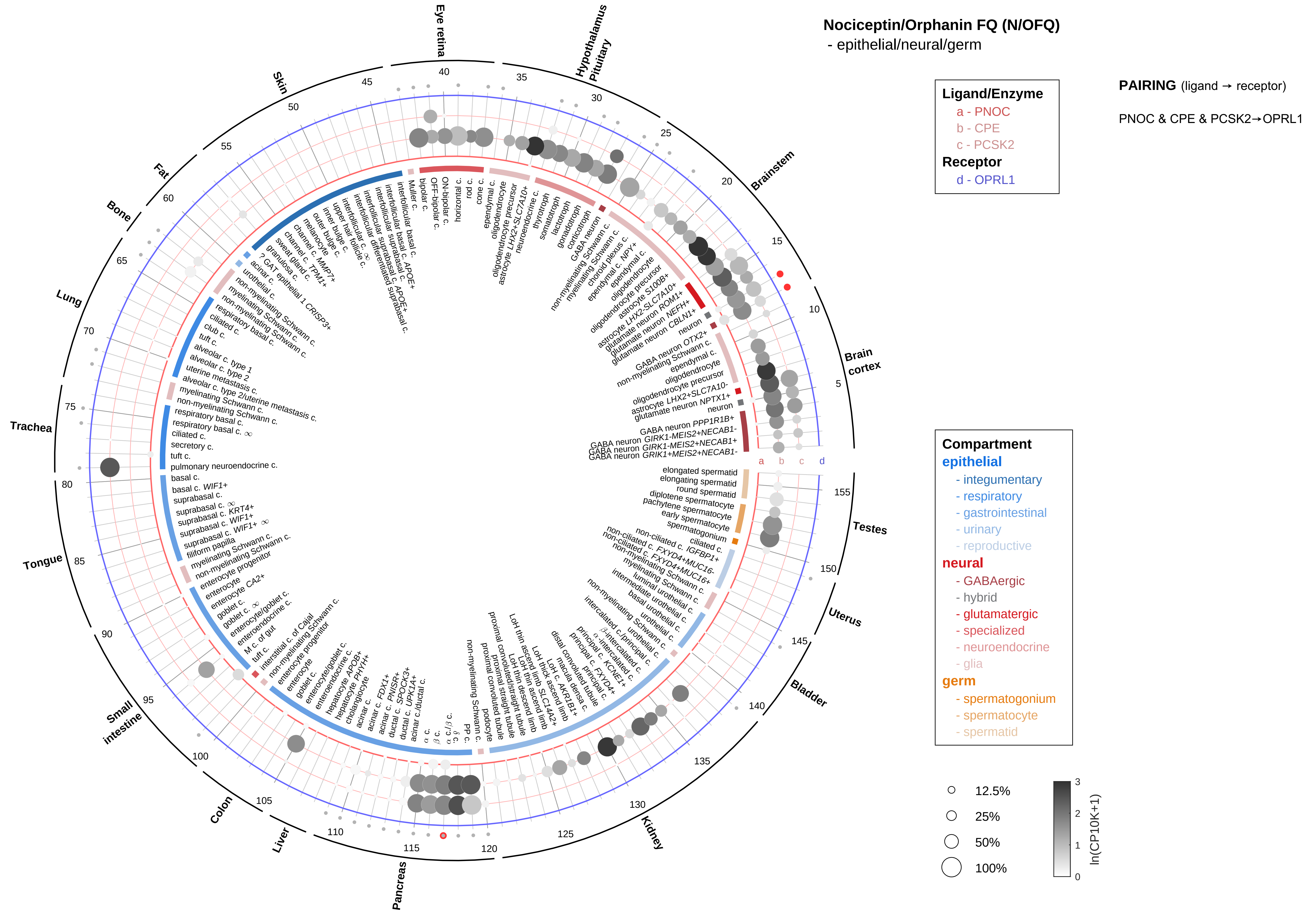

Nociceptin/Orphanin FQ (N/OFQ)
- epithelial/neural/germ
Ligand/Enzyme
a - PNOC
b - CPE
c - PCSK2
Receptor
d - OPRL1
PAIRING (ligand → receptor)
PNOC & CPE & PCSK2→OPRL1
Compartment
epithelial
- integumentary
- respiratory
- gastrointestinal
- urinary
- reproductive
neural
- GABAergic
- hybrid
- glutamatergic
- specialized
- neuroendocrine
- glia
germ
- spermatogonium
- spermatocyte
- spermatid
12.5%
25%
50%
100%
ln(CP10K+1)
Eye retina
Hypothalamus Pituitary
Brainstem
Brain cortex
Testes
Uterus
Bladder
Kidney
Pancreas
Liver
Colon
Small intestine
Tongue
Trachea
Lung
Bone
Fat
Skin

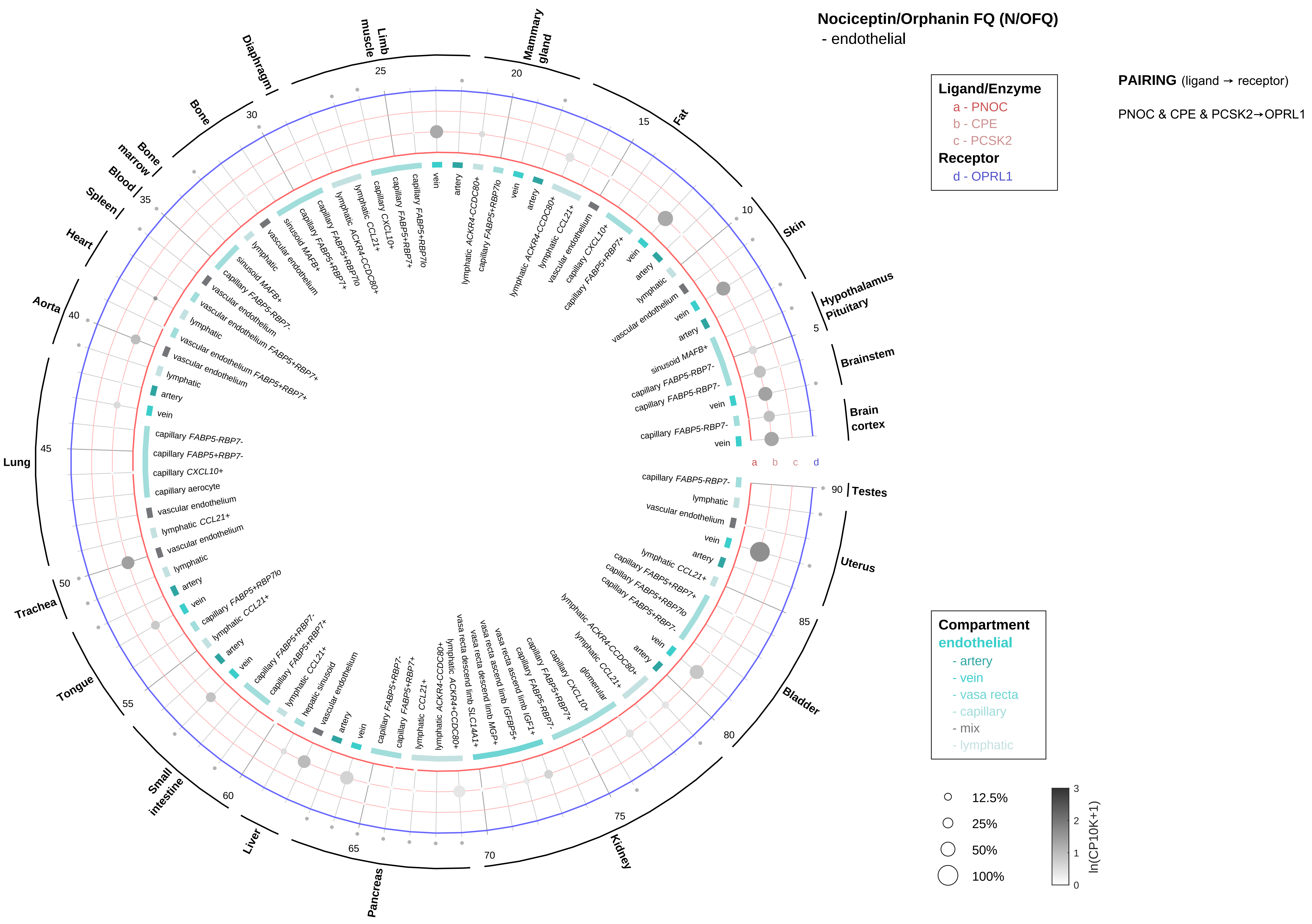
Nociceptin/Orphanin FQ (N/OFQ)
- endothelial
Ligand/Enzyme
a - PNOC
b - CPE
c - PCSK2
Receptor
d - OPRL1
PAIRING (ligand → receptor)
PNOC & CPE & PCSK2→OPRL1
Compartment
endothelial
- artery
- vein
- vasa recta
- capillary
- mix
- lymphatic
12.5%
25%
50%
100%
ln(CP10K+1)
Brain cortex
Brainstem
Hypothalamus Pituitary
Skin
Fat
Mammary gland
Limb muscle
Diaphragm
Bone
Bone marrow
Blood
Spleen
Heart
Aorta
Lung
Trachea
Tongue
Small intestine
Liver
Pancreas
Kidney
Bladder
Uterus
Testes

Nociceptin/Orphanin FQ (N/OFQ)
- stromal

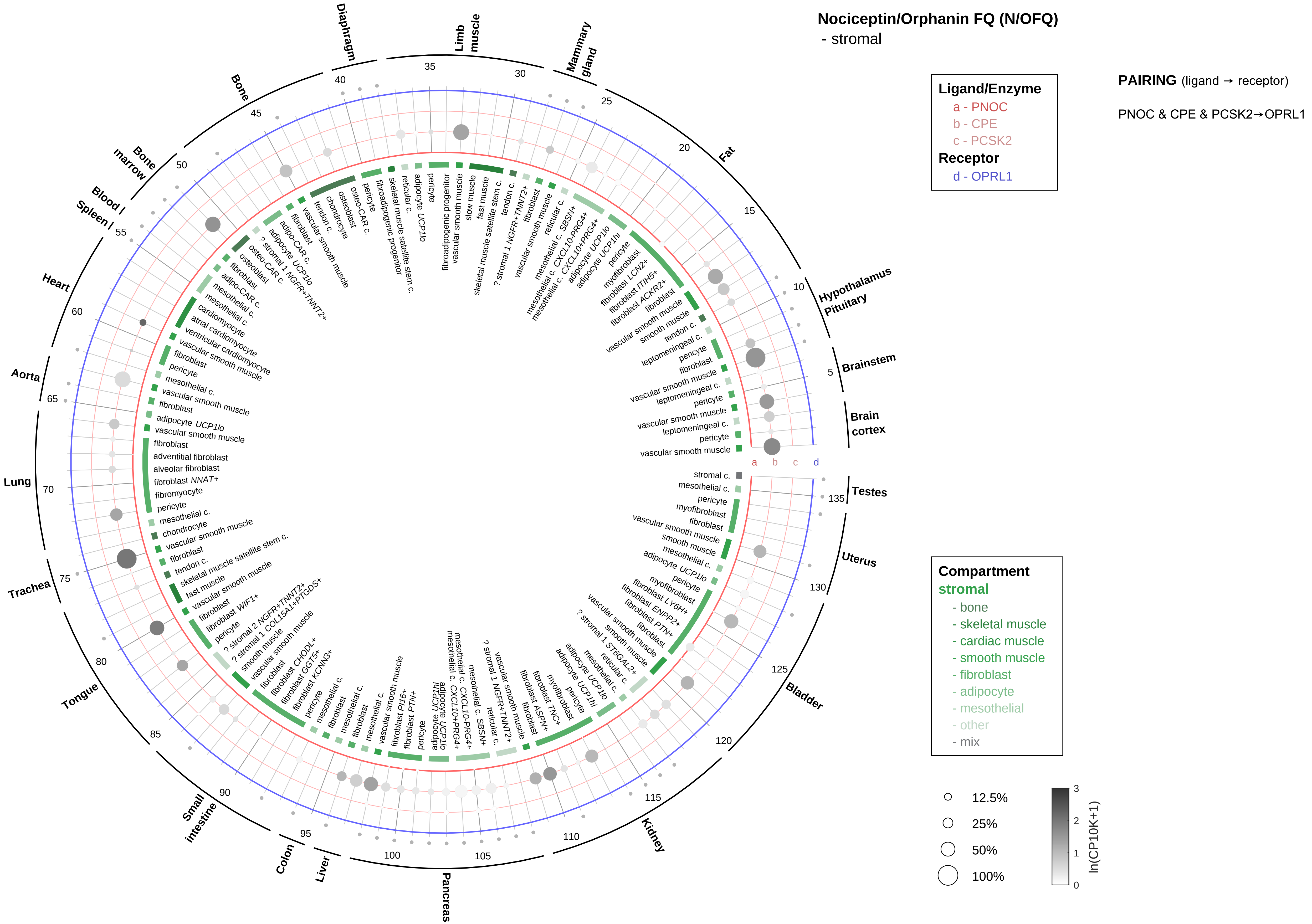

Nociceptin/Orphanin FQ (N/OFQ)
- lymphoid

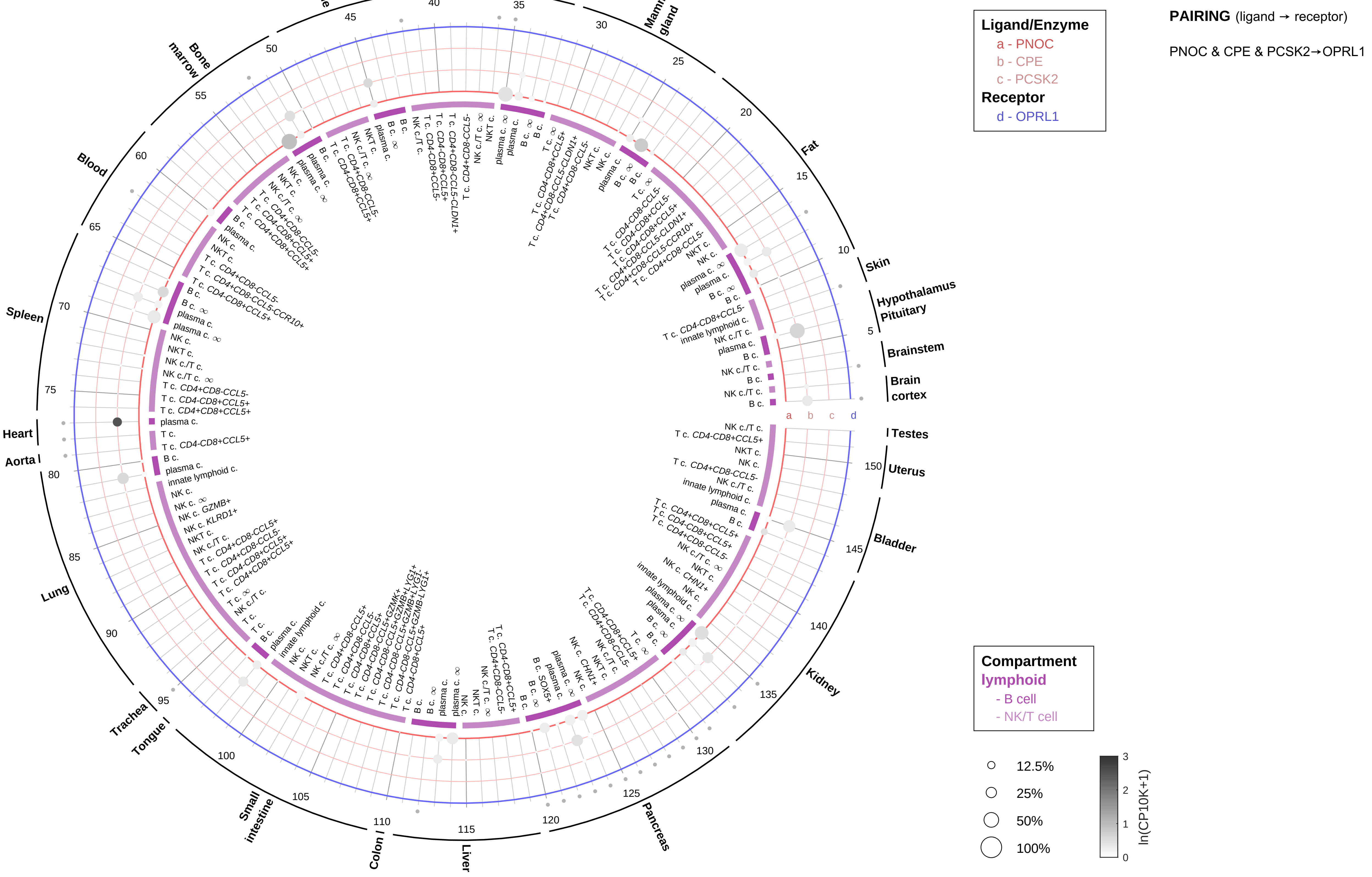

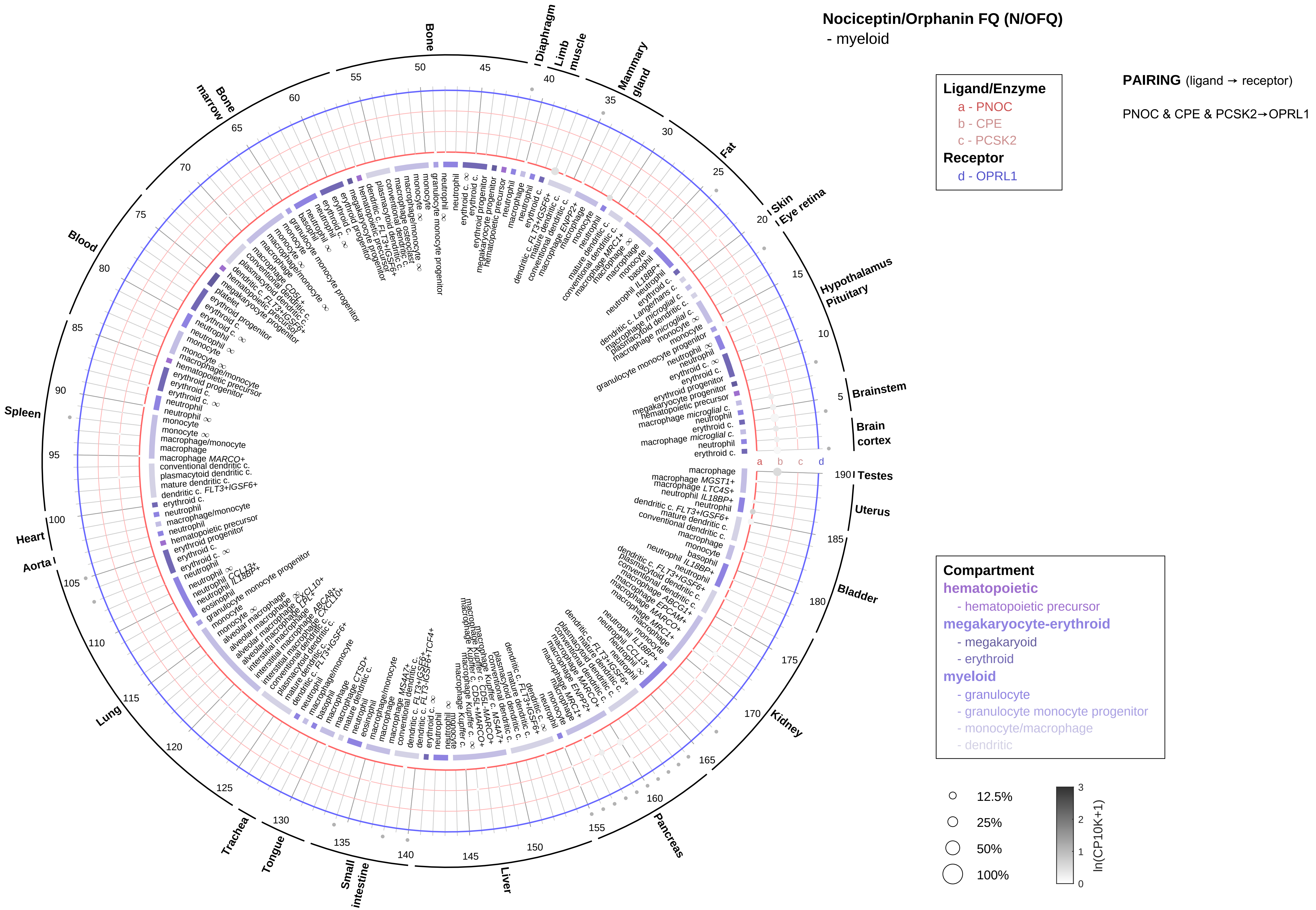
Nociceptin/Orphanin FQ (N/OFQ)
- myeloid
PAIRING (ligand → receptor)
PNOC & CPE & PCSK2→OPRL1
Ligand/Enzyme
a - PNOC
b - CPE
c - PCSK2
Receptor
d - OPRL1
Compartment
hematopoietic
- hematopoietic precursor
megakaryocyte-erythroid
- megakaryoid
- erythroid
myeloid
- granulocyte
- granulocyte monocyte progenitor
- monocyte/macrophage
- dendritic
12.5%
25%
50%
100%
ln(CP10K+1)
Bone
Diaphragm
Limb muscle
Mammary gland
Fat
Skin
Eye retina
Hypothalamus
Pituitary
Brainstem
Brain cortex
Testes
Uterus
Bladder
Kidney
Pancreas
Liver
Small intestine
Tongue
Trachea
Lung
Aorta
Heart
Spleen
Blood
Bone marrow

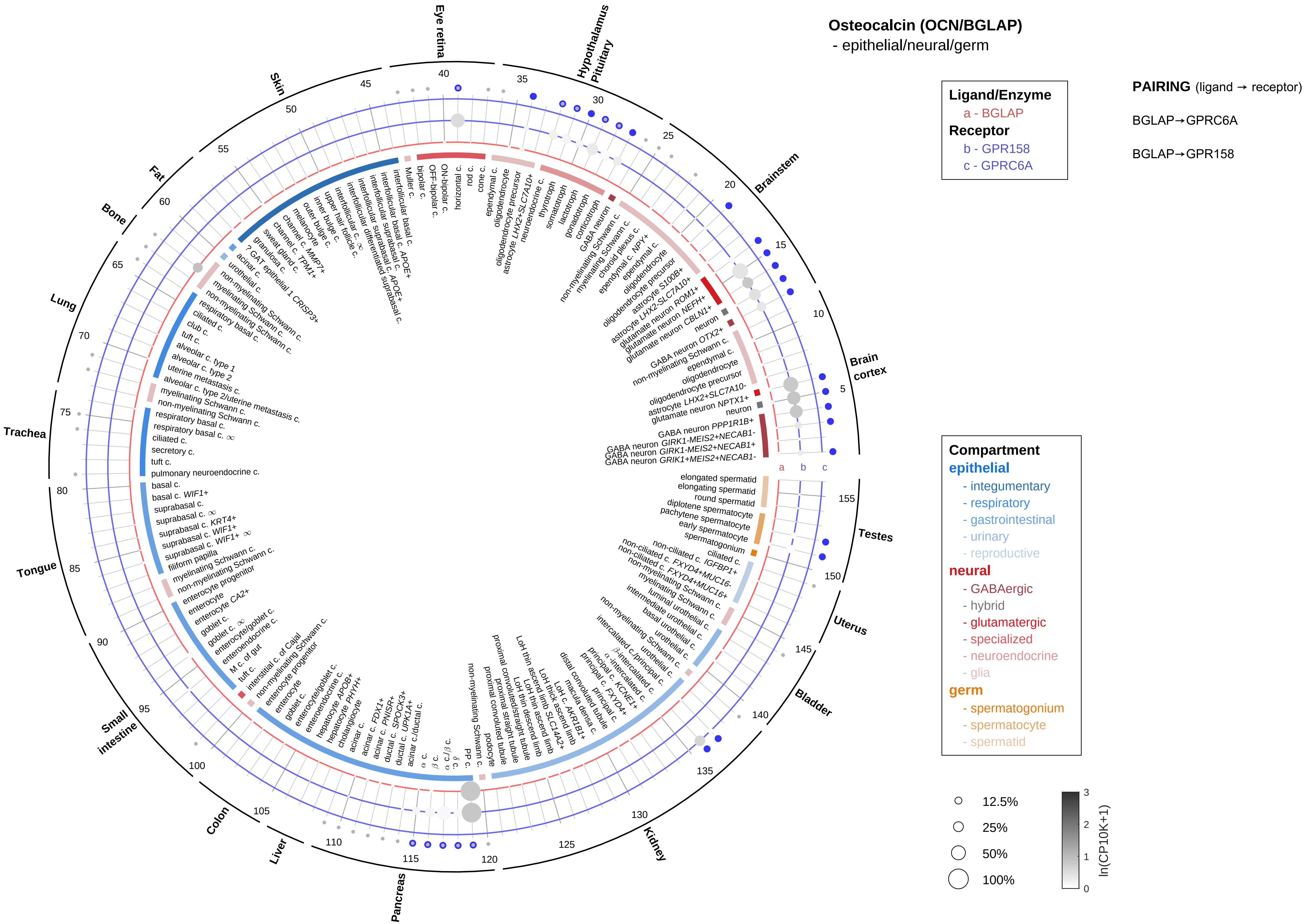
Osteocalcin (OCN/BGLAP)
- epithelial/neural/germ
Ligand/Enzyme
a - BGLAP
Receptor
b - GPR158
c - GPRC6A
PAIRING (ligand → receptor)
BGLAP→GPRC6A
BGLAP→GPR158
Compartment
epithelial
- integumentary
- respiratory
- gastrointestinal
- urinary
- reproductive
neural
- GABAergic
- hybrid
- glutamatergic
- specialized
- neuroendocrine
- glia
germ
- spermatogonium
- spermatocyte
- spermatid
12.5%
25%
50%
100%
ln(CP10K+1)
Eye retina
Hypothalamus Pituitary
Brainstem
Brain cortex
Testes
Uterus
Bladder
Kidney
Pancreas
Liver
Colon
Small intestine
Tongue
Trachea
Lung
Bone
Fat
Skin

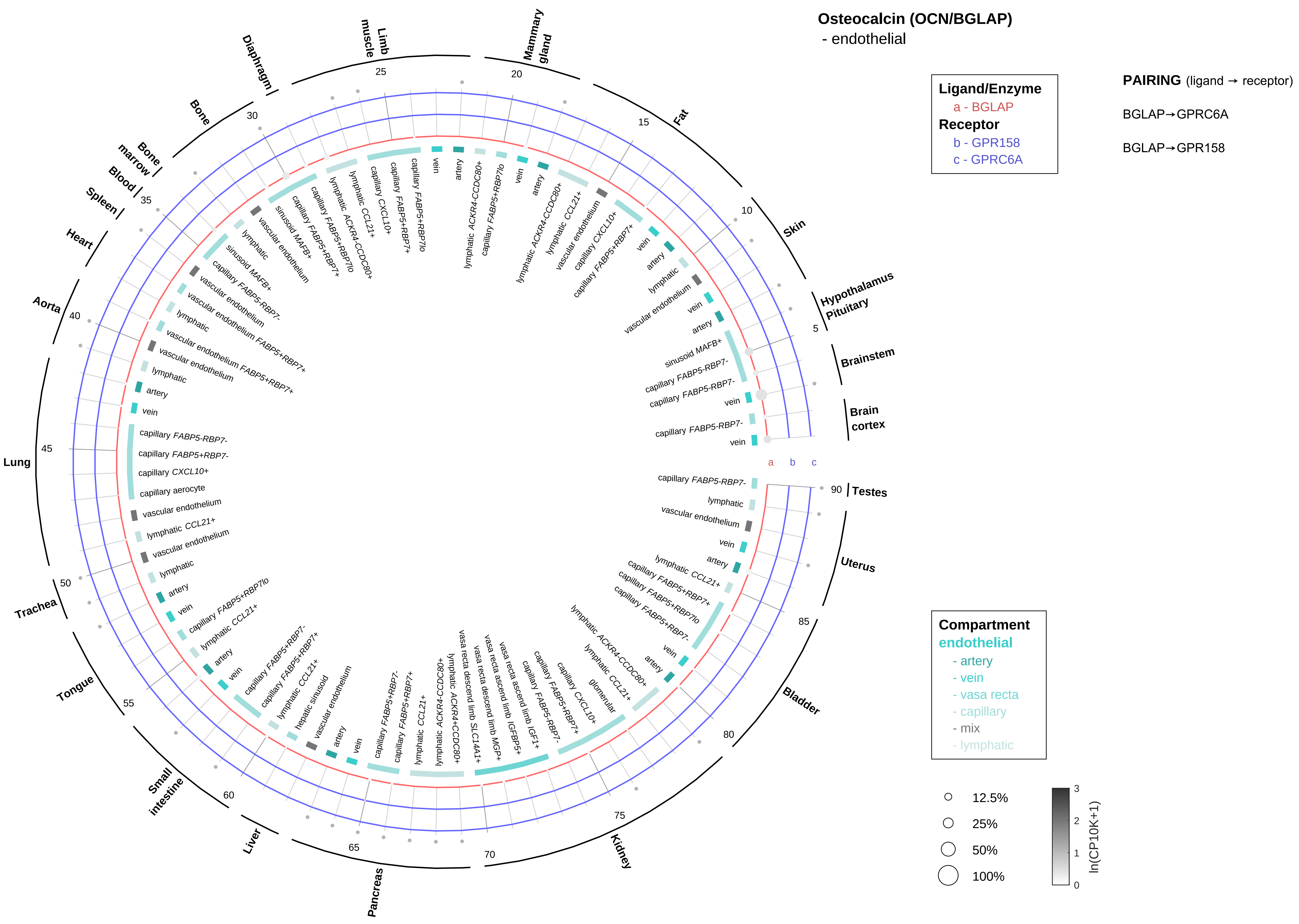

Osteocalcin (OCN/BGLAP)
- endothelial
Ligand/Enzyme
a - BGLAP
Receptor
b - GPR158
c - GPRC6A
PAIRING (ligand → receptor)
BGLAP→GPRC6A
BGLAP→GPR158
Compartment
endothelial
- artery
- vein
- vasa recta
- capillary
- mix
- lymphatic
12.5%
25%
50%
100%
ln(CP10K+1)
Brain cortex
Brainstem
Hypothalamus Pituitary
Skin
Fat
Mammary gland
Limb muscle
Diaphragm
Bone
Bone marrow
Blood
Spleen
Heart
Aorta
Lung
Trachea
Tongue
Small intestine
Liver
Pancreas
Kidney
Bladder
Uterus
Testes

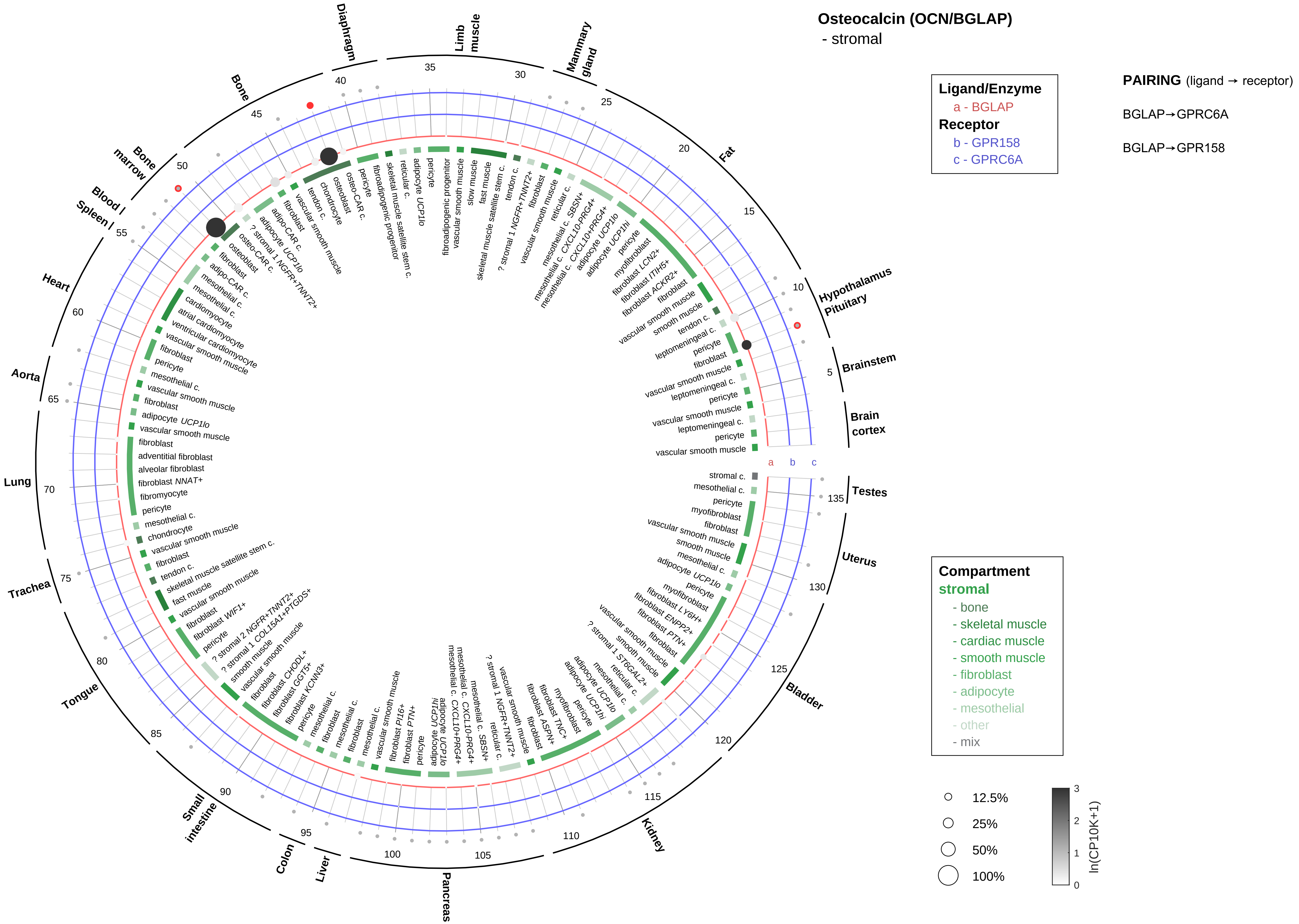

Osteocalcin (OCN/BGLAP)
- stromal
Ligand/Enzyme
a - BGLAP
Receptor
b - GPR158
c - GPRC6A
PAIRING (ligand → receptor)
BGLAP→GPRC6A
BGLAP→GPR158
Compartment
stromal
- bone
- skeletal muscle
- cardiac muscle
- smooth muscle
- fibroblast
- adipocyte
- mesothelial
- other
- mix
12.5%
25%
50%
100%
ln(CP10K+1)
Brain cortex
Brainstem
Hypothalamus Pituitary
Fat
Mammary gland
Limb muscle
Diaphragm
Bone
Bone marrow
Blood
Spleen
Heart
Aorta
Lung
Trachea
Tongue
Small intestine
Colon
Liver
Pancreas
Kidney
Bladder
Uterus
Testes

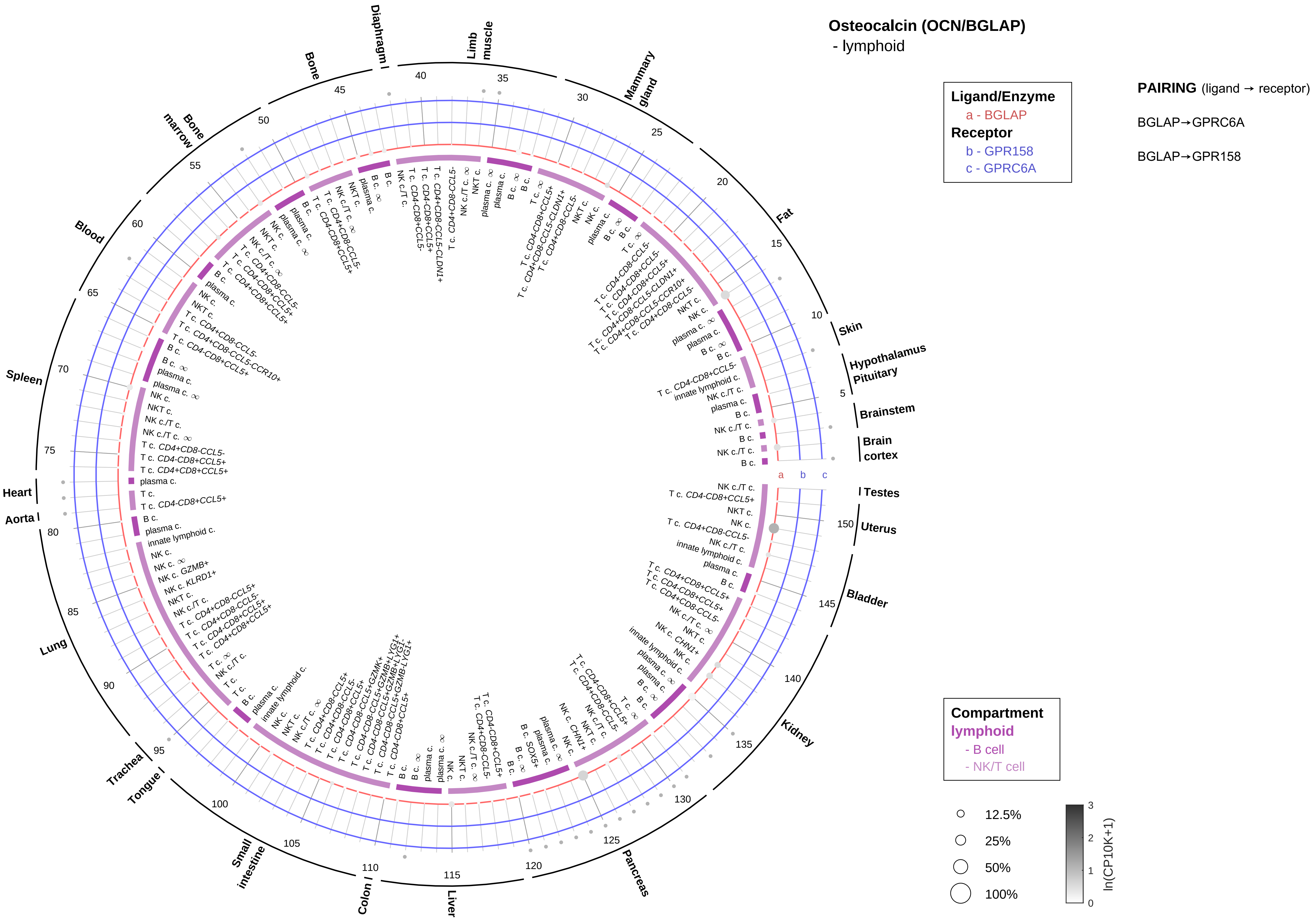

Osteocalcin (OCN/BGLAP)
- lymphoid
Ligand/Enzyme
a - BGLAP
Receptor
b - GPR158
c - GPRC6A
PAIRING (ligand → receptor)
BGLAP→GPRC6A
BGLAP→GPR158
Compartment
lymphoid
- B cell
- NK/T cell
12.5%
25%
50%
100%
ln(CP10K+1)
Skin
Hypothalamus
Pituitary
Brainstem
Brain cortex
Testes
Uterus
Bladder
Kidney
Pancreas
Liver
Colon
Small intestine
Tongue
Trachea
Lung
Aorta
Heart
Spleen
Blood
Bone marrow
Bone
Diaphragm
Limb muscle
Mammary gland
Fat

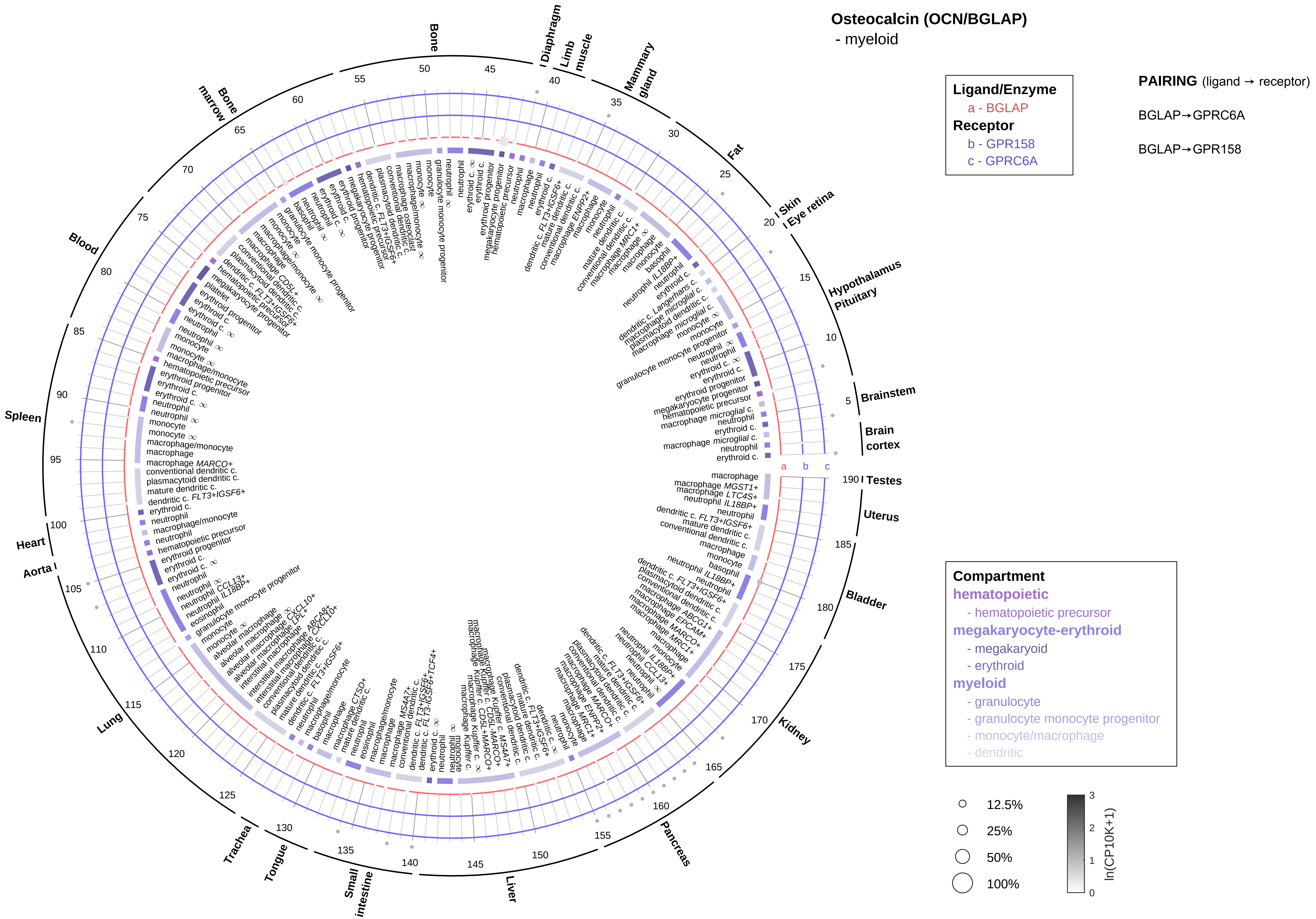

Osteocalcin (OCN/BGLAP)
- myeloid
Ligand/Enzyme
a - BGLAP
Receptor
b - GPR158
c - GPRC6A
PAIRING (ligand → receptor)
BGLAP→GPRC6A
BGLAP→GPR158
Compartment
hematopoietic
- hematopoietic precursor
megakaryocyte-erythroid
- megakaryoid
- erythroid
myeloid
- granulocyte
- granulocyte monocyte progenitor
- monocyte/macrophage
- dendritic
12.5%
25%
50%
100%
ln(CP10K+1)
Bone
Diaphragm
Limb muscle
Mammary gland
Fat
Skin
Eye retina
Hypothalamus
Pituitary
Brainstem
Brain cortex
Testes
Uterus
Bladder
Kidney
Pancreas
Liver
Small intestine
Tongue
Trachea
Lung
Aorta
Heart
Spleen
Blood
Bone marrow

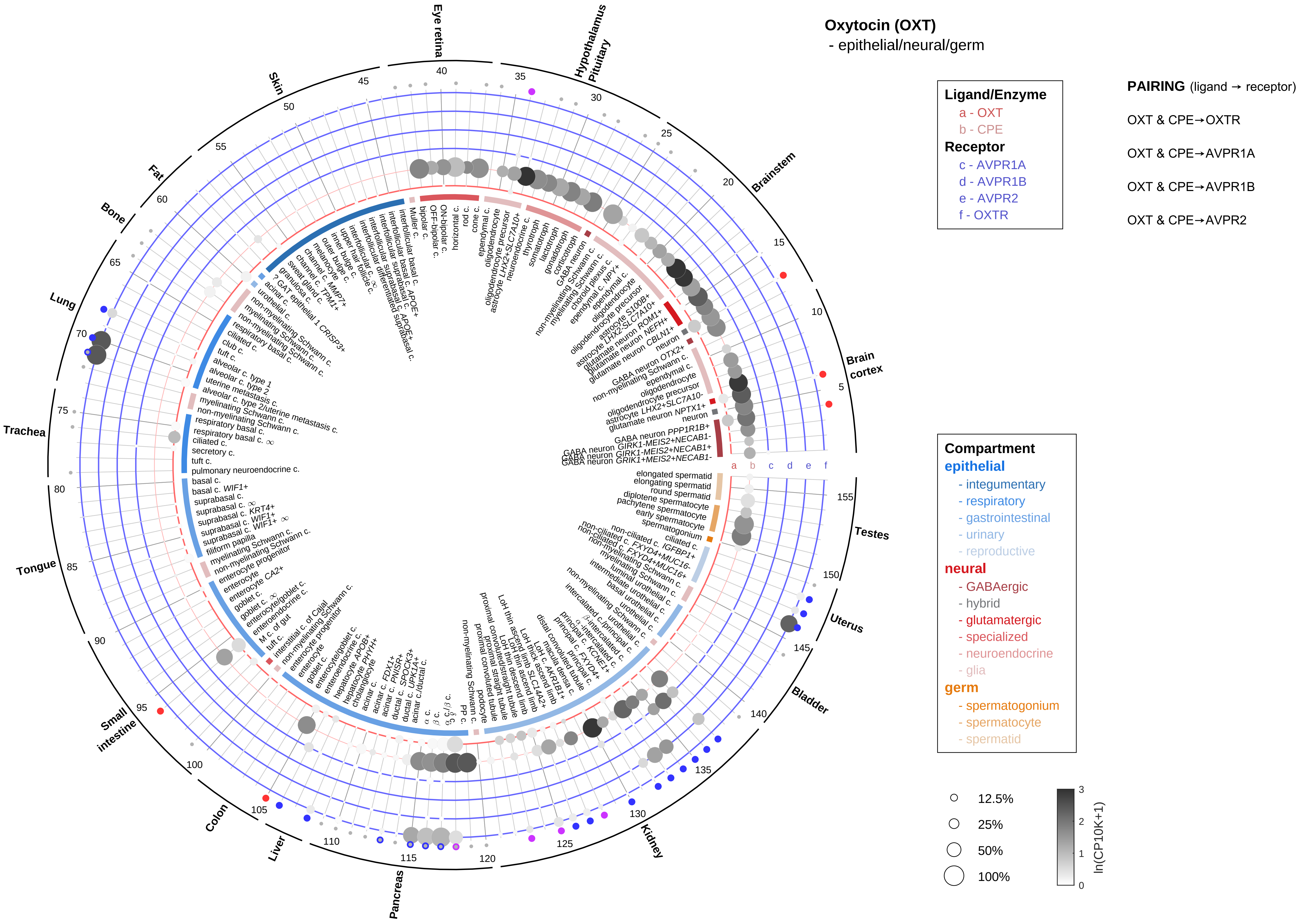

Oxytocin (OXT)
- epithelial/neural/germ
Ligand/Enzyme
a - OXT
b - CPE
Receptor
c - AVPR1A
d - AVPR1B
e - AVPR2
f - OXTR
PAIRING (ligand → receptor)
OXT & CPE→OXTR
OXT & CPE→AVPR1A
OXT & CPE→AVPR1B
OXT & CPE→AVPR2
Compartment
epithelial
- integumentary
- respiratory
- gastrointestinal
- urinary
- reproductive
neural
- GABAergic
- hybrid
- glutamatergic
- specialized
- neuroendocrine
- glia
germ
- spermatogonium
- spermatocyte
- spermatid
12.5%
25%
50%
100%
ln(CP10K+1)
Eye retina
Hypothalamus Pituitary
Brainstem
Brain cortex
Testes
Uterus
Bladder
Kidney
Pancreas
Liver
Colon
Small intestine
Tongue
Trachea
Lung
Bone
Fat
Skin

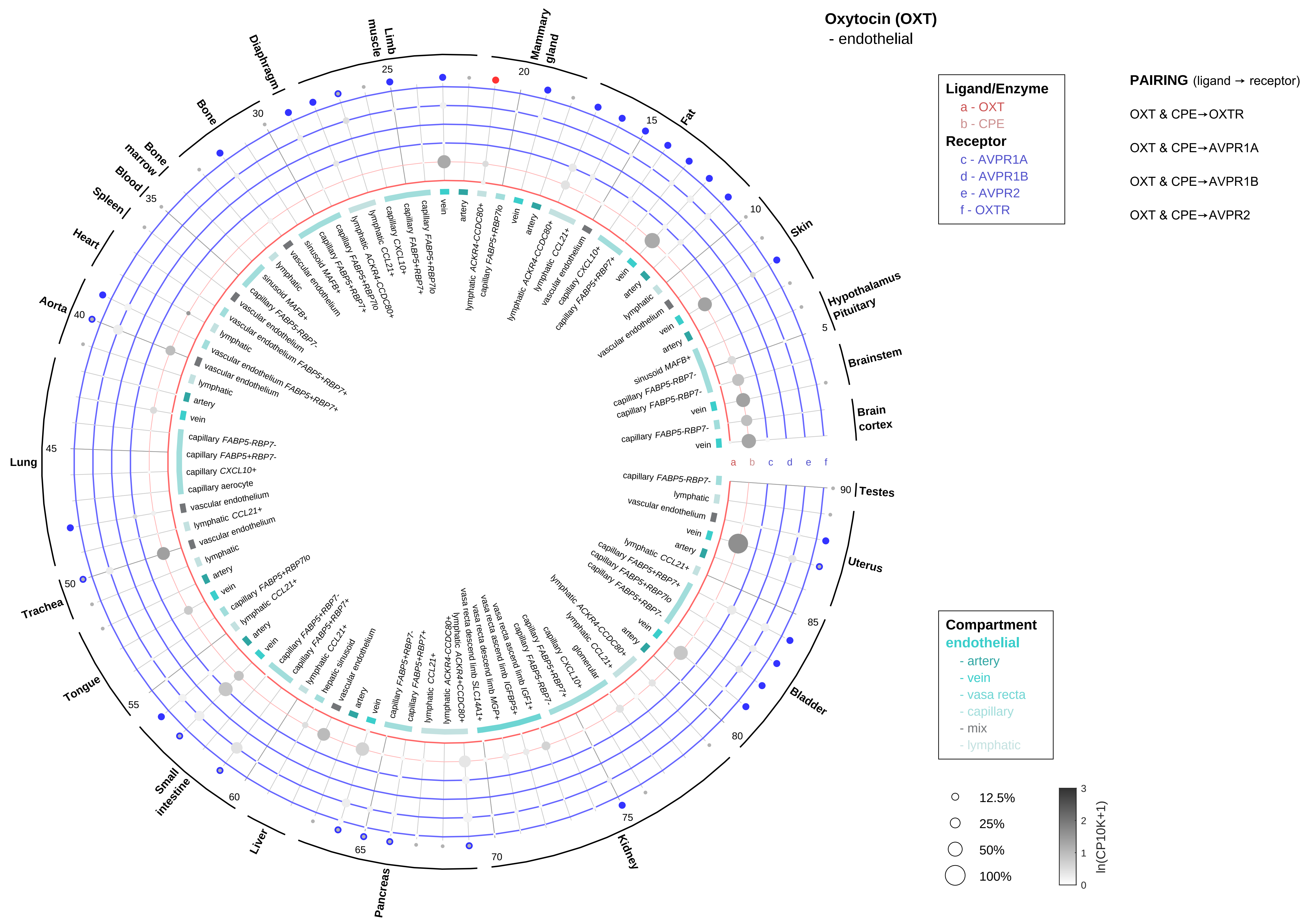

Oxytocin (OXT)
- endothelial
Ligand/Enzyme
a - OXT
b - CPE
Receptor
c - AVPR1A
d - AVPR1B
e - AVPR2
f - OXTR
PAIRING (ligand → receptor)
OXT & CPE→OXTR
OXT & CPE→AVPR1A
OXT & CPE→AVPR1B
OXT & CPE→AVPR2
Compartment
endothelial
- artery
- vein
- vasa recta
- capillary
- mix
- lymphatic
12.5%
25%
50%
100%
ln(CP10K+1)
Brain cortex
Brainstem
Hypothalamus Pituitary
Skin
Fat
Mammary gland
Limb muscle
Diaphragm
Bone
Bone marrow
Blood
Spleen
Heart
Aorta
Lung
Trachea
Tongue
Small intestine
Liver
Pancreas
Kidney
Bladder
Uterus
Testes
a b c d e f

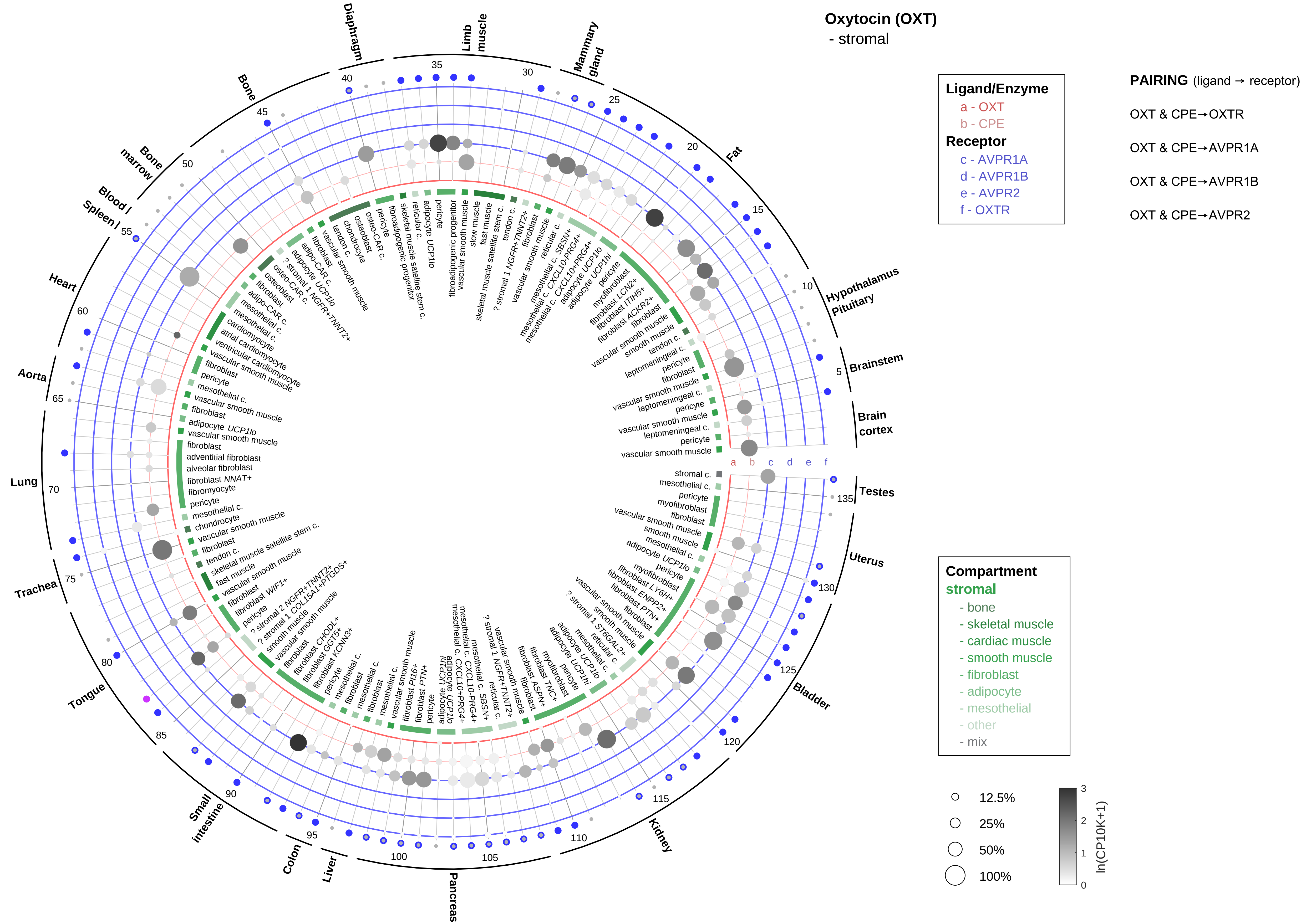

Oxytocin (OXT)
- stromal
Ligand/Enzyme
a - OXT
b - CPE
Receptor
c - AVPR1A
d - AVPR1B
e - AVPR2
f - OXTR
PAIRING (ligand → receptor)
OXT & CPE→OXTR
OXT & CPE→AVPR1A
OXT & CPE→AVPR1B
OXT & CPE→AVPR2
Compartment
stromal
- bone
- skeletal muscle
- cardiac muscle
- smooth muscle
- fibroblast
- adipocyte
- mesothelial
- other
- mix
12.5%
25%
50%
100%
ln(CP10K+1)
Brain cortex
Brainstem
Hypothalamus Pituitary
Fat
Mammary gland
Limb muscle
Diaphragm
Bone
Bone marrow
Blood
Spleen
Heart
Aorta
Lung
Trachea
Tongue
Small intestine
Colon
Liver
Pancreas
Kidney
Bladder
Uterus
Testes

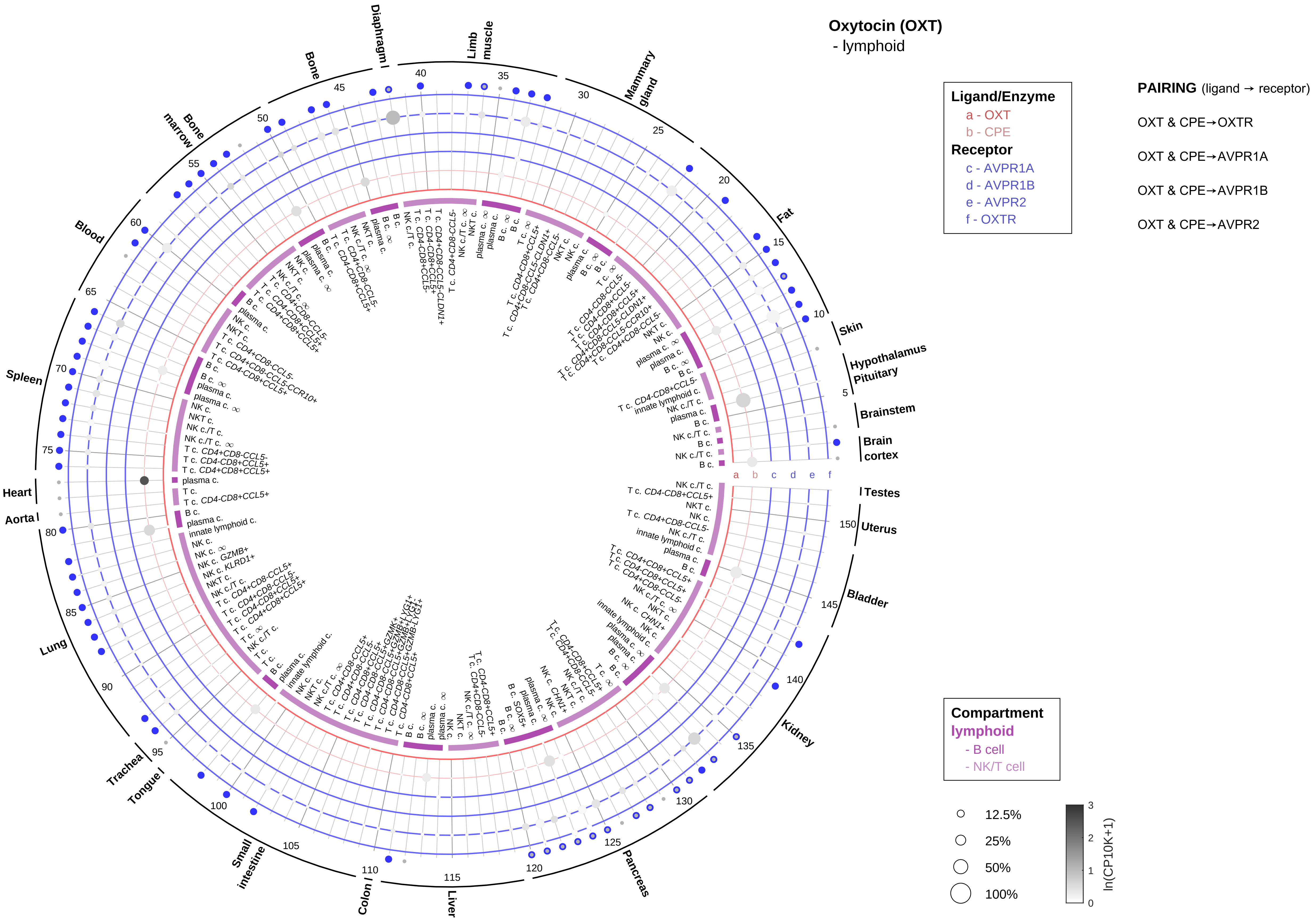
Oxytocin (OXT)
- lymphoid
Ligand/Enzyme
a - OXT
b - CPE
Receptor
c - AVPR1A
d - AVPR1B
e - AVPR2
f - OXTR
PAIRING (ligand → receptor)
OXT & CPE→OXTR
OXT & CPE→AVPR1A
OXT & CPE→AVPR1B
OXT & CPE→AVPR2
Compartment
lymphoid
- B cell
- NK/T cell
12.5%
25%
50%
100%
ln(CP10K+1)
Diaphragm
Limb muscle
Mammary gland
Fat
Skin
Hypothalamus Pituitary
Brainstem
Brain cortex
Testes
Uterus
Bladder
Kidney
Pancreas
Liver
Colon
Small intestine
Tongue
Trachea
Lung
Aorta
Heart
Spleen
Blood
Bone marrow
Bone

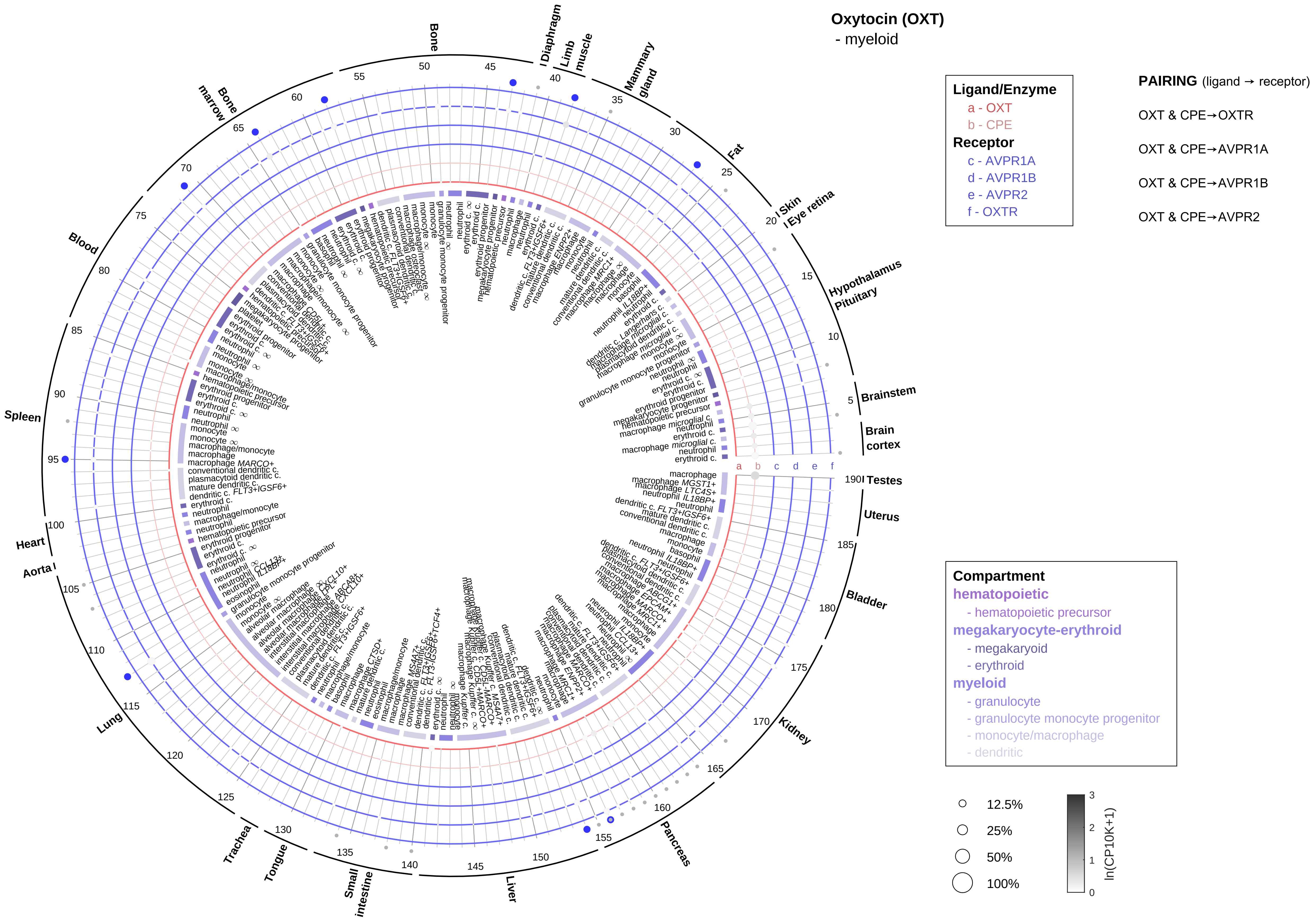
Oxytocin (OXT)
- myeloid
Ligand/Enzyme
a - OXT
b - CPE
Receptor
c - AVPR1A
d - AVPR1B
e - AVPR2
f - OXTR
PAIRING (ligand → receptor)
OXT & CPE→OXTR
OXT & CPE→AVPR1A
OXT & CPE→AVPR1B
OXT & CPE→AVPR2
Compartment
hematopoietic
- hematopoietic precursor
megakaryocyte-erythroid
- megakaryoid
- erythroid
myeloid
- granulocyte
- granulocyte monocyte progenitor
- monocyte/macrophage
- dendritic
12.5%
25%
50%
100%
ln(CP10K+1)
Brainstem
Brain cortex
Testes
Uterus
Bladder
Kidney
Pancreas
Liver
Small intestine
Tongue
Trachea
Lung
Aorta
Heart
Spleen
Blood
Bone marrow
Bone
Diaphragm
Limb muscle
Mammary gland
Fat
Skin
Eye retina
Hypothalamus
Pituitary

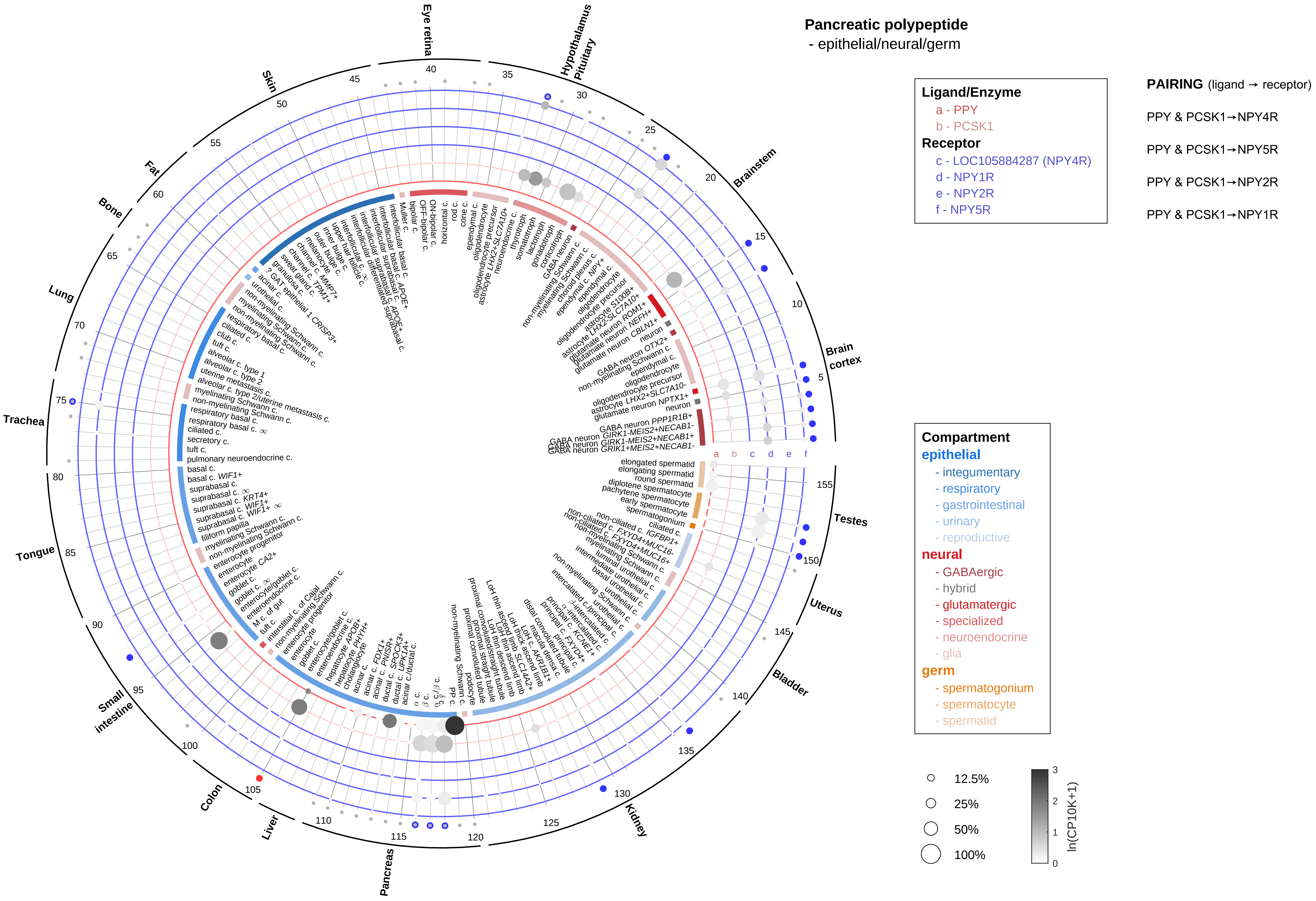
Pancreatic polypeptide
- epithelial/neural/germ
Ligand/Enzyme
a - PPY
b - PCSK1
Receptor
c - LOC105884287 (NPY4R)
d - NPY1R
e - NPY2R
f - NPY5R
PAIRING (ligand → receptor)
PPY & PCSK1→NPY4R
PPY & PCSK1→NPY5R
PPY & PCSK1→NPY2R
PPY & PCSK1→NPY1R
Compartment
epithelial
- integumentary
- respiratory
- gastrointestinal
- urinary
- reproductive
neural
- GABAergic
- hybrid
- glutamatergic
- specialized
- neuroendocrine
- glia
germ
- spermatogonium
- spermatocyte
- spermatid
12.5%
25%
50%
100%
ln(CP10K+1)
Eye retina
Hypothalamus Pituitary
Brainstem
Brain cortex
Testes
Uterus
Bladder
Kidney
Pancreas
Liver
Colon
Small intestine
Tongue
Trachea
Lung
Bone
Fat
Skin
a b c d e f

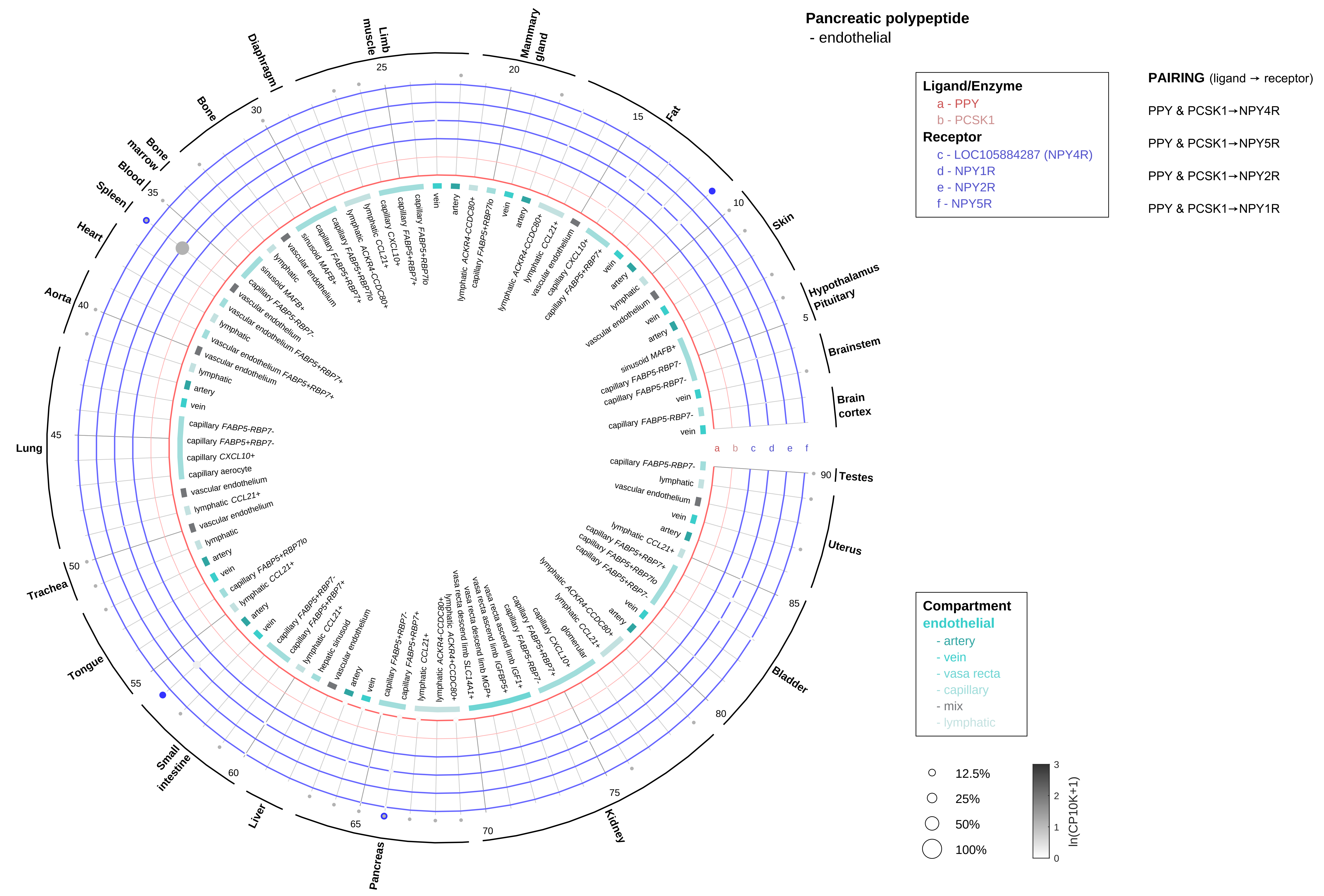

Pancreatic polypeptide
- endothelial
Ligand/Enzyme
a - PPY
b - PCSK1
Receptor
c - LOC105884287 (NPY4R)
d - NPY1R
e - NPY2R
f - NPY5R
PAIRING (ligand → receptor)
PPY & PCSK1→NPY4R
PPY & PCSK1→NPY5R
PPY & PCSK1→NPY2R
PPY & PCSK1→NPY1R
Compartment
endothelial
- artery
- vein
- vasa recta
- capillary
- mix
- lymphatic
12.5%
25%
50%
100%
ln(CP10K+1)
Brain cortex
Brainstem
Hypothalamus Pituitary
Skin
Fat
Mammary gland
Limb muscle
Diaphragm
Bone
Bone marrow
Blood
Spleen
Heart
Aorta
Lung
Trachea
Tongue
Small intestine
Liver
Pancreas
Kidney
Bladder
Uterus
Testes

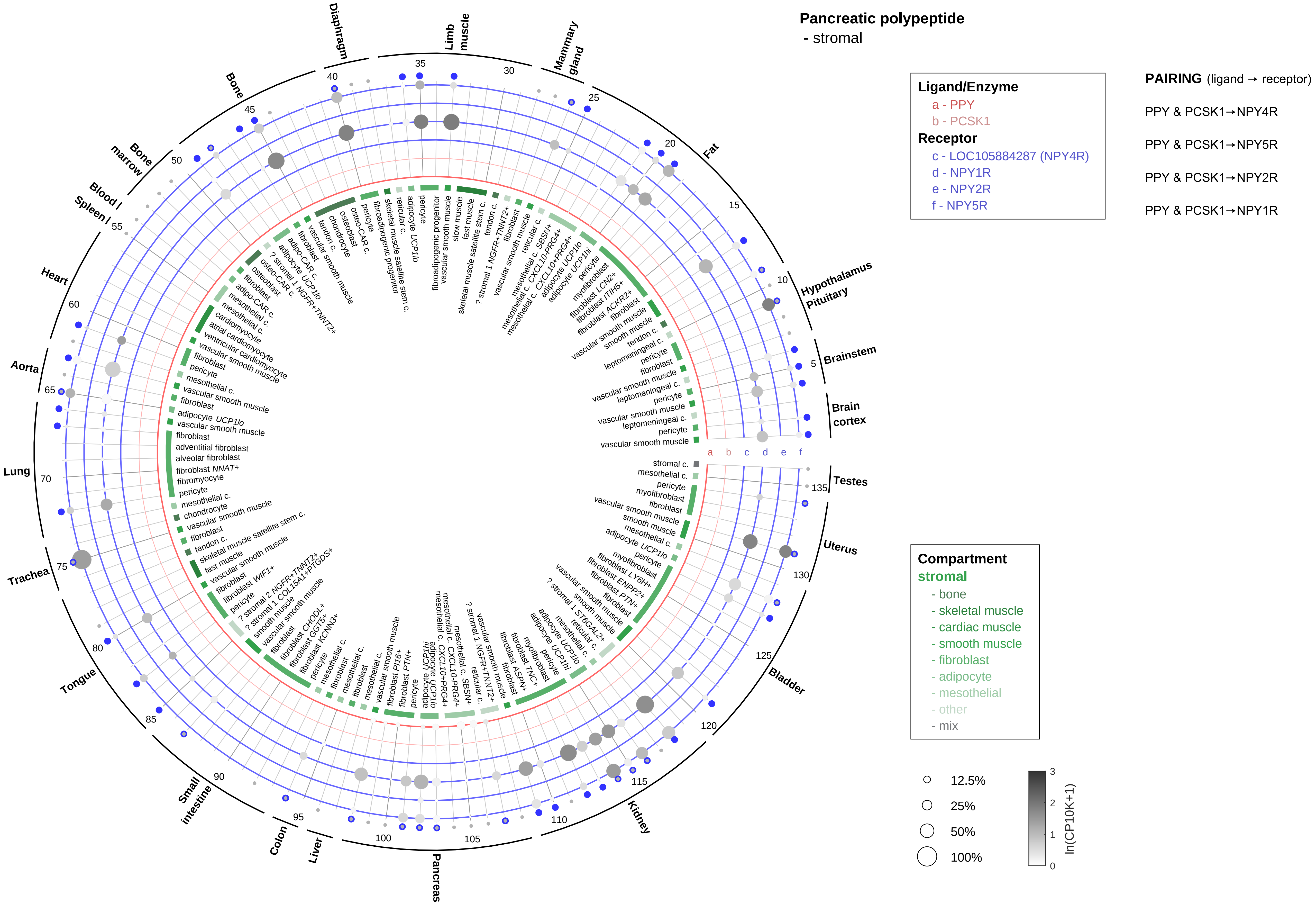
Pancreatic polypeptide
- stromal
Ligand/Enzyme
a - PPY
b - PCSK1
Receptor
c - LOC105884287 (NPY4R)
d - NPY1R
e - NPY2R
f - NPY5R
PAIRING (ligand → receptor)
PPY & PCSK1→NPY4R
PPY & PCSK1→NPY5R
PPY & PCSK1→NPY2R
PPY & PCSK1→NPY1R
Compartment
stromal
- bone
- skeletal muscle
- cardiac muscle
- smooth muscle
- fibroblast
- adipocyte
- mesothelial
- other
- mix
12.5%
25%
50%
100%
ln(CP10K+1)
Diaphragm
Limb muscle
Mammary gland
Fat
Hypothalamus Pituitary
Brainstem
Brain cortex
Testes
Uterus
Bladder
Kidney
Pancreas
Liver
Colon
Small intestine
Tongue
Trachea
Lung
Aorta
Heart
Spleen
Blood
Bone marrow
Bone

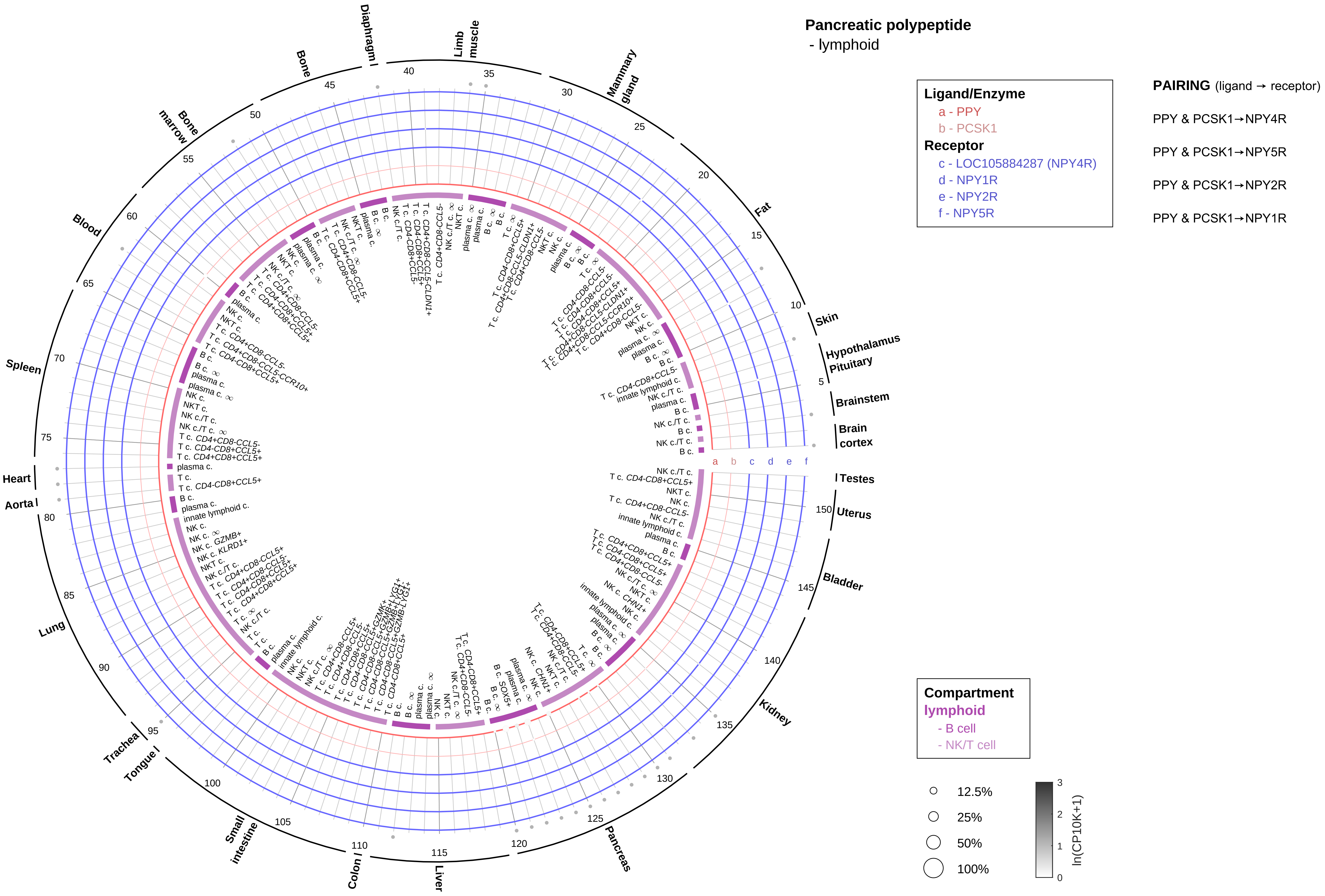

Pancreatic polypeptide
- lymphoid
Ligand/Enzyme
a - PPY
b - PCSK1
Receptor
c - LOC105884287 (NPY4R)
d - NPY1R
e - NPY2R
f - NPY5R
PAIRING (ligand → receptor)
PPY & PCSK1→NPY4R
PPY & PCSK1→NPY5R
PPY & PCSK1→NPY2R
PPY & PCSK1→NPY1R
Compartment
lymphoid
- B cell
- NK/T cell
12.5%
25%
50%
100%
ln(CP10K+1)
Brain cortex
Brainstem
Hypothalamus
Pituitary
Skin
Fat
Mammary gland
Limb muscle
Diaphragm
Bone
Bone marrow
Blood
Spleen
Heart
Aorta
Lung
Trachea
Tongue
Small intestine
Colon
Liver
Pancreas
Kidney
Bladder
Uterus
Testes

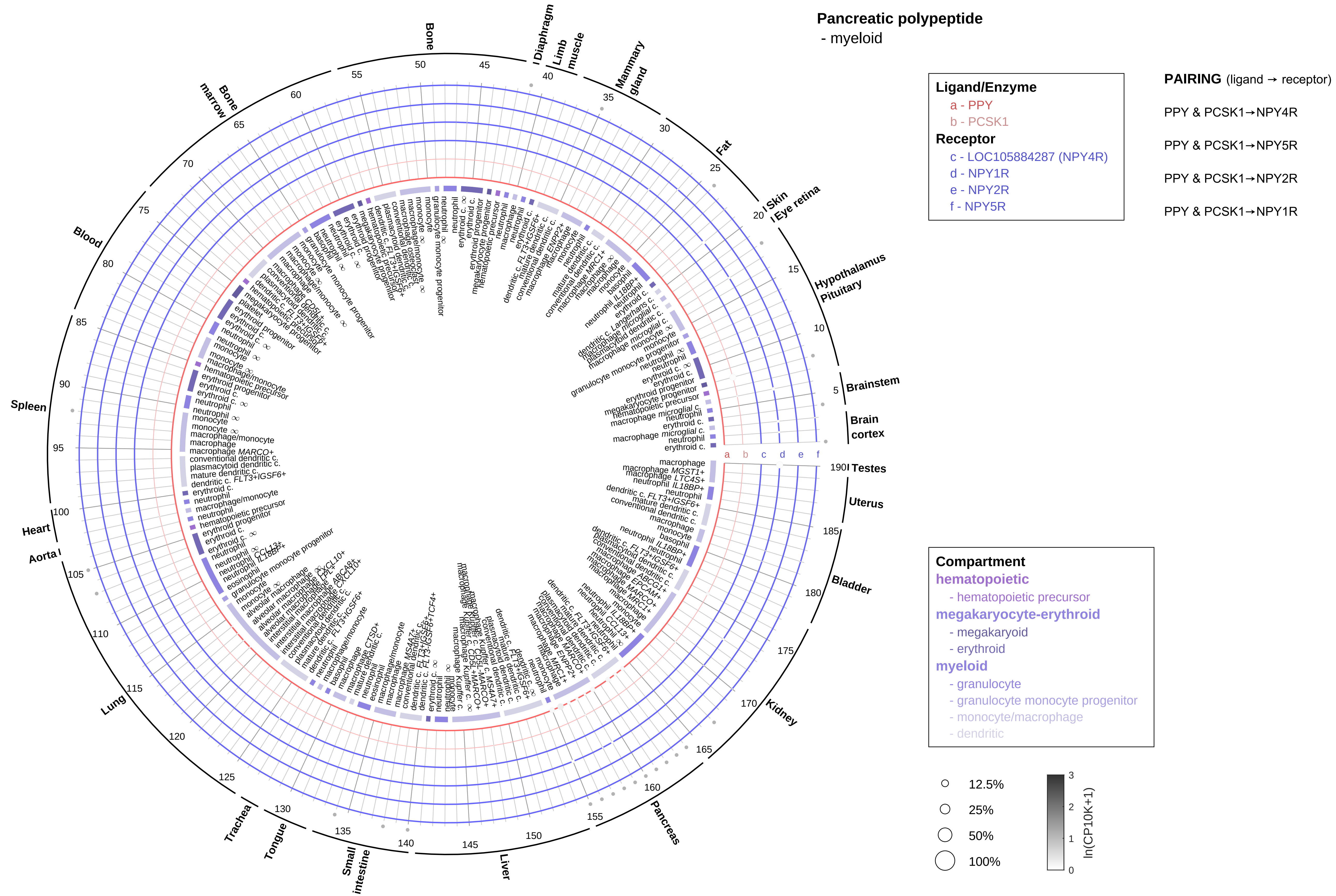
Pancreatic polypeptide
- myeloid
Ligand/Enzyme
a - PPY
b - PCSK1
Receptor
c - LOC105884287 (NPY4R)
d - NPY1R
e - NPY2R
f - NPY5R
PAIRING (ligand → receptor)
PPY & PCSK1→NPY4R
PPY & PCSK1→NPY5R
PPY & PCSK1→NPY2R
PPY & PCSK1→NPY1R
Compartment
hematopoietic
- hematopoietic precursor
megakaryocyte-erythroid
- megakaryoid
- erythroid
myeloid
- granulocyte
- granulocyte monocyte progenitor
- monocyte/macrophage
- dendritic
12.5%
25%
50%
100%
ln(CP10K+1)
Bone
Diaphragm
Limb muscle
Mammary gland
Fat
Skin
Eye retina
Hypothalamus
Pituitary
Brainstem
Brain cortex
Testes
Uterus
Bladder
Kidney
Pancreas
Liver
Small intestine
Tongue
Trachea
Lung
Aorta
Heart
Spleen
Blood
Bone marrow

Parathyroid hormone/Parathyroid hormone-like hormone
- epithelial/neural/germ

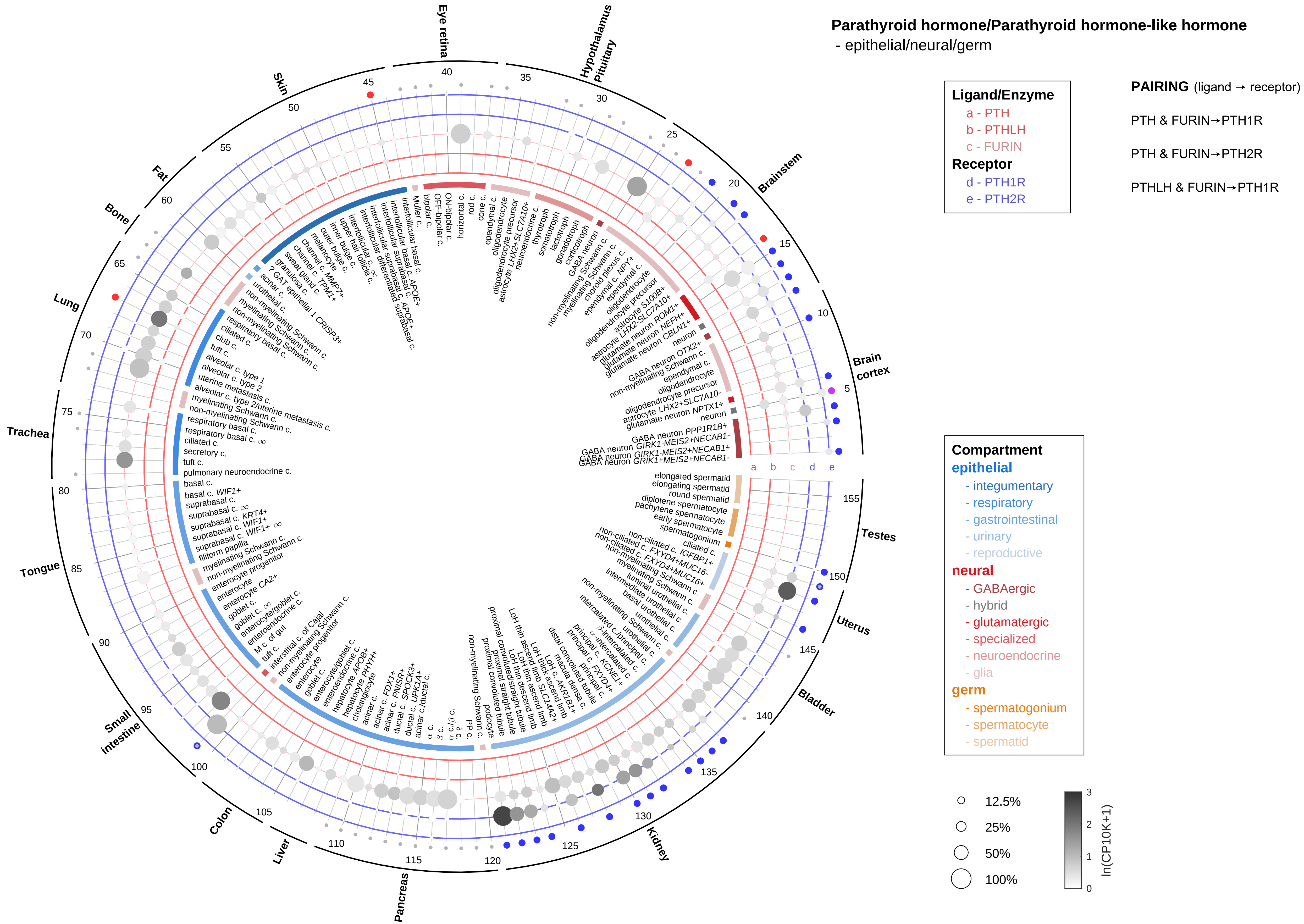

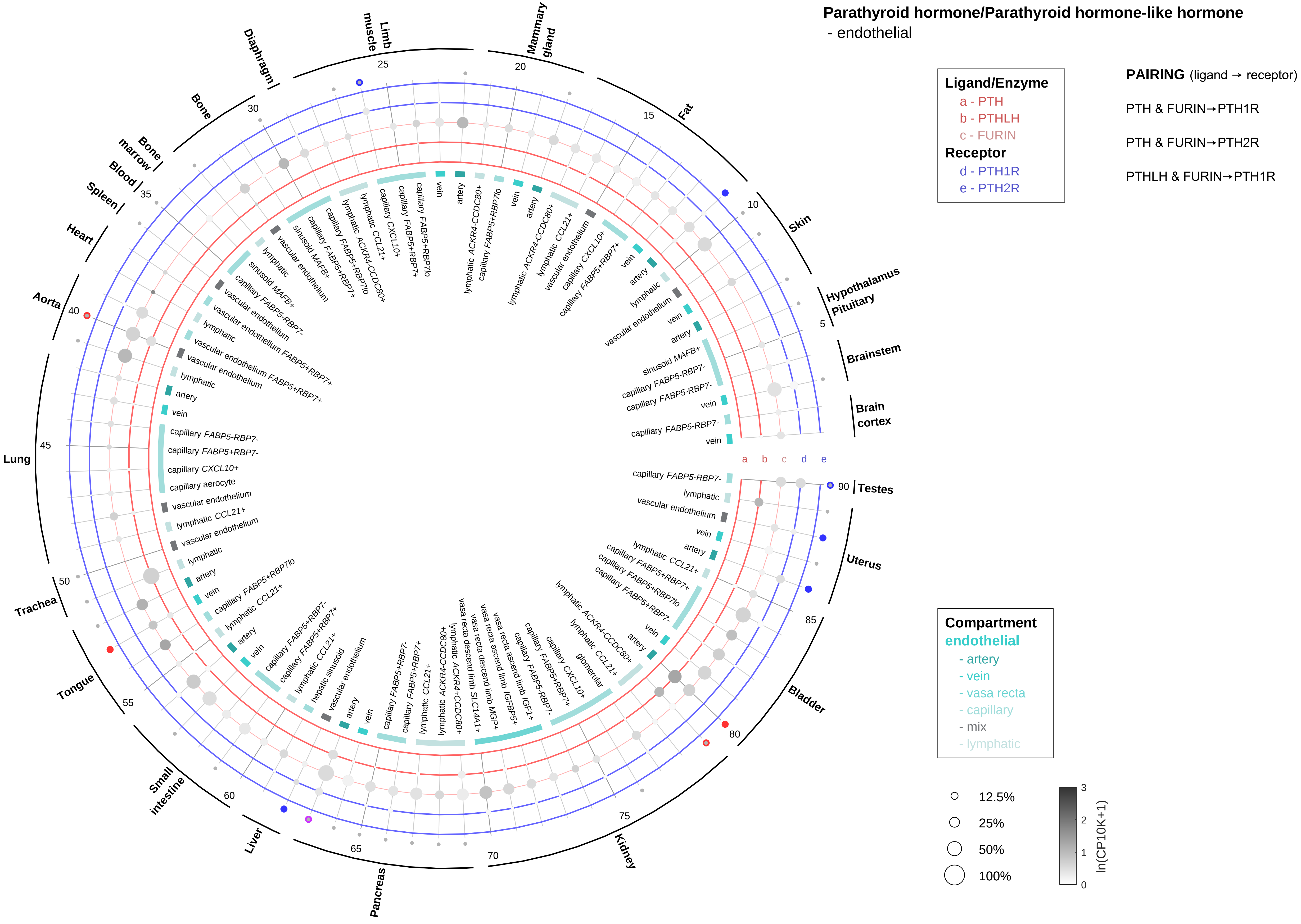

Parathyroid hormone/Parathyroid hormone-like hormone
- endothelial
Ligand/Enzyme
a - PTH
b - PTHLH
c - FURIN
Receptor
d - PTH1R
e - PTH2R
PAIRING (ligand → receptor)
PTH & FURIN→PTH1R
PTH & FURIN→PTH2R
PTHLH & FURIN→PTH1R
Compartment
endothelial
- artery
- vein
- vasa recta
- capillary
- mix
- lymphatic
12.5%
25%
50%
100%
ln(CP10K+1)
Brain cortex
Brainstem
Hypothalamus Pituitary
Skin
Fat
Mammary gland
Limb muscle
Diaphragm
Bone
Bone marrow
Blood
Spleen
Heart
Aorta
Lung
Trachea
Tongue
Small intestine
Liver
Pancreas
Kidney
Bladder
Uterus
Testes

Parathyroid hormone/Parathyroid hormone-like hormone
- stromal

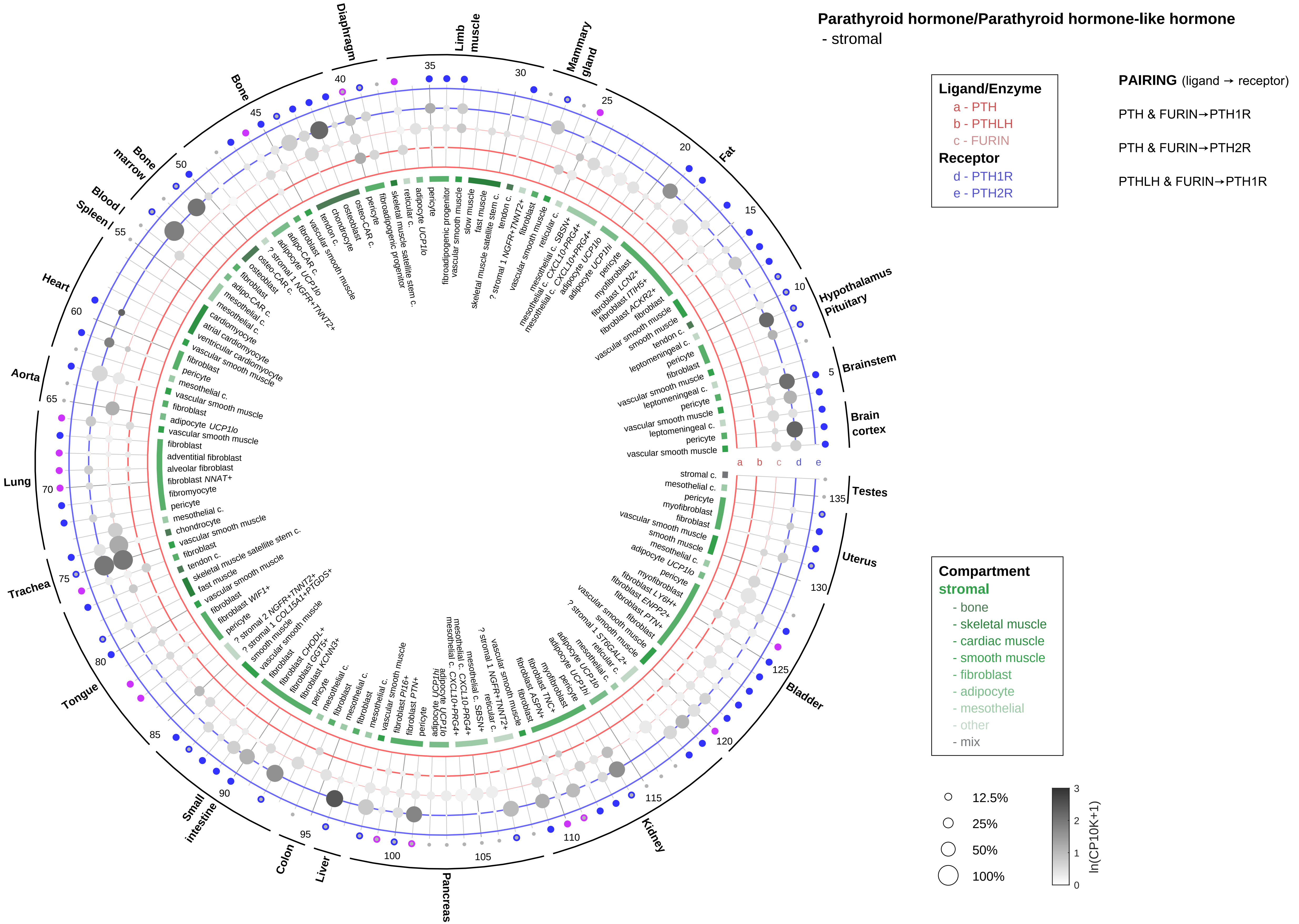

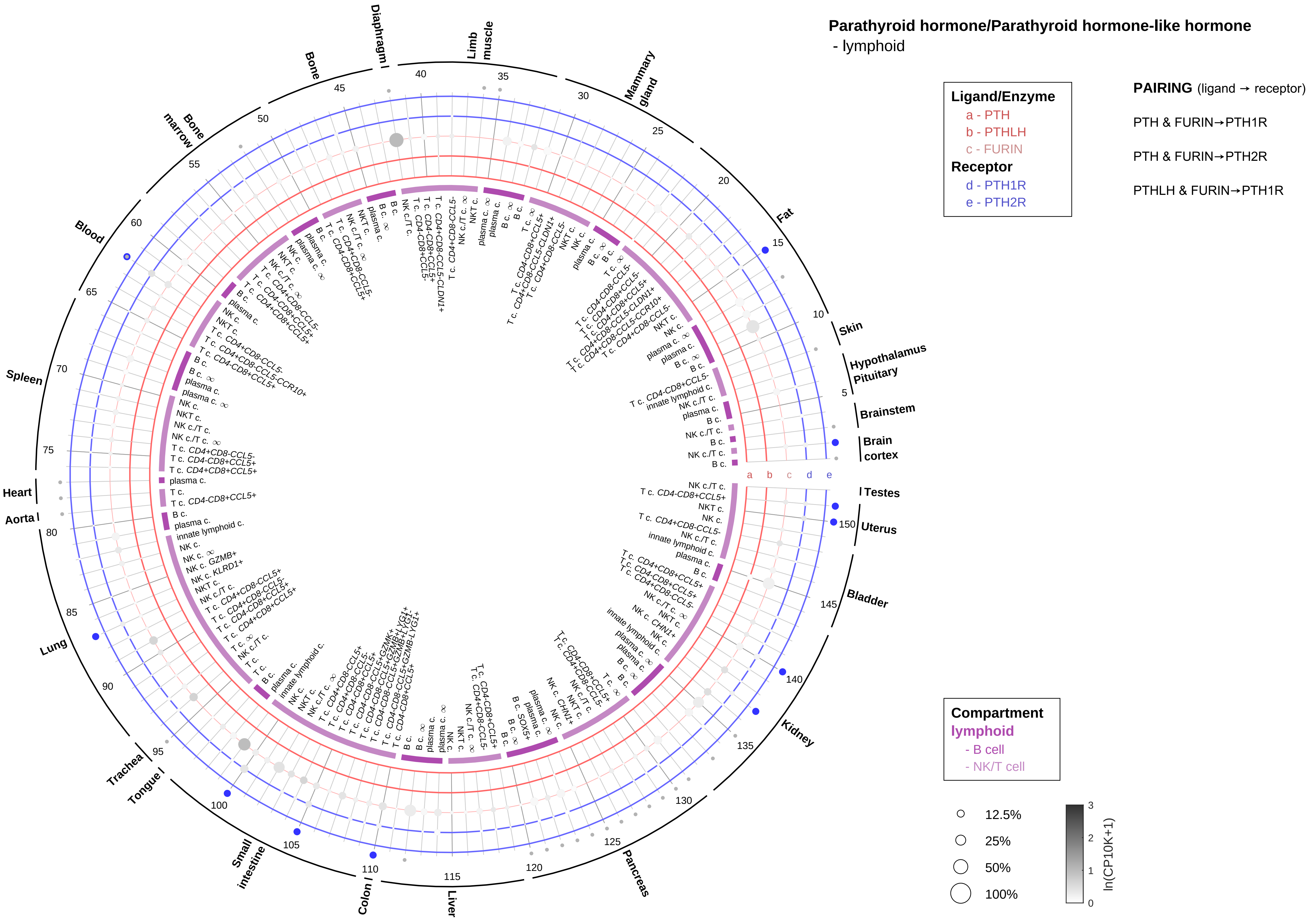

Parathyroid hormone/Parathyroid hormone-like hormone
- lymphoid
Ligand/Enzyme
a - PTH
b - PTHLH
c - FURIN
Receptor
d - PTH1R
e - PTH2R
PAIRING (ligand → receptor)
PTH & FURIN→PTH1R
PTH & FURIN→PTH2R
PTHLH & FURIN→PTH1R
Compartment
lymphoid
- B cell
- NK/T cell
12.5%
25%
50%
100%
ln(CP10K+1)
Skin
Hypothalamus
Pituitary
Brainstem
Brain cortex
Testes
Uterus
Bladder
Kidney
Pancreas
Liver
Colon
Small intestine
Tongue
Trachea
Lung
Aorta
Heart
Spleen
Blood
Bone marrow
Bone
Diaphragm
Limb muscle
Mammary gland
Fat

Parathyroid hormone/Parathyroid hormone-like hormone
- myeloid

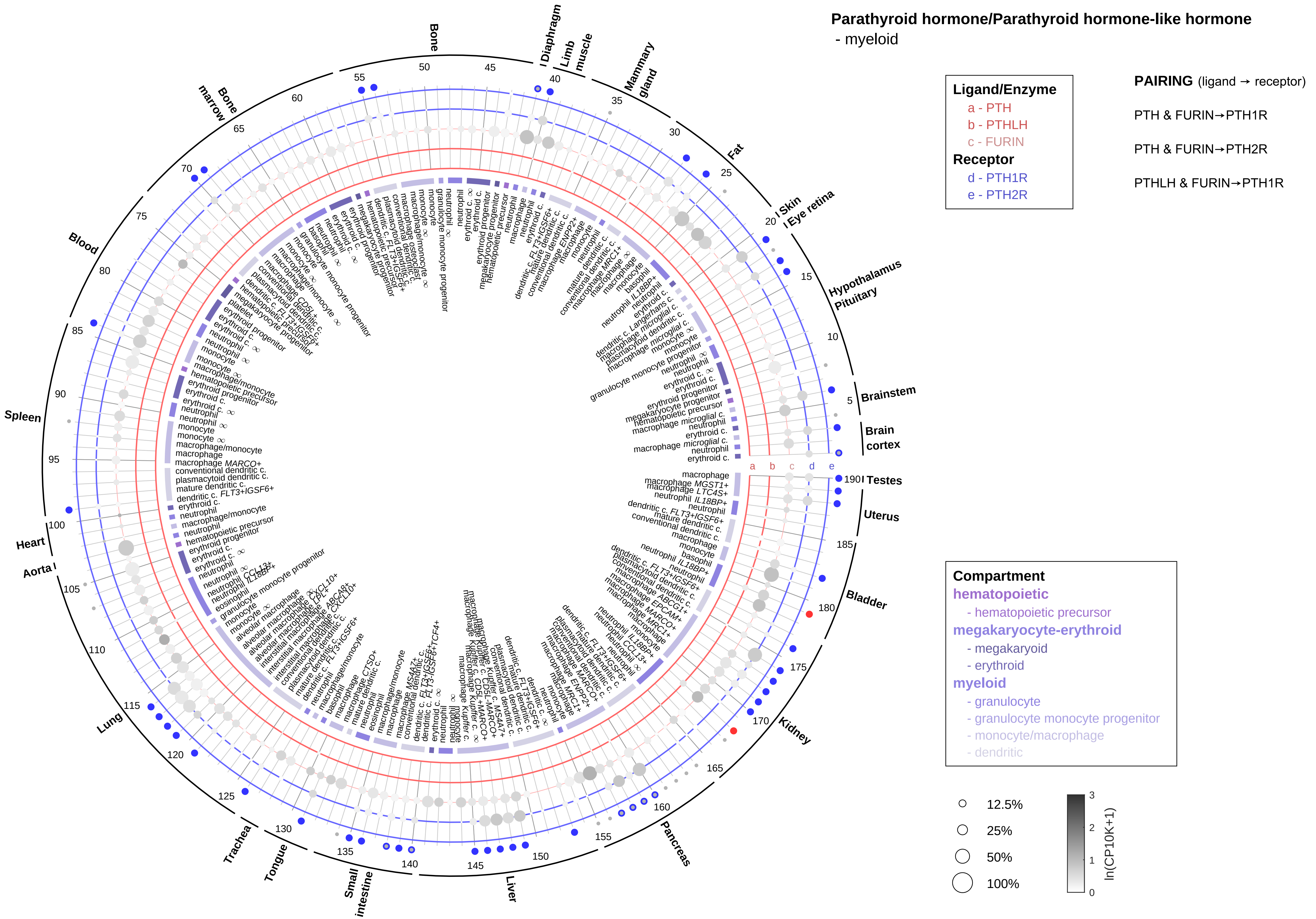

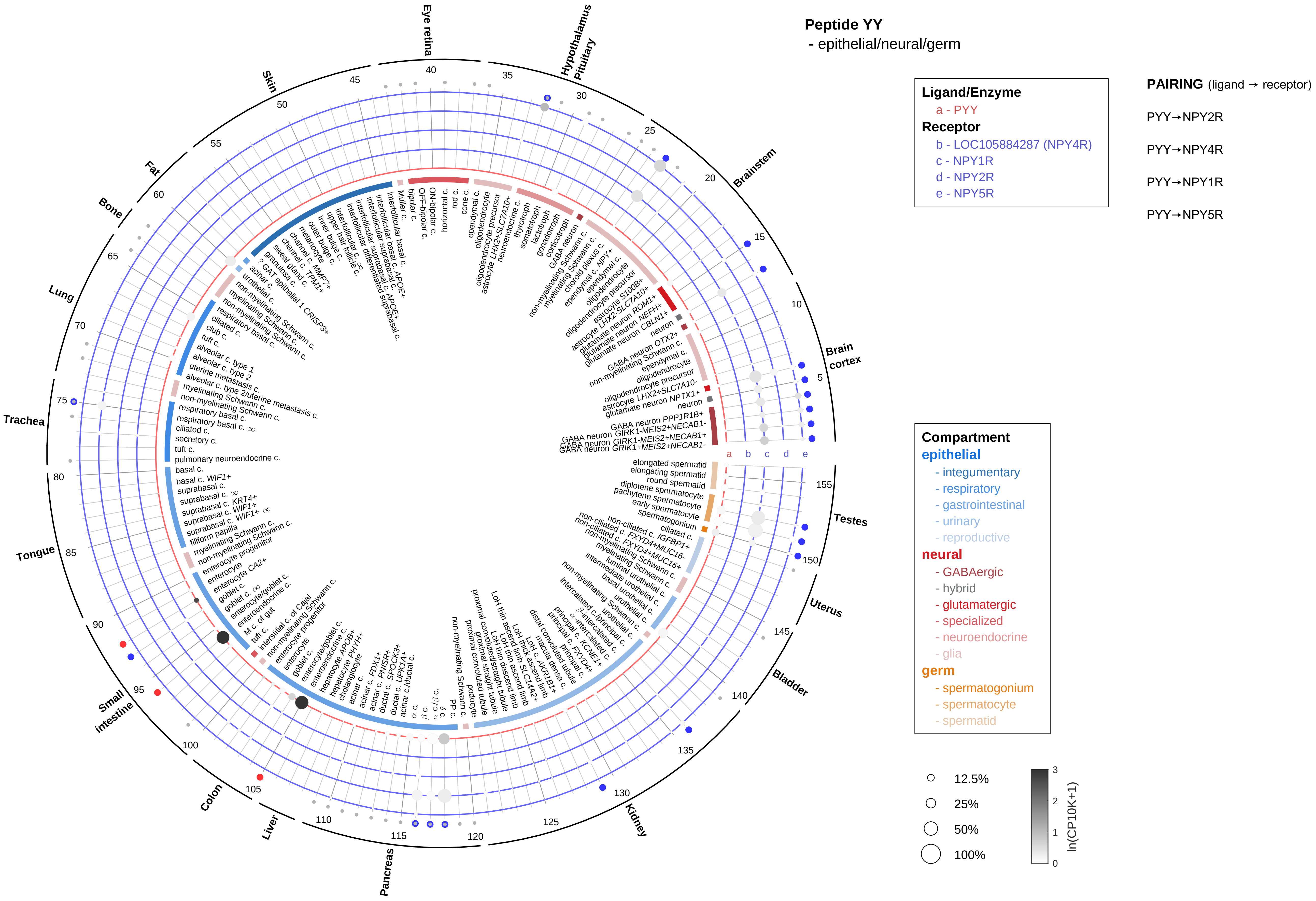
Peptide YY
- epithelial/neural/germ
Ligand/Enzyme
a - PYY
Receptor
b - LOC105884287 (NPY4R)
c - NPY1R
d - NPY2R
e - NPY5R
PAIRING (ligand → receptor)
PYY→NPY2R
PYY→NPY4R
PYY→NPY1R
PYY→NPY5R
Compartment
epithelial
- integumentary
- respiratory
- gastrointestinal
- urinary
- reproductive
neural
- GABAergic
- hybrid
- glutamatergic
- specialized
- neuroendocrine
- glia
germ
- spermatogonium
- spermatocyte
- spermatid
12.5%
25%
50%
100%
ln(CP10K+1)
Eye retina
Hypothalamus Pituitary
Brainstem
Brain cortex
Testes
Uterus
Bladder
Kidney
Pancreas
Liver
Colon
Small intestine
Tongue
Trachea
Lung
Bone
Fat
Skin

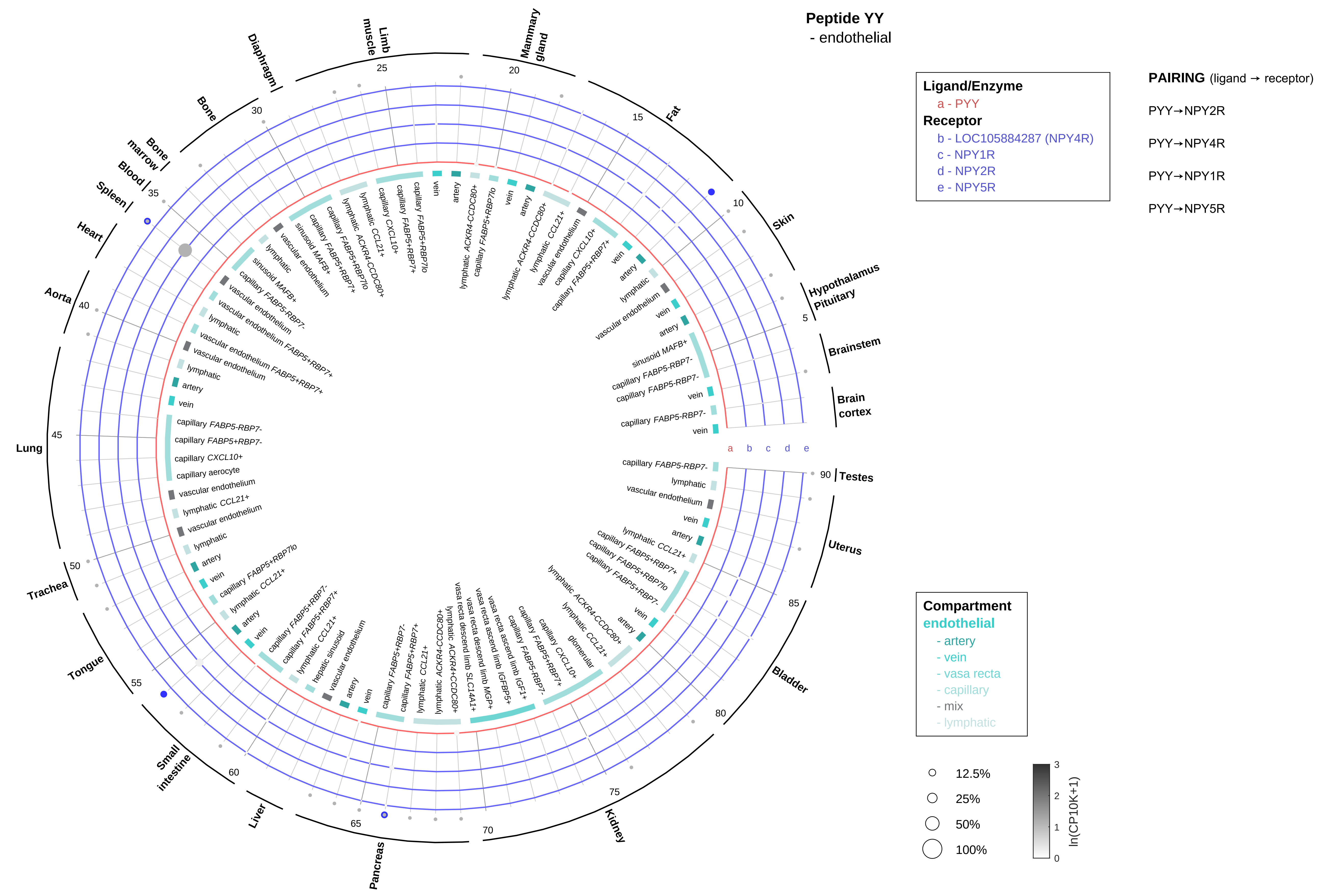
Peptide YY
- endothelial
Ligand/Enzyme
a - PYY
Receptor
b - LOC105884287 (NPY4R)
c - NPY1R
d - NPY2R
e - NPY5R
PAIRING (ligand → receptor)
PYY→NPY2R
PYY→NPY4R
PYY→NPY1R
PYY→NPY5R
Compartment
endothelial
- artery
- vein
- vasa recta
- capillary
- mix
- lymphatic
12.5%
25%
50%
100%
ln(CP10K+1)
Brain cortex
Brainstem
Hypothalamus Pituitary
Skin
Fat
Mammary gland
Limb muscle
Diaphragm
Bone
Bone marrow
Blood
Spleen
Heart
Aorta
Lung
Trachea
Tongue
Small intestine
Liver
Pancreas
Kidney
Bladder
Uterus
Testes

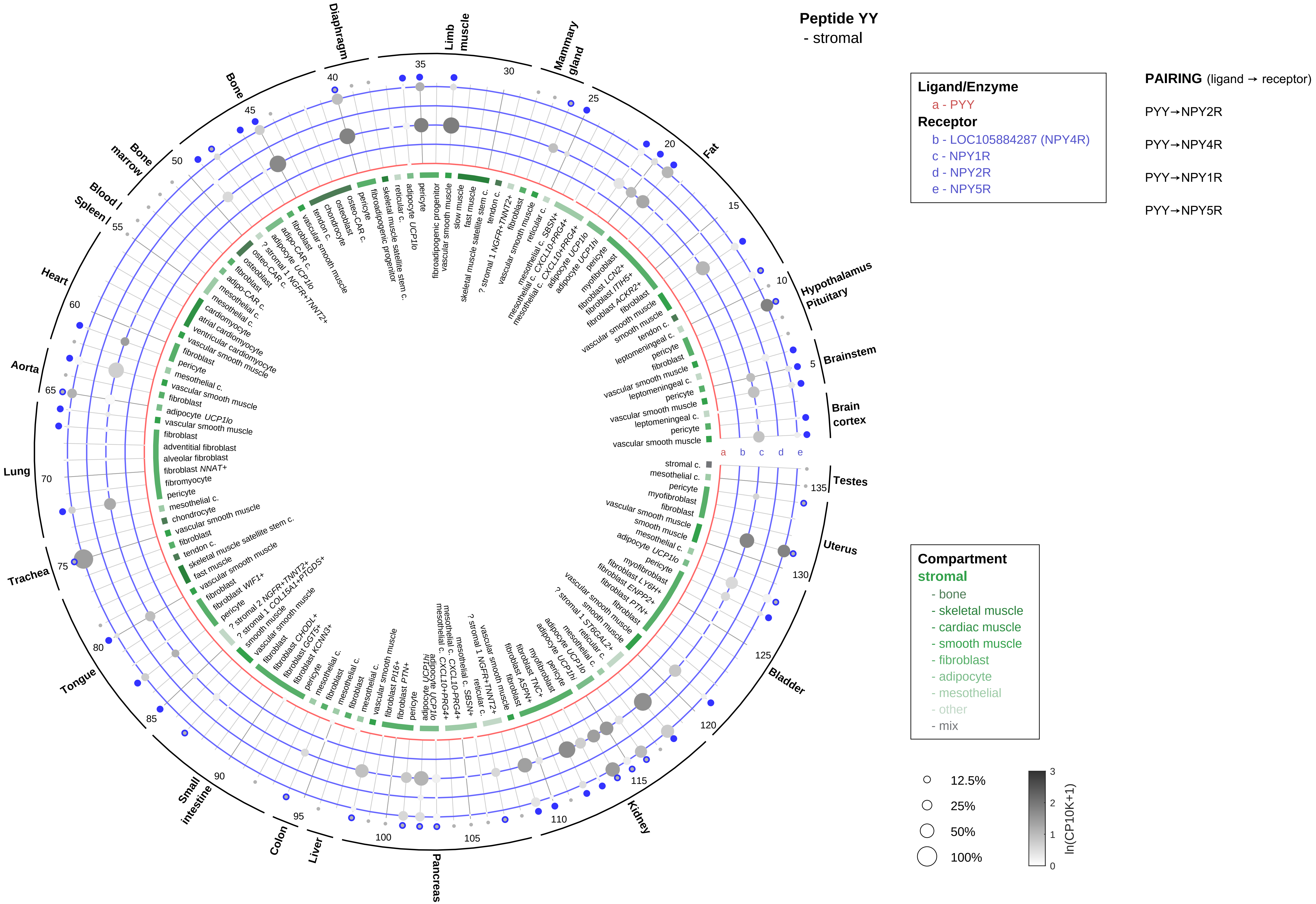
Peptide YY
- stromal
Ligand/Enzyme
a - PYY
Receptor
b - LOC105884287 (NPY4R)
c - NPY1R
d - NPY2R
e - NPY5R
PAIRING (ligand → receptor)
PYY→NPY2R
PYY→NPY4R
PYY→NPY1R
PYY→NPY5R
Compartment
stromal
- bone
- skeletal muscle
- cardiac muscle
- smooth muscle
- fibroblast
- adipocyte
- mesothelial
- other
- mix
12.5%
25%
50%
100%
ln(CP10K+1)
Diaphragm
Limb muscle
Mammary gland
Fat
Hypothalamus Pituitary
Brainstem
Brain cortex
Testes
Uterus
Bladder
Kidney
Pancreas
Liver
Colon
Small intestine
Tongue
Trachea
Lung
Aorta
Heart
Spleen
Blood
Bone marrow
Bone

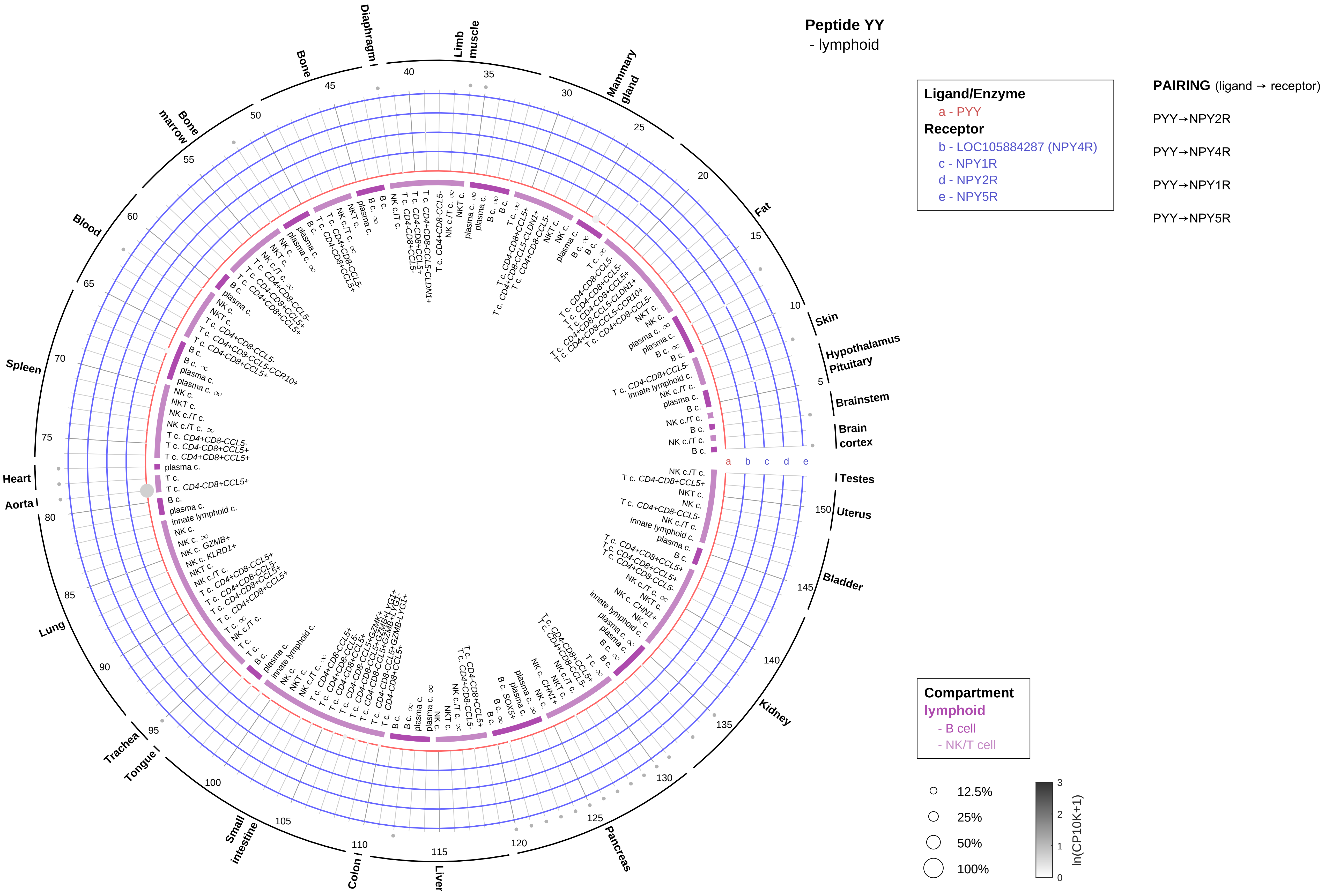
Peptide YY
- lymphoid
Ligand/Enzyme
a - PYY
Receptor
b - LOC105884287 (NPY4R)
c - NPY1R
d - NPY2R
e - NPY5R
PAIRING (ligand → receptor)
PYY→NPY2R
PYY→NPY4R
PYY→NPY1R
PYY→NPY5R
Compartment
lymphoid
- B cell
- NK/T cell
12.5%
25%
50%
100%
ln(CP10K+1)
Skin
Hypothalamus
Pituitary
Brainstem
Brain cortex
Testes
Uterus
Bladder
Kidney
Pancreas
Liver
Colon
Small intestine
Tongue
Trachea
Lung
Aorta
Heart
Spleen
Blood
Bone marrow
Bone
Diaphragm
Limb muscle
Mammary gland
Fat

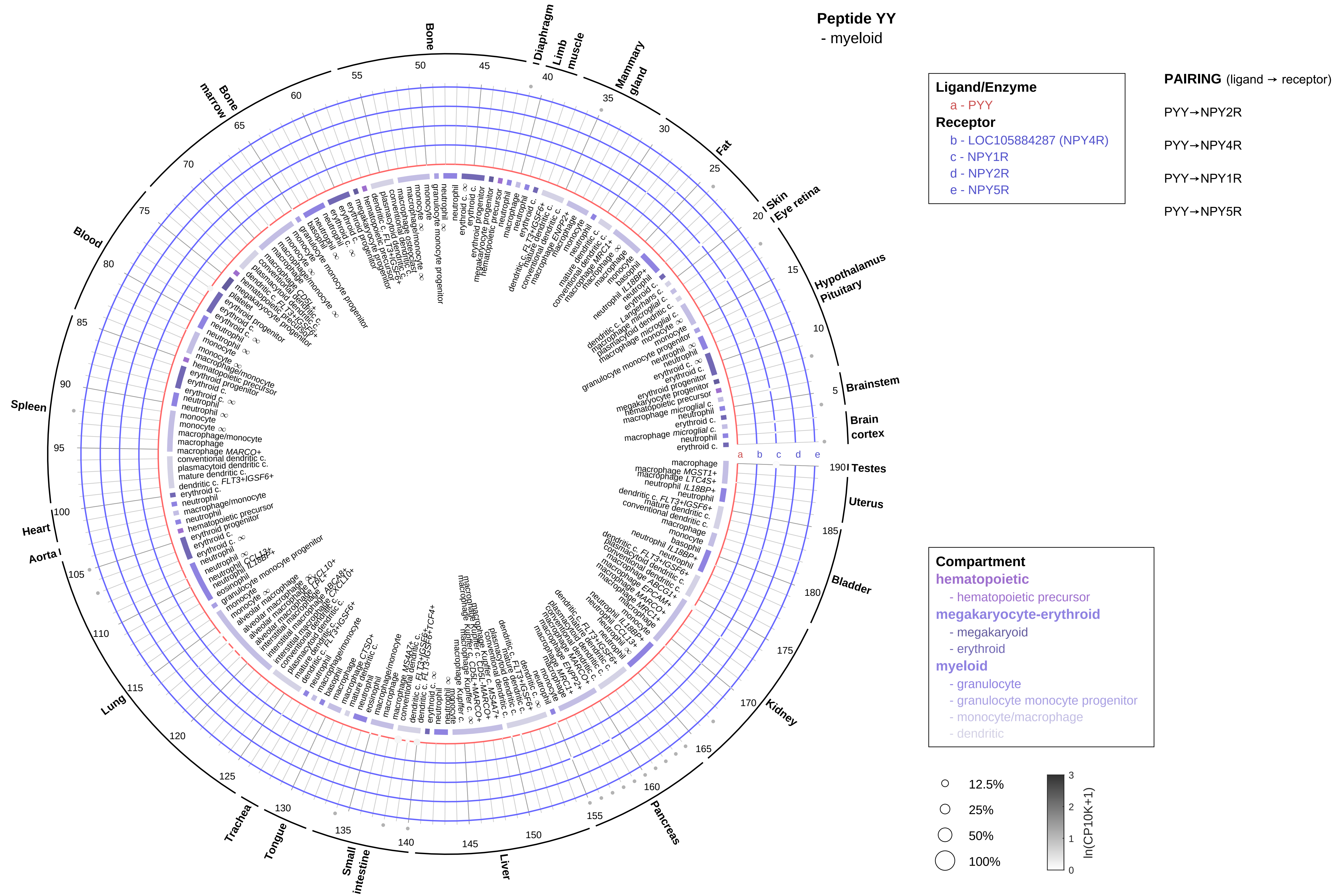

Peptide YY
- myeloid
Ligand/Enzyme
a - PYY
Receptor
b - LOC105884287 (NPY4R)
c - NPY1R
d - NPY2R
e - NPY5R
PAIRING (ligand → receptor)
PYY→NPY2R
PYY→NPY4R
PYY→NPY1R
PYY→NPY5R
Compartment
hematopoietic
- hematopoietic precursor
megakaryocyte-erythroid
- megakaryoid
- erythroid
myeloid
- granulocyte
- granulocyte monocyte progenitor
- monocyte/macrophage
- dendritic
12.5%
25%
50%
100%
ln(CP10K+1)
Bone
Diaphragm
Limb muscle
Mammary gland
Fat
Skin
Eye retina
Hypothalamus
Pituitary
Brainstem
Brain cortex
Testes
Uterus
Bladder
Kidney
Pancreas
Liver
Small intestine
Tongue
Trachea
Lung
Aorta
Heart
Spleen
Blood
Bone marrow

Pituitary adenylate cyclase-activating polypeptide (PACAP)
- epithelial/neural/germ

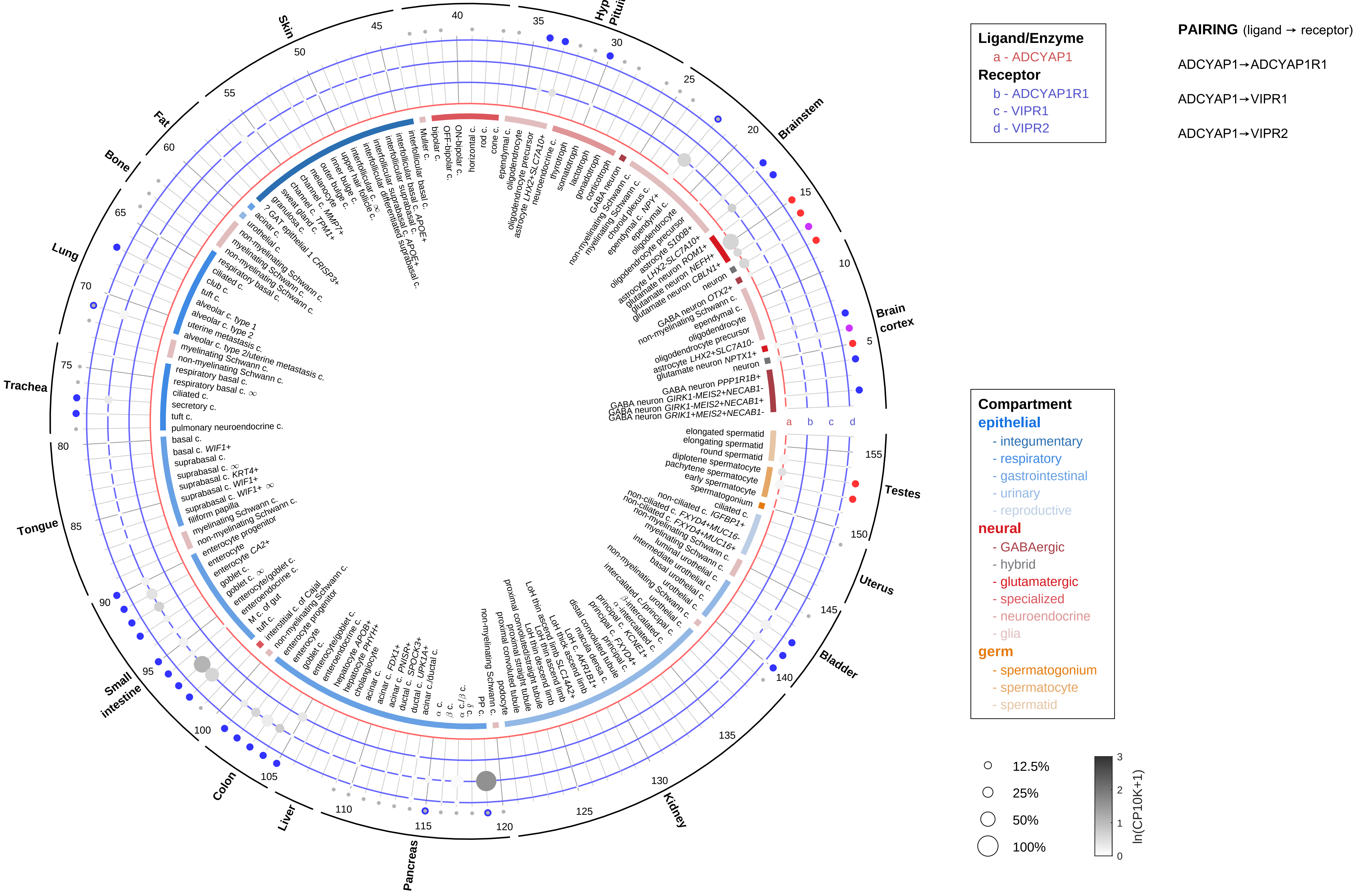

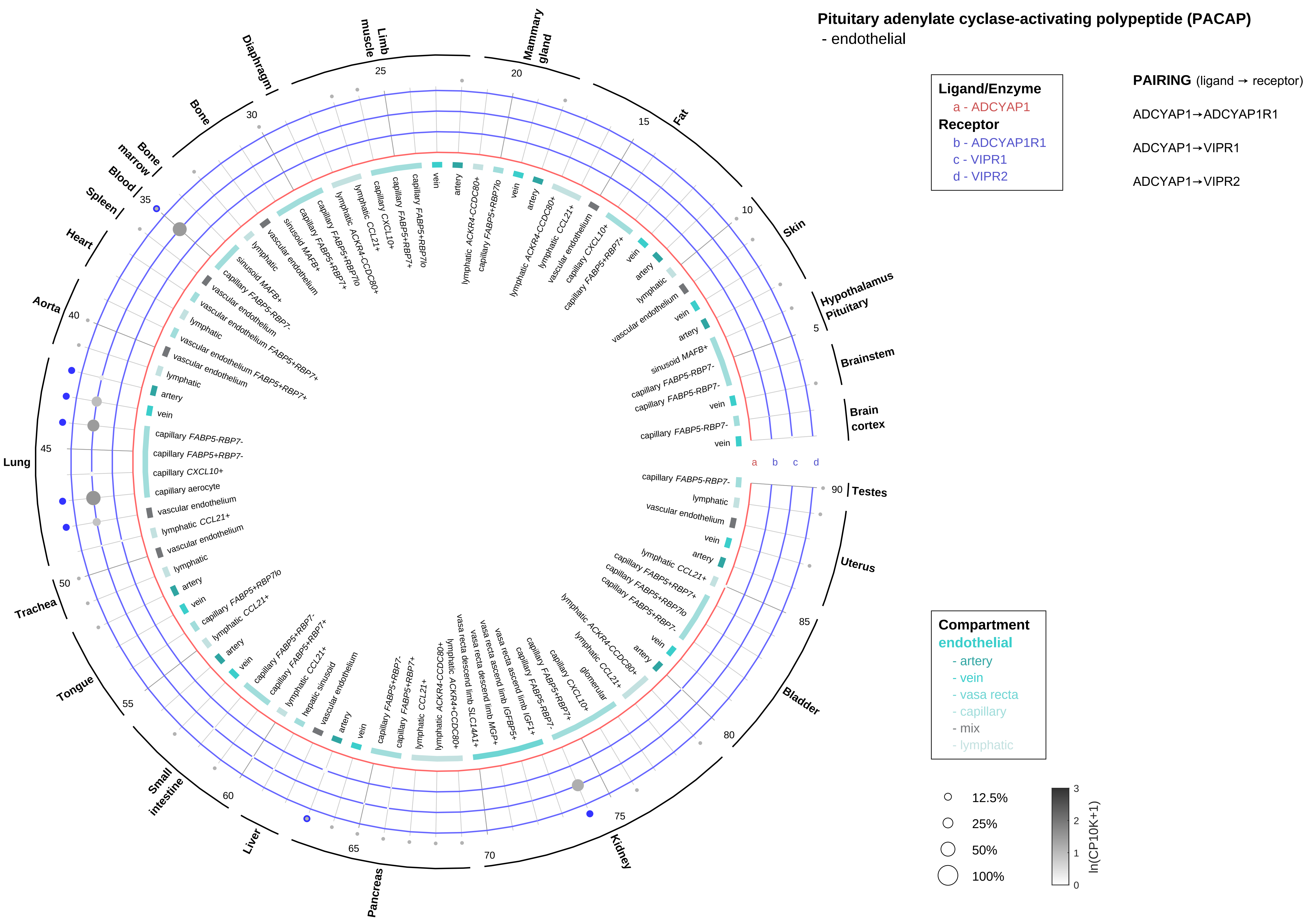

Pituitary adenylate cyclase-activating polypeptide (PACAP)
- endothelial
Ligand/Enzyme
a - ADCYAP1
Receptor
b - ADCYAP1R1
c - VIPR1
d - VIPR2
PAIRING (ligand → receptor)
ADCYAP1→ADCYAP1R1
ADCYAP1→VIPR1
ADCYAP1→VIPR2
Compartment
endothelial
- artery
- vein
- vasa recta
- capillary
- mix
- lymphatic
12.5%
25%
50%
100%
ln(CP10K+1)
Hypothalamus Pituitary
Brainstem
Brain cortex
Testes
Uterus
Bladder
Kidney
Pancreas
Liver
Small intestine
Tongue
Trachea
Lung
Aorta
Heart
Spleen
Blood
Bone marrow
Bone
Diaphragm
Limb muscle
Mammary gland
Fat
Skin
a
b
c
d

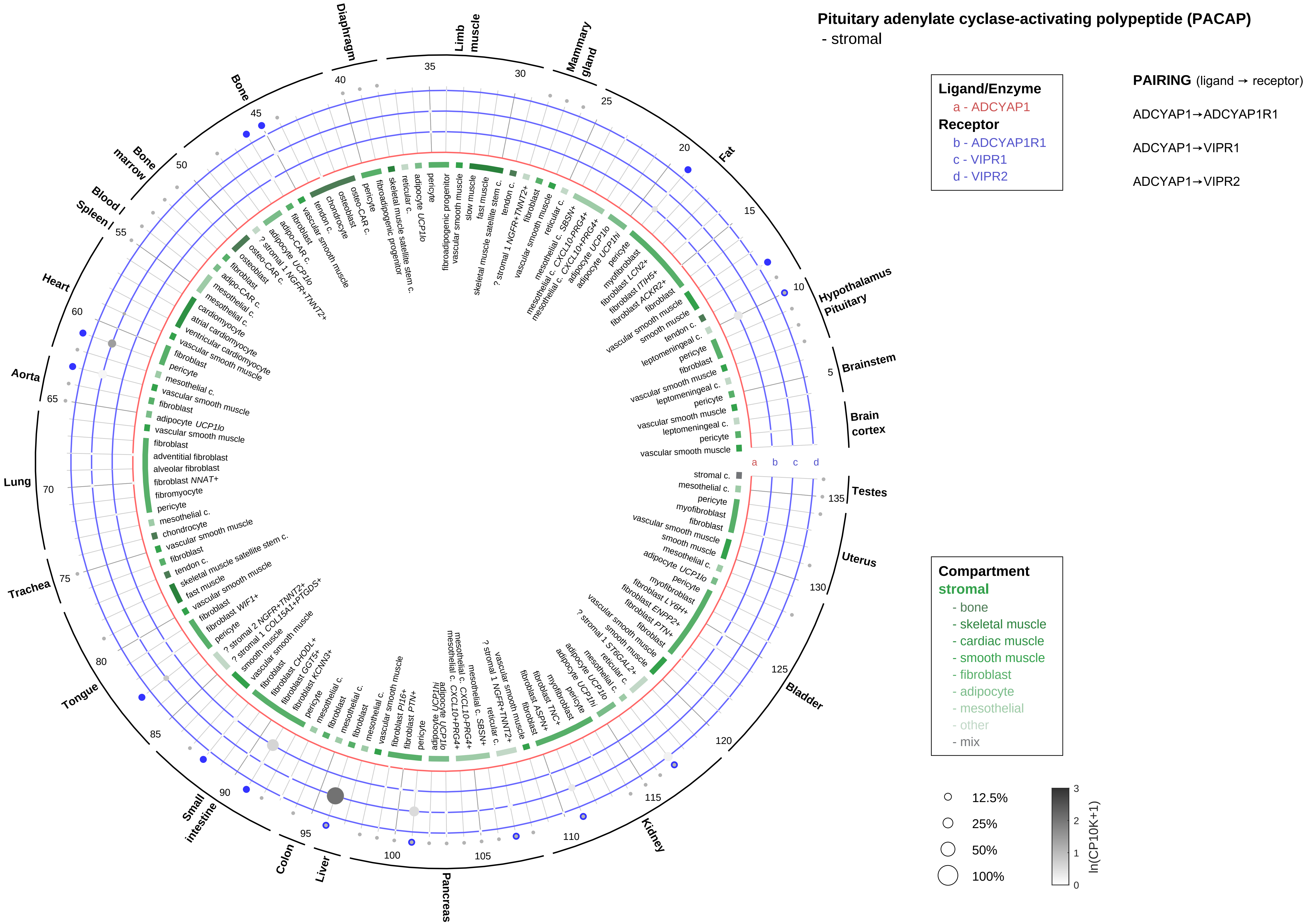

Pituitary adenylate cyclase-activating polypeptide (PACAP)
- stromal
Ligand/Enzyme
a - ADCYAP1
Receptor
b - ADCYAP1R1
c - VIPR1
d - VIPR2
PAIRING (ligand → receptor)
ADCYAP1→ADCYAP1R1
ADCYAP1→VIPR1
ADCYAP1→VIPR2
Compartment
stromal
- bone
- skeletal muscle
- cardiac muscle
- smooth muscle
- fibroblast
- adipocyte
- mesothelial
- other
- mix
ln(CP10K+1)
Brain cortex
Brainstem
Hypothalamus
Pituitary
Fat
Mammary gland
Limb muscle
Diaphragm
Bone
Bone marrow
Blood
Spleen
Heart
Aorta
Lung
Trachea
Tongue
Small intestine
Colon
Liver
Pancreas
Kidney
Bladder
Uterus
Testes
vascular smooth muscle
pericyte
leptomeningeal c.
fibroblast
tendon c.
smooth muscle
fibroblast ACKR2+
fibroblast ITIH5+
fibroblast LCN2+
myofibroblast
adipocyte UCP1hi
adipocyte UCP1lo
mesothelial c. CXCL10+PRG4+
mesothelial c. SBSN+
reticular c.
? stromal 1 NGFR+TNNT2+
skeletal muscle satellite stem c.
fast muscle
slow muscle
fibroadipogenic progenitor
osteo-CAR c.
osteoblast
chondrocyte
adipo-CAR c.
mesothelial c.
cardiomyocyte
atrial cardiomyocyte
ventricular cardiomyocyte
adventitial fibroblast
alveolar fibroblast
fibroblast NNAT+
fibromyocyte
fibroblast WIF1+
? stromal 2 NGFR+TNNT2+
? stromal 1 COL15A1+PTGDS+
fibroblast CHODL+
fibroblast GGT5+
fibroblast KCNN3+
fibroblast PI16+
fibroblast PTN+
fibroblast ASPN+
fibroblast TNC+
? stromal 1 ST6GAL2+
fibroblast ENPP2+
fibroblast LY6H+
stromal c.

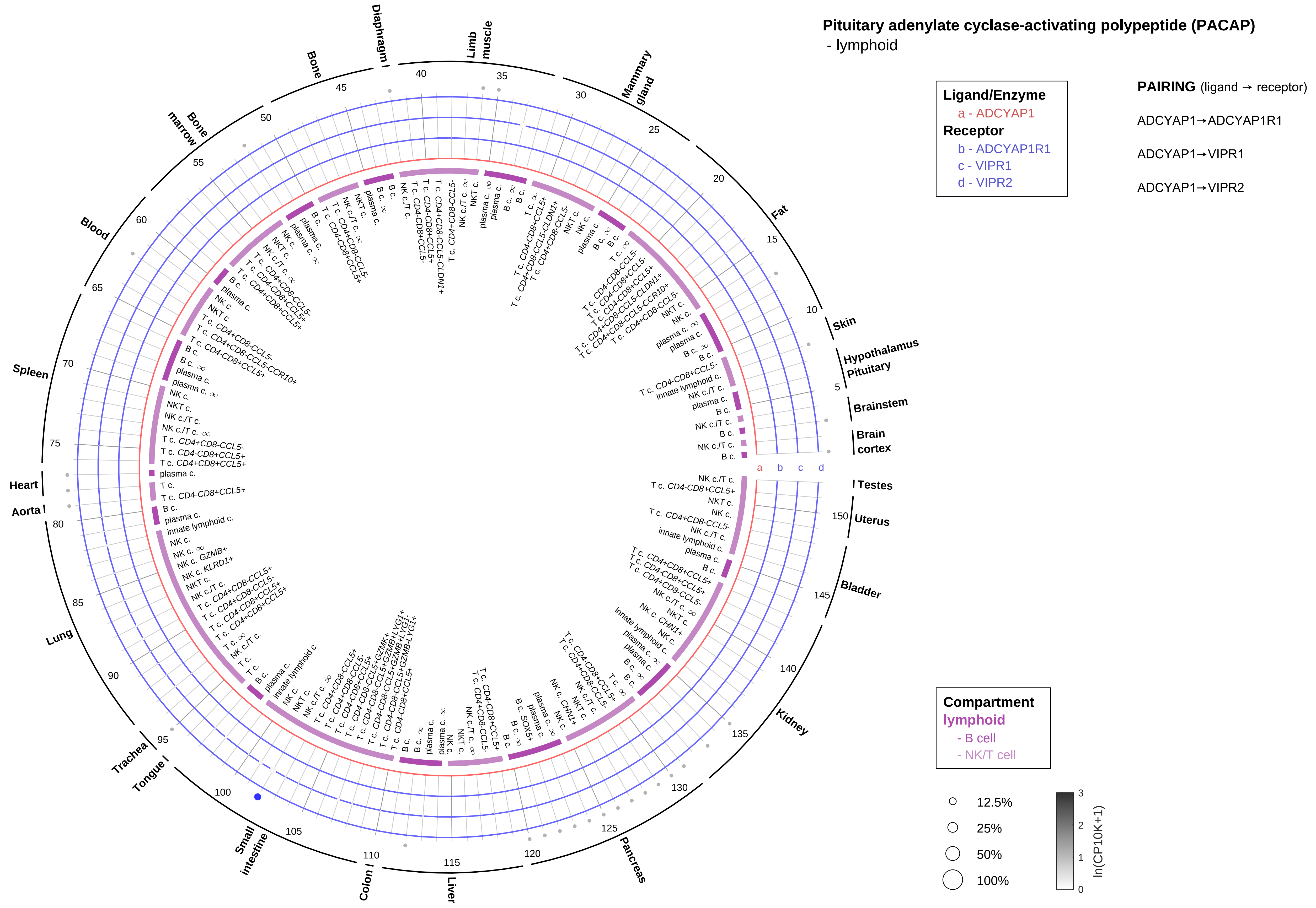

Pituitary adenylate cyclase-activating polypeptide (PACAP)
- lymphoid
Ligand/Enzyme
a - ADCYAP1
Receptor
b - ADCYAP1R1
c - VIPR1
d - VIPR2
PAIRING (ligand → receptor)
ADCYAP1→ADCYAP1R1
ADCYAP1→VIPR1
ADCYAP1→VIPR2
Compartment
lymphoid
- B cell
- NK/T cell
12.5%
25%
50%
100%
ln(CP10K+1)

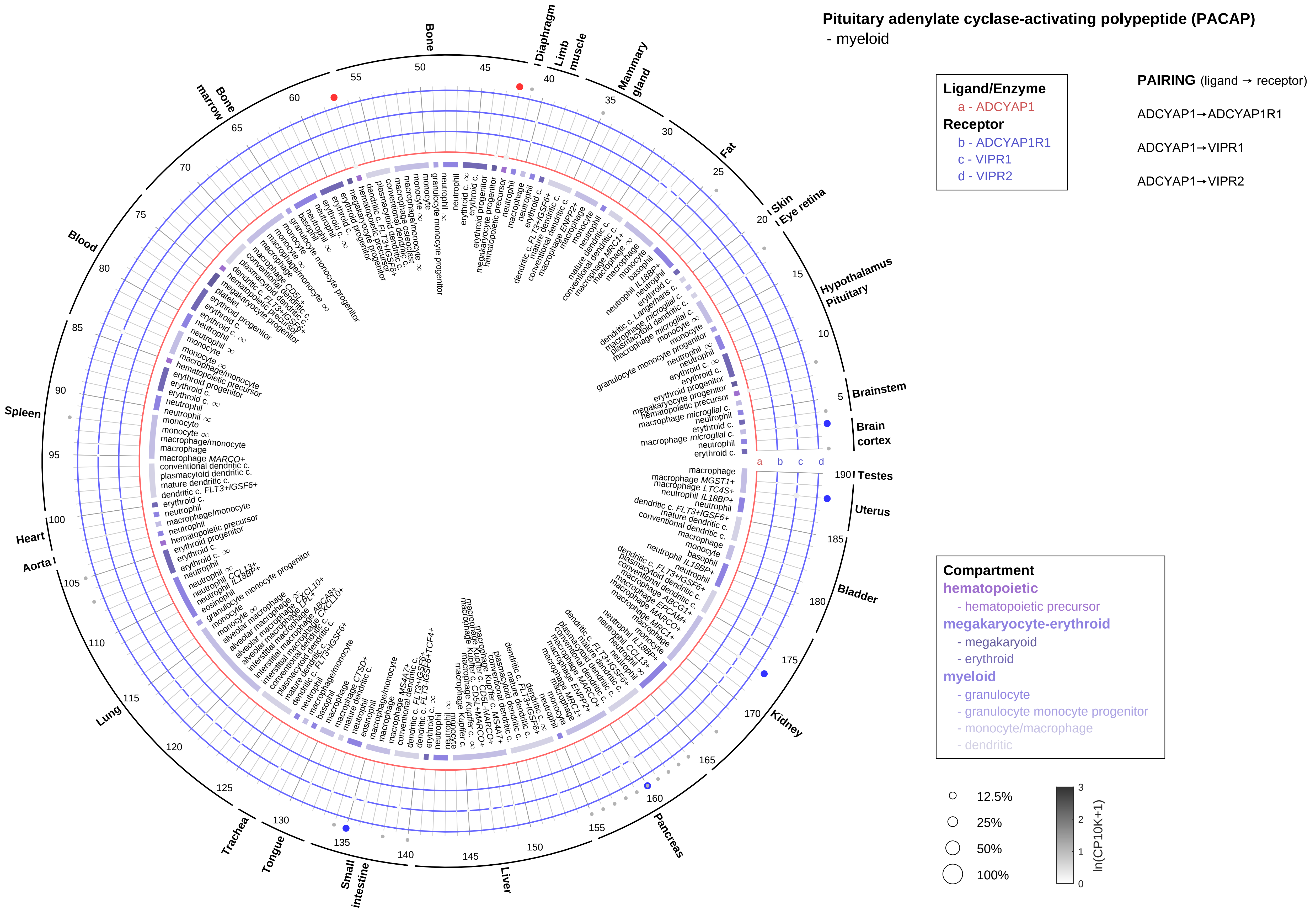

Pituitary adenylate cyclase-activating polypeptide (PACAP)
- myeloid
Ligand/Enzyme
a - ADCYAP1
Receptor
b - ADCYAP1R1
c - VIPR1
d - VIPR2
PAIRING (ligand → receptor)
ADCYAP1→ADCYAP1R1
ADCYAP1→VIPR1
ADCYAP1→VIPR2
Compartment
hematopoietic
- hematopoietic precursor
megakaryocyte-erythroid
- megakaryoid
- erythroid
myeloid
- granulocyte
- granulocyte monocyte progenitor
- monocyte/macrophage
- dendritic
12.5%
25%
50%
100%
ln(CP10K+1)
Bone
Diaphragm
Limb muscle
Mammary gland
Fat
Skin
Eye retina
Hypothalamus
Pituitary
Brainstem
Brain cortex
Testes
Uterus
Bladder
Kidney
Pancreas
Liver
Small intestine
Tongue
Trachea
Lung
Aorta
Heart
Spleen
Blood
Bone marrow

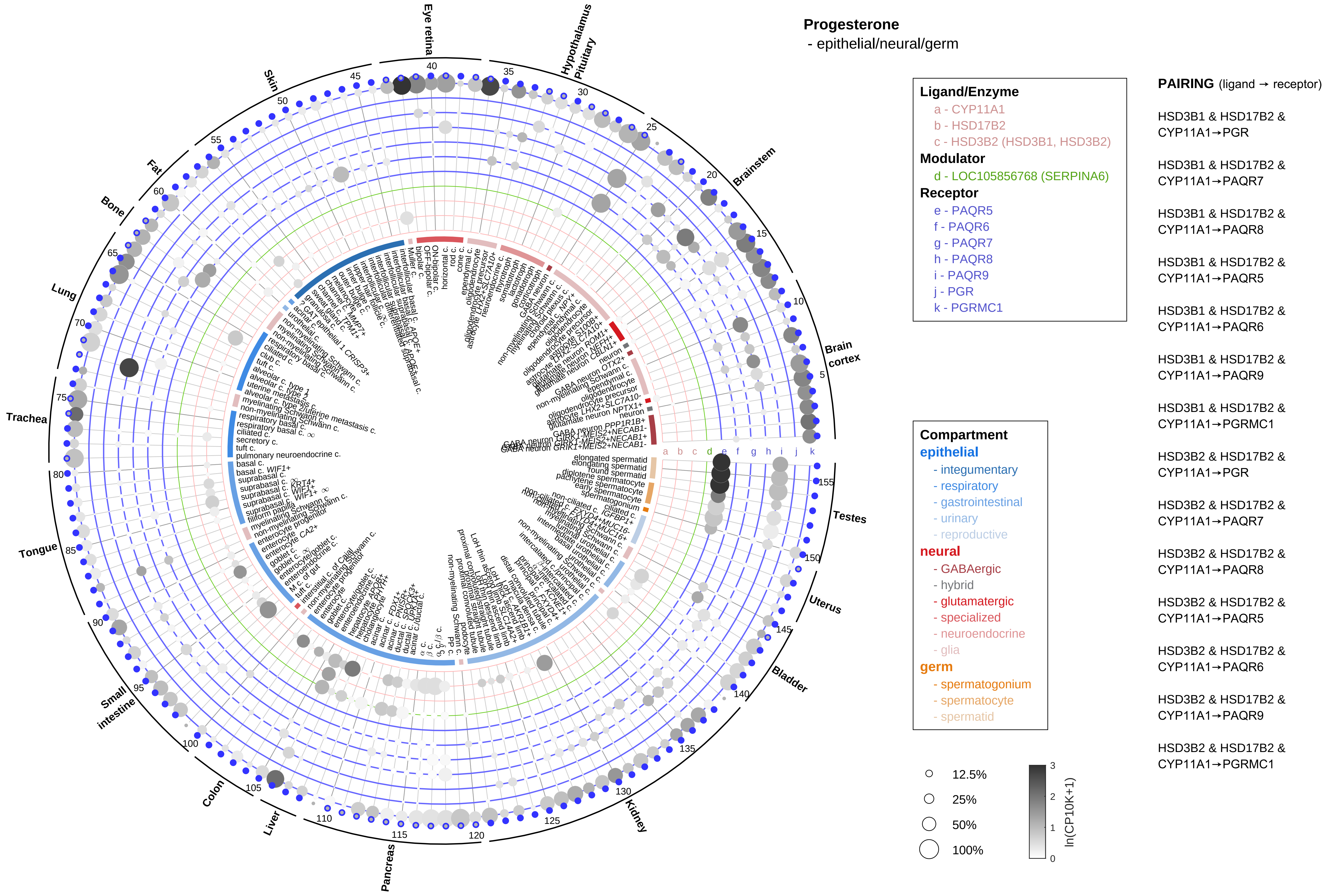
Progesterone
- epithelial/neural/germ
Ligand/Enzyme
a - CYP11A1
b - HSD17B2
c - HSD3B2 (HSD3B1, HSD3B2)
Modulator
d - LOC105856768 (SERPINA6)
Receptor
e - PAQR5
f - PAQR6
g - PAQR7
h - PAQR8
i - PAQR9
j - PGR
k - PGRMC1
Compartment
epithelial
- integumentary
- respiratory
- gastrointestinal
- urinary
- reproductive
neural
- GABAergic
- hybrid
- glutamatergic
- specialized
- neuroendocrine
- glia
germ
- spermatogonium
- spermatocyte
- spermatid
12.5%
25%
50%
100%
ln(CP10K+1)
PAIRING (ligand → receptor)
HSD3B1 & HSD17B2 & CYP11A1→PGR
HSD3B1 & HSD17B2 & CYP11A1→PAQR7
HSD3B1 & HSD17B2 & CYP11A1→PAQR8
HSD3B1 & HSD17B2 & CYP11A1→PAQR5
HSD3B1 & HSD17B2 & CYP11A1→PAQR6
HSD3B1 & HSD17B2 & CYP11A1→PAQR9
HSD3B1 & HSD17B2 & CYP11A1→PGRMC1
HSD3B2 & HSD17B2 & CYP11A1→PGR
HSD3B2 & HSD17B2 & CYP11A1→PAQR7
HSD3B2 & HSD17B2 & CYP11A1→PAQR8
HSD3B2 & HSD17B2 & CYP11A1→PAQR5
HSD3B2 & HSD17B2 & CYP11A1→PAQR6
HSD3B2 & HSD17B2 & CYP11A1→PAQR9
HSD3B2 & HSD17B2 & CYP11A1→PGRMC1
Eye retina
Hypothalamus Pituitary
Brainstem
Brain cortex
Testes
Uterus
Bladder
Kidney
Pancreas
Liver
Colon
Small intestine
Tongue
Trachea
Lung
Bone
Fat
Skin

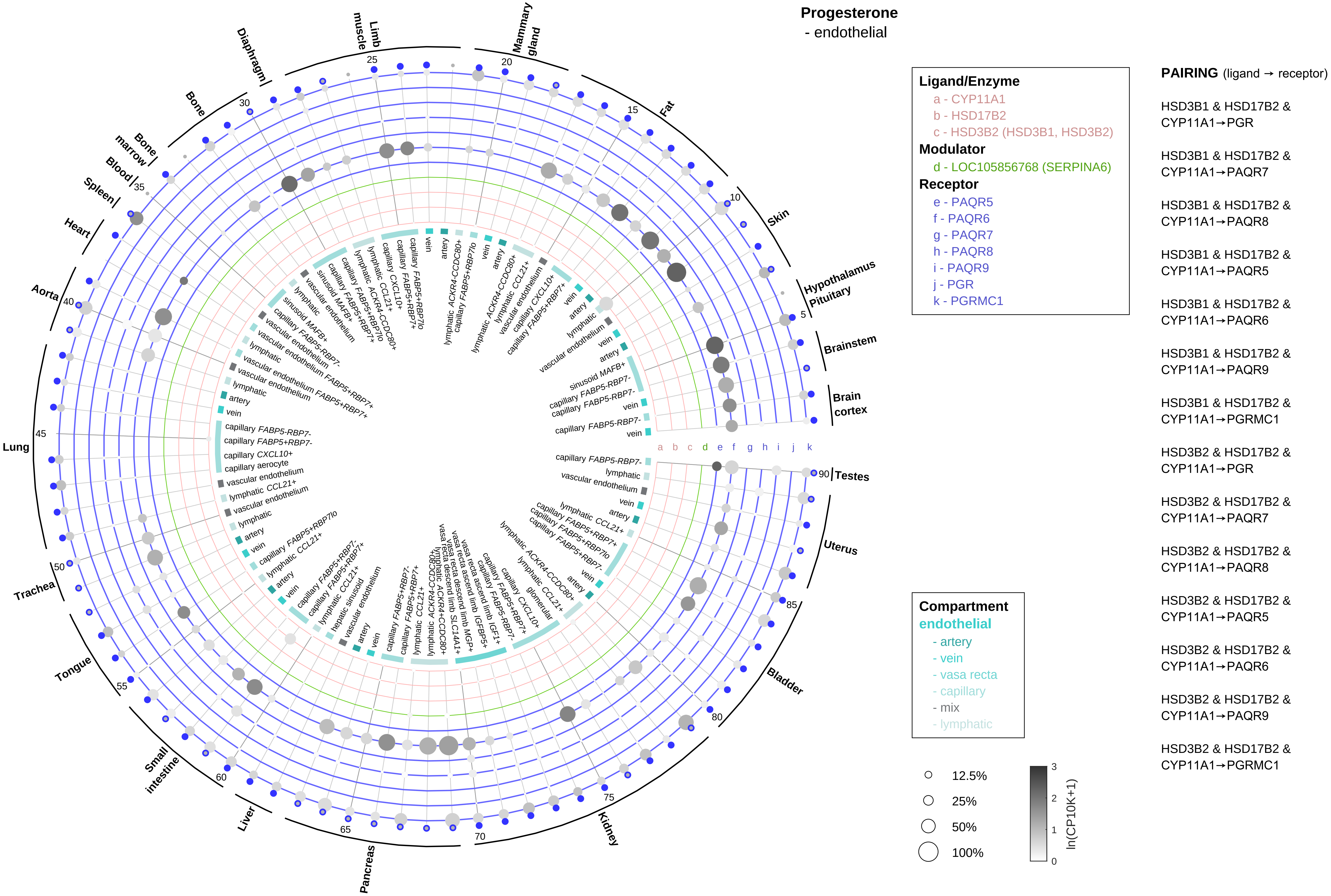
Progesterone
- endothelial
Ligand/Enzyme
a - CYP11A1
b - HSD17B2
c - HSD3B2 (HSD3B1, HSD3B2)
Modulator
d - LOC105856768 (SERPINA6)
Receptor
e - PAQR5
f - PAQR6
g - PAQR7
h - PAQR8
i - PAQR9
j - PGR
k - PGRMC1
PAIRING (ligand → receptor)
HSD3B1 & HSD17B2 & CYP11A1→PGR
HSD3B1 & HSD17B2 & CYP11A1→PAQR7
HSD3B1 & HSD17B2 & CYP11A1→PAQR8
HSD3B1 & HSD17B2 & CYP11A1→PAQR5
HSD3B1 & HSD17B2 & CYP11A1→PAQR6
HSD3B1 & HSD17B2 & CYP11A1→PAQR9
HSD3B1 & HSD17B2 & CYP11A1→PGRMC1
HSD3B2 & HSD17B2 & CYP11A1→PGR
HSD3B2 & HSD17B2 & CYP11A1→PAQR7
HSD3B2 & HSD17B2 & CYP11A1→PAQR8
HSD3B2 & HSD17B2 & CYP11A1→PAQR5
HSD3B2 & HSD17B2 & CYP11A1→PAQR6
HSD3B2 & HSD17B2 & CYP11A1→PAQR9
HSD3B2 & HSD17B2 & CYP11A1→PGRMC1
Compartment
endothelial
- artery
- vein
- vasa recta
- capillary
- mix
- lymphatic
12.5%
25%
50%
100%
ln(CP10K+1)
0
1
2
3
Mammary gland
Fat
Skin
Hypothalamus
Pituitary
Brainstem
Brain cortex
Testes
Uterus
Bladder
Kidney
Pancreas
Liver
Small intestine
Tongue
Trachea
Lung
Aorta
Heart
Spleen
Blood
Bone marrow
Bone
Diaphragm
Limb muscle
a b c d e f g h i j k

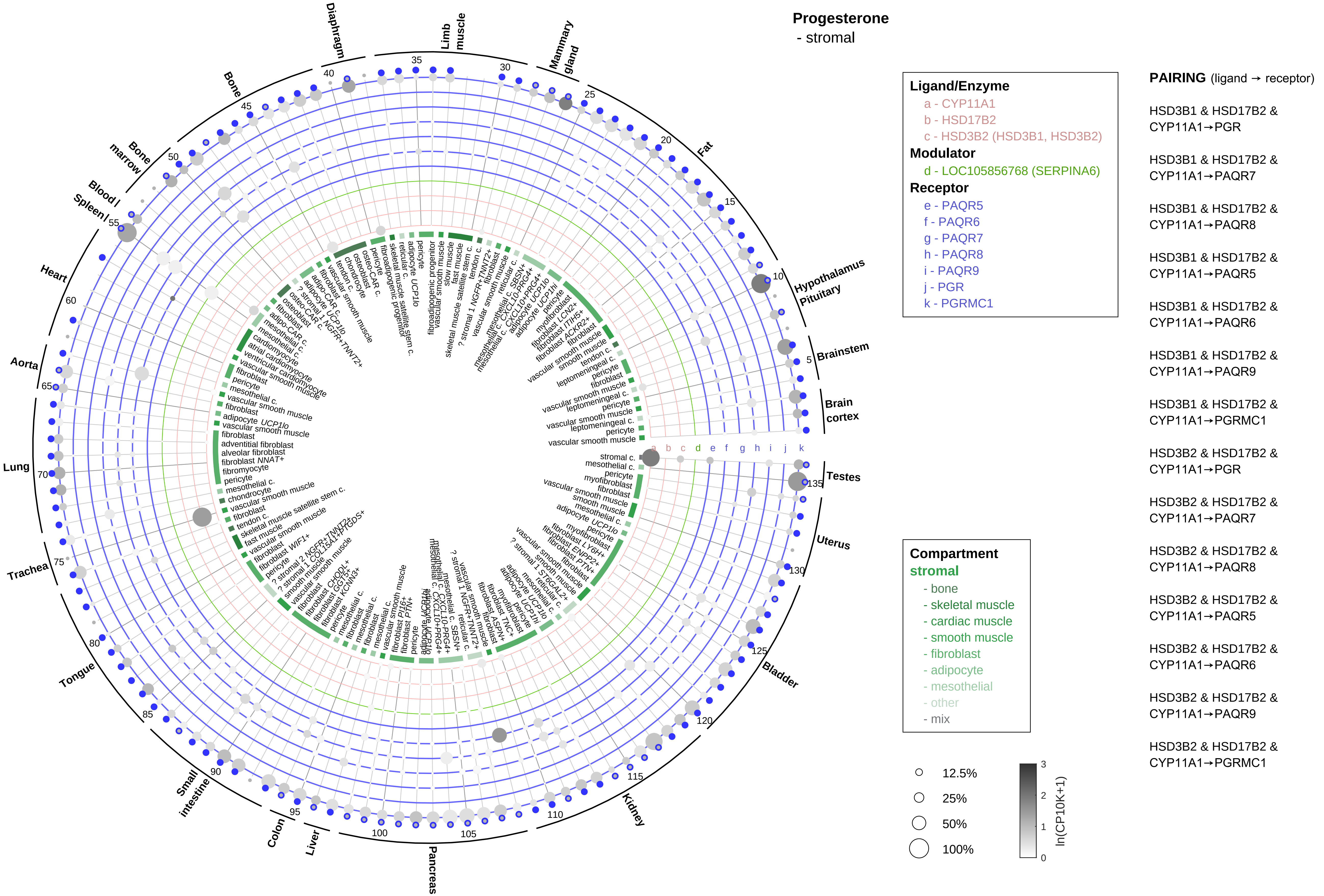

Progesterone
- stromal
Ligand/Enzyme
a - CYP11A1
b - HSD17B2
c - HSD3B2 (HSD3B1, HSD3B2)
Modulator
d - LOC105856768 (SERPINA6)
Receptor
e - PAQR5
f - PAQR6
g - PAQR7
h - PAQR8
i - PAQR9
j - PGR
k - PGRMC1
Compartment
stromal
- bone
- skeletal muscle
- cardiac muscle
- smooth muscle
- fibroblast
- adipocyte
- mesothelial
- other
- mix
12.5%
25%
50%
100%
ln(CP10K+1)
PAIRING (ligand → receptor)
HSD3B1 & HSD17B2 & CYP11A1→PGR
HSD3B1 & HSD17B2 & CYP11A1→PAQR7
HSD3B1 & HSD17B2 & CYP11A1→PAQR8
HSD3B1 & HSD17B2 & CYP11A1→PAQR5
HSD3B1 & HSD17B2 & CYP11A1→PAQR6
HSD3B1 & HSD17B2 & CYP11A1→PAQR9
HSD3B1 & HSD17B2 & CYP11A1→PGRMC1
HSD3B2 & HSD17B2 & CYP11A1→PGR
HSD3B2 & HSD17B2 & CYP11A1→PAQR7
HSD3B2 & HSD17B2 & CYP11A1→PAQR8
HSD3B2 & HSD17B2 & CYP11A1→PAQR5
HSD3B2 & HSD17B2 & CYP11A1→PAQR6
HSD3B2 & HSD17B2 & CYP11A1→PAQR9
HSD3B2 & HSD17B2 & CYP11A1→PGRMC1
Diaphragm
Limb muscle
Mammary gland
Fat
Hypothalamus
Pituitary
Brainstem
Brain cortex
Testes
Uterus
Bladder
Kidney
Pancreas
Liver
Colon
Small intestine
Tongue
Trachea
Lung
Aorta
Heart
Spleen
Blood
Bone marrow
Bone

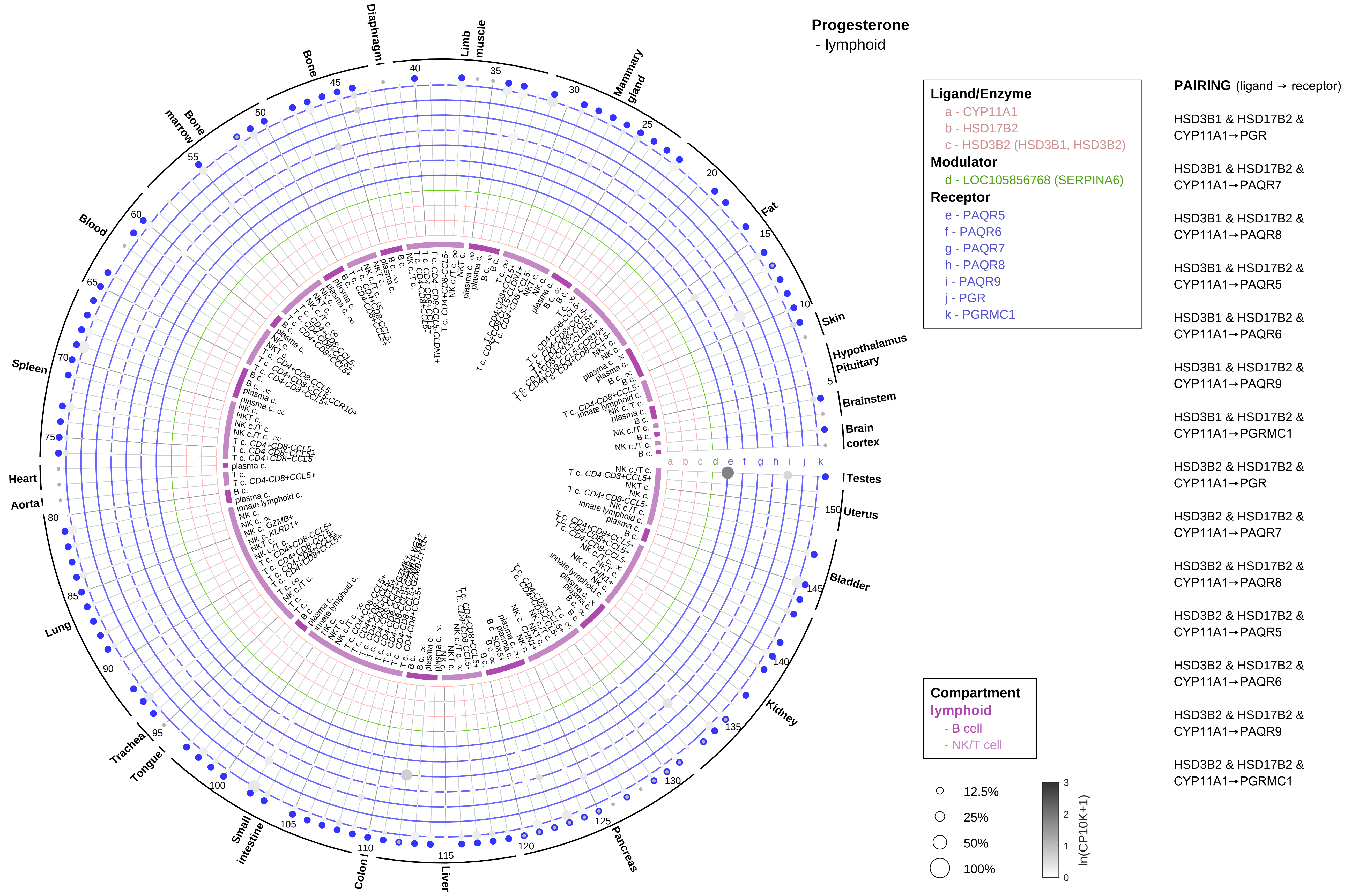

Progesterone
- lymphoid
Ligand/Enzyme
a - CYP11A1
b - HSD17B2
c - HSD3B2 (HSD3B1, HSD3B2)
Modulator
d - LOC105856768 (SERPINA6)
Receptor
e - PAQR5
f - PAQR6
g - PAQR7
h - PAQR8
i - PAQR9
j - PGR
k - PGRMC1
PAIRING (ligand → receptor)
HSD3B1 & HSD17B2 & CYP11A1→PGR
HSD3B1 & HSD17B2 & CYP11A1→PAQR7
HSD3B1 & HSD17B2 & CYP11A1→PAQR8
HSD3B1 & HSD17B2 & CYP11A1→PAQR5
HSD3B1 & HSD17B2 & CYP11A1→PAQR6
HSD3B1 & HSD17B2 & CYP11A1→PAQR9
HSD3B1 & HSD17B2 & CYP11A1→PGRMC1
HSD3B2 & HSD17B2 & CYP11A1→PGR
HSD3B2 & HSD17B2 & CYP11A1→PAQR7
HSD3B2 & HSD17B2 & CYP11A1→PAQR8
HSD3B2 & HSD17B2 & CYP11A1→PAQR5
HSD3B2 & HSD17B2 & CYP11A1→PAQR6
HSD3B2 & HSD17B2 & CYP11A1→PAQR9
HSD3B2 & HSD17B2 & CYP11A1→PGRMC1
Compartment
lymphoid
- B cell
- NK/T cell
12.5%
25%
50%
100%
ln(CP10K+1)
Testes
Uterus
Bladder
Kidney
Pancreas
Liver
Colon
Small intestine
Tongue
Trachea
Lung
Aorta
Heart
Spleen
Blood
Bone marrow
Bone
Diaphragm
Limb muscle
Mammary gland
Fat
Skin
Hypothalamus
Pituitary
Brainstem
Brain cortex

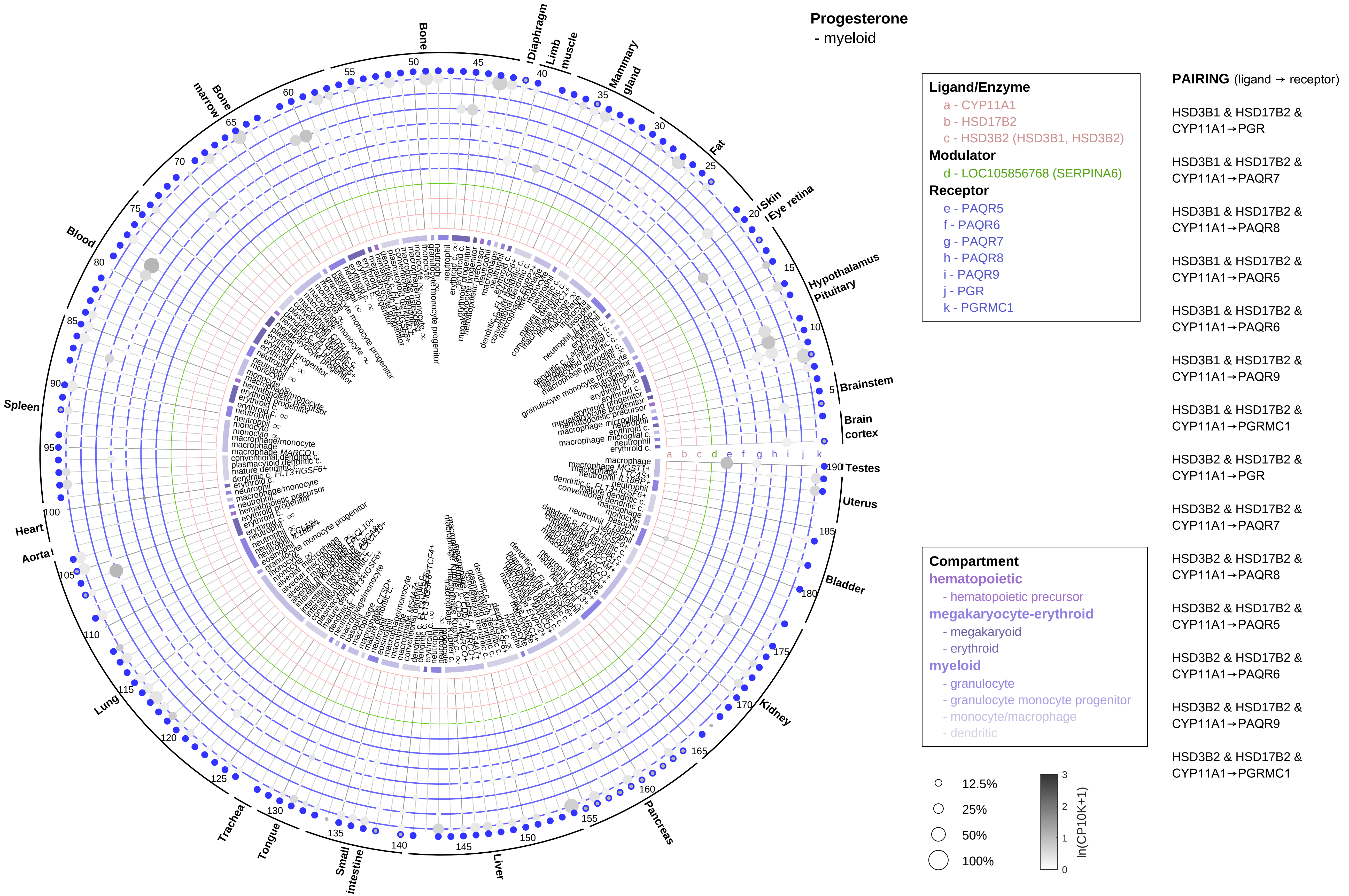

Progesterone
- myeloid
Ligand/Enzyme
a - CYP11A1
b - HSD17B2
c - HSD3B2 (HSD3B1, HSD3B2)
Modulator
d - LOC105856768 (SERPINA6)
Receptor
e - PAQR5
f - PAQR6
g - PAQR7
h - PAQR8
i - PAQR9
j - PGR
k - PGRMC1
Compartment
hematopoietic
- hematopoietic precursor
megakaryocyte-erythroid
- megakaryoid
- erythroid
myeloid
- granulocyte
- granulocyte monocyte progenitor
- monocyte/macrophage
- dendritic
12.5%
25%
50%
100%
ln(CP10K+1)
PAIRING (ligand → receptor)
HSD3B1 & HSD17B2 & CYP11A1→PGR
HSD3B1 & HSD17B2 & CYP11A1→PAQR7
HSD3B1 & HSD17B2 & CYP11A1→PAQR8
HSD3B1 & HSD17B2 & CYP11A1→PAQR5
HSD3B1 & HSD17B2 & CYP11A1→PAQR6
HSD3B1 & HSD17B2 & CYP11A1→PAQR9
HSD3B1 & HSD17B2 & CYP11A1→PGRMC1
HSD3B2 & HSD17B2 & CYP11A1→PGR
HSD3B2 & HSD17B2 & CYP11A1→PAQR7
HSD3B2 & HSD17B2 & CYP11A1→PAQR8
HSD3B2 & HSD17B2 & CYP11A1→PAQR5
HSD3B2 & HSD17B2 & CYP11A1→PAQR6
HSD3B2 & HSD17B2 & CYP11A1→PAQR9
HSD3B2 & HSD17B2 & CYP11A1→PGRMC1
Testes
Uterus
Bladder
Kidney
Pancreas
Liver
Small intestine
Tongue
Trachea
Lung
Aorta
Heart
Spleen
Blood
Bone marrow
Bone
Diaphragm
Limb muscle
Mammary gland
Fat
Skin
Eye retina
Hypothalamus
Pituitary
Brainstem
Brain cortex

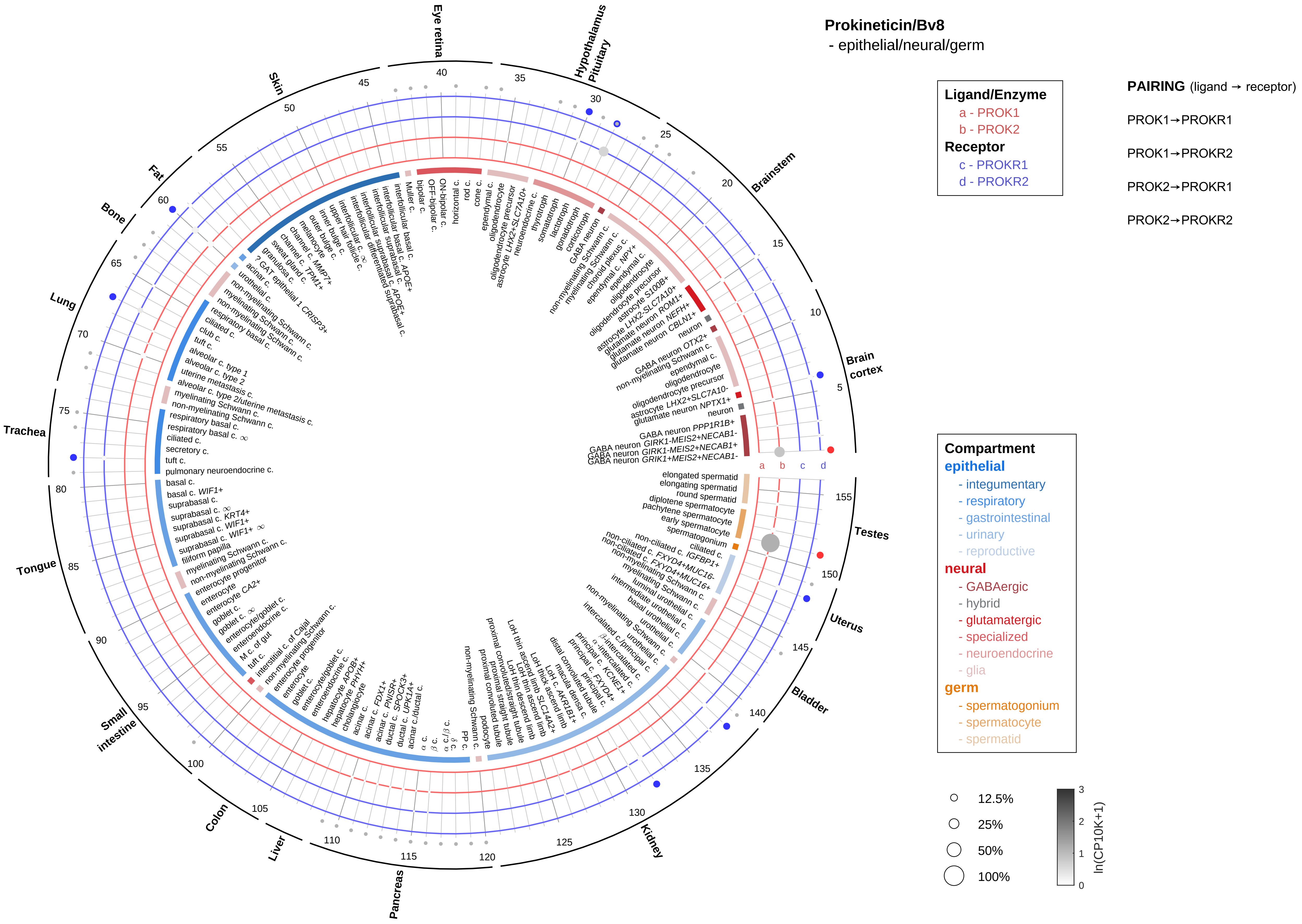

Prokineticin/Bv8
- epithelial/neural/germ
Ligand/Enzyme
a - PROK1
b - PROK2
Receptor
c - PROKR1
d - PROKR2
PAIRING (ligand → receptor)
PROK1→PROKR1
PROK1→PROKR2
PROK2→PROKR1
PROK2→PROKR2
Compartment
epithelial
- integumentary
- respiratory
- gastrointestinal
- urinary
- reproductive
neural
- GABAergic
- hybrid
- glutamatergic
- specialized
- neuroendocrine
- glia
germ
- spermatogonium
- spermatocyte
- spermatid
12.5%
25%
50%
100%
ln(CP10K+1)
Eye retina
Hypothalamus Pituitary
Brainstem
Brain cortex
Testes
Uterus
Bladder
Kidney
Pancreas
Liver
Colon
Small intestine
Tongue
Trachea
Lung
Bone
Fat
Skin

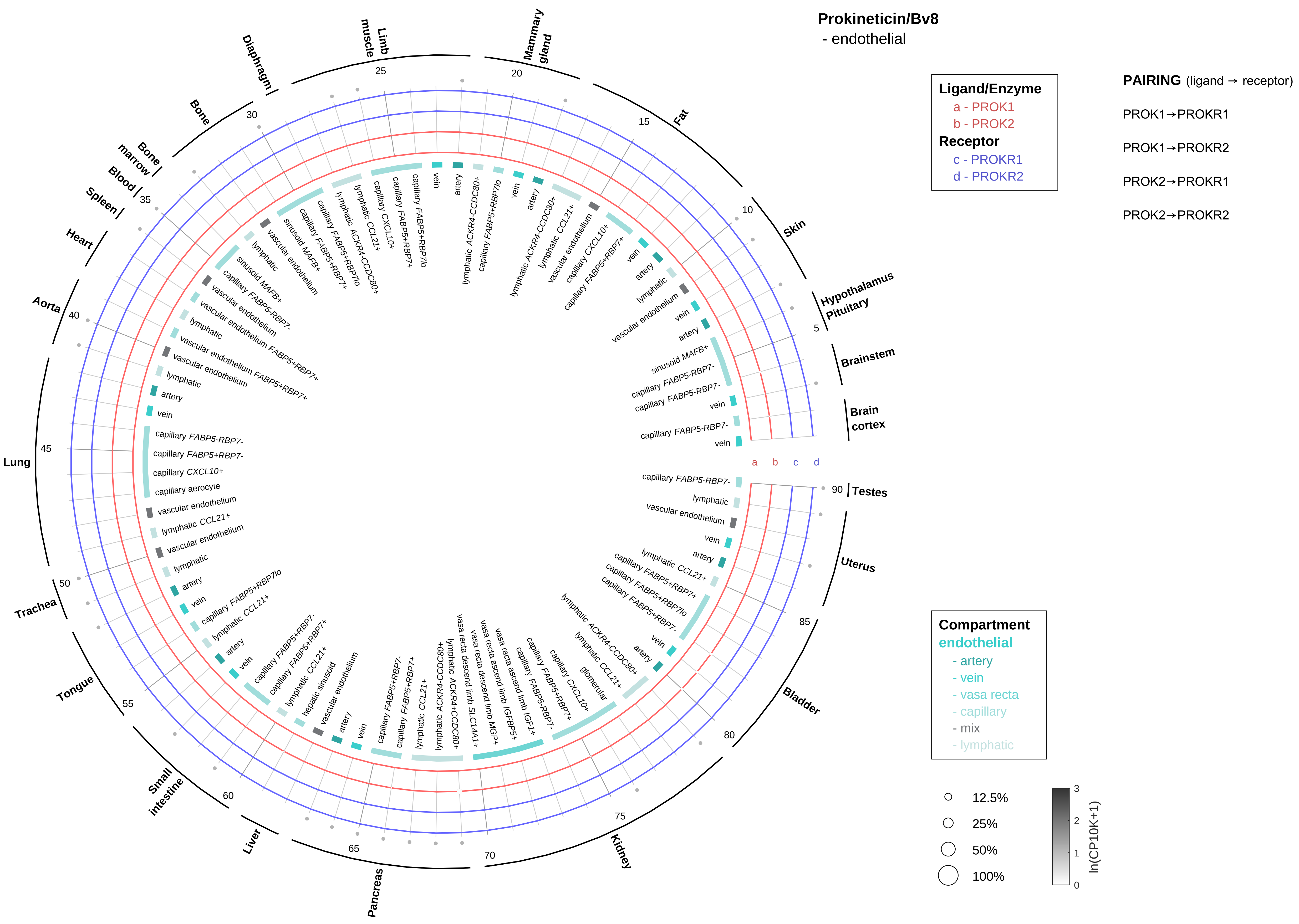

Prokineticin/Bv8
- endothelial
Ligand/Enzyme
a - PROK1
b - PROK2
Receptor
c - PROKR1
d - PROKR2
PAIRING (ligand → receptor)
PROK1→PROKR1
PROK1→PROKR2
PROK2→PROKR1
PROK2→PROKR2
Compartment
endothelial
- artery
- vein
- vasa recta
- capillary
- mix
- lymphatic
12.5%
25%
50%
100%
ln(CP10K+1)
Brain cortex
Brainstem
Hypothalamus Pituitary
Skin
Fat
Mammary gland
Limb muscle
Diaphragm
Bone
Bone marrow
Blood
Spleen
Heart
Aorta
Lung
Trachea
Tongue
Small intestine
Liver
Pancreas
Kidney
Bladder
Uterus
Testes

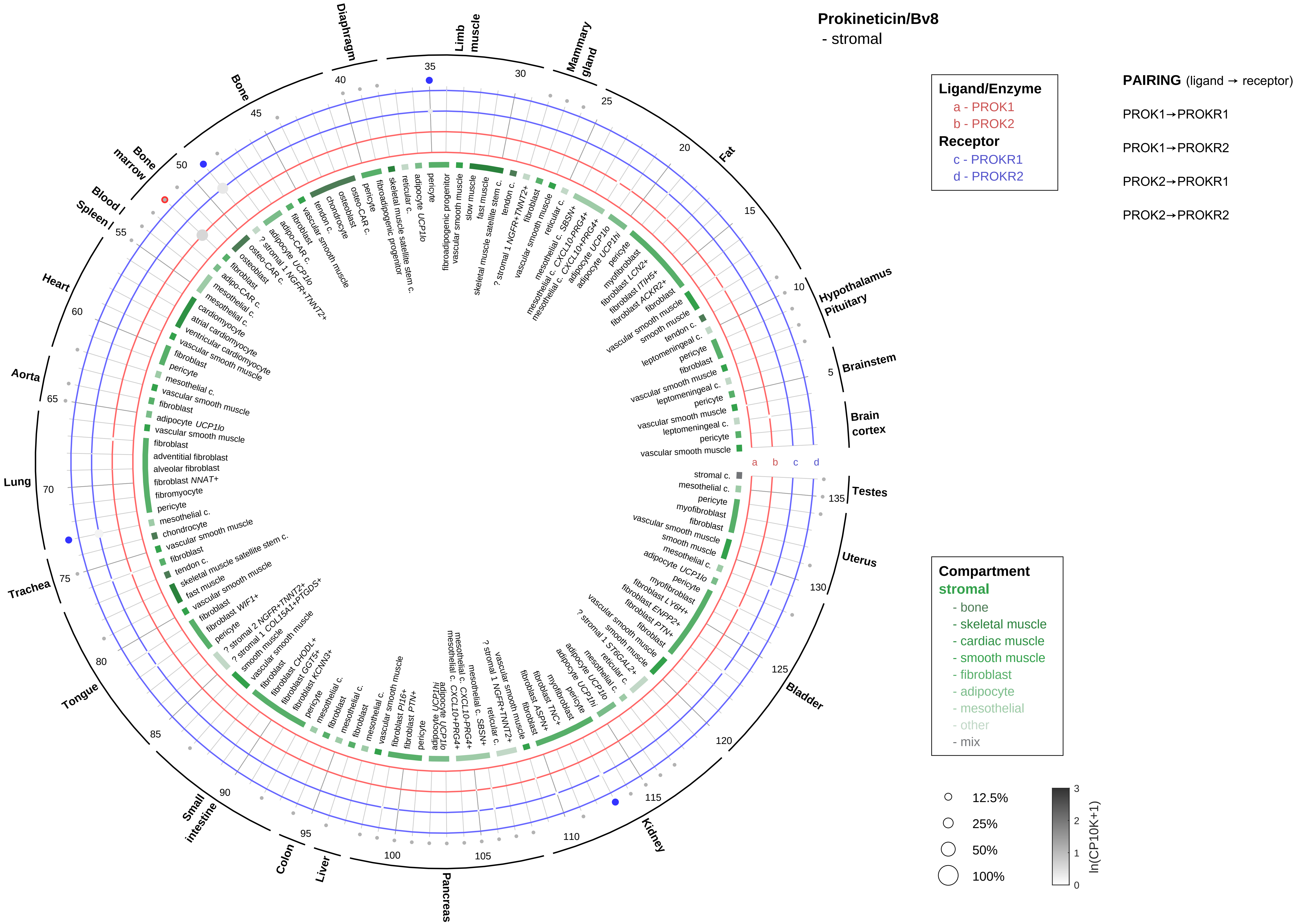

Prokineticin/Bv8
- stromal
Ligand/Enzyme
a - PROK1
b - PROK2
Receptor
c - PROKR1
d - PROKR2
PAIRING (ligand → receptor)
PROK1→PROKR1
PROK1→PROKR2
PROK2→PROKR1
PROK2→PROKR2
Compartment
stromal
- bone
- skeletal muscle
- cardiac muscle
- smooth muscle
- fibroblast
- adipocyte
- mesothelial
- other
- mix
12.5%
25%
50%
100%
ln(CP10K+1)
Hypothalamus Pituitary
Brainstem
Brain cortex
Testes
Uterus
Bladder
Kidney
Pancreas
Liver
Colon
Small intestine
Tongue
Trachea
Lung
Aorta
Heart
Spleen
Blood
Bone marrow
Bone
Diaphragm
Limb muscle
Mammary gland
Fat

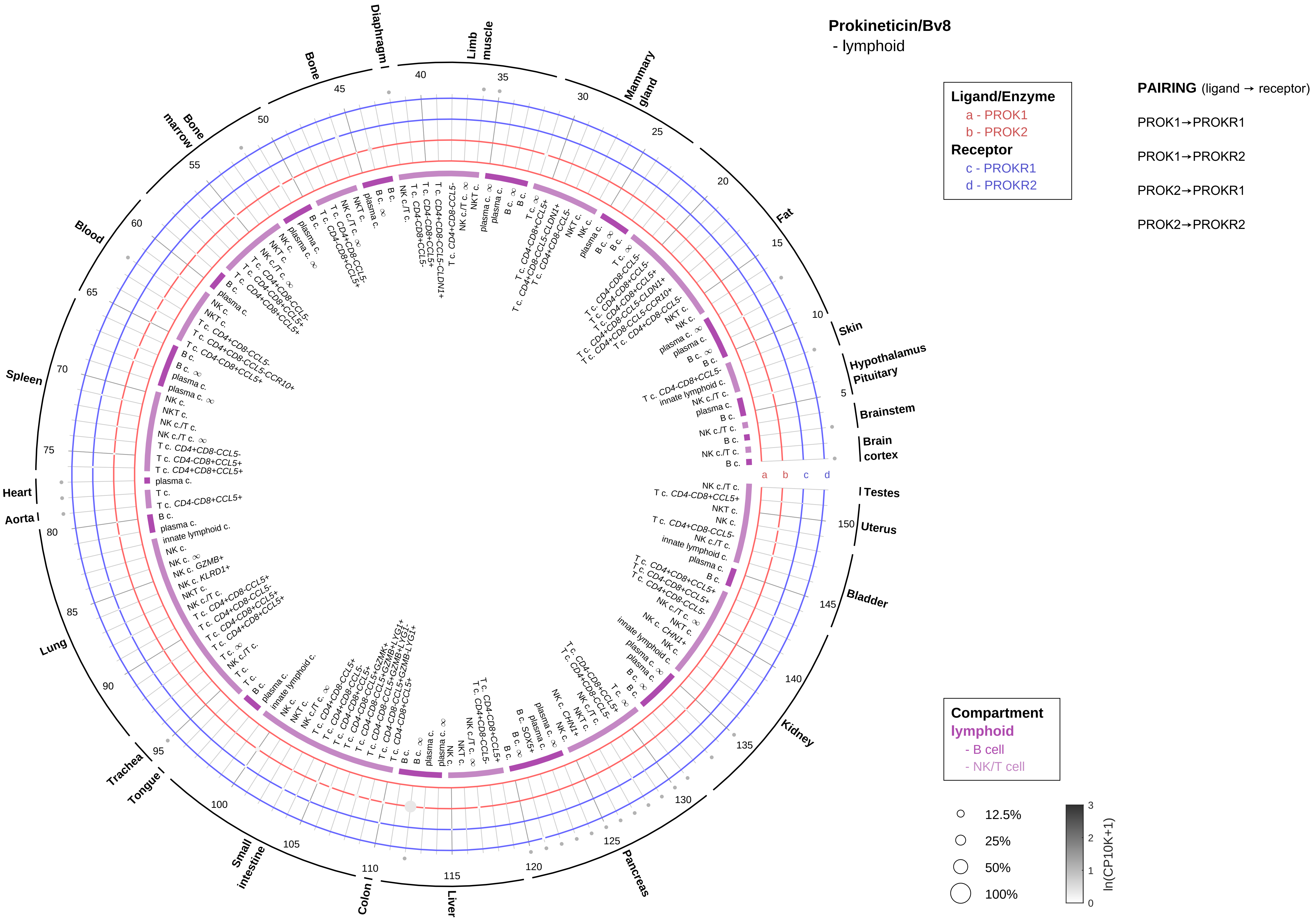

Prokineticin/Bv8
- lymphoid
Ligand/Enzyme
a - PROK1
b - PROK2
Receptor
c - PROKR1
d - PROKR2
PAIRING (ligand → receptor)
PROK1→PROKR1
PROK1→PROKR2
PROK2→PROKR1
PROK2→PROKR2
Compartment
lymphoid
- B cell
- NK/T cell
12.5%
25%
50%
100%
ln(CP10K+1)
Skin
Hypothalamus
Pituitary
Brainstem
Brain cortex
Testes
Uterus
Bladder
Kidney
Pancreas
Liver
Colon
Small intestine
Tongue
Trachea
Lung
Aorta
Heart
Spleen
Blood
Bone marrow
Bone
Diaphragm
Limb muscle
Mammary gland
Fat

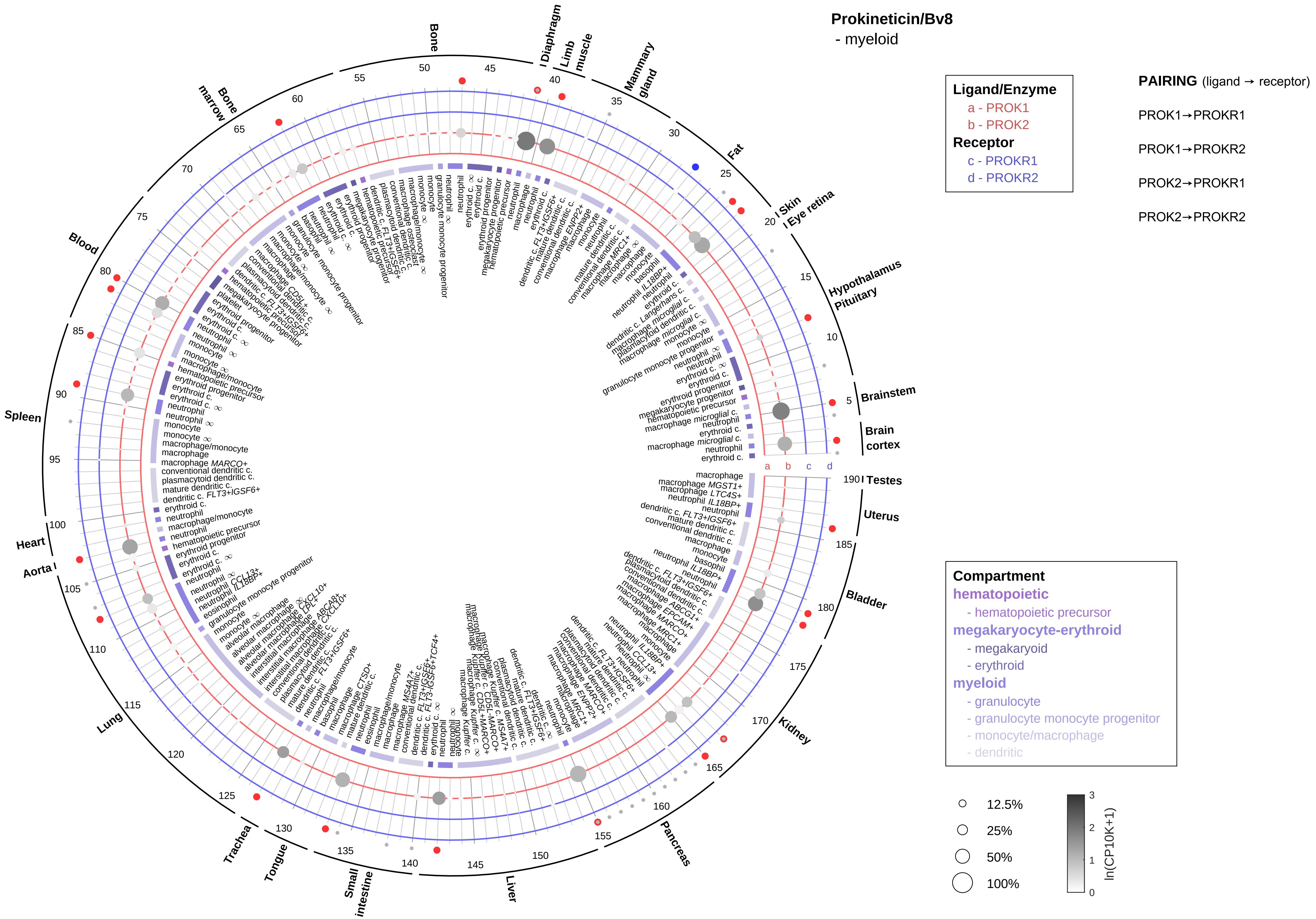

Prokineticin/Bv8
- myeloid
Ligand/Enzyme
a - PROK1
b - PROK2
Receptor
c - PROKR1
d - PROKR2
PAIRING (ligand → receptor)
PROK1→PROKR1
PROK1→PROKR2
PROK2→PROKR1
PROK2→PROKR2
Compartment
hematopoietic
- hematopoietic precursor
megakaryocyte-erythroid
- megakaryoid
- erythroid
myeloid
- granulocyte
- granulocyte monocyte progenitor
- monocyte/macrophage
- dendritic
12.5%
25%
50%
100%
ln(CP10K+1)
Brain cortex
Brainstem
Hypothalamus Pituitary
Eye retina
Skin
Fat
Mammary gland
Limb muscle
Diaphragm
Bone
Bone marrow
Blood
Spleen
Heart
Aorta
Lung
Trachea
Tongue
Small intestine
Liver
Pancreas
Kidney
Bladder
Uterus
Testes

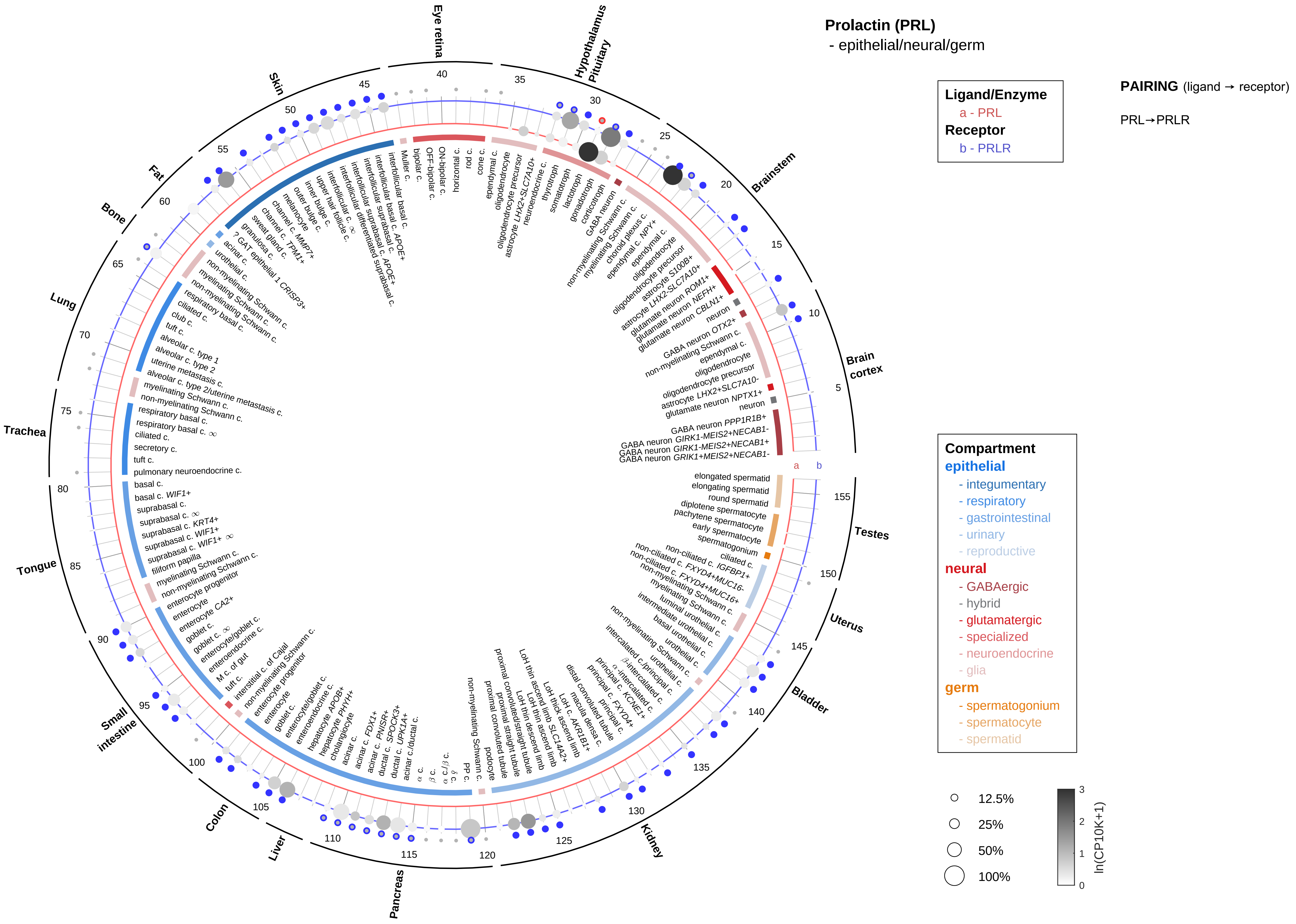
Prolactin (PRL)
- epithelial/neural/germ
Ligand/Enzyme
a - PRL
Receptor
b - PRLR
PAIRING (ligand → receptor)
PRL→PRLR
Compartment
epithelial
- integumentary
- respiratory
- gastrointestinal
- urinary
- reproductive
neural
- GABAergic
- hybrid
- glutamatergic
- specialized
- neuroendocrine
- glia
germ
- spermatogonium
- spermatocyte
- spermatid
12.5%
25%
50%
100%
ln(CP10K+1)
0
1
2
3
a
b
Brain cortex
Brainstem
Hypothalamus Pituitary
Eye retina
Skin
Fat
Bone
Lung
Trachea
Tongue
Small intestine
Colon
Liver
Pancreas
Kidney
Bladder
Uterus
Testes
5
10
15
20
25
30
35
40
45
50
55
60
65
70
75
80
85
90
95
100
105
110
115
120
125
130
135
140
145
150
155
GABA neuron GRIK1+MEIS2+NECAB1-
GABA neuron GIRK1-MEIS2+NECAB1+
GABA neuron GIRK1-MEIS2+NECAB1-
GABA neuron PPP1R1B+
neuron
glutamate neuron NPTX1+
astrocyte LHX2+SLC7A10-
oligodendrocyte precursor
oligodendrocyte
ependymal c.
non-myelinating Schwann c.
GABA neuron OTX2+
neuron
glutamate neuron CBLN1+
glutamate neuron NEFH+
glutamate neuron ROM1+
astrocyte LHX2-SLC7A10+
astrocyte S100B+
oligodendrocyte precursor
oligodendrocyte
ependymal c.
ependymal c. NPY+
choroid plexus c.
myelinating Schwann c.
non-myelinating Schwann c.
GABA neuron
corticotroph
gonadotroph
lactotroph
somatotroph
thyrotroph
neuroendocrine c.
astrocyte LHX2+SLC7A10+
oligodendrocyte precursor
oligodendrocyte
ependymal c.
cone c.
rod c.
horizontal c.
ON-bipolar c.
OFF-bipolar c.
bipolar c.
Muller c.
interfollicular basal c.
interfollicular basal c. APOE+
interfollicular suprabasal c. APOE+
interfollicular differentiated suprabasal c.
interfollicular c. ∞
upper hair follicle c.
inner bulge c.
outer bulge c.
melanocyte
channel c. MMP7+
channel c. TPM1+
sweat gland c.
granulosa c.
? GAT epithelial 1 CRISP3+
acinar c.
urothelial c.
non-myelinating Schwann c.
myelinating Schwann c.
non-myelinating Schwann c.
respiratory basal c.
ciliated c.
club c.
tuft c.
alveolar c. type 1
alveolar c. type 2
uterine metastasis c.
alveolar c. type 2/uterine metastasis c.
myelinating Schwann c.
non-myelinating Schwann c.
respiratory basal c.
respiratory basal c. ∞
ciliated c.
secretory c.
tuft c.
pulmonary neuroendocrine c.
basal c.
basal c. WIF1+
suprabasal c.
suprabasal c. ∞
suprabasal c. KRT4+
suprabasal c. WIF1+
suprabasal c. WIF1+ ∞
filiform papilla
myelinating Schwann c.
non-myelinating Schwann c.
enterocyte progenitor
enterocyte
enterocyte CA2+
goblet c.
goblet c. ∞
enterocyte/goblet c.
enteroendocrine c.
M c. of gut
tuft c.
interstitial c. of Cajal
non-myelinating Schwann c.
enterocyte progenitor
enterocyte
goblet c.
enterocyte/goblet c.
enteroendocrine c.
hepatocyte APOB+
hepatocyte PHYH+
cholangiocyte
acinar c.
acinar c. FDX1+
acinar c. PNISR+
ductal c. SPOCK3+
ductal c. UPK1A+
acinar c./ductal c.
α c.
β c.
α c./β c.
δ c.
PP c.
non-myelinating Schwann c.
podocyte
proximal convoluted tubule
proximal convoluted/straight tubule
proximal straight tubule
LoH thin descend limb
LoH thin descend limb
LoH thin ascend limb
LoH thick ascend limb SLC14A2+
LoH c. AKR1B1+
macula densa c.
distal convoluted tubule
principal c.
principal c. FXYD4+
principal c. KCNE1+
α-intercalated c.
β-intercalated c.
intercalated c./principal c.
urothelial c.
non-myelinating Schwann c.
urothelial c.
basal urothelial c.
intermediate urothelial c.
luminal urothelial c.
myelinating Schwann c.
non-myelinating Schwann c.
non-ciliated c. FXYD4+MUC16+
non-ciliated c. FXYD4+MUC16-
non-ciliated c. IGFBP1+
ciliated c.
spermatogonium
early spermatocyte
pachytene spermatocyte
diplotene spermatocyte
round spermatid
elongating spermatid
elongated spermatid

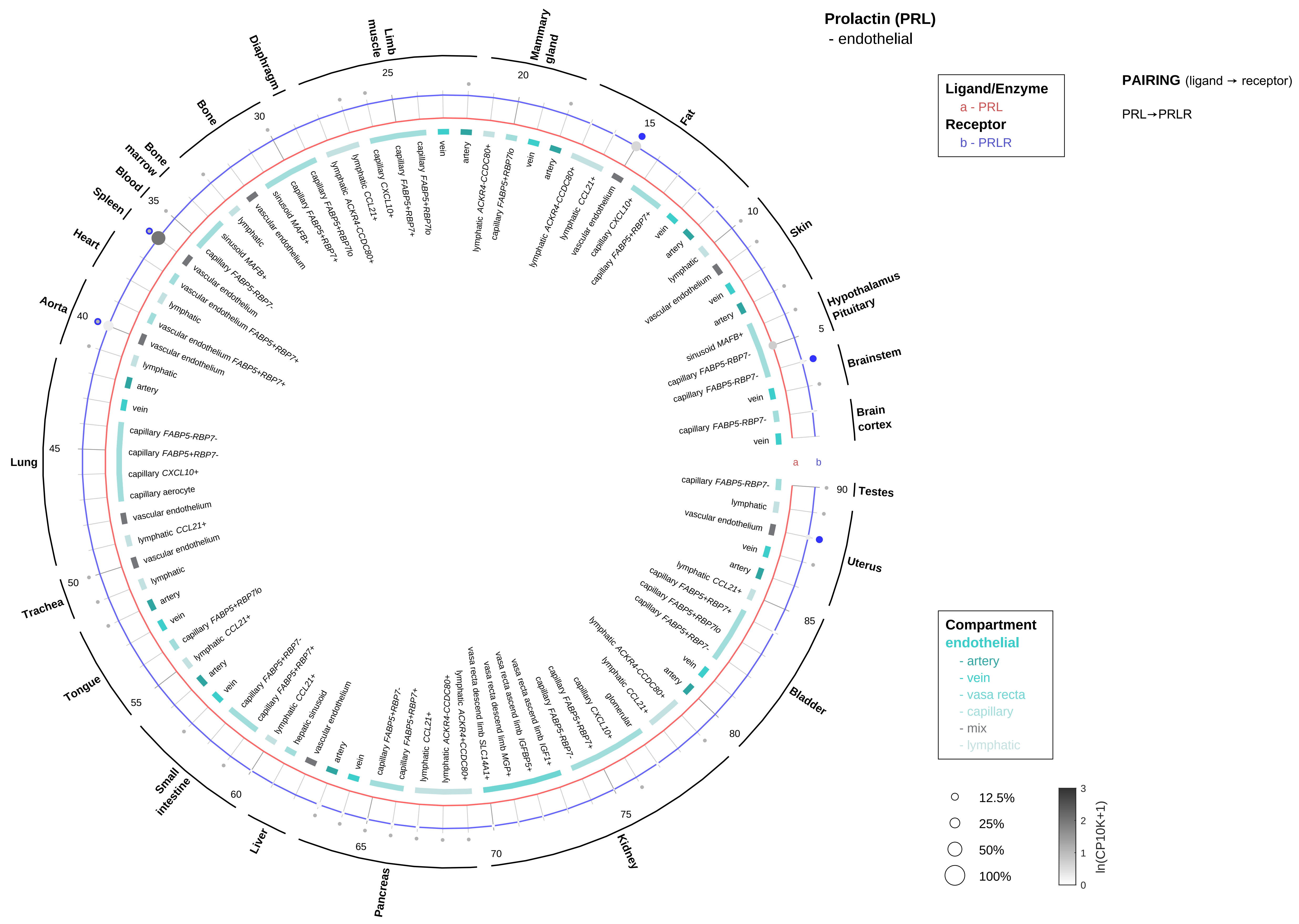
Prolactin (PRL)
- endothelial
Ligand/Enzyme
a - PRL
Receptor
b - PRLR
PAIRING (ligand → receptor)
PRL→PRLR
Compartment
endothelial
- artery
- vein
- vasa recta
- capillary
- mix
- lymphatic
12.5%
25%
50%
100%
ln(CP10K+1)
Brain cortex
Brainstem
Hypothalamus Pituitary
Skin
Fat
Mammary gland
Limb muscle
Diaphragm
Bone
Bone marrow
Blood
Spleen
Heart
Aorta
Lung
Trachea
Tongue
Small intestine
Liver
Pancreas
Kidney
Bladder
Uterus
Testes

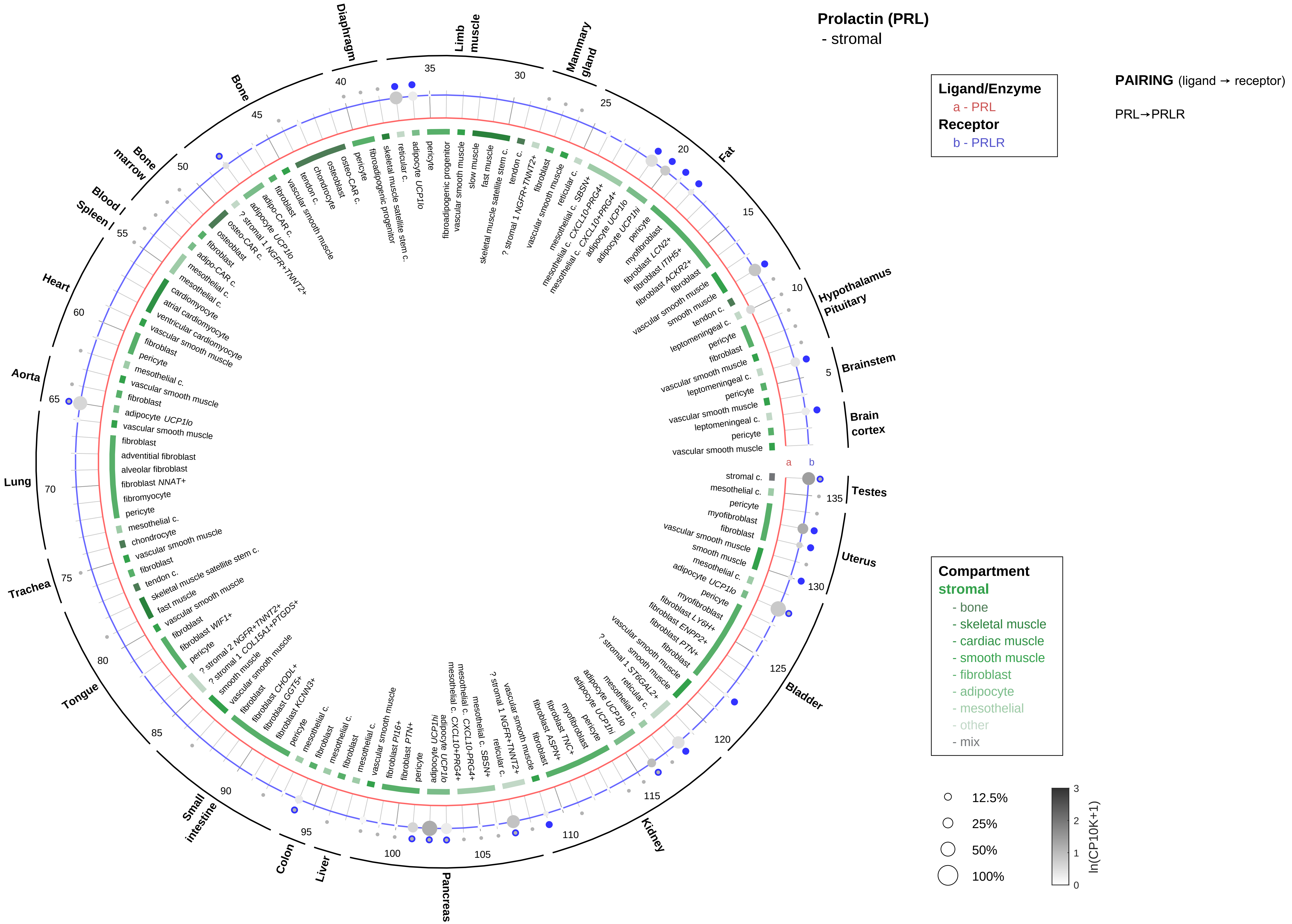

Prolactin (PRL)
- stromal
Ligand/Enzyme
a - PRL
Receptor
b - PRLR
PAIRING (ligand → receptor)
PRL→PRLR
Compartment
stromal
- bone
- skeletal muscle
- cardiac muscle
- smooth muscle
- fibroblast
- adipocyte
- mesothelial
- other
- mix
12.5%
25%
50%
100%
ln(CP10K+1)
Brain cortex
Brainstem
Hypothalamus Pituitary
Fat
Mammary gland
Limb muscle
Diaphragm
Bone
Bone marrow
Blood
Spleen
Heart
Aorta
Lung
Trachea
Tongue
Small intestine
Colon
Liver
Pancreas
Kidney
Bladder
Uterus
Testes

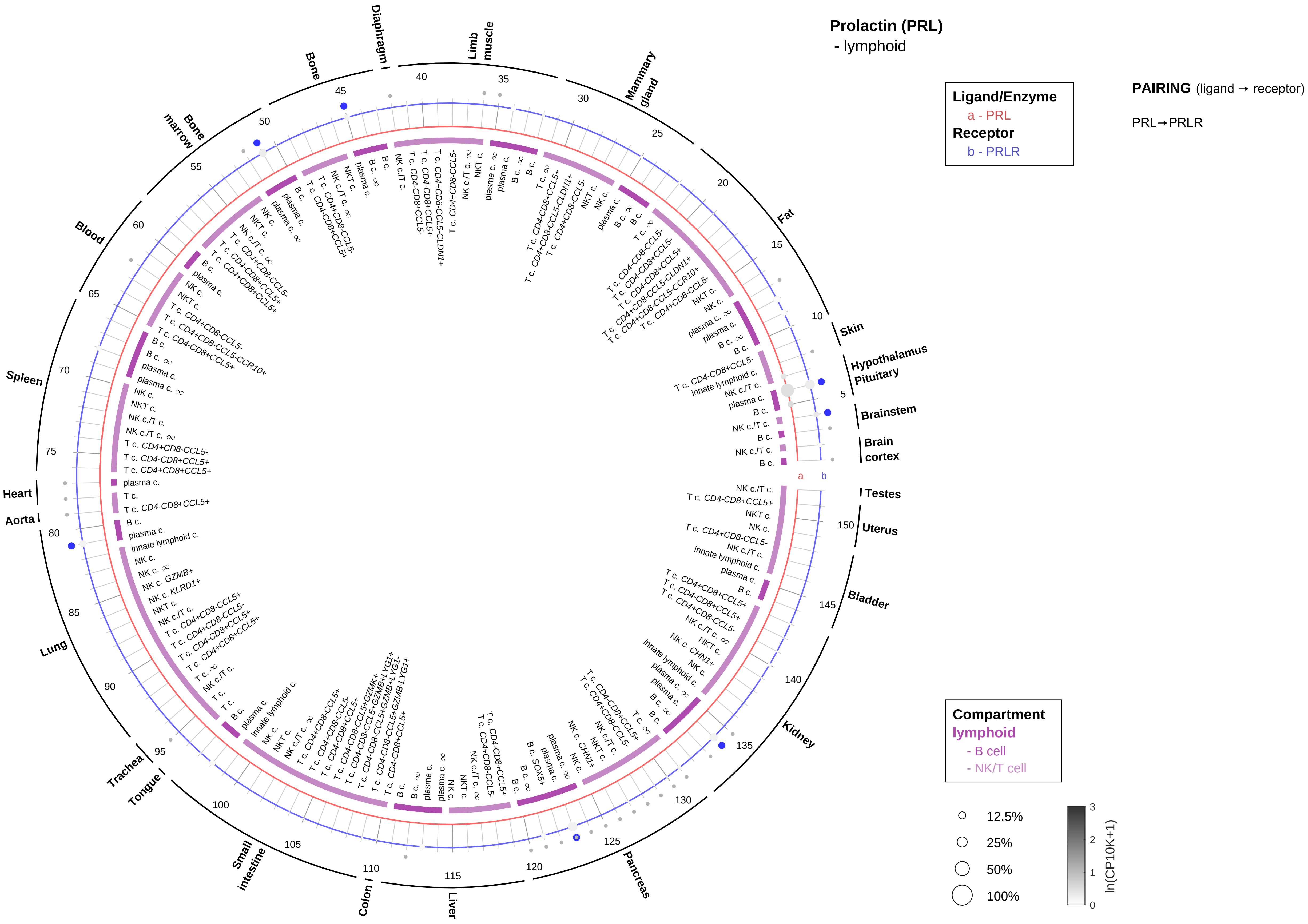

Prolactin (PRL)
- lymphoid
Ligand/Enzyme
a - PRL
Receptor
b - PRLR
PAIRING (ligand → receptor)
PRL→PRLR
Compartment
lymphoid
- B cell
- NK/T cell
12.5%
25%
50%
100%
ln(CP10K+1)
Skin
Hypothalamus
Pituitary
Brainstem
Brain cortex
Testes
Uterus
Bladder
Kidney
Pancreas
Liver
Colon
Small intestine
Tongue
Trachea
Lung
Aorta
Heart
Spleen
Blood
Bone marrow
Bone
Diaphragm
Limb muscle
Mammary gland
Fat

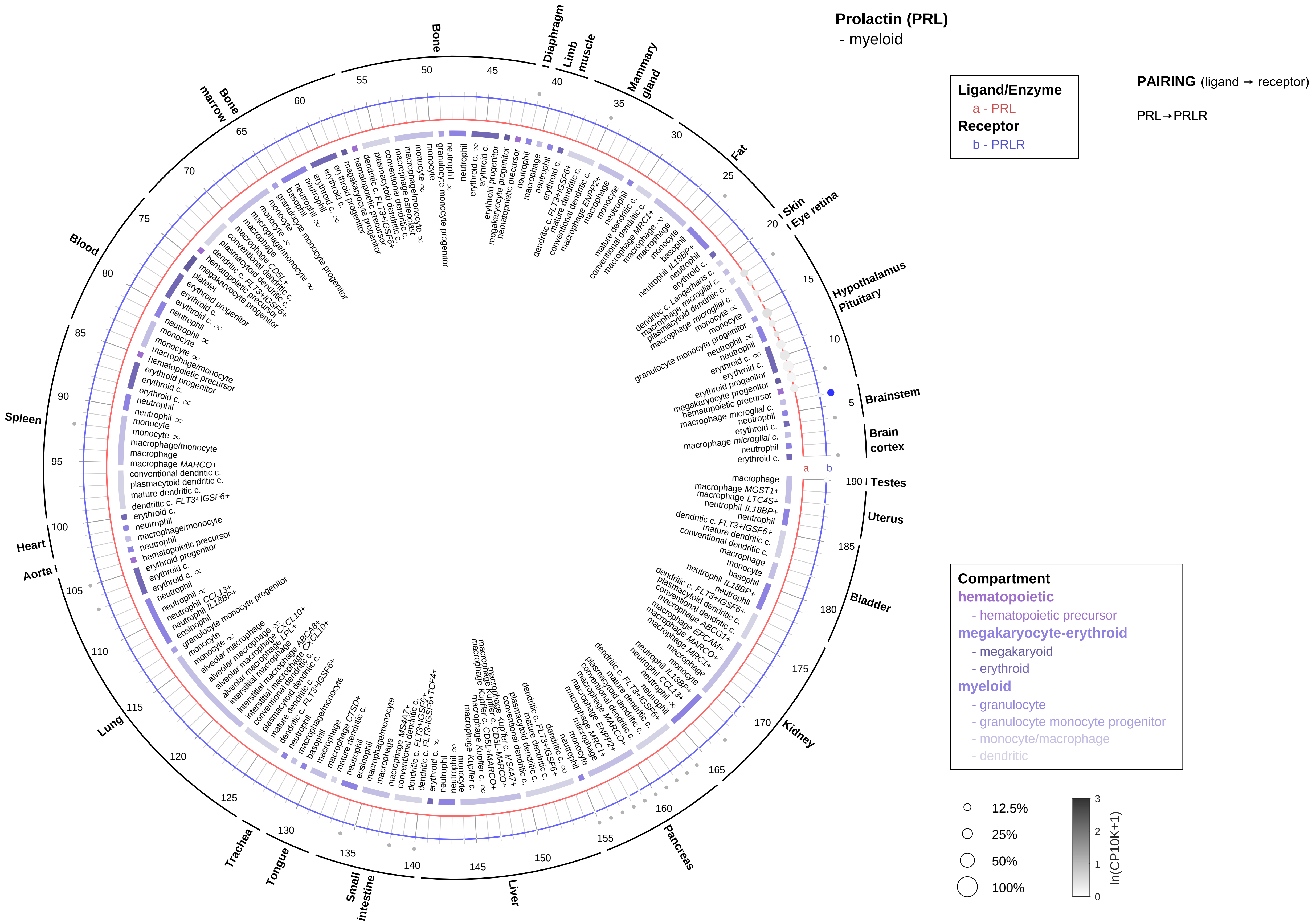
Prolactin (PRL)
- myeloid
Ligand/Enzyme
a - PRL
Receptor
b - PRLR
PAIRING (ligand → receptor)
PRL→PRLR
Compartment
hematopoietic
- hematopoietic precursor
megakaryocyte-erythroid
- megakaryoid
- erythroid
myeloid
- granulocyte
- granulocyte monocyte progenitor
- monocyte/macrophage
- dendritic
12.5%
25%
50%
100%
ln(CP10K+1)
Bone
Diaphragm
Limb muscle
Mammary gland
Fat
Skin
Eye retina
Hypothalamus
Pituitary
Brainstem
Brain cortex
Testes
Uterus
Bladder
Kidney
Pancreas
Liver
Small intestine
Tongue
Trachea
Lung
Aorta
Heart
Spleen
Blood
Bone marrow

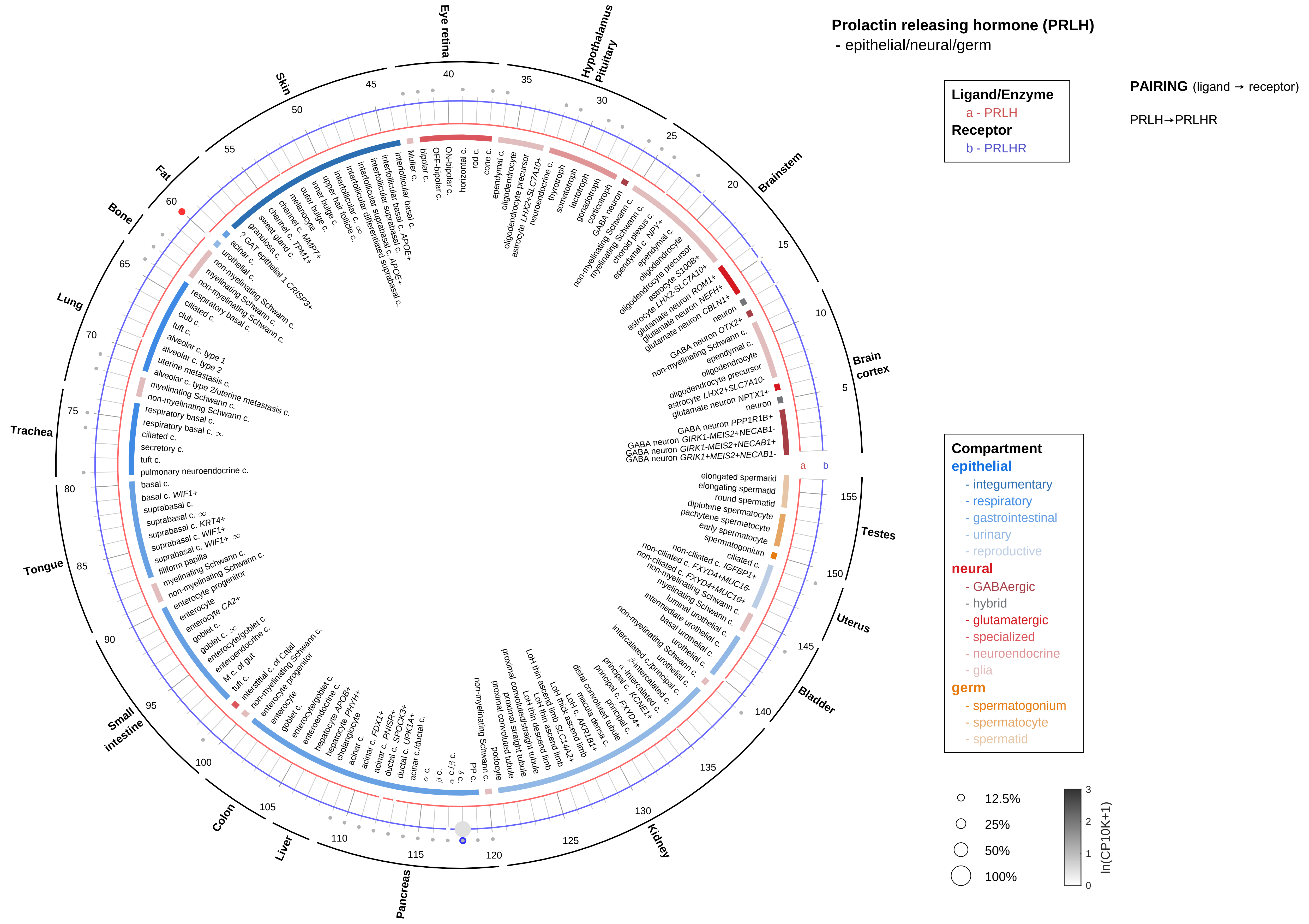

Prolactin releasing hormone (PRLH)
- epithelial/neural/germ
Ligand/Enzyme
a - PRLH
Receptor
b - PRLHR
PAIRING (ligand → receptor)
PRLH→PRLHR
Compartment
epithelial
- integumentary
- respiratory
- gastrointestinal
- urinary
- reproductive
neural
- GABAergic
- hybrid
- glutamatergic
- specialized
- neuroendocrine
- glia
germ
- spermatogonium
- spermatocyte
- spermatid
12.5%
25%
50%
100%
ln(CP10K+1)
Eye retina
Hypothalamus Pituitary
Brainstem
Brain cortex
Testes
Uterus
Bladder
Kidney
Pancreas
Liver
Colon
Small intestine
Tongue
Trachea
Lung
Bone
Fat
Skin

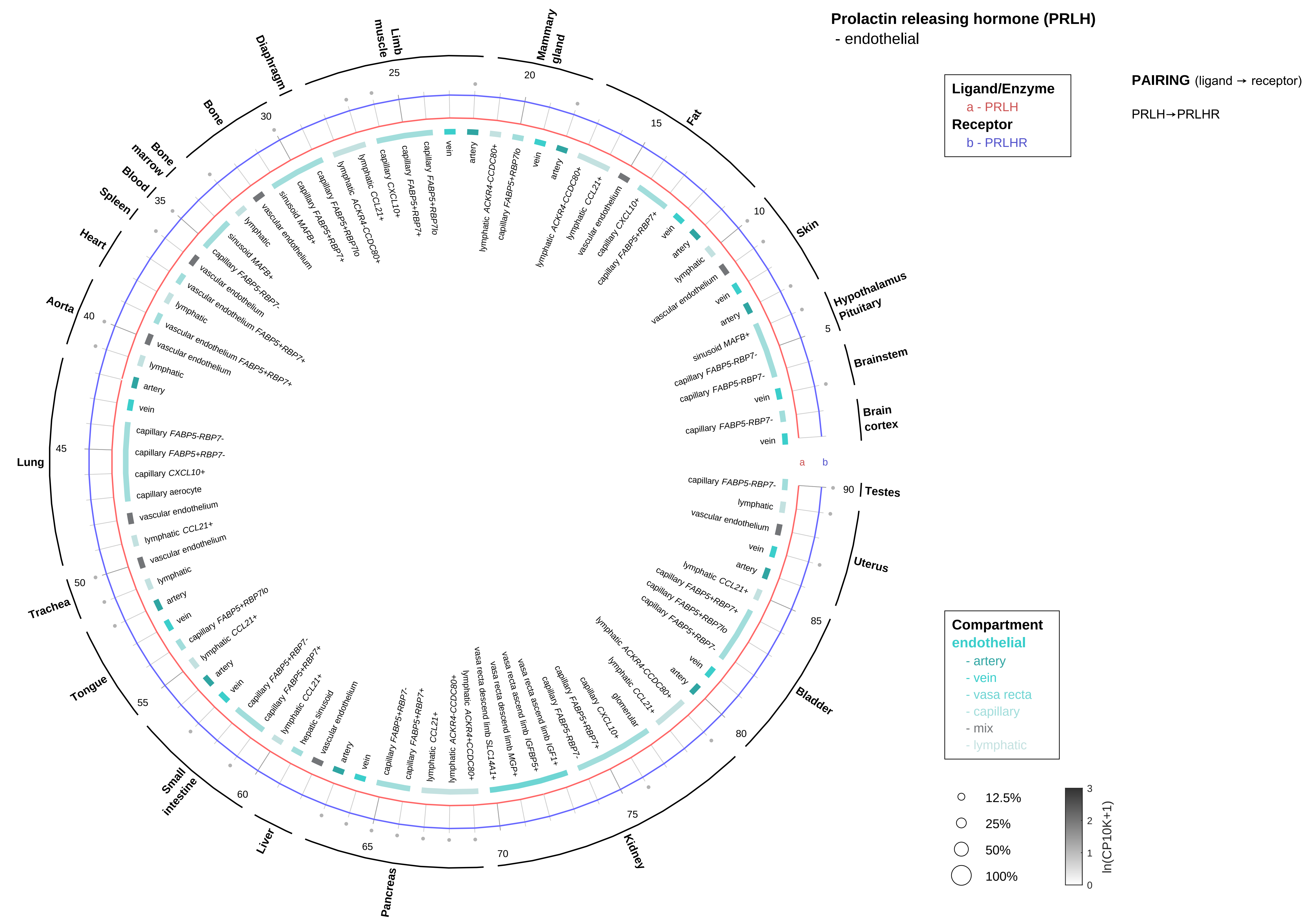
Prolactin releasing hormone (PRLH)
- endothelial
Ligand/Enzyme
a - PRLH
Receptor
b - PRLHR
PAIRING (ligand → receptor)
PRLH→PRLHR
Compartment
endothelial
- artery
- vein
- vasa recta
- capillary
- mix
- lymphatic
12.5%
25%
50%
100%
ln(CP10K+1)
Brain cortex
Brainstem
Hypothalamus Pituitary
Skin
Fat
Mammary gland
Limb muscle
Diaphragm
Bone
Bone marrow
Blood
Spleen
Heart
Aorta
Lung
Trachea
Tongue
Small intestine
Liver
Pancreas
Kidney
Bladder
Uterus
Testes

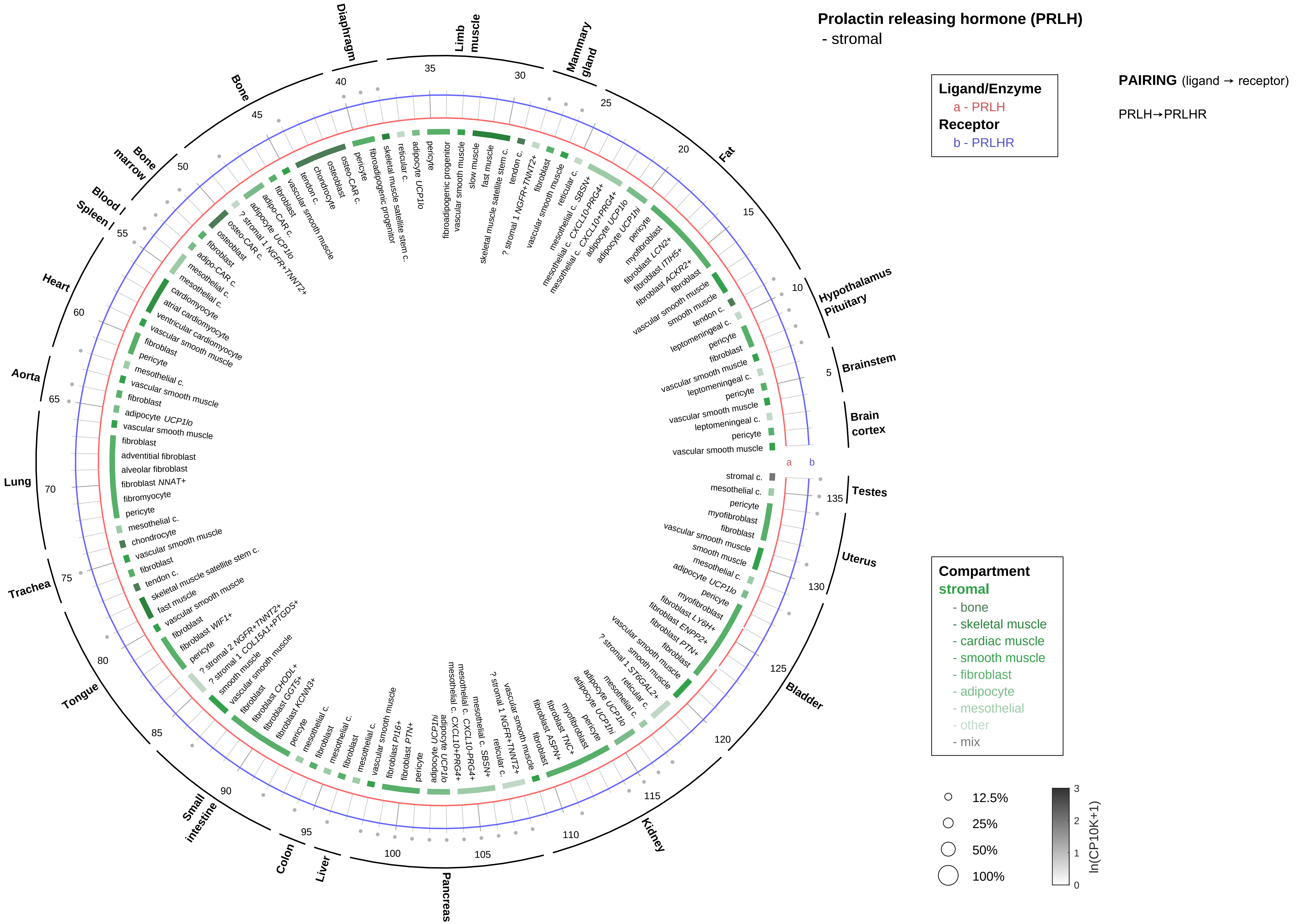

Prolactin releasing hormone (PRLH)
- stromal
PAIRING (ligand → receptor)
PRLH→PRLHR
Ligand/Enzyme
a - PRLH
Receptor
b - PRLHR
Compartment
stromal
- bone
- skeletal muscle
- cardiac muscle
- smooth muscle
- fibroblast
- adipocyte
- mesothelial
- other
- mix
12.5%
25%
50%
100%
ln(CP10K+1)
Testes
Uterus
Bladder
Kidney
Pancreas
Liver
Colon
Small intestine
Tongue
Trachea
Lung
Aorta
Heart
Spleen
Blood
Bone marrow
Bone
Diaphragm
Limb muscle
Mammary gland
Fat
Hypothalamus
Pituitary
Brainstem
Brain cortex

Prolactin releasing hormone (PRLH)
- lymphoid

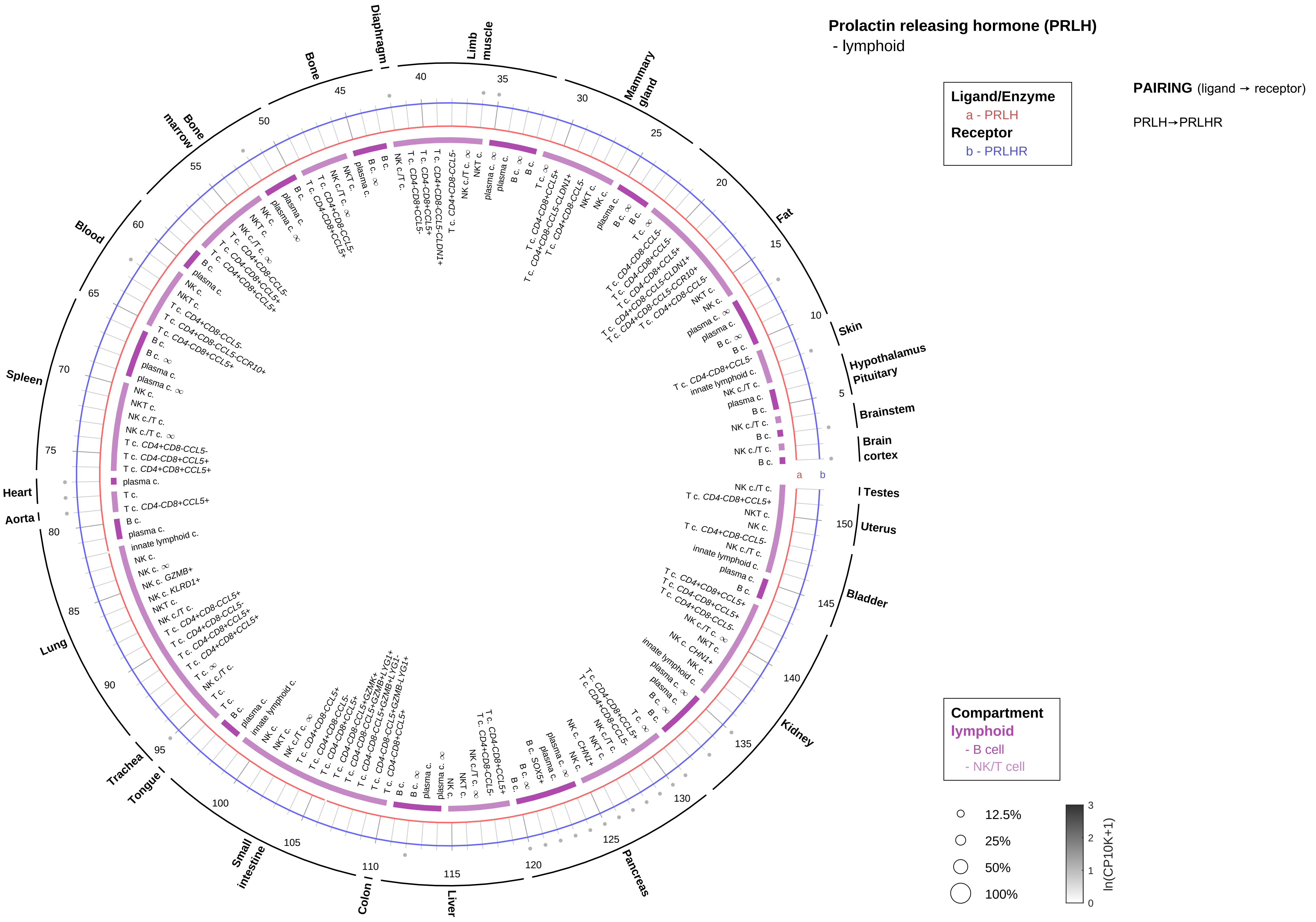

Prolactin releasing hormone (PRLH)
- myeloid

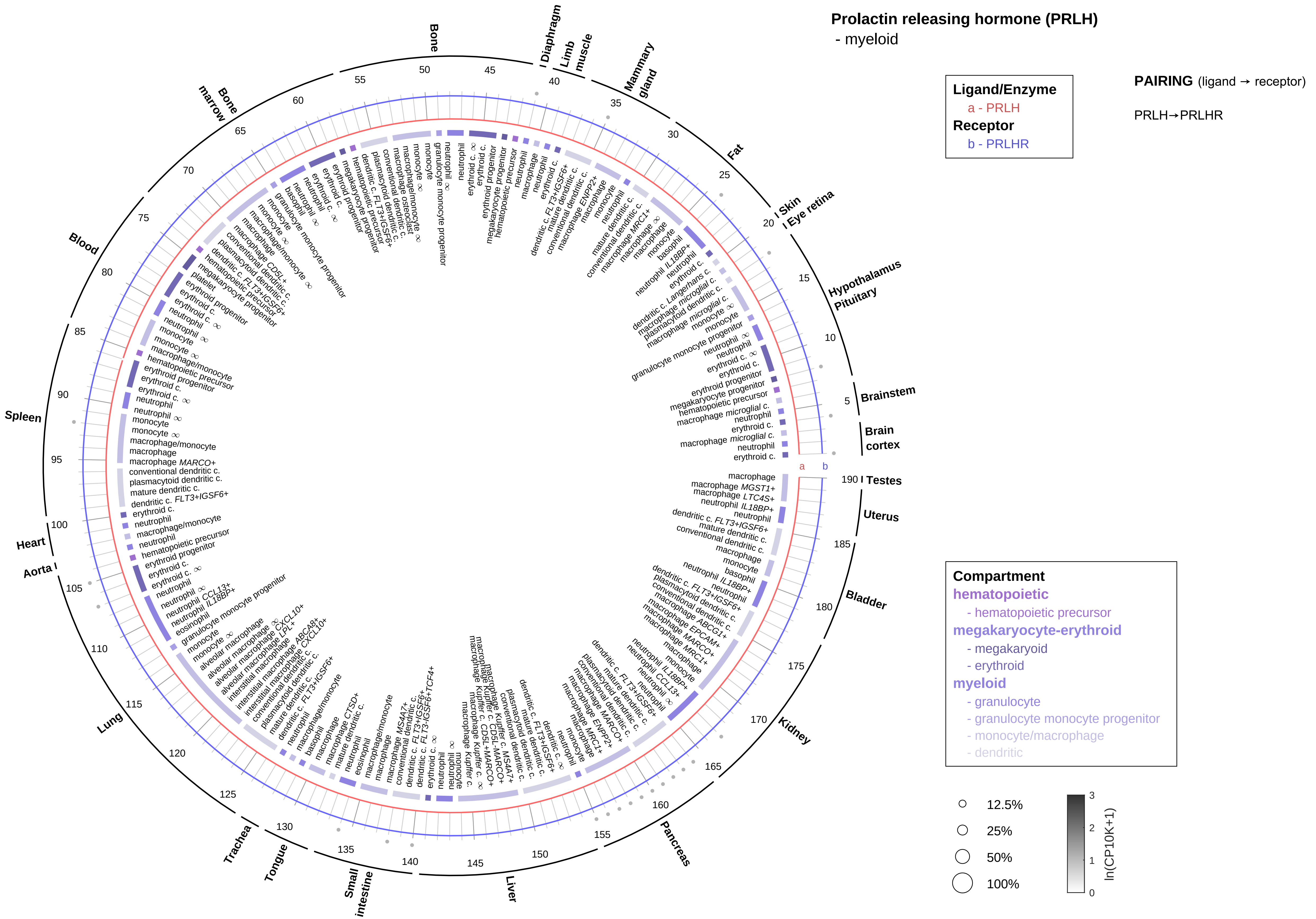

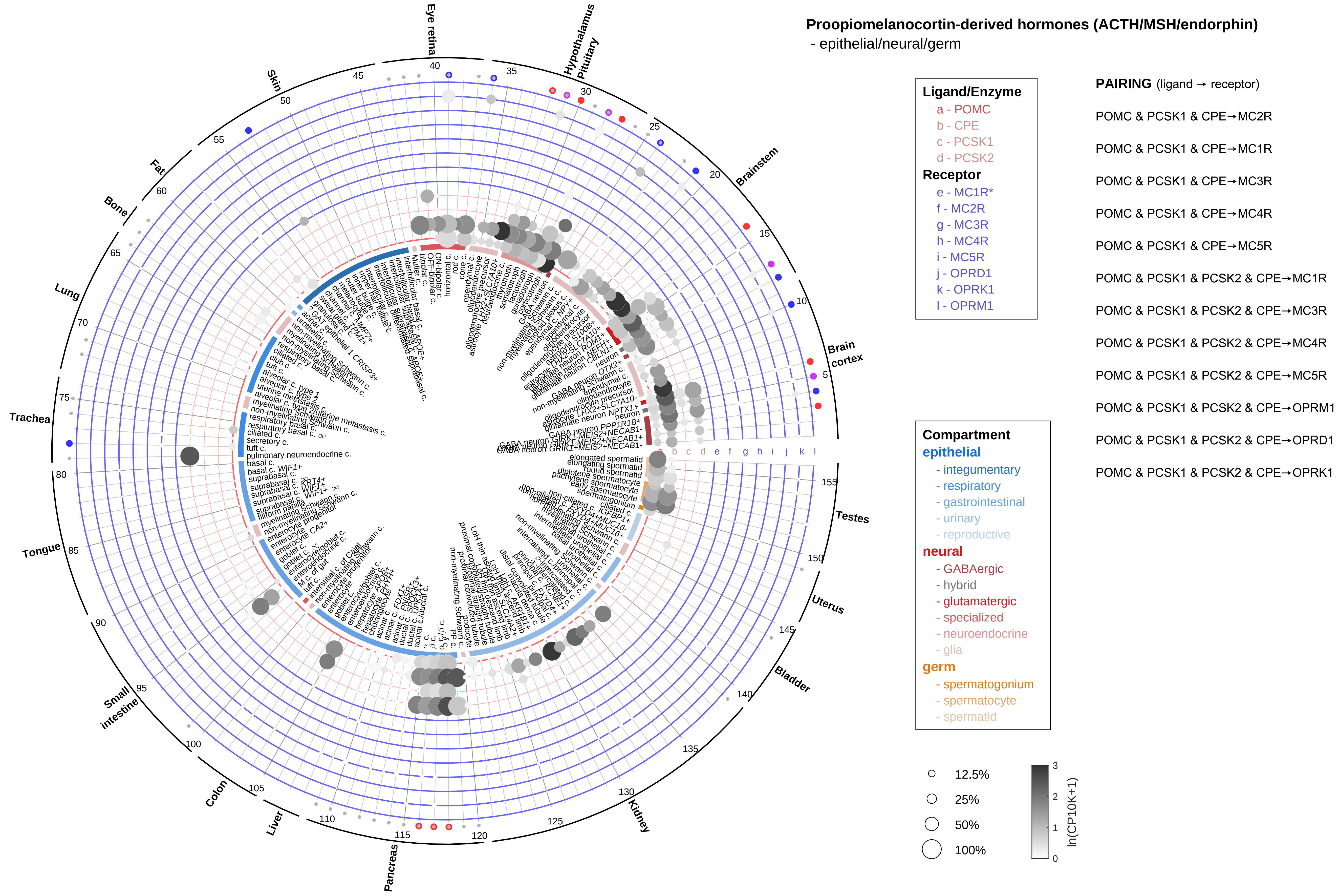

Proopiomelanocortin-derived hormones (ACTH/MSH/endorphin)
- epithelial/neural/germ
Ligand/Enzyme
a - POMC
b - CPE
c - PCSK1
d - PCSK2
Receptor
e - MC1R*
f - MC2R
g - MC3R
h - MC4R
i - MC5R
j - OPRD1
k - OPRK1
l - OPRM1
PAIRING (ligand → receptor)
POMC & PCSK1 & CPE→MC2R
POMC & PCSK1 & CPE→MC1R
POMC & PCSK1 & CPE→MC3R
POMC & PCSK1 & CPE→MC4R
POMC & PCSK1 & CPE→MC5R
POMC & PCSK1 & PCSK2 & CPE→MC1R
POMC & PCSK1 & PCSK2 & CPE→MC3R
POMC & PCSK1 & PCSK2 & CPE→MC4R
POMC & PCSK1 & PCSK2 & CPE→MC5R
POMC & PCSK1 & PCSK2 & CPE→OPRM1
POMC & PCSK1 & PCSK2 & CPE→OPRD1
POMC & PCSK1 & PCSK2 & CPE→OPRK1
Compartment
epithelial
- integumentary
- respiratory
- gastrointestinal
- urinary
- reproductive
neural
- GABAergic
- hybrid
- glutamatergic
- specialized
- neuroendocrine
- glia
germ
- spermatogonium
- spermatocyte
- spermatid
12.5%
25%
50%
100%
ln(CP10K+1)
Eye retina
Hypothalamus Pituitary
Brainstem
Brain cortex
Testes
Uterus
Bladder
Kidney
Pancreas
Liver
Colon
Small intestine
Tongue
Trachea
Lung
Bone
Fat
Skin

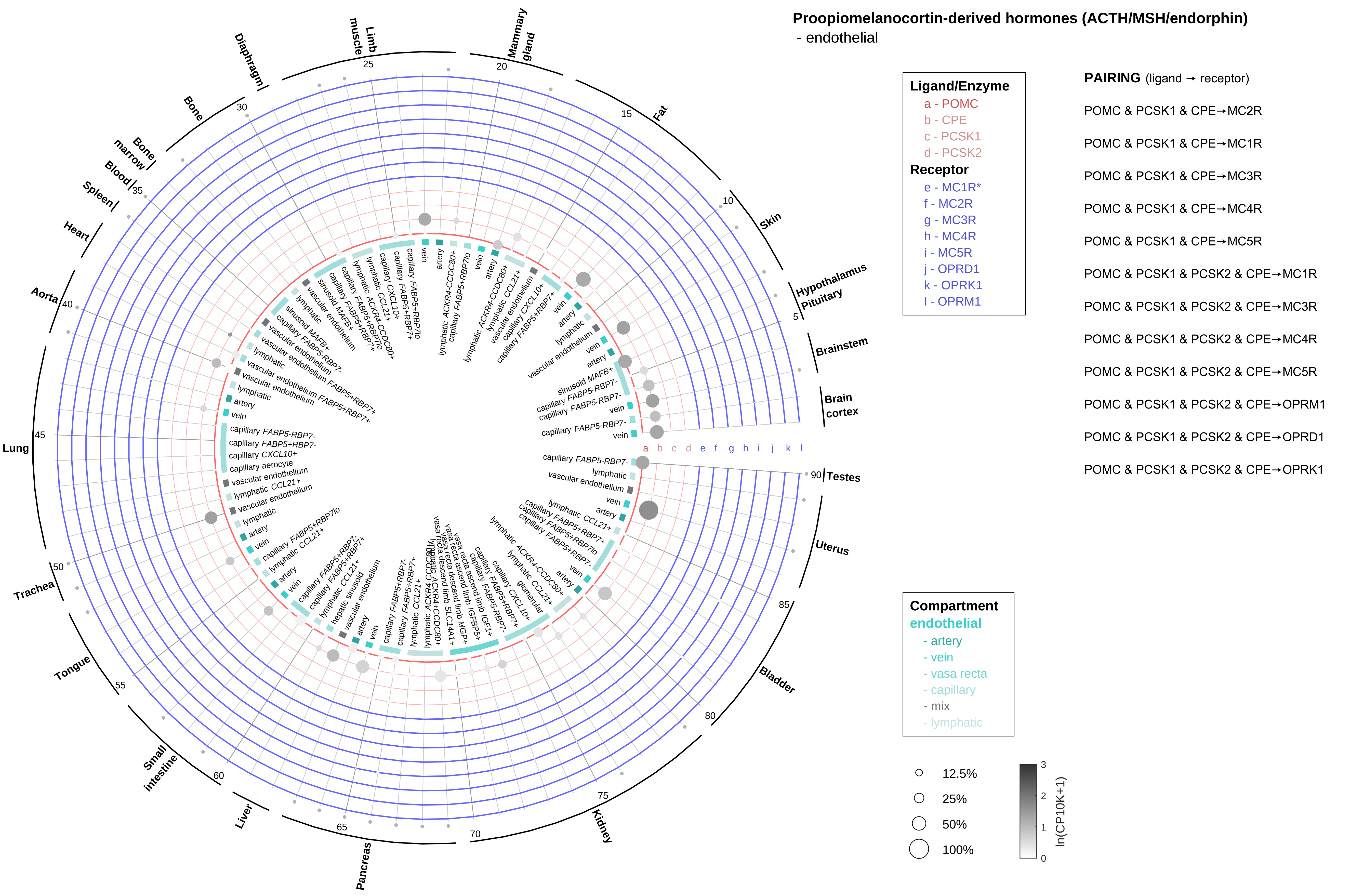

Proopiomelanocortin-derived hormones (ACTH/MSH/endorphin)
- endothelial
Ligand/Enzyme
a - POMC
b - CPE
c - PCSK1
d - PCSK2
Receptor
e - MC1R*
f - MC2R
g - MC3R
h - MC4R
i - MC5R
j - OPRD1
k - OPRK1
l - OPRM1
PAIRING (ligand → receptor)
POMC & PCSK1 & CPE→MC2R
POMC & PCSK1 & CPE→MC1R
POMC & PCSK1 & CPE→MC3R
POMC & PCSK1 & CPE→MC4R
POMC & PCSK1 & CPE→MC5R
POMC & PCSK1 & PCSK2 & CPE→MC1R
POMC & PCSK1 & PCSK2 & CPE→MC3R
POMC & PCSK1 & PCSK2 & CPE→MC4R
POMC & PCSK1 & PCSK2 & CPE→MC5R
POMC & PCSK1 & PCSK2 & CPE→OPRM1
POMC & PCSK1 & PCSK2 & CPE→OPRD1
POMC & PCSK1 & PCSK2 & CPE→OPRK1
Compartment
endothelial
- artery
- vein
- vasa recta
- capillary
- mix
- lymphatic
12.5%
25%
50%
100%
ln(CP10K+1)
Brain cortex
Brainstem
Hypothalamus
Pituitary
Skin
Fat
Mammary gland
Limb muscle
Diaphragm
Bone
Bone marrow
Blood
Spleen
Heart
Aorta
Lung
Trachea
Tongue
Small intestine
Liver
Pancreas
Kidney
Bladder
Uterus
Testes

Proopiomelanocortin-derived hormones (ACTH/MSH/endorphin)
- stromal

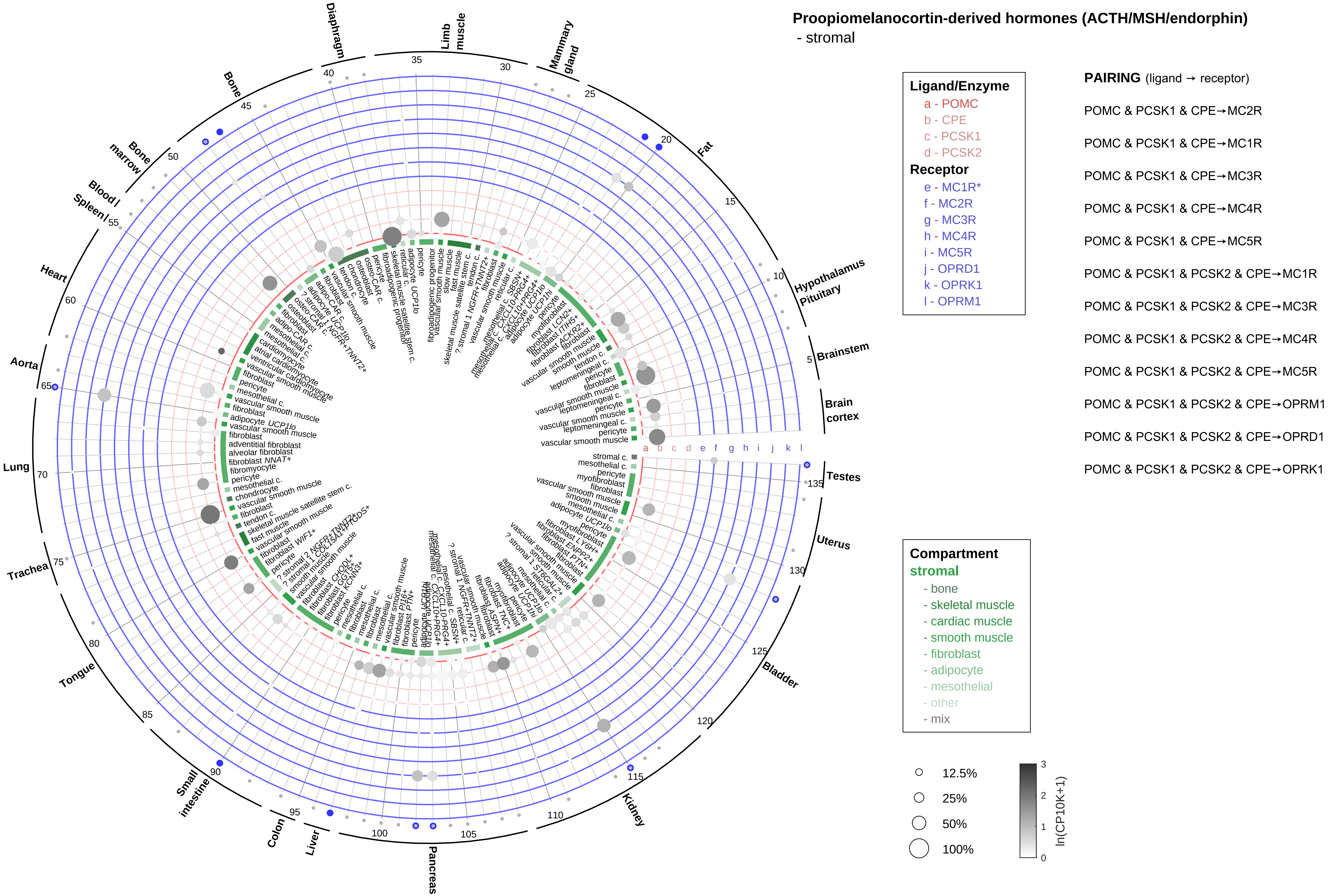

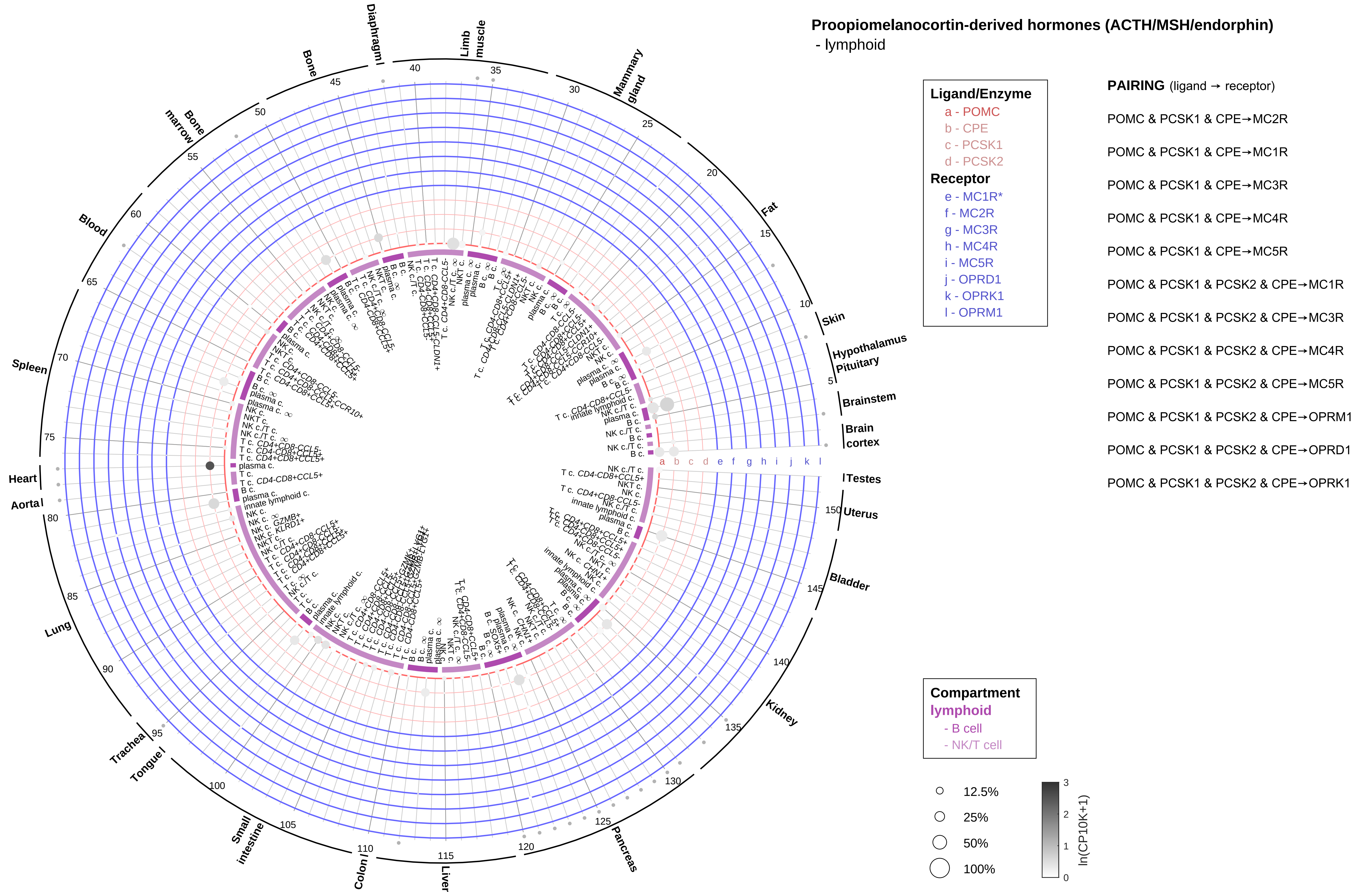

Proopiomelanocortin-derived hormones (ACTH/MSH/endorphin)
- lymphoid
Ligand/Enzyme
a - POMC
b - CPE
c - PCSK1
d - PCSK2
Receptor
e - MC1R*
f - MC2R
g - MC3R
h - MC4R
i - MC5R
j - OPRD1
k - OPRK1
l - OPRM1
PAIRING (ligand → receptor)
POMC & PCSK1 & CPE→MC2R
POMC & PCSK1 & CPE→MC1R
POMC & PCSK1 & CPE→MC3R
POMC & PCSK1 & CPE→MC4R
POMC & PCSK1 & CPE→MC5R
POMC & PCSK1 & PCSK2 & CPE→MC1R
POMC & PCSK1 & PCSK2 & CPE→MC3R
POMC & PCSK1 & PCSK2 & CPE→MC4R
POMC & PCSK1 & PCSK2 & CPE→MC5R
POMC & PCSK1 & PCSK2 & CPE→OPRM1
POMC & PCSK1 & PCSK2 & CPE→OPRD1
POMC & PCSK1 & PCSK2 & CPE→OPRK1
Compartment
lymphoid
- B cell
- NK/T cell
12.5%
25%
50%
100%
ln(CP10K+1)
Skin
Hypothalamus
Pituitary
Brainstem
Brain cortex
Testes
Uterus
Bladder
Kidney
Pancreas
Liver
Colon
Small intestine
Tongue
Trachea
Lung
Aorta
Heart
Spleen
Blood
Bone marrow
Bone
Diaphragm
Limb muscle
Mammary gland
Fat

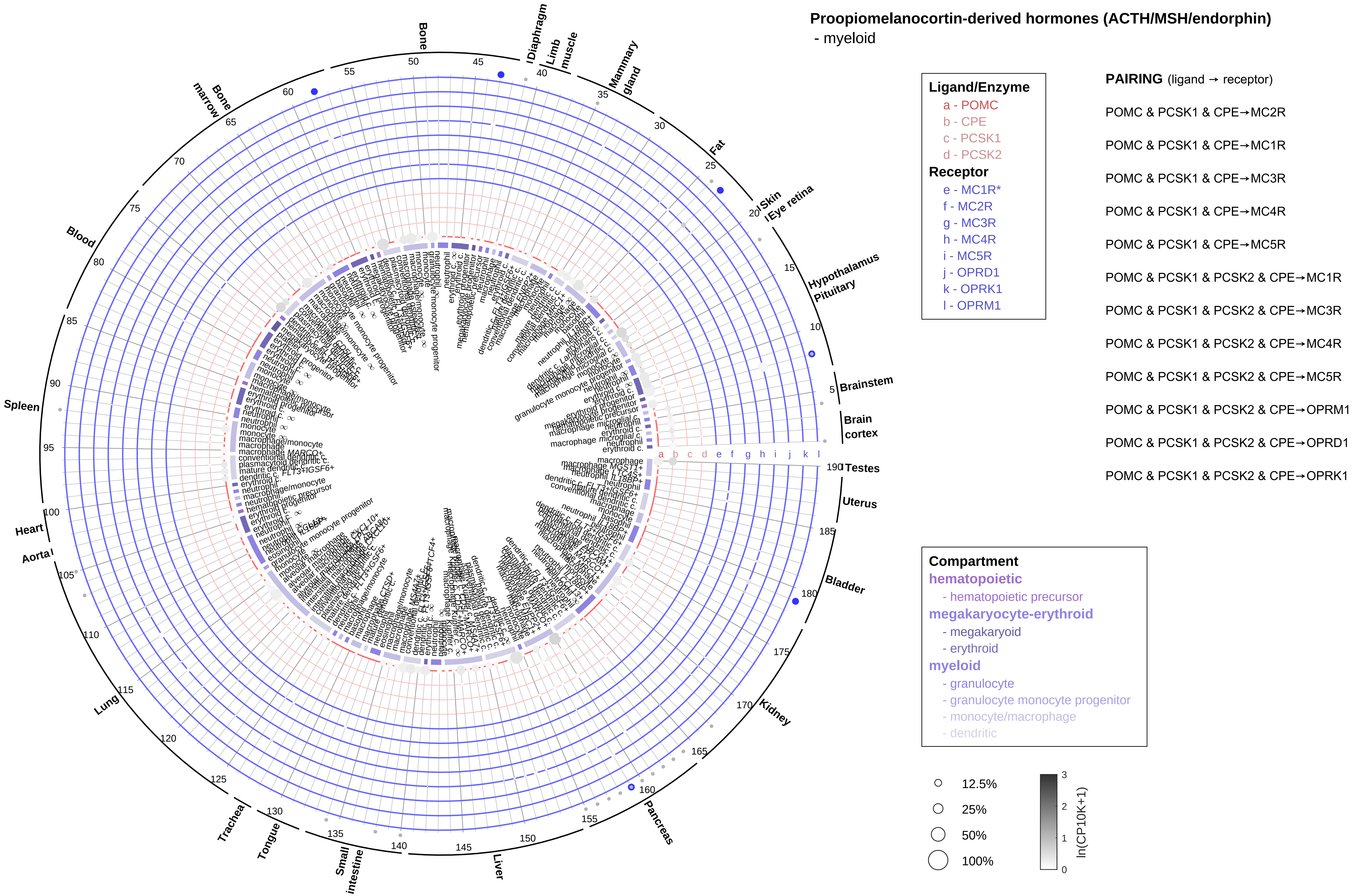

Proopiomelanocortin-derived hormones (ACTH/MSH/endorphin)
- myeloid
Ligand/Enzyme
a - POMC
b - CPE
c - PCSK1
d - PCSK2
Receptor
e - MC1R*
f - MC2R
g - MC3R
h - MC4R
i - MC5R
j - OPRD1
k - OPRK1
l - OPRM1
PAIRING (ligand → receptor)
POMC & PCSK1 & CPE→MC2R
POMC & PCSK1 & CPE→MC1R
POMC & PCSK1 & CPE→MC3R
POMC & PCSK1 & CPE→MC4R
POMC & PCSK1 & CPE→MC5R
POMC & PCSK1 & PCSK2 & CPE→MC1R
POMC & PCSK1 & PCSK2 & CPE→MC3R
POMC & PCSK1 & PCSK2 & CPE→MC4R
POMC & PCSK1 & PCSK2 & CPE→MC5R
POMC & PCSK1 & PCSK2 & CPE→OPRM1
POMC & PCSK1 & PCSK2 & CPE→OPRD1
POMC & PCSK1 & PCSK2 & CPE→OPRK1
Compartment
hematopoietic
- hematopoietic precursor
megakaryocyte-erythroid
- megakaryoid
- erythroid
myeloid
- granulocyte
- granulocyte monocyte progenitor
- monocyte/macrophage
- dendritic
12.5%
25%
50%
100%
ln(CP10K+1)
0
1
2
3
Bone
Diaphragm
Limb muscle
Mammary gland
Fat
Skin
Eye retina
Hypothalamus
Pituitary
Brainstem
Brain cortex
Testes
Uterus
Bladder
Kidney
Pancreas
Liver
Small intestine
Tongue
Trachea
Lung
Aorta
Heart
Spleen
Blood
Bone marrow
a b c d e f g h i j k l

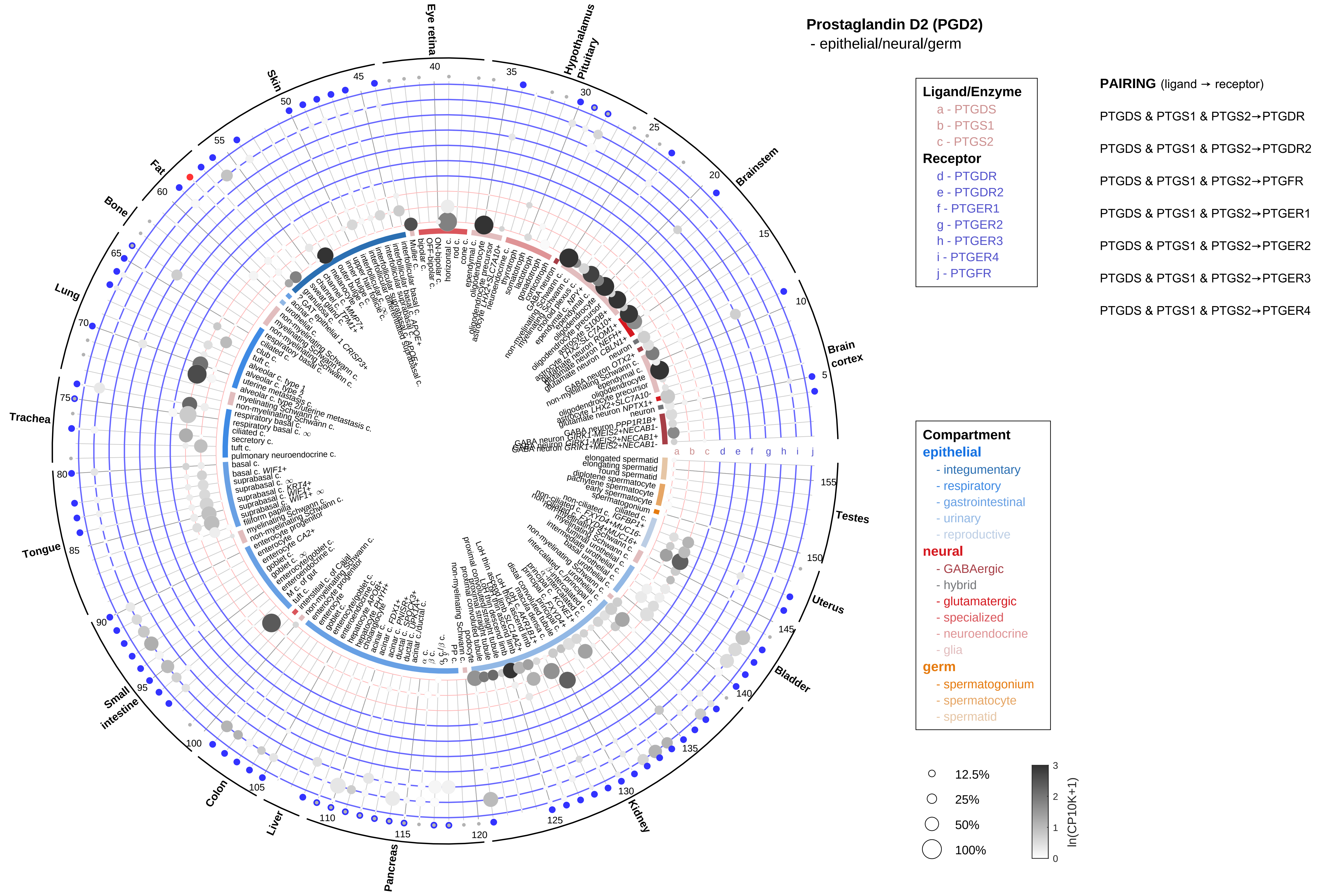

Prostaglandin D2 (PGD2)
- epithelial/neural/germ
Ligand/Enzyme
a - PTGDS
b - PTGS1
c - PTGS2
Receptor
d - PTGDR
e - PTGDR2
f - PTGER1
g - PTGER2
h - PTGER3
i - PTGER4
j - PTGFR
PAIRING (ligand → receptor)
PTGDS & PTGS1 & PTGS2→PTGDR
PTGDS & PTGS1 & PTGS2→PTGDR2
PTGDS & PTGS1 & PTGS2→PTGFR
PTGDS & PTGS1 & PTGS2→PTGER1
PTGDS & PTGS1 & PTGS2→PTGER2
PTGDS & PTGS1 & PTGS2→PTGER3
PTGDS & PTGS1 & PTGS2→PTGER4
Compartment
epithelial
- integumentary
- respiratory
- gastrointestinal
- urinary
- reproductive
neural
- GABAergic
- hybrid
- glutamatergic
- specialized
- neuroendocrine
- glia
germ
- spermatogonium
- spermatocyte
- spermatid
12.5%
25%
50%
100%
ln(CP10K+1)
Eye retina
Hypothalamus
Pituitary
Brainstem
Brain cortex
Testes
Uterus
Bladder
Kidney
Pancreas
Liver
Colon
Small intestine
Tongue
Trachea
Lung
Bone
Fat
Skin

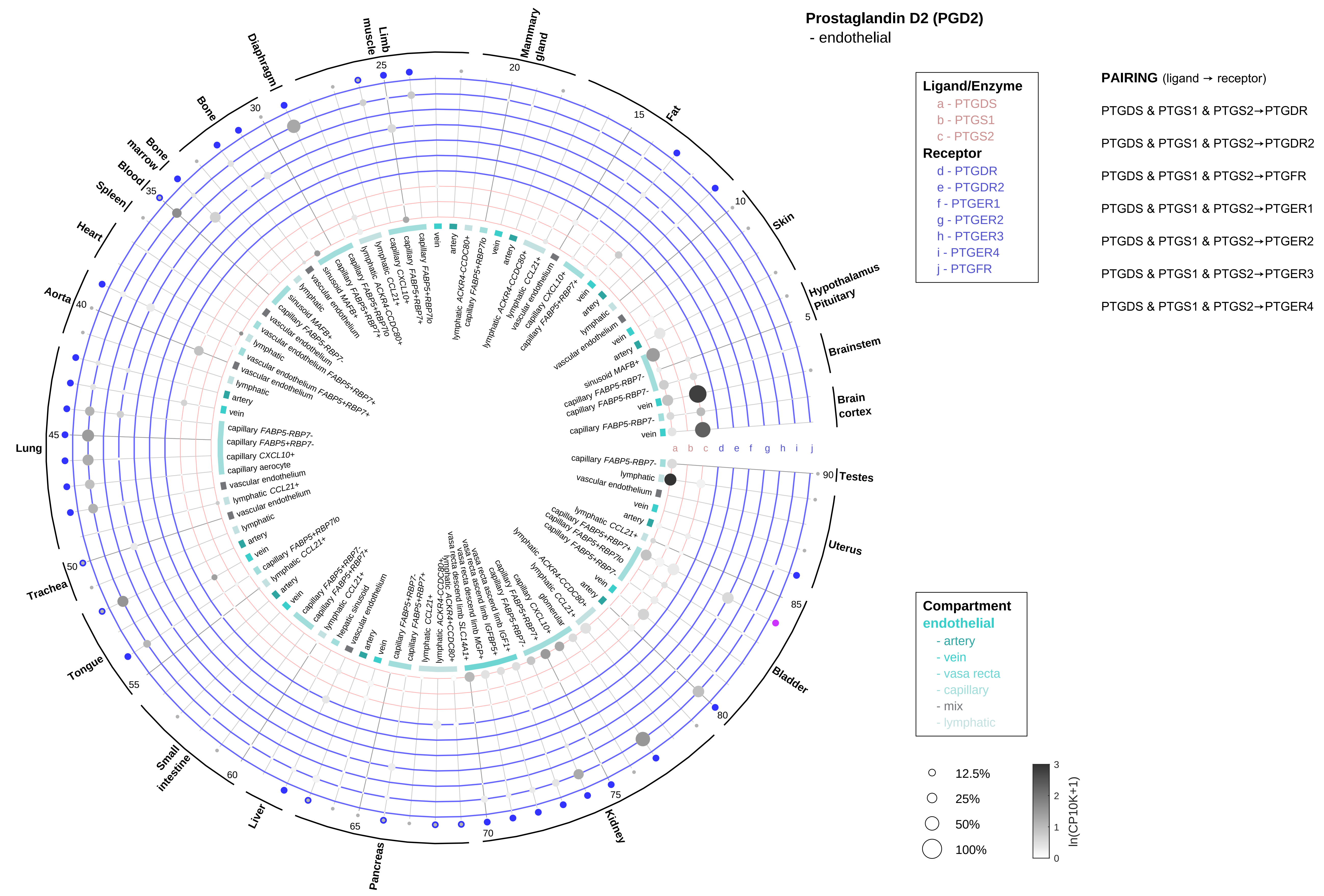

Prostaglandin D2 (PGD2)
- endothelial
Ligand/Enzyme
a - PTGDS
b - PTGS1
c - PTGS2
Receptor
d - PTGDR
e - PTGDR2
f - PTGER1
g - PTGER2
h - PTGER3
i - PTGER4
j - PTGFR
PAIRING (ligand → receptor)
PTGDS & PTGS1 & PTGS2→PTGDR
PTGDS & PTGS1 & PTGS2→PTGDR2
PTGDS & PTGS1 & PTGS2→PTGFR
PTGDS & PTGS1 & PTGS2→PTGER1
PTGDS & PTGS1 & PTGS2→PTGER2
PTGDS & PTGS1 & PTGS2→PTGER3
PTGDS & PTGS1 & PTGS2→PTGER4
Compartment
endothelial
- artery
- vein
- vasa recta
- capillary
- mix
- lymphatic
12.5%
25%
50%
100%
ln(CP10K+1)
Brain cortex
Brainstem
Hypothalamus Pituitary
Skin
Fat
Mammary gland
Limb muscle
Diaphragm
Bone
Bone marrow
Blood
Spleen
Heart
Aorta
Lung
Trachea
Tongue
Small intestine
Liver
Pancreas
Kidney
Bladder
Uterus
Testes

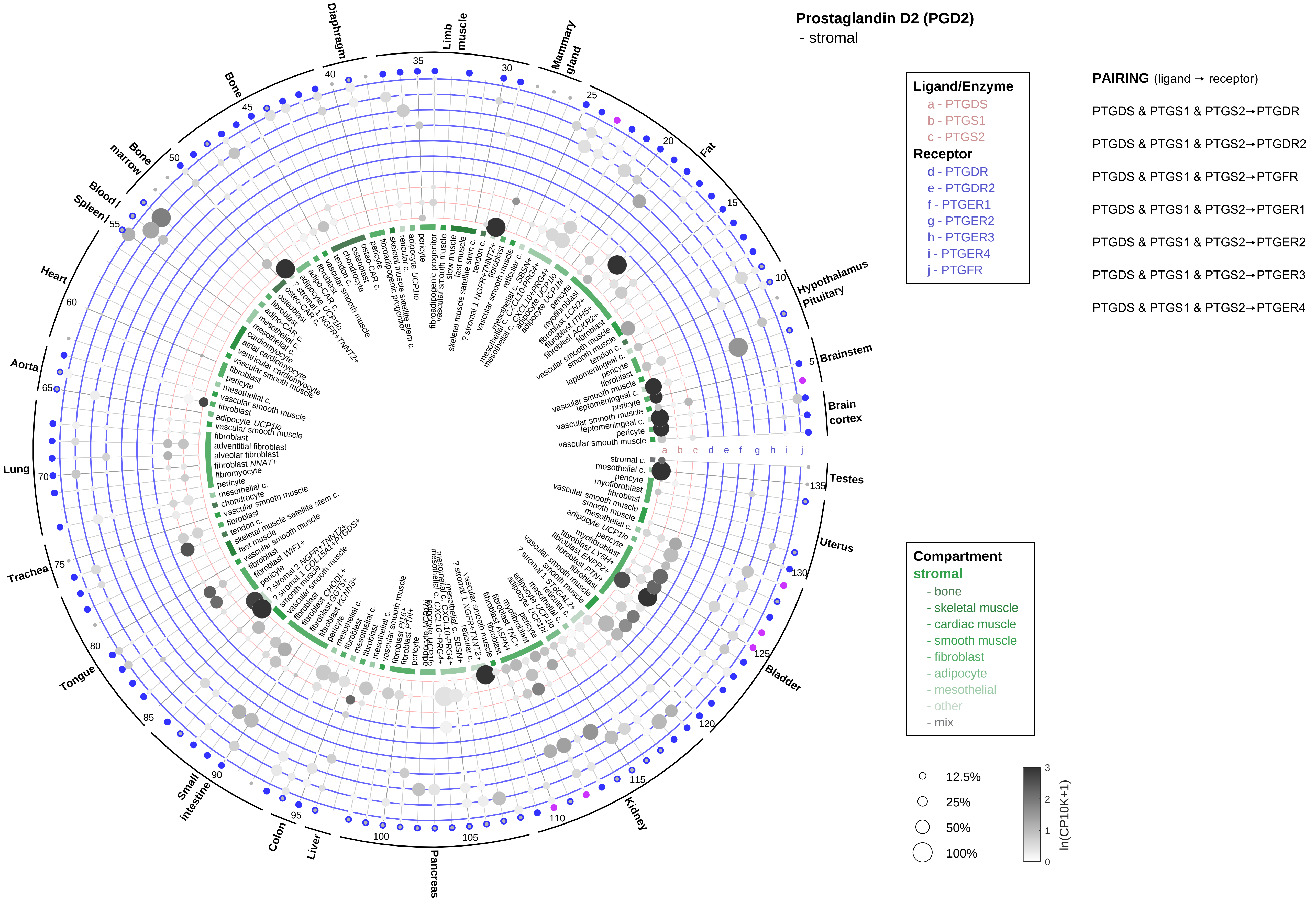
Prostaglandin D2 (PGD2)
- stromal
Ligand/Enzyme
a - PTGDS
b - PTGS1
c - PTGS2
Receptor
d - PTGDR
e - PTGDR2
f - PTGER1
g - PTGER2
h - PTGER3
i - PTGER4
j - PTGFR
PAIRING (ligand → receptor)
PTGDS & PTGS1 & PTGS2→PTGDR
PTGDS & PTGS1 & PTGS2→PTGDR2
PTGDS & PTGS1 & PTGS2→PTGFR
PTGDS & PTGS1 & PTGS2→PTGER1
PTGDS & PTGS1 & PTGS2→PTGER2
PTGDS & PTGS1 & PTGS2→PTGER3
PTGDS & PTGS1 & PTGS2→PTGER4
Compartment
stromal
- bone
- skeletal muscle
- cardiac muscle
- smooth muscle
- fibroblast
- adipocyte
- mesothelial
- other
- mix
12.5%
25%
50%
100%
ln(CP10K+1)
Limb muscle
Mammary gland
Fat
Hypothalamus Pituitary
Brainstem
Brain cortex
Testes
Uterus
Bladder
Kidney
Pancreas
Liver
Colon
Small intestine
Tongue
Trachea
Lung
Aorta
Heart
Spleen
Blood
Bone marrow
Bone
Diaphragm

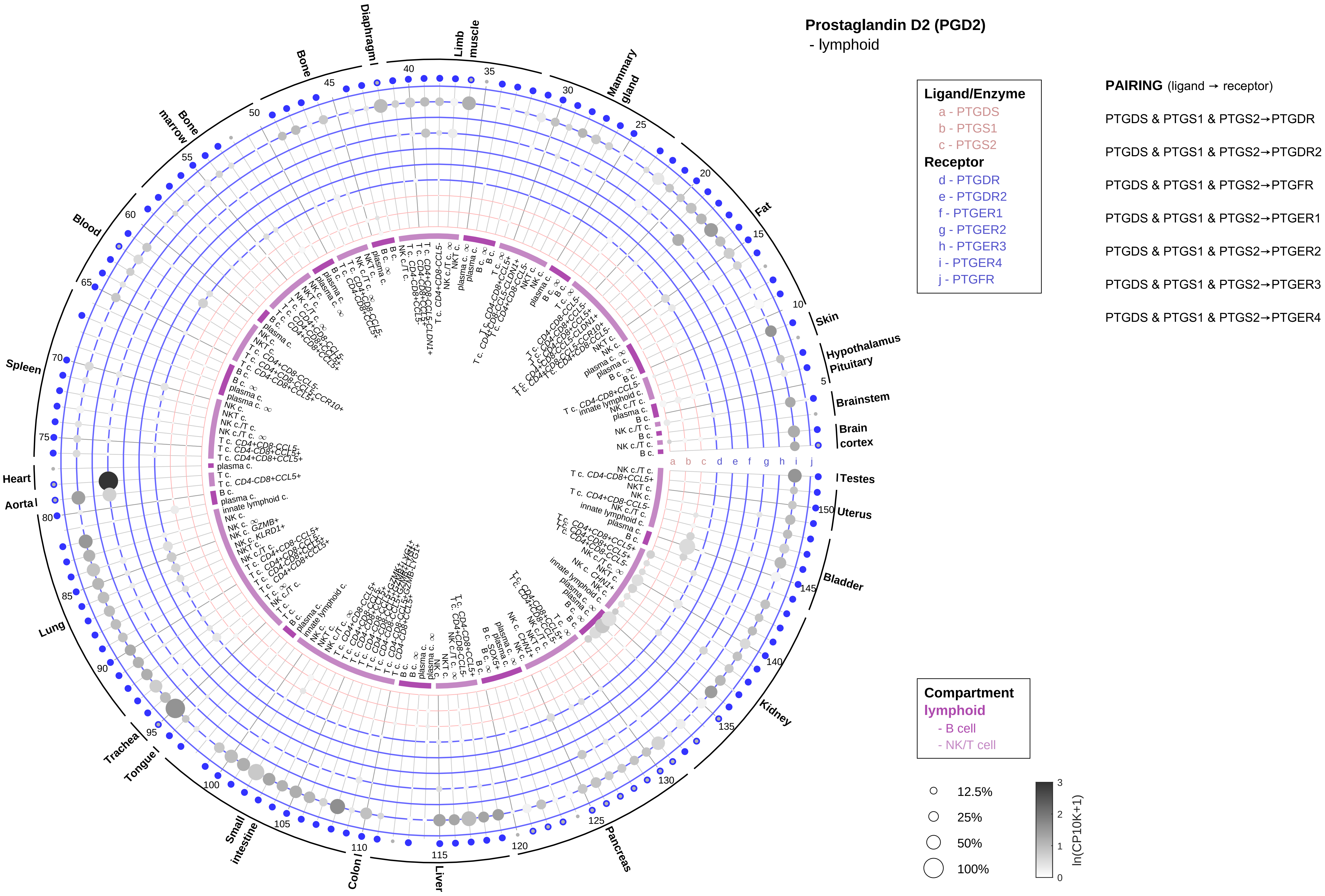
Prostaglandin D2 (PGD2)
- lymphoid
Ligand/Enzyme
a - PTGDS
b - PTGS1
c - PTGS2
Receptor
d - PTGDR
e - PTGDR2
f - PTGER1
g - PTGER2
h - PTGER3
i - PTGER4
j - PTGFR
PAIRING (ligand → receptor)
PTGDS & PTGS1 & PTGS2→PTGDR
PTGDS & PTGS1 & PTGS2→PTGDR2
PTGDS & PTGS1 & PTGS2→PTGFR
PTGDS & PTGS1 & PTGS2→PTGER1
PTGDS & PTGS1 & PTGS2→PTGER2
PTGDS & PTGS1 & PTGS2→PTGER3
PTGDS & PTGS1 & PTGS2→PTGER4
Compartment
lymphoid
- B cell
- NK/T cell
12.5%
25%
50%
100%
ln(CP10K+1)
Skin
Hypothalamus
Pituitary
Brainstem
Brain cortex
Testes
Uterus
Bladder
Kidney
Pancreas
Liver
Colon
Small intestine
Tongue
Trachea
Lung
Aorta
Heart
Spleen
Blood
Bone marrow
Bone
Diaphragm
Limb muscle
Mammary gland
Fat

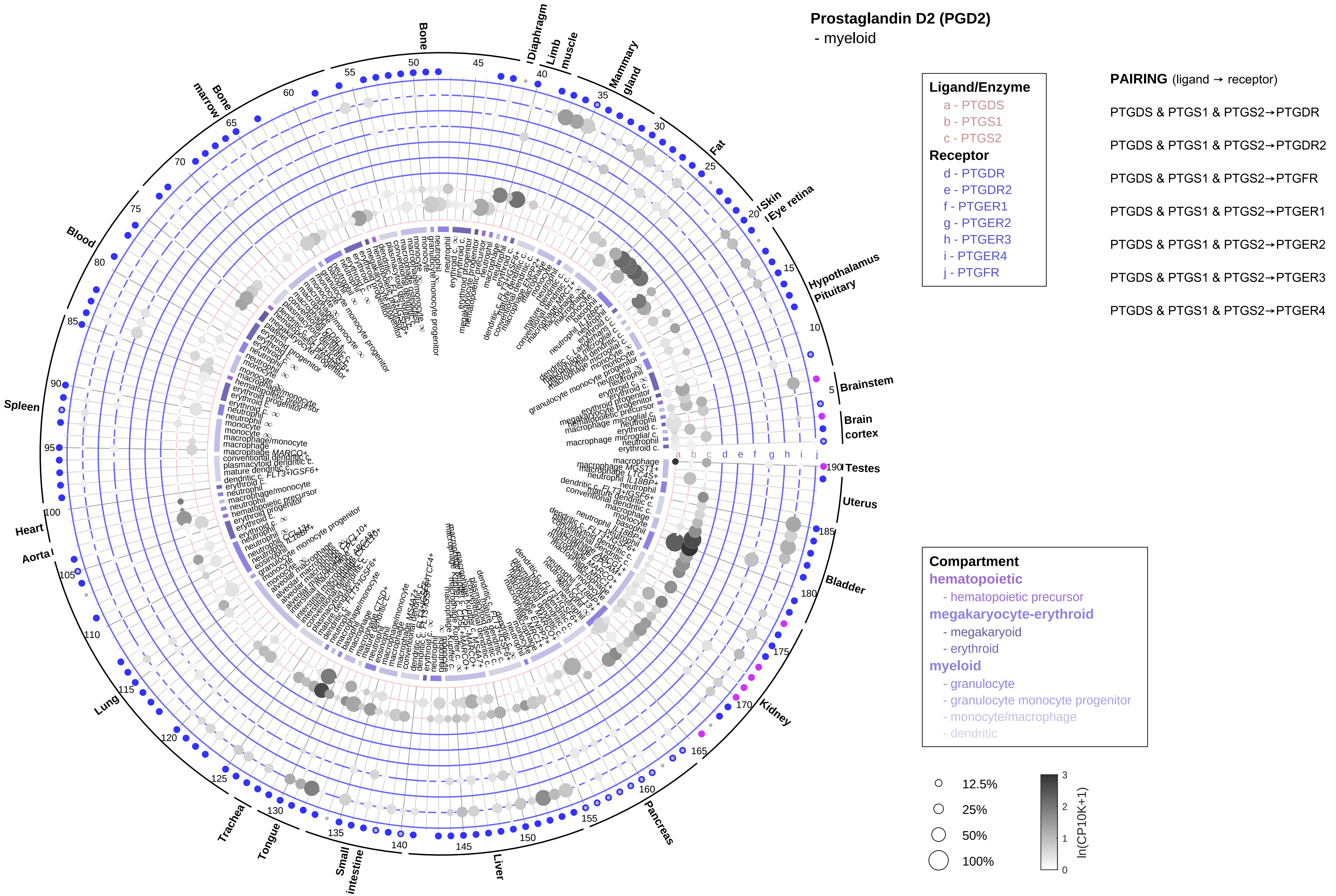
Prostaglandin D2 (PGD2)
- myeloid
Ligand/Enzyme
a - PTGDS
b - PTGS1
c - PTGS2
Receptor
d - PTGDR
e - PTGDR2
f - PTGER1
g - PTGER2
h - PTGER3
i - PTGER4
j - PTGFR
PAIRING (ligand → receptor)
PTGDS & PTGS1 & PTGS2→PTGDR
PTGDS & PTGS1 & PTGS2→PTGDR2
PTGDS & PTGS1 & PTGS2→PTGFR
PTGDS & PTGS1 & PTGS2→PTGER1
PTGDS & PTGS1 & PTGS2→PTGER2
PTGDS & PTGS1 & PTGS2→PTGER3
PTGDS & PTGS1 & PTGS2→PTGER4
Compartment
hematopoietic
- hematopoietic precursor
megakaryocyte-erythroid
- megakaryoid
- erythroid
myeloid
- granulocyte
- granulocyte monocyte progenitor
- monocyte/macrophage
- dendritic
12.5%
25%
50%
100%
ln(CP10K+1)
Brainstem
Brain cortex
Testes
Uterus
Bladder
Kidney
Pancreas
Liver
Small intestine
Tongue
Trachea
Lung
Aorta
Heart
Spleen
Blood
Bone marrow
Bone
Diaphragm
Limb muscle
Mammary gland
Fat
Skin
Eye retina
Hypothalamus
Pituitary

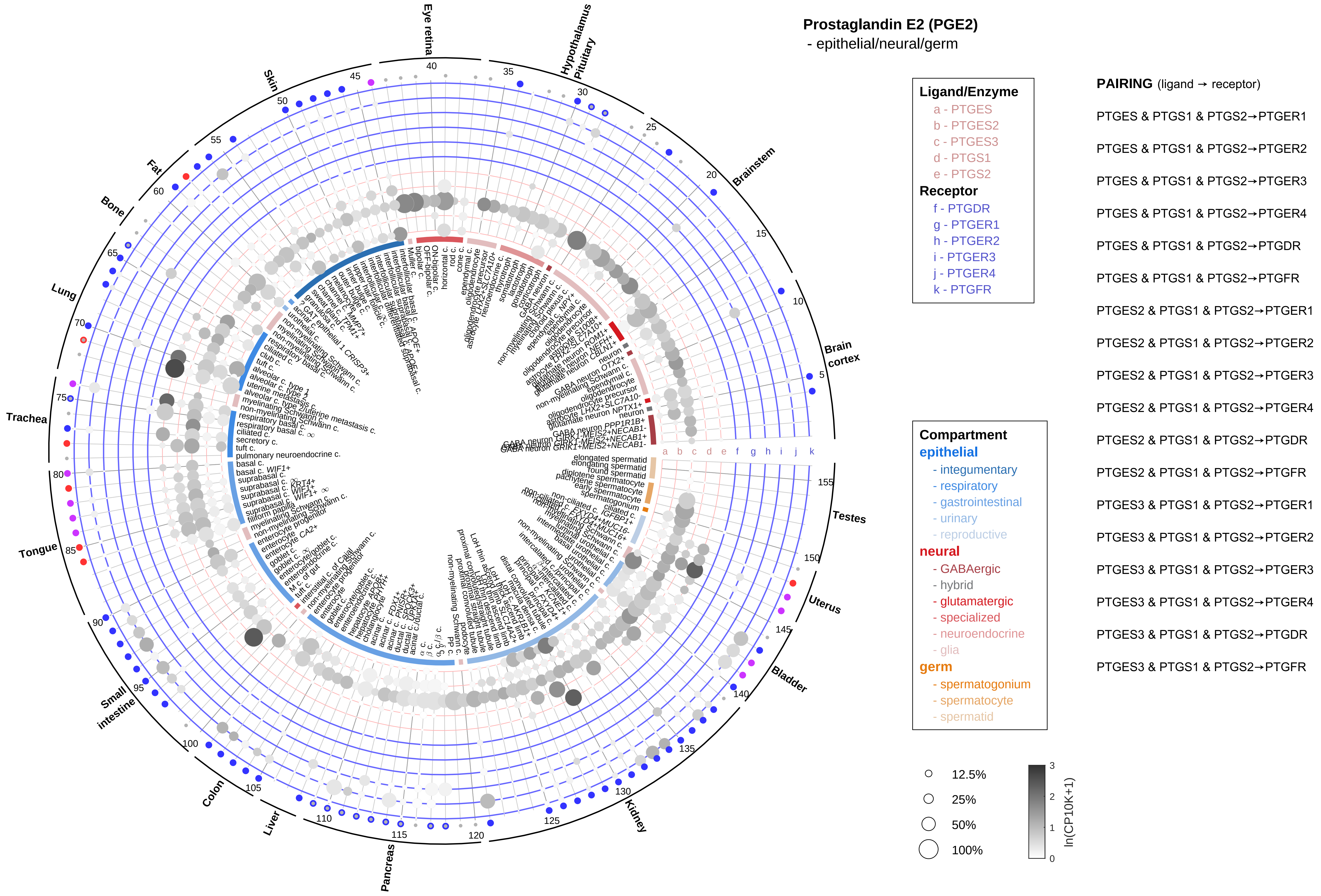

Prostaglandin E2 (PGE2)
- epithelial/neural/germ
Ligand/Enzyme
a - PTGES
b - PTGES2
c - PTGES3
d - PTGS1
e - PTGS2
Receptor
f - PTGDR
g - PTGER1
h - PTGER2
i - PTGER3
j - PTGER4
k - PTGFR
PAIRING (ligand → receptor)
PTGES & PTGS1 & PTGS2→PTGER1
PTGES & PTGS1 & PTGS2→PTGER2
PTGES & PTGS1 & PTGS2→PTGER3
PTGES & PTGS1 & PTGS2→PTGER4
PTGES & PTGS1 & PTGS2→PTGDR
PTGES & PTGS1 & PTGS2→PTGFR
PTGES2 & PTGS1 & PTGS2→PTGER1
PTGES2 & PTGS1 & PTGS2→PTGER2
PTGES2 & PTGS1 & PTGS2→PTGER3
PTGES2 & PTGS1 & PTGS2→PTGER4
PTGES2 & PTGS1 & PTGS2→PTGDR
PTGES2 & PTGS1 & PTGS2→PTGFR
PTGES3 & PTGS1 & PTGS2→PTGER1
PTGES3 & PTGS1 & PTGS2→PTGER2
PTGES3 & PTGS1 & PTGS2→PTGER3
PTGES3 & PTGS1 & PTGS2→PTGER4
PTGES3 & PTGS1 & PTGS2→PTGDR
PTGES3 & PTGS1 & PTGS2→PTGFR
Compartment
epithelial
- integumentary
- respiratory
- gastrointestinal
- urinary
- reproductive
neural
- GABAergic
- hybrid
- glutamatergic
- specialized
- neuroendocrine
- glia
germ
- spermatogonium
- spermatocyte
- spermatid
12.5%
25%
50%
100%
ln(CP10K+1)
Eye retina
Hypothalamus Pituitary
Brainstem
Brain cortex
Testes
Uterus
Bladder
Kidney
Pancreas
Liver
Colon
Small intestine
Tongue
Trachea
Lung
Bone
Fat
Skin

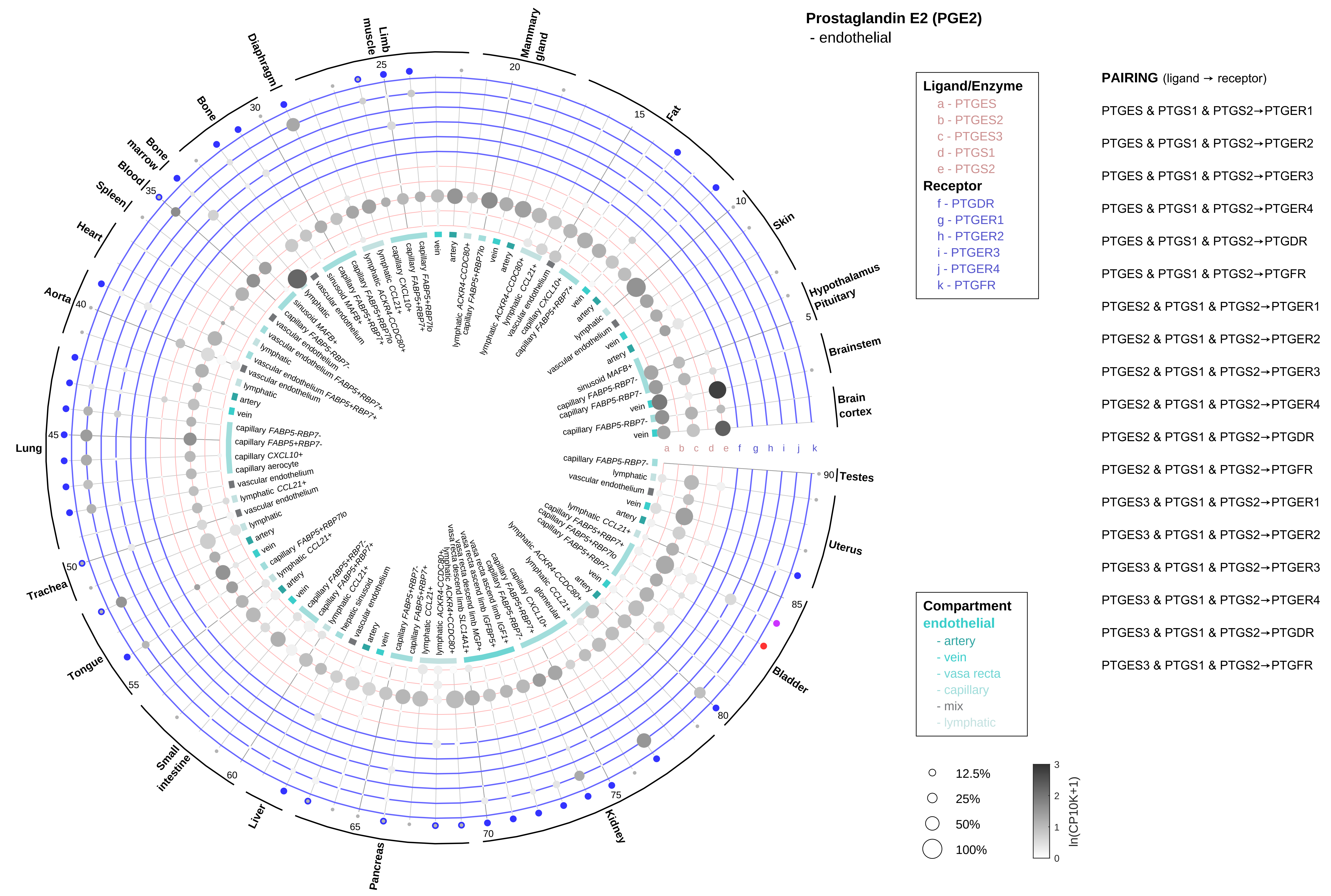

Prostaglandin E2 (PGE2)
- endothelial
Ligand/Enzyme
a - PTGES
b - PTGES2
c - PTGES3
d - PTGS1
e - PTGS2
Receptor
f - PTGDR
g - PTGER1
h - PTGER2
i - PTGER3
j - PTGER4
k - PTGFR
PAIRING (ligand → receptor)
PTGES & PTGS1 & PTGS2→PTGER1
PTGES & PTGS1 & PTGS2→PTGER2
PTGES & PTGS1 & PTGS2→PTGER3
PTGES & PTGS1 & PTGS2→PTGER4
PTGES & PTGS1 & PTGS2→PTGDR
PTGES & PTGS1 & PTGS2→PTGFR
PTGES2 & PTGS1 & PTGS2→PTGER1
PTGES2 & PTGS1 & PTGS2→PTGER2
PTGES2 & PTGS1 & PTGS2→PTGER3
PTGES2 & PTGS1 & PTGS2→PTGER4
PTGES2 & PTGS1 & PTGS2→PTGDR
PTGES2 & PTGS1 & PTGS2→PTGFR
PTGES3 & PTGS1 & PTGS2→PTGER1
PTGES3 & PTGS1 & PTGS2→PTGER2
PTGES3 & PTGS1 & PTGS2→PTGER3
PTGES3 & PTGS1 & PTGS2→PTGER4
PTGES3 & PTGS1 & PTGS2→PTGDR
PTGES3 & PTGS1 & PTGS2→PTGFR
Compartment
endothelial
- artery
- vein
- vasa recta
- capillary
- mix
- lymphatic
12.5%
25%
50%
100%
ln(CP10K+1)
0
1
2
3
Brain cortex
Brainstem
Hypothalamus Pituitary
Skin
Fat
Mammary gland
Limb muscle
Diaphragm
Bone
Bone marrow
Blood
Spleen
Heart
Aorta
Lung
Trachea
Tongue
Small intestine
Liver
Pancreas
Kidney
Bladder
Uterus
Testes
a b c d e f g h i j k

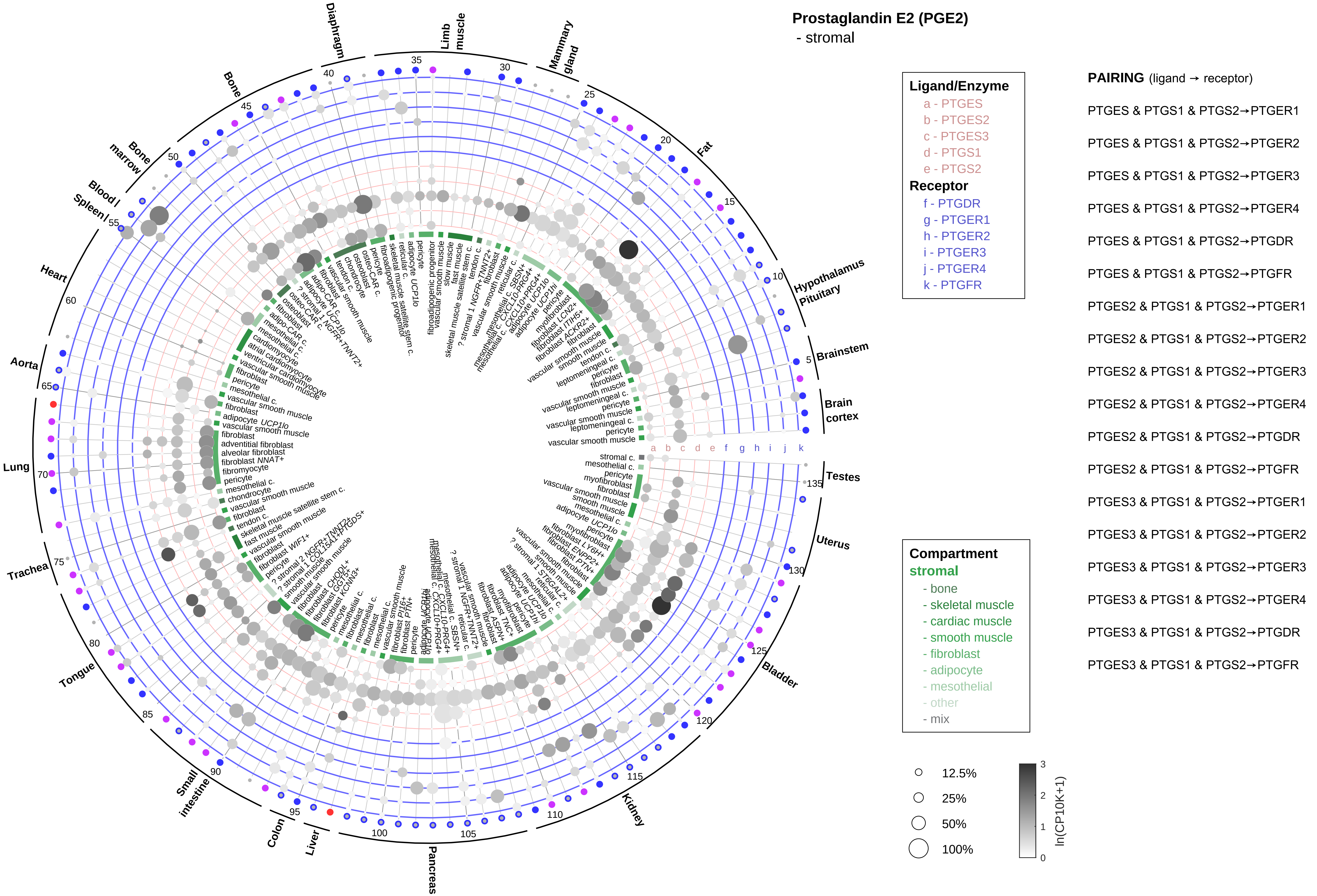
Prostaglandin E2 (PGE2)
- stromal
Ligand/Enzyme
a - PTGES
b - PTGES2
c - PTGES3
d - PTGS1
e - PTGS2
Receptor
f - PTGDR
g - PTGER1
h - PTGER2
i - PTGER3
j - PTGER4
k - PTGFR
PAIRING (ligand → receptor)
PTGES & PTGS1 & PTGS2→PTGER1
PTGES & PTGS1 & PTGS2→PTGER2
PTGES & PTGS1 & PTGS2→PTGER3
PTGES & PTGS1 & PTGS2→PTGER4
PTGES & PTGS1 & PTGS2→PTGDR
PTGES & PTGS1 & PTGS2→PTGFR
PTGES2 & PTGS1 & PTGS2→PTGER1
PTGES2 & PTGS1 & PTGS2→PTGER2
PTGES2 & PTGS1 & PTGS2→PTGER3
PTGES2 & PTGS1 & PTGS2→PTGER4
PTGES2 & PTGS1 & PTGS2→PTGDR
PTGES2 & PTGS1 & PTGS2→PTGFR
PTGES3 & PTGS1 & PTGS2→PTGER1
PTGES3 & PTGS1 & PTGS2→PTGER2
PTGES3 & PTGS1 & PTGS2→PTGER3
PTGES3 & PTGS1 & PTGS2→PTGER4
PTGES3 & PTGS1 & PTGS2→PTGDR
PTGES3 & PTGS1 & PTGS2→PTGFR
Compartment
stromal
- bone
- skeletal muscle
- cardiac muscle
- smooth muscle
- fibroblast
- adipocyte
- mesothelial
- other
- mix
12.5%
25%
50%
100%
ln(CP10K+1)
0
1
2
3
Hypothalamus
Pituitary
Brainstem
Brain cortex
Testes
Uterus
Bladder
Kidney
Pancreas
Liver
Colon
Small intestine
Tongue
Trachea
Lung
Aorta
Heart
Spleen
Blood
Bone marrow
Bone
Diaphragm
Limb muscle
Mammary gland
Fat
a b c d e f g h i j k

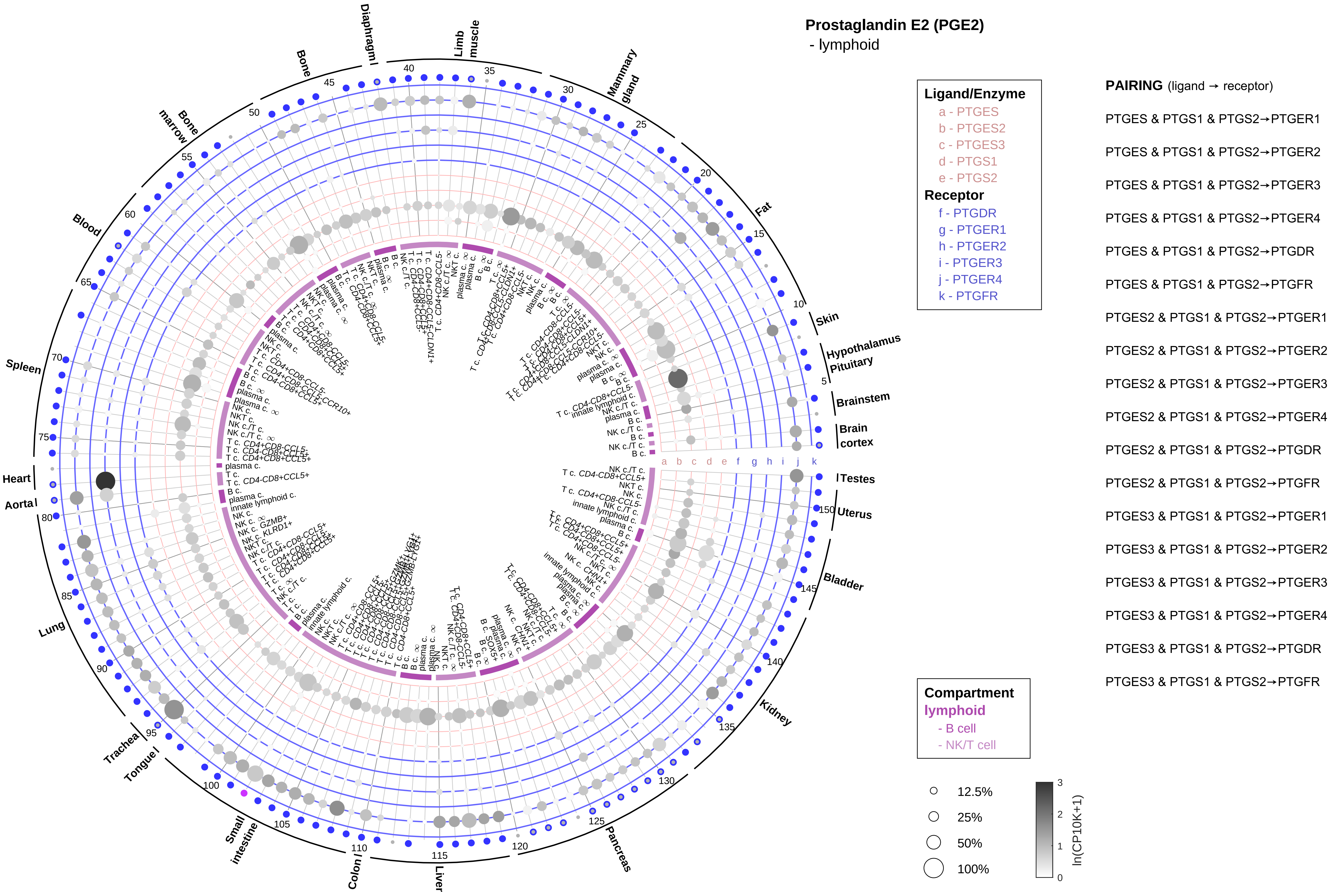

Prostaglandin E2 (PGE2)
- lymphoid
Ligand/Enzyme
a - PTGES
b - PTGES2
c - PTGES3
d - PTGS1
e - PTGS2
Receptor
f - PTGDR
g - PTGER1
h - PTGER2
i - PTGER3
j - PTGER4
k - PTGFR
PAIRING (ligand → receptor)
PTGES & PTGS1 & PTGS2→PTGER1
PTGES & PTGS1 & PTGS2→PTGER2
PTGES & PTGS1 & PTGS2→PTGER3
PTGES & PTGS1 & PTGS2→PTGER4
PTGES & PTGS1 & PTGS2→PTGDR
PTGES & PTGS1 & PTGS2→PTGFR
PTGES2 & PTGS1 & PTGS2→PTGER1
PTGES2 & PTGS1 & PTGS2→PTGER2
PTGES2 & PTGS1 & PTGS2→PTGER3
PTGES2 & PTGS1 & PTGS2→PTGER4
PTGES2 & PTGS1 & PTGS2→PTGDR
PTGES2 & PTGS1 & PTGS2→PTGFR
PTGES3 & PTGS1 & PTGS2→PTGER1
PTGES3 & PTGS1 & PTGS2→PTGER2
PTGES3 & PTGS1 & PTGS2→PTGER3
PTGES3 & PTGS1 & PTGS2→PTGER4
PTGES3 & PTGS1 & PTGS2→PTGDR
PTGES3 & PTGS1 & PTGS2→PTGFR
Compartment
lymphoid
- B cell
- NK/T cell
12.5%
25%
50%
100%
ln(CP10K+1)
0
1
2
3
Skin
Hypothalamus
Pituitary
Brainstem
Brain cortex
Testes
Uterus
Bladder
Kidney
Pancreas
Liver
Colon
Small intestine
Tongue
Trachea
Lung
Aorta
Heart
Spleen
Blood
Bone marrow
Bone
Diaphragm
Limb muscle
Mammary gland
Fat
a
b
c
d
e
f
g
h
i
j
k

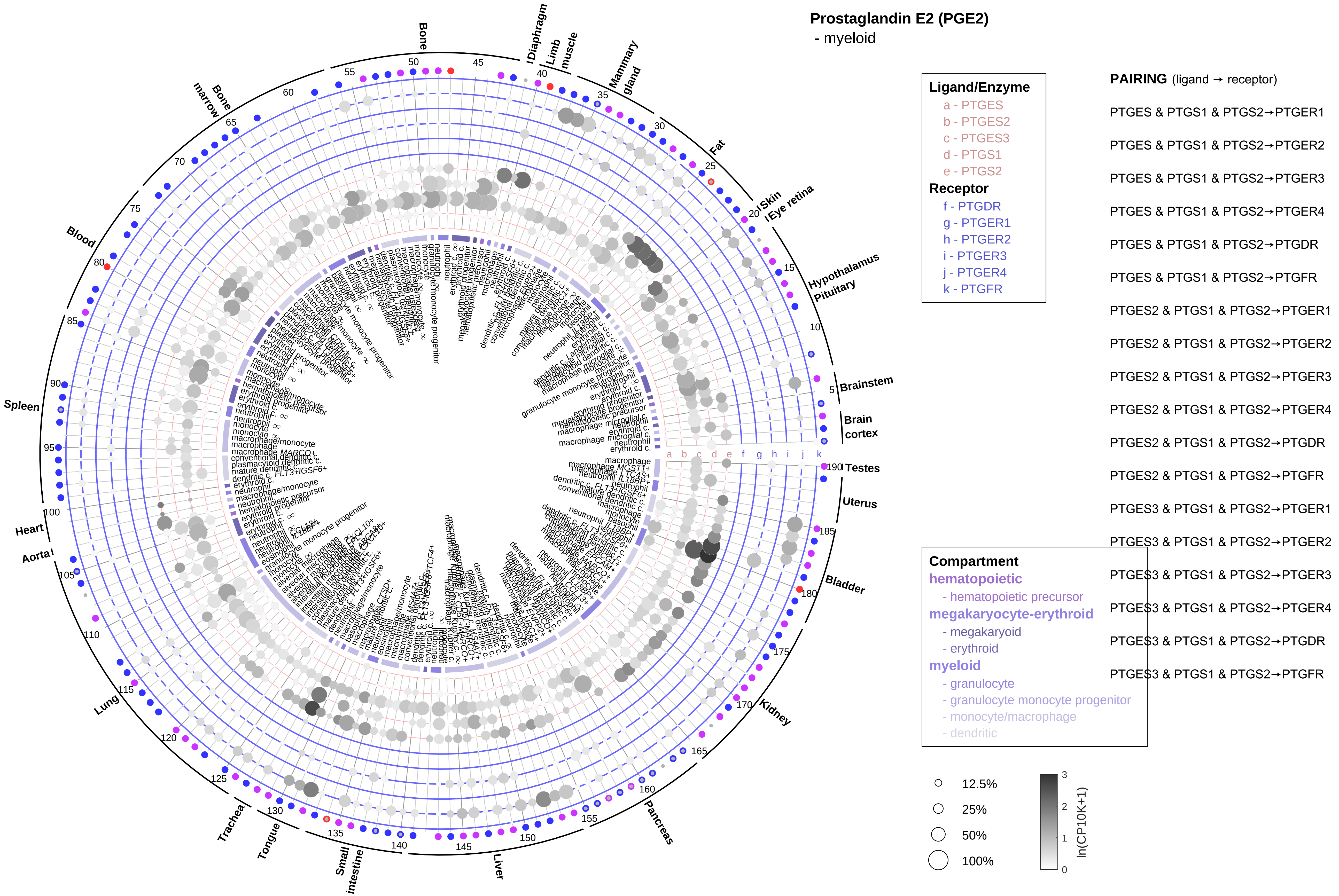

Prostaglandin E2 (PGE2)
- myeloid
Ligand/Enzyme
a - PTGES
b - PTGES2
c - PTGES3
d - PTGS1
e - PTGS2
Receptor
f - PTGDR
g - PTGER1
h - PTGER2
i - PTGER3
j - PTGER4
k - PTGFR
PAIRING (ligand → receptor)
PTGES & PTGS1 & PTGS2→PTGER1
PTGES & PTGS1 & PTGS2→PTGER2
PTGES & PTGS1 & PTGS2→PTGER3
PTGES & PTGS1 & PTGS2→PTGER4
PTGES & PTGS1 & PTGS2→PTGDR
PTGES & PTGS1 & PTGS2→PTGFR
PTGES2 & PTGS1 & PTGS2→PTGER1
PTGES2 & PTGS1 & PTGS2→PTGER2
PTGES2 & PTGS1 & PTGS2→PTGER3
PTGES2 & PTGS1 & PTGS2→PTGER4
PTGES2 & PTGS1 & PTGS2→PTGDR
PTGES2 & PTGS1 & PTGS2→PTGFR
PTGES3 & PTGS1 & PTGS2→PTGER1
PTGES3 & PTGS1 & PTGS2→PTGER2
PTGES3 & PTGS1 & PTGS2→PTGER3
PTGES3 & PTGS1 & PTGS2→PTGER4
PTGES3 & PTGS1 & PTGS2→PTGDR
PTGES3 & PTGS1 & PTGS2→PTGFR
Compartment
hematopoietic
- hematopoietic precursor
megakaryocyte-erythroid
- megakaryoid
- erythroid
myeloid
- granulocyte
- granulocyte monocyte progenitor
- monocyte/macrophage
- dendritic
12.5%
25%
50%
100%
ln(CP10K+1)
Bone
Diaphragm
Limb muscle
Mammary gland
Fat
Skin
Eye retina
Hypothalamus
Pituitary
Brainstem
Brain cortex
Testes
Uterus
Bladder
Kidney
Pancreas
Liver
Small intestine
Tongue
Trachea
Lung
Aorta
Heart
Spleen
Blood
Bone marrow

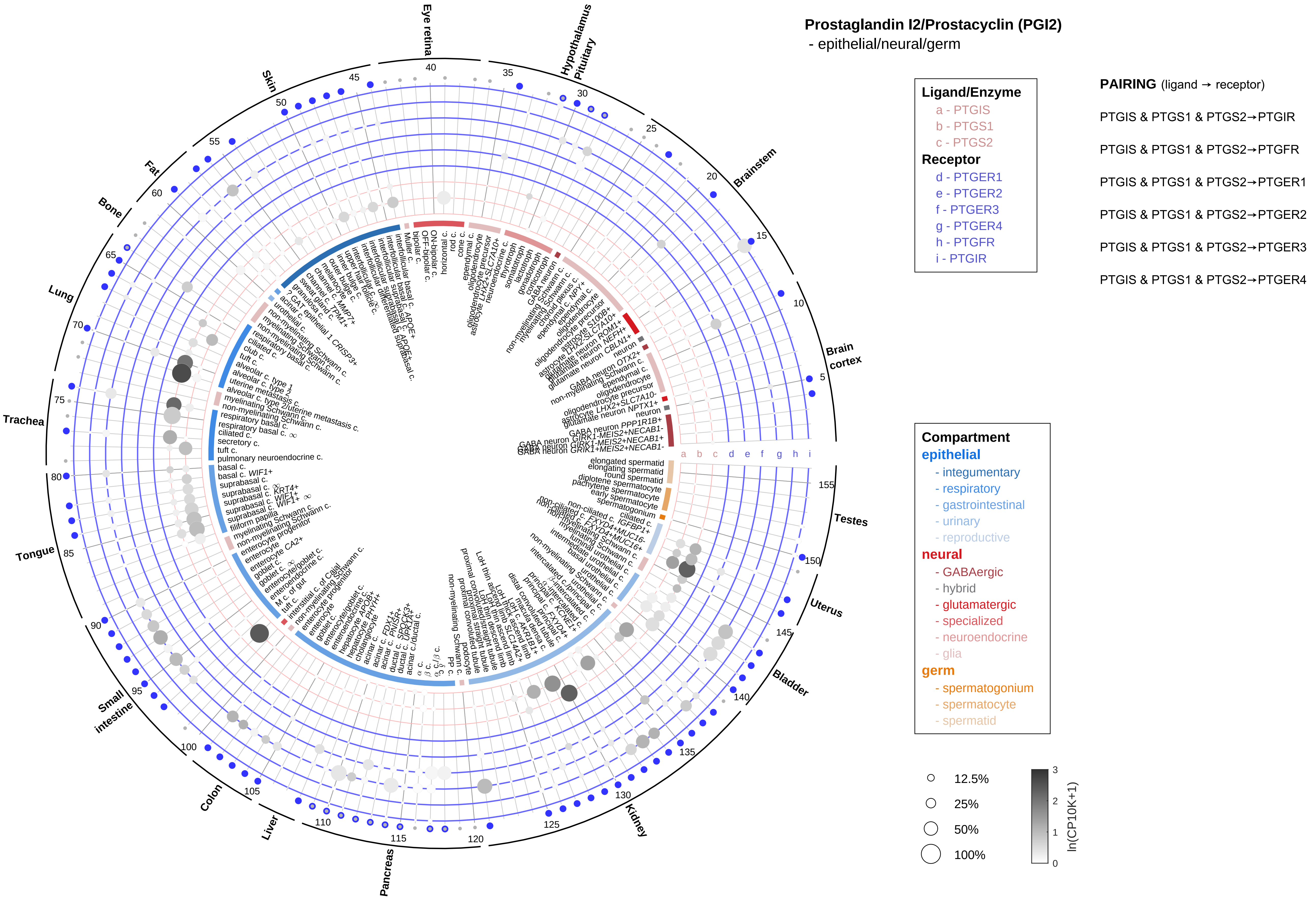
Prostaglandin I2/Prostacyclin (PGI2)
- epithelial/neural/germ
Ligand/Enzyme
a - PTGIS
b - PTGS1
c - PTGS2
Receptor
d - PTGER1
e - PTGER2
f - PTGER3
g - PTGER4
h - PTGFR
i - PTGIR
PAIRING (ligand → receptor)
PTGIS & PTGS1 & PTGS2→PTGIR
PTGIS & PTGS1 & PTGS2→PTGFR
PTGIS & PTGS1 & PTGS2→PTGER1
PTGIS & PTGS1 & PTGS2→PTGER2
PTGIS & PTGS1 & PTGS2→PTGER3
PTGIS & PTGS1 & PTGS2→PTGER4
Compartment
epithelial
- integumentary
- respiratory
- gastrointestinal
- urinary
- reproductive
neural
- GABAergic
- hybrid
- glutamatergic
- specialized
- neuroendocrine
- glia
germ
- spermatogonium
- spermatocyte
- spermatid
12.5%
25%
50%
100%
ln(CP10K+1)
Eye retina
Hypothalamus Pituitary
Brainstem
Brain cortex
Testes
Uterus
Bladder
Kidney
Pancreas
Liver
Colon
Small intestine
Tongue
Trachea
Lung
Bone
Fat
Skin

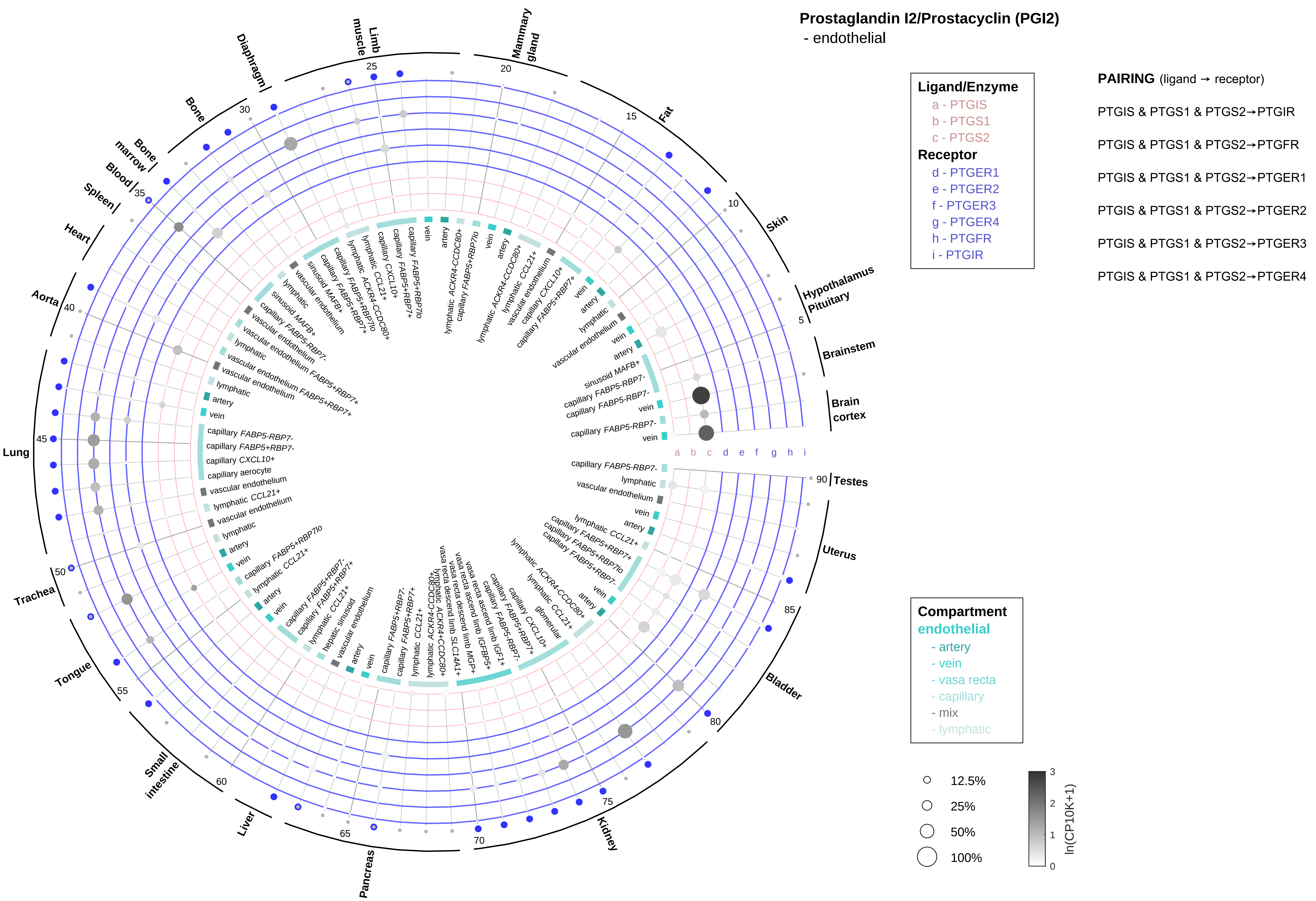
Prostaglandin I2/Prostacyclin (PGI2)
- endothelial
Ligand/Enzyme
a - PTGIS
b - PTGS1
c - PTGS2
Receptor
d - PTGER1
e - PTGER2
f - PTGER3
g - PTGER4
h - PTGFR
i - PTGIR
PAIRING (ligand → receptor)
PTGIS & PTGS1 & PTGS2→PTGIR
PTGIS & PTGS1 & PTGS2→PTGFR
PTGIS & PTGS1 & PTGS2→PTGER1
PTGIS & PTGS1 & PTGS2→PTGER2
PTGIS & PTGS1 & PTGS2→PTGER3
PTGIS & PTGS1 & PTGS2→PTGER4
Compartment
endothelial
- artery
- vein
- vasa recta
- capillary
- mix
- lymphatic
12.5%
25%
50%
100%
ln(CP10K+1)
0
1
2
3
Brain cortex
Brainstem
Hypothalamus Pituitary
Skin
Fat
Mammary gland
Limb muscle
Diaphragm
Bone
Bone marrow
Blood
Spleen
Heart
Aorta
Lung
Trachea
Tongue
Small intestine
Liver
Pancreas
Kidney
Bladder
Uterus
Testes
a
b
c
d
e
f
g
h
i

Prostaglandin I2/Prostacyclin (PGI2)
- stromal

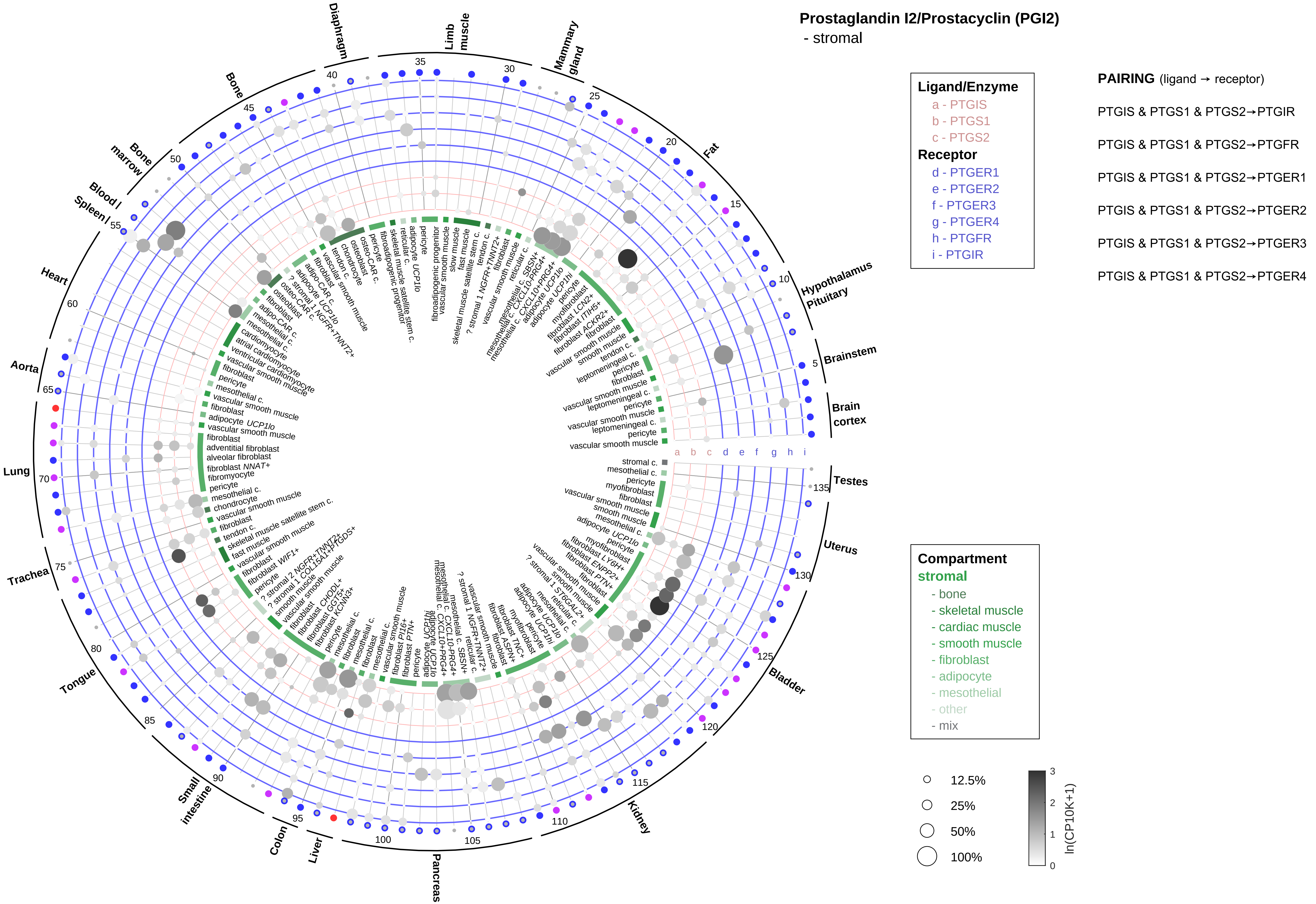

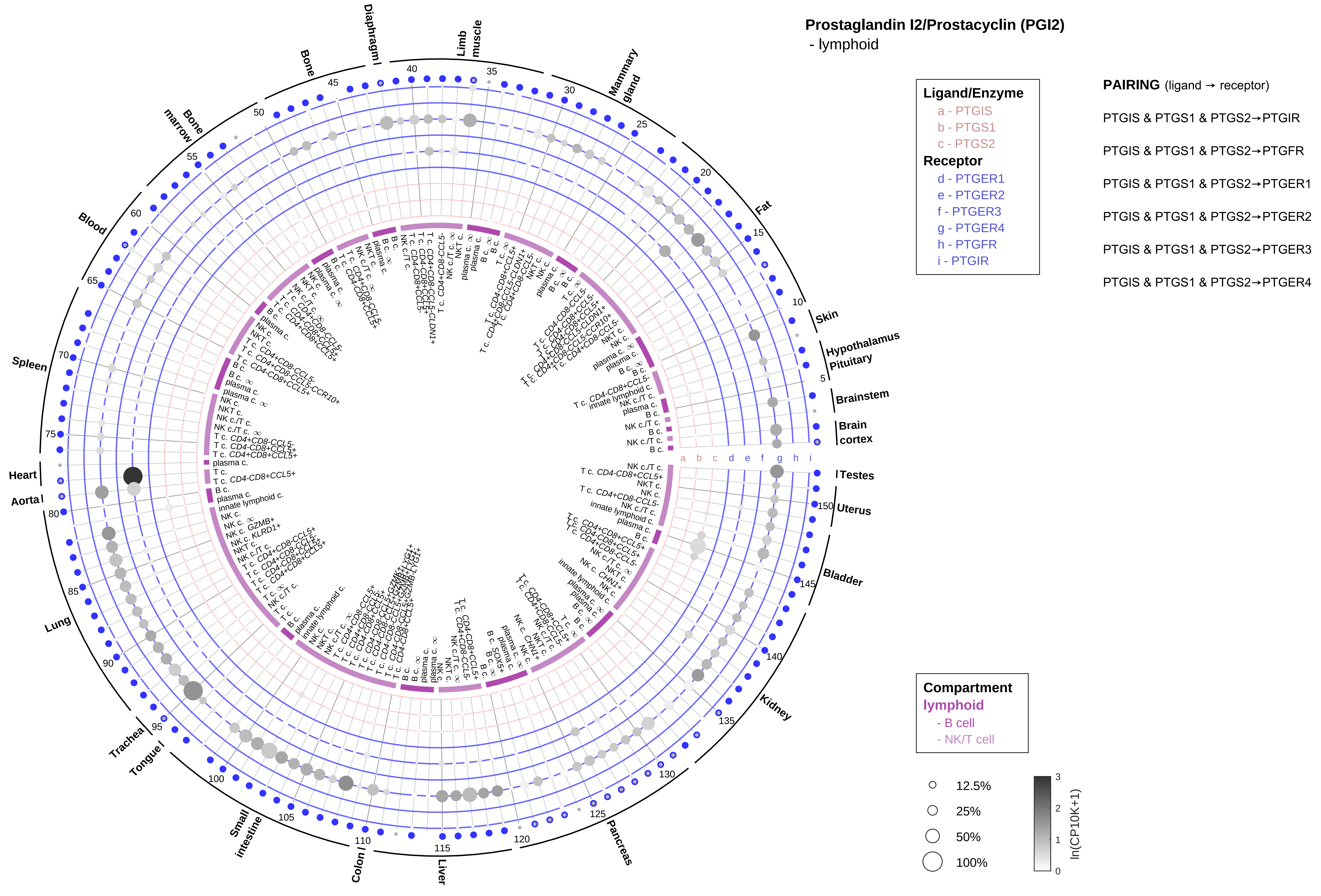
Prostaglandin I2/Prostacyclin (PGI2)
- lymphoid
Ligand/Enzyme
a - PTGIS
b - PTGS1
c - PTGS2
Receptor
d - PTGER1
e - PTGER2
f - PTGER3
g - PTGER4
h - PTGFR
i - PTGIR
PAIRING (ligand → receptor)
PTGIS & PTGS1 & PTGS2→PTGIR
PTGIS & PTGS1 & PTGS2→PTGFR
PTGIS & PTGS1 & PTGS2→PTGER1
PTGIS & PTGS1 & PTGS2→PTGER2
PTGIS & PTGS1 & PTGS2→PTGER3
PTGIS & PTGS1 & PTGS2→PTGER4
Compartment
lymphoid
- B cell
- NK/T cell
12.5%
25%
50%
100%
ln(CP10K+1)
Diaphragm
Limb muscle
Mammary gland
Fat
Skin
Hypothalamus
Pituitary
Brainstem
Brain cortex
Testes
Uterus
Bladder
Kidney
Pancreas
Liver
Colon
Small intestine
Tongue
Trachea
Lung
Aorta
Heart
Spleen
Blood
Bone marrow
Bone

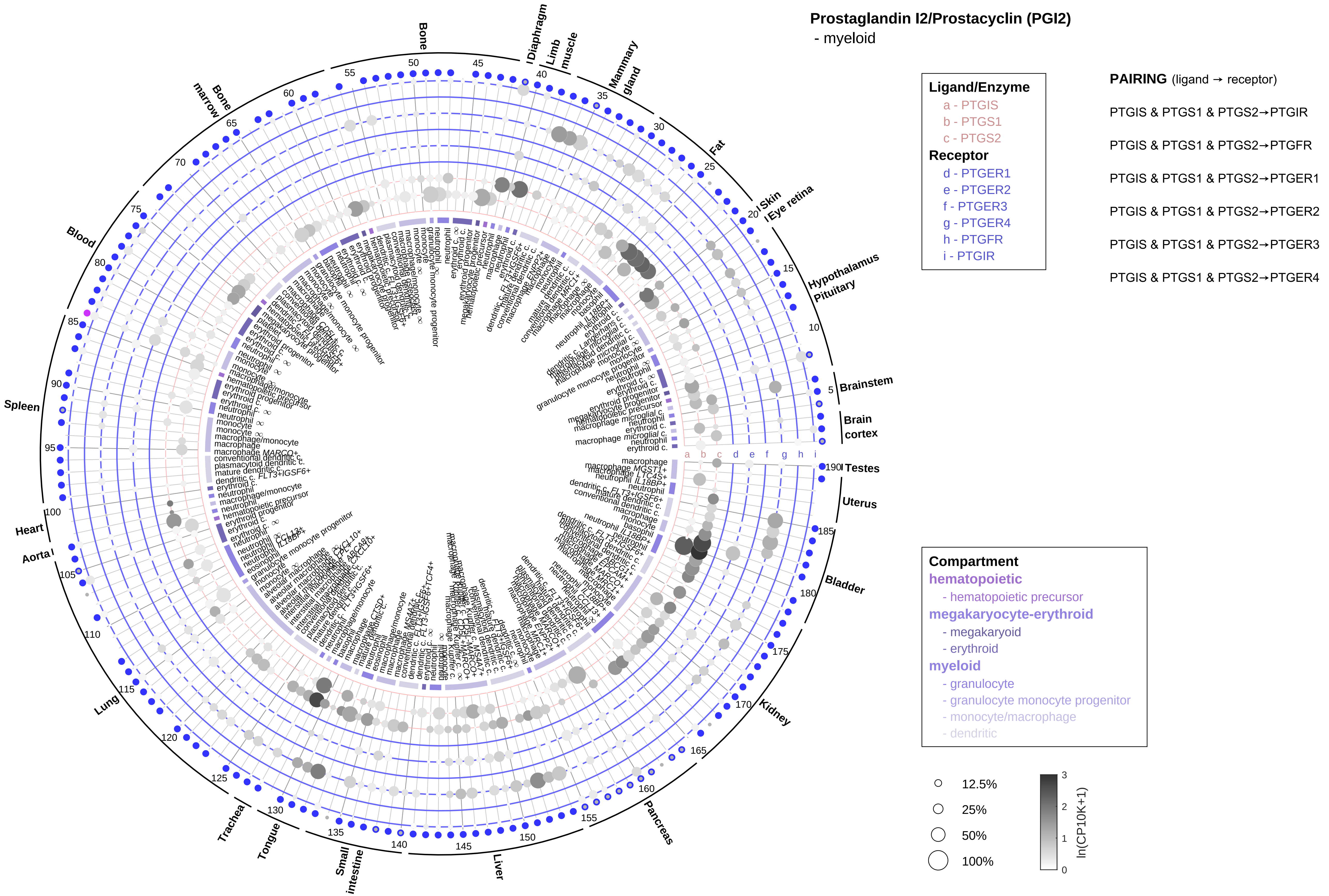

Prostaglandin I2/Prostacyclin (PGI2)
- myeloid
Ligand/Enzyme
a - PTGIS
b - PTGS1
c - PTGS2
Receptor
d - PTGER1
e - PTGER2
f - PTGER3
g - PTGER4
h - PTGFR
i - PTGIR
PAIRING (ligand → receptor)
PTGIS & PTGS1 & PTGS2→PTGIR
PTGIS & PTGS1 & PTGS2→PTGFR
PTGIS & PTGS1 & PTGS2→PTGER1
PTGIS & PTGS1 & PTGS2→PTGER2
PTGIS & PTGS1 & PTGS2→PTGER3
PTGIS & PTGS1 & PTGS2→PTGER4
Compartment
hematopoietic
- hematopoietic precursor
megakaryocyte-erythroid
- megakaryoid
- erythroid
myeloid
- granulocyte
- granulocyte monocyte progenitor
- monocyte/macrophage
- dendritic
12.5%
25%
50%
100%
ln(CP10K+1)
Diaphragm
Limb muscle
Mammary gland
Fat
Skin
Eye retina
Hypothalamus
Pituitary
Brainstem
Brain cortex
Testes
Uterus
Bladder
Kidney
Pancreas
Liver
Small intestine
Tongue
Trachea
Lung
Aorta
Heart
Spleen
Blood
Bone marrow
Bone

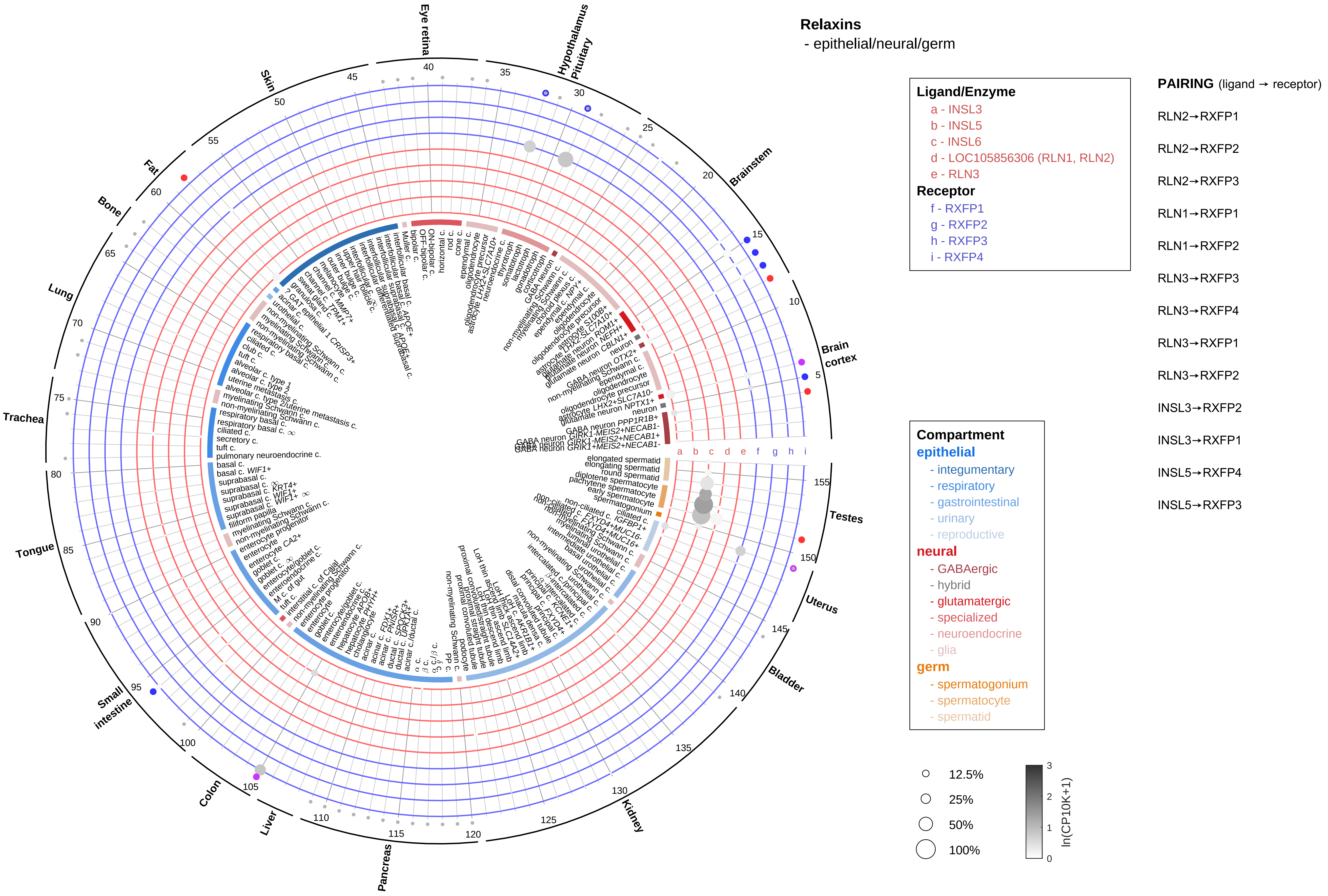
Relaxins
- epithelial/neural/germ
Ligand/Enzyme
a - INSL3
b - INSL5
c - INSL6
d - LOC105856306 (RLN1, RLN2)
e - RLN3
Receptor
f - RXFP1
g - RXFP2
h - RXFP3
i - RXFP4
PAIRING (ligand → receptor)
RLN2→RXFP1
RLN2→RXFP2
RLN2→RXFP3
RLN1→RXFP1
RLN1→RXFP2
RLN3→RXFP3
RLN3→RXFP4
RLN3→RXFP1
RLN3→RXFP2
INSL3→RXFP2
INSL3→RXFP1
INSL5→RXFP4
INSL5→RXFP3
Compartment
epithelial
- integumentary
- respiratory
- gastrointestinal
- urinary
- reproductive
neural
- GABAergic
- hybrid
- glutamatergic
- specialized
- neuroendocrine
- glia
germ
- spermatogonium
- spermatocyte
- spermatid
12.5%
25%
50%
100%
ln(CP10K+1)
0
1
2
3
a b c d e f g h i
Eye retina
Hypothalamus
Pituitary
Brainstem
Brain cortex
Testes
Uterus
Bladder
Kidney
Pancreas
Liver
Colon
Small intestine
Tongue
Trachea
Lung
Bone
Fat
Skin

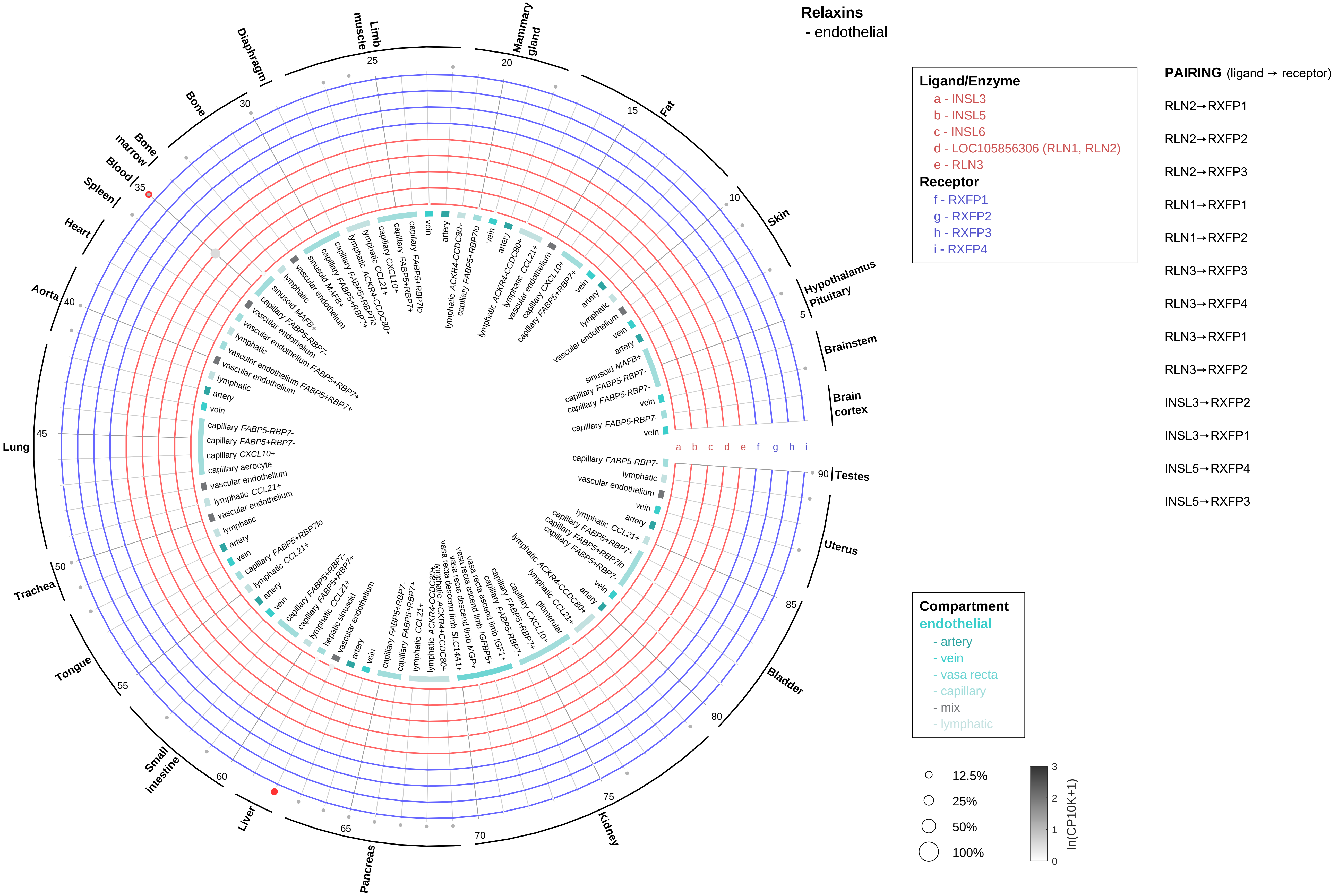

Relaxins
- endothelial
Ligand/Enzyme
a - INSL3
b - INSL5
c - INSL6
d - LOC105856306 (RLN1, RLN2)
e - RLN3
Receptor
f - RXFP1
g - RXFP2
h - RXFP3
i - RXFP4
PAIRING (ligand → receptor)
RLN2→RXFP1
RLN2→RXFP2
RLN2→RXFP3
RLN1→RXFP1
RLN1→RXFP2
RLN3→RXFP3
RLN3→RXFP4
RLN3→RXFP1
RLN3→RXFP2
INSL3→RXFP2
INSL3→RXFP1
INSL5→RXFP4
INSL5→RXFP3
Compartment
endothelial
- artery
- vein
- vasa recta
- capillary
- mix
- lymphatic
12.5%
25%
50%
100%
ln(CP10K+1)
Brain cortex
Brainstem
Hypothalamus Pituitary
Skin
Fat
Mammary gland
Limb muscle
Diaphragm
Bone
Bone marrow
Blood
Spleen
Heart
Aorta
Lung
Trachea
Tongue
Small intestine
Liver
Pancreas
Kidney
Bladder
Uterus
Testes
a b c d e f g h i

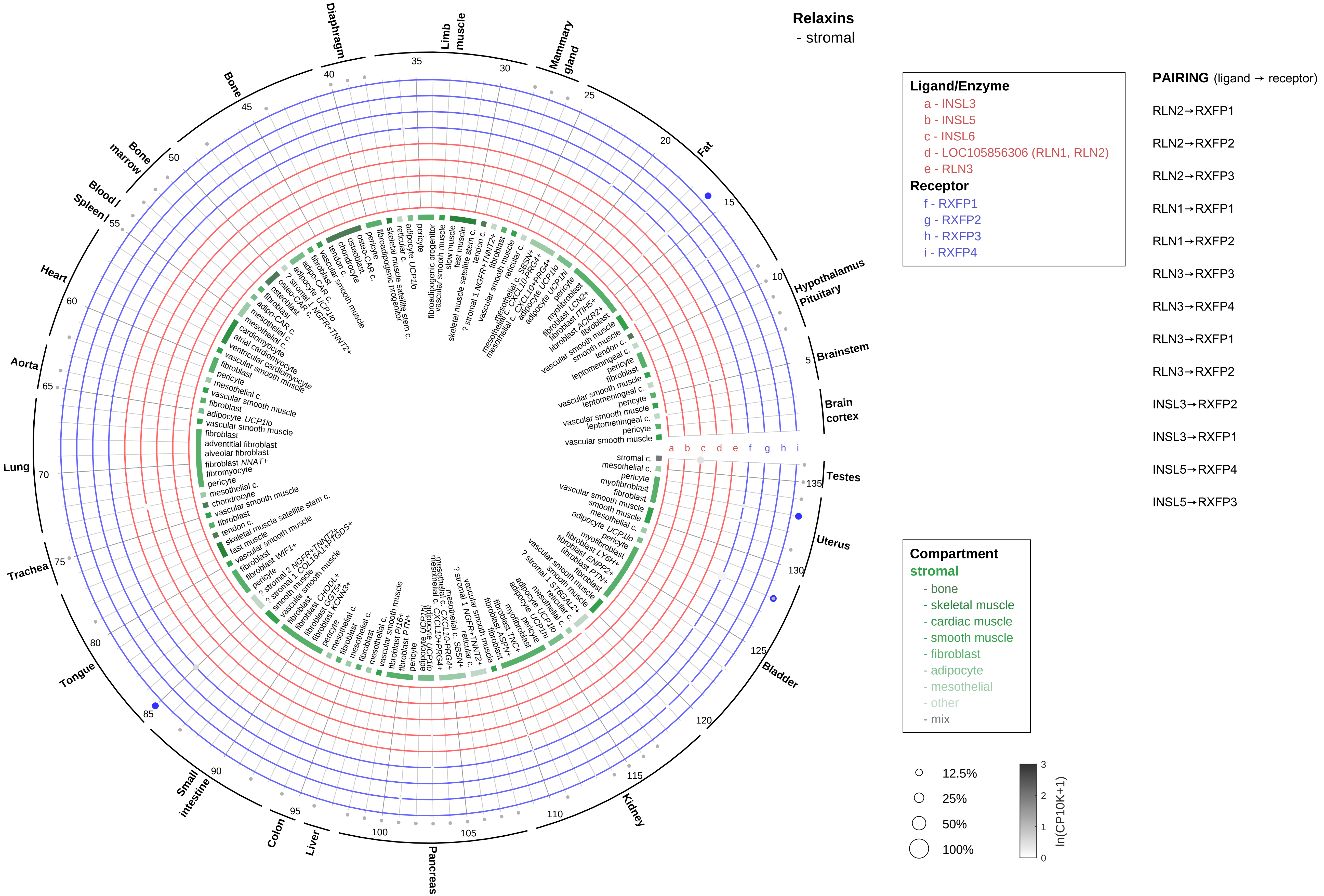
Relaxins
- stromal
Ligand/Enzyme
a - INSL3
b - INSL5
c - INSL6
d - LOC105856306 (RLN1, RLN2)
e - RLN3
Receptor
f - RXFP1
g - RXFP2
h - RXFP3
i - RXFP4
PAIRING (ligand → receptor)
RLN2→RXFP1
RLN2→RXFP2
RLN2→RXFP3
RLN1→RXFP1
RLN1→RXFP2
RLN3→RXFP3
RLN3→RXFP4
RLN3→RXFP1
RLN3→RXFP2
INSL3→RXFP2
INSL3→RXFP1
INSL5→RXFP4
INSL5→RXFP3
Compartment
stromal
- bone
- skeletal muscle
- cardiac muscle
- smooth muscle
- fibroblast
- adipocyte
- mesothelial
- other
- mix
12.5%
25%
50%
100%
ln(CP10K+1)
Diaphragm
Limb muscle
Mammary gland
Fat
Hypothalamus
Pituitary
Brainstem
Brain cortex
Testes
Uterus
Bladder
Kidney
Pancreas
Liver
Colon
Small intestine
Tongue
Trachea
Lung
Aorta
Heart
Spleen
Blood
Bone marrow
Bone

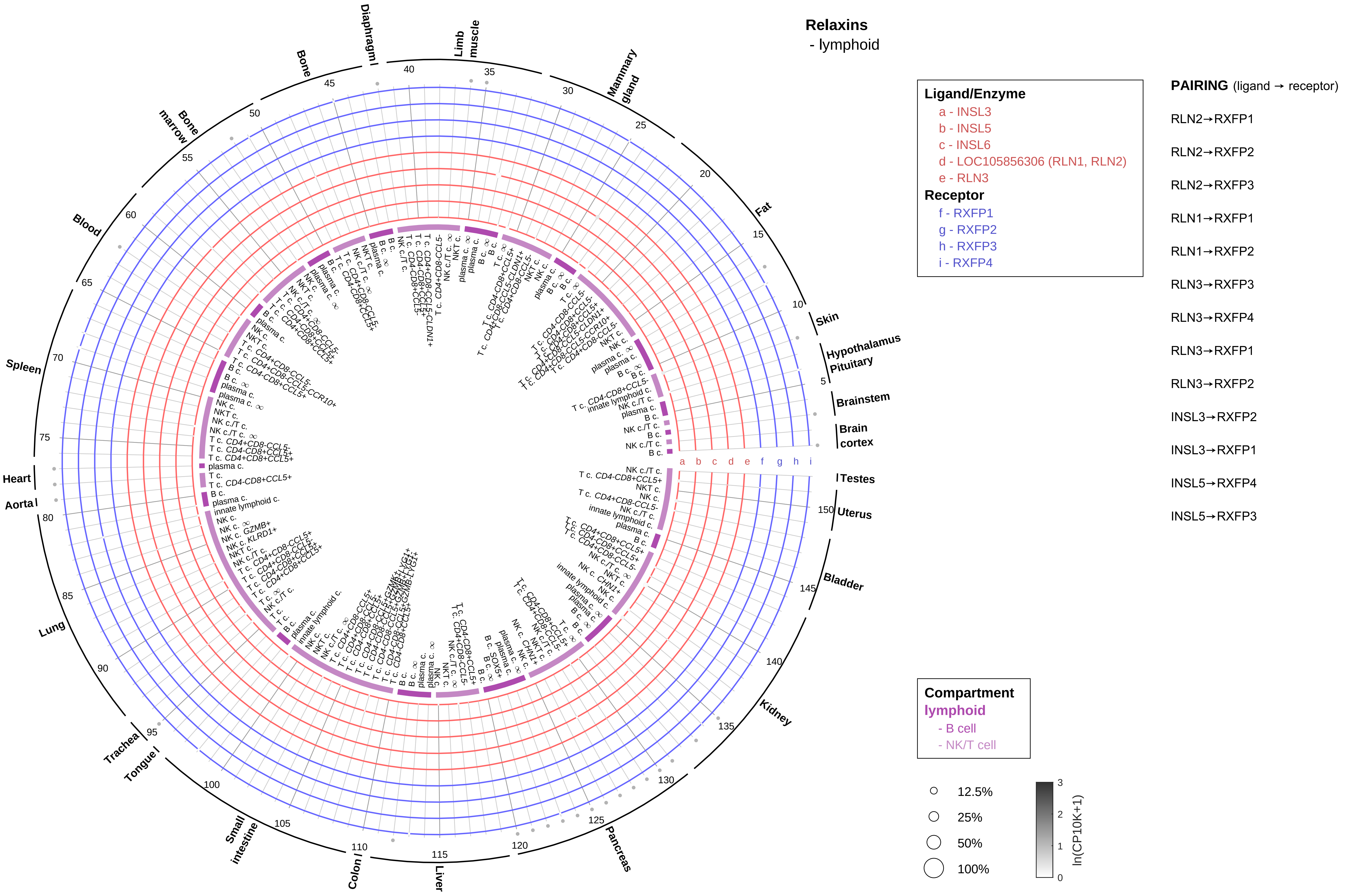

Relaxins
- lymphoid
Ligand/Enzyme
a - INSL3
b - INSL5
c - INSL6
d - LOC105856306 (RLN1, RLN2)
e - RLN3
Receptor
f - RXFP1
g - RXFP2
h - RXFP3
i - RXFP4
PAIRING (ligand → receptor)
RLN2→RXFP1
RLN2→RXFP2
RLN2→RXFP3
RLN1→RXFP1
RLN1→RXFP2
RLN3→RXFP3
RLN3→RXFP4
RLN3→RXFP1
RLN3→RXFP2
INSL3→RXFP2
INSL3→RXFP1
INSL5→RXFP4
INSL5→RXFP3
Compartment
lymphoid
- B cell
- NK/T cell
12.5%
25%
50%
100%
ln(CP10K+1)
Bone
Diaphragm
Limb muscle
Mammary gland
Fat
Skin
Hypothalamus
Pituitary
Brainstem
Brain cortex
Testes
Uterus
Bladder
Kidney
Pancreas
Liver
Colon
Small intestine
Tongue
Trachea
Lung
Aorta
Heart
Spleen
Blood
Bone marrow

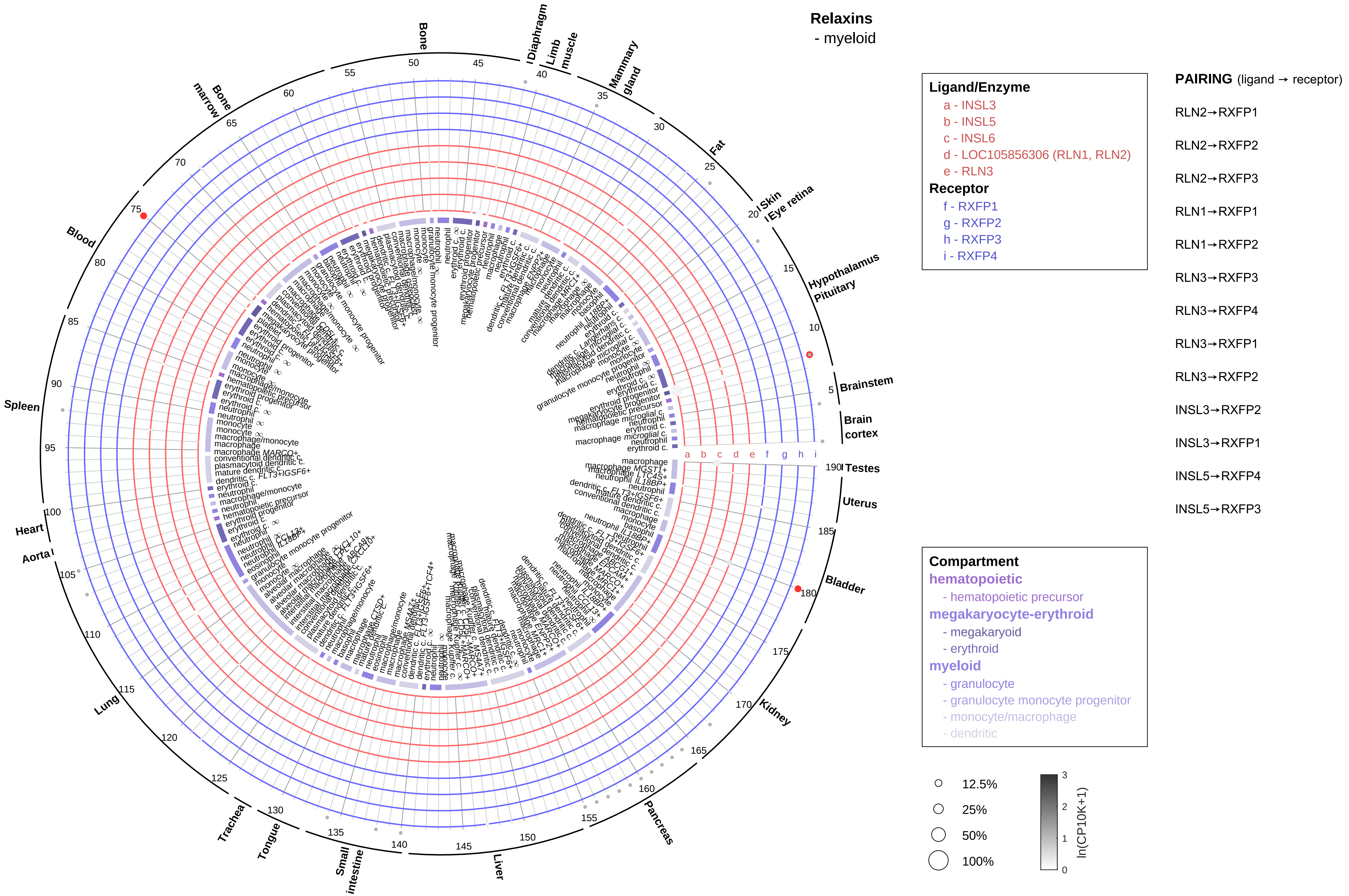

Relaxins
- myeloid
Ligand/Enzyme
a - INSL3
b - INSL5
c - INSL6
d - LOC105856306 (RLN1, RLN2)
e - RLN3
Receptor
f - RXFP1
g - RXFP2
h - RXFP3
i - RXFP4
PAIRING (ligand → receptor)
RLN2→RXFP1
RLN2→RXFP2
RLN2→RXFP3
RLN1→RXFP1
RLN1→RXFP2
RLN3→RXFP3
RLN3→RXFP4
RLN3→RXFP1
RLN3→RXFP2
INSL3→RXFP2
INSL3→RXFP1
INSL5→RXFP4
INSL5→RXFP3
Compartment
hematopoietic
- hematopoietic precursor
megakaryocyte-erythroid
- megakaryoid
- erythroid
myeloid
- granulocyte
- granulocyte monocyte progenitor
- monocyte/macrophage
- dendritic
12.5%
25%
50%
100%
ln(CP10K+1)
Bone
Diaphragm
Limb muscle
Mammary gland
Fat
Skin
Eye retina
Hypothalamus
Pituitary
Brainstem
Brain cortex
Testes
Uterus
Bladder
Kidney
Pancreas
Liver
Small intestine
Tongue
Trachea
Lung
Aorta
Heart
Spleen
Blood
Bone marrow

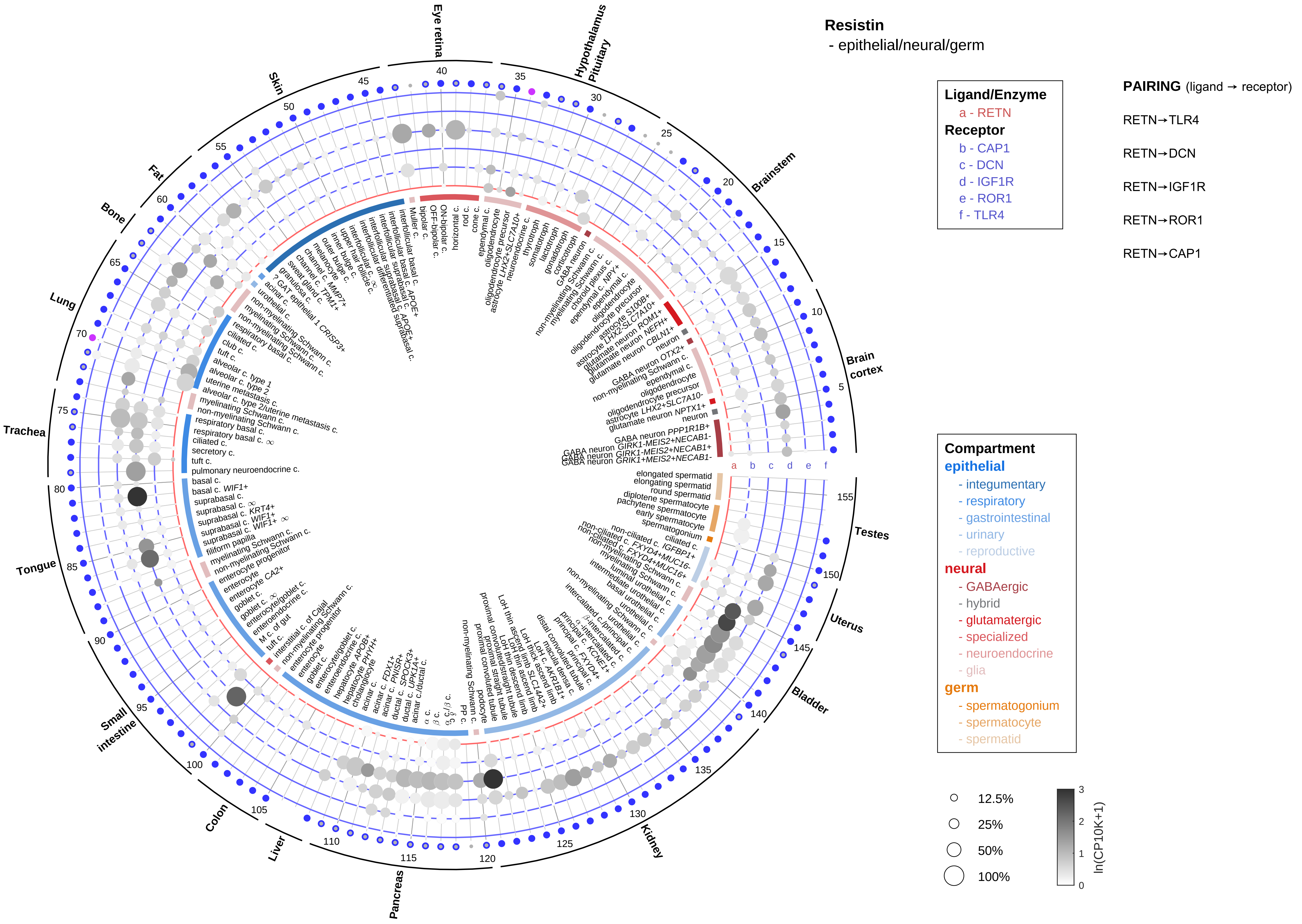

Resistin
- epithelial/neural/germ
Ligand/Enzyme
a - RETN
Receptor
b - CAP1
c - DCN
d - IGF1R
e - ROR1
f - TLR4
PAIRING (ligand → receptor)
RETN→TLR4
RETN→DCN
RETN→IGF1R
RETN→ROR1
RETN→CAP1
Compartment
epithelial
- integumentary
- respiratory
- gastrointestinal
- urinary
- reproductive
neural
- GABAergic
- hybrid
- glutamatergic
- specialized
- neuroendocrine
- glia
germ
- spermatogonium
- spermatocyte
- spermatid
12.5%
25%
50%
100%
ln(CP10K+1)
0
1
2
3
Eye retina
Hypothalamus
Pituitary
Brainstem
Brain cortex
Testes
Uterus
Bladder
Kidney
Pancreas
Liver
Colon
Small intestine
Tongue
Trachea
Lung
Bone
Fat
Skin
a
b
c
d
e
f

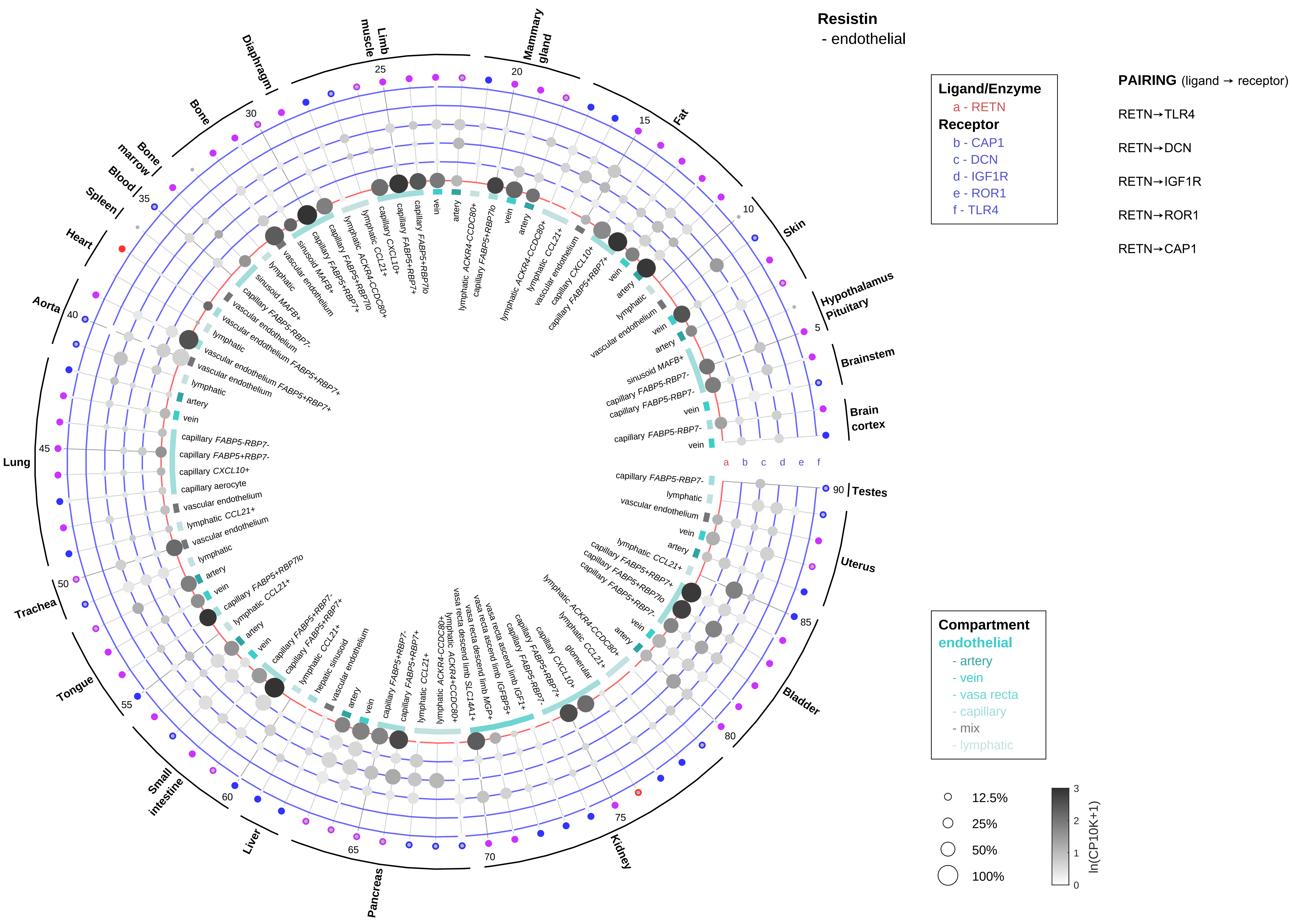

Resistin
- endothelial
Ligand/Enzyme
a - RETN
Receptor
b - CAP1
c - DCN
d - IGF1R
e - ROR1
f - TLR4
PAIRING (ligand → receptor)
RETN→TLR4
RETN→DCN
RETN→IGF1R
RETN→ROR1
RETN→CAP1
Compartment
endothelial
- artery
- vein
- vasa recta
- capillary
- mix
- lymphatic
12.5%
25%
50%
100%
ln(CP10K+1)
Brain cortex
Brainstem
Hypothalamus Pituitary
Skin
Fat
Mammary gland
Limb muscle
Diaphragm
Bone
Bone marrow
Blood
Spleen
Heart
Aorta
Lung
Trachea
Tongue
Small intestine
Liver
Pancreas
Kidney
Bladder
Uterus
Testes

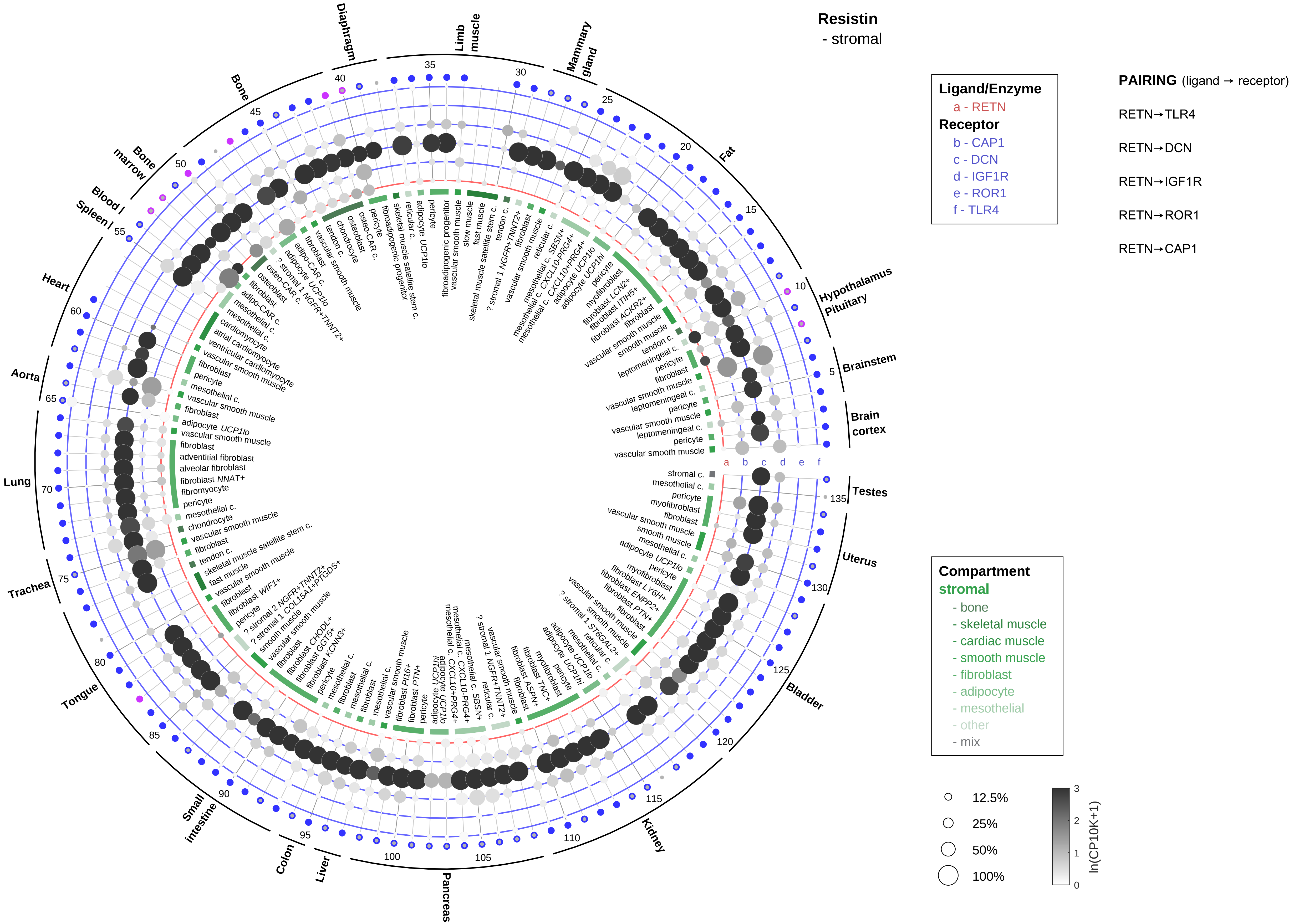

Resistin
- stromal
Ligand/Enzyme
a - RETN
Receptor
b - CAP1
c - DCN
d - IGF1R
e - ROR1
f - TLR4
PAIRING (ligand → receptor)
RETN→TLR4
RETN→DCN
RETN→IGF1R
RETN→ROR1
RETN→CAP1
Compartment
stromal
- bone
- skeletal muscle
- cardiac muscle
- smooth muscle
- fibroblast
- adipocyte
- mesothelial
- other
- mix
12.5%
25%
50%
100%
ln(CP10K+1)
Brain cortex
Brainstem
Hypothalamus Pituitary
Fat
Mammary gland
Limb muscle
Diaphragm
Bone
Bone marrow
Blood
Spleen
Heart
Aorta
Lung
Trachea
Tongue
Small intestine
Colon
Liver
Pancreas
Kidney
Bladder
Uterus
Testes

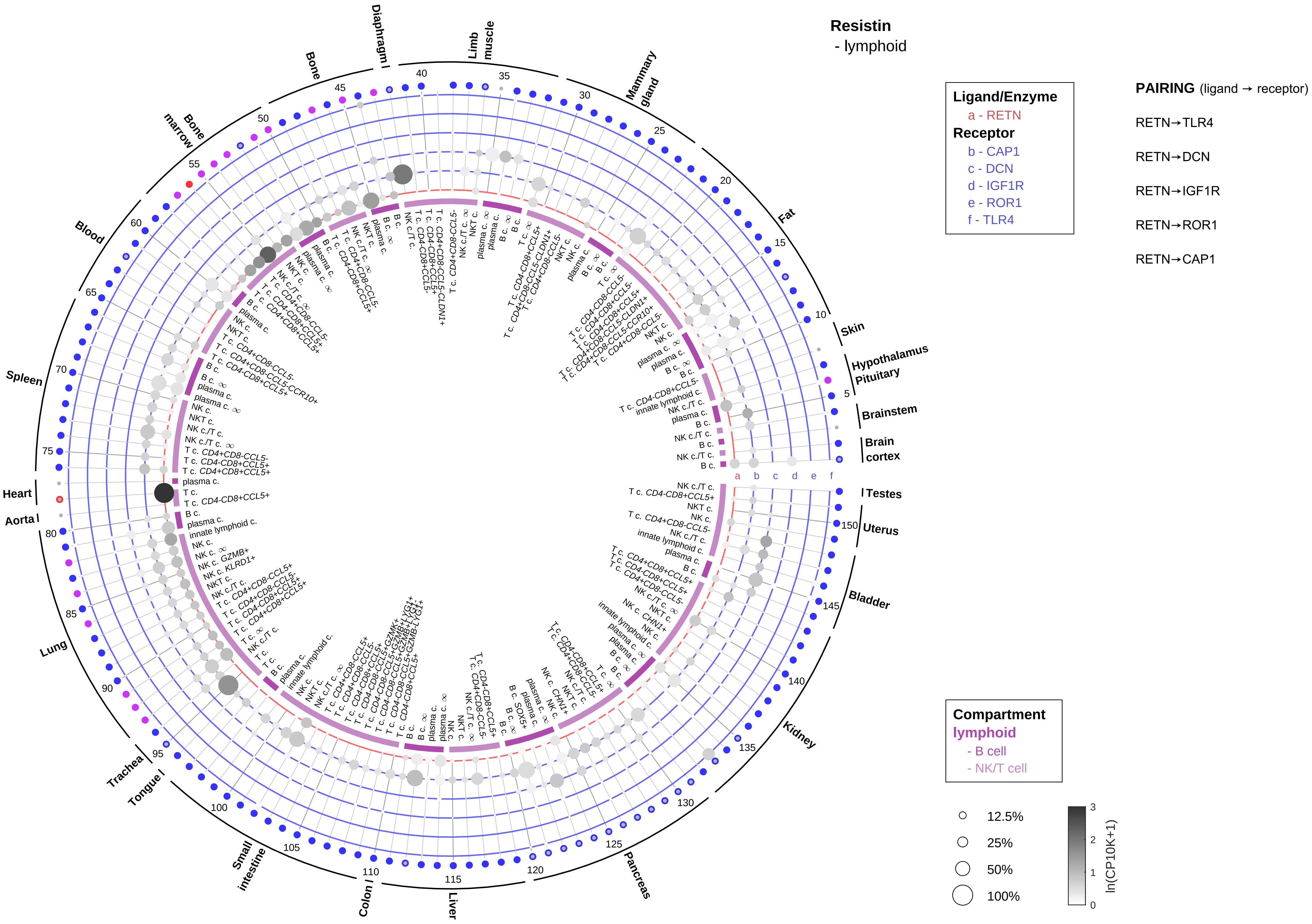
Resistin
- lymphoid
Ligand/Enzyme
a - RETN
Receptor
b - CAP1
c - DCN
d - IGF1R
e - ROR1
f - TLR4
PAIRING (ligand → receptor)
RETN→TLR4
RETN→DCN
RETN→IGF1R
RETN→ROR1
RETN→CAP1
Compartment
lymphoid
- B cell
- NK/T cell
12.5%
25%
50%
100%
ln(CP10K+1)
Skin
Hypothalamus
Pituitary
Brainstem
Brain cortex
Testes
Uterus
Bladder
Kidney
Pancreas
Liver
Colon
Small intestine
Tongue
Trachea
Lung
Aorta
Heart
Spleen
Blood
Bone marrow
Bone
Diaphragm
Limb muscle
Mammary gland
Fat
a
b
c
d
e
f

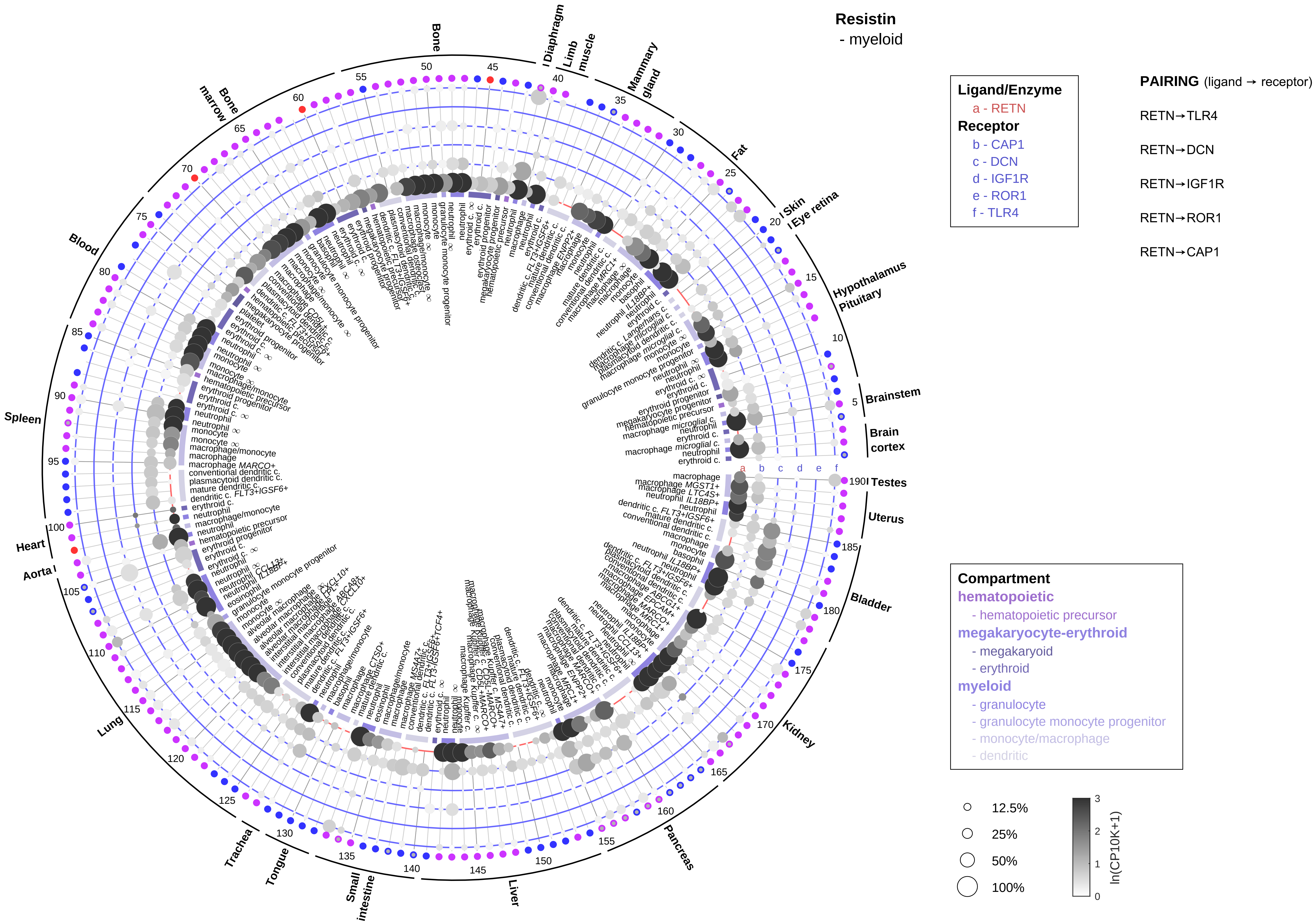
Resistin
- myeloid
Ligand/Enzyme
a - RETN
Receptor
b - CAP1
c - DCN
d - IGF1R
e - ROR1
f - TLR4
PAIRING (ligand → receptor)
RETN→TLR4
RETN→DCN
RETN→IGF1R
RETN→ROR1
RETN→CAP1
Compartment
hematopoietic
- hematopoietic precursor
megakaryocyte-erythroid
- megakaryoid
- erythroid
myeloid
- granulocyte
- granulocyte monocyte progenitor
- monocyte/macrophage
- dendritic
12.5%
25%
50%
100%
ln(CP10K+1)
Bone
Diaphragm
Limb muscle
Mammary gland
Fat
Skin
Eye retina
Hypothalamus
Pituitary
Brainstem
Brain cortex
Testes
Uterus
Bladder
Kidney
Pancreas
Liver
Small intestine
Tongue
Trachea
Lung
Aorta
Heart
Spleen
Blood
Bone marrow

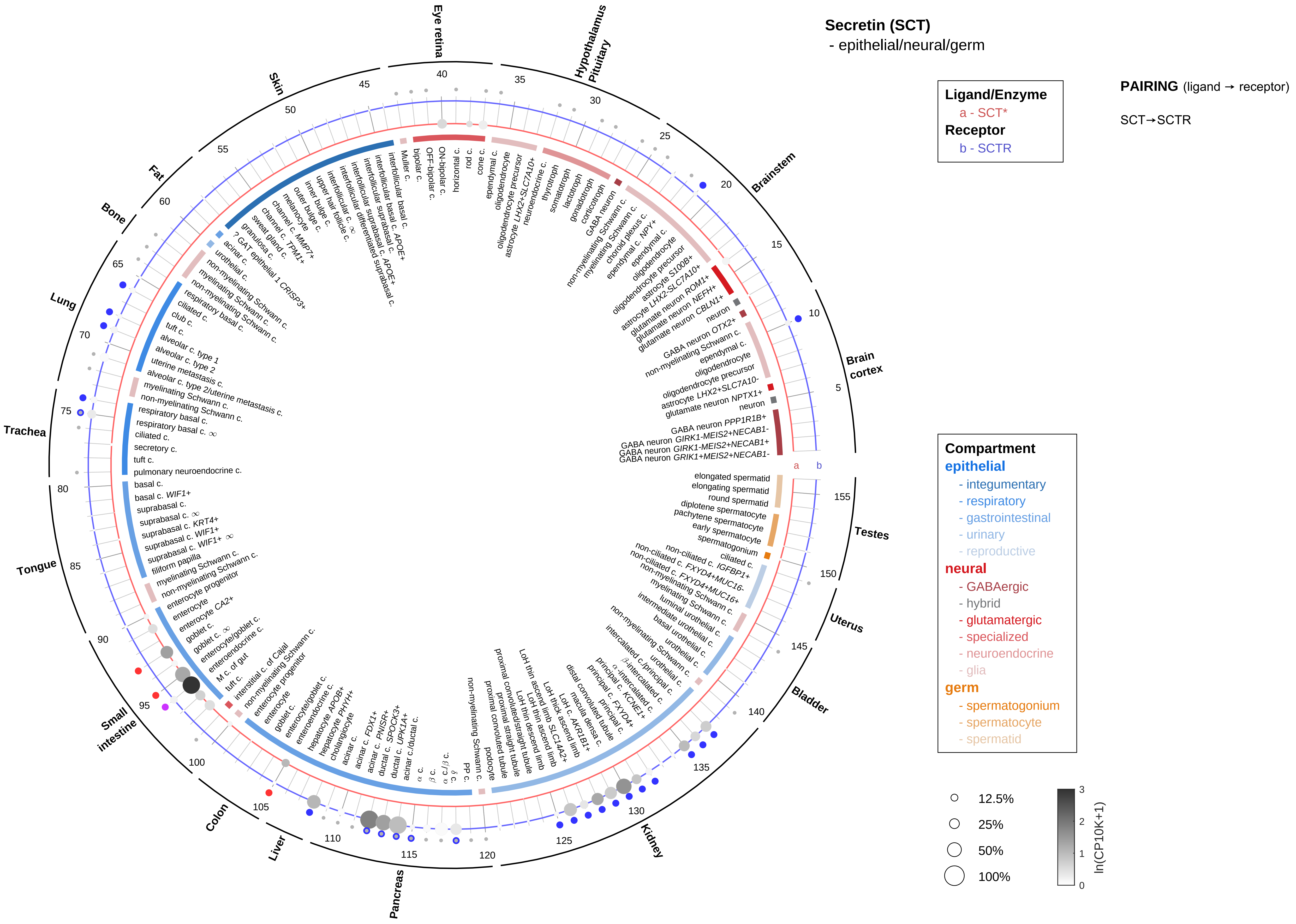
Secretin (SCT)
- epithelial/neural/germ
Ligand/Enzyme
a - SCT*
Receptor
b - SCTR
PAIRING (ligand → receptor)
SCT→SCTR
Compartment
epithelial
- integumentary
- respiratory
- gastrointestinal
- urinary
- reproductive
neural
- GABAergic
- hybrid
- glutamatergic
- specialized
- neuroendocrine
- glia
germ
- spermatogonium
- spermatocyte
- spermatid
12.5%
25%
50%
100%
ln(CP10K+1)
Eye retina
Hypothalamus Pituitary
Brainstem
Brain cortex
Testes
Uterus
Bladder
Kidney
Pancreas
Liver
Colon
Small intestine
Tongue
Trachea
Lung
Bone
Fat
Skin

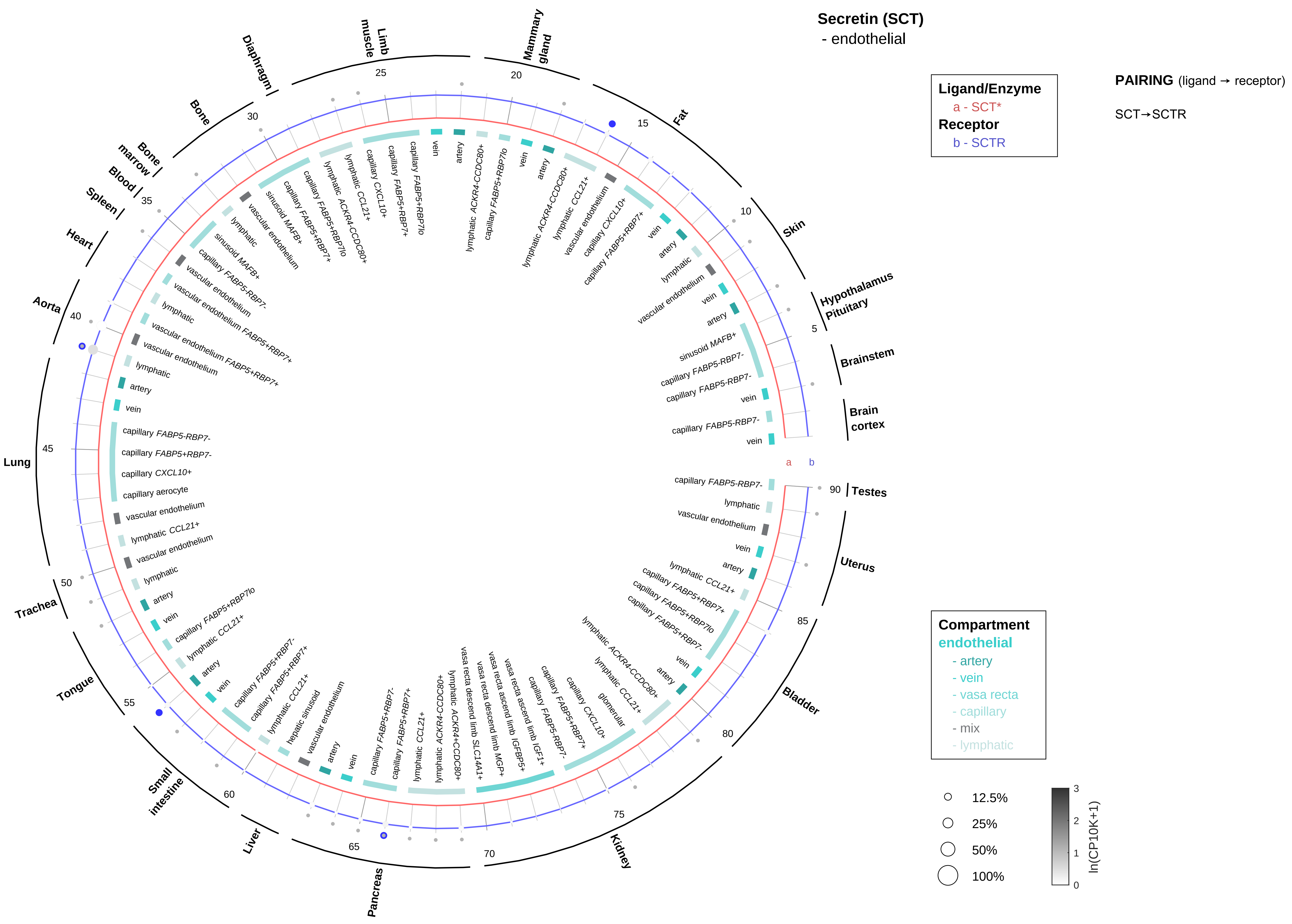
Secretin (SCT)
- endothelial
Ligand/Enzyme
a - SCT*
Receptor
b - SCTR
PAIRING (ligand → receptor)
SCT→SCTR
Compartment
endothelial
- artery
- vein
- vasa recta
- capillary
- mix
- lymphatic
12.5%
25%
50%
100%
ln(CP10K+1)
0
1
2
3
a
b
Brain cortex
Brainstem
Hypothalamus Pituitary
Skin
Fat
Mammary gland
Limb muscle
Diaphragm
Bone
Bone marrow
Blood
Spleen
Heart
Aorta
Lung
Trachea
Tongue
Small intestine
Liver
Pancreas
Kidney
Bladder
Uterus
Testes

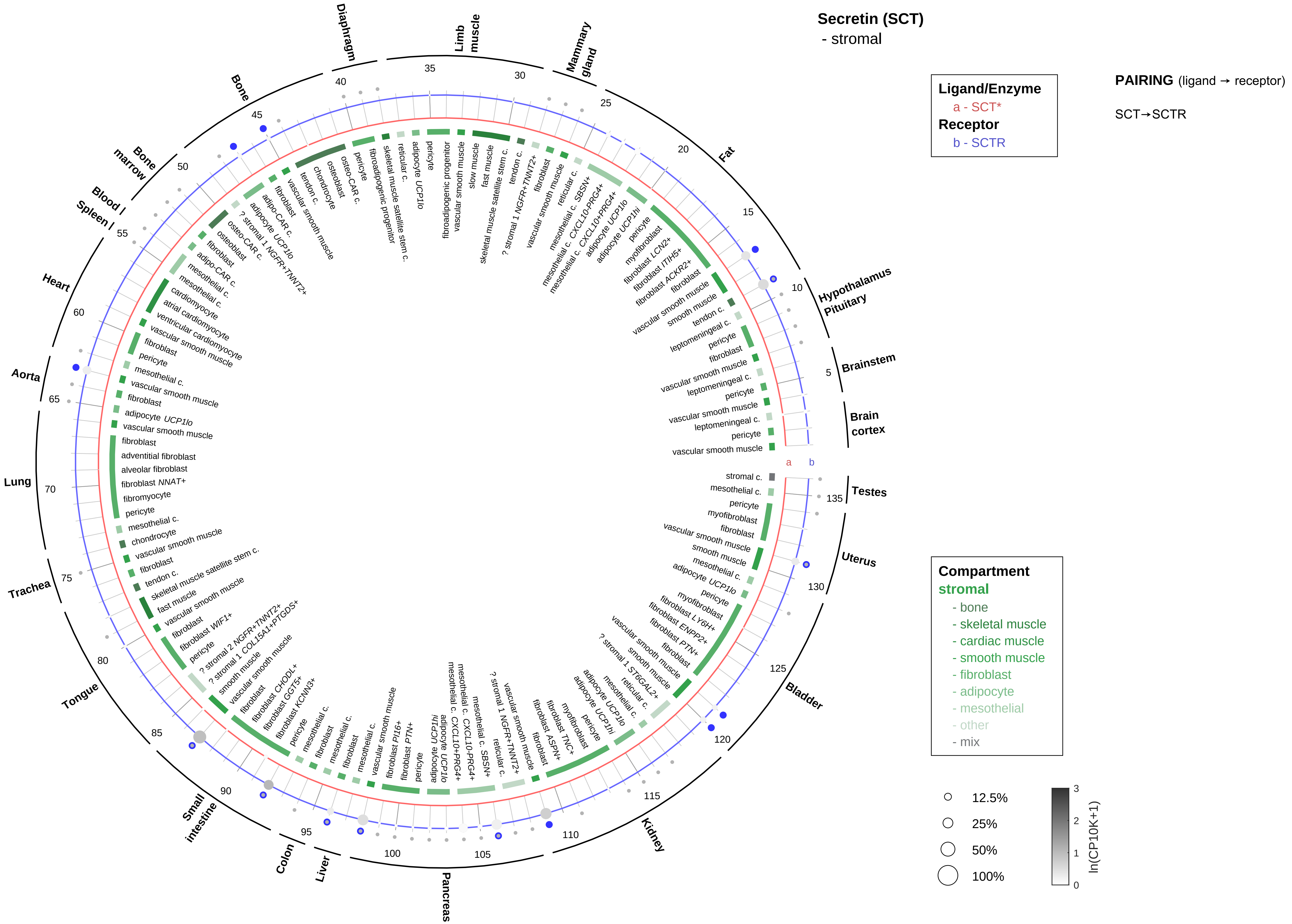

Secretin (SCT)
- stromal
Ligand/Enzyme
a - SCT*
Receptor
b - SCTR
PAIRING (ligand → receptor)
SCT→SCTR
Compartment
stromal
- bone
- skeletal muscle
- cardiac muscle
- smooth muscle
- fibroblast
- adipocyte
- mesothelial
- other
- mix
12.5%
25%
50%
100%
ln(CP10K+1)
Brain cortex
Brainstem
Hypothalamus Pituitary
Fat
Mammary gland
Limb muscle
Diaphragm
Bone
Bone marrow
Blood
Spleen
Heart
Aorta
Lung
Trachea
Tongue
Small intestine
Colon
Liver
Pancreas
Kidney
Bladder
Uterus
Testes

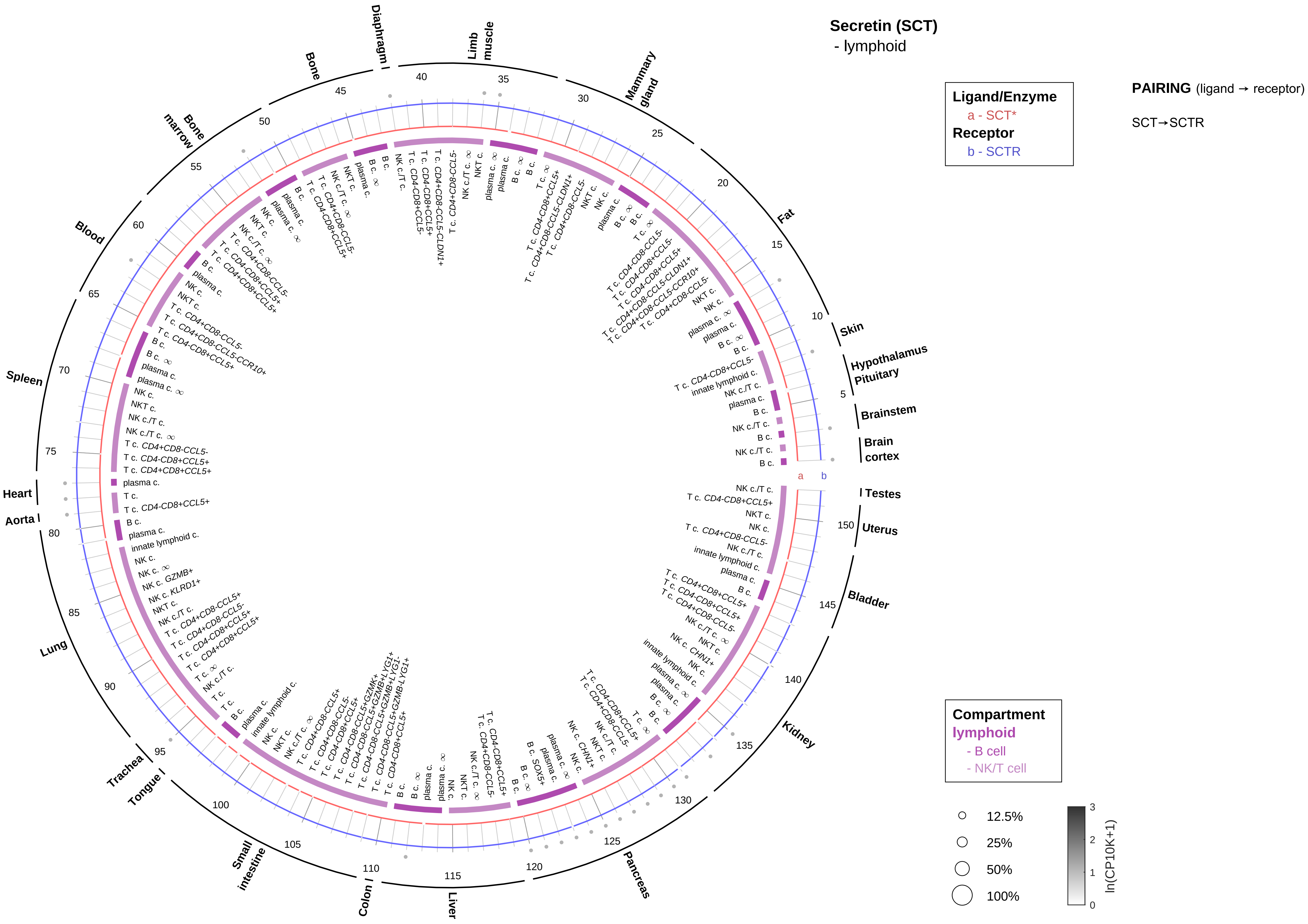

Secretin (SCT)
- lymphoid
Ligand/Enzyme
a - SCT*
Receptor
b - SCTR
PAIRING (ligand → receptor)
SCT→SCTR
Compartment
lymphoid
- B cell
- NK/T cell
12.5%
25%
50%
100%
ln(CP10K+1)
Skin
Hypothalamus
Pituitary
Brainstem
Brain cortex
Testes
Uterus
Bladder
Kidney
Pancreas
Liver
Colon
Small intestine
Tongue
Trachea
Lung
Aorta
Heart
Spleen
Blood
Bone marrow
Bone
Diaphragm
Limb muscle
Mammary gland
Fat

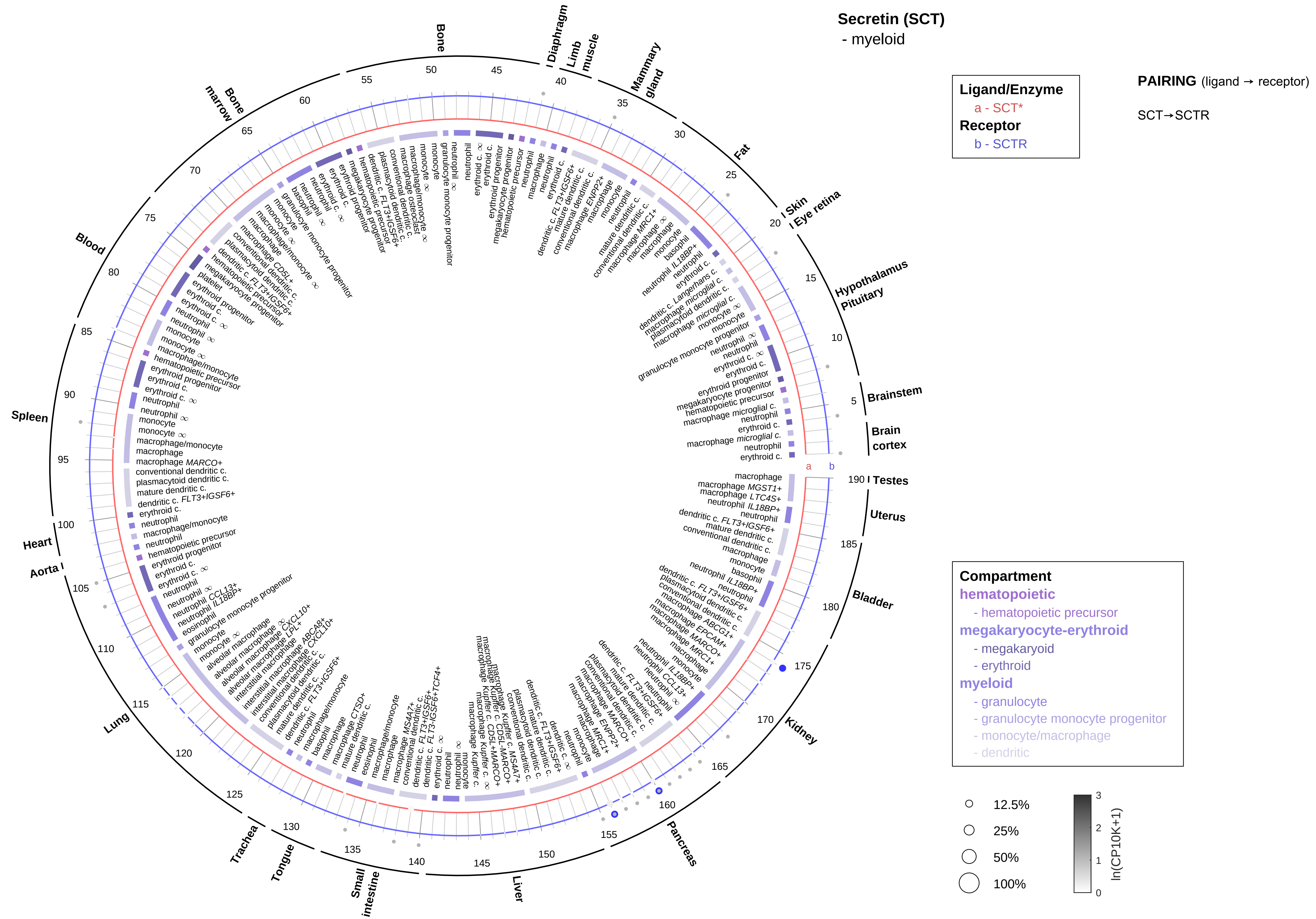

Secretin (SCT)
- myeloid
Ligand/Enzyme
a - SCT*
Receptor
b - SCTR
PAIRING (ligand → receptor)
SCT→SCTR
Compartment
hematopoietic
- hematopoietic precursor
megakaryocyte-erythroid
- megakaryoid
- erythroid
myeloid
- granulocyte
- granulocyte monocyte progenitor
- monocyte/macrophage
- dendritic
12.5%
25%
50%
100%
ln(CP10K+1)
Bone
Diaphragm
Limb muscle
Mammary gland
Fat
Skin
Eye retina
Hypothalamus
Pituitary
Brainstem
Brain cortex
Testes
Uterus
Bladder
Kidney
Pancreas
Liver
Small intestine
Tongue
Trachea
Lung
Aorta
Heart
Spleen
Blood
Bone marrow

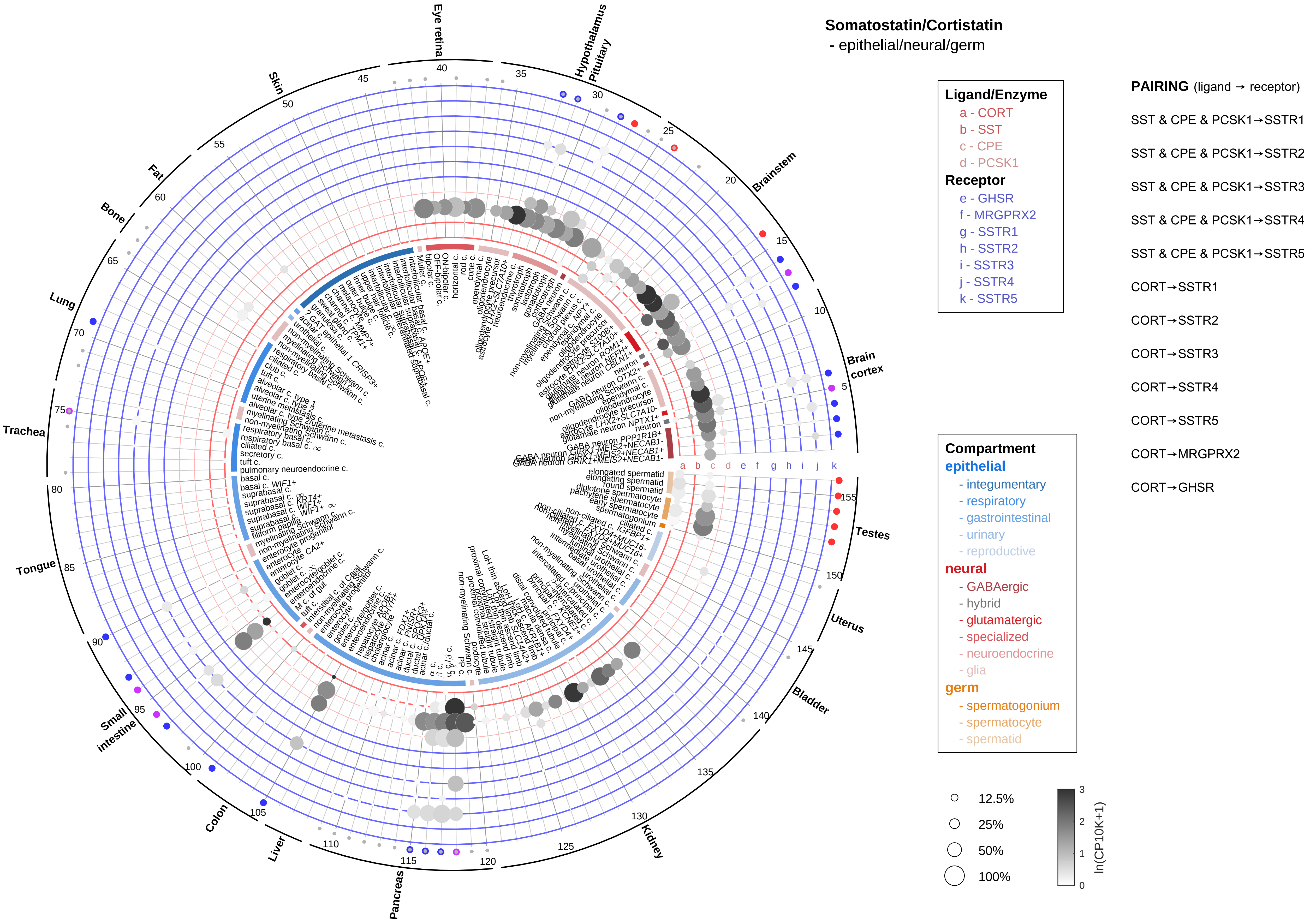

Somatostatin/Cortistatin
- epithelial/neural/germ
Ligand/Enzyme
a - CORT
b - SST
c - CPE
d - PCSK1
Receptor
e - GHSR
f - MRGPRX2
g - SSTR1
h - SSTR2
i - SSTR3
j - SSTR4
k - SSTR5
PAIRING (ligand → receptor)
SST & CPE & PCSK1→SSTR1
SST & CPE & PCSK1→SSTR2
SST & CPE & PCSK1→SSTR3
SST & CPE & PCSK1→SSTR4
SST & CPE & PCSK1→SSTR5
CORT→SSTR1
CORT→SSTR2
CORT→SSTR3
CORT→SSTR4
CORT→SSTR5
CORT→MRGPRX2
CORT→GHSR
Compartment
epithelial
- integumentary
- respiratory
- gastrointestinal
- urinary
- reproductive
neural
- GABAergic
- hybrid
- glutamatergic
- specialized
- neuroendocrine
- glia
germ
- spermatogonium
- spermatocyte
- spermatid
12.5%
25%
50%
100%
ln(CP10K+1)
Eye retina
Hypothalamus
Pituitary
Brainstem
Brain cortex
Testes
Uterus
Bladder
Kidney
Pancreas
Liver
Colon
Small intestine
Tongue
Trachea
Lung
Bone
Fat
Skin

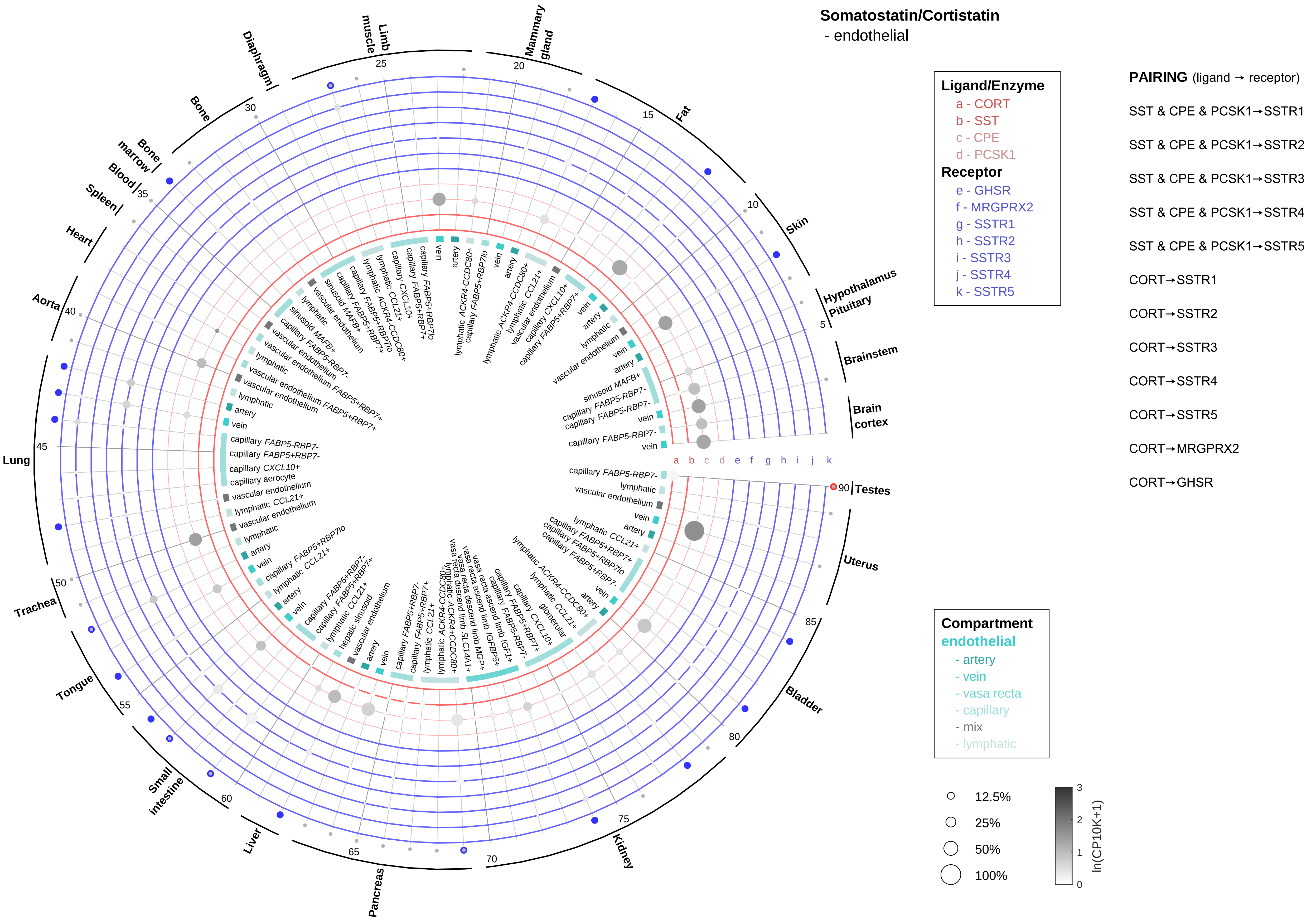
Somatostatin/Cortistatin
- endothelial
Ligand/Enzyme
a - CORT
b - SST
c - CPE
d - PCSK1
Receptor
e - GHSR
f - MRGPRX2
g - SSTR1
h - SSTR2
i - SSTR3
j - SSTR4
k - SSTR5
PAIRING (ligand → receptor)
SST & CPE & PCSK1→SSTR1
SST & CPE & PCSK1→SSTR2
SST & CPE & PCSK1→SSTR3
SST & CPE & PCSK1→SSTR4
SST & CPE & PCSK1→SSTR5
CORT→SSTR1
CORT→SSTR2
CORT→SSTR3
CORT→SSTR4
CORT→SSTR5
CORT→MRGPRX2
CORT→GHSR
Compartment
endothelial
- artery
- vein
- vasa recta
- capillary
- mix
- lymphatic
12.5%
25%
50%
100%
ln(CP10K+1)
Brain cortex
Brainstem
Hypothalamus Pituitary
Skin
Fat
Mammary gland
Limb muscle
Diaphragm
Bone
Bone marrow
Blood
Spleen
Heart
Aorta
Lung
Trachea
Tongue
Small intestine
Liver
Pancreas
Kidney
Bladder
Uterus
Testes
a b c d e f g h i j k

Somatostatin/Cortistatin
- stromal

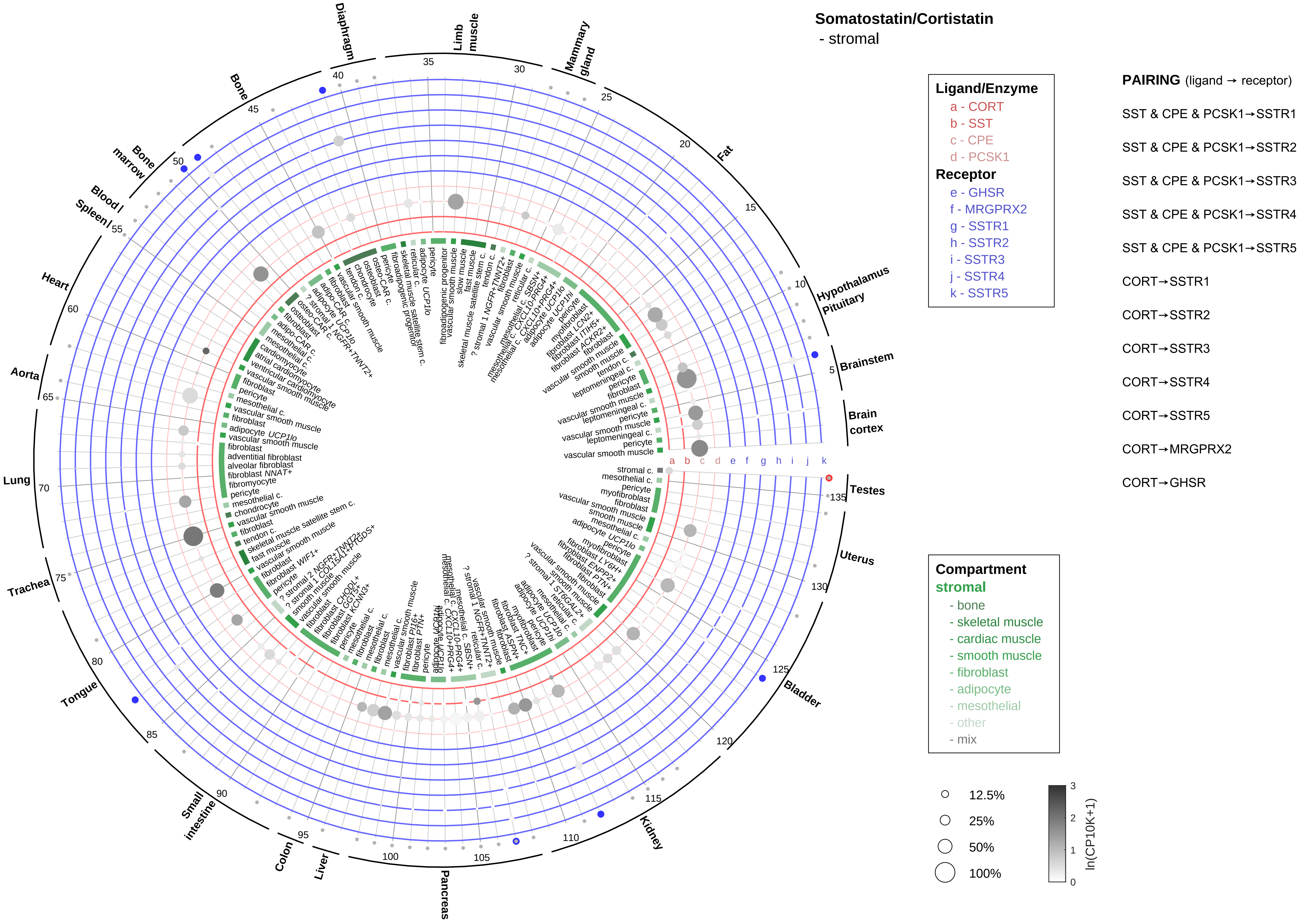

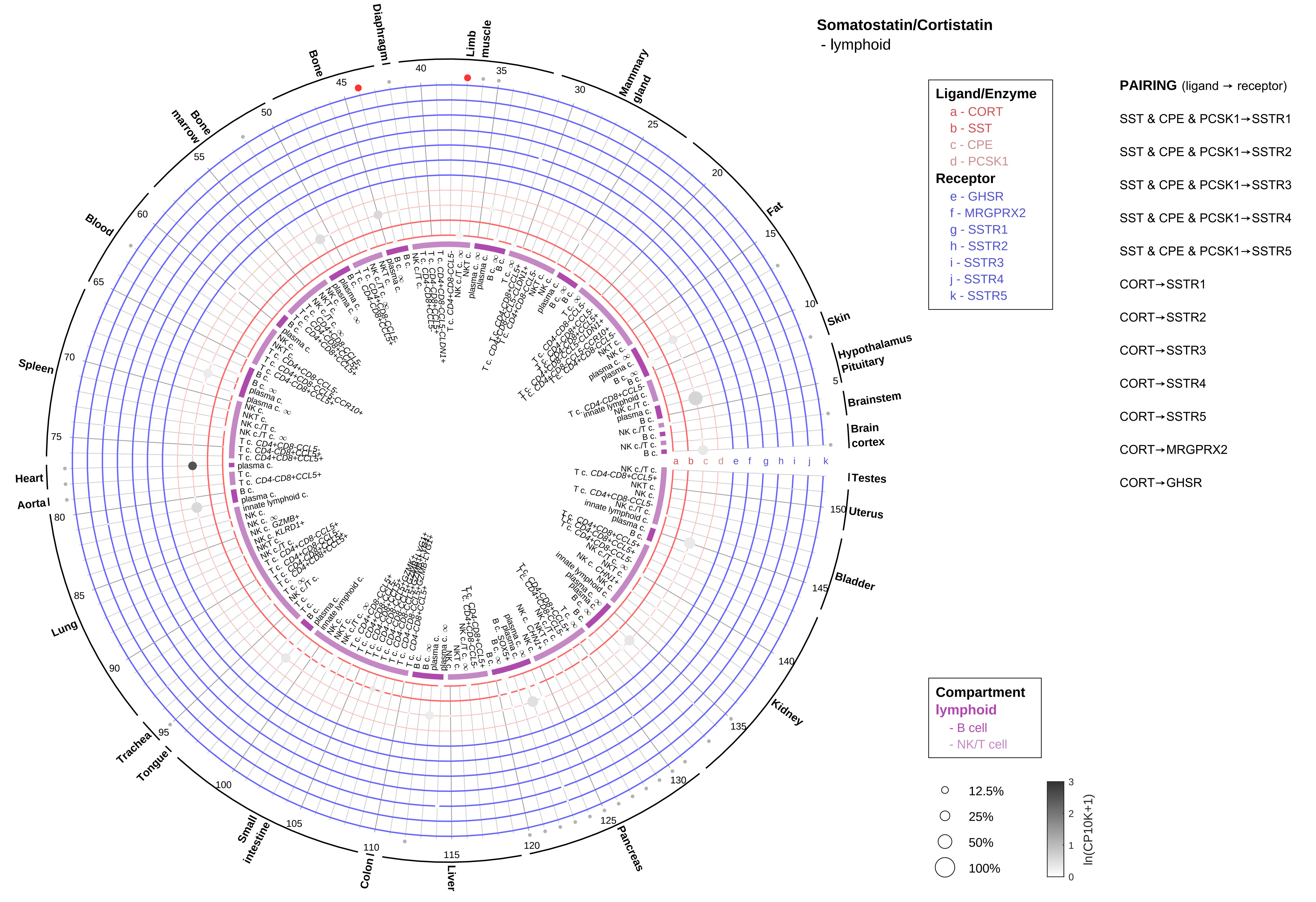

Somatostatin/Cortistatin
- lymphoid
Ligand/Enzyme
a - CORT
b - SST
c - CPE
d - PCSK1
Receptor
e - GHSR
f - MRGPRX2
g - SSTR1
h - SSTR2
i - SSTR3
j - SSTR4
k - SSTR5
PAIRING (ligand → receptor)
SST & CPE & PCSK1→SSTR1
SST & CPE & PCSK1→SSTR2
SST & CPE & PCSK1→SSTR3
SST & CPE & PCSK1→SSTR4
SST & CPE & PCSK1→SSTR5
CORT→SSTR1
CORT→SSTR2
CORT→SSTR3
CORT→SSTR4
CORT→SSTR5
CORT→MRGPRX2
CORT→GHSR
Compartment
lymphoid
- B cell
- NK/T cell
12.5%
25%
50%
100%
ln(CP10K+1)
Diaphragm
Limb muscle
Mammary gland
Fat
Skin
Hypothalamus
Pituitary
Brainstem
Brain cortex
Testes
Uterus
Bladder
Kidney
Pancreas
Liver
Colon
Small intestine
Tongue
Trachea
Lung
Aorta
Heart
Spleen
Blood
Bone marrow
Bone

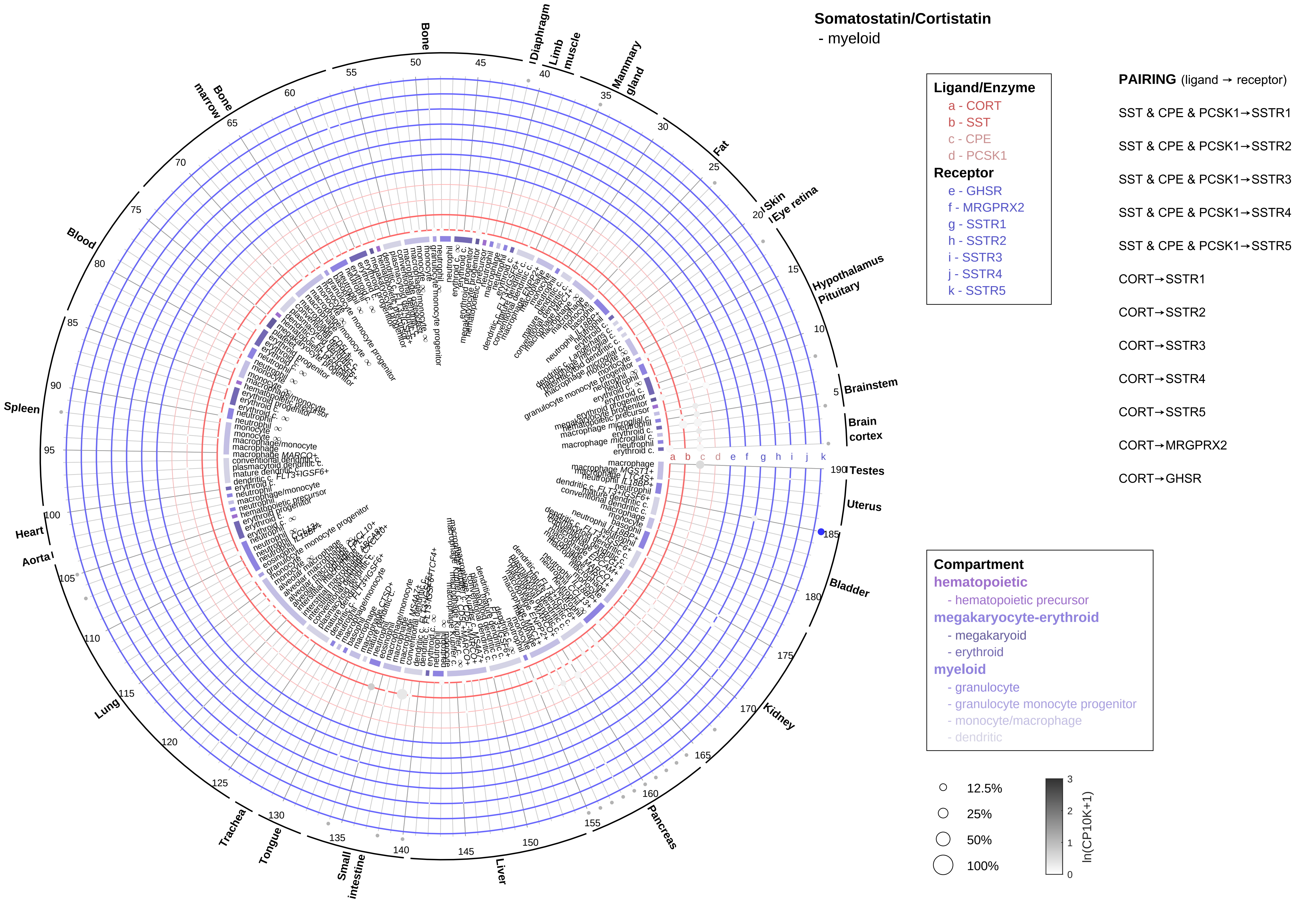

Somatostatin/Cortistatin
- myeloid
Ligand/Enzyme
a - CORT
b - SST
c - CPE
d - PCSK1
Receptor
e - GHSR
f - MRGPRX2
g - SSTR1
h - SSTR2
i - SSTR3
j - SSTR4
k - SSTR5
PAIRING (ligand → receptor)
SST & CPE & PCSK1→SSTR1
SST & CPE & PCSK1→SSTR2
SST & CPE & PCSK1→SSTR3
SST & CPE & PCSK1→SSTR4
SST & CPE & PCSK1→SSTR5
CORT→SSTR1
CORT→SSTR2
CORT→SSTR3
CORT→SSTR4
CORT→SSTR5
CORT→MRGPRX2
CORT→GHSR
Compartment
hematopoietic
- hematopoietic precursor
megakaryocyte-erythroid
- megakaryoid
- erythroid
myeloid
- granulocyte
- granulocyte monocyte progenitor
- monocyte/macrophage
- dendritic
12.5%
25%
50%
100%
ln(CP10K+1)
Bone
Diaphragm
Limb muscle
Mammary gland
Fat
Skin
Eye retina
Hypothalamus
Pituitary
Brainstem
Brain cortex
Testes
Uterus
Bladder
Kidney
Pancreas
Liver
Small intestine
Tongue
Trachea
Lung
Aorta
Heart
Spleen
Blood
Bone marrow

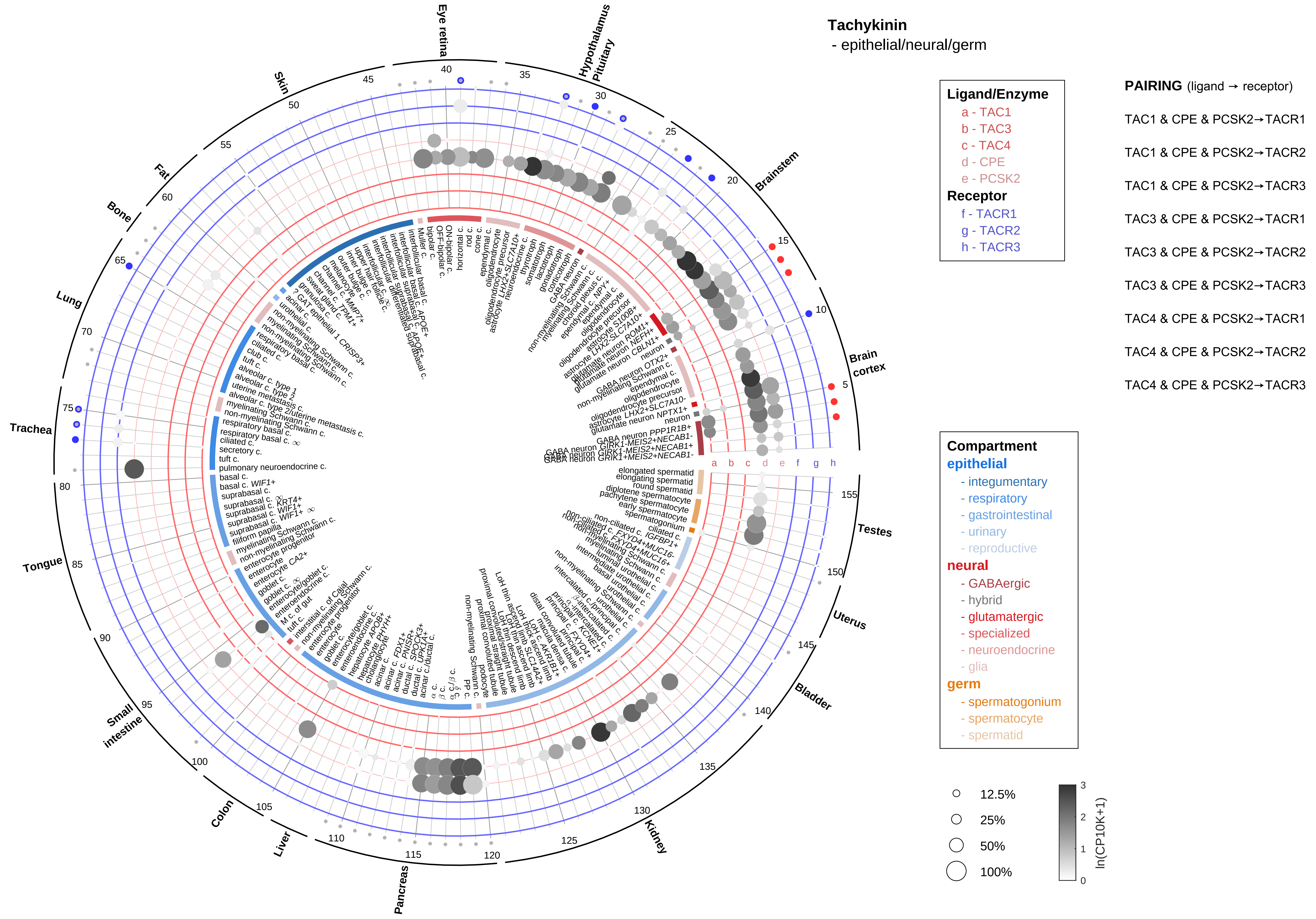
Tachykinin
- epithelial/neural/germ
Ligand/Enzyme
a - TAC1
b - TAC3
c - TAC4
d - CPE
e - PCSK2
Receptor
f - TACR1
g - TACR2
h - TACR3
PAIRING (ligand → receptor)
TAC1 & CPE & PCSK2→TACR1
TAC1 & CPE & PCSK2→TACR2
TAC1 & CPE & PCSK2→TACR3
TAC3 & CPE & PCSK2→TACR1
TAC3 & CPE & PCSK2→TACR2
TAC3 & CPE & PCSK2→TACR3
TAC4 & CPE & PCSK2→TACR1
TAC4 & CPE & PCSK2→TACR2
TAC4 & CPE & PCSK2→TACR3
Compartment
epithelial
- integumentary
- respiratory
- gastrointestinal
- urinary
- reproductive
neural
- GABAergic
- hybrid
- glutamatergic
- specialized
- neuroendocrine
- glia
germ
- spermatogonium
- spermatocyte
- spermatid
12.5%
25%
50%
100%
ln(CP10K+1)
Eye retina
Hypothalamus Pituitary
Brainstem
Brain cortex
Testes
Uterus
Bladder
Kidney
Pancreas
Liver
Colon
Small intestine
Tongue
Trachea
Lung
Bone
Fat
Skin

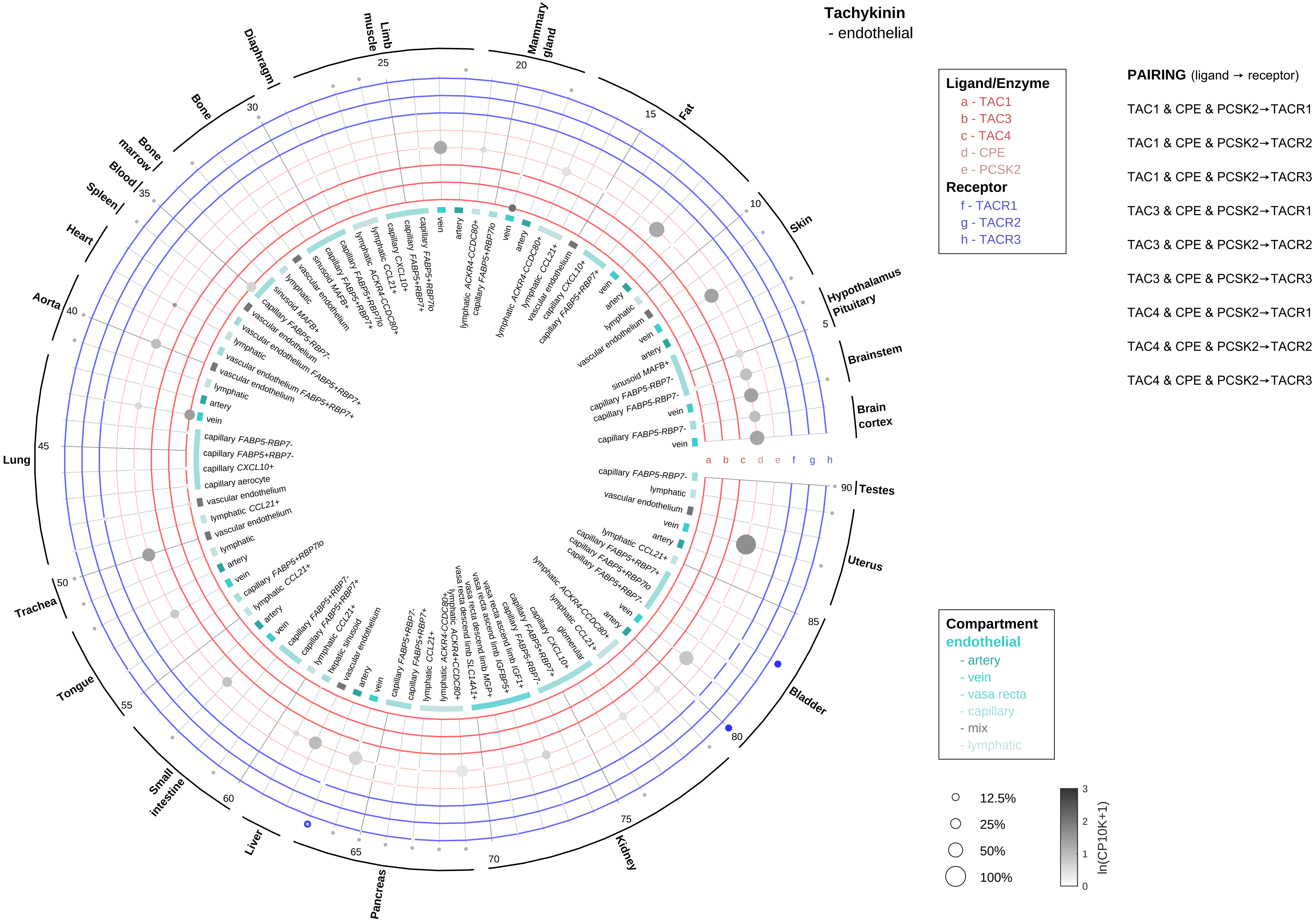
Tachykinin
- endothelial
Ligand/Enzyme
a - TAC1
b - TAC3
c - TAC4
d - CPE
e - PCSK2
Receptor
f - TACR1
g - TACR2
h - TACR3
PAIRING (ligand → receptor)
TAC1 & CPE & PCSK2→TACR1
TAC1 & CPE & PCSK2→TACR2
TAC1 & CPE & PCSK2→TACR3
TAC3 & CPE & PCSK2→TACR1
TAC3 & CPE & PCSK2→TACR2
TAC3 & CPE & PCSK2→TACR3
TAC4 & CPE & PCSK2→TACR1
TAC4 & CPE & PCSK2→TACR2
TAC4 & CPE & PCSK2→TACR3
Compartment
endothelial
- artery
- vein
- vasa recta
- capillary
- mix
- lymphatic
12.5%
25%
50%
100%
ln(CP10K+1)
Brain cortex
Brainstem
Hypothalamus Pituitary
Skin
Fat
Mammary gland
Limb muscle
Diaphragm
Bone
Bone marrow
Blood
Spleen
Heart
Aorta
Lung
Trachea
Tongue
Small intestine
Liver
Pancreas
Kidney
Bladder
Uterus
Testes

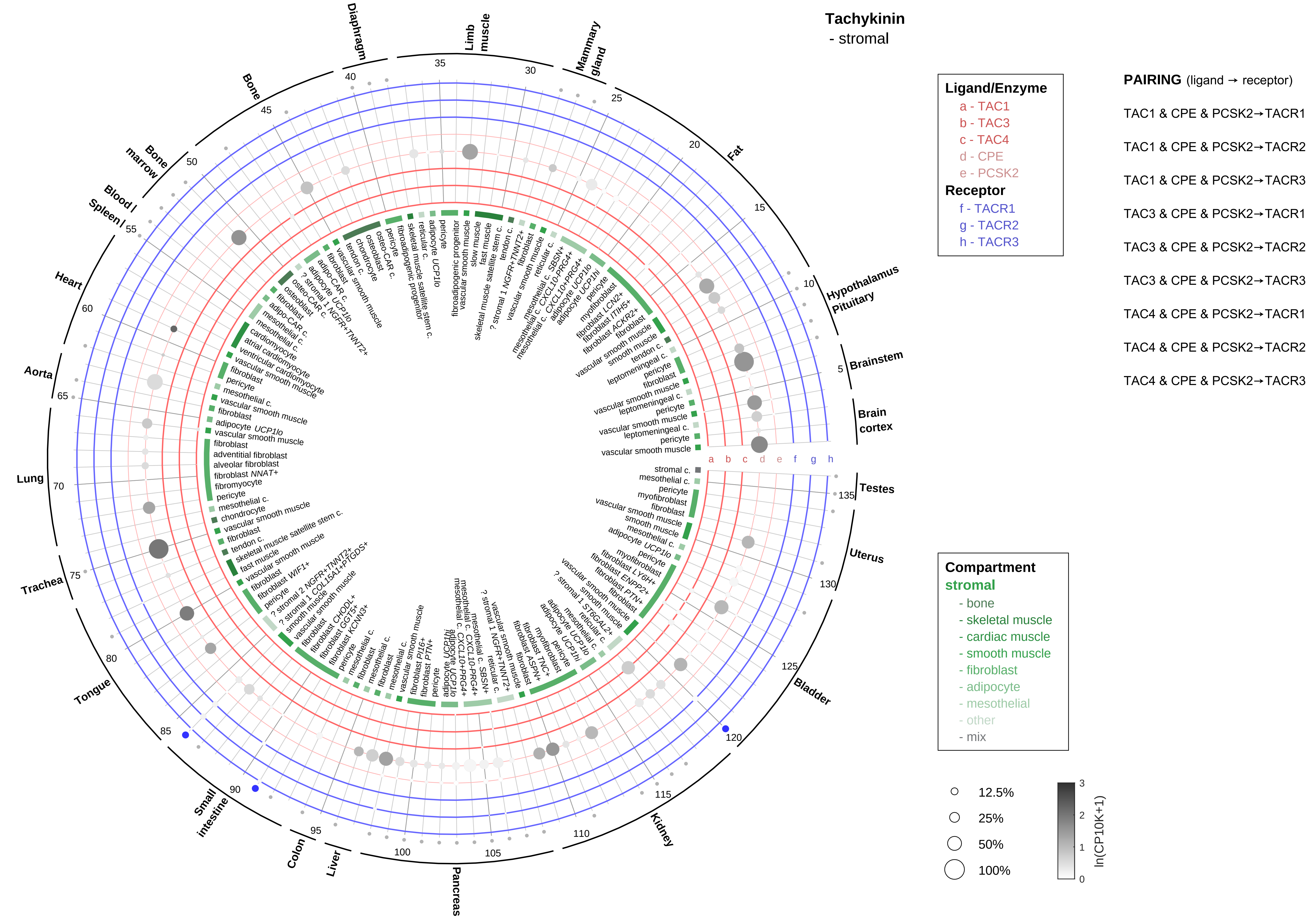
Tachykinin
- stromal
Ligand/Enzyme
a - TAC1
b - TAC3
c - TAC4
d - CPE
e - PCSK2
Receptor
f - TACR1
g - TACR2
h - TACR3
PAIRING (ligand → receptor)
TAC1 & CPE & PCSK2→TACR1
TAC1 & CPE & PCSK2→TACR2
TAC1 & CPE & PCSK2→TACR3
TAC3 & CPE & PCSK2→TACR1
TAC3 & CPE & PCSK2→TACR2
TAC3 & CPE & PCSK2→TACR3
TAC4 & CPE & PCSK2→TACR1
TAC4 & CPE & PCSK2→TACR2
TAC4 & CPE & PCSK2→TACR3
Compartment
stromal
- bone
- skeletal muscle
- cardiac muscle
- smooth muscle
- fibroblast
- adipocyte
- mesothelial
- other
- mix
12.5%
25%
50%
100%
ln(CP10K+1)
Diaphragm
Limb muscle
Mammary gland
Fat
Hypothalamus Pituitary
Brainstem
Brain cortex
Testes
Uterus
Bladder
Kidney
Pancreas
Liver
Colon
Small intestine
Tongue
Trachea
Lung
Aorta
Heart
Spleen
Blood
Bone marrow
Bone

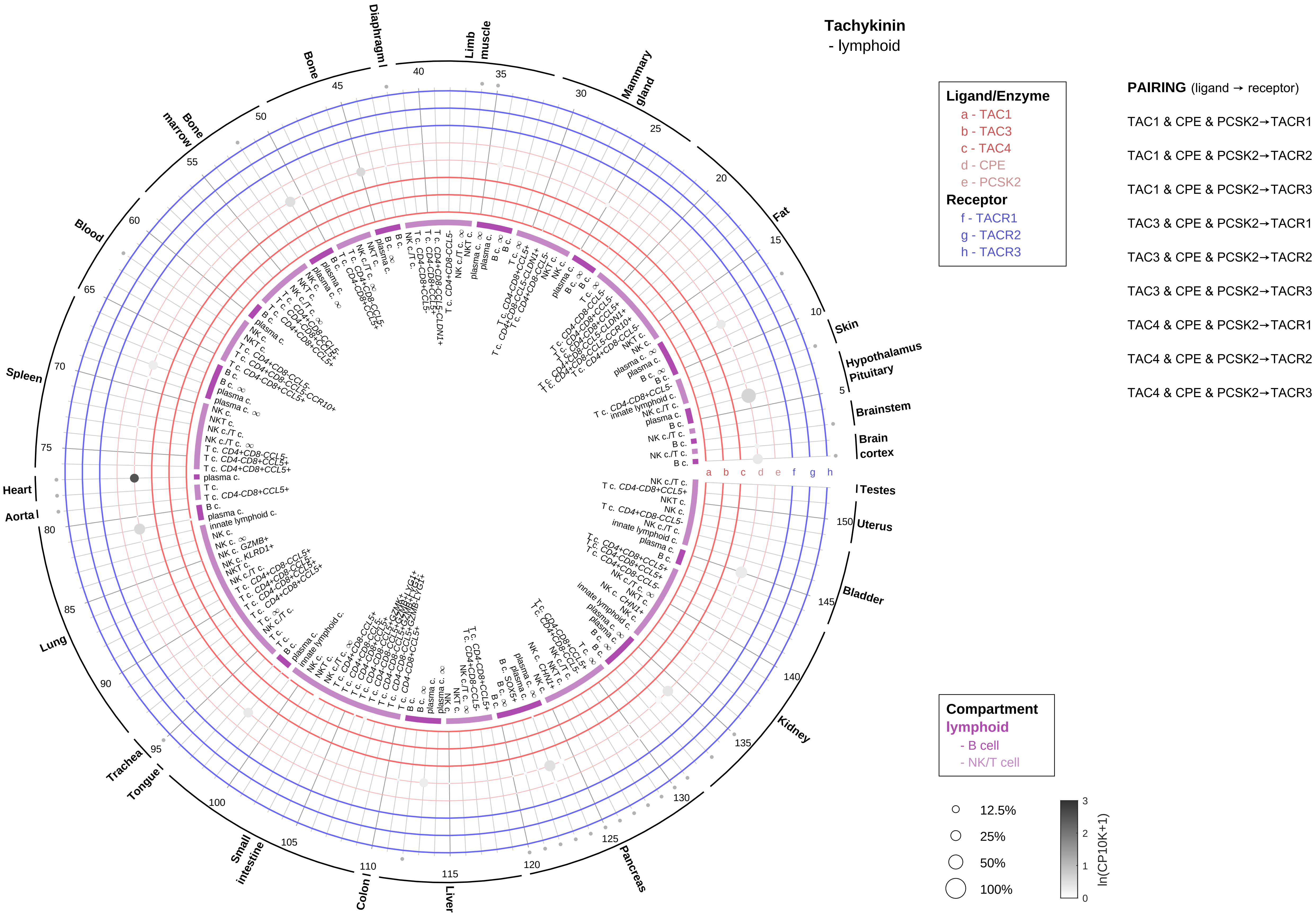

Tachykinin
- lymphoid
Ligand/Enzyme
a - TAC1
b - TAC3
c - TAC4
d - CPE
e - PCSK2
Receptor
f - TACR1
g - TACR2
h - TACR3
PAIRING (ligand → receptor)
TAC1 & CPE & PCSK2→TACR1
TAC1 & CPE & PCSK2→TACR2
TAC1 & CPE & PCSK2→TACR3
TAC3 & CPE & PCSK2→TACR1
TAC3 & CPE & PCSK2→TACR2
TAC3 & CPE & PCSK2→TACR3
TAC4 & CPE & PCSK2→TACR1
TAC4 & CPE & PCSK2→TACR2
TAC4 & CPE & PCSK2→TACR3
Compartment
lymphoid
- B cell
- NK/T cell
12.5%
25%
50%
100%
ln(CP10K+1)
Diaphragm
Limb muscle
Mammary gland
Fat
Skin
Hypothalamus
Pituitary
Brainstem
Brain cortex
Testes
Uterus
Bladder
Kidney
Pancreas
Liver
Colon
Small intestine
Tongue
Trachea
Lung
Aorta
Heart
Spleen
Blood
Bone marrow
Bone

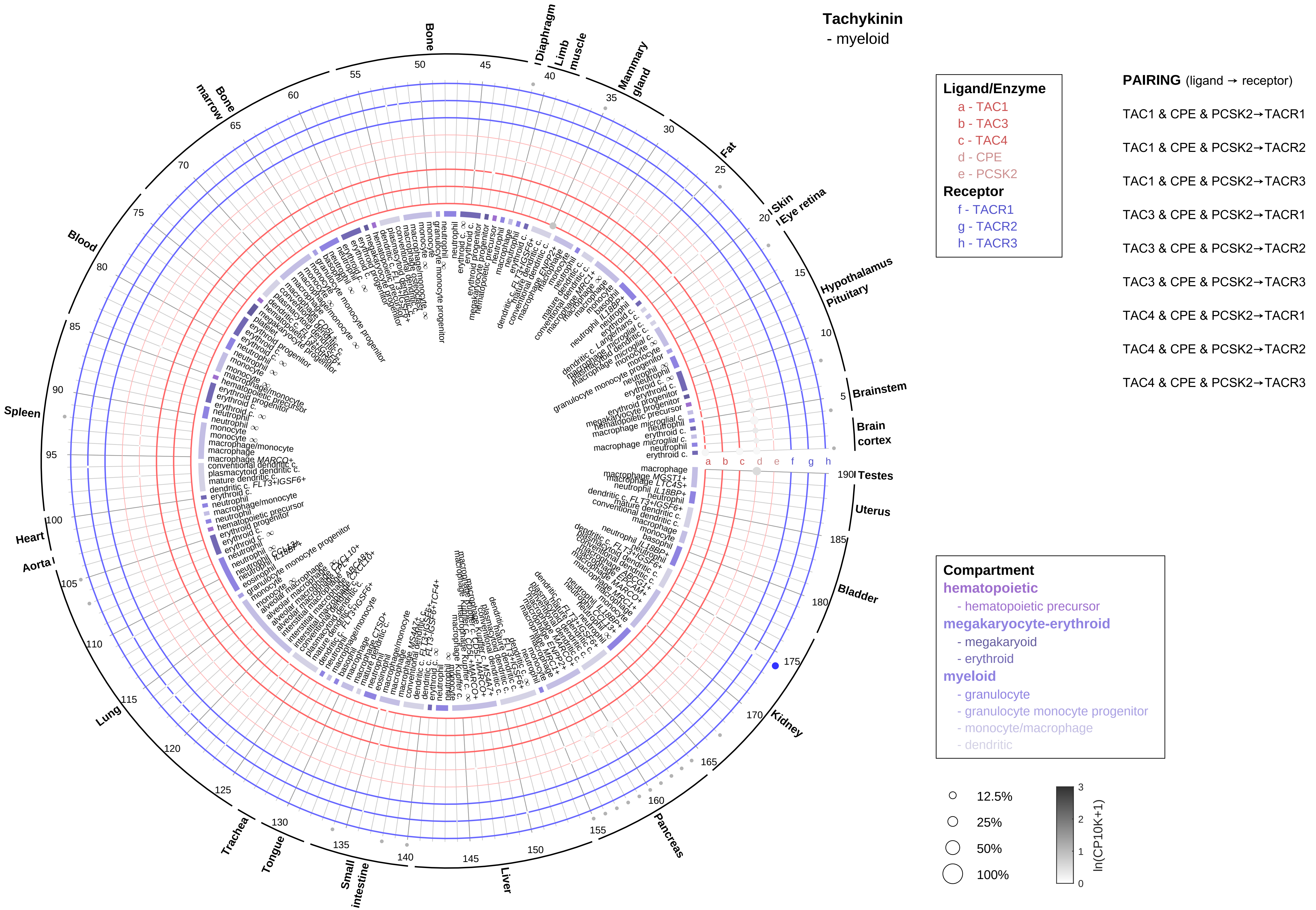

Tachykinin
- myeloid
Ligand/Enzyme
a - TAC1
b - TAC3
c - TAC4
d - CPE
e - PCSK2
Receptor
f - TACR1
g - TACR2
h - TACR3
PAIRING (ligand → receptor)
TAC1 & CPE & PCSK2→TACR1
TAC1 & CPE & PCSK2→TACR2
TAC1 & CPE & PCSK2→TACR3
TAC3 & CPE & PCSK2→TACR1
TAC3 & CPE & PCSK2→TACR2
TAC3 & CPE & PCSK2→TACR3
TAC4 & CPE & PCSK2→TACR1
TAC4 & CPE & PCSK2→TACR2
TAC4 & CPE & PCSK2→TACR3
Compartment
hematopoietic
- hematopoietic precursor
megakaryocyte-erythroid
- megakaryoid
- erythroid
myeloid
- granulocyte
- granulocyte monocyte progenitor
- monocyte/macrophage
- dendritic
12.5%
25%
50%
100%
ln(CP10K+1)
Bone
Diaphragm
Limb muscle
Mammary gland
Fat
Skin
Eye retina
Hypothalamus
Pituitary
Brainstem
Brain cortex
Testes
Uterus
Bladder
Kidney
Pancreas
Liver
Small intestine
Tongue
Trachea
Lung
Aorta
Heart
Spleen
Blood
Bone marrow

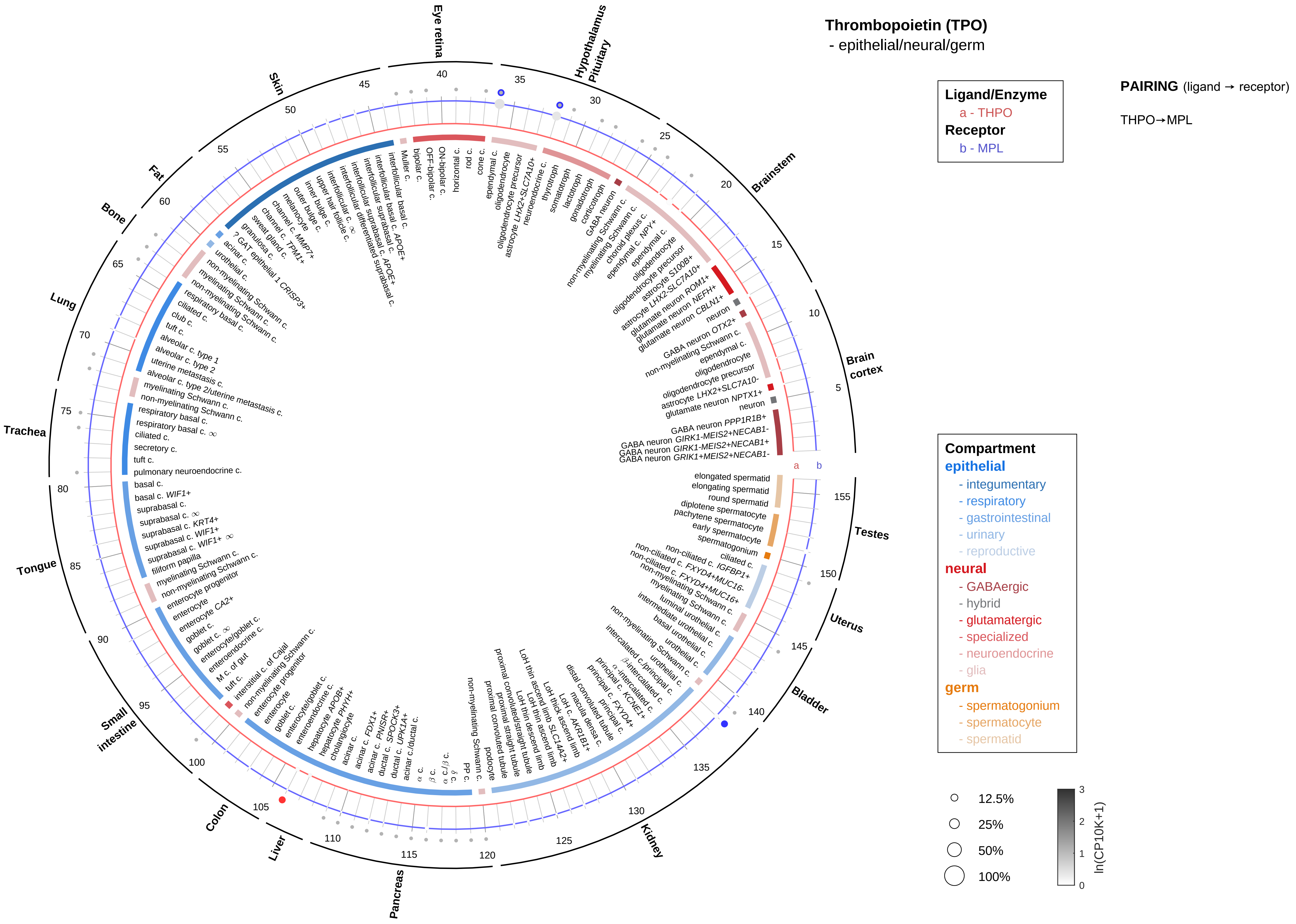

Thrombopoietin (TPO)
- epithelial/neural/germ
Ligand/Enzyme
a - THPO
Receptor
b - MPL
PAIRING (ligand → receptor)
THPO→MPL
Compartment
epithelial
- integumentary
- respiratory
- gastrointestinal
- urinary
- reproductive
neural
- GABAergic
- hybrid
- glutamatergic
- specialized
- neuroendocrine
- glia
germ
- spermatogonium
- spermatocyte
- spermatid
12.5%
25%
50%
100%
ln(CP10K+1)
Eye retina
Hypothalamus
Pituitary
Brainstem
Brain cortex
Testes
Uterus
Bladder
Kidney
Pancreas
Liver
Colon
Small intestine
Tongue
Trachea
Lung
Bone
Fat
Skin

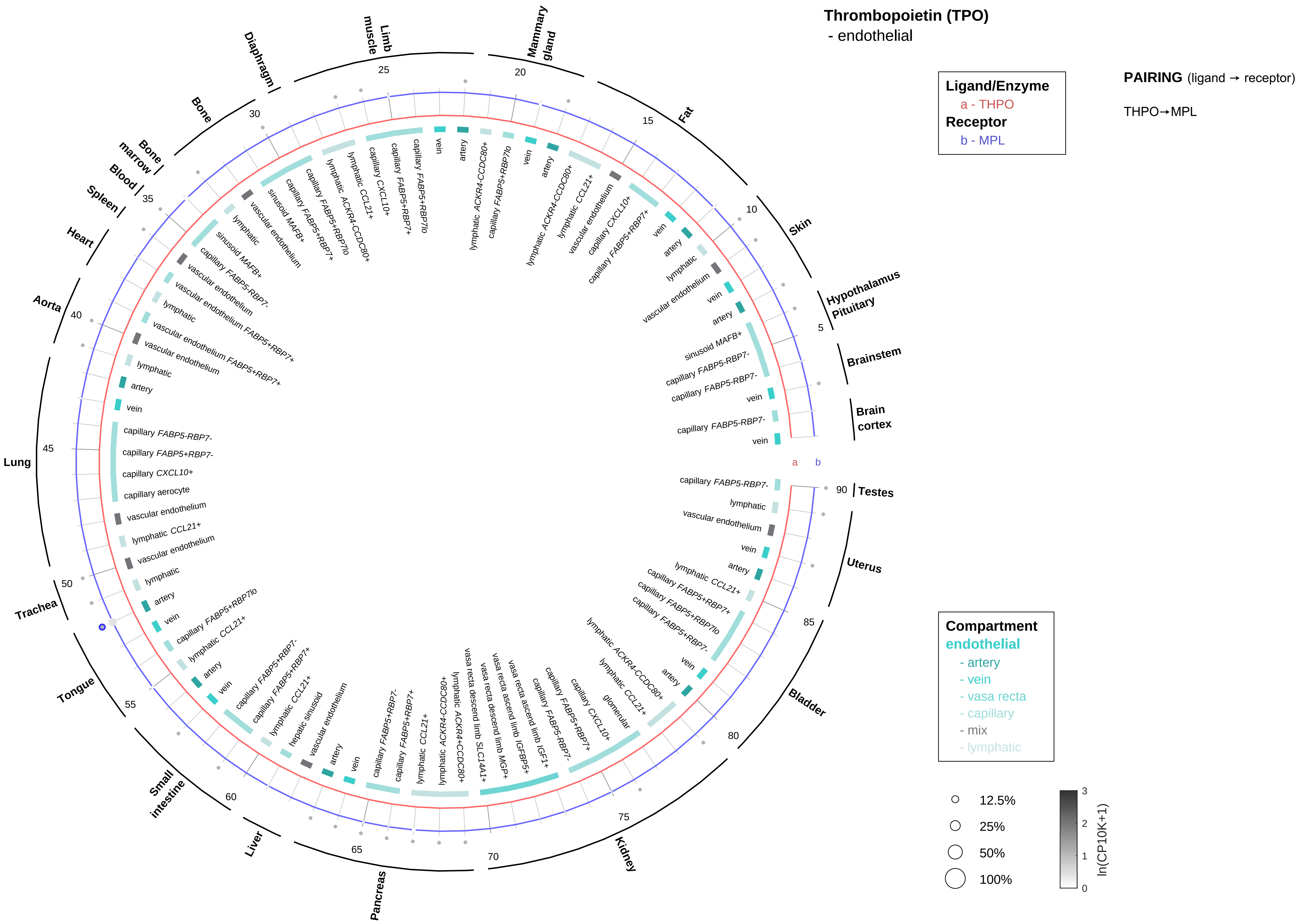
Thrombopoietin (TPO)
- endothelial
Ligand/Enzyme
a - THPO
Receptor
b - MPL
PAIRING (ligand → receptor)
THPO→MPL
Compartment
endothelial
- artery
- vein
- vasa recta
- capillary
- mix
- lymphatic
12.5%
25%
50%
100%
ln(CP10K+1)
Brain cortex
Brainstem
Hypothalamus Pituitary
Skin
Fat
Mammary gland
Limb muscle
Diaphragm
Bone
Bone marrow
Blood
Spleen
Heart
Aorta
Lung
Trachea
Tongue
Small intestine
Liver
Pancreas
Kidney
Bladder
Uterus
Testes

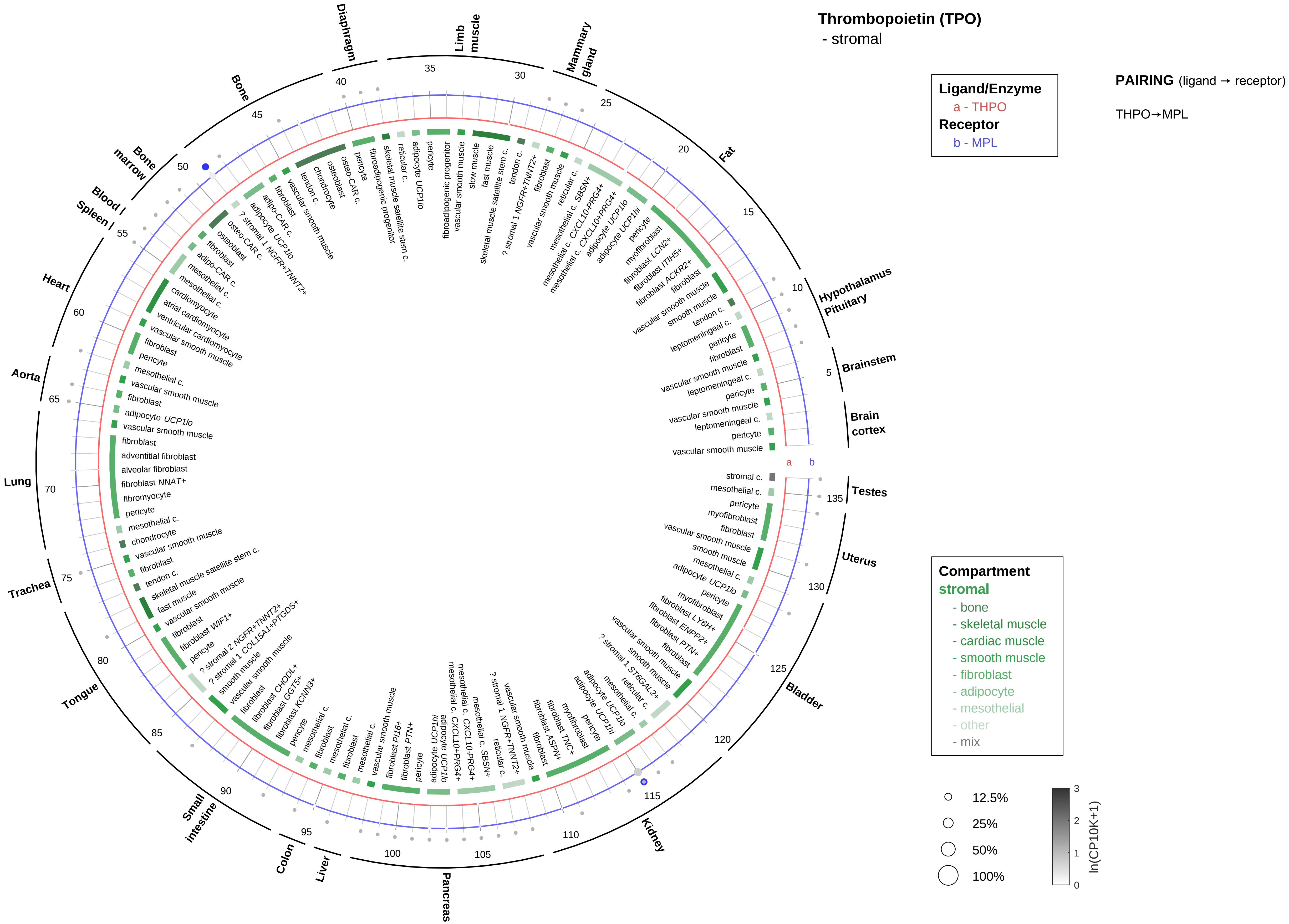

Thrombopoietin (TPO)
- stromal
Ligand/Enzyme
a - THPO
Receptor
b - MPL
PAIRING (ligand → receptor)
THPO→MPL
Compartment
stromal
- bone
- skeletal muscle
- cardiac muscle
- smooth muscle
- fibroblast
- adipocyte
- mesothelial
- other
- mix
12.5%
25%
50%
100%
ln(CP10K+1)
0
1
2
3
a
b
Brain cortex
Brainstem
Hypothalamus Pituitary
Fat
Mammary gland
Limb muscle
Diaphragm
Bone
Bone marrow
Blood
Spleen
Heart
Aorta
Lung
Trachea
Tongue
Small intestine
Colon
Liver
Pancreas
Kidney
Bladder
Uterus
Testes
vascular smooth muscle
pericyte
leptomeningeal c.
vascular smooth muscle
pericyte
leptomeningeal c.
vascular smooth muscle
fibroblast
pericyte
leptomeningeal c.
tendon c.
smooth muscle
vascular smooth muscle
fibroblast
fibroblast ACKR2+
fibroblast ITIH5+
fibroblast LCN2+
myofibroblast
pericyte
adipocyte UCP1hi
adipocyte UCP1lo
mesothelial c. CXCL10-PRG4+
mesothelial c. CXCL10+PRG4+
mesothelial c. SBSN+
reticular c.
vascular smooth muscle
fibroblast
? stromal 1 NGFR+TNNT2+
tendon c.
skeletal muscle satellite stem c.
fast muscle
slow muscle
vascular smooth muscle
fibroadipogenic progenitor
pericyte
adipocyte UCP1lo
reticular c.
skeletal muscle satellite stem c.
fibroadipogenic progenitor
pericyte
osteo-CAR c.
osteoblast
chondrocyte
tendon c.
vascular smooth muscle
fibroblast
adipo-CAR c.
adipocyte UCP1lo
? stromal 1 NGFR+TNNT2+
osteo-CAR c.
osteoblast
fibroblast
adipo-CAR c.
mesothelial c.
mesothelial c.
cardiomyocyte
atrial cardiomyocyte
ventricular cardiomyocyte
vascular smooth muscle
fibroblast
pericyte
mesothelial c.
vascular smooth muscle
fibroblast
adipocyte UCP1lo
vascular smooth muscle
fibroblast
adventitial fibroblast
alveolar fibroblast
fibroblast NNAT+
fibromyocyte
pericyte
mesothelial c.
chondrocyte
vascular smooth muscle
fibroblast
tendon c.
skeletal muscle satellite stem c.
fast muscle
vascular smooth muscle
fibroblast
fibroblast WIF1+
pericyte
? stromal 2 NGFR+TNNT2+
? stromal 1 COL15A1+PTGDS+
smooth muscle
vascular smooth muscle
fibroblast
fibroblast CHODL+
fibroblast GGT5+
fibroblast KCNN3+
pericyte
mesothelial c.
fibroblast
mesothelial c.
fibroblast
mesothelial c.
vascular smooth muscle
fibroblast PI16+
fibroblast PTN+
pericyte
adipocyte UCP1hi
adipocyte UCP1lo
mesothelial c. CXCL10+PRG4+
mesothelial c. CXCL10-PRG4+
mesothelial c. SBSN+
reticular c.
? stromal 1 NGFR+TNNT2+
vascular smooth muscle
fibroblast
fibroblast ASPN+
fibroblast TNC+
myofibroblast
pericyte
adipocyte UCP1lo
adipocyte UCP1hi
mesothelial c.
reticular c.
? stromal 1 ST6GAL2+
smooth muscle
vascular smooth muscle
fibroblast
fibroblast PTN+
fibroblast ENPP2+
fibroblast LY6H+
myofibroblast
pericyte
adipocyte UCP1lo
mesothelial c.
smooth muscle
vascular smooth muscle
fibroblast
myofibroblast
pericyte
mesothelial c.
stromal c.
5
10
15
20
25
30
35
40
45
50
55
60
65
70
75
80
85
90
95
100
105
110
115
120
125
130
135

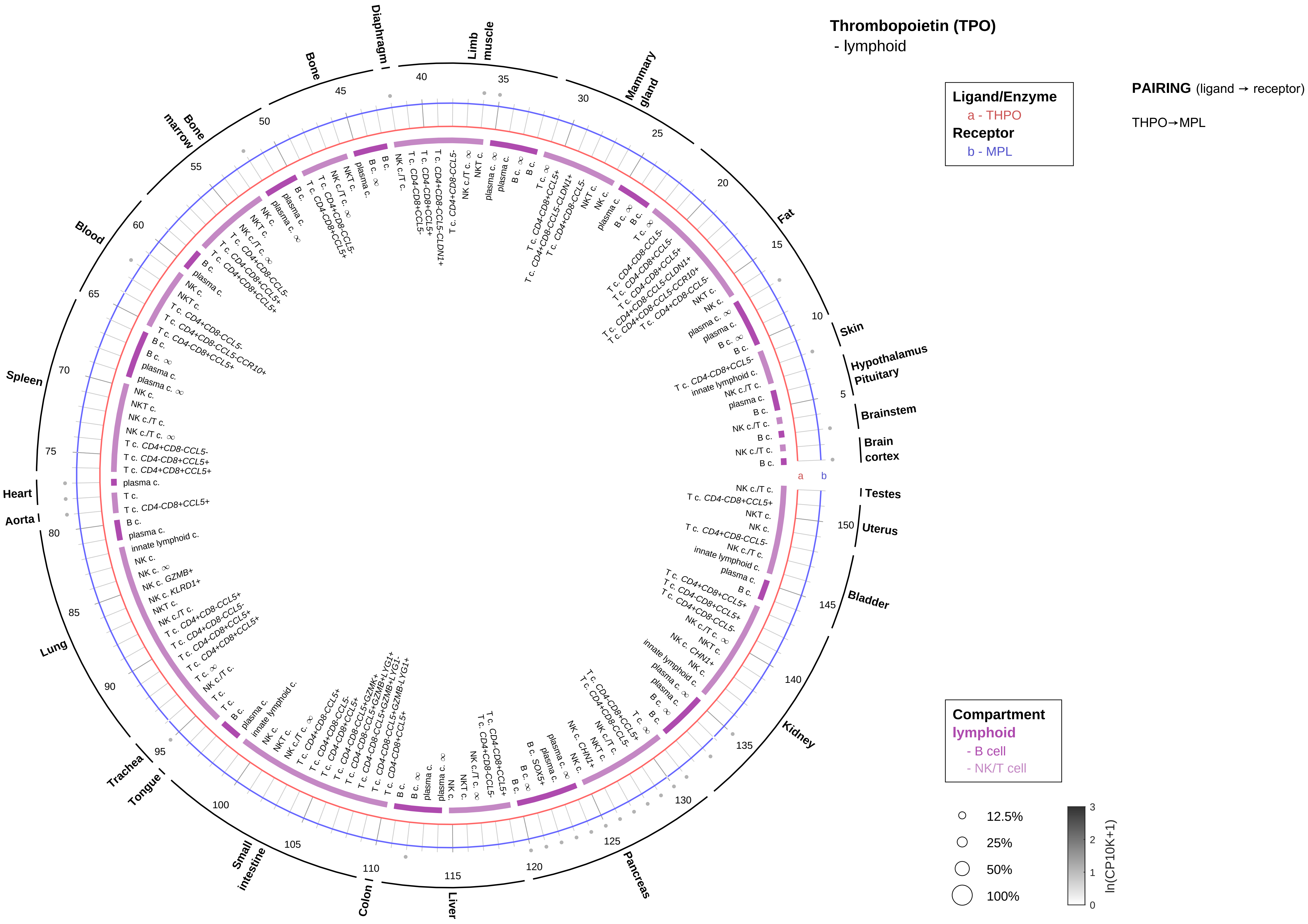

Thrombopoietin (TPO)
- lymphoid
Ligand/Enzyme
a - THPO
Receptor
b - MPL
PAIRING (ligand → receptor)
THPO→MPL
Compartment
lymphoid
- B cell
- NK/T cell
12.5%
25%
50%
100%
ln(CP10K+1)
Brain cortex
Brainstem
Hypothalamus Pituitary
Skin
Fat
Mammary gland
Limb muscle
Diaphragm
Bone
Bone marrow
Blood
Spleen
Heart
Aorta
Lung
Trachea
Tongue
Small intestine
Colon
Liver
Pancreas
Kidney
Bladder
Uterus
Testes

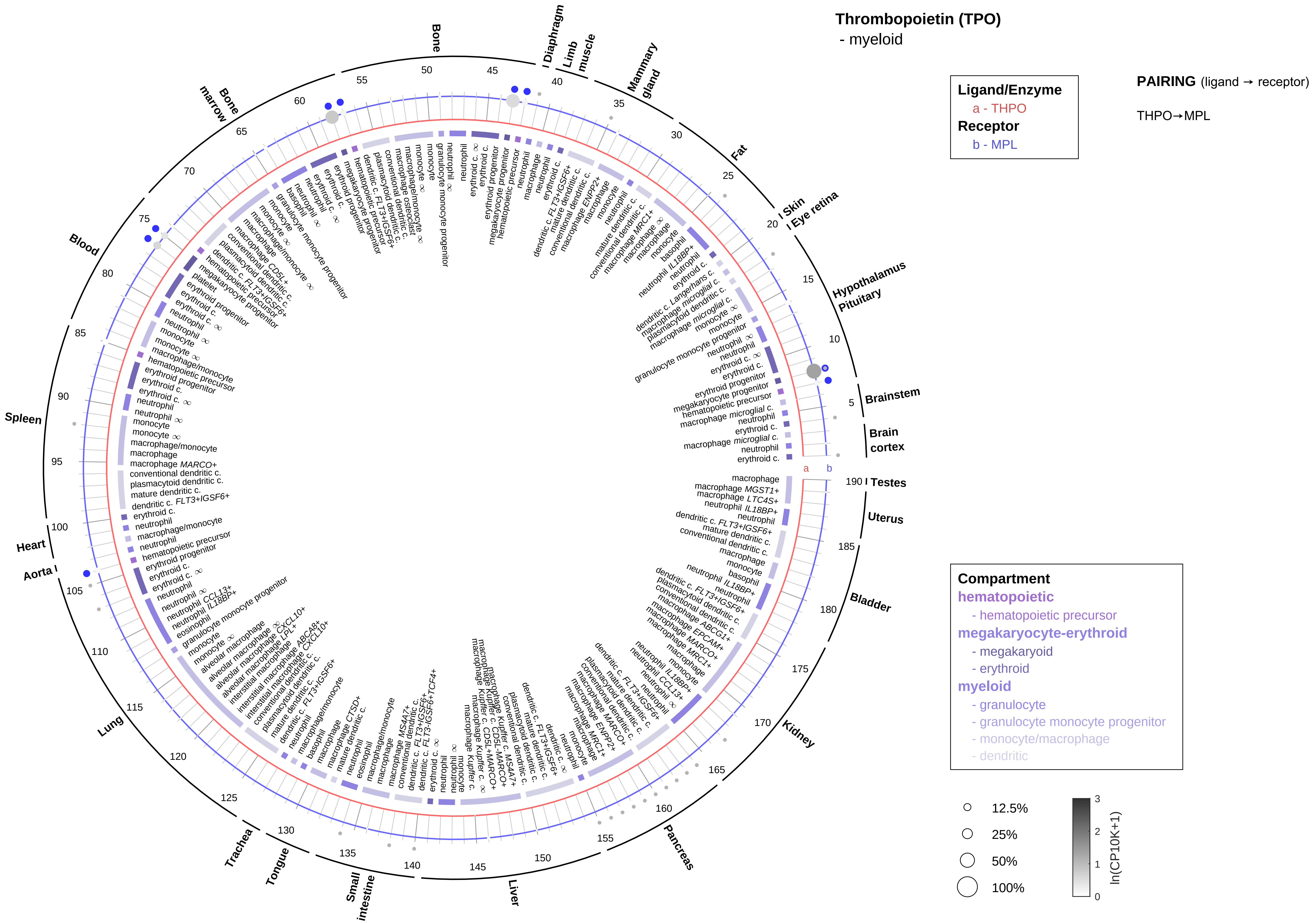

Thrombopoietin (TPO)
- myeloid
Ligand/Enzyme
a - THPO
Receptor
b - MPL
PAIRING (ligand → receptor)
THPO→MPL
Compartment
hematopoietic
- hematopoietic precursor
megakaryocyte-erythroid
- megakaryoid
- erythroid
myeloid
- granulocyte
- granulocyte monocyte progenitor
- monocyte/macrophage
- dendritic
12.5%
25%
50%
100%
ln(CP10K+1)
Bone
Diaphragm
Limb muscle
Mammary gland
Fat
Skin
Eye retina
Hypothalamus
Pituitary
Brainstem
Brain cortex
Testes
Uterus
Bladder
Kidney
Pancreas
Liver
Small intestine
Tongue
Trachea
Lung
Aorta
Heart
Spleen
Blood
Bone marrow

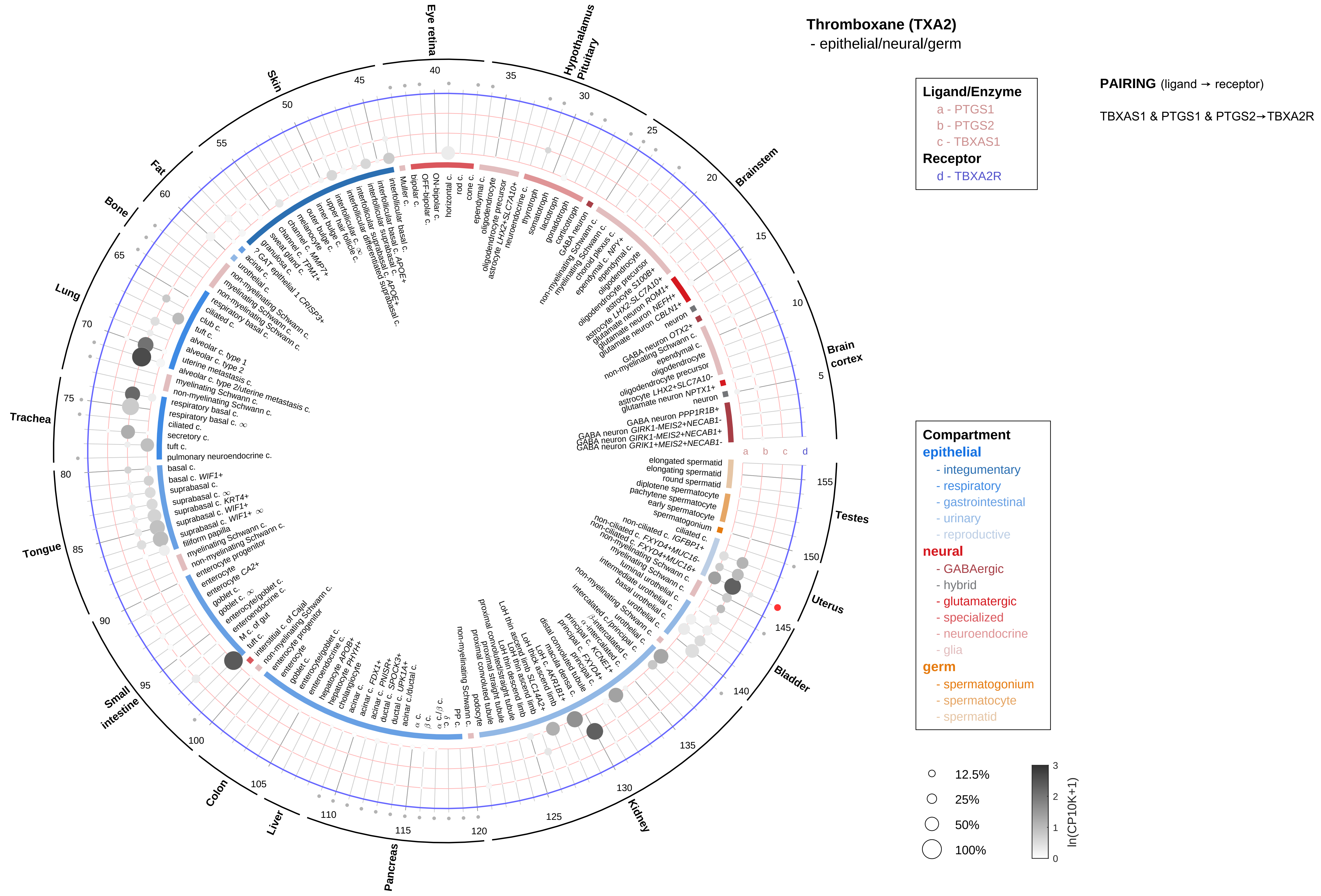

Thromboxane (TXA2)
- epithelial/neural/germ
Ligand/Enzyme
a - PTGS1
b - PTGS2
c - TBXAS1
Receptor
d - TBXA2R
PAIRING (ligand → receptor)
TBXAS1 & PTGS1 & PTGS2→TBXA2R
Compartment
epithelial
- integumentary
- respiratory
- gastrointestinal
- urinary
- reproductive
neural
- GABAergic
- hybrid
- glutamatergic
- specialized
- neuroendocrine
- glia
germ
- spermatogonium
- spermatocyte
- spermatid
12.5%
25%
50%
100%
ln(CP10K+1)
Eye retina
Hypothalamus Pituitary
Brainstem
Brain cortex
Testes
Uterus
Bladder
Kidney
Pancreas
Liver
Colon
Small intestine
Tongue
Trachea
Lung
Bone
Fat
Skin

Thromboxane (TXA2)
- endothelial

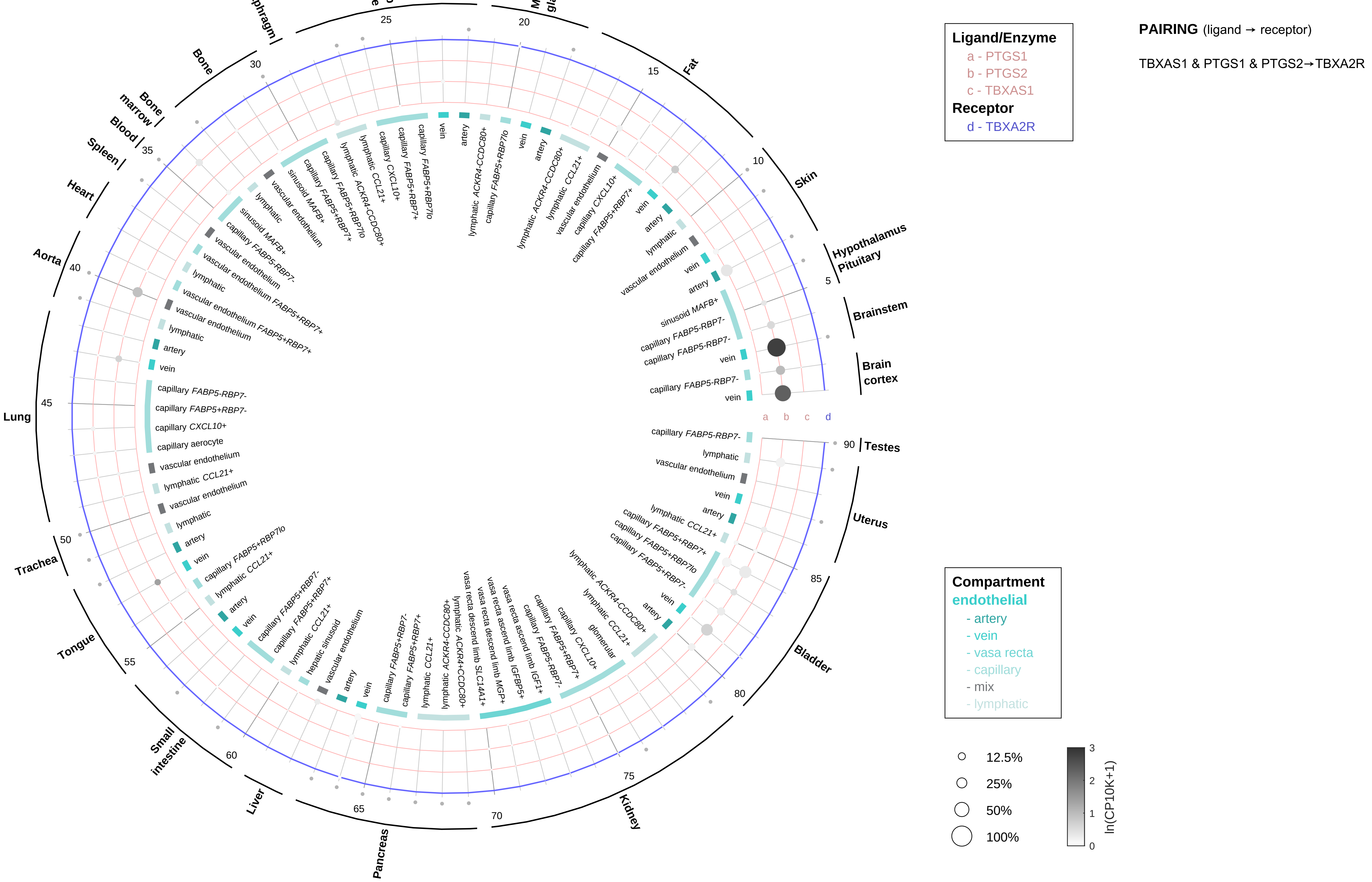

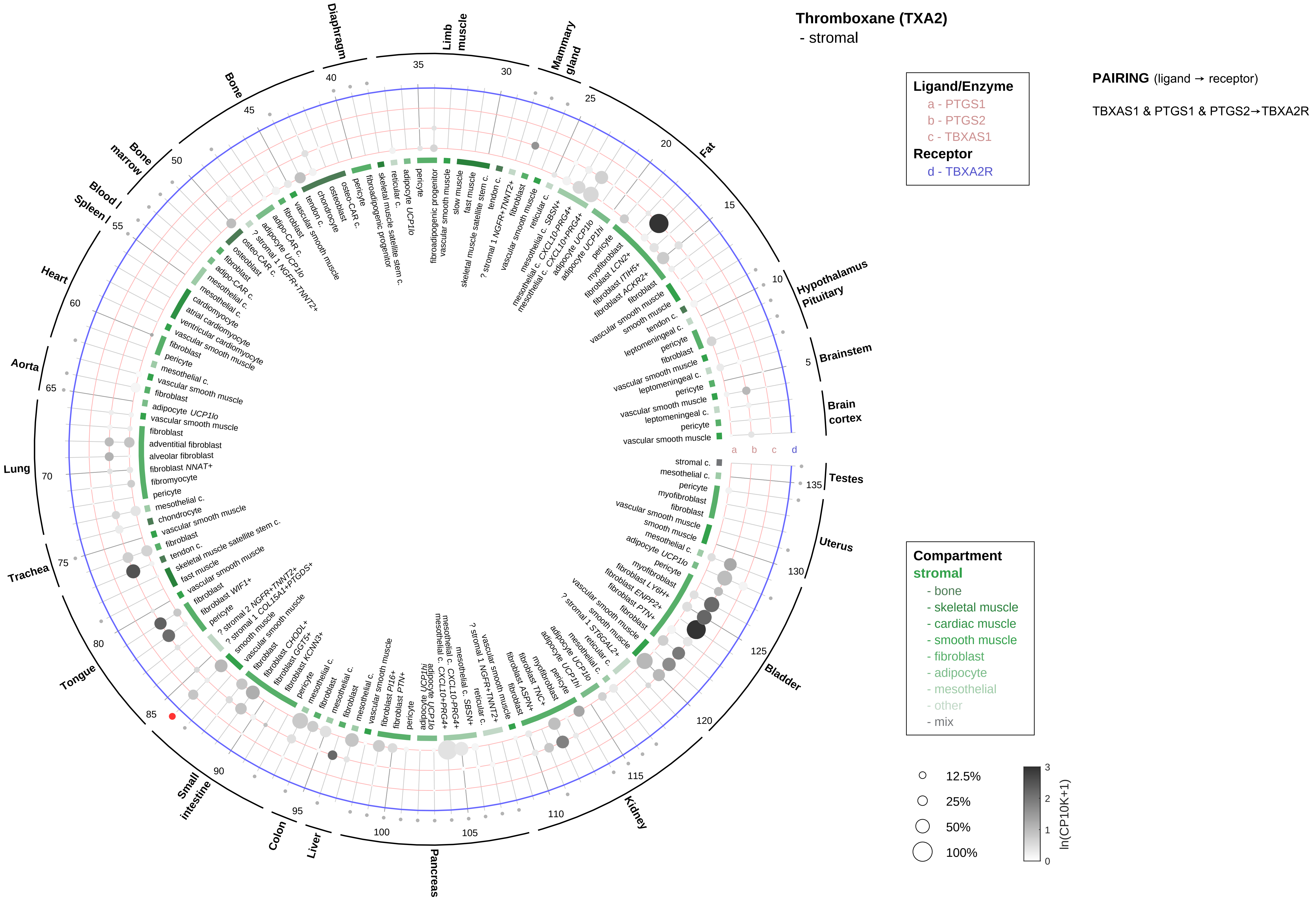
Thromboxane (TXA2)
- stromal
PAIRING (ligand → receptor)
TBXAS1 & PTGS1 & PTGS2→TBXA2R
Ligand/Enzyme
a - PTGS1
b - PTGS2
c - TBXAS1
Receptor
d - TBXA2R
Compartment
stromal
- bone
- skeletal muscle
- cardiac muscle
- smooth muscle
- fibroblast
- adipocyte
- mesothelial
- other
- mix
12.5%
25%
50%
100%
ln(CP10K+1)
Hypothalamus
Pituitary
Brainstem
Brain cortex
Testes
Uterus
Bladder
Kidney
Pancreas
Liver
Colon
Small intestine
Tongue
Trachea
Lung
Aorta
Heart
Spleen
Blood
Bone marrow
Bone
Diaphragm
Limb muscle
Mammary gland
Fat

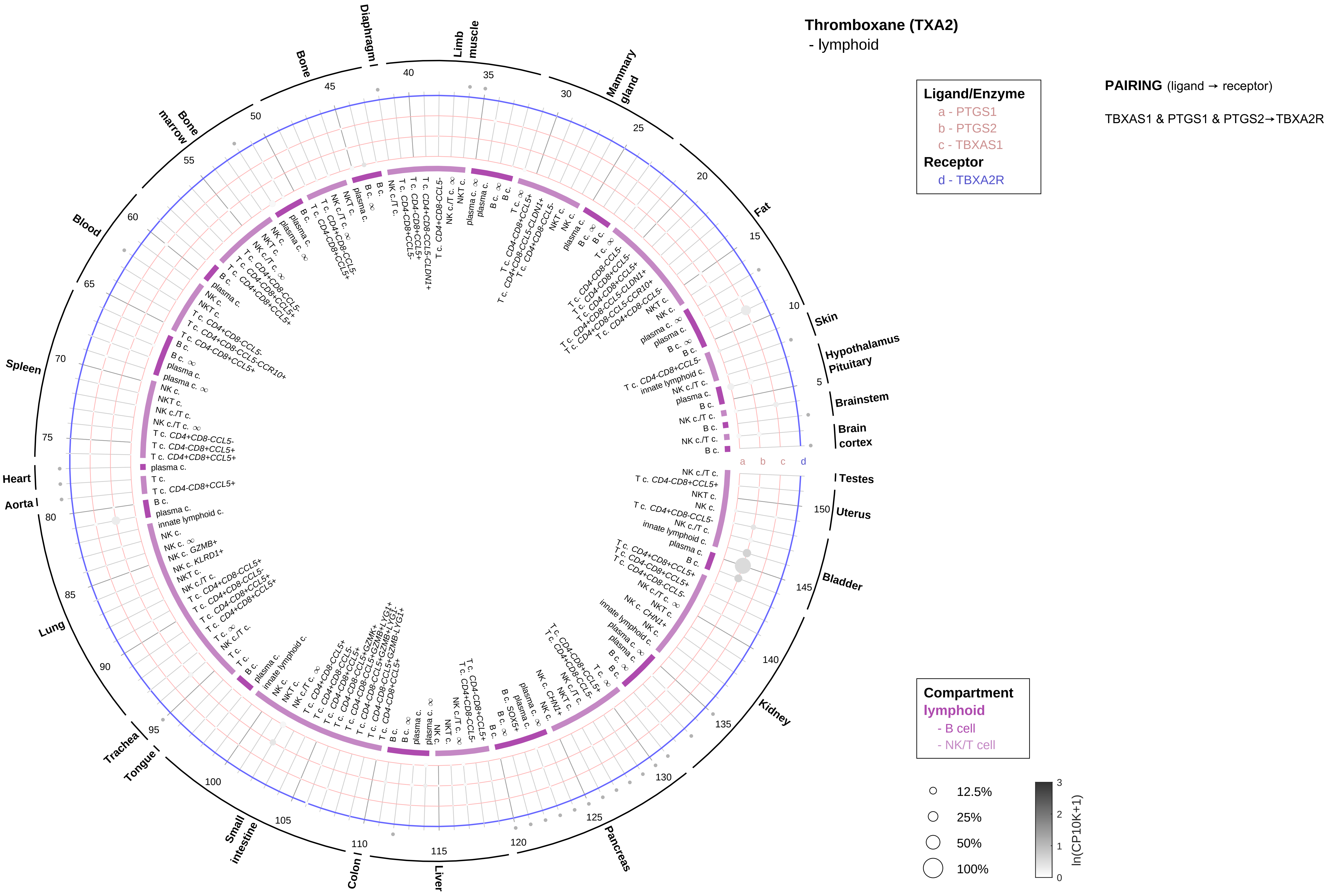

Thromboxane (TXA2)
- lymphoid
Ligand/Enzyme
a - PTGS1
b - PTGS2
c - TBXAS1
Receptor
d - TBXA2R
PAIRING (ligand → receptor)
TBXAS1 & PTGS1 & PTGS2→TBXA2R
Compartment
lymphoid
- B cell
- NK/T cell
12.5%
25%
50%
100%
ln(CP10K+1)

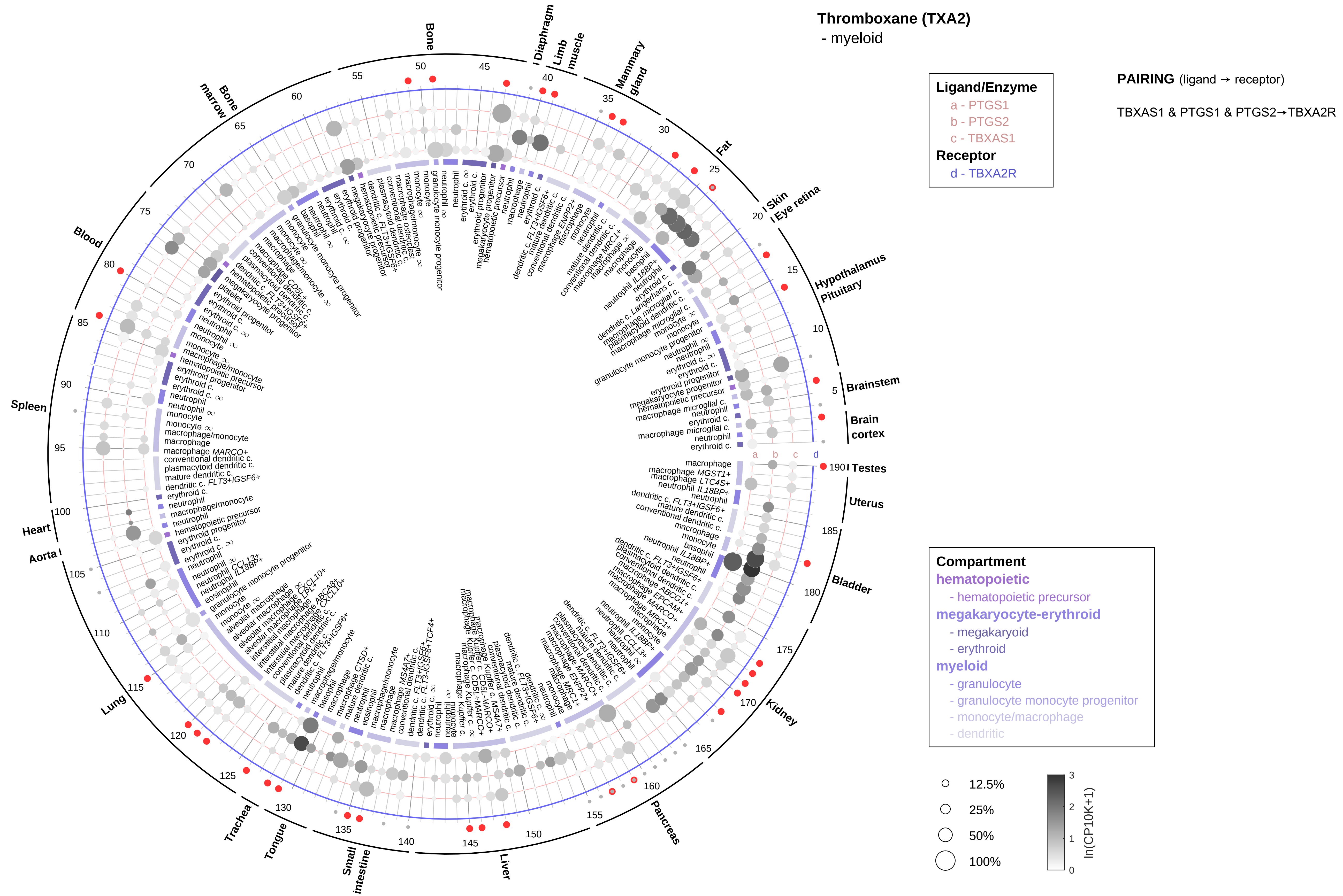

Thromboxane (TXA2)
- myeloid
Ligand/Enzyme
a - PTGS1
b - PTGS2
c - TBXAS1
Receptor
d - TBXA2R
PAIRING (ligand → receptor)
TBXAS1 & PTGS1 & PTGS2→TBXA2R
Compartment
hematopoietic
- hematopoietic precursor
megakaryocyte-erythroid
- megakaryoid
- erythroid
myeloid
- granulocyte
- granulocyte monocyte progenitor
- monocyte/macrophage
- dendritic
12.5%
25%
50%
100%
ln(CP10K+1)
Bone
Diaphragm
Limb muscle
Mammary gland
Fat
Skin
Eye retina
Hypothalamus
Pituitary
Brainstem
Brain cortex
Testes
Uterus
Bladder
Kidney
Pancreas
Liver
Small intestine
Tongue
Trachea
Lung
Aorta
Heart
Spleen
Blood
Bone marrow

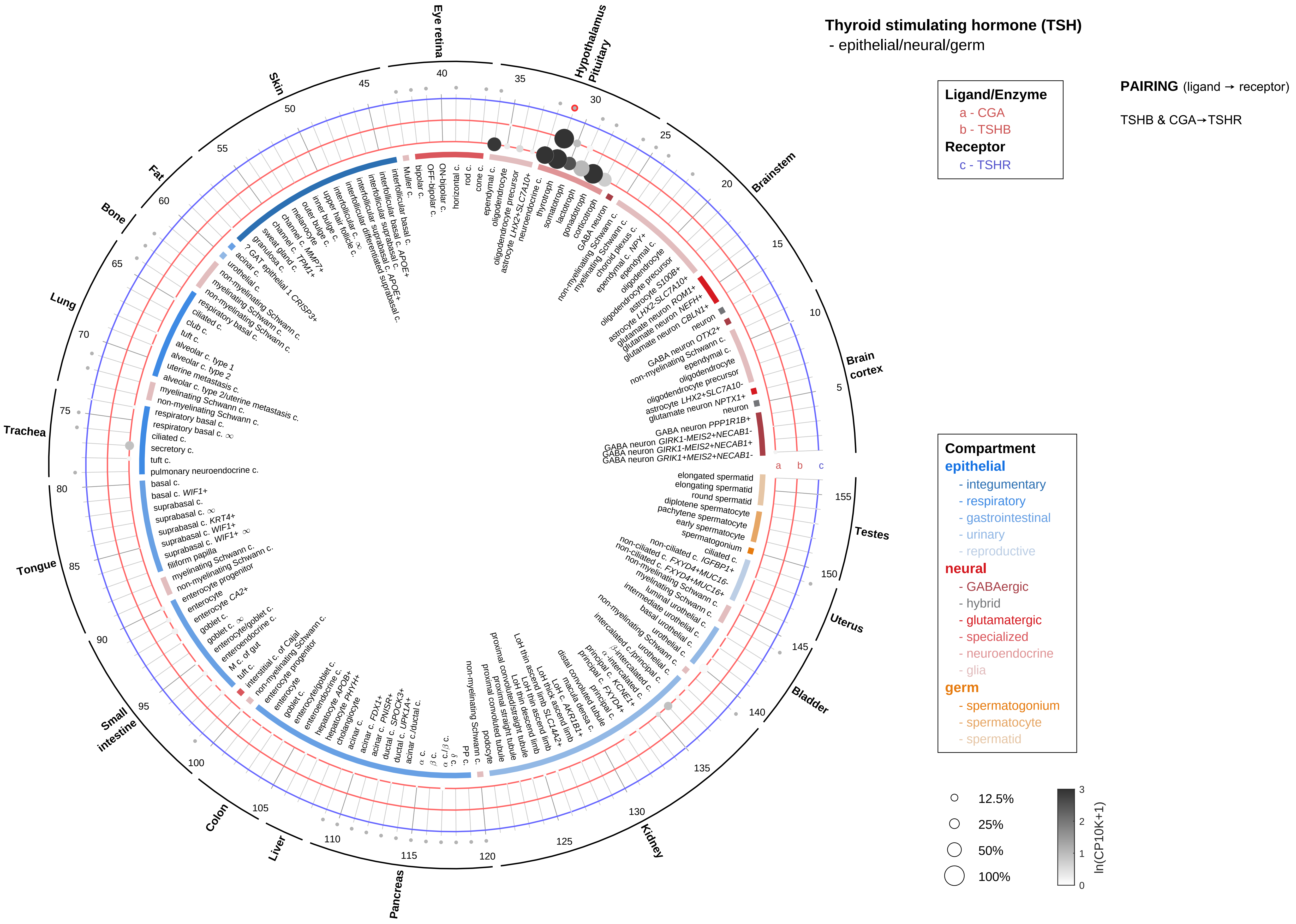
Thyroid stimulating hormone (TSH)
- epithelial/neural/germ
Ligand/Enzyme
a - CGA
b - TSHB
Receptor
c - TSHR
PAIRING (ligand → receptor)
TSHB & CGA→TSHR
Compartment
epithelial
- integumentary
- respiratory
- gastrointestinal
- urinary
- reproductive
neural
- GABAergic
- hybrid
- glutamatergic
- specialized
- neuroendocrine
- glia
germ
- spermatogonium
- spermatocyte
- spermatid
12.5%
25%
50%
100%
ln(CP10K+1)
Eye retina
Hypothalamus
Pituitary
Brainstem
Brain cortex
Testes
Uterus
Bladder
Kidney
Pancreas
Liver
Colon
Small intestine
Tongue
Trachea
Lung
Bone
Fat
Skin

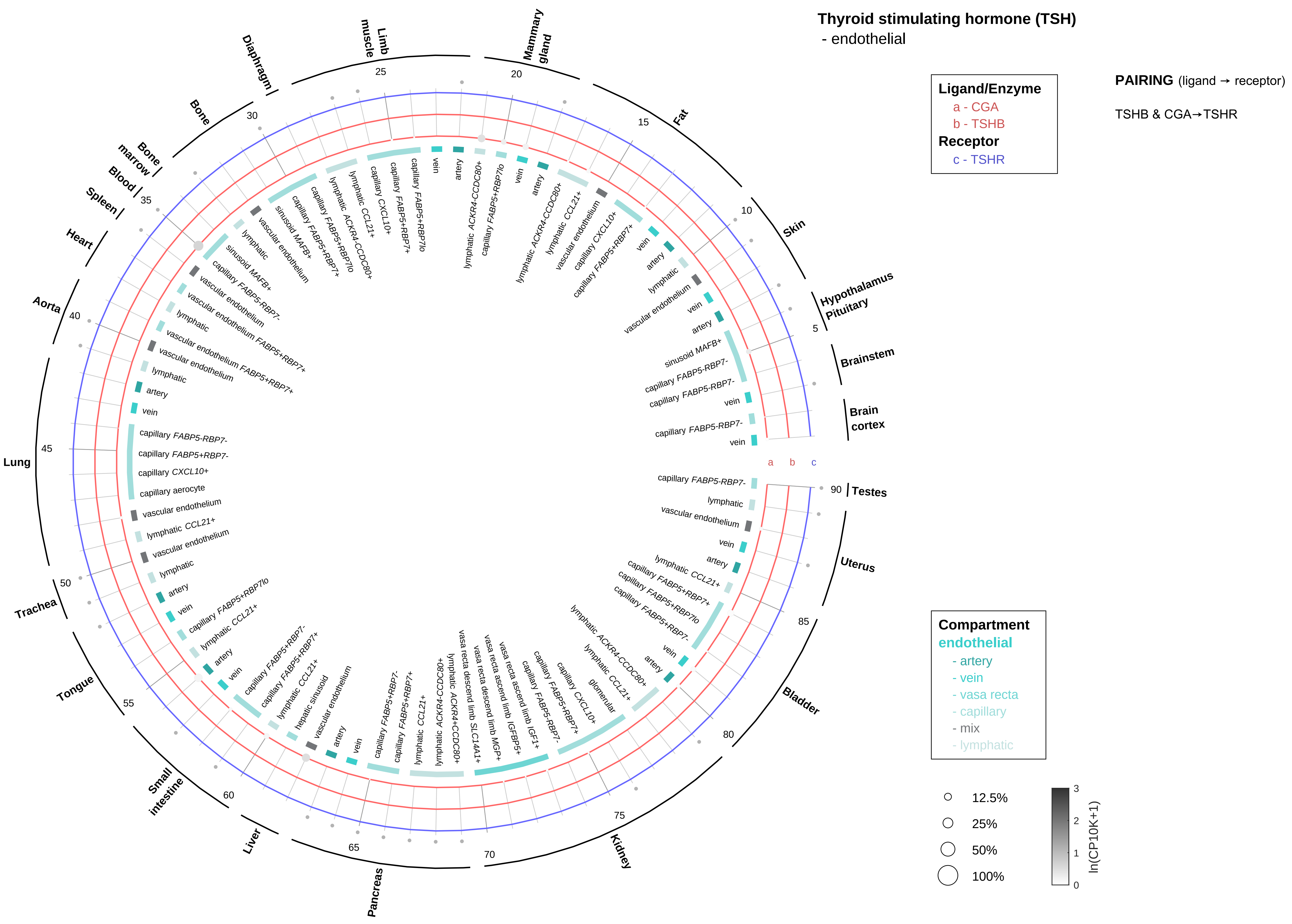
Thyroid stimulating hormone (TSH)
- endothelial
Ligand/Enzyme
a - CGA
b - TSHB
Receptor
c - TSHR
PAIRING (ligand → receptor)
TSHB & CGA→TSHR
Compartment
endothelial
- artery
- vein
- vasa recta
- capillary
- mix
- lymphatic
12.5%
25%
50%
100%
ln(CP10K+1)
Brain cortex
Brainstem
Hypothalamus Pituitary
Skin
Fat
Mammary gland
Limb muscle
Diaphragm
Bone
Bone marrow
Blood
Spleen
Heart
Aorta
Lung
Trachea
Tongue
Small intestine
Liver
Pancreas
Kidney
Bladder
Uterus
Testes

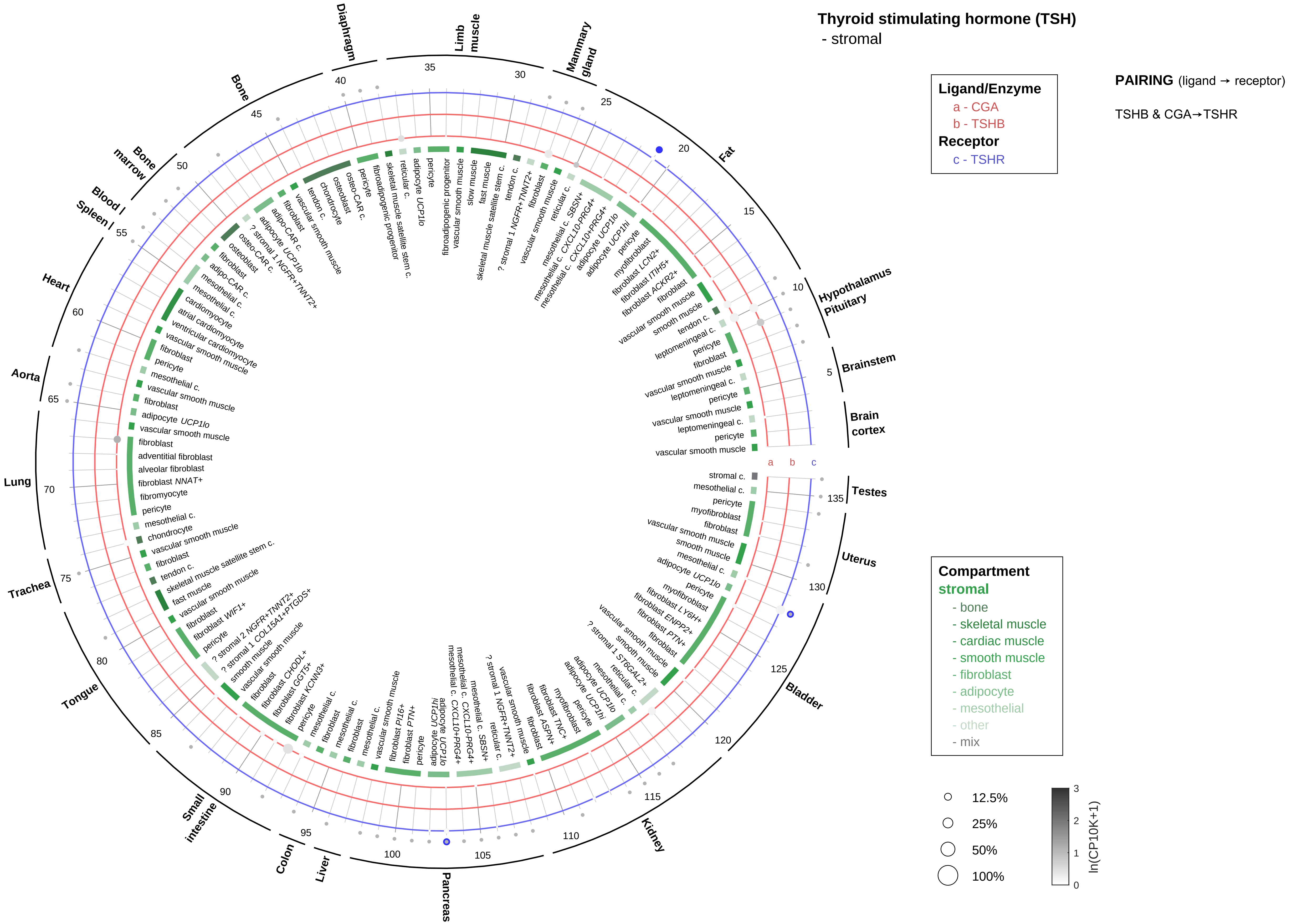

Thyroid stimulating hormone (TSH)
- stromal
Ligand/Enzyme
a - CGA
b - TSHB
Receptor
c - TSHR
PAIRING (ligand → receptor)
TSHB & CGA→TSHR
Compartment
stromal
- bone
- skeletal muscle
- cardiac muscle
- smooth muscle
- fibroblast
- adipocyte
- mesothelial
- other
- mix
12.5%
25%
50%
100%
ln(CP10K+1)
Brain cortex
Brainstem
Hypothalamus Pituitary
Fat
Mammary gland
Limb muscle
Diaphragm
Bone
Bone marrow
Blood / Spleen /
Heart
Aorta
Lung
Trachea
Tongue
Small intestine
Colon
Liver
Pancreas
Kidney
Bladder
Uterus
Testes

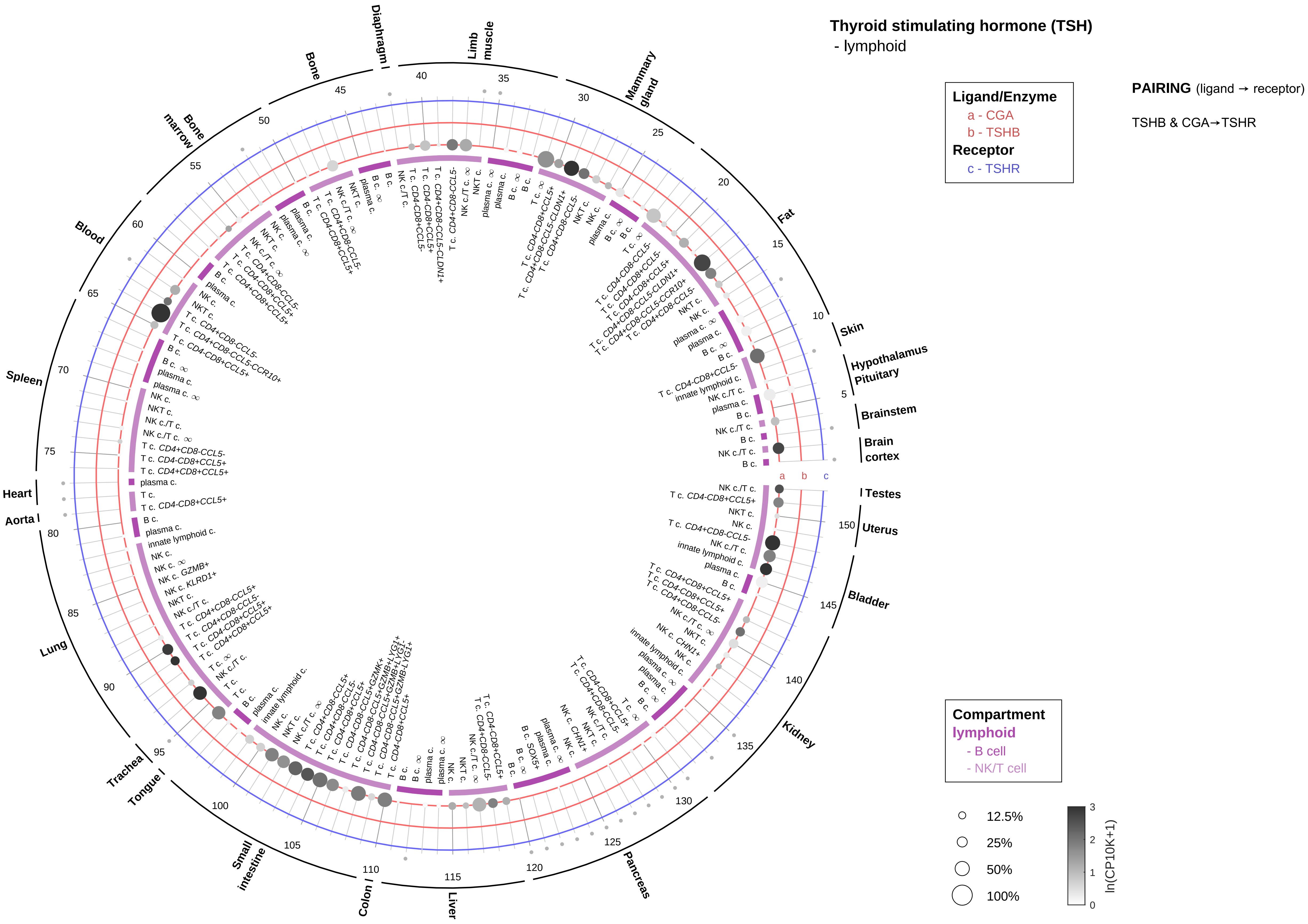

Thyroid stimulating hormone (TSH)
- lymphoid
Ligand/Enzyme
a - CGA
b - TSHB
Receptor
c - TSHR
PAIRING (ligand → receptor)
TSHB & CGA→TSHR
Compartment
lymphoid
- B cell
- NK/T cell
12.5%
25%
50%
100%
ln(CP10K+1)
Skin
Hypothalamus
Pituitary
Brainstem
Brain cortex
Testes
Uterus
Bladder
Kidney
Pancreas
Liver
Colon
Small intestine
Tongue
Trachea
Lung
Aorta
Heart
Spleen
Blood
Bone marrow
Bone
Diaphragm
Limb muscle
Mammary gland
Fat

Thyroid stimulating hormone (TSH)
- myeloid

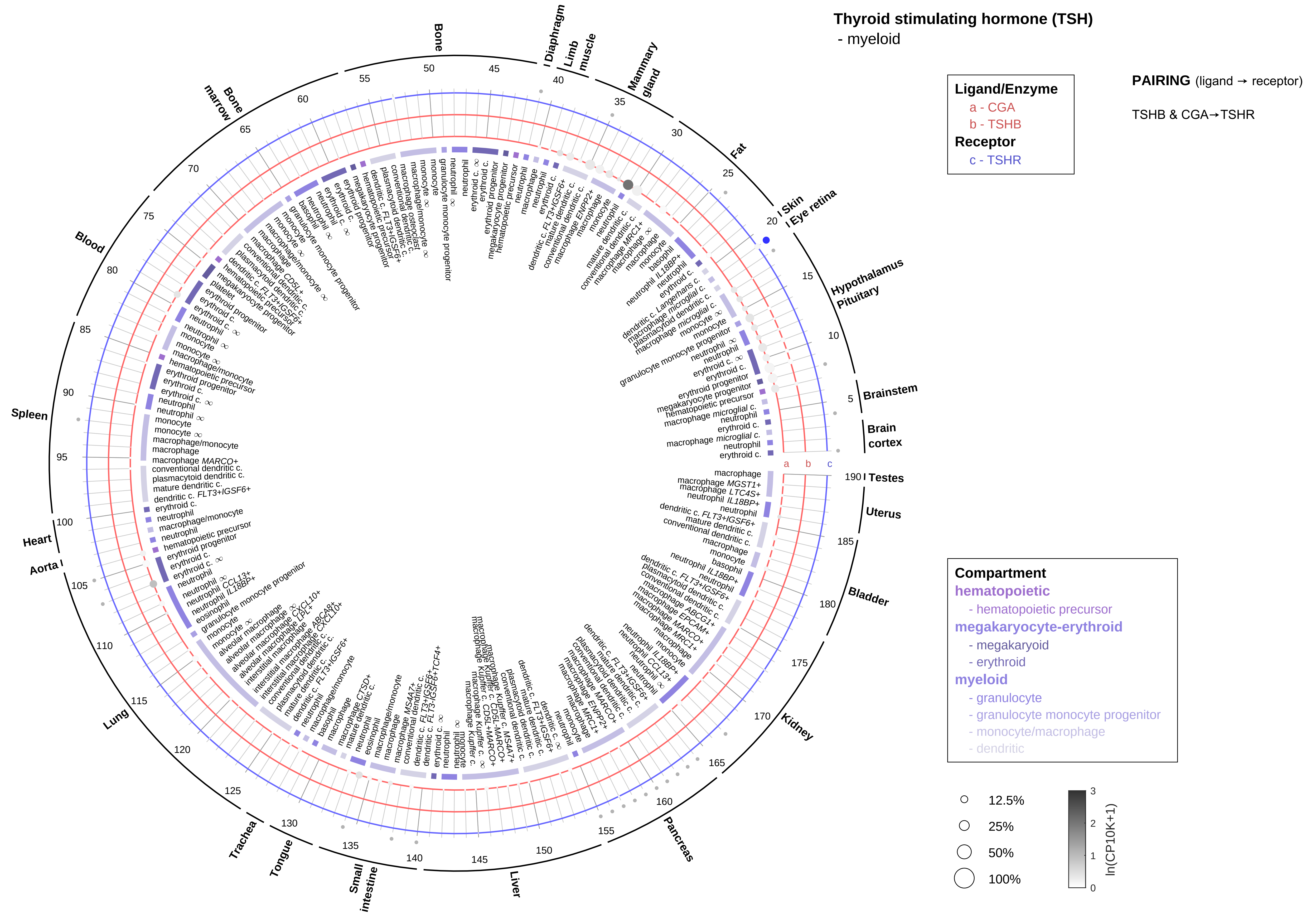

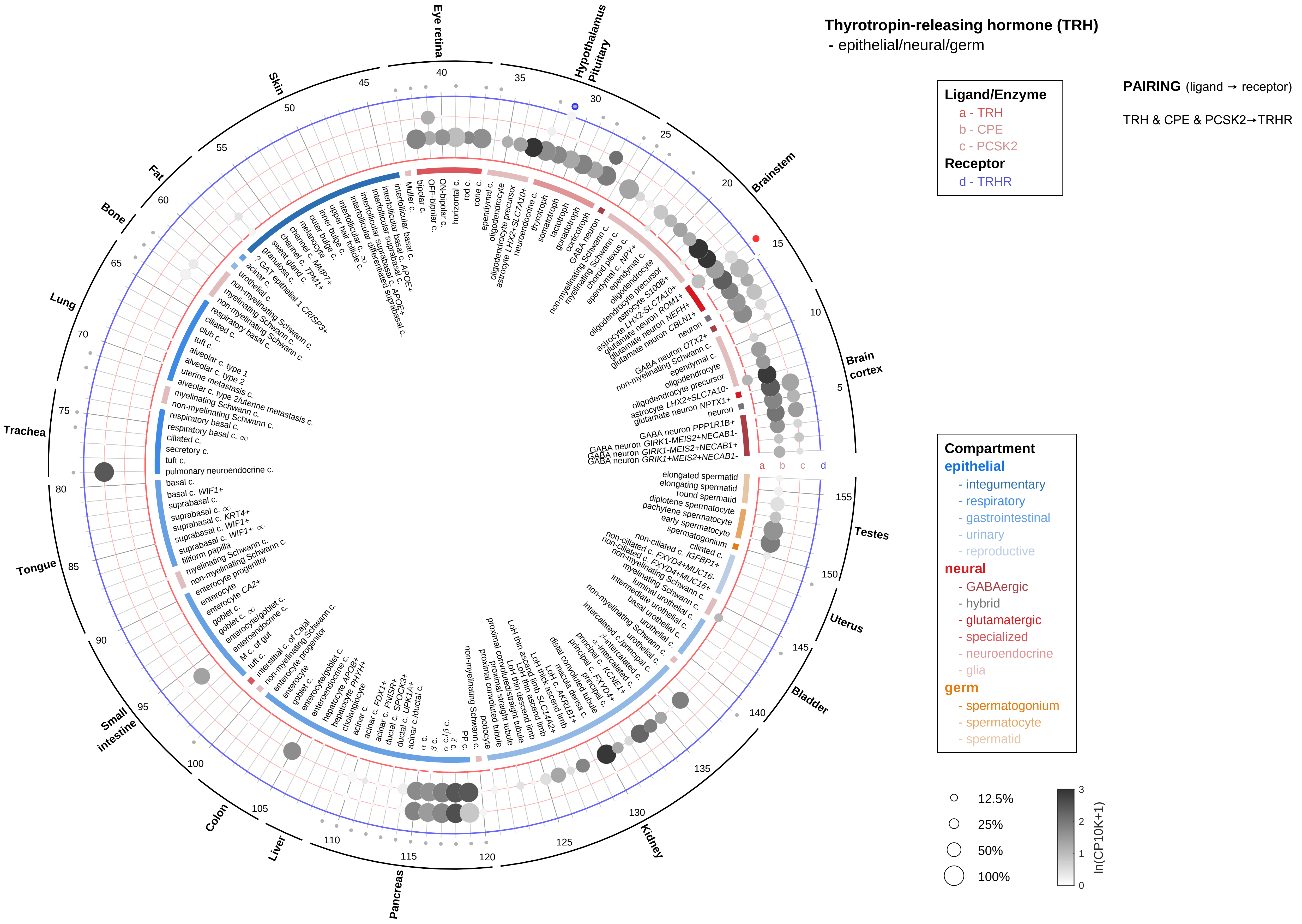

Thyrotropin-releasing hormone (TRH)
- epithelial/neural/germ
Ligand/Enzyme
a - TRH
b - CPE
c - PCSK2
Receptor
d - TRHR
PAIRING (ligand → receptor)
TRH & CPE & PCSK2→TRHR
Compartment
epithelial
- integumentary
- respiratory
- gastrointestinal
- urinary
- reproductive
neural
- GABAergic
- hybrid
- glutamatergic
- specialized
- neuroendocrine
- glia
germ
- spermatogonium
- spermatocyte
- spermatid
12.5%
25%
50%
100%
ln(CP10K+1)
Eye retina
Hypothalamus Pituitary
Brainstem
Brain cortex
Testes
Uterus
Bladder
Kidney
Pancreas
Liver
Colon
Small intestine
Tongue
Trachea
Lung
Bone
Fat
Skin
a
b
c
d

Thyrotropin-releasing hormone (TRH)
- endothelial

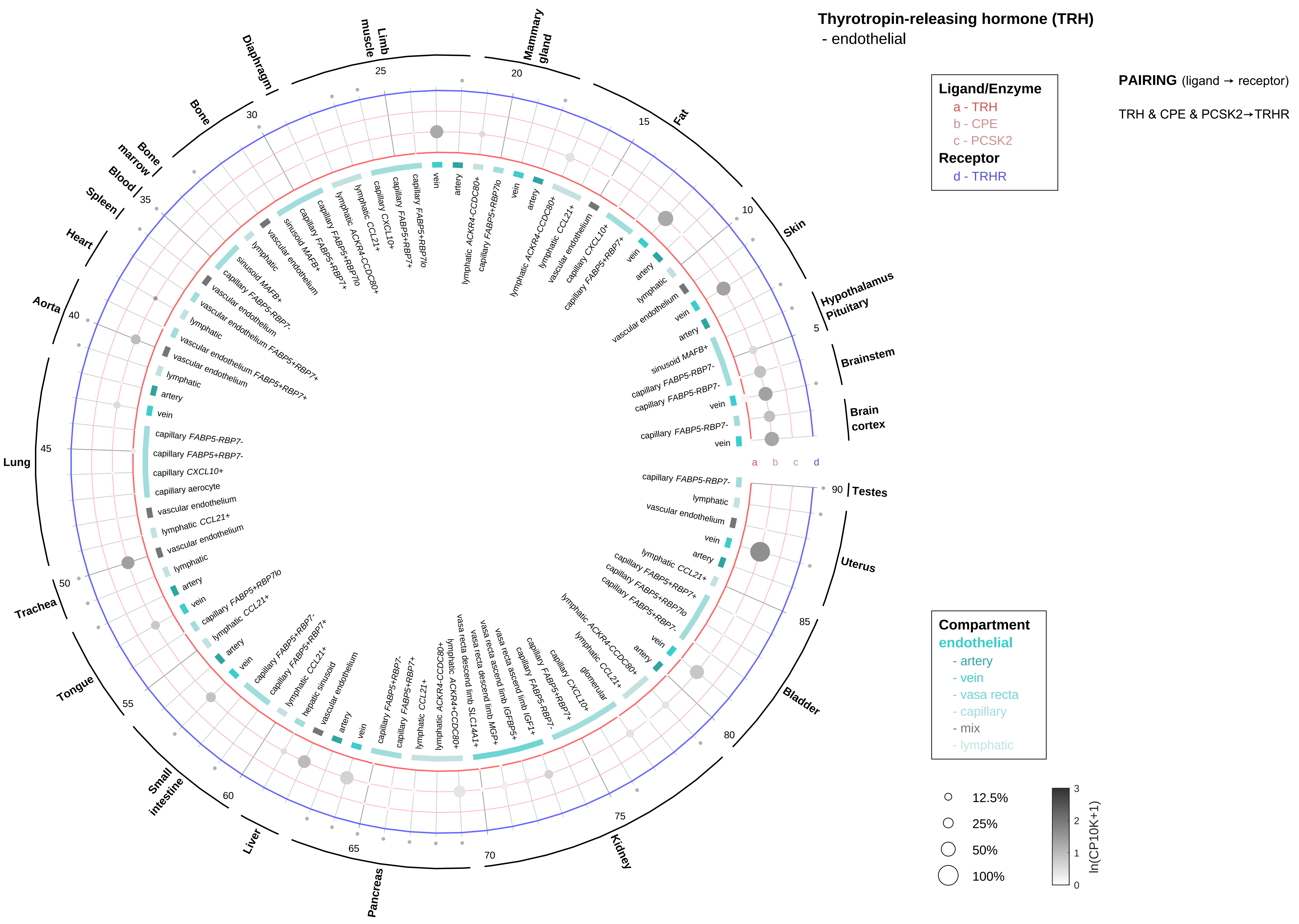

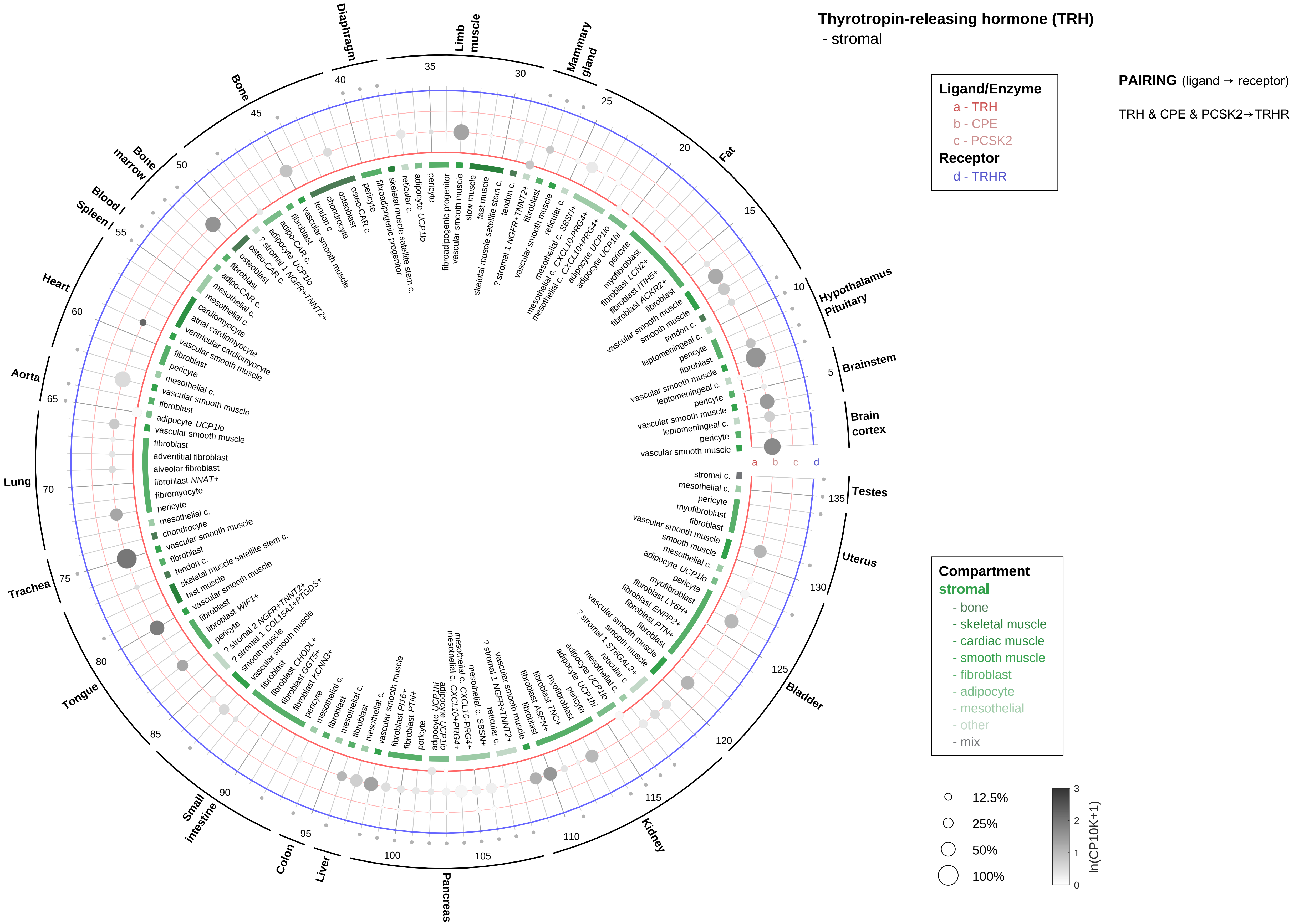

Thyrotropin-releasing hormone (TRH)
- stromal
Ligand/Enzyme
a - TRH
b - CPE
c - PCSK2
Receptor
d - TRHR
PAIRING (ligand → receptor)
TRH & CPE & PCSK2→TRHR
Compartment
stromal
- bone
- skeletal muscle
- cardiac muscle
- smooth muscle
- fibroblast
- adipocyte
- mesothelial
- other
- mix
12.5%
25%
50%
100%
ln(CP10K+1)
Brain cortex
Brainstem
Hypothalamus Pituitary
Fat
Mammary gland
Limb muscle
Diaphragm
Bone
Bone marrow
Blood / Spleen /
Heart
Aorta
Lung
Trachea
Tongue
Small intestine
Colon
Liver
Pancreas
Kidney
Bladder
Uterus
Testes

Thyrotropin-releasing hormone (TRH)
- lymphoid

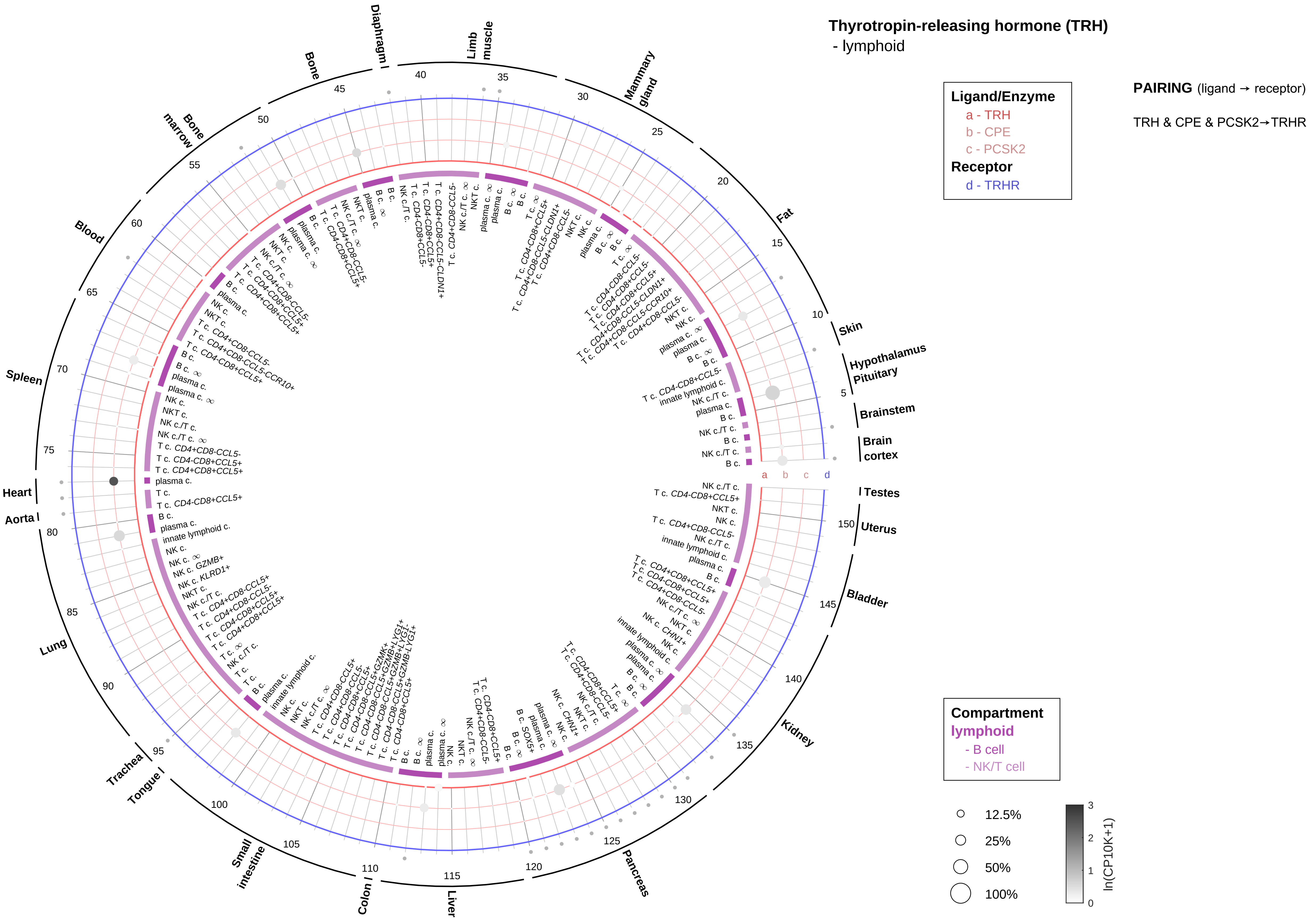

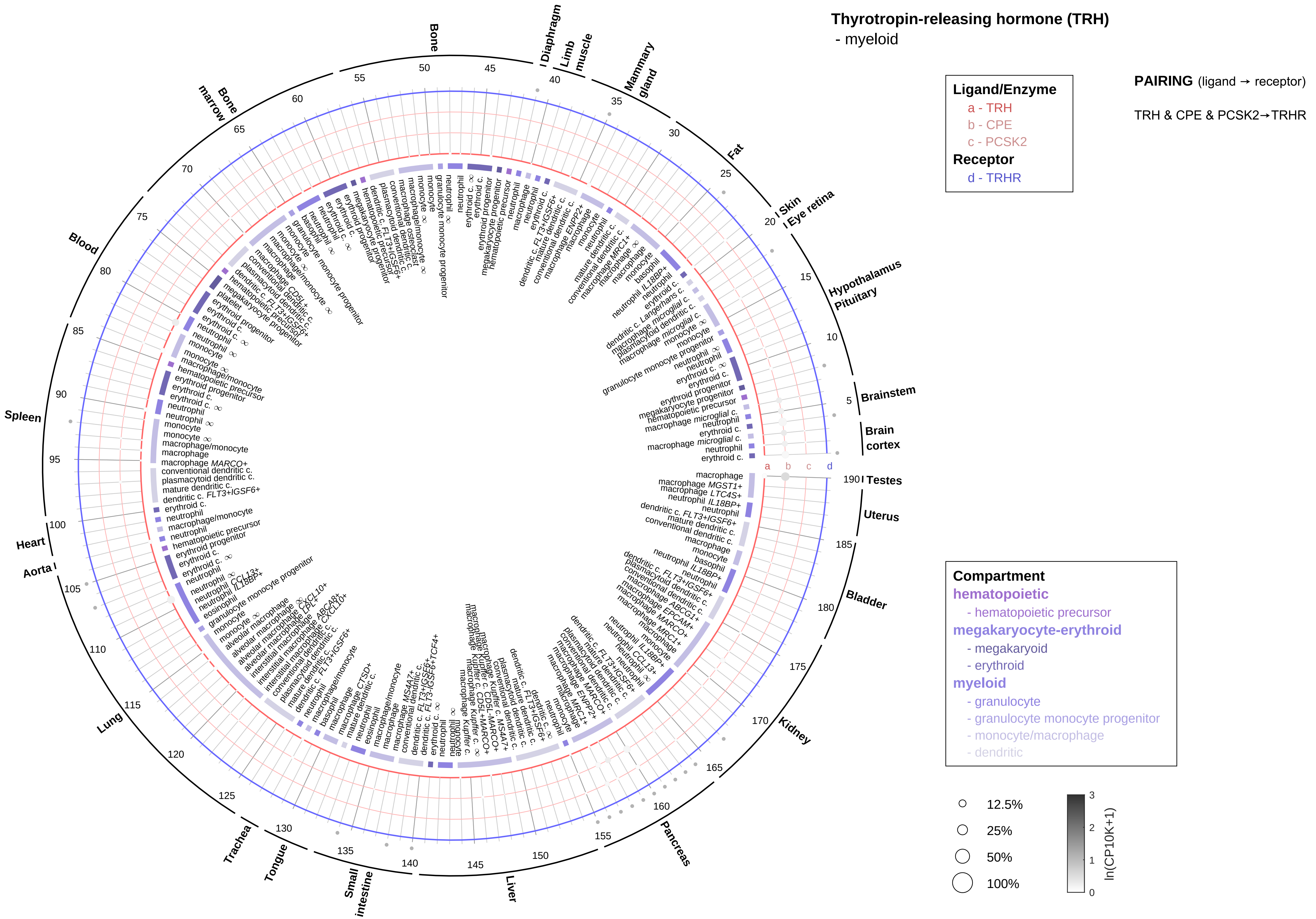
Thyrotropin-releasing hormone (TRH)
- myeloid
Ligand/Enzyme
a - TRH
b - CPE
c - PCSK2
Receptor
d - TRHR
PAIRING (ligand → receptor)
TRH & CPE & PCSK2→TRHR
Compartment
hematopoietic
- hematopoietic precursor
megakaryocyte-erythroid
- megakaryoid
- erythroid
myeloid
- granulocyte
- granulocyte monocyte progenitor
- monocyte/macrophage
- dendritic
12.5%
25%
50%
100%
ln(CP10K+1)
Bone
Diaphragm
Limb muscle
Mammary gland
Fat
Skin
Eye retina
Hypothalamus
Pituitary
Brainstem
Brain cortex
Testes
Uterus
Bladder
Kidney
Pancreas
Liver
Small intestine
Tongue
Trachea
Lung
Aorta
Heart
Spleen
Blood
Bone marrow

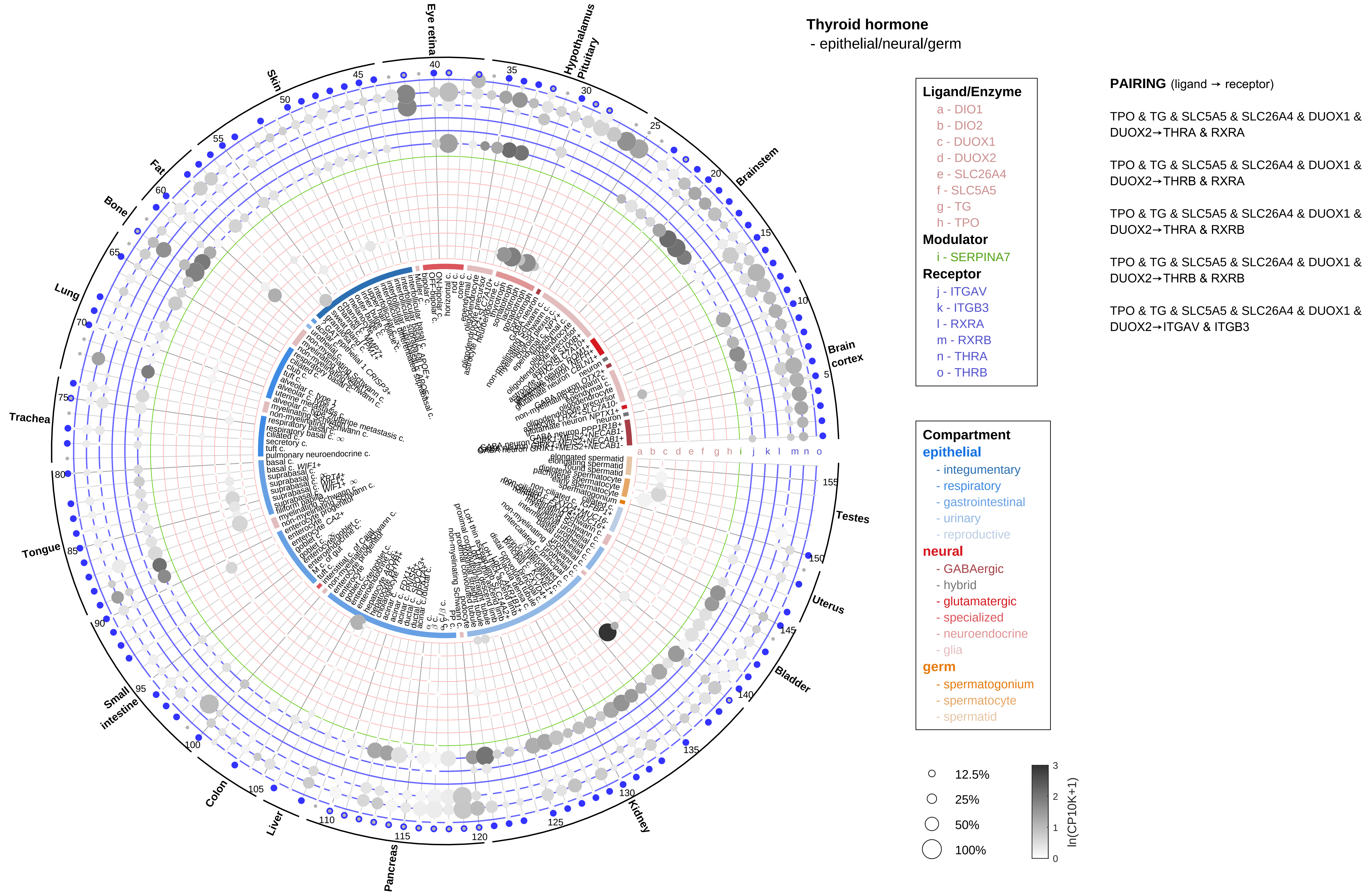
Thyroid hormone
- epithelial/neural/germ
Ligand/Enzyme
a - DIO1
b - DIO2
c - DUOX1
d - DUOX2
e - SLC26A4
f - SLC5A5
g - TG
h - TPO
Modulator
i - SERPINA7
Receptor
j - ITGAV
k - ITGB3
l - RXRA
m - RXRB
n - THRA
o - THRB
PAIRING (ligand → receptor)
TPO & TG & SLC5A5 & SLC26A4 & DUOX1 & DUOX2→THRA & RXRA
TPO & TG & SLC5A5 & SLC26A4 & DUOX1 & DUOX2→THRB & RXRA
TPO & TG & SLC5A5 & SLC26A4 & DUOX1 & DUOX2→THRA & RXRB
TPO & TG & SLC5A5 & SLC26A4 & DUOX1 & DUOX2→THRB & RXRB
TPO & TG & SLC5A5 & SLC26A4 & DUOX1 & DUOX2→ITGAV & ITGB3
Compartment
epithelial
- integumentary
- respiratory
- gastrointestinal
- urinary
- reproductive
neural
- GABAergic
- hybrid
- glutamatergic
- specialized
- neuroendocrine
- glia
germ
- spermatogonium
- spermatocyte
- spermatid
12.5%
25%
50%
100%
ln(CP10K+1)
Eye retina
Hypothalamus Pituitary
Brainstem
Brain cortex
Testes
Uterus
Bladder
Kidney
Pancreas
Liver
Colon
Small intestine
Tongue
Trachea
Lung
Bone
Fat
Skin

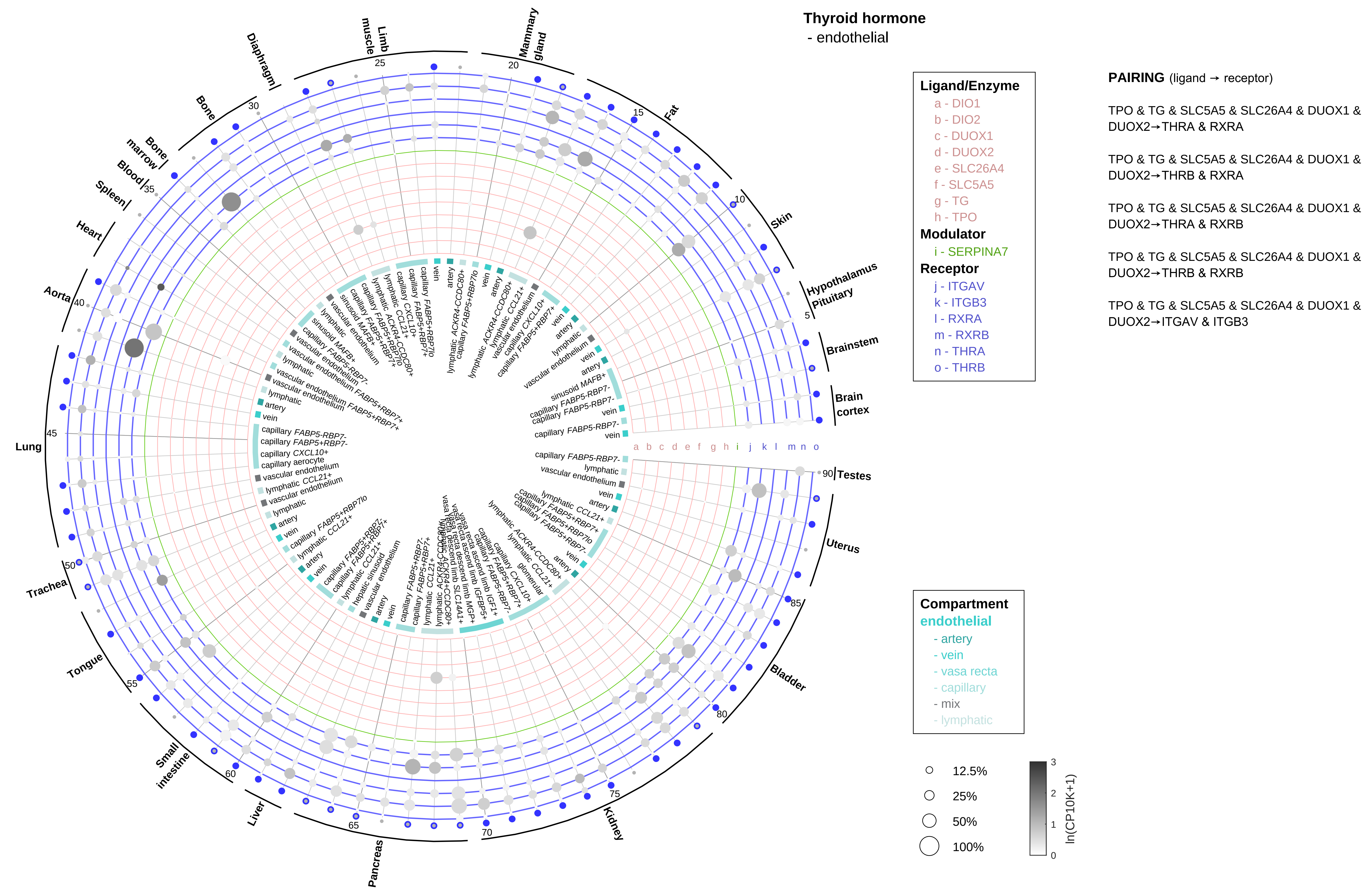

Thyroid hormone
- endothelial
Ligand/Enzyme
a - DIO1
b - DIO2
c - DUOX1
d - DUOX2
e - SLC26A4
f - SLC5A5
g - TG
h - TPO
Modulator
i - SERPINA7
Receptor
j - ITGAV
k - ITGB3
l - RXRA
m - RXRB
n - THRA
o - THRB
PAIRING (ligand → receptor)
TPO & TG & SLC5A5 & SLC26A4 & DUOX1 & DUOX2→THRA & RXRA
TPO & TG & SLC5A5 & SLC26A4 & DUOX1 & DUOX2→THRB & RXRA
TPO & TG & SLC5A5 & SLC26A4 & DUOX1 & DUOX2→THRA & RXRB
TPO & TG & SLC5A5 & SLC26A4 & DUOX1 & DUOX2→THRB & RXRB
TPO & TG & SLC5A5 & SLC26A4 & DUOX1 & DUOX2→ITGAV & ITGB3
Compartment
endothelial
- artery
- vein
- vasa recta
- capillary
- mix
- lymphatic
12.5%
25%
50%
100%
ln(CP10K+1)
0
1
2
3
Brain cortex
Brainstem
Hypothalamus Pituitary
Skin
Fat
Mammary gland
Limb muscle
Diaphragm
Bone
Bone marrow
Blood
Spleen
Heart
Aorta
Lung
Trachea
Tongue
Small intestine
Liver
Pancreas
Kidney
Bladder
Uterus
Testes
a b c d e f g h i j k l m n o

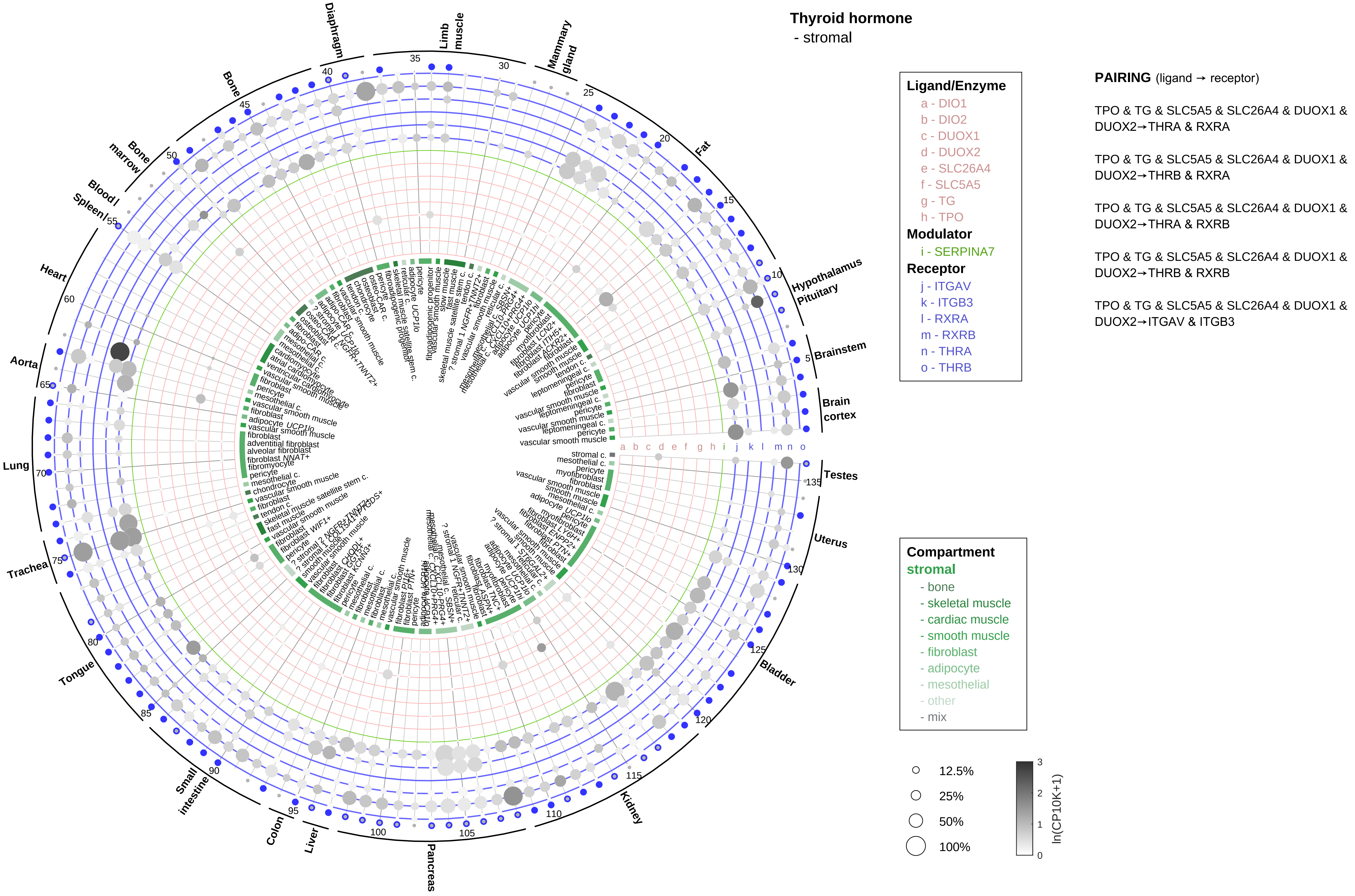

Thyroid hormone
- stromal
Ligand/Enzyme
a - DIO1
b - DIO2
c - DUOX1
d - DUOX2
e - SLC26A4
f - SLC5A5
g - TG
h - TPO
Modulator
i - SERPINA7
Receptor
j - ITGAV
k - ITGB3
l - RXRA
m - RXRB
n - THRA
o - THRB
PAIRING (ligand → receptor)
TPO & TG & SLC5A5 & SLC26A4 & DUOX1 & DUOX2→THRA & RXRA
TPO & TG & SLC5A5 & SLC26A4 & DUOX1 & DUOX2→THRB & RXRA
TPO & TG & SLC5A5 & SLC26A4 & DUOX1 & DUOX2→THRA & RXRB
TPO & TG & SLC5A5 & SLC26A4 & DUOX1 & DUOX2→THRB & RXRB
TPO & TG & SLC5A5 & SLC26A4 & DUOX1 & DUOX2→ITGAV & ITGB3
Compartment
stromal
- bone
- skeletal muscle
- cardiac muscle
- smooth muscle
- fibroblast
- adipocyte
- mesothelial
- other
- mix
12.5%
25%
50%
100%
ln(CP10K+1)
0
1
2
3
Limb muscle
Mammary gland
Fat
Hypothalamus Pituitary
Brainstem
Brain cortex
Testes
Uterus
Bladder
Kidney
Pancreas
Liver
Colon
Small intestine
Tongue
Trachea
Lung
Aorta
Heart
Spleen
Blood
Bone marrow
Bone
Diaphragm

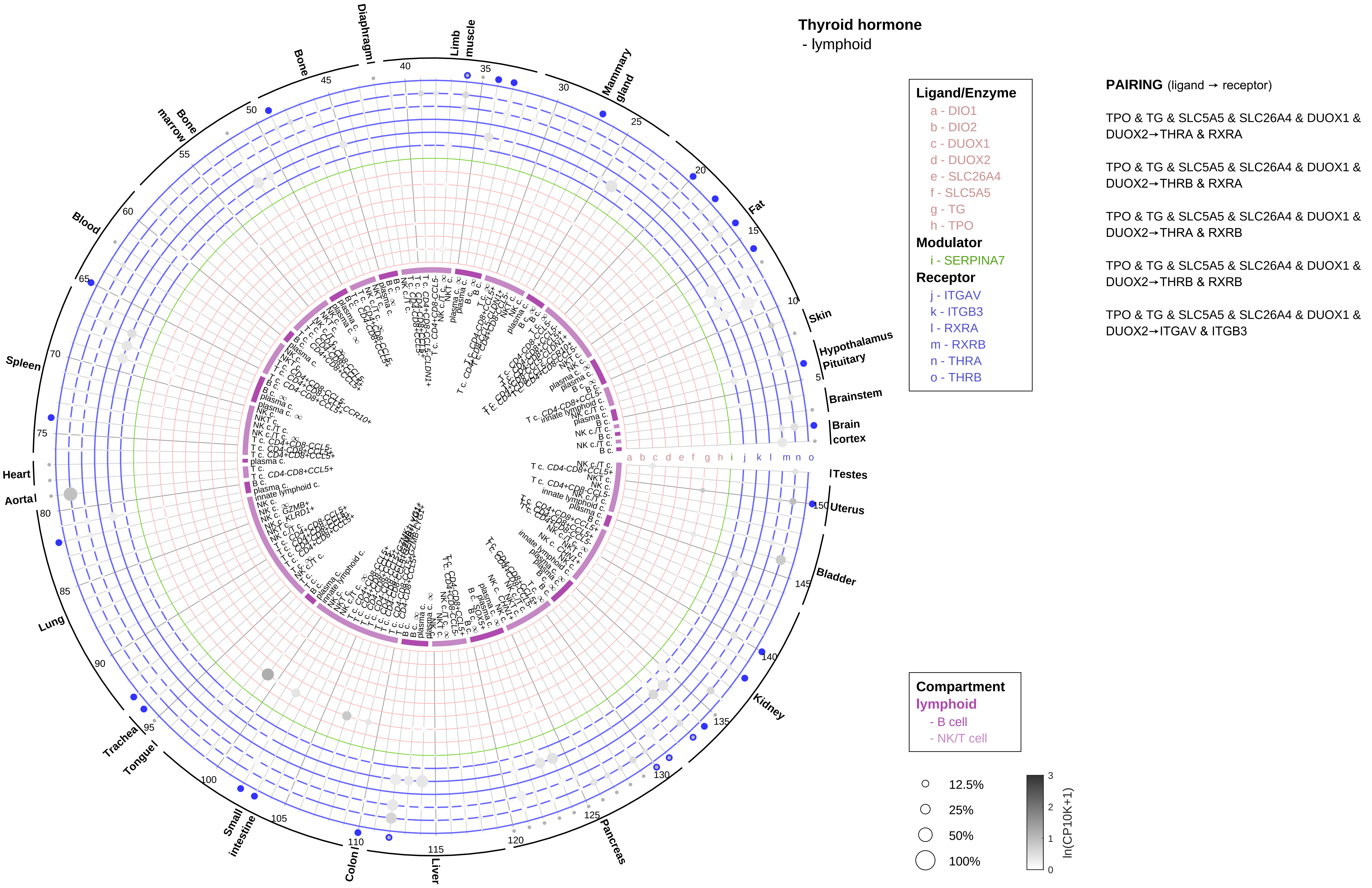

Thyroid hormone
- lymphoid
Ligand/Enzyme
a - DIO1
b - DIO2
c - DUOX1
d - DUOX2
e - SLC26A4
f - SLC5A5
g - TG
h - TPO
Modulator
i - SERPINA7
Receptor
j - ITGAV
k - ITGB3
l - RXRA
m - RXRB
n - THRA
o - THRB
PAIRING (ligand → receptor)
TPO & TG & SLC5A5 & SLC26A4 & DUOX1 & DUOX2→THRA & RXRA
TPO & TG & SLC5A5 & SLC26A4 & DUOX1 & DUOX2→THRB & RXRA
TPO & TG & SLC5A5 & SLC26A4 & DUOX1 & DUOX2→THRA & RXRB
TPO & TG & SLC5A5 & SLC26A4 & DUOX1 & DUOX2→THRB & RXRB
TPO & TG & SLC5A5 & SLC26A4 & DUOX1 & DUOX2→ITGAV & ITGB3
Compartment
lymphoid
- B cell
- NK/T cell
12.5%
25%
50%
100%
ln(CP10K+1)
0
1
2
3
Skin
Hypothalamus
Pituitary
Brainstem
Brain cortex
Testes
Uterus
Bladder
Kidney
Pancreas
Liver
Colon
Small intestine
Tongue
Trachea
Lung
Aortal
Heart
Spleen
Blood
Bone marrow
Bone
Diaphragm
Limb muscle
Mammary gland
Fat

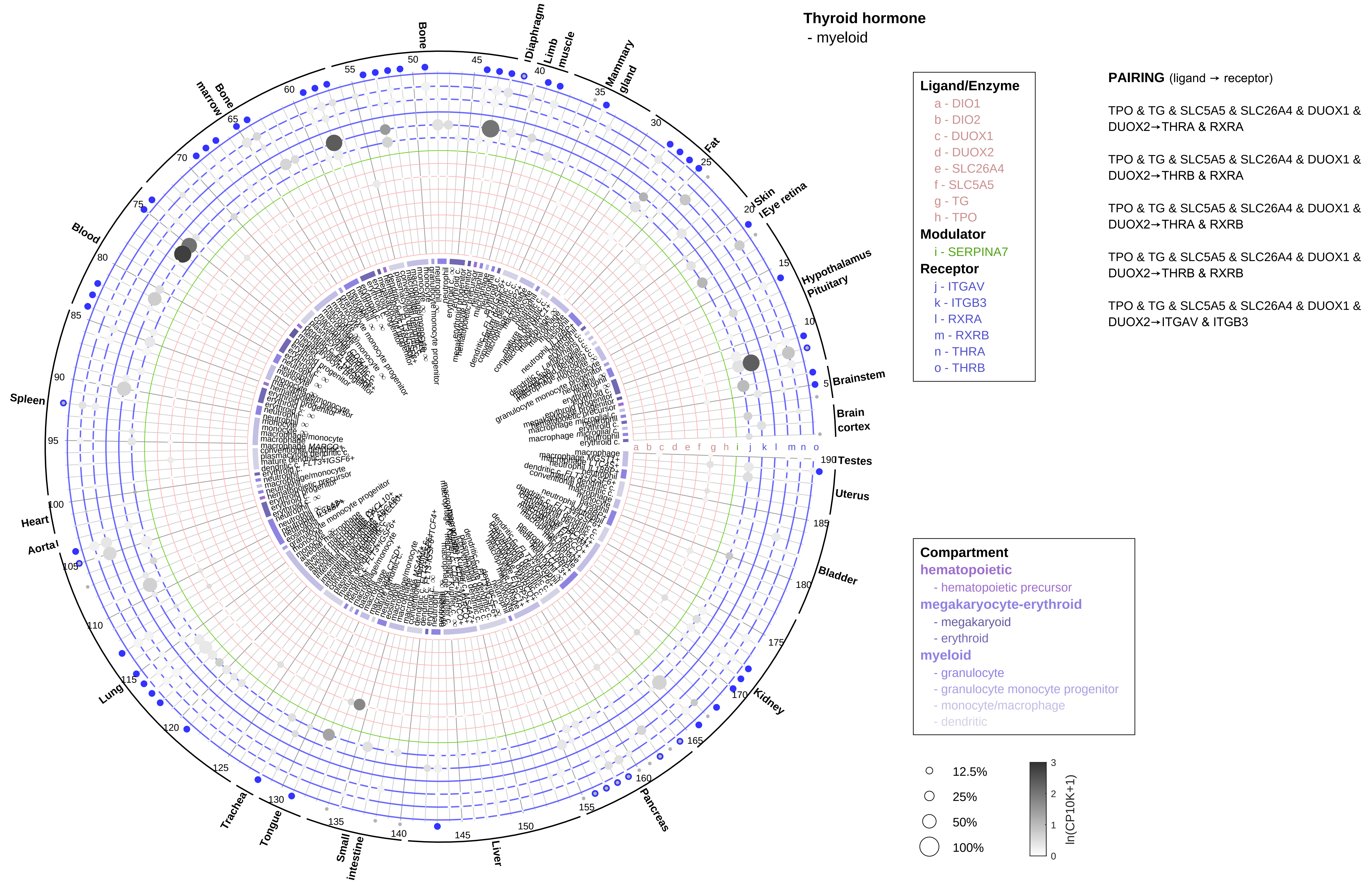

Thyroid hormone
- myeloid
Ligand/Enzyme
a - DIO1
b - DIO2
c - DUOX1
d - DUOX2
e - SLC26A4
f - SLC5A5
g - TG
h - TPO
Modulator
i - SERPINA7
Receptor
j - ITGAV
k - ITGB3
l - RXRA
m - RXRB
n - THRA
o - THRB
PAIRING (ligand → receptor)
TPO & TG & SLC5A5 & SLC26A4 & DUOX1 & DUOX2→THRA & RXRA
TPO & TG & SLC5A5 & SLC26A4 & DUOX1 & DUOX2→THRB & RXRA
TPO & TG & SLC5A5 & SLC26A4 & DUOX1 & DUOX2→THRA & RXRB
TPO & TG & SLC5A5 & SLC26A4 & DUOX1 & DUOX2→THRB & RXRB
TPO & TG & SLC5A5 & SLC26A4 & DUOX1 & DUOX2→ITGAV & ITGB3
Compartment
hematopoietic
- hematopoietic precursor
megakaryocyte-erythroid
- megakaryoid
- erythroid
myeloid
- granulocyte
- granulocyte monocyte progenitor
- monocyte/macrophage
- dendritic
12.5%
25%
50%
100%
ln(CP10K+1)
0
1
2
3
Bone
Diaphragm
Limb muscle
Mammary gland
Fat
Skin
Eye retina
Hypothalamus
Pituitary
Brainstem
Brain cortex
Testes
Uterus
Bladder
Kidney
Pancreas
Liver
Small intestine
Tongue
Trachea
Lung
Aorta
Heart
Spleen
Blood
Bone marrow

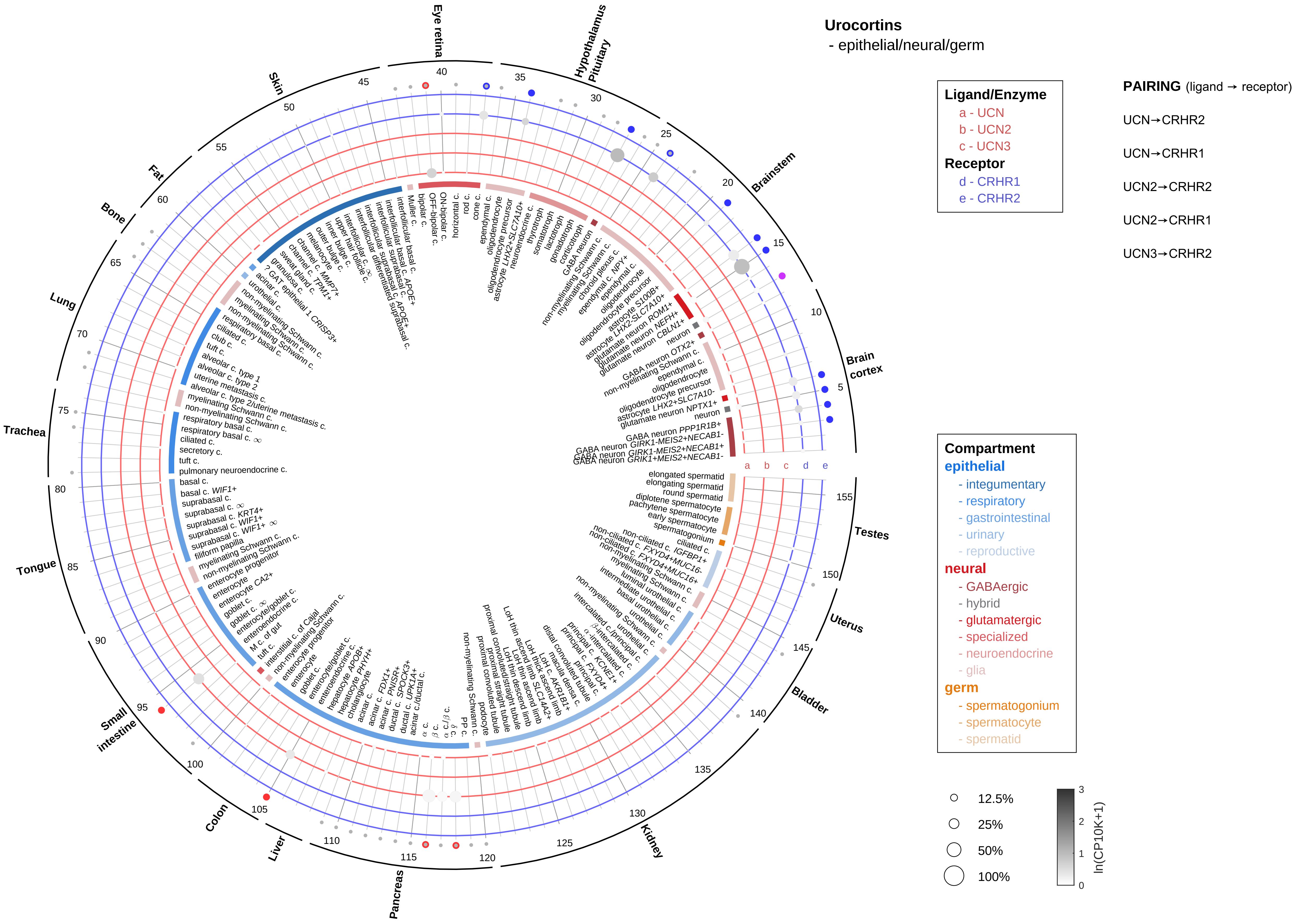
Urocortins
- epithelial/neural/germ
Ligand/Enzyme
a - UCN
b - UCN2
c - UCN3
Receptor
d - CRHR1
e - CRHR2
PAIRING (ligand → receptor)
UCN→CRHR2
UCN→CRHR1
UCN2→CRHR2
UCN2→CRHR1
UCN3→CRHR2
Compartment
epithelial
- integumentary
- respiratory
- gastrointestinal
- urinary
- reproductive
neural
- GABAergic
- hybrid
- glutamatergic
- specialized
- neuroendocrine
- glia
germ
- spermatogonium
- spermatocyte
- spermatid
12.5%
25%
50%
100%
ln(CP10K+1)
Brain cortex
Brainstem
Hypothalamus Pituitary
Eye retina
Skin
Fat
Bone
Lung
Trachea
Tongue
Small intestine
Colon
Liver
Pancreas
Kidney
Bladder
Uterus
Testes

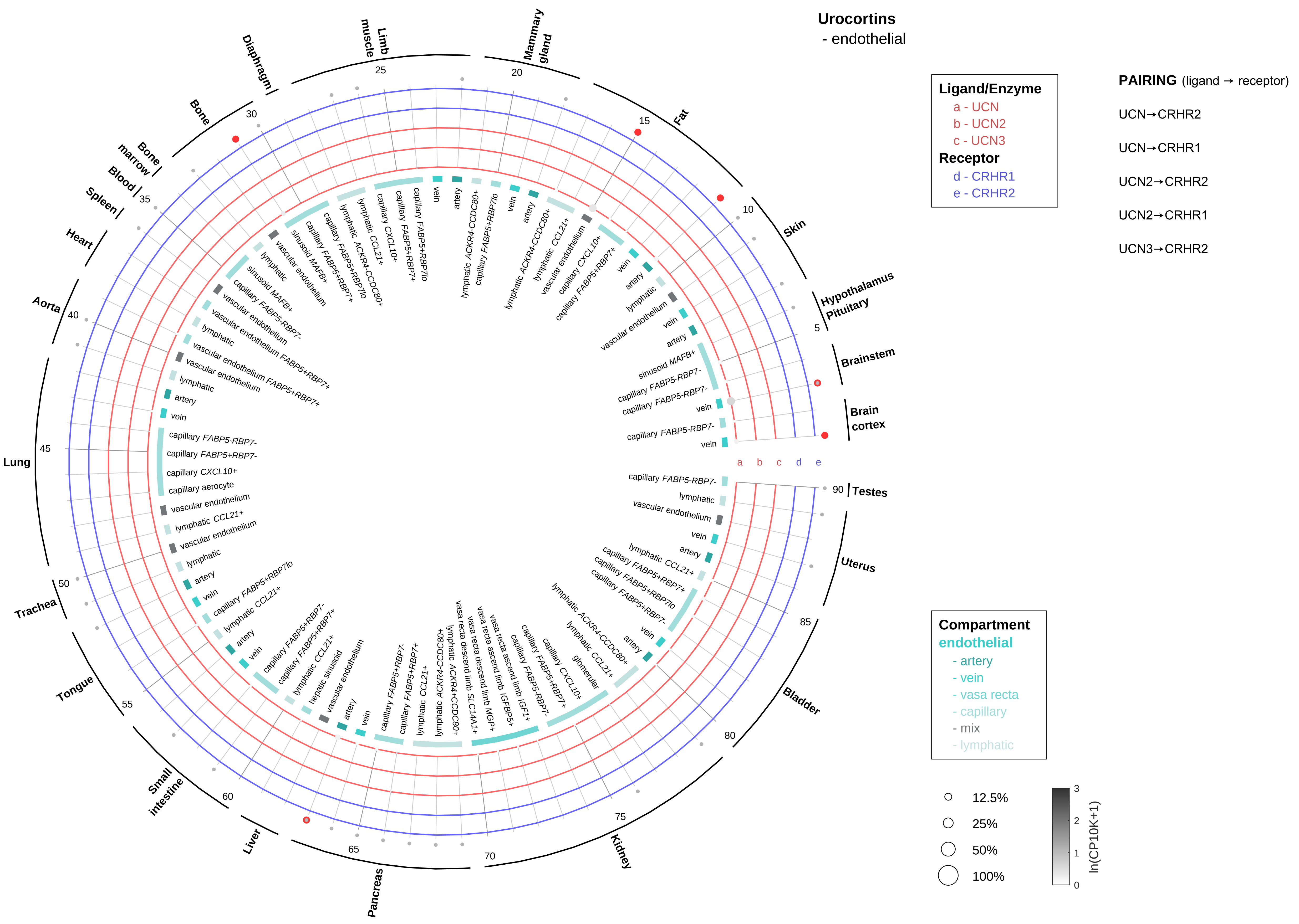

Urocortins
- endothelial
Ligand/Enzyme
a - UCN
b - UCN2
c - UCN3
Receptor
d - CRHR1
e - CRHR2
PAIRING (ligand → receptor)
UCN→CRHR2
UCN→CRHR1
UCN2→CRHR2
UCN2→CRHR1
UCN3→CRHR2
Compartment
endothelial
- artery
- vein
- vasa recta
- capillary
- mix
- lymphatic
12.5%
25%
50%
100%
ln(CP10K+1)
Hypothalamus
Pituitary
Brainstem
Brain cortex
Testes
Uterus
Bladder
Kidney
Pancreas
Liver
Small intestine
Tongue
Trachea
Lung
Aorta
Heart
Spleen
Blood
Bone marrow
Bone
Diaphragm
Limb muscle
Mammary gland
Fat
Skin

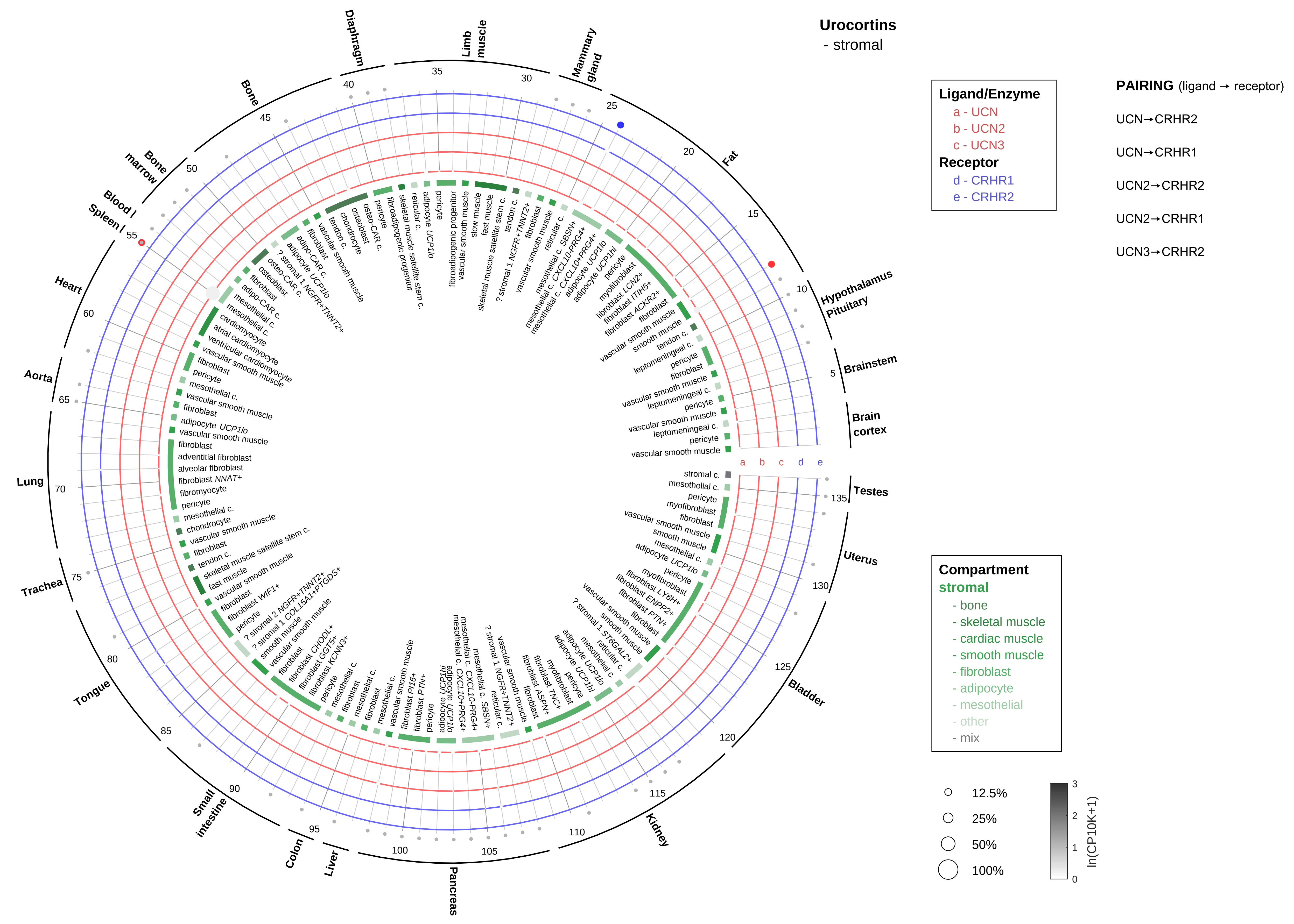

Urocortins
- stromal
Ligand/Enzyme
a - UCN
b - UCN2
c - UCN3
Receptor
d - CRHR1
e - CRHR2
PAIRING (ligand → receptor)
UCN→CRHR2
UCN→CRHR1
UCN2→CRHR2
UCN2→CRHR1
UCN3→CRHR2
Compartment
stromal
- bone
- skeletal muscle
- cardiac muscle
- smooth muscle
- fibroblast
- adipocyte
- mesothelial
- other
- mix
12.5%
25%
50%
100%
ln(CP10K+1)
Hypothalamus Pituitary
Brainstem
Brain cortex
Testes
Uterus
Bladder
Kidney
Pancreas
Liver
Colon
Small intestine
Tongue
Trachea
Lung
Aorta
Heart
Spleen
Blood
Bone marrow
Bone
Diaphragm
Limb muscle
Mammary gland
Fat

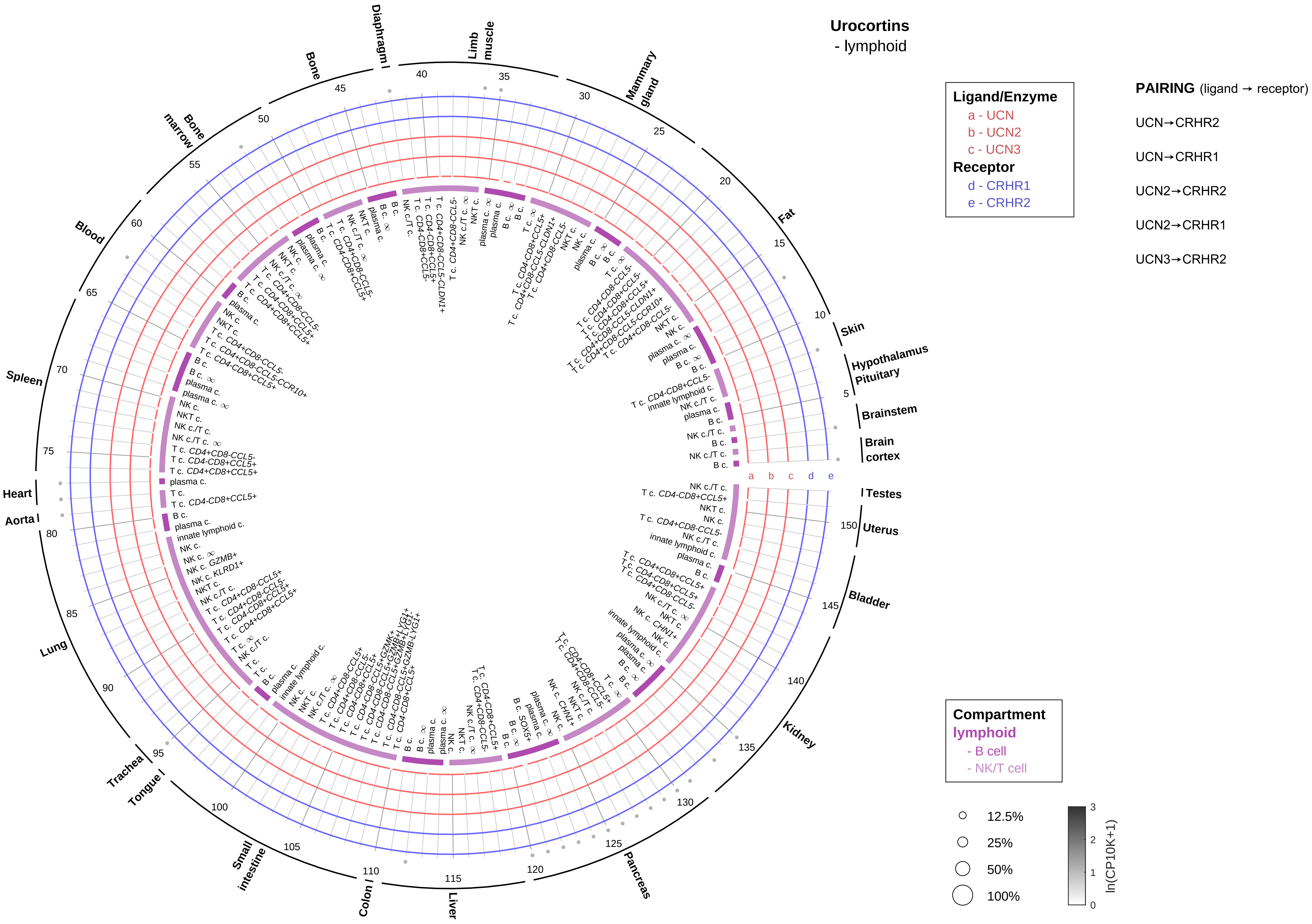
Urocortins
- lymphoid
Ligand/Enzyme
a - UCN
b - UCN2
c - UCN3
Receptor
d - CRHR1
e - CRHR2
PAIRING (ligand → receptor)
UCN→CRHR2
UCN→CRHR1
UCN2→CRHR2
UCN2→CRHR1
UCN3→CRHR2
Compartment
lymphoid
- B cell
- NK/T cell
12.5%
25%
50%
100%
ln(CP10K+1)
Skin
Hypothalamus
Pituitary
Brainstem
Brain cortex
Testes
Uterus
Bladder
Kidney
Pancreas
Liver
Colon
Small intestine
Tongue
Trachea
Lung
Aorta
Heart
Spleen
Blood
Bone marrow
Bone
Diaphragm
Limb muscle
Mammary gland
Fat

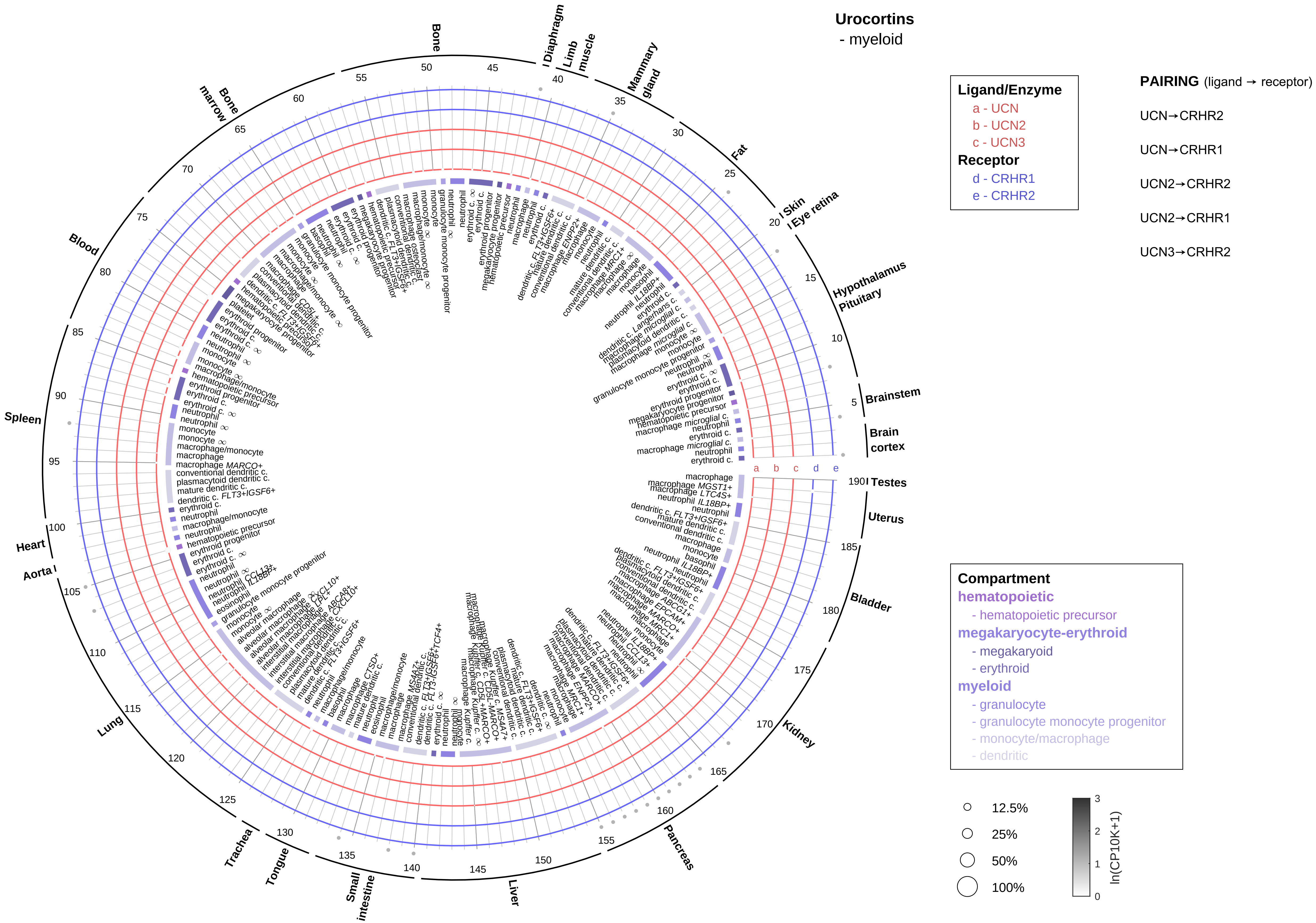
Urocortins
- myeloid
Ligand/Enzyme
a - UCN
b - UCN2
c - UCN3
Receptor
d - CRHR1
e - CRHR2
PAIRING (ligand → receptor)
UCN→CRHR2
UCN→CRHR1
UCN2→CRHR2
UCN2→CRHR1
UCN3→CRHR2
Compartment
hematopoietic
- hematopoietic precursor
megakaryocyte-erythroid
- megakaryoid
- erythroid
myeloid
- granulocyte
- granulocyte monocyte progenitor
- monocyte/macrophage
- dendritic
12.5%
25%
50%
100%
ln(CP10K+1)
Bone
Diaphragm
Limb muscle
Mammary gland
Fat
Skin
Eye retina
Hypothalamus
Pituitary
Brainstem
Brain cortex
Testes
Uterus
Bladder
Kidney
Pancreas
Liver
Small intestine
Tongue
Trachea
Lung
Aorta
Heart
Spleen
Blood
Bone marrow

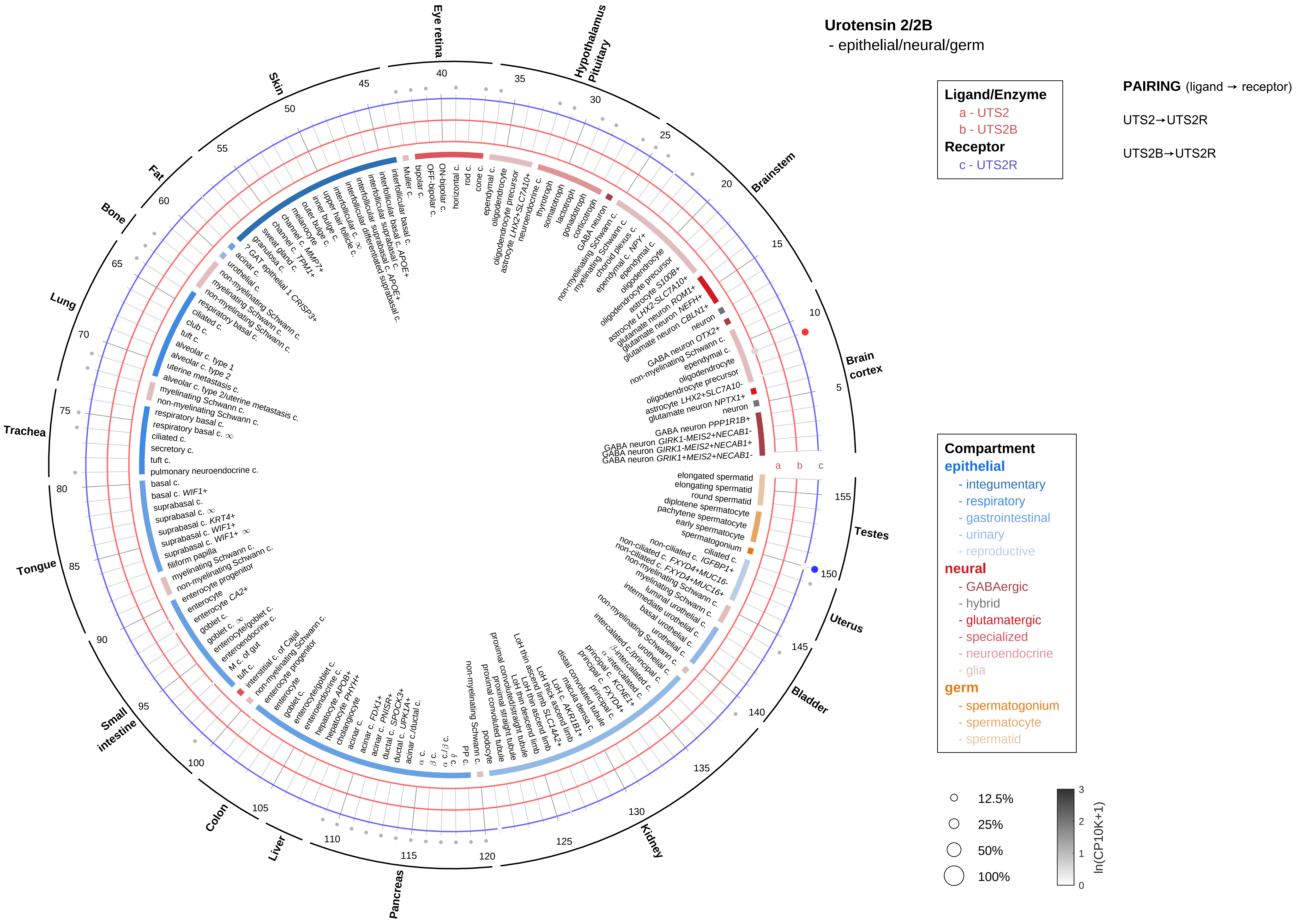

Urotensin 2/2B
- epithelial/neural/germ
Ligand/Enzyme
a - UTS2
b - UTS2B
Receptor
c - UTS2R
PAIRING (ligand → receptor)
UTS2→UTS2R
UTS2B→UTS2R
Compartment
epithelial
- integumentary
- respiratory
- gastrointestinal
- urinary
- reproductive
neural
- GABAergic
- hybrid
- glutamatergic
- specialized
- neuroendocrine
- glia
germ
- spermatogonium
- spermatocyte
- spermatid
12.5%
25%
50%
100%
ln(CP10K+1)
Eye retina
Hypothalamus Pituitary
Brainstem
Brain cortex
Testes
Uterus
Bladder
Kidney
Pancreas
Liver
Colon
Small intestine
Tongue
Trachea
Lung
Bone
Fat
Skin

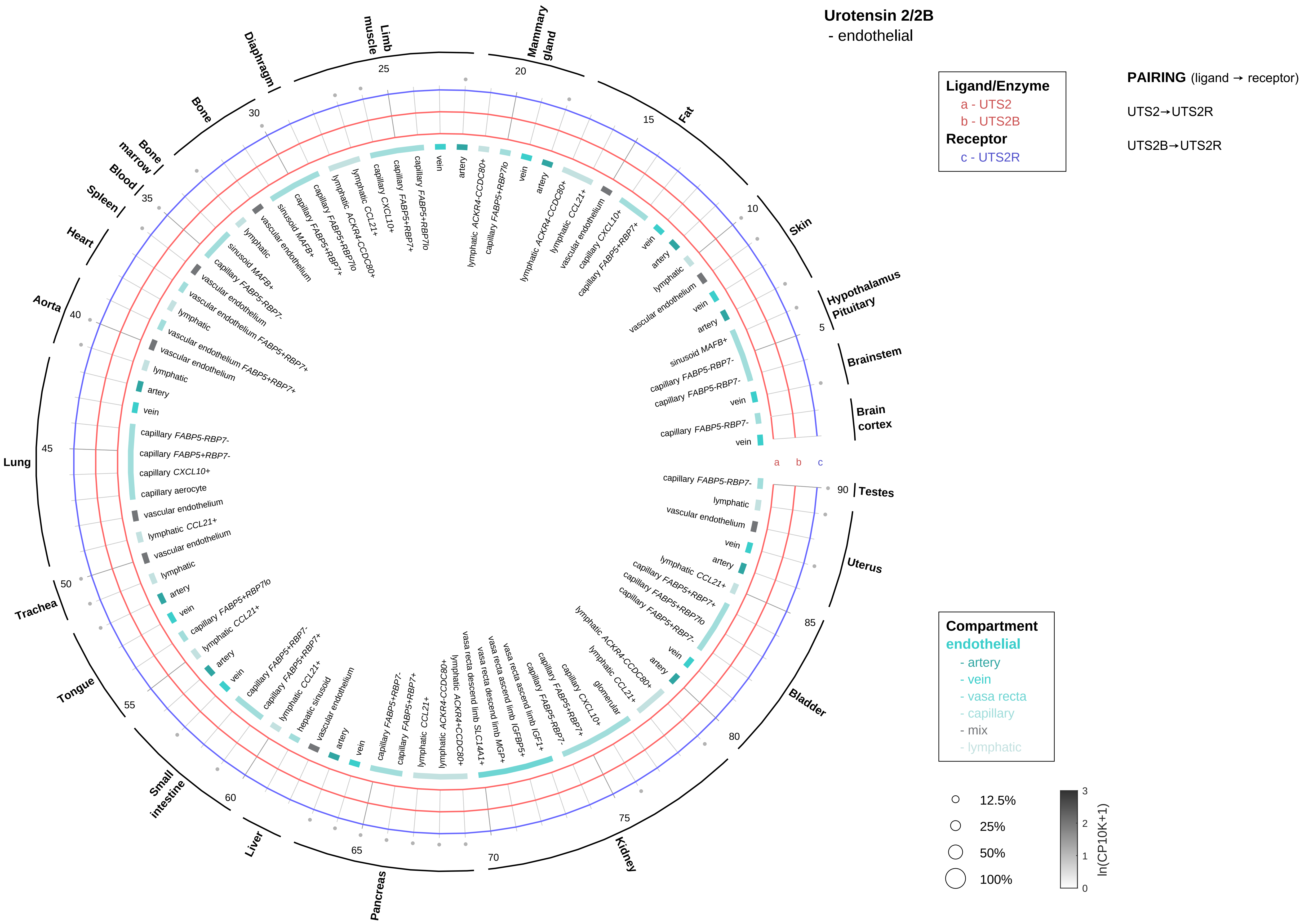

Urotensin 2/2B
- endothelial
Ligand/Enzyme
a - UTS2
b - UTS2B
Receptor
c - UTS2R
PAIRING (ligand → receptor)
UTS2→UTS2R
UTS2B→UTS2R
Compartment
endothelial
- artery
- vein
- vasa recta
- capillary
- mix
- lymphatic
12.5%
25%
50%
100%
ln(CP10K+1)
Brain cortex
Brainstem
Hypothalamus
Pituitary
Skin
Fat
Mammary gland
Limb muscle
Diaphragm
Bone
Bone marrow
Blood
Spleen
Heart
Aorta
Lung
Trachea
Tongue
Small intestine
Liver
Pancreas
Kidney
Bladder
Uterus
Testes

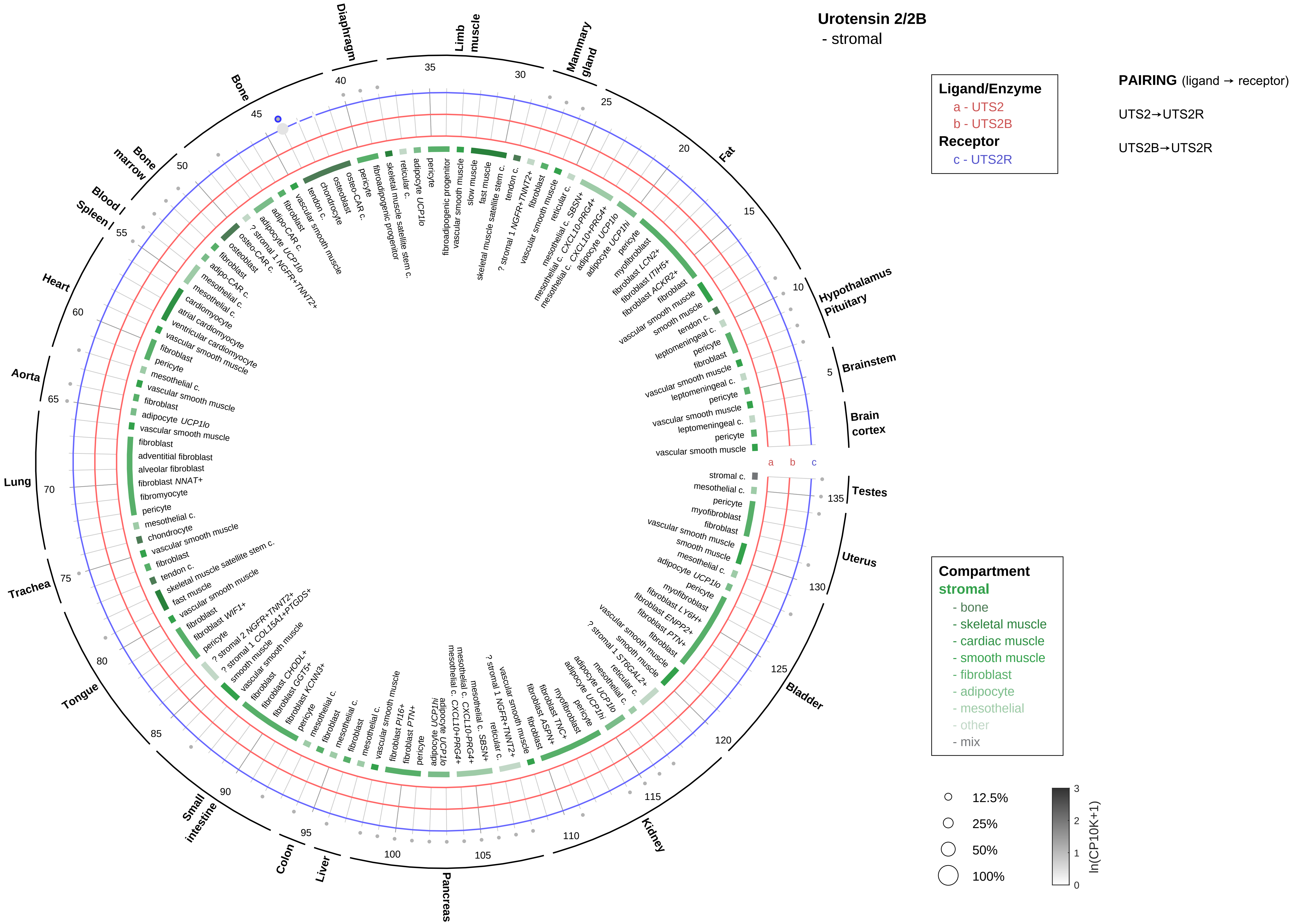

Urotensin 2/2B
- stromal
Ligand/Enzyme
a - UTS2
b - UTS2B
Receptor
c - UTS2R
PAIRING (ligand → receptor)
UTS2→UTS2R
UTS2B→UTS2R
Compartment
stromal
- bone
- skeletal muscle
- cardiac muscle
- smooth muscle
- fibroblast
- adipocyte
- mesothelial
- other
- mix
12.5%
25%
50%
100%
ln(CP10K+1)
Brain cortex
Brainstem
Hypothalamus Pituitary
Fat
Mammary gland
Limb muscle
Diaphragm
Bone
Bone marrow
Blood / Spleen /
Heart
Aorta
Lung
Trachea
Tongue
Small intestine
Colon
Liver
Pancreas
Kidney
Bladder
Uterus
Testes

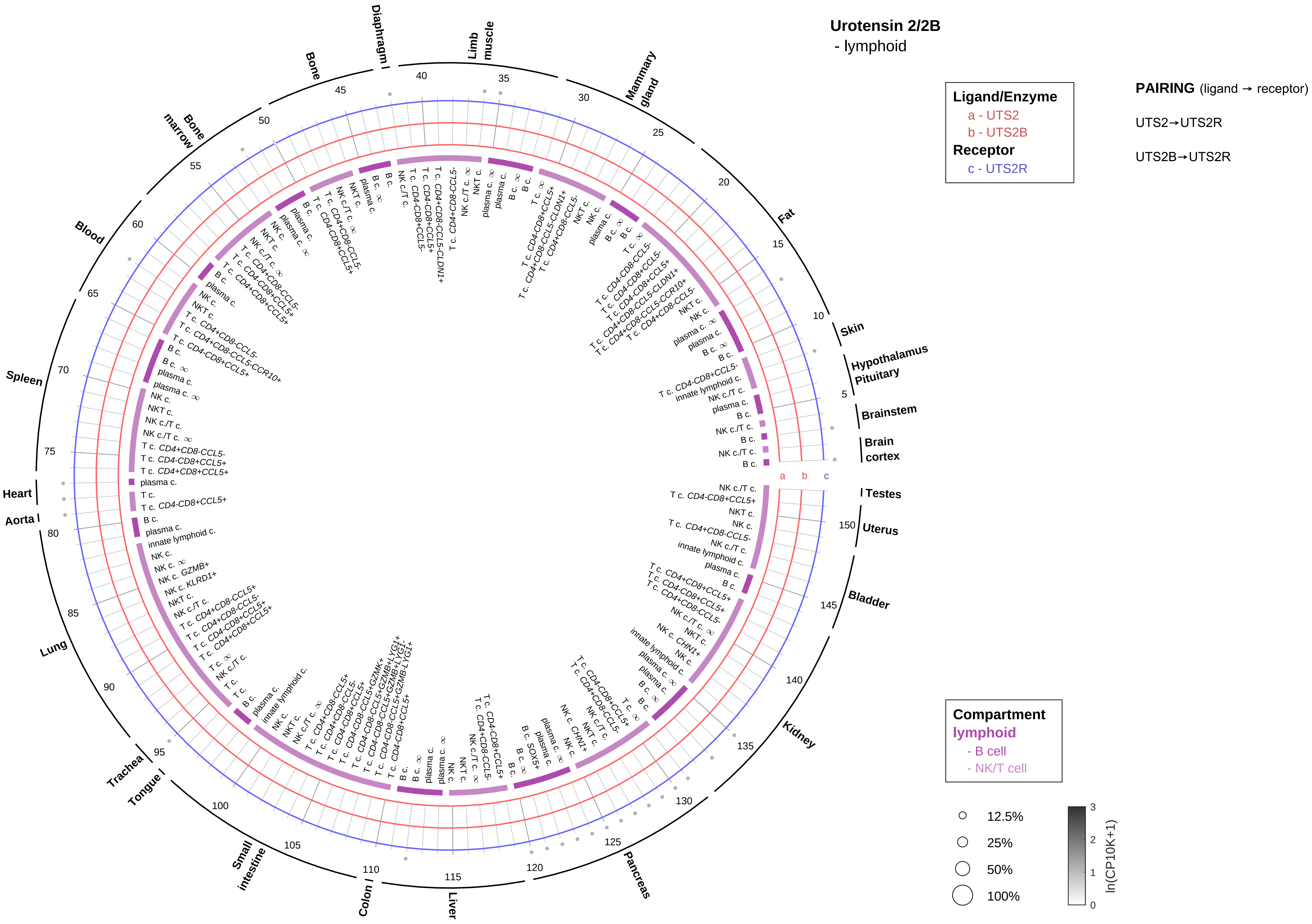
Urotensin 2/2B
- lymphoid
Ligand/Enzyme
a - UTS2
b - UTS2B
Receptor
c - UTS2R
PAIRING (ligand → receptor)
UTS2→UTS2R
UTS2B→UTS2R
Compartment
lymphoid
- B cell
- NK/T cell
12.5%
25%
50%
100%
ln(CP10K+1)
0
1
2
3
Brain cortex
Brainstem
Hypothalamus Pituitary
Skin
Fat
Mammary gland
Limb muscle
Diaphragm
Bone
Bone marrow
Blood
Spleen
Heart
Aorta
Lung
Trachea
Tongue
Small intestine
Colon
Liver
Pancreas
Kidney
Bladder
Uterus
Testes
5
10
15
20
25
30
35
40
45
50
55
60
65
70
75
80
85
90
95
100
105
110
115
120
125
130
135
140
145
150
a
b
c

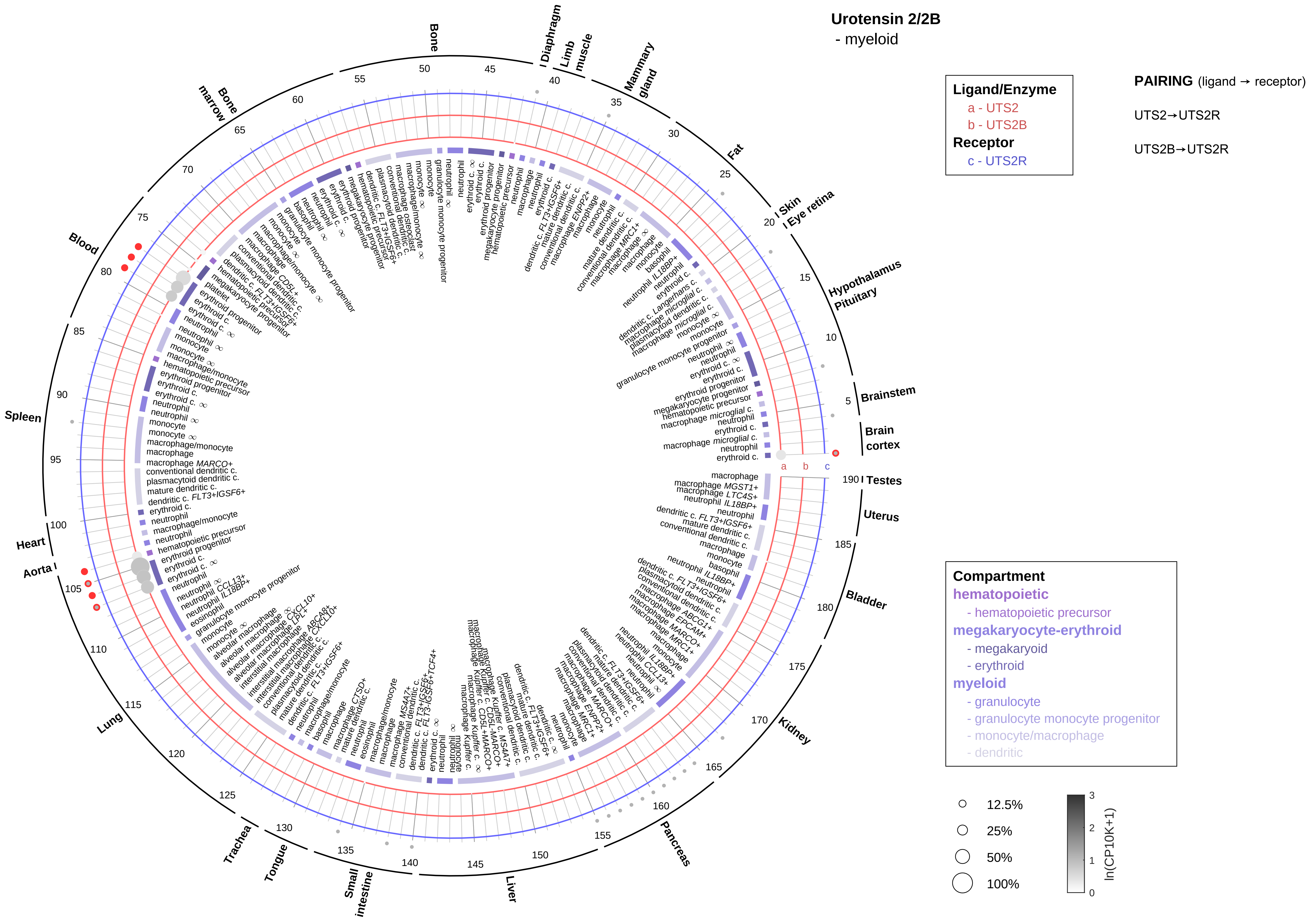
Urotensin 2/2B
- myeloid
Ligand/Enzyme
a - UTS2
b - UTS2B
Receptor
c - UTS2R
PAIRING (ligand → receptor)
UTS2→UTS2R
UTS2B→UTS2R
Compartment
hematopoietic
- hematopoietic precursor
megakaryocyte-erythroid
- megakaryoid
- erythroid
myeloid
- granulocyte
- granulocyte monocyte progenitor
- monocyte/macrophage
- dendritic
12.5%
25%
50%
100%
ln(CP10K+1)
Bone
Diaphragm
Limb muscle
Mammary gland
Fat
Skin
Eye retina
Hypothalamus
Pituitary
Brainstem
Brain cortex
Testes
Uterus
Bladder
Kidney
Pancreas
Liver
Small intestine
Tongue
Trachea
Lung
Aorta
Heart
Spleen
Blood
Bone marrow

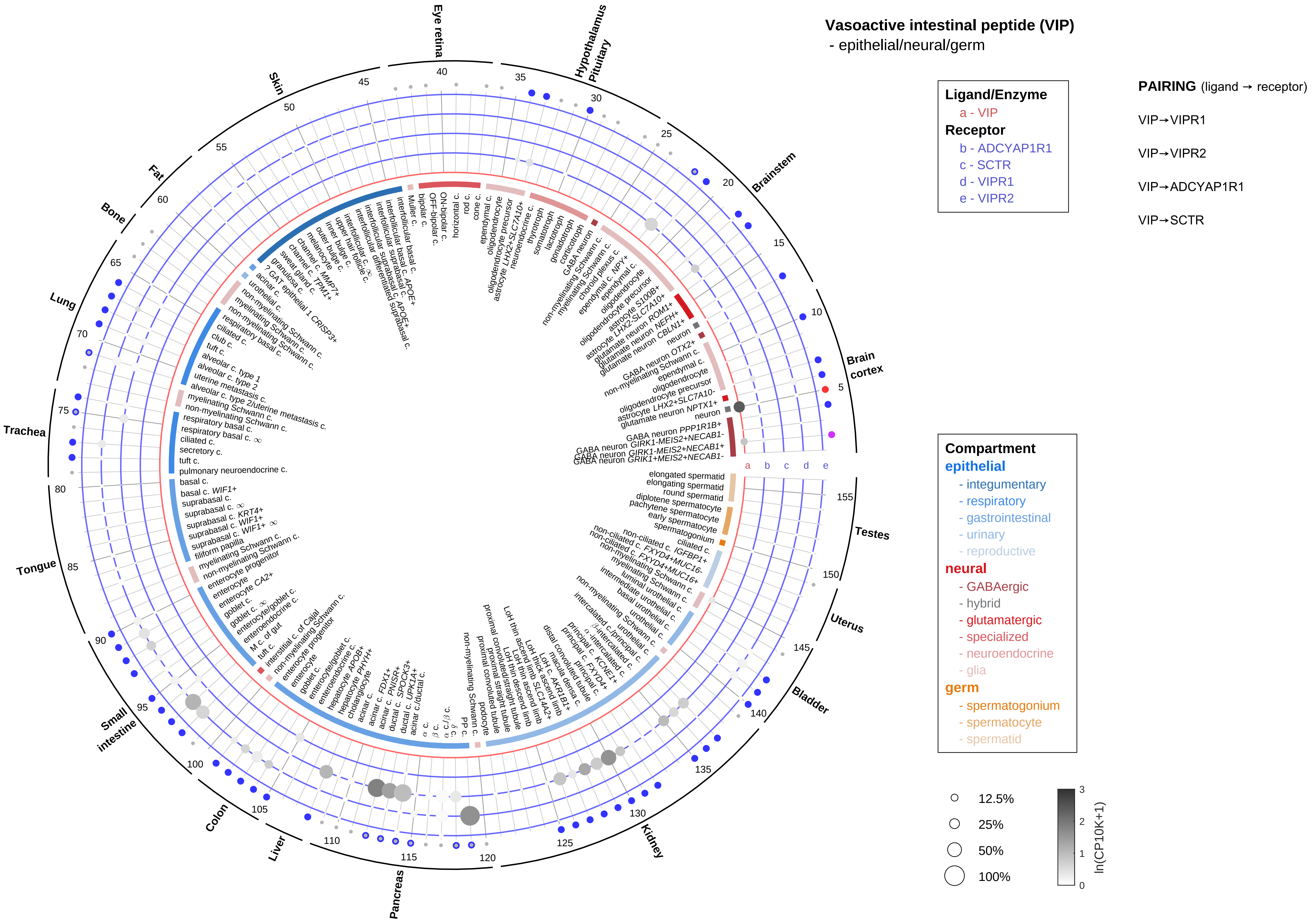
Vasoactive intestinal peptide (VIP)
- epithelial/neural/germ
Ligand/Enzyme
a - VIP
Receptor
b - ADCYAP1R1
c - SCTR
d - VIPR1
e - VIPR2
PAIRING (ligand → receptor)
VIP→VIPR1
VIP→VIPR2
VIP→ADCYAP1R1
VIP→SCTR
Compartment
epithelial
- integumentary
- respiratory
- gastrointestinal
- urinary
- reproductive
neural
- GABAergic
- hybrid
- glutamatergic
- specialized
- neuroendocrine
- glia
germ
- spermatogonium
- spermatocyte
- spermatid
12.5%
25%
50%
100%
ln(CP10K+1)
Eye retina
Hypothalamus
Pituitary
Brainstem
Brain cortex
Testes
Uterus
Bladder
Kidney
Pancreas
Liver
Colon
Small intestine
Tongue
Trachea
Lung
Bone
Fat
Skin

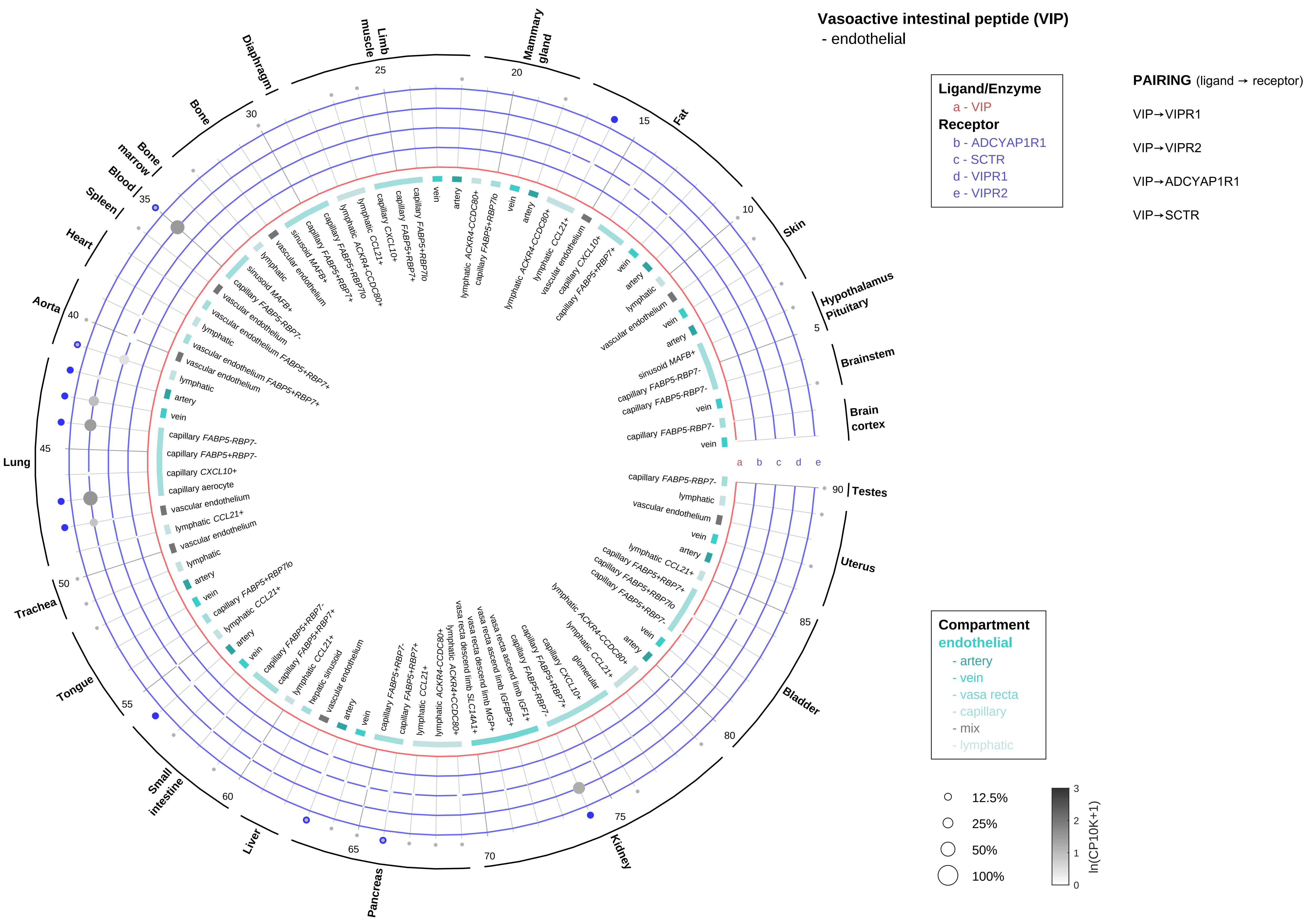

Vasoactive intestinal peptide (VIP)
- endothelial
Ligand/Enzyme
a - VIP
Receptor
b - ADCYAP1R1
c - SCTR
d - VIPR1
e - VIPR2
PAIRING (ligand → receptor)
VIP→VIPR1
VIP→VIPR2
VIP→ADCYAP1R1
VIP→SCTR
Compartment
endothelial
- artery
- vein
- vasa recta
- capillary
- mix
- lymphatic
12.5%
25%
50%
100%
ln(CP10K+1)
Brain cortex
Brainstem
Hypothalamus Pituitary
Skin
Fat
Mammary gland
Limb muscle
Diaphragm
Bone
Bone marrow
Blood
Spleen
Heart
Aorta
Lung
Trachea
Tongue
Small intestine
Liver
Pancreas
Kidney
Bladder
Uterus
Testes

Vasoactive intestinal peptide (VIP)
- stromal

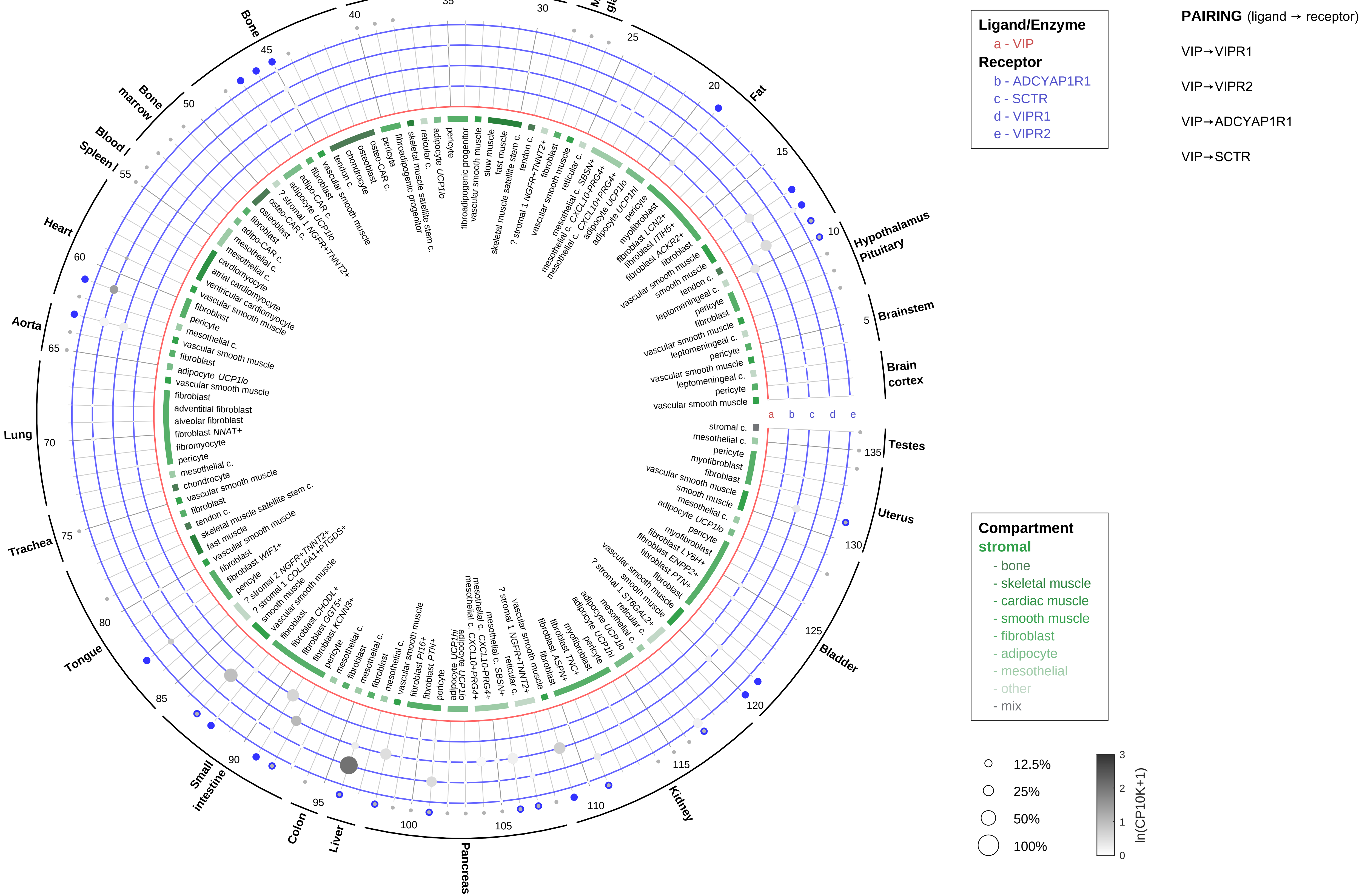

Vasoactive intestinal peptide (VIP)
- lymphoid

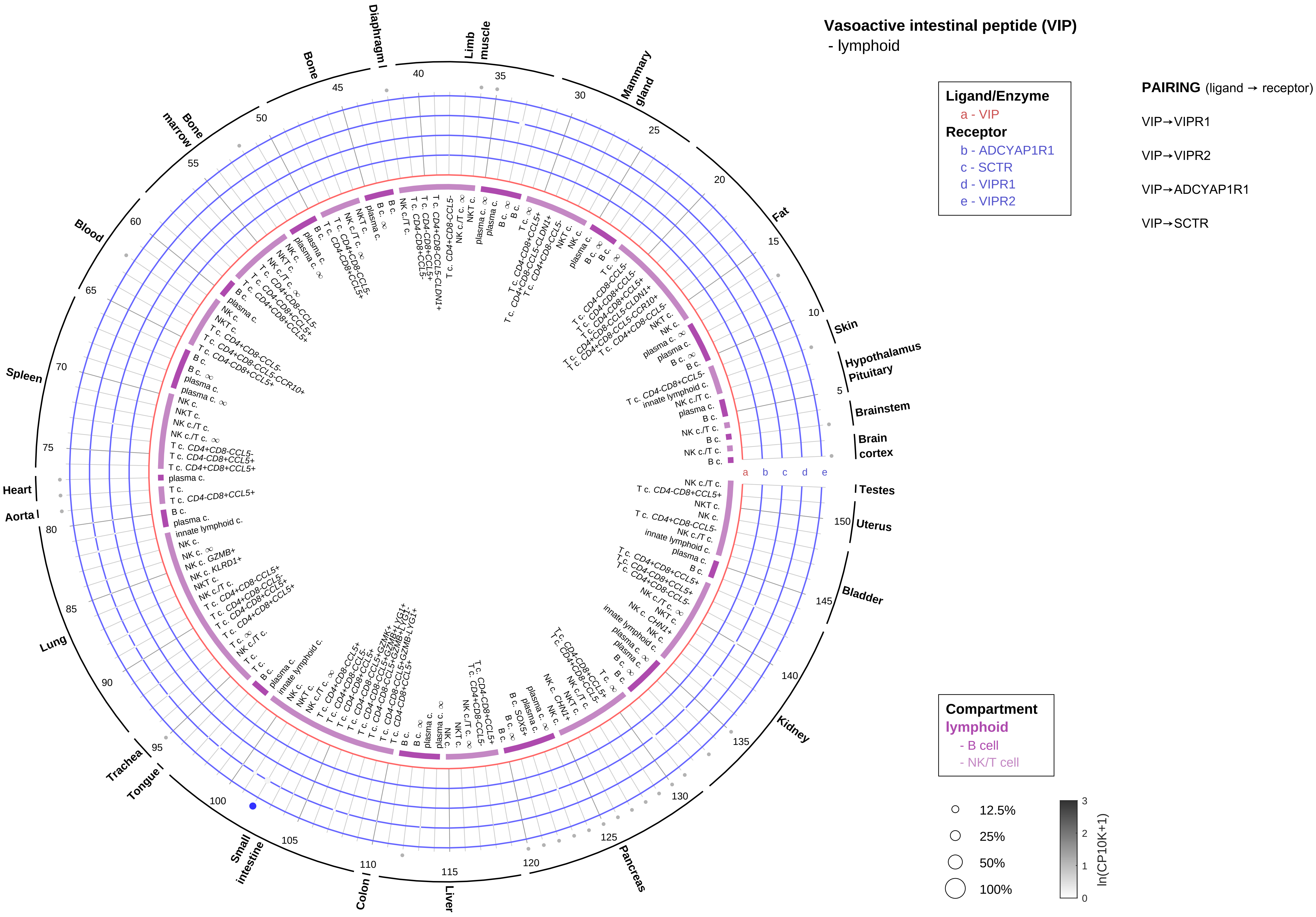

Vasoactive intestinal peptide (VIP)
- myeloid

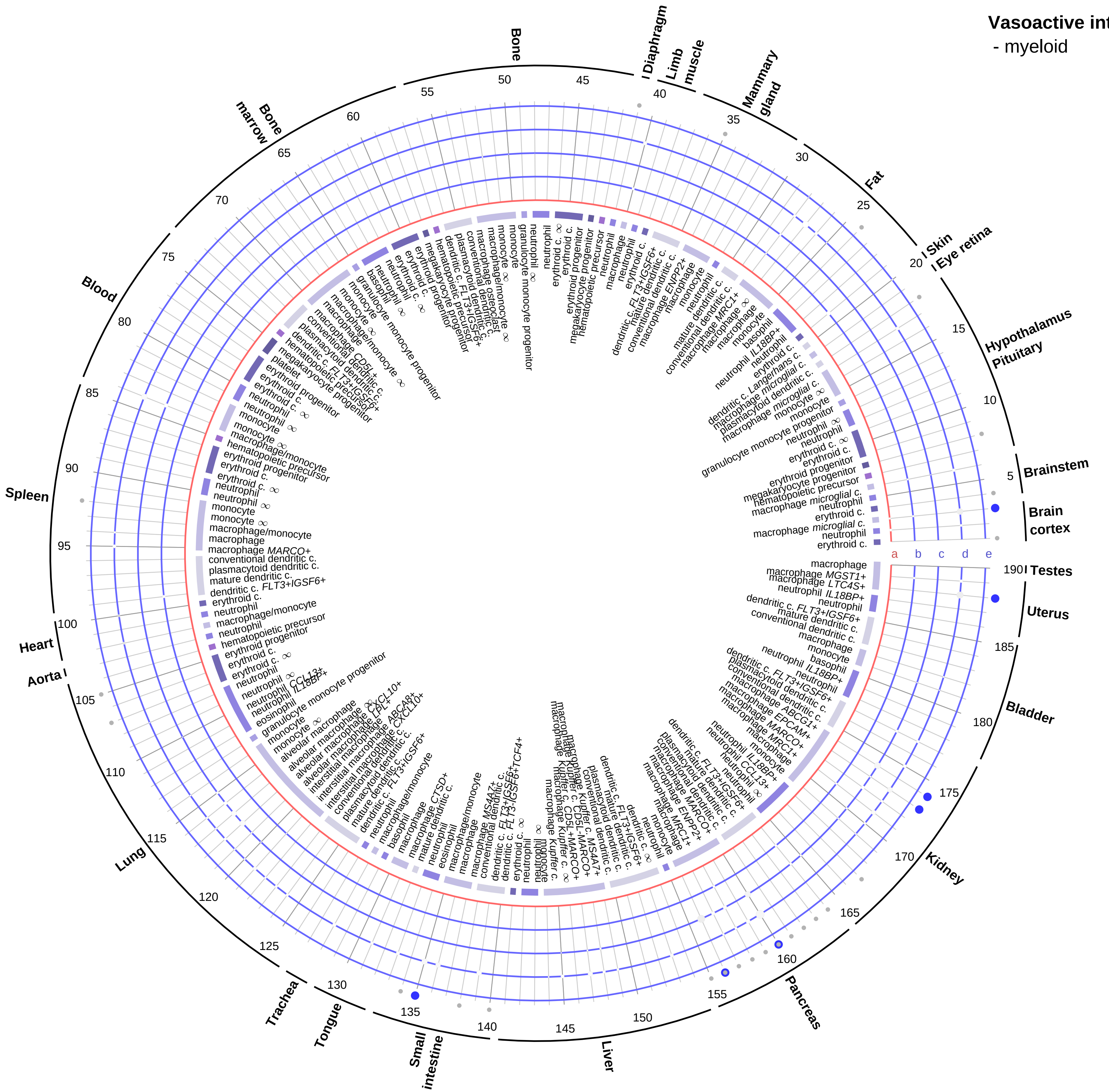

Ligand/Enzyme
- a - VIP

Receptor
- b - ADCYAP1R1
- c - SCTR
- d - VIPR1
- e - VIPR2

PAIRING (ligand → receptor)

VIP→VIPR1

VIP→VIPR2

VIP→ADCYAP1R1

VIP→SCTR

Compartment

hematopoietic
- hematopoietic precursor

megakaryocyte-erythroid
- megakaryoid
- erythroid

myeloid
- granulocyte
- granulocyte monocyte progenitor
- monocyte/macrophage
- dendritic

12.5%

25%

50%

100%

ln(CP10K+1)

0 1 2 3

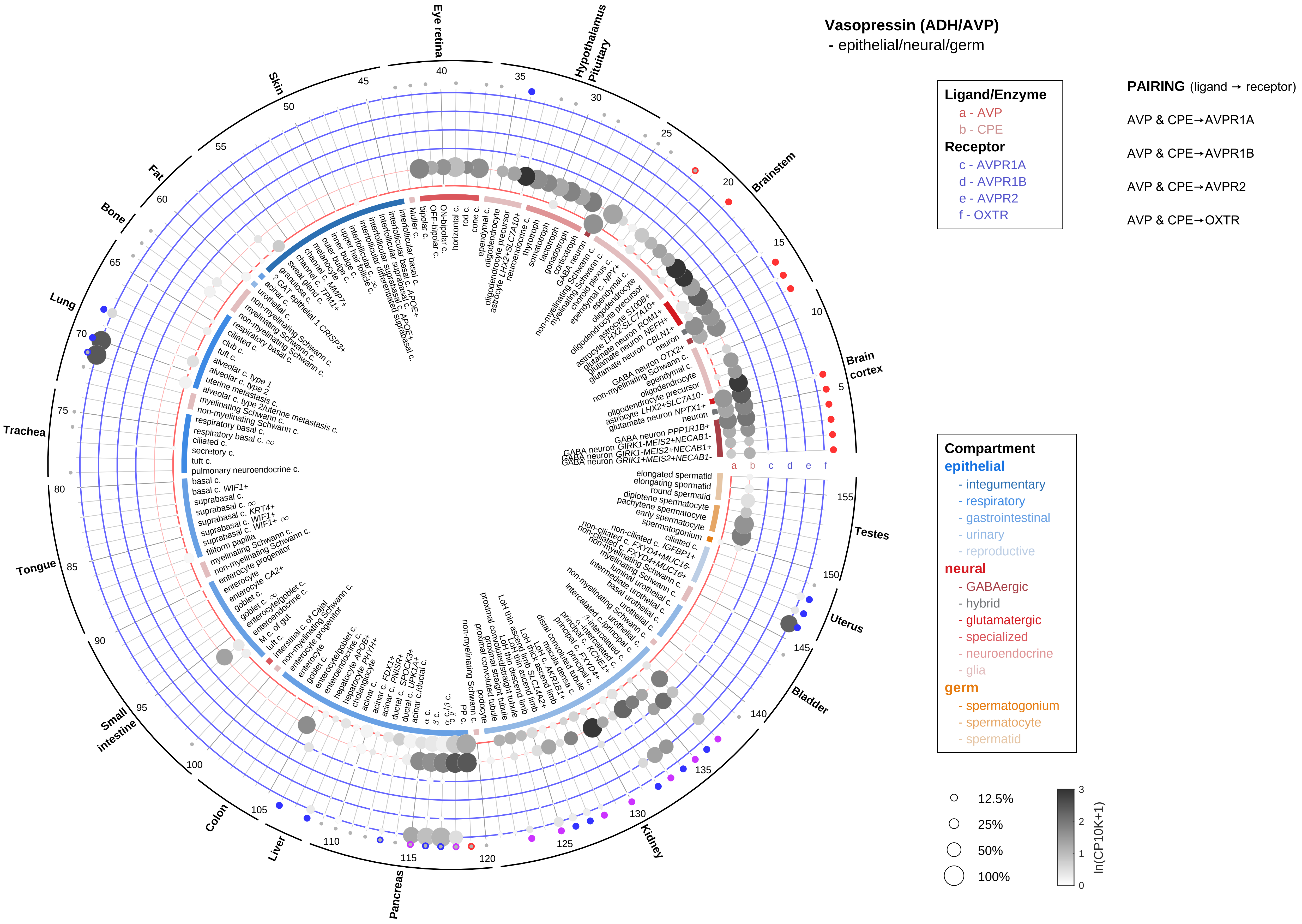

Vasopressin (ADH/AVP)
- epithelial/neural/germ
Ligand/Enzyme
a - AVP
b - CPE
Receptor
c - AVPR1A
d - AVPR1B
e - AVPR2
f - OXTR
PAIRING (ligand → receptor)
AVP & CPE→AVPR1A
AVP & CPE→AVPR1B
AVP & CPE→AVPR2
AVP & CPE→OXTR
Compartment
epithelial
- integumentary
- respiratory
- gastrointestinal
- urinary
- reproductive
neural
- GABAergic
- hybrid
- glutamatergic
- specialized
- neuroendocrine
- glia
germ
- spermatogonium
- spermatocyte
- spermatid
12.5%
25%
50%
100%
ln(CP10K+1)
Eye retina
Hypothalamus Pituitary
Brainstem
Brain cortex
Testes
Uterus
Bladder
Kidney
Pancreas
Liver
Colon
Small intestine
Tongue
Trachea
Lung
Bone
Fat
Skin

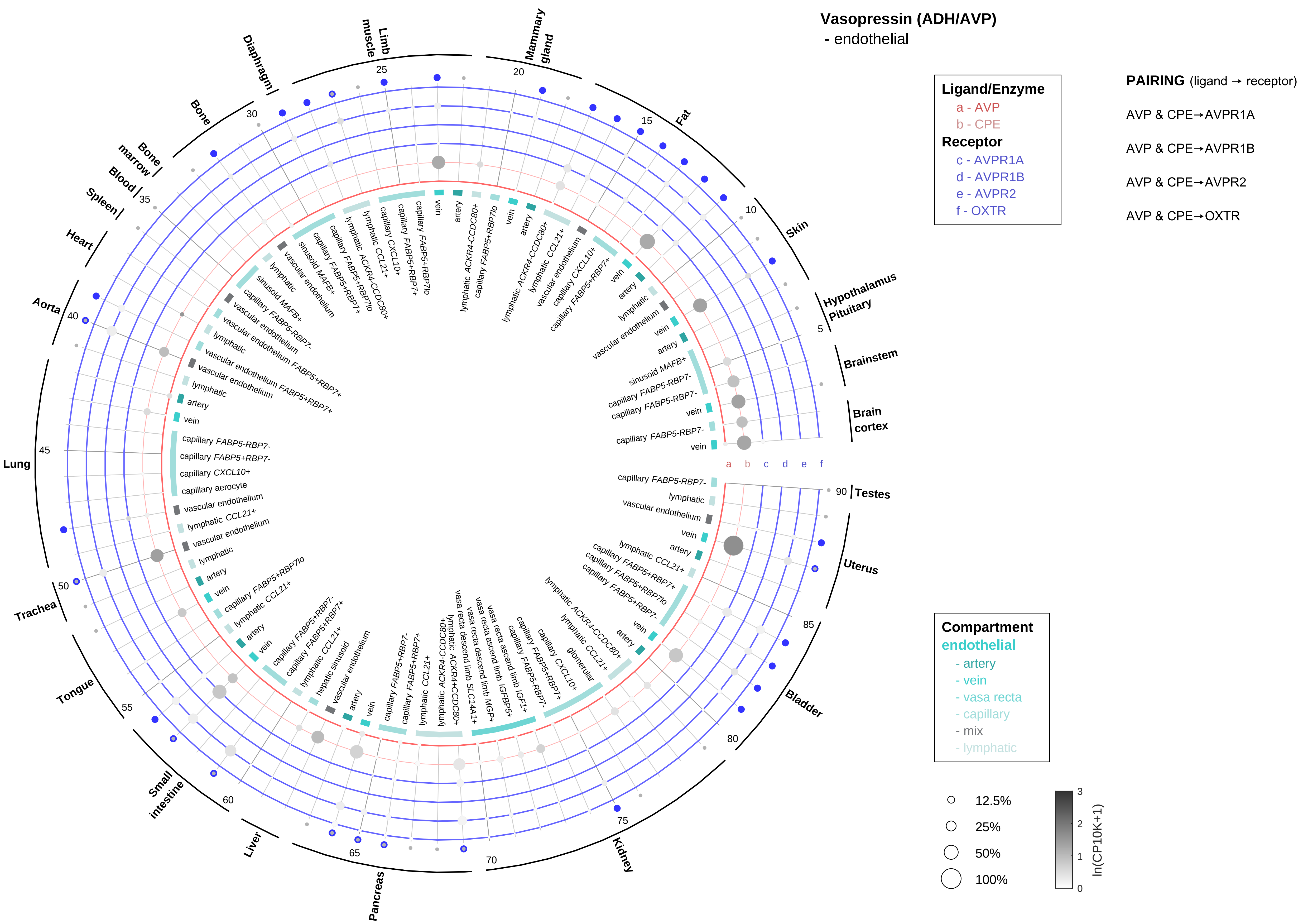

Vasopressin (ADH/AVP)
- endothelial
Ligand/Enzyme
a - AVP
b - CPE
Receptor
c - AVPR1A
d - AVPR1B
e - AVPR2
f - OXTR
PAIRING (ligand → receptor)
AVP & CPE→AVPR1A
AVP & CPE→AVPR1B
AVP & CPE→AVPR2
AVP & CPE→OXTR
Compartment
endothelial
- artery
- vein
- vasa recta
- capillary
- mix
- lymphatic
12.5%
25%
50%
100%
ln(CP10K+1)
Brain cortex
Brainstem
Hypothalamus Pituitary
Skin
Fat
Mammary gland
Limb muscle
Diaphragm
Bone
Bone marrow
Blood
Spleen
Heart
Aorta
Lung
Trachea
Tongue
Small intestine
Liver
Pancreas
Kidney
Bladder
Uterus
Testes

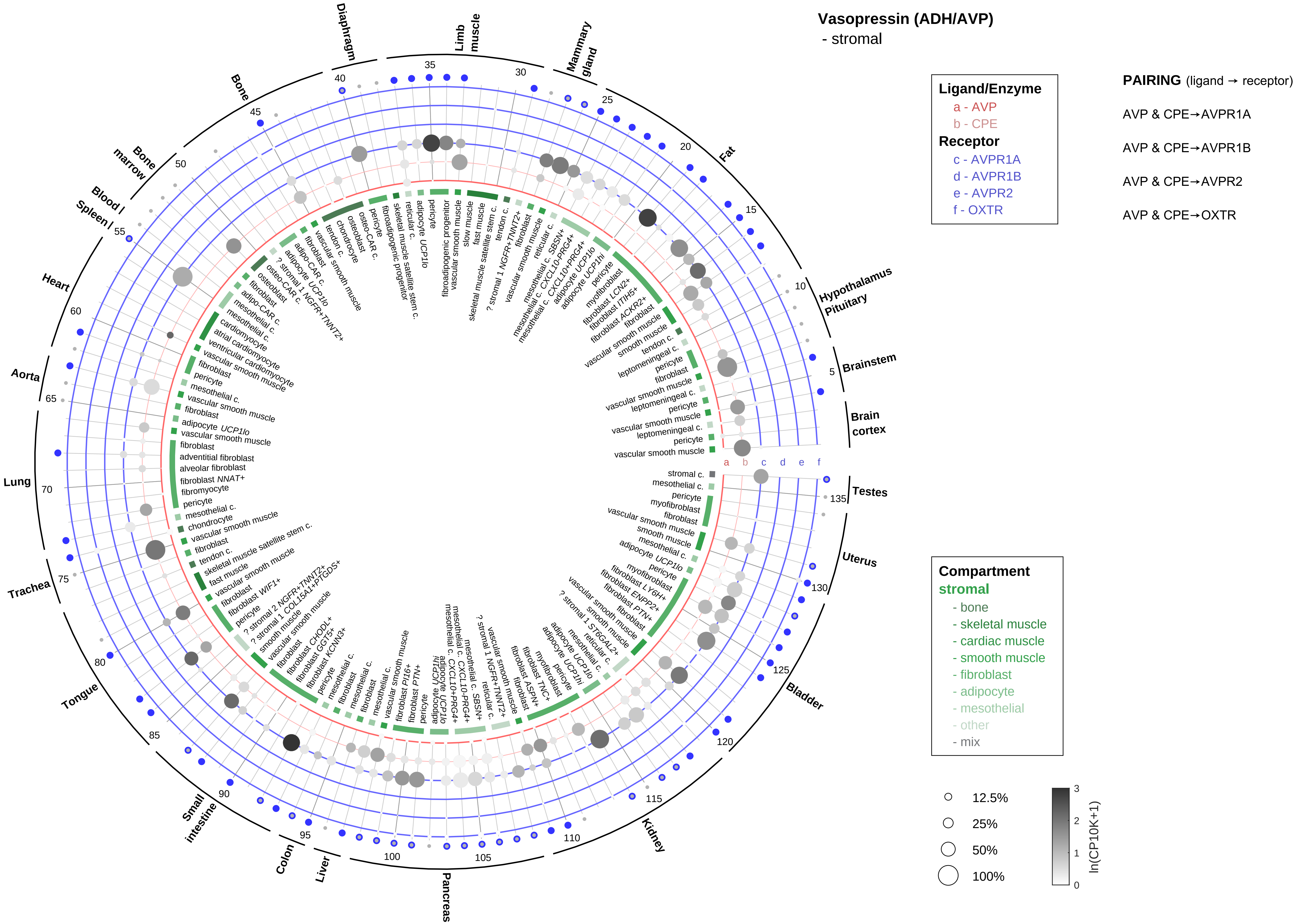

Vasopressin (ADH/AVP)
- stromal
Ligand/Enzyme
a - AVP
b - CPE
Receptor
c - AVPR1A
d - AVPR1B
e - AVPR2
f - OXTR
PAIRING (ligand → receptor)
AVP & CPE→AVPR1A
AVP & CPE→AVPR1B
AVP & CPE→AVPR2
AVP & CPE→OXTR
Compartment
stromal
- bone
- skeletal muscle
- cardiac muscle
- smooth muscle
- fibroblast
- adipocyte
- mesothelial
- other
- mix
12.5%
25%
50%
100%
ln(CP10K+1)
Diaphragm
Limb muscle
Mammary gland
Fat
Hypothalamus Pituitary
Brainstem
Brain cortex
Testes
Uterus
Bladder
Kidney
Pancreas
Liver
Colon
Small intestine
Tongue
Trachea
Lung
Aorta
Heart
Spleen
Blood
Bone marrow
Bone

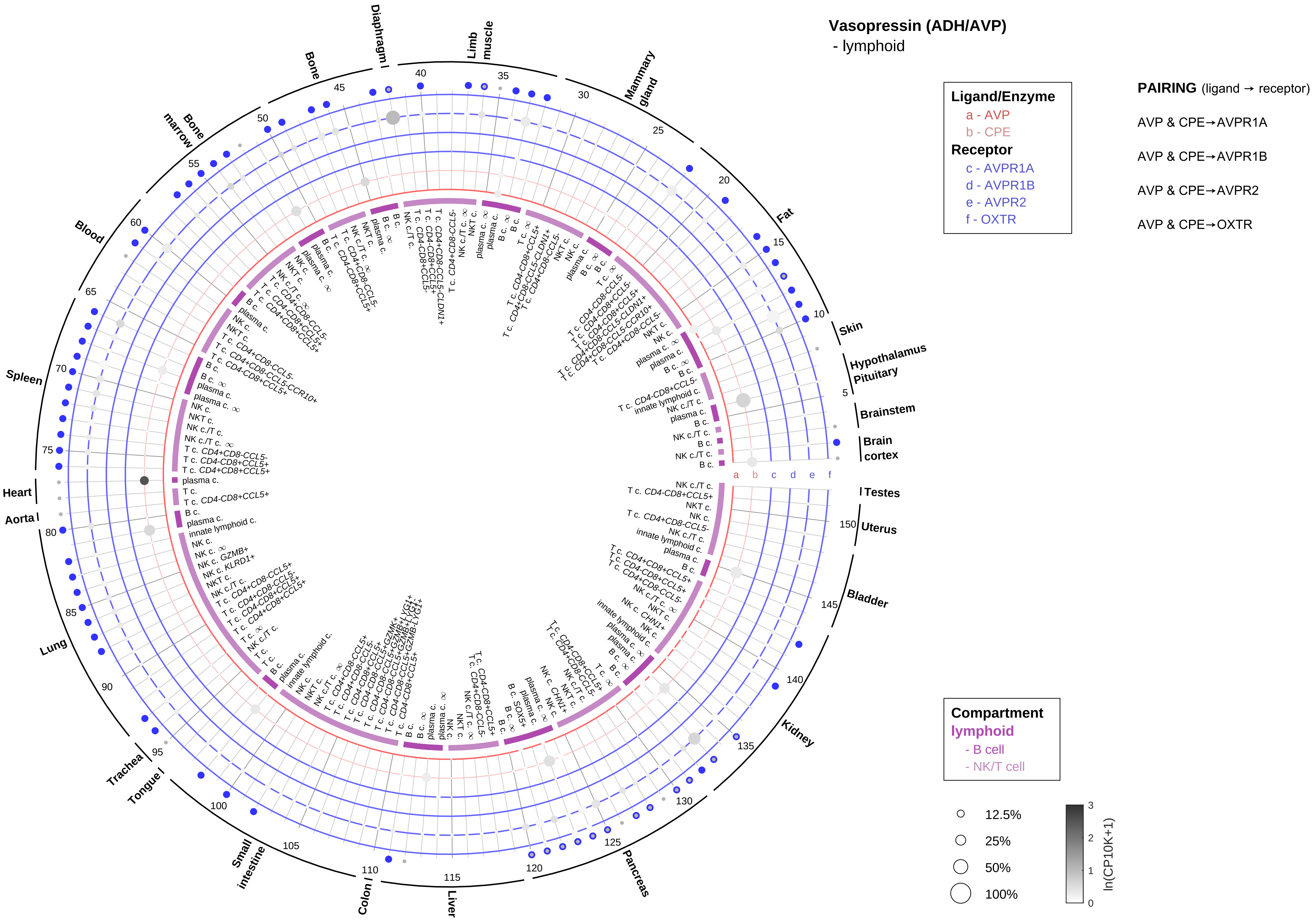

Vasopressin (ADH/AVP)
- lymphoid
Ligand/Enzyme
a - AVP
b - CPE
Receptor
c - AVPR1A
d - AVPR1B
e - AVPR2
f - OXTR
PAIRING (ligand → receptor)
AVP & CPE→AVPR1A
AVP & CPE→AVPR1B
AVP & CPE→AVPR2
AVP & CPE→OXTR
Compartment
lymphoid
- B cell
- NK/T cell
12.5%
25%
50%
100%
ln(CP10K+1)
Diaphragm
Limb muscle
Mammary gland
Fat
Skin
Hypothalamus
Pituitary
Brainstem
Brain cortex
Testes
Uterus
Bladder
Kidney
Pancreas
Liver
Colon
Small intestine
Tongue
Trachea
Lung
Aorta
Heart
Spleen
Blood
Bone marrow
Bone

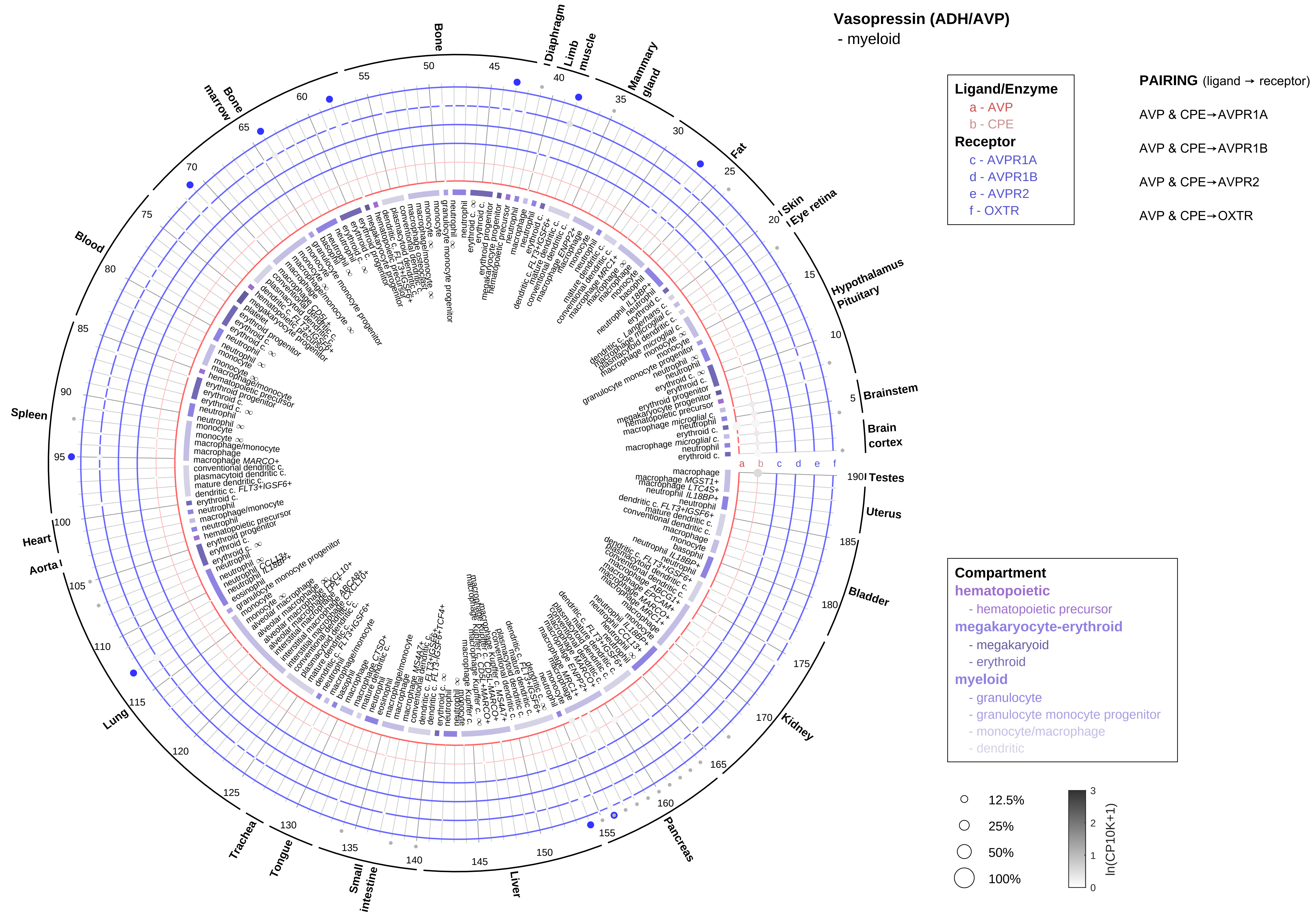

Vasopressin (ADH/AVP)
- myeloid
Ligand/Enzyme
a - AVP
b - CPE
Receptor
c - AVPR1A
d - AVPR1B
e - AVPR2
f - OXTR
PAIRING (ligand → receptor)
AVP & CPE→AVPR1A
AVP & CPE→AVPR1B
AVP & CPE→AVPR2
AVP & CPE→OXTR
Compartment
hematopoietic
- hematopoietic precursor
megakaryocyte-erythroid
- megakaryoid
- erythroid
myeloid
- granulocyte
- granulocyte monocyte progenitor
- monocyte/macrophage
- dendritic
12.5%
25%
50%
100%
ln(CP10K+1)
Brainstem
Brain cortex
Testes
Uterus
Bladder
Kidney
Pancreas
Liver
Small intestine
Tongue
Trachea
Lung
Aorta
Heart
Spleen
Blood
Bone marrow
Bone
Diaphragm
Limb muscle
Mammary gland
Fat
Skin
Eye retina
Hypothalamus
Pituitary

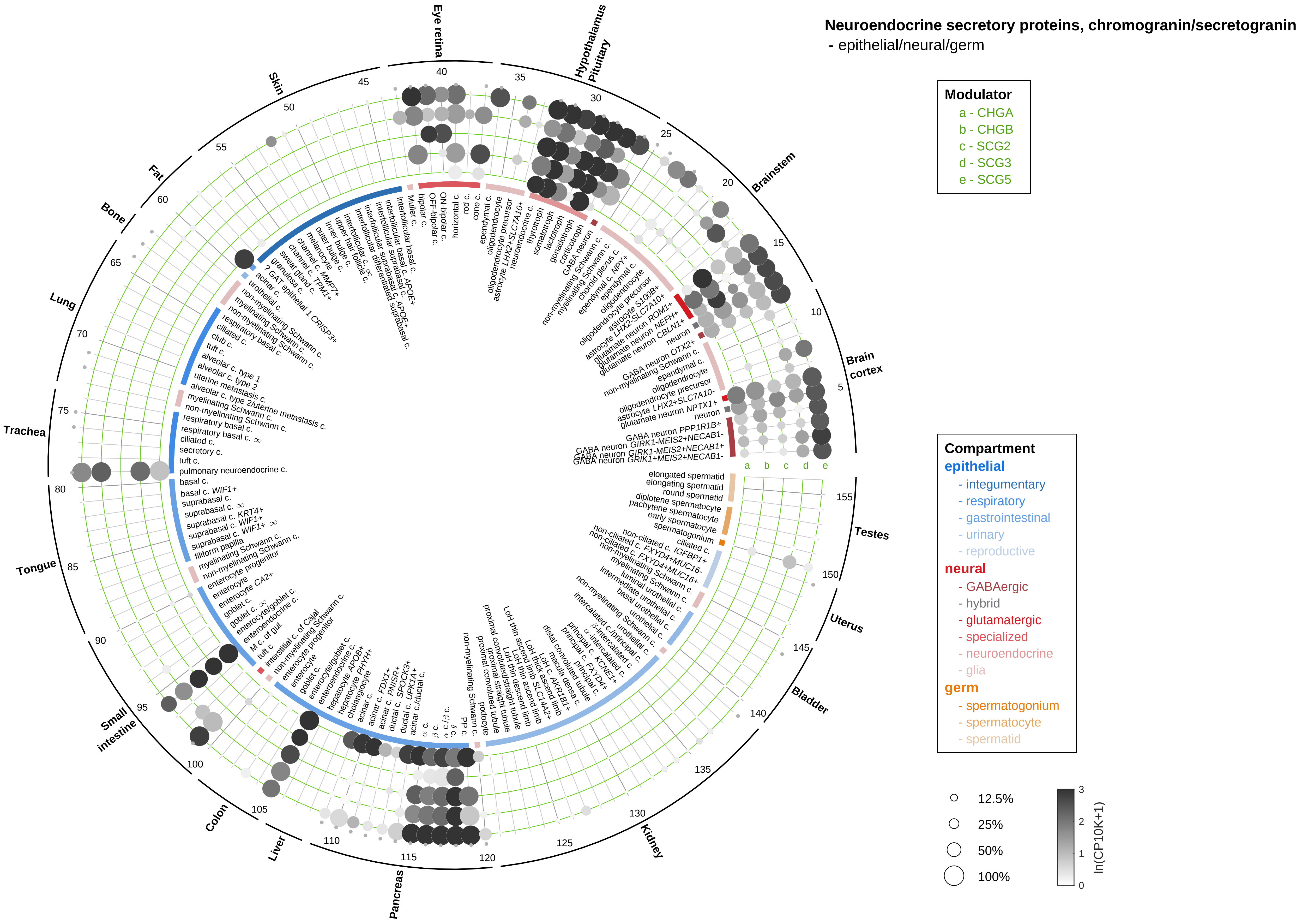

Neuroendocrine secretory proteins, chromogranin/secretogranin
- epithelial/neural/germ
Modulator
a - CHGA
b - CHGB
c - SCG2
d - SCG3
e - SCG5
Compartment
epithelial
- integumentary
- respiratory
- gastrointestinal
- urinary
- reproductive
neural
- GABAergic
- hybrid
- glutamatergic
- specialized
- neuroendocrine
- glia
germ
- spermatogonium
- spermatocyte
- spermatid
12.5%
25%
50%
100%
ln(CP10K+1)
Eye retina
Hypothalamus
Pituitary
Brainstem
Brain cortex
Testes
Uterus
Bladder
Kidney
Pancreas
Liver
Colon
Small intestine
Tongue
Trachea
Lung
Bone
Fat
Skin

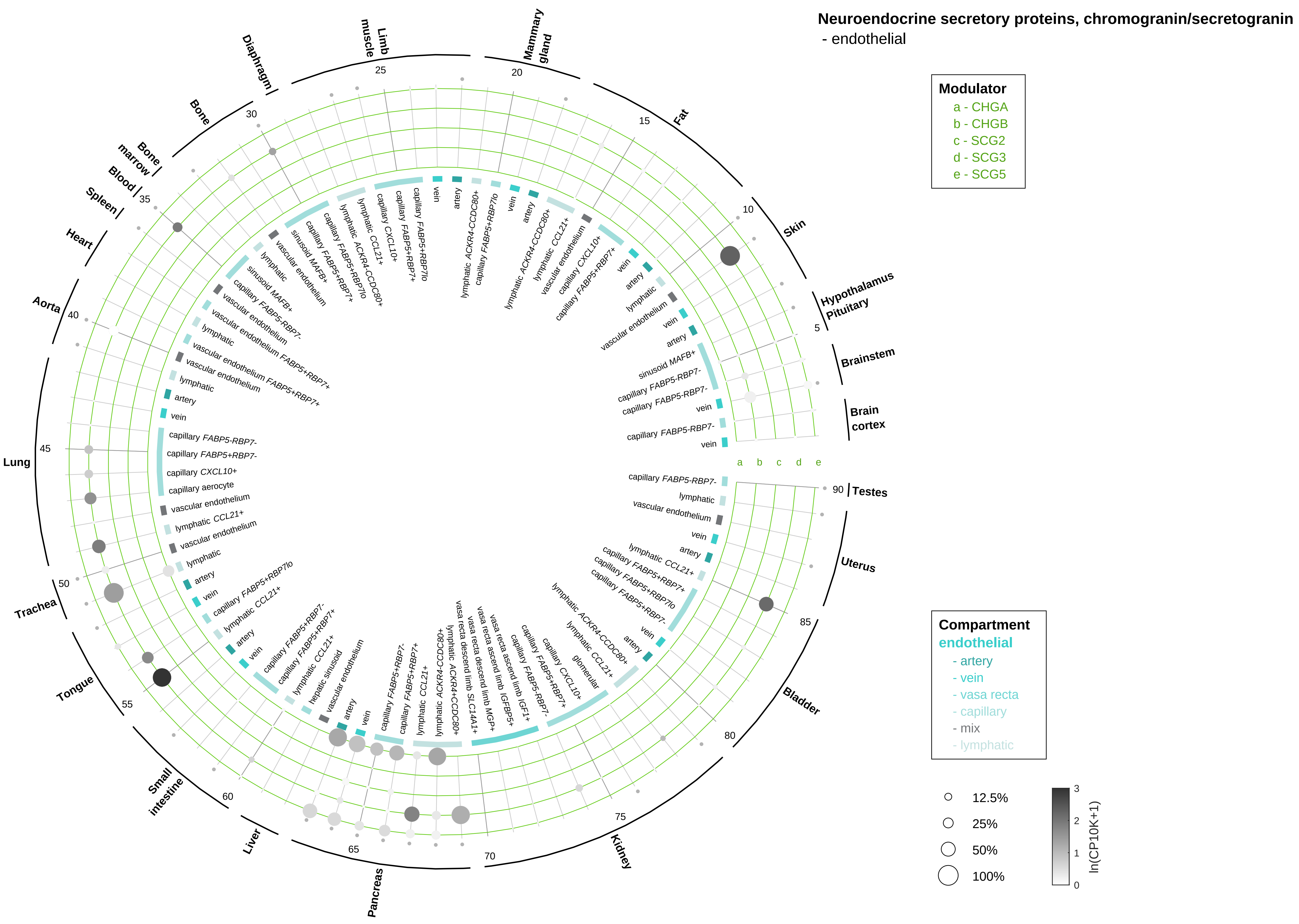

Neuroendocrine secretory proteins, chromogranin/secretogranin
- endothelial
Modulator
a - CHGA
b - CHGB
c - SCG2
d - SCG3
e - SCG5
Compartment
endothelial
- artery
- vein
- vasa recta
- capillary
- mix
- lymphatic
12.5%
25%
50%
100%
ln(CP10K+1)
Testes
Uterus
Bladder
Kidney
Pancreas
Liver
Small intestine
Tongue
Trachea
Lung
Aorta
Heart
Spleen
Blood
Bone marrow
Bone
Diaphragm
Limb muscle
Mammary gland
Fat
Skin
Hypothalamus Pituitary
Brainstem
Brain cortex

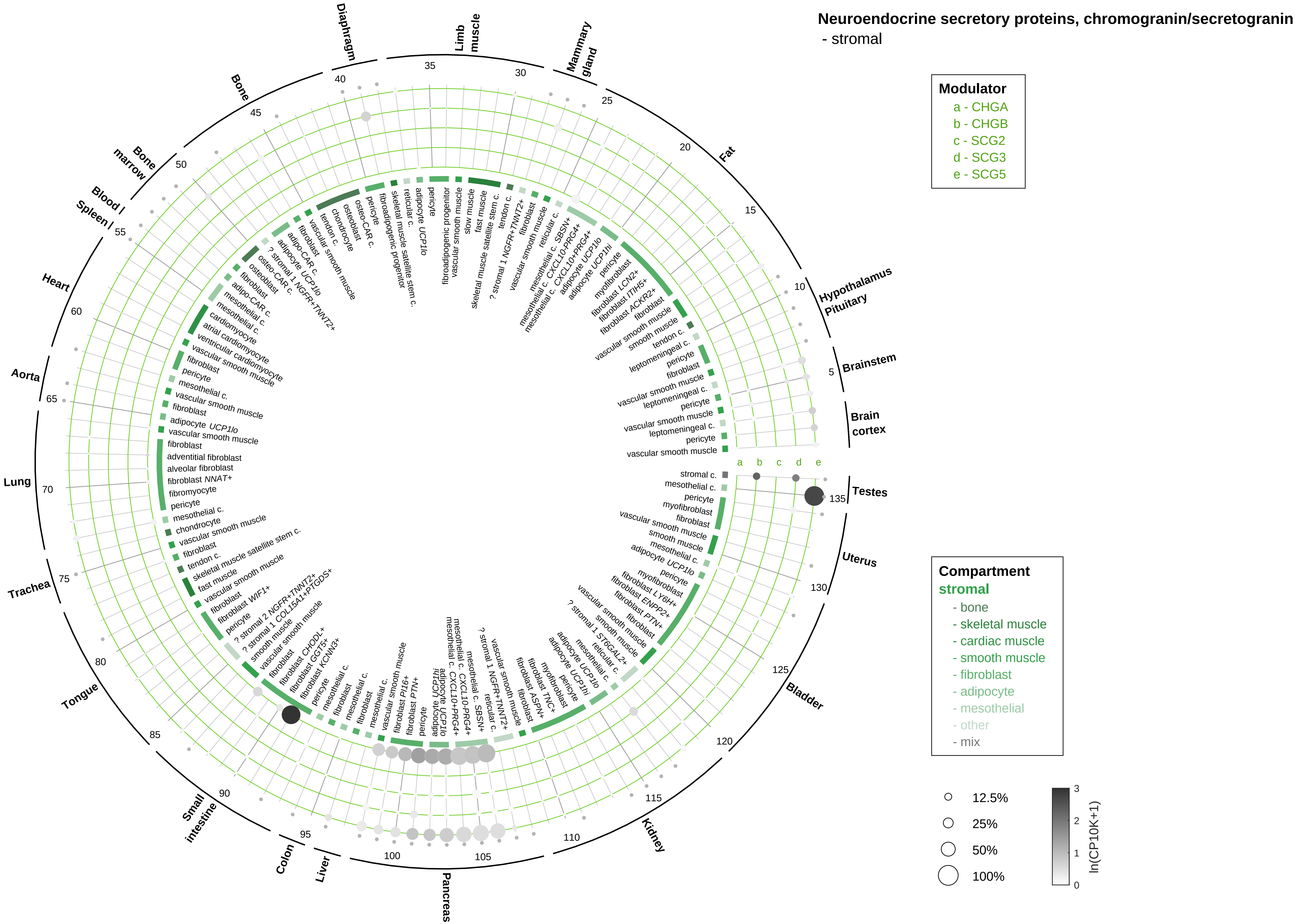
Neuroendocrine secretory proteins, chromogranin/secretogranin
- stromal
Modulator
a - CHGA
b - CHGB
c - SCG2
d - SCG3
e - SCG5
Compartment
stromal
- bone
- skeletal muscle
- cardiac muscle
- smooth muscle
- fibroblast
- adipocyte
- mesothelial
- other
- mix
12.5%
25%
50%
100%
ln(CP10K+1)
Brain cortex
Brainstem
Hypothalamus Pituitary
Fat
Mammary gland
Limb muscle
Diaphragm
Bone
Bone marrow
Blood
Spleen
Heart
Aorta
Lung
Trachea
Tongue
Small intestine
Colon
Liver
Pancreas
Kidney
Bladder
Uterus
Testes

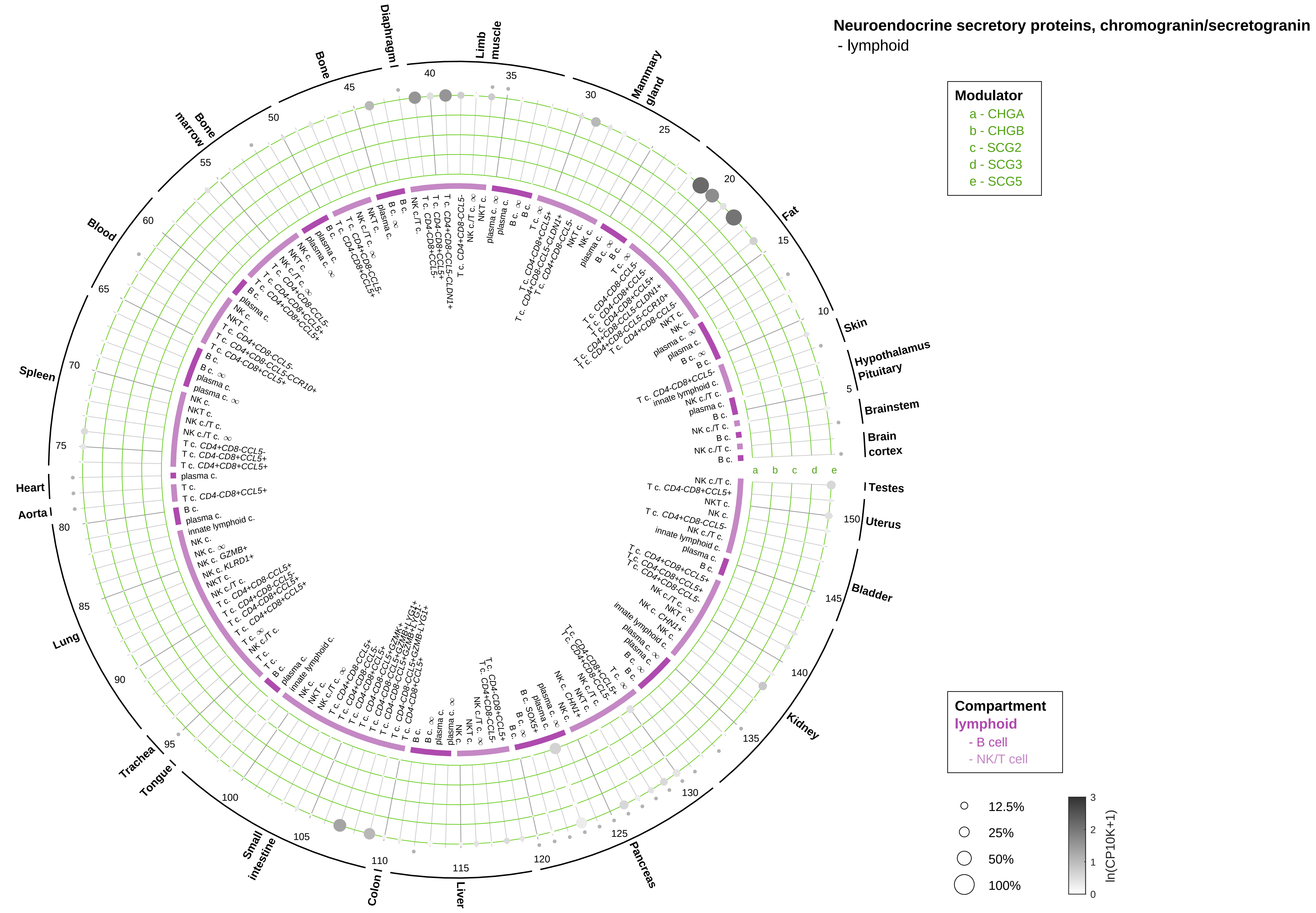
Neuroendocrine secretory proteins, chromogranin/secretogranin
- lymphoid
Modulator
a - CHGA
b - CHGB
c - SCG2
d - SCG3
e - SCG5
Compartment
lymphoid
- B cell
- NK/T cell
12.5%
25%
50%
100%
ln(CP10K+1)
Brain cortex
Brainstem
Hypothalamus Pituitary
Skin
Fat
Mammary gland
Limb muscle
Diaphragm
Bone
Bone marrow
Blood
Spleen
Heart
Aorta
Lung
Trachea
Tongue
Small intestine
Colon
Liver
Pancreas
Kidney
Bladder
Uterus
Testes

Neuroendocrine secretory proteins, chromogranin/secretogranin - myeloid

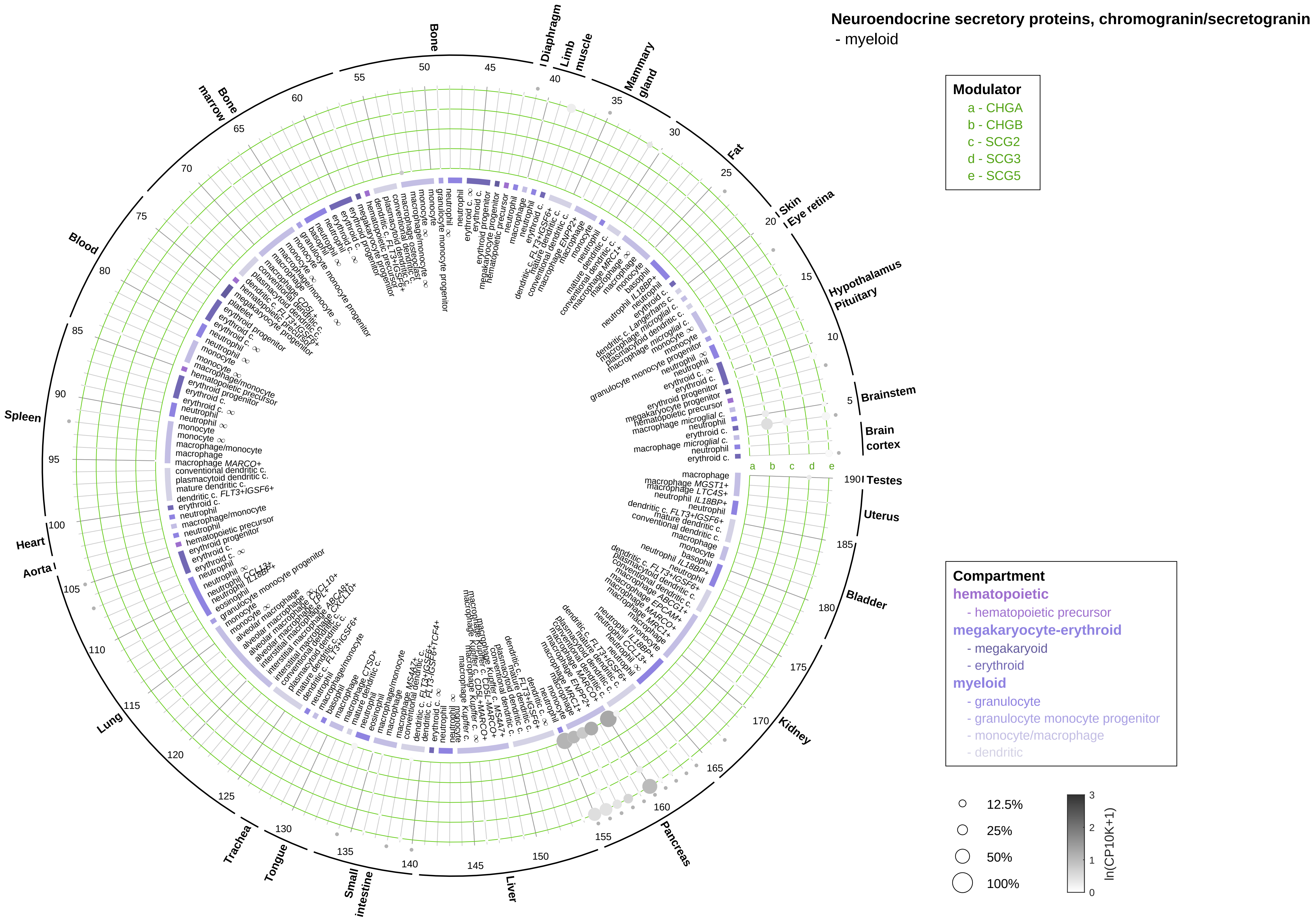

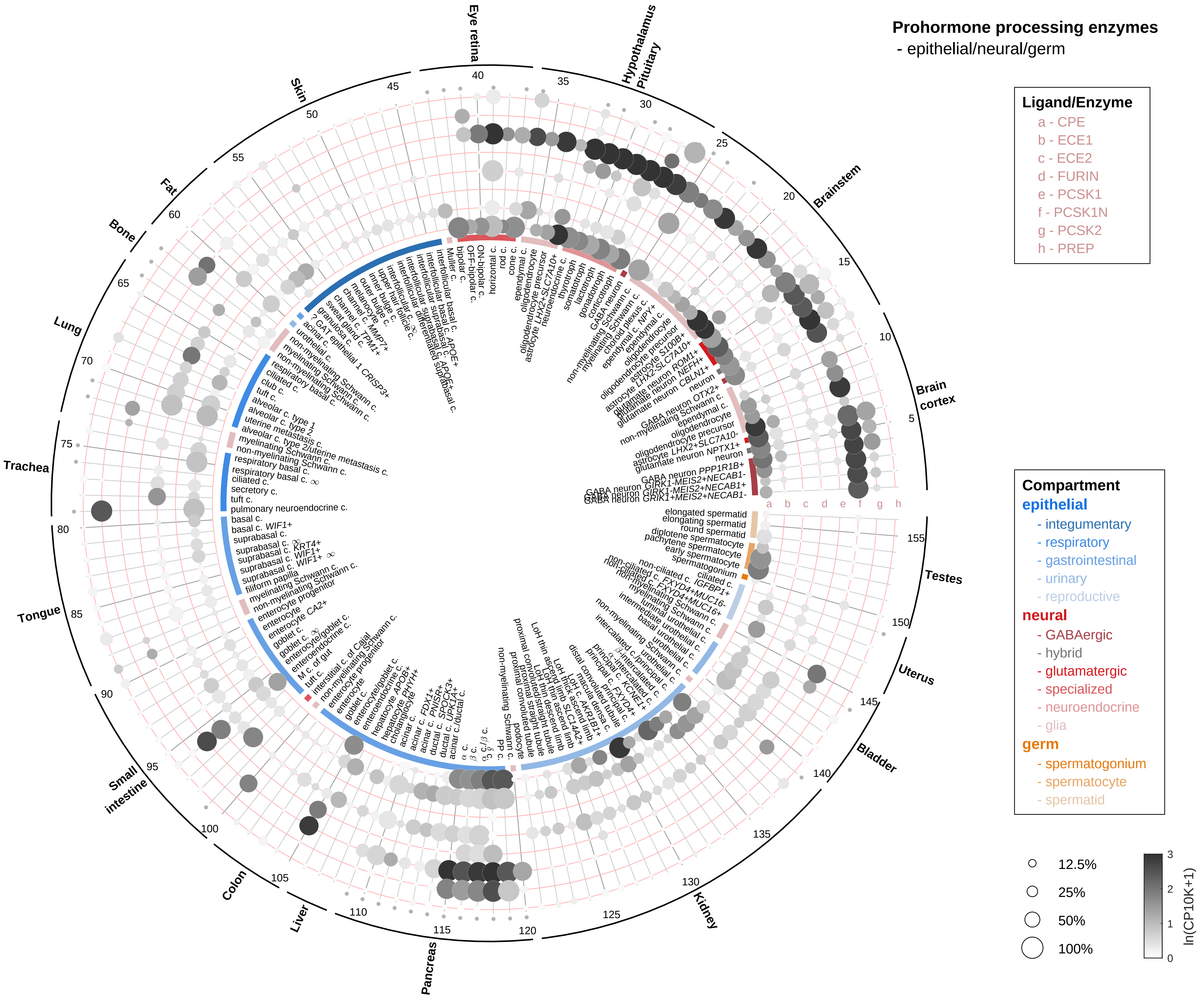

Prohormone processing enzymes
- epithelial/neural/germ
Ligand/Enzyme
a - CPE
b - ECE1
c - ECE2
d - FURIN
e - PCSK1
f - PCSK1N
g - PCSK2
h - PREP
Compartment
epithelial
- integumentary
- respiratory
- gastrointestinal
- urinary
- reproductive
neural
- GABAergic
- hybrid
- glutamatergic
- specialized
- neuroendocrine
- glia
germ
- spermatogonium
- spermatocyte
- spermatid
12.5%
25%
50%
100%
ln(CP10K+1)
Eye retina
Hypothalamus Pituitary
Brainstem
Brain cortex
Testes
Uterus
Bladder
Kidney
Pancreas
Liver
Colon
Small intestine
Tongue
Trachea
Lung
Bone
Fat
Skin

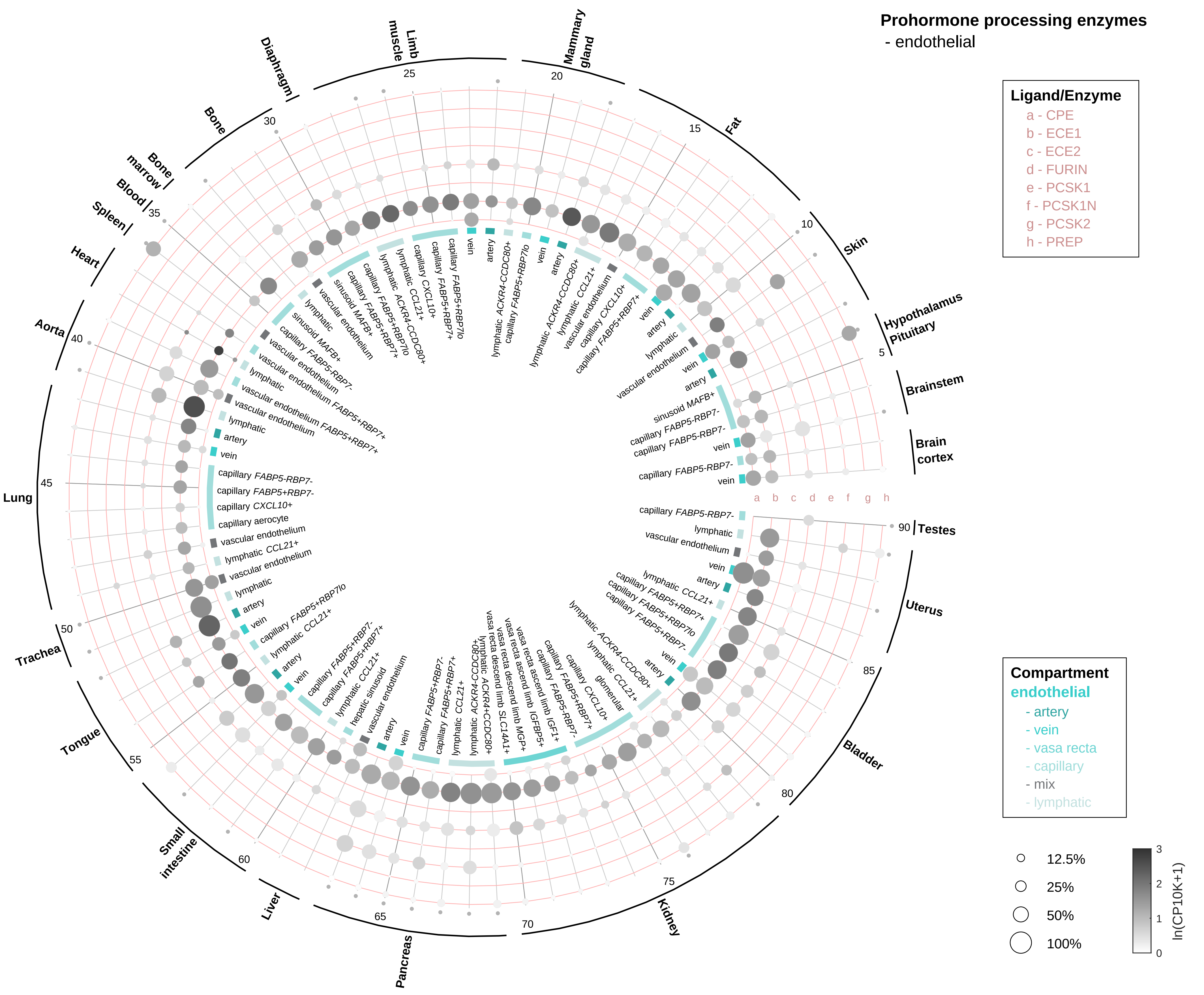
Prohormone processing enzymes
- endothelial
Ligand/Enzyme
a - CPE
b - ECE1
c - ECE2
d - FURIN
e - PCSK1
f - PCSK1N
g - PCSK2
h - PREP
Compartment
endothelial
- artery
- vein
- vasa recta
- capillary
- mix
- lymphatic
12.5%
25%
50%
100%
ln(CP10K+1)
Brain cortex
Brainstem
Hypothalamus Pituitary
Skin
Fat
Mammary gland
Limb muscle
Diaphragm
Bone
Bone marrow
Blood
Spleen
Heart
Aorta
Lung
Trachea
Tongue
Small intestine
Liver
Pancreas
Kidney
Bladder
Uterus
Testes

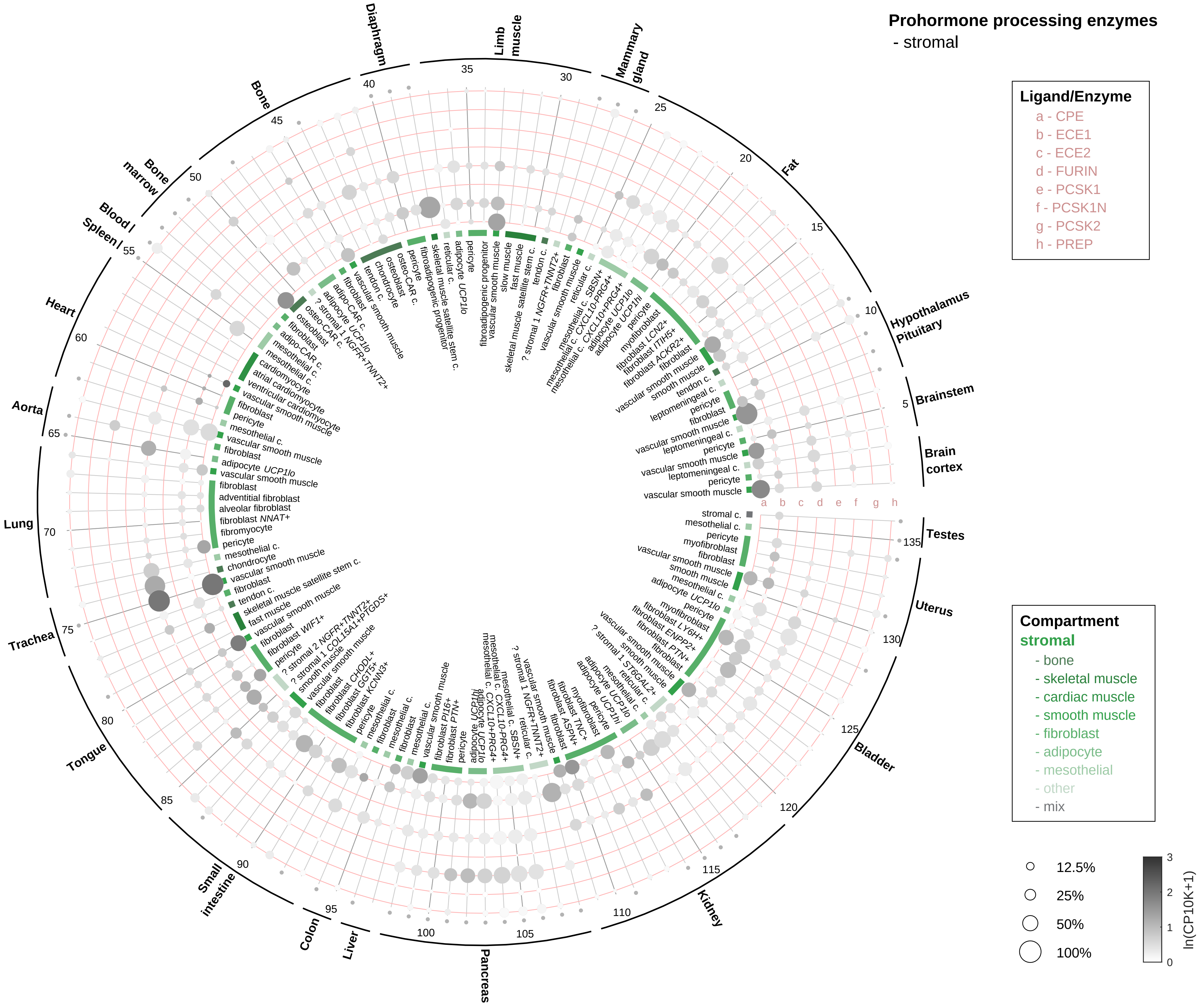
Prohormone processing enzymes
- stromal
Ligand/Enzyme
a - CPE
b - ECE1
c - ECE2
d - FURIN
e - PCSK1
f - PCSK1N
g - PCSK2
h - PREP
Compartment
stromal
- bone
- skeletal muscle
- cardiac muscle
- smooth muscle
- fibroblast
- adipocyte
- mesothelial
- other
- mix
12.5%
25%
50%
100%
ln(CP10K+1)
Hypothalamus
Pituitary
Brainstem
Brain cortex
Testes
Uterus
Bladder
Kidney
Pancreas
Liver
Colon
Small intestine
Tongue
Trachea
Lung
Aorta
Heart
Blood / Spleen
Bone marrow
Bone
Diaphragm
Limb muscle
Mammary gland
Fat

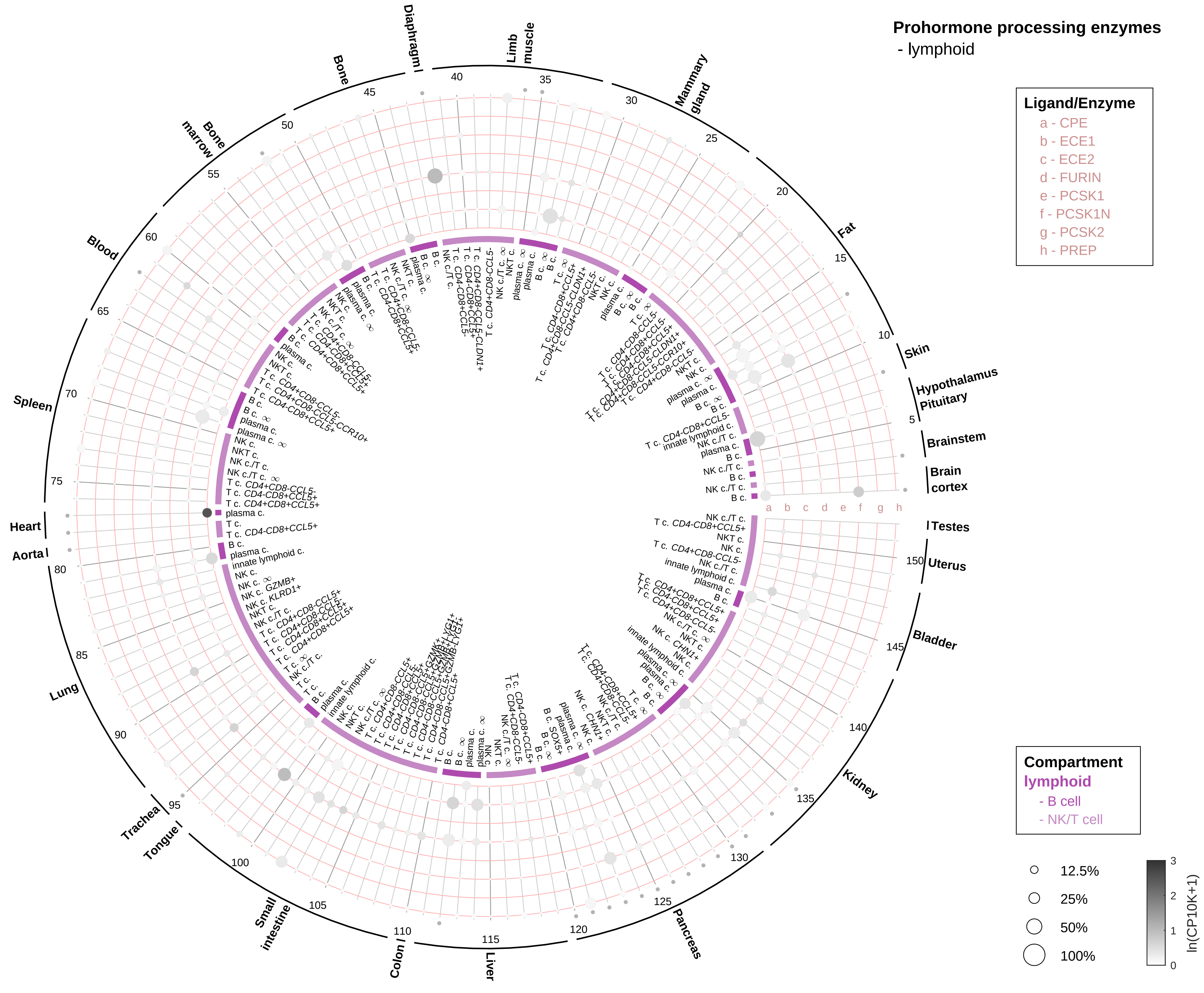

Prohormone processing enzymes
- lymphoid
Ligand/Enzyme
a - CPE
b - ECE1
c - ECE2
d - FURIN
e - PCSK1
f - PCSK1N
g - PCSK2
h - PREP
Compartment
lymphoid
- B cell
- NK/T cell
12.5%
25%
50%
100%
ln(CP10K+1)
Diaphragm
Limb muscle
Mammary gland
Fat
Skin
Hypothalamus
Pituitary
Brainstem
Brain cortex
Testes
Uterus
Bladder
Kidney
Pancreas
Liver
Colon
Small intestine
Tongue
Trachea
Lung
Aorta
Heart
Spleen
Blood
Bone marrow
Bone

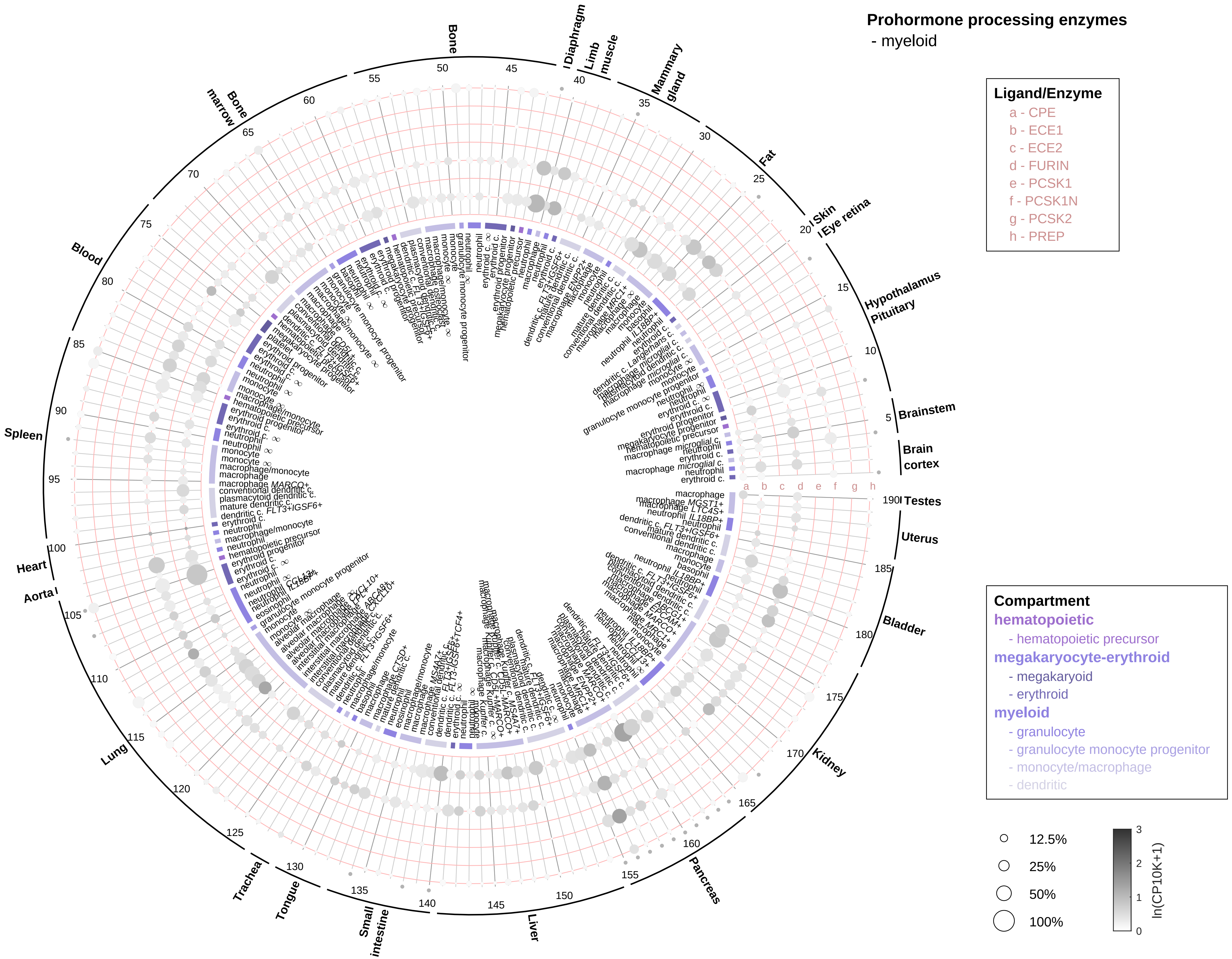
Prohormone processing enzymes
- myeloid
Ligand/Enzyme
a - CPE
b - ECE1
c - ECE2
d - FURIN
e - PCSK1
f - PCSK1N
g - PCSK2
h - PREP
Compartment
hematopoietic
- hematopoietic precursor
megakaryocyte-erythroid
- megakaryoid
- erythroid
myeloid
- granulocyte
- granulocyte monocyte progenitor
- monocyte/macrophage
- dendritic
12.5%
25%
50%
100%
ln(CP10K+1)
Bone
Diaphragm
Limb muscle
Mammary gland
Fat
Skin
Eye retina
Hypothalamus
Pituitary
Brainstem
Brain cortex
Testes
Uterus
Bladder
Kidney
Pancreas
Liver
Small intestine
Tongue
Trachea
Lung
Aorta
Heart
Spleen
Blood
Bone marrow

Fig. S1b

Acylation stimulating protein (ASP)
- epithelial/neural/germ

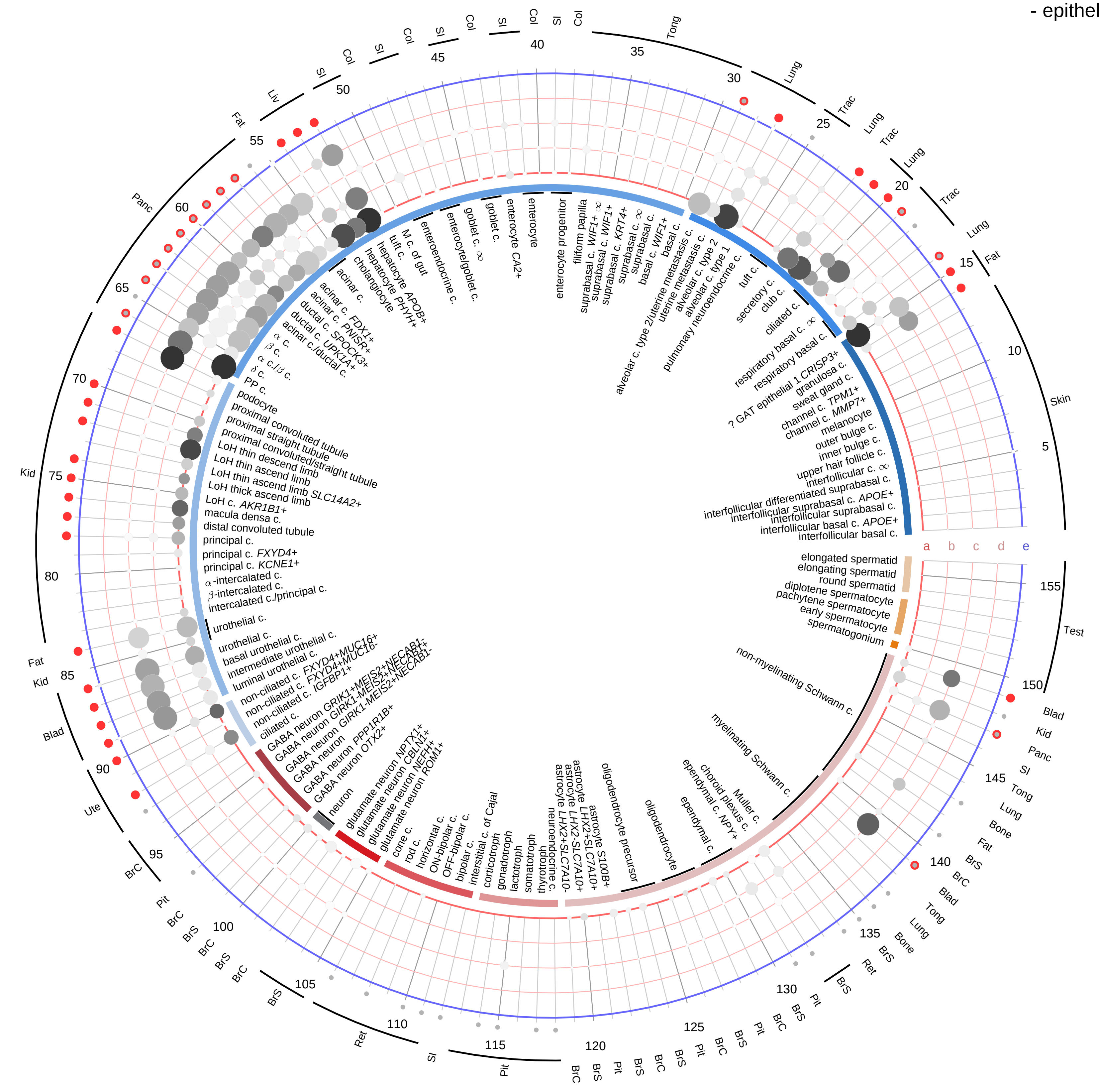

Ligand/Enzyme
a - C3
b - CFB
c - CFD
d - CPB2
Receptor
e - C5AR2

PAIRING (ligand → receptor)

C3→C5AR2

Compartment
epithelial
- integumentary
- respiratory
- gastrointestinal
- urinary
- reproductive
neural
- GABAergic
- hybrid
- glutamatergic
- specialized
- neuroendocrine
- glia
germ
- spermatogonium
- spermatocyte
- spermatid

12.5%
25%
50%
100%

ln(CP10K+1): 0, 1, 2, 3

Abbrev.	Tissue
Aort	Aorta
Blad	Bladder
Blo	Blood
Bone	Bone
BM	Bone marrow
BrC	Brain cortex
BrS	Brainstem
Col	Colon
Dia	Diaphragm
Ret	Eye retina
Fat	Fat
Hrt	Heart
Pit	Hypothalamus/Pituitary
Kid	Kidney
LM	Limb muscle
Liv	Liver
Lung	Lung
MG	Mammary gland
Panc	Pancreas
Skin	Skin
SI	Small intestine
Spl	Spleen
Test	Testes
Thym	Thymus
Tong	Tongue
Trac	Trachea
Ute	Uterus

Acylation stimulating protein (ASP)
- endothelial

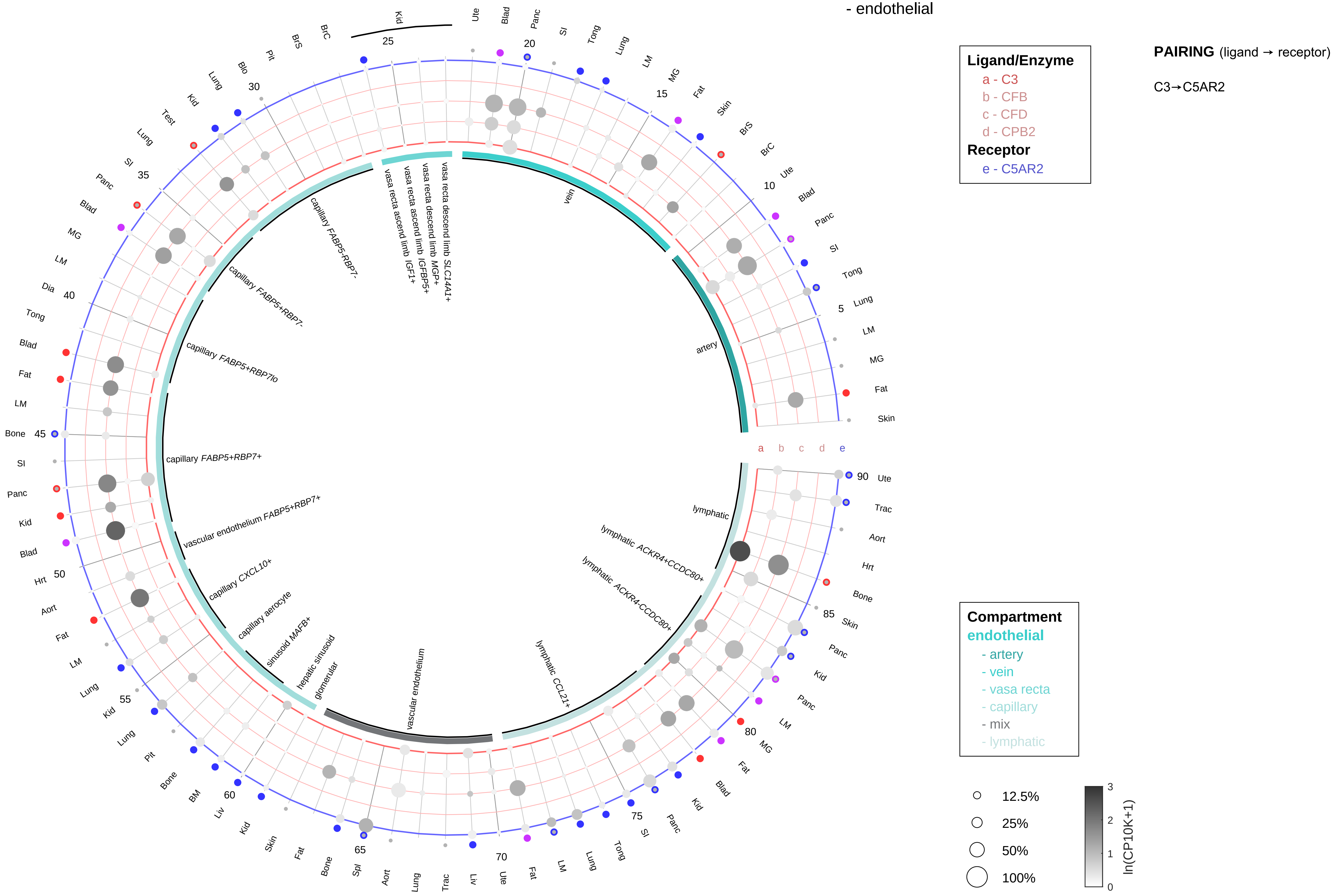

Abbrev.	Tissue
Aort	Aorta
Blad	Bladder
Blo	Blood
Bone	Bone
BM	Bone marrow
BrC	Brain cortex
BrS	Brainstem
Col	Colon
Dia	Diaphragm
Ret	Eye retina
Fat	Fat
Hrt	Heart
Pit	Hypothalamus/Pituitary
Kid	Kidney
LM	Limb muscle
Liv	Liver
Lung	Lung
MG	Mammary gland
Panc	Pancreas
Skin	Skin
SI	Small intestine
Spl	Spleen
Test	Testes
Thym	Thymus
Tong	Tongue
Trac	Trachea
Ute	Uterus

Acylation stimulating protein (ASP)
- stromal

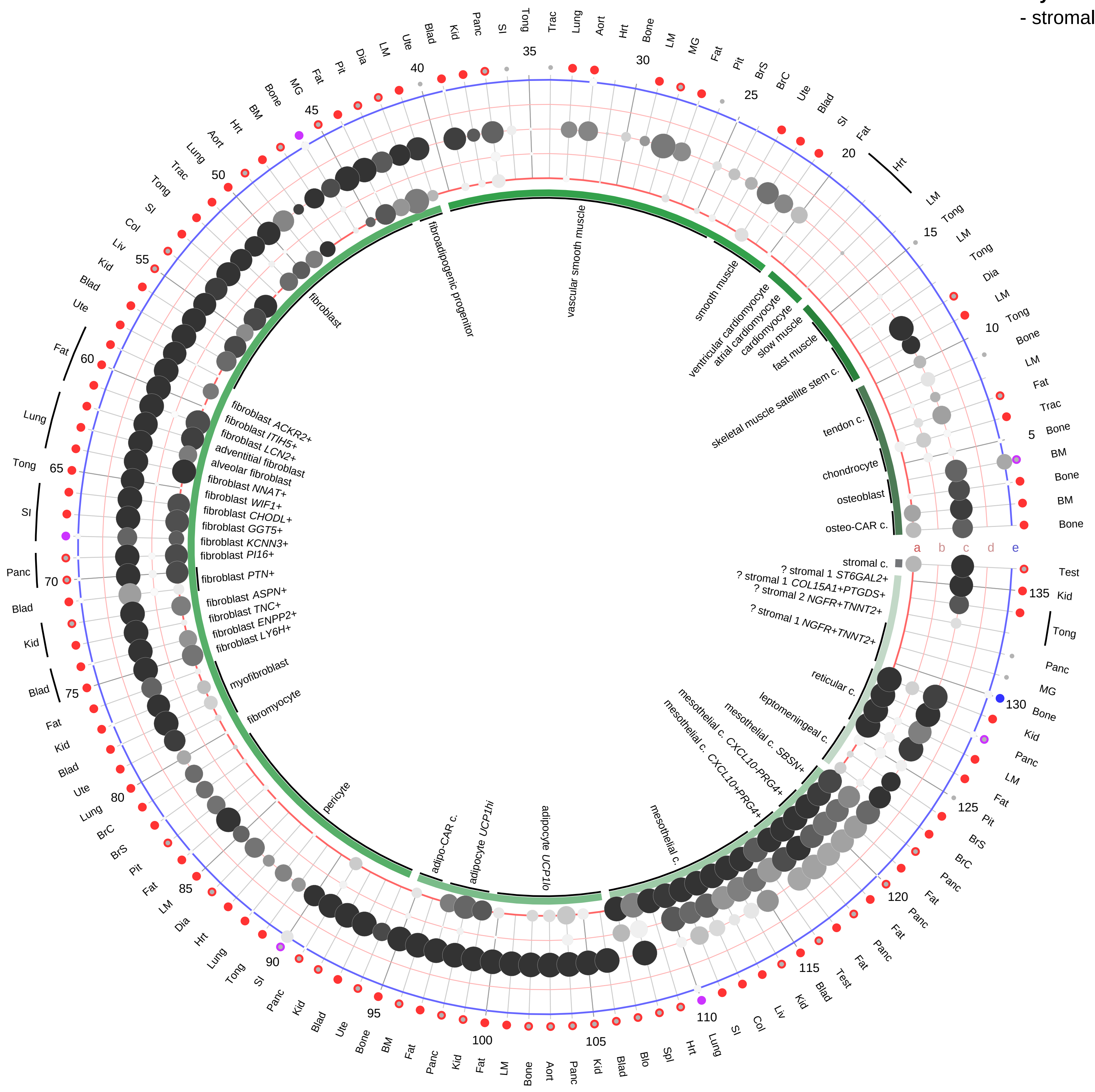

Ligand/Enzyme
a - C3
b - CFB
c - CFD
d - CPB2
Receptor
e - C5AR2

PAIRING (ligand → receptor)

C3→C5AR2

Compartment
stromal
- bone
- skeletal muscle
- cardiac muscle
- smooth muscle
- fibroblast
- adipocyte
- mesothelial
- other
- mix

○ 12.5%
○ 25%
○ 50%
○ 100%

ln(CP10K+1): 0, 1, 2, 3

Abbrev.	Tissue
Aort	Aorta
Blad	Bladder
Blo	Blood
Bone	Bone
BM	Bone marrow
BrC	Brain cortex
BrS	Brainstem
Col	Colon
Dia	Diaphragm
Ret	Eye retina
Fat	Fat
Hrt	Heart
Pit	Hypothalamus/Pituitary
Kid	Kidney
LM	Limb muscle
Liv	Liver
Lung	Lung
MG	Mammary gland
Panc	Pancreas
Skin	Skin
SI	Small intestine
Spl	Spleen
Test	Testes
Thym	Thymus
Tong	Tongue
Trac	Trachea
Ute	Uterus

Acylation stimulating protein (ASP)
- lymphoid

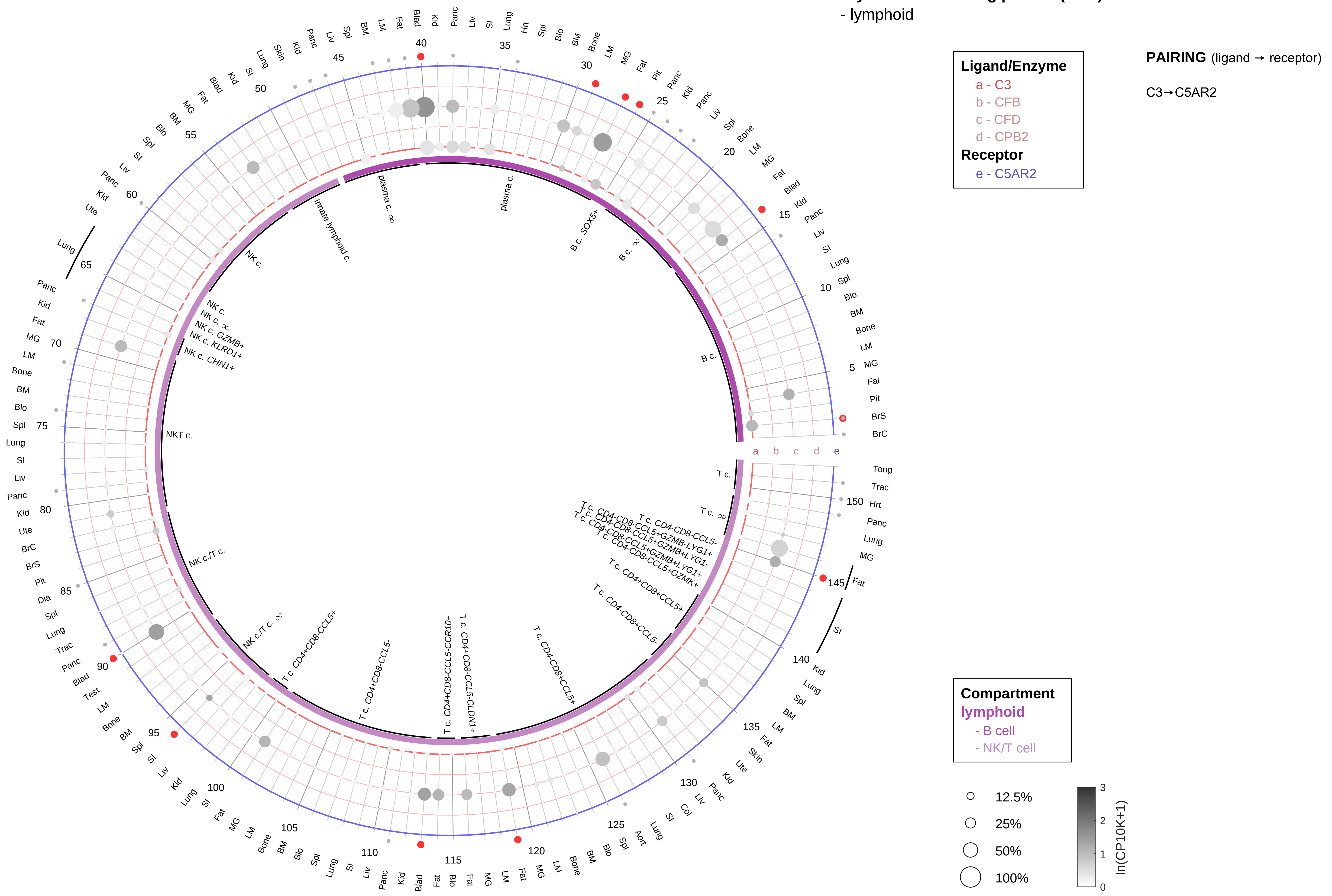

Abbrev.	Tissue
Aort	Aorta
Blad	Bladder
Blo	Blood
Bone	Bone
BM	Bone marrow
BrC	Brain cortex
BrS	Brainstem
Col	Colon
Dia	Diaphragm
Ret	Eye retina
Fat	Fat
Hrt	Heart
Pit	Hypothalamus/Pituitary
Kid	Kidney
LM	Limb muscle
Liv	Liver
Lung	Lung
MG	Mammary gland
Panc	Pancreas
Skin	Skin
SI	Small intestine
Spl	Spleen
Test	Testes
Thym	Thymus
Tong	Tongue
Trac	Trachea
Ute	Uterus

Acylation stimulating protein (ASP)
- myeloid

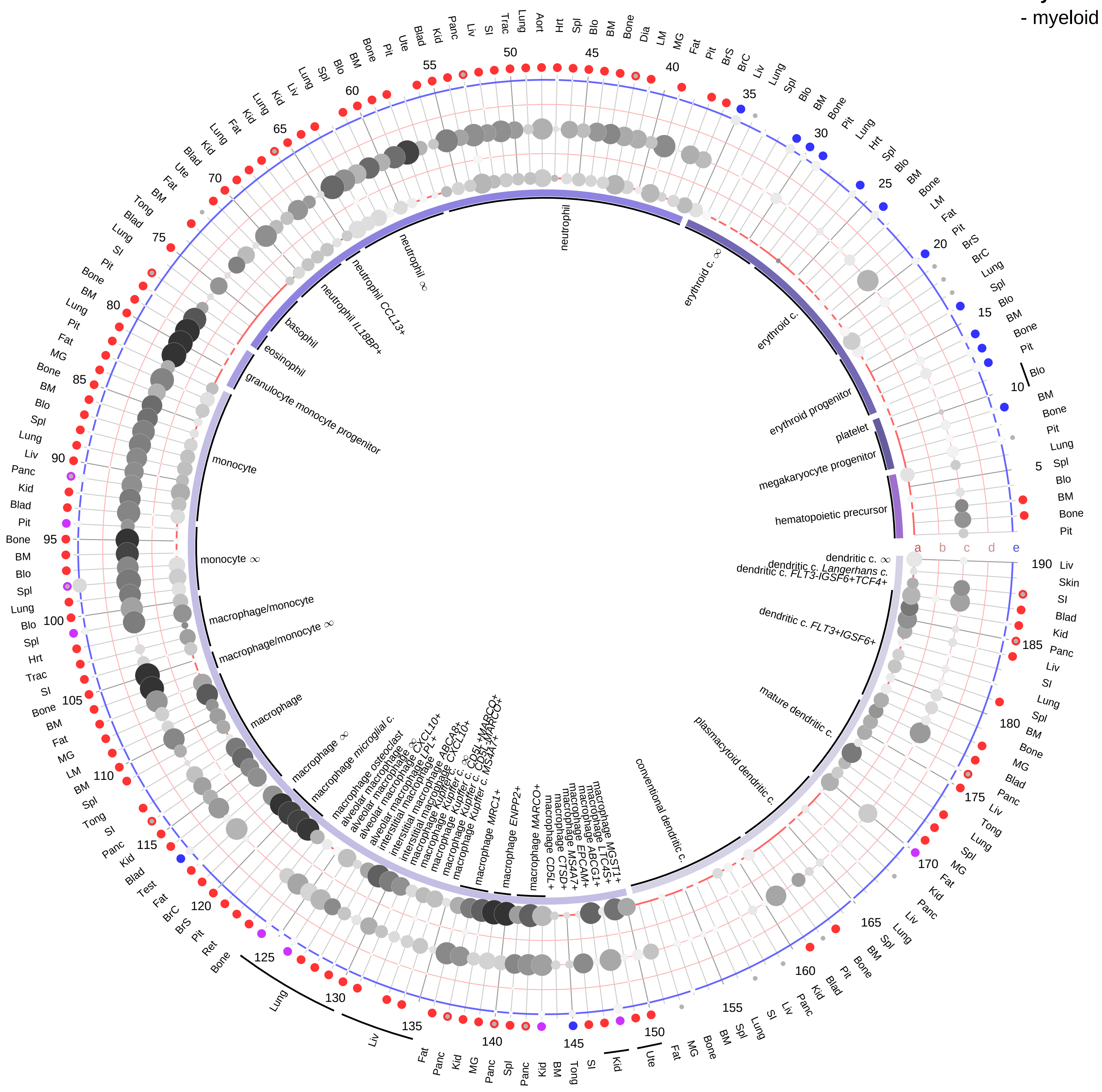

Ligand/Enzyme
- a - C3
- b - CFB
- c - CFD
- d - CPB2

Receptor
- e - C5AR2

PAIRING (ligand → receptor)

C3→C5AR2

**Abbrev.**	**Tissue**
Aort	Aorta
Blad	Bladder
Blo	Blood
Bone	Bone
BM	Bone marrow
BrC	Brain cortex
BrS	Brainstem
Col	Colon
Dia	Diaphragm
Ret	Eye retina
Fat	Fat
Hrt	Heart
Pit	Hypothalamus/Pituitary
Kid	Kidney
LM	Limb muscle
Liv	Liver
Lung	Lung
MG	Mammary gland
Panc	Pancreas
Skin	Skin
SI	Small intestine
Spl	Spleen
Test	Testes
Thym	Thymus
Tong	Tongue
Trac	Trachea
Ute	Uterus

Compartment

hematopoietic
- hematopoietic precursor

megakaryocyte-erythroid
- megakaryoid
- erythroid

myeloid
- granulocyte
- granulocyte monocyte progenitor
- monocyte/macrophage
- dendritic

12.5%
25%
50%
100%

ln(CP10K+1): 0, 1, 2, 3

Adiponectin (AdipoQ)
- epithelial/neural/germ

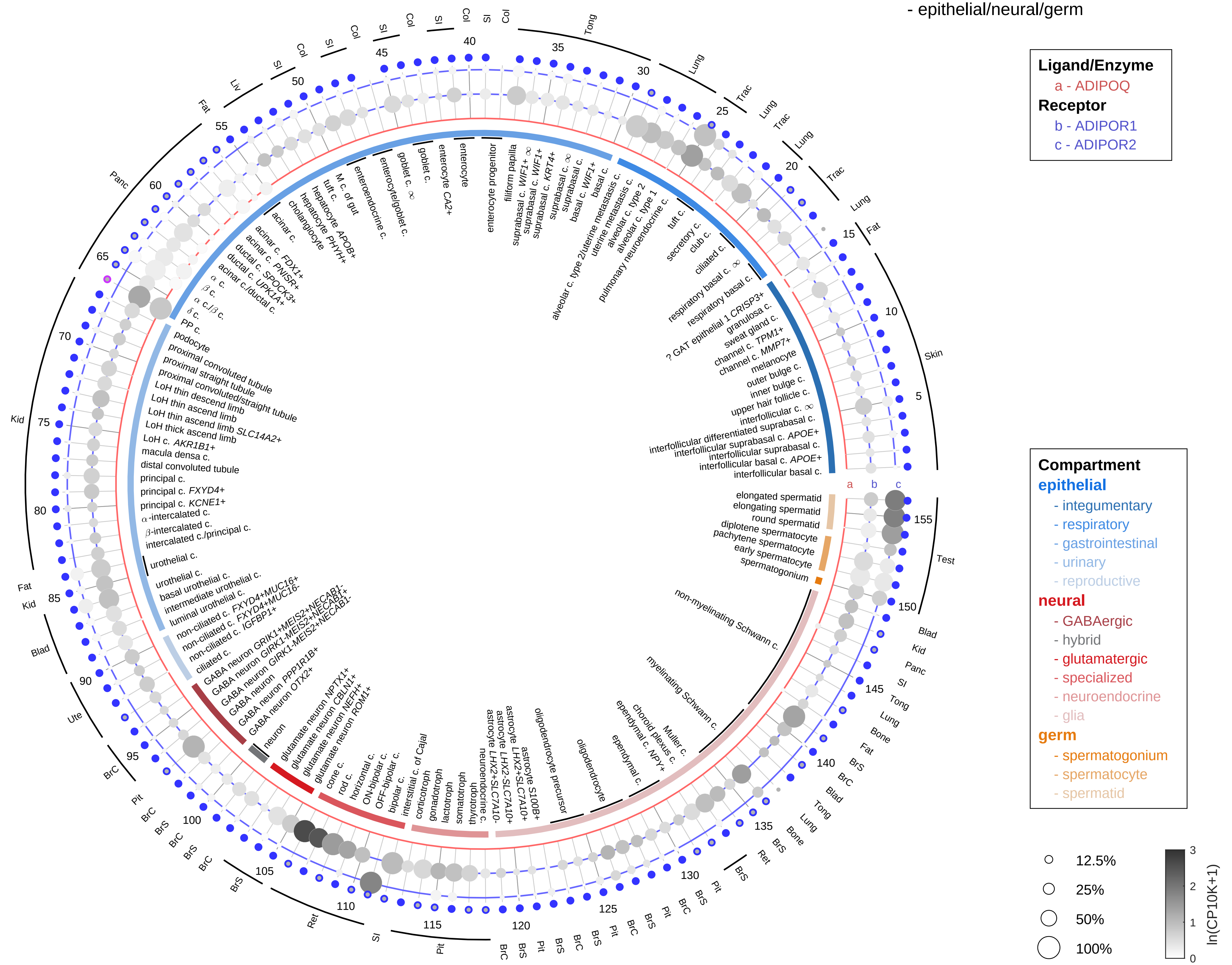

Abbrev.	Tissue
Aort	Aorta
Blad	Bladder
Blo	Blood
Bone	Bone
BM	Bone marrow
BrC	Brain cortex
BrS	Brainstem
Col	Colon
Dia	Diaphragm
Ret	Eye retina
Fat	Fat
Hrt	Heart
Pit	Hypothalamus/Pituitary
Kid	Kidney
LM	Limb muscle
Liv	Liver
Lung	Lung
MG	Mammary gland
Panc	Pancreas
Skin	Skin
SI	Small intestine
Spl	Spleen
Test	Testes
Thym	Thymus
Tong	Tongue
Trac	Trachea
Ute	Uterus

Adiponectin (AdipoQ)
- endothelial

PAIRING (ligand → receptor)

ADIPOQ→ADIPOR1

ADIPOQ→ADIPOR2

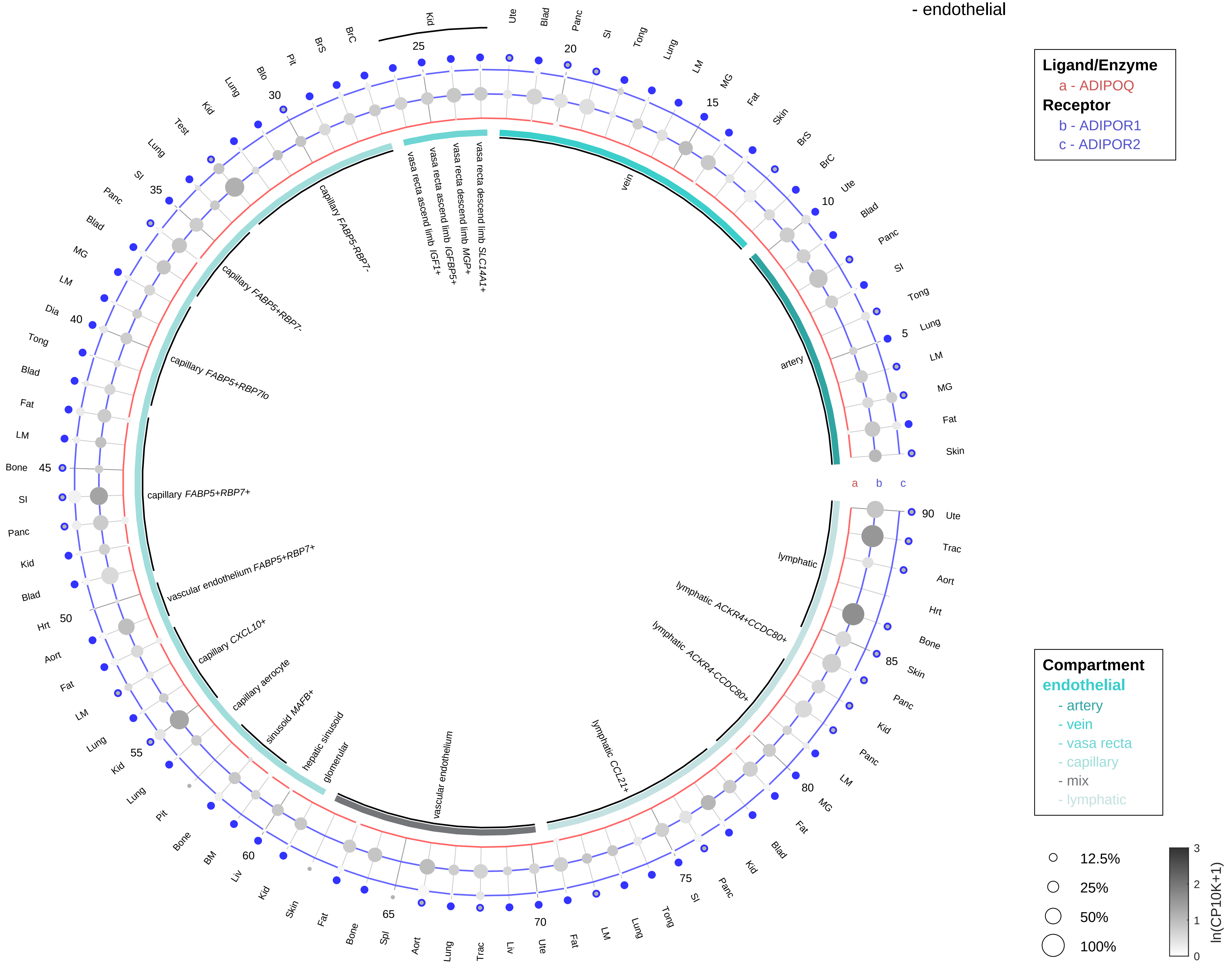

Abbrev.	Tissue
Aort	Aorta
Blad	Bladder
Blo	Blood
Bone	Bone
BM	Bone marrow
BrC	Brain cortex
BrS	Brainstem
Col	Colon
Dia	Diaphragm
Ret	Eye retina
Fat	Fat
Hrt	Heart
Pit	Hypothalamus/Pituitary
Kid	Kidney
LM	Limb muscle
Liv	Liver
Lung	Lung
MG	Mammary gland
Panc	Pancreas
Skin	Skin
SI	Small intestine
Spl	Spleen
Test	Testes
Thym	Thymus
Tong	Tongue
Trac	Trachea
Ute	Uterus

Adiponectin (AdipoQ)
- stromal

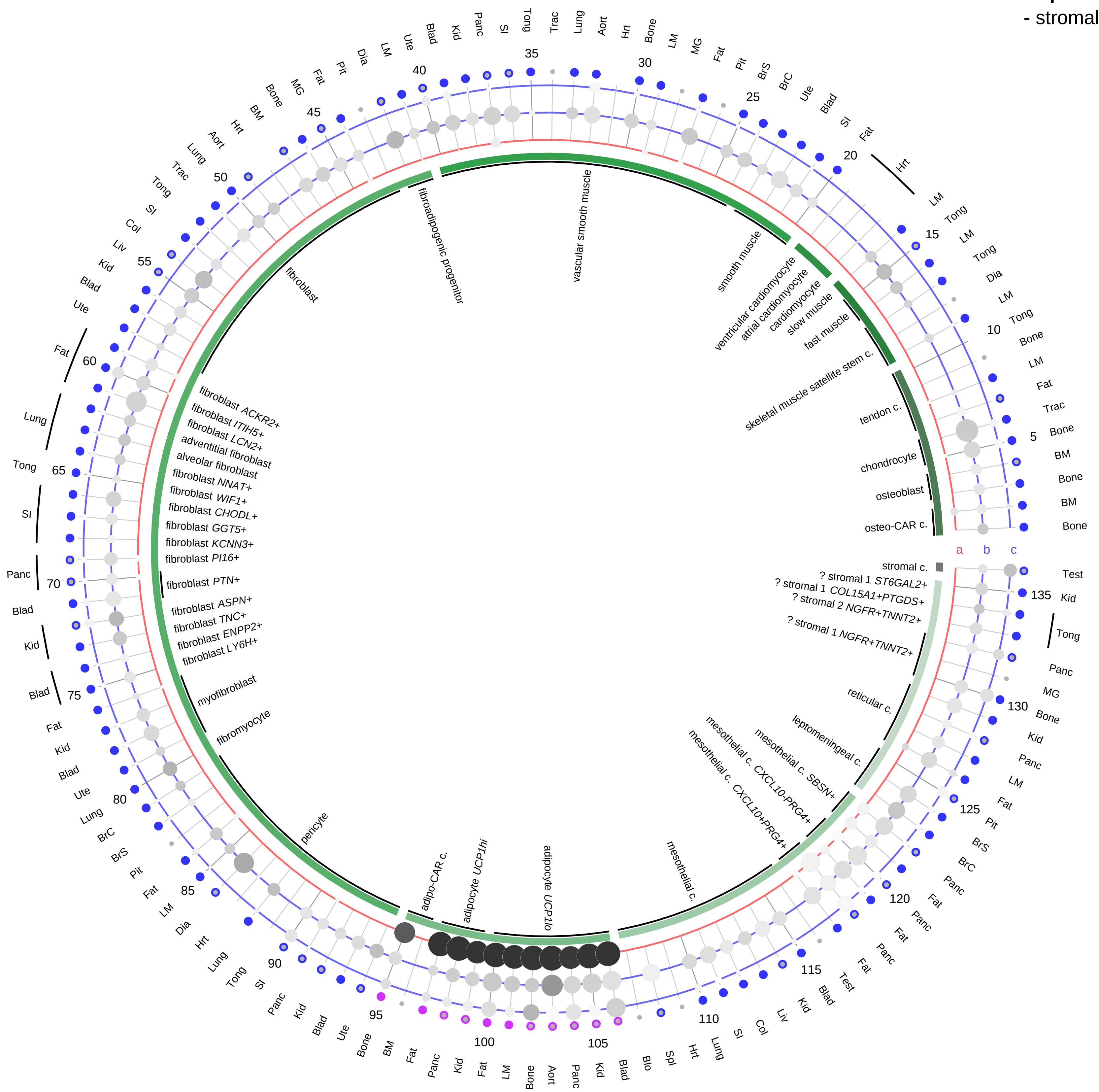

Ligand/Enzyme
a - ADIPOQ
Receptor
b - ADIPOR1
c - ADIPOR2

PAIRING (ligand → receptor)

ADIPOQ→ADIPOR1

ADIPOQ→ADIPOR2

Compartment
stromal
- bone
- skeletal muscle
- cardiac muscle
- smooth muscle
- fibroblast
- adipocyte
- mesothelial
- other
- mix

12.5%
25%
50%
100%

ln(CP10K+1): 0, 1, 2, 3

Abbrev.	Tissue
Aort	Aorta
Blad	Bladder
Blo	Blood
Bone	Bone
BM	Bone marrow
BrC	Brain cortex
BrS	Brainstem
Col	Colon
Dia	Diaphragm
Ret	Eye retina
Fat	Fat
Hrt	Heart
Pit	Hypothalamus/Pituitary
Kid	Kidney
LM	Limb muscle
Liv	Liver
Lung	Lung
MG	Mammary gland
Panc	Pancreas
Skin	Skin
SI	Small intestine
Spl	Spleen
Test	Testes
Thym	Thymus
Tong	Tongue
Trac	Trachea
Ute	Uterus

Adiponectin (AdipoQ)
- lymphoid

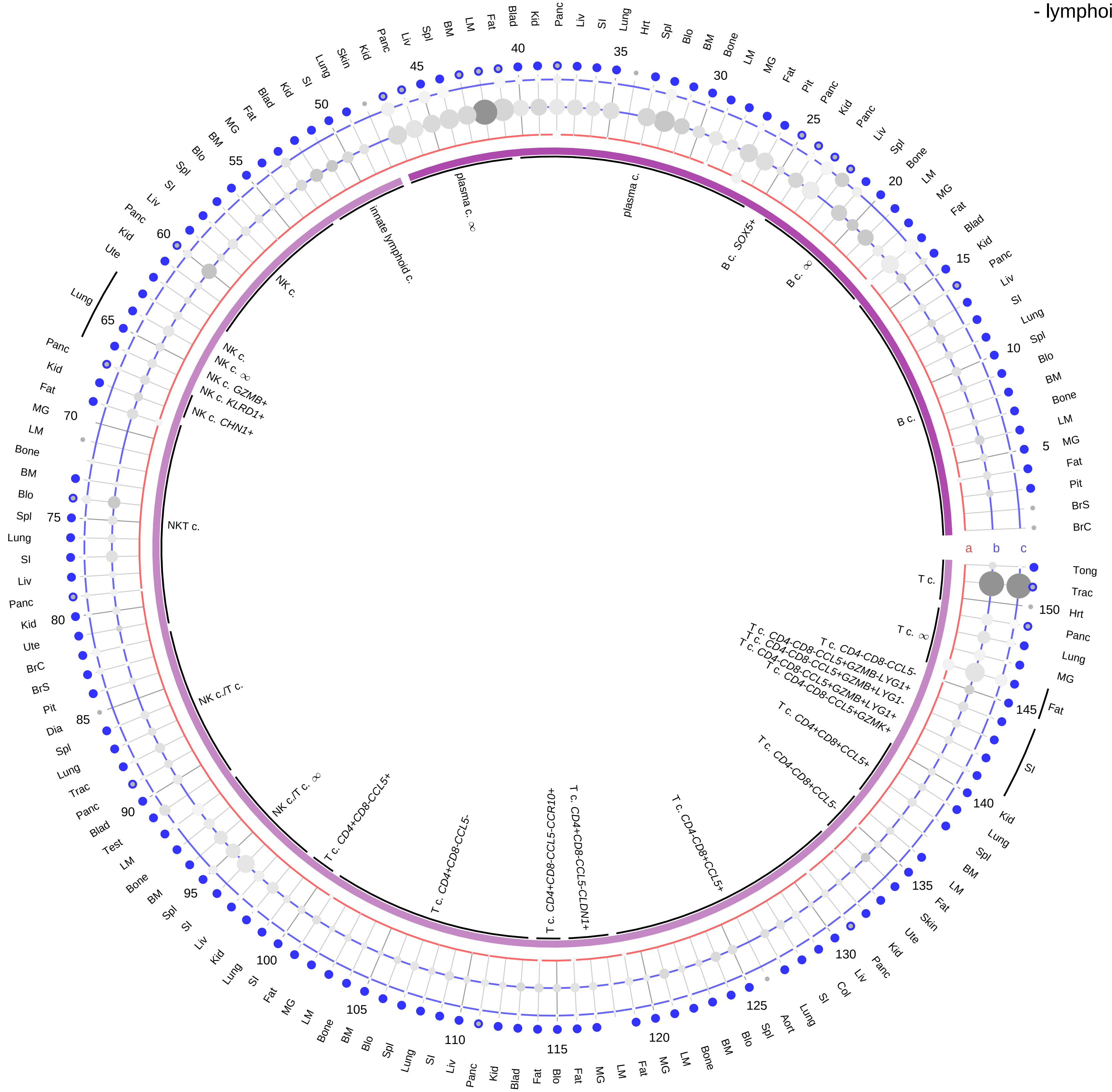

Ligand/Enzyme
a - ADIPOQ
Receptor
b - ADIPOR1
c - ADIPOR2

PAIRING (ligand → receptor)

ADIPOQ→ADIPOR1

ADIPOQ→ADIPOR2

Compartment
lymphoid
- B cell
- NK/T cell

12.5%
25%
50%
100%

ln(CP10K+1): 0, 1, 2, 3

**Abbrev.**	**Tissue**
Aort	Aorta
Blad	Bladder
Blo	Blood
Bone	Bone
BM	Bone marrow
BrC	Brain cortex
BrS	Brainstem
Col	Colon
Dia	Diaphragm
Ret	Eye retina
Fat	Fat
Hrt	Heart
Pit	Hypothalamus/Pituitary
Kid	Kidney
LM	Limb muscle
Liv	Liver
Lung	Lung
MG	Mammary gland
Panc	Pancreas
Skin	Skin
SI	Small intestine
Spl	Spleen
Test	Testes
Thym	Thymus
Tong	Tongue
Trac	Trachea
Ute	Uterus

Adiponectin (AdipoQ)
- myeloid

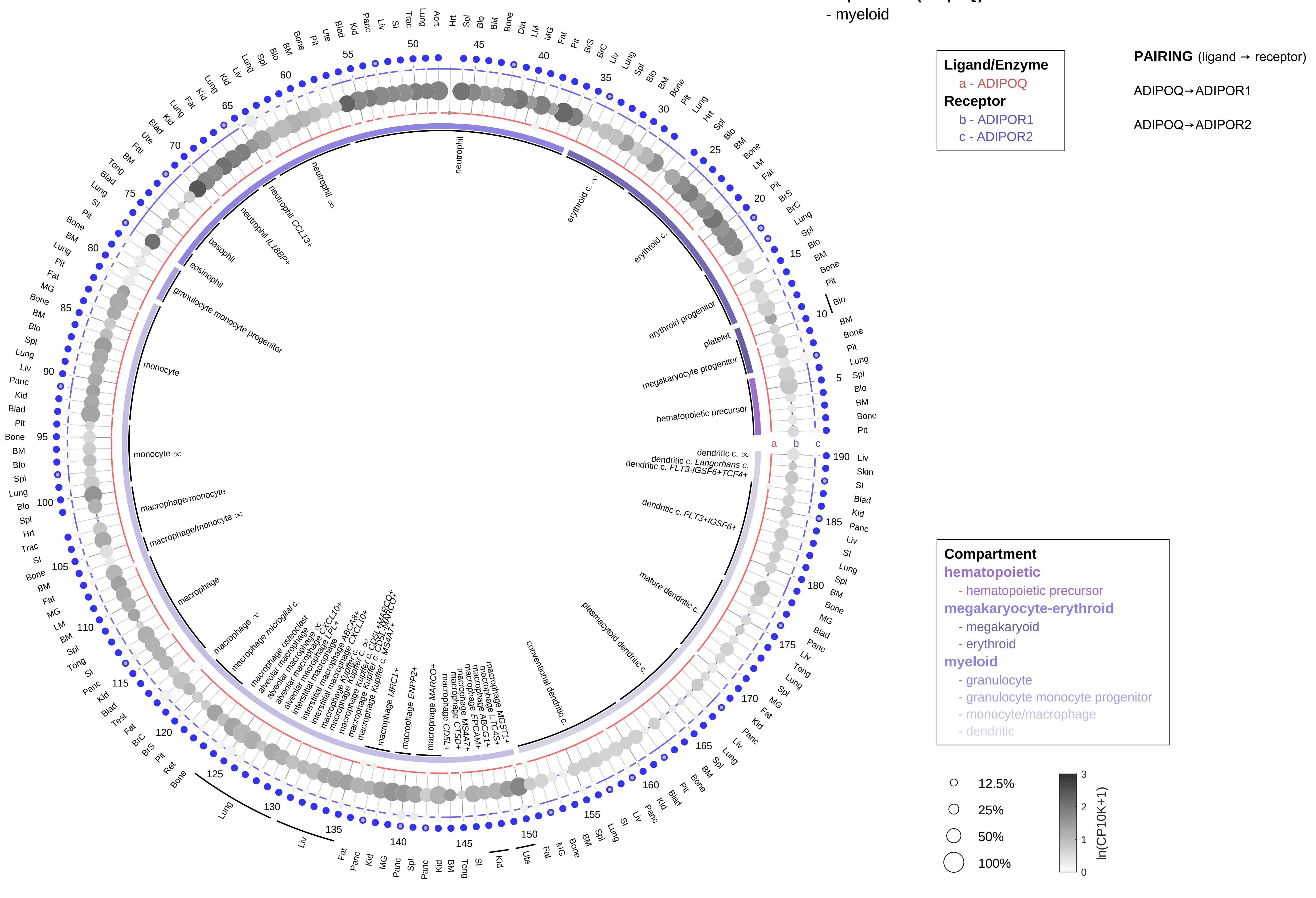

Abbrev.	Tissue
Aort	Aorta
Blad	Bladder
Blo	Blood
Bone	Bone
BM	Bone marrow
BrC	Brain cortex
BrS	Brainstem
Col	Colon
Dia	Diaphragm
Ret	Eye retina
Fat	Fat
Hrt	Heart
Pit	Hypothalamus/Pituitary
Kid	Kidney
LM	Limb muscle
Liv	Liver
Lung	Lung
MG	Mammary gland
Panc	Pancreas
Skin	Skin
SI	Small intestine
Spl	Spleen
Test	Testes
Thym	Thymus
Tong	Tongue
Trac	Trachea
Ute	Uterus

Adrenomedullin (ADM/ADM2)
- epithelial/neural/germ

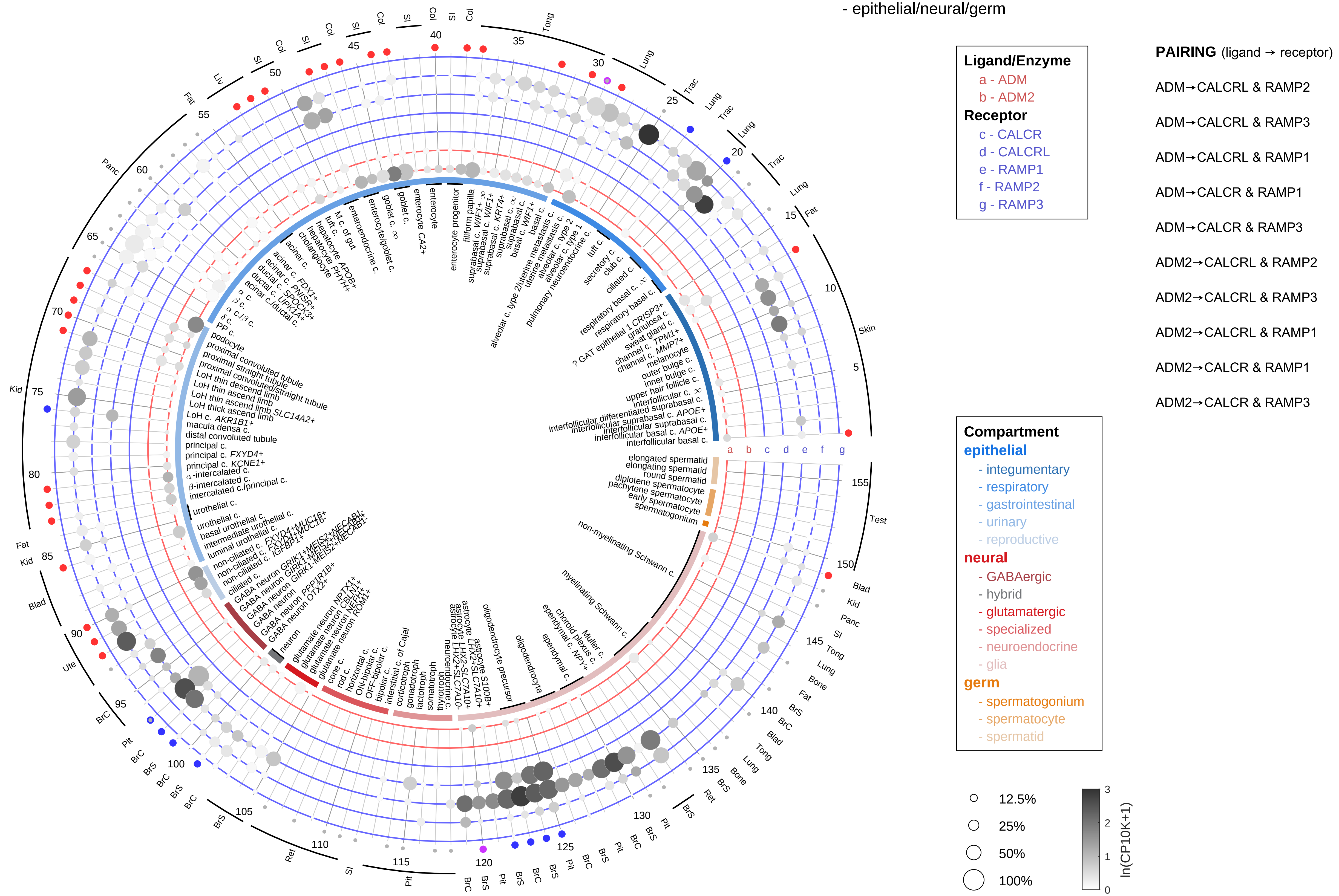

Abbrev.	Tissue
Aort	Aorta
Blad	Bladder
Blo	Blood
Bone	Bone
BM	Bone marrow
BrC	Brain cortex
BrS	Brainstem
Col	Colon
Dia	Diaphragm
Ret	Eye retina
Fat	Fat
Hrt	Heart
Pit	Hypothalamus/Pituitary
Kid	Kidney
LM	Limb muscle
Liv	Liver
Lung	Lung
MG	Mammary gland
Panc	Pancreas
Skin	Skin
SI	Small intestine
Spl	Spleen
Test	Testes
Thym	Thymus
Tong	Tongue
Trac	Trachea
Ute	Uterus

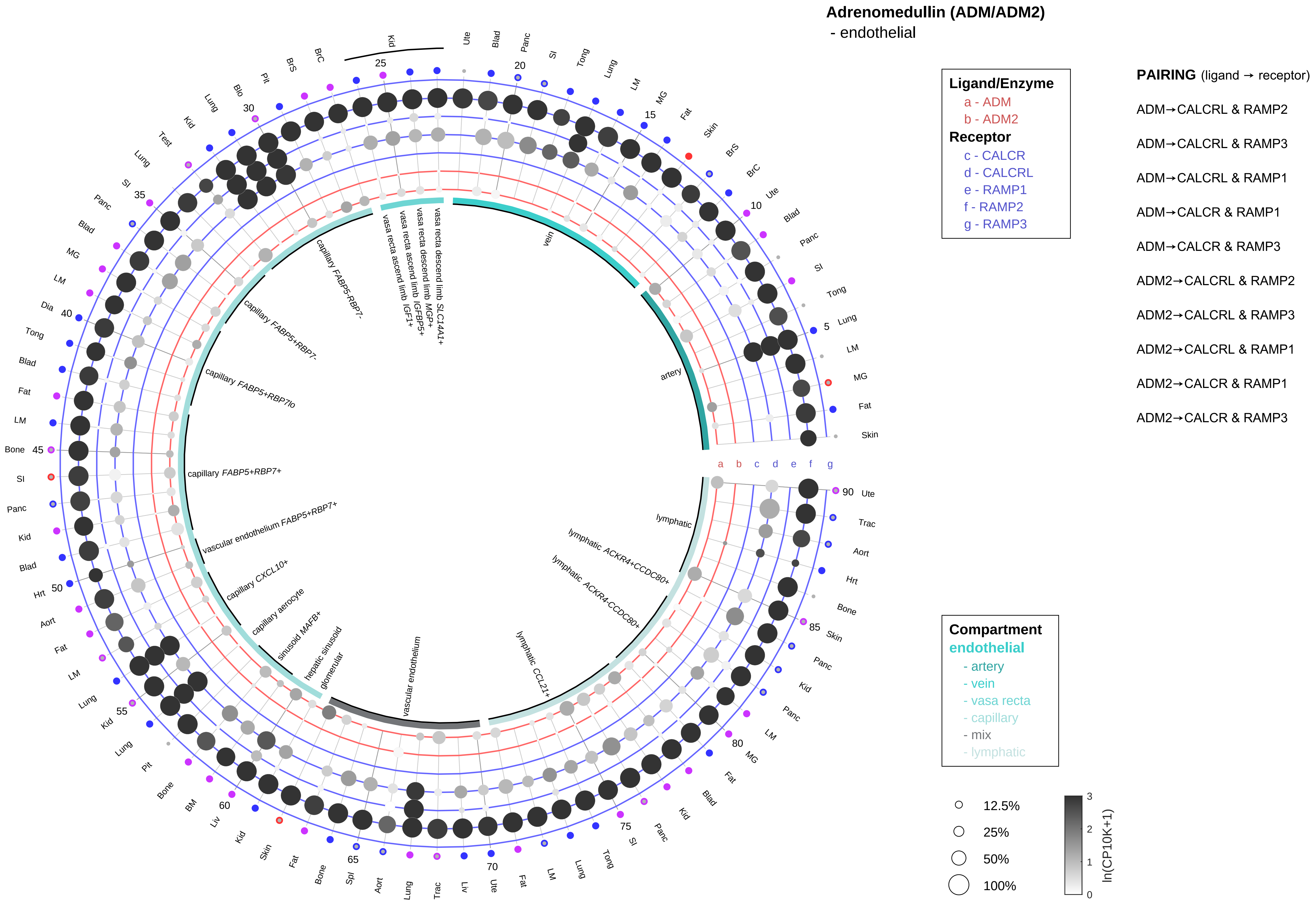

Abbrev.	Tissue
Aort	Aorta
Blad	Bladder
Blo	Blood
Bone	Bone
BM	Bone marrow
BrC	Brain cortex
BrS	Brainstem
Col	Colon
Dia	Diaphragm
Ret	Eye retina
Fat	Fat
Hrt	Heart
Pit	Hypothalamus/Pituitary
Kid	Kidney
LM	Limb muscle
Liv	Liver
Lung	Lung
MG	Mammary gland
Panc	Pancreas
Skin	Skin
SI	Small intestine
Spl	Spleen
Test	Testes
Thym	Thymus
Tong	Tongue
Trac	Trachea
Ute	Uterus

Adrenomedullin (ADM/ADM2)
- stromal

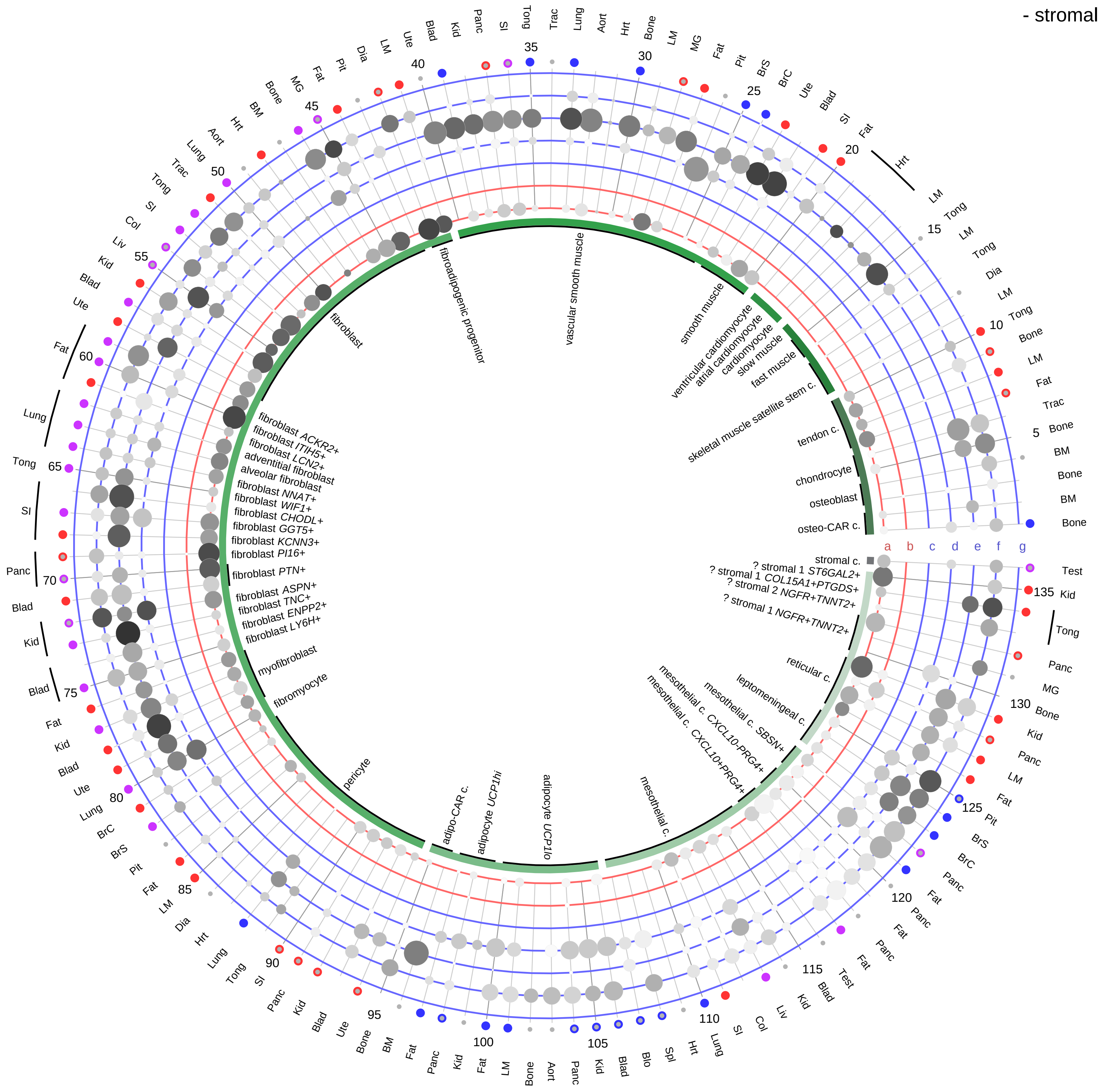

Ligand/Enzyme
- a - ADM
- b - ADM2

Receptor
- c - CALCR
- d - CALCRL
- e - RAMP1
- f - RAMP2
- g - RAMP3

PAIRING (ligand → receptor)

ADM→CALCRL & RAMP2

ADM→CALCRL & RAMP3

ADM→CALCRL & RAMP1

ADM→CALCR & RAMP1

ADM→CALCR & RAMP3

ADM2→CALCRL & RAMP2

ADM2→CALCRL & RAMP3

ADM2→CALCRL & RAMP1

ADM2→CALCR & RAMP1

ADM2→CALCR & RAMP3

Compartment
stromal
- bone
- skeletal muscle
- cardiac muscle
- smooth muscle
- fibroblast
- adipocyte
- mesothelial
- other
- mix

12.5%
25%
50%
100%

ln(CP10K+1): 0, 1, 2, 3

Abbrev.	Tissue
Aort	Aorta
Blad	Bladder
Blo	Blood
Bone	Bone
BM	Bone marrow
BrC	Brain cortex
BrS	Brainstem
Col	Colon
Dia	Diaphragm
Ret	Eye retina
Fat	Fat
Hrt	Heart
Pit	Hypothalamus/Pituitary
Kid	Kidney
LM	Limb muscle
Liv	Liver
Lung	Lung
MG	Mammary gland
Panc	Pancreas
Skin	Skin
SI	Small intestine
Spl	Spleen
Test	Testes
Thym	Thymus
Tong	Tongue
Trac	Trachea
Ute	Uterus

Adrenomedullin (ADM/ADM2)
- lymphoid

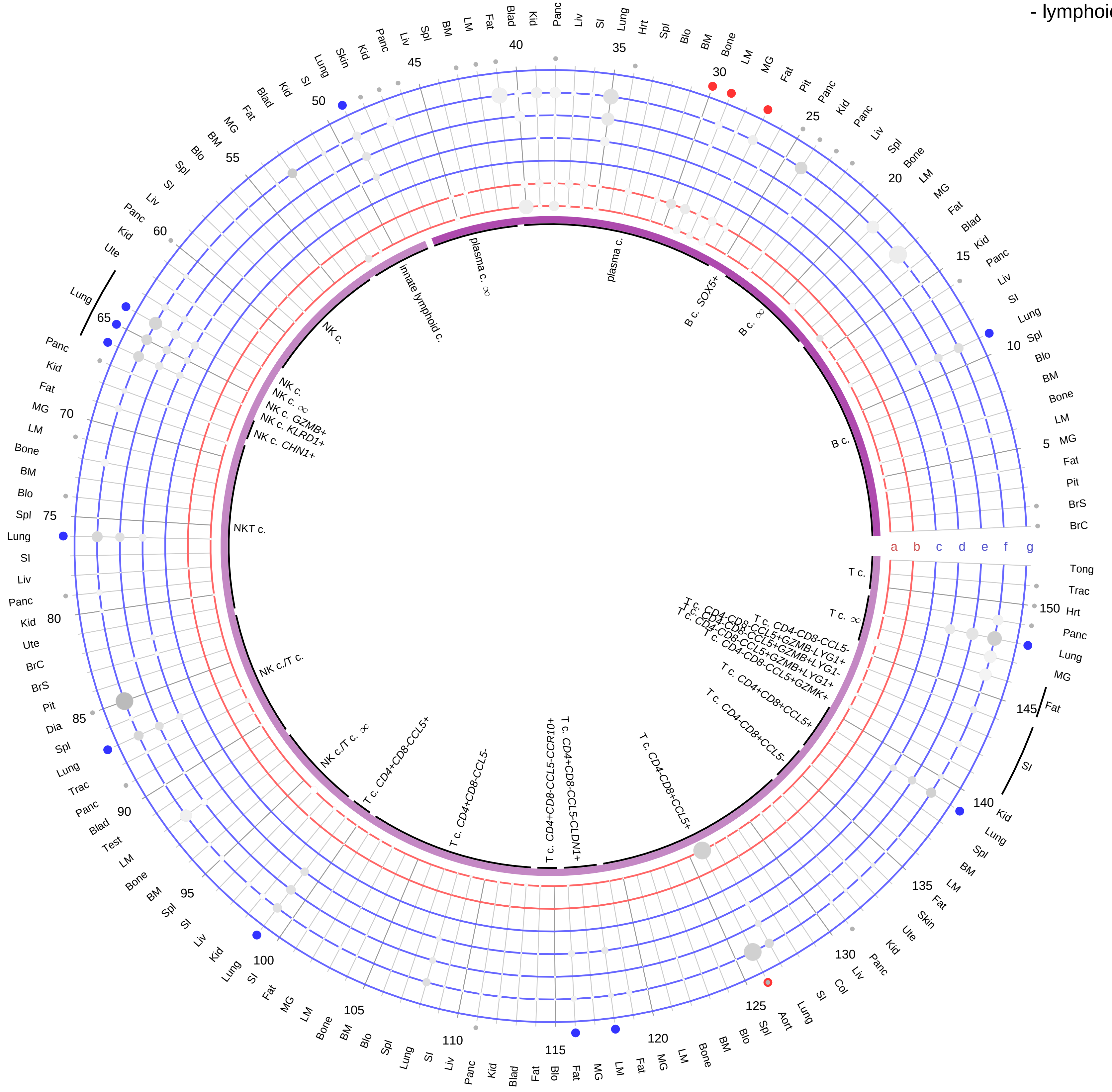

Ligand/Enzyme
a - ADM
b - ADM2
**Receptor**
c - CALCR
d - CALCRL
e - RAMP1
f - RAMP2
g - RAMP3

PAIRING (ligand → receptor)

ADM→CALCRL & RAMP2
ADM→CALCRL & RAMP3
ADM→CALCRL & RAMP1
ADM→CALCR & RAMP1
ADM→CALCR & RAMP3
ADM2→CALCRL & RAMP2
ADM2→CALCRL & RAMP3
ADM2→CALCRL & RAMP1
ADM2→CALCR & RAMP1
ADM2→CALCR & RAMP3

Abbrev.	Tissue
Aort	Aorta
Blad	Bladder
Blo	Blood
Bone	Bone
BM	Bone marrow
BrC	Brain cortex
BrS	Brainstem
Col	Colon
Dia	Diaphragm
Ret	Eye retina
Fat	Fat
Hrt	Heart
Pit	Hypothalamus/Pituitary
Kid	Kidney
LM	Limb muscle
Liv	Liver
Lung	Lung
MG	Mammary gland
Panc	Pancreas
Skin	Skin
SI	Small intestine
Spl	Spleen
Test	Testes
Thym	Thymus
Tong	Tongue
Trac	Trachea
Ute	Uterus

Compartment
lymphoid
- B cell
- NK/T cell

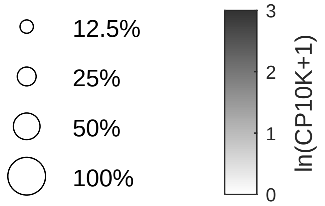

Adrenomedullin (ADM/ADM2)
- myeloid

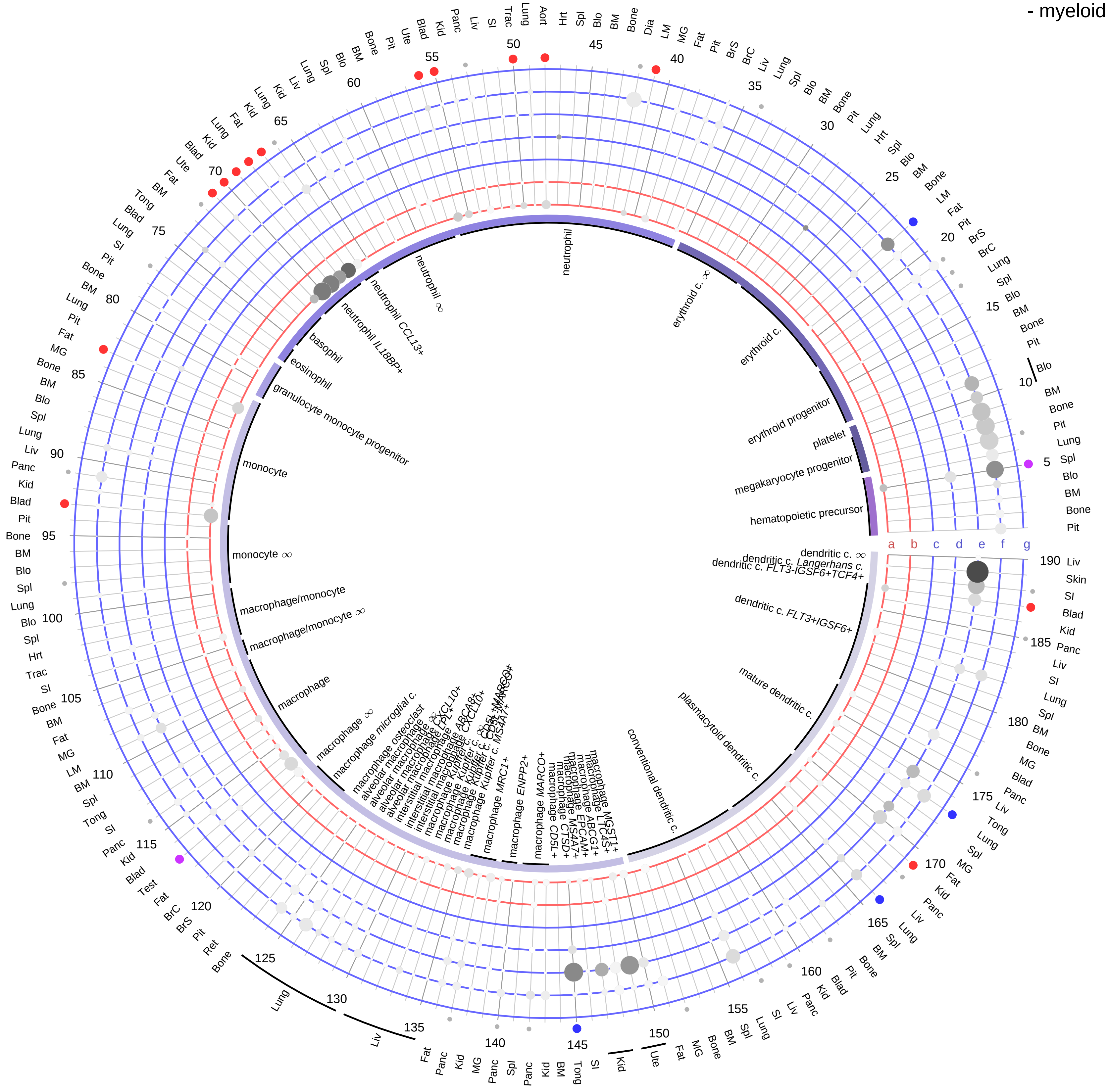

Ligand/Enzyme
- a - ADM
- b - ADM2

Receptor
- c - CALCR
- d - CALCRL
- e - RAMP1
- f - RAMP2
- g - RAMP3

PAIRING (ligand → receptor)

ADM→CALCRL & RAMP2

ADM→CALCRL & RAMP3

ADM→CALCRL & RAMP1

ADM→CALCR & RAMP1

ADM→CALCR & RAMP3

ADM2→CALCRL & RAMP2

ADM2→CALCRL & RAMP3

ADM2→CALCRL & RAMP1

ADM2→CALCR & RAMP1

ADM2→CALCR & RAMP3

Abbrev.	Tissue
Aort	Aorta
Blad	Bladder
Blo	Blood
Bone	Bone
BM	Bone marrow
BrC	Brain cortex
BrS	Brainstem
Col	Colon
Dia	Diaphragm
Ret	Eye retina
Fat	Fat
Hrt	Heart
Pit	Hypothalamus/Pituitary
Kid	Kidney
LM	Limb muscle
Liv	Liver
Lung	Lung
MG	Mammary gland
Panc	Pancreas
Skin	Skin
SI	Small intestine
Spl	Spleen
Test	Testes
Thym	Thymus
Tong	Tongue
Trac	Trachea
Ute	Uterus

Compartment

hematopoietic
- hematopoietic precursor

megakaryocyte-erythroid
- megakaryoid
- erythroid

myeloid
- granulocyte
- granulocyte monocyte progenitor
- monocyte/macrophage
- dendritic

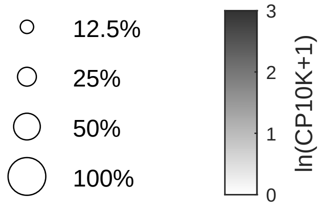

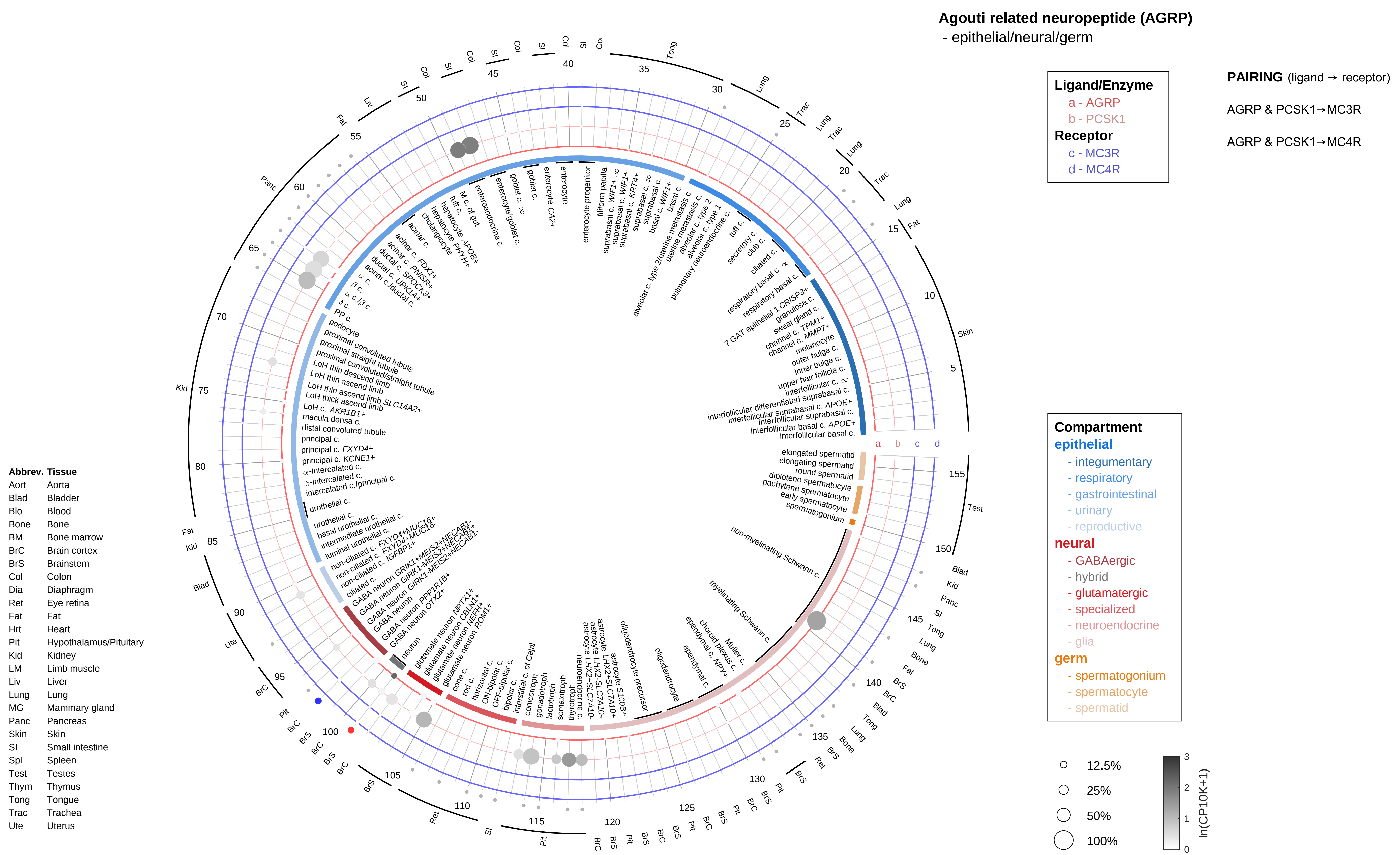
Agouti related neuropeptide (AGRP)
- epithelial/neural/germ
Ligand/Enzyme
a - AGRP
b - PCSK1
Receptor
c - MC3R
d - MC4R
PAIRING (ligand → receptor)
AGRP & PCSK1→MC3R
AGRP & PCSK1→MC4R
Compartment
epithelial
- integumentary
- respiratory
- gastrointestinal
- urinary
- reproductive
neural
- GABAergic
- hybrid
- glutamatergic
- specialized
- neuroendocrine
- glia
germ
- spermatogonium
- spermatocyte
- spermatid
12.5%
25%
50%
100%
ln(CP10K+1)
Abbrev. Tissue
Aort Aorta
Blad Bladder
Blo Blood
Bone Bone
BM Bone marrow
BrC Brain cortex
BrS Brainstem
Col Colon
Dia Diaphragm
Ret Eye retina
Fat Fat
Hrt Heart
Pit Hypothalamus/Pituitary
Kid Kidney
LM Limb muscle
Liv Liver
Lung Lung
MG Mammary gland
Panc Pancreas
Skin Skin
SI Small intestine
Spl Spleen
Test Testes
Thym Thymus
Tong Tongue
Trac Trachea
Ute Uterus

Agouti related neuropeptide (AGRP)
- endothelial

PAIRING (ligand → receptor)

AGRP & PCSK1→MC3R

AGRP & PCSK1→MC4R

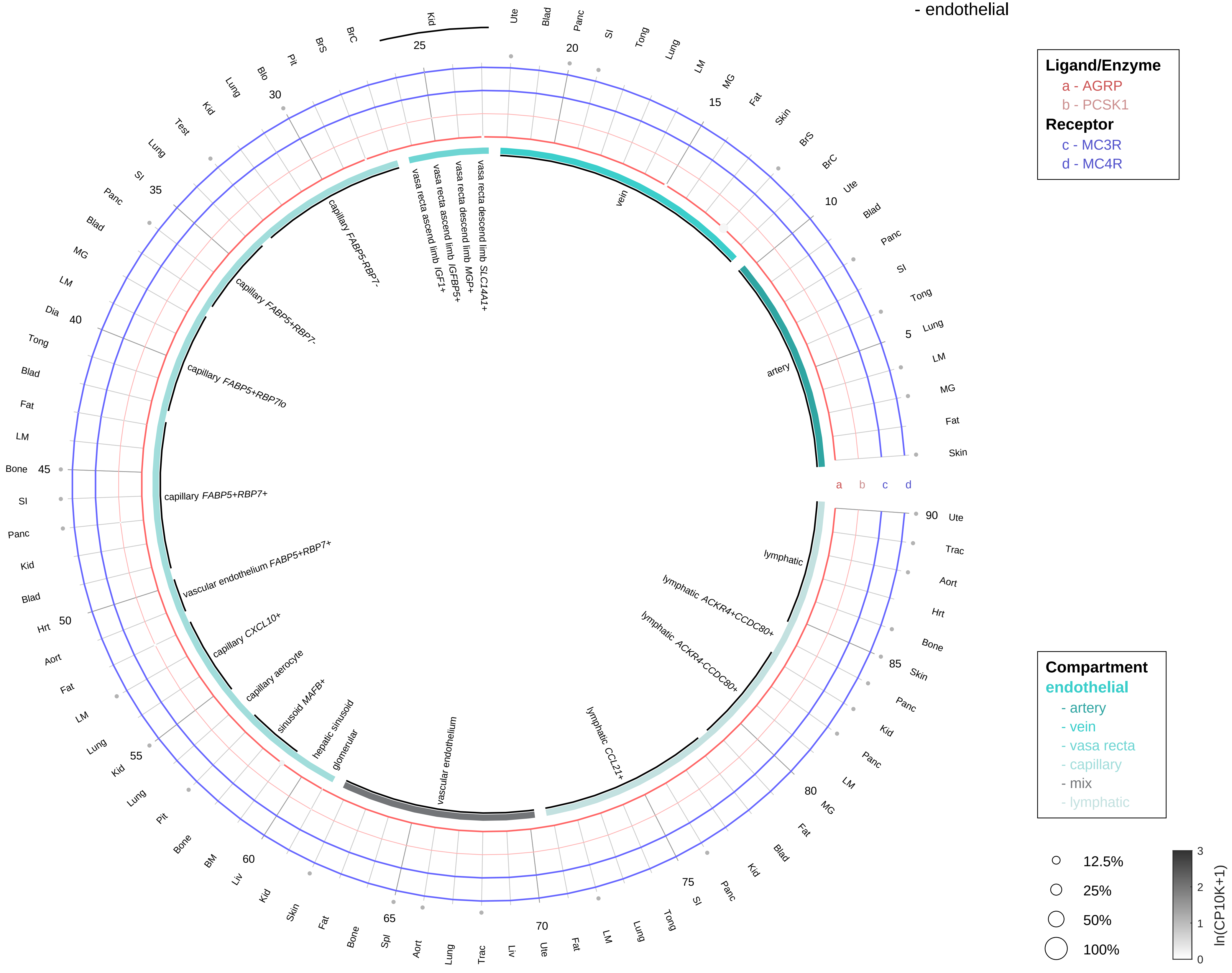

**Abbrev.**	**Tissue**
Aort	Aorta
Blad	Bladder
Blo	Blood
Bone	Bone
BM	Bone marrow
BrC	Brain cortex
BrS	Brainstem
Col	Colon
Dia	Diaphragm
Ret	Eye retina
Fat	Fat
Hrt	Heart
Pit	Hypothalamus/Pituitary
Kid	Kidney
LM	Limb muscle
Liv	Liver
Lung	Lung
MG	Mammary gland
Panc	Pancreas
Skin	Skin
SI	Small intestine
Spl	Spleen
Test	Testes
Thym	Thymus
Tong	Tongue
Trac	Trachea
Ute	Uterus

Agouti related neuropeptide (AGRP)
- stromal

Ligand/Enzyme
- a - AGRP
- b - PCSK1

Receptor
- c - MC3R
- d - MC4R

PAIRING (ligand → receptor)

AGRP & PCSK1→MC3R

AGRP & PCSK1→MC4R

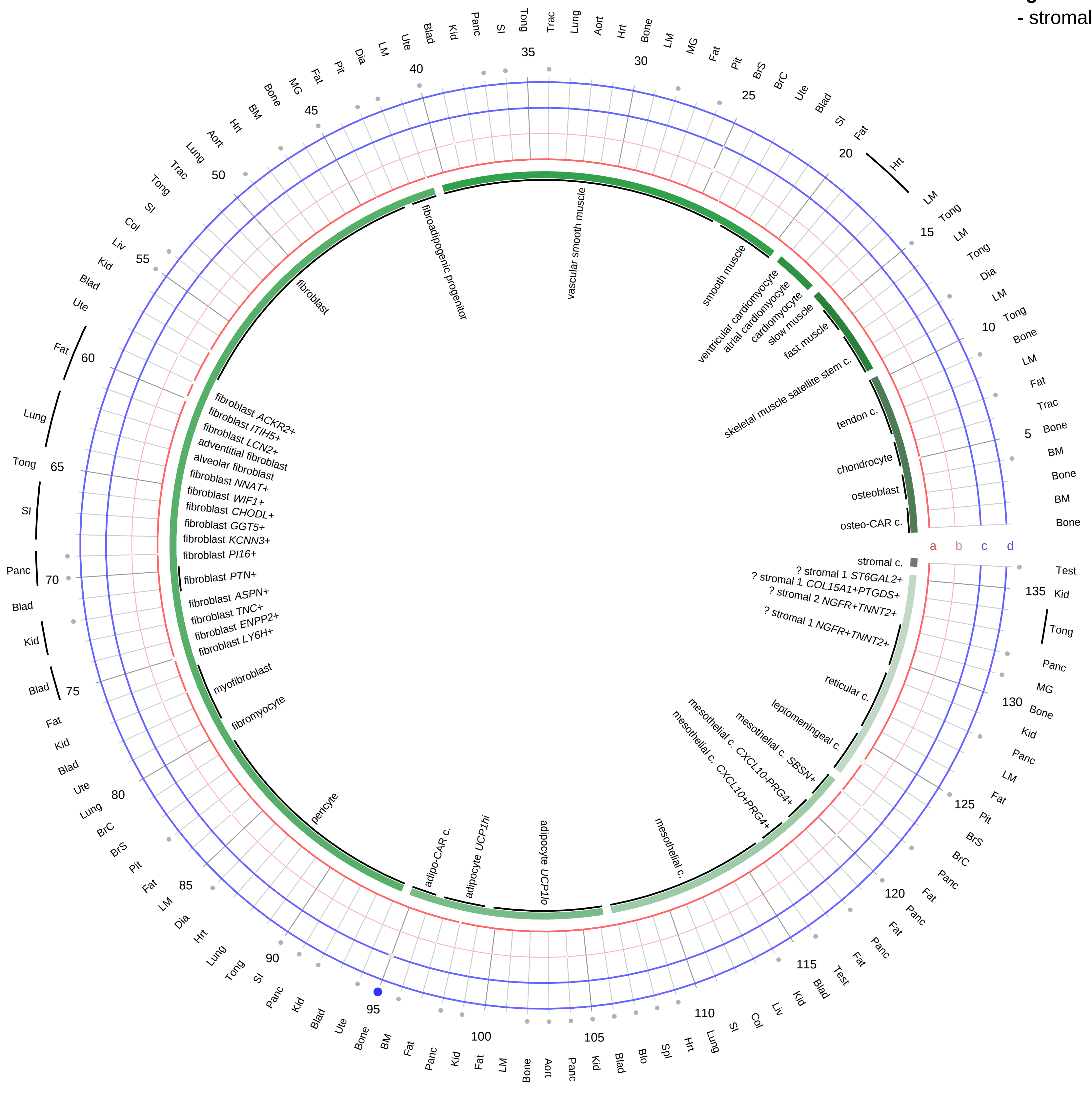

Abbrev. Tissue

Abbrev.	Tissue
Aort	Aorta
Blad	Bladder
Blo	Blood
Bone	Bone
BM	Bone marrow
BrC	Brain cortex
BrS	Brainstem
Col	Colon
Dia	Diaphragm
Ret	Eye retina
Fat	Fat
Hrt	Heart
Pit	Hypothalamus/Pituitary
Kid	Kidney
LM	Limb muscle
Liv	Liver
Lung	Lung
MG	Mammary gland
Panc	Pancreas
Skin	Skin
SI	Small intestine
Spl	Spleen
Test	Testes
Thym	Thymus
Tong	Tongue
Trac	Trachea
Ute	Uterus

Compartment

stromal
- bone
- skeletal muscle
- cardiac muscle
- smooth muscle
- fibroblast
- adipocyte
- mesothelial
- other
- mix

12.5%
25%
50%
100%

ln(CP10K+1): 0, 1, 2, 3

Agouti related neuropeptide (AGRP)
- lymphoid

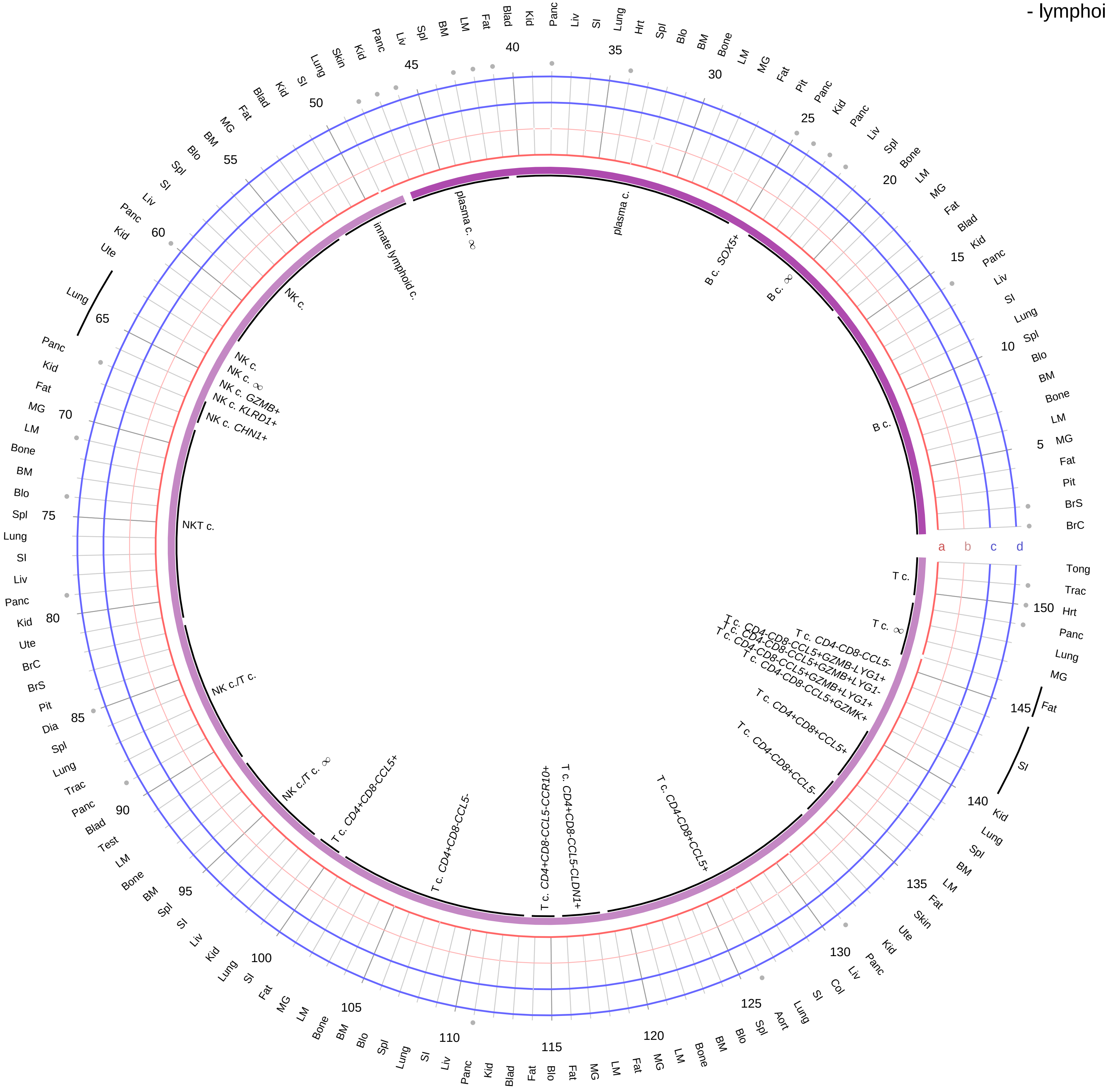

Ligand/Enzyme
a - AGRP
b - PCSK1
Receptor
c - MC3R
d - MC4R

PAIRING (ligand → receptor)

AGRP & PCSK1→MC3R

AGRP & PCSK1→MC4R

Abbrev.	Tissue
Aort	Aorta
Blad	Bladder
Blo	Blood
Bone	Bone
BM	Bone marrow
BrC	Brain cortex
BrS	Brainstem
Col	Colon
Dia	Diaphragm
Ret	Eye retina
Fat	Fat
Hrt	Heart
Pit	Hypothalamus/Pituitary
Kid	Kidney
LM	Limb muscle
Liv	Liver
Lung	Lung
MG	Mammary gland
Panc	Pancreas
Skin	Skin
SI	Small intestine
Spl	Spleen
Test	Testes
Thym	Thymus
Tong	Tongue
Trac	Trachea
Ute	Uterus

Compartment
lymphoid
- B cell
- NK/T cell

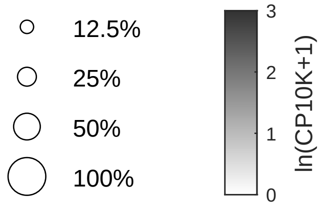

Agouti related neuropeptide (AGRP)
- myeloid

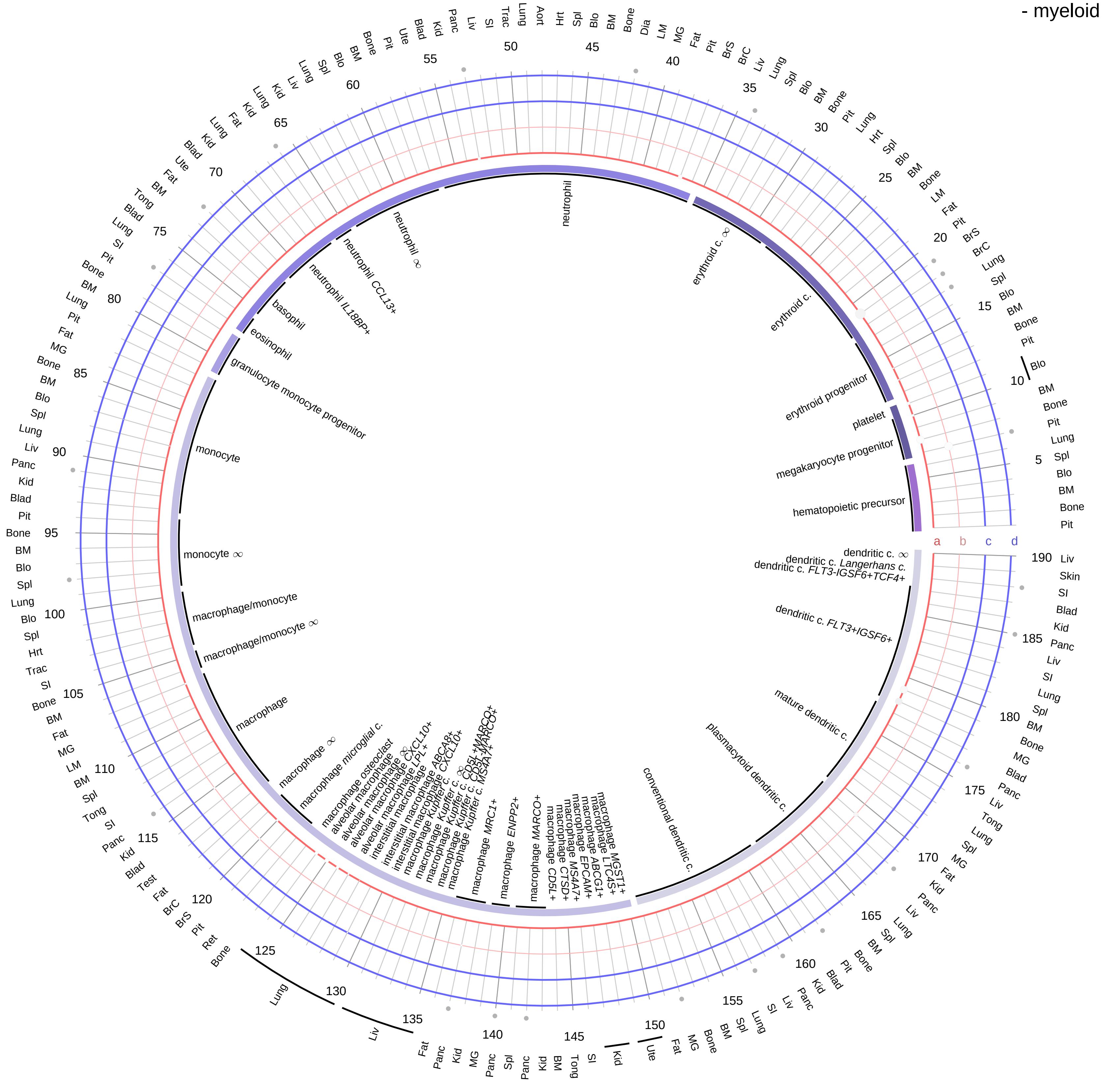

Ligand/Enzyme
- a - AGRP
- b - PCSK1

Receptor
- c - MC3R
- d - MC4R

PAIRING (ligand → receptor)

AGRP & PCSK1→MC3R

AGRP & PCSK1→MC4R

Compartment
- **hematopoietic**
  - hematopoietic precursor
- **megakaryocyte-erythroid**
  - megakaryoid
  - erythroid
- **myeloid**
  - granulocyte
  - granulocyte monocyte progenitor
  - monocyte/macrophage
  - dendritic

12.5%
25%
50%
100%

ln(CP10K+1): 0, 1, 2, 3

Abbrev.	Tissue
Aort	Aorta
Blad	Bladder
Blo	Blood
Bone	Bone
BM	Bone marrow
BrC	Brain cortex
BrS	Brainstem
Col	Colon
Dia	Diaphragm
Ret	Eye retina
Fat	Fat
Hrt	Heart
Pit	Hypothalamus/Pituitary
Kid	Kidney
LM	Limb muscle
Liv	Liver
Lung	Lung
MG	Mammary gland
Panc	Pancreas
Skin	Skin
SI	Small intestine
Spl	Spleen
Test	Testes
Thym	Thymus
Tong	Tongue
Trac	Trachea
Ute	Uterus

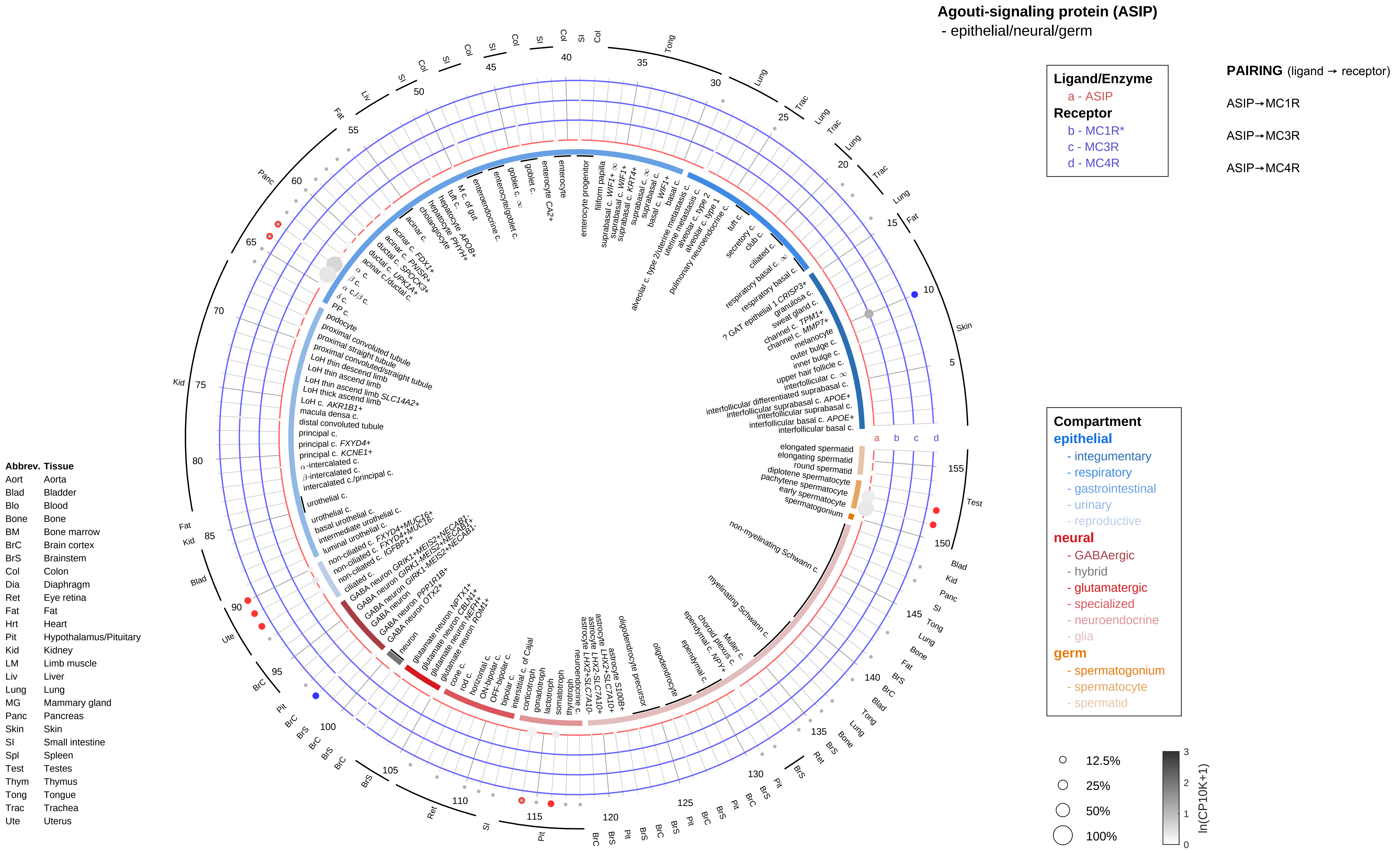

Agouti-signaling protein (ASIP)
- epithelial/neural/germ
Ligand/Enzyme
a - ASIP
Receptor
b - MC1R*
c - MC3R
d - MC4R
PAIRING (ligand → receptor)
ASIP→MC1R
ASIP→MC3R
ASIP→MC4R
Compartment
epithelial
- integumentary
- respiratory
- gastrointestinal
- urinary
- reproductive
neural
- GABAergic
- hybrid
- glutamatergic
- specialized
- neuroendocrine
- glia
germ
- spermatogonium
- spermatocyte
- spermatid
12.5%
25%
50%
100%
ln(CP10K+1)
Abbrev. Tissue
Aort Aorta
Blad Bladder
Blo Blood
Bone Bone
BM Bone marrow
BrC Brain cortex
BrS Brainstem
Col Colon
Dia Diaphragm
Ret Eye retina
Fat Fat
Hrt Heart
Pit Hypothalamus/Pituitary
Kid Kidney
LM Limb muscle
Liv Liver
Lung Lung
MG Mammary gland
Panc Pancreas
Skin Skin
SI Small intestine
Spl Spleen
Test Testes
Thym Thymus
Tong Tongue
Trac Trachea
Ute Uterus

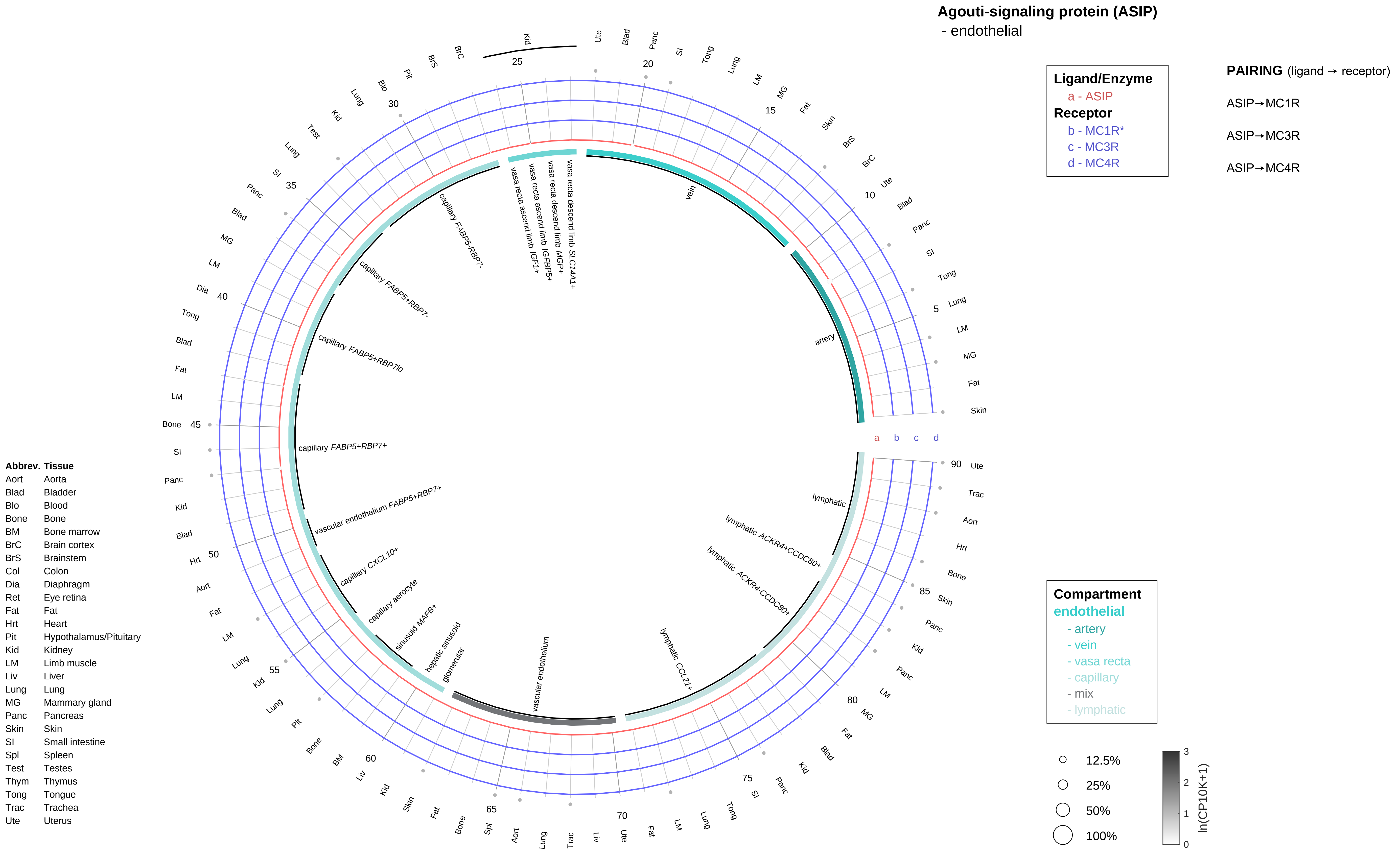
Agouti-signaling protein (ASIP)
- endothelial
Ligand/Enzyme
a - ASIP
Receptor
b - MC1R*
c - MC3R
d - MC4R
PAIRING (ligand → receptor)
ASIP→MC1R
ASIP→MC3R
ASIP→MC4R
vein
artery
vasa recta descend limb SLC14A1+
vasa recta descend limb MGP+
vasa recta ascend limb IGFBP5+
vasa recta ascend limb IGF1+
capillary FABP5-RBP7-
capillary FABP5+RBP7-
capillary FABP5+RBP7lo
capillary FABP5+RBP7+
vascular endothelium FABP5+RBP7+
capillary CXCL10+
capillary aerocyte
sinusoid MAFB+
hepatic sinusoid
glomerular
vascular endothelium
lymphatic CCL21+
lymphatic ACKR4-CCDC80+
lymphatic ACKR4+CCDC80+
lymphatic
a
b
c
d
Compartment
endothelial
- artery
- vein
- vasa recta
- capillary
- mix
- lymphatic
12.5%
25%
50%
100%
ln(CP10K+1)
0
1
2
3
Abbrev. Tissue
Aort Aorta
Blad Bladder
Blo Blood
Bone Bone
BM Bone marrow
BrC Brain cortex
BrS Brainstem
Col Colon
Dia Diaphragm
Ret Eye retina
Fat Fat
Hrt Heart
Pit Hypothalamus/Pituitary
Kid Kidney
LM Limb muscle
Liv Liver
Lung Lung
MG Mammary gland
Panc Pancreas
Skin Skin
SI Small intestine
Spl Spleen
Test Testes
Thym Thymus
Tong Tongue
Trac Trachea
Ute Uterus

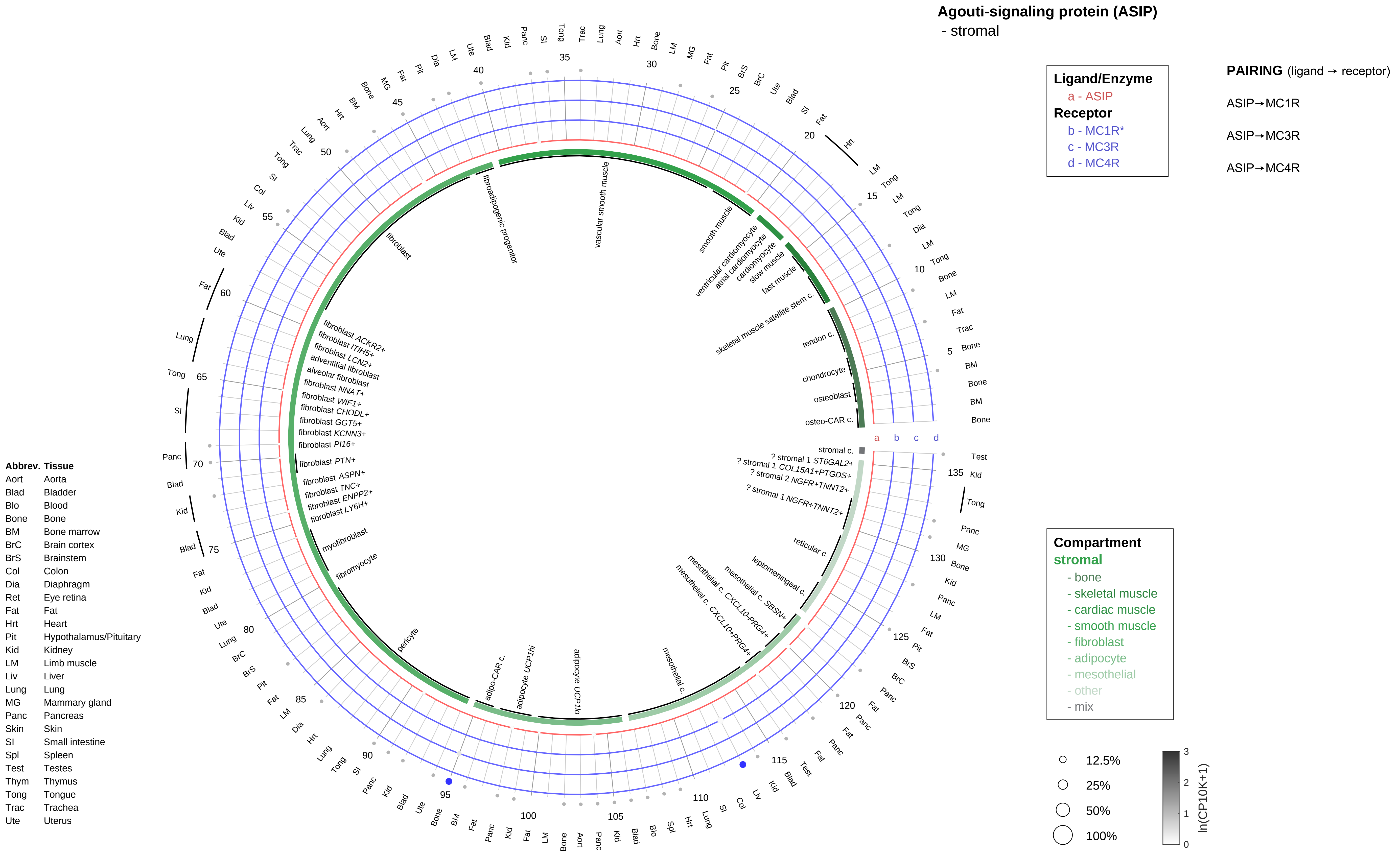

Agouti-signaling protein (ASIP)
- stromal
Ligand/Enzyme
a - ASIP
Receptor
b - MC1R*
c - MC3R
d - MC4R
PAIRING (ligand → receptor)
ASIP→MC1R
ASIP→MC3R
ASIP→MC4R
vascular smooth muscle
fibroadipogenic progenitor
fibroblast
smooth muscle
ventricular cardiomyocyte
atrial cardiomyocyte
cardiomyocyte
slow muscle
fast muscle
skeletal muscle satellite stem c.
tendon c.
chondrocyte
osteoblast
osteo-CAR c.
stromal c.
? stromal 1 ST6GAL2+
? stromal 1 COL15A1+PTGDS+
? stromal 2 NGFR+TNNT2+
? stromal 1 NGFR+TNNT2+
reticular c.
leptomeningeal c.
mesothelial c. SBSN+
mesothelial c. CXCL10-PRG4+
mesothelial c. CXCL10+PRG4+
mesothelial c.
adipocyte UCP1lo
adipocyte UCP1hi
adipo-CAR c.
pericyte
fibromyocyte
myofibroblast
fibroblast LY6H+
fibroblast ENPP2+
fibroblast TNC+
fibroblast ASPN+
fibroblast PTN+
fibroblast PI16+
fibroblast KCNN3+
fibroblast GGT5+
fibroblast CHODL+
fibroblast WIF1+
fibroblast NNAT+
alveolar fibroblast
adventitial fibroblast
fibroblast LCN2+
fibroblast ITIH5+
fibroblast ACKR2+
a b c d
Abbrev. Tissue
Aort Aorta
Blad Bladder
Blo Blood
Bone Bone
BM Bone marrow
BrC Brain cortex
BrS Brainstem
Col Colon
Dia Diaphragm
Ret Eye retina
Fat Fat
Hrt Heart
Pit Hypothalamus/Pituitary
Kid Kidney
LM Limb muscle
Liv Liver
Lung Lung
MG Mammary gland
Panc Pancreas
Skin Skin
SI Small intestine
Spl Spleen
Test Testes
Thym Thymus
Tong Tongue
Trac Trachea
Ute Uterus
Compartment
stromal
- bone
- skeletal muscle
- cardiac muscle
- smooth muscle
- fibroblast
- adipocyte
- mesothelial
- other
- mix
12.5%
25%
50%
100%
ln(CP10K+1)
0
1
2
3

Agouti-signaling protein (ASIP)
- lymphoid

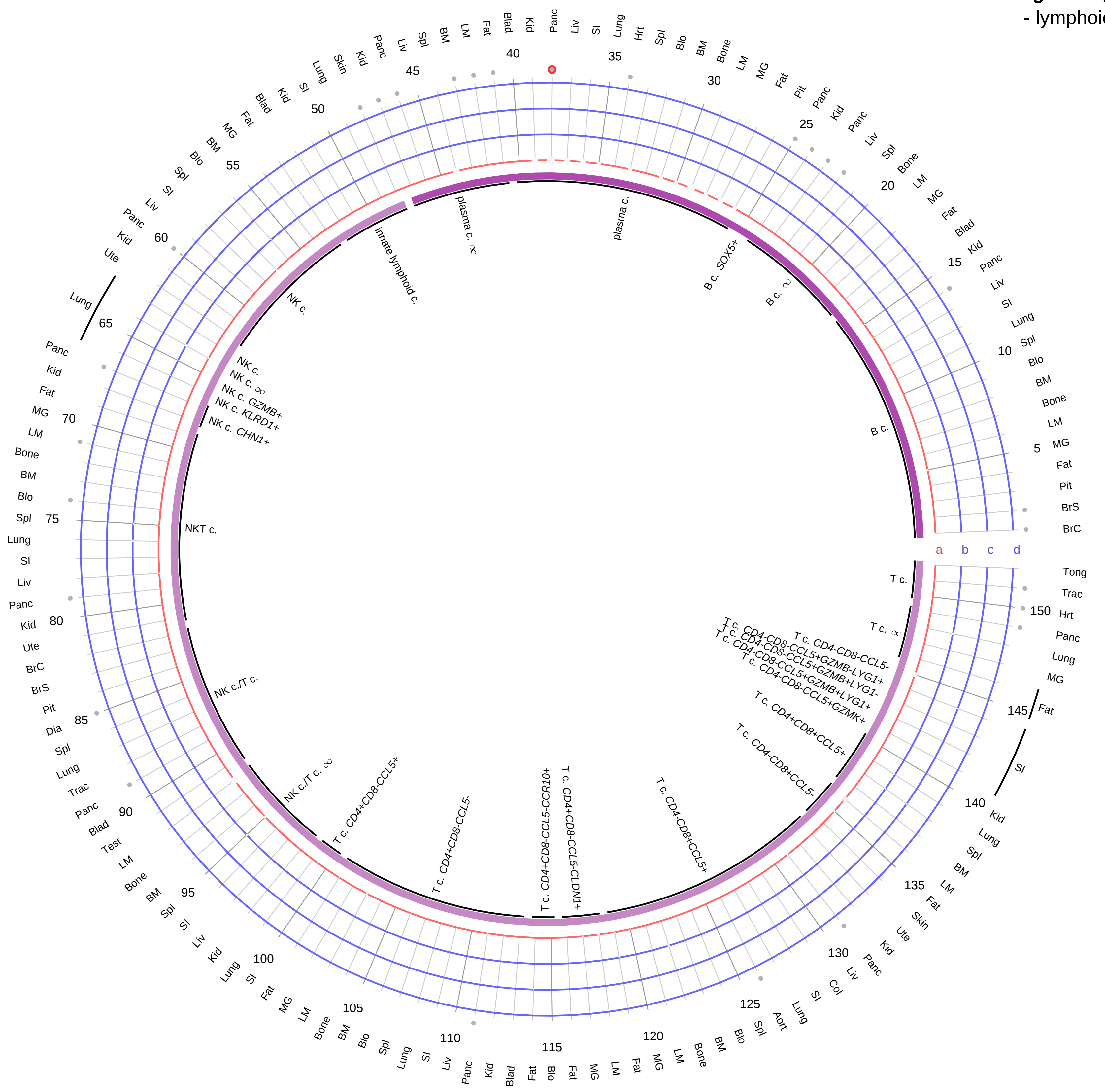

Ligand/Enzyme
a - ASIP
Receptor
b - MC1R*
c - MC3R
d - MC4R

PAIRING (ligand → receptor)

ASIP→MC1R

ASIP→MC3R

ASIP→MC4R

Compartment
lymphoid
- B cell
- NK/T cell

12.5%
25%
50%
100%

ln(CP10K+1) 0 1 2 3

**Abbrev.**	**Tissue**
Aort	Aorta
Blad	Bladder
Blo	Blood
Bone	Bone
BM	Bone marrow
BrC	Brain cortex
BrS	Brainstem
Col	Colon
Dia	Diaphragm
Ret	Eye retina
Fat	Fat
Hrt	Heart
Pit	Hypothalamus/Pituitary
Kid	Kidney
LM	Limb muscle
Liv	Liver
Lung	Lung
MG	Mammary gland
Panc	Pancreas
Skin	Skin
SI	Small intestine
Spl	Spleen
Test	Testes
Thym	Thymus
Tong	Tongue
Trac	Trachea
Ute	Uterus

Agouti-signaling protein (ASIP)
- myeloid

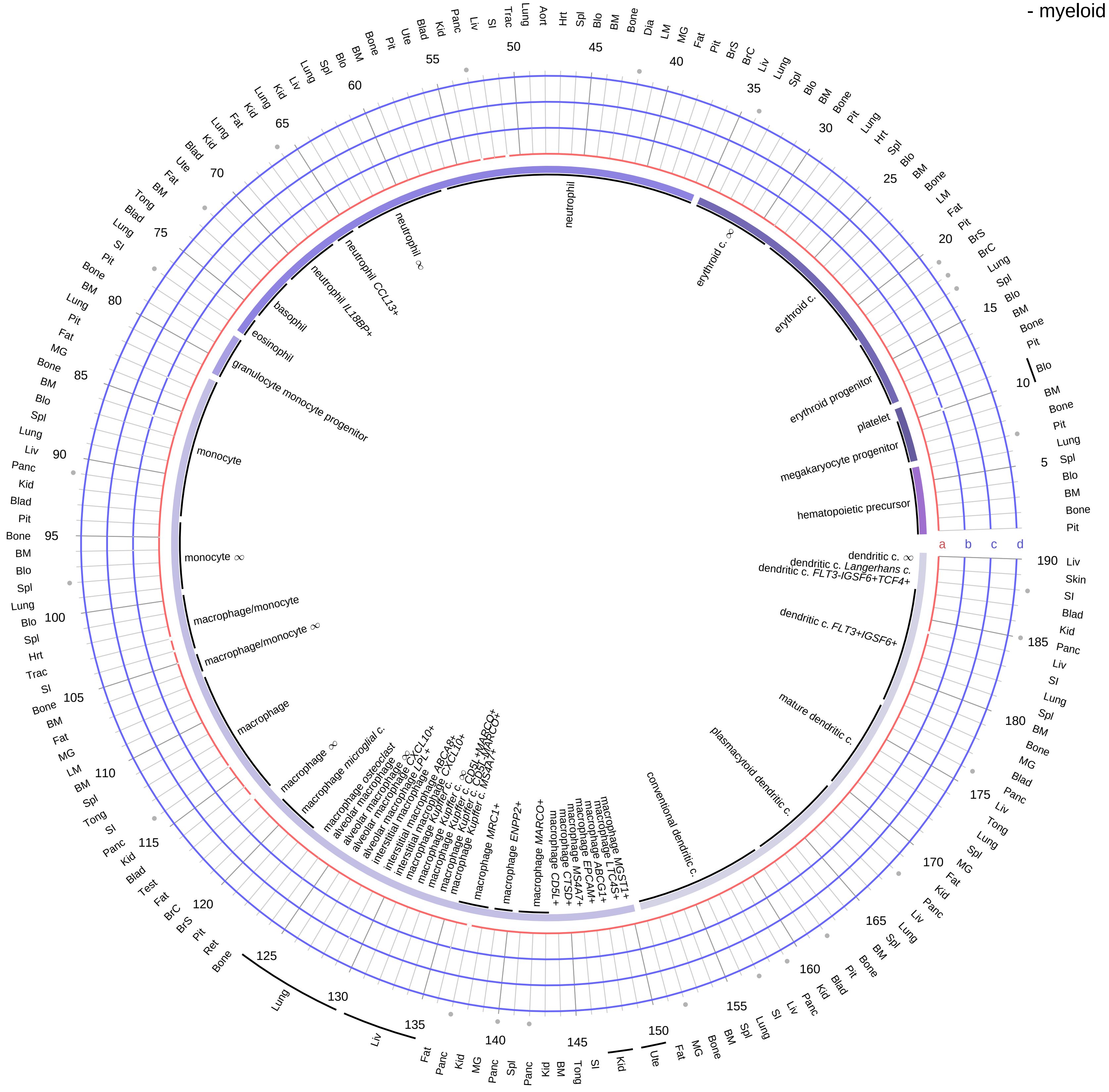

Ligand/Enzyme
a - ASIP
Receptor
b - MC1R*
c - MC3R
d - MC4R

PAIRING (ligand → receptor)

ASIP→MC1R

ASIP→MC3R

ASIP→MC4R

Compartment
hematopoietic
- hematopoietic precursor
megakaryocyte-erythroid
- megakaryoid
- erythroid
myeloid
- granulocyte
- granulocyte monocyte progenitor
- monocyte/macrophage
- dendritic

12.5%
25%
50%
100%

ln(CP10K+1): 0, 1, 2, 3

Abbrev.	Tissue
Aort	Aorta
Blad	Bladder
Blo	Blood
Bone	Bone
BM	Bone marrow
BrC	Brain cortex
BrS	Brainstem
Col	Colon
Dia	Diaphragm
Ret	Eye retina
Fat	Fat
Hrt	Heart
Pit	Hypothalamus/Pituitary
Kid	Kidney
LM	Limb muscle
Liv	Liver
Lung	Lung
MG	Mammary gland
Panc	Pancreas
Skin	Skin
SI	Small intestine
Spl	Spleen
Test	Testes
Thym	Thymus
Tong	Tongue
Trac	Trachea
Ute	Uterus

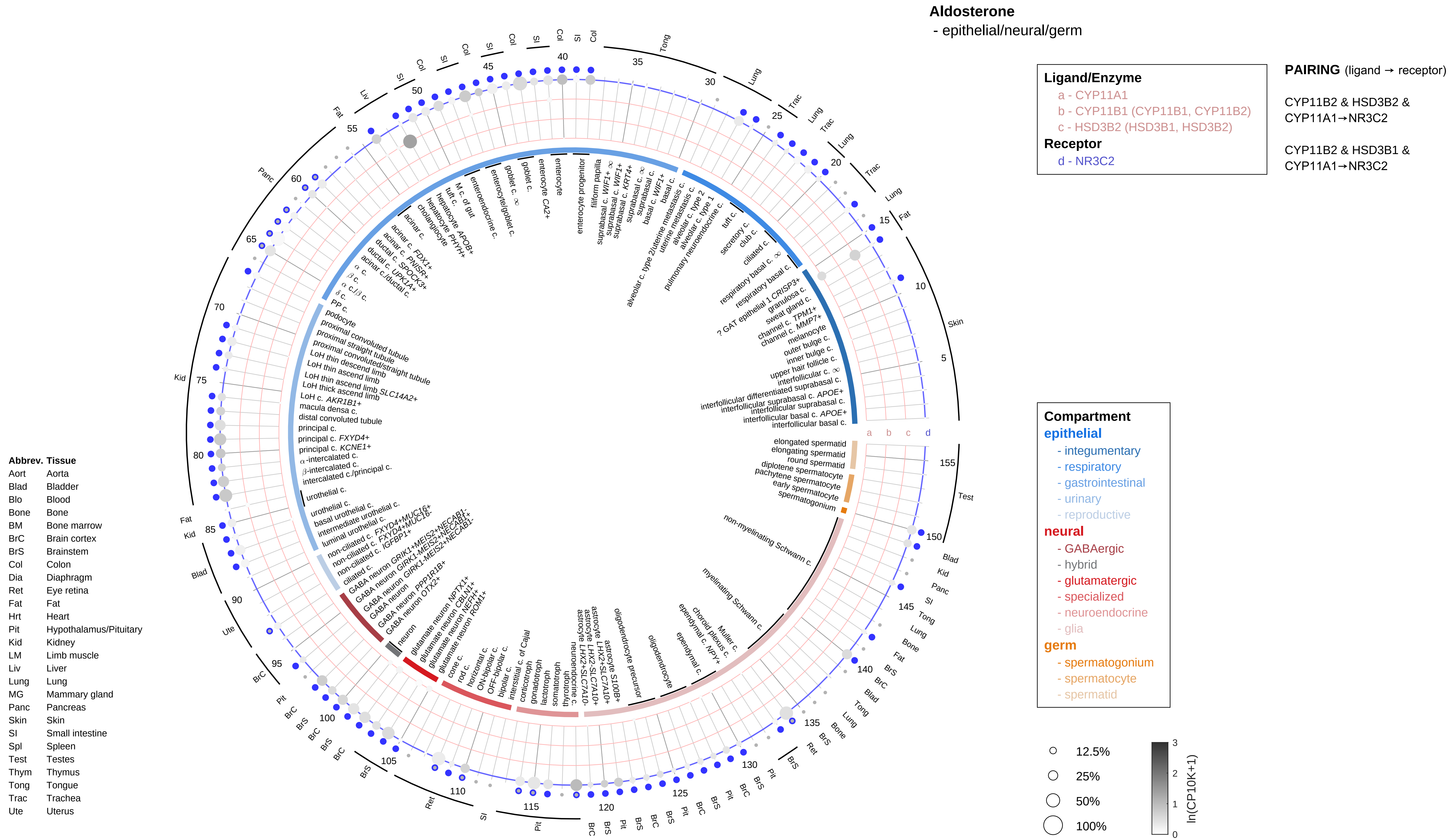
Aldosterone
- epithelial/neural/germ
Ligand/Enzyme
a - CYP11A1
b - CYP11B1 (CYP11B1, CYP11B2)
c - HSD3B2 (HSD3B1, HSD3B2)
Receptor
d - NR3C2
PAIRING (ligand → receptor)
CYP11B2 & HSD3B2 & CYP11A1→NR3C2
CYP11B2 & HSD3B1 & CYP11A1→NR3C2
Compartment
epithelial
- integumentary
- respiratory
- gastrointestinal
- urinary
- reproductive
neural
- GABAergic
- hybrid
- glutamatergic
- specialized
- neuroendocrine
- glia
germ
- spermatogonium
- spermatocyte
- spermatid
12.5%
25%
50%
100%
ln(CP10K+1)
Abbrev. Tissue
Aort Aorta
Blad Bladder
Blo Blood
Bone Bone
BM Bone marrow
BrC Brain cortex
BrS Brainstem
Col Colon
Dia Diaphragm
Ret Eye retina
Fat Fat
Hrt Heart
Pit Hypothalamus/Pituitary
Kid Kidney
LM Limb muscle
Liv Liver
Lung Lung
MG Mammary gland
Panc Pancreas
Skin Skin
SI Small intestine
Spl Spleen
Test Testes
Thym Thymus
Tong Tongue
Trac Trachea
Ute Uterus

Aldosterone
- endothelial

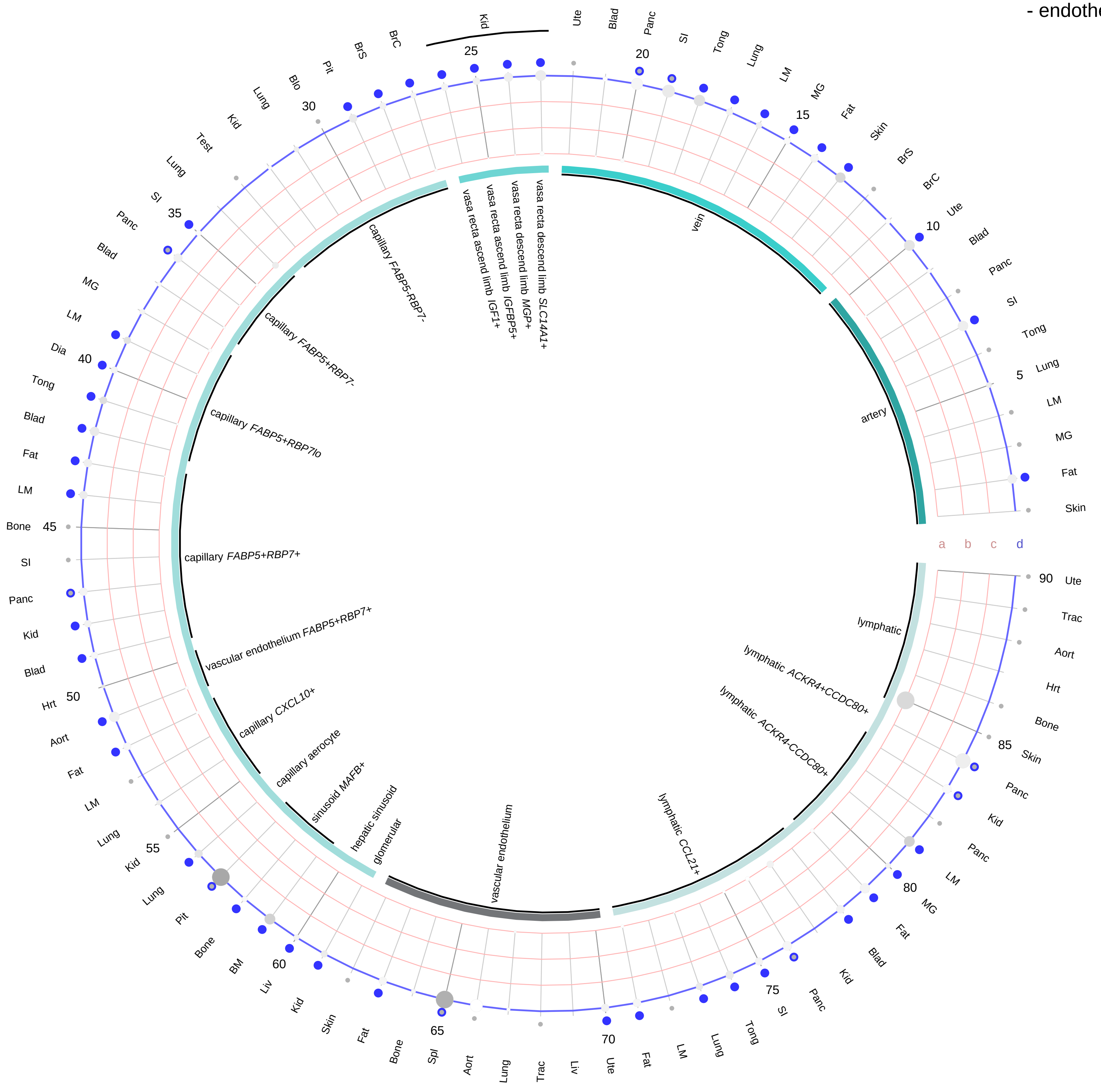

Ligand/Enzyme
- a - CYP11A1
- b - CYP11B1 (CYP11B1, CYP11B2)
- c - HSD3B2 (HSD3B1, HSD3B2)

Receptor
- d - NR3C2

PAIRING (ligand → receptor)

CYP11B2 & HSD3B2 & CYP11A1→NR3C2

CYP11B2 & HSD3B1 & CYP11A1→NR3C2

Abbrev.	Tissue
Aort	Aorta
Blad	Bladder
Blo	Blood
Bone	Bone
BM	Bone marrow
BrC	Brain cortex
BrS	Brainstem
Col	Colon
Dia	Diaphragm
Ret	Eye retina
Fat	Fat
Hrt	Heart
Pit	Hypothalamus/Pituitary
Kid	Kidney
LM	Limb muscle
Liv	Liver
Lung	Lung
MG	Mammary gland
Panc	Pancreas
Skin	Skin
SI	Small intestine
Spl	Spleen
Test	Testes
Thym	Thymus
Tong	Tongue
Trac	Trachea
Ute	Uterus

Compartment
endothelial
- artery
- vein
- vasa recta
- capillary
- mix
- lymphatic

12.5%
25%
50%
100%

ln(CP10K+1): 0 1 2 3

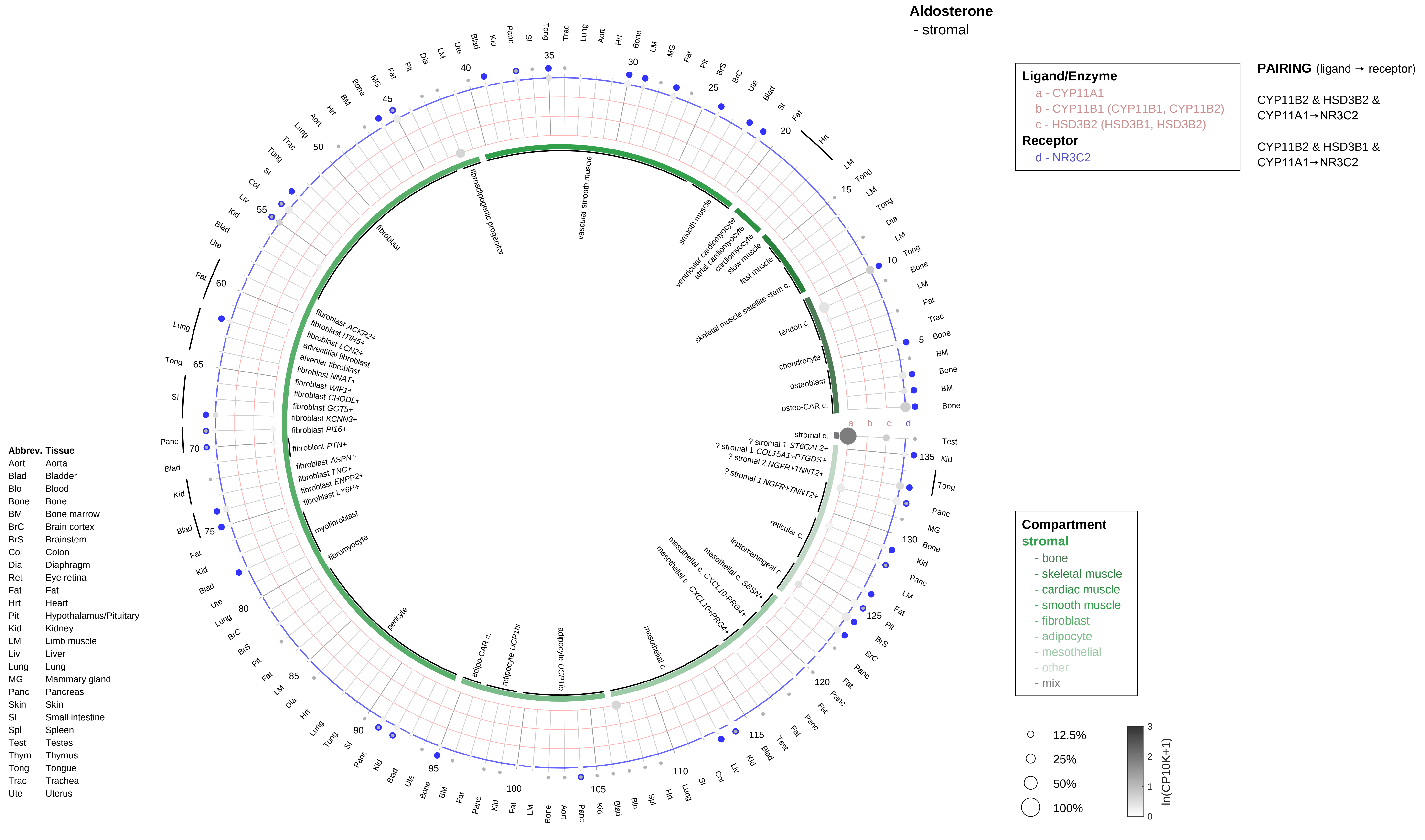

Aldosterone
- stromal
Ligand/Enzyme
a - CYP11A1
b - CYP11B1 (CYP11B1, CYP11B2)
c - HSD3B2 (HSD3B1, HSD3B2)
Receptor
d - NR3C2
PAIRING (ligand → receptor)
CYP11B2 & HSD3B2 & CYP11A1→NR3C2
CYP11B2 & HSD3B1 & CYP11A1→NR3C2
Compartment
stromal
- bone
- skeletal muscle
- cardiac muscle
- smooth muscle
- fibroblast
- adipocyte
- mesothelial
- other
- mix
12.5%
25%
50%
100%
ln(CP10K+1)
0
1
2
3
Abbrev. Tissue
Aort Aorta
Blad Bladder
Blo Blood
Bone Bone
BM Bone marrow
BrC Brain cortex
BrS Brainstem
Col Colon
Dia Diaphragm
Ret Eye retina
Fat Fat
Hrt Heart
Pit Hypothalamus/Pituitary
Kid Kidney
LM Limb muscle
Liv Liver
Lung Lung
MG Mammary gland
Panc Pancreas
Skin Skin
SI Small intestine
Spl Spleen
Test Testes
Thym Thymus
Tong Tongue
Trac Trachea
Ute Uterus
fibroadipogenic progenitor
vascular smooth muscle
smooth muscle
ventricular cardiomyocyte
atrial cardiomyocyte
cardiomyocyte
slow muscle
fast muscle
skeletal muscle satellite stem c.
tendon c.
chondrocyte
osteoblast
osteo-CAR c.
stromal c.
? stromal 1 ST6GAL2+
? stromal 1 COL15A1+PTGDS+
? stromal 2 NGFR+TNNT2+
? stromal 1 NGFR+TNNT2+
reticular c.
leptomeningeal c.
mesothelial c. SBSN+
mesothelial c. CXCL10-PRG4+
mesothelial c. CXCL10+PRG4+
mesothelial c.
adipocyte UCP1lo
adipocyte UCP1hi
adipo-CAR c.
pericyte
fibromyocyte
myofibroblast
fibroblast LY6H+
fibroblast ENPP2+
fibroblast TNC+
fibroblast ASPN+
fibroblast PTN+
fibroblast PI16+
fibroblast KCNN3+
fibroblast GGT5+
fibroblast CHODL+
fibroblast WIF1+
fibroblast NNAT+
alveolar fibroblast
adventitial fibroblast
fibroblast LCN2+
fibroblast ITIH5+
fibroblast ACKR2+
fibroblast
a b c d

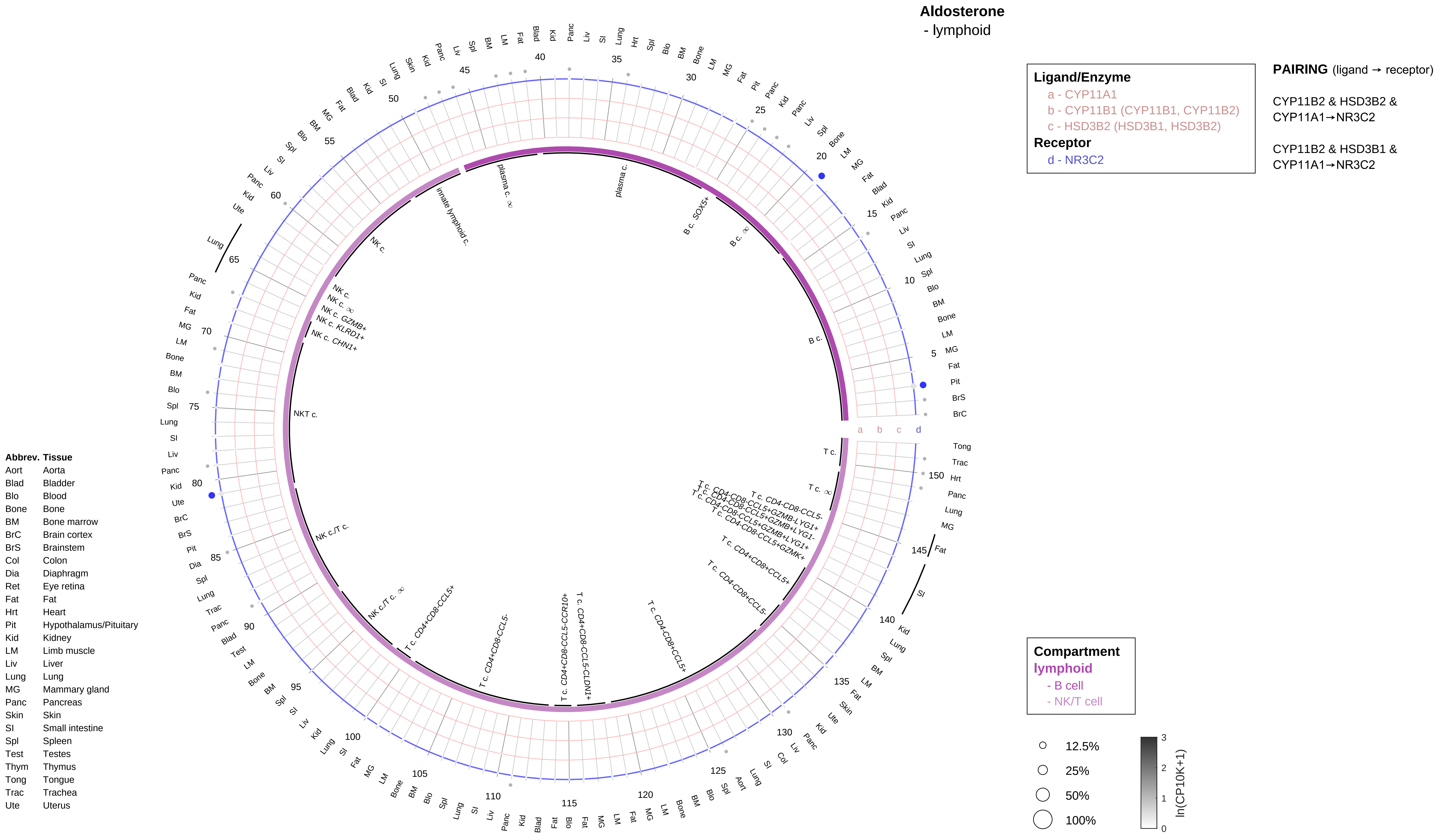
Aldosterone
- lymphoid
Ligand/Enzyme
a - CYP11A1
b - CYP11B1 (CYP11B1, CYP11B2)
c - HSD3B2 (HSD3B1, HSD3B2)
Receptor
d - NR3C2
PAIRING (ligand → receptor)
CYP11B2 & HSD3B2 & CYP11A1→NR3C2
CYP11B2 & HSD3B1 & CYP11A1→NR3C2
Compartment
lymphoid
- B cell
- NK/T cell
12.5%
25%
50%
100%
ln(CP10K+1)
Abbrev. Tissue
Aort Aorta
Blad Bladder
Blo Blood
Bone Bone
BM Bone marrow
BrC Brain cortex
BrS Brainstem
Col Colon
Dia Diaphragm
Ret Eye retina
Fat Fat
Hrt Heart
Pit Hypothalamus/Pituitary
Kid Kidney
LM Limb muscle
Liv Liver
Lung Lung
MG Mammary gland
Panc Pancreas
Skin Skin
SI Small intestine
Spl Spleen
Test Testes
Thym Thymus
Tong Tongue
Trac Trachea
Ute Uterus

Aldosterone
- myeloid

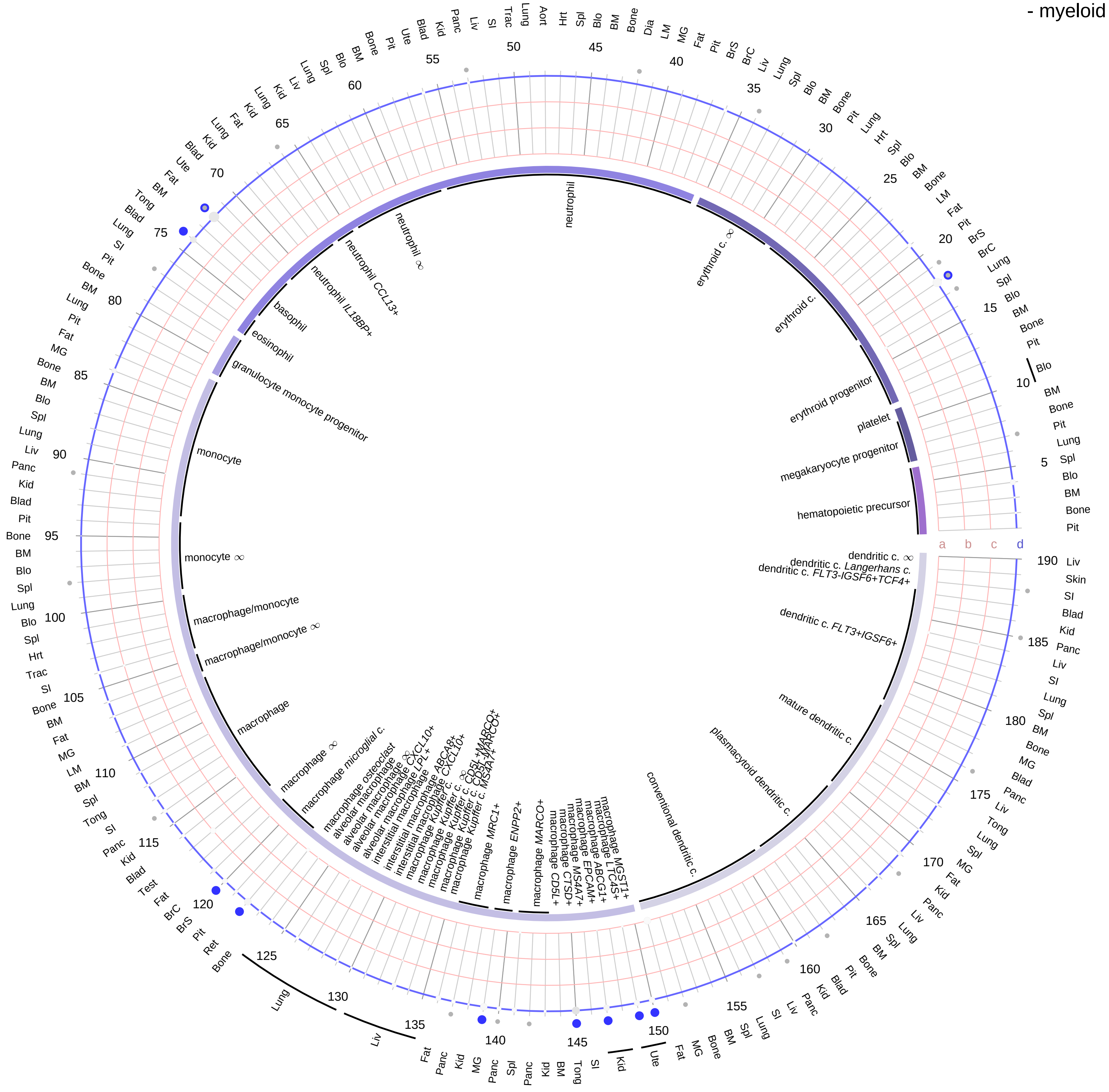

**Ligand/Enzyme**
a - CYP11A1
b - CYP11B1 (CYP11B1, CYP11B2)
c - HSD3B2 (HSD3B1, HSD3B2)
**Receptor**
d - NR3C2

PAIRING (ligand → receptor)

CYP11B2 & HSD3B2 & CYP11A1→NR3C2

CYP11B2 & HSD3B1 & CYP11A1→NR3C2

**Abbrev.**	**Tissue**
Aort	Aorta
Blad	Bladder
Blo	Blood
Bone	Bone
BM	Bone marrow
BrC	Brain cortex
BrS	Brainstem
Col	Colon
Dia	Diaphragm
Ret	Eye retina
Fat	Fat
Hrt	Heart
Pit	Hypothalamus/Pituitary
Kid	Kidney
LM	Limb muscle
Liv	Liver
Lung	Lung
MG	Mammary gland
Panc	Pancreas
Skin	Skin
SI	Small intestine
Spl	Spleen
Test	Testes
Thym	Thymus
Tong	Tongue
Trac	Trachea
Ute	Uterus

**Compartment**
**hematopoietic**
- hematopoietic precursor
**megakaryocyte-erythroid**
- megakaryoid
- erythroid
**myeloid**
- granulocyte
- granulocyte monocyte progenitor
- monocyte/macrophage
- dendritic

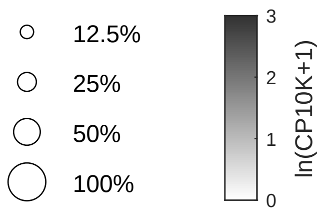

Amylin/IAPP
- epithelial/neural/germ

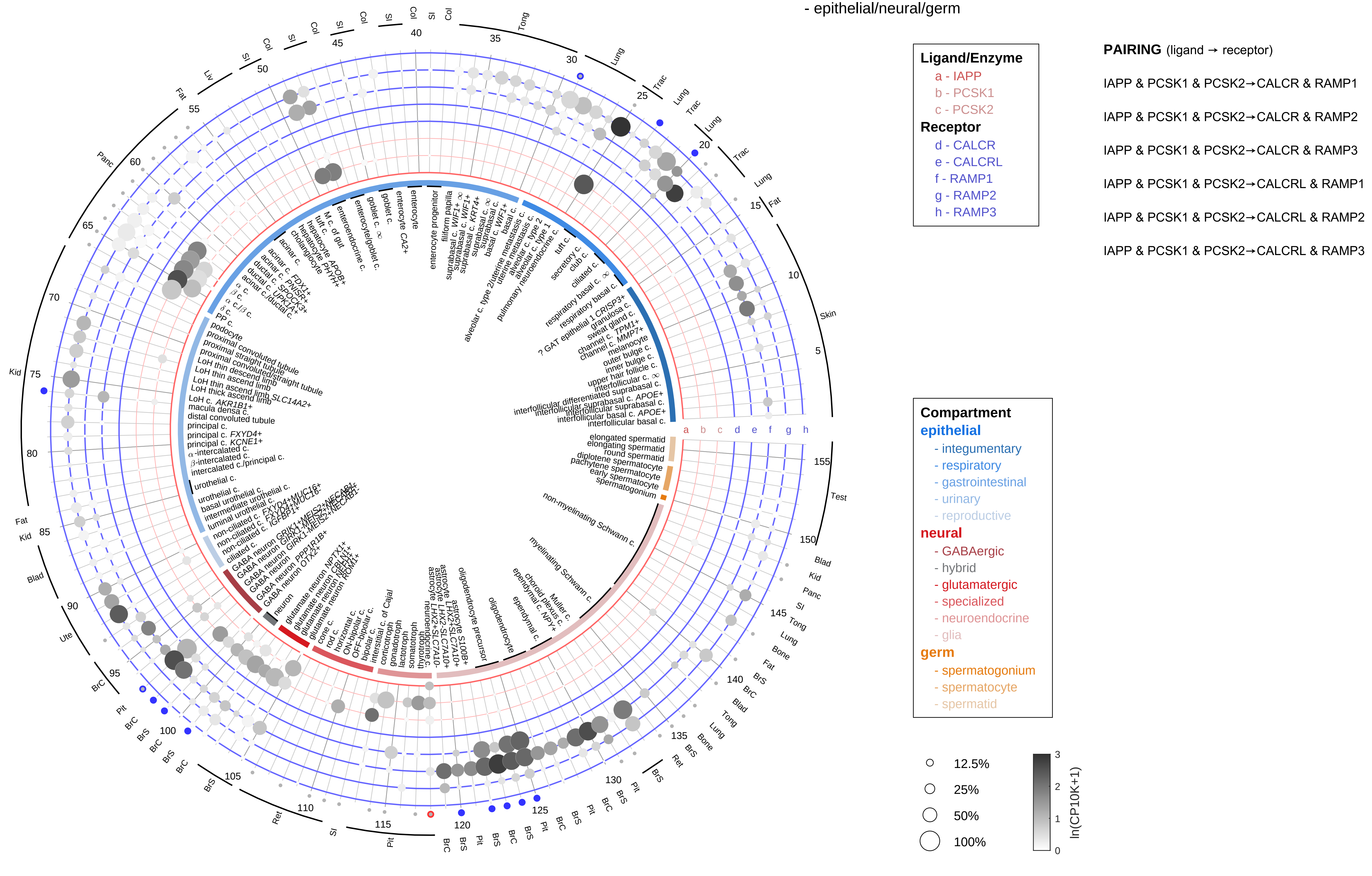

Abbrev.	Tissue
Aort	Aorta
Blad	Bladder
Blo	Blood
Bone	Bone
BM	Bone marrow
BrC	Brain cortex
BrS	Brainstem
Col	Colon
Dia	Diaphragm
Ret	Eye retina
Fat	Fat
Hrt	Heart
Pit	Hypothalamus/Pituitary
Kid	Kidney
LM	Limb muscle
Liv	Liver
Lung	Lung
MG	Mammary gland
Panc	Pancreas
Skin	Skin
SI	Small intestine
Spl	Spleen
Test	Testes
Thym	Thymus
Tong	Tongue
Trac	Trachea
Ute	Uterus

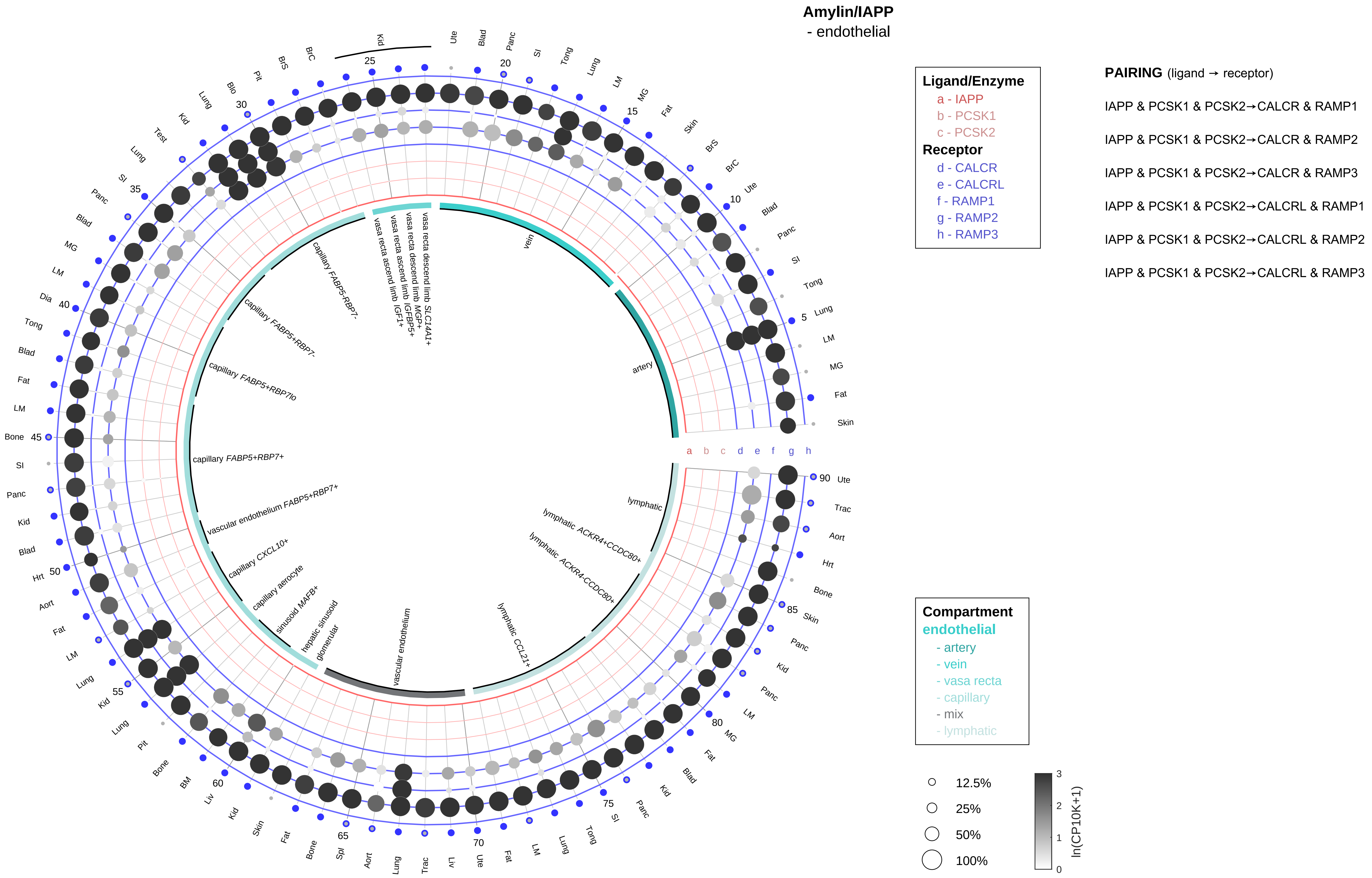

Abbrev.	Tissue
Aort	Aorta
Blad	Bladder
Blo	Blood
Bone	Bone
BM	Bone marrow
BrC	Brain cortex
BrS	Brainstem
Col	Colon
Dia	Diaphragm
Ret	Eye retina
Fat	Fat
Hrt	Heart
Pit	Hypothalamus/Pituitary
Kid	Kidney
LM	Limb muscle
Liv	Liver
Lung	Lung
MG	Mammary gland
Panc	Pancreas
Skin	Skin
SI	Small intestine
Spl	Spleen
Test	Testes
Thym	Thymus
Tong	Tongue
Trac	Trachea
Ute	Uterus

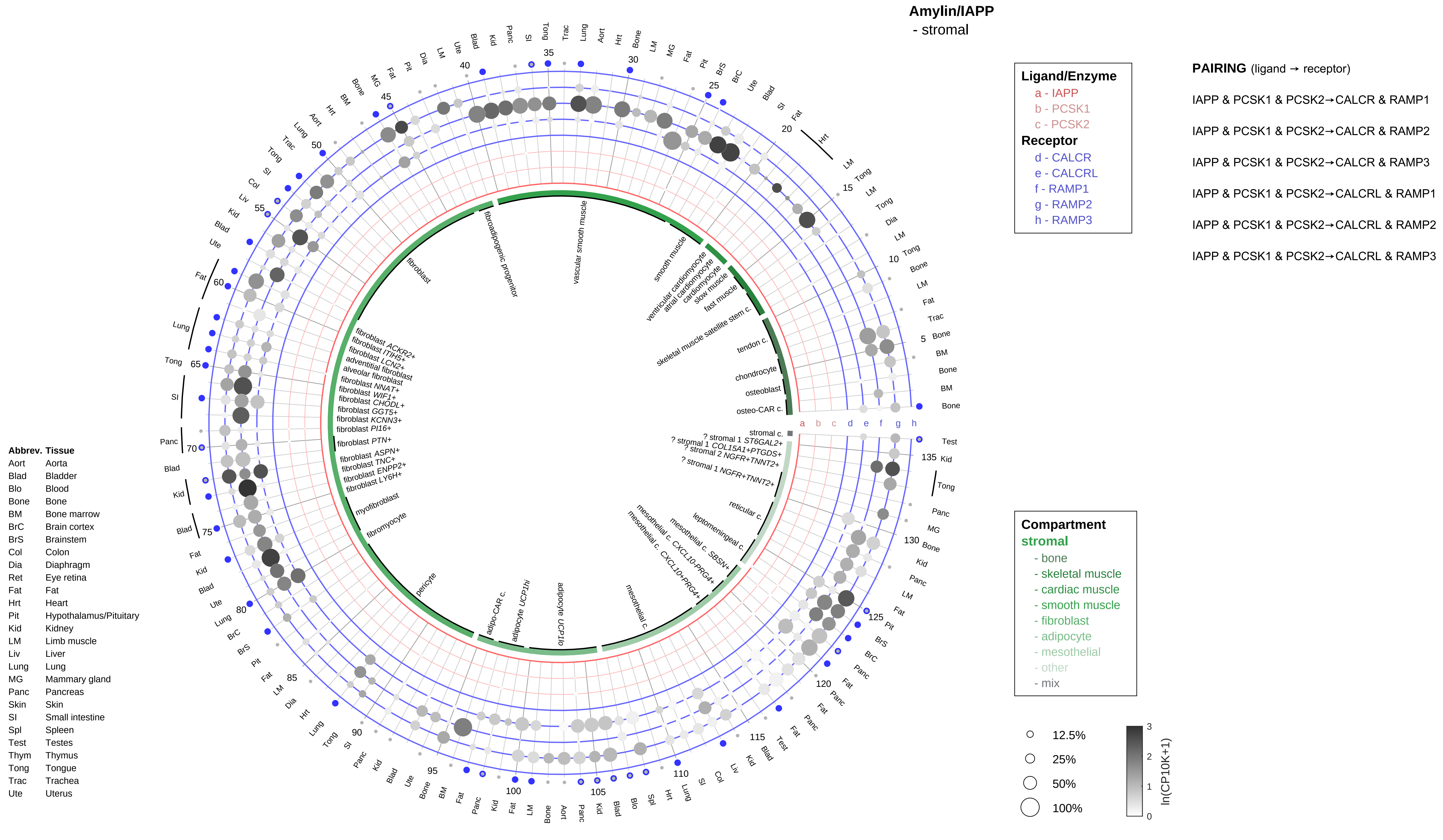
Amylin/IAPP
- stromal
Ligand/Enzyme
a - IAPP
b - PCSK1
c - PCSK2
Receptor
d - CALCR
e - CALCRL
f - RAMP1
g - RAMP2
h - RAMP3
PAIRING (ligand → receptor)
IAPP & PCSK1 & PCSK2→CALCR & RAMP1
IAPP & PCSK1 & PCSK2→CALCR & RAMP2
IAPP & PCSK1 & PCSK2→CALCR & RAMP3
IAPP & PCSK1 & PCSK2→CALCRL & RAMP1
IAPP & PCSK1 & PCSK2→CALCRL & RAMP2
IAPP & PCSK1 & PCSK2→CALCRL & RAMP3
Compartment
stromal
- bone
- skeletal muscle
- cardiac muscle
- smooth muscle
- fibroblast
- adipocyte
- mesothelial
- other
- mix
12.5%
25%
50%
100%
ln(CP10K+1)
0
1
2
3
Abbrev. Tissue
Aort Aorta
Blad Bladder
Blo Blood
Bone Bone
BM Bone marrow
BrC Brain cortex
BrS Brainstem
Col Colon
Dia Diaphragm
Ret Eye retina
Fat Fat
Hrt Heart
Pit Hypothalamus/Pituitary
Kid Kidney
LM Limb muscle
Liv Liver
Lung Lung
MG Mammary gland
Panc Pancreas
Skin Skin
SI Small intestine
Spl Spleen
Test Testes
Thym Thymus
Tong Tongue
Trac Trachea
Ute Uterus

Amylin/IAPP
- lymphoid

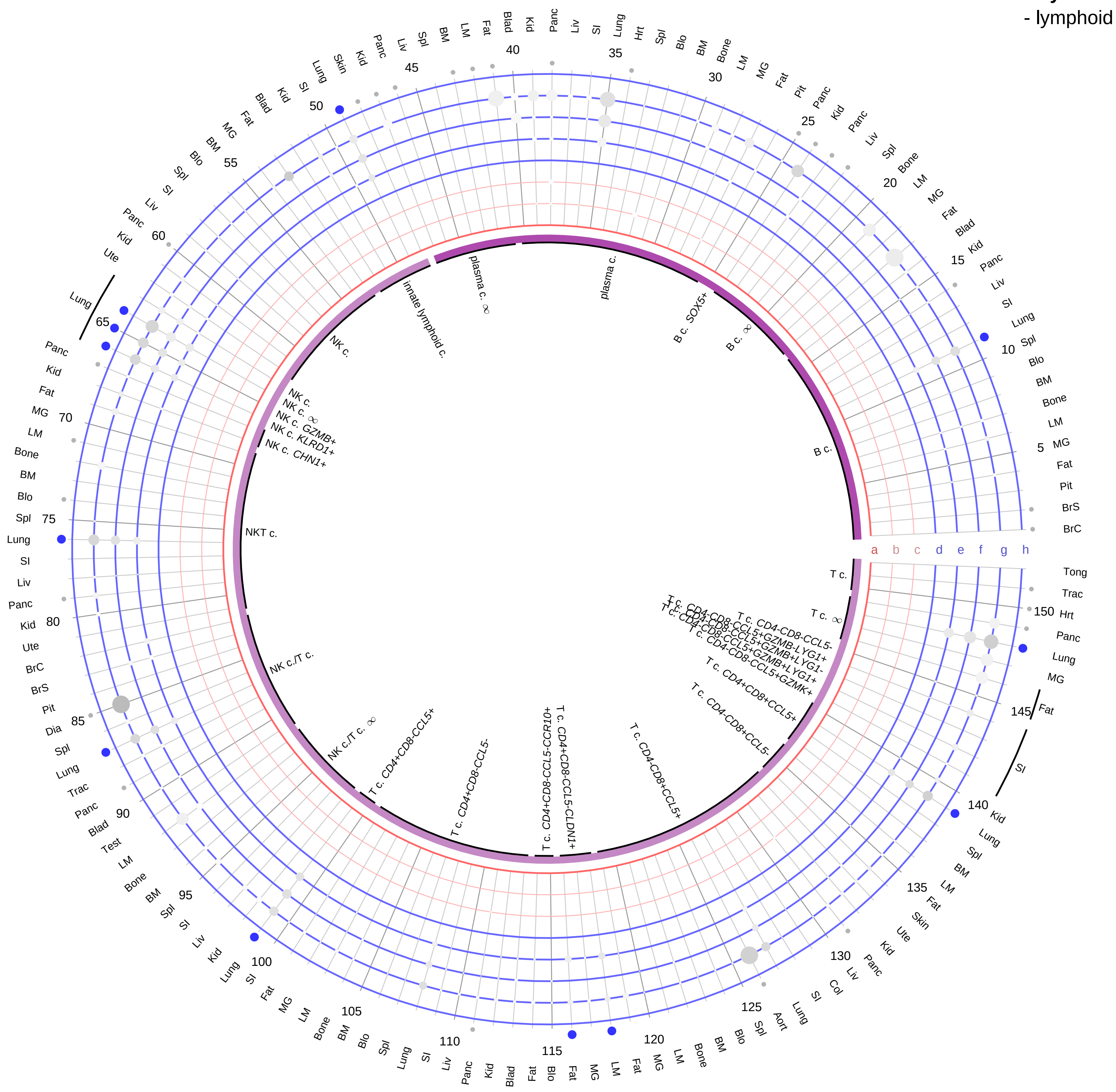

Ligand/Enzyme
- a - IAPP
- b - PCSK1
- c - PCSK2

Receptor
- d - CALCR
- e - CALCRL
- f - RAMP1
- g - RAMP2
- h - RAMP3

PAIRING (ligand → receptor)

IAPP & PCSK1 & PCSK2→CALCR & RAMP1

IAPP & PCSK1 & PCSK2→CALCR & RAMP2

IAPP & PCSK1 & PCSK2→CALCR & RAMP3

IAPP & PCSK1 & PCSK2→CALCRL & RAMP1

IAPP & PCSK1 & PCSK2→CALCRL & RAMP2

IAPP & PCSK1 & PCSK2→CALCRL & RAMP3

Compartment

lymphoid
- B cell
- NK/T cell

12.5%
25%
50%
100%

ln(CP10K+1): 0 1 2 3

Abbrev.	Tissue
Aort	Aorta
Blad	Bladder
Blo	Blood
Bone	Bone
BM	Bone marrow
BrC	Brain cortex
BrS	Brainstem
Col	Colon
Dia	Diaphragm
Ret	Eye retina
Fat	Fat
Hrt	Heart
Pit	Hypothalamus/Pituitary
Kid	Kidney
LM	Limb muscle
Liv	Liver
Lung	Lung
MG	Mammary gland
Panc	Pancreas
Skin	Skin
SI	Small intestine
Spl	Spleen
Test	Testes
Thym	Thymus
Tong	Tongue
Trac	Trachea
Ute	Uterus

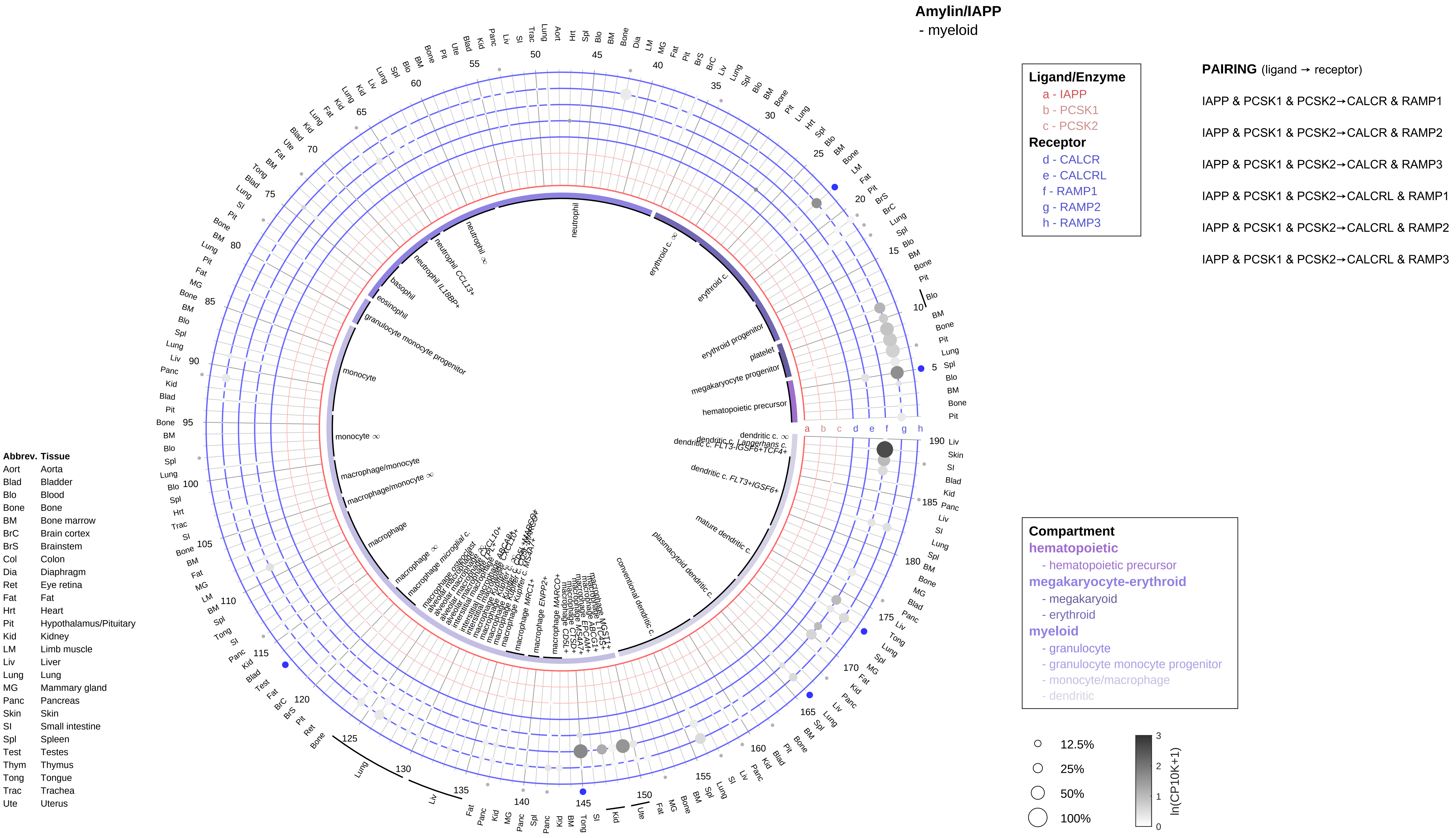

Amylin/IAPP
- myeloid
Ligand/Enzyme
a - IAPP
b - PCSK1
c - PCSK2
Receptor
d - CALCR
e - CALCRL
f - RAMP1
g - RAMP2
h - RAMP3
PAIRING (ligand → receptor)
IAPP & PCSK1 & PCSK2→CALCR & RAMP1
IAPP & PCSK1 & PCSK2→CALCR & RAMP2
IAPP & PCSK1 & PCSK2→CALCR & RAMP3
IAPP & PCSK1 & PCSK2→CALCRL & RAMP1
IAPP & PCSK1 & PCSK2→CALCRL & RAMP2
IAPP & PCSK1 & PCSK2→CALCRL & RAMP3
Abbrev. Tissue
Aort Aorta
Blad Bladder
Blo Blood
Bone Bone
BM Bone marrow
BrC Brain cortex
BrS Brainstem
Col Colon
Dia Diaphragm
Ret Eye retina
Fat Fat
Hrt Heart
Pit Hypothalamus/Pituitary
Kid Kidney
LM Limb muscle
Liv Liver
Lung Lung
MG Mammary gland
Panc Pancreas
Skin Skin
SI Small intestine
Spl Spleen
Test Testes
Thym Thymus
Tong Tongue
Trac Trachea
Ute Uterus
Compartment
hematopoietic
- hematopoietic precursor
megakaryocyte-erythroid
- megakaryoid
- erythroid
myeloid
- granulocyte
- granulocyte monocyte progenitor
- monocyte/macrophage
- dendritic
12.5%
25%
50%
100%
ln(CP10K+1)
0
1
2
3

Androgen
- epithelial/neural/germ

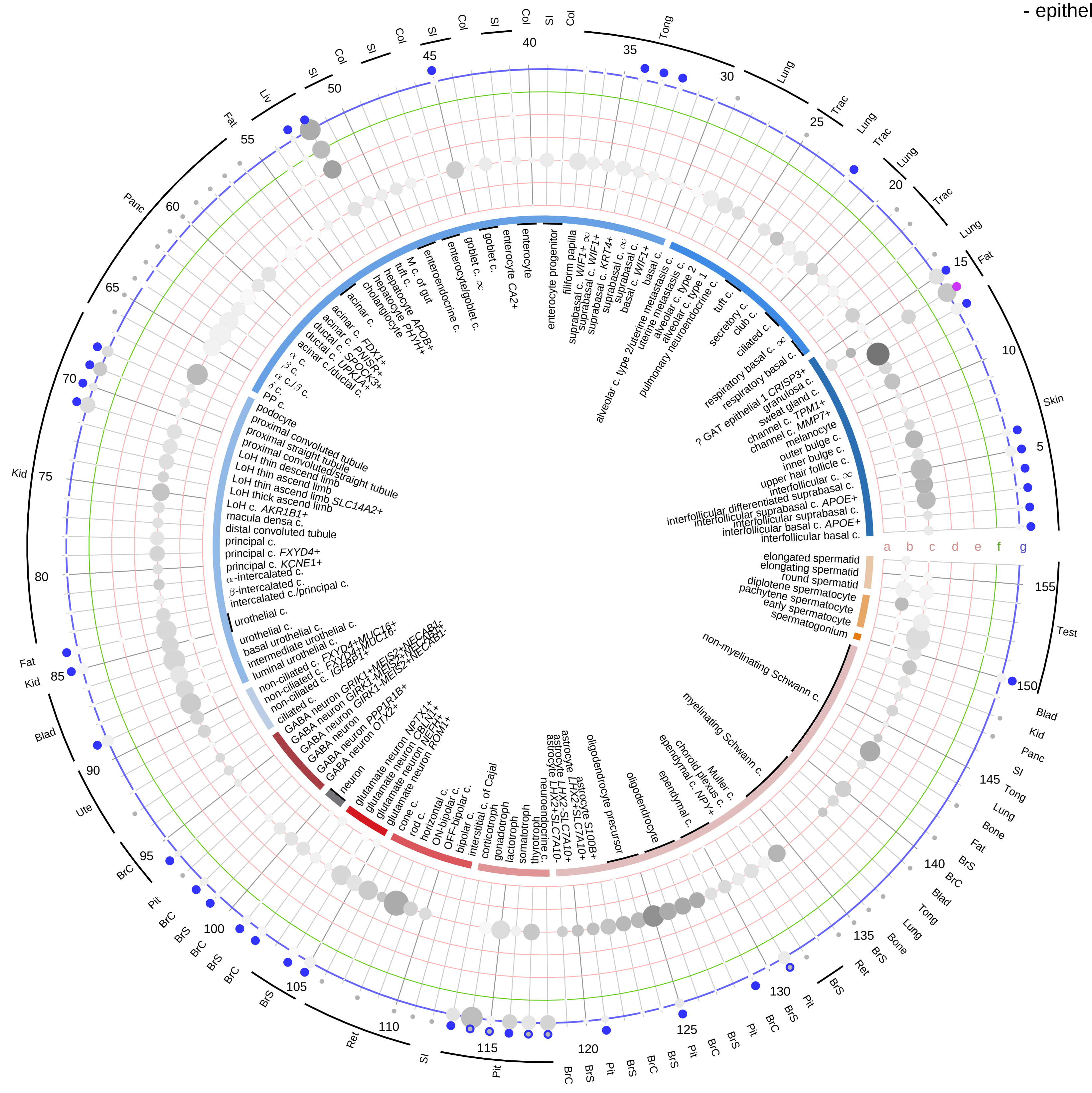

Ligand/Enzyme
- a - CYP11A1
- b - CYP17A1
- c - HSD17B12
- d - HSD17B3
- e - HSD3B2 (HSD3B1, HSD3B2)

Modulator
- f - SHBG

Receptor
- g - AR

PAIRING (ligand → receptor)

HSD3B2 & CYP17A1 & HSD17B12 & CYP11A1→AR

HSD3B1 & CYP17A1 & HSD17B12 & CYP11A1→AR

HSD3B2 & CYP17A1 & HSD17B3 & CYP11A1→AR

HSD3B1 & CYP17A1 & HSD17B3 & CYP11A1→AR

Compartment

epithelial
- integumentary
- respiratory
- gastrointestinal
- urinary
- reproductive

neural
- GABAergic
- hybrid
- glutamatergic
- specialized
- neuroendocrine
- glia

germ
- spermatogonium
- spermatocyte
- spermatid

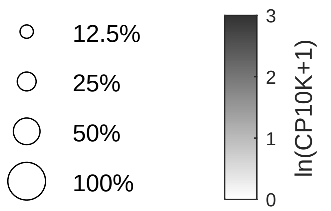

Abbrev.	Tissue
Aort	Aorta
Blad	Bladder
Blo	Blood
Bone	Bone
BM	Bone marrow
BrC	Brain cortex
BrS	Brainstem
Col	Colon
Dia	Diaphragm
Ret	Eye retina
Fat	Fat
Hrt	Heart
Pit	Hypothalamus/Pituitary
Kid	Kidney
LM	Limb muscle
Liv	Liver
Lung	Lung
MG	Mammary gland
Panc	Pancreas
Skin	Skin
SI	Small intestine
Spl	Spleen
Test	Testes
Thym	Thymus
Tong	Tongue
Trac	Trachea
Ute	Uterus

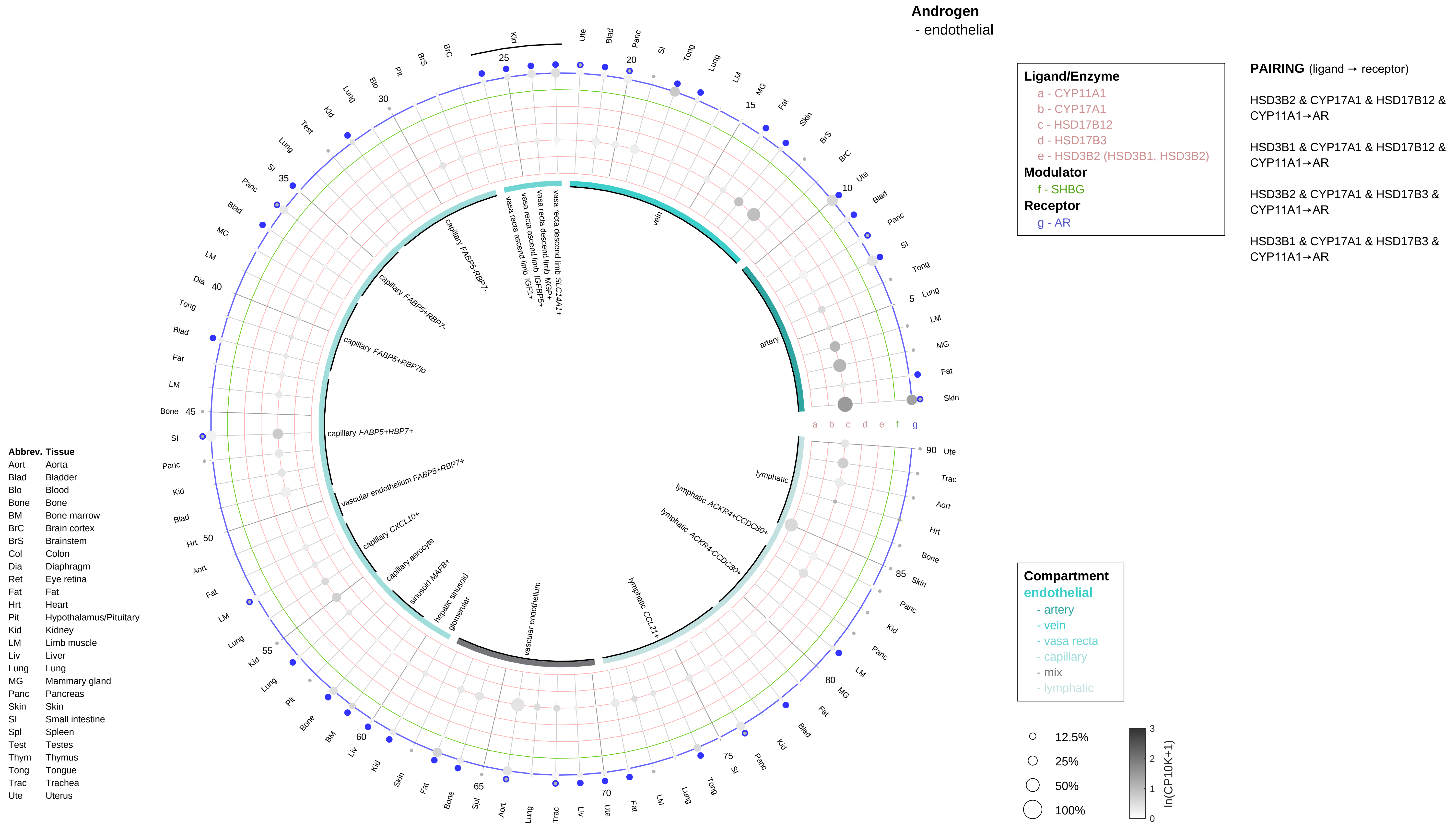

Androgen
- endothelial
Ligand/Enzyme
a - CYP11A1
b - CYP17A1
c - HSD17B12
d - HSD17B3
e - HSD3B2 (HSD3B1, HSD3B2)
Modulator
f - SHBG
Receptor
g - AR
PAIRING (ligand → receptor)
HSD3B2 & CYP17A1 & HSD17B12 & CYP11A1→AR
HSD3B1 & CYP17A1 & HSD17B12 & CYP11A1→AR
HSD3B2 & CYP17A1 & HSD17B3 & CYP11A1→AR
HSD3B1 & CYP17A1 & HSD17B3 & CYP11A1→AR
artery
vein
vasa recta descend limb SLC14A1+
vasa recta descend limb MGP+
vasa recta ascend limb IGFBP5+
vasa recta ascend limb IGF1+
capillary FABP5-RBP7-
capillary FABP5+RBP7-
capillary FABP5+RBP7lo
capillary FABP5+RBP7+
vascular endothelium FABP5+RBP7+
capillary CXCL10+
capillary aerocyte
sinusoid MAFB+
hepatic sinusoid
glomerular
vascular endothelium
lymphatic CCL21+
lymphatic ACKR4-CCDC80+
lymphatic ACKR4+CCDC80+
lymphatic
Abbrev. Tissue
Aort Aorta
Blad Bladder
Blo Blood
Bone Bone
BM Bone marrow
BrC Brain cortex
BrS Brainstem
Col Colon
Dia Diaphragm
Ret Eye retina
Fat Fat
Hrt Heart
Pit Hypothalamus/Pituitary
Kid Kidney
LM Limb muscle
Liv Liver
Lung Lung
MG Mammary gland
Panc Pancreas
Skin Skin
SI Small intestine
Spl Spleen
Test Testes
Thym Thymus
Tong Tongue
Trac Trachea
Ute Uterus
Compartment
endothelial
- artery
- vein
- vasa recta
- capillary
- mix
- lymphatic
12.5%
25%
50%
100%
ln(CP10K+1)

Androgen
- stromal

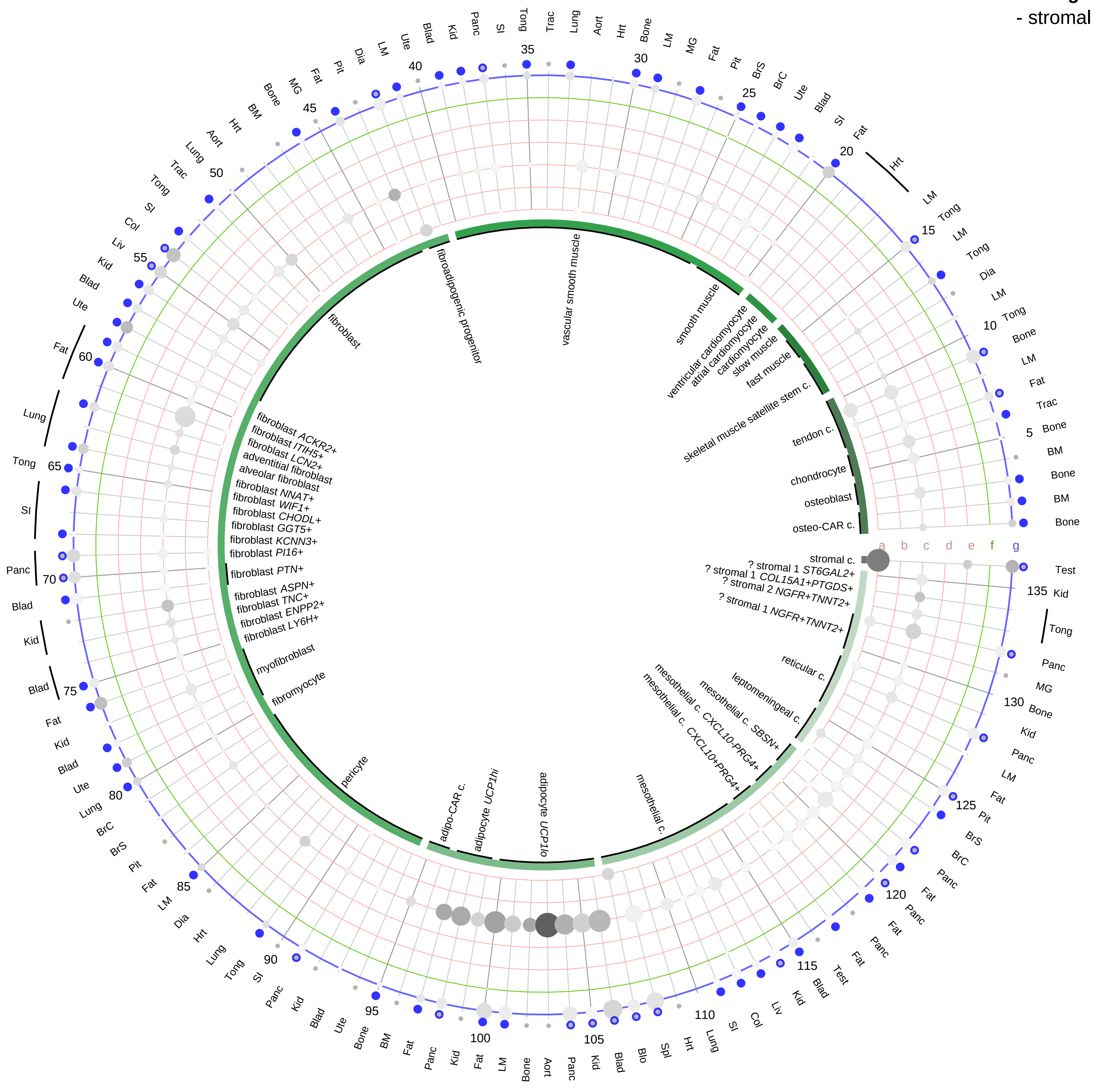

Ligand/Enzyme
- a - CYP11A1
- b - CYP17A1
- c - HSD17B12
- d - HSD17B3
- e - HSD3B2 (HSD3B1, HSD3B2)

Modulator
- f - SHBG

Receptor
- g - AR

PAIRING (ligand → receptor)

HSD3B2 & CYP17A1 & HSD17B12 & CYP11A1→AR

HSD3B1 & CYP17A1 & HSD17B12 & CYP11A1→AR

HSD3B2 & CYP17A1 & HSD17B3 & CYP11A1→AR

HSD3B1 & CYP17A1 & HSD17B3 & CYP11A1→AR

Abbrev.	Tissue
Aort	Aorta
Blad	Bladder
Blo	Blood
Bone	Bone
BM	Bone marrow
BrC	Brain cortex
BrS	Brainstem
Col	Colon
Dia	Diaphragm
Ret	Eye retina
Fat	Fat
Hrt	Heart
Pit	Hypothalamus/Pituitary
Kid	Kidney
LM	Limb muscle
Liv	Liver
Lung	Lung
MG	Mammary gland
Panc	Pancreas
Skin	Skin
SI	Small intestine
Spl	Spleen
Test	Testes
Thym	Thymus
Tong	Tongue
Trac	Trachea
Ute	Uterus

Compartment
stromal
- bone
- skeletal muscle
- cardiac muscle
- smooth muscle
- fibroblast
- adipocyte
- mesothelial
- other
- mix

12.5%
25%
50%
100%

ln(CP10K+1): 0 1 2 3

Androgen
- lymphoid

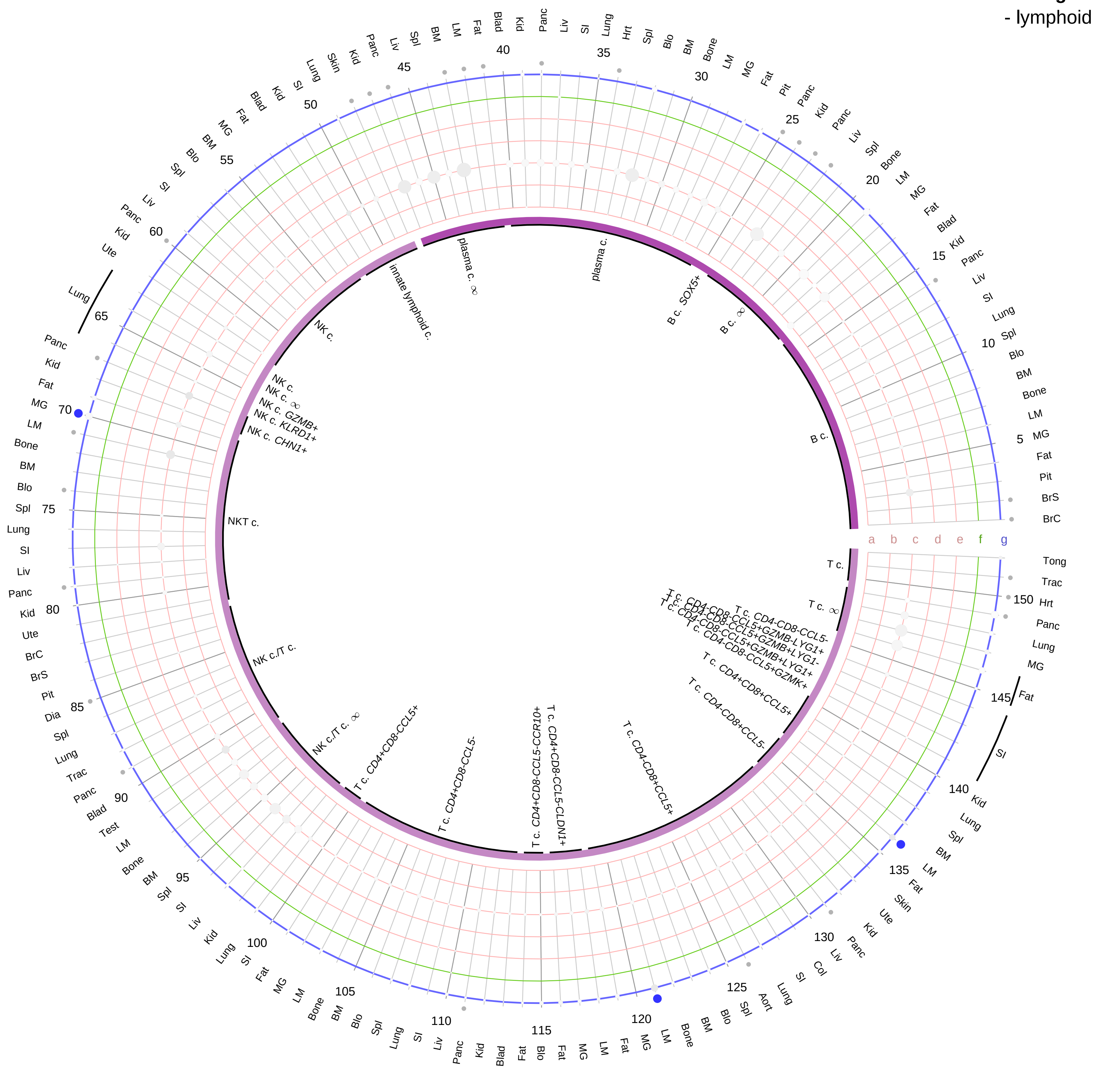

Ligand/Enzyme
- a - CYP11A1
- b - CYP17A1
- c - HSD17B12
- d - HSD17B3
- e - HSD3B2 (HSD3B1, HSD3B2)

Modulator
- f - SHBG

Receptor
- g - AR

PAIRING (ligand → receptor)

HSD3B2 & CYP17A1 & HSD17B12 & CYP11A1→AR

HSD3B1 & CYP17A1 & HSD17B12 & CYP11A1→AR

HSD3B2 & CYP17A1 & HSD17B3 & CYP11A1→AR

HSD3B1 & CYP17A1 & HSD17B3 & CYP11A1→AR

Compartment
lymphoid
- B cell
- NK/T cell

12.5% 25% 50% 100%

ln(CP10K+1) 0 1 2 3

Abbrev.	Tissue
Aort	Aorta
Blad	Bladder
Blo	Blood
Bone	Bone
BM	Bone marrow
BrC	Brain cortex
BrS	Brainstem
Col	Colon
Dia	Diaphragm
Ret	Eye retina
Fat	Fat
Hrt	Heart
Pit	Hypothalamus/Pituitary
Kid	Kidney
LM	Limb muscle
Liv	Liver
Lung	Lung
MG	Mammary gland
Panc	Pancreas
Skin	Skin
SI	Small intestine
Spl	Spleen
Test	Testes
Thym	Thymus
Tong	Tongue
Trac	Trachea
Ute	Uterus

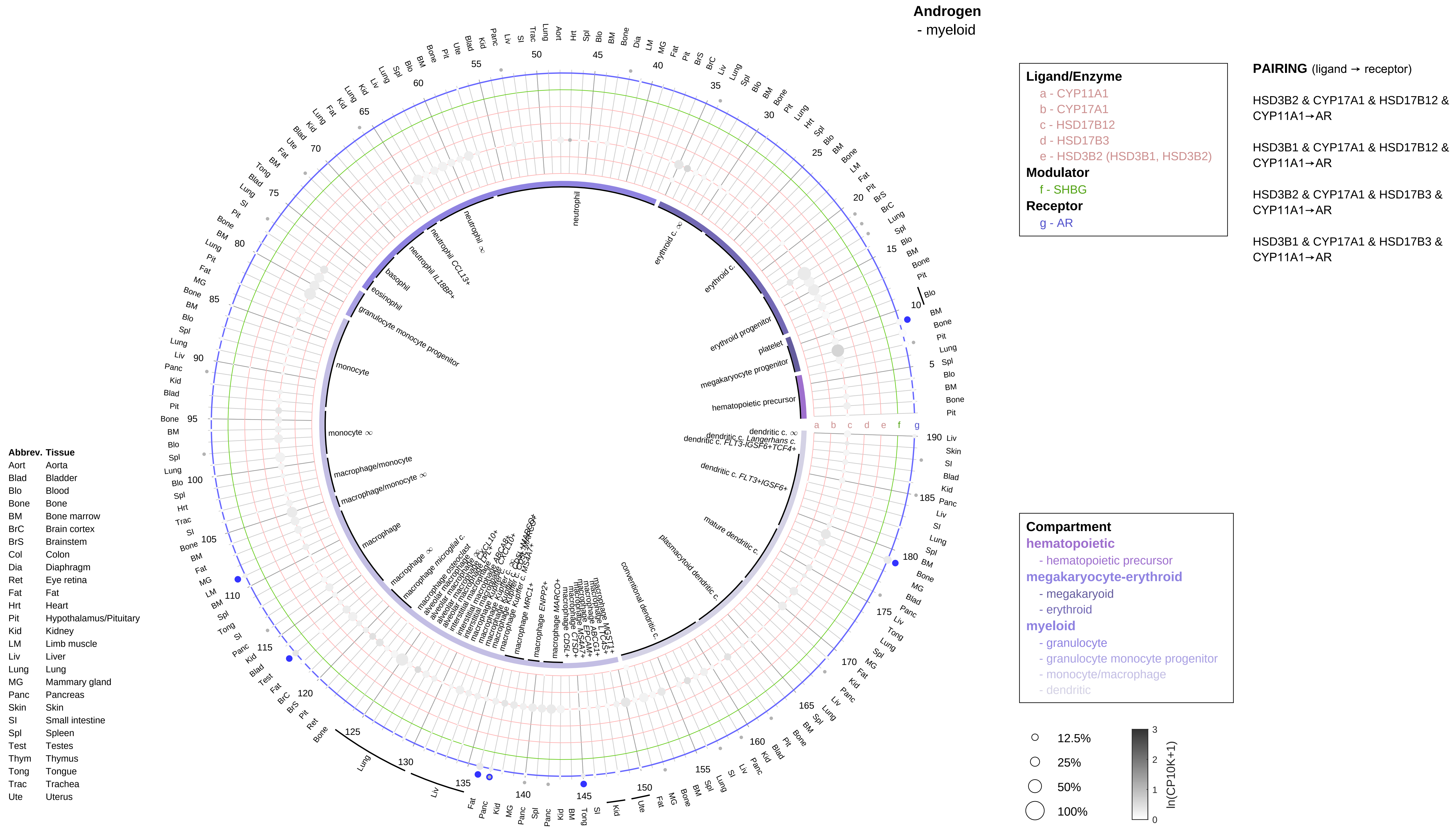

Androgen
- myeloid
Ligand/Enzyme
a - CYP11A1
b - CYP17A1
c - HSD17B12
d - HSD17B3
e - HSD3B2 (HSD3B1, HSD3B2)
Modulator
f - SHBG
Receptor
g - AR
PAIRING (ligand → receptor)
HSD3B2 & CYP17A1 & HSD17B12 & CYP11A1→AR
HSD3B1 & CYP17A1 & HSD17B12 & CYP11A1→AR
HSD3B2 & CYP17A1 & HSD17B3 & CYP11A1→AR
HSD3B1 & CYP17A1 & HSD17B3 & CYP11A1→AR
Compartment
hematopoietic
- hematopoietic precursor
megakaryocyte-erythroid
- megakaryoid
- erythroid
myeloid
- granulocyte
- granulocyte monocyte progenitor
- monocyte/macrophage
- dendritic
12.5%
25%
50%
100%
ln(CP10K+1)
Abbrev. Tissue
Aort Aorta
Blad Bladder
Blo Blood
Bone Bone
BM Bone marrow
BrC Brain cortex
BrS Brainstem
Col Colon
Dia Diaphragm
Ret Eye retina
Fat Fat
Hrt Heart
Pit Hypothalamus/Pituitary
Kid Kidney
LM Limb muscle
Liv Liver
Lung Lung
MG Mammary gland
Panc Pancreas
Skin Skin
SI Small intestine
Spl Spleen
Test Testes
Thym Thymus
Tong Tongue
Trac Trachea
Ute Uterus

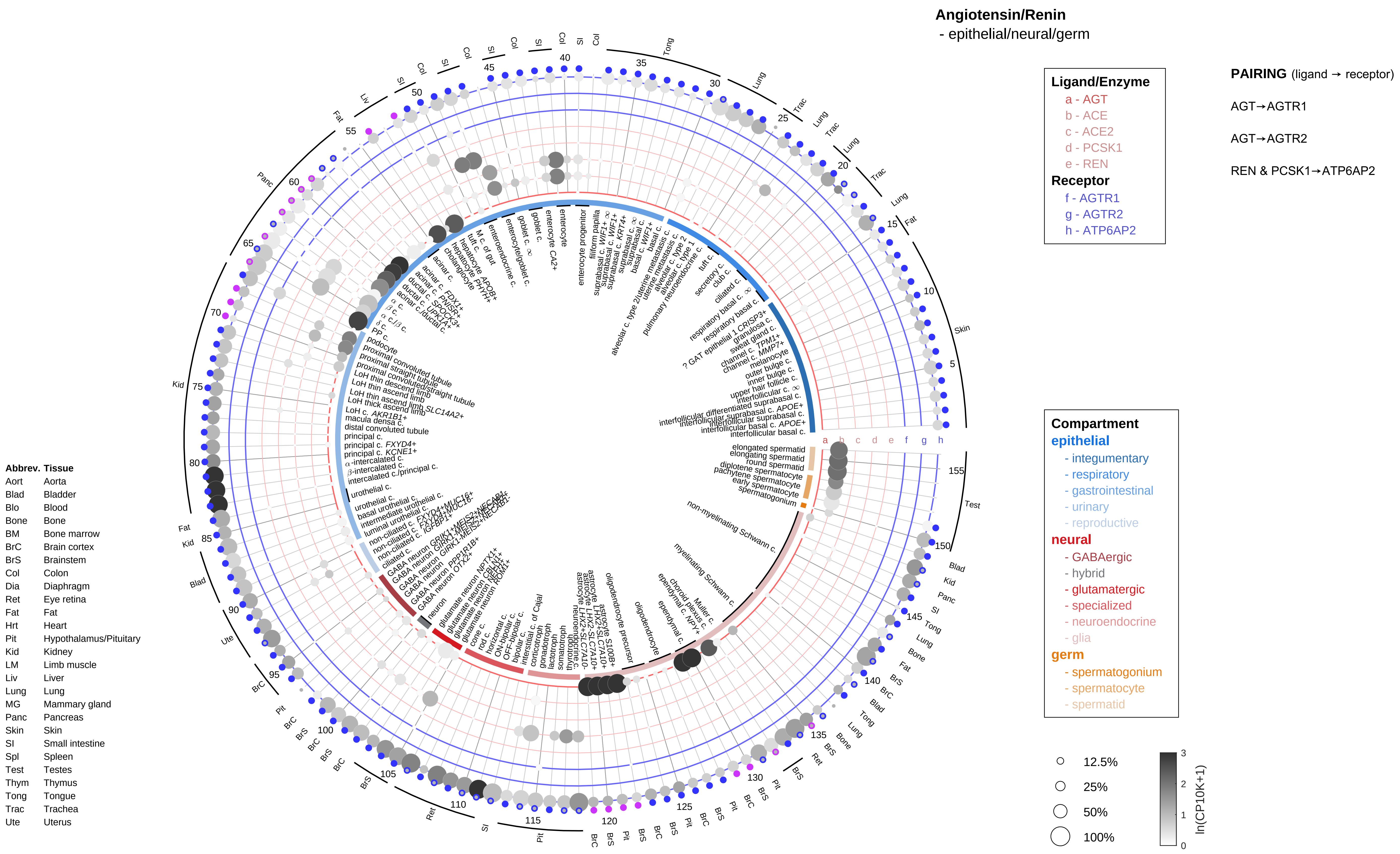
Angiotensin/Renin
- epithelial/neural/germ
Ligand/Enzyme
a - AGT
b - ACE
c - ACE2
d - PCSK1
e - REN
Receptor
f - AGTR1
g - AGTR2
h - ATP6AP2
PAIRING (ligand → receptor)
AGT→AGTR1
AGT→AGTR2
REN & PCSK1→ATP6AP2
Compartment
epithelial
- integumentary
- respiratory
- gastrointestinal
- urinary
- reproductive
neural
- GABAergic
- hybrid
- glutamatergic
- specialized
- neuroendocrine
- glia
germ
- spermatogonium
- spermatocyte
- spermatid
12.5%
25%
50%
100%
ln(CP10K+1)
0
1
2
3
Abbrev. Tissue
Aort Aorta
Blad Bladder
Blo Blood
Bone Bone
BM Bone marrow
BrC Brain cortex
BrS Brainstem
Col Colon
Dia Diaphragm
Ret Eye retina
Fat Fat
Hrt Heart
Pit Hypothalamus/Pituitary
Kid Kidney
LM Limb muscle
Liv Liver
Lung Lung
MG Mammary gland
Panc Pancreas
Skin Skin
SI Small intestine
Spl Spleen
Test Testes
Thym Thymus
Tong Tongue
Trac Trachea
Ute Uterus

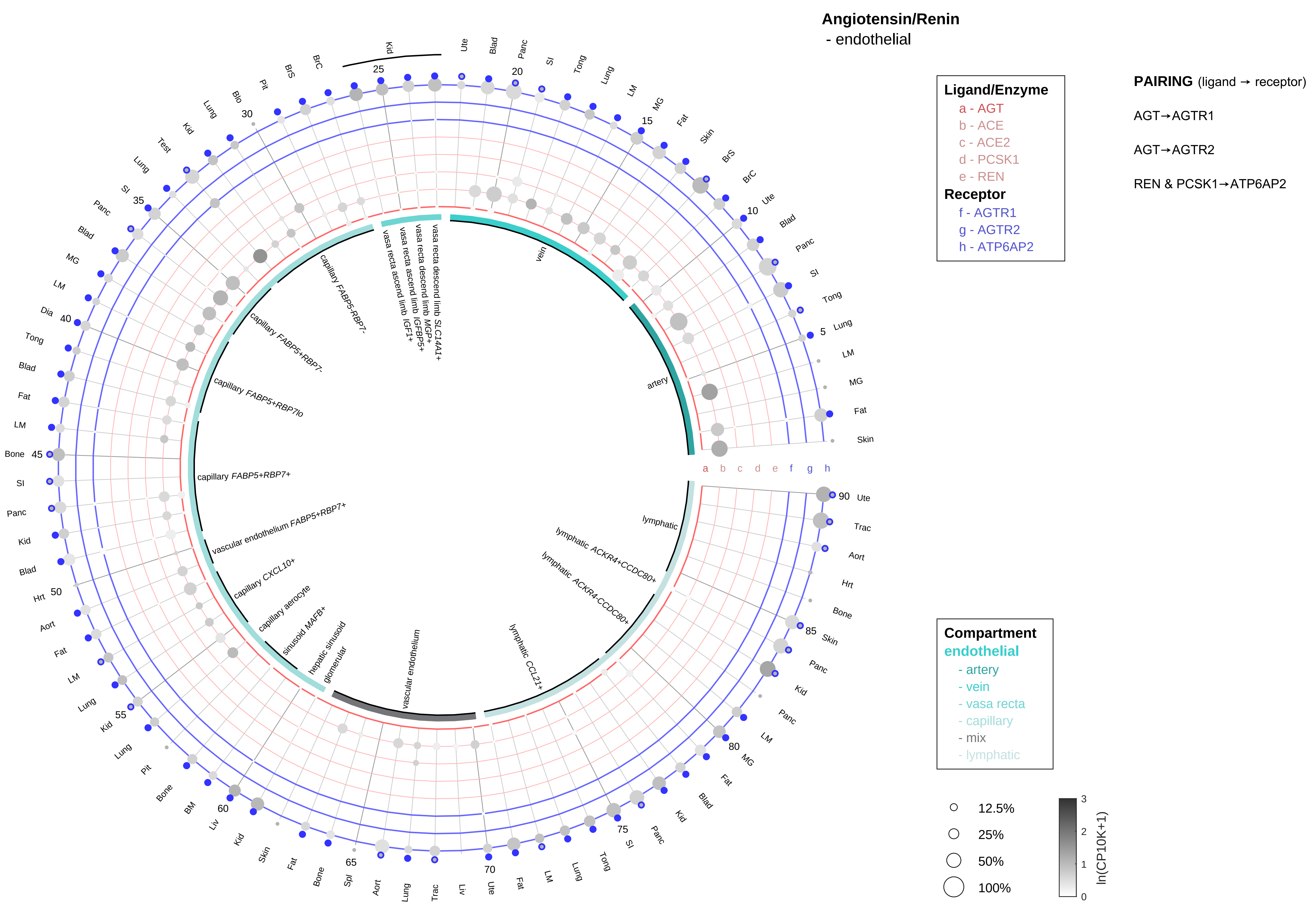

Abbrev.	Tissue
Aort	Aorta
Blad	Bladder
Blo	Blood
Bone	Bone
BM	Bone marrow
BrC	Brain cortex
BrS	Brainstem
Col	Colon
Dia	Diaphragm
Ret	Eye retina
Fat	Fat
Hrt	Heart
Pit	Hypothalamus/Pituitary
Kid	Kidney
LM	Limb muscle
Liv	Liver
Lung	Lung
MG	Mammary gland
Panc	Pancreas
Skin	Skin
SI	Small intestine
Spl	Spleen
Test	Testes
Thym	Thymus
Tong	Tongue
Trac	Trachea
Ute	Uterus

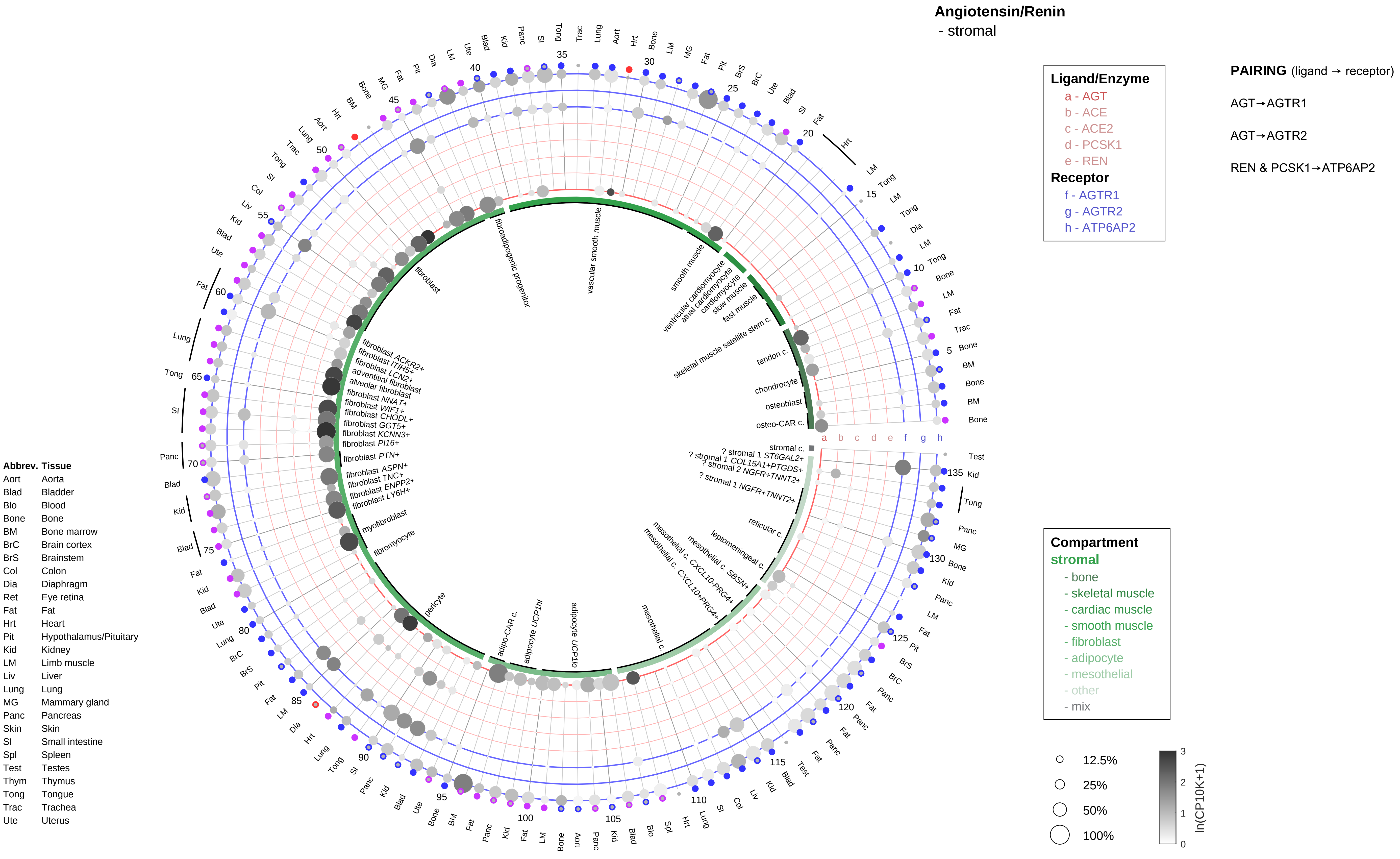

Angiotensin/Renin
- stromal
Ligand/Enzyme
a - AGT
b - ACE
c - ACE2
d - PCSK1
e - REN
Receptor
f - AGTR1
g - AGTR2
h - ATP6AP2
PAIRING (ligand → receptor)
AGT→AGTR1
AGT→AGTR2
REN & PCSK1→ATP6AP2
Compartment
stromal
- bone
- skeletal muscle
- cardiac muscle
- smooth muscle
- fibroblast
- adipocyte
- mesothelial
- other
- mix
12.5%
25%
50%
100%
ln(CP10K+1)
Abbrev. Tissue
Aort Aorta
Blad Bladder
Blo Blood
Bone Bone
BM Bone marrow
BrC Brain cortex
BrS Brainstem
Col Colon
Dia Diaphragm
Ret Eye retina
Fat Fat
Hrt Heart
Pit Hypothalamus/Pituitary
Kid Kidney
LM Limb muscle
Liv Liver
Lung Lung
MG Mammary gland
Panc Pancreas
Skin Skin
SI Small intestine
Spl Spleen
Test Testes
Thym Thymus
Tong Tongue
Trac Trachea
Ute Uterus

Angiotensin/Renin
- lymphoid

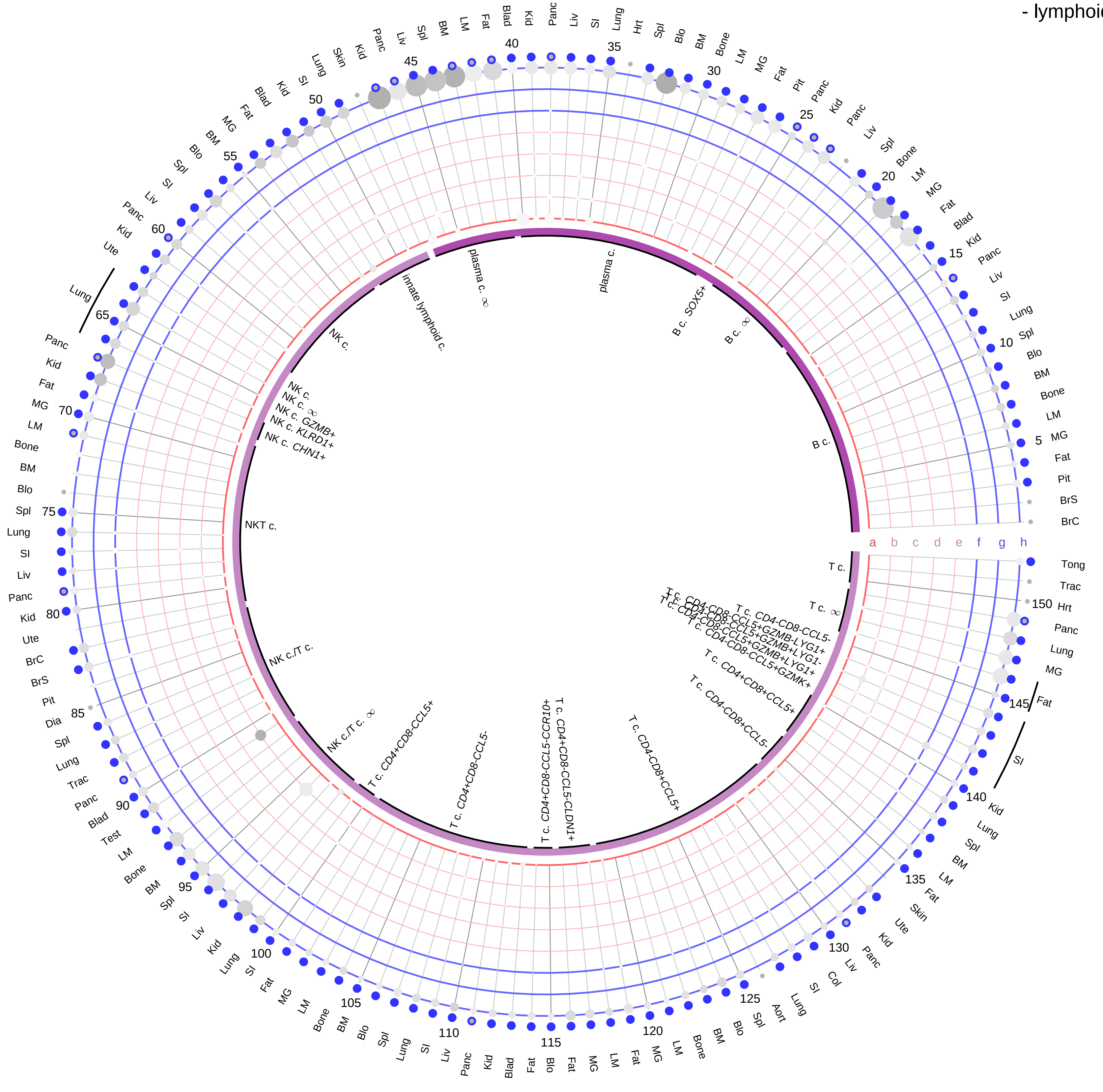

Ligand/Enzyme
- a - AGT
- b - ACE
- c - ACE2
- d - PCSK1
- e - REN

Receptor
- f - AGTR1
- g - AGTR2
- h - ATP6AP2

PAIRING (ligand → receptor)

AGT→AGTR1

AGT→AGTR2

REN & PCSK1→ATP6AP2

Compartment

lymphoid
- B cell
- NK/T cell

12.5%
25%
50%
100%

ln(CP10K+1): 0, 1, 2, 3

Abbrev.	Tissue
Aort	Aorta
Blad	Bladder
Blo	Blood
Bone	Bone
BM	Bone marrow
BrC	Brain cortex
BrS	Brainstem
Col	Colon
Dia	Diaphragm
Ret	Eye retina
Fat	Fat
Hrt	Heart
Pit	Hypothalamus/Pituitary
Kid	Kidney
LM	Limb muscle
Liv	Liver
Lung	Lung
MG	Mammary gland
Panc	Pancreas
Skin	Skin
SI	Small intestine
Spl	Spleen
Test	Testes
Thym	Thymus
Tong	Tongue
Trac	Trachea
Ute	Uterus

Angiotensin/Renin
- myeloid

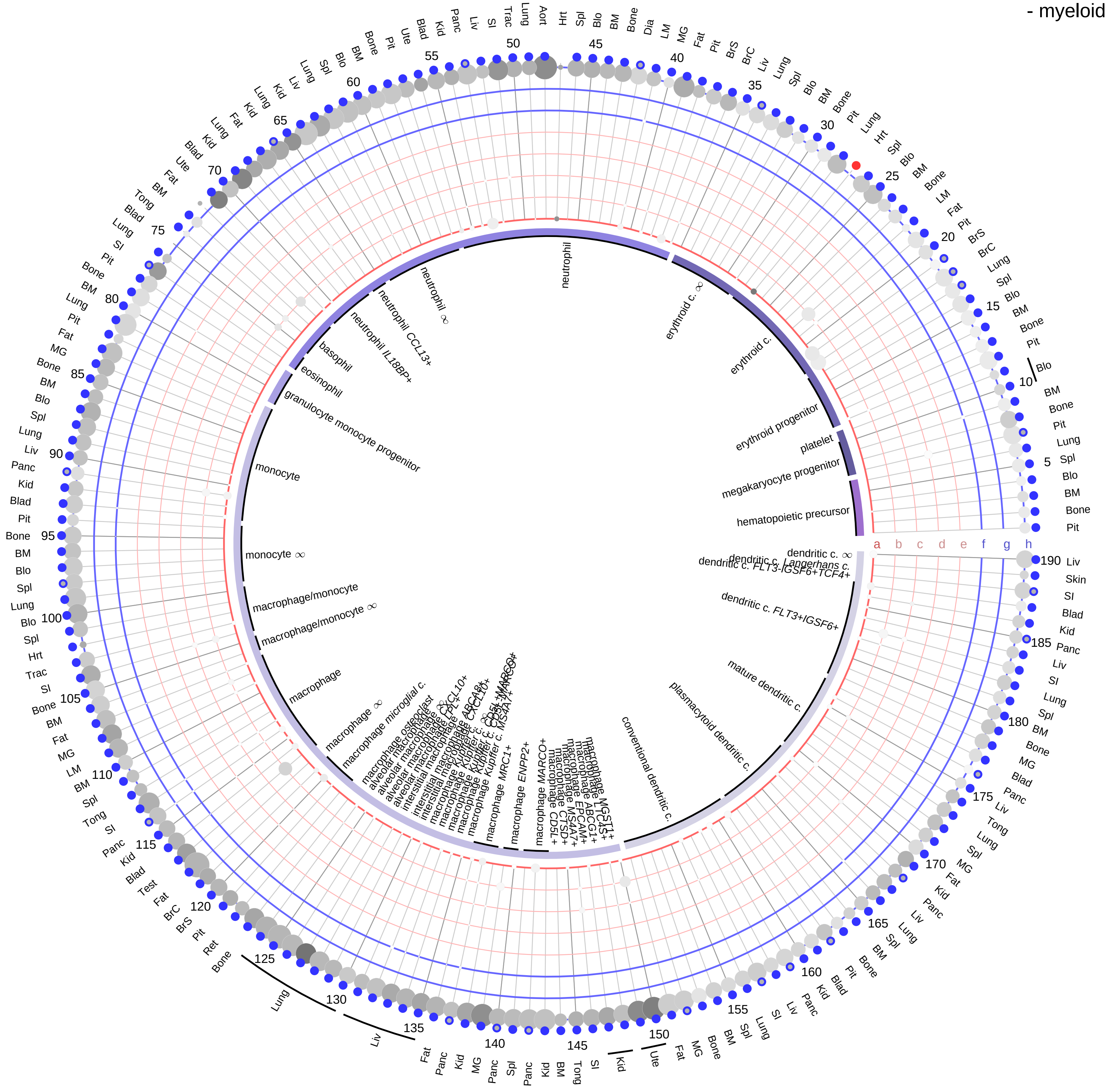

Ligand/Enzyme
- a - AGT
- b - ACE
- c - ACE2
- d - PCSK1
- e - REN

Receptor
- f - AGTR1
- g - AGTR2
- h - ATP6AP2

PAIRING (ligand → receptor)

AGT→AGTR1

AGT→AGTR2

REN & PCSK1→ATP6AP2

Abbrev.	Tissue
Aort	Aorta
Blad	Bladder
Blo	Blood
Bone	Bone
BM	Bone marrow
BrC	Brain cortex
BrS	Brainstem
Col	Colon
Dia	Diaphragm
Ret	Eye retina
Fat	Fat
Hrt	Heart
Pit	Hypothalamus/Pituitary
Kid	Kidney
LM	Limb muscle
Liv	Liver
Lung	Lung
MG	Mammary gland
Panc	Pancreas
Skin	Skin
SI	Small intestine
Spl	Spleen
Test	Testes
Thym	Thymus
Tong	Tongue
Trac	Trachea
Ute	Uterus

Compartment

hematopoietic
- hematopoietic precursor

megakaryocyte-erythroid
- megakaryoid
- erythroid

myeloid
- granulocyte
- granulocyte monocyte progenitor
- monocyte/macrophage
- dendritic

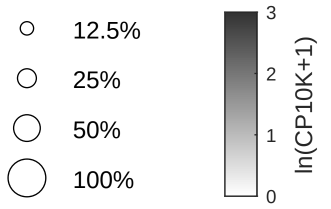

Anti-Müllerian hormone
- epithelial/neural/germ

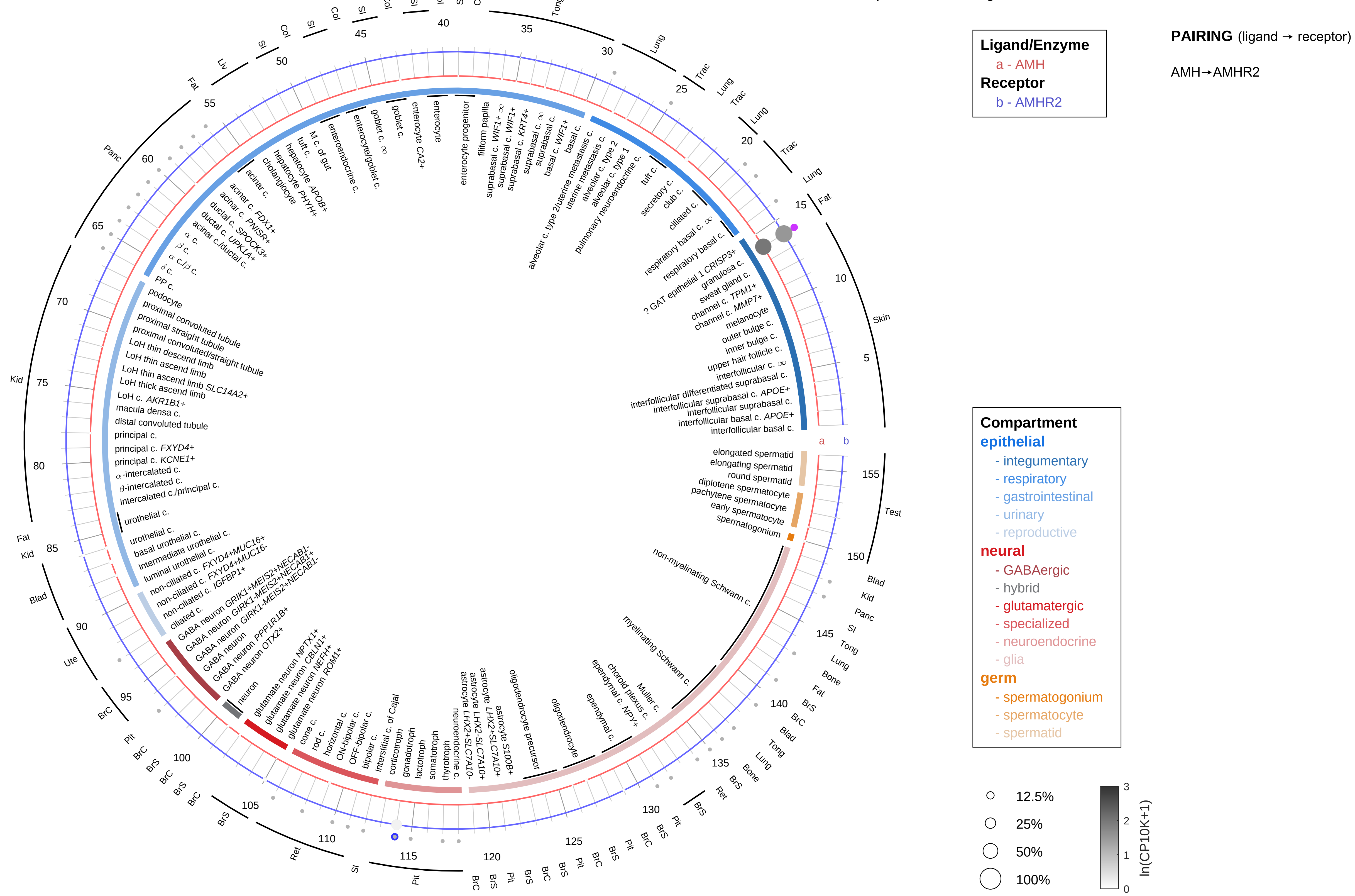

Abbrev. Tissue

Abbrev.	Tissue
Aort	Aorta
Blad	Bladder
Blo	Blood
Bone	Bone
BM	Bone marrow
BrC	Brain cortex
BrS	Brainstem
Col	Colon
Dia	Diaphragm
Ret	Eye retina
Fat	Fat
Hrt	Heart
Pit	Hypothalamus/Pituitary
Kid	Kidney
LM	Limb muscle
Liv	Liver
Lung	Lung
MG	Mammary gland
Panc	Pancreas
Skin	Skin
SI	Small intestine
Spl	Spleen
Test	Testes
Thym	Thymus
Tong	Tongue
Trac	Trachea
Ute	Uterus

Anti-Müllerian hormone
- endothelial

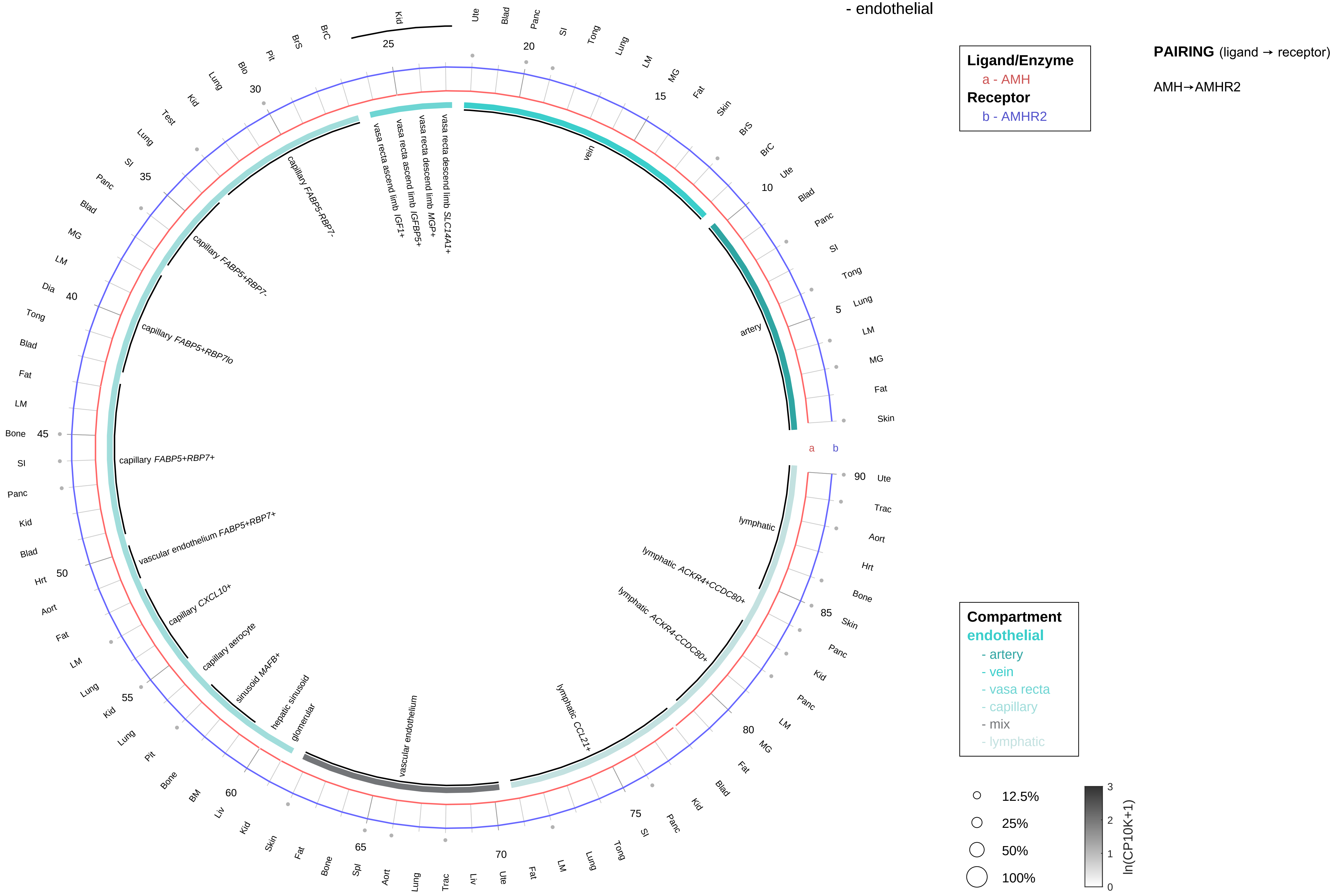

Abbrev.	Tissue
Aort	Aorta
Blad	Bladder
Blo	Blood
Bone	Bone
BM	Bone marrow
BrC	Brain cortex
BrS	Brainstem
Col	Colon
Dia	Diaphragm
Ret	Eye retina
Fat	Fat
Hrt	Heart
Pit	Hypothalamus/Pituitary
Kid	Kidney
LM	Limb muscle
Liv	Liver
Lung	Lung
MG	Mammary gland
Panc	Pancreas
Skin	Skin
SI	Small intestine
Spl	Spleen
Test	Testes
Thym	Thymus
Tong	Tongue
Trac	Trachea
Ute	Uterus

Anti-Müllerian hormone
- stromal

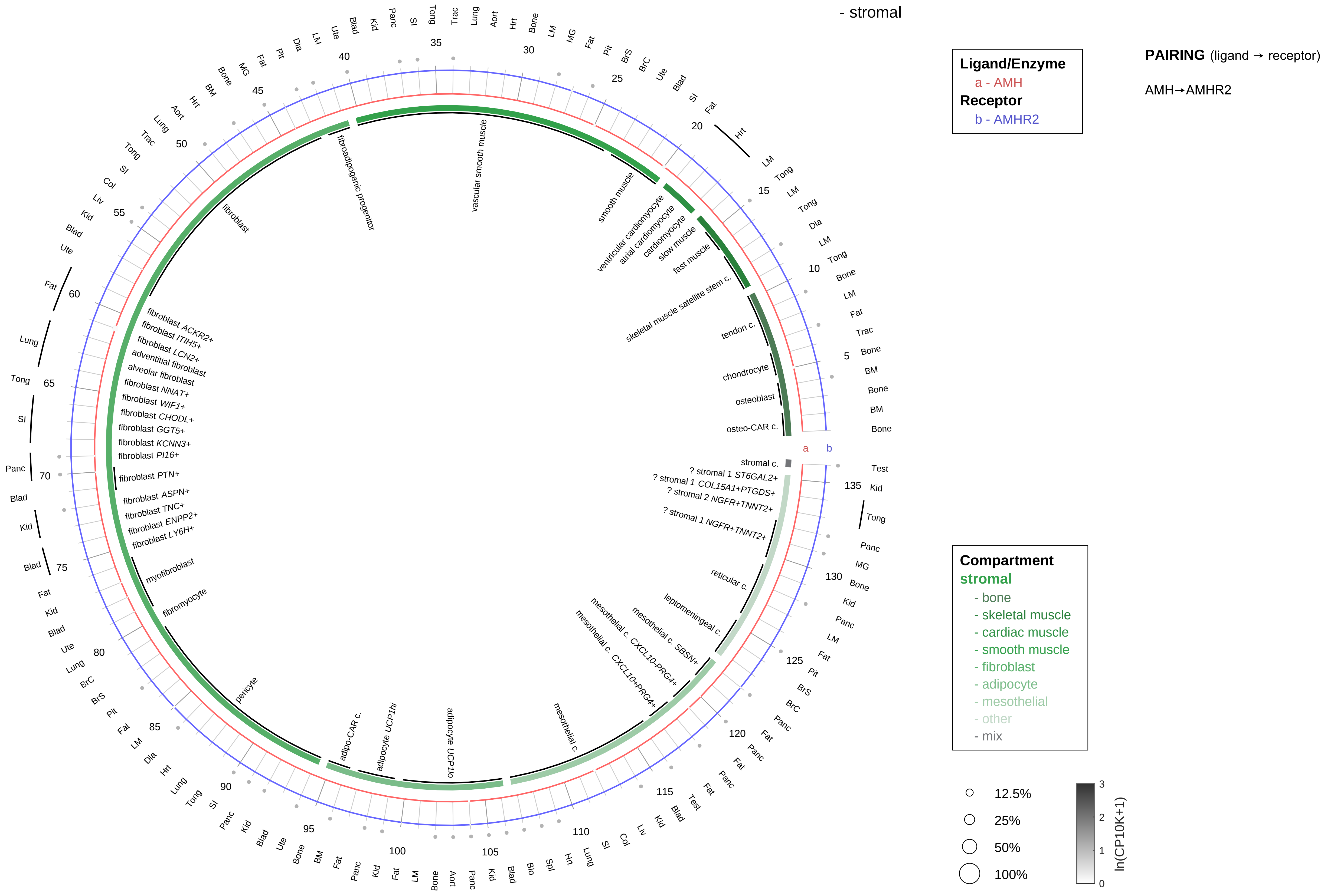

Abbrev.	Tissue
Aort	Aorta
Blad	Bladder
Blo	Blood
Bone	Bone
BM	Bone marrow
BrC	Brain cortex
BrS	Brainstem
Col	Colon
Dia	Diaphragm
Ret	Eye retina
Fat	Fat
Hrt	Heart
Pit	Hypothalamus/Pituitary
Kid	Kidney
LM	Limb muscle
Liv	Liver
Lung	Lung
MG	Mammary gland
Panc	Pancreas
Skin	Skin
SI	Small intestine
Spl	Spleen
Test	Testes
Thym	Thymus
Tong	Tongue
Trac	Trachea
Ute	Uterus

Anti-Müllerian hormone
- lymphoid

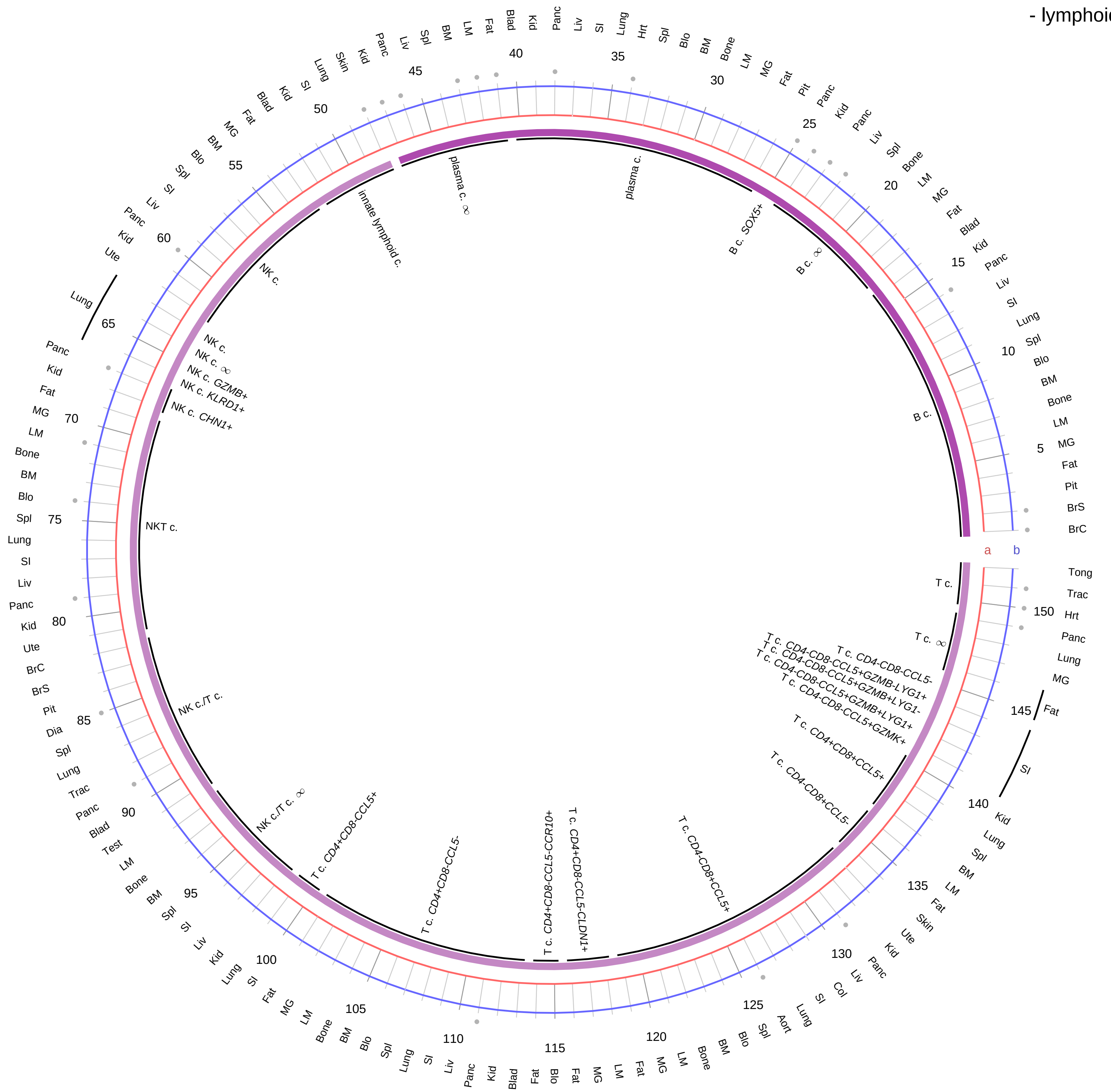

Ligand/Enzyme
a - AMH
Receptor
b - AMHR2

PAIRING (ligand → receptor)

AMH→AMHR2

Compartment
lymphoid
- B cell
- NK/T cell

12.5%
25%
50%
100%

ln(CP10K+1): 0, 1, 2, 3

Abbrev.	Tissue
Aort	Aorta
Blad	Bladder
Blo	Blood
Bone	Bone
BM	Bone marrow
BrC	Brain cortex
BrS	Brainstem
Col	Colon
Dia	Diaphragm
Ret	Eye retina
Fat	Fat
Hrt	Heart
Pit	Hypothalamus/Pituitary
Kid	Kidney
LM	Limb muscle
Liv	Liver
Lung	Lung
MG	Mammary gland
Panc	Pancreas
Skin	Skin
SI	Small intestine
Spl	Spleen
Test	Testes
Thym	Thymus
Tong	Tongue
Trac	Trachea
Ute	Uterus

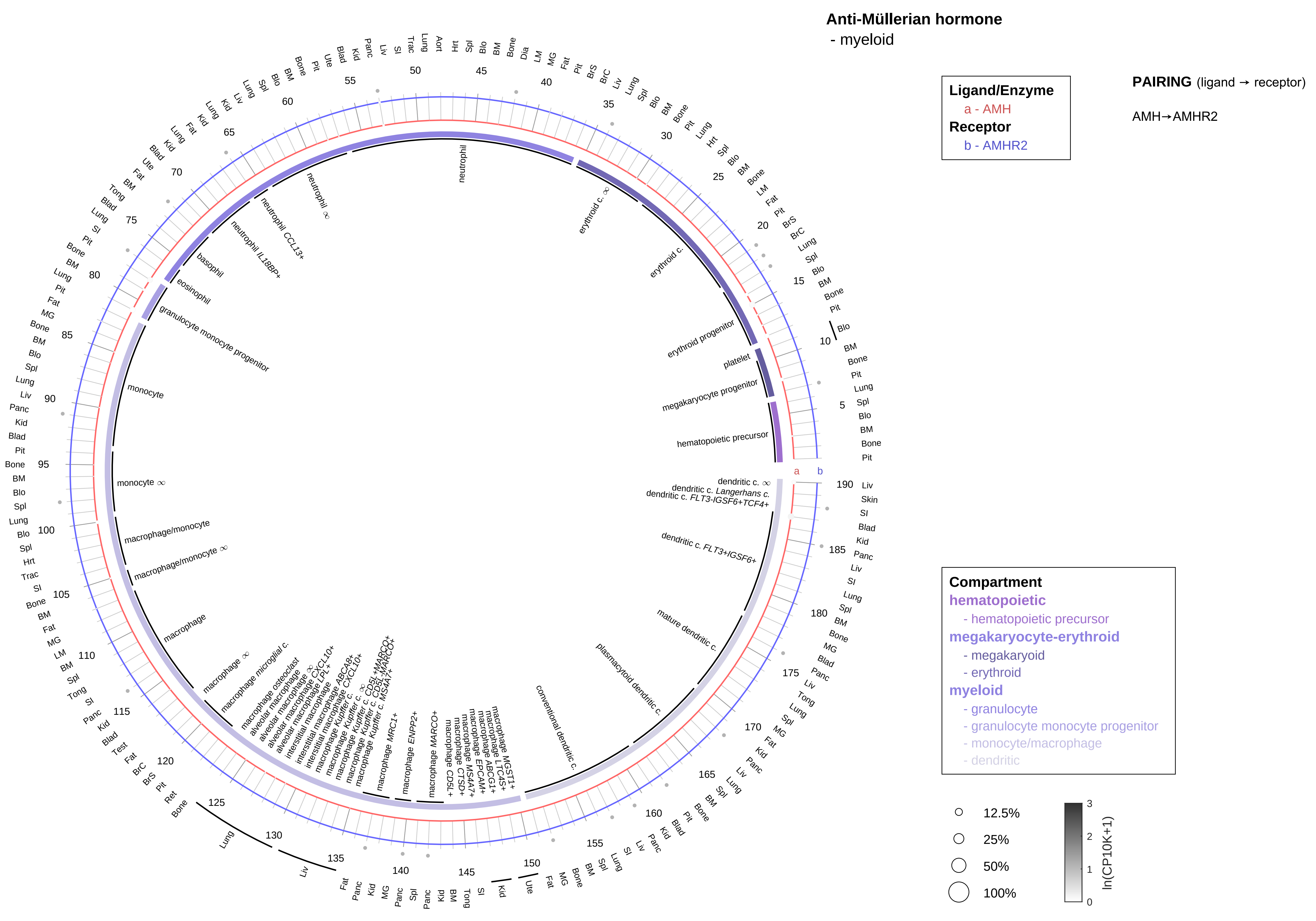

Abbrev.	Tissue
Aort	Aorta
Blad	Bladder
Blo	Blood
Bone	Bone
BM	Bone marrow
BrC	Brain cortex
BrS	Brainstem
Col	Colon
Dia	Diaphragm
Ret	Eye retina
Fat	Fat
Hrt	Heart
Pit	Hypothalamus/Pituitary
Kid	Kidney
LM	Limb muscle
Liv	Liver
Lung	Lung
MG	Mammary gland
Panc	Pancreas
Skin	Skin
SI	Small intestine
Spl	Spleen
Test	Testes
Thym	Thymus
Tong	Tongue
Trac	Trachea
Ute	Uterus

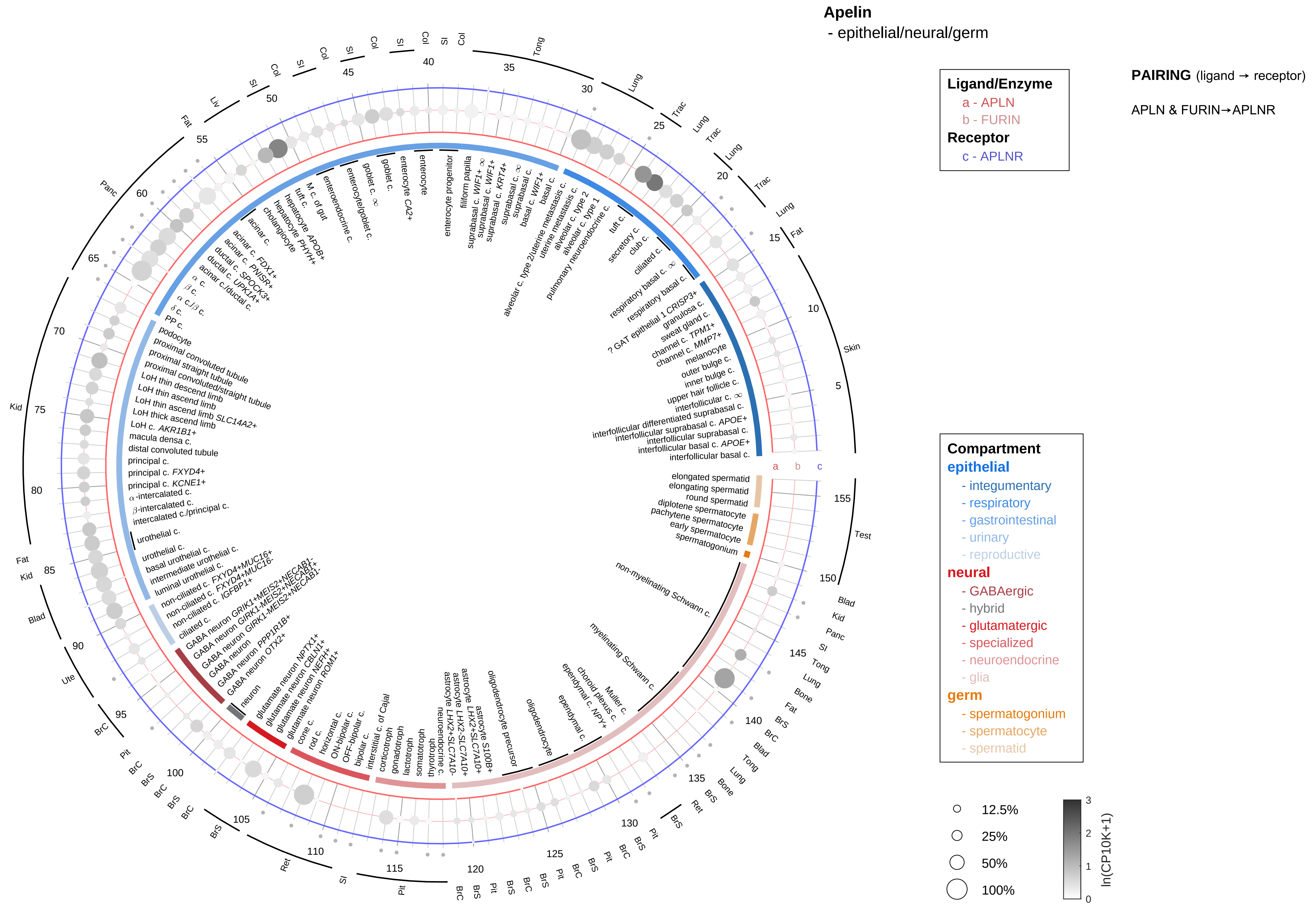

Abbrev.	Tissue
Aort	Aorta
Blad	Bladder
Blo	Blood
Bone	Bone
BM	Bone marrow
BrC	Brain cortex
BrS	Brainstem
Col	Colon
Dia	Diaphragm
Ret	Eye retina
Fat	Fat
Hrt	Heart
Pit	Hypothalamus/Pituitary
Kid	Kidney
LM	Limb muscle
Liv	Liver
Lung	Lung
MG	Mammary gland
Panc	Pancreas
Skin	Skin
SI	Small intestine
Spl	Spleen
Test	Testes
Thym	Thymus
Tong	Tongue
Trac	Trachea
Ute	Uterus

Apelin
- endothelial

Ligand/Enzyme
a - APLN
b - FURIN
Receptor
c - APLNR

PAIRING (ligand → receptor)

APLN & FURIN→APLNR

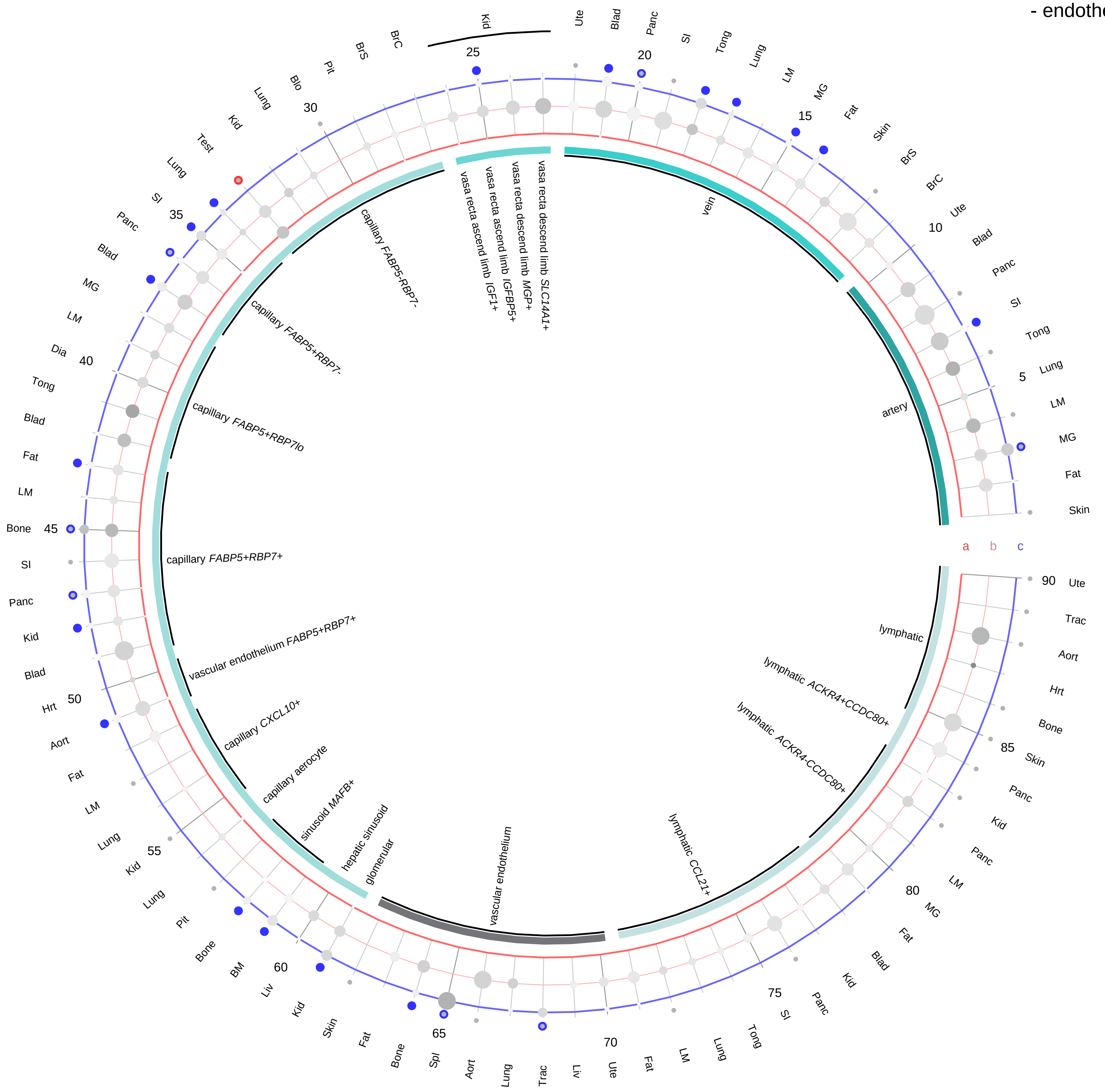

Abbrev.	Tissue
Aort	Aorta
Blad	Bladder
Blo	Blood
Bone	Bone
BM	Bone marrow
BrC	Brain cortex
BrS	Brainstem
Col	Colon
Dia	Diaphragm
Ret	Eye retina
Fat	Fat
Hrt	Heart
Pit	Hypothalamus/Pituitary
Kid	Kidney
LM	Limb muscle
Liv	Liver
Lung	Lung
MG	Mammary gland
Panc	Pancreas
Skin	Skin
SI	Small intestine
Spl	Spleen
Test	Testes
Thym	Thymus
Tong	Tongue
Trac	Trachea
Ute	Uterus

Compartment
endothelial
- artery
- vein
- vasa recta
- capillary
- mix
- lymphatic

12.5%
25%
50%
100%

ln(CP10K+1)
0
1
2
3

Apelin
- stromal

Ligand/Enzyme
- a - APLN
- b - FURIN

Receptor
- c - APLNR

PAIRING (ligand → receptor)

APLN & FURIN→APLNR

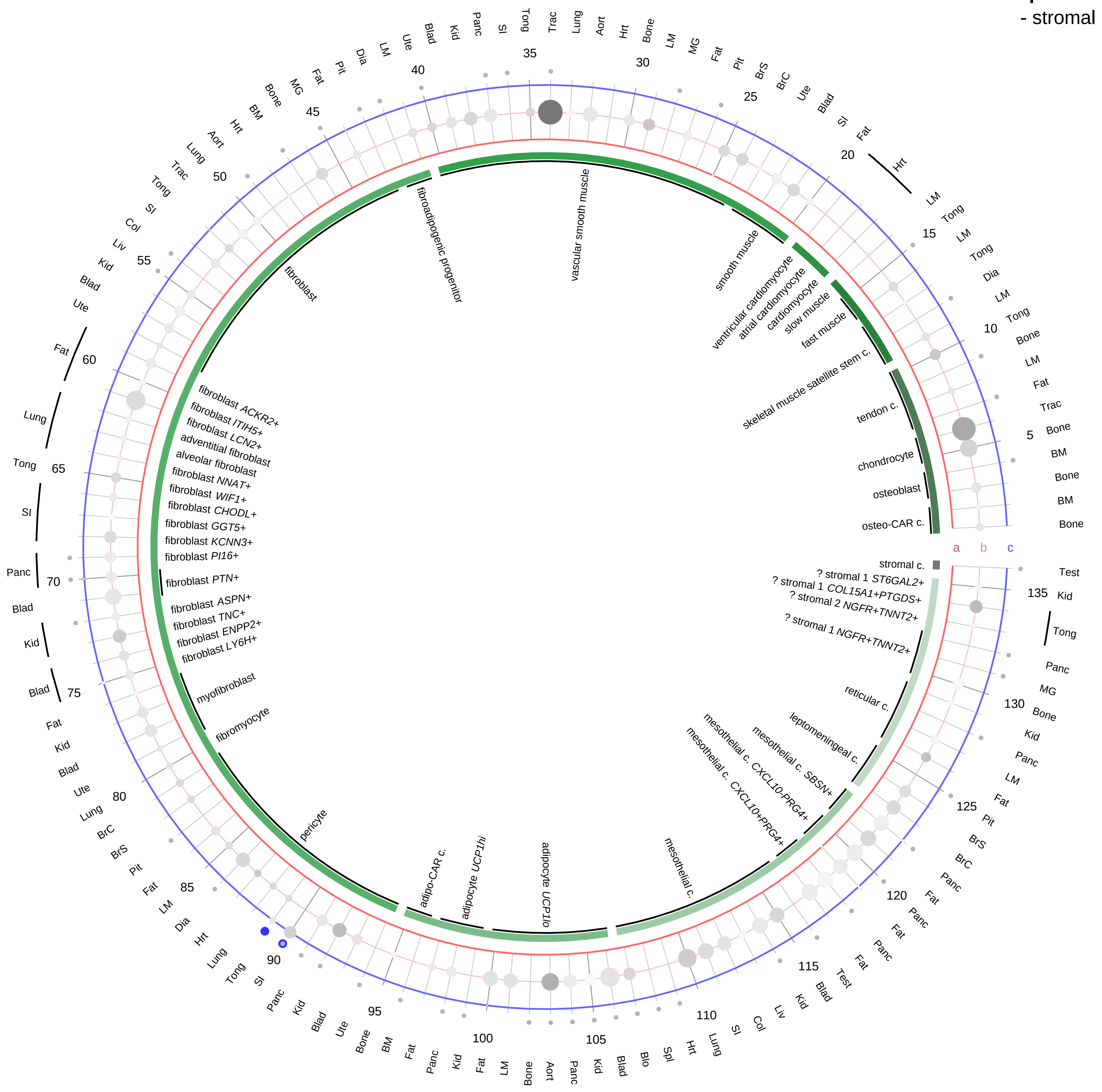

Abbrev.	Tissue
Aort	Aorta
Blad	Bladder
Blo	Blood
Bone	Bone
BM	Bone marrow
BrC	Brain cortex
BrS	Brainstem
Col	Colon
Dia	Diaphragm
Ret	Eye retina
Fat	Fat
Hrt	Heart
Pit	Hypothalamus/Pituitary
Kid	Kidney
LM	Limb muscle
Liv	Liver
Lung	Lung
MG	Mammary gland
Panc	Pancreas
Skin	Skin
SI	Small intestine
Spl	Spleen
Test	Testes
Thym	Thymus
Tong	Tongue
Trac	Trachea
Ute	Uterus

Compartment

stromal
- bone
- skeletal muscle
- cardiac muscle
- smooth muscle
- fibroblast
- adipocyte
- mesothelial
- other
- mix

12.5%
25%
50%
100%

ln(CP10K+1): 0, 1, 2, 3

Apelin
- lymphoid

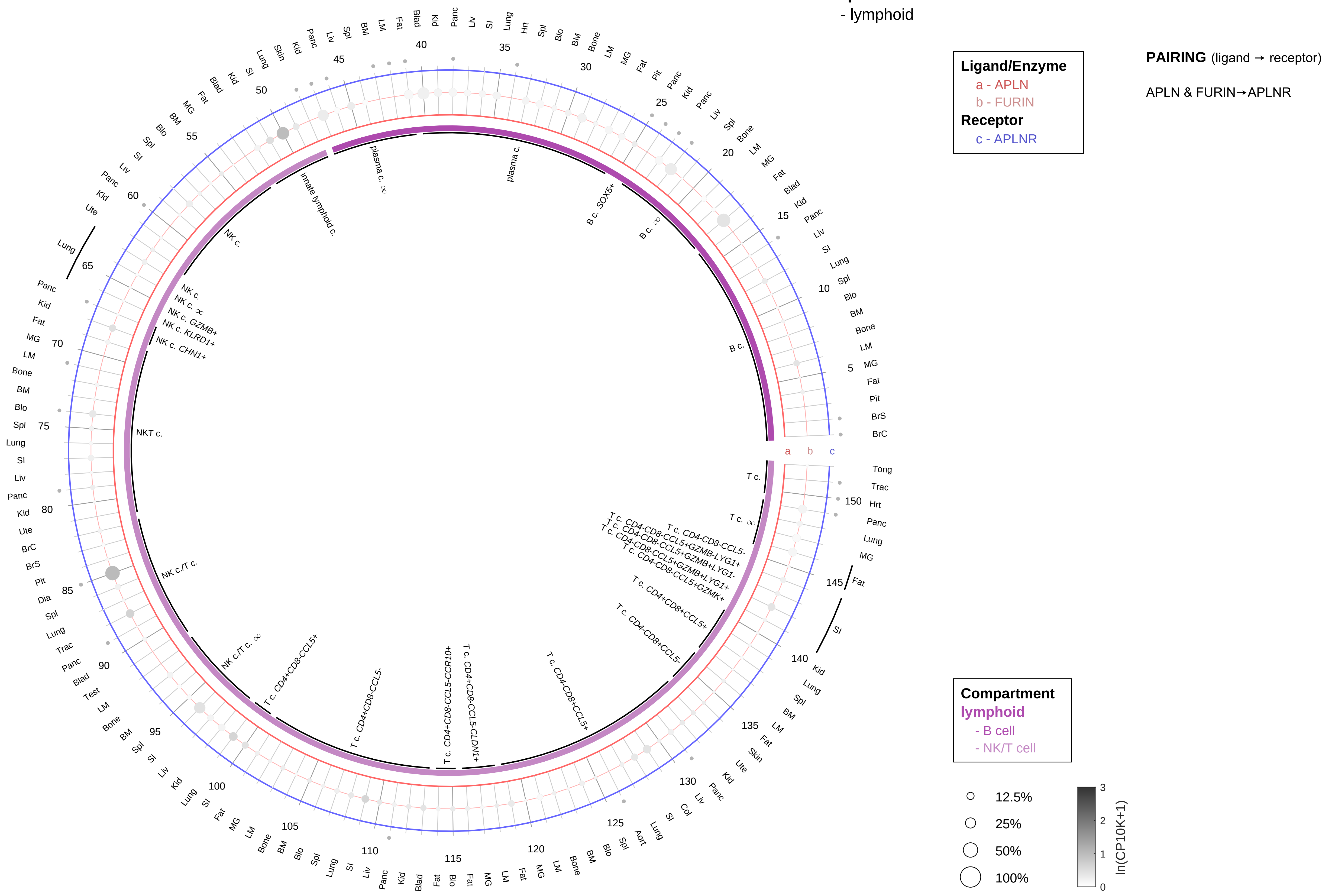

Abbrev.	Tissue
Aort	Aorta
Blad	Bladder
Blo	Blood
Bone	Bone
BM	Bone marrow
BrC	Brain cortex
BrS	Brainstem
Col	Colon
Dia	Diaphragm
Ret	Eye retina
Fat	Fat
Hrt	Heart
Pit	Hypothalamus/Pituitary
Kid	Kidney
LM	Limb muscle
Liv	Liver
Lung	Lung
MG	Mammary gland
Panc	Pancreas
Skin	Skin
SI	Small intestine
Spl	Spleen
Test	Testes
Thym	Thymus
Tong	Tongue
Trac	Trachea
Ute	Uterus

Apelin
- myeloid

Ligand/Enzyme
- a - APLN
- b - FURIN

Receptor
- c - APLNR

PAIRING (ligand → receptor)

APLN & FURIN→APLNR

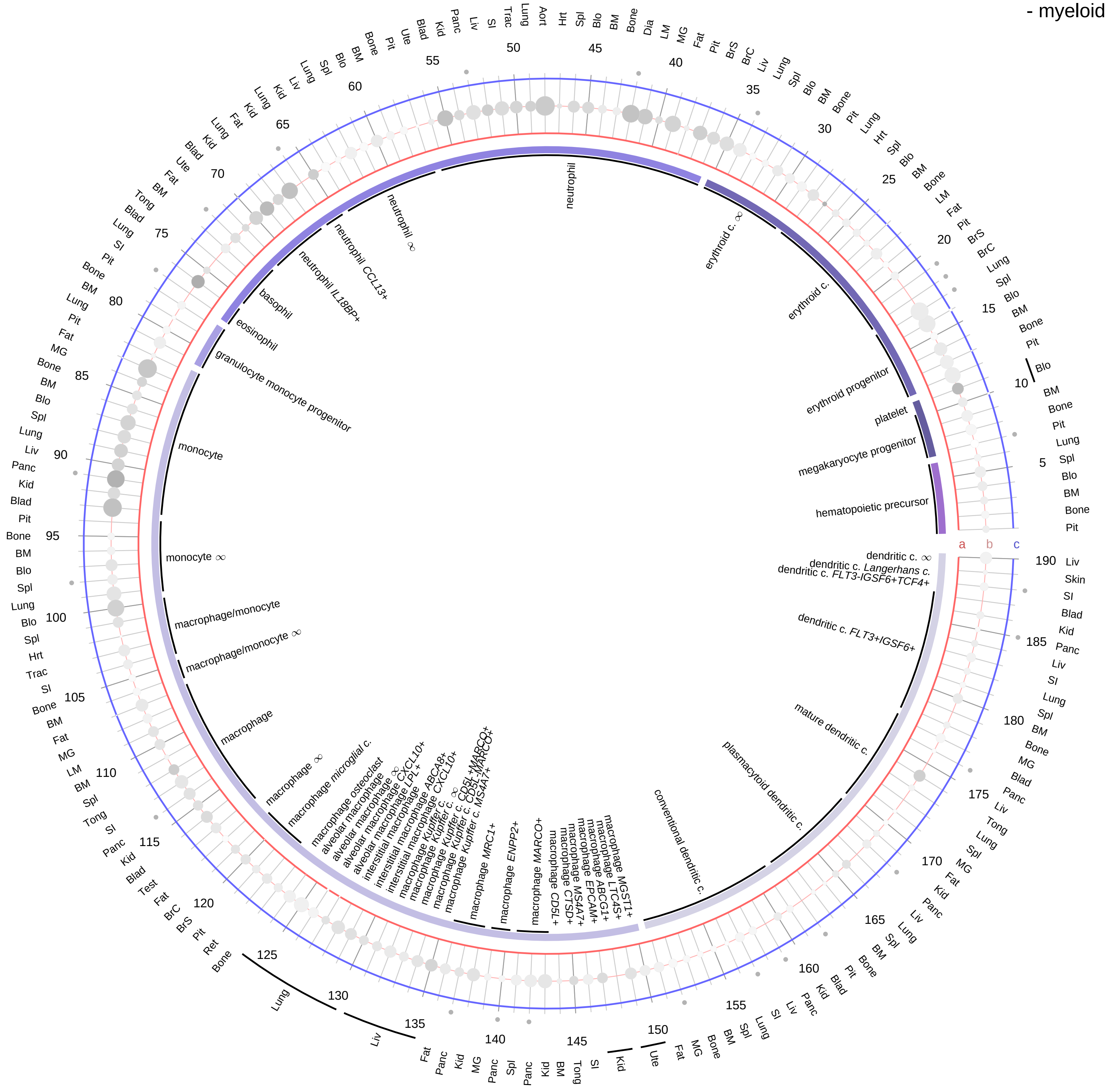

Compartment
- **hematopoietic**
  - hematopoietic precursor
- **megakaryocyte-erythroid**
  - megakaryoid
  - erythroid
- **myeloid**
  - granulocyte
  - granulocyte monocyte progenitor
  - monocyte/macrophage
  - dendritic

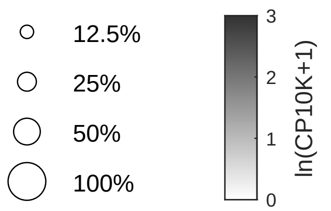

Abbrev.	Tissue
Aort	Aorta
Blad	Bladder
Blo	Blood
Bone	Bone
BM	Bone marrow
BrC	Brain cortex
BrS	Brainstem
Col	Colon
Dia	Diaphragm
Ret	Eye retina
Fat	Fat
Hrt	Heart
Pit	Hypothalamus/Pituitary
Kid	Kidney
LM	Limb muscle
Liv	Liver
Lung	Lung
MG	Mammary gland
Panc	Pancreas
Skin	Skin
SI	Small intestine
Spl	Spleen
Test	Testes
Thym	Thymus
Tong	Tongue
Trac	Trachea
Ute	Uterus

Asprosin
- epithelial/neural/germ

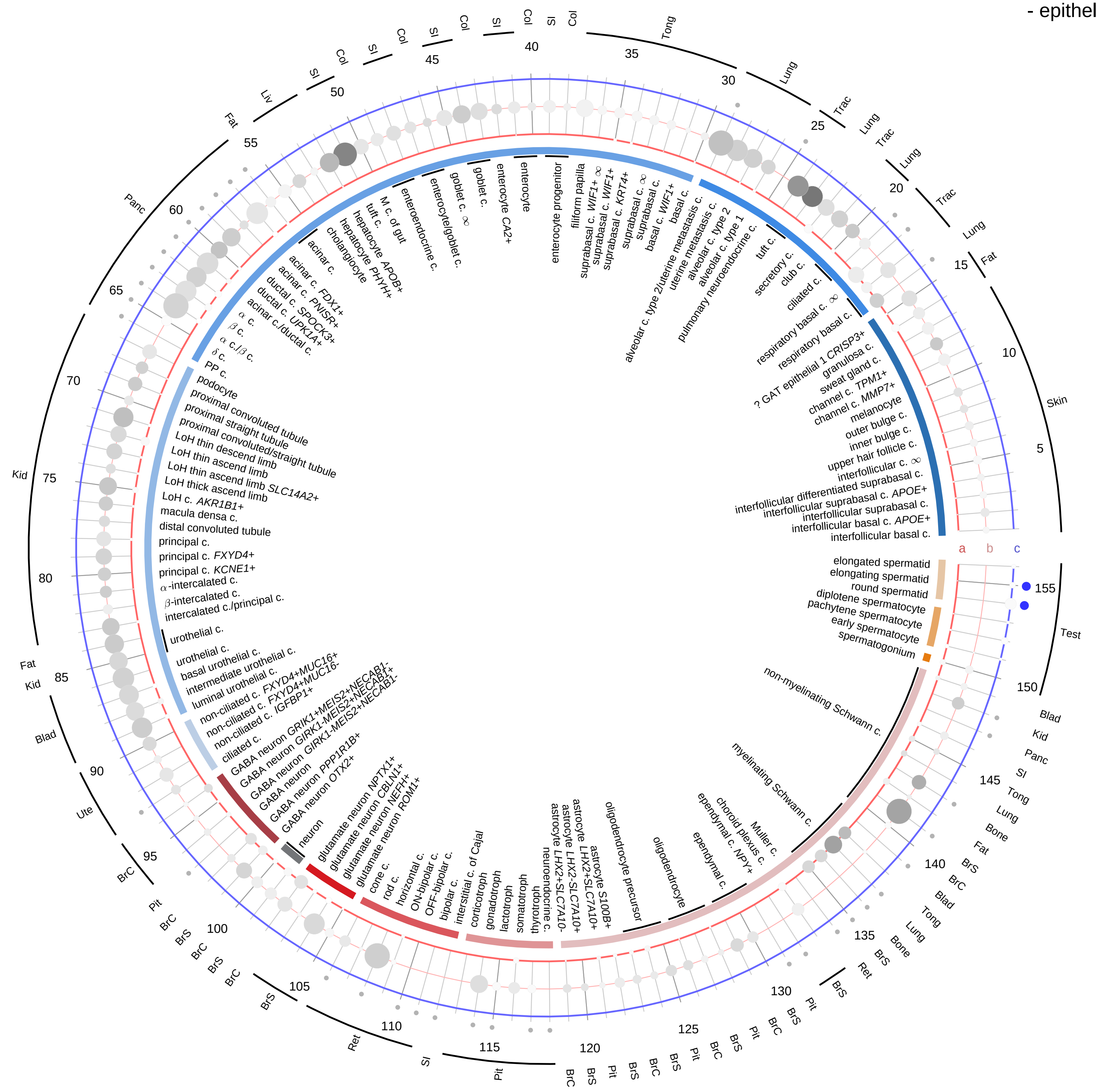

Ligand/Enzyme
- a - FBN1
- b - FURIN

Receptor
- c - LOC105864759 (OR4M1)

PAIRING (ligand → receptor)

FBN1 & FURIN→OR4M1

Compartment

epithelial
- integumentary
- respiratory
- gastrointestinal
- urinary
- reproductive

neural
- GABAergic
- hybrid
- glutamatergic
- specialized
- neuroendocrine
- glia

germ
- spermatogonium
- spermatocyte
- spermatid

12.5%
25%
50%
100%

ln(CP10K+1): 0, 1, 2, 3

Abbrev.	Tissue
Aort	Aorta
Blad	Bladder
Blo	Blood
Bone	Bone
BM	Bone marrow
BrC	Brain cortex
BrS	Brainstem
Col	Colon
Dia	Diaphragm
Ret	Eye retina
Fat	Fat
Hrt	Heart
Pit	Hypothalamus/Pituitary
Kid	Kidney
LM	Limb muscle
Liv	Liver
Lung	Lung
MG	Mammary gland
Panc	Pancreas
Skin	Skin
SI	Small intestine
Spl	Spleen
Test	Testes
Thym	Thymus
Tong	Tongue
Trac	Trachea
Ute	Uterus

Asprosin
- endothelial

PAIRING (ligand → receptor)

FBN1 & FURIN→OR4M1

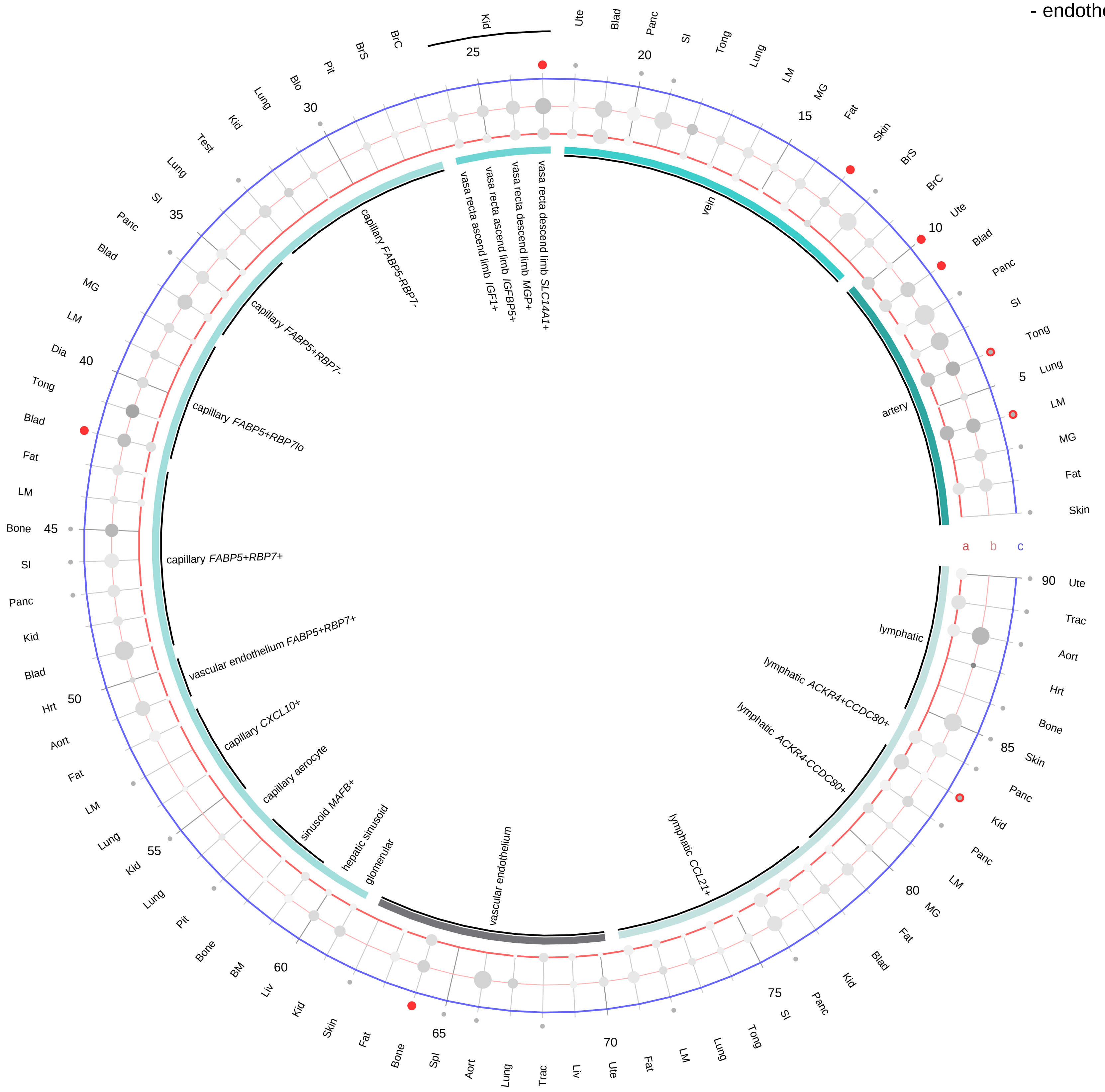

Ligand/Enzyme
a - FBN1
b - FURIN
Receptor
c - LOC105864759 (OR4M1)

Compartment
endothelial
- artery
- vein
- vasa recta
- capillary
- mix
- lymphatic

12.5%
25%
50%
100%

ln(CP10K+1): 0, 1, 2, 3

**Abbrev.**	**Tissue**
Aort	Aorta
Blad	Bladder
Blo	Blood
Bone	Bone
BM	Bone marrow
BrC	Brain cortex
BrS	Brainstem
Col	Colon
Dia	Diaphragm
Ret	Eye retina
Fat	Fat
Hrt	Heart
Pit	Hypothalamus/Pituitary
Kid	Kidney
LM	Limb muscle
Liv	Liver
Lung	Lung
MG	Mammary gland
Panc	Pancreas
Skin	Skin
SI	Small intestine
Spl	Spleen
Test	Testes
Thym	Thymus
Tong	Tongue
Trac	Trachea
Ute	Uterus

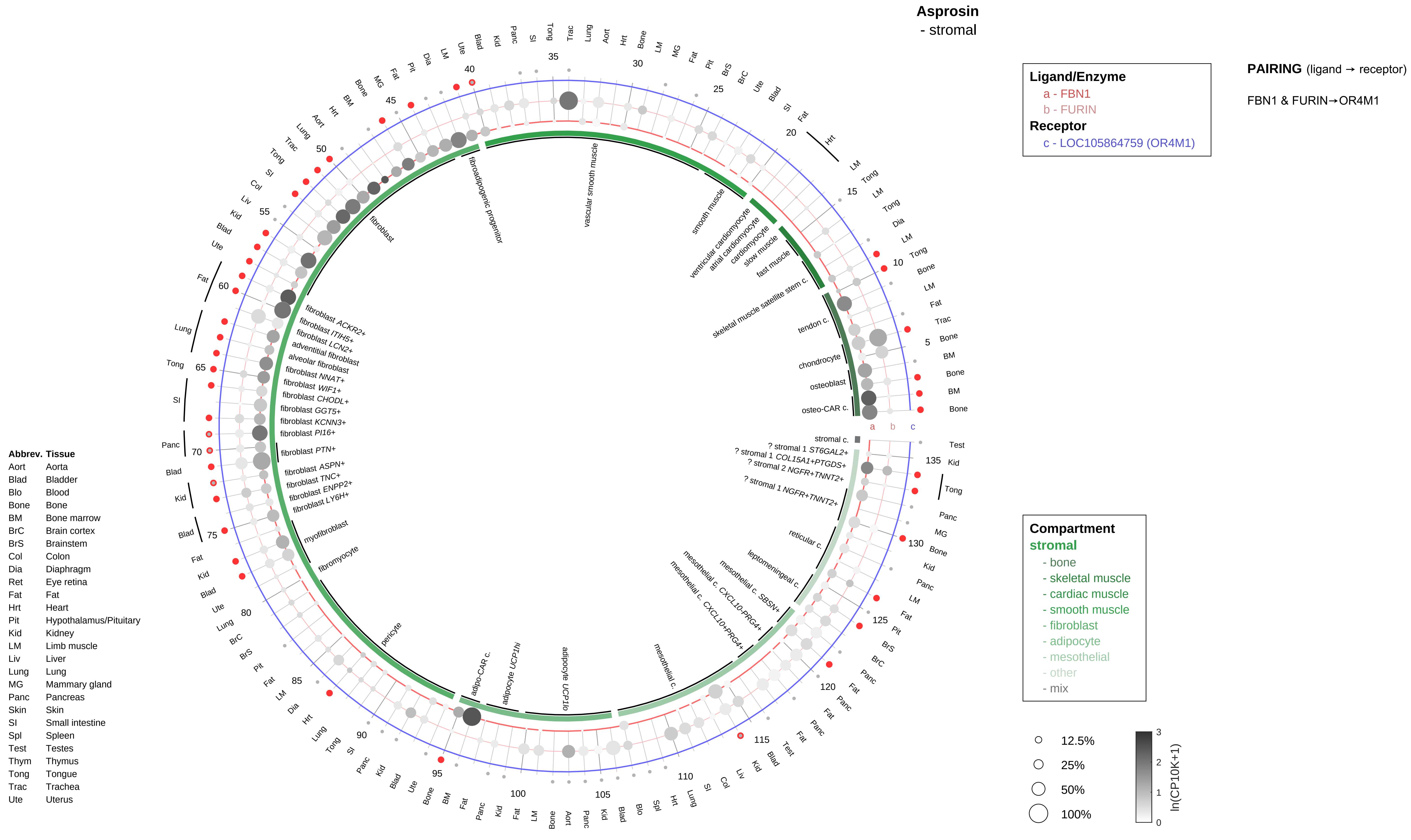
Asprosin
- stromal
Ligand/Enzyme
a - FBN1
b - FURIN
Receptor
c - LOC105864759 (OR4M1)
PAIRING (ligand → receptor)
FBN1 & FURIN→OR4M1
Compartment
stromal
- bone
- skeletal muscle
- cardiac muscle
- smooth muscle
- fibroblast
- adipocyte
- mesothelial
- other
- mix
12.5%
25%
50%
100%
ln(CP10K+1)
0
1
2
3
a
b
c
osteo-CAR c.
osteoblast
chondrocyte
tendon c.
skeletal muscle satellite stem c.
fast muscle
slow muscle
cardiomyocyte
atrial cardiomyocyte
ventricular cardiomyocyte
smooth muscle
vascular smooth muscle
fibroadipogenic progenitor
fibroblast
fibroblast ACKR2+
fibroblast ITIH5+
fibroblast LCN2+
adventitial fibroblast
alveolar fibroblast
fibroblast NNAT+
fibroblast WIF1+
fibroblast CHODL+
fibroblast GGT5+
fibroblast KCNN3+
fibroblast PI16+
fibroblast PTN+
fibroblast ASPN+
fibroblast TNC+
fibroblast ENPP2+
fibroblast LY6H+
myofibroblast
fibromyocyte
pericyte
adipo-CAR c.
adipocyte UCP1hi
adipocyte UCP1lo
mesothelial c.
mesothelial c. CXCL10+PRG4+
mesothelial c. CXCL10-PRG4+
mesothelial c. SBSN+
leptomeningeal c.
reticular c.
? stromal 1 NGFR+TNNT2+
? stromal 2 NGFR+TNNT2+
? stromal 1 COL15A1+PTGDS+
? stromal 1 ST6GAL2+
stromal c.
Abbrev. Tissue
Aort Aorta
Blad Bladder
Blo Blood
Bone Bone
BM Bone marrow
BrC Brain cortex
BrS Brainstem
Col Colon
Dia Diaphragm
Ret Eye retina
Fat Fat
Hrt Heart
Pit Hypothalamus/Pituitary
Kid Kidney
LM Limb muscle
Liv Liver
Lung Lung
MG Mammary gland
Panc Pancreas
Skin Skin
SI Small intestine
Spl Spleen
Test Testes
Thym Thymus
Tong Tongue
Trac Trachea
Ute Uterus

Asprosin
- lymphoid

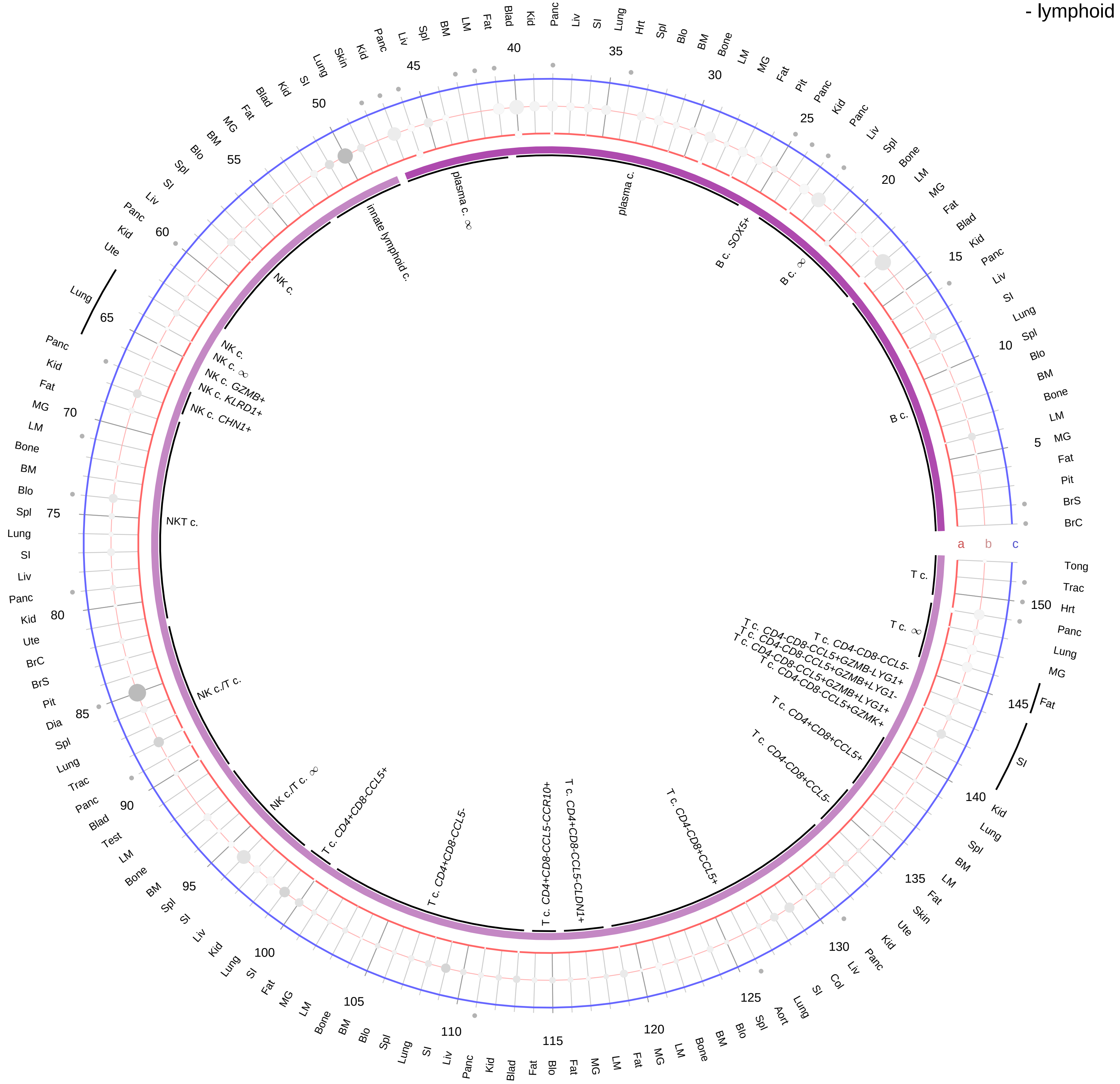

Ligand/Enzyme
a - FBN1
b - FURIN
Receptor
c - LOC105864759 (OR4M1)

PAIRING (ligand → receptor)

FBN1 & FURIN→OR4M1

Compartment
lymphoid
- B cell
- NK/T cell

12.5%
25%
50%
100%

ln(CP10K+1): 0, 1, 2, 3

Abbrev.	Tissue
Aort	Aorta
Blad	Bladder
Blo	Blood
Bone	Bone
BM	Bone marrow
BrC	Brain cortex
BrS	Brainstem
Col	Colon
Dia	Diaphragm
Ret	Eye retina
Fat	Fat
Hrt	Heart
Pit	Hypothalamus/Pituitary
Kid	Kidney
LM	Limb muscle
Liv	Liver
Lung	Lung
MG	Mammary gland
Panc	Pancreas
Skin	Skin
SI	Small intestine
Spl	Spleen
Test	Testes
Thym	Thymus
Tong	Tongue
Trac	Trachea
Ute	Uterus

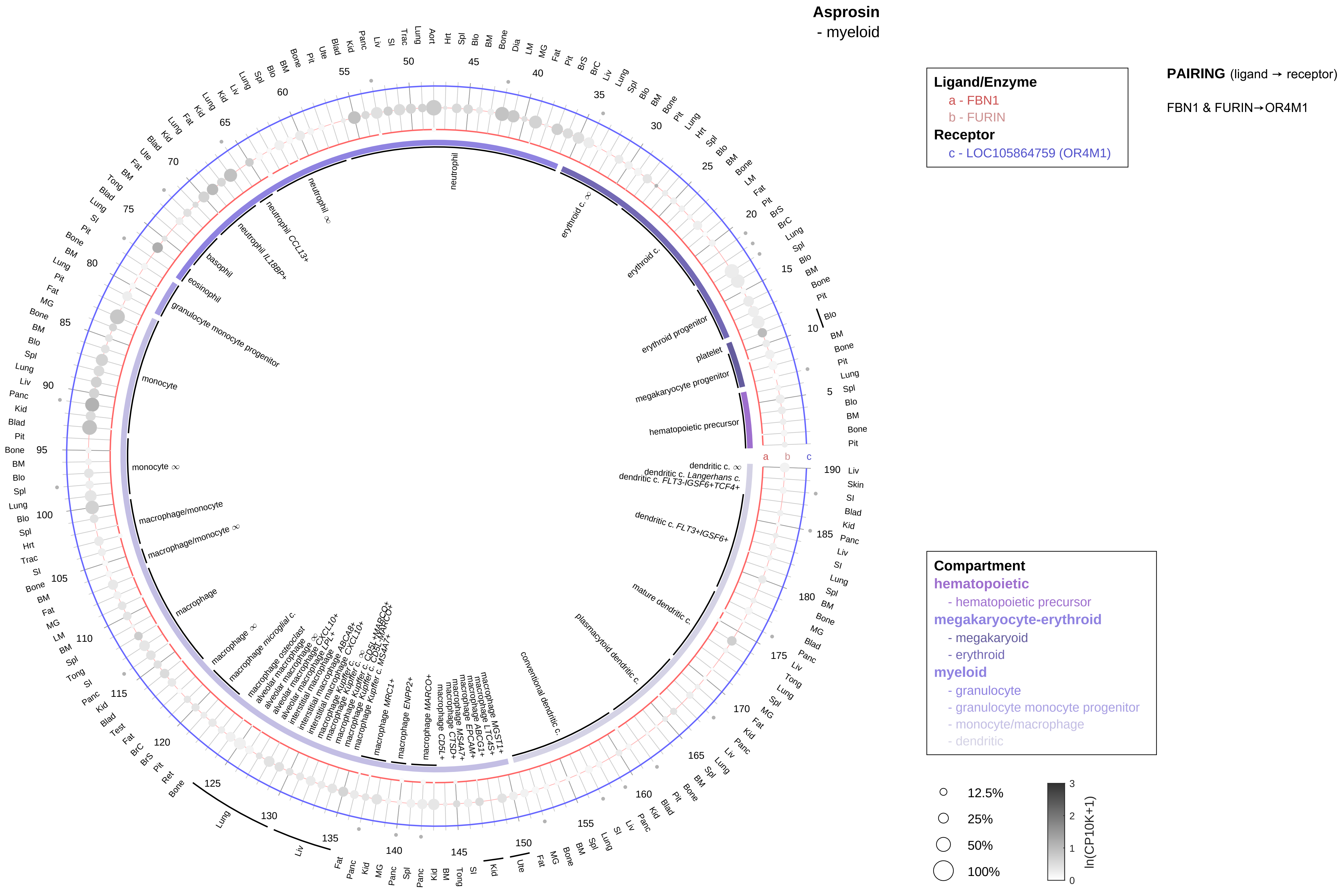

Abbrev.	Tissue
Aort	Aorta
Blad	Bladder
Blo	Blood
Bone	Bone
BM	Bone marrow
BrC	Brain cortex
BrS	Brainstem
Col	Colon
Dia	Diaphragm
Ret	Eye retina
Fat	Fat
Hrt	Heart
Pit	Hypothalamus/Pituitary
Kid	Kidney
LM	Limb muscle
Liv	Liver
Lung	Lung
MG	Mammary gland
Panc	Pancreas
Skin	Skin
SI	Small intestine
Spl	Spleen
Test	Testes
Thym	Thymus
Tong	Tongue
Trac	Trachea
Ute	Uterus

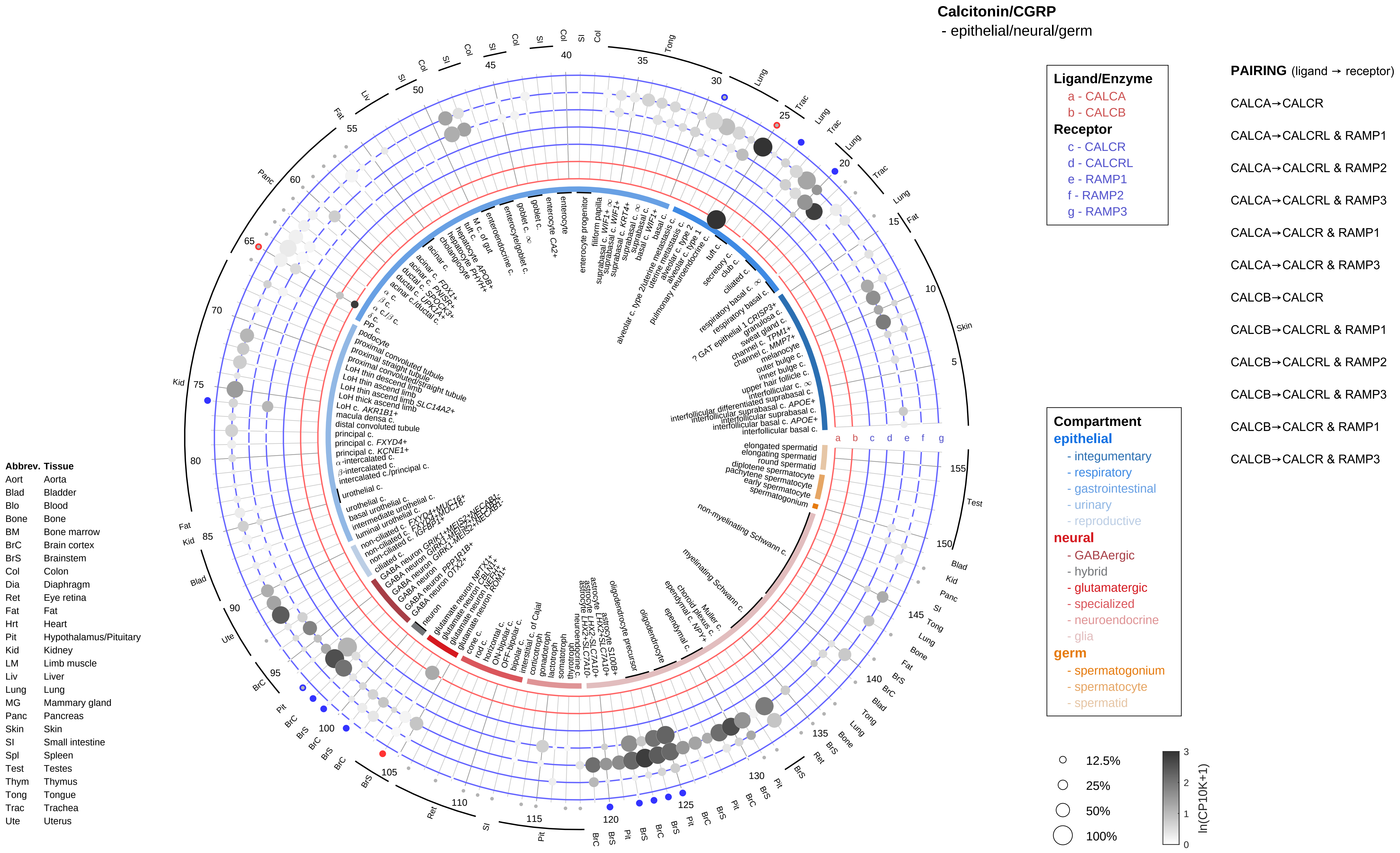
Calcitonin/CGRP
- epithelial/neural/germ
Ligand/Enzyme
a - CALCA
b - CALCB
Receptor
c - CALCR
d - CALCRL
e - RAMP1
f - RAMP2
g - RAMP3
PAIRING (ligand → receptor)
CALCA→CALCR
CALCA→CALCRL & RAMP1
CALCA→CALCRL & RAMP2
CALCA→CALCRL & RAMP3
CALCA→CALCR & RAMP1
CALCA→CALCR & RAMP3
CALCB→CALCR
CALCB→CALCRL & RAMP1
CALCB→CALCRL & RAMP2
CALCB→CALCRL & RAMP3
CALCB→CALCR & RAMP1
CALCB→CALCR & RAMP3
Compartment
epithelial
- integumentary
- respiratory
- gastrointestinal
- urinary
- reproductive
neural
- GABAergic
- hybrid
- glutamatergic
- specialized
- neuroendocrine
- glia
germ
- spermatogonium
- spermatocyte
- spermatid
12.5%
25%
50%
100%
ln(CP10K+1)
Abbrev. Tissue
Aort Aorta
Blad Bladder
Blo Blood
Bone Bone
BM Bone marrow
BrC Brain cortex
BrS Brainstem
Col Colon
Dia Diaphragm
Ret Eye retina
Fat Fat
Hrt Heart
Pit Hypothalamus/Pituitary
Kid Kidney
LM Limb muscle
Liv Liver
Lung Lung
MG Mammary gland
Panc Pancreas
Skin Skin
SI Small intestine
Spl Spleen
Test Testes
Thym Thymus
Tong Tongue
Trac Trachea
Ute Uterus

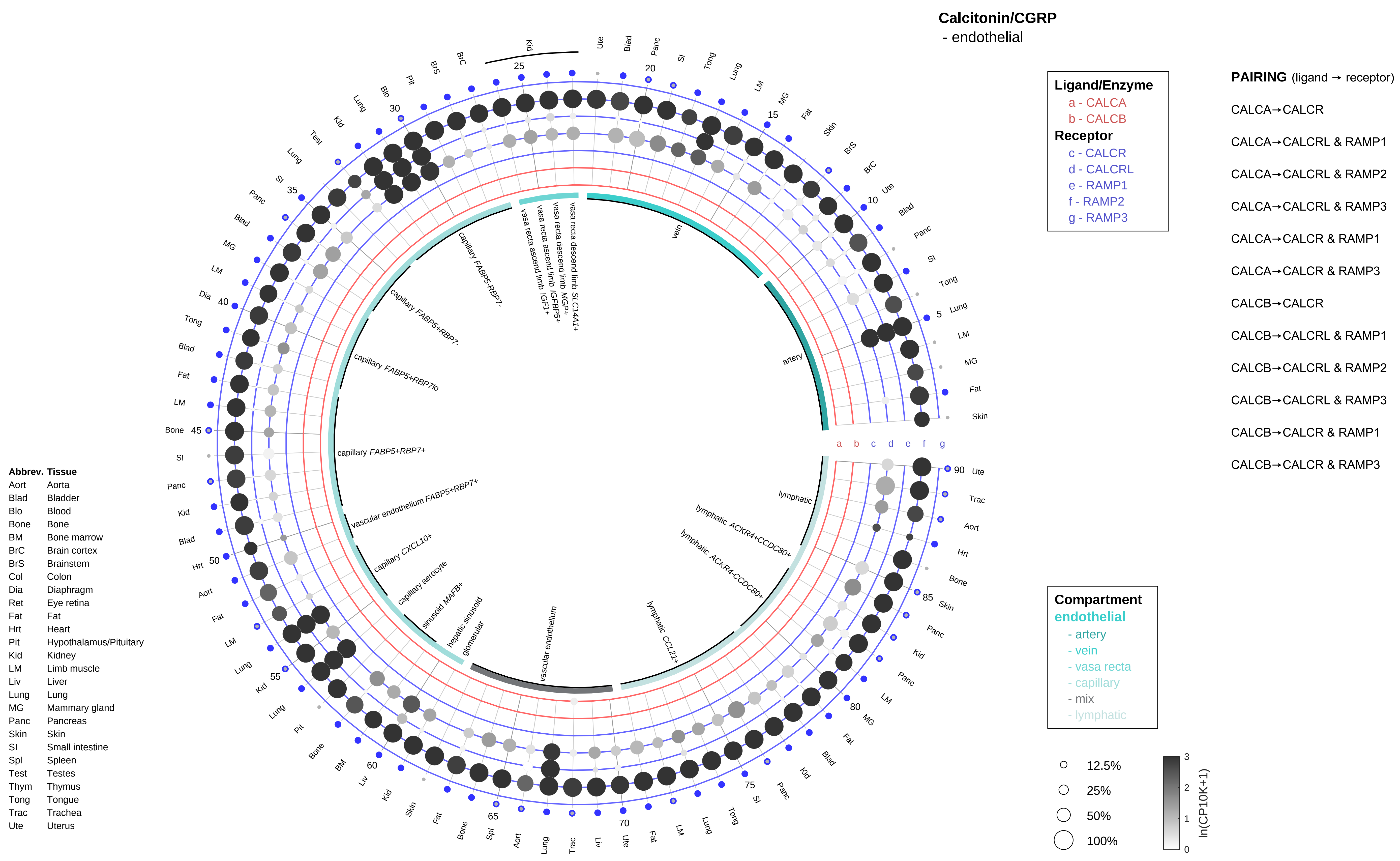
Calcitonin/CGRP
- endothelial
Ligand/Enzyme
a - CALCA
b - CALCB
Receptor
c - CALCR
d - CALCRL
e - RAMP1
f - RAMP2
g - RAMP3
PAIRING (ligand → receptor)
CALCA→CALCR
CALCA→CALCRL & RAMP1
CALCA→CALCRL & RAMP2
CALCA→CALCRL & RAMP3
CALCA→CALCR & RAMP1
CALCA→CALCR & RAMP3
CALCB→CALCR
CALCB→CALCRL & RAMP1
CALCB→CALCRL & RAMP2
CALCB→CALCRL & RAMP3
CALCB→CALCR & RAMP1
CALCB→CALCR & RAMP3
Compartment
endothelial
- artery
- vein
- vasa recta
- capillary
- mix
- lymphatic
12.5%
25%
50%
100%
ln(CP10K+1)
0
1
2
3
Abbrev. Tissue
Aort Aorta
Blad Bladder
Blo Blood
Bone Bone
BM Bone marrow
BrC Brain cortex
BrS Brainstem
Col Colon
Dia Diaphragm
Ret Eye retina
Fat Fat
Hrt Heart
Pit Hypothalamus/Pituitary
Kid Kidney
LM Limb muscle
Liv Liver
Lung Lung
MG Mammary gland
Panc Pancreas
Skin Skin
SI Small intestine
Spl Spleen
Test Testes
Thym Thymus
Tong Tongue
Trac Trachea
Ute Uterus
a b c d e f g
artery
vein
vasa recta descend limb SLC14A1+
vasa recta descend limb MGP+
vasa recta ascend limb IGFBP5+
vasa recta ascend limb IGF1+
capillary FABP5-RBP7-
capillary FABP5+RBP7-
capillary FABP5+RBP7lo
capillary FABP5+RBP7+
vascular endothelium FABP5+RBP7+
capillary CXCL10+
capillary aerocyte
sinusoid MAFB+
hepatic sinusoid
glomerular
vascular endothelium
lymphatic CCL21+
lymphatic ACKR4-CCDC80+
lymphatic ACKR4+CCDC80+
lymphatic

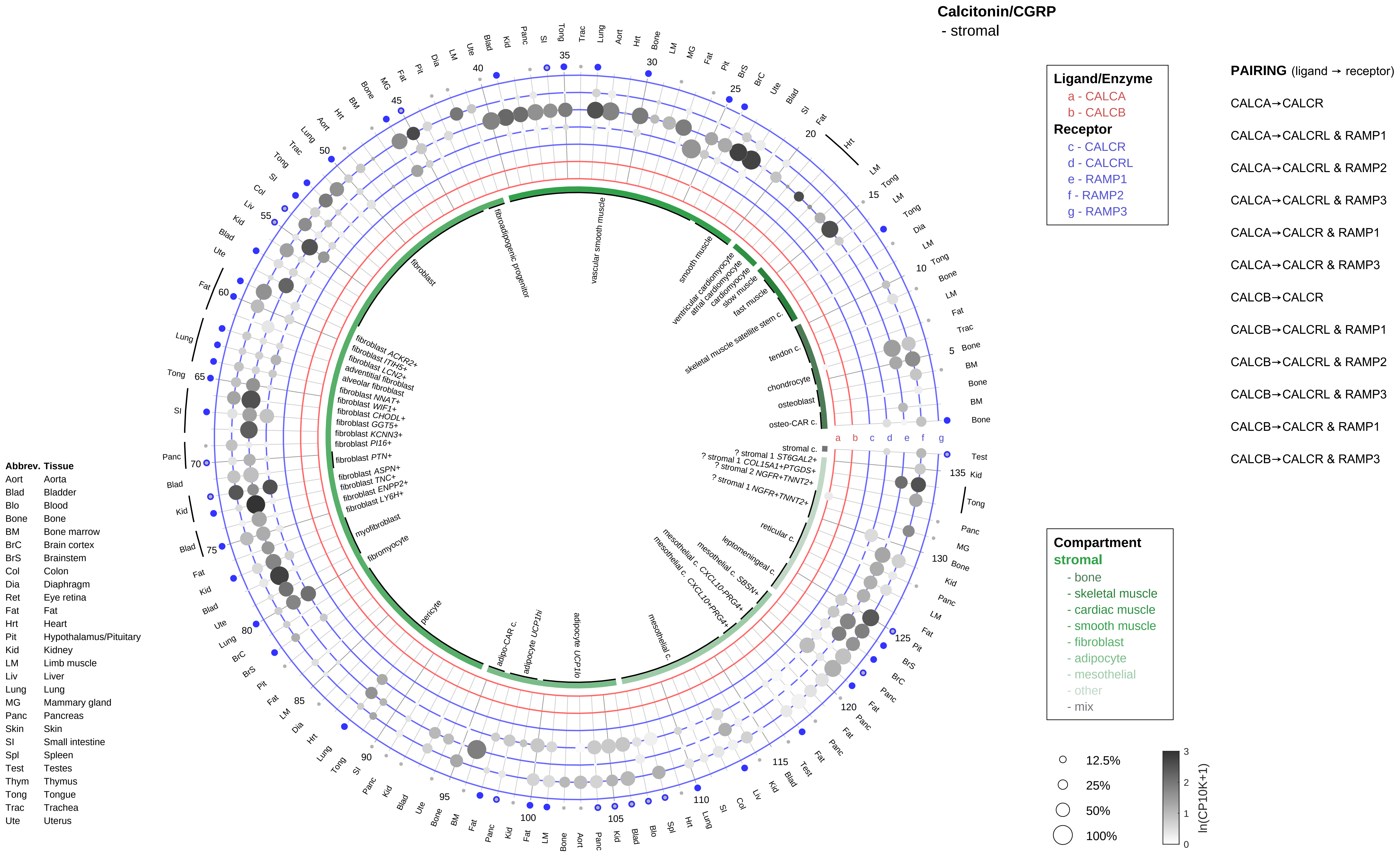

Calcitonin/CGRP
- stromal
Ligand/Enzyme
a - CALCA
b - CALCB
Receptor
c - CALCR
d - CALCRL
e - RAMP1
f - RAMP2
g - RAMP3
PAIRING (ligand → receptor)
CALCA→CALCR
CALCA→CALCRL & RAMP1
CALCA→CALCRL & RAMP2
CALCA→CALCRL & RAMP3
CALCA→CALCR & RAMP1
CALCA→CALCR & RAMP3
CALCB→CALCR
CALCB→CALCRL & RAMP1
CALCB→CALCRL & RAMP2
CALCB→CALCRL & RAMP3
CALCB→CALCR & RAMP1
CALCB→CALCR & RAMP3
Compartment
stromal
- bone
- skeletal muscle
- cardiac muscle
- smooth muscle
- fibroblast
- adipocyte
- mesothelial
- other
- mix
12.5%
25%
50%
100%
ln(CP10K+1)
0
1
2
3
fibroadipogenic progenitor
vascular smooth muscle
smooth muscle
ventricular cardiomyocyte
atrial cardiomyocyte
cardiomyocyte
slow muscle
fast muscle
skeletal muscle satellite stem c.
tendon c.
chondrocyte
osteoblast
osteo-CAR c.
stromal c.
? stromal 1 ST6GAL2+
? stromal 1 COL15A1+PTGDS+
? stromal 2 NGFR+TNNT2+
? stromal 1 NGFR+TNNT2+
reticular c.
leptomeningeal c.
mesothelial c. SBSN+
mesothelial c. CXCL10-PRG4+
mesothelial c. CXCL10+PRG4+
mesothelial c.
adipocyte UCP1lo
adipocyte UCP1hi
adipo-CAR c.
pericyte
fibromyocyte
myofibroblast
fibroblast LY6H+
fibroblast ENPP2+
fibroblast TNC+
fibroblast ASPN+
fibroblast PTN+
fibroblast PI16+
fibroblast KCNN3+
fibroblast GGT5+
fibroblast CHODL+
fibroblast WIF1+
fibroblast NNAT+
alveolar fibroblast
adventitial fibroblast
fibroblast LCN2+
fibroblast ITIH5+
fibroblast ACKR2+
fibroblast
Abbrev. Tissue
Aort Aorta
Blad Bladder
Blo Blood
Bone Bone
BM Bone marrow
BrC Brain cortex
BrS Brainstem
Col Colon
Dia Diaphragm
Ret Eye retina
Fat Fat
Hrt Heart
Pit Hypothalamus/Pituitary
Kid Kidney
LM Limb muscle
Liv Liver
Lung Lung
MG Mammary gland
Panc Pancreas
Skin Skin
SI Small intestine
Spl Spleen
Test Testes
Thym Thymus
Tong Tongue
Trac Trachea
Ute Uterus

Calcitonin/CGRP
- lymphoid

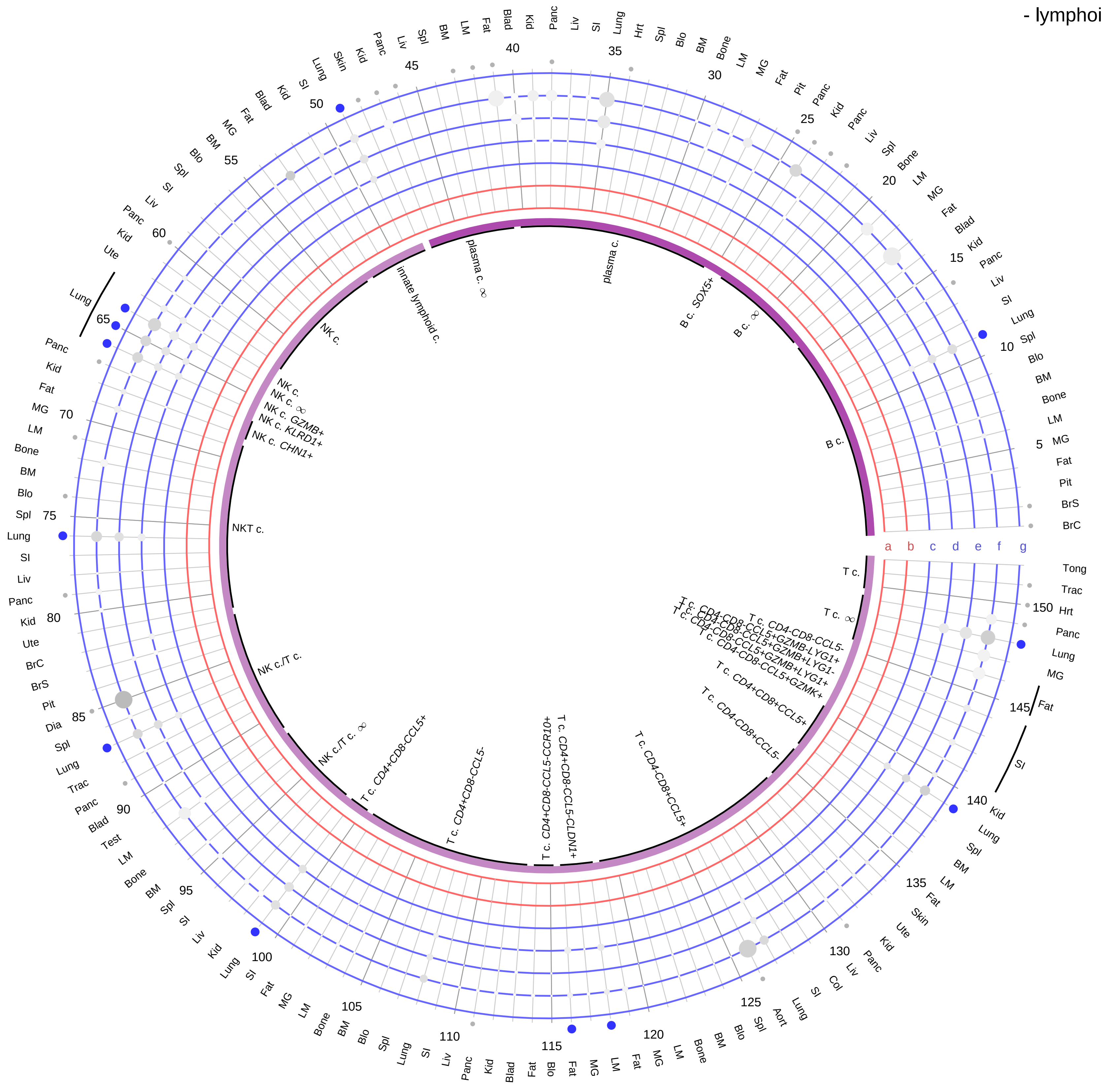

Ligand/Enzyme
- a - CALCA
- b - CALCB

Receptor
- c - CALCR
- d - CALCRL
- e - RAMP1
- f - RAMP2
- g - RAMP3

PAIRING (ligand → receptor)

CALCA→CALCR

CALCA→CALCRL & RAMP1

CALCA→CALCRL & RAMP2

CALCA→CALCRL & RAMP3

CALCA→CALCR & RAMP1

CALCA→CALCR & RAMP3

CALCB→CALCR

CALCB→CALCRL & RAMP1

CALCB→CALCRL & RAMP2

CALCB→CALCRL & RAMP3

CALCB→CALCR & RAMP1

CALCB→CALCR & RAMP3

Compartment
lymphoid
- B cell
- NK/T cell

12.5%
25%
50%
100%

ln(CP10K+1): 0, 1, 2, 3

**Abbrev.**	**Tissue**
Aort	Aorta
Blad	Bladder
Blo	Blood
Bone	Bone
BM	Bone marrow
BrC	Brain cortex
BrS	Brainstem
Col	Colon
Dia	Diaphragm
Ret	Eye retina
Fat	Fat
Hrt	Heart
Pit	Hypothalamus/Pituitary
Kid	Kidney
LM	Limb muscle
Liv	Liver
Lung	Lung
MG	Mammary gland
Panc	Pancreas
Skin	Skin
SI	Small intestine
Spl	Spleen
Test	Testes
Thym	Thymus
Tong	Tongue
Trac	Trachea
Ute	Uterus

Calcitonin/CGRP
- myeloid

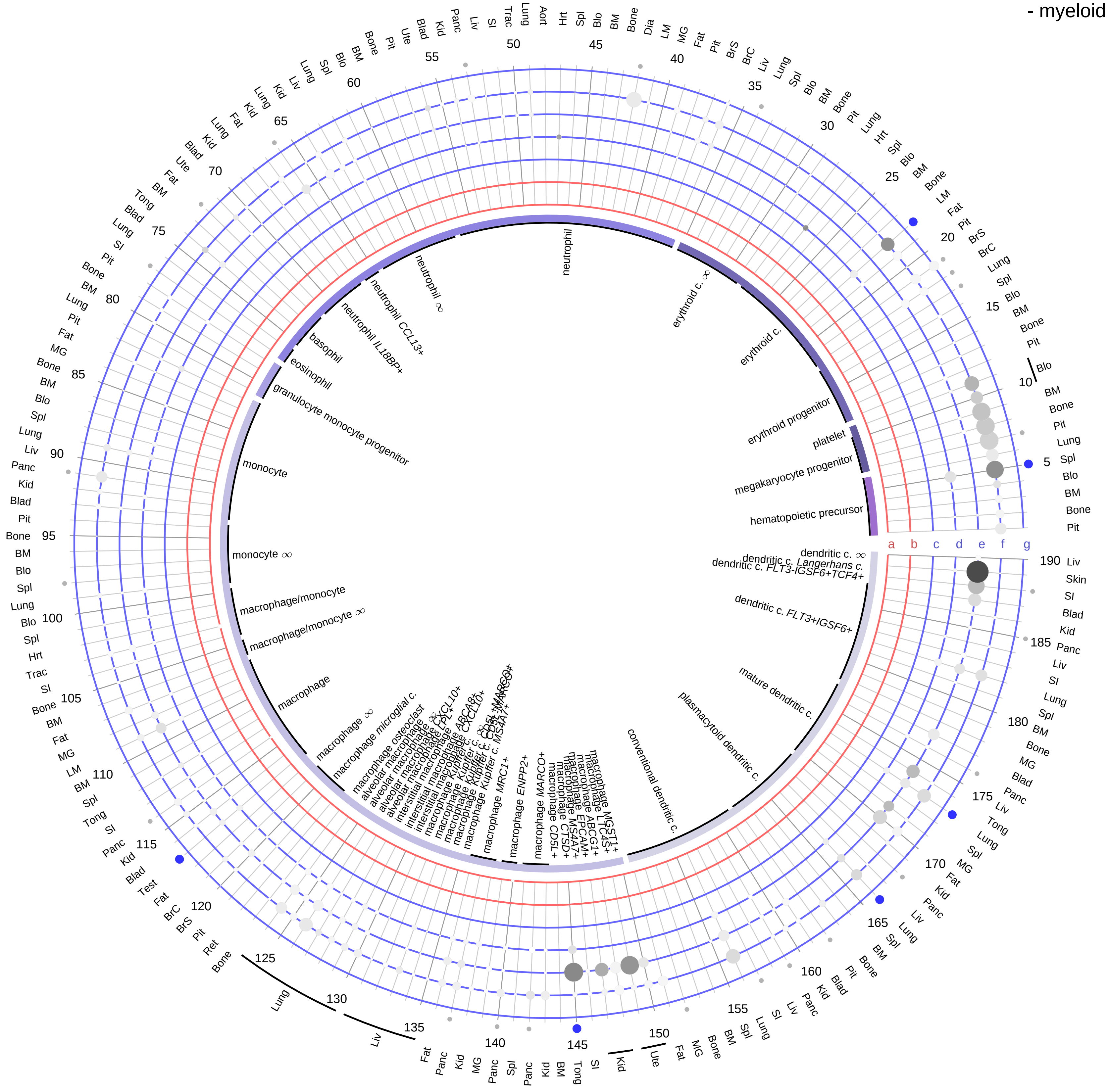

Ligand/Enzyme
- a - CALCA
- b - CALCB

Receptor
- c - CALCR
- d - CALCRL
- e - RAMP1
- f - RAMP2
- g - RAMP3

PAIRING (ligand → receptor)

CALCA→CALCR

CALCA→CALCRL & RAMP1

CALCA→CALCRL & RAMP2

CALCA→CALCRL & RAMP3

CALCA→CALCR & RAMP1

CALCA→CALCR & RAMP3

CALCB→CALCR

CALCB→CALCRL & RAMP1

CALCB→CALCRL & RAMP2

CALCB→CALCRL & RAMP3

CALCB→CALCR & RAMP1

CALCB→CALCR & RAMP3

Compartment

hematopoietic
- hematopoietic precursor

megakaryocyte-erythroid
- megakaryoid
- erythroid

myeloid
- granulocyte
- granulocyte monocyte progenitor
- monocyte/macrophage
- dendritic

12.5%
25%
50%
100%

ln(CP10K+1): 0, 1, 2, 3

Abbrev.	Tissue
Aort	Aorta
Blad	Bladder
Blo	Blood
Bone	Bone
BM	Bone marrow
BrC	Brain cortex
BrS	Brainstem
Col	Colon
Dia	Diaphragm
Ret	Eye retina
Fat	Fat
Hrt	Heart
Pit	Hypothalamus/Pituitary
Kid	Kidney
LM	Limb muscle
Liv	Liver
Lung	Lung
MG	Mammary gland
Panc	Pancreas
Skin	Skin
SI	Small intestine
Spl	Spleen
Test	Testes
Thym	Thymus
Tong	Tongue
Trac	Trachea
Ute	Uterus

Cholecalciferol/Calcidiol/Calcitriol
- epithelial/neural/germ

PAIRING (ligand → receptor)

CYP27B1→VDR & RXRA

CYP27B1→VDR & RXRB

CYP27B1→VDR & RXRG

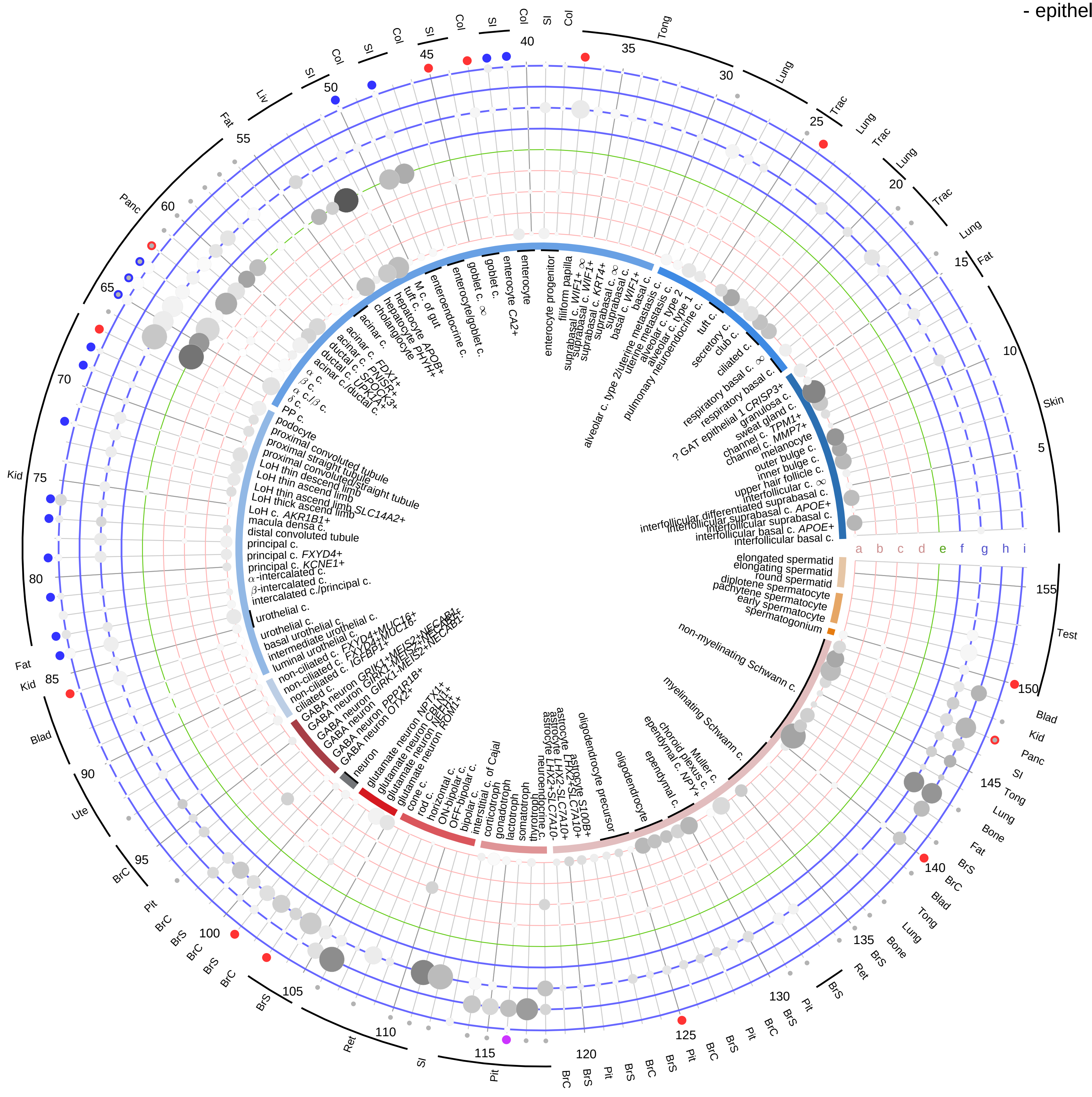

Ligand/Enzyme
- a - CYP27A1
- b - CYP27B1
- c - CYP2R1
- d - DHCR7

Modulator
- e - GC

Receptor
- f - RXRA
- g - RXRB
- h - RXRG
- i - VDR

Compartment

epithelial
- integumentary
- respiratory
- gastrointestinal
- urinary
- reproductive

neural
- GABAergic
- hybrid
- glutamatergic
- specialized
- neuroendocrine
- glia

germ
- spermatogonium
- spermatocyte
- spermatid

12.5%
25%
50%
100%

ln(CP10K+1): 0, 1, 2, 3

Abbrev.	Tissue
Aort	Aorta
Blad	Bladder
Blo	Blood
Bone	Bone
BM	Bone marrow
BrC	Brain cortex
BrS	Brainstem
Col	Colon
Dia	Diaphragm
Ret	Eye retina
Fat	Fat
Hrt	Heart
Pit	Hypothalamus/Pituitary
Kid	Kidney
LM	Limb muscle
Liv	Liver
Lung	Lung
MG	Mammary gland
Panc	Pancreas
Skin	Skin
SI	Small intestine
Spl	Spleen
Test	Testes
Thym	Thymus
Tong	Tongue
Trac	Trachea
Ute	Uterus

Cholecalciferol/Calcidiol/Calcitriol
- endothelial

PAIRING (ligand → receptor)

CYP27B1→VDR & RXRA

CYP27B1→VDR & RXRB

CYP27B1→VDR & RXRG

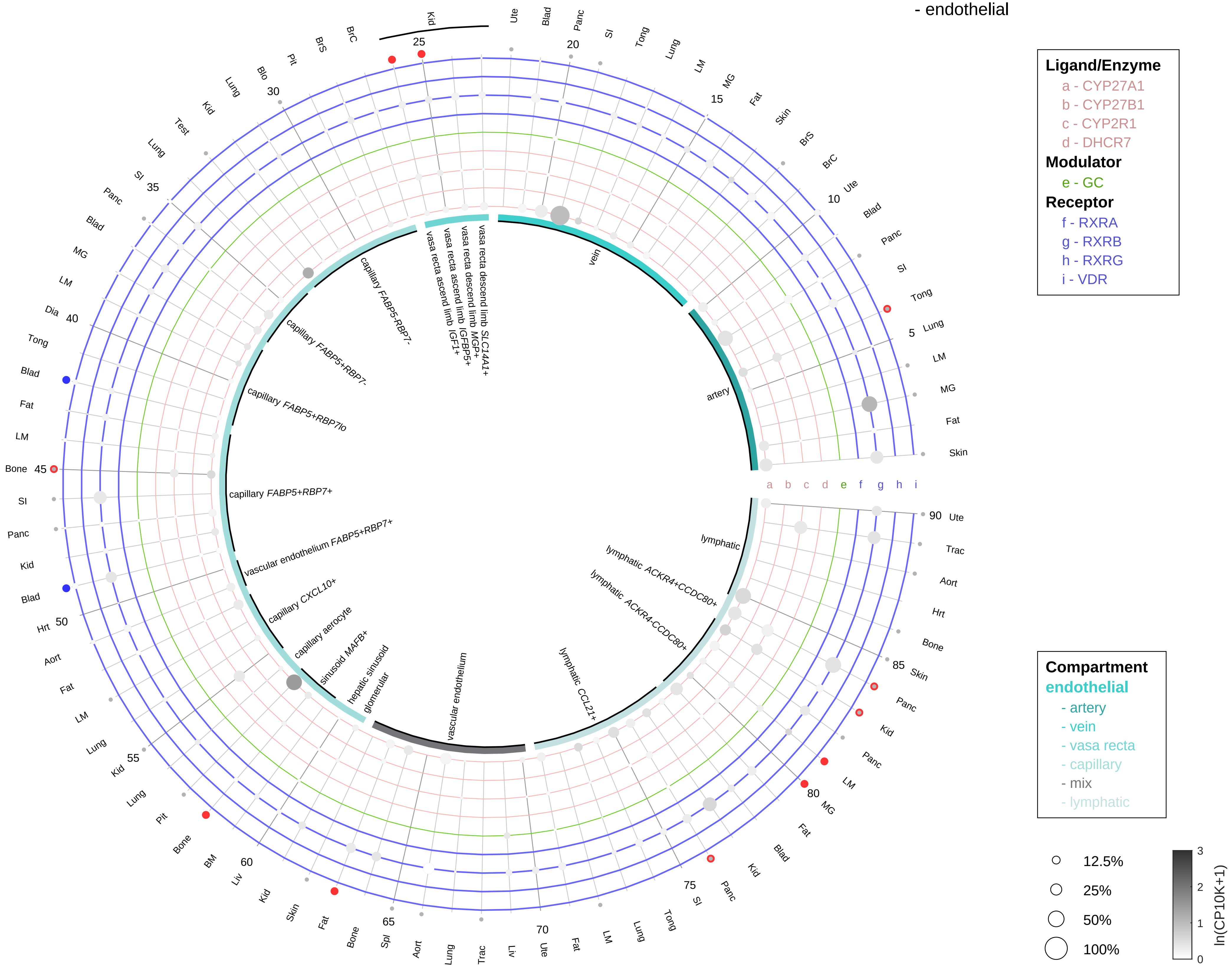

Abbrev.	Tissue
Aort	Aorta
Blad	Bladder
Blo	Blood
Bone	Bone
BM	Bone marrow
BrC	Brain cortex
BrS	Brainstem
Col	Colon
Dia	Diaphragm
Ret	Eye retina
Fat	Fat
Hrt	Heart
Pit	Hypothalamus/Pituitary
Kid	Kidney
LM	Limb muscle
Liv	Liver
Lung	Lung
MG	Mammary gland
Panc	Pancreas
Skin	Skin
SI	Small intestine
Spl	Spleen
Test	Testes
Thym	Thymus
Tong	Tongue
Trac	Trachea
Ute	Uterus

Cholecalciferol/Calcidiol/Calcitriol

- stromal

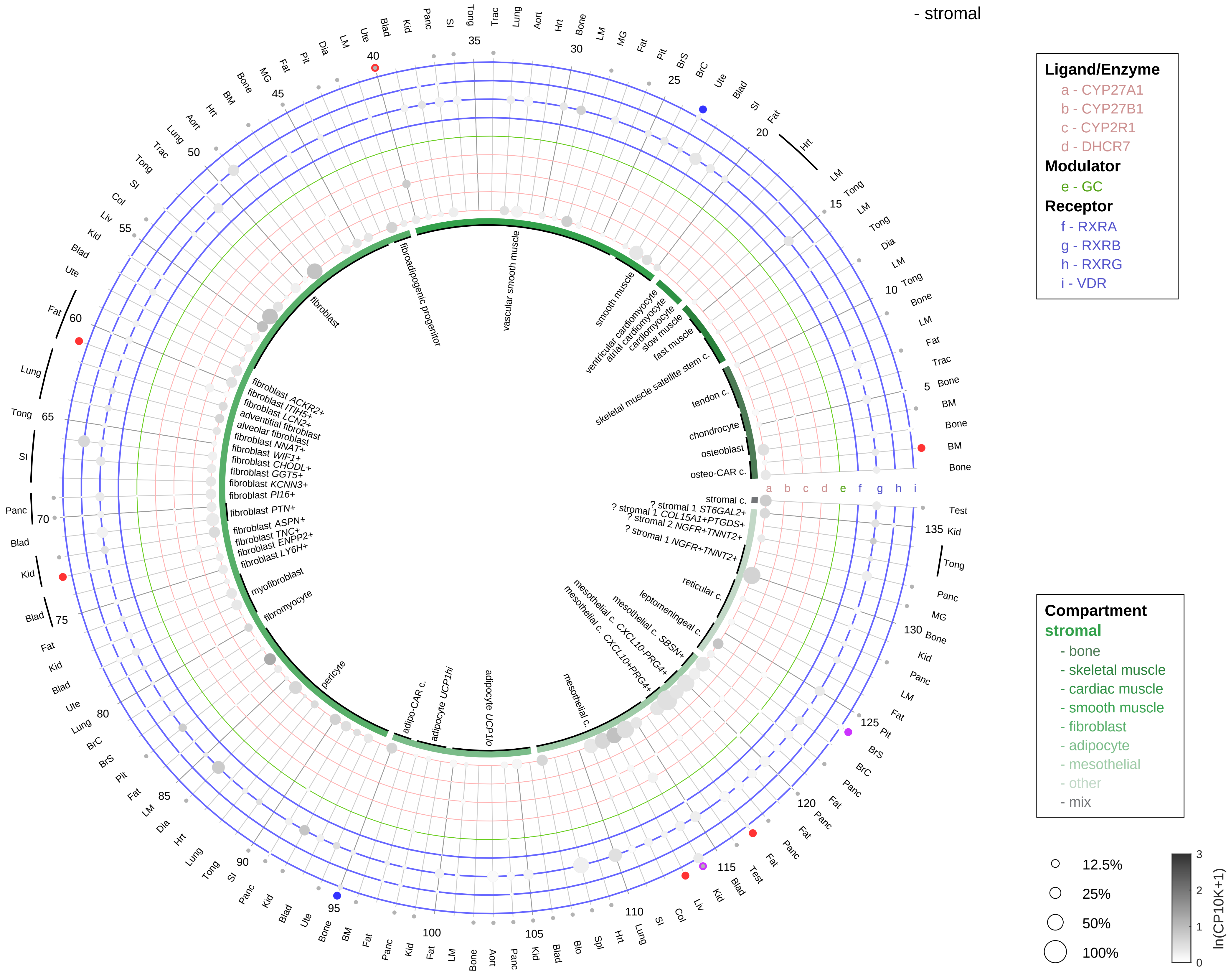

PAIRING (ligand → receptor)

CYP27B1→VDR & RXRA

CYP27B1→VDR & RXRB

CYP27B1→VDR & RXRG

Abbrev.	Tissue
Aort	Aorta
Blad	Bladder
Blo	Blood
Bone	Bone
BM	Bone marrow
BrC	Brain cortex
BrS	Brainstem
Col	Colon
Dia	Diaphragm
Ret	Eye retina
Fat	Fat
Hrt	Heart
Pit	Hypothalamus/Pituitary
Kid	Kidney
LM	Limb muscle
Liv	Liver
Lung	Lung
MG	Mammary gland
Panc	Pancreas
Skin	Skin
SI	Small intestine
Spl	Spleen
Test	Testes
Thym	Thymus
Tong	Tongue
Trac	Trachea
Ute	Uterus

Cholecalciferol/Calcidiol/Calcitriol

- lymphoid

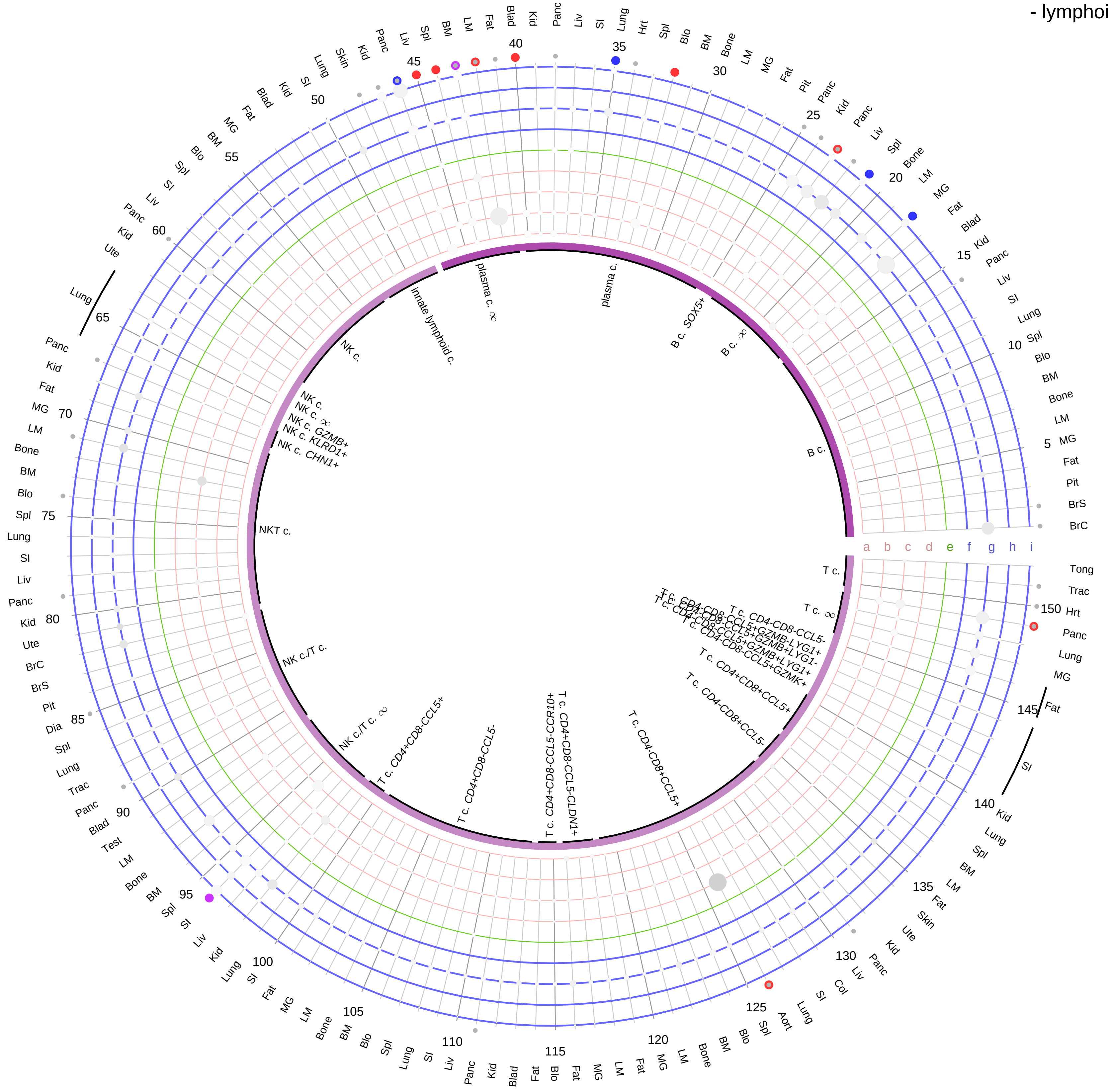

Ligand/Enzyme
a - CYP27A1
b - CYP27B1
c - CYP2R1
d - DHCR7
Modulator
e - GC
Receptor
f - RXRA
g - RXRB
h - RXRG
i - VDR

PAIRING (ligand → receptor)

CYP27B1→VDR & RXRA

CYP27B1→VDR & RXRB

CYP27B1→VDR & RXRG

Compartment
lymphoid
- B cell
- NK/T cell

12.5%
25%
50%
100%

ln(CP10K+1): 0, 1, 2, 3

Abbrev.	Tissue
Aort	Aorta
Blad	Bladder
Blo	Blood
Bone	Bone
BM	Bone marrow
BrC	Brain cortex
BrS	Brainstem
Col	Colon
Dia	Diaphragm
Ret	Eye retina
Fat	Fat
Hrt	Heart
Pit	Hypothalamus/Pituitary
Kid	Kidney
LM	Limb muscle
Liv	Liver
Lung	Lung
MG	Mammary gland
Panc	Pancreas
Skin	Skin
SI	Small intestine
Spl	Spleen
Test	Testes
Thym	Thymus
Tong	Tongue
Trac	Trachea
Ute	Uterus

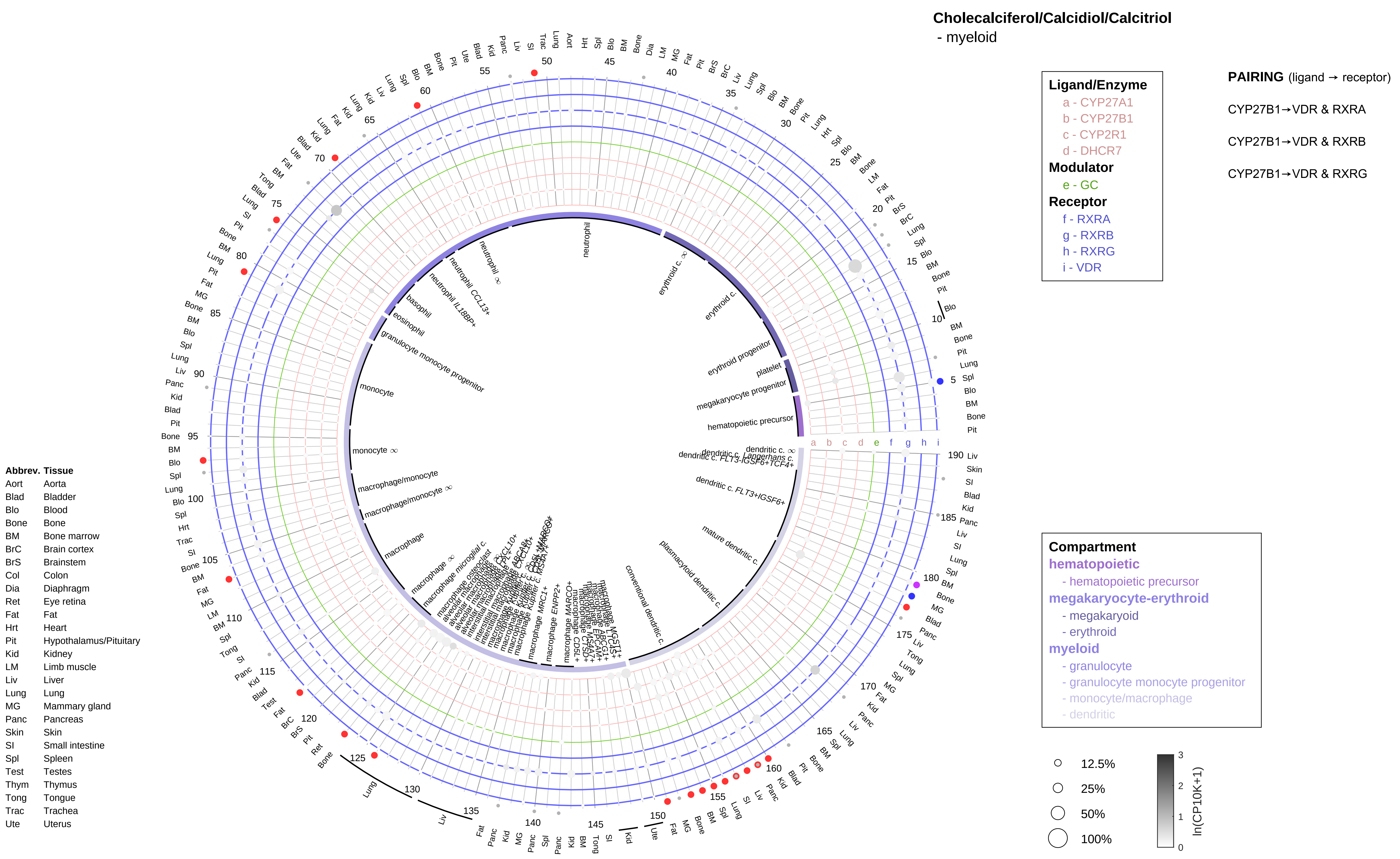
Cholecalciferol/Calcidiol/Calcitriol
- myeloid
Ligand/Enzyme
a - CYP27A1
b - CYP27B1
c - CYP2R1
d - DHCR7
Modulator
e - GC
Receptor
f - RXRA
g - RXRB
h - RXRG
i - VDR
PAIRING (ligand → receptor)
CYP27B1→VDR & RXRA
CYP27B1→VDR & RXRB
CYP27B1→VDR & RXRG
Compartment
hematopoietic
- hematopoietic precursor
megakaryocyte-erythroid
- megakaryoid
- erythroid
myeloid
- granulocyte
- granulocyte monocyte progenitor
- monocyte/macrophage
- dendritic
12.5%
25%
50%
100%
ln(CP10K+1)
0
1
2
3
Abbrev. Tissue
Aort Aorta
Blad Bladder
Blo Blood
Bone Bone
BM Bone marrow
BrC Brain cortex
BrS Brainstem
Col Colon
Dia Diaphragm
Ret Eye retina
Fat Fat
Hrt Heart
Pit Hypothalamus/Pituitary
Kid Kidney
LM Limb muscle
Liv Liver
Lung Lung
MG Mammary gland
Panc Pancreas
Skin Skin
SI Small intestine
Spl Spleen
Test Testes
Thym Thymus
Tong Tongue
Trac Trachea
Ute Uterus

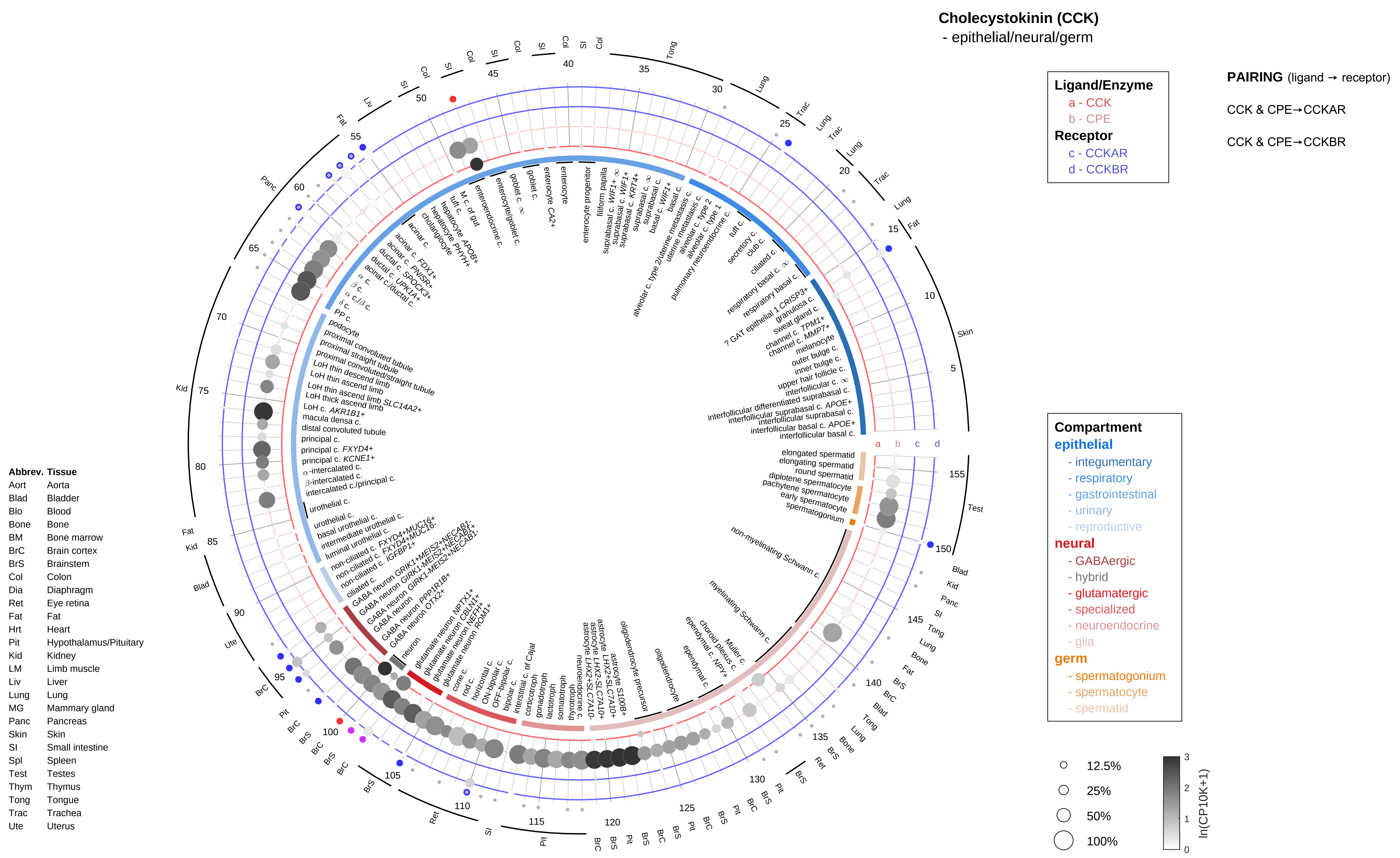

Cholecystokinin (CCK)
- epithelial/neural/germ
Ligand/Enzyme
a - CCK
b - CPE
Receptor
c - CCKAR
d - CCKBR
PAIRING (ligand → receptor)
CCK & CPE→CCKAR
CCK & CPE→CCKBR
Compartment
epithelial
- integumentary
- respiratory
- gastrointestinal
- urinary
- reproductive
neural
- GABAergic
- hybrid
- glutamatergic
- specialized
- neuroendocrine
- glia
germ
- spermatogonium
- spermatocyte
- spermatid
12.5%
25%
50%
100%
ln(CP10K+1)
Abbrev. Tissue
Aort Aorta
Blad Bladder
Blo Blood
Bone Bone
BM Bone marrow
BrC Brain cortex
BrS Brainstem
Col Colon
Dia Diaphragm
Ret Eye retina
Fat Fat
Hrt Heart
Pit Hypothalamus/Pituitary
Kid Kidney
LM Limb muscle
Liv Liver
Lung Lung
MG Mammary gland
Panc Pancreas
Skin Skin
SI Small intestine
Spl Spleen
Test Testes
Thym Thymus
Tong Tongue
Trac Trachea
Ute Uterus

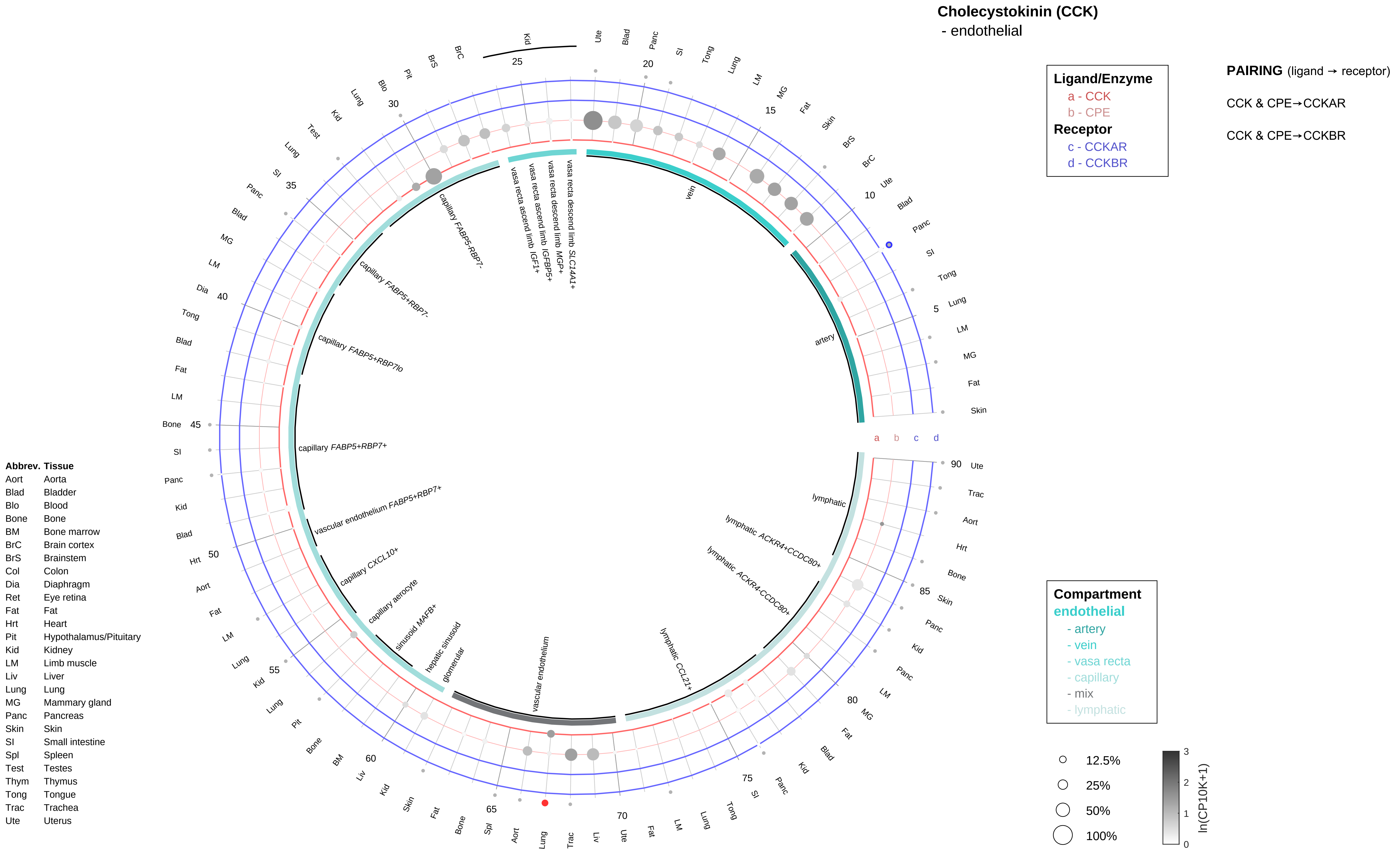
Cholecystokinin (CCK)
- endothelial
Ligand/Enzyme
a - CCK
b - CPE
Receptor
c - CCKAR
d - CCKBR
PAIRING (ligand → receptor)
CCK & CPE→CCKAR
CCK & CPE→CCKBR
vein
artery
lymphatic
lymphatic ACKR4+CCDC80+
lymphatic ACKR4-CCDC80+
lymphatic CCL21+
vascular endothelium
glomerular
hepatic sinusoid
sinusoid MAFB+
capillary aerocyte
capillary CXCL10+
vascular endothelium FABP5+RBP7+
capillary FABP5+RBP7+
capillary FABP5+RBP7lo
capillary FABP5+RBP7-
capillary FABP5-RBP7-
vasa recta ascend limb IGF1+
vasa recta ascend limb IGFBP5+
vasa recta descend limb MGP+
vasa recta descend limb SLC14A1+
a b c d
Compartment
endothelial
- artery
- vein
- vasa recta
- capillary
- mix
- lymphatic
12.5%
25%
50%
100%
ln(CP10K+1)
0
1
2
3
Abbrev. Tissue
Aort Aorta
Blad Bladder
Blo Blood
Bone Bone
BM Bone marrow
BrC Brain cortex
BrS Brainstem
Col Colon
Dia Diaphragm
Ret Eye retina
Fat Fat
Hrt Heart
Pit Hypothalamus/Pituitary
Kid Kidney
LM Limb muscle
Liv Liver
Lung Lung
MG Mammary gland
Panc Pancreas
Skin Skin
SI Small intestine
Spl Spleen
Test Testes
Thym Thymus
Tong Tongue
Trac Trachea
Ute Uterus

Cholecystokinin (CCK)
- stromal

Ligand/Enzyme
- a - CCK
- b - CPE

Receptor
- c - CCKAR
- d - CCKBR

PAIRING (ligand → receptor)

CCK & CPE→CCKAR

CCK & CPE→CCKBR

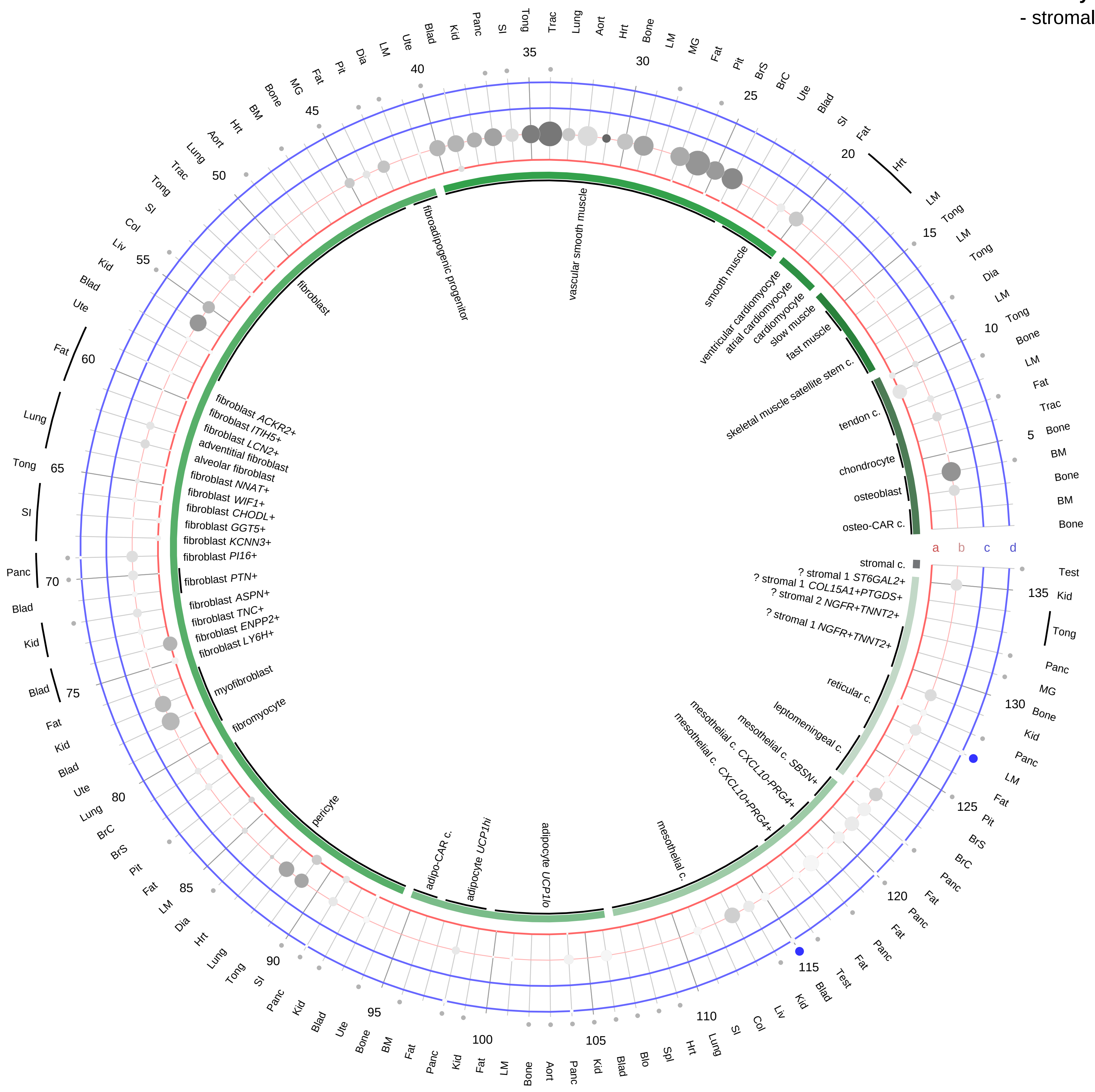

Abbrev.	Tissue
Aort	Aorta
Blad	Bladder
Blo	Blood
Bone	Bone
BM	Bone marrow
BrC	Brain cortex
BrS	Brainstem
Col	Colon
Dia	Diaphragm
Ret	Eye retina
Fat	Fat
Hrt	Heart
Pit	Hypothalamus/Pituitary
Kid	Kidney
LM	Limb muscle
Liv	Liver
Lung	Lung
MG	Mammary gland
Panc	Pancreas
Skin	Skin
SI	Small intestine
Spl	Spleen
Test	Testes
Thym	Thymus
Tong	Tongue
Trac	Trachea
Ute	Uterus

Compartment
stromal
- bone
- skeletal muscle
- cardiac muscle
- smooth muscle
- fibroblast
- adipocyte
- mesothelial
- other
- mix

12.5%
25%
50%
100%

ln(CP10K+1): 0, 1, 2, 3

Cholecystokinin (CCK)
- lymphoid

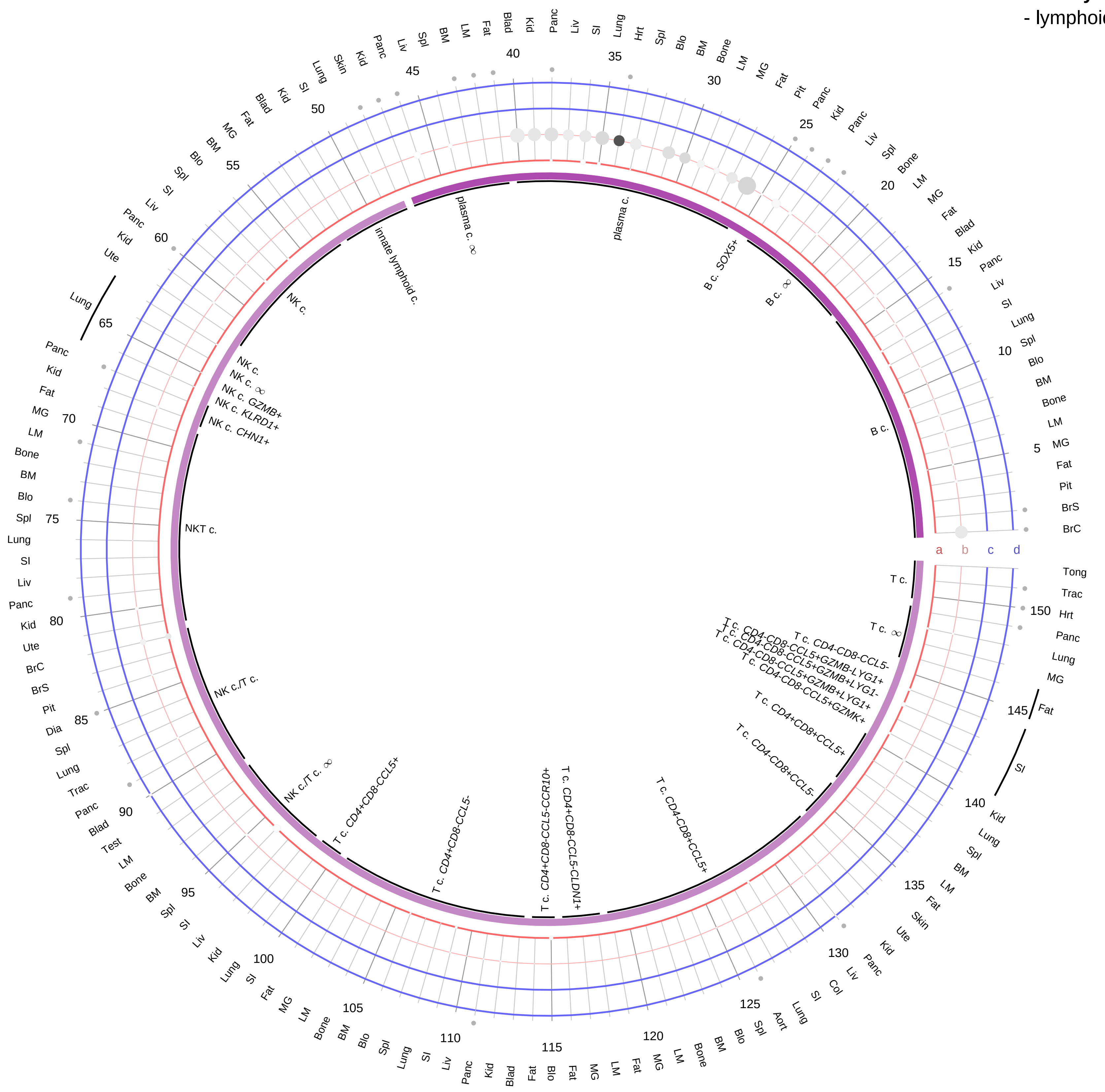

Ligand/Enzyme
- a - CCK
- b - CPE

Receptor
- c - CCKAR
- d - CCKBR

PAIRING (ligand → receptor)

CCK & CPE→CCKAR

CCK & CPE→CCKBR

Compartment
lymphoid
- B cell
- NK/T cell

12.5%
25%
50%
100%

ln(CP10K+1): 0, 1, 2, 3

Abbrev.	Tissue
Aort	Aorta
Blad	Bladder
Blo	Blood
Bone	Bone
BM	Bone marrow
BrC	Brain cortex
BrS	Brainstem
Col	Colon
Dia	Diaphragm
Ret	Eye retina
Fat	Fat
Hrt	Heart
Pit	Hypothalamus/Pituitary
Kid	Kidney
LM	Limb muscle
Liv	Liver
Lung	Lung
MG	Mammary gland
Panc	Pancreas
Skin	Skin
SI	Small intestine
Spl	Spleen
Test	Testes
Thym	Thymus
Tong	Tongue
Trac	Trachea
Ute	Uterus

Cholecystokinin (CCK)
- myeloid

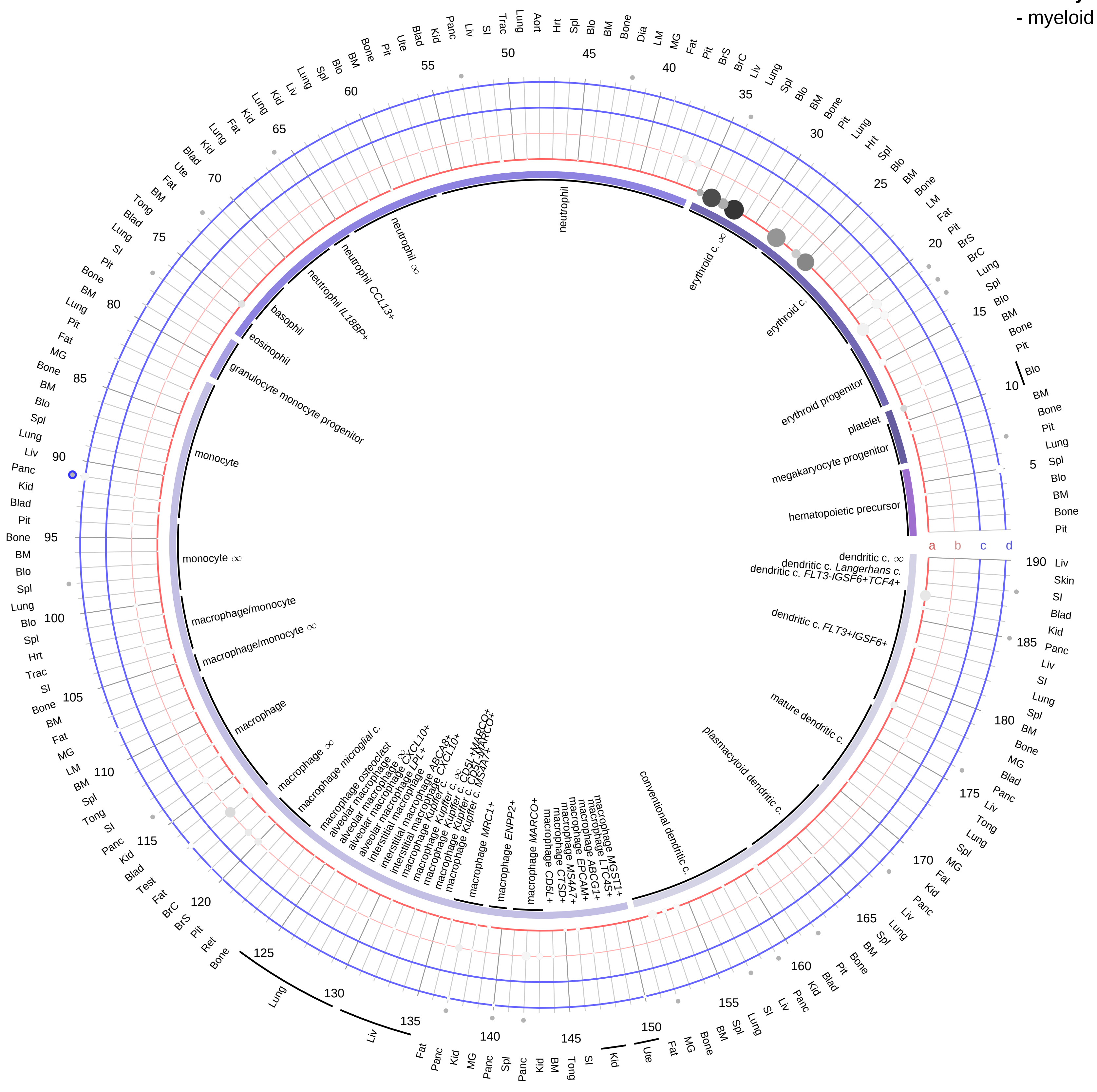

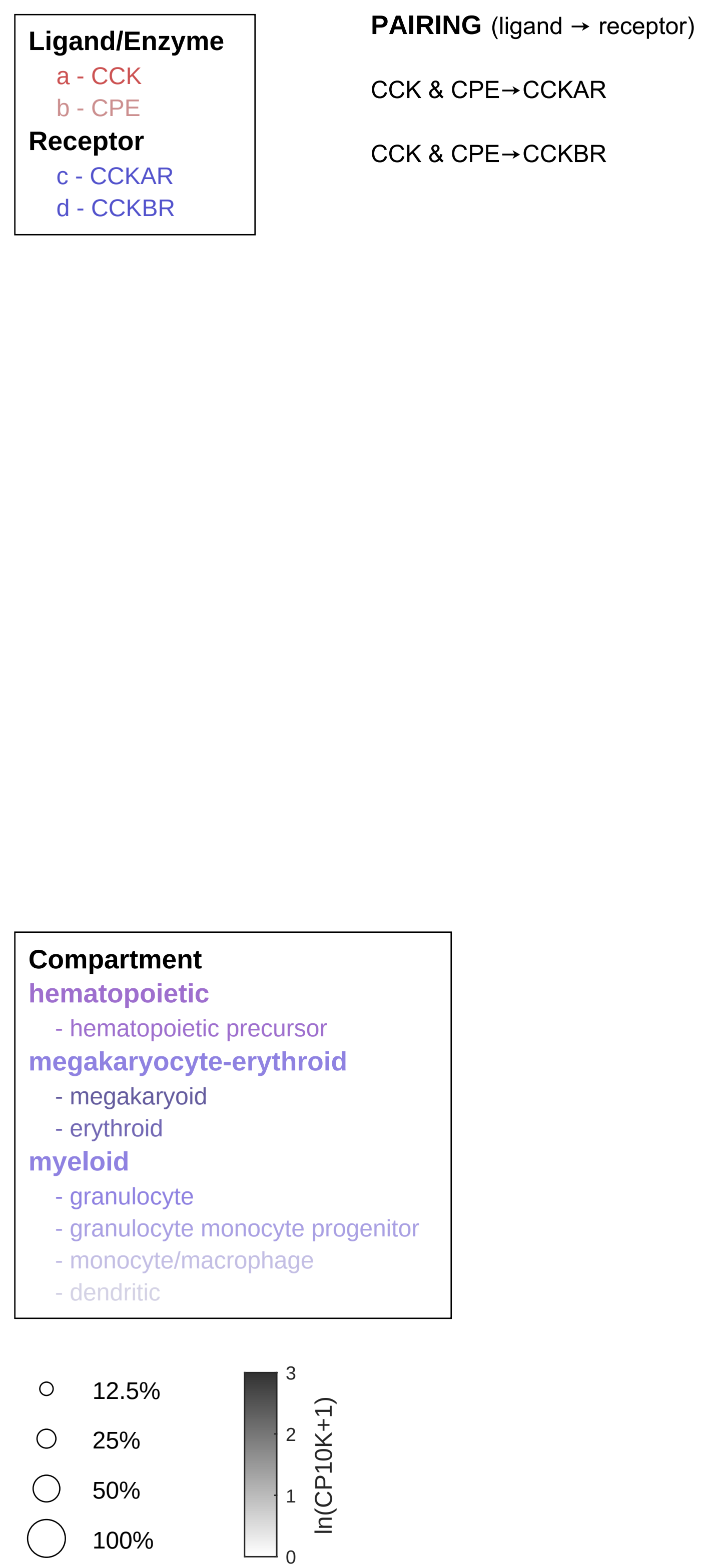

Abbrev.	Tissue
Aort	Aorta
Blad	Bladder
Blo	Blood
Bone	Bone
BM	Bone marrow
BrC	Brain cortex
BrS	Brainstem
Col	Colon
Dia	Diaphragm
Ret	Eye retina
Fat	Fat
Hrt	Heart
Pit	Hypothalamus/Pituitary
Kid	Kidney
LM	Limb muscle
Liv	Liver
Lung	Lung
MG	Mammary gland
Panc	Pancreas
Skin	Skin
SI	Small intestine
Spl	Spleen
Test	Testes
Thym	Thymus
Tong	Tongue
Trac	Trachea
Ute	Uterus

Cocaine- and amphetamine-regulated transcript (CART)
- epithelial/neural/germ

Ligand/Enzyme
- a - LOC105881981 (CARTPT)
- b - CPE

Receptor
- c - GPR68

PAIRING (ligand → receptor)

CARTPT & CPE→GPR68

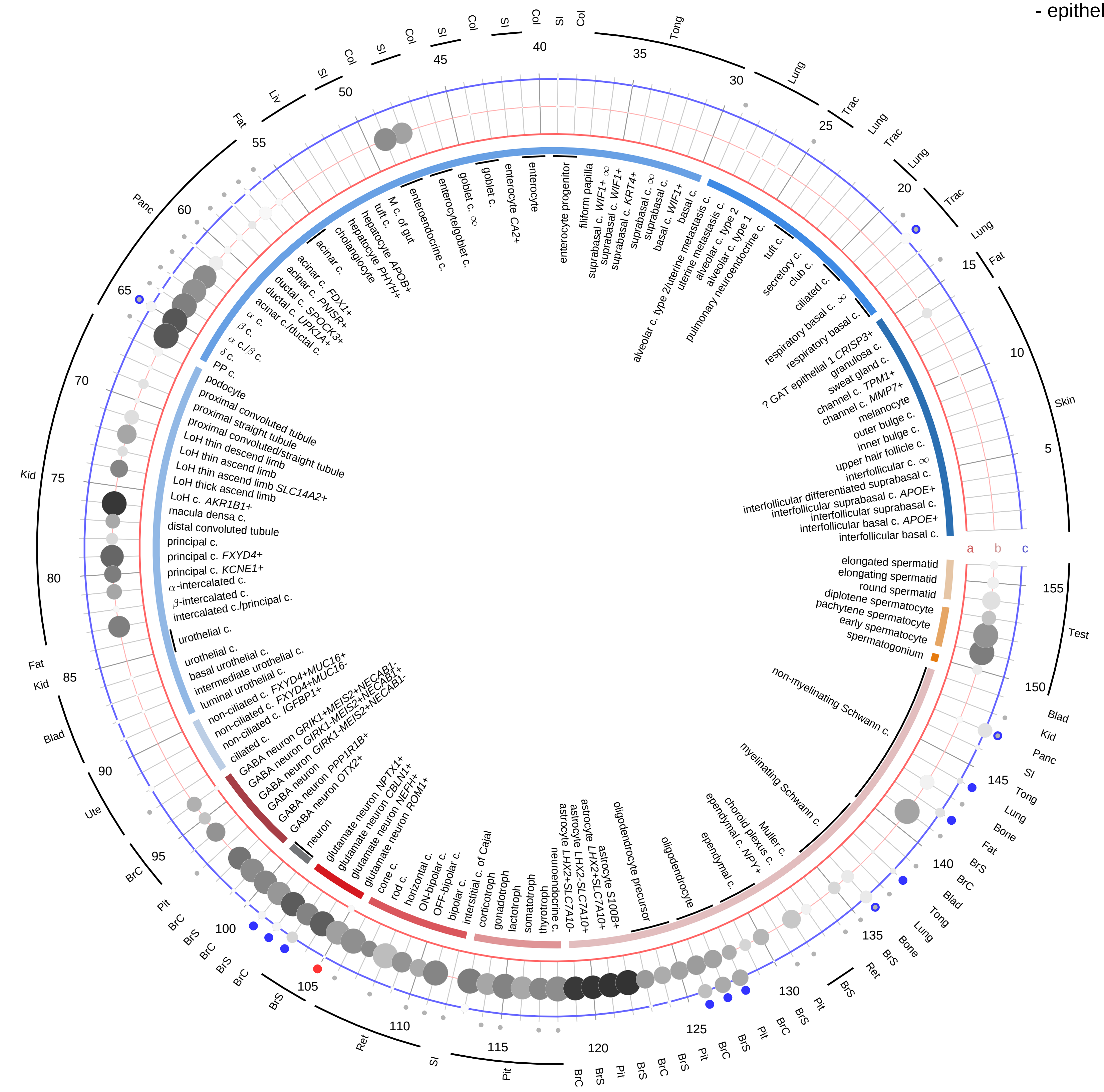

Abbrev.	Tissue
Aort	Aorta
Blad	Bladder
Blo	Blood
Bone	Bone
BM	Bone marrow
BrC	Brain cortex
BrS	Brainstem
Col	Colon
Dia	Diaphragm
Ret	Eye retina
Fat	Fat
Hrt	Heart
Pit	Hypothalamus/Pituitary
Kid	Kidney
LM	Limb muscle
Liv	Liver
Lung	Lung
MG	Mammary gland
Panc	Pancreas
Skin	Skin
SI	Small intestine
Spl	Spleen
Test	Testes
Thym	Thymus
Tong	Tongue
Trac	Trachea
Ute	Uterus

Compartment

epithelial
- integumentary
- respiratory
- gastrointestinal
- urinary
- reproductive

neural
- GABAergic
- hybrid
- glutamatergic
- specialized
- neuroendocrine
- glia

germ
- spermatogonium
- spermatocyte
- spermatid

12.5%
25%
50%
100%

ln(CP10K+1): 0, 1, 2, 3

Cocaine- and amphetamine-regulated transcript (CART)
- endothelial

Ligand/Enzyme
a - LOC105881981 (CARTPT)
b - CPE
Receptor
c - GPR68

PAIRING (ligand → receptor)

CARTPT & CPE→GPR68

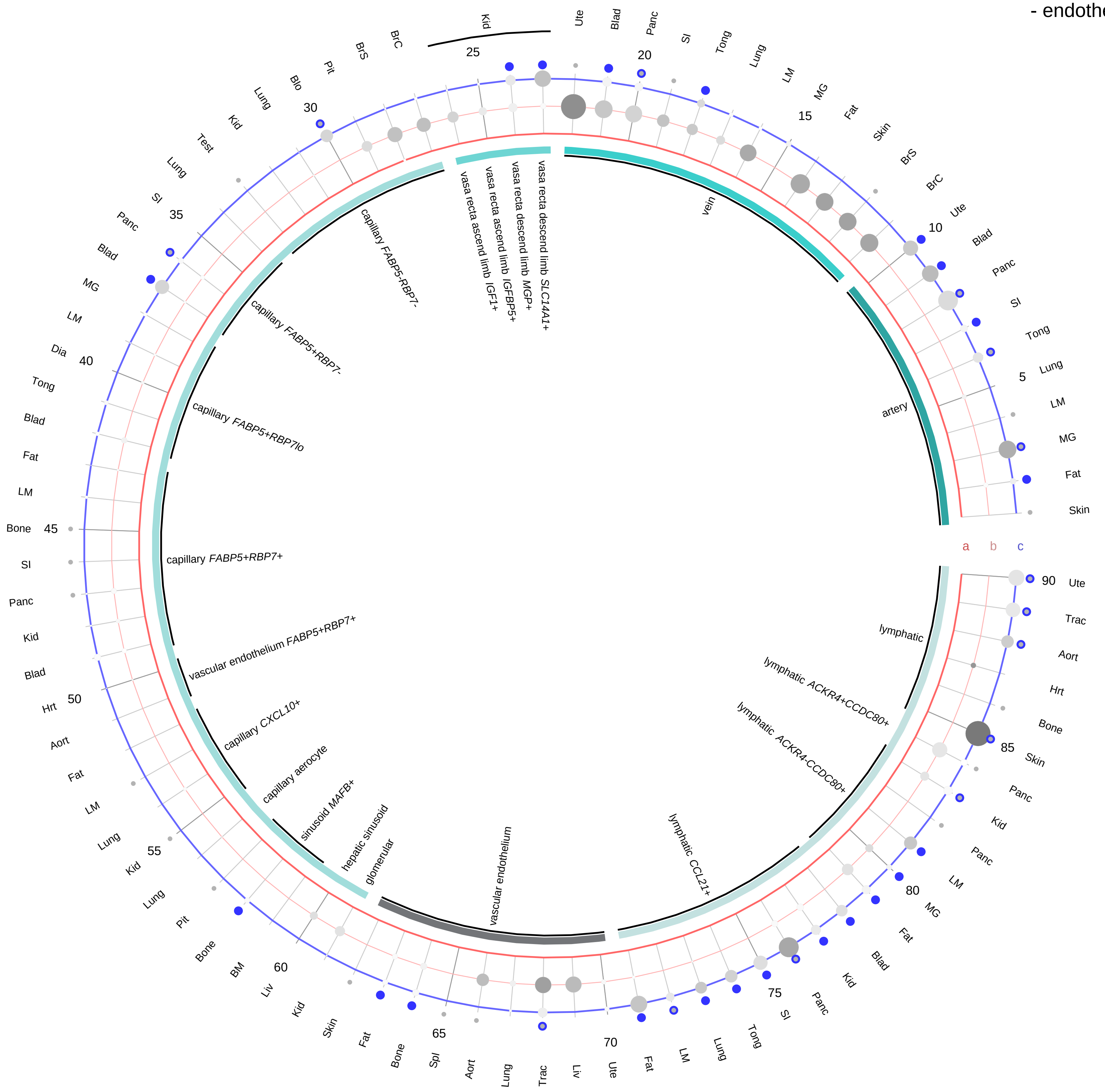

Abbrev.	Tissue
Aort	Aorta
Blad	Bladder
Blo	Blood
Bone	Bone
BM	Bone marrow
BrC	Brain cortex
BrS	Brainstem
Col	Colon
Dia	Diaphragm
Ret	Eye retina
Fat	Fat
Hrt	Heart
Pit	Hypothalamus/Pituitary
Kid	Kidney
LM	Limb muscle
Liv	Liver
Lung	Lung
MG	Mammary gland
Panc	Pancreas
Skin	Skin
SI	Small intestine
Spl	Spleen
Test	Testes
Thym	Thymus
Tong	Tongue
Trac	Trachea
Ute	Uterus

Compartment
endothelial
- artery
- vein
- vasa recta
- capillary
- mix
- lymphatic

12.5%
25%
50%
100%

ln(CP10K+1): 0, 1, 2, 3

Cocaine- and amphetamine-regulated transcript (CART)
- stromal

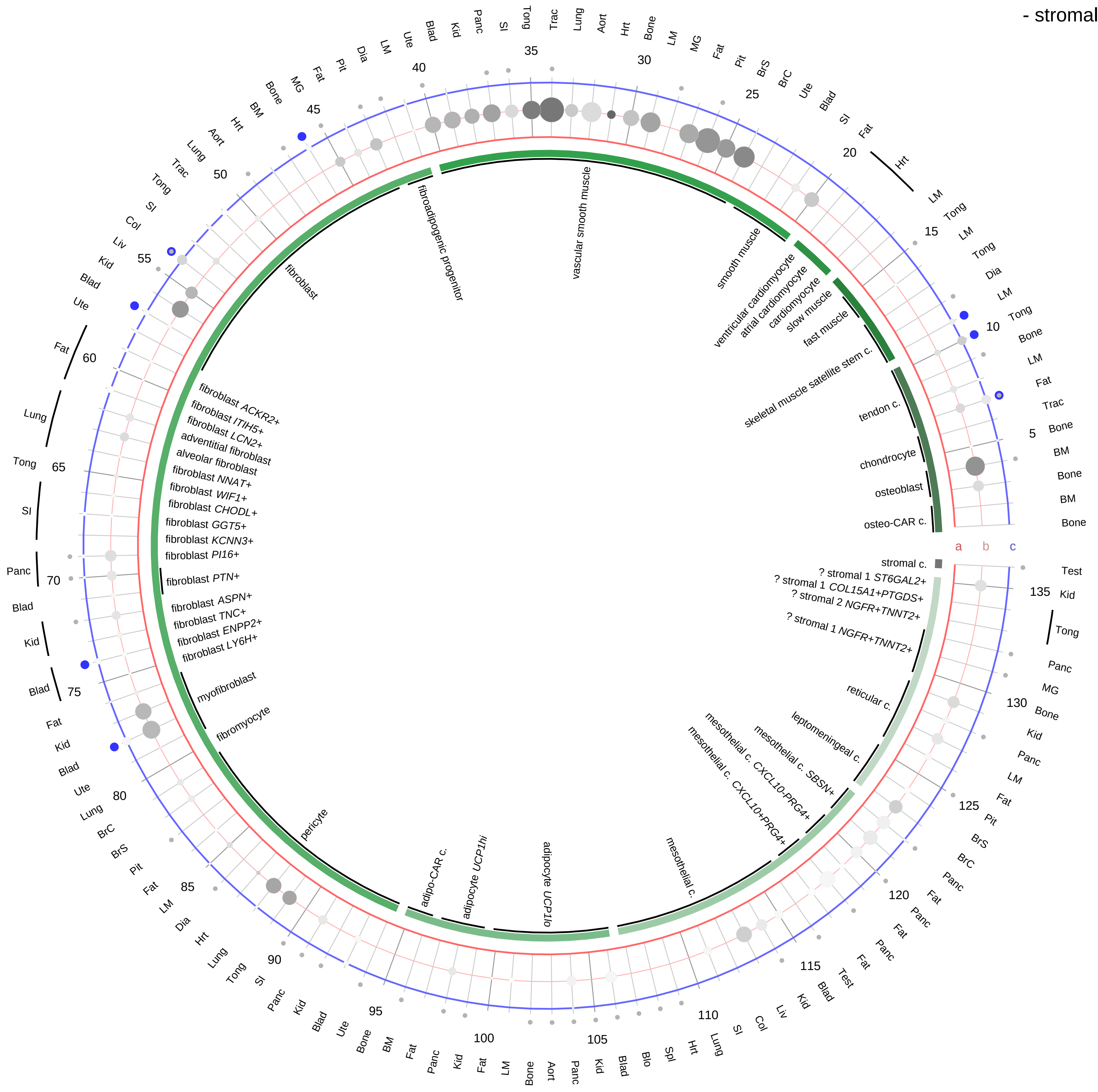

Ligand/Enzyme
- a - LOC105881981 (CARTPT)
- b - CPE

Receptor
- c - GPR68

PAIRING (ligand → receptor)

CARTPT & CPE→GPR68

Compartment
stromal
- bone
- skeletal muscle
- cardiac muscle
- smooth muscle
- fibroblast
- adipocyte
- mesothelial
- other
- mix

12.5% 25% 50% 100%

ln(CP10K+1) 0 1 2 3

Abbrev.	Tissue
Aort	Aorta
Blad	Bladder
Blo	Blood
Bone	Bone
BM	Bone marrow
BrC	Brain cortex
BrS	Brainstem
Col	Colon
Dia	Diaphragm
Ret	Eye retina
Fat	Fat
Hrt	Heart
Pit	Hypothalamus/Pituitary
Kid	Kidney
LM	Limb muscle
Liv	Liver
Lung	Lung
MG	Mammary gland
Panc	Pancreas
Skin	Skin
SI	Small intestine
Spl	Spleen
Test	Testes
Thym	Thymus
Tong	Tongue
Trac	Trachea
Ute	Uterus

Cocaine- and amphetamine-regulated transcript (CART)
- lymphoid

PAIRING (ligand → receptor)

CARTPT & CPE→GPR68

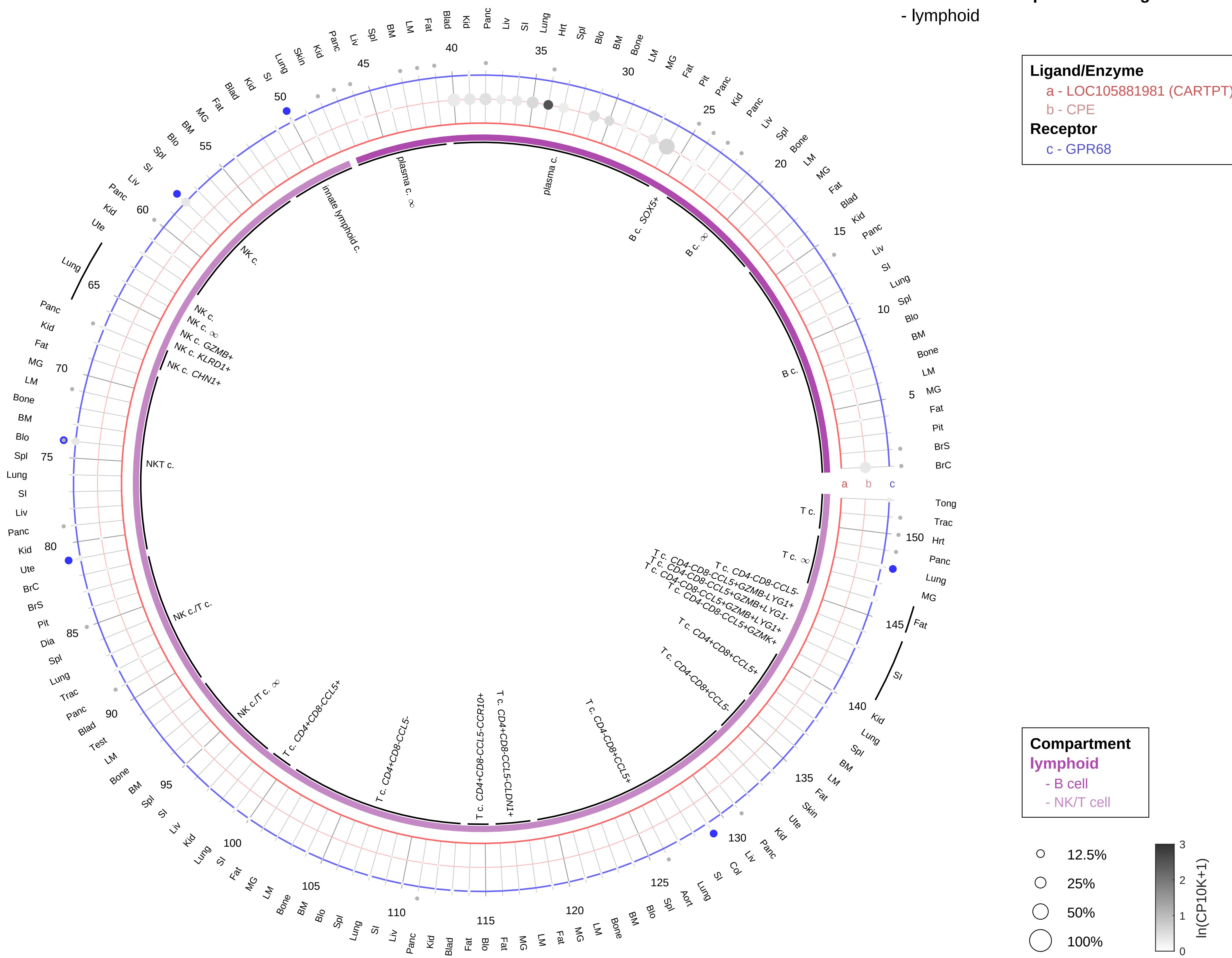

Abbrev.	Tissue
Aort	Aorta
Blad	Bladder
Blo	Blood
Bone	Bone
BM	Bone marrow
BrC	Brain cortex
BrS	Brainstem
Col	Colon
Dia	Diaphragm
Ret	Eye retina
Fat	Fat
Hrt	Heart
Pit	Hypothalamus/Pituitary
Kid	Kidney
LM	Limb muscle
Liv	Liver
Lung	Lung
MG	Mammary gland
Panc	Pancreas
Skin	Skin
SI	Small intestine
Spl	Spleen
Test	Testes
Thym	Thymus
Tong	Tongue
Trac	Trachea
Ute	Uterus

Cocaine- and amphetamine-regulated transcript (CART)
- myeloid

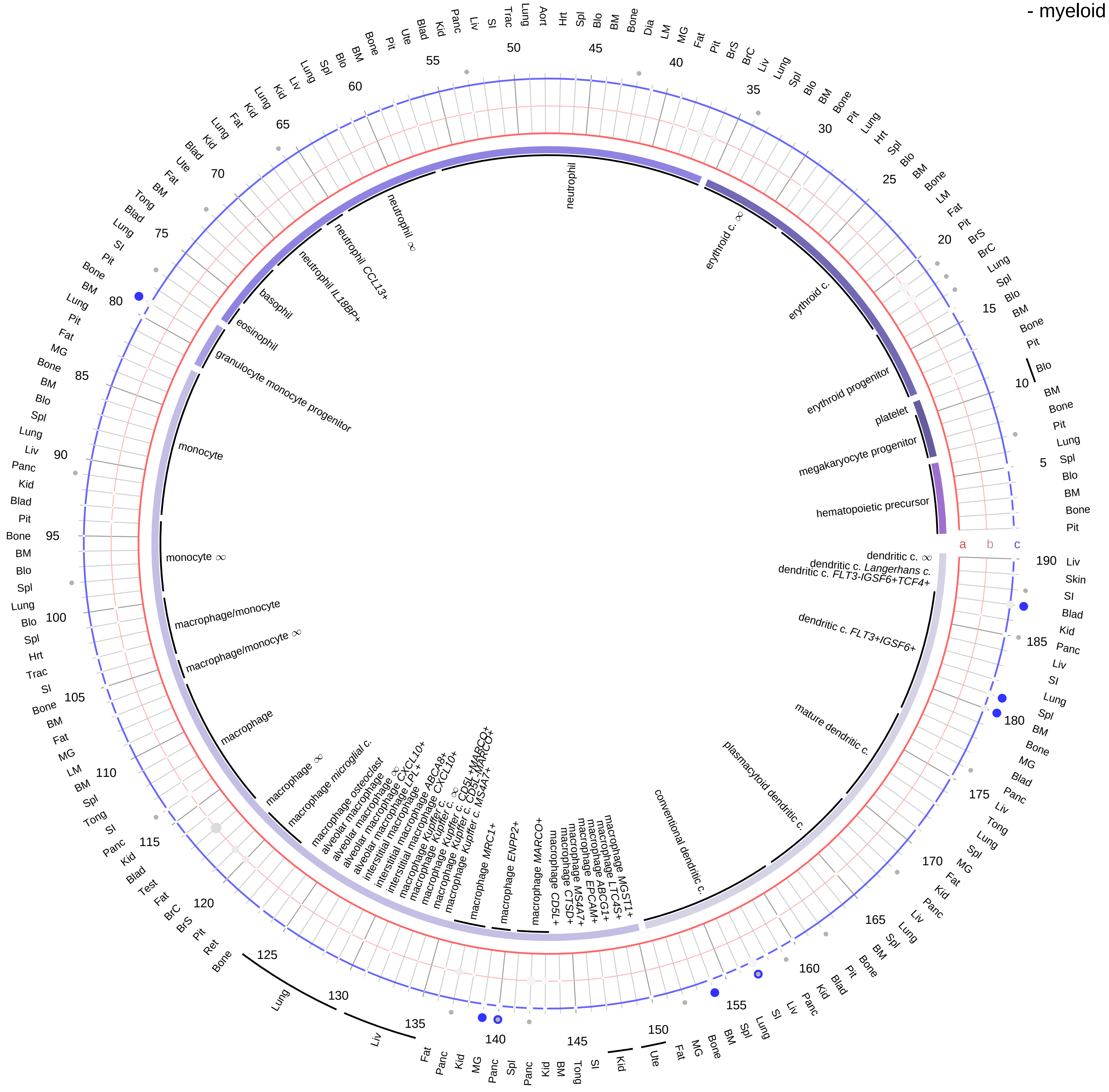

Ligand/Enzyme
- a - LOC105881981 (CARTPT)
- b - CPE

Receptor
- c - GPR68

PAIRING (ligand → receptor)

CARTPT & CPE→GPR68

Abbrev.	Tissue
Aort	Aorta
Blad	Bladder
Blo	Blood
Bone	Bone
BM	Bone marrow
BrC	Brain cortex
BrS	Brainstem
Col	Colon
Dia	Diaphragm
Ret	Eye retina
Fat	Fat
Hrt	Heart
Pit	Hypothalamus/Pituitary
Kid	Kidney
LM	Limb muscle
Liv	Liver
Lung	Lung
MG	Mammary gland
Panc	Pancreas
Skin	Skin
SI	Small intestine
Spl	Spleen
Test	Testes
Thym	Thymus
Tong	Tongue
Trac	Trachea
Ute	Uterus

Compartment

hematopoietic
- hematopoietic precursor

megakaryocyte-erythroid
- megakaryoid
- erythroid

myeloid
- granulocyte
- granulocyte monocyte progenitor
- monocyte/macrophage
- dendritic

12.5%
25%
50%
100%

ln(CP10K+1): 0, 1, 2, 3

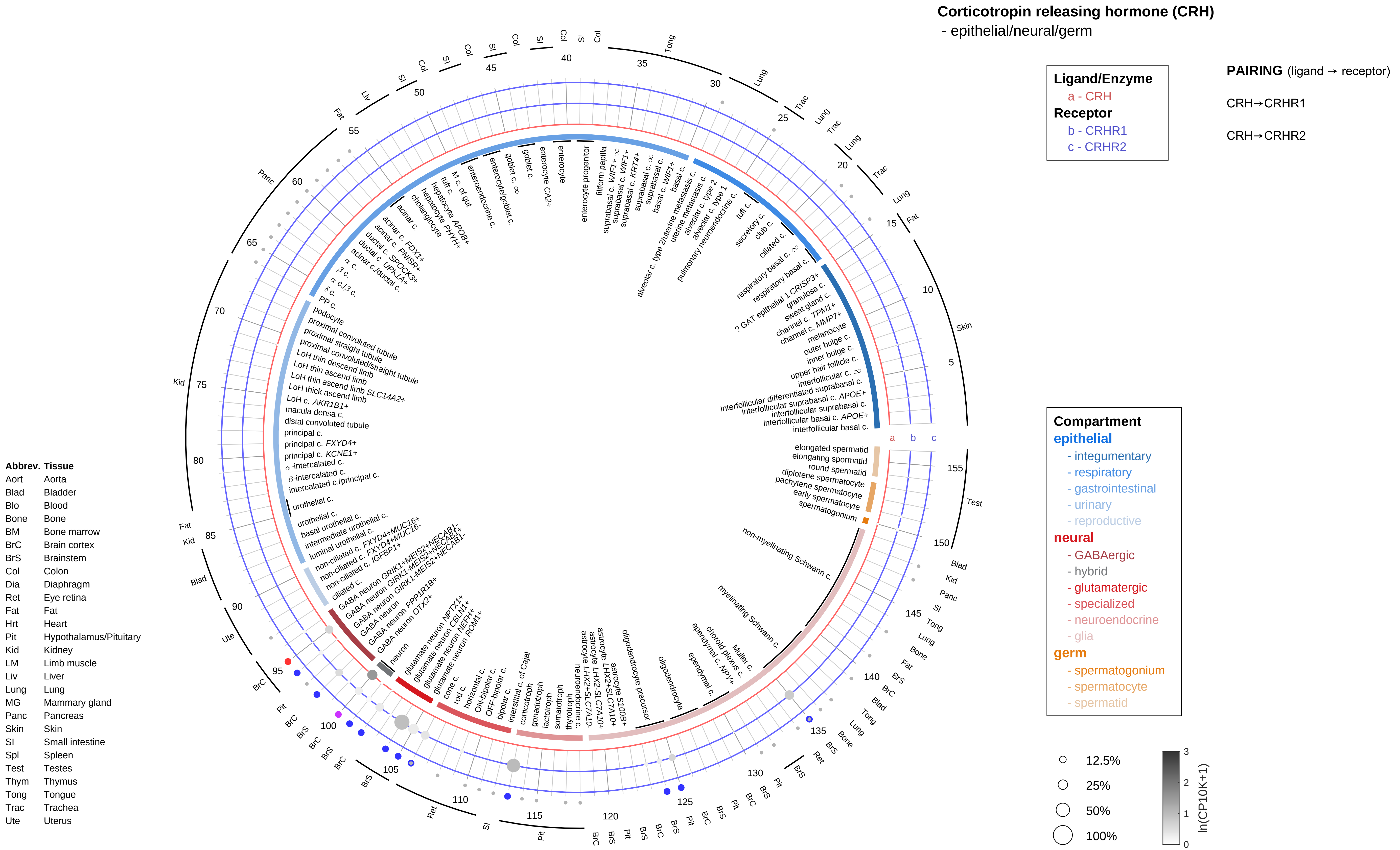
Corticotropin releasing hormone (CRH)
- epithelial/neural/germ
Ligand/Enzyme
a - CRH
Receptor
b - CRHR1
c - CRHR2
PAIRING (ligand → receptor)
CRH→CRHR1
CRH→CRHR2
Compartment
epithelial
- integumentary
- respiratory
- gastrointestinal
- urinary
- reproductive
neural
- GABAergic
- hybrid
- glutamatergic
- specialized
- neuroendocrine
- glia
germ
- spermatogonium
- spermatocyte
- spermatid
12.5%
25%
50%
100%
ln(CP10K+1)
Abbrev. Tissue
Aort Aorta
Blad Bladder
Blo Blood
Bone Bone
BM Bone marrow
BrC Brain cortex
BrS Brainstem
Col Colon
Dia Diaphragm
Ret Eye retina
Fat Fat
Hrt Heart
Pit Hypothalamus/Pituitary
Kid Kidney
LM Limb muscle
Liv Liver
Lung Lung
MG Mammary gland
Panc Pancreas
Skin Skin
SI Small intestine
Spl Spleen
Test Testes
Thym Thymus
Tong Tongue
Trac Trachea
Ute Uterus

Corticotropin releasing hormone (CRH)
- endothelial

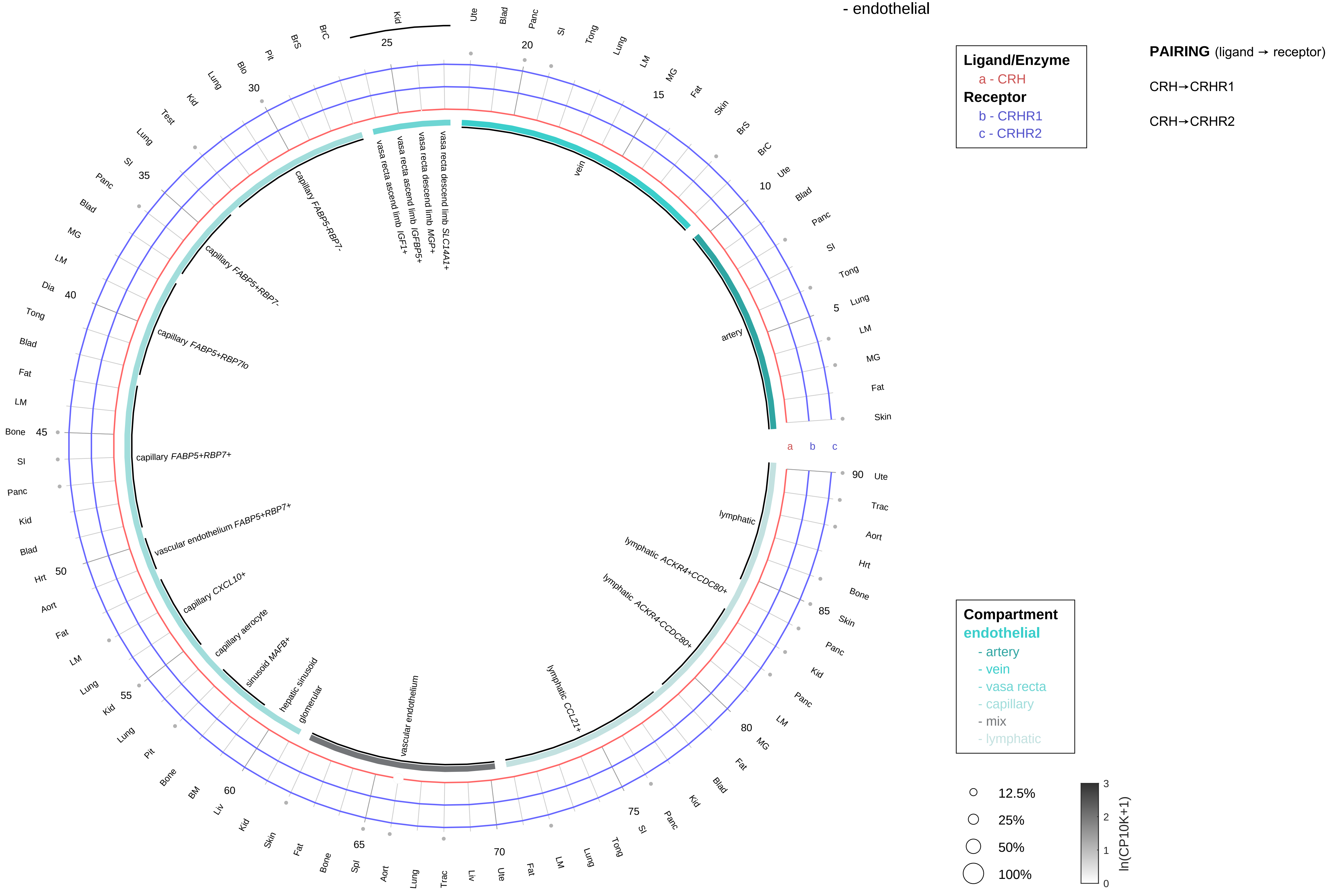

Abbrev.	Tissue
Aort	Aorta
Blad	Bladder
Blo	Blood
Bone	Bone
BM	Bone marrow
BrC	Brain cortex
BrS	Brainstem
Col	Colon
Dia	Diaphragm
Ret	Eye retina
Fat	Fat
Hrt	Heart
Pit	Hypothalamus/Pituitary
Kid	Kidney
LM	Limb muscle
Liv	Liver
Lung	Lung
MG	Mammary gland
Panc	Pancreas
Skin	Skin
SI	Small intestine
Spl	Spleen
Test	Testes
Thym	Thymus
Tong	Tongue
Trac	Trachea
Ute	Uterus

Corticotropin releasing hormone (CRH)
- stromal

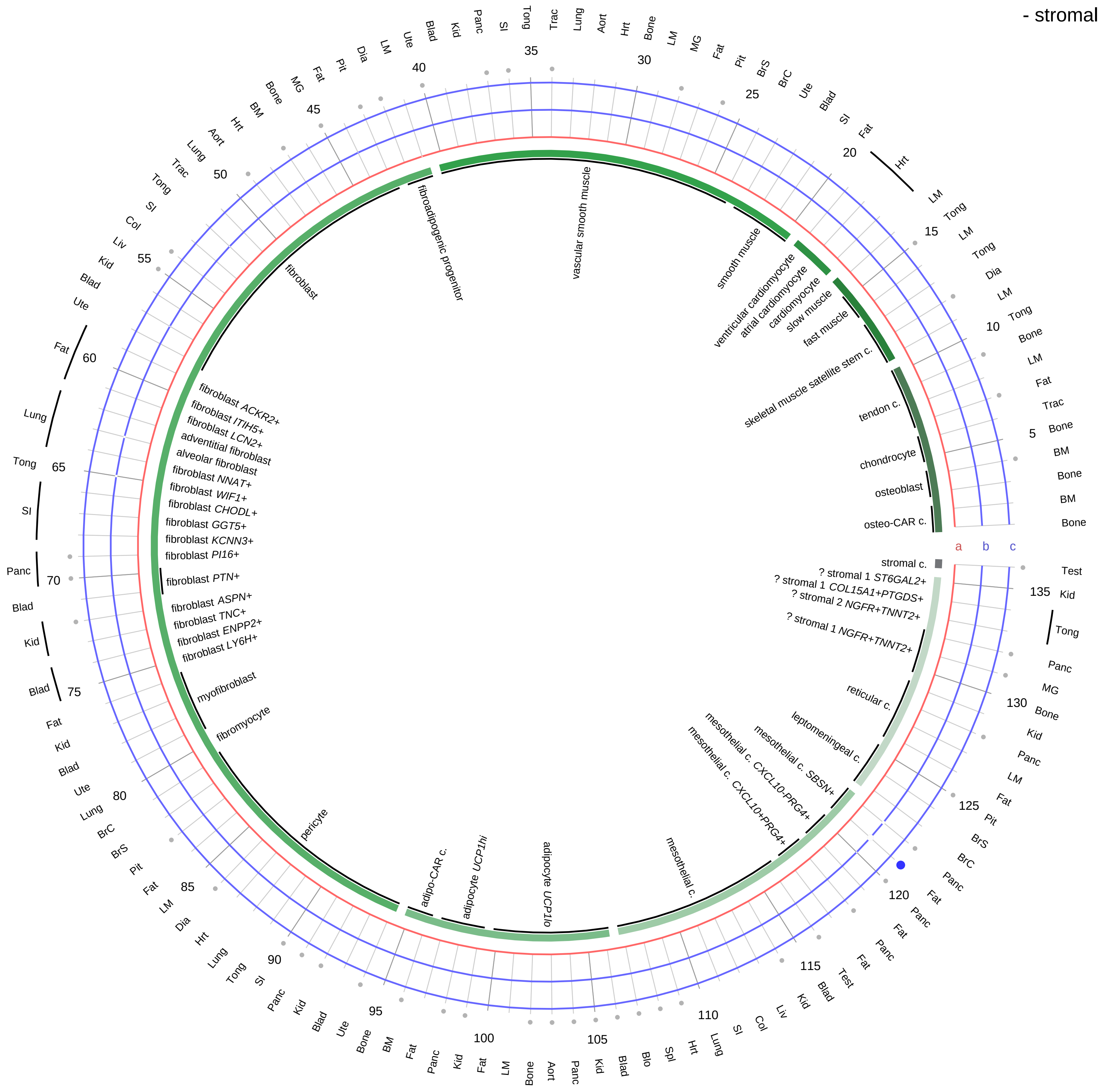

Ligand/Enzyme
a - CRH
Receptor
b - CRHR1
c - CRHR2

PAIRING (ligand → receptor)

CRH→CRHR1

CRH→CRHR2

Compartment
stromal
- bone
- skeletal muscle
- cardiac muscle
- smooth muscle
- fibroblast
- adipocyte
- mesothelial
- other
- mix

12.5%
25%
50%
100%

ln(CP10K+1): 0, 1, 2, 3

Abbrev.	Tissue
Aort	Aorta
Blad	Bladder
Blo	Blood
Bone	Bone
BM	Bone marrow
BrC	Brain cortex
BrS	Brainstem
Col	Colon
Dia	Diaphragm
Ret	Eye retina
Fat	Fat
Hrt	Heart
Pit	Hypothalamus/Pituitary
Kid	Kidney
LM	Limb muscle
Liv	Liver
Lung	Lung
MG	Mammary gland
Panc	Pancreas
Skin	Skin
SI	Small intestine
Spl	Spleen
Test	Testes
Thym	Thymus
Tong	Tongue
Trac	Trachea
Ute	Uterus

Corticotropin releasing hormone (CRH)
- lymphoid

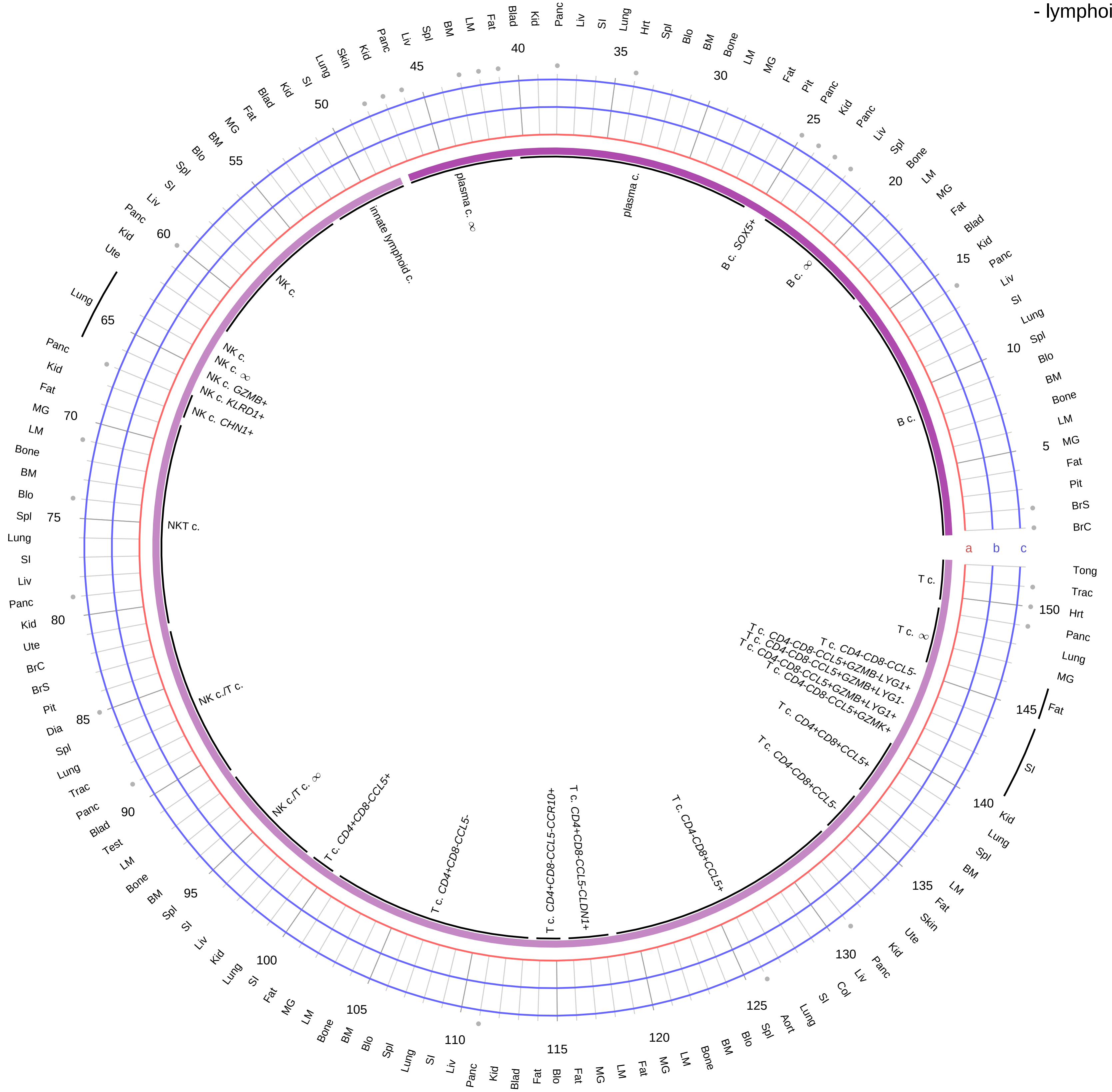

Ligand/Enzyme
a - CRH
Receptor
b - CRHR1
c - CRHR2

PAIRING (ligand → receptor)

CRH→CRHR1

CRH→CRHR2

Compartment
lymphoid
- B cell
- NK/T cell

12.5%
25%
50%
100%

ln(CP10K+1): 0, 1, 2, 3

Abbrev.	Tissue
Aort	Aorta
Blad	Bladder
Blo	Blood
Bone	Bone
BM	Bone marrow
BrC	Brain cortex
BrS	Brainstem
Col	Colon
Dia	Diaphragm
Ret	Eye retina
Fat	Fat
Hrt	Heart
Pit	Hypothalamus/Pituitary
Kid	Kidney
LM	Limb muscle
Liv	Liver
Lung	Lung
MG	Mammary gland
Panc	Pancreas
Skin	Skin
SI	Small intestine
Spl	Spleen
Test	Testes
Thym	Thymus
Tong	Tongue
Trac	Trachea
Ute	Uterus

Corticotropin releasing hormone (CRH)
- myeloid

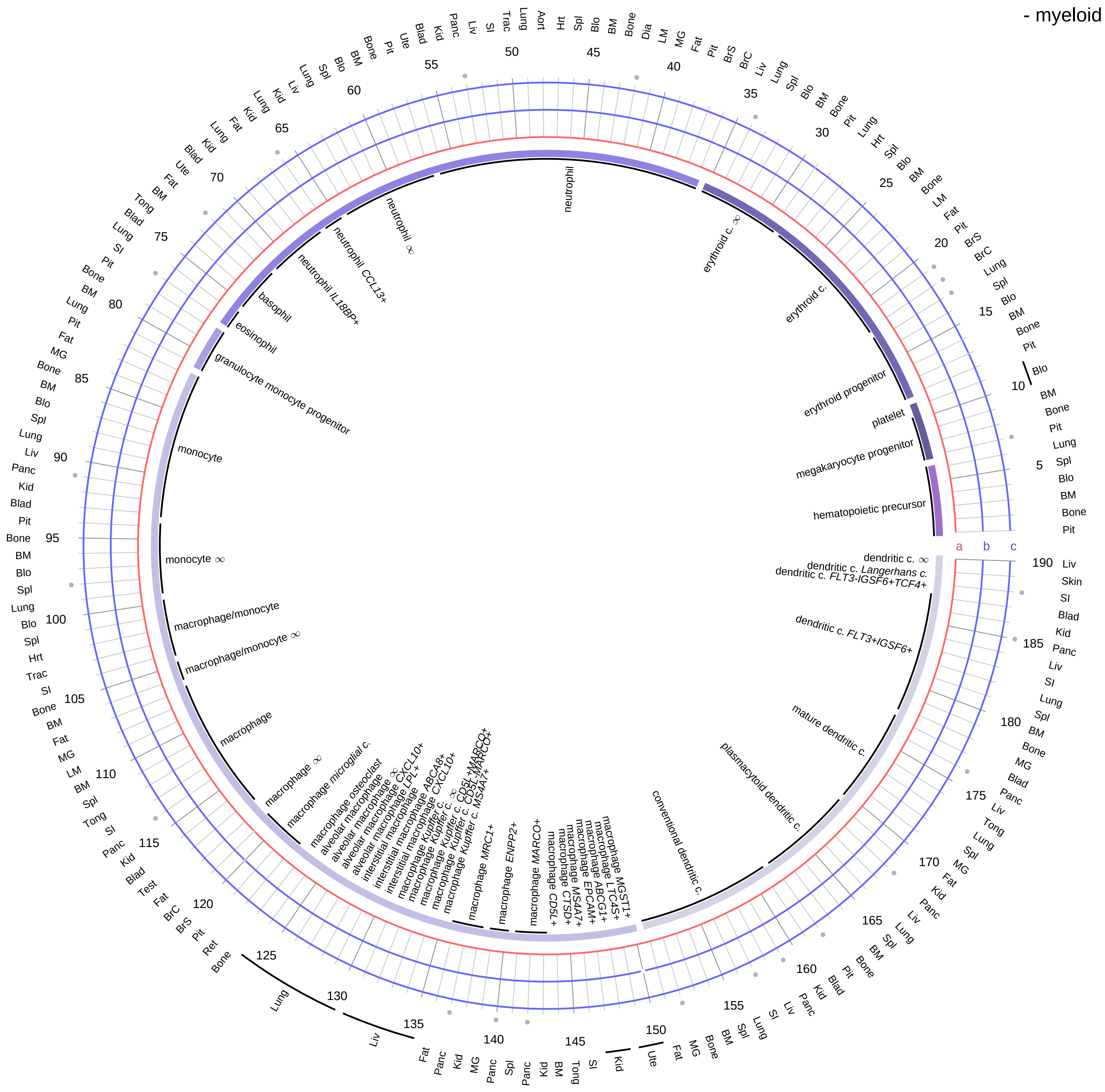

Ligand/Enzyme
a - CRH
Receptor
b - CRHR1
c - CRHR2

PAIRING (ligand → receptor)

CRH→CRHR1

CRH→CRHR2

Compartment
hematopoietic
- hematopoietic precursor
megakaryocyte-erythroid
- megakaryoid
- erythroid
myeloid
- granulocyte
- granulocyte monocyte progenitor
- monocyte/macrophage
- dendritic

12.5%
25%
50%
100%

ln(CP10K+1): 0, 1, 2, 3

Abbrev.	Tissue
Aort	Aorta
Blad	Bladder
Blo	Blood
Bone	Bone
BM	Bone marrow
BrC	Brain cortex
BrS	Brainstem
Col	Colon
Dia	Diaphragm
Ret	Eye retina
Fat	Fat
Hrt	Heart
Pit	Hypothalamus/Pituitary
Kid	Kidney
LM	Limb muscle
Liv	Liver
Lung	Lung
MG	Mammary gland
Panc	Pancreas
Skin	Skin
SI	Small intestine
Spl	Spleen
Test	Testes
Thym	Thymus
Tong	Tongue
Trac	Trachea
Ute	Uterus

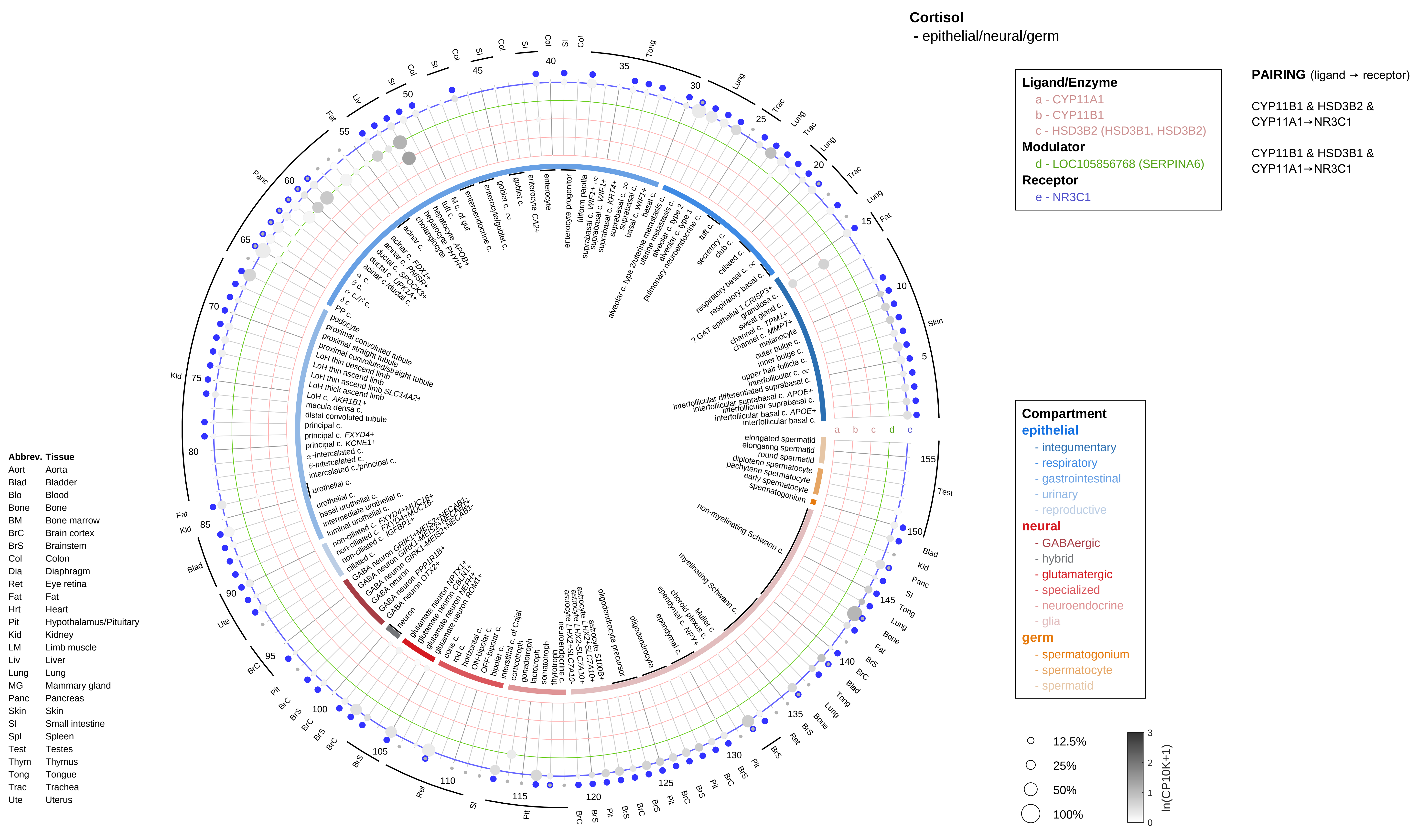

Cortisol
- epithelial/neural/germ
Ligand/Enzyme
a - CYP11A1
b - CYP11B1
c - HSD3B2 (HSD3B1, HSD3B2)
Modulator
d - LOC105856768 (SERPINA6)
Receptor
e - NR3C1
PAIRING (ligand → receptor)
CYP11B1 & HSD3B2 & CYP11A1→NR3C1
CYP11B1 & HSD3B1 & CYP11A1→NR3C1
Compartment
epithelial
- integumentary
- respiratory
- gastrointestinal
- urinary
- reproductive
neural
- GABAergic
- hybrid
- glutamatergic
- specialized
- neuroendocrine
- glia
germ
- spermatogonium
- spermatocyte
- spermatid
12.5%
25%
50%
100%
ln(CP10K+1)
0
1
2
3
Abbrev. Tissue
Aort Aorta
Blad Bladder
Blo Blood
Bone Bone
BM Bone marrow
BrC Brain cortex
BrS Brainstem
Col Colon
Dia Diaphragm
Ret Eye retina
Fat Fat
Hrt Heart
Pit Hypothalamus/Pituitary
Kid Kidney
LM Limb muscle
Liv Liver
Lung Lung
MG Mammary gland
Panc Pancreas
Skin Skin
SI Small intestine
Spl Spleen
Test Testes
Thym Thymus
Tong Tongue
Trac Trachea
Ute Uterus

Cortisol
- endothelial

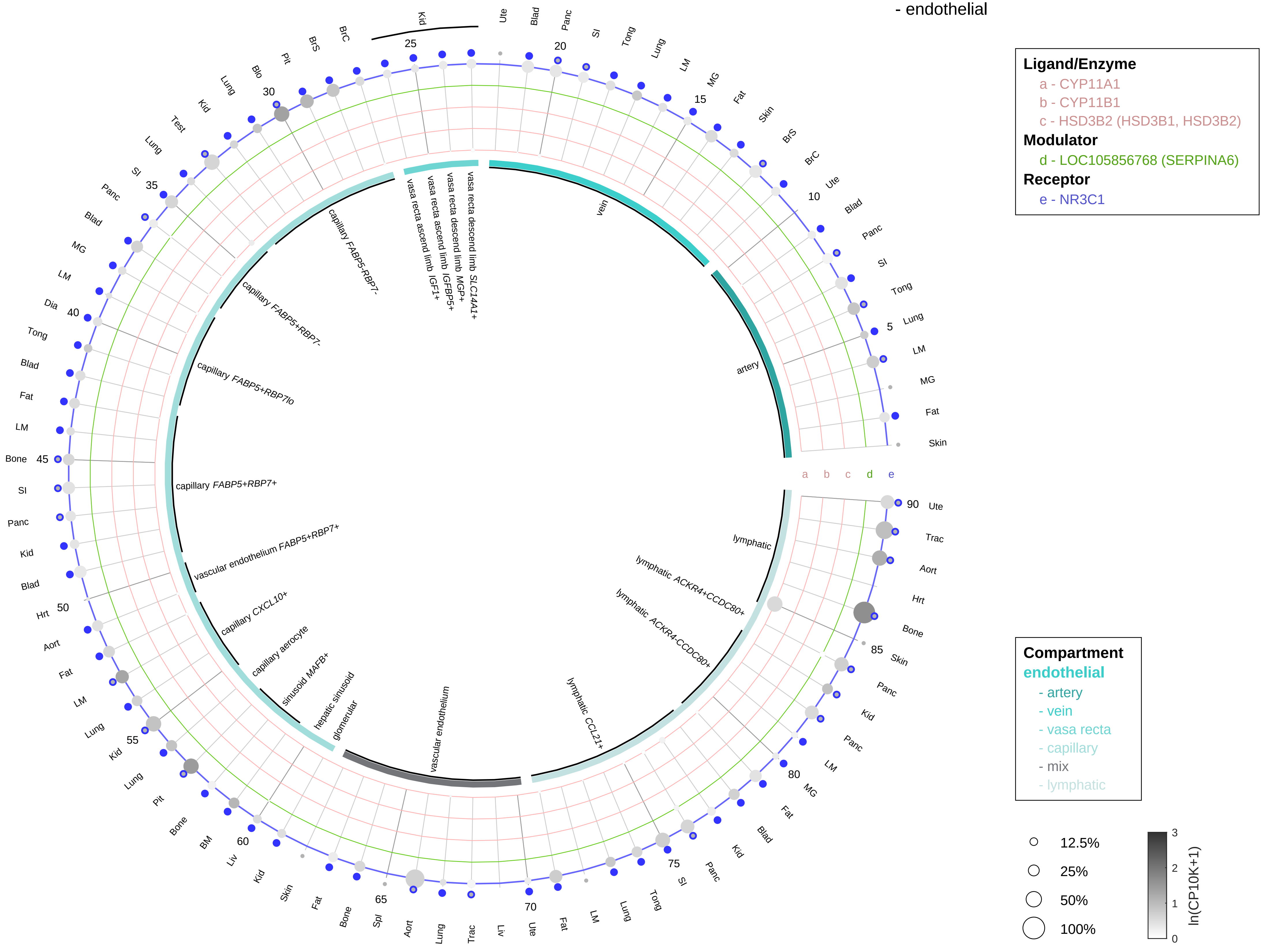

PAIRING (ligand → receptor)

CYP11B1 & HSD3B2 & CYP11A1→NR3C1

CYP11B1 & HSD3B1 & CYP11A1→NR3C1

Abbrev.	Tissue
Aort	Aorta
Blad	Bladder
Blo	Blood
Bone	Bone
BM	Bone marrow
BrC	Brain cortex
BrS	Brainstem
Col	Colon
Dia	Diaphragm
Ret	Eye retina
Fat	Fat
Hrt	Heart
Pit	Hypothalamus/Pituitary
Kid	Kidney
LM	Limb muscle
Liv	Liver
Lung	Lung
MG	Mammary gland
Panc	Pancreas
Skin	Skin
SI	Small intestine
Spl	Spleen
Test	Testes
Thym	Thymus
Tong	Tongue
Trac	Trachea
Ute	Uterus

Cortisol
- stromal

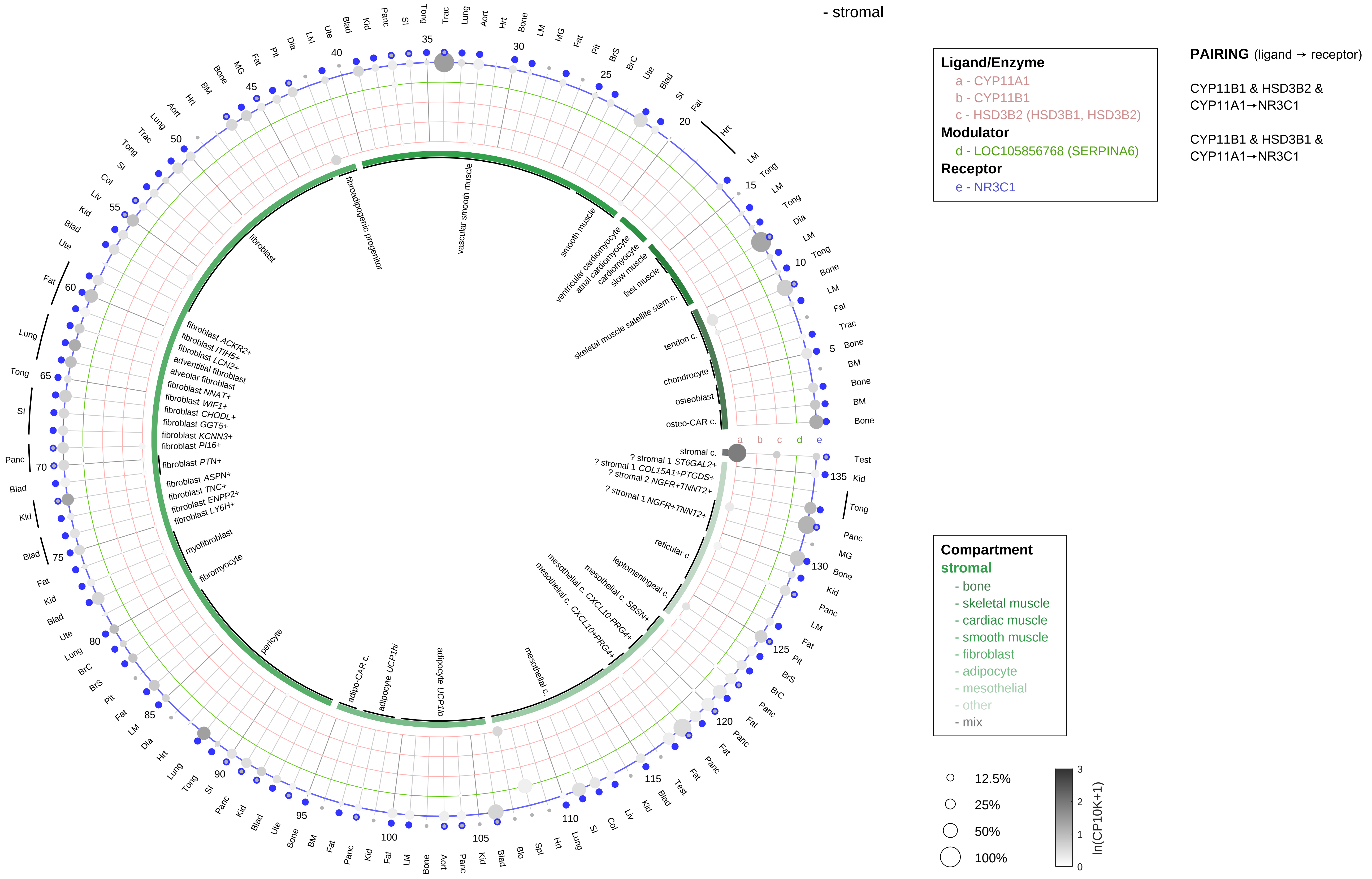

Abbrev.	Tissue
Aort	Aorta
Blad	Bladder
Blo	Blood
Bone	Bone
BM	Bone marrow
BrC	Brain cortex
BrS	Brainstem
Col	Colon
Dia	Diaphragm
Ret	Eye retina
Fat	Fat
Hrt	Heart
Pit	Hypothalamus/Pituitary
Kid	Kidney
LM	Limb muscle
Liv	Liver
Lung	Lung
MG	Mammary gland
Panc	Pancreas
Skin	Skin
SI	Small intestine
Spl	Spleen
Test	Testes
Thym	Thymus
Tong	Tongue
Trac	Trachea
Ute	Uterus

Cortisol
- lymphoid

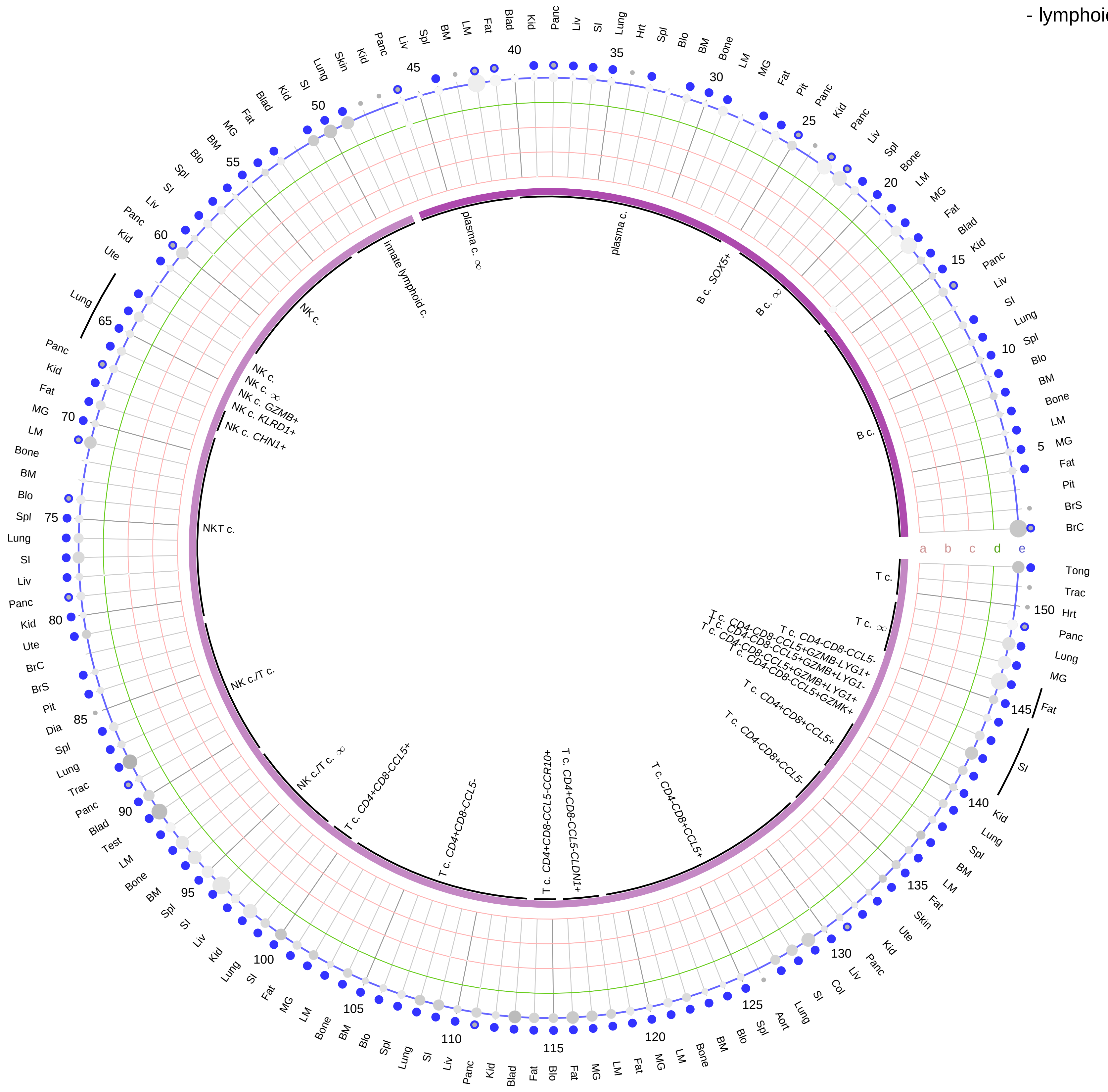

Ligand/Enzyme
- a - CYP11A1
- b - CYP11B1
- c - HSD3B2 (HSD3B1, HSD3B2)

Modulator
- d - LOC105856768 (SERPINA6)

Receptor
- e - NR3C1

PAIRING (ligand → receptor)

CYP11B1 & HSD3B2 & CYP11A1→NR3C1

CYP11B1 & HSD3B1 & CYP11A1→NR3C1

Compartment
lymphoid
- B cell
- NK/T cell

12.5%
25%
50%
100%

ln(CP10K+1): 0, 1, 2, 3

Abbrev.	Tissue
Aort	Aorta
Blad	Bladder
Blo	Blood
Bone	Bone
BM	Bone marrow
BrC	Brain cortex
BrS	Brainstem
Col	Colon
Dia	Diaphragm
Ret	Eye retina
Fat	Fat
Hrt	Heart
Pit	Hypothalamus/Pituitary
Kid	Kidney
LM	Limb muscle
Liv	Liver
Lung	Lung
MG	Mammary gland
Panc	Pancreas
Skin	Skin
SI	Small intestine
Spl	Spleen
Test	Testes
Thym	Thymus
Tong	Tongue
Trac	Trachea
Ute	Uterus

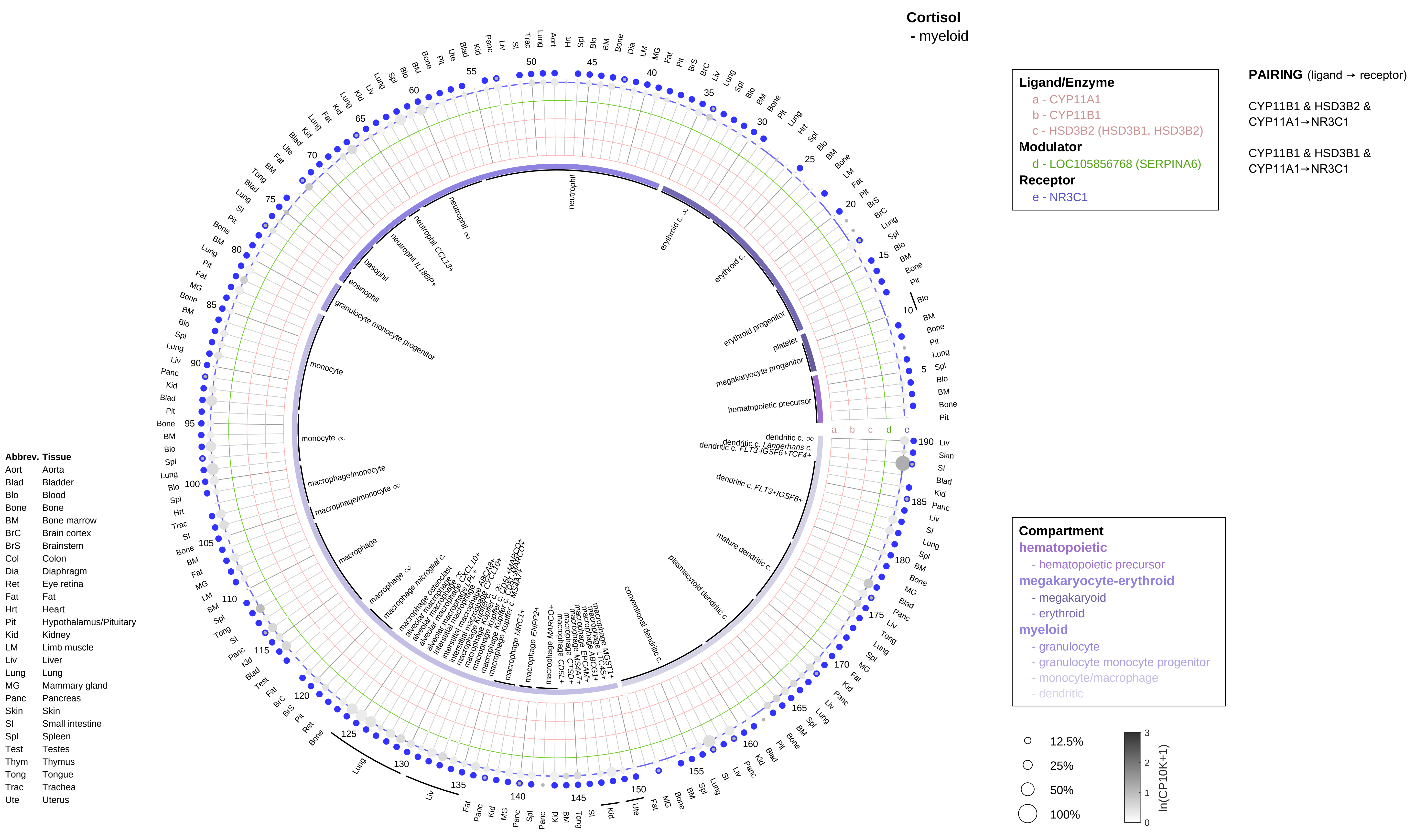
Cortisol
- myeloid
Ligand/Enzyme
a - CYP11A1
b - CYP11B1
c - HSD3B2 (HSD3B1, HSD3B2)
Modulator
d - LOC105856768 (SERPINA6)
Receptor
e - NR3C1
PAIRING (ligand → receptor)
CYP11B1 & HSD3B2 & CYP11A1→NR3C1
CYP11B1 & HSD3B1 & CYP11A1→NR3C1
Compartment
hematopoietic
- hematopoietic precursor
megakaryocyte-erythroid
- megakaryoid
- erythroid
myeloid
- granulocyte
- granulocyte monocyte progenitor
- monocyte/macrophage
- dendritic
12.5%
25%
50%
100%
ln(CP10K+1)
Abbrev. Tissue
Aort Aorta
Blad Bladder
Blo Blood
Bone Bone
BM Bone marrow
BrC Brain cortex
BrS Brainstem
Col Colon
Dia Diaphragm
Ret Eye retina
Fat Fat
Hrt Heart
Pit Hypothalamus/Pituitary
Kid Kidney
LM Limb muscle
Liv Liver
Lung Lung
MG Mammary gland
Panc Pancreas
Skin Skin
SI Small intestine
Spl Spleen
Test Testes
Thym Thymus
Tong Tongue
Trac Trachea
Ute Uterus

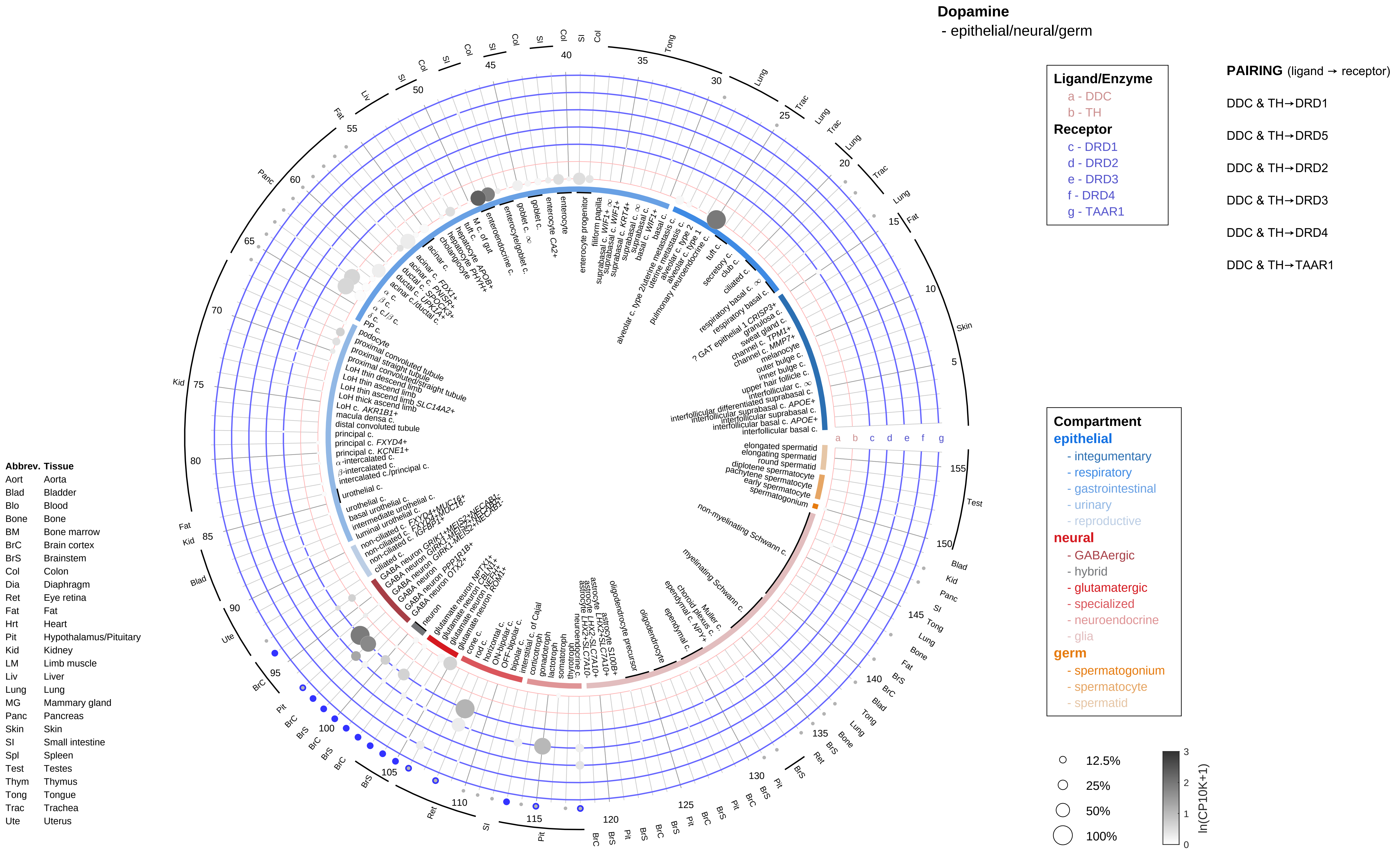
Dopamine
- epithelial/neural/germ
Ligand/Enzyme
a - DDC
b - TH
Receptor
c - DRD1
d - DRD2
e - DRD3
f - DRD4
g - TAAR1
PAIRING (ligand → receptor)
DDC & TH→DRD1
DDC & TH→DRD5
DDC & TH→DRD2
DDC & TH→DRD3
DDC & TH→DRD4
DDC & TH→TAAR1
Compartment
epithelial
- integumentary
- respiratory
- gastrointestinal
- urinary
- reproductive
neural
- GABAergic
- hybrid
- glutamatergic
- specialized
- neuroendocrine
- glia
germ
- spermatogonium
- spermatocyte
- spermatid
12.5%
25%
50%
100%
ln(CP10K+1)
Abbrev. Tissue
Aort Aorta
Blad Bladder
Blo Blood
Bone Bone
BM Bone marrow
BrC Brain cortex
BrS Brainstem
Col Colon
Dia Diaphragm
Ret Eye retina
Fat Fat
Hrt Heart
Pit Hypothalamus/Pituitary
Kid Kidney
LM Limb muscle
Liv Liver
Lung Lung
MG Mammary gland
Panc Pancreas
Skin Skin
SI Small intestine
Spl Spleen
Test Testes
Thym Thymus
Tong Tongue
Trac Trachea
Ute Uterus

Dopamine
- endothelial

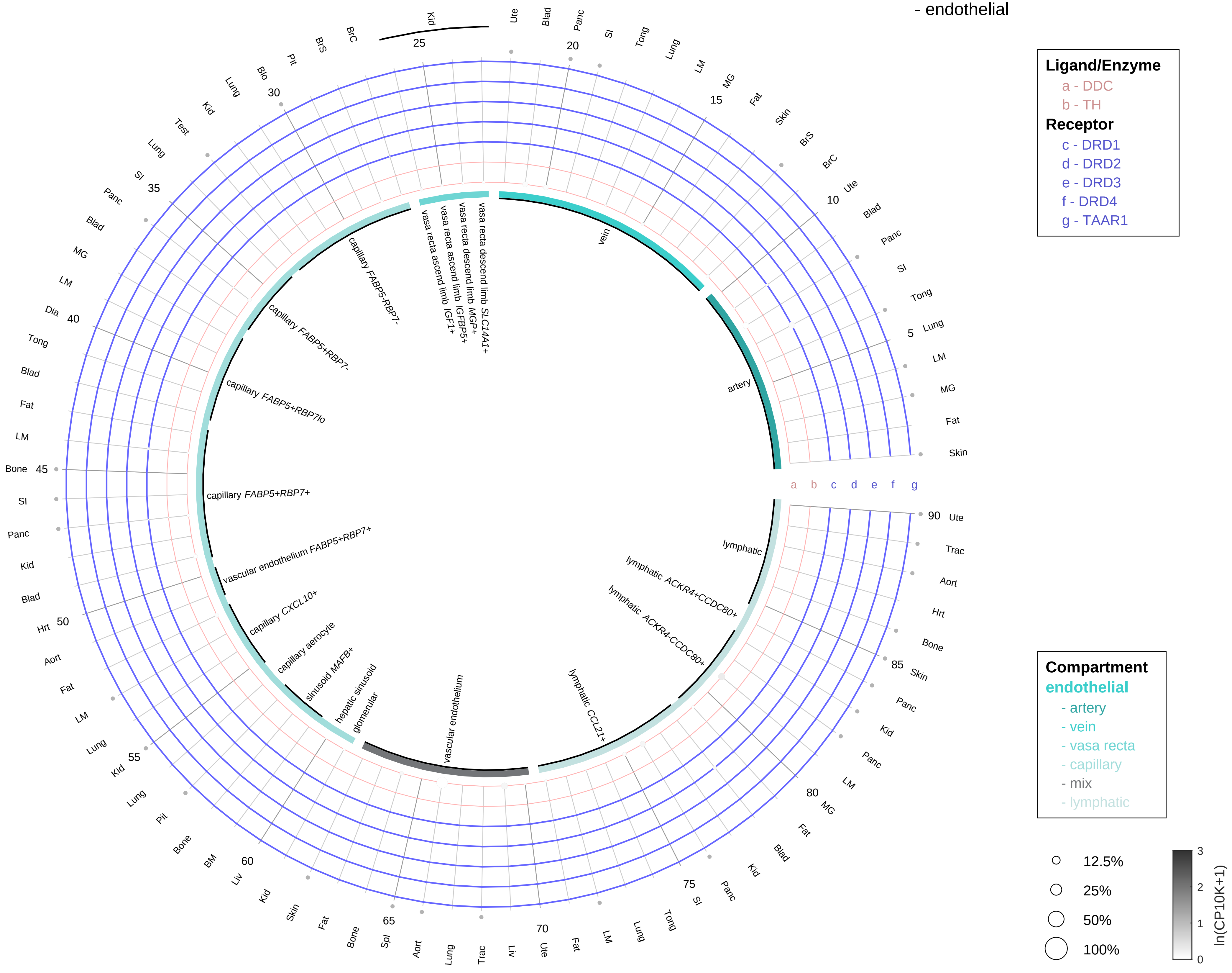

PAIRING (ligand → receptor)

DDC & TH→DRD1

DDC & TH→DRD5

DDC & TH→DRD2

DDC & TH→DRD3

DDC & TH→DRD4

DDC & TH→TAAR1

Abbrev.	Tissue
Aort	Aorta
Blad	Bladder
Blo	Blood
Bone	Bone
BM	Bone marrow
BrC	Brain cortex
BrS	Brainstem
Col	Colon
Dia	Diaphragm
Ret	Eye retina
Fat	Fat
Hrt	Heart
Pit	Hypothalamus/Pituitary
Kid	Kidney
LM	Limb muscle
Liv	Liver
Lung	Lung
MG	Mammary gland
Panc	Pancreas
Skin	Skin
SI	Small intestine
Spl	Spleen
Test	Testes
Thym	Thymus
Tong	Tongue
Trac	Trachea
Ute	Uterus

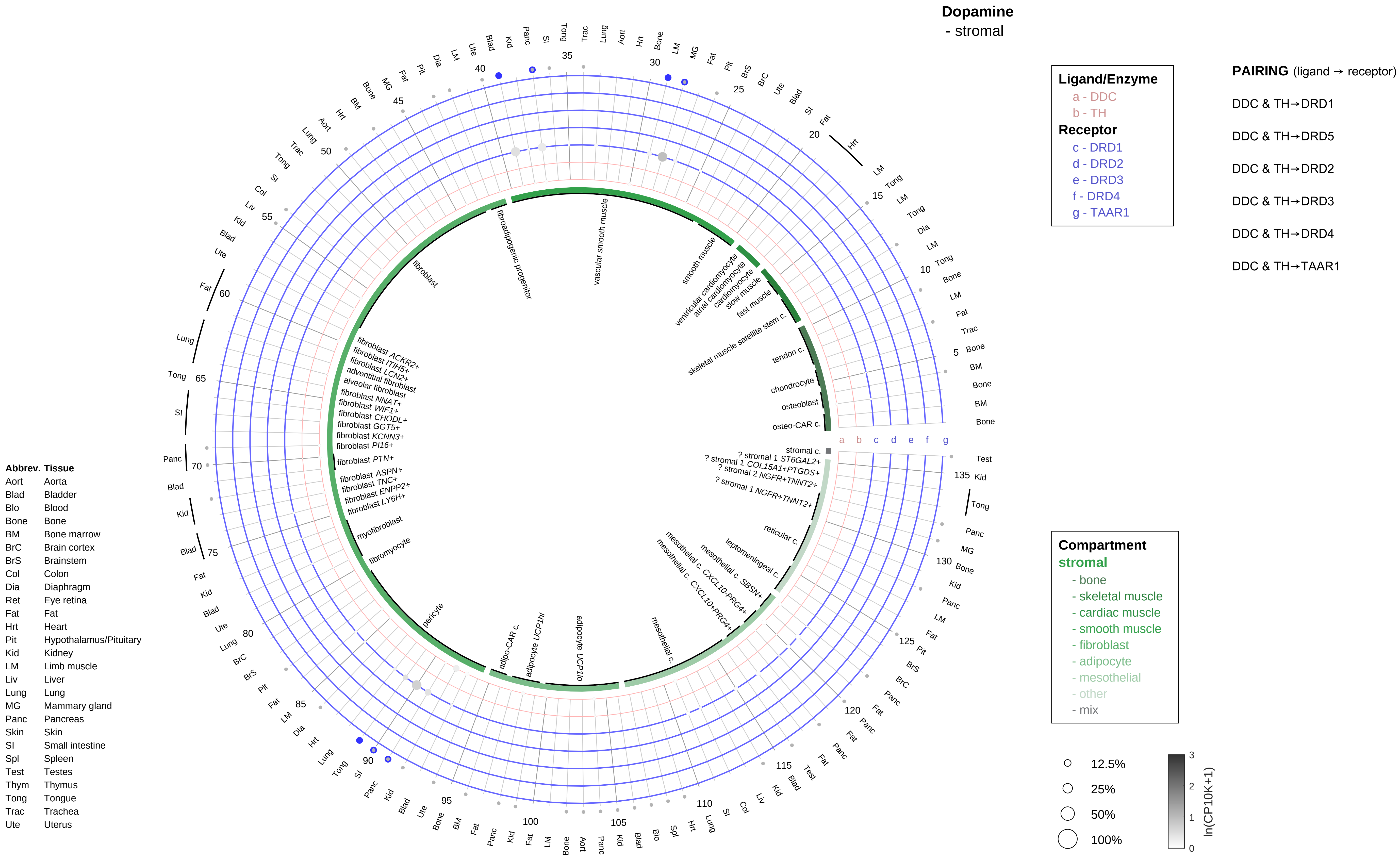

Dopamine
- stromal
Ligand/Enzyme
a - DDC
b - TH
Receptor
c - DRD1
d - DRD2
e - DRD3
f - DRD4
g - TAAR1
PAIRING (ligand → receptor)
DDC & TH→DRD1
DDC & TH→DRD5
DDC & TH→DRD2
DDC & TH→DRD3
DDC & TH→DRD4
DDC & TH→TAAR1
Compartment
stromal
- bone
- skeletal muscle
- cardiac muscle
- smooth muscle
- fibroblast
- adipocyte
- mesothelial
- other
- mix
12.5%
25%
50%
100%
ln(CP10K+1)
Abbrev. Tissue
Aort Aorta
Blad Bladder
Blo Blood
Bone Bone
BM Bone marrow
BrC Brain cortex
BrS Brainstem
Col Colon
Dia Diaphragm
Ret Eye retina
Fat Fat
Hrt Heart
Pit Hypothalamus/Pituitary
Kid Kidney
LM Limb muscle
Liv Liver
Lung Lung
MG Mammary gland
Panc Pancreas
Skin Skin
SI Small intestine
Spl Spleen
Test Testes
Thym Thymus
Tong Tongue
Trac Trachea
Ute Uterus
vascular smooth muscle
fibroadipogenic progenitor
fibroblast
smooth muscle
ventricular cardiomyocyte
atrial cardiomyocyte
cardiomyocyte
slow muscle
fast muscle
skeletal muscle satellite stem c.
tendon c.
chondrocyte
osteoblast
osteo-CAR c.
stromal c.
? stromal 1 ST6GAL2+
? stromal 1 COL15A1+PTGDS+
? stromal 2 NGFR+TNNT2+
? stromal 1 NGFR+TNNT2+
reticular c.
leptomeningeal c.
mesothelial c. SBSN+
mesothelial c. CXCL10-PRG4+
mesothelial c. CXCL10+PRG4+
mesothelial c.
adipocyte UCP1lo
adipocyte UCP1hi
adipo-CAR c.
pericyte
fibromyocyte
myofibroblast
fibroblast LY6H+
fibroblast ENPP2+
fibroblast TNC+
fibroblast ASPN+
fibroblast PTN+
fibroblast PI16+
fibroblast KCNN3+
fibroblast GGT5+
fibroblast CHODL+
fibroblast WIF1+
fibroblast NNAT+
alveolar fibroblast
adventitial fibroblast
fibroblast LCN2+
fibroblast ITIH5+
fibroblast ACKR2+

Dopamine
- lymphoid

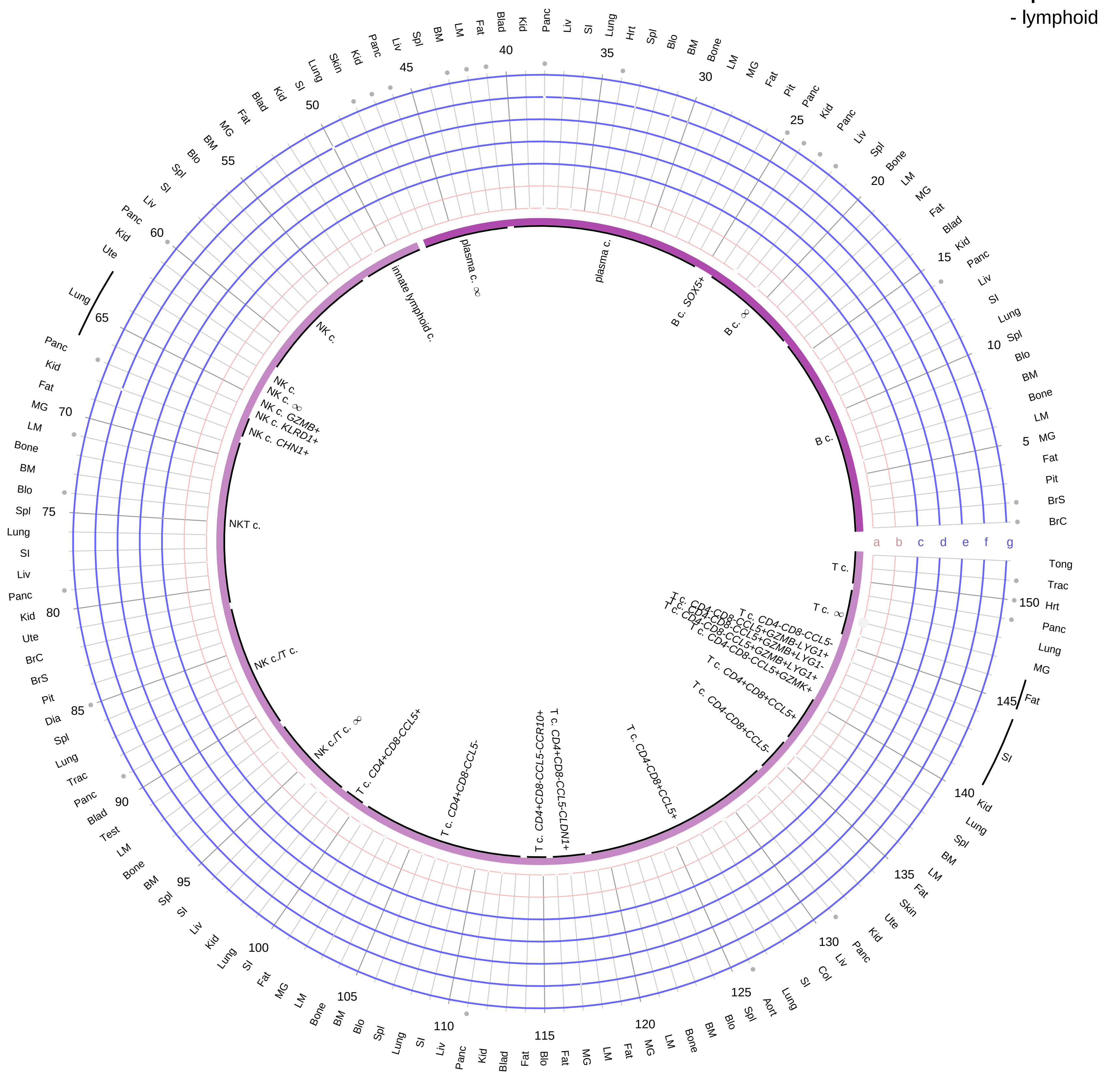

Ligand/Enzyme
- a - DDC
- b - TH

Receptor
- c - DRD1
- d - DRD2
- e - DRD3
- f - DRD4
- g - TAAR1

PAIRING (ligand → receptor)

DDC & TH→DRD1

DDC & TH→DRD5

DDC & TH→DRD2

DDC & TH→DRD3

DDC & TH→DRD4

DDC & TH→TAAR1

Abbrev.	Tissue
Aort	Aorta
Blad	Bladder
Blo	Blood
Bone	Bone
BM	Bone marrow
BrC	Brain cortex
BrS	Brainstem
Col	Colon
Dia	Diaphragm
Ret	Eye retina
Fat	Fat
Hrt	Heart
Pit	Hypothalamus/Pituitary
Kid	Kidney
LM	Limb muscle
Liv	Liver
Lung	Lung
MG	Mammary gland
Panc	Pancreas
Skin	Skin
SI	Small intestine
Spl	Spleen
Test	Testes
Thym	Thymus
Tong	Tongue
Trac	Trachea
Ute	Uterus

Compartment
lymphoid
- B cell
- NK/T cell

12.5%
25%
50%
100%

ln(CP10K+1) 0 1 2 3

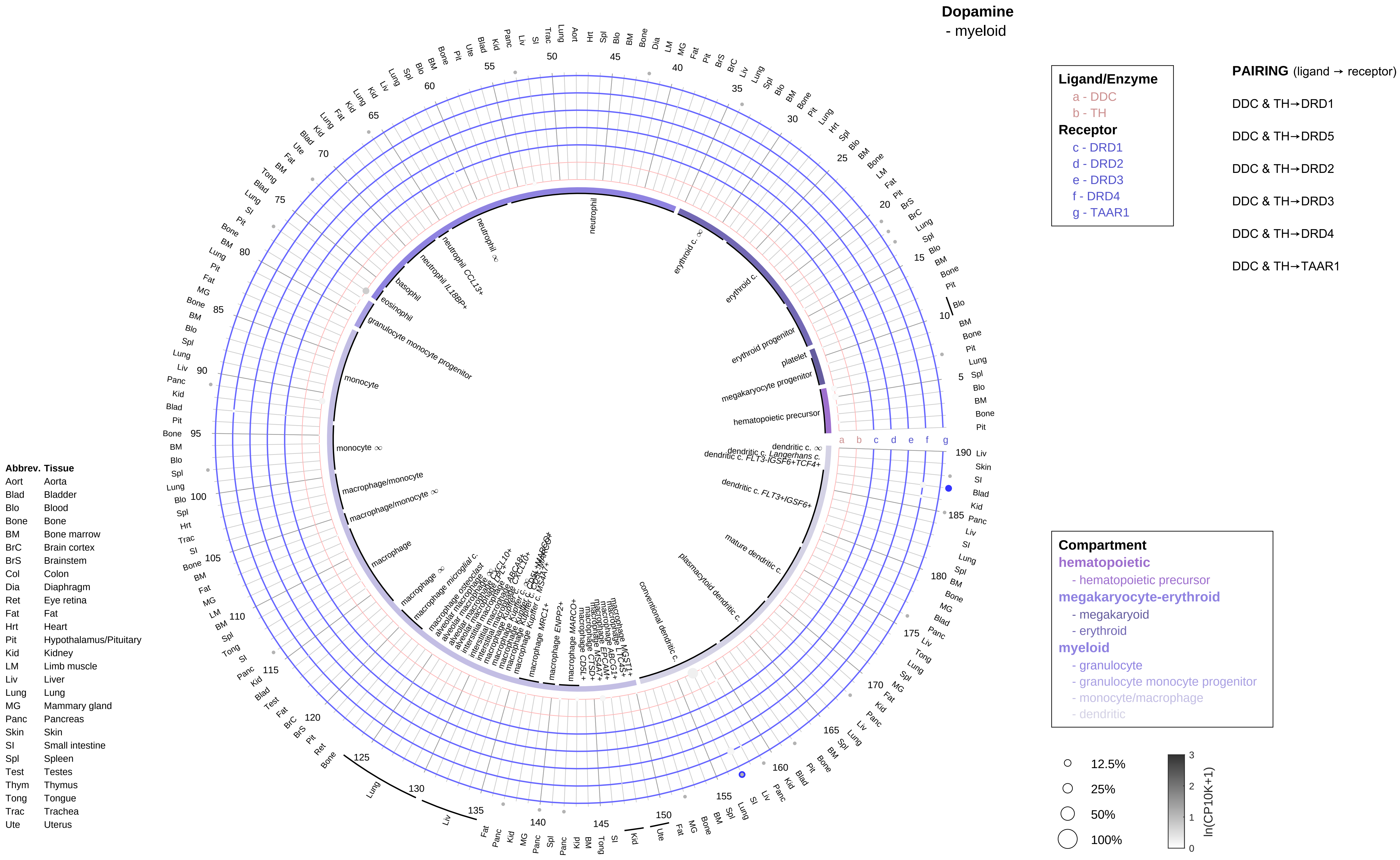
Dopamine
- myeloid
Ligand/Enzyme
a - DDC
b - TH
Receptor
c - DRD1
d - DRD2
e - DRD3
f - DRD4
g - TAAR1
PAIRING (ligand → receptor)
DDC & TH→DRD1
DDC & TH→DRD5
DDC & TH→DRD2
DDC & TH→DRD3
DDC & TH→DRD4
DDC & TH→TAAR1
Compartment
hematopoietic
- hematopoietic precursor
megakaryocyte-erythroid
- megakaryoid
- erythroid
myeloid
- granulocyte
- granulocyte monocyte progenitor
- monocyte/macrophage
- dendritic
12.5%
25%
50%
100%
ln(CP10K+1)
Abbrev. Tissue
Aort Aorta
Blad Bladder
Blo Blood
Bone Bone
BM Bone marrow
BrC Brain cortex
BrS Brainstem
Col Colon
Dia Diaphragm
Ret Eye retina
Fat Fat
Hrt Heart
Pit Hypothalamus/Pituitary
Kid Kidney
LM Limb muscle
Liv Liver
Lung Lung
MG Mammary gland
Panc Pancreas
Skin Skin
SI Small intestine
Spl Spleen
Test Testes
Thym Thymus
Tong Tongue
Trac Trachea
Ute Uterus

Dynorphin/Enkephalin
- epithelial/neural/germ

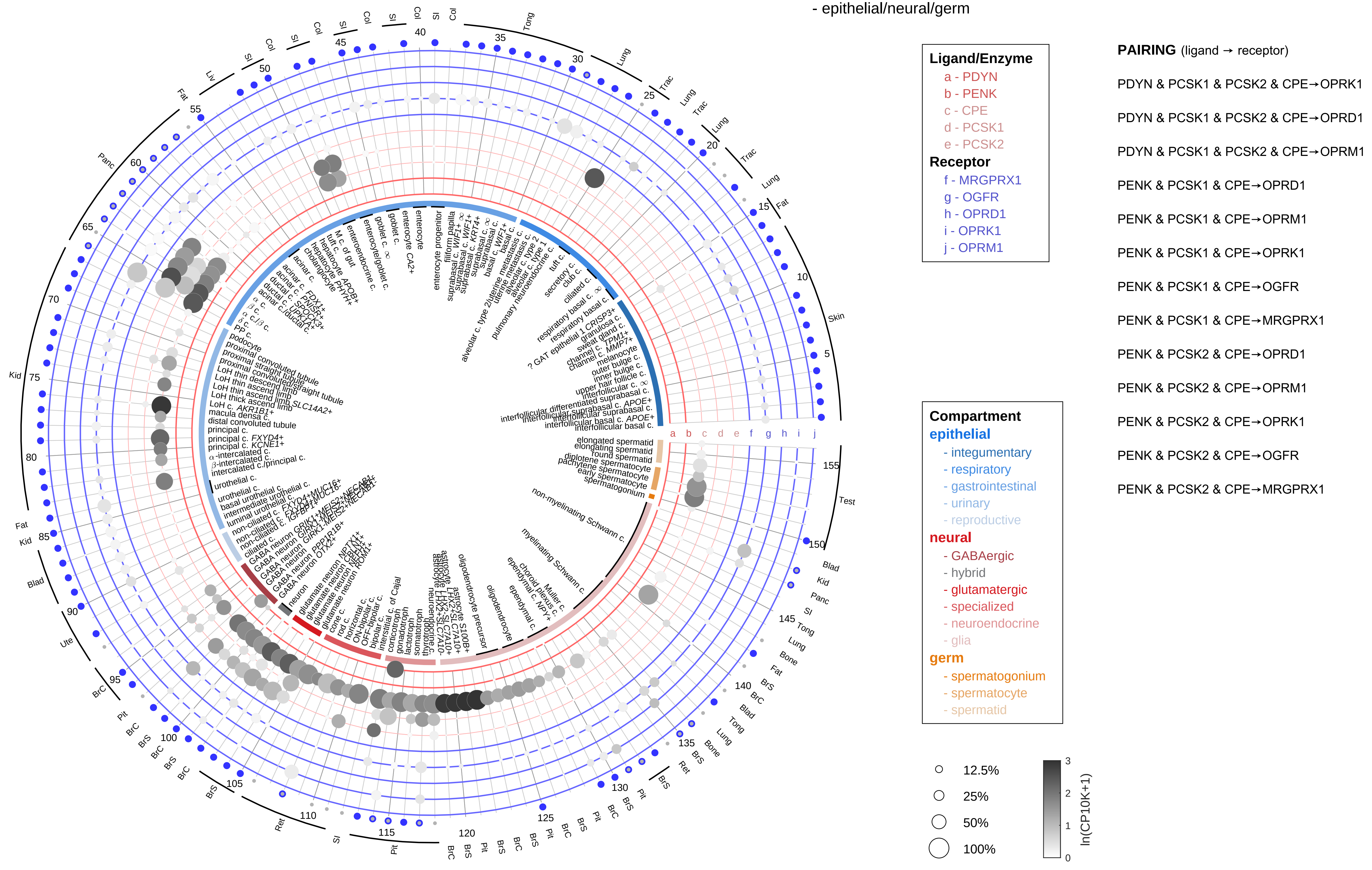

Abbrev.	Tissue
Aort	Aorta
Blad	Bladder
Blo	Blood
Bone	Bone
BM	Bone marrow
BrC	Brain cortex
BrS	Brainstem
Col	Colon
Dia	Diaphragm
Ret	Eye retina
Fat	Fat
Hrt	Heart
Pit	Hypothalamus/Pituitary
Kid	Kidney
LM	Limb muscle
Liv	Liver
Lung	Lung
MG	Mammary gland
Panc	Pancreas
Skin	Skin
SI	Small intestine
Spl	Spleen
Test	Testes
Thym	Thymus
Tong	Tongue
Trac	Trachea
Ute	Uterus

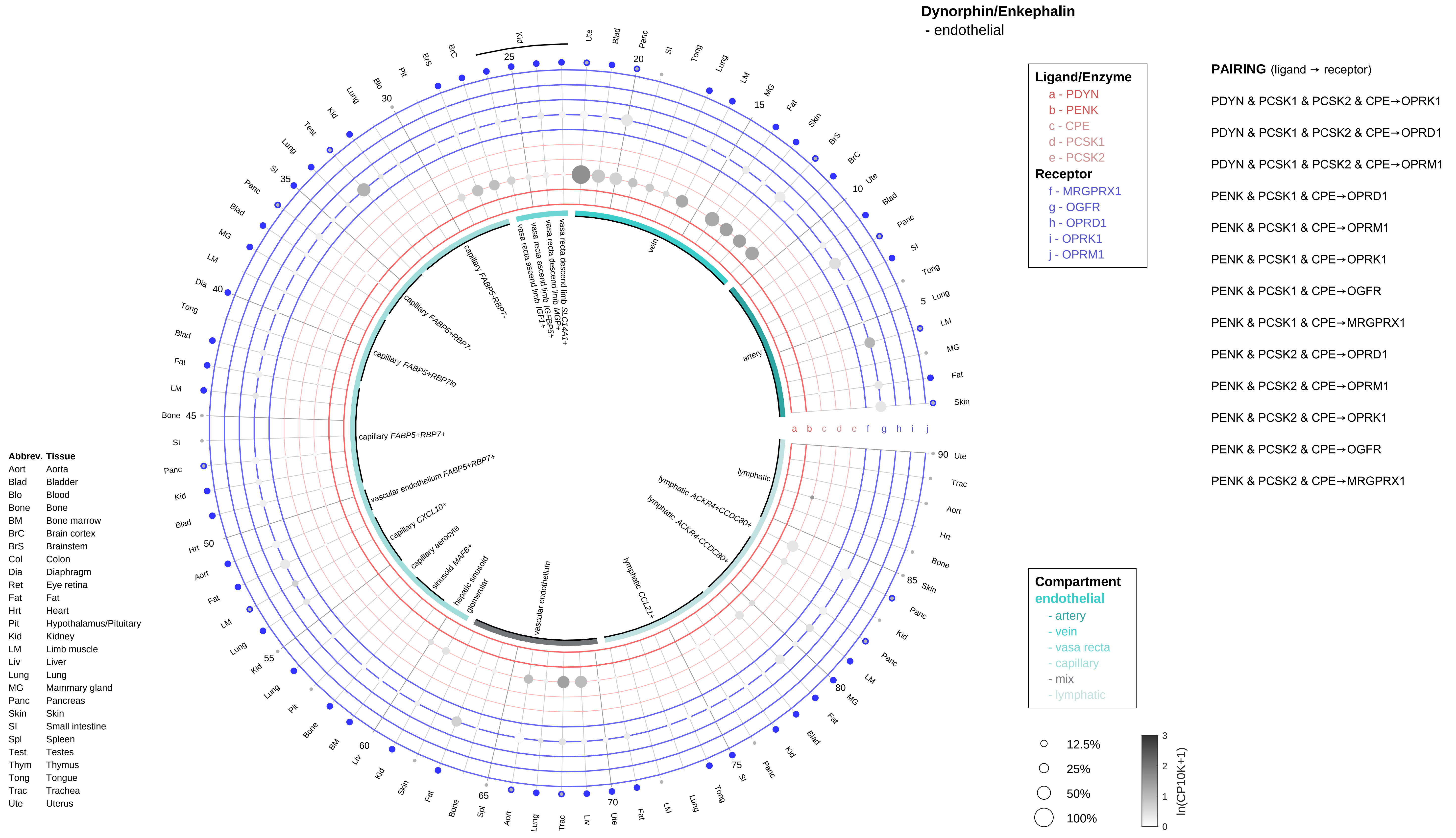

Dynorphin/Enkephalin
- endothelial
Ligand/Enzyme
a - PDYN
b - PENK
c - CPE
d - PCSK1
e - PCSK2
Receptor
f - MRGPRX1
g - OGFR
h - OPRD1
i - OPRK1
j - OPRM1
PAIRING (ligand → receptor)
PDYN & PCSK1 & PCSK2 & CPE→OPRK1
PDYN & PCSK1 & PCSK2 & CPE→OPRD1
PDYN & PCSK1 & PCSK2 & CPE→OPRM1
PENK & PCSK1 & CPE→OPRD1
PENK & PCSK1 & CPE→OPRM1
PENK & PCSK1 & CPE→OPRK1
PENK & PCSK1 & CPE→OGFR
PENK & PCSK1 & CPE→MRGPRX1
PENK & PCSK2 & CPE→OPRD1
PENK & PCSK2 & CPE→OPRM1
PENK & PCSK2 & CPE→OPRK1
PENK & PCSK2 & CPE→OGFR
PENK & PCSK2 & CPE→MRGPRX1
Compartment
endothelial
- artery
- vein
- vasa recta
- capillary
- mix
- lymphatic
12.5%
25%
50%
100%
ln(CP10K+1)
Abbrev. Tissue
Aort Aorta
Blad Bladder
Blo Blood
Bone Bone
BM Bone marrow
BrC Brain cortex
BrS Brainstem
Col Colon
Dia Diaphragm
Ret Eye retina
Fat Fat
Hrt Heart
Pit Hypothalamus/Pituitary
Kid Kidney
LM Limb muscle
Liv Liver
Lung Lung
MG Mammary gland
Panc Pancreas
Skin Skin
SI Small intestine
Spl Spleen
Test Testes
Thym Thymus
Tong Tongue
Trac Trachea
Ute Uterus

Dynorphin/Enkephalin
- stromal

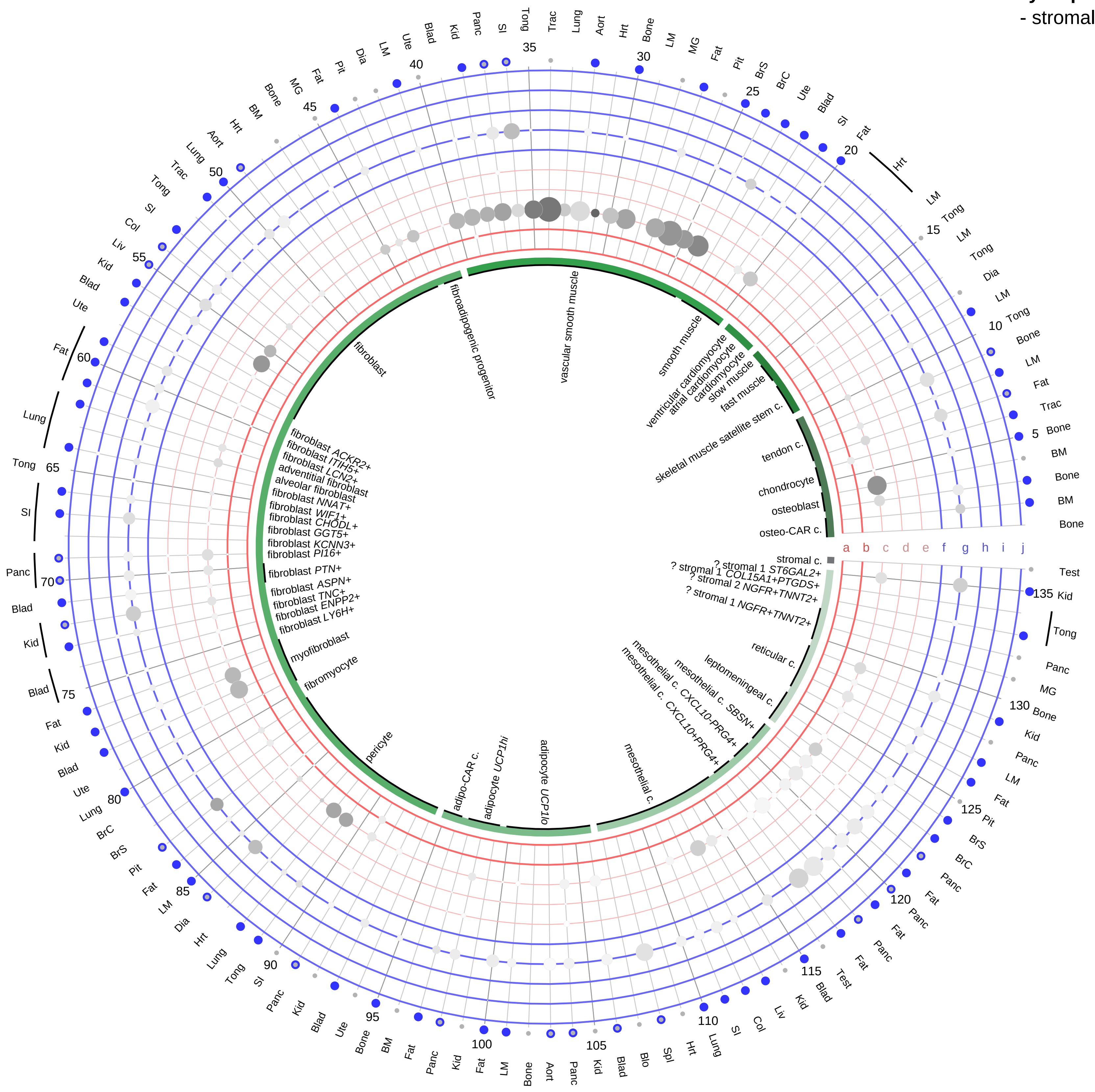

Ligand/Enzyme
- a - PDYN
- b - PENK
- c - CPE
- d - PCSK1
- e - PCSK2

Receptor
- f - MRGPRX1
- g - OGFR
- h - OPRD1
- i - OPRK1
- j - OPRM1

PAIRING (ligand → receptor)

PDYN & PCSK1 & PCSK2 & CPE→OPRK1

PDYN & PCSK1 & PCSK2 & CPE→OPRD1

PDYN & PCSK1 & PCSK2 & CPE→OPRM1

PENK & PCSK1 & CPE→OPRD1

PENK & PCSK1 & CPE→OPRM1

PENK & PCSK1 & CPE→OPRK1

PENK & PCSK1 & CPE→OGFR

PENK & PCSK1 & CPE→MRGPRX1

PENK & PCSK2 & CPE→OPRD1

PENK & PCSK2 & CPE→OPRM1

PENK & PCSK2 & CPE→OPRK1

PENK & PCSK2 & CPE→OGFR

PENK & PCSK2 & CPE→MRGPRX1

Compartment
stromal
- bone
- skeletal muscle
- cardiac muscle
- smooth muscle
- fibroblast
- adipocyte
- mesothelial
- other
- mix

12.5%
25%
50%
100%

ln(CP10K+1): 0 1 2 3

Abbrev.	Tissue
Aort	Aorta
Blad	Bladder
Blo	Blood
Bone	Bone
BM	Bone marrow
BrC	Brain cortex
BrS	Brainstem
Col	Colon
Dia	Diaphragm
Ret	Eye retina
Fat	Fat
Hrt	Heart
Pit	Hypothalamus/Pituitary
Kid	Kidney
LM	Limb muscle
Liv	Liver
Lung	Lung
MG	Mammary gland
Panc	Pancreas
Skin	Skin
SI	Small intestine
Spl	Spleen
Test	Testes
Thym	Thymus
Tong	Tongue
Trac	Trachea
Ute	Uterus

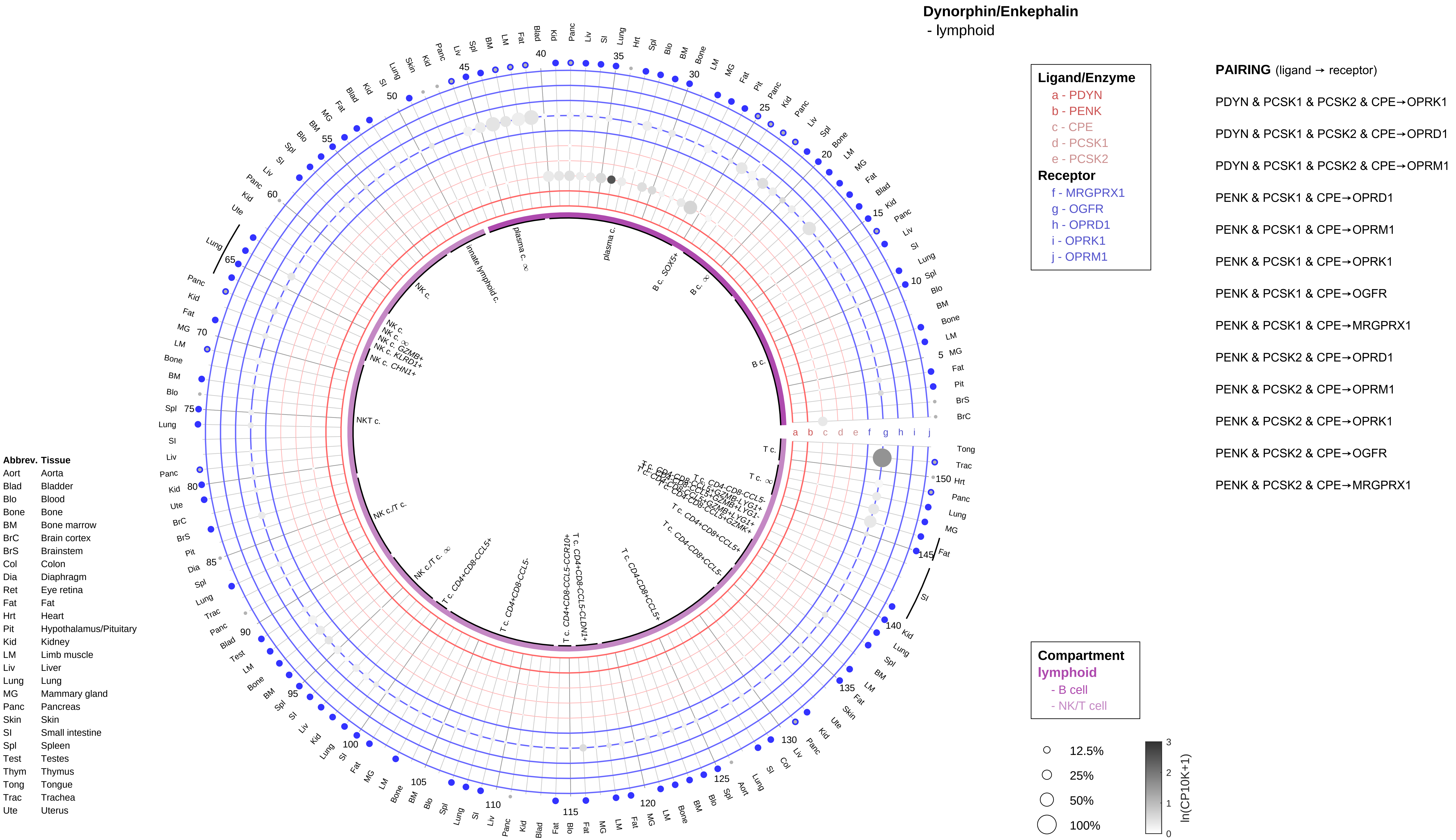
Dynorphin/Enkephalin
- lymphoid
Ligand/Enzyme
a - PDYN
b - PENK
c - CPE
d - PCSK1
e - PCSK2
Receptor
f - MRGPRX1
g - OGFR
h - OPRD1
i - OPRK1
j - OPRM1
PAIRING (ligand → receptor)
PDYN & PCSK1 & PCSK2 & CPE→OPRK1
PDYN & PCSK1 & PCSK2 & CPE→OPRD1
PDYN & PCSK1 & PCSK2 & CPE→OPRM1
PENK & PCSK1 & CPE→OPRD1
PENK & PCSK1 & CPE→OPRM1
PENK & PCSK1 & CPE→OPRK1
PENK & PCSK1 & CPE→OGFR
PENK & PCSK1 & CPE→MRGPRX1
PENK & PCSK2 & CPE→OPRD1
PENK & PCSK2 & CPE→OPRM1
PENK & PCSK2 & CPE→OPRK1
PENK & PCSK2 & CPE→OGFR
PENK & PCSK2 & CPE→MRGPRX1
Compartment
lymphoid
- B cell
- NK/T cell
12.5%
25%
50%
100%
ln(CP10K+1)
Abbrev. Tissue
Aort Aorta
Blad Bladder
Blo Blood
Bone Bone
BM Bone marrow
BrC Brain cortex
BrS Brainstem
Col Colon
Dia Diaphragm
Ret Eye retina
Fat Fat
Hrt Heart
Pit Hypothalamus/Pituitary
Kid Kidney
LM Limb muscle
Liv Liver
Lung Lung
MG Mammary gland
Panc Pancreas
Skin Skin
SI Small intestine
Spl Spleen
Test Testes
Thym Thymus
Tong Tongue
Trac Trachea
Ute Uterus

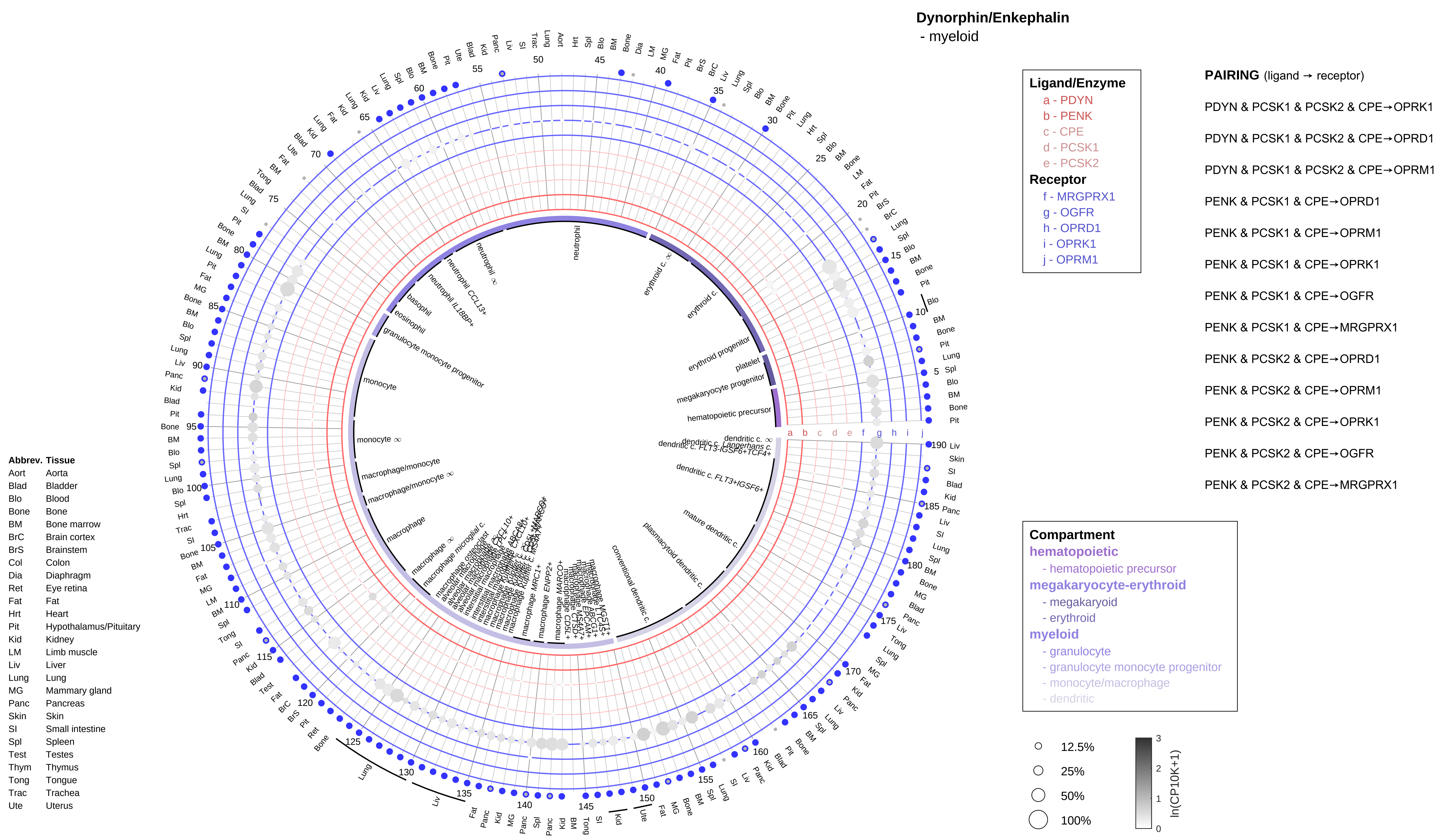
Dynorphin/Enkephalin
- myeloid
Ligand/Enzyme
a - PDYN
b - PENK
c - CPE
d - PCSK1
e - PCSK2
Receptor
f - MRGPRX1
g - OGFR
h - OPRD1
i - OPRK1
j - OPRM1
PAIRING (ligand → receptor)
PDYN & PCSK1 & PCSK2 & CPE→OPRK1
PDYN & PCSK1 & PCSK2 & CPE→OPRD1
PDYN & PCSK1 & PCSK2 & CPE→OPRM1
PENK & PCSK1 & CPE→OPRD1
PENK & PCSK1 & CPE→OPRM1
PENK & PCSK1 & CPE→OPRK1
PENK & PCSK1 & CPE→OGFR
PENK & PCSK1 & CPE→MRGPRX1
PENK & PCSK2 & CPE→OPRD1
PENK & PCSK2 & CPE→OPRM1
PENK & PCSK2 & CPE→OPRK1
PENK & PCSK2 & CPE→OGFR
PENK & PCSK2 & CPE→MRGPRX1
Compartment
hematopoietic
- hematopoietic precursor
megakaryocyte-erythroid
- megakaryoid
- erythroid
myeloid
- granulocyte
- granulocyte monocyte progenitor
- monocyte/macrophage
- dendritic
12.5%
25%
50%
100%
ln(CP10K+1)
Abbrev. Tissue
Aort Aorta
Blad Bladder
Blo Blood
Bone Bone
BM Bone marrow
BrC Brain cortex
BrS Brainstem
Col Colon
Dia Diaphragm
Ret Eye retina
Fat Fat
Hrt Heart
Pit Hypothalamus/Pituitary
Kid Kidney
LM Limb muscle
Liv Liver
Lung Lung
MG Mammary gland
Panc Pancreas
Skin Skin
SI Small intestine
Spl Spleen
Test Testes
Thym Thymus
Tong Tongue
Trac Trachea
Ute Uterus

Endomorphin
- epithelial/neural/germ

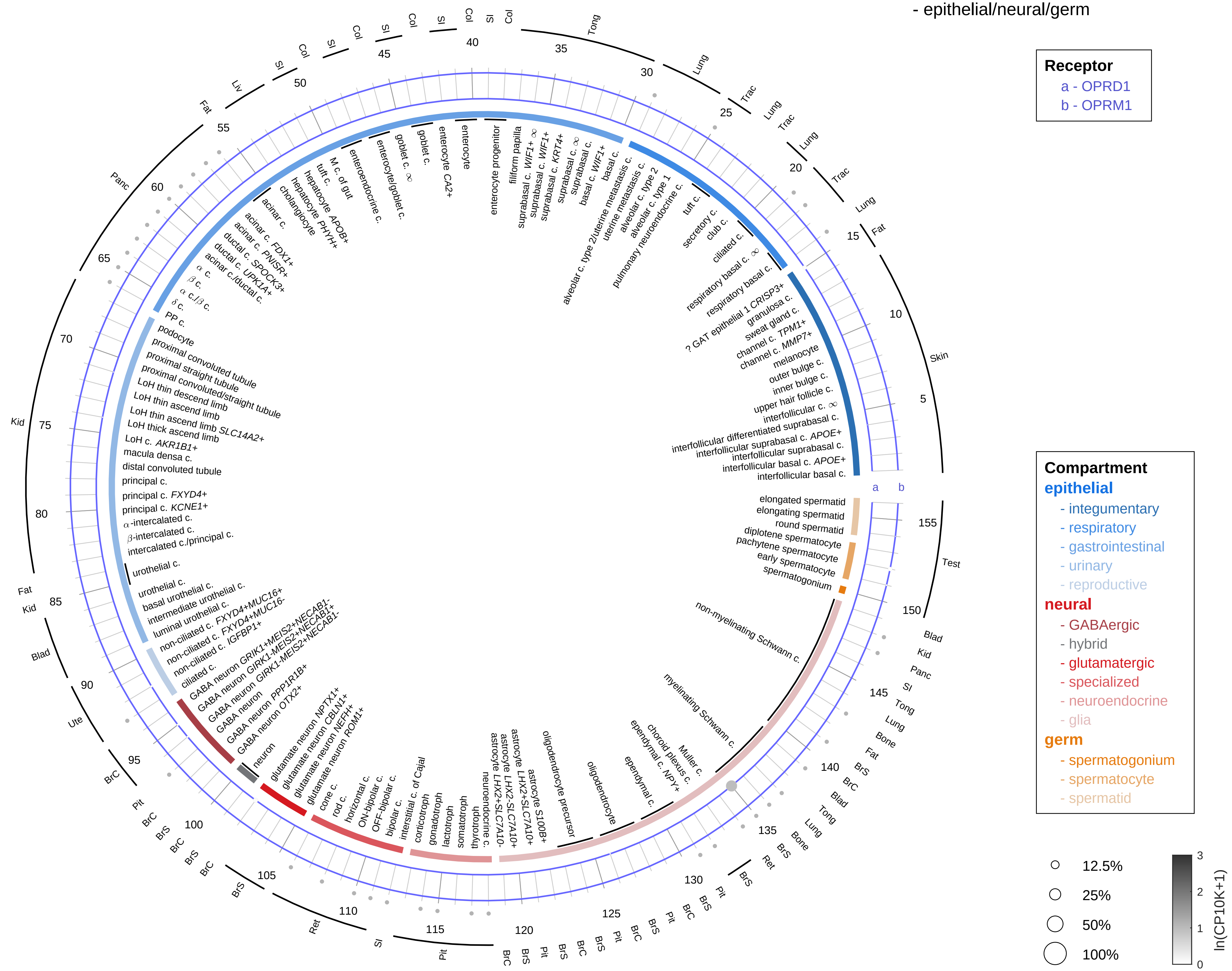

Abbrev.	Tissue
Aort	Aorta
Blad	Bladder
Blo	Blood
Bone	Bone
BM	Bone marrow
BrC	Brain cortex
BrS	Brainstem
Col	Colon
Dia	Diaphragm
Ret	Eye retina
Fat	Fat
Hrt	Heart
Pit	Hypothalamus/Pituitary
Kid	Kidney
LM	Limb muscle
Liv	Liver
Lung	Lung
MG	Mammary gland
Panc	Pancreas
Skin	Skin
SI	Small intestine
Spl	Spleen
Test	Testes
Thym	Thymus
Tong	Tongue
Trac	Trachea
Ute	Uterus

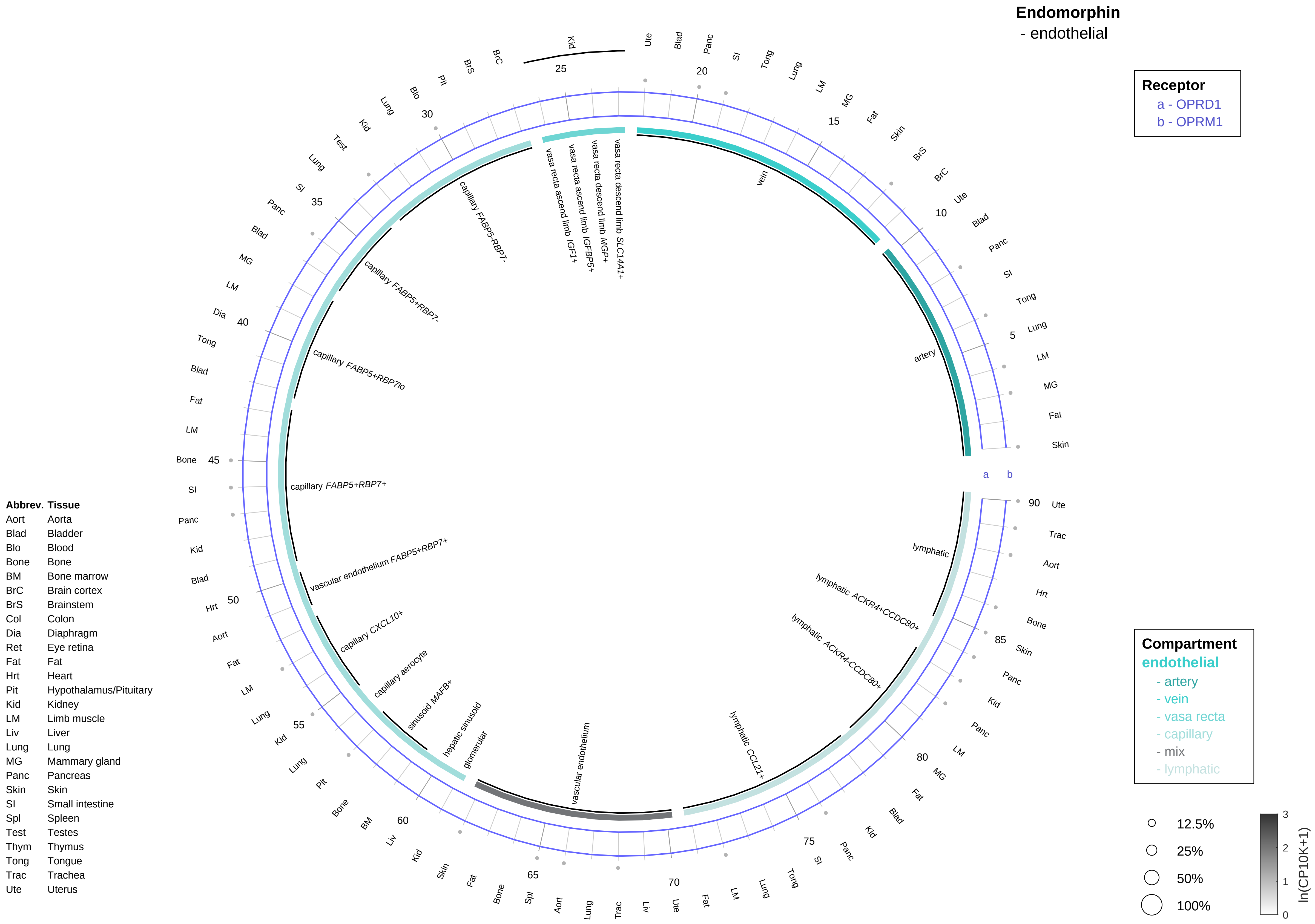

Endomorphin
- endothelial
Receptor
a - OPRD1
b - OPRM1
Compartment
endothelial
- artery
- vein
- vasa recta
- capillary
- mix
- lymphatic
12.5%
25%
50%
100%
ln(CP10K+1)
0
1
2
3
artery
vein
vasa recta descend limb SLC14A1+
vasa recta descend limb MGP+
vasa recta descend limb IGFBP5+
vasa recta ascend limb IGF1+
capillary FABP5-RBP7-
capillary FABP5+RBP7-
capillary FABP5+RBP7lo
capillary FABP5+RBP7+
vascular endothelium FABP5+RBP7+
capillary CXCL10+
capillary aerocyte
sinusoid MAFB+
hepatic sinusoid
glomerular
vascular endothelium
lymphatic CCL21+
lymphatic ACKR4-CCDC80+
lymphatic ACKR4+CCDC80+
lymphatic
Abbrev. Tissue
Aort Aorta
Blad Bladder
Blo Blood
Bone Bone
BM Bone marrow
BrC Brain cortex
BrS Brainstem
Col Colon
Dia Diaphragm
Ret Eye retina
Fat Fat
Hrt Heart
Pit Hypothalamus/Pituitary
Kid Kidney
LM Limb muscle
Liv Liver
Lung Lung
MG Mammary gland
Panc Pancreas
Skin Skin
SI Small intestine
Spl Spleen
Test Testes
Thym Thymus
Tong Tongue
Trac Trachea
Ute Uterus

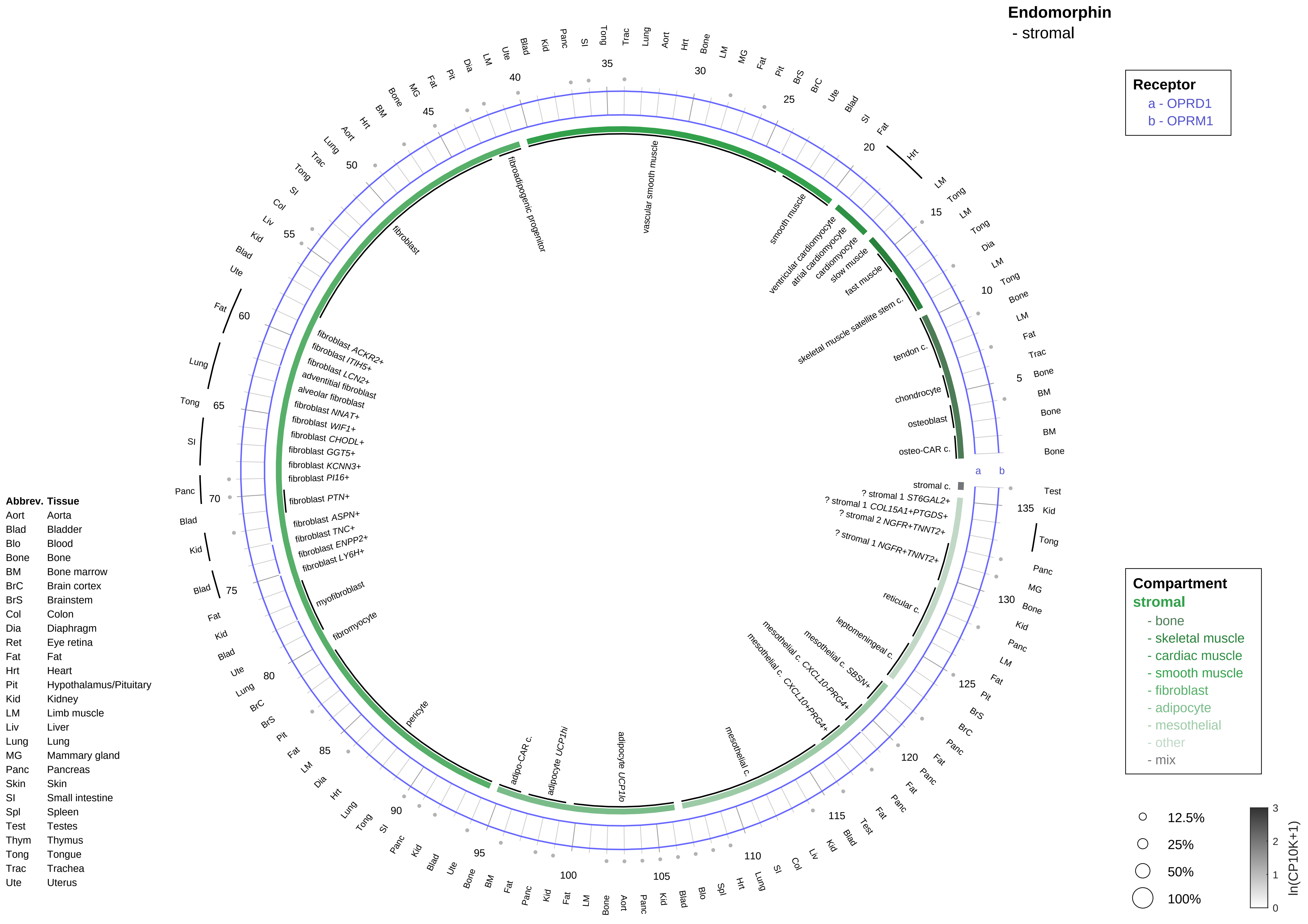
Endomorphin
- stromal
Receptor
a - OPRD1
b - OPRM1
Compartment
stromal
- bone
- skeletal muscle
- cardiac muscle
- smooth muscle
- fibroblast
- adipocyte
- mesothelial
- other
- mix
12.5%
25%
50%
100%
ln(CP10K+1)
0
1
2
3
Abbrev. Tissue
Aort Aorta
Blad Bladder
Blo Blood
Bone Bone
BM Bone marrow
BrC Brain cortex
BrS Brainstem
Col Colon
Dia Diaphragm
Ret Eye retina
Fat Fat
Hrt Heart
Pit Hypothalamus/Pituitary
Kid Kidney
LM Limb muscle
Liv Liver
Lung Lung
MG Mammary gland
Panc Pancreas
Skin Skin
SI Small intestine
Spl Spleen
Test Testes
Thym Thymus
Tong Tongue
Trac Trachea
Ute Uterus
osteo-CAR c.
osteoblast
chondrocyte
tendon c.
skeletal muscle satellite stem c.
fast muscle
slow muscle
cardiomyocyte
atrial cardiomyocyte
ventricular cardiomyocyte
smooth muscle
vascular smooth muscle
fibroadipogenic progenitor
fibroblast
fibroblast ACKR2+
fibroblast ITIH5+
fibroblast LCN2+
adventitial fibroblast
alveolar fibroblast
fibroblast NNAT+
fibroblast WIF1+
fibroblast CHODL+
fibroblast GGT5+
fibroblast KCNN3+
fibroblast PI16+
fibroblast PTN+
fibroblast ASPN+
fibroblast TNC+
fibroblast ENPP2+
fibroblast LY6H+
myofibroblast
fibromyocyte
pericyte
adipo-CAR c.
adipocyte UCP1hi
adipocyte UCP1lo
mesothelial c.
mesothelial c. CXCL10+PRG4+
mesothelial c. CXCL10-PRG4+
mesothelial c. SBSN+
leptomeningeal c.
reticular c.
? stromal 1 NGFR+TNNT2+
? stromal 2 NGFR+TNNT2+
? stromal 1 COL15A1+PTGDS+
? stromal 1 ST6GAL2+
stromal c.
a
b

Endomorphin
- lymphoid

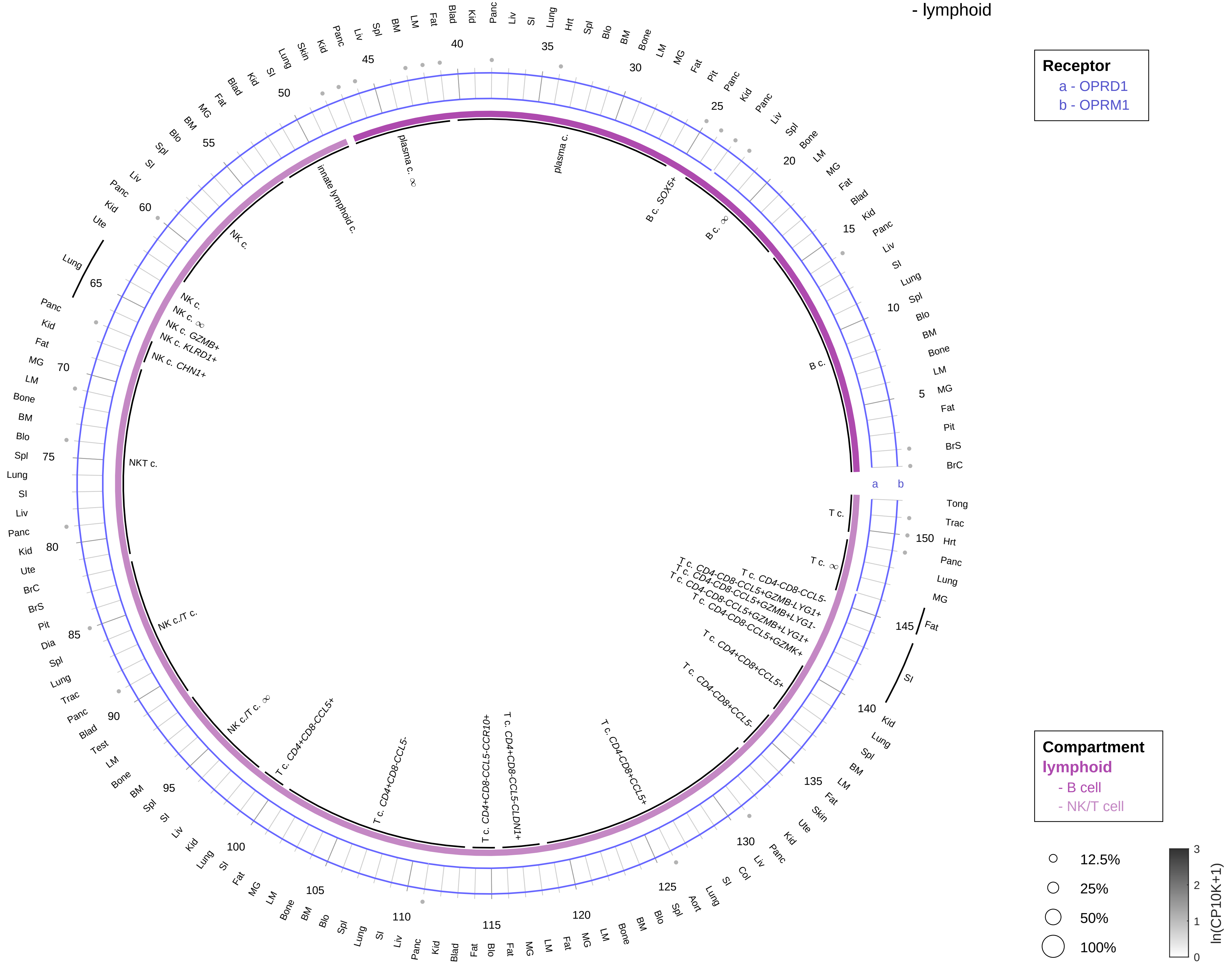

Abbrev.	Tissue
Aort	Aorta
Blad	Bladder
Blo	Blood
Bone	Bone
BM	Bone marrow
BrC	Brain cortex
BrS	Brainstem
Col	Colon
Dia	Diaphragm
Ret	Eye retina
Fat	Fat
Hrt	Heart
Pit	Hypothalamus/Pituitary
Kid	Kidney
LM	Limb muscle
Liv	Liver
Lung	Lung
MG	Mammary gland
Panc	Pancreas
Skin	Skin
SI	Small intestine
Spl	Spleen
Test	Testes
Thym	Thymus
Tong	Tongue
Trac	Trachea
Ute	Uterus

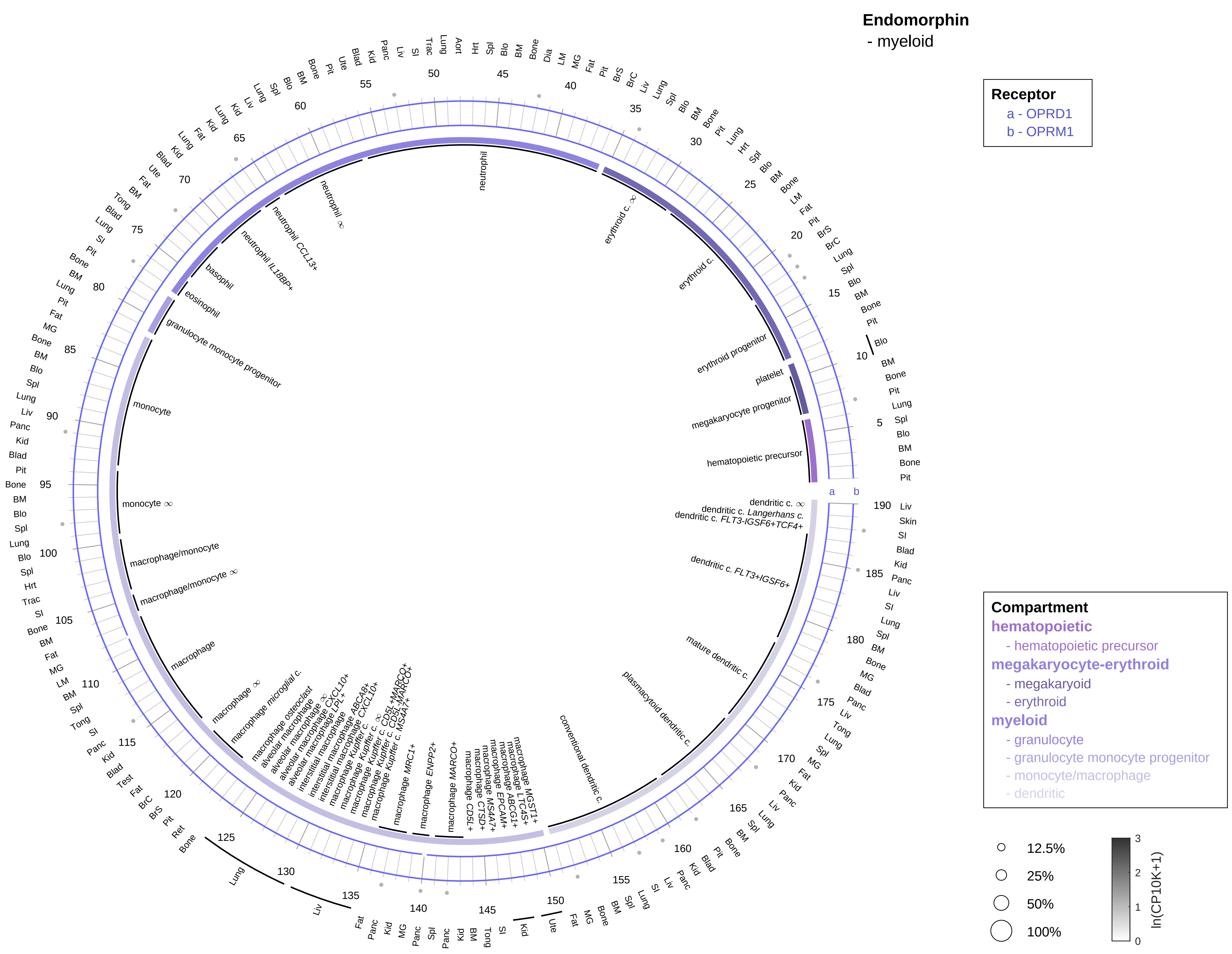

Abbrev.	Tissue
Aort	Aorta
Blad	Bladder
Blo	Blood
Bone	Bone
BM	Bone marrow
BrC	Brain cortex
BrS	Brainstem
Col	Colon
Dia	Diaphragm
Ret	Eye retina
Fat	Fat
Hrt	Heart
Pit	Hypothalamus/Pituitary
Kid	Kidney
LM	Limb muscle
Liv	Liver
Lung	Lung
MG	Mammary gland
Panc	Pancreas
Skin	Skin
SI	Small intestine
Spl	Spleen
Test	Testes
Thym	Thymus
Tong	Tongue
Trac	Trachea
Ute	Uterus

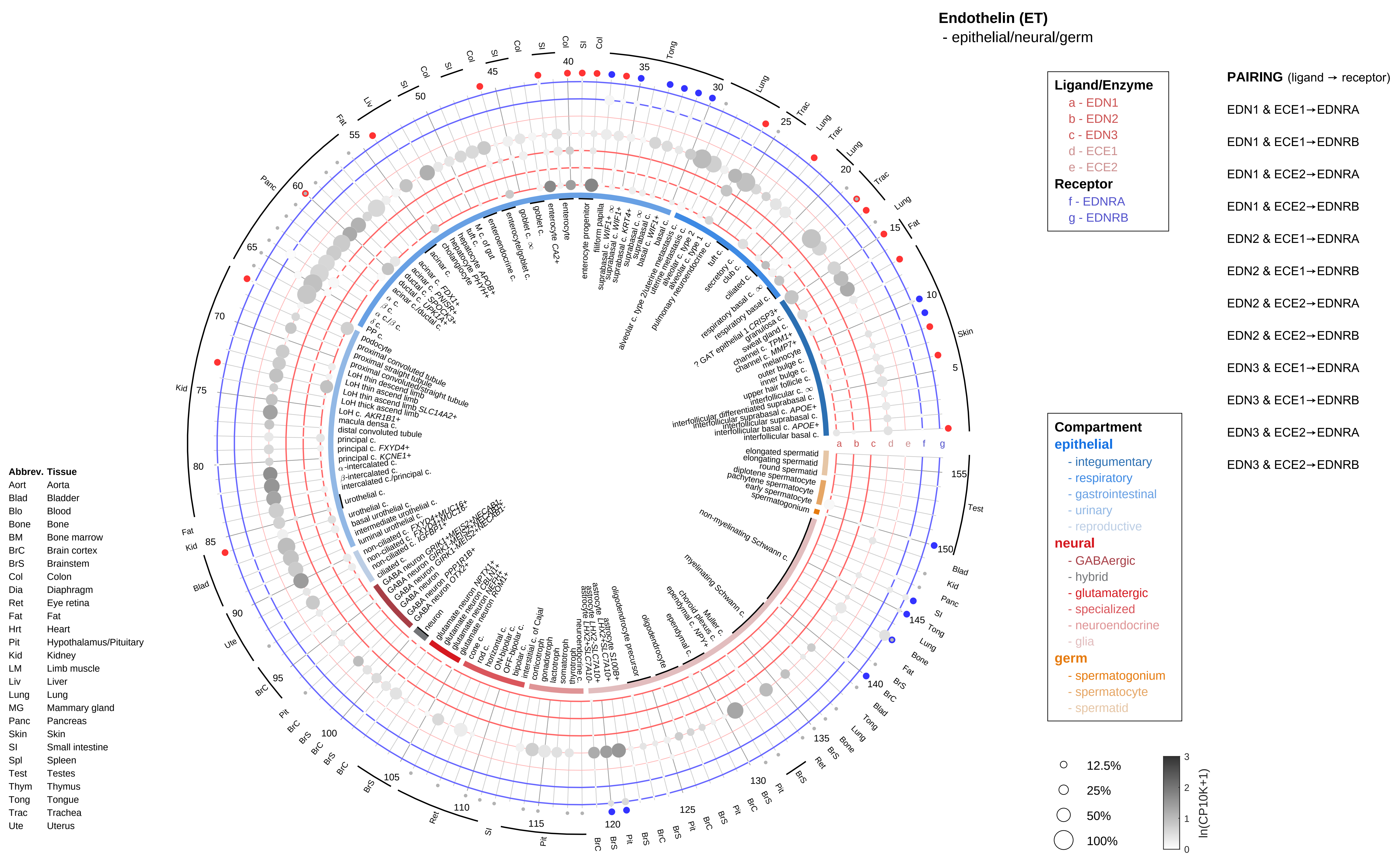
Endothelin (ET)
- epithelial/neural/germ
Ligand/Enzyme
a - EDN1
b - EDN2
c - EDN3
d - ECE1
e - ECE2
Receptor
f - EDNRA
g - EDNRB
PAIRING (ligand → receptor)
EDN1 & ECE1→EDNRA
EDN1 & ECE1→EDNRB
EDN1 & ECE2→EDNRA
EDN1 & ECE2→EDNRB
EDN2 & ECE1→EDNRA
EDN2 & ECE1→EDNRB
EDN2 & ECE2→EDNRA
EDN2 & ECE2→EDNRB
EDN3 & ECE1→EDNRA
EDN3 & ECE1→EDNRB
EDN3 & ECE2→EDNRA
EDN3 & ECE2→EDNRB
Compartment
epithelial
- integumentary
- respiratory
- gastrointestinal
- urinary
- reproductive
neural
- GABAergic
- hybrid
- glutamatergic
- specialized
- neuroendocrine
- glia
germ
- spermatogonium
- spermatocyte
- spermatid
12.5%
25%
50%
100%
ln(CP10K+1)
Abbrev. Tissue
Aort Aorta
Blad Bladder
Blo Blood
Bone Bone
BM Bone marrow
BrC Brain cortex
BrS Brainstem
Col Colon
Dia Diaphragm
Ret Eye retina
Fat Fat
Hrt Heart
Pit Hypothalamus/Pituitary
Kid Kidney
LM Limb muscle
Liv Liver
Lung Lung
MG Mammary gland
Panc Pancreas
Skin Skin
SI Small intestine
Spl Spleen
Test Testes
Thym Thymus
Tong Tongue
Trac Trachea
Ute Uterus

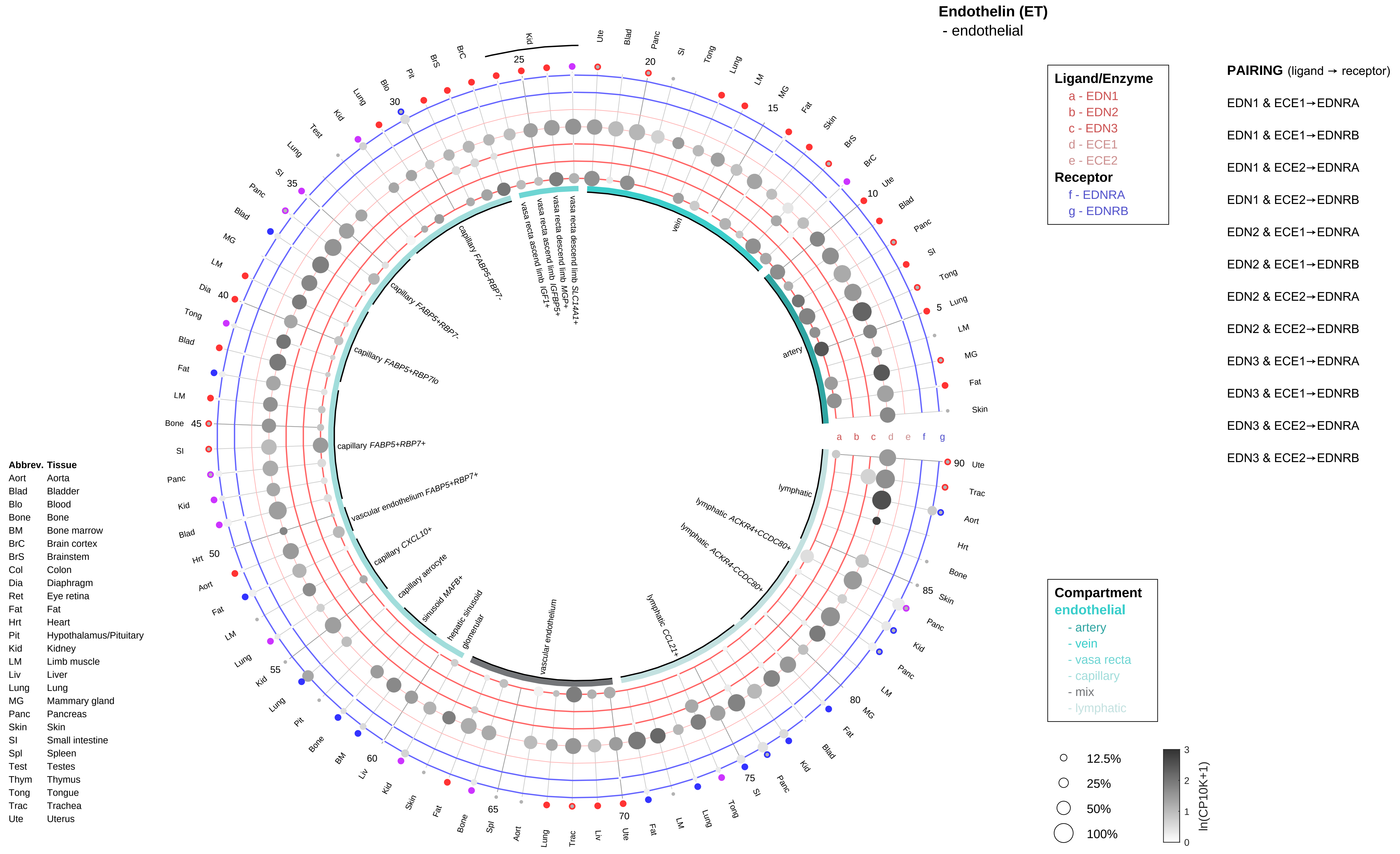
Endothelin (ET)
- endothelial
Ligand/Enzyme
a - EDN1
b - EDN2
c - EDN3
d - ECE1
e - ECE2
Receptor
f - EDNRA
g - EDNRB
PAIRING (ligand → receptor)
EDN1 & ECE1→EDNRA
EDN1 & ECE1→EDNRB
EDN1 & ECE2→EDNRA
EDN1 & ECE2→EDNRB
EDN2 & ECE1→EDNRA
EDN2 & ECE1→EDNRB
EDN2 & ECE2→EDNRA
EDN2 & ECE2→EDNRB
EDN3 & ECE1→EDNRA
EDN3 & ECE1→EDNRB
EDN3 & ECE2→EDNRA
EDN3 & ECE2→EDNRB
Compartment
endothelial
- artery
- vein
- vasa recta
- capillary
- mix
- lymphatic
12.5%
25%
50%
100%
ln(CP10K+1)
0
1
2
3
artery
vein
vasa recta descend limb SLC14A1+
vasa recta descend limb MGP+
vasa recta ascend limb IGFBP5+
vasa recta ascend limb IGF1+
capillary FABP5-RBP7-
capillary FABP5+RBP7-
capillary FABP5+RBP7lo
capillary FABP5+RBP7+
vascular endothelium FABP5+RBP7+
capillary CXCL10+
capillary aerocyte
sinusoid MAFB+
hepatic sinusoid
glomerular
vascular endothelium
lymphatic CCL21+
lymphatic ACKR4-CCDC80+
lymphatic ACKR4+CCDC80+
lymphatic
Abbrev. Tissue
Aort Aorta
Blad Bladder
Blo Blood
Bone Bone
BM Bone marrow
BrC Brain cortex
BrS Brainstem
Col Colon
Dia Diaphragm
Ret Eye retina
Fat Fat
Hrt Heart
Pit Hypothalamus/Pituitary
Kid Kidney
LM Limb muscle
Liv Liver
Lung Lung
MG Mammary gland
Panc Pancreas
Skin Skin
SI Small intestine
Spl Spleen
Test Testes
Thym Thymus
Tong Tongue
Trac Trachea
Ute Uterus

Endothelin (ET)
- stromal

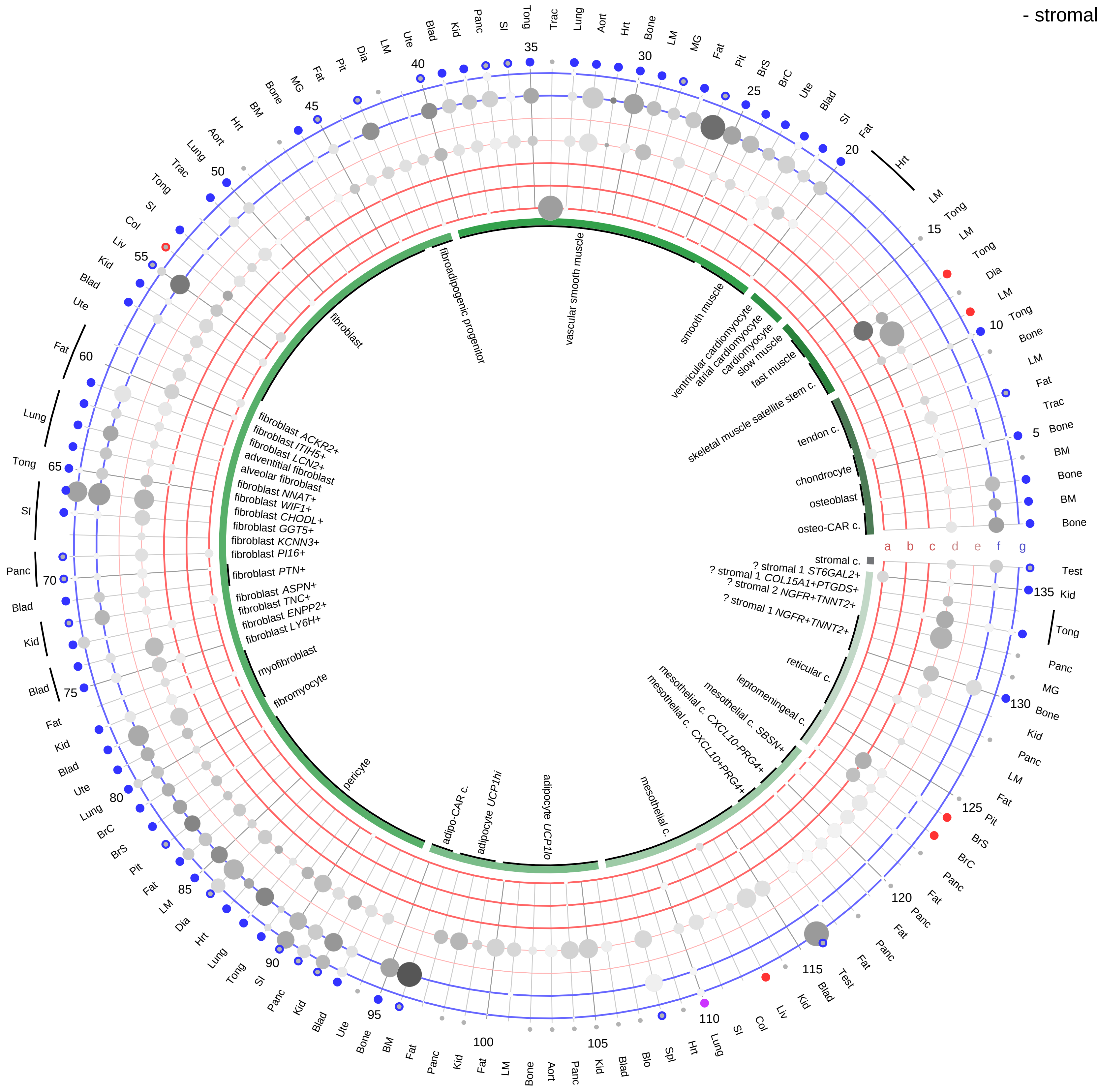

Ligand/Enzyme
- a - EDN1
- b - EDN2
- c - EDN3
- d - ECE1
- e - ECE2

Receptor
- f - EDNRA
- g - EDNRB

PAIRING (ligand → receptor)

EDN1 & ECE1→EDNRA
EDN1 & ECE1→EDNRB
EDN1 & ECE2→EDNRA
EDN1 & ECE2→EDNRB
EDN2 & ECE1→EDNRA
EDN2 & ECE1→EDNRB
EDN2 & ECE2→EDNRA
EDN2 & ECE2→EDNRB
EDN3 & ECE1→EDNRA
EDN3 & ECE1→EDNRB
EDN3 & ECE2→EDNRA
EDN3 & ECE2→EDNRB

Compartment
stromal
- bone
- skeletal muscle
- cardiac muscle
- smooth muscle
- fibroblast
- adipocyte
- mesothelial
- other
- mix

12.5%
25%
50%
100%

ln(CP10K+1): 0, 1, 2, 3

**Abbrev.**	**Tissue**
Aort	Aorta
Blad	Bladder
Blo	Blood
Bone	Bone
BM	Bone marrow
BrC	Brain cortex
BrS	Brainstem
Col	Colon
Dia	Diaphragm
Ret	Eye retina
Fat	Fat
Hrt	Heart
Pit	Hypothalamus/Pituitary
Kid	Kidney
LM	Limb muscle
Liv	Liver
Lung	Lung
MG	Mammary gland
Panc	Pancreas
Skin	Skin
SI	Small intestine
Spl	Spleen
Test	Testes
Thym	Thymus
Tong	Tongue
Trac	Trachea
Ute	Uterus

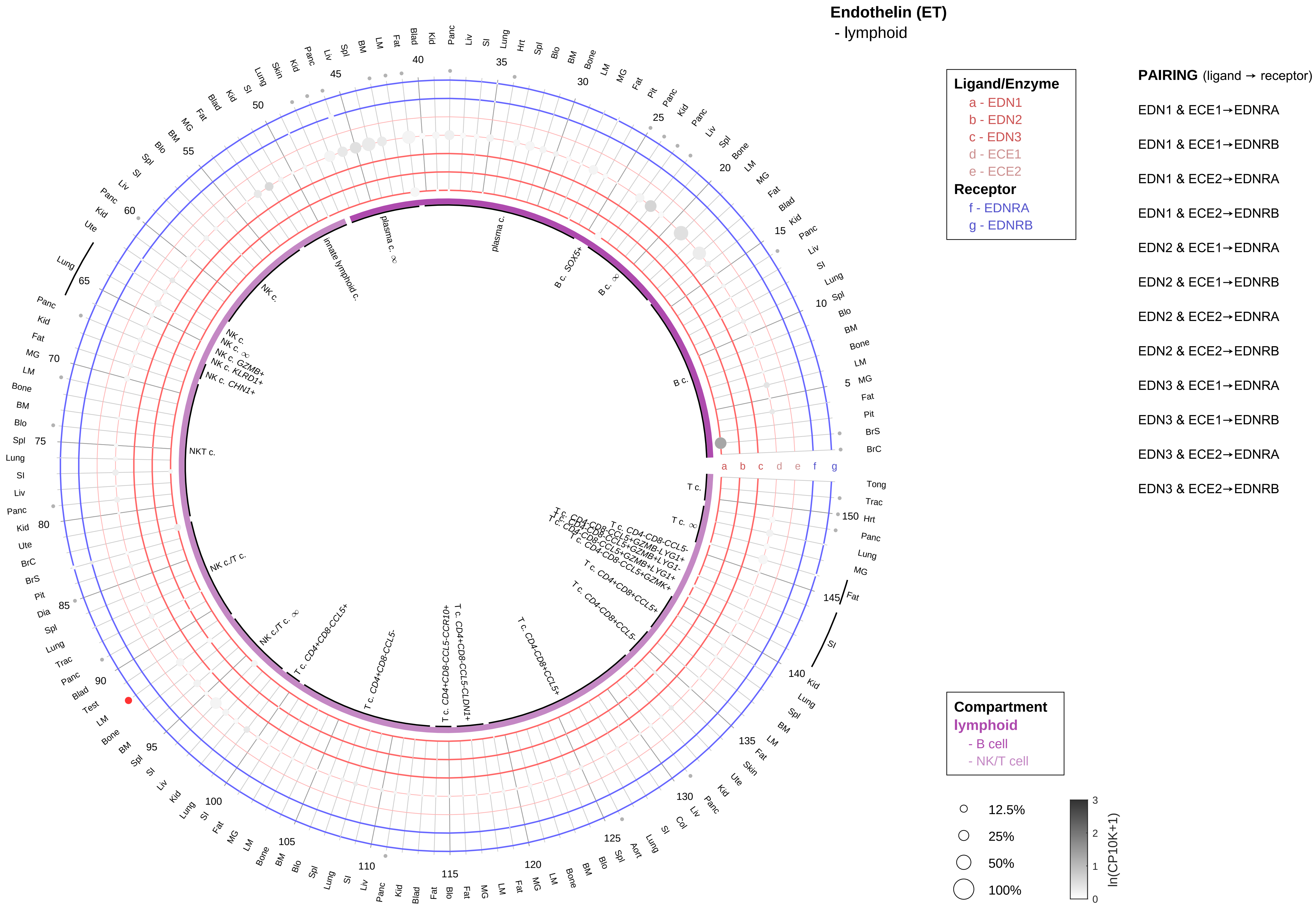

Abbrev.	Tissue
Aort	Aorta
Blad	Bladder
Blo	Blood
Bone	Bone
BM	Bone marrow
BrC	Brain cortex
BrS	Brainstem
Col	Colon
Dia	Diaphragm
Ret	Eye retina
Fat	Fat
Hrt	Heart
Pit	Hypothalamus/Pituitary
Kid	Kidney
LM	Limb muscle
Liv	Liver
Lung	Lung
MG	Mammary gland
Panc	Pancreas
Skin	Skin
SI	Small intestine
Spl	Spleen
Test	Testes
Thym	Thymus
Tong	Tongue
Trac	Trachea
Ute	Uterus

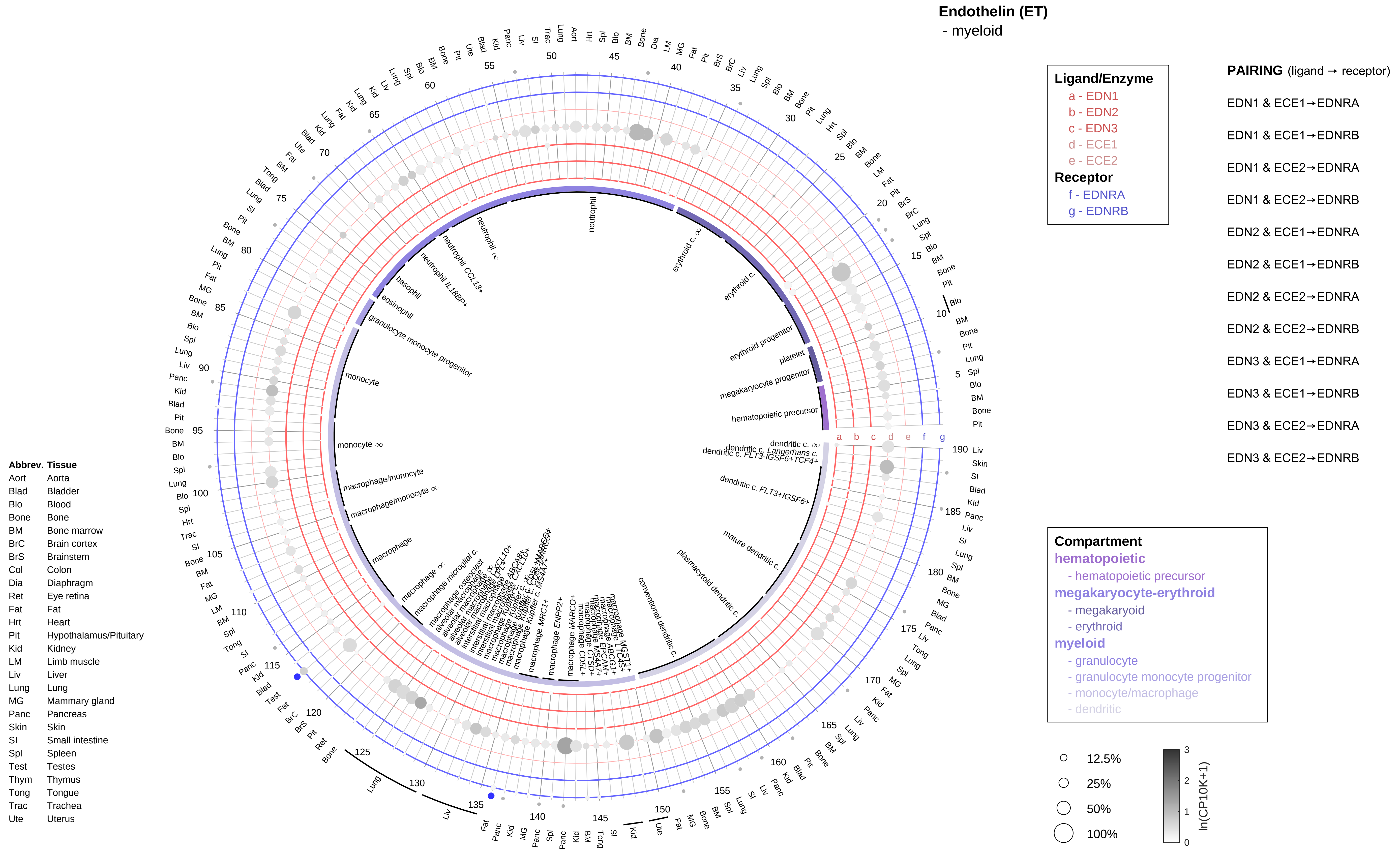

Endothelin (ET)
- myeloid
Ligand/Enzyme
a - EDN1
b - EDN2
c - EDN3
d - ECE1
e - ECE2
Receptor
f - EDNRA
g - EDNRB
PAIRING (ligand → receptor)
EDN1 & ECE1→EDNRA
EDN1 & ECE1→EDNRB
EDN1 & ECE2→EDNRA
EDN1 & ECE2→EDNRB
EDN2 & ECE1→EDNRA
EDN2 & ECE1→EDNRB
EDN2 & ECE2→EDNRA
EDN2 & ECE2→EDNRB
EDN3 & ECE1→EDNRA
EDN3 & ECE1→EDNRB
EDN3 & ECE2→EDNRA
EDN3 & ECE2→EDNRB
Compartment
hematopoietic
- hematopoietic precursor
megakaryocyte-erythroid
- megakaryoid
- erythroid
myeloid
- granulocyte
- granulocyte monocyte progenitor
- monocyte/macrophage
- dendritic
12.5%
25%
50%
100%
ln(CP10K+1)
0
1
2
3
Abbrev. Tissue
Aort Aorta
Blad Bladder
Blo Blood
Bone Bone
BM Bone marrow
BrC Brain cortex
BrS Brainstem
Col Colon
Dia Diaphragm
Ret Eye retina
Fat Fat
Hrt Heart
Pit Hypothalamus/Pituitary
Kid Kidney
LM Limb muscle
Liv Liver
Lung Lung
MG Mammary gland
Panc Pancreas
Skin Skin
SI Small intestine
Spl Spleen
Test Testes
Thym Thymus
Tong Tongue
Trac Trachea
Ute Uterus
neutrophil
erythroid c. ∞
erythroid c.
erythroid progenitor
platelet
megakaryocyte progenitor
hematopoietic precursor
dendritic c. ∞
dendritic c. Langerhans c.
dendritic c. FLT3-IGSF6+TCF4+
dendritic c. FLT3+IGSF6+
mature dendritic c.
plasmacytoid dendritic c.
conventional dendritic c.
macrophage
macrophage ∞
macrophage/monocyte
macrophage/monocyte ∞
monocyte ∞
monocyte
granulocyte monocyte progenitor
eosinophil
basophil
neutrophil IL18BP+
neutrophil CCL13+
neutrophil ∞
a
b
c
d
e
f
g

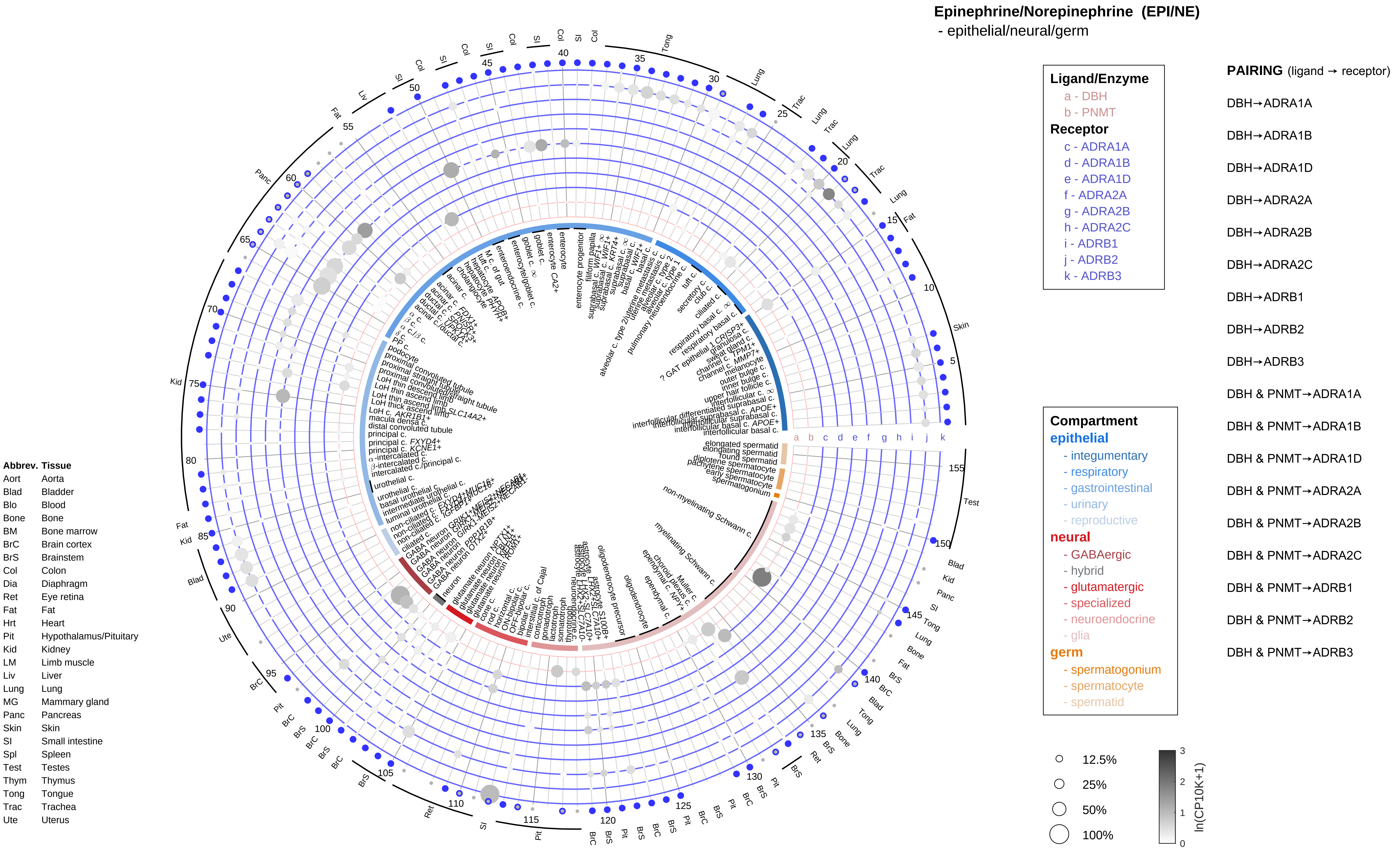
Epinephrine/Norepinephrine (EPI/NE)
- epithelial/neural/germ
Ligand/Enzyme
a - DBH
b - PNMT
Receptor
c - ADRA1A
d - ADRA1B
e - ADRA1D
f - ADRA2A
g - ADRA2B
h - ADRA2C
i - ADRB1
j - ADRB2
k - ADRB3
PAIRING (ligand → receptor)
DBH→ADRA1A
DBH→ADRA1B
DBH→ADRA1D
DBH→ADRA2A
DBH→ADRA2B
DBH→ADRA2C
DBH→ADRB1
DBH→ADRB2
DBH→ADRB3
DBH & PNMT→ADRA1A
DBH & PNMT→ADRA1B
DBH & PNMT→ADRA1D
DBH & PNMT→ADRA2A
DBH & PNMT→ADRA2B
DBH & PNMT→ADRA2C
DBH & PNMT→ADRB1
DBH & PNMT→ADRB2
DBH & PNMT→ADRB3
Compartment
epithelial
- integumentary
- respiratory
- gastrointestinal
- urinary
- reproductive
neural
- GABAergic
- hybrid
- glutamatergic
- specialized
- neuroendocrine
- glia
germ
- spermatogonium
- spermatocyte
- spermatid
12.5%
25%
50%
100%
ln(CP10K+1)
0
1
2
3
Abbrev. Tissue
Aort Aorta
Blad Bladder
Blo Blood
Bone Bone
BM Bone marrow
BrC Brain cortex
BrS Brainstem
Col Colon
Dia Diaphragm
Ret Eye retina
Fat Fat
Hrt Heart
Pit Hypothalamus/Pituitary
Kid Kidney
LM Limb muscle
Liv Liver
Lung Lung
MG Mammary gland
Panc Pancreas
Skin Skin
SI Small intestine
Spl Spleen
Test Testes
Thym Thymus
Tong Tongue
Trac Trachea
Ute Uterus

Epinephrine/Norepinephrine (EPI/NE)
- endothelial

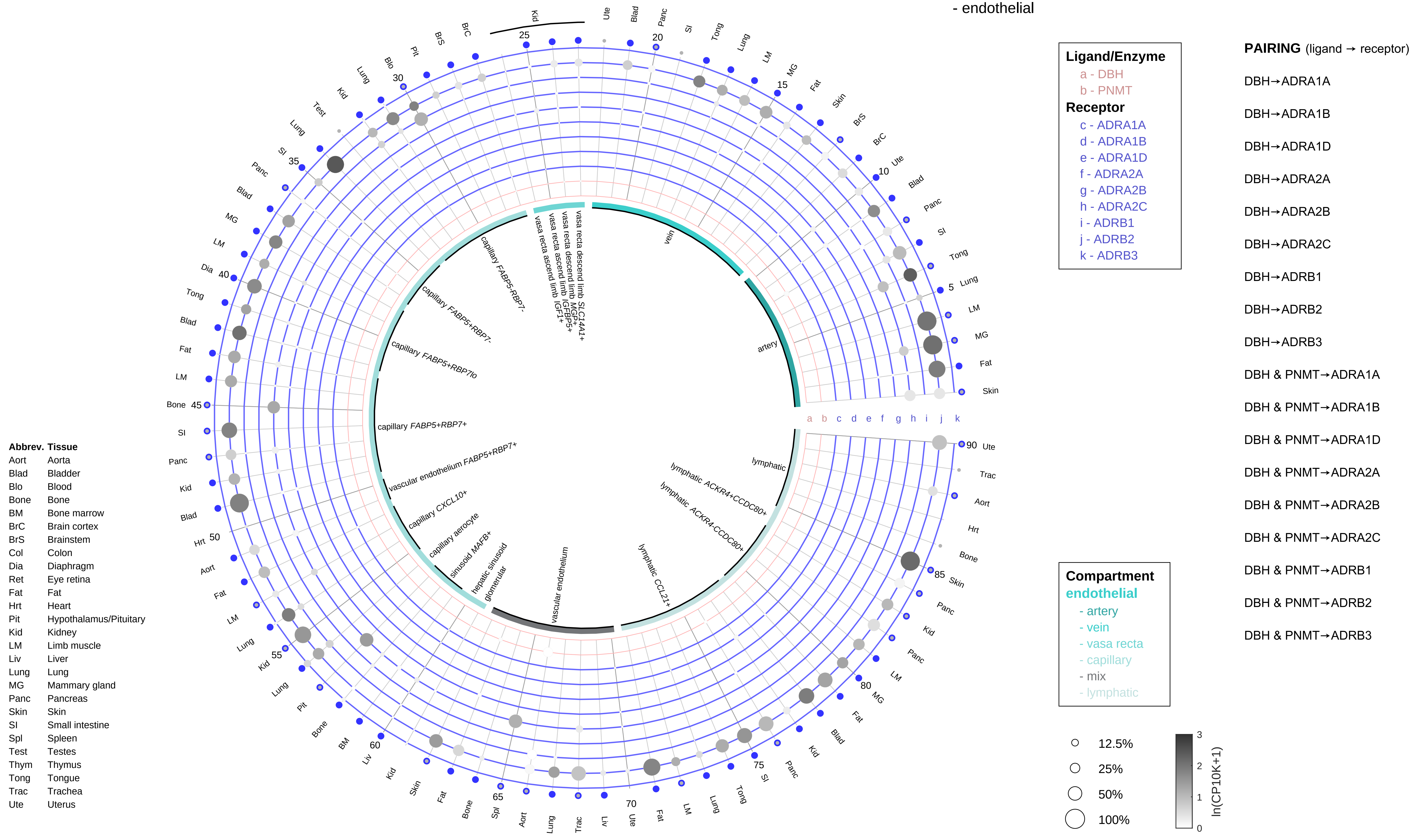

Epinephrine/Norepinephrine (EPI/NE)
- stromal

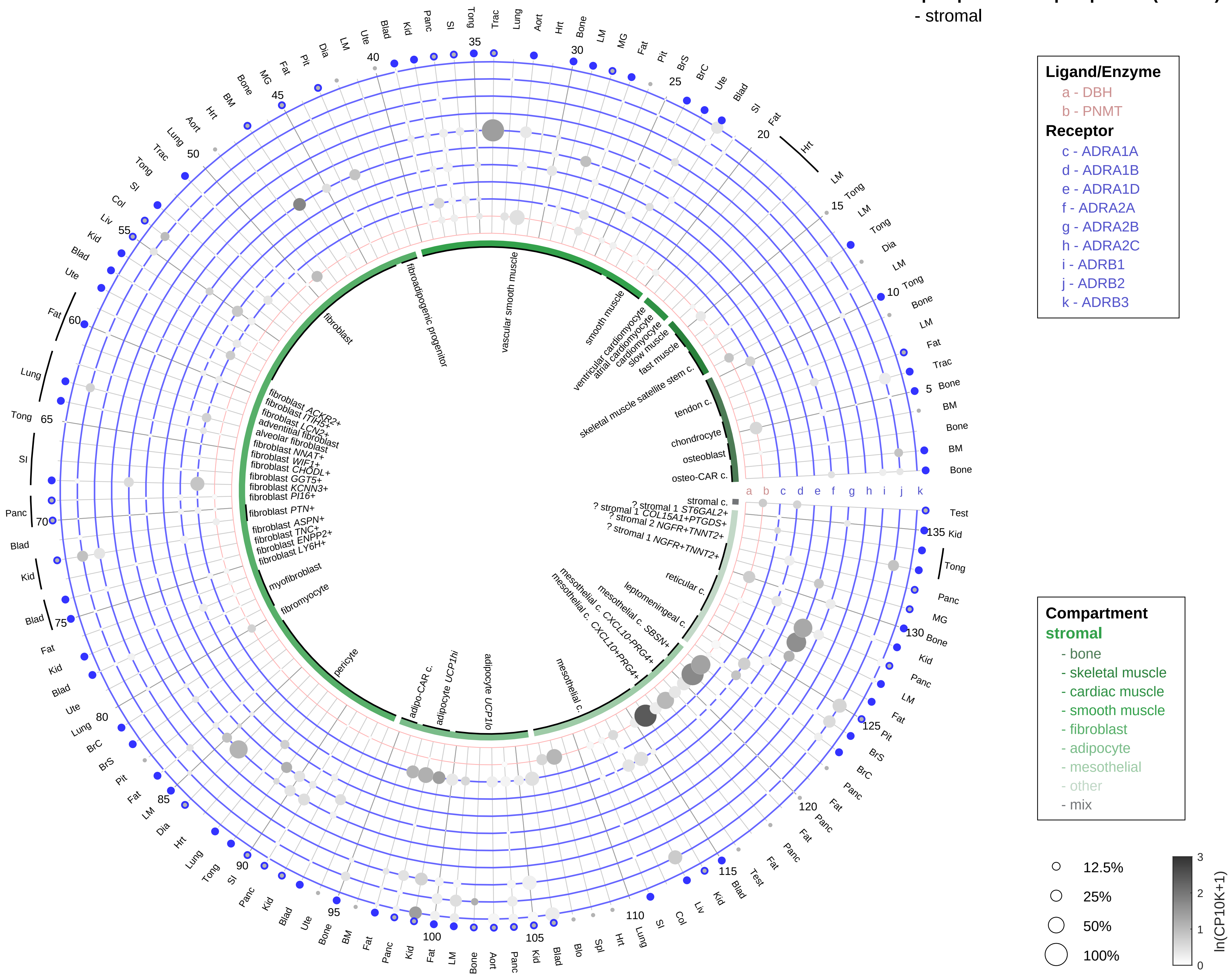

PAIRING (ligand → receptor)

DBH→ADRA1A

DBH→ADRA1B

DBH→ADRA1D

DBH→ADRA2A

DBH→ADRA2B

DBH→ADRA2C

DBH→ADRB1

DBH→ADRB2

DBH→ADRB3

DBH & PNMT→ADRA1A

DBH & PNMT→ADRA1B

DBH & PNMT→ADRA1D

DBH & PNMT→ADRA2A

DBH & PNMT→ADRA2B

DBH & PNMT→ADRA2C

DBH & PNMT→ADRB1

DBH & PNMT→ADRB2

DBH & PNMT→ADRB3

Abbrev.	Tissue
Aort	Aorta
Blad	Bladder
Blo	Blood
Bone	Bone
BM	Bone marrow
BrC	Brain cortex
BrS	Brainstem
Col	Colon
Dia	Diaphragm
Ret	Eye retina
Fat	Fat
Hrt	Heart
Pit	Hypothalamus/Pituitary
Kid	Kidney
LM	Limb muscle
Liv	Liver
Lung	Lung
MG	Mammary gland
Panc	Pancreas
Skin	Skin
SI	Small intestine
Spl	Spleen
Test	Testes
Thym	Thymus
Tong	Tongue
Trac	Trachea
Ute	Uterus

Epinephrine/Norepinephrine (EPI/NE)
- lymphoid

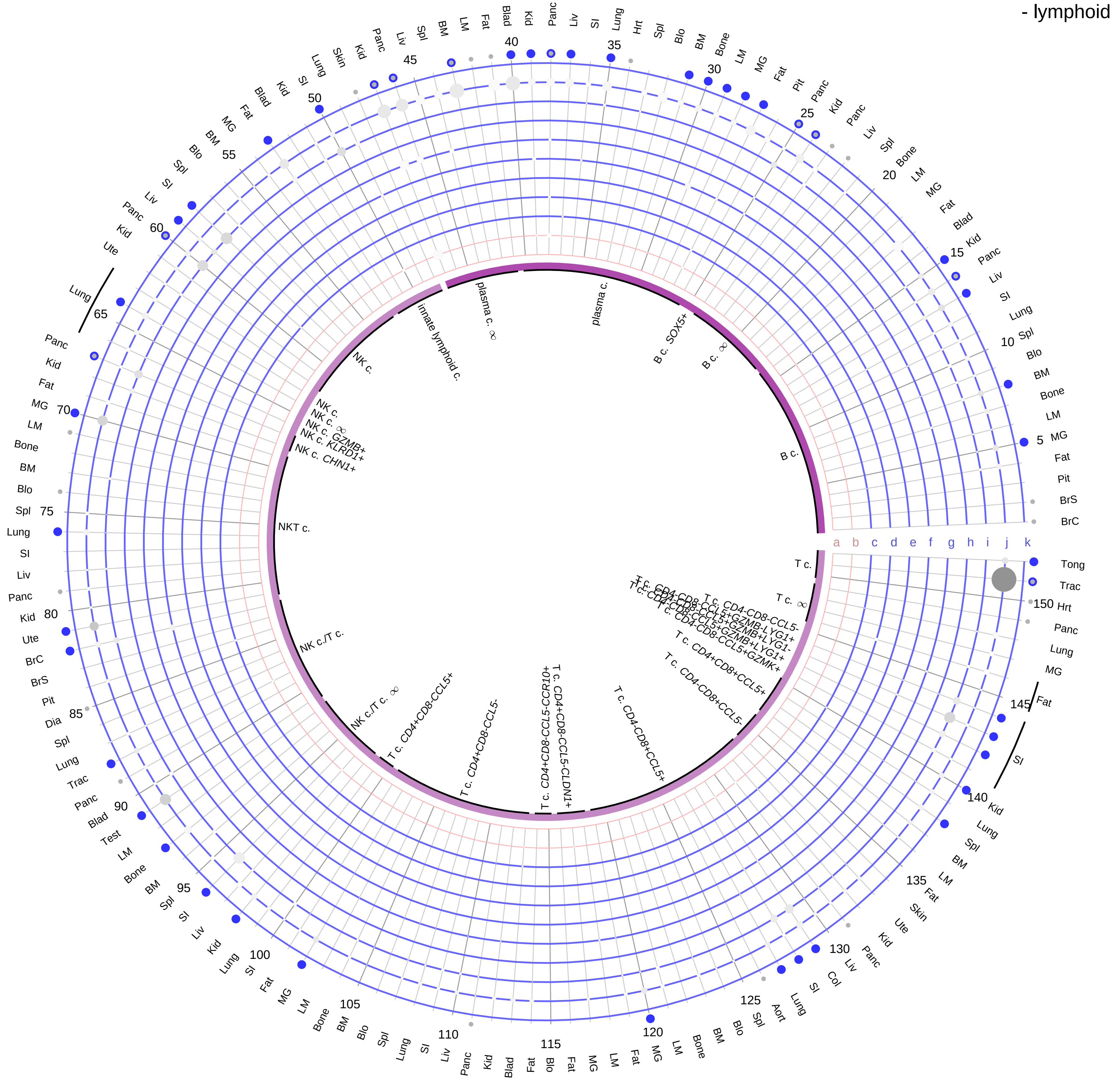

Ligand/Enzyme
a - DBH
b - PNMT

Receptor
c - ADRA1A
d - ADRA1B
e - ADRA1D
f - ADRA2A
g - ADRA2B
h - ADRA2C
i - ADRB1
j - ADRB2
k - ADRB3

PAIRING (ligand → receptor)

DBH→ADRA1A
DBH→ADRA1B
DBH→ADRA1D
DBH→ADRA2A
DBH→ADRA2B
DBH→ADRA2C
DBH→ADRB1
DBH→ADRB2
DBH→ADRB3
DBH & PNMT→ADRA1A
DBH & PNMT→ADRA1B
DBH & PNMT→ADRA1D
DBH & PNMT→ADRA2A
DBH & PNMT→ADRA2B
DBH & PNMT→ADRA2C
DBH & PNMT→ADRB1
DBH & PNMT→ADRB2
DBH & PNMT→ADRB3

Compartment
lymphoid
- B cell
- NK/T cell

12.5%
25%
50%
100%

ln(CP10K+1): 0, 1, 2, 3

Abbrev.	Tissue
Aort	Aorta
Blad	Bladder
Blo	Blood
Bone	Bone
BM	Bone marrow
BrC	Brain cortex
BrS	Brainstem
Col	Colon
Dia	Diaphragm
Ret	Eye retina
Fat	Fat
Hrt	Heart
Pit	Hypothalamus/Pituitary
Kid	Kidney
LM	Limb muscle
Liv	Liver
Lung	Lung
MG	Mammary gland
Panc	Pancreas
Skin	Skin
SI	Small intestine
Spl	Spleen
Test	Testes
Thym	Thymus
Tong	Tongue
Trac	Trachea
Ute	Uterus

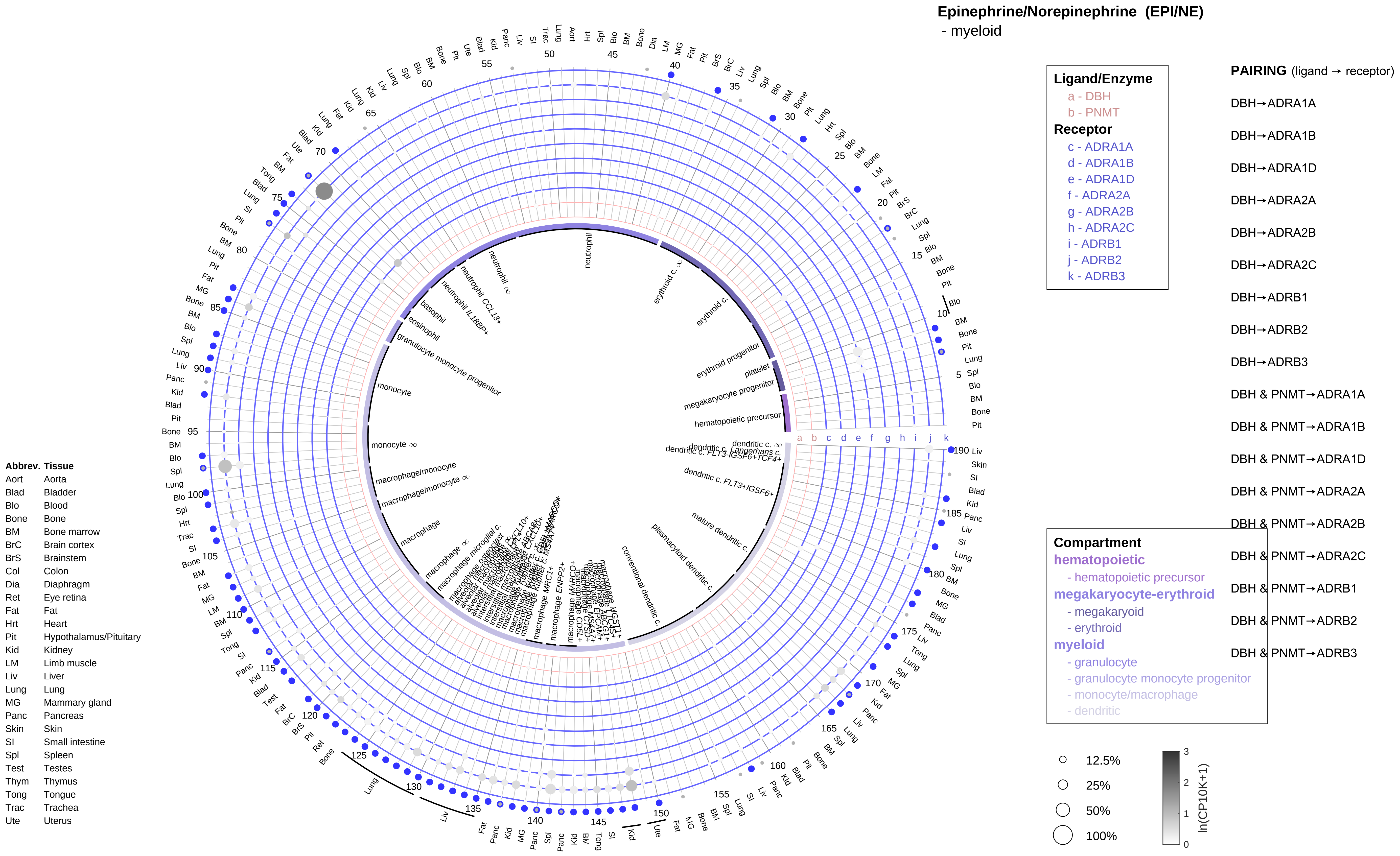
Epinephrine/Norepinephrine (EPI/NE)
- myeloid
Ligand/Enzyme
a - DBH
b - PNMT
Receptor
c - ADRA1A
d - ADRA1B
e - ADRA1D
f - ADRA2A
g - ADRA2B
h - ADRA2C
i - ADRB1
j - ADRB2
k - ADRB3
PAIRING (ligand → receptor)
DBH→ADRA1A
DBH→ADRA1B
DBH→ADRA1D
DBH→ADRA2A
DBH→ADRA2B
DBH→ADRA2C
DBH→ADRB1
DBH→ADRB2
DBH→ADRB3
DBH & PNMT→ADRA1A
DBH & PNMT→ADRA1B
DBH & PNMT→ADRA1D
DBH & PNMT→ADRA2A
DBH & PNMT→ADRA2B
DBH & PNMT→ADRA2C
DBH & PNMT→ADRB1
DBH & PNMT→ADRB2
DBH & PNMT→ADRB3
Compartment
hematopoietic
- hematopoietic precursor
megakaryocyte-erythroid
- megakaryoid
- erythroid
myeloid
- granulocyte
- granulocyte monocyte progenitor
- monocyte/macrophage
- dendritic
12.5%
25%
50%
100%
ln(CP10K+1)
0
1
2
3
Abbrev. Tissue
Aort Aorta
Blad Bladder
Blo Blood
Bone Bone
BM Bone marrow
BrC Brain cortex
BrS Brainstem
Col Colon
Dia Diaphragm
Ret Eye retina
Fat Fat
Hrt Heart
Pit Hypothalamus/Pituitary
Kid Kidney
LM Limb muscle
Liv Liver
Lung Lung
MG Mammary gland
Panc Pancreas
Skin Skin
SI Small intestine
Spl Spleen
Test Testes
Thym Thymus
Tong Tongue
Trac Trachea
Ute Uterus
neutrophil
erythroid c. ∞
erythroid c.
erythroid progenitor
platelet
megakaryocyte progenitor
hematopoietic precursor
dendritic c. ∞
dendritic c. Langerhans c.
dendritic c. FLT3-IGSF6+TCF4+
dendritic c. FLT3+IGSF6+
mature dendritic c.
plasmacytoid dendritic c.
conventional dendritic c.
macrophage ENPP2+
macrophage MRC1+
macrophage
macrophage ∞
macrophage/monocyte ∞
macrophage/monocyte
monocyte ∞
monocyte
granulocyte monocyte progenitor
eosinophil
basophil
neutrophil IL18BP+
neutrophil CCL13+
neutrophil ∞
a b c d e f g h i j k

Erythropoietin (EPO)
- epithelial/neural/germ

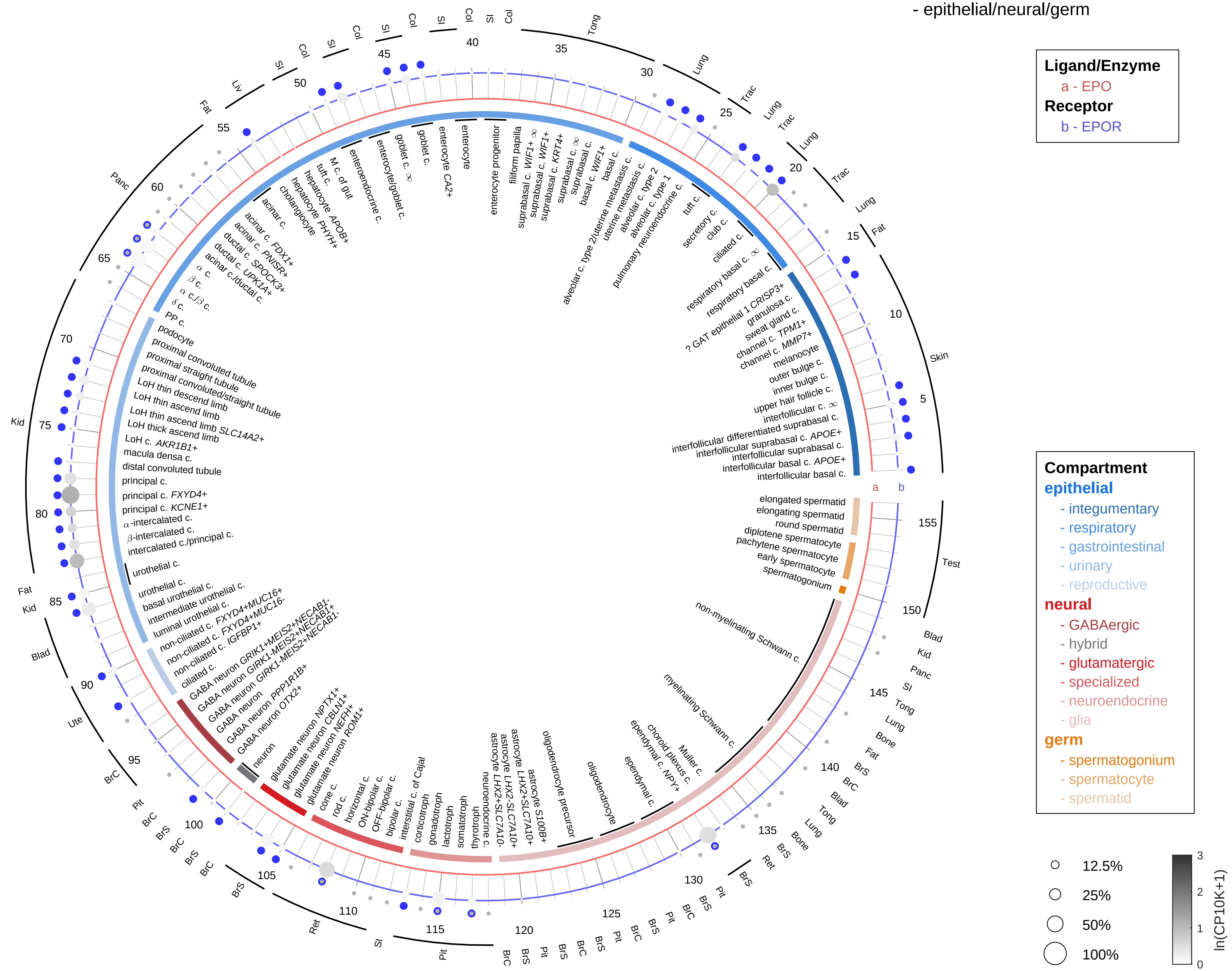

Abbrev.	Tissue
Aort	Aorta
Blad	Bladder
Blo	Blood
Bone	Bone
BM	Bone marrow
BrC	Brain cortex
BrS	Brainstem
Col	Colon
Dia	Diaphragm
Ret	Eye retina
Fat	Fat
Hrt	Heart
Pit	Hypothalamus/Pituitary
Kid	Kidney
LM	Limb muscle
Liv	Liver
Lung	Lung
MG	Mammary gland
Panc	Pancreas
Skin	Skin
SI	Small intestine
Spl	Spleen
Test	Testes
Thym	Thymus
Tong	Tongue
Trac	Trachea
Ute	Uterus

Erythropoietin (EPO)
- endothelial

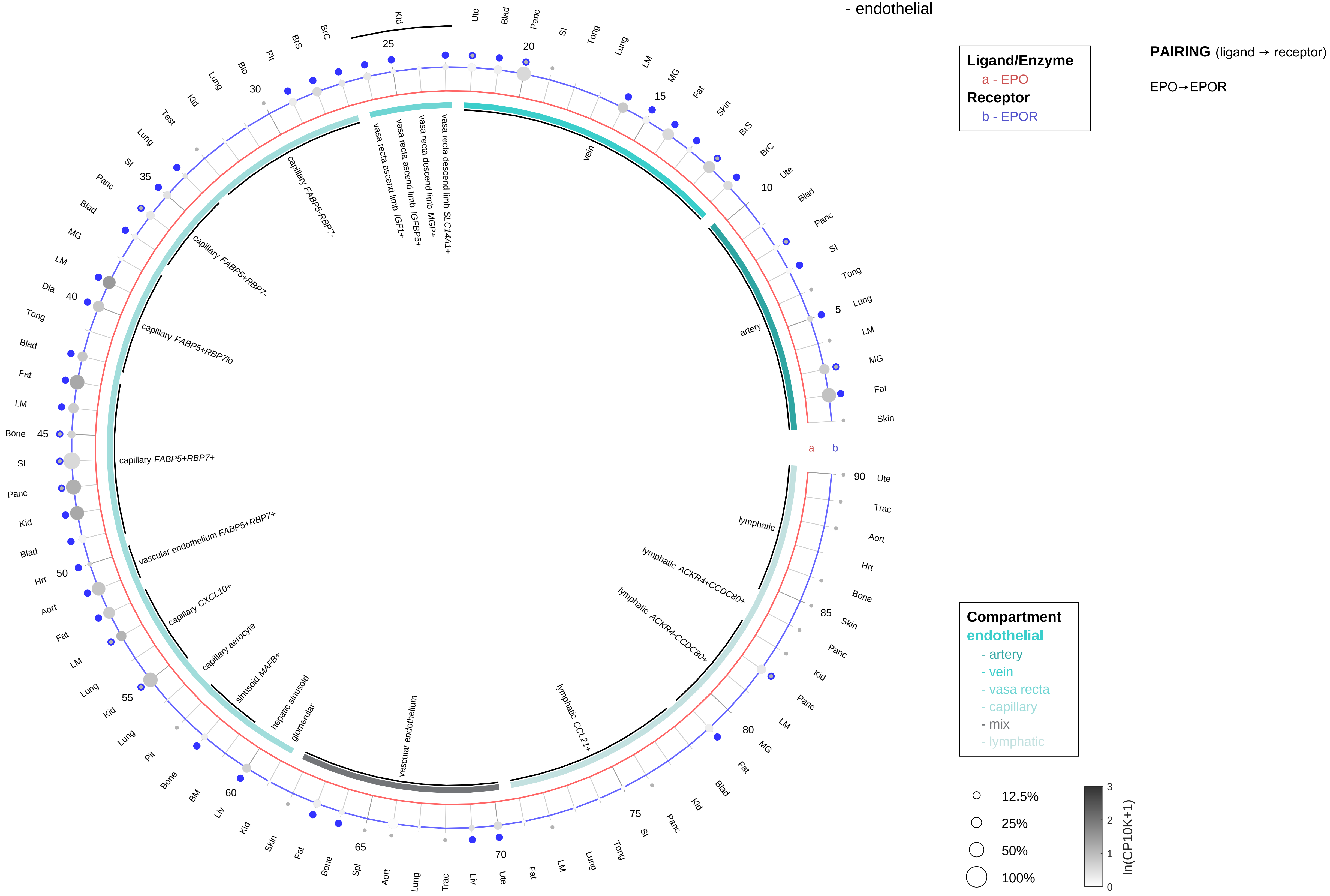

Abbrev.	Tissue
Aort	Aorta
Blad	Bladder
Blo	Blood
Bone	Bone
BM	Bone marrow
BrC	Brain cortex
BrS	Brainstem
Col	Colon
Dia	Diaphragm
Ret	Eye retina
Fat	Fat
Hrt	Heart
Pit	Hypothalamus/Pituitary
Kid	Kidney
LM	Limb muscle
Liv	Liver
Lung	Lung
MG	Mammary gland
Panc	Pancreas
Skin	Skin
SI	Small intestine
Spl	Spleen
Test	Testes
Thym	Thymus
Tong	Tongue
Trac	Trachea
Ute	Uterus

Erythropoietin (EPO)
- stromal

Ligand/Enzyme
a - EPO
Receptor
b - EPOR

PAIRING (ligand → receptor)

EPO→EPOR

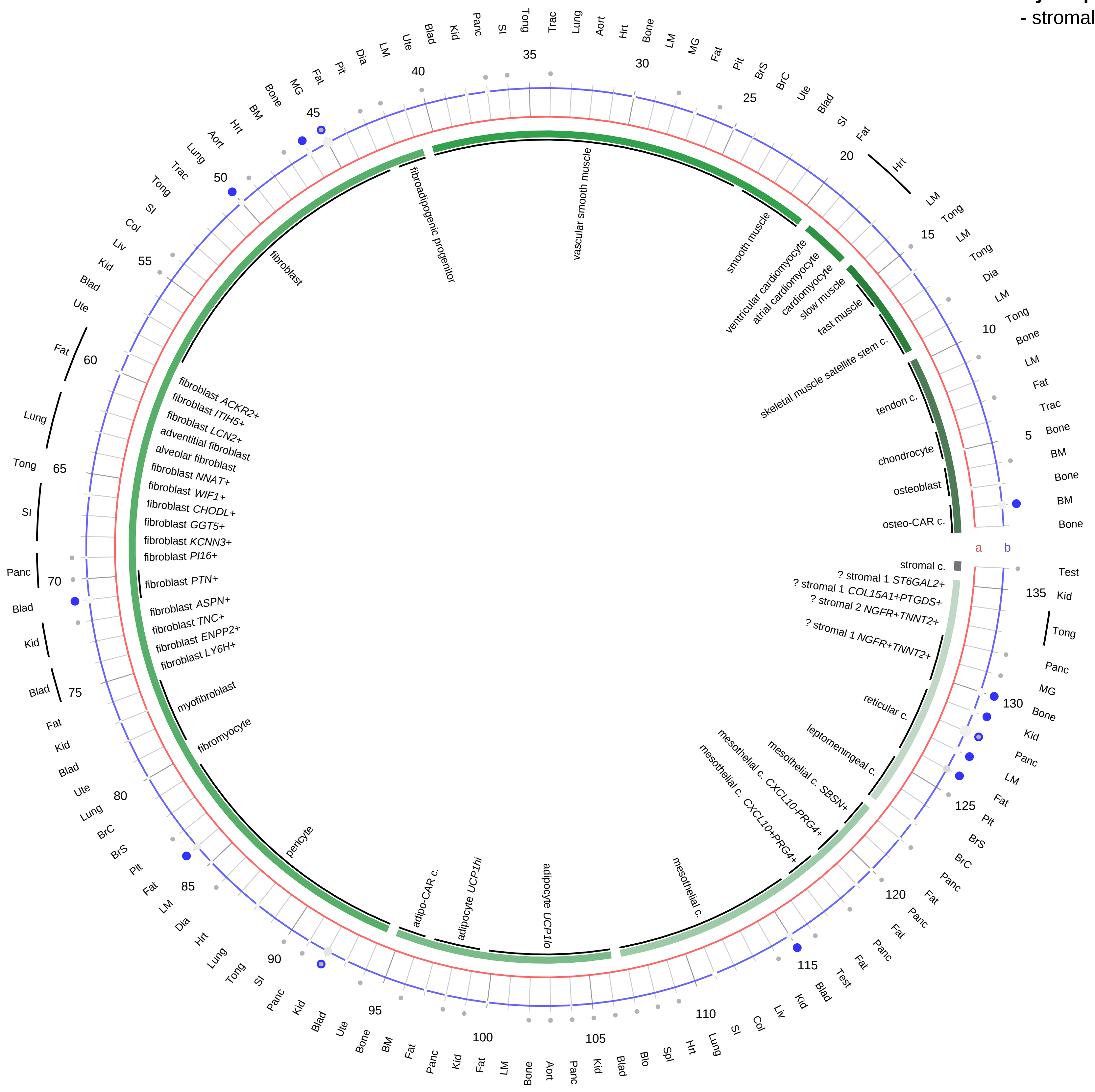

Compartment
stromal
- bone
- skeletal muscle
- cardiac muscle
- smooth muscle
- fibroblast
- adipocyte
- mesothelial
- other
- mix

12.5%
25%
50%
100%

ln(CP10K+1): 0, 1, 2, 3

Abbrev.	Tissue
Aort	Aorta
Blad	Bladder
Blo	Blood
Bone	Bone
BM	Bone marrow
BrC	Brain cortex
BrS	Brainstem
Col	Colon
Dia	Diaphragm
Ret	Eye retina
Fat	Fat
Hrt	Heart
Pit	Hypothalamus/Pituitary
Kid	Kidney
LM	Limb muscle
Liv	Liver
Lung	Lung
MG	Mammary gland
Panc	Pancreas
Skin	Skin
SI	Small intestine
Spl	Spleen
Test	Testes
Thym	Thymus
Tong	Tongue
Trac	Trachea
Ute	Uterus

Erythropoietin (EPO)
- lymphoid

Ligand/Enzyme
a - EPO
Receptor
b - EPOR

PAIRING (ligand → receptor)

EPO→EPOR

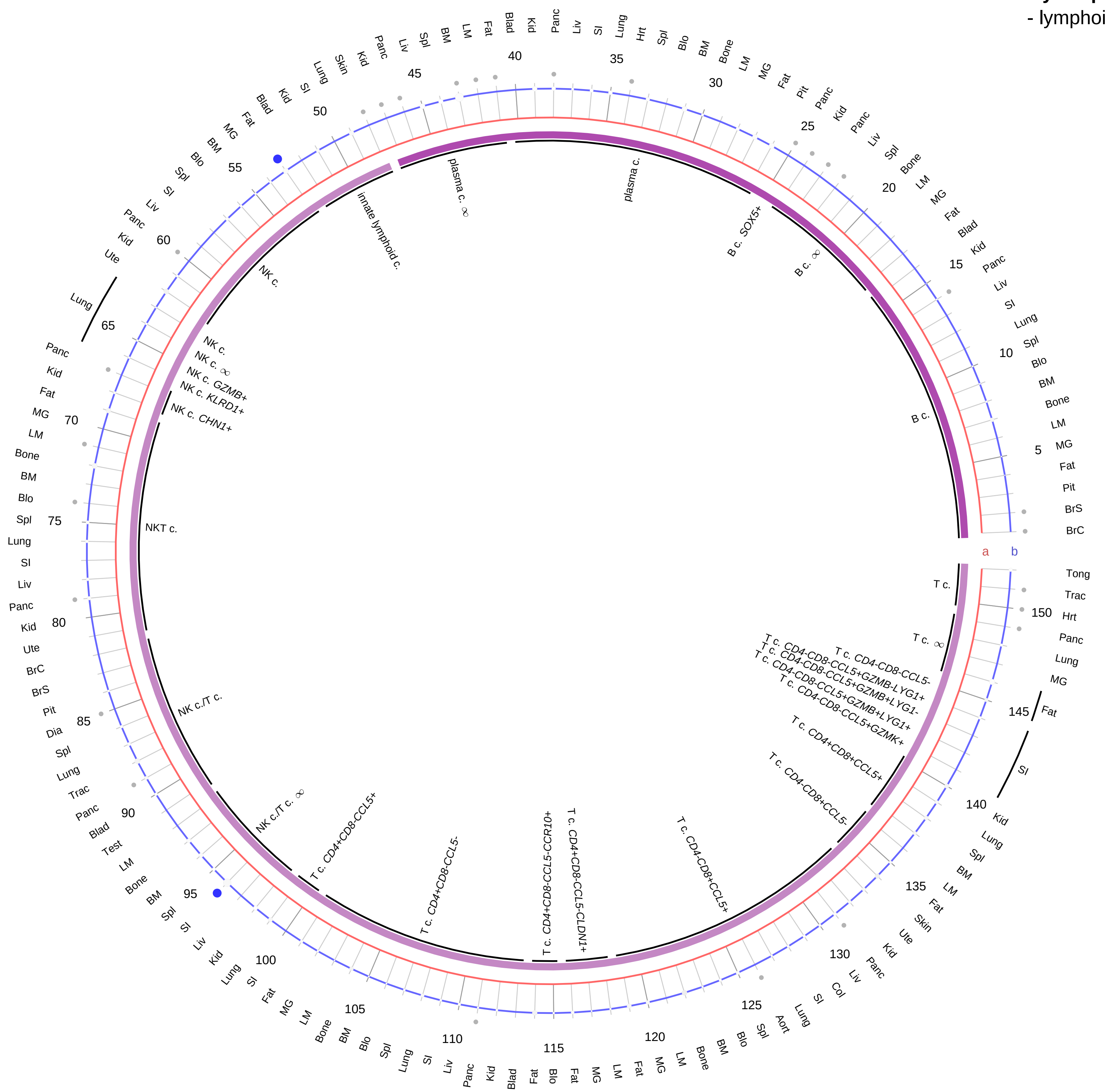

Compartment
lymphoid
- B cell
- NK/T cell

12.5%
25%
50%
100%

ln(CP10K+1): 0, 1, 2, 3

Abbrev.	Tissue
Aort	Aorta
Blad	Bladder
Blo	Blood
Bone	Bone
BM	Bone marrow
BrC	Brain cortex
BrS	Brainstem
Col	Colon
Dia	Diaphragm
Ret	Eye retina
Fat	Fat
Hrt	Heart
Pit	Hypothalamus/Pituitary
Kid	Kidney
LM	Limb muscle
Liv	Liver
Lung	Lung
MG	Mammary gland
Panc	Pancreas
Skin	Skin
SI	Small intestine
Spl	Spleen
Test	Testes
Thym	Thymus
Tong	Tongue
Trac	Trachea
Ute	Uterus

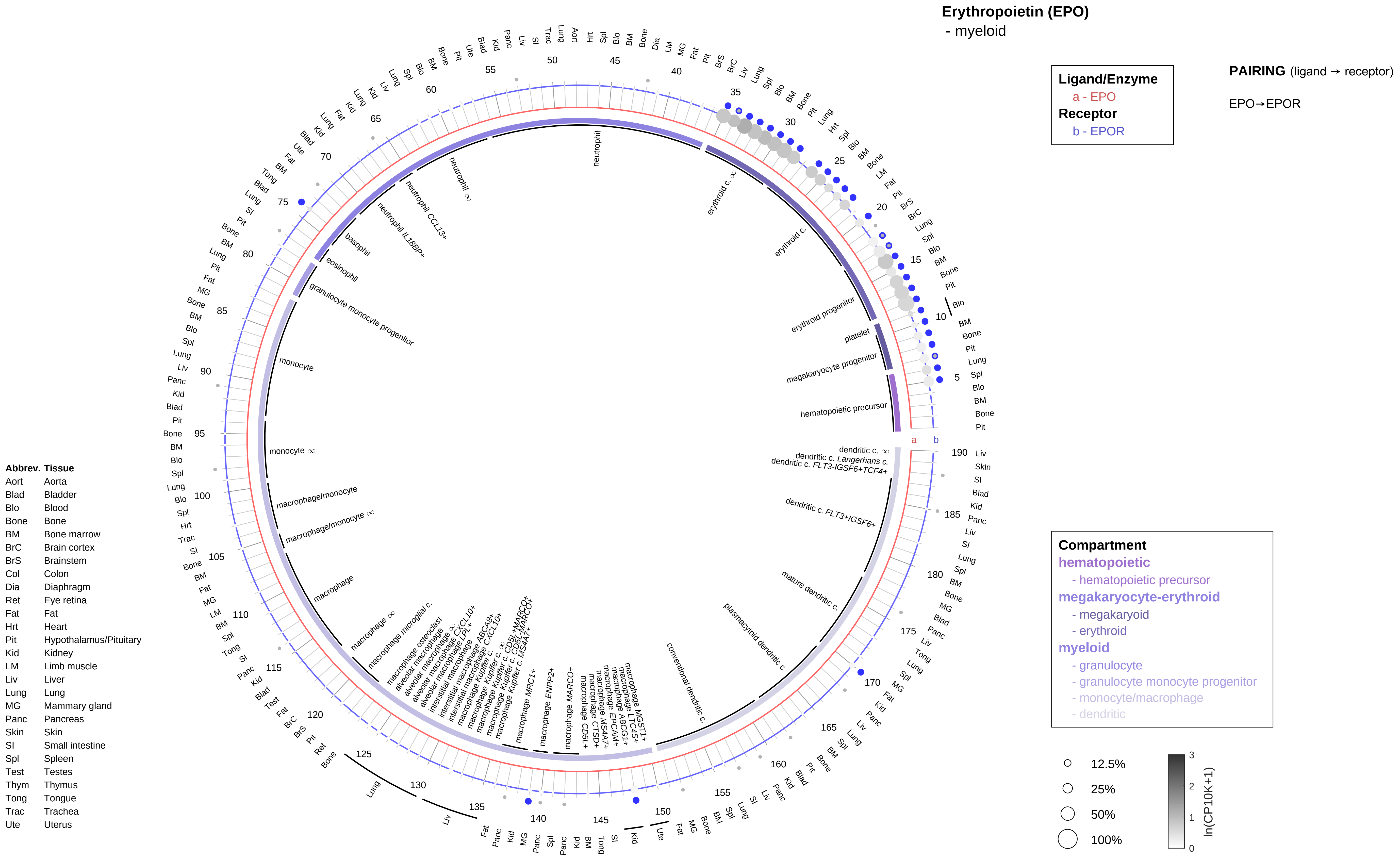
Erythropoietin (EPO)
- myeloid
Ligand/Enzyme
a - EPO
Receptor
b - EPOR
PAIRING (ligand → receptor)
EPO→EPOR
Compartment
hematopoietic
- hematopoietic precursor
megakaryocyte-erythroid
- megakaryoid
- erythroid
myeloid
- granulocyte
- granulocyte monocyte progenitor
- monocyte/macrophage
- dendritic
12.5%
25%
50%
100%
ln(CP10K+1)
Abbrev. Tissue
Aort Aorta
Blad Bladder
Blo Blood
Bone Bone
BM Bone marrow
BrC Brain cortex
BrS Brainstem
Col Colon
Dia Diaphragm
Ret Eye retina
Fat Fat
Hrt Heart
Pit Hypothalamus/Pituitary
Kid Kidney
LM Limb muscle
Liv Liver
Lung Lung
MG Mammary gland
Panc Pancreas
Skin Skin
SI Small intestine
Spl Spleen
Test Testes
Thym Thymus
Tong Tongue
Trac Trachea
Ute Uterus

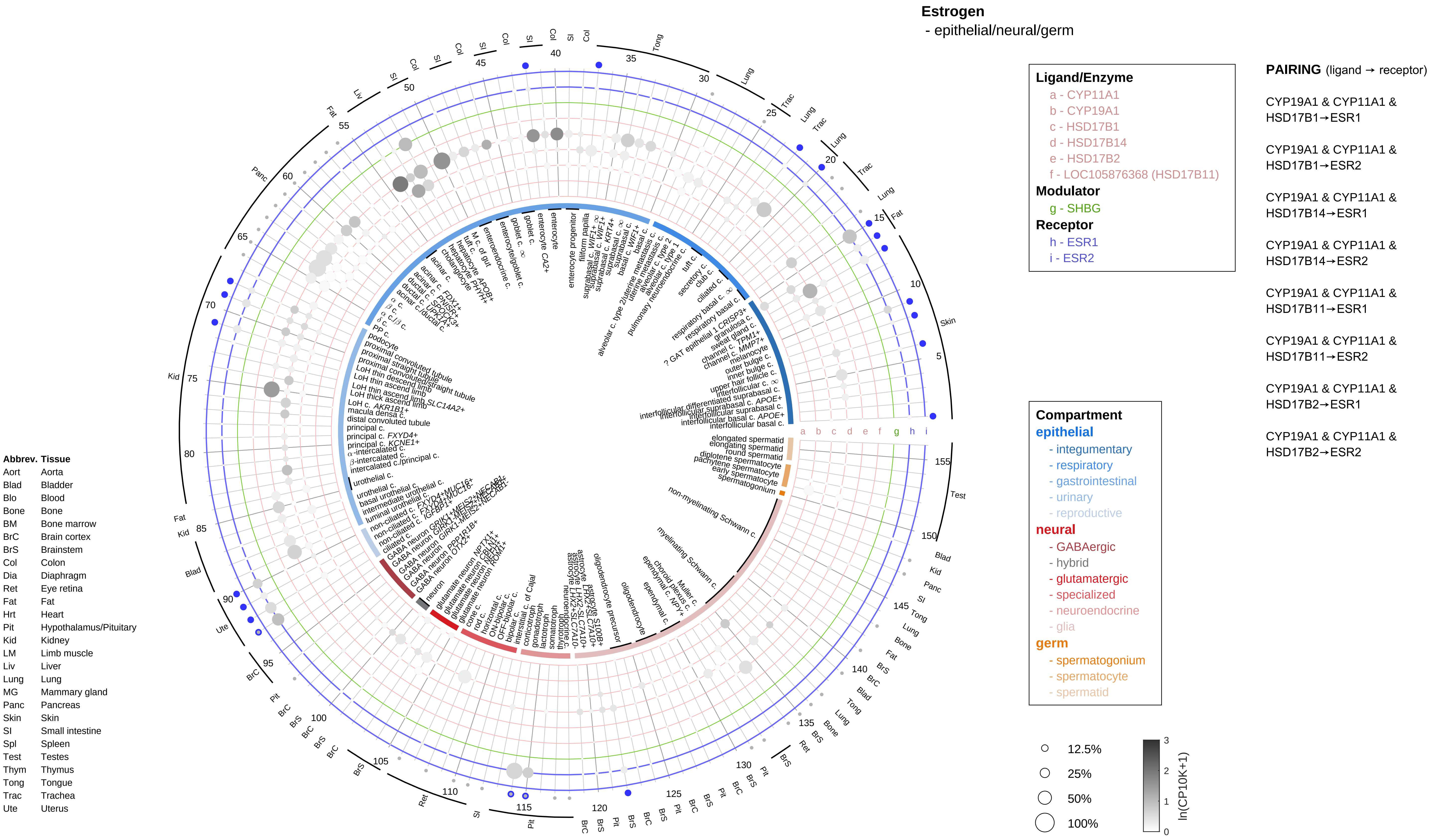

Estrogen
- epithelial/neural/germ
Ligand/Enzyme
a - CYP11A1
b - CYP19A1
c - HSD17B1
d - HSD17B14
e - HSD17B2
f - LOC105876368 (HSD17B11)
Modulator
g - SHBG
Receptor
h - ESR1
i - ESR2
PAIRING (ligand → receptor)
CYP19A1 & CYP11A1 & HSD17B1→ESR1
CYP19A1 & CYP11A1 & HSD17B1→ESR2
CYP19A1 & CYP11A1 & HSD17B14→ESR1
CYP19A1 & CYP11A1 & HSD17B14→ESR2
CYP19A1 & CYP11A1 & HSD17B11→ESR1
CYP19A1 & CYP11A1 & HSD17B11→ESR2
CYP19A1 & CYP11A1 & HSD17B2→ESR1
CYP19A1 & CYP11A1 & HSD17B2→ESR2
Compartment
epithelial
- integumentary
- respiratory
- gastrointestinal
- urinary
- reproductive
neural
- GABAergic
- hybrid
- glutamatergic
- specialized
- neuroendocrine
- glia
germ
- spermatogonium
- spermatocyte
- spermatid
12.5%
25%
50%
100%
ln(CP10K+1)
Abbrev. Tissue
Aort Aorta
Blad Bladder
Blo Blood
Bone Bone
BM Bone marrow
BrC Brain cortex
BrS Brainstem
Col Colon
Dia Diaphragm
Ret Eye retina
Fat Fat
Hrt Heart
Pit Hypothalamus/Pituitary
Kid Kidney
LM Limb muscle
Liv Liver
Lung Lung
MG Mammary gland
Panc Pancreas
Skin Skin
SI Small intestine
Spl Spleen
Test Testes
Thym Thymus
Tong Tongue
Trac Trachea
Ute Uterus

Estrogen
- endothelial

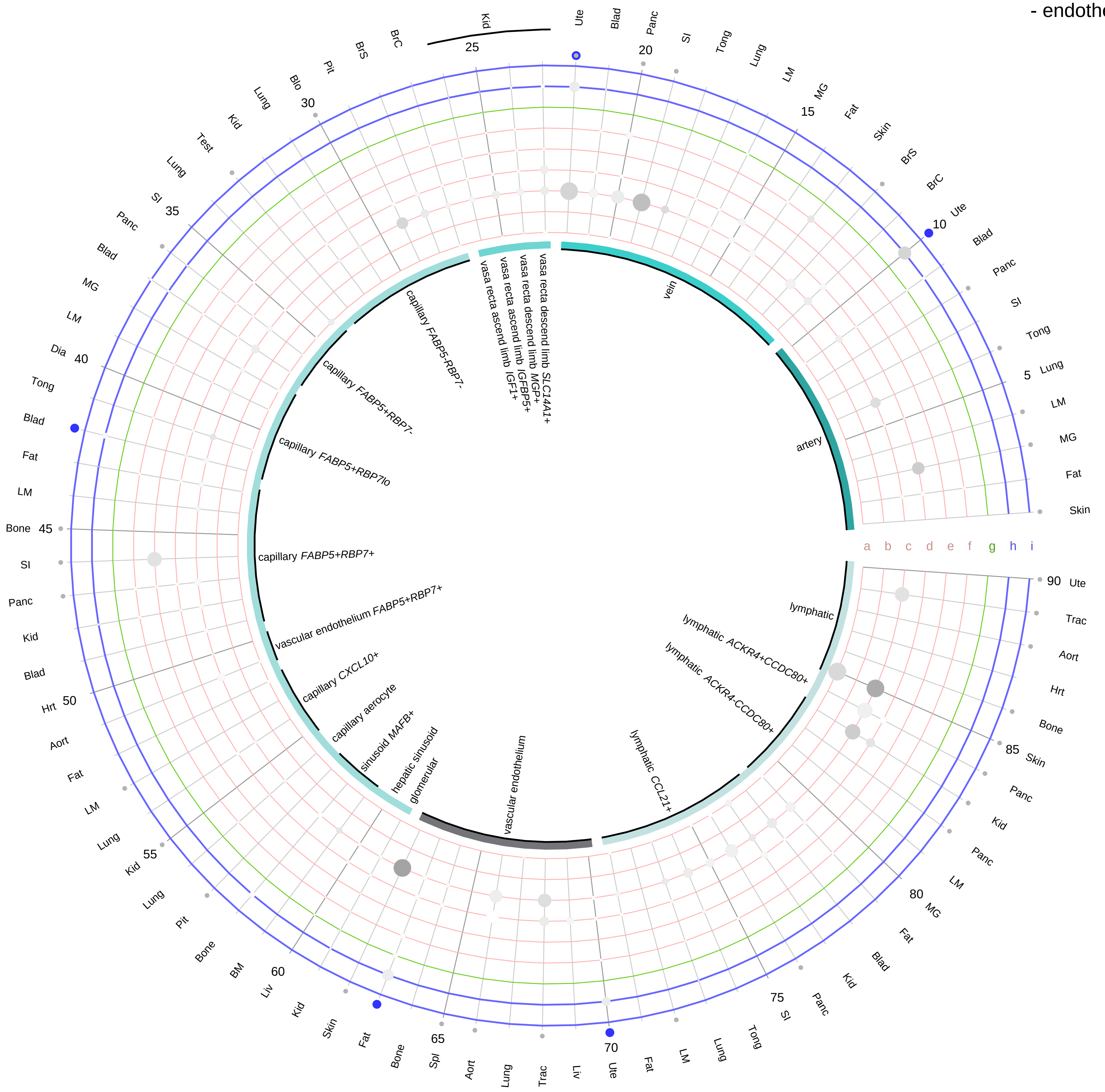

Ligand/Enzyme
- a - CYP11A1
- b - CYP19A1
- c - HSD17B1
- d - HSD17B14
- e - HSD17B2
- f - LOC105876368 (HSD17B11)

Modulator
- g - SHBG

Receptor
- h - ESR1
- i - ESR2

PAIRING (ligand → receptor)

CYP19A1 & CYP11A1 & HSD17B1→ESR1

CYP19A1 & CYP11A1 & HSD17B1→ESR2

CYP19A1 & CYP11A1 & HSD17B14→ESR1

CYP19A1 & CYP11A1 & HSD17B14→ESR2

CYP19A1 & CYP11A1 & HSD17B11→ESR1

CYP19A1 & CYP11A1 & HSD17B11→ESR2

CYP19A1 & CYP11A1 & HSD17B2→ESR1

CYP19A1 & CYP11A1 & HSD17B2→ESR2

Compartment
endothelial
- artery
- vein
- vasa recta
- capillary
- mix
- lymphatic

○ 12.5%
○ 25%
○ 50%
○ 100%

ln(CP10K+1): 0, 1, 2, 3

Abbrev.	Tissue
Aort	Aorta
Blad	Bladder
Blo	Blood
Bone	Bone
BM	Bone marrow
BrC	Brain cortex
BrS	Brainstem
Col	Colon
Dia	Diaphragm
Ret	Eye retina
Fat	Fat
Hrt	Heart
Pit	Hypothalamus/Pituitary
Kid	Kidney
LM	Limb muscle
Liv	Liver
Lung	Lung
MG	Mammary gland
Panc	Pancreas
Skin	Skin
SI	Small intestine
Spl	Spleen
Test	Testes
Thym	Thymus
Tong	Tongue
Trac	Trachea
Ute	Uterus

Estrogen
- stromal

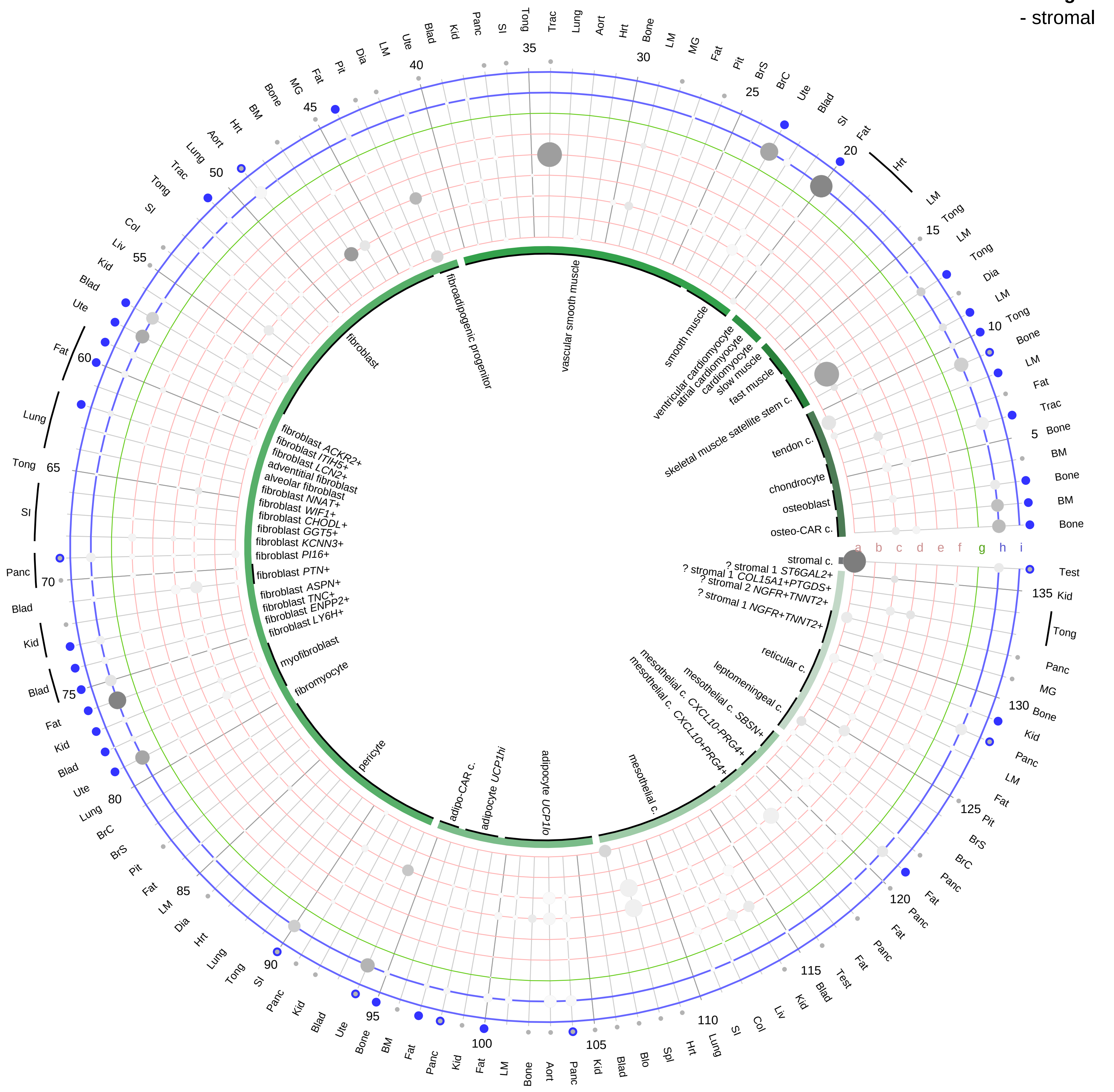

Ligand/Enzyme
- a - CYP11A1
- b - CYP19A1
- c - HSD17B1
- d - HSD17B14
- e - HSD17B2
- f - LOC105876368 (HSD17B11)

Modulator
- g - SHBG

Receptor
- h - ESR1
- i - ESR2

PAIRING (ligand → receptor)

CYP19A1 & CYP11A1 & HSD17B1→ESR1

CYP19A1 & CYP11A1 & HSD17B1→ESR2

CYP19A1 & CYP11A1 & HSD17B14→ESR1

CYP19A1 & CYP11A1 & HSD17B14→ESR2

CYP19A1 & CYP11A1 & HSD17B11→ESR1

CYP19A1 & CYP11A1 & HSD17B11→ESR2

CYP19A1 & CYP11A1 & HSD17B2→ESR1

CYP19A1 & CYP11A1 & HSD17B2→ESR2

Abbrev.	Tissue
Aort	Aorta
Blad	Bladder
Blo	Blood
Bone	Bone
BM	Bone marrow
BrC	Brain cortex
BrS	Brainstem
Col	Colon
Dia	Diaphragm
Ret	Eye retina
Fat	Fat
Hrt	Heart
Pit	Hypothalamus/Pituitary
Kid	Kidney
LM	Limb muscle
Liv	Liver
Lung	Lung
MG	Mammary gland
Panc	Pancreas
Skin	Skin
SI	Small intestine
Spl	Spleen
Test	Testes
Thym	Thymus
Tong	Tongue
Trac	Trachea
Ute	Uterus

Compartment
stromal
- bone
- skeletal muscle
- cardiac muscle
- smooth muscle
- fibroblast
- adipocyte
- mesothelial
- other
- mix

12.5%
25%
50%
100%

ln(CP10K+1): 0 1 2 3

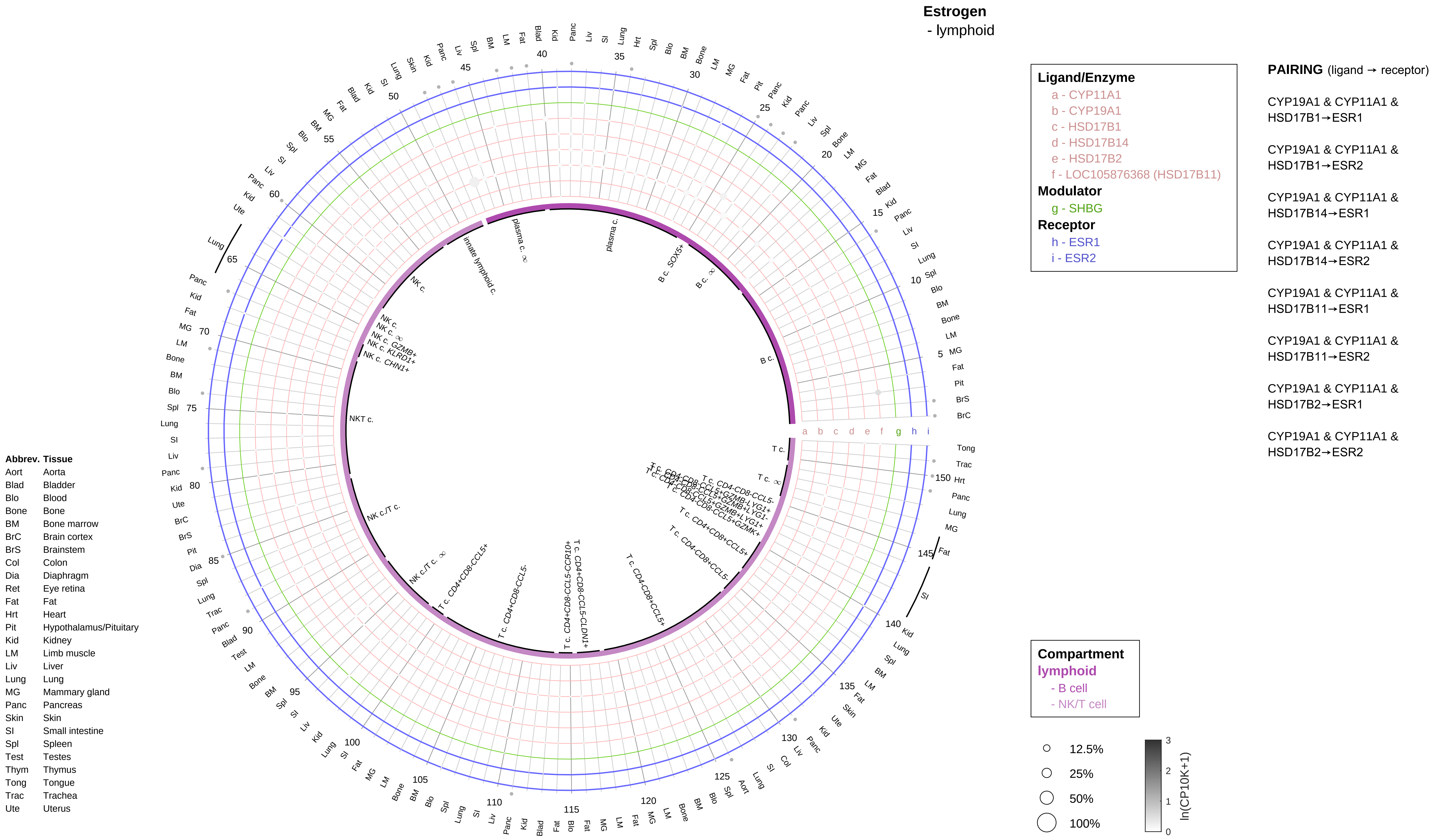
Estrogen
- lymphoid
Ligand/Enzyme
a - CYP11A1
b - CYP19A1
c - HSD17B1
d - HSD17B14
e - HSD17B2
f - LOC105876368 (HSD17B11)
Modulator
g - SHBG
Receptor
h - ESR1
i - ESR2
PAIRING (ligand → receptor)
CYP19A1 & CYP11A1 & HSD17B1→ESR1
CYP19A1 & CYP11A1 & HSD17B1→ESR2
CYP19A1 & CYP11A1 & HSD17B14→ESR1
CYP19A1 & CYP11A1 & HSD17B14→ESR2
CYP19A1 & CYP11A1 & HSD17B11→ESR1
CYP19A1 & CYP11A1 & HSD17B11→ESR2
CYP19A1 & CYP11A1 & HSD17B2→ESR1
CYP19A1 & CYP11A1 & HSD17B2→ESR2
Compartment
lymphoid
- B cell
- NK/T cell
12.5%
25%
50%
100%
ln(CP10K+1)
0
1
2
3
Abbrev. Tissue
Aort Aorta
Blad Bladder
Blo Blood
Bone Bone
BM Bone marrow
BrC Brain cortex
BrS Brainstem
Col Colon
Dia Diaphragm
Ret Eye retina
Fat Fat
Hrt Heart
Pit Hypothalamus/Pituitary
Kid Kidney
LM Limb muscle
Liv Liver
Lung Lung
MG Mammary gland
Panc Pancreas
Skin Skin
SI Small intestine
Spl Spleen
Test Testes
Thym Thymus
Tong Tongue
Trac Trachea
Ute Uterus

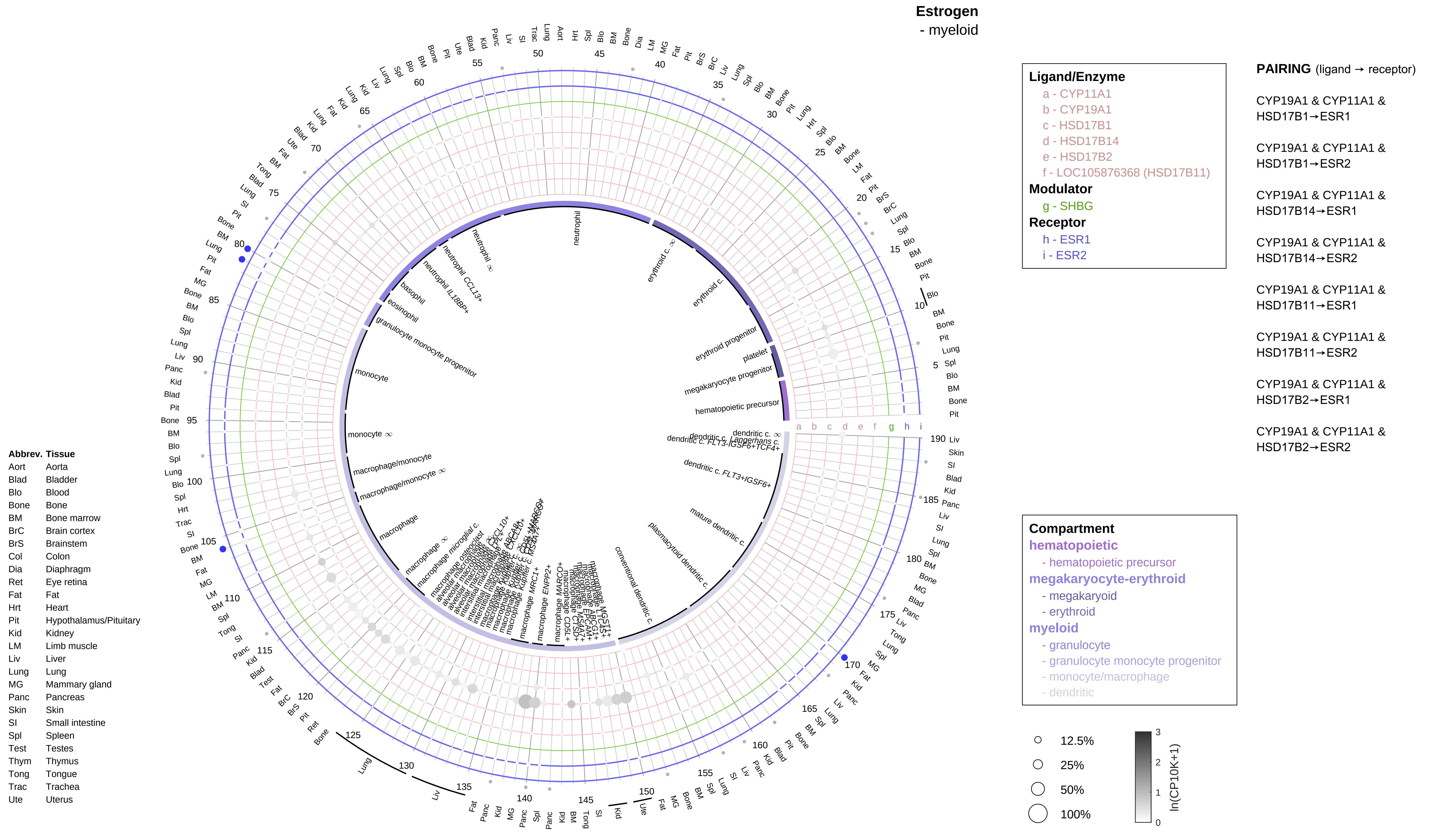

Estrogen
- myeloid
Ligand/Enzyme
a - CYP11A1
b - CYP19A1
c - HSD17B1
d - HSD17B14
e - HSD17B2
f - LOC105876368 (HSD17B11)
Modulator
g - SHBG
Receptor
h - ESR1
i - ESR2
PAIRING (ligand → receptor)
CYP19A1 & CYP11A1 & HSD17B1→ESR1
CYP19A1 & CYP11A1 & HSD17B1→ESR2
CYP19A1 & CYP11A1 & HSD17B14→ESR1
CYP19A1 & CYP11A1 & HSD17B14→ESR2
CYP19A1 & CYP11A1 & HSD17B11→ESR1
CYP19A1 & CYP11A1 & HSD17B11→ESR2
CYP19A1 & CYP11A1 & HSD17B2→ESR1
CYP19A1 & CYP11A1 & HSD17B2→ESR2
Compartment
hematopoietic
- hematopoietic precursor
megakaryocyte-erythroid
- megakaryoid
- erythroid
myeloid
- granulocyte
- granulocyte monocyte progenitor
- monocyte/macrophage
- dendritic
12.5%
25%
50%
100%
ln(CP10K+1)
Abbrev. Tissue
Aort Aorta
Blad Bladder
Blo Blood
Bone Bone
BM Bone marrow
BrC Brain cortex
BrS Brainstem
Col Colon
Dia Diaphragm
Ret Eye retina
Fat Fat
Hrt Heart
Pit Hypothalamus/Pituitary
Kid Kidney
LM Limb muscle
Liv Liver
Lung Lung
MG Mammary gland
Panc Pancreas
Skin Skin
SI Small intestine
Spl Spleen
Test Testes
Thym Thymus
Tong Tongue
Trac Trachea
Ute Uterus

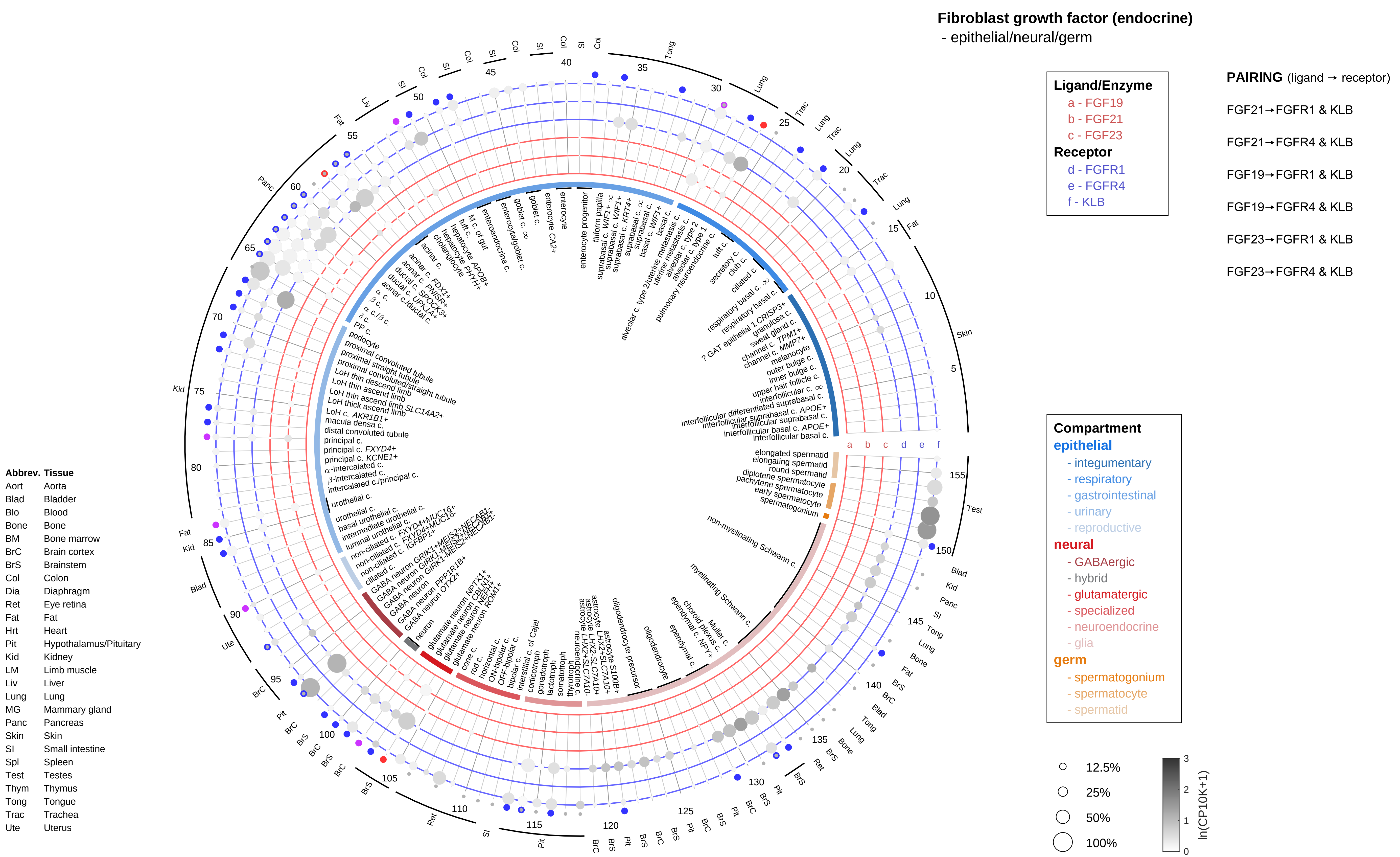

Fibroblast growth factor (endocrine)
- epithelial/neural/germ
Ligand/Enzyme
a - FGF19
b - FGF21
c - FGF23
Receptor
d - FGFR1
e - FGFR4
f - KLB
PAIRING (ligand → receptor)
FGF21→FGFR1 & KLB
FGF21→FGFR4 & KLB
FGF19→FGFR1 & KLB
FGF19→FGFR4 & KLB
FGF23→FGFR1 & KLB
FGF23→FGFR4 & KLB
Compartment
epithelial
- integumentary
- respiratory
- gastrointestinal
- urinary
- reproductive
neural
- GABAergic
- hybrid
- glutamatergic
- specialized
- neuroendocrine
- glia
germ
- spermatogonium
- spermatocyte
- spermatid
12.5%
25%
50%
100%
ln(CP10K+1)
Abbrev. Tissue
Aort Aorta
Blad Bladder
Blo Blood
Bone Bone
BM Bone marrow
BrC Brain cortex
BrS Brainstem
Col Colon
Dia Diaphragm
Ret Eye retina
Fat Fat
Hrt Heart
Pit Hypothalamus/Pituitary
Kid Kidney
LM Limb muscle
Liv Liver
Lung Lung
MG Mammary gland
Panc Pancreas
Skin Skin
SI Small intestine
Spl Spleen
Test Testes
Thym Thymus
Tong Tongue
Trac Trachea
Ute Uterus

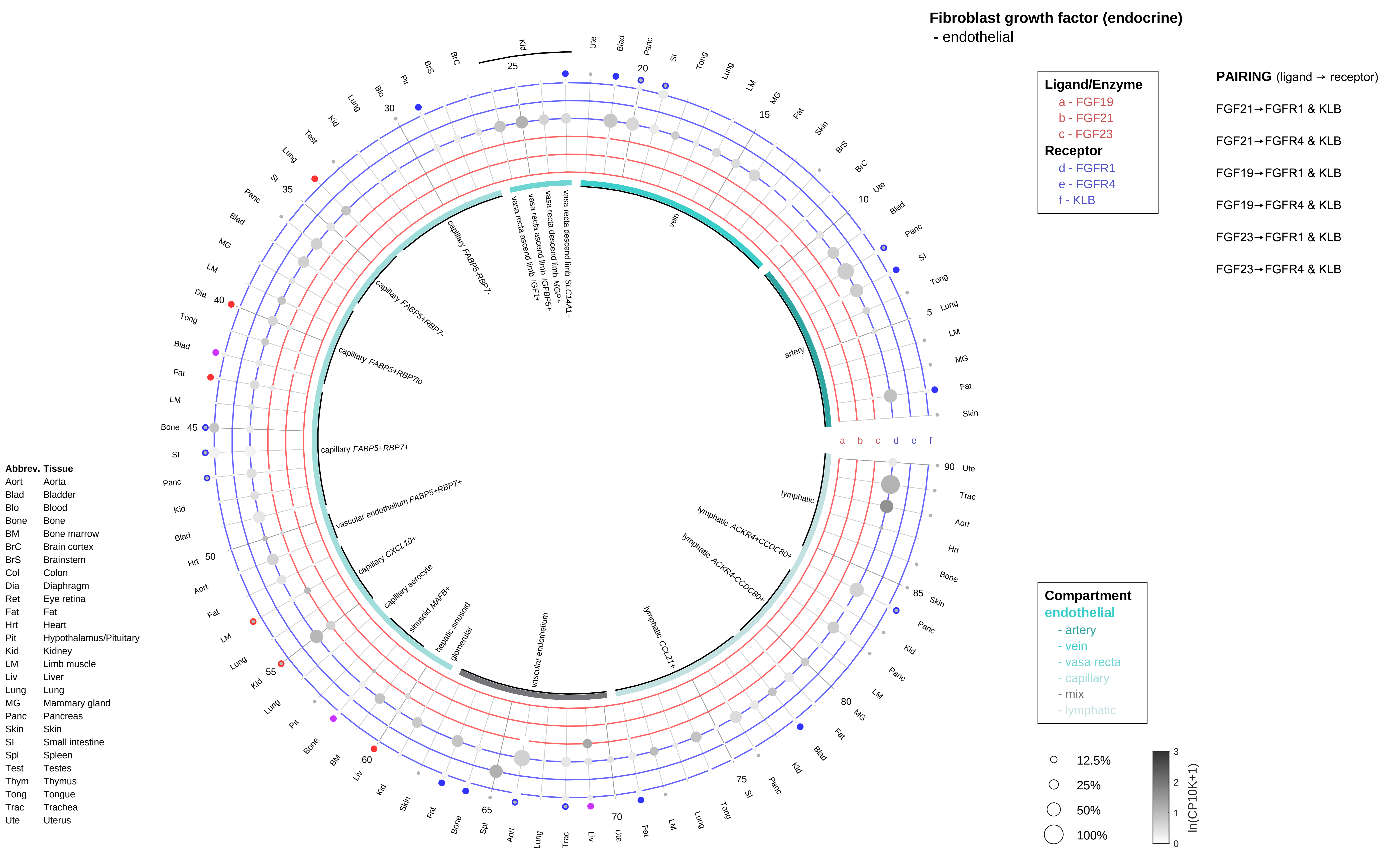

Fibroblast growth factor (endocrine)
- endothelial
Ligand/Enzyme
a - FGF19
b - FGF21
c - FGF23
Receptor
d - FGFR1
e - FGFR4
f - KLB
PAIRING (ligand → receptor)
FGF21→FGFR1 & KLB
FGF21→FGFR4 & KLB
FGF19→FGFR1 & KLB
FGF19→FGFR4 & KLB
FGF23→FGFR1 & KLB
FGF23→FGFR4 & KLB
Compartment
endothelial
- artery
- vein
- vasa recta
- capillary
- mix
- lymphatic
12.5%
25%
50%
100%
ln(CP10K+1)
vein
artery
lymphatic
lymphatic ACKR4+CCDC80+
lymphatic ACKR4-CCDC80+
lymphatic CCL21+
vascular endothelium
glomerular
hepatic sinusoid
sinusoid MAFB+
capillary aerocyte
capillary CXCL10+
vascular endothelium FABP5+RBP7+
capillary FABP5+RBP7+
capillary FABP5+RBP7lo
capillary FABP5+RBP7-
capillary FABP5-RBP7-
vasa recta ascend limb IGF1+
vasa recta ascend limb IGFBP5+
vasa recta descend limb MGP+
vasa recta descend limb SLC14A1+
Abbrev. Tissue
Aort Aorta
Blad Bladder
Blo Blood
Bone Bone
BM Bone marrow
BrC Brain cortex
BrS Brainstem
Col Colon
Dia Diaphragm
Ret Eye retina
Fat Fat
Hrt Heart
Pit Hypothalamus/Pituitary
Kid Kidney
LM Limb muscle
Liv Liver
Lung Lung
MG Mammary gland
Panc Pancreas
Skin Skin
SI Small intestine
Spl Spleen
Test Testes
Thym Thymus
Tong Tongue
Trac Trachea
Ute Uterus

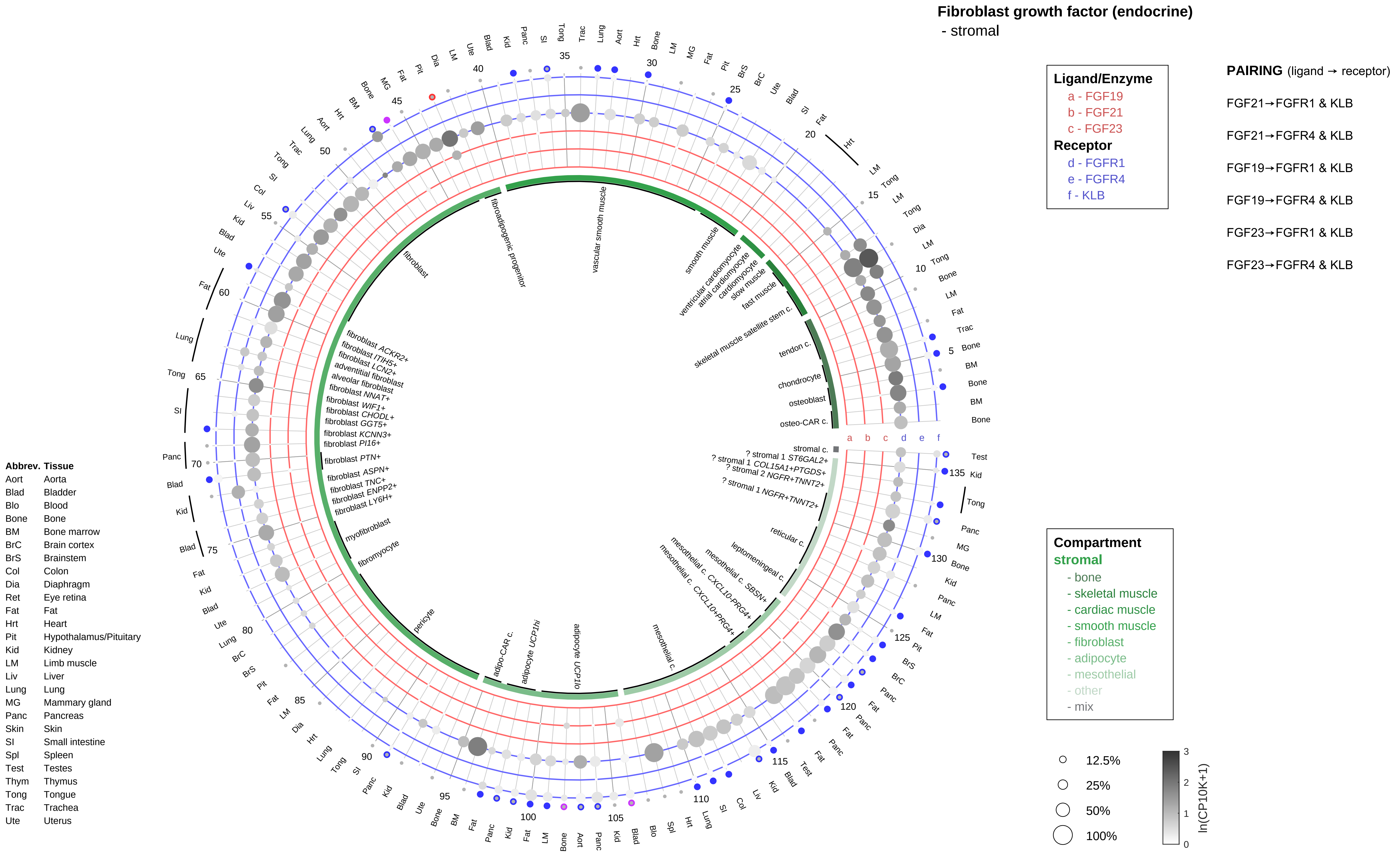
Fibroblast growth factor (endocrine)
- stromal
Ligand/Enzyme
a - FGF19
b - FGF21
c - FGF23
Receptor
d - FGFR1
e - FGFR4
f - KLB
PAIRING (ligand → receptor)
FGF21→FGFR1 & KLB
FGF21→FGFR4 & KLB
FGF19→FGFR1 & KLB
FGF19→FGFR4 & KLB
FGF23→FGFR1 & KLB
FGF23→FGFR4 & KLB
Compartment
stromal
- bone
- skeletal muscle
- cardiac muscle
- smooth muscle
- fibroblast
- adipocyte
- mesothelial
- other
- mix
12.5%
25%
50%
100%
ln(CP10K+1)
0
1
2
3
Abbrev. Tissue
Aort Aorta
Blad Bladder
Blo Blood
Bone Bone
BM Bone marrow
BrC Brain cortex
BrS Brainstem
Col Colon
Dia Diaphragm
Ret Eye retina
Fat Fat
Hrt Heart
Pit Hypothalamus/Pituitary
Kid Kidney
LM Limb muscle
Liv Liver
Lung Lung
MG Mammary gland
Panc Pancreas
Skin Skin
SI Small intestine
Spl Spleen
Test Testes
Thym Thymus
Tong Tongue
Trac Trachea
Ute Uterus
a
b
c
d
e
f
osteo-CAR c.
osteoblast
chondrocyte
tendon c.
skeletal muscle satellite stem c.
fast muscle
slow muscle
cardiomyocyte
atrial cardiomyocyte
ventricular cardiomyocyte
smooth muscle
vascular smooth muscle
fibroadipogenic progenitor
fibroblast
fibroblast ACKR2+
fibroblast ITIH5+
fibroblast LCN2+
adventitial fibroblast
alveolar fibroblast
fibroblast NNAT+
fibroblast WIF1+
fibroblast CHODL+
fibroblast GGT5+
fibroblast KCNN3+
fibroblast PI16+
fibroblast PTN+
fibroblast ASPN+
fibroblast TNC+
fibroblast ENPP2+
fibroblast LY6H+
myofibroblast
fibromyocyte
pericyte
adipo-CAR c.
adipocyte UCP1hi
adipocyte UCP1lo
mesothelial c.
mesothelial c. CXCL10+PRG4+
mesothelial c. CXCL10-PRG4+
mesothelial c. SBSN+
leptomeningeal c.
reticular c.
? stromal 1 NGFR+TNNT2+
? stromal 2 NGFR+TNNT2+
? stromal 1 COL15A1+PTGDS+
? stromal 1 ST6GAL2+
stromal c.

Fibroblast growth factor (endocrine)
- lymphoid

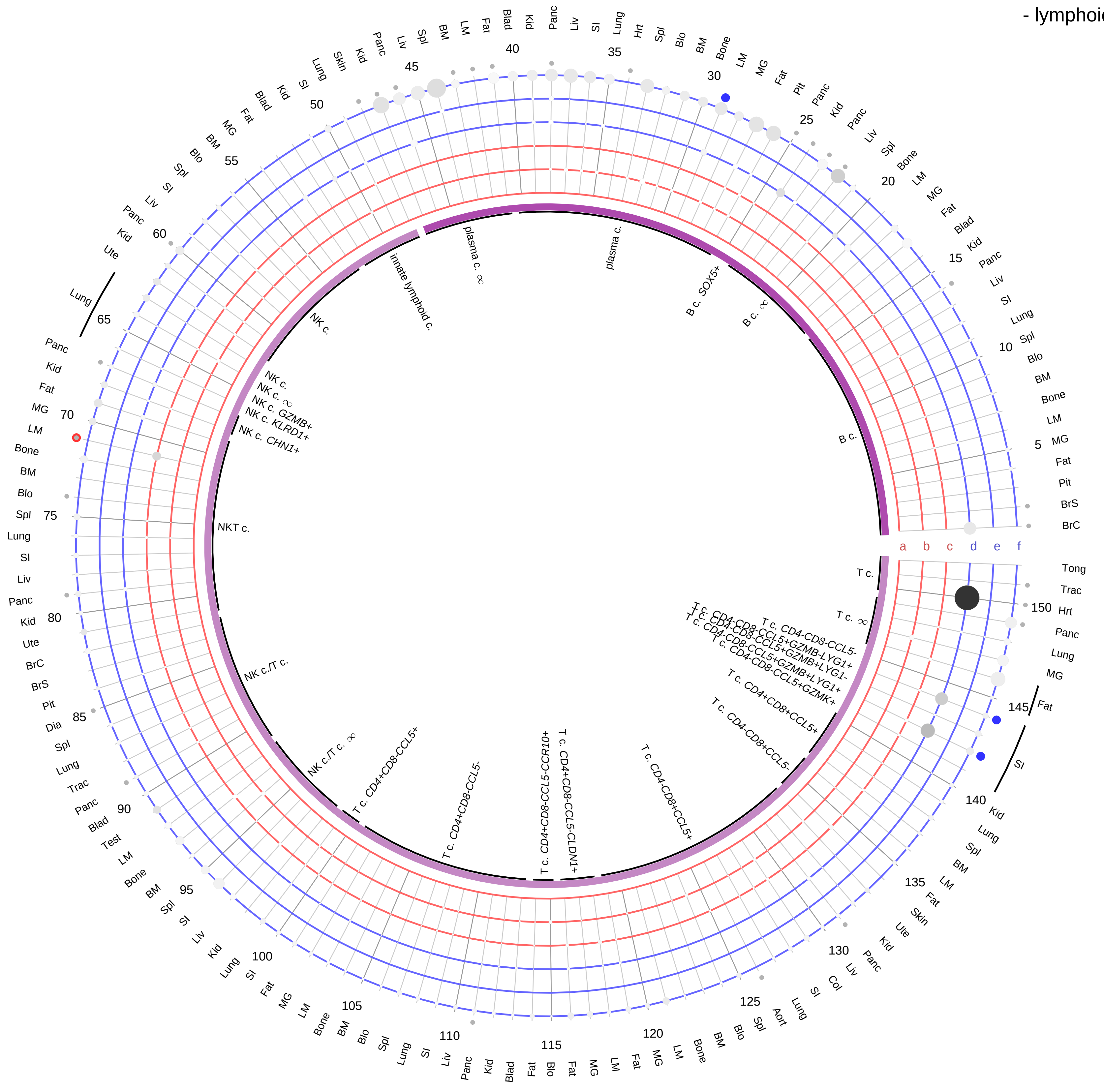

**Ligand/Enzyme**
a - FGF19
b - FGF21
c - FGF23
**Receptor**
d - FGFR1
e - FGFR4
f - KLB

PAIRING (ligand → receptor)

FGF21→FGFR1 & KLB

FGF21→FGFR4 & KLB

FGF19→FGFR1 & KLB

FGF19→FGFR4 & KLB

FGF23→FGFR1 & KLB

FGF23→FGFR4 & KLB

**Abbrev.**	**Tissue**
Aort	Aorta
Blad	Bladder
Blo	Blood
Bone	Bone
BM	Bone marrow
BrC	Brain cortex
BrS	Brainstem
Col	Colon
Dia	Diaphragm
Ret	Eye retina
Fat	Fat
Hrt	Heart
Pit	Hypothalamus/Pituitary
Kid	Kidney
LM	Limb muscle
Liv	Liver
Lung	Lung
MG	Mammary gland
Panc	Pancreas
Skin	Skin
SI	Small intestine
Spl	Spleen
Test	Testes
Thym	Thymus
Tong	Tongue
Trac	Trachea
Ute	Uterus

**Compartment**
**lymphoid**
- B cell
- NK/T cell

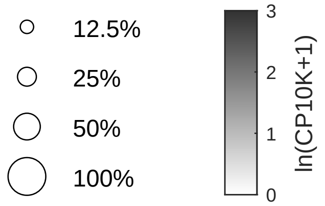

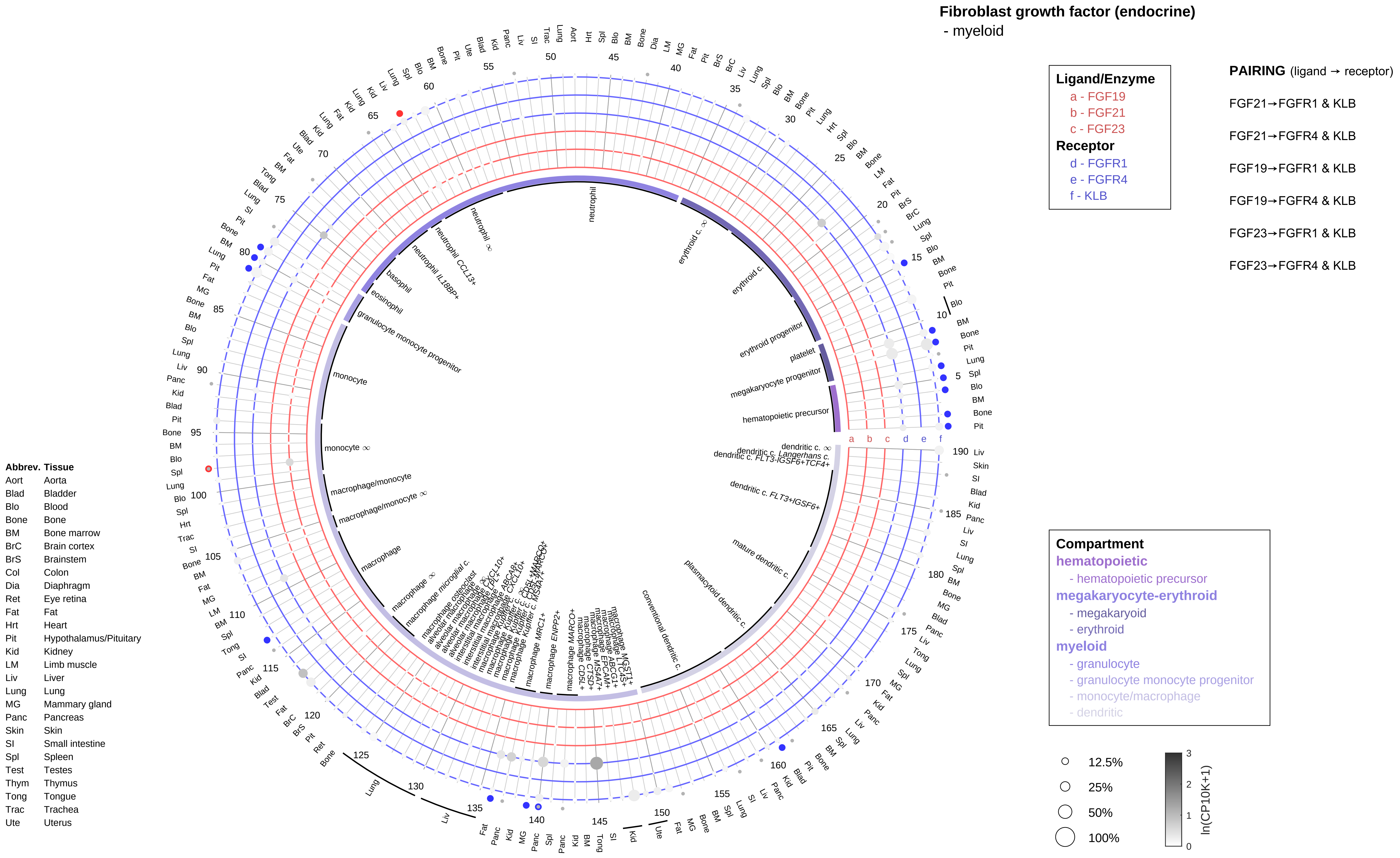

Fibroblast growth factor (endocrine)
- myeloid
Ligand/Enzyme
a - FGF19
b - FGF21
c - FGF23
Receptor
d - FGFR1
e - FGFR4
f - KLB
PAIRING (ligand → receptor)
FGF21→FGFR1 & KLB
FGF21→FGFR4 & KLB
FGF19→FGFR1 & KLB
FGF19→FGFR4 & KLB
FGF23→FGFR1 & KLB
FGF23→FGFR4 & KLB
Compartment
hematopoietic
- hematopoietic precursor
megakaryocyte-erythroid
- megakaryoid
- erythroid
myeloid
- granulocyte
- granulocyte monocyte progenitor
- monocyte/macrophage
- dendritic
12.5%
25%
50%
100%
ln(CP10K+1)
Abbrev. Tissue
Aort Aorta
Blad Bladder
Blo Blood
Bone Bone
BM Bone marrow
BrC Brain cortex
BrS Brainstem
Col Colon
Dia Diaphragm
Ret Eye retina
Fat Fat
Hrt Heart
Pit Hypothalamus/Pituitary
Kid Kidney
LM Limb muscle
Liv Liver
Lung Lung
MG Mammary gland
Panc Pancreas
Skin Skin
SI Small intestine
Spl Spleen
Test Testes
Thym Thymus
Tong Tongue
Trac Trachea
Ute Uterus
neutrophil
erythroid c. ∞
erythroid c.
erythroid progenitor
platelet
megakaryocyte progenitor
hematopoietic precursor
dendritic c. ∞
dendritic c. Langerhans c.
dendritic c. FLT3-IGSF6+TCF4+
dendritic c. FLT3+IGSF6+
mature dendritic c.
plasmacytoid dendritic c.
conventional dendritic c.
macrophage
macrophage ∞
macrophage/monocyte
macrophage/monocyte ∞
monocyte ∞
monocyte
granulocyte monocyte progenitor
eosinophil
basophil
neutrophil IL18BP+
neutrophil CCL13+
neutrophil ∞

Follicle stimulating hormone (FSH)
- epithelial/neural/germ

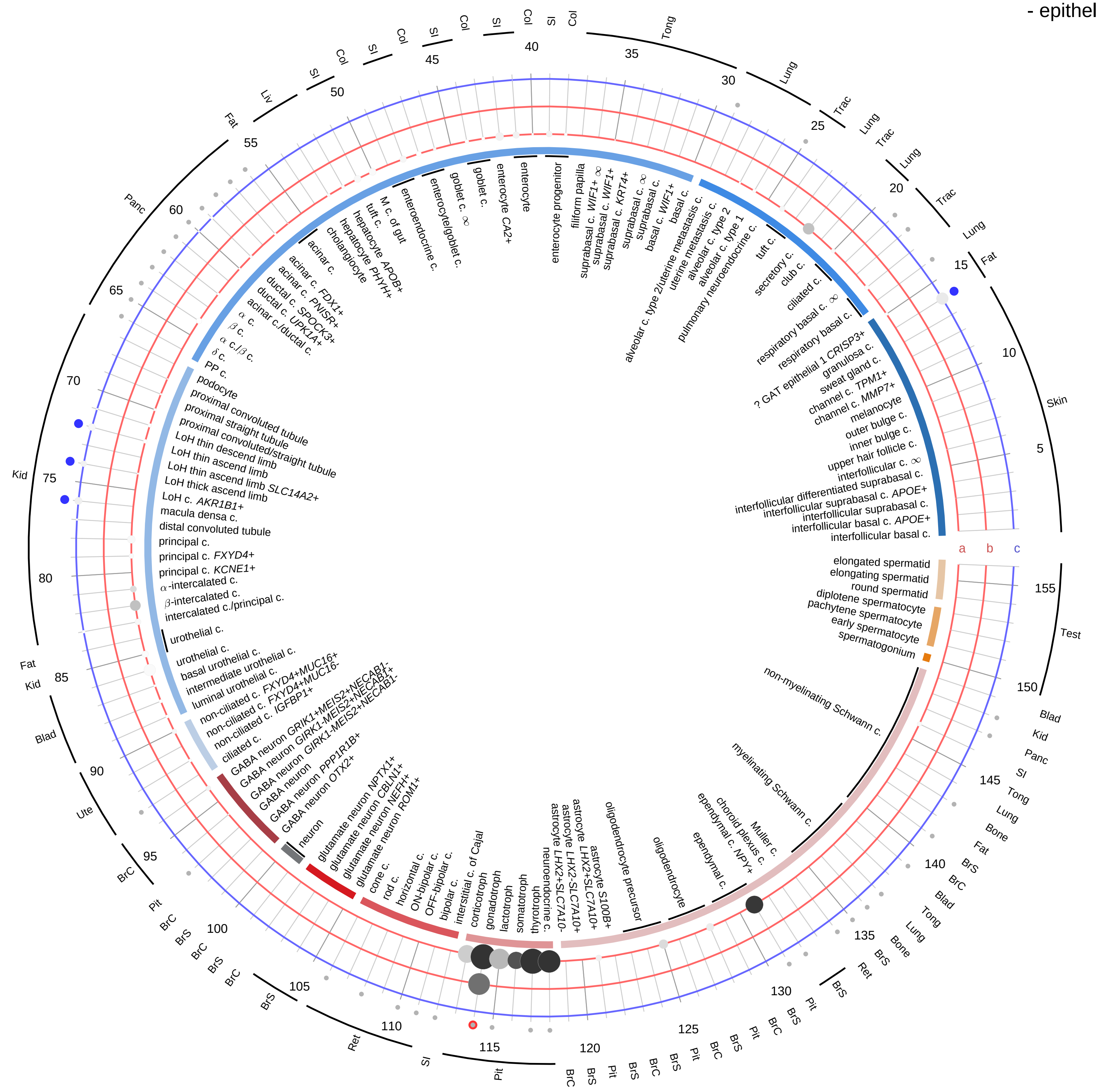

Ligand/Enzyme
a - CGA
b - FSHB
Receptor
c - FSHR

PAIRING (ligand → receptor)

FSHB & CGA→FSHR

Compartment
epithelial
- integumentary
- respiratory
- gastrointestinal
- urinary
- reproductive
neural
- GABAergic
- hybrid
- glutamatergic
- specialized
- neuroendocrine
- glia
germ
- spermatogonium
- spermatocyte
- spermatid

12.5%
25%
50%
100%

ln(CP10K+1): 0, 1, 2, 3

Abbrev.	Tissue
Aort	Aorta
Blad	Bladder
Blo	Blood
Bone	Bone
BM	Bone marrow
BrC	Brain cortex
BrS	Brainstem
Col	Colon
Dia	Diaphragm
Ret	Eye retina
Fat	Fat
Hrt	Heart
Pit	Hypothalamus/Pituitary
Kid	Kidney
LM	Limb muscle
Liv	Liver
Lung	Lung
MG	Mammary gland
Panc	Pancreas
Skin	Skin
SI	Small intestine
Spl	Spleen
Test	Testes
Thym	Thymus
Tong	Tongue
Trac	Trachea
Ute	Uterus

Follicle stimulating hormone (FSH)
- endothelial

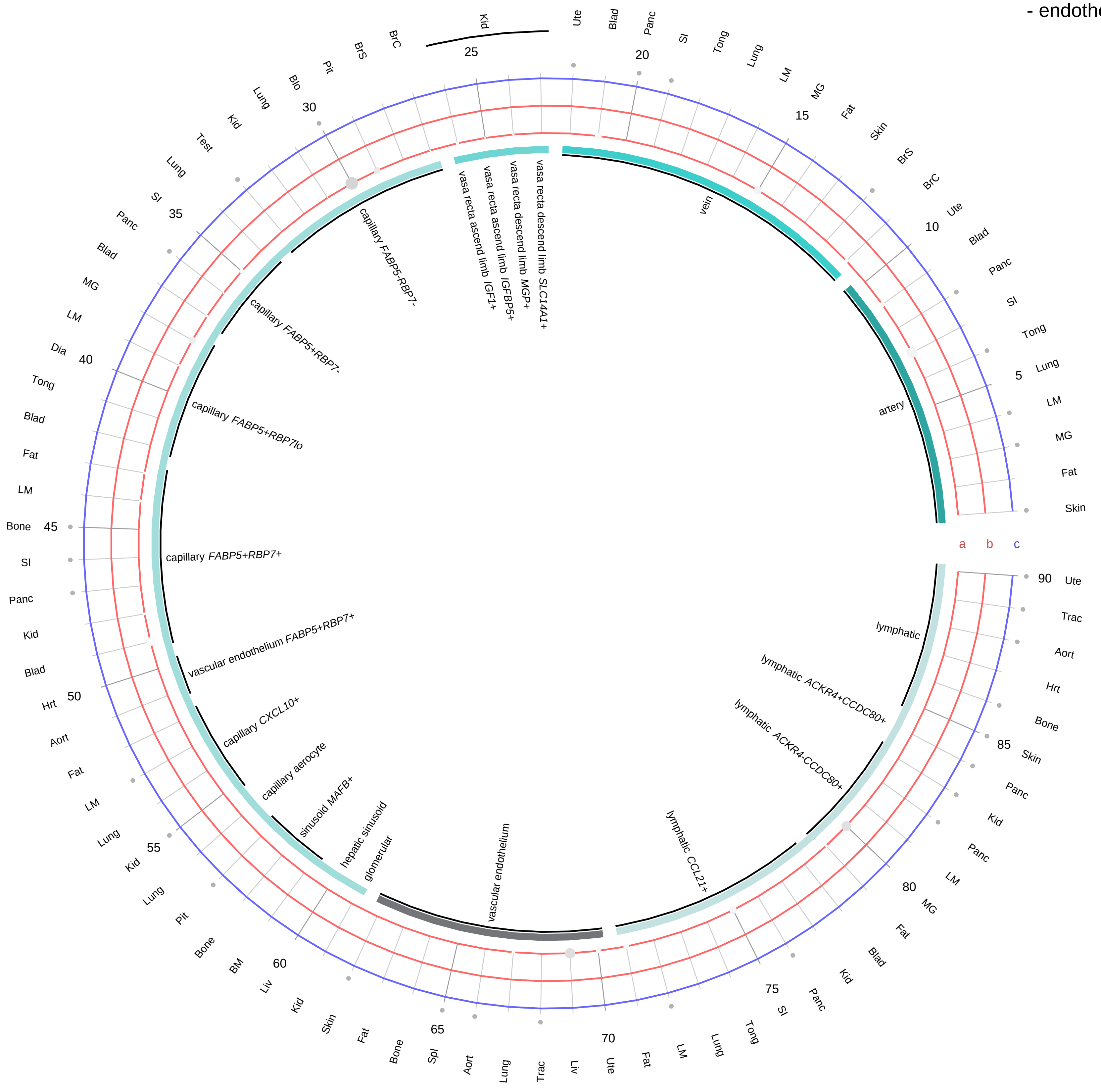

Ligand/Enzyme
- a - CGA
- b - FSHB

Receptor
- c - FSHR

PAIRING (ligand → receptor)

FSHB & CGA→FSHR

Abbrev.	Tissue
Aort	Aorta
Blad	Bladder
Blo	Blood
Bone	Bone
BM	Bone marrow
BrC	Brain cortex
BrS	Brainstem
Col	Colon
Dia	Diaphragm
Ret	Eye retina
Fat	Fat
Hrt	Heart
Pit	Hypothalamus/Pituitary
Kid	Kidney
LM	Limb muscle
Liv	Liver
Lung	Lung
MG	Mammary gland
Panc	Pancreas
Skin	Skin
SI	Small intestine
Spl	Spleen
Test	Testes
Thym	Thymus
Tong	Tongue
Trac	Trachea
Ute	Uterus

Compartment
endothelial
- artery
- vein
- vasa recta
- capillary
- mix
- lymphatic

12.5%
25%
50%
100%

ln(CP10K+1): 0, 1, 2, 3

Follicle stimulating hormone (FSH)
- stromal

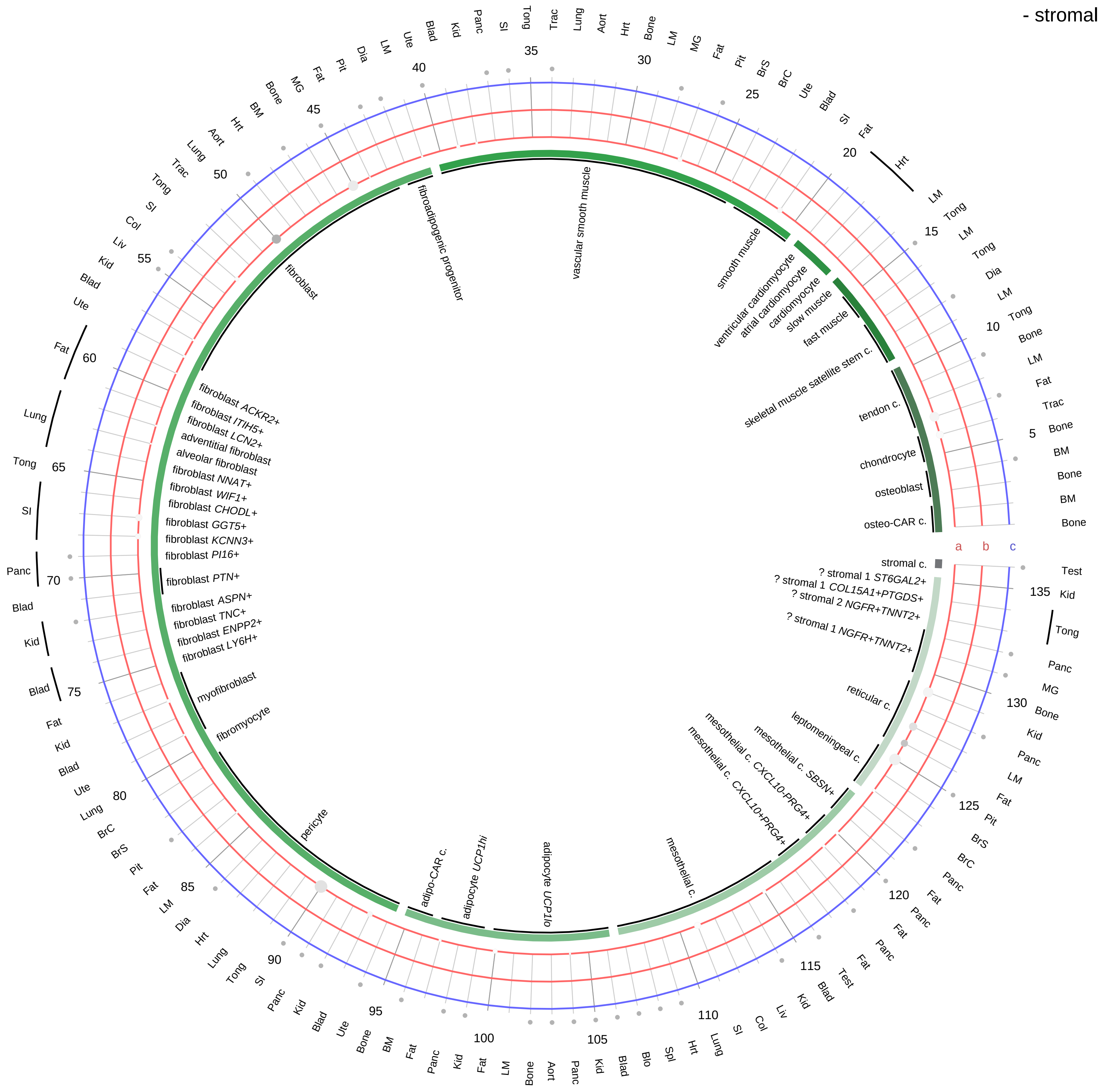

Ligand/Enzyme
- a - CGA
- b - FSHB

Receptor
- c - FSHR

PAIRING (ligand → receptor)

FSHB & CGA→FSHR

**Abbrev.**	**Tissue**
Aort	Aorta
Blad	Bladder
Blo	Blood
Bone	Bone
BM	Bone marrow
BrC	Brain cortex
BrS	Brainstem
Col	Colon
Dia	Diaphragm
Ret	Eye retina
Fat	Fat
Hrt	Heart
Pit	Hypothalamus/Pituitary
Kid	Kidney
LM	Limb muscle
Liv	Liver
Lung	Lung
MG	Mammary gland
Panc	Pancreas
Skin	Skin
SI	Small intestine
Spl	Spleen
Test	Testes
Thym	Thymus
Tong	Tongue
Trac	Trachea
Ute	Uterus

Compartment

stromal
- bone
- skeletal muscle
- cardiac muscle
- smooth muscle
- fibroblast
- adipocyte
- mesothelial
- other
- mix

12.5%
25%
50%
100%

ln(CP10K+1): 0, 1, 2, 3

Follicle stimulating hormone (FSH)
- lymphoid

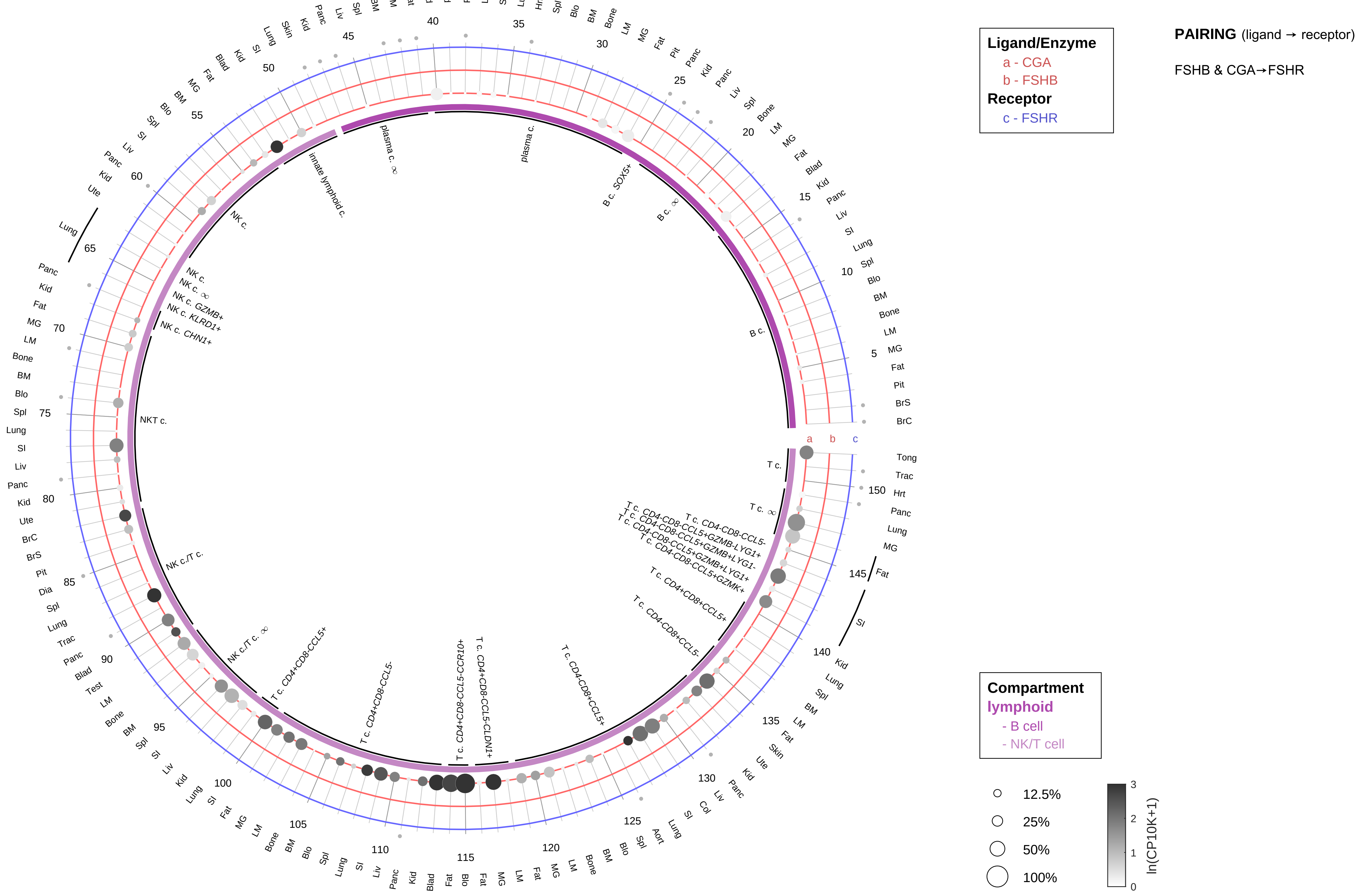

Abbrev.	Tissue
Aort	Aorta
Blad	Bladder
Blo	Blood
Bone	Bone
BM	Bone marrow
BrC	Brain cortex
BrS	Brainstem
Col	Colon
Dia	Diaphragm
Ret	Eye retina
Fat	Fat
Hrt	Heart
Pit	Hypothalamus/Pituitary
Kid	Kidney
LM	Limb muscle
Liv	Liver
Lung	Lung
MG	Mammary gland
Panc	Pancreas
Skin	Skin
SI	Small intestine
Spl	Spleen
Test	Testes
Thym	Thymus
Tong	Tongue
Trac	Trachea
Ute	Uterus

Follicle stimulating hormone (FSH)
- myeloid

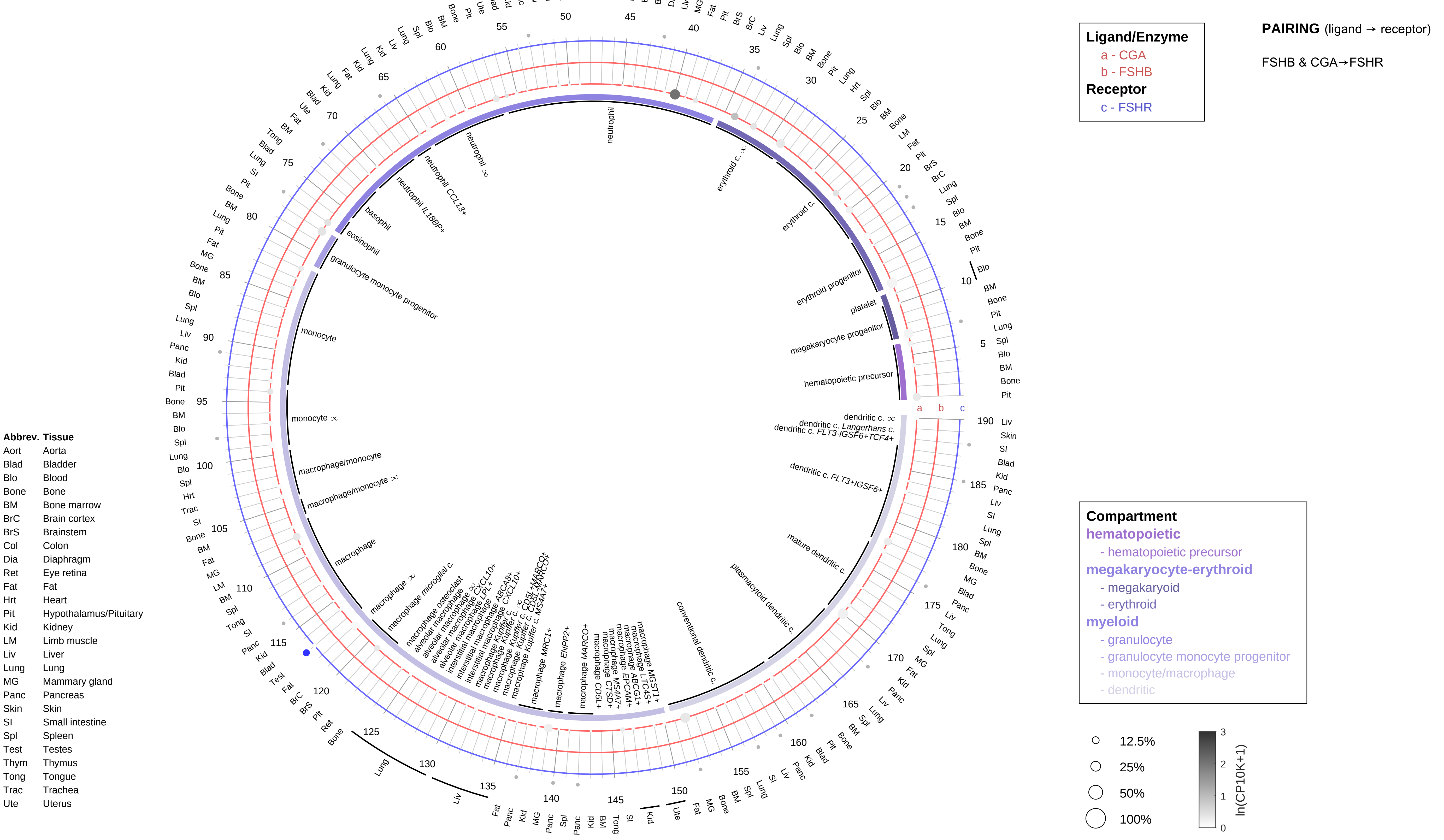

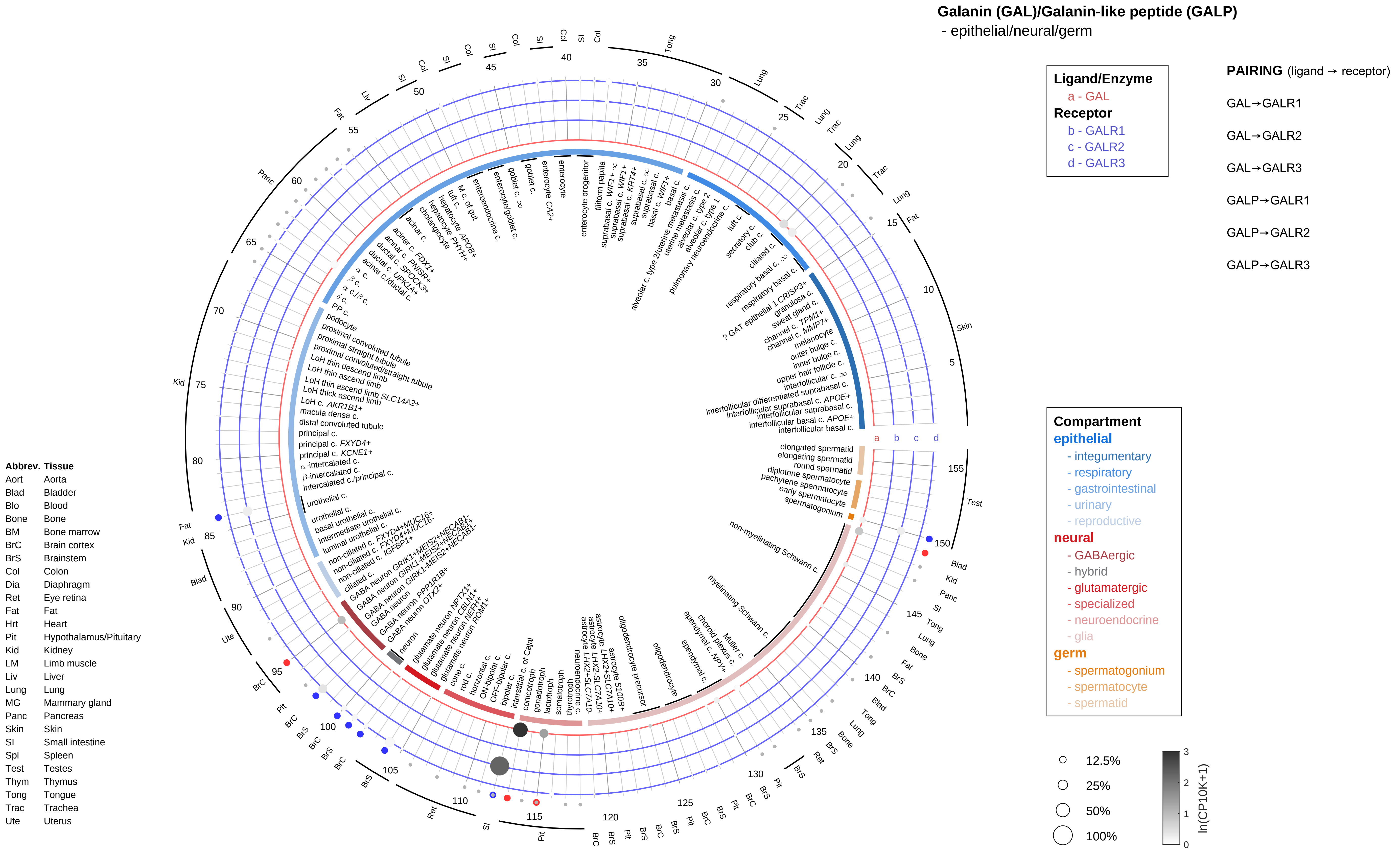

Galanin (GAL)/Galanin-like peptide (GALP)
- epithelial/neural/germ
Ligand/Enzyme
a - GAL
Receptor
b - GALR1
c - GALR2
d - GALR3
PAIRING (ligand → receptor)
GAL→GALR1
GAL→GALR2
GAL→GALR3
GALP→GALR1
GALP→GALR2
GALP→GALR3
Compartment
epithelial
- integumentary
- respiratory
- gastrointestinal
- urinary
- reproductive
neural
- GABAergic
- hybrid
- glutamatergic
- specialized
- neuroendocrine
- glia
germ
- spermatogonium
- spermatocyte
- spermatid
12.5%
25%
50%
100%
ln(CP10K+1)
Abbrev. Tissue
Aort Aorta
Blad Bladder
Blo Blood
Bone Bone
BM Bone marrow
BrC Brain cortex
BrS Brainstem
Col Colon
Dia Diaphragm
Ret Eye retina
Fat Fat
Hrt Heart
Pit Hypothalamus/Pituitary
Kid Kidney
LM Limb muscle
Liv Liver
Lung Lung
MG Mammary gland
Panc Pancreas
Skin Skin
SI Small intestine
Spl Spleen
Test Testes
Thym Thymus
Tong Tongue
Trac Trachea
Ute Uterus

Galanin (GAL)/Galanin-like peptide (GALP)
- endothelial

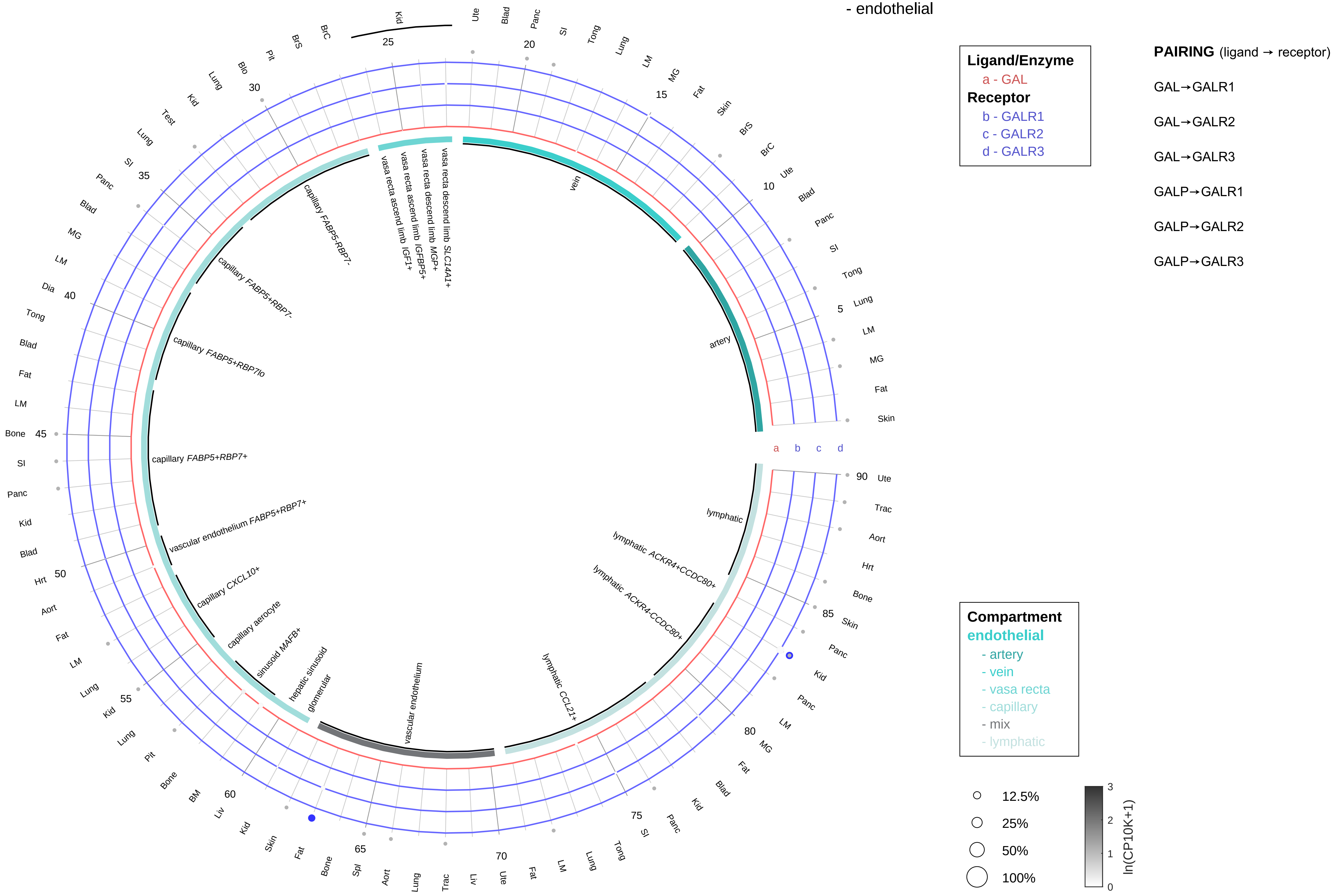

Abbrev.	Tissue
Aort	Aorta
Blad	Bladder
Blo	Blood
Bone	Bone
BM	Bone marrow
BrC	Brain cortex
BrS	Brainstem
Col	Colon
Dia	Diaphragm
Ret	Eye retina
Fat	Fat
Hrt	Heart
Pit	Hypothalamus/Pituitary
Kid	Kidney
LM	Limb muscle
Liv	Liver
Lung	Lung
MG	Mammary gland
Panc	Pancreas
Skin	Skin
SI	Small intestine
Spl	Spleen
Test	Testes
Thym	Thymus
Tong	Tongue
Trac	Trachea
Ute	Uterus

Galanin (GAL)/Galanin-like peptide (GALP)
- stromal

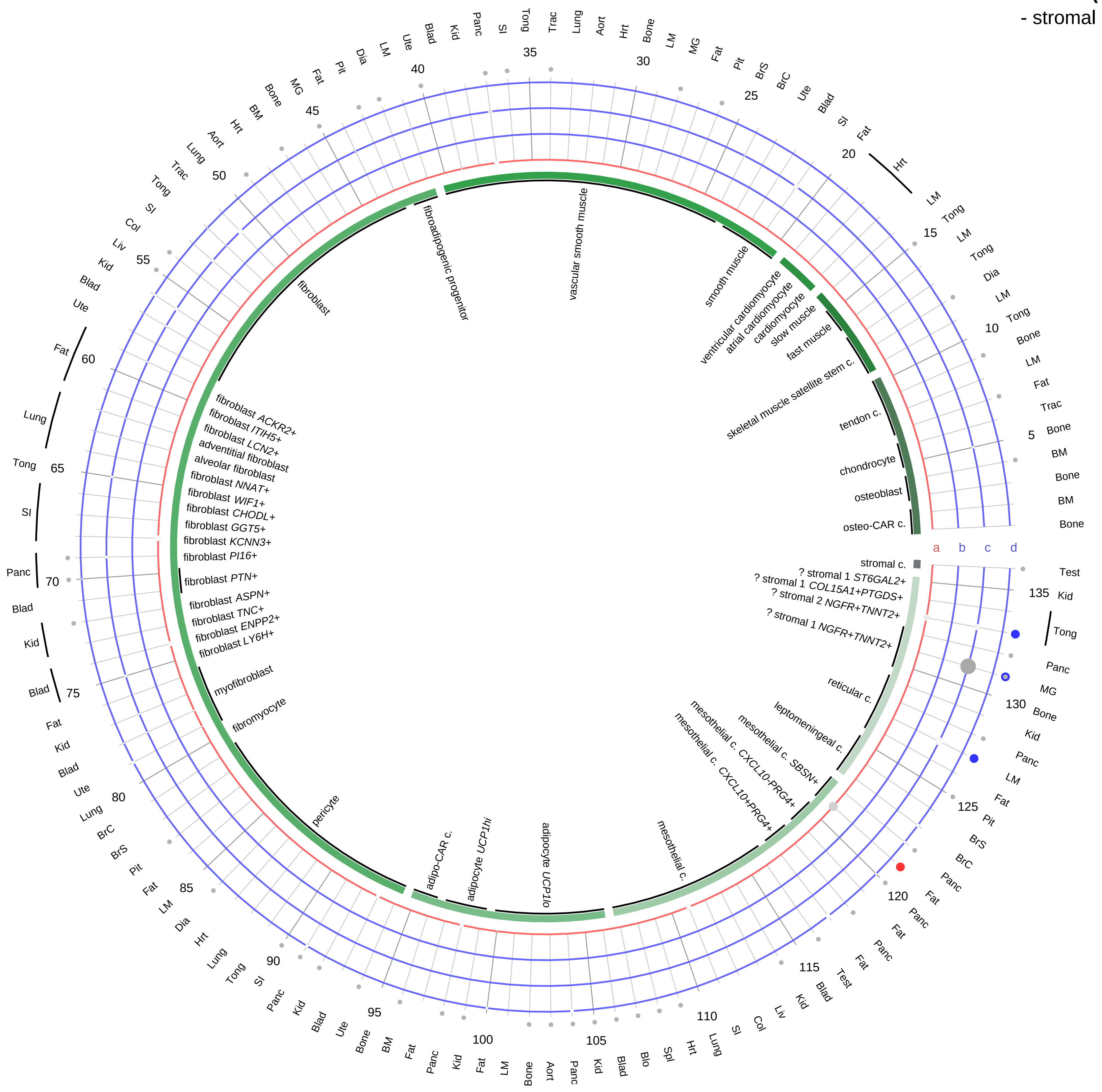

Ligand/Enzyme
a - GAL
Receptor
b - GALR1
c - GALR2
d - GALR3

PAIRING (ligand → receptor)

GAL→GALR1

GAL→GALR2

GAL→GALR3

GALP→GALR1

GALP→GALR2

GALP→GALR3

Abbrev.	Tissue
Aort	Aorta
Blad	Bladder
Blo	Blood
Bone	Bone
BM	Bone marrow
BrC	Brain cortex
BrS	Brainstem
Col	Colon
Dia	Diaphragm
Ret	Eye retina
Fat	Fat
Hrt	Heart
Pit	Hypothalamus/Pituitary
Kid	Kidney
LM	Limb muscle
Liv	Liver
Lung	Lung
MG	Mammary gland
Panc	Pancreas
Skin	Skin
SI	Small intestine
Spl	Spleen
Test	Testes
Thym	Thymus
Tong	Tongue
Trac	Trachea
Ute	Uterus

Compartment
stromal
- bone
- skeletal muscle
- cardiac muscle
- smooth muscle
- fibroblast
- adipocyte
- mesothelial
- other
- mix

12.5%
25%
50%
100%

ln(CP10K+1): 0, 1, 2, 3

Galanin (GAL)/Galanin-like peptide (GALP)
- lymphoid

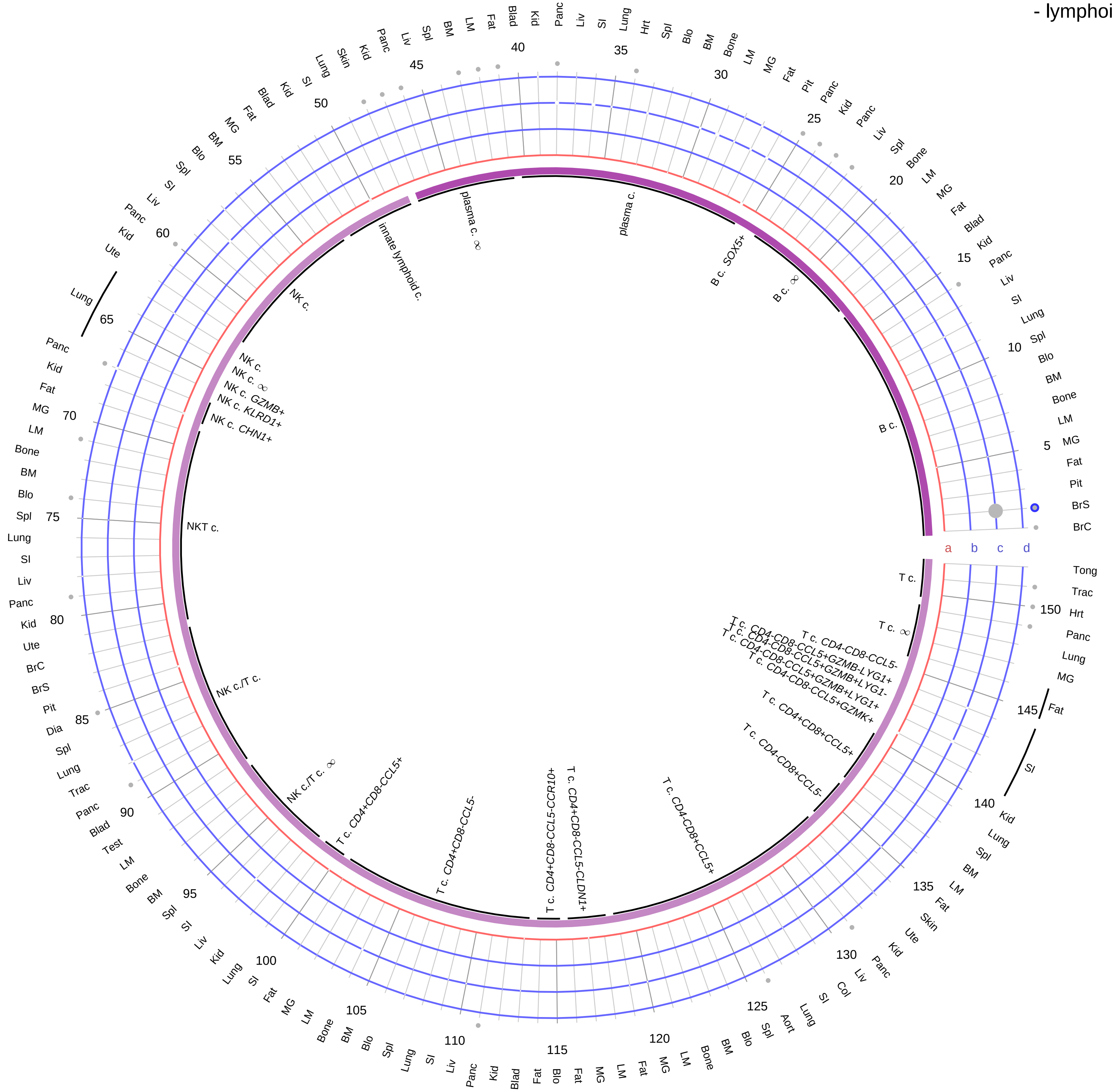

Ligand/Enzyme
- a - GAL

Receptor
- b - GALR1
- c - GALR2
- d - GALR3

PAIRING (ligand → receptor)

GAL→GALR1

GAL→GALR2

GAL→GALR3

GALP→GALR1

GALP→GALR2

GALP→GALR3

**Abbrev.**	**Tissue**
Aort	Aorta
Blad	Bladder
Blo	Blood
Bone	Bone
BM	Bone marrow
BrC	Brain cortex
BrS	Brainstem
Col	Colon
Dia	Diaphragm
Ret	Eye retina
Fat	Fat
Hrt	Heart
Pit	Hypothalamus/Pituitary
Kid	Kidney
LM	Limb muscle
Liv	Liver
Lung	Lung
MG	Mammary gland
Panc	Pancreas
Skin	Skin
SI	Small intestine
Spl	Spleen
Test	Testes
Thym	Thymus
Tong	Tongue
Trac	Trachea
Ute	Uterus

Compartment

lymphoid
- B cell
- NK/T cell

12.5%

25%

50%

100%

ln(CP10K+1): 0 1 2 3

Galanin (GAL)/Galanin-like peptide (GALP)
- myeloid

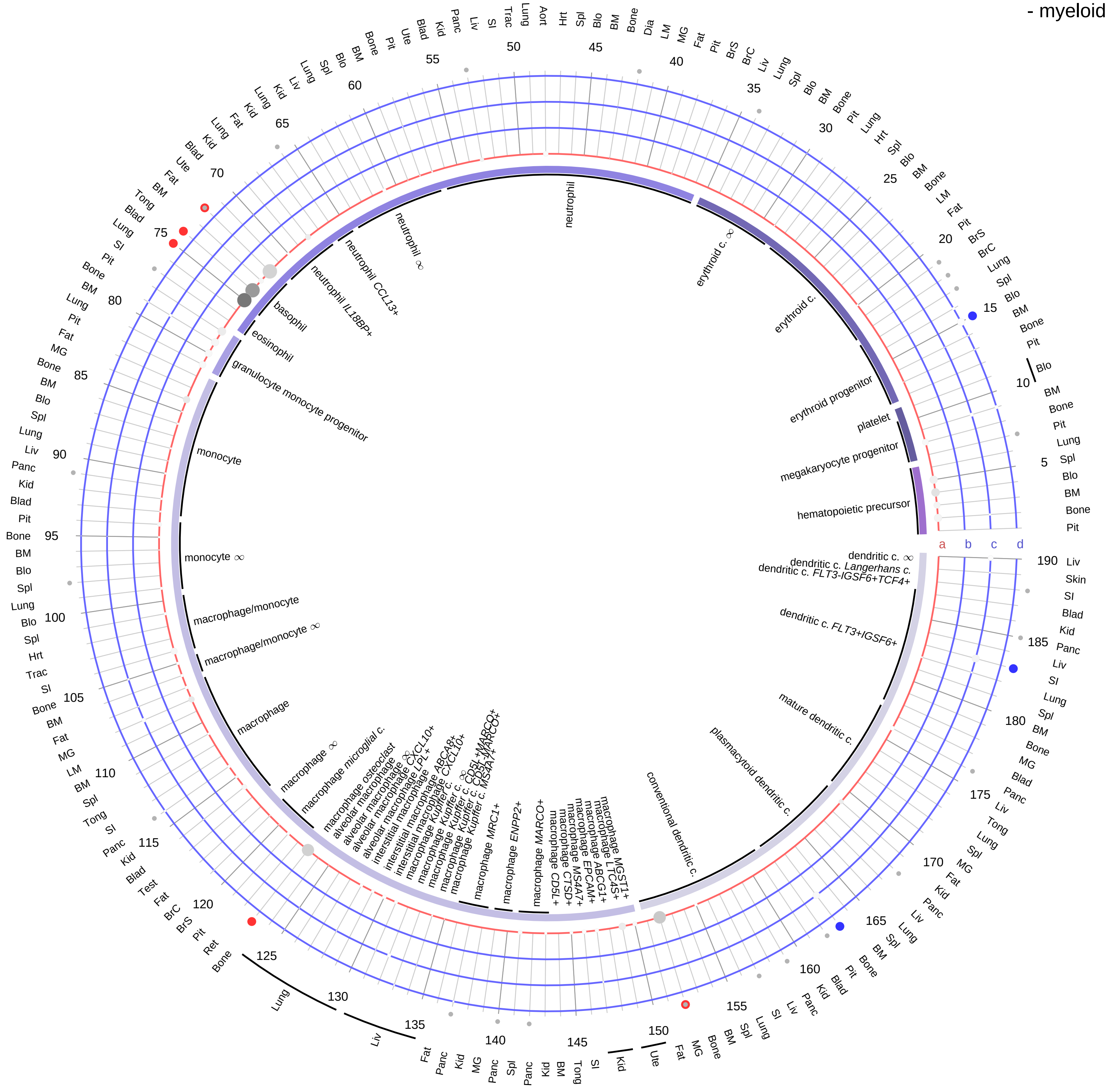

Ligand/Enzyme	
a - GAL	
**Receptor**	
b - GALR1	
c - GALR2	
d - GALR3	

PAIRING (ligand → receptor)

GAL→GALR1

GAL→GALR2

GAL→GALR3

GALP→GALR1

GALP→GALR2

GALP→GALR3

Abbrev.	Tissue
Aort	Aorta
Blad	Bladder
Blo	Blood
Bone	Bone
BM	Bone marrow
BrC	Brain cortex
BrS	Brainstem
Col	Colon
Dia	Diaphragm
Ret	Eye retina
Fat	Fat
Hrt	Heart
Pit	Hypothalamus/Pituitary
Kid	Kidney
LM	Limb muscle
Liv	Liver
Lung	Lung
MG	Mammary gland
Panc	Pancreas
Skin	Skin
SI	Small intestine
Spl	Spleen
Test	Testes
Thym	Thymus
Tong	Tongue
Trac	Trachea
Ute	Uterus

Compartment
hematopoietic
- hematopoietic precursor

megakaryocyte-erythroid
- megakaryoid
- erythroid

myeloid
- granulocyte
- granulocyte monocyte progenitor
- monocyte/macrophage
- dendritic

12.5%
25%
50%
100%

ln(CP10K+1): 0, 1, 2, 3

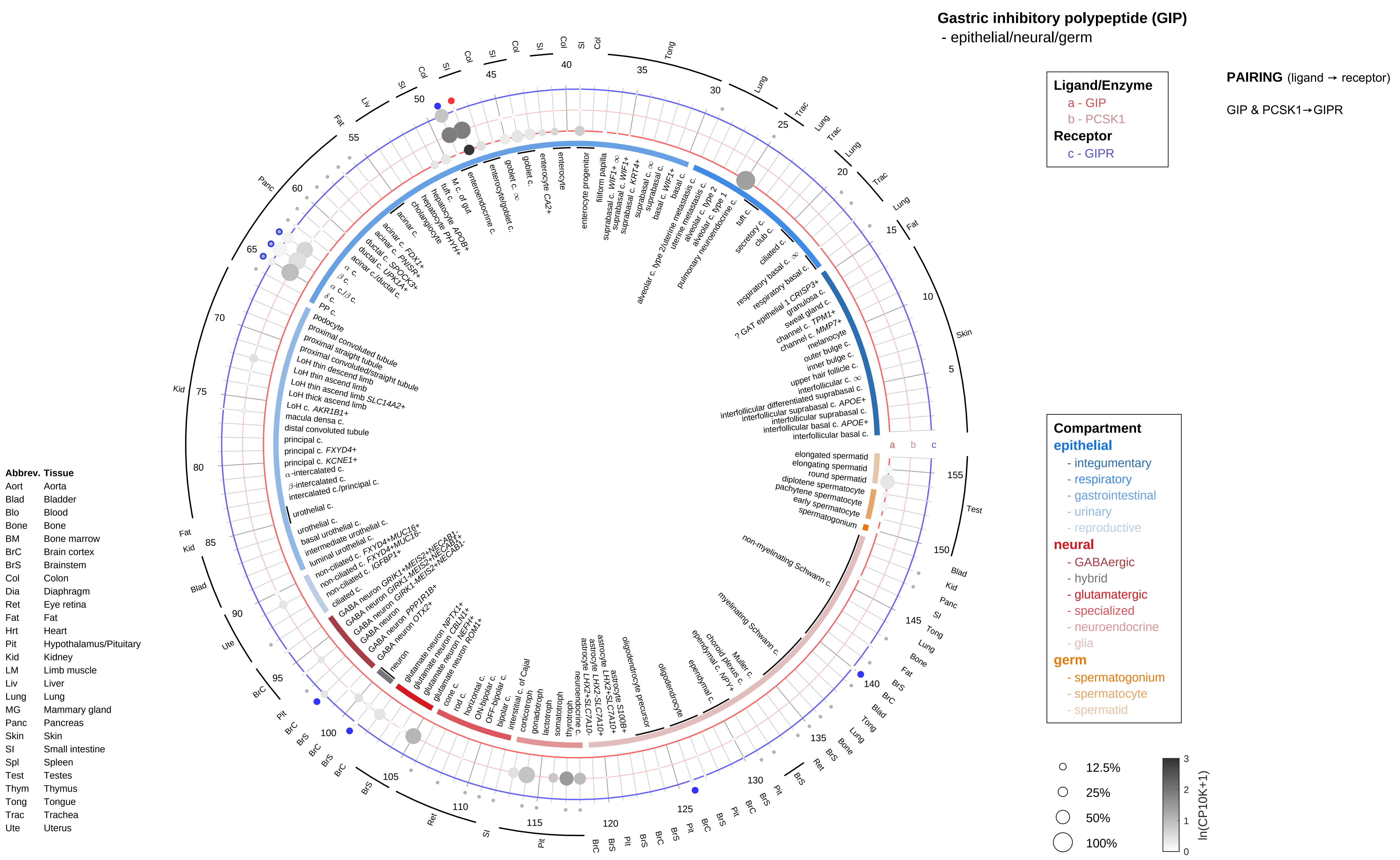
Gastric inhibitory polypeptide (GIP)
- epithelial/neural/germ
PAIRING (ligand → receptor)
GIP & PCSK1→GIPR
Ligand/Enzyme
a - GIP
b - PCSK1
Receptor
c - GIPR
Compartment
epithelial
- integumentary
- respiratory
- gastrointestinal
- urinary
- reproductive
neural
- GABAergic
- hybrid
- glutamatergic
- specialized
- neuroendocrine
- glia
germ
- spermatogonium
- spermatocyte
- spermatid
12.5%
25%
50%
100%
ln(CP10K+1)
Abbrev. Tissue
Aort Aorta
Blad Bladder
Blo Blood
Bone Bone
BM Bone marrow
BrC Brain cortex
BrS Brainstem
Col Colon
Dia Diaphragm
Ret Eye retina
Fat Fat
Hrt Heart
Pit Hypothalamus/Pituitary
Kid Kidney
LM Limb muscle
Liv Liver
Lung Lung
MG Mammary gland
Panc Pancreas
Skin Skin
SI Small intestine
Spl Spleen
Test Testes
Thym Thymus
Tong Tongue
Trac Trachea
Ute Uterus

Gastric inhibitory polypeptide (GIP)
- endothelial

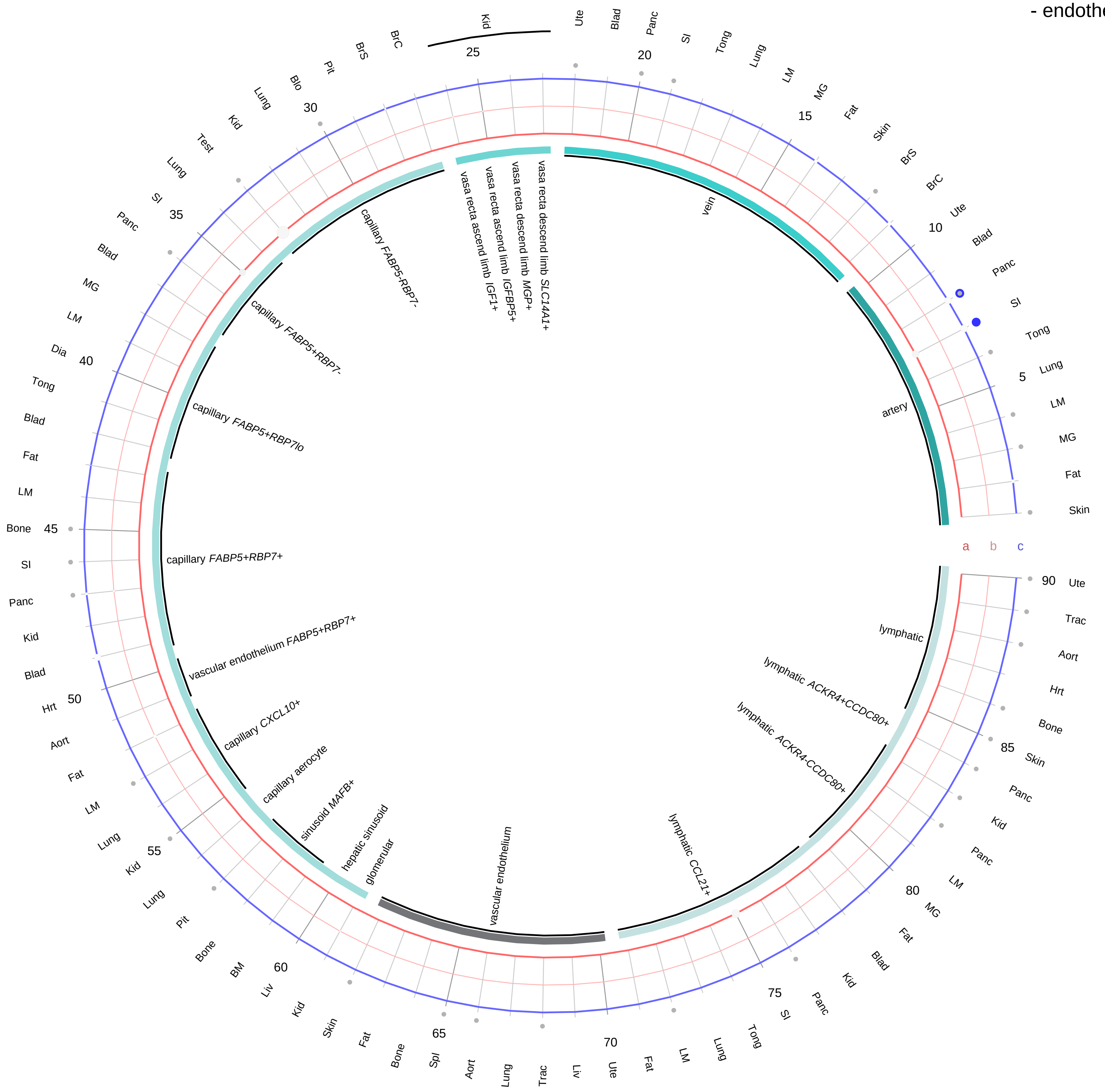

Ligand/Enzyme
a - GIP
b - PCSK1
Receptor
c - GIPR

PAIRING (ligand → receptor)

GIP & PCSK1→GIPR

Compartment
endothelial
- artery
- vein
- vasa recta
- capillary
- mix
- lymphatic

12.5%
25%
50%
100%

ln(CP10K+1): 0, 1, 2, 3

Abbrev.	Tissue
Aort	Aorta
Blad	Bladder
Blo	Blood
Bone	Bone
BM	Bone marrow
BrC	Brain cortex
BrS	Brainstem
Col	Colon
Dia	Diaphragm
Ret	Eye retina
Fat	Fat
Hrt	Heart
Pit	Hypothalamus/Pituitary
Kid	Kidney
LM	Limb muscle
Liv	Liver
Lung	Lung
MG	Mammary gland
Panc	Pancreas
Skin	Skin
SI	Small intestine
Spl	Spleen
Test	Testes
Thym	Thymus
Tong	Tongue
Trac	Trachea
Ute	Uterus

Gastric inhibitory polypeptide (GIP)
- stromal

Ligand/Enzyme
a - GIP
b - PCSK1
Receptor
c - GIPR

PAIRING (ligand → receptor)

GIP & PCSK1→GIPR

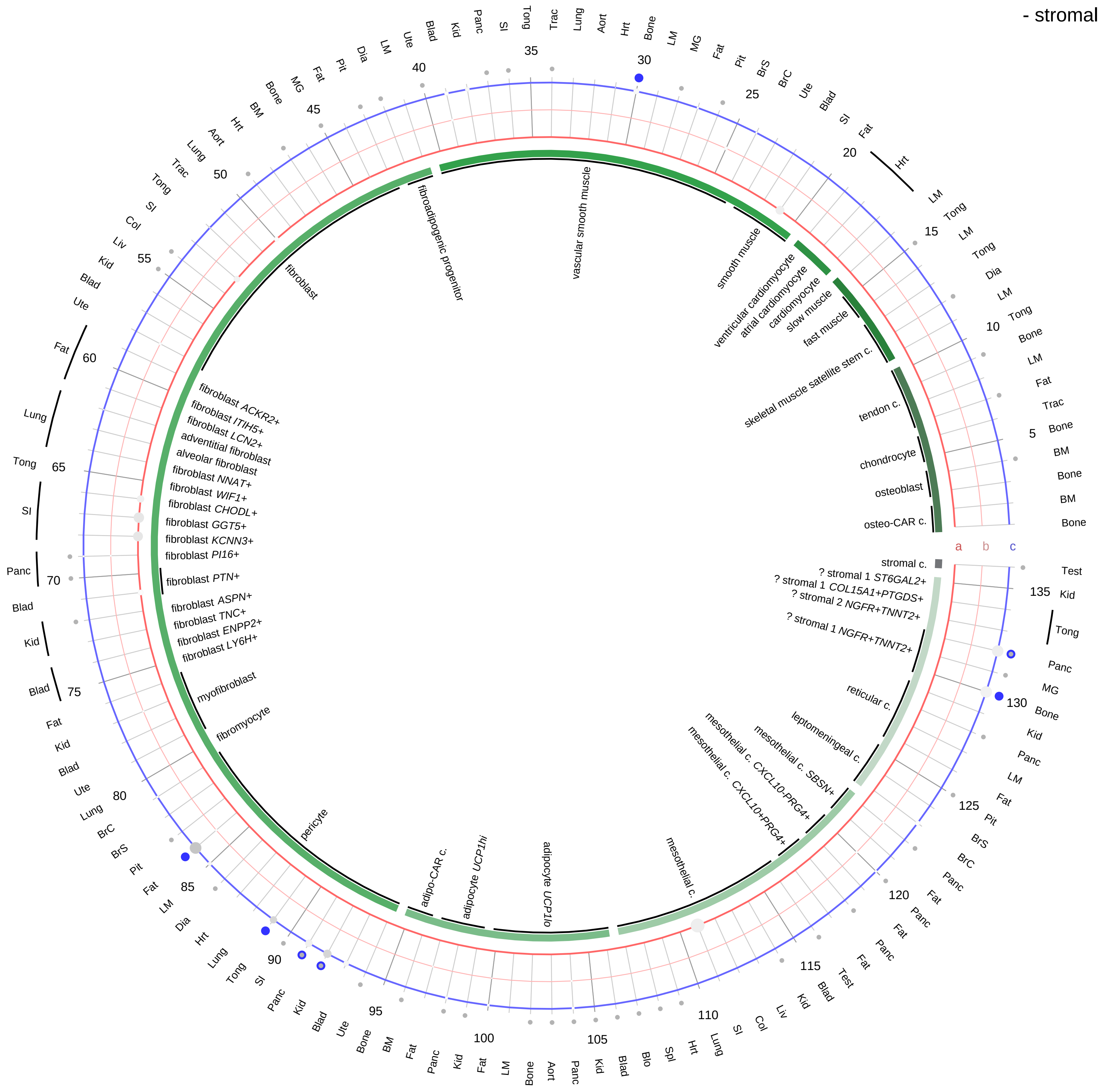

Compartment
stromal
- bone
- skeletal muscle
- cardiac muscle
- smooth muscle
- fibroblast
- adipocyte
- mesothelial
- other
- mix

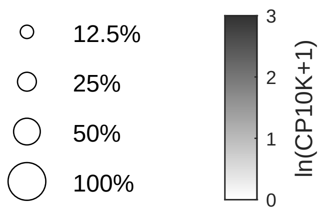

Abbrev.	Tissue
Aort	Aorta
Blad	Bladder
Blo	Blood
Bone	Bone
BM	Bone marrow
BrC	Brain cortex
BrS	Brainstem
Col	Colon
Dia	Diaphragm
Ret	Eye retina
Fat	Fat
Hrt	Heart
Pit	Hypothalamus/Pituitary
Kid	Kidney
LM	Limb muscle
Liv	Liver
Lung	Lung
MG	Mammary gland
Panc	Pancreas
Skin	Skin
SI	Small intestine
Spl	Spleen
Test	Testes
Thym	Thymus
Tong	Tongue
Trac	Trachea
Ute	Uterus

Gastric inhibitory polypeptide (GIP)
- lymphoid

Ligand/Enzyme
a - GIP
b - PCSK1
Receptor
c - GIPR

PAIRING (ligand → receptor)

GIP & PCSK1→GIPR

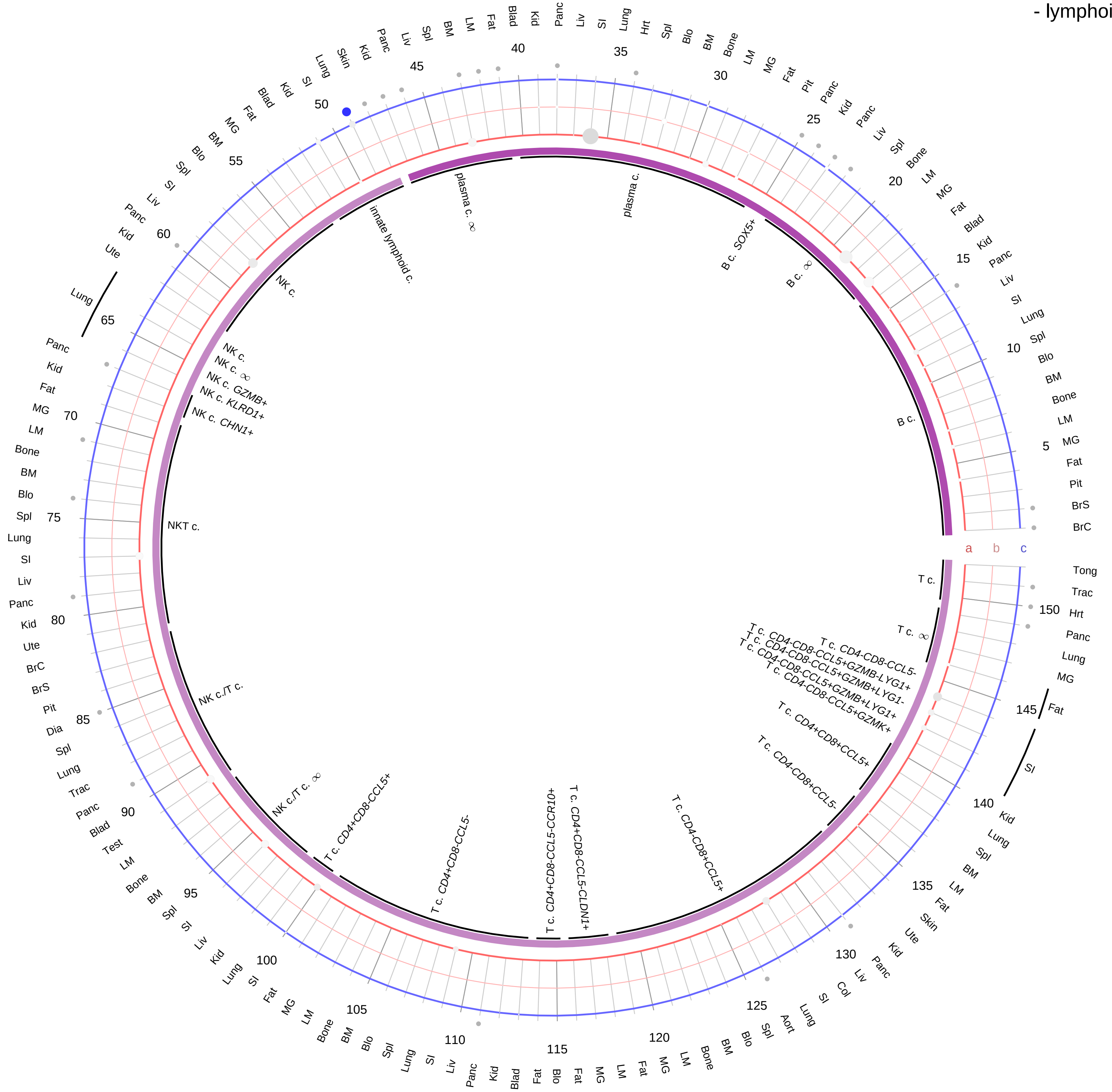

Abbrev.	Tissue
Aort	Aorta
Blad	Bladder
Blo	Blood
Bone	Bone
BM	Bone marrow
BrC	Brain cortex
BrS	Brainstem
Col	Colon
Dia	Diaphragm
Ret	Eye retina
Fat	Fat
Hrt	Heart
Pit	Hypothalamus/Pituitary
Kid	Kidney
LM	Limb muscle
Liv	Liver
Lung	Lung
MG	Mammary gland
Panc	Pancreas
Skin	Skin
SI	Small intestine
Spl	Spleen
Test	Testes
Thym	Thymus
Tong	Tongue
Trac	Trachea
Ute	Uterus

Compartment
lymphoid
- B cell
- NK/T cell

12.5%
25%
50%
100%

ln(CP10K+1): 0, 1, 2, 3

Gastric inhibitory polypeptide (GIP)
- myeloid

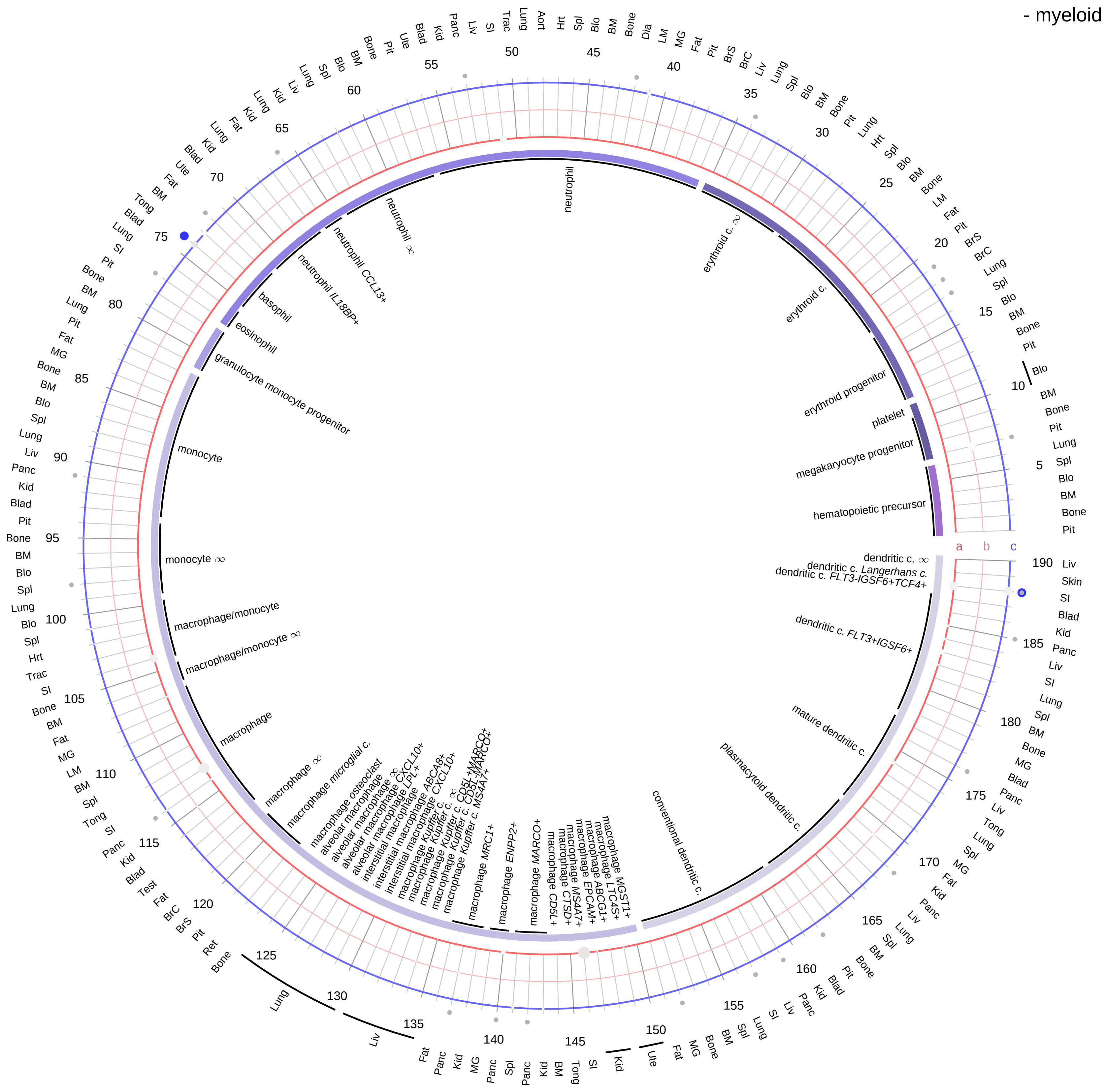

Ligand/Enzyme	
a - GIP	
b - PCSK1	
**Receptor**	
c - GIPR	

PAIRING (ligand → receptor)

GIP & PCSK1→GIPR

**Abbrev.**	**Tissue**
Aort	Aorta
Blad	Bladder
Blo	Blood
Bone	Bone
BM	Bone marrow
BrC	Brain cortex
BrS	Brainstem
Col	Colon
Dia	Diaphragm
Ret	Eye retina
Fat	Fat
Hrt	Heart
Pit	Hypothalamus/Pituitary
Kid	Kidney
LM	Limb muscle
Liv	Liver
Lung	Lung
MG	Mammary gland
Panc	Pancreas
Skin	Skin
SI	Small intestine
Spl	Spleen
Test	Testes
Thym	Thymus
Tong	Tongue
Trac	Trachea
Ute	Uterus

Compartment
hematopoietic
- hematopoietic precursor
megakaryocyte-erythroid
- megakaryoid
- erythroid
myeloid
- granulocyte
- granulocyte monocyte progenitor
- monocyte/macrophage
- dendritic

12.5%
25%
50%
100%

ln(CP10K+1) 0 1 2 3

Gastrin (GAS)
- epithelial/neural/germ

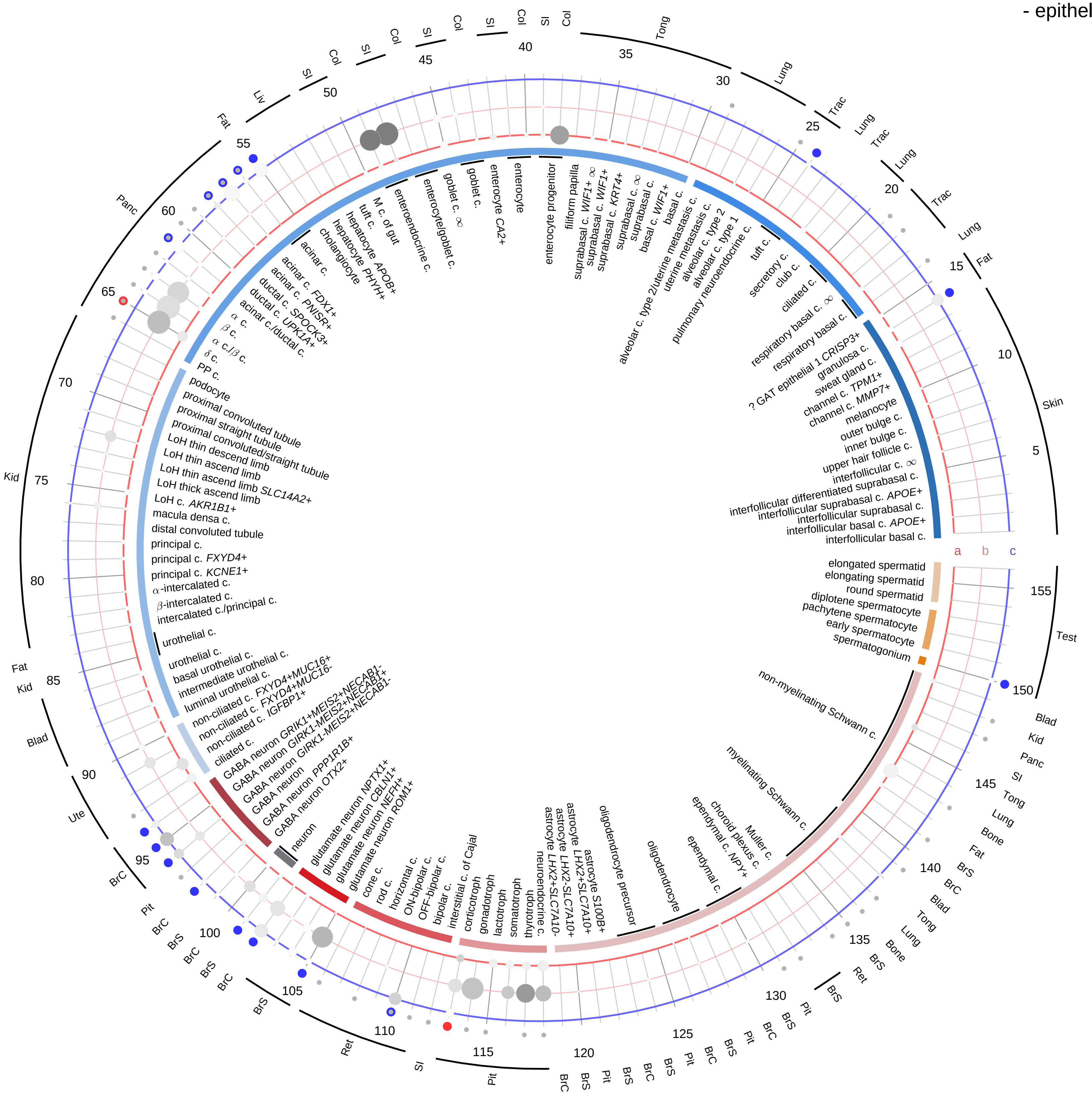

Ligand/Enzyme
a - GAST
b - PCSK1
**Receptor**
c - CCKBR

PAIRING (ligand → receptor)

GAST & PCSK1→CCKBR

Abbrev.	Tissue
Aort	Aorta
Blad	Bladder
Blo	Blood
Bone	Bone
BM	Bone marrow
BrC	Brain cortex
BrS	Brainstem
Col	Colon
Dia	Diaphragm
Ret	Eye retina
Fat	Fat
Hrt	Heart
Pit	Hypothalamus/Pituitary
Kid	Kidney
LM	Limb muscle
Liv	Liver
Lung	Lung
MG	Mammary gland
Panc	Pancreas
Skin	Skin
SI	Small intestine
Spl	Spleen
Test	Testes
Thym	Thymus
Tong	Tongue
Trac	Trachea
Ute	Uterus

Compartment

epithelial
- integumentary
- respiratory
- gastrointestinal
- urinary
- reproductive

neural
- GABAergic
- hybrid
- glutamatergic
- specialized
- neuroendocrine
- glia

germ
- spermatogonium
- spermatocyte
- spermatid

12.5%
25%
50%
100%

ln(CP10K+1): 0, 1, 2, 3

Gastrin (GAS)
- endothelial

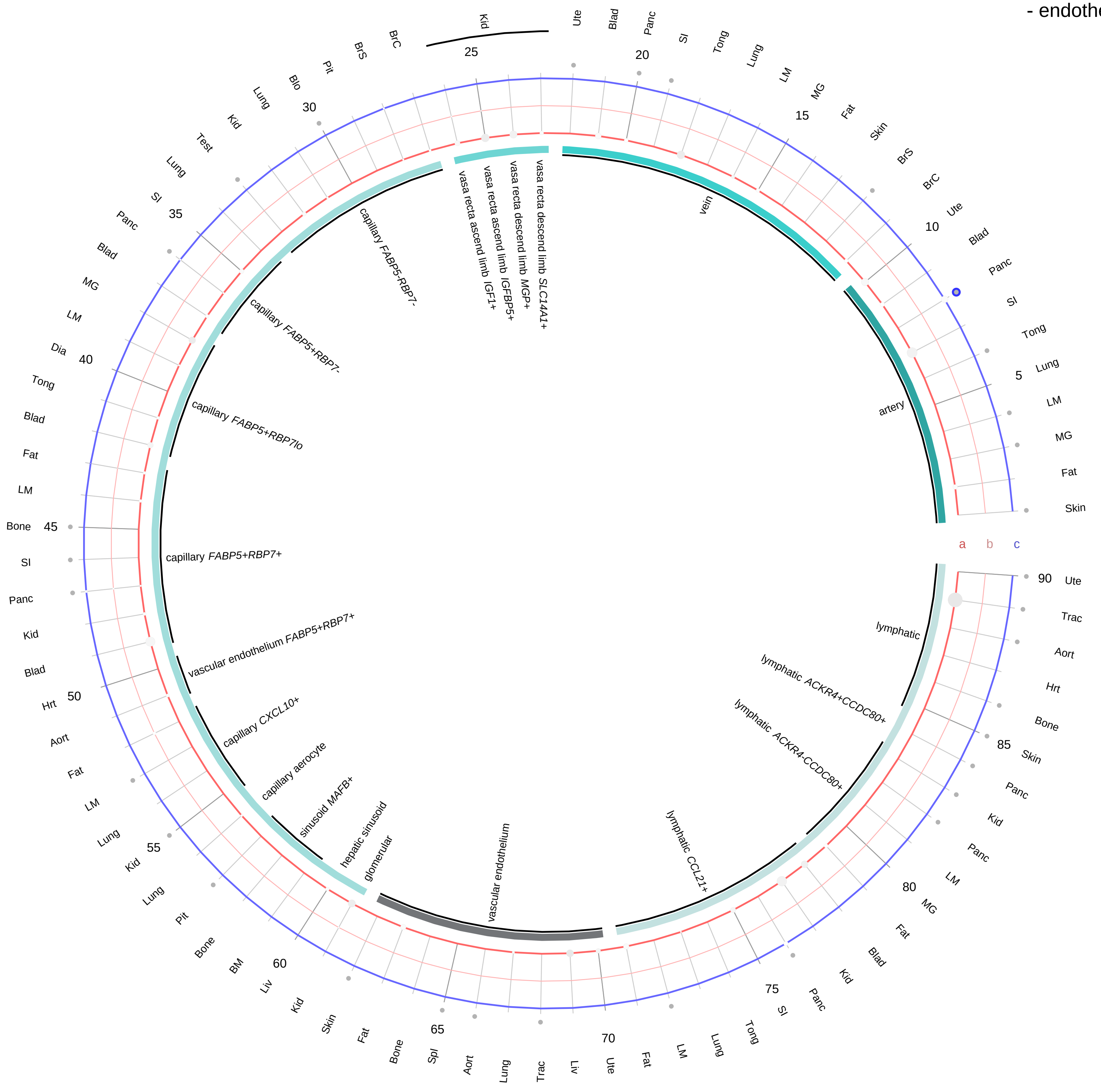

Ligand/Enzyme
- a - GAST
- b - PCSK1

Receptor
- c - CCKBR

PAIRING (ligand → receptor)

GAST & PCSK1→CCKBR

Compartment
endothelial
- artery
- vein
- vasa recta
- capillary
- mix
- lymphatic

12.5%
25%
50%
100%

ln(CP10K+1): 0, 1, 2, 3

Abbrev.	Tissue
Aort	Aorta
Blad	Bladder
Blo	Blood
Bone	Bone
BM	Bone marrow
BrC	Brain cortex
BrS	Brainstem
Col	Colon
Dia	Diaphragm
Ret	Eye retina
Fat	Fat
Hrt	Heart
Pit	Hypothalamus/Pituitary
Kid	Kidney
LM	Limb muscle
Liv	Liver
Lung	Lung
MG	Mammary gland
Panc	Pancreas
Skin	Skin
SI	Small intestine
Spl	Spleen
Test	Testes
Thym	Thymus
Tong	Tongue
Trac	Trachea
Ute	Uterus

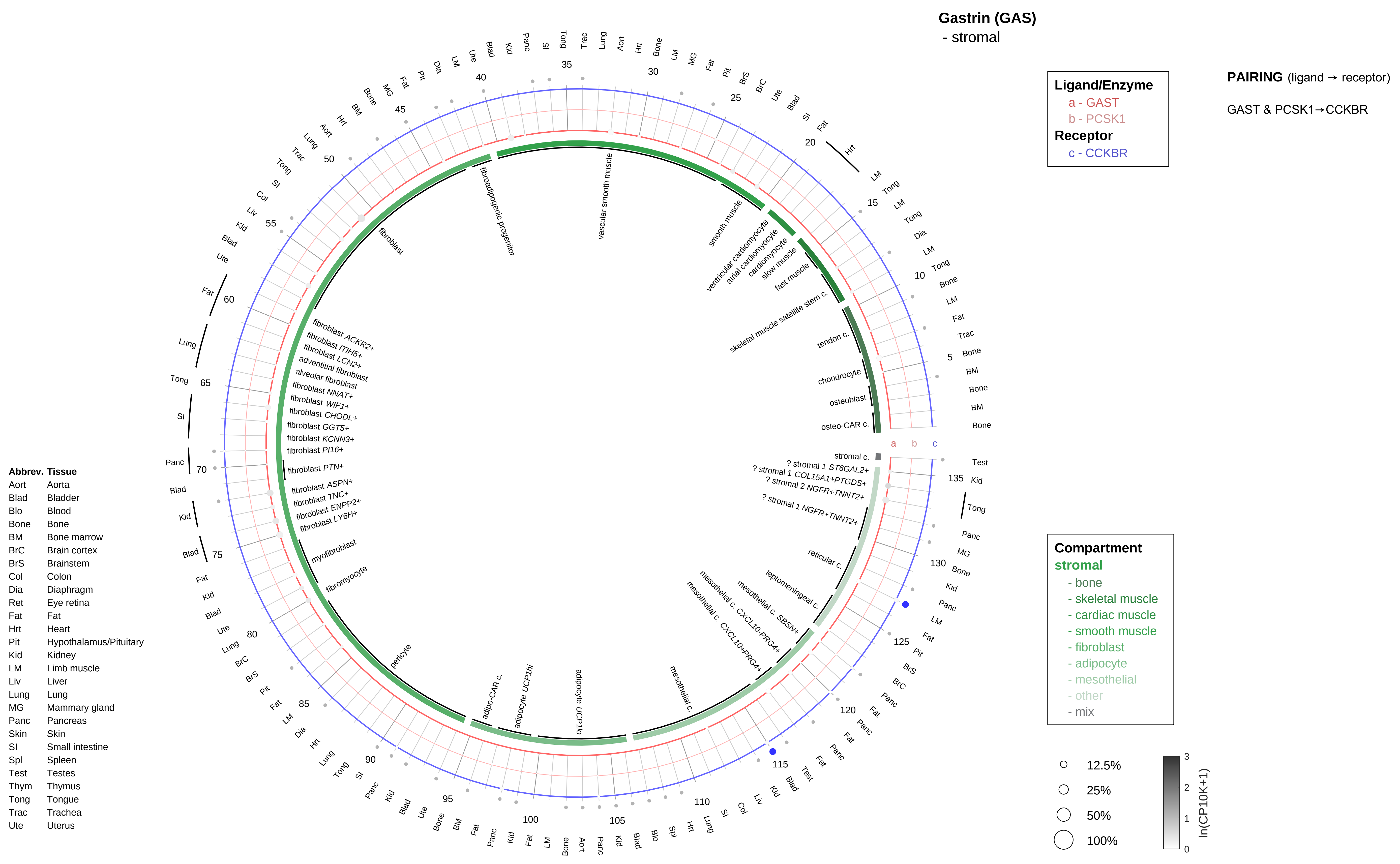

Gastrin (GAS)
- stromal
Ligand/Enzyme
a - GAST
b - PCSK1
Receptor
c - CCKBR
PAIRING (ligand → receptor)
GAST & PCSK1→CCKBR
Compartment
stromal
- bone
- skeletal muscle
- cardiac muscle
- smooth muscle
- fibroblast
- adipocyte
- mesothelial
- other
- mix
12.5%
25%
50%
100%
ln(CP10K+1)
Abbrev. Tissue
Aort Aorta
Blad Bladder
Blo Blood
Bone Bone
BM Bone marrow
BrC Brain cortex
BrS Brainstem
Col Colon
Dia Diaphragm
Ret Eye retina
Fat Fat
Hrt Heart
Pit Hypothalamus/Pituitary
Kid Kidney
LM Limb muscle
Liv Liver
Lung Lung
MG Mammary gland
Panc Pancreas
Skin Skin
SI Small intestine
Spl Spleen
Test Testes
Thym Thymus
Tong Tongue
Trac Trachea
Ute Uterus
osteo-CAR c.
osteoblast
chondrocyte
tendon c.
skeletal muscle satellite stem c.
fast muscle
slow muscle
cardiomyocyte
atrial cardiomyocyte
ventricular cardiomyocyte
smooth muscle
vascular smooth muscle
fibroadipogenic progenitor
fibroblast
fibroblast ACKR2+
fibroblast ITIH5+
fibroblast LCN2+
adventitial fibroblast
alveolar fibroblast
fibroblast NNAT+
fibroblast WIF1+
fibroblast CHODL+
fibroblast GGT5+
fibroblast KCNN3+
fibroblast PI16+
fibroblast PTN+
fibroblast ASPN+
fibroblast TNC+
fibroblast ENPP2+
fibroblast LY6H+
myofibroblast
fibromyocyte
pericyte
adipo-CAR c.
adipocyte UCP1hi
adipocyte UCP1lo
mesothelial c.
mesothelial c. CXCL10-PRG4+
mesothelial c. CXCL10+PRG4+
mesothelial c. SBSN+
leptomeningeal c.
reticular c.
? stromal 1 NGFR+TNNT2+
? stromal 2 NGFR+TNNT2+
? stromal 1 COL15A1+PTGDS+
? stromal 1 ST6GAL2+
stromal c.

Gastrin (GAS)
- lymphoid

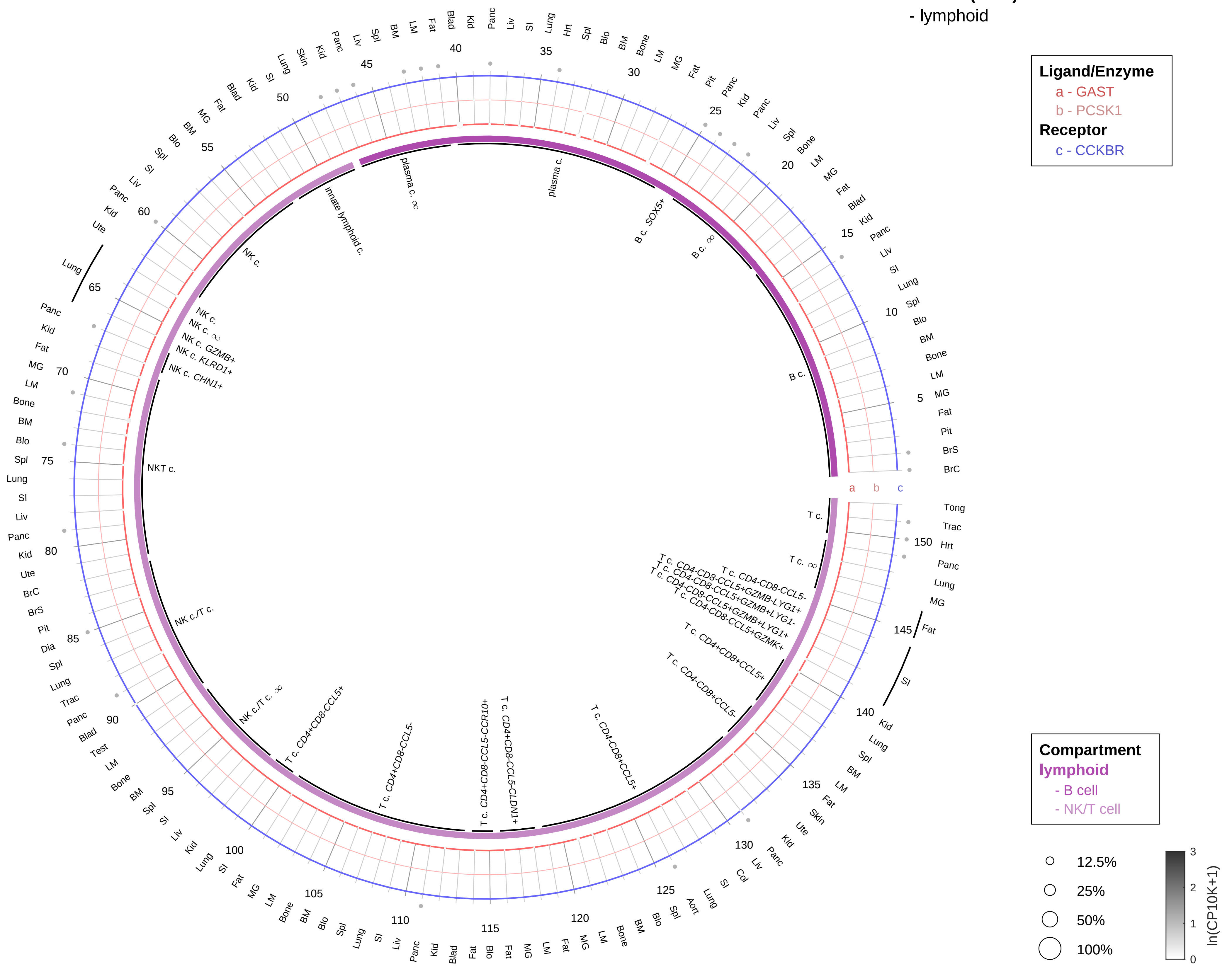

Abbrev.	Tissue
Aort	Aorta
Blad	Bladder
Blo	Blood
Bone	Bone
BM	Bone marrow
BrC	Brain cortex
BrS	Brainstem
Col	Colon
Dia	Diaphragm
Ret	Eye retina
Fat	Fat
Hrt	Heart
Pit	Hypothalamus/Pituitary
Kid	Kidney
LM	Limb muscle
Liv	Liver
Lung	Lung
MG	Mammary gland
Panc	Pancreas
Skin	Skin
SI	Small intestine
Spl	Spleen
Test	Testes
Thym	Thymus
Tong	Tongue
Trac	Trachea
Ute	Uterus

Gastrin (GAS)
- myeloid

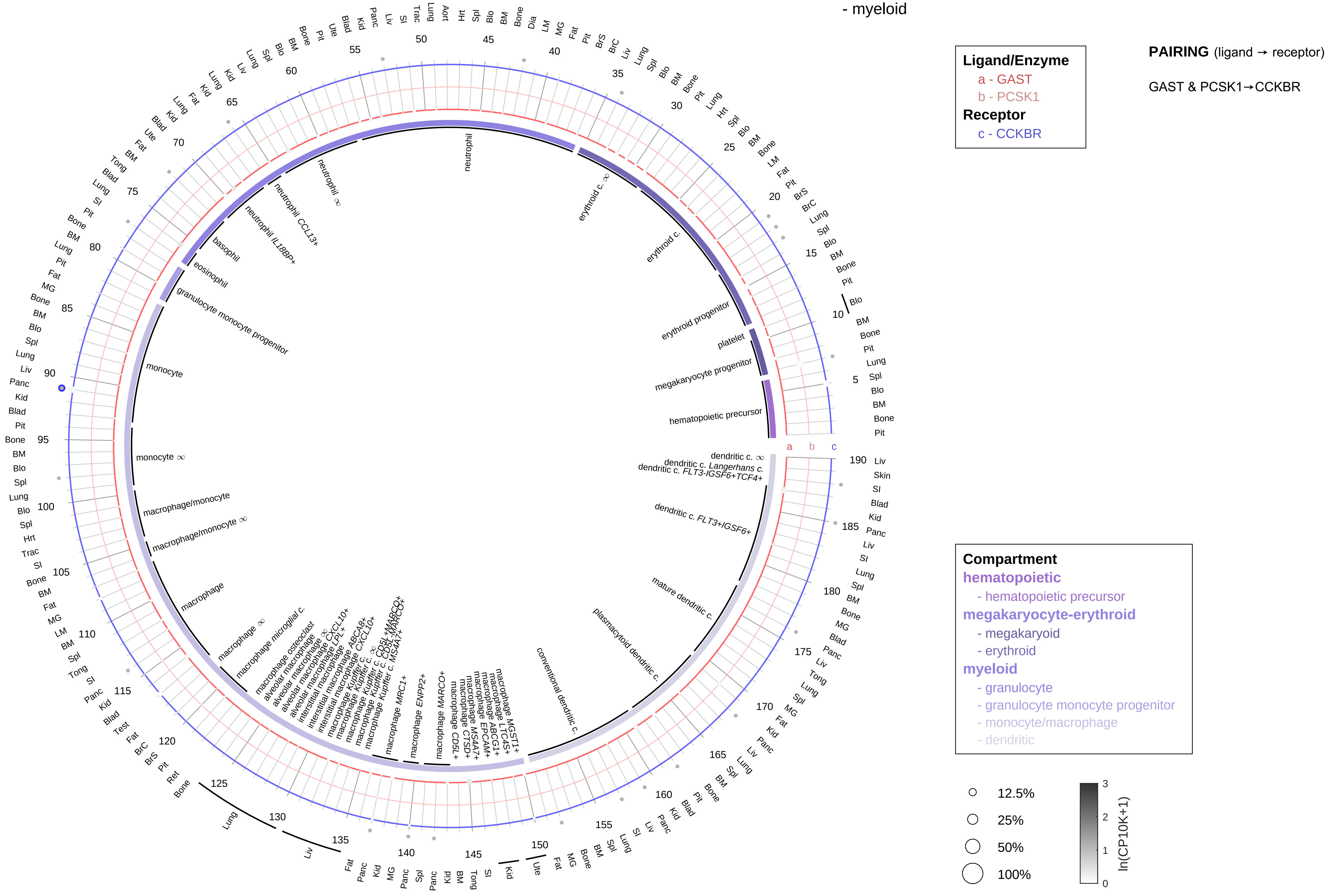

Abbrev.	Tissue
Aort	Aorta
Blad	Bladder
Blo	Blood
Bone	Bone
BM	Bone marrow
BrC	Brain cortex
BrS	Brainstem
Col	Colon
Dia	Diaphragm
Ret	Eye retina
Fat	Fat
Hrt	Heart
Pit	Hypothalamus/Pituitary
Kid	Kidney
LM	Limb muscle
Liv	Liver
Lung	Lung
MG	Mammary gland
Panc	Pancreas
Skin	Skin
SI	Small intestine
Spl	Spleen
Test	Testes
Thym	Thymus
Tong	Tongue
Trac	Trachea
Ute	Uterus

Gastrin-releasing peptide (GRP)
- epithelial/neural/germ

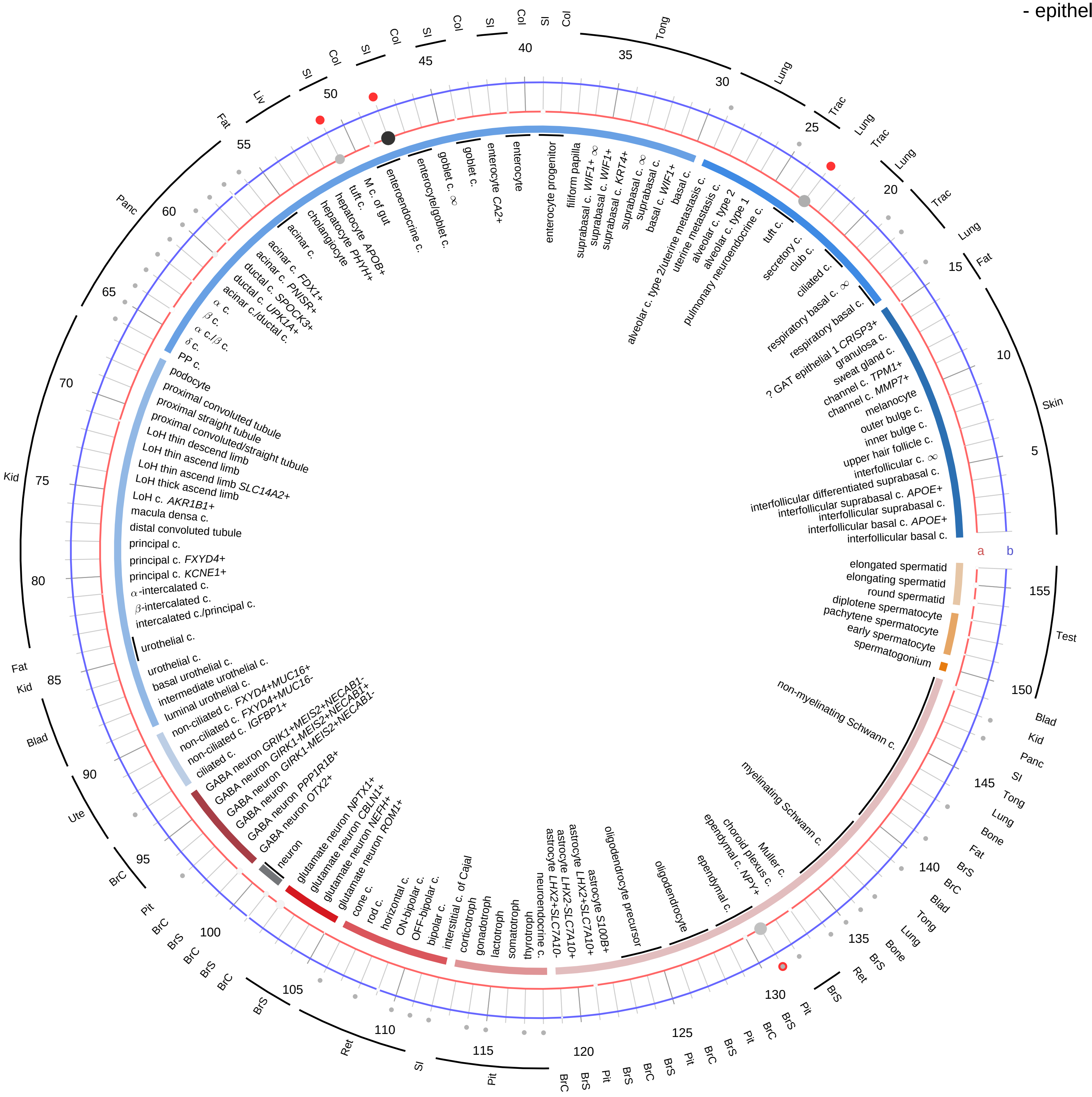

Ligand/Enzyme
a - GRP
Receptor
b - GRPR

PAIRING (ligand → receptor)

GRP→GRPR

Compartment
epithelial
- integumentary
- respiratory
- gastrointestinal
- urinary
- reproductive

neural
- GABAergic
- hybrid
- glutamatergic
- specialized
- neuroendocrine
- glia

germ
- spermatogonium
- spermatocyte
- spermatid

12.5%
25%
50%
100%

ln(CP10K+1): 0, 1, 2, 3

Abbrev.	Tissue
Aort	Aorta
Blad	Bladder
Blo	Blood
Bone	Bone
BM	Bone marrow
BrC	Brain cortex
BrS	Brainstem
Col	Colon
Dia	Diaphragm
Ret	Eye retina
Fat	Fat
Hrt	Heart
Pit	Hypothalamus/Pituitary
Kid	Kidney
LM	Limb muscle
Liv	Liver
Lung	Lung
MG	Mammary gland
Panc	Pancreas
Skin	Skin
SI	Small intestine
Spl	Spleen
Test	Testes
Thym	Thymus
Tong	Tongue
Trac	Trachea
Ute	Uterus

Gastrin-releasing peptide (GRP)
- endothelial

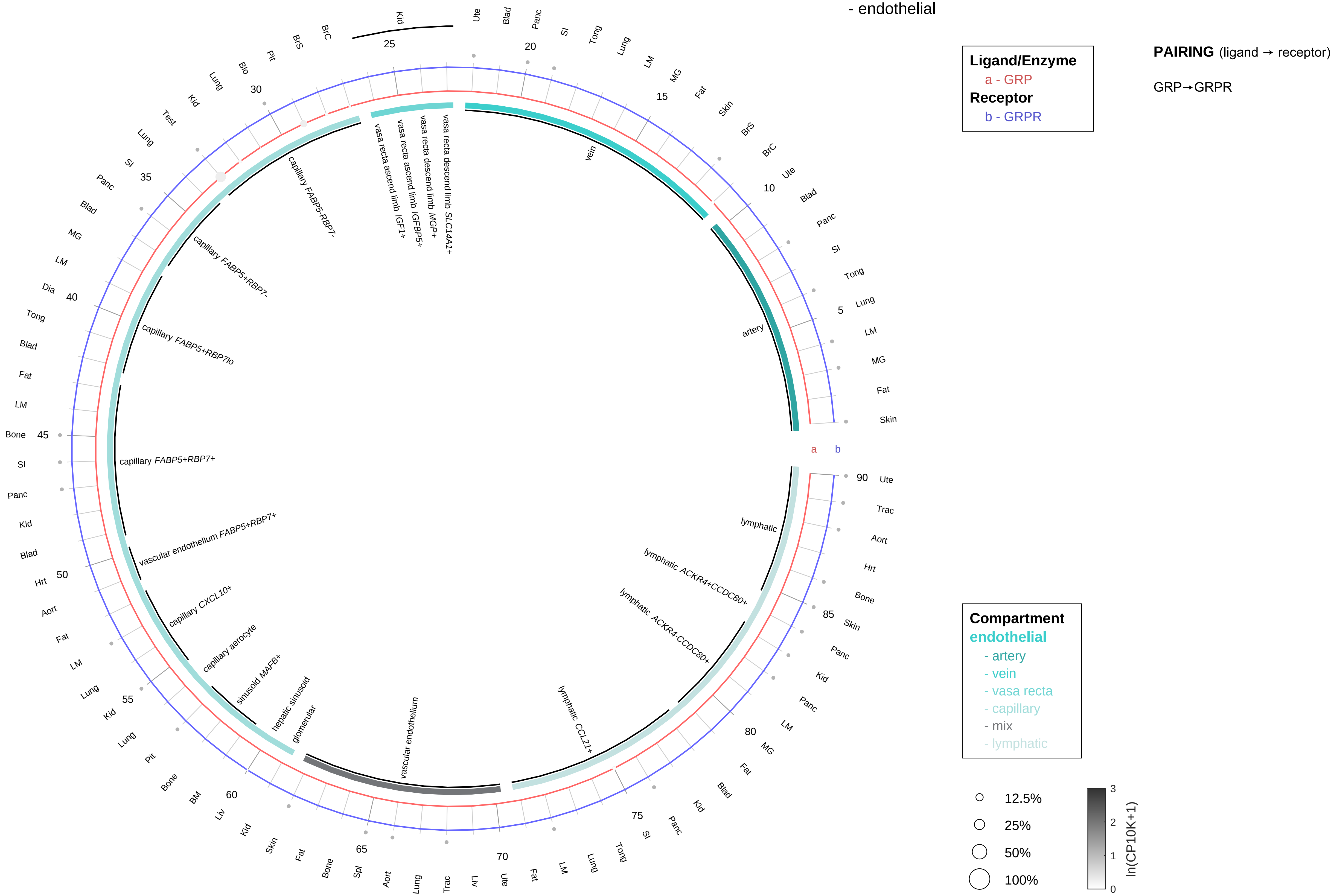

Abbrev.	Tissue
Aort	Aorta
Blad	Bladder
Blo	Blood
Bone	Bone
BM	Bone marrow
BrC	Brain cortex
BrS	Brainstem
Col	Colon
Dia	Diaphragm
Ret	Eye retina
Fat	Fat
Hrt	Heart
Pit	Hypothalamus/Pituitary
Kid	Kidney
LM	Limb muscle
Liv	Liver
Lung	Lung
MG	Mammary gland
Panc	Pancreas
Skin	Skin
SI	Small intestine
Spl	Spleen
Test	Testes
Thym	Thymus
Tong	Tongue
Trac	Trachea
Ute	Uterus

Gastrin-releasing peptide (GRP)
- stromal

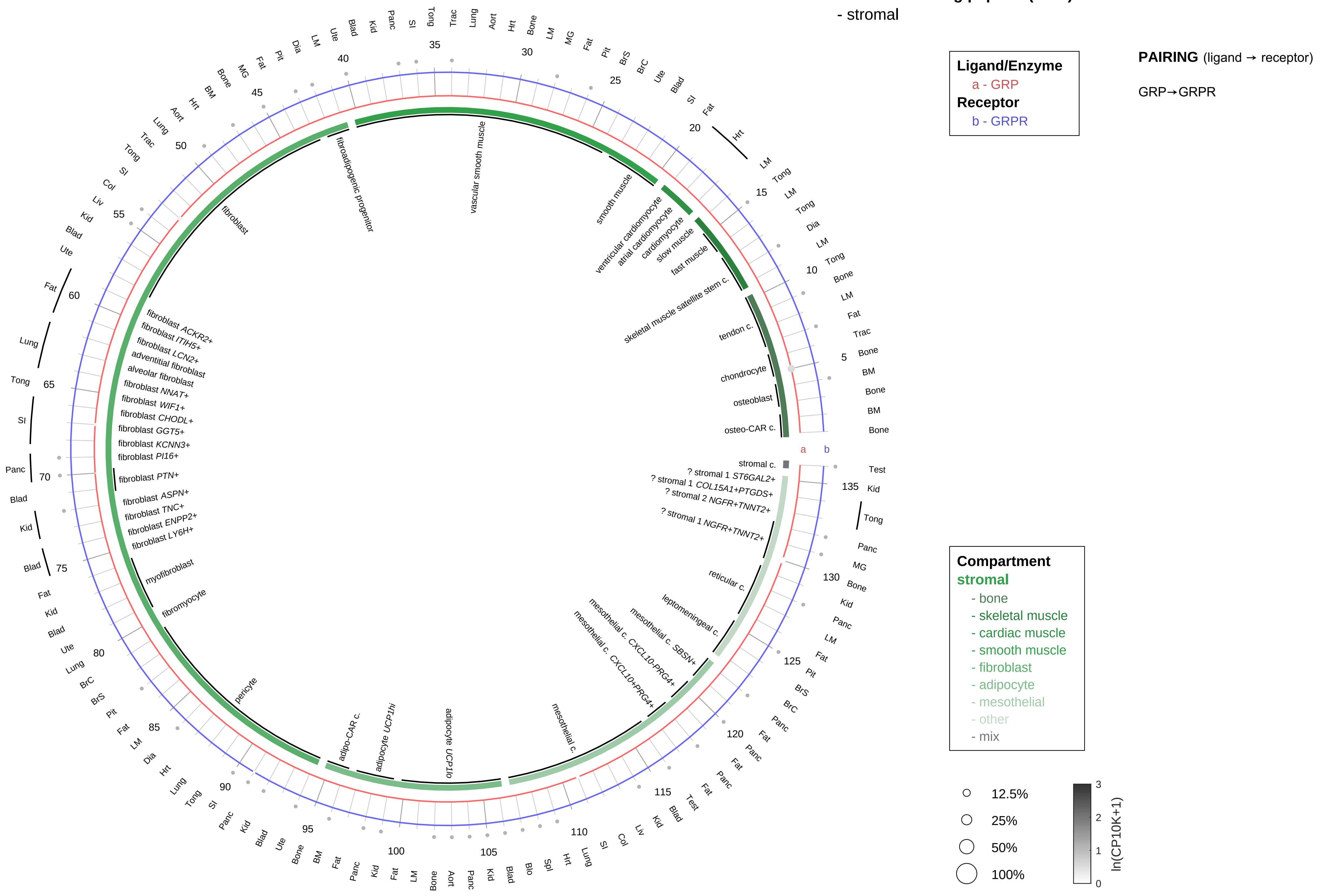

Abbrev.	Tissue
Aort	Aorta
Blad	Bladder
Blo	Blood
Bone	Bone
BM	Bone marrow
BrC	Brain cortex
BrS	Brainstem
Col	Colon
Dia	Diaphragm
Ret	Eye retina
Fat	Fat
Hrt	Heart
Pit	Hypothalamus/Pituitary
Kid	Kidney
LM	Limb muscle
Liv	Liver
Lung	Lung
MG	Mammary gland
Panc	Pancreas
Skin	Skin
SI	Small intestine
Spl	Spleen
Test	Testes
Thym	Thymus
Tong	Tongue
Trac	Trachea
Ute	Uterus

Gastrin-releasing peptide (GRP)
- lymphoid

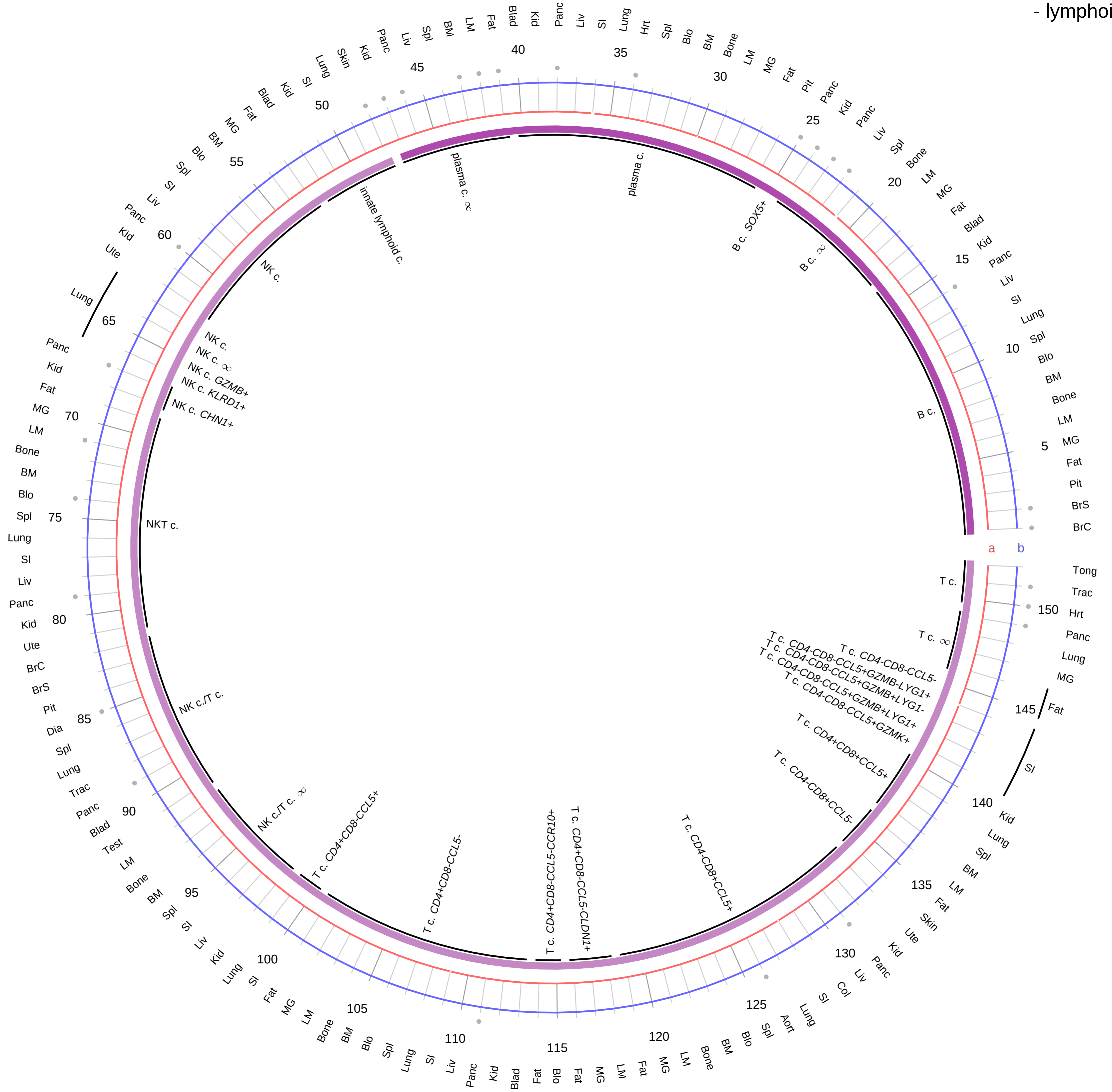

Ligand/Enzyme
a - GRP
Receptor
b - GRPR

PAIRING (ligand → receptor)

GRP→GRPR

Compartment
lymphoid
- B cell
- NK/T cell

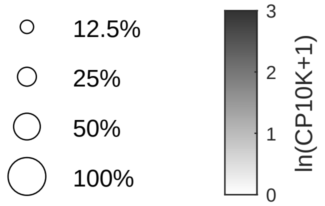

Abbrev.	Tissue
Aort	Aorta
Blad	Bladder
Blo	Blood
Bone	Bone
BM	Bone marrow
BrC	Brain cortex
BrS	Brainstem
Col	Colon
Dia	Diaphragm
Ret	Eye retina
Fat	Fat
Hrt	Heart
Pit	Hypothalamus/Pituitary
Kid	Kidney
LM	Limb muscle
Liv	Liver
Lung	Lung
MG	Mammary gland
Panc	Pancreas
Skin	Skin
SI	Small intestine
Spl	Spleen
Test	Testes
Thym	Thymus
Tong	Tongue
Trac	Trachea
Ute	Uterus

Gastrin-releasing peptide (GRP)
- myeloid

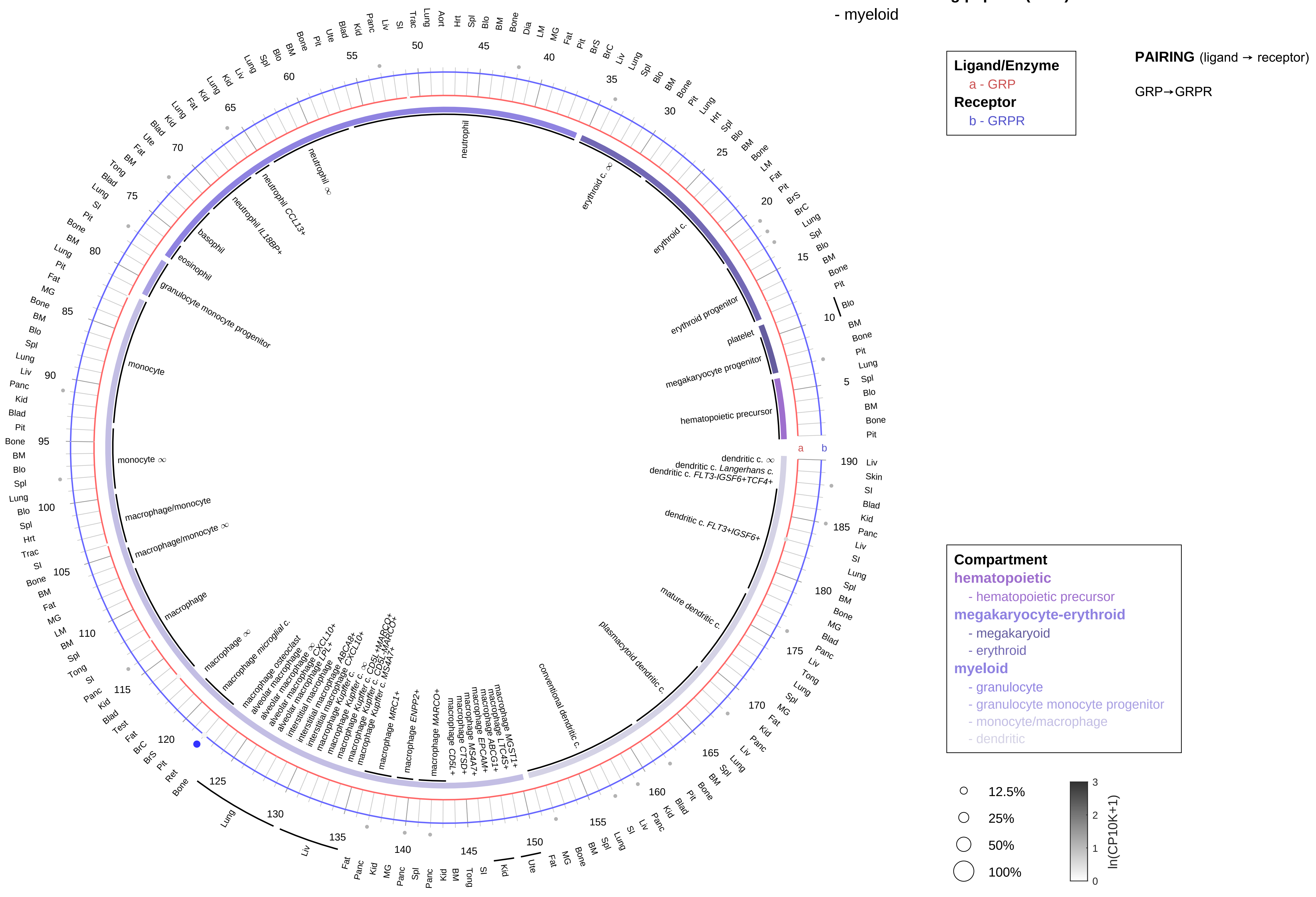

Abbrev.	Tissue
Aort	Aorta
Blad	Bladder
Blo	Blood
Bone	Bone
BM	Bone marrow
BrC	Brain cortex
BrS	Brainstem
Col	Colon
Dia	Diaphragm
Ret	Eye retina
Fat	Fat
Hrt	Heart
Pit	Hypothalamus/Pituitary
Kid	Kidney
LM	Limb muscle
Liv	Liver
Lung	Lung
MG	Mammary gland
Panc	Pancreas
Skin	Skin
SI	Small intestine
Spl	Spleen
Test	Testes
Thym	Thymus
Tong	Tongue
Trac	Trachea
Ute	Uterus

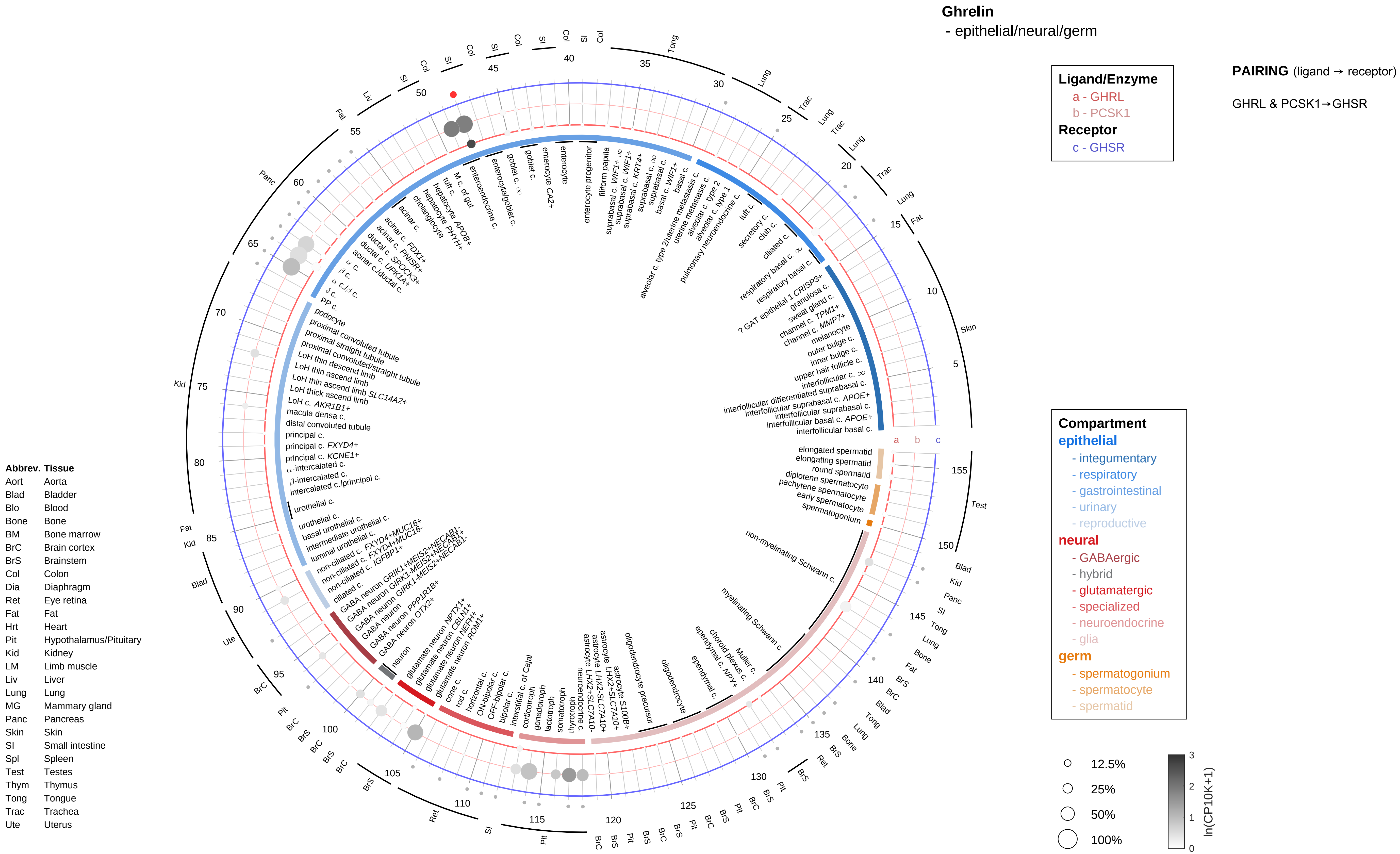

Ghrelin
- epithelial/neural/germ
Ligand/Enzyme
a - GHRL
b - PCSK1
Receptor
c - GHSR
PAIRING (ligand → receptor)
GHRL & PCSK1→GHSR
Compartment
epithelial
- integumentary
- respiratory
- gastrointestinal
- urinary
- reproductive
neural
- GABAergic
- hybrid
- glutamatergic
- specialized
- neuroendocrine
- glia
germ
- spermatogonium
- spermatocyte
- spermatid
12.5%
25%
50%
100%
ln(CP10K+1)
Abbrev. Tissue
Aort Aorta
Blad Bladder
Blo Blood
Bone Bone
BM Bone marrow
BrC Brain cortex
BrS Brainstem
Col Colon
Dia Diaphragm
Ret Eye retina
Fat Fat
Hrt Heart
Pit Hypothalamus/Pituitary
Kid Kidney
LM Limb muscle
Liv Liver
Lung Lung
MG Mammary gland
Panc Pancreas
Skin Skin
SI Small intestine
Spl Spleen
Test Testes
Thym Thymus
Tong Tongue
Trac Trachea
Ute Uterus

Ghrelin
- endothelial

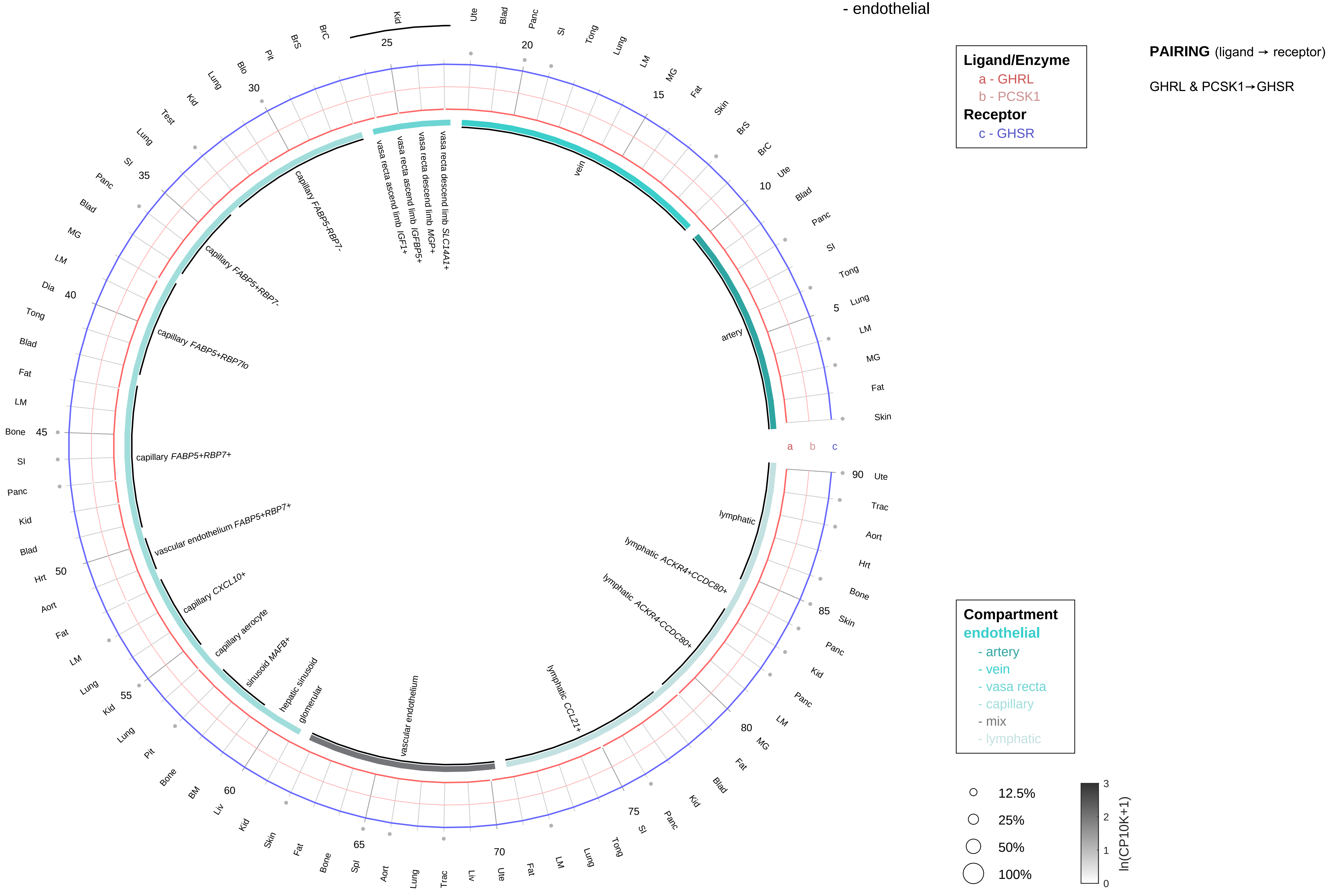

Abbrev.	Tissue
Aort	Aorta
Blad	Bladder
Blo	Blood
Bone	Bone
BM	Bone marrow
BrC	Brain cortex
BrS	Brainstem
Col	Colon
Dia	Diaphragm
Ret	Eye retina
Fat	Fat
Hrt	Heart
Pit	Hypothalamus/Pituitary
Kid	Kidney
LM	Limb muscle
Liv	Liver
Lung	Lung
MG	Mammary gland
Panc	Pancreas
Skin	Skin
SI	Small intestine
Spl	Spleen
Test	Testes
Thym	Thymus
Tong	Tongue
Trac	Trachea
Ute	Uterus

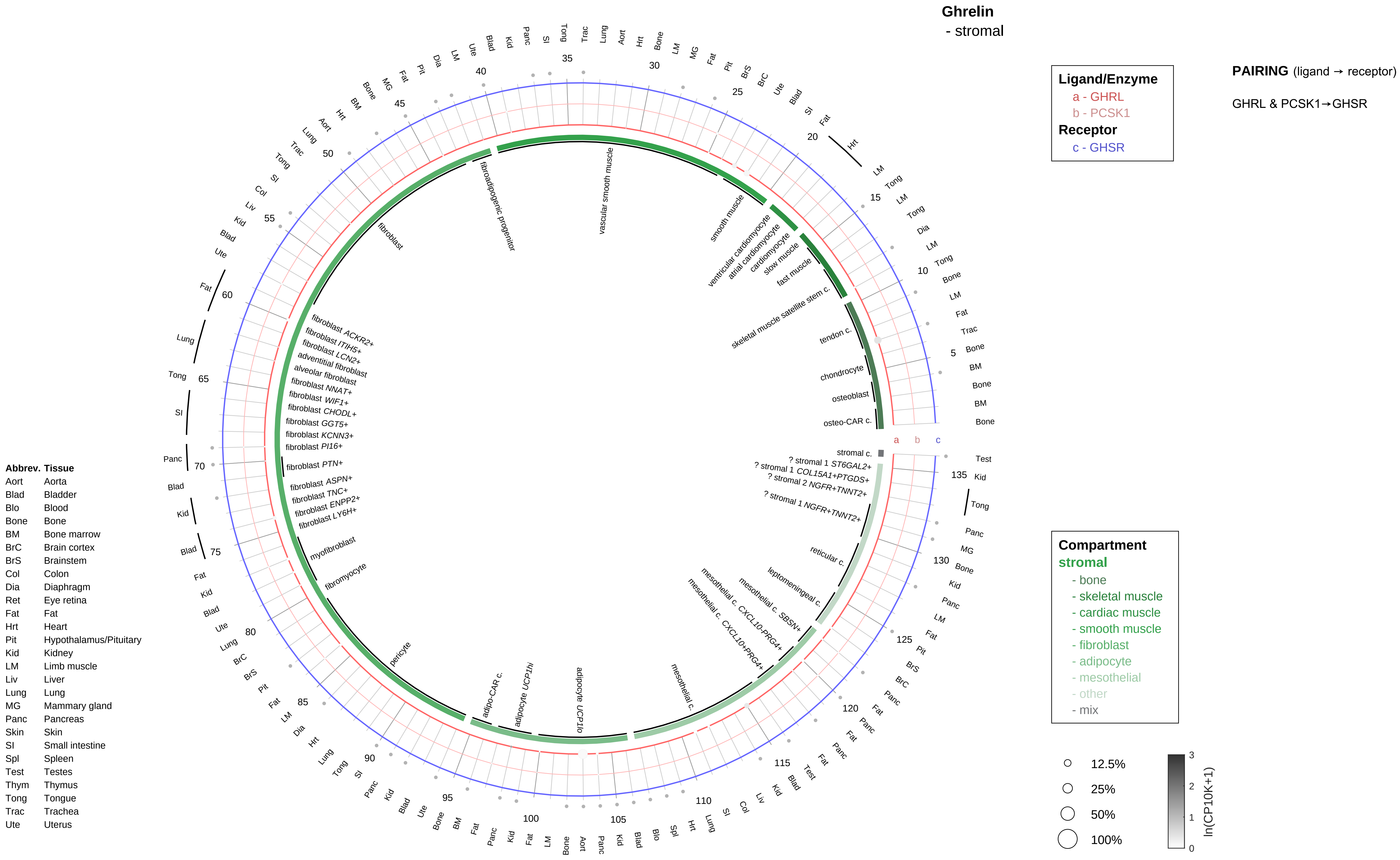
Ghrelin
- stromal
Ligand/Enzyme
a - GHRL
b - PCSK1
Receptor
c - GHSR
PAIRING (ligand → receptor)
GHRL & PCSK1→GHSR
Compartment
stromal
- bone
- skeletal muscle
- cardiac muscle
- smooth muscle
- fibroblast
- adipocyte
- mesothelial
- other
- mix
12.5%
25%
50%
100%
ln(CP10K+1)
Abbrev. Tissue
Aort Aorta
Blad Bladder
Blo Blood
Bone Bone
BM Bone marrow
BrC Brain cortex
BrS Brainstem
Col Colon
Dia Diaphragm
Ret Eye retina
Fat Fat
Hrt Heart
Pit Hypothalamus/Pituitary
Kid Kidney
LM Limb muscle
Liv Liver
Lung Lung
MG Mammary gland
Panc Pancreas
Skin Skin
SI Small intestine
Spl Spleen
Test Testes
Thym Thymus
Tong Tongue
Trac Trachea
Ute Uterus
osteo-CAR c.
osteoblast
chondrocyte
tendon c.
skeletal muscle satellite stem c.
fast muscle
slow muscle
cardiomyocyte
atrial cardiomyocyte
ventricular cardiomyocyte
smooth muscle
vascular smooth muscle
fibroadipogenic progenitor
fibroblast
fibroblast ACKR2+
fibroblast ITIH5+
fibroblast LCN2+
adventitial fibroblast
alveolar fibroblast
fibroblast NNAT+
fibroblast WIF1+
fibroblast CHODL+
fibroblast GGT5+
fibroblast KCNN3+
fibroblast PI16+
fibroblast PTN+
fibroblast ASPN+
fibroblast TNC+
fibroblast ENPP2+
fibroblast LY6H+
myofibroblast
fibromyocyte
pericyte
adipo-CAR c.
adipocyte UCP1hi
adipocyte UCP1lo
mesothelial c.
mesothelial c. CXCL10+PRG4+
mesothelial c. CXCL10-PRG4+
mesothelial c. SBSN+
leptomeningeal c.
reticular c.
? stromal 1 NGFR+TNNT2+
? stromal 2 NGFR+TNNT2+
? stromal 1 COL15A1+PTGDS+
? stromal 1 ST6GAL2+
stromal c.

Ghrelin
- lymphoid

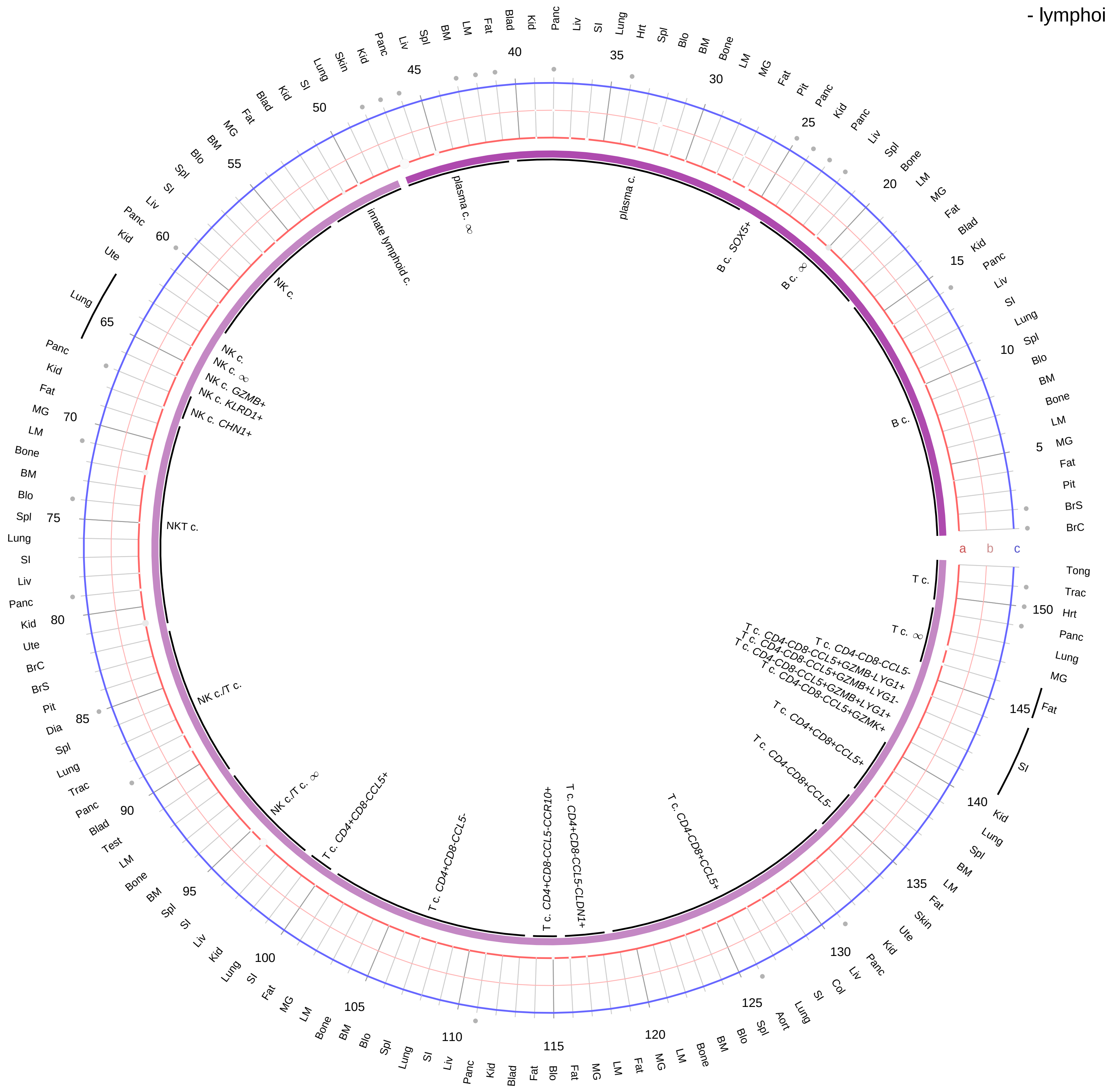

Ligand/Enzyme
- a - GHRL
- b - PCSK1

Receptor
- c - GHSR

PAIRING (ligand → receptor)

GHRL & PCSK1→GHSR

Compartment
lymphoid
- B cell
- NK/T cell

12.5%
25%
50%
100%

ln(CP10K+1): 0, 1, 2, 3

Abbrev.	Tissue
Aort	Aorta
Blad	Bladder
Blo	Blood
Bone	Bone
BM	Bone marrow
BrC	Brain cortex
BrS	Brainstem
Col	Colon
Dia	Diaphragm
Ret	Eye retina
Fat	Fat
Hrt	Heart
Pit	Hypothalamus/Pituitary
Kid	Kidney
LM	Limb muscle
Liv	Liver
Lung	Lung
MG	Mammary gland
Panc	Pancreas
Skin	Skin
SI	Small intestine
Spl	Spleen
Test	Testes
Thym	Thymus
Tong	Tongue
Trac	Trachea
Ute	Uterus

Ghrelin
- myeloid

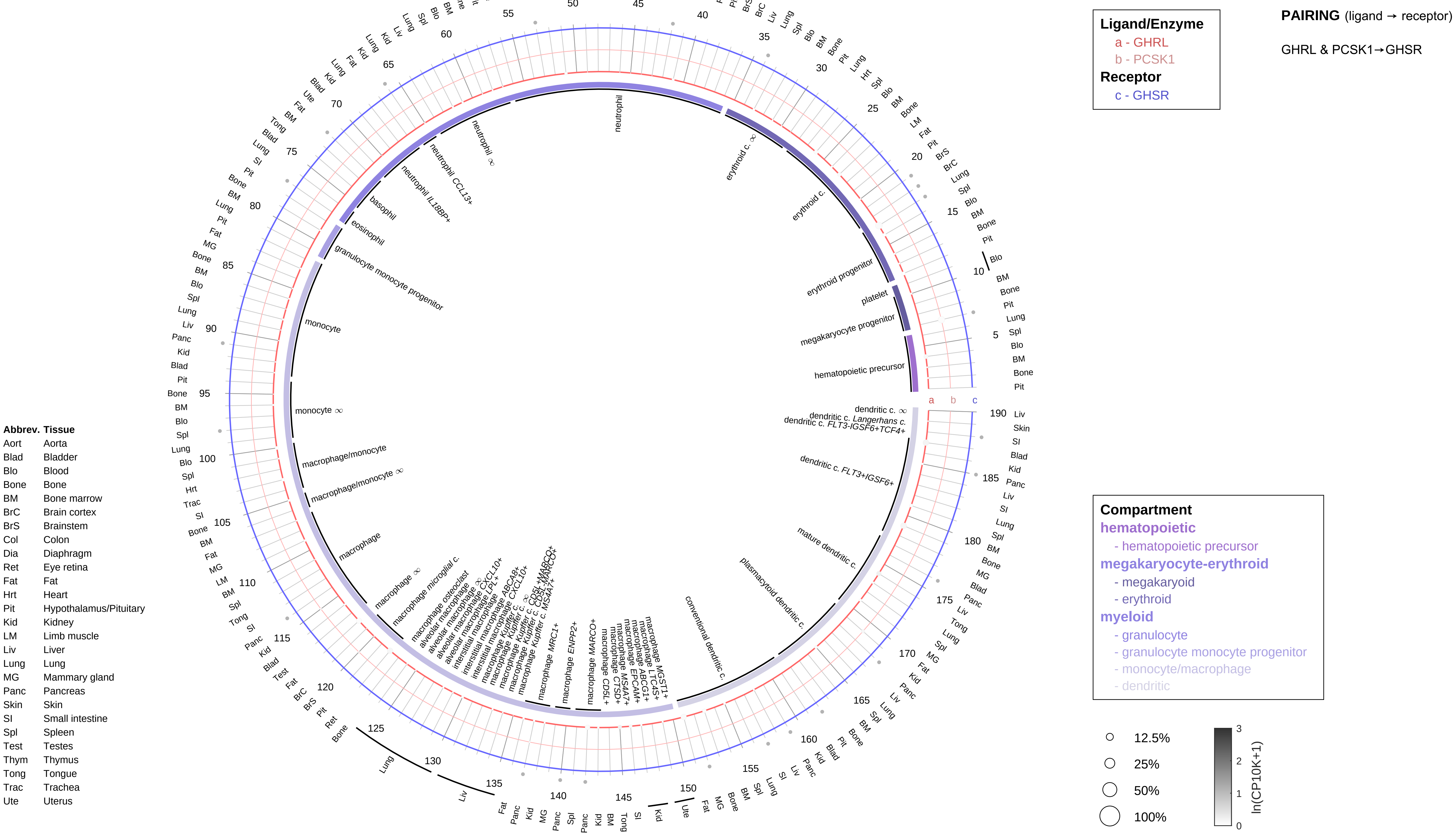

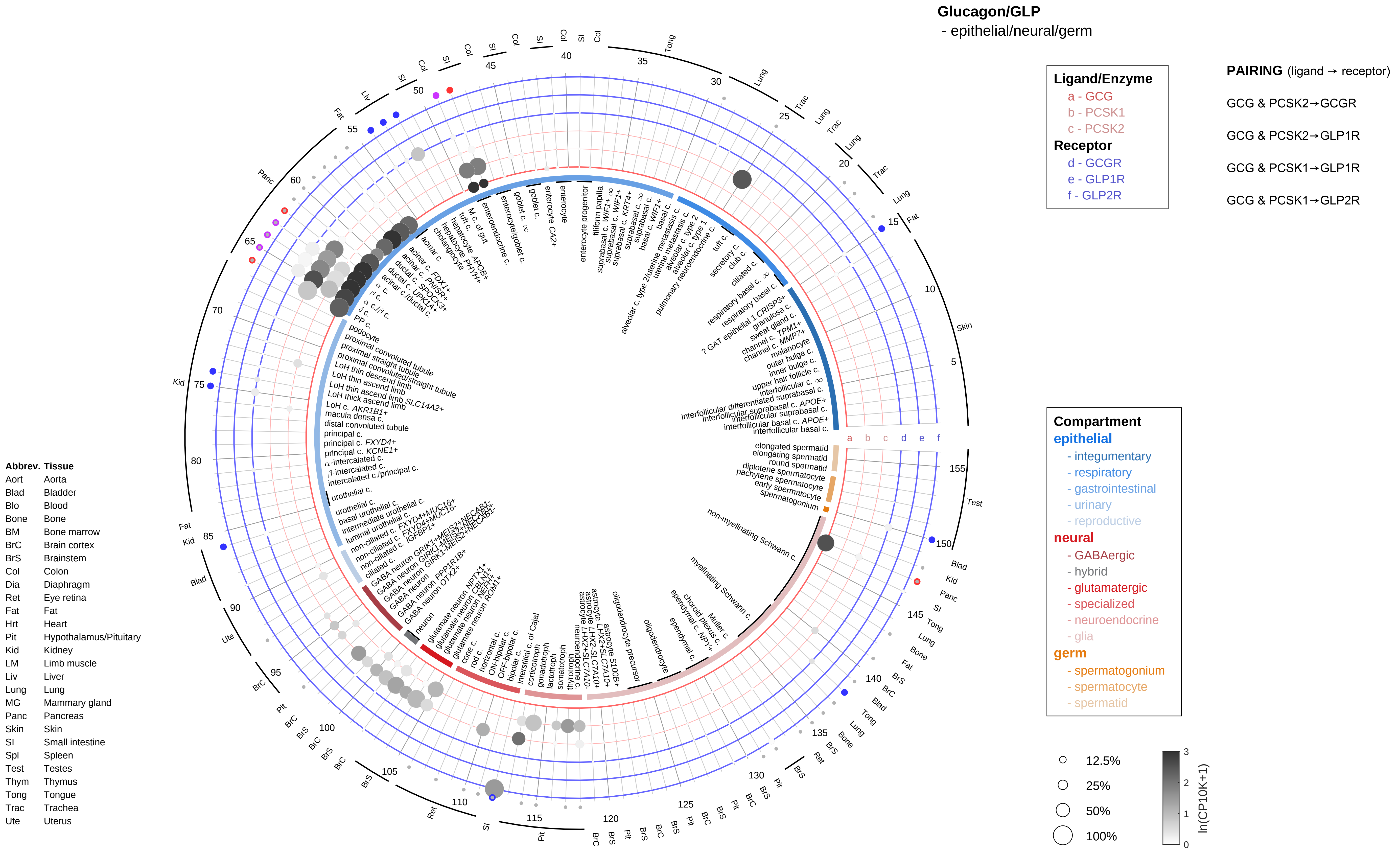
Glucagon/GLP
- epithelial/neural/germ
Ligand/Enzyme
a - GCG
b - PCSK1
c - PCSK2
Receptor
d - GCGR
e - GLP1R
f - GLP2R
PAIRING (ligand → receptor)
GCG & PCSK2→GCGR
GCG & PCSK2→GLP1R
GCG & PCSK1→GLP1R
GCG & PCSK1→GLP2R
Compartment
epithelial
- integumentary
- respiratory
- gastrointestinal
- urinary
- reproductive
neural
- GABAergic
- hybrid
- glutamatergic
- specialized
- neuroendocrine
- glia
germ
- spermatogonium
- spermatocyte
- spermatid
12.5%
25%
50%
100%
ln(CP10K+1)
Abbrev. Tissue
Aort Aorta
Blad Bladder
Blo Blood
Bone Bone
BM Bone marrow
BrC Brain cortex
BrS Brainstem
Col Colon
Dia Diaphragm
Ret Eye retina
Fat Fat
Hrt Heart
Pit Hypothalamus/Pituitary
Kid Kidney
LM Limb muscle
Liv Liver
Lung Lung
MG Mammary gland
Panc Pancreas
Skin Skin
SI Small intestine
Spl Spleen
Test Testes
Thym Thymus
Tong Tongue
Trac Trachea
Ute Uterus

Glucagon/GLP
- endothelial

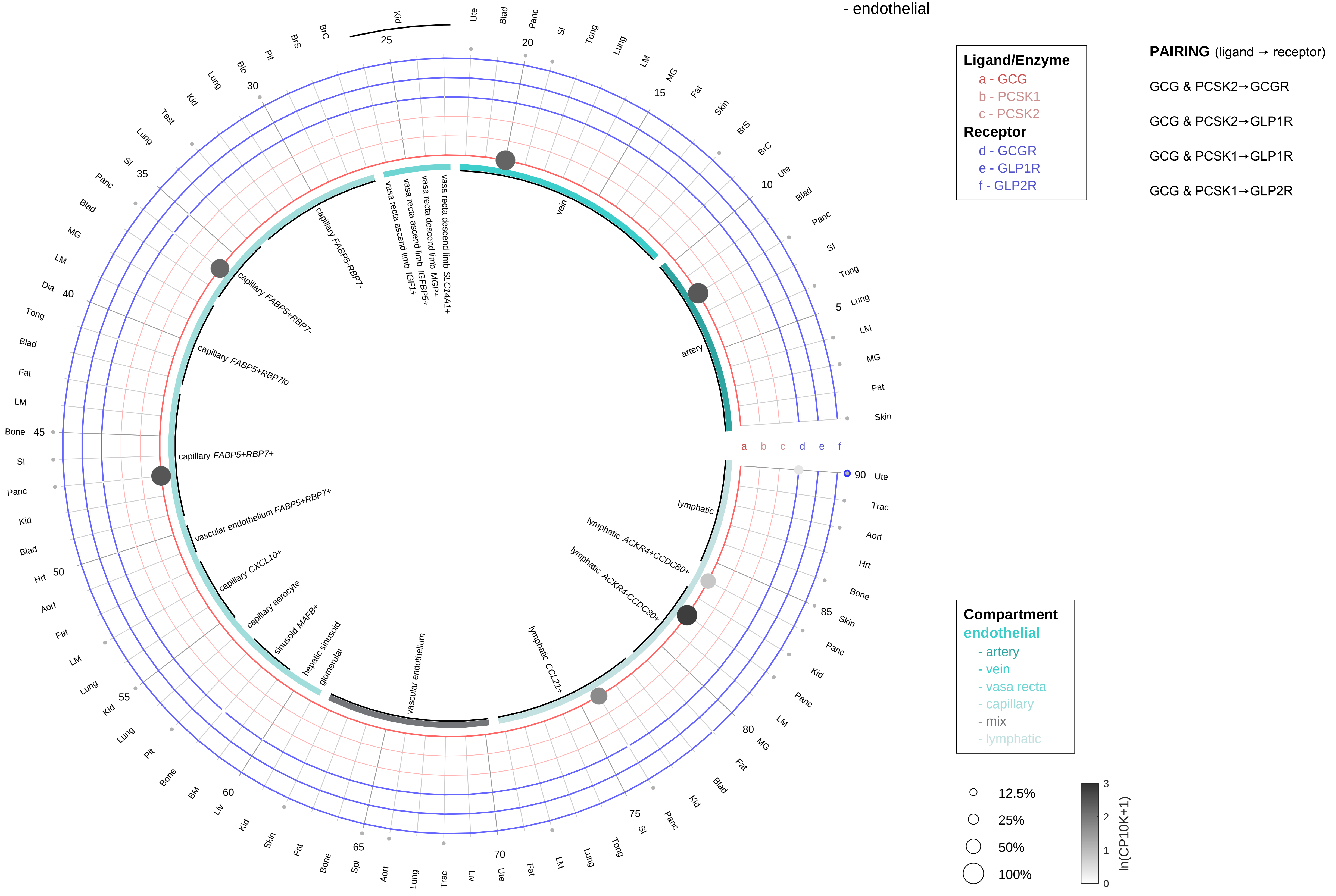

Abbrev.	Tissue
Aort	Aorta
Blad	Bladder
Blo	Blood
Bone	Bone
BM	Bone marrow
BrC	Brain cortex
BrS	Brainstem
Col	Colon
Dia	Diaphragm
Ret	Eye retina
Fat	Fat
Hrt	Heart
Pit	Hypothalamus/Pituitary
Kid	Kidney
LM	Limb muscle
Liv	Liver
Lung	Lung
MG	Mammary gland
Panc	Pancreas
Skin	Skin
SI	Small intestine
Spl	Spleen
Test	Testes
Thym	Thymus
Tong	Tongue
Trac	Trachea
Ute	Uterus

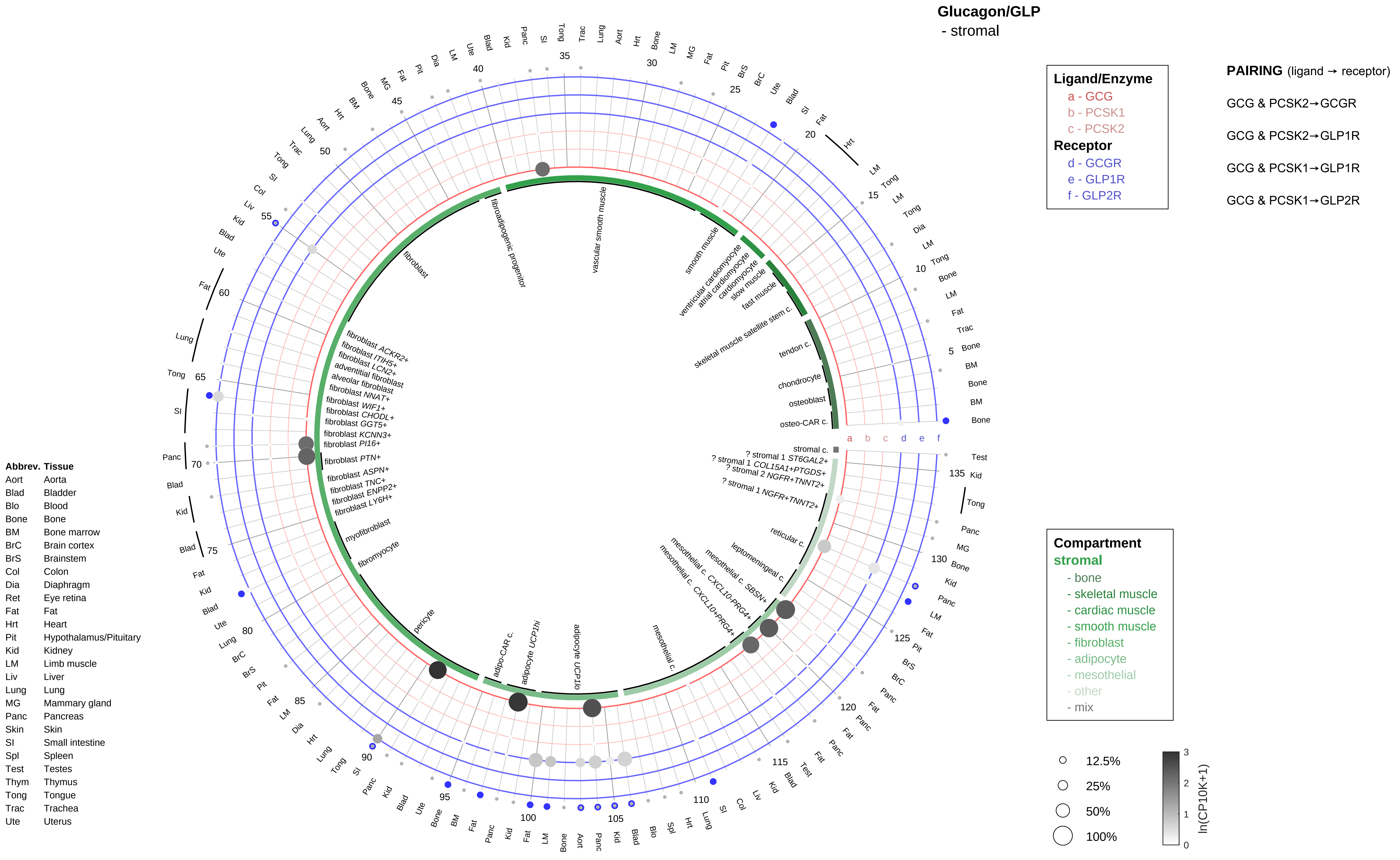
Glucagon/GLP
- stromal
Ligand/Enzyme
a - GCG
b - PCSK1
c - PCSK2
Receptor
d - GCGR
e - GLP1R
f - GLP2R
PAIRING (ligand → receptor)
GCG & PCSK2→GCGR
GCG & PCSK2→GLP1R
GCG & PCSK1→GLP1R
GCG & PCSK1→GLP2R
Compartment
stromal
- bone
- skeletal muscle
- cardiac muscle
- smooth muscle
- fibroblast
- adipocyte
- mesothelial
- other
- mix
12.5%
25%
50%
100%
ln(CP10K+1)
Abbrev. Tissue
Aort Aorta
Blad Bladder
Blo Blood
Bone Bone
BM Bone marrow
BrC Brain cortex
BrS Brainstem
Col Colon
Dia Diaphragm
Ret Eye retina
Fat Fat
Hrt Heart
Pit Hypothalamus/Pituitary
Kid Kidney
LM Limb muscle
Liv Liver
Lung Lung
MG Mammary gland
Panc Pancreas
Skin Skin
SI Small intestine
Spl Spleen
Test Testes
Thym Thymus
Tong Tongue
Trac Trachea
Ute Uterus
fibroblast
fibroadipogenic progenitor
vascular smooth muscle
smooth muscle
ventricular cardiomyocyte
atrial cardiomyocyte
cardiomyocyte
slow muscle
fast muscle
skeletal muscle satellite stem c.
tendon c.
chondrocyte
osteoblast
osteo-CAR c.
stromal c.
? stromal 1 ST6GAL2+
? stromal 1 COL15A1+PTGDS+
? stromal 2 NGFR+TNNT2+
? stromal 1 NGFR+TNNT2+
reticular c.
leptomeningeal c.
mesothelial c. SBSN+
mesothelial c. CXCL10-PRG4+
mesothelial c. CXCL10+PRG4+
mesothelial c.
adipocyte UCP1lo
adipocyte UCP1hi
adipo-CAR c.
pericyte
fibromyocyte
myofibroblast
fibroblast LY6H+
fibroblast ENPP2+
fibroblast TNC+
fibroblast ASPN+
fibroblast PTN+
fibroblast PI16+
fibroblast KCNN3+
fibroblast GGT5+
fibroblast CHODL+
fibroblast WIF1+
fibroblast NNAT+
alveolar fibroblast
adventitial fibroblast
fibroblast LCN2+
fibroblast ITIH5+
fibroblast ACKR2+

Glucagon/GLP
- lymphoid

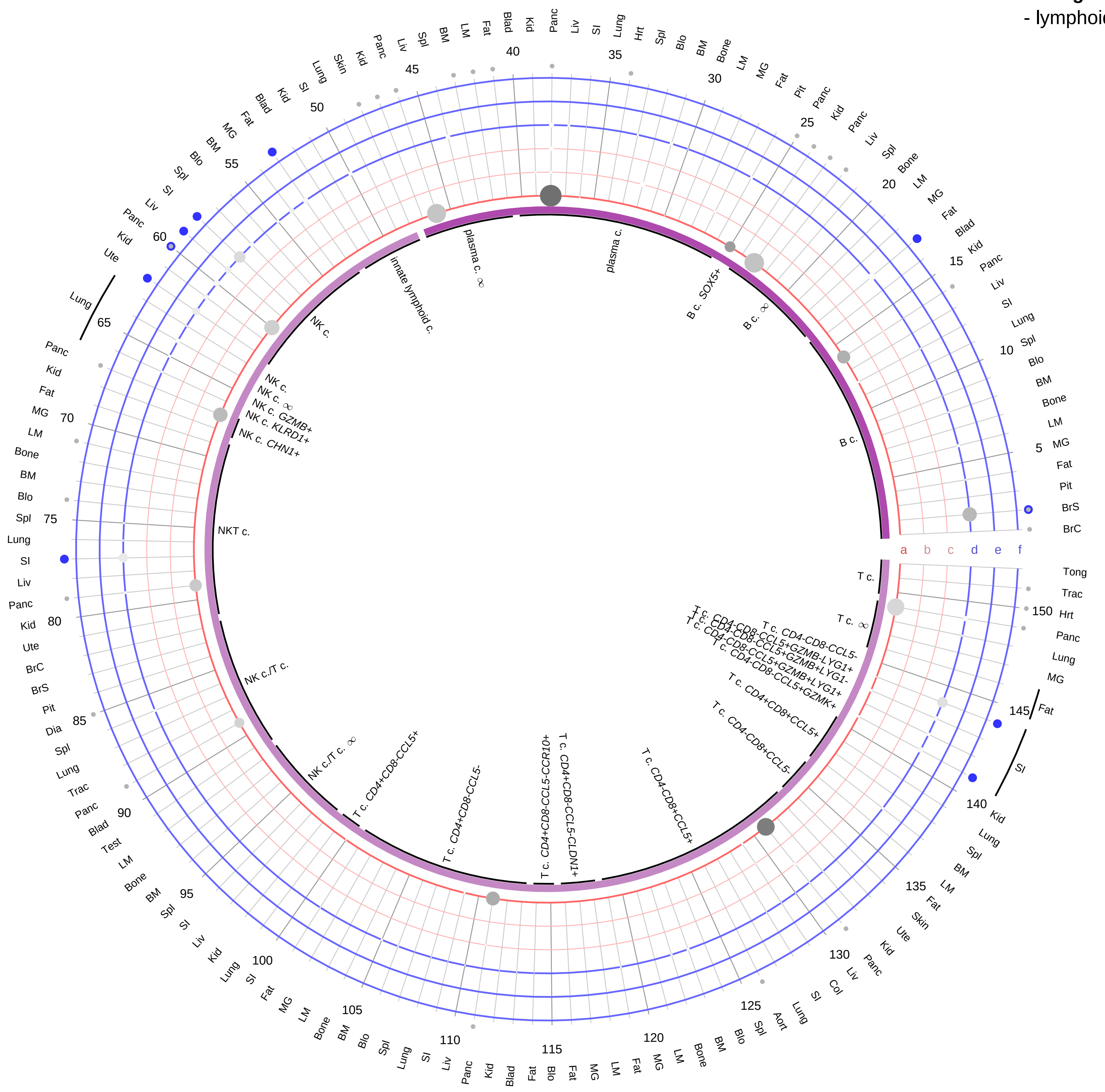

Ligand/Enzyme
- a - GCG
- b - PCSK1
- c - PCSK2

Receptor
- d - GCGR
- e - GLP1R
- f - GLP2R

PAIRING (ligand → receptor)

GCG & PCSK2→GCGR

GCG & PCSK2→GLP1R

GCG & PCSK1→GLP1R

GCG & PCSK1→GLP2R

Abbrev.	Tissue
Aort	Aorta
Blad	Bladder
Blo	Blood
Bone	Bone
BM	Bone marrow
BrC	Brain cortex
BrS	Brainstem
Col	Colon
Dia	Diaphragm
Ret	Eye retina
Fat	Fat
Hrt	Heart
Pit	Hypothalamus/Pituitary
Kid	Kidney
LM	Limb muscle
Liv	Liver
Lung	Lung
MG	Mammary gland
Panc	Pancreas
Skin	Skin
SI	Small intestine
Spl	Spleen
Test	Testes
Thym	Thymus
Tong	Tongue
Trac	Trachea
Ute	Uterus

Compartment
lymphoid
- B cell
- NK/T cell

12.5%
25%
50%
100%

ln(CP10K+1): 0 1 2 3

Glucagon/GLP
- myeloid

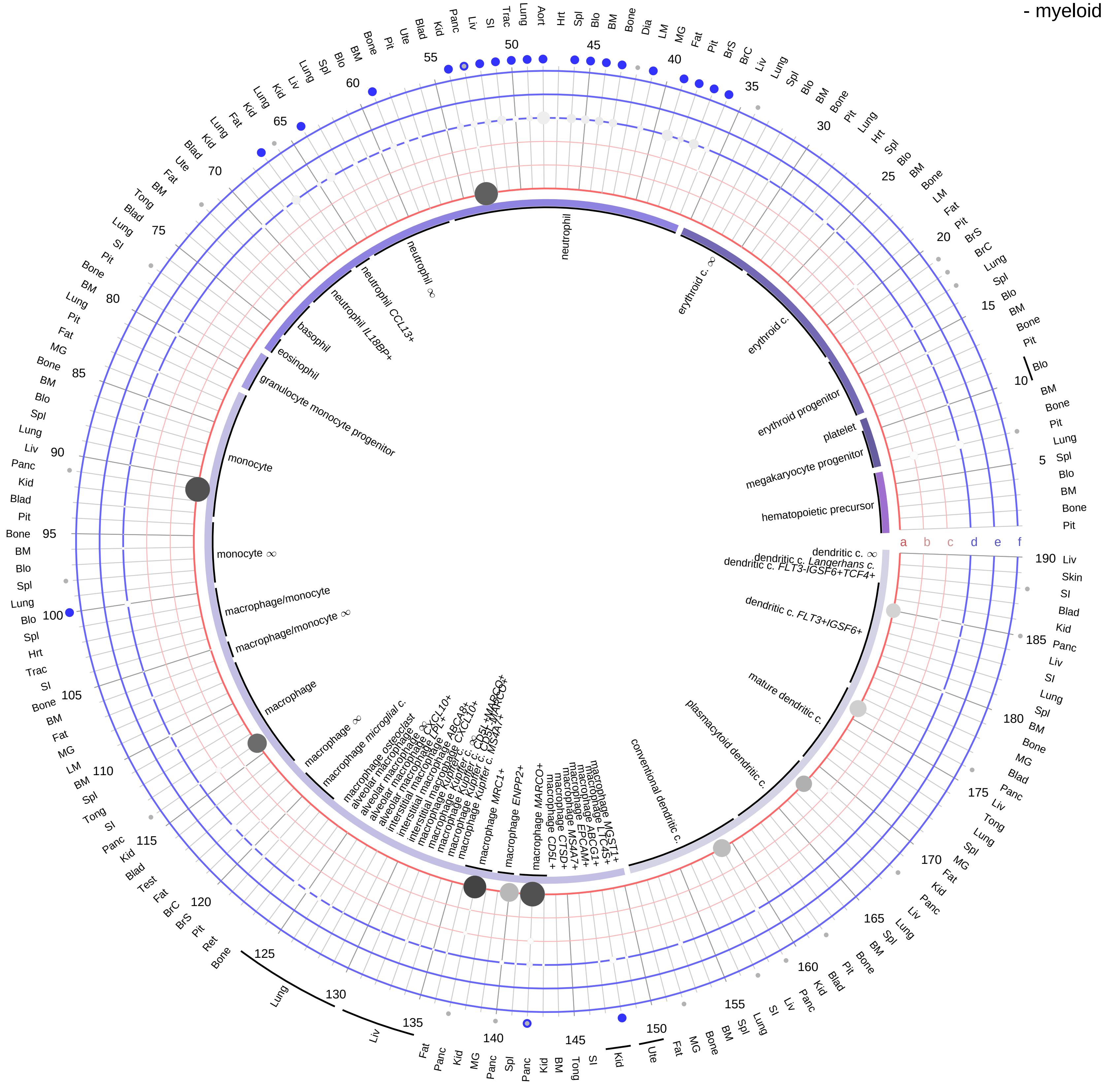

Ligand/Enzyme
- a - GCG
- b - PCSK1
- c - PCSK2

Receptor
- d - GCGR
- e - GLP1R
- f - GLP2R

PAIRING (ligand → receptor)

GCG & PCSK2→GCGR

GCG & PCSK2→GLP1R

GCG & PCSK1→GLP1R

GCG & PCSK1→GLP2R

Compartment
- **hematopoietic**
  - hematopoietic precursor
- **megakaryocyte-erythroid**
  - megakaryoid
  - erythroid
- **myeloid**
  - granulocyte
  - granulocyte monocyte progenitor
  - monocyte/macrophage
  - dendritic

12.5%
25%
50%
100%

ln(CP10K+1): 0, 1, 2, 3

Abbrev.	Tissue
Aort	Aorta
Blad	Bladder
Blo	Blood
Bone	Bone
BM	Bone marrow
BrC	Brain cortex
BrS	Brainstem
Col	Colon
Dia	Diaphragm
Ret	Eye retina
Fat	Fat
Hrt	Heart
Pit	Hypothalamus/Pituitary
Kid	Kidney
LM	Limb muscle
Liv	Liver
Lung	Lung
MG	Mammary gland
Panc	Pancreas
Skin	Skin
SI	Small intestine
Spl	Spleen
Test	Testes
Thym	Thymus
Tong	Tongue
Trac	Trachea
Ute	Uterus

Gonadotropin-releasing hormone (GnRH)
- epithelial/neural/germ

Ligand/Enzyme
- a - GNRH1
- b - GNRH2
- c - CPE
- d - PCSK1

Receptor
- e - GNRHR

PAIRING (ligand → receptor)

GNRH1 & CPE & PCSK1→GNRHR

GNRH2 & CPE & PCSK1→GNRHR

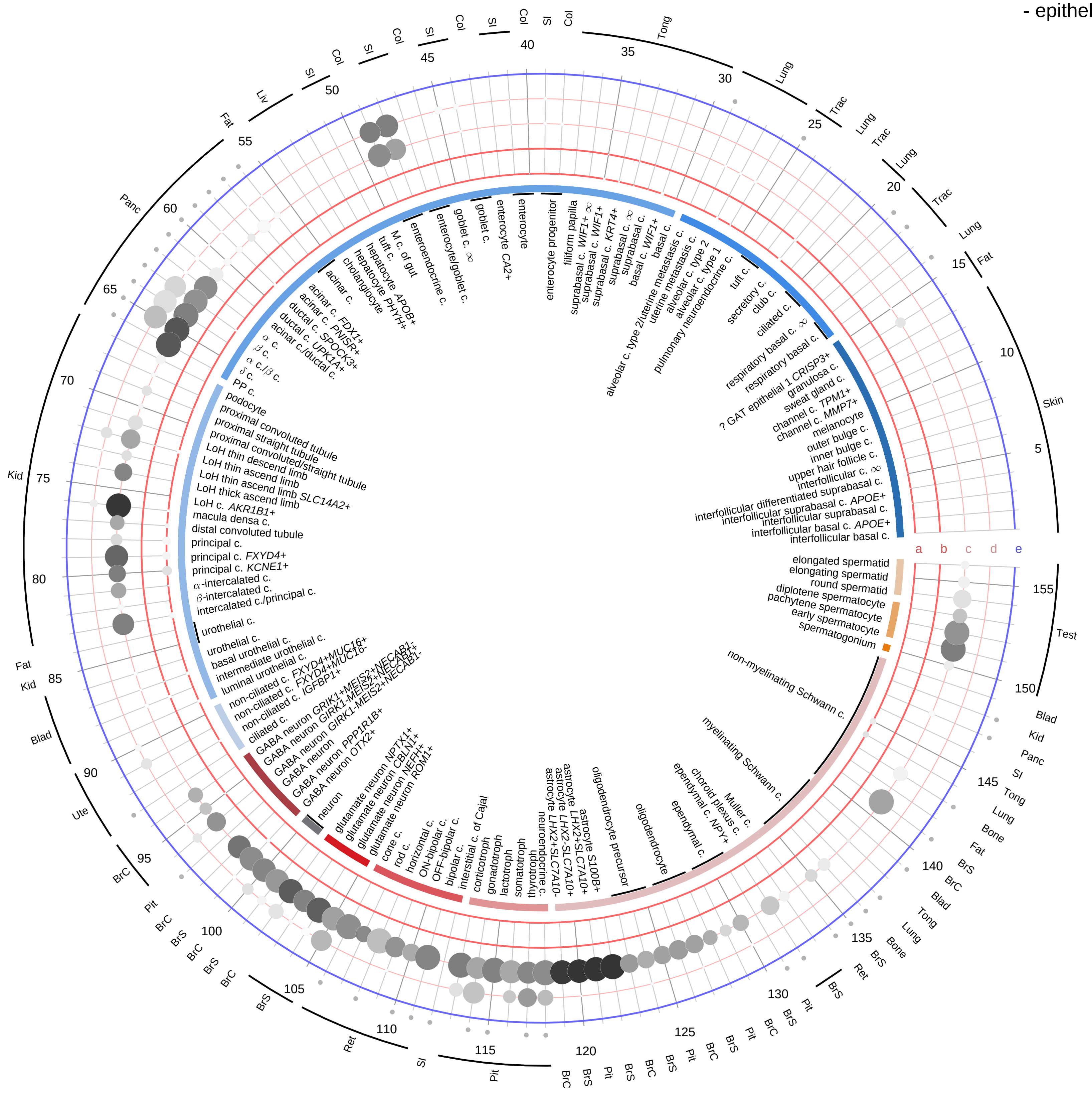

Compartment
- **epithelial**
  - integumentary
  - respiratory
  - gastrointestinal
  - urinary
  - reproductive
- **neural**
  - GABAergic
  - hybrid
  - glutamatergic
  - specialized
  - neuroendocrine
  - glia
- **germ**
  - spermatogonium
  - spermatocyte
  - spermatid

12.5%
25%
50%
100%

ln(CP10K+1): 0 1 2 3

Abbrev.	Tissue
Aort	Aorta
Blad	Bladder
Blo	Blood
Bone	Bone
BM	Bone marrow
BrC	Brain cortex
BrS	Brainstem
Col	Colon
Dia	Diaphragm
Ret	Eye retina
Fat	Fat
Hrt	Heart
Pit	Hypothalamus/Pituitary
Kid	Kidney
LM	Limb muscle
Liv	Liver
Lung	Lung
MG	Mammary gland
Panc	Pancreas
Skin	Skin
SI	Small intestine
Spl	Spleen
Test	Testes
Thym	Thymus
Tong	Tongue
Trac	Trachea
Ute	Uterus

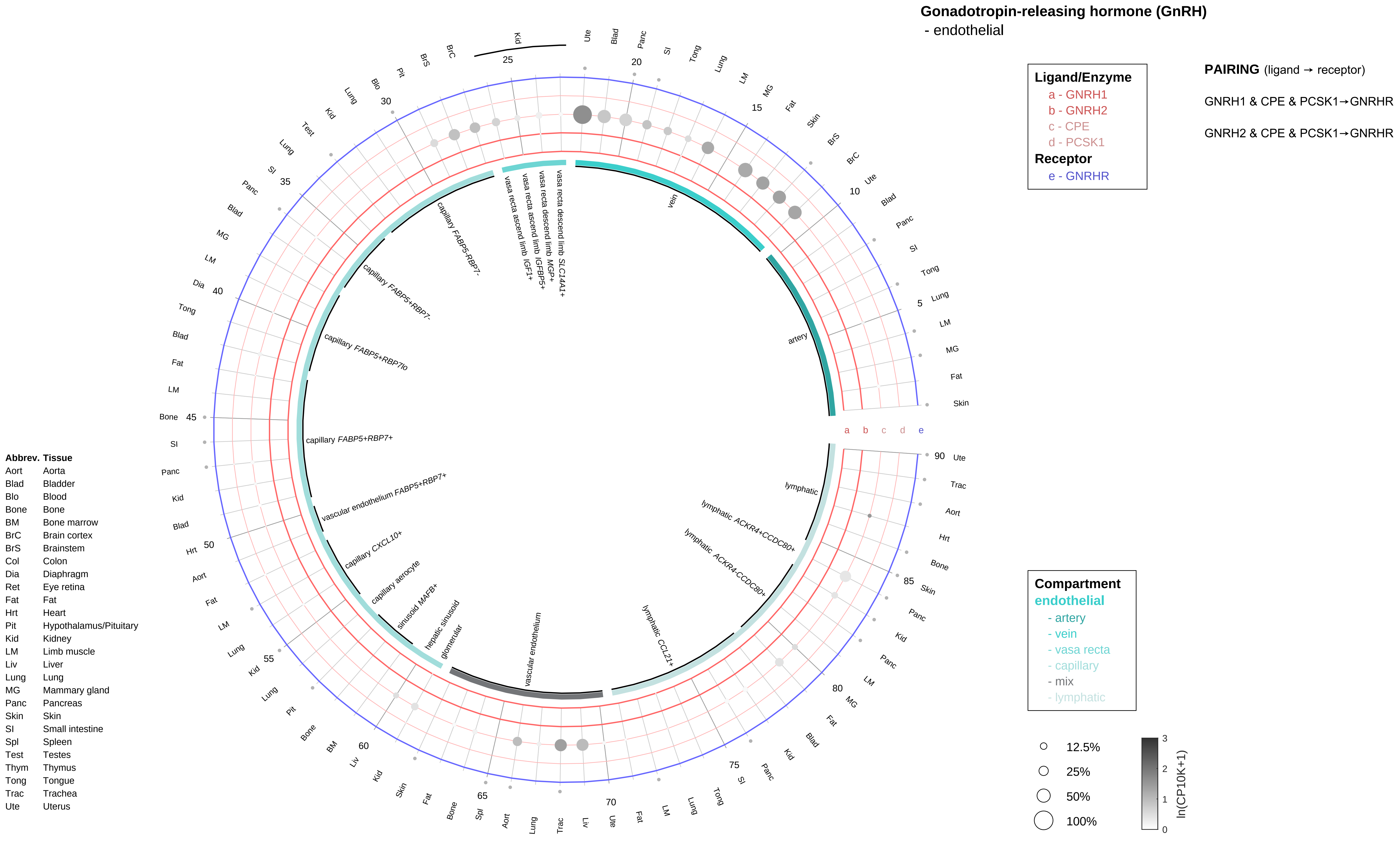

Gonadotropin-releasing hormone (GnRH)
- endothelial
Ligand/Enzyme
a - GNRH1
b - GNRH2
c - CPE
d - PCSK1
Receptor
e - GNRHR
PAIRING (ligand → receptor)
GNRH1 & CPE & PCSK1→GNRHR
GNRH2 & CPE & PCSK1→GNRHR
artery
vein
vasa recta descend limb SLC14A1+
vasa recta descend limb MGP+
vasa recta descend limb IGFBP5+
vasa recta ascend limb IGF1+
capillary FABP5-RBP7-
capillary FABP5+RBP7-
capillary FABP5+RBP7lo
capillary FABP5+RBP7+
vascular endothelium FABP5+RBP7+
capillary CXCL10+
capillary aerocyte
sinusoid MAFB+
hepatic sinusoid
glomerular
vascular endothelium
lymphatic CCL21+
lymphatic ACKR4-CCDC80+
lymphatic ACKR4+CCDC80+
lymphatic
a b c d e
Compartment
endothelial
- artery
- vein
- vasa recta
- capillary
- mix
- lymphatic
12.5%
25%
50%
100%
ln(CP10K+1)
0
1
2
3
Abbrev. Tissue
Aort Aorta
Blad Bladder
Blo Blood
Bone Bone
BM Bone marrow
BrC Brain cortex
BrS Brainstem
Col Colon
Dia Diaphragm
Ret Eye retina
Fat Fat
Hrt Heart
Pit Hypothalamus/Pituitary
Kid Kidney
LM Limb muscle
Liv Liver
Lung Lung
MG Mammary gland
Panc Pancreas
Skin Skin
SI Small intestine
Spl Spleen
Test Testes
Thym Thymus
Tong Tongue
Trac Trachea
Ute Uterus

Gonadotropin-releasing hormone (GnRH)
- stromal

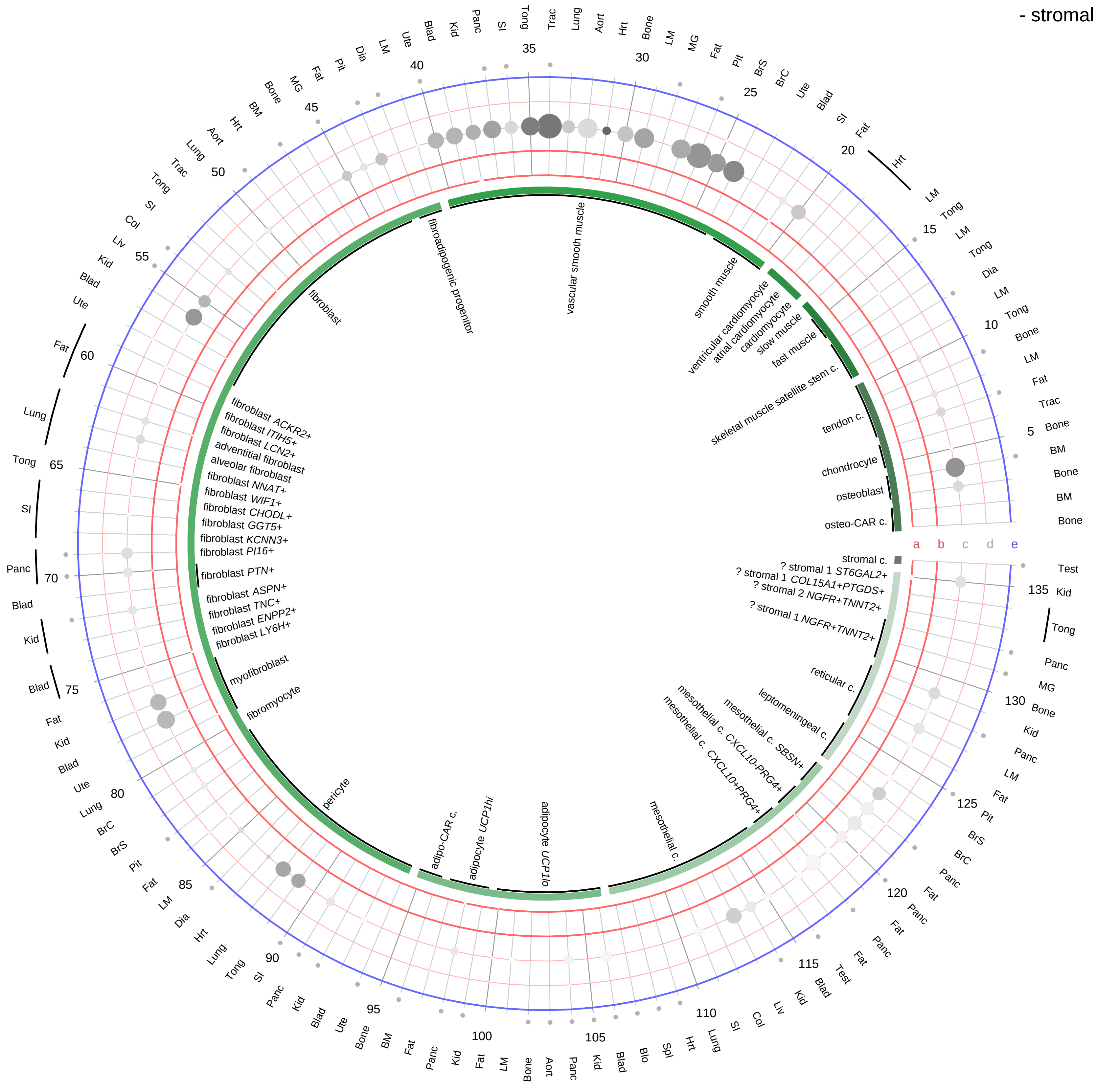

Ligand/Enzyme
- a - GNRH1
- b - GNRH2
- c - CPE
- d - PCSK1

Receptor
- e - GNRHR

PAIRING (ligand → receptor)

GNRH1 & CPE & PCSK1→GNRHR

GNRH2 & CPE & PCSK1→GNRHR

Compartment
stromal
- bone
- skeletal muscle
- cardiac muscle
- smooth muscle
- fibroblast
- adipocyte
- mesothelial
- other
- mix

12.5%
25%
50%
100%

ln(CP10K+1): 0, 1, 2, 3

Abbrev.	Tissue
Aort	Aorta
Blad	Bladder
Blo	Blood
Bone	Bone
BM	Bone marrow
BrC	Brain cortex
BrS	Brainstem
Col	Colon
Dia	Diaphragm
Ret	Eye retina
Fat	Fat
Hrt	Heart
Pit	Hypothalamus/Pituitary
Kid	Kidney
LM	Limb muscle
Liv	Liver
Lung	Lung
MG	Mammary gland
Panc	Pancreas
Skin	Skin
SI	Small intestine
Spl	Spleen
Test	Testes
Thym	Thymus
Tong	Tongue
Trac	Trachea
Ute	Uterus

Gonadotropin-releasing hormone (GnRH)
- lymphoid

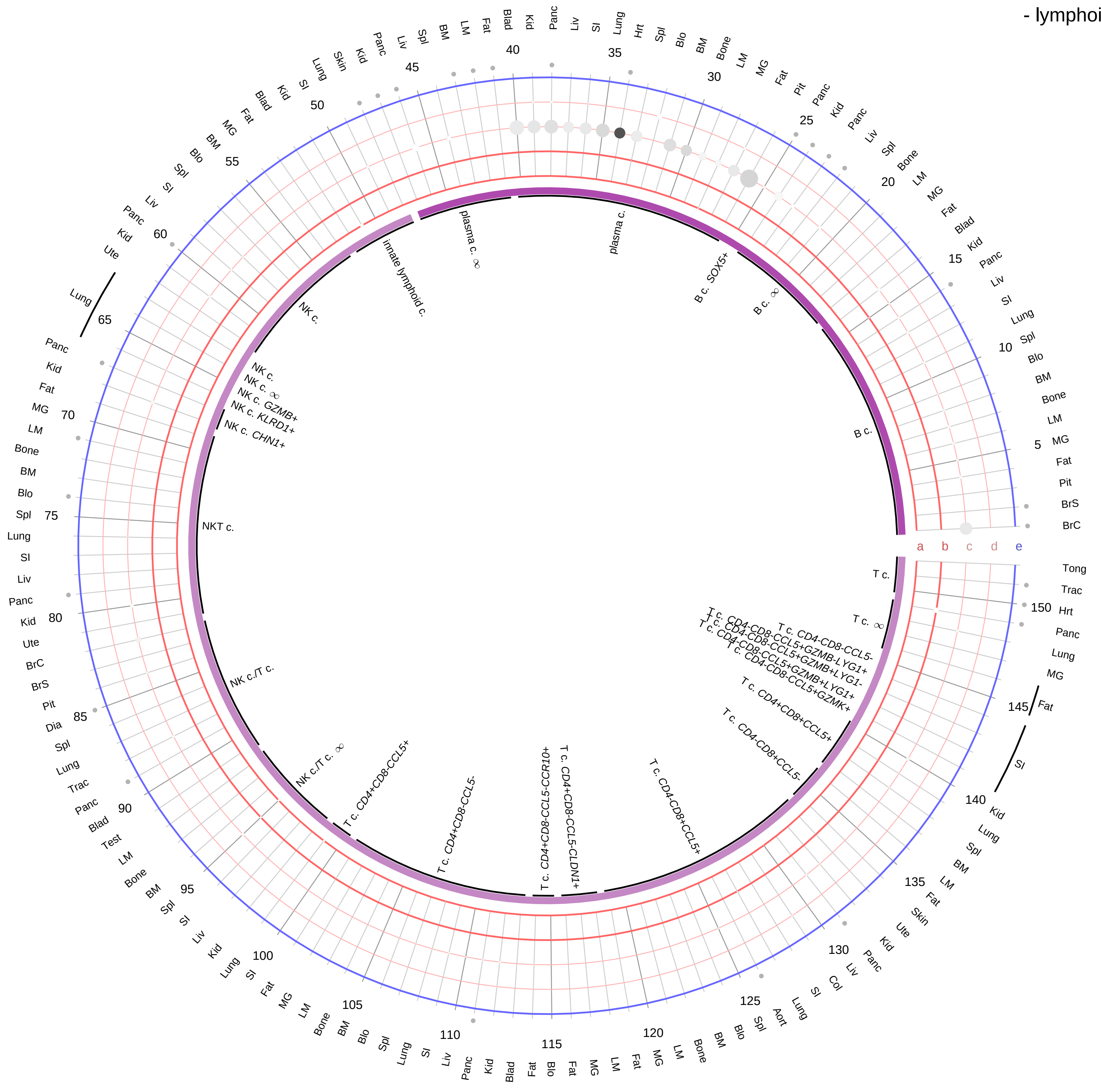

Ligand/Enzyme
- a - GNRH1
- b - GNRH2
- c - CPE
- d - PCSK1

Receptor
- e - GNRHR

PAIRING (ligand → receptor)

GNRH1 & CPE & PCSK1→GNRHR

GNRH2 & CPE & PCSK1→GNRHR

Compartment
lymphoid
- B cell
- NK/T cell

12.5%
25%
50%
100%

ln(CP10K+1): 0, 1, 2, 3

**Abbrev.**	**Tissue**
Aort	Aorta
Blad	Bladder
Blo	Blood
Bone	Bone
BM	Bone marrow
BrC	Brain cortex
BrS	Brainstem
Col	Colon
Dia	Diaphragm
Ret	Eye retina
Fat	Fat
Hrt	Heart
Pit	Hypothalamus/Pituitary
Kid	Kidney
LM	Limb muscle
Liv	Liver
Lung	Lung
MG	Mammary gland
Panc	Pancreas
Skin	Skin
SI	Small intestine
Spl	Spleen
Test	Testes
Thym	Thymus
Tong	Tongue
Trac	Trachea
Ute	Uterus

Gonadotropin-releasing hormone (GnRH)
- myeloid

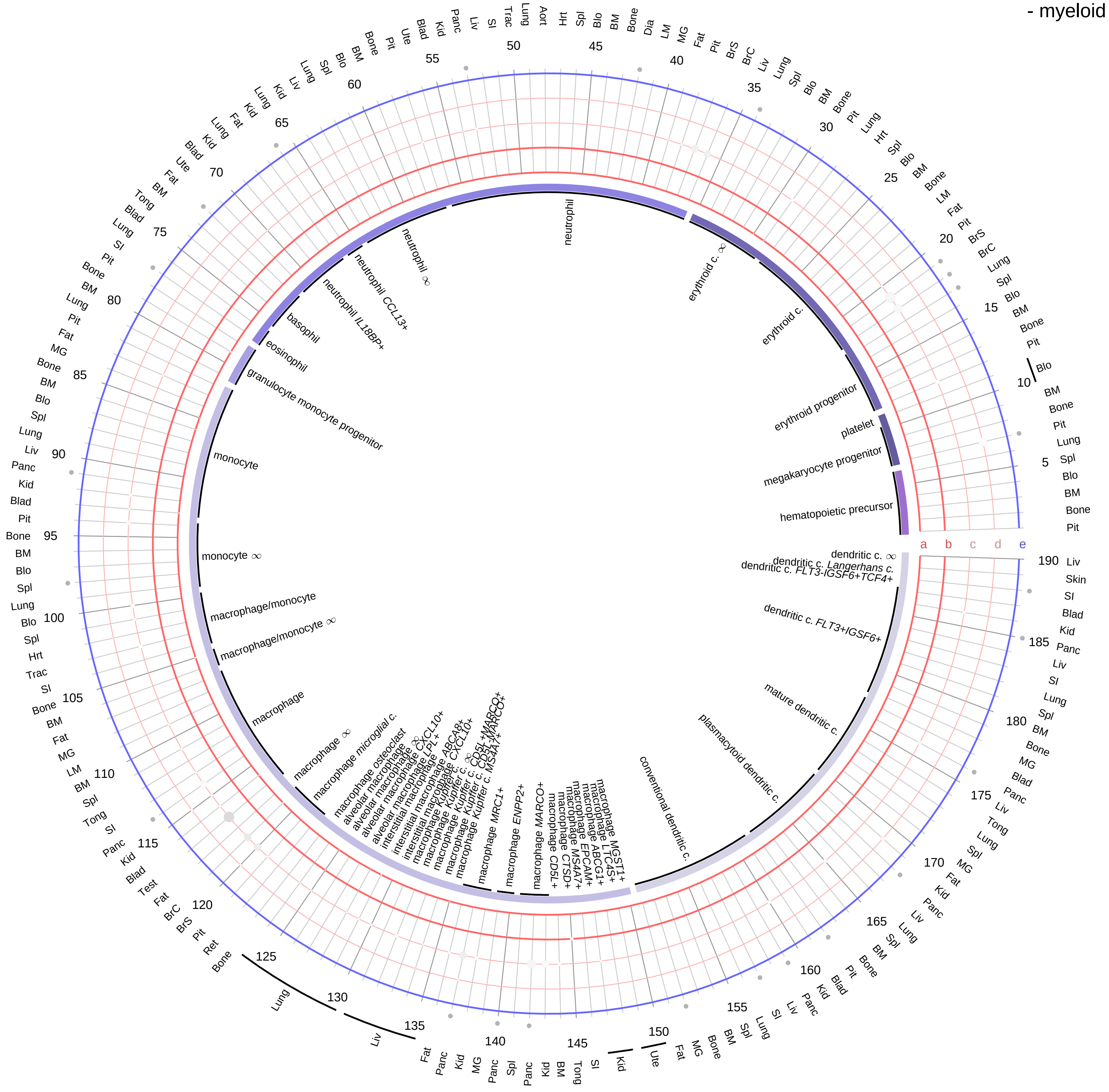

Ligand/Enzyme
- a - GNRH1
- b - GNRH2
- c - CPE
- d - PCSK1

Receptor
- e - GNRHR

PAIRING (ligand → receptor)

GNRH1 & CPE & PCSK1→GNRHR

GNRH2 & CPE & PCSK1→GNRHR

Compartment
- **hematopoietic**
  - hematopoietic precursor
- **megakaryocyte-erythroid**
  - megakaryoid
  - erythroid
- **myeloid**
  - granulocyte
  - granulocyte monocyte progenitor
  - monocyte/macrophage
  - dendritic

12.5%
25%
50%
100%

ln(CP10K+1): 0, 1, 2, 3

Abbrev.	Tissue
Aort	Aorta
Blad	Bladder
Blo	Blood
Bone	Bone
BM	Bone marrow
BrC	Brain cortex
BrS	Brainstem
Col	Colon
Dia	Diaphragm
Ret	Eye retina
Fat	Fat
Hrt	Heart
Pit	Hypothalamus/Pituitary
Kid	Kidney
LM	Limb muscle
Liv	Liver
Lung	Lung
MG	Mammary gland
Panc	Pancreas
Skin	Skin
SI	Small intestine
Spl	Spleen
Test	Testes
Thym	Thymus
Tong	Tongue
Trac	Trachea
Ute	Uterus

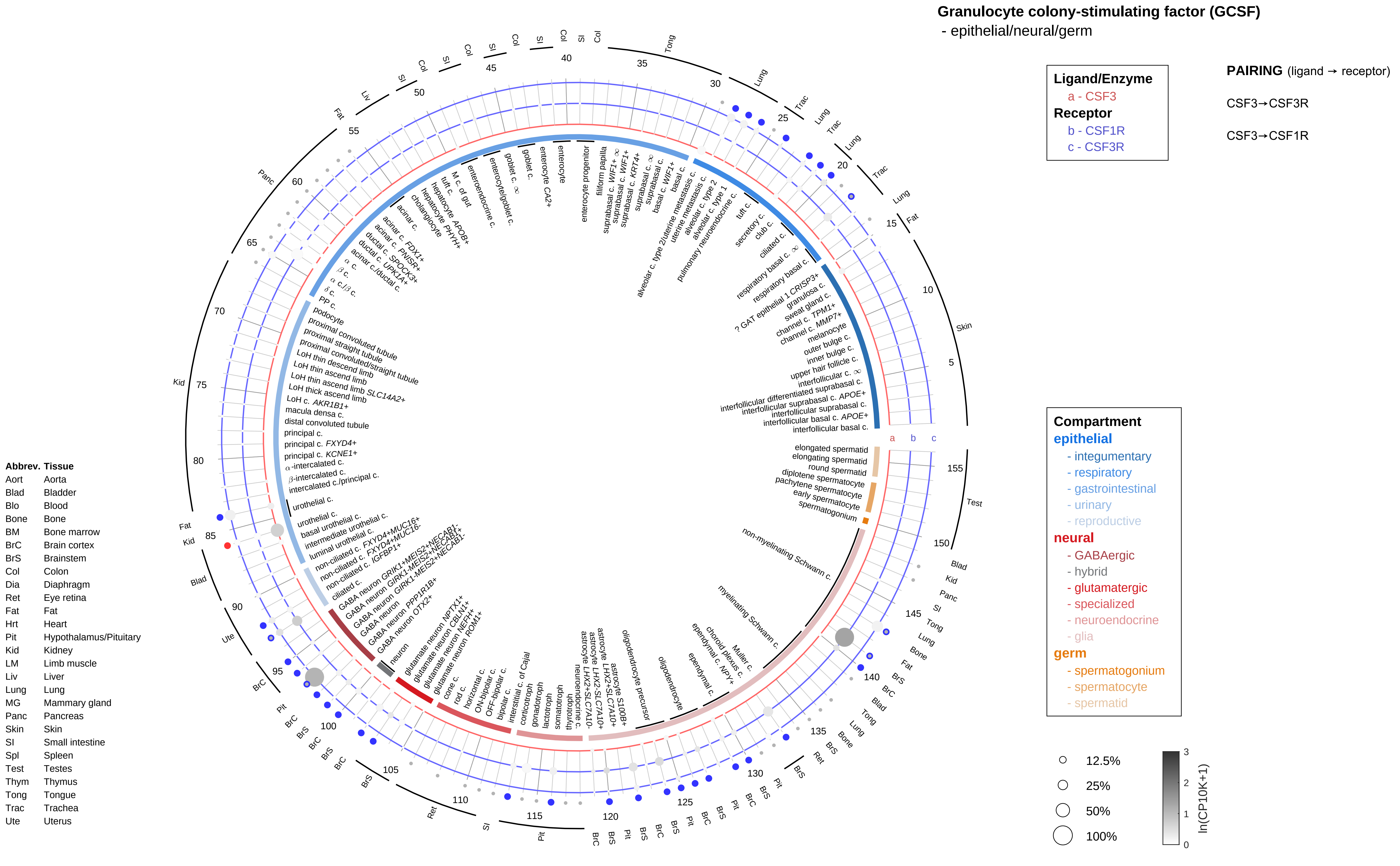

Granulocyte colony-stimulating factor (GCSF)
- epithelial/neural/germ
Ligand/Enzyme
a - CSF3
Receptor
b - CSF1R
c - CSF3R
PAIRING (ligand → receptor)
CSF3→CSF3R
CSF3→CSF1R
Compartment
epithelial
- integumentary
- respiratory
- gastrointestinal
- urinary
- reproductive
neural
- GABAergic
- hybrid
- glutamatergic
- specialized
- neuroendocrine
- glia
germ
- spermatogonium
- spermatocyte
- spermatid
12.5%
25%
50%
100%
ln(CP10K+1)
Abbrev. Tissue
Aort Aorta
Blad Bladder
Blo Blood
Bone Bone
BM Bone marrow
BrC Brain cortex
BrS Brainstem
Col Colon
Dia Diaphragm
Ret Eye retina
Fat Fat
Hrt Heart
Pit Hypothalamus/Pituitary
Kid Kidney
LM Limb muscle
Liv Liver
Lung Lung
MG Mammary gland
Panc Pancreas
Skin Skin
SI Small intestine
Spl Spleen
Test Testes
Thym Thymus
Tong Tongue
Trac Trachea
Ute Uterus

Granulocyte colony-stimulating factor (GCSF)
- endothelial

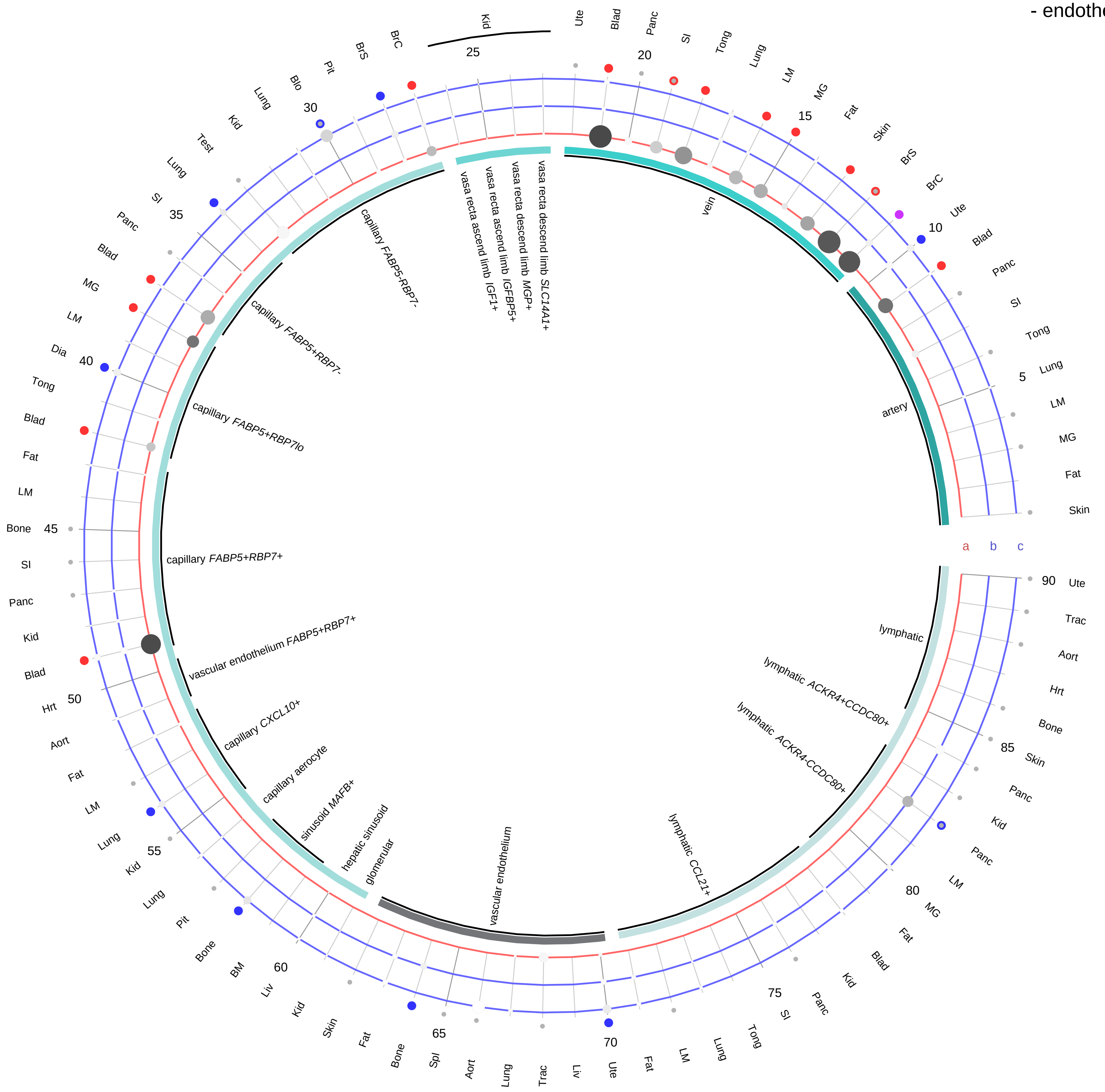

Ligand/Enzyme
a - CSF3
Receptor
b - CSF1R
c - CSF3R

PAIRING (ligand → receptor)

CSF3→CSF3R

CSF3→CSF1R

Compartment
endothelial
- artery
- vein
- vasa recta
- capillary
- mix
- lymphatic

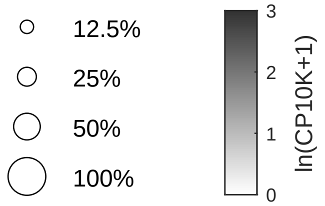

Abbrev.	Tissue
Aort	Aorta
Blad	Bladder
Blo	Blood
Bone	Bone
BM	Bone marrow
BrC	Brain cortex
BrS	Brainstem
Col	Colon
Dia	Diaphragm
Ret	Eye retina
Fat	Fat
Hrt	Heart
Pit	Hypothalamus/Pituitary
Kid	Kidney
LM	Limb muscle
Liv	Liver
Lung	Lung
MG	Mammary gland
Panc	Pancreas
Skin	Skin
SI	Small intestine
Spl	Spleen
Test	Testes
Thym	Thymus
Tong	Tongue
Trac	Trachea
Ute	Uterus

Granulocyte colony-stimulating factor (GCSF)
 - stromal

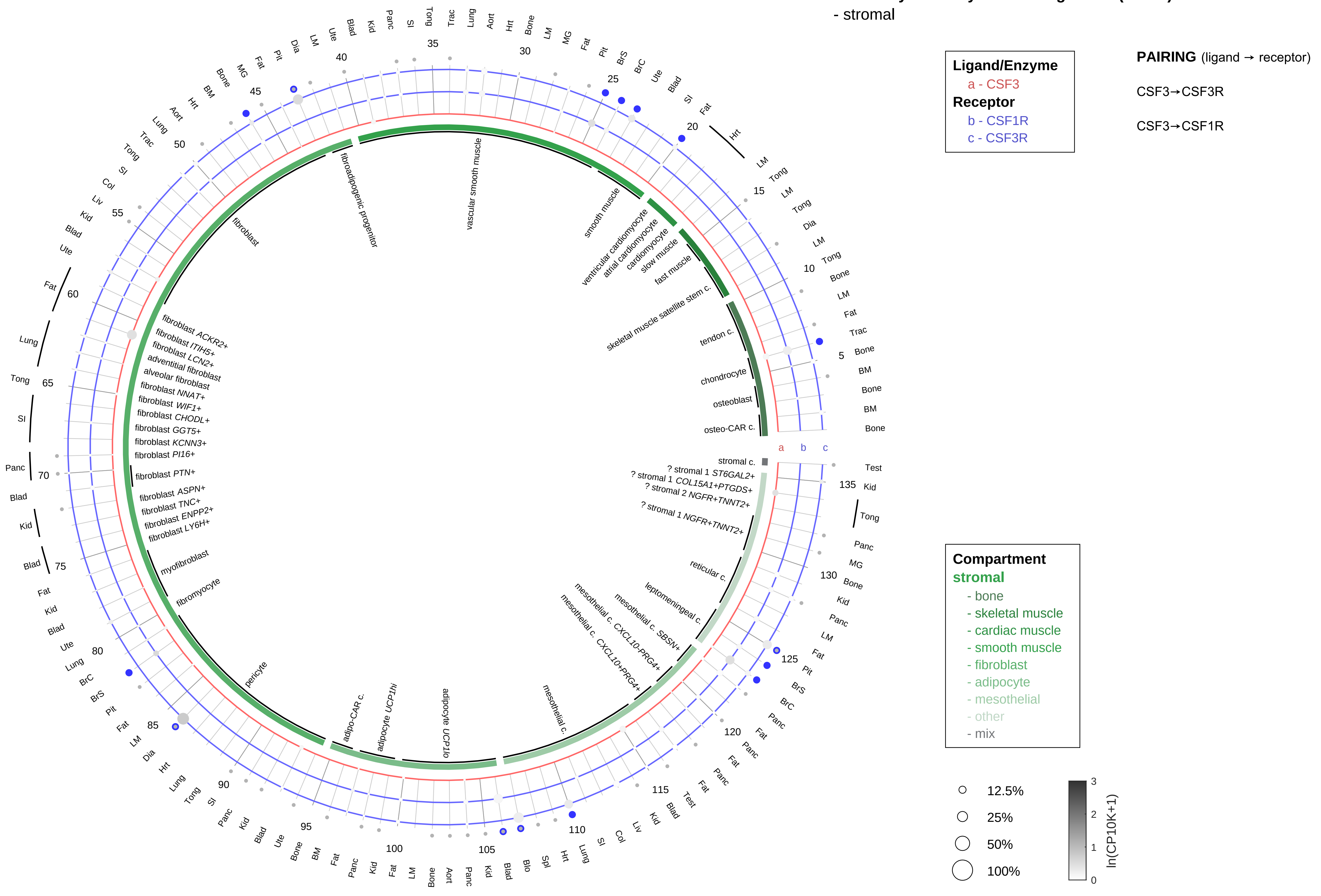

Abbrev.	Tissue
Aort	Aorta
Blad	Bladder
Blo	Blood
Bone	Bone
BM	Bone marrow
BrC	Brain cortex
BrS	Brainstem
Col	Colon
Dia	Diaphragm
Ret	Eye retina
Fat	Fat
Hrt	Heart
Pit	Hypothalamus/Pituitary
Kid	Kidney
LM	Limb muscle
Liv	Liver
Lung	Lung
MG	Mammary gland
Panc	Pancreas
Skin	Skin
SI	Small intestine
Spl	Spleen
Test	Testes
Thym	Thymus
Tong	Tongue
Trac	Trachea
Ute	Uterus

Granulocyte colony-stimulating factor (GCSF)
- lymphoid

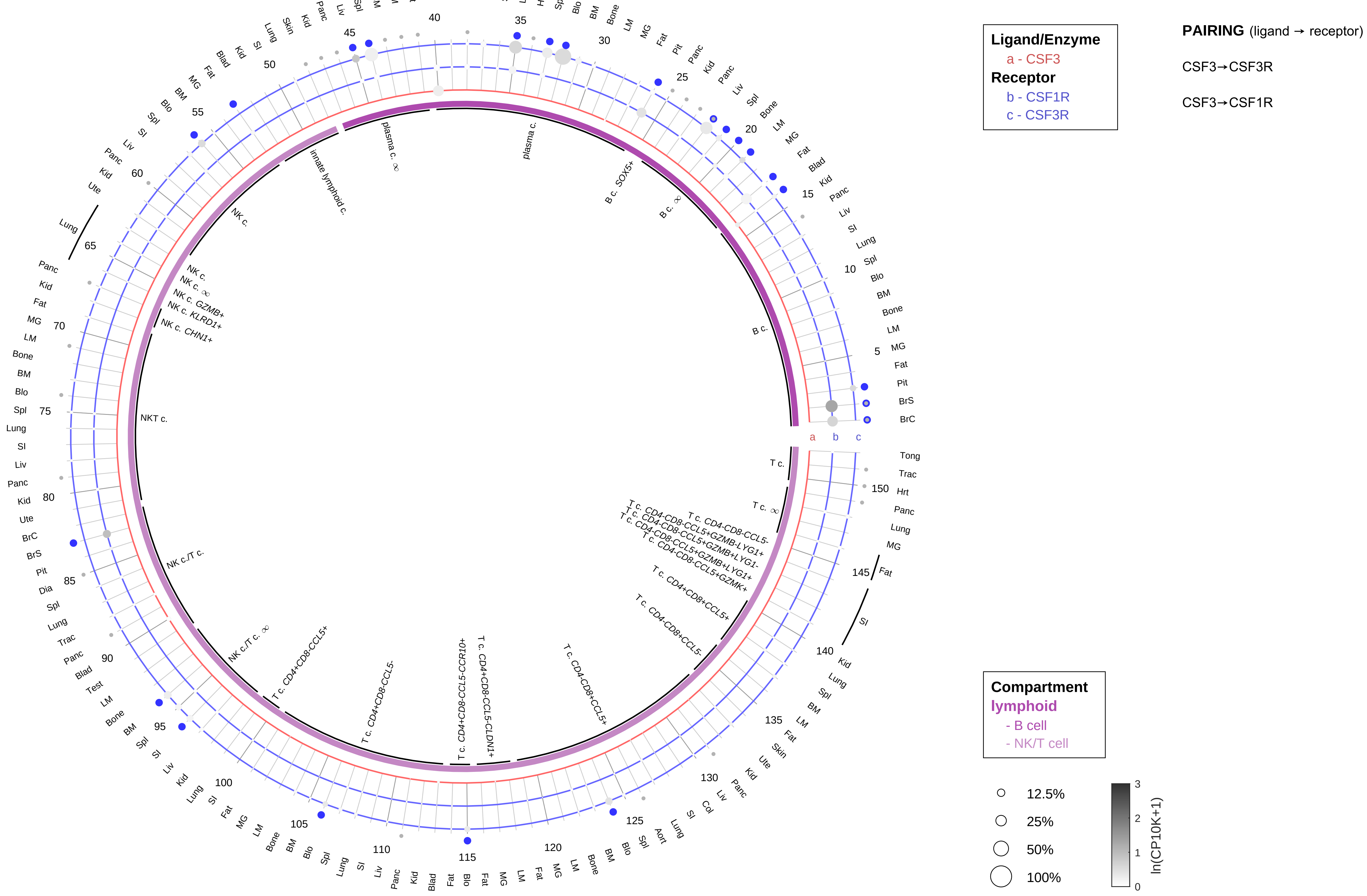

Abbrev.	Tissue
Aort	Aorta
Blad	Bladder
Blo	Blood
Bone	Bone
BM	Bone marrow
BrC	Brain cortex
BrS	Brainstem
Col	Colon
Dia	Diaphragm
Ret	Eye retina
Fat	Fat
Hrt	Heart
Pit	Hypothalamus/Pituitary
Kid	Kidney
LM	Limb muscle
Liv	Liver
Lung	Lung
MG	Mammary gland
Panc	Pancreas
Skin	Skin
SI	Small intestine
Spl	Spleen
Test	Testes
Thym	Thymus
Tong	Tongue
Trac	Trachea
Ute	Uterus

Granulocyte colony-stimulating factor (GCSF)
- myeloid

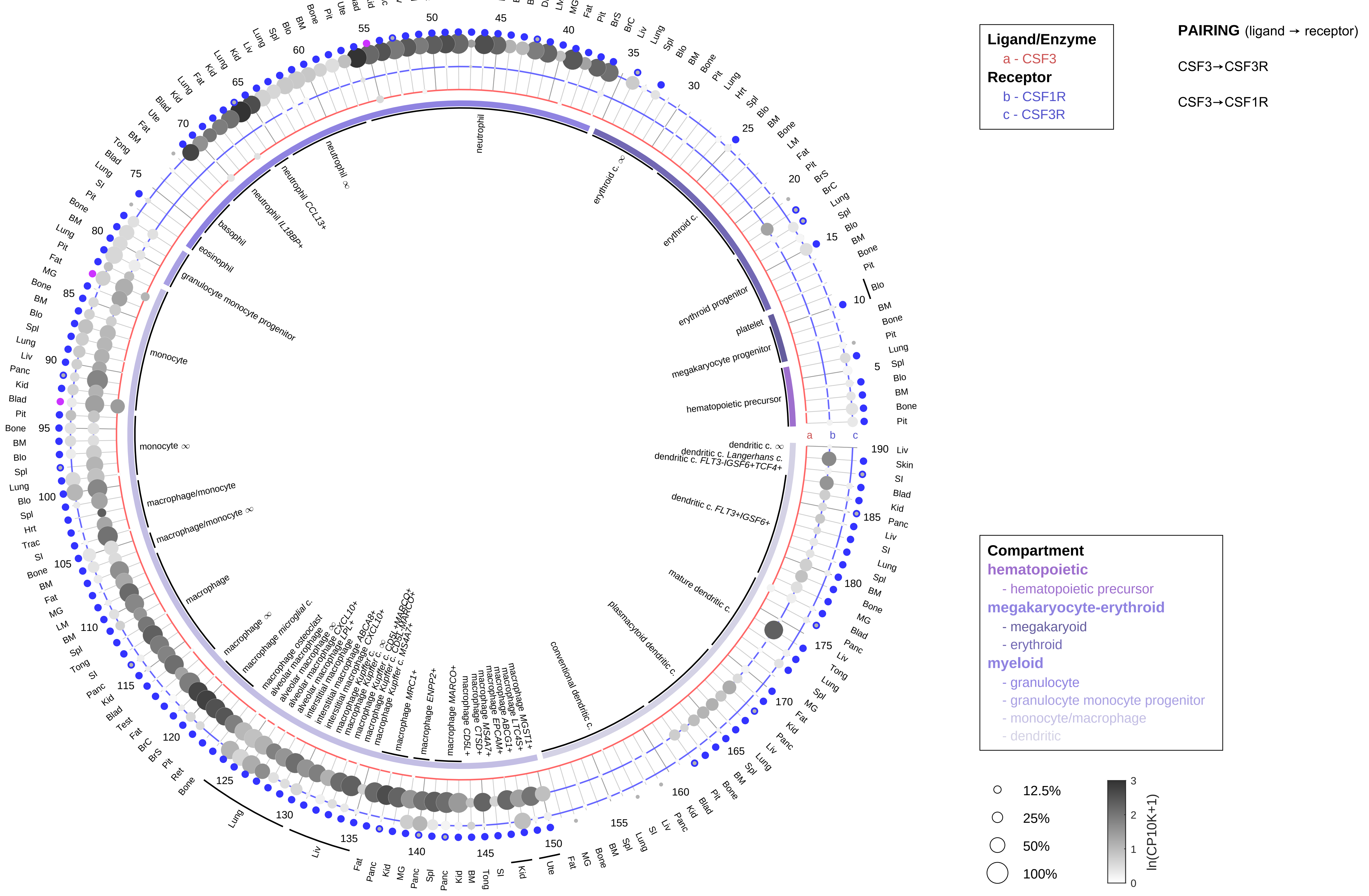

Abbrev.	Tissue
Aort	Aorta
Blad	Bladder
Blo	Blood
Bone	Bone
BM	Bone marrow
BrC	Brain cortex
BrS	Brainstem
Col	Colon
Dia	Diaphragm
Ret	Eye retina
Fat	Fat
Hrt	Heart
Pit	Hypothalamus/Pituitary
Kid	Kidney
LM	Limb muscle
Liv	Liver
Lung	Lung
MG	Mammary gland
Panc	Pancreas
Skin	Skin
SI	Small intestine
Spl	Spleen
Test	Testes
Thym	Thymus
Tong	Tongue
Trac	Trachea
Ute	Uterus

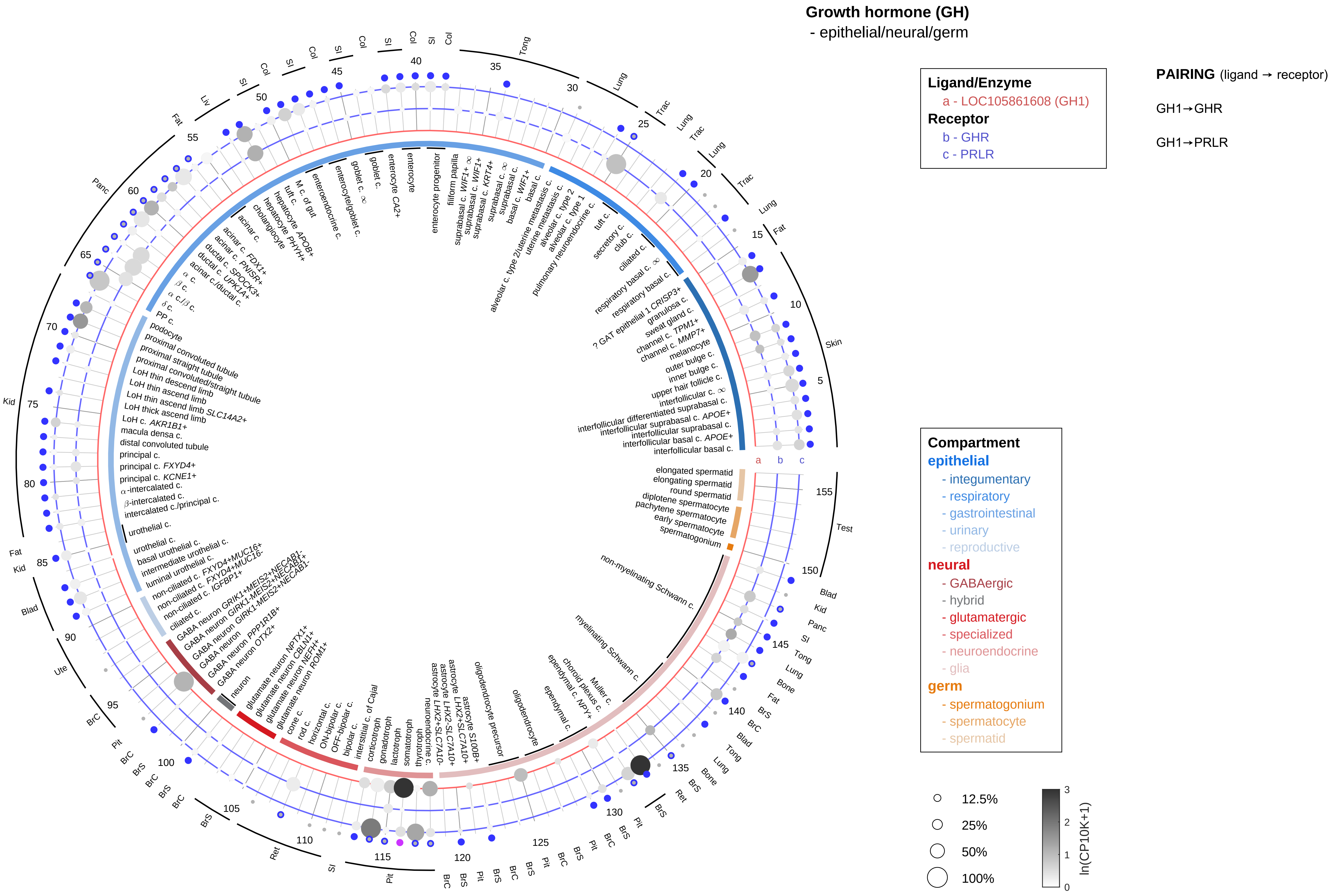

Abbrev.	Tissue
Aort	Aorta
Blad	Bladder
Blo	Blood
Bone	Bone
BM	Bone marrow
BrC	Brain cortex
BrS	Brainstem
Col	Colon
Dia	Diaphragm
Ret	Eye retina
Fat	Fat
Hrt	Heart
Pit	Hypothalamus/Pituitary
Kid	Kidney
LM	Limb muscle
Liv	Liver
Lung	Lung
MG	Mammary gland
Panc	Pancreas
Skin	Skin
SI	Small intestine
Spl	Spleen
Test	Testes
Thym	Thymus
Tong	Tongue
Trac	Trachea
Ute	Uterus

Growth hormone (GH)
- endothelial

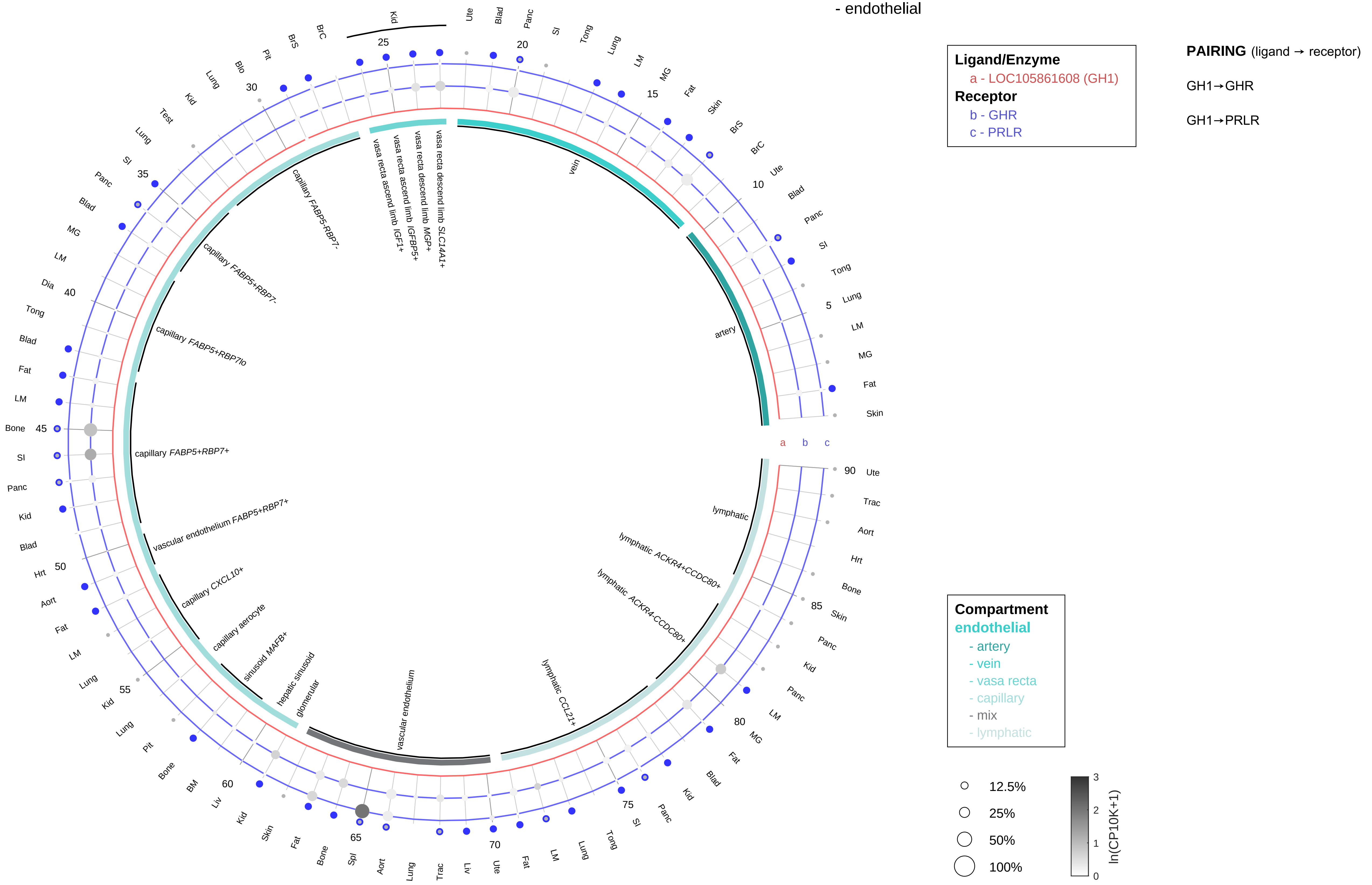

Abbrev.	Tissue
Aort	Aorta
Blad	Bladder
Blo	Blood
Bone	Bone
BM	Bone marrow
BrC	Brain cortex
BrS	Brainstem
Col	Colon
Dia	Diaphragm
Ret	Eye retina
Fat	Fat
Hrt	Heart
Pit	Hypothalamus/Pituitary
Kid	Kidney
LM	Limb muscle
Liv	Liver
Lung	Lung
MG	Mammary gland
Panc	Pancreas
Skin	Skin
SI	Small intestine
Spl	Spleen
Test	Testes
Thym	Thymus
Tong	Tongue
Trac	Trachea
Ute	Uterus

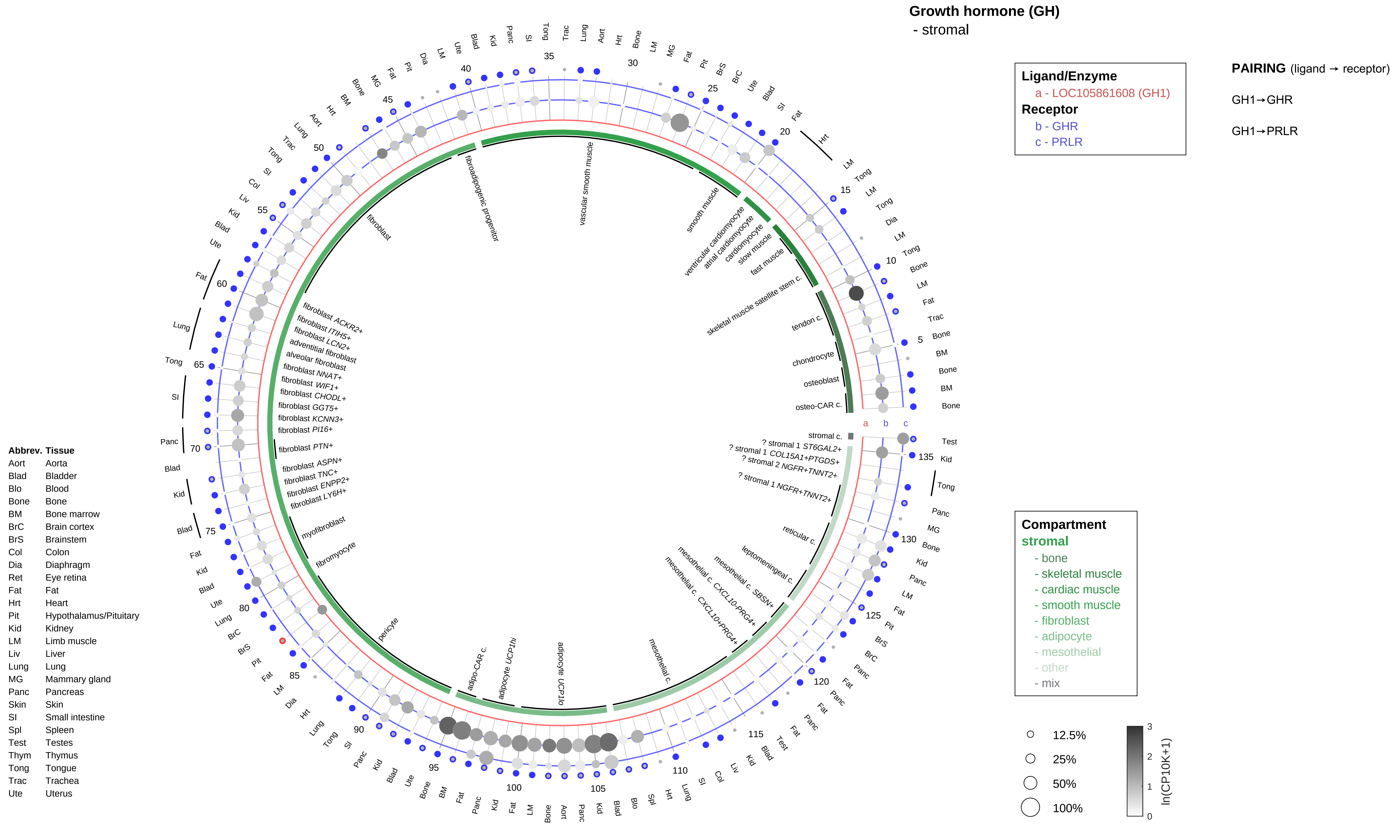

Growth hormone (GH)
- stromal
Ligand/Enzyme
a - LOC105861608 (GH1)
Receptor
b - GHR
c - PRLR
PAIRING (ligand → receptor)
GH1→GHR
GH1→PRLR
Compartment
stromal
- bone
- skeletal muscle
- cardiac muscle
- smooth muscle
- fibroblast
- adipocyte
- mesothelial
- other
- mix
12.5%
25%
50%
100%
ln(CP10K+1)
Abbrev. Tissue
Aort Aorta
Blad Bladder
Blo Blood
Bone Bone
BM Bone marrow
BrC Brain cortex
BrS Brainstem
Col Colon
Dia Diaphragm
Ret Eye retina
Fat Fat
Hrt Heart
Pit Hypothalamus/Pituitary
Kid Kidney
LM Limb muscle
Liv Liver
Lung Lung
MG Mammary gland
Panc Pancreas
Skin Skin
SI Small intestine
Spl Spleen
Test Testes
Thym Thymus
Tong Tongue
Trac Trachea
Ute Uterus
fibroadipogenic progenitor
vascular smooth muscle
smooth muscle
ventricular cardiomyocyte
atrial cardiomyocyte
cardiomyocyte
slow muscle
fast muscle
skeletal muscle satellite stem c.
tendon c.
chondrocyte
osteoblast
osteo-CAR c.
stromal c.
? stromal 1 ST6GAL2+
? stromal 1 COL15A1+PTGDS+
? stromal 2 NGFR+TNNT2+
? stromal 1 NGFR+TNNT2+
reticular c.
leptomeningeal c.
mesothelial c. SBSN+
mesothelial c. CXCL10+PRG4+
mesothelial c. CXCL10+PRG4+
mesothelial c.
adipocyte UCP1lo
adipocyte UCP1hi
adipo-CAR c.
pericyte
fibromyocyte
myofibroblast
fibroblast LY6H+
fibroblast ENPP2+
fibroblast TNC+
fibroblast ASPN+
fibroblast PTN+
fibroblast PI16+
fibroblast KCNN3+
fibroblast GGT5+
fibroblast CHODL+
fibroblast WIF1+
fibroblast NNAT+
alveolar fibroblast
adventitial fibroblast
fibroblast LCN2+
fibroblast ITIH5+
fibroblast ACKR2+
fibroblast

Growth hormone (GH)
- lymphoid

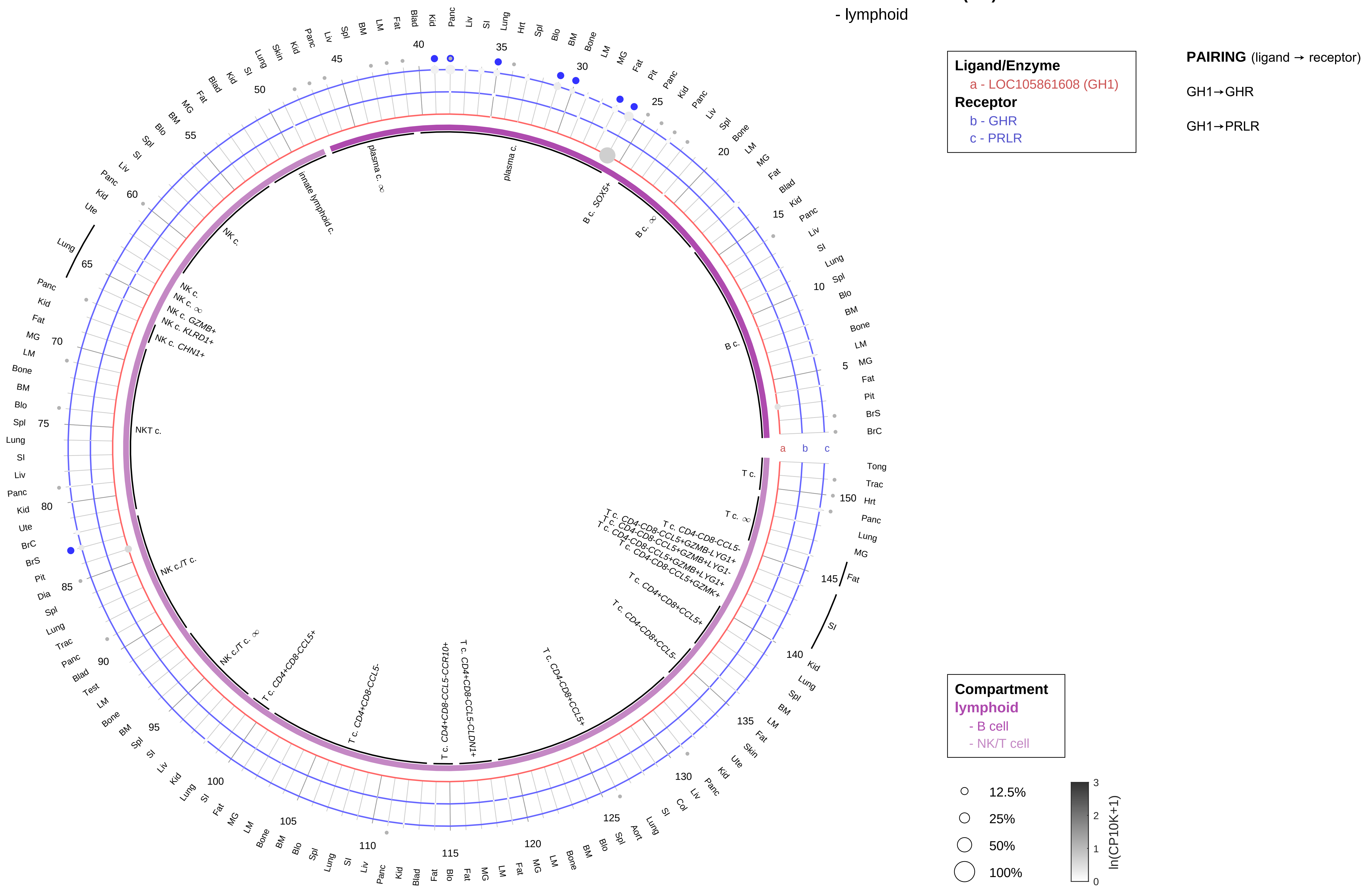

Abbrev.	Tissue
Aort	Aorta
Blad	Bladder
Blo	Blood
Bone	Bone
BM	Bone marrow
BrC	Brain cortex
BrS	Brainstem
Col	Colon
Dia	Diaphragm
Ret	Eye retina
Fat	Fat
Hrt	Heart
Pit	Hypothalamus/Pituitary
Kid	Kidney
LM	Limb muscle
Liv	Liver
Lung	Lung
MG	Mammary gland
Panc	Pancreas
Skin	Skin
SI	Small intestine
Spl	Spleen
Test	Testes
Thym	Thymus
Tong	Tongue
Trac	Trachea
Ute	Uterus

Growth hormone (GH)
- myeloid

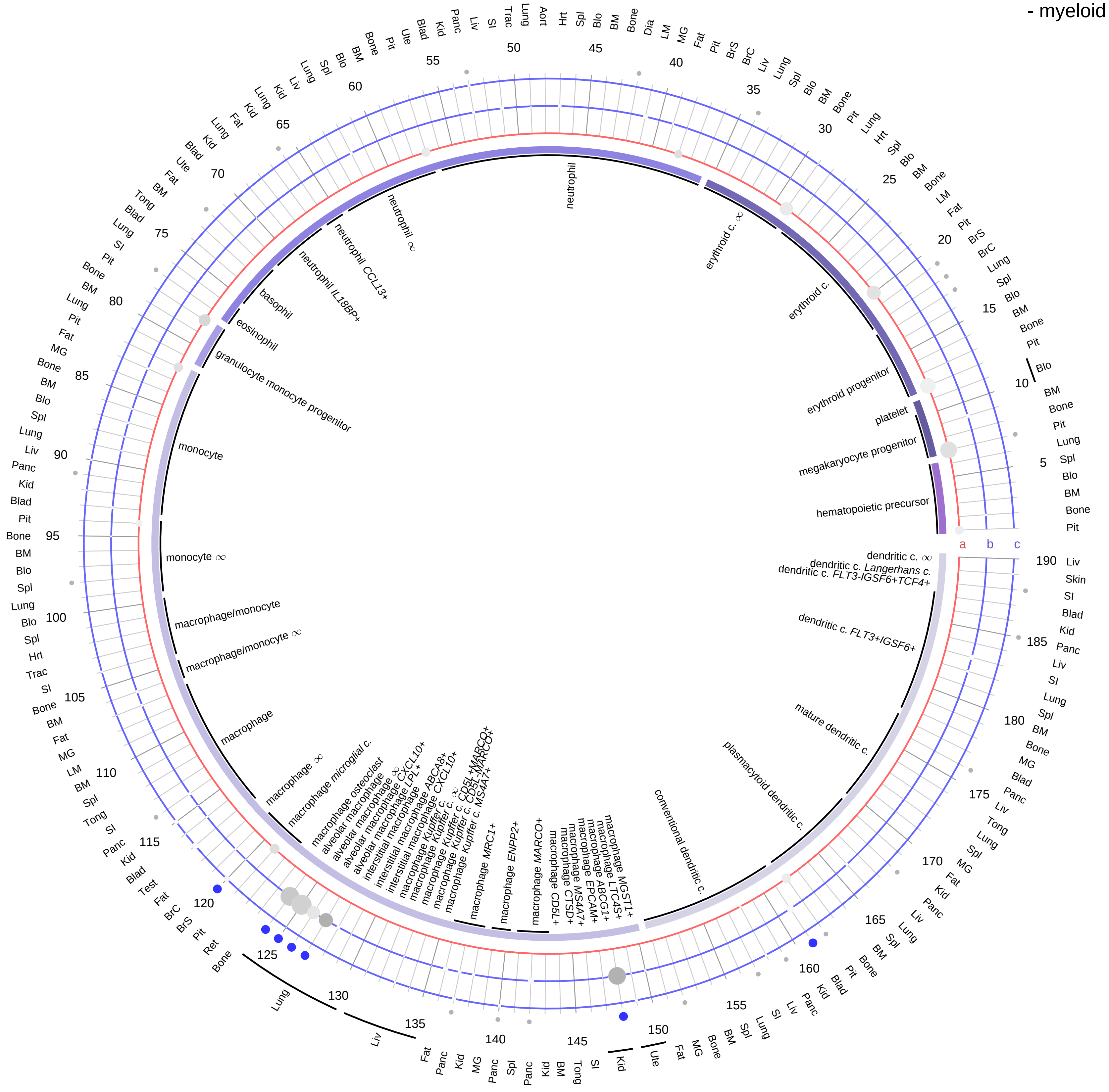

Ligand/Enzyme
a - LOC105861608 (GH1)
Receptor
b - GHR
c - PRLR

PAIRING (ligand → receptor)

GH1→GHR

GH1→PRLR

**Abbrev.**	**Tissue**
Aort	Aorta
Blad	Bladder
Blo	Blood
Bone	Bone
BM	Bone marrow
BrC	Brain cortex
BrS	Brainstem
Col	Colon
Dia	Diaphragm
Ret	Eye retina
Fat	Fat
Hrt	Heart
Pit	Hypothalamus/Pituitary
Kid	Kidney
LM	Limb muscle
Liv	Liver
Lung	Lung
MG	Mammary gland
Panc	Pancreas
Skin	Skin
SI	Small intestine
Spl	Spleen
Test	Testes
Thym	Thymus
Tong	Tongue
Trac	Trachea
Ute	Uterus

Compartment
hematopoietic
- hematopoietic precursor
megakaryocyte-erythroid
- megakaryoid
- erythroid
myeloid
- granulocyte
- granulocyte monocyte progenitor
- monocyte/macrophage
- dendritic

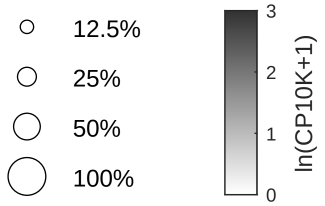

Growth hormone releasing hormone (GHRH)
- epithelial/neural/germ

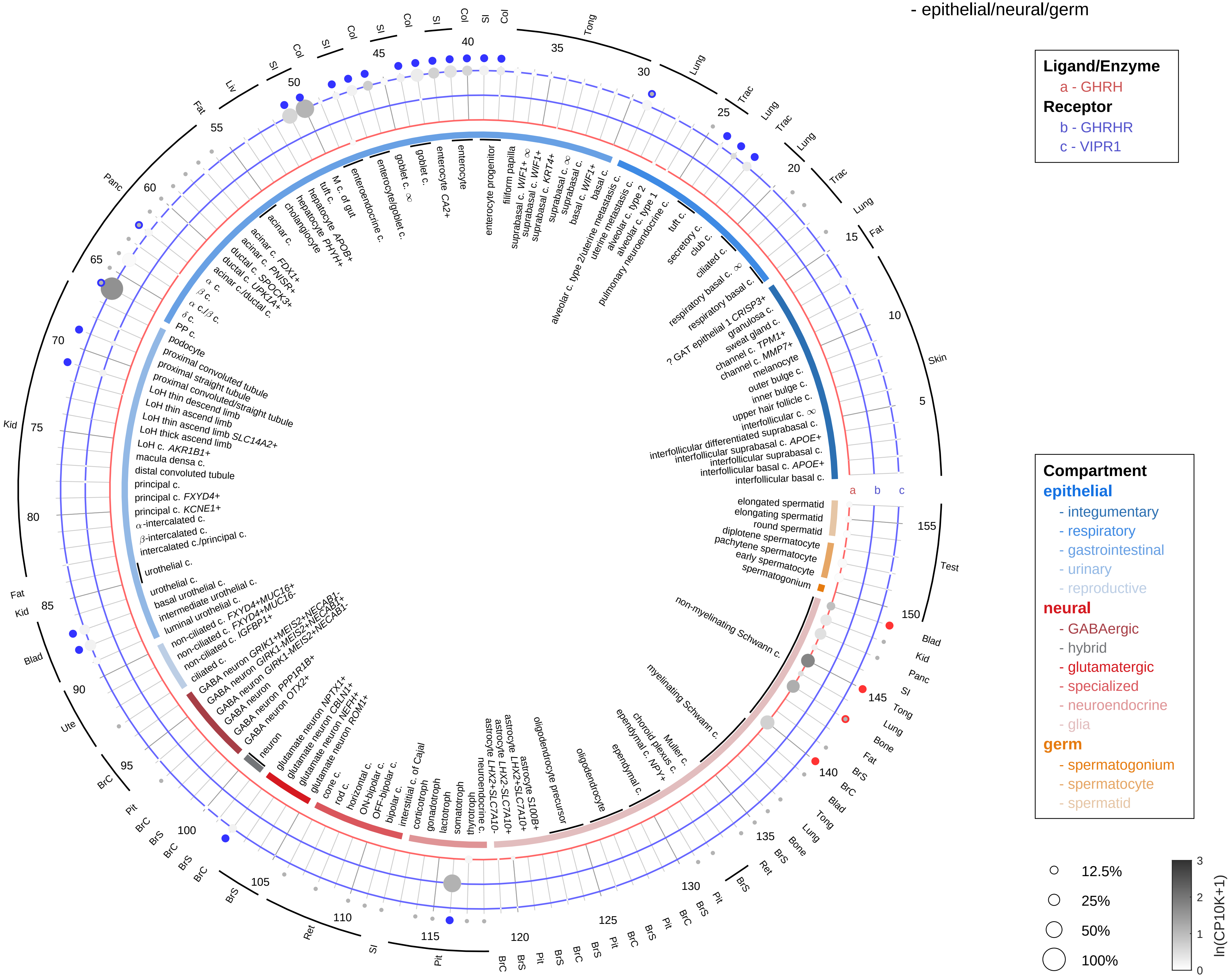

Abbrev.	Tissue
Aort	Aorta
Blad	Bladder
Blo	Blood
Bone	Bone
BM	Bone marrow
BrC	Brain cortex
BrS	Brainstem
Col	Colon
Dia	Diaphragm
Ret	Eye retina
Fat	Fat
Hrt	Heart
Pit	Hypothalamus/Pituitary
Kid	Kidney
LM	Limb muscle
Liv	Liver
Lung	Lung
MG	Mammary gland
Panc	Pancreas
Skin	Skin
SI	Small intestine
Spl	Spleen
Test	Testes
Thym	Thymus
Tong	Tongue
Trac	Trachea
Ute	Uterus

Growth hormone releasing hormone (GHRH)
- endothelial

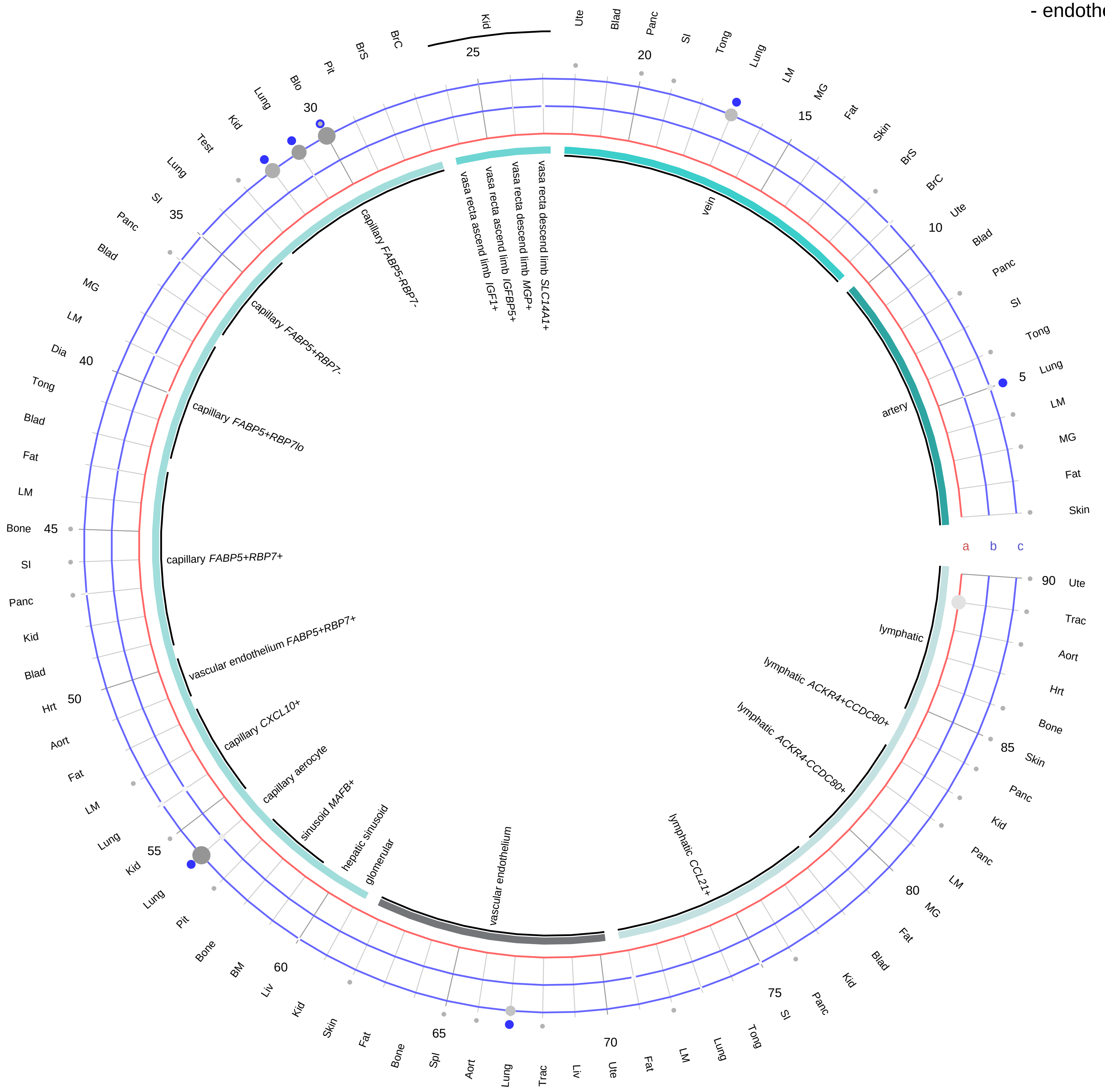

Ligand/Enzyme
a - GHRH
Receptor
b - GHRHR
c - VIPR1

PAIRING (ligand → receptor)

GHRH→GHRHR

GHRH→VIPR1

Compartment
endothelial
- artery
- vein
- vasa recta
- capillary
- mix
- lymphatic

12.5%
25%
50%
100%

ln(CP10K+1): 0, 1, 2, 3

Abbrev.	Tissue
Aort	Aorta
Blad	Bladder
Blo	Blood
Bone	Bone
BM	Bone marrow
BrC	Brain cortex
BrS	Brainstem
Col	Colon
Dia	Diaphragm
Ret	Eye retina
Fat	Fat
Hrt	Heart
Pit	Hypothalamus/Pituitary
Kid	Kidney
LM	Limb muscle
Liv	Liver
Lung	Lung
MG	Mammary gland
Panc	Pancreas
Skin	Skin
SI	Small intestine
Spl	Spleen
Test	Testes
Thym	Thymus
Tong	Tongue
Trac	Trachea
Ute	Uterus

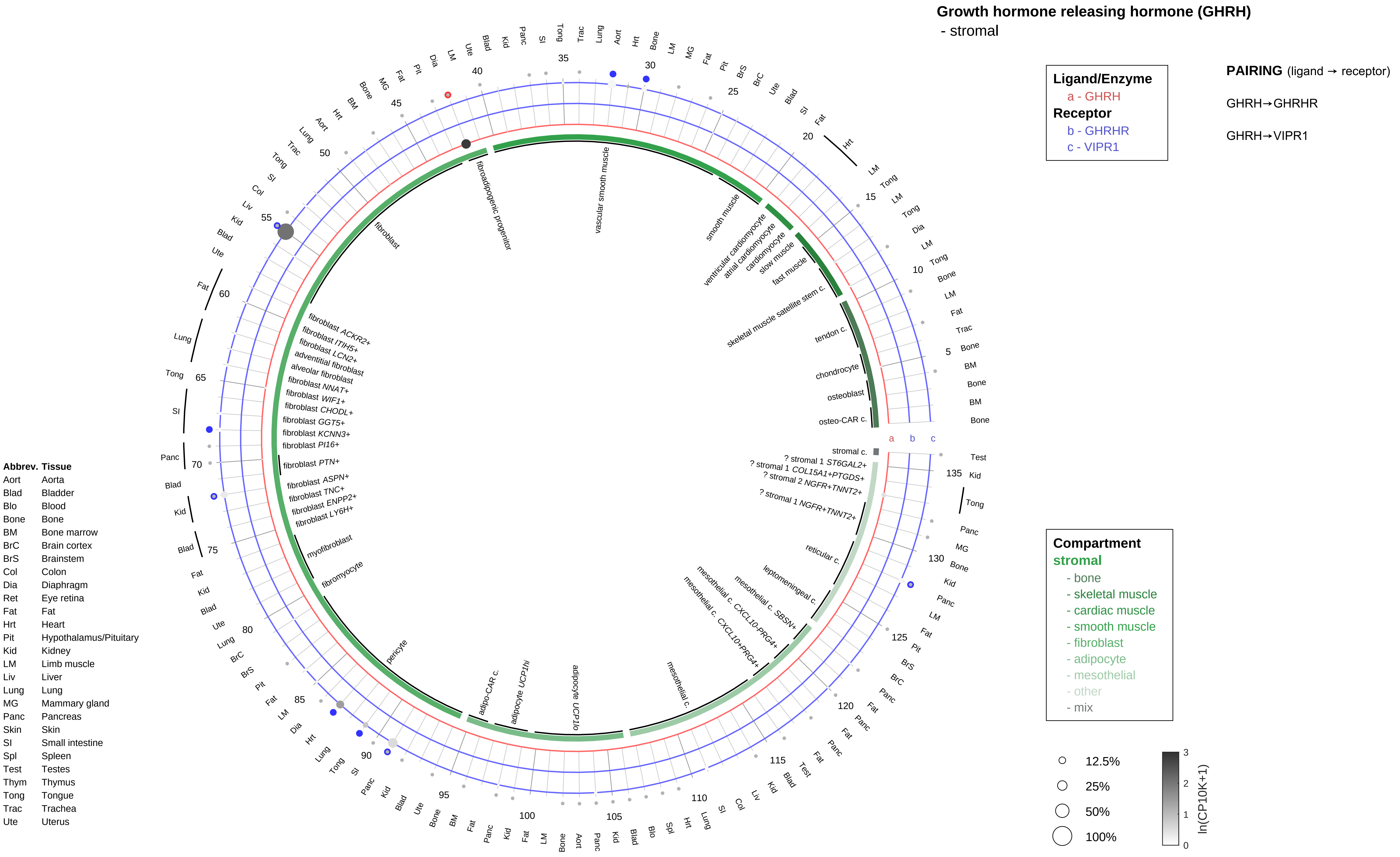

Growth hormone releasing hormone (GHRH)
- stromal
Ligand/Enzyme
a - GHRH
Receptor
b - GHRHR
c - VIPR1
PAIRING (ligand → receptor)
GHRH→GHRHR
GHRH→VIPR1
Compartment
stromal
- bone
- skeletal muscle
- cardiac muscle
- smooth muscle
- fibroblast
- adipocyte
- mesothelial
- other
- mix
12.5%
25%
50%
100%
ln(CP10K+1)
a
b
c
osteo-CAR c.
osteoblast
chondrocyte
tendon c.
skeletal muscle satellite stem c.
fast muscle
slow muscle
cardiomyocyte
atrial cardiomyocyte
ventricular cardiomyocyte
smooth muscle
vascular smooth muscle
fibroadipogenic progenitor
fibroblast
fibroblast ACKR2+
fibroblast ITIH5+
fibroblast LCN2+
adventitial fibroblast
alveolar fibroblast
fibroblast NNAT+
fibroblast WIF1+
fibroblast CHODL+
fibroblast GGT5+
fibroblast KCNN3+
fibroblast PI16+
fibroblast PTN+
fibroblast ASPN+
fibroblast TNC+
fibroblast ENPP2+
fibroblast LY6H+
myofibroblast
fibromyocyte
pericyte
adipo-CAR c.
adipocyte UCP1hi
adipocyte UCP1lo
mesothelial c.
mesothelial c. CXCL10+PRG4+
mesothelial c. CXCL10-PRG4+
mesothelial c. SBSN+
leptomeningeal c.
reticular c.
? stromal 1 NGFR+TNNT2+
? stromal 2 NGFR+TNNT2+
? stromal 1 COL15A1+PTGDS+
? stromal 1 ST6GAL2+
stromal c.
Abbrev. Tissue
Aort Aorta
Blad Bladder
Blo Blood
Bone Bone
BM Bone marrow
BrC Brain cortex
BrS Brainstem
Col Colon
Dia Diaphragm
Ret Eye retina
Fat Fat
Hrt Heart
Pit Hypothalamus/Pituitary
Kid Kidney
LM Limb muscle
Liv Liver
Lung Lung
MG Mammary gland
Panc Pancreas
Skin Skin
SI Small intestine
Spl Spleen
Test Testes
Thym Thymus
Tong Tongue
Trac Trachea
Ute Uterus

Growth hormone releasing hormone (GHRH)
- lymphoid

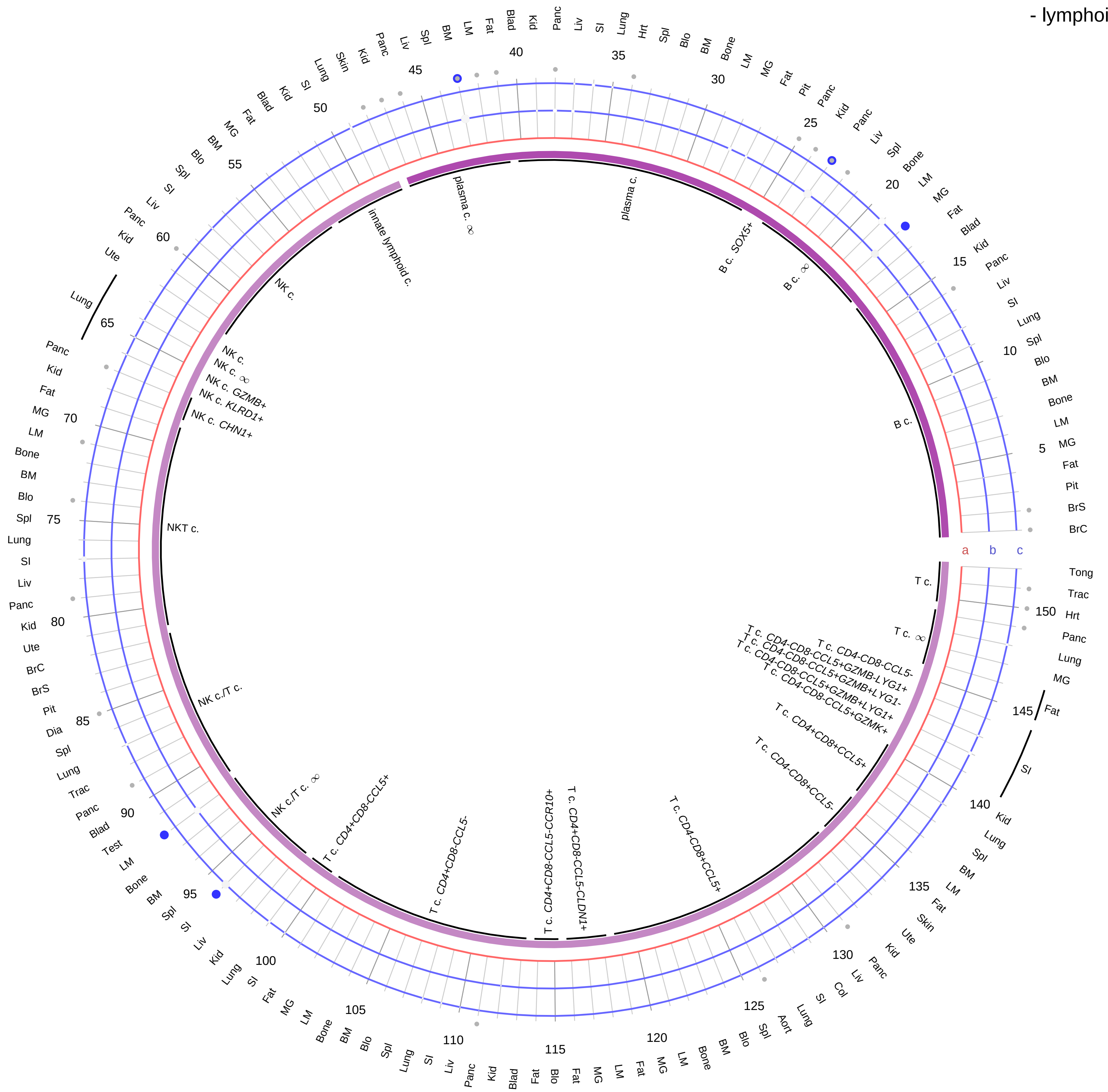

Ligand/Enzyme
a - GHRH
Receptor
b - GHRHR
c - VIPR1

PAIRING (ligand → receptor)

GHRH→GHRHR

GHRH→VIPR1

Compartment
lymphoid
- B cell
- NK/T cell

12.5%
25%
50%
100%

ln(CP10K+1): 0, 1, 2, 3

Abbrev.	Tissue
Aort	Aorta
Blad	Bladder
Blo	Blood
Bone	Bone
BM	Bone marrow
BrC	Brain cortex
BrS	Brainstem
Col	Colon
Dia	Diaphragm
Ret	Eye retina
Fat	Fat
Hrt	Heart
Pit	Hypothalamus/Pituitary
Kid	Kidney
LM	Limb muscle
Liv	Liver
Lung	Lung
MG	Mammary gland
Panc	Pancreas
Skin	Skin
SI	Small intestine
Spl	Spleen
Test	Testes
Thym	Thymus
Tong	Tongue
Trac	Trachea
Ute	Uterus

Growth hormone releasing hormone (GHRH)
- myeloid

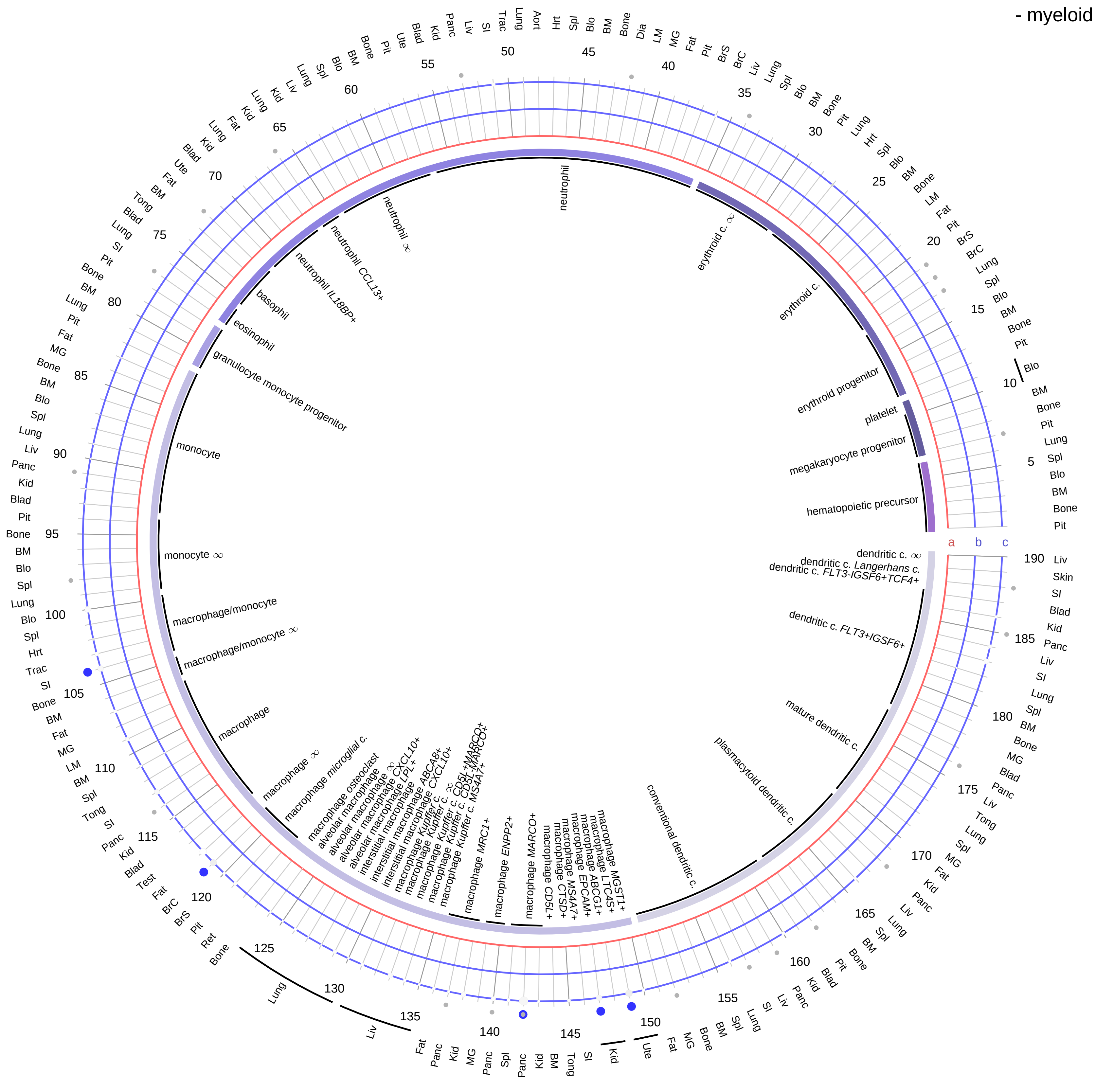

Ligand/Enzyme
- a - GHRH

Receptor
- b - GHRHR
- c - VIPR1

PAIRING (ligand → receptor)

GHRH→GHRHR

GHRH→VIPR1

Compartment

hematopoietic
- hematopoietic precursor

megakaryocyte-erythroid
- megakaryoid
- erythroid

myeloid
- granulocyte
- granulocyte monocyte progenitor
- monocyte/macrophage
- dendritic

12.5%
25%
50%
100%

ln(CP10K+1): 0, 1, 2, 3

Abbrev.	Tissue
Aort	Aorta
Blad	Bladder
Blo	Blood
Bone	Bone
BM	Bone marrow
BrC	Brain cortex
BrS	Brainstem
Col	Colon
Dia	Diaphragm
Ret	Eye retina
Fat	Fat
Hrt	Heart
Pit	Hypothalamus/Pituitary
Kid	Kidney
LM	Limb muscle
Liv	Liver
Lung	Lung
MG	Mammary gland
Panc	Pancreas
Skin	Skin
SI	Small intestine
Spl	Spleen
Test	Testes
Thym	Thymus
Tong	Tongue
Trac	Trachea
Ute	Uterus

Guanylin/Uroguanylin (GN/UGN)
- epithelial/neural/germ

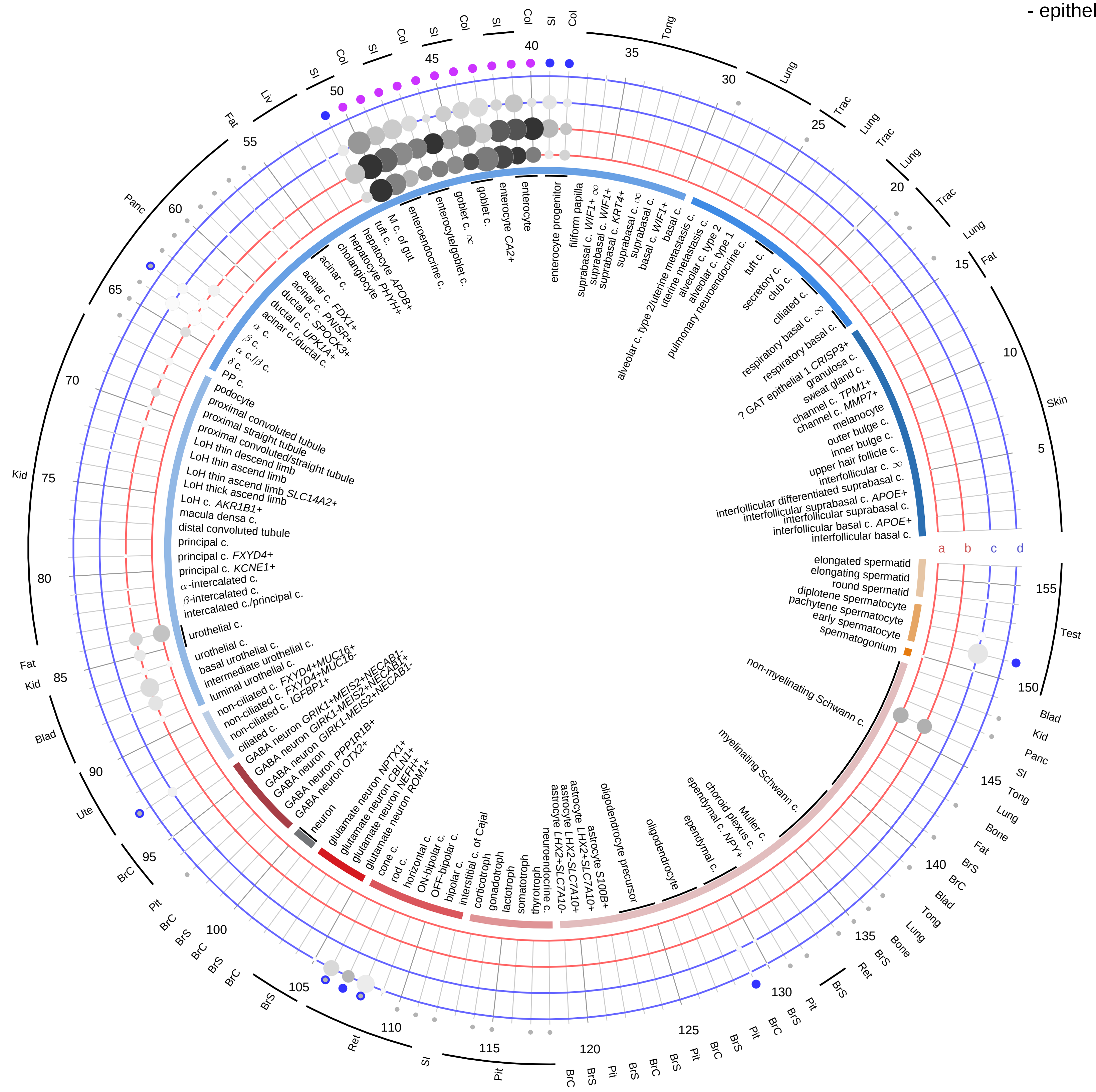

Ligand/Enzyme
- a - GUCA2A
- b - GUCA2B

Receptor
- c - GUCY2C
- d - GUCY2D

PAIRING (ligand → receptor)

GUCA2A→GUCY2C

GUCA2B→GUCY2C

GUCA2B→GUCY2D

Compartment

epithelial
- integumentary
- respiratory
- gastrointestinal
- urinary
- reproductive

neural
- GABAergic
- hybrid
- glutamatergic
- specialized
- neuroendocrine
- glia

germ
- spermatogonium
- spermatocyte
- spermatid

12.5%
25%
50%
100%

ln(CP10K+1): 0, 1, 2, 3

Abbrev.	Tissue
Aort	Aorta
Blad	Bladder
Blo	Blood
Bone	Bone
BM	Bone marrow
BrC	Brain cortex
BrS	Brainstem
Col	Colon
Dia	Diaphragm
Ret	Eye retina
Fat	Fat
Hrt	Heart
Pit	Hypothalamus/Pituitary
Kid	Kidney
LM	Limb muscle
Liv	Liver
Lung	Lung
MG	Mammary gland
Panc	Pancreas
Skin	Skin
SI	Small intestine
Spl	Spleen
Test	Testes
Thym	Thymus
Tong	Tongue
Trac	Trachea
Ute	Uterus

Guanylin/Uroguanylin (GN/UGN)
- endothelial

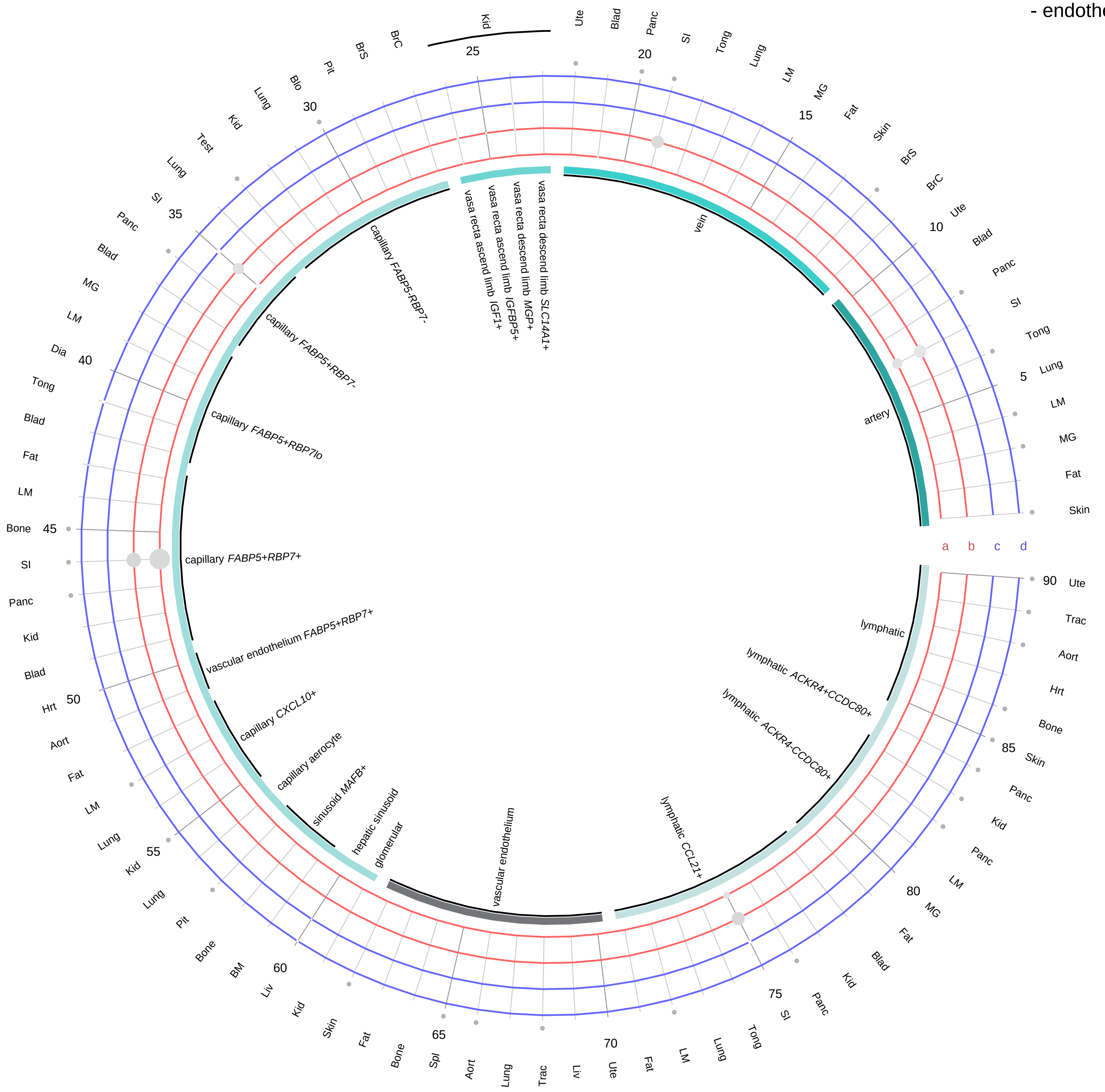

Ligand/Enzyme
- a - GUCA2A
- b - GUCA2B

Receptor
- c - GUCY2C
- d - GUCY2D

PAIRING (ligand → receptor)

GUCA2A→GUCY2C

GUCA2B→GUCY2C

GUCA2B→GUCY2D

Compartment

endothelial
- artery
- vein
- vasa recta
- capillary
- mix
- lymphatic

12.5%
25%
50%
100%

ln(CP10K+1): 0, 1, 2, 3

Abbrev.	Tissue
Aort	Aorta
Blad	Bladder
Blo	Blood
Bone	Bone
BM	Bone marrow
BrC	Brain cortex
BrS	Brainstem
Col	Colon
Dia	Diaphragm
Ret	Eye retina
Fat	Fat
Hrt	Heart
Pit	Hypothalamus/Pituitary
Kid	Kidney
LM	Limb muscle
Liv	Liver
Lung	Lung
MG	Mammary gland
Panc	Pancreas
Skin	Skin
SI	Small intestine
Spl	Spleen
Test	Testes
Thym	Thymus
Tong	Tongue
Trac	Trachea
Ute	Uterus

Guanylin/Uroguanylin (GN/UGN)
- stromal

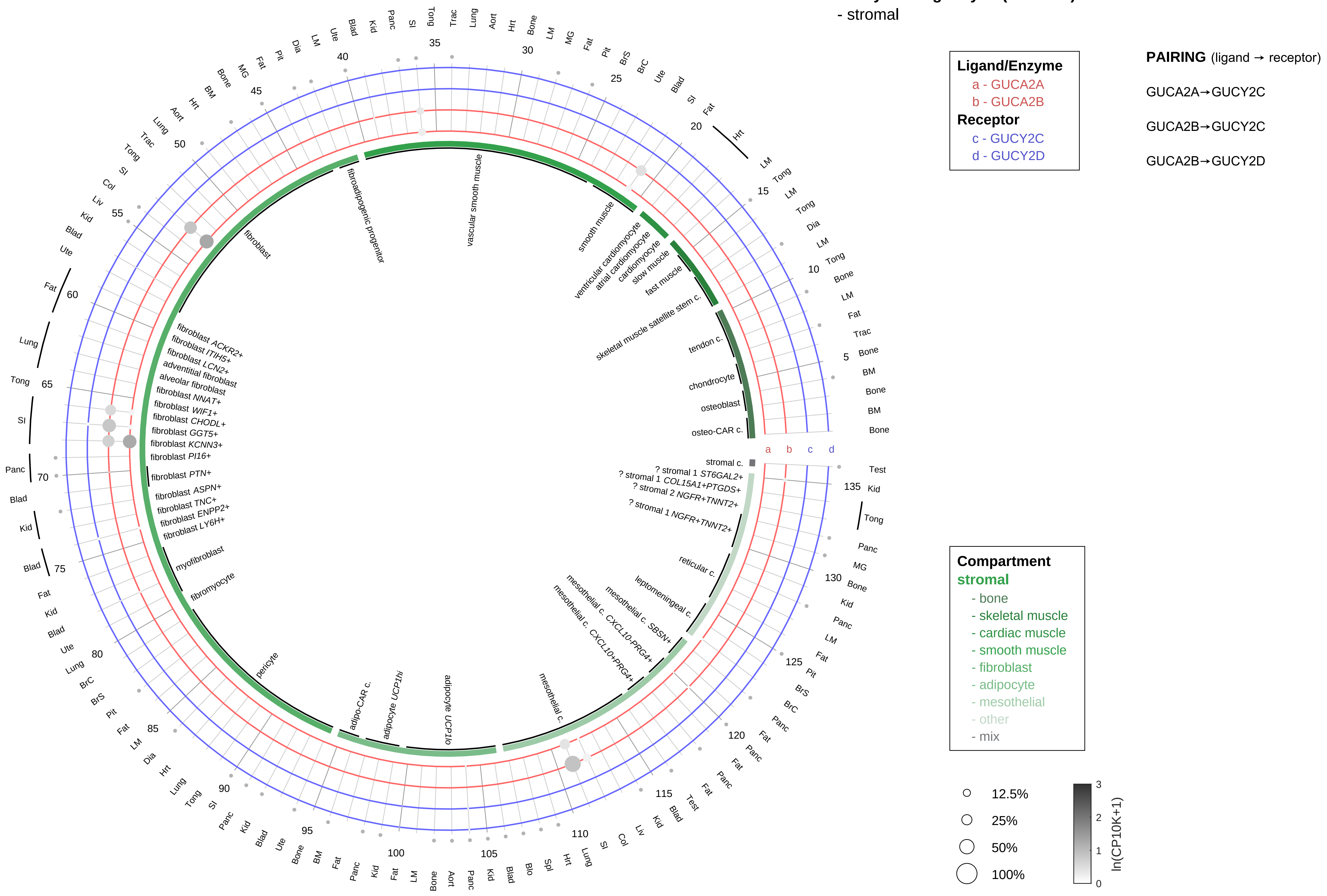

Abbrev.	Tissue
Aort	Aorta
Blad	Bladder
Blo	Blood
Bone	Bone
BM	Bone marrow
BrC	Brain cortex
BrS	Brainstem
Col	Colon
Dia	Diaphragm
Ret	Eye retina
Fat	Fat
Hrt	Heart
Pit	Hypothalamus/Pituitary
Kid	Kidney
LM	Limb muscle
Liv	Liver
Lung	Lung
MG	Mammary gland
Panc	Pancreas
Skin	Skin
SI	Small intestine
Spl	Spleen
Test	Testes
Thym	Thymus
Tong	Tongue
Trac	Trachea
Ute	Uterus

Guanylin/Uroguanylin (GN/UGN)
- lymphoid

PAIRING (ligand → receptor)

GUCA2A→GUCY2C

GUCA2B→GUCY2C

GUCA2B→GUCY2D

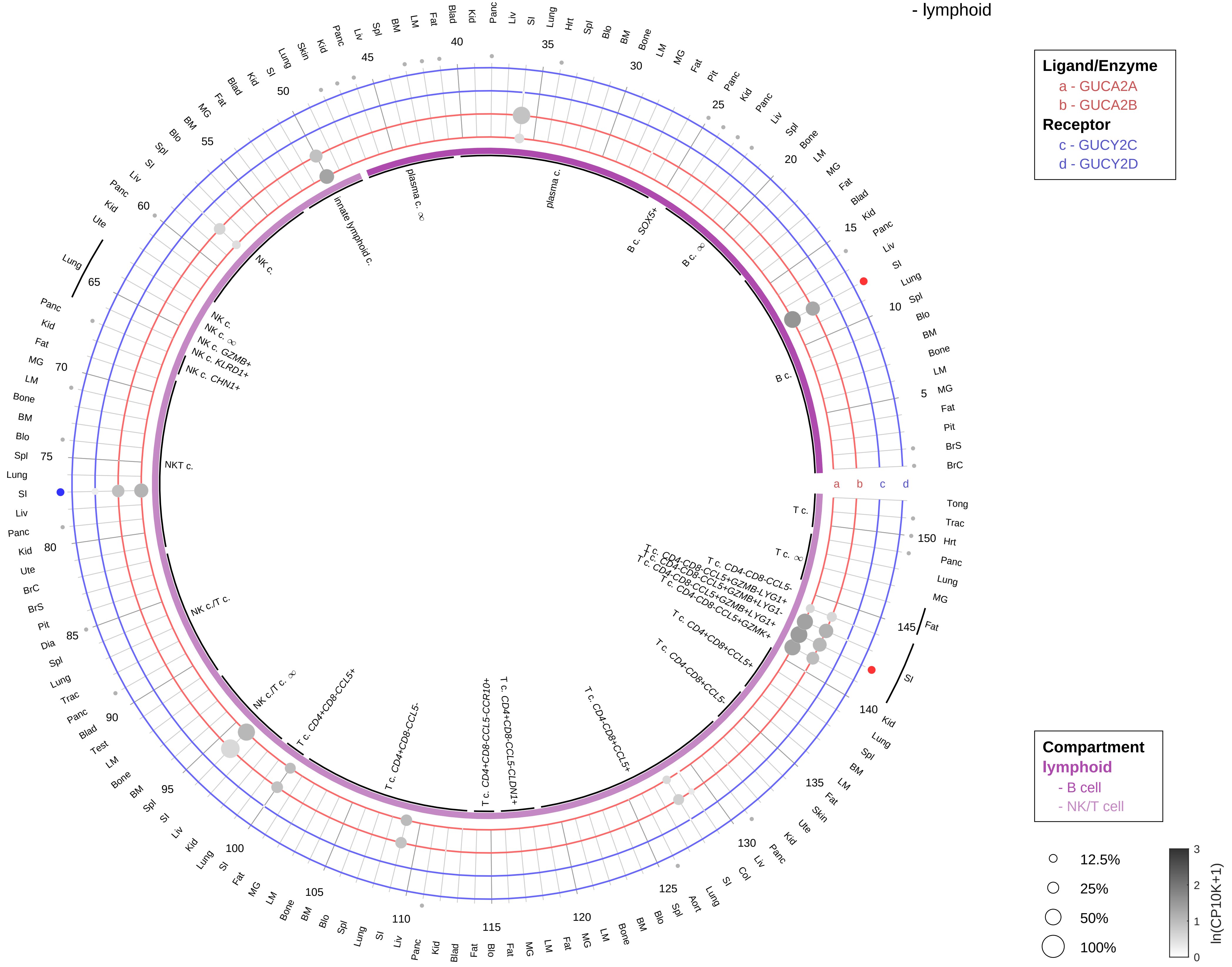

Abbrev.	Tissue
Aort	Aorta
Blad	Bladder
Blo	Blood
Bone	Bone
BM	Bone marrow
BrC	Brain cortex
BrS	Brainstem
Col	Colon
Dia	Diaphragm
Ret	Eye retina
Fat	Fat
Hrt	Heart
Pit	Hypothalamus/Pituitary
Kid	Kidney
LM	Limb muscle
Liv	Liver
Lung	Lung
MG	Mammary gland
Panc	Pancreas
Skin	Skin
SI	Small intestine
Spl	Spleen
Test	Testes
Thym	Thymus
Tong	Tongue
Trac	Trachea
Ute	Uterus

Guanylin/Uroguanylin (GN/UGN)
- myeloid

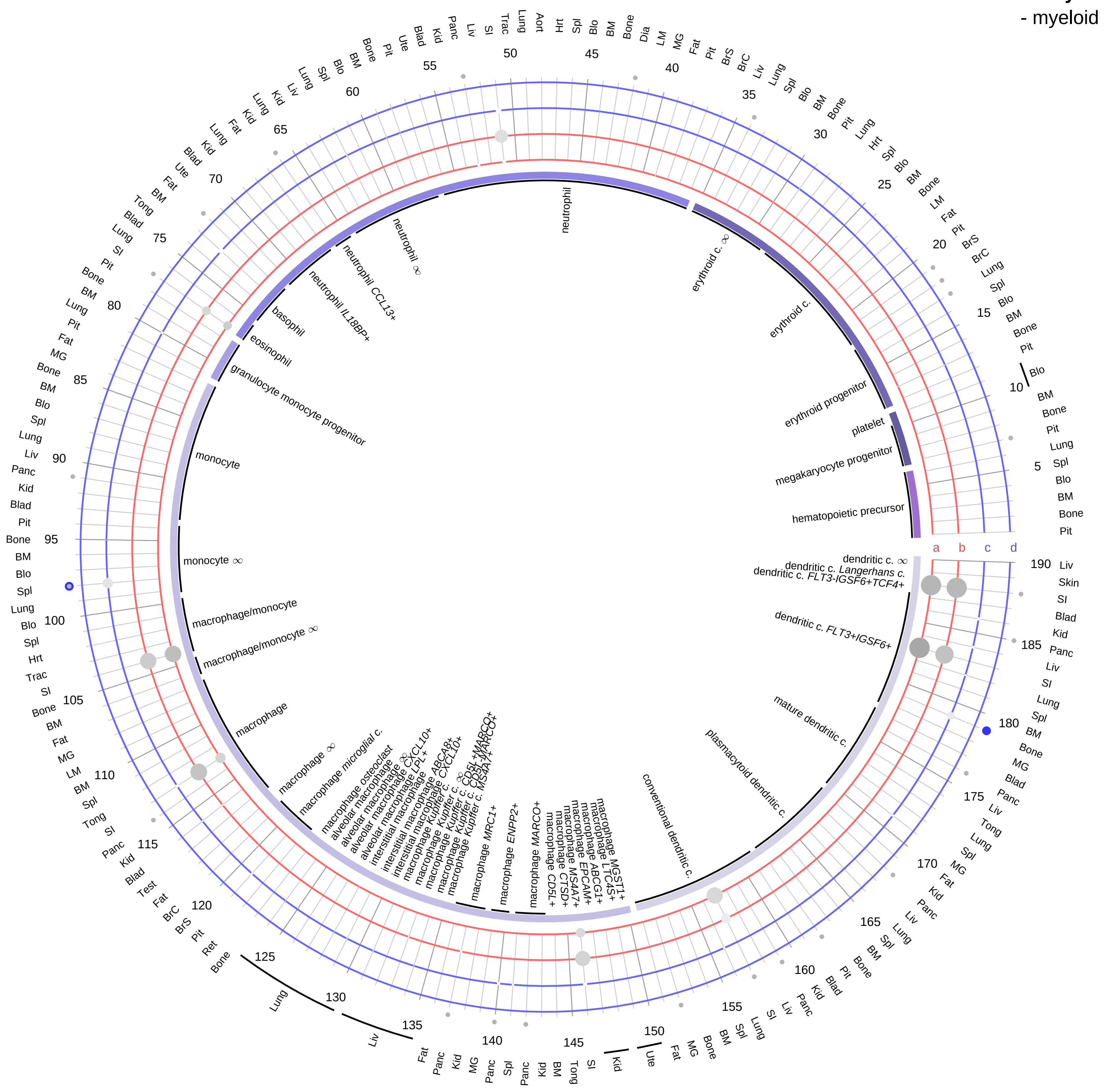

Ligand/Enzyme
a - GUCA2A
b - GUCA2B
**Receptor**
c - GUCY2C
d - GUCY2D

PAIRING (ligand → receptor)

GUCA2A→GUCY2C

GUCA2B→GUCY2C

GUCA2B→GUCY2D

Compartment

hematopoietic
- hematopoietic precursor

megakaryocyte-erythroid
- megakaryoid
- erythroid

myeloid
- granulocyte
- granulocyte monocyte progenitor
- monocyte/macrophage
- dendritic

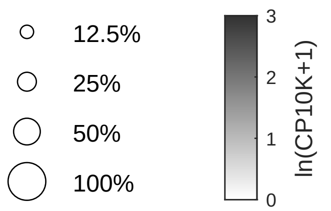

Abbrev.	Tissue
Aort	Aorta
Blad	Bladder
Blo	Blood
Bone	Bone
BM	Bone marrow
BrC	Brain cortex
BrS	Brainstem
Col	Colon
Dia	Diaphragm
Ret	Eye retina
Fat	Fat
Hrt	Heart
Pit	Hypothalamus/Pituitary
Kid	Kidney
LM	Limb muscle
Liv	Liver
Lung	Lung
MG	Mammary gland
Panc	Pancreas
Skin	Skin
SI	Small intestine
Spl	Spleen
Test	Testes
Thym	Thymus
Tong	Tongue
Trac	Trachea
Ute	Uterus

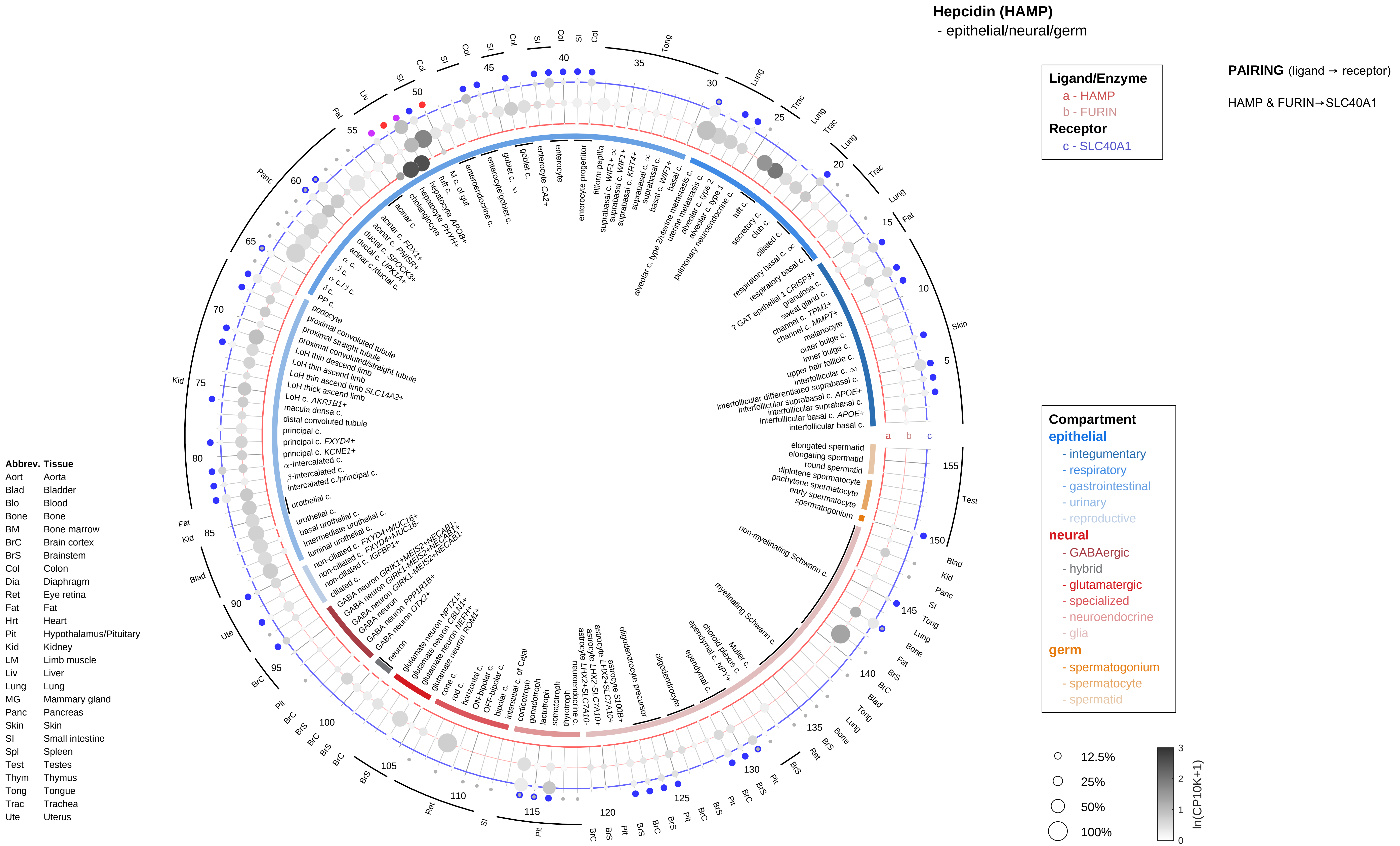
Hepcidin (HAMP)
- epithelial/neural/germ
Ligand/Enzyme
a - HAMP
b - FURIN
Receptor
c - SLC40A1
PAIRING (ligand → receptor)
HAMP & FURIN→SLC40A1
Compartment
epithelial
- integumentary
- respiratory
- gastrointestinal
- urinary
- reproductive
neural
- GABAergic
- hybrid
- glutamatergic
- specialized
- neuroendocrine
- glia
germ
- spermatogonium
- spermatocyte
- spermatid
12.5%
25%
50%
100%
ln(CP10K+1)
Abbrev. Tissue
Aort Aorta
Blad Bladder
Blo Blood
Bone Bone
BM Bone marrow
BrC Brain cortex
BrS Brainstem
Col Colon
Dia Diaphragm
Ret Eye retina
Fat Fat
Hrt Heart
Pit Hypothalamus/Pituitary
Kid Kidney
LM Limb muscle
Liv Liver
Lung Lung
MG Mammary gland
Panc Pancreas
Skin Skin
SI Small intestine
Spl Spleen
Test Testes
Thym Thymus
Tong Tongue
Trac Trachea
Ute Uterus

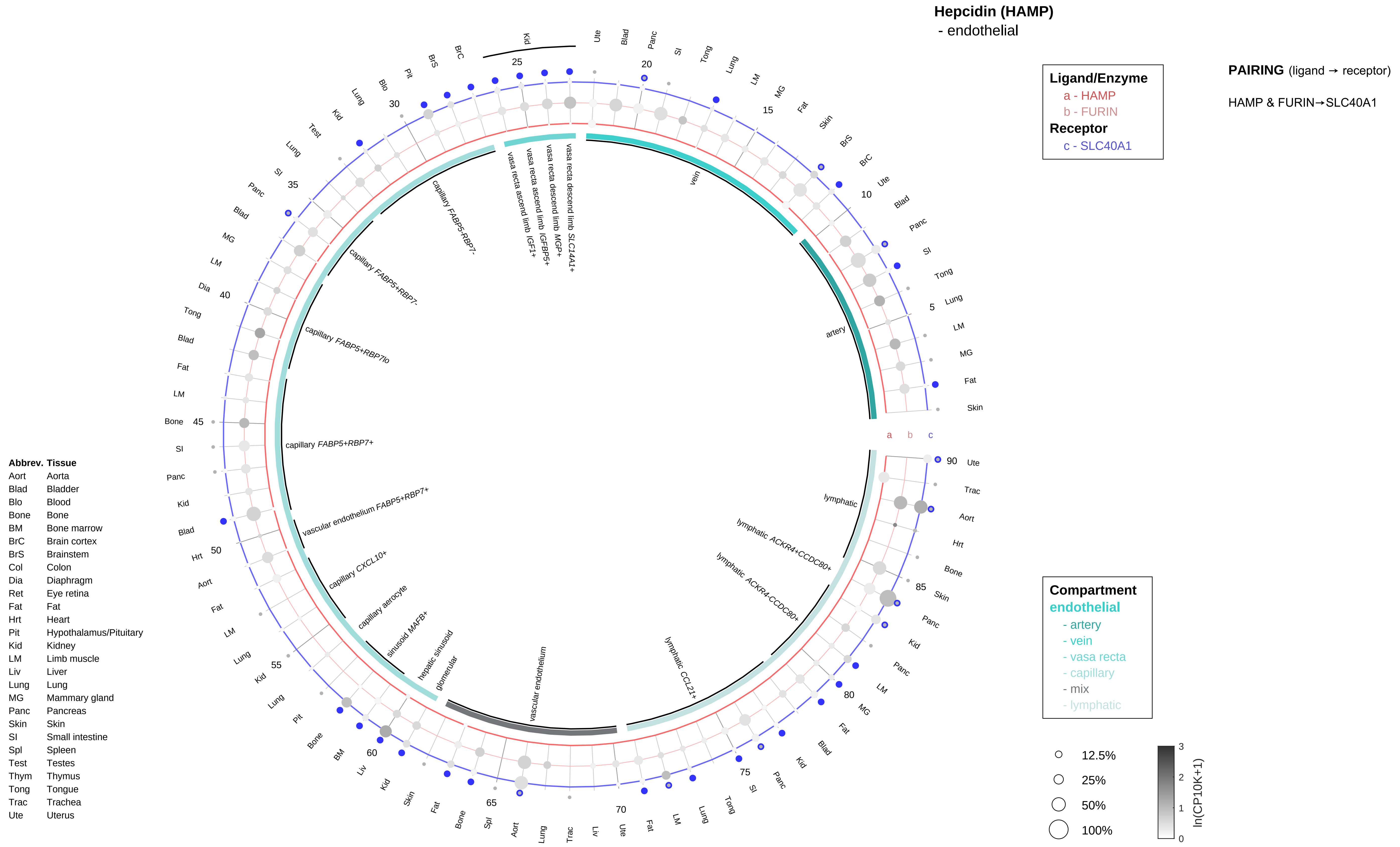

Hepcidin (HAMP)
- endothelial
Ligand/Enzyme
a - HAMP
b - FURIN
Receptor
c - SLC40A1
PAIRING (ligand → receptor)
HAMP & FURIN→SLC40A1
Compartment
endothelial
- artery
- vein
- vasa recta
- capillary
- mix
- lymphatic
12.5%
25%
50%
100%
ln(CP10K+1)
vein
artery
vasa recta descend limb SLC14A1+
vasa recta descend limb MGP+
vasa recta ascend limb IGFBP5+
vasa recta ascend limb IGF1+
capillary FABP5-RBP7-
capillary FABP5+RBP7-
capillary FABP5+RBP7lo
capillary FABP5+RBP7+
vascular endothelium FABP5+RBP7+
capillary CXCL10+
capillary aerocyte
sinusoid MAFB+
hepatic sinusoid
glomerular
vascular endothelium
lymphatic CCL21+
lymphatic ACKR4-CCDC80+
lymphatic ACKR4+CCDC80+
lymphatic
Abbrev. Tissue
Aort Aorta
Blad Bladder
Blo Blood
Bone Bone
BM Bone marrow
BrC Brain cortex
BrS Brainstem
Col Colon
Dia Diaphragm
Ret Eye retina
Fat Fat
Hrt Heart
Pit Hypothalamus/Pituitary
Kid Kidney
LM Limb muscle
Liv Liver
Lung Lung
MG Mammary gland
Panc Pancreas
Skin Skin
SI Small intestine
Spl Spleen
Test Testes
Thym Thymus
Tong Tongue
Trac Trachea
Ute Uterus

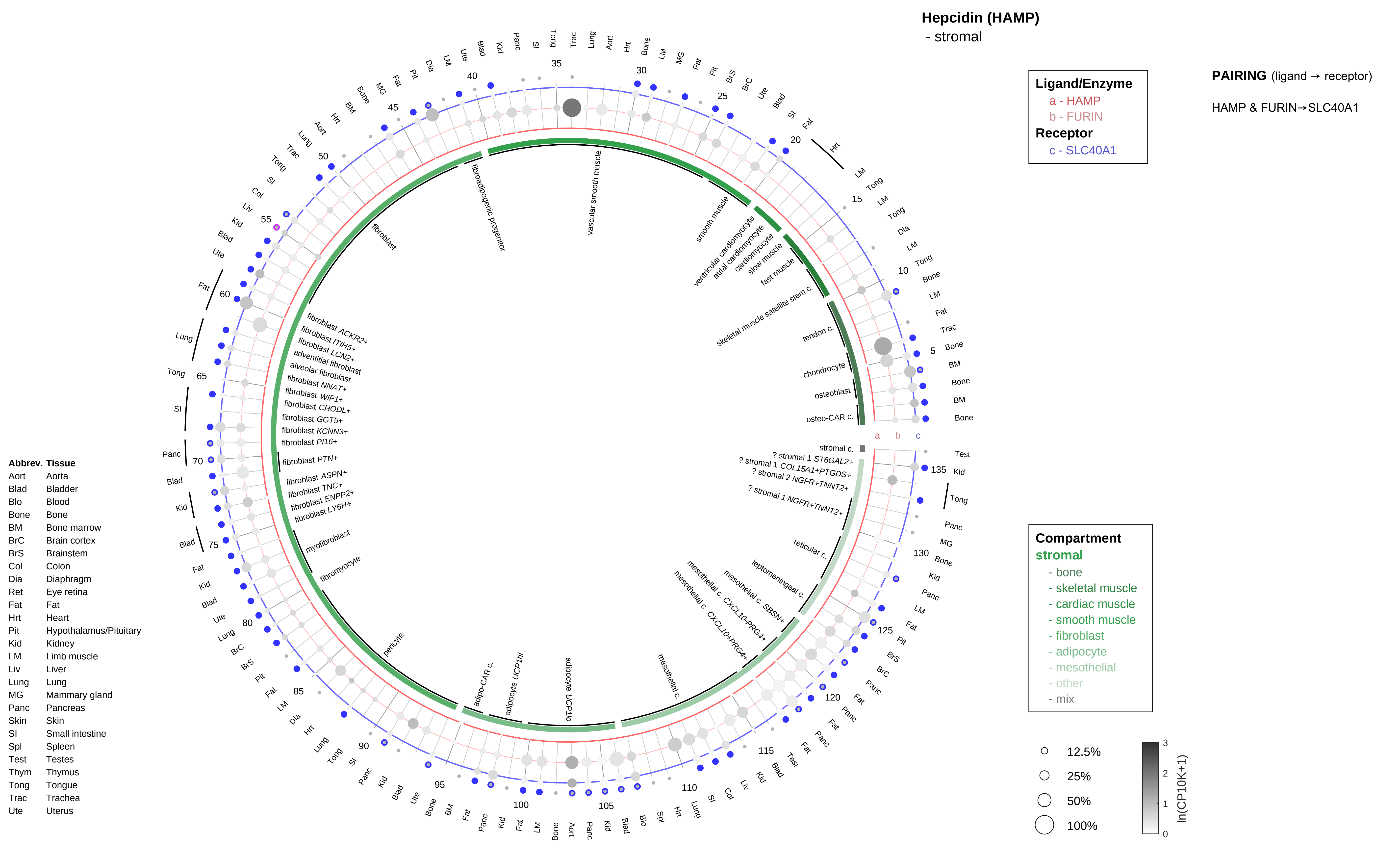
Hepcidin (HAMP)
- stromal
Ligand/Enzyme
a - HAMP
b - FURIN
Receptor
c - SLC40A1
PAIRING (ligand → receptor)
HAMP & FURIN→SLC40A1
Compartment
stromal
- bone
- skeletal muscle
- cardiac muscle
- smooth muscle
- fibroblast
- adipocyte
- mesothelial
- other
- mix
12.5%
25%
50%
100%
ln(CP10K+1)
Abbrev. Tissue
Aort Aorta
Blad Bladder
Blo Blood
Bone Bone
BM Bone marrow
BrC Brain cortex
BrS Brainstem
Col Colon
Dia Diaphragm
Ret Eye retina
Fat Fat
Hrt Heart
Pit Hypothalamus/Pituitary
Kid Kidney
LM Limb muscle
Liv Liver
Lung Lung
MG Mammary gland
Panc Pancreas
Skin Skin
SI Small intestine
Spl Spleen
Test Testes
Thym Thymus
Tong Tongue
Trac Trachea
Ute Uterus
fibroadipogenic progenitor
vascular smooth muscle
smooth muscle
ventricular cardiomyocyte
atrial cardiomyocyte
cardiomyocyte
slow muscle
fast muscle
skeletal muscle satellite stem c.
tendon c.
chondrocyte
osteoblast
osteo-CAR c.
stromal c.
? stromal 1 ST6GAL2+
? stromal 1 COL15A1+PTGDS+
? stromal 2 NGFR+TNNT2+
? stromal 1 NGFR+TNNT2+
reticular c.
leptomeningeal c.
mesothelial c. SBSN+
mesothelial c. CXCL10-PRG4+
mesothelial c. CXCL10+PRG4+
mesothelial c.
adipocyte UCP1lo
adipocyte UCP1hi
adipo-CAR c.
pericyte
fibromyocyte
myofibroblast
fibroblast LY6H+
fibroblast ENPP2+
fibroblast TNC+
fibroblast ASPN+
fibroblast PTN+
fibroblast PI16+
fibroblast KCNN3+
fibroblast GGT5+
fibroblast CHODL+
fibroblast WIF1+
fibroblast NNAT+
alveolar fibroblast
adventitial fibroblast
fibroblast LCN2+
fibroblast ITIH5+
fibroblast ACKR2+
fibroblast

Hepcidin (HAMP)
- lymphoid

PAIRING (ligand → receptor)

HAMP & FURIN→SLC40A1

Ligand/Enzyme
a - HAMP
b - FURIN
Receptor
c - SLC40A1

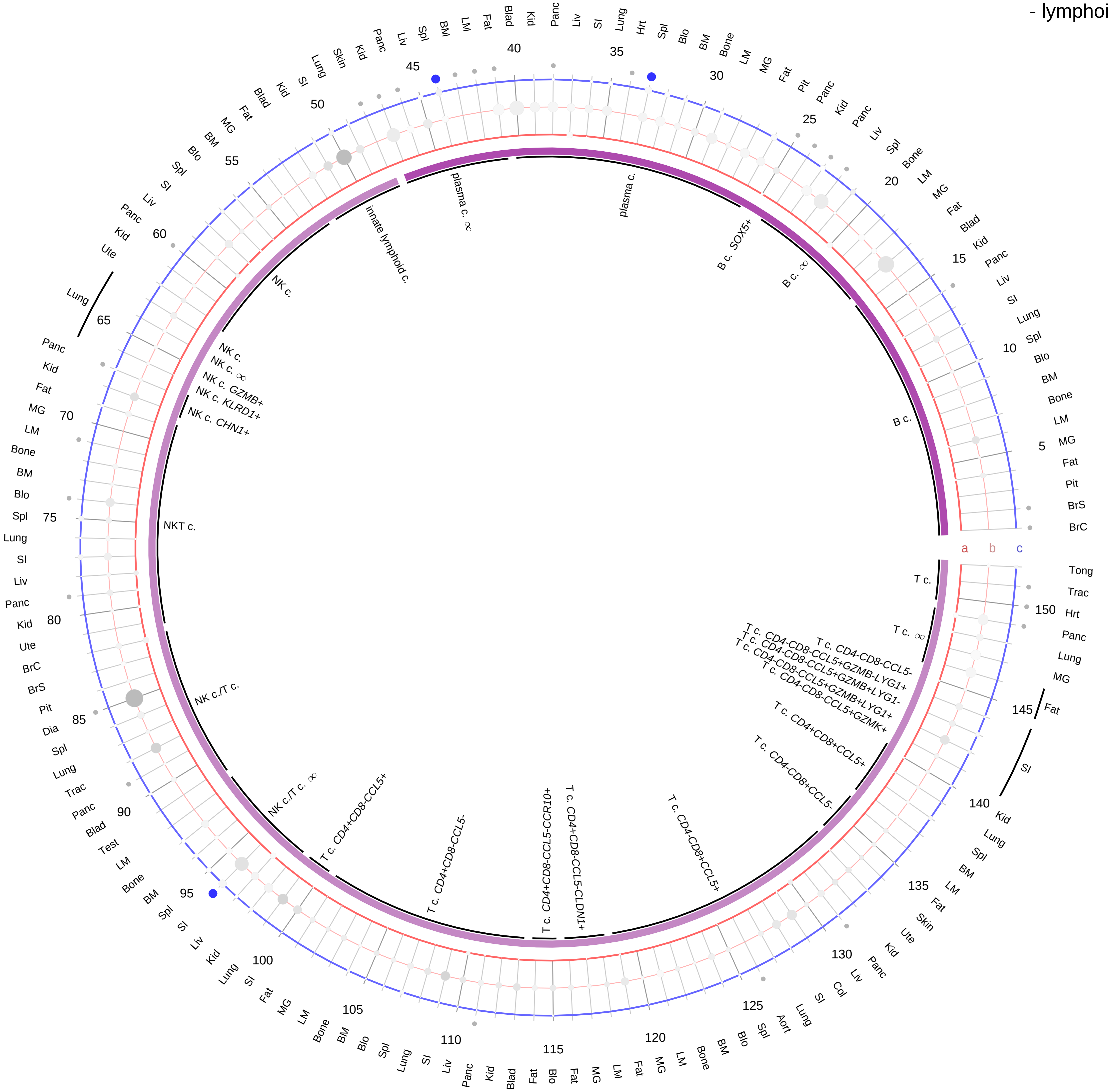

Compartment
lymphoid
- B cell
- NK/T cell

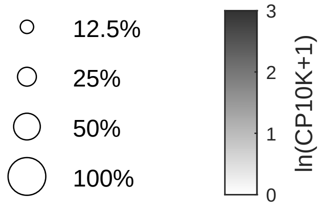

**Abbrev.**	**Tissue**
Aort	Aorta
Blad	Bladder
Blo	Blood
Bone	Bone
BM	Bone marrow
BrC	Brain cortex
BrS	Brainstem
Col	Colon
Dia	Diaphragm
Ret	Eye retina
Fat	Fat
Hrt	Heart
Pit	Hypothalamus/Pituitary
Kid	Kidney
LM	Limb muscle
Liv	Liver
Lung	Lung
MG	Mammary gland
Panc	Pancreas
Skin	Skin
SI	Small intestine
Spl	Spleen
Test	Testes
Thym	Thymus
Tong	Tongue
Trac	Trachea
Ute	Uterus

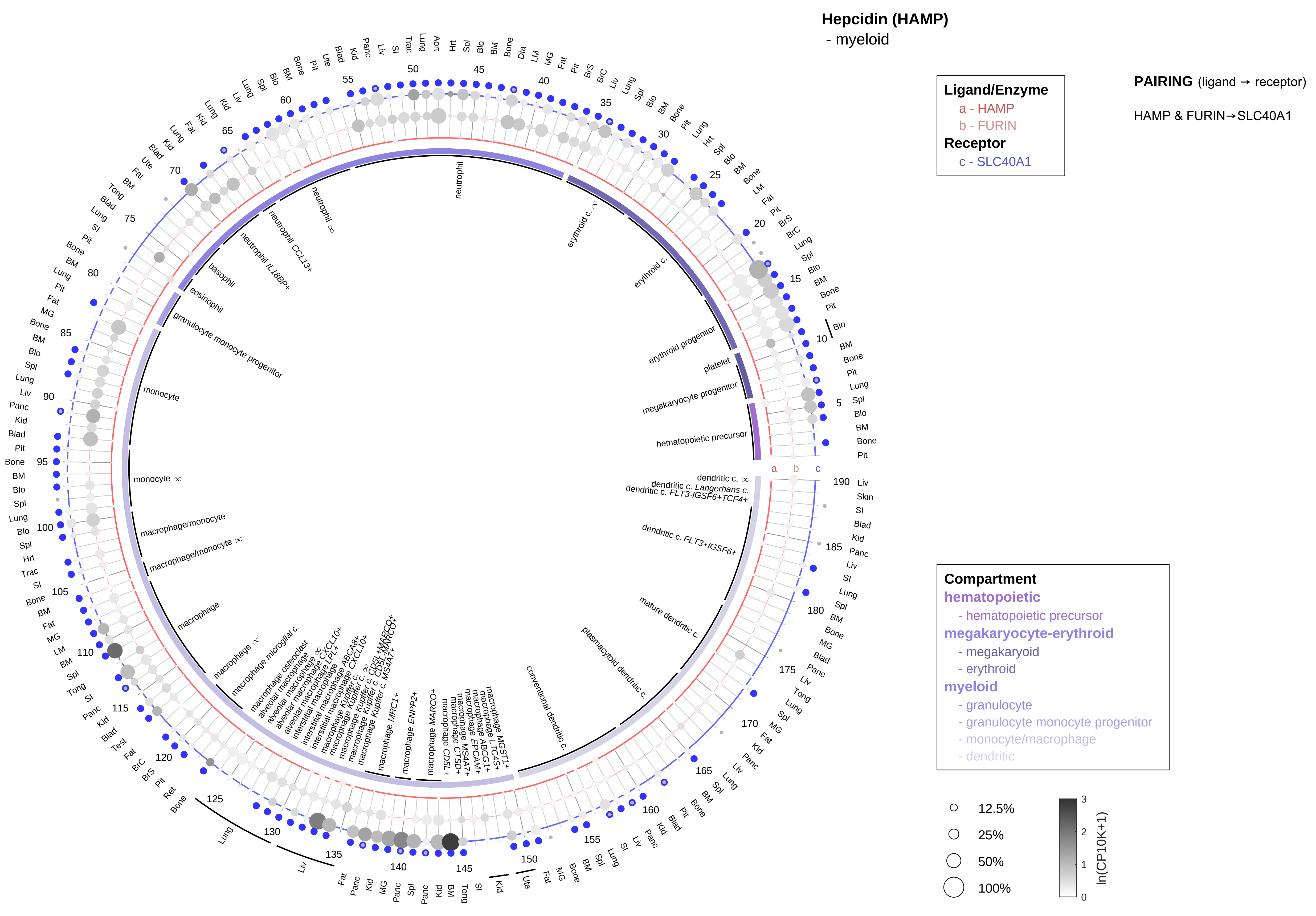

Abbrev.	Tissue
Aort	Aorta
Blad	Bladder
Blo	Blood
Bone	Bone
BM	Bone marrow
BrC	Brain cortex
BrS	Brainstem
Col	Colon
Dia	Diaphragm
Ret	Eye retina
Fat	Fat
Hrt	Heart
Pit	Hypothalamus/Pituitary
Kid	Kidney
LM	Limb muscle
Liv	Liver
Lung	Lung
MG	Mammary gland
Panc	Pancreas
Skin	Skin
SI	Small intestine
Spl	Spleen
Test	Testes
Thym	Thymus
Tong	Tongue
Trac	Trachea
Ute	Uterus

Hypocretin (Orexin)
- epithelial/neural/germ

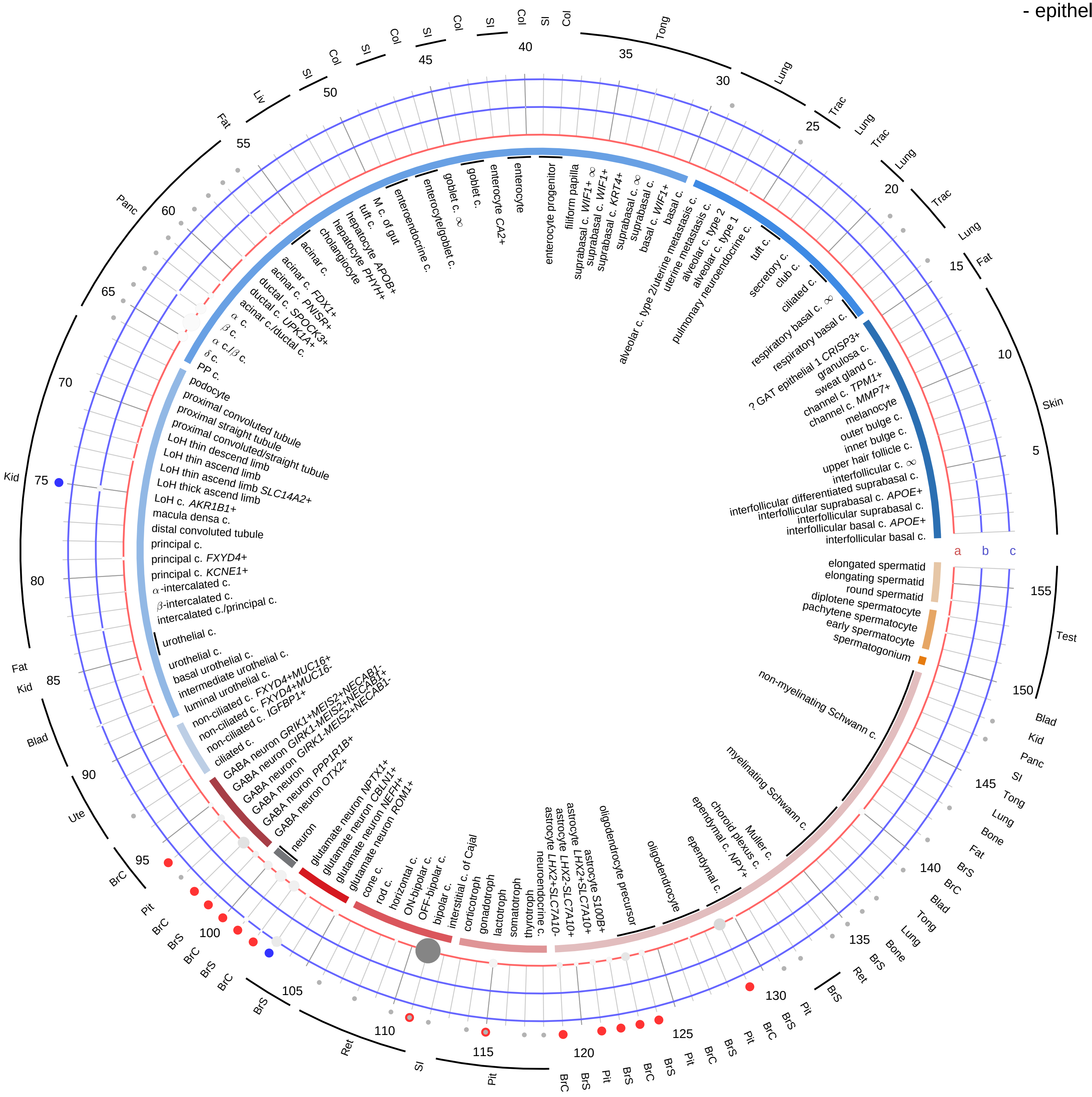

Ligand/Enzyme
a - HCRT
Receptor
b - HCRTR1
c - HCRTR2

PAIRING (ligand → receptor)

HCRT→HCRTR1

HCRT→HCRTR2

Compartment
epithelial
- integumentary
- respiratory
- gastrointestinal
- urinary
- reproductive

neural
- GABAergic
- hybrid
- glutamatergic
- specialized
- neuroendocrine
- glia

germ
- spermatogonium
- spermatocyte
- spermatid

12.5%
25%
50%
100%

ln(CP10K+1): 0, 1, 2, 3

Abbrev.	Tissue
Aort	Aorta
Blad	Bladder
Blo	Blood
Bone	Bone
BM	Bone marrow
BrC	Brain cortex
BrS	Brainstem
Col	Colon
Dia	Diaphragm
Ret	Eye retina
Fat	Fat
Hrt	Heart
Pit	Hypothalamus/Pituitary
Kid	Kidney
LM	Limb muscle
Liv	Liver
Lung	Lung
MG	Mammary gland
Panc	Pancreas
Skin	Skin
SI	Small intestine
Spl	Spleen
Test	Testes
Thym	Thymus
Tong	Tongue
Trac	Trachea
Ute	Uterus

Hypocretin (Orexin)
- endothelial

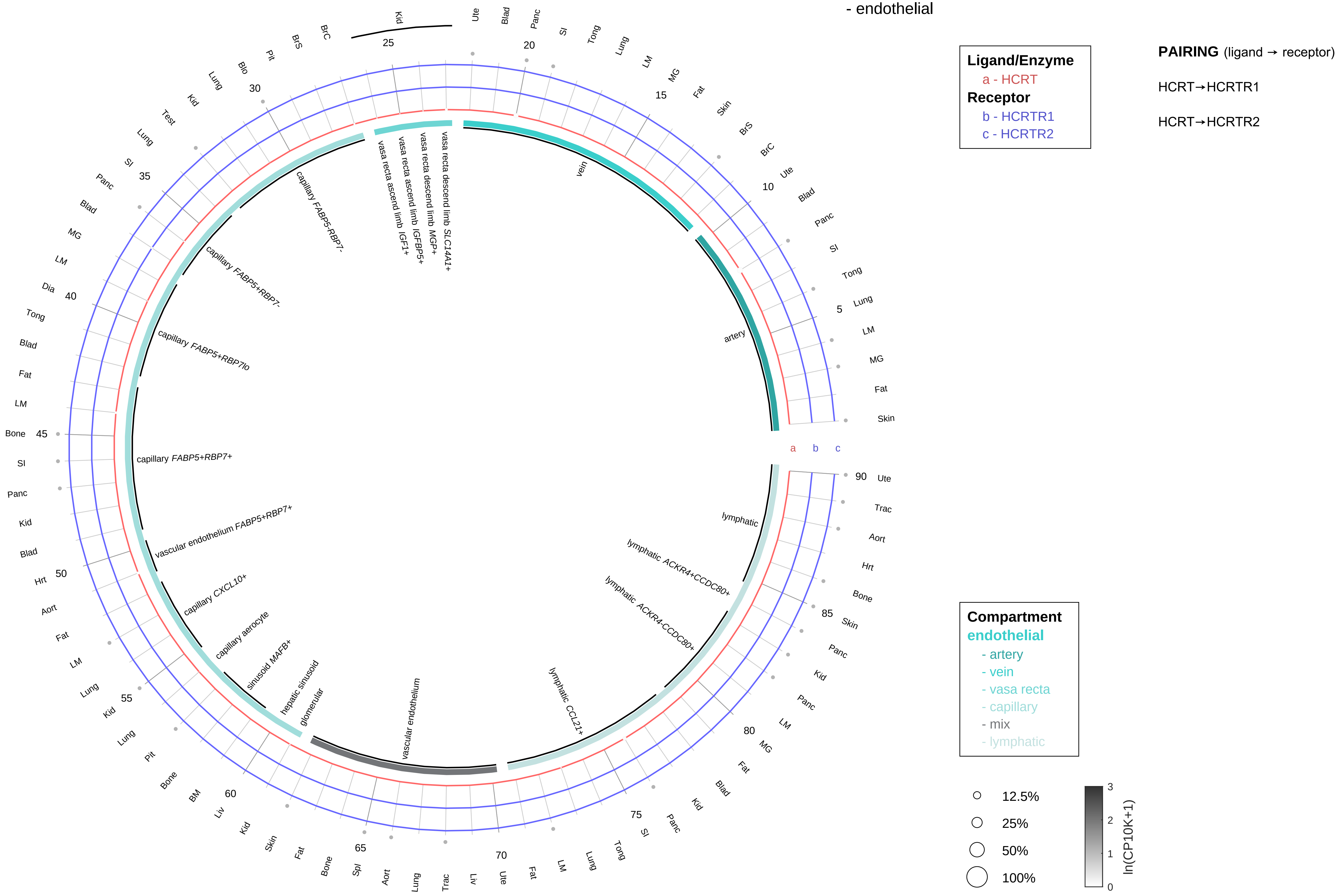

Abbrev.	Tissue
Aort	Aorta
Blad	Bladder
Blo	Blood
Bone	Bone
BM	Bone marrow
BrC	Brain cortex
BrS	Brainstem
Col	Colon
Dia	Diaphragm
Ret	Eye retina
Fat	Fat
Hrt	Heart
Pit	Hypothalamus/Pituitary
Kid	Kidney
LM	Limb muscle
Liv	Liver
Lung	Lung
MG	Mammary gland
Panc	Pancreas
Skin	Skin
SI	Small intestine
Spl	Spleen
Test	Testes
Thym	Thymus
Tong	Tongue
Trac	Trachea
Ute	Uterus

Hypocretin (Orexin)
- stromal

Ligand/Enzyme
a - HCRT
Receptor
b - HCRTR1
c - HCRTR2

PAIRING (ligand → receptor)

HCRT→HCRTR1

HCRT→HCRTR2

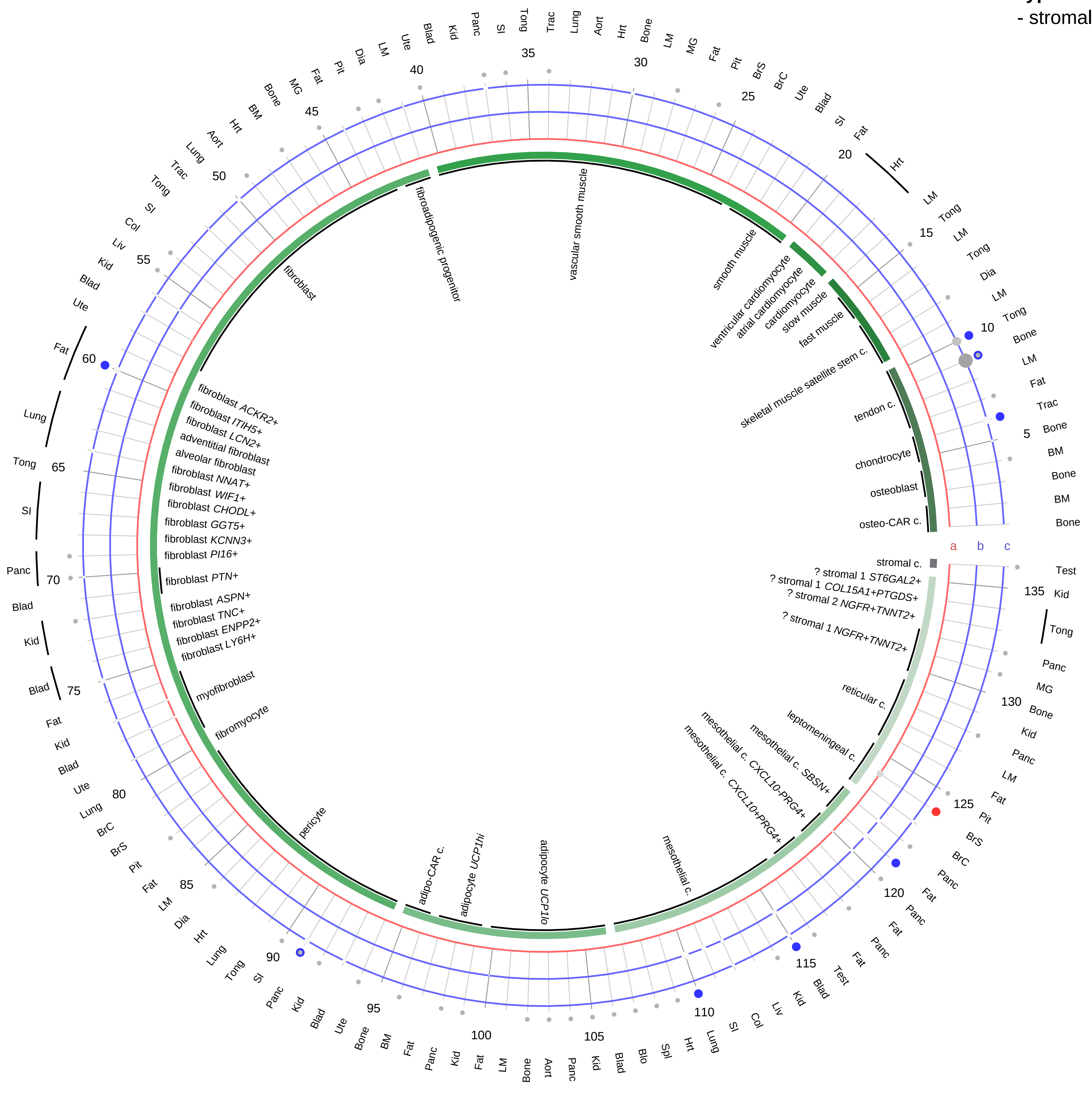

Abbrev.	Tissue
Aort	Aorta
Blad	Bladder
Blo	Blood
Bone	Bone
BM	Bone marrow
BrC	Brain cortex
BrS	Brainstem
Col	Colon
Dia	Diaphragm
Ret	Eye retina
Fat	Fat
Hrt	Heart
Pit	Hypothalamus/Pituitary
Kid	Kidney
LM	Limb muscle
Liv	Liver
Lung	Lung
MG	Mammary gland
Panc	Pancreas
Skin	Skin
SI	Small intestine
Spl	Spleen
Test	Testes
Thym	Thymus
Tong	Tongue
Trac	Trachea
Ute	Uterus

Compartment
stromal
- bone
- skeletal muscle
- cardiac muscle
- smooth muscle
- fibroblast
- adipocyte
- mesothelial
- other
- mix

12.5%
25%
50%
100%

ln(CP10K+1): 0, 1, 2, 3

Hypocretin (Orexin)
- lymphoid

Ligand/Enzyme
a - HCRT
Receptor
b - HCRTR1
c - HCRTR2

PAIRING (ligand → receptor)

HCRT→HCRTR1

HCRT→HCRTR2

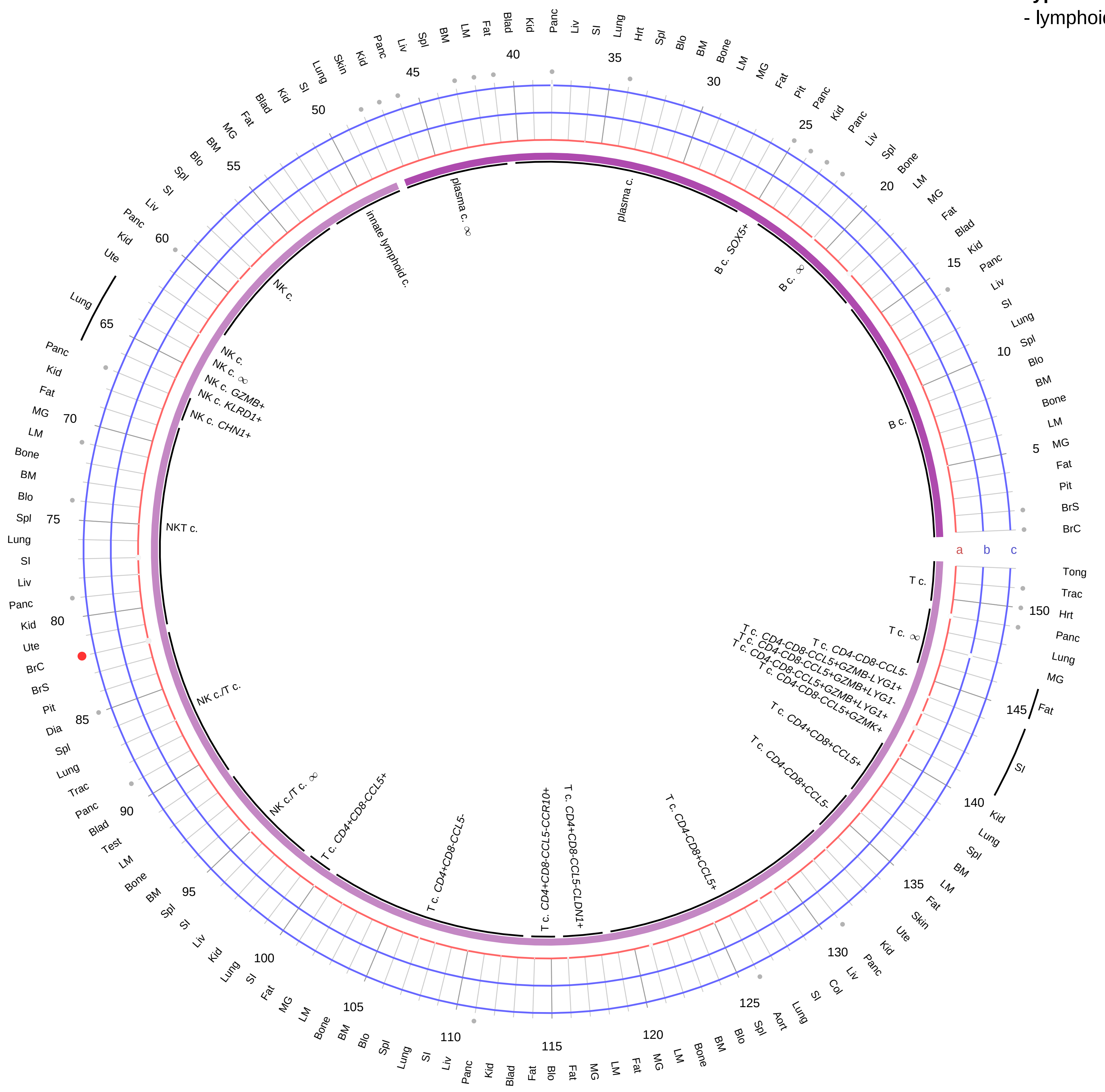

Compartment
lymphoid
- B cell
- NK/T cell

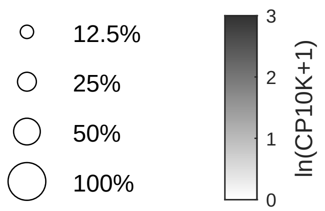

Abbrev.	Tissue
Aort	Aorta
Blad	Bladder
Blo	Blood
Bone	Bone
BM	Bone marrow
BrC	Brain cortex
BrS	Brainstem
Col	Colon
Dia	Diaphragm
Ret	Eye retina
Fat	Fat
Hrt	Heart
Pit	Hypothalamus/Pituitary
Kid	Kidney
LM	Limb muscle
Liv	Liver
Lung	Lung
MG	Mammary gland
Panc	Pancreas
Skin	Skin
SI	Small intestine
Spl	Spleen
Test	Testes
Thym	Thymus
Tong	Tongue
Trac	Trachea
Ute	Uterus

Hypocretin (Orexin)
- myeloid

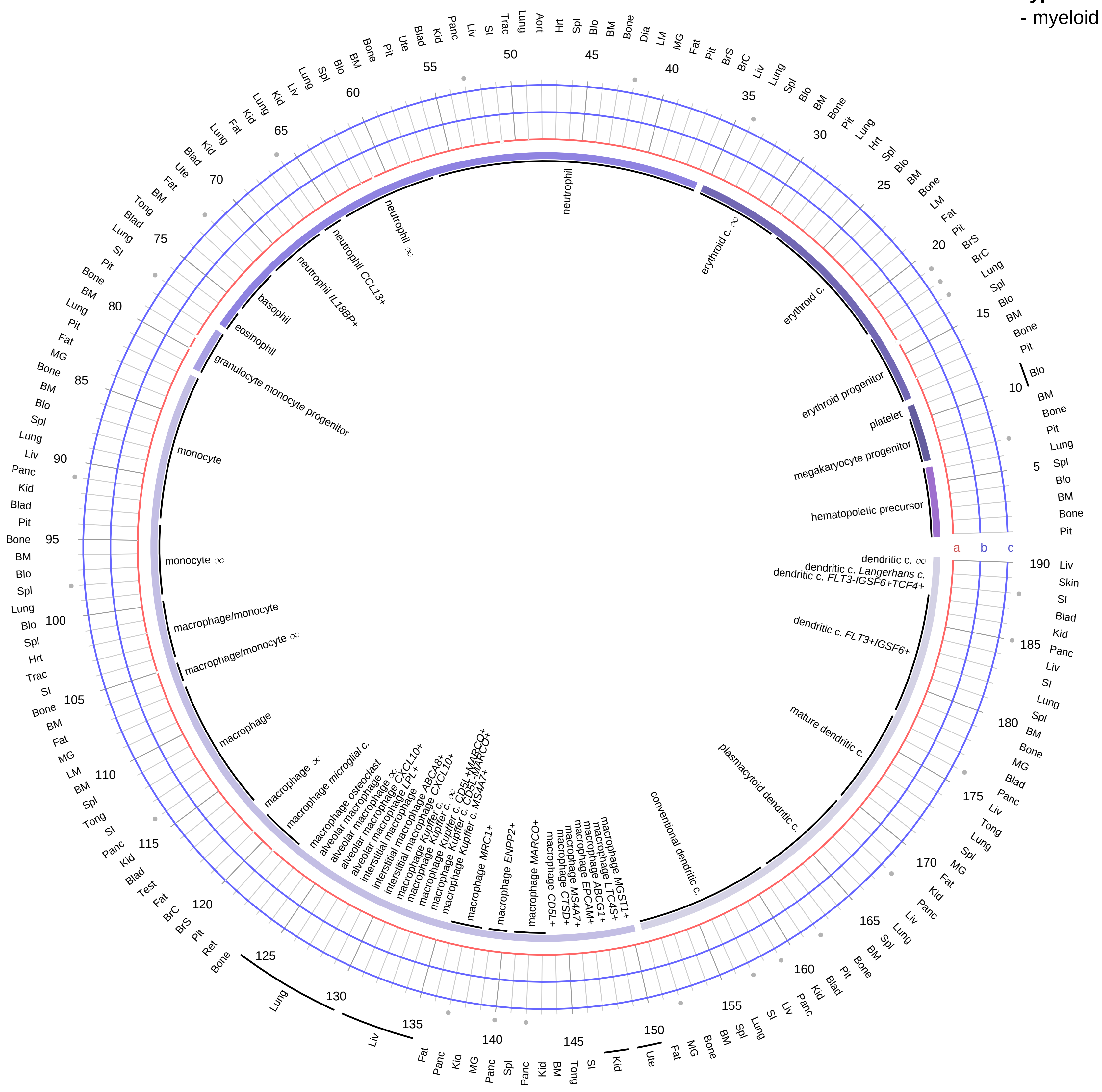

Ligand/Enzyme
a - HCRT
Receptor
b - HCRTR1
c - HCRTR2

PAIRING (ligand → receptor)

HCRT→HCRTR1

HCRT→HCRTR2

Abbrev.	Tissue
Aort	Aorta
Blad	Bladder
Blo	Blood
Bone	Bone
BM	Bone marrow
BrC	Brain cortex
BrS	Brainstem
Col	Colon
Dia	Diaphragm
Ret	Eye retina
Fat	Fat
Hrt	Heart
Pit	Hypothalamus/Pituitary
Kid	Kidney
LM	Limb muscle
Liv	Liver
Lung	Lung
MG	Mammary gland
Panc	Pancreas
Skin	Skin
SI	Small intestine
Spl	Spleen
Test	Testes
Thym	Thymus
Tong	Tongue
Trac	Trachea
Ute	Uterus

Compartment
hematopoietic
- hematopoietic precursor
megakaryocyte-erythroid
- megakaryoid
- erythroid
myeloid
- granulocyte
- granulocyte monocyte progenitor
- monocyte/macrophage
- dendritic

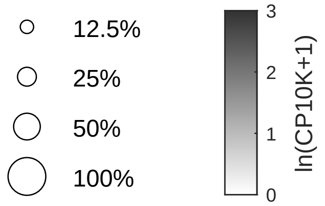

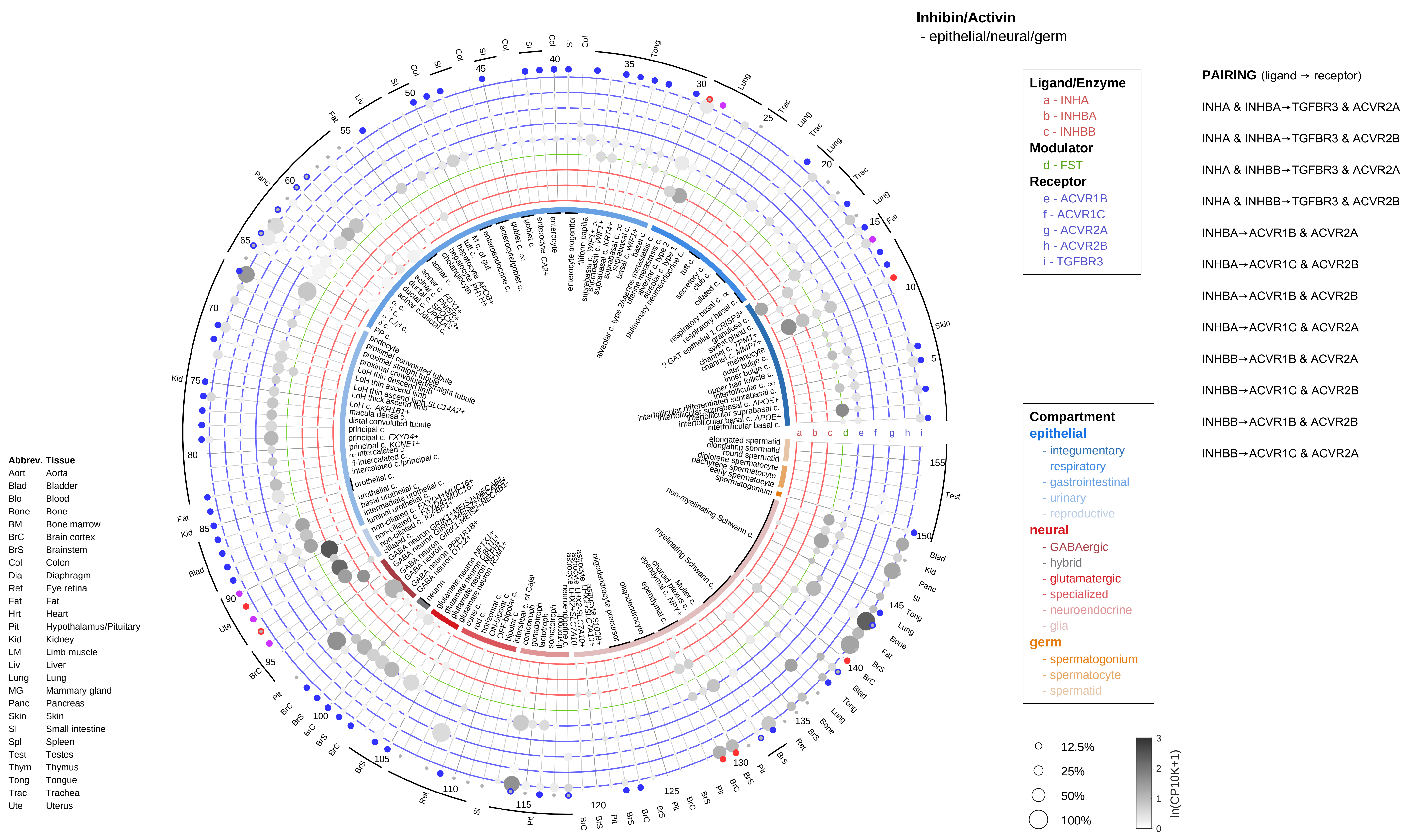

Inhibin/Activin
- epithelial/neural/germ
Ligand/Enzyme
a - INHA
b - INHBA
c - INHBB
Modulator
d - FST
Receptor
e - ACVR1B
f - ACVR1C
g - ACVR2A
h - ACVR2B
i - TGFBR3
PAIRING (ligand → receptor)
INHA & INHBA→TGFBR3 & ACVR2A
INHA & INHBA→TGFBR3 & ACVR2B
INHA & INHBB→TGFBR3 & ACVR2A
INHA & INHBB→TGFBR3 & ACVR2B
INHBA→ACVR1B & ACVR2A
INHBA→ACVR1C & ACVR2B
INHBA→ACVR1B & ACVR2B
INHBA→ACVR1C & ACVR2A
INHBB→ACVR1B & ACVR2A
INHBB→ACVR1C & ACVR2B
INHBB→ACVR1B & ACVR2B
INHBB→ACVR1C & ACVR2A
Compartment
epithelial
- integumentary
- respiratory
- gastrointestinal
- urinary
- reproductive
neural
- GABAergic
- hybrid
- glutamatergic
- specialized
- neuroendocrine
- glia
germ
- spermatogonium
- spermatocyte
- spermatid
12.5%
25%
50%
100%
ln(CP10K+1)
0
1
2
3
Abbrev. Tissue
Aort Aorta
Blad Bladder
Blo Blood
Bone Bone
BM Bone marrow
BrC Brain cortex
BrS Brainstem
Col Colon
Dia Diaphragm
Ret Eye retina
Fat Fat
Hrt Heart
Pit Hypothalamus/Pituitary
Kid Kidney
LM Limb muscle
Liv Liver
Lung Lung
MG Mammary gland
Panc Pancreas
Skin Skin
SI Small intestine
Spl Spleen
Test Testes
Thym Thymus
Tong Tongue
Trac Trachea
Ute Uterus

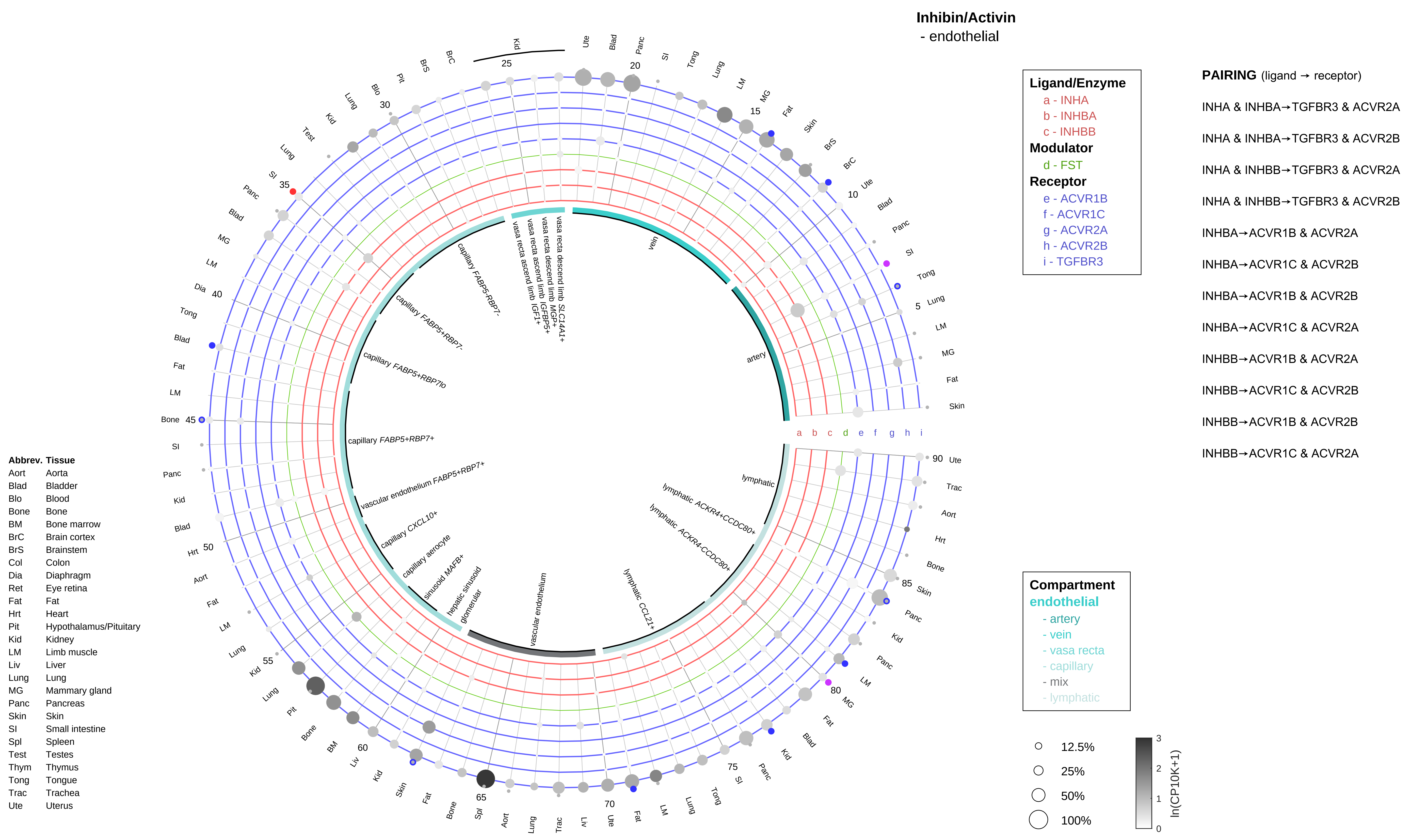

Inhibin/Activin
- endothelial
Ligand/Enzyme
a - INHA
b - INHBA
c - INHBB
Modulator
d - FST
Receptor
e - ACVR1B
f - ACVR1C
g - ACVR2A
h - ACVR2B
i - TGFBR3
PAIRING (ligand → receptor)
INHA & INHBA→TGFBR3 & ACVR2A
INHA & INHBA→TGFBR3 & ACVR2B
INHA & INHBB→TGFBR3 & ACVR2A
INHA & INHBB→TGFBR3 & ACVR2B
INHBA→ACVR1B & ACVR2A
INHBA→ACVR1C & ACVR2B
INHBA→ACVR1B & ACVR2B
INHBA→ACVR1C & ACVR2A
INHBB→ACVR1B & ACVR2A
INHBB→ACVR1C & ACVR2B
INHBB→ACVR1B & ACVR2B
INHBB→ACVR1C & ACVR2A
Compartment
endothelial
- artery
- vein
- vasa recta
- capillary
- mix
- lymphatic
12.5%
25%
50%
100%
ln(CP10K+1)
0
1
2
3
vein
artery
lymphatic
lymphatic ACKR4+CCDC80+
lymphatic ACKR4-CCDC80+
lymphatic CCL21+
vascular endothelium
glomerular
hepatic sinusoid
sinusoid MAFB+
capillary aerocyte
capillary CXCL10+
vascular endothelium FABP5+RBP7+
capillary FABP5+RBP7+
capillary FABP5+RBP7lo
capillary FABP5+RBP7-
capillary FABP5-RBP7-
vasa recta ascend limb IGF1+
vasa recta ascend limb IGFBP5+
vasa recta descend limb MGP+
vasa recta descend limb SLC14A1+
a b c d e f g h i
Abbrev. Tissue
Aort Aorta
Blad Bladder
Blo Blood
Bone Bone
BM Bone marrow
BrC Brain cortex
BrS Brainstem
Col Colon
Dia Diaphragm
Ret Eye retina
Fat Fat
Hrt Heart
Pit Hypothalamus/Pituitary
Kid Kidney
LM Limb muscle
Liv Liver
Lung Lung
MG Mammary gland
Panc Pancreas
Skin Skin
SI Small intestine
Spl Spleen
Test Testes
Thym Thymus
Tong Tongue
Trac Trachea
Ute Uterus

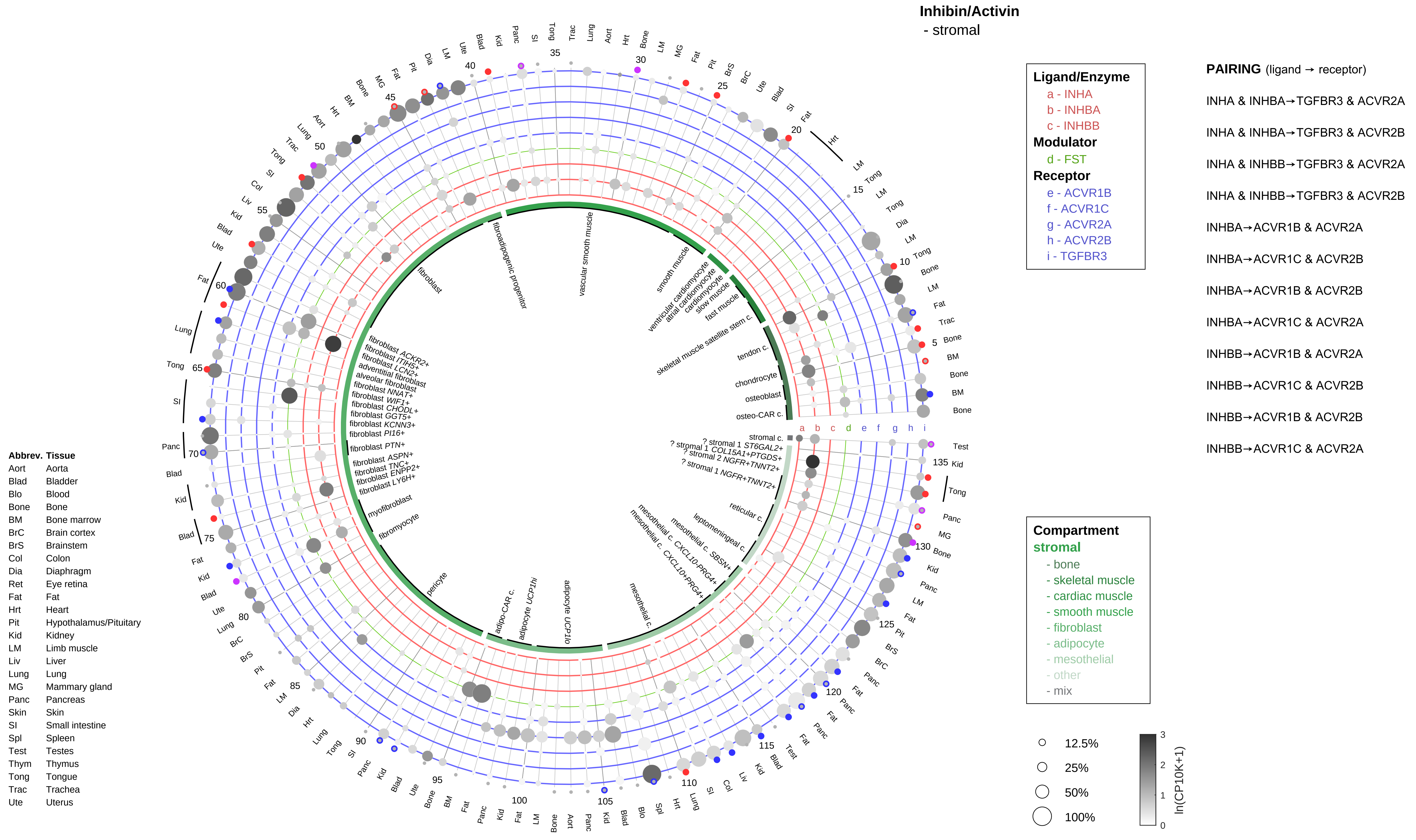
Inhibin/Activin
- stromal
Ligand/Enzyme
a - INHA
b - INHBA
c - INHBB
Modulator
d - FST
Receptor
e - ACVR1B
f - ACVR1C
g - ACVR2A
h - ACVR2B
i - TGFBR3
PAIRING (ligand → receptor)
INHA & INHBA→TGFBR3 & ACVR2A
INHA & INHBA→TGFBR3 & ACVR2B
INHA & INHBB→TGFBR3 & ACVR2A
INHA & INHBB→TGFBR3 & ACVR2B
INHBA→ACVR1B & ACVR2A
INHBA→ACVR1C & ACVR2B
INHBA→ACVR1B & ACVR2B
INHBA→ACVR1C & ACVR2A
INHBB→ACVR1B & ACVR2A
INHBB→ACVR1C & ACVR2B
INHBB→ACVR1B & ACVR2B
INHBB→ACVR1C & ACVR2A
Compartment
stromal
- bone
- skeletal muscle
- cardiac muscle
- smooth muscle
- fibroblast
- adipocyte
- mesothelial
- other
- mix
12.5%
25%
50%
100%
ln(CP10K+1)
Abbrev. Tissue
Aort Aorta
Blad Bladder
Blo Blood
Bone Bone
BM Bone marrow
BrC Brain cortex
BrS Brainstem
Col Colon
Dia Diaphragm
Ret Eye retina
Fat Fat
Hrt Heart
Pit Hypothalamus/Pituitary
Kid Kidney
LM Limb muscle
Liv Liver
Lung Lung
MG Mammary gland
Panc Pancreas
Skin Skin
SI Small intestine
Spl Spleen
Test Testes
Thym Thymus
Tong Tongue
Trac Trachea
Ute Uterus
fibroblast
fibroadipogenic progenitor
vascular smooth muscle
smooth muscle
ventricular cardiomyocyte
atrial cardiomyocyte
cardiomyocyte
slow muscle
fast muscle
skeletal muscle satellite stem c.
tendon c.
chondrocyte
osteoblast
osteo-CAR c.
stromal c.
? stromal 1 ST6GAL2+
? stromal 1 COL15A1+PTGDS+
? stromal 2 NGFR+TNNT2+
? stromal 1 NGFR+TNNT2+
reticular c.
leptomeningeal c.
mesothelial c. SBSN+
mesothelial c. CXCL10+PRG4+
mesothelial c. CXCL10+PRG4+
mesothelial c.
adipocyte UCP1lo
adipocyte UCP1hi
adipo-CAR c.
pericyte
fibromyocyte
myofibroblast
fibroblast LY6H+
fibroblast ENPP2+
fibroblast TNC+
fibroblast ASPN+
fibroblast PTN+
fibroblast PI16+
fibroblast KCNN3+
fibroblast GGT5+
fibroblast CHODL+
fibroblast WIF1+
fibroblast NNAT+
alveolar fibroblast
adventitial fibroblast
fibroblast LCN2+
fibroblast ITIH5+
fibroblast ACKR2+

Inhibin/Activin
- lymphoid

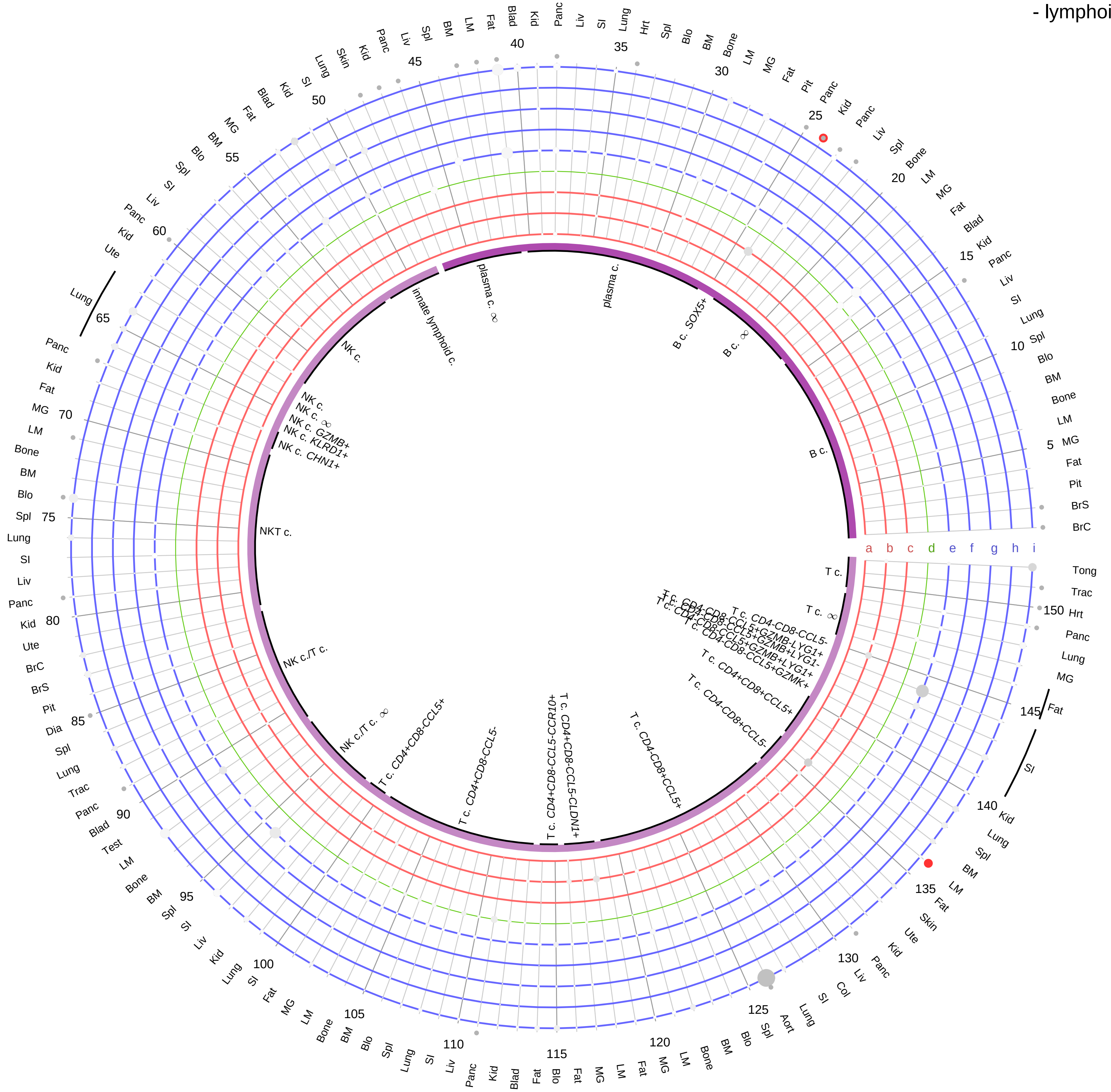

Ligand/Enzyme
a - INHA
b - INHBA
c - INHBB
Modulator
d - FST
Receptor
e - ACVR1B
f - ACVR1C
g - ACVR2A
h - ACVR2B
i - TGFBR3

PAIRING (ligand → receptor)

INHA & INHBA→TGFBR3 & ACVR2A
INHA & INHBA→TGFBR3 & ACVR2B
INHA & INHBB→TGFBR3 & ACVR2A
INHA & INHBB→TGFBR3 & ACVR2B
INHBA→ACVR1B & ACVR2A
INHBA→ACVR1C & ACVR2B
INHBA→ACVR1B & ACVR2B
INHBA→ACVR1C & ACVR2A
INHBB→ACVR1B & ACVR2A
INHBB→ACVR1C & ACVR2B
INHBB→ACVR1B & ACVR2B
INHBB→ACVR1C & ACVR2A

Abbrev.	Tissue
Aort	Aorta
Blad	Bladder
Blo	Blood
Bone	Bone
BM	Bone marrow
BrC	Brain cortex
BrS	Brainstem
Col	Colon
Dia	Diaphragm
Ret	Eye retina
Fat	Fat
Hrt	Heart
Pit	Hypothalamus/Pituitary
Kid	Kidney
LM	Limb muscle
Liv	Liver
Lung	Lung
MG	Mammary gland
Panc	Pancreas
Skin	Skin
SI	Small intestine
Spl	Spleen
Test	Testes
Thym	Thymus
Tong	Tongue
Trac	Trachea
Ute	Uterus

Compartment
lymphoid
- B cell
- NK/T cell

12.5%
25%
50%
100%

ln(CP10K+1): 0, 1, 2, 3

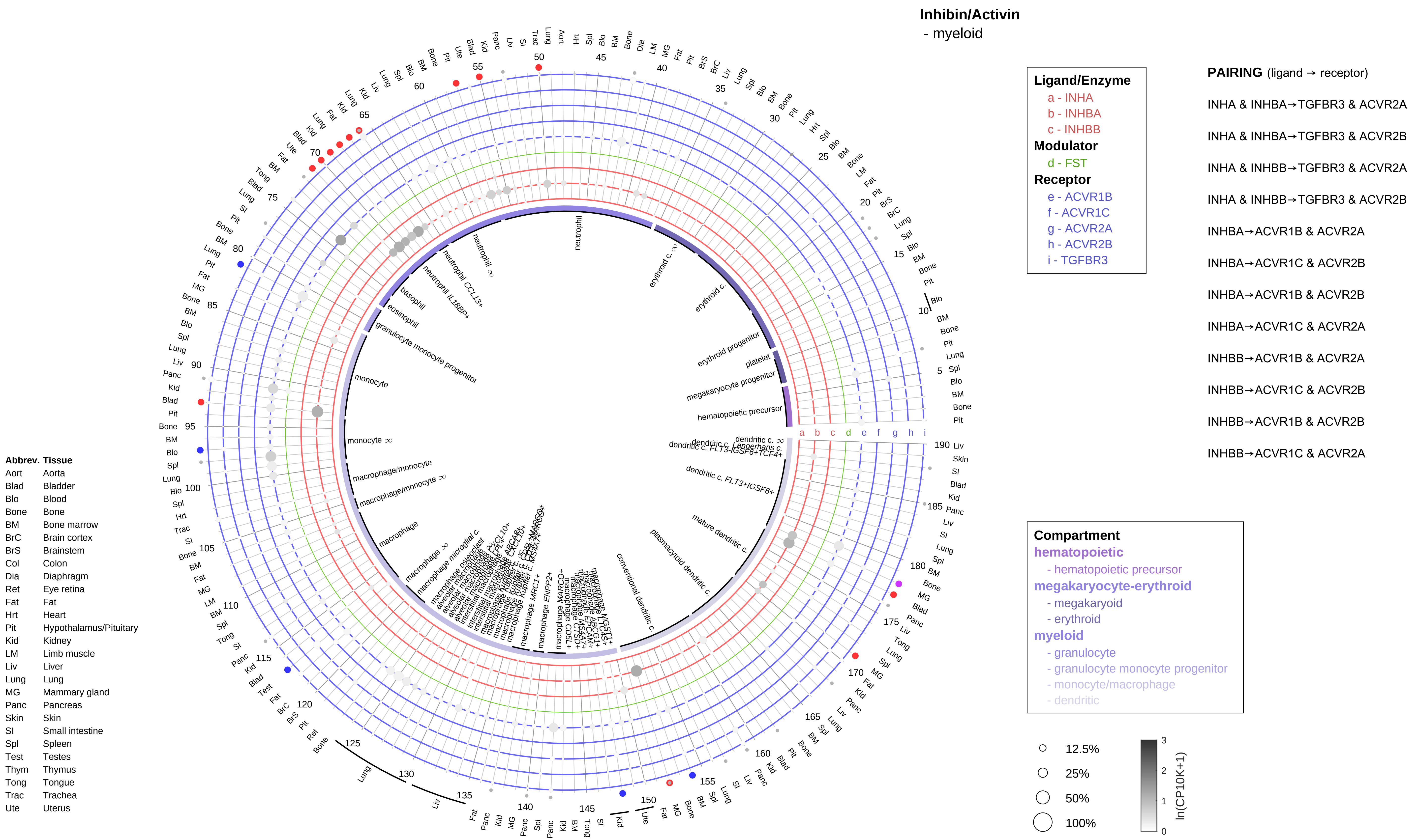

Inhibin/Activin
- myeloid
Ligand/Enzyme
a - INHA
b - INHBA
c - INHBB
Modulator
d - FST
Receptor
e - ACVR1B
f - ACVR1C
g - ACVR2A
h - ACVR2B
i - TGFBR3
PAIRING (ligand → receptor)
INHA & INHBA→TGFBR3 & ACVR2A
INHA & INHBA→TGFBR3 & ACVR2B
INHA & INHBB→TGFBR3 & ACVR2A
INHA & INHBB→TGFBR3 & ACVR2B
INHBA→ACVR1B & ACVR2A
INHBA→ACVR1C & ACVR2B
INHBA→ACVR1B & ACVR2B
INHBA→ACVR1C & ACVR2A
INHBB→ACVR1B & ACVR2A
INHBB→ACVR1C & ACVR2B
INHBB→ACVR1B & ACVR2B
INHBB→ACVR1C & ACVR2A
Compartment
hematopoietic
- hematopoietic precursor
megakaryocyte-erythroid
- megakaryoid
- erythroid
myeloid
- granulocyte
- granulocyte monocyte progenitor
- monocyte/macrophage
- dendritic
12.5%
25%
50%
100%
ln(CP10K+1)
0
1
2
3
neutrophil
erythroid c. ∞
erythroid c.
erythroid progenitor
platelet
megakaryocyte progenitor
hematopoietic precursor
dendritic c. ∞
dendritic c. Langerhans c.
dendritic c. FLT3-IGSF6+TCF4+
dendritic c. FLT3+IGSF6+
mature dendritic c.
plasmacytoid dendritic c.
conventional dendritic c.
neutrophil ∞
neutrophil CCL13+
neutrophil IL18BP+
basophil
eosinophil
granulocyte monocyte progenitor
monocyte
monocyte ∞
macrophage/monocyte
macrophage/monocyte ∞
macrophage
macrophage ∞
macrophage microglial c.
macrophage MRC1+
macrophage ENPP2+
macrophage MARCO+
a
b
c
d
e
f
g
h
i
Abbrev. Tissue
Aort Aorta
Blad Bladder
Blo Blood
Bone Bone
BM Bone marrow
BrC Brain cortex
BrS Brainstem
Col Colon
Dia Diaphragm
Ret Eye retina
Fat Fat
Hrt Heart
Pit Hypothalamus/Pituitary
Kid Kidney
LM Limb muscle
Liv Liver
Lung Lung
MG Mammary gland
Panc Pancreas
Skin Skin
SI Small intestine
Spl Spleen
Test Testes
Thym Thymus
Tong Tongue
Trac Trachea
Ute Uterus

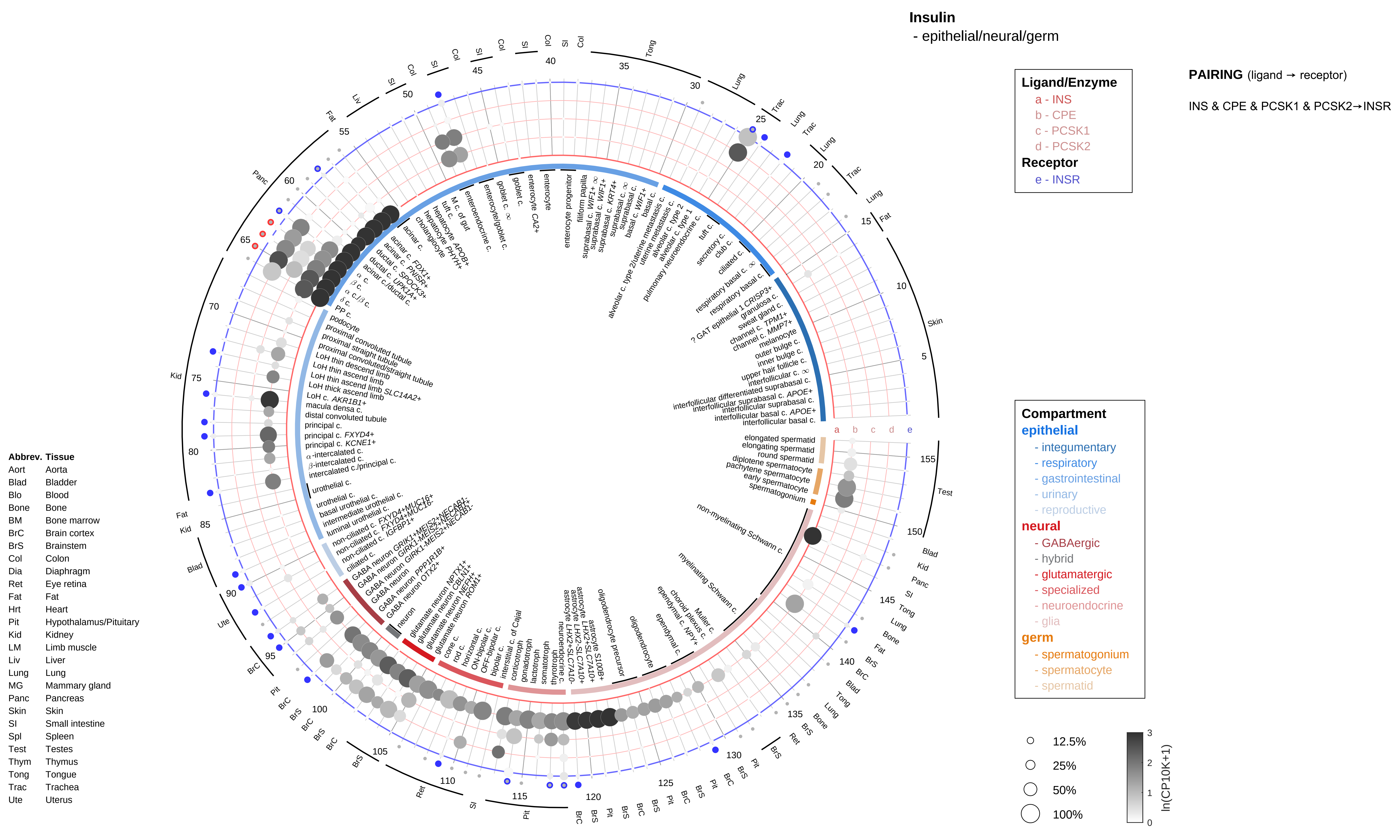

Insulin
- epithelial/neural/germ
Ligand/Enzyme
a - INS
b - CPE
c - PCSK1
d - PCSK2
Receptor
e - INSR
PAIRING (ligand → receptor)
INS & CPE & PCSK1 & PCSK2→INSR
Compartment
epithelial
- integumentary
- respiratory
- gastrointestinal
- urinary
- reproductive
neural
- GABAergic
- hybrid
- glutamatergic
- specialized
- neuroendocrine
- glia
germ
- spermatogonium
- spermatocyte
- spermatid
12.5%
25%
50%
100%
ln(CP10K+1)
0
1
2
3
Abbrev. Tissue
Aort Aorta
Blad Bladder
Blo Blood
Bone Bone
BM Bone marrow
BrC Brain cortex
BrS Brainstem
Col Colon
Dia Diaphragm
Ret Eye retina
Fat Fat
Hrt Heart
Pit Hypothalamus/Pituitary
Kid Kidney
LM Limb muscle
Liv Liver
Lung Lung
MG Mammary gland
Panc Pancreas
Skin Skin
SI Small intestine
Spl Spleen
Test Testes
Thym Thymus
Tong Tongue
Trac Trachea
Ute Uterus

Insulin
- endothelial

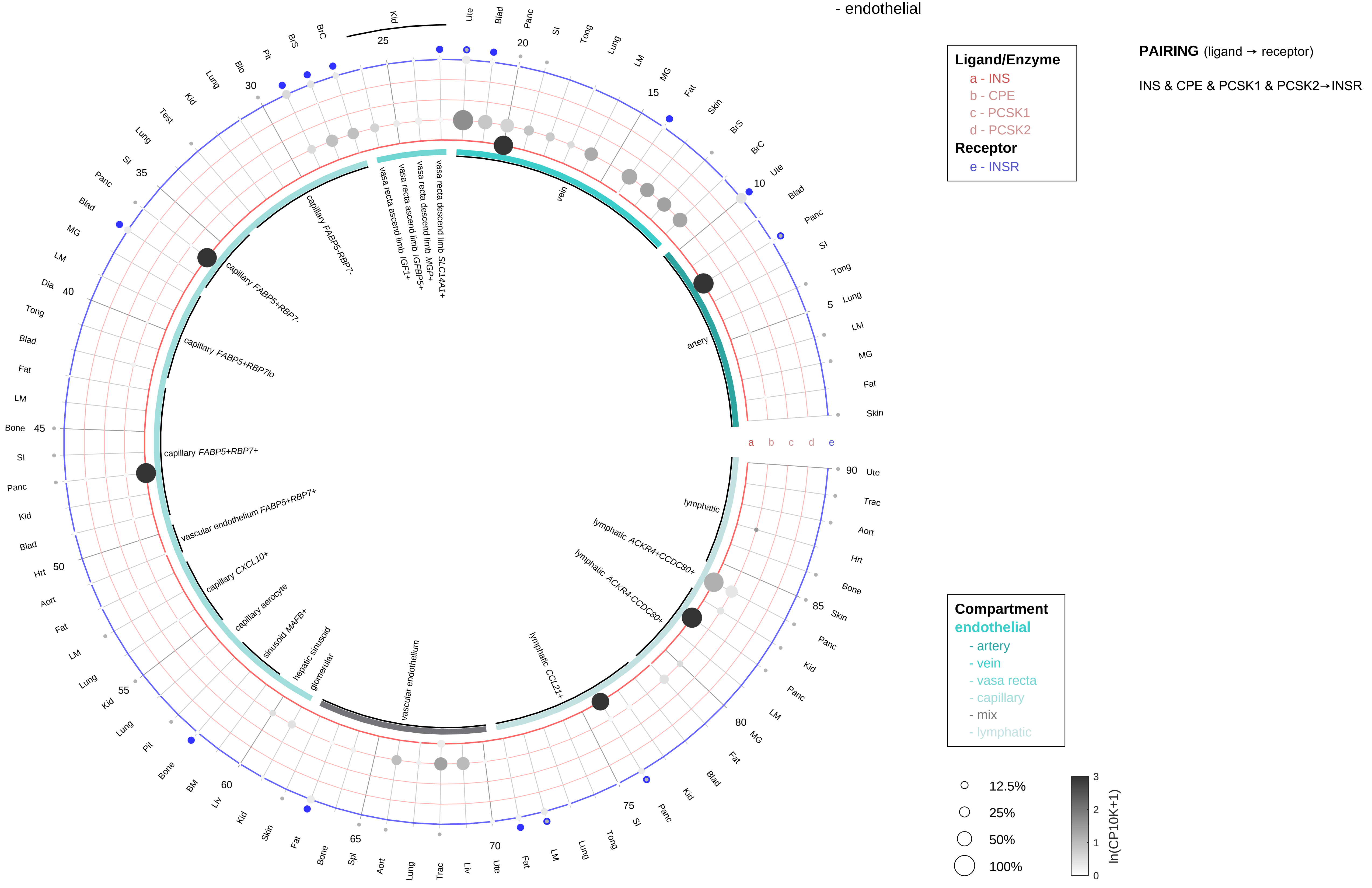

Abbrev.	Tissue
Aort	Aorta
Blad	Bladder
Blo	Blood
Bone	Bone
BM	Bone marrow
BrC	Brain cortex
BrS	Brainstem
Col	Colon
Dia	Diaphragm
Ret	Eye retina
Fat	Fat
Hrt	Heart
Pit	Hypothalamus/Pituitary
Kid	Kidney
LM	Limb muscle
Liv	Liver
Lung	Lung
MG	Mammary gland
Panc	Pancreas
Skin	Skin
SI	Small intestine
Spl	Spleen
Test	Testes
Thym	Thymus
Tong	Tongue
Trac	Trachea
Ute	Uterus

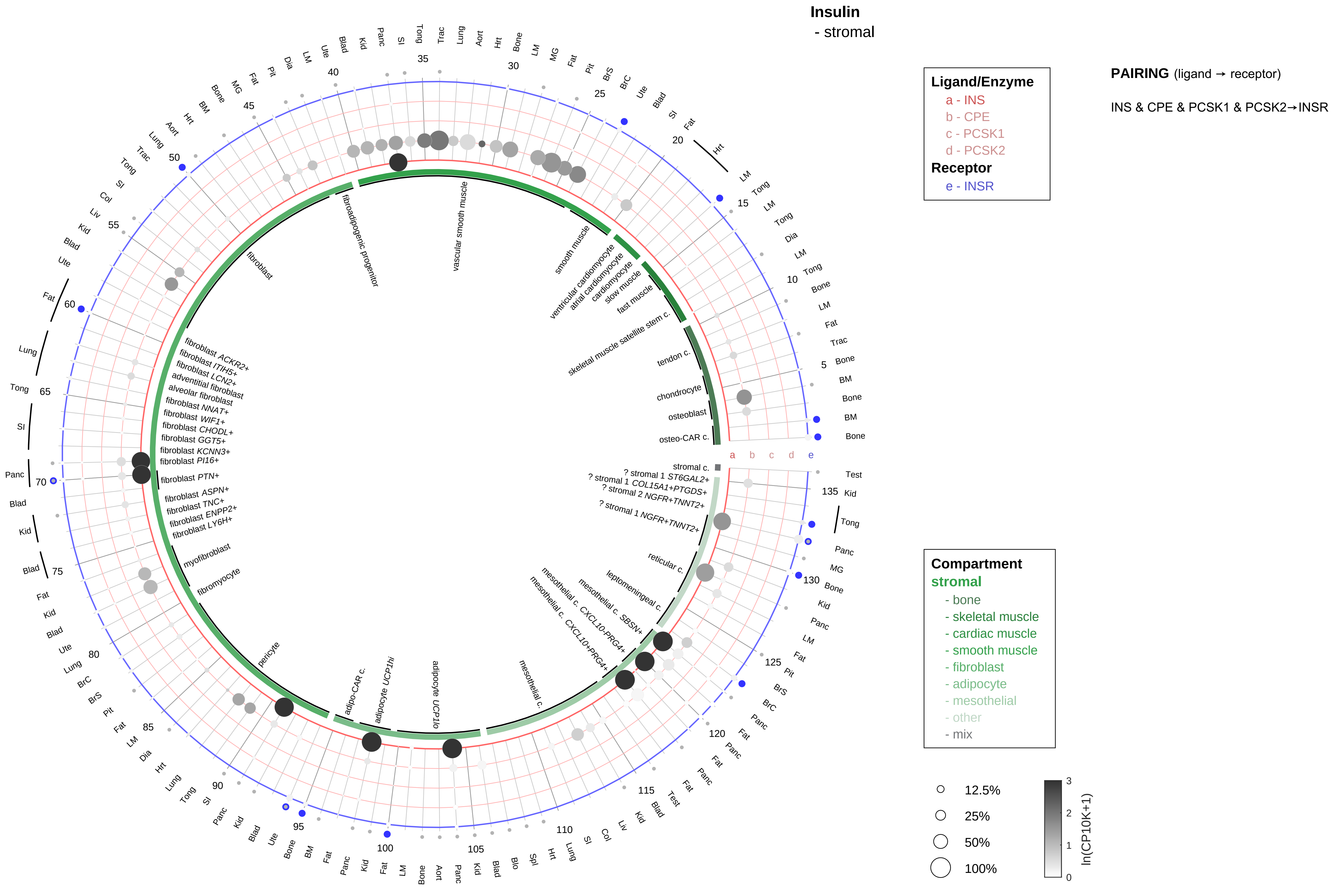

Abbrev.	Tissue
Aort	Aorta
Blad	Bladder
Blo	Blood
Bone	Bone
BM	Bone marrow
BrC	Brain cortex
BrS	Brainstem
Col	Colon
Dia	Diaphragm
Ret	Eye retina
Fat	Fat
Hrt	Heart
Pit	Hypothalamus/Pituitary
Kid	Kidney
LM	Limb muscle
Liv	Liver
Lung	Lung
MG	Mammary gland
Panc	Pancreas
Skin	Skin
SI	Small intestine
Spl	Spleen
Test	Testes
Thym	Thymus
Tong	Tongue
Trac	Trachea
Ute	Uterus

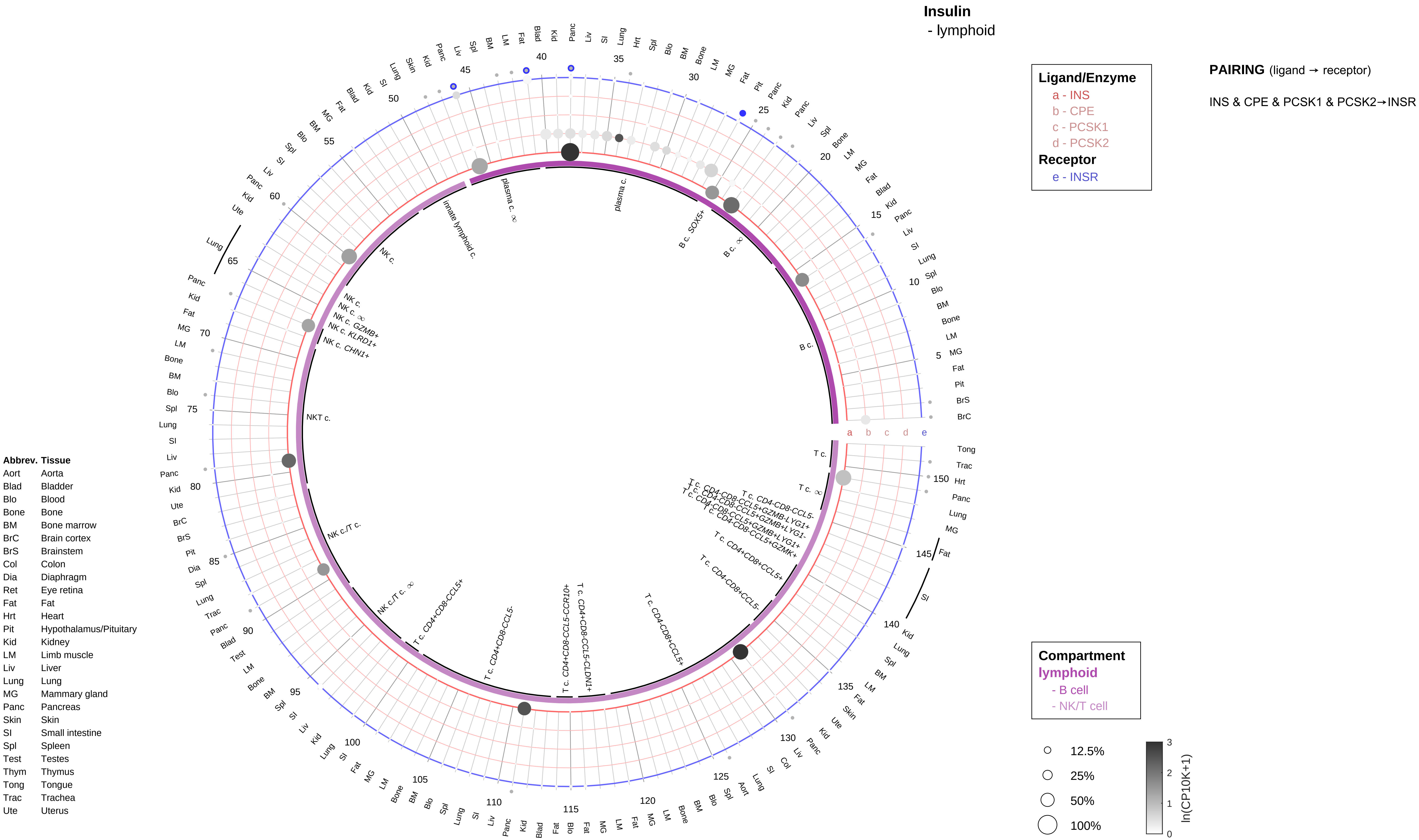

Insulin
- lymphoid
Ligand/Enzyme
a - INS
b - CPE
c - PCSK1
d - PCSK2
Receptor
e - INSR
PAIRING (ligand → receptor)
INS & CPE & PCSK1 & PCSK2→INSR
Compartment
lymphoid
- B cell
- NK/T cell
12.5%
25%
50%
100%
ln(CP10K+1)
0
1
2
3
a
b
c
d
e
plasma c.
plasma c. ∞
innate lymphoid c.
B c. SOX5+
B c. ∞
B c.
NK c.
NK c.
NK c. ∞
NK c. GZMB+
NK c. KLRD1+
NK c. CHN1+
NKT c.
NK c./T c.
NK c./T c. ∞
T c.
T c. ∞
T c. CD4-CD8-CCL5-
T c. CD4-CD8-CCL5+GZMB-LYG1+
T c. CD4-CD8-CCL5+GZMB+LYG1-
T c. CD4-CD8-CCL5+GZMK+
T c. CD4+CD8+CCL5+
T c. CD4-CD8-CCL5-
T c. CD4-CD8+CCL5+
T c. CD4+CD8-CCL5-CLDN1+
T c. CD4+CD8-CCL5-CCR10+
T c. CD4+CD8-CCL5-
T c. CD4+CD8-CCL5+
Abbrev. Tissue
Aort Aorta
Blad Bladder
Blo Blood
Bone Bone
BM Bone marrow
BrC Brain cortex
BrS Brainstem
Col Colon
Dia Diaphragm
Ret Eye retina
Fat Fat
Hrt Heart
Pit Hypothalamus/Pituitary
Kid Kidney
LM Limb muscle
Liv Liver
Lung Lung
MG Mammary gland
Panc Pancreas
Skin Skin
SI Small intestine
Spl Spleen
Test Testes
Thym Thymus
Tong Tongue
Trac Trachea
Ute Uterus

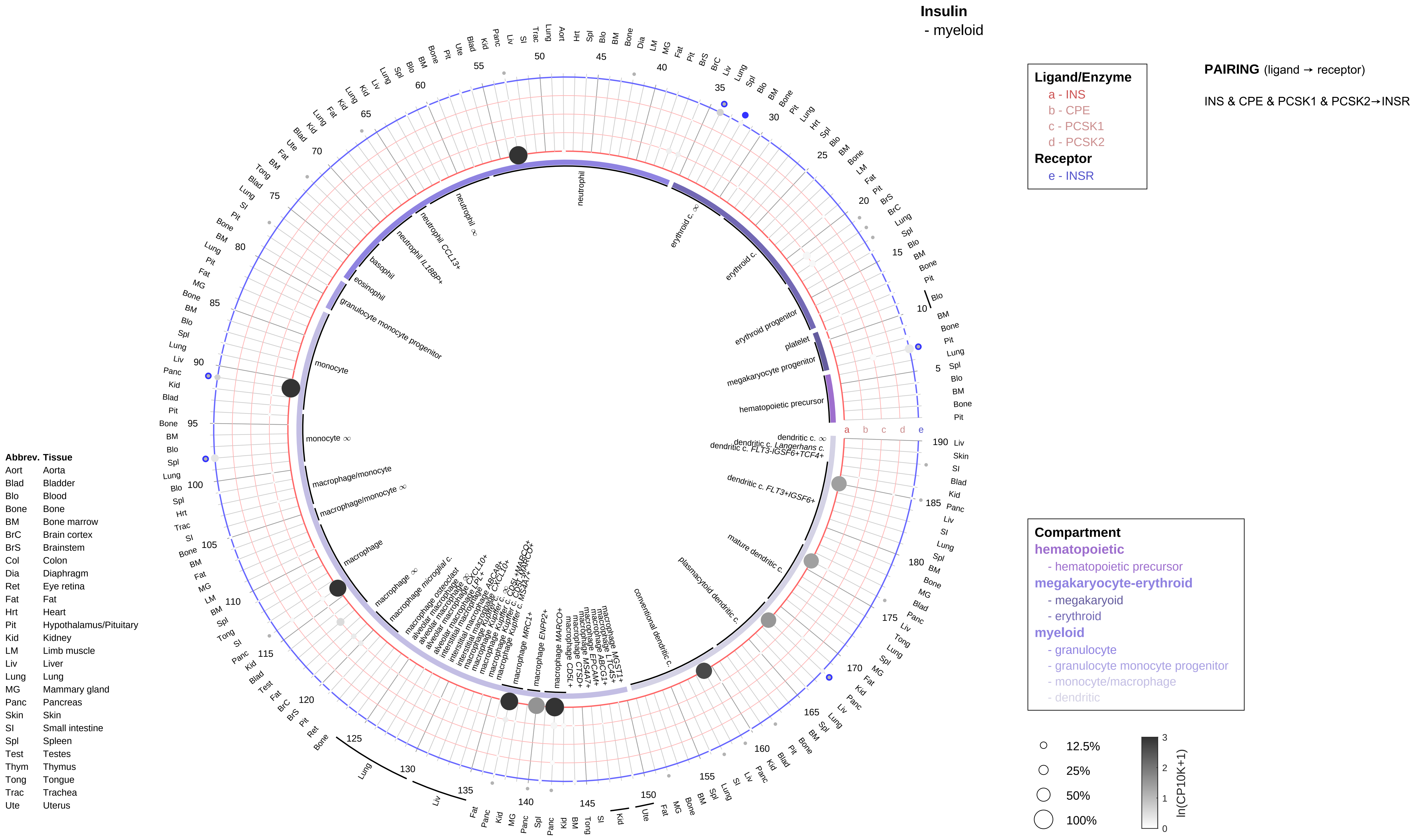
Insulin
- myeloid
Ligand/Enzyme
a - INS
b - CPE
c - PCSK1
d - PCSK2
Receptor
e - INSR
PAIRING (ligand → receptor)
INS & CPE & PCSK1 & PCSK2→INSR
Compartment
hematopoietic
- hematopoietic precursor
megakaryocyte-erythroid
- megakaryoid
- erythroid
myeloid
- granulocyte
- granulocyte monocyte progenitor
- monocyte/macrophage
- dendritic
12.5%
25%
50%
100%
ln(CP10K+1)
Abbrev. Tissue
Aort Aorta
Blad Bladder
Blo Blood
Bone Bone
BM Bone marrow
BrC Brain cortex
BrS Brainstem
Col Colon
Dia Diaphragm
Ret Eye retina
Fat Fat
Hrt Heart
Pit Hypothalamus/Pituitary
Kid Kidney
LM Limb muscle
Liv Liver
Lung Lung
MG Mammary gland
Panc Pancreas
Skin Skin
SI Small intestine
Spl Spleen
Test Testes
Thym Thymus
Tong Tongue
Trac Trachea
Ute Uterus
neutrophil
erythroid c. ∞
erythroid c.
erythroid progenitor
platelet
megakaryocyte progenitor
hematopoietic precursor
dendritic c. ∞
dendritic c. Langerhans c.
dendritic c. FLT3-IGSF6+TCF4+
dendritic c. FLT3+IGSF6+
mature dendritic c.
plasmacytoid dendritic c.
conventional dendritic c.
macrophage MRC1+
macrophage ENPP2+
macrophage MARCO+
macrophage CD5L+
macrophage CTSD+
macrophage MS4A7+
macrophage EPCAM+
macrophage ABCG1+
macrophage LTC4S+
macrophage MGST1+
macrophage ∞
macrophage microglial c.
macrophage osteoclast
macrophage
macrophage/monocyte ∞
macrophage/monocyte
monocyte ∞
monocyte
granulocyte monocyte progenitor
eosinophil
basophil
neutrophil IL18BP+
neutrophil CCL13+
neutrophil ∞

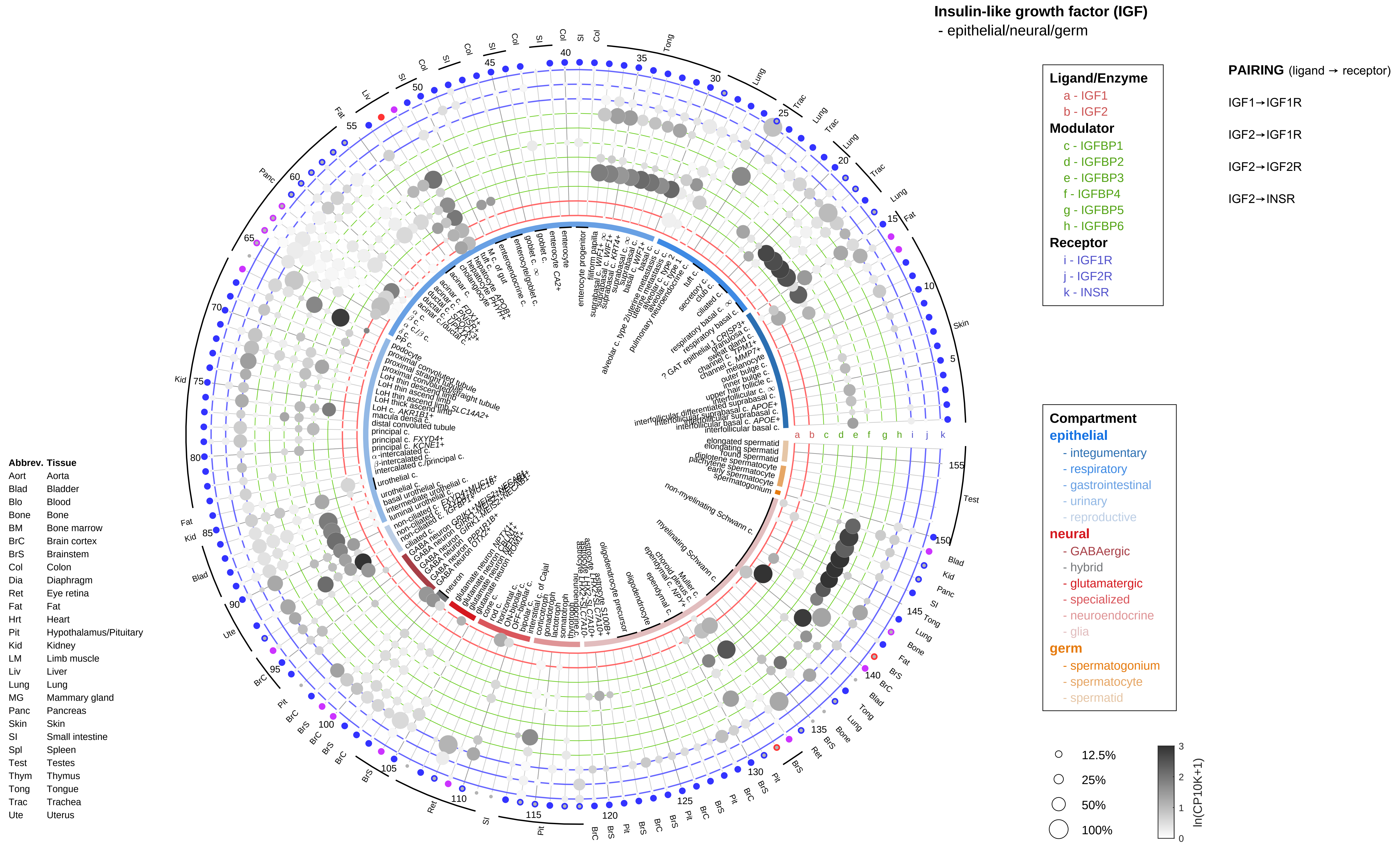

Insulin-like growth factor (IGF)
- epithelial/neural/germ
Ligand/Enzyme
a - IGF1
b - IGF2
Modulator
c - IGFBP1
d - IGFBP2
e - IGFBP3
f - IGFBP4
g - IGFBP5
h - IGFBP6
Receptor
i - IGF1R
j - IGF2R
k - INSR
PAIRING (ligand → receptor)
IGF1→IGF1R
IGF2→IGF1R
IGF2→IGF2R
IGF2→INSR
Compartment
epithelial
- integumentary
- respiratory
- gastrointestinal
- urinary
- reproductive
neural
- GABAergic
- hybrid
- glutamatergic
- specialized
- neuroendocrine
- glia
germ
- spermatogonium
- spermatocyte
- spermatid
12.5%
25%
50%
100%
ln(CP10K+1)
Abbrev. Tissue
Aort Aorta
Blad Bladder
Blo Blood
Bone Bone
BM Bone marrow
BrC Brain cortex
BrS Brainstem
Col Colon
Dia Diaphragm
Ret Eye retina
Fat Fat
Hrt Heart
Pit Hypothalamus/Pituitary
Kid Kidney
LM Limb muscle
Liv Liver
Lung Lung
MG Mammary gland
Panc Pancreas
Skin Skin
SI Small intestine
Spl Spleen
Test Testes
Thym Thymus
Tong Tongue
Trac Trachea
Ute Uterus

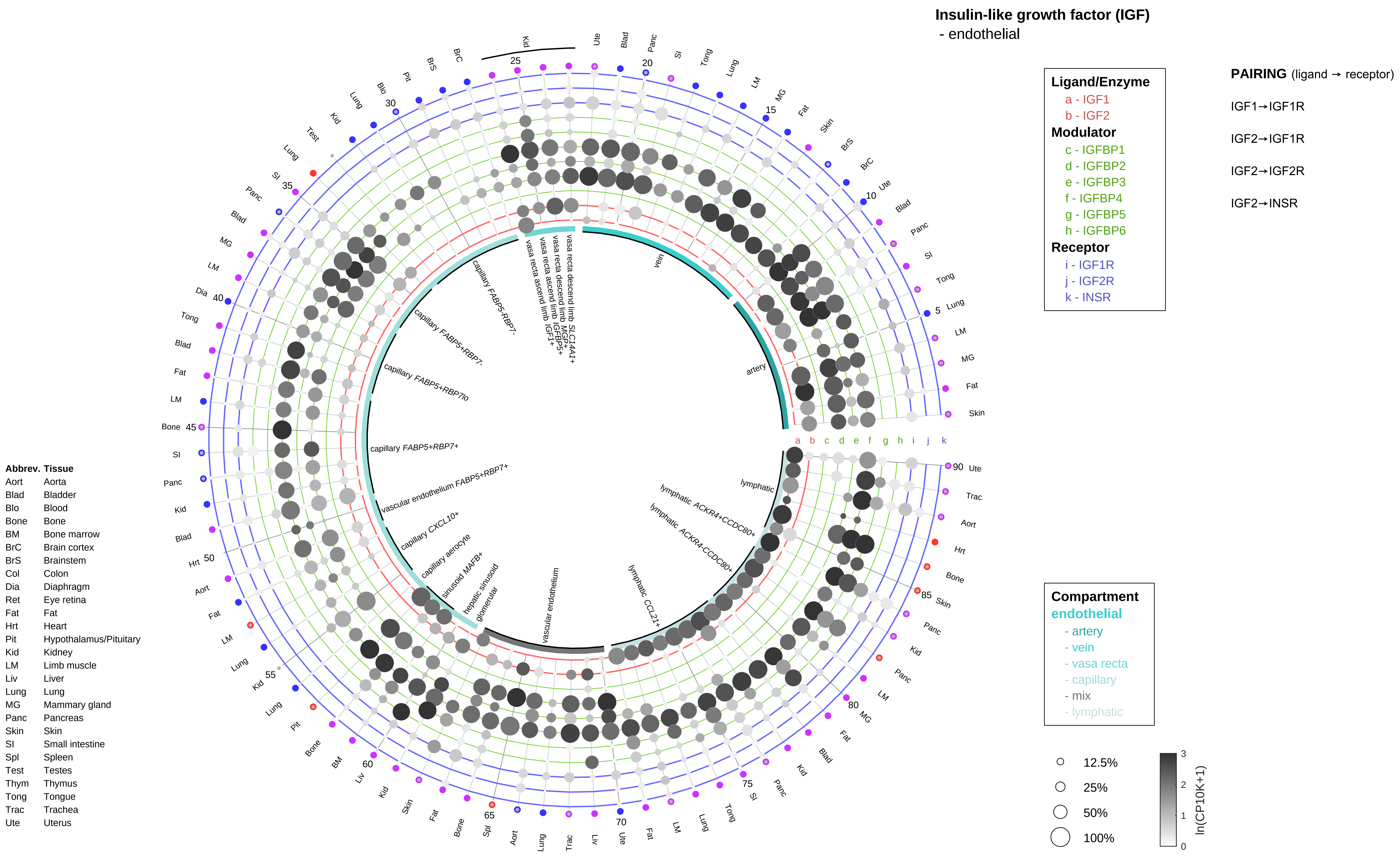

Insulin-like growth factor (IGF)
- endothelial
Ligand/Enzyme
a - IGF1
b - IGF2
Modulator
c - IGFBP1
d - IGFBP2
e - IGFBP3
f - IGFBP4
g - IGFBP5
h - IGFBP6
Receptor
i - IGF1R
j - IGF2R
k - INSR
PAIRING (ligand → receptor)
IGF1→IGF1R
IGF2→IGF1R
IGF2→IGF2R
IGF2→INSR
Compartment
endothelial
- artery
- vein
- vasa recta
- capillary
- mix
- lymphatic
12.5%
25%
50%
100%
ln(CP10K+1)
0
1
2
3
Abbrev. Tissue
Aort Aorta
Blad Bladder
Blo Blood
Bone Bone
BM Bone marrow
BrC Brain cortex
BrS Brainstem
Col Colon
Dia Diaphragm
Ret Eye retina
Fat Fat
Hrt Heart
Pit Hypothalamus/Pituitary
Kid Kidney
LM Limb muscle
Liv Liver
Lung Lung
MG Mammary gland
Panc Pancreas
Skin Skin
SI Small intestine
Spl Spleen
Test Testes
Thym Thymus
Tong Tongue
Trac Trachea
Ute Uterus
vein
artery
vasa recta descend limb SLC14A1+
vasa recta descend limb MGP+
vasa recta ascend limb IGFBP5+
vasa recta ascend limb IGF1+
capillary FABP5-RBP7-
capillary FABP5+RBP7-
capillary FABP5+RBP7lo
capillary FABP5+RBP7+
vascular endothelium FABP5+RBP7+
capillary CXCL10+
capillary aerocyte
sinusoid MAFB+
hepatic sinusoid
glomerular
vascular endothelium
lymphatic CCL21+
lymphatic ACKR4-CCDC80+
lymphatic ACKR4+CCDC80+
lymphatic
a b c d e f g h i j k

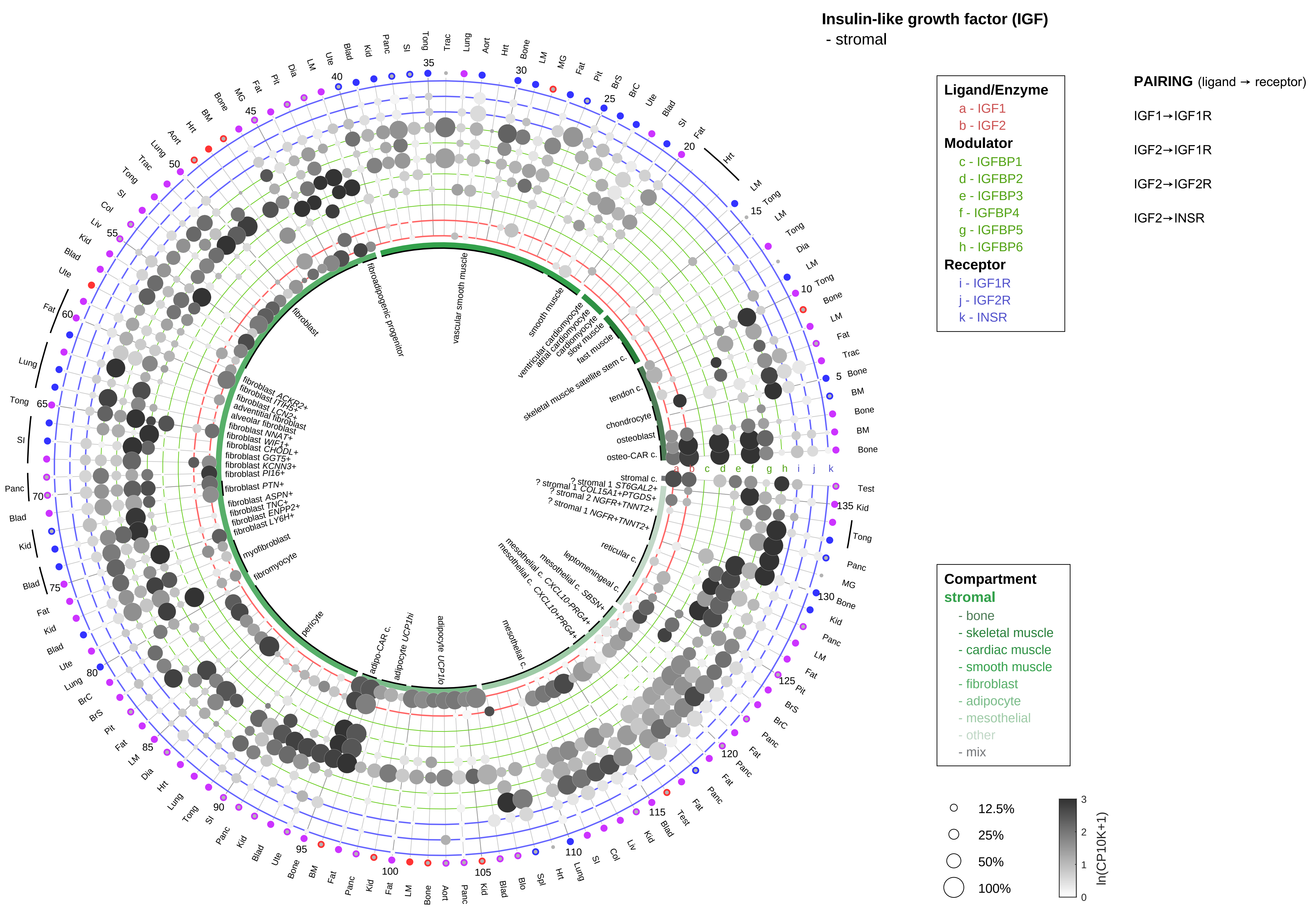

Abbrev.	Tissue
Aort	Aorta
Blad	Bladder
Blo	Blood
Bone	Bone
BM	Bone marrow
BrC	Brain cortex
BrS	Brainstem
Col	Colon
Dia	Diaphragm
Ret	Eye retina
Fat	Fat
Hrt	Heart
Pit	Hypothalamus/Pituitary
Kid	Kidney
LM	Limb muscle
Liv	Liver
Lung	Lung
MG	Mammary gland
Panc	Pancreas
Skin	Skin
SI	Small intestine
Spl	Spleen
Test	Testes
Thym	Thymus
Tong	Tongue
Trac	Trachea
Ute	Uterus

Insulin-like growth factor (IGF)
- lymphoid

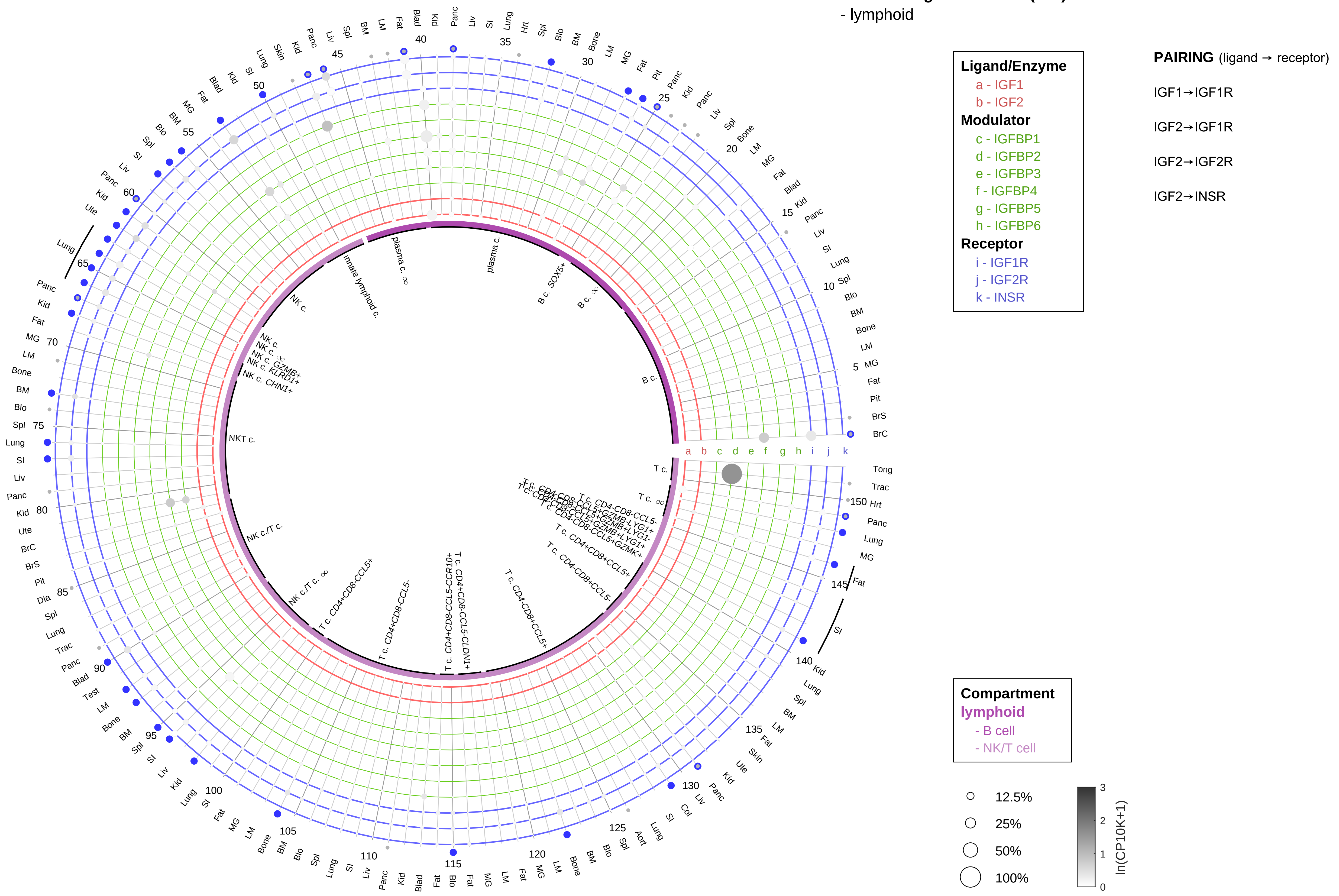

Abbrev.	Tissue
Aort	Aorta
Blad	Bladder
Blo	Blood
Bone	Bone
BM	Bone marrow
BrC	Brain cortex
BrS	Brainstem
Col	Colon
Dia	Diaphragm
Ret	Eye retina
Fat	Fat
Hrt	Heart
Pit	Hypothalamus/Pituitary
Kid	Kidney
LM	Limb muscle
Liv	Liver
Lung	Lung
MG	Mammary gland
Panc	Pancreas
Skin	Skin
SI	Small intestine
Spl	Spleen
Test	Testes
Thym	Thymus
Tong	Tongue
Trac	Trachea
Ute	Uterus

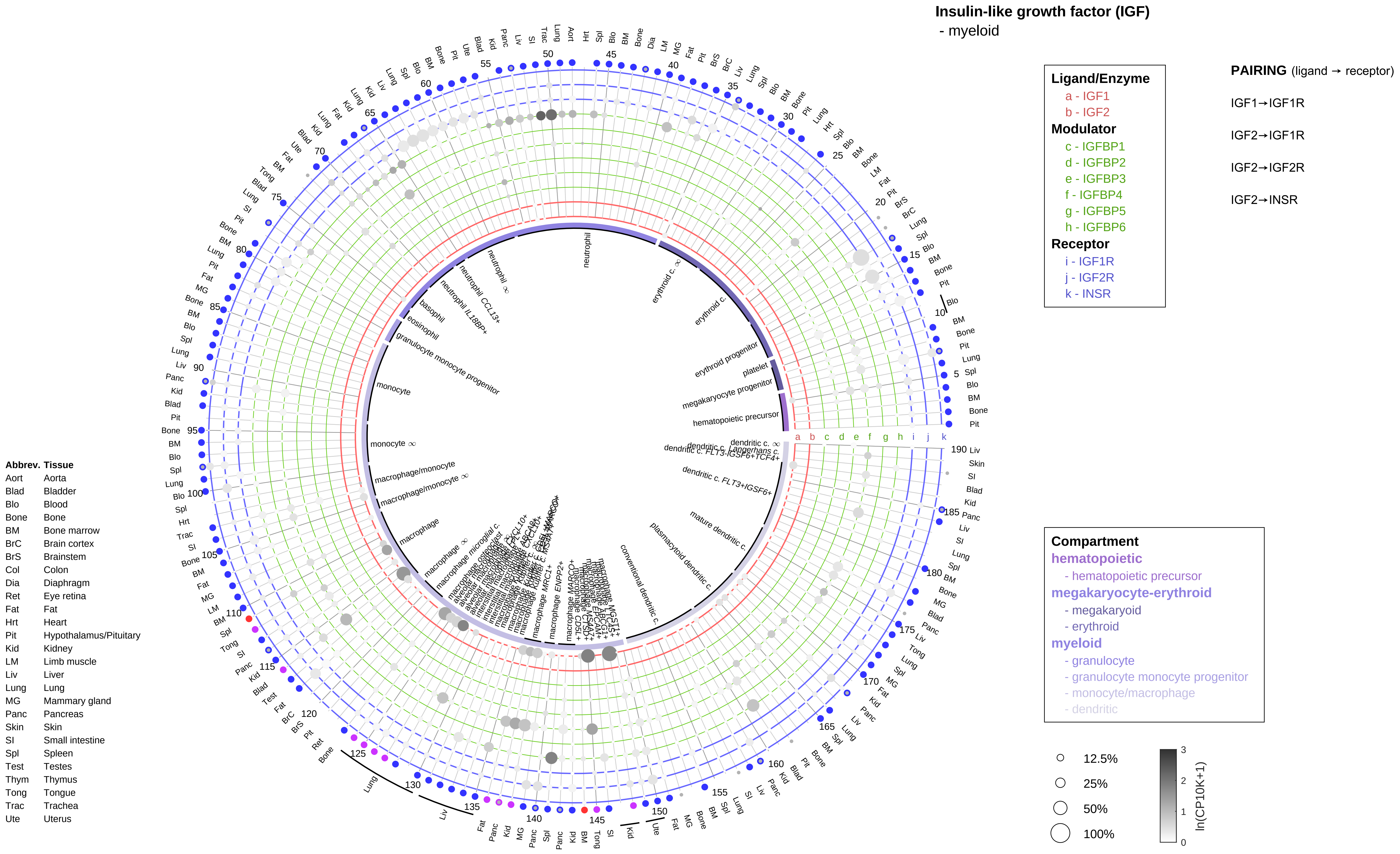
Insulin-like growth factor (IGF)
- myeloid
Ligand/Enzyme
a - IGF1
b - IGF2
Modulator
c - IGFBP1
d - IGFBP2
e - IGFBP3
f - IGFBP4
g - IGFBP5
h - IGFBP6
Receptor
i - IGF1R
j - IGF2R
k - INSR
PAIRING (ligand → receptor)
IGF1→IGF1R
IGF2→IGF1R
IGF2→IGF2R
IGF2→INSR
Compartment
hematopoietic
- hematopoietic precursor
megakaryocyte-erythroid
- megakaryoid
- erythroid
myeloid
- granulocyte
- granulocyte monocyte progenitor
- monocyte/macrophage
- dendritic
12.5%
25%
50%
100%
ln(CP10K+1)
Abbrev. Tissue
Aort Aorta
Blad Bladder
Blo Blood
Bone Bone
BM Bone marrow
BrC Brain cortex
BrS Brainstem
Col Colon
Dia Diaphragm
Ret Eye retina
Fat Fat
Hrt Heart
Pit Hypothalamus/Pituitary
Kid Kidney
LM Limb muscle
Liv Liver
Lung Lung
MG Mammary gland
Panc Pancreas
Skin Skin
SI Small intestine
Spl Spleen
Test Testes
Thym Thymus
Tong Tongue
Trac Trachea
Ute Uterus
neutrophil
erythroid c.
erythroid progenitor
platelet
megakaryocyte progenitor
hematopoietic precursor
dendritic c. Langerhans c.
dendritic c. FLT3+IGSF6+
mature dendritic c.
plasmacytoid dendritic c.
conventional dendritic c.
macrophage
macrophage/monocyte
monocyte
granulocyte monocyte progenitor
eosinophil
basophil
neutrophil IL18BP+
neutrophil CCL13+

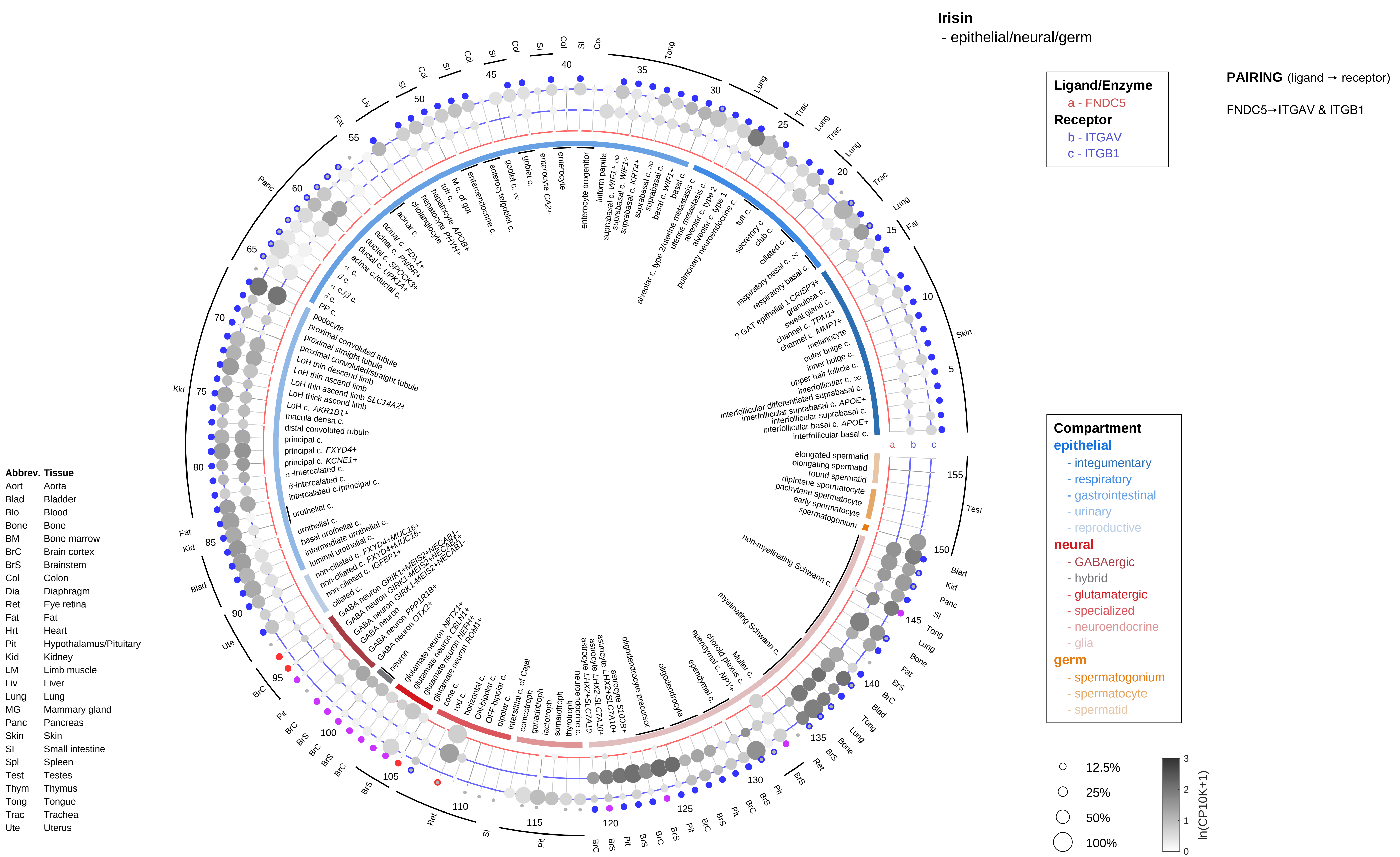

Irisin
- epithelial/neural/germ
Ligand/Enzyme
a - FNDC5
Receptor
b - ITGAV
c - ITGB1
PAIRING (ligand → receptor)
FNDC5→ITGAV & ITGB1
Compartment
epithelial
- integumentary
- respiratory
- gastrointestinal
- urinary
- reproductive
neural
- GABAergic
- hybrid
- glutamatergic
- specialized
- neuroendocrine
- glia
germ
- spermatogonium
- spermatocyte
- spermatid
12.5%
25%
50%
100%
ln(CP10K+1)
Abbrev. Tissue
Aort Aorta
Blad Bladder
Blo Blood
Bone Bone
BM Bone marrow
BrC Brain cortex
BrS Brainstem
Col Colon
Dia Diaphragm
Ret Eye retina
Fat Fat
Hrt Heart
Pit Hypothalamus/Pituitary
Kid Kidney
LM Limb muscle
Liv Liver
Lung Lung
MG Mammary gland
Panc Pancreas
Skin Skin
SI Small intestine
Spl Spleen
Test Testes
Thym Thymus
Tong Tongue
Trac Trachea
Ute Uterus

Irisin
- endothelial

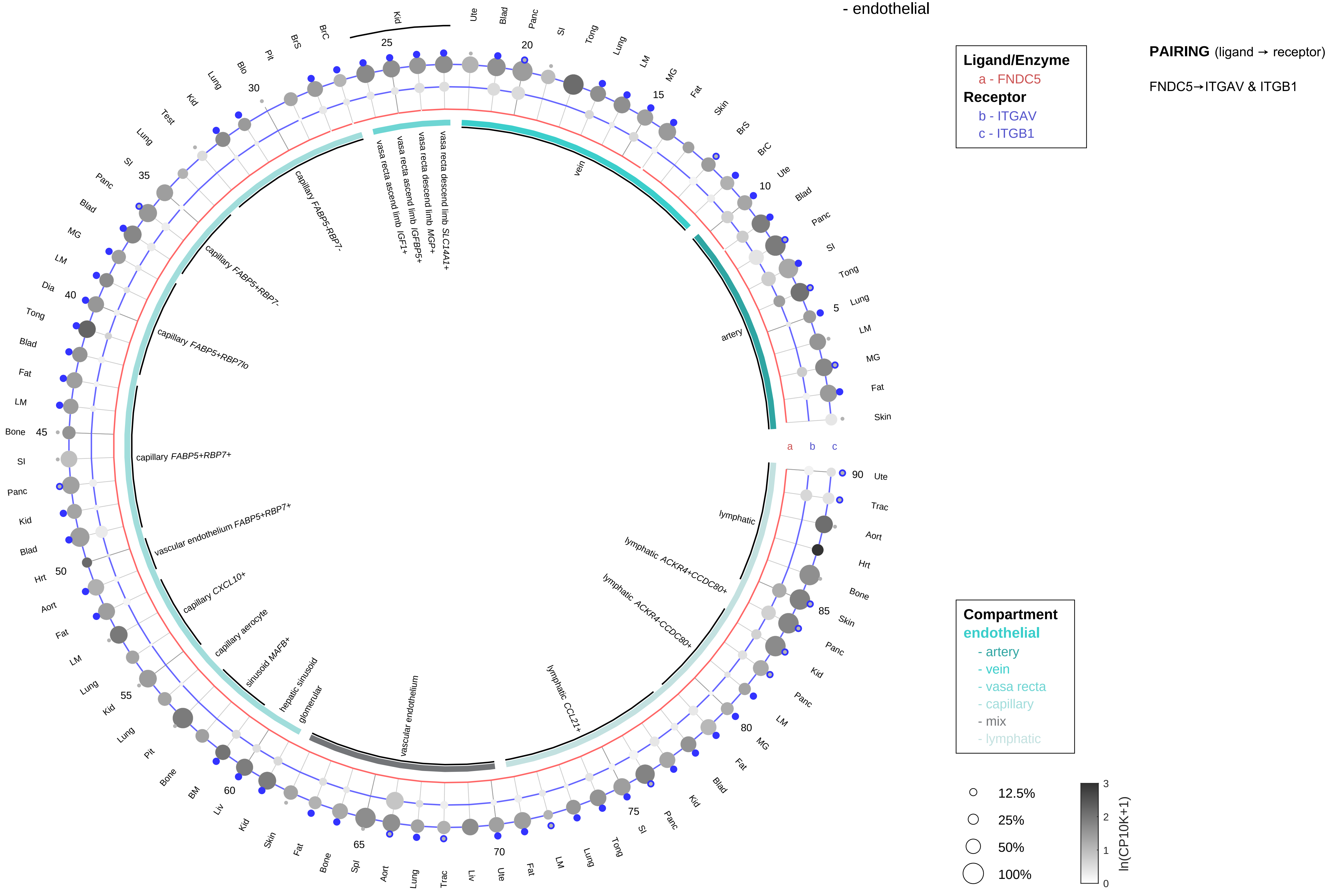

Abbrev.	Tissue
Aort	Aorta
Blad	Bladder
Blo	Blood
Bone	Bone
BM	Bone marrow
BrC	Brain cortex
BrS	Brainstem
Col	Colon
Dia	Diaphragm
Ret	Eye retina
Fat	Fat
Hrt	Heart
Pit	Hypothalamus/Pituitary
Kid	Kidney
LM	Limb muscle
Liv	Liver
Lung	Lung
MG	Mammary gland
Panc	Pancreas
Skin	Skin
SI	Small intestine
Spl	Spleen
Test	Testes
Thym	Thymus
Tong	Tongue
Trac	Trachea
Ute	Uterus

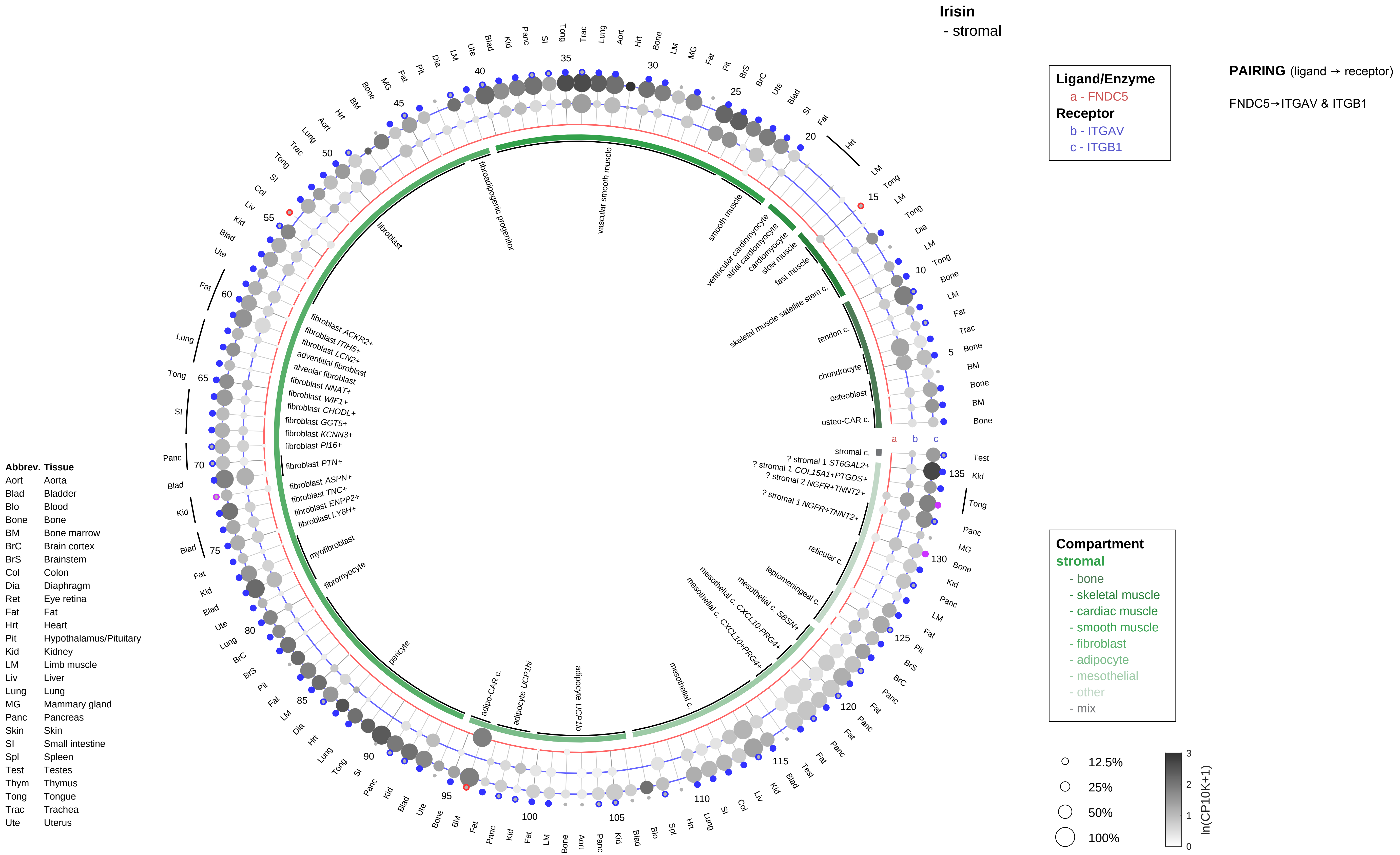
Irisin
- stromal
Ligand/Enzyme
a - FNDC5
Receptor
b - ITGAV
c - ITGB1
PAIRING (ligand → receptor)
FNDC5→ITGAV & ITGB1
Compartment
stromal
- bone
- skeletal muscle
- cardiac muscle
- smooth muscle
- fibroblast
- adipocyte
- mesothelial
- other
- mix
12.5%
25%
50%
100%
ln(CP10K+1)
Abbrev. Tissue
Aort Aorta
Blad Bladder
Blo Blood
Bone Bone
BM Bone marrow
BrC Brain cortex
BrS Brainstem
Col Colon
Dia Diaphragm
Ret Eye retina
Fat Fat
Hrt Heart
Pit Hypothalamus/Pituitary
Kid Kidney
LM Limb muscle
Liv Liver
Lung Lung
MG Mammary gland
Panc Pancreas
Skin Skin
SI Small intestine
Spl Spleen
Test Testes
Thym Thymus
Tong Tongue
Trac Trachea
Ute Uterus
vascular smooth muscle
fibroadipogenic progenitor
fibroblast
smooth muscle
ventricular cardiomyocyte
atrial cardiomyocyte
cardiomyocyte
slow muscle
fast muscle
skeletal muscle satellite stem c.
tendon c.
chondrocyte
osteoblast
osteo-CAR c.
stromal c.
? stromal 1 ST6GAL2+
? stromal 1 COL15A1+PTGDS+
? stromal 2 NGFR+TNNT2+
? stromal 1 NGFR+TNNT2+
reticular c.
leptomeningeal c.
mesothelial c. SBSN+
mesothelial c. CXCL10-PRG4+
mesothelial c. CXCL10+PRG4+
mesothelial c.
adipocyte UCP1lo
adipocyte UCP1hi
adipo-CAR c.
pericyte
fibromyocyte
myofibroblast
fibroblast LY6H+
fibroblast ENPP2+
fibroblast TNC+
fibroblast ASPN+
fibroblast PTN+
fibroblast PI16+
fibroblast KCNN3+
fibroblast GGT5+
fibroblast CHODL+
fibroblast WIF1+
fibroblast NNAT+
alveolar fibroblast
adventitial fibroblast
fibroblast LCN2+
fibroblast ITIH5+
fibroblast ACKR2+

Irisin
- lymphoid

Ligand/Enzyme
- a - FNDC5

Receptor
- b - ITGAV
- c - ITGB1

PAIRING (ligand → receptor)

FNDC5→ITGAV & ITGB1

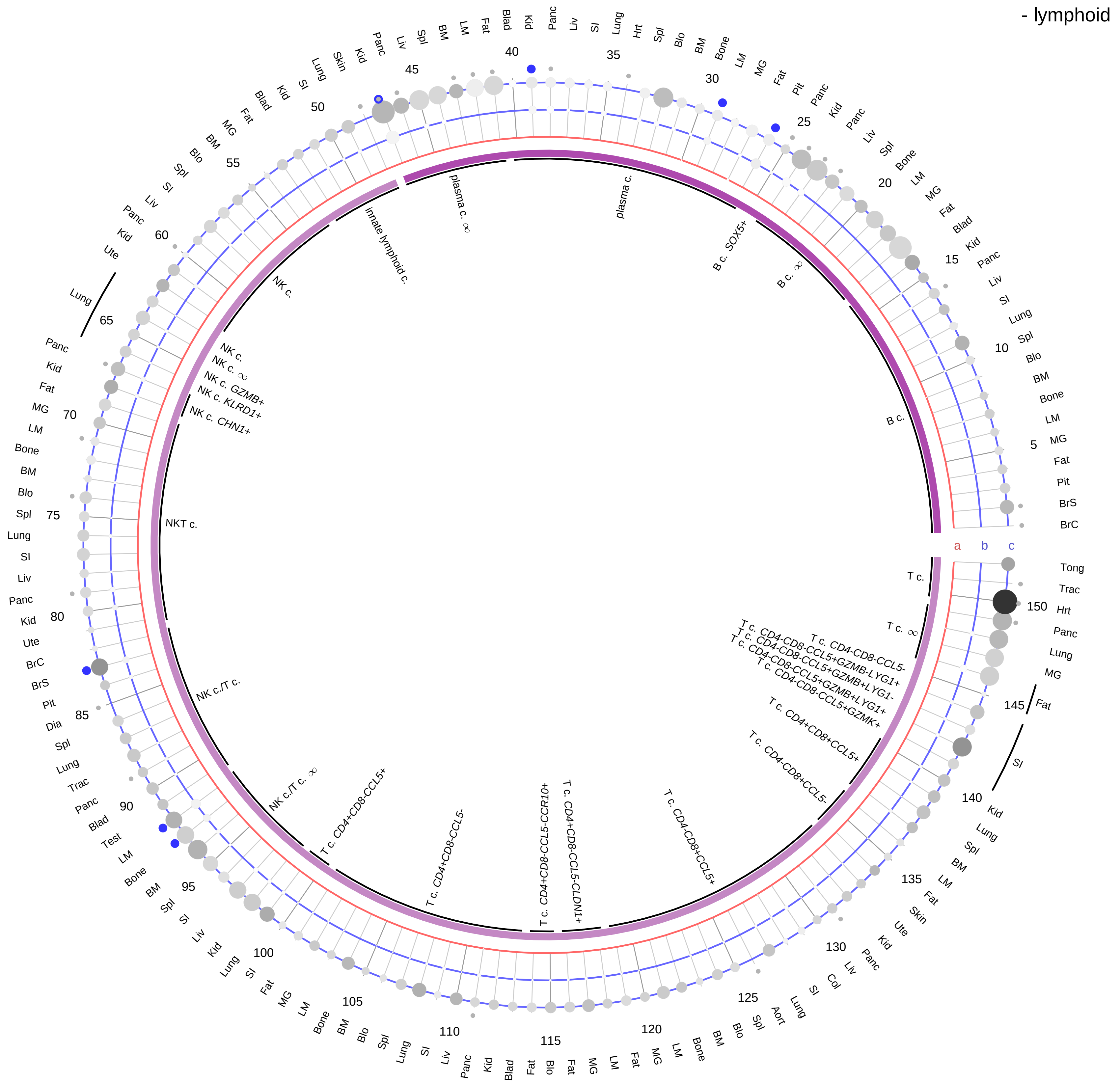

Compartment

lymphoid
- B cell
- NK/T cell

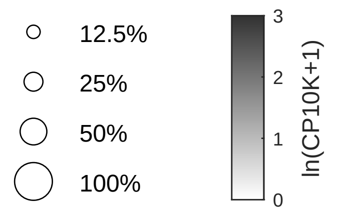

Abbrev.	Tissue
Aort	Aorta
Blad	Bladder
Blo	Blood
Bone	Bone
BM	Bone marrow
BrC	Brain cortex
BrS	Brainstem
Col	Colon
Dia	Diaphragm
Ret	Eye retina
Fat	Fat
Hrt	Heart
Pit	Hypothalamus/Pituitary
Kid	Kidney
LM	Limb muscle
Liv	Liver
Lung	Lung
MG	Mammary gland
Panc	Pancreas
Skin	Skin
SI	Small intestine
Spl	Spleen
Test	Testes
Thym	Thymus
Tong	Tongue
Trac	Trachea
Ute	Uterus

Irisin
- myeloid

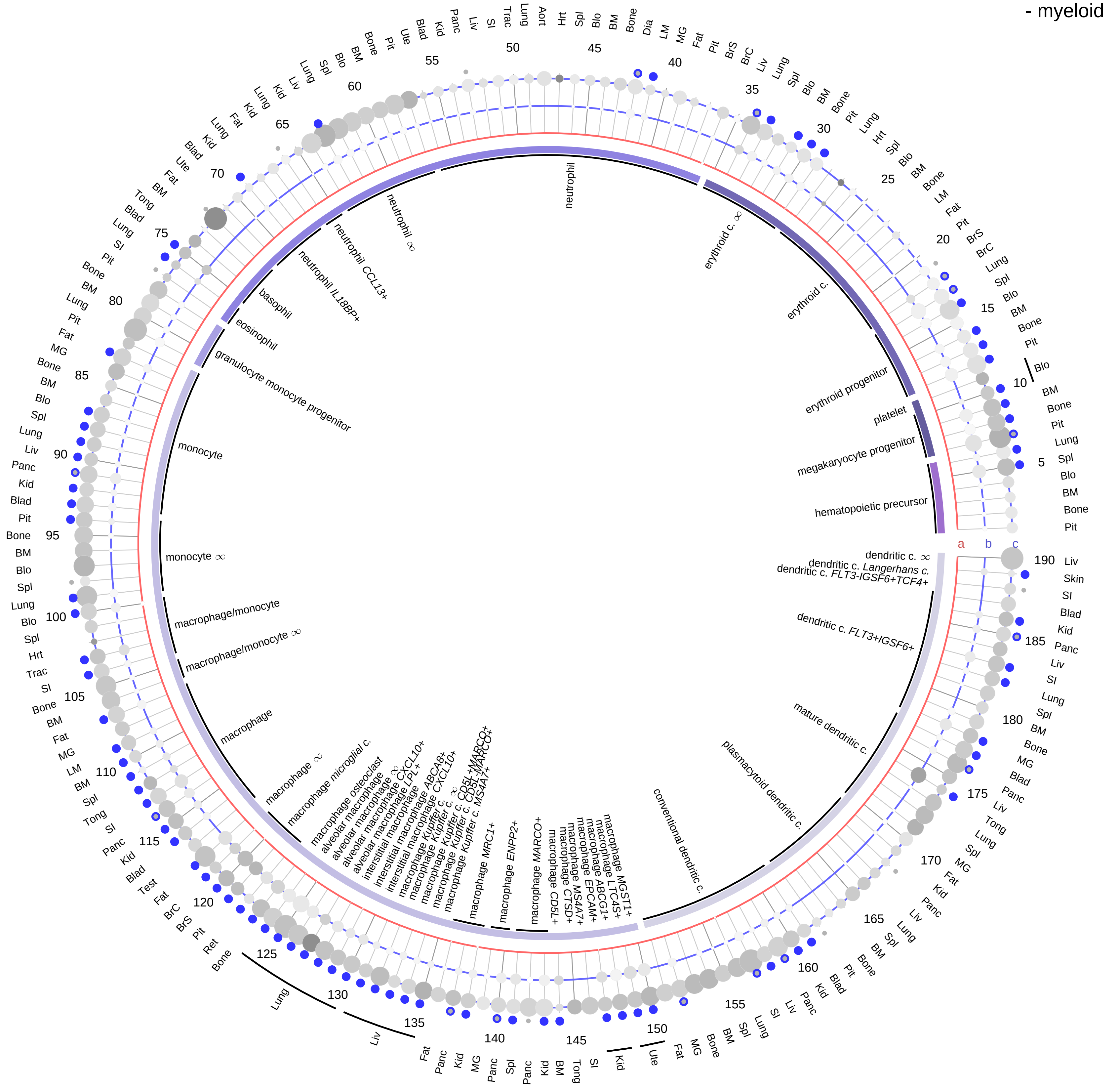

Ligand/Enzyme
a - FNDC5
Receptor
b - ITGAV
c - ITGB1

PAIRING (ligand → receptor)

FNDC5→ITGAV & ITGB1

Abbrev.	Tissue
Aort	Aorta
Blad	Bladder
Blo	Blood
Bone	Bone
BM	Bone marrow
BrC	Brain cortex
BrS	Brainstem
Col	Colon
Dia	Diaphragm
Ret	Eye retina
Fat	Fat
Hrt	Heart
Pit	Hypothalamus/Pituitary
Kid	Kidney
LM	Limb muscle
Liv	Liver
Lung	Lung
MG	Mammary gland
Panc	Pancreas
Skin	Skin
SI	Small intestine
Spl	Spleen
Test	Testes
Thym	Thymus
Tong	Tongue
Trac	Trachea
Ute	Uterus

Compartment
hematopoietic
- hematopoietic precursor
megakaryocyte-erythroid
- megakaryoid
- erythroid
myeloid
- granulocyte
- granulocyte monocyte progenitor
- monocyte/macrophage
- dendritic

12.5%
25%
50%
100%

ln(CP10K+1): 0, 1, 2, 3

Kininogen/Bradykinin/Kallikrein
- epithelial/neural/germ

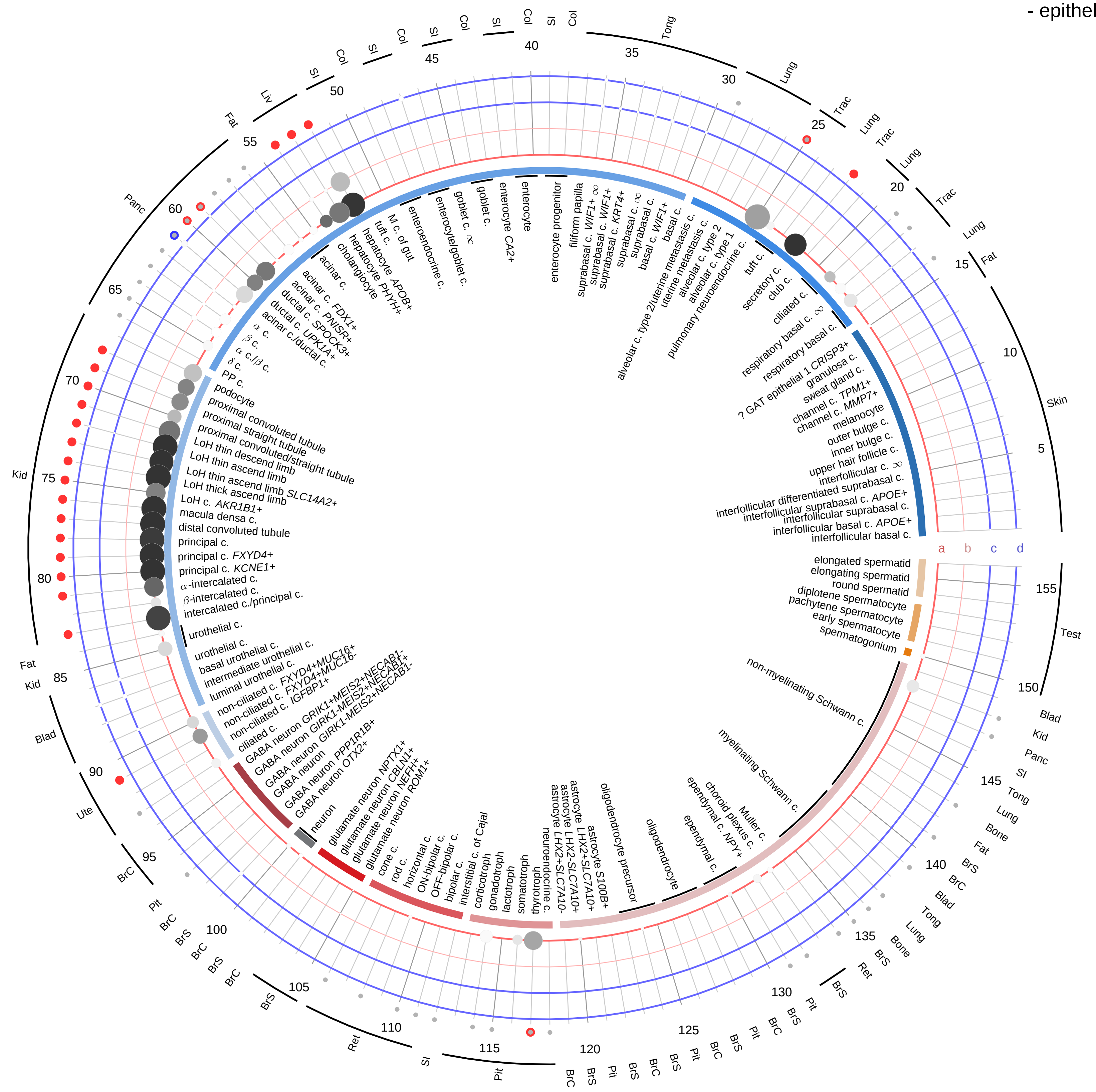

Ligand/Enzyme
- a - KNG1
- b - KLKB1

Receptor
- c - BDKRB1
- d - BDKRB2

PAIRING (ligand → receptor)

KNG1→BDKRB1

KNG1→BDKRB2

Compartment

epithelial
- integumentary
- respiratory
- gastrointestinal
- urinary
- reproductive

neural
- GABAergic
- hybrid
- glutamatergic
- specialized
- neuroendocrine
- glia

germ
- spermatogonium
- spermatocyte
- spermatid

12.5%
25%
50%
100%

ln(CP10K+1): 0, 1, 2, 3

Abbrev.	Tissue
Aort	Aorta
Blad	Bladder
Blo	Blood
Bone	Bone
BM	Bone marrow
BrC	Brain cortex
BrS	Brainstem
Col	Colon
Dia	Diaphragm
Ret	Eye retina
Fat	Fat
Hrt	Heart
Pit	Hypothalamus/Pituitary
Kid	Kidney
LM	Limb muscle
Liv	Liver
Lung	Lung
MG	Mammary gland
Panc	Pancreas
Skin	Skin
SI	Small intestine
Spl	Spleen
Test	Testes
Thym	Thymus
Tong	Tongue
Trac	Trachea
Ute	Uterus

Kininogen/Bradykinin/Kallikrein
- endothelial

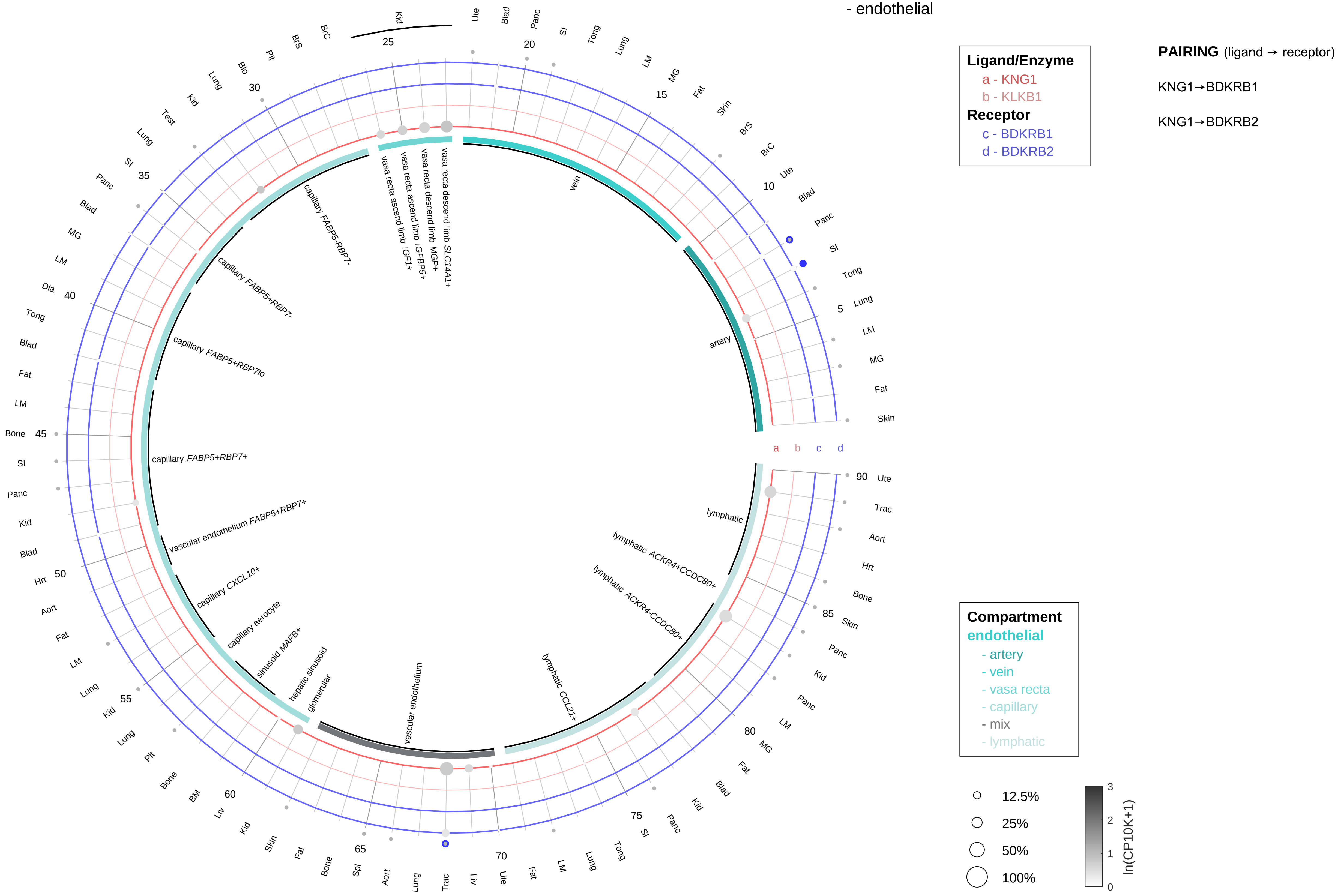

Abbrev.	Tissue
Aort	Aorta
Blad	Bladder
Blo	Blood
Bone	Bone
BM	Bone marrow
BrC	Brain cortex
BrS	Brainstem
Col	Colon
Dia	Diaphragm
Ret	Eye retina
Fat	Fat
Hrt	Heart
Pit	Hypothalamus/Pituitary
Kid	Kidney
LM	Limb muscle
Liv	Liver
Lung	Lung
MG	Mammary gland
Panc	Pancreas
Skin	Skin
SI	Small intestine
Spl	Spleen
Test	Testes
Thym	Thymus
Tong	Tongue
Trac	Trachea
Ute	Uterus

Kininogen/Bradykinin/Kallikrein
- stromal

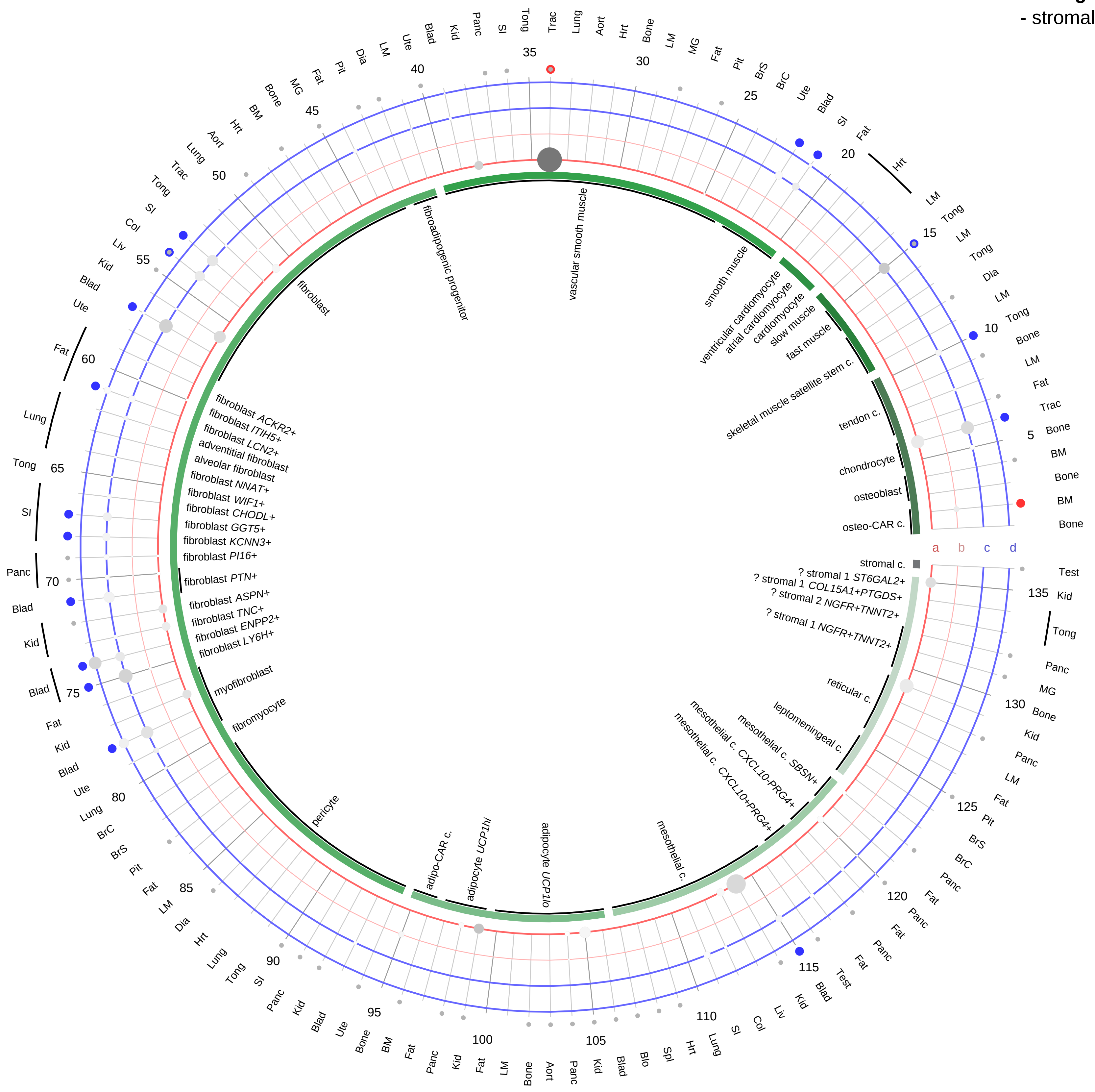

Ligand/Enzyme
- a - KNG1
- b - KLKB1

Receptor
- c - BDKRB1
- d - BDKRB2

PAIRING (ligand → receptor)

KNG1→BDKRB1

KNG1→BDKRB2

Compartment
stromal
- bone
- skeletal muscle
- cardiac muscle
- smooth muscle
- fibroblast
- adipocyte
- mesothelial
- other
- mix

12.5%
25%
50%
100%

ln(CP10K+1): 0, 1, 2, 3

Abbrev.	Tissue
Aort	Aorta
Blad	Bladder
Blo	Blood
Bone	Bone
BM	Bone marrow
BrC	Brain cortex
BrS	Brainstem
Col	Colon
Dia	Diaphragm
Ret	Eye retina
Fat	Fat
Hrt	Heart
Pit	Hypothalamus/Pituitary
Kid	Kidney
LM	Limb muscle
Liv	Liver
Lung	Lung
MG	Mammary gland
Panc	Pancreas
Skin	Skin
SI	Small intestine
Spl	Spleen
Test	Testes
Thym	Thymus
Tong	Tongue
Trac	Trachea
Ute	Uterus

Kininogen/Bradykinin/Kallikrein
- lymphoid

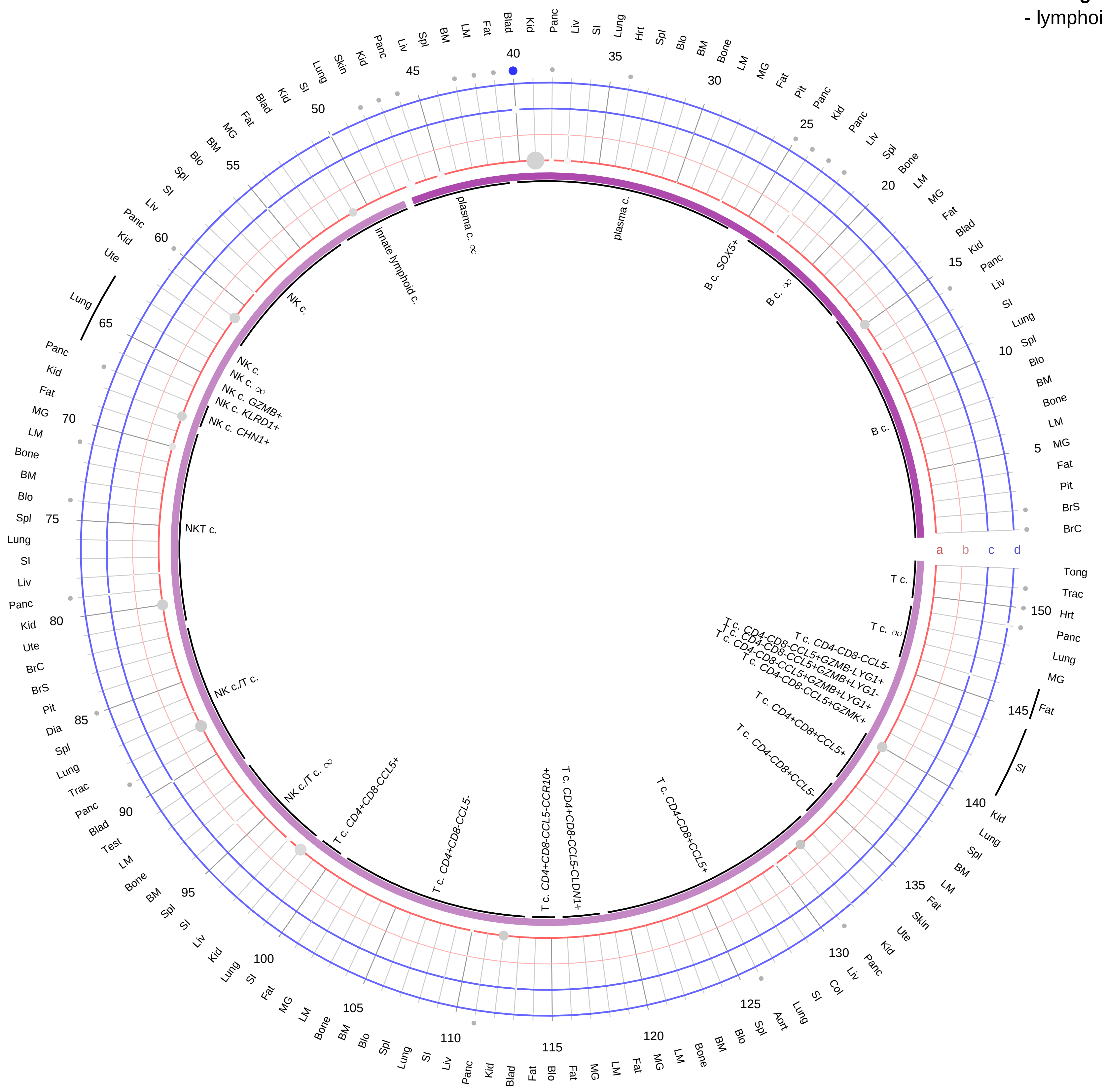

Ligand/Enzyme
- a - KNG1
- b - KLKB1

Receptor
- c - BDKRB1
- d - BDKRB2

PAIRING (ligand → receptor)

KNG1→BDKRB1

KNG1→BDKRB2

**Abbrev.**	**Tissue**
Aort	Aorta
Blad	Bladder
Blo	Blood
Bone	Bone
BM	Bone marrow
BrC	Brain cortex
BrS	Brainstem
Col	Colon
Dia	Diaphragm
Ret	Eye retina
Fat	Fat
Hrt	Heart
Pit	Hypothalamus/Pituitary
Kid	Kidney
LM	Limb muscle
Liv	Liver
Lung	Lung
MG	Mammary gland
Panc	Pancreas
Skin	Skin
SI	Small intestine
Spl	Spleen
Test	Testes
Thym	Thymus
Tong	Tongue
Trac	Trachea
Ute	Uterus

Compartment
lymphoid
- B cell
- NK/T cell

12.5%
25%
50%
100%

ln(CP10K+1): 0, 1, 2, 3

Kininogen/Bradykinin/Kallikrein
- myeloid

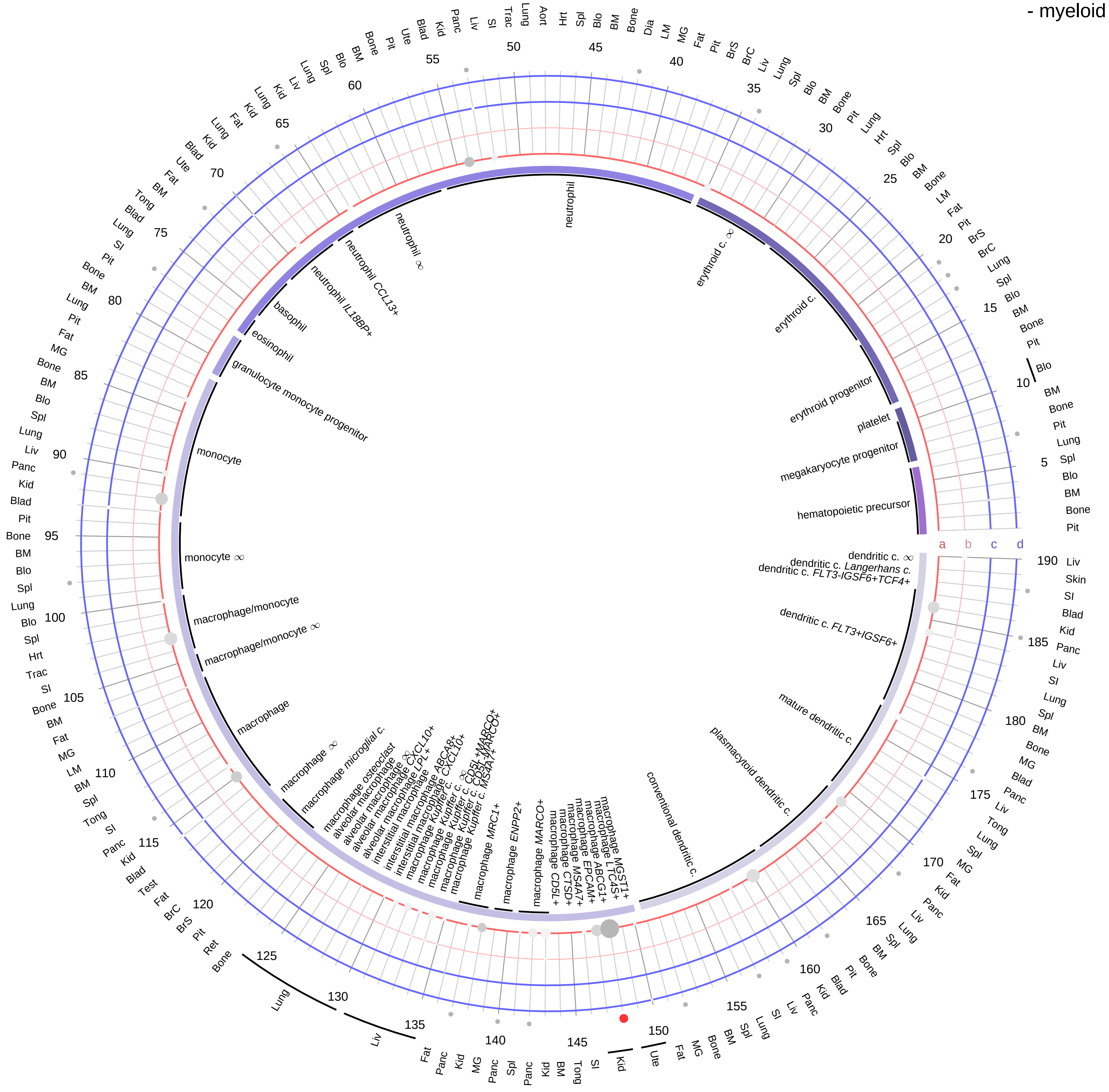

Ligand/Enzyme
- a - KNG1
- b - KLKB1

Receptor
- c - BDKRB1
- d - BDKRB2

PAIRING (ligand → receptor)

KNG1→BDKRB1

KNG1→BDKRB2

Compartment
- **hematopoietic**
  - hematopoietic precursor
- **megakaryocyte-erythroid**
  - megakaryoid
  - erythroid
- **myeloid**
  - granulocyte
  - granulocyte monocyte progenitor
  - monocyte/macrophage
  - dendritic

○ 12.5%
○ 25%
○ 50%
○ 100%

ln(CP10K+1): 0, 1, 2, 3

Abbrev.	Tissue
Aort	Aorta
Blad	Bladder
Blo	Blood
Bone	Bone
BM	Bone marrow
BrC	Brain cortex
BrS	Brainstem
Col	Colon
Dia	Diaphragm
Ret	Eye retina
Fat	Fat
Hrt	Heart
Pit	Hypothalamus/Pituitary
Kid	Kidney
LM	Limb muscle
Liv	Liver
Lung	Lung
MG	Mammary gland
Panc	Pancreas
Skin	Skin
SI	Small intestine
Spl	Spleen
Test	Testes
Thym	Thymus
Tong	Tongue
Trac	Trachea
Ute	Uterus

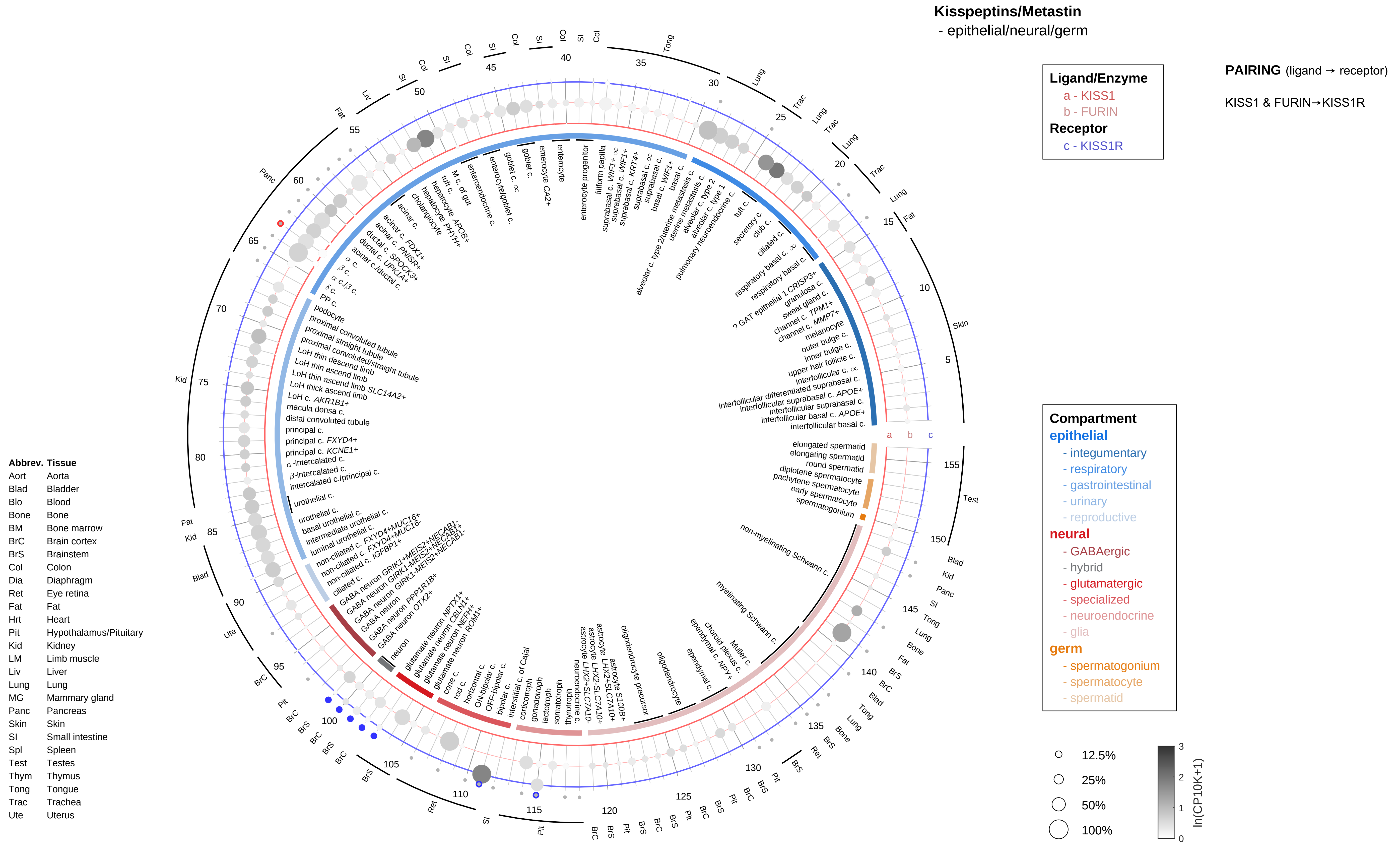

Kisspeptins/Metastin
- epithelial/neural/germ
Ligand/Enzyme
a - KISS1
b - FURIN
Receptor
c - KISS1R
PAIRING (ligand → receptor)
KISS1 & FURIN→KISS1R
Compartment
epithelial
- integumentary
- respiratory
- gastrointestinal
- urinary
- reproductive
neural
- GABAergic
- hybrid
- glutamatergic
- specialized
- neuroendocrine
- glia
germ
- spermatogonium
- spermatocyte
- spermatid
12.5%
25%
50%
100%
ln(CP10K+1)
Abbrev. Tissue
Aort Aorta
Blad Bladder
Blo Blood
Bone Bone
BM Bone marrow
BrC Brain cortex
BrS Brainstem
Col Colon
Dia Diaphragm
Ret Eye retina
Fat Fat
Hrt Heart
Pit Hypothalamus/Pituitary
Kid Kidney
LM Limb muscle
Liv Liver
Lung Lung
MG Mammary gland
Panc Pancreas
Skin Skin
SI Small intestine
Spl Spleen
Test Testes
Thym Thymus
Tong Tongue
Trac Trachea
Ute Uterus

Kisspeptins/Metastin
- endothelial

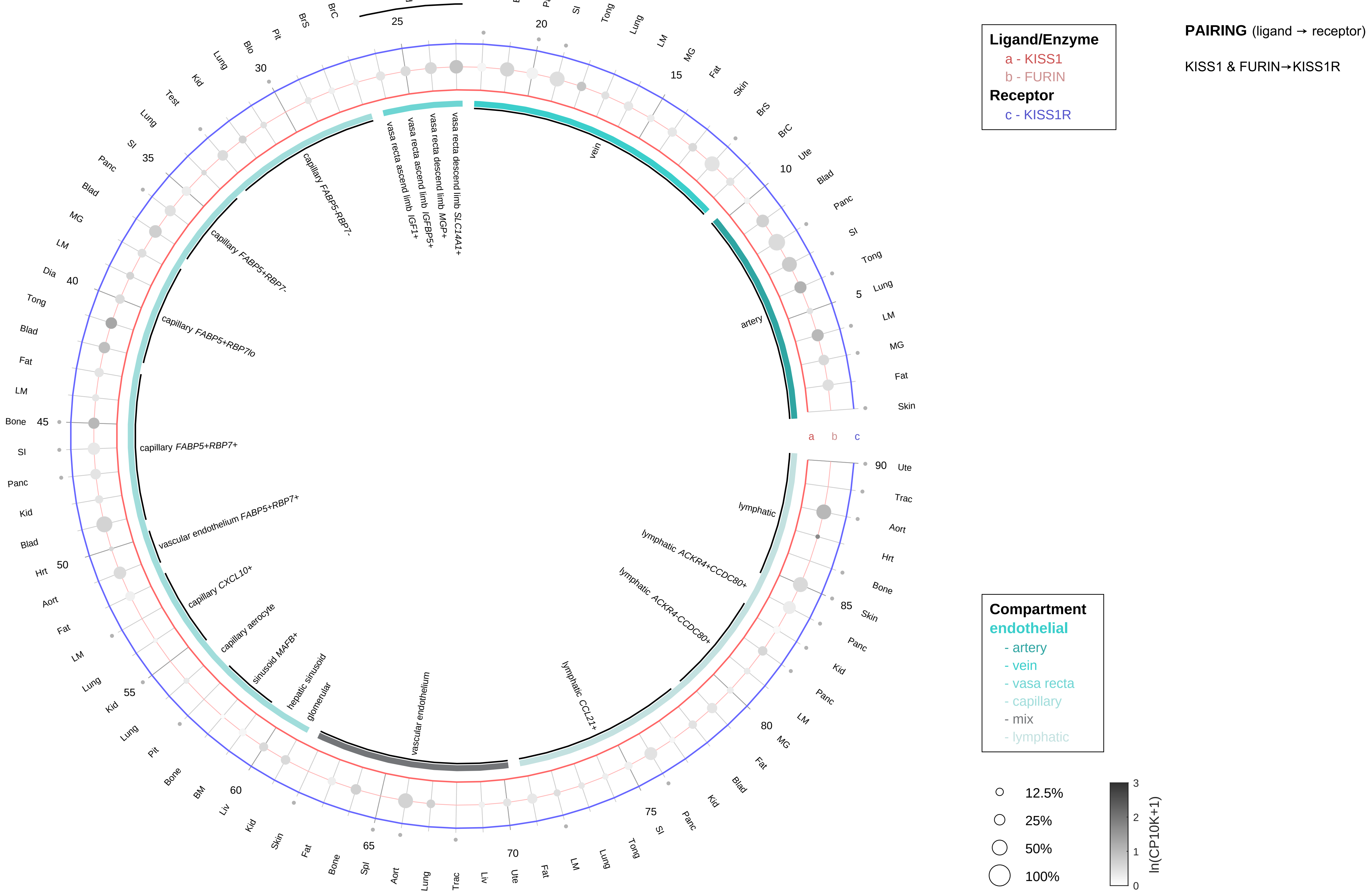

Abbrev.	Tissue
Aort	Aorta
Blad	Bladder
Blo	Blood
Bone	Bone
BM	Bone marrow
BrC	Brain cortex
BrS	Brainstem
Col	Colon
Dia	Diaphragm
Ret	Eye retina
Fat	Fat
Hrt	Heart
Pit	Hypothalamus/Pituitary
Kid	Kidney
LM	Limb muscle
Liv	Liver
Lung	Lung
MG	Mammary gland
Panc	Pancreas
Skin	Skin
SI	Small intestine
Spl	Spleen
Test	Testes
Thym	Thymus
Tong	Tongue
Trac	Trachea
Ute	Uterus

Kisspeptins/Metastin
- stromal

PAIRING (ligand → receptor)

KISS1 & FURIN→KISS1R

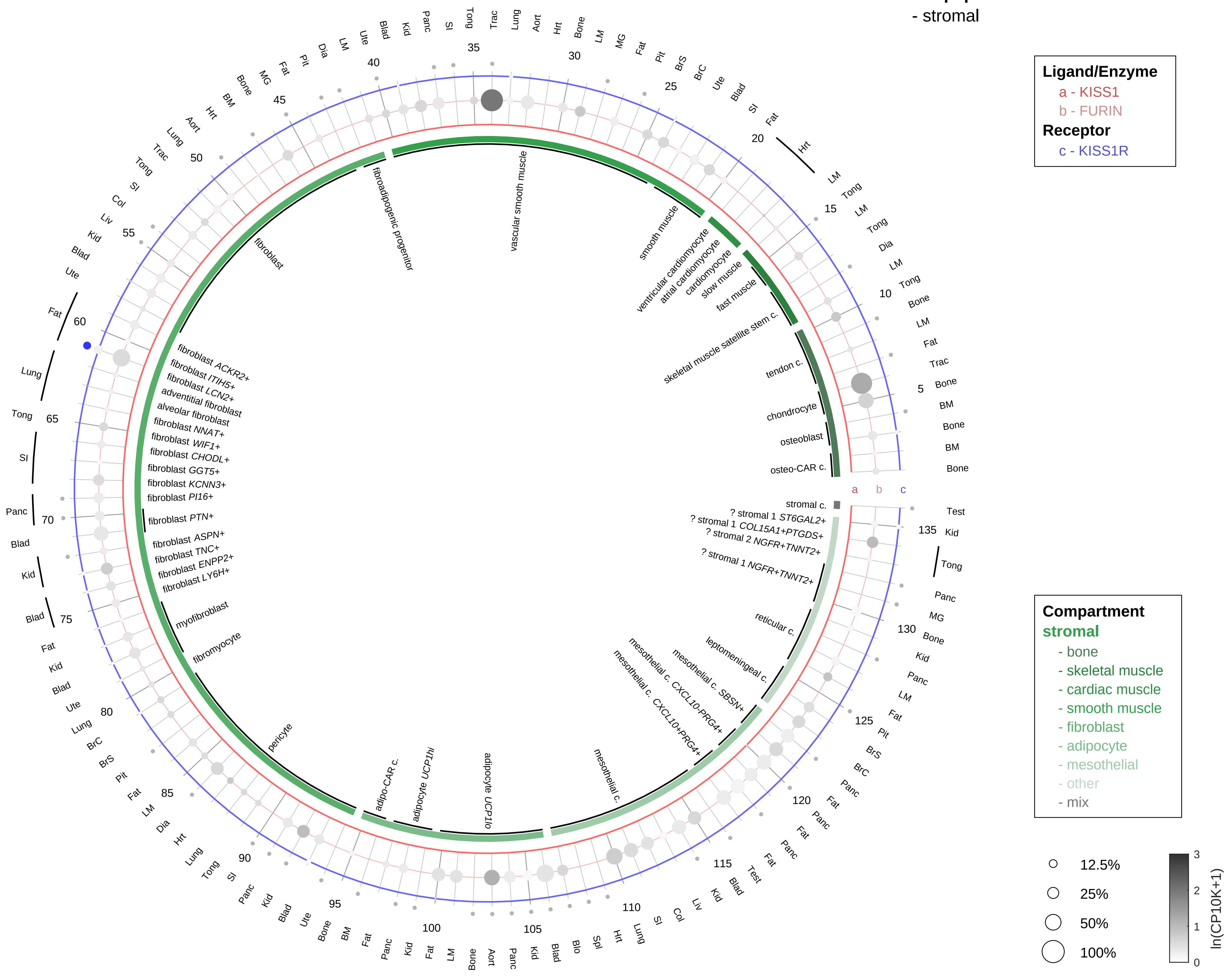

**Abbrev.**	**Tissue**
Aort	Aorta
Blad	Bladder
Blo	Blood
Bone	Bone
BM	Bone marrow
BrC	Brain cortex
BrS	Brainstem
Col	Colon
Dia	Diaphragm
Ret	Eye retina
Fat	Fat
Hrt	Heart
Pit	Hypothalamus/Pituitary
Kid	Kidney
LM	Limb muscle
Liv	Liver
Lung	Lung
MG	Mammary gland
Panc	Pancreas
Skin	Skin
SI	Small intestine
Spl	Spleen
Test	Testes
Thym	Thymus
Tong	Tongue
Trac	Trachea
Ute	Uterus

Kisspeptins/Metastin
- lymphoid

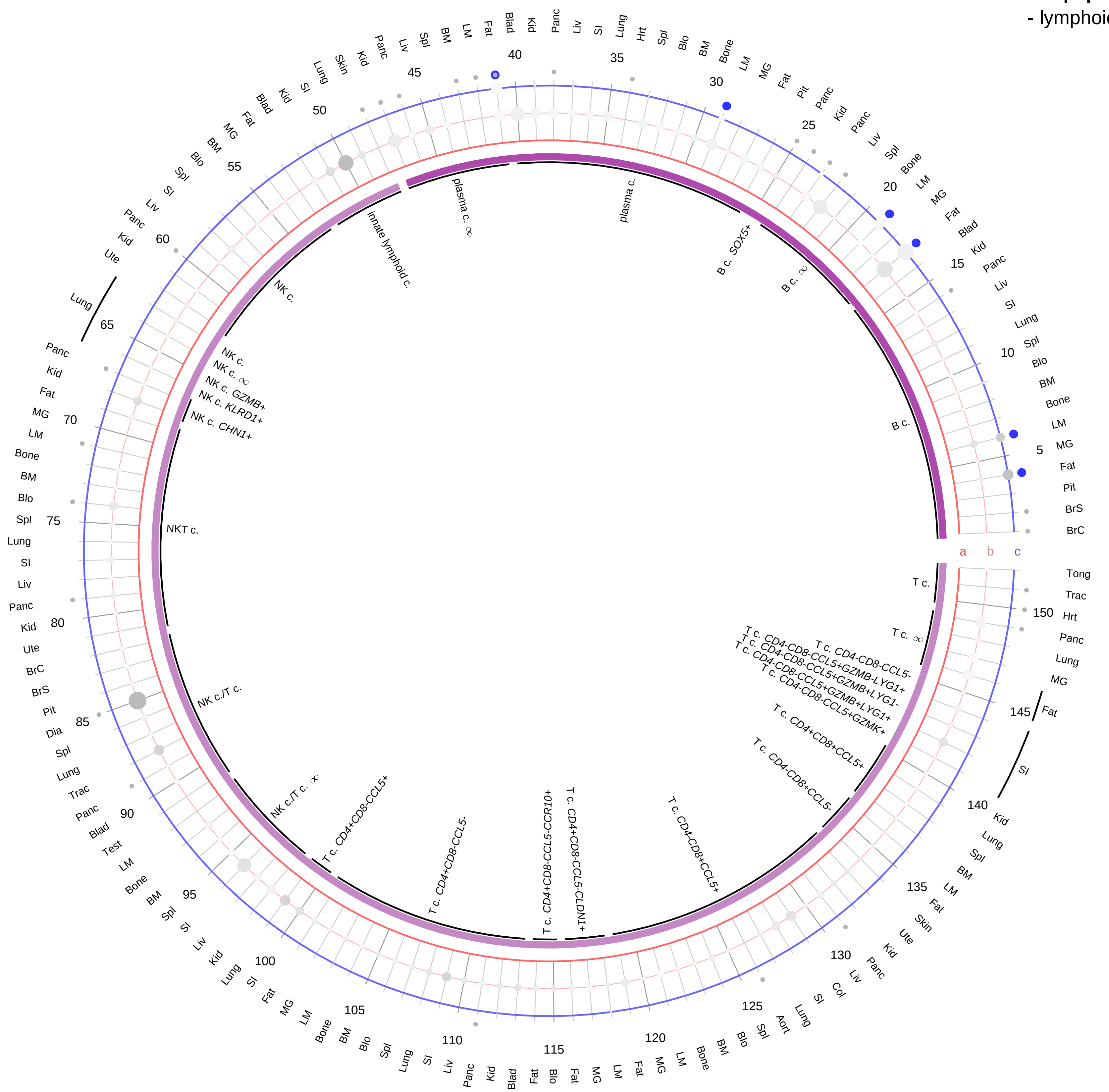

Ligand/Enzyme
- a - KISS1
- b - FURIN

Receptor
- c - KISS1R

PAIRING (ligand → receptor)

KISS1 & FURIN→KISS1R

Compartment
lymphoid
- B cell
- NK/T cell

12.5%
25%
50%
100%

ln(CP10K+1): 0, 1, 2, 3

Abbrev.	Tissue
Aort	Aorta
Blad	Bladder
Blo	Blood
Bone	Bone
BM	Bone marrow
BrC	Brain cortex
BrS	Brainstem
Col	Colon
Dia	Diaphragm
Ret	Eye retina
Fat	Fat
Hrt	Heart
Pit	Hypothalamus/Pituitary
Kid	Kidney
LM	Limb muscle
Liv	Liver
Lung	Lung
MG	Mammary gland
Panc	Pancreas
Skin	Skin
SI	Small intestine
Spl	Spleen
Test	Testes
Thym	Thymus
Tong	Tongue
Trac	Trachea
Ute	Uterus

Kisspeptins/Metastin
- myeloid

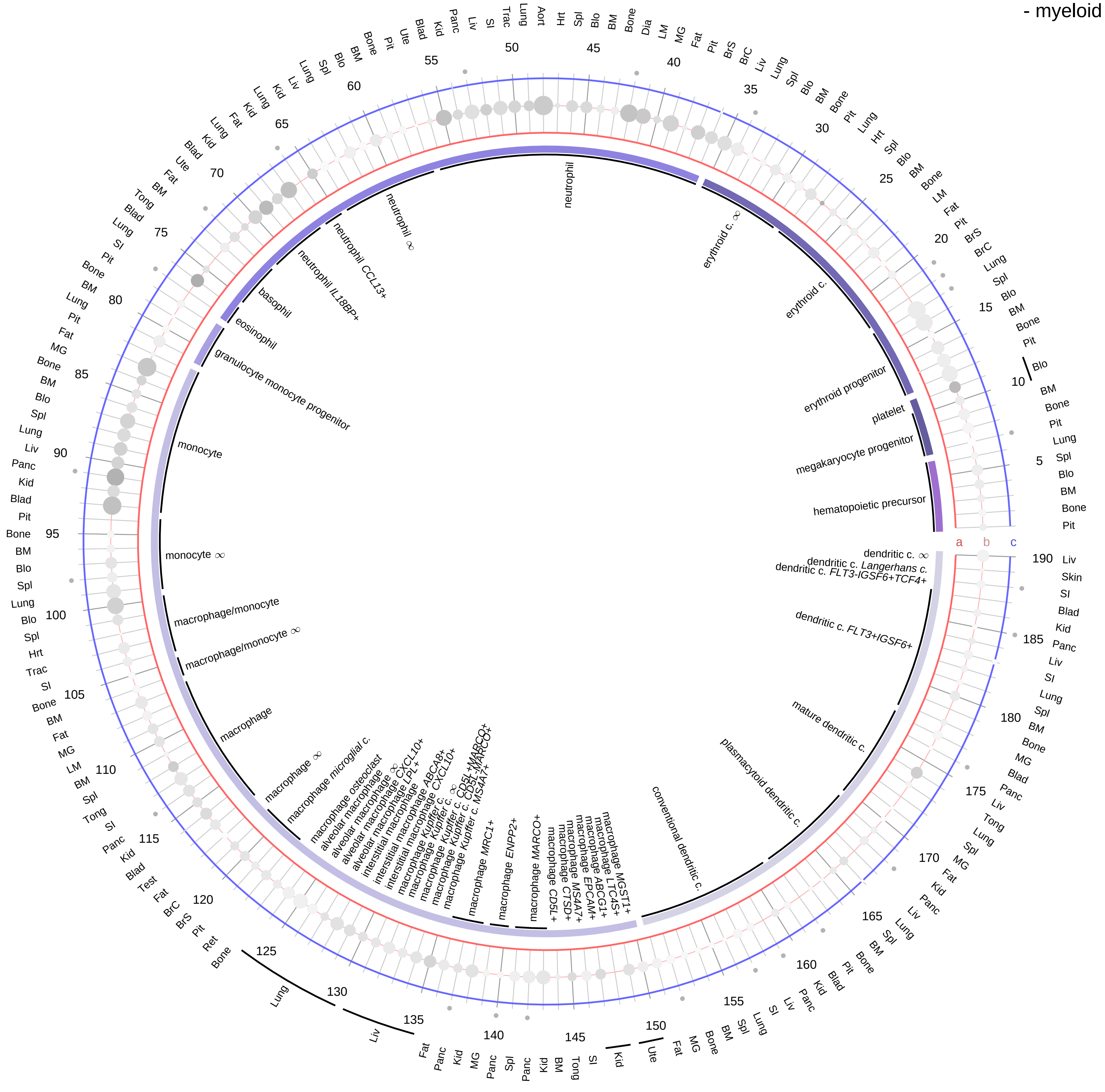

Ligand/Enzyme
- a - KISS1
- b - FURIN

Receptor
- c - KISS1R

PAIRING (ligand → receptor)

KISS1 & FURIN→KISS1R

Compartment
- **hematopoietic**
  - hematopoietic precursor
- **megakaryocyte-erythroid**
  - megakaryoid
  - erythroid
- **myeloid**
  - granulocyte
  - granulocyte monocyte progenitor
  - monocyte/macrophage
  - dendritic

12.5%
25%
50%
100%

ln(CP10K+1): 0, 1, 2, 3

Abbrev.	Tissue
Aort	Aorta
Blad	Bladder
Blo	Blood
Bone	Bone
BM	Bone marrow
BrC	Brain cortex
BrS	Brainstem
Col	Colon
Dia	Diaphragm
Ret	Eye retina
Fat	Fat
Hrt	Heart
Pit	Hypothalamus/Pituitary
Kid	Kidney
LM	Limb muscle
Liv	Liver
Lung	Lung
MG	Mammary gland
Panc	Pancreas
Skin	Skin
SI	Small intestine
Spl	Spleen
Test	Testes
Thym	Thymus
Tong	Tongue
Trac	Trachea
Ute	Uterus

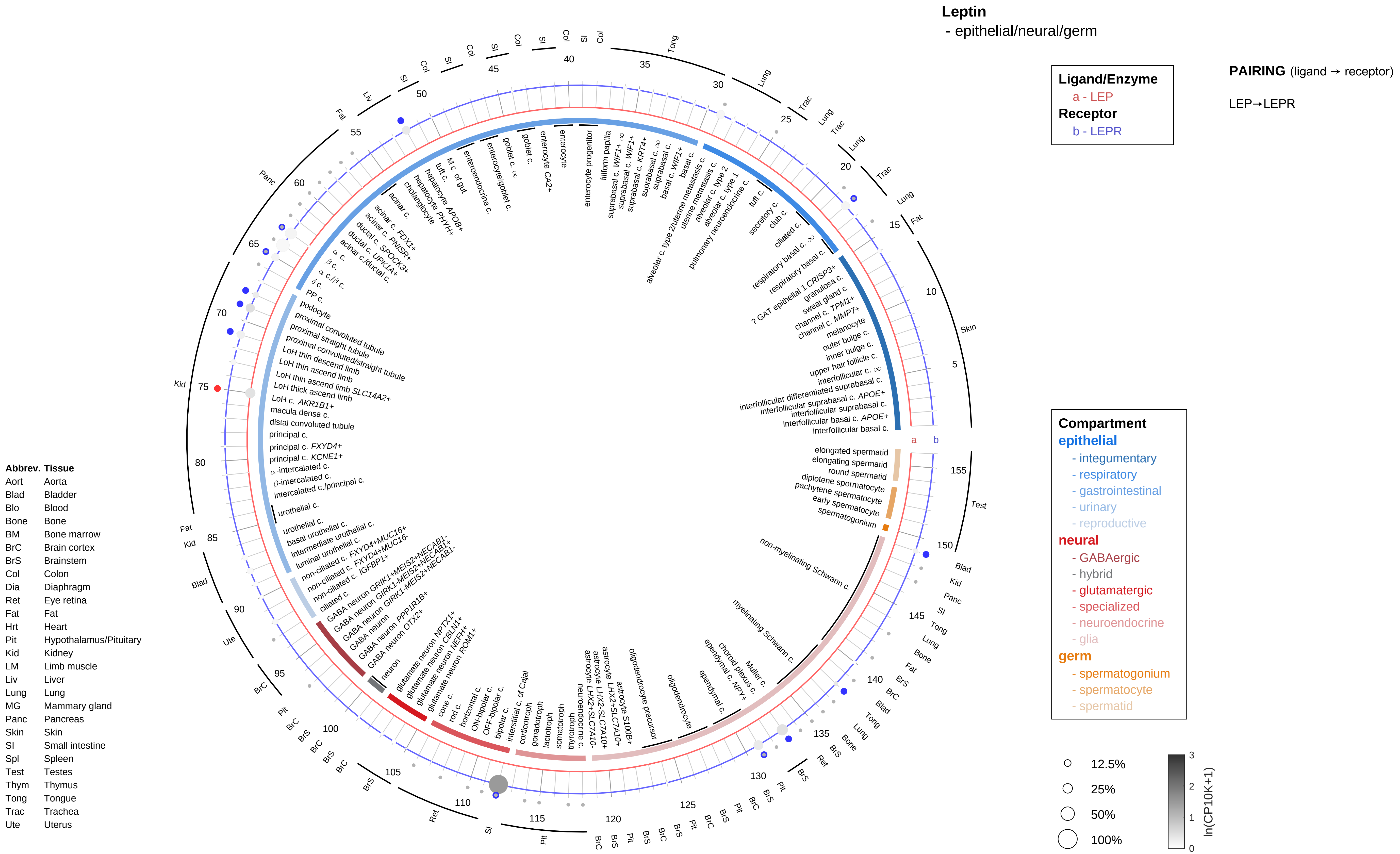

Leptin
- epithelial/neural/germ
Ligand/Enzyme
a - LEP
Receptor
b - LEPR
PAIRING (ligand → receptor)
LEP→LEPR
Compartment
epithelial
- integumentary
- respiratory
- gastrointestinal
- urinary
- reproductive
neural
- GABAergic
- hybrid
- glutamatergic
- specialized
- neuroendocrine
- glia
germ
- spermatogonium
- spermatocyte
- spermatid
12.5%
25%
50%
100%
ln(CP10K+1)
Abbrev. Tissue
Aort Aorta
Blad Bladder
Blo Blood
Bone Bone
BM Bone marrow
BrC Brain cortex
BrS Brainstem
Col Colon
Dia Diaphragm
Ret Eye retina
Fat Fat
Hrt Heart
Pit Hypothalamus/Pituitary
Kid Kidney
LM Limb muscle
Liv Liver
Lung Lung
MG Mammary gland
Panc Pancreas
Skin Skin
SI Small intestine
Spl Spleen
Test Testes
Thym Thymus
Tong Tongue
Trac Trachea
Ute Uterus

Leptin
- endothelial

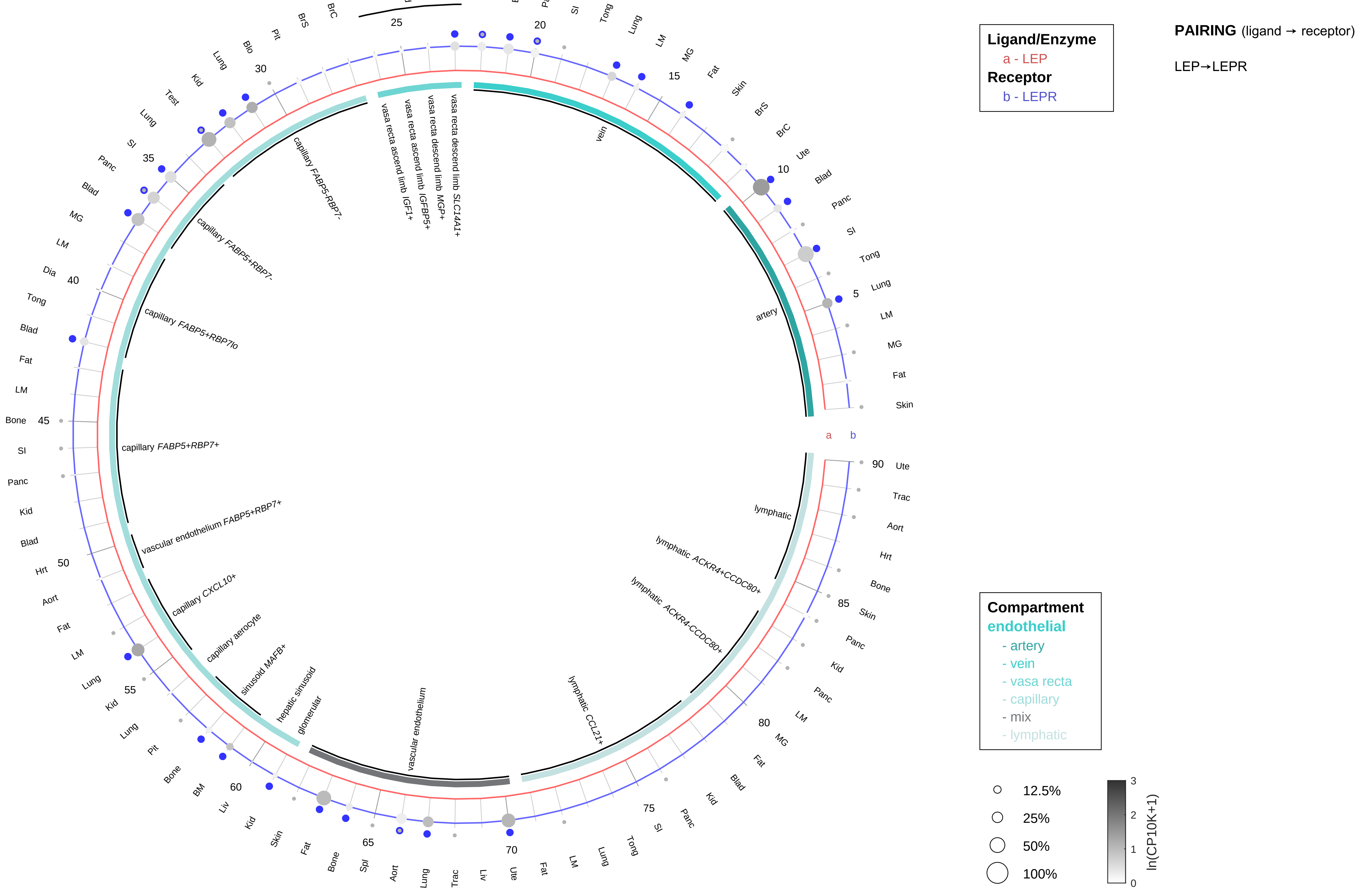

Abbrev.	Tissue
Aort	Aorta
Blad	Bladder
Blo	Blood
Bone	Bone
BM	Bone marrow
BrC	Brain cortex
BrS	Brainstem
Col	Colon
Dia	Diaphragm
Ret	Eye retina
Fat	Fat
Hrt	Heart
Pit	Hypothalamus/Pituitary
Kid	Kidney
LM	Limb muscle
Liv	Liver
Lung	Lung
MG	Mammary gland
Panc	Pancreas
Skin	Skin
SI	Small intestine
Spl	Spleen
Test	Testes
Thym	Thymus
Tong	Tongue
Trac	Trachea
Ute	Uterus

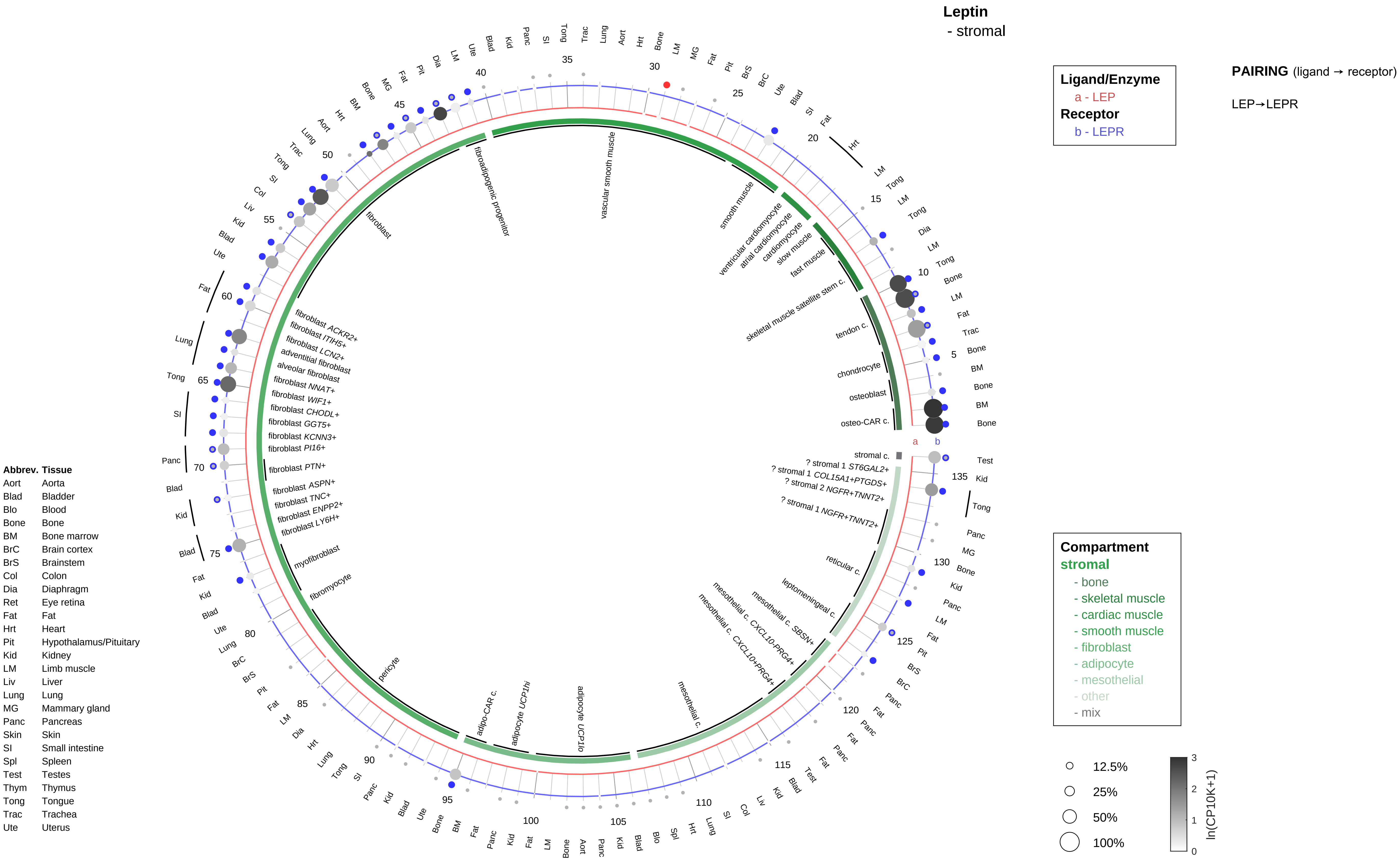

Leptin
- stromal
Ligand/Enzyme
a - LEP
Receptor
b - LEPR
PAIRING (ligand → receptor)
LEP→LEPR
Compartment
stromal
- bone
- skeletal muscle
- cardiac muscle
- smooth muscle
- fibroblast
- adipocyte
- mesothelial
- other
- mix
12.5%
25%
50%
100%
ln(CP10K+1)
0
1
2
3
Abbrev. Tissue
Aort Aorta
Blad Bladder
Blo Blood
Bone Bone
BM Bone marrow
BrC Brain cortex
BrS Brainstem
Col Colon
Dia Diaphragm
Ret Eye retina
Fat Fat
Hrt Heart
Pit Hypothalamus/Pituitary
Kid Kidney
LM Limb muscle
Liv Liver
Lung Lung
MG Mammary gland
Panc Pancreas
Skin Skin
SI Small intestine
Spl Spleen
Test Testes
Thym Thymus
Tong Tongue
Trac Trachea
Ute Uterus
osteo-CAR c.
osteoblast
chondrocyte
tendon c.
skeletal muscle satellite stem c.
fast muscle
slow muscle
cardiomyocyte
atrial cardiomyocyte
ventricular cardiomyocyte
smooth muscle
vascular smooth muscle
fibroadipogenic progenitor
fibroblast
fibroblast ACKR2+
fibroblast ITIH5+
fibroblast LCN2+
adventitial fibroblast
alveolar fibroblast
fibroblast NNAT+
fibroblast WIF1+
fibroblast CHODL+
fibroblast GGT5+
fibroblast KCNN3+
fibroblast PI16+
fibroblast PTN+
fibroblast ASPN+
fibroblast TNC+
fibroblast ENPP2+
fibroblast LY6H+
myofibroblast
fibromyocyte
pericyte
adipo-CAR c.
adipocyte UCP1hi
adipocyte UCP1lo
mesothelial c.
mesothelial c. CXCL10+PRG4+
mesothelial c. CXCL10-PRG4+
mesothelial c. SBSN+
leptomeningeal c.
reticular c.
? stromal 1 NGFR+TNNT2+
? stromal 2 NGFR+TNNT2+
? stromal 1 COL15A1+PTGDS+
? stromal 1 ST6GAL2+
stromal c.
a
b

Leptin
- lymphoid

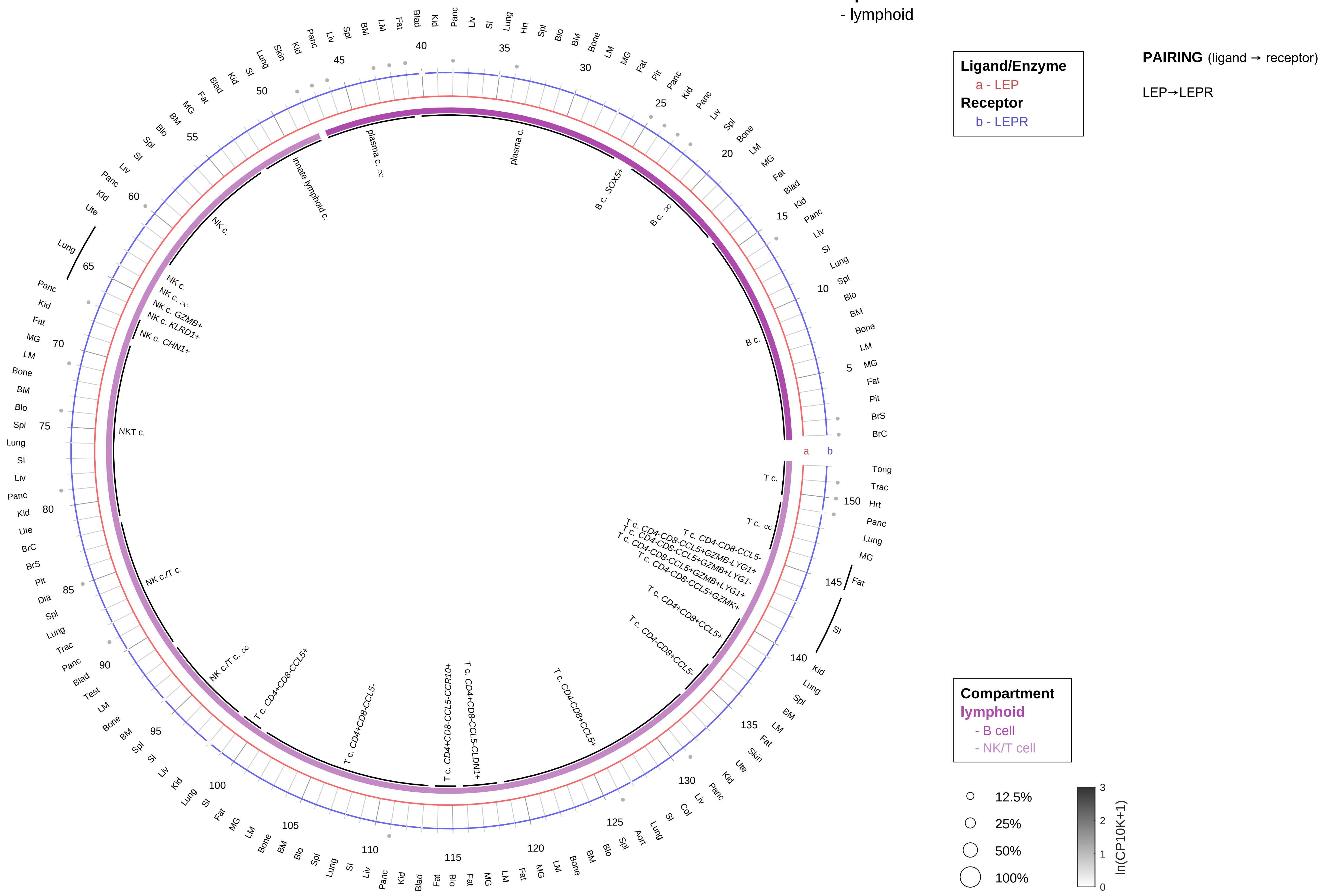

Abbrev.	Tissue
Aort	Aorta
Blad	Bladder
Blo	Blood
Bone	Bone
BM	Bone marrow
BrC	Brain cortex
BrS	Brainstem
Col	Colon
Dia	Diaphragm
Ret	Eye retina
Fat	Fat
Hrt	Heart
Pit	Hypothalamus/Pituitary
Kid	Kidney
LM	Limb muscle
Liv	Liver
Lung	Lung
MG	Mammary gland
Panc	Pancreas
Skin	Skin
SI	Small intestine
Spl	Spleen
Test	Testes
Thym	Thymus
Tong	Tongue
Trac	Trachea
Ute	Uterus

Leptin
- myeloid

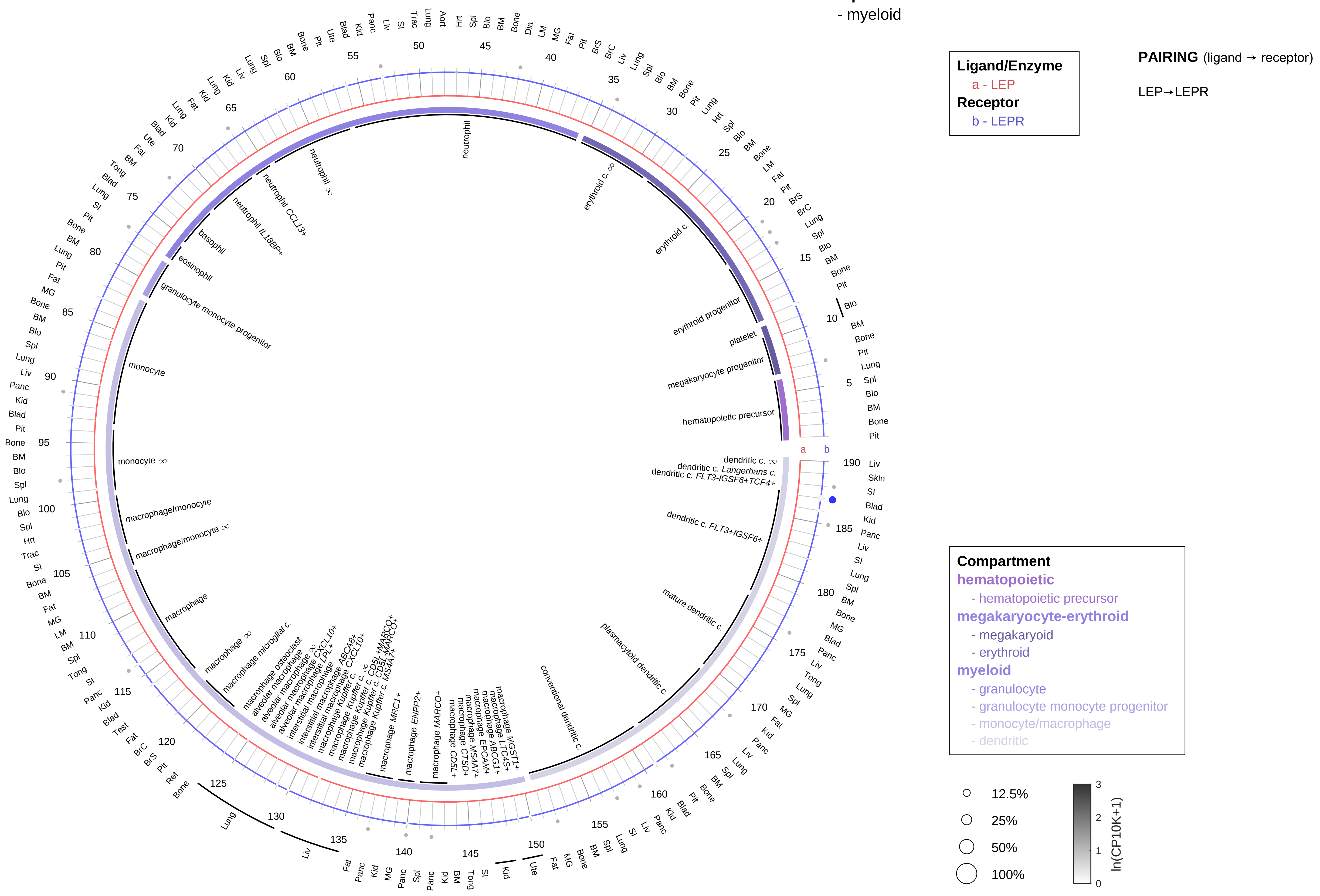

Abbrev.	Tissue
Aort	Aorta
Blad	Bladder
Blo	Blood
Bone	Bone
BM	Bone marrow
BrC	Brain cortex
BrS	Brainstem
Col	Colon
Dia	Diaphragm
Ret	Eye retina
Fat	Fat
Hrt	Heart
Pit	Hypothalamus/Pituitary
Kid	Kidney
LM	Limb muscle
Liv	Liver
Lung	Lung
MG	Mammary gland
Panc	Pancreas
Skin	Skin
SI	Small intestine
Spl	Spleen
Test	Testes
Thym	Thymus
Tong	Tongue
Trac	Trachea
Ute	Uterus

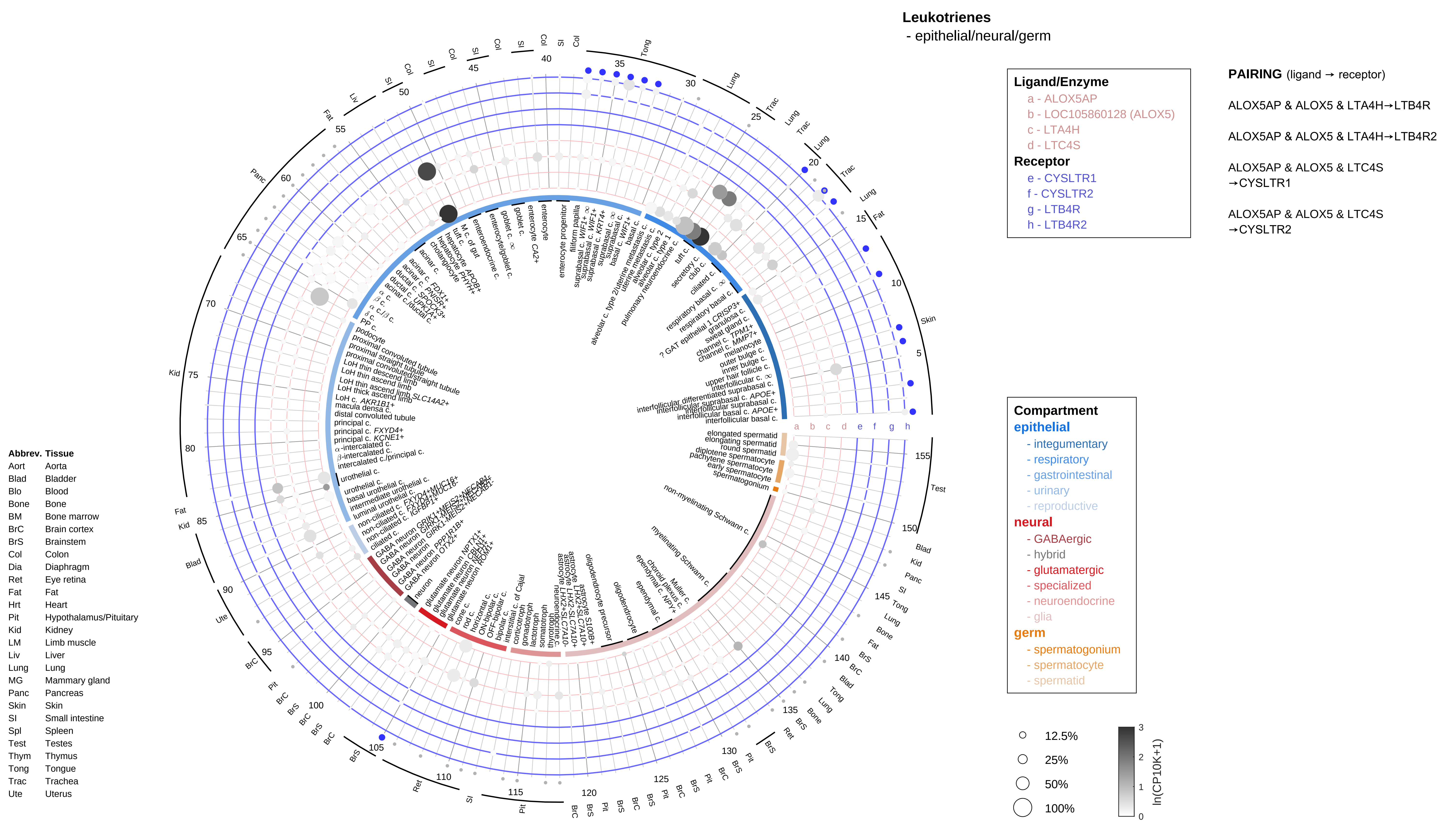

Leukotrienes
- epithelial/neural/germ
Ligand/Enzyme
a - ALOX5AP
b - LOC105860128 (ALOX5)
c - LTA4H
d - LTC4S
Receptor
e - CYSLTR1
f - CYSLTR2
g - LTB4R
h - LTB4R2
PAIRING (ligand → receptor)
ALOX5AP & ALOX5 & LTA4H→LTB4R
ALOX5AP & ALOX5 & LTA4H→LTB4R2
ALOX5AP & ALOX5 & LTC4S →CYSLTR1
ALOX5AP & ALOX5 & LTC4S →CYSLTR2
Compartment
epithelial
- integumentary
- respiratory
- gastrointestinal
- urinary
- reproductive
neural
- GABAergic
- hybrid
- glutamatergic
- specialized
- neuroendocrine
- glia
germ
- spermatogonium
- spermatocyte
- spermatid
12.5%
25%
50%
100%
ln(CP10K+1)
0
1
2
3
Abbrev. Tissue
Aort Aorta
Blad Bladder
Blo Blood
Bone Bone
BM Bone marrow
BrC Brain cortex
BrS Brainstem
Col Colon
Dia Diaphragm
Ret Eye retina
Fat Fat
Hrt Heart
Pit Hypothalamus/Pituitary
Kid Kidney
LM Limb muscle
Liv Liver
Lung Lung
MG Mammary gland
Panc Pancreas
Skin Skin
SI Small intestine
Spl Spleen
Test Testes
Thym Thymus
Tong Tongue
Trac Trachea
Ute Uterus

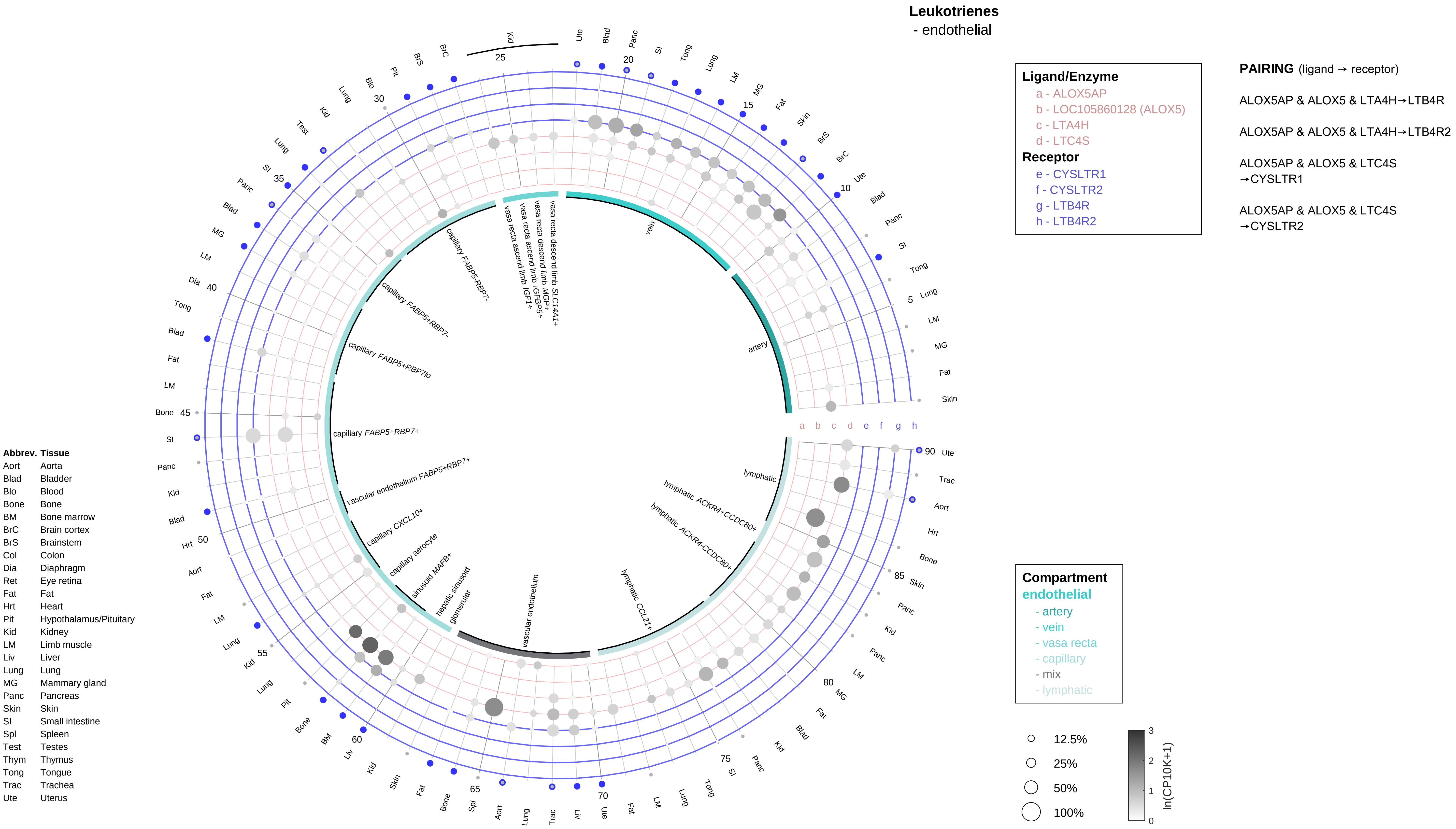
Leukotrienes
- endothelial
Ligand/Enzyme
a - ALOX5AP
b - LOC105860128 (ALOX5)
c - LTA4H
d - LTC4S
Receptor
e - CYSLTR1
f - CYSLTR2
g - LTB4R
h - LTB4R2
PAIRING (ligand → receptor)
ALOX5AP & ALOX5 & LTA4H→LTB4R
ALOX5AP & ALOX5 & LTA4H→LTB4R2
ALOX5AP & ALOX5 & LTC4S →CYSLTR1
ALOX5AP & ALOX5 & LTC4S →CYSLTR2
Compartment
endothelial
- artery
- vein
- vasa recta
- capillary
- mix
- lymphatic
12.5%
25%
50%
100%
ln(CP10K+1)
0
1
2
3
artery
vein
vasa recta descend limb SLC14A1+
vasa recta descend limb MGP+
vasa recta ascend limb IGFBP5+
vasa recta ascend limb IGF1+
capillary FABP5-RBP7-
capillary FABP5+RBP7-
capillary FABP5+RBP7lo
capillary FABP5+RBP7+
vascular endothelium FABP5+RBP7+
capillary CXCL10+
capillary aerocyte
sinusoid MAFB+
hepatic sinusoid
glomerular
vascular endothelium
lymphatic CCL21+
lymphatic ACKR4-CCDC80+
lymphatic ACKR4+CCDC80+
lymphatic
a b c d e f g h
Abbrev. Tissue
Aort Aorta
Blad Bladder
Blo Blood
Bone Bone
BM Bone marrow
BrC Brain cortex
BrS Brainstem
Col Colon
Dia Diaphragm
Ret Eye retina
Fat Fat
Hrt Heart
Pit Hypothalamus/Pituitary
Kid Kidney
LM Limb muscle
Liv Liver
Lung Lung
MG Mammary gland
Panc Pancreas
Skin Skin
SI Small intestine
Spl Spleen
Test Testes
Thym Thymus
Tong Tongue
Trac Trachea
Ute Uterus

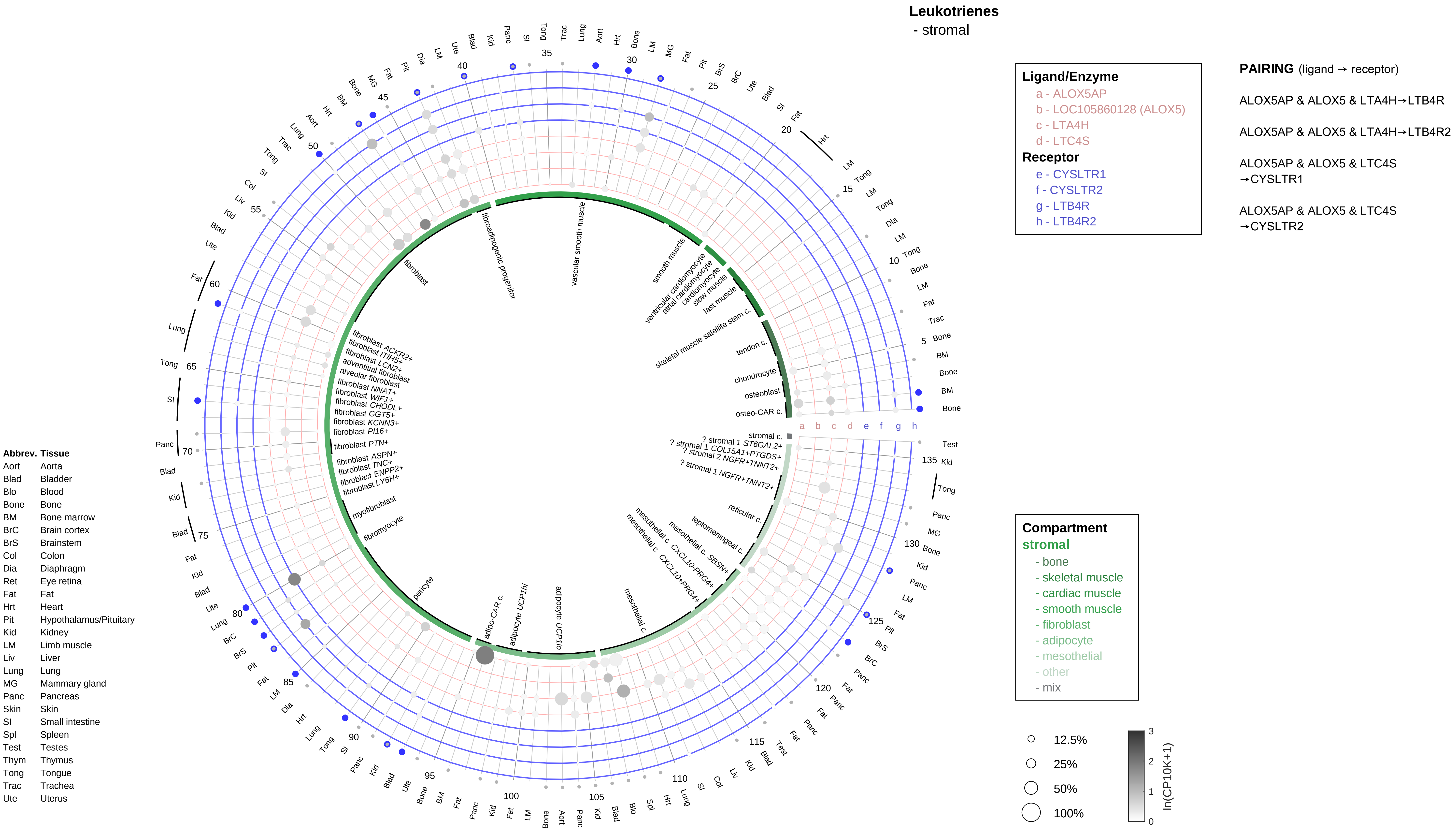
Leukotrienes
- stromal
Ligand/Enzyme
a - ALOX5AP
b - LOC105860128 (ALOX5)
c - LTA4H
d - LTC4S
Receptor
e - CYSLTR1
f - CYSLTR2
g - LTB4R
h - LTB4R2
PAIRING (ligand → receptor)
ALOX5AP & ALOX5 & LTA4H→LTB4R
ALOX5AP & ALOX5 & LTA4H→LTB4R2
ALOX5AP & ALOX5 & LTC4S →CYSLTR1
ALOX5AP & ALOX5 & LTC4S →CYSLTR2
Compartment
stromal
- bone
- skeletal muscle
- cardiac muscle
- smooth muscle
- fibroblast
- adipocyte
- mesothelial
- other
- mix
12.5%
25%
50%
100%
ln(CP10K+1)
0
1
2
3
Abbrev. Tissue
Aort Aorta
Blad Bladder
Blo Blood
Bone Bone
BM Bone marrow
BrC Brain cortex
BrS Brainstem
Col Colon
Dia Diaphragm
Ret Eye retina
Fat Fat
Hrt Heart
Pit Hypothalamus/Pituitary
Kid Kidney
LM Limb muscle
Liv Liver
Lung Lung
MG Mammary gland
Panc Pancreas
Skin Skin
SI Small intestine
Spl Spleen
Test Testes
Thym Thymus
Tong Tongue
Trac Trachea
Ute Uterus
a b c d e f g h
osteo-CAR c.
osteoblast
chondrocyte
tendon c.
skeletal muscle satellite stem c.
fast muscle
slow muscle
cardiomyocyte
atrial cardiomyocyte
ventricular cardiomyocyte
smooth muscle
vascular smooth muscle
fibroadipogenic progenitor
fibroblast
fibroblast ACKR2+
fibroblast ITIH5+
fibroblast LCN2+
adventitial fibroblast
alveolar fibroblast
fibroblast NNAT+
fibroblast WIF1+
fibroblast CHODL+
fibroblast GGT5+
fibroblast KCNN3+
fibroblast PI16+
fibroblast PTN+
fibroblast ASPN+
fibroblast TNC+
fibroblast ENPP2+
fibroblast LY6H+
myofibroblast
fibromyocyte
pericyte
adipo-CAR c.
adipocyte UCP1hi
adipocyte UCP1lo
mesothelial c.
mesothelial c. CXCL10+PRG4+
mesothelial c. CXCL10-PRG4+
mesothelial c. SBSN+
leptomeningeal c.
reticular c.
? stromal 1 NGFR+TNNT2+
? stromal 2 NGFR+TNNT2+
? stromal 1 COL15A1+PTGDS+
? stromal 1 ST6GAL2+
stromal c.
5
10
15
20
25
30
35
40
45
50
55
60
65
70
75
80
85
90
95
100
105
110
115
120
125
130
135

Leukotrienes
- lymphoid

Ligand/Enzyme
- a - ALOX5AP
- b - LOC105860128 (ALOX5)
- c - LTA4H
- d - LTC4S

Receptor
- e - CYSLTR1
- f - CYSLTR2
- g - LTB4R
- h - LTB4R2

PAIRING (ligand → receptor)

ALOX5AP & ALOX5 & LTA4H→LTB4R

ALOX5AP & ALOX5 & LTA4H→LTB4R2

ALOX5AP & ALOX5 & LTC4S →CYSLTR1

ALOX5AP & ALOX5 & LTC4S →CYSLTR2

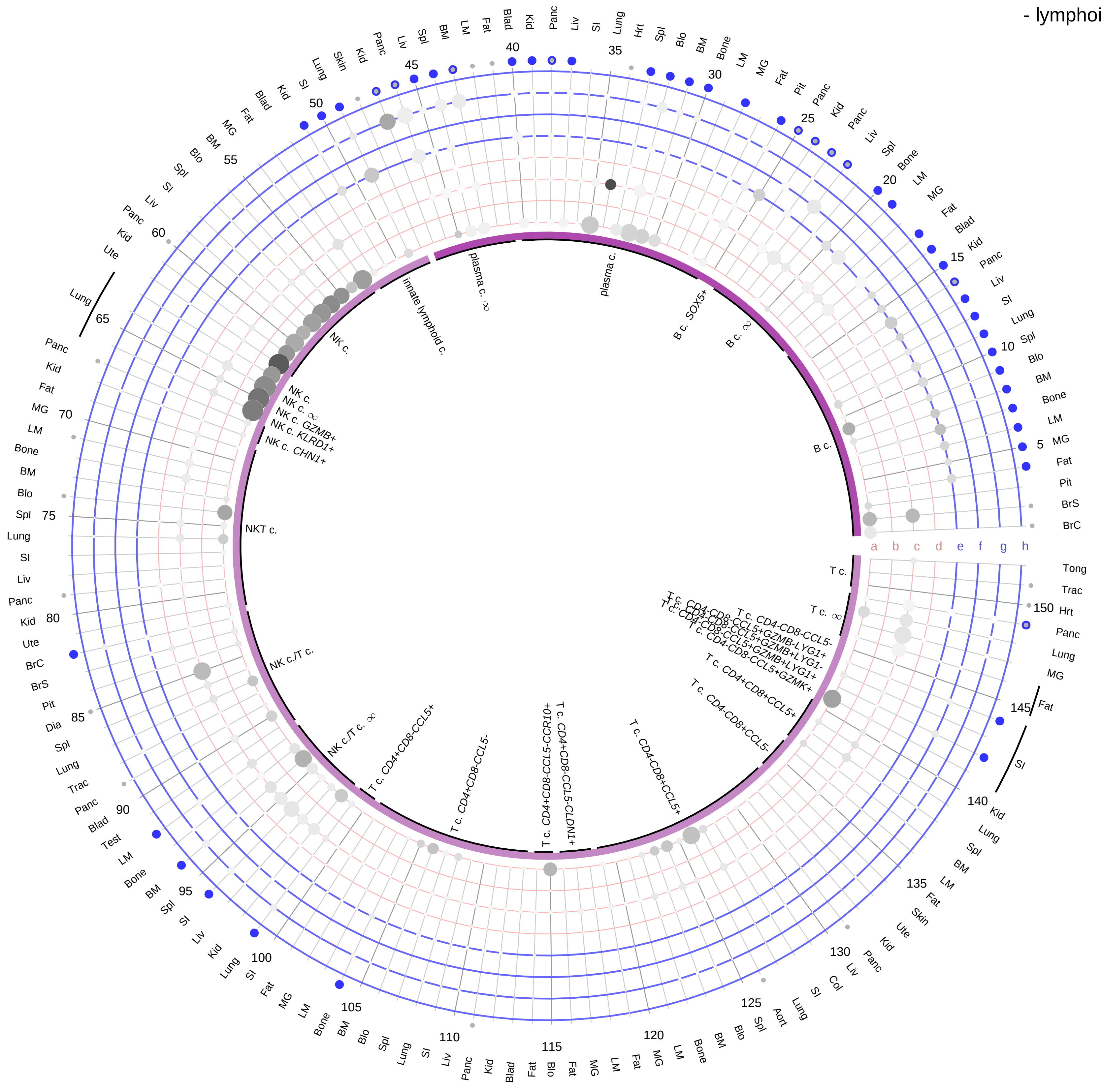

Abbrev.	Tissue
Aort	Aorta
Blad	Bladder
Blo	Blood
Bone	Bone
BM	Bone marrow
BrC	Brain cortex
BrS	Brainstem
Col	Colon
Dia	Diaphragm
Ret	Eye retina
Fat	Fat
Hrt	Heart
Pit	Hypothalamus/Pituitary
Kid	Kidney
LM	Limb muscle
Liv	Liver
Lung	Lung
MG	Mammary gland
Panc	Pancreas
Skin	Skin
SI	Small intestine
Spl	Spleen
Test	Testes
Thym	Thymus
Tong	Tongue
Trac	Trachea
Ute	Uterus

Compartment
lymphoid
- B cell
- NK/T cell

12.5%
25%
50%
100%

ln(CP10K+1) 0 1 2 3

Leukotrienes
- myeloid

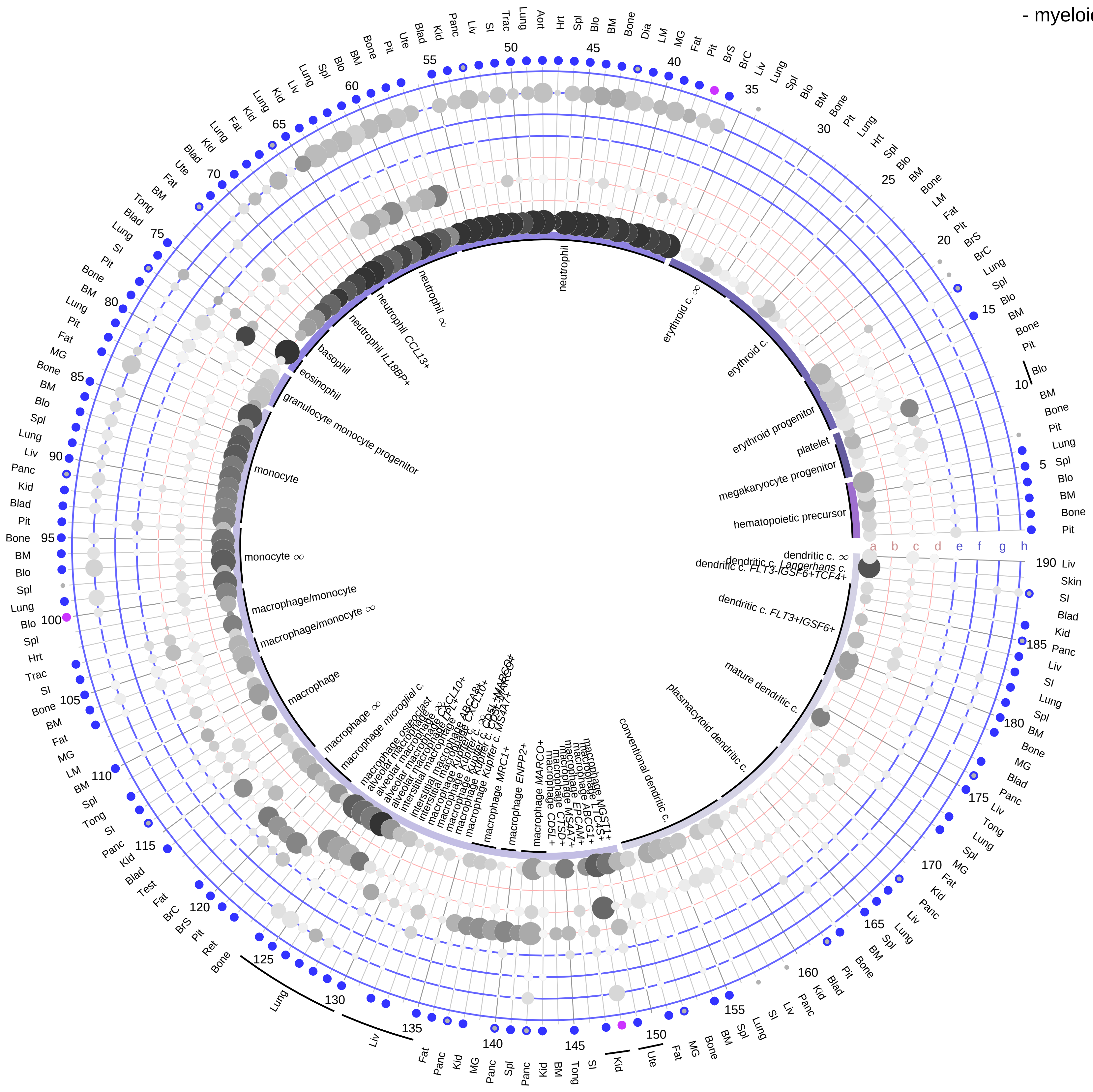

Ligand/Enzyme
- a - ALOX5AP
- b - LOC105860128 (ALOX5)
- c - LTA4H
- d - LTC4S

Receptor
- e - CYSLTR1
- f - CYSLTR2
- g - LTB4R
- h - LTB4R2

PAIRING (ligand → receptor)

ALOX5AP & ALOX5 & LTA4H→LTB4R

ALOX5AP & ALOX5 & LTA4H→LTB4R2

ALOX5AP & ALOX5 & LTC4S →CYSLTR1

ALOX5AP & ALOX5 & LTC4S →CYSLTR2

Abbrev.	Tissue
Aort	Aorta
Blad	Bladder
Blo	Blood
Bone	Bone
BM	Bone marrow
BrC	Brain cortex
BrS	Brainstem
Col	Colon
Dia	Diaphragm
Ret	Eye retina
Fat	Fat
Hrt	Heart
Pit	Hypothalamus/Pituitary
Kid	Kidney
LM	Limb muscle
Liv	Liver
Lung	Lung
MG	Mammary gland
Panc	Pancreas
Skin	Skin
SI	Small intestine
Spl	Spleen
Test	Testes
Thym	Thymus
Tong	Tongue
Trac	Trachea
Ute	Uterus

Compartment

hematopoietic
- hematopoietic precursor

megakaryocyte-erythroid
- megakaryoid
- erythroid

myeloid
- granulocyte
- granulocyte monocyte progenitor
- monocyte/macrophage
- dendritic

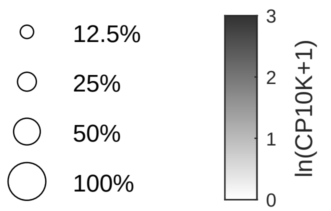

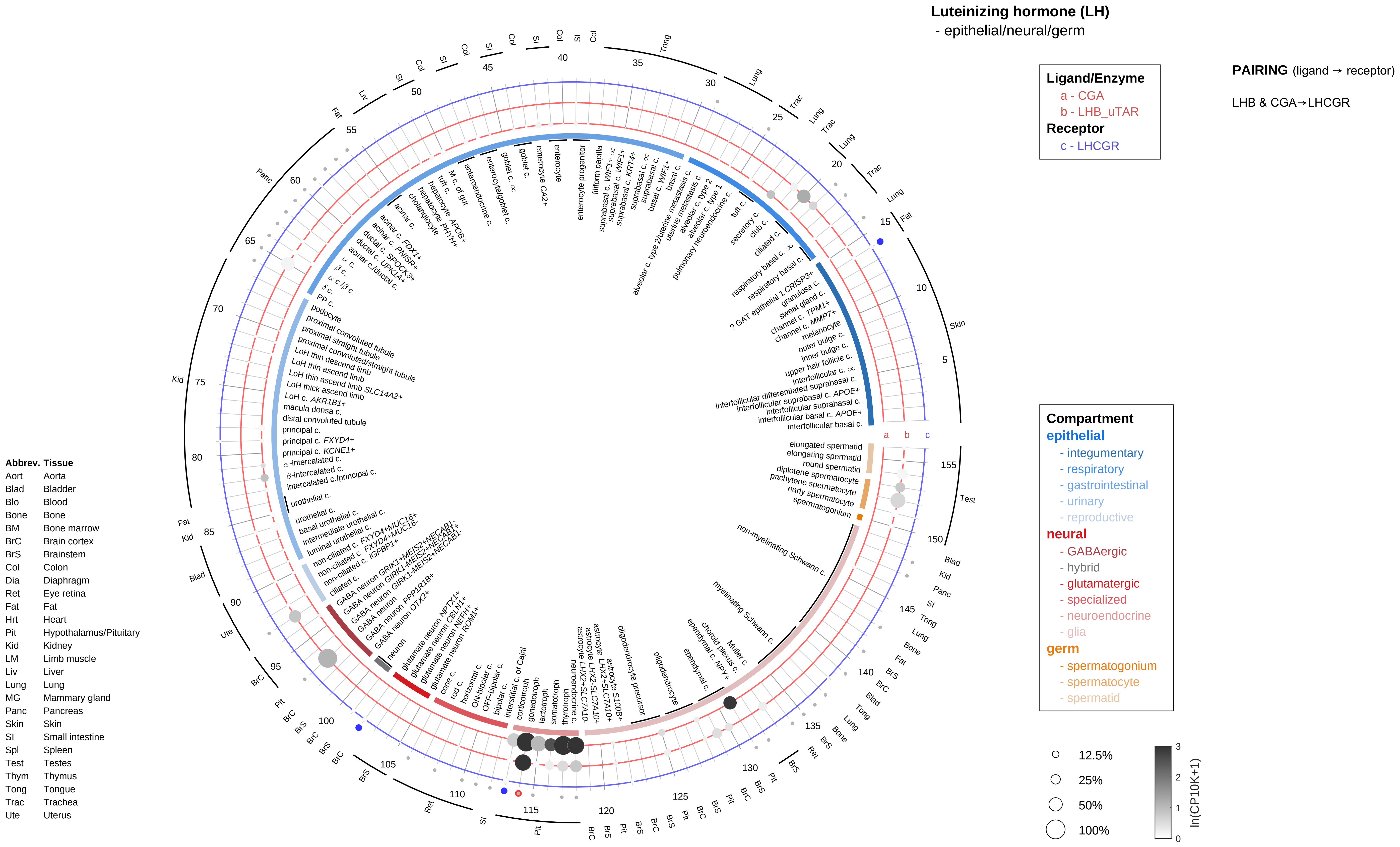

Luteinizing hormone (LH)
- epithelial/neural/germ
Ligand/Enzyme
a - CGA
b - LHB_uTAR
Receptor
c - LHCGR
PAIRING (ligand → receptor)
LHB & CGA→LHCGR
Compartment
epithelial
- integumentary
- respiratory
- gastrointestinal
- urinary
- reproductive
neural
- GABAergic
- hybrid
- glutamatergic
- specialized
- neuroendocrine
- glia
germ
- spermatogonium
- spermatocyte
- spermatid
12.5%
25%
50%
100%
ln(CP10K+1)
0
1
2
3
Abbrev. Tissue
Aort Aorta
Blad Bladder
Blo Blood
Bone Bone
BM Bone marrow
BrC Brain cortex
BrS Brainstem
Col Colon
Dia Diaphragm
Ret Eye retina
Fat Fat
Hrt Heart
Pit Hypothalamus/Pituitary
Kid Kidney
LM Limb muscle
Liv Liver
Lung Lung
MG Mammary gland
Panc Pancreas
Skin Skin
SI Small intestine
Spl Spleen
Test Testes
Thym Thymus
Tong Tongue
Trac Trachea
Ute Uterus

Luteinizing hormone (LH)
- endothelial

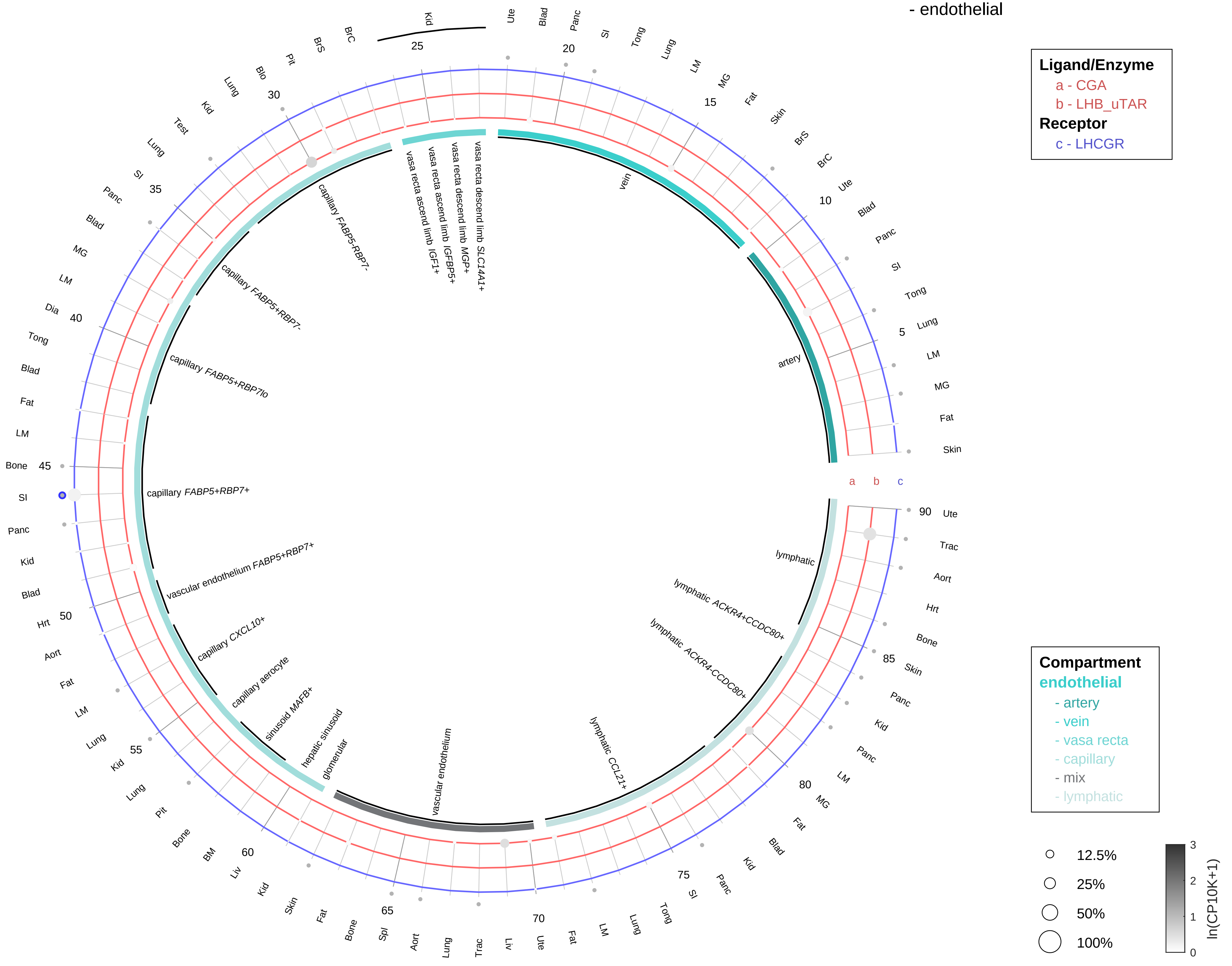

Abbrev.	Tissue
Aort	Aorta
Blad	Bladder
Blo	Blood
Bone	Bone
BM	Bone marrow
BrC	Brain cortex
BrS	Brainstem
Col	Colon
Dia	Diaphragm
Ret	Eye retina
Fat	Fat
Hrt	Heart
Pit	Hypothalamus/Pituitary
Kid	Kidney
LM	Limb muscle
Liv	Liver
Lung	Lung
MG	Mammary gland
Panc	Pancreas
Skin	Skin
SI	Small intestine
Spl	Spleen
Test	Testes
Thym	Thymus
Tong	Tongue
Trac	Trachea
Ute	Uterus

Luteinizing hormone (LH)
- stromal

Ligand/Enzyme
a - CGA
b - LHB_uTAR
Receptor
c - LHCGR

PAIRING (ligand → receptor)

LHB & CGA→LHCGR

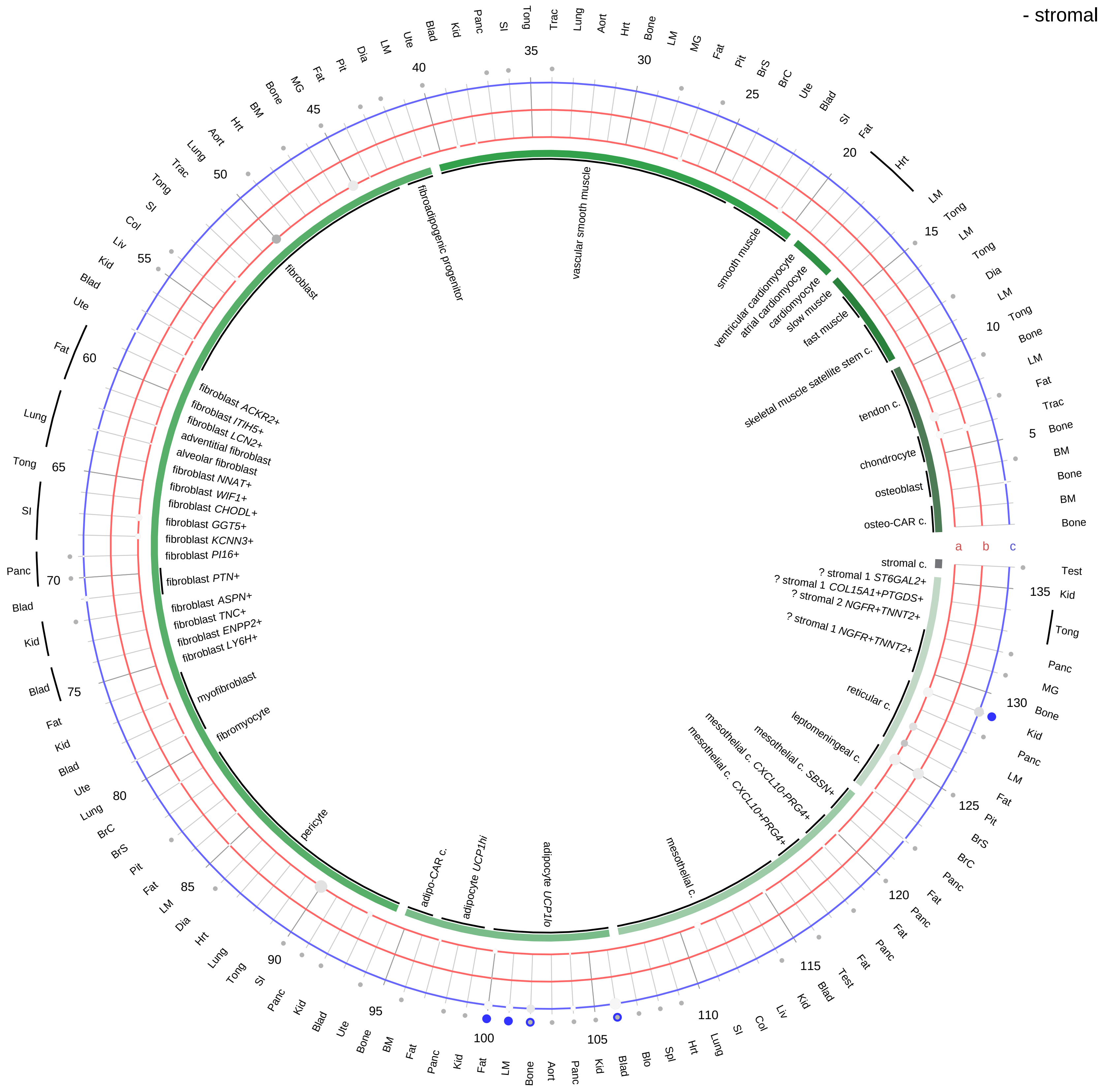

Abbrev. Tissue

Abbrev.	Tissue
Aort	Aorta
Blad	Bladder
Blo	Blood
Bone	Bone
BM	Bone marrow
BrC	Brain cortex
BrS	Brainstem
Col	Colon
Dia	Diaphragm
Ret	Eye retina
Fat	Fat
Hrt	Heart
Pit	Hypothalamus/Pituitary
Kid	Kidney
LM	Limb muscle
Liv	Liver
Lung	Lung
MG	Mammary gland
Panc	Pancreas
Skin	Skin
SI	Small intestine
Spl	Spleen
Test	Testes
Thym	Thymus
Tong	Tongue
Trac	Trachea
Ute	Uterus

Compartment
stromal
- bone
- skeletal muscle
- cardiac muscle
- smooth muscle
- fibroblast
- adipocyte
- mesothelial
- other
- mix

12.5%
25%
50%
100%

ln(CP10K+1): 0, 1, 2, 3

Luteinizing hormone (LH)
- lymphoid

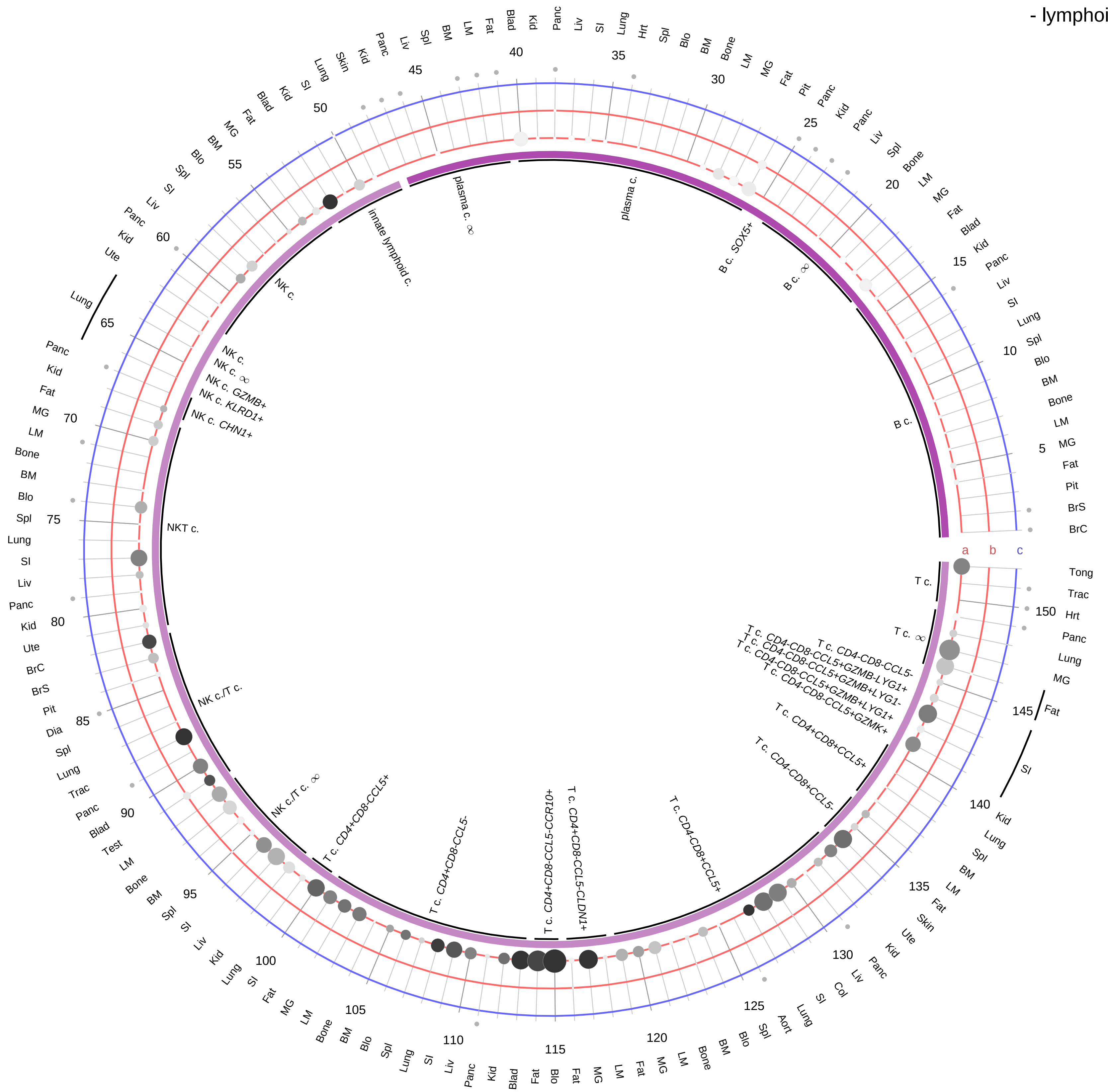

Ligand/Enzyme
- a - CGA
- b - LHB_uTAR

Receptor
- c - LHCGR

PAIRING (ligand → receptor)

LHB & CGA→LHCGR

Compartment
lymphoid
- B cell
- NK/T cell

12.5%
25%
50%
100%

ln(CP10K+1): 0, 1, 2, 3

Abbrev.	Tissue
Aort	Aorta
Blad	Bladder
Blo	Blood
Bone	Bone
BM	Bone marrow
BrC	Brain cortex
BrS	Brainstem
Col	Colon
Dia	Diaphragm
Ret	Eye retina
Fat	Fat
Hrt	Heart
Pit	Hypothalamus/Pituitary
Kid	Kidney
LM	Limb muscle
Liv	Liver
Lung	Lung
MG	Mammary gland
Panc	Pancreas
Skin	Skin
SI	Small intestine
Spl	Spleen
Test	Testes
Thym	Thymus
Tong	Tongue
Trac	Trachea
Ute	Uterus

Luteinizing hormone (LH)
- myeloid

PAIRING (ligand → receptor)

LHB & CGA→LHCGR

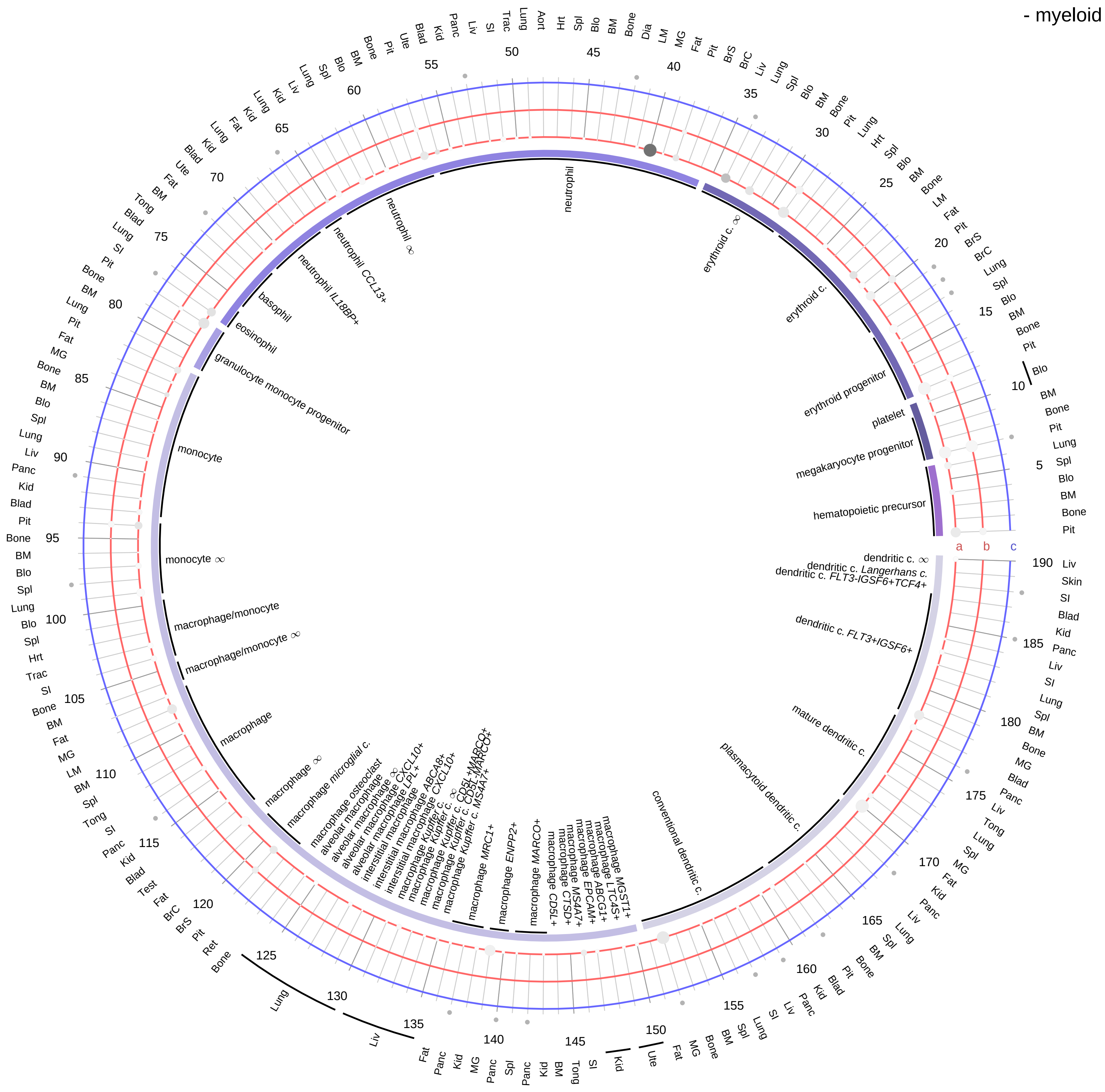

Ligand/Enzyme
- a - CGA
- b - LHB_uTAR

Receptor
- c - LHCGR

Compartment
- **hematopoietic**
  - hematopoietic precursor
- **megakaryocyte-erythroid**
  - megakaryoid
  - erythroid
- **myeloid**
  - granulocyte
  - granulocyte monocyte progenitor
  - monocyte/macrophage
  - dendritic

12.5%
25%
50%
100%

ln(CP10K+1): 0, 1, 2, 3

Abbrev.	Tissue
Aort	Aorta
Blad	Bladder
Blo	Blood
Bone	Bone
BM	Bone marrow
BrC	Brain cortex
BrS	Brainstem
Col	Colon
Dia	Diaphragm
Ret	Eye retina
Fat	Fat
Hrt	Heart
Pit	Hypothalamus/Pituitary
Kid	Kidney
LM	Limb muscle
Liv	Liver
Lung	Lung
MG	Mammary gland
Panc	Pancreas
Skin	Skin
SI	Small intestine
Spl	Spleen
Test	Testes
Thym	Thymus
Tong	Tongue
Trac	Trachea
Ute	Uterus

Melanin-concentrating hormone (MCH)
- epithelial/neural/germ

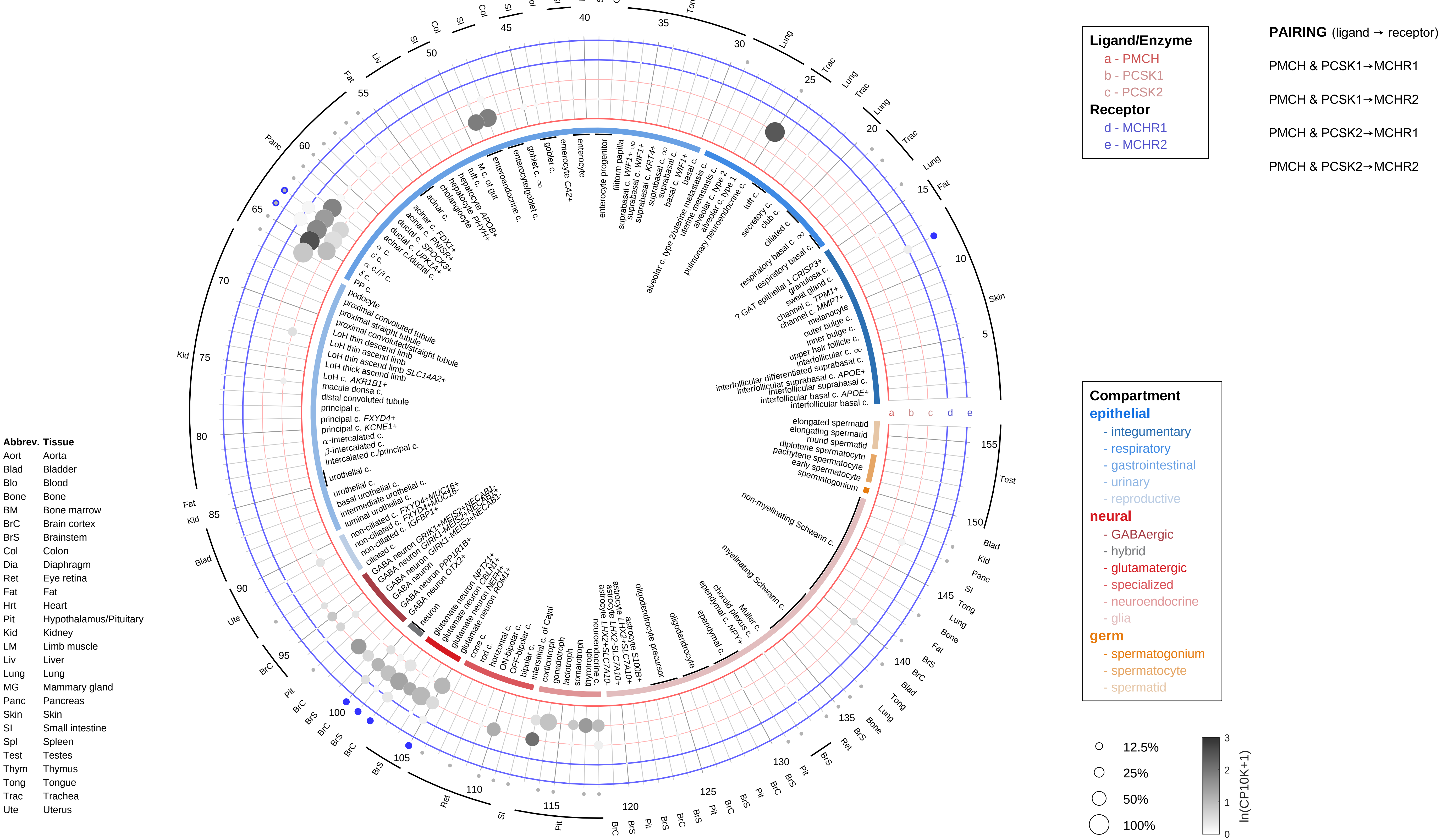

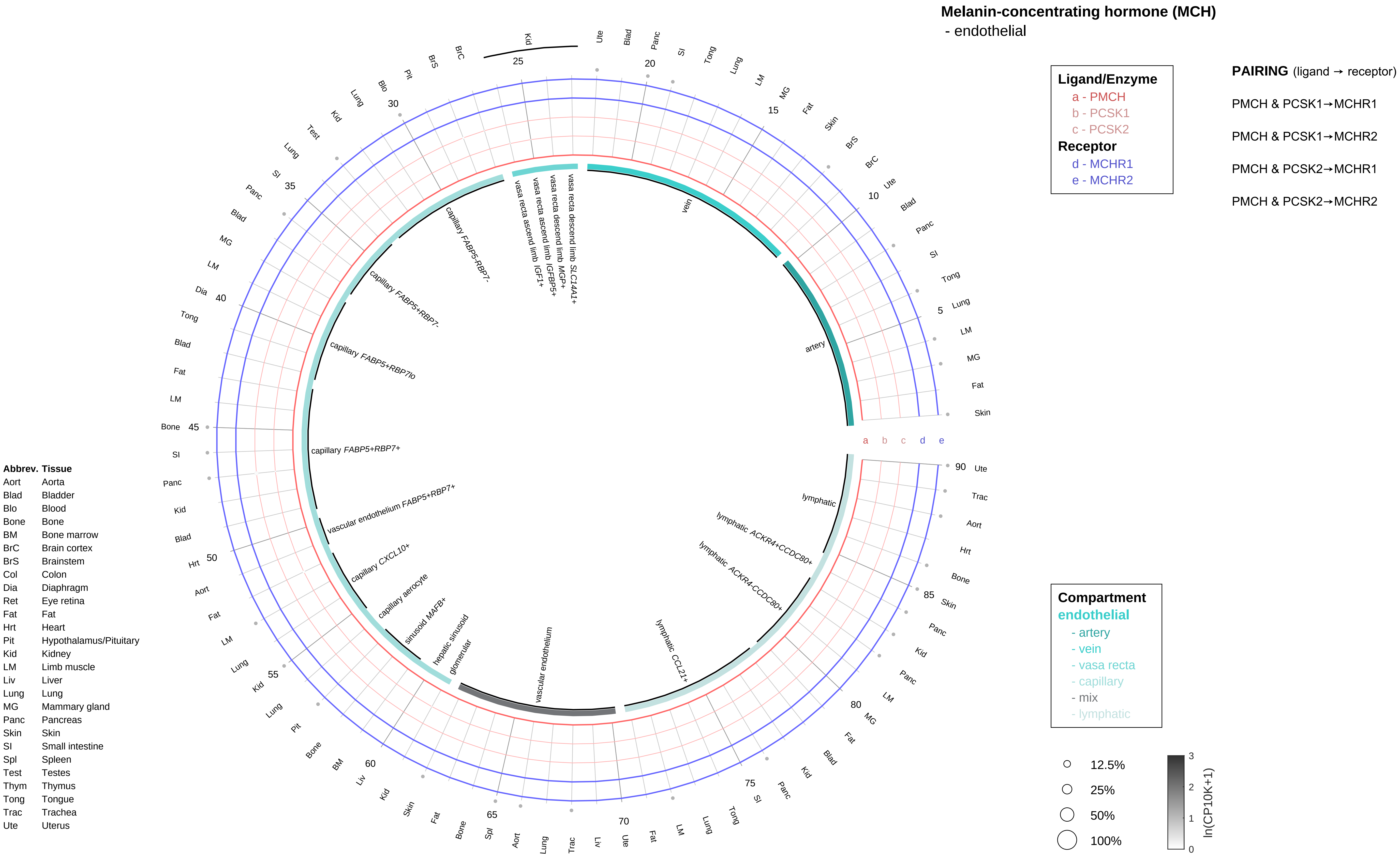

Melanin-concentrating hormone (MCH)
- endothelial
Ligand/Enzyme
a - PMCH
b - PCSK1
c - PCSK2
Receptor
d - MCHR1
e - MCHR2
PAIRING (ligand → receptor)
PMCH & PCSK1→MCHR1
PMCH & PCSK1→MCHR2
PMCH & PCSK2→MCHR1
PMCH & PCSK2→MCHR2
Compartment
endothelial
- artery
- vein
- vasa recta
- capillary
- mix
- lymphatic
12.5%
25%
50%
100%
ln(CP10K+1)
Abbrev. Tissue
Aort Aorta
Blad Bladder
Blo Blood
Bone Bone
BM Bone marrow
BrC Brain cortex
BrS Brainstem
Col Colon
Dia Diaphragm
Ret Eye retina
Fat Fat
Hrt Heart
Pit Hypothalamus/Pituitary
Kid Kidney
LM Limb muscle
Liv Liver
Lung Lung
MG Mammary gland
Panc Pancreas
Skin Skin
SI Small intestine
Spl Spleen
Test Testes
Thym Thymus
Tong Tongue
Trac Trachea
Ute Uterus
vein
artery
vasa recta descend limb SLC14A1+
vasa recta descend limb MGP+
vasa recta descend limb IGFBP5+
vasa recta ascend limb IGF1+
capillary FABP5-RBP7-
capillary FABP5+RBP7-
capillary FABP5+RBP7lo
capillary FABP5+RBP7+
vascular endothelium FABP5+RBP7+
capillary CXCL10+
capillary aerocyte
sinusoid MAFB+
hepatic sinusoid
glomerular
vascular endothelium
lymphatic CCL21+
lymphatic ACKR4-CCDC80+
lymphatic ACKR4+CCDC80+
lymphatic

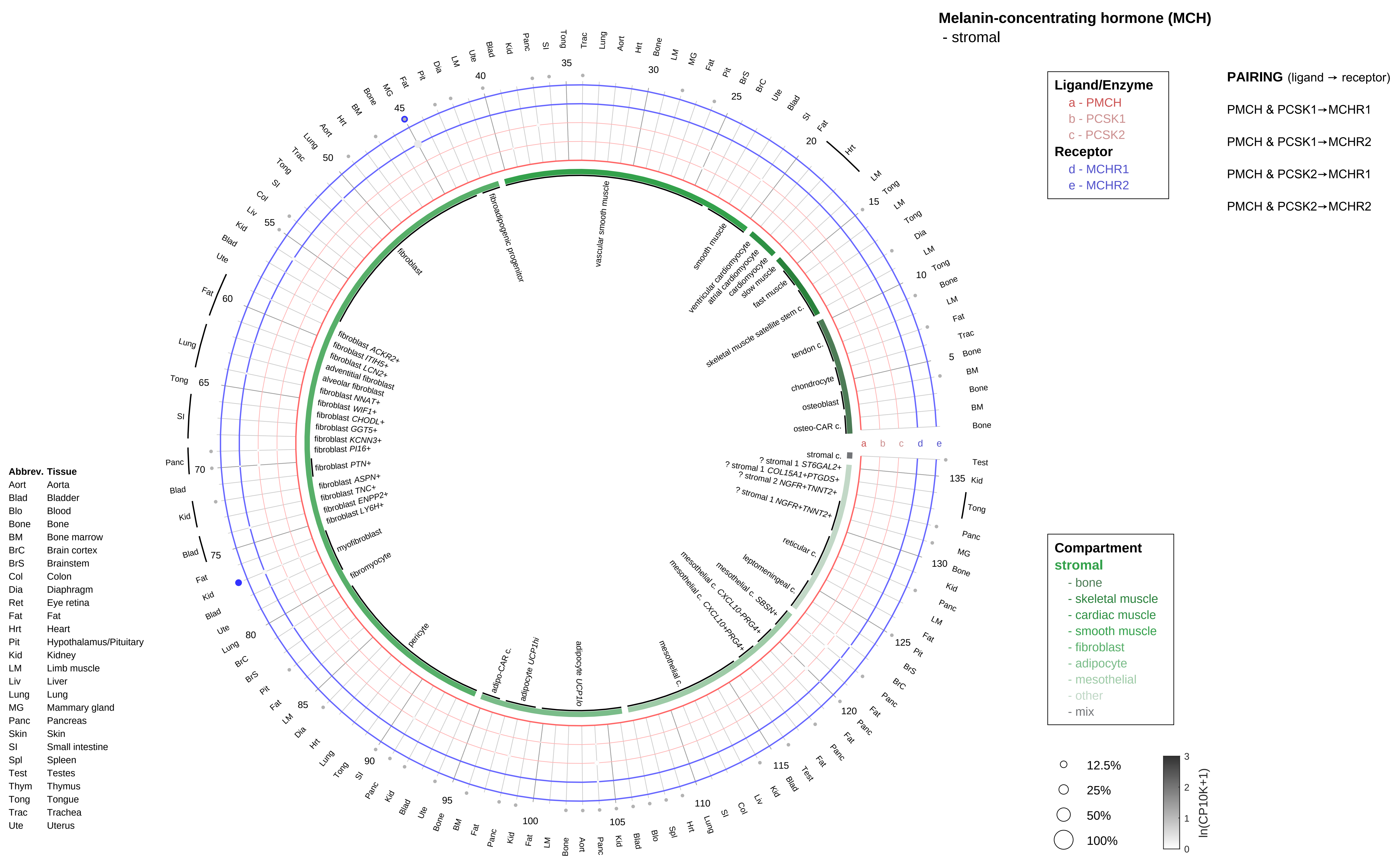
Melanin-concentrating hormone (MCH)
- stromal
Ligand/Enzyme
a - PMCH
b - PCSK1
c - PCSK2
Receptor
d - MCHR1
e - MCHR2
PAIRING (ligand → receptor)
PMCH & PCSK1→MCHR1
PMCH & PCSK1→MCHR2
PMCH & PCSK2→MCHR1
PMCH & PCSK2→MCHR2
fibroadipogenic progenitor
vascular smooth muscle
smooth muscle
ventricular cardiomyocyte
atrial cardiomyocyte
cardiomyocyte
slow muscle
fast muscle
skeletal muscle satellite stem c.
tendon c.
chondrocyte
osteoblast
osteo-CAR c.
stromal c.
? stromal 1 ST6GAL2+
? stromal 1 COL15A1+PTGDS+
? stromal 2 NGFR+TNNT2+
? stromal 1 NGFR+TNNT2+
reticular c.
leptomeningeal c.
mesothelial c. SBSN+
mesothelial c. CXCL10+PRG4+
mesothelial c. CXCL10+PRG4+
mesothelial c.
adipocyte UCP1lo
adipocyte UCP1hi
adipo-CAR c.
pericyte
fibromyocyte
myofibroblast
fibroblast LY6H+
fibroblast ENPP2+
fibroblast TNC+
fibroblast ASPN+
fibroblast PTN+
fibroblast PI16+
fibroblast KCNN3+
fibroblast GGT5+
fibroblast CHODL+
fibroblast WIF1+
fibroblast NNAT+
alveolar fibroblast
adventitial fibroblast
fibroblast LCN2+
fibroblast ITIH5+
fibroblast ACKR2+
fibroblast
Abbrev. Tissue
Aort Aorta
Blad Bladder
Blo Blood
Bone Bone
BM Bone marrow
BrC Brain cortex
BrS Brainstem
Col Colon
Dia Diaphragm
Ret Eye retina
Fat Fat
Hrt Heart
Pit Hypothalamus/Pituitary
Kid Kidney
LM Limb muscle
Liv Liver
Lung Lung
MG Mammary gland
Panc Pancreas
Skin Skin
SI Small intestine
Spl Spleen
Test Testes
Thym Thymus
Tong Tongue
Trac Trachea
Ute Uterus
Compartment
stromal
- bone
- skeletal muscle
- cardiac muscle
- smooth muscle
- fibroblast
- adipocyte
- mesothelial
- other
- mix
12.5%
25%
50%
100%
ln(CP10K+1)

Melanin-concentrating hormone (MCH)
- lymphoid

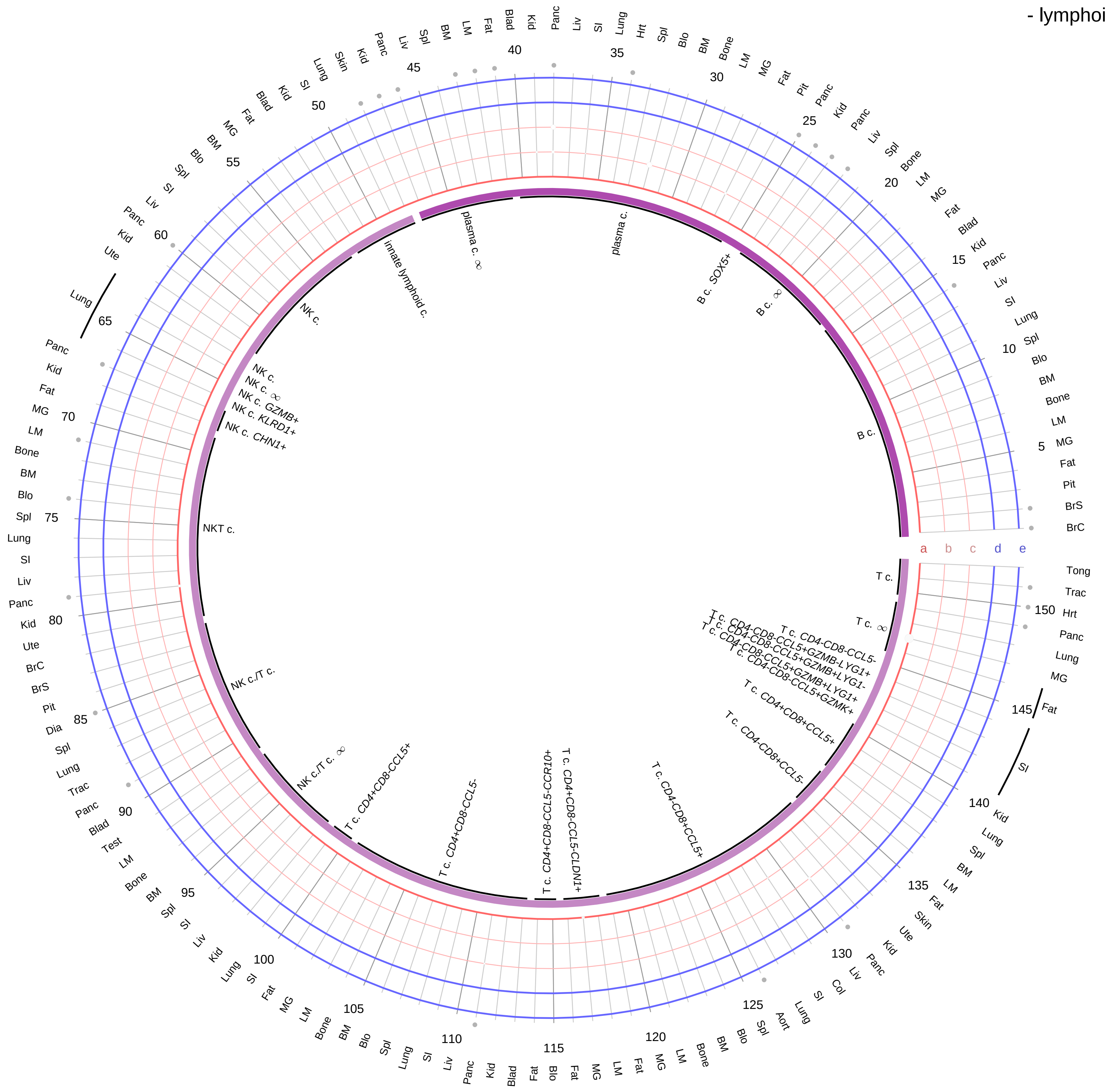

Ligand/Enzyme
- a - PMCH
- b - PCSK1
- c - PCSK2

Receptor
- d - MCHR1
- e - MCHR2

PAIRING (ligand → receptor)

PMCH & PCSK1→MCHR1

PMCH & PCSK1→MCHR2

PMCH & PCSK2→MCHR1

PMCH & PCSK2→MCHR2

Compartment

lymphoid
- B cell
- NK/T cell

12.5%
25%
50%
100%

ln(CP10K+1): 0, 1, 2, 3

Abbrev.	Tissue
Aort	Aorta
Blad	Bladder
Blo	Blood
Bone	Bone
BM	Bone marrow
BrC	Brain cortex
BrS	Brainstem
Col	Colon
Dia	Diaphragm
Ret	Eye retina
Fat	Fat
Hrt	Heart
Pit	Hypothalamus/Pituitary
Kid	Kidney
LM	Limb muscle
Liv	Liver
Lung	Lung
MG	Mammary gland
Panc	Pancreas
Skin	Skin
SI	Small intestine
Spl	Spleen
Test	Testes
Thym	Thymus
Tong	Tongue
Trac	Trachea
Ute	Uterus

Melanin-concentrating hormone (MCH)
- myeloid

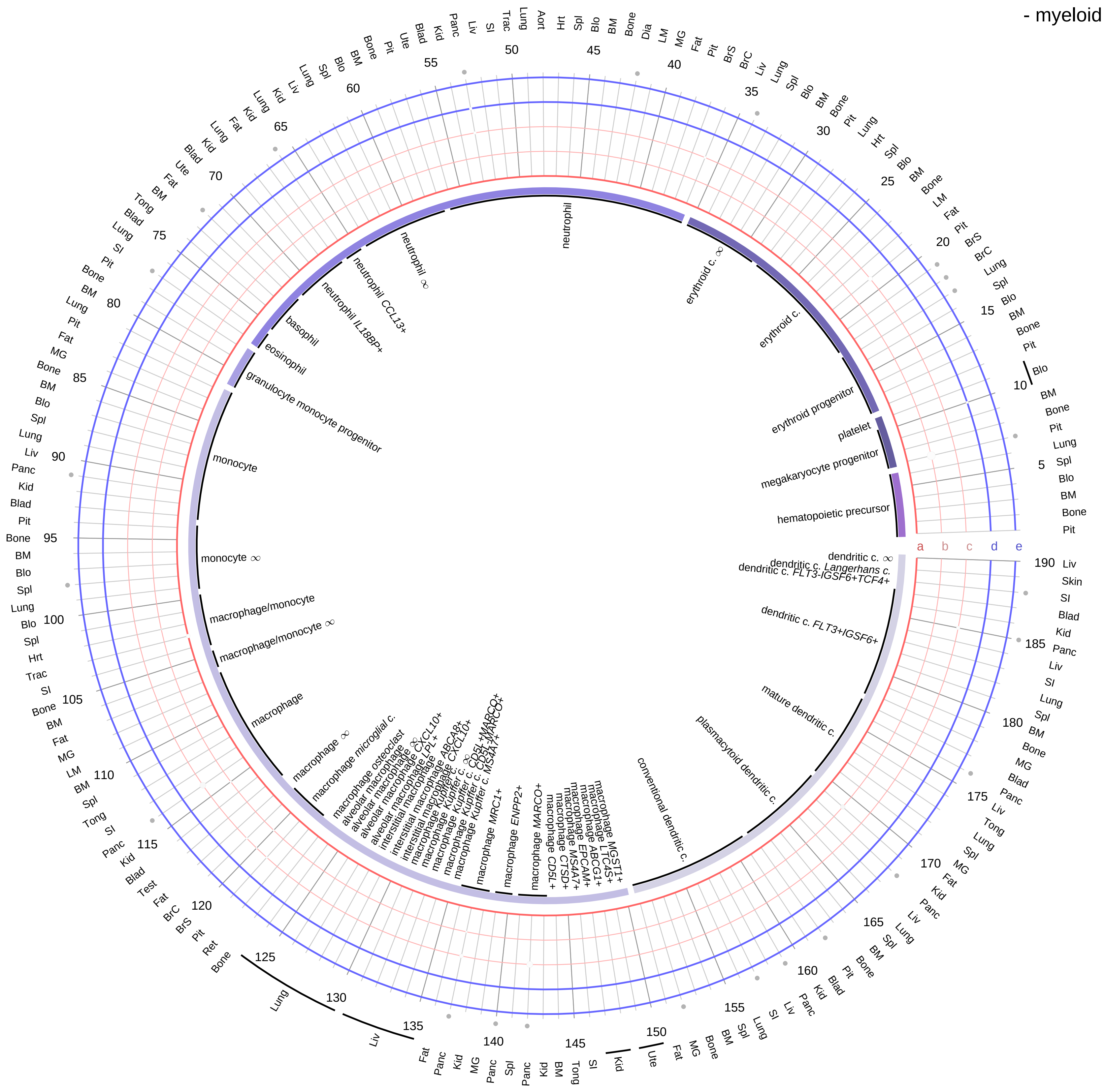

Ligand/Enzyme
a - PMCH
b - PCSK1
c - PCSK2
**Receptor**
d - MCHR1
e - MCHR2

PAIRING (ligand → receptor)

PMCH & PCSK1→MCHR1

PMCH & PCSK1→MCHR2

PMCH & PCSK2→MCHR1

PMCH & PCSK2→MCHR2

Abbrev.	Tissue
Aort	Aorta
Blad	Bladder
Blo	Blood
Bone	Bone
BM	Bone marrow
BrC	Brain cortex
BrS	Brainstem
Col	Colon
Dia	Diaphragm
Ret	Eye retina
Fat	Fat
Hrt	Heart
Pit	Hypothalamus/Pituitary
Kid	Kidney
LM	Limb muscle
Liv	Liver
Lung	Lung
MG	Mammary gland
Panc	Pancreas
Skin	Skin
SI	Small intestine
Spl	Spleen
Test	Testes
Thym	Thymus
Tong	Tongue
Trac	Trachea
Ute	Uterus

Compartment
hematopoietic
- hematopoietic precursor

megakaryocyte-erythroid
- megakaryoid
- erythroid

myeloid
- granulocyte
- granulocyte monocyte progenitor
- monocyte/macrophage
- dendritic

12.5%
25%
50%
100%

ln(CP10K+1): 0 1 2 3

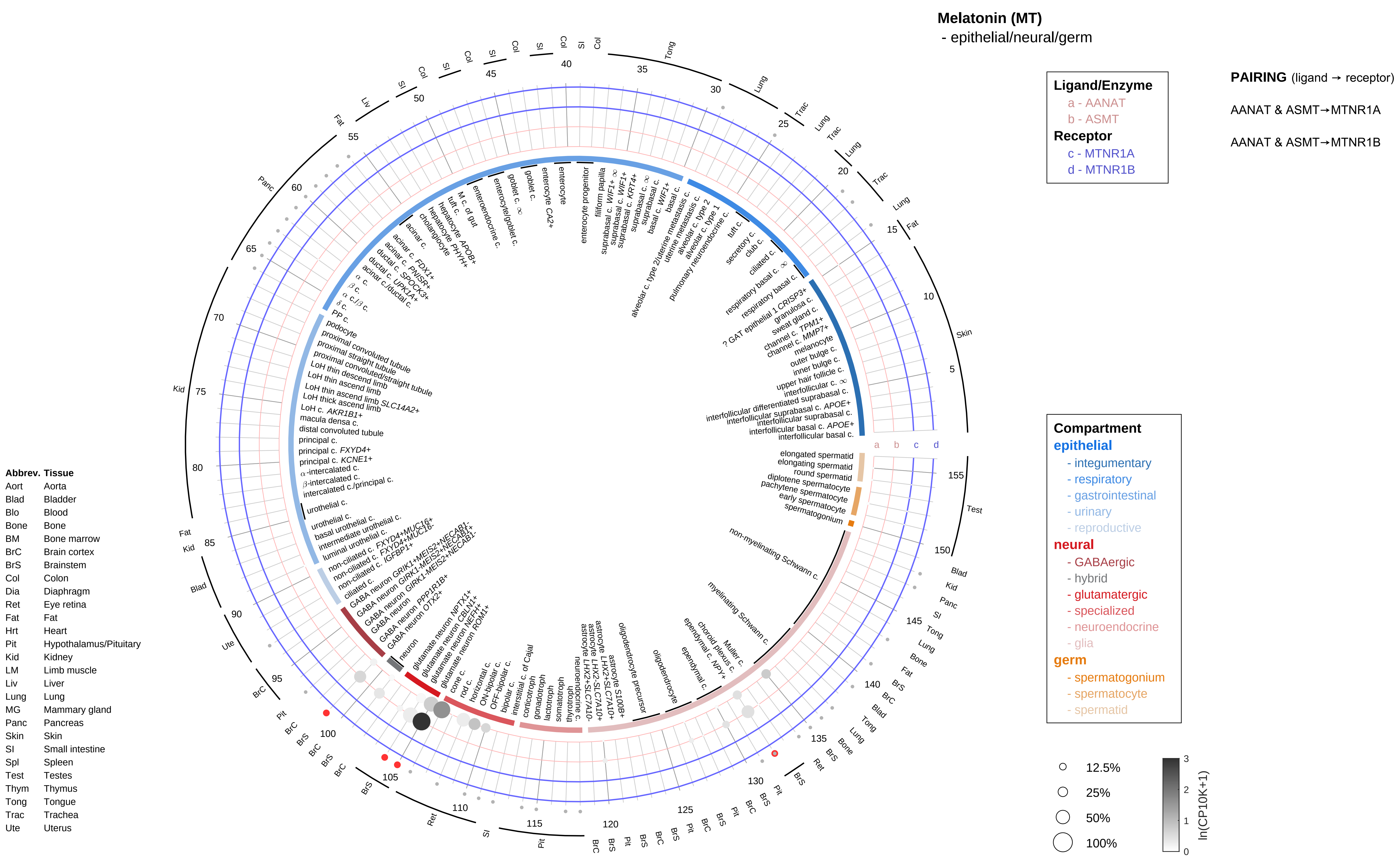

Melatonin (MT)
- epithelial/neural/germ
Ligand/Enzyme
a - AANAT
b - ASMT
Receptor
c - MTNR1A
d - MTNR1B
PAIRING (ligand → receptor)
AANAT & ASMT→MTNR1A
AANAT & ASMT→MTNR1B
Compartment
epithelial
- integumentary
- respiratory
- gastrointestinal
- urinary
- reproductive
neural
- GABAergic
- hybrid
- glutamatergic
- specialized
- neuroendocrine
- glia
germ
- spermatogonium
- spermatocyte
- spermatid
12.5%
25%
50%
100%
ln(CP10K+1)
0
1
2
3
Abbrev. Tissue
Aort Aorta
Blad Bladder
Blo Blood
Bone Bone
BM Bone marrow
BrC Brain cortex
BrS Brainstem
Col Colon
Dia Diaphragm
Ret Eye retina
Fat Fat
Hrt Heart
Pit Hypothalamus/Pituitary
Kid Kidney
LM Limb muscle
Liv Liver
Lung Lung
MG Mammary gland
Panc Pancreas
Skin Skin
SI Small intestine
Spl Spleen
Test Testes
Thym Thymus
Tong Tongue
Trac Trachea
Ute Uterus

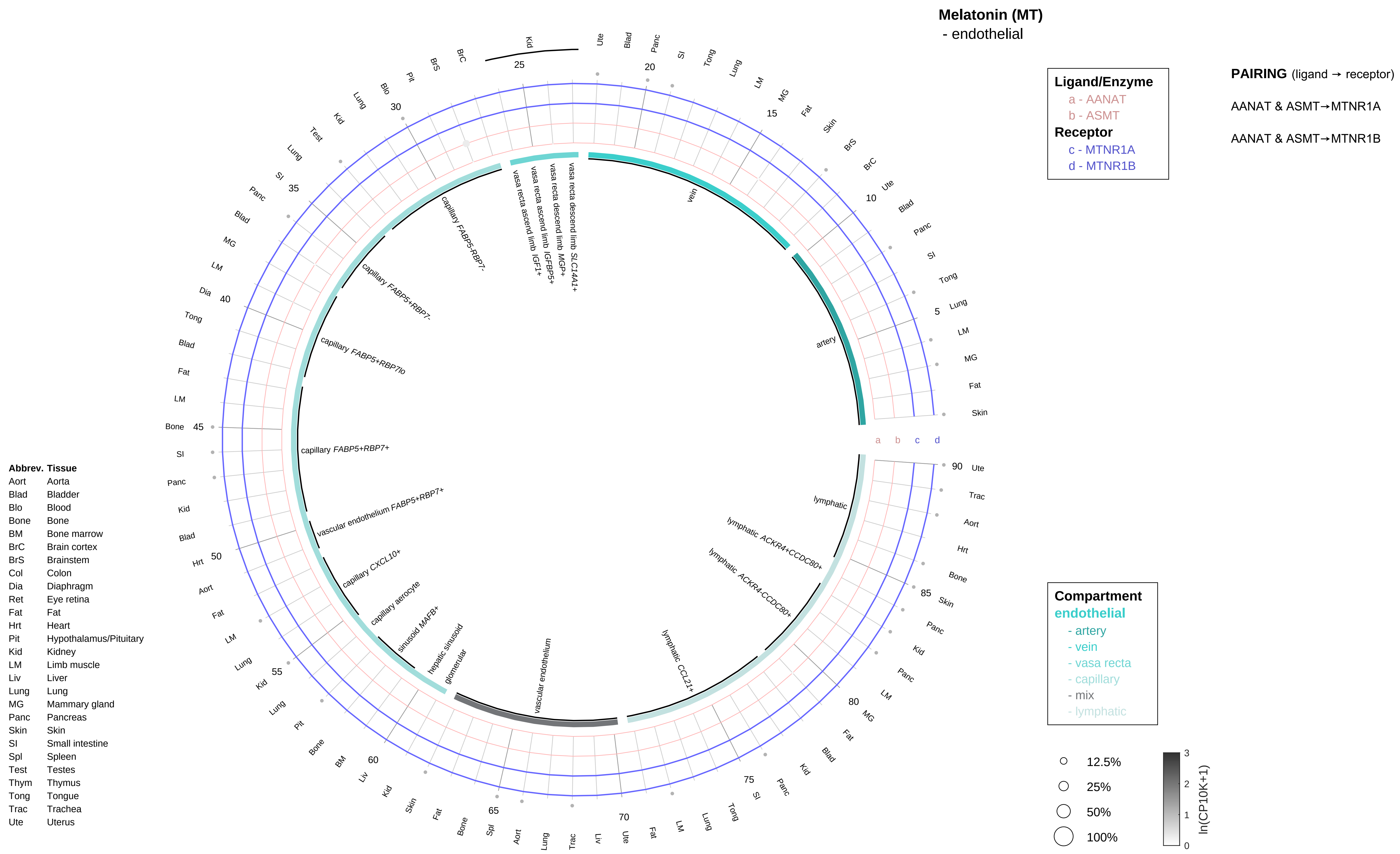
Melatonin (MT)
- endothelial
Ligand/Enzyme
a - AANAT
b - ASMT
Receptor
c - MTNR1A
d - MTNR1B
PAIRING (ligand → receptor)
AANAT & ASMT→MTNR1A
AANAT & ASMT→MTNR1B
Compartment
endothelial
- artery
- vein
- vasa recta
- capillary
- mix
- lymphatic
12.5%
25%
50%
100%
ln(CP10K+1)
0
1
2
3
a
b
c
d
vein
artery
lymphatic
lymphatic ACKR4+CCDC80+
lymphatic ACKR4-CCDC80+
lymphatic CCL21+
vascular endothelium
glomerular
hepatic sinusoid
sinusoid MAFB+
capillary aerocyte
capillary CXCL10+
vascular endothelium FABP5+RBP7+
capillary FABP5+RBP7+
capillary FABP5+RBP7lo
capillary FABP5+RBP7-
capillary FABP5-RBP7-
vasa recta ascend limb IGF1+
vasa recta ascend limb IGFBP5+
vasa recta descend limb MGP+
vasa recta descend limb SLC14A1+
Abbrev. Tissue
Aort Aorta
Blad Bladder
Blo Blood
Bone Bone
BM Bone marrow
BrC Brain cortex
BrS Brainstem
Col Colon
Dia Diaphragm
Ret Eye retina
Fat Fat
Hrt Heart
Pit Hypothalamus/Pituitary
Kid Kidney
LM Limb muscle
Liv Liver
Lung Lung
MG Mammary gland
Panc Pancreas
Skin Skin
SI Small intestine
Spl Spleen
Test Testes
Thym Thymus
Tong Tongue
Trac Trachea
Ute Uterus

Melatonin (MT)
- stromal

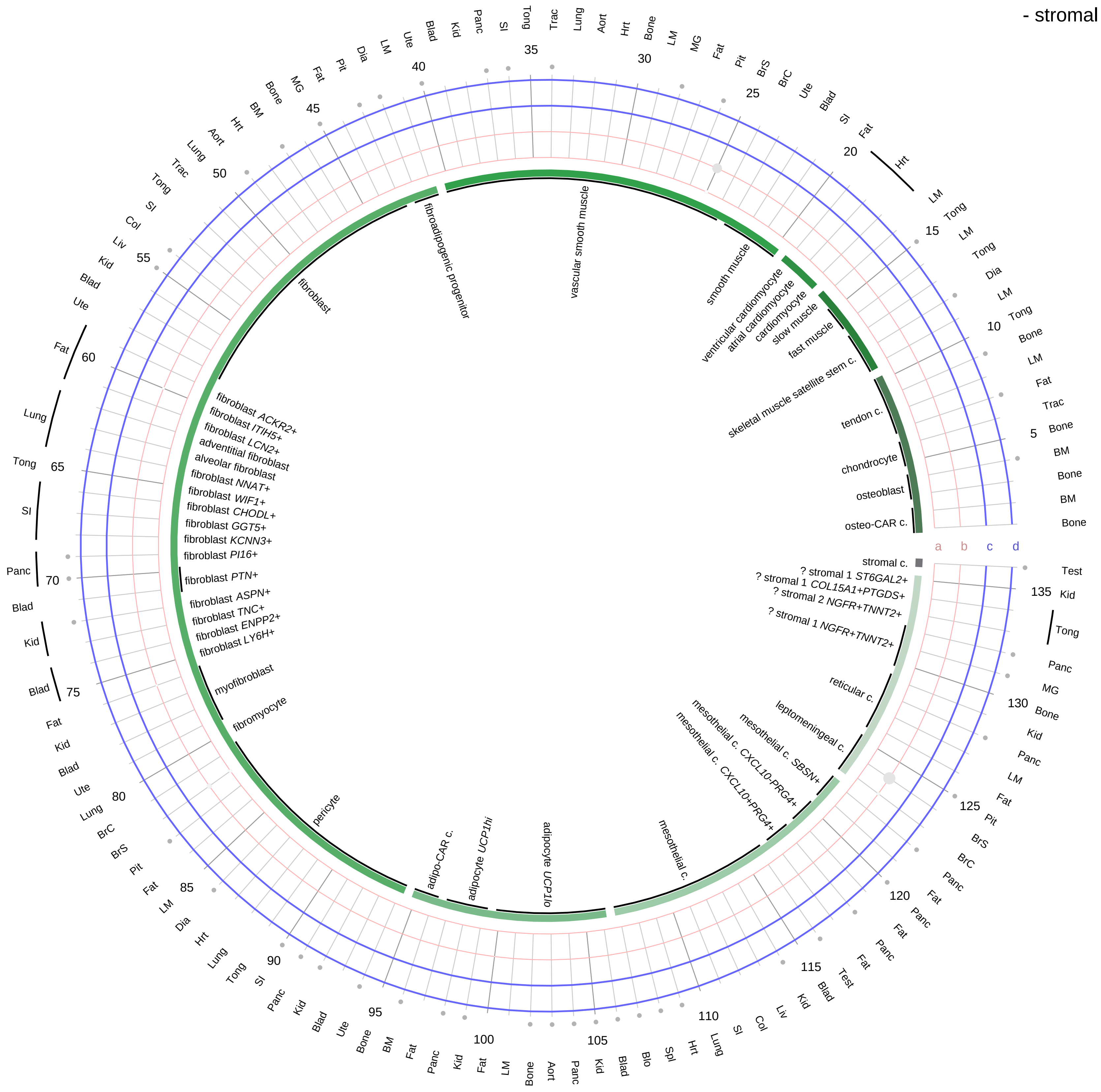

Ligand/Enzyme
- a - AANAT
- b - ASMT

Receptor
- c - MTNR1A
- d - MTNR1B

PAIRING (ligand → receptor)

AANAT & ASMT→MTNR1A

AANAT & ASMT→MTNR1B

Compartment
stromal
- bone
- skeletal muscle
- cardiac muscle
- smooth muscle
- fibroblast
- adipocyte
- mesothelial
- other
- mix

12.5%
25%
50%
100%

ln(CP10K+1): 0, 1, 2, 3

Abbrev.	Tissue
Aort	Aorta
Blad	Bladder
Blo	Blood
Bone	Bone
BM	Bone marrow
BrC	Brain cortex
BrS	Brainstem
Col	Colon
Dia	Diaphragm
Ret	Eye retina
Fat	Fat
Hrt	Heart
Pit	Hypothalamus/Pituitary
Kid	Kidney
LM	Limb muscle
Liv	Liver
Lung	Lung
MG	Mammary gland
Panc	Pancreas
Skin	Skin
SI	Small intestine
Spl	Spleen
Test	Testes
Thym	Thymus
Tong	Tongue
Trac	Trachea
Ute	Uterus

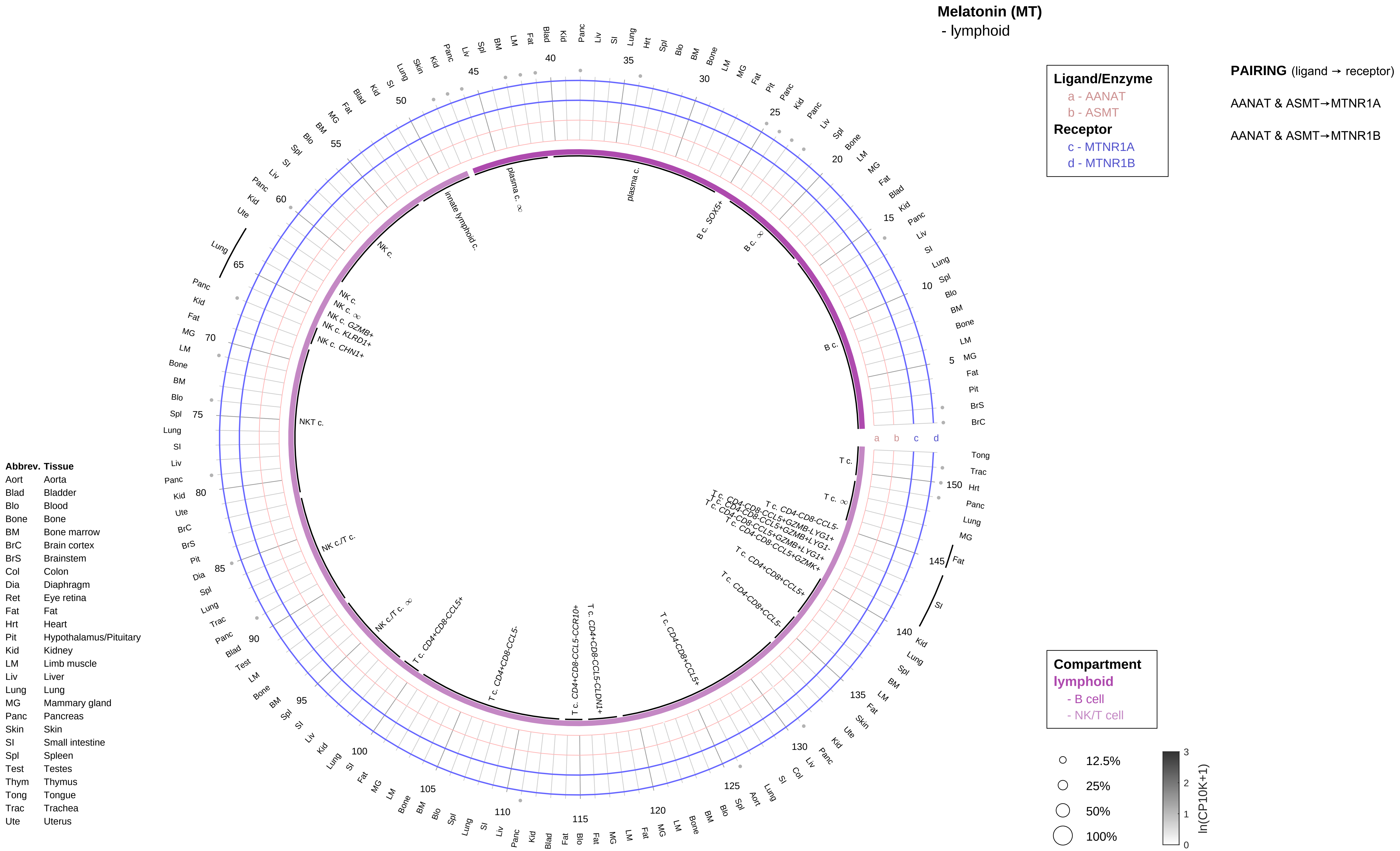
Melatonin (MT)
- lymphoid
Ligand/Enzyme
a - AANAT
b - ASMT
Receptor
c - MTNR1A
d - MTNR1B
PAIRING (ligand → receptor)
AANAT & ASMT→MTNR1A
AANAT & ASMT→MTNR1B
Compartment
lymphoid
- B cell
- NK/T cell
12.5%
25%
50%
100%
ln(CP10K+1)
Abbrev. Tissue
Aort Aorta
Blad Bladder
Blo Blood
Bone Bone
BM Bone marrow
BrC Brain cortex
BrS Brainstem
Col Colon
Dia Diaphragm
Ret Eye retina
Fat Fat
Hrt Heart
Pit Hypothalamus/Pituitary
Kid Kidney
LM Limb muscle
Liv Liver
Lung Lung
MG Mammary gland
Panc Pancreas
Skin Skin
SI Small intestine
Spl Spleen
Test Testes
Thym Thymus
Tong Tongue
Trac Trachea
Ute Uterus
plasma c.
plasma c. ∞
innate lymphoid c.
B c. SOX5+
B c. ∞
B c.
NK c.
NK c. GZMB+
NK c. KLRD1+
NK c. CHN1+
NKT c.
NK c./T c.
NK c./T c. ∞
T c.
T c. ∞
T c. CD4-CD8-CCL5-
T c. CD4-CD8-CCL5+GZMB-LYG1+
T c. CD4-CD8-CCL5+GZMB-LYG1-
T c. CD4-CD8-CCL5+GZMB+LYG1+
T c. CD4-CD8-CCL5+GZMK+
T c. CD4+CD8+CCL5+
T c. CD4-CD8+CCL5-
T c. CD4-CD8+CCL5+
T c. CD4+CD8-CCL5-CLDN1+
T c. CD4+CD8-CCL5-CCR10+
T c. CD4+CD8-CCL5-
T c. CD4+CD8-CCL5+

Melatonin (MT)
- myeloid

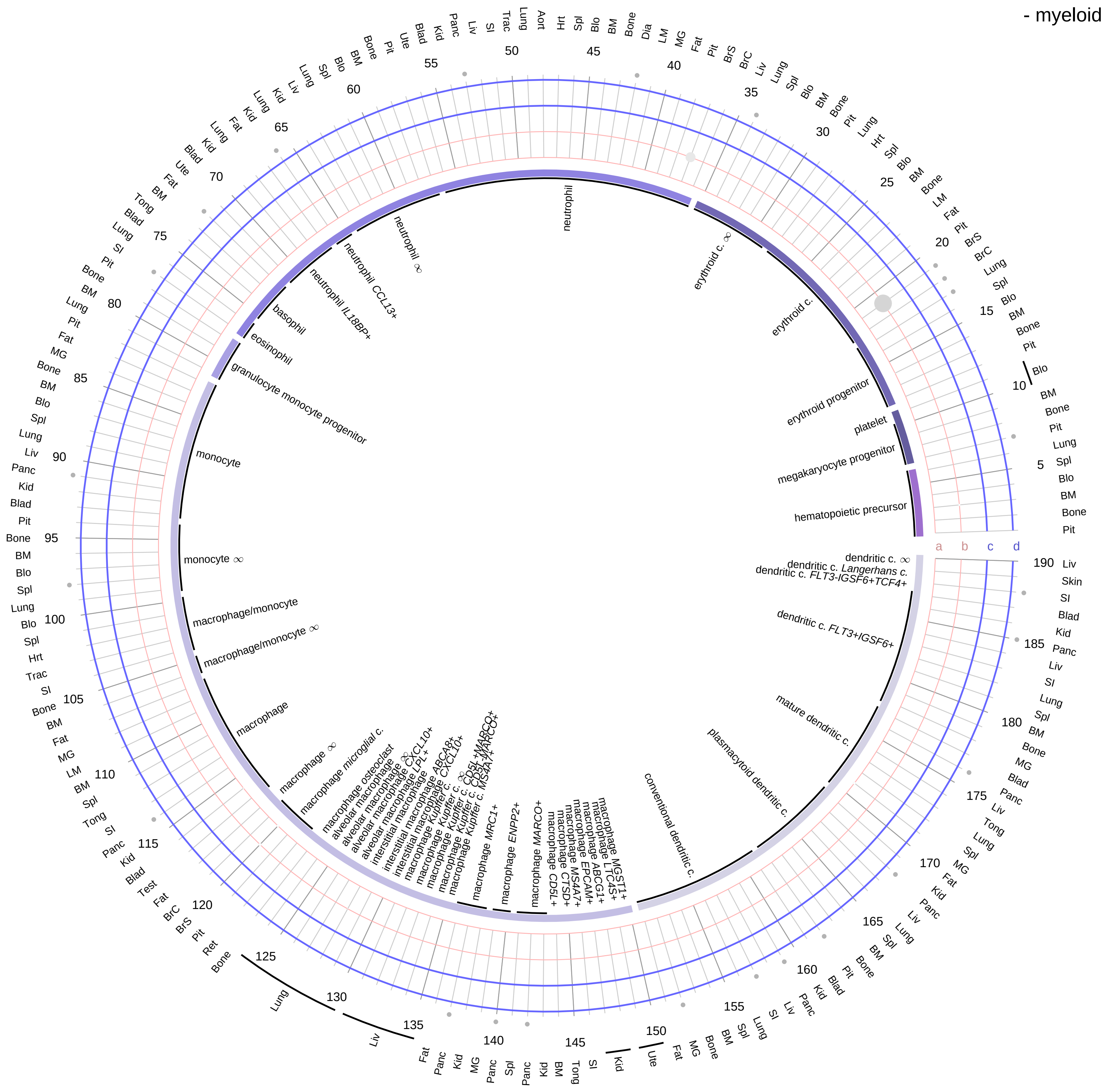

Ligand/Enzyme
- a - AANAT
- b - ASMT

Receptor
- c - MTNR1A
- d - MTNR1B

PAIRING (ligand → receptor)

AANAT & ASMT→MTNR1A

AANAT & ASMT→MTNR1B

Compartment

hematopoietic
- hematopoietic precursor

megakaryocyte-erythroid
- megakaryoid
- erythroid

myeloid
- granulocyte
- granulocyte monocyte progenitor
- monocyte/macrophage
- dendritic

12.5%
25%
50%
100%

ln(CP10K+1): 0, 1, 2, 3

Abbrev.	Tissue
Aort	Aorta
Blad	Bladder
Blo	Blood
Bone	Bone
BM	Bone marrow
BrC	Brain cortex
BrS	Brainstem
Col	Colon
Dia	Diaphragm
Ret	Eye retina
Fat	Fat
Hrt	Heart
Pit	Hypothalamus/Pituitary
Kid	Kidney
LM	Limb muscle
Liv	Liver
Lung	Lung
MG	Mammary gland
Panc	Pancreas
Skin	Skin
SI	Small intestine
Spl	Spleen
Test	Testes
Thym	Thymus
Tong	Tongue
Trac	Trachea
Ute	Uterus

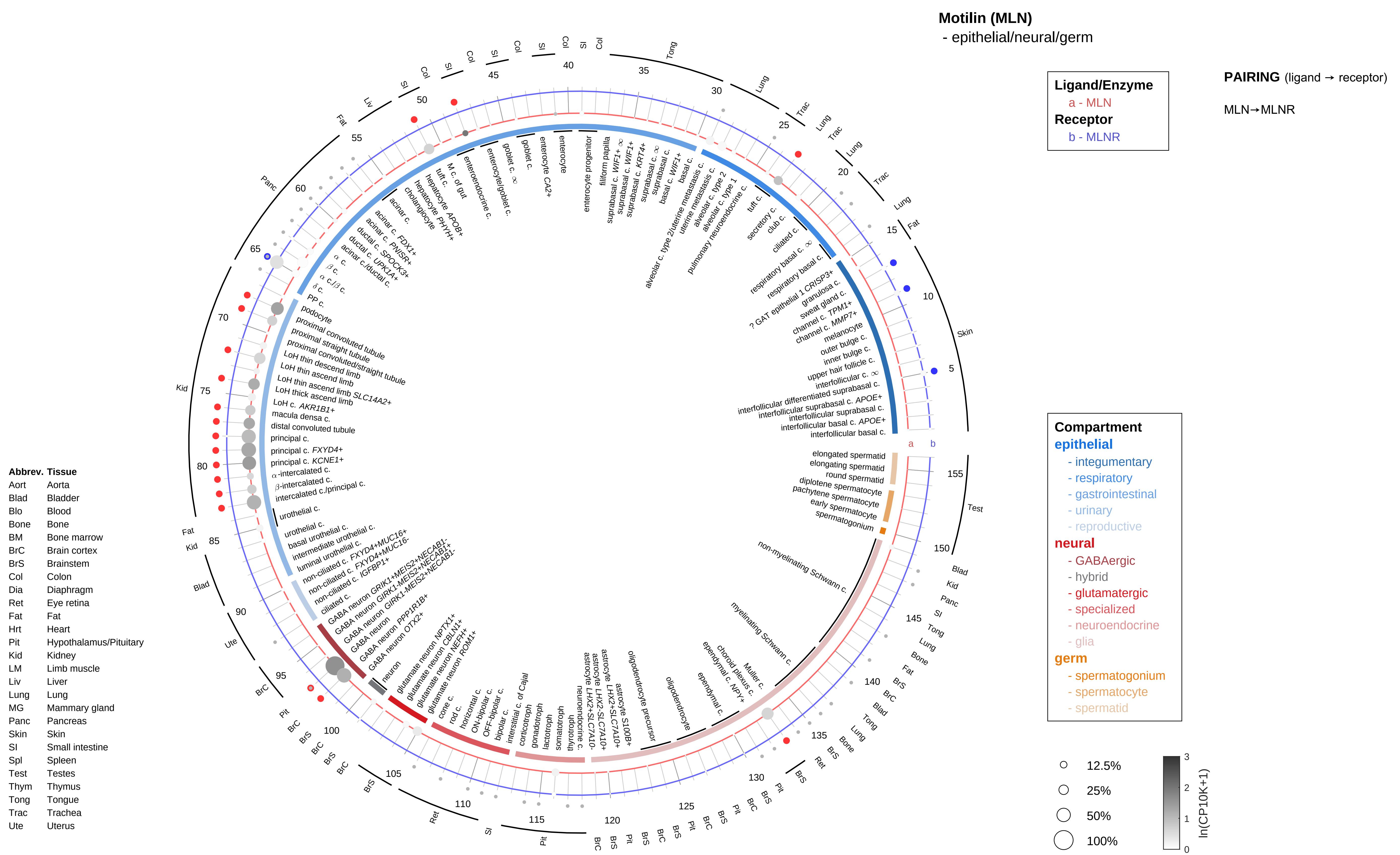

Motilin (MLN)
- epithelial/neural/germ
Ligand/Enzyme
a - MLN
Receptor
b - MLNR
PAIRING (ligand → receptor)
MLN→MLNR
Compartment
epithelial
- integumentary
- respiratory
- gastrointestinal
- urinary
- reproductive
neural
- GABAergic
- hybrid
- glutamatergic
- specialized
- neuroendocrine
- glia
germ
- spermatogonium
- spermatocyte
- spermatid
12.5%
25%
50%
100%
ln(CP10K+1)
0
1
2
3
Abbrev. Tissue
Aort Aorta
Blad Bladder
Blo Blood
Bone Bone
BM Bone marrow
BrC Brain cortex
BrS Brainstem
Col Colon
Dia Diaphragm
Ret Eye retina
Fat Fat
Hrt Heart
Pit Hypothalamus/Pituitary
Kid Kidney
LM Limb muscle
Liv Liver
Lung Lung
MG Mammary gland
Panc Pancreas
Skin Skin
SI Small intestine
Spl Spleen
Test Testes
Thym Thymus
Tong Tongue
Trac Trachea
Ute Uterus
interfollicular basal c.
interfollicular basal c. APOE+
interfollicular suprabasal c.
interfollicular suprabasal c. APOE+
interfollicular differentiated suprabasal c.
interfollicular c.
upper hair follicle c.
inner bulge c.
outer bulge c.
melanocyte
channel c. MMP7+
channel c. TPM1+
sweat gland c.
granulosa c.
? GAT epithelial 1 CRISP3+
respiratory basal c.
respiratory basal c.
ciliated c.
club c.
secretory c.
tuft c.
pulmonary neuroendocrine c.
alveolar c. type 1
alveolar c. type 2
uterine metastasis c.
alveolar c. type 2/uterine metastasis c.
basal c.
basal c. WIF1+
suprabasal c.
suprabasal c. KRT4+
suprabasal c. WIF1+
suprabasal c. WIF1+
filiform papilla
enterocyte progenitor
enterocyte
enterocyte CA2+
goblet c.
goblet c.
enterocyte/goblet c.
enteroendocrine c.
M c. of gut
tuft c.
hepatocyte APOB+
hepatocyte PHYH+
cholangiocyte
acinar c.
acinar c. FDX1+
acinar c. PNISR+
ductal c. SPOCK3+
ductal c. UPK1A+
acinar c./ductal c.
α c.
β c.
α c./β c.
δ c.
PP c.
podocyte
proximal convoluted tubule
proximal straight tubule
proximal convoluted/straight tubule
LoH thin descend limb
LoH thin ascend limb
LoH thin ascend limb SLC14A2+
LoH thick ascend limb
LoH c. AKR1B1+
macula densa c.
distal convoluted tubule
principal c.
principal c. FXYD4+
principal c. KCNE1+
α-intercalated c.
β-intercalated c.
intercalated c./principal c.
urothelial c.
urothelial c.
basal urothelial c.
intermediate urothelial c.
luminal urothelial c.
non-ciliated c. FXYD4+MUC16+
non-ciliated c. FXYD4+MUC16-
non-ciliated c. IGFBP1+
ciliated c.
GABA neuron GRIK1+MEIS2+NECAB1-
GABA neuron GIRK1+MEIS2+NECAB1+
GABA neuron GIRK1-MEIS2+NECAB1-
GABA neuron
GABA neuron PPP1R1B+
GABA neuron OTX2+
neuron
glutamate neuron NPTX1+
glutamate neuron CBLN1+
glutamate neuron NEFH+
glutamate neuron ROM1+
cone c.
rod c.
horizontal c.
ON-bipolar c.
OFF-bipolar c.
bipolar c.
interstitial c. of Cajal
corticotroph
gonadotroph
lactotroph
somatotroph
thyrotroph
neuroendocrine c.
astrocyte LHX2-SLC7A10-
astrocyte LHX2+SLC7A10+
astrocyte LHX2-SLC7A10+
astrocyte S100B+
oligodendrocyte precursor
oligodendrocyte
ependymal c.
ependymal c. NPY+
choroid plexus c.
Muller c.
myelinating Schwann c.
non-myelinating Schwann c.
spermatogonium
early spermatocyte
pachytene spermatocyte
diplotene spermatocyte
round spermatid
elongating spermatid
elongated spermatid
a
b

Motilin (MLN)
- endothelial

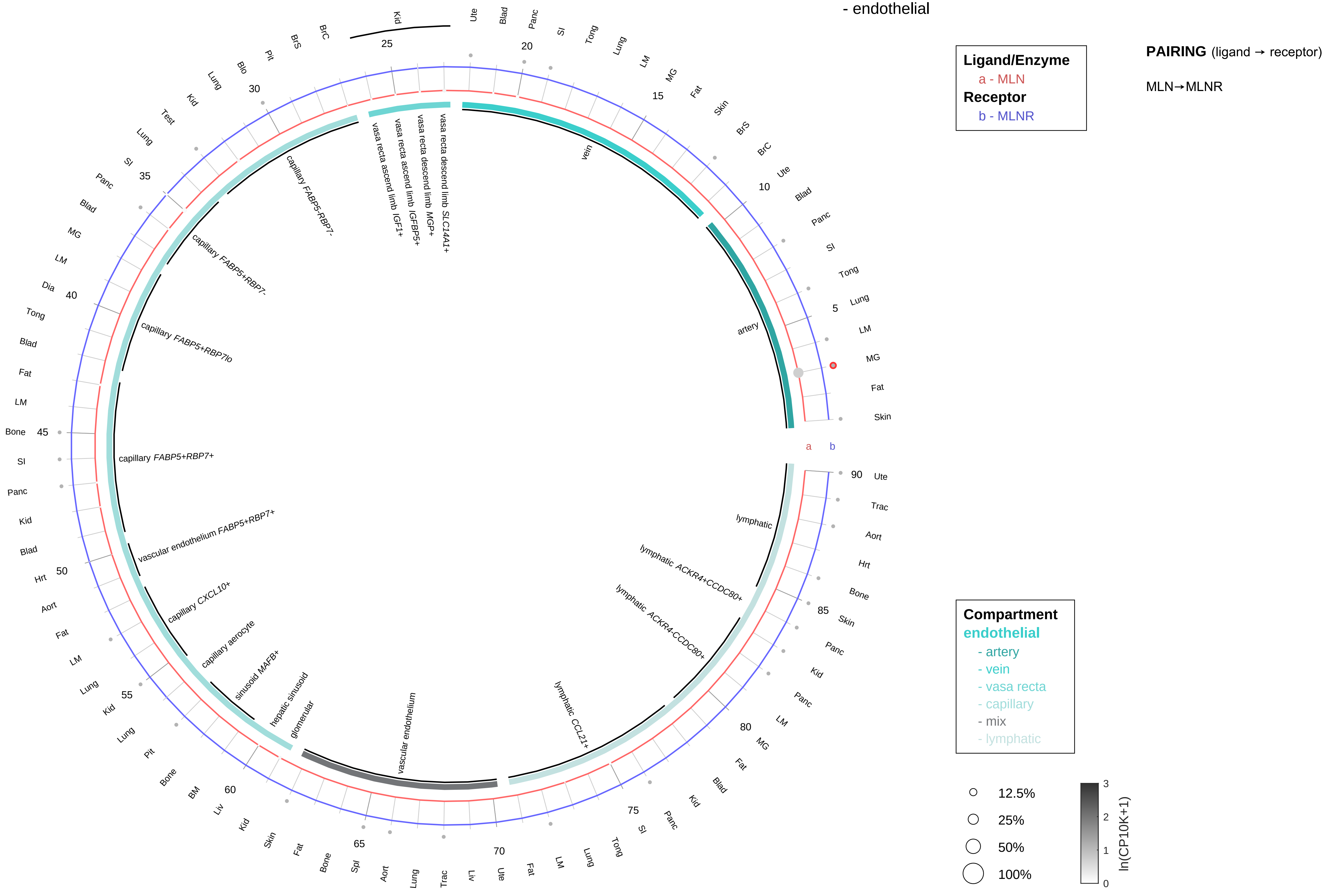

Abbrev.	Tissue
Aort	Aorta
Blad	Bladder
Blo	Blood
Bone	Bone
BM	Bone marrow
BrC	Brain cortex
BrS	Brainstem
Col	Colon
Dia	Diaphragm
Ret	Eye retina
Fat	Fat
Hrt	Heart
Pit	Hypothalamus/Pituitary
Kid	Kidney
LM	Limb muscle
Liv	Liver
Lung	Lung
MG	Mammary gland
Panc	Pancreas
Skin	Skin
SI	Small intestine
Spl	Spleen
Test	Testes
Thym	Thymus
Tong	Tongue
Trac	Trachea
Ute	Uterus

Motilin (MLN)
- stromal

Ligand/Enzyme
a - MLN
Receptor
b - MLNR

PAIRING (ligand → receptor)

MLN→MLNR

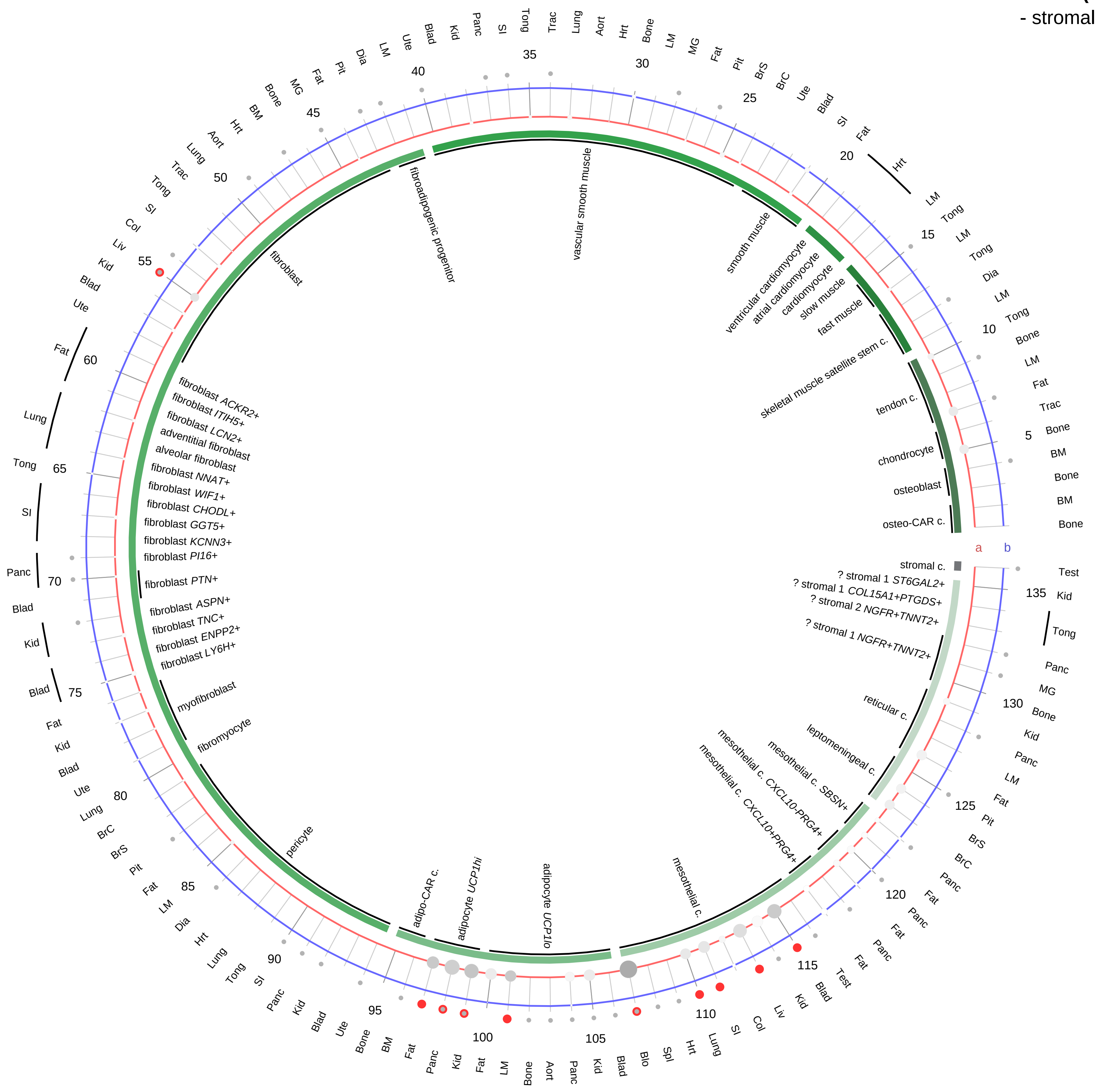

Compartment
stromal
- bone
- skeletal muscle
- cardiac muscle
- smooth muscle
- fibroblast
- adipocyte
- mesothelial
- other
- mix

12.5%
25%
50%
100%

ln(CP10K+1): 0, 1, 2, 3

Abbrev.	Tissue
Aort	Aorta
Blad	Bladder
Blo	Blood
Bone	Bone
BM	Bone marrow
BrC	Brain cortex
BrS	Brainstem
Col	Colon
Dia	Diaphragm
Ret	Eye retina
Fat	Fat
Hrt	Heart
Pit	Hypothalamus/Pituitary
Kid	Kidney
LM	Limb muscle
Liv	Liver
Lung	Lung
MG	Mammary gland
Panc	Pancreas
Skin	Skin
SI	Small intestine
Spl	Spleen
Test	Testes
Thym	Thymus
Tong	Tongue
Trac	Trachea
Ute	Uterus

Motilin (MLN)
- lymphoid

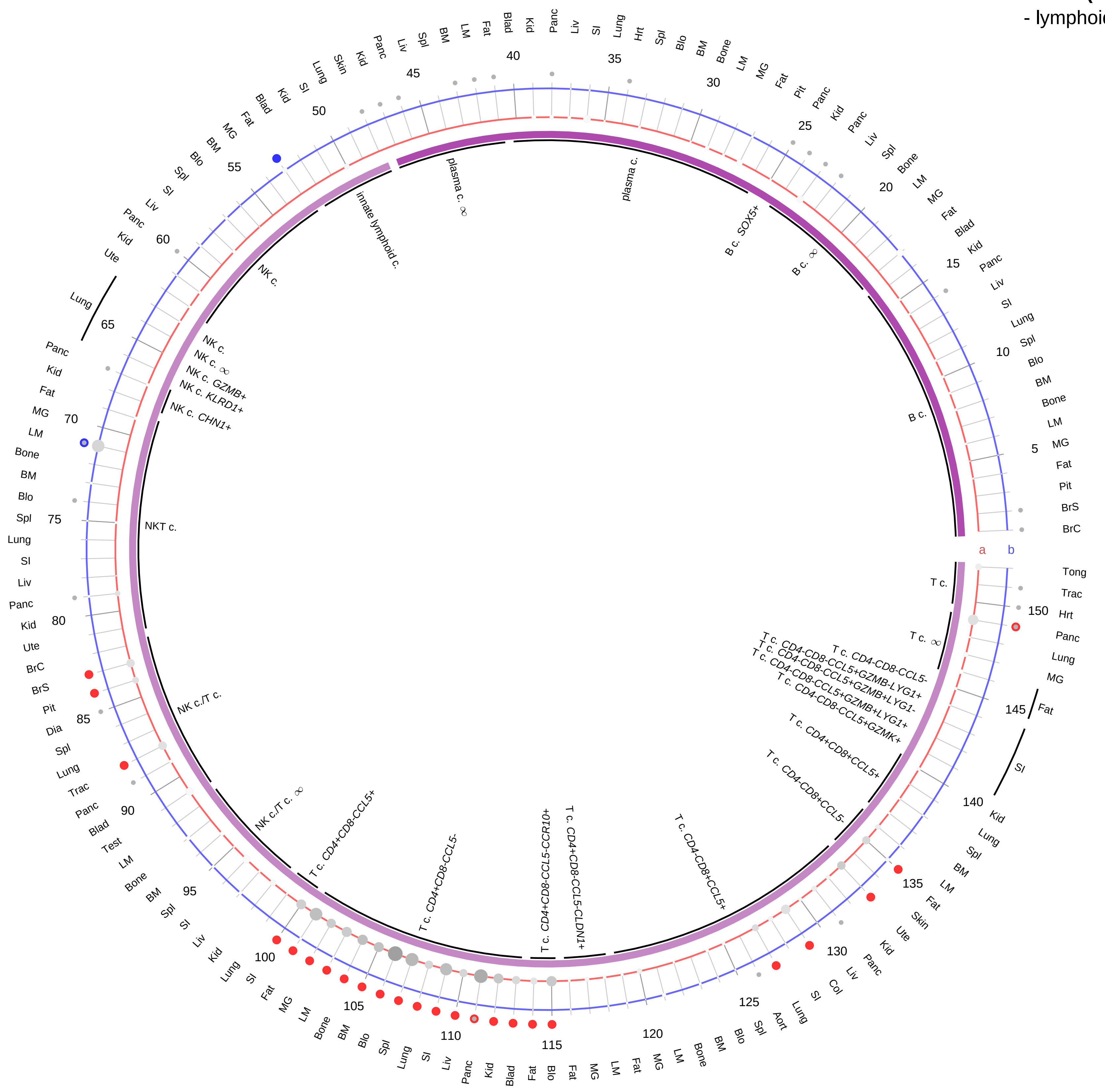

Ligand/Enzyme
a - MLN
Receptor
b - MLNR

PAIRING (ligand → receptor)

MLN→MLNR

Compartment
lymphoid
- B cell
- NK/T cell

12.5%
25%
50%
100%

ln(CP10K+1): 0, 1, 2, 3

Abbrev.	Tissue
Aort	Aorta
Blad	Bladder
Blo	Blood
Bone	Bone
BM	Bone marrow
BrC	Brain cortex
BrS	Brainstem
Col	Colon
Dia	Diaphragm
Ret	Eye retina
Fat	Fat
Hrt	Heart
Pit	Hypothalamus/Pituitary
Kid	Kidney
LM	Limb muscle
Liv	Liver
Lung	Lung
MG	Mammary gland
Panc	Pancreas
Skin	Skin
SI	Small intestine
Spl	Spleen
Test	Testes
Thym	Thymus
Tong	Tongue
Trac	Trachea
Ute	Uterus

Motilin (MLN)
- myeloid

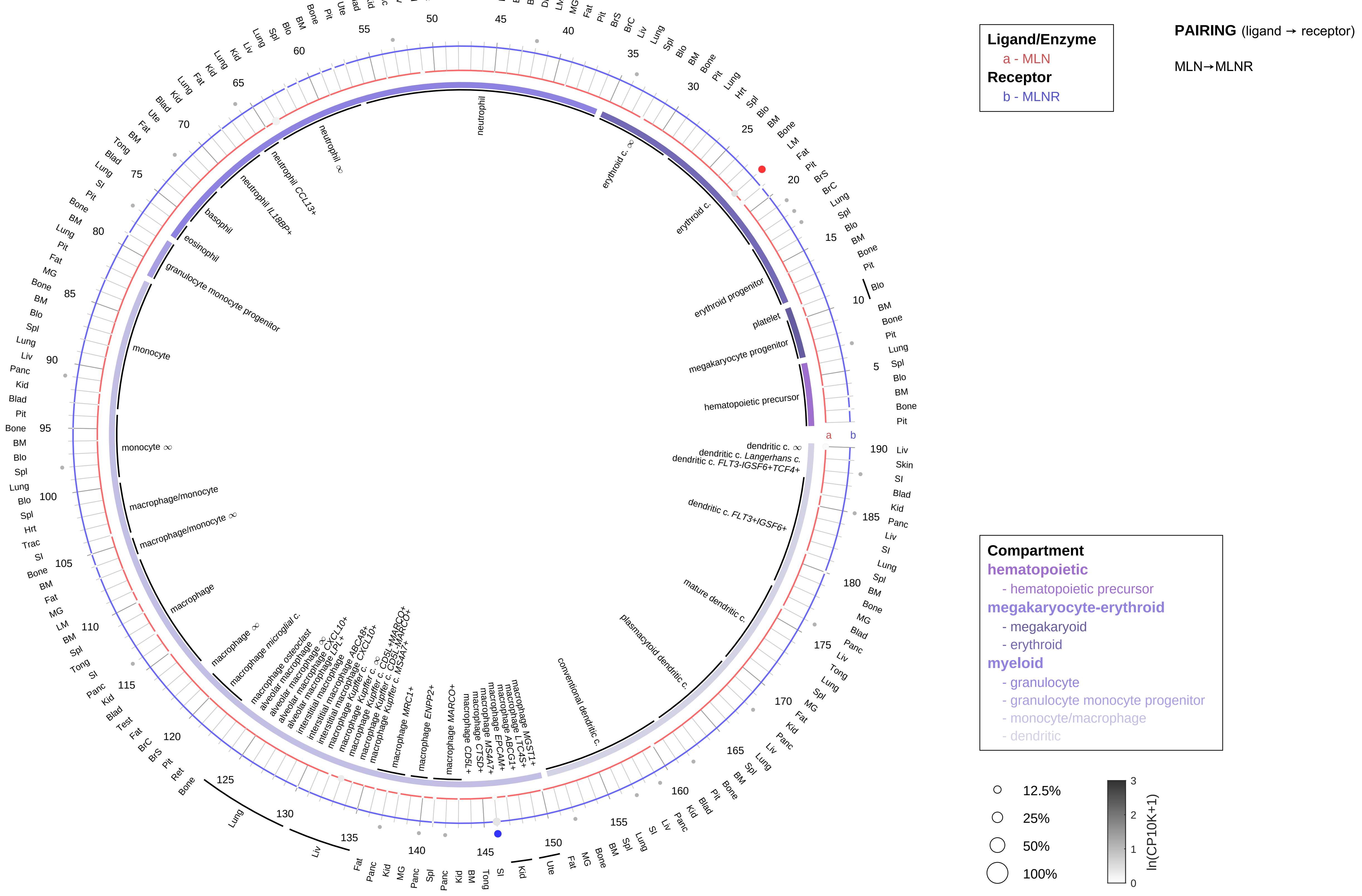

Abbrev.	Tissue
Aort	Aorta
Blad	Bladder
Blo	Blood
Bone	Bone
BM	Bone marrow
BrC	Brain cortex
BrS	Brainstem
Col	Colon
Dia	Diaphragm
Ret	Eye retina
Fat	Fat
Hrt	Heart
Pit	Hypothalamus/Pituitary
Kid	Kidney
LM	Limb muscle
Liv	Liver
Lung	Lung
MG	Mammary gland
Panc	Pancreas
Skin	Skin
SI	Small intestine
Spl	Spleen
Test	Testes
Thym	Thymus
Tong	Tongue
Trac	Trachea
Ute	Uterus

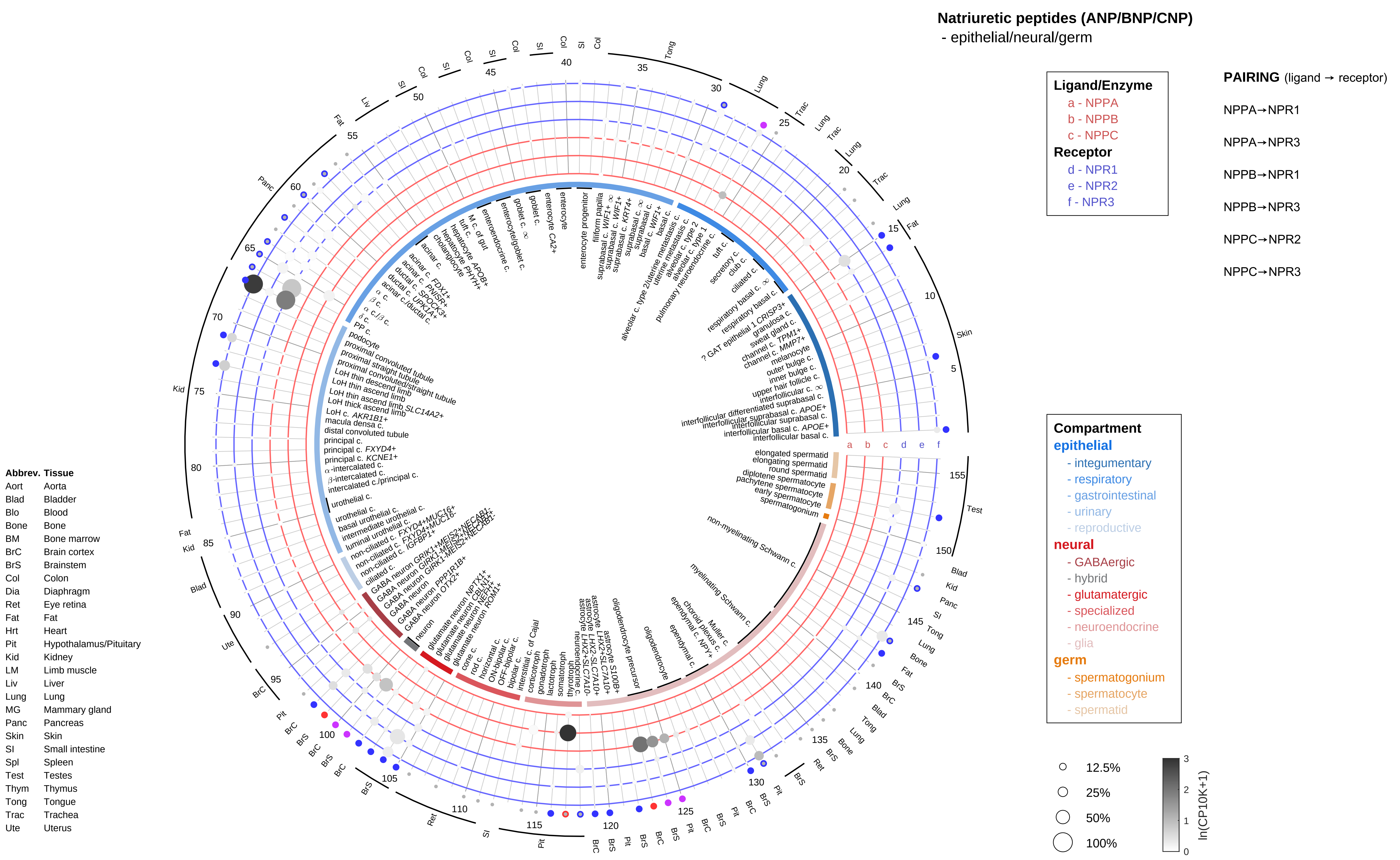
Natriuretic peptides (ANP/BNP/CNP)
- epithelial/neural/germ
Ligand/Enzyme
a - NPPA
b - NPPB
c - NPPC
Receptor
d - NPR1
e - NPR2
f - NPR3
PAIRING (ligand → receptor)
NPPA→NPR1
NPPA→NPR3
NPPB→NPR1
NPPB→NPR3
NPPC→NPR2
NPPC→NPR3
Compartment
epithelial
- integumentary
- respiratory
- gastrointestinal
- urinary
- reproductive
neural
- GABAergic
- hybrid
- glutamatergic
- specialized
- neuroendocrine
- glia
germ
- spermatogonium
- spermatocyte
- spermatid
12.5%
25%
50%
100%
ln(CP10K+1)
Abbrev. Tissue
Aort Aorta
Blad Bladder
Blo Blood
Bone Bone
BM Bone marrow
BrC Brain cortex
BrS Brainstem
Col Colon
Dia Diaphragm
Ret Eye retina
Fat Fat
Hrt Heart
Pit Hypothalamus/Pituitary
Kid Kidney
LM Limb muscle
Liv Liver
Lung Lung
MG Mammary gland
Panc Pancreas
Skin Skin
SI Small intestine
Spl Spleen
Test Testes
Thym Thymus
Tong Tongue
Trac Trachea
Ute Uterus

Natriuretic peptides (ANP/BNP/CNP)
- endothelial

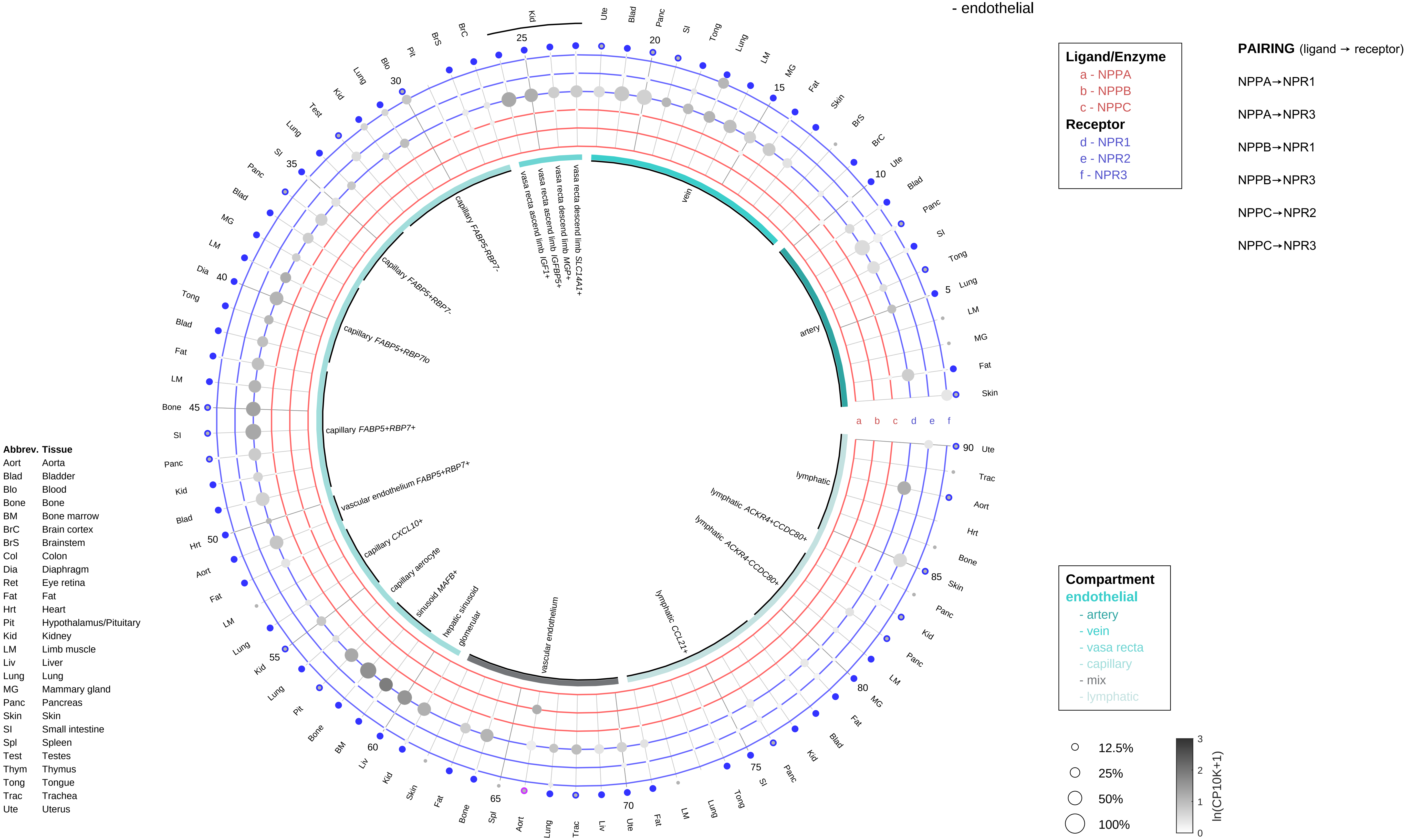

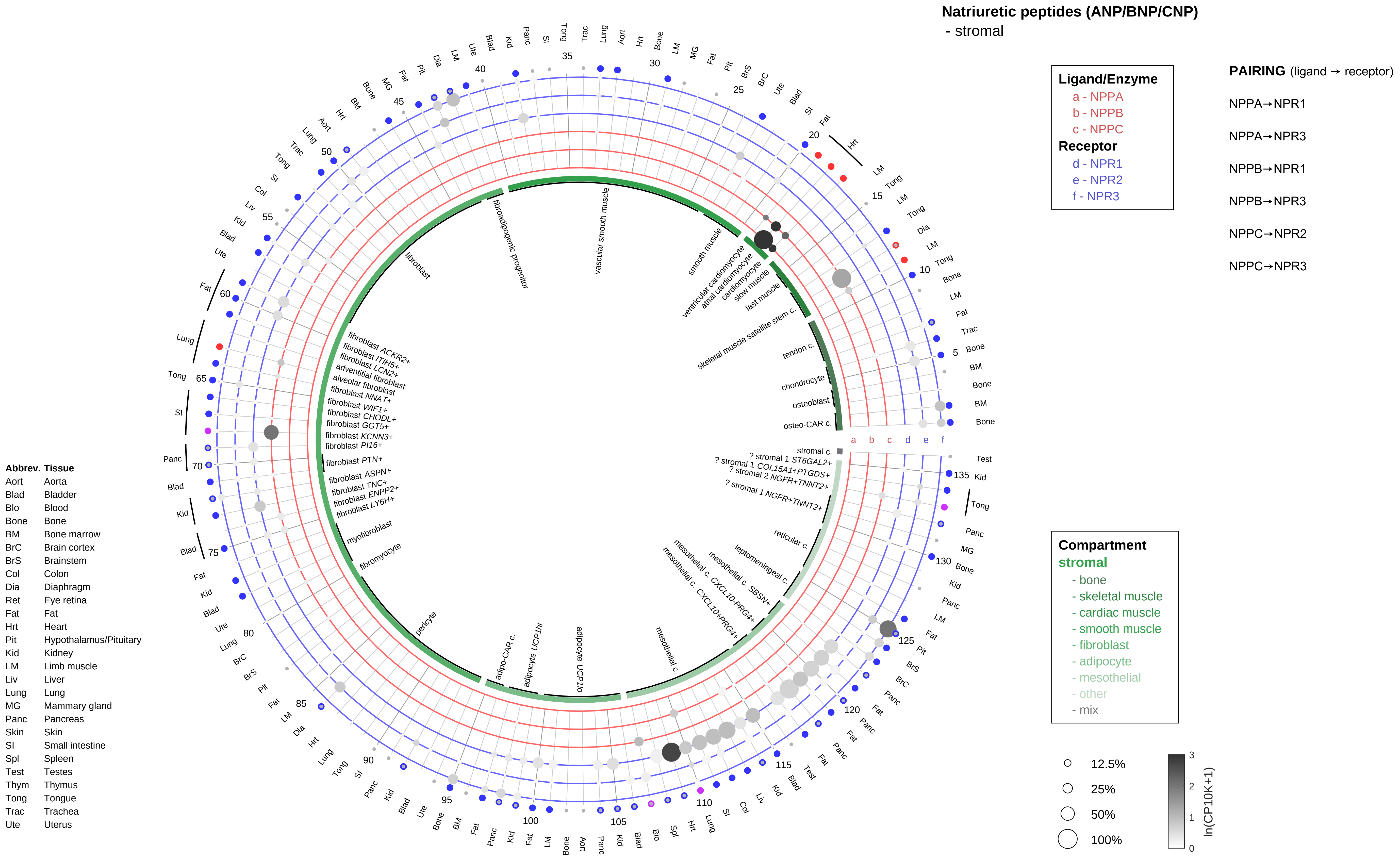

Natriuretic peptides (ANP/BNP/CNP)
- stromal
Ligand/Enzyme
a - NPPA
b - NPPB
c - NPPC
Receptor
d - NPR1
e - NPR2
f - NPR3
PAIRING (ligand → receptor)
NPPA→NPR1
NPPA→NPR3
NPPB→NPR1
NPPB→NPR3
NPPC→NPR2
NPPC→NPR3
Compartment
stromal
- bone
- skeletal muscle
- cardiac muscle
- smooth muscle
- fibroblast
- adipocyte
- mesothelial
- other
- mix
12.5%
25%
50%
100%
ln(CP10K+1)
Abbrev. Tissue
Aort Aorta
Blad Bladder
Blo Blood
Bone Bone
BM Bone marrow
BrC Brain cortex
BrS Brainstem
Col Colon
Dia Diaphragm
Ret Eye retina
Fat Fat
Hrt Heart
Pit Hypothalamus/Pituitary
Kid Kidney
LM Limb muscle
Liv Liver
Lung Lung
MG Mammary gland
Panc Pancreas
Skin Skin
SI Small intestine
Spl Spleen
Test Testes
Thym Thymus
Tong Tongue
Trac Trachea
Ute Uterus
vascular smooth muscle
smooth muscle
ventricular cardiomyocyte
atrial cardiomyocyte
cardiomyocyte
slow muscle
fast muscle
skeletal muscle satellite stem c.
tendon c.
chondrocyte
osteoblast
osteo-CAR c.
stromal c.
? stromal 1 ST6GAL2+
? stromal 1 COL15A1+PTGDS+
? stromal 2 NGFR+TNNT2+
? stromal 1 NGFR+TNNT2+
reticular c.
leptomeningeal c.
mesothelial c. SBSN+
mesothelial c. CXCL10-PRG4+
mesothelial c. CXCL10+PRG4+
mesothelial c.
adipocyte UCP1lo
adipocyte UCP1hi
adipo-CAR c.
pericyte
fibromyocyte
myofibroblast
fibroblast LY6H+
fibroblast ENPP2+
fibroblast TNC+
fibroblast ASPN+
fibroblast PTN+
fibroblast PI16+
fibroblast KCNN3+
fibroblast GGT5+
fibroblast CHODL+
fibroblast WIF1+
fibroblast NNAT+
alveolar fibroblast
adventitial fibroblast
fibroblast LCN2+
fibroblast ITIH5+
fibroblast ACKR2+
fibroblast
fibroadipogenic progenitor

Natriuretic peptides (ANP/BNP/CNP)
- lymphoid

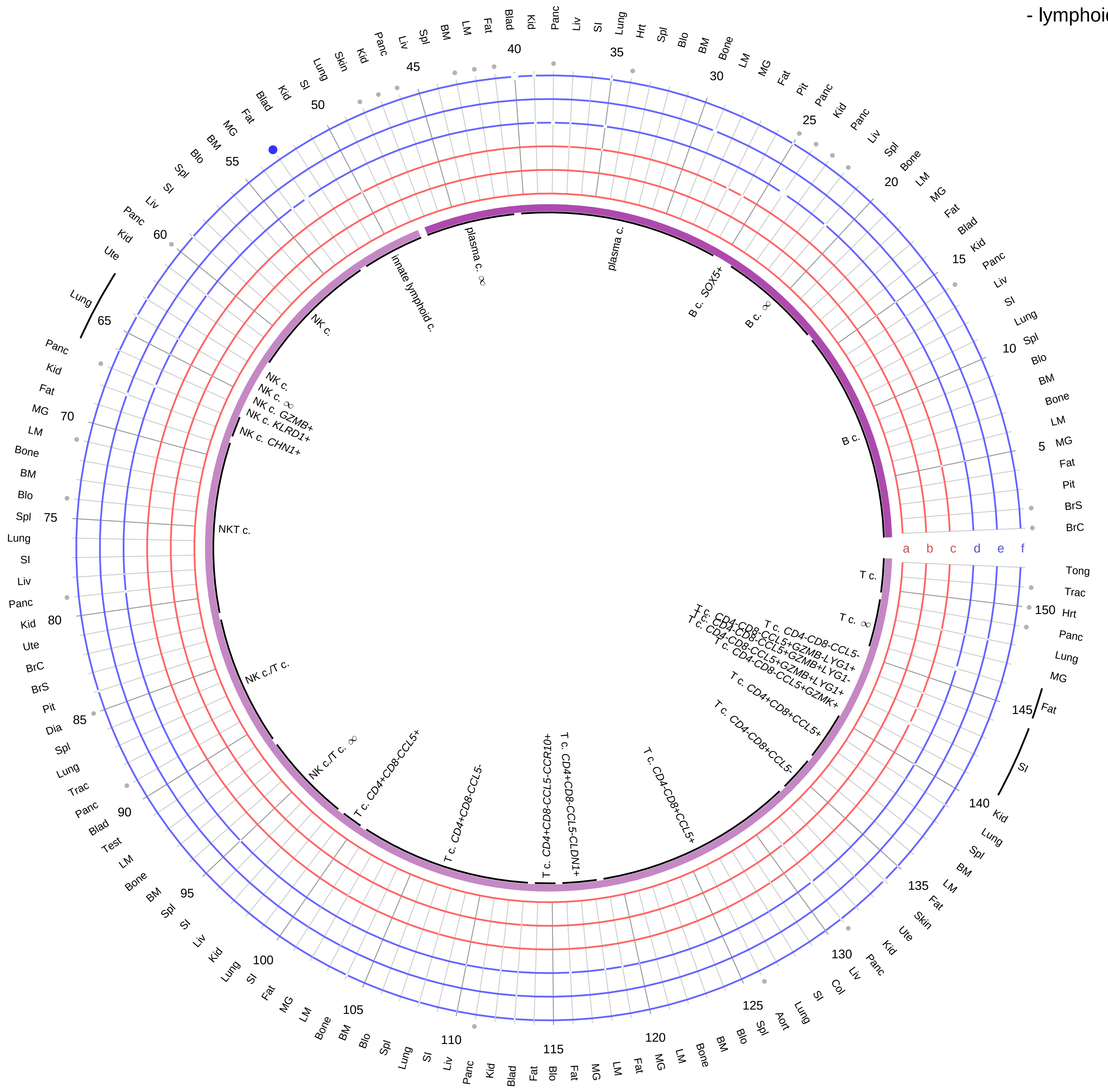

Ligand/Enzyme
- a - NPPA
- b - NPPB
- c - NPPC

Receptor
- d - NPR1
- e - NPR2
- f - NPR3

PAIRING (ligand → receptor)

NPPA→NPR1

NPPA→NPR3

NPPB→NPR1

NPPB→NPR3

NPPC→NPR2

NPPC→NPR3

Compartment

lymphoid
- B cell
- NK/T cell

12.5%
25%
50%
100%

ln(CP10K+1): 0, 1, 2, 3

Abbrev.	Tissue
Aort	Aorta
Blad	Bladder
Blo	Blood
Bone	Bone
BM	Bone marrow
BrC	Brain cortex
BrS	Brainstem
Col	Colon
Dia	Diaphragm
Ret	Eye retina
Fat	Fat
Hrt	Heart
Pit	Hypothalamus/Pituitary
Kid	Kidney
LM	Limb muscle
Liv	Liver
Lung	Lung
MG	Mammary gland
Panc	Pancreas
Skin	Skin
SI	Small intestine
Spl	Spleen
Test	Testes
Thym	Thymus
Tong	Tongue
Trac	Trachea
Ute	Uterus

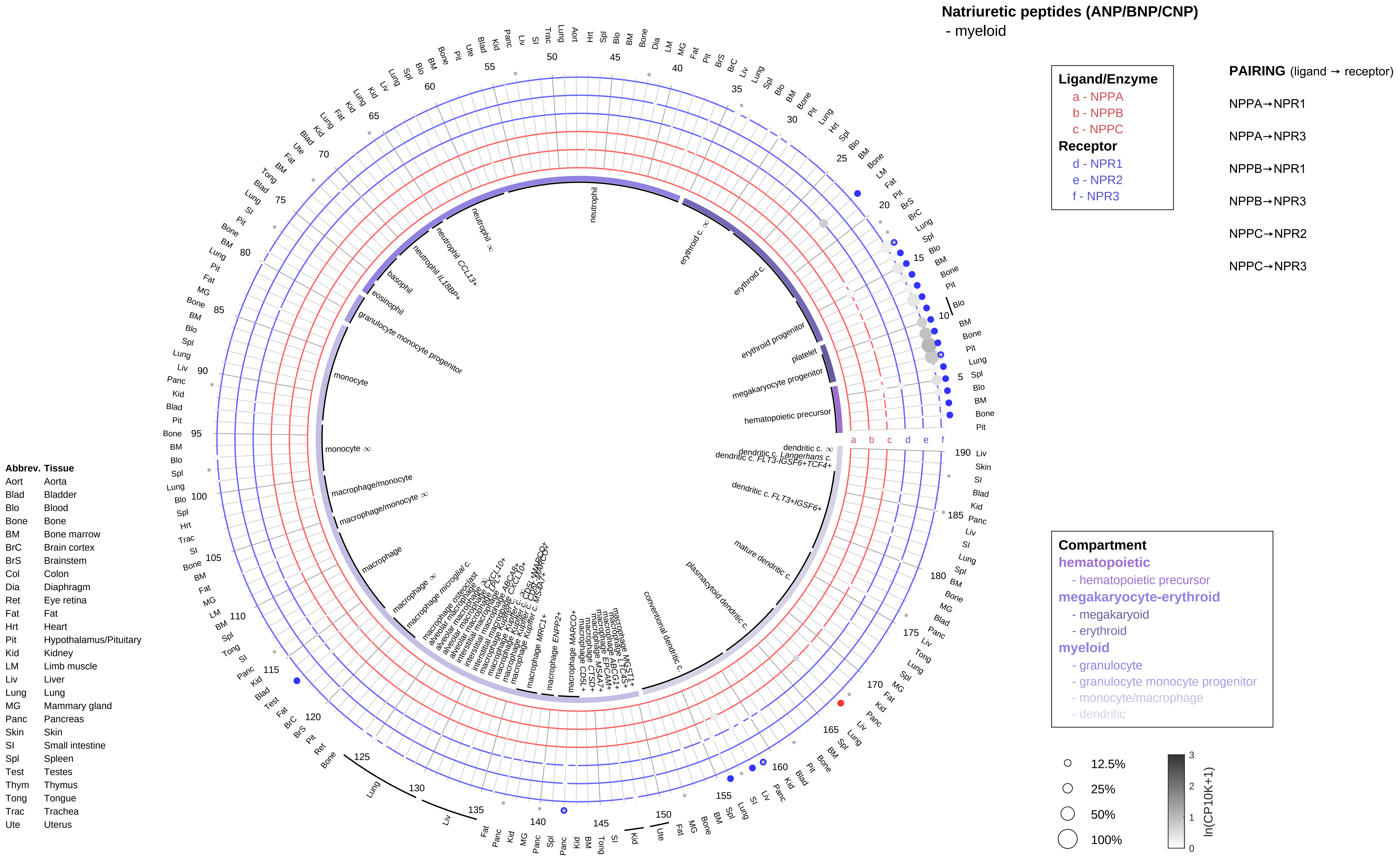
Natriuretic peptides (ANP/BNP/CNP)
- myeloid
Ligand/Enzyme
a - NPPA
b - NPPB
c - NPPC
Receptor
d - NPR1
e - NPR2
f - NPR3
PAIRING (ligand → receptor)
NPPA→NPR1
NPPA→NPR3
NPPB→NPR1
NPPB→NPR3
NPPC→NPR2
NPPC→NPR3
Compartment
hematopoietic
- hematopoietic precursor
megakaryocyte-erythroid
- megakaryoid
- erythroid
myeloid
- granulocyte
- granulocyte monocyte progenitor
- monocyte/macrophage
- dendritic
12.5%
25%
50%
100%
ln(CP10K+1)
Abbrev. Tissue
Aort Aorta
Blad Bladder
Blo Blood
Bone Bone
BM Bone marrow
BrC Brain cortex
BrS Brainstem
Col Colon
Dia Diaphragm
Ret Eye retina
Fat Fat
Hrt Heart
Pit Hypothalamus/Pituitary
Kid Kidney
LM Limb muscle
Liv Liver
Lung Lung
MG Mammary gland
Panc Pancreas
Skin Skin
SI Small intestine
Spl Spleen
Test Testes
Thym Thymus
Tong Tongue
Trac Trachea
Ute Uterus
neutrophil
erythroid c. ∞
erythroid c.
erythroid progenitor
platelet
megakaryocyte progenitor
hematopoietic precursor
dendritic c. ∞
dendritic c. Langerhans c.
dendritic c. FLT3-IGSF6+TCF4+
dendritic c. FLT3+IGSF6+
mature dendritic c.
plasmacytoid dendritic c.
conventional dendritic c.
macrophage MARCO+
macrophage ENPP2+
macrophage MRC1+
macrophage
macrophage ∞
macrophage microglial c.
macrophage/monocyte ∞
macrophage/monocyte
monocyte ∞
monocyte
granulocyte monocyte progenitor
eosinophil
basophil
neutrophil IL18BP+
neutrophil CCL13+
neutrophil ∞

Neuropeptide B/W
- epithelial/neural/germ

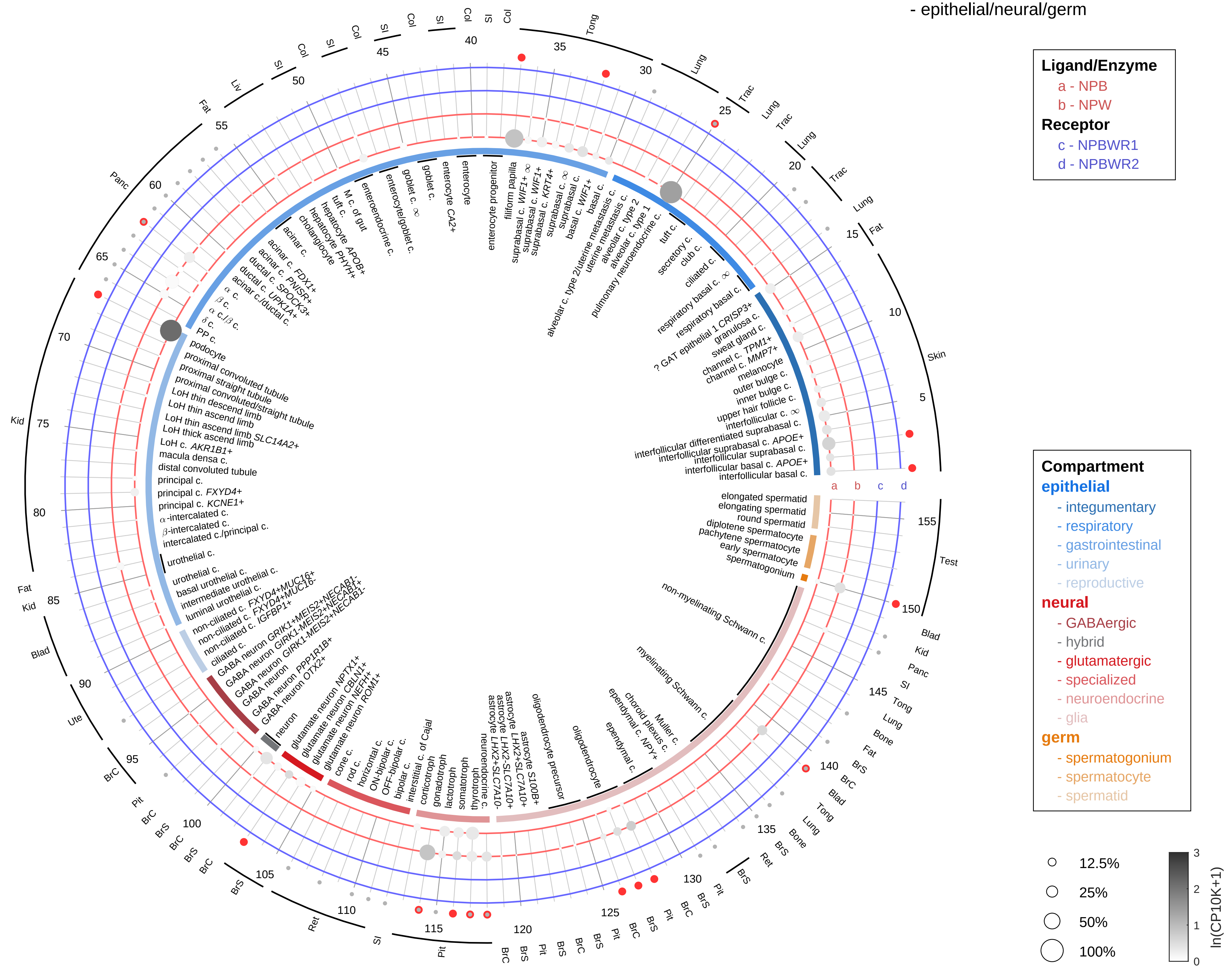

PAIRING (ligand → receptor)

NPB→NPBWR1

NPB→NPBWR2

NPW→NPBWR1

NPW→NPBWR2

Abbrev.	Tissue
Aort	Aorta
Blad	Bladder
Blo	Blood
Bone	Bone
BM	Bone marrow
BrC	Brain cortex
BrS	Brainstem
Col	Colon
Dia	Diaphragm
Ret	Eye retina
Fat	Fat
Hrt	Heart
Pit	Hypothalamus/Pituitary
Kid	Kidney
LM	Limb muscle
Liv	Liver
Lung	Lung
MG	Mammary gland
Panc	Pancreas
Skin	Skin
SI	Small intestine
Spl	Spleen
Test	Testes
Thym	Thymus
Tong	Tongue
Trac	Trachea
Ute	Uterus

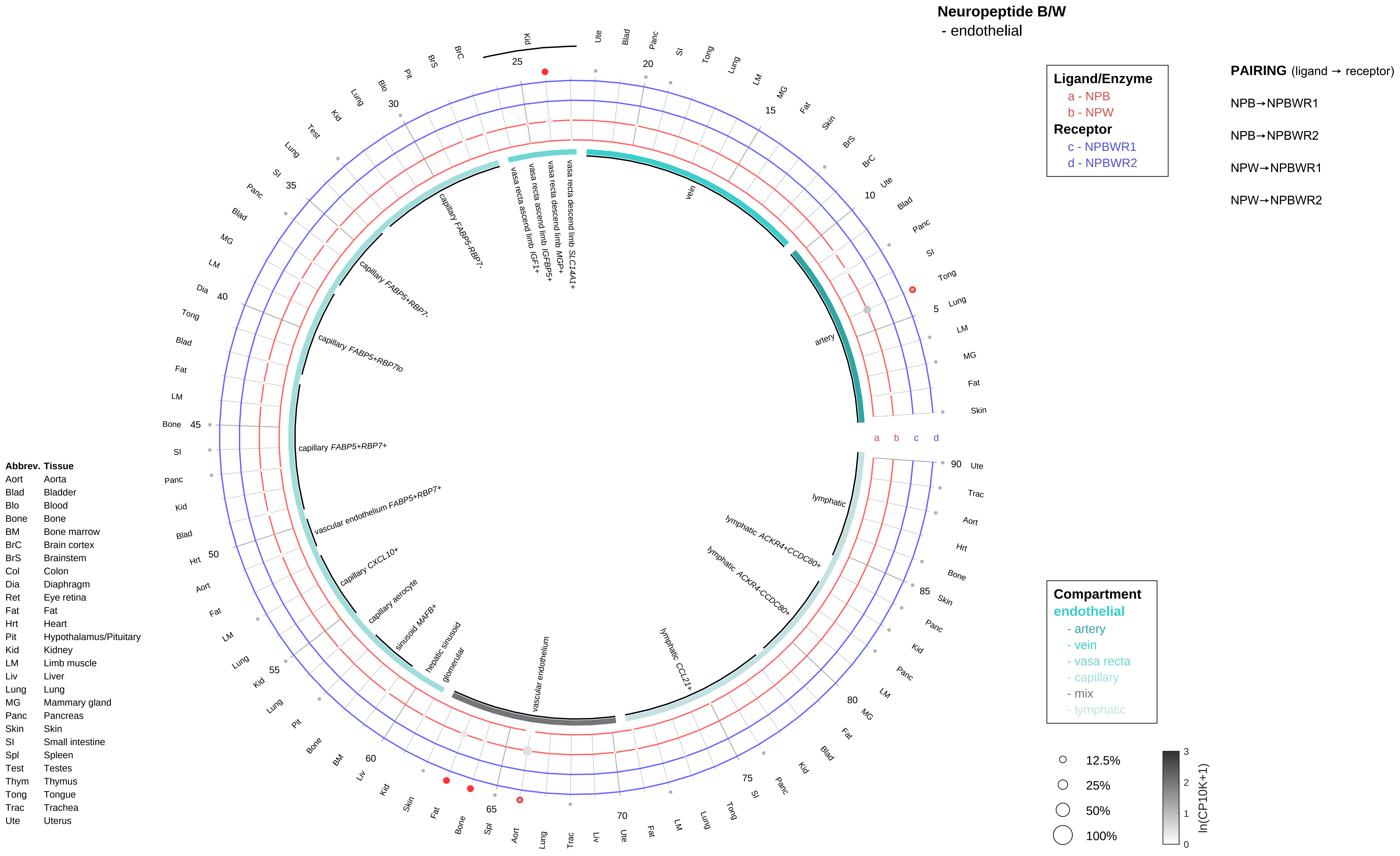

Neuropeptide B/W
- endothelial
Ligand/Enzyme
a - NPB
b - NPW
Receptor
c - NPBWR1
d - NPBWR2
PAIRING (ligand → receptor)
NPB→NPBWR1
NPB→NPBWR2
NPW→NPBWR1
NPW→NPBWR2
vein
artery
lymphatic
lymphatic ACKR4+CCDC80+
lymphatic ACKR4-CCDC80+
lymphatic CCL21+
vascular endothelium
glomerular
hepatic sinusoid
sinusoid MAFB+
capillary aerocyte
capillary CXCL10+
vascular endothelium FABP5+RBP7+
capillary FABP5+RBP7+
capillary FABP5+RBP7lo
capillary FABP5+RBP7-
capillary FABP5-RBP7-
vasa recta ascend limb IGF1+
vasa recta ascend limb IGFBP5+
vasa recta descend limb MGP+
vasa recta descend limb SLC14A1+
Abbrev. Tissue
Aort Aorta
Blad Bladder
Blo Blood
Bone Bone
BM Bone marrow
BrC Brain cortex
BrS Brainstem
Col Colon
Dia Diaphragm
Ret Eye retina
Fat Fat
Hrt Heart
Pit Hypothalamus/Pituitary
Kid Kidney
LM Limb muscle
Liv Liver
Lung Lung
MG Mammary gland
Panc Pancreas
Skin Skin
SI Small intestine
Spl Spleen
Test Testes
Thym Thymus
Tong Tongue
Trac Trachea
Ute Uterus
Compartment
endothelial
- artery
- vein
- vasa recta
- capillary
- mix
- lymphatic
12.5%
25%
50%
100%
ln(CP10K+1)

Neuropeptide B/W

- stromal

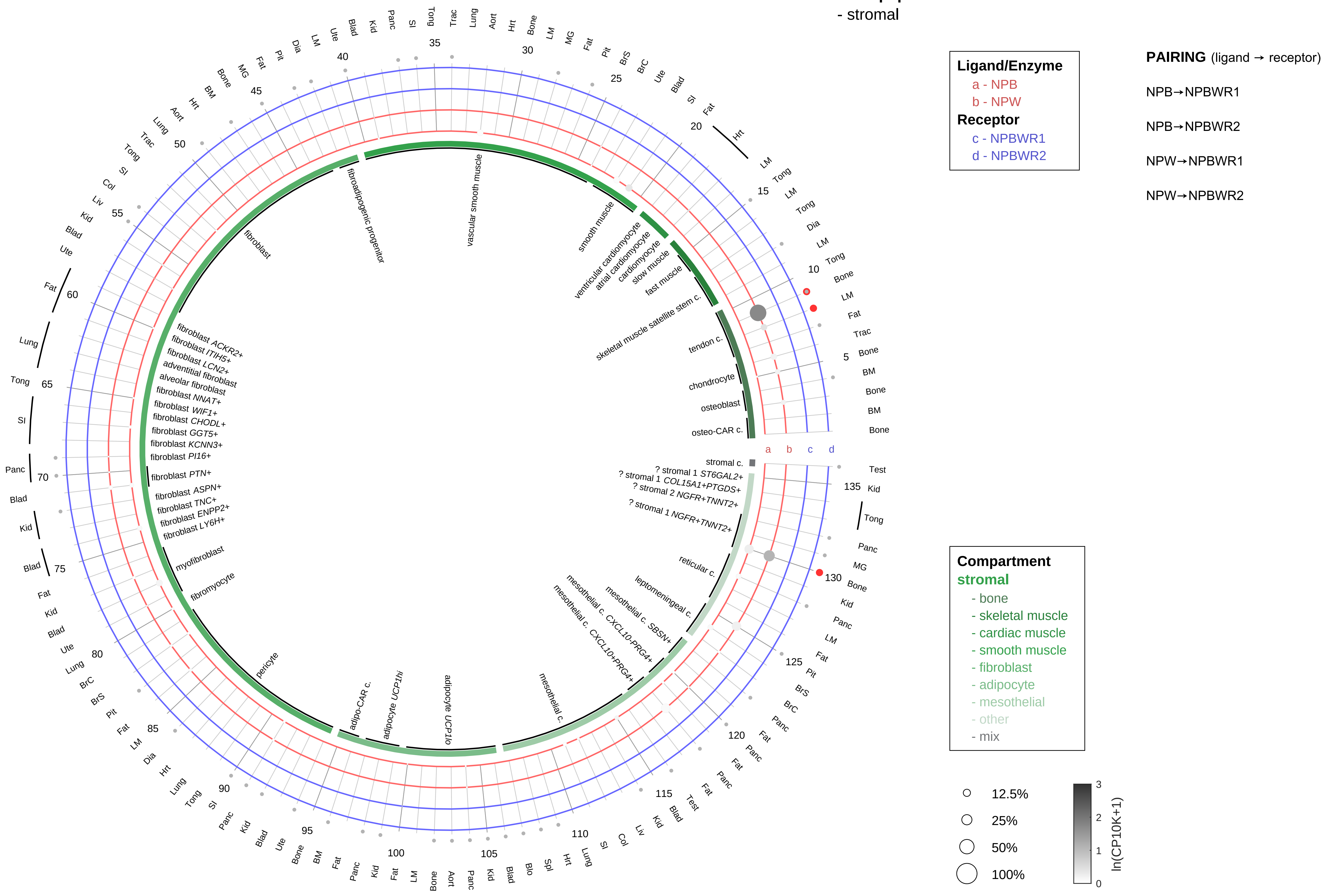

Abbrev.	Tissue
Aort	Aorta
Blad	Bladder
Blo	Blood
Bone	Bone
BM	Bone marrow
BrC	Brain cortex
BrS	Brainstem
Col	Colon
Dia	Diaphragm
Ret	Eye retina
Fat	Fat
Hrt	Heart
Pit	Hypothalamus/Pituitary
Kid	Kidney
LM	Limb muscle
Liv	Liver
Lung	Lung
MG	Mammary gland
Panc	Pancreas
Skin	Skin
SI	Small intestine
Spl	Spleen
Test	Testes
Thym	Thymus
Tong	Tongue
Trac	Trachea
Ute	Uterus

Neuropeptide B/W
- lymphoid

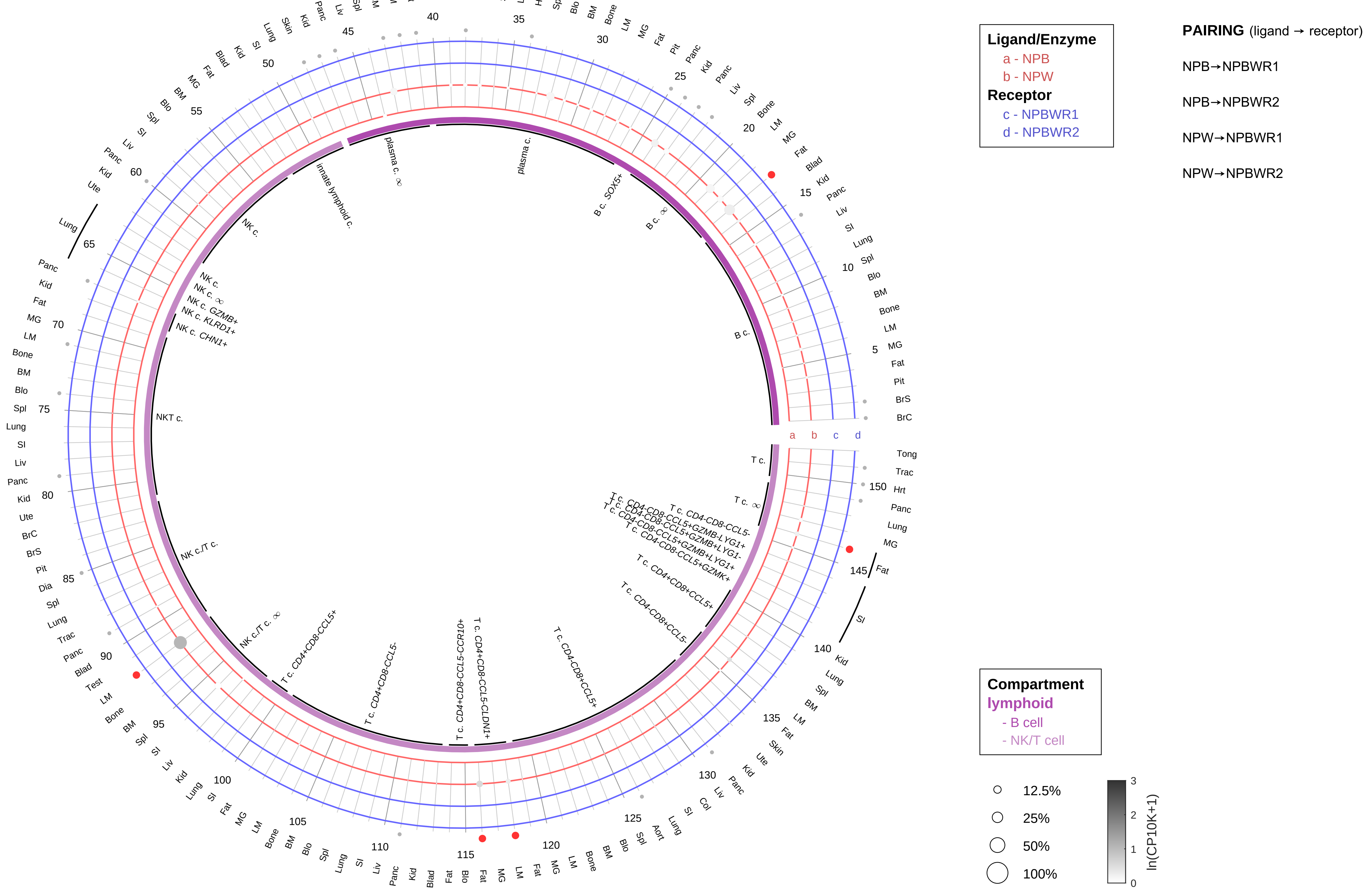

Abbrev.	Tissue
Aort	Aorta
Blad	Bladder
Blo	Blood
Bone	Bone
BM	Bone marrow
BrC	Brain cortex
BrS	Brainstem
Col	Colon
Dia	Diaphragm
Ret	Eye retina
Fat	Fat
Hrt	Heart
Pit	Hypothalamus/Pituitary
Kid	Kidney
LM	Limb muscle
Liv	Liver
Lung	Lung
MG	Mammary gland
Panc	Pancreas
Skin	Skin
SI	Small intestine
Spl	Spleen
Test	Testes
Thym	Thymus
Tong	Tongue
Trac	Trachea
Ute	Uterus

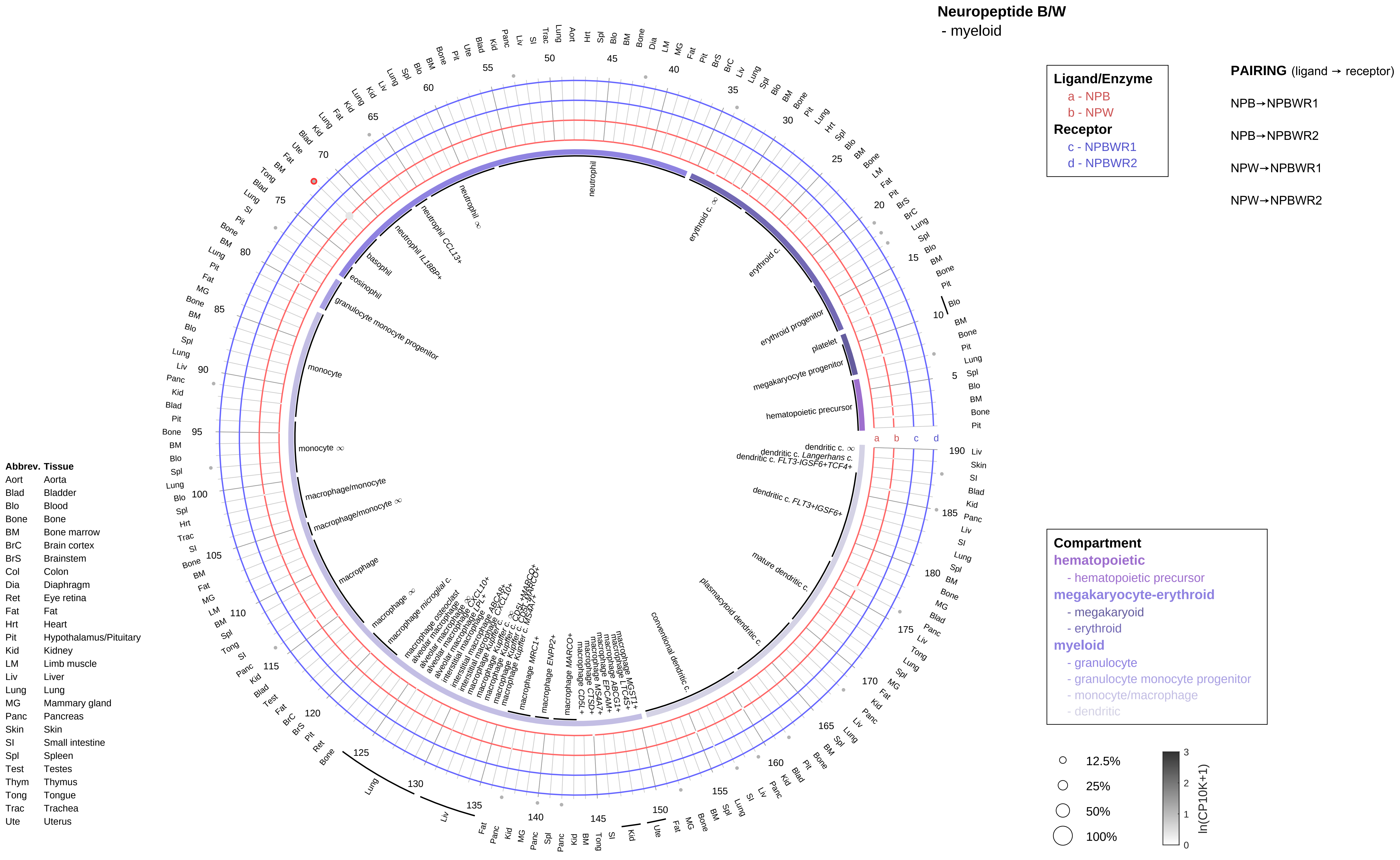
Neuropeptide B/W
- myeloid
Ligand/Enzyme
a - NPB
b - NPW
Receptor
c - NPBWR1
d - NPBWR2
PAIRING (ligand → receptor)
NPB→NPBWR1
NPB→NPBWR2
NPW→NPBWR1
NPW→NPBWR2
Compartment
hematopoietic
- hematopoietic precursor
megakaryocyte-erythroid
- megakaryoid
- erythroid
myeloid
- granulocyte
- granulocyte monocyte progenitor
- monocyte/macrophage
- dendritic
12.5%
25%
50%
100%
ln(CP10K+1)
0
1
2
3
Abbrev. Tissue
Aort Aorta
Blad Bladder
Blo Blood
Bone Bone
BM Bone marrow
BrC Brain cortex
BrS Brainstem
Col Colon
Dia Diaphragm
Ret Eye retina
Fat Fat
Hrt Heart
Pit Hypothalamus/Pituitary
Kid Kidney
LM Limb muscle
Liv Liver
Lung Lung
MG Mammary gland
Panc Pancreas
Skin Skin
SI Small intestine
Spl Spleen
Test Testes
Thym Thymus
Tong Tongue
Trac Trachea
Ute Uterus

Neuropeptide FF/VF
- epithelial/neural/germ

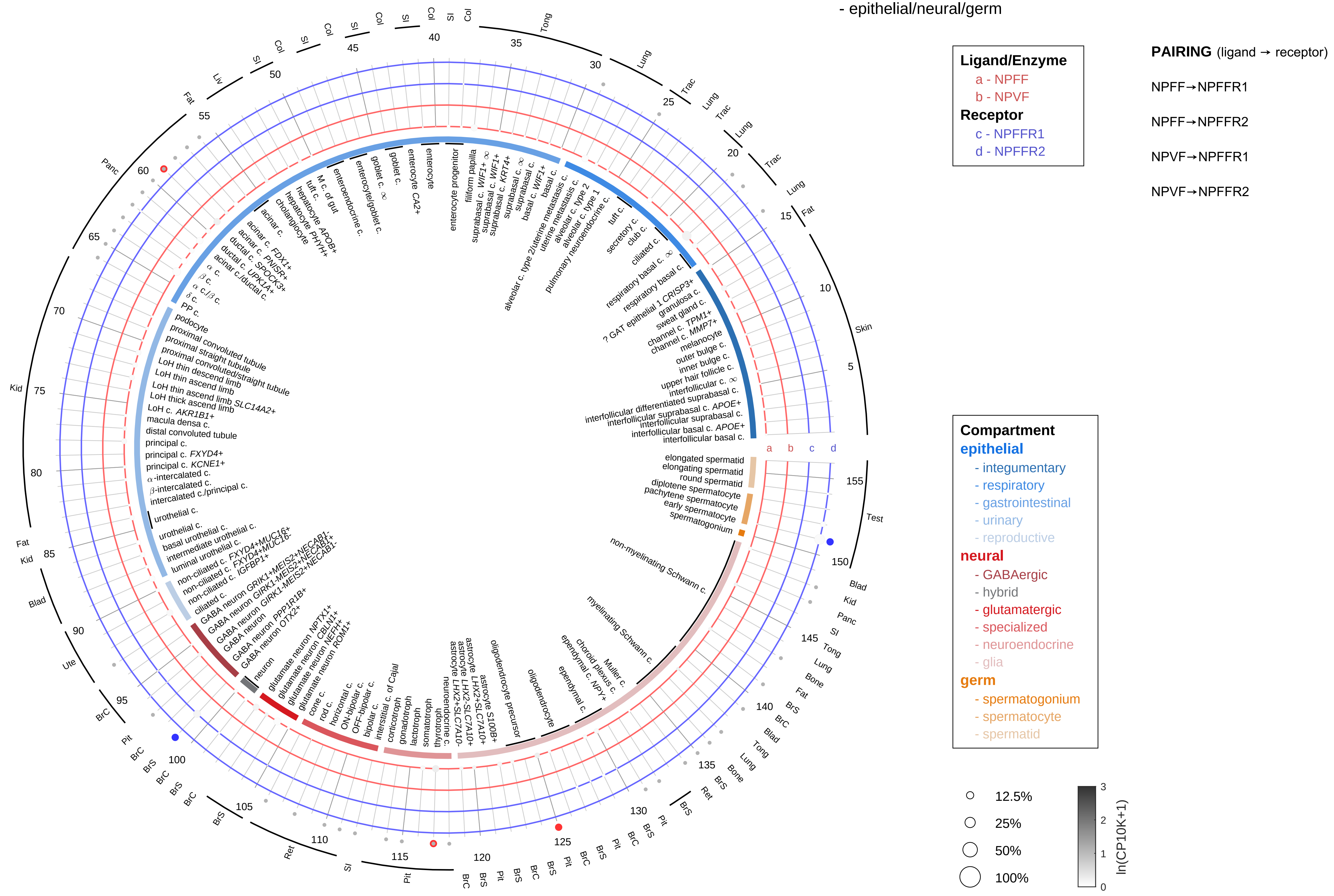

Abbrev.	Tissue
Aort	Aorta
Blad	Bladder
Blo	Blood
Bone	Bone
BM	Bone marrow
BrC	Brain cortex
BrS	Brainstem
Col	Colon
Dia	Diaphragm
Ret	Eye retina
Fat	Fat
Hrt	Heart
Pit	Hypothalamus/Pituitary
Kid	Kidney
LM	Limb muscle
Liv	Liver
Lung	Lung
MG	Mammary gland
Panc	Pancreas
Skin	Skin
SI	Small intestine
Spl	Spleen
Test	Testes
Thym	Thymus
Tong	Tongue
Trac	Trachea
Ute	Uterus

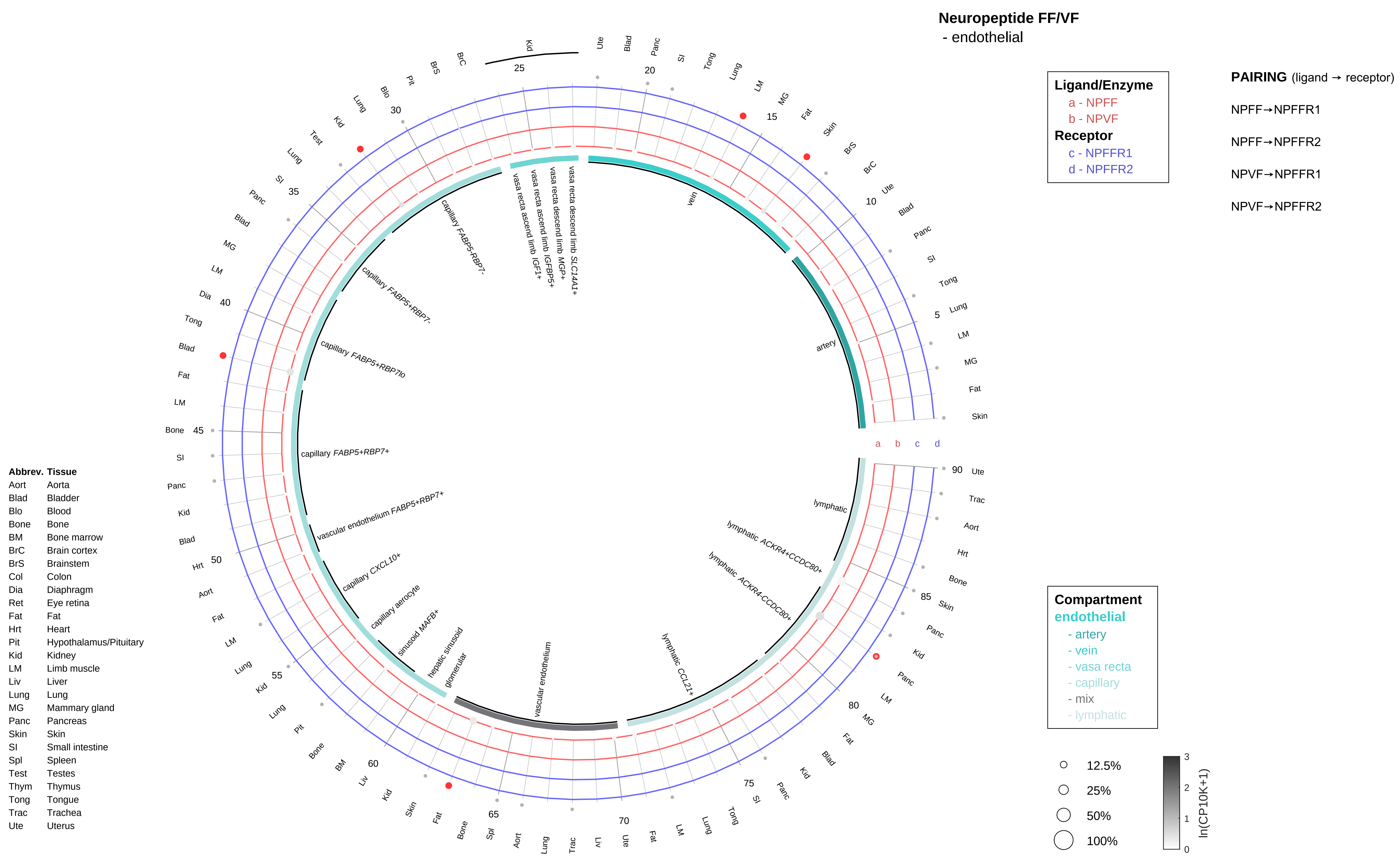
Neuropeptide FF/VF
- endothelial
Ligand/Enzyme
a - NPFF
b - NPVF
Receptor
c - NPFFR1
d - NPFFR2
PAIRING (ligand → receptor)
NPFF→NPFFR1
NPFF→NPFFR2
NPVF→NPFFR1
NPVF→NPFFR2
Compartment
endothelial
- artery
- vein
- vasa recta
- capillary
- mix
- lymphatic
12.5%
25%
50%
100%
ln(CP10K+1)
a b c d
artery
vein
vasa recta descend limb SLC14A1+
vasa recta descend limb MGP+
vasa recta ascend limb IGFBP5+
vasa recta ascend limb IGF1+
capillary FABP5-RBP7-
capillary FABP5+RBP7-
capillary FABP5+RBP7lo
capillary FABP5+RBP7+
vascular endothelium FABP5+RBP7+
capillary CXCL10+
capillary aerocyte
sinusoid MAFB+
hepatic sinusoid
glomerular
vascular endothelium
lymphatic CCL21+
lymphatic ACKR4-CCDC80+
lymphatic ACKR4+CCDC80+
lymphatic
Abbrev. Tissue
Aort Aorta
Blad Bladder
Blo Blood
Bone Bone
BM Bone marrow
BrC Brain cortex
BrS Brainstem
Col Colon
Dia Diaphragm
Ret Eye retina
Fat Fat
Hrt Heart
Pit Hypothalamus/Pituitary
Kid Kidney
LM Limb muscle
Liv Liver
Lung Lung
MG Mammary gland
Panc Pancreas
Skin Skin
SI Small intestine
Spl Spleen
Test Testes
Thym Thymus
Tong Tongue
Trac Trachea
Ute Uterus

Neuropeptide FF/VF
- stromal

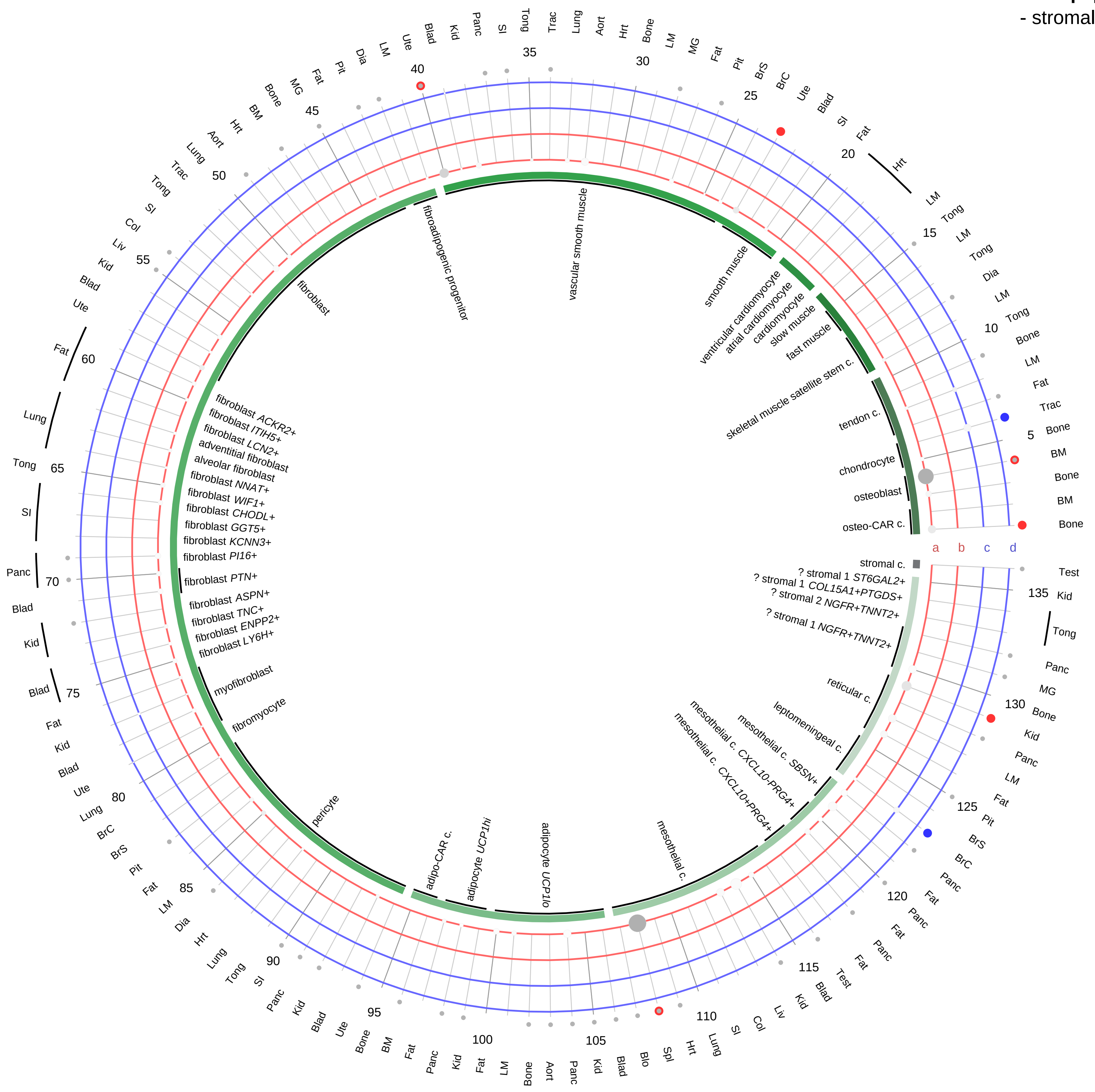

Ligand/Enzyme
- a - NPFF
- b - NPVF

Receptor
- c - NPFFR1
- d - NPFFR2

PAIRING (ligand → receptor)

NPFF→NPFFR1

NPFF→NPFFR2

NPVF→NPFFR1

NPVF→NPFFR2

Compartment

stromal
- bone
- skeletal muscle
- cardiac muscle
- smooth muscle
- fibroblast
- adipocyte
- mesothelial
- other
- mix

12.5%

25%

50%

100%

ln(CP10K+1): 0, 1, 2, 3

Abbrev.	Tissue
Aort	Aorta
Blad	Bladder
Blo	Blood
Bone	Bone
BM	Bone marrow
BrC	Brain cortex
BrS	Brainstem
Col	Colon
Dia	Diaphragm
Ret	Eye retina
Fat	Fat
Hrt	Heart
Pit	Hypothalamus/Pituitary
Kid	Kidney
LM	Limb muscle
Liv	Liver
Lung	Lung
MG	Mammary gland
Panc	Pancreas
Skin	Skin
SI	Small intestine
Spl	Spleen
Test	Testes
Thym	Thymus
Tong	Tongue
Trac	Trachea
Ute	Uterus

Neuropeptide FF/VF
- lymphoid

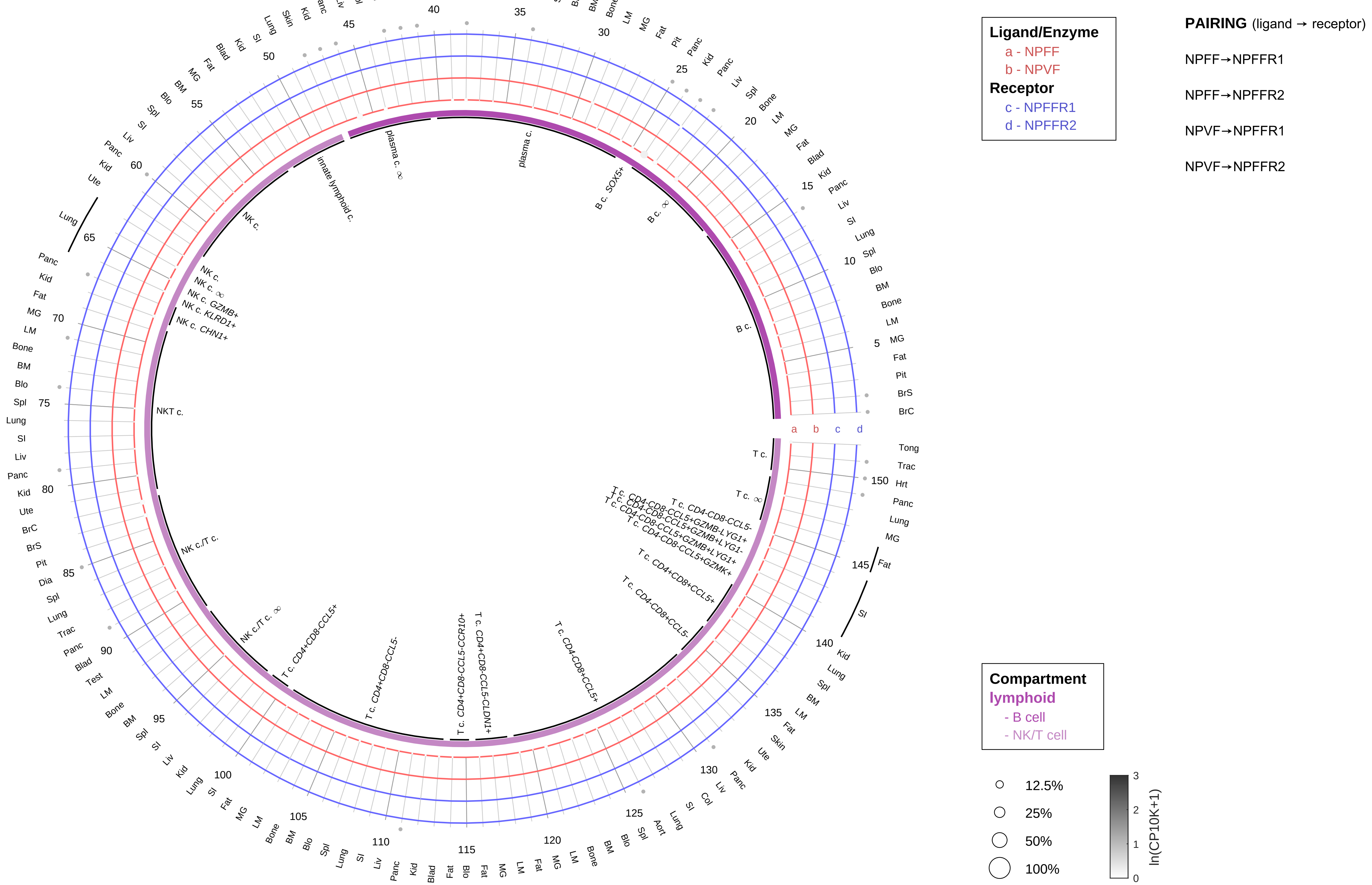

Abbrev.	Tissue
Aort	Aorta
Blad	Bladder
Blo	Blood
Bone	Bone
BM	Bone marrow
BrC	Brain cortex
BrS	Brainstem
Col	Colon
Dia	Diaphragm
Ret	Eye retina
Fat	Fat
Hrt	Heart
Pit	Hypothalamus/Pituitary
Kid	Kidney
LM	Limb muscle
Liv	Liver
Lung	Lung
MG	Mammary gland
Panc	Pancreas
Skin	Skin
SI	Small intestine
Spl	Spleen
Test	Testes
Thym	Thymus
Tong	Tongue
Trac	Trachea
Ute	Uterus

Neuropeptide FF/VF
- myeloid

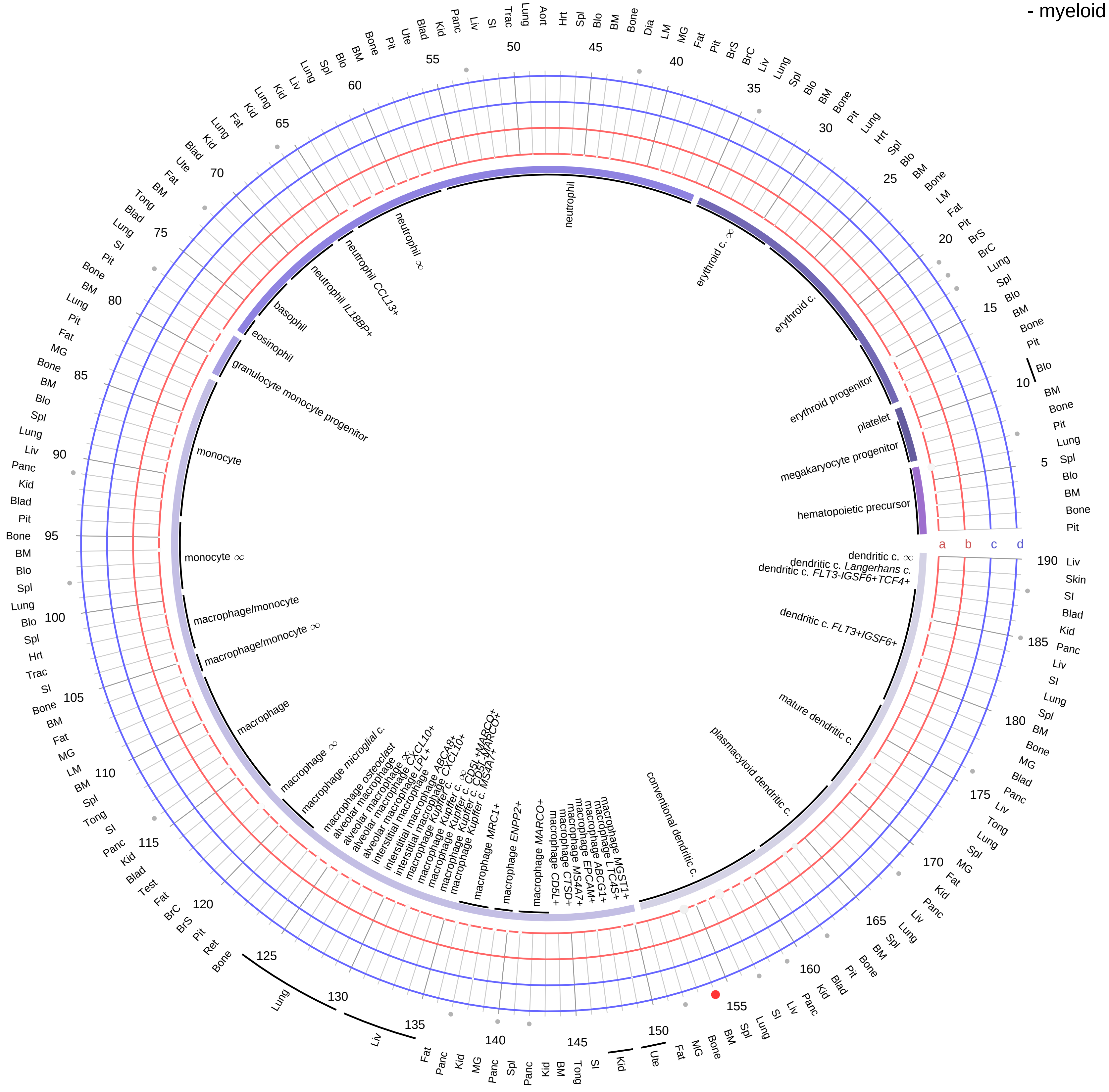

Ligand/Enzyme
- a - NPFF
- b - NPVF

Receptor
- c - NPFFR1
- d - NPFFR2

PAIRING (ligand → receptor)

NPFF→NPFFR1

NPFF→NPFFR2

NPVF→NPFFR1

NPVF→NPFFR2

Compartment

hematopoietic
- hematopoietic precursor

megakaryocyte-erythroid
- megakaryoid
- erythroid

myeloid
- granulocyte
- granulocyte monocyte progenitor
- monocyte/macrophage
- dendritic

12.5%

25%

50%

100%

ln(CP10K+1): 0, 1, 2, 3

Abbrev.	Tissue
Aort	Aorta
Blad	Bladder
Blo	Blood
Bone	Bone
BM	Bone marrow
BrC	Brain cortex
BrS	Brainstem
Col	Colon
Dia	Diaphragm
Ret	Eye retina
Fat	Fat
Hrt	Heart
Pit	Hypothalamus/Pituitary
Kid	Kidney
LM	Limb muscle
Liv	Liver
Lung	Lung
MG	Mammary gland
Panc	Pancreas
Skin	Skin
SI	Small intestine
Spl	Spleen
Test	Testes
Thym	Thymus
Tong	Tongue
Trac	Trachea
Ute	Uterus

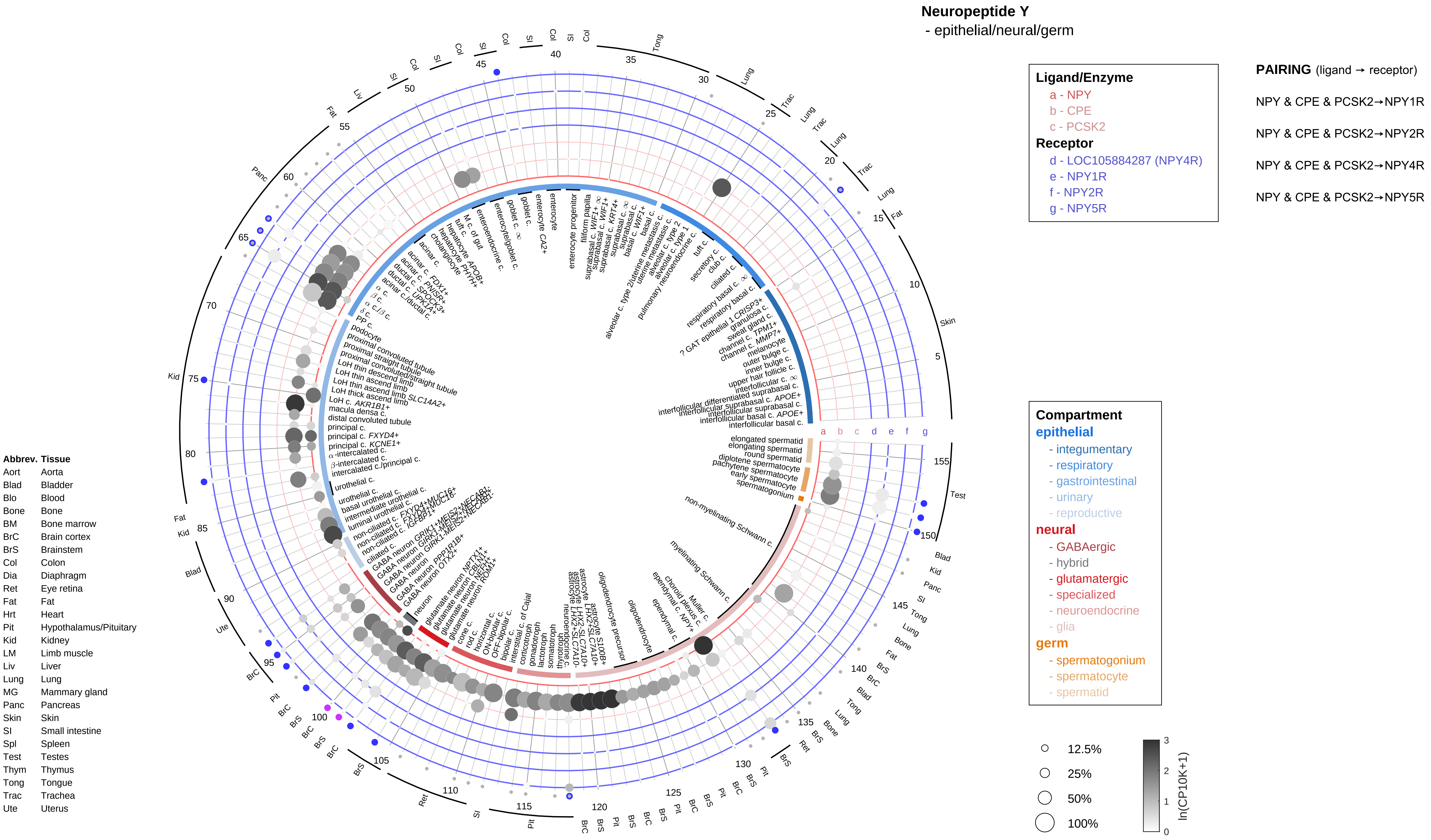

Neuropeptide Y
- epithelial/neural/germ
Ligand/Enzyme
a - NPY
b - CPE
c - PCSK2
Receptor
d - LOC105884287 (NPY4R)
e - NPY1R
f - NPY2R
g - NPY5R
PAIRING (ligand → receptor)
NPY & CPE & PCSK2→NPY1R
NPY & CPE & PCSK2→NPY2R
NPY & CPE & PCSK2→NPY4R
NPY & CPE & PCSK2→NPY5R
Compartment
epithelial
- integumentary
- respiratory
- gastrointestinal
- urinary
- reproductive
neural
- GABAergic
- hybrid
- glutamatergic
- specialized
- neuroendocrine
- glia
germ
- spermatogonium
- spermatocyte
- spermatid
12.5%
25%
50%
100%
ln(CP10K+1)
Abbrev. Tissue
Aort Aorta
Blad Bladder
Blo Blood
Bone Bone
BM Bone marrow
BrC Brain cortex
BrS Brainstem
Col Colon
Dia Diaphragm
Ret Eye retina
Fat Fat
Hrt Heart
Pit Hypothalamus/Pituitary
Kid Kidney
LM Limb muscle
Liv Liver
Lung Lung
MG Mammary gland
Panc Pancreas
Skin Skin
SI Small intestine
Spl Spleen
Test Testes
Thym Thymus
Tong Tongue
Trac Trachea
Ute Uterus

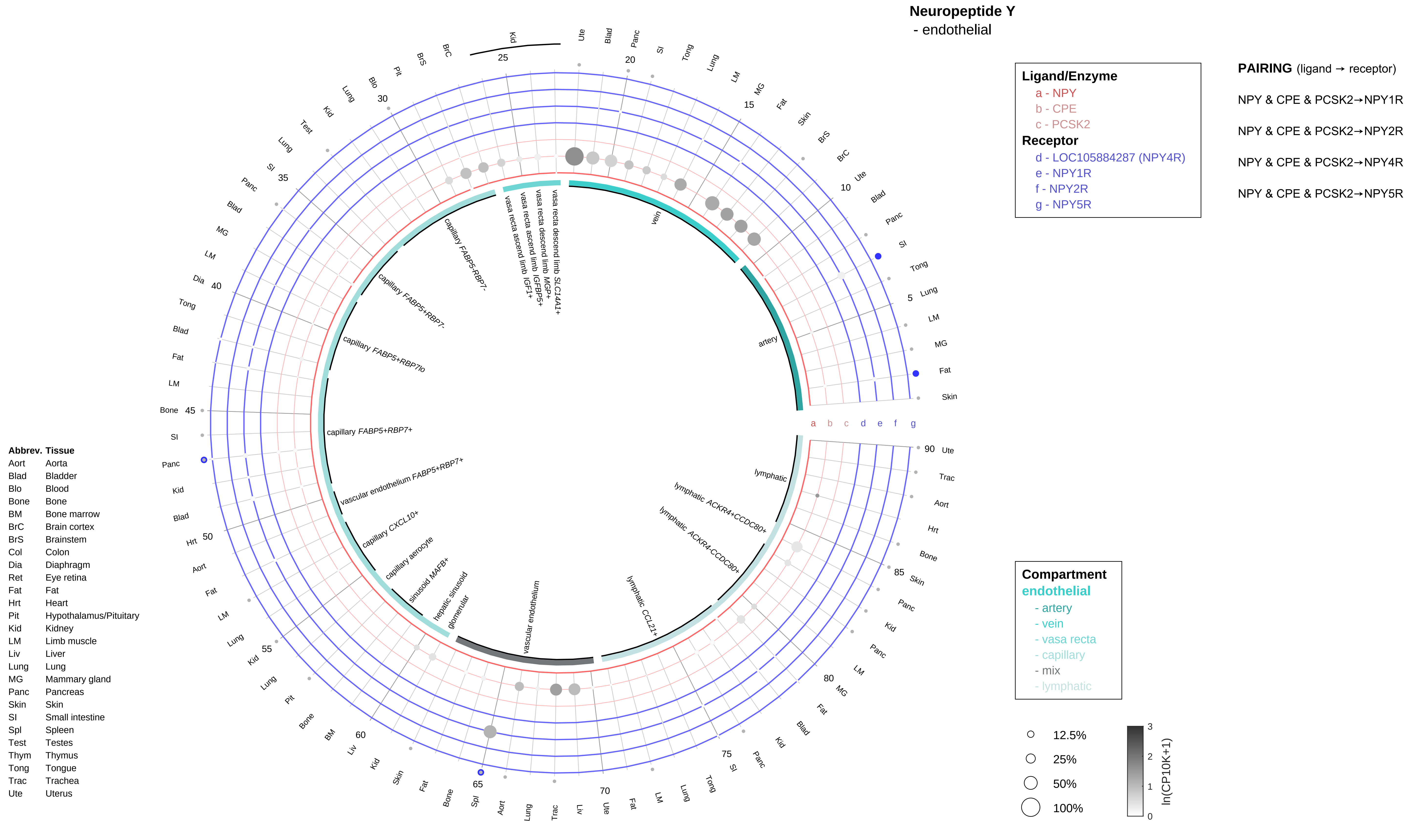
Neuropeptide Y
- endothelial
Ligand/Enzyme
a - NPY
b - CPE
c - PCSK2
Receptor
d - LOC105884287 (NPY4R)
e - NPY1R
f - NPY2R
g - NPY5R
PAIRING (ligand → receptor)
NPY & CPE & PCSK2→NPY1R
NPY & CPE & PCSK2→NPY2R
NPY & CPE & PCSK2→NPY4R
NPY & CPE & PCSK2→NPY5R
Compartment
endothelial
- artery
- vein
- vasa recta
- capillary
- mix
- lymphatic
12.5%
25%
50%
100%
ln(CP10K+1)
vein
artery
vasa recta descend limb SLC14A1+
vasa recta descend limb MGP+
vasa recta ascend limb IGFBP5+
vasa recta ascend limb IGF1+
capillary FABP5-RBP7-
capillary FABP5+RBP7-
capillary FABP5+RBP7lo
capillary FABP5+RBP7+
vascular endothelium FABP5+RBP7+
capillary CXCL10+
capillary aerocyte
sinusoid MAFB+
hepatic sinusoid
glomerular
vascular endothelium
lymphatic CCL21+
lymphatic ACKR4-CCDC80+
lymphatic ACKR4+CCDC80+
lymphatic
Abbrev. Tissue
Aort Aorta
Blad Bladder
Blo Blood
Bone Bone
BM Bone marrow
BrC Brain cortex
BrS Brainstem
Col Colon
Dia Diaphragm
Ret Eye retina
Fat Fat
Hrt Heart
Pit Hypothalamus/Pituitary
Kid Kidney
LM Limb muscle
Liv Liver
Lung Lung
MG Mammary gland
Panc Pancreas
Skin Skin
SI Small intestine
Spl Spleen
Test Testes
Thym Thymus
Tong Tongue
Trac Trachea
Ute Uterus

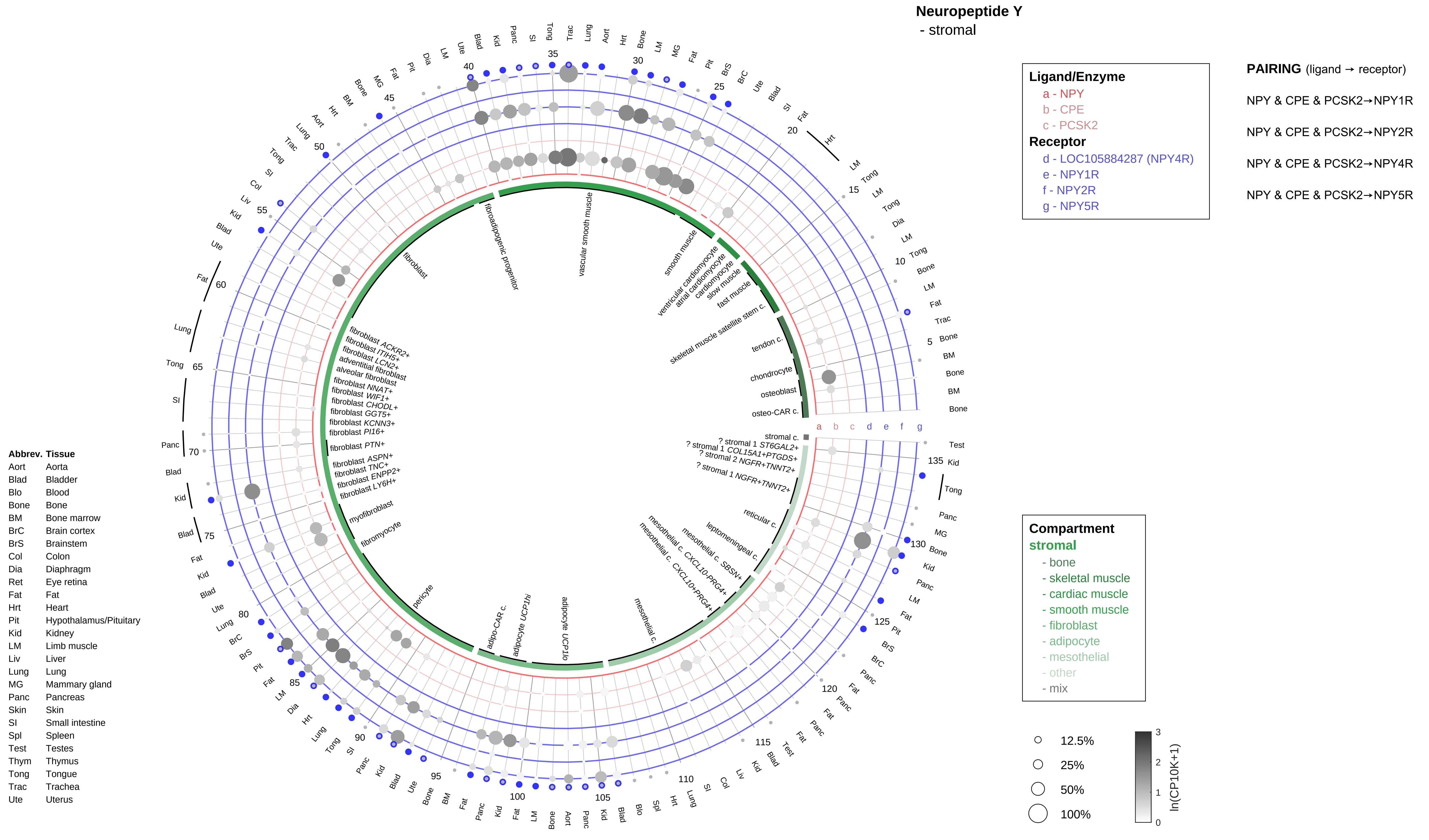

Neuropeptide Y
- stromal
Ligand/Enzyme
a - NPY
b - CPE
c - PCSK2
Receptor
d - LOC105884287 (NPY4R)
e - NPY1R
f - NPY2R
g - NPY5R
PAIRING (ligand → receptor)
NPY & CPE & PCSK2→NPY1R
NPY & CPE & PCSK2→NPY2R
NPY & CPE & PCSK2→NPY4R
NPY & CPE & PCSK2→NPY5R
Compartment
stromal
- bone
- skeletal muscle
- cardiac muscle
- smooth muscle
- fibroblast
- adipocyte
- mesothelial
- other
- mix
12.5%
25%
50%
100%
ln(CP10K+1)
0
1
2
3
Abbrev. Tissue
Aort Aorta
Blad Bladder
Blo Blood
Bone Bone
BM Bone marrow
BrC Brain cortex
BrS Brainstem
Col Colon
Dia Diaphragm
Ret Eye retina
Fat Fat
Hrt Heart
Pit Hypothalamus/Pituitary
Kid Kidney
LM Limb muscle
Liv Liver
Lung Lung
MG Mammary gland
Panc Pancreas
Skin Skin
SI Small intestine
Spl Spleen
Test Testes
Thym Thymus
Tong Tongue
Trac Trachea
Ute Uterus
vascular smooth muscle
smooth muscle
ventricular cardiomyocyte
atrial cardiomyocyte
cardiomyocyte
slow muscle
fast muscle
skeletal muscle satellite stem c.
tendon c.
chondrocyte
osteoblast
osteo-CAR c.
stromal c.
? stromal 1 ST6GAL2+
? stromal 1 COL15A1+PTGDS+
? stromal 2 NGFR+TNNT2+
? stromal 1 NGFR+TNNT2+
reticular c.
leptomeningeal c.
mesothelial c. SBSN+
mesothelial c. CXCL10-PRG4+
mesothelial c. CXCL10+PRG4+
mesothelial c.
adipocyte UCP1lo
adipocyte UCP1hi
adipo-CAR c.
pericyte
fibromyocyte
myofibroblast
fibroblast LY6H+
fibroblast ENPP2+
fibroblast TNC+
fibroblast ASPN+
fibroblast PTN+
fibroblast PI16+
fibroblast KCNN3+
fibroblast GGT5+
fibroblast CHODL+
fibroblast WIF1+
fibroblast NNAT+
alveolar fibroblast
adventitial fibroblast
fibroblast LCN2+
fibroblast ITIH5+
fibroblast ACKR2+
fibroblast
fibroadipogenic progenitor
a
b
c
d
e
f
g

Neuropeptide Y
- lymphoid

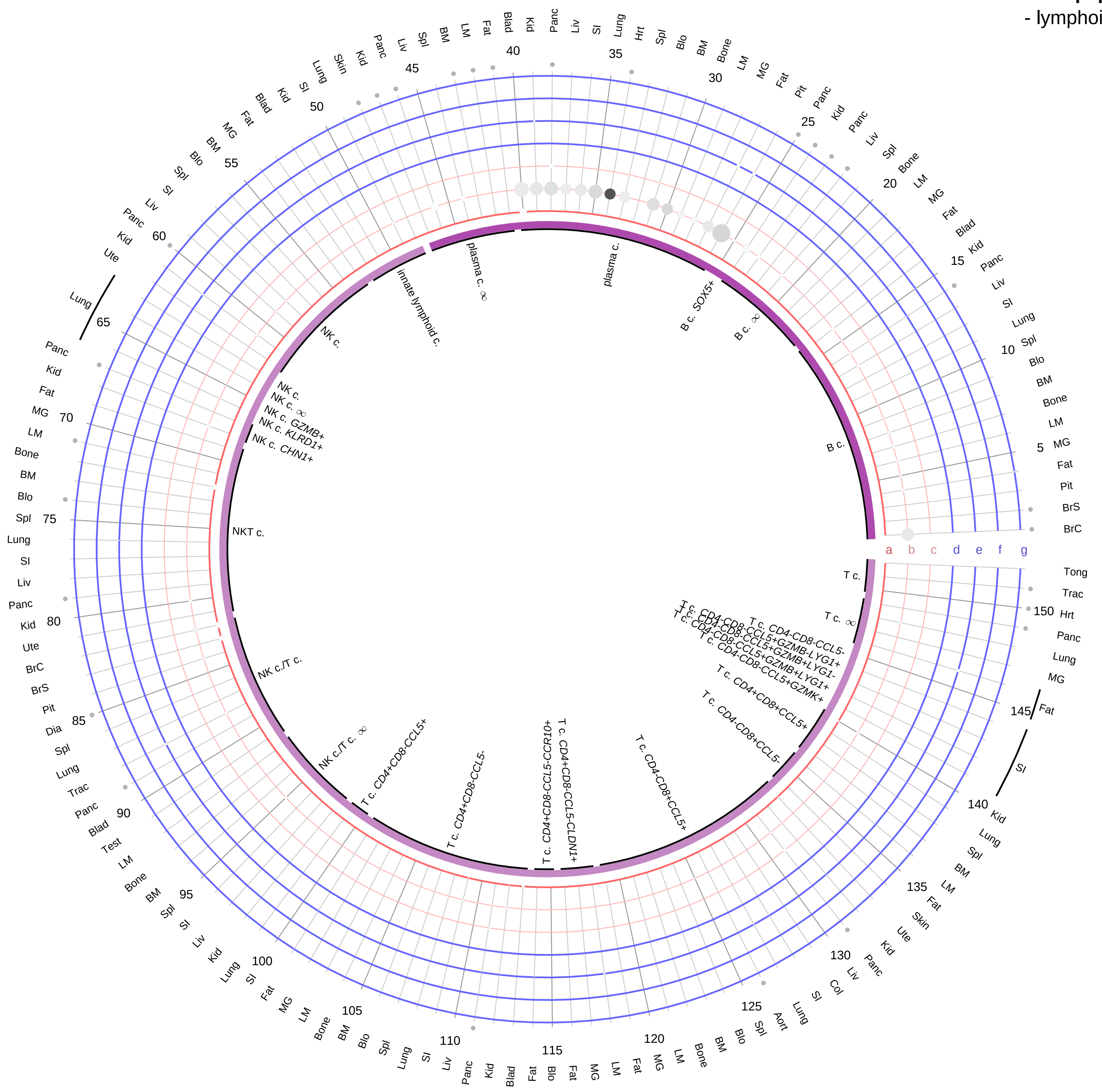

Ligand/Enzyme
- a - NPY
- b - CPE
- c - PCSK2

Receptor
- d - LOC105884287 (NPY4R)
- e - NPY1R
- f - NPY2R
- g - NPY5R

PAIRING (ligand → receptor)

NPY & CPE & PCSK2→NPY1R

NPY & CPE & PCSK2→NPY2R

NPY & CPE & PCSK2→NPY4R

NPY & CPE & PCSK2→NPY5R

Abbrev.	Tissue
Aort	Aorta
Blad	Bladder
Blo	Blood
Bone	Bone
BM	Bone marrow
BrC	Brain cortex
BrS	Brainstem
Col	Colon
Dia	Diaphragm
Ret	Eye retina
Fat	Fat
Hrt	Heart
Pit	Hypothalamus/Pituitary
Kid	Kidney
LM	Limb muscle
Liv	Liver
Lung	Lung
MG	Mammary gland
Panc	Pancreas
Skin	Skin
SI	Small intestine
Spl	Spleen
Test	Testes
Thym	Thymus
Tong	Tongue
Trac	Trachea
Ute	Uterus

Compartment

lymphoid
- B cell
- NK/T cell

12.5%
25%
50%
100%

ln(CP10K+1): 0, 1, 2, 3

Neuropeptide Y
- myeloid

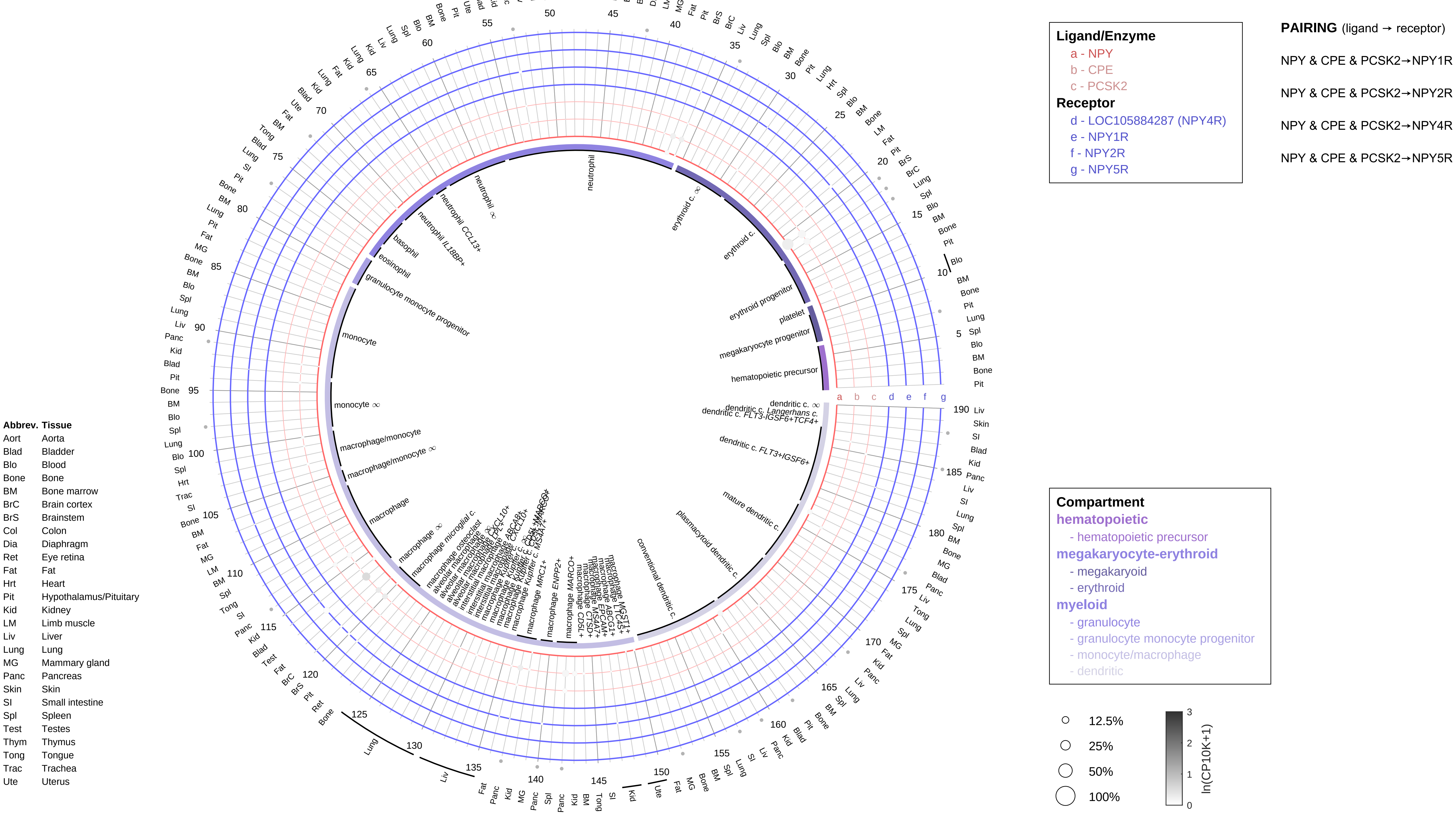

Neurotensin/Neuromedin N
- epithelial/neural/germ

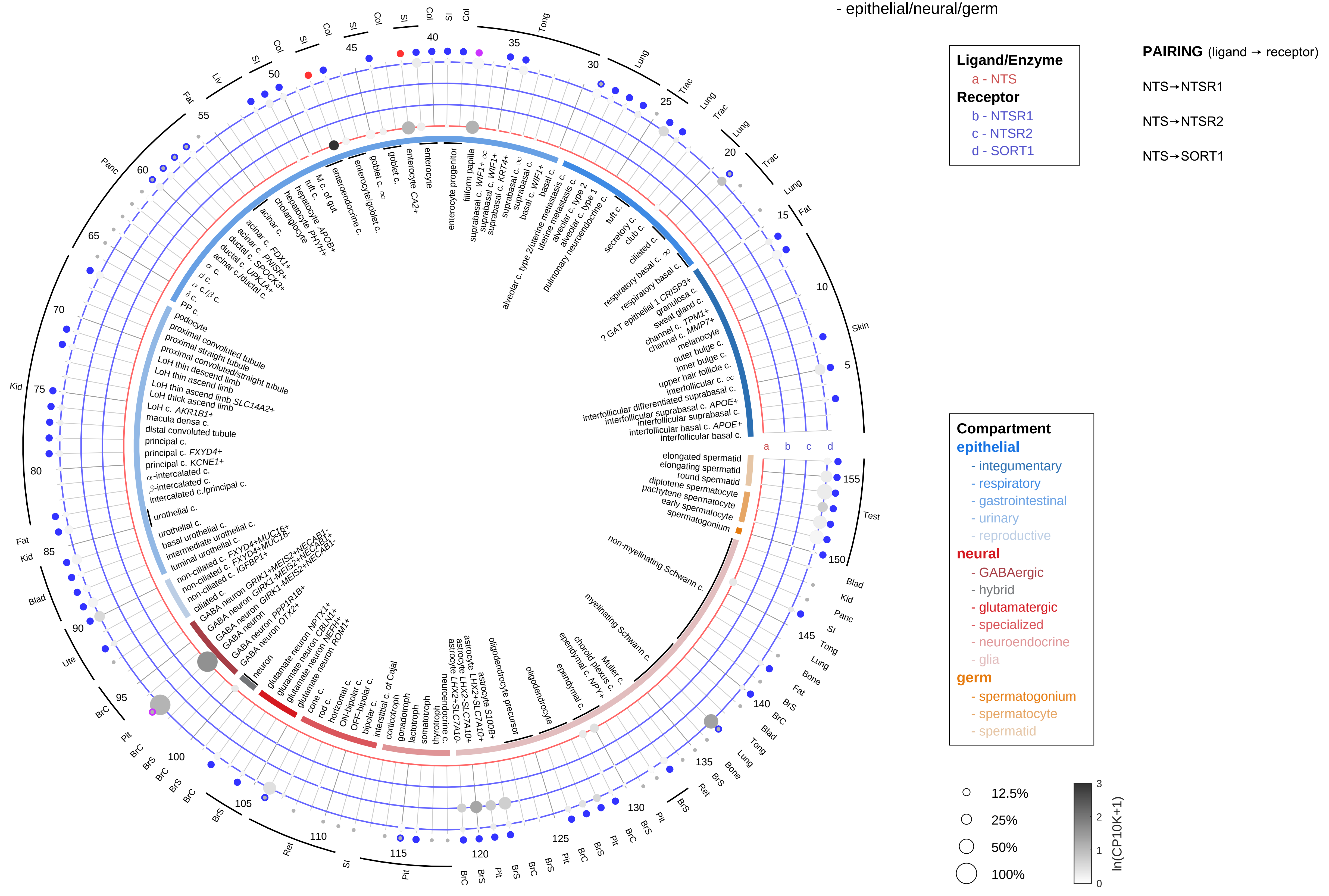

Abbrev.	Tissue
Aort	Aorta
Blad	Bladder
Blo	Blood
Bone	Bone
BM	Bone marrow
BrC	Brain cortex
BrS	Brainstem
Col	Colon
Dia	Diaphragm
Ret	Eye retina
Fat	Fat
Hrt	Heart
Pit	Hypothalamus/Pituitary
Kid	Kidney
LM	Limb muscle
Liv	Liver
Lung	Lung
MG	Mammary gland
Panc	Pancreas
Skin	Skin
SI	Small intestine
Spl	Spleen
Test	Testes
Thym	Thymus
Tong	Tongue
Trac	Trachea
Ute	Uterus

Neurotensin/Neuromedin N
- endothelial

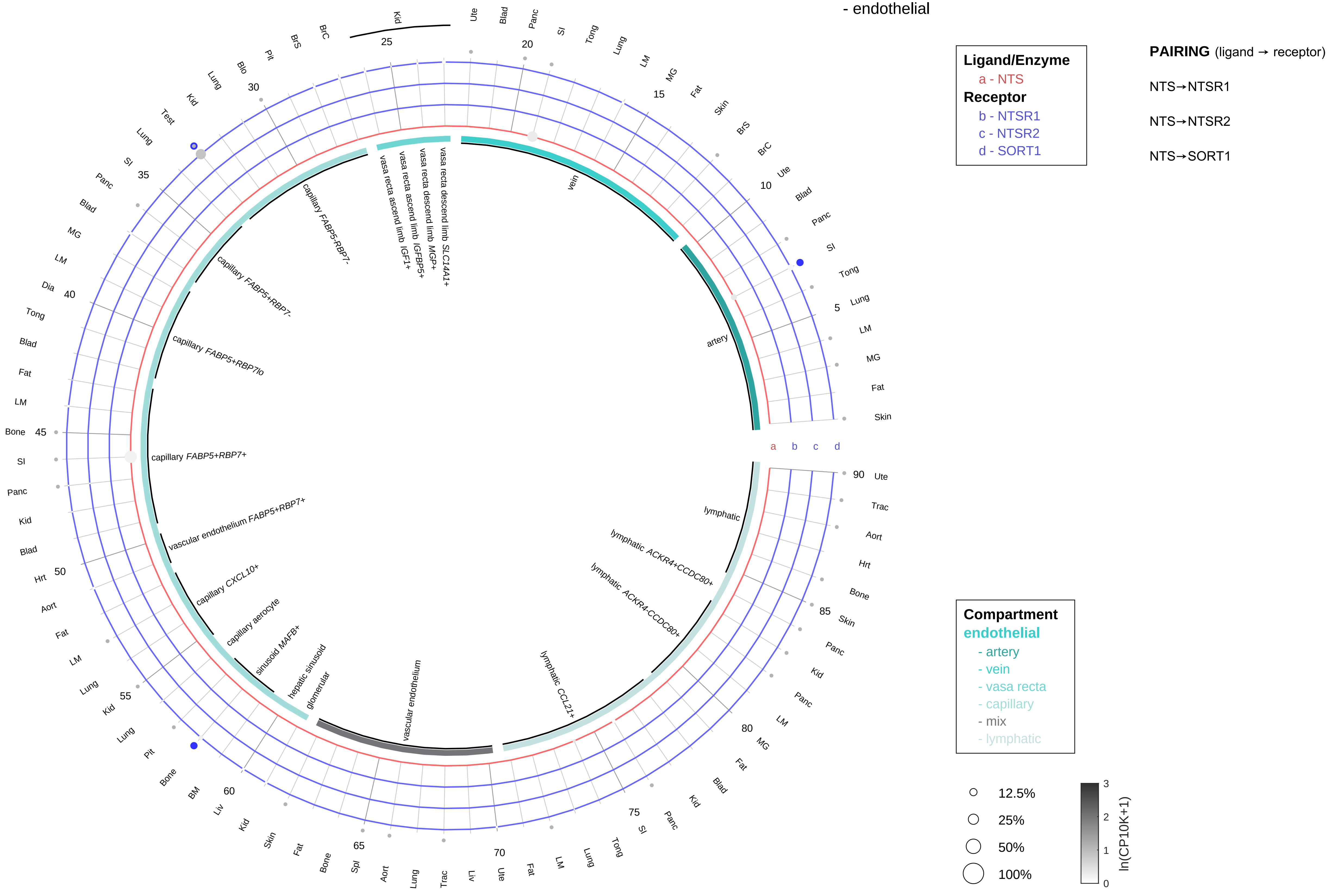

Abbrev.	Tissue
Aort	Aorta
Blad	Bladder
Blo	Blood
Bone	Bone
BM	Bone marrow
BrC	Brain cortex
BrS	Brainstem
Col	Colon
Dia	Diaphragm
Ret	Eye retina
Fat	Fat
Hrt	Heart
Pit	Hypothalamus/Pituitary
Kid	Kidney
LM	Limb muscle
Liv	Liver
Lung	Lung
MG	Mammary gland
Panc	Pancreas
Skin	Skin
SI	Small intestine
Spl	Spleen
Test	Testes
Thym	Thymus
Tong	Tongue
Trac	Trachea
Ute	Uterus

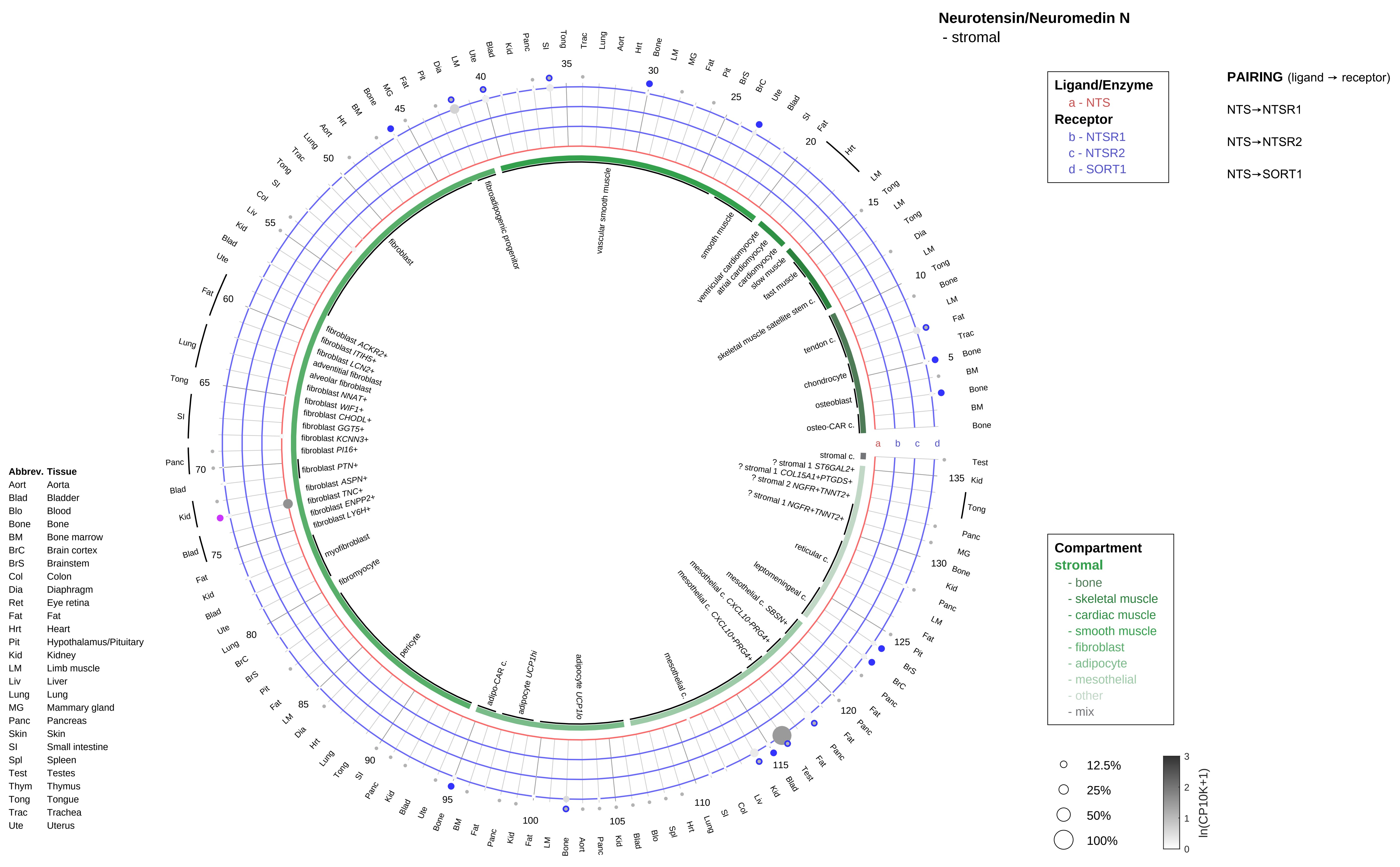

Neurotensin/Neuromedin N
- stromal
Ligand/Enzyme
a - NTS
Receptor
b - NTSR1
c - NTSR2
d - SORT1
PAIRING (ligand → receptor)
NTS→NTSR1
NTS→NTSR2
NTS→SORT1
Compartment
stromal
- bone
- skeletal muscle
- cardiac muscle
- smooth muscle
- fibroblast
- adipocyte
- mesothelial
- other
- mix
12.5%
25%
50%
100%
ln(CP10K+1)
Abbrev. Tissue
Aort Aorta
Blad Bladder
Blo Blood
Bone Bone
BM Bone marrow
BrC Brain cortex
BrS Brainstem
Col Colon
Dia Diaphragm
Ret Eye retina
Fat Fat
Hrt Heart
Pit Hypothalamus/Pituitary
Kid Kidney
LM Limb muscle
Liv Liver
Lung Lung
MG Mammary gland
Panc Pancreas
Skin Skin
SI Small intestine
Spl Spleen
Test Testes
Thym Thymus
Tong Tongue
Trac Trachea
Ute Uterus

Neurotensin/Neuromedin N
- lymphoid

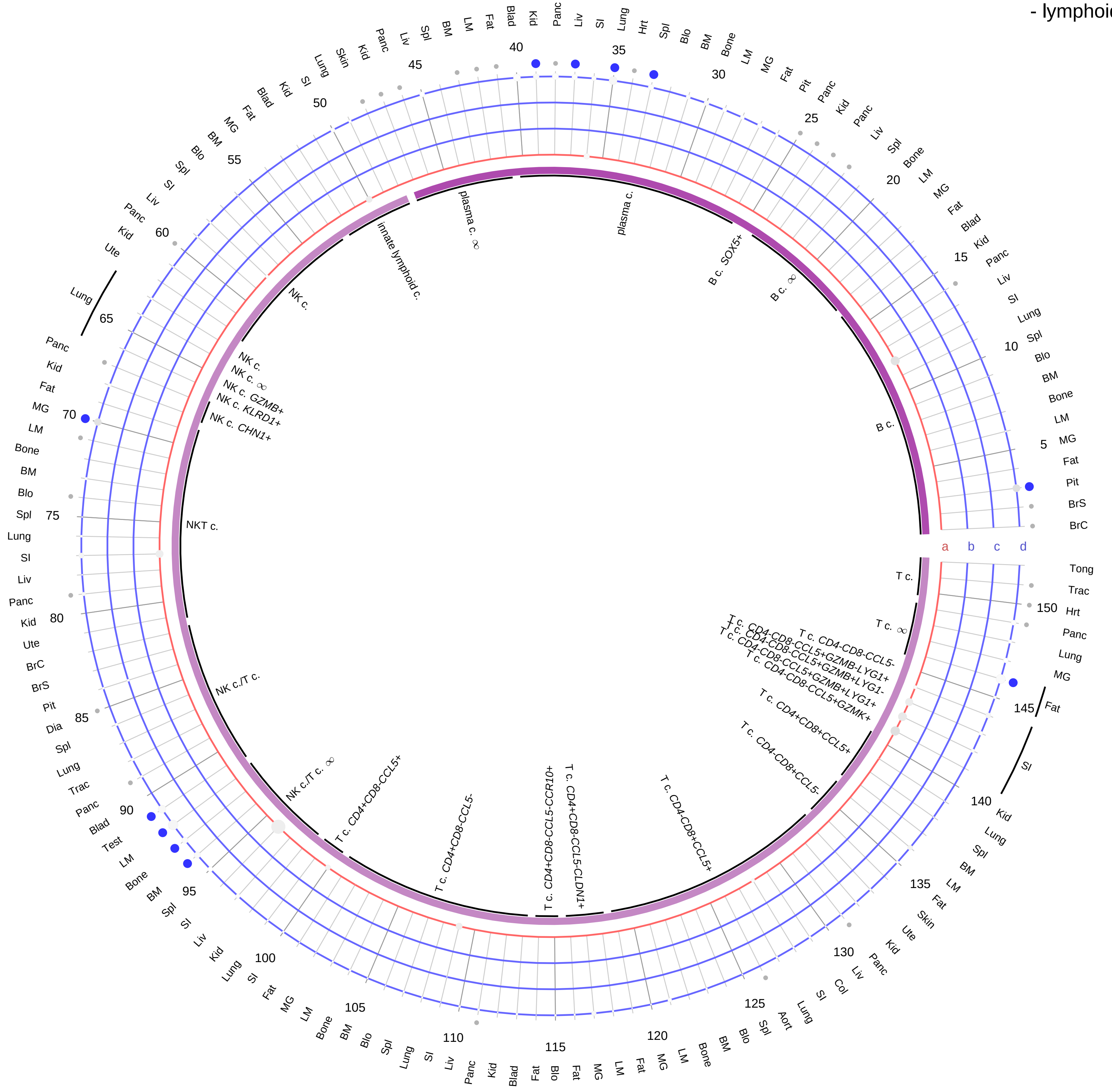

Ligand/Enzyme
a - NTS
Receptor
b - NTSR1
c - NTSR2
d - SORT1

PAIRING (ligand → receptor)

NTS→NTSR1

NTS→NTSR2

NTS→SORT1

**Abbrev.**	**Tissue**
Aort	Aorta
Blad	Bladder
Blo	Blood
Bone	Bone
BM	Bone marrow
BrC	Brain cortex
BrS	Brainstem
Col	Colon
Dia	Diaphragm
Ret	Eye retina
Fat	Fat
Hrt	Heart
Pit	Hypothalamus/Pituitary
Kid	Kidney
LM	Limb muscle
Liv	Liver
Lung	Lung
MG	Mammary gland
Panc	Pancreas
Skin	Skin
SI	Small intestine
Spl	Spleen
Test	Testes
Thym	Thymus
Tong	Tongue
Trac	Trachea
Ute	Uterus

Compartment
lymphoid
- B cell
- NK/T cell

12.5%
25%
50%
100%

ln(CP10K+1): 0, 1, 2, 3

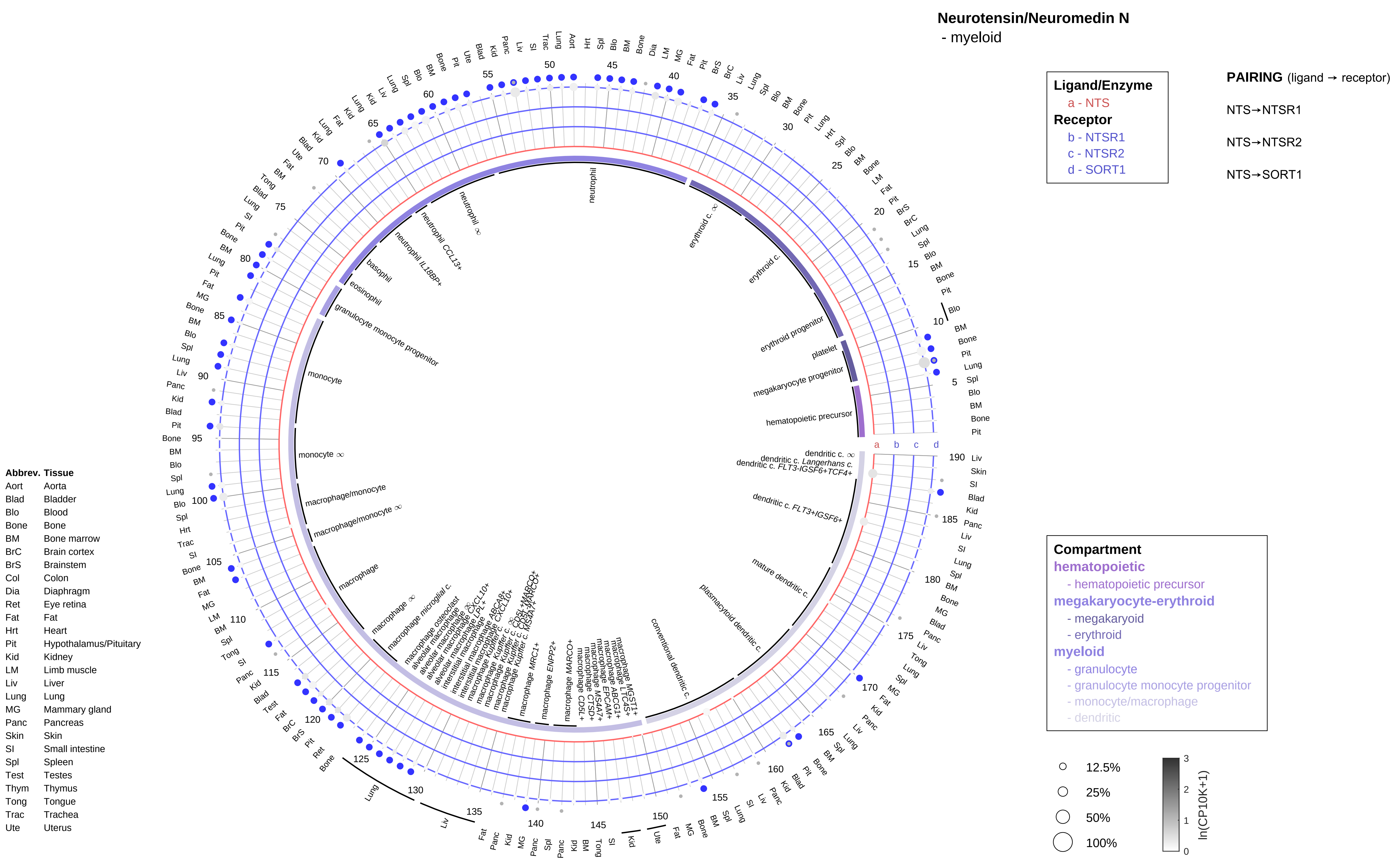

Neurotensin/Neuromedin N
- myeloid
Ligand/Enzyme
a - NTS
Receptor
b - NTSR1
c - NTSR2
d - SORT1
PAIRING (ligand → receptor)
NTS→NTSR1
NTS→NTSR2
NTS→SORT1
Compartment
hematopoietic
- hematopoietic precursor
megakaryocyte-erythroid
- megakaryoid
- erythroid
myeloid
- granulocyte
- granulocyte monocyte progenitor
- monocyte/macrophage
- dendritic
12.5%
25%
50%
100%
ln(CP10K+1)
Abbrev. Tissue
Aort Aorta
Blad Bladder
Blo Blood
Bone Bone
BM Bone marrow
BrC Brain cortex
BrS Brainstem
Col Colon
Dia Diaphragm
Ret Eye retina
Fat Fat
Hrt Heart
Pit Hypothalamus/Pituitary
Kid Kidney
LM Limb muscle
Liv Liver
Lung Lung
MG Mammary gland
Panc Pancreas
Skin Skin
SI Small intestine
Spl Spleen
Test Testes
Thym Thymus
Tong Tongue
Trac Trachea
Ute Uterus

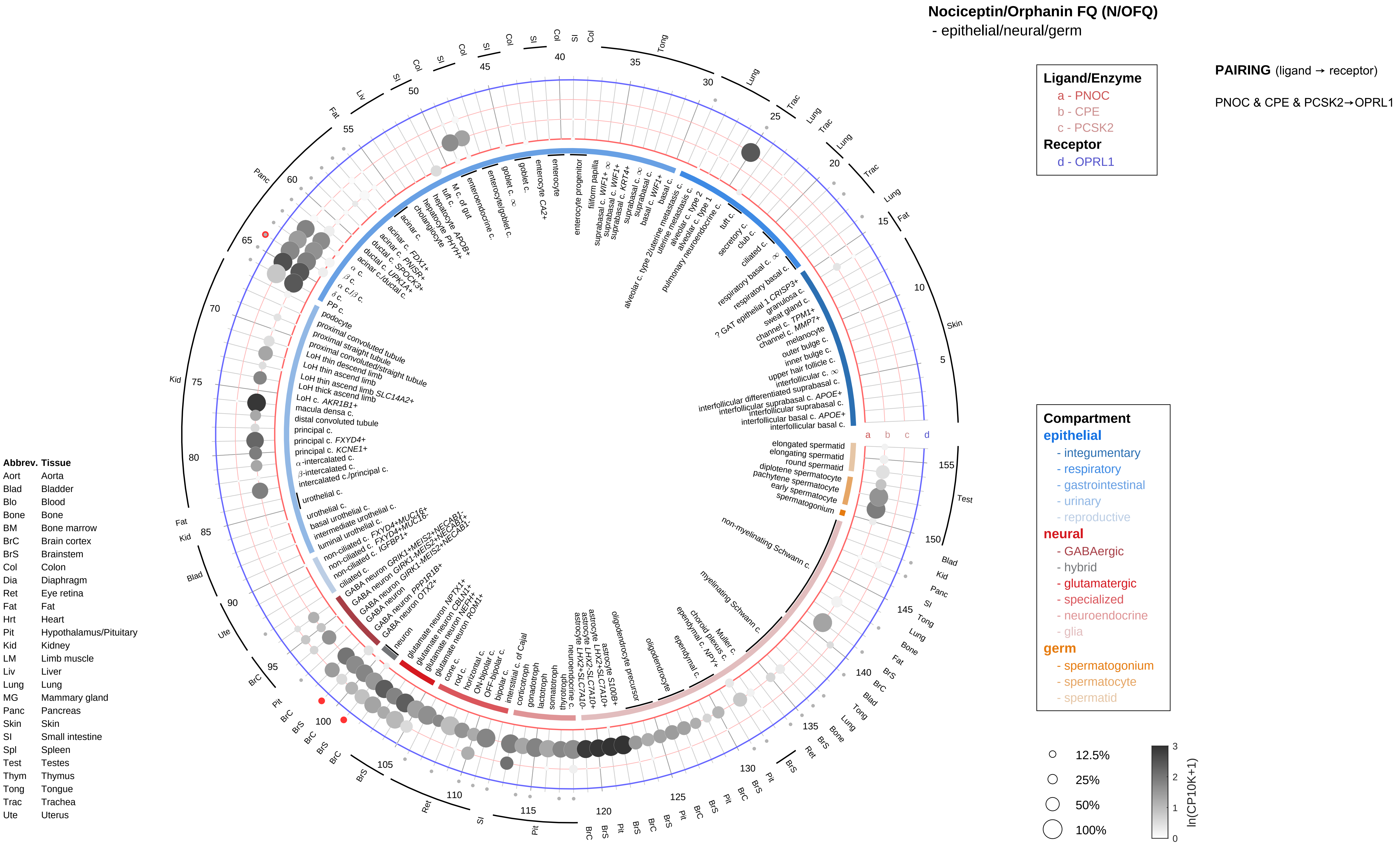

Nociceptin/Orphanin FQ (N/OFQ)
- epithelial/neural/germ
Ligand/Enzyme
a - PNOC
b - CPE
c - PCSK2
Receptor
d - OPRL1
PAIRING (ligand → receptor)
PNOC & CPE & PCSK2→OPRL1
Compartment
epithelial
- integumentary
- respiratory
- gastrointestinal
- urinary
- reproductive
neural
- GABAergic
- hybrid
- glutamatergic
- specialized
- neuroendocrine
- glia
germ
- spermatogonium
- spermatocyte
- spermatid
12.5%
25%
50%
100%
ln(CP10K+1)
Abbrev. Tissue
Aort Aorta
Blad Bladder
Blo Blood
Bone Bone
BM Bone marrow
BrC Brain cortex
BrS Brainstem
Col Colon
Dia Diaphragm
Ret Eye retina
Fat Fat
Hrt Heart
Pit Hypothalamus/Pituitary
Kid Kidney
LM Limb muscle
Liv Liver
Lung Lung
MG Mammary gland
Panc Pancreas
Skin Skin
SI Small intestine
Spl Spleen
Test Testes
Thym Thymus
Tong Tongue
Trac Trachea
Ute Uterus

Nociceptin/Orphanin FQ (N/OFQ)
- endothelial

PAIRING (ligand → receptor)

PNOC & CPE & PCSK2→OPRL1

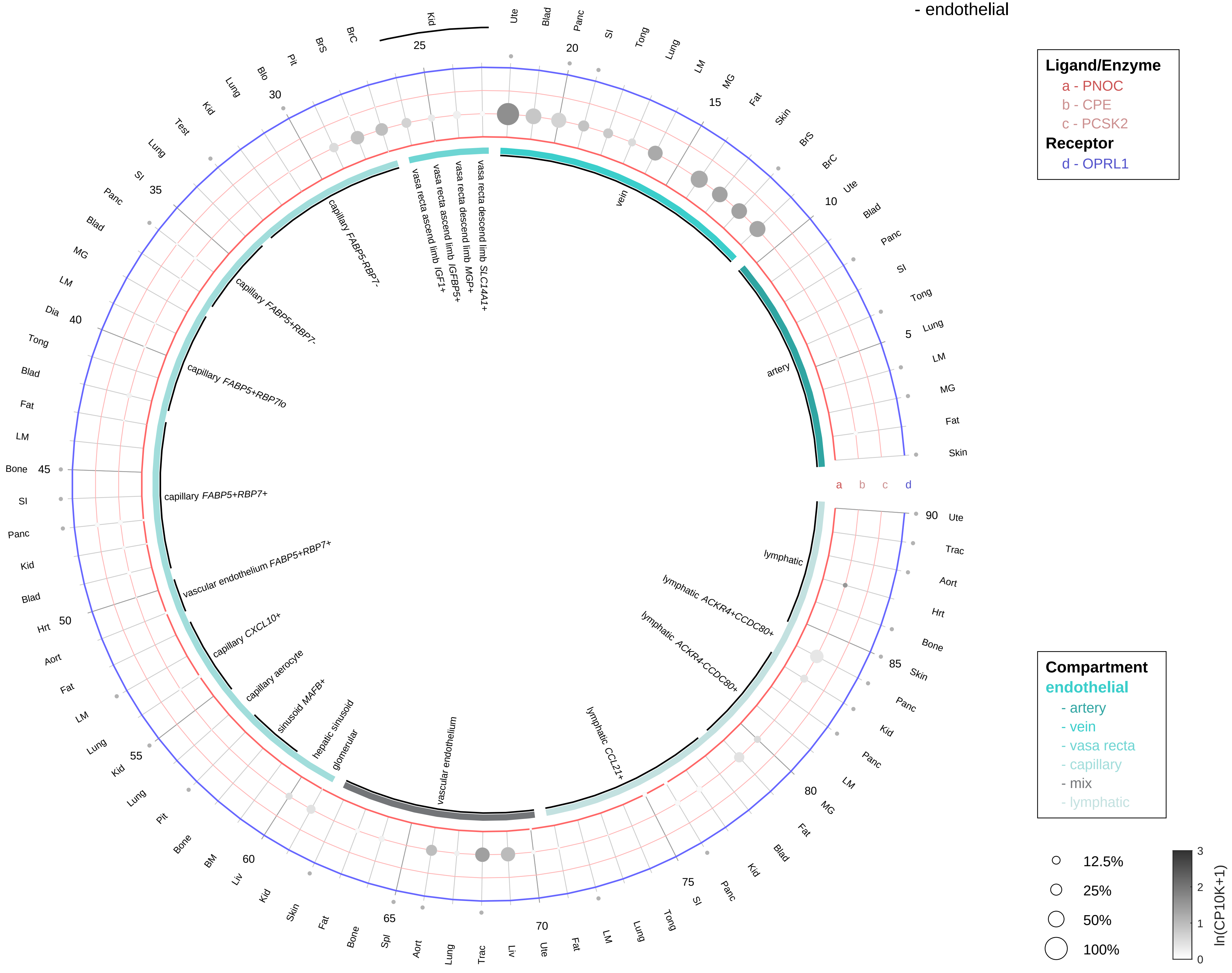

Abbrev.	Tissue
Aort	Aorta
Blad	Bladder
Blo	Blood
Bone	Bone
BM	Bone marrow
BrC	Brain cortex
BrS	Brainstem
Col	Colon
Dia	Diaphragm
Ret	Eye retina
Fat	Fat
Hrt	Heart
Pit	Hypothalamus/Pituitary
Kid	Kidney
LM	Limb muscle
Liv	Liver
Lung	Lung
MG	Mammary gland
Panc	Pancreas
Skin	Skin
SI	Small intestine
Spl	Spleen
Test	Testes
Thym	Thymus
Tong	Tongue
Trac	Trachea
Ute	Uterus

Nociceptin/Orphanin FQ (N/OFQ)
- stromal

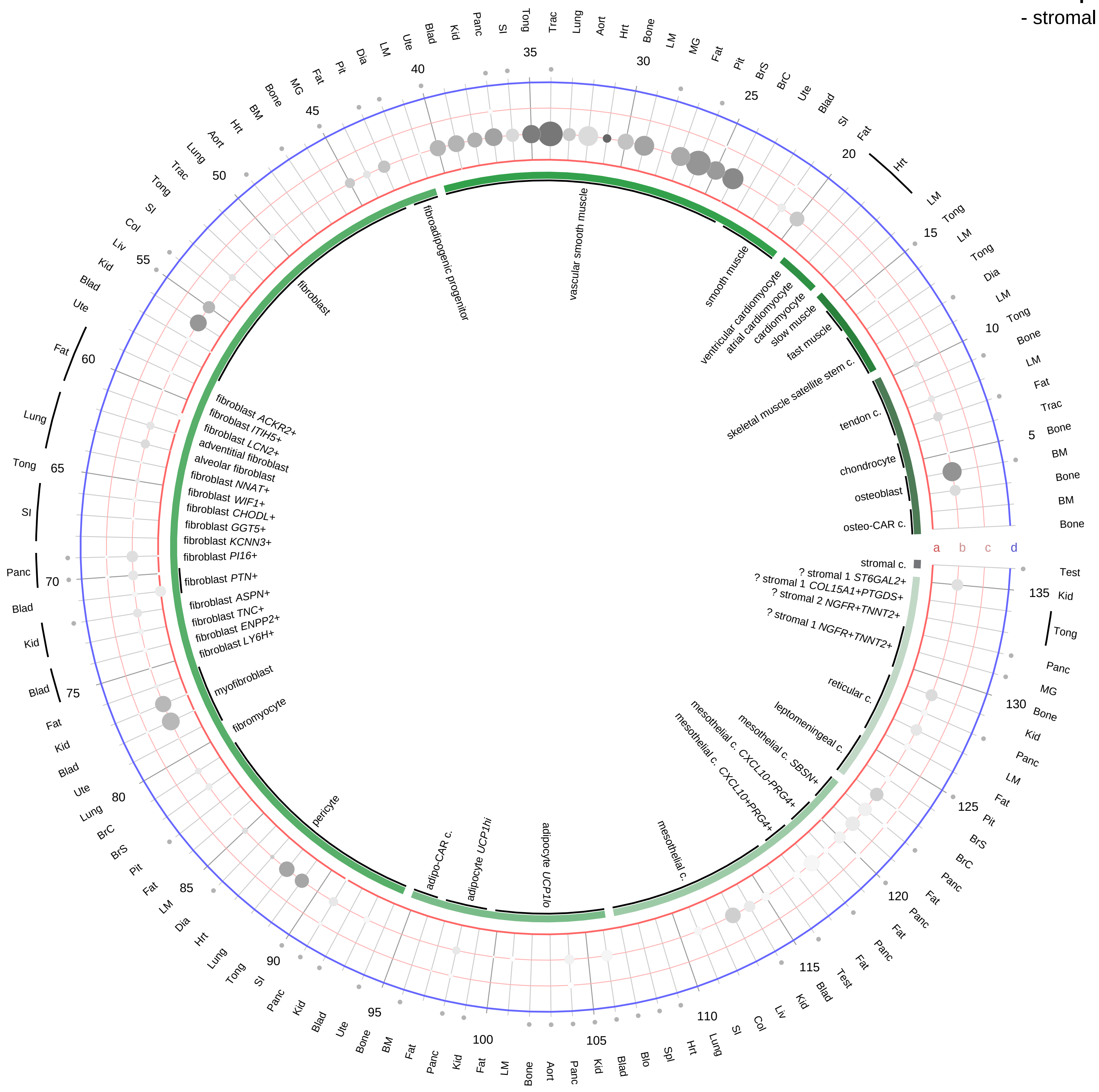

Ligand/Enzyme
- a - PNOC
- b - CPE
- c - PCSK2

Receptor
- d - OPRL1

PAIRING (ligand → receptor)

PNOC & CPE & PCSK2→OPRL1

**Abbrev.**	**Tissue**
Aort	Aorta
Blad	Bladder
Blo	Blood
Bone	Bone
BM	Bone marrow
BrC	Brain cortex
BrS	Brainstem
Col	Colon
Dia	Diaphragm
Ret	Eye retina
Fat	Fat
Hrt	Heart
Pit	Hypothalamus/Pituitary
Kid	Kidney
LM	Limb muscle
Liv	Liver
Lung	Lung
MG	Mammary gland
Panc	Pancreas
Skin	Skin
SI	Small intestine
Spl	Spleen
Test	Testes
Thym	Thymus
Tong	Tongue
Trac	Trachea
Ute	Uterus

Compartment
stromal
- bone
- skeletal muscle
- cardiac muscle
- smooth muscle
- fibroblast
- adipocyte
- mesothelial
- other
- mix

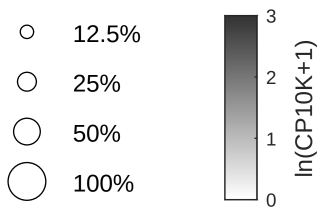

Nociceptin/Orphanin FQ (N/OFQ)
- lymphoid

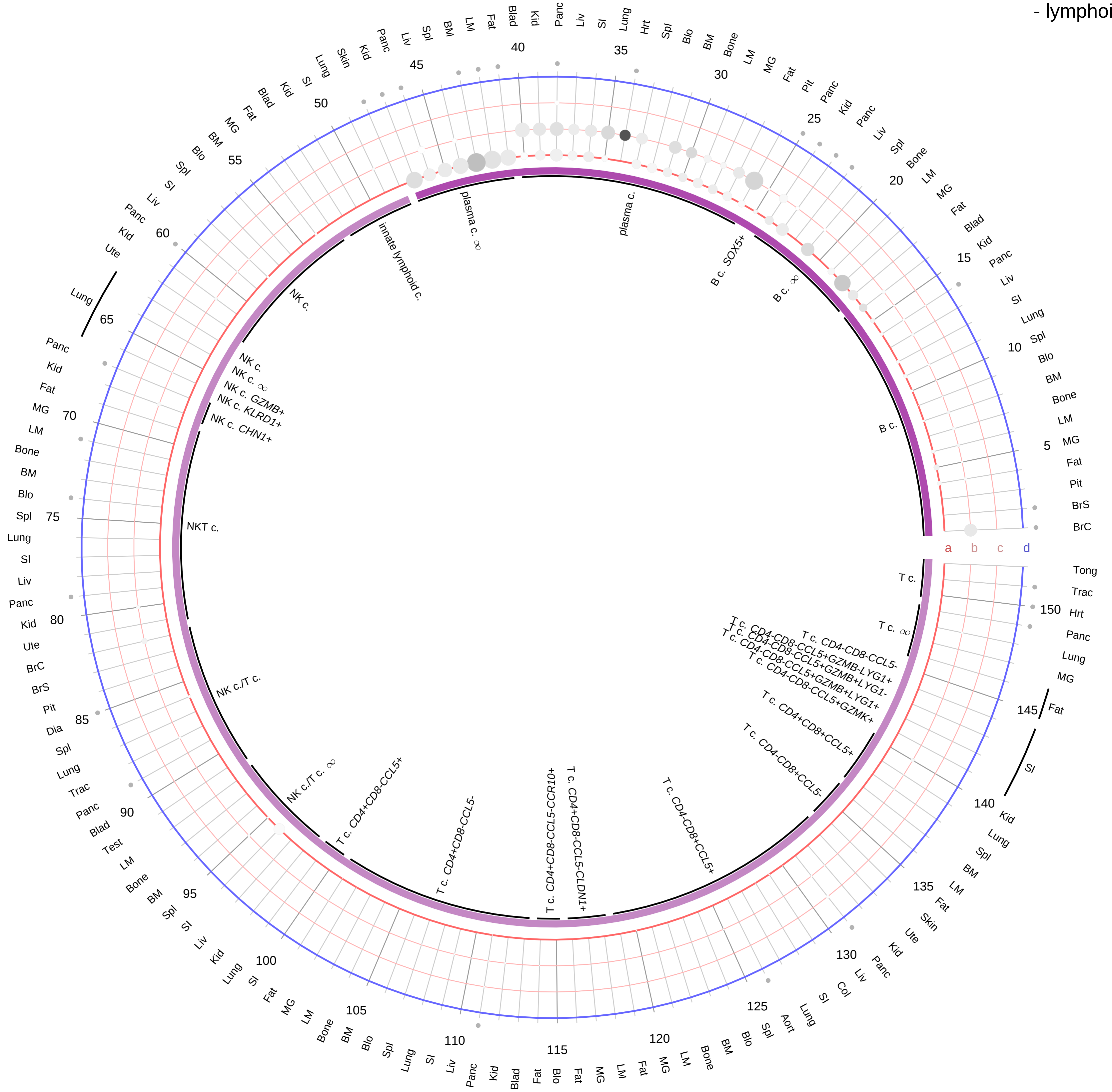

Ligand/Enzyme
- a - PNOC
- b - CPE
- c - PCSK2

Receptor
- d - OPRL1

PAIRING (ligand → receptor)

PNOC & CPE & PCSK2→OPRL1

Compartment

lymphoid
- B cell
- NK/T cell

○ 12.5%
○ 25%
○ 50%
○ 100%

ln(CP10K+1): 0, 1, 2, 3

**Abbrev.**	**Tissue**
Aort	Aorta
Blad	Bladder
Blo	Blood
Bone	Bone
BM	Bone marrow
BrC	Brain cortex
BrS	Brainstem
Col	Colon
Dia	Diaphragm
Ret	Eye retina
Fat	Fat
Hrt	Heart
Pit	Hypothalamus/Pituitary
Kid	Kidney
LM	Limb muscle
Liv	Liver
Lung	Lung
MG	Mammary gland
Panc	Pancreas
Skin	Skin
SI	Small intestine
Spl	Spleen
Test	Testes
Thym	Thymus
Tong	Tongue
Trac	Trachea
Ute	Uterus

Nociceptin/Orphanin FQ (N/OFQ)
- myeloid

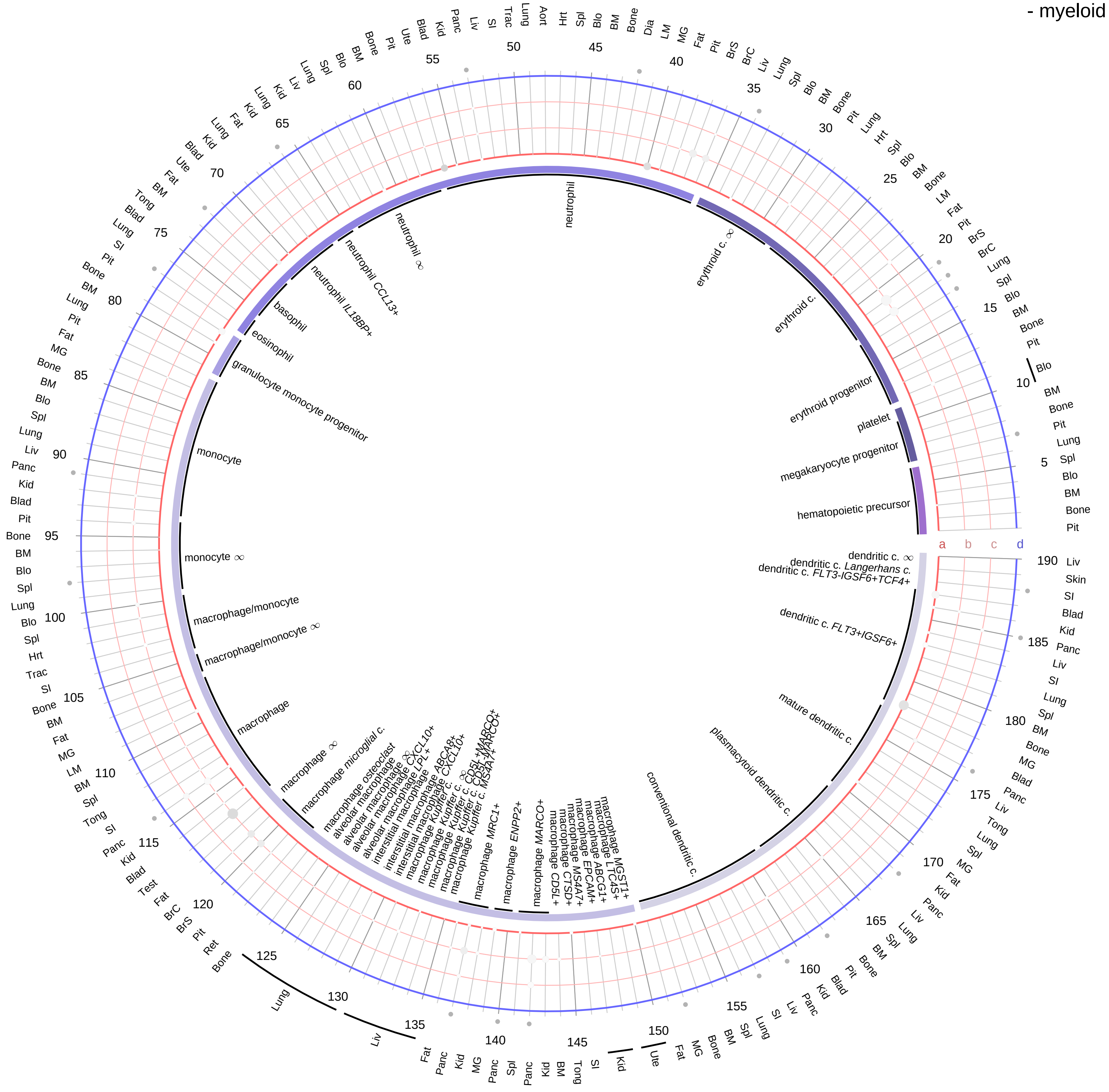

Ligand/Enzyme
- a - PNOC
- b - CPE
- c - PCSK2

Receptor
- d - OPRL1

PAIRING (ligand → receptor)

PNOC & CPE & PCSK2→OPRL1

Compartment

hematopoietic
- hematopoietic precursor

megakaryocyte-erythroid
- megakaryoid
- erythroid

myeloid
- granulocyte
- granulocyte monocyte progenitor
- monocyte/macrophage
- dendritic

Abbrev.	Tissue
Aort	Aorta
Blad	Bladder
Blo	Blood
Bone	Bone
BM	Bone marrow
BrC	Brain cortex
BrS	Brainstem
Col	Colon
Dia	Diaphragm
Ret	Eye retina
Fat	Fat
Hrt	Heart
Pit	Hypothalamus/Pituitary
Kid	Kidney
LM	Limb muscle
Liv	Liver
Lung	Lung
MG	Mammary gland
Panc	Pancreas
Skin	Skin
SI	Small intestine
Spl	Spleen
Test	Testes
Thym	Thymus
Tong	Tongue
Trac	Trachea
Ute	Uterus

12.5%
25%
50%
100%

ln(CP10K+1): 0, 1, 2, 3

Osteocalcin (OCN/BGLAP)
- epithelial/neural/germ

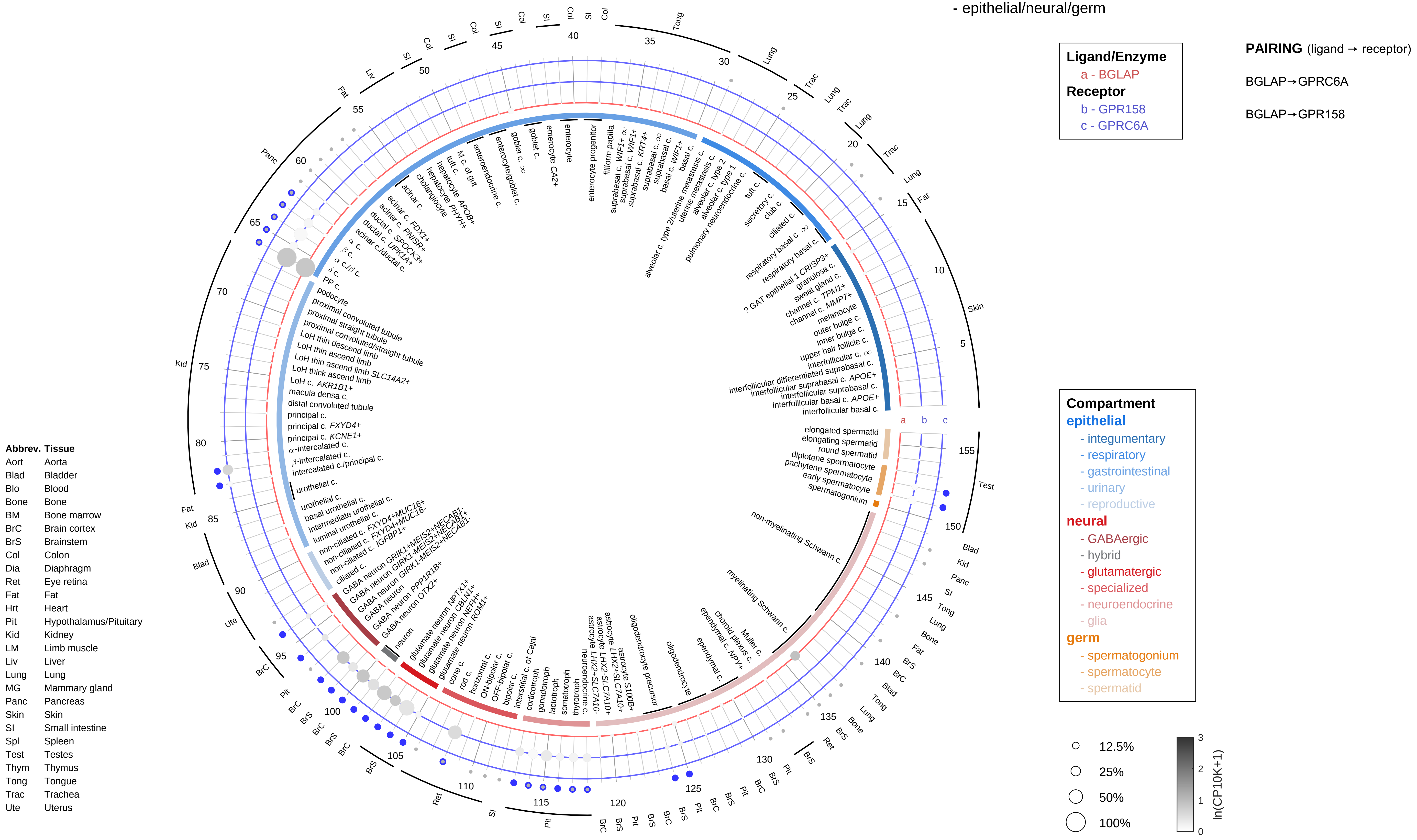

Osteocalcin (OCN/BGLAP)
- endothelial

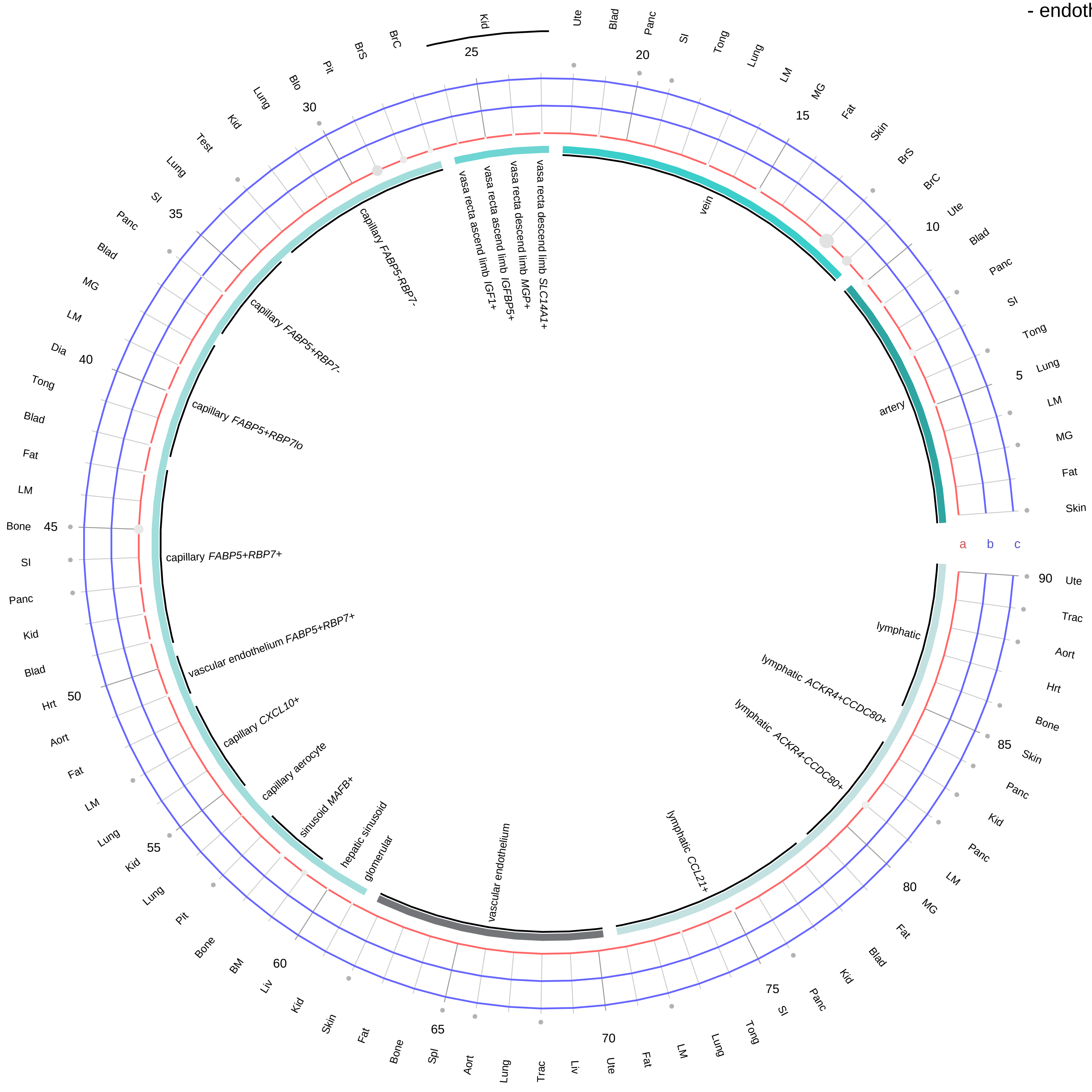

Ligand/Enzyme
a - BGLAP
Receptor
b - GPR158
c - GPRC6A

PAIRING (ligand → receptor)

BGLAP→GPRC6A

BGLAP→GPR158

**Abbrev.**	**Tissue**
Aort	Aorta
Blad	Bladder
Blo	Blood
Bone	Bone
BM	Bone marrow
BrC	Brain cortex
BrS	Brainstem
Col	Colon
Dia	Diaphragm
Ret	Eye retina
Fat	Fat
Hrt	Heart
Pit	Hypothalamus/Pituitary
Kid	Kidney
LM	Limb muscle
Liv	Liver
Lung	Lung
MG	Mammary gland
Panc	Pancreas
Skin	Skin
SI	Small intestine
Spl	Spleen
Test	Testes
Thym	Thymus
Tong	Tongue
Trac	Trachea
Ute	Uterus

Compartment
endothelial
- artery
- vein
- vasa recta
- capillary
- mix
- lymphatic

12.5%
25%
50%
100%

ln(CP10K+1): 0, 1, 2, 3

Osteocalcin (OCN/BGLAP)
- stromal

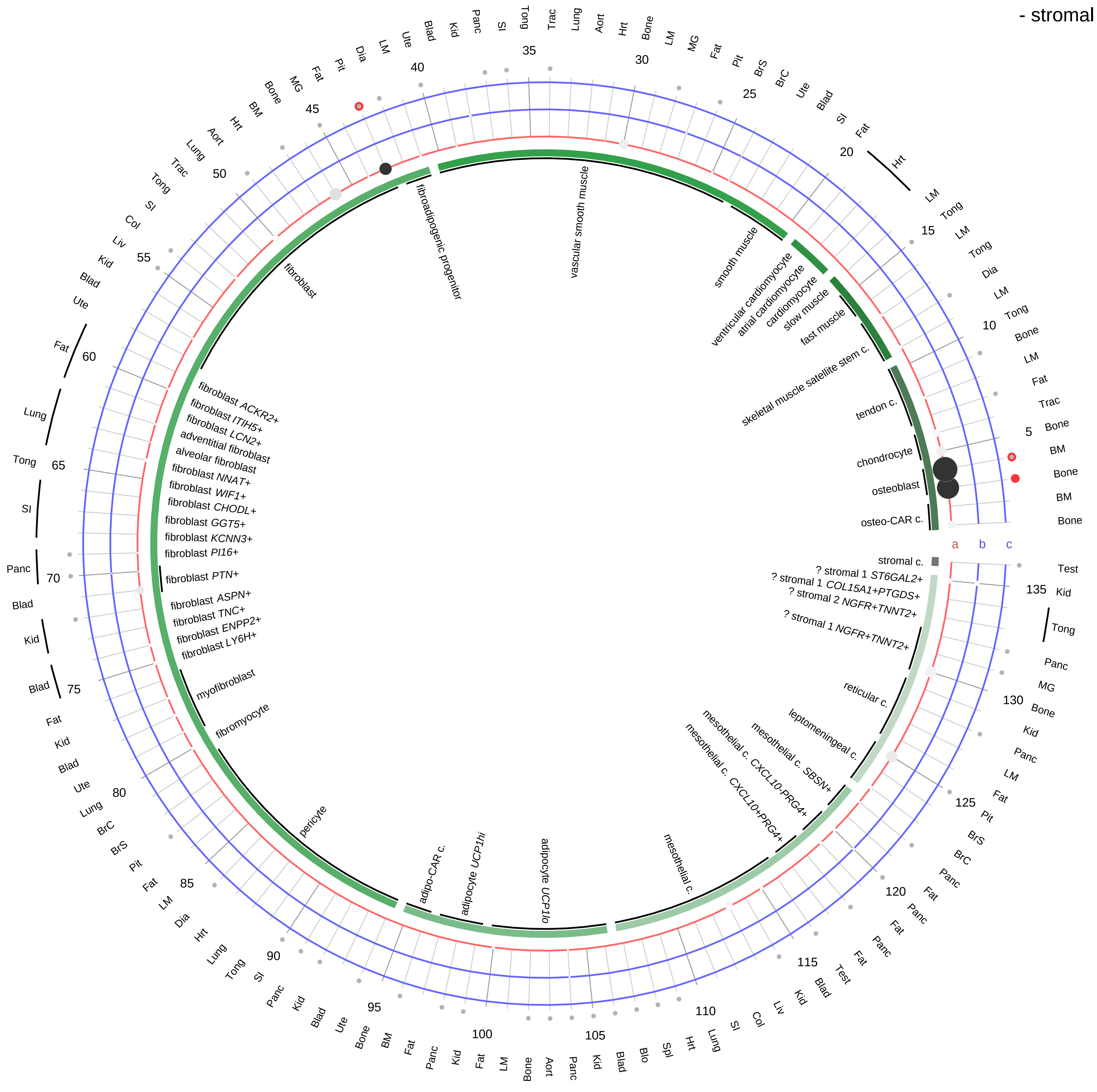

Ligand/Enzyme
a - BGLAP
Receptor
b - GPR158
c - GPRC6A

PAIRING (ligand → receptor)

BGLAP→GPRC6A

BGLAP→GPR158

Abbrev.	Tissue
Aort	Aorta
Blad	Bladder
Blo	Blood
Bone	Bone
BM	Bone marrow
BrC	Brain cortex
BrS	Brainstem
Col	Colon
Dia	Diaphragm
Ret	Eye retina
Fat	Fat
Hrt	Heart
Pit	Hypothalamus/Pituitary
Kid	Kidney
LM	Limb muscle
Liv	Liver
Lung	Lung
MG	Mammary gland
Panc	Pancreas
Skin	Skin
SI	Small intestine
Spl	Spleen
Test	Testes
Thym	Thymus
Tong	Tongue
Trac	Trachea
Ute	Uterus

Compartment
stromal
- bone
- skeletal muscle
- cardiac muscle
- smooth muscle
- fibroblast
- adipocyte
- mesothelial
- other
- mix

12.5%
25%
50%
100%

ln(CP10K+1)
0
1
2
3

Osteocalcin (OCN/BGLAP)
- lymphoid

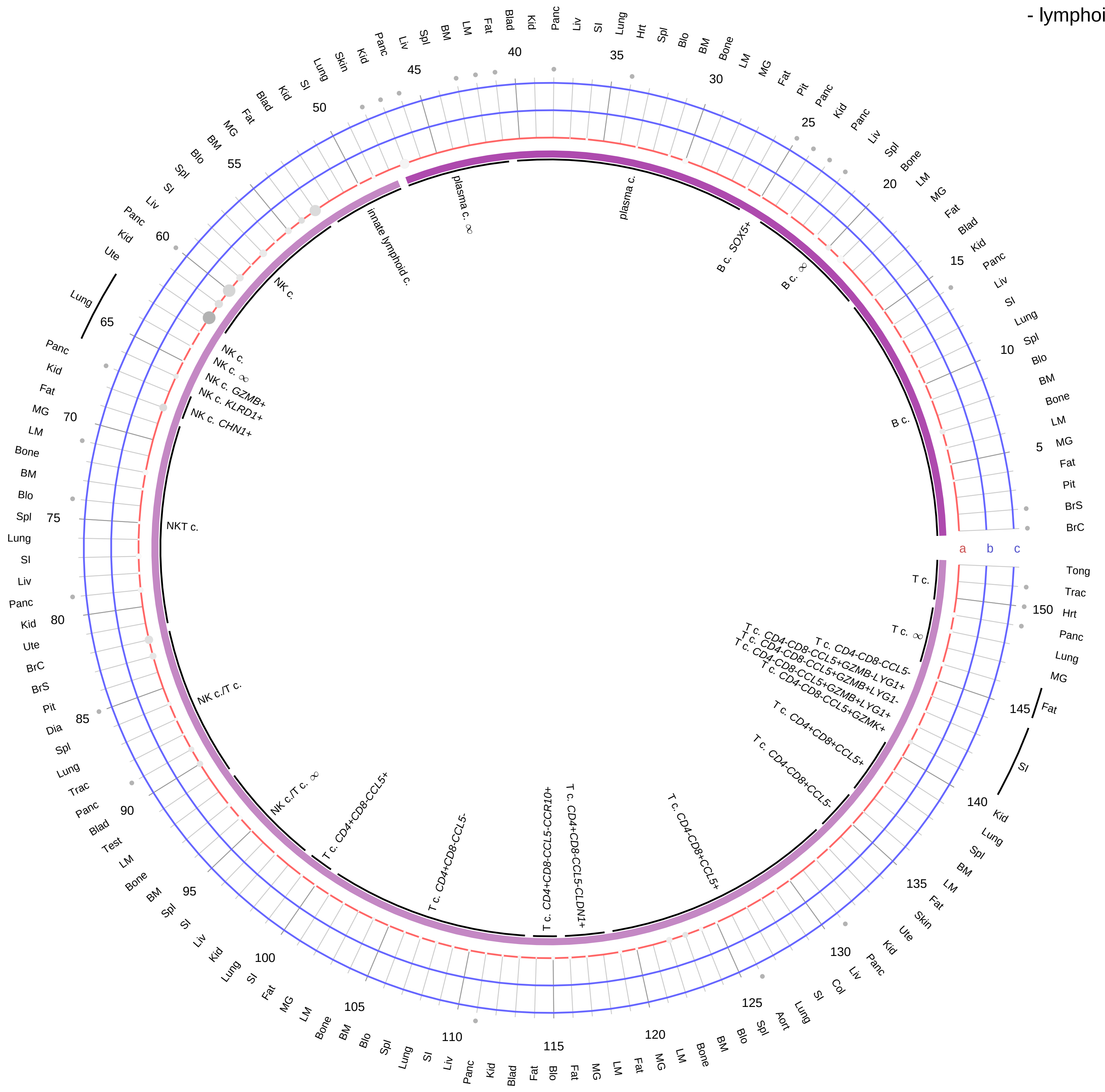

Ligand/Enzyme
a - BGLAP
Receptor
b - GPR158
c - GPRC6A

PAIRING (ligand → receptor)

BGLAP→GPRC6A

BGLAP→GPR158

Compartment
lymphoid
- B cell
- NK/T cell

12.5%
25%
50%
100%

ln(CP10K+1): 0, 1, 2, 3

Abbrev.	Tissue
Aort	Aorta
Blad	Bladder
Blo	Blood
Bone	Bone
BM	Bone marrow
BrC	Brain cortex
BrS	Brainstem
Col	Colon
Dia	Diaphragm
Ret	Eye retina
Fat	Fat
Hrt	Heart
Pit	Hypothalamus/Pituitary
Kid	Kidney
LM	Limb muscle
Liv	Liver
Lung	Lung
MG	Mammary gland
Panc	Pancreas
Skin	Skin
SI	Small intestine
Spl	Spleen
Test	Testes
Thym	Thymus
Tong	Tongue
Trac	Trachea
Ute	Uterus

Osteocalcin (OCN/BGLAP)
- myeloid

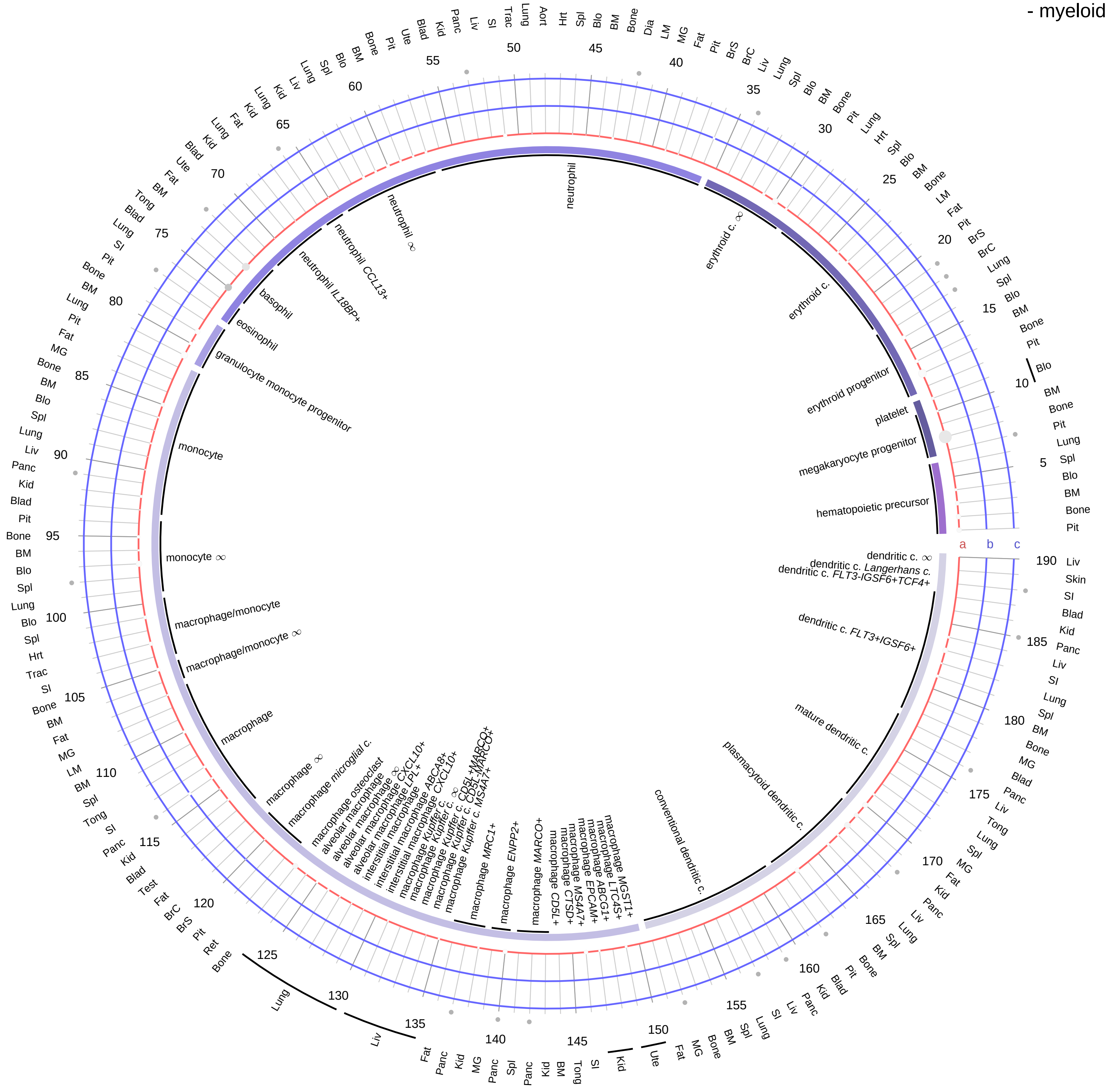

Ligand/Enzyme
a - BGLAP
**Receptor**
b - GPR158
c - GPRC6A

PAIRING (ligand → receptor)

BGLAP→GPRC6A

BGLAP→GPR158

Abbrev.	Tissue
Aort	Aorta
Blad	Bladder
Blo	Blood
Bone	Bone
BM	Bone marrow
BrC	Brain cortex
BrS	Brainstem
Col	Colon
Dia	Diaphragm
Ret	Eye retina
Fat	Fat
Hrt	Heart
Pit	Hypothalamus/Pituitary
Kid	Kidney
LM	Limb muscle
Liv	Liver
Lung	Lung
MG	Mammary gland
Panc	Pancreas
Skin	Skin
SI	Small intestine
Spl	Spleen
Test	Testes
Thym	Thymus
Tong	Tongue
Trac	Trachea
Ute	Uterus

Compartment

hematopoietic
- hematopoietic precursor

megakaryocyte-erythroid
- megakaryoid
- erythroid

myeloid
- granulocyte
- granulocyte monocyte progenitor
- monocyte/macrophage
- dendritic

12.5%

25%

50%

100%

ln(CP10K+1): 0, 1, 2, 3

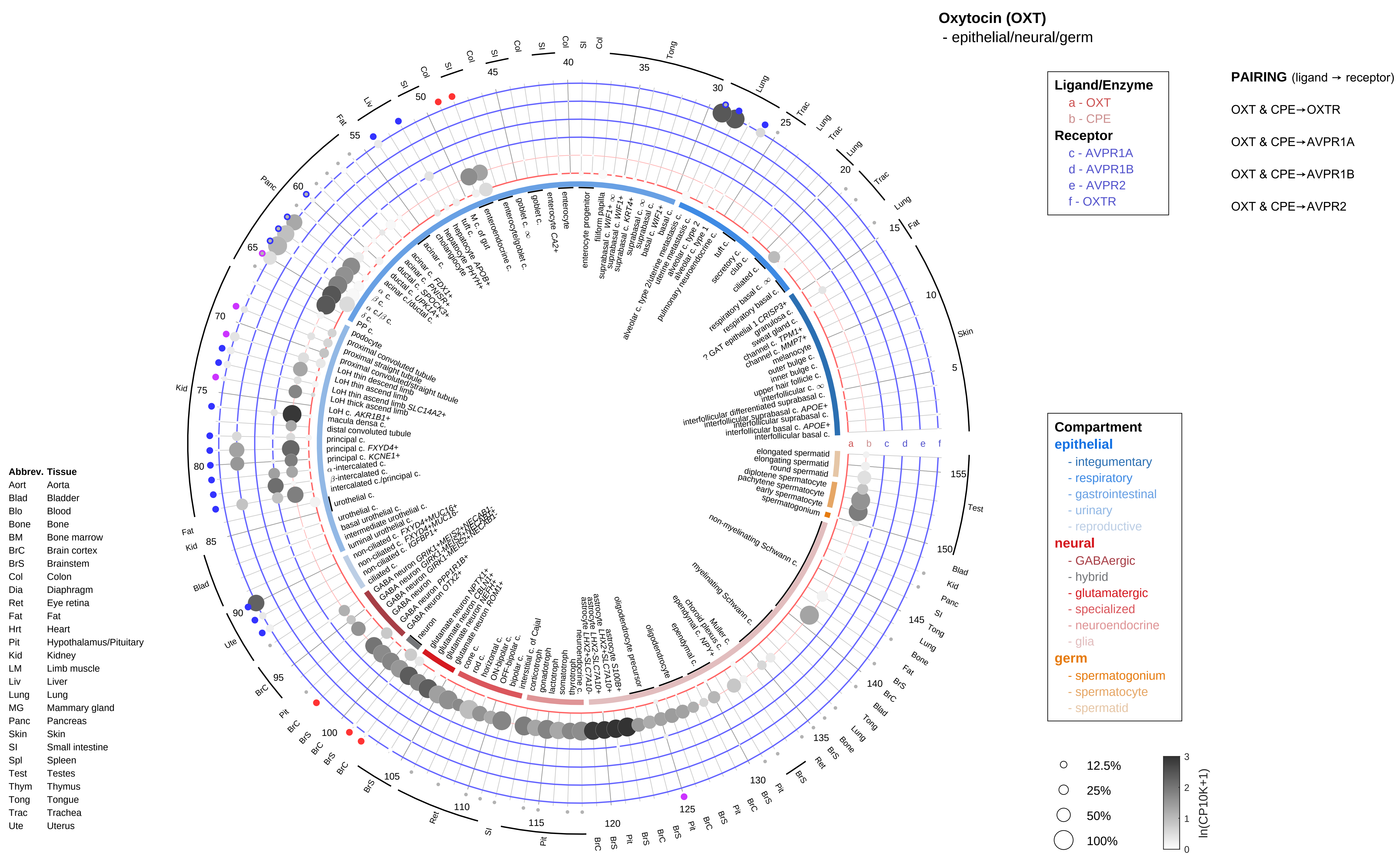
Oxytocin (OXT)
- epithelial/neural/germ
Ligand/Enzyme
a - OXT
b - CPE
Receptor
c - AVPR1A
d - AVPR1B
e - AVPR2
f - OXTR
PAIRING (ligand → receptor)
OXT & CPE→OXTR
OXT & CPE→AVPR1A
OXT & CPE→AVPR1B
OXT & CPE→AVPR2
Compartment
epithelial
- integumentary
- respiratory
- gastrointestinal
- urinary
- reproductive
neural
- GABAergic
- hybrid
- glutamatergic
- specialized
- neuroendocrine
- glia
germ
- spermatogonium
- spermatocyte
- spermatid
12.5%
25%
50%
100%
ln(CP10K+1)
Abbrev. Tissue
Aort Aorta
Blad Bladder
Blo Blood
Bone Bone
BM Bone marrow
BrC Brain cortex
BrS Brainstem
Col Colon
Dia Diaphragm
Ret Eye retina
Fat Fat
Hrt Heart
Pit Hypothalamus/Pituitary
Kid Kidney
LM Limb muscle
Liv Liver
Lung Lung
MG Mammary gland
Panc Pancreas
Skin Skin
SI Small intestine
Spl Spleen
Test Testes
Thym Thymus
Tong Tongue
Trac Trachea
Ute Uterus

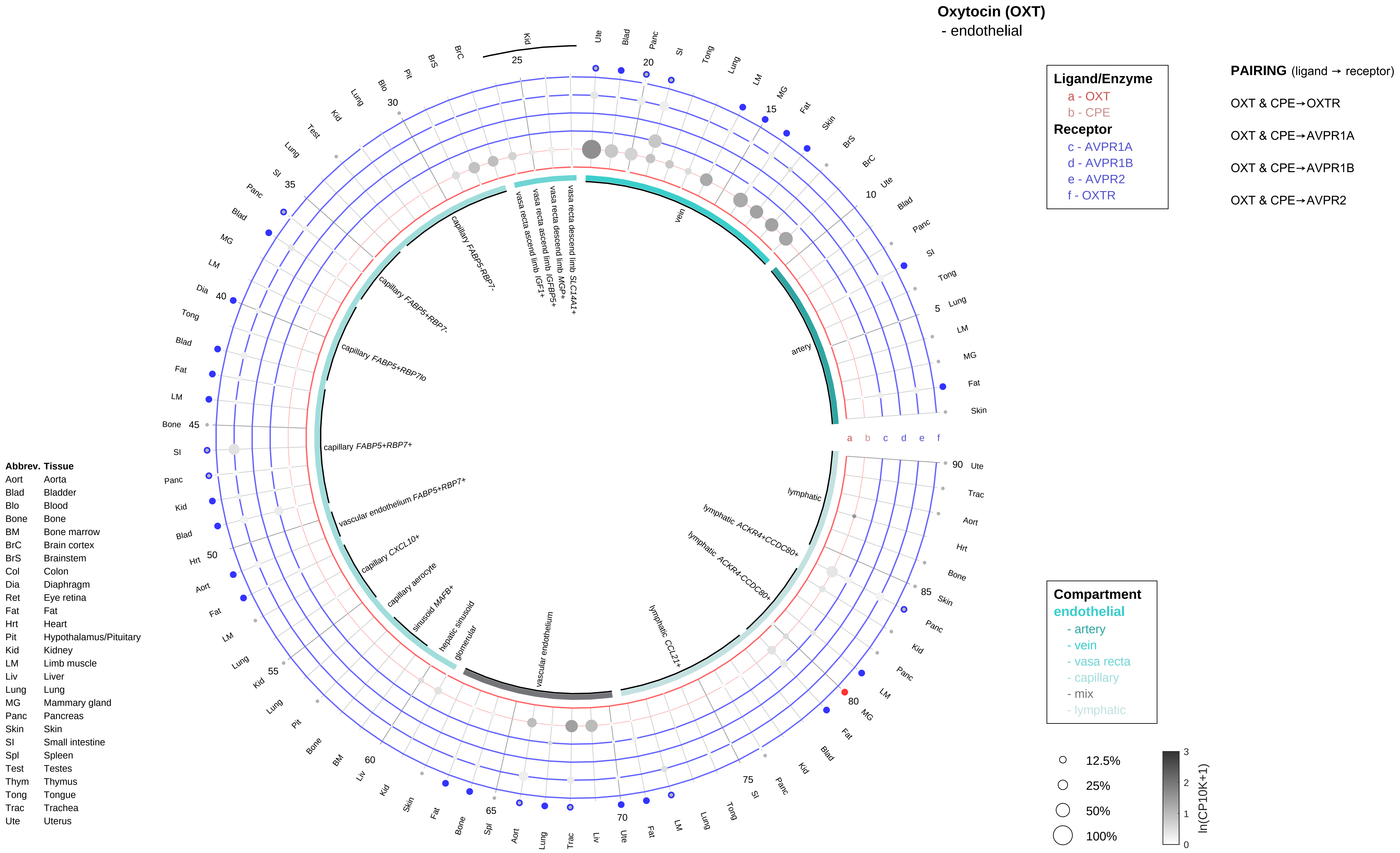

Oxytocin (OXT)
- endothelial
Ligand/Enzyme
a - OXT
b - CPE
Receptor
c - AVPR1A
d - AVPR1B
e - AVPR2
f - OXTR
PAIRING (ligand → receptor)
OXT & CPE→OXTR
OXT & CPE→AVPR1A
OXT & CPE→AVPR1B
OXT & CPE→AVPR2
Compartment
endothelial
- artery
- vein
- vasa recta
- capillary
- mix
- lymphatic
12.5%
25%
50%
100%
ln(CP10K+1)
0
1
2
3
artery
vein
vasa recta descend limb SLC14A1+
vasa recta descend limb MGP+
vasa recta ascend limb IGFBP5+
vasa recta ascend limb IGF1+
capillary FABP5-RBP7-
capillary FABP5+RBP7-
capillary FABP5+RBP7lo
capillary FABP5+RBP7+
vascular endothelium FABP5+RBP7+
capillary CXCL10+
capillary aerocyte
sinusoid MAFB+
hepatic sinusoid
glomerular
vascular endothelium
lymphatic CCL21+
lymphatic ACKR4-CCDC80+
lymphatic ACKR4+CCDC80+
lymphatic
a
b
c
d
e
f
Abbrev. Tissue
Aort Aorta
Blad Bladder
Blo Blood
Bone Bone
BM Bone marrow
BrC Brain cortex
BrS Brainstem
Col Colon
Dia Diaphragm
Ret Eye retina
Fat Fat
Hrt Heart
Pit Hypothalamus/Pituitary
Kid Kidney
LM Limb muscle
Liv Liver
Lung Lung
MG Mammary gland
Panc Pancreas
Skin Skin
SI Small intestine
Spl Spleen
Test Testes
Thym Thymus
Tong Tongue
Trac Trachea
Ute Uterus

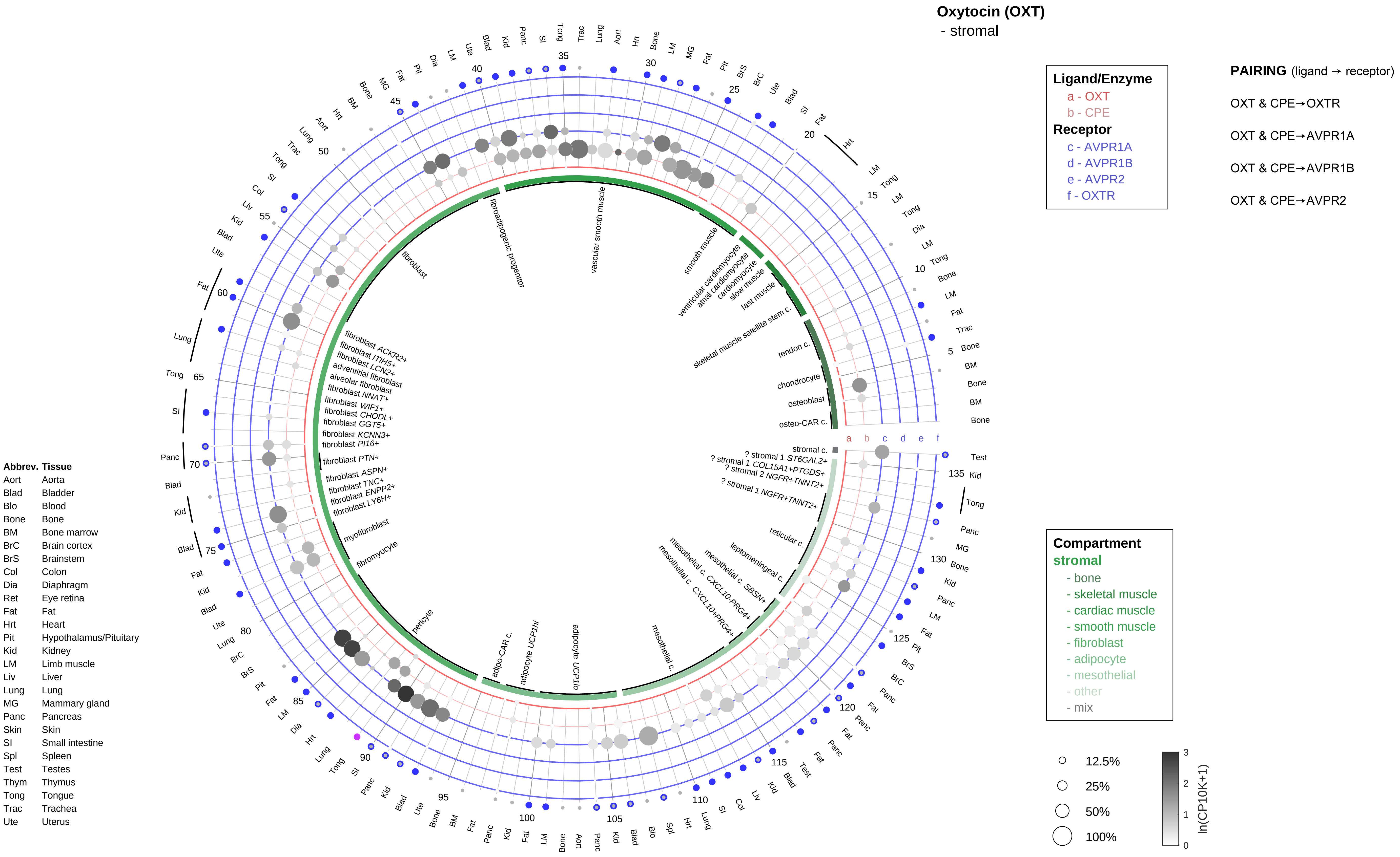
Oxytocin (OXT)
- stromal
Ligand/Enzyme
a - OXT
b - CPE
Receptor
c - AVPR1A
d - AVPR1B
e - AVPR2
f - OXTR
PAIRING (ligand → receptor)
OXT & CPE→OXTR
OXT & CPE→AVPR1A
OXT & CPE→AVPR1B
OXT & CPE→AVPR2
Compartment
stromal
- bone
- skeletal muscle
- cardiac muscle
- smooth muscle
- fibroblast
- adipocyte
- mesothelial
- other
- mix
12.5%
25%
50%
100%
ln(CP10K+1)
0
1
2
3
Abbrev. Tissue
Aort Aorta
Blad Bladder
Blo Blood
Bone Bone
BM Bone marrow
BrC Brain cortex
BrS Brainstem
Col Colon
Dia Diaphragm
Ret Eye retina
Fat Fat
Hrt Heart
Pit Hypothalamus/Pituitary
Kid Kidney
LM Limb muscle
Liv Liver
Lung Lung
MG Mammary gland
Panc Pancreas
Skin Skin
SI Small intestine
Spl Spleen
Test Testes
Thym Thymus
Tong Tongue
Trac Trachea
Ute Uterus
fibroblast
fibroadipogenic progenitor
vascular smooth muscle
smooth muscle
ventricular cardiomyocyte
atrial cardiomyocyte
cardiomyocyte
slow muscle
fast muscle
skeletal muscle satellite stem c.
tendon c.
chondrocyte
osteoblast
osteo-CAR c.
stromal c.
? stromal 1 ST6GAL2+
? stromal 1 COL15A1+PTGDS+
? stromal 2 NGFR+TNNT2+
? stromal 1 NGFR+TNNT2+
reticular c.
leptomeningeal c.
mesothelial c. SBSN+
mesothelial c. CXCL10+PRG4+
mesothelial c. CXCL10+PRG4+
mesothelial c.
adipocyte UCP1lo
adipocyte UCP1hi
adipo-CAR c.
pericyte
fibromyocyte
myofibroblast
fibroblast LY6H+
fibroblast ENPP2+
fibroblast TNC+
fibroblast ASPN+
fibroblast PTN+
fibroblast PI16+
fibroblast KCNN3+
fibroblast GGT5+
fibroblast CHODL+
fibroblast WIF1+
fibroblast NNAT+
alveolar fibroblast
adventitial fibroblast
fibroblast LCN2+
fibroblast ITIH5+
fibroblast ACKR2+
a
b
c
d
e
f

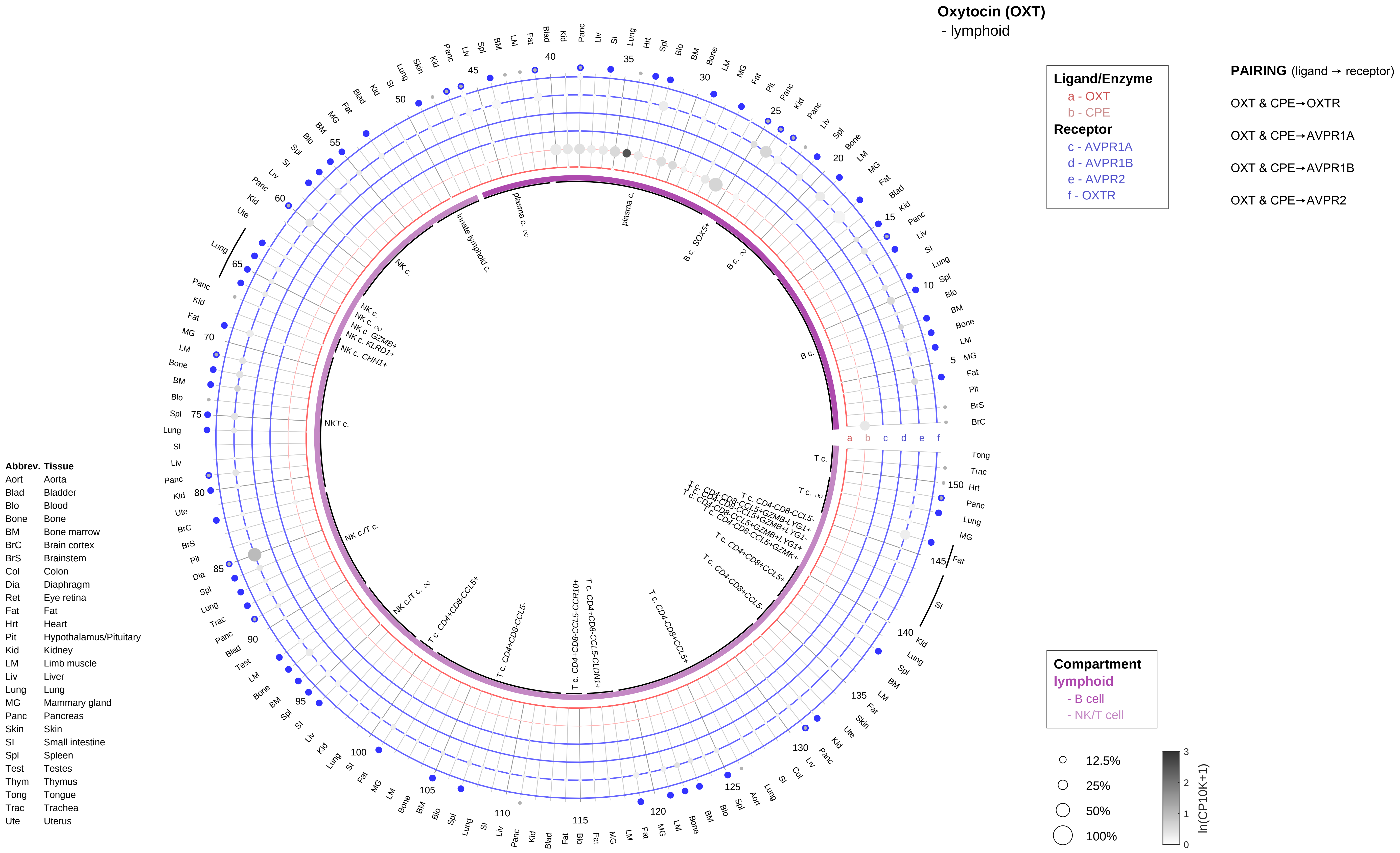

Oxytocin (OXT)
- lymphoid
Ligand/Enzyme
a - OXT
b - CPE
Receptor
c - AVPR1A
d - AVPR1B
e - AVPR2
f - OXTR
PAIRING (ligand → receptor)
OXT & CPE→OXTR
OXT & CPE→AVPR1A
OXT & CPE→AVPR1B
OXT & CPE→AVPR2
Compartment
lymphoid
- B cell
- NK/T cell
12.5%
25%
50%
100%
ln(CP10K+1)
0
1
2
3
plasma c.
plasma c. ∞
innate lymphoid c.
NK c.
NK c. ∞
NK c. GZMB+
NK c. KLRD1+
NK c. CHN1+
NKT c.
NK c./T c.
NK c./T c. ∞
T c. CD4+CD8-CCL5+
T c. CD4+CD8-CCL5-
T c. CD4+CD8-CCL5-CCR10+
T c. CD4+CD8-CCL5-CLDN1+
T c. CD4-CD8+CCL5+
T c. CD4-CD8+CCL5-
T c. CD4+CD8+CCL5+
T c. CD4-CD8-CCL5+GZMK+
T c. CD4-CD8-CCL5+GZMB+LYG1-
T c. CD4-CD8-CCL5+GZMB+LYG1+
T c. CD4-CD8-CCL5-
T c. ∞
T c.
B c.
B c. ∞
B c. SOX5+
Abbrev. Tissue
Aort Aorta
Blad Bladder
Blo Blood
Bone Bone
BM Bone marrow
BrC Brain cortex
BrS Brainstem
Col Colon
Dia Diaphragm
Ret Eye retina
Fat Fat
Hrt Heart
Pit Hypothalamus/Pituitary
Kid Kidney
LM Limb muscle
Liv Liver
Lung Lung
MG Mammary gland
Panc Pancreas
Skin Skin
SI Small intestine
Spl Spleen
Test Testes
Thym Thymus
Tong Tongue
Trac Trachea
Ute Uterus

Oxytocin (OXT)
- myeloid

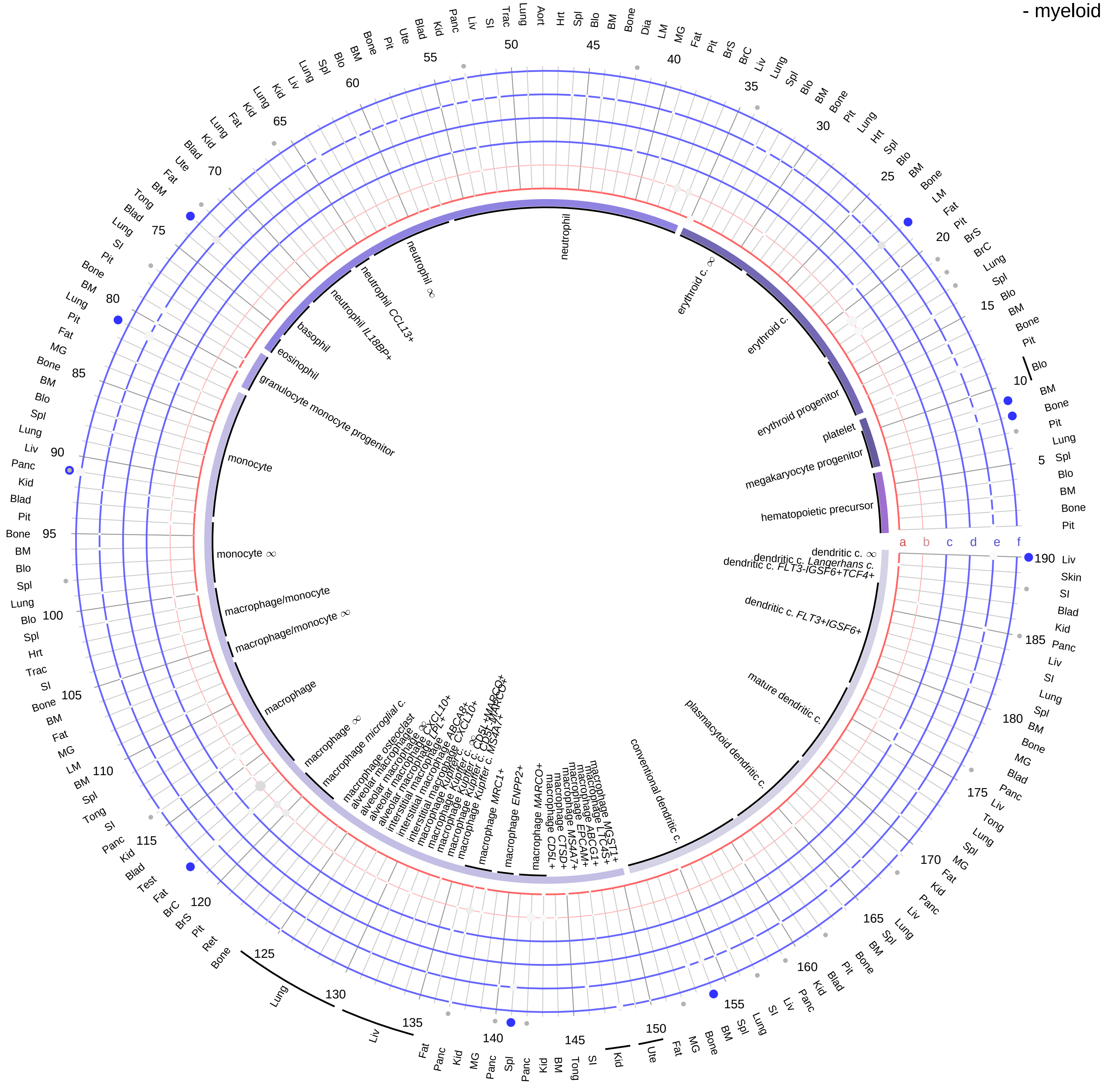

Ligand/Enzyme
- a - OXT
- b - CPE

Receptor
- c - AVPR1A
- d - AVPR1B
- e - AVPR2
- f - OXTR

PAIRING (ligand → receptor)

OXT & CPE→OXTR

OXT & CPE→AVPR1A

OXT & CPE→AVPR1B

OXT & CPE→AVPR2

Compartment
- **hematopoietic**
  - hematopoietic precursor
- **megakaryocyte-erythroid**
  - megakaryoid
  - erythroid
- **myeloid**
  - granulocyte
  - granulocyte monocyte progenitor
  - monocyte/macrophage
  - dendritic

12.5% 25% 50% 100%

ln(CP10K+1) 0 1 2 3

Abbrev.	Tissue
Aort	Aorta
Blad	Bladder
Blo	Blood
Bone	Bone
BM	Bone marrow
BrC	Brain cortex
BrS	Brainstem
Col	Colon
Dia	Diaphragm
Ret	Eye retina
Fat	Fat
Hrt	Heart
Pit	Hypothalamus/Pituitary
Kid	Kidney
LM	Limb muscle
Liv	Liver
Lung	Lung
MG	Mammary gland
Panc	Pancreas
Skin	Skin
SI	Small intestine
Spl	Spleen
Test	Testes
Thym	Thymus
Tong	Tongue
Trac	Trachea
Ute	Uterus

Pancreatic polypeptide
- epithelial/neural/germ

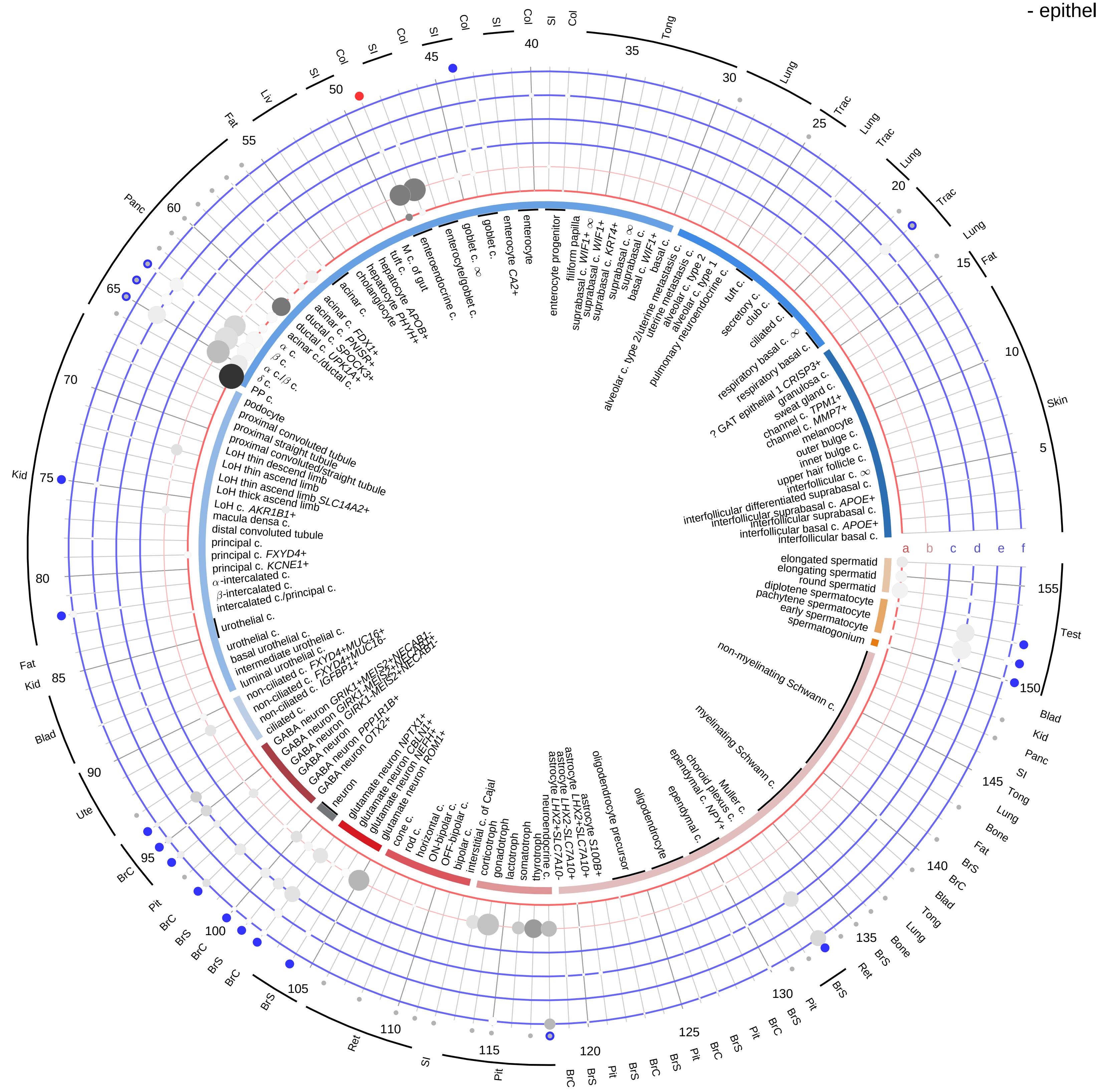

Ligand/Enzyme
- a - PPY
- b - PCSK1

Receptor
- c - LOC105884287 (NPY4R)
- d - NPY1R
- e - NPY2R
- f - NPY5R

PAIRING (ligand → receptor)

PPY & PCSK1→NPY4R

PPY & PCSK1→NPY5R

PPY & PCSK1→NPY2R

PPY & PCSK1→NPY1R

Compartment

epithelial
- integumentary
- respiratory
- gastrointestinal
- urinary
- reproductive

neural
- GABAergic
- hybrid
- glutamatergic
- specialized
- neuroendocrine
- glia

germ
- spermatogonium
- spermatocyte
- spermatid

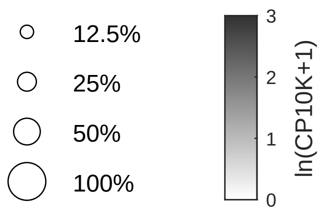

Abbrev.	Tissue
Aort	Aorta
Blad	Bladder
Blo	Blood
Bone	Bone
BM	Bone marrow
BrC	Brain cortex
BrS	Brainstem
Col	Colon
Dia	Diaphragm
Ret	Eye retina
Fat	Fat
Hrt	Heart
Pit	Hypothalamus/Pituitary
Kid	Kidney
LM	Limb muscle
Liv	Liver
Lung	Lung
MG	Mammary gland
Panc	Pancreas
Skin	Skin
SI	Small intestine
Spl	Spleen
Test	Testes
Thym	Thymus
Tong	Tongue
Trac	Trachea
Ute	Uterus

Pancreatic polypeptide
- endothelial

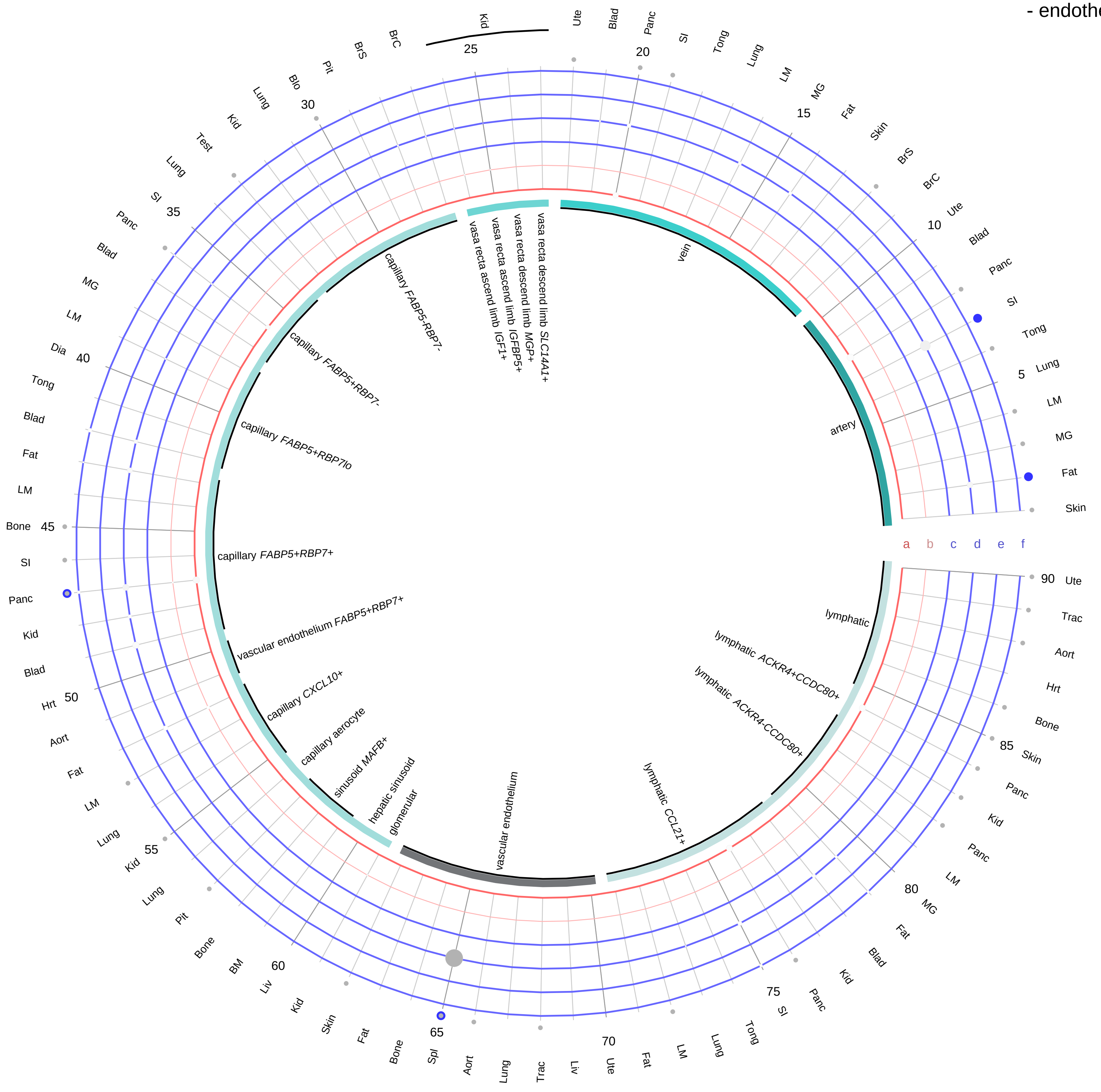

Ligand/Enzyme
- a - PPY
- b - PCSK1

Receptor
- c - LOC105884287 (NPY4R)
- d - NPY1R
- e - NPY2R
- f - NPY5R

PAIRING (ligand → receptor)

PPY & PCSK1→NPY4R

PPY & PCSK1→NPY5R

PPY & PCSK1→NPY2R

PPY & PCSK1→NPY1R

Abbrev.	Tissue
Aort	Aorta
Blad	Bladder
Blo	Blood
Bone	Bone
BM	Bone marrow
BrC	Brain cortex
BrS	Brainstem
Col	Colon
Dia	Diaphragm
Ret	Eye retina
Fat	Fat
Hrt	Heart
Pit	Hypothalamus/Pituitary
Kid	Kidney
LM	Limb muscle
Liv	Liver
Lung	Lung
MG	Mammary gland
Panc	Pancreas
Skin	Skin
SI	Small intestine
Spl	Spleen
Test	Testes
Thym	Thymus
Tong	Tongue
Trac	Trachea
Ute	Uterus

Compartment

endothelial
- artery
- vein
- vasa recta
- capillary
- mix
- lymphatic

12.5%
25%
50%
100%

ln(CP10K+1): 0 1 2 3

Pancreatic polypeptide
- stromal

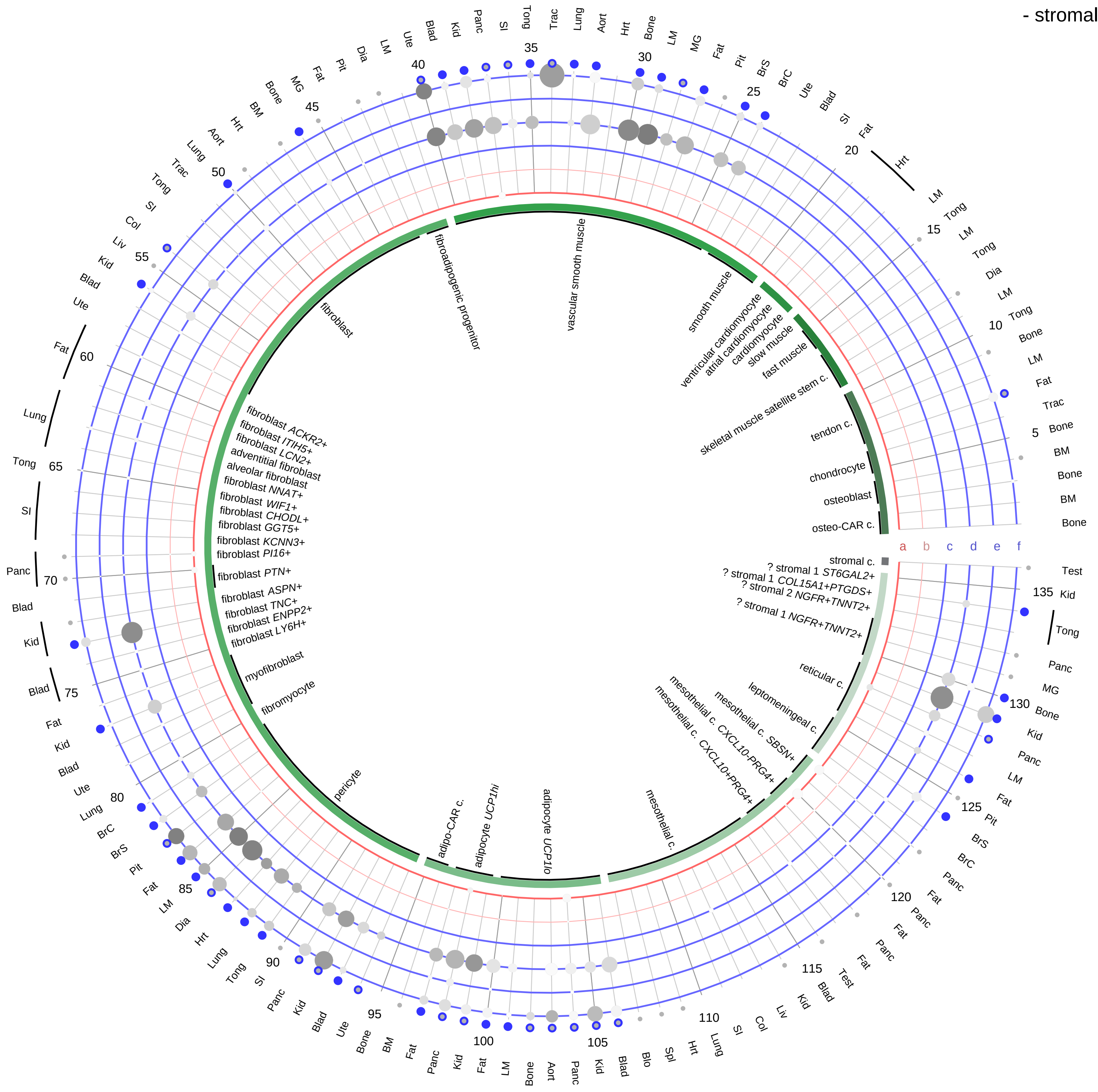

Abbrev.	Tissue
Aort	Aorta
Blad	Bladder
Blo	Blood
Bone	Bone
BM	Bone marrow
BrC	Brain cortex
BrS	Brainstem
Col	Colon
Dia	Diaphragm
Ret	Eye retina
Fat	Fat
Hrt	Heart
Pit	Hypothalamus/Pituitary
Kid	Kidney
LM	Limb muscle
Liv	Liver
Lung	Lung
MG	Mammary gland
Panc	Pancreas
Skin	Skin
SI	Small intestine
Spl	Spleen
Test	Testes
Thym	Thymus
Tong	Tongue
Trac	Trachea
Ute	Uterus

Ligand/Enzyme
a - PPY
b - PCSK1
Receptor
c - LOC105884287 (NPY4R)
d - NPY1R
e - NPY2R
f - NPY5R

PAIRING (ligand → receptor)

PPY & PCSK1→NPY4R

PPY & PCSK1→NPY5R

PPY & PCSK1→NPY2R

PPY & PCSK1→NPY1R

Compartment
stromal
- bone
- skeletal muscle
- cardiac muscle
- smooth muscle
- fibroblast
- adipocyte
- mesothelial
- other
- mix

12.5%
25%
50%
100%

ln(CP10K+1) 0 1 2 3

Pancreatic polypeptide
- lymphoid

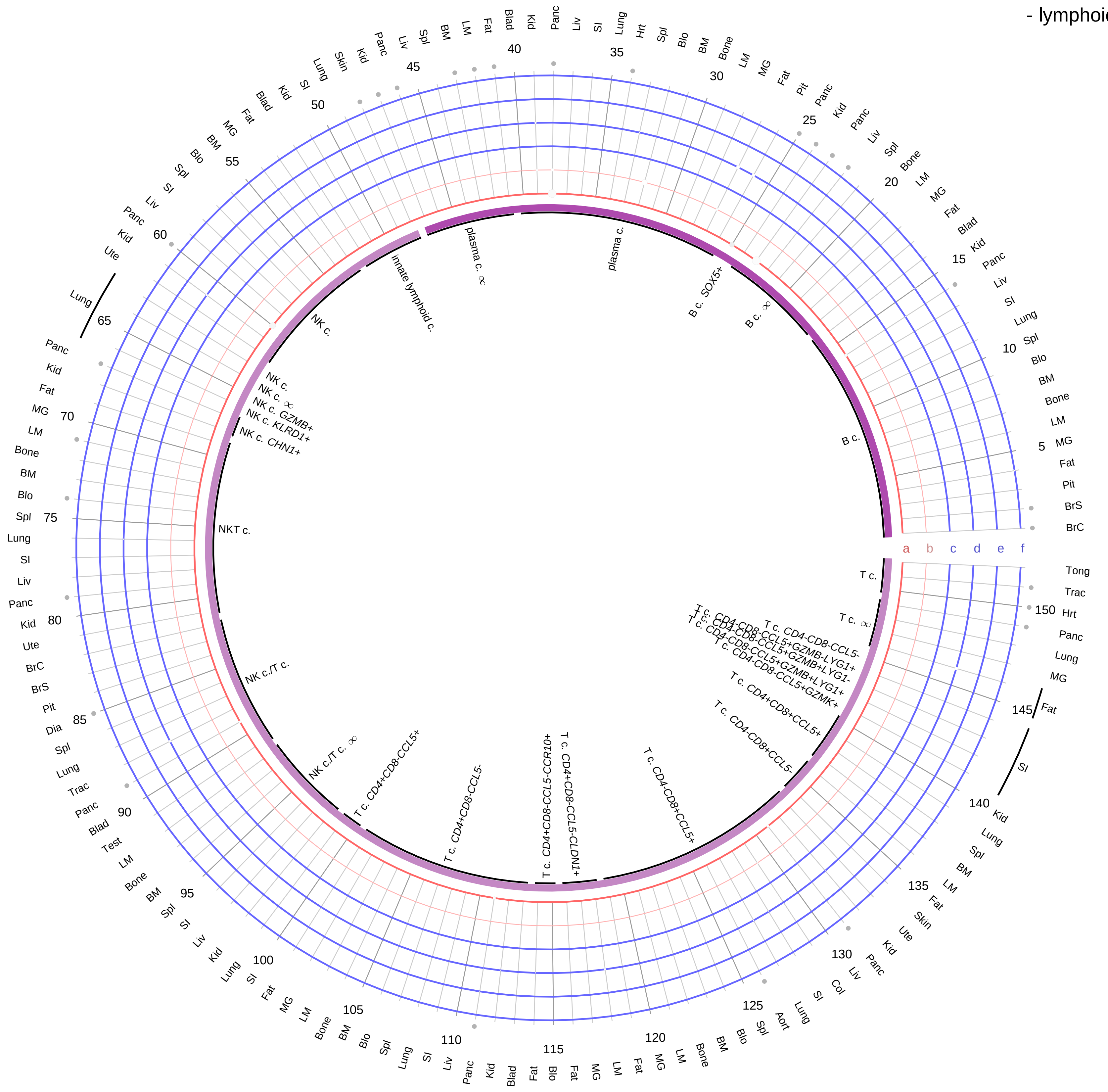

Ligand/Enzyme
- a - PPY
- b - PCSK1

Receptor
- c - LOC105884287 (NPY4R)
- d - NPY1R
- e - NPY2R
- f - NPY5R

PAIRING (ligand → receptor)

PPY & PCSK1→NPY4R

PPY & PCSK1→NPY5R

PPY & PCSK1→NPY2R

PPY & PCSK1→NPY1R

Abbrev.	Tissue
Aort	Aorta
Blad	Bladder
Blo	Blood
Bone	Bone
BM	Bone marrow
BrC	Brain cortex
BrS	Brainstem
Col	Colon
Dia	Diaphragm
Ret	Eye retina
Fat	Fat
Hrt	Heart
Pit	Hypothalamus/Pituitary
Kid	Kidney
LM	Limb muscle
Liv	Liver
Lung	Lung
MG	Mammary gland
Panc	Pancreas
Skin	Skin
SI	Small intestine
Spl	Spleen
Test	Testes
Thym	Thymus
Tong	Tongue
Trac	Trachea
Ute	Uterus

Compartment
lymphoid
- B cell
- NK/T cell

12.5%
25%
50%
100%

ln(CP10K+1): 0 1 2 3

Pancreatic polypeptide
- myeloid

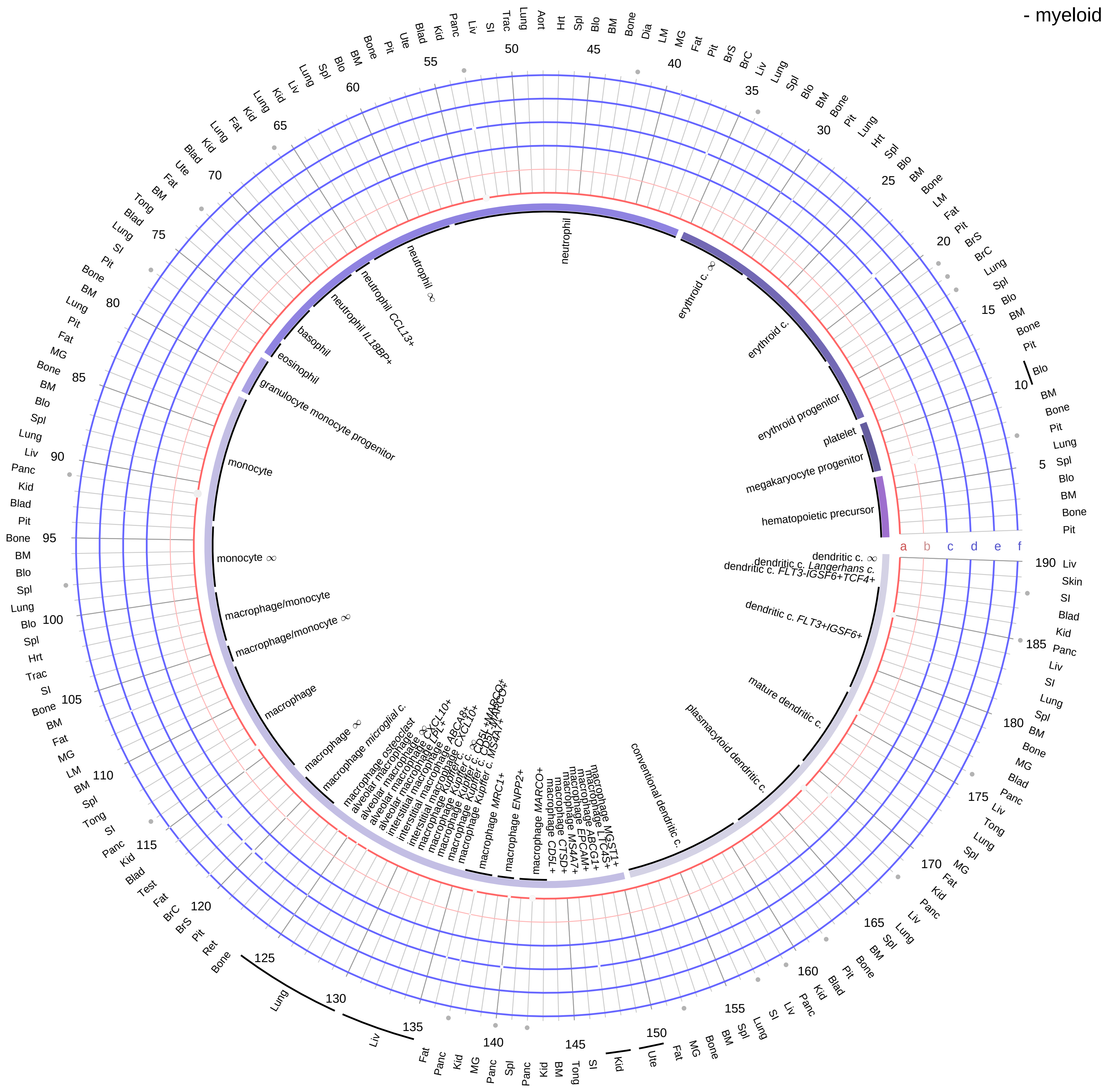

Ligand/Enzyme
- a - PPY
- b - PCSK1

Receptor
- c - LOC105884287 (NPY4R)
- d - NPY1R
- e - NPY2R
- f - NPY5R

PAIRING (ligand → receptor)

PPY & PCSK1→NPY4R

PPY & PCSK1→NPY5R

PPY & PCSK1→NPY2R

PPY & PCSK1→NPY1R

Compartment

hematopoietic
- hematopoietic precursor

megakaryocyte-erythroid
- megakaryoid
- erythroid

myeloid
- granulocyte
- granulocyte monocyte progenitor
- monocyte/macrophage
- dendritic

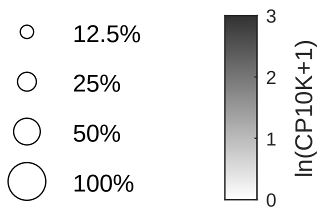

Abbrev.	Tissue
Aort	Aorta
Blad	Bladder
Blo	Blood
Bone	Bone
BM	Bone marrow
BrC	Brain cortex
BrS	Brainstem
Col	Colon
Dia	Diaphragm
Ret	Eye retina
Fat	Fat
Hrt	Heart
Pit	Hypothalamus/Pituitary
Kid	Kidney
LM	Limb muscle
Liv	Liver
Lung	Lung
MG	Mammary gland
Panc	Pancreas
Skin	Skin
SI	Small intestine
Spl	Spleen
Test	Testes
Thym	Thymus
Tong	Tongue
Trac	Trachea
Ute	Uterus

Parathyroid hormone/Parathyroid hormone-like hormone
- epithelial/neural/germ

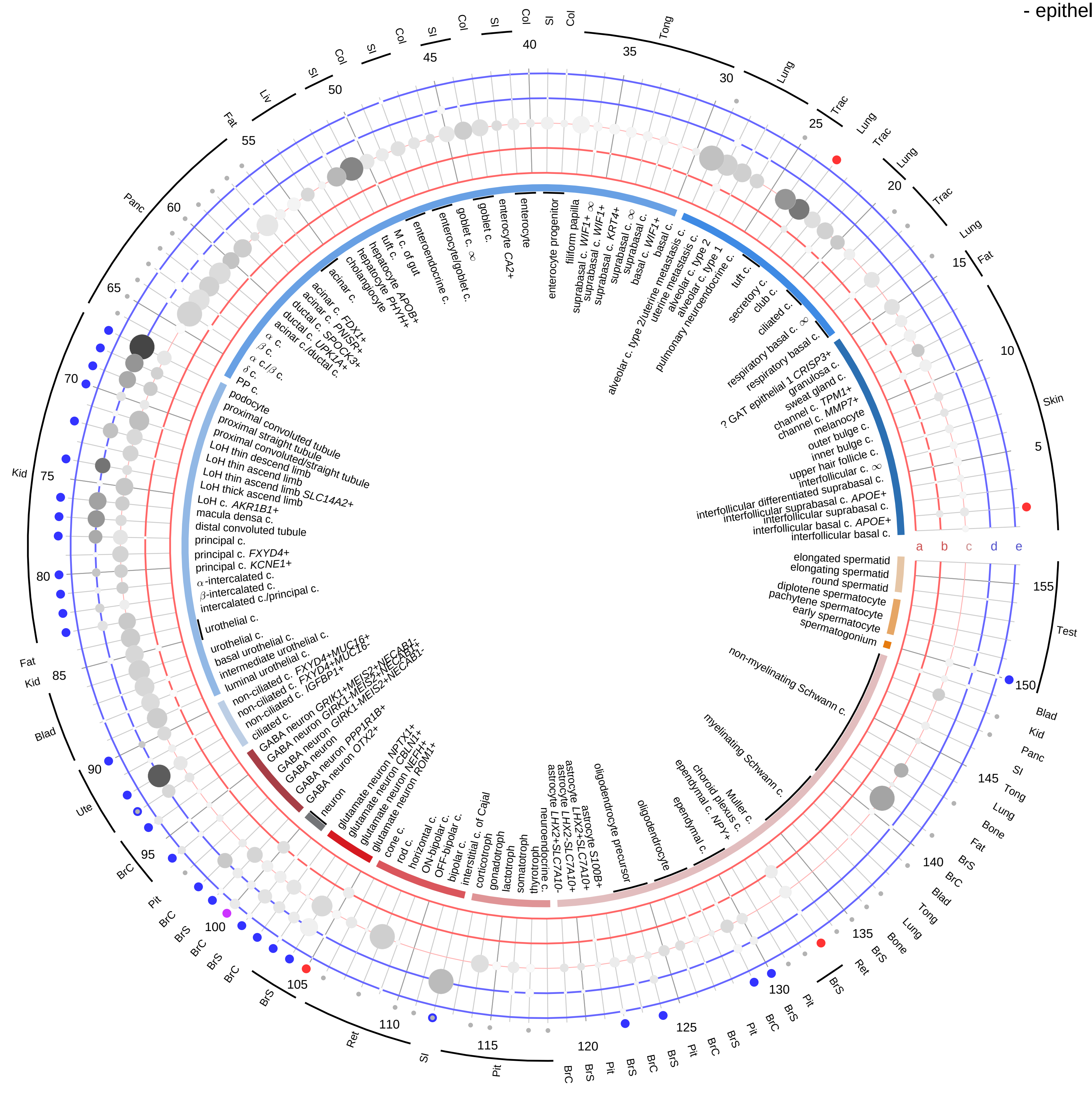

Ligand/Enzyme
- a - PTH
- b - PTHLH
- c - FURIN

Receptor
- d - PTH1R
- e - PTH2R

PAIRING (ligand → receptor)

PTH & FURIN→PTH1R

PTH & FURIN→PTH2R

PTHLH & FURIN→PTH1R

Compartment

epithelial
- integumentary
- respiratory
- gastrointestinal
- urinary
- reproductive

neural
- GABAergic
- hybrid
- glutamatergic
- specialized
- neuroendocrine
- glia

germ
- spermatogonium
- spermatocyte
- spermatid

12.5%
25%
50%
100%

ln(CP10K+1): 0, 1, 2, 3

Abbrev.	Tissue
Aort	Aorta
Blad	Bladder
Blo	Blood
Bone	Bone
BM	Bone marrow
BrC	Brain cortex
BrS	Brainstem
Col	Colon
Dia	Diaphragm
Ret	Eye retina
Fat	Fat
Hrt	Heart
Pit	Hypothalamus/Pituitary
Kid	Kidney
LM	Limb muscle
Liv	Liver
Lung	Lung
MG	Mammary gland
Panc	Pancreas
Skin	Skin
SI	Small intestine
Spl	Spleen
Test	Testes
Thym	Thymus
Tong	Tongue
Trac	Trachea
Ute	Uterus

Parathyroid hormone/Parathyroid hormone-like hormone
- endothelial

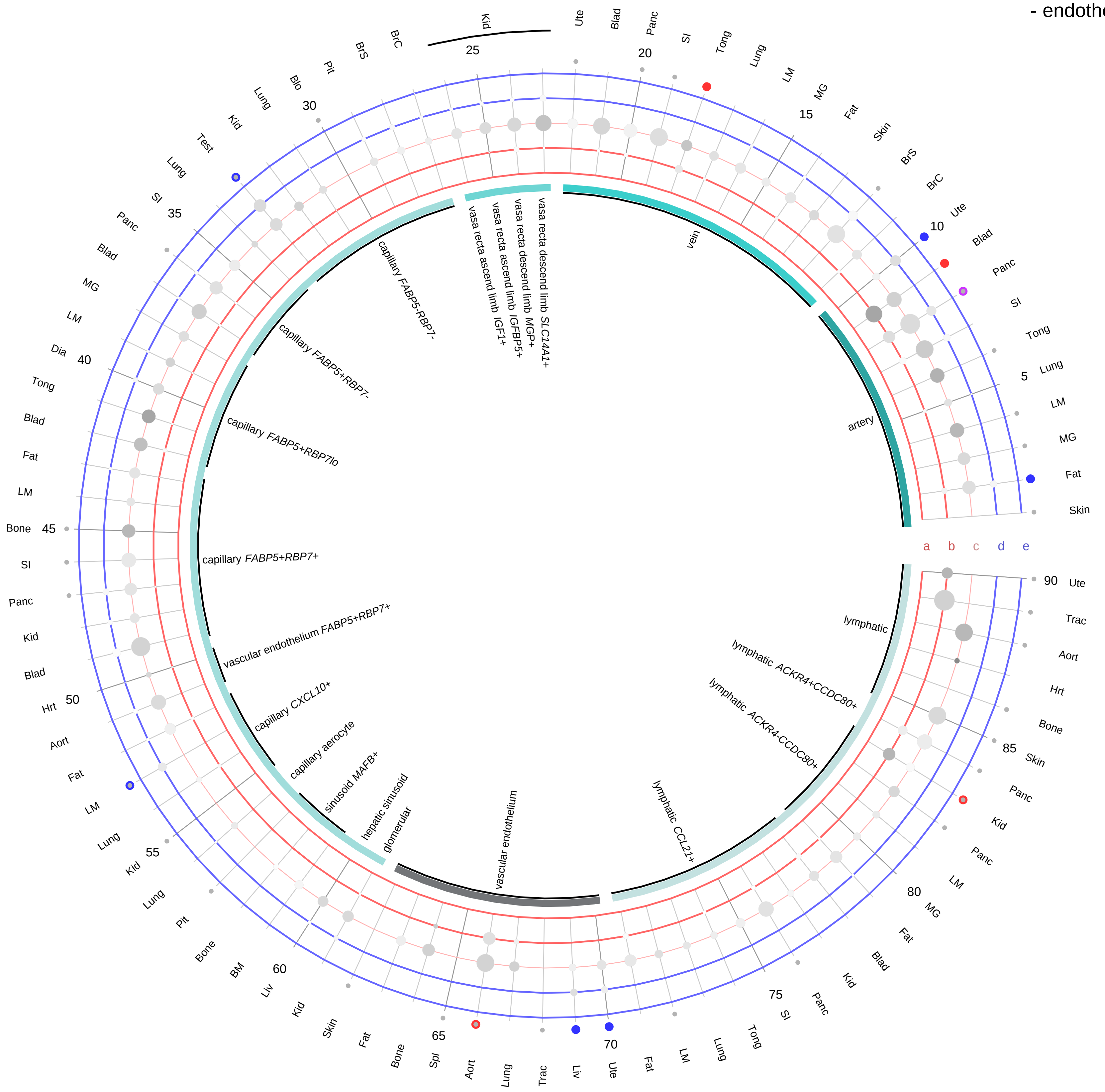

Ligand/Enzyme
- a - PTH
- b - PTHLH
- c - FURIN

Receptor
- d - PTH1R
- e - PTH2R

PAIRING (ligand → receptor)

PTH & FURIN→PTH1R

PTH & FURIN→PTH2R

PTHLH & FURIN→PTH1R

Abbrev.	Tissue
Aort	Aorta
Blad	Bladder
Blo	Blood
Bone	Bone
BM	Bone marrow
BrC	Brain cortex
BrS	Brainstem
Col	Colon
Dia	Diaphragm
Ret	Eye retina
Fat	Fat
Hrt	Heart
Pit	Hypothalamus/Pituitary
Kid	Kidney
LM	Limb muscle
Liv	Liver
Lung	Lung
MG	Mammary gland
Panc	Pancreas
Skin	Skin
SI	Small intestine
Spl	Spleen
Test	Testes
Thym	Thymus
Tong	Tongue
Trac	Trachea
Ute	Uterus

Compartment

endothelial
- artery
- vein
- vasa recta
- capillary
- mix
- lymphatic

12.5%
25%
50%
100%

ln(CP10K+1) 0 1 2 3

Parathyroid hormone/Parathyroid hormone-like hormone
- stromal

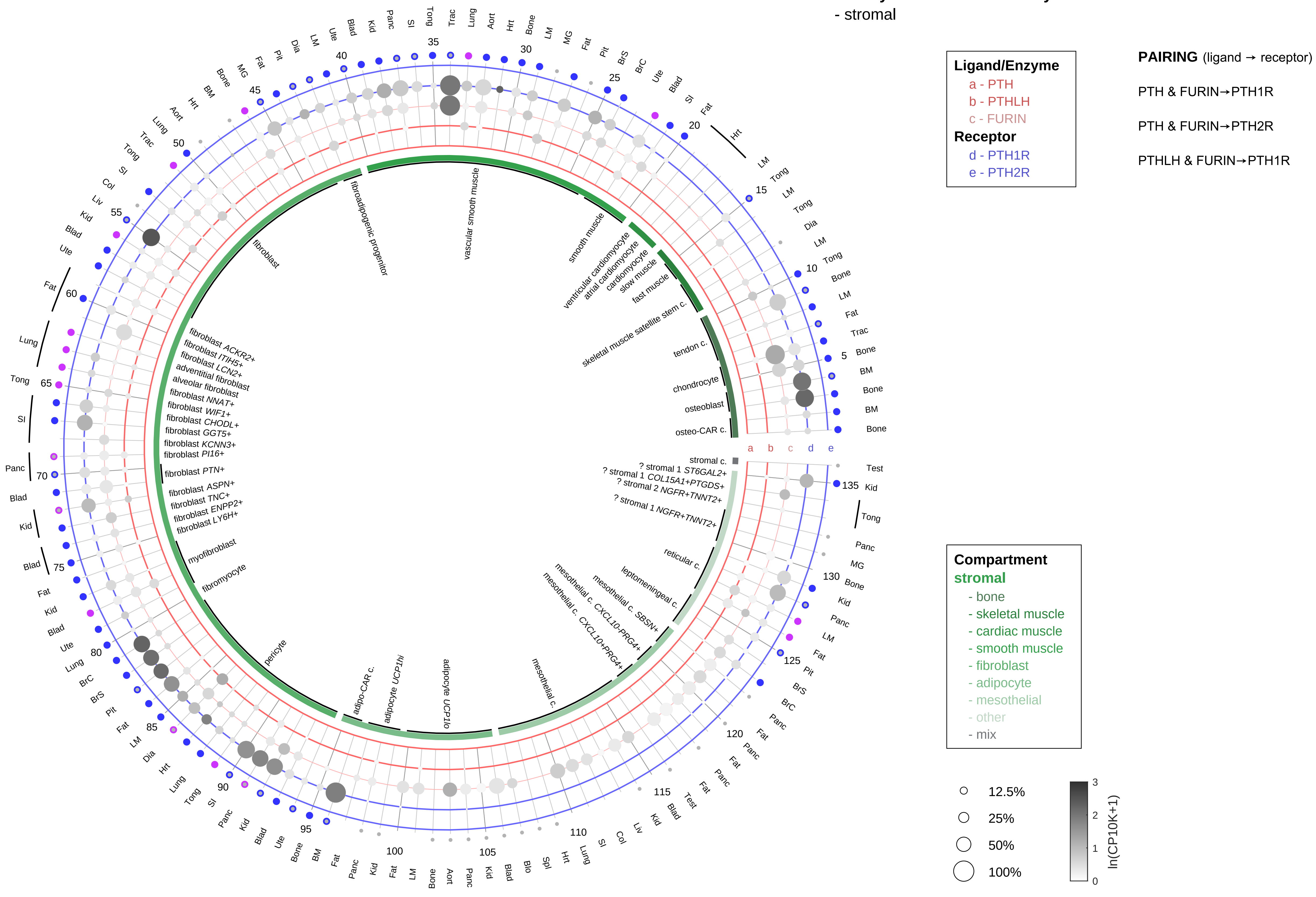

Abbrev.	Tissue
Aort	Aorta
Blad	Bladder
Blo	Blood
Bone	Bone
BM	Bone marrow
BrC	Brain cortex
BrS	Brainstem
Col	Colon
Dia	Diaphragm
Ret	Eye retina
Fat	Fat
Hrt	Heart
Pit	Hypothalamus/Pituitary
Kid	Kidney
LM	Limb muscle
Liv	Liver
Lung	Lung
MG	Mammary gland
Panc	Pancreas
Skin	Skin
SI	Small intestine
Spl	Spleen
Test	Testes
Thym	Thymus
Tong	Tongue
Trac	Trachea
Ute	Uterus

Parathyroid hormone/Parathyroid hormone-like hormone
- lymphoid

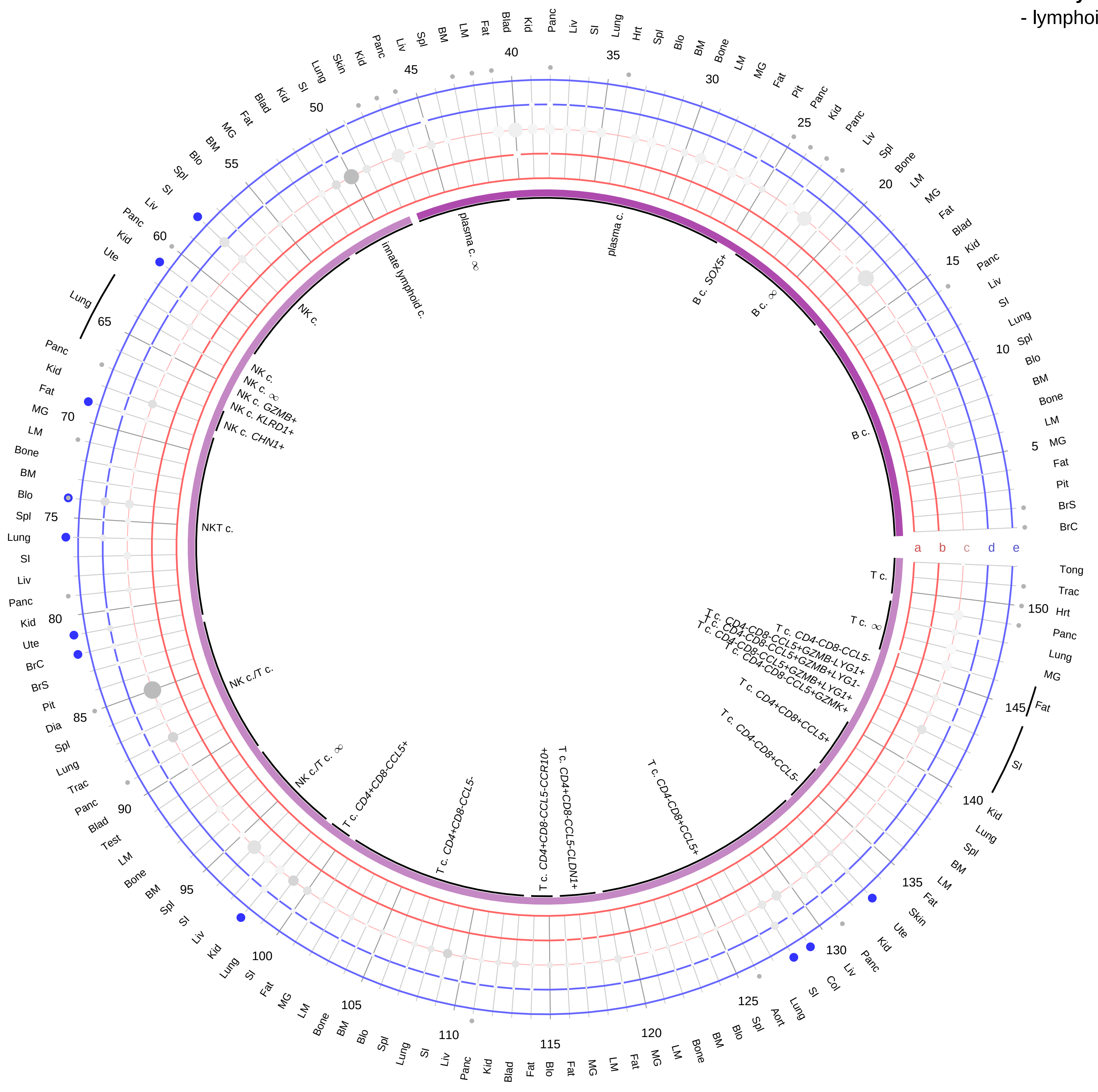

Ligand/Enzyme
- a - PTH
- b - PTHLH
- c - FURIN

Receptor
- d - PTH1R
- e - PTH2R

PAIRING (ligand → receptor)

PTH & FURIN→PTH1R

PTH & FURIN→PTH2R

PTHLH & FURIN→PTH1R

Compartment
lymphoid
- B cell
- NK/T cell

12.5%
25%
50%
100%

ln(CP10K+1): 0, 1, 2, 3

Abbrev.	Tissue
Aort	Aorta
Blad	Bladder
Blo	Blood
Bone	Bone
BM	Bone marrow
BrC	Brain cortex
BrS	Brainstem
Col	Colon
Dia	Diaphragm
Ret	Eye retina
Fat	Fat
Hrt	Heart
Pit	Hypothalamus/Pituitary
Kid	Kidney
LM	Limb muscle
Liv	Liver
Lung	Lung
MG	Mammary gland
Panc	Pancreas
Skin	Skin
SI	Small intestine
Spl	Spleen
Test	Testes
Thym	Thymus
Tong	Tongue
Trac	Trachea
Ute	Uterus

Parathyroid hormone/Parathyroid hormone-like hormone
- myeloid

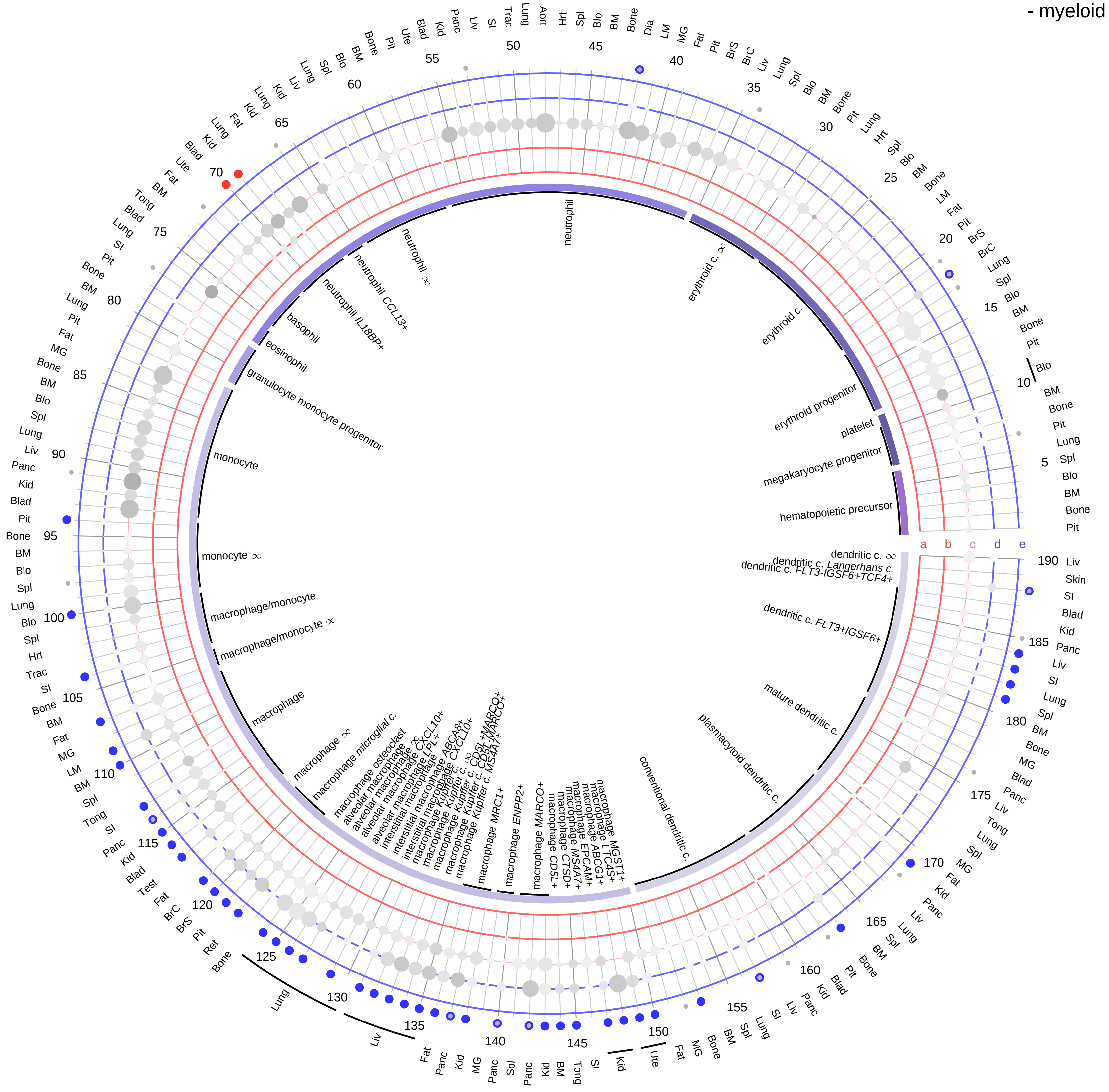

Ligand/Enzyme
- a - PTH
- b - PTHLH
- c - FURIN

Receptor
- d - PTH1R
- e - PTH2R

PAIRING (ligand → receptor)

PTH & FURIN→PTH1R

PTH & FURIN→PTH2R

PTHLH & FURIN→PTH1R

Compartment
- **hematopoietic**
  - hematopoietic precursor
- **megakaryocyte-erythroid**
  - megakaryoid
  - erythroid
- **myeloid**
  - granulocyte
  - granulocyte monocyte progenitor
  - monocyte/macrophage
  - dendritic

12.5%
25%
50%
100%

ln(CP10K+1): 0, 1, 2, 3

Abbrev.	Tissue
Aort	Aorta
Blad	Bladder
Blo	Blood
Bone	Bone
BM	Bone marrow
BrC	Brain cortex
BrS	Brainstem
Col	Colon
Dia	Diaphragm
Ret	Eye retina
Fat	Fat
Hrt	Heart
Pit	Hypothalamus/Pituitary
Kid	Kidney
LM	Limb muscle
Liv	Liver
Lung	Lung
MG	Mammary gland
Panc	Pancreas
Skin	Skin
SI	Small intestine
Spl	Spleen
Test	Testes
Thym	Thymus
Tong	Tongue
Trac	Trachea
Ute	Uterus

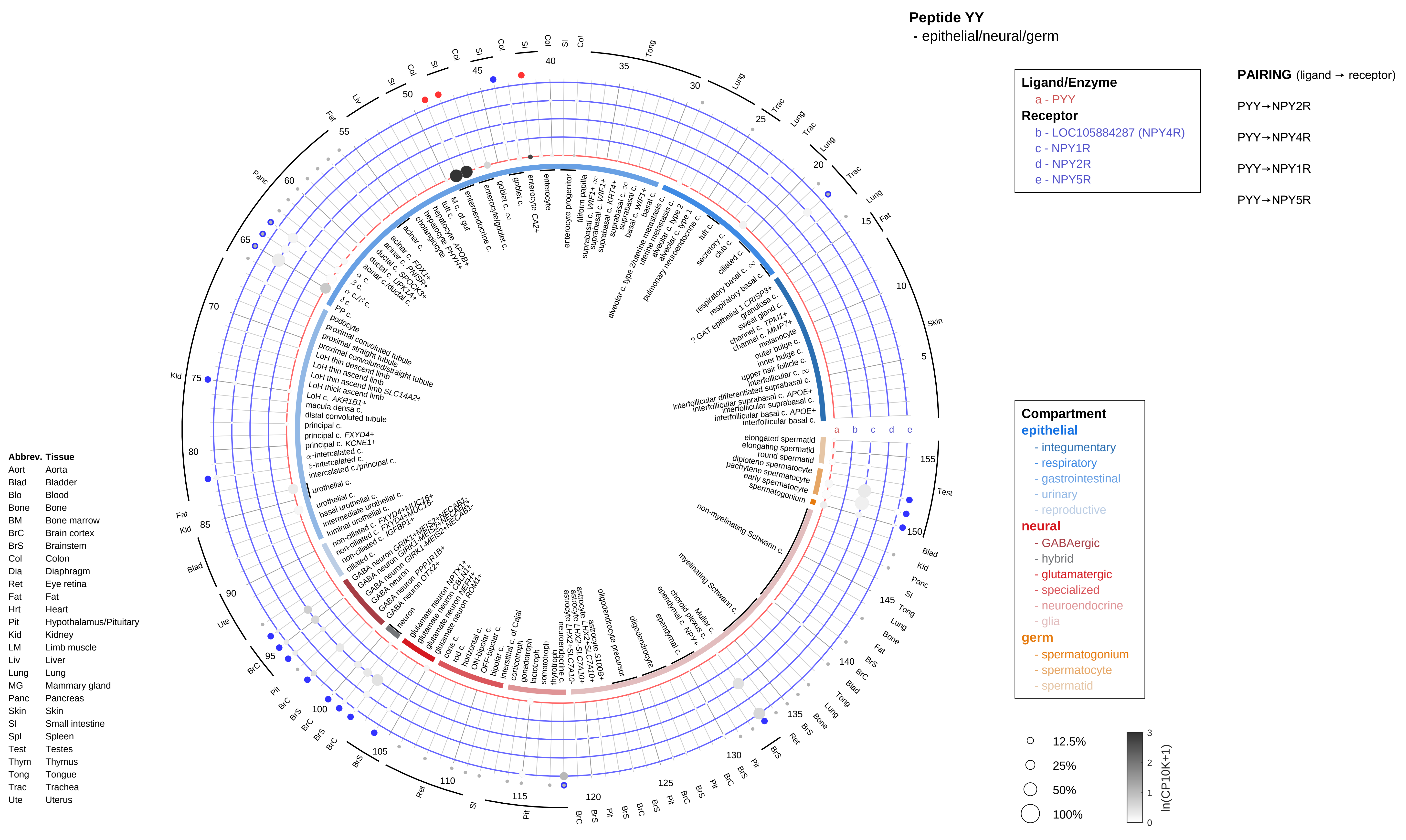

Peptide YY
- epithelial/neural/germ
Ligand/Enzyme
a - PYY
Receptor
b - LOC105884287 (NPY4R)
c - NPY1R
d - NPY2R
e - NPY5R
PAIRING (ligand → receptor)
PYY→NPY2R
PYY→NPY4R
PYY→NPY1R
PYY→NPY5R
Compartment
epithelial
- integumentary
- respiratory
- gastrointestinal
- urinary
- reproductive
neural
- GABAergic
- hybrid
- glutamatergic
- specialized
- neuroendocrine
- glia
germ
- spermatogonium
- spermatocyte
- spermatid
12.5%
25%
50%
100%
ln(CP10K+1)
Abbrev. Tissue
Aort Aorta
Blad Bladder
Blo Blood
Bone Bone
BM Bone marrow
BrC Brain cortex
BrS Brainstem
Col Colon
Dia Diaphragm
Ret Eye retina
Fat Fat
Hrt Heart
Pit Hypothalamus/Pituitary
Kid Kidney
LM Limb muscle
Liv Liver
Lung Lung
MG Mammary gland
Panc Pancreas
Skin Skin
SI Small intestine
Spl Spleen
Test Testes
Thym Thymus
Tong Tongue
Trac Trachea
Ute Uterus

Peptide YY
- endothelial

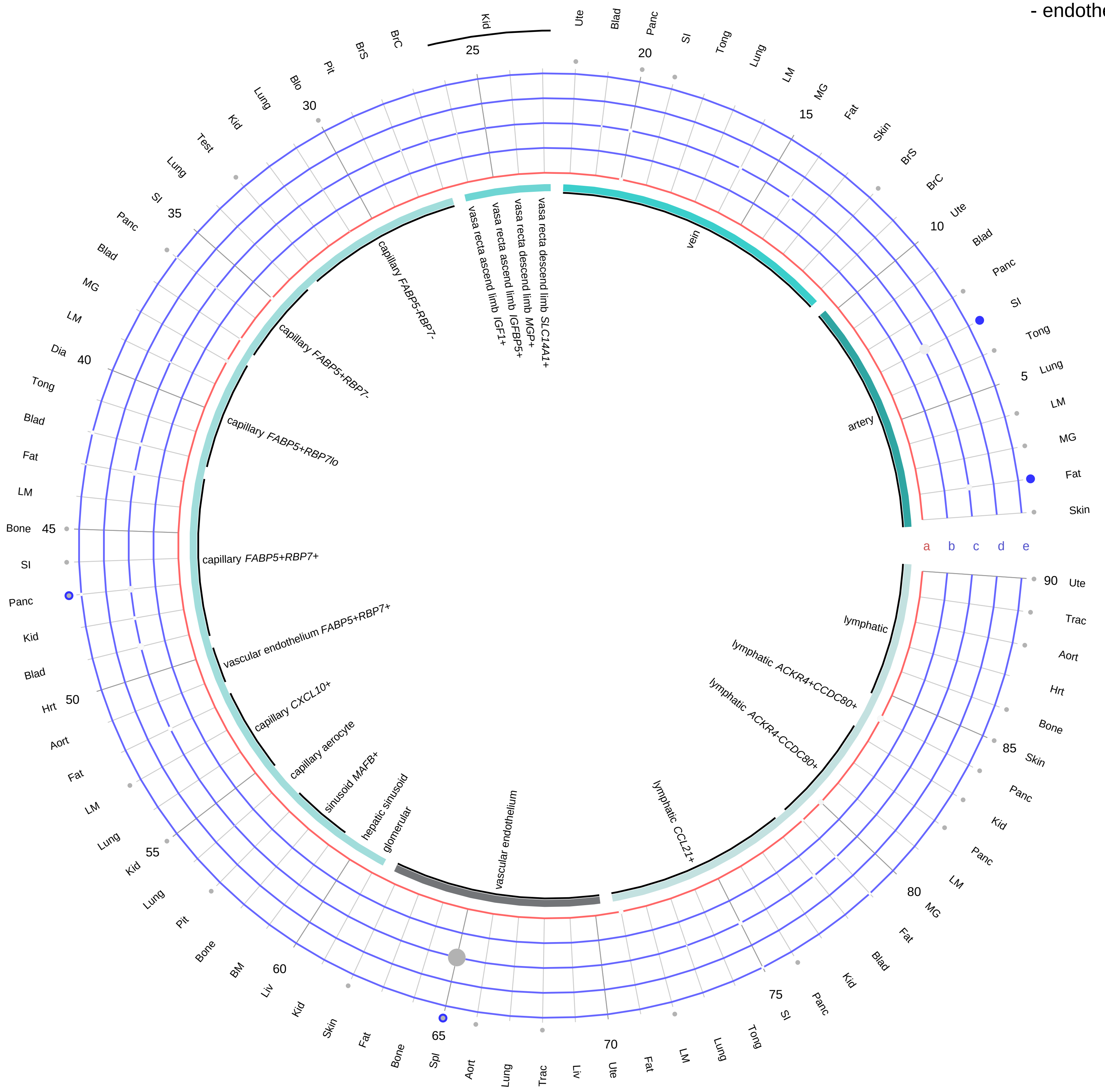

Ligand/Enzyme
a - PYY
Receptor
b - LOC105884287 (NPY4R)
c - NPY1R
d - NPY2R
e - NPY5R

PAIRING (ligand → receptor)

PYY→NPY2R

PYY→NPY4R

PYY→NPY1R

PYY→NPY5R

Abbrev.	Tissue
Aort	Aorta
Blad	Bladder
Blo	Blood
Bone	Bone
BM	Bone marrow
BrC	Brain cortex
BrS	Brainstem
Col	Colon
Dia	Diaphragm
Ret	Eye retina
Fat	Fat
Hrt	Heart
Pit	Hypothalamus/Pituitary
Kid	Kidney
LM	Limb muscle
Liv	Liver
Lung	Lung
MG	Mammary gland
Panc	Pancreas
Skin	Skin
SI	Small intestine
Spl	Spleen
Test	Testes
Thym	Thymus
Tong	Tongue
Trac	Trachea
Ute	Uterus

Compartment
endothelial
- artery
- vein
- vasa recta
- capillary
- mix
- lymphatic

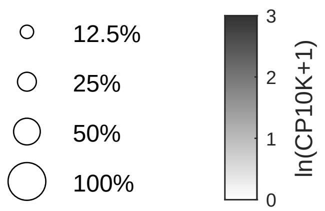

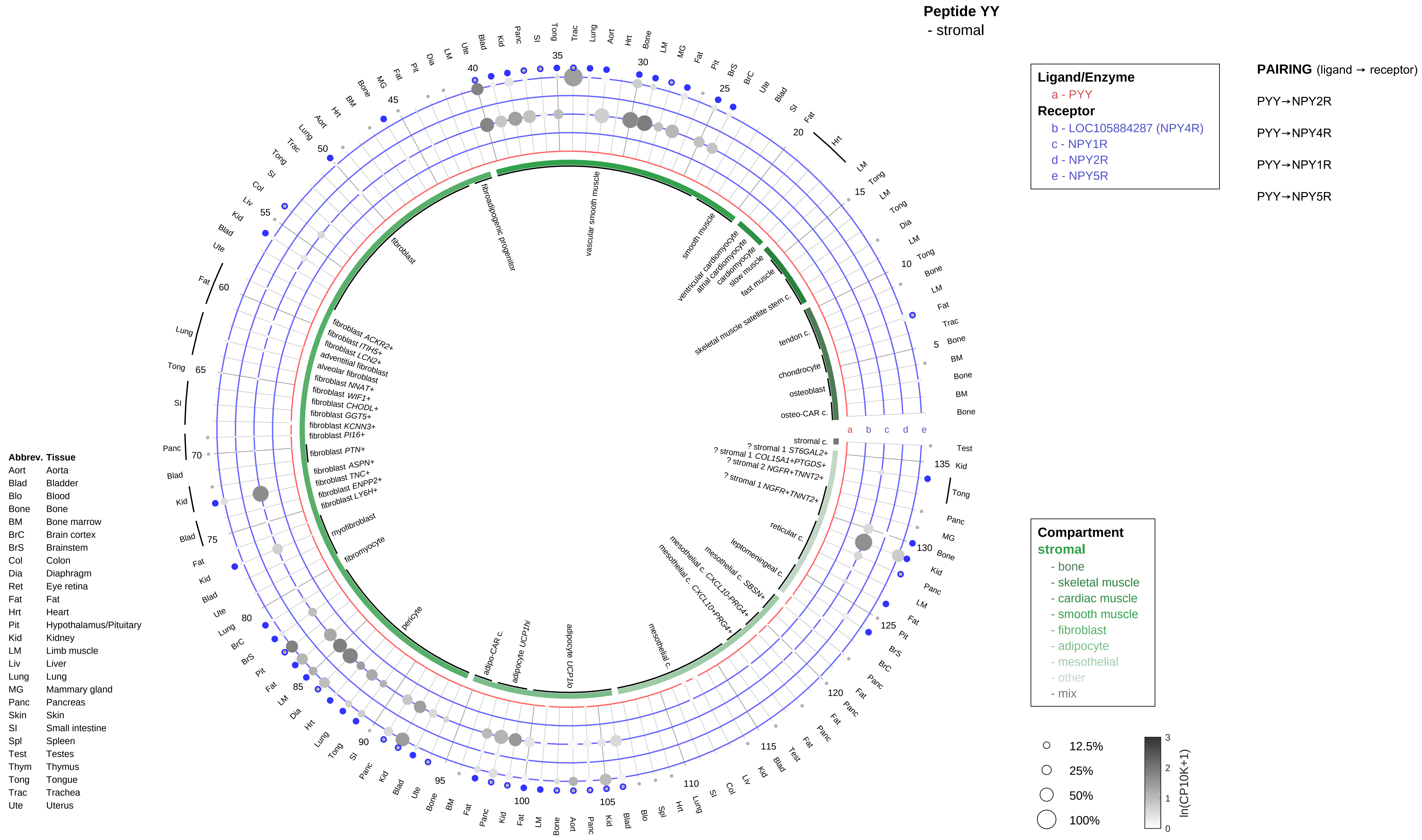
Peptide YY
- stromal
Ligand/Enzyme
a - PYY
Receptor
b - LOC105884287 (NPY4R)
c - NPY1R
d - NPY2R
e - NPY5R
PAIRING (ligand → receptor)
PYY→NPY2R
PYY→NPY4R
PYY→NPY1R
PYY→NPY5R
Compartment
stromal
- bone
- skeletal muscle
- cardiac muscle
- smooth muscle
- fibroblast
- adipocyte
- mesothelial
- other
- mix
12.5%
25%
50%
100%
ln(CP10K+1)
Abbrev. Tissue
Aort Aorta
Blad Bladder
Blo Blood
Bone Bone
BM Bone marrow
BrC Brain cortex
BrS Brainstem
Col Colon
Dia Diaphragm
Ret Eye retina
Fat Fat
Hrt Heart
Pit Hypothalamus/Pituitary
Kid Kidney
LM Limb muscle
Liv Liver
Lung Lung
MG Mammary gland
Panc Pancreas
Skin Skin
SI Small intestine
Spl Spleen
Test Testes
Thym Thymus
Tong Tongue
Trac Trachea
Ute Uterus
vascular smooth muscle
fibroadipogenic progenitor
fibroblast
smooth muscle
ventricular cardiomyocyte
atrial cardiomyocyte
cardiomyocyte
slow muscle
fast muscle
skeletal muscle satellite stem c.
tendon c.
chondrocyte
osteoblast
osteo-CAR c.
stromal c.
? stromal 1 ST6GAL2+
? stromal 1 COL15A1+PTGDS+
? stromal 2 NGFR+TNNT2+
? stromal 1 NGFR+TNNT2+
reticular c.
leptomeningeal c.
mesothelial c. SBSN+
mesothelial c. CXCL10+PRG4+
mesothelial c. CXCL10+PRG4+
mesothelial c.
adipocyte UCP1lo
adipocyte UCP1hi
adipo-CAR c.
pericyte
fibromyocyte
myofibroblast
fibroblast ACKR2+
fibroblast ITIH5+
fibroblast LCN2+
adventitial fibroblast
alveolar fibroblast
fibroblast NNAT+
fibroblast WIF1+
fibroblast CHODL+
fibroblast GGT5+
fibroblast KCNN3+
fibroblast PI16+
fibroblast PTN+
fibroblast ASPN+
fibroblast TNC+
fibroblast ENPP2+
fibroblast LY6H+

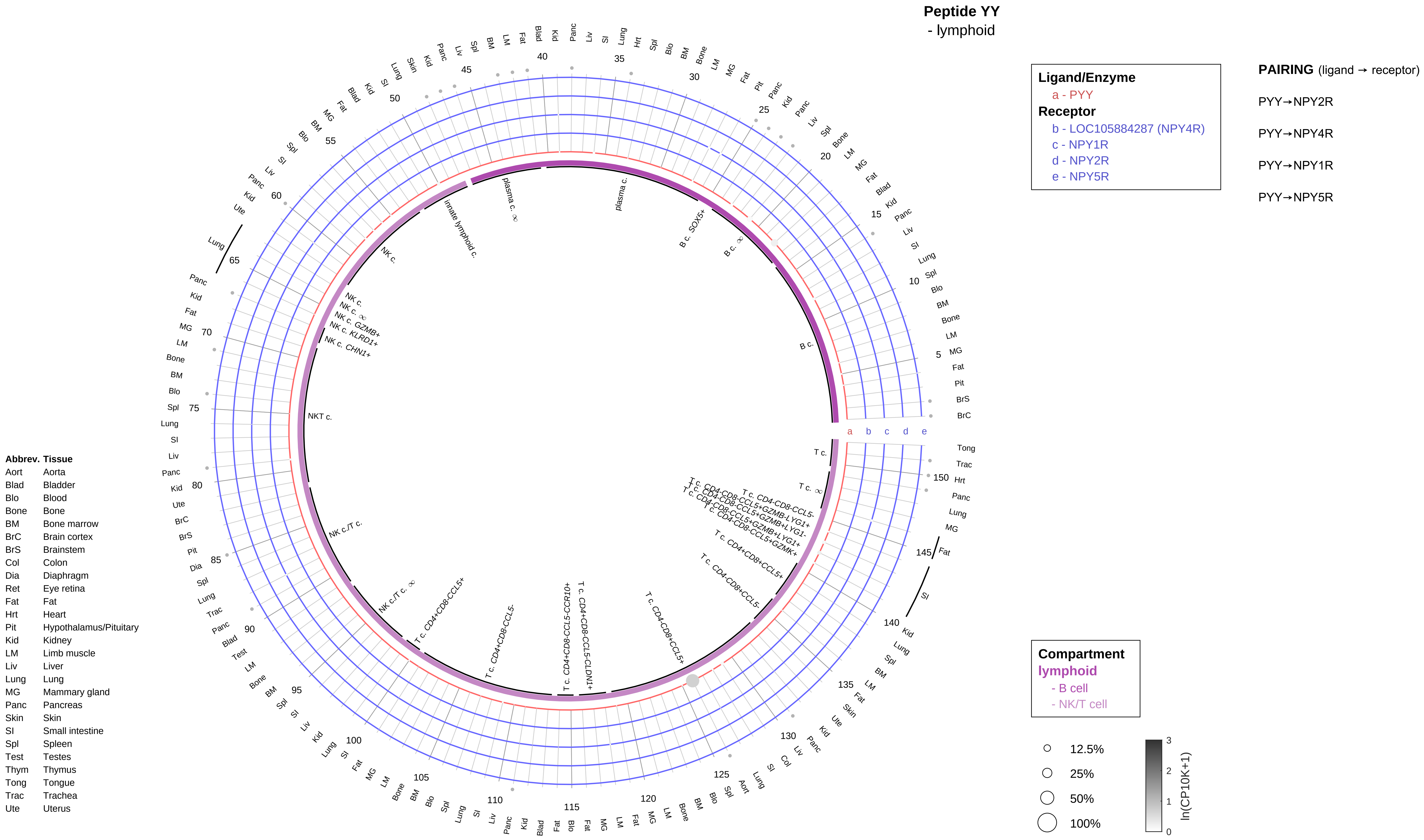
Peptide YY
- lymphoid
Ligand/Enzyme
a - PYY
Receptor
b - LOC105884287 (NPY4R)
c - NPY1R
d - NPY2R
e - NPY5R
PAIRING (ligand → receptor)
PYY→NPY2R
PYY→NPY4R
PYY→NPY1R
PYY→NPY5R
Compartment
lymphoid
- B cell
- NK/T cell
12.5%
25%
50%
100%
ln(CP10K+1)
Abbrev. Tissue
Aort Aorta
Blad Bladder
Blo Blood
Bone Bone
BM Bone marrow
BrC Brain cortex
BrS Brainstem
Col Colon
Dia Diaphragm
Ret Eye retina
Fat Fat
Hrt Heart
Pit Hypothalamus/Pituitary
Kid Kidney
LM Limb muscle
Liv Liver
Lung Lung
MG Mammary gland
Panc Pancreas
Skin Skin
SI Small intestine
Spl Spleen
Test Testes
Thym Thymus
Tong Tongue
Trac Trachea
Ute Uterus
plasma c.
B c. SOX5+
B c. ∞
B c.
T c.
T c. ∞
T c. CD4-CD8-CCL5-
T c. CD4-CD8-CCL5+GZMB-LYG1+
T c. CD4-CD8-CCL5+GZMB+LYG1-
T c. CD4-CD8-CCL5+GZMB+LYG1+
T c. CD4-CD8-CCL5+GZMK+
T c. CD4+CD8+CCL5+
T c. CD4-CD8+CCL5-
T c. CD4-CD8+CCL5+
T c. CD4+CD8-CCL5-CLDN1+
T c. CD4+CD8-CCL5-CCR10+
T c. CD4+CD8-CCL5-
T c. CD4+CD8-CCL5+
NK c./T c. ∞
NK c./T c.
NKT c.
NK c. CHN1+
NK c. KLRD1+
NK c. GZMB+
NK c. ∞
NK c.
innate lymphoid c.
plasma c. ∞

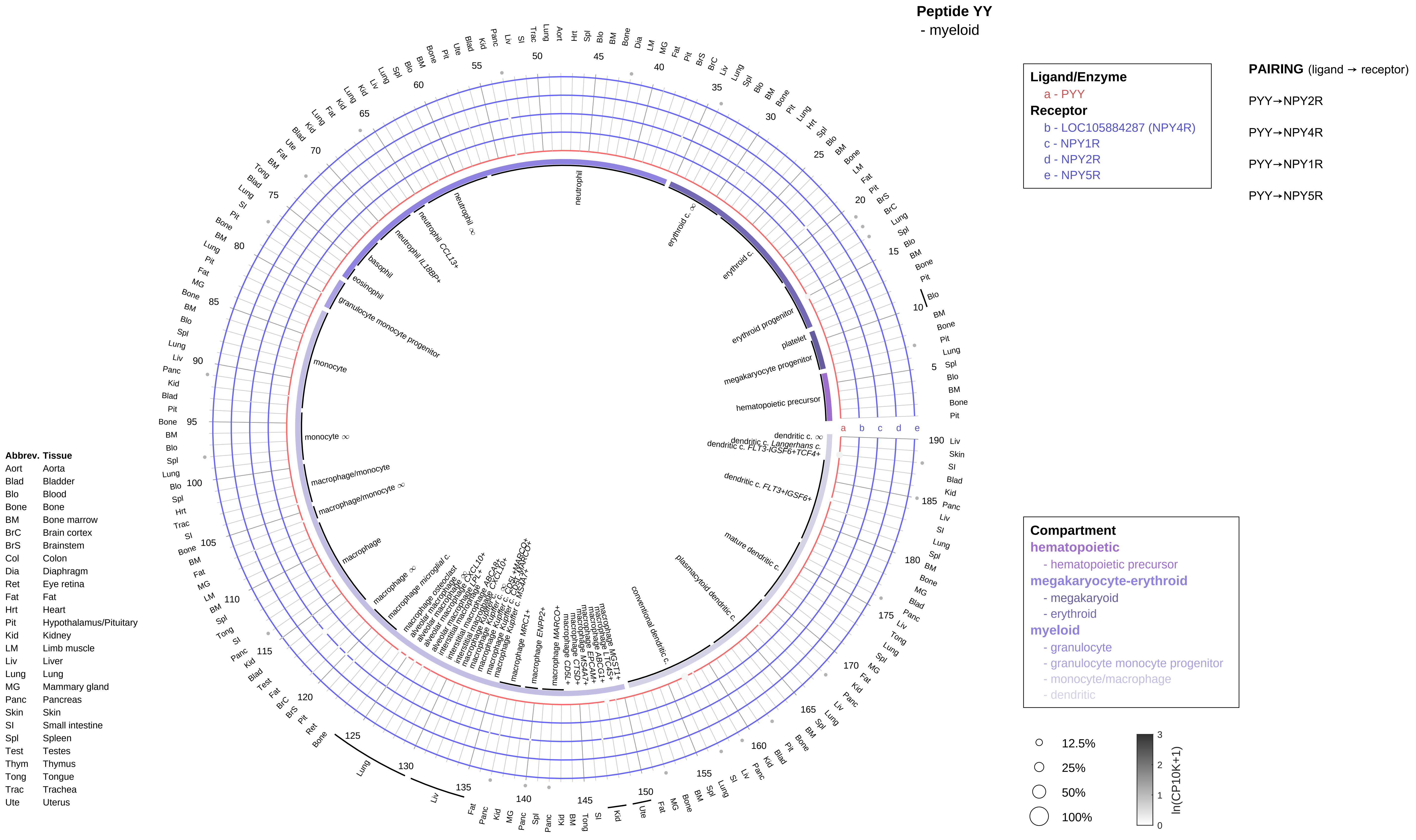
Peptide YY
- myeloid
Ligand/Enzyme
a - PYY
Receptor
b - LOC105884287 (NPY4R)
c - NPY1R
d - NPY2R
e - NPY5R
PAIRING (ligand → receptor)
PYY→NPY2R
PYY→NPY4R
PYY→NPY1R
PYY→NPY5R
Compartment
hematopoietic
- hematopoietic precursor
megakaryocyte-erythroid
- megakaryoid
- erythroid
myeloid
- granulocyte
- granulocyte monocyte progenitor
- monocyte/macrophage
- dendritic
12.5%
25%
50%
100%
ln(CP10K+1)
Abbrev. Tissue
Aort Aorta
Blad Bladder
Blo Blood
Bone Bone
BM Bone marrow
BrC Brain cortex
BrS Brainstem
Col Colon
Dia Diaphragm
Ret Eye retina
Fat Fat
Hrt Heart
Pit Hypothalamus/Pituitary
Kid Kidney
LM Limb muscle
Liv Liver
Lung Lung
MG Mammary gland
Panc Pancreas
Skin Skin
SI Small intestine
Spl Spleen
Test Testes
Thym Thymus
Tong Tongue
Trac Trachea
Ute Uterus
neutrophil
erythroid c. ∞
erythroid c.
erythroid progenitor
platelet
megakaryocyte progenitor
hematopoietic precursor
dendritic c. ∞
dendritic c. Langerhans c.
dendritic c. FLT3-IGSF6+TCF4+
dendritic c. FLT3+IGSF6+
mature dendritic c.
plasmacytoid dendritic c.
conventional dendritic c.
macrophage MARCO+
macrophage ENPP2+
macrophage MRC1+
macrophage
macrophage ∞
macrophage/monocyte
macrophage/monocyte ∞
monocyte
monocyte ∞
granulocyte monocyte progenitor
eosinophil
basophil
neutrophil IL18BP+
neutrophil CCL13+
neutrophil ∞

Pituitary adenylate cyclase-activating polypeptide (PACAP)
- epithelial/neural/germ

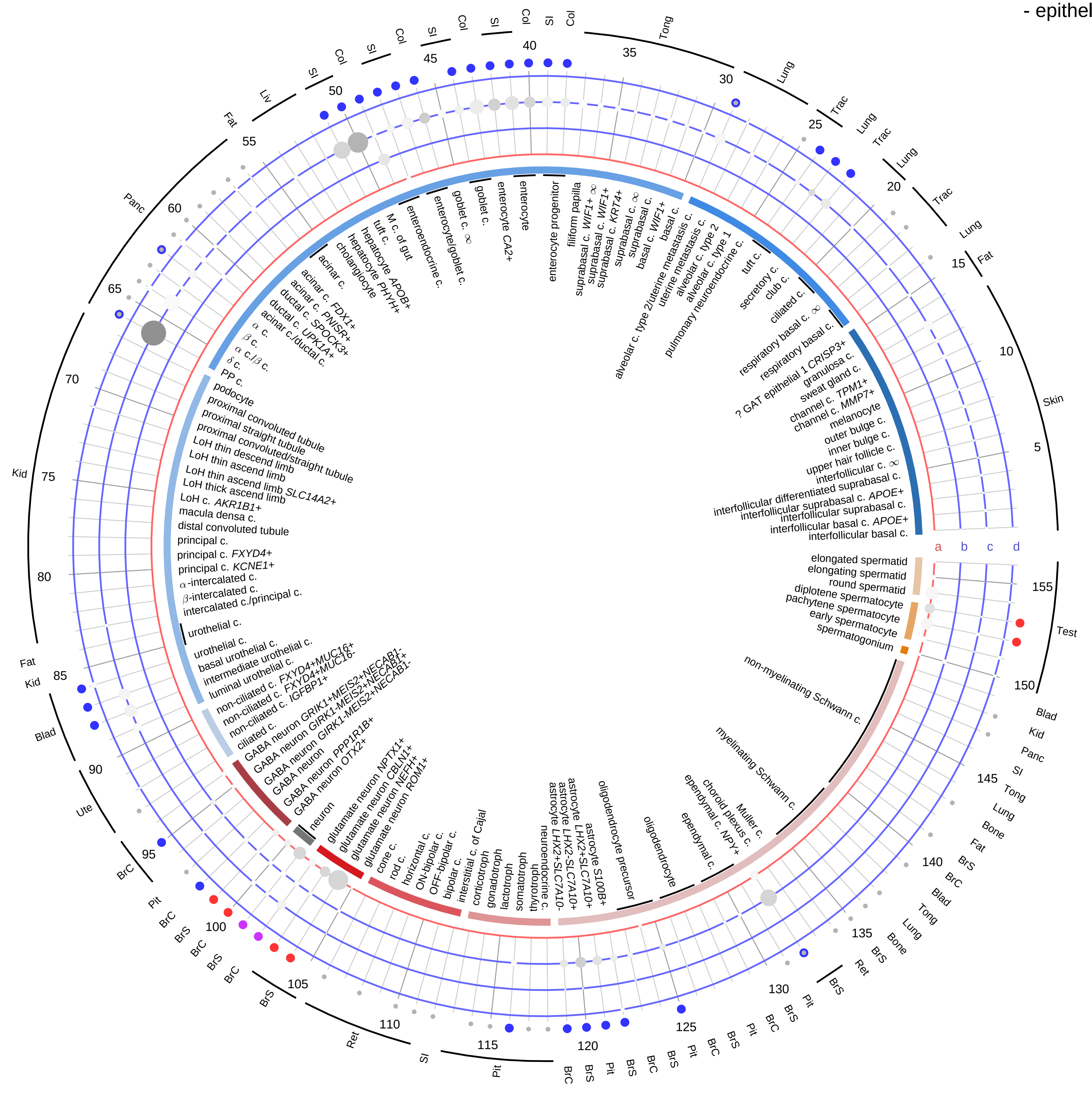

Ligand/Enzyme
a - ADCYAP1
Receptor
b - ADCYAP1R1
c - VIPR1
d - VIPR2

PAIRING (ligand → receptor)

ADCYAP1→ADCYAP1R1

ADCYAP1→VIPR1

ADCYAP1→VIPR2

Compartment
epithelial
- integumentary
- respiratory
- gastrointestinal
- urinary
- reproductive
neural
- GABAergic
- hybrid
- glutamatergic
- specialized
- neuroendocrine
- glia
germ
- spermatogonium
- spermatocyte
- spermatid

12.5%
25%
50%
100%

ln(CP10K+1): 0, 1, 2, 3

Abbrev.	Tissue
Aort	Aorta
Blad	Bladder
Blo	Blood
Bone	Bone
BM	Bone marrow
BrC	Brain cortex
BrS	Brainstem
Col	Colon
Dia	Diaphragm
Ret	Eye retina
Fat	Fat
Hrt	Heart
Pit	Hypothalamus/Pituitary
Kid	Kidney
LM	Limb muscle
Liv	Liver
Lung	Lung
MG	Mammary gland
Panc	Pancreas
Skin	Skin
SI	Small intestine
Spl	Spleen
Test	Testes
Thym	Thymus
Tong	Tongue
Trac	Trachea
Ute	Uterus

Pituitary adenylate cyclase-activating polypeptide (PACAP)
- endothelial

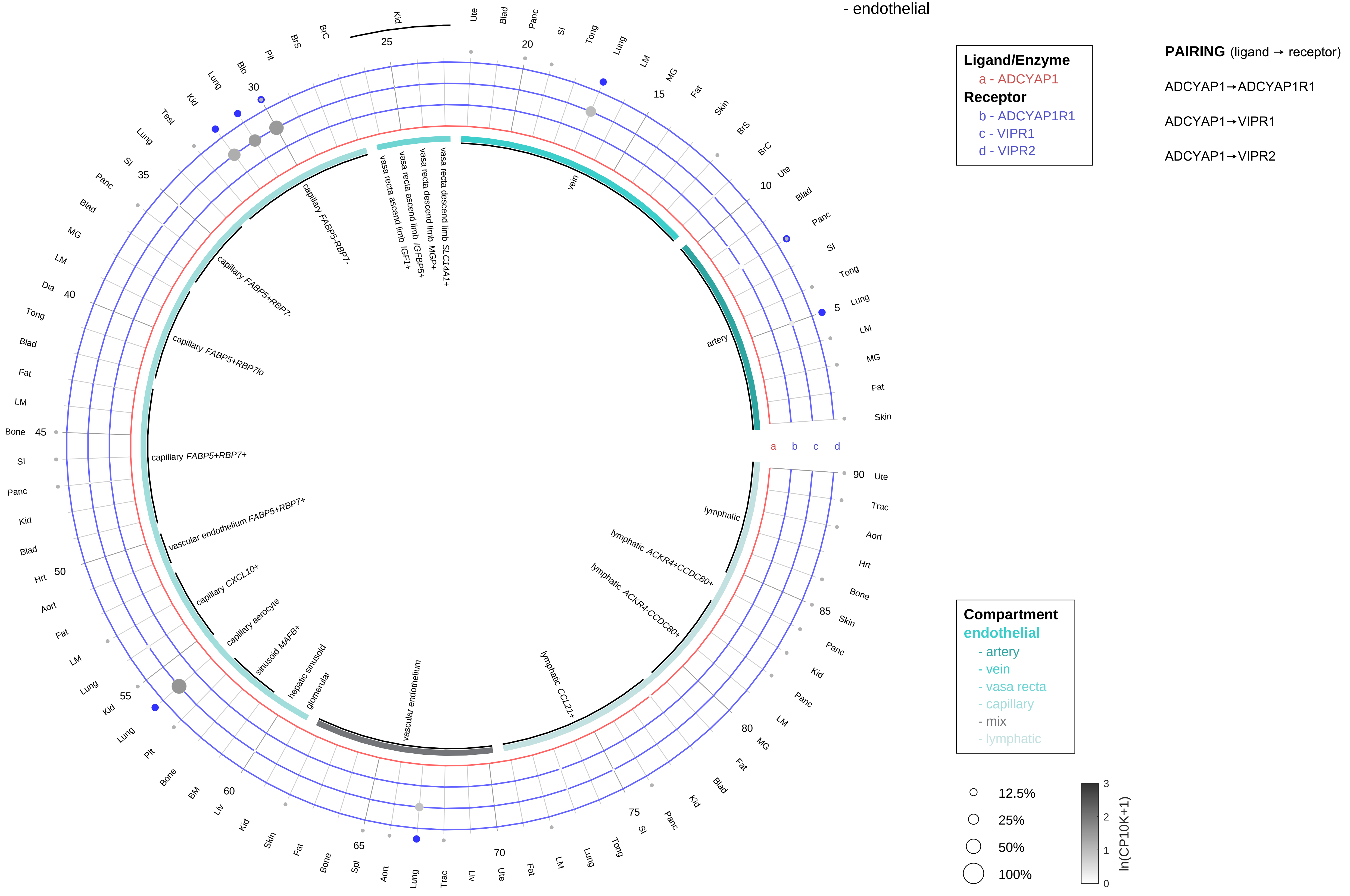

Abbrev.	Tissue
Aort	Aorta
Blad	Bladder
Blo	Blood
Bone	Bone
BM	Bone marrow
BrC	Brain cortex
BrS	Brainstem
Col	Colon
Dia	Diaphragm
Ret	Eye retina
Fat	Fat
Hrt	Heart
Pit	Hypothalamus/Pituitary
Kid	Kidney
LM	Limb muscle
Liv	Liver
Lung	Lung
MG	Mammary gland
Panc	Pancreas
Skin	Skin
SI	Small intestine
Spl	Spleen
Test	Testes
Thym	Thymus
Tong	Tongue
Trac	Trachea
Ute	Uterus

Pituitary adenylate cyclase-activating polypeptide (PACAP)
- stromal

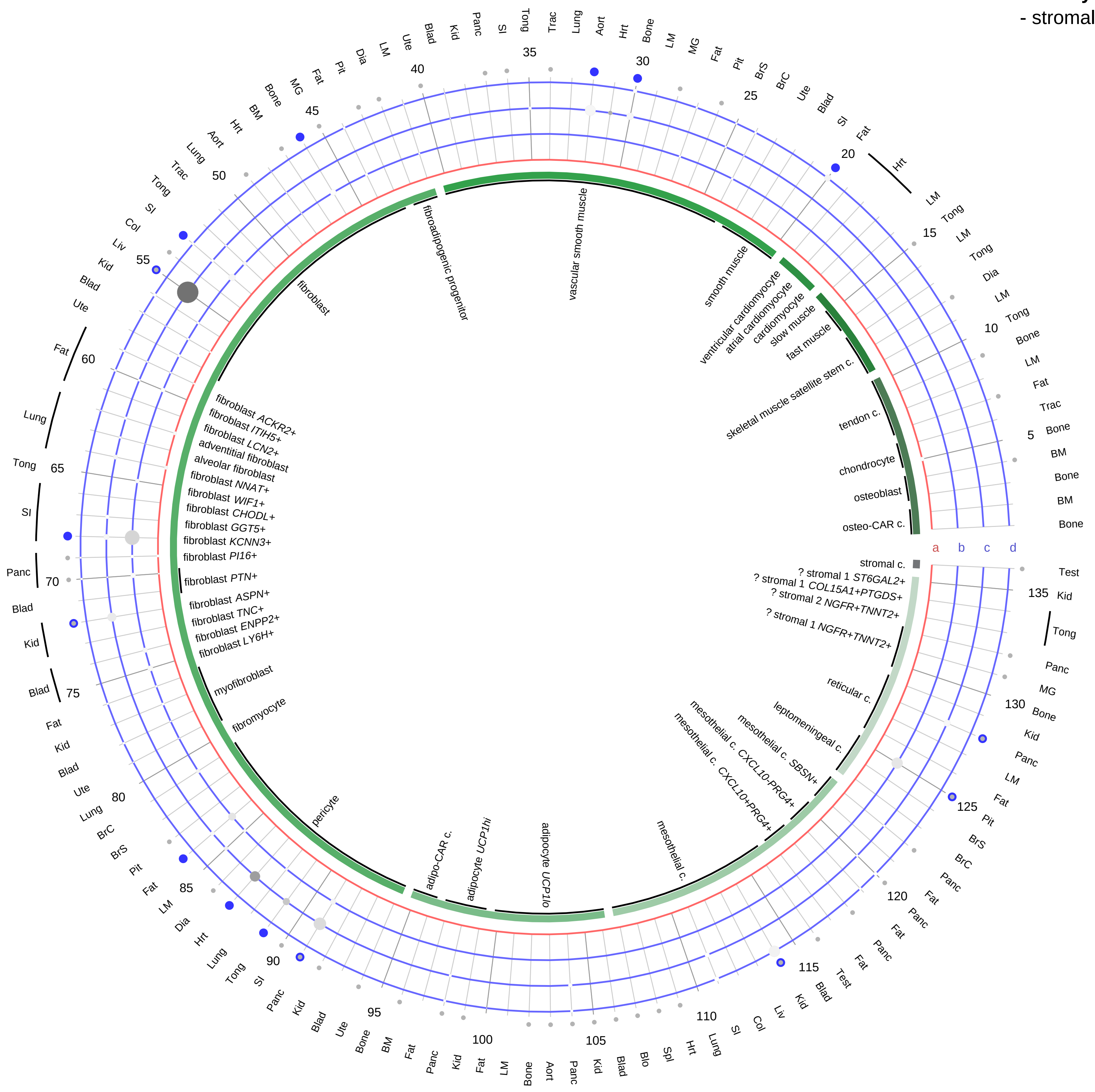

Ligand/Enzyme
a - ADCYAP1
Receptor
b - ADCYAP1R1
c - VIPR1
d - VIPR2

PAIRING (ligand → receptor)

ADCYAP1→ADCYAP1R1

ADCYAP1→VIPR1

ADCYAP1→VIPR2

Abbrev.	Tissue
Aort	Aorta
Blad	Bladder
Blo	Blood
Bone	Bone
BM	Bone marrow
BrC	Brain cortex
BrS	Brainstem
Col	Colon
Dia	Diaphragm
Ret	Eye retina
Fat	Fat
Hrt	Heart
Pit	Hypothalamus/Pituitary
Kid	Kidney
LM	Limb muscle
Liv	Liver
Lung	Lung
MG	Mammary gland
Panc	Pancreas
Skin	Skin
SI	Small intestine
Spl	Spleen
Test	Testes
Thym	Thymus
Tong	Tongue
Trac	Trachea
Ute	Uterus

Compartment
stromal
- bone
- skeletal muscle
- cardiac muscle
- smooth muscle
- fibroblast
- adipocyte
- mesothelial
- other
- mix

12.5%
25%
50%
100%

ln(CP10K+1): 0, 1, 2, 3

Pituitary adenylate cyclase-activating polypeptide (PACAP)
- lymphoid

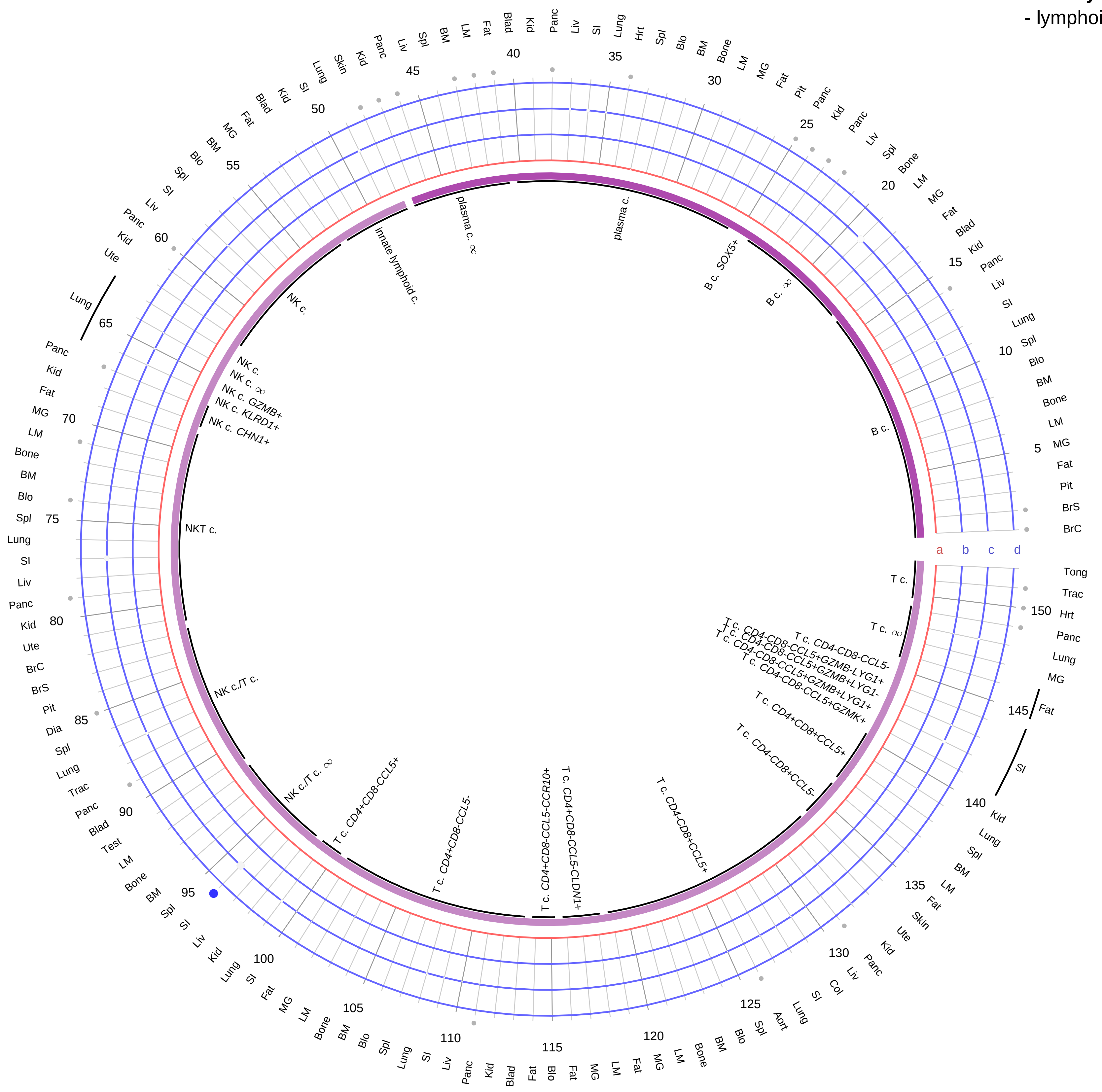

Ligand/Enzyme
a - ADCYAP1
Receptor
b - ADCYAP1R1
c - VIPR1
d - VIPR2

PAIRING (ligand → receptor)

ADCYAP1→ADCYAP1R1

ADCYAP1→VIPR1

ADCYAP1→VIPR2

Abbrev.	Tissue
Aort	Aorta
Blad	Bladder
Blo	Blood
Bone	Bone
BM	Bone marrow
BrC	Brain cortex
BrS	Brainstem
Col	Colon
Dia	Diaphragm
Ret	Eye retina
Fat	Fat
Hrt	Heart
Pit	Hypothalamus/Pituitary
Kid	Kidney
LM	Limb muscle
Liv	Liver
Lung	Lung
MG	Mammary gland
Panc	Pancreas
Skin	Skin
SI	Small intestine
Spl	Spleen
Test	Testes
Thym	Thymus
Tong	Tongue
Trac	Trachea
Ute	Uterus

Compartment
lymphoid
- B cell
- NK/T cell

12.5%
25%
50%
100%

ln(CP10K+1): 0, 1, 2, 3

Pituitary adenylate cyclase-activating polypeptide (PACAP)
- myeloid

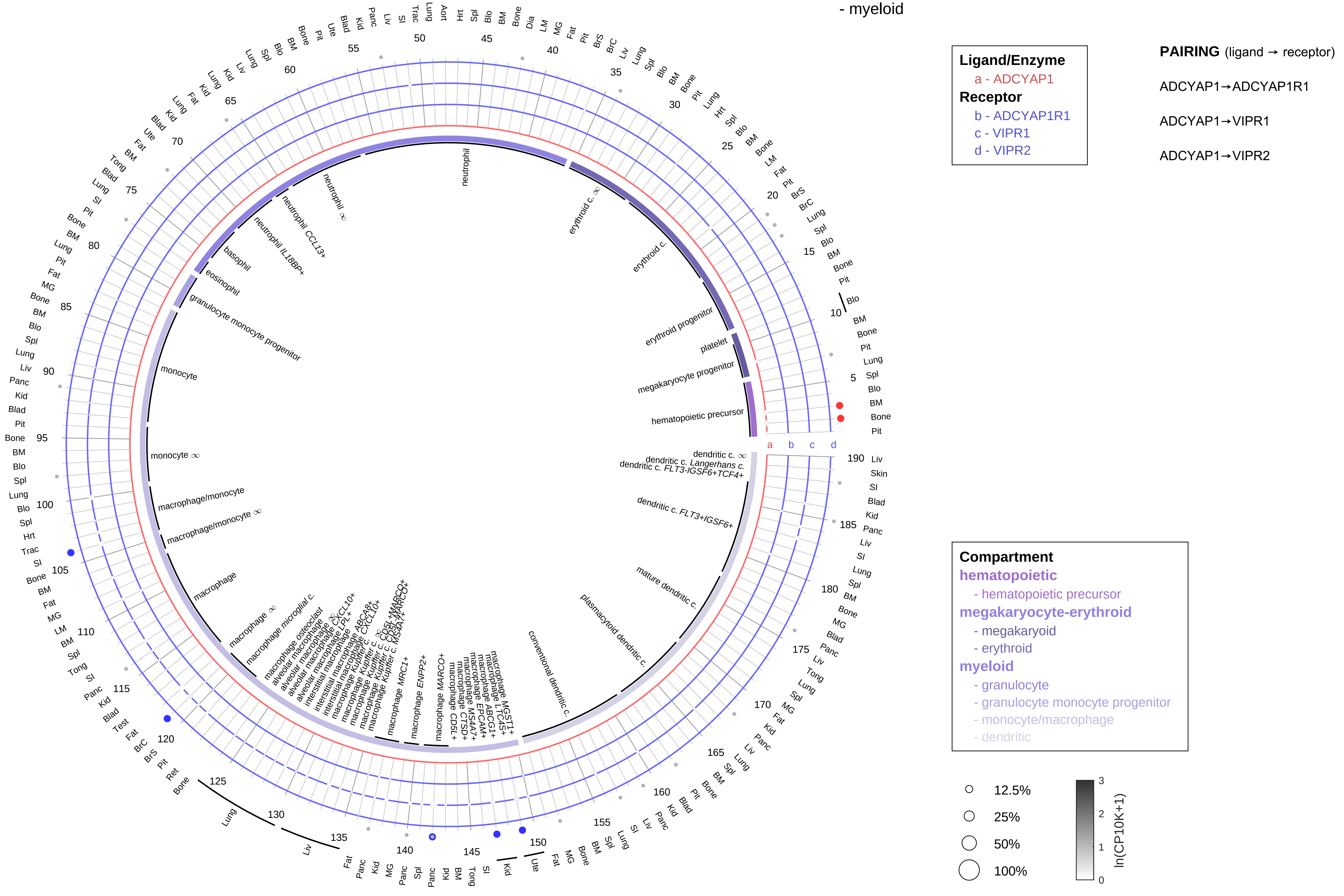

Abbrev.	Tissue
Aort	Aorta
Blad	Bladder
Blo	Blood
Bone	Bone
BM	Bone marrow
BrC	Brain cortex
BrS	Brainstem
Col	Colon
Dia	Diaphragm
Ret	Eye retina
Fat	Fat
Hrt	Heart
Pit	Hypothalamus/Pituitary
Kid	Kidney
LM	Limb muscle
Liv	Liver
Lung	Lung
MG	Mammary gland
Panc	Pancreas
Skin	Skin
SI	Small intestine
Spl	Spleen
Test	Testes
Thym	Thymus
Tong	Tongue
Trac	Trachea
Ute	Uterus

Progesterone
- epithelial/neural/germ

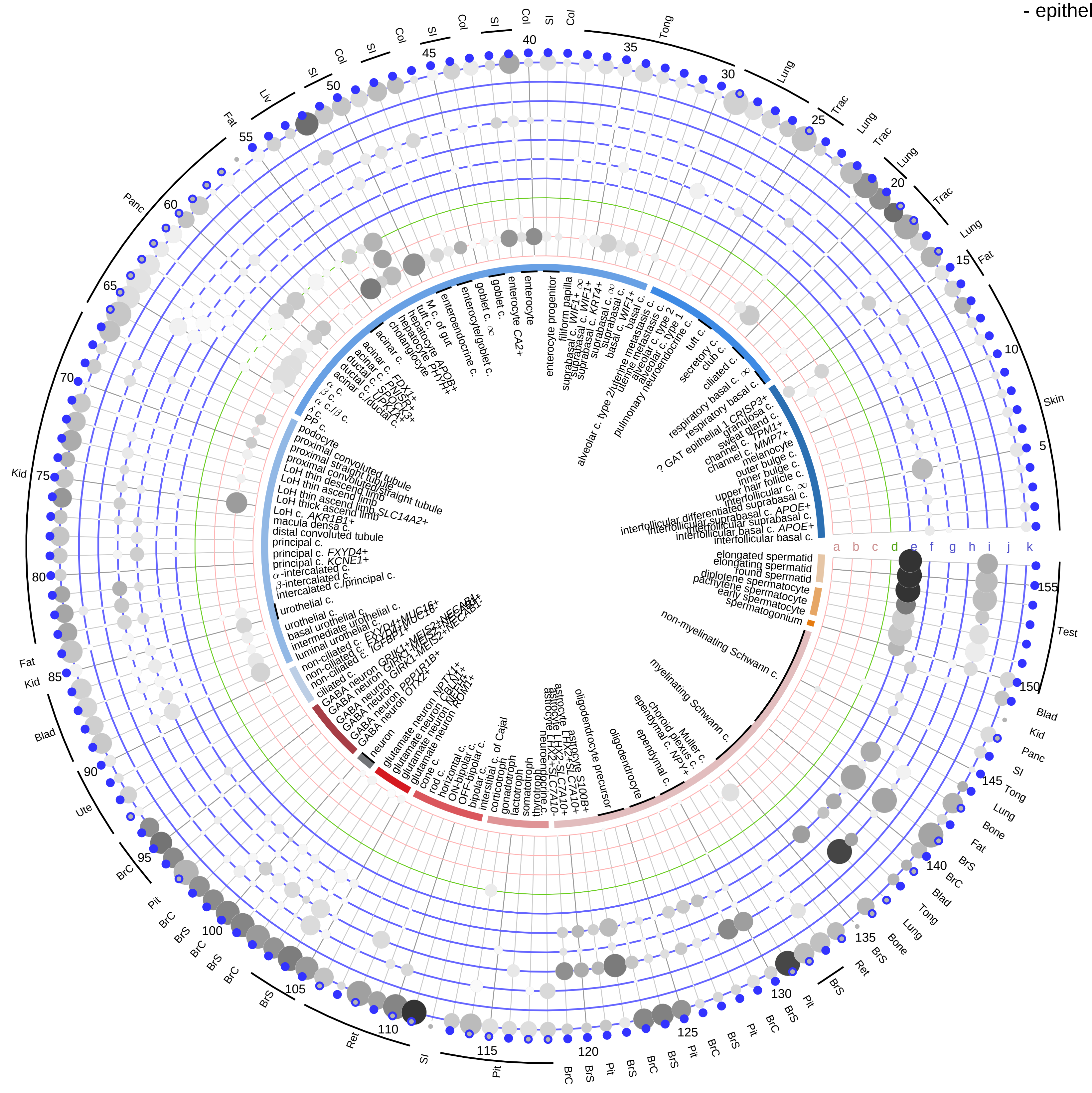

Ligand/Enzyme
- a - CYP11A1
- b - HSD17B2
- c - HSD3B2 (HSD3B1, HSD3B2)

Modulator
- d - LOC105856768 (SERPINA6)

Receptor
- e - PAQR5
- f - PAQR6
- g - PAQR7
- h - PAQR8
- i - PAQR9
- j - PGR
- k - PGRMC1

Compartment

epithelial
- integumentary
- respiratory
- gastrointestinal
- urinary
- reproductive

neural
- GABAergic
- hybrid
- glutamatergic
- specialized
- neuroendocrine
- glia

germ
- spermatogonium
- spermatocyte
- spermatid

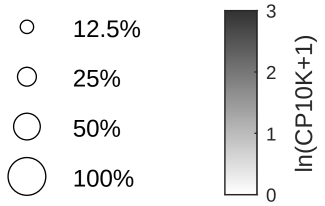

PAIRING (ligand → receptor)

HSD3B1 & HSD17B2 & CYP11A1→PGR

HSD3B1 & HSD17B2 & CYP11A1→PAQR7

HSD3B1 & HSD17B2 & CYP11A1→PAQR8

HSD3B1 & HSD17B2 & CYP11A1→PAQR5

HSD3B1 & HSD17B2 & CYP11A1→PAQR6

HSD3B1 & HSD17B2 & CYP11A1→PAQR9

HSD3B1 & HSD17B2 & CYP11A1→PGRMC1

HSD3B2 & HSD17B2 & CYP11A1→PGR

HSD3B2 & HSD17B2 & CYP11A1→PAQR7

HSD3B2 & HSD17B2 & CYP11A1→PAQR8

HSD3B2 & HSD17B2 & CYP11A1→PAQR5

HSD3B2 & HSD17B2 & CYP11A1→PAQR6

HSD3B2 & HSD17B2 & CYP11A1→PAQR9

HSD3B2 & HSD17B2 & CYP11A1→PGRMC1

Abbrev.	Tissue
Aort	Aorta
Blad	Bladder
Blo	Blood
Bone	Bone
BM	Bone marrow
BrC	Brain cortex
BrS	Brainstem
Col	Colon
Dia	Diaphragm
Ret	Eye retina
Fat	Fat
Hrt	Heart
Pit	Hypothalamus/Pituitary
Kid	Kidney
LM	Limb muscle
Liv	Liver
Lung	Lung
MG	Mammary gland
Panc	Pancreas
Skin	Skin
SI	Small intestine
Spl	Spleen
Test	Testes
Thym	Thymus
Tong	Tongue
Trac	Trachea
Ute	Uterus

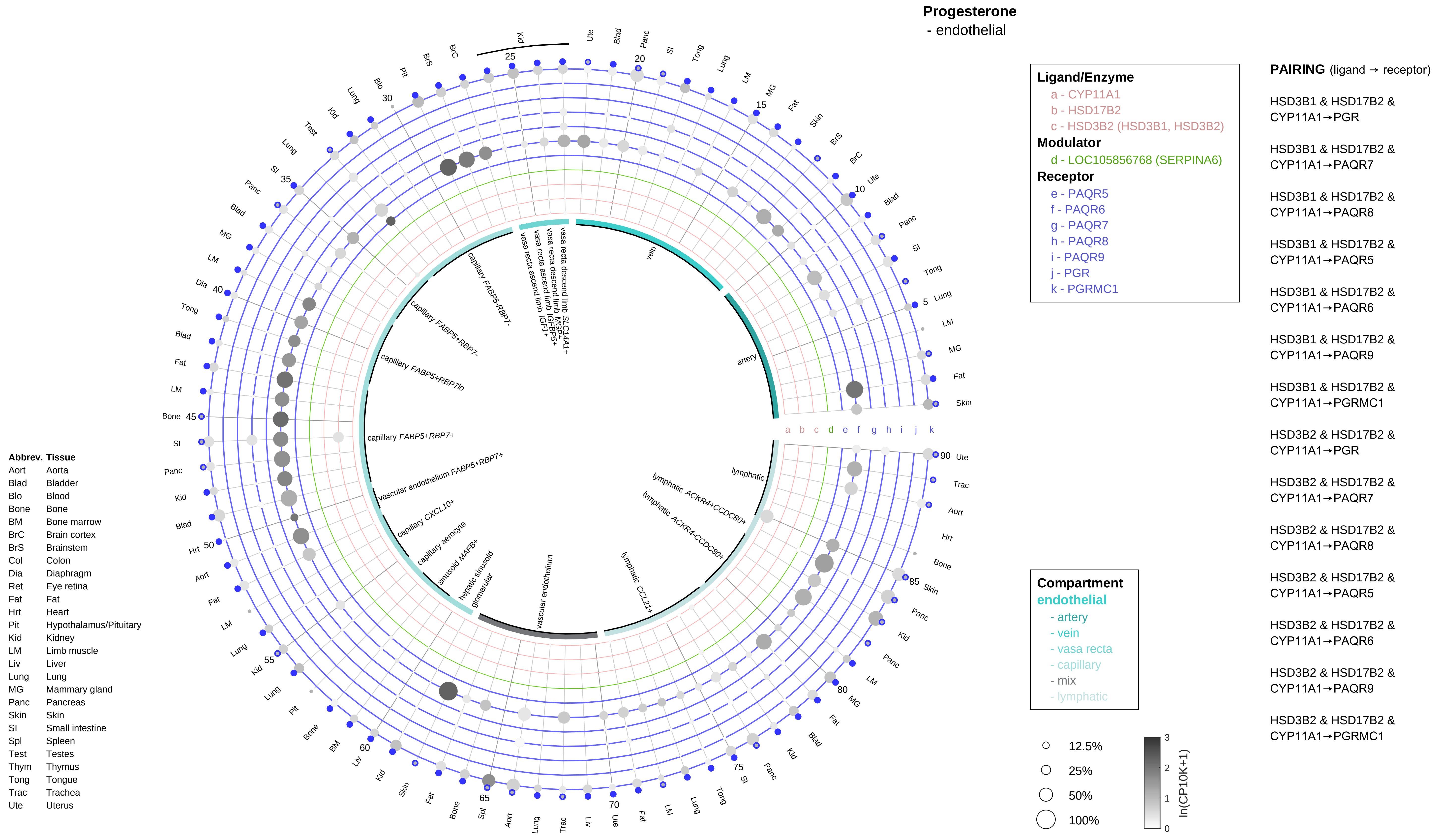

Progesterone
- endothelial
Ligand/Enzyme
a - CYP11A1
b - HSD17B2
c - HSD3B2 (HSD3B1, HSD3B2)
Modulator
d - LOC105856768 (SERPINA6)
Receptor
e - PAQR5
f - PAQR6
g - PAQR7
h - PAQR8
i - PAQR9
j - PGR
k - PGRMC1
PAIRING (ligand → receptor)
HSD3B1 & HSD17B2 & CYP11A1→PGR
HSD3B1 & HSD17B2 & CYP11A1→PAQR7
HSD3B1 & HSD17B2 & CYP11A1→PAQR8
HSD3B1 & HSD17B2 & CYP11A1→PAQR5
HSD3B1 & HSD17B2 & CYP11A1→PAQR6
HSD3B1 & HSD17B2 & CYP11A1→PAQR9
HSD3B1 & HSD17B2 & CYP11A1→PGRMC1
HSD3B2 & HSD17B2 & CYP11A1→PGR
HSD3B2 & HSD17B2 & CYP11A1→PAQR7
HSD3B2 & HSD17B2 & CYP11A1→PAQR8
HSD3B2 & HSD17B2 & CYP11A1→PAQR5
HSD3B2 & HSD17B2 & CYP11A1→PAQR6
HSD3B2 & HSD17B2 & CYP11A1→PAQR9
HSD3B2 & HSD17B2 & CYP11A1→PGRMC1
Compartment
endothelial
- artery
- vein
- vasa recta
- capillary
- mix
- lymphatic
12.5%
25%
50%
100%
ln(CP10K+1)
0
1
2
3
Abbrev. Tissue
Aort Aorta
Blad Bladder
Blo Blood
Bone Bone
BM Bone marrow
BrC Brain cortex
BrS Brainstem
Col Colon
Dia Diaphragm
Ret Eye retina
Fat Fat
Hrt Heart
Pit Hypothalamus/Pituitary
Kid Kidney
LM Limb muscle
Liv Liver
Lung Lung
MG Mammary gland
Panc Pancreas
Skin Skin
SI Small intestine
Spl Spleen
Test Testes
Thym Thymus
Tong Tongue
Trac Trachea
Ute Uterus
vein
artery
lymphatic
lymphatic ACKR4+CCDC80+
lymphatic ACKR4-CCDC80+
lymphatic CCL21+
vascular endothelium
glomerular
hepatic sinusoid
sinusoid MAFB+
capillary aerocyte
capillary CXCL10+
vascular endothelium FABP5+RBP7+
capillary FABP5+RBP7+
capillary FABP5+RBP7lo
capillary FABP5+RBP7-
capillary FABP5-RBP7-
vasa recta ascend limb IGF1+
vasa recta ascend limb IGFBP5+
vasa recta descend limb MGP+
vasa recta descend limb SLC14A1+

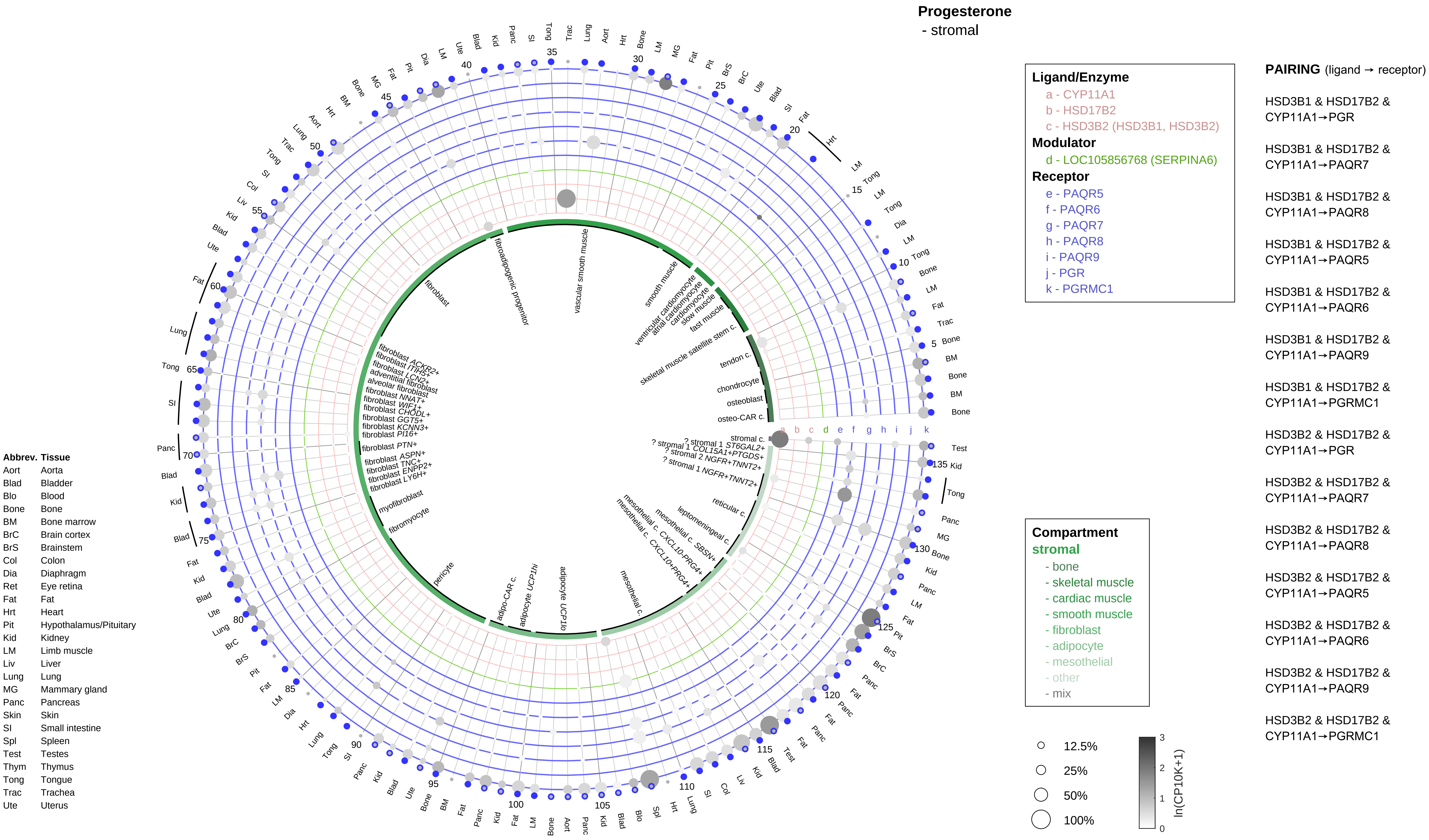
Progesterone
- stromal
Ligand/Enzyme
a - CYP11A1
b - HSD17B2
c - HSD3B2 (HSD3B1, HSD3B2)
Modulator
d - LOC105856768 (SERPINA6)
Receptor
e - PAQR5
f - PAQR6
g - PAQR7
h - PAQR8
i - PAQR9
j - PGR
k - PGRMC1
PAIRING (ligand → receptor)
HSD3B1 & HSD17B2 & CYP11A1→PGR
HSD3B1 & HSD17B2 & CYP11A1→PAQR7
HSD3B1 & HSD17B2 & CYP11A1→PAQR8
HSD3B1 & HSD17B2 & CYP11A1→PAQR5
HSD3B1 & HSD17B2 & CYP11A1→PAQR6
HSD3B1 & HSD17B2 & CYP11A1→PAQR9
HSD3B1 & HSD17B2 & CYP11A1→PGRMC1
HSD3B2 & HSD17B2 & CYP11A1→PGR
HSD3B2 & HSD17B2 & CYP11A1→PAQR7
HSD3B2 & HSD17B2 & CYP11A1→PAQR8
HSD3B2 & HSD17B2 & CYP11A1→PAQR5
HSD3B2 & HSD17B2 & CYP11A1→PAQR6
HSD3B2 & HSD17B2 & CYP11A1→PAQR9
HSD3B2 & HSD17B2 & CYP11A1→PGRMC1
Compartment
stromal
- bone
- skeletal muscle
- cardiac muscle
- smooth muscle
- fibroblast
- adipocyte
- mesothelial
- other
- mix
12.5%
25%
50%
100%
ln(CP10K+1)
0
1
2
3
Abbrev. Tissue
Aort Aorta
Blad Bladder
Blo Blood
Bone Bone
BM Bone marrow
BrC Brain cortex
BrS Brainstem
Col Colon
Dia Diaphragm
Ret Eye retina
Fat Fat
Hrt Heart
Pit Hypothalamus/Pituitary
Kid Kidney
LM Limb muscle
Liv Liver
Lung Lung
MG Mammary gland
Panc Pancreas
Skin Skin
SI Small intestine
Spl Spleen
Test Testes
Thym Thymus
Tong Tongue
Trac Trachea
Ute Uterus
fibroblast
fibroadipogenic progenitor
vascular smooth muscle
smooth muscle
ventricular cardiomyocyte
atrial cardiomyocyte
slow muscle
fast muscle
skeletal muscle satellite stem c.
tendon c.
chondrocyte
osteoblast
osteo-CAR c.
stromal c.
? stromal 1 ST6GAL2+
? stromal 1 COL15A1+PTGDS+
? stromal 2 NGFR+TNNT2+
? stromal 1 NGFR+TNNT2+
reticular c.
leptomeningeal c.
mesothelial c. SBSN+
mesothelial c. CXCL10+PRG4+
mesothelial c. CXCL10+PRG4+
mesothelial c.
adipocyte UCP1lo
adipocyte UCP1hi
adipo-CAR c.
pericyte
fibromyocyte
myofibroblast
fibroblast LY6H+
fibroblast ENPP2+
fibroblast TNC+
fibroblast ASPN+
fibroblast PTN+
fibroblast PI16+
fibroblast KCNN3+
fibroblast GGT5+
fibroblast CHODL+
fibroblast WIF1+
fibroblast NNAT+
alveolar fibroblast
adventitial fibroblast
fibroblast LCN2+
fibroblast ITIH5+
fibroblast ACKR2+
a b c d e f g h i j k

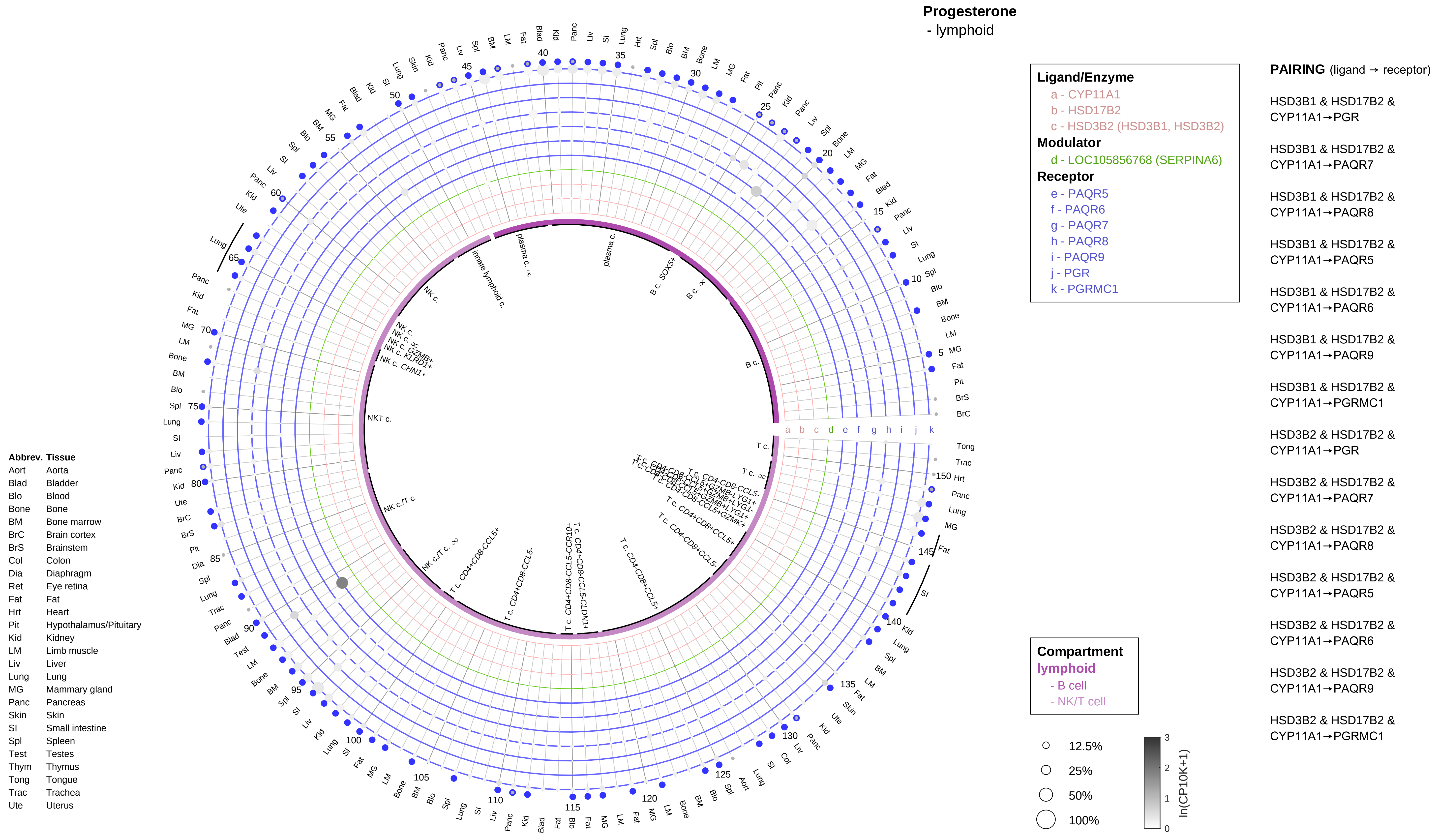
Progesterone
- lymphoid
Ligand/Enzyme
a - CYP11A1
b - HSD17B2
c - HSD3B2 (HSD3B1, HSD3B2)
Modulator
d - LOC105856768 (SERPINA6)
Receptor
e - PAQR5
f - PAQR6
g - PAQR7
h - PAQR8
i - PAQR9
j - PGR
k - PGRMC1
PAIRING (ligand → receptor)
HSD3B1 & HSD17B2 & CYP11A1→PGR
HSD3B1 & HSD17B2 & CYP11A1→PAQR7
HSD3B1 & HSD17B2 & CYP11A1→PAQR8
HSD3B1 & HSD17B2 & CYP11A1→PAQR5
HSD3B1 & HSD17B2 & CYP11A1→PAQR6
HSD3B1 & HSD17B2 & CYP11A1→PAQR9
HSD3B1 & HSD17B2 & CYP11A1→PGRMC1
HSD3B2 & HSD17B2 & CYP11A1→PGR
HSD3B2 & HSD17B2 & CYP11A1→PAQR7
HSD3B2 & HSD17B2 & CYP11A1→PAQR8
HSD3B2 & HSD17B2 & CYP11A1→PAQR5
HSD3B2 & HSD17B2 & CYP11A1→PAQR6
HSD3B2 & HSD17B2 & CYP11A1→PAQR9
HSD3B2 & HSD17B2 & CYP11A1→PGRMC1
Compartment
lymphoid
- B cell
- NK/T cell
12.5%
25%
50%
100%
ln(CP10K+1)
0
1
2
3
Abbrev. Tissue
Aort Aorta
Blad Bladder
Blo Blood
Bone Bone
BM Bone marrow
BrC Brain cortex
BrS Brainstem
Col Colon
Dia Diaphragm
Ret Eye retina
Fat Fat
Hrt Heart
Pit Hypothalamus/Pituitary
Kid Kidney
LM Limb muscle
Liv Liver
Lung Lung
MG Mammary gland
Panc Pancreas
Skin Skin
SI Small intestine
Spl Spleen
Test Testes
Thym Thymus
Tong Tongue
Trac Trachea
Ute Uterus
a b c d e f g h i j k
B c.
B c. SOX5+
B c. ∞
plasma c.
plasma c. ∞
innate lymphoid c.
NK c.
NK c.
NK c. ∞
NK c. GZMB+
NK c. KLRD1+
NK c. CHN1+
NKT c.
NK c./T c.
NK c./T c. ∞
T c. CD4+CD8-CCL5+
T c. CD4+CD8-CCL5-
T c. CD4+CD8-CCL5-CCR10+
T c. CD4+CD8-CCL5-CLDN1+
T c. CD4-CD8+CCL5+
T c. CD4-CD8+CCL5-
T c. CD4+CD8+CCL5+
T c. CD4-CD8-CCL5+GZMK+
T c.
T c. ∞
Lung
SI

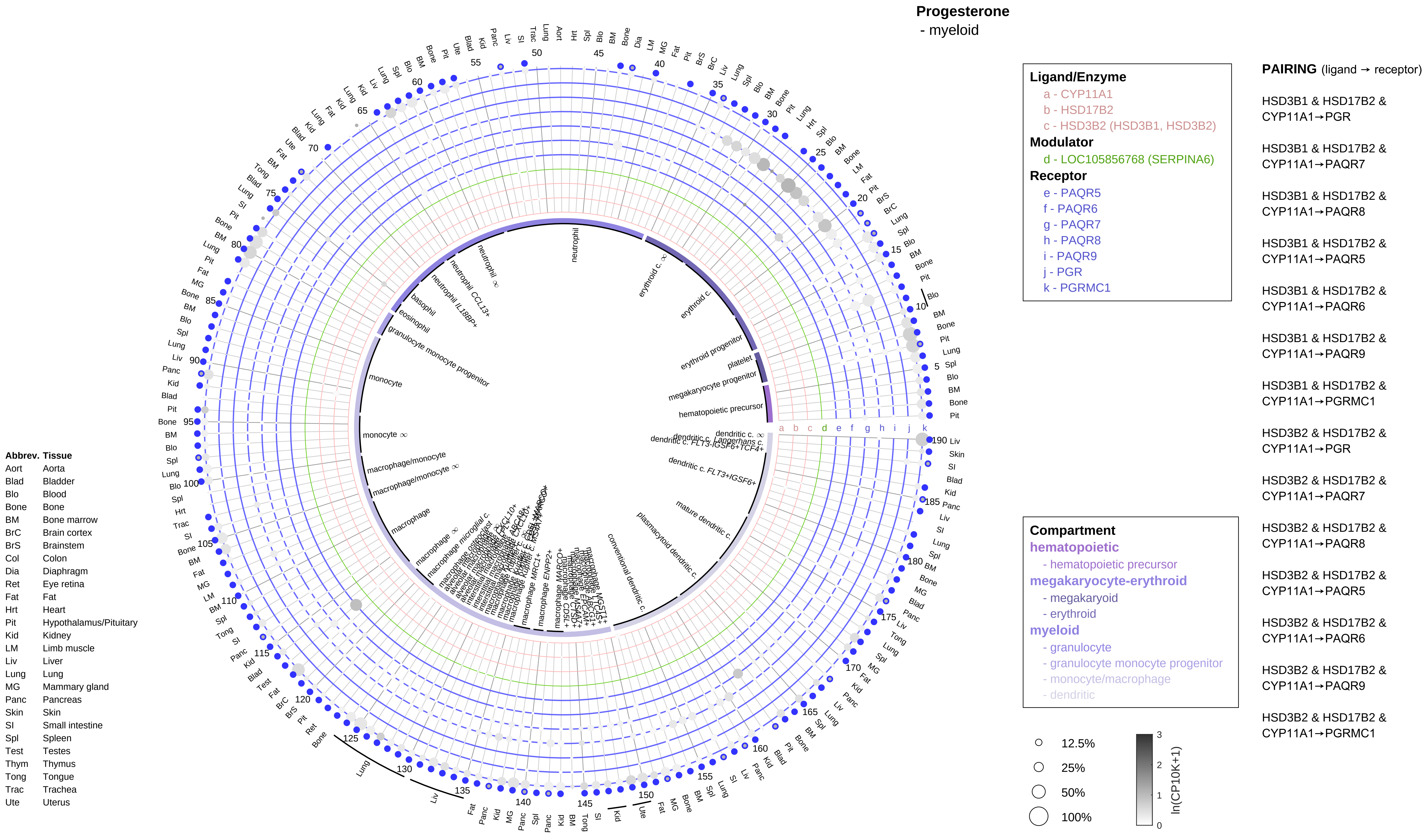

Progesterone
- myeloid
Ligand/Enzyme
a - CYP11A1
b - HSD17B2
c - HSD3B2 (HSD3B1, HSD3B2)
Modulator
d - LOC105856768 (SERPINA6)
Receptor
e - PAQR5
f - PAQR6
g - PAQR7
h - PAQR8
i - PAQR9
j - PGR
k - PGRMC1
PAIRING (ligand → receptor)
HSD3B1 & HSD17B2 & CYP11A1→PGR
HSD3B1 & HSD17B2 & CYP11A1→PAQR7
HSD3B1 & HSD17B2 & CYP11A1→PAQR8
HSD3B1 & HSD17B2 & CYP11A1→PAQR5
HSD3B1 & HSD17B2 & CYP11A1→PAQR6
HSD3B1 & HSD17B2 & CYP11A1→PAQR9
HSD3B1 & HSD17B2 & CYP11A1→PGRMC1
HSD3B2 & HSD17B2 & CYP11A1→PGR
HSD3B2 & HSD17B2 & CYP11A1→PAQR7
HSD3B2 & HSD17B2 & CYP11A1→PAQR8
HSD3B2 & HSD17B2 & CYP11A1→PAQR5
HSD3B2 & HSD17B2 & CYP11A1→PAQR6
HSD3B2 & HSD17B2 & CYP11A1→PAQR9
HSD3B2 & HSD17B2 & CYP11A1→PGRMC1
Compartment
hematopoietic
- hematopoietic precursor
megakaryocyte-erythroid
- megakaryoid
- erythroid
myeloid
- granulocyte
- granulocyte monocyte progenitor
- monocyte/macrophage
- dendritic
12.5%
25%
50%
100%
ln(CP10K+1)
0
1
2
3
Abbrev. Tissue
Aort Aorta
Blad Bladder
Blo Blood
Bone Bone
BM Bone marrow
BrC Brain cortex
BrS Brainstem
Col Colon
Dia Diaphragm
Ret Eye retina
Fat Fat
Hrt Heart
Pit Hypothalamus/Pituitary
Kid Kidney
LM Limb muscle
Liv Liver
Lung Lung
MG Mammary gland
Panc Pancreas
Skin Skin
SI Small intestine
Spl Spleen
Test Testes
Thym Thymus
Tong Tongue
Trac Trachea
Ute Uterus

Prokineticin/Bv8
- epithelial/neural/germ

Ligand/Enzyme
- a - PROK1
- b - PROK2

Receptor
- c - PROKR1
- d - PROKR2

PAIRING (ligand → receptor)

PROK1→PROKR1

PROK1→PROKR2

PROK2→PROKR1

PROK2→PROKR2

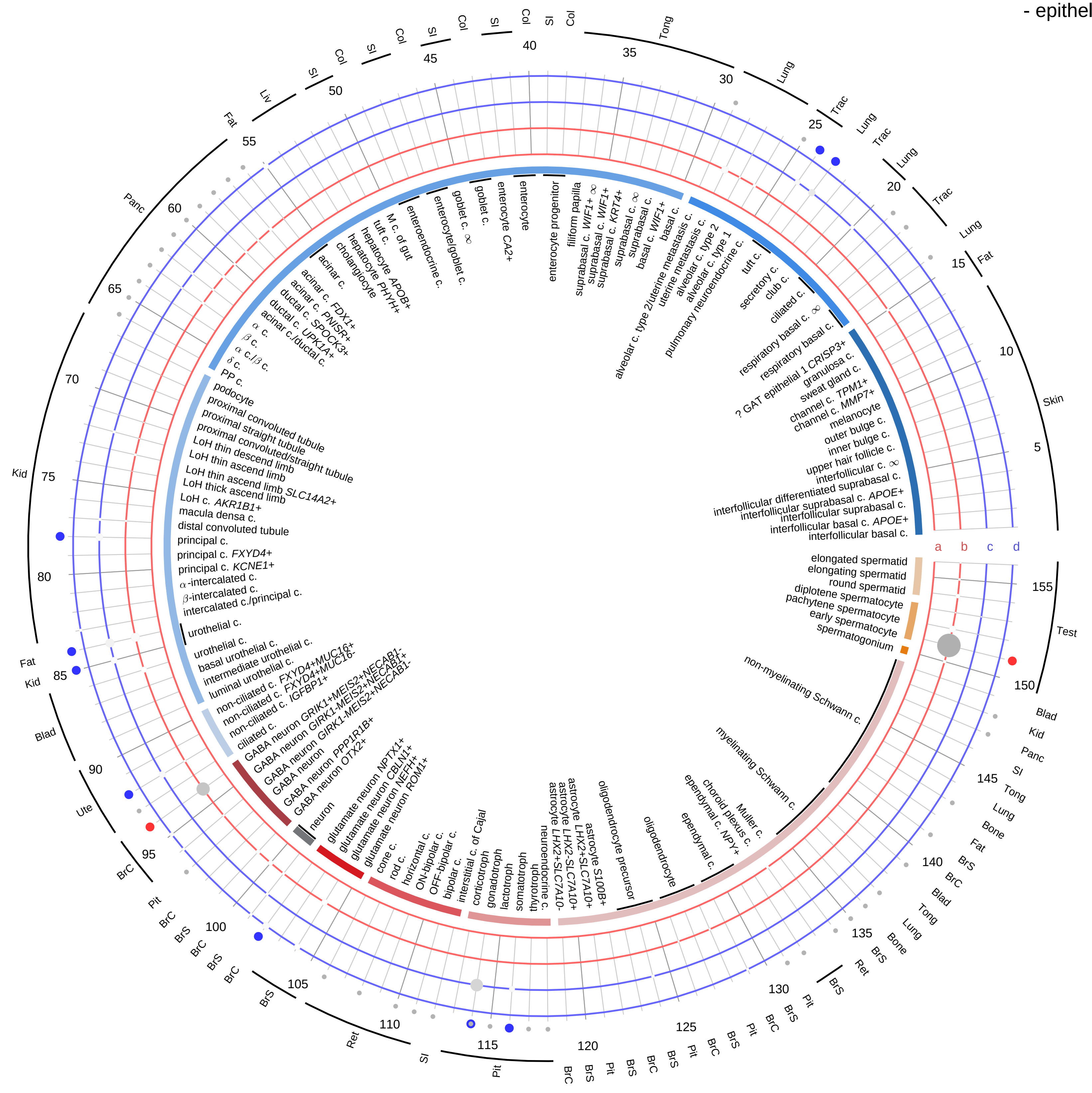

Compartment

epithelial
- integumentary
- respiratory
- gastrointestinal
- urinary
- reproductive

neural
- GABAergic
- hybrid
- glutamatergic
- specialized
- neuroendocrine
- glia

germ
- spermatogonium
- spermatocyte
- spermatid

12.5%
25%
50%
100%

ln(CP10K+1): 0, 1, 2, 3

Abbrev.	Tissue
Aort	Aorta
Blad	Bladder
Blo	Blood
Bone	Bone
BM	Bone marrow
BrC	Brain cortex
BrS	Brainstem
Col	Colon
Dia	Diaphragm
Ret	Eye retina
Fat	Fat
Hrt	Heart
Pit	Hypothalamus/Pituitary
Kid	Kidney
LM	Limb muscle
Liv	Liver
Lung	Lung
MG	Mammary gland
Panc	Pancreas
Skin	Skin
SI	Small intestine
Spl	Spleen
Test	Testes
Thym	Thymus
Tong	Tongue
Trac	Trachea
Ute	Uterus

Prokineticin/Bv8
- endothelial

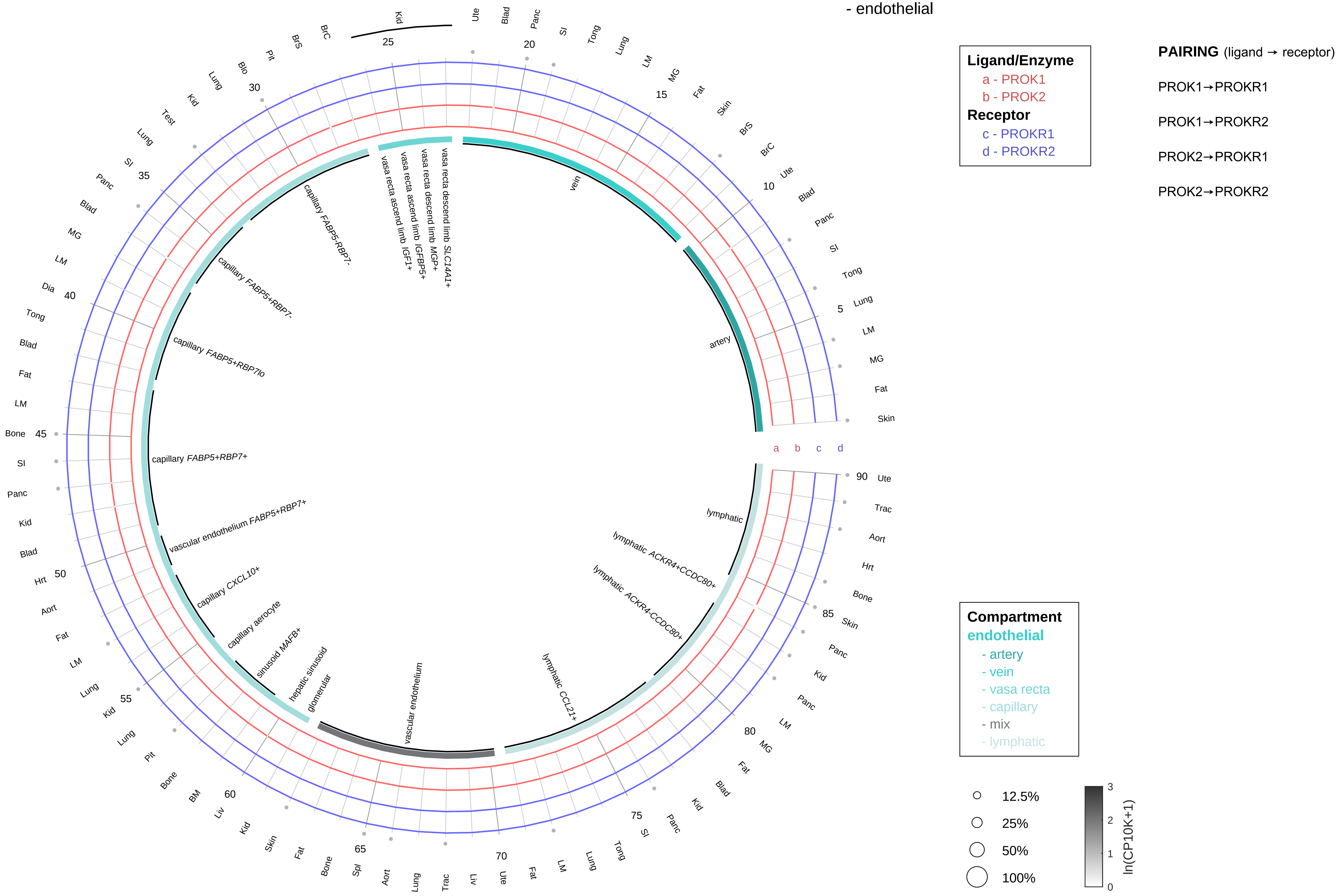

Abbrev.	Tissue
Aort	Aorta
Blad	Bladder
Blo	Blood
Bone	Bone
BM	Bone marrow
BrC	Brain cortex
BrS	Brainstem
Col	Colon
Dia	Diaphragm
Ret	Eye retina
Fat	Fat
Hrt	Heart
Pit	Hypothalamus/Pituitary
Kid	Kidney
LM	Limb muscle
Liv	Liver
Lung	Lung
MG	Mammary gland
Panc	Pancreas
Skin	Skin
SI	Small intestine
Spl	Spleen
Test	Testes
Thym	Thymus
Tong	Tongue
Trac	Trachea
Ute	Uterus

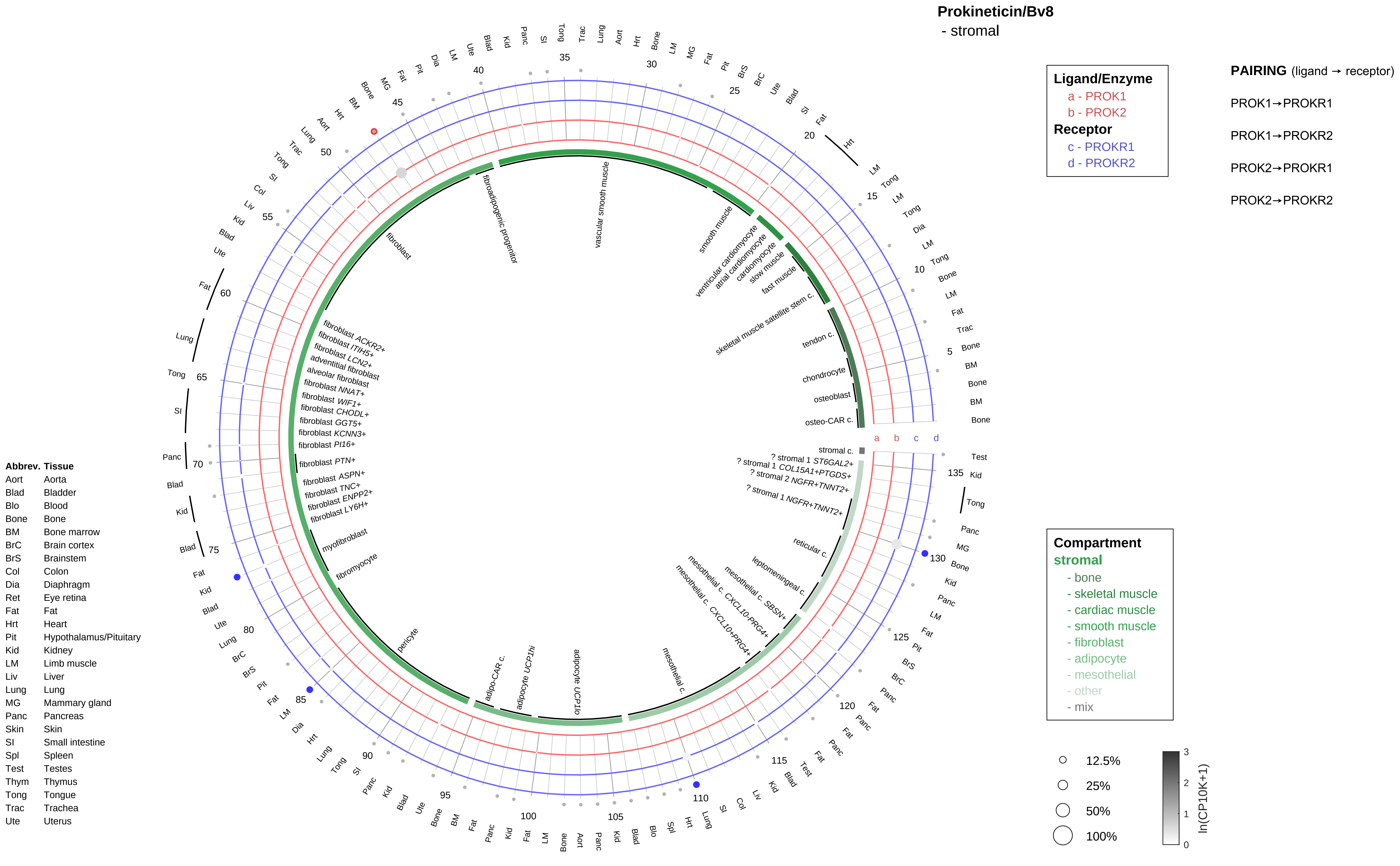
Prokineticin/Bv8
- stromal
Ligand/Enzyme
a - PROK1
b - PROK2
Receptor
c - PROKR1
d - PROKR2
PAIRING (ligand → receptor)
PROK1→PROKR1
PROK1→PROKR2
PROK2→PROKR1
PROK2→PROKR2
Compartment
stromal
- bone
- skeletal muscle
- cardiac muscle
- smooth muscle
- fibroblast
- adipocyte
- mesothelial
- other
- mix
12.5%
25%
50%
100%
ln(CP10K+1)
0
1
2
3
a
b
c
d
osteo-CAR c.
osteoblast
chondrocyte
tendon c.
skeletal muscle satellite stem c.
fast muscle
slow muscle
cardiomyocyte
atrial cardiomyocyte
ventricular cardiomyocyte
smooth muscle
vascular smooth muscle
fibroadipogenic progenitor
fibroblast
fibroblast ACKR2+
fibroblast ITIH5+
fibroblast LCN2+
adventitial fibroblast
alveolar fibroblast
fibroblast NNAT+
fibroblast WIF1+
fibroblast CHODL+
fibroblast GGT5+
fibroblast KCNN3+
fibroblast PI16+
fibroblast PTN+
fibroblast ASPN+
fibroblast TNC+
fibroblast ENPP2+
fibroblast LY6H+
myofibroblast
fibromyocyte
pericyte
adipo-CAR c.
adipocyte UCP1hi
adipocyte UCP1lo
mesothelial c.
mesothelial c. CXCL10+PRG4+
mesothelial c. CXCL10-PRG4+
mesothelial c. SBSN+
leptomeningeal c.
reticular c.
? stromal 1 NGFR+TNNT2+
? stromal 2 NGFR+TNNT2+
? stromal 1 COL15A1+PTGDS+
? stromal 1 ST6GAL2+
stromal c.
Abbrev. Tissue
Aort Aorta
Blad Bladder
Blo Blood
Bone Bone
BM Bone marrow
BrC Brain cortex
BrS Brainstem
Col Colon
Dia Diaphragm
Ret Eye retina
Fat Fat
Hrt Heart
Pit Hypothalamus/Pituitary
Kid Kidney
LM Limb muscle
Liv Liver
Lung Lung
MG Mammary gland
Panc Pancreas
Skin Skin
SI Small intestine
Spl Spleen
Test Testes
Thym Thymus
Tong Tongue
Trac Trachea
Ute Uterus

Prokineticin/Bv8
- lymphoid

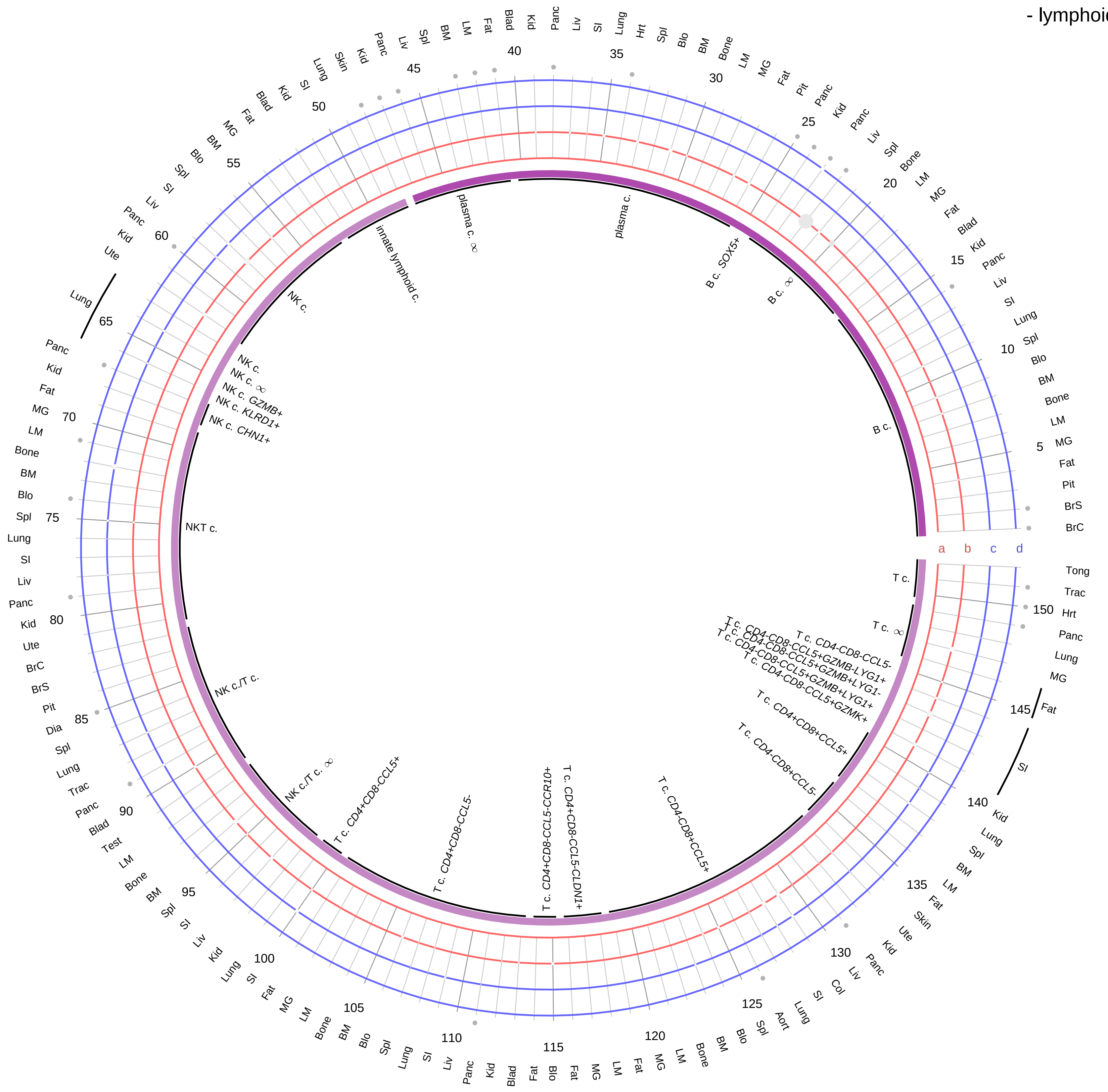

Ligand/Enzyme
- a - PROK1
- b - PROK2

Receptor
- c - PROKR1
- d - PROKR2

PAIRING (ligand → receptor)

PROK1→PROKR1

PROK1→PROKR2

PROK2→PROKR1

PROK2→PROKR2

Compartment
lymphoid
- B cell
- NK/T cell

12.5%
25%
50%
100%

ln(CP10K+1): 0, 1, 2, 3

Abbrev.	Tissue
Aort	Aorta
Blad	Bladder
Blo	Blood
Bone	Bone
BM	Bone marrow
BrC	Brain cortex
BrS	Brainstem
Col	Colon
Dia	Diaphragm
Ret	Eye retina
Fat	Fat
Hrt	Heart
Pit	Hypothalamus/Pituitary
Kid	Kidney
LM	Limb muscle
Liv	Liver
Lung	Lung
MG	Mammary gland
Panc	Pancreas
Skin	Skin
SI	Small intestine
Spl	Spleen
Test	Testes
Thym	Thymus
Tong	Tongue
Trac	Trachea
Ute	Uterus

Prokineticin/Bv8

- myeloid

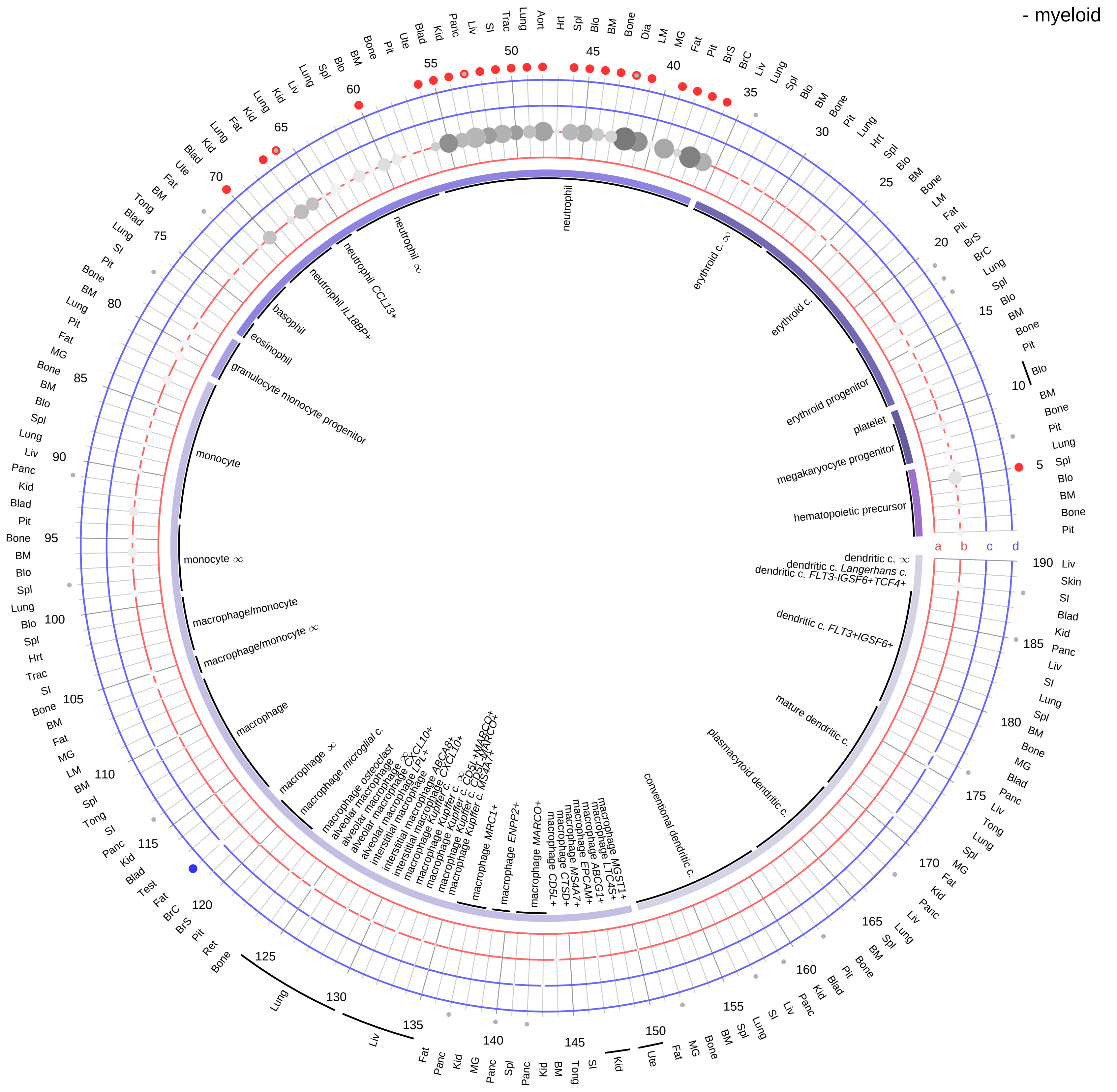

Abbrev.	Tissue
Aort	Aorta
Blad	Bladder
Blo	Blood
Bone	Bone
BM	Bone marrow
BrC	Brain cortex
BrS	Brainstem
Col	Colon
Dia	Diaphragm
Ret	Eye retina
Fat	Fat
Hrt	Heart
Pit	Hypothalamus/Pituitary
Kid	Kidney
LM	Limb muscle
Liv	Liver
Lung	Lung
MG	Mammary gland
Panc	Pancreas
Skin	Skin
SI	Small intestine
Spl	Spleen
Test	Testes
Thym	Thymus
Tong	Tongue
Trac	Trachea
Ute	Uterus

Ligand/Enzyme
- a - PROK1
- b - PROK2

Receptor
- c - PROKR1
- d - PROKR2

PAIRING (ligand → receptor)

PROK1→PROKR1

PROK1→PROKR2

PROK2→PROKR1

PROK2→PROKR2

Compartment

hematopoietic
- hematopoietic precursor

megakaryocyte-erythroid
- megakaryoid
- erythroid

myeloid
- granulocyte
- granulocyte monocyte progenitor
- monocyte/macrophage
- dendritic

12.5%

25%

50%

100%

ln(CP10K+1): 0, 1, 2, 3

Prolactin (PRL)
- epithelial/neural/germ

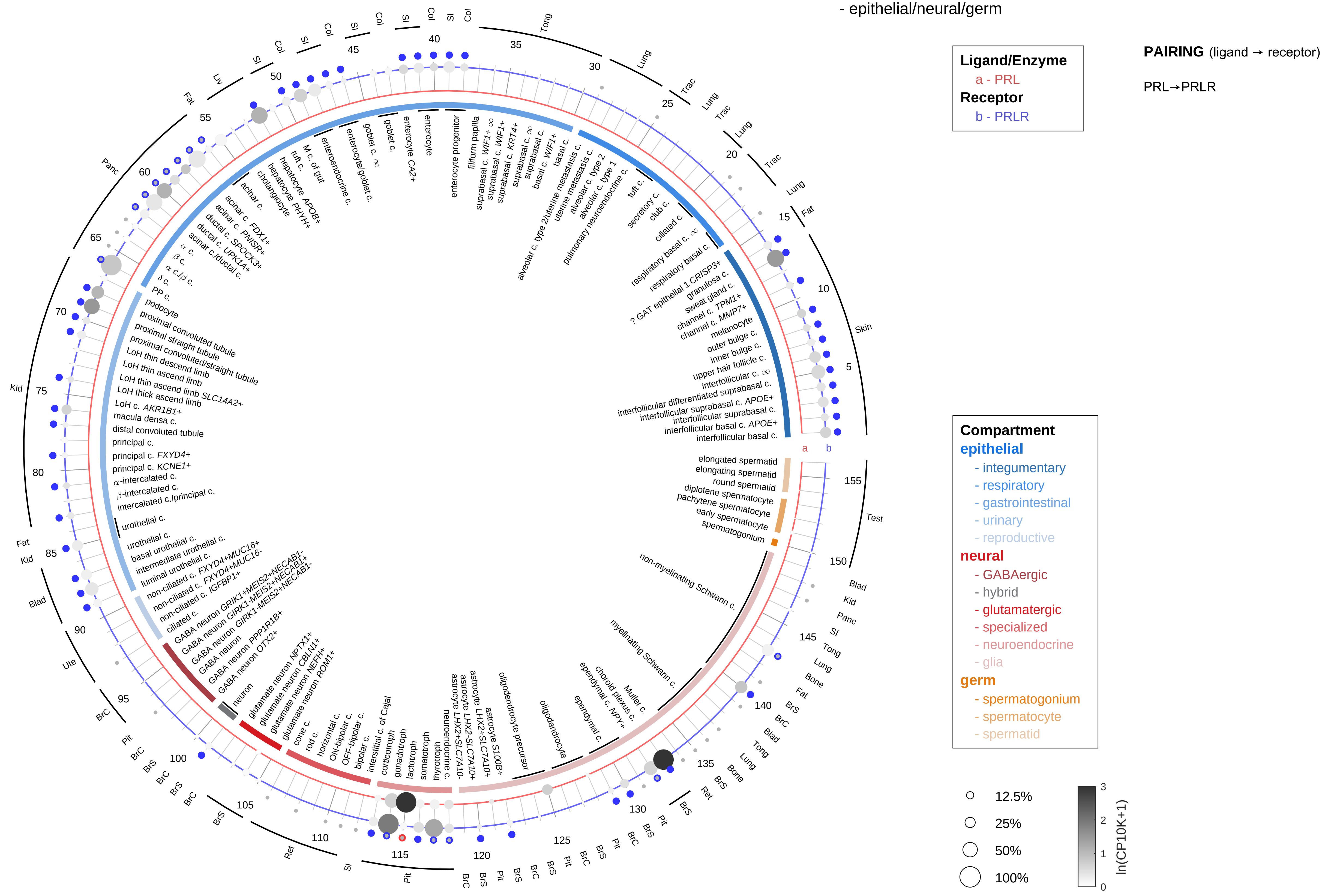

Abbrev.	Tissue
Aort	Aorta
Blad	Bladder
Blo	Blood
Bone	Bone
BM	Bone marrow
BrC	Brain cortex
BrS	Brainstem
Col	Colon
Dia	Diaphragm
Ret	Eye retina
Fat	Fat
Hrt	Heart
Pit	Hypothalamus/Pituitary
Kid	Kidney
LM	Limb muscle
Liv	Liver
Lung	Lung
MG	Mammary gland
Panc	Pancreas
Skin	Skin
SI	Small intestine
Spl	Spleen
Test	Testes
Thym	Thymus
Tong	Tongue
Trac	Trachea
Ute	Uterus

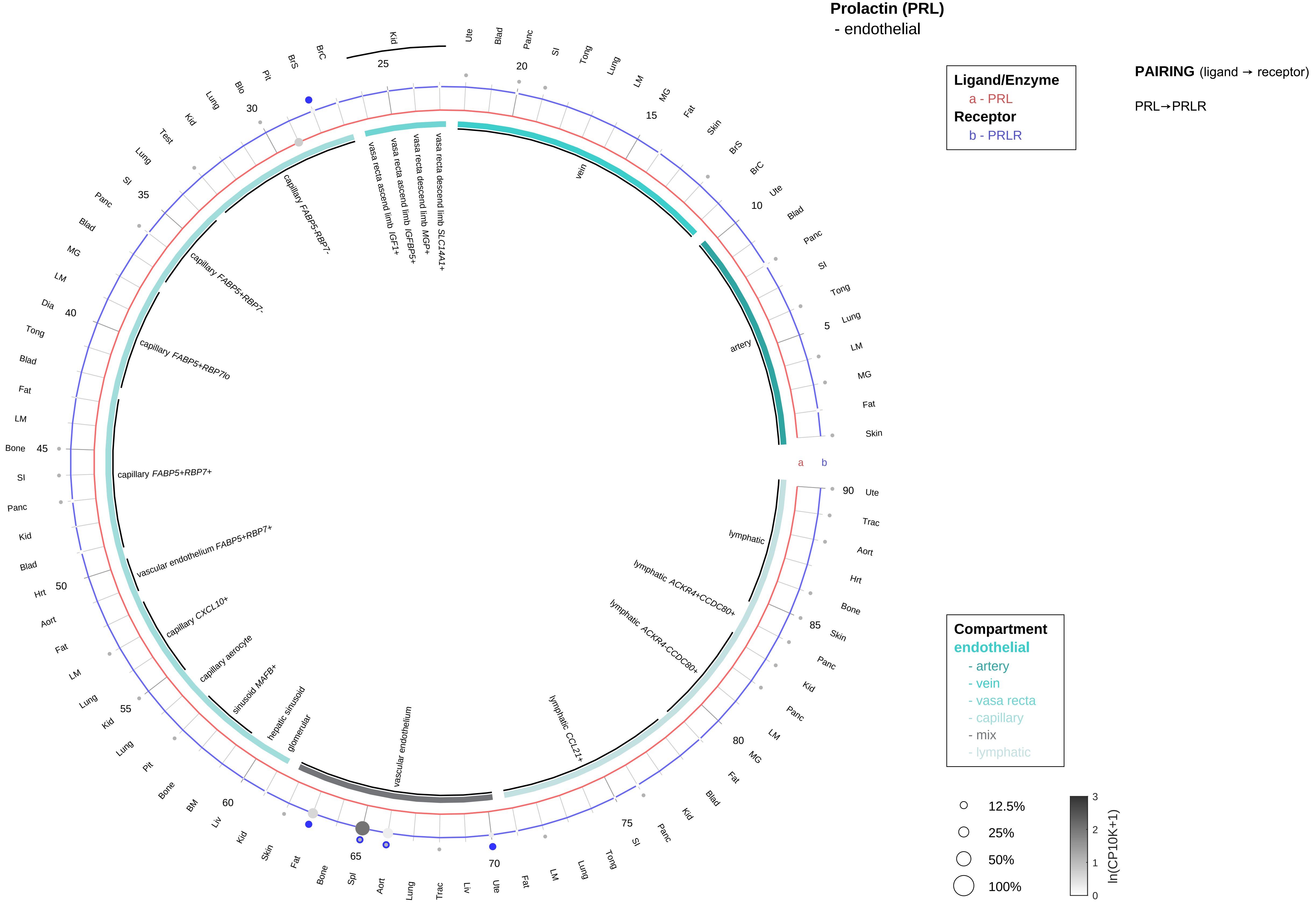

Abbrev.	Tissue
Aort	Aorta
Blad	Bladder
Blo	Blood
Bone	Bone
BM	Bone marrow
BrC	Brain cortex
BrS	Brainstem
Col	Colon
Dia	Diaphragm
Ret	Eye retina
Fat	Fat
Hrt	Heart
Pit	Hypothalamus/Pituitary
Kid	Kidney
LM	Limb muscle
Liv	Liver
Lung	Lung
MG	Mammary gland
Panc	Pancreas
Skin	Skin
SI	Small intestine
Spl	Spleen
Test	Testes
Thym	Thymus
Tong	Tongue
Trac	Trachea
Ute	Uterus

Prolactin (PRL)
- stromal

Ligand/Enzyme
a - PRL
Receptor
b - PRLR

PAIRING (ligand → receptor)

PRL→PRLR

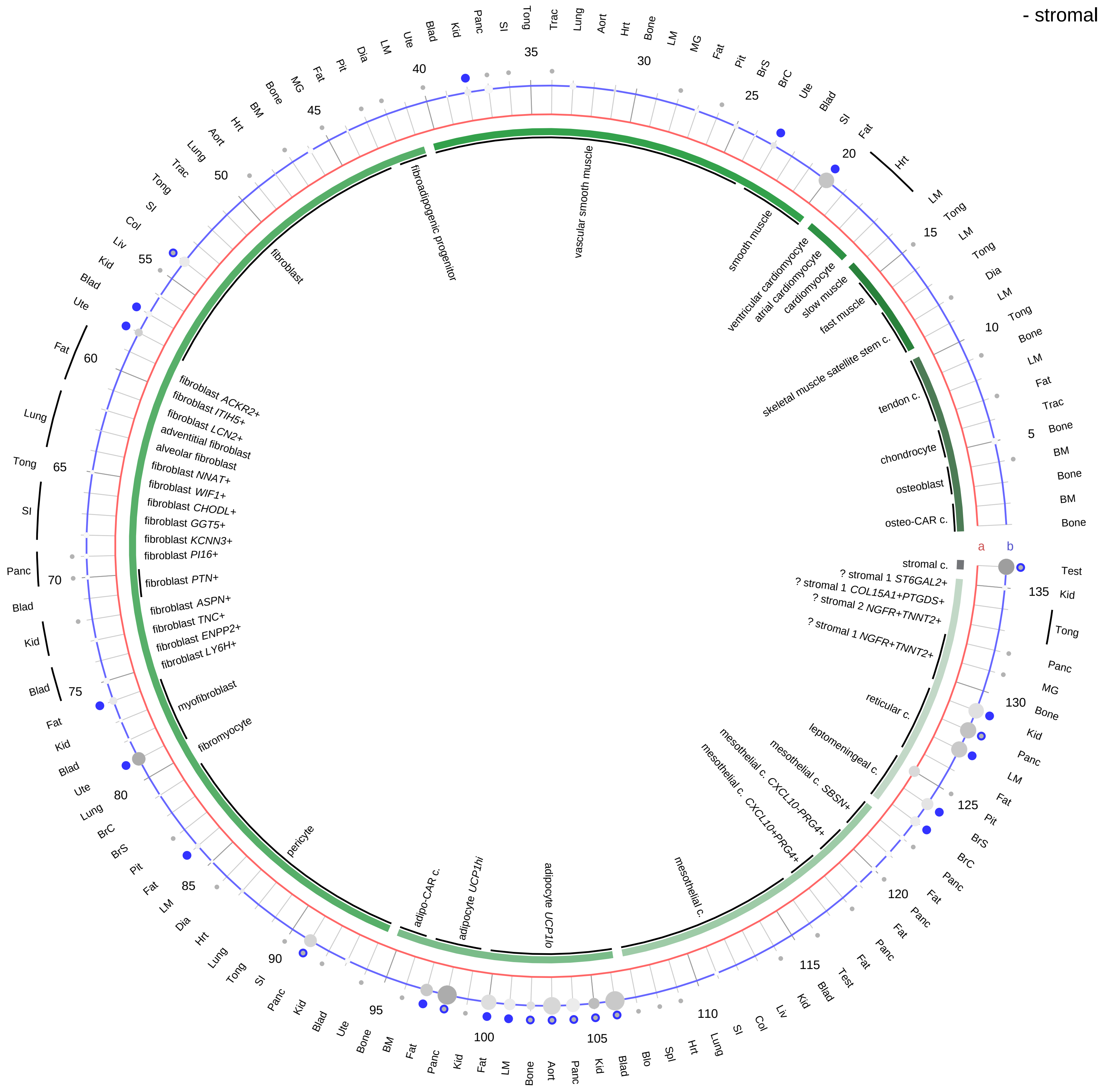

Compartment
stromal
- bone
- skeletal muscle
- cardiac muscle
- smooth muscle
- fibroblast
- adipocyte
- mesothelial
- other
- mix

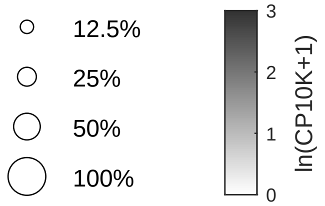

Abbrev.	Tissue
Aort	Aorta
Blad	Bladder
Blo	Blood
Bone	Bone
BM	Bone marrow
BrC	Brain cortex
BrS	Brainstem
Col	Colon
Dia	Diaphragm
Ret	Eye retina
Fat	Fat
Hrt	Heart
Pit	Hypothalamus/Pituitary
Kid	Kidney
LM	Limb muscle
Liv	Liver
Lung	Lung
MG	Mammary gland
Panc	Pancreas
Skin	Skin
SI	Small intestine
Spl	Spleen
Test	Testes
Thym	Thymus
Tong	Tongue
Trac	Trachea
Ute	Uterus

Prolactin (PRL)
- lymphoid

Ligand/Enzyme
a - PRL
Receptor
b - PRLR

PAIRING (ligand → receptor)

PRL→PRLR

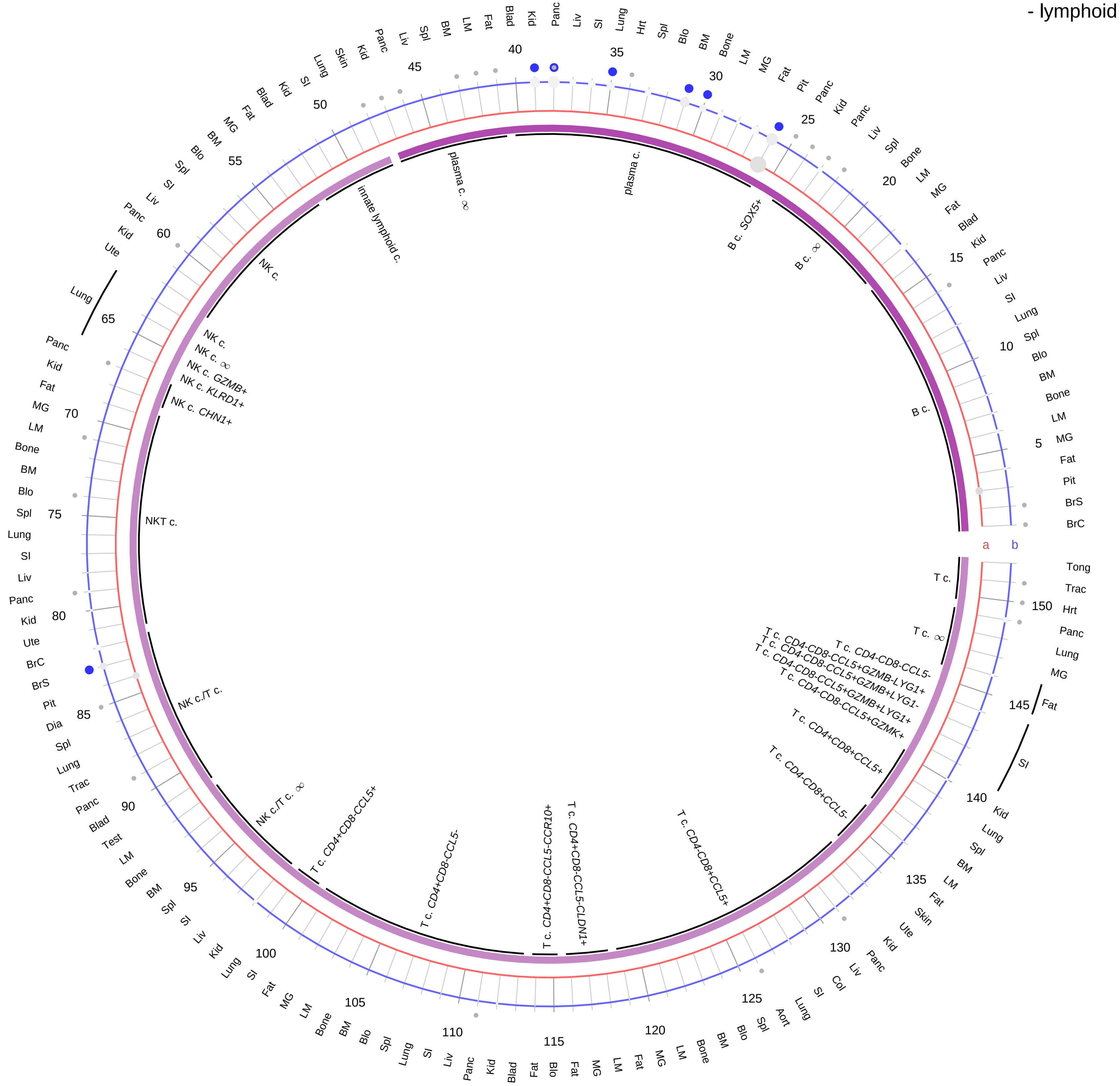

Compartment
lymphoid
- B cell
- NK/T cell

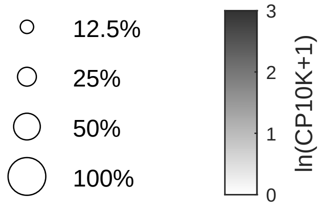

Abbrev.	Tissue
Aort	Aorta
Blad	Bladder
Blo	Blood
Bone	Bone
BM	Bone marrow
BrC	Brain cortex
BrS	Brainstem
Col	Colon
Dia	Diaphragm
Ret	Eye retina
Fat	Fat
Hrt	Heart
Pit	Hypothalamus/Pituitary
Kid	Kidney
LM	Limb muscle
Liv	Liver
Lung	Lung
MG	Mammary gland
Panc	Pancreas
Skin	Skin
SI	Small intestine
Spl	Spleen
Test	Testes
Thym	Thymus
Tong	Tongue
Trac	Trachea
Ute	Uterus

Prolactin (PRL)
- myeloid

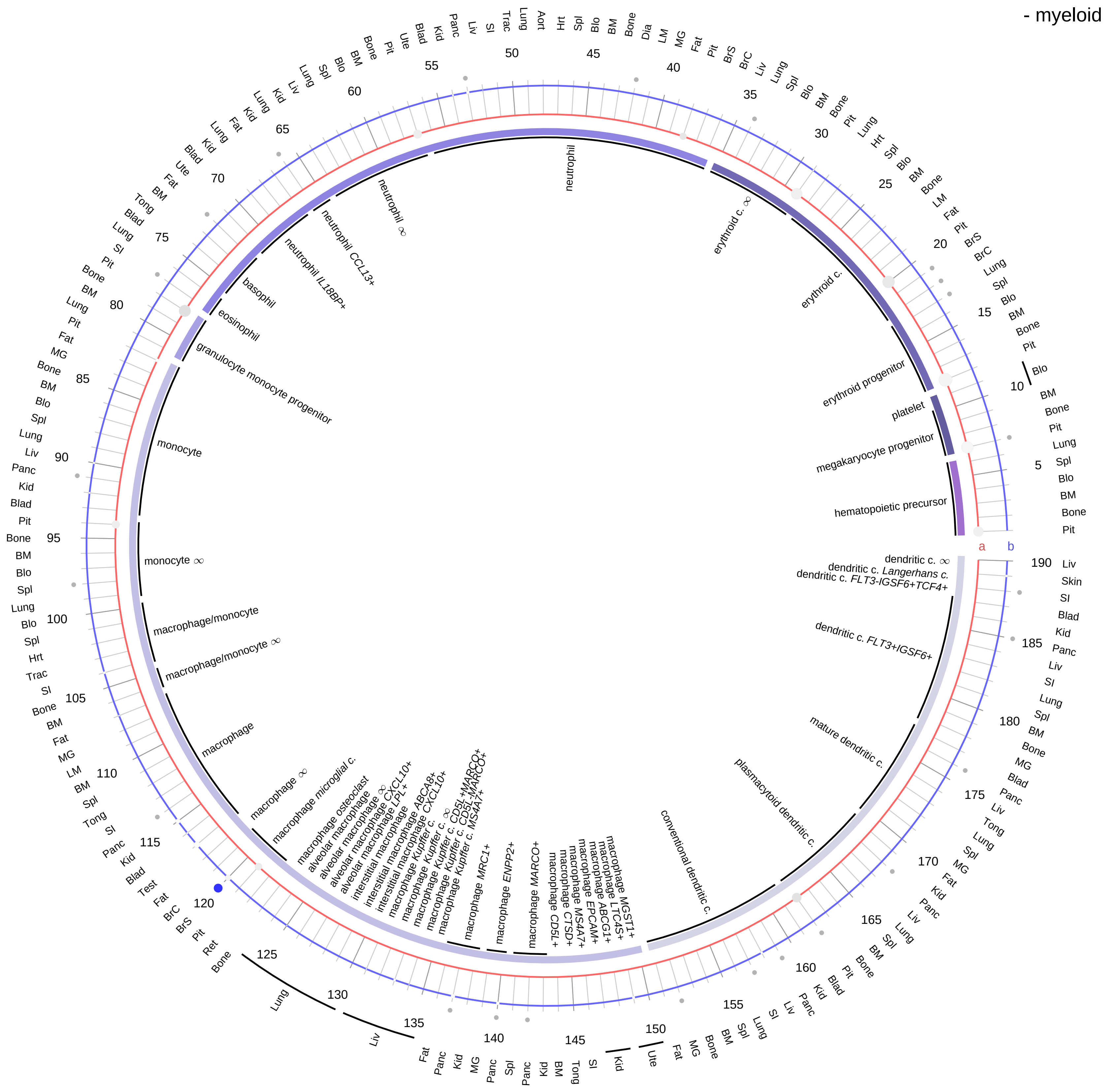

Ligand/Enzyme
a - PRL
Receptor
b - PRLR

PAIRING (ligand → receptor)

PRL→PRLR

Compartment
hematopoietic
- hematopoietic precursor

megakaryocyte-erythroid
- megakaryoid
- erythroid

myeloid
- granulocyte
- granulocyte monocyte progenitor
- monocyte/macrophage
- dendritic

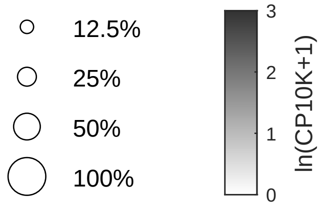

Abbrev.	Tissue
Aort	Aorta
Blad	Bladder
Blo	Blood
Bone	Bone
BM	Bone marrow
BrC	Brain cortex
BrS	Brainstem
Col	Colon
Dia	Diaphragm
Ret	Eye retina
Fat	Fat
Hrt	Heart
Pit	Hypothalamus/Pituitary
Kid	Kidney
LM	Limb muscle
Liv	Liver
Lung	Lung
MG	Mammary gland
Panc	Pancreas
Skin	Skin
SI	Small intestine
Spl	Spleen
Test	Testes
Thym	Thymus
Tong	Tongue
Trac	Trachea
Ute	Uterus

Prolactin releasing hormone (PRLH)
- epithelial/neural/germ

Ligand/Enzyme
a - PRLH
Receptor
b - PRLHR

PAIRING (ligand → receptor)

PRLH→PRLHR

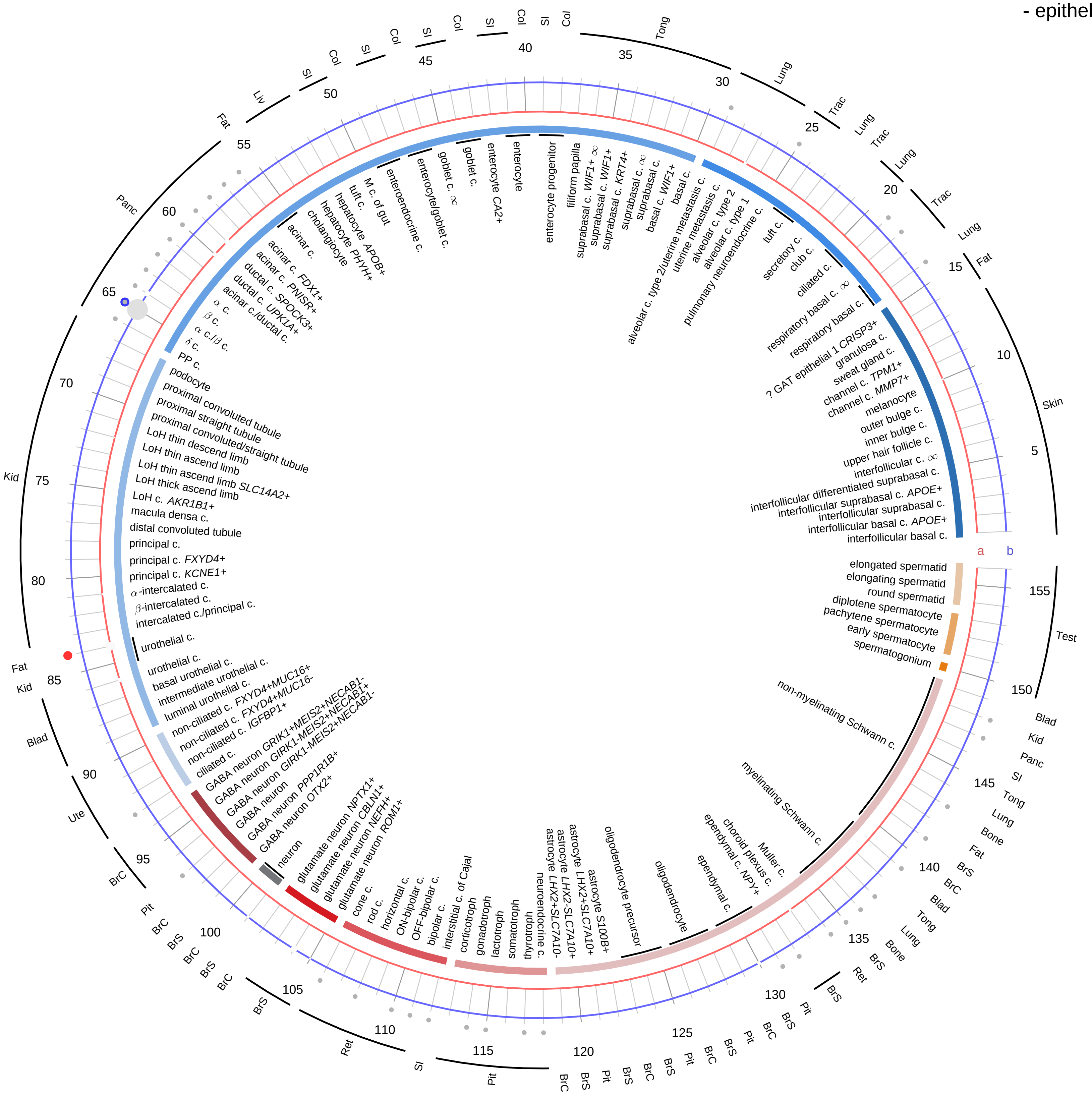

Compartment
epithelial
- integumentary
- respiratory
- gastrointestinal
- urinary
- reproductive

neural
- GABAergic
- hybrid
- glutamatergic
- specialized
- neuroendocrine
- glia

germ
- spermatogonium
- spermatocyte
- spermatid

12.5%
25%
50%
100%

ln(CP10K+1): 0, 1, 2, 3

Abbrev.	Tissue
Aort	Aorta
Blad	Bladder
Blo	Blood
Bone	Bone
BM	Bone marrow
BrC	Brain cortex
BrS	Brainstem
Col	Colon
Dia	Diaphragm
Ret	Eye retina
Fat	Fat
Hrt	Heart
Pit	Hypothalamus/Pituitary
Kid	Kidney
LM	Limb muscle
Liv	Liver
Lung	Lung
MG	Mammary gland
Panc	Pancreas
Skin	Skin
SI	Small intestine
Spl	Spleen
Test	Testes
Thym	Thymus
Tong	Tongue
Trac	Trachea
Ute	Uterus

Prolactin releasing hormone (PRLH)
- endothelial

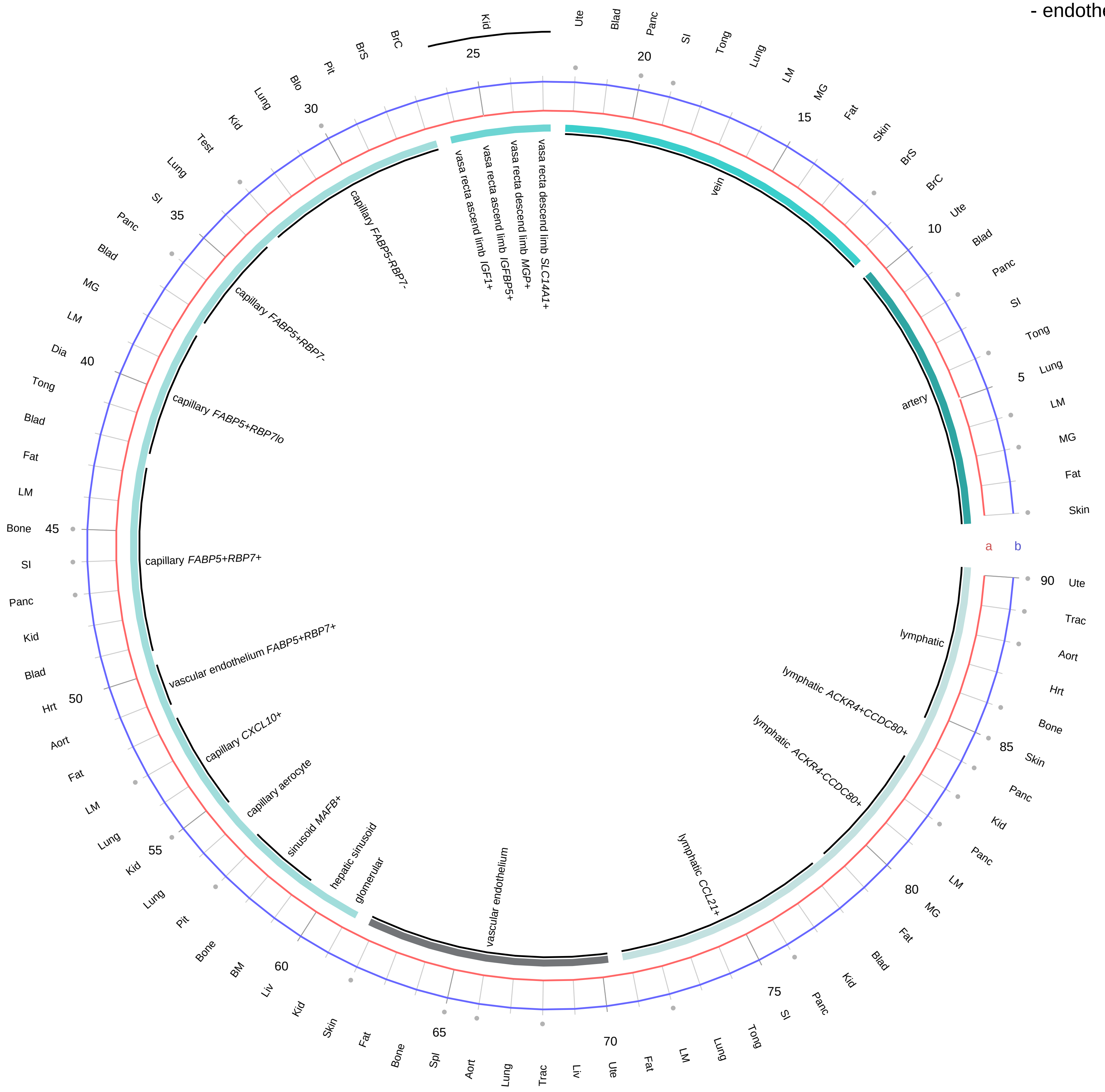

Ligand/Enzyme
a - PRLH
Receptor
b - PRLHR

PAIRING (ligand → receptor)

PRLH→PRLHR

Compartment
endothelial
- artery
- vein
- vasa recta
- capillary
- mix
- lymphatic

○ 12.5%
○ 25%
○ 50%
○ 100%

ln(CP10K+1): 0, 1, 2, 3

Abbrev.	Tissue
Aort	Aorta
Blad	Bladder
Blo	Blood
Bone	Bone
BM	Bone marrow
BrC	Brain cortex
BrS	Brainstem
Col	Colon
Dia	Diaphragm
Ret	Eye retina
Fat	Fat
Hrt	Heart
Pit	Hypothalamus/Pituitary
Kid	Kidney
LM	Limb muscle
Liv	Liver
Lung	Lung
MG	Mammary gland
Panc	Pancreas
Skin	Skin
SI	Small intestine
Spl	Spleen
Test	Testes
Thym	Thymus
Tong	Tongue
Trac	Trachea
Ute	Uterus

Prolactin releasing hormone (PRLH)
- stromal

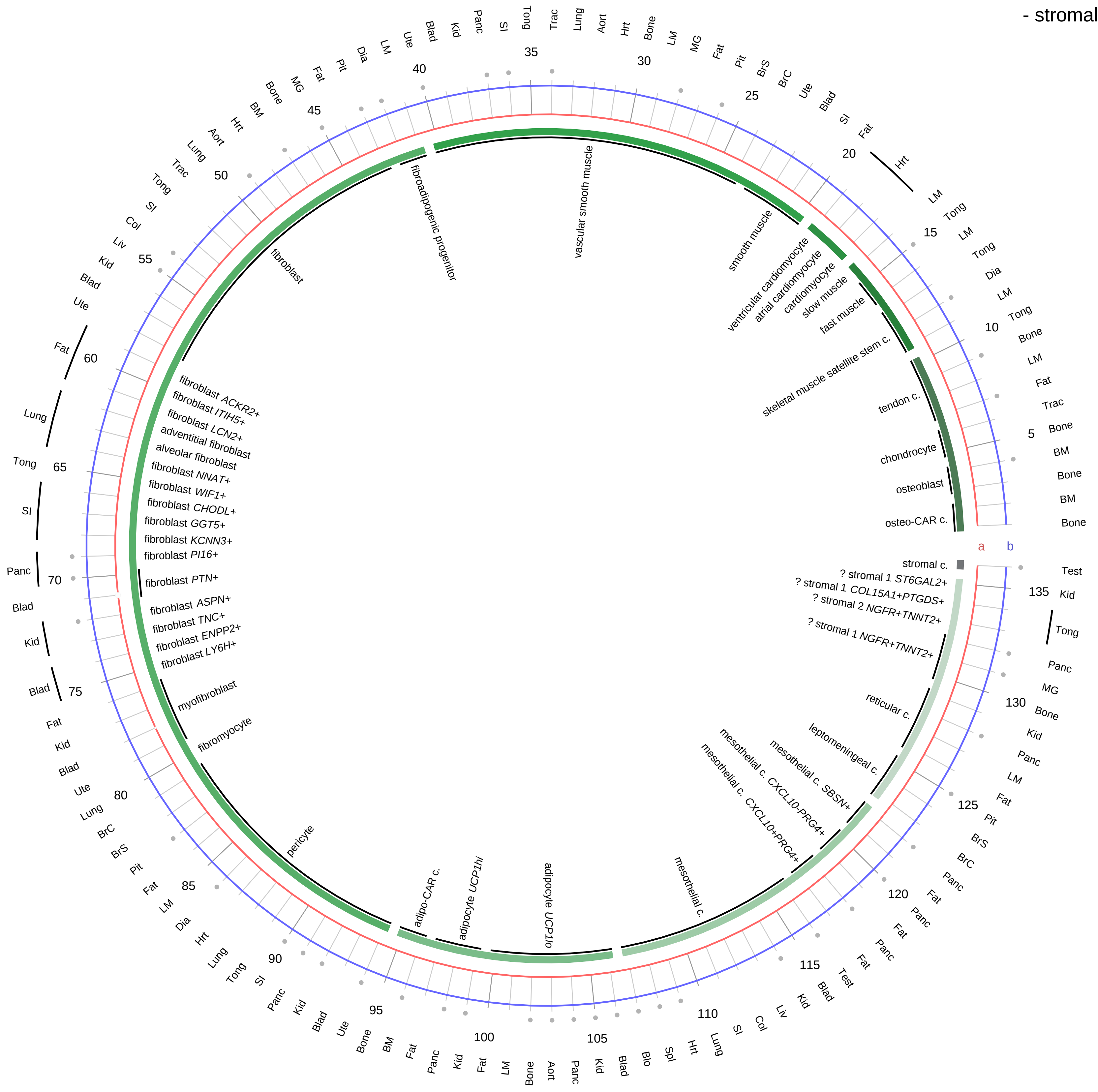

Ligand/Enzyme
a - PRLH
Receptor
b - PRLHR

PAIRING (ligand → receptor)

PRLH→PRLHR

Compartment
stromal
- bone
- skeletal muscle
- cardiac muscle
- smooth muscle
- fibroblast
- adipocyte
- mesothelial
- other
- mix

12.5%
25%
50%
100%

ln(CP10K+1): 0, 1, 2, 3

Abbrev.	Tissue
Aort	Aorta
Blad	Bladder
Blo	Blood
Bone	Bone
BM	Bone marrow
BrC	Brain cortex
BrS	Brainstem
Col	Colon
Dia	Diaphragm
Ret	Eye retina
Fat	Fat
Hrt	Heart
Pit	Hypothalamus/Pituitary
Kid	Kidney
LM	Limb muscle
Liv	Liver
Lung	Lung
MG	Mammary gland
Panc	Pancreas
Skin	Skin
SI	Small intestine
Spl	Spleen
Test	Testes
Thym	Thymus
Tong	Tongue
Trac	Trachea
Ute	Uterus

Prolactin releasing hormone (PRLH)
- lymphoid

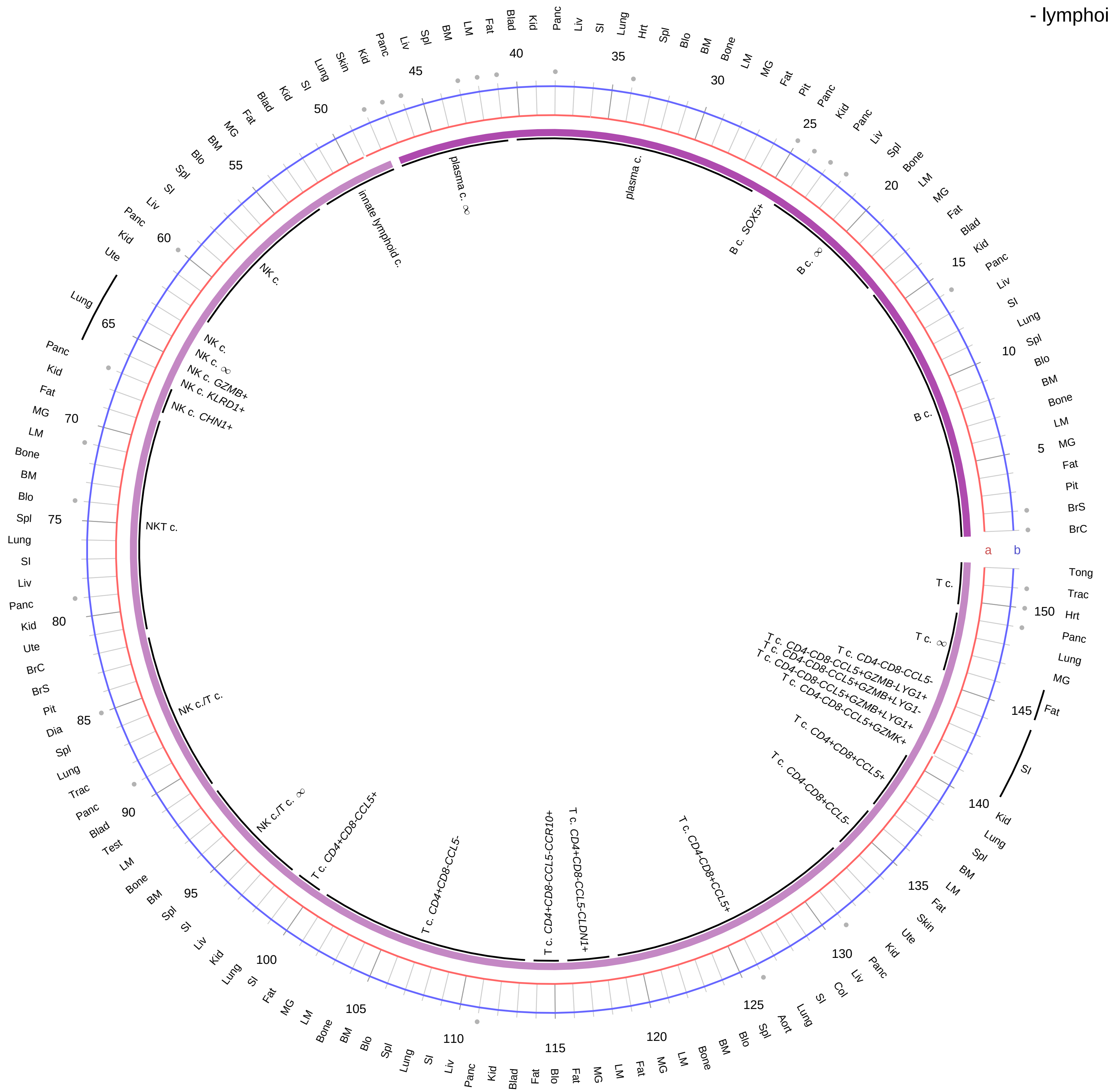

Ligand/Enzyme
a - PRLH
Receptor
b - PRLHR

PAIRING (ligand → receptor)

PRLH→PRLHR

Compartment
lymphoid
- B cell
- NK/T cell

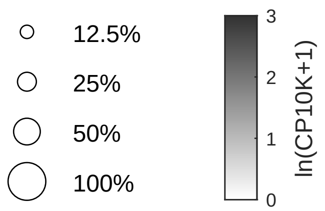

Abbrev.	Tissue
Aort	Aorta
Blad	Bladder
Blo	Blood
Bone	Bone
BM	Bone marrow
BrC	Brain cortex
BrS	Brainstem
Col	Colon
Dia	Diaphragm
Ret	Eye retina
Fat	Fat
Hrt	Heart
Pit	Hypothalamus/Pituitary
Kid	Kidney
LM	Limb muscle
Liv	Liver
Lung	Lung
MG	Mammary gland
Panc	Pancreas
Skin	Skin
SI	Small intestine
Spl	Spleen
Test	Testes
Thym	Thymus
Tong	Tongue
Trac	Trachea
Ute	Uterus

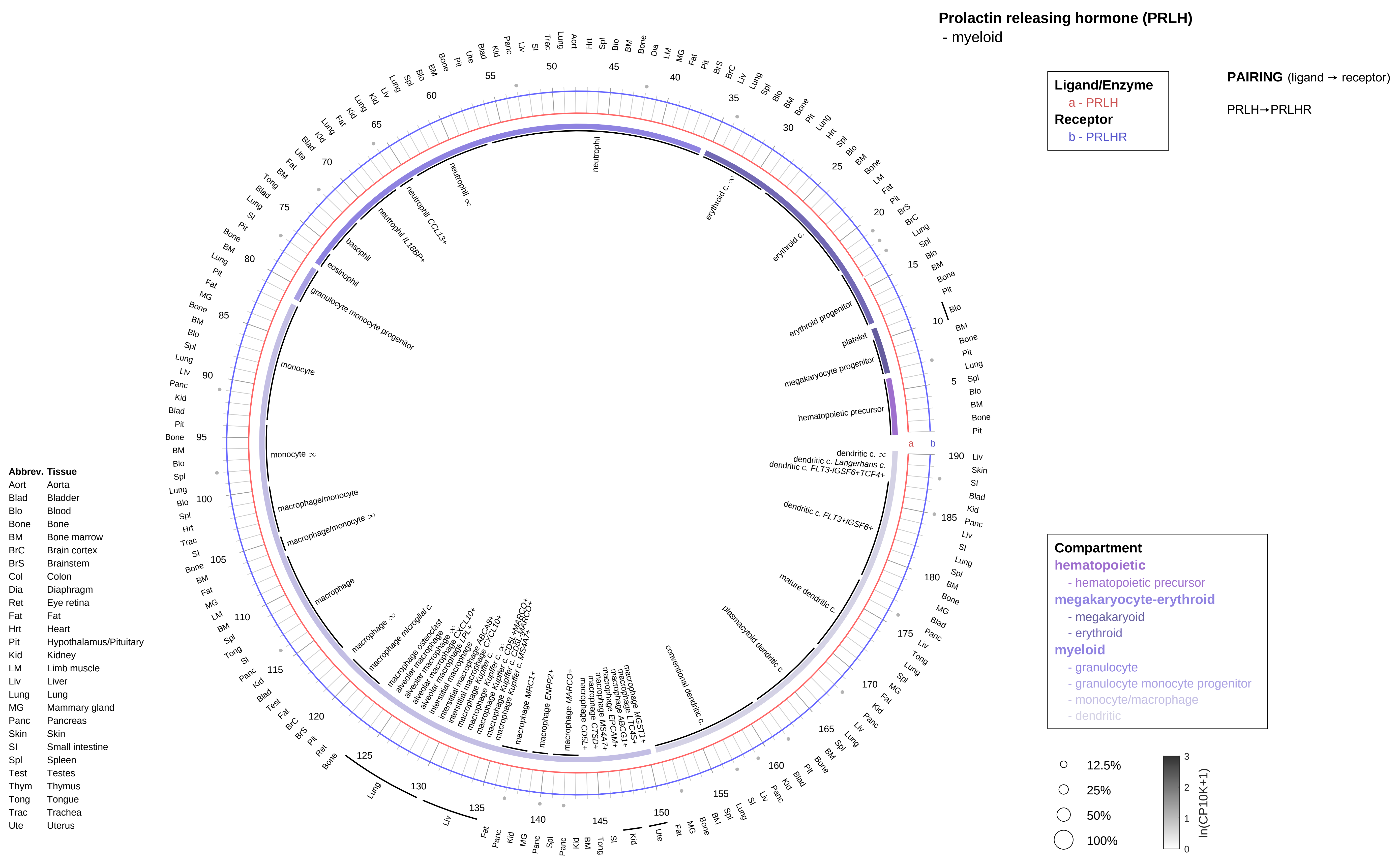

Prolactin releasing hormone (PRLH)
- myeloid
Ligand/Enzyme
a - PRLH
Receptor
b - PRLHR
PAIRING (ligand → receptor)
PRLH→PRLHR
Compartment
hematopoietic
- hematopoietic precursor
megakaryocyte-erythroid
- megakaryoid
- erythroid
myeloid
- granulocyte
- granulocyte monocyte progenitor
- monocyte/macrophage
- dendritic
12.5%
25%
50%
100%
ln(CP10K+1)
Abbrev. Tissue
Aort Aorta
Blad Bladder
Blo Blood
Bone Bone
BM Bone marrow
BrC Brain cortex
BrS Brainstem
Col Colon
Dia Diaphragm
Ret Eye retina
Fat Fat
Hrt Heart
Pit Hypothalamus/Pituitary
Kid Kidney
LM Limb muscle
Liv Liver
Lung Lung
MG Mammary gland
Panc Pancreas
Skin Skin
SI Small intestine
Spl Spleen
Test Testes
Thym Thymus
Tong Tongue
Trac Trachea
Ute Uterus
neutrophil
erythroid c. ∞
erythroid c.
erythroid progenitor
platelet
megakaryocyte progenitor
hematopoietic precursor
dendritic c. ∞
dendritic c. Langerhans c.
dendritic c. FLT3-IGSF6+TCF4+
dendritic c. FLT3+IGSF6+
mature dendritic c.
plasmacytoid dendritic c.
conventional dendritic c.
macrophage MGST1+
macrophage LTC4S+
macrophage ABCG1+
macrophage EPCAM+
macrophage MS4A7+
macrophage CTSD+
macrophage CD5L+
macrophage MARCO+
macrophage ENPP2+
macrophage MRC1+
macrophage Kupffer c. MS4A7+
macrophage Kupffer c. CD5L-MARCO+
macrophage Kupffer c. CD5L+MARCO+
macrophage Kupffer c. ∞
macrophage Kupffer c.
interstitial macrophage CXCL10+
interstitial macrophage ABCA8+
interstitial macrophage LPL+
alveolar macrophage CXCL10+
alveolar macrophage ∞
alveolar macrophage
macrophage osteoclast
macrophage microglial c.
macrophage ∞
macrophage
macrophage/monocyte ∞
macrophage/monocyte
monocyte ∞
monocyte
granulocyte monocyte progenitor
eosinophil
basophil
neutrophil IL18BP+
neutrophil CCL13+
neutrophil ∞

Proopiomelanocortin-derived hormones (ACTH/MSH/endorphin)
- epithelial/neural/germ

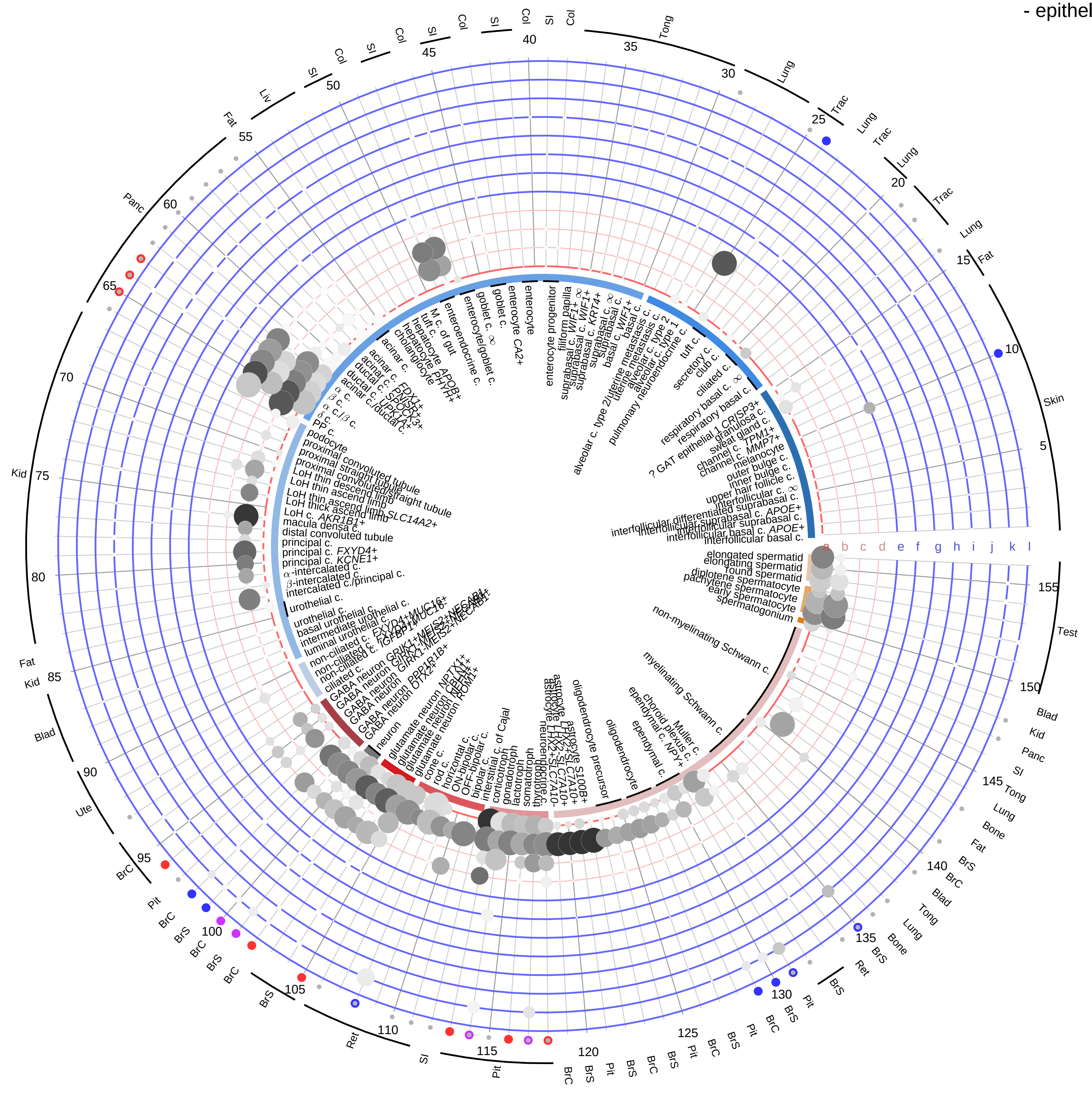

Ligand/Enzyme
- a - POMC
- b - CPE
- c - PCSK1
- d - PCSK2

Receptor
- e - MC1R*
- f - MC2R
- g - MC3R
- h - MC4R
- i - MC5R
- j - OPRD1
- k - OPRK1
- l - OPRM1

PAIRING (ligand → receptor)

POMC & PCSK1 & CPE→MC2R

POMC & PCSK1 & CPE→MC1R

POMC & PCSK1 & CPE→MC3R

POMC & PCSK1 & CPE→MC4R

POMC & PCSK1 & CPE→MC5R

POMC & PCSK1 & PCSK2 & CPE→MC1R

POMC & PCSK1 & PCSK2 & CPE→MC3R

POMC & PCSK1 & PCSK2 & CPE→MC4R

POMC & PCSK1 & PCSK2 & CPE→MC5R

POMC & PCSK1 & PCSK2 & CPE→OPRM1

POMC & PCSK1 & PCSK2 & CPE→OPRD1

POMC & PCSK1 & PCSK2 & CPE→OPRK1

Compartment

epithelial
- integumentary
- respiratory
- gastrointestinal
- urinary
- reproductive

neural
- GABAergic
- hybrid
- glutamatergic
- specialized
- neuroendocrine
- glia

germ
- spermatogonium
- spermatocyte
- spermatid

Abbrev.	Tissue
Aort	Aorta
Blad	Bladder
Blo	Blood
Bone	Bone
BM	Bone marrow
BrC	Brain cortex
BrS	Brainstem
Col	Colon
Dia	Diaphragm
Ret	Eye retina
Fat	Fat
Hrt	Heart
Pit	Hypothalamus/Pituitary
Kid	Kidney
LM	Limb muscle
Liv	Liver
Lung	Lung
MG	Mammary gland
Panc	Pancreas
Skin	Skin
SI	Small intestine
Spl	Spleen
Test	Testes
Thym	Thymus
Tong	Tongue
Trac	Trachea
Ute	Uterus

12.5%
25%
50%
100%

ln(CP10K+1): 0, 1, 2, 3

Proopiomelanocortin-derived hormones (ACTH/MSH/endorphin)
- endothelial

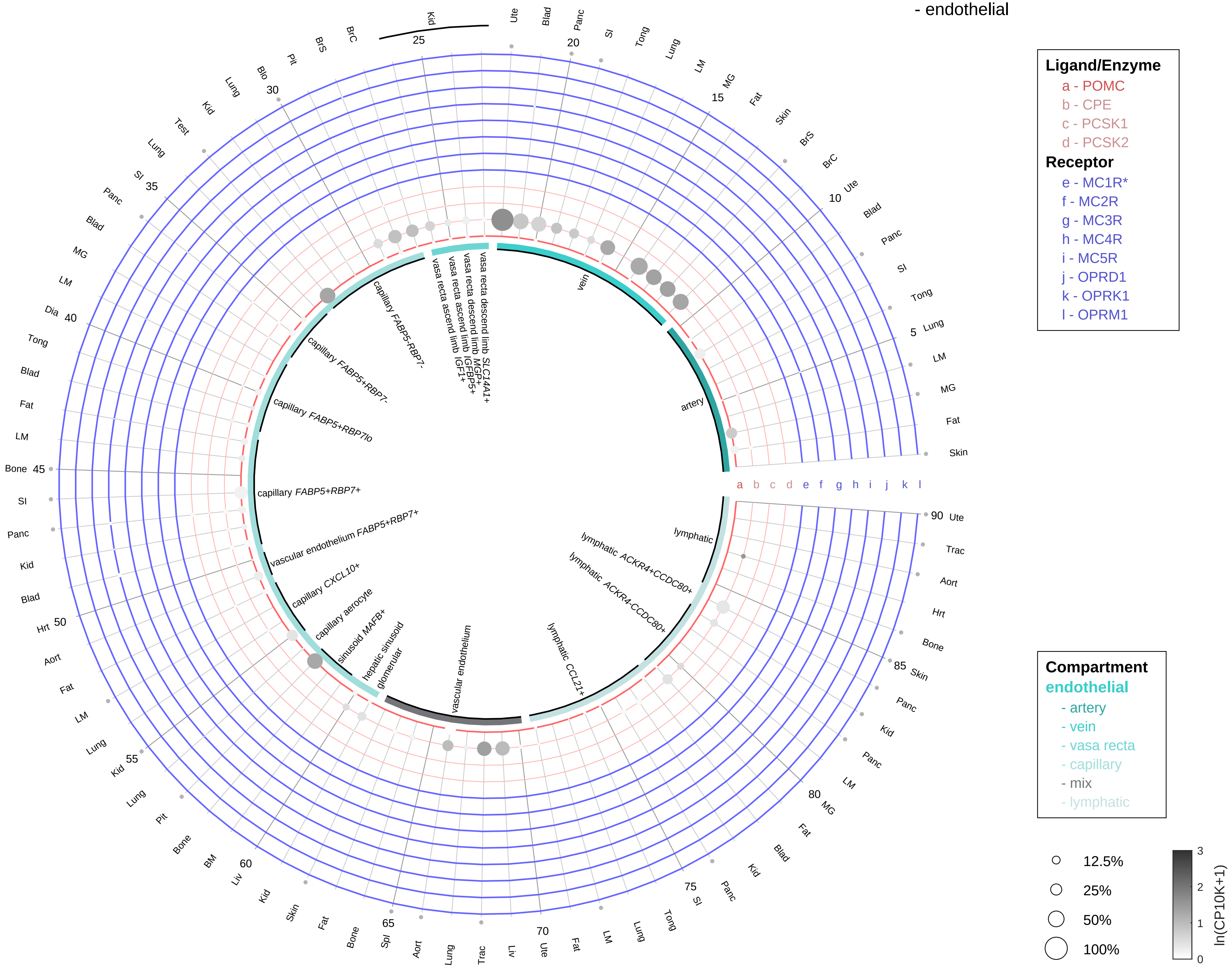

PAIRING (ligand → receptor)

POMC & PCSK1 & CPE→MC2R

POMC & PCSK1 & CPE→MC1R

POMC & PCSK1 & CPE→MC3R

POMC & PCSK1 & CPE→MC4R

POMC & PCSK1 & CPE→MC5R

POMC & PCSK1 & PCSK2 & CPE→MC1R

POMC & PCSK1 & PCSK2 & CPE→MC3R

POMC & PCSK1 & PCSK2 & CPE→MC4R

POMC & PCSK1 & PCSK2 & CPE→MC5R

POMC & PCSK1 & PCSK2 & CPE→OPRM1

POMC & PCSK1 & PCSK2 & CPE→OPRD1

POMC & PCSK1 & PCSK2 & CPE→OPRK1

Abbrev.	Tissue
Aort	Aorta
Blad	Bladder
Blo	Blood
Bone	Bone
BM	Bone marrow
BrC	Brain cortex
BrS	Brainstem
Col	Colon
Dia	Diaphragm
Ret	Eye retina
Fat	Fat
Hrt	Heart
Pit	Hypothalamus/Pituitary
Kid	Kidney
LM	Limb muscle
Liv	Liver
Lung	Lung
MG	Mammary gland
Panc	Pancreas
Skin	Skin
SI	Small intestine
Spl	Spleen
Test	Testes
Thym	Thymus
Tong	Tongue
Trac	Trachea
Ute	Uterus

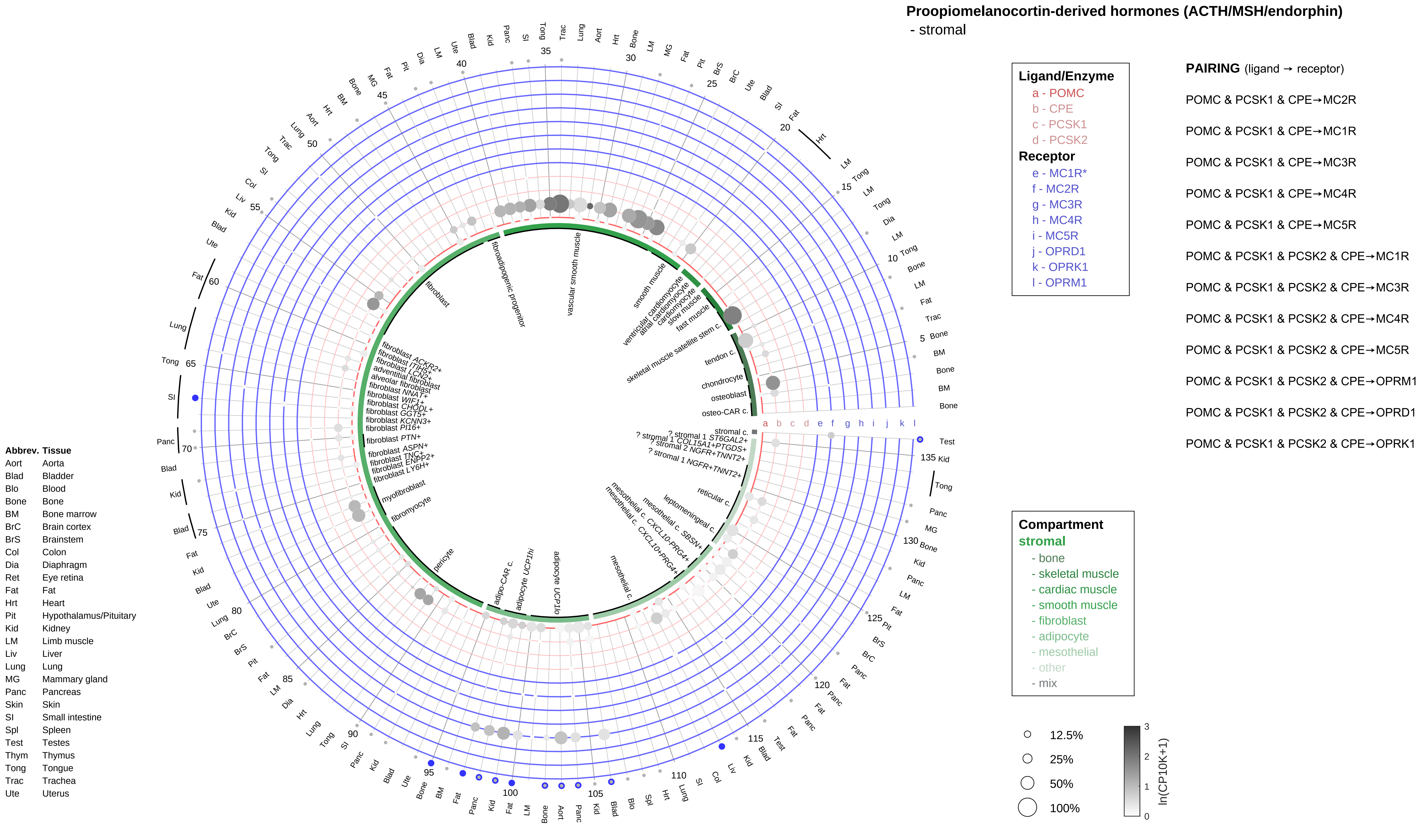

Proopiomelanocortin-derived hormones (ACTH/MSH/endorphin)
- stromal
Ligand/Enzyme
a - POMC
b - CPE
c - PCSK1
d - PCSK2
Receptor
e - MC1R*
f - MC2R
g - MC3R
h - MC4R
i - MC5R
j - OPRD1
k - OPRK1
l - OPRM1
PAIRING (ligand → receptor)
POMC & PCSK1 & CPE→MC2R
POMC & PCSK1 & CPE→MC1R
POMC & PCSK1 & CPE→MC3R
POMC & PCSK1 & CPE→MC4R
POMC & PCSK1 & CPE→MC5R
POMC & PCSK1 & PCSK2 & CPE→MC1R
POMC & PCSK1 & PCSK2 & CPE→MC3R
POMC & PCSK1 & PCSK2 & CPE→MC4R
POMC & PCSK1 & PCSK2 & CPE→MC5R
POMC & PCSK1 & PCSK2 & CPE→OPRM1
POMC & PCSK1 & PCSK2 & CPE→OPRD1
POMC & PCSK1 & PCSK2 & CPE→OPRK1
Compartment
stromal
- bone
- skeletal muscle
- cardiac muscle
- smooth muscle
- fibroblast
- adipocyte
- mesothelial
- other
- mix
12.5%
25%
50%
100%
ln(CP10K+1)
0
1
2
3
Abbrev. Tissue
Aort Aorta
Blad Bladder
Blo Blood
Bone Bone
BM Bone marrow
BrC Brain cortex
BrS Brainstem
Col Colon
Dia Diaphragm
Ret Eye retina
Fat Fat
Hrt Heart
Pit Hypothalamus/Pituitary
Kid Kidney
LM Limb muscle
Liv Liver
Lung Lung
MG Mammary gland
Panc Pancreas
Skin Skin
SI Small intestine
Spl Spleen
Test Testes
Thym Thymus
Tong Tongue
Trac Trachea
Ute Uterus
fibroblast
fibroadipogenic progenitor
vascular smooth muscle
smooth muscle
ventricular cardiomyocyte
atrial cardiomyocyte
slow muscle
fast muscle
skeletal muscle satellite stem c.
tendon c.
chondrocyte
osteoblast
osteo-CAR c.
stromal c.
? stromal 1 ST6GAL2+
? stromal 1 COL15A1+PTGDS+
? stromal 2 NGFR+TNNT2+
? stromal 1 NGFR+TNNT2+
reticular c.
leptomeningeal c.
mesothelial c. SBSN+
mesothelial c. CXCL10-PRG4+
mesothelial c. CXCL10+PRG4+
mesothelial c.
adipocyte UCP1lo
adipocyte UCP1hi
adipo-CAR c.
pericyte
fibromyocyte
myofibroblast
fibroblast LY6H+
fibroblast ENPP2+
fibroblast TNC+
fibroblast ASPN+
fibroblast PTN+
fibroblast PI16+
fibroblast KCNN3+
fibroblast GGT5+
fibroblast CHODL+
fibroblast WIF1+
fibroblast NNAT+
alveolar fibroblast
adventitial fibroblast
fibroblast LCN2+
fibroblast ITIH5+
fibroblast ACKR2+

Proopiomelanocortin-derived hormones (ACTH/MSH/endorphin)
- lymphoid

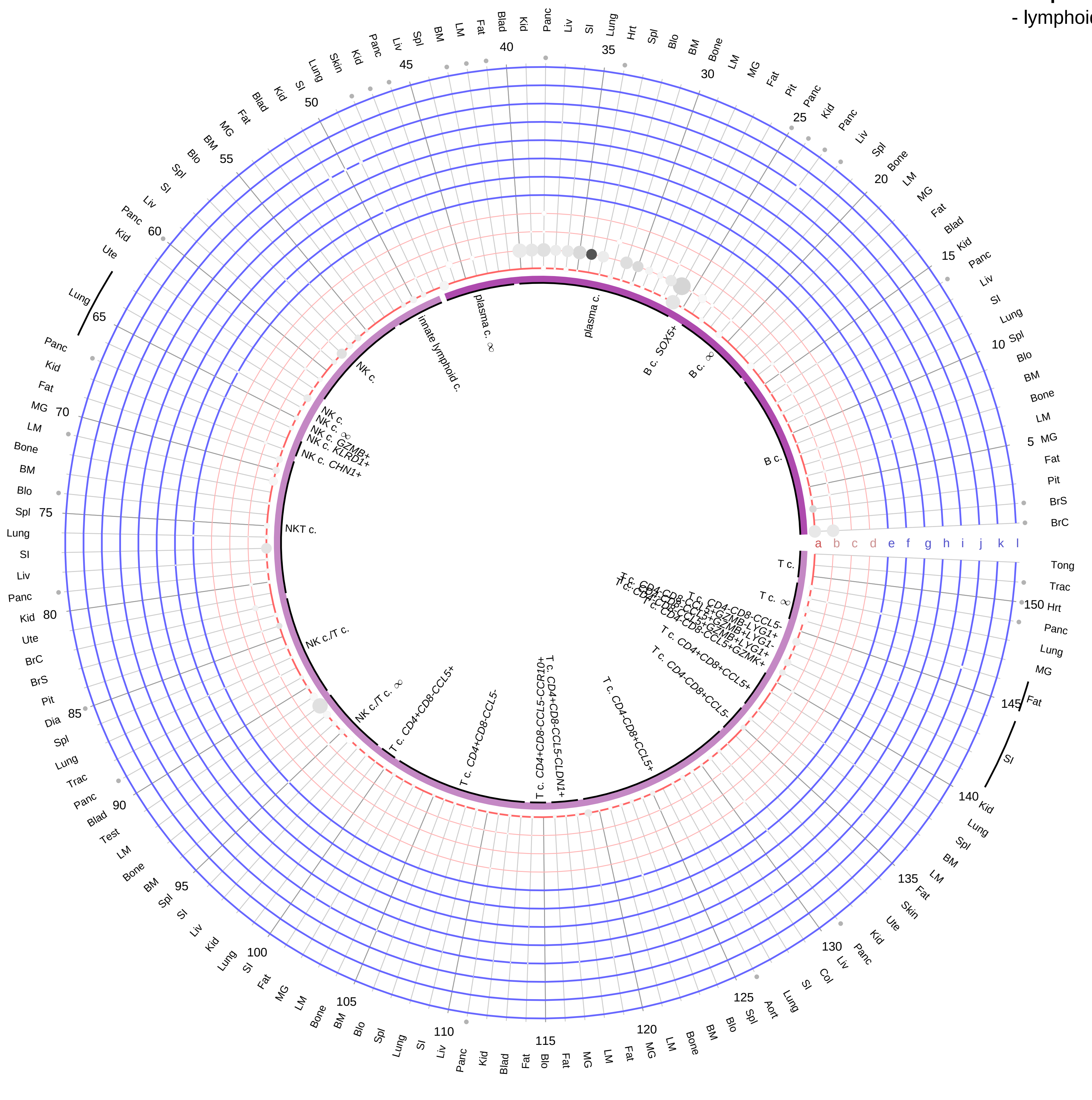

Ligand/Enzyme
a - POMC
b - CPE
c - PCSK1
d - PCSK2

Receptor
e - MC1R*
f - MC2R
g - MC3R
h - MC4R
i - MC5R
j - OPRD1
k - OPRK1
l - OPRM1

PAIRING (ligand → receptor)

POMC & PCSK1 & CPE→MC2R

POMC & PCSK1 & CPE→MC1R

POMC & PCSK1 & CPE→MC3R

POMC & PCSK1 & CPE→MC4R

POMC & PCSK1 & CPE→MC5R

POMC & PCSK1 & PCSK2 & CPE→MC1R

POMC & PCSK1 & PCSK2 & CPE→MC3R

POMC & PCSK1 & PCSK2 & CPE→MC4R

POMC & PCSK1 & PCSK2 & CPE→MC5R

POMC & PCSK1 & PCSK2 & CPE→OPRM1

POMC & PCSK1 & PCSK2 & CPE→OPRD1

POMC & PCSK1 & PCSK2 & CPE→OPRK1

Compartment
lymphoid
- B cell
- NK/T cell

12.5%
25%
50%
100%

ln(CP10K+1): 0, 1, 2, 3

Abbrev.	Tissue
Aort	Aorta
Blad	Bladder
Blo	Blood
Bone	Bone
BM	Bone marrow
BrC	Brain cortex
BrS	Brainstem
Col	Colon
Dia	Diaphragm
Ret	Eye retina
Fat	Fat
Hrt	Heart
Pit	Hypothalamus/Pituitary
Kid	Kidney
LM	Limb muscle
Liv	Liver
Lung	Lung
MG	Mammary gland
Panc	Pancreas
Skin	Skin
SI	Small intestine
Spl	Spleen
Test	Testes
Thym	Thymus
Tong	Tongue
Trac	Trachea
Ute	Uterus

Proopiomelanocortin-derived hormones (ACTH/MSH/endorphin)
- myeloid

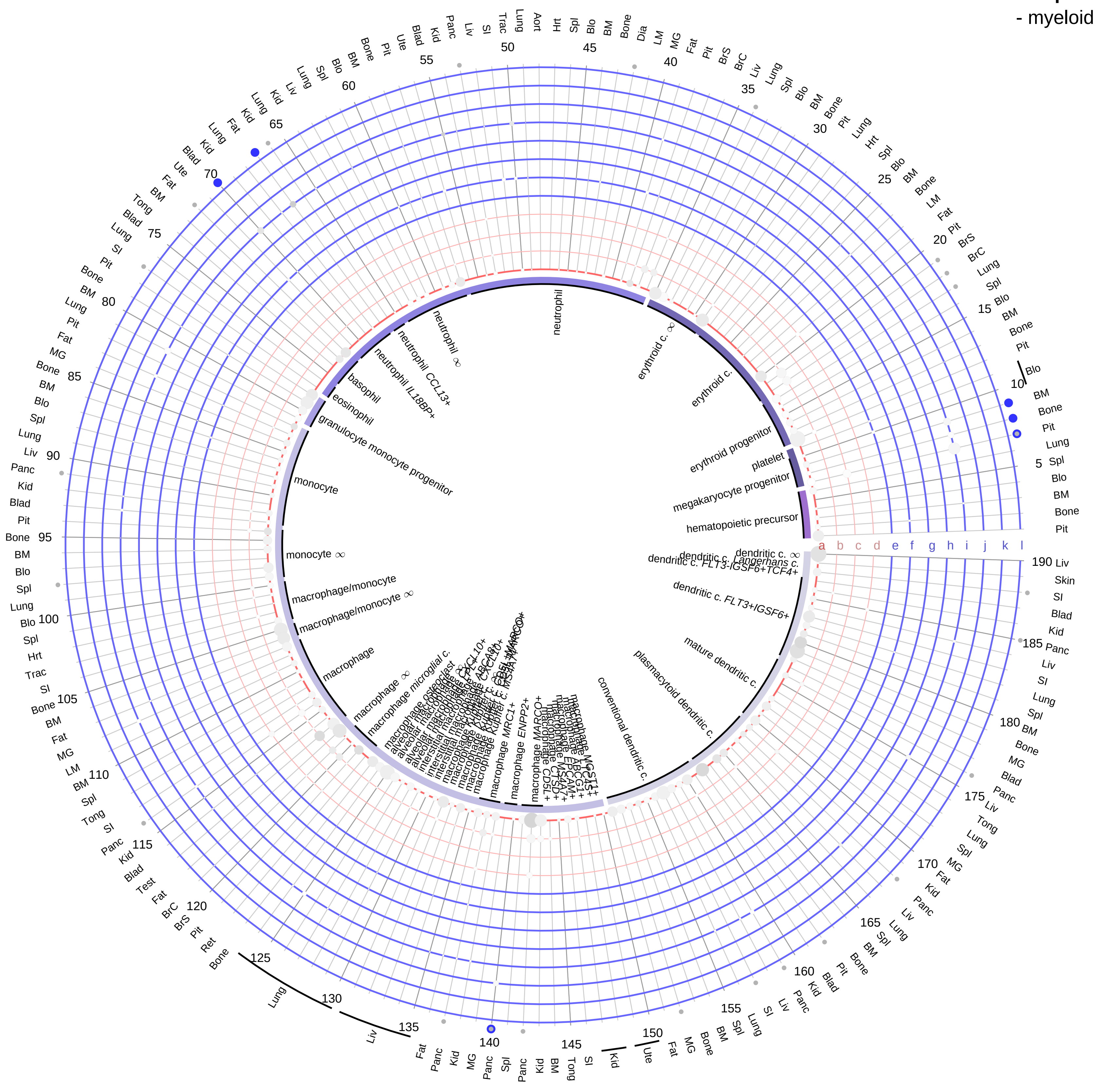

Ligand/Enzyme
a - POMC
b - CPE
c - PCSK1
d - PCSK2
**Receptor**
e - MC1R*
f - MC2R
g - MC3R
h - MC4R
i - MC5R
j - OPRD1
k - OPRK1
l - OPRM1

PAIRING (ligand → receptor)

POMC & PCSK1 & CPE→MC2R

POMC & PCSK1 & CPE→MC1R

POMC & PCSK1 & CPE→MC3R

POMC & PCSK1 & CPE→MC4R

POMC & PCSK1 & CPE→MC5R

POMC & PCSK1 & PCSK2 & CPE→MC1R

POMC & PCSK1 & PCSK2 & CPE→MC3R

POMC & PCSK1 & PCSK2 & CPE→MC4R

POMC & PCSK1 & PCSK2 & CPE→MC5R

POMC & PCSK1 & PCSK2 & CPE→OPRM1

POMC & PCSK1 & PCSK2 & CPE→OPRD1

POMC & PCSK1 & PCSK2 & CPE→OPRK1

Compartment

hematopoietic
- hematopoietic precursor

megakaryocyte-erythroid
- megakaryoid
- erythroid

myeloid
- granulocyte
- granulocyte monocyte progenitor
- monocyte/macrophage
- dendritic

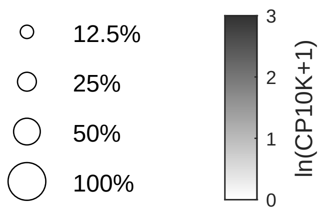

Abbrev.	Tissue
Aort	Aorta
Blad	Bladder
Blo	Blood
Bone	Bone
BM	Bone marrow
BrC	Brain cortex
BrS	Brainstem
Col	Colon
Dia	Diaphragm
Ret	Eye retina
Fat	Fat
Hrt	Heart
Pit	Hypothalamus/Pituitary
Kid	Kidney
LM	Limb muscle
Liv	Liver
Lung	Lung
MG	Mammary gland
Panc	Pancreas
Skin	Skin
SI	Small intestine
Spl	Spleen
Test	Testes
Thym	Thymus
Tong	Tongue
Trac	Trachea
Ute	Uterus

Prostaglandin D2 (PGD2)
- epithelial/neural/germ

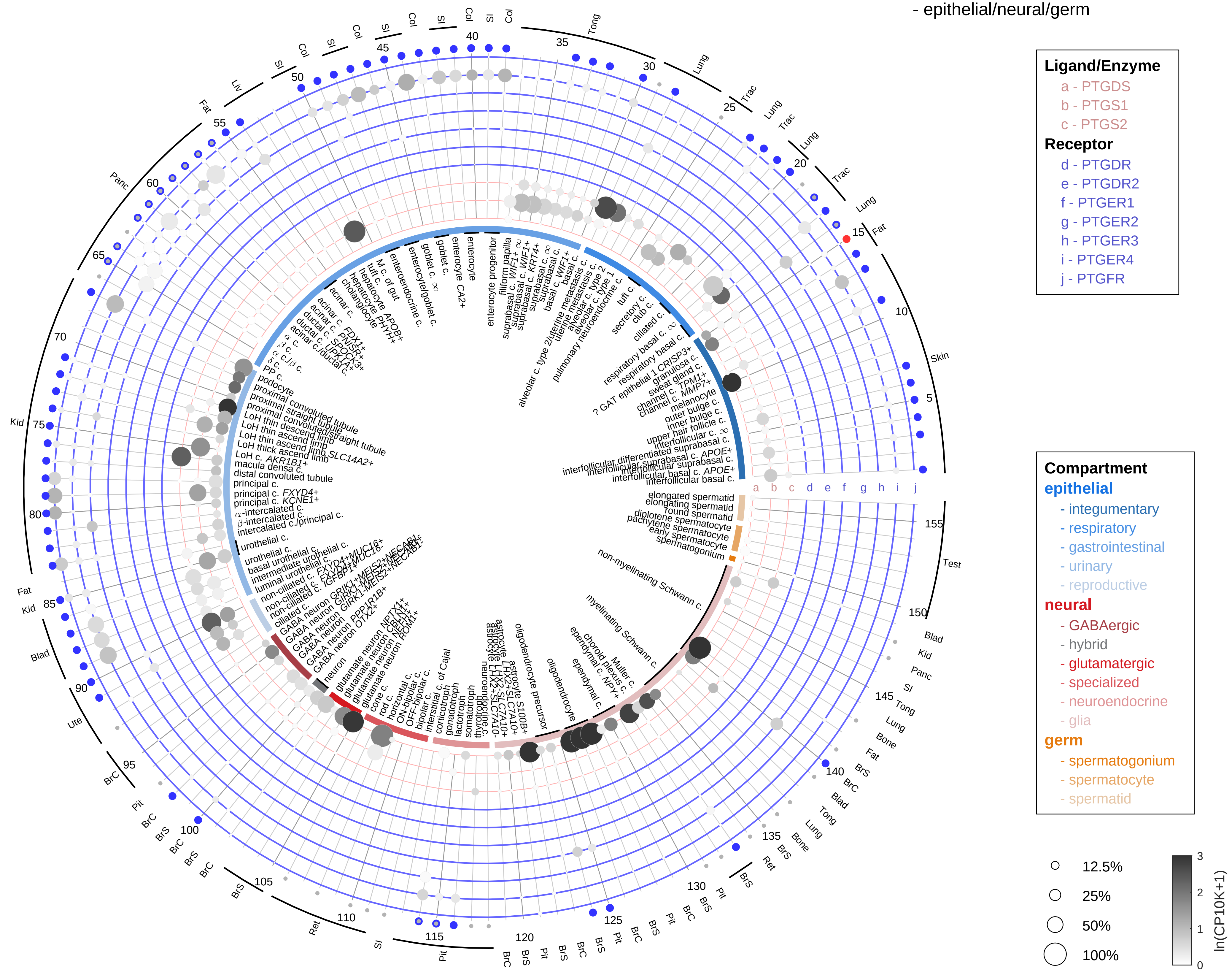

Abbrev.	Tissue
Aort	Aorta
Blad	Bladder
Blo	Blood
Bone	Bone
BM	Bone marrow
BrC	Brain cortex
BrS	Brainstem
Col	Colon
Dia	Diaphragm
Ret	Eye retina
Fat	Fat
Hrt	Heart
Pit	Hypothalamus/Pituitary
Kid	Kidney
LM	Limb muscle
Liv	Liver
Lung	Lung
MG	Mammary gland
Panc	Pancreas
Skin	Skin
SI	Small intestine
Spl	Spleen
Test	Testes
Thym	Thymus
Tong	Tongue
Trac	Trachea
Ute	Uterus

Prostaglandin D2 (PGD2)
- endothelial

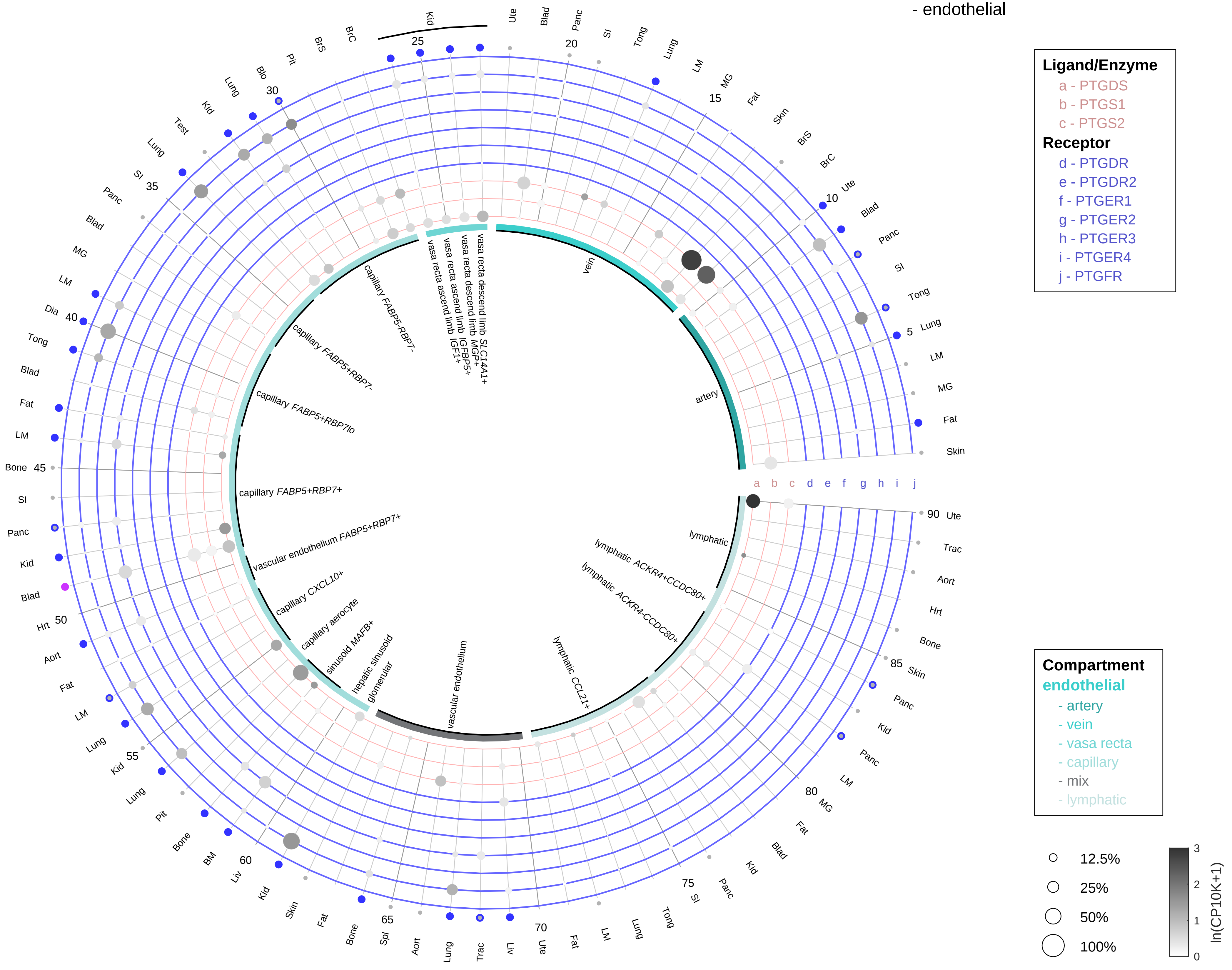

PAIRING (ligand → receptor)

PTGDS & PTGS1 & PTGS2→PTGDR

PTGDS & PTGS1 & PTGS2→PTGDR2

PTGDS & PTGS1 & PTGS2→PTGFR

PTGDS & PTGS1 & PTGS2→PTGER1

PTGDS & PTGS1 & PTGS2→PTGER2

PTGDS & PTGS1 & PTGS2→PTGER3

PTGDS & PTGS1 & PTGS2→PTGER4

Abbrev.	Tissue
Aort	Aorta
Blad	Bladder
Blo	Blood
Bone	Bone
BM	Bone marrow
BrC	Brain cortex
BrS	Brainstem
Col	Colon
Dia	Diaphragm
Ret	Eye retina
Fat	Fat
Hrt	Heart
Pit	Hypothalamus/Pituitary
Kid	Kidney
LM	Limb muscle
Liv	Liver
Lung	Lung
MG	Mammary gland
Panc	Pancreas
Skin	Skin
SI	Small intestine
Spl	Spleen
Test	Testes
Thym	Thymus
Tong	Tongue
Trac	Trachea
Ute	Uterus

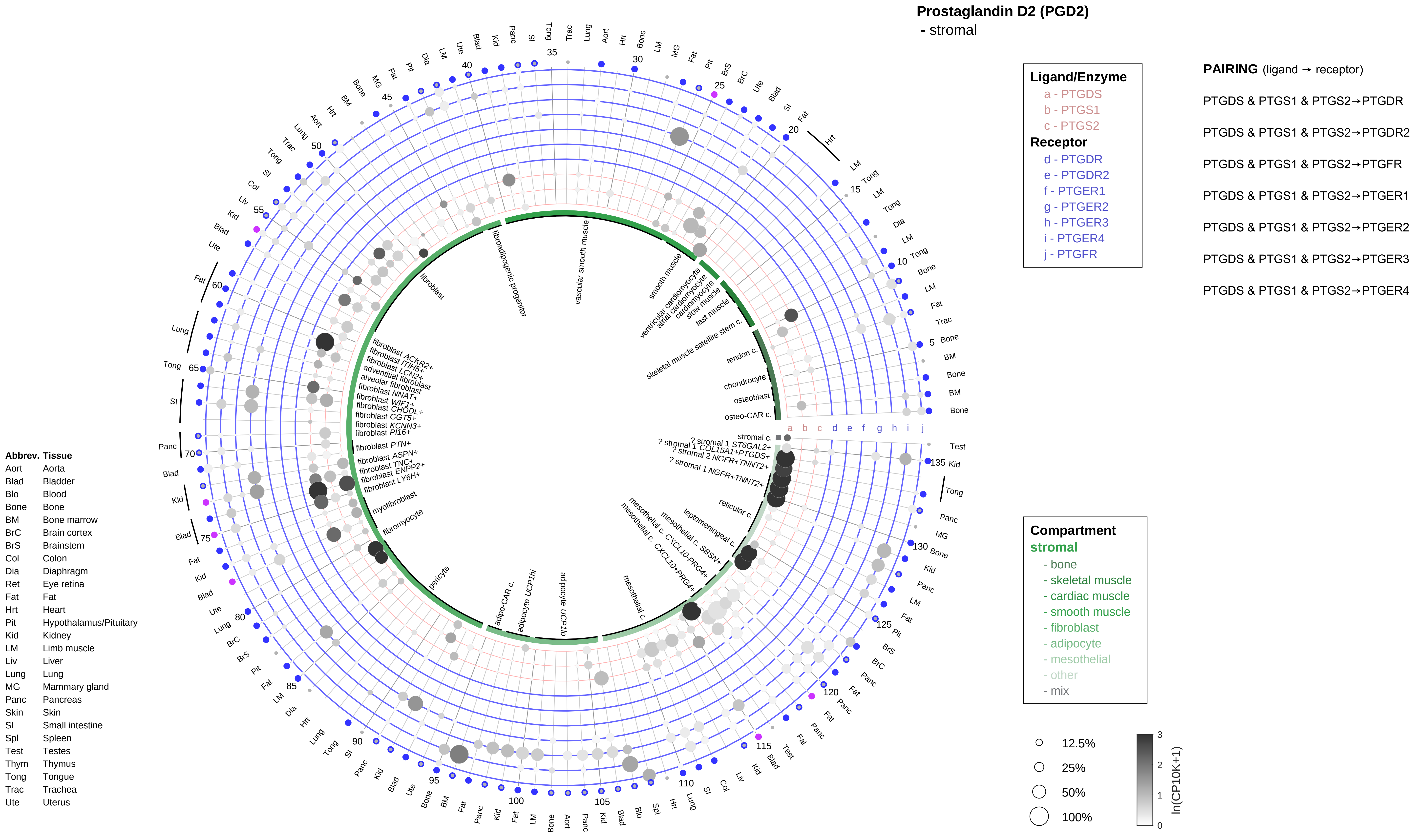

Prostaglandin D2 (PGD2)
- stromal
Ligand/Enzyme
a - PTGDS
b - PTGS1
c - PTGS2
Receptor
d - PTGDR
e - PTGDR2
f - PTGER1
g - PTGER2
h - PTGER3
i - PTGER4
j - PTGFR
PAIRING (ligand → receptor)
PTGDS & PTGS1 & PTGS2→PTGDR
PTGDS & PTGS1 & PTGS2→PTGDR2
PTGDS & PTGS1 & PTGS2→PTGFR
PTGDS & PTGS1 & PTGS2→PTGER1
PTGDS & PTGS1 & PTGS2→PTGER2
PTGDS & PTGS1 & PTGS2→PTGER3
PTGDS & PTGS1 & PTGS2→PTGER4
Compartment
stromal
- bone
- skeletal muscle
- cardiac muscle
- smooth muscle
- fibroblast
- adipocyte
- mesothelial
- other
- mix
12.5%
25%
50%
100%
ln(CP10K+1)
Abbrev. Tissue
Aort Aorta
Blad Bladder
Blo Blood
Bone Bone
BM Bone marrow
BrC Brain cortex
BrS Brainstem
Col Colon
Dia Diaphragm
Ret Eye retina
Fat Fat
Hrt Heart
Pit Hypothalamus/Pituitary
Kid Kidney
LM Limb muscle
Liv Liver
Lung Lung
MG Mammary gland
Panc Pancreas
Skin Skin
SI Small intestine
Spl Spleen
Test Testes
Thym Thymus
Tong Tongue
Trac Trachea
Ute Uterus

Prostaglandin D2 (PGD2)
- lymphoid

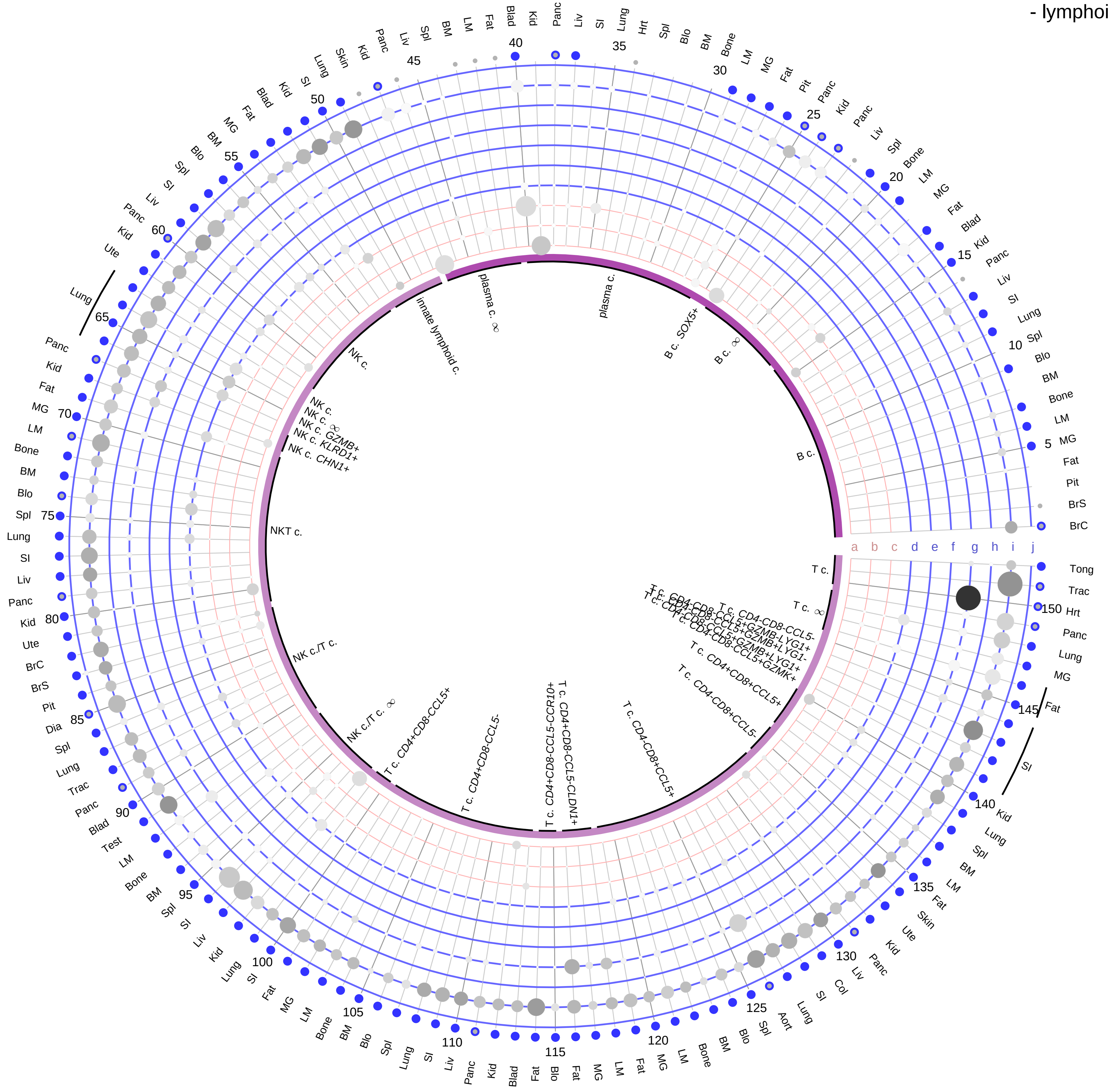

Ligand/Enzyme
- a - PTGDS
- b - PTGS1
- c - PTGS2

Receptor
- d - PTGDR
- e - PTGDR2
- f - PTGER1
- g - PTGER2
- h - PTGER3
- i - PTGER4
- j - PTGFR

PAIRING (ligand → receptor)

PTGDS & PTGS1 & PTGS2→PTGDR

PTGDS & PTGS1 & PTGS2→PTGDR2

PTGDS & PTGS1 & PTGS2→PTGFR

PTGDS & PTGS1 & PTGS2→PTGER1

PTGDS & PTGS1 & PTGS2→PTGER2

PTGDS & PTGS1 & PTGS2→PTGER3

PTGDS & PTGS1 & PTGS2→PTGER4

Abbrev.	Tissue
Aort	Aorta
Blad	Bladder
Blo	Blood
Bone	Bone
BM	Bone marrow
BrC	Brain cortex
BrS	Brainstem
Col	Colon
Dia	Diaphragm
Ret	Eye retina
Fat	Fat
Hrt	Heart
Pit	Hypothalamus/Pituitary
Kid	Kidney
LM	Limb muscle
Liv	Liver
Lung	Lung
MG	Mammary gland
Panc	Pancreas
Skin	Skin
SI	Small intestine
Spl	Spleen
Test	Testes
Thym	Thymus
Tong	Tongue
Trac	Trachea
Ute	Uterus

Compartment
lymphoid
- B cell
- NK/T cell

12.5%
25%
50%
100%

ln(CP10K+1): 0, 1, 2, 3

Prostaglandin D2 (PGD2)
- myeloid

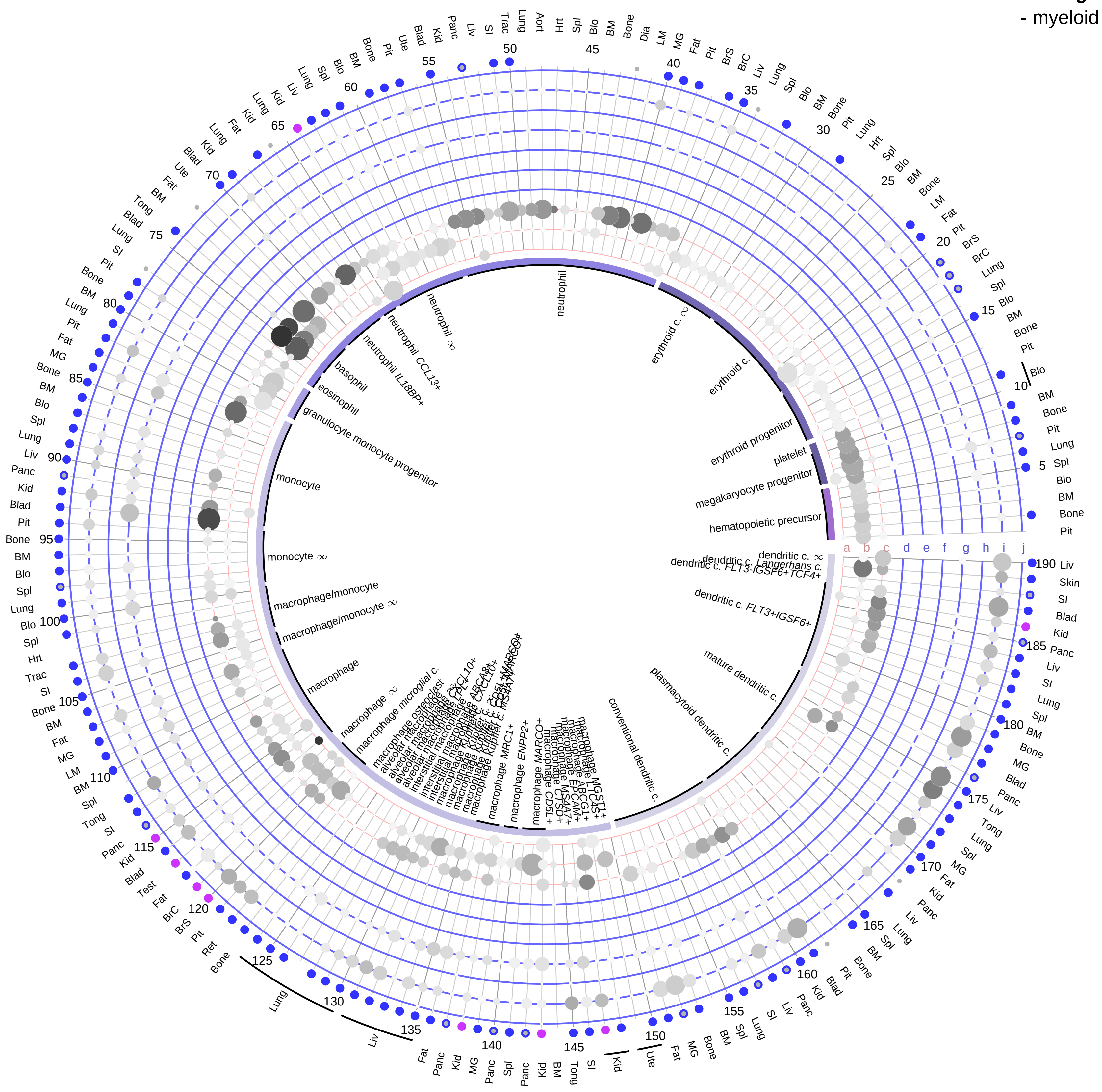

Ligand/Enzyme
- a - PTGDS
- b - PTGS1
- c - PTGS2

Receptor
- d - PTGDR
- e - PTGDR2
- f - PTGER1
- g - PTGER2
- h - PTGER3
- i - PTGER4
- j - PTGFR

PAIRING (ligand → receptor)

PTGDS & PTGS1 & PTGS2→PTGDR

PTGDS & PTGS1 & PTGS2→PTGDR2

PTGDS & PTGS1 & PTGS2→PTGFR

PTGDS & PTGS1 & PTGS2→PTGER1

PTGDS & PTGS1 & PTGS2→PTGER2

PTGDS & PTGS1 & PTGS2→PTGER3

PTGDS & PTGS1 & PTGS2→PTGER4

Compartment
- **hematopoietic**
  - hematopoietic precursor
- **megakaryocyte-erythroid**
  - megakaryoid
  - erythroid
- **myeloid**
  - granulocyte
  - granulocyte monocyte progenitor
  - monocyte/macrophage
  - dendritic

12.5%
25%
50%
100%

ln(CP10K+1): 0, 1, 2, 3

Abbrev.	Tissue
Aort	Aorta
Blad	Bladder
Blo	Blood
Bone	Bone
BM	Bone marrow
BrC	Brain cortex
BrS	Brainstem
Col	Colon
Dia	Diaphragm
Ret	Eye retina
Fat	Fat
Hrt	Heart
Pit	Hypothalamus/Pituitary
Kid	Kidney
LM	Limb muscle
Liv	Liver
Lung	Lung
MG	Mammary gland
Panc	Pancreas
Skin	Skin
SI	Small intestine
Spl	Spleen
Test	Testes
Thym	Thymus
Tong	Tongue
Trac	Trachea
Ute	Uterus

Prostaglandin E2 (PGE2)
- epithelial/neural/germ

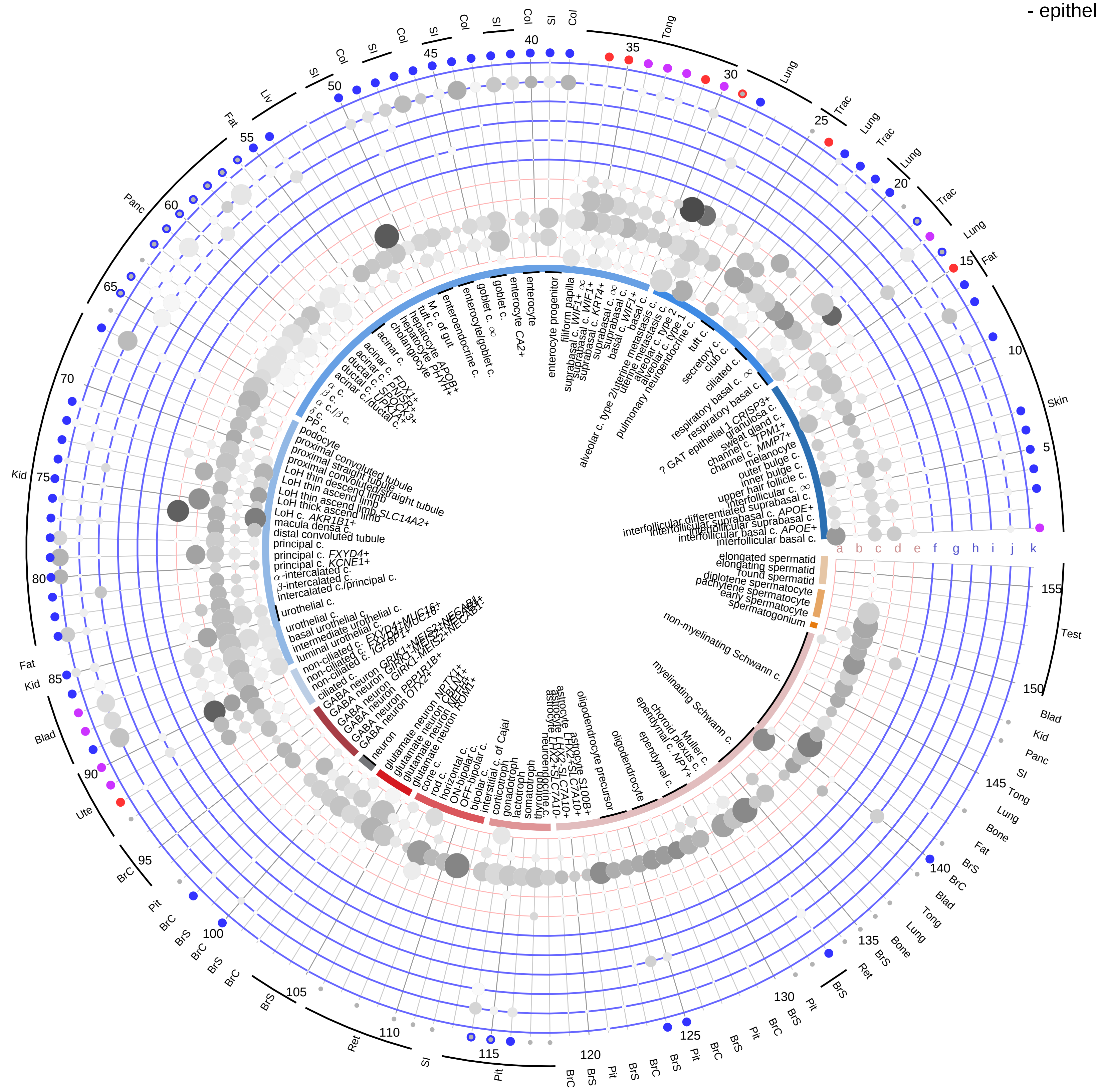

Ligand/Enzyme
- a - PTGES
- b - PTGES2
- c - PTGES3
- d - PTGS1
- e - PTGS2

Receptor
- f - PTGDR
- g - PTGER1
- h - PTGER2
- i - PTGER3
- j - PTGER4
- k - PTGFR

PAIRING (ligand → receptor)

PTGES & PTGS1 & PTGS2→PTGER1
PTGES & PTGS1 & PTGS2→PTGER2
PTGES & PTGS1 & PTGS2→PTGER3
PTGES & PTGS1 & PTGS2→PTGER4
PTGES & PTGS1 & PTGS2→PTGDR
PTGES & PTGS1 & PTGS2→PTGFR
PTGES2 & PTGS1 & PTGS2→PTGER1
PTGES2 & PTGS1 & PTGS2→PTGER2
PTGES2 & PTGS1 & PTGS2→PTGER3
PTGES2 & PTGS1 & PTGS2→PTGER4
PTGES2 & PTGS1 & PTGS2→PTGDR
PTGES2 & PTGS1 & PTGS2→PTGFR
PTGES3 & PTGS1 & PTGS2→PTGER1
PTGES3 & PTGS1 & PTGS2→PTGER2
PTGES3 & PTGS1 & PTGS2→PTGER3
PTGES3 & PTGS1 & PTGS2→PTGER4
PTGES3 & PTGS1 & PTGS2→PTGDR
PTGES3 & PTGS1 & PTGS2→PTGFR

Compartment

epithelial
- integumentary
- respiratory
- gastrointestinal
- urinary
- reproductive

neural
- GABAergic
- hybrid
- glutamatergic
- specialized
- neuroendocrine
- glia

germ
- spermatogonium
- spermatocyte
- spermatid

12.5% | 25% | 50% | 100%

ln(CP10K+1): 0 1 2 3

Abbrev.	Tissue
Aort	Aorta
Blad	Bladder
Blo	Blood
Bone	Bone
BM	Bone marrow
BrC	Brain cortex
BrS	Brainstem
Col	Colon
Dia	Diaphragm
Ret	Eye retina
Fat	Fat
Hrt	Heart
Pit	Hypothalamus/Pituitary
Kid	Kidney
LM	Limb muscle
Liv	Liver
Lung	Lung
MG	Mammary gland
Panc	Pancreas
Skin	Skin
SI	Small intestine
Spl	Spleen
Test	Testes
Thym	Thymus
Tong	Tongue
Trac	Trachea
Ute	Uterus

Prostaglandin E2 (PGE2)
- endothelial

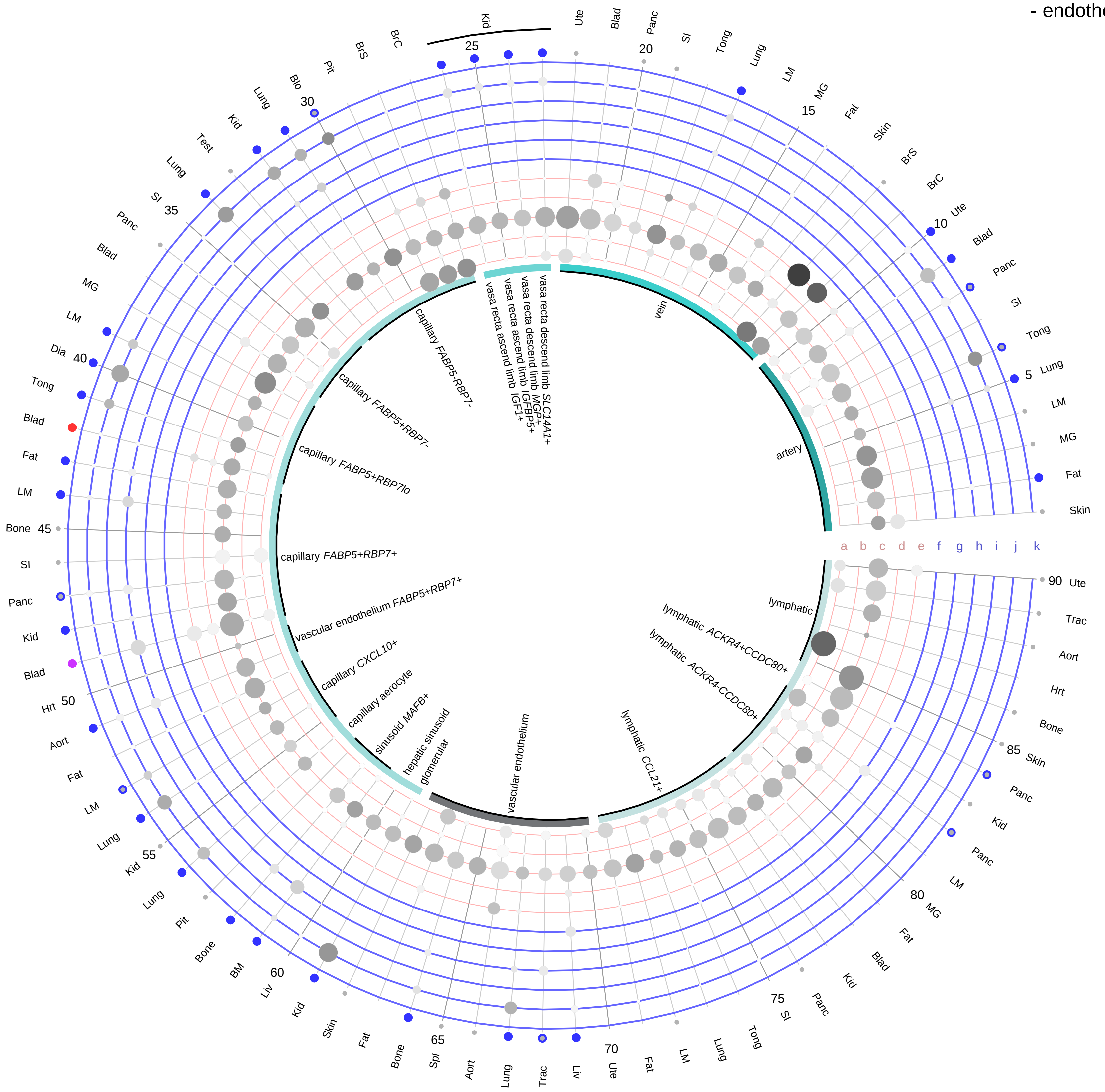

Abbrev. **Tissue**
Aort Aorta
Blad Bladder
Blo Blood
Bone Bone
BM Bone marrow
BrC Brain cortex
BrS Brainstem
Col Colon
Dia Diaphragm
Ret Eye retina
Fat Fat
Hrt Heart
Pit Hypothalamus/Pituitary
Kid Kidney
LM Limb muscle
Liv Liver
Lung Lung
MG Mammary gland
Panc Pancreas
Skin Skin
SI Small intestine
Spl Spleen
Test Testes
Thym Thymus
Tong Tongue
Trac Trachea
Ute Uterus

Ligand/Enzyme
a - PTGES
b - PTGES2
c - PTGES3
d - PTGS1
e - PTGS2
Receptor
f - PTGDR
g - PTGER1
h - PTGER2
i - PTGER3
j - PTGER4
k - PTGFR

PAIRING (ligand → receptor)
PTGES & PTGS1 & PTGS2→PTGER1
PTGES & PTGS1 & PTGS2→PTGER2
PTGES & PTGS1 & PTGS2→PTGER3
PTGES & PTGS1 & PTGS2→PTGER4
PTGES & PTGS1 & PTGS2→PTGDR
PTGES & PTGS1 & PTGS2→PTGFR
PTGES2 & PTGS1 & PTGS2→PTGER1
PTGES2 & PTGS1 & PTGS2→PTGER2
PTGES2 & PTGS1 & PTGS2→PTGER3
PTGES2 & PTGS1 & PTGS2→PTGER4
PTGES2 & PTGS1 & PTGS2→PTGDR
PTGES2 & PTGS1 & PTGS2→PTGFR
PTGES3 & PTGS1 & PTGS2→PTGER1
PTGES3 & PTGS1 & PTGS2→PTGER2
PTGES3 & PTGS1 & PTGS2→PTGER3
PTGES3 & PTGS1 & PTGS2→PTGER4
PTGES3 & PTGS1 & PTGS2→PTGDR
PTGES3 & PTGS1 & PTGS2→PTGFR

Compartment
endothelial
- artery
- vein
- vasa recta
- capillary
- mix
- lymphatic

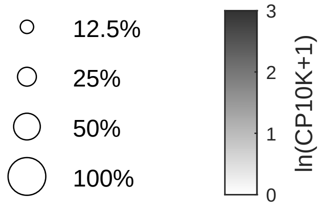

Prostaglandin E2 (PGE2)
- stromal

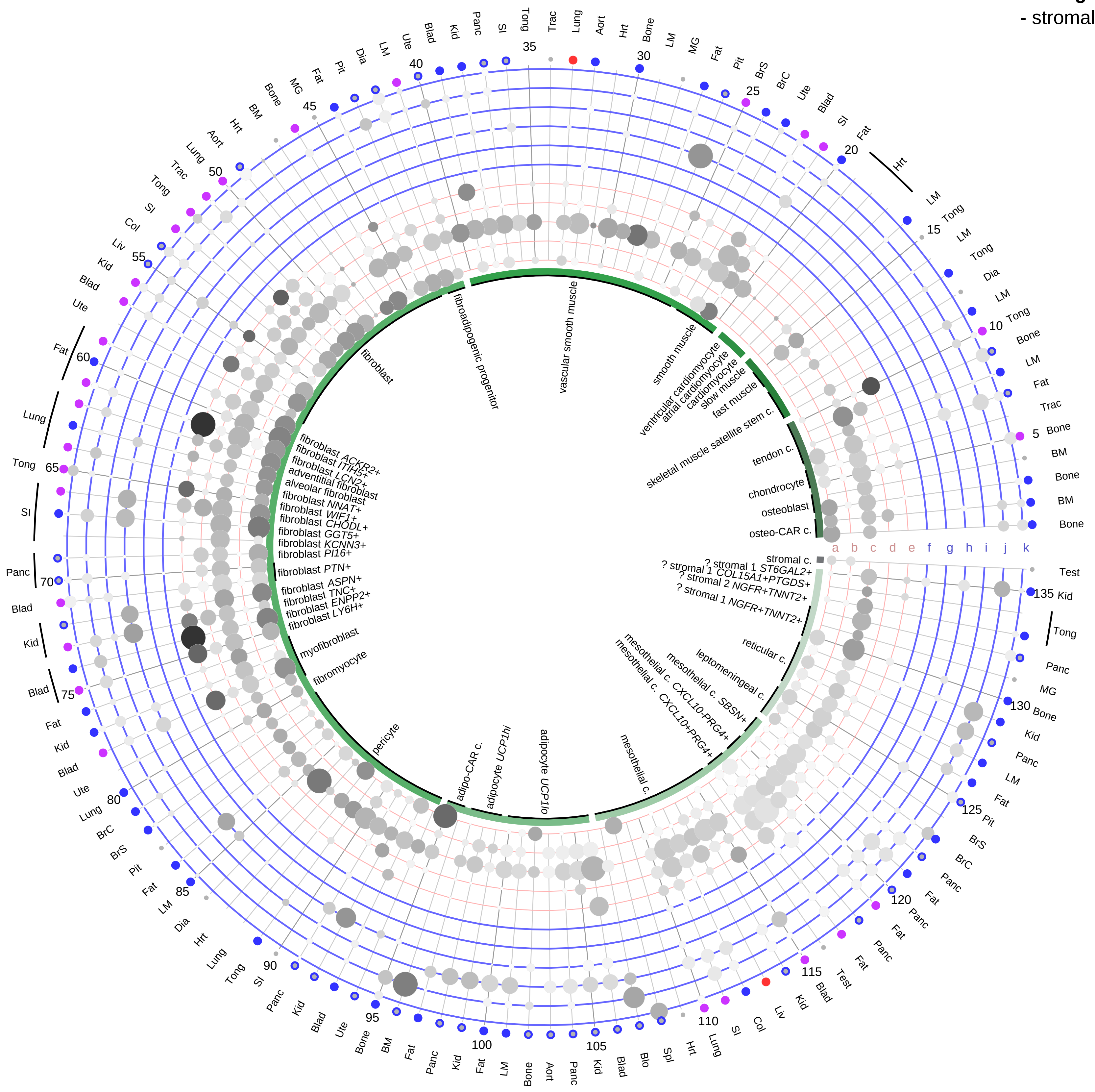

Ligand/Enzyme
- a - PTGES
- b - PTGES2
- c - PTGES3
- d - PTGS1
- e - PTGS2

Receptor
- f - PTGDR
- g - PTGER1
- h - PTGER2
- i - PTGER3
- j - PTGER4
- k - PTGFR

PAIRING (ligand → receptor)

PTGES & PTGS1 & PTGS2→PTGER1
PTGES & PTGS1 & PTGS2→PTGER2
PTGES & PTGS1 & PTGS2→PTGER3
PTGES & PTGS1 & PTGS2→PTGER4
PTGES & PTGS1 & PTGS2→PTGDR
PTGES & PTGS1 & PTGS2→PTGFR
PTGES2 & PTGS1 & PTGS2→PTGER1
PTGES2 & PTGS1 & PTGS2→PTGER2
PTGES2 & PTGS1 & PTGS2→PTGER3
PTGES2 & PTGS1 & PTGS2→PTGER4
PTGES2 & PTGS1 & PTGS2→PTGDR
PTGES2 & PTGS1 & PTGS2→PTGFR
PTGES3 & PTGS1 & PTGS2→PTGER1
PTGES3 & PTGS1 & PTGS2→PTGER2
PTGES3 & PTGS1 & PTGS2→PTGER3
PTGES3 & PTGS1 & PTGS2→PTGER4
PTGES3 & PTGS1 & PTGS2→PTGDR
PTGES3 & PTGS1 & PTGS2→PTGFR

Compartment
stromal
- bone
- skeletal muscle
- cardiac muscle
- smooth muscle
- fibroblast
- adipocyte
- mesothelial
- other
- mix

12.5%
25%
50%
100%

ln(CP10K+1): 0 1 2 3

Abbrev.	Tissue
Aort	Aorta
Blad	Bladder
Blo	Blood
Bone	Bone
BM	Bone marrow
BrC	Brain cortex
BrS	Brainstem
Col	Colon
Dia	Diaphragm
Ret	Eye retina
Fat	Fat
Hrt	Heart
Pit	Hypothalamus/Pituitary
Kid	Kidney
LM	Limb muscle
Liv	Liver
Lung	Lung
MG	Mammary gland
Panc	Pancreas
Skin	Skin
SI	Small intestine
Spl	Spleen
Test	Testes
Thym	Thymus
Tong	Tongue
Trac	Trachea
Ute	Uterus

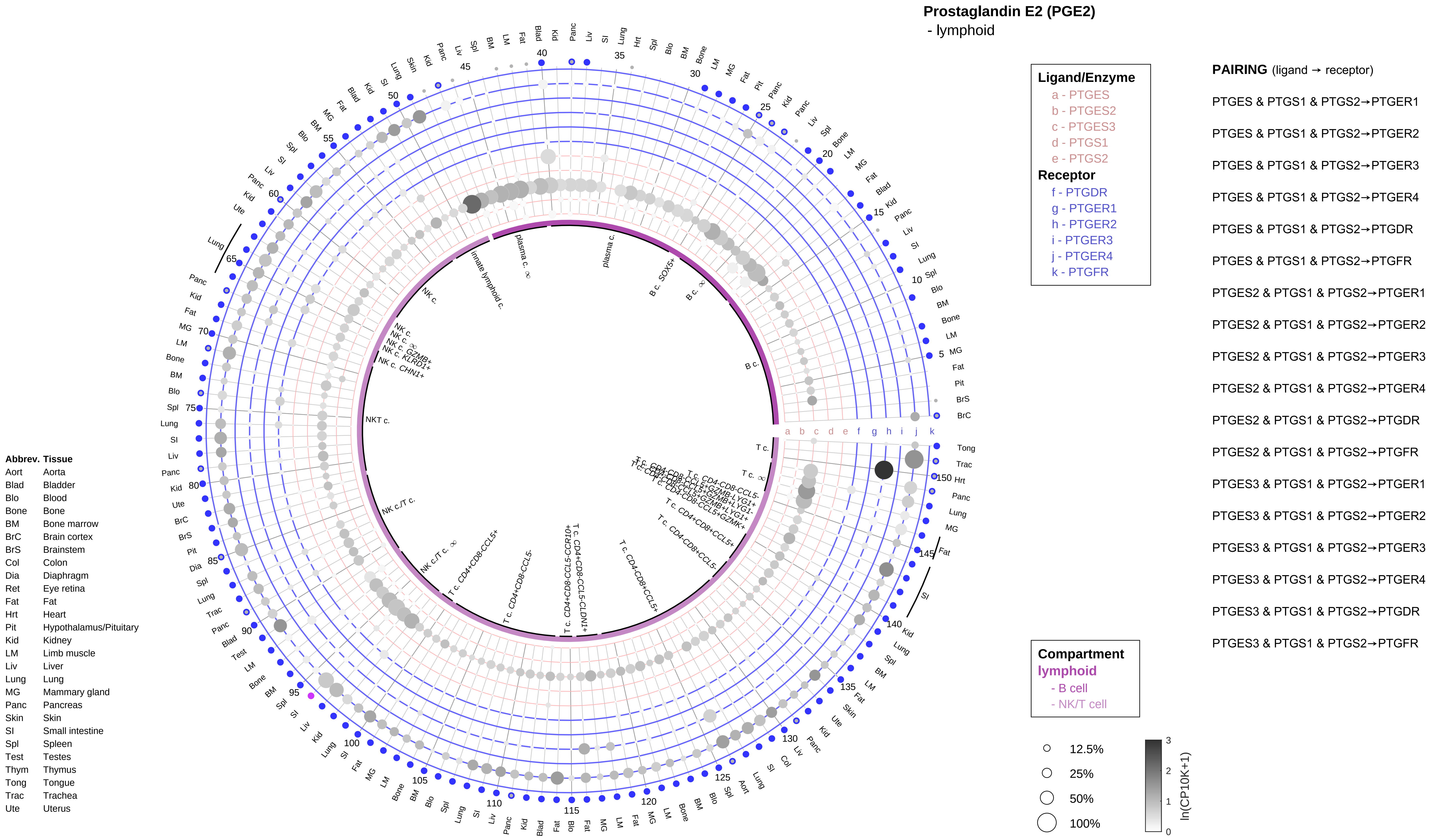
Prostaglandin E2 (PGE2)
- lymphoid
Ligand/Enzyme
a - PTGES
b - PTGES2
c - PTGES3
d - PTGS1
e - PTGS2
Receptor
f - PTGDR
g - PTGER1
h - PTGER2
i - PTGER3
j - PTGER4
k - PTGFR
PAIRING (ligand → receptor)
PTGES & PTGS1 & PTGS2→PTGER1
PTGES & PTGS1 & PTGS2→PTGER2
PTGES & PTGS1 & PTGS2→PTGER3
PTGES & PTGS1 & PTGS2→PTGER4
PTGES & PTGS1 & PTGS2→PTGDR
PTGES & PTGS1 & PTGS2→PTGFR
PTGES2 & PTGS1 & PTGS2→PTGER1
PTGES2 & PTGS1 & PTGS2→PTGER2
PTGES2 & PTGS1 & PTGS2→PTGER3
PTGES2 & PTGS1 & PTGS2→PTGER4
PTGES2 & PTGS1 & PTGS2→PTGDR
PTGES2 & PTGS1 & PTGS2→PTGFR
PTGES3 & PTGS1 & PTGS2→PTGER1
PTGES3 & PTGS1 & PTGS2→PTGER2
PTGES3 & PTGS1 & PTGS2→PTGER3
PTGES3 & PTGS1 & PTGS2→PTGER4
PTGES3 & PTGS1 & PTGS2→PTGDR
PTGES3 & PTGS1 & PTGS2→PTGFR
Compartment
lymphoid
- B cell
- NK/T cell
12.5%
25%
50%
100%
ln(CP10K+1)
0
1
2
3
Abbrev. Tissue
Aort Aorta
Blad Bladder
Blo Blood
Bone Bone
BM Bone marrow
BrC Brain cortex
BrS Brainstem
Col Colon
Dia Diaphragm
Ret Eye retina
Fat Fat
Hrt Heart
Pit Hypothalamus/Pituitary
Kid Kidney
LM Limb muscle
Liv Liver
Lung Lung
MG Mammary gland
Panc Pancreas
Skin Skin
SI Small intestine
Spl Spleen
Test Testes
Thym Thymus
Tong Tongue
Trac Trachea
Ute Uterus
plasma c.
innate lymphoid c.
B c. SOX5+
B c.
NK c.
NKT c.
NK c./T c.
T c.

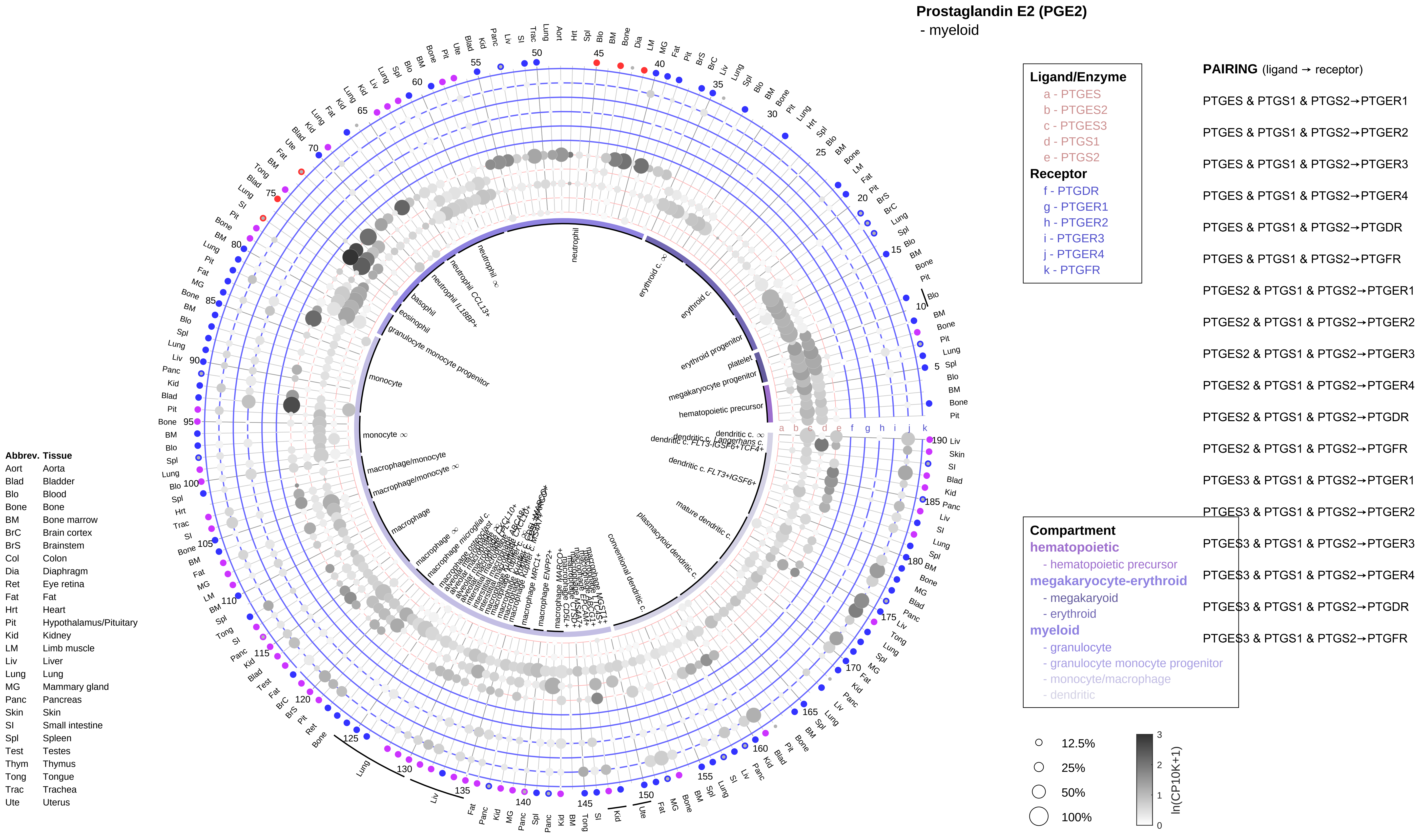
Prostaglandin E2 (PGE2)
- myeloid
Ligand/Enzyme
a - PTGES
b - PTGES2
c - PTGES3
d - PTGS1
e - PTGS2
Receptor
f - PTGDR
g - PTGER1
h - PTGER2
i - PTGER3
j - PTGER4
k - PTGFR
PAIRING (ligand → receptor)
PTGES & PTGS1 & PTGS2→PTGER1
PTGES & PTGS1 & PTGS2→PTGER2
PTGES & PTGS1 & PTGS2→PTGER3
PTGES & PTGS1 & PTGS2→PTGER4
PTGES & PTGS1 & PTGS2→PTGDR
PTGES & PTGS1 & PTGS2→PTGFR
PTGES2 & PTGS1 & PTGS2→PTGER1
PTGES2 & PTGS1 & PTGS2→PTGER2
PTGES2 & PTGS1 & PTGS2→PTGER3
PTGES2 & PTGS1 & PTGS2→PTGER4
PTGES2 & PTGS1 & PTGS2→PTGDR
PTGES2 & PTGS1 & PTGS2→PTGFR
PTGES3 & PTGS1 & PTGS2→PTGER1
PTGES3 & PTGS1 & PTGS2→PTGER2
PTGES3 & PTGS1 & PTGS2→PTGER3
PTGES3 & PTGS1 & PTGS2→PTGER4
PTGES3 & PTGS1 & PTGS2→PTGDR
PTGES3 & PTGS1 & PTGS2→PTGFR
Compartment
hematopoietic
- hematopoietic precursor
megakaryocyte-erythroid
- megakaryoid
- erythroid
myeloid
- granulocyte
- granulocyte monocyte progenitor
- monocyte/macrophage
- dendritic
12.5%
25%
50%
100%
ln(CP10K+1)
0
1
2
3
Abbrev. Tissue
Aort Aorta
Blad Bladder
Blo Blood
Bone Bone
BM Bone marrow
BrC Brain cortex
BrS Brainstem
Col Colon
Dia Diaphragm
Ret Eye retina
Fat Fat
Hrt Heart
Pit Hypothalamus/Pituitary
Kid Kidney
LM Limb muscle
Liv Liver
Lung Lung
MG Mammary gland
Panc Pancreas
Skin Skin
SI Small intestine
Spl Spleen
Test Testes
Thym Thymus
Tong Tongue
Trac Trachea
Ute Uterus
neutrophil
erythroid c. ∞
erythroid c.
erythroid progenitor
platelet
megakaryocyte progenitor
hematopoietic precursor
dendritic c. ∞
dendritic c. Langerhans c.
dendritic c. FLT3-IGSF6+TCF4+
dendritic c. FLT3+IGSF6+
mature dendritic c.
plasmacytoid dendritic c.
conventional dendritic c.
neutrophil ∞
neutrophil CCL13+
neutrophil IL18BP+
basophil
eosinophil
granulocyte monocyte progenitor
monocyte
monocyte ∞
macrophage/monocyte
macrophage/monocyte ∞
macrophage
macrophage ∞
macrophage microglial c.
macrophage MRC1+
macrophage ENPP2+
macrophage MARCO+
a
b
c
d
e
f
g
h
i
j
k

Prostaglandin I2/Prostacyclin (PGI2)
- epithelial/neural/germ

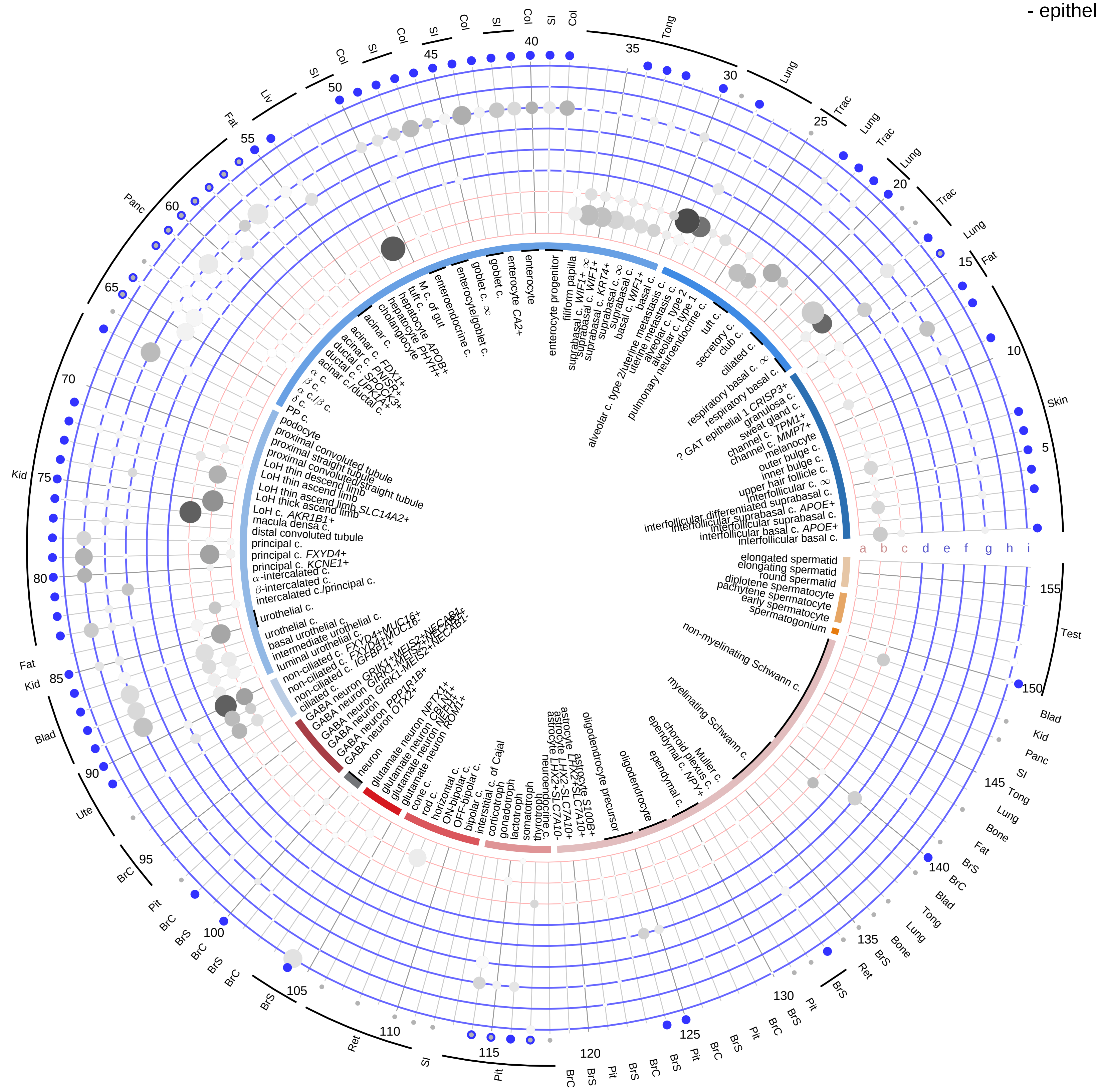

Ligand/Enzyme
- a - PTGIS
- b - PTGS1
- c - PTGS2

Receptor
- d - PTGER1
- e - PTGER2
- f - PTGER3
- g - PTGER4
- h - PTGFR
- i - PTGIR

PAIRING (ligand → receptor)

PTGIS & PTGS1 & PTGS2→PTGIR

PTGIS & PTGS1 & PTGS2→PTGFR

PTGIS & PTGS1 & PTGS2→PTGER1

PTGIS & PTGS1 & PTGS2→PTGER2

PTGIS & PTGS1 & PTGS2→PTGER3

PTGIS & PTGS1 & PTGS2→PTGER4

Compartment

epithelial
- integumentary
- respiratory
- gastrointestinal
- urinary
- reproductive

neural
- GABAergic
- hybrid
- glutamatergic
- specialized
- neuroendocrine
- glia

germ
- spermatogonium
- spermatocyte
- spermatid

12.5%
25%
50%
100%

ln(CP10K+1): 0, 1, 2, 3

Abbrev.	Tissue
Aort	Aorta
Blad	Bladder
Blo	Blood
Bone	Bone
BM	Bone marrow
BrC	Brain cortex
BrS	Brainstem
Col	Colon
Dia	Diaphragm
Ret	Eye retina
Fat	Fat
Hrt	Heart
Pit	Hypothalamus/Pituitary
Kid	Kidney
LM	Limb muscle
Liv	Liver
Lung	Lung
MG	Mammary gland
Panc	Pancreas
Skin	Skin
SI	Small intestine
Spl	Spleen
Test	Testes
Thym	Thymus
Tong	Tongue
Trac	Trachea
Ute	Uterus

Prostaglandin I2/Prostacyclin (PGI2)
- endothelial

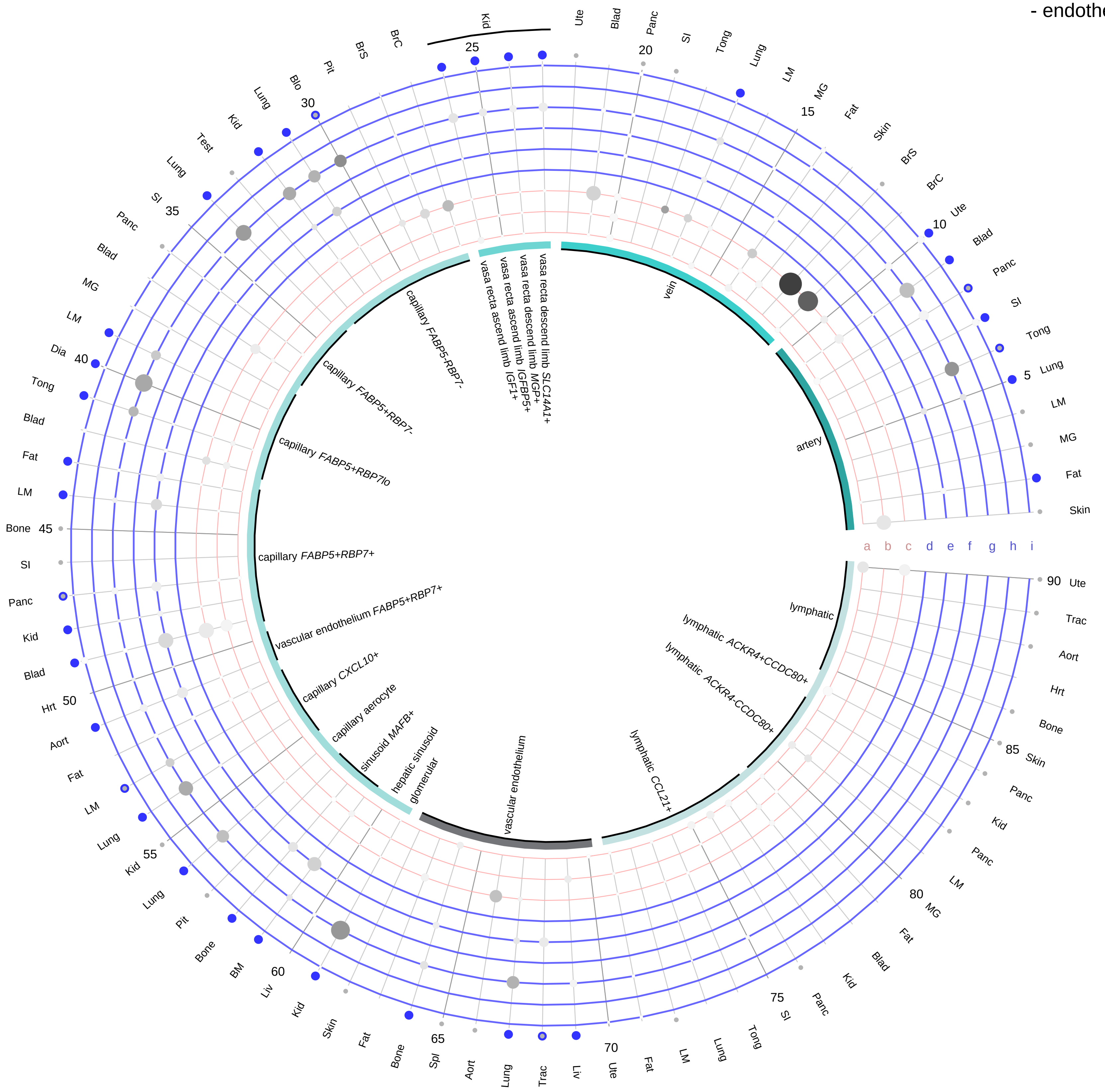

Ligand/Enzyme
- a - PTGIS
- b - PTGS1
- c - PTGS2

Receptor
- d - PTGER1
- e - PTGER2
- f - PTGER3
- g - PTGER4
- h - PTGFR
- i - PTGIR

PAIRING (ligand → receptor)

PTGIS & PTGS1 & PTGS2→PTGIR

PTGIS & PTGS1 & PTGS2→PTGFR

PTGIS & PTGS1 & PTGS2→PTGER1

PTGIS & PTGS1 & PTGS2→PTGER2

PTGIS & PTGS1 & PTGS2→PTGER3

PTGIS & PTGS1 & PTGS2→PTGER4

Abbrev.	Tissue
Aort	Aorta
Blad	Bladder
Blo	Blood
Bone	Bone
BM	Bone marrow
BrC	Brain cortex
BrS	Brainstem
Col	Colon
Dia	Diaphragm
Ret	Eye retina
Fat	Fat
Hrt	Heart
Pit	Hypothalamus/Pituitary
Kid	Kidney
LM	Limb muscle
Liv	Liver
Lung	Lung
MG	Mammary gland
Panc	Pancreas
Skin	Skin
SI	Small intestine
Spl	Spleen
Test	Testes
Thym	Thymus
Tong	Tongue
Trac	Trachea
Ute	Uterus

Compartment

endothelial
- artery
- vein
- vasa recta
- capillary
- mix
- lymphatic

12.5%
25%
50%
100%

ln(CP10K+1): 0, 1, 2, 3

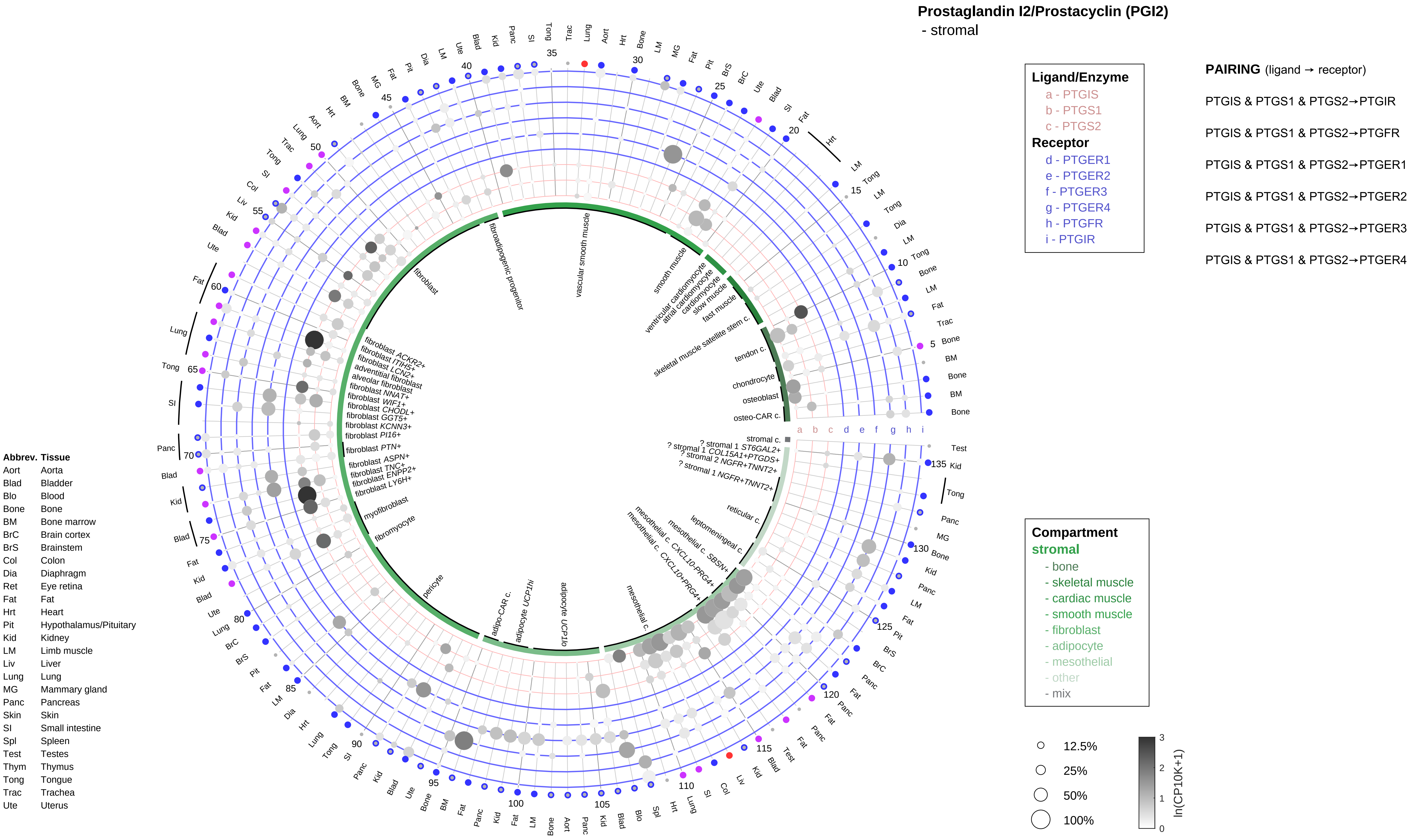

Prostaglandin I2/Prostacyclin (PGI2)
- stromal
Ligand/Enzyme
a - PTGIS
b - PTGS1
c - PTGS2
Receptor
d - PTGER1
e - PTGER2
f - PTGER3
g - PTGER4
h - PTGFR
i - PTGIR
PAIRING (ligand → receptor)
PTGIS & PTGS1 & PTGS2→PTGIR
PTGIS & PTGS1 & PTGS2→PTGFR
PTGIS & PTGS1 & PTGS2→PTGER1
PTGIS & PTGS1 & PTGS2→PTGER2
PTGIS & PTGS1 & PTGS2→PTGER3
PTGIS & PTGS1 & PTGS2→PTGER4
Compartment
stromal
- bone
- skeletal muscle
- cardiac muscle
- smooth muscle
- fibroblast
- adipocyte
- mesothelial
- other
- mix
12.5%
25%
50%
100%
ln(CP10K+1)
0
1
2
3
Abbrev. Tissue
Aort Aorta
Blad Bladder
Blo Blood
Bone Bone
BM Bone marrow
BrC Brain cortex
BrS Brainstem
Col Colon
Dia Diaphragm
Ret Eye retina
Fat Fat
Hrt Heart
Pit Hypothalamus/Pituitary
Kid Kidney
LM Limb muscle
Liv Liver
Lung Lung
MG Mammary gland
Panc Pancreas
Skin Skin
SI Small intestine
Spl Spleen
Test Testes
Thym Thymus
Tong Tongue
Trac Trachea
Ute Uterus
vascular smooth muscle
smooth muscle
ventricular cardiomyocyte
atrial cardiomyocyte
cardiomyocyte
slow muscle
fast muscle
skeletal muscle satellite stem c.
tendon c.
chondrocyte
osteoblast
osteo-CAR c.
stromal c.
? stromal 1 ST6GAL2+
? stromal 1 COL15A1+PTGDS+
? stromal 2 NGFR+TNNT2+
? stromal 1 NGFR+TNNT2+
reticular c.
leptomeningeal c.
mesothelial c. SBSN+
mesothelial c. CXCL10+PRG4+
mesothelial c. CXCL10-PRG4+
mesothelial c.
adipocyte UCP1lo
adipocyte UCP1hi
adipo-CAR c.
pericyte
fibromyocyte
myofibroblast
fibroblast LY6H+
fibroblast ENPP2+
fibroblast TNC+
fibroblast ASPN+
fibroblast PTN+
fibroblast PI16+
fibroblast KCNN3+
fibroblast GGT5+
fibroblast CHODL+
fibroblast WIF1+
fibroblast NNAT+
alveolar fibroblast
adventitial fibroblast
fibroblast LCN2+
fibroblast ITIH5+
fibroblast ACKR2+
fibroblast
fibroadipogenic progenitor

Prostaglandin I2/Prostacyclin (PGI2)
- lymphoid

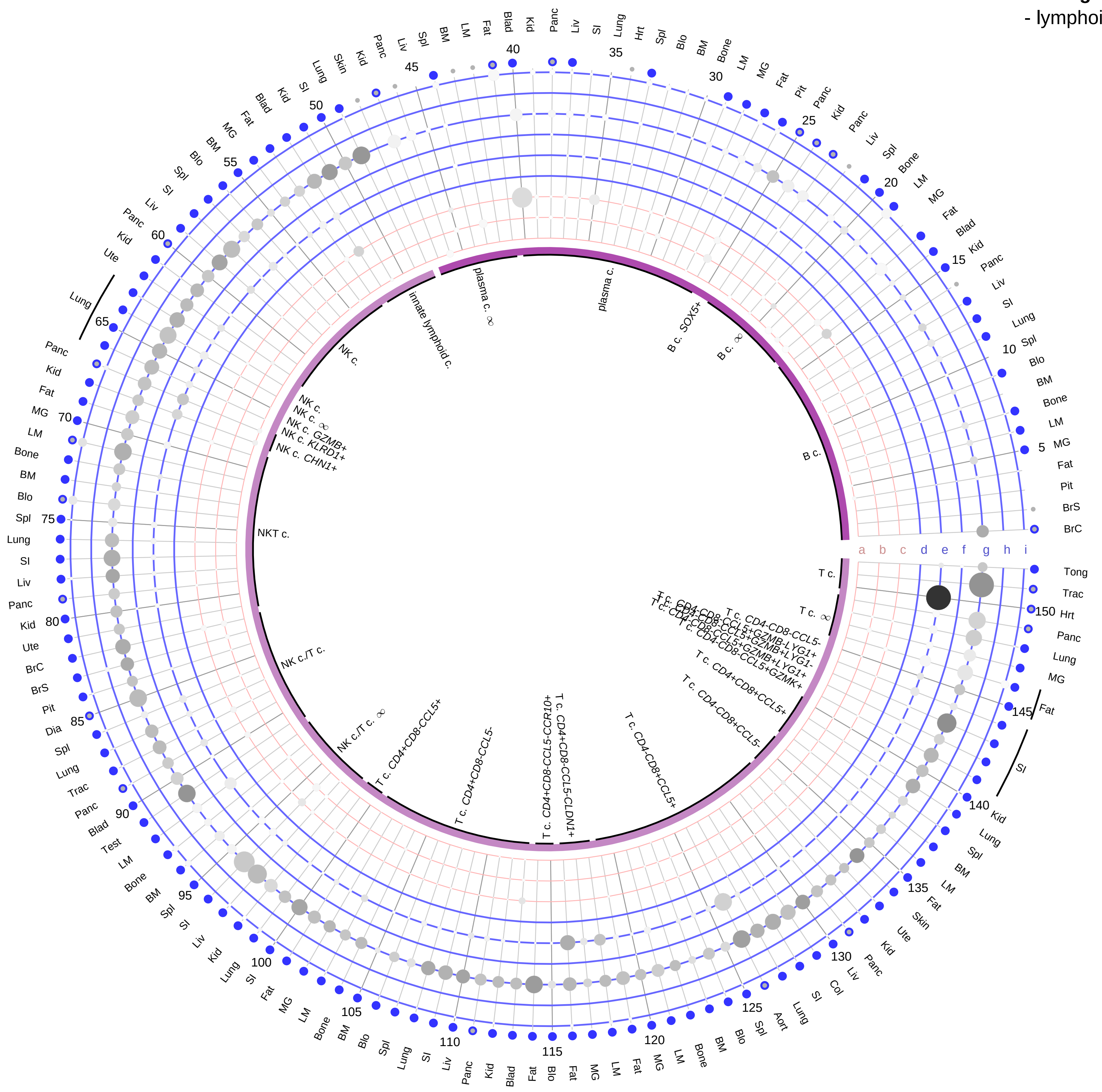

Ligand/Enzyme
- a - PTGIS
- b - PTGS1
- c - PTGS2

Receptor
- d - PTGER1
- e - PTGER2
- f - PTGER3
- g - PTGER4
- h - PTGFR
- i - PTGIR

PAIRING (ligand → receptor)

PTGIS & PTGS1 & PTGS2→PTGIR

PTGIS & PTGS1 & PTGS2→PTGFR

PTGIS & PTGS1 & PTGS2→PTGER1

PTGIS & PTGS1 & PTGS2→PTGER2

PTGIS & PTGS1 & PTGS2→PTGER3

PTGIS & PTGS1 & PTGS2→PTGER4

Abbrev.	Tissue
Aort	Aorta
Blad	Bladder
Blo	Blood
Bone	Bone
BM	Bone marrow
BrC	Brain cortex
BrS	Brainstem
Col	Colon
Dia	Diaphragm
Ret	Eye retina
Fat	Fat
Hrt	Heart
Pit	Hypothalamus/Pituitary
Kid	Kidney
LM	Limb muscle
Liv	Liver
Lung	Lung
MG	Mammary gland
Panc	Pancreas
Skin	Skin
SI	Small intestine
Spl	Spleen
Test	Testes
Thym	Thymus
Tong	Tongue
Trac	Trachea
Ute	Uterus

Compartment
lymphoid
- B cell
- NK/T cell

12.5%
25%
50%
100%

ln(CP10K+1): 0, 1, 2, 3

Prostaglandin I2/Prostacyclin (PGI2)
- myeloid

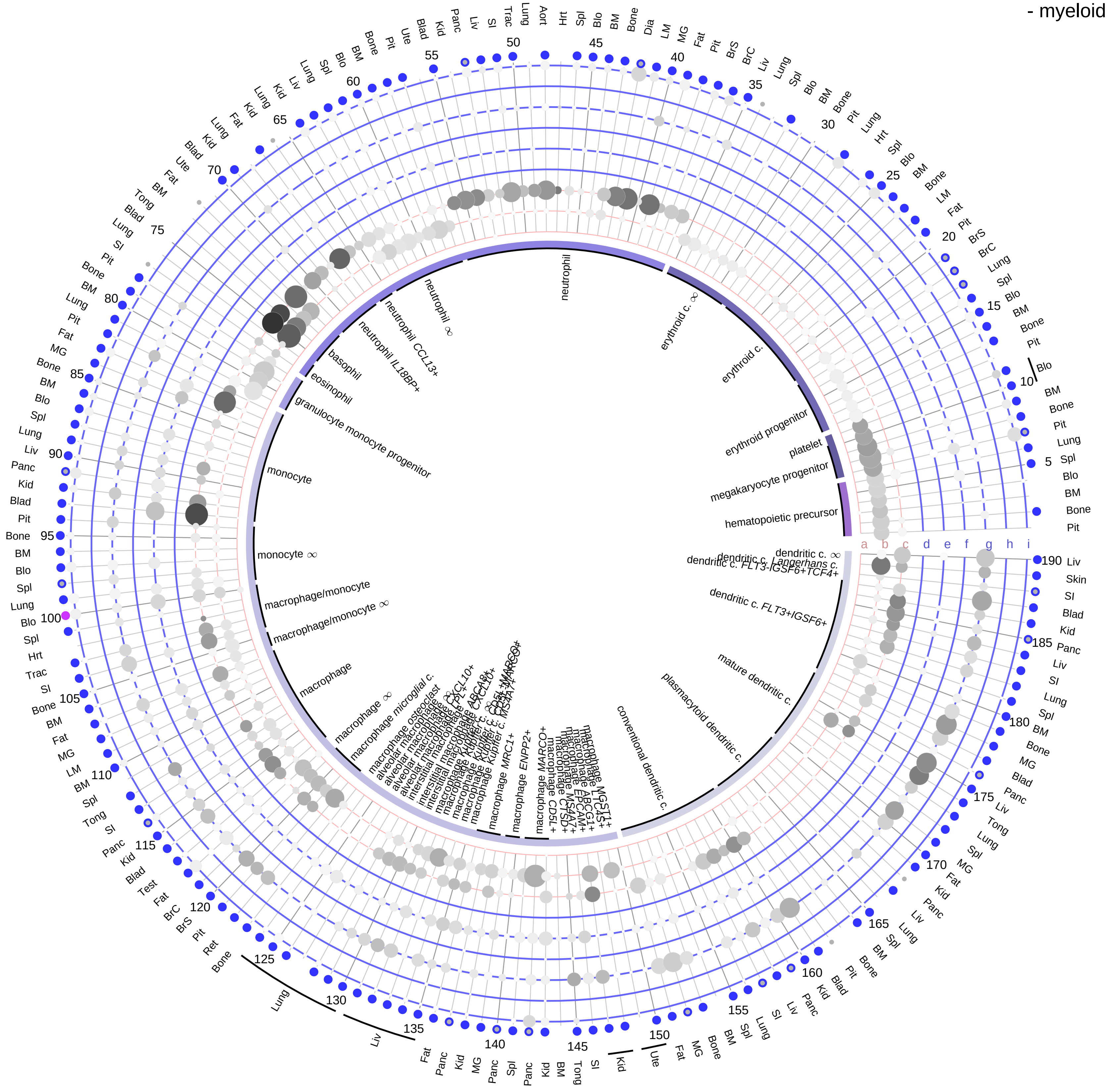

Ligand/Enzyme
- a - PTGIS
- b - PTGS1
- c - PTGS2

Receptor
- d - PTGER1
- e - PTGER2
- f - PTGER3
- g - PTGER4
- h - PTGFR
- i - PTGIR

PAIRING (ligand → receptor)

PTGIS & PTGS1 & PTGS2→PTGIR

PTGIS & PTGS1 & PTGS2→PTGFR

PTGIS & PTGS1 & PTGS2→PTGER1

PTGIS & PTGS1 & PTGS2→PTGER2

PTGIS & PTGS1 & PTGS2→PTGER3

PTGIS & PTGS1 & PTGS2→PTGER4

Abbrev.	Tissue
Aort	Aorta
Blad	Bladder
Blo	Blood
Bone	Bone
BM	Bone marrow
BrC	Brain cortex
BrS	Brainstem
Col	Colon
Dia	Diaphragm
Ret	Eye retina
Fat	Fat
Hrt	Heart
Pit	Hypothalamus/Pituitary
Kid	Kidney
LM	Limb muscle
Liv	Liver
Lung	Lung
MG	Mammary gland
Panc	Pancreas
Skin	Skin
SI	Small intestine
Spl	Spleen
Test	Testes
Thym	Thymus
Tong	Tongue
Trac	Trachea
Ute	Uterus

Compartment

hematopoietic
- hematopoietic precursor

megakaryocyte-erythroid
- megakaryoid
- erythroid

myeloid
- granulocyte
- granulocyte monocyte progenitor
- monocyte/macrophage
- dendritic

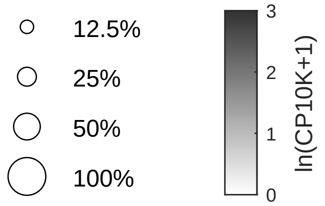

Relaxins
- epithelial/neural/germ

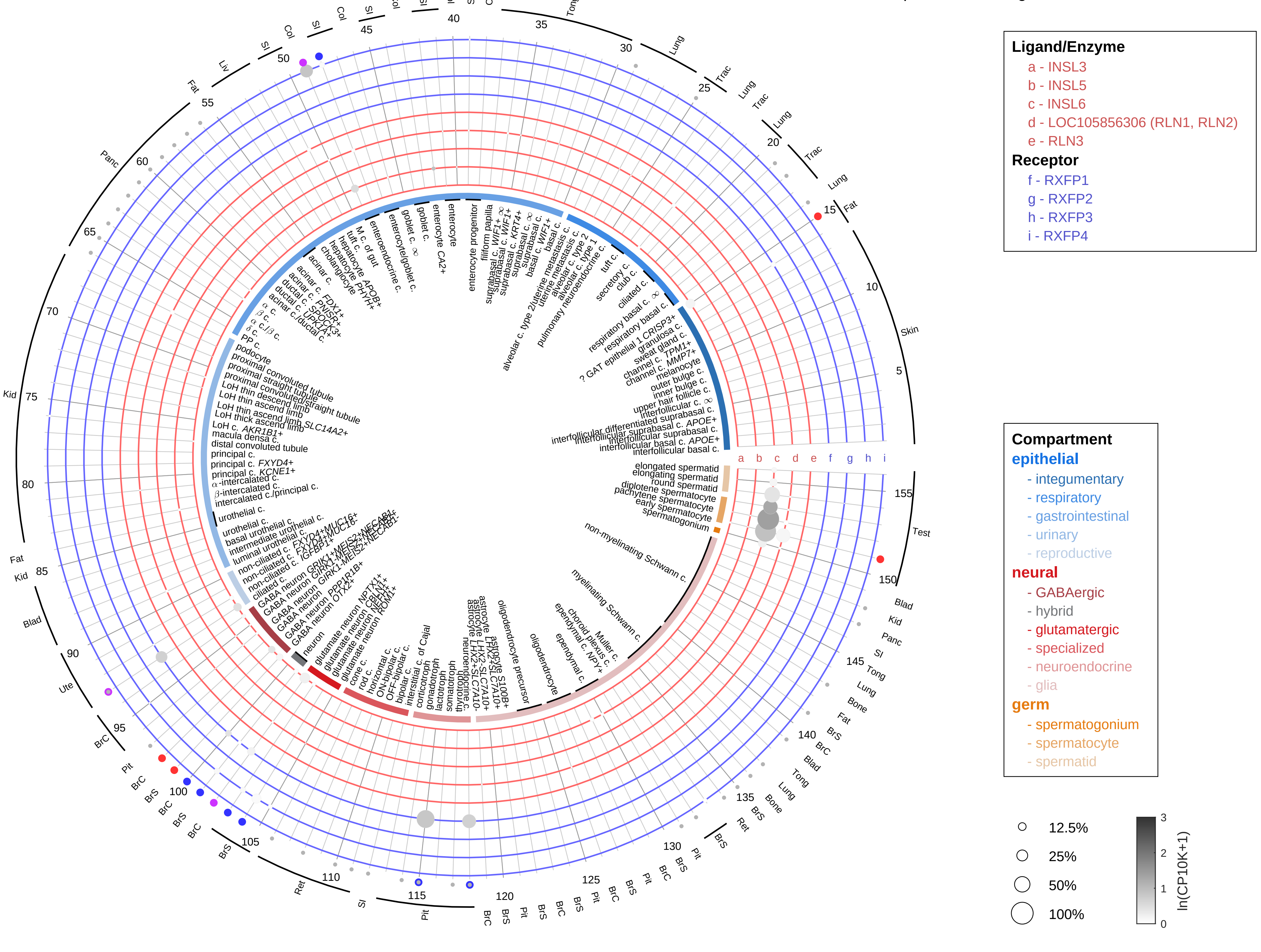

Abbrev.	Tissue
Aort	Aorta
Blad	Bladder
Blo	Blood
Bone	Bone
BM	Bone marrow
BrC	Brain cortex
BrS	Brainstem
Col	Colon
Dia	Diaphragm
Ret	Eye retina
Fat	Fat
Hrt	Heart
Pit	Hypothalamus/Pituitary
Kid	Kidney
LM	Limb muscle
Liv	Liver
Lung	Lung
MG	Mammary gland
Panc	Pancreas
Skin	Skin
SI	Small intestine
Spl	Spleen
Test	Testes
Thym	Thymus
Tong	Tongue
Trac	Trachea
Ute	Uterus

PAIRING (ligand → receptor)

RLN2→RXFP1

RLN2→RXFP2

RLN2→RXFP3

RLN1→RXFP1

RLN1→RXFP2

RLN3→RXFP3

RLN3→RXFP4

RLN3→RXFP1

RLN3→RXFP2

INSL3→RXFP2

INSL3→RXFP1

INSL5→RXFP4

INSL5→RXFP3

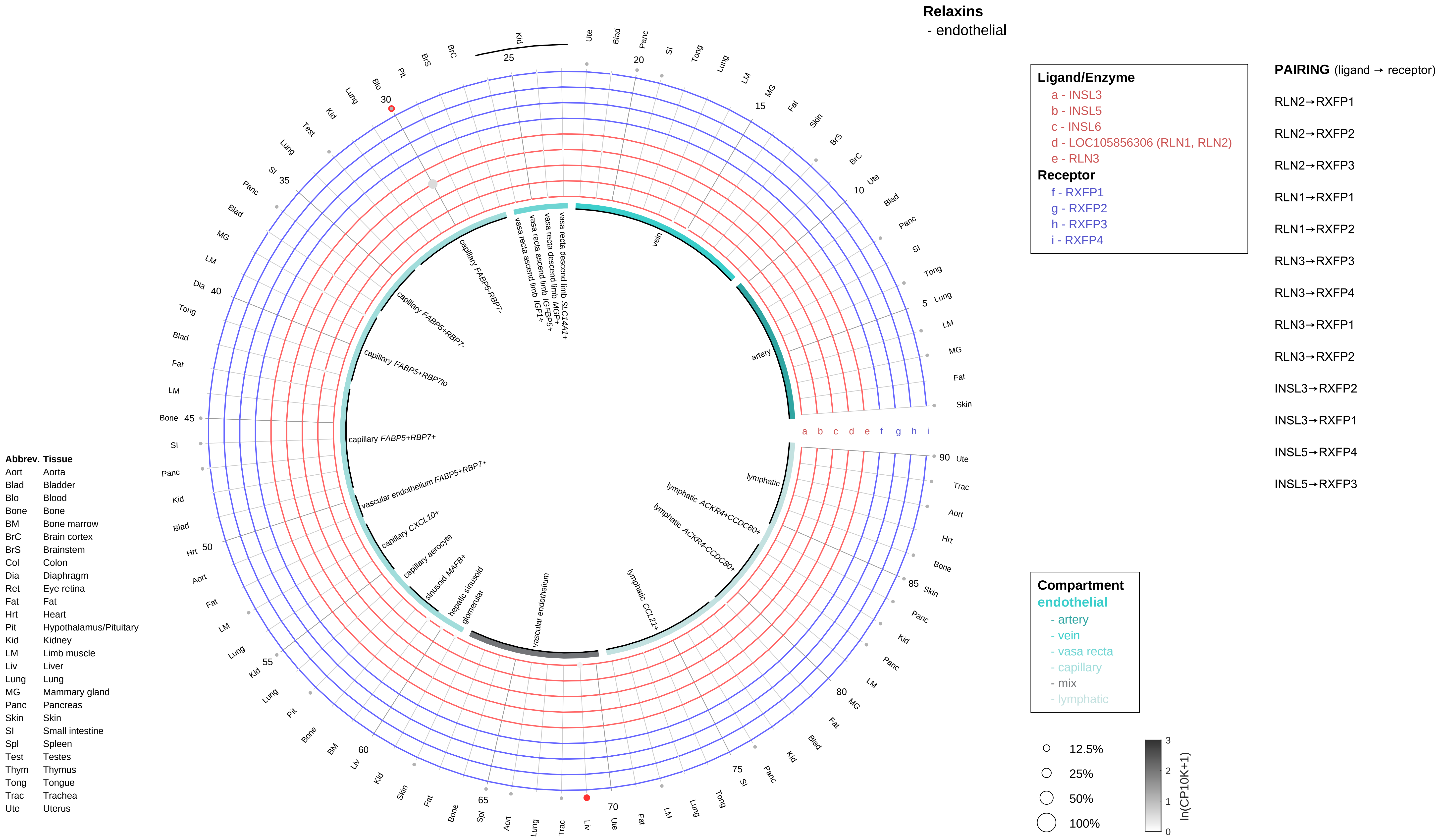

Relaxins
- endothelial
Ligand/Enzyme
a - INSL3
b - INSL5
c - INSL6
d - LOC105856306 (RLN1, RLN2)
e - RLN3
Receptor
f - RXFP1
g - RXFP2
h - RXFP3
i - RXFP4
PAIRING (ligand → receptor)
RLN2→RXFP1
RLN2→RXFP2
RLN2→RXFP3
RLN1→RXFP1
RLN1→RXFP2
RLN3→RXFP3
RLN3→RXFP4
RLN3→RXFP1
RLN3→RXFP2
INSL3→RXFP2
INSL3→RXFP1
INSL5→RXFP4
INSL5→RXFP3
Compartment
endothelial
- artery
- vein
- vasa recta
- capillary
- mix
- lymphatic
12.5%
25%
50%
100%
ln(CP10K+1)
0
1
2
3
vein
artery
vasa recta descend limb SLC14A1+
vasa recta descend limb MGP+
vasa recta ascend limb IGFBP5+
vasa recta ascend limb IGF1+
capillary FABP5-RBP7-
capillary FABP5+RBP7-
capillary FABP5+RBP7lo
capillary FABP5+RBP7+
vascular endothelium FABP5+RBP7+
capillary CXCL10+
capillary aerocyte
sinusoid MAFB+
hepatic sinusoid
glomerular
vascular endothelium
lymphatic CCL21+
lymphatic ACKR4-CCDC80+
lymphatic ACKR4+CCDC80+
lymphatic
a b c d e f g h i
Abbrev. Tissue
Aort Aorta
Blad Bladder
Blo Blood
Bone Bone
BM Bone marrow
BrC Brain cortex
BrS Brainstem
Col Colon
Dia Diaphragm
Ret Eye retina
Fat Fat
Hrt Heart
Pit Hypothalamus/Pituitary
Kid Kidney
LM Limb muscle
Liv Liver
Lung Lung
MG Mammary gland
Panc Pancreas
Skin Skin
SI Small intestine
Spl Spleen
Test Testes
Thym Thymus
Tong Tongue
Trac Trachea
Ute Uterus

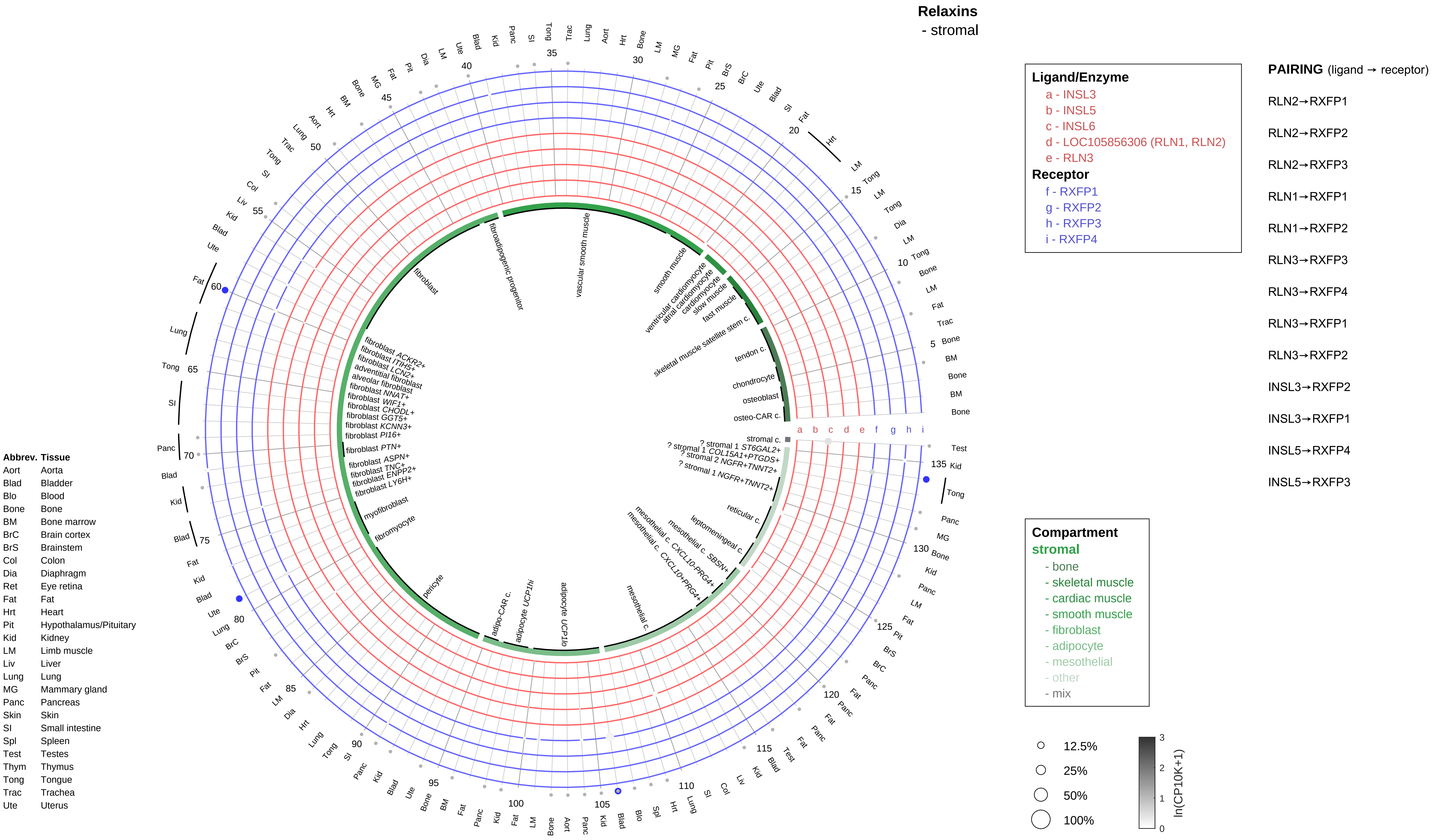
Relaxins
- stromal
Ligand/Enzyme
a - INSL3
b - INSL5
c - INSL6
d - LOC105856306 (RLN1, RLN2)
e - RLN3
Receptor
f - RXFP1
g - RXFP2
h - RXFP3
i - RXFP4
PAIRING (ligand → receptor)
RLN2→RXFP1
RLN2→RXFP2
RLN2→RXFP3
RLN1→RXFP1
RLN1→RXFP2
RLN3→RXFP3
RLN3→RXFP4
RLN3→RXFP1
RLN3→RXFP2
INSL3→RXFP2
INSL3→RXFP1
INSL5→RXFP4
INSL5→RXFP3
Compartment
stromal
- bone
- skeletal muscle
- cardiac muscle
- smooth muscle
- fibroblast
- adipocyte
- mesothelial
- other
- mix
12.5%
25%
50%
100%
ln(CP10K+1)
Abbrev. Tissue
Aort Aorta
Blad Bladder
Blo Blood
Bone Bone
BM Bone marrow
BrC Brain cortex
BrS Brainstem
Col Colon
Dia Diaphragm
Ret Eye retina
Fat Fat
Hrt Heart
Pit Hypothalamus/Pituitary
Kid Kidney
LM Limb muscle
Liv Liver
Lung Lung
MG Mammary gland
Panc Pancreas
Skin Skin
SI Small intestine
Spl Spleen
Test Testes
Thym Thymus
Tong Tongue
Trac Trachea
Ute Uterus
vascular smooth muscle
fibroadipogenic progenitor
fibroblast
smooth muscle
ventricular cardiomyocyte
atrial cardiomyocyte
cardiomyocyte
slow muscle
fast muscle
skeletal muscle satellite stem c.
tendon c.
chondrocyte
osteoblast
osteo-CAR c.
stromal c.
? stromal 1 ST6GAL2+
? stromal 1 COL15A1+PTGDS+
? stromal 2 NGFR+TNNT2+
? stromal 1 NGFR+TNNT2+
reticular c.
leptomeningeal c.
mesothelial c. SBSN+
mesothelial c. CXCL10-PRG4+
mesothelial c. CXCL10-PRG4+
mesothelial c.
adipocyte UCP1lo
adipocyte UCP1hi
adipo-CAR c.
pericyte
fibromyocyte
myofibroblast
fibroblast LY6H+
fibroblast ENPP2+
fibroblast TNC+
fibroblast ASPN+
fibroblast PTN+
fibroblast PI16+
fibroblast KCNN3+
fibroblast GGT5+
fibroblast CHODL+
fibroblast WIF1+
fibroblast NNAT+
alveolar fibroblast
adventitial fibroblast
fibroblast LCN2+
fibroblast ITIH5+
fibroblast ACKR2+

Relaxins
- lymphoid

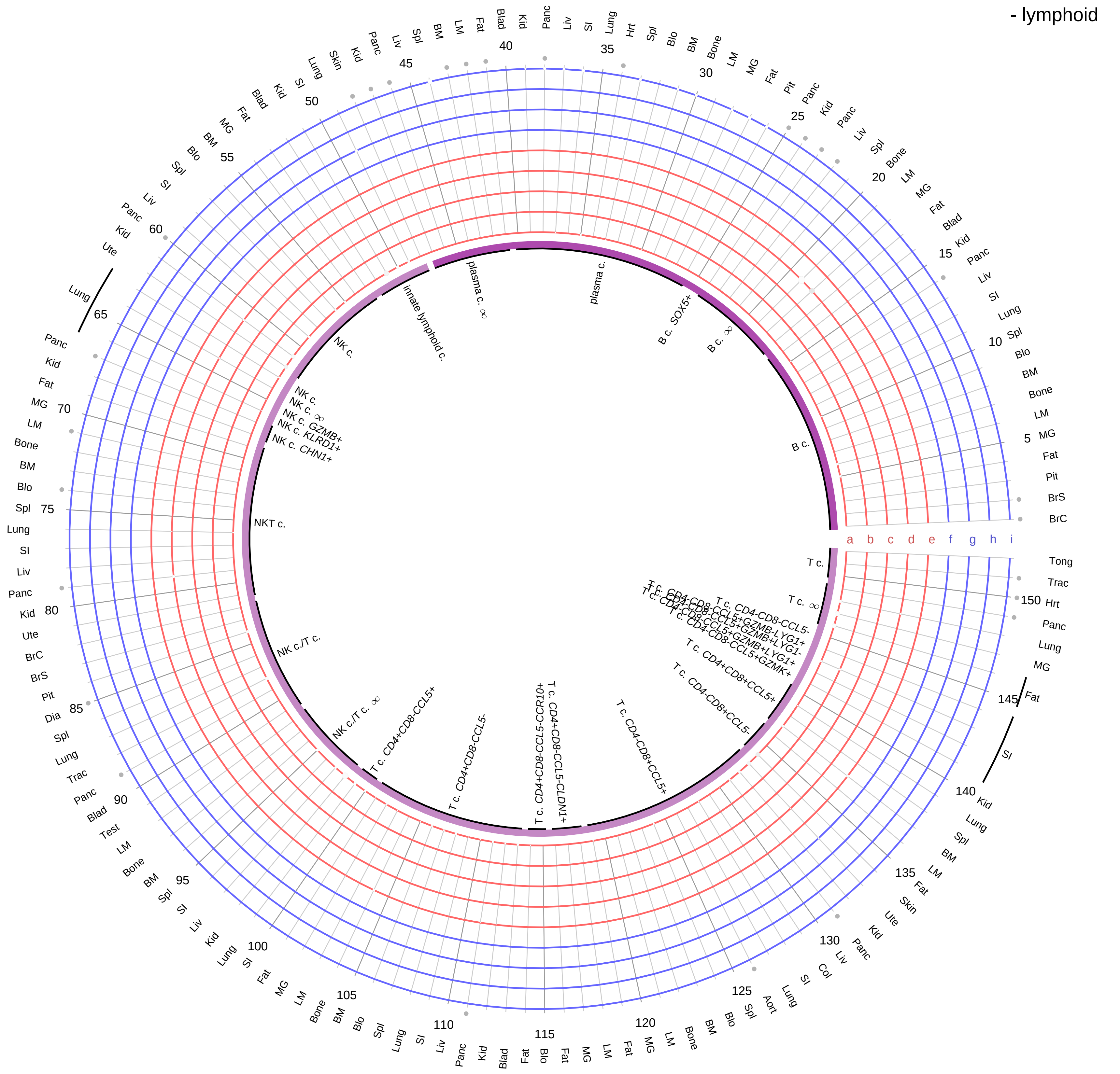

Ligand/Enzyme
- a - INSL3
- b - INSL5
- c - INSL6
- d - LOC105856306 (RLN1, RLN2)
- e - RLN3

Receptor
- f - RXFP1
- g - RXFP2
- h - RXFP3
- i - RXFP4

PAIRING (ligand → receptor)

RLN2→RXFP1
RLN2→RXFP2
RLN2→RXFP3
RLN1→RXFP1
RLN1→RXFP2
RLN3→RXFP3
RLN3→RXFP4
RLN3→RXFP1
RLN3→RXFP2
INSL3→RXFP2
INSL3→RXFP1
INSL5→RXFP4
INSL5→RXFP3

Compartment
lymphoid
- B cell
- NK/T cell

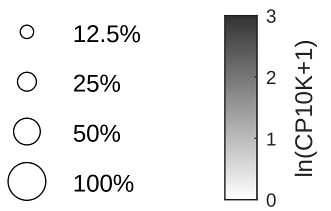

Abbrev.	Tissue
Aort	Aorta
Blad	Bladder
Blo	Blood
Bone	Bone
BM	Bone marrow
BrC	Brain cortex
BrS	Brainstem
Col	Colon
Dia	Diaphragm
Ret	Eye retina
Fat	Fat
Hrt	Heart
Pit	Hypothalamus/Pituitary
Kid	Kidney
LM	Limb muscle
Liv	Liver
Lung	Lung
MG	Mammary gland
Panc	Pancreas
Skin	Skin
SI	Small intestine
Spl	Spleen
Test	Testes
Thym	Thymus
Tong	Tongue
Trac	Trachea
Ute	Uterus

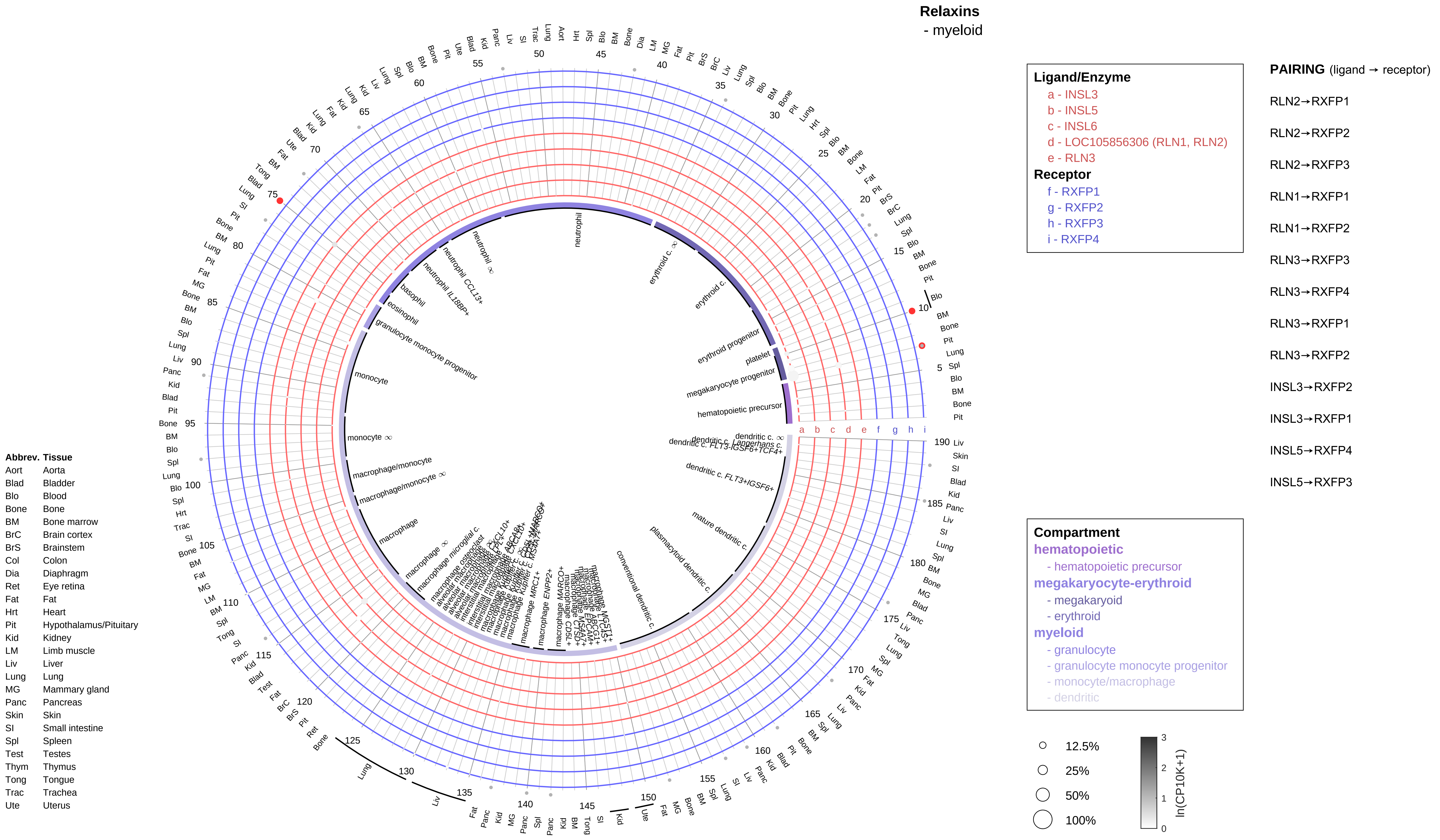

Relaxins
- myeloid
Ligand/Enzyme
a - INSL3
b - INSL5
c - INSL6
d - LOC105856306 (RLN1, RLN2)
e - RLN3
Receptor
f - RXFP1
g - RXFP2
h - RXFP3
i - RXFP4
PAIRING (ligand → receptor)
RLN2→RXFP1
RLN2→RXFP2
RLN2→RXFP3
RLN1→RXFP1
RLN1→RXFP2
RLN3→RXFP3
RLN3→RXFP4
RLN3→RXFP1
RLN3→RXFP2
INSL3→RXFP2
INSL3→RXFP1
INSL5→RXFP4
INSL5→RXFP3
Compartment
hematopoietic
- hematopoietic precursor
megakaryocyte-erythroid
- megakaryoid
- erythroid
myeloid
- granulocyte
- granulocyte monocyte progenitor
- monocyte/macrophage
- dendritic
12.5%
25%
50%
100%
ln(CP10K+1)
Abbrev. Tissue
Aort Aorta
Blad Bladder
Blo Blood
Bone Bone
BM Bone marrow
BrC Brain cortex
BrS Brainstem
Col Colon
Dia Diaphragm
Ret Eye retina
Fat Fat
Hrt Heart
Pit Hypothalamus/Pituitary
Kid Kidney
LM Limb muscle
Liv Liver
Lung Lung
MG Mammary gland
Panc Pancreas
Skin Skin
SI Small intestine
Spl Spleen
Test Testes
Thym Thymus
Tong Tongue
Trac Trachea
Ute Uterus

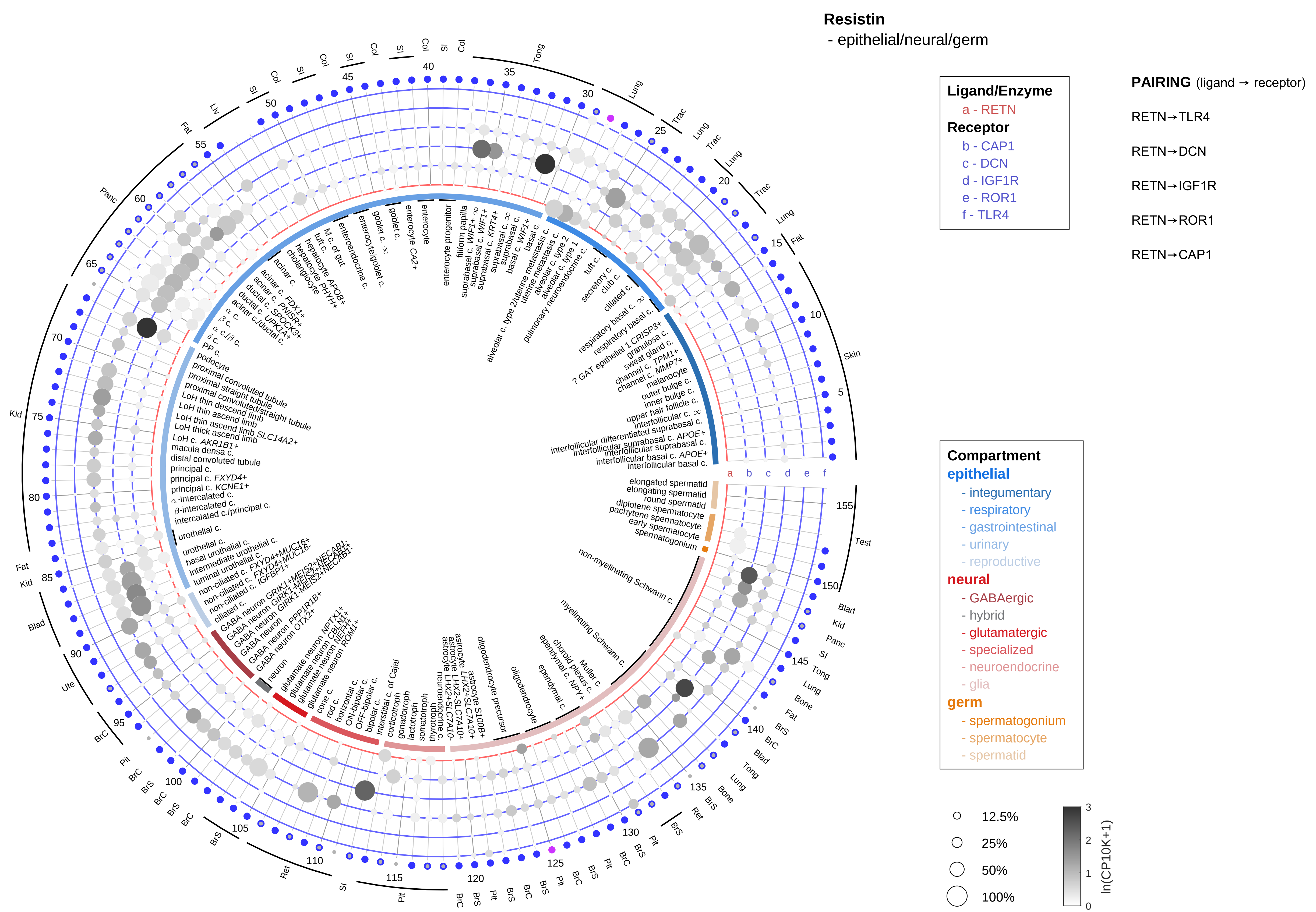

Abbrev.	Tissue
Aort	Aorta
Blad	Bladder
Blo	Blood
Bone	Bone
BM	Bone marrow
BrC	Brain cortex
BrS	Brainstem
Col	Colon
Dia	Diaphragm
Ret	Eye retina
Fat	Fat
Hrt	Heart
Pit	Hypothalamus/Pituitary
Kid	Kidney
LM	Limb muscle
Liv	Liver
Lung	Lung
MG	Mammary gland
Panc	Pancreas
Skin	Skin
SI	Small intestine
Spl	Spleen
Test	Testes
Thym	Thymus
Tong	Tongue
Trac	Trachea
Ute	Uterus

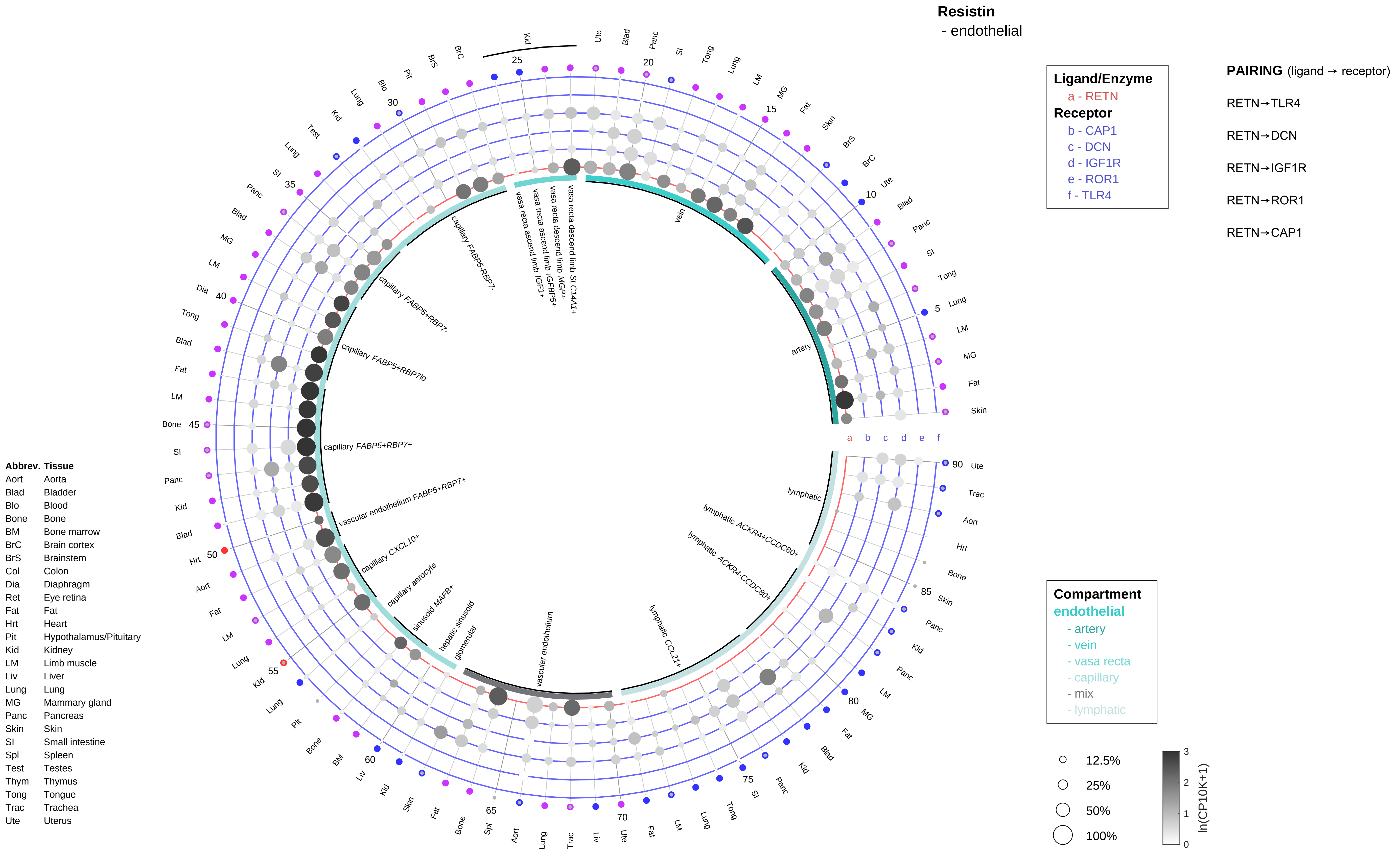
Resistin
- endothelial
Ligand/Enzyme
a - RETN
Receptor
b - CAP1
c - DCN
d - IGF1R
e - ROR1
f - TLR4
PAIRING (ligand → receptor)
RETN→TLR4
RETN→DCN
RETN→IGF1R
RETN→ROR1
RETN→CAP1
Compartment
endothelial
- artery
- vein
- vasa recta
- capillary
- mix
- lymphatic
12.5%
25%
50%
100%
ln(CP10K+1)
0
1
2
3
a b c d e f
artery
vein
vasa recta descend limb SLC14A1+
vasa recta descend limb MGP+
vasa recta ascend limb IGFBP5+
vasa recta ascend limb IGF1+
capillary FABP5-RBP7-
capillary FABP5+RBP7-
capillary FABP5+RBP7lo
capillary FABP5+RBP7+
vascular endothelium FABP5+RBP7+
capillary CXCL10+
capillary aerocyte
sinusoid MAFB+
hepatic sinusoid
glomerular
vascular endothelium
lymphatic CCL21+
lymphatic ACKR4-CCDC80+
lymphatic ACKR4+CCDC80+
lymphatic
Abbrev. Tissue
Aort Aorta
Blad Bladder
Blo Blood
Bone Bone
BM Bone marrow
BrC Brain cortex
BrS Brainstem
Col Colon
Dia Diaphragm
Ret Eye retina
Fat Fat
Hrt Heart
Pit Hypothalamus/Pituitary
Kid Kidney
LM Limb muscle
Liv Liver
Lung Lung
MG Mammary gland
Panc Pancreas
Skin Skin
SI Small intestine
Spl Spleen
Test Testes
Thym Thymus
Tong Tongue
Trac Trachea
Ute Uterus

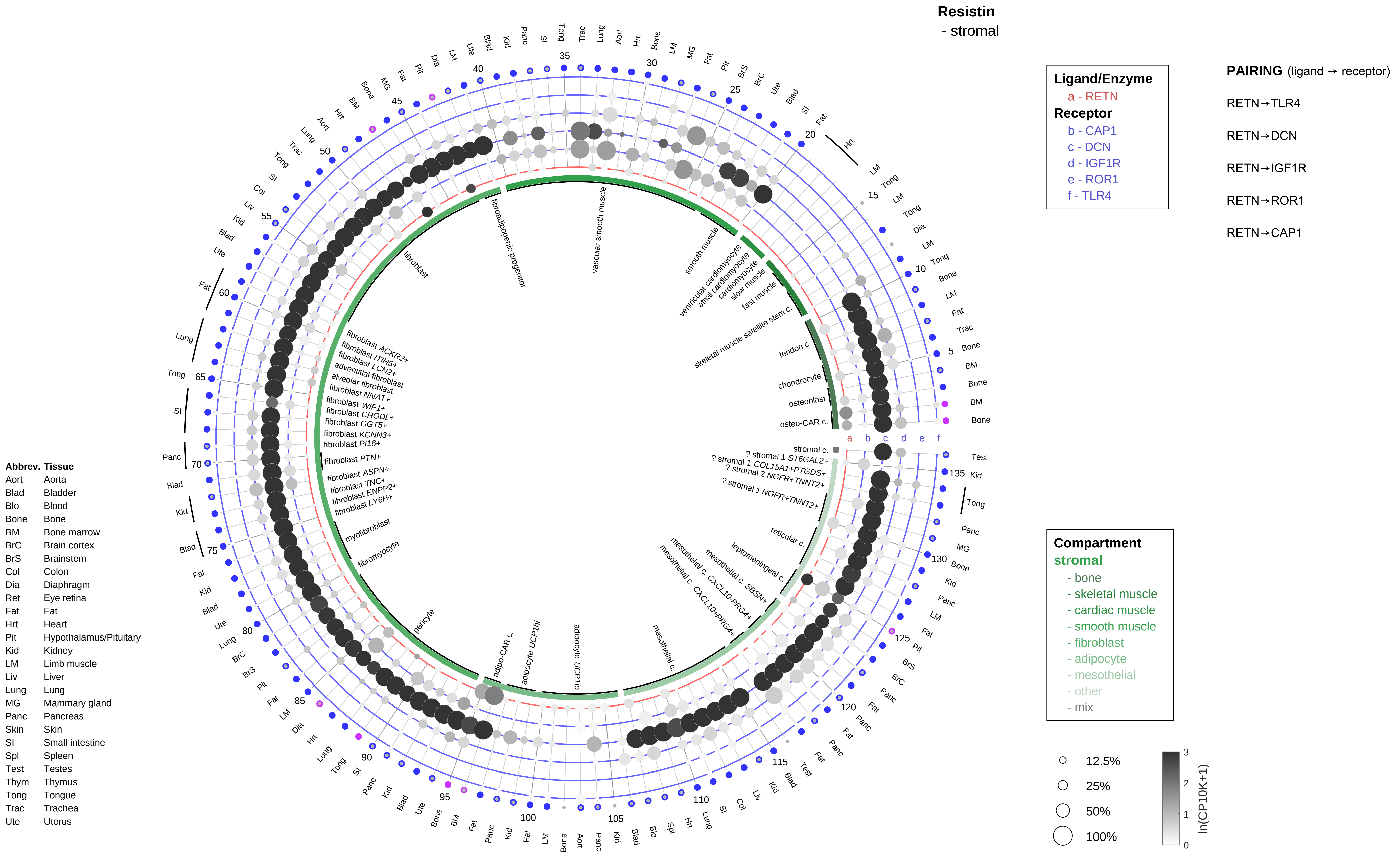

Resistin
- stromal
Ligand/Enzyme
a - RETN
Receptor
b - CAP1
c - DCN
d - IGF1R
e - ROR1
f - TLR4
PAIRING (ligand → receptor)
RETN→TLR4
RETN→DCN
RETN→IGF1R
RETN→ROR1
RETN→CAP1
Compartment
stromal
- bone
- skeletal muscle
- cardiac muscle
- smooth muscle
- fibroblast
- adipocyte
- mesothelial
- other
- mix
12.5%
25%
50%
100%
ln(CP10K+1)
Abbrev. Tissue
Aort Aorta
Blad Bladder
Blo Blood
Bone Bone
BM Bone marrow
BrC Brain cortex
BrS Brainstem
Col Colon
Dia Diaphragm
Ret Eye retina
Fat Fat
Hrt Heart
Pit Hypothalamus/Pituitary
Kid Kidney
LM Limb muscle
Liv Liver
Lung Lung
MG Mammary gland
Panc Pancreas
Skin Skin
SI Small intestine
Spl Spleen
Test Testes
Thym Thymus
Tong Tongue
Trac Trachea
Ute Uterus
fibroblast
fibroadipogenic progenitor
vascular smooth muscle
smooth muscle
ventricular cardiomyocyte
atrial cardiomyocyte
cardiomyocyte
slow muscle
fast muscle
skeletal muscle satellite stem c.
tendon c.
chondrocyte
osteoblast
osteo-CAR c.
stromal c.
? stromal 1 ST6GAL2+
? stromal 1 COL15A1+PTGDS+
? stromal 2 NGFR+TNNT2+
? stromal 1 NGFR+TNNT2+
reticular c.
leptomeningeal c.
mesothelial c. SBSN+
mesothelial c. CXCL10-PRG4+
mesothelial c. CXCL10+PRG4+
mesothelial c.
adipocyte UCP1lo
adipocyte UCP1hi
adipo-CAR c.
pericyte
fibromyocyte
myofibroblast
fibroblast LY6H+
fibroblast ENPP2+
fibroblast TNC+
fibroblast ASPN+
fibroblast PTN+
fibroblast PI16+
fibroblast KCNN3+
fibroblast GGT5+
fibroblast CHODL+
fibroblast WIF1+
fibroblast NNAT+
alveolar fibroblast
adventitial fibroblast
fibroblast LCN2+
fibroblast ITIH5+
fibroblast ACKR2+

Resistin
- lymphoid

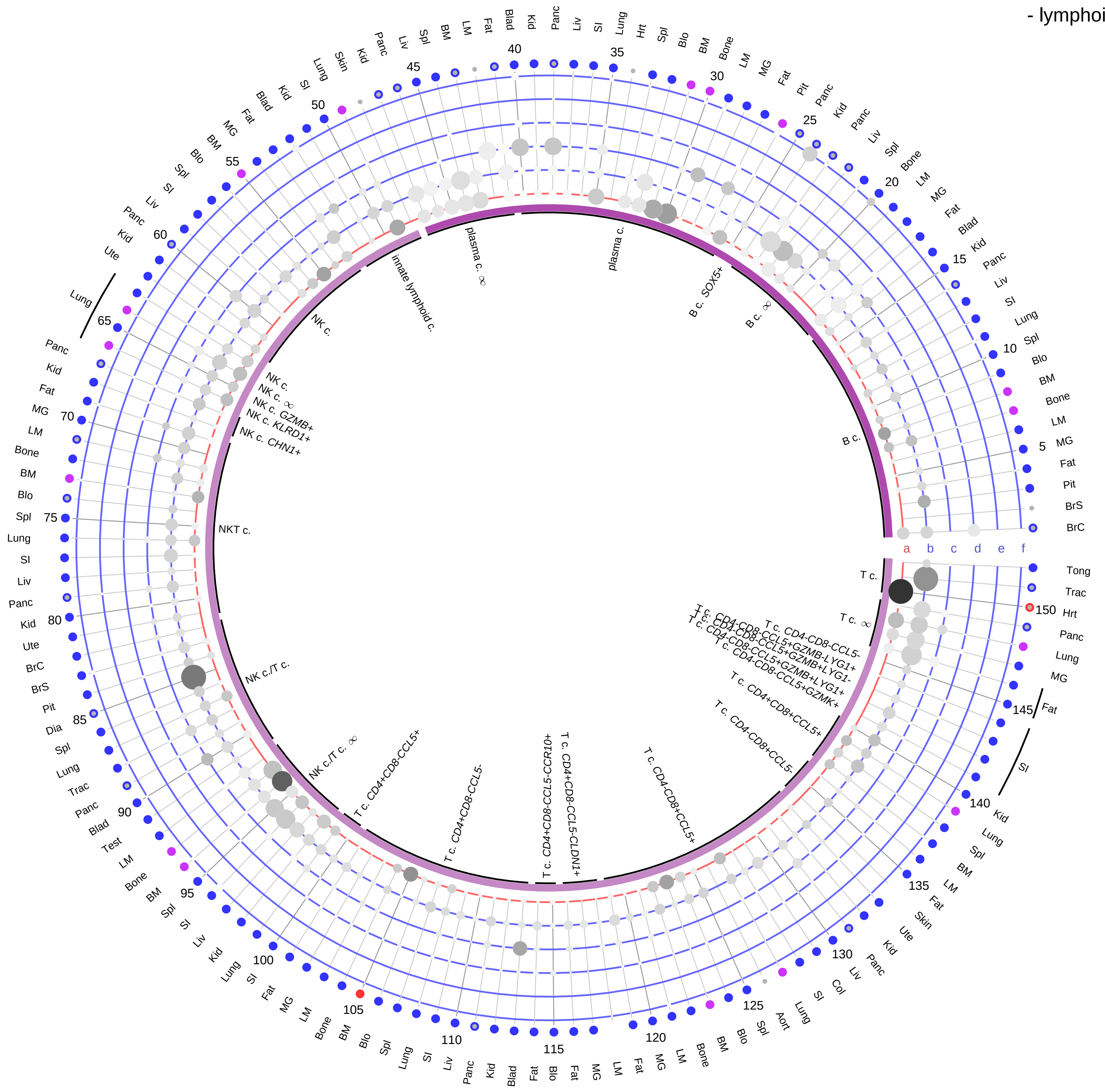

Ligand/Enzyme
a - RETN

Receptor
b - CAP1
c - DCN
d - IGF1R
e - ROR1
f - TLR4

PAIRING (ligand → receptor)

RETN→TLR4

RETN→DCN

RETN→IGF1R

RETN→ROR1

RETN→CAP1

Abbrev.	Tissue
Aort	Aorta
Blad	Bladder
Blo	Blood
Bone	Bone
BM	Bone marrow
BrC	Brain cortex
BrS	Brainstem
Col	Colon
Dia	Diaphragm
Ret	Eye retina
Fat	Fat
Hrt	Heart
Pit	Hypothalamus/Pituitary
Kid	Kidney
LM	Limb muscle
Liv	Liver
Lung	Lung
MG	Mammary gland
Panc	Pancreas
Skin	Skin
SI	Small intestine
Spl	Spleen
Test	Testes
Thym	Thymus
Tong	Tongue
Trac	Trachea
Ute	Uterus

Compartment
lymphoid
- B cell
- NK/T cell

12.5%
25%
50%
100%

ln(CP10K+1): 0, 1, 2, 3

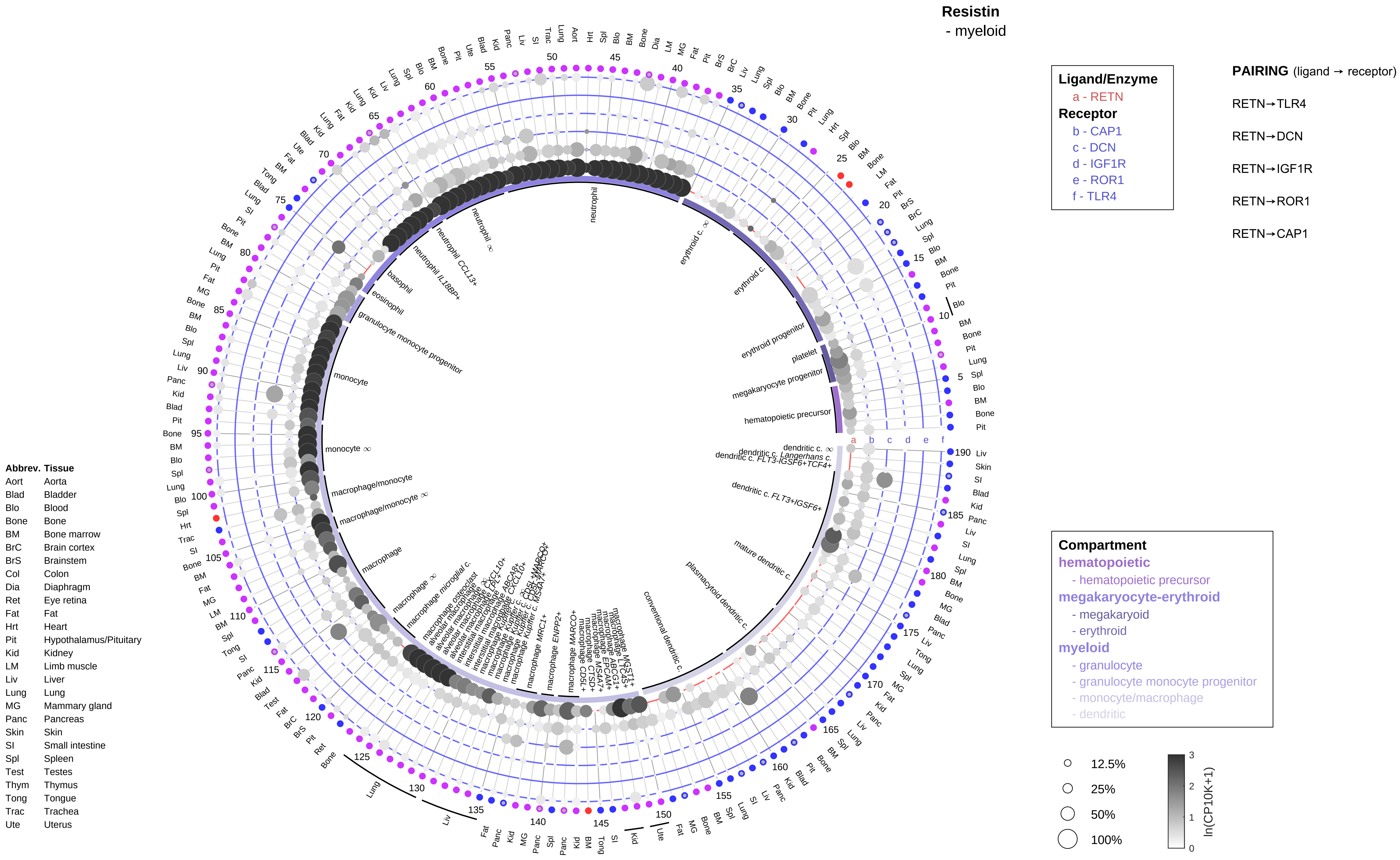

Resistin
- myeloid
Ligand/Enzyme
a - RETN
Receptor
b - CAP1
c - DCN
d - IGF1R
e - ROR1
f - TLR4
PAIRING (ligand → receptor)
RETN→TLR4
RETN→DCN
RETN→IGF1R
RETN→ROR1
RETN→CAP1
Compartment
hematopoietic
- hematopoietic precursor
megakaryocyte-erythroid
- megakaryoid
- erythroid
myeloid
- granulocyte
- granulocyte monocyte progenitor
- monocyte/macrophage
- dendritic
12.5%
25%
50%
100%
ln(CP10K+1)
Abbrev. Tissue
Aort Aorta
Blad Bladder
Blo Blood
Bone Bone
BM Bone marrow
BrC Brain cortex
BrS Brainstem
Col Colon
Dia Diaphragm
Ret Eye retina
Fat Fat
Hrt Heart
Pit Hypothalamus/Pituitary
Kid Kidney
LM Limb muscle
Liv Liver
Lung Lung
MG Mammary gland
Panc Pancreas
Skin Skin
SI Small intestine
Spl Spleen
Test Testes
Thym Thymus
Tong Tongue
Trac Trachea
Ute Uterus

Secretin (SCT)
- epithelial/neural/germ

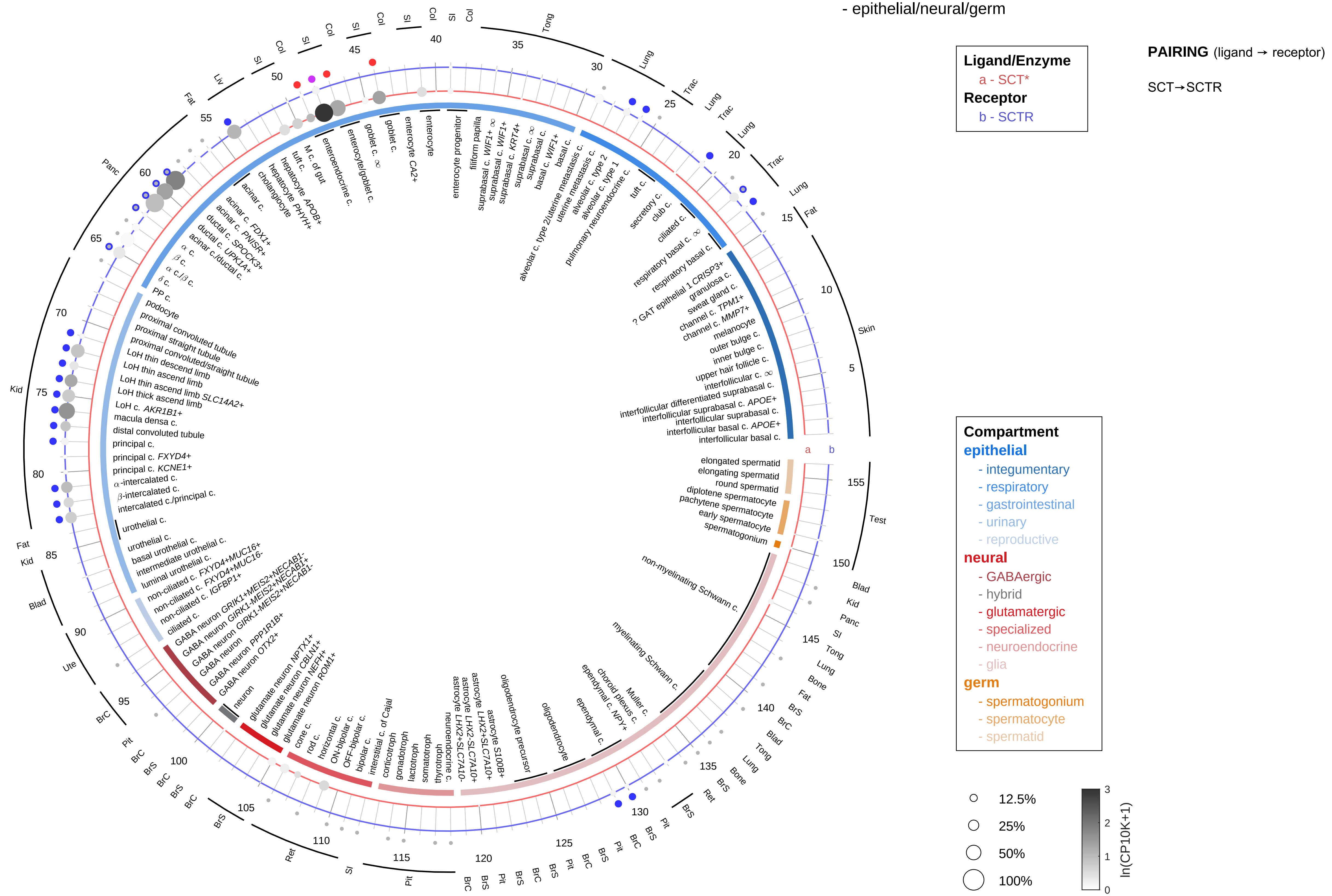

Abbrev.	Tissue
Aort	Aorta
Blad	Bladder
Blo	Blood
Bone	Bone
BM	Bone marrow
BrC	Brain cortex
BrS	Brainstem
Col	Colon
Dia	Diaphragm
Ret	Eye retina
Fat	Fat
Hrt	Heart
Pit	Hypothalamus/Pituitary
Kid	Kidney
LM	Limb muscle
Liv	Liver
Lung	Lung
MG	Mammary gland
Panc	Pancreas
Skin	Skin
SI	Small intestine
Spl	Spleen
Test	Testes
Thym	Thymus
Tong	Tongue
Trac	Trachea
Ute	Uterus

Secretin (SCT)
- endothelial

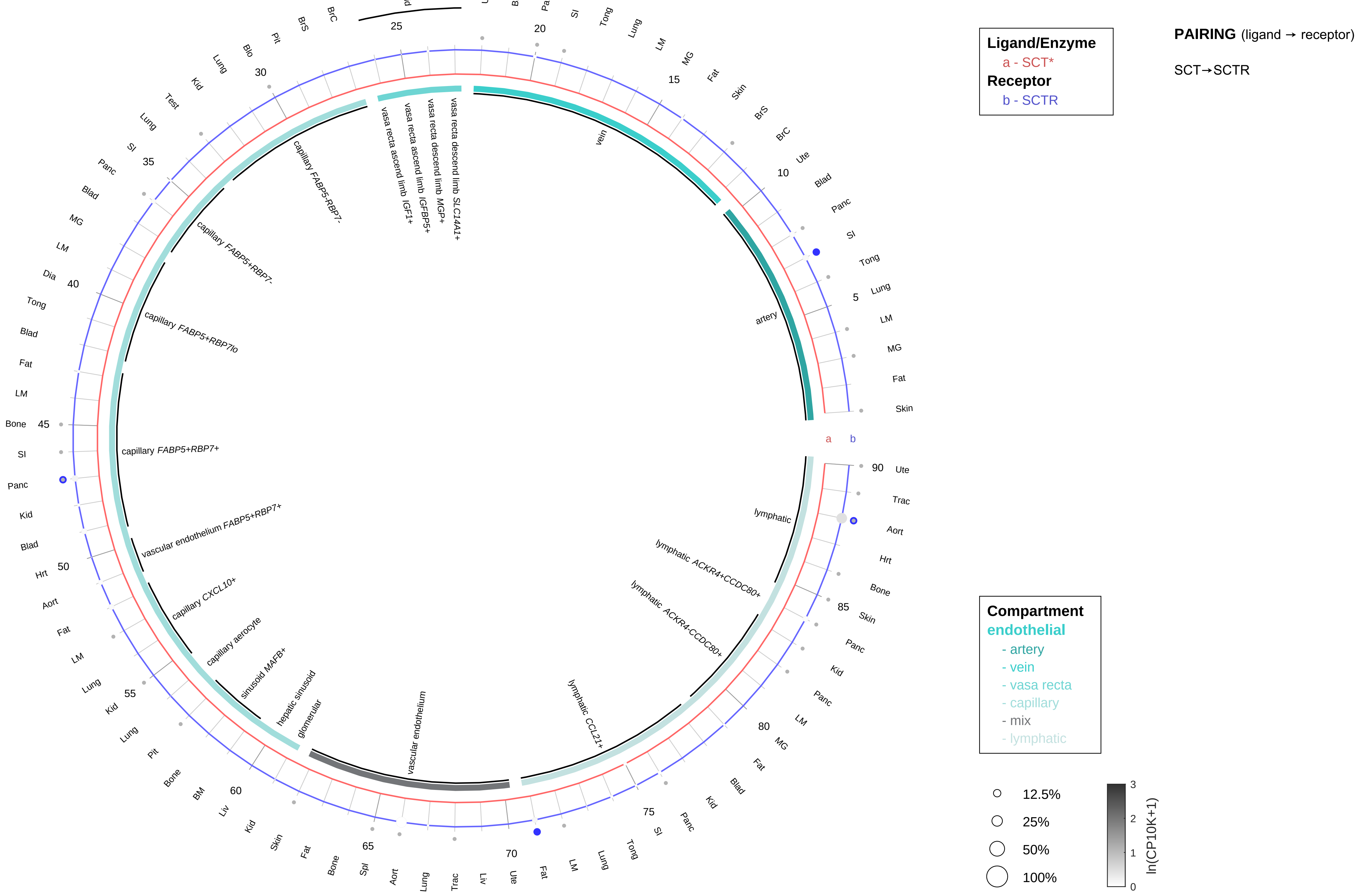

Abbrev.	Tissue
Aort	Aorta
Blad	Bladder
Blo	Blood
Bone	Bone
BM	Bone marrow
BrC	Brain cortex
BrS	Brainstem
Col	Colon
Dia	Diaphragm
Ret	Eye retina
Fat	Fat
Hrt	Heart
Pit	Hypothalamus/Pituitary
Kid	Kidney
LM	Limb muscle
Liv	Liver
Lung	Lung
MG	Mammary gland
Panc	Pancreas
Skin	Skin
SI	Small intestine
Spl	Spleen
Test	Testes
Thym	Thymus
Tong	Tongue
Trac	Trachea
Ute	Uterus

Secretin (SCT)
- stromal

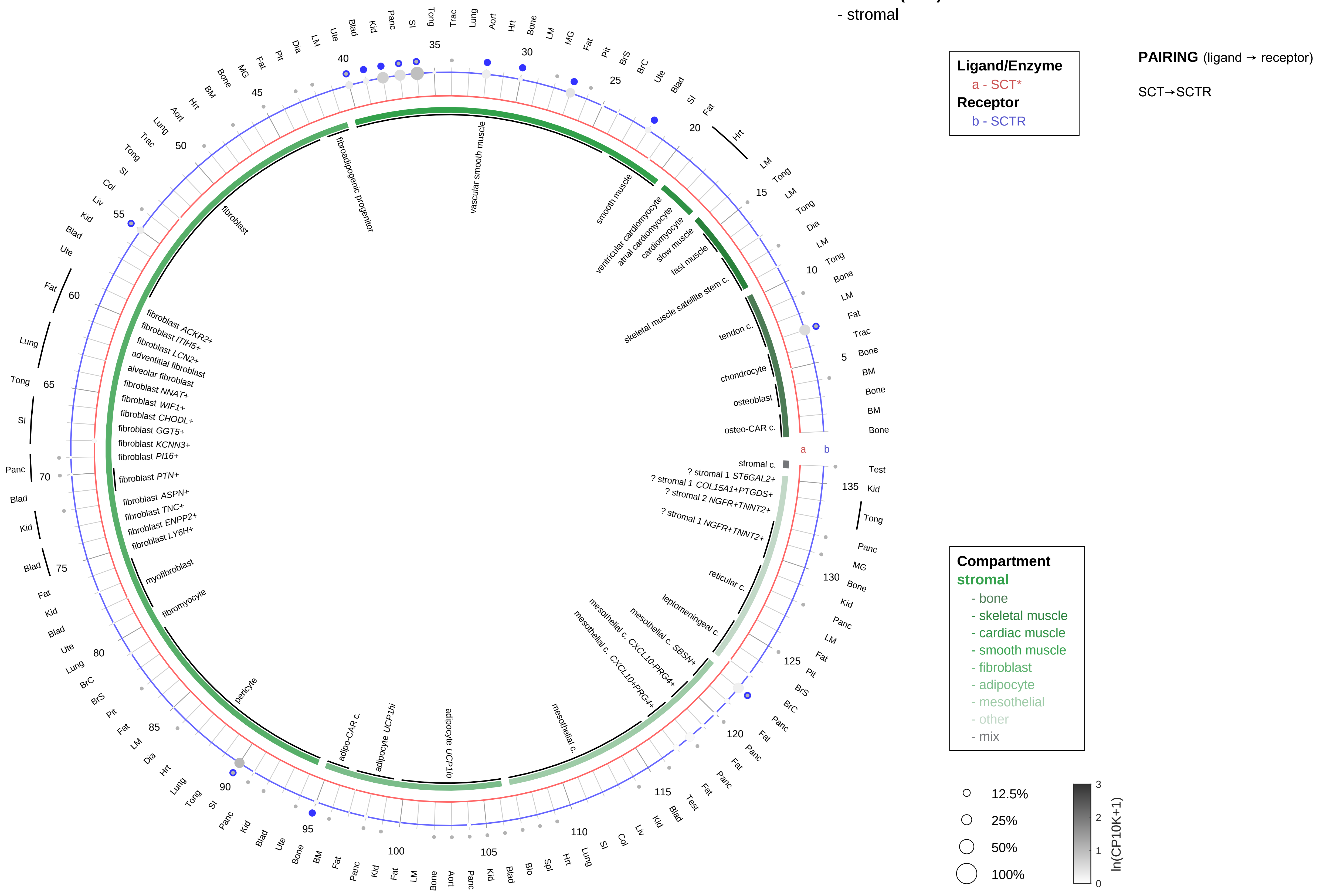

Abbrev.	Tissue
Aort	Aorta
Blad	Bladder
Blo	Blood
Bone	Bone
BM	Bone marrow
BrC	Brain cortex
BrS	Brainstem
Col	Colon
Dia	Diaphragm
Ret	Eye retina
Fat	Fat
Hrt	Heart
Pit	Hypothalamus/Pituitary
Kid	Kidney
LM	Limb muscle
Liv	Liver
Lung	Lung
MG	Mammary gland
Panc	Pancreas
Skin	Skin
SI	Small intestine
Spl	Spleen
Test	Testes
Thym	Thymus
Tong	Tongue
Trac	Trachea
Ute	Uterus

Secretin (SCT)
- lymphoid

Ligand/Enzyme
a - SCT*
Receptor
b - SCTR

PAIRING (ligand → receptor)

SCT→SCTR

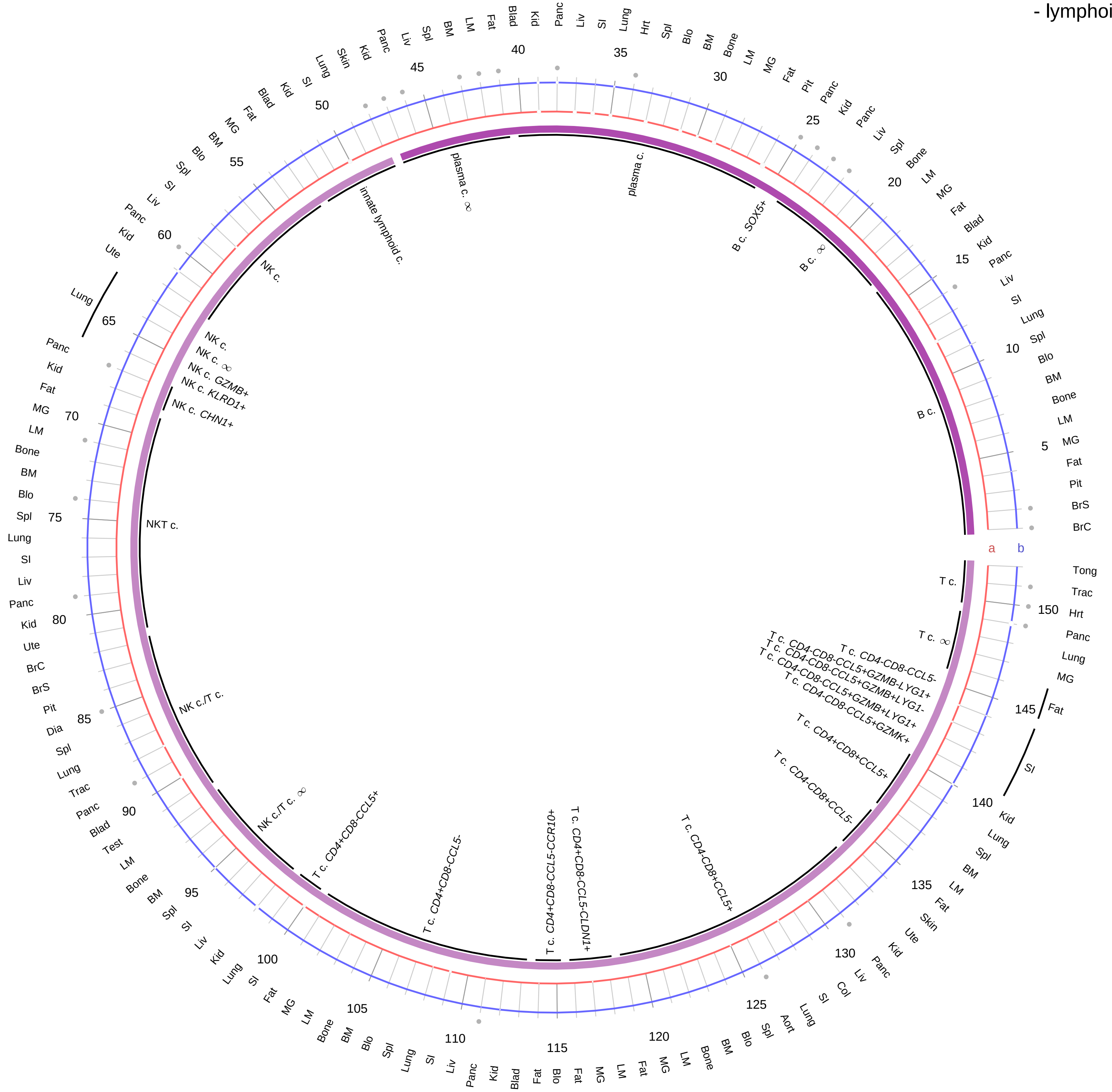

Abbrev.	Tissue
Aort	Aorta
Blad	Bladder
Blo	Blood
Bone	Bone
BM	Bone marrow
BrC	Brain cortex
BrS	Brainstem
Col	Colon
Dia	Diaphragm
Ret	Eye retina
Fat	Fat
Hrt	Heart
Pit	Hypothalamus/Pituitary
Kid	Kidney
LM	Limb muscle
Liv	Liver
Lung	Lung
MG	Mammary gland
Panc	Pancreas
Skin	Skin
SI	Small intestine
Spl	Spleen
Test	Testes
Thym	Thymus
Tong	Tongue
Trac	Trachea
Ute	Uterus

Compartment
lymphoid
- B cell
- NK/T cell

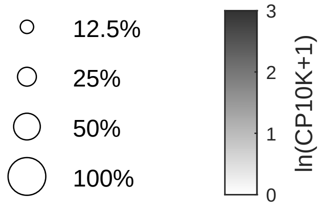

Secretin (SCT)
- myeloid

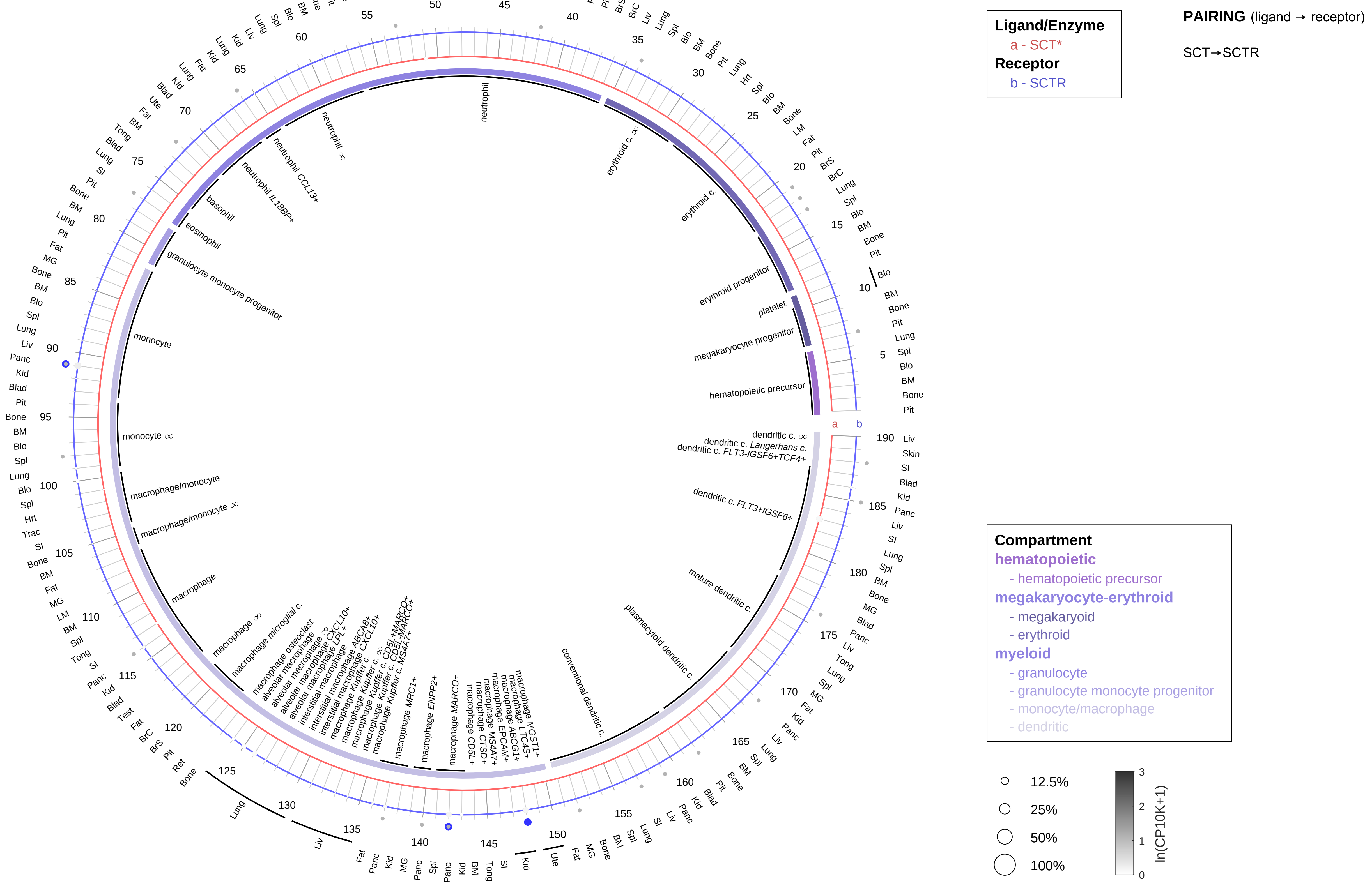

Abbrev.	Tissue
Aort	Aorta
Blad	Bladder
Blo	Blood
Bone	Bone
BM	Bone marrow
BrC	Brain cortex
BrS	Brainstem
Col	Colon
Dia	Diaphragm
Ret	Eye retina
Fat	Fat
Hrt	Heart
Pit	Hypothalamus/Pituitary
Kid	Kidney
LM	Limb muscle
Liv	Liver
Lung	Lung
MG	Mammary gland
Panc	Pancreas
Skin	Skin
SI	Small intestine
Spl	Spleen
Test	Testes
Thym	Thymus
Tong	Tongue
Trac	Trachea
Ute	Uterus

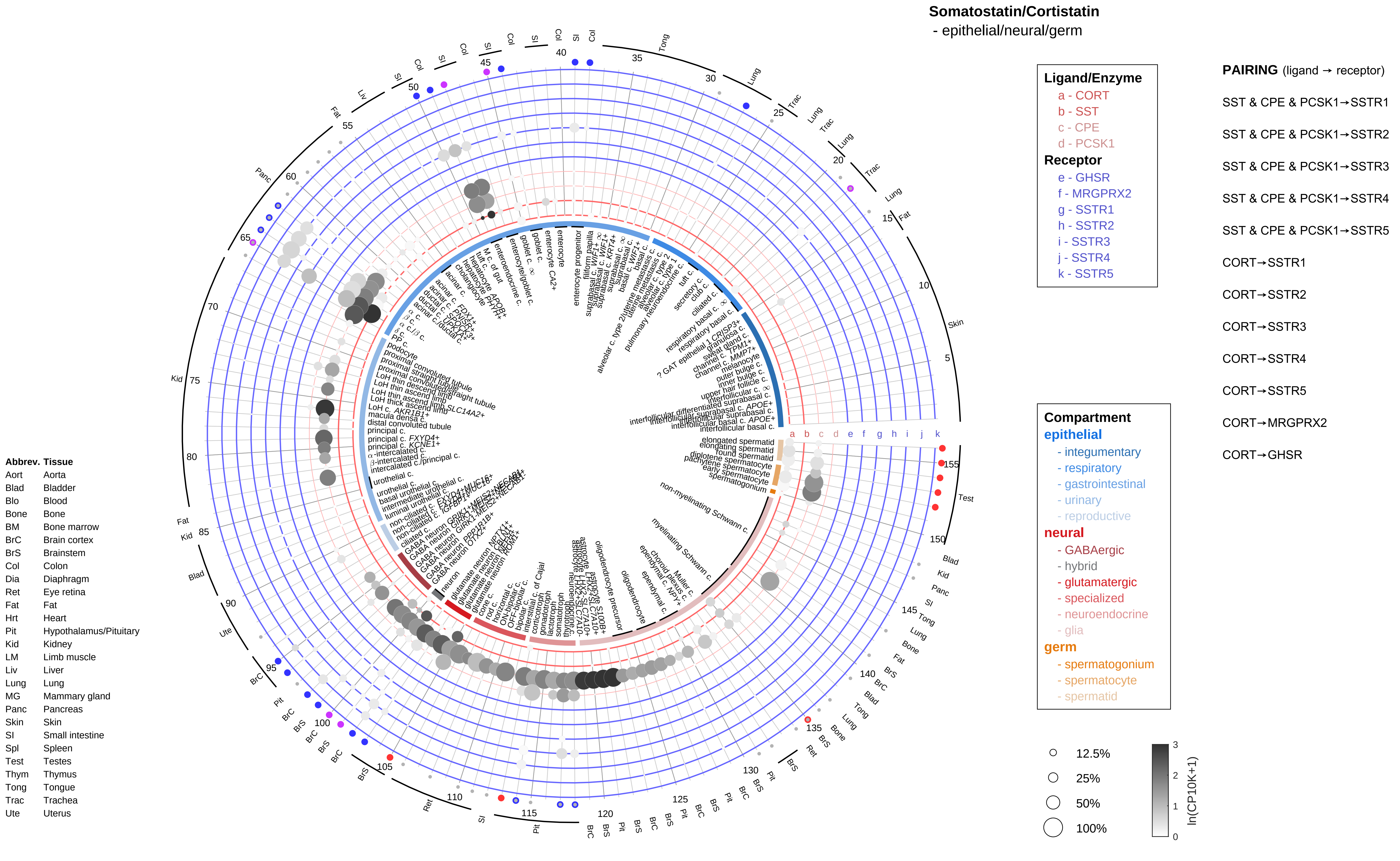

Somatostatin/Cortistatin
- epithelial/neural/germ
Ligand/Enzyme
a - CORT
b - SST
c - CPE
d - PCSK1
Receptor
e - GHSR
f - MRGPRX2
g - SSTR1
h - SSTR2
i - SSTR3
j - SSTR4
k - SSTR5
PAIRING (ligand → receptor)
SST & CPE & PCSK1→SSTR1
SST & CPE & PCSK1→SSTR2
SST & CPE & PCSK1→SSTR3
SST & CPE & PCSK1→SSTR4
SST & CPE & PCSK1→SSTR5
CORT→SSTR1
CORT→SSTR2
CORT→SSTR3
CORT→SSTR4
CORT→SSTR5
CORT→MRGPRX2
CORT→GHSR
Compartment
epithelial
- integumentary
- respiratory
- gastrointestinal
- urinary
- reproductive
neural
- GABAergic
- hybrid
- glutamatergic
- specialized
- neuroendocrine
- glia
germ
- spermatogonium
- spermatocyte
- spermatid
12.5%
25%
50%
100%
ln(CP10K+1)
0
1
2
3
Abbrev. Tissue
Aort Aorta
Blad Bladder
Blo Blood
Bone Bone
BM Bone marrow
BrC Brain cortex
BrS Brainstem
Col Colon
Dia Diaphragm
Ret Eye retina
Fat Fat
Hrt Heart
Pit Hypothalamus/Pituitary
Kid Kidney
LM Limb muscle
Liv Liver
Lung Lung
MG Mammary gland
Panc Pancreas
Skin Skin
SI Small intestine
Spl Spleen
Test Testes
Thym Thymus
Tong Tongue
Trac Trachea
Ute Uterus

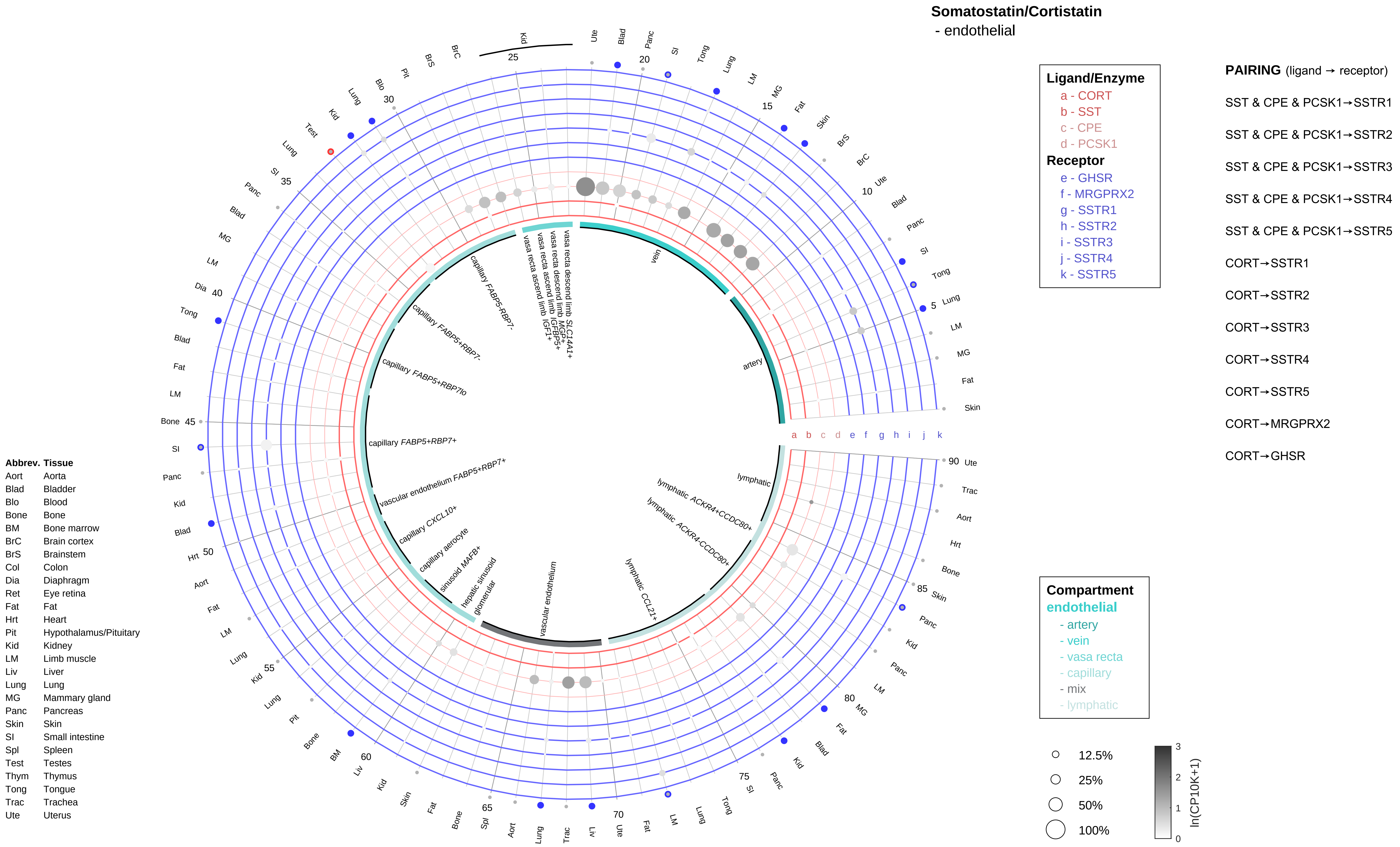

Somatostatin/Cortistatin
- endothelial
Ligand/Enzyme
a - CORT
b - SST
c - CPE
d - PCSK1
Receptor
e - GHSR
f - MRGPRX2
g - SSTR1
h - SSTR2
i - SSTR3
j - SSTR4
k - SSTR5
PAIRING (ligand → receptor)
SST & CPE & PCSK1→SSTR1
SST & CPE & PCSK1→SSTR2
SST & CPE & PCSK1→SSTR3
SST & CPE & PCSK1→SSTR4
SST & CPE & PCSK1→SSTR5
CORT→SSTR1
CORT→SSTR2
CORT→SSTR3
CORT→SSTR4
CORT→SSTR5
CORT→MRGPRX2
CORT→GHSR
Compartment
endothelial
- artery
- vein
- vasa recta
- capillary
- mix
- lymphatic
12.5%
25%
50%
100%
ln(CP10K+1)
0
1
2
3
Abbrev. Tissue
Aort Aorta
Blad Bladder
Blo Blood
Bone Bone
BM Bone marrow
BrC Brain cortex
BrS Brainstem
Col Colon
Dia Diaphragm
Ret Eye retina
Fat Fat
Hrt Heart
Pit Hypothalamus/Pituitary
Kid Kidney
LM Limb muscle
Liv Liver
Lung Lung
MG Mammary gland
Panc Pancreas
Skin Skin
SI Small intestine
Spl Spleen
Test Testes
Thym Thymus
Tong Tongue
Trac Trachea
Ute Uterus
a b c d e f g h i j k
vein
artery
lymphatic
lymphatic ACKR4+CCDC80+
lymphatic ACKR4-CCDC80+
lymphatic CCL21+
vascular endothelium
glomerular
hepatic sinusoid
sinusoid MAFB+
capillary aerocyte
capillary CXCL10+
vascular endothelium FABP5+RBP7+
capillary FABP5+RBP7+
capillary FABP5+RBP7lo
capillary FABP5+RBP7-
capillary FABP5-RBP7-
vasa recta ascend limb IGF1+
vasa recta ascend limb IGFBP5+
vasa recta descend limb MGP+
vasa recta descend limb SLC14A1+

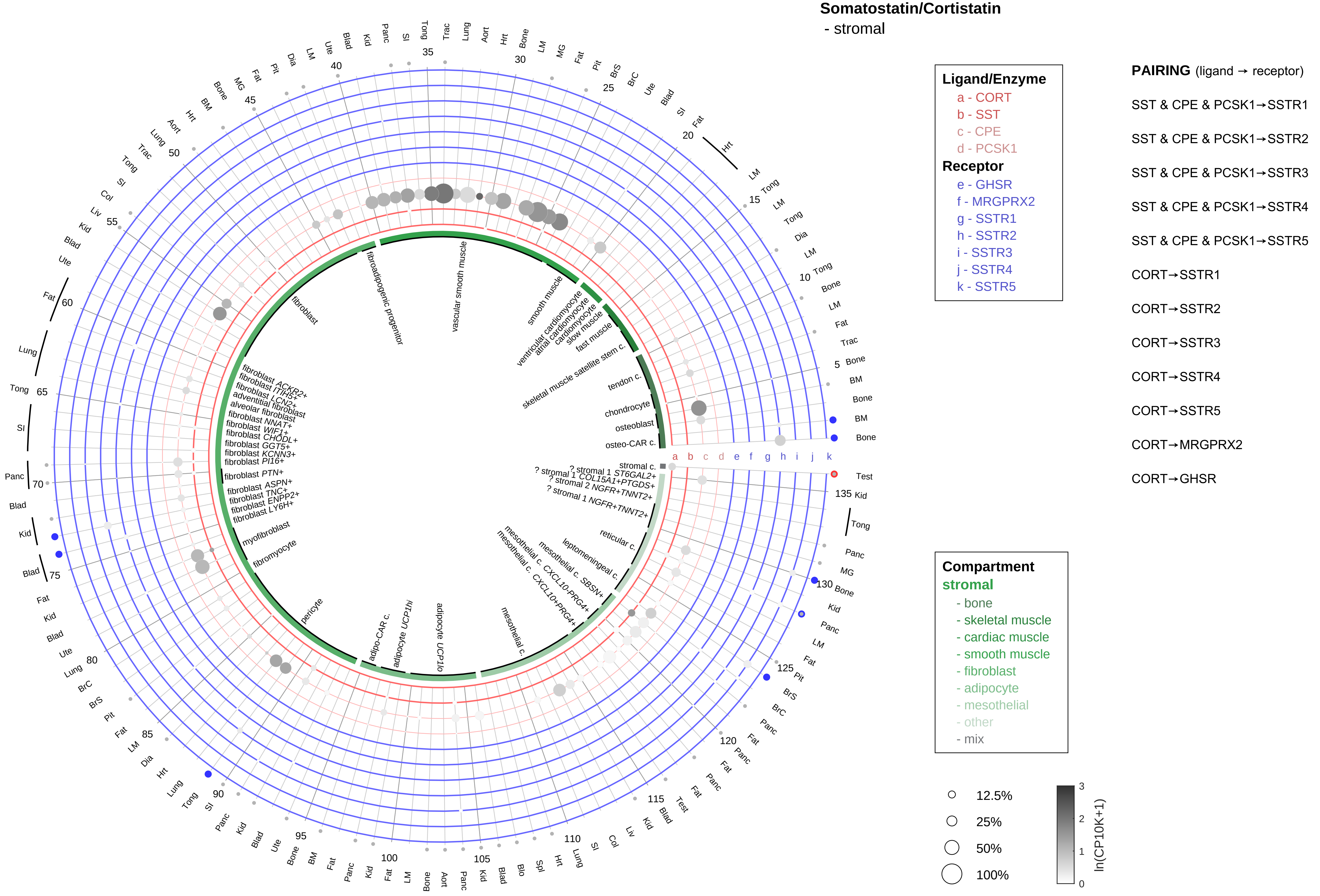

Abbrev.	Tissue
Aort	Aorta
Blad	Bladder
Blo	Blood
Bone	Bone
BM	Bone marrow
BrC	Brain cortex
BrS	Brainstem
Col	Colon
Dia	Diaphragm
Ret	Eye retina
Fat	Fat
Hrt	Heart
Pit	Hypothalamus/Pituitary
Kid	Kidney
LM	Limb muscle
Liv	Liver
Lung	Lung
MG	Mammary gland
Panc	Pancreas
Skin	Skin
SI	Small intestine
Spl	Spleen
Test	Testes
Thym	Thymus
Tong	Tongue
Trac	Trachea
Ute	Uterus

Somatostatin/Cortistatin

- lymphoid

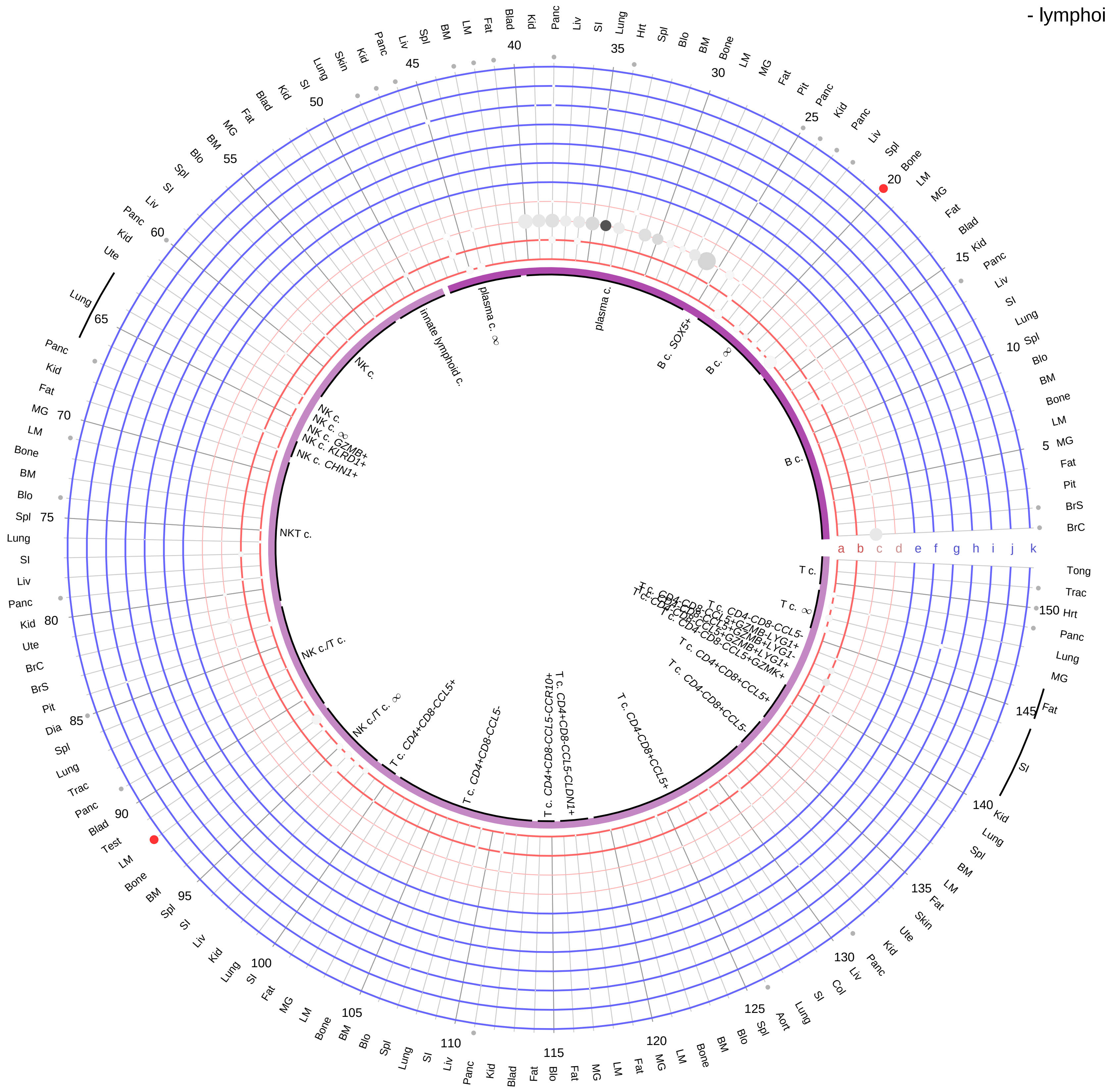

Ligand/Enzyme
- a - CORT
- b - SST
- c - CPE
- d - PCSK1

Receptor
- e - GHSR
- f - MRGPRX2
- g - SSTR1
- h - SSTR2
- i - SSTR3
- j - SSTR4
- k - SSTR5

PAIRING (ligand → receptor)

SST & CPE & PCSK1→SSTR1

SST & CPE & PCSK1→SSTR2

SST & CPE & PCSK1→SSTR3

SST & CPE & PCSK1→SSTR4

SST & CPE & PCSK1→SSTR5

CORT→SSTR1

CORT→SSTR2

CORT→SSTR3

CORT→SSTR4

CORT→SSTR5

CORT→MRGPRX2

CORT→GHSR

Abbrev.	Tissue
Aort	Aorta
Blad	Bladder
Blo	Blood
Bone	Bone
BM	Bone marrow
BrC	Brain cortex
BrS	Brainstem
Col	Colon
Dia	Diaphragm
Ret	Eye retina
Fat	Fat
Hrt	Heart
Pit	Hypothalamus/Pituitary
Kid	Kidney
LM	Limb muscle
Liv	Liver
Lung	Lung
MG	Mammary gland
Panc	Pancreas
Skin	Skin
SI	Small intestine
Spl	Spleen
Test	Testes
Thym	Thymus
Tong	Tongue
Trac	Trachea
Ute	Uterus

Compartment

lymphoid
- B cell
- NK/T cell

12.5%

25%

50%

100%

ln(CP10K+1): 0, 1, 2, 3

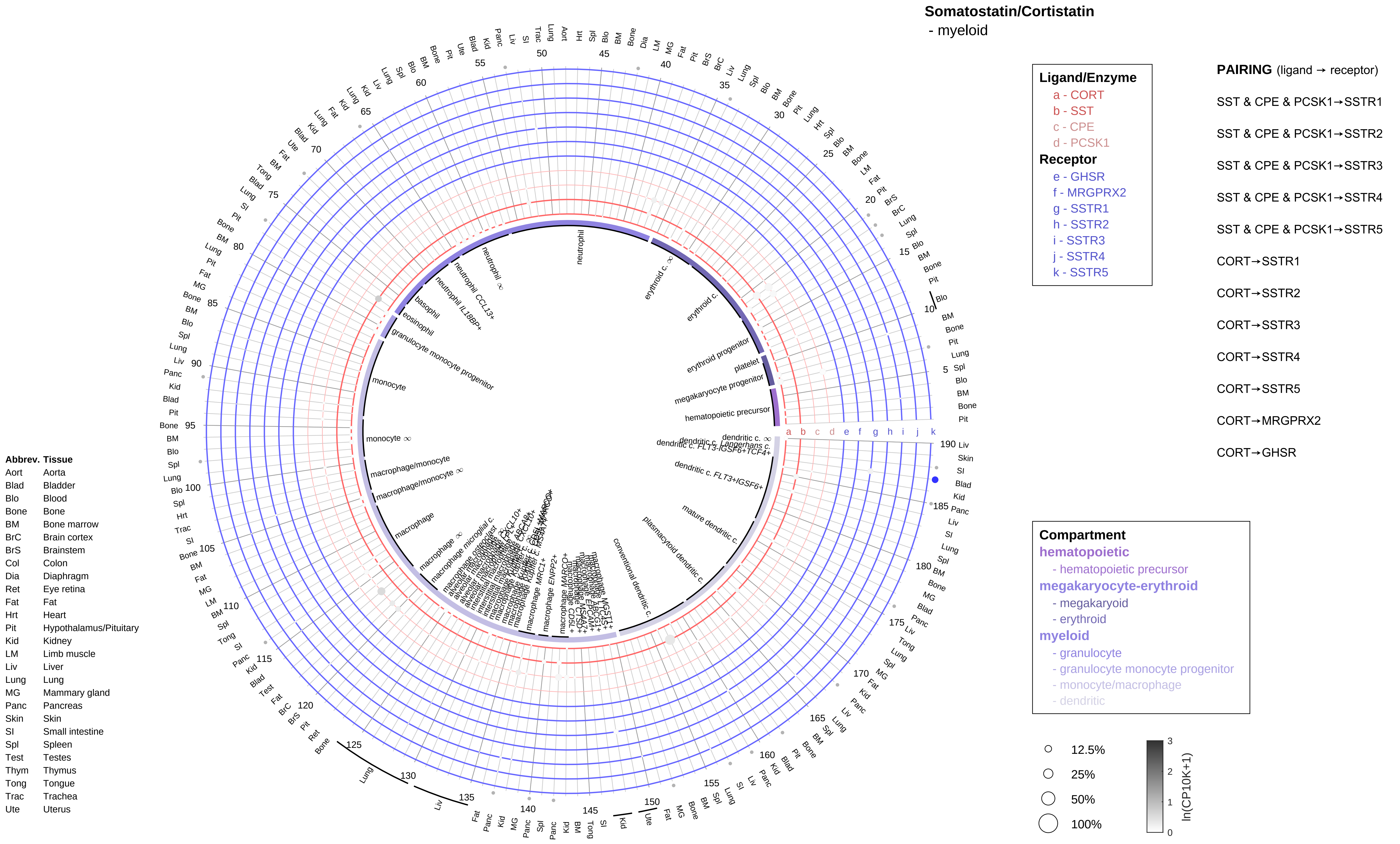
Somatostatin/Cortistatin
- myeloid
Ligand/Enzyme
a - CORT
b - SST
c - CPE
d - PCSK1
Receptor
e - GHSR
f - MRGPRX2
g - SSTR1
h - SSTR2
i - SSTR3
j - SSTR4
k - SSTR5
PAIRING (ligand → receptor)
SST & CPE & PCSK1→SSTR1
SST & CPE & PCSK1→SSTR2
SST & CPE & PCSK1→SSTR3
SST & CPE & PCSK1→SSTR4
SST & CPE & PCSK1→SSTR5
CORT→SSTR1
CORT→SSTR2
CORT→SSTR3
CORT→SSTR4
CORT→SSTR5
CORT→MRGPRX2
CORT→GHSR
Compartment
hematopoietic
- hematopoietic precursor
megakaryocyte-erythroid
- megakaryoid
- erythroid
myeloid
- granulocyte
- granulocyte monocyte progenitor
- monocyte/macrophage
- dendritic
12.5%
25%
50%
100%
ln(CP10K+1)
0
1
2
3
Abbrev. Tissue
Aort Aorta
Blad Bladder
Blo Blood
Bone Bone
BM Bone marrow
BrC Brain cortex
BrS Brainstem
Col Colon
Dia Diaphragm
Ret Eye retina
Fat Fat
Hrt Heart
Pit Hypothalamus/Pituitary
Kid Kidney
LM Limb muscle
Liv Liver
Lung Lung
MG Mammary gland
Panc Pancreas
Skin Skin
SI Small intestine
Spl Spleen
Test Testes
Thym Thymus
Tong Tongue
Trac Trachea
Ute Uterus

Tachykinin
- epithelial/neural/germ

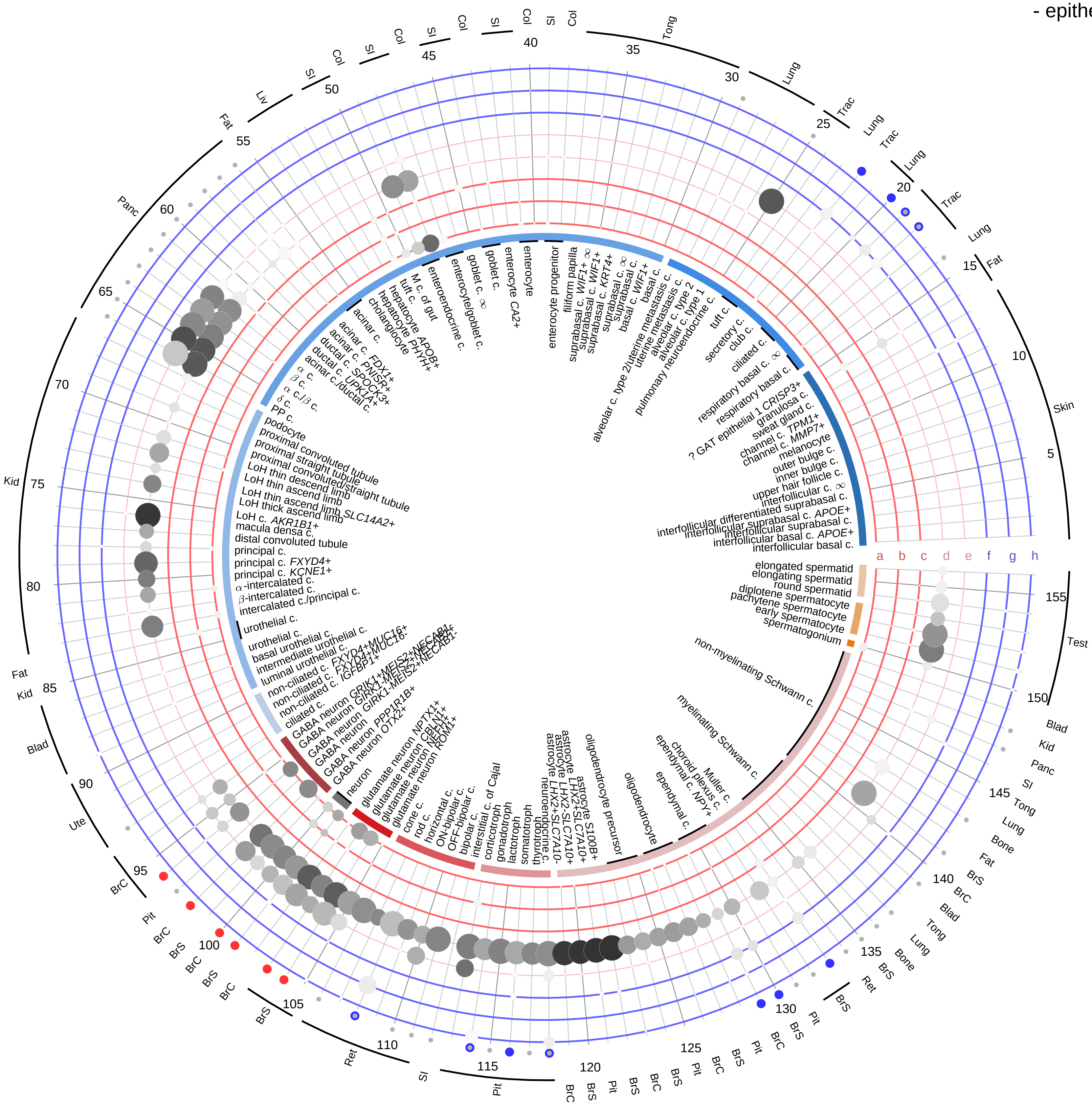

Ligand/Enzyme
- a - TAC1
- b - TAC3
- c - TAC4
- d - CPE
- e - PCSK2

Receptor
- f - TACR1
- g - TACR2
- h - TACR3

PAIRING (ligand → receptor)

TAC1 & CPE & PCSK2→TACR1

TAC1 & CPE & PCSK2→TACR2

TAC1 & CPE & PCSK2→TACR3

TAC3 & CPE & PCSK2→TACR1

TAC3 & CPE & PCSK2→TACR2

TAC3 & CPE & PCSK2→TACR3

TAC4 & CPE & PCSK2→TACR1

TAC4 & CPE & PCSK2→TACR2

TAC4 & CPE & PCSK2→TACR3

Compartment

epithelial
- integumentary
- respiratory
- gastrointestinal
- urinary
- reproductive

neural
- GABAergic
- hybrid
- glutamatergic
- specialized
- neuroendocrine
- glia

germ
- spermatogonium
- spermatocyte
- spermatid

12.5%
25%
50%
100%

ln(CP10K+1): 0, 1, 2, 3

Abbrev.	Tissue
Aort	Aorta
Blad	Bladder
Blo	Blood
Bone	Bone
BM	Bone marrow
BrC	Brain cortex
BrS	Brainstem
Col	Colon
Dia	Diaphragm
Ret	Eye retina
Fat	Fat
Hrt	Heart
Pit	Hypothalamus/Pituitary
Kid	Kidney
LM	Limb muscle
Liv	Liver
Lung	Lung
MG	Mammary gland
Panc	Pancreas
Skin	Skin
SI	Small intestine
Spl	Spleen
Test	Testes
Thym	Thymus
Tong	Tongue
Trac	Trachea
Ute	Uterus

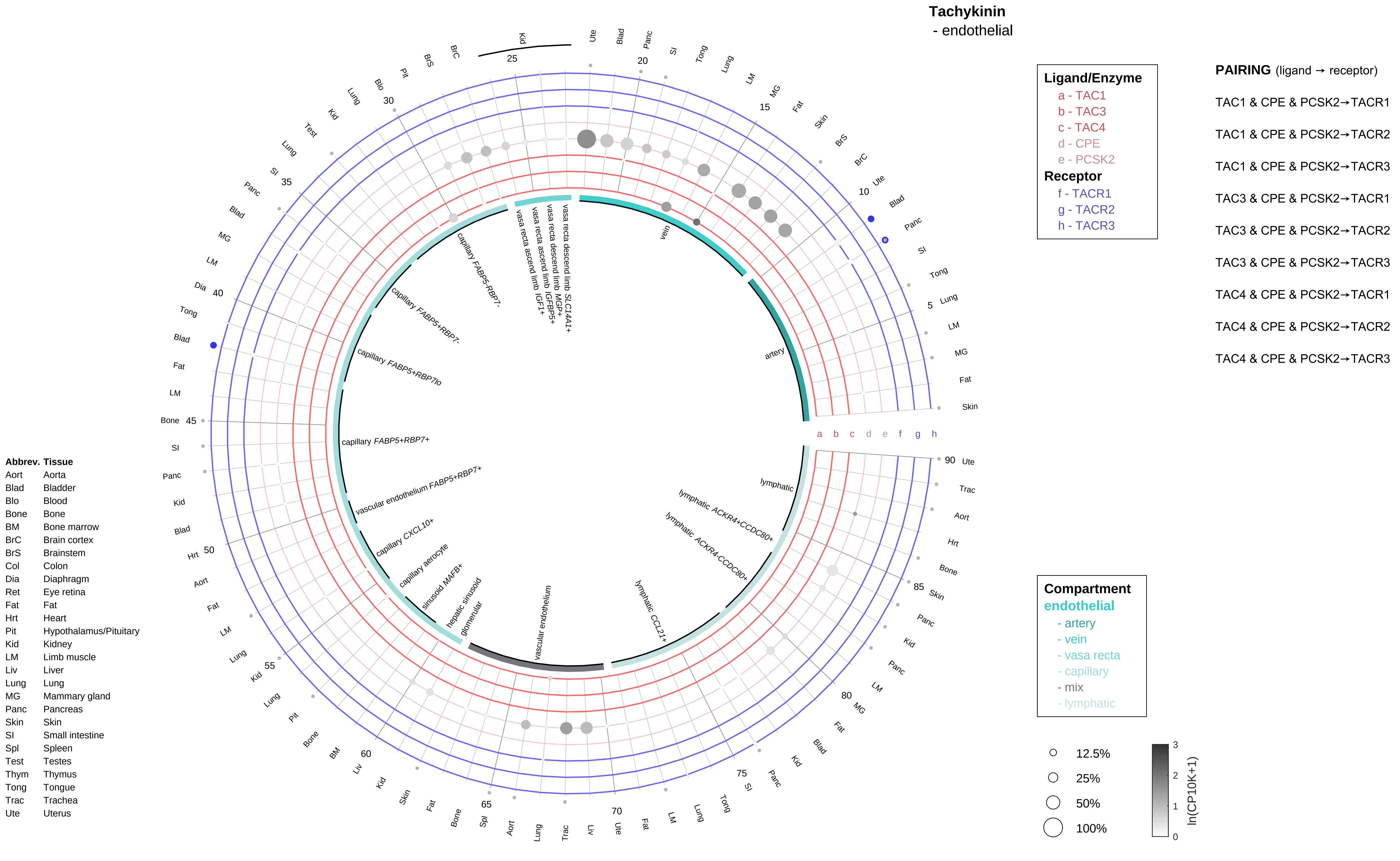

Tachykinin
- endothelial
Ligand/Enzyme
a - TAC1
b - TAC3
c - TAC4
d - CPE
e - PCSK2
Receptor
f - TACR1
g - TACR2
h - TACR3
PAIRING (ligand → receptor)
TAC1 & CPE & PCSK2→TACR1
TAC1 & CPE & PCSK2→TACR2
TAC1 & CPE & PCSK2→TACR3
TAC3 & CPE & PCSK2→TACR1
TAC3 & CPE & PCSK2→TACR2
TAC3 & CPE & PCSK2→TACR3
TAC4 & CPE & PCSK2→TACR1
TAC4 & CPE & PCSK2→TACR2
TAC4 & CPE & PCSK2→TACR3
vasa recta descend limb SLC14A1+
vasa recta descend limb MGP+
vasa recta ascend limb IGFBP5+
vasa recta ascend limb IGF1+
vein
artery
capillary FABP5-RBP7-
capillary FABP5+RBP7-
capillary FABP5+RBP7lo
capillary FABP5+RBP7+
vascular endothelium FABP5+RBP7+
capillary CXCL10+
capillary aerocyte
sinusoid MAFB+
hepatic sinusoid
glomerular
vascular endothelium
lymphatic CCL21+
lymphatic ACKR4-CCDC80+
lymphatic ACKR4+CCDC80+
lymphatic
Abbrev. Tissue
Aort Aorta
Blad Bladder
Blo Blood
Bone Bone
BM Bone marrow
BrC Brain cortex
BrS Brainstem
Col Colon
Dia Diaphragm
Ret Eye retina
Fat Fat
Hrt Heart
Pit Hypothalamus/Pituitary
Kid Kidney
LM Limb muscle
Liv Liver
Lung Lung
MG Mammary gland
Panc Pancreas
Skin Skin
SI Small intestine
Spl Spleen
Test Testes
Thym Thymus
Tong Tongue
Trac Trachea
Ute Uterus
Compartment
endothelial
- artery
- vein
- vasa recta
- capillary
- mix
- lymphatic
12.5%
25%
50%
100%
ln(CP10K+1)

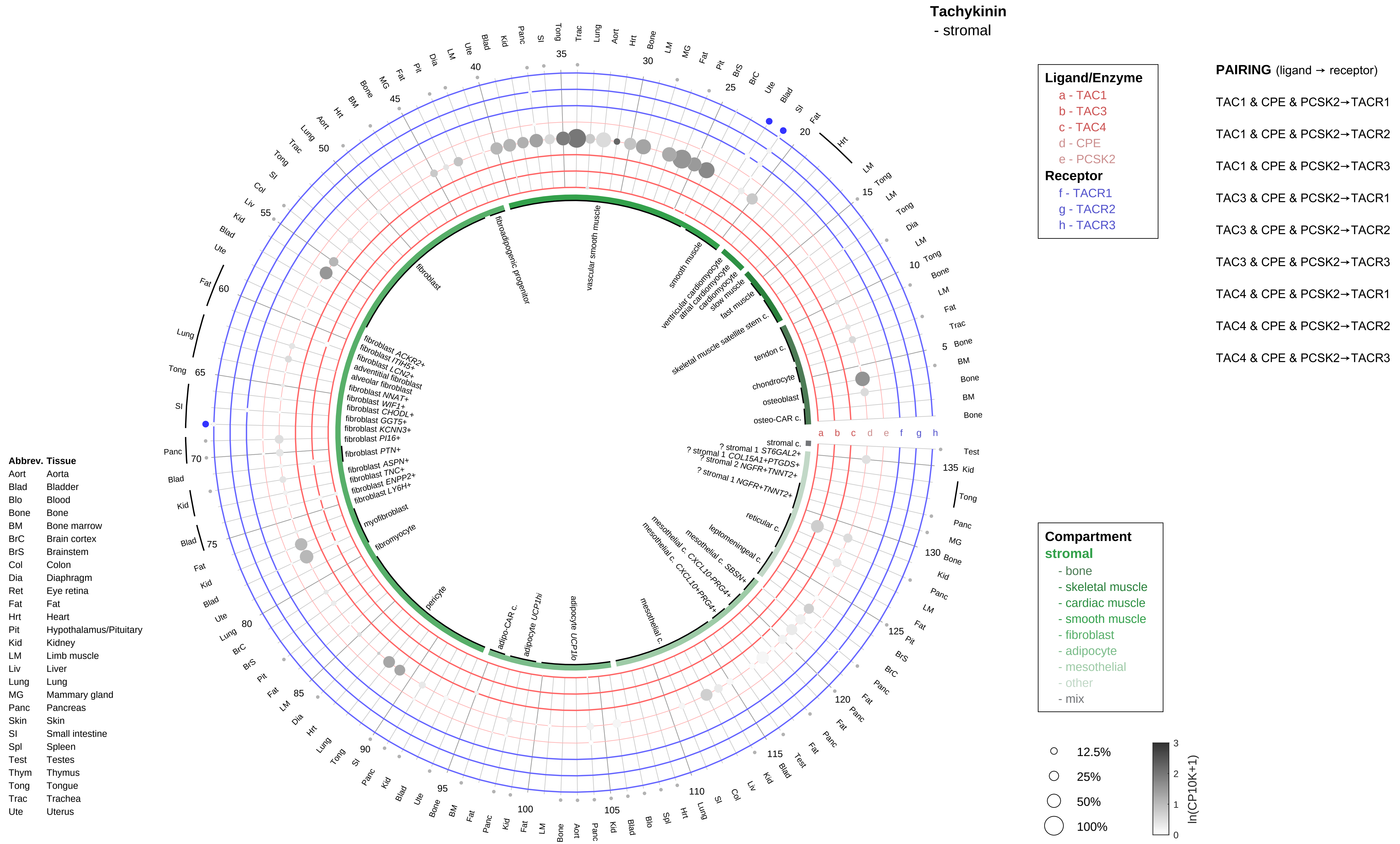

Tachykinin
- stromal
Ligand/Enzyme
a - TAC1
b - TAC3
c - TAC4
d - CPE
e - PCSK2
Receptor
f - TACR1
g - TACR2
h - TACR3
PAIRING (ligand → receptor)
TAC1 & CPE & PCSK2→TACR1
TAC1 & CPE & PCSK2→TACR2
TAC1 & CPE & PCSK2→TACR3
TAC3 & CPE & PCSK2→TACR1
TAC3 & CPE & PCSK2→TACR2
TAC3 & CPE & PCSK2→TACR3
TAC4 & CPE & PCSK2→TACR1
TAC4 & CPE & PCSK2→TACR2
TAC4 & CPE & PCSK2→TACR3
Compartment
stromal
- bone
- skeletal muscle
- cardiac muscle
- smooth muscle
- fibroblast
- adipocyte
- mesothelial
- other
- mix
12.5%
25%
50%
100%
ln(CP10K+1)
Abbrev. Tissue
Aort Aorta
Blad Bladder
Blo Blood
Bone Bone
BM Bone marrow
BrC Brain cortex
BrS Brainstem
Col Colon
Dia Diaphragm
Ret Eye retina
Fat Fat
Hrt Heart
Pit Hypothalamus/Pituitary
Kid Kidney
LM Limb muscle
Liv Liver
Lung Lung
MG Mammary gland
Panc Pancreas
Skin Skin
SI Small intestine
Spl Spleen
Test Testes
Thym Thymus
Tong Tongue
Trac Trachea
Ute Uterus
vascular smooth muscle
smooth muscle
ventricular cardiomyocyte
atrial cardiomyocyte
cardiomyocyte
slow muscle
fast muscle
skeletal muscle satellite stem c.
tendon c.
chondrocyte
osteoblast
osteo-CAR c.
stromal c.
? stromal 1 ST6GAL2+
? stromal 1 COL15A1+PTGDS+
? stromal 2 NGFR+TNNT2+
? stromal 1 NGFR+TNNT2+
reticular c.
leptomeningeal c.
mesothelial c. SBSN+
mesothelial c. CXCL10-PRG4+
mesothelial c. CXCL10+PRG4+
mesothelial c.
adipocyte UCP1lo
adipocyte UCP1hi
adipo-CAR c.
pericyte
fibromyocyte
myofibroblast
fibroblast LY6H+
fibroblast ENPP2+
fibroblast TNC+
fibroblast ASPN+
fibroblast PTN+
fibroblast PI16+
fibroblast KCNN3+
fibroblast GGT5+
fibroblast CHODL+
fibroblast WIF1+
fibroblast NNAT+
alveolar fibroblast
adventitial fibroblast
fibroblast LCN2+
fibroblast ITIH5+
fibroblast ACKR2+
fibroblast
fibroadipogenic progenitor

Tachykinin
- lymphoid

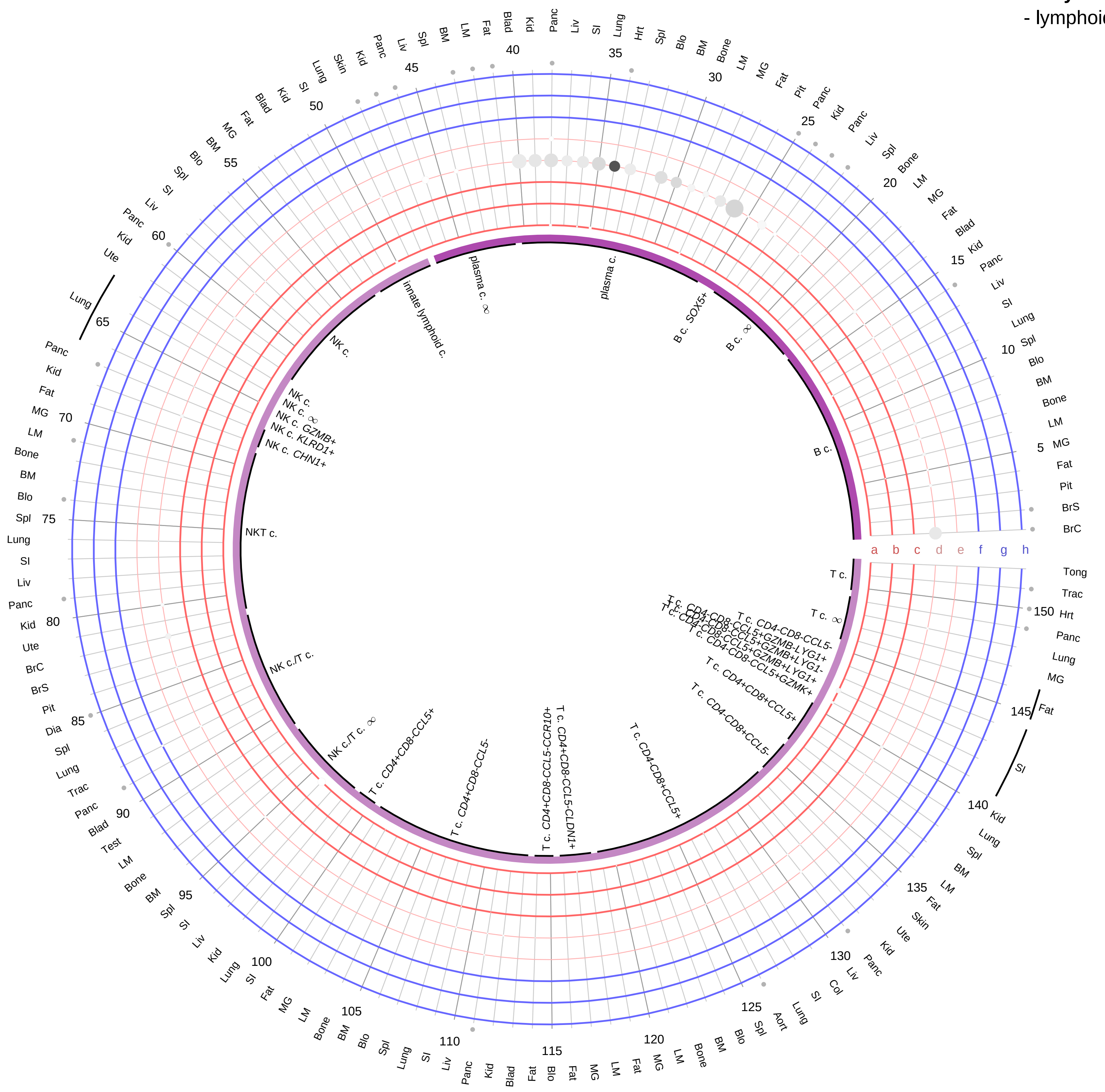

Ligand/Enzyme
- a - TAC1
- b - TAC3
- c - TAC4
- d - CPE
- e - PCSK2

Receptor
- f - TACR1
- g - TACR2
- h - TACR3

PAIRING (ligand → receptor)

TAC1 & CPE & PCSK2→TACR1

TAC1 & CPE & PCSK2→TACR2

TAC1 & CPE & PCSK2→TACR3

TAC3 & CPE & PCSK2→TACR1

TAC3 & CPE & PCSK2→TACR2

TAC3 & CPE & PCSK2→TACR3

TAC4 & CPE & PCSK2→TACR1

TAC4 & CPE & PCSK2→TACR2

TAC4 & CPE & PCSK2→TACR3

Compartment
lymphoid
- B cell
- NK/T cell

12.5%
25%
50%
100%

ln(CP10K+1): 0, 1, 2, 3

Abbrev.	Tissue
Aort	Aorta
Blad	Bladder
Blo	Blood
Bone	Bone
BM	Bone marrow
BrC	Brain cortex
BrS	Brainstem
Col	Colon
Dia	Diaphragm
Ret	Eye retina
Fat	Fat
Hrt	Heart
Pit	Hypothalamus/Pituitary
Kid	Kidney
LM	Limb muscle
Liv	Liver
Lung	Lung
MG	Mammary gland
Panc	Pancreas
Skin	Skin
SI	Small intestine
Spl	Spleen
Test	Testes
Thym	Thymus
Tong	Tongue
Trac	Trachea
Ute	Uterus

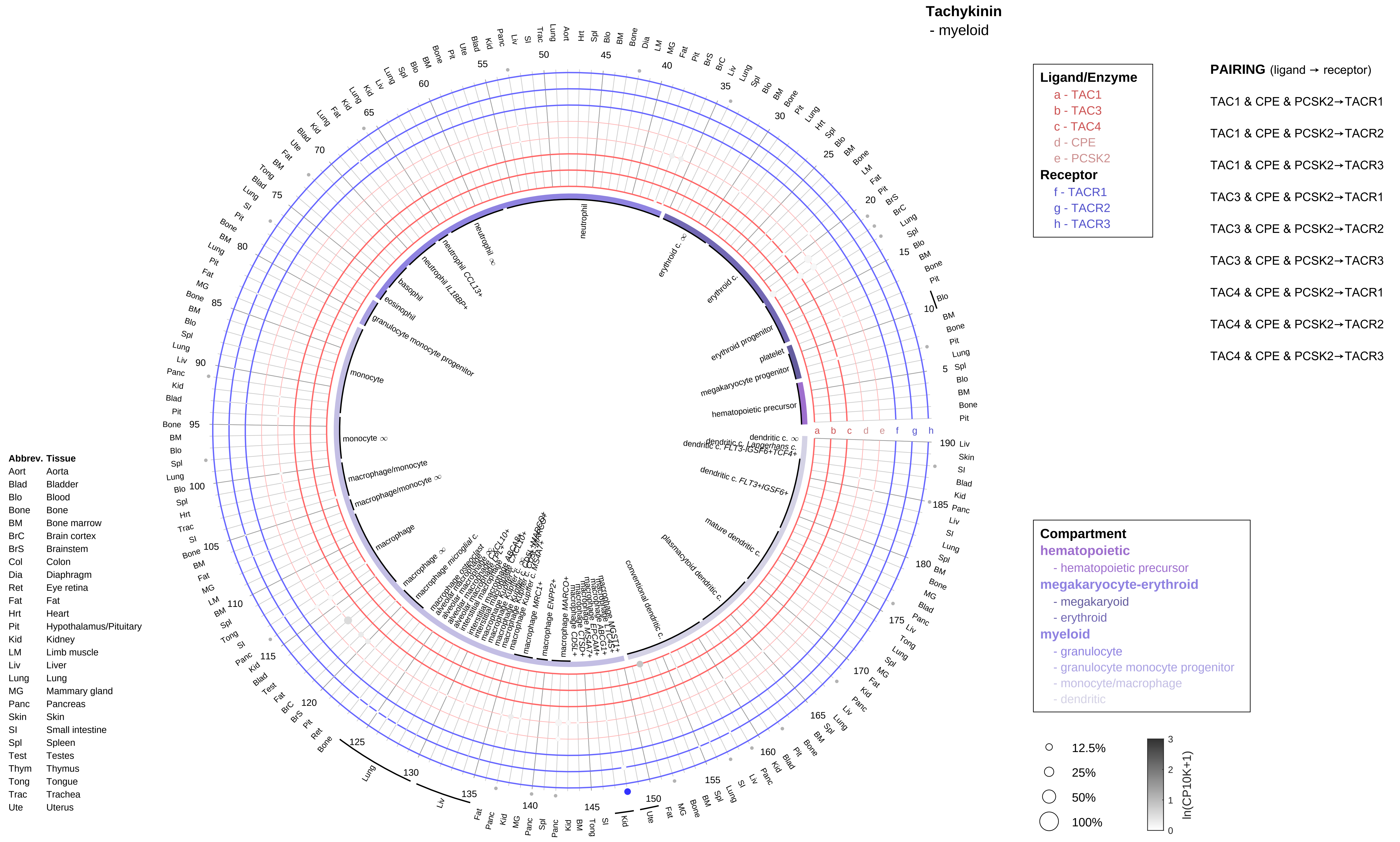

Tachykinin
- myeloid
Ligand/Enzyme
a - TAC1
b - TAC3
c - TAC4
d - CPE
e - PCSK2
Receptor
f - TACR1
g - TACR2
h - TACR3
PAIRING (ligand → receptor)
TAC1 & CPE & PCSK2→TACR1
TAC1 & CPE & PCSK2→TACR2
TAC1 & CPE & PCSK2→TACR3
TAC3 & CPE & PCSK2→TACR1
TAC3 & CPE & PCSK2→TACR2
TAC3 & CPE & PCSK2→TACR3
TAC4 & CPE & PCSK2→TACR1
TAC4 & CPE & PCSK2→TACR2
TAC4 & CPE & PCSK2→TACR3
Compartment
hematopoietic
- hematopoietic precursor
megakaryocyte-erythroid
- megakaryoid
- erythroid
myeloid
- granulocyte
- granulocyte monocyte progenitor
- monocyte/macrophage
- dendritic
12.5%
25%
50%
100%
ln(CP10K+1)
Abbrev. Tissue
Aort Aorta
Blad Bladder
Blo Blood
Bone Bone
BM Bone marrow
BrC Brain cortex
BrS Brainstem
Col Colon
Dia Diaphragm
Ret Eye retina
Fat Fat
Hrt Heart
Pit Hypothalamus/Pituitary
Kid Kidney
LM Limb muscle
Liv Liver
Lung Lung
MG Mammary gland
Panc Pancreas
Skin Skin
SI Small intestine
Spl Spleen
Test Testes
Thym Thymus
Tong Tongue
Trac Trachea
Ute Uterus

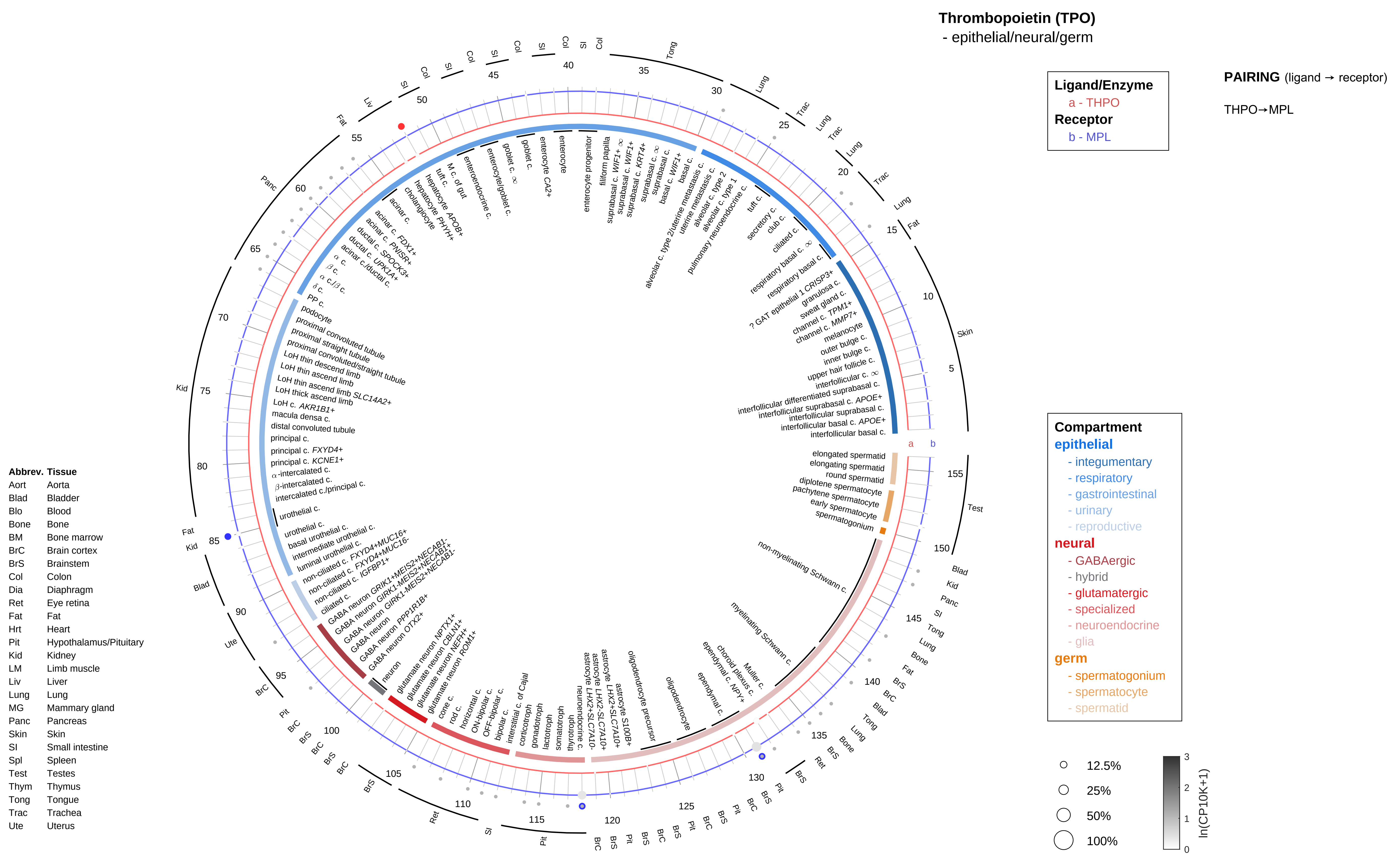

Thrombopoietin (TPO)
- epithelial/neural/germ
Ligand/Enzyme
a - THPO
Receptor
b - MPL
PAIRING (ligand → receptor)
THPO→MPL
Compartment
epithelial
- integumentary
- respiratory
- gastrointestinal
- urinary
- reproductive
neural
- GABAergic
- hybrid
- glutamatergic
- specialized
- neuroendocrine
- glia
germ
- spermatogonium
- spermatocyte
- spermatid
12.5%
25%
50%
100%
ln(CP10K+1)
Abbrev. Tissue
Aort Aorta
Blad Bladder
Blo Blood
Bone Bone
BM Bone marrow
BrC Brain cortex
BrS Brainstem
Col Colon
Dia Diaphragm
Ret Eye retina
Fat Fat
Hrt Heart
Pit Hypothalamus/Pituitary
Kid Kidney
LM Limb muscle
Liv Liver
Lung Lung
MG Mammary gland
Panc Pancreas
Skin Skin
SI Small intestine
Spl Spleen
Test Testes
Thym Thymus
Tong Tongue
Trac Trachea
Ute Uterus
interfollicular basal c.
interfollicular basal c. APOE+
interfollicular suprabasal c.
interfollicular suprabasal c. APOE+
interfollicular differentiated suprabasal c.
interfollicular c. ∞
upper hair follicle c.
inner bulge c.
outer bulge c.
melanocyte
channel c. MMP7+
channel c. TPM1+
sweat gland c.
granulosa c.
? GAT epithelial 1 CRISP3+
respiratory basal c.
respiratory basal c. ∞
ciliated c.
club c.
secretory c.
tuft c.
pulmonary neuroendocrine c.
alveolar c. type 1
alveolar c. type 2
uterine metastasis c.
alveolar c. type 2/uterine metastasis c.
basal c.
basal c. WIF1+
suprabasal c.
suprabasal c. ∞
suprabasal c. KRT4+
suprabasal c. WIF1+
suprabasal c. WIF1+ ∞
filiform papilla
enterocyte progenitor
enterocyte
enterocyte CA2+
goblet c.
goblet c. ∞
enterocyte/goblet c.
enteroendocrine c.
M c. of gut
tuft c.
hepatocyte APOB+
hepatocyte PHYH+
cholangiocyte
acinar c.
acinar c. FDX1+
acinar c. PNISR+
ductal c. SPOCK3+
ductal c. UPK1A+
acinar c./ductal c.
α c.
β c.
α c./β c.
δ c.
PP c.
podocyte
proximal convoluted tubule
proximal straight tubule
proximal convoluted/straight tubule
LoH thin descend limb
LoH thin ascend limb
LoH thin ascend limb SLC14A2+
LoH thick ascend limb
LoH c. AKR1B1+
macula densa c.
distal convoluted tubule
principal c.
principal c. FXYD4+
principal c. KCNE1+
α-intercalated c.
β-intercalated c.
intercalated c./principal c.
urothelial c.
urothelial c.
basal urothelial c.
intermediate urothelial c.
luminal urothelial c.
non-ciliated c. FXYD4+MUC16+
non-ciliated c. FXYD4+MUC16-
non-ciliated c. IGFBP1+
ciliated c.
GABA neuron GRIK1+MEIS2+NECAB1-
GABA neuron GIRK1-MEIS2+NECAB1+
GABA neuron GIRK1-MEIS2+NECAB1-
GABA neuron
GABA neuron PPP1R1B+
GABA neuron OTX2+
neuron
glutamate neuron NPTX1+
glutamate neuron CBLN1+
glutamate neuron NEFH+
glutamate neuron ROM1+
cone c.
rod c.
horizontal c.
ON-bipolar c.
OFF-bipolar c.
bipolar c.
interstitial c. of Cajal
corticotroph
gonadotroph
lactotroph
somatotroph
thyrotroph
neuroendocrine c.
astrocyte LHX2-SLC7A10-
astrocyte LHX2+SLC7A10+
astrocyte LHX2-SLC7A10+
astrocyte S100B+
oligodendrocyte precursor
oligodendrocyte
ependymal c.
ependymal c. NPY+
choroid plexus c.
Müller c.
myelinating Schwann c.
non-myelinating Schwann c.
spermatogonium
early spermatocyte
pachytene spermatocyte
diplotene spermatocyte
round spermatid
elongating spermatid
elongated spermatid

Thrombopoietin (TPO)
- endothelial

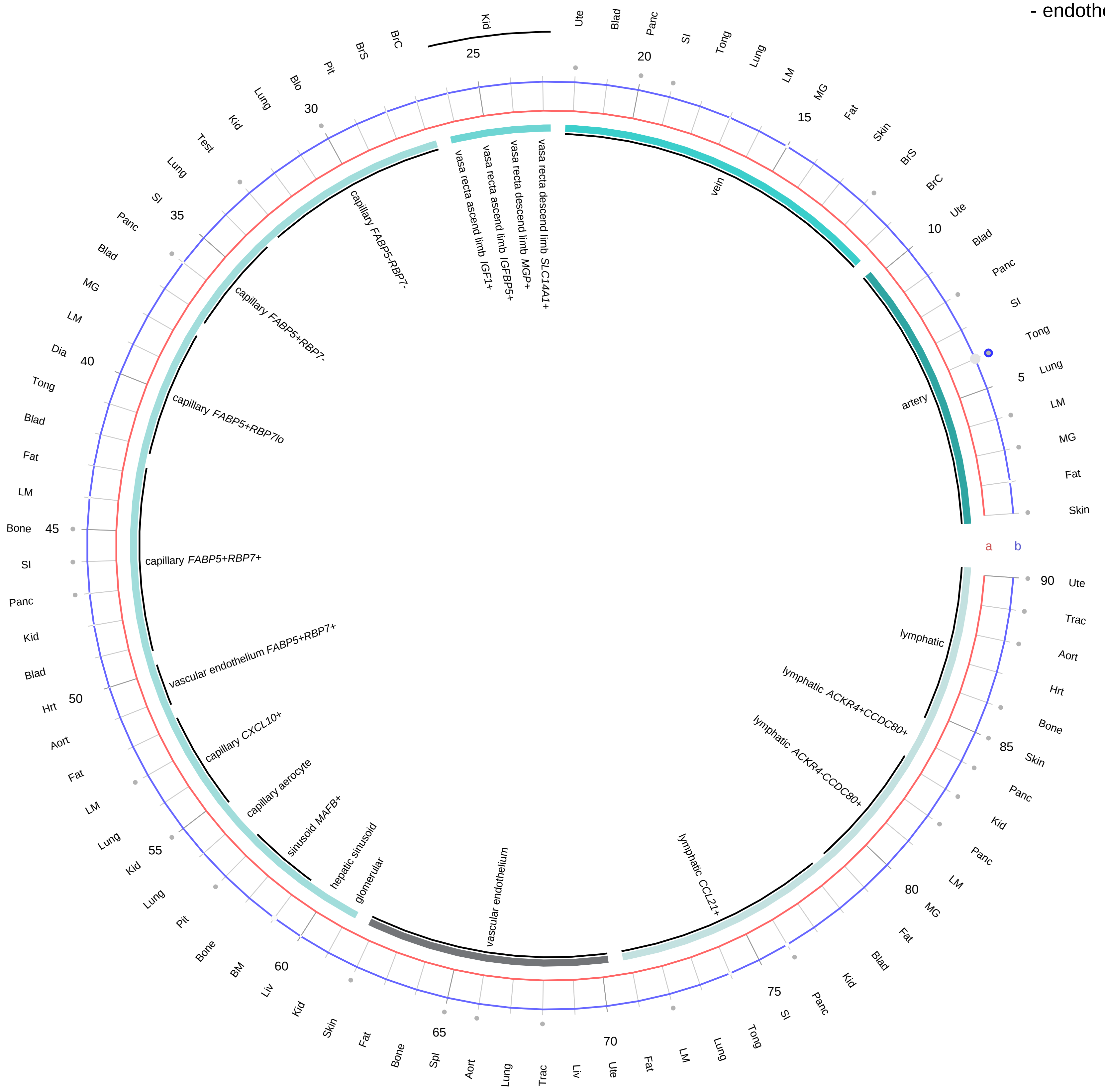

Ligand/Enzyme
a - THPO
Receptor
b - MPL

PAIRING (ligand → receptor)

THPO→MPL

Compartment
endothelial
- artery
- vein
- vasa recta
- capillary
- mix
- lymphatic

12.5%
25%
50%
100%

ln(CP10K+1): 0, 1, 2, 3

Abbrev.	Tissue
Aort	Aorta
Blad	Bladder
Blo	Blood
Bone	Bone
BM	Bone marrow
BrC	Brain cortex
BrS	Brainstem
Col	Colon
Dia	Diaphragm
Ret	Eye retina
Fat	Fat
Hrt	Heart
Pit	Hypothalamus/Pituitary
Kid	Kidney
LM	Limb muscle
Liv	Liver
Lung	Lung
MG	Mammary gland
Panc	Pancreas
Skin	Skin
SI	Small intestine
Spl	Spleen
Test	Testes
Thym	Thymus
Tong	Tongue
Trac	Trachea
Ute	Uterus

Thrombopoietin (TPO)
- stromal

PAIRING (ligand → receptor)

THPO→MPL

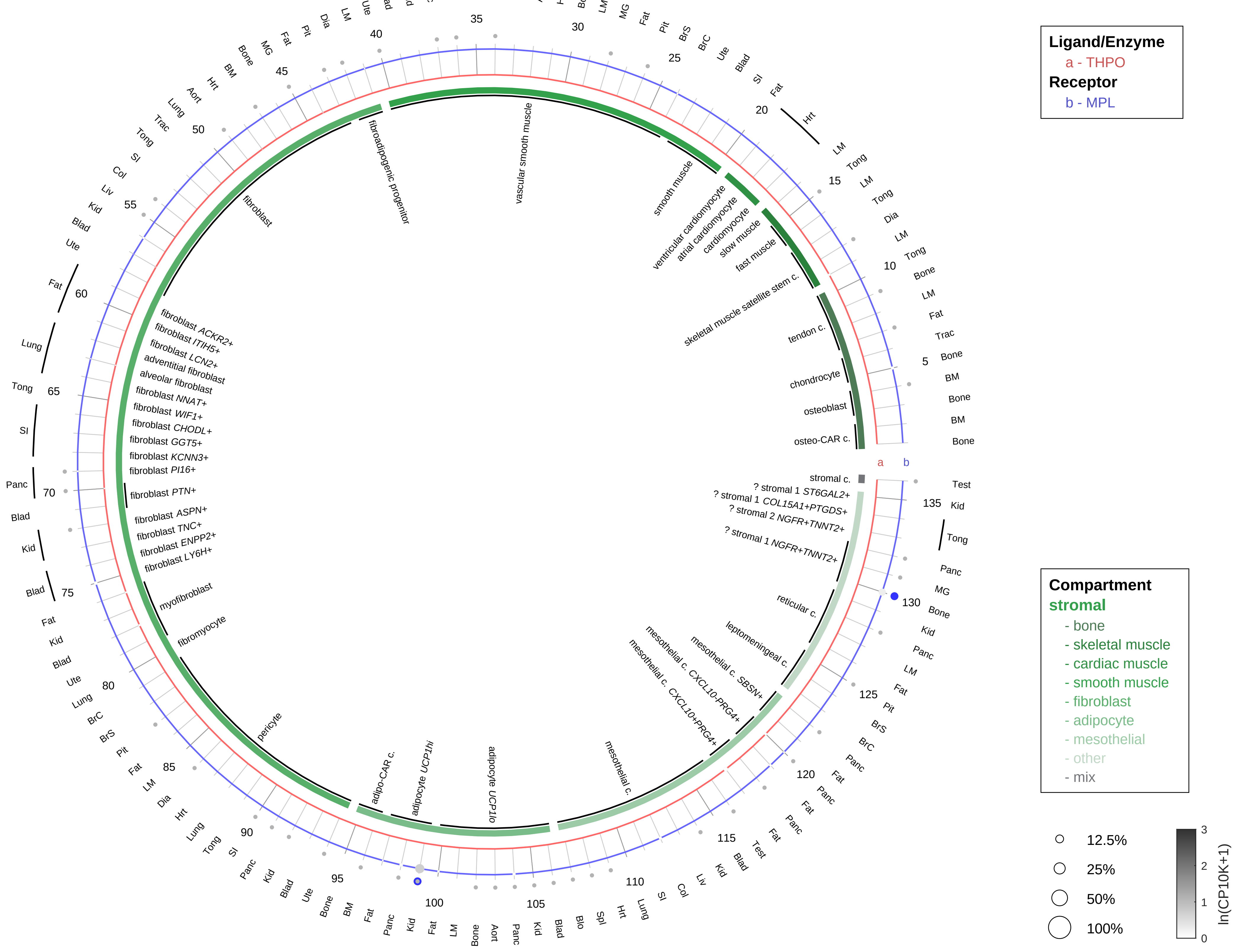

Abbrev.	Tissue
Aort	Aorta
Blad	Bladder
Blo	Blood
Bone	Bone
BM	Bone marrow
BrC	Brain cortex
BrS	Brainstem
Col	Colon
Dia	Diaphragm
Ret	Eye retina
Fat	Fat
Hrt	Heart
Pit	Hypothalamus/Pituitary
Kid	Kidney
LM	Limb muscle
Liv	Liver
Lung	Lung
MG	Mammary gland
Panc	Pancreas
Skin	Skin
SI	Small intestine
Spl	Spleen
Test	Testes
Thym	Thymus
Tong	Tongue
Trac	Trachea
Ute	Uterus

Thrombopoietin (TPO)
- lymphoid

Ligand/Enzyme
a - THPO
Receptor
b - MPL

PAIRING (ligand → receptor)

THPO→MPL

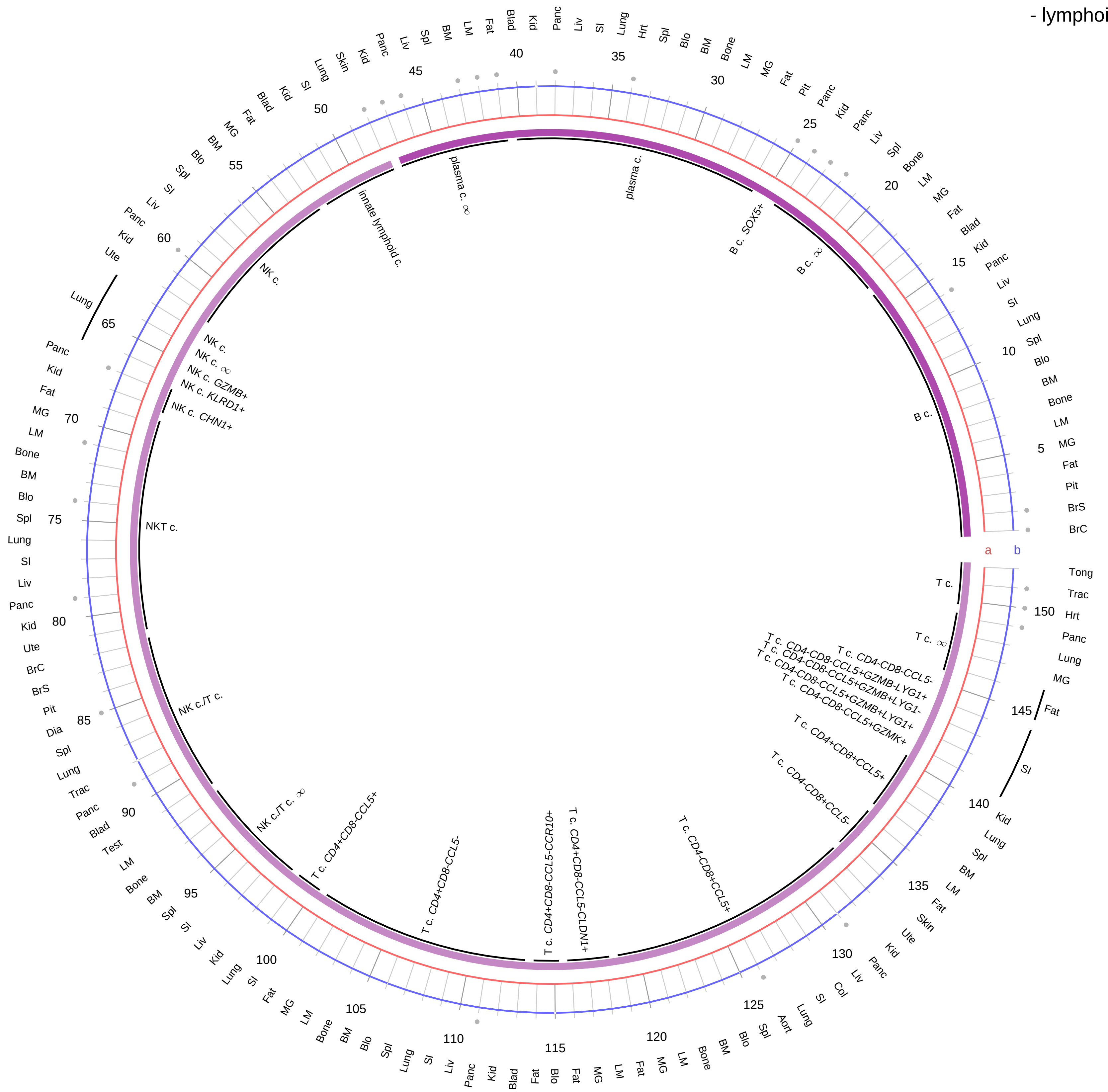

Abbrev.	Tissue
Aort	Aorta
Blad	Bladder
Blo	Blood
Bone	Bone
BM	Bone marrow
BrC	Brain cortex
BrS	Brainstem
Col	Colon
Dia	Diaphragm
Ret	Eye retina
Fat	Fat
Hrt	Heart
Pit	Hypothalamus/Pituitary
Kid	Kidney
LM	Limb muscle
Liv	Liver
Lung	Lung
MG	Mammary gland
Panc	Pancreas
Skin	Skin
SI	Small intestine
Spl	Spleen
Test	Testes
Thym	Thymus
Tong	Tongue
Trac	Trachea
Ute	Uterus

Compartment
lymphoid
- B cell
- NK/T cell

12.5%
25%
50%
100%

ln(CP10K+1): 0 1 2 3

Thrombopoietin (TPO)
- myeloid

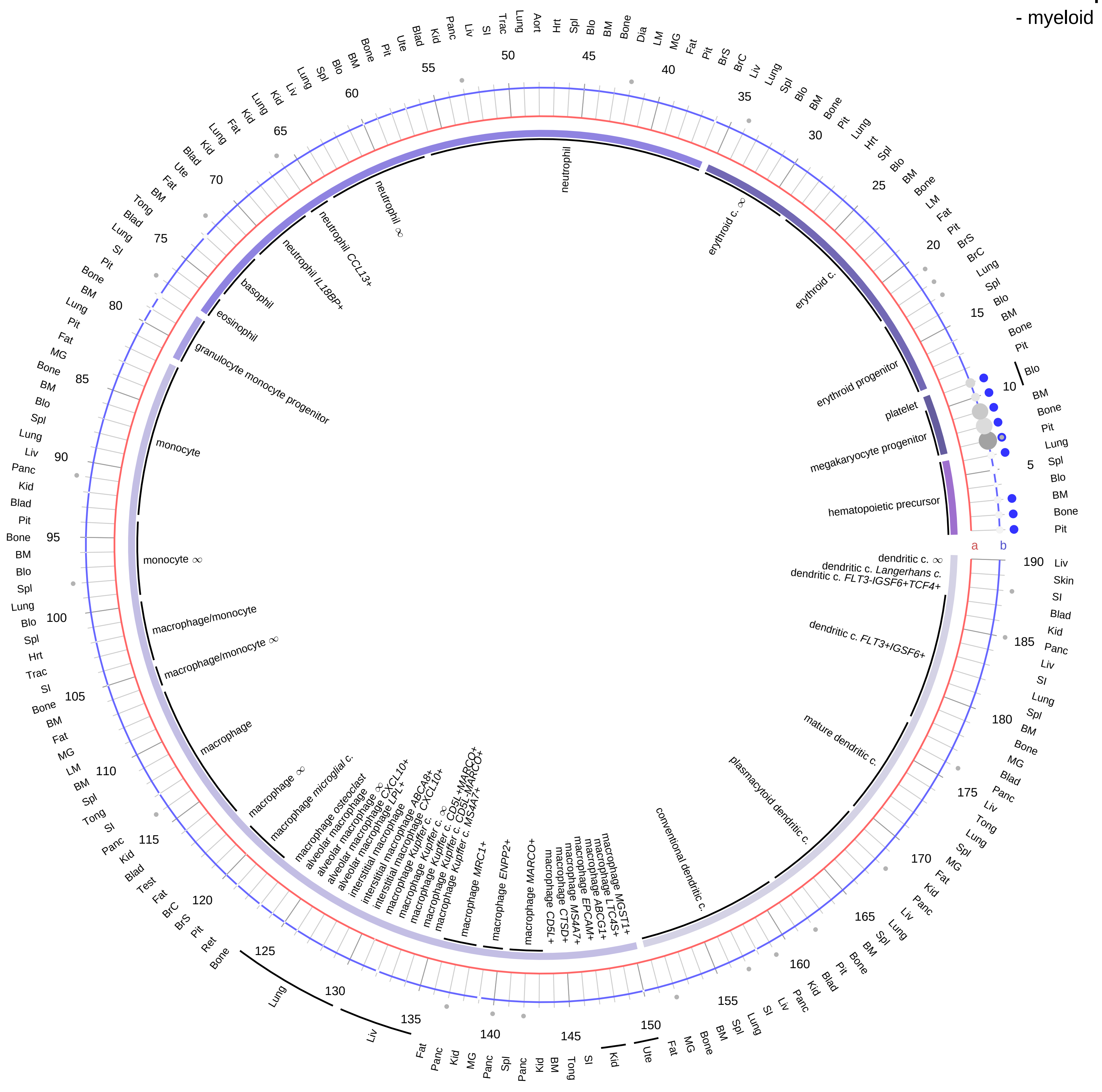

Ligand/Enzyme
a - THPO
Receptor
b - MPL

PAIRING (ligand → receptor)

THPO→MPL

Compartment
hematopoietic
- hematopoietic precursor

megakaryocyte-erythroid
- megakaryoid
- erythroid

myeloid
- granulocyte
- granulocyte monocyte progenitor
- monocyte/macrophage
- dendritic

12.5%
25%
50%
100%

ln(CP10K+1): 0, 1, 2, 3

Abbrev.	Tissue
Aort	Aorta
Blad	Bladder
Blo	Blood
Bone	Bone
BM	Bone marrow
BrC	Brain cortex
BrS	Brainstem
Col	Colon
Dia	Diaphragm
Ret	Eye retina
Fat	Fat
Hrt	Heart
Pit	Hypothalamus/Pituitary
Kid	Kidney
LM	Limb muscle
Liv	Liver
Lung	Lung
MG	Mammary gland
Panc	Pancreas
Skin	Skin
SI	Small intestine
Spl	Spleen
Test	Testes
Thym	Thymus
Tong	Tongue
Trac	Trachea
Ute	Uterus

Thromboxane (TXA2)
- epithelial/neural/germ

PAIRING (ligand → receptor)

TBXAS1 & PTGS1 & PTGS2→TBXA2R

Ligand/Enzyme
- a - PTGS1
- b - PTGS2
- c - TBXAS1

Receptor
- d - TBXA2R

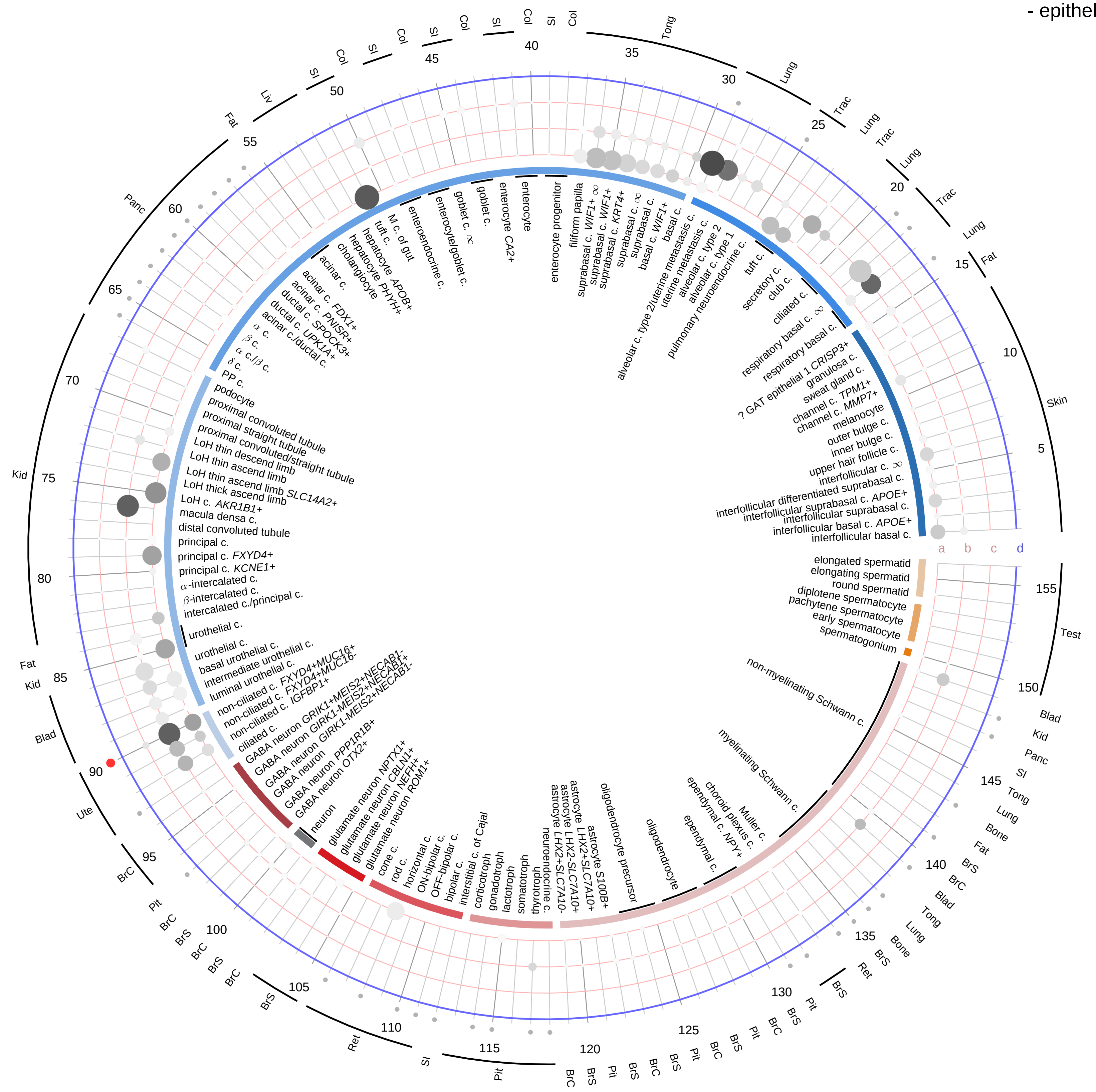

Abbrev.	Tissue
Aort	Aorta
Blad	Bladder
Blo	Blood
Bone	Bone
BM	Bone marrow
BrC	Brain cortex
BrS	Brainstem
Col	Colon
Dia	Diaphragm
Ret	Eye retina
Fat	Fat
Hrt	Heart
Pit	Hypothalamus/Pituitary
Kid	Kidney
LM	Limb muscle
Liv	Liver
Lung	Lung
MG	Mammary gland
Panc	Pancreas
Skin	Skin
SI	Small intestine
Spl	Spleen
Test	Testes
Thym	Thymus
Tong	Tongue
Trac	Trachea
Ute	Uterus

Compartment

epithelial
- integumentary
- respiratory
- gastrointestinal
- urinary
- reproductive

neural
- GABAergic
- hybrid
- glutamatergic
- specialized
- neuroendocrine
- glia

germ
- spermatogonium
- spermatocyte
- spermatid

○ 12.5%
○ 25%
○ 50%
○ 100%

ln(CP10K+1): 0, 1, 2, 3

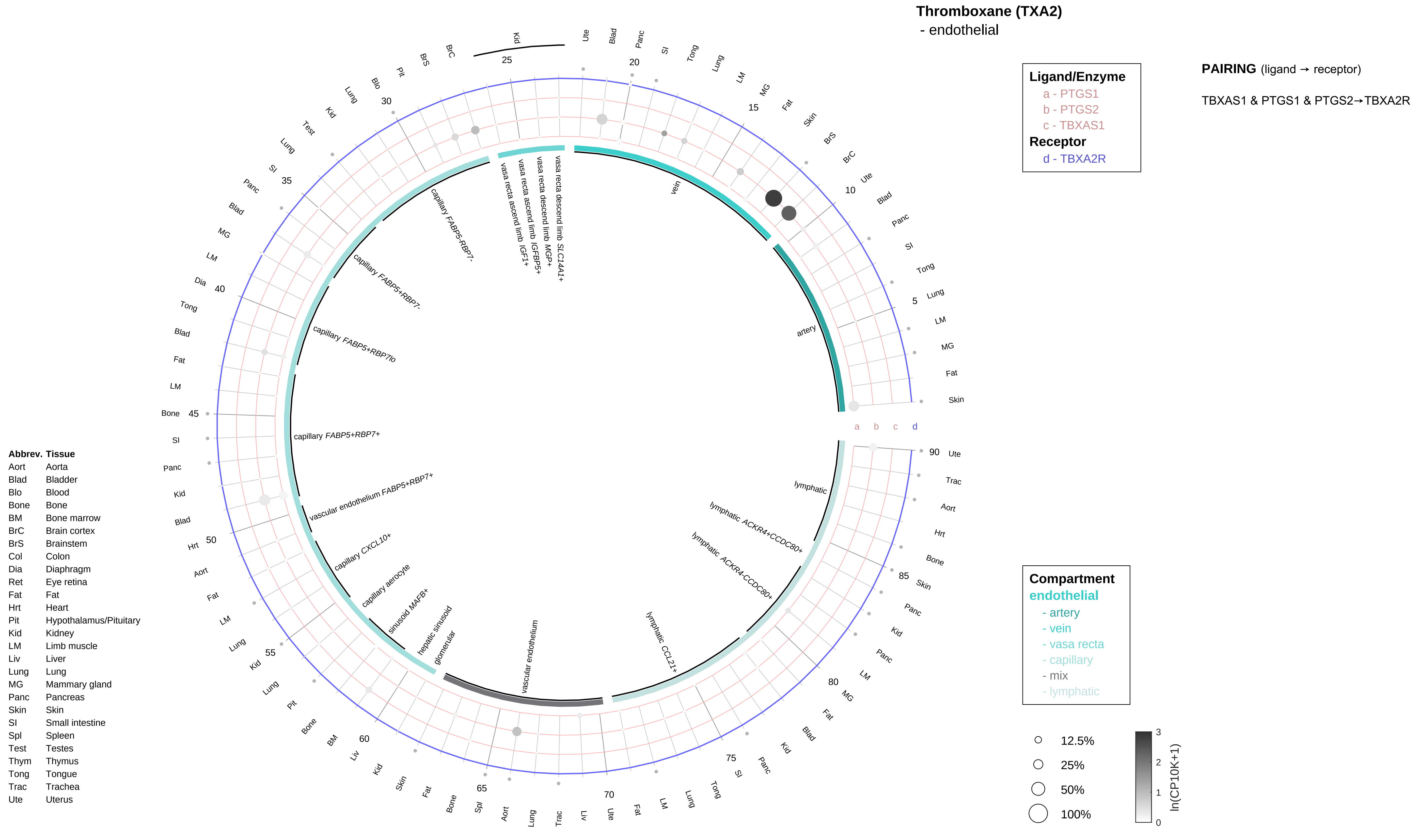

Thromboxane (TXA2)
- endothelial
Ligand/Enzyme
a - PTGS1
b - PTGS2
c - TBXAS1
Receptor
d - TBXA2R
PAIRING (ligand → receptor)
TBXAS1 & PTGS1 & PTGS2→TBXA2R
Compartment
endothelial
- artery
- vein
- vasa recta
- capillary
- mix
- lymphatic
12.5%
25%
50%
100%
ln(CP10K+1)
vein
artery
lymphatic
lymphatic ACKR4+CCDC80+
lymphatic ACKR4-CCDC80+
lymphatic CCL21+
vascular endothelium
glomerular
hepatic sinusoid
sinusoid MAFB+
capillary aerocyte
capillary CXCL10+
vascular endothelium FABP5+RBP7+
capillary FABP5+RBP7+
capillary FABP5+RBP7lo
capillary FABP5+RBP7-
capillary FABP5-RBP7-
vasa recta ascend limb IGF1+
vasa recta ascend limb IGFBP5+
vasa recta descend limb MGP+
vasa recta descend limb SLC14A1+
Abbrev. Tissue
Aort Aorta
Blad Bladder
Blo Blood
Bone Bone
BM Bone marrow
BrC Brain cortex
BrS Brainstem
Col Colon
Dia Diaphragm
Ret Eye retina
Fat Fat
Hrt Heart
Pit Hypothalamus/Pituitary
Kid Kidney
LM Limb muscle
Liv Liver
Lung Lung
MG Mammary gland
Panc Pancreas
Skin Skin
SI Small intestine
Spl Spleen
Test Testes
Thym Thymus
Tong Tongue
Trac Trachea
Ute Uterus

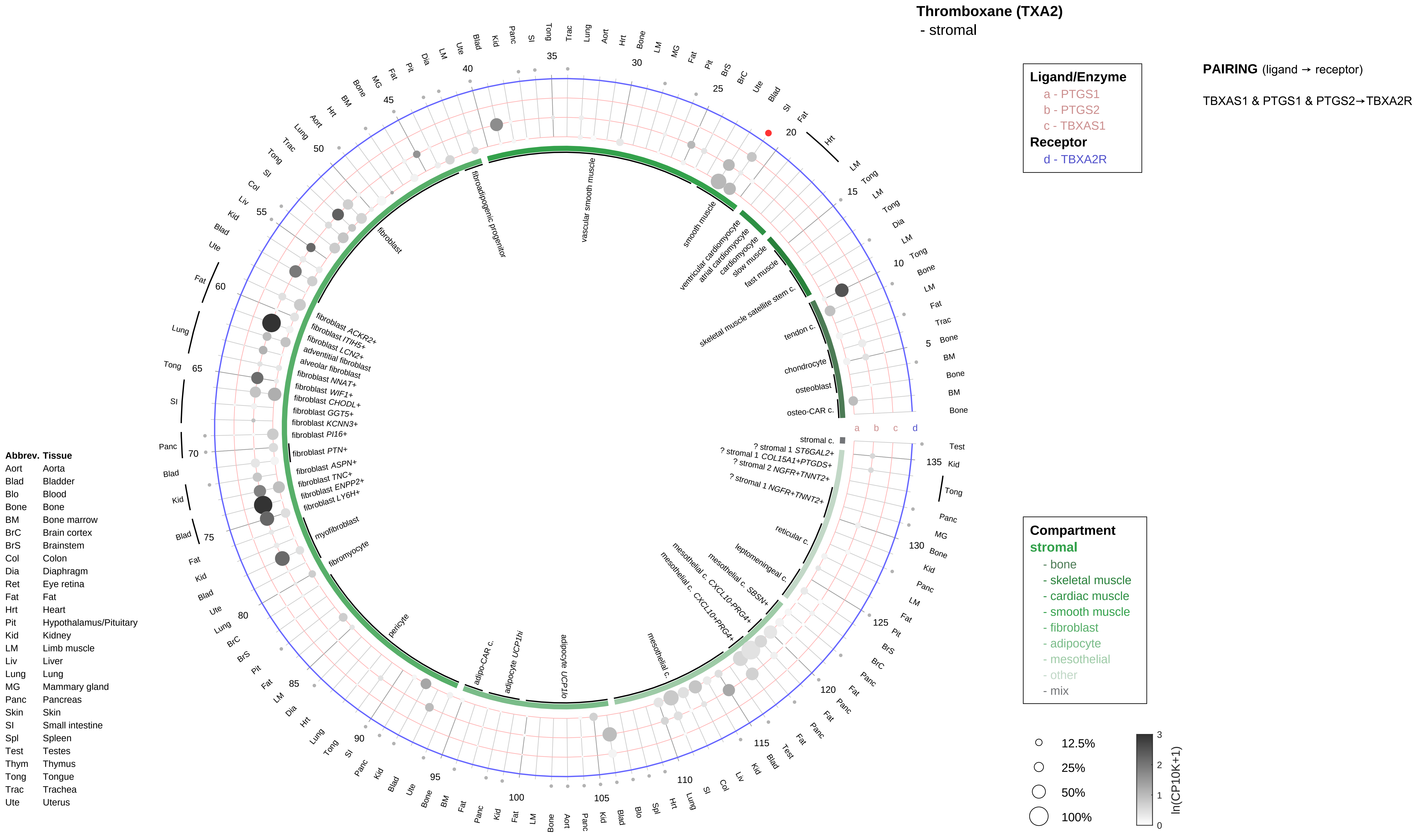

Thromboxane (TXA2)
- stromal
PAIRING (ligand → receptor)
TBXAS1 & PTGS1 & PTGS2→TBXA2R
Ligand/Enzyme
a - PTGS1
b - PTGS2
c - TBXAS1
Receptor
d - TBXA2R
Compartment
stromal
- bone
- skeletal muscle
- cardiac muscle
- smooth muscle
- fibroblast
- adipocyte
- mesothelial
- other
- mix
12.5%
25%
50%
100%
ln(CP10K+1)
0
1
2
3
Abbrev. Tissue
Aort Aorta
Blad Bladder
Blo Blood
Bone Bone
BM Bone marrow
BrC Brain cortex
BrS Brainstem
Col Colon
Dia Diaphragm
Ret Eye retina
Fat Fat
Hrt Heart
Pit Hypothalamus/Pituitary
Kid Kidney
LM Limb muscle
Liv Liver
Lung Lung
MG Mammary gland
Panc Pancreas
Skin Skin
SI Small intestine
Spl Spleen
Test Testes
Thym Thymus
Tong Tongue
Trac Trachea
Ute Uterus
fibroadipogenic progenitor
vascular smooth muscle
smooth muscle
ventricular cardiomyocyte
atrial cardiomyocyte
cardiomyocyte
slow muscle
fast muscle
skeletal muscle satellite stem c.
tendon c.
chondrocyte
osteoblast
osteo-CAR c.
stromal c.
? stromal 1 ST6GAL2+
? stromal 1 COL15A1+PTGDS+
? stromal 2 NGFR+TNNT2+
? stromal 1 NGFR+TNNT2+
reticular c.
leptomeningeal c.
mesothelial c. SBSN+
mesothelial c. CXCL10-PRG4+
mesothelial c. CXCL10+PRG4+
mesothelial c.
adipocyte UCP1lo
adipocyte UCP1hi
adipo-CAR c.
pericyte
fibromyocyte
myofibroblast
fibroblast
fibroblast ACKR2+
fibroblast ITIH5+
fibroblast LCN2+
adventitial fibroblast
alveolar fibroblast
fibroblast NNAT+
fibroblast WIF1+
fibroblast CHODL+
fibroblast GGT5+
fibroblast KCNN3+
fibroblast PI16+
fibroblast PTN+
fibroblast ASPN+
fibroblast TNC+
fibroblast ENPP2+
fibroblast LY6H+
a
b
c
d

Thromboxane (TXA2)
- lymphoid

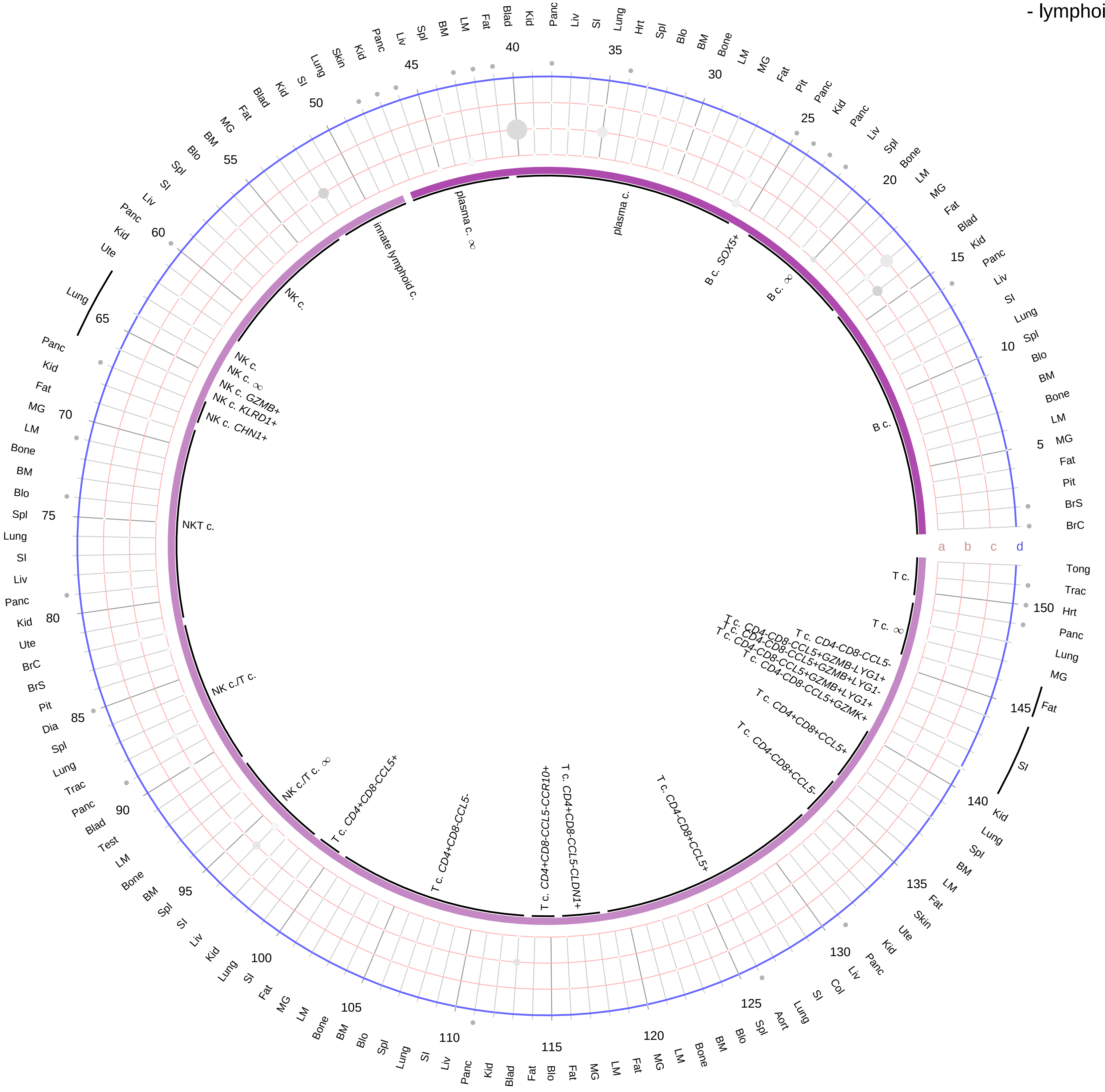

Ligand/Enzyme
- a - PTGS1
- b - PTGS2
- c - TBXAS1

Receptor
- d - TBXA2R

PAIRING (ligand → receptor)

TBXAS1 & PTGS1 & PTGS2→TBXA2R

Compartment
lymphoid
- B cell
- NK/T cell

12.5%
25%
50%
100%

ln(CP10K+1): 0, 1, 2, 3

Abbrev.	Tissue
Aort	Aorta
Blad	Bladder
Blo	Blood
Bone	Bone
BM	Bone marrow
BrC	Brain cortex
BrS	Brainstem
Col	Colon
Dia	Diaphragm
Ret	Eye retina
Fat	Fat
Hrt	Heart
Pit	Hypothalamus/Pituitary
Kid	Kidney
LM	Limb muscle
Liv	Liver
Lung	Lung
MG	Mammary gland
Panc	Pancreas
Skin	Skin
SI	Small intestine
Spl	Spleen
Test	Testes
Thym	Thymus
Tong	Tongue
Trac	Trachea
Ute	Uterus

Thromboxane (TXA2)
- myeloid

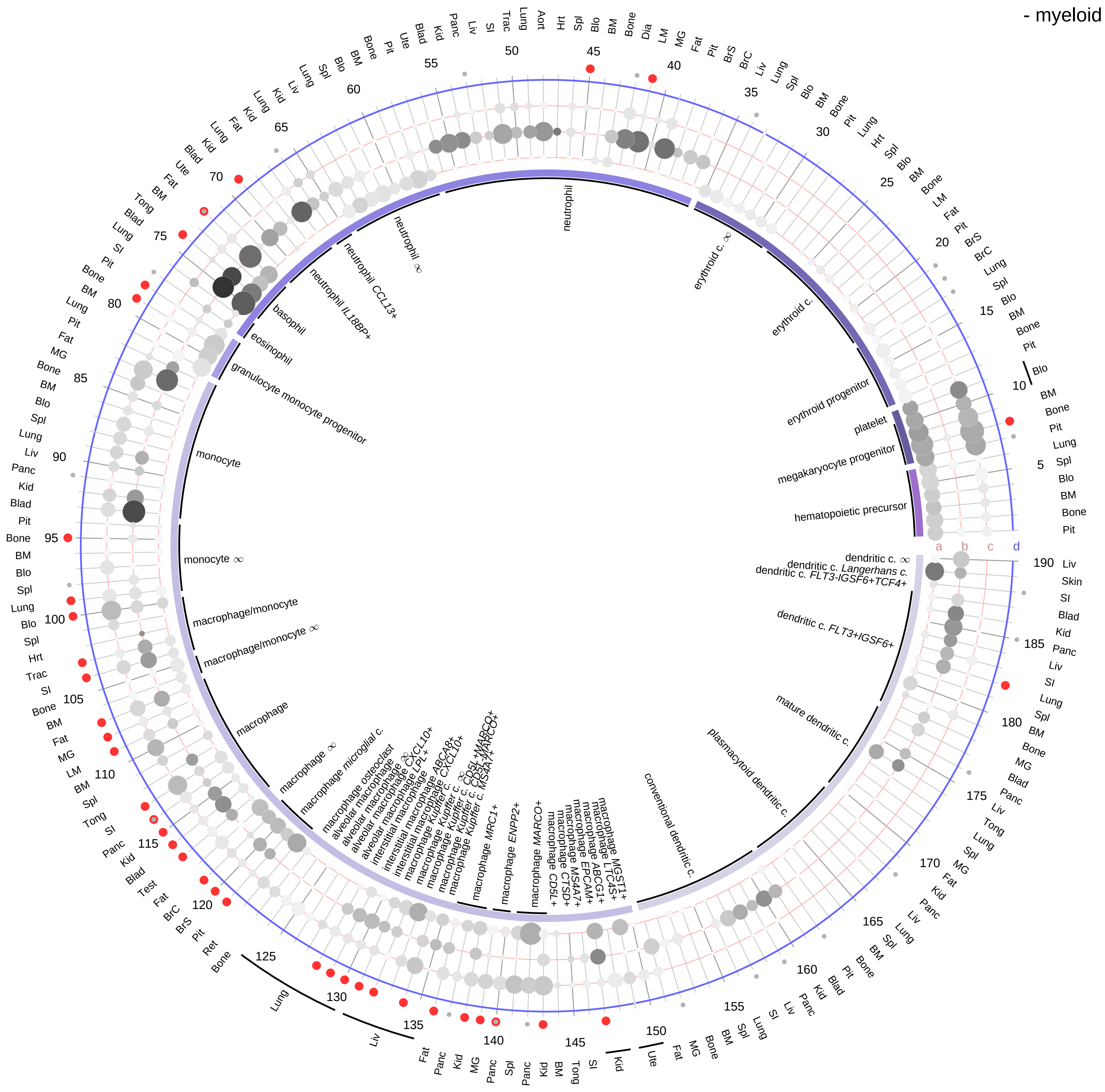

Ligand/Enzyme
a - PTGS1
b - PTGS2
c - TBXAS1
**Receptor**
d - TBXA2R

PAIRING (ligand → receptor)

TBXAS1 & PTGS1 & PTGS2→TBXA2R

Abbrev.	Tissue
Aort	Aorta
Blad	Bladder
Blo	Blood
Bone	Bone
BM	Bone marrow
BrC	Brain cortex
BrS	Brainstem
Col	Colon
Dia	Diaphragm
Ret	Eye retina
Fat	Fat
Hrt	Heart
Pit	Hypothalamus/Pituitary
Kid	Kidney
LM	Limb muscle
Liv	Liver
Lung	Lung
MG	Mammary gland
Panc	Pancreas
Skin	Skin
SI	Small intestine
Spl	Spleen
Test	Testes
Thym	Thymus
Tong	Tongue
Trac	Trachea
Ute	Uterus

Compartment
- **hematopoietic**
  - hematopoietic precursor
- **megakaryocyte-erythroid**
  - megakaryoid
  - erythroid
- **myeloid**
  - granulocyte
  - granulocyte monocyte progenitor
  - monocyte/macrophage
  - dendritic

12.5%
25%
50%
100%

ln(CP10K+1): 0, 1, 2, 3

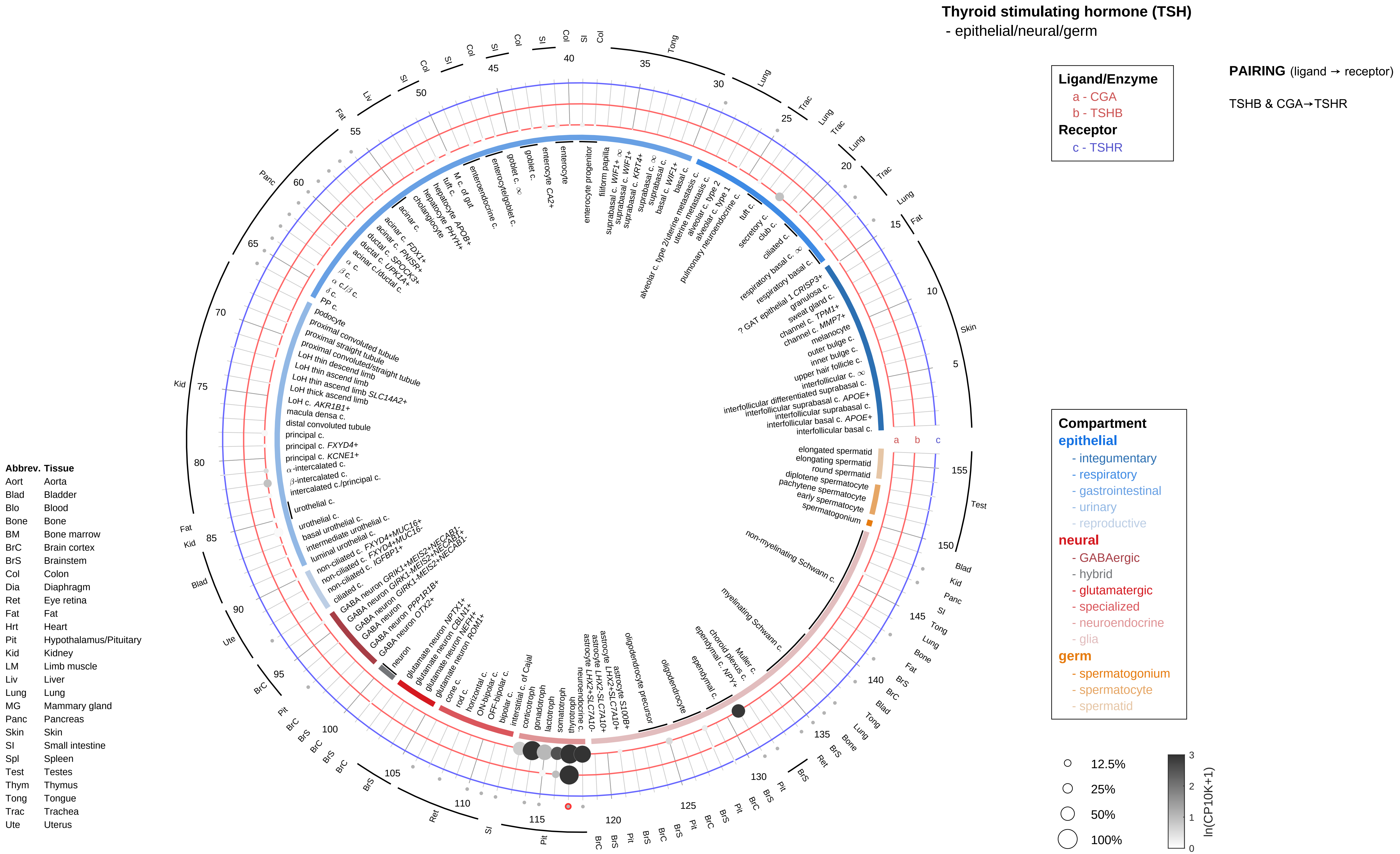
Thyroid stimulating hormone (TSH)
- epithelial/neural/germ
Ligand/Enzyme
a - CGA
b - TSHB
Receptor
c - TSHR
PAIRING (ligand → receptor)
TSHB & CGA→TSHR
Compartment
epithelial
- integumentary
- respiratory
- gastrointestinal
- urinary
- reproductive
neural
- GABAergic
- hybrid
- glutamatergic
- specialized
- neuroendocrine
- glia
germ
- spermatogonium
- spermatocyte
- spermatid
12.5%
25%
50%
100%
ln(CP10K+1)
Abbrev. Tissue
Aort Aorta
Blad Bladder
Blo Blood
Bone Bone
BM Bone marrow
BrC Brain cortex
BrS Brainstem
Col Colon
Dia Diaphragm
Ret Eye retina
Fat Fat
Hrt Heart
Pit Hypothalamus/Pituitary
Kid Kidney
LM Limb muscle
Liv Liver
Lung Lung
MG Mammary gland
Panc Pancreas
Skin Skin
SI Small intestine
Spl Spleen
Test Testes
Thym Thymus
Tong Tongue
Trac Trachea
Ute Uterus

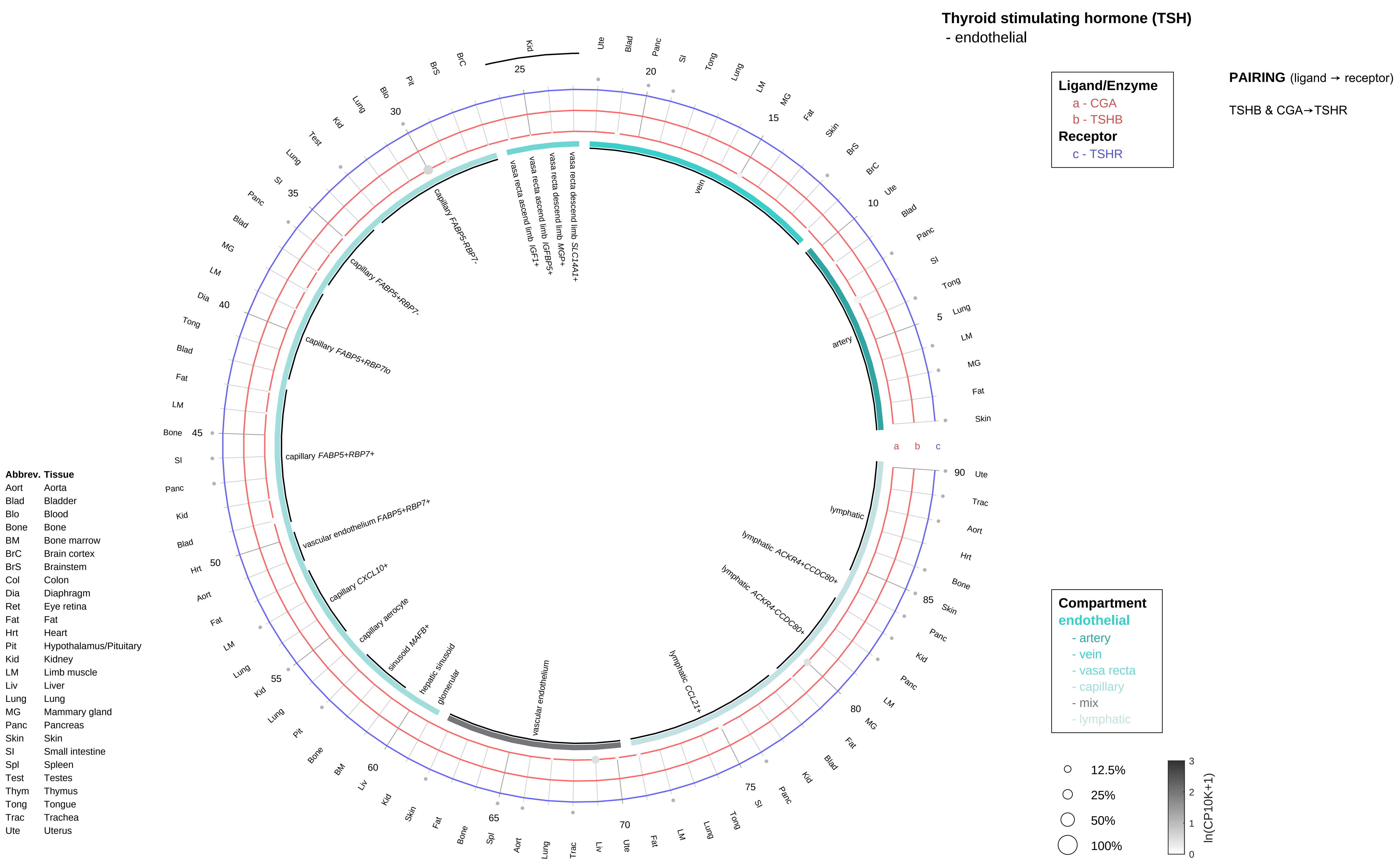
Thyroid stimulating hormone (TSH)
- endothelial
Ligand/Enzyme
a - CGA
b - TSHB
Receptor
c - TSHR
PAIRING (ligand → receptor)
TSHB & CGA→TSHR
artery
vein
vasa recta descend limb SLC14A1+
vasa recta descend limb MGP+
vasa recta ascend limb IGFBP5+
vasa recta ascend limb IGF1+
capillary FABP5-RBP7-
capillary FABP5+RBP7-
capillary FABP5+RBP7lo
capillary FABP5+RBP7+
vascular endothelium FABP5+RBP7+
capillary CXCL10+
capillary aerocyte
sinusoid MAFB+
hepatic sinusoid
glomerular
vascular endothelium
lymphatic CCL21+
lymphatic ACKR4-CCDC80+
lymphatic ACKR4+CCDC80+
lymphatic
Abbrev. Tissue
Aort Aorta
Blad Bladder
Blo Blood
Bone Bone
BM Bone marrow
BrC Brain cortex
BrS Brainstem
Col Colon
Dia Diaphragm
Ret Eye retina
Fat Fat
Hrt Heart
Pit Hypothalamus/Pituitary
Kid Kidney
LM Limb muscle
Liv Liver
Lung Lung
MG Mammary gland
Panc Pancreas
Skin Skin
SI Small intestine
Spl Spleen
Test Testes
Thym Thymus
Tong Tongue
Trac Trachea
Ute Uterus
Compartment
endothelial
- artery
- vein
- vasa recta
- capillary
- mix
- lymphatic
12.5%
25%
50%
100%
ln(CP10K+1)
3
2
1
0

Thyroid stimulating hormone (TSH)
- stromal

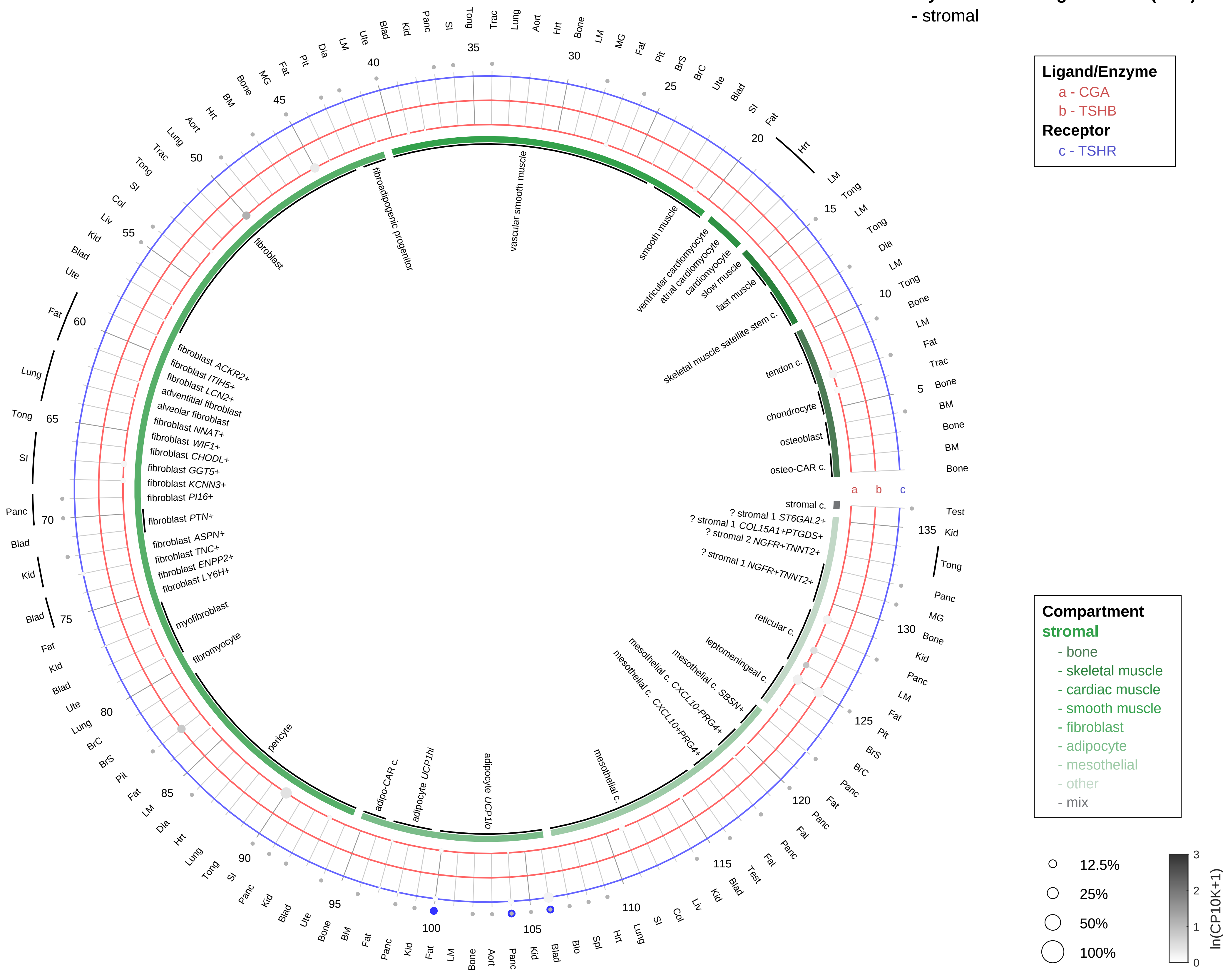

Abbrev.	Tissue
Aort	Aorta
Blad	Bladder
Blo	Blood
Bone	Bone
BM	Bone marrow
BrC	Brain cortex
BrS	Brainstem
Col	Colon
Dia	Diaphragm
Ret	Eye retina
Fat	Fat
Hrt	Heart
Pit	Hypothalamus/Pituitary
Kid	Kidney
LM	Limb muscle
Liv	Liver
Lung	Lung
MG	Mammary gland
Panc	Pancreas
Skin	Skin
SI	Small intestine
Spl	Spleen
Test	Testes
Thym	Thymus
Tong	Tongue
Trac	Trachea
Ute	Uterus

Thyroid stimulating hormone (TSH)
- lymphoid

PAIRING (ligand → receptor)

TSHB & CGA→TSHR

Ligand/Enzyme
a - CGA
b - TSHB
Receptor
c - TSHR

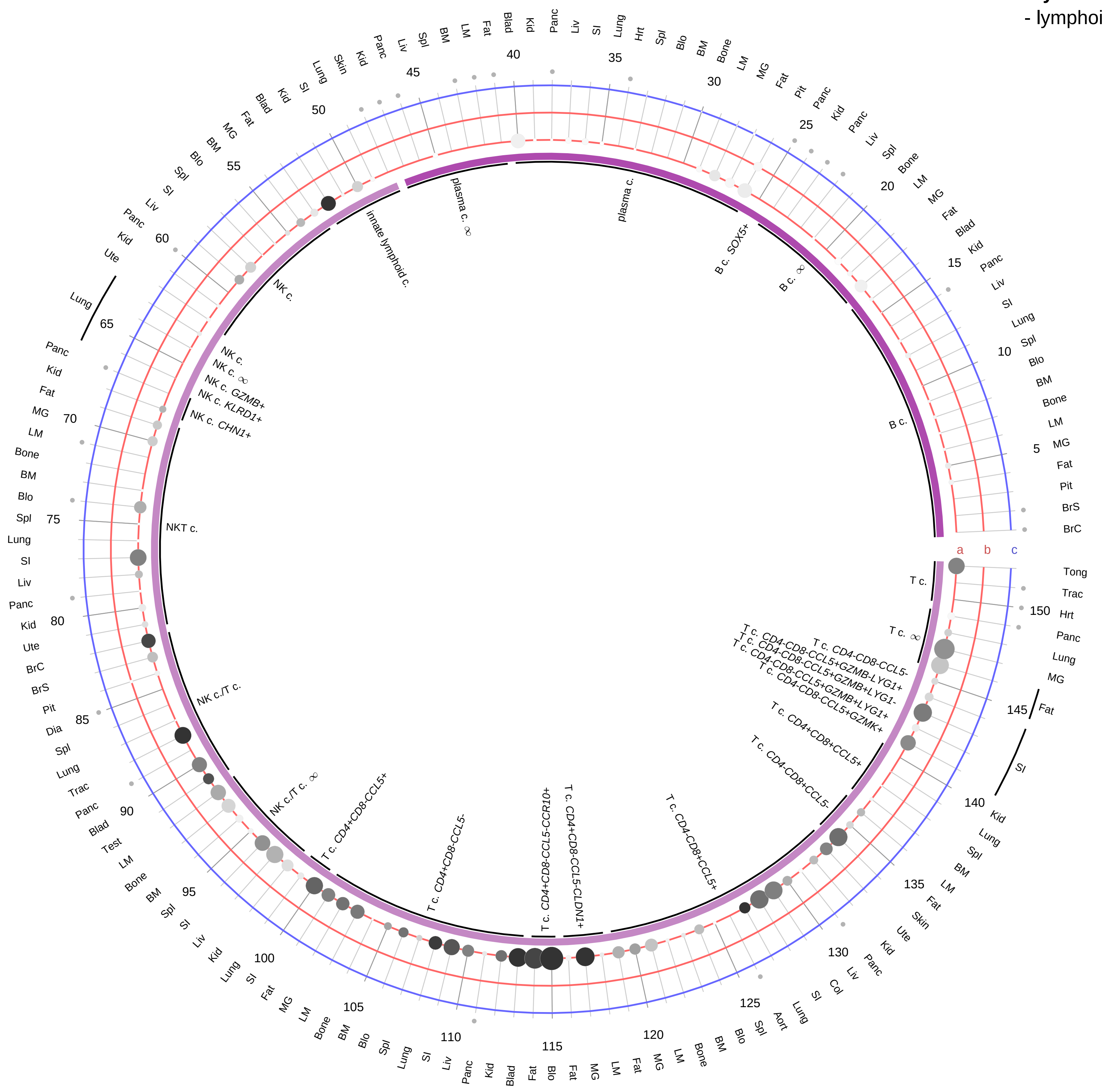

Compartment
lymphoid
- B cell
- NK/T cell

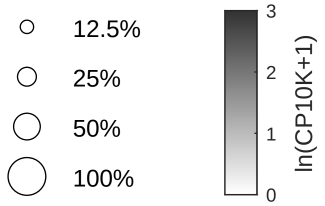

Abbrev.	Tissue
Aort	Aorta
Blad	Bladder
Blo	Blood
Bone	Bone
BM	Bone marrow
BrC	Brain cortex
BrS	Brainstem
Col	Colon
Dia	Diaphragm
Ret	Eye retina
Fat	Fat
Hrt	Heart
Pit	Hypothalamus/Pituitary
Kid	Kidney
LM	Limb muscle
Liv	Liver
Lung	Lung
MG	Mammary gland
Panc	Pancreas
Skin	Skin
SI	Small intestine
Spl	Spleen
Test	Testes
Thym	Thymus
Tong	Tongue
Trac	Trachea
Ute	Uterus

Thyroid stimulating hormone (TSH)
- myeloid

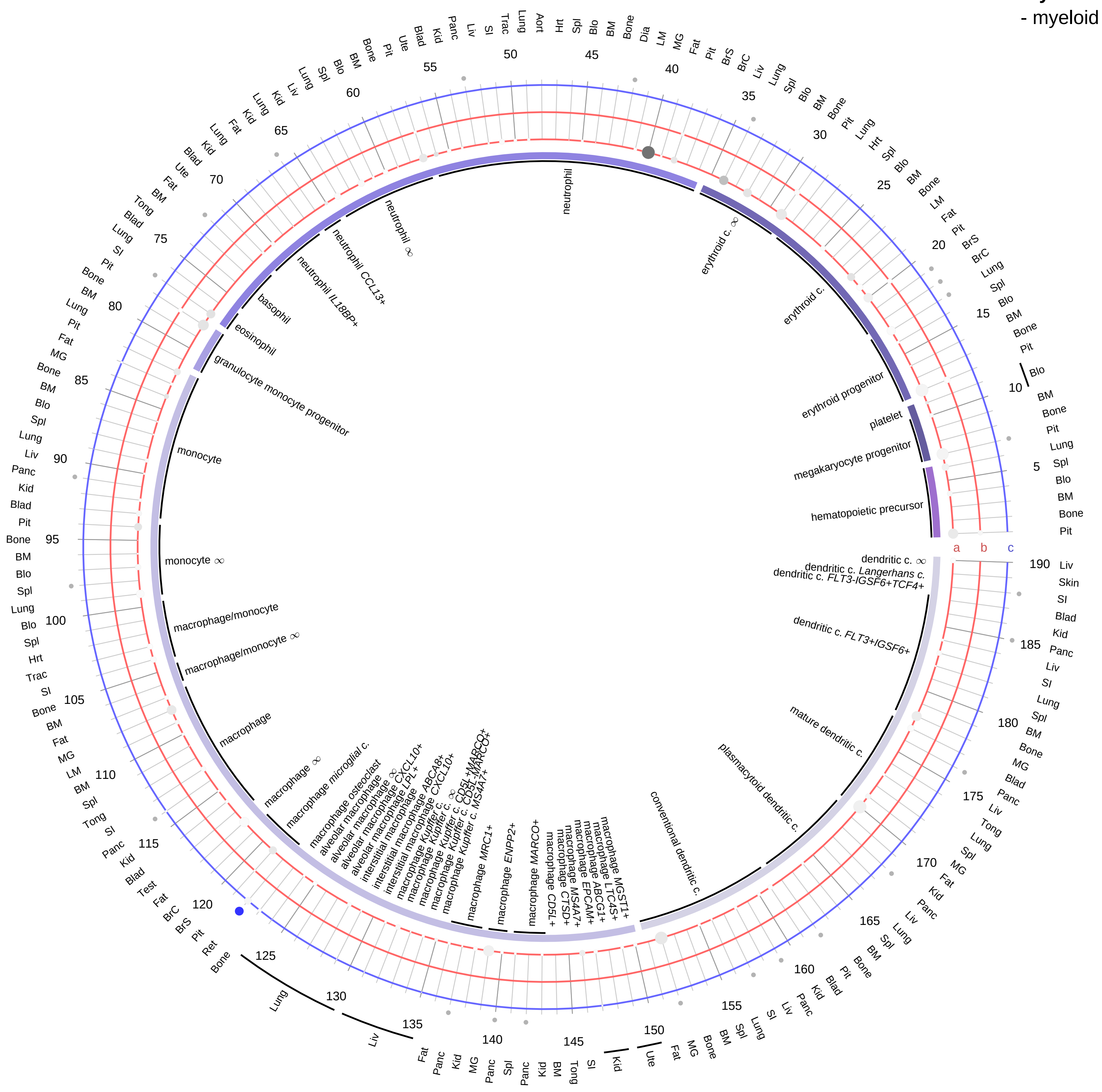

Ligand/Enzyme
a - CGA
b - TSHB
Receptor
c - TSHR

PAIRING (ligand → receptor)

TSHB & CGA→TSHR

Abbrev.	Tissue
Aort	Aorta
Blad	Bladder
Blo	Blood
Bone	Bone
BM	Bone marrow
BrC	Brain cortex
BrS	Brainstem
Col	Colon
Dia	Diaphragm
Ret	Eye retina
Fat	Fat
Hrt	Heart
Pit	Hypothalamus/Pituitary
Kid	Kidney
LM	Limb muscle
Liv	Liver
Lung	Lung
MG	Mammary gland
Panc	Pancreas
Skin	Skin
SI	Small intestine
Spl	Spleen
Test	Testes
Thym	Thymus
Tong	Tongue
Trac	Trachea
Ute	Uterus

Compartment
hematopoietic
- hematopoietic precursor
megakaryocyte-erythroid
- megakaryoid
- erythroid
myeloid
- granulocyte
- granulocyte monocyte progenitor
- monocyte/macrophage
- dendritic

12.5%
25%
50%
100%

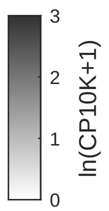

Thyrotropin-releasing hormone (TRH)
- epithelial/neural/germ

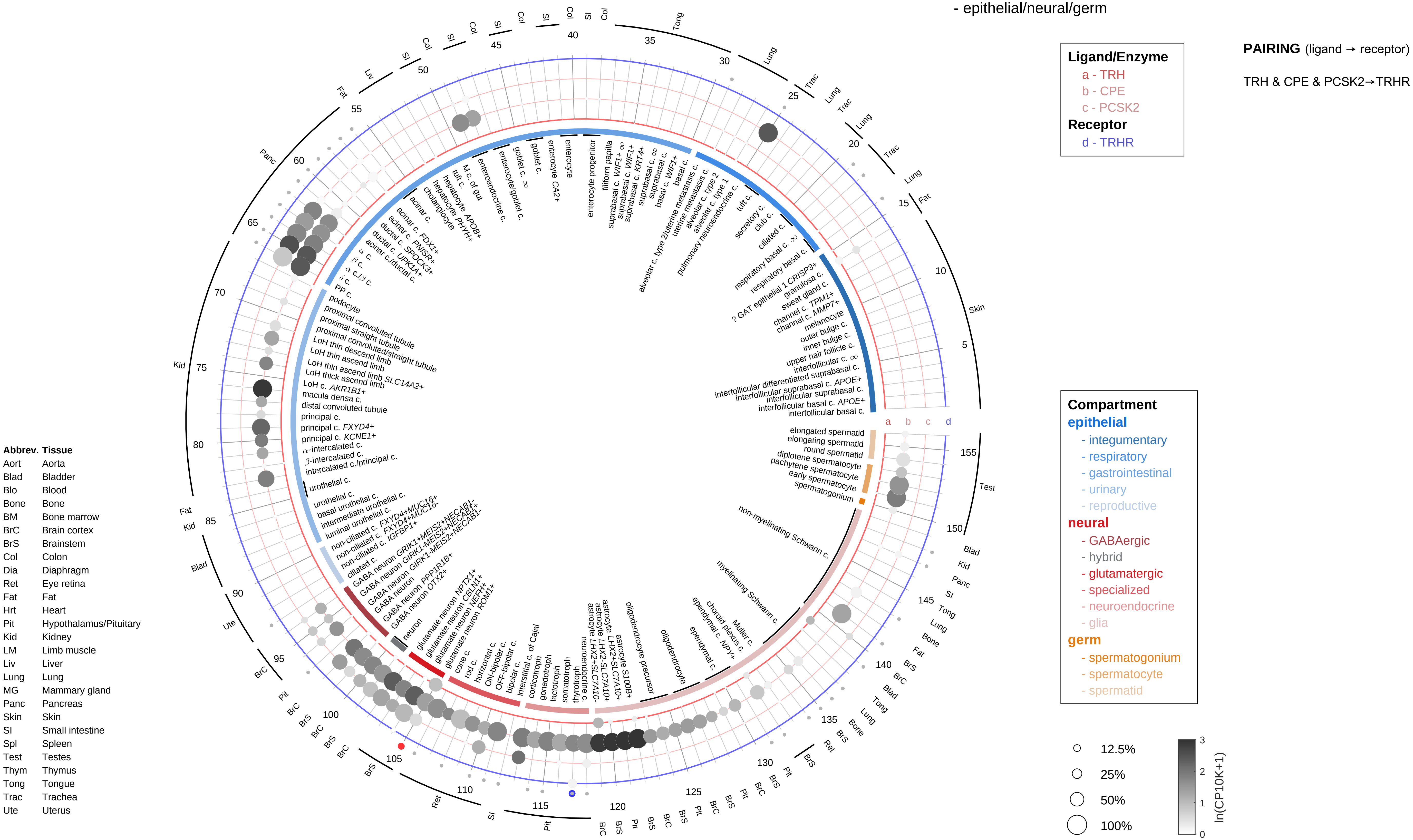

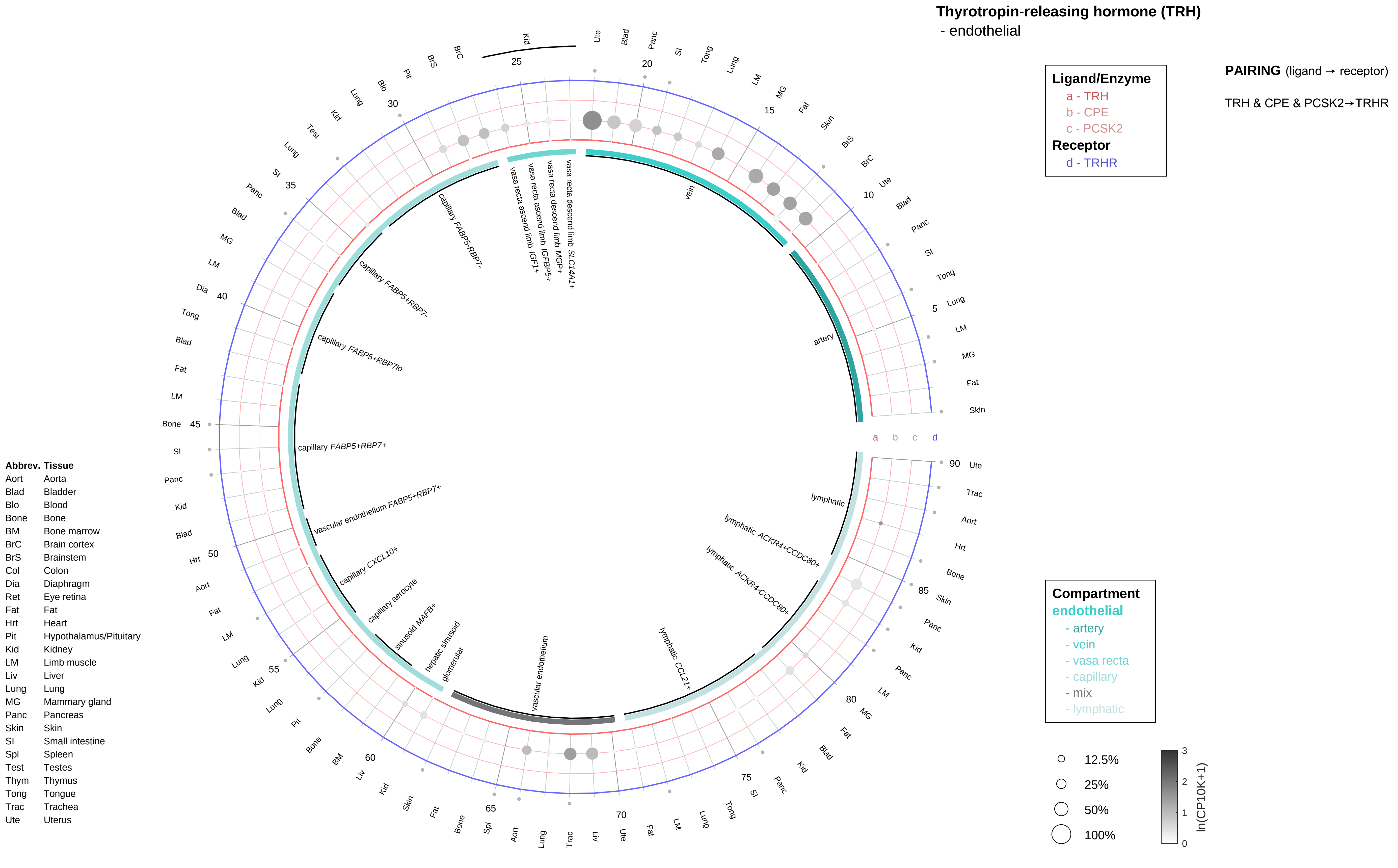

Thyrotropin-releasing hormone (TRH)
- endothelial
PAIRING (ligand → receptor)
TRH & CPE & PCSK2→TRHR
Ligand/Enzyme
a - TRH
b - CPE
c - PCSK2
Receptor
d - TRHR
a b c d
Compartment
endothelial
- artery
- vein
- vasa recta
- capillary
- mix
- lymphatic
12.5%
25%
50%
100%
ln(CP10K+1)
0
1
2
3
artery
vein
vasa recta descend limb SLC14A1+
vasa recta descend limb MGP+
vasa recta ascend limb IGFBP5+
vasa recta ascend limb IGF1+
capillary FABP5-RBP7-
capillary FABP5+RBP7-
capillary FABP5+RBP7lo
capillary FABP5+RBP7+
vascular endothelium FABP5+RBP7+
capillary CXCL10+
capillary aerocyte
sinusoid MAFB+
hepatic sinusoid
glomerular
vascular endothelium
lymphatic CCL21+
lymphatic ACKR4-CCDC80+
lymphatic ACKR4+CCDC80+
lymphatic
Abbrev. Tissue
Aort Aorta
Blad Bladder
Blo Blood
Bone Bone
BM Bone marrow
BrC Brain cortex
BrS Brainstem
Col Colon
Dia Diaphragm
Ret Eye retina
Fat Fat
Hrt Heart
Pit Hypothalamus/Pituitary
Kid Kidney
LM Limb muscle
Liv Liver
Lung Lung
MG Mammary gland
Panc Pancreas
Skin Skin
SI Small intestine
Spl Spleen
Test Testes
Thym Thymus
Tong Tongue
Trac Trachea
Ute Uterus

Thyrotropin-releasing hormone (TRH)
- stromal

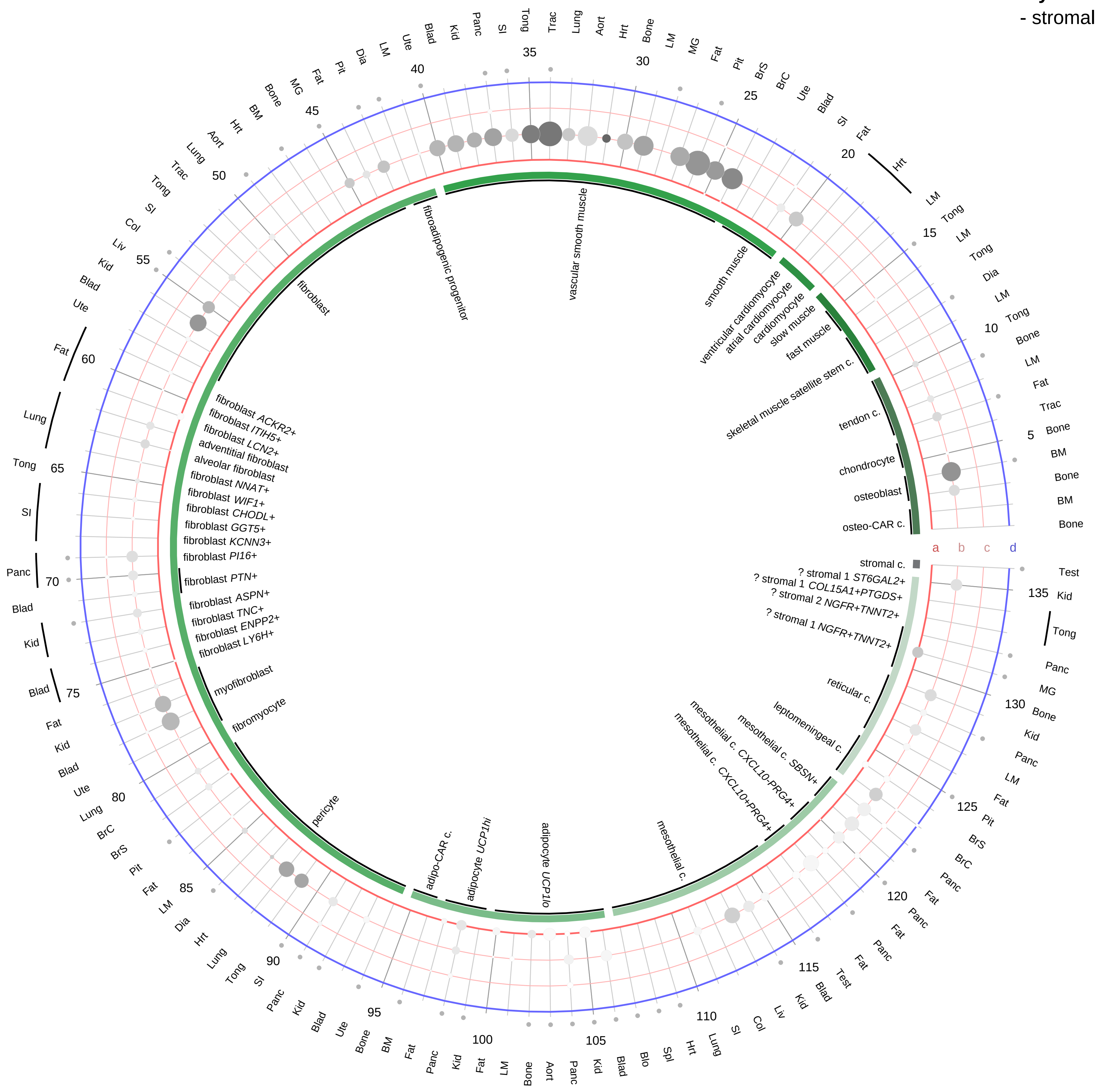

Ligand/Enzyme
- a - TRH
- b - CPE
- c - PCSK2

Receptor
- d - TRHR

PAIRING (ligand → receptor)

TRH & CPE & PCSK2→TRHR

Compartment
stromal
- bone
- skeletal muscle
- cardiac muscle
- smooth muscle
- fibroblast
- adipocyte
- mesothelial
- other
- mix

12.5%
25%
50%
100%

ln(CP10K+1): 0, 1, 2, 3

Abbrev.	Tissue
Aort	Aorta
Blad	Bladder
Blo	Blood
Bone	Bone
BM	Bone marrow
BrC	Brain cortex
BrS	Brainstem
Col	Colon
Dia	Diaphragm
Ret	Eye retina
Fat	Fat
Hrt	Heart
Pit	Hypothalamus/Pituitary
Kid	Kidney
LM	Limb muscle
Liv	Liver
Lung	Lung
MG	Mammary gland
Panc	Pancreas
Skin	Skin
SI	Small intestine
Spl	Spleen
Test	Testes
Thym	Thymus
Tong	Tongue
Trac	Trachea
Ute	Uterus

Thyrotropin-releasing hormone (TRH)
- lymphoid

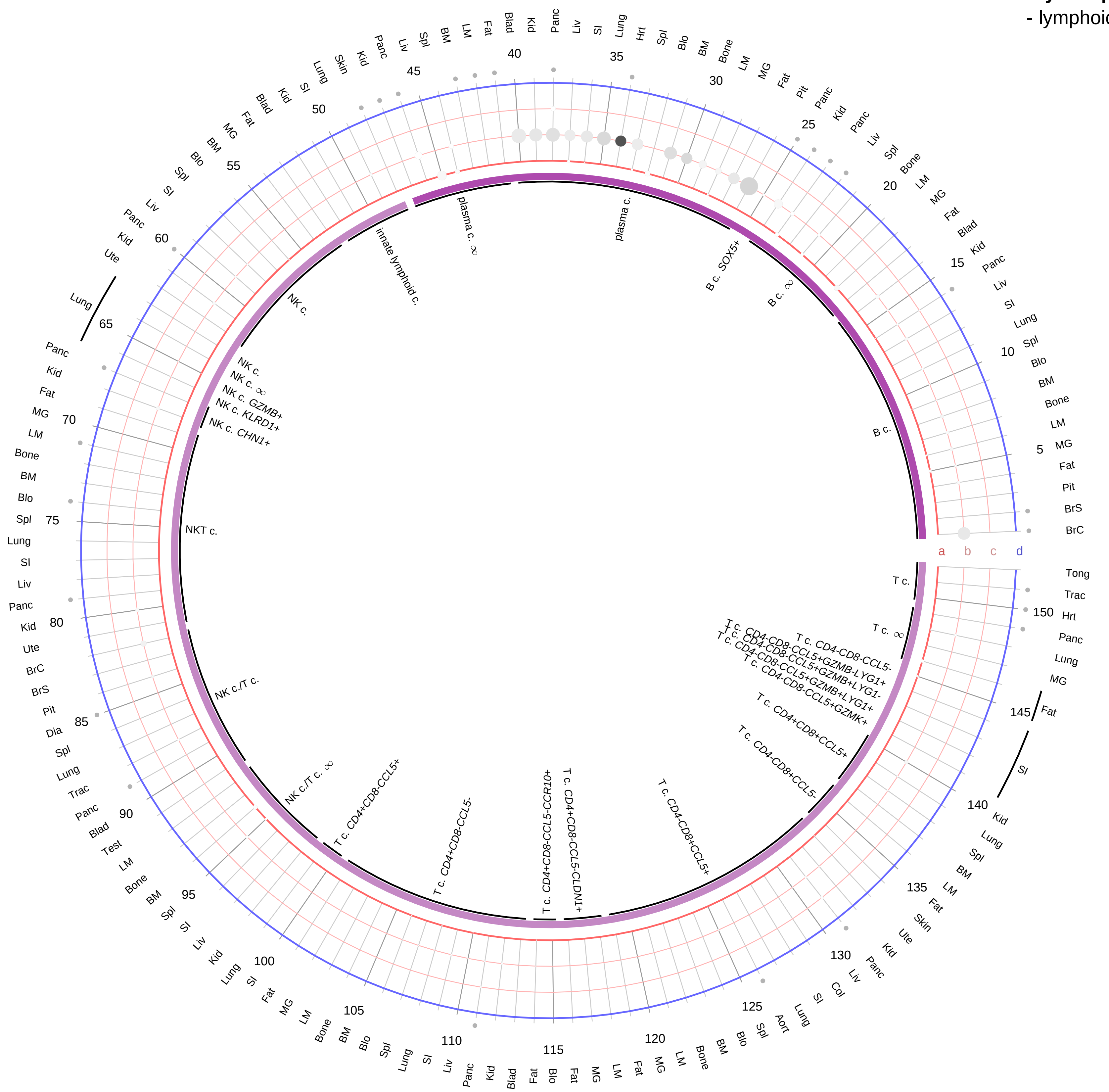

Ligand/Enzyme
- a - TRH
- b - CPE
- c - PCSK2

Receptor
- d - TRHR

PAIRING (ligand → receptor)

TRH & CPE & PCSK2→TRHR

Compartment

lymphoid
- B cell
- NK/T cell

12.5%
25%
50%
100%

ln(CP10K+1): 0, 1, 2, 3

Abbrev.	Tissue
Aort	Aorta
Blad	Bladder
Blo	Blood
Bone	Bone
BM	Bone marrow
BrC	Brain cortex
BrS	Brainstem
Col	Colon
Dia	Diaphragm
Ret	Eye retina
Fat	Fat
Hrt	Heart
Pit	Hypothalamus/Pituitary
Kid	Kidney
LM	Limb muscle
Liv	Liver
Lung	Lung
MG	Mammary gland
Panc	Pancreas
Skin	Skin
SI	Small intestine
Spl	Spleen
Test	Testes
Thym	Thymus
Tong	Tongue
Trac	Trachea
Ute	Uterus

Thyrotropin-releasing hormone (TRH)
- myeloid

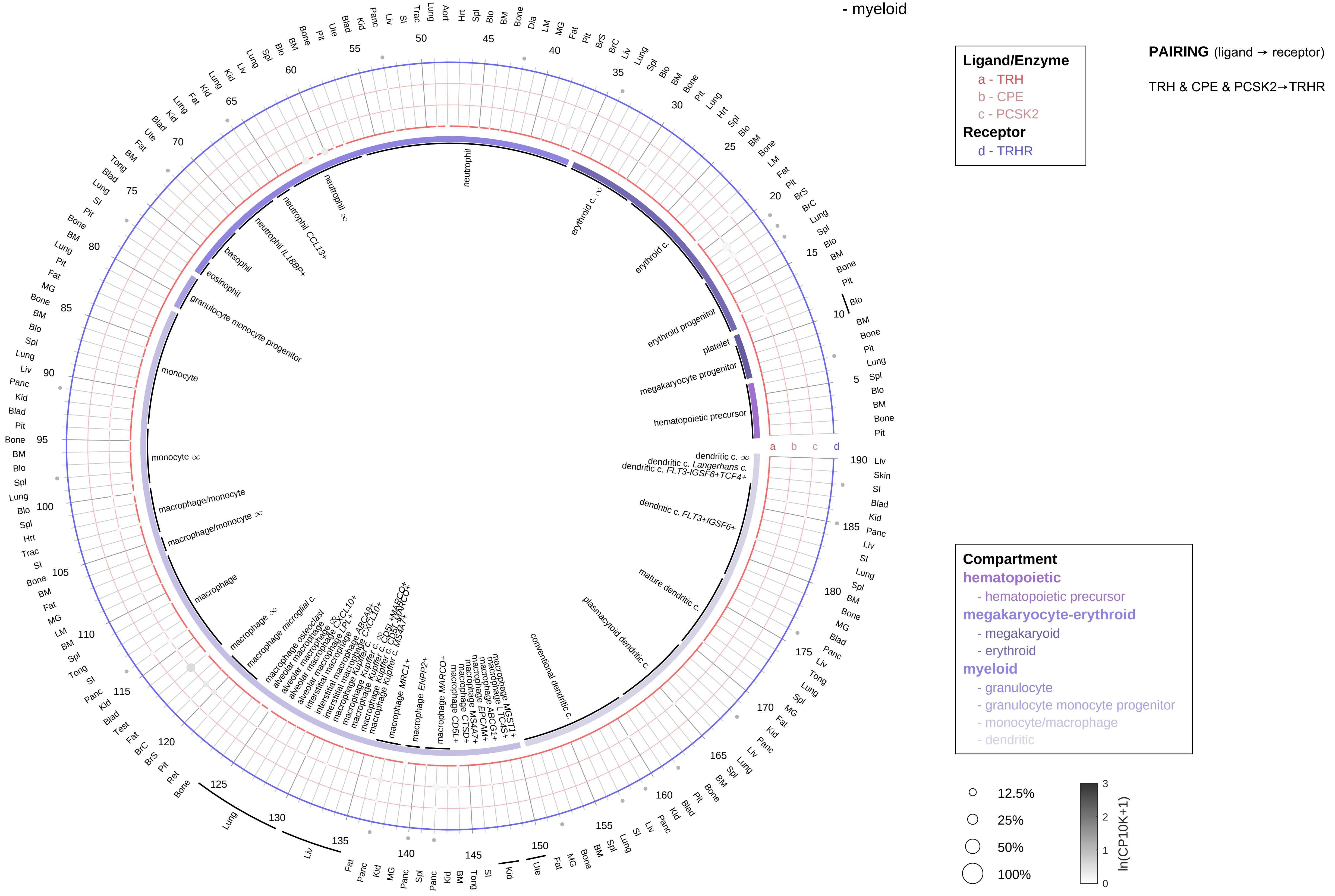

Abbrev.	Tissue
Aort	Aorta
Blad	Bladder
Blo	Blood
Bone	Bone
BM	Bone marrow
BrC	Brain cortex
BrS	Brainstem
Col	Colon
Dia	Diaphragm
Ret	Eye retina
Fat	Fat
Hrt	Heart
Pit	Hypothalamus/Pituitary
Kid	Kidney
LM	Limb muscle
Liv	Liver
Lung	Lung
MG	Mammary gland
Panc	Pancreas
Skin	Skin
SI	Small intestine
Spl	Spleen
Test	Testes
Thym	Thymus
Tong	Tongue
Trac	Trachea
Ute	Uterus

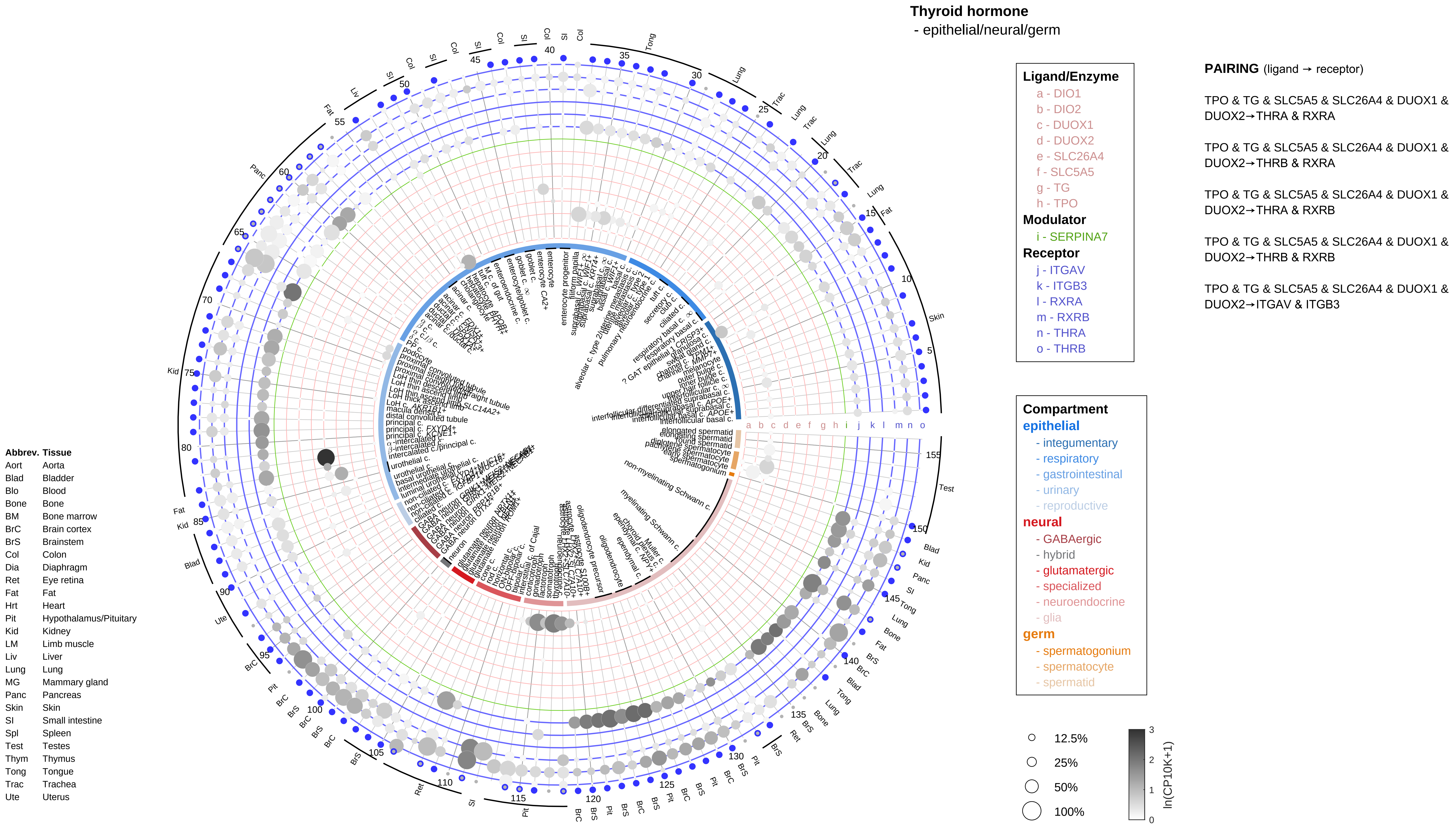

Thyroid hormone
- epithelial/neural/germ
Ligand/Enzyme
a - DIO1
b - DIO2
c - DUOX1
d - DUOX2
e - SLC26A4
f - SLC5A5
g - TG
h - TPO
Modulator
i - SERPINA7
Receptor
j - ITGAV
k - ITGB3
l - RXRA
m - RXRB
n - THRA
o - THRB
PAIRING (ligand → receptor)
TPO & TG & SLC5A5 & SLC26A4 & DUOX1 & DUOX2→THRA & RXRA
TPO & TG & SLC5A5 & SLC26A4 & DUOX1 & DUOX2→THRB & RXRA
TPO & TG & SLC5A5 & SLC26A4 & DUOX1 & DUOX2→THRA & RXRB
TPO & TG & SLC5A5 & SLC26A4 & DUOX1 & DUOX2→THRB & RXRB
TPO & TG & SLC5A5 & SLC26A4 & DUOX1 & DUOX2→ITGAV & ITGB3
Compartment
epithelial
- integumentary
- respiratory
- gastrointestinal
- urinary
- reproductive
neural
- GABAergic
- hybrid
- glutamatergic
- specialized
- neuroendocrine
- glia
germ
- spermatogonium
- spermatocyte
- spermatid
12.5%
25%
50%
100%
ln(CP10K+1)
0
1
2
3
Abbrev. Tissue
Aort Aorta
Blad Bladder
Blo Blood
Bone Bone
BM Bone marrow
BrC Brain cortex
BrS Brainstem
Col Colon
Dia Diaphragm
Ret Eye retina
Fat Fat
Hrt Heart
Pit Hypothalamus/Pituitary
Kid Kidney
LM Limb muscle
Liv Liver
Lung Lung
MG Mammary gland
Panc Pancreas
Skin Skin
SI Small intestine
Spl Spleen
Test Testes
Thym Thymus
Tong Tongue
Trac Trachea
Ute Uterus

Thyroid hormone
- endothelial

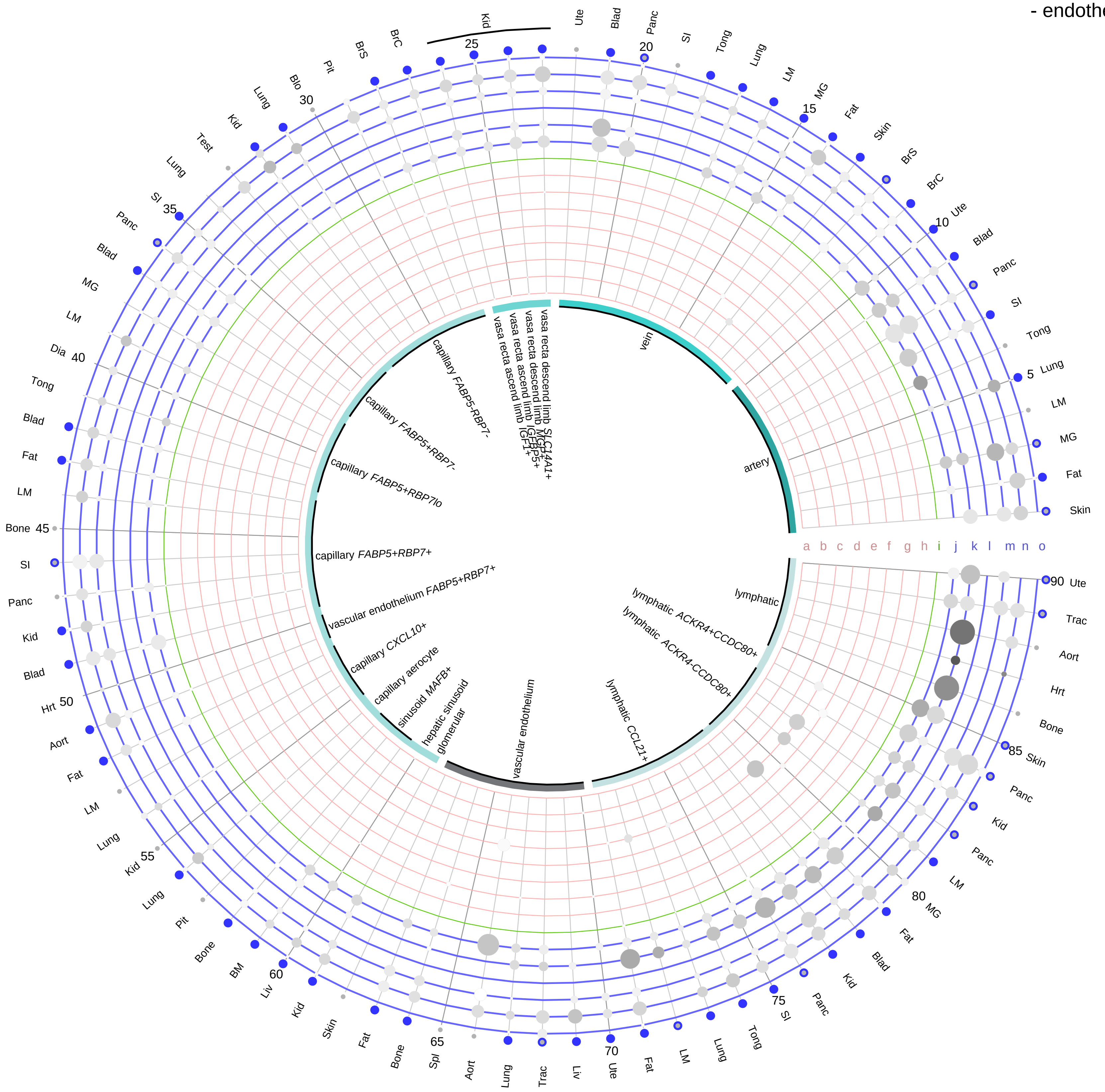

Ligand/Enzyme
- a - DIO1
- b - DIO2
- c - DUOX1
- d - DUOX2
- e - SLC26A4
- f - SLC5A5
- g - TG
- h - TPO

Modulator
- i - SERPINA7

Receptor
- j - ITGAV
- k - ITGB3
- l - RXRA
- m - RXRB
- n - THRA
- o - THRB

PAIRING (ligand → receptor)

TPO & TG & SLC5A5 & SLC26A4 & DUOX1 & DUOX2→THRA & RXRA

TPO & TG & SLC5A5 & SLC26A4 & DUOX1 & DUOX2→THRB & RXRA

TPO & TG & SLC5A5 & SLC26A4 & DUOX1 & DUOX2→THRA & RXRB

TPO & TG & SLC5A5 & SLC26A4 & DUOX1 & DUOX2→THRB & RXRB

TPO & TG & SLC5A5 & SLC26A4 & DUOX1 & DUOX2→ITGAV & ITGB3

Compartment
endothelial
- artery
- vein
- vasa recta
- capillary
- mix
- lymphatic

12.5%
25%
50%
100%

ln(CP10K+1): 0, 1, 2, 3

Abbrev.	Tissue
Aort	Aorta
Blad	Bladder
Blo	Blood
Bone	Bone
BM	Bone marrow
BrC	Brain cortex
BrS	Brainstem
Col	Colon
Dia	Diaphragm
Ret	Eye retina
Fat	Fat
Hrt	Heart
Pit	Hypothalamus/Pituitary
Kid	Kidney
LM	Limb muscle
Liv	Liver
Lung	Lung
MG	Mammary gland
Panc	Pancreas
Skin	Skin
SI	Small intestine
Spl	Spleen
Test	Testes
Thym	Thymus
Tong	Tongue
Trac	Trachea
Ute	Uterus

Thyroid hormone
- stromal

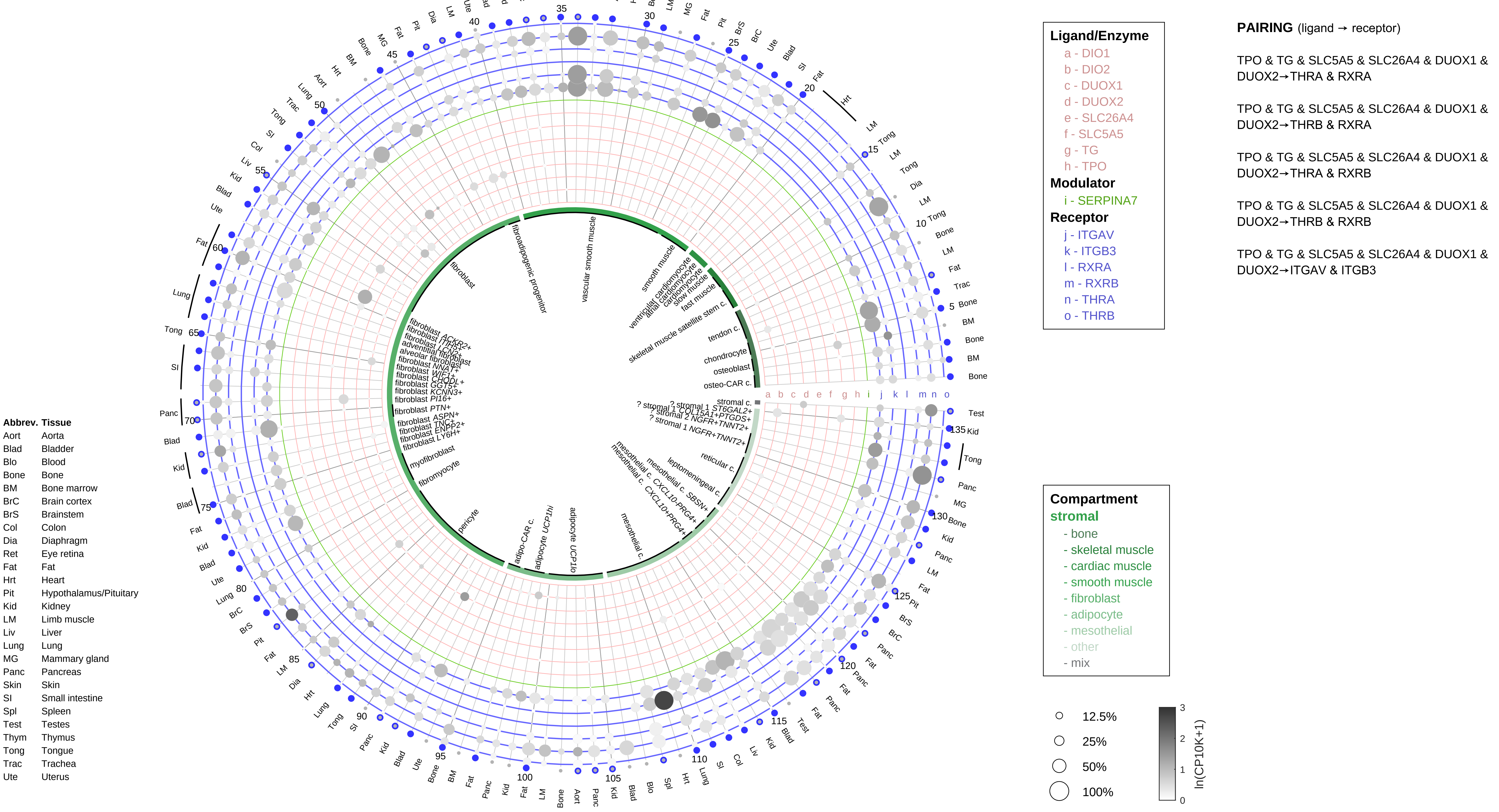

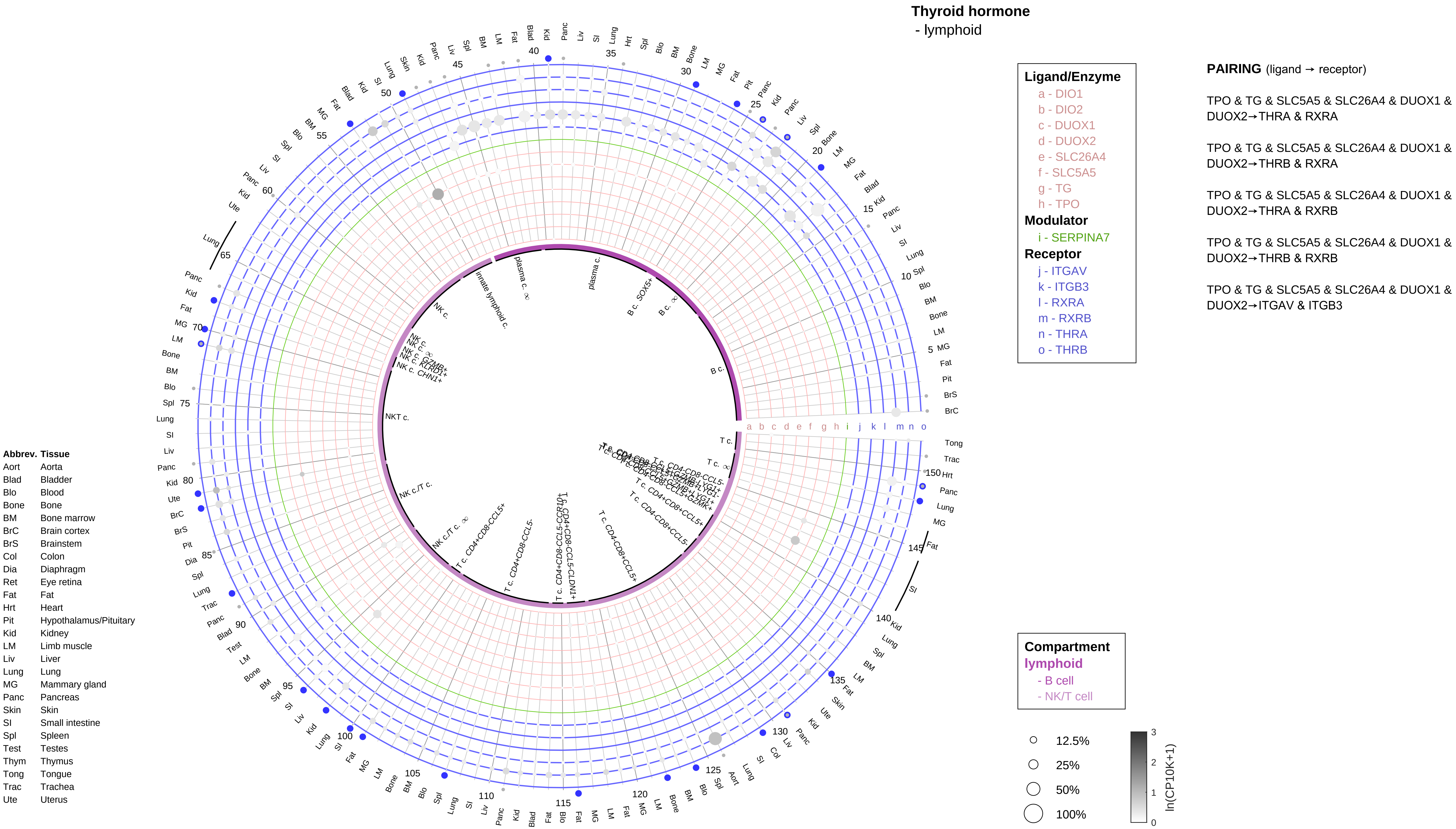
Thyroid hormone
- lymphoid
Ligand/Enzyme
a - DIO1
b - DIO2
c - DUOX1
d - DUOX2
e - SLC26A4
f - SLC5A5
g - TG
h - TPO
Modulator
i - SERPINA7
Receptor
j - ITGAV
k - ITGB3
l - RXRA
m - RXRB
n - THRA
o - THRB
PAIRING (ligand → receptor)
TPO & TG & SLC5A5 & SLC26A4 & DUOX1 & DUOX2→THRA & RXRA
TPO & TG & SLC5A5 & SLC26A4 & DUOX1 & DUOX2→THRB & RXRA
TPO & TG & SLC5A5 & SLC26A4 & DUOX1 & DUOX2→THRA & RXRB
TPO & TG & SLC5A5 & SLC26A4 & DUOX1 & DUOX2→THRB & RXRB
TPO & TG & SLC5A5 & SLC26A4 & DUOX1 & DUOX2→ITGAV & ITGB3
Compartment
lymphoid
- B cell
- NK/T cell
12.5%
25%
50%
100%
ln(CP10K+1)
0
1
2
3
Abbrev. Tissue
Aort Aorta
Blad Bladder
Blo Blood
Bone Bone
BM Bone marrow
BrC Brain cortex
BrS Brainstem
Col Colon
Dia Diaphragm
Ret Eye retina
Fat Fat
Hrt Heart
Pit Hypothalamus/Pituitary
Kid Kidney
LM Limb muscle
Liv Liver
Lung Lung
MG Mammary gland
Panc Pancreas
Skin Skin
SI Small intestine
Spl Spleen
Test Testes
Thym Thymus
Tong Tongue
Trac Trachea
Ute Uterus
plasma c.
B c. SOX5+
B c.
T c.
NKT c.
NK c.
NK/T c.
innate lymphoid c.

Thyroid hormone
- myeloid

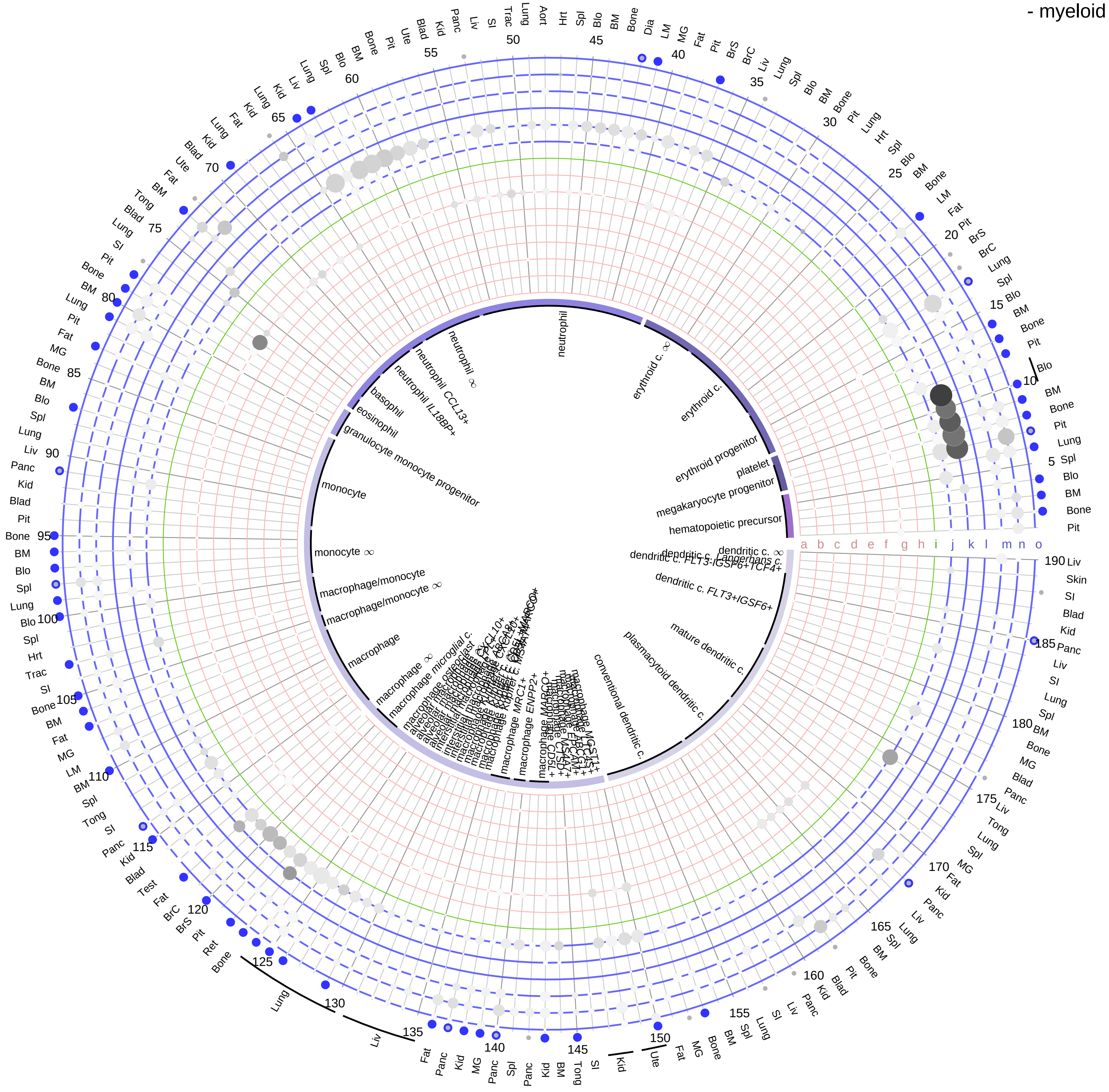

Ligand/Enzyme
a - DIO1
b - DIO2
c - DUOX1
d - DUOX2
e - SLC26A4
f - SLC5A5
g - TG
h - TPO

Modulator
i - SERPINA7

Receptor
j - ITGAV
k - ITGB3
l - RXRA
m - RXRB
n - THRA
o - THRB

PAIRING (ligand → receptor)

TPO & TG & SLC5A5 & SLC26A4 & DUOX1 & DUOX2→THRA & RXRA

TPO & TG & SLC5A5 & SLC26A4 & DUOX1 & DUOX2→THRB & RXRA

TPO & TG & SLC5A5 & SLC26A4 & DUOX1 & DUOX2→THRA & RXRB

TPO & TG & SLC5A5 & SLC26A4 & DUOX1 & DUOX2→THRB & RXRB

TPO & TG & SLC5A5 & SLC26A4 & DUOX1 & DUOX2→ITGAV & ITGB3

Compartment
hematopoietic
- hematopoietic precursor
megakaryocyte-erythroid
- megakaryoid
- erythroid
myeloid
- granulocyte
- granulocyte monocyte progenitor
- monocyte/macrophage
- dendritic

12.5%
25%
50%
100%

ln(CP10K+1): 0, 1, 2, 3

Abbrev.	Tissue
Aort	Aorta
Blad	Bladder
Blo	Blood
Bone	Bone
BM	Bone marrow
BrC	Brain cortex
BrS	Brainstem
Col	Colon
Dia	Diaphragm
Ret	Eye retina
Fat	Fat
Hrt	Heart
Pit	Hypothalamus/Pituitary
Kid	Kidney
LM	Limb muscle
Liv	Liver
Lung	Lung
MG	Mammary gland
Panc	Pancreas
Skin	Skin
SI	Small intestine
Spl	Spleen
Test	Testes
Thym	Thymus
Tong	Tongue
Trac	Trachea
Ute	Uterus

Urocortins
- epithelial/neural/germ

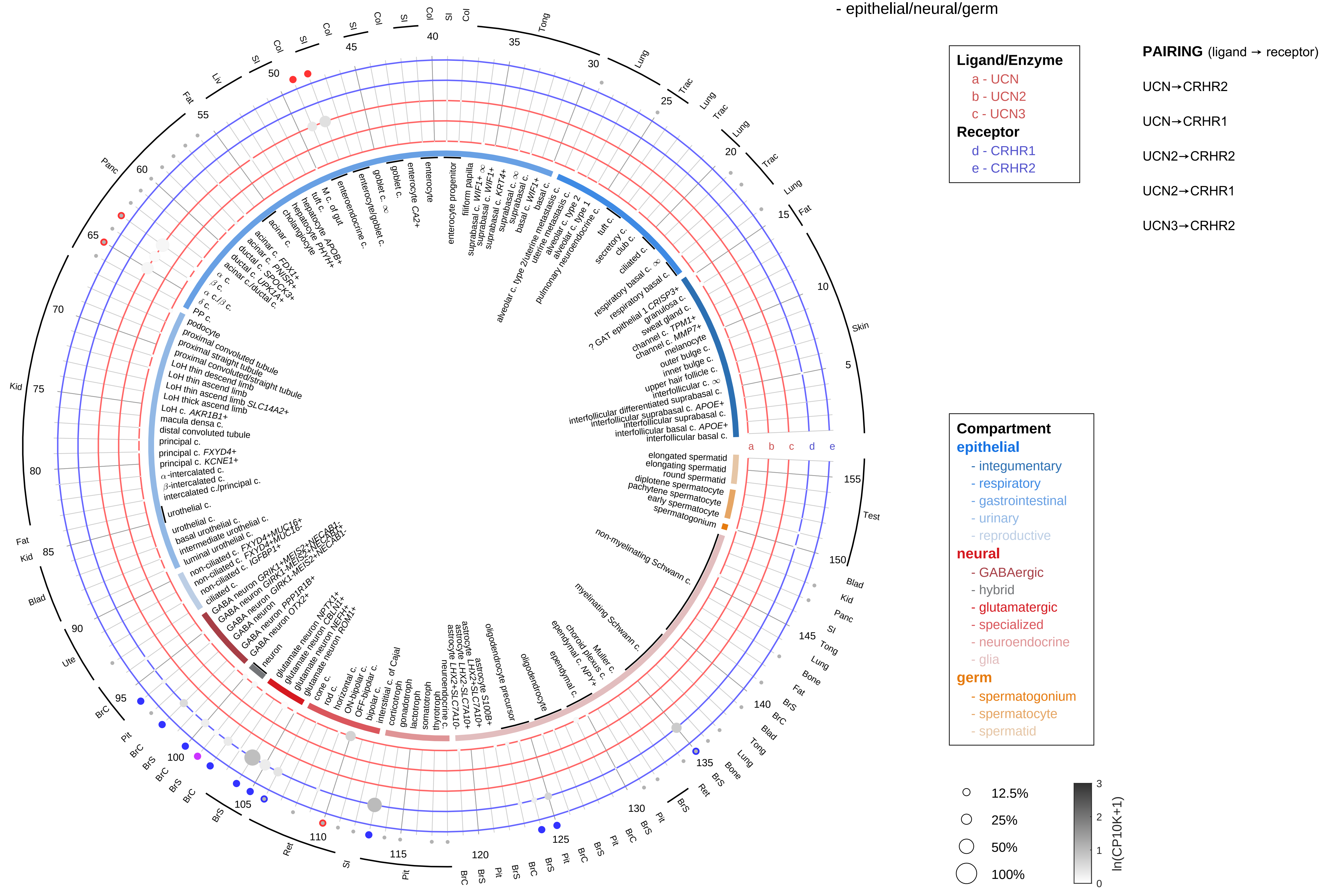

Abbrev.	Tissue
Aort	Aorta
Blad	Bladder
Blo	Blood
Bone	Bone
BM	Bone marrow
BrC	Brain cortex
BrS	Brainstem
Col	Colon
Dia	Diaphragm
Ret	Eye retina
Fat	Fat
Hrt	Heart
Pit	Hypothalamus/Pituitary
Kid	Kidney
LM	Limb muscle
Liv	Liver
Lung	Lung
MG	Mammary gland
Panc	Pancreas
Skin	Skin
SI	Small intestine
Spl	Spleen
Test	Testes
Thym	Thymus
Tong	Tongue
Trac	Trachea
Ute	Uterus

Urocortins
- endothelial

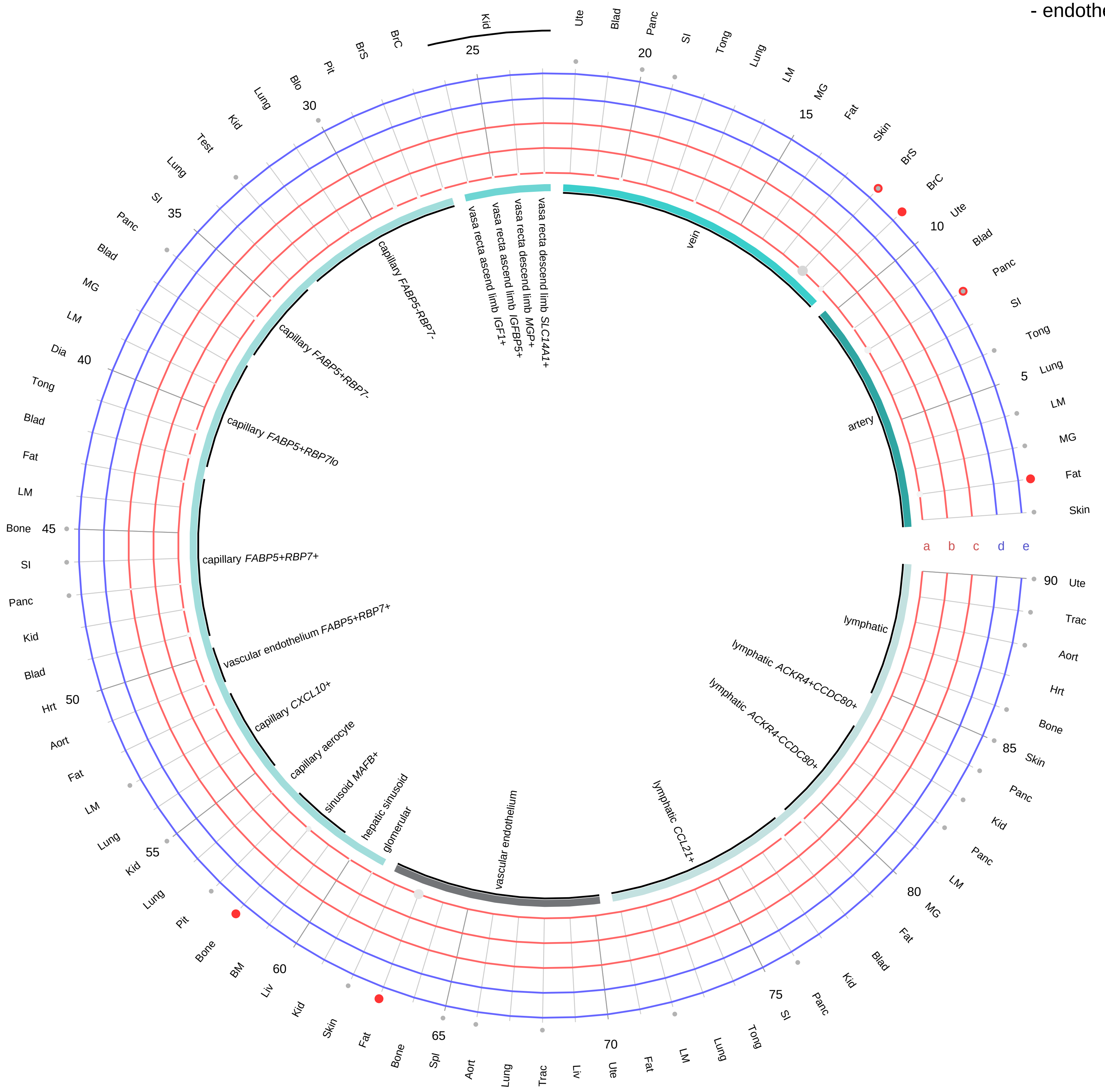

Ligand/Enzyme
- a - UCN
- b - UCN2
- c - UCN3

Receptor
- d - CRHR1
- e - CRHR2

PAIRING (ligand → receptor)

UCN→CRHR2

UCN→CRHR1

UCN2→CRHR2

UCN2→CRHR1

UCN3→CRHR2

Abbrev.	Tissue
Aort	Aorta
Blad	Bladder
Blo	Blood
Bone	Bone
BM	Bone marrow
BrC	Brain cortex
BrS	Brainstem
Col	Colon
Dia	Diaphragm
Ret	Eye retina
Fat	Fat
Hrt	Heart
Pit	Hypothalamus/Pituitary
Kid	Kidney
LM	Limb muscle
Liv	Liver
Lung	Lung
MG	Mammary gland
Panc	Pancreas
Skin	Skin
SI	Small intestine
Spl	Spleen
Test	Testes
Thym	Thymus
Tong	Tongue
Trac	Trachea
Ute	Uterus

Compartment

endothelial
- artery
- vein
- vasa recta
- capillary
- mix
- lymphatic

12.5%
25%
50%
100%

ln(CP10K+1): 0, 1, 2, 3

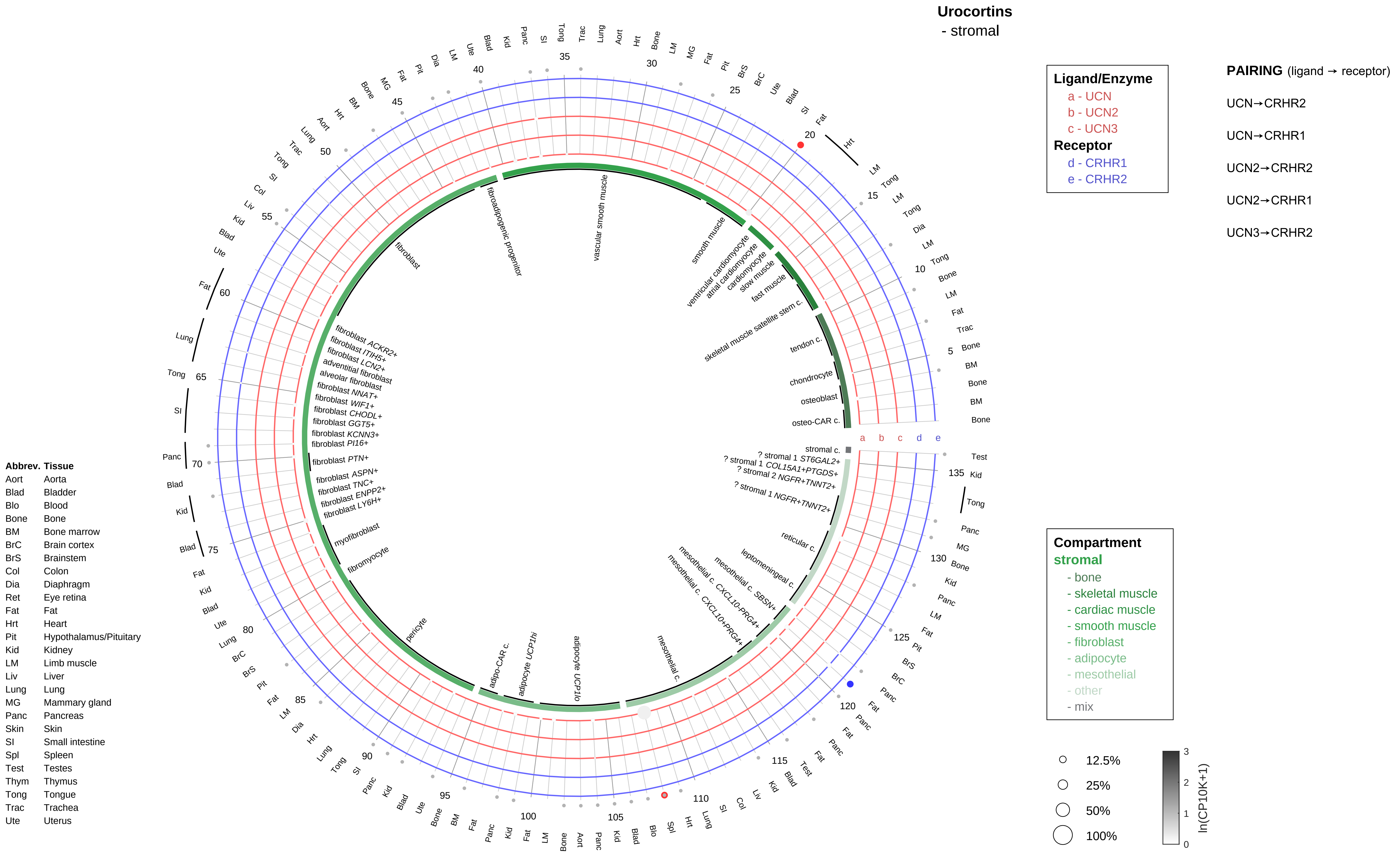

Urocortins
- stromal
Ligand/Enzyme
a - UCN
b - UCN2
c - UCN3
Receptor
d - CRHR1
e - CRHR2
PAIRING (ligand → receptor)
UCN→CRHR2
UCN→CRHR1
UCN2→CRHR2
UCN2→CRHR1
UCN3→CRHR2
Compartment
stromal
- bone
- skeletal muscle
- cardiac muscle
- smooth muscle
- fibroblast
- adipocyte
- mesothelial
- other
- mix
12.5%
25%
50%
100%
ln(CP10K+1)
Abbrev. Tissue
Aort Aorta
Blad Bladder
Blo Blood
Bone Bone
BM Bone marrow
BrC Brain cortex
BrS Brainstem
Col Colon
Dia Diaphragm
Ret Eye retina
Fat Fat
Hrt Heart
Pit Hypothalamus/Pituitary
Kid Kidney
LM Limb muscle
Liv Liver
Lung Lung
MG Mammary gland
Panc Pancreas
Skin Skin
SI Small intestine
Spl Spleen
Test Testes
Thym Thymus
Tong Tongue
Trac Trachea
Ute Uterus

Urocortins
- lymphoid

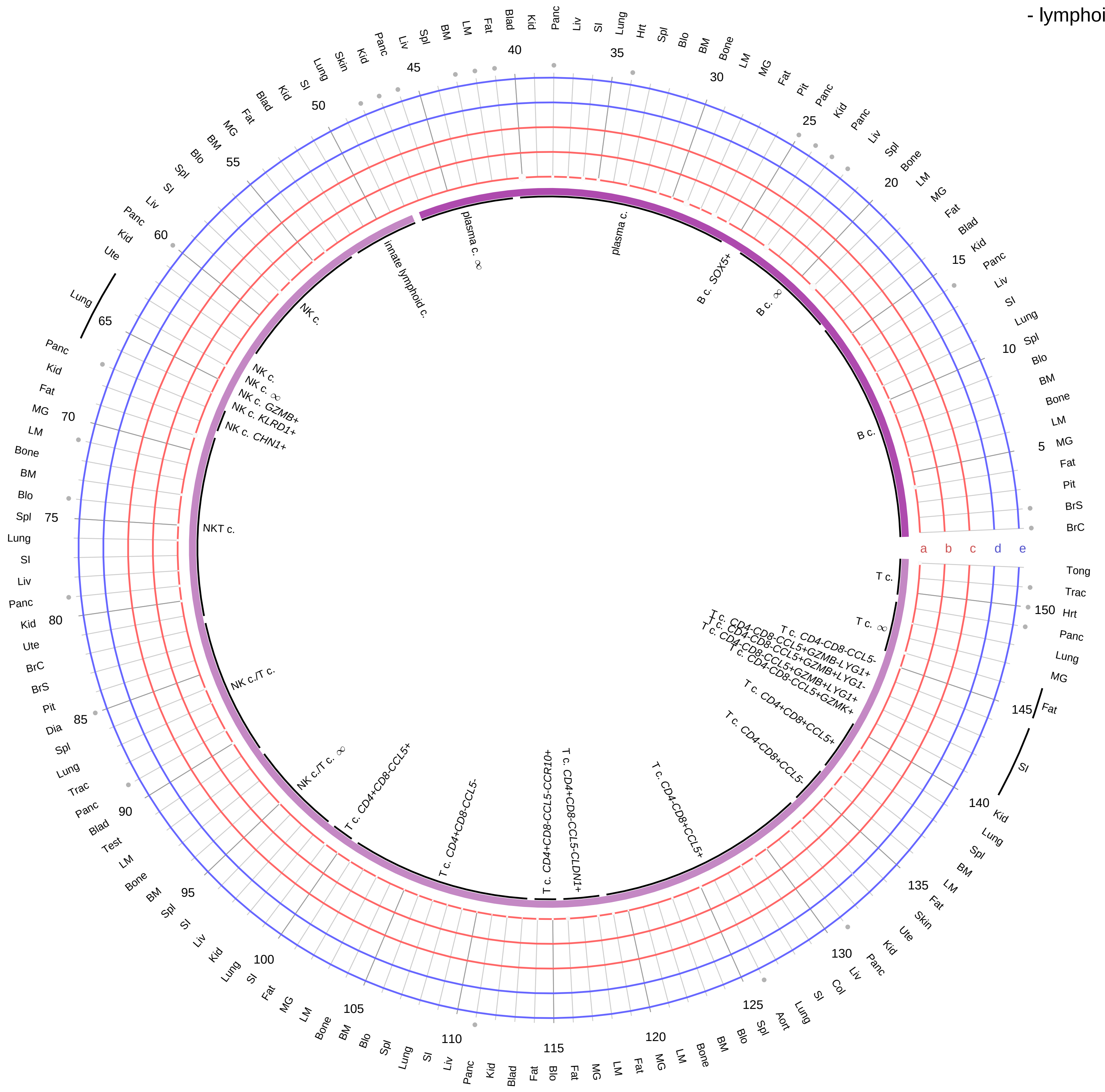

Ligand/Enzyme
- a - UCN
- b - UCN2
- c - UCN3

Receptor
- d - CRHR1
- e - CRHR2

PAIRING (ligand → receptor)

UCN→CRHR2

UCN→CRHR1

UCN2→CRHR2

UCN2→CRHR1

UCN3→CRHR2

**Abbrev.**	**Tissue**
Aort	Aorta
Blad	Bladder
Blo	Blood
Bone	Bone
BM	Bone marrow
BrC	Brain cortex
BrS	Brainstem
Col	Colon
Dia	Diaphragm
Ret	Eye retina
Fat	Fat
Hrt	Heart
Pit	Hypothalamus/Pituitary
Kid	Kidney
LM	Limb muscle
Liv	Liver
Lung	Lung
MG	Mammary gland
Panc	Pancreas
Skin	Skin
SI	Small intestine
Spl	Spleen
Test	Testes
Thym	Thymus
Tong	Tongue
Trac	Trachea
Ute	Uterus

Compartment
lymphoid
- B cell
- NK/T cell

12.5%
25%
50%
100%

ln(CP10K+1): 0, 1, 2, 3

Urocortins
- myeloid

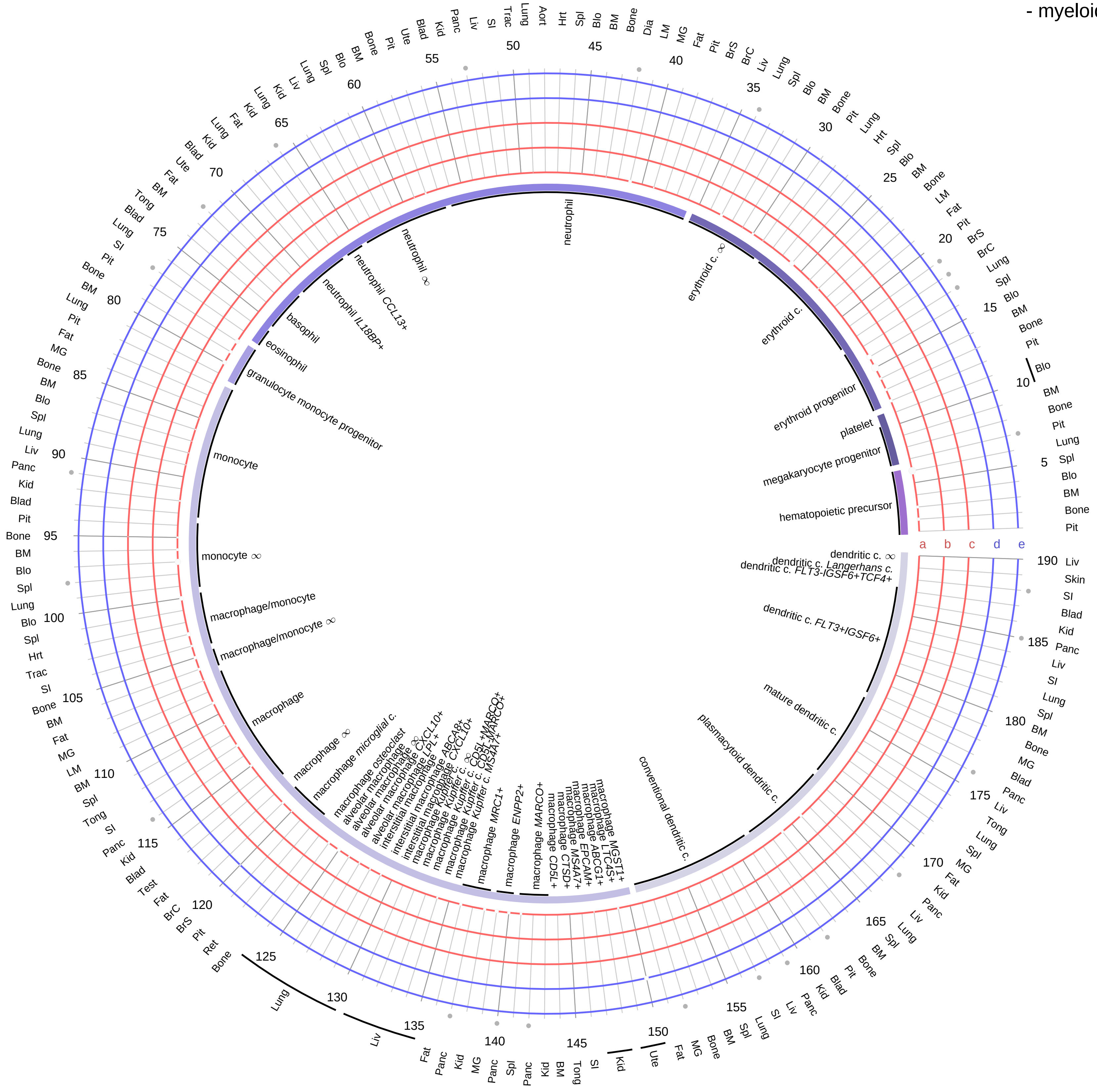

Ligand/Enzyme
- a - UCN
- b - UCN2
- c - UCN3

Receptor
- d - CRHR1
- e - CRHR2

PAIRING (ligand → receptor)

UCN→CRHR2

UCN→CRHR1

UCN2→CRHR2

UCN2→CRHR1

UCN3→CRHR2

**Abbrev.**	**Tissue**
Aort	Aorta
Blad	Bladder
Blo	Blood
Bone	Bone
BM	Bone marrow
BrC	Brain cortex
BrS	Brainstem
Col	Colon
Dia	Diaphragm
Ret	Eye retina
Fat	Fat
Hrt	Heart
Pit	Hypothalamus/Pituitary
Kid	Kidney
LM	Limb muscle
Liv	Liver
Lung	Lung
MG	Mammary gland
Panc	Pancreas
Skin	Skin
SI	Small intestine
Spl	Spleen
Test	Testes
Thym	Thymus
Tong	Tongue
Trac	Trachea
Ute	Uterus

Compartment

hematopoietic
- hematopoietic precursor

megakaryocyte-erythroid
- megakaryoid
- erythroid

myeloid
- granulocyte
- granulocyte monocyte progenitor
- monocyte/macrophage
- dendritic

12.5%
25%
50%
100%

ln(CP10K+1): 0, 1, 2, 3

Urotensin 2/2B
- epithelial/neural/germ

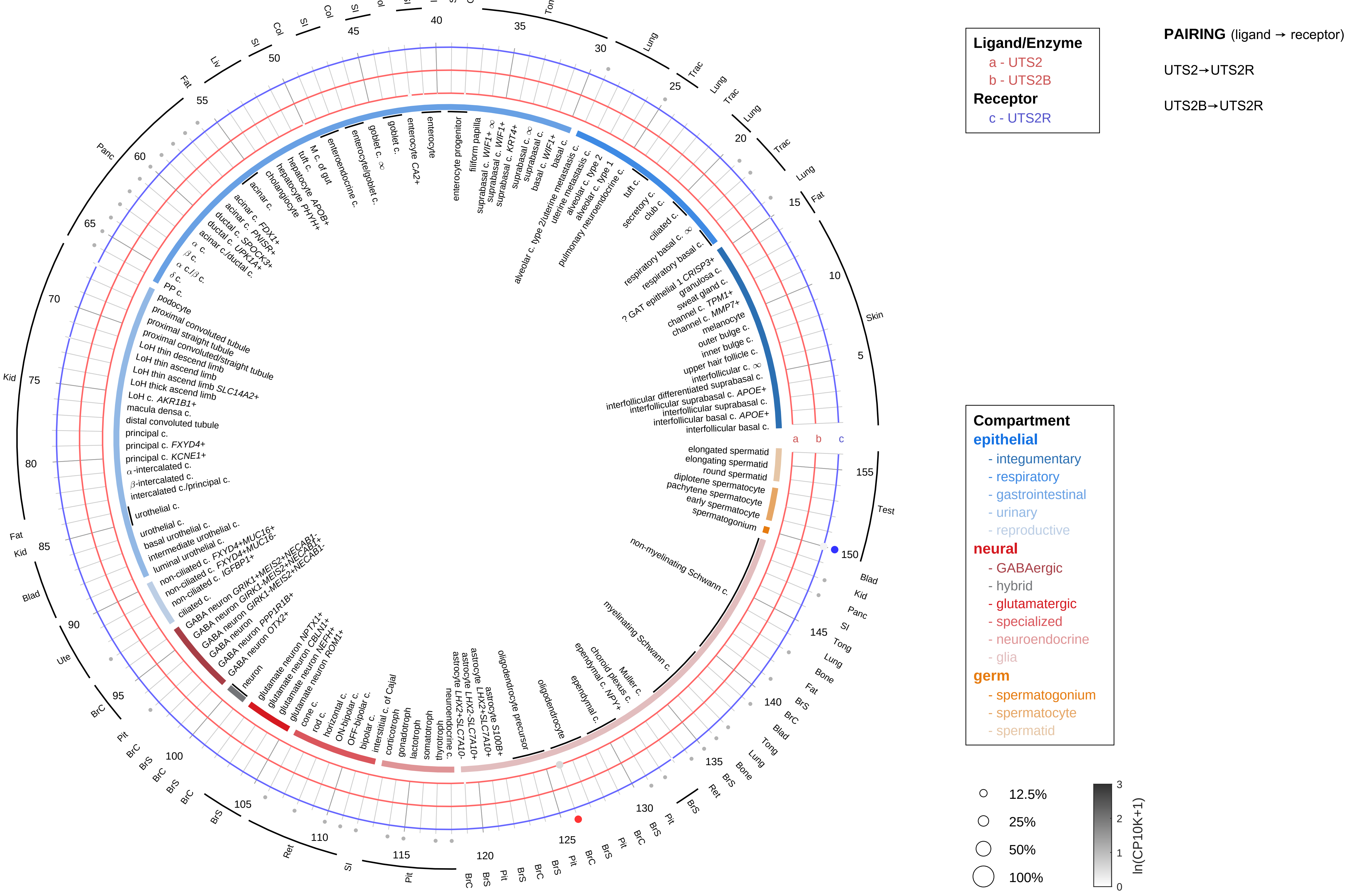

Abbrev.	Tissue
Aort	Aorta
Blad	Bladder
Blo	Blood
Bone	Bone
BM	Bone marrow
BrC	Brain cortex
BrS	Brainstem
Col	Colon
Dia	Diaphragm
Ret	Eye retina
Fat	Fat
Hrt	Heart
Pit	Hypothalamus/Pituitary
Kid	Kidney
LM	Limb muscle
Liv	Liver
Lung	Lung
MG	Mammary gland
Panc	Pancreas
Skin	Skin
SI	Small intestine
Spl	Spleen
Test	Testes
Thym	Thymus
Tong	Tongue
Trac	Trachea
Ute	Uterus

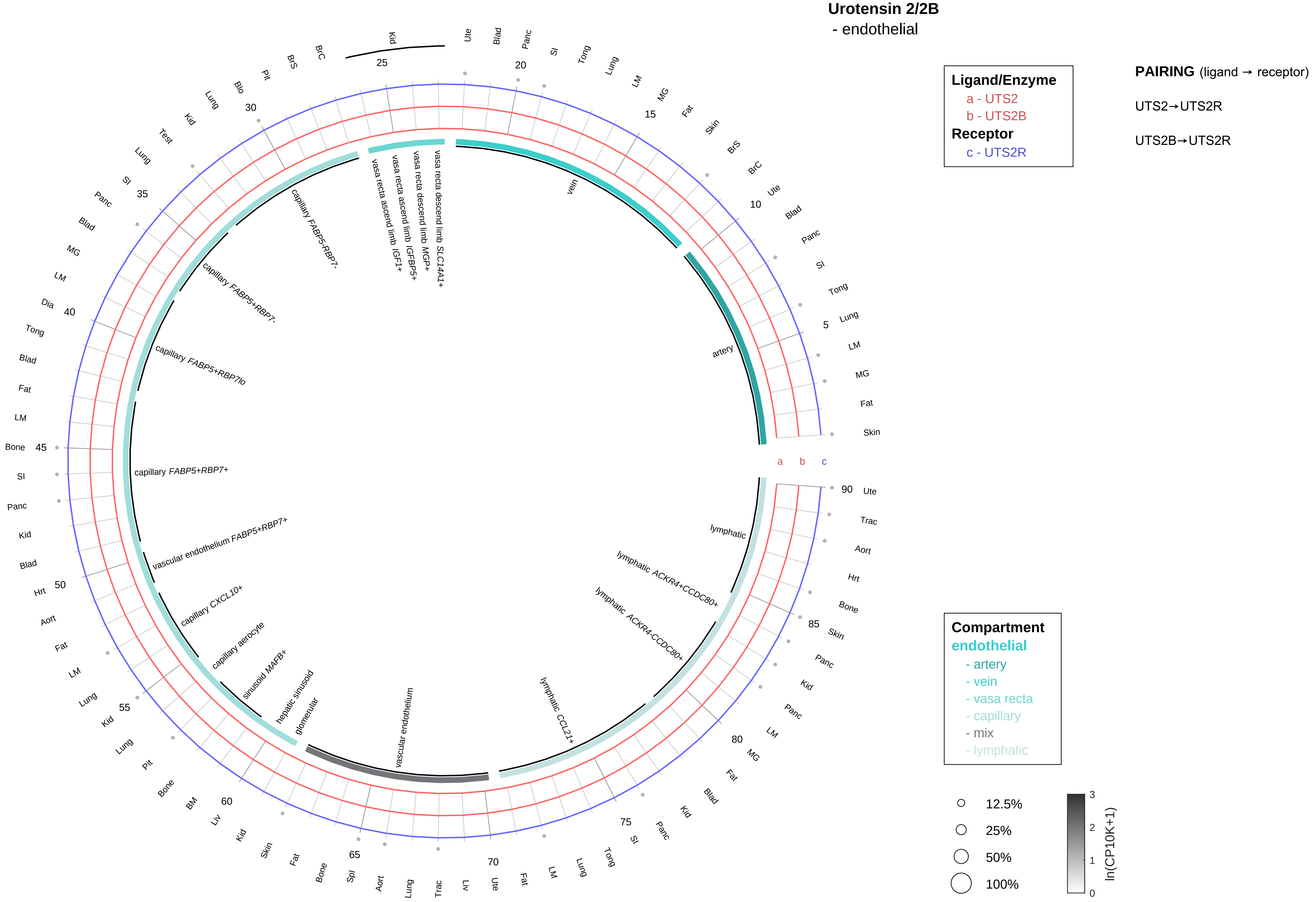

Abbrev.	Tissue
Aort	Aorta
Blad	Bladder
Blo	Blood
Bone	Bone
BM	Bone marrow
BrC	Brain cortex
BrS	Brainstem
Col	Colon
Dia	Diaphragm
Ret	Eye retina
Fat	Fat
Hrt	Heart
Pit	Hypothalamus/Pituitary
Kid	Kidney
LM	Limb muscle
Liv	Liver
Lung	Lung
MG	Mammary gland
Panc	Pancreas
Skin	Skin
SI	Small intestine
Spl	Spleen
Test	Testes
Thym	Thymus
Tong	Tongue
Trac	Trachea
Ute	Uterus

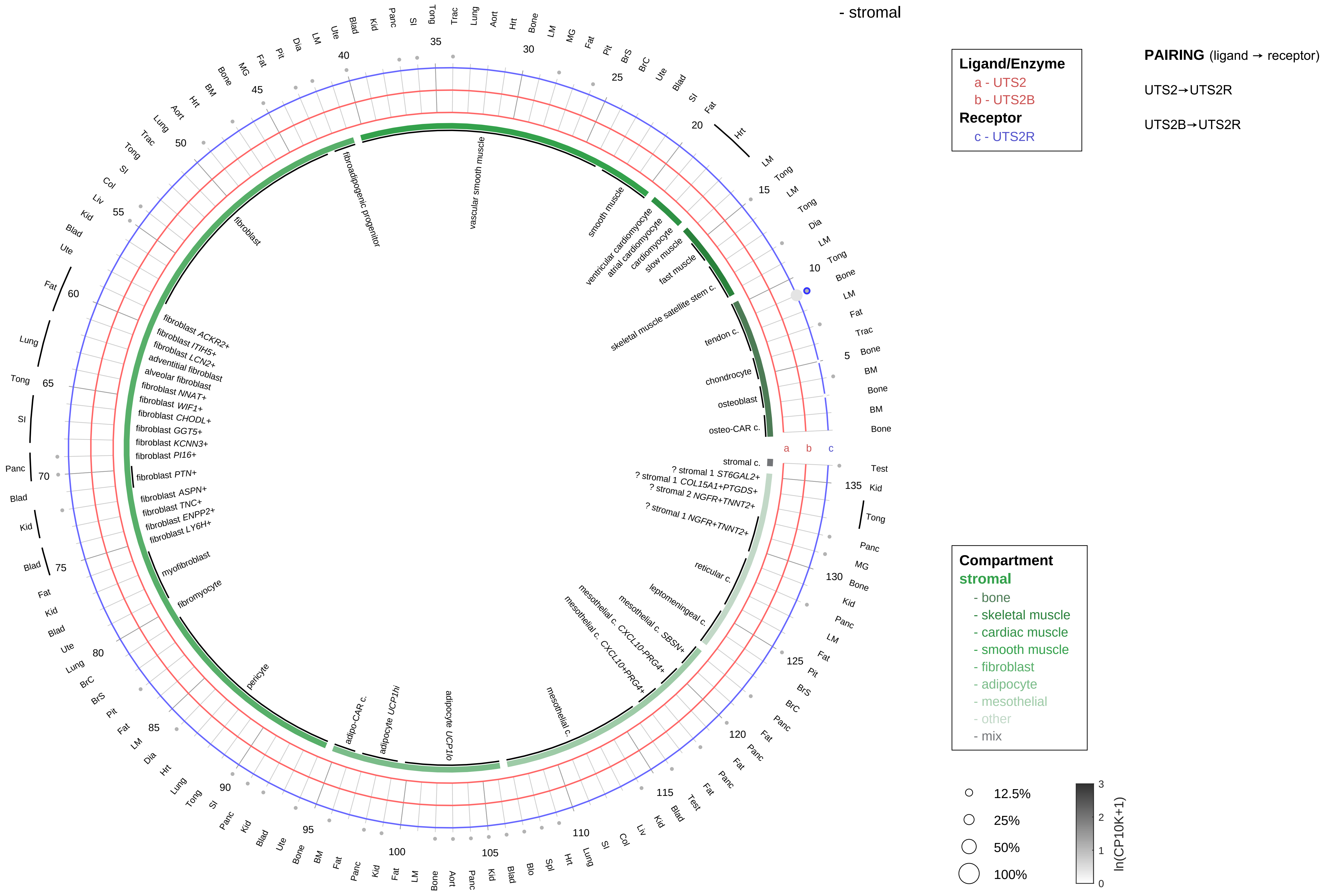

Abbrev.	Tissue
Aort	Aorta
Blad	Bladder
Blo	Blood
Bone	Bone
BM	Bone marrow
BrC	Brain cortex
BrS	Brainstem
Col	Colon
Dia	Diaphragm
Ret	Eye retina
Fat	Fat
Hrt	Heart
Pit	Hypothalamus/Pituitary
Kid	Kidney
LM	Limb muscle
Liv	Liver
Lung	Lung
MG	Mammary gland
Panc	Pancreas
Skin	Skin
SI	Small intestine
Spl	Spleen
Test	Testes
Thym	Thymus
Tong	Tongue
Trac	Trachea
Ute	Uterus

Urotensin 2/2B
- lymphoid

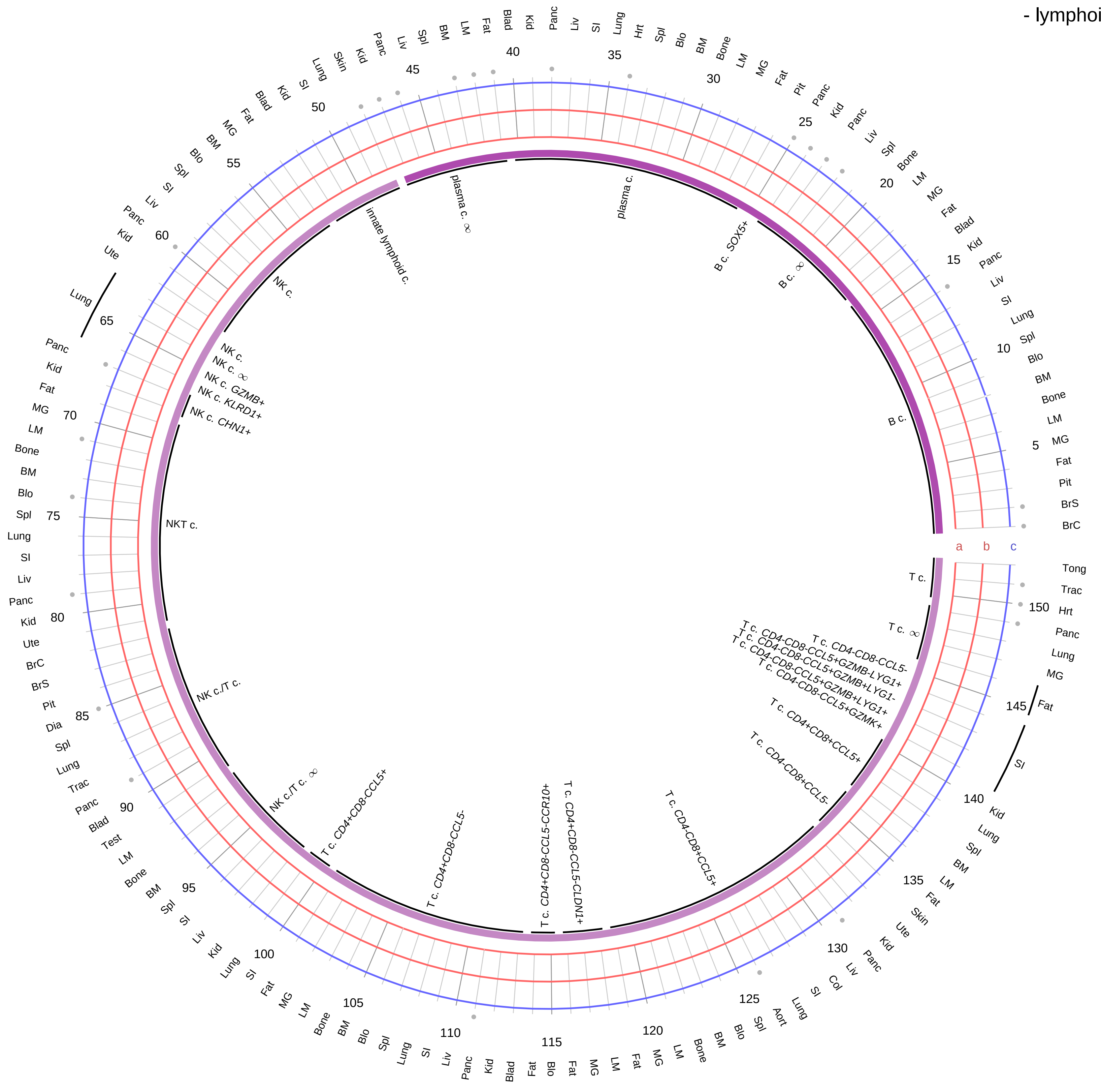

Ligand/Enzyme
- a - UTS2
- b - UTS2B

Receptor
- c - UTS2R

PAIRING (ligand → receptor)

UTS2→UTS2R

UTS2B→UTS2R

Compartment
lymphoid
- B cell
- NK/T cell

12.5%
25%
50%
100%

ln(CP10K+1): 0, 1, 2, 3

Abbrev.	Tissue
Aort	Aorta
Blad	Bladder
Blo	Blood
Bone	Bone
BM	Bone marrow
BrC	Brain cortex
BrS	Brainstem
Col	Colon
Dia	Diaphragm
Ret	Eye retina
Fat	Fat
Hrt	Heart
Pit	Hypothalamus/Pituitary
Kid	Kidney
LM	Limb muscle
Liv	Liver
Lung	Lung
MG	Mammary gland
Panc	Pancreas
Skin	Skin
SI	Small intestine
Spl	Spleen
Test	Testes
Thym	Thymus
Tong	Tongue
Trac	Trachea
Ute	Uterus

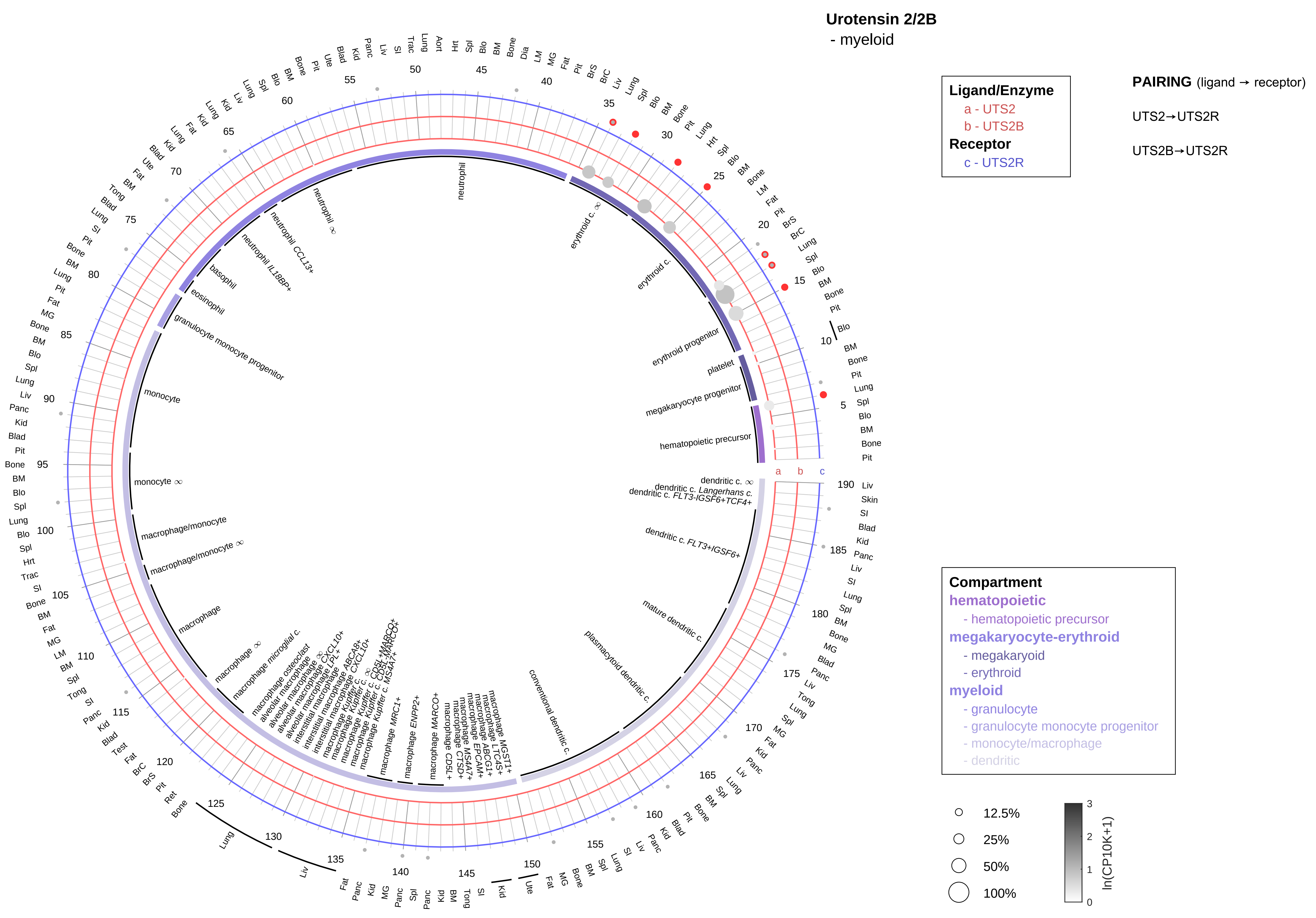

Abbrev.	Tissue
Aort	Aorta
Blad	Bladder
Blo	Blood
Bone	Bone
BM	Bone marrow
BrC	Brain cortex
BrS	Brainstem
Col	Colon
Dia	Diaphragm
Ret	Eye retina
Fat	Fat
Hrt	Heart
Pit	Hypothalamus/Pituitary
Kid	Kidney
LM	Limb muscle
Liv	Liver
Lung	Lung
MG	Mammary gland
Panc	Pancreas
Skin	Skin
SI	Small intestine
Spl	Spleen
Test	Testes
Thym	Thymus
Tong	Tongue
Trac	Trachea
Ute	Uterus

Vasoactive intestinal peptide (VIP)
- epithelial/neural/germ

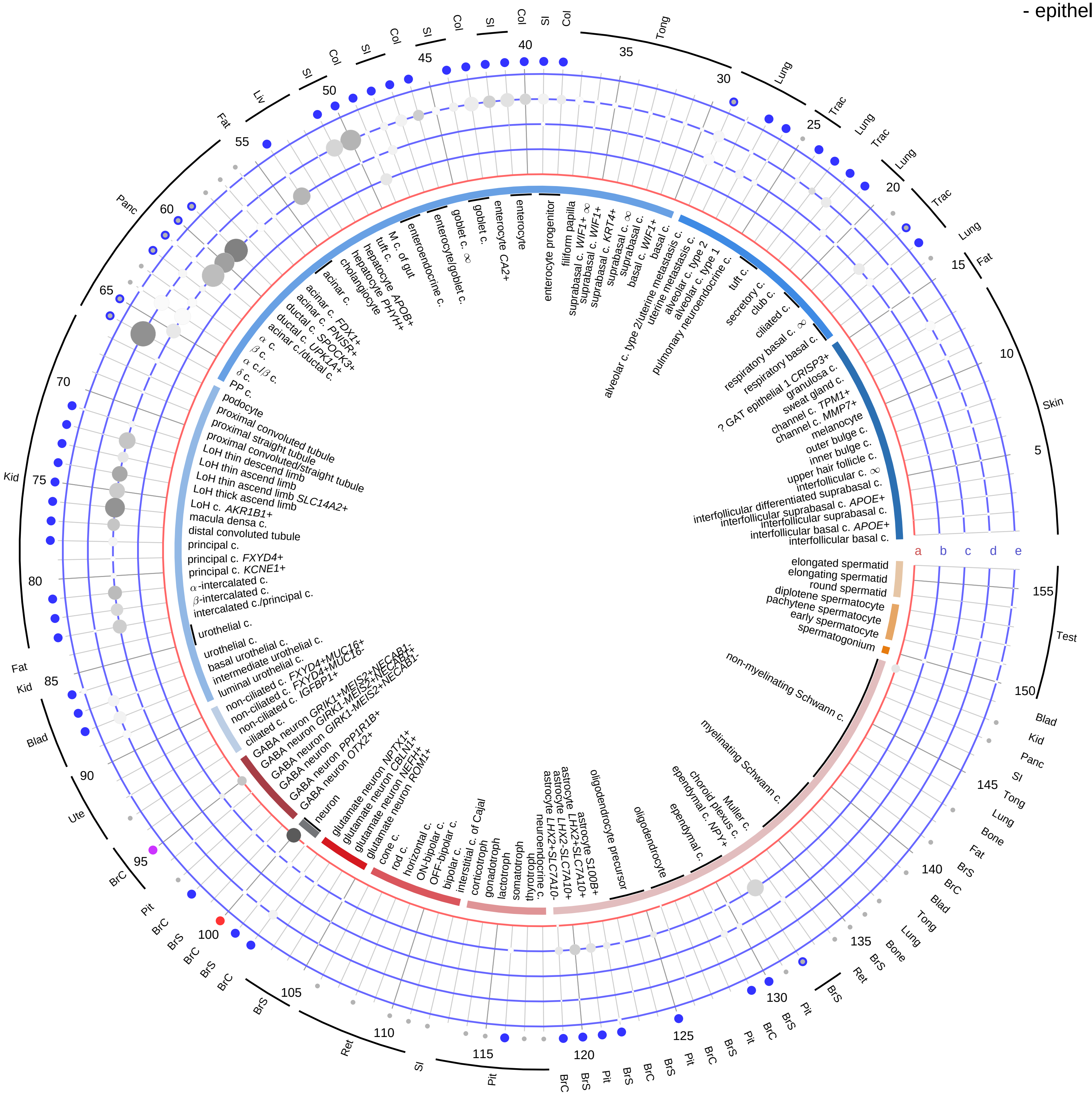

Ligand/Enzyme
a - VIP
Receptor
b - ADCYAP1R1
c - SCTR
d - VIPR1
e - VIPR2

PAIRING (ligand → receptor)

VIP→VIPR1

VIP→VIPR2

VIP→ADCYAP1R1

VIP→SCTR

Compartment
epithelial
- integumentary
- respiratory
- gastrointestinal
- urinary
- reproductive

neural
- GABAergic
- hybrid
- glutamatergic
- specialized
- neuroendocrine
- glia

germ
- spermatogonium
- spermatocyte
- spermatid

12.5%
25%
50%
100%

ln(CP10K+1): 0, 1, 2, 3

Abbrev.	Tissue
Aort	Aorta
Blad	Bladder
Blo	Blood
Bone	Bone
BM	Bone marrow
BrC	Brain cortex
BrS	Brainstem
Col	Colon
Dia	Diaphragm
Ret	Eye retina
Fat	Fat
Hrt	Heart
Pit	Hypothalamus/Pituitary
Kid	Kidney
LM	Limb muscle
Liv	Liver
Lung	Lung
MG	Mammary gland
Panc	Pancreas
Skin	Skin
SI	Small intestine
Spl	Spleen
Test	Testes
Thym	Thymus
Tong	Tongue
Trac	Trachea
Ute	Uterus

Vasoactive intestinal peptide (VIP)
- endothelial

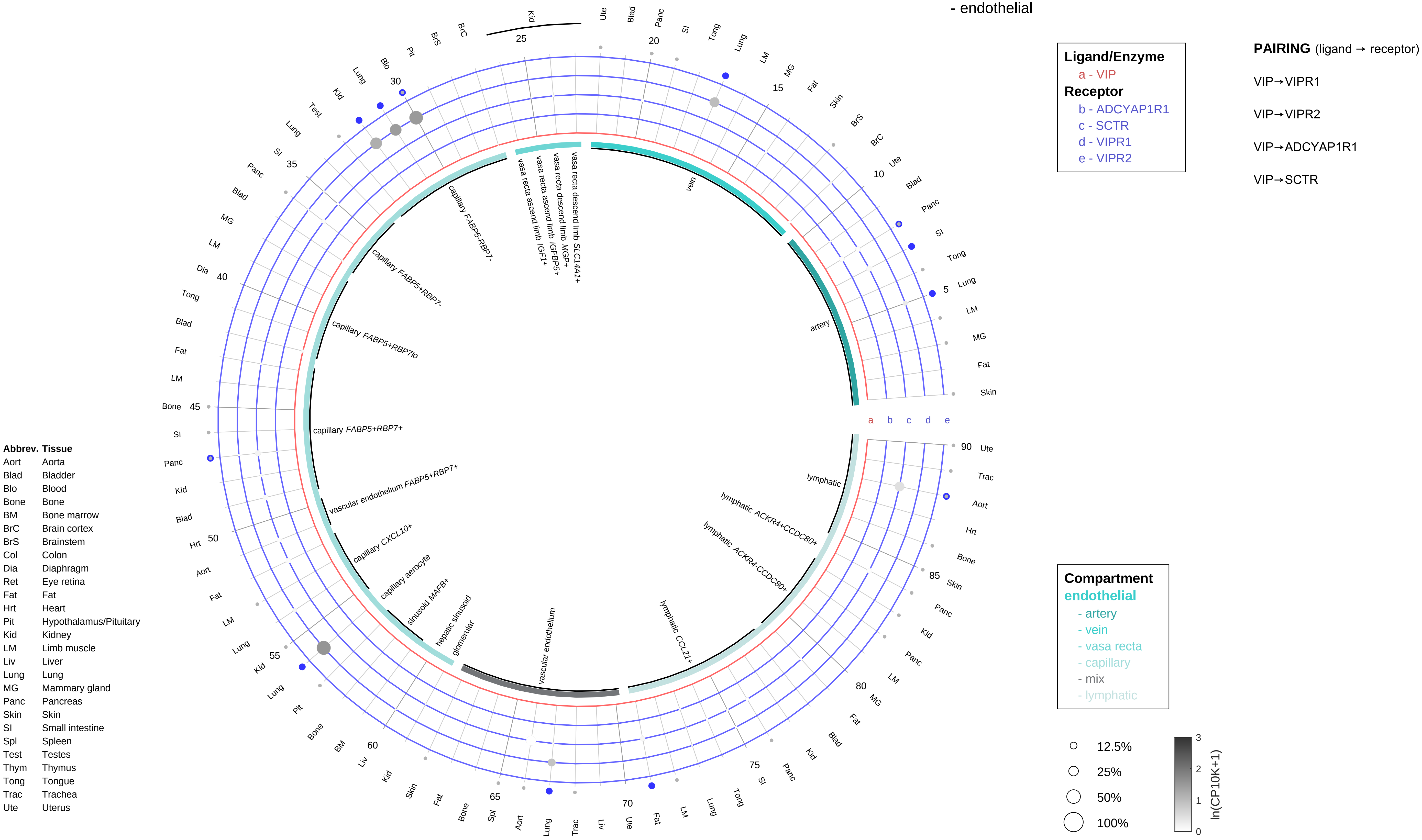

Vasoactive intestinal peptide (VIP)
- stromal

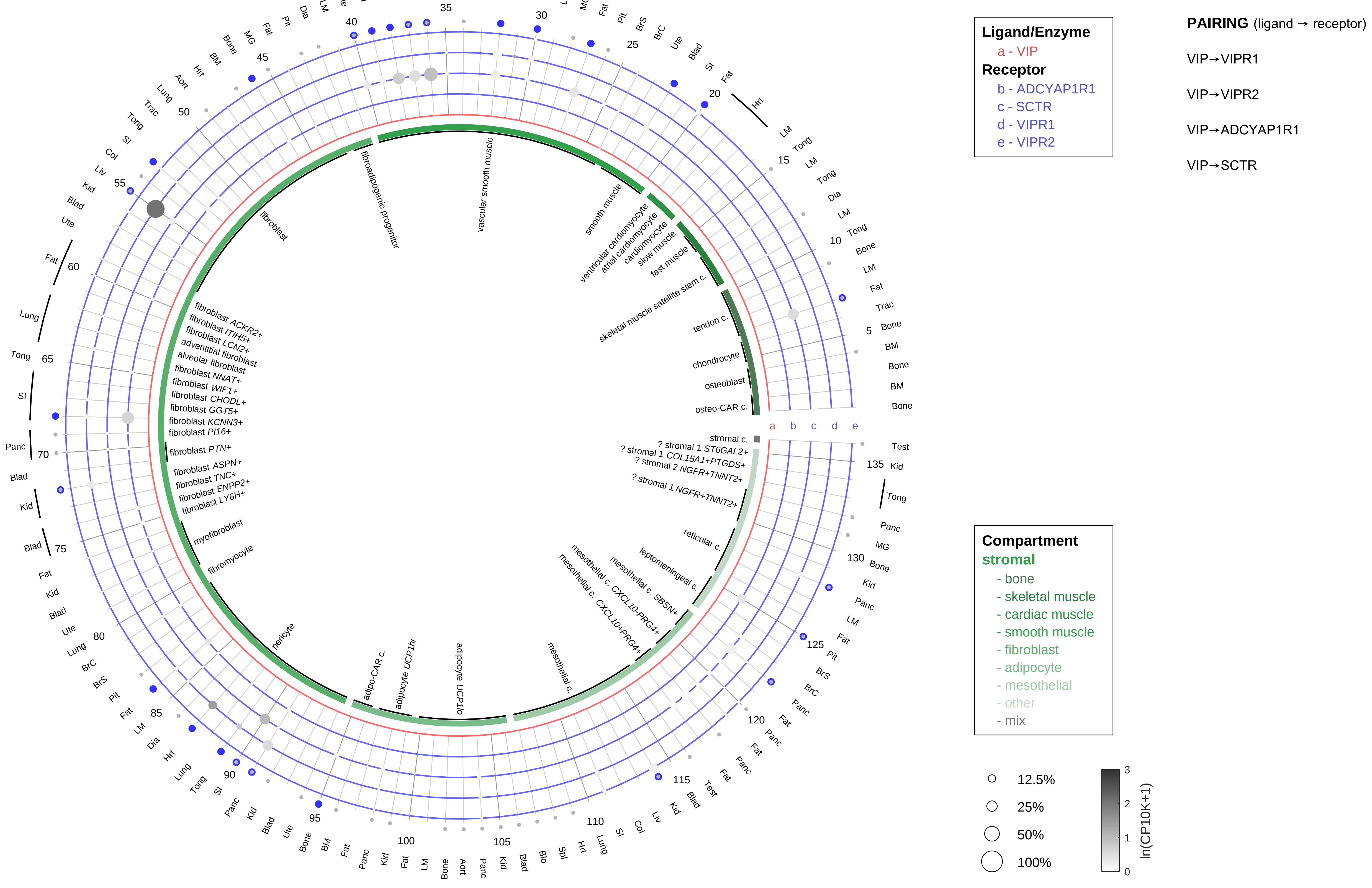

Abbrev.	Tissue
Aort	Aorta
Blad	Bladder
Blo	Blood
Bone	Bone
BM	Bone marrow
BrC	Brain cortex
BrS	Brainstem
Col	Colon
Dia	Diaphragm
Ret	Eye retina
Fat	Fat
Hrt	Heart
Pit	Hypothalamus/Pituitary
Kid	Kidney
LM	Limb muscle
Liv	Liver
Lung	Lung
MG	Mammary gland
Panc	Pancreas
Skin	Skin
SI	Small intestine
Spl	Spleen
Test	Testes
Thym	Thymus
Tong	Tongue
Trac	Trachea
Ute	Uterus

Vasoactive intestinal peptide (VIP)
- lymphoid

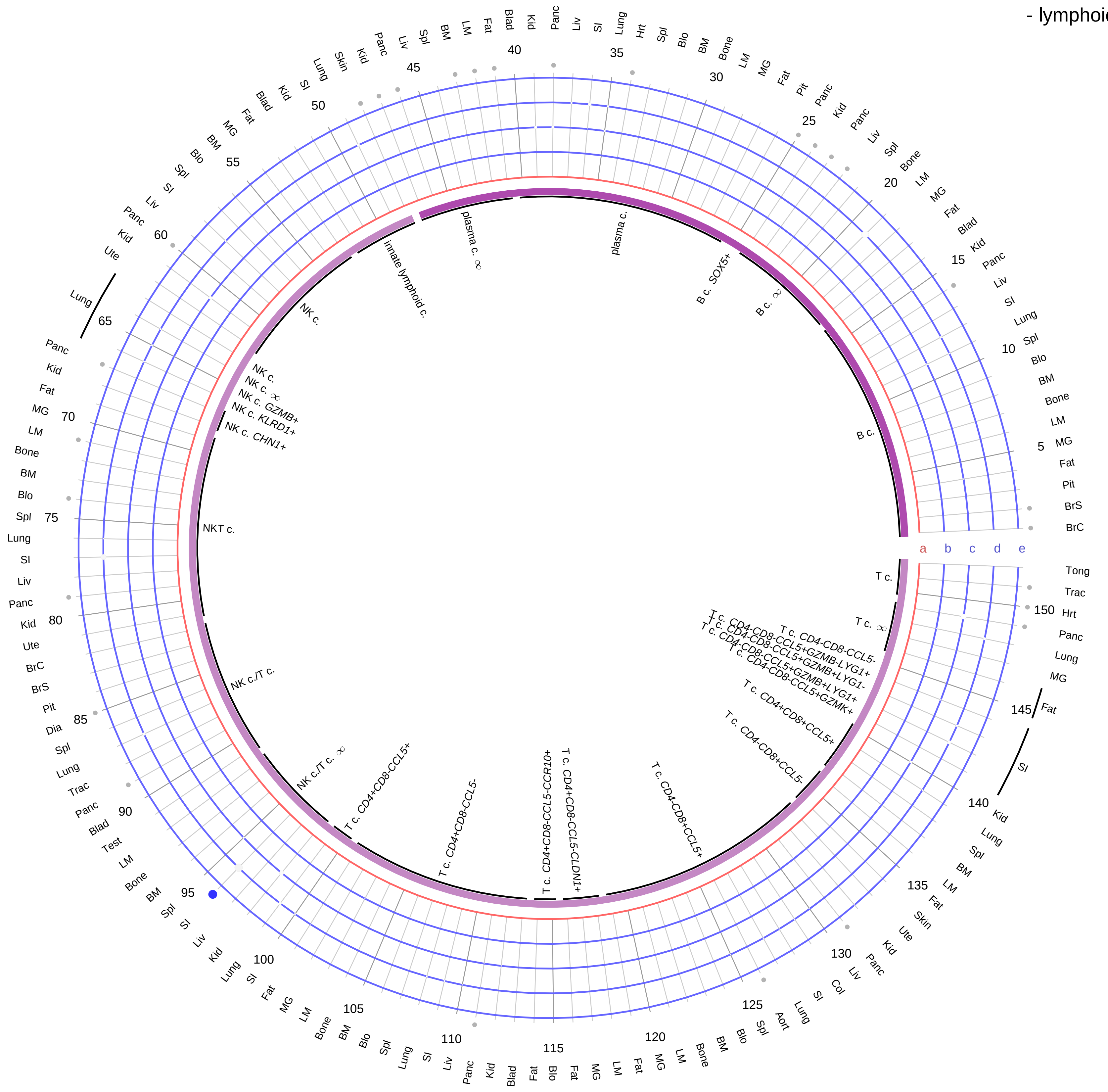

Ligand/Enzyme
a - VIP
Receptor
b - ADCYAP1R1
c - SCTR
d - VIPR1
e - VIPR2

PAIRING (ligand → receptor)

VIP→VIPR1

VIP→VIPR2

VIP→ADCYAP1R1

VIP→SCTR

**Abbrev.**	**Tissue**
Aort	Aorta
Blad	Bladder
Blo	Blood
Bone	Bone
BM	Bone marrow
BrC	Brain cortex
BrS	Brainstem
Col	Colon
Dia	Diaphragm
Ret	Eye retina
Fat	Fat
Hrt	Heart
Pit	Hypothalamus/Pituitary
Kid	Kidney
LM	Limb muscle
Liv	Liver
Lung	Lung
MG	Mammary gland
Panc	Pancreas
Skin	Skin
SI	Small intestine
Spl	Spleen
Test	Testes
Thym	Thymus
Tong	Tongue
Trac	Trachea
Ute	Uterus

Compartment
lymphoid
- B cell
- NK/T cell

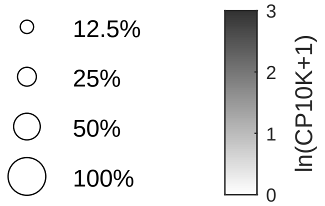

Vasoactive intestinal peptide (VIP)
- myeloid

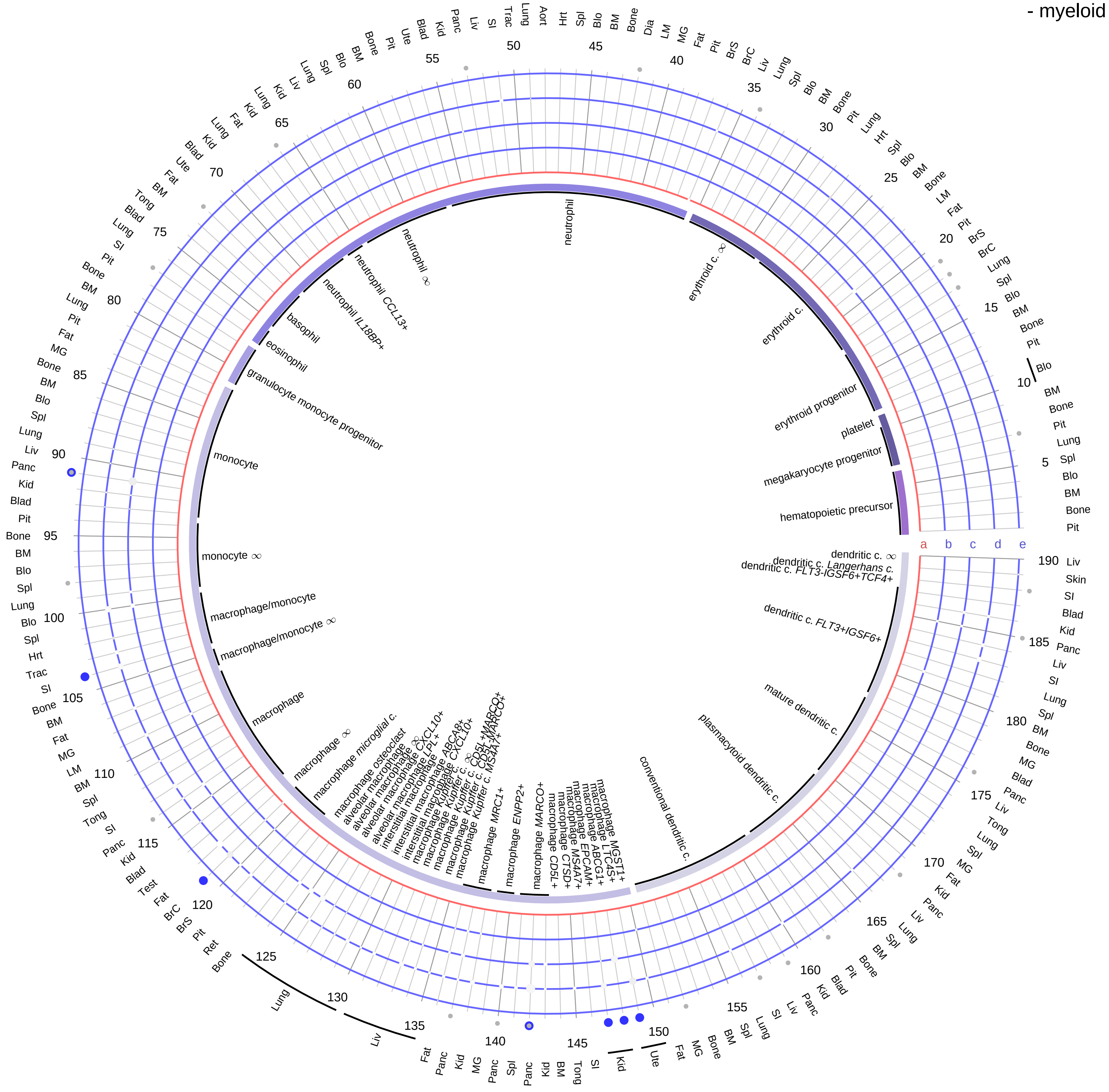

Ligand/Enzyme
- a - VIP

Receptor
- b - ADCYAP1R1
- c - SCTR
- d - VIPR1
- e - VIPR2

PAIRING (ligand → receptor)

VIP→VIPR1

VIP→VIPR2

VIP→ADCYAP1R1

VIP→SCTR

Compartment
- **hematopoietic**
  - hematopoietic precursor
- **megakaryocyte-erythroid**
  - megakaryoid
  - erythroid
- **myeloid**
  - granulocyte
  - granulocyte monocyte progenitor
  - monocyte/macrophage
  - dendritic

12.5%
25%
50%
100%

ln(CP10K+1): 0, 1, 2, 3

Abbrev.	Tissue
Aort	Aorta
Blad	Bladder
Blo	Blood
Bone	Bone
BM	Bone marrow
BrC	Brain cortex
BrS	Brainstem
Col	Colon
Dia	Diaphragm
Ret	Eye retina
Fat	Fat
Hrt	Heart
Pit	Hypothalamus/Pituitary
Kid	Kidney
LM	Limb muscle
Liv	Liver
Lung	Lung
MG	Mammary gland
Panc	Pancreas
Skin	Skin
SI	Small intestine
Spl	Spleen
Test	Testes
Thym	Thymus
Tong	Tongue
Trac	Trachea
Ute	Uterus

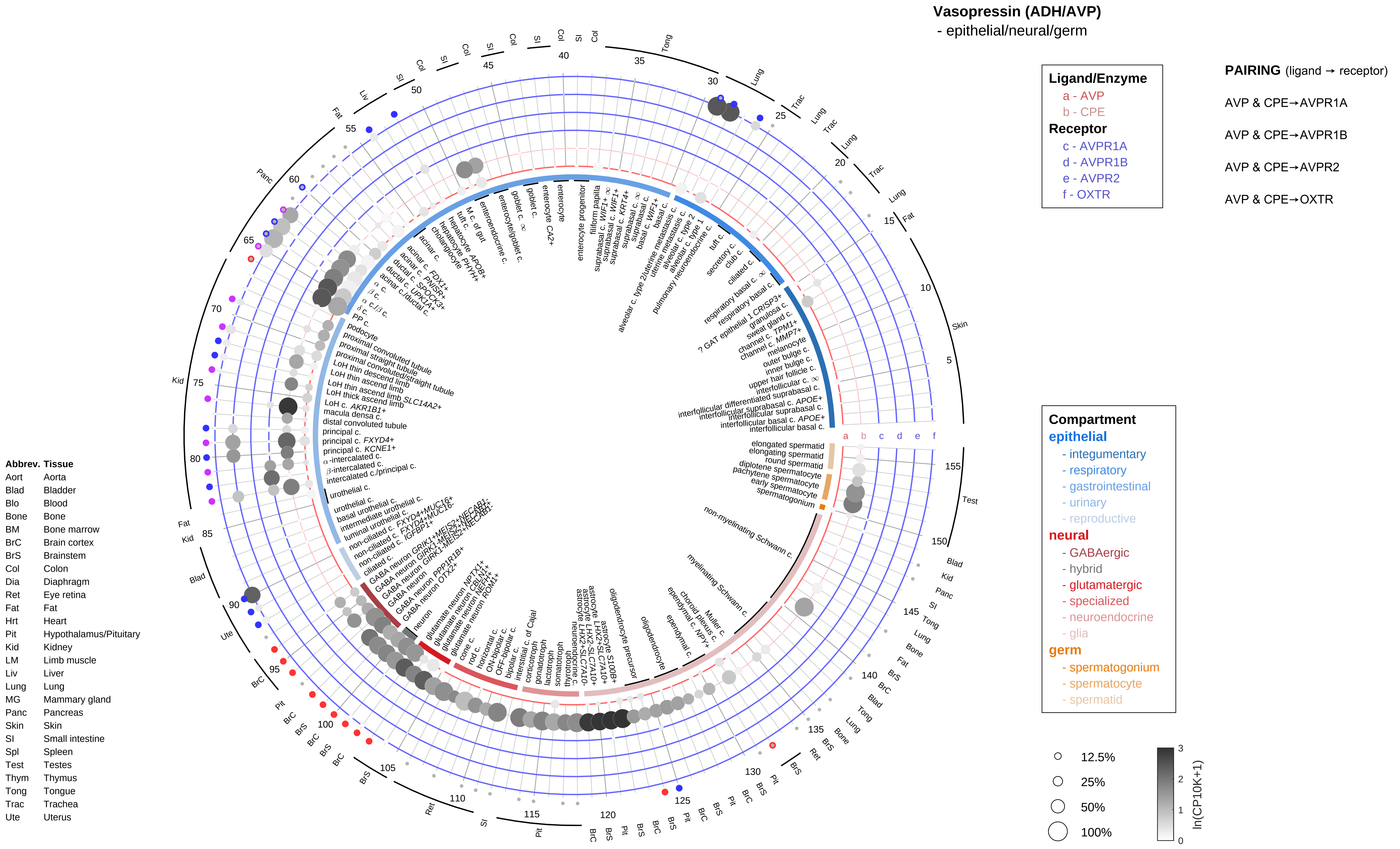
Vasopressin (ADH/AVP)
- epithelial/neural/germ
Ligand/Enzyme
a - AVP
b - CPE
Receptor
c - AVPR1A
d - AVPR1B
e - AVPR2
f - OXTR
PAIRING (ligand → receptor)
AVP & CPE→AVPR1A
AVP & CPE→AVPR1B
AVP & CPE→AVPR2
AVP & CPE→OXTR
Compartment
epithelial
- integumentary
- respiratory
- gastrointestinal
- urinary
- reproductive
neural
- GABAergic
- hybrid
- glutamatergic
- specialized
- neuroendocrine
- glia
germ
- spermatogonium
- spermatocyte
- spermatid
12.5%
25%
50%
100%
ln(CP10K+1)
0
1
2
3
Abbrev. Tissue
Aort Aorta
Blad Bladder
Blo Blood
Bone Bone
BM Bone marrow
BrC Brain cortex
BrS Brainstem
Col Colon
Dia Diaphragm
Ret Eye retina
Fat Fat
Hrt Heart
Pit Hypothalamus/Pituitary
Kid Kidney
LM Limb muscle
Liv Liver
Lung Lung
MG Mammary gland
Panc Pancreas
Skin Skin
SI Small intestine
Spl Spleen
Test Testes
Thym Thymus
Tong Tongue
Trac Trachea
Ute Uterus

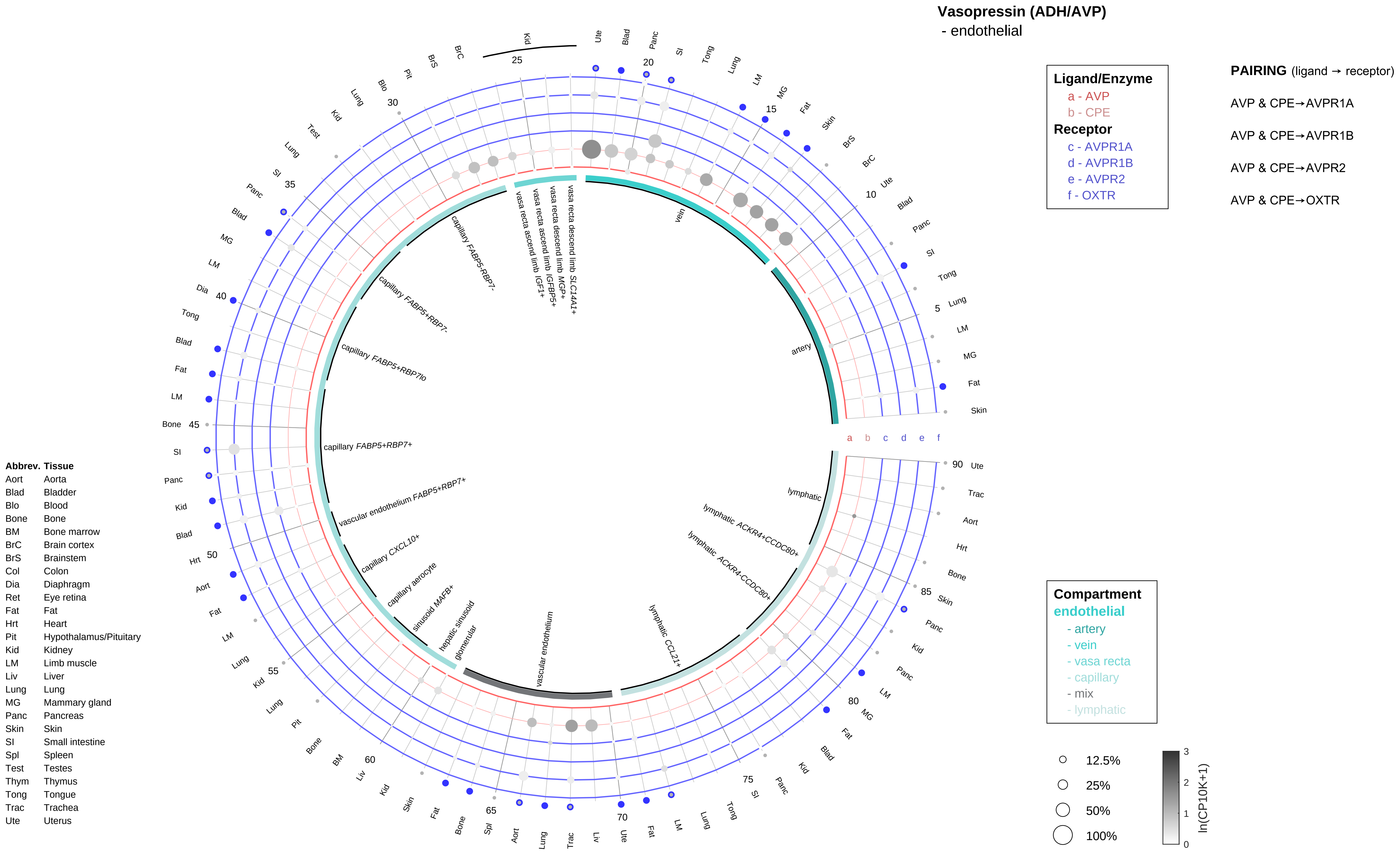
Vasopressin (ADH/AVP)
- endothelial
Ligand/Enzyme
a - AVP
b - CPE
Receptor
c - AVPR1A
d - AVPR1B
e - AVPR2
f - OXTR
PAIRING (ligand → receptor)
AVP & CPE→AVPR1A
AVP & CPE→AVPR1B
AVP & CPE→AVPR2
AVP & CPE→OXTR
Compartment
endothelial
- artery
- vein
- vasa recta
- capillary
- mix
- lymphatic
12.5%
25%
50%
100%
ln(CP10K+1)
vein
artery
lymphatic
lymphatic ACKR4+CCDC80+
lymphatic ACKR4-CCDC80+
lymphatic CCL21+
vascular endothelium
glomerular
hepatic sinusoid
sinusoid MAFB+
capillary aerocyte
capillary CXCL10+
vascular endothelium FABP5+RBP7+
capillary FABP5+RBP7+
capillary FABP5+RBP7lo
capillary FABP5+RBP7-
capillary FABP5-RBP7-
vasa recta ascend limb IGF1+
vasa recta ascend limb IGFBP5+
vasa recta descend limb MGP+
vasa recta descend limb SLC14A1+
Abbrev. Tissue
Aort Aorta
Blad Bladder
Blo Blood
Bone Bone
BM Bone marrow
BrC Brain cortex
BrS Brainstem
Col Colon
Dia Diaphragm
Ret Eye retina
Fat Fat
Hrt Heart
Pit Hypothalamus/Pituitary
Kid Kidney
LM Limb muscle
Liv Liver
Lung Lung
MG Mammary gland
Panc Pancreas
Skin Skin
SI Small intestine
Spl Spleen
Test Testes
Thym Thymus
Tong Tongue
Trac Trachea
Ute Uterus

Vasopressin (ADH/AVP)
- stromal

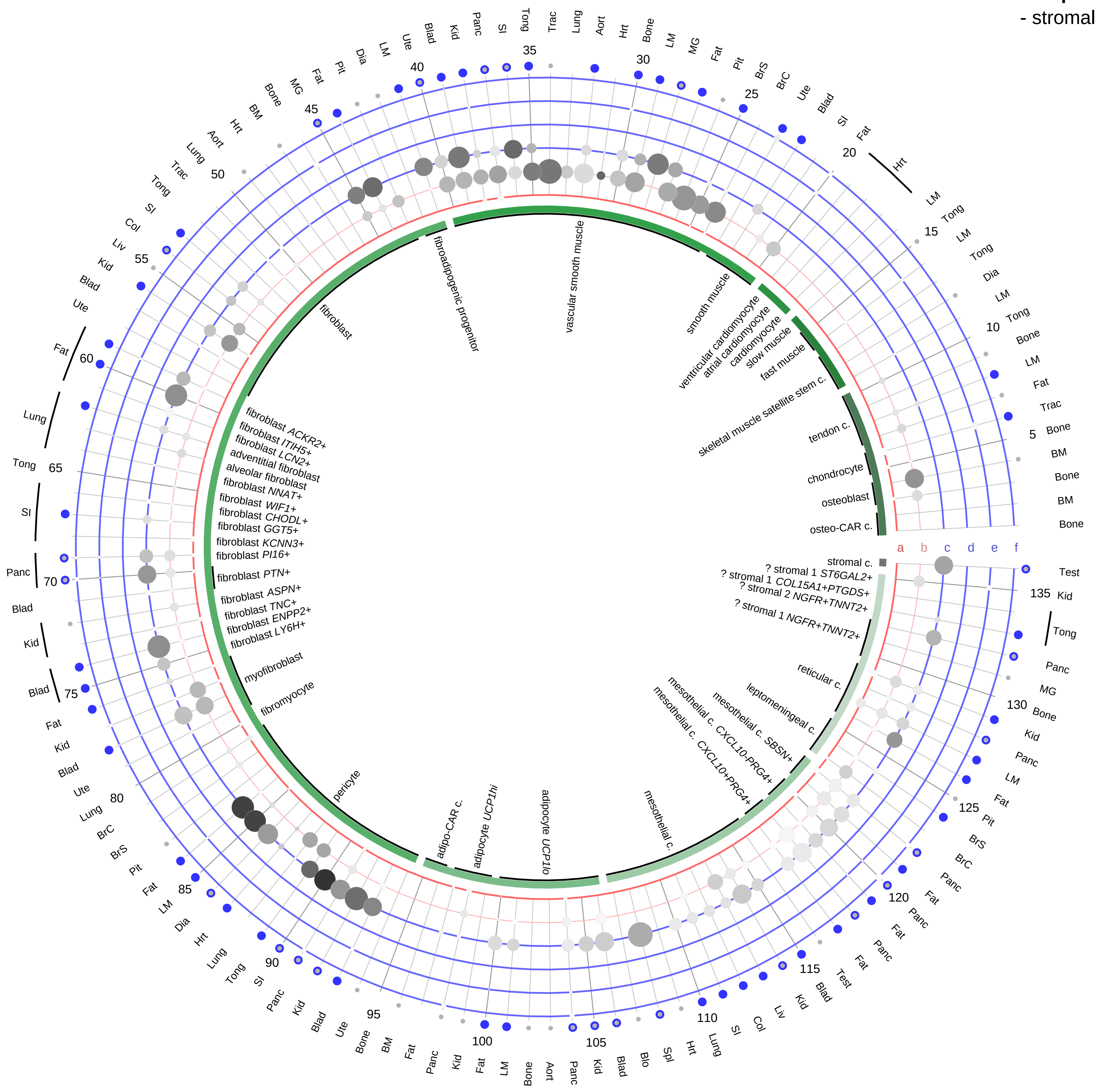

Ligand/Enzyme
- a - AVP
- b - CPE

Receptor
- c - AVPR1A
- d - AVPR1B
- e - AVPR2
- f - OXTR

PAIRING (ligand → receptor)

AVP & CPE→AVPR1A

AVP & CPE→AVPR1B

AVP & CPE→AVPR2

AVP & CPE→OXTR

Abbrev.	Tissue
Aort	Aorta
Blad	Bladder
Blo	Blood
Bone	Bone
BM	Bone marrow
BrC	Brain cortex
BrS	Brainstem
Col	Colon
Dia	Diaphragm
Ret	Eye retina
Fat	Fat
Hrt	Heart
Pit	Hypothalamus/Pituitary
Kid	Kidney
LM	Limb muscle
Liv	Liver
Lung	Lung
MG	Mammary gland
Panc	Pancreas
Skin	Skin
SI	Small intestine
Spl	Spleen
Test	Testes
Thym	Thymus
Tong	Tongue
Trac	Trachea
Ute	Uterus

Compartment

stromal
- bone
- skeletal muscle
- cardiac muscle
- smooth muscle
- fibroblast
- adipocyte
- mesothelial
- other
- mix

12.5%

25%

50%

100%

ln(CP10K+1): 0, 1, 2, 3

Vasopressin (ADH/AVP)
- lymphoid

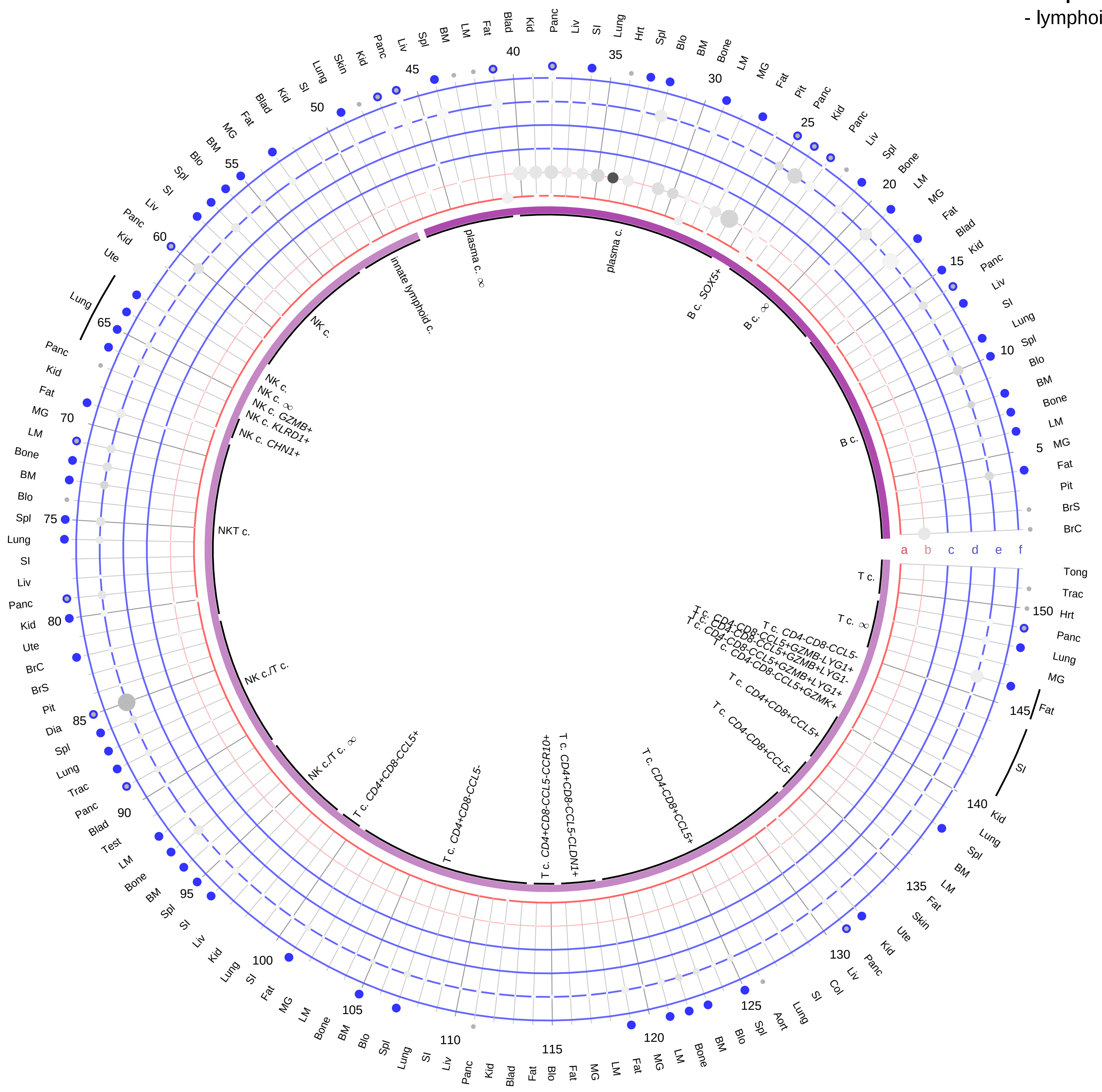

Ligand/Enzyme
- a - AVP
- b - CPE

Receptor
- c - AVPR1A
- d - AVPR1B
- e - AVPR2
- f - OXTR

PAIRING (ligand → receptor)

AVP & CPE→AVPR1A

AVP & CPE→AVPR1B

AVP & CPE→AVPR2

AVP & CPE→OXTR

Abbrev.	Tissue
Aort	Aorta
Blad	Bladder
Blo	Blood
Bone	Bone
BM	Bone marrow
BrC	Brain cortex
BrS	Brainstem
Col	Colon
Dia	Diaphragm
Ret	Eye retina
Fat	Fat
Hrt	Heart
Pit	Hypothalamus/Pituitary
Kid	Kidney
LM	Limb muscle
Liv	Liver
Lung	Lung
MG	Mammary gland
Panc	Pancreas
Skin	Skin
SI	Small intestine
Spl	Spleen
Test	Testes
Thym	Thymus
Tong	Tongue
Trac	Trachea
Ute	Uterus

Compartment
lymphoid
- B cell
- NK/T cell

12.5%
25%
50%
100%

ln(CP10K+1) 0 1 2 3

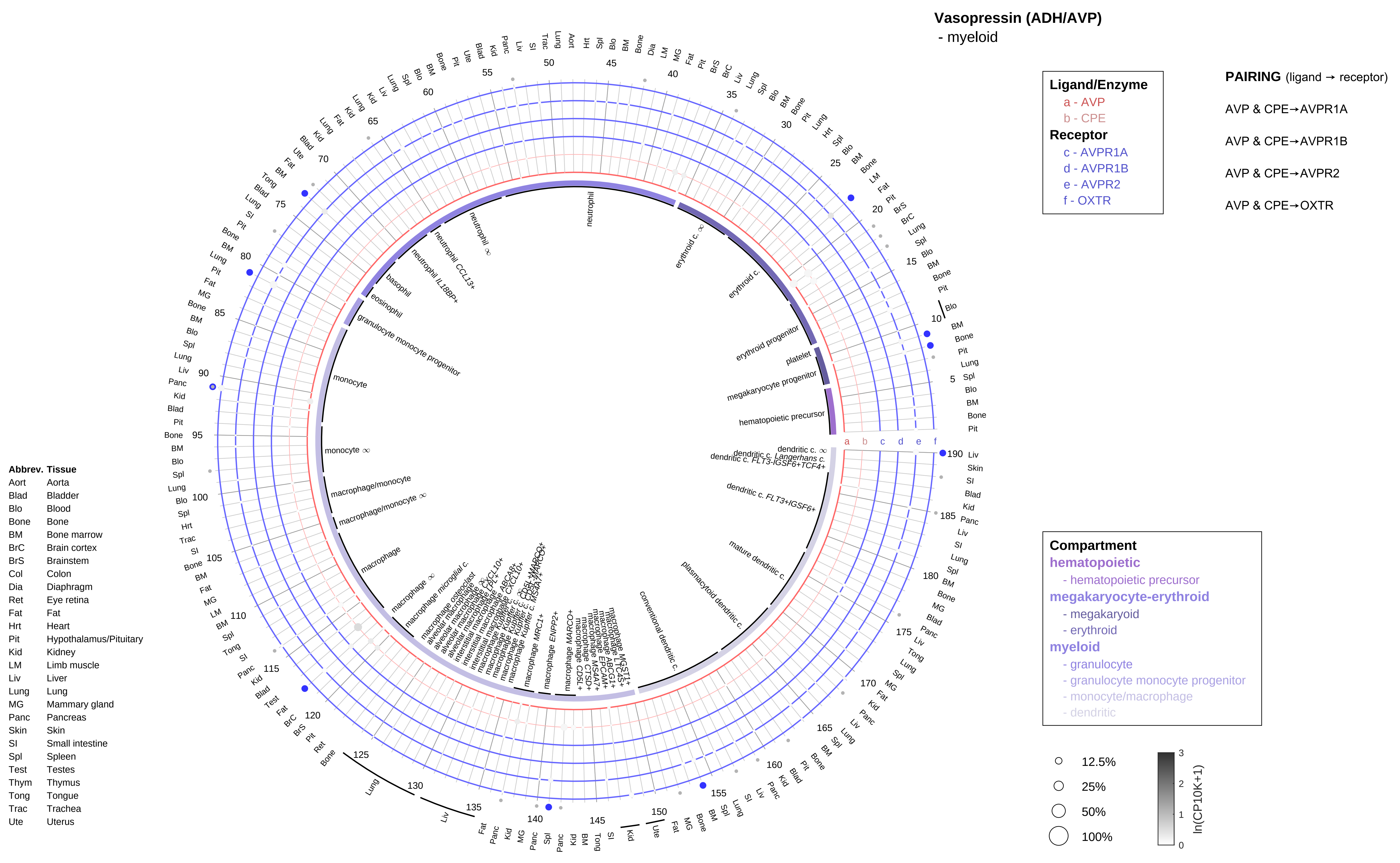

Vasopressin (ADH/AVP)
- myeloid
Ligand/Enzyme
a - AVP
b - CPE
Receptor
c - AVPR1A
d - AVPR1B
e - AVPR2
f - OXTR
PAIRING (ligand → receptor)
AVP & CPE→AVPR1A
AVP & CPE→AVPR1B
AVP & CPE→AVPR2
AVP & CPE→OXTR
Compartment
hematopoietic
- hematopoietic precursor
megakaryocyte-erythroid
- megakaryoid
- erythroid
myeloid
- granulocyte
- granulocyte monocyte progenitor
- monocyte/macrophage
- dendritic
12.5%
25%
50%
100%
ln(CP10K+1)
0
1
2
3
Abbrev. Tissue
Aort Aorta
Blad Bladder
Blo Blood
Bone Bone
BM Bone marrow
BrC Brain cortex
BrS Brainstem
Col Colon
Dia Diaphragm
Ret Eye retina
Fat Fat
Hrt Heart
Pit Hypothalamus/Pituitary
Kid Kidney
LM Limb muscle
Liv Liver
Lung Lung
MG Mammary gland
Panc Pancreas
Skin Skin
SI Small intestine
Spl Spleen
Test Testes
Thym Thymus
Tong Tongue
Trac Trachea
Ute Uterus
neutrophil
erythroid c. ∞
erythroid c.
erythroid progenitor
platelet
megakaryocyte progenitor
hematopoietic precursor
dendritic c. ∞
dendritic c. Langerhans c.
dendritic c. FLT3-IGSF6+TCF4+
dendritic c. FLT3+IGSF6+
mature dendritic c.
plasmacytoid dendritic c.
conventional dendritic c.
macrophage
macrophage ∞
macrophage/monocyte
macrophage/monocyte ∞
monocyte
monocyte ∞
granulocyte monocyte progenitor
eosinophil
basophil
neutrophil IL18BP+
neutrophil CCL13+
neutrophil ∞

Neuroendocrine secretory proteins, chromogranin/secretogranin
- epithelial/neural/germ

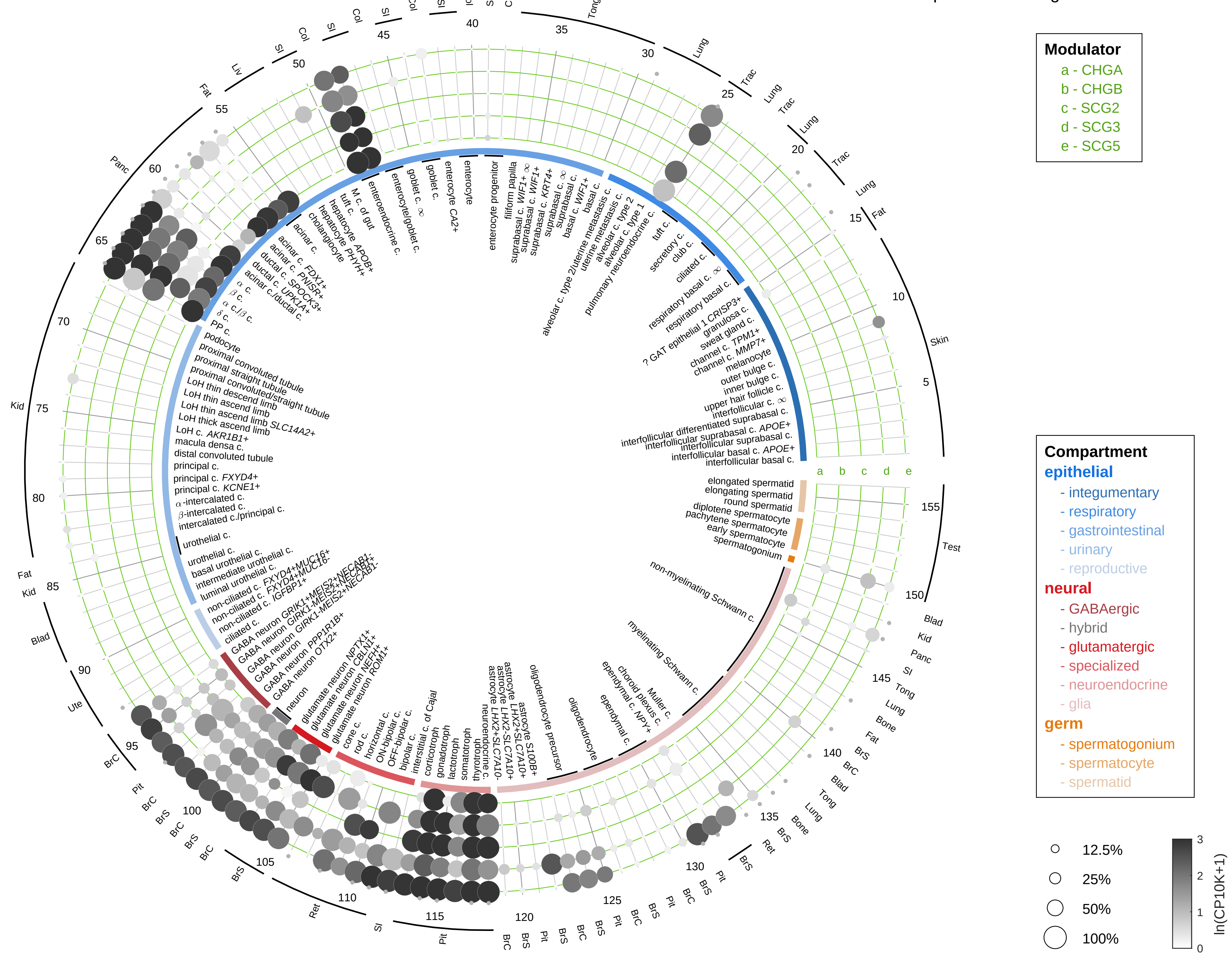

Abbrev.	Tissue
Aort	Aorta
Blad	Bladder
Blo	Blood
Bone	Bone
BM	Bone marrow
BrC	Brain cortex
BrS	Brainstem
Col	Colon
Dia	Diaphragm
Ret	Eye retina
Fat	Fat
Hrt	Heart
Pit	Hypothalamus/Pituitary
Kid	Kidney
LM	Limb muscle
Liv	Liver
Lung	Lung
MG	Mammary gland
Panc	Pancreas
Skin	Skin
SI	Small intestine
Spl	Spleen
Test	Testes
Thym	Thymus
Tong	Tongue
Trac	Trachea
Ute	Uterus

Neuroendocrine secretory proteins, chromogranin/secretogranin
- endothelial

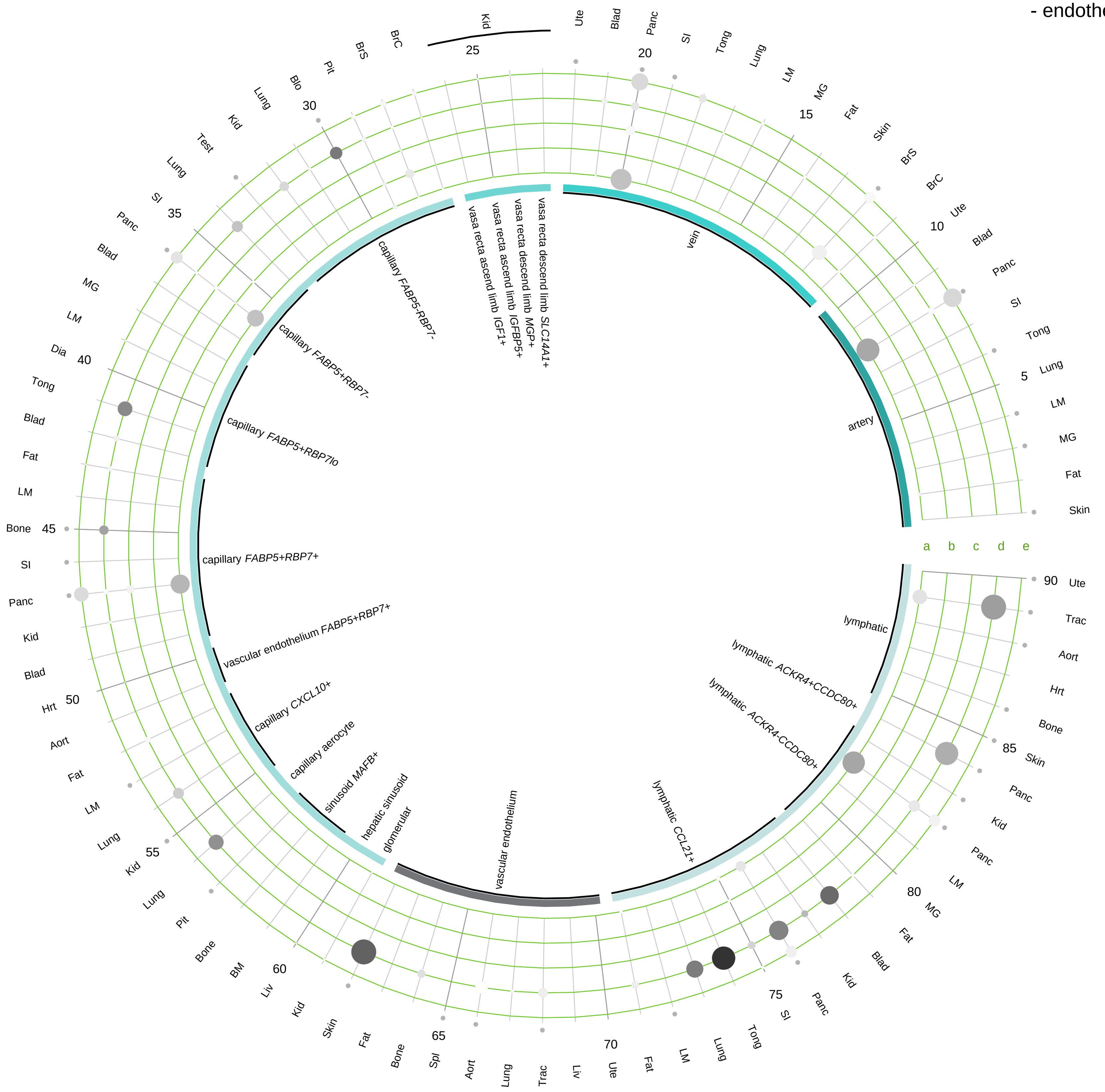

Modulator
a - CHGA
b - CHGB
c - SCG2
d - SCG3
e - SCG5

Compartment
endothelial
- artery
- vein
- vasa recta
- capillary
- mix
- lymphatic

12.5%
25%
50%
100%

ln(CP10K+1) 0 1 2 3

Abbrev.	Tissue
Aort	Aorta
Blad	Bladder
Blo	Blood
Bone	Bone
BM	Bone marrow
BrC	Brain cortex
BrS	Brainstem
Col	Colon
Dia	Diaphragm
Ret	Eye retina
Fat	Fat
Hrt	Heart
Pit	Hypothalamus/Pituitary
Kid	Kidney
LM	Limb muscle
Liv	Liver
Lung	Lung
MG	Mammary gland
Panc	Pancreas
Skin	Skin
SI	Small intestine
Spl	Spleen
Test	Testes
Thym	Thymus
Tong	Tongue
Trac	Trachea
Ute	Uterus

Neuroendocrine secretory proteins, chromogranin/secretogranin
- stromal

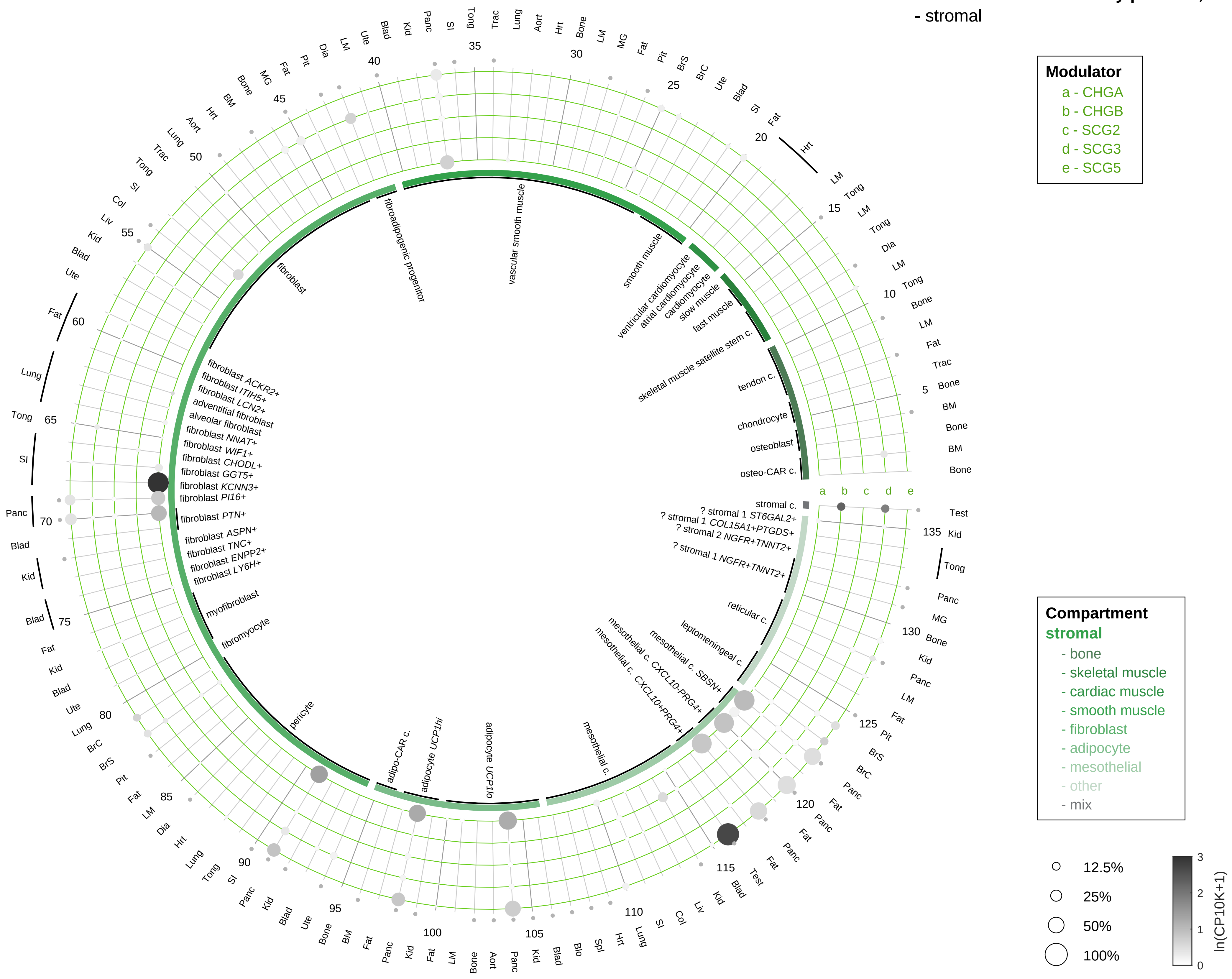

**Abbrev.**	**Tissue**
Aort	Aorta
Blad	Bladder
Blo	Blood
Bone	Bone
BM	Bone marrow
BrC	Brain cortex
BrS	Brainstem
Col	Colon
Dia	Diaphragm
Ret	Eye retina
Fat	Fat
Hrt	Heart
Pit	Hypothalamus/Pituitary
Kid	Kidney
LM	Limb muscle
Liv	Liver
Lung	Lung
MG	Mammary gland
Panc	Pancreas
Skin	Skin
SI	Small intestine
Spl	Spleen
Test	Testes
Thym	Thymus
Tong	Tongue
Trac	Trachea
Ute	Uterus

Neuroendocrine secretory proteins, chromogranin/secretogranin
- lymphoid

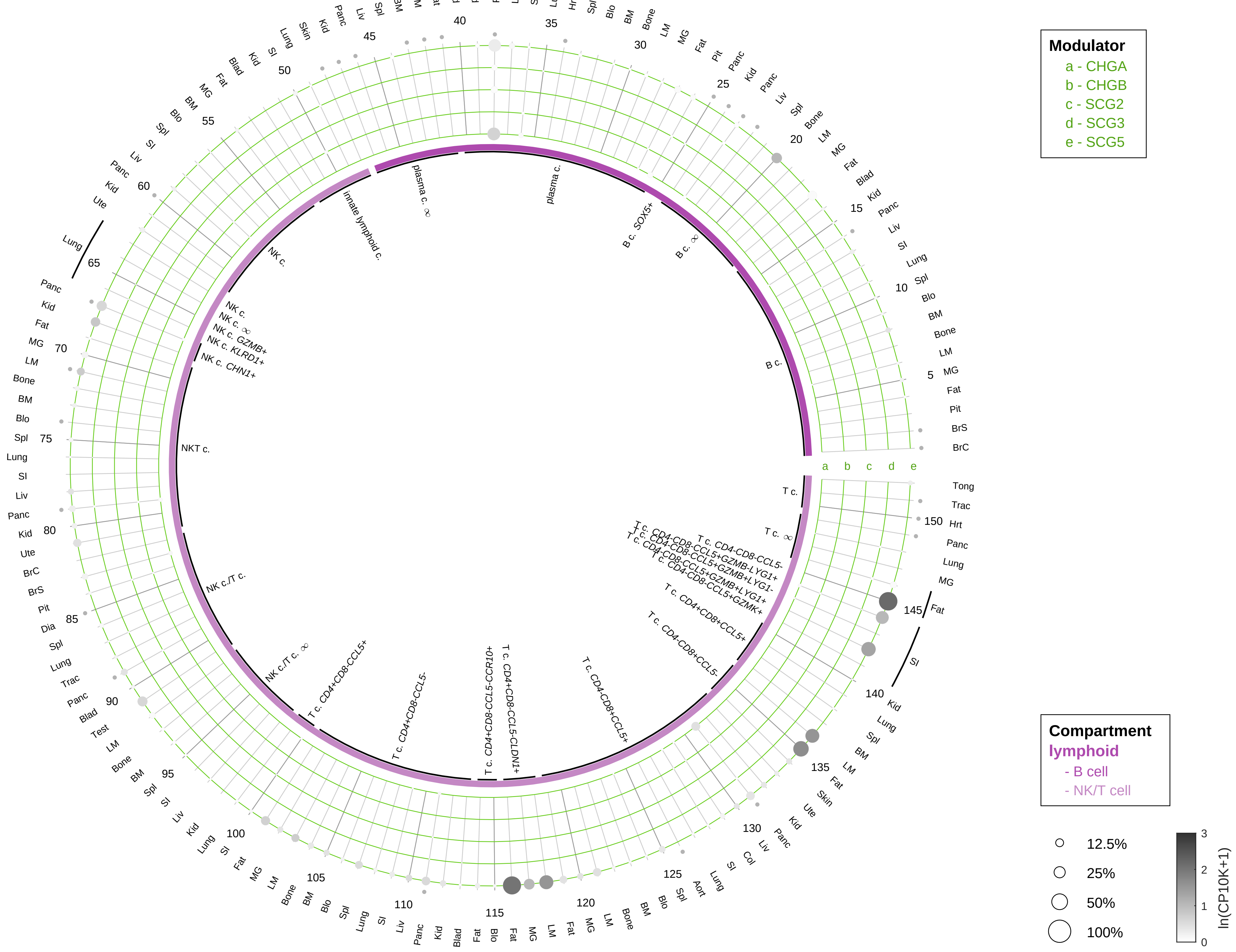

Abbrev.	Tissue
Aort	Aorta
Blad	Bladder
Blo	Blood
Bone	Bone
BM	Bone marrow
BrC	Brain cortex
BrS	Brainstem
Col	Colon
Dia	Diaphragm
Ret	Eye retina
Fat	Fat
Hrt	Heart
Pit	Hypothalamus/Pituitary
Kid	Kidney
LM	Limb muscle
Liv	Liver
Lung	Lung
MG	Mammary gland
Panc	Pancreas
Skin	Skin
SI	Small intestine
Spl	Spleen
Test	Testes
Thym	Thymus
Tong	Tongue
Trac	Trachea
Ute	Uterus

Neuroendocrine secretory proteins, chromogranin/secretogranin
- myeloid

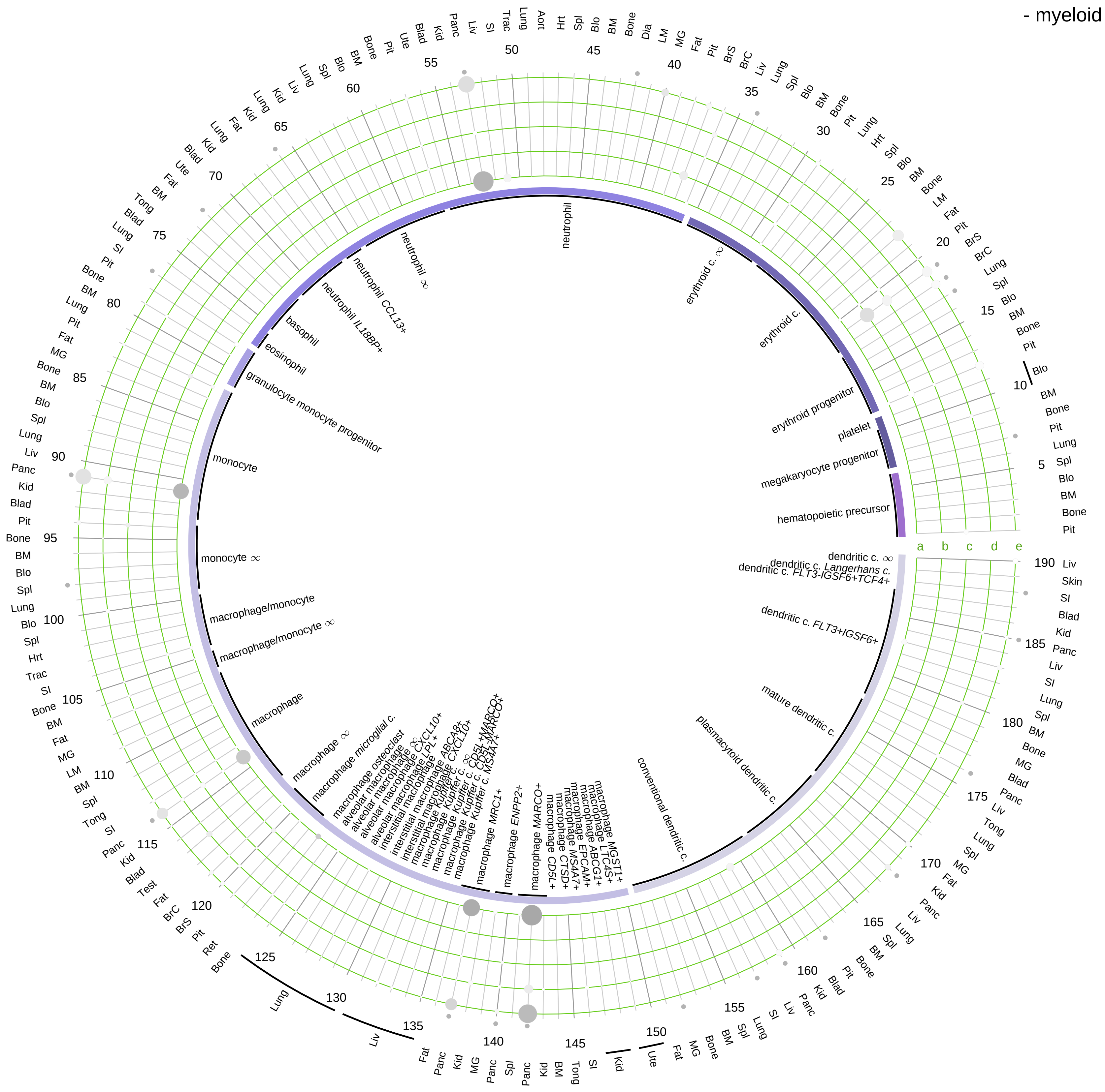

Modulator

- a - CHGA
- b - CHGB
- c - SCG2
- d - SCG3
- e - SCG5

Compartment

hematopoietic
- hematopoietic precursor

megakaryocyte-erythroid
- megakaryoid
- erythroid

myeloid
- granulocyte
- granulocyte monocyte progenitor
- monocyte/macrophage
- dendritic

12.5%
25%
50%
100%

ln(CP10K+1): 0, 1, 2, 3

Abbrev.	Tissue
Aort	Aorta
Blad	Bladder
Blo	Blood
Bone	Bone
BM	Bone marrow
BrC	Brain cortex
BrS	Brainstem
Col	Colon
Dia	Diaphragm
Ret	Eye retina
Fat	Fat
Hrt	Heart
Pit	Hypothalamus/Pituitary
Kid	Kidney
LM	Limb muscle
Liv	Liver
Lung	Lung
MG	Mammary gland
Panc	Pancreas
Skin	Skin
SI	Small intestine
Spl	Spleen
Test	Testes
Thym	Thymus
Tong	Tongue
Trac	Trachea
Ute	Uterus

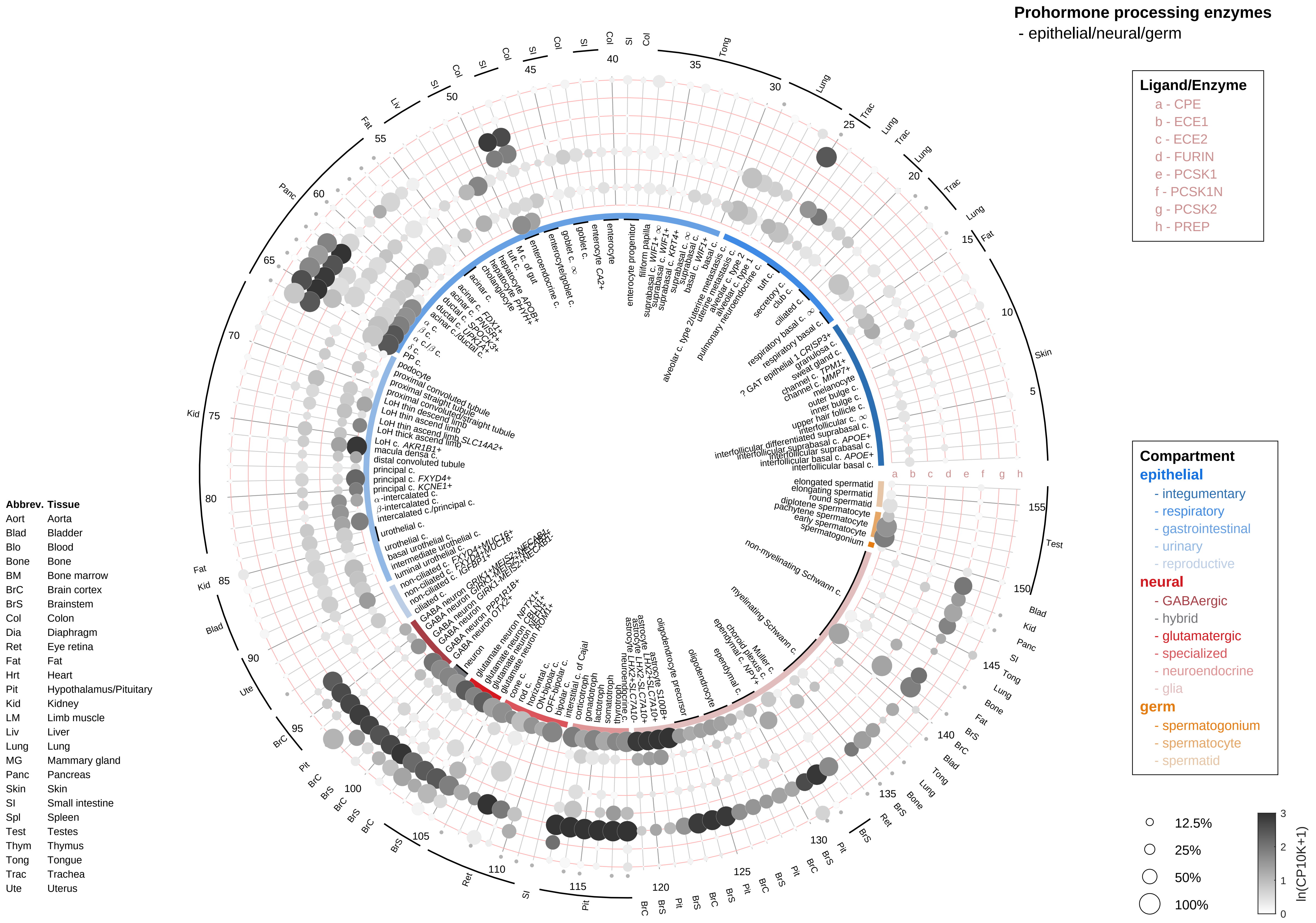

Prohormone processing enzymes
- epithelial/neural/germ
Ligand/Enzyme
a - CPE
b - ECE1
c - ECE2
d - FURIN
e - PCSK1
f - PCSK1N
g - PCSK2
h - PREP
Compartment
epithelial
- integumentary
- respiratory
- gastrointestinal
- urinary
- reproductive
neural
- GABAergic
- hybrid
- glutamatergic
- specialized
- neuroendocrine
- glia
germ
- spermatogonium
- spermatocyte
- spermatid
12.5%
25%
50%
100%
ln(CP10K+1)
Abbrev. Tissue
Aort Aorta
Blad Bladder
Blo Blood
Bone Bone
BM Bone marrow
BrC Brain cortex
BrS Brainstem
Col Colon
Dia Diaphragm
Ret Eye retina
Fat Fat
Hrt Heart
Pit Hypothalamus/Pituitary
Kid Kidney
LM Limb muscle
Liv Liver
Lung Lung
MG Mammary gland
Panc Pancreas
Skin Skin
SI Small intestine
Spl Spleen
Test Testes
Thym Thymus
Tong Tongue
Trac Trachea
Ute Uterus

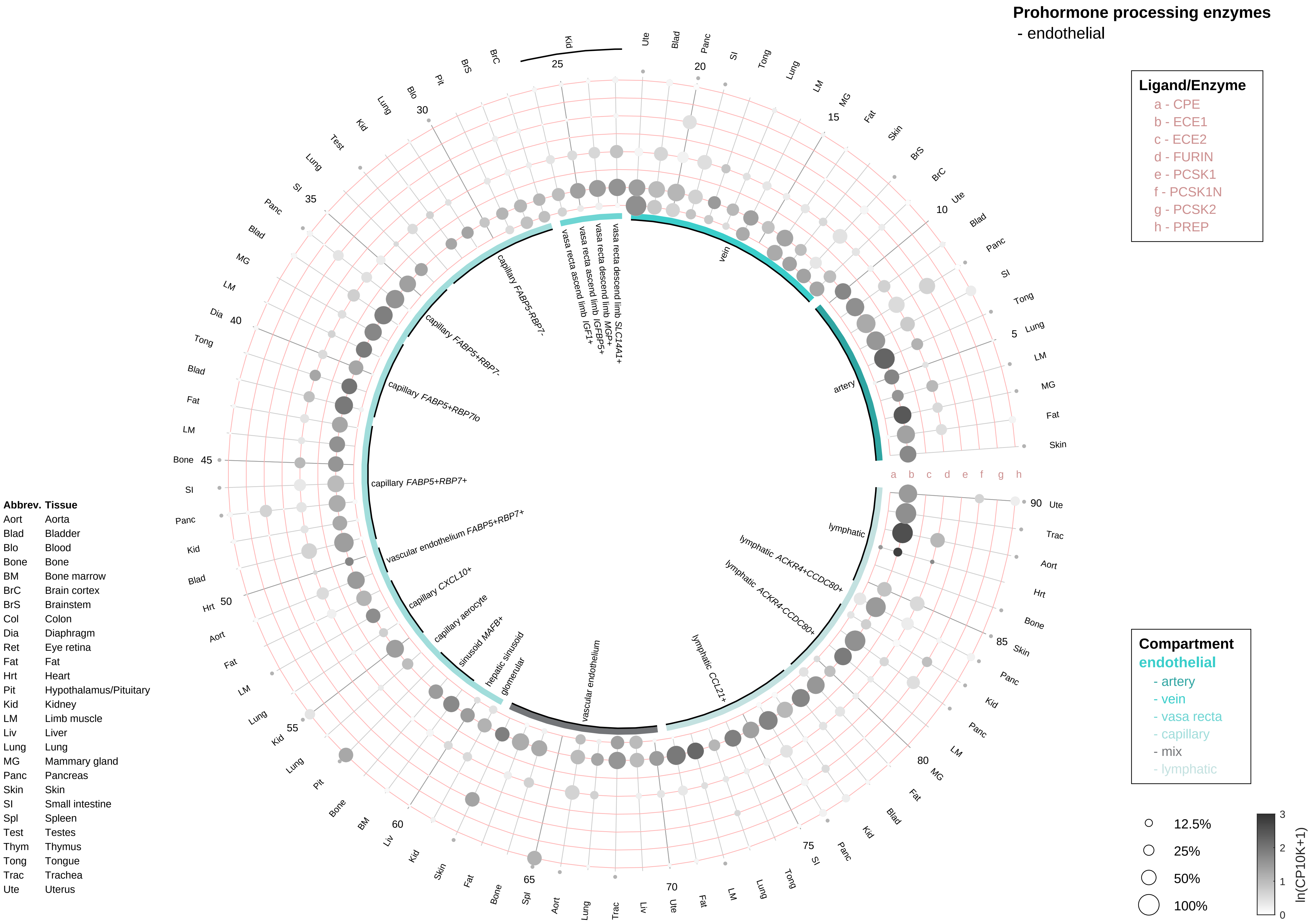
Prohormone processing enzymes
- endothelial
Ligand/Enzyme
a - CPE
b - ECE1
c - ECE2
d - FURIN
e - PCSK1
f - PCSK1N
g - PCSK2
h - PREP
Compartment
endothelial
- artery
- vein
- vasa recta
- capillary
- mix
- lymphatic
12.5%
25%
50%
100%
ln(CP10K+1)
0
1
2
3
vein
artery
vasa recta descend limb SLC14A1+
vasa recta descend limb MGP+
vasa recta ascend limb IGFBP5+
vasa recta ascend limb IGF1+
capillary FABP5-RBP7-
capillary FABP5+RBP7-
capillary FABP5+RBP7lo
capillary FABP5+RBP7+
vascular endothelium FABP5+RBP7+
capillary CXCL10+
capillary aerocyte
sinusoid MAFB+
hepatic sinusoid
glomerular
vascular endothelium
lymphatic CCL21+
lymphatic ACKR4-CCDC80+
lymphatic ACKR4+CCDC80+
lymphatic
Abbrev. Tissue
Aort Aorta
Blad Bladder
Blo Blood
Bone Bone
BM Bone marrow
BrC Brain cortex
BrS Brainstem
Col Colon
Dia Diaphragm
Ret Eye retina
Fat Fat
Hrt Heart
Pit Hypothalamus/Pituitary
Kid Kidney
LM Limb muscle
Liv Liver
Lung Lung
MG Mammary gland
Panc Pancreas
Skin Skin
SI Small intestine
Spl Spleen
Test Testes
Thym Thymus
Tong Tongue
Trac Trachea
Ute Uterus

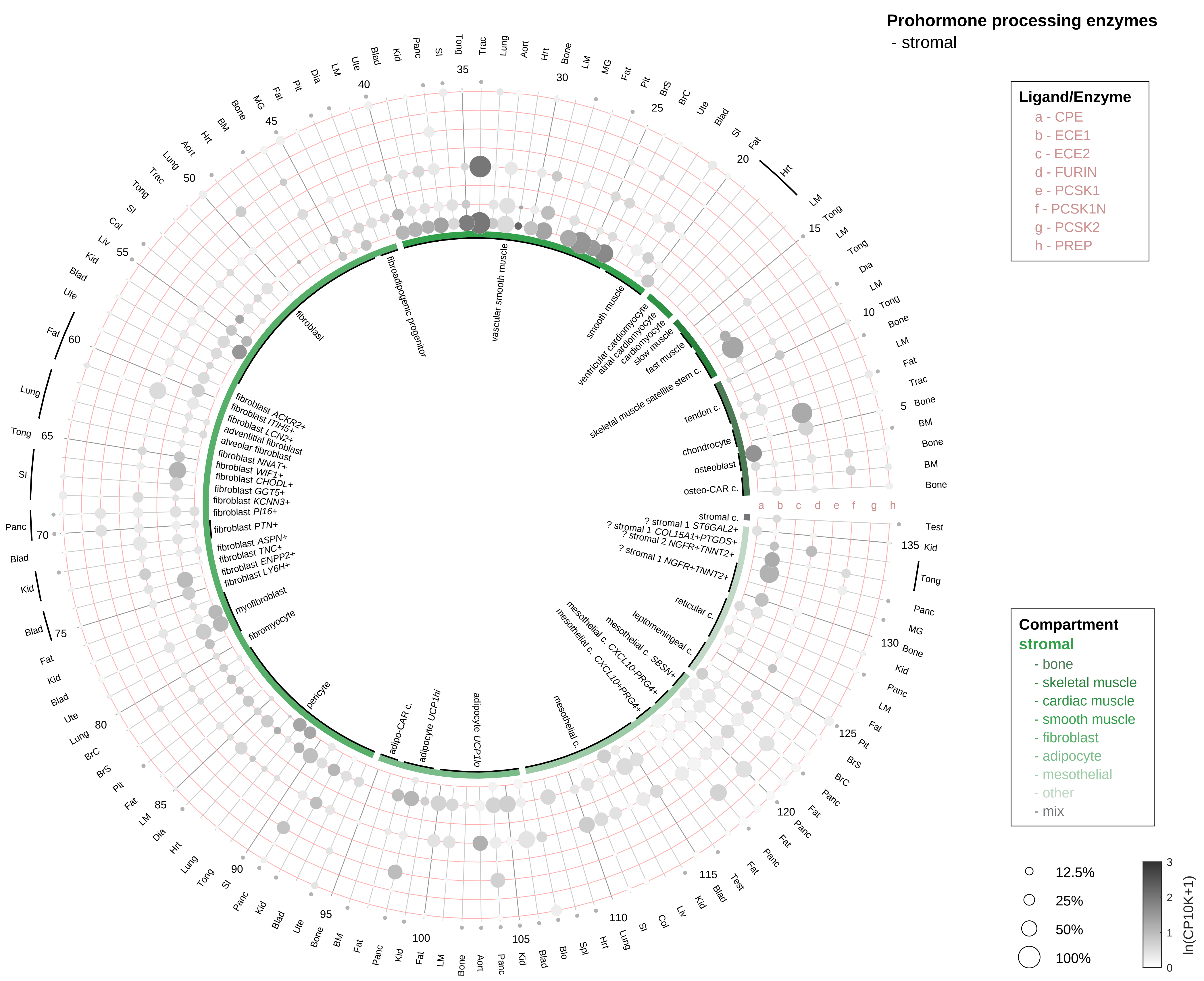

Abbrev.	Tissue
Aort	Aorta
Blad	Bladder
Blo	Blood
Bone	Bone
BM	Bone marrow
BrC	Brain cortex
BrS	Brainstem
Col	Colon
Dia	Diaphragm
Ret	Eye retina
Fat	Fat
Hrt	Heart
Pit	Hypothalamus/Pituitary
Kid	Kidney
LM	Limb muscle
Liv	Liver
Lung	Lung
MG	Mammary gland
Panc	Pancreas
Skin	Skin
SI	Small intestine
Spl	Spleen
Test	Testes
Thym	Thymus
Tong	Tongue
Trac	Trachea
Ute	Uterus

Prohormone processing enzymes
- lymphoid

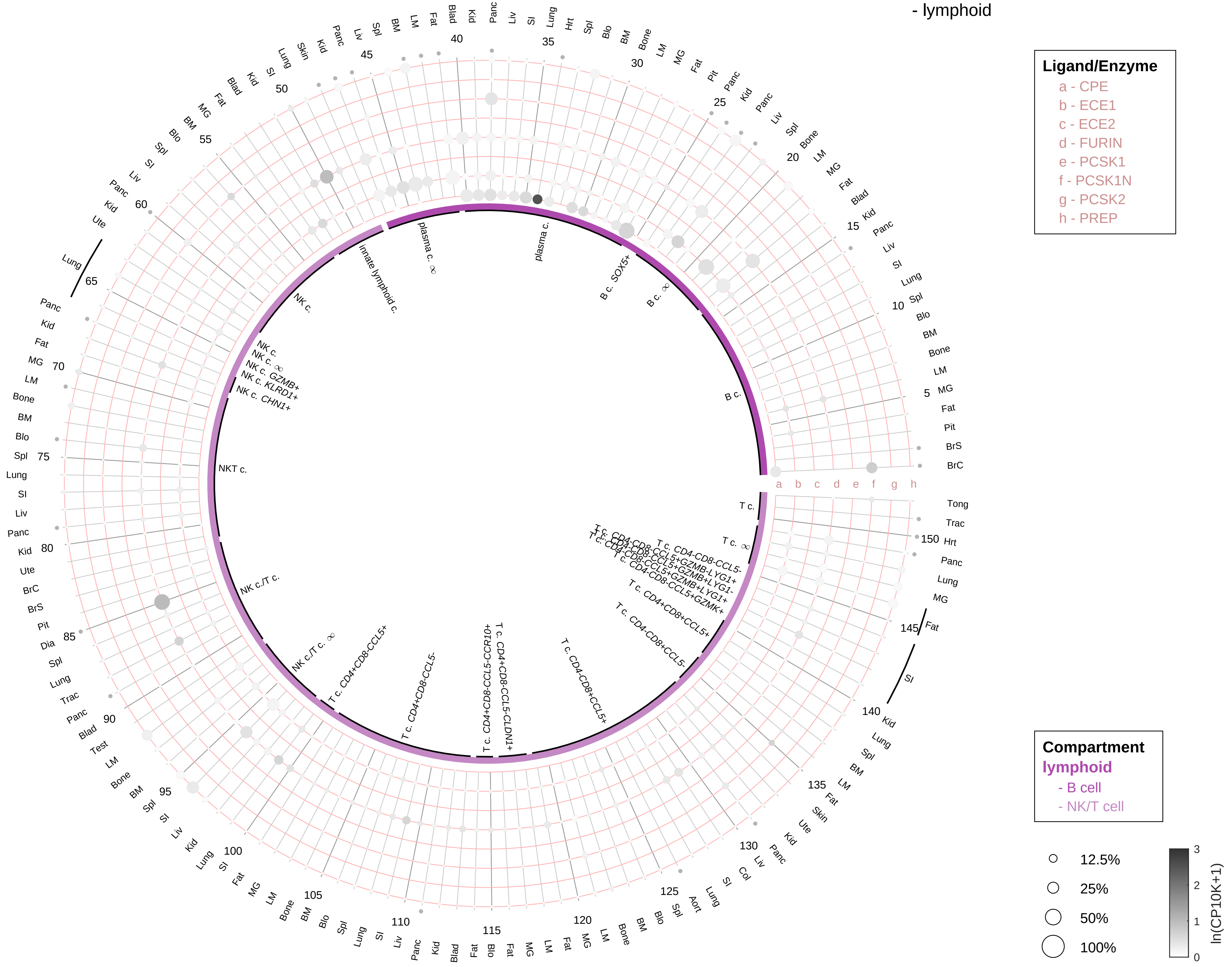

Abbrev.	Tissue
Aort	Aorta
Blad	Bladder
Blo	Blood
Bone	Bone
BM	Bone marrow
BrC	Brain cortex
BrS	Brainstem
Col	Colon
Dia	Diaphragm
Ret	Eye retina
Fat	Fat
Hrt	Heart
Pit	Hypothalamus/Pituitary
Kid	Kidney
LM	Limb muscle
Liv	Liver
Lung	Lung
MG	Mammary gland
Panc	Pancreas
Skin	Skin
SI	Small intestine
Spl	Spleen
Test	Testes
Thym	Thymus
Tong	Tongue
Trac	Trachea
Ute	Uterus

Prohormone processing enzymes
- myeloid

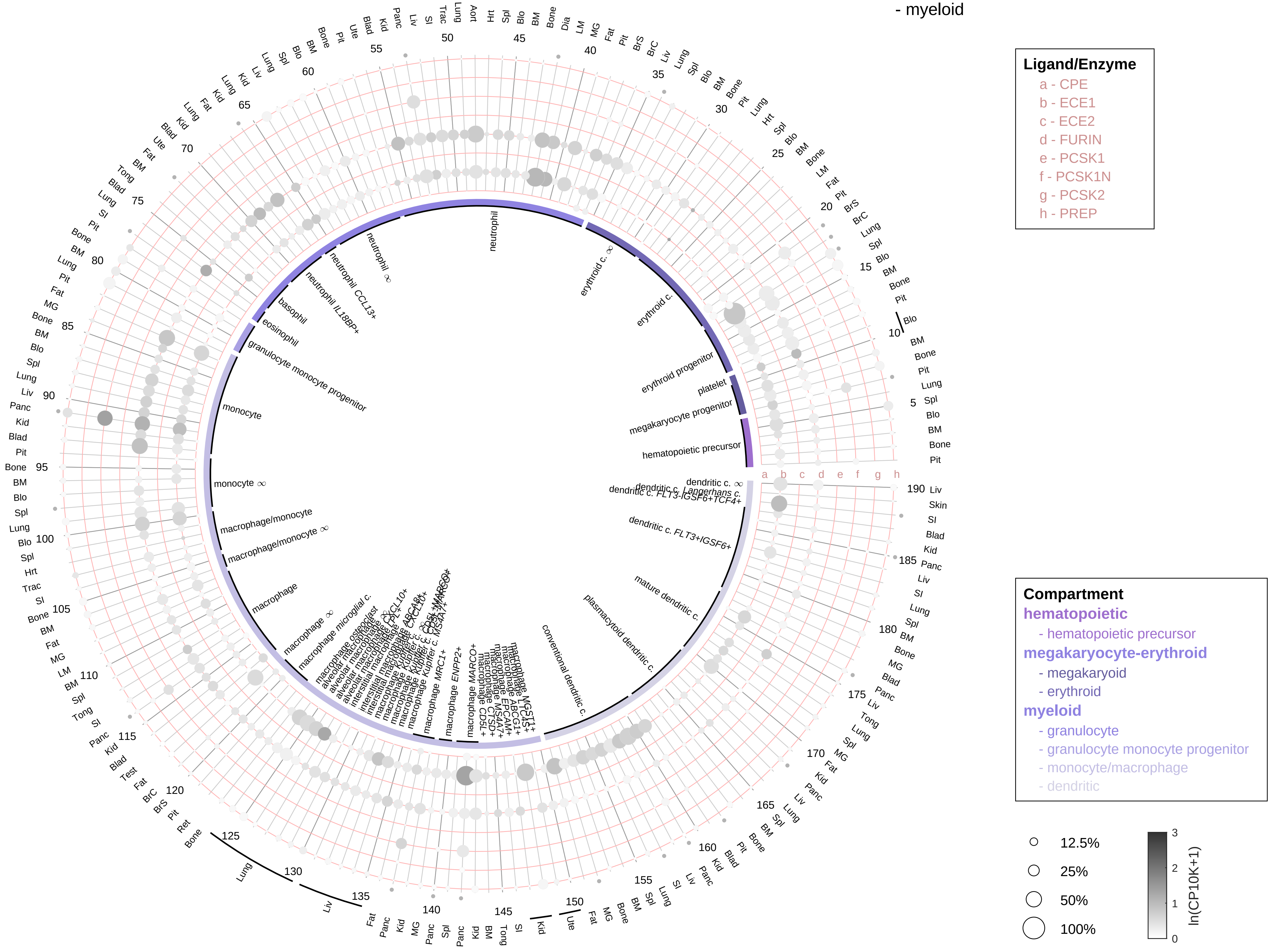

Abbrev.	Tissue
Aort	Aorta
Blad	Bladder
Blo	Blood
Bone	Bone
BM	Bone marrow
BrC	Brain cortex
BrS	Brainstem
Col	Colon
Dia	Diaphragm
Ret	Eye retina
Fat	Fat
Hrt	Heart
Pit	Hypothalamus/Pituitary
Kid	Kidney
LM	Limb muscle
Liv	Liver
Lung	Lung
MG	Mammary gland
Panc	Pancreas
Skin	Skin
SI	Small intestine
Spl	Spleen
Test	Testes
Thym	Thymus
Tong	Tongue
Trac	Trachea
Ute	Uterus
